# circRNA circ_102049 Implicates in Pancreatic Ductal Adenocarcinoma Progression through Activating CD80 by Targeting miR-455-3p

**DOI:** 10.1155/2021/8819990

**Published:** 2021-01-07

**Authors:** Jie Zhu, Yong Zhou, Shanshan Zhu, Fei Li, Jiajia Xu, Liming Zhang, Hairong Shu

**Affiliations:** ^1^Medical Laboratory, Taizhou Central Hospital (Taizhou University Hospital), Taizhou, Zhejiang, China; ^2^Department of Medical Service, Taizhou Central Hospital (Taizhou University Hospital), Taizhou, Zhejiang, China

## Abstract

Emerging evidence has shown that circular RNAs (circRNAs) and DNA methylation play important roles in the causation and progression of cancers. However, the roles of circRNAs and abnormal methylation genes in the tumorigenesis of pancreatic ductal adenocarcinoma (PDAC) are still largely unknown. Expression profiles of circRNA, gene methylation, and mRNA were downloaded from the GEO database, and differentially expressed genes were obtained via GEO2R, and a ceRNA network was constructed based on circRNA-miRNA pairs and miRNA-mRNA pairs. Inflammation-associated genes were collected from the GeneCards database. Then, functional enrichment analysis and protein-protein interaction (PPI) networks of inflammation-associated methylated expressed genes were investigated using Metascape and STRING databases, respectively, and visualized in Cytoscape. Hub genes of PPI networks were identified using the NetworkAnalyzer plugin. Also, we analyzed the methylation, protein expression levels, and prognostic value of hub genes in PDAC patients through the UALCAN, Human Protein Atlas (HPA), and Kaplan-Meier plotter databases, respectively. The circRNA_102049/miR-455-3p/CD80 axis was identified by the ceRNA network and hub genes. In vitro and in vivo experiments were performed to evaluate the functions of circRNA_102049. The regulatory mechanisms of circRNA_102049 and miR-455-3p were explored by RT-PCR, western blot, and dual-luciferase assays. In the present study, twelve hub genes (STAT1, CCND1, KRAS, CD80, ICAM1, ESR1, RAF1, RPS6KA2, KDM6B, TNRC6A, FOSB, and DNM1) were determined from the PPI networks. Additionally, the circRNA_102049 was upregulated in PDAC cell lines. Functionally, the knockdown of circRNA_102049 by siRNAs inhibited cell growth, inflammatory factors, and migratory and invasive potential and promoted cell apoptosis. Mechanistically, circRNA_102049 functioned as a sponge of miR-455-3p and partially reversed the effect of miR-455-3p and consequently upregulated CD80 expression. Our findings showed that circRNA_102049 and methylated hub genes play an important role in the proliferation, apoptosis, migration, invasion, and inflammatory response of PDAC, which might be selected as a promising prognostic marker and therapeutic target for PDAC.

## 1. Introduction

Pancreatic cancer is the seventh leading cause of global cancer-associated death, with an estimated 432,242 deaths in 2018 [1], and is predicted to become in 2030 the second leading cause of cancer-related death [2, 3]. Pancreatic ductal adenocarcinoma (PDAC) is the most common class of aggressive malignancies in the exocrine pancreas, accounting for approximately 90% of cases, with an unsatisfactory prognosis [4]. This fatal disease is characterized by a few obvious clinical presentation, metastasis, and recurrence that contribute to the poor quality of life of patients [5]. At present, curative resection, classical chemotherapy, and radiotherapy are the main therapeutic strategies [6]. Despite the great advances of clinical technology, the outcomes remain unsatisfactory and the five-year survival rate of PDAC patients remains below 5% [5]. Therefore, there is an urgent need to increase the understanding of underlying molecular mechanisms of PDAC that might result in discovering distinct diagnostic and therapeutic targets for PDAC treatment.

Accumulating studies demonstrated that the inflammatory microenvironment plays a significant role in the development of various cancers, including PDAC [7, 8]. Additionally, the inflammatory cytokines widely participate in various biological processes such as cell growth, differentiation, and metabolism by directly affecting cancer cells [9]. Moreover, previous research studies have reported that cancer cells are also produced and secrete many different kinds of cytokines, including IL-6, IL-1*β*, IL-8, IL-17, and TNF-*α* [8, 10]. However, the pathogenesis of PDAC patients has not been fully investigated. Recently, it has been reported that abnormal DNA methylation, one of the epigenetic modifications, is an important mechanism helping the initiation and development of cancers [11, 12]. Besides, a study by Yang et al. demonstrated that DNA methylation frequently appears in early cancers and might be used as the biological marker for early diagnosis and screening of tumors [13]. The clinical potential of DNA methylation in the pathogenesis of cancer has gradually been valued. Significantly, recent studies show that chronic inflammation promotes the genetic and epigenetic disturbances with external stimuli [14], and PDAC is an epigenetic disease characterized by widespread and profound alterations in DNA methylation [15]. According to the finding above, potential therapeutic strategies associated with inflammation and DNA methylation are urgently needed.

Currently, noncoding RNAs, including circular RNAs (circRNAs), long noncoding RNAs (lncRNAs), and microRNAs (miRNAs), all have an important role in physiological and pathological events, and some of which have emerged as promising novel biomarkers for the detection of PDAC [16–18]. circRNAs are formed by the linking of the 3′ end of the exon back to its 5′ end, forming a covalently closed loop with no or limited protein-coding potential [19]. miRNAs are a class of small endogenous noncoding RNAs that consisted of 18~22 nucleotides, which mainly regulate the expression of the target genes posttranscriptionally to affect the PDAC progression [20]. Mechanistically, the competing endogenous RNA (ceRNA) mechanism is identified as one of the vital mechanisms of circRNAs to function as a “sponge” by binding the miRNA response elements (MREs) for mediating the pathological processes in PDAC. For instance, upregulated circ_0030235 facilitates PDAC cell growth and migratory and invasive potential, partly by inhibiting and sponging miR-1253 and miR-1294 [21]. Highly expressed circBFAR is also correlated with the poorer prognosis of patients with PDAC and promoted the progression of PDAC cells by sponging miR-34b-5p to activate the mesenchymal-epithelial transition factor (MET)/PI3K/Akt signaling pathway [22]. Hence, the evidence has been obtained to support the clinical significance of investigating the functional roles of circRNAs in PDAC and many circRNAs remain to be discovered.

With the great advances of high-throughput sequencing technologies and highly developed bioinformatics tools, the differentially expressed genes (DEGs) or differentially methylated genes (DMGs) in cancers have laid a favorable foundation for personalized target therapy. In this study, we integrated and analyzed the circRNA profiling microarrays (GSE69362, GSE79634), gene methylation profiling microarray (GSE49149), and seven microarray datasets (GSE14245, GSE27890, GSE32676, GSE41372, GSE62165, GSE62452, and GSE71989) to identify the circRNA biomarkers and the abnormal inflammation-associated methylation target genes of PDAC patients. Subsequently, we screened the hub genes in the protein-protein interaction (PPI) networks. Moreover, we estimated the promoter methylation level and protein expression level of hub genes by using the UALCAN platform and the Human Protein Atlas database, respectively. And the prognostic value of hub genes was assessed through the Kaplan-Meier plotter. After that, we focused on an oncogenic circRNA (hsa_circRNA_102049, also known as hsa_circ_0043278) generated from exon 16 of the TADA2A gene, termed circTADA2A, located at chromosome 17: 35797838-35800763. In the current study, we measured the expression levels of hsa_circRNA_102049 in PDAC cell lines. Then, loss-of-function experiments were performed in two selected PDAC cells to measure the specific impacts of circRNA_102049 on cell growth, apoptosis, migration, and invasion characteristics. Importantly, we revealed that circRNA_102049 sponged hsa-miR-455-3p to promote CD80 expression and partially reversed the effect of miR-455-3p. Collectively, the results indicated that the circRNA_102049/miR-455-3p/CD80 axis had an important role in the development of PDAC, which suggested that circRNA_102049 might serve as a novel therapeutic target in PDAC.

## 2. Materials and Methods

### 2.1. Microarray Datasets and Data Processing

We retrieved the National Center for Biotechnology Information Gene Expression Omnibus (GEO, https://www.ncbi.nlm.nih.gov/geo/) database with the keyword “Pancreatic ductal adenocarcinoma.” Then, the circRNA expression profiling datasets (GSE69362, GSE79634), gene methylation profiling microarray (GSE49149), and gene expression datasets (GSE14245, GSE27890, GSE32676, GSE41372, GSE62165, GSE62452, and GSE71989) were downloaded for further analysis. All included datasets met the following criteria: (1) samples consisted of both PDAC tissues and normal control tissues and (2) gene expression profiling of circRNA or mRNA. Subsequently, we employed the GEO2R (https://www.ncbi.nlm.nih.gov/geo/geo2r/), an R programming language-based web tool that allows users to compare two or more groups of samples in a GEO series to identify DEGs and DMGs between the PDAC and normal tissue samples. *P* value < 0.05 and ∣log2 fold change (FC) | >1 were set as the thresholds for screening DEGs. And the criteria for identifying DMGs were ∣*t* | >2 and *q* value < 0.05 (*q* value is the *P* value adjusted by FDR (false discovery rate)). To screen the intersectional circRNAs significantly expressed in GSE69362 and GSE79634, an online tool Venny2.1 (https://bioinfogp.cnb.csic.es/tools/venny/) was used to plot the Venn diagrams.

### 2.2. Prediction of MRE and miRNA Targets

The screened differentially expressed circRNA- (DEC-) miRNA interactions were predicted using the Circular RNA Interactome (CircInteractome, https://circinteractome.nia.nih.gov/) [23], which predicts the miRNAs which can potentially target the circRNAs using the TargetScan prediction tool [24]. Additionally, the genes targeted by miRNAs were predicted using TargetScanHuman (http://www.targetscan.org/vert_72/) [25] and miRDB (http://mirdb.org/) [26] online databases. The target genes of miRNAs identified simultaneously by both the TargetScan and miRDB databases were chosen. According to these selected DEC-miRNA and miRNA-mRNA interactions, we constructed a circRNA-miRNA-mRNA regulatory network, which was visualized using Cytoscape (version 3.8.0, https://cytoscape.org/), an open-source software platform for visualizing molecular interaction networks.

### 2.3. Identification of Inflammation-Associated Methylation Genes

In this study, we collected different genes associated with inflammation according to GeneCards (version 5.0; https://www.genecards.org/), which is a searchable, integrative online platform that provides comprehensive, user-friendly information on all annotated and predicted human genes [27]. The performing keyword “inflammation” was input into GeneCards to obtain targets associated with PDAC. Finally, we obtained inflammation-associated hypomethylation-high expression genes after overlapping of upregulated DEGs and hypomethylation genes and obtained hypermethylation-low expression genes after overlapping of downregulated DEGs and hypermethylation genes through drawing a Venn diagram.

### 2.4. Functional Enrichment Analysis

The Metascape database (http://metascape.org/gp/index.html#/main/step1) was used to perform functional enrichment analysis of the hypomethylation-high expression genes and hypermethylation-low expression genes, respectively. Metascape is an integrated and user-friendly web tool that combines functional enrichment, interactome analysis, and gene annotation to aid in revealing the biological meaning of the offered gene list, including the Gene Ontology (GO) and Kyoto Encyclopedia of Genes and Genomes (KEGG) pathway terms [28]. The inflammation-associated hypomethylation-high expression genes and hypermethylation-low expression genes of PDAC were input into Metascape for GO-biological processes (BP) and KEGG analysis, and the species was selected as “*Homo sapiens*.”*P* < 0.05 was considered statistically significant.

### 2.5. Protein-Protein Interaction (PPI) Network of Inflammation-Associated Methylation Genes

The inflammation-associated hypomethylation-high expression genes and hypermethylation-low expression genes of PDAC were imported into Search Tool for the Retrieval of Interacting Genes (STRING) (version 11.0, https://string-db.org/) to construct the PPI networks, respectively. STRING is an online tool that is aimed at collecting, scoring, and integrating the direct and indirect interaction between proteins and proteins [29], and interaction associations with a combined score of ≥0.7 were retained. In search of hub genes in the two networks, Cytoscape was utilized to analyze the degree value of nodes. Using Cytoscape's plugin NetworkAnalyzer, we selected a total of 12 DEGs with high connectivity in the inflammation-associated methylation DEG networks as the hub genes based on the degree algorithm.

### 2.6. Methylation, Immunohistochemical, and Survival Analyses of Hub Genes

To further confirm the bioinformatics results of the hub genes, we used UALCAN (http://ualcan.path.uab.edu/index.html) for methylation level analysis. UALCAN is a comprehensive, user-friendly, and interactive web portal that is extremely helpful in gene-level querying of The Cancer Genome Atlas (TCGA) data [30]. The gene symbol of hub genes was imported into the UALCAN, and TCGA dataset was selected as “Pancreatic adenocarcinoma.” The beta value indicates the level of DNA methylation ranging from 0 (unmethylated) to 1 (fully methylated). Different cutoff beta values have been considered to indicate hypermethylation (beta value: 0.7-0.5) or hypomethylation (beta value: 0.3-0.25) [31, 32], and differences were regarded as significant with *P* value less than 0.05.

For immunohistochemical analysis, Human Protein Atlas (HPA) (version 19.3, https://www.proteinatlas.org/), which is aimed at mapping all the human proteins in cells, tissues, and organs using an integration of various omics technologies [33], was used to assess the protein expressions of hub genes in normal tissues and pancreatic cancer tissues. To further elucidate the relationship between DEC targets and hub gene expression and PDAC prognosis, the web server Kaplan-Meier (KM) plotter (http://kmplot.com/analysis/) was utilized for overall survival (OS) analyses using the log-rank test [34]. *P* < 0.05 was considered statistically significant.

### 2.7. Cell Culture and Transfection

The human PDAC cell lines AsPC-1, CFPAC-1, BxPC-3, SW1990, and Panc-1 were purchased from the cell bank of the Chinese Academy of Science, Shanghai, and maintained in Dulbecco's Modified Eagle's Medium (DMEM, Thermo Fisher Scientific, USA) supplemented with 10% FBS, 100 units/mL penicillin, and 100 *μ*g/mL streptomycin in a humidified incubator with 5% CO_2_ at 37°C. HPDE cells, the immortalized pancreatic duct cells, are used as the normal control subject in this study. Two siRNAs against circRNA_102049 (si-circ_102049_#1 and si-circ_102049_#2) were constructed to block the expression of circRNA_102049 in SW1990 and Panc-1 cells, respectively. Negative control siRNA (si-NC) was utilized as a control. The target sequence of si-circ_102049_#1 and si-circ_102049_#2 was 5′-ATTCCATTTCACTACT TCAGA-3′and 5′-TCCATTTCACTACTTCAGATT-3′, respectively. In addition, the miR-455-3p inhibitor and mimic were utilized to silence and enhance the expression of miR-455-3p, respectively. For transfection, SW1990 and Panc-1 cells were placed in 6-well plates with DMEM. After growth to 80% confluence, SW1990 and Panc-1 cells were transfected with 50 nM si-circ_102049_#1 or si-circ_102049_#2 and 100 nM miR-455-3p inhibitor or mimic by using Lipofectamine 3000 (Thermo Fisher Scientific, USA). The circ_102049 expression in the transfected cells was measured by real-time quantitative PCR (RT-qPCR) with specially appointed primers.

### 2.8. RNA Isolation, Treatment with RNase R, and RT-qPCR

Total RNA was extracted from the PDAC cell lines using the TRIzol reagent (Beyotime, Shanghai, China) following the manufacturer's protocol. The quality of RNA was assessed by NanoDrop 2000 spectrophotometry (Thermo Fisher Scientific, USA). RNase R treatment was performed for 20 min at 37°C using 3 U/*μ*g RNase R (Sigma-Aldrich, St. Louis, MO, USA). Then, total RNAs were reverse transcribed into cDNA through PrimeScript RT Master Mix (Takara, Japan). Reverse transcription of miRNA was performed using the Mir-X™ miRNA First-Strand Synthesis Kit (Clontech Laboratories, Inc.), and RT-qPCR analysis was performed using the SYBR Green qPCR Kit (Takara Biotechnology Co., Dalian, China) on an ABI 7300 Real-Time PCR System (Applied Biosystems, Thermo Fisher Scientific). Primers used in the current study were all obtained from Sangon Biotech (Shanghai, China). The sequence of primers was as follows: circ_102049, F: 5′-ACCCTGCTGAACCTGAAACA-3′; R: 5′-TCCTGCCAATTTCCAAAGCC-3′; miR-455-3p, F: 5′-TGCGCCAAACCACACTGTGGTG-3′; R: 5′-CCAGTGCAGGGTCCGAGGTATT-3′; TADA2A, F: 5′-CCTTTTTTCCTCTGCTTGCA-3′; R: 5′-ATCCTGCCAATTTCCAAAGC-3′; CD80, F: 5′-CCTCTCCATTGTGATCCTGG-3′; R: 5′-GGCGTACACTTTCCCTTCTC-3′; TNF-*α*, F: 5′-GTGACAAGCCTGTAGCCCAT-3′; R: 5′-CAGACTCGGCAAAGTCGAGA-3′; IL-6, F: 5′-TGAACTCCTTCTCCACAAGCG-3′; R: 5′-ATTTGTGGTTGGGTCAGGGG-3′; IL-1*β*, F: 5′-CTTTCCCGTGGACCTTCCAG-3′; R: 5′-AATGGGAACGTCACACACCA-3′; IL-8, F: 5′-AAGGTGCAGTTTTGCCAAGG-3′; R: 5′-CAACCCTCTGCACCCAGTTT-3′; IL-17, F: 5′-CTGTCCCCATCCAGCAAGAG-3′; R: 5′-AGGCCACATGGTGGACAATC-3′; GAPDH, F: 5′-GAAGGTGAAGGTCGGAGTC-3′; R: 5′-GAAGATGGTGATGGGATTTC-3′; and U6, F: 5′-CTCGCTTCGGCAGCACA-3′; R: 5′-AACGCTTCACGAATT TGCGT-3′. The expression of circ_102049, TADA2A, CD80, TNF-*α*, IL-6, IL-1*β*, IL-8, and IL-17 were normalized to GAPDH, and miR-455-3p expression was normalized to U6, using the 2^−*ΔΔ*Ct^ method [35].

### 2.9. circRNA Localization

For circRNA localization, the nuclear and cytoplasmic RNAs of SW1990 and Panc-1 cells were isolated and extracted using the Cytoplasmic & Nuclear RNA Purification Kit (Ambion, Austin, TX, USA), and the expression of circ_102049 in nuclear and cytoplasmic RNAs was detected by qRT-PCR. GAPDH and U6 served as the cytoplasm and nuclear controls, respectively.

### 2.10. Actinomycin D Assay

To assess the stability of circ_102049 and its linear isoform, SW1990 and Panc-1 cells were seeded at 5 × 10^4^ cells per well in 24-well plates. After 24 h, the cells were treated with 2 mg/L actinomycin D (Sigma-Aldrich, St. Louis, MO, USA) for 0 h, 6 h, 12 h, and 24 h. After treatment with actinomycin D, qRT-PCR was performed to determine the expression levels of circ_102049 and TADA2A mRNA.

### 2.11. Cell Viability and Colony Formation Assay

After transfection, SW1990 and Panc-1 cells were seeded at 4 × 10^3^ cells per well in a 96-well plate and incubated for 24 h, 48 h, 72 h, and 96 h, and then, 10 *μ*L of Cell Counting Kit- (CCK-) 8 (Beyotime, China) was added to each well. The plates were then incubated at 37°C for 2 hours. A microplate reader (Tecan, Mannedorf, Switzerland) was used to measure the OD value of each well at 450 nm. For colony formation assay, SW1990 and Panc-1 cells were seeded into 6-well plates. After transfection, cells were cultured for 2 weeks until most colonies were visible to the naked eye. Then, SW1990 and Panc-1 cells were fixed with 4% paraformaldehyde for 20 minutes and stained with 0.1% crystal violet to observe and count the number of colonies.

### 2.12. Flow Cytometry Assay

For the cell apoptosis analysis, the cell apoptosis was assessed with the Annexin V Apoptosis Detection Kit (BD Biosciences, NJ, USA). SW1990 and Panc-1 cells were collected and washed with ice-cold PBS, followed by staining with 5 *μ*L fluorescein isothiocyanate (FITC) and 5 *μ*L propidium iodide (PI), and then incubated for 15 min in the dark at room temperature. Subsequently, a BD FACSCalibur (Beckman Coulter, USA) was utilized to measure the cell apoptotic rate.

### 2.13. Wound Healing and Invasion Assays

Transfected SW1990 and Panc-1 cells were cultured in 6-well plates, and the cell monolayer was scratched with a 200 *μ*L pipette tip when cells were grown to approximately 80% confluence, and PBS was used to remove the detached cells. Representative images of cell migration were captured at 0 h and 24 h after injury using an Olympus light microscope (Olympus Optical Co., Ltd., Tokyo, Japan), and the wound width was calculated using ImageJ software (National Institutes of Health, USA). For the invasion assay, the chambers were coated with Matrigel. SW1990 and Panc-1 cells were seeded into the upper chamber (4 × 10^3^ cells) with a serum-free medium, while a medium containing 10% FBS was added into the lower chamber. After incubation for 24 h, cotton wool was used to remove the cells in the upper chamber, and cells on the lower side of the filter were fixed with 4% paraformaldehyde for 30 min and then stained with 0.5% crystal violet for 10 min. The PDAC cells of the invasion were counted and photographed in at least five random fields under a light microscope.

### 2.14. Tumor Xenograft Assay

Six-week-old female BALB/c male nude mice were purchased from Shanghai Slac Laboratory Animals, Ltd., and randomly divided into two groups (*n* = 3 for each group). Control cells and circ_102049 knockdown Panc-1 cells (1 × 10^7^) were suspended in 100 *μ*L DMEM without FBS and then subcutaneously inoculated into the right flank of the mice, respectively. Mice were monitored every 5 days for tumor growth, and tumor size was measured using a caliper and calculated as follows: volume = (length × width^2^)/2. After 25 days, mice were sacrificed, and then, the primary tumors were removed and harvested and weighed. All experiments were performed in accordance with the *Guide for the Care and Use of Laboratory Animals* (NIH publication 80-23, revised 1996), with the approval of the Animal Care and Welfare Committee of Zhejiang Chinese Medical University.

### 2.15. Dual-Luciferase Reporter Assay

The constructs containing wild-type (wt) or mutant (mut) circ_102049-miR-455-3p and CD80-miR-455-3p were subcloned into the luciferase gene by the psiCHECK2 Vector (Promega Corporation, Madison, WI, USA) or the pmirGLO Vector (Promega Corporation, Madison, WI, USA), respectively. The above reporter vectors were cotransfected with miR-455-3p mimic or miR-455-3p NC into SW1990 and Panc-1 cells using Lipofectamine 3000. After 24 h of transfection, activities were analyzed using a dual-luciferase reporter assay system (Promega Corporation, Madison, WI) according to the manufacturer's instructions. Relative firefly luciferase activity was normalized to Renilla luciferase activity.

### 2.16. Western Blot

The total protein of transfected SW1990 and Panc-1 cells was lysed with ice-cold RIPA buffer (Beyotime, Shanghai, China). And the protein concentration was quantified by the bicinchoninic acid (BCA) kit (Beyotime, Shanghai, China) according to the manufacturer's instructions. Afterward, equal amounts of protein (30 *μ*g) were separated via sodium dodecyl sulfate-polyacrylamide gel electrophoresis (SDS-PAGE) gel and transferred onto polyvinylidene fluoride (PVDF) membranes (Millipore). After blocking with 5% nonfat milk in TBST, the membranes were incubated with primary antibodies against CD80 (1 : 1000, #54521, Cell Signaling Technology, Inc., USA) and GAPDH (1 : 5000, P04406, Sangon Biotech, China) at 4°C overnight. Then, the membranes were incubated with the horseradish peroxidase-labeled IgG goat anti-rabbit or goat anti-mouse antibody (1 : 5000, Invitrogen, USA) for 1 h at room temperature. Finally, the membranes were treated with enhanced chemiluminescence (ECL) solution (Beyotime, China) to detect the protein blots.

### 2.17. Statistical Analysis

All statistical data were expressed as the mean ± standard deviation. Two-tailed Student's *t*-test was utilized to make a comparison in two groups, and one-way ANOVA was used to compare multiple groups, respectively. All analyses were performed using SPSS 22.0 (SPSS Inc., Chicago). *P* < 0.05 was considered statistically significant.

## 3. Results

### 3.1. Identification of DEGs and DMGs in PDAC

**Figure 1:**
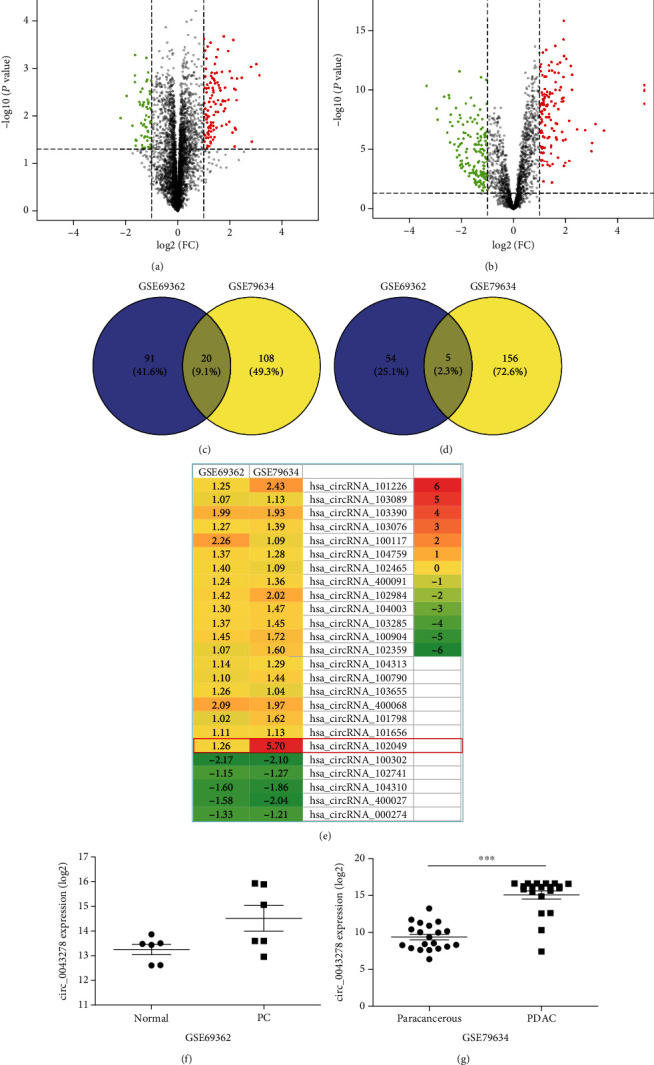
Identification of differentially expressed circRNAs (DECs) between the PDAC and nonmalignant tissues. The volcano plots of DECs for GSE69362 (a) and GSE79634 (b) datasets. The Venn diagrams of the overlapping DECs, including 20 upregulated (c) and 5 downregulated (d), among the two datasets. (e) Heatmap of the twenty-five DECs in the two microarray datasets. The expression levels of hsa_circRNA_102049 (circ_0043278) in GSE69362 (f) and GSE79634 (g) datasets, respectively.

To identify the DEGs between PDAC tissue samples and normal tissue samples, we obtained the publicly available microarray datasets GSE69362 and GSE79634 from the GEO database. There were 170 DECs (111 upregulated and 59 downregulated) in GSE69362 and 289 DECs (128 upregulated and 161 downregulated) in GSE79634, which were differentially expressed between PDAC tissues and noncancerous tissues as shown by volcano plots in Figures 1(a) and 1(b) (Supplementary Table S1 and Table S2). Among them, 25 DECs overlapped, and 20 were upregulated (Figure 1(c)), and 5 were downregulated (Figure 1(d)). The DECs from each of the two datasets were ranked and are shown by a heatmap in Figure 1(e). Further, we found that hsa_circRNA_102049 (circ_0043278) was the most significantly upregulated circRNA in GSE69362 and GSE79634, respectively (Figures 1(f) and 1(g)).

### 3.2. Construction of the circRNA-miRNA-mRNA Network in PDAC

**Figure 2:**
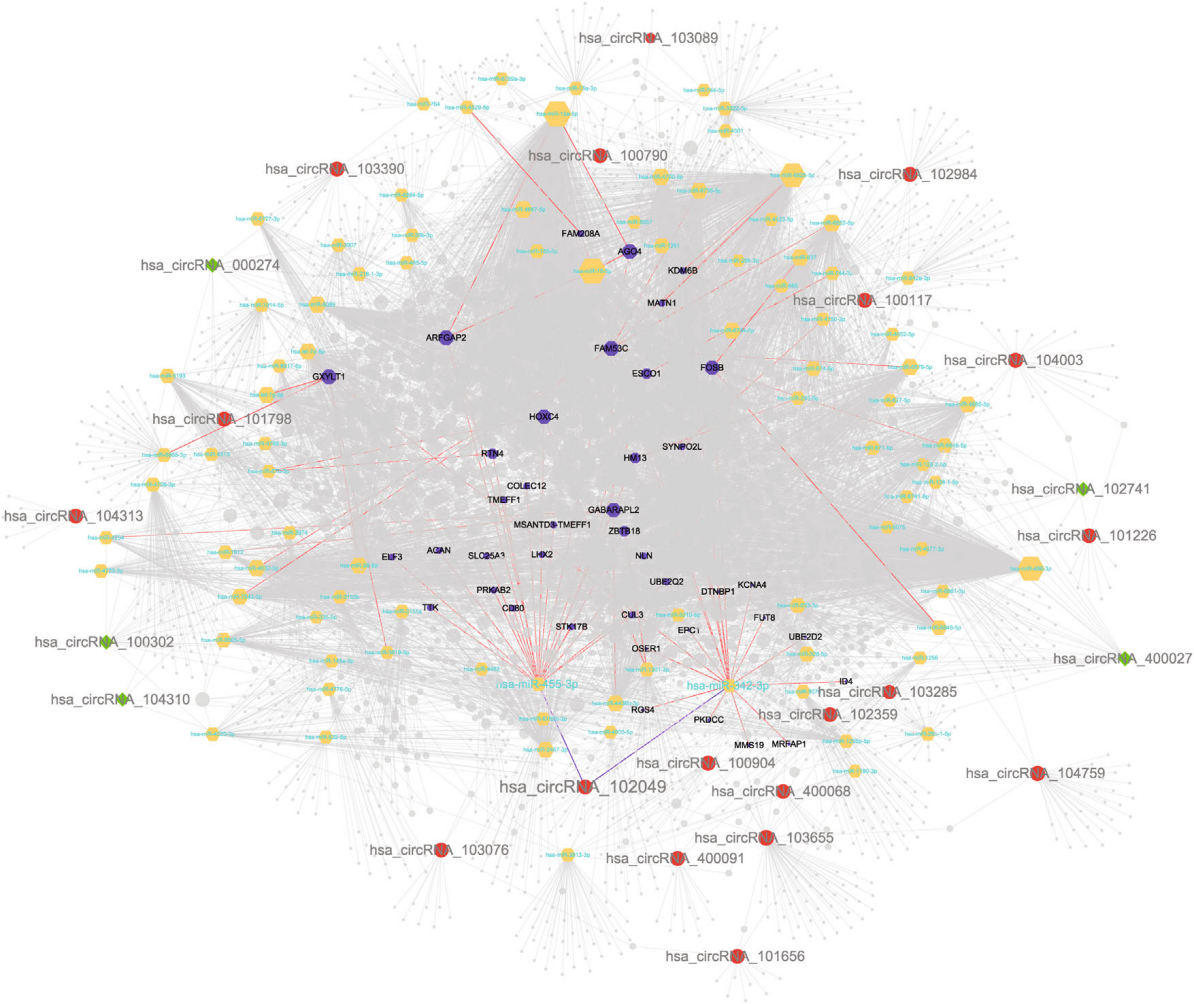
The circRNA-miRNA-mRNA network in PDAC. Red ellipse indicates upregulated circRNAs, green diamond indicates downregulated circRNAs, yellow hexagon indicates predicted miRNAs, and purple hexagon indicates target genes of hsa-miR-455-3p, as well as hsa-miR-342-3p.

To better explore the role of overlapped DECs and miRNAs in the ceRNA mechanism of PDAC, we established a circRNA-miRNA-mRNA network. The CircInteractome database was used for overlapped circRNA-miRNA prediction, and a total of 372 target miRNAs were obtained (Supplementary Table S3). Furthermore, we conducted an overall survival analysis to detect the potential prognostic value. The results are shown in Supplementary Figure S1 and Table S4. The 53 highly expressed miRNAs and 38 lowly expressed miRNAs in the KM plotter may be associated with poor survival of PDAC patients. Besides, we then identified mRNAs targeted by these 91 miRNAs in TargetScan and miRDB databases. These results indicated that 7871 mRNAs were involved in the ceRNA network (Supplementary Table S5 and Table S6). Finally, we used 22 overlapped DECs, 91 miRNAs, and 7871 mRNAs in Cytoscape 3.6.1 to construct a circRNA-miRNA-mRNA network (Figure 2). Significantly, we found that hsa-miR-455-3p and hsa-miR-342-3p were predicted to be the downstream of hsa_circRNA_102049, which suggested that there could be some biological relationships between them in the development of PDAC.

### 3.3. Identification of Inflammation-Associated Methylated Expressed Genes

**Figure 3:**
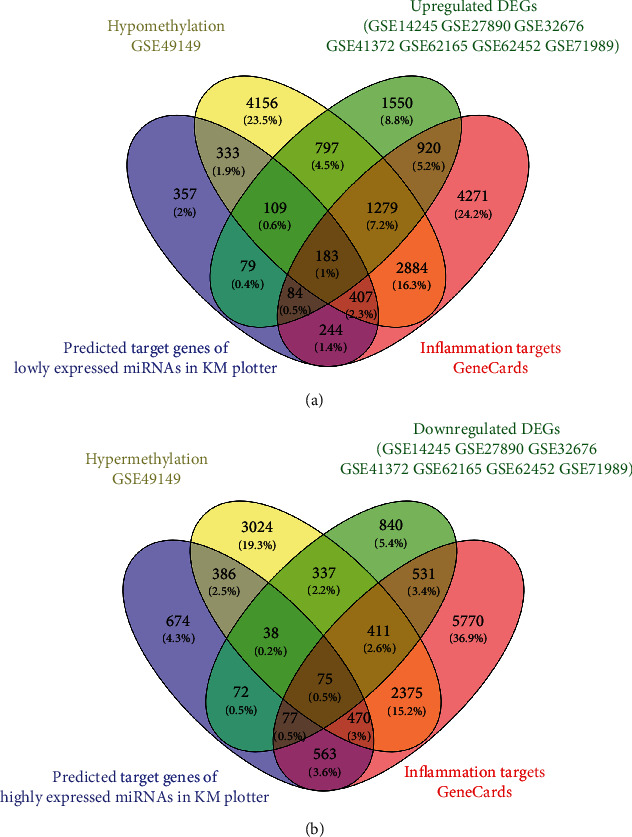
Identification of overlapping inflammation-associated methylated expressed genes. (a) Highly expressed inflammation-associated genes with low methylation. (b) Lowly expressed inflammation-associated genes with high methylation.

We used the GEO2R to identify the data of each microarray separately and screened the DEGs and DMGs. The detailed platform and number of samples for microarray datasets are shown in Table 1, respectively. A total of 65,535 DMGs were extracted from GSE49149 (Supplementary Table S7). And a total of 1981, 2375, 864, 1948, 817, 321, and 1375 DEGs were extracted from GSE14245, GSE27890, GSE32676, GSE41372, GSE62165, GSE62452, and GSE71989, respectively (Supplementary Table S8). Also, a total of 10,272 potential inflammation-associated genes were collected in the GeneCards database (Supplementary Table S9). Finally, we further identified 183 overlapping inflammation-associated hypomethylation-high expression genes by intersecting the results of integrated microarray and the predicted target genes of lowly expressed miRNAs in the KM plotter (Figure 3(a)). Similarly, 75 overlapping inflammation-associated hypermethylation-low expression genes were selected by intersecting the results of integrated microarray and the predicted target genes of highly expressed miRNAs in the KM plotter (Figure 3(b)).

### 3.4. Functional Enrichment Analysis

**Figure 4:**
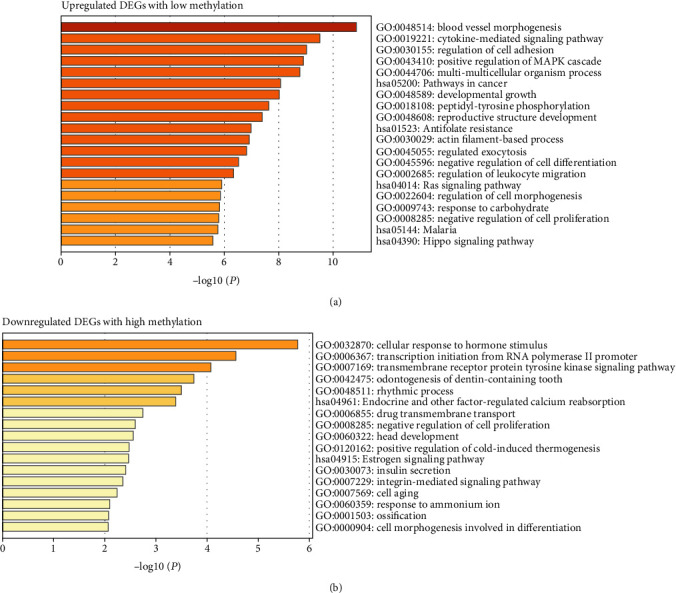
GO and KEGG pathway enrichment analysis for the inflammation-associated methylated expressed genes. GO-BP terms and KEGG pathway enrichment analysis of upregulated DEGs with low methylation (a) and downregulated DEGs with high methylation (b).

To further investigate the biological functions of the abovementioned potential overlapping inflammation-associated methylated expressed genes, GO-BP terms and KEGG pathway enrichment analysis were conducted via the Metascape platform. As shown in Figure 4(a), the upregulated DEGs with low methylation were significantly enriched in GO:0048514~blood vessel morphogenesis, GO:0019221~cytokine-mediated signaling pathway, and GO:0030155~regulation of cell adhesion; additionally, KEGG analysis proved that the upregulated DEGs were significantly enriched in hsa05200~pathways in cancer (Figure 4(a)). And again, Metascape was used to analyze the roles of identified downregulated DEGs with high methylation. Downregulated DEGs with high methylation were mainly involved in BP: GO:0032870~cellular response to hormone stimulus, GO:0006367~transcription initiation from RNA polymerase II promoter, and GO:0007169~transmembrane receptor protein tyrosine kinase signaling pathway. Based on the KEGG pathway enrichment analysis, the specific downregulated DEGs mainly participated in hsa04961~endocrine and other factor-regulated calcium reabsorption and hsa04915~estrogen signaling pathway (Figure 4(b)).

### 3.5. PPI Network of Inflammation-Associated Methylated Expressed Genes

**Figure 5:**
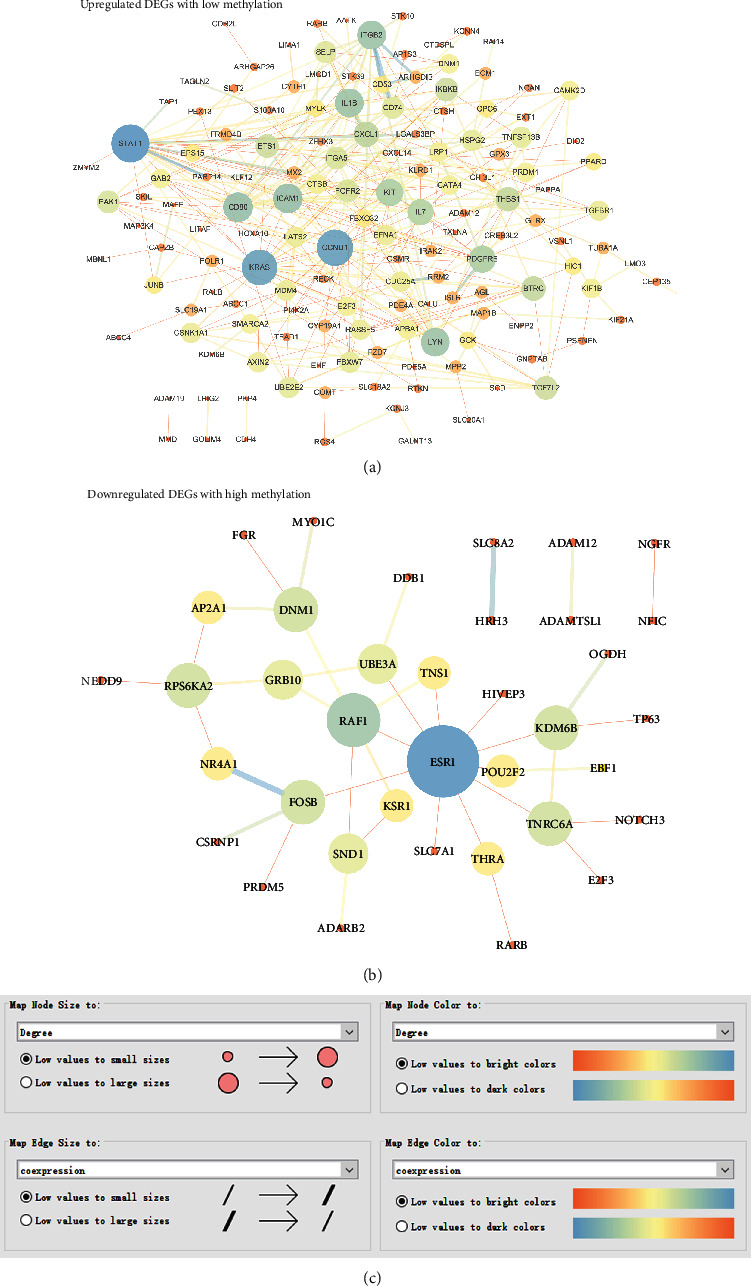
Identification of hub genes in the PPI network of inflammation-associated methylated expressed genes. (a) PPI network of upregulated DEGs with low methylation. (b) PPI network of downregulated DEGs with high methylation. (c) The node size and color were mapped with the degree value. And the edge size and color were mapped with the coexpression value.

The PPI network was built to analyze interactions between the 183 upregulated DEGs with low methylation, as well as 75 downregulated DEGs with high methylation, respectively. After removing the isolated nodes, 146 nodes and 320 edges remained in the upregulated DEG PPI network (Figure 5(a)). Similarly, the PPI network of downregulated DEGs with 37 nodes and 39 edges was constructed using the Cytoscape software (Figure 5(b)). To identify hub genes associated with the pathogenesis of PDAC, the NetworkAnalyzer plugin was used to analyze the degree value of nodes. Five genes with degree > 16 were chosen as hub genes in the upregulated DEG PPI network, and seven genes with degree > 3 were selected as hub genes in the downregulated DEG PPI network also, including signal transducer and activator of transcription 1 (STAT1); cyclin D1 (CCND1); KRAS proto-oncogene, GTPase (KRAS); CD80 molecule (CD80); intercellular adhesion molecule 1 (ICAM1); estrogen receptor 1 (ESR1); Raf-1 proto-oncogene, serine/threonine kinase (RAF1); ribosomal protein S6 kinase A2 (RPS6KA2); lysine demethylase 6B (KDM6B); trinucleotide repeat-containing adaptor 6A (TNRC6A); FosB proto-oncogene, AP-1 transcription factor subunit (FOSB); and dynamin 1 (DNM1).

### 3.6. Validation of Hub Genes according to Various Pathophysiological Characteristics

**Figure 6:**
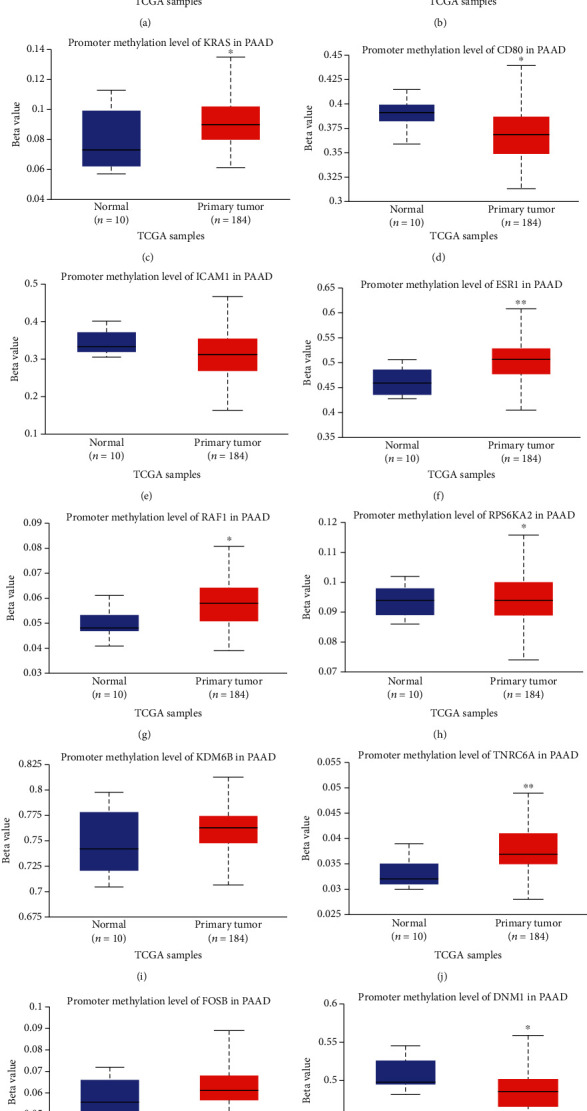
Verification of methylation levels of hub genes in pancreatic adenocarcinoma (PAAD). Promoter methylation level of STAT1 (a), CCND1 (b), KRAS (c), CD80 (d), ICAM1 (e), ESR1 (f), RAF1 (g), RPS6KA2 (h), KDM6B (i), TNRC6A (j), FOSB (k), and DNM1 (l) in PAAD. ^∗^*P* < 0.05, ^∗∗^*P* < 0.01. Primary tumor tissues vs. normal tissues.

**Figure 7:**
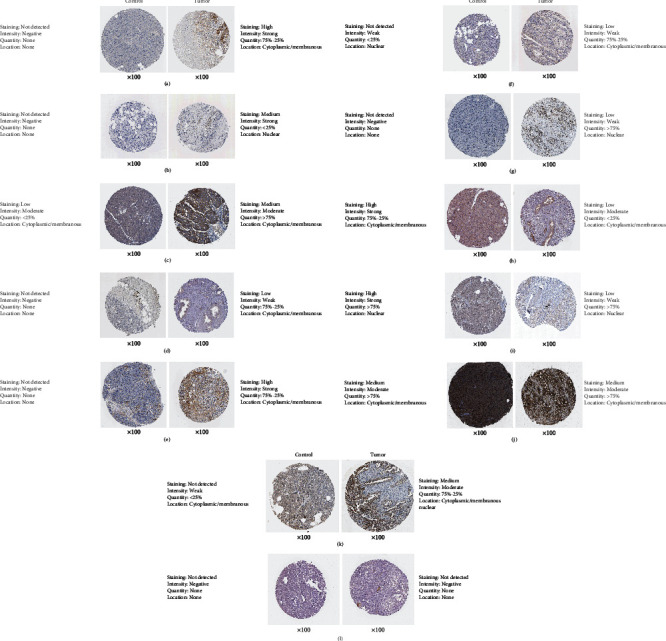
Comparison of protein expression of hub genes between normal pancreatic tissues and pancreatic cancer tissues. (a) STAT1, (b) CCND1, (c) KRAS, (d) CD80, (e) ICAM, (f) ESR1, (g) RAF1, (h) RPS6KA2, (i) KDM6B, (j) TNRC6A, (k) FOSB, and (l) DNM1.

**Figure 8:**
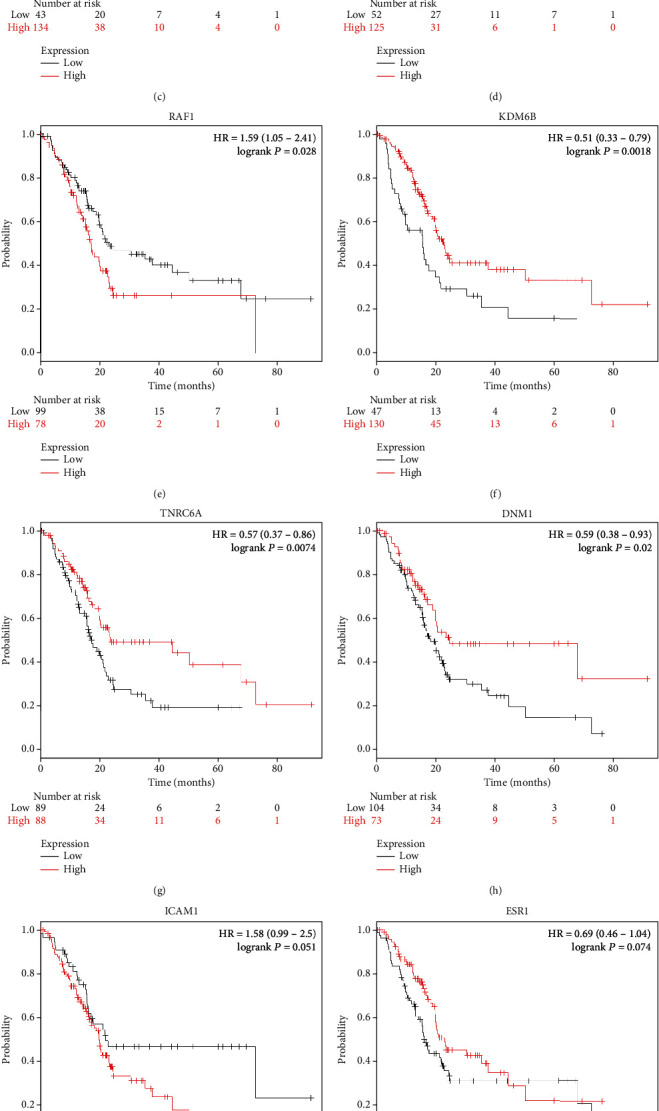
Kaplan-Meier survival curves of twelve hub genes in pancreatic adenocarcinoma patients. Overall survival by low and high (a) STAT1, (b) CCND1, (c) KRAS, (d) CD80, (e) RAF1, (f) KDM6B, (g) TNRC6A, (h) DNM1, (i) ICAM1, (j) ESR1, (k) RPS6KA2, and (l) FOSB expression.

Next, UALCAN was used to validate the methylation levels of 12 hub genes in pancreatic adenocarcinoma. The methylation levels of KRAS, ESR1, RAF1, RPS6KA2, and TNRC6A were significantly higher in primary tumor tissues compared with those in normal tissues, while the methylation levels of CD80 and DNM1 were significantly decreased in tumor samples than adjacent normal samples; but, there is no statistical difference in methylation levels of STAT1, CCND1, ICAM1, KDM6B, and FOSB (Figures 6(a)–6(l)). Additionally, the clinical stage analysis of hub genes was investigated by using the GEPIA2 database (http://gepia2.cancer-pku.cn/#index), and the results suggest that the expression levels of CD80 and ICAM1 were significantly increased in various tumor stages (Supplementary Figure S2). Further analysis of hub genes in the HPA database showed that the protein expression levels of STAT1, CCND1, KRAS, CD80, ICAM1, ESR1, RAF1, FOSB, and DNM1 were upregulated in PDAC tissues compared with normal tissues, and the RPS6KA2 protein level was significantly decreased in PDAC tissues; however, the expression of RPS6KA2, KDM6B, and TNRC6A was not significantly different between pancreatic cancer samples and normal control tissues (Figures 7(a)–7(l)). Additionally, we used the KM plotter to analyze the correlation between hub gene expression and PDAC prognosis. We found that high mRNA expression of STAT1, CCND1, KRAS, CD80, and RAF1 and low mRNA expression of KDM6B, TNRC6A, and DNM1 were associated with worse overall survival (Figures 8(a)–8(h)). There was no significant association between ICAM1, ESR1, RPS6KA2, and FOSB expression and overall survival (Figures 8(i)–8(l)). Considering the ceRNA mechanisms and the signature value of hub genes, the circ_102049/miR-455-3p/CD80 axis was selected for further circRNA functional verification in PDAC cell lines.

### 3.7. Characteristics of circ_102049 in PDAC

**Figure 9:**
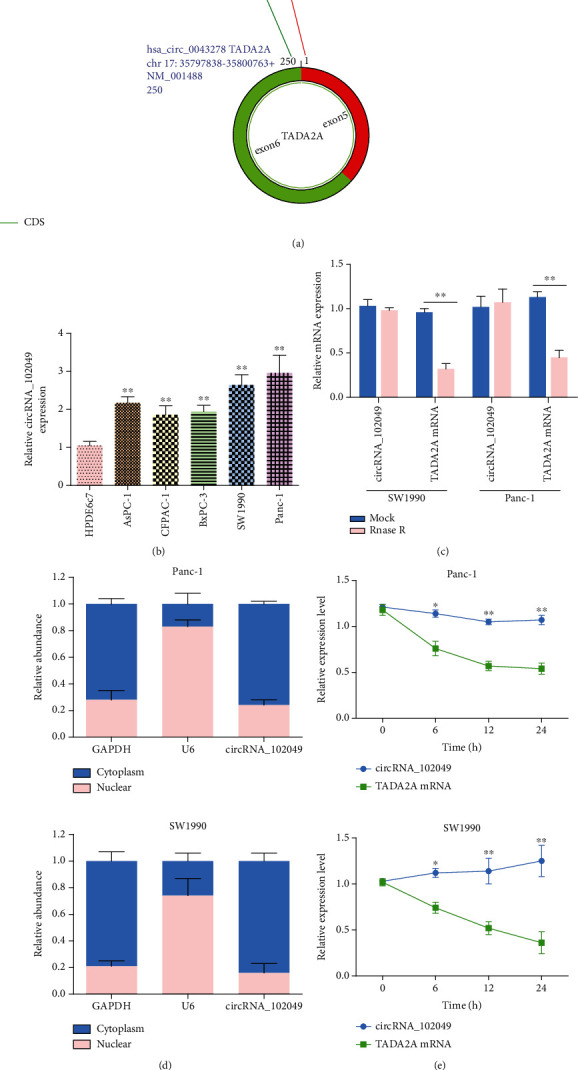
hsa_circRNA_102049 is upregulated in PDAC cell lines. (a) Schematic illustration showed that the circularization of TADA2A exons 5 and 6 formed circ_102049. (b) Measurement of circ_102049 expression in HPDE6c7 cells and PDAC cell lines. (c) RT-PCR analysis of circ_102049, liner TADA2A in SW1990 and Panc-1 cells treated with RNase R. (d) qPCR for the abundance of circ_102049 in the cytoplasm of SW1990 and Panc-1 cells. GAPDH and U6 were endogenous controls. (e) RT-PCR analysis of circ_102049 and TADA2A RNA after treatment with actinomycin D at the indicated time points in SW1990 and Panc-1 cells. ^∗^*P* < 0.05, ^∗∗^*P* < 0.01.

The basic structural molds of hsa_circ_102049 (alias: hsa_circ_0043278) are displayed in Figure 9(a). circ_102049 originates from exons 5 and 6 of the transcriptional adaptor 2A (TADA2A) gene, and its mature length after splicing is 250 nt. RT-qPCR results confirmed that Panc-1 cells had the highest circ_102049 expression, followed by SW1990 cells (Figure 9(b)). Therefore, the SW1990 and Panc-1 cell lines were selected for subsequent experiments in this study. To confirm the stability of circ_102049, RNase R was used in the current study. As illustrated in Figure 9(c), circ_102049 was resistant to RNase R, whereas the linear forms of TADA2A were digested by RNase R treatment. Moreover, we also determined that circ_102049 is mainly located in the cytoplasm by performing nuclear and cytoplasmic separation RT-PCR in SW1990 and Panc-1 cells, respectively (Figure 9(d)). Additionally, we further found that circ_102049 was more stable than the linear forms of TADA2A in SW1990 and Panc-1 cells under treatment with the transcription inhibitor actinomycin D (Figure 9(e)). Taken together, these results indicated that circ_102049 is a stable cytoplasmic circRNA.

### 3.8. hsa_circ_102049 Exerts Oncogenic Effects in the SW1990 and Panc-1 Cells

**Figure 10:**
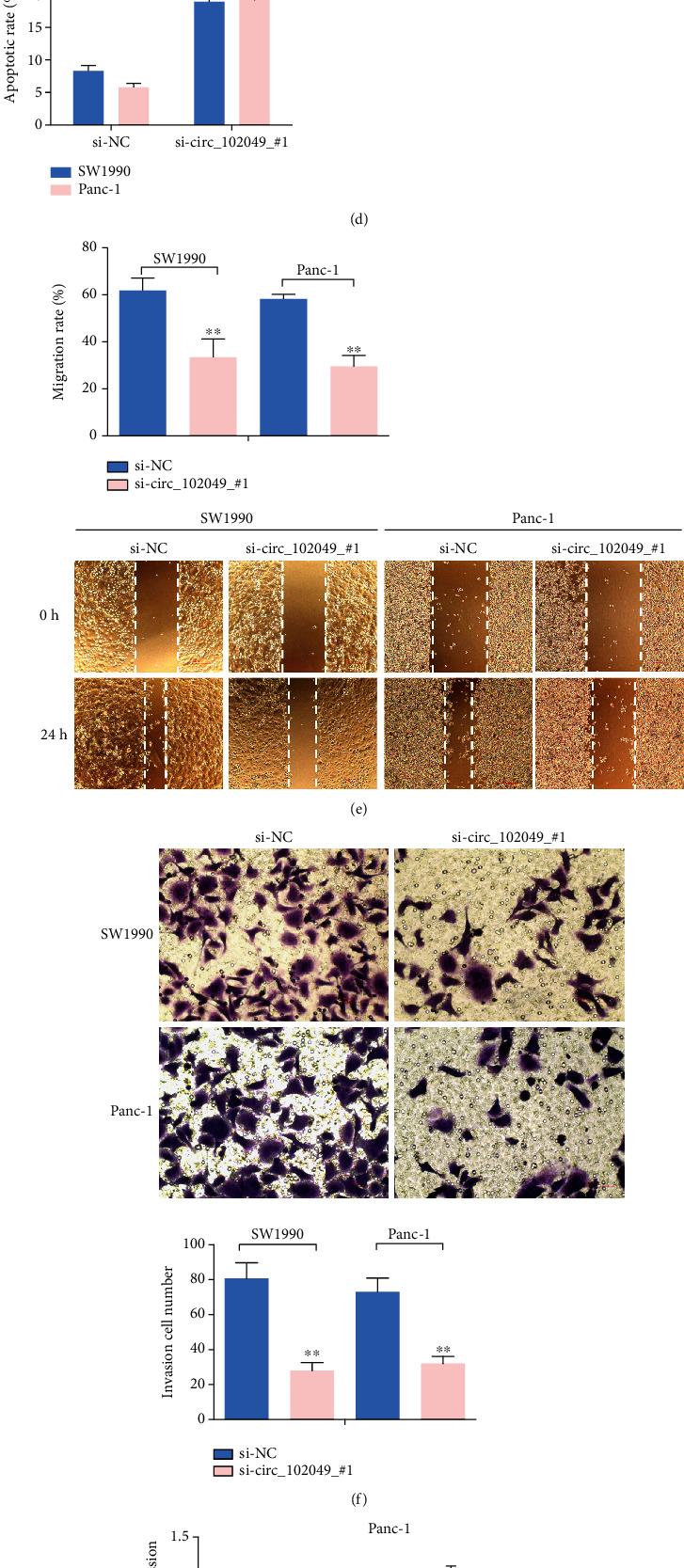
The role of circ_102049 knockdown in cell growth, apoptosis, inflammation, invasion, and migration in PDAC cells. (a) circ_102049 expression was measured after transfection in SW1990 and Panc-1 cells by RT-PCR. (b) CCK-8 assays were used to detect cell viability of SW1990 and Panc-1 cells after transfection. (c) Numbers of clone formation were decreased when transfected with si-circ_102049_#1. (d) The apoptosis of SW1990 and Panc-1 cells examined by flow cytometry after transfection. (e) Wound healing assays were performed in SW1990 and Panc-1 cells treated with si-NC or si-circ_102049_#1. (f) Cell invasion ability of SW1990 and Panc-1 cells transfected with si-NC or si-circ_102049_#1 was evaluated by the transwell assays. (g) mRNA expression levels of TNF-*α*, IL-6, IL-1*β*, IL-8, and IL-17 were detected in SW1990 and Panc-1 cells transfected with si-NC or si-circ_102049_#1 by RT-PCR. (h) circ_102049 knockdown inhibited tumor growth in vivo. Tumor weight and volumes were significantly reduced in the si-circ_102049_#1 group compared with the si-NC group. ^∗^*P* < 0.05, ^∗∗^*P* < 0.01.

Due to a high expression of circ_102049 in the SW1990 and Panc-1 cells, we depleted the expression of circ_102049 in the SW1990 and Panc-1 cell lines by treating cells with specific circ_102049 siRNAs (si-circ_102049_#1, si-circ_102049_#2) and si-NC as a control. Results showed that circ_102049 was apparently decreased in si-circ_102049_#1- and si-circ_102049_#2-treated PDAC cells compared to those cells treated with si-NC; in particular, si-circ_102049_#1-treated cells are more obvious (Figure 10(a)). Then, we selected the siRNA, si-circ_102049_#1, for further functional experiments. CCK-8 assay showed that cell viability was significantly reduced in circ_102049 knockdown SW1990 and Panc-1 cells (Figure 10(b)), and the colony formation assay showed a decrease of the colony number in si-circ_102049_#1-treated SW1990 and Panc-1 cells (Figure 10(c)). Also, as shown in Figure 10(d), after the knockdown of circ_102049 in SW1990 and Panc-1 cells, the cell apoptotic rate significantly increased, respectively. Moreover, the wound healing assay demonstrated that circ_102049 knockdown led to decreased migration ability in PDAC cells (Figure 10(e)), and transwell analysis also demonstrated that circ_102049 knockdown dramatically inhibited the invasion of SW1990 and Panc-1 cells (Figure 10(f)). Meanwhile, RT-qPCR results demonstrated that circ_102049 knockdown significantly inhibited the mRNA expression levels of TNF-*α*, IL-6, IL-1*β*, IL-8, and IL-17 in the PDAC cell lines (Figure 10(g)). To further assess the effect of circ_102049 knockdown in vivo, we inoculated stable circ_102049 knockdown with Panc-1 cells subcutaneously into 6-week-old nude mice. Our results indicate that the silencing of circ_102049 decreased the weight and sizes of PDAC tumors (Figure 10(h)). These results demonstrated that blocking circ_102049 inhibited PDAC progression in vitro and in vivo.

### 3.9. Identification of miR-455-3p as a circ_102049 Target Gene

**Figure 11:**
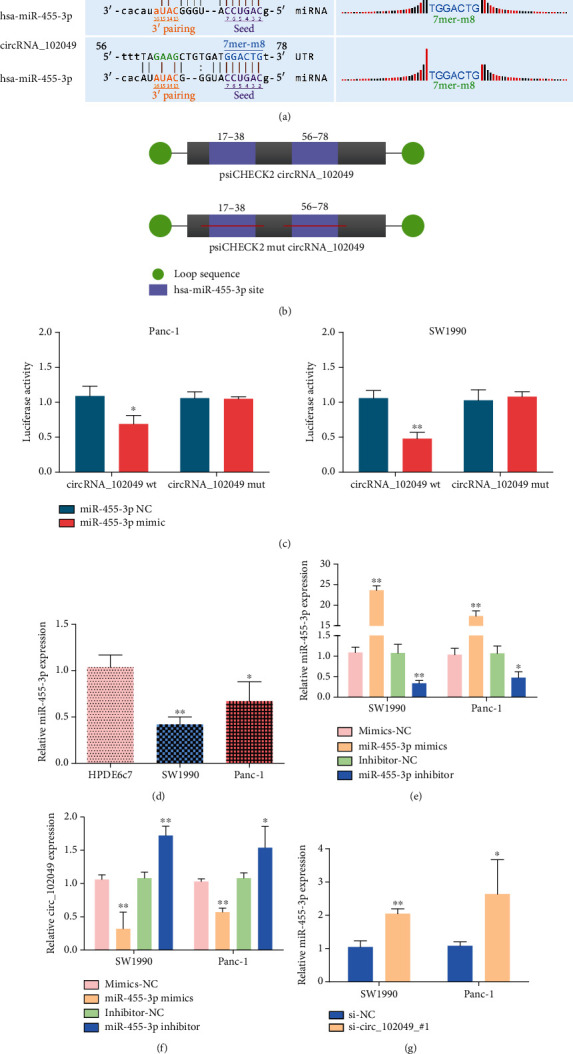
circ_102049 functions as a miRNA sponge for miR-455-3p. (a) The binding site for miR-455-3p and circ_102049 was analyzed with the bioinformatics tool. (b) Schematic outlining the wild-type and mutant circ_102049 luciferase plasmid. (c) Luciferase reporter assay was performed to analyze the interaction between circ_102049 and miR-455-3p in PDAC cells. (d) miR-455-3p expression was confirmed by using RT-PCR in PDAC cells. (e) Overexpression and silencing of miR-455-3p in SW1990 and Panc-1 cells via miR-455-3p mimic or miR-455-3p inhibitor, respectively. (f) circ_102049 expression was detected in transfected PDAC cells with miR-455-3p mimic or miR-455-3p inhibitor, respectively. (g) miR-455-3p expression was measured in SW1990 and Panc-1 cells that were transfected with si-NC or si-circ_102049_#1. ^∗^*P* < 0.05, ^∗∗^*P* < 0.01.

Increasingly, circRNAs have been reported to function as a miRNA sponge. To confirm the ability of hsa_circ_102049 to regulate the expression of miR-455-3p, the CircInteractome database was used to predict the miRNAs with potential circ_102049 binding sites (Figures 11(a) and 11(b)). Luciferase assays confirmed that miR-455-3p overexpression inhibited the luciferase of wild-type circ_102049 in SW1990 and Panc-1 cells, respectively (Figure 11(c)). In addition, the mRNA expression level of miR-455-3p was observably lower in SW1990 and Panc-1 cells than in HPDE6c7 cells (Figure 11(d)). Furthermore, SW1990 and Panc-1 cell lines were transfected with miR-455-3p mimic or miR-455-3p inhibitor to regulate miR-455-3p expression, as confirmed using RT-PCR (Figure 11(e)). Interestingly enough, the expression of circ_102049 was negatively regulated by miR-455-3p in both PDAC cell lines (Figure 11(f)). Similarly, circ_102049 knockdown significantly increased miR-455-3p expression (Figure 11(g)). From the results above, we can confirm that miR-455-3p acts as a downstream target of circ_102049 in PDAC.

### 3.10. circ_102049 Regulates CD80 Expression in PDAC Cells by Sponging miR-455-3p

**Figure 12:**
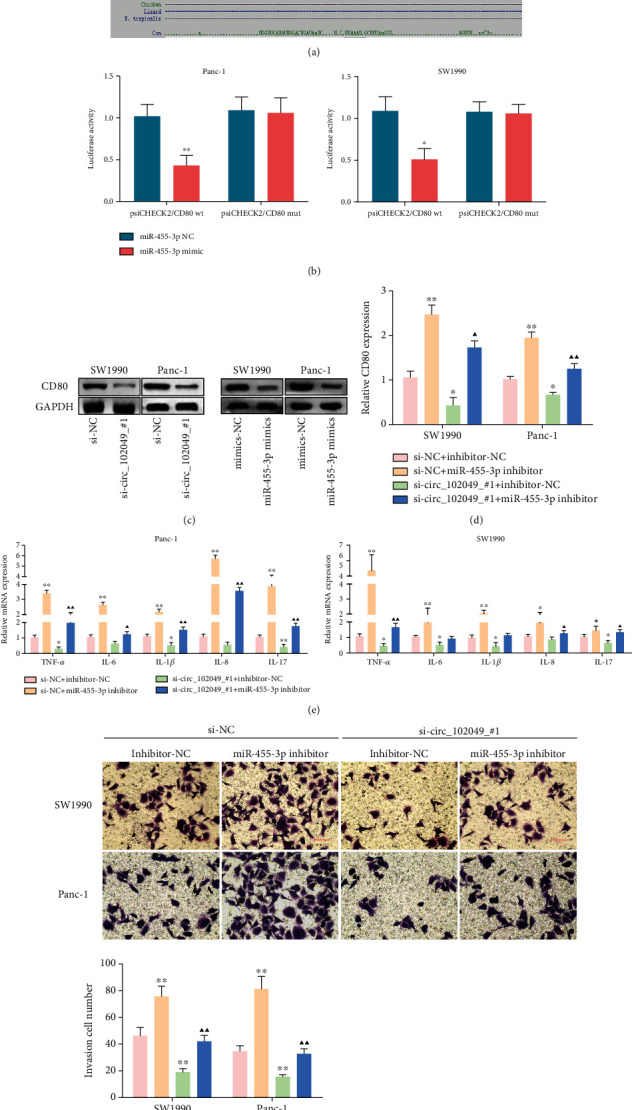
circ_102049 regulates CD80 expression in PDAC cells by sponging miR-455-3p. (a) A predicted binding site of miR-455-3p within the CD80 3′-UTR. (b) Luciferase reporter assays were employed to assess the interaction between CD80 3′-UTR and miR-455-3p. (c) circ_102049 knockdown and miR-455-3p overexpression suppressed CD80 protein expression in SW1990 and Panc-1 cells. Knockdown of circ_102049 reduced (d) CD80; (e) TNF-*α*, IL-6, IL-1*β*, IL-8, and IL-17 expression; and (f) invasion ability in SW1990 and Panc-1 cells while inhibition of miR-455-3p in the meantime reversed it. The results were measured and expressed as mean ± SD. ^∗^*P* < 0.05, ^∗∗^*P* < 0.01 vs. the si-NC+inhibitor-NC group. ^▲^*P* < 0.05, ^▲▲^*P* < 0.01 vs. the si-circ_102049_#1+inhibitor-NC group.

Based on the theory of ceRNA, there should be a positive correlation between the circ_102049 expression and the expression of its potential target genes. In the circRNA-miRNA-mRNA network of circ_102049 within miR-455-3p and the hub genes, we found that miR-455-3p could target CD80. Also, bioinformatics analysis showed there are putative binding sites between miR-455-3p and CD80, and it is conserved among species (Figure 12(a)). Hence, we selected CD80 for further validation. Then, luciferase assays confirmed the binding activity of miR-455-3p to CD80, and miR-455-3p mimic significantly attenuated the luciferase activity of SW1990 and Panc-1 cells driven by psiCHECK2 CD80 wt, but not that driven by psiCHECK2 CD80 mut, compared to the miR-455-3p NC group (Figure 12(b)). Notably, the results of western blot assay indicate that circ_102049 knockdown and miR-455-3p overexpression resulted in a decrease of CD80 protein expression in SW1990 and Panc-1 cells (Figure 12(c)). Then, we investigated the roles of circ_102049 in the miR-455-3p/CD80 axis, and relative mRNA expression of CD80; TNF-*α*, IL-6, IL-1*β*, IL-8, and IL-17 expression; and invasion cell number were estimated in SW1990 and Panc-1 cells treated with si-circ_102049_#1 and miR-455-3p inhibitor. Our results showed that miR-455-3p knockdown significantly increased the mRNA expression of CD80; TNF-*α*, IL-6, IL-1*β*, IL-8, and IL-17 expression; and invasion ability of PDAC cells, while cotransfection of si-circ_102049_#1 and miR-455-3p inhibitor could partially reverse the suppressive role of circ_102049 deficiency in CD80; TNF-*α*, IL-6, IL-1*β*, IL-8, and IL-17 expression; and invasion of SW1990 and Panc-1 cells (Figures 12(d)–12(f)). These results indicate that circ_102049 promoted the tumorigenesis of PDAC by increasing the expression of CD80 via miR-455-3p.

## 4. Discussion

Pancreatic ductal adenocarcinoma (PDAC) is a typically malignant tumor of the digestive system, with a 5-year overall survival rate of less than 6% [36]. It is reported that approximately 80% of patients have lost the chance for surgical resection because of its complexity and invisibility [36, 37]. Hence, it is imperative to promote the development of early biomarkers and novel therapeutic methods according to the molecular mechanisms for the prognosis of PDAC. Great advances in high-throughput gene expression profiling and the development of integrated bioinformatics analysis are providing remarkable perspectives for us to perform methylation research that is associated with the pathogenesis of cancers. Also, aberrations of methylation are extremely common in the cancer genome. It is widely accepted that high methylation of tumor suppressor genes and low methylation of cancer genes play significant roles in the occurrence and development of cancers [38]. Recently, a number of studies have elucidated that circRNAs, a type of covalent closed circular noncoding RNAs, are strongly implicated in tumor growth, metastasis, and proliferation through the circRNA-miRNA-mRNA axis in a wide variety of tumors [39, 40]. circRNAs are highly stable and tissue-specific compared with linear RNAs that might be used as promising biomarkers for the detection of clinicopathological status in PDAC. Meanwhile, systemic inflammation is a hallmark of PDAC, which is associated with poor clinical outcomes by supporting malignant cells escape from immune elimination [41]. Thus, exploring further strategies to ameliorate inflammatory reaction is necessary for increasing the effectiveness of cancer immunotherapy in PDAC.

In the current study, the circRNA expression profiling datasets (GSE69362, GSE79634), gene methylation profiling microarray (GSE49149), and gene expression datasets (GSE14245, GSE27890, GSE32676, GSE41372, GSE62165, GSE62452, and GSE71989) were downloaded from the GEO database to identify DECs and DMGs with GEO2R, and *P* value < 0.05 was considered statistically significant. Then, we comprehensively analyzed the ceRNA regulatory network consisting of circRNAs, miRNAs, and mRNAs in PDAC. Twenty upregulated and five downregulated DECs were screened for further research. Particularly, hsa_circ_102049 was significantly upregulated in GSE69362 and GSE79634 datasets. We predicted potential circRNA-miRNA via the CircInteractome database, and TargetScan and miRDB databases were used to identify miRNA-mRNA pairs. Finally, we confirmed 53 highly expressed miRNAs and 38 lowly expressed miRNAs in the KM plotter, associated with poor survival of PDAC patients, and 7871 mRNAs to construct a circRNA-miRNA-mRNA regulatory network preliminarily. In addition, we also found that hsa-miR-455-3p and hsa-miR-342-3p may be the target genes of circ_102049. Next, we identified and investigated the inflammation-associated hypomethylation-high expression genes and hypermethylation-low expression genes and their potential upstream regulatory mechanisms in PDAC, respectively. Functional enrichment analysis showed that these hypomethylation-high expression genes were involved in blood vessel morphogenesis, cytokine-mediated signaling pathway, regulation of cell adhesion, and pathways in cancer, which are well known in regulating the progression of PDAC [42, 43].

The PPI network revealed the intercommunication of the hypomethylation-high expression genes/hypermethylation-low expression genes, among which the hub genes were STAT1, CCND1, KRAS, CD80, ICAM1, ESR1, RAF1, RPS6KA2, KDM6B, TNRC6A, FOSB, and DNM1. Among them, KRAS, ESR1, RAF1, RPS6KA2, and TNRC6A have higher methylation levels, and in particular, CD80 and DNM1 have low methylation levels; furthermore, the results of the Kaplan-Meier analyses demonstrate that the STAT1, CCND1, KRAS, CD80, ICAM1, KDM6B, TNRC6A, and DNM1 were associated with the prognostic outcomes of patients with PDAC. STAT1 is activated by interferon-*γ* (IFN*γ*) in pancreatic stellate cells (PSCs), which plays an important role in chronic pancreatitis and pancreatic cancer [44]. Besides, a recent study showed that STAT1 inhibition significantly suppressed the upregulation of PD-L1 in stimulated B cells for the immunology regulation of PDAC patients [45]. Radulovich et al. uncovered that the overexpression of CCND1 is most common in PDAC and occurred mainly in late-stage pancreatic intraepithelial neoplastic (PanIN) lesions [46]. In addition, Chen et al. revealed that CCND1 was targeted by miR-193a-3p, and overexpression of miR-193a-3p significantly decreased CCND1 expression to inhibit the tumor growth in PDAC cells [47]. CCND1 may be one of the abnormally methylated genes, which have been indicated as proto-oncogenes that are positively associated with lymph node metastasis, regulating G1-to-S phase progression in human tumors [48–50]. KRAS is the predominant mutated RAS gene in human cancer and the initiating genetic event for PDAC [51]. CD80 (also known as B7-1) encodes a membrane receptor that is activated by the binding of CD28 or CTLA-4, then further induces T cell proliferation and cytokine production [52]. Additionally, Bengsch et al. found that the CTLA-4/CD80 pathway was implicated in T cell immunotherapy in PDAC [53]. Moreover, Das et al. reported that the potential immunosuppressive effect in gingivobuccal oral squamous cell carcinoma (OSCC-GB) may be associated with promoter hypomethylation, which was driven by the upregulation of CD80 [54]. To our knowledge, cytokines have a vital function in angiogenesis, tumor carcinogenesis, and metastasis in the tumor microenvironment. A study by Huang et al. found that interleukin 35 (IL-35) promotes PDAC metastasis by mediating ICAM1 expression [55]. KDM6B, also known as JMJD3, is a histone demethylase and located on the short arm of chromosome 17 (17p13.1) that removes the H3K27me3 methyl marks [56]. Also, the loss of KDM6B enhances the aggressiveness of PDAC cells by inhibiting the expression of C/EBP*α* [57]. TNRC6A, an essential mRNA-binding protein in miRISC, is inducing the DDRNA expression and functionality; and miR-30 promotes cancer by suppressing TNRC6A [58]. Shukla et al. discussed DNM1 may participate in driving long-term radiation toxicity in the brain [59]. Even though the results of hub genes were according to computational biology, in vitro and in vivo biological and molecular experiments need to be performed to further verify our hypothesis. Generally, the identification of the above hub genes could improve the understanding of the pathogenesis and also provide prognostic markers and therapeutic targets for PDAC.

Herein, circ_102049, also known as hsa_circ_0043278 according to circBase, was found to be the common circRNA in the GSE69362 and GSE79634 datasets and was upregulated in PDAC cell lines. The expression of circ_102049 was resistant to RNase R degradation, and the subcellular distribution of circ_102049 was overwhelming in the cytoplasm. All these results suggested an important role of circ_102049 in PDAC cell progression. Functionally, circ_102049 knockdown significantly decreased the growth, inflammatory factors, and migratory and invasive potential and promoted cell apoptosis in SW1990 and Panc-1 cells, whereas these effects could be reversed by downregulation of miR-455-3p. Importantly, we found that miR-455-3p was sponged by circ_102049, leading to the increased CD80 expression and the inflammatory reaction and invasion of PDAC. Therefore, the identification of the circ_102049/miR-455-3p/CD80 axis increases our understanding of the regulatory mechanism underlying PDAC progression.

## 5. Conclusion

In conclusion, twelve hub genes (STAT1, CCND1, KRAS, CD80, ICAM1, ESR1, RAF1, RPS6KA2, KDM6B, TNRC6A, FOSB, and DNM1) were identified as abnormal methylation genes in PDAC with integrated bioinformatics analysis. We further indicated that circ_102049 knockdown could effectively inhibit cell growth, inflammatory state, migration, and invasion of PDAC cells in vivo and in vitro via the miR-455-3p/CD80 axis. Our results suggest that the circ_102049/miR-455-3p/CD80 axis may potentially be used as a promising prognostic biomarker for PDAC.

## Figures and Tables

**Table 1:** Information of the microarray datasets in GEO for integrated analysis in this study.

Dataset	PMID	Platform	Number of samples (tumor/control)	circRNA/mRNA	Number of DEGs/DMGs
GSE69362	27997903	GPL19978 Agilent-069978 Arraystar Human CircRNA microarray V1	12 (6/6)	circRNA	170
GSE79634	29620241	GPL19978 Agilent-069978 Arraystar Human CircRNA microarray V1	40 (20/20)	circRNA	289
GSE49149	24500968	GPL13534 Illumina Human Methylation450 BeadChip (HumanMethylation450_15017482)	196 (167/29)	mRNA	65,531
GSE14245	19931263	GPL570 [HG-U133_Plus_2] Affymetrix Human Genome U133 Plus 2.0 Array	24 (12/12)	mRNA	1981
GSE27890	—	GPL570 [HG-U133_Plus_2] Affymetrix Human Genome U133 Plus 2.0 Array	8 (4/4)	mRNA	2375
GSE32676	25846727	GPL570 [HG-U133_Plus_2] Affymetrix Human Genome U133 Plus 2.0 Array	32 (25/7)	mRNA	864
GSE41372	24120476	GPL6244 [HuGene-1_0-st] Affymetrix Human Gene 1.0 ST Array [transcript (gene) version]	30 (15/15)	mRNA	1948
GSE62165	27520560	GPL13667 [HG-U219] Affymetrix Human Genome U219 Array	131 (118/13)	mRNA	817
GSE62452	27197190	GPL6244 [HuGene-1_0-st] Affymetrix Human Gene 1.0 ST Array [transcript (gene) version]	130 (69/61)	mRNA	321
GSE71989	27363020	GPL570 [HG-U133_Plus_2] Affymetrix Human Genome U133 Plus 2.0 Array	21 (13/8)	mRNA	1375

## Data Availability

The data used to support the findings of this study are available from the corresponding author upon request.

## Supplementary Materials

Supplementary 1 Figure S1 The survival analysis of target miRNAs of selected DECs.

Supplementary 2 Figure S2 The clinical stage analysis of hub genes by using the GEPIA database.

Supplementary 3 Table S1 The differentially expressed circRNAs in the GSE69362 dataset.

Supplementary 4 Table S2 The differentially expressed circRNAs in the GSE79634 dataset.

Supplementary 5 Table S3 The target miRNAs of overlapped DECs according to the CircInteractome database.

Supplementary 6 Table S4 The target miRNAs of overlapped DECs according to the KM plotter analysis.

Supplementary 7 Table S5 The potential target genes of 53 highly expressed miRNAs with poor survival of PDAC patients.

Supplementary 8 Table S6 The potential target genes of 38 lowly expressed miRNAs with poor survival of PDAC patients.

Supplementary 9 Table S7 The differentially methylated genes in the GSE14245 dataset.

Supplementary 10 Table S8 The differentially expressed genes of GSE14245, GSE27890, GSE32676, GSE41372, GSE62165, GSE62452, and GSE71989.

Supplementary 11 Table S9 The inflammation-associated genes in the GeneCards database.
