# Supplementary figures and images for: circRNA circ_102049 Implicates in Pancreatic Ductal Adenocarcinoma Progression through Activating CD80 by Targeting miR-455-3p
Source: Mediators Inflamm. 2021 Jan 7;2021:8819990. doi: 10.1155/2021/8819990 (PMC7811564; doi:10.1155/2021/8819990)

**Supplementary Figure S1** The survival analysis of target miRNAs of selected DECircRNAs

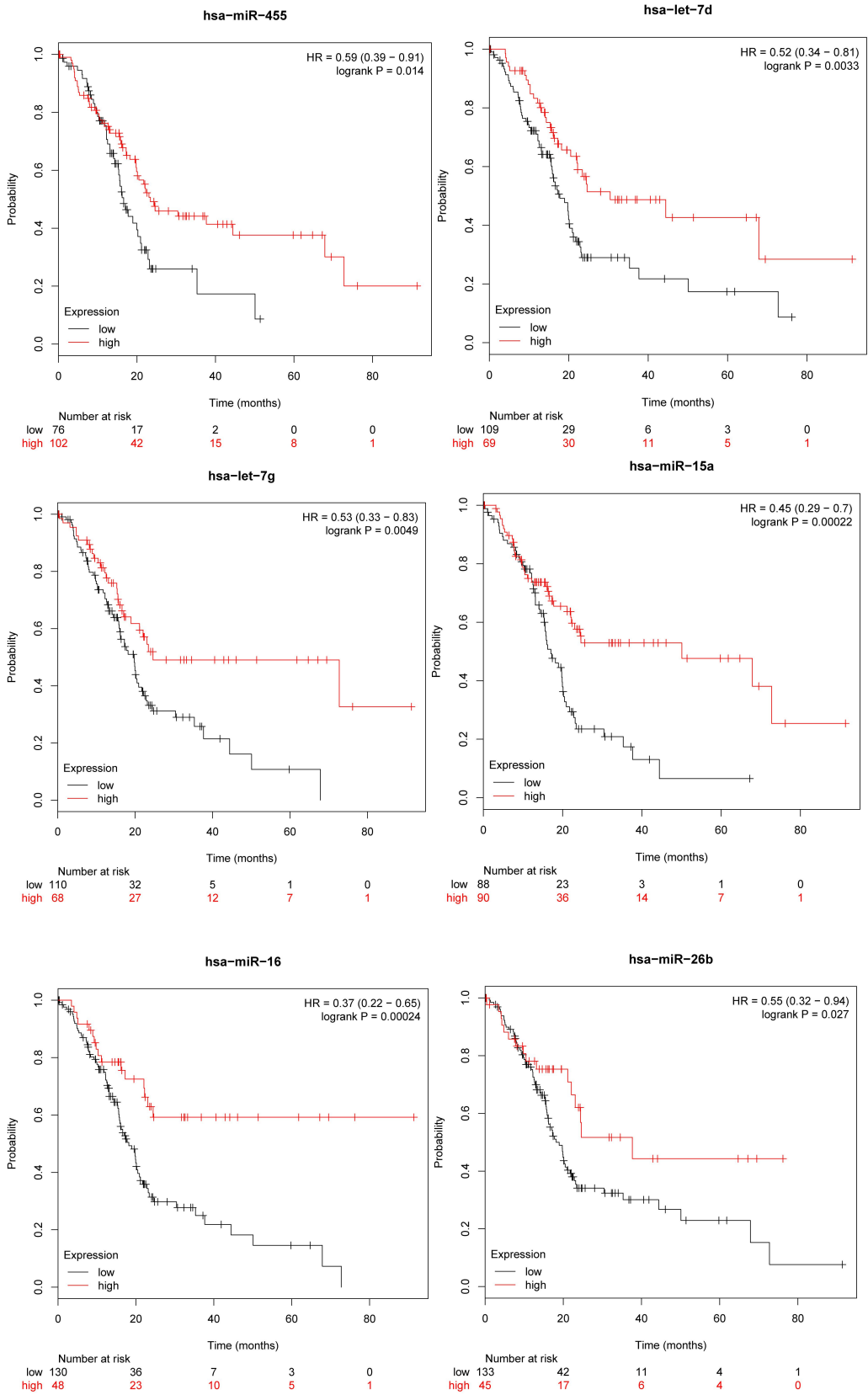

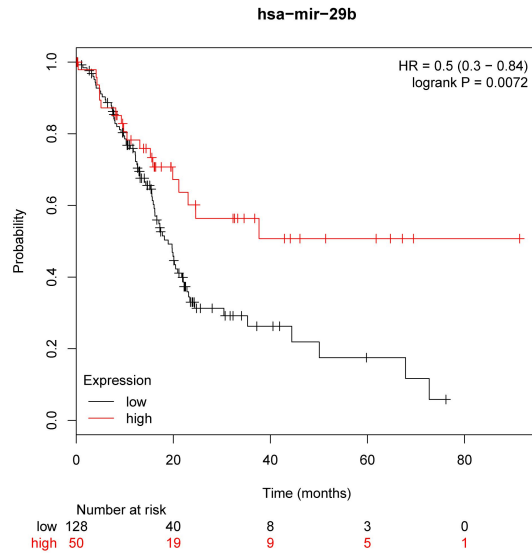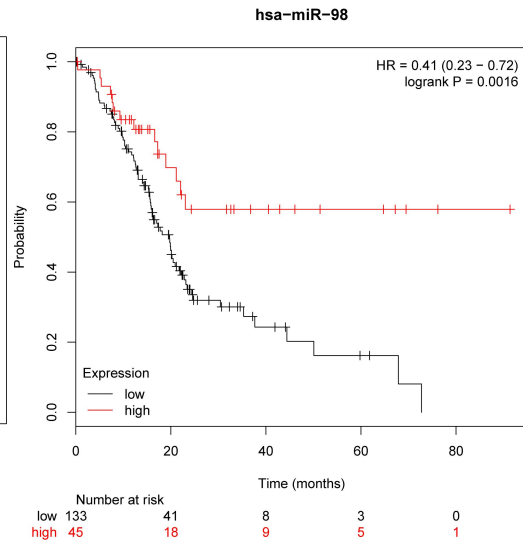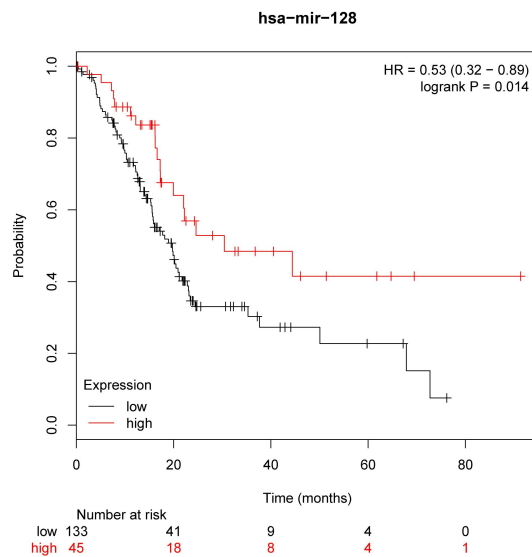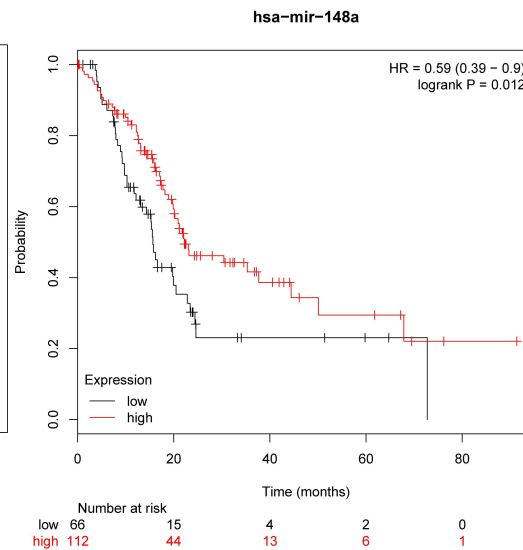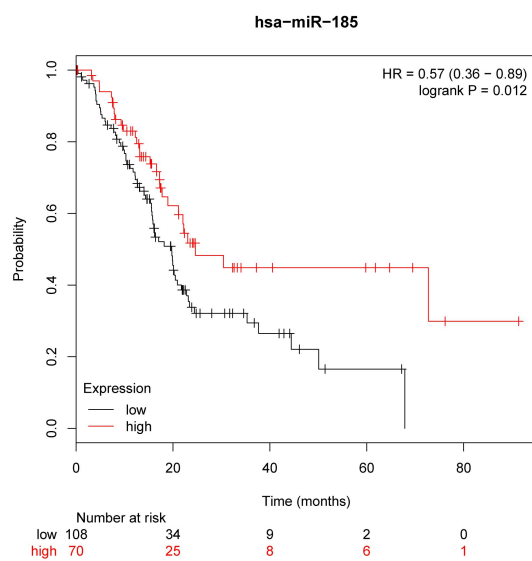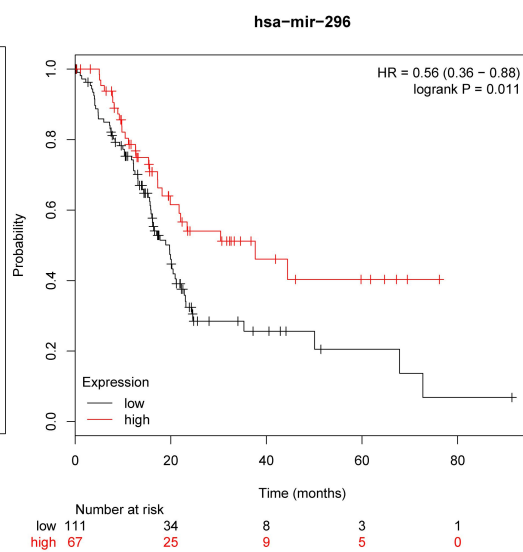

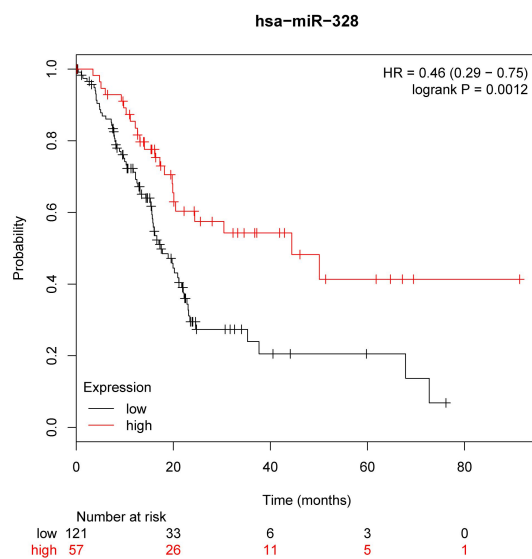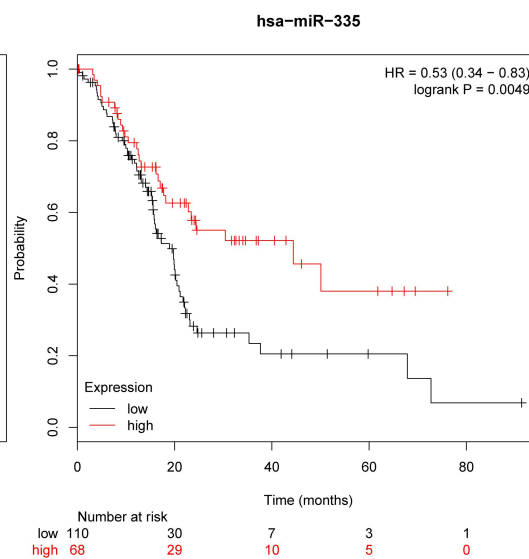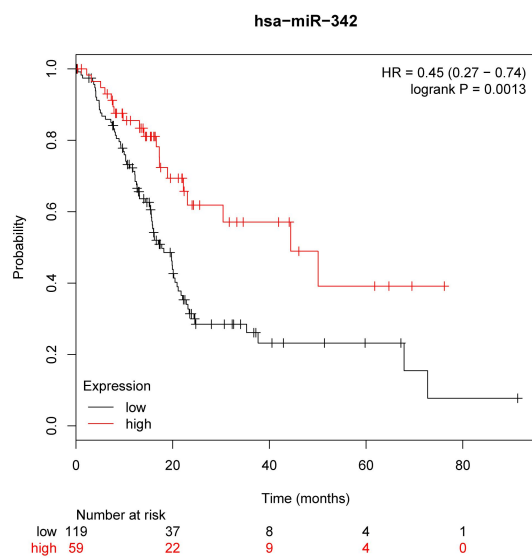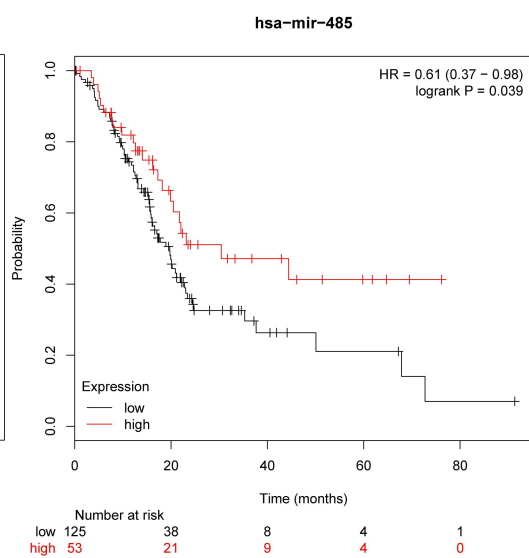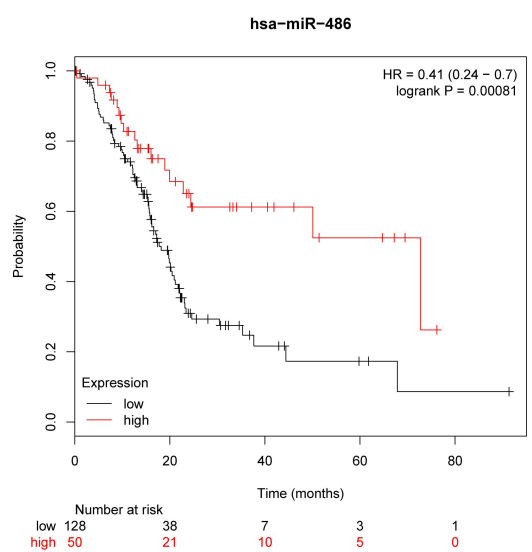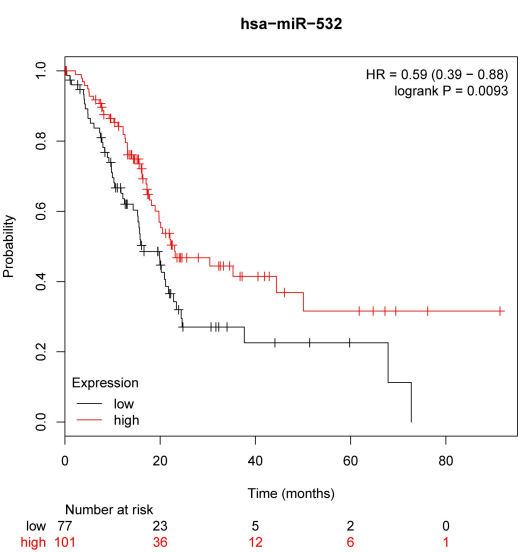

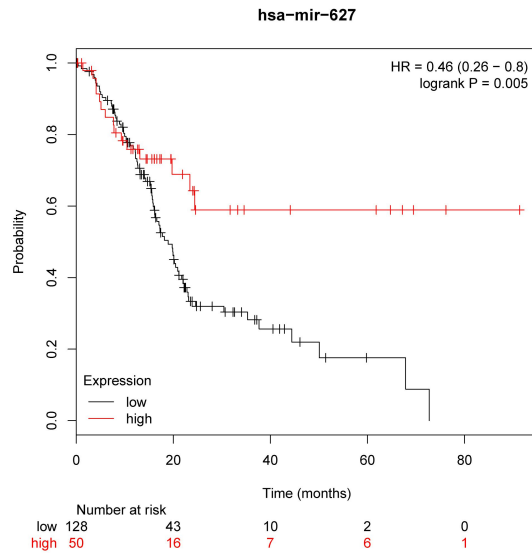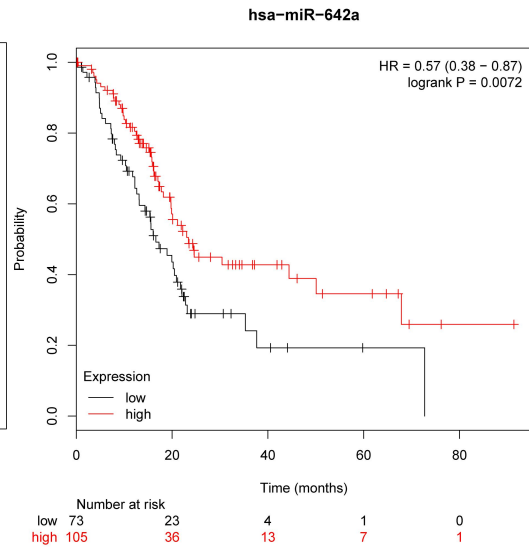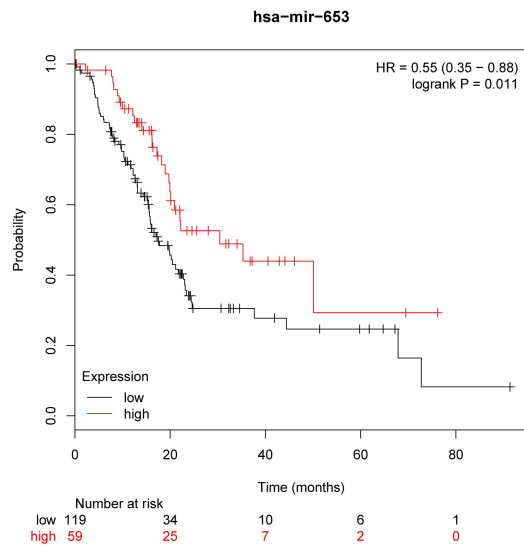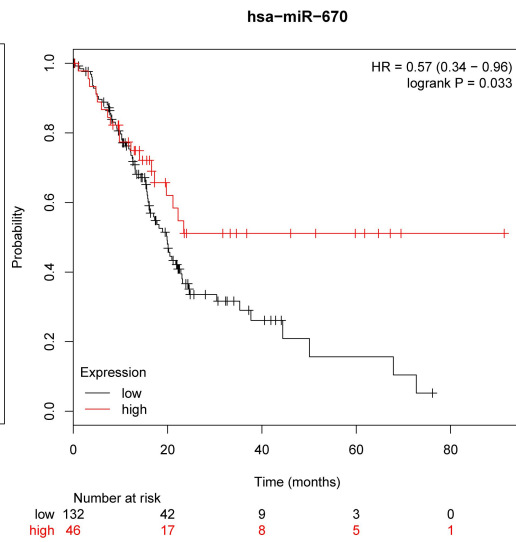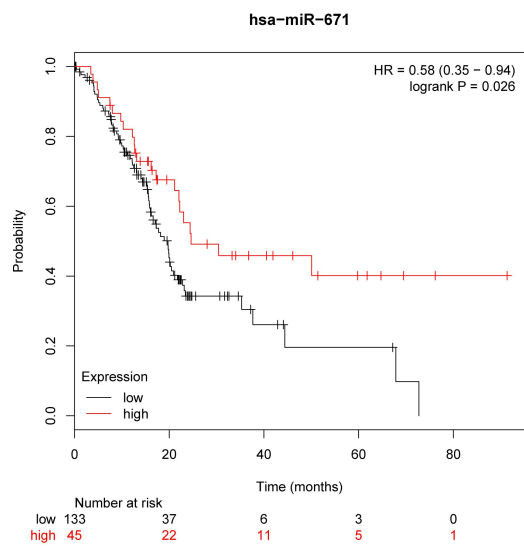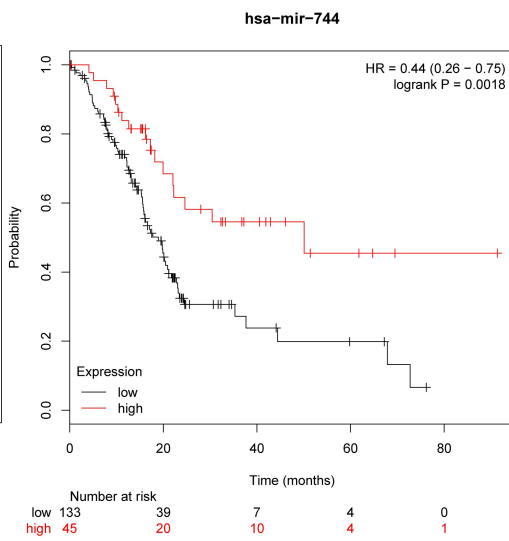

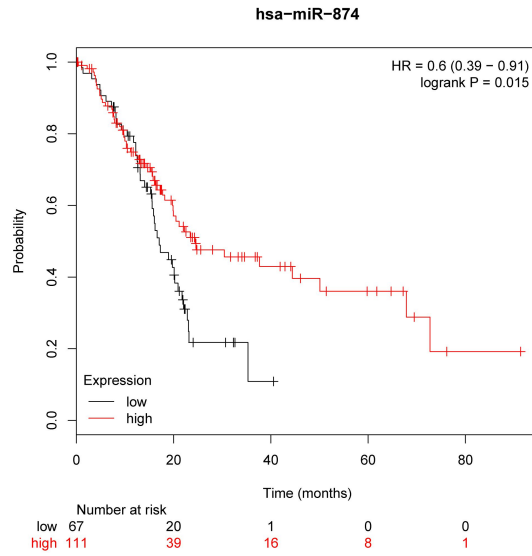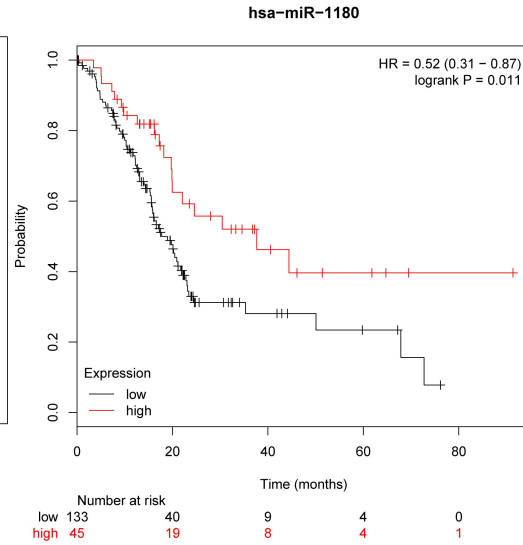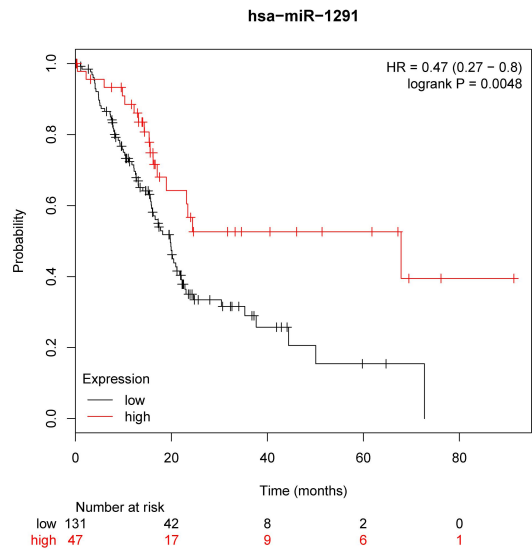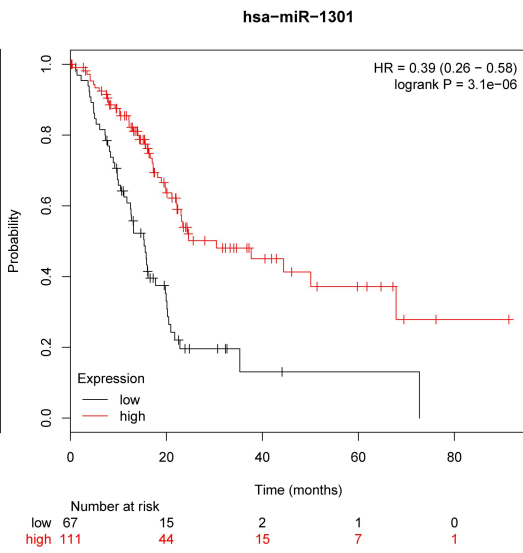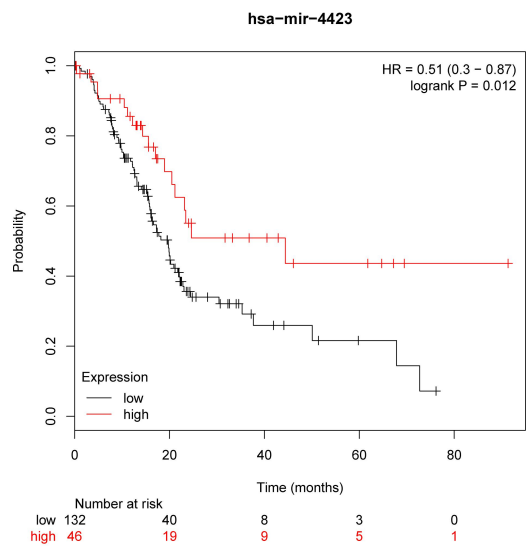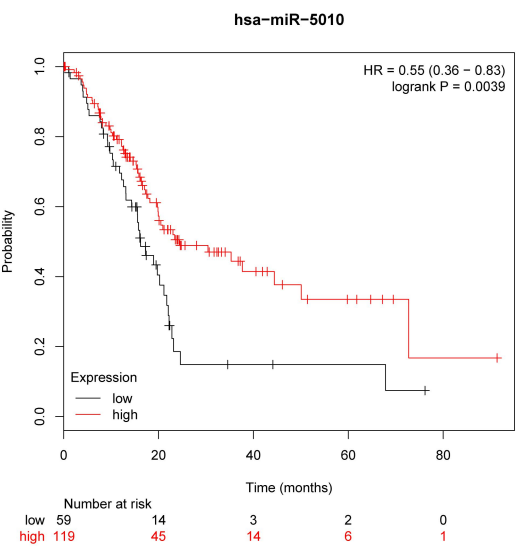

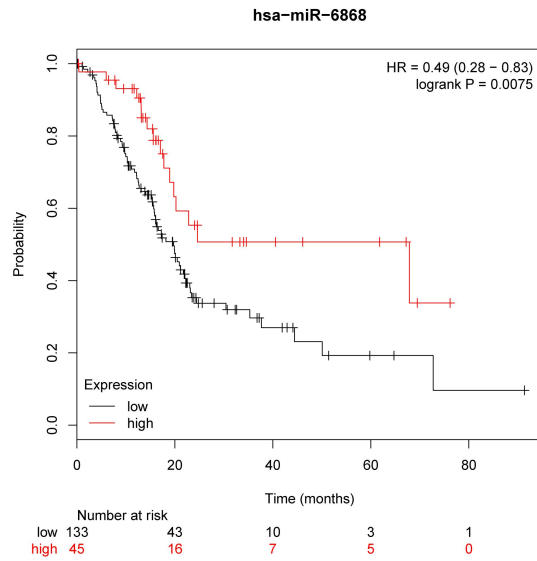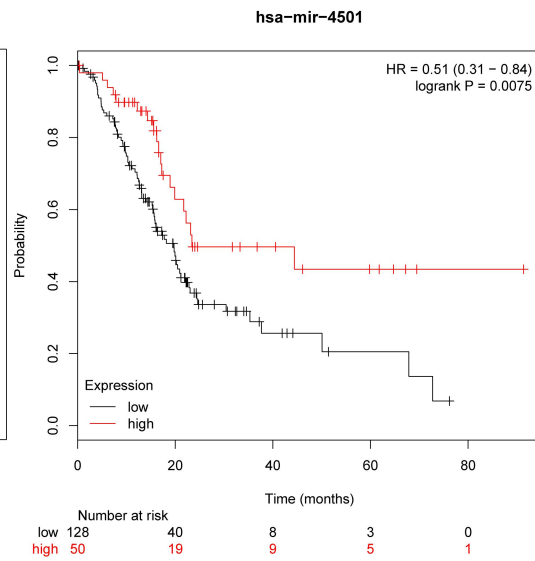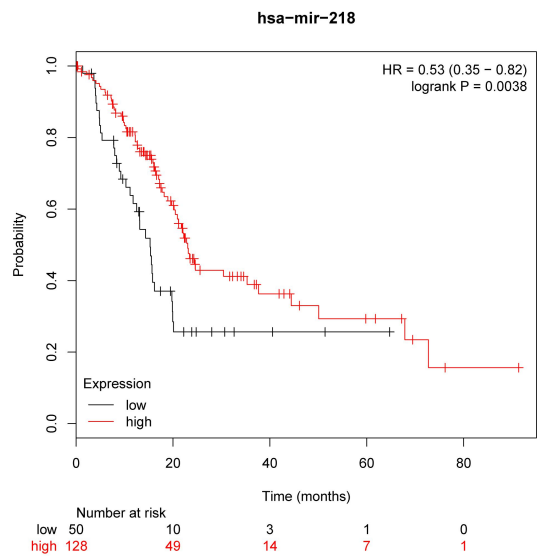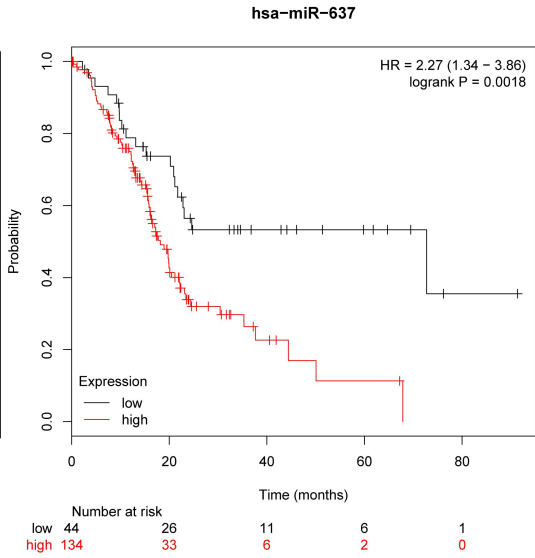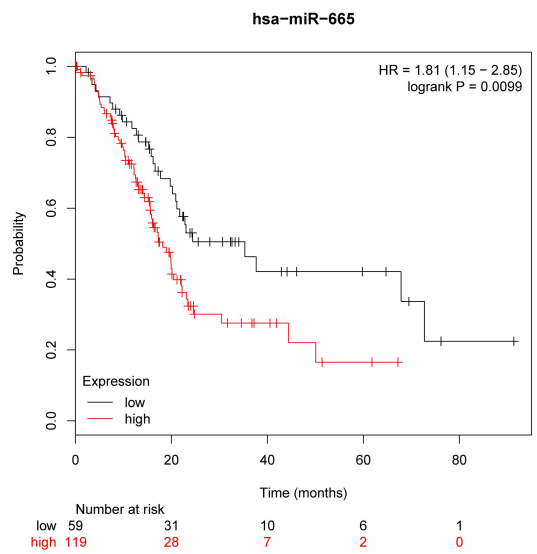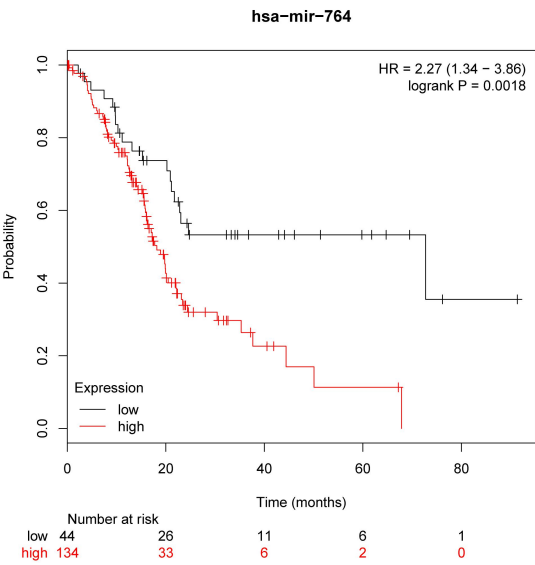

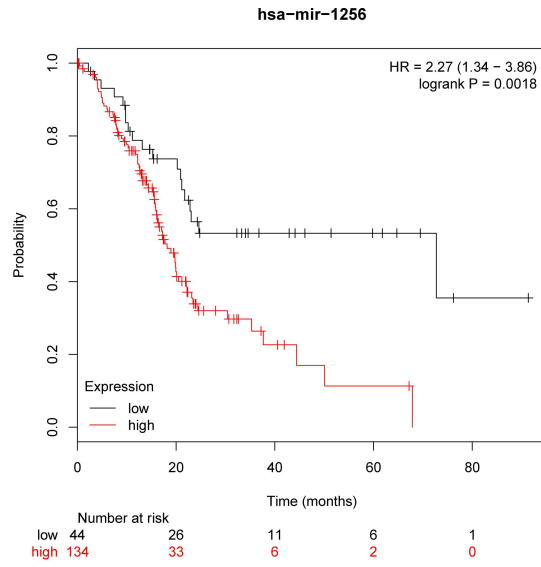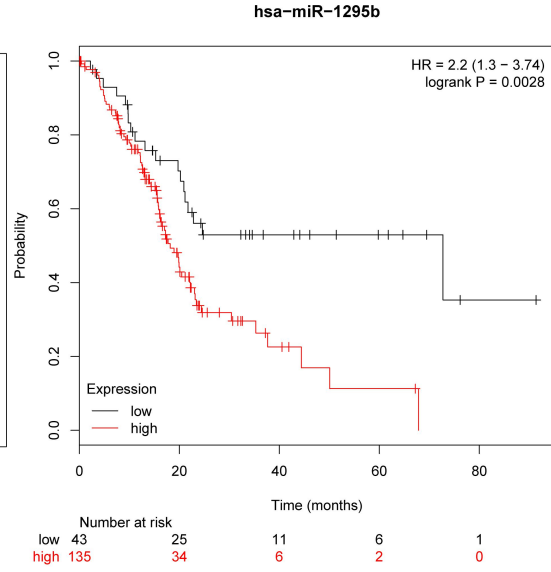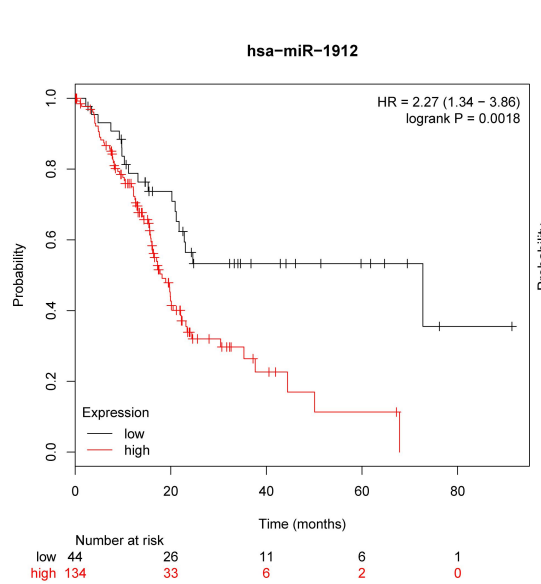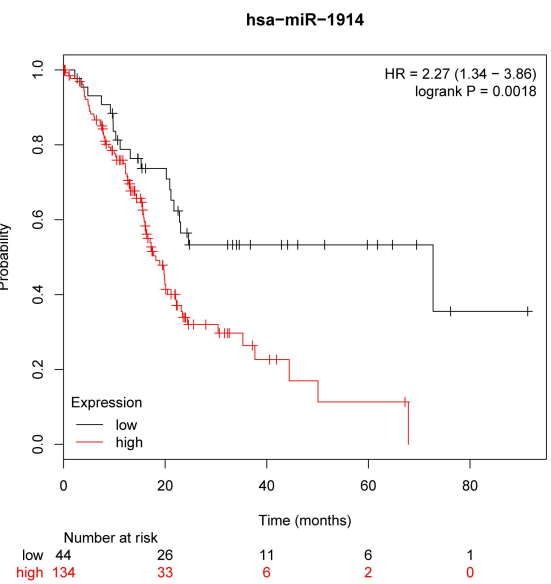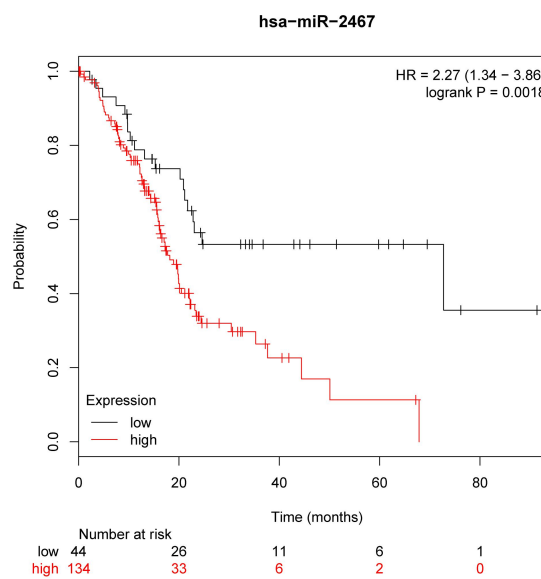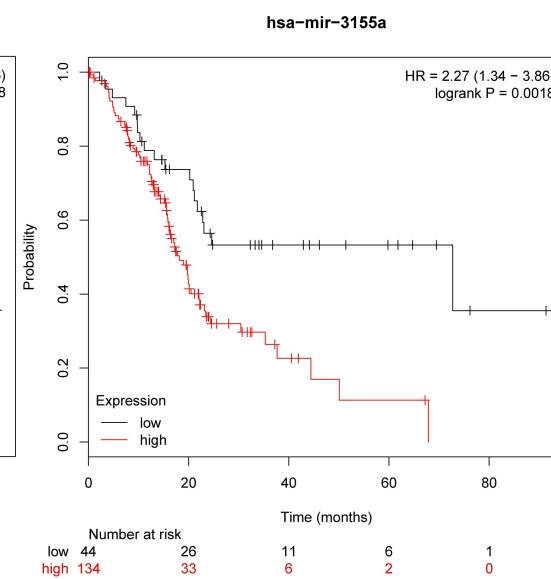

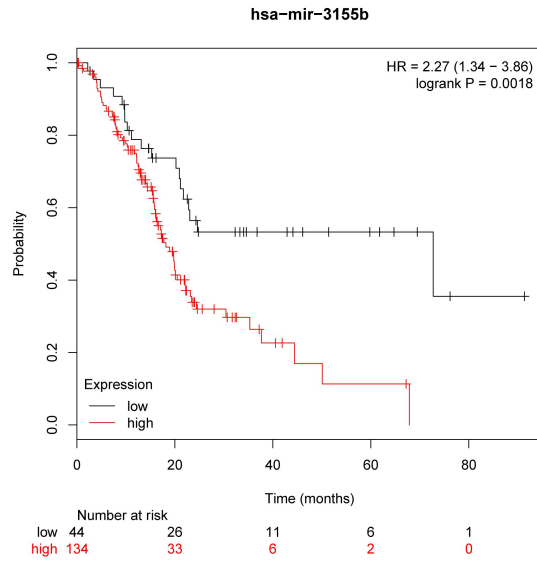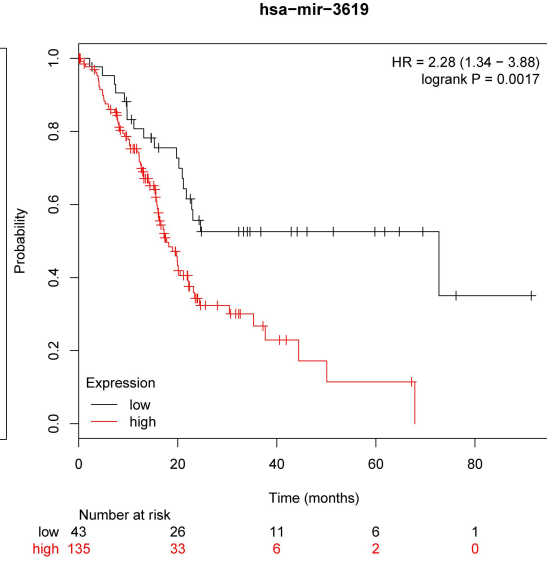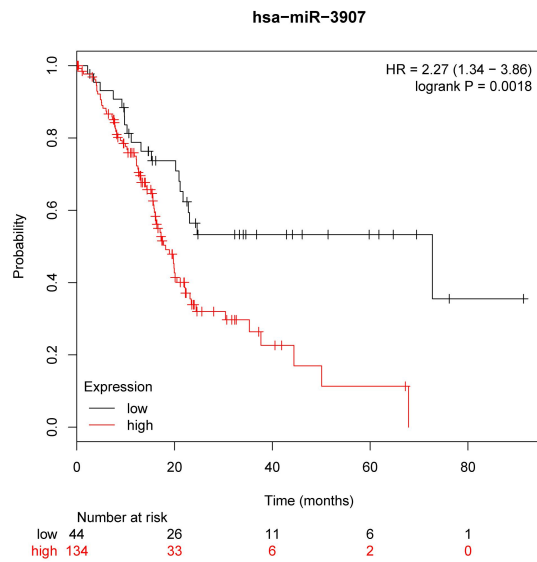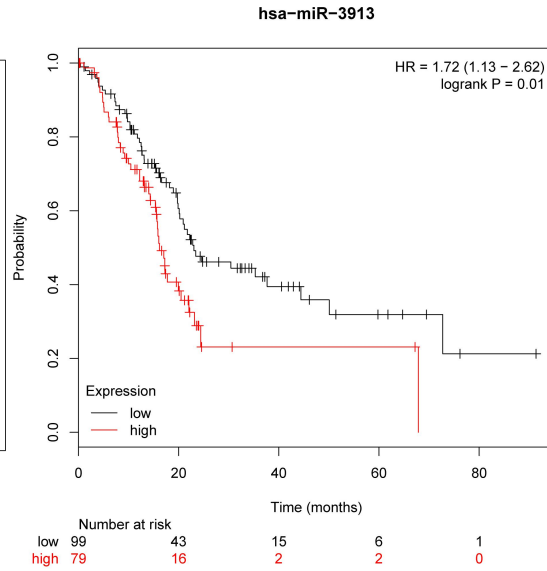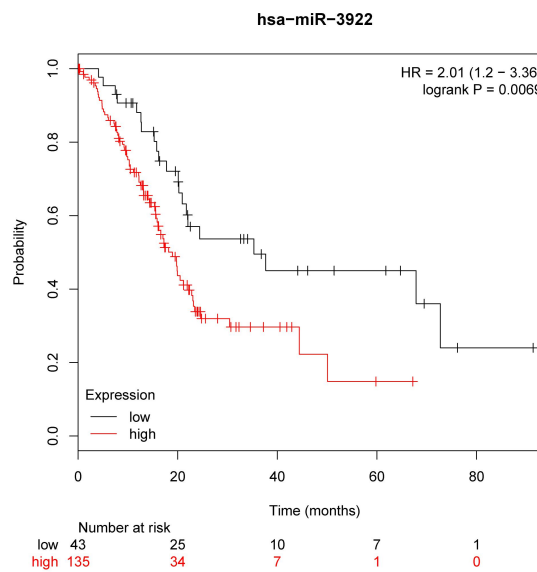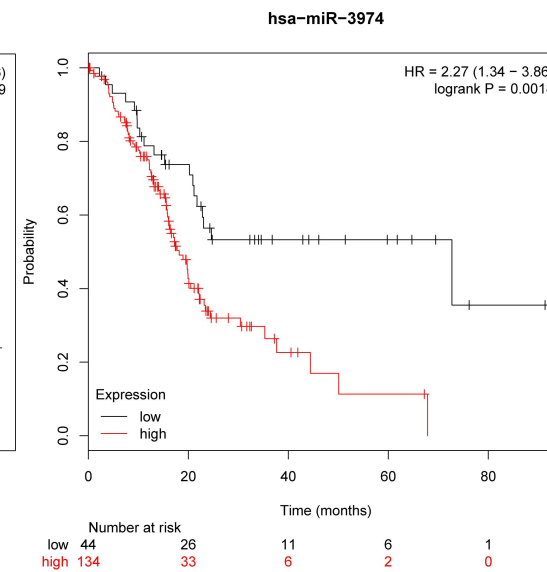

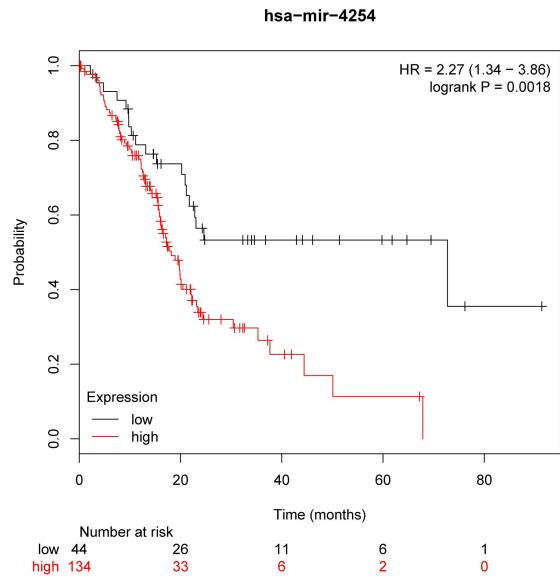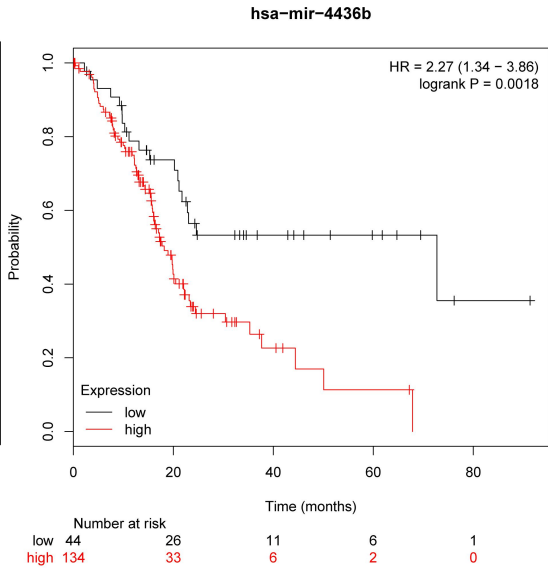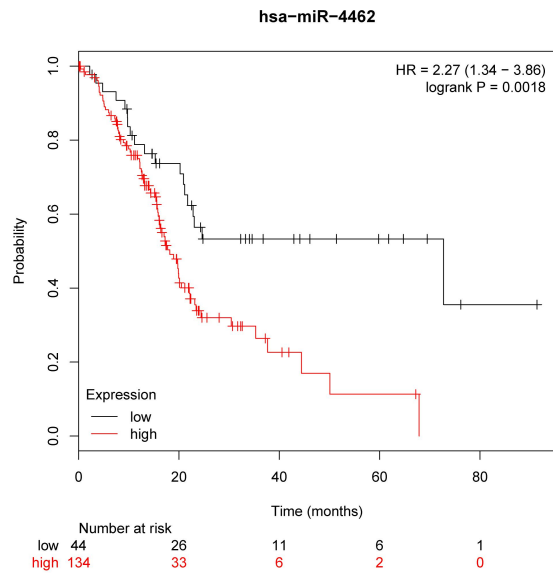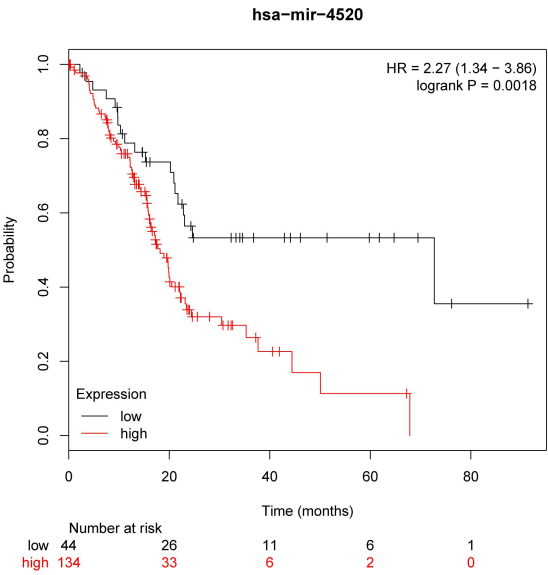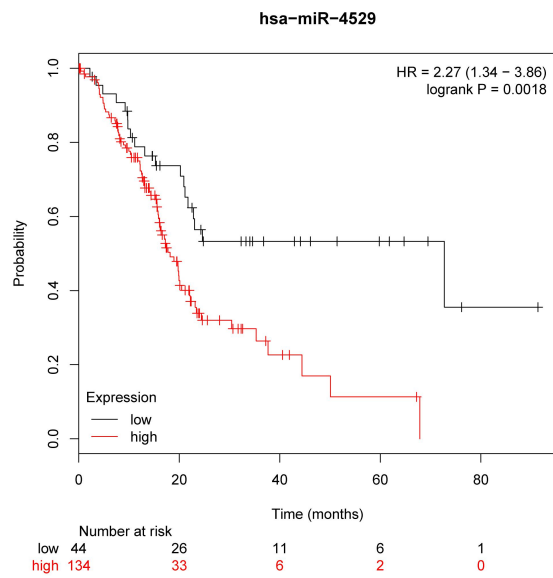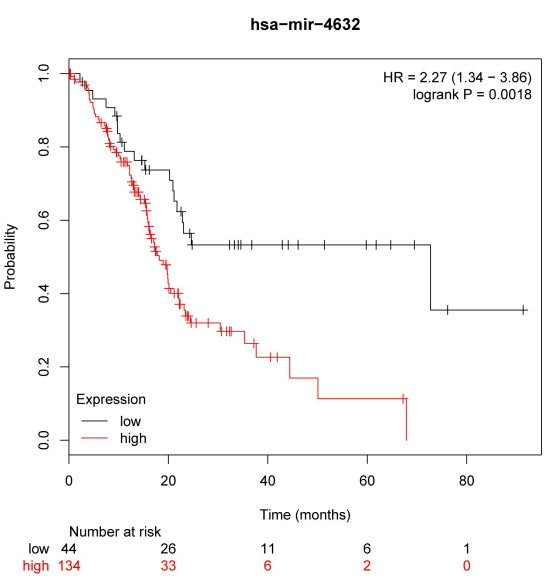

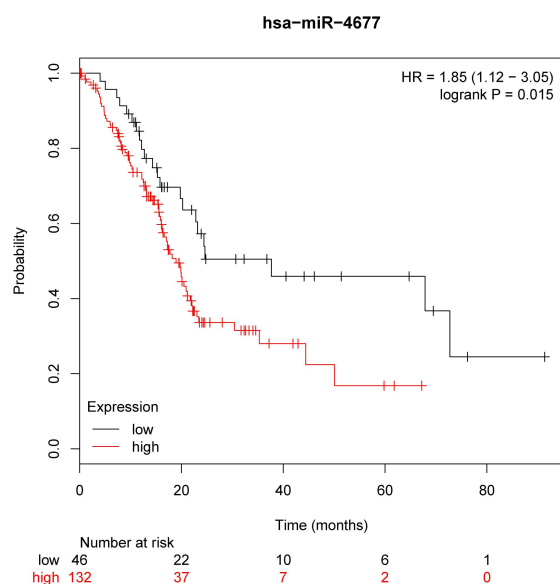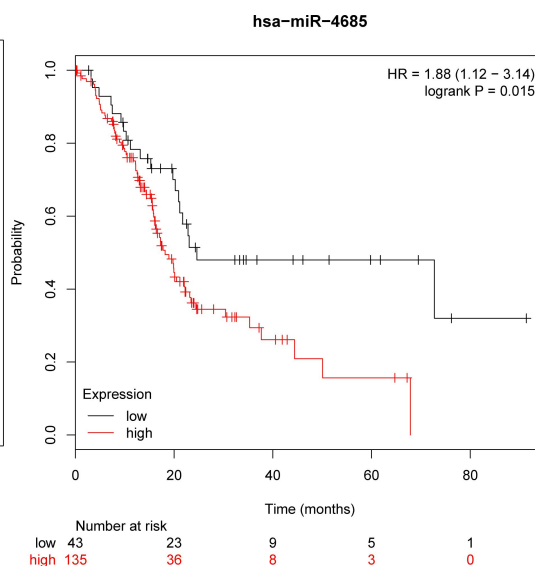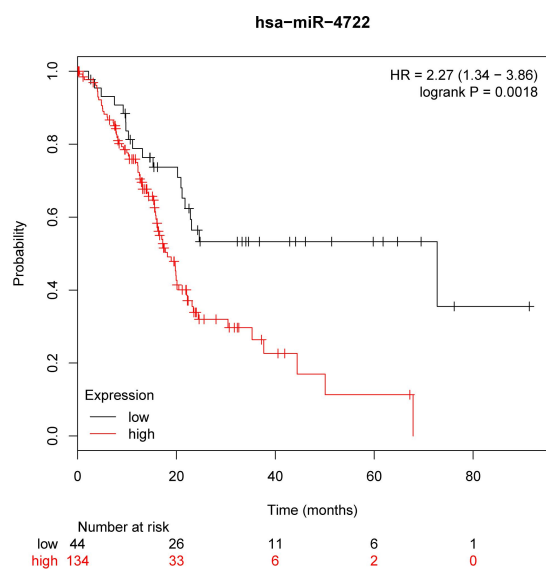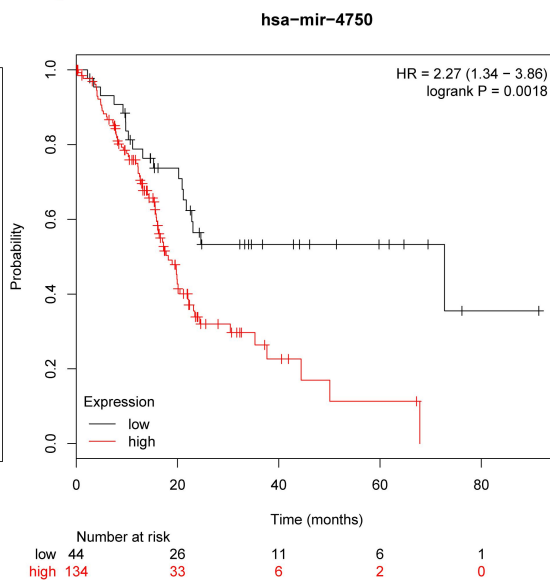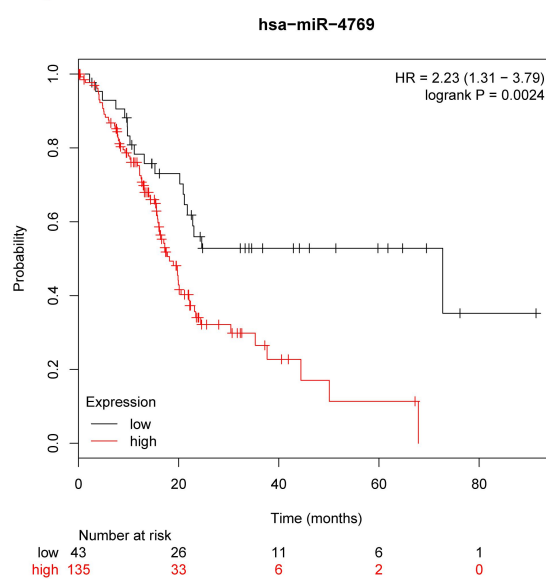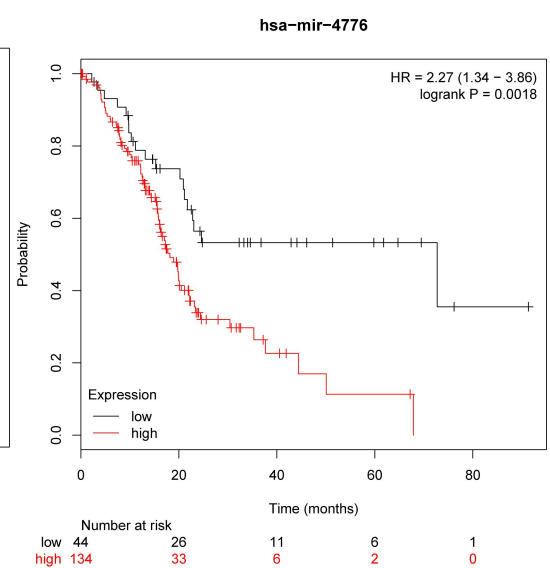

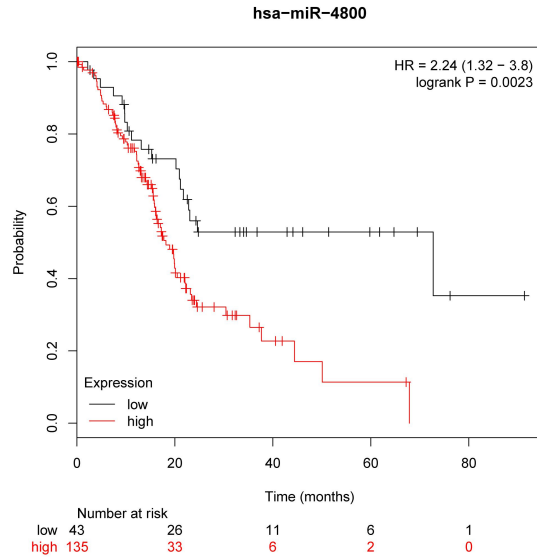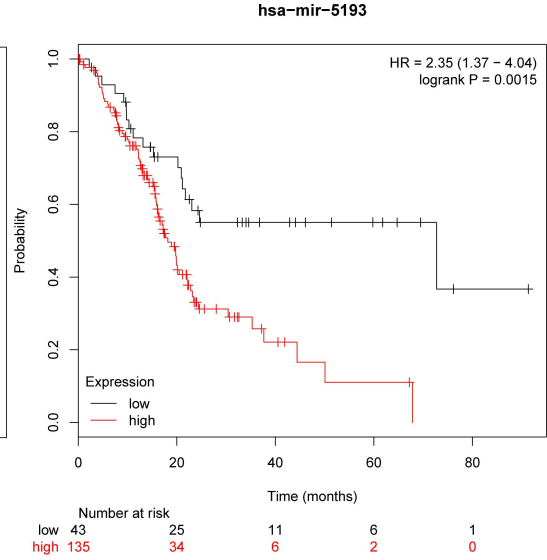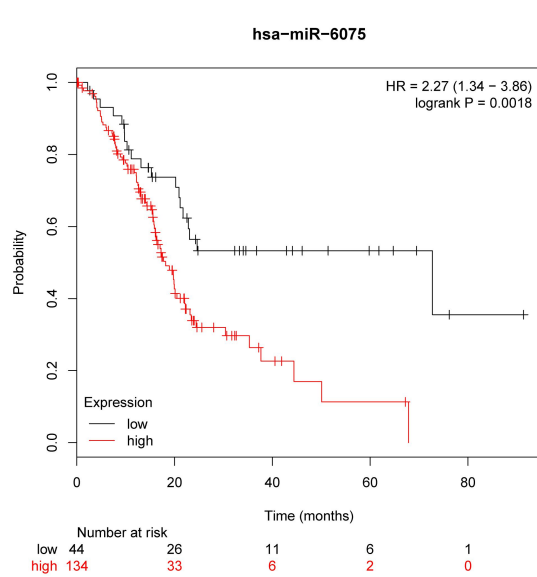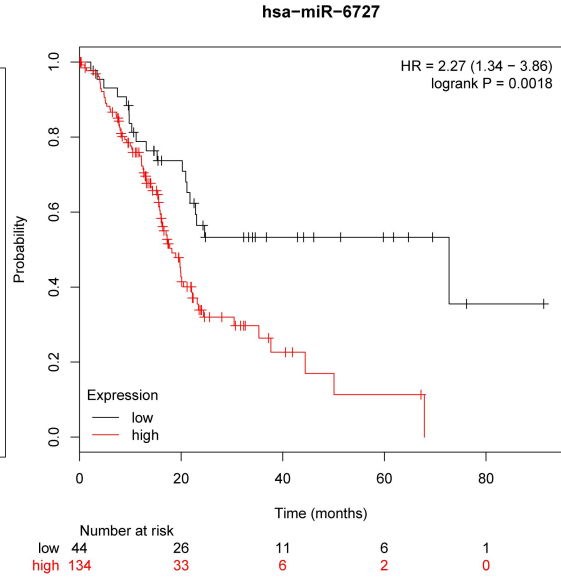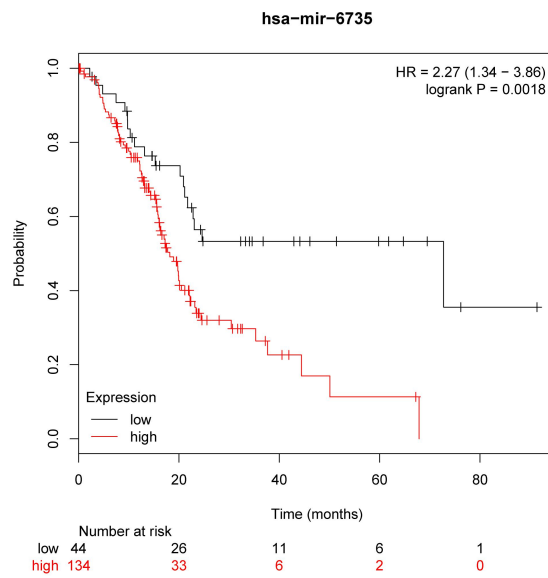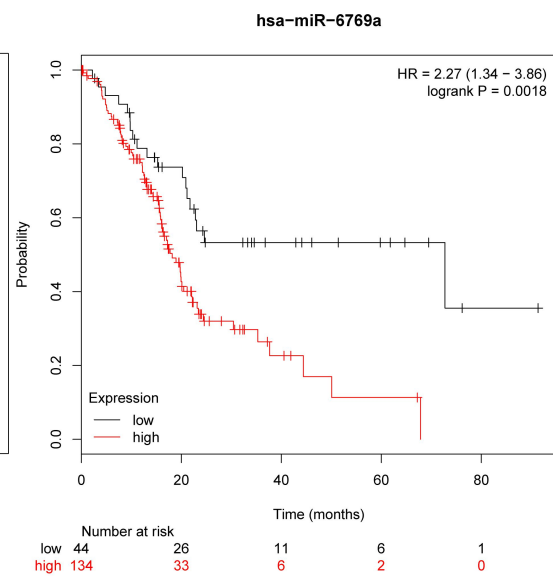

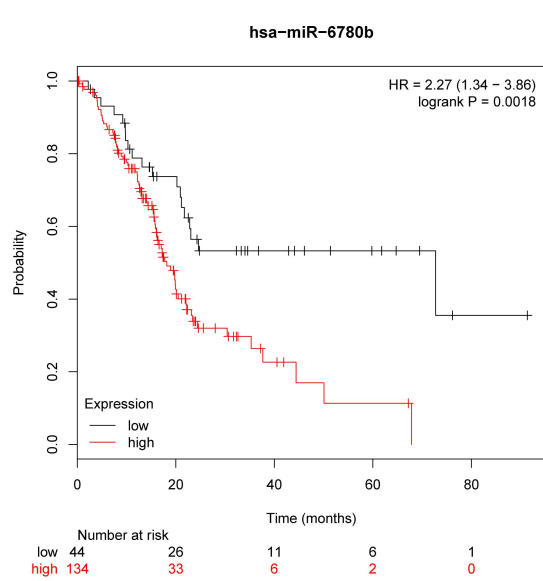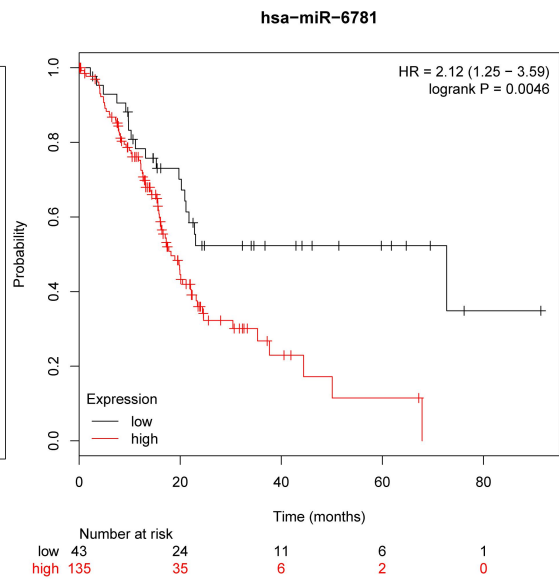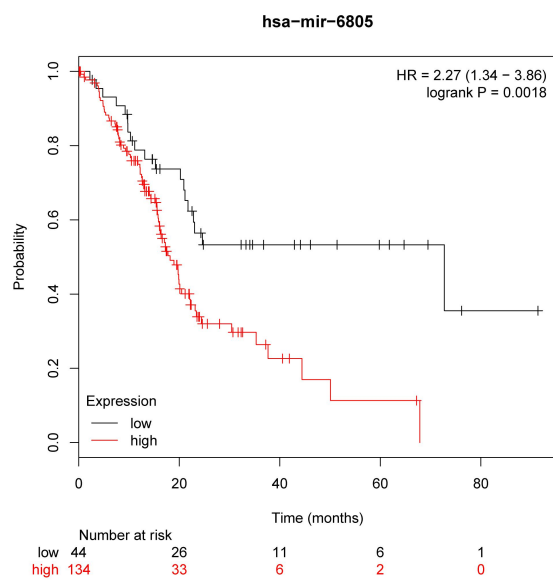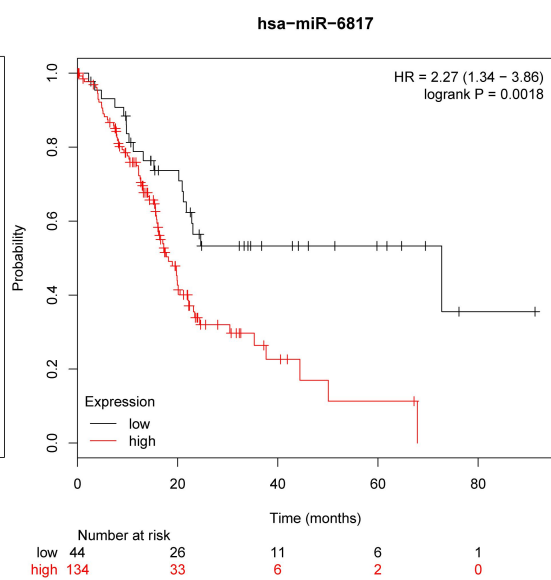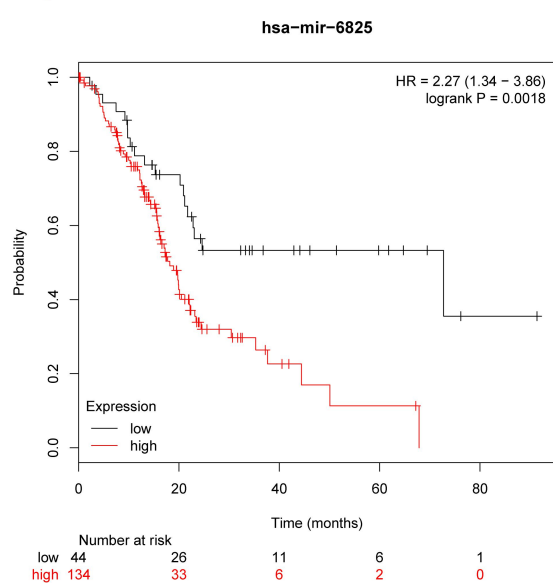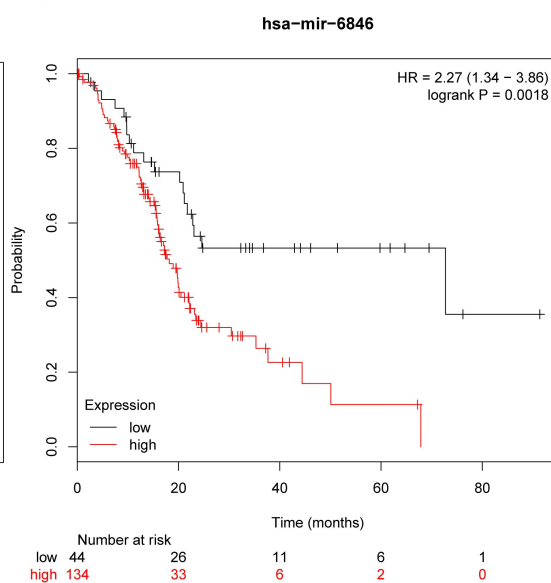

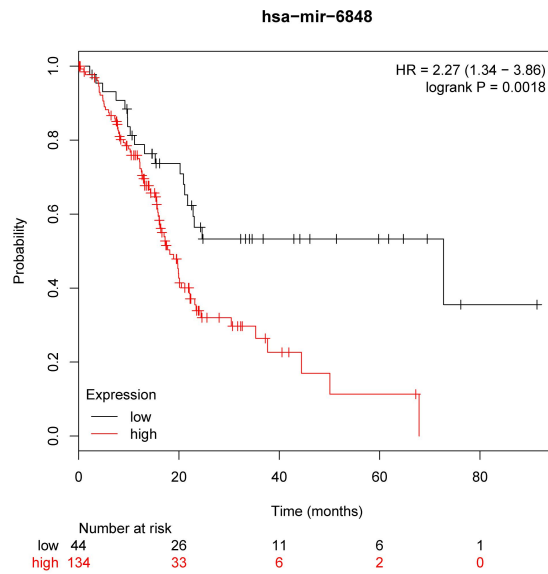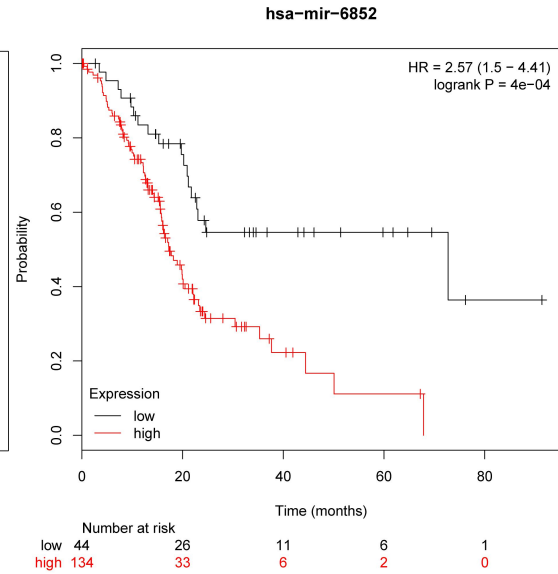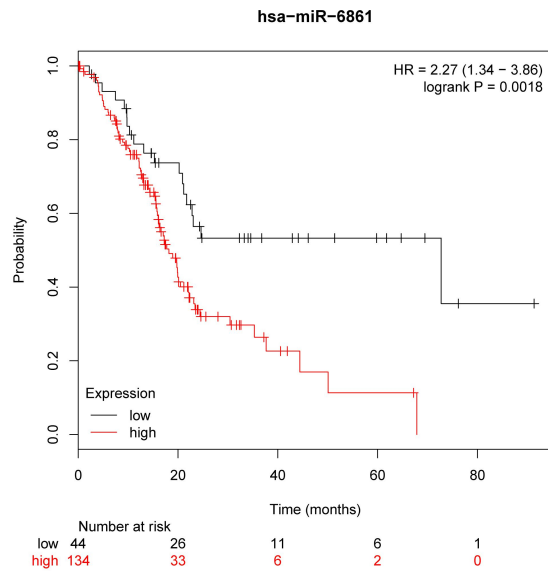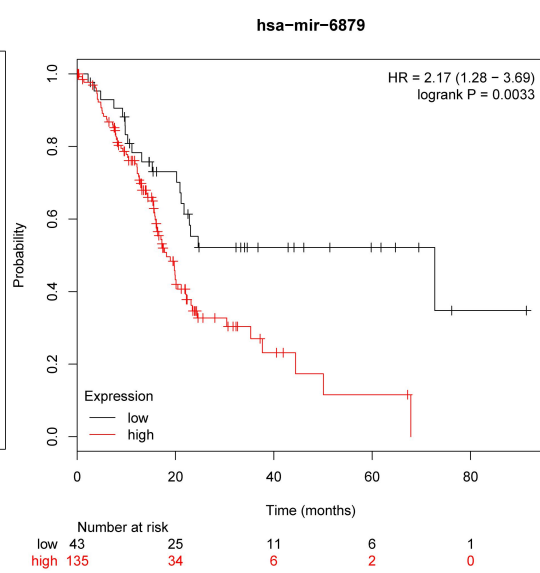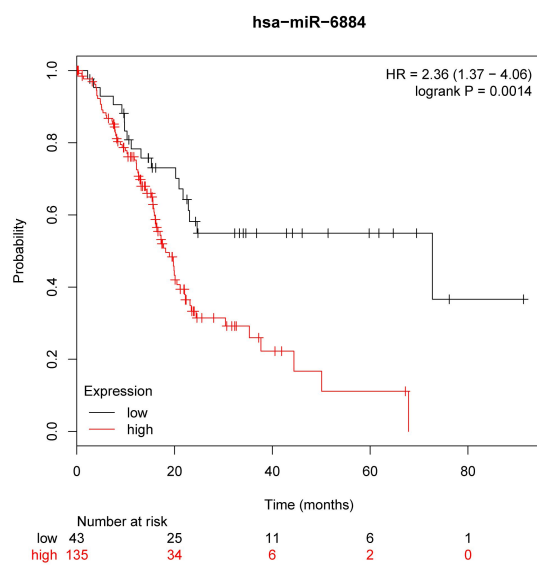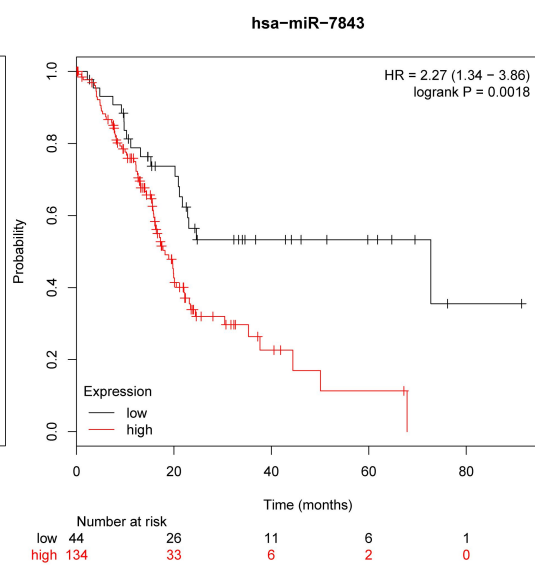

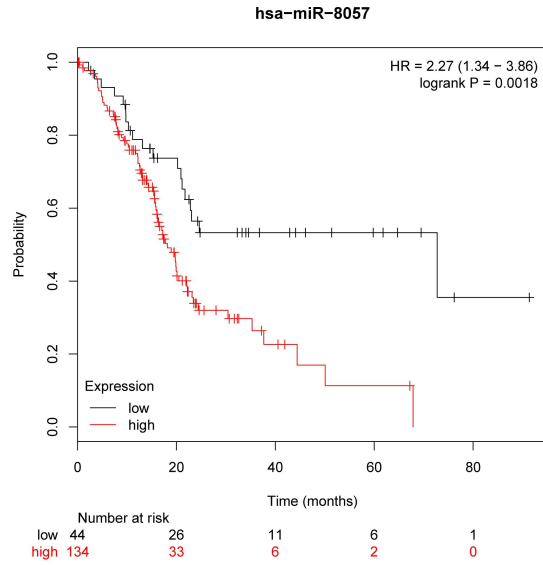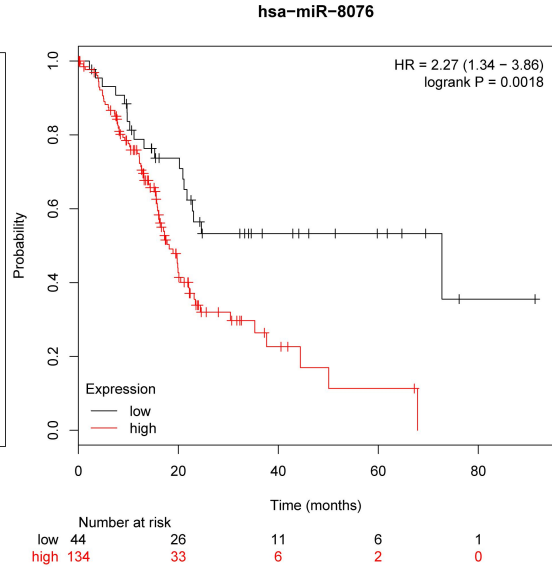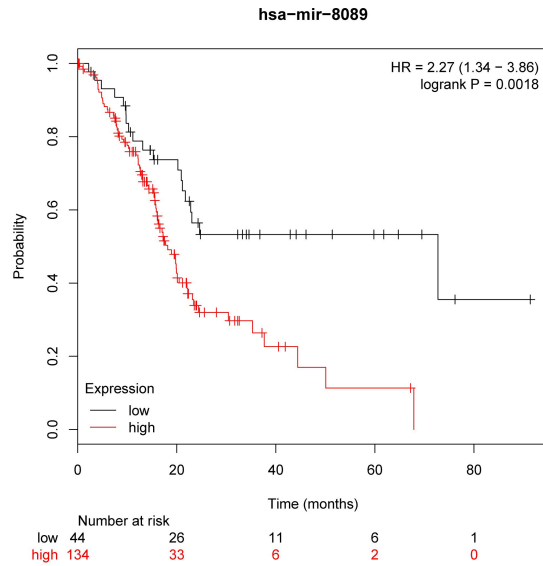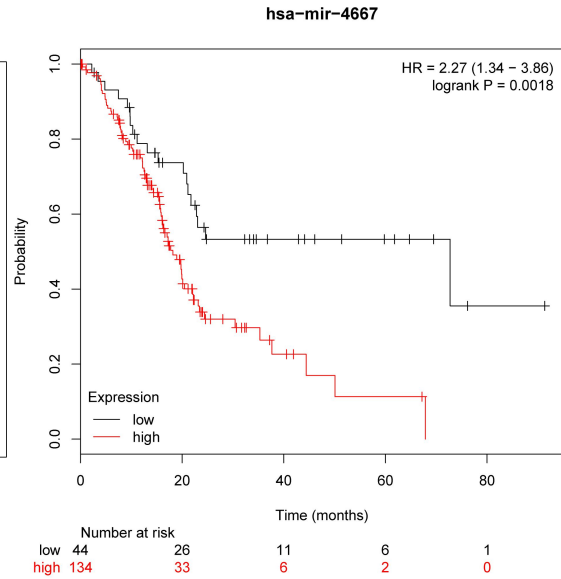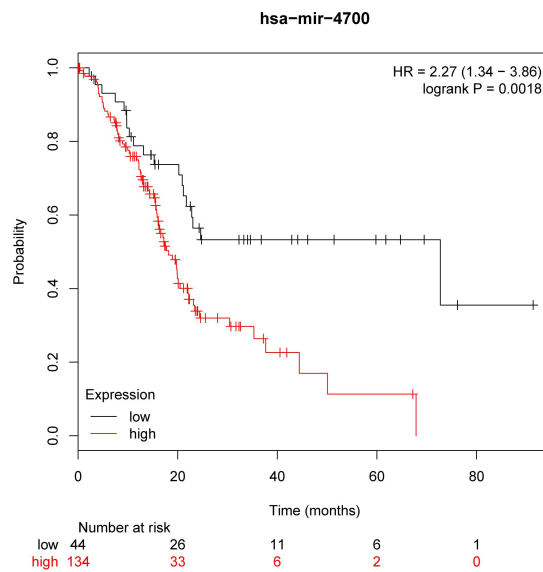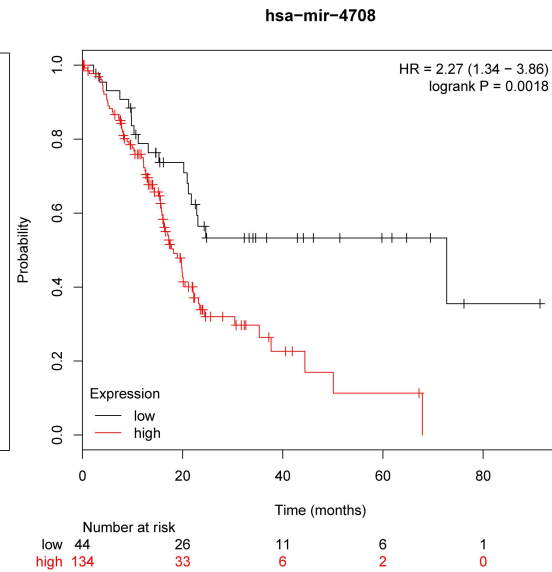

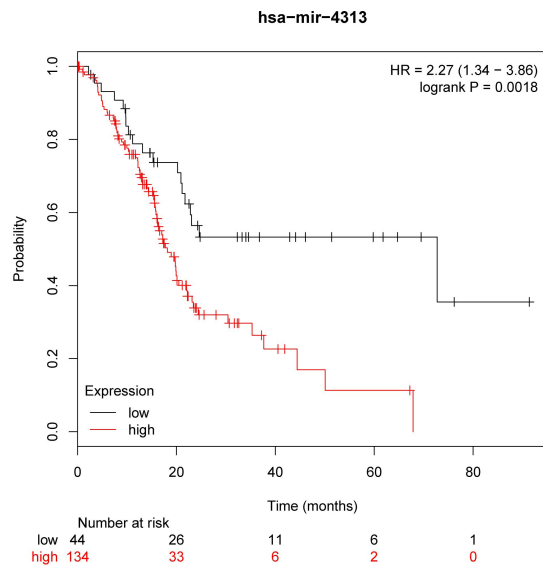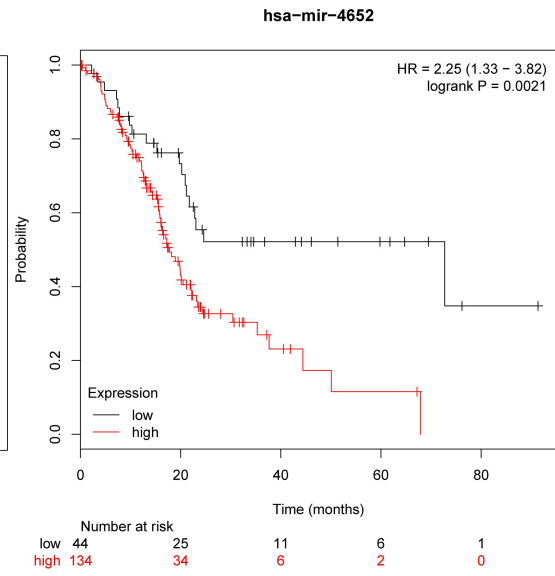

Supplement: Supplementary 1 — Figure S1 The survival analysis of target miRNAs of selected DECs. [file 8819990.f1.pdf]

**Supplementary Figure S2** The clinical stage analysis of hub genes by using the GEPIA database.


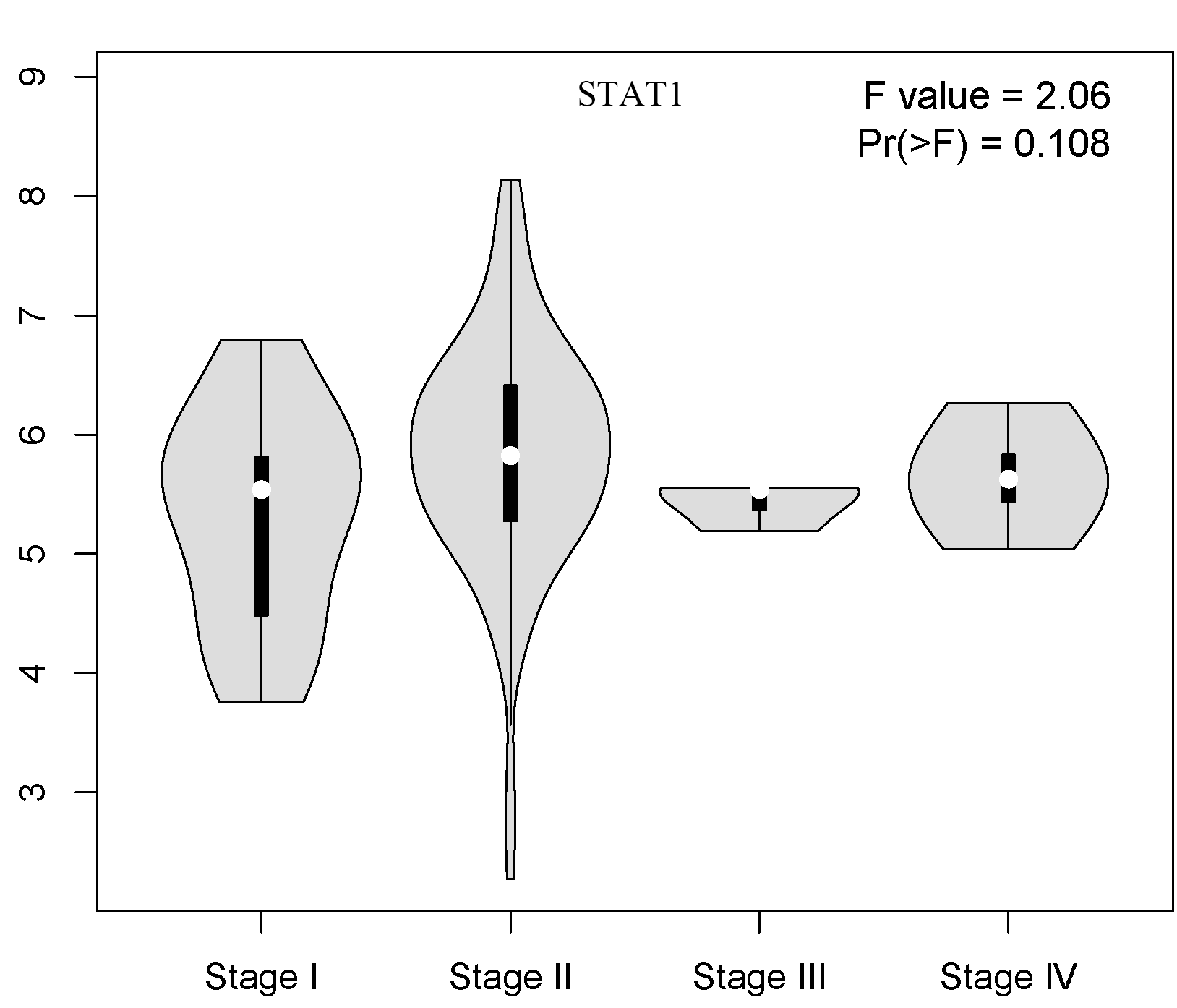


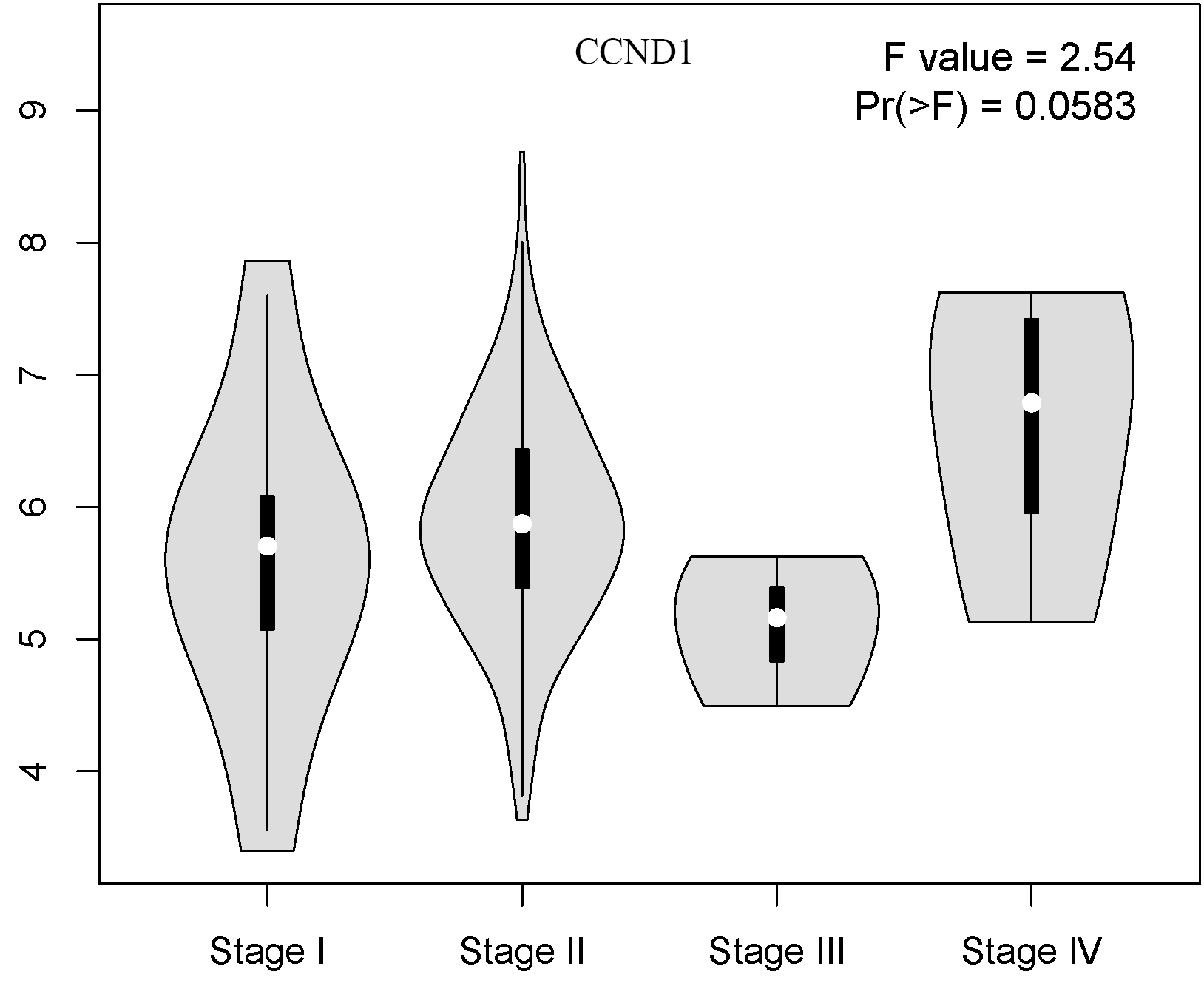


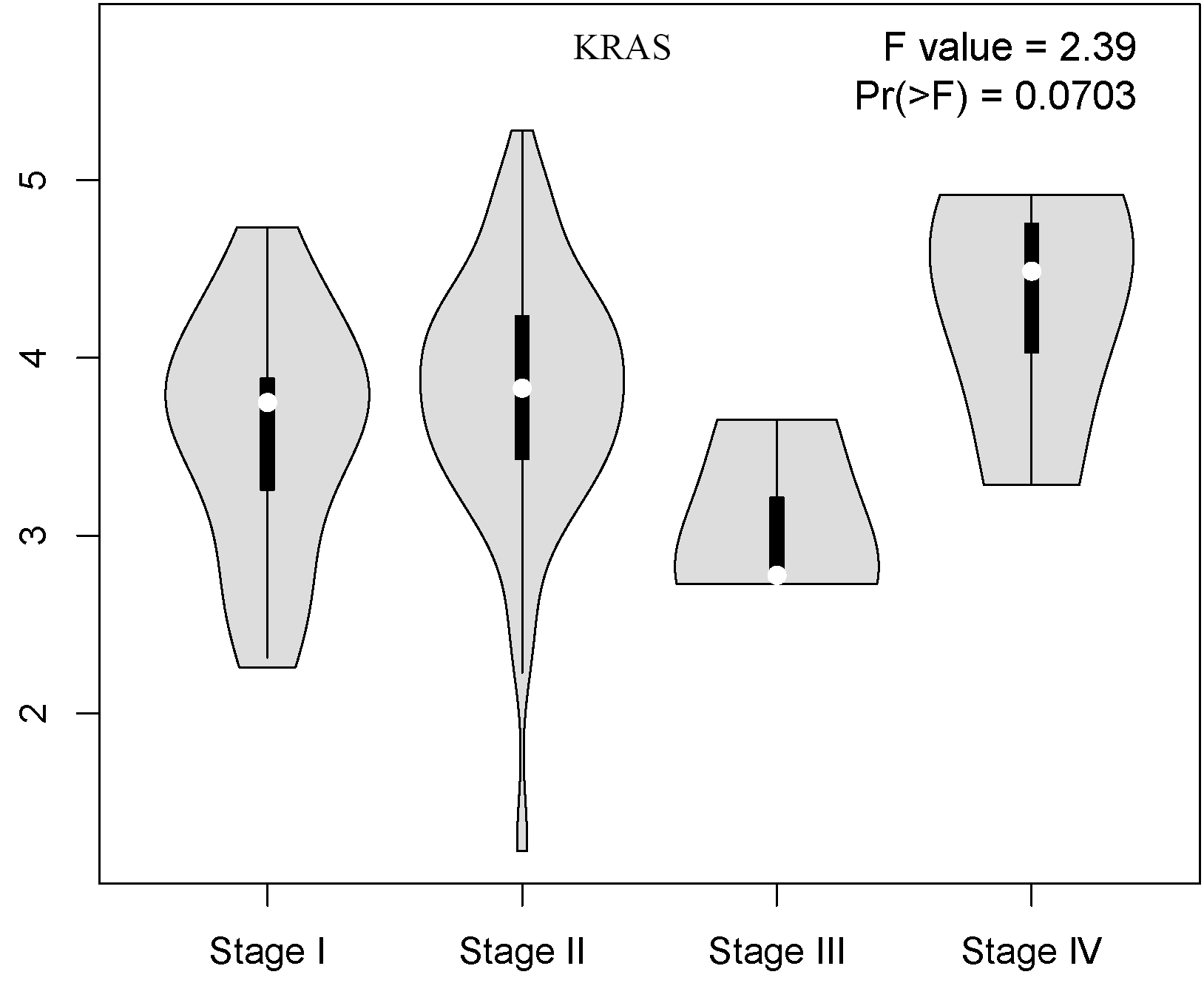


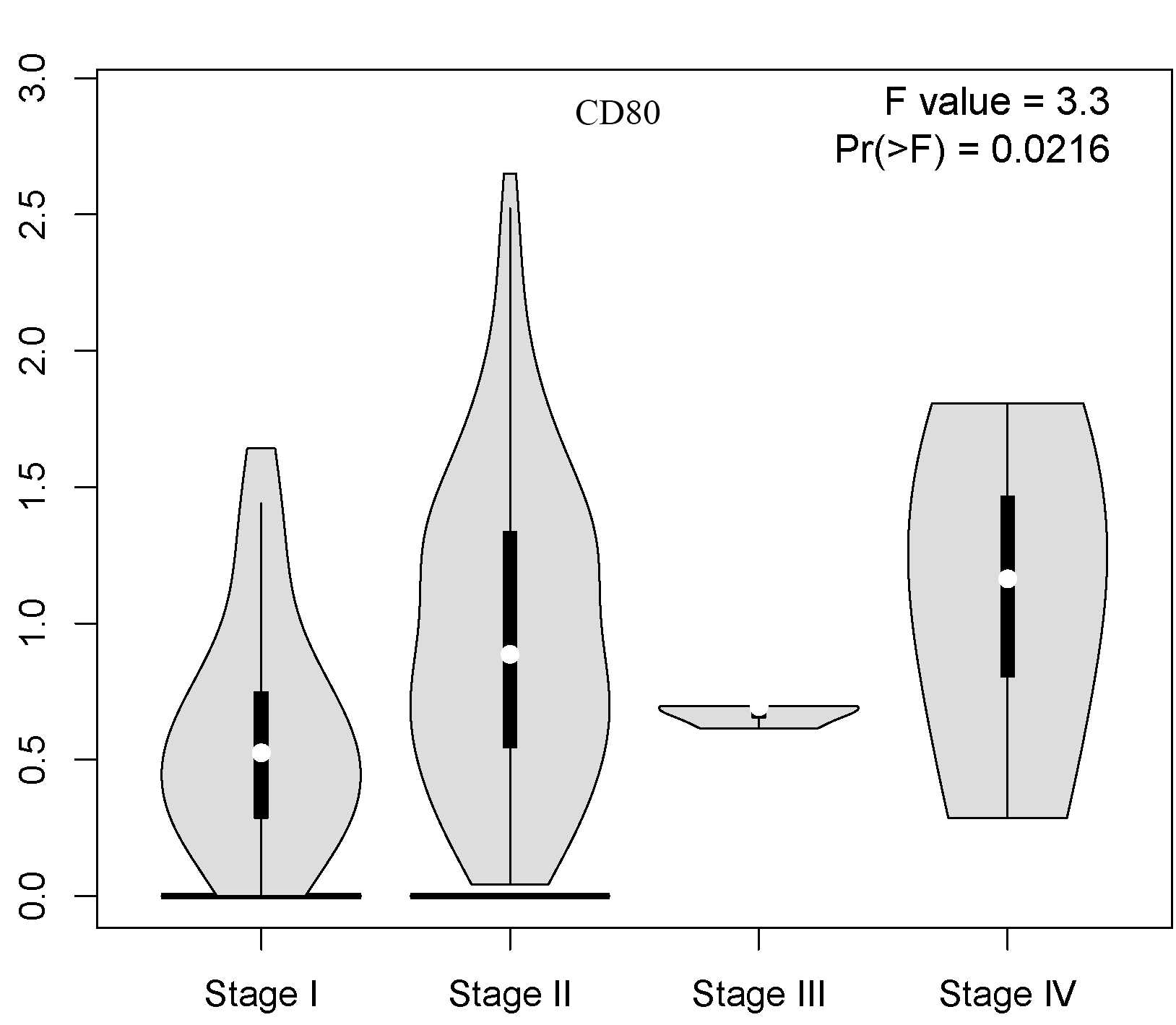


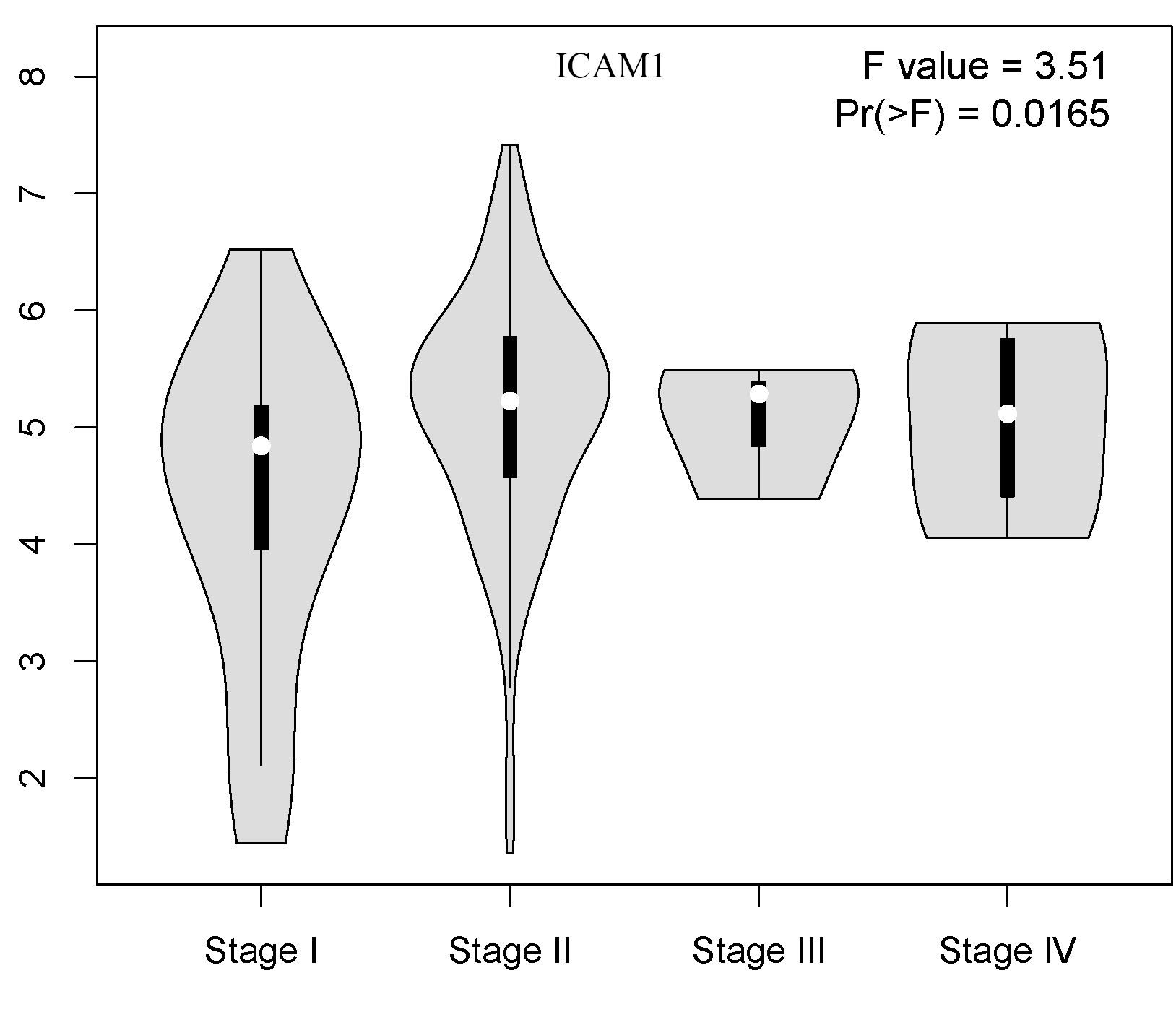


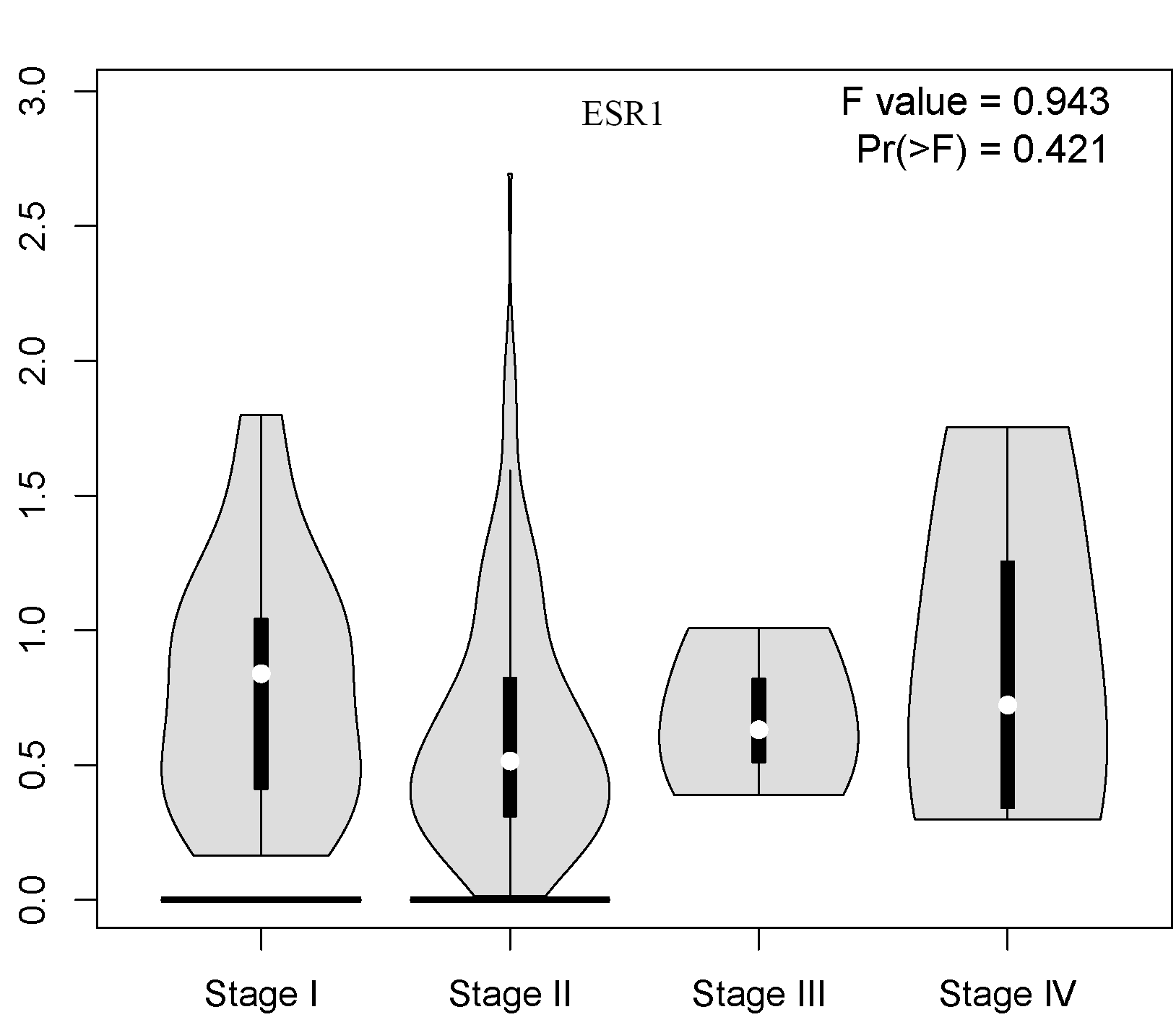


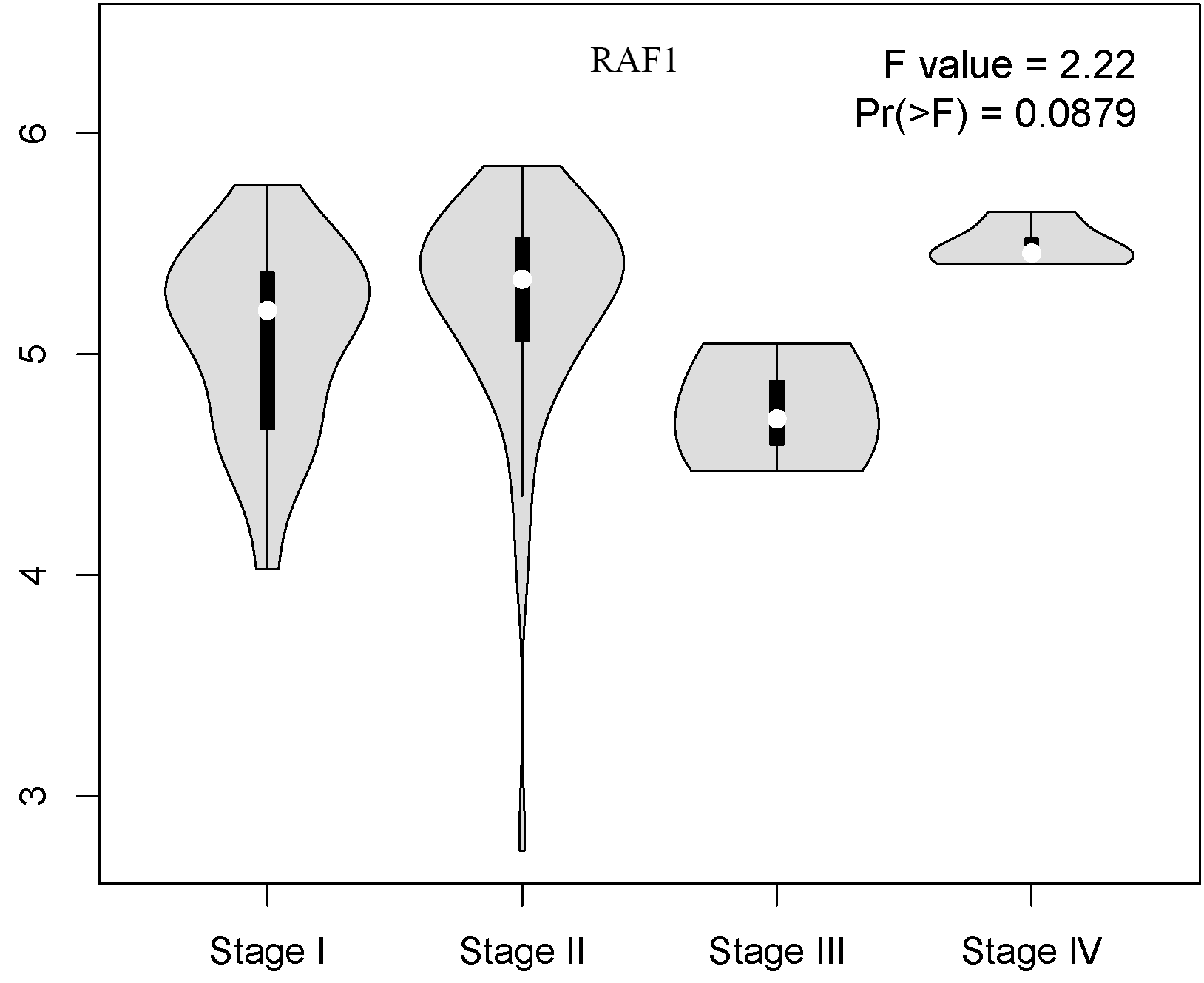


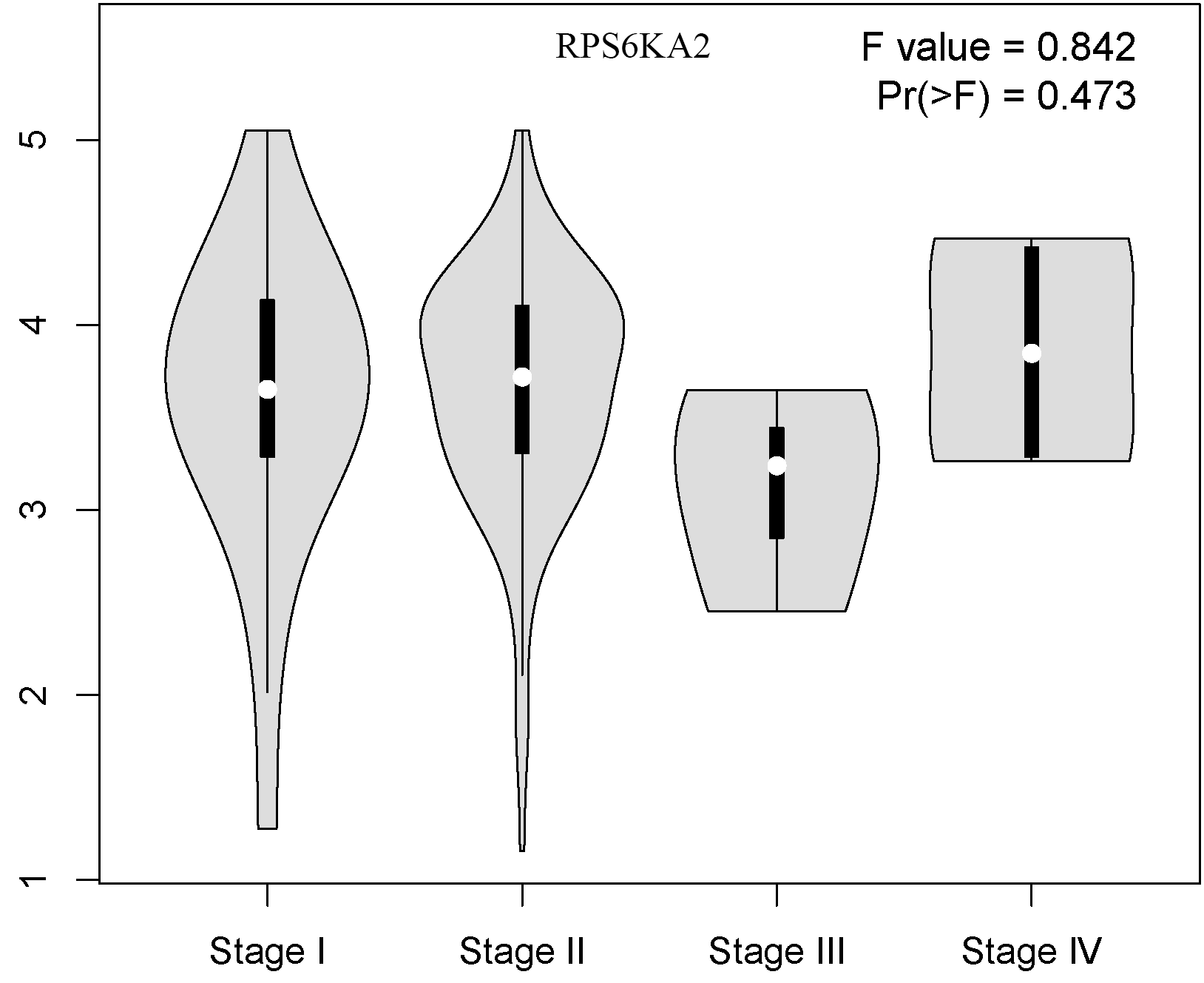


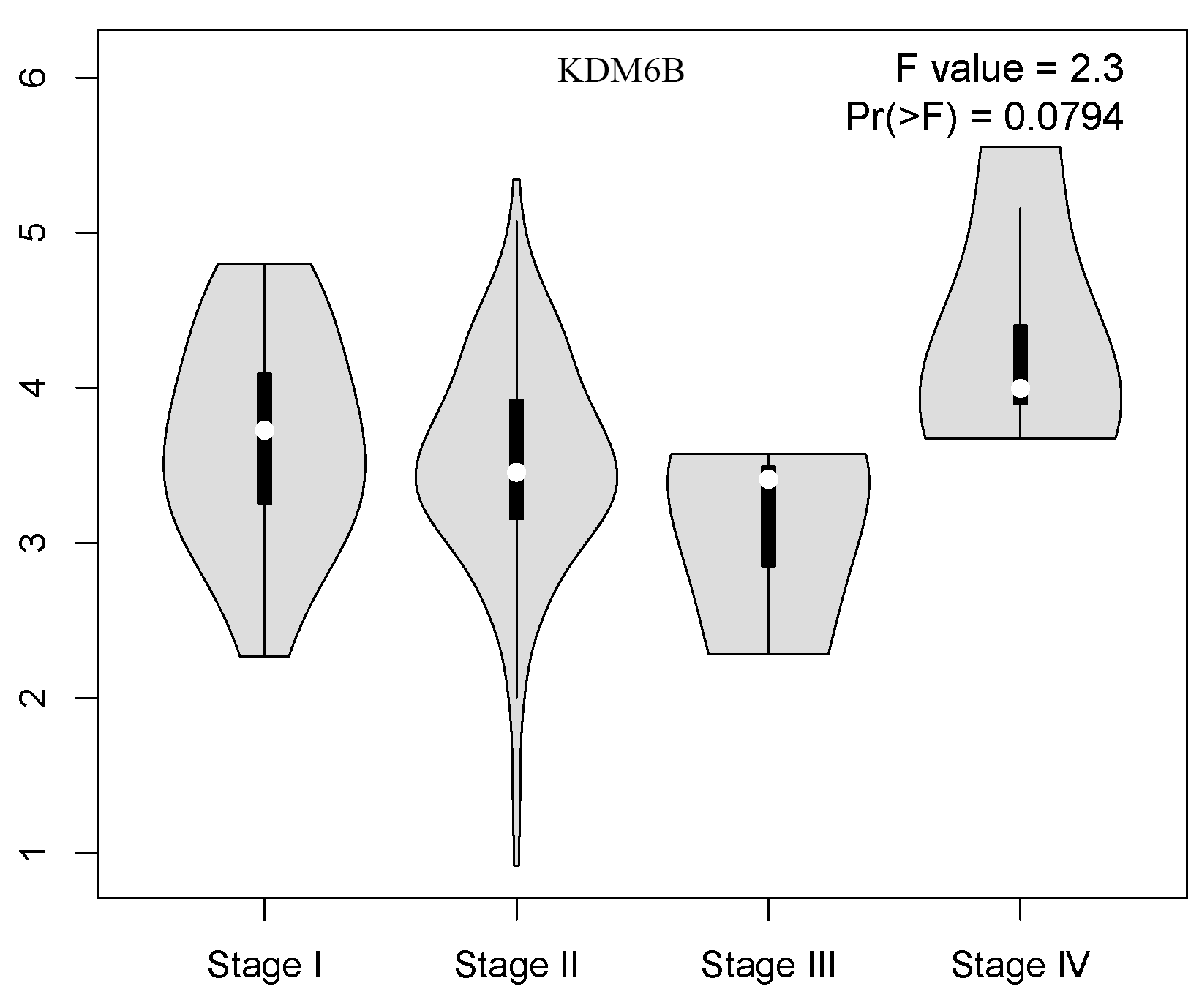


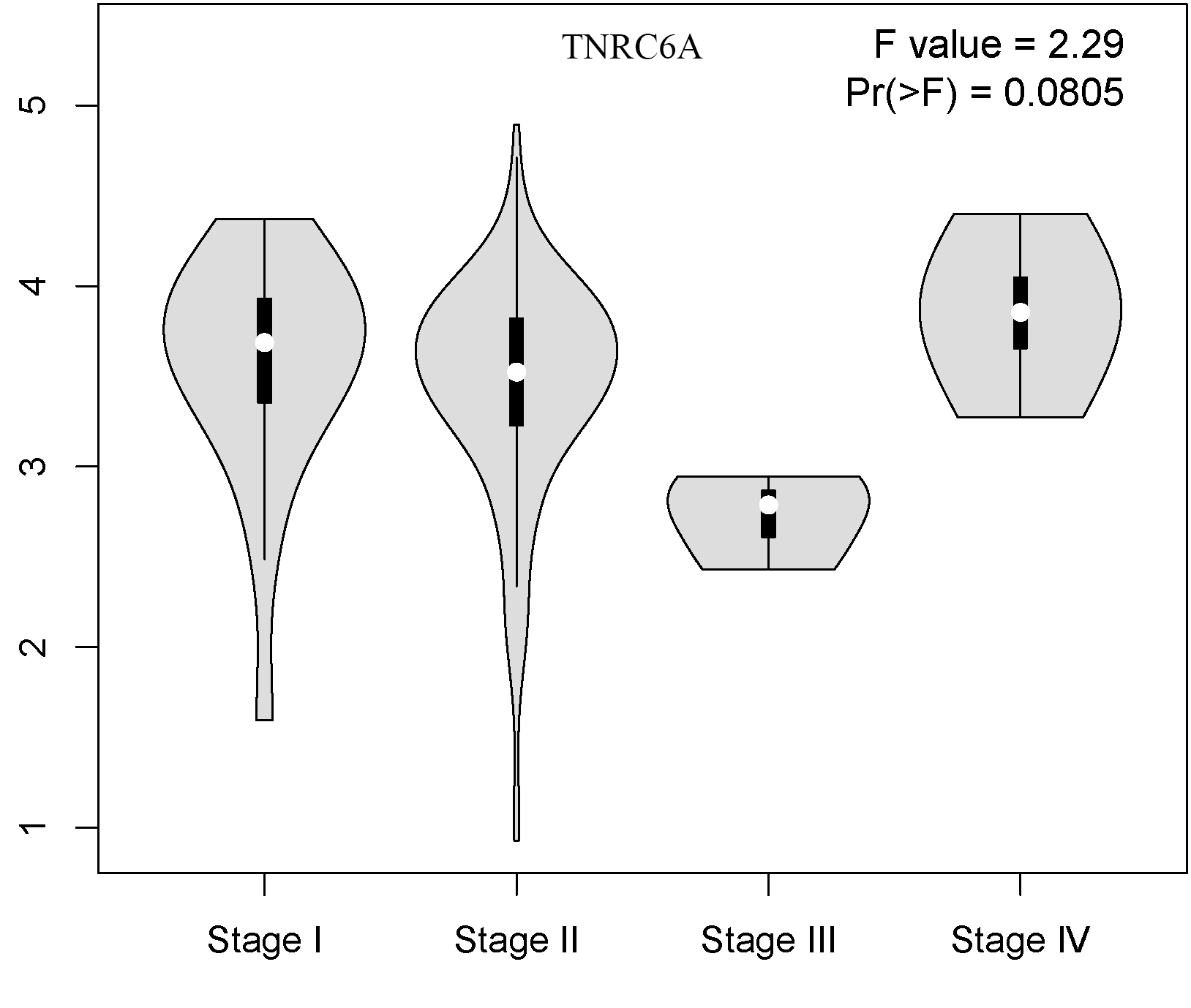


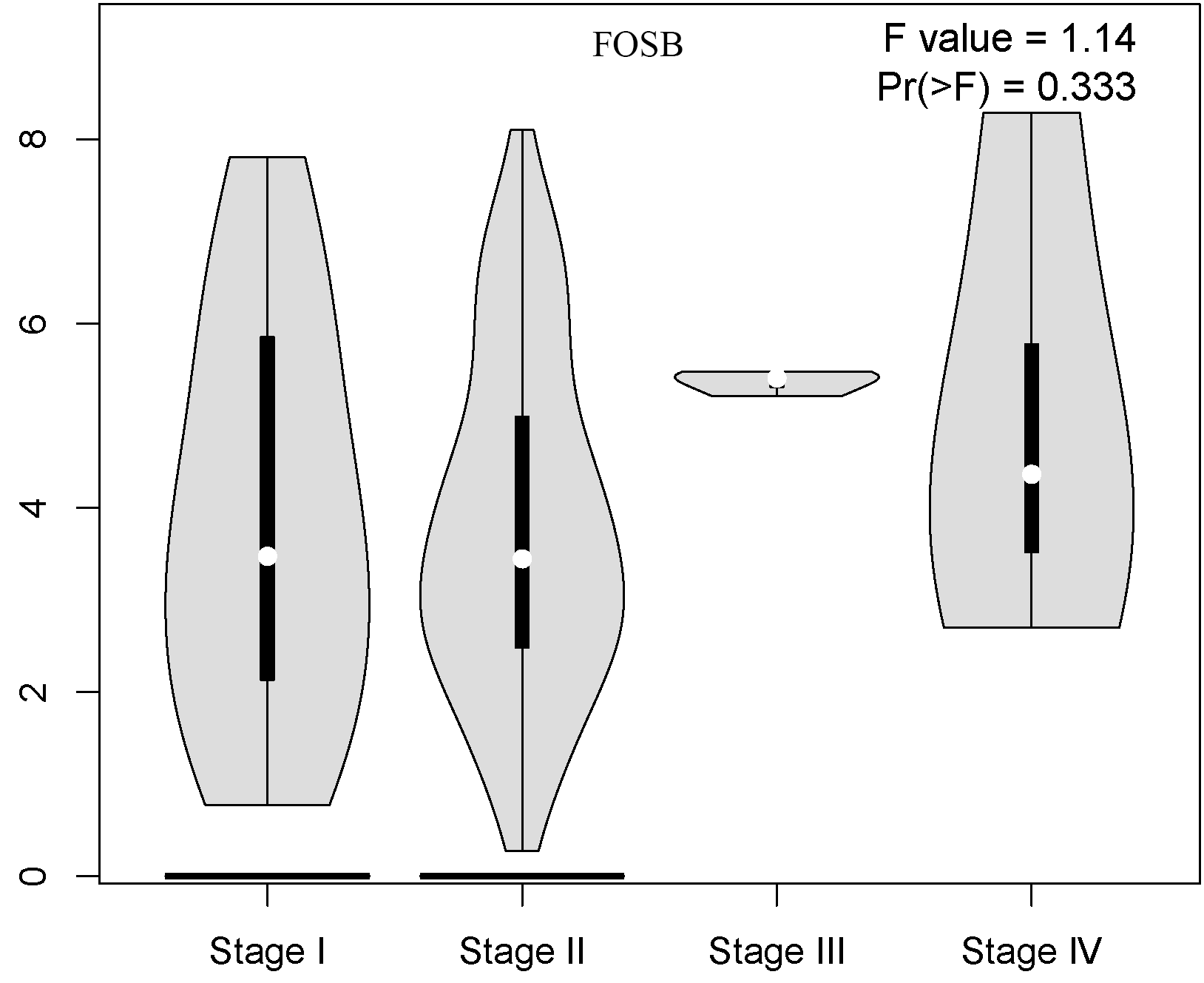


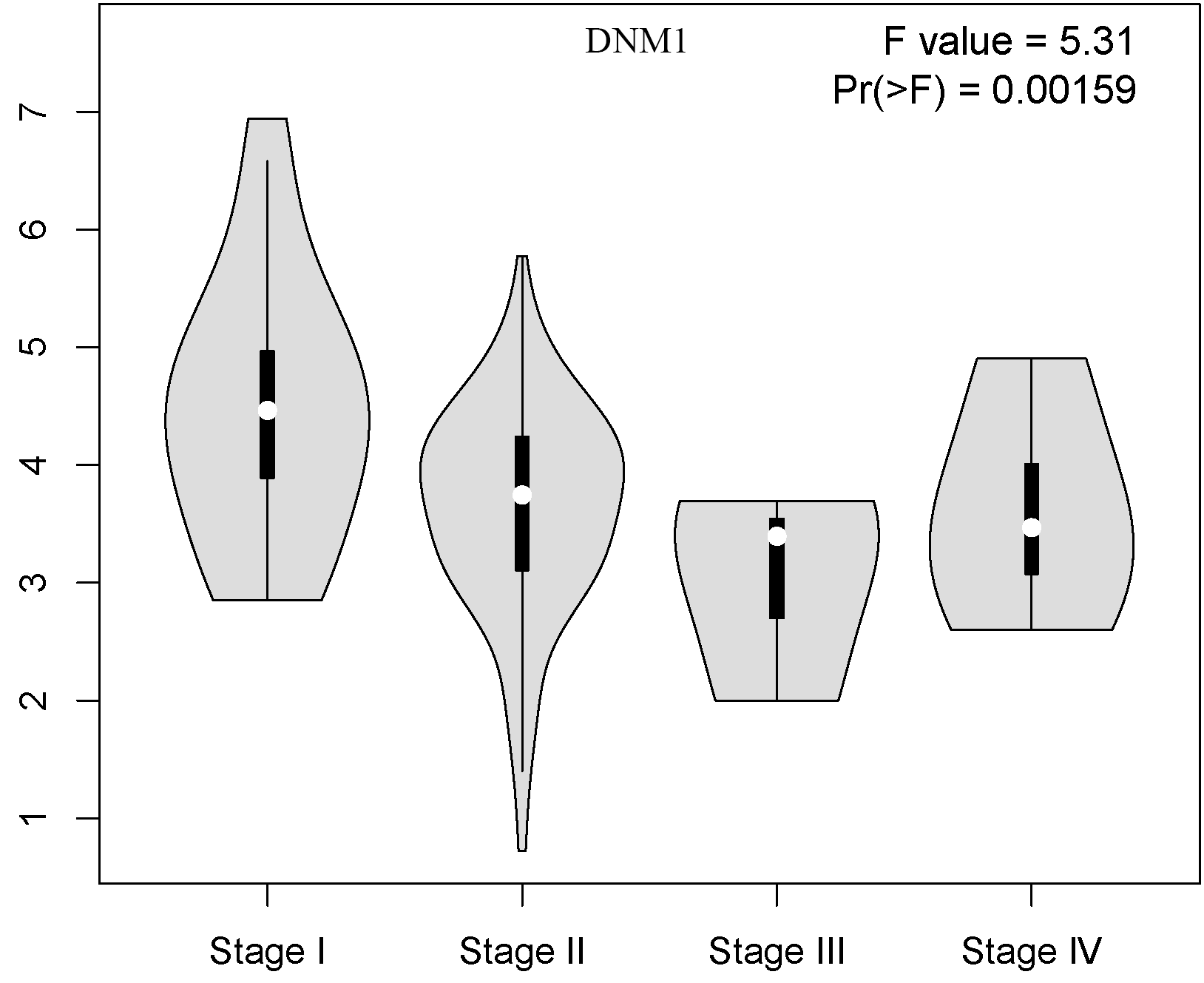

Supplement: Supplementary 2 — Figure S2 The clinical stage analysis of hub genes by using the GEPIA database. [file 8819990.f2.doc]
