# Supplementary material for: circRNA circ_102049 Implicates in Pancreatic Ductal Adenocarcinoma Progression through Activating CD80 by Targeting miR-455-3p
Source: Mediators Inflamm. 2021 Jan 7;2021:8819990. doi: 10.1155/2021/8819990 (PMC7811564; doi:10.1155/2021/8819990)
Supplement: Supplementary 3 — Table S1 The differentially expressed circRNAs in the GSE69362 dataset. [file 8819990.f3.doc]

**Supplementary Table S1 The differentially expressed circRNAs in the GSE69362 dataset**

| ID | adj.P.Val | P.Value | t | B | logFC | SPOT_ID | SEQUENCE |
| --- | --- | --- | --- | --- | --- | --- | --- |
| ASCRP003058 | 0.0625 | 0.00022 | 5.344 | 0.88748 | 1.7550218 | hsa_circRNA_102746 | GTGTCCTGGATTATGTGAAACCTGTTTCCCTCCAATCTTGTGGTTG |
| ASCRP002715 | 0.0625 | 0.000249 | 5.263801 | 0.77783 | 1.002811 | hsa_circRNA_102396 | TCCTGGCCCTCGTCGTGGACCTGCTGCAGATGCTGGAGATGAACAT |
| ASCRP000950 | 0.0625 | 0.000263 | 5.229721 | 0.73089 | 2.1171295 | hsa_circRNA_100571 | TGTTTGCCTGTCAGCAGGTCTCTGTTCTAGGATGTCCCGACCCAGT |
| ASCRP001589 | 0.0625 | 0.0003 | 5.145607 | 0.6142 | 1.2537145 | hsa_circRNA_101226 | ATATTCCTGTTCCTACTACAGTTCCTGTTCCTGGTTCAGCACCACC |
| ASCRP002069 | 0.0625 | 0.000359 | 5.032506 | 0.45537 | 1.1379253 | hsa_circRNA_101722 | GCCCTTTCTGAATCTGGGCCCTTCCGTCCTGGCTGGAGTGGCGGTG |
| ASCRP004222 | 0.0625 | 0.000414 | 4.943394 | 0.32869 | 1.5292973 | hsa_circRNA_103942 | GGCTTATTGGGTCCTTCTGTACCTCCCTTAGTGAATCCACCTCTGC |
| ASCRP001808 | 0.0625 | 0.000435 | 4.912781 | 0.28486 | 1.0485152 | hsa_circRNA_101457 | GTCTCATAAGCTTTTGTCTGGTCCTCTGAGCCCCAGTGAGAGTTTC |
| ASCRP002062 | 0.0625 | 0.000446 | 4.897991 | 0.26362 | 1.9240145 | hsa_circRNA_101715 | TTCTCCTCAGATTAATGATGTTTTGCAGCAGTTTTCTACGTCTGAA |
| ASCRP002357 | 0.0625 | 0.000598 | 4.717876 | 0.00213 | 1.468878 | hsa_circRNA_102021 | TTCTCCTCTAATGACAGCATCAGCGAGAGCTTTCTAACTGTCAAAG |
| ASCRP003361 | 0.0625 | 0.000641 | 4.674907 | -0.06105 | 1.095995 | hsa_circRNA_103056 | TACCTCCCAGGTGAACAGCATGAAGGAGCTGTACTTGCTGATGGAG |
| ASCRP003076 | 0.0625 | 0.000832 | 4.517823 | -0.29453 | 1.4145092 | hsa_circRNA_102765 | CTCCATTGCTACGTCCTTCTATGGCCGCTTCAGGCACTTCTTGGAT |
| ASCRP001625 | 0.0625 | 0.00084 | 4.512079 | -0.30315 | 2.9981763 | hsa_circRNA_101263 | AAAGTCCTTCTCAAGATTCGGATTATCGAGATTGCAGCCTGTCACT |
| ASCRP003344 | 0.0625 | 0.000957 | 4.434054 | -0.42064 | 2.7884452 | hsa_circRNA_103038 | AAAGTGCAGTTCCCTCCGGTGTGTTCGAGGGCGAGCTCTCCGATAC |
| ASCRP003393 | 0.0625 | 0.00105 | 4.378863 | -0.50431 | 1.0741992 | hsa_circRNA_103089 | CTATACCTACTGGGTTCCCCTGGTGCTGCGAGTGGCTGAGATGCTG |
| ASCRP003688 | 0.0625 | 0.001193 | 4.303257 | -0.61968 | 1.9850347 | hsa_circRNA_103390 | CTTCCCTCCGGTTTTTGATGAGAAGTACTTTCTGGTGGAAATGGAT |
| ASCRP001778 | 0.0625 | 0.001218 | 4.290976 | -0.63849 | 1.6873439 | hsa_circRNA_101419 | GACTCAACTGCCCTACCATTGACCATGCCCTGCTGGACATGCCCTT |
| ASCRP000239 | 0.0625 | 0.001263 | 4.269383 | -0.67164 | 1.2896531 | hsa_circRNA_001259 | GGCCATCTATTTTCCTGTTTTCCAGCACCCAACATAACTTGTAAGG |
| ASCRP004548 | 0.0625 | 0.001269 | 4.266532 | -0.67602 | 1.4893747 | hsa_circRNA_104283 | AGAAACTCATCAGGATTTATCCTGGCCCTGCCCTGGGAGAAGCCCT |
| ASCRP003826 | 0.0625 | 0.001323 | 4.24197 | -0.71381 | 1.4095176 | hsa_circRNA_103528 | GCCCTCCAAGCTCGAGATGCTGCTGTGCAGAAGATTGAGACTATTA |
| ASCRP004099 | 0.0625 | 0.001429 | 4.196842 | -0.78347 | 3.1215138 | hsa_circRNA_103809 | TCCAAGCTGGCCCTTACGTCGTCCTGACTCATCTGATGACCGTTAT |
| ASCRP003380 | 0.0625 | 0.001481 | 4.175815 | -0.81602 | 1.2674373 | hsa_circRNA_103076 | CCTTTGCTTTGCCTTTGACTAATCTTTTTAAGGTTGAAGATGAACC |
| ASCRP001062 | 0.0625 | 0.001521 | 4.16059 | -0.83963 | 1.0507483 | hsa_circRNA_100688 | ACCTTCTTCAGACAGCCTTTTGATTGATTATCAATTTACCTTCAGC |
| ASCRP002369 | 0.0625 | 0.001617 | 4.124811 | -0.89524 | 2.4375377 | hsa_circRNA_102034 | AAGACAGCAGGTTCCTCCCCGGGCAGAAGAAATCACCATTCCAGCT |
| ASCRP002911 | 0.0625 | 0.001652 | 4.112057 | -0.91511 | 1.3164396 | hsa_circRNA_102598 | TCTCTGGCATCTTCTTCATACTATCGGAGACAGTGCGCACGGCCAC |
| ASCRP004028 | 0.0625 | 0.001653 | 4.111833 | -0.91546 | 2.0563114 | hsa_circRNA_103737 | AAGCGTCAAATAATTCGGTTCCCTCAAAATTATAAAGGCAGGAAAG |
| ASCRP000507 | 0.0625 | 0.00168 | 4.102603 | -0.92985 | 2.2585815 | hsa_circRNA_100117 | CCTCCTTTTGATTGTGAAAACTGCTGAGAGAGACTTGCAATCCAGT |
| ASCRP005009 | 0.0625 | 0.001717 | 4.089604 | -0.95013 | 1.3735866 | hsa_circRNA_104759 | AGTCCTCAGTCCTCAGTCATCTTGCTTTCTGAAACAAAGGTCTTCA |
| ASCRP002782 | 0.0625 | 0.001846 | 4.047717 | -1.01565 | 1.3956664 | hsa_circRNA_102465 | CCTCCTCAACTGGGTTCAAGTGCTCCACCAAGACCTCCAAGGGCAG |
| ASCRP002219 | 0.0625 | 0.002026 | 3.993941 | -1.10011 | 1.5140642 | hsa_circRNA_101877 | CTCTTTTCTTCCAGTTCCCTACTGAAGGAACAGATGCTAAGGAAAC |
| ASCRP005384 | 0.0625 | 0.002059 | 3.984596 | -1.11482 | 1.2438745 | hsa_circRNA_400091 | ATCTCCATCTGGGCCCTTCCTCCAGCTTTGCTTTGTTTGGCTAACA |
| ASCRP003291 | 0.0625 | 0.002118 | 3.968126 | -1.14078 | 1.4236274 | hsa_circRNA_102984 | TTCCCTCCTTCAGATAAGCATGGATTCCTGGTTCATTCTTGTTCTG |
| ASCRP003713 | 0.0625 | 0.002257 | 3.931609 | -1.19845 | 1.1243985 | hsa_circRNA_103415 | CACTCCCAAAAGGGAAAGGTTCCCGTGTCAGTGGCTATGATGACAC |
| ASCRP003221 | 0.0625 | 0.002295 | 3.921903 | -1.2138 | 1.5588267 | hsa_circRNA_102913 | TTAGCCTTGAAGCCTTATTTAGTGTCTCTGACAAAACCGGCCTTGT |
| ASCRP005164 | 0.0633 | 0.002597 | 3.851205 | -1.32599 | 1.79381 | hsa_circRNA_104917 | CACTCCCTAGCAACTGAACCACCCAAATATCATCAAGTATTTGGAC |
| ASCRP003063 | 0.0633 | 0.002638 | 3.842051 | -1.34056 | 1.1747788 | hsa_circRNA_102751 | TGTGCAGGAGCTGGCCCGGATTGCGGACAGTAAGGATCATGTGTTT |
| ASCRP002042 | 0.0633 | 0.002641 | 3.841492 | -1.34145 | 1.5967734 | hsa_circRNA_101695 | AGAACTCTCCTTTTTCACCAGCCAGGACAACATGTGGCGCTGTCCC |
| ASCRP005008 | 0.0633 | 0.002649 | 3.839814 | -1.34412 | 1.3283072 | hsa_circRNA_104758 | AGTCATCTTGTCATCCGTCCTTGGCTCAGGATTTGGAGAGCTTGCA |
| ASCRP000282 | 0.0633 | 0.002658 | 3.837905 | -1.34716 | 1.0328666 | hsa_circRNA_001486 | TCAATGTCTTCTTCATGGCTCAGCCCAAGGATGGCATGTTGAAAAC |
| ASCRP004753 | 0.0636 | 0.00273 | 3.822574 | -1.37159 | 1.0951885 | hsa_circRNA_104499 | GCCTCCGTGGGACTCTCCTTCAGCAACTCCAGAAGCTTCAGACTTT |
| ASCRP000535 | 0.0636 | 0.002766 | 3.81512 | -1.38347 | 2.1525883 | hsa_circRNA_100146 | TAGTGCCAAGGAAAGCAGCTGGCCCTTCTCAAGACCAATTCGGCTG |
| ASCRP004441 | 0.0648 | 0.002865 | 3.794996 | -1.41559 | 2.1981401 | hsa_circRNA_104168 | ATGACATTGCAGAACTGGTGGATGCGGGAAAGTGTTCTAATCTTAG |
| ASCRP003890 | 0.0659 | 0.002986 | 3.771324 | -1.45343 | 1.3579303 | hsa_circRNA_103595 | GCCCTCGCAGGTCCAGCACCATGATGCCATCACTGGGACTGAGTCC |
| ASCRP004407 | 0.0659 | 0.002988 | 3.771087 | -1.45381 | 1.6310848 | hsa_circRNA_104134 | AAAGCCCTTCTGTGTTATAGTTTTCTCCTTGTCTTTGGTTGCTTGA |
| ASCRP003782 | 0.0659 | 0.003277 | 3.718478 | -1.53811 | 1.1040017 | hsa_circRNA_103484 | CTTGCTGTTGATATTTCACAGTTCTTGCCGAGTGCTTCACTTTTTC |
| ASCRP002067 | 0.0659 | 0.003283 | 3.717489 | -1.5397 | 1.3596221 | hsa_circRNA_101720 | AGAAGGAATGCGCCAAGACTAGGAAAATCCCTGCCCAGAGTCCAGC |
| ASCRP004283 | 0.0666 | 0.003794 | 3.63551 | -1.67162 | 1.3031152 | hsa_circRNA_104003 | GAGTTCCCCTCCAACCACAGTGTACAGTGGACCCTTCTGTGAACAC |
| ASCRP000594 | 0.0685 | 0.004343 | 3.559097 | -1.79513 | 1.2473638 | hsa_circRNA_100205 | TCCTTCCAAGTCACCTACTGCCACCTTCGAAAAACACGGAGAGCAC |
| ASCRP003740 | 0.0685 | 0.004661 | 3.519403 | -1.85948 | 1.9342077 | hsa_circRNA_103442 | GCAGCGTCAGGTCTTCCGACCCCGAACTCCACCGGAGGCAATTGCA |
| ASCRP000343 | 0.0685 | 0.004779 | 3.505287 | -1.88239 | 2.2454076 | hsa_circRNA_001846 | CTCCCTGAGCTTCGGGGAGGGAAGCTCATCAGTGGGGCCACGAGCT |
| ASCRP000737 | 0.0685 | 0.004786 | 3.504435 | -1.88377 | 1.5346376 | hsa_circRNA_100352 | ATAATAAAAGTAAACGGAGTTCCCTCTCGAAGTCCAAGATTAGTTG |
| ASCRP003300 | 0.0685 | 0.004798 | 3.503092 | -1.88596 | 1.7152861 | hsa_circRNA_102993 | GGTTCATTTCCTTTACTCTCTATCTAATAGGTGATGTGCGTTCAGA |
| ASCRP003057 | 0.0685 | 0.004968 | 3.483473 | -1.91783 | 1.8043983 | hsa_circRNA_102745 | ATGTAACCCATGAAAAGAAACCTGTTTCCCTCCAATCTTGTGGTTG |
| ASCRP000051 | 0.0685 | 0.005023 | 3.477314 | -1.92784 | 1.7085989 | hsa_circRNA_000455 | GCCTTCAACTTCCAGCCCTATTACTGAATGAGAAAATGTATTATGA |
| ASCRP003583 | 0.0685 | 0.005137 | 3.464761 | -1.94825 | 1.3673728 | hsa_circRNA_103285 | TACTCCTCAACAAGTTCCTCCTCTTGACATTTGCAGAGTGCTCCCA |
| ASCRP003406 | 0.0685 | 0.005165 | 3.461628 | -1.95335 | 1.2038675 | hsa_circRNA_103102 | TTGTATAAATGAGCTTACTCCACGGGAACAGCCTCTAGATAATCTG |
| ASCRP003682 | 0.0685 | 0.005214 | 3.456345 | -1.96195 | 1.1909085 | hsa_circRNA_103384 | CATTGCCCACATTCCGTCCTATTTCAGTGTTCCGGGAAGCCAATGA |
| ASCRP002565 | 0.0691 | 0.005561 | 3.420302 | -2.02064 | 1.0165585 | hsa_circRNA_102237 | GCCTGTCCTGGAATGTAGTAAGAAAACAAAAACTGATGACCAAGAG |
| ASCRP004349 | 0.0694 | 0.00573 | 3.403443 | -2.04812 | 1.438743 | hsa_circRNA_104075 | CTCCTCTGAATTCTGCACCAGTTAGAAGACAAATGAAAGCTAAGCA |
| ASCRP002009 | 0.0696 | 0.00585 | 3.391872 | -2.06699 | 1.6363141 | hsa_circRNA_101661 | CTGCCCTTTGGCTACTTGAAATTGACATTTCCAGCAACAAGTTGTC |
| ASCRP001746 | 0.0716 | 0.006559 | 3.327933 | -2.17139 | 1.2604827 | hsa_circRNA_101387 | GCCCTGTTTGATAGATTCGATTGAATAGACTATTGACCATTGATAC |
| ASCRP004572 | 0.0733 | 0.007094 | 3.284199 | -2.24292 | 1.8035023 | hsa_circRNA_104308 | GTCTTTCTTATTCAGGTATTCTGCCTTTGACTGCAACTCTTGTCGT |
| ASCRP005171 | 0.0733 | 0.007148 | 3.279988 | -2.24981 | 1.0532891 | hsa_circRNA_104924 | ACCTTCCCAGGACCAGTCCACCATGCCTGAAGTCAAAGACCTCTCA |
| ASCRP003283 | 0.0733 | 0.007215 | 3.274734 | -2.25841 | 1.0365472 | hsa_circRNA_102976 | TGTCGCCCAGGTCCTGAAATTGGAGGTGGACACAAAGGAAGAGGCA |
| ASCRP004360 | 0.0753 | 0.007807 | 3.230854 | -2.33027 | 1.1466721 | hsa_circRNA_104086 | TCTCTCCAGTGTCCTTACATCATGCCGAGCCTTCCTTGTAACAGCG |
| ASCRP005010 | 0.0762 | 0.008098 | 3.210465 | -2.36368 | 1.7596948 | hsa_circRNA_104760 | GTTCTCCTCAGAGCCTGCTTTCTGAAACAAAGGTCTTCACTGCCTC |
| ASCRP001277 | 0.0771 | 0.008399 | 3.190168 | -2.39695 | 1.4467232 | hsa_circRNA_100904 | GGGTCCTTTCCTGGCCTTGGTGCTGTAAATGCATTGATGGAAAAAA |
| ASCRP000657 | 0.0771 | 0.008453 | 3.186596 | -2.40281 | 1.5337574 | hsa_circRNA_100269 | CCCCGATGCCTTCAAAATTATGACTCAAAGATATGAAGATCAATGA |
| ASCRP002678 | 0.0793 | 0.009419 | 3.126488 | -2.50139 | 1.0654358 | hsa_circRNA_102359 | TTTCTTTCCCTTATTCCAGTTGATCCCCAGGGTTCTCATGTATCAG |
| ASCRP005195 | 0.0803 | 0.009749 | 3.10739 | -2.53273 | 1.3771764 | hsa_circRNA_104948 | ATTGGAGCAGTTTTGACGAAGTATATTATGGGCAGTACATCTCTTT |
| ASCRP004576 | 0.0823 | 0.010262 | 3.078896 | -2.57948 | 1.1365509 | hsa_circRNA_104313 | TTCTTCATCTTCCAAGAGAACCAGAGGACGAAAACGAAGCTTCGTT |
| ASCRP000860 | 0.0827 | 0.010379 | 3.072634 | -2.58976 | 1.1866159 | hsa_circRNA_100477 | CCATCTCCAGAGCCTTAGATCGTCTGTCAGTGAAGGAATTCGGGCA |
| ASCRP004851 | 0.0844 | 0.010799 | 3.050616 | -2.62589 | 1.0092499 | hsa_circRNA_104598 | CCTTCTTCCACATGAAAAGGATAAGGATTCTTTCTACTAATCCAGA |
| ASCRP003795 | 0.0852 | 0.010967 | 3.042064 | -2.63992 | 1.1342732 | hsa_circRNA_103497 | TGCTGTTGAGATTTCCTTTTTTTCTGGGGAACTGTATTTTTAATAA |
| ASCRP001164 | 0.0883 | 0.012101 | 2.98751 | -2.72943 | 1.1009202 | hsa_circRNA_100790 | ATTCCAATCCATGCAAACGGTTCCTTTGGTATCATCCACAAGTGAG |
| ASCRP004827 | 0.0918 | 0.013531 | 2.925579 | -2.83098 | 1.9639417 | hsa_circRNA_104574 | ACGTTTCTTTTCTCATGGTGGAGGCTGCATGGCTGGAAGGCCGGAT |
| ASCRP003949 | 0.0929 | 0.013929 | 2.909548 | -2.85725 | 1.2641106 | hsa_circRNA_103655 | ACCTGCTCCAAGTCCCTCAGGGGTGGCACTGAATCAACAAGACAAG |
| ASCRP000566 | 0.0929 | 0.013959 | 2.908334 | -2.85924 | 1.348887 | hsa_circRNA_100177 | CCTCTTCCAGGTCCCCGTCTGGGCAACTCACCGGTGCCAAGCATAG |
| ASCRP000601 | 0.0929 | 0.013974 | 2.907743 | -2.8602 | 1.1197416 | hsa_circRNA_100213 | ACTACTTCAGGCACAGGTCTTCCCAAAAGTTATCTTCTGGGAAGAT |
| ASCRP003465 | 0.0954 | 0.015359 | 2.855387 | -2.94593 | 1.8521402 | hsa_circRNA_103164 | CTGTCTGCAGACCTCCCGACCTCTTTTGTGAAGGAGATCCATGATT |
| ASCRP002736 | 0.0967 | 0.015767 | 2.840854 | -2.9697 | 1.2623582 | hsa_circRNA_102417 | CCTCCGCAGACCCGTCTGCCGTTTAAGCGCCTGAATCTTGTCCCAA |
| ASCRP000083 | 0.0979 | 0.016502 | 2.815624 | -3.01095 | 1.3562256 | hsa_circRNA_000624 | CCCCGTCTCGATGGGGGTGGGGCTGGATCCGAAAAGCGCGGACGGG |
| ASCRP000100 | 0.0979 | 0.016531 | 2.81463 | -3.01257 | 1.6794541 | hsa_circRNA_000695 | TAGTTCTTTTTGATCTTTGTGAAAGTGGAGAGCAAGGTGGCTGTGC |
| ASCRP001986 | 0.0999 | 0.01711 | 2.795554 | -3.04373 | 1.2014516 | hsa_circRNA_101637 | TGACCAAAGACTTTTAGGTTTGTTTGAATAAGAGATCTGACCTGAC |
| ASCRP003853 | 0.1013 | 0.017971 | 2.768347 | -3.08814 | 1.4752626 | hsa_circRNA_103555 | TTCTTTCTGTTTTTGCGAGGTACCAGCAACTTCAAGGTTTCTGCCA |
| ASCRP004878 | 0.1013 | 0.017985 | 2.7679 | -3.08887 | 1.1732152 | hsa_circRNA_104625 | GCTTGCATTGTTTTCTGAGTTTAACCAGTAATGCCATTCAGTTGCC |
| ASCRP005325 | 0.1019 | 0.018238 | 2.760184 | -3.10145 | 2.1749197 | hsa_circRNA_400029 | TAGATAACTGTCTCGTCCGTCCTGGTGCGCGGGGACAGTGAGGCTT |
| ASCRP004228 | 0.1026 | 0.018437 | 2.754141 | -3.1113 | 1.2556206 | hsa_circRNA_103948 | TGTTGGCTCTCCTTAAACAGGATATACACATGACACCATCCACTGA |
| ASCRP000062 | 0.1057 | 0.019691 | 2.717651 | -3.17074 | 2.2119587 | hsa_circRNA_000543 | CTGGAGTAACTGGCATGTGAACAAGCTTTTTCTGTATTTACATACA |
| ASCRP005163 | 0.1062 | 0.02001 | 2.708749 | -3.18522 | 1.3684508 | hsa_circRNA_104916 | CACTCCCTAGTTCGTGCCCTCGTGAGGCTGGCATGCAGGATGGCAG |
| ASCRP005315 | 0.108 | 0.020891 | 2.684831 | -3.22411 | 1.1879237 | hsa_circRNA_400019 | TCTCTGTTCTGTAAGTTAATTGGATGTTTTTCTGTACTTCCATACC |
| ASCRP004273 | 0.1094 | 0.021519 | 2.668371 | -3.25084 | 1.2710323 | hsa_circRNA_103993 | GTCAAGTATGTAGCAGCTGATGTTCTAATCATGTCAGATAAAGATG |
| ASCRP003152 | 0.1112 | 0.022437 | 2.645165 | -3.28847 | 1.1571654 | hsa_circRNA_102844 | TTCCTCTCTGACCCCCTGGTTGTGGAATACGAGGCATTGCTGGTAC |
| ASCRP005362 | 0.1117 | 0.022768 | 2.637036 | -3.30165 | 2.0891649 | hsa_circRNA_400068 | TCAGAGGTCTTTCCAGTCCAGATCGTCCATGCTGTTTGGGGGCCAT |
| ASCRP002075 | 0.1119 | 0.023046 | 2.63029 | -3.31257 | 1.1287109 | hsa_circRNA_101728 | TGAAAATCGAGGATGTCCTGGGCTGCAATGTCTCACCAGTGCCTGG |
| ASCRP002537 | 0.1166 | 0.024984 | 2.585362 | -3.38521 | 1.1796155 | hsa_circRNA_102209 | GCAGCAGCAGCTTCACCAGGTCACCATGGCTGTTATTGGCTCCCTT |
| ASCRP002143 | 0.1172 | 0.025333 | 2.577641 | -3.39767 | 1.0203881 | hsa_circRNA_101798 | ACTTCTCATTTCATTTCCCGCTCCCGGCCCGTGGTGGATATGGTCA |
| ASCRP004439 | 0.1176 | 0.025822 | 2.566998 | -3.41483 | 1.0384312 | hsa_circRNA_104166 | TGTCCTCTCTTCGTAAAGATTGAAAAGGAACTCAGTGGCATGTACC |
| ASCRP000390 | 0.1219 | 0.028027 | 2.521332 | -3.48831 | 1.2598369 | hsa_circRNA_002178 | CTTCGGGGAGCTGAGTGCGTCCTGTCACTCCACTCCCATGTCCCTT |
| ASCRP000339 | 0.1225 | 0.028348 | 2.514964 | -3.49854 | 1.3976463 | hsa_circRNA_001826 | AAGTAGATGCTCACTTTCGACTGAGTGTTATAGTTTTGCCGCTGGA |
| ASCRP002004 | 0.1225 | 0.028809 | 2.505966 | -3.51297 | 1.105871 | hsa_circRNA_101656 | CCTTTCTTTGGTCCCAGTTCCTCTGGGAATCGTCCTGTATGCAACA |
| ASCRP002196 | 0.1225 | 0.028876 | 2.50468 | -3.51504 | 1.1644747 | hsa_circRNA_101853 | AGTCTCTCTAGGTTGGTTCATTCTGGCTCCGGATGTCGATCCCCCT |
| ASCRP002793 | 0.123 | 0.029262 | 2.497266 | -3.52692 | 1.0895587 | hsa_circRNA_102476 | GCTGGAGCAGGTGAAGCGAGAAATCTTGGTGGAGGTTCTGACCAAA |
| ASCRP003808 | 0.1283 | 0.031872 | 2.449491 | -3.60332 | 1.3401338 | hsa_circRNA_103510 | CCCGTTCTTCCGAAATGTTGATTGGGATATGTTATTTTGGAAAAAC |
| ASCRP003861 | 0.1341 | 0.034954 | 2.397767 | -3.68561 | 1.01941 | hsa_circRNA_103563 | GATCCCTCTATGTCAGCTTCTTCTCAGCCTGTTGATAACCATGTTA |
| ASCRP005397 | 0.1342 | 0.034998 | 2.397063 | -3.68673 | 2.8221019 | hsa_circRNA_105055 | AGGTCTTCCAGTGTCTGCAATATCCAGGGTTTCCGATGGCACCTGT |
| ASCRP003855 | 0.1351 | 0.035537 | 2.388491 | -3.70032 | 1.2518548 | hsa_circRNA_103557 | CTTTTTCTTGATTGGTTCTTCTGTGTGGCAGTTCAGAATGATGGAT |
| ASCRP002384 | 0.1396 | 0.038314 | 2.346198 | -3.76719 | 1.2588936 | hsa_circRNA_102049 | CCATTCCATTTCACTACTTCAGATTTTCCTGTCCTTGATCCCAGCT |
| ASCRP002195 | 0.1477 | 0.042963 | 2.281602 | -3.86864 | 1.1300791 | hsa_circRNA_101852 | AGTCTCTCTAGTCAAGTTCATCCGAGAAGTAACACCATATATCAAG |
| ASCRP004031 | 0.149 | 0.043785 | 2.270876 | -3.8854 | 1.2006721 | hsa_circRNA_103740 | GAAAAAAACTTAGGTTGCACAGTGAATTAAAAGATGCTGTTGAAAA |
| ASCRP003766 | 0.1511 | 0.044627 | 2.260104 | -3.9022 | 2.1695938 | hsa_circRNA_103468 | GTCTTCCTTGCCTAACAAAAGACATGACTGGGAAAGAAGACAACTA |
| ASCRP003364 | 0.1554 | 0.047927 | 2.219645 | -3.96509 | 1.1170216 | hsa_circRNA_103059 | AGATGCAAAGGTTCAAGCACTGTCTTCCTGTTGGCCCTGACAATCA |
| ASCRP000459 | 0.0625 | 0.000541 | -4.779202 | 0.09177 | -1.627049 | hsa_circRNA_100069 | ACAGACTGGGAAGGATGCCCCTTACCTAGACCTGGCCCCCTACATG |
| ASCRP003569 | 0.0625 | 0.00062 | -4.694964 | -0.03152 | -1.1925831 | hsa_circRNA_103271 | CTGCATCATGACAGTGACAATACCCTCCCCTCCCTTGGGCTGGACC |
| ASCRP001100 | 0.0625 | 0.001246 | -4.277424 | -0.65929 | -1.1251937 | hsa_circRNA_100726 | AGATTCTGAACTCATTACTGCAGAGAGATCCTGCTTCCCATGATGA |
| ASCRP001048 | 0.0625 | 0.001441 | -4.191959 | -0.79102 | -1.6321233 | hsa_circRNA_100674 | TAAAGCTCAGACTAAAGCCCCTCCCAAACCAGACACAGCAGGATGC |
| ASCRP002248 | 0.0625 | 0.001773 | -4.071023 | -0.97917 | -1.1647969 | hsa_circRNA_101906 | ATGACAAGTATGACCGCATGAAGATCGCACCCCCTCCCTCCCTGGC |
| ASCRP000479 | 0.0625 | 0.001857 | -4.044124 | -1.02128 | -1.0134274 | hsa_circRNA_100089 | TTTGATGATCAAGGTGGAGCATGACCCCGCCCATGGTGAACGCCTA |
| ASCRP000054 | 0.0625 | 0.001915 | -4.026453 | -1.049 | -1.189082 | hsa_circRNA_000481 | TCTTGGGCAGGTGCCCTGGAGCAGCGCCCTGCTCCCCTCCCCTCTT |
| ASCRP004668 | 0.0625 | 0.001998 | -4.001983 | -1.08745 | -1.63323 | hsa_circRNA_104412 | AAGGCCATCGGCGAGATCACCTCCTCTCCACGGTCACATCCATTTC |
| ASCRP000500 | 0.0636 | 0.002747 | -3.819027 | -1.37724 | -1.3475792 | hsa_circRNA_100110 | TATCCAAAACAGGATCTATCTGGTTCAATAGATGACCTCCCCATGG |
| ASCRP005006 | 0.0659 | 0.003081 | -3.753547 | -1.48189 | -1.0613713 | hsa_circRNA_104756 | GTCACACACACTCCAAAGACAACAGGCCCTCCCTCTGCCCTCCCGT |
| ASCRP002266 | 0.0659 | 0.003185 | -3.734603 | -1.51224 | -1.4105866 | hsa_circRNA_101924 | TAAGATCTGCAGTGAGCAAAGAGAGATGTGACTCCCCACCCCCAGC |
| ASCRP003900 | 0.066 | 0.003464 | -3.68705 | -1.58861 | -1.0556009 | hsa_circRNA_103605 | CTACCTCAGGCTATCGGCAGGACCCTCATCCCCCGTTACTTTAGCA |
| ASCRP001842 | 0.0663 | 0.003601 | -3.664927 | -1.62421 | -1.0513786 | hsa_circRNA_101491 | GGAGCTGAGGGTGTGTGGTTGTGTTGTTCAATCCCCGGAAACACAA |
| ASCRP004315 | 0.0666 | 0.003874 | -3.623705 | -1.69067 | -1.9449955 | hsa_circRNA_104040 | CCTATGTTGGAGATCCTGCCCGGCCTGTACATCGGCAACTTCAAAG |
| ASCRP001507 | 0.0685 | 0.004675 | -3.517663 | -1.8623 | -1.1496051 | hsa_circRNA_101141 | CTTAGATTGTTATGAAGTGAGTTATTCTCCCCACCTCAGAAGTACC |
| ASCRP000828 | 0.0685 | 0.005213 | -3.45653 | -1.96164 | -1.4600592 | hsa_circRNA_100445 | TTTTACAAGAGCAAGGTTGGACTAGATTTCTCCCCCAGATTTGCTG |
| ASCRP000581 | 0.0685 | 0.005258 | -3.45163 | -1.96962 | -1.1080194 | hsa_circRNA_100192 | TATGCTTCAGCCTTGATGACGGCCATCTTCCCCCGCTCACCGAGGA |
| ASCRP001482 | 0.0685 | 0.005295 | -3.447688 | -1.97603 | -1.2935094 | hsa_circRNA_101115 | TGGGAAGACATATCAAGCAAAAGTTCAAACAGACCCTCCCTCAGTT |
| ASCRP005386 | 0.0693 | 0.005668 | -3.409623 | -2.03804 | -1.124062 | hsa_circRNA_400093 | TTTAGTAGGGTCTTCCTGTGGAAGCACAGGAGCTGCCTCCCTAGGT |
| ASCRP005221 | 0.0696 | 0.005907 | -3.386508 | -2.07574 | -1.3151714 | hsa_circRNA_104974 | TAAAAGAAGAAACCTTGAACGACAAAGTTTTCCCCCCACGACCTGA |
| ASCRP005211 | 0.0696 | 0.005946 | -3.382736 | -2.08189 | -1.4281556 | hsa_circRNA_104964 | TAAGATCCTCAACTGCCAAAAGGCAATGGGGGGCGACGATGGCCTT |
| ASCRP004108 | 0.0706 | 0.006356 | -3.345539 | -2.14262 | -1.3312252 | hsa_circRNA_103818 | TTGCAAACAGAGGAGACCTTGCCTATTTTGATGGACTTAGTGAGAC |
| ASCRP003298 | 0.0716 | 0.006606 | -3.323946 | -2.17791 | -1.1861879 | hsa_circRNA_102991 | TCAGCAAAGGGCTGTGTCAGCGTGTTATGATGCCGTCCCGTACCAA |
| ASCRP001195 | 0.0732 | 0.007018 | -3.290172 | -2.23315 | -1.0553264 | hsa_circRNA_100821 | GAGATGCCAGGGTCTGTCCCCTTACGCAAGAGCCTCTGCTCCCCCA |
| ASCRP000463 | 0.0733 | 0.007235 | -3.273257 | -2.26083 | -1.1502405 | hsa_circRNA_100073 | TGCATCGTTCACCAGATGCTGTCCCACACCCCAGATTCTACTGTGT |
| ASCRP000199 | 0.0737 | 0.007461 | -3.25608 | -2.28895 | -1.0100099 | hsa_circRNA_001096 | CGCGGAGACCATGCCCACCACCACCCTGCCTGGAAAGGACCCAGCG |
| ASCRP005317 | 0.0811 | 0.010002 | -3.093139 | -2.55611 | -1.2011242 | hsa_circRNA_400021 | GCTGTGATATAACTAGTTGTTTACGAAACCCCATCTCTACTAACAA |
| ASCRP002246 | 0.0842 | 0.0107 | -3.055738 | -2.61749 | -1.0981404 | hsa_circRNA_101904 | GGACGGTCAGAGCCGATGATGAGCACCCCACCTCCTGCCAGCGAGC |
| ASCRP000689 | 0.0858 | 0.011316 | -3.024674 | -2.66846 | -2.1731689 | hsa_circRNA_100302 | ATGAGCGGTGGTGGCACTGGTACCCGGGTGATTTGCTCAGATTGTT |
| ASCRP002783 | 0.0892 | 0.012311 | -2.977972 | -2.74507 | -1.240188 | hsa_circRNA_102466 | ATCCCTGGCCGCAAAGGAAACAGAGAAACCCCCACTTCCTTGCAAG |
| ASCRP001223 | 0.0918 | 0.013172 | -2.940503 | -2.80651 | -1.0358364 | hsa_circRNA_100849 | AGAGCTGAGGTACTTCGTGAAGCTCCGGCTCCCACCTTGTCTTTGT |
| ASCRP000143 | 0.0918 | 0.013614 | -2.922228 | -2.83647 | -1.0054552 | hsa_circRNA_000872 | ATGATCTTCACCTGGGCTGCCACCTCCCGCACCAGCTTGGCAAACT |
| ASCRP001182 | 0.0929 | 0.013976 | -2.907685 | -2.8603 | -1.0513147 | hsa_circRNA_100808 | TTGAGGACTTTGAGAGGCTGCTGGAATCTTCCCTCATTTCATTATC |
| ASCRP002860 | 0.0939 | 0.014445 | -2.889381 | -2.89028 | -1.0209248 | hsa_circRNA_102547 | ATTCTCCAGACGAGAGACTGTGCCCTGTCCACGGTGCCTCCTGCAT |
| ASCRP001444 | 0.0939 | 0.014486 | -2.887803 | -2.89286 | -1.389585 | hsa_circRNA_101076 | TCCAAACGATTCCCCCCCGAAGGGCGTGACCATCCCGTACCGGCCC |
| ASCRP001590 | 0.0954 | 0.015354 | -2.855586 | -2.9456 | -1.2808562 | hsa_circRNA_101227 | TTTGCCTTGGAATACAGGAGGGTTCAGCACCACCCCCTTCTCCAAC |
| ASCRP003203 | 0.0979 | 0.016192 | -2.82612 | -2.99379 | -1.3248344 | hsa_circRNA_102895 | AACACCACTCACTTCGCAGAATCAAGAACGGCTATGTGCGTTTAAA |
| ASCRP002928 | 0.0979 | 0.016342 | -2.821012 | -3.00214 | -1.7247682 | hsa_circRNA_102615 | AAACCCAACCCAGGCAGCGACATGCTAACCGGCCTCCTGCAAAGCT |
| ASCRP000300 | 0.1005 | 0.017279 | -2.790101 | -3.05264 | -1.2270057 | hsa_circRNA_001579 | TTTTGTAAAATGTCCCCCAACGAACCCACATGCCATTGACTCCCGA |
| ASCRP005220 | 0.1008 | 0.017428 | -2.785367 | -3.06037 | -1.1957441 | hsa_circRNA_104973 | AACATGGATGACCTTCAGAGCAGTTTTCCCCCCACGACCTGACCGT |
| ASCRP000994 | 0.108 | 0.020913 | -2.684229 | -3.22508 | -1.1413352 | hsa_circRNA_100620 | TTACCCTAGTCAGAATGTCATACCCAGGCTATCCCCCAACAGGCTA |
| ASCRP003872 | 0.111 | 0.022322 | -2.648031 | -3.28383 | -1.1286042 | hsa_circRNA_103574 | AATTGGTGTACCTCAGGTCATGCTTCACCCCTTCCTCCATCTGCTG |
| ASCRP002918 | 0.1182 | 0.026161 | -2.559734 | -3.42654 | -1.3836913 | hsa_circRNA_102605 | TATGCTGGCCACCAGCTCCCAAGAACTCAACGAAGCGGTACTGCAA |
| ASCRP001375 | 0.1209 | 0.027417 | -2.533607 | -3.46859 | -1.241346 | hsa_circRNA_101004 | CCAGGGAGAGGTCCCATCCATGGGGGGGCGGAAGCGGGAGCGCAAG |
| ASCRP003053 | 0.1216 | 0.027814 | -2.525583 | -3.48148 | -1.152531 | hsa_circRNA_102741 | AAGACTAGAGCTCCTTTTTGGTTCAGGTGTGCTGGTGTCTCTAAGC |
| ASCRP005081 | 0.1225 | 0.028823 | -2.505689 | -3.51342 | -1.2801641 | hsa_circRNA_104833 | GGATGGCCAGGCCATGTGTTTATACTGTGGGATCTCCCATAAACAA |
| ASCRP000969 | 0.1231 | 0.029336 | -2.495851 | -3.52919 | -1.0542815 | hsa_circRNA_100593 | GTTGTATAAATGTACAGTAACTGGTATGAACCCTCTGTCTCCTTAT |
| ASCRP004574 | 0.1288 | 0.032193 | -2.443882 | -3.61226 | -1.6019583 | hsa_circRNA_104310 | TTATGGACACGGTCTTTCTTATTCAGGTGTGTGTAACTGGTGTGTG |
| ASCRP005323 | 0.1309 | 0.033565 | -2.420516 | -3.64947 | -1.5753154 | hsa_circRNA_400027 | TGGATCGATGCATTTGCAGAAACAAAGATTGTGTGTGGATCGATGA |
| ASCRP004026 | 0.1309 | 0.033568 | -2.420457 | -3.64957 | -1.1568894 | hsa_circRNA_103735 | TTTCCAAAAACAAGTACAGATGAAGCTCAGGTTCCCCAAGGAAATA |
| ASCRP002233 | 0.1435 | 0.040201 | -2.319111 | -3.80983 | -1.028692 | hsa_circRNA_101891 | TCCAACATGGAGTACATGAGCTCAAGCGCCCAGCTCTGCCCGAGGA |
| ASCRP002125 | 0.1456 | 0.041658 | -2.299018 | -3.84137 | -1.3569376 | hsa_circRNA_101779 | GAACTTAGCTCTGCTTCCCATCACCACGGCGACTACTACTCAGGAG |
| ASCRP000803 | 0.1469 | 0.042528 | -2.287349 | -3.85964 | -1.6005823 | hsa_circRNA_100420 | ACTACTGCTGCATTCCTACTTCATTGCCCCTGATGTAACTGGACTC |
| ASCRP000789 | 0.1518 | 0.045285 | -2.251812 | -3.91512 | -1.0150846 | hsa_circRNA_100405 | ACTAAACTGGATGTGAAAGGACCACCCACCCACCGTCTGTCTTGTG |
| ASCRP002505 | 0.1518 | 0.045425 | -2.250063 | -3.91784 | -1.1134219 | hsa_circRNA_102173 | TTTGACACCACAATCAGGCTGCTCCCGGCAGATCGGCTTCTCCTTT |
| ASCRP001820 | 0.1531 | 0.046304 | -2.239199 | -3.93474 | -1.0205035 | hsa_circRNA_101469 | AGACCTGCTGGAAGGTGGCAGTGACGAAAGAAGAGACTCTCCCGGC |
| ASCRP000311 | 0.1548 | 0.047236 | -2.227888 | -3.95231 | -1.2504491 | hsa_circRNA_001654 | GGAAGGTCAGGCCTGCATGTATCTTAGTGTGTCTGCTGGTGTGAGT |
| ASCRP000027 | 0.1553 | 0.047505 | -2.224676 | -3.95729 | -1.3291847 | hsa_circRNA_000274 | GGGTAGGGAGGTGAGGGCCTGGTGTGGGGTTTCCCAAGGAGAGCGC |
| ASCRP002480 | 0.1561 | 0.048616 | -2.211536 | -3.97765 | -1.3090598 | hsa_circRNA_102148 | TTCCACAGTGATTGATGCTGCCTCAGTGGTTCCCCCTGGAAGTCCT |
