# Supplementary material for: circRNA circ_102049 Implicates in Pancreatic Ductal Adenocarcinoma Progression through Activating CD80 by Targeting miR-455-3p
Source: Mediators Inflamm. 2021 Jan 7;2021:8819990. doi: 10.1155/2021/8819990 (PMC7811564; doi:10.1155/2021/8819990)
Supplement: Supplementary 4 — Table S2 The differentially expressed circRNAs in the GSE79634 dataset. [file 8819990.f4.doc]

**Supplementary Table S2 The differentially expressed circRNAs in the GSE79634 dataset**

| ID | adj.P.Val | P.Value | t | B | logFC | SPOT_ID | SEQUENCE |
| --- | --- | --- | --- | --- | --- | --- | --- |
| ASCRP004752 | 2.86E-13 | 1.56E-16 | 13.4883 | 27.51806 | 1.917328 | hsa_circRNA_104498 | TCTCCTACAGATTTATTCAGGAGATTGAGCATGCTCTGGGTCTTGG |
| ASCRP001626 | 5.09E-12 | 5.54E-15 | 12.08429 | 24.02438 | 1.910676 | hsa_circRNA_101264 | ATCCTCCTACAGGTGAGCTTTCCGTTGAAGAGGCGCAGGACCCTTT |
| ASCRP002678 | 1.01E-11 | 1.95E-14 | 11.61234 | 22.79156 | 1.596466 | hsa_circRNA_102359 | TTTCTTTCCCTTATTCCAGTTGATCCCCAGGGTTCTCATGTATCAG |
| ASCRP001250 | 1.55E-11 | 4.21E-14 | 11.32785 | 22.03391 | 1.249961 | hsa_circRNA_100877 | CTCCTACAATGGTCGAGCACGCACGCATGCATGCCAAGCACCGTGG |
| ASCRP002004 | 1.92E-11 | 7.03E-14 | 11.14089 | 21.53002 | 1.126229 | hsa_circRNA_101656 | CCTTTCTTTGGTCCCAGTTCCTCTGGGAATCGTCCTGTATGCAACA |
| ASCRP003688 | 2.88E-11 | 1.35E-13 | 10.9047 | 20.88666 | 1.933834 | hsa_circRNA_103390 | CTTCCCTCCGGTTTTTGATGAGAAGTACTTTCTGGTGGAAATGGAT |
| ASCRP001070 | 2.88E-11 | 1.41E-13 | 10.88838 | 20.84195 | 1.925537 | hsa_circRNA_100696 | CATTCCTCCTGCAATTTCTTTCCTCACACCCCTGGCTGTTATTTGT |
| ASCRP003291 | 4.39E-11 | 2.39E-13 | 10.7003 | 20.32378 | 2.017896 | hsa_circRNA_102984 | TTCCCTCCTTCAGATAAGCATGGATTCCTGGTTCATTCTTGTTCTG |
| ASCRP002315 | 6.69E-11 | 4.37E-13 | 10.487 | 19.73035 | 1.454436 | hsa_circRNA_101975 | TCATCCACAGGCTCCTCCTGCTGGACCCTCCAGCCCCGGGCCTCAG |
| ASCRP002892 | 6.71E-11 | 4.75E-13 | 10.45774 | 19.64846 | 1.778128 | hsa_circRNA_102579 | GGATCATGTGAAATATCTTCCCTTCCAACCTGGTGTCAGCAGCCTT |
| ASCRP000906 | 8.14E-11 | 6.62E-13 | 10.34109 | 19.32086 | 1.053672 | hsa_circRNA_100525 | TGACACTAGTGATTCTAAACTGCCTTCCTCGGTTCGCAGTACACTT |
| ASCRP004869 | 8.14E-11 | 6.65E-13 | 10.33927 | 19.31574 | 1.68795 | hsa_circRNA_104616 | TCCCTCCCTGAGGTGGACTAGCAGAAAGACTAAATGGACTGCAGAA |
| ASCRP001413 | 9.05E-11 | 8.38E-13 | 10.25858 | 19.08802 | 1.349962 | hsa_circRNA_101043 | AACTGAAAGAGCTGCTATGCCTTTCTTGCCTCCTTCTATGACAATC |
| ASCRP003290 | 9.36E-11 | 9.18E-13 | 10.22707 | 18.99886 | 2.188693 | hsa_circRNA_102983 | CTTTCCCTCCTTCAGGACACATGTTCAAAGAGCATAATTAACTTTT |
| ASCRP004072 | 1.07E-10 | 1.10E-12 | 10.16297 | 18.81708 | 1.018559 | hsa_circRNA_103782 | ATGAATGTCCCCTTTCCAGGTATCCTGAAGAACTTGCCTGGCACAC |
| ASCRP004283 | 1.92E-10 | 2.29E-12 | 9.91004 | 18.0944 | 1.469002 | hsa_circRNA_104003 | GAGTTCCCCTCCAACCACAGTGTACAGTGGACCCTTCTGTGAACAC |
| ASCRP005384 | 1.92E-10 | 2.41E-12 | 9.89371 | 18.04746 | 1.357601 | hsa_circRNA_400091 | ATCTCCATCTGGGCCCTTCCTCCAGCTTTGCTTTGTTTGGCTAACA |
| ASCRP003949 | 1.94E-10 | 2.53E-12 | 9.8766 | 17.99822 | 1.037054 | hsa_circRNA_103655 | ACCTGCTCCAAGTCCCTCAGGGGTGGCACTGAATCAACAAGACAAG |
| ASCRP000774 | 2.36E-10 | 3.34E-12 | 9.78191 | 17.72508 | 1.570803 | hsa_circRNA_100389 | ACTCCTACAGCATCACTATCAGGAGTCTGTAACAGACTATACTACA |
| ASCRP001277 | 2.37E-10 | 3.61E-12 | 9.75485 | 17.6468 | 1.718792 | hsa_circRNA_100904 | GGGTCCTTTCCTGGCCTTGGTGCTGTAAATGCATTGATGGAAAAAA |
| ASCRP004224 | 3.23E-10 | 5.10E-12 | 9.6372 | 17.30533 | 2.244627 | hsa_circRNA_103944 | GATATTGAAGTTACGACCACCTCAGCCTCCAGTGTATCTCTTTGTA |
| ASCRP003287 | 3.36E-10 | 5.49E-12 | 9.6127 | 17.23403 | 1.095378 | hsa_circRNA_102980 | AGCCTCCCTGGACCTACAAGAACAGTGAGGAGCTTCGGTCTCGTAT |
| ASCRP002889 | 3.96E-10 | 7.08E-12 | 9.52653 | 16.98251 | 1.239889 | hsa_circRNA_102576 | TATCCCTGTCACTCAGCCCCGGTGTAGAGCAGACCAAACAGTGCAA |
| ASCRP002237 | 3.96E-10 | 7.26E-12 | 9.51803 | 16.95764 | 1.190348 | hsa_circRNA_101895 | CATGGAGAAGGTCCTGGCAATCCCTCCCTTTCTAAAGTAGGTACCG |
| ASCRP000085 | 4.11E-10 | 7.83E-12 | 9.49227 | 16.88224 | 1.906236 | hsa_circRNA_000629 | AGGCCTCTTCCTTTCTTCCTTTCTCAGATACATCCCGGCCTCAGCT |
| ASCRP001620 | 8.78E-10 | 2.12E-11 | 9.15927 | 15.89991 | 1.611421 | hsa_circRNA_101258 | GTTACTAAAGCCTGCCTTTCCAGATCCATTTTTCCTATCCATCCCT |
| ASCRP002386 | 1.46E-09 | 3.81E-11 | 8.96494 | 15.32025 | 6.301989 | hsa_circRNA_102051 | TGACACAGCCATTCCATTTCACTGCAGGATGTAGCCAATCAAATGT |
| ASCRP003092 | 2.05E-09 | 5.80E-11 | 8.82631 | 14.9039 | 1.807339 | hsa_circRNA_102782 | ACACTCCTGCTTCCTTCCTTCCCTGCAGTTGGTGCTGAACCTGAGA |
| ASCRP003776 | 2.65E-09 | 7.97E-11 | 8.72219 | 14.58969 | 1.203097 | hsa_circRNA_103478 | AGACACCTTGATGAAGTGGCCATTCTGCCTGCCCCTCAGAACCTCT |
| ASCRP000384 | 2.98E-09 | 9.25E-11 | 8.67336 | 14.44189 | 2.234757 | hsa_circRNA_002143 | ATTTGTGGAATCCTCAGTCATCGACACACAAGACAGAGACAAGGTA |
| ASCRP003002 | 3.01E-09 | 9.51E-11 | 8.66443 | 14.41482 | 1.606651 | hsa_circRNA_102690 | AACTTATGATTGGAAGCATTGACATTTCCTCCTTCTTCTGGAAAGT |
| ASCRP004073 | 3.05E-09 | 9.97E-11 | 8.64903 | 14.36812 | 1.765504 | hsa_circRNA_103783 | CGTGCGGCCTCATCATAAGGTATCCTGAAGAACTTGCCTGGCACAC |
| ASCRP002384 | 3.05E-09 | 9.98E-11 | 8.6488 | 14.36742 | 5.702157 | hsa_circRNA_102049 | CCATTCCATTTCACTACTTCAGATTTTCCTGTCCTTGATCCCAGCT |
| ASCRP004540 | 3.23E-09 | 1.13E-10 | 8.60686 | 14.24016 | 5.730049 | hsa_circRNA_104270 | TGCAGATGACCATTCCAGATCCTTTCCCGGAGTTCAGTTATGGGTG |
| ASCRP005009 | 3.67E-09 | 1.37E-10 | 8.54446 | 14.05041 | 1.280919 | hsa_circRNA_104759 | AGTCCTCAGTCCTCAGTCATCTTGCTTTCTGAAACAAAGGTCTTCA |
| ASCRP002345 | 3.91E-09 | 1.53E-10 | 8.51052 | 13.94702 | 1.079041 | hsa_circRNA_102009 | TAGCTGCTGGGAAAACTCCTCCTTAACTTGGTAGATCATACTGAAG |
| ASCRP003380 | 3.91E-09 | 1.53E-10 | 8.50909 | 13.94265 | 1.39176 | hsa_circRNA_103076 | CCTTTGCTTTGCCTTTGACTAATCTTTTTAAGGTTGAAGATGAACC |
| ASCRP002790 | 4.80E-09 | 1.93E-10 | 8.43369 | 13.7125 | 1.484043 | hsa_circRNA_102473 | AGCTTATCCTTCCCTACAGCCTCAGCGAGGATTTTTGCCGTCCTCT |
| ASCRP004135 | 5.31E-09 | 2.19E-10 | 8.39393 | 13.59087 | 1.387282 | hsa_circRNA_103846 | ATGCTTTTGTCAGTGTACTGGATTTCCTCCTTCTTCACCCCTGAAA |
| ASCRP000230 | 5.31E-09 | 2.20E-10 | 8.39243 | 13.58628 | 1.177012 | hsa_circRNA_001216 | TCCGAGAAAATGCTCTTTAACTCCTCCTTATTCTTTTCACTGGCAG |
| ASCRP003703 | 5.83E-09 | 2.48E-10 | 8.3538 | 13.46793 | 1.108615 | hsa_circRNA_103405 | TGTCCTGCTGTTCCTTCCAAAGAACCTGCACTTGGTCTGCGTGGAC |
| ASCRP005005 | 6.05E-09 | 2.64E-10 | 8.33379 | 13.40658 | 1.257755 | hsa_circRNA_104755 | TACAGTACAGGTCCCTCCAGCCTCAGCCAAGCAACATGGGGTGAAC |
| ASCRP005047 | 7.79E-09 | 3.69E-10 | 8.22557 | 13.07397 | 1.181941 | hsa_circRNA_104798 | CTCCATCAAGCAACAACTCCAGGCCCAGCATTTATCACATGGACAT |
| ASCRP003235 | 7.79E-09 | 3.69E-10 | 8.22534 | 13.07325 | 1.340047 | hsa_circRNA_102928 | TTACAAGCCAGCCTCTGGGACCGAGGTTCAGCCGTCTGTATATCTC |
| ASCRP001740 | 8.75E-09 | 4.24E-10 | 8.18089 | 12.93628 | 1.906279 | hsa_circRNA_101381 | GAAGAACAAGGCCCTTCCTTCTACCATGATTATAGTAGCAGTTTAT |
| ASCRP003983 | 9.60E-09 | 4.81E-10 | 8.14071 | 12.81229 | 1.180394 | hsa_circRNA_103689 | TGGTATTGAAAGCAACAAGTAATCCTTCCCCTCTACCACCGGATAT |
| ASCRP001241 | 1.02E-08 | 5.19E-10 | 8.11654 | 12.73761 | 1.854209 | hsa_circRNA_100868 | CCTTTCCTTAAGGAAGTGGAACAGAGGCTGAAGTTGTTCAAGTTGG |
| ASCRP003393 | 1.07E-08 | 5.53E-10 | 8.09617 | 12.67463 | 1.127665 | hsa_circRNA_103089 | CTATACCTACTGGGTTCCCCTGGTGCTGCGAGTGGCTGAGATGCTG |
| ASCRP001410 | 1.35E-08 | 7.20E-10 | 8.01183 | 12.41346 | 1.13794 | hsa_circRNA_101040 | ACTTGAGGGAGGCTTCCTTTCTGTCTGACCAGCCTGAGCCTTACCT |
| ASCRP000314 | 1.57E-08 | 8.46E-10 | 7.9602 | 12.25321 | 1.895234 | hsa_circRNA_001676 | AAAGGCCACCCAGCCCTGTAAGACCAGTAATAAATTTCTGTCTACT |
| ASCRP003583 | 1.59E-08 | 8.86E-10 | 7.94565 | 12.20802 | 1.447016 | hsa_circRNA_103285 | TACTCCTCAACAAGTTCCTCCTCTTGACATTTGCAGAGTGCTCCCA |
| ASCRP004757 | 1.60E-08 | 9.17E-10 | 7.93452 | 12.17344 | 1.844889 | hsa_circRNA_104503 | TAATCTTCCCTCTCTTCCGGATAGGCAGTTGGTGTCACCAGCCAGC |
| ASCRP002541 | 1.81E-08 | 1.07E-09 | 7.88651 | 12.02407 | 1.14683 | hsa_circRNA_102213 | GCTGTGCCAAGCCACCAGGGAAGCTTCTGCATGCTGTGTGTCATGC |
| ASCRP004165 | 2.07E-08 | 1.23E-09 | 7.84115 | 11.88277 | 1.084745 | hsa_circRNA_103885 | CTGCCTGCAGGAAAATGTTCTGCTGCTGCCCTGGATGTTCTTGCAA |
| ASCRP001059 | 2.18E-08 | 1.32E-09 | 7.81988 | 11.81645 | 1.607201 | hsa_circRNA_100685 | TCATAGGCAGCTTGCACCTTCTCTTCCTTTACAAGAAGATTTTGTT |
| ASCRP002932 | 2.24E-08 | 1.39E-09 | 7.80154 | 11.75923 | 6.156615 | hsa_circRNA_102619 | CCCAAGATGGGCATCTATTACATTCCATTCTGAAGTTTATAGGTTA |
| ASCRP005370 | 2.47E-08 | 1.57E-09 | 7.76329 | 11.6398 | 1.030715 | hsa_circRNA_400076 | TGCTGCACACCTAGGAACCTTGTGCTTGCCTGCCAGTAGGCACAGG |
| ASCRP003001 | 2.54E-08 | 1.65E-09 | 7.74924 | 11.59587 | 1.169347 | hsa_circRNA_102689 | TACTGCATAGATCAACCTTTTCGGCCTTCCTTGTCCCGCAATAACA |
| ASCRP004576 | 2.56E-08 | 1.70E-09 | 7.73882 | 11.56332 | 1.294757 | hsa_circRNA_104313 | TTCTTCATCTTCCAAGAGAACCAGAGGACGAAAACGAAGCTTCGTT |
| ASCRP002547 | 2.56E-08 | 1.72E-09 | 7.73566 | 11.55344 | 1.128278 | hsa_circRNA_102219 | TTCCTCCCAGCCAGGTGCTGCCCACCTACGACAGCCTGGATGAGCC |
| ASCRP004825 | 2.92E-08 | 2.00E-09 | 7.68686 | 11.40074 | 1.018686 | hsa_circRNA_104572 | AGCCACCAGGACCCTAACTCAATCTTGGAGCCACTTTTGGAGCGCA |
| ASCRP005305 | 3.01E-08 | 2.11E-09 | 7.67132 | 11.35206 | 1.547805 | hsa_circRNA_400009 | ATAGATAACTGTCTCTTGATTATGGAATCTCTGTCTCTGCCCAGGC |
| ASCRP002435 | 3.01E-08 | 2.15E-09 | 7.66531 | 11.33324 | 1.279249 | hsa_circRNA_102101 | ATGTATATTGTACACTCAGAAGAAGCCTTGTTTTTACTGGCAACCT |
| ASCRP001219 | 3.08E-08 | 2.22E-09 | 7.65502 | 11.30099 | 1.25409 | hsa_circRNA_100845 | AACTGAACTCCTCCAGCCTCCAGAAACCCACCTTGGGACACCTTGA |
| ASCRP000591 | 4.88E-08 | 3.88E-09 | 7.47869 | 10.74711 | 1.713381 | hsa_circRNA_100202 | TGGAGGAAGGGTGCCCAAATTGCTGTCTGCTCTCTGTCCAGAAGAA |
| ASCRP003982 | 4.98E-08 | 3.99E-09 | 7.47039 | 10.721 | 1.171367 | hsa_circRNA_103688 | GTCACTGAAGGAATCTTCCTCTTCAGTGAATACATCCAACAAGATG |
| ASCRP002424 | 5.26E-08 | 4.27E-09 | 7.44884 | 10.65311 | 1.186094 | hsa_circRNA_102089 | AGCCCTTCCCCAAGGCAGCCCTGAAGCAGCTTCCTAGGAACAAGGT |
| ASCRP000507 | 7.02E-08 | 5.81E-09 | 7.3523 | 10.34861 | 1.091306 | hsa_circRNA_100117 | CCTCCTTTTGATTGTGAAAACTGCTGAGAGAGACTTGCAATCCAGT |
| ASCRP000719 | 8.15E-08 | 6.84E-09 | 7.30126 | 10.18734 | 1.037627 | hsa_circRNA_100334 | TAACTACAAGTGAAGCCTTCTCCTTCCAAAAAGTTTCGGTCTGGCT |
| ASCRP000818 | 1.13E-07 | 9.92E-09 | 7.18526 | 9.82019 | 1.097089 | hsa_circRNA_100435 | TCTCTTACAGGCCAACTGAGCTGCATTTCCTTCCCACCTAAGGAAG |
| ASCRP005308 | 1.15E-07 | 1.01E-08 | 7.17893 | 9.80012 | 1.355977 | hsa_circRNA_400012 | AAGTTTTTTGTAGAGAAACCTCCACCTCCCAAGCTCAAGCAATTCT |
| ASCRP001401 | 1.80E-07 | 1.79E-08 | 7.00166 | 9.23735 | 1.364851 | hsa_circRNA_101031 | GTGCTTCCCTAGACTCTCCTTTCCAATTCCTCGGAAACAGGCACTG |
| ASCRP004906 | 1.84E-07 | 1.85E-08 | 6.99178 | 9.20592 | 1.246124 | hsa_circRNA_104653 | AAGGGACAAAAGCTGACTGAGTTGAATACCTTCTTCTTGAAGCATA |
| ASCRP002653 | 1.87E-07 | 1.88E-08 | 6.98576 | 9.18677 | 1.325895 | hsa_circRNA_102334 | ATATGACCAGCCACCCACACAGAATATGCCTATGGGTCCTGGAGGG |
| ASCRP004406 | 1.90E-07 | 1.94E-08 | 6.97621 | 9.15641 | 1.023491 | hsa_circRNA_104133 | TACTTTGAGGGTATGACTTTAGCCTTTCCAGCCTCAGTTCATGATT |
| ASCRP000611 | 2.55E-07 | 2.90E-08 | 6.85161 | 8.75966 | 1.075602 | hsa_circRNA_100223 | CTGCCCTCTACCACCTGCCACAGACCCCTTTGCTTCTGTTTTTGGG |
| ASCRP002964 | 2.68E-07 | 3.10E-08 | 6.83141 | 8.69529 | 1.419253 | hsa_circRNA_102651 | TGGTACCTGATGATCGAAAGATATCCTTCTTTCCAGAGTCAGGACA |
| ASCRP002802 | 3.20E-07 | 3.79E-08 | 6.76931 | 8.49721 | 2.125059 | hsa_circRNA_102485 | CAGTCCACAGAGATGGGATCTCACTATATTGCCCTGGCTGGTCTTG |
| ASCRP004839 | 3.64E-07 | 4.44E-08 | 6.7202 | 8.3405 | 1.080665 | hsa_circRNA_104586 | ATACTTCTTCATCCTTGCCTCCCCGATTGAAAGAGATGAAAAGCCA |
| ASCRP002143 | 4.76E-07 | 6.07E-08 | 6.62322 | 8.03075 | 1.615995 | hsa_circRNA_101798 | ACTTCTCATTTCATTTCCCGCTCCCGGCCCGTGGTGGATATGGTCA |
| ASCRP005353 | 4.83E-07 | 6.21E-08 | 6.61617 | 8.00822 | 1.031048 | hsa_circRNA_400059 | GCCCTCCTGCGCTGTCGAAATGTGAAGCATGTAGAACGTGAAGTGC |
| ASCRP001505 | 5.11E-07 | 6.65E-08 | 6.59528 | 7.94145 | 1.668256 | hsa_circRNA_101139 | AAGTTGTCTGGTGAATCTGTCTCTGTCATCAAGCACACTGATCCCG |
| ASCRP000816 | 5.33E-07 | 7.03E-08 | 6.57821 | 7.8869 | 3.135898 | hsa_circRNA_100433 | ATTCAGCCCATTCCCTGCAAACCAGGATGTCCATGAGGAGCCCCAT |
| ASCRP000205 | 6.73E-07 | 9.05E-08 | 6.50009 | 7.63709 | 1.208522 | hsa_circRNA_001109 | CCCCTTCCCGCTGGGAGTGGAGTATGAAGACCAAGGAATTCTCTTA |
| ASCRP000212 | 1.12E-06 | 1.62E-07 | 6.32145 | 7.06549 | 1.468117 | hsa_circRNA_001153 | GGTTTTATAAGCTCATCCTCAACCATGACTTCCTTCCAATTTTGTC |
| ASCRP001589 | 1.33E-06 | 1.97E-07 | 6.2597 | 6.86782 | 2.427013 | hsa_circRNA_101226 | ATATTCCTGTTCCTACTACAGTTCCTGTTCCTGGTTCAGCACCACC |
| ASCRP002451 | 1.50E-06 | 2.26E-07 | 6.21761 | 6.73311 | 2.73586 | hsa_circRNA_102119 | AACTCTCTAGATTCCAGTCCTGTTCGAAATCTGCAGTCTTTTGGCA |
| ASCRP004499 | 1.60E-06 | 2.44E-07 | 6.19504 | 6.66086 | 3.455038 | hsa_circRNA_104227 | AAAGAAGCCGATTCCAGTTCCTGTATGATGTTCAGAACATCCAGCT |
| ASCRP000266 | 1.60E-06 | 2.44E-07 | 6.1941 | 6.65785 | 1.06545 | hsa_circRNA_001401 | GAAAAGTTCATCCACTCAGGATTCCATTGTATCCCACCAGAAACAG |
| ASCRP000890 | 1.65E-06 | 2.54E-07 | 6.1818 | 6.61847 | 1.550561 | hsa_circRNA_100508 | GCCGCCTCCTTCTTTGCACGGACCCTGCTTTCTCGGCTGTGATTCA |
| ASCRP001788 | 1.96E-06 | 3.11E-07 | 6.11994 | 6.4205 | 1.061615 | hsa_circRNA_101429 | TCCTGAAGAGCAATCCCACTCCAGGAATTGTGATCAACAGGCCCAA |
| ASCRP000064 | 2.00E-06 | 3.19E-07 | 6.11239 | 6.39635 | 1.85836 | hsa_circRNA_000554 | ACTCCATCTATCCATATGAGAGTTGGATTCTCACTCTGTTGTCCAC |
| ASCRP002779 | 2.21E-06 | 3.62E-07 | 6.07281 | 6.26969 | 1.046069 | hsa_circRNA_102462 | AGCGTCATCAAGTGCCGCCTGCCCCTGTCAACAAGGACGACTTTGC |
| ASCRP002072 | 2.23E-06 | 3.69E-07 | 6.06764 | 6.25316 | 1.100039 | hsa_circRNA_101725 | ATTTCACAAGACAGCTCAGCAGCTCCTCCTCCTATAGTGGGGACAT |
| ASCRP003871 | 4.01E-06 | 7.61E-07 | 5.84443 | 5.53955 | 1.133058 | hsa_circRNA_103573 | ACCAGCTGTATTCATCATTCCCCTGCATATTCTTTTCCTGCTGCTA |
| ASCRP004170 | 4.41E-06 | 8.53E-07 | 5.80938 | 5.42765 | 1.364815 | hsa_circRNA_103890 | ACTGTCTTCTCTCATGTATACAGAGGATGGCATTCCCTGTGGATAT |
| ASCRP002064 | 4.76E-06 | 9.32E-07 | 5.78202 | 5.34032 | 1.175762 | hsa_circRNA_101717 | GGAAACCATCCACGACCCTGACTGGAATGTCACGTGGAATACCAGC |
| ASCRP001020 | 4.80E-06 | 9.44E-07 | 5.77834 | 5.32857 | 1.624357 | hsa_circRNA_100646 | ATTTTCCTCCTAAAGGTCGATCTTCTCTCTTTCCCTTTGAAGATGC |
| ASCRP000741 | 5.29E-06 | 1.06E-06 | 5.74253 | 5.21433 | 1.304437 | hsa_circRNA_100356 | TTCTGTCCCAGAACTCCAGGCCAGTCTGCCATCCATCCAGGAAGAA |
| ASCRP003643 | 6.66E-06 | 1.39E-06 | 5.65935 | 4.94924 | 1.64702 | hsa_circRNA_103345 | GGGCTTGAGAGCTGCCAAAGACCTTCCTTCAGAAGAAGAAAATGAG |
| ASCRP000265 | 6.69E-06 | 1.40E-06 | 5.65664 | 4.94063 | 1.989261 | hsa_circRNA_001396 | GGTGAAGCACCCATCTCATCACACCATATCAAAGCACCCATGGGAC |
| ASCRP000098 | 7.54E-06 | 1.63E-06 | 5.60959 | 4.79086 | 1.891929 | hsa_circRNA_000684 | AAGGAAGCAAAAGAAAGTCTTGTTCTGTCATCCAGGCTGGAGTGTA |
| ASCRP000706 | 1.20E-05 | 2.69E-06 | 5.45481 | 4.2992 | 3.004105 | hsa_circRNA_100319 | ATTGTCTTCCAATGAGCCCATTCCAGATGATGGGATTTATTGGCAG |
| ASCRP001164 | 1.88E-05 | 4.48E-06 | 5.29736 | 3.80104 | 1.43894 | hsa_circRNA_100790 | ATTCCAATCCATGCAAACGGTTCCTTTGGTATCATCCACAAGTGAG |
| ASCRP000391 | 2.17E-05 | 5.25E-06 | 5.24809 | 3.64564 | 1.039849 | hsa_circRNA_100001 | CCCAGCCACGCCAGGACGGCAACCTTCCCACCCTCATATCCAGCGT |
| ASCRP004358 | 2.33E-05 | 5.76E-06 | 5.21893 | 3.55378 | 1.43542 | hsa_circRNA_104084 | AGAGCCAGCCAGGATCTGCATTTACTGCTCAACCACATCTAATTTG |
| ASCRP004071 | 2.47E-05 | 6.23E-06 | 5.19485 | 3.47801 | 1.125623 | hsa_circRNA_103781 | CTGCTCCACGCCCATCGATTTGAGTGCATGCACTGTTGCACTTCAC |
| ASCRP001388 | 2.66E-05 | 6.72E-06 | 5.17134 | 3.40408 | 1.181796 | hsa_circRNA_101018 | GTGAATACAGCATCCACCCAACCCACCAGTATCACCAGGAAAAACT |
| ASCRP002782 | 3.01E-05 | 7.75E-06 | 5.12682 | 3.26427 | 1.090936 | hsa_circRNA_102465 | CCTCCTCAACTGGGTTCAAGTGCTCCACCAAGACCTCCAAGGGCAG |
| ASCRP001007 | 4.85E-05 | 1.32E-05 | 4.95945 | 2.74095 | 2.978418 | hsa_circRNA_100633 | AAATATCAGTTGGATCCCACTGCTTCCATTTCTATTCCCCTCTTCC |
| ASCRP002809 | 6.16E-05 | 1.75E-05 | 4.8728 | 2.47159 | 1.063026 | hsa_circRNA_102492 | CATCCTCGAGACAAAGATCATTCCACTCAGCCTGGGACGATGGGGA |
| ASCRP000614 | 6.26E-05 | 1.78E-05 | 4.86642 | 2.45182 | 1.147175 | hsa_circRNA_100226 | TTTTGTTGGCAATCTCTTCTCTGAAAGCTGAATTAACTAGTCAGGA |
| ASCRP000075 | 6.49E-05 | 1.86E-05 | 4.85359 | 2.41203 | 1.119338 | hsa_circRNA_000598 | CACCCTGGAGCCCAGCCCCCTGGCCCAGGGTCCCTTCCCTGTCACT |
| ASCRP001538 | 7.20E-05 | 2.11E-05 | 4.81365 | 2.2884 | 1.431316 | hsa_circRNA_101175 | AATGTAGACCATCCCATTCATCTAGGAGCGCATTTGTAGCCATGAA |
| ASCRP000010 | 1.46E-04 | 4.61E-05 | 4.56526 | 1.52587 | 1.697145 | hsa_circRNA_000094 | ACATTCGCAGGTCTTTCACTACCATTCCTACAGTGATTGGCAAGAT |
| ASCRP003607 | 2.30E-04 | 7.54E-05 | 4.40793 | 1.04933 | 1.089017 | hsa_circRNA_103309 | GCAACCAACACCCCCAGTGAGCCTTTGCTGTGCAAATTCGCTGATG |
| ASCRP000615 | 2.42E-04 | 7.97E-05 | 4.39003 | 0.99544 | 1.060014 | hsa_circRNA_100227 | AGGAATCTCCAATCTCTTCTCTGAAAGCTGAATTAACTAGTCAGGA |
| ASCRP000096 | 2.64E-04 | 8.83E-05 | 4.35701 | 0.89629 | 2.128482 | hsa_circRNA_000676 | GGCCCCAGAACTGTCCCATTCCTTTAAGGTACGAGCAGTCTACACA |
| ASCRP002846 | 2.94E-04 | 1.00E-04 | 4.31684 | 0.776 | 1.178506 | hsa_circRNA_102533 | CTCCACCTCCACAGGAGAAGAAACGAATGCATCAGATCAACAGAAT |
| ASCRP000716 | 3.17E-04 | 1.10E-04 | 4.28692 | 0.68669 | 1.273675 | hsa_circRNA_100331 | TAACAGTTGCTGCCGCCGGAAGTACAGCTTTGCTTGTGCCATCTGC |
| ASCRP005362 | 4.00E-04 | 1.42E-04 | 4.20207 | 0.43462 | 1.971595 | hsa_circRNA_400068 | TCAGAGGTCTTTCCAGTCCAGATCGTCCATGCTGTTTGGGGGCCAT |
| ASCRP001053 | 5.37E-04 | 1.97E-04 | 4.0957 | 0.12139 | 1.488197 | hsa_circRNA_100679 | CACAGCCGAGGTGCCGACGCCACCGCCGAGCCCATGATCCTGGAAC |
| ASCRP002856 | 5.93E-04 | 2.20E-04 | 4.05917 | 0.01455 | 1.302966 | hsa_circRNA_102543 | TCTCATGTCTCTCTCAGACCAGCACCTGCTCCTGGGGGCAGAGGAA |
| ASCRP002712 | 6.57E-04 | 2.46E-04 | 4.02329 | -0.08999 | 1.115353 | hsa_circRNA_102393 | CCCGCCAACGCCCGACCCTGCCCGCCCTGGACTGGCAGCTGCCGTC |
| ASCRP004636 | 7.24E-04 | 2.76E-04 | 3.98452 | -0.20249 | 1.927708 | hsa_circRNA_104374 | GCCGCCGCAAAGCAGATATTCTGGAGGAAGGTGTGAACGCAACTTT |
| ASCRP004797 | 7.39E-04 | 2.83E-04 | 3.97617 | -0.22669 | 1.384834 | hsa_circRNA_104543 | GACTTCTTTGGACCCATTCCAACAATCTCGTAAAACATGGTGGATT |
| ASCRP000624 | 9.93E-03 | 5.30E-03 | 2.94774 | -2.9995 | 1.144013 | hsa_circRNA_100236 | TTTTAAGATGCCTCTCTCTCTTATGTCCCTGATGCCAAAAATGCAC |
| ASCRP003708 | 1.20E-02 | 6.58E-03 | 2.8659 | -3.19904 | 1.468899 | hsa_circRNA_103410 | TCTCTCTTTCTAAACCTGAGTTACAACAAACTCTCTGAGATTGACC |
| ASCRP004842 | 1.94E-10 | 2.65E-12 | -9.86106 | 17.95348 | -2.067104 | hsa_circRNA_104589 | CGGCCTCGGATGGTGGTATAAGTTGAAATCCAAACCAGGAAAGAAG |
| ASCRP004828 | 4.13E-10 | 8.32E-12 | -9.47176 | 16.82214 | -1.238223 | hsa_circRNA_104575 | AAGCGATGAGGGCGGTGGCCAGTTTGAATACTCCCTTCATACCAGC |
| ASCRP001666 | 5.73E-10 | 1.34E-11 | -9.31138 | 16.35037 | -1.044619 | hsa_circRNA_101307 | GCGCCAGTGCATCTTCTAGGTGTGGAAGATTTCCTTACAAGGGCCG |
| ASCRP000018 | 1.63E-09 | 4.34E-11 | -8.92144 | 15.18985 | -3.323448 | hsa_circRNA_000167 | TCCGCGCGAGCTCATCAGTGGGGCCACGAGCTGAGTGCGTCCTGTC |
| ASCRP000385 | 6.05E-09 | 2.60E-10 | -8.33767 | 13.41847 | -2.479662 | hsa_circRNA_002144 | AGTGAGTTCAATGGCTGAGGTGAGTTCCCAGAGAACGGGGCTCCGC |
| ASCRP000343 | 7.68E-09 | 3.52E-10 | -8.24112 | 13.12182 | -2.651249 | hsa_circRNA_001846 | CTCCCTGAGCTTCGGGGAGGGAAGCTCATCAGTGGGGCCACGAGCT |
| ASCRP004827 | 8.83E-09 | 4.33E-10 | -8.17429 | 12.91593 | -1.623898 | hsa_circRNA_104574 | ACGTTTCTTTTCTCATGGTGGAGGCTGCATGGCTGGAAGGCCGGAT |
| ASCRP000315 | 9.34E-09 | 4.63E-10 | -8.15269 | 12.84926 | -2.454084 | hsa_circRNA_001678 | TCCGCGCGAGGTCTGAGACTAGGGCCAGAGGCGGCCCTAACAGGGC |
| ASCRP000294 | 2.15E-08 | 1.29E-09 | -7.82704 | 11.83877 | -1.822016 | hsa_circRNA_001547 | AGGGCAGTGGGTGGGAGGGTGCAGGCACTGGCCTGGGGCTGCTCTT |
| ASCRP004441 | 2.24E-08 | 1.41E-09 | -7.799 | 11.75129 | -1.033055 | hsa_circRNA_104168 | ATGACATTGCAGAACTGGTGGATGCGGGAAAGTGTTCTAATCTTAG |
| ASCRP001906 | 2.92E-08 | 2.02E-09 | -7.68439 | 11.39299 | -1.500188 | hsa_circRNA_101555 | TCGAAATCAGGTGAAGGTCTCCCACAGGTGTATTACTTTGGACCAT |
| ASCRP000062 | 4.21E-08 | 3.19E-09 | -7.54069 | 10.94216 | -2.19811 | hsa_circRNA_000543 | CTGGAGTAACTGGCATGTGAACAAGCTTTTTCTGTATTTACATACA |
| ASCRP004099 | 4.61E-08 | 3.56E-09 | -7.50572 | 10.83219 | -2.937258 | hsa_circRNA_103809 | TCCAAGCTGGCCCTTACGTCGTCCTGACTCATCTGATGACCGTTAT |
| ASCRP001226 | 5.21E-08 | 4.20E-09 | -7.45379 | 10.6687 | -1.277969 | hsa_circRNA_100852 | TGTGCTTGGCTCAGGAAAAGCTGGAACGGGTGATCCTAGGGAGTGA |
| ASCRP003040 | 1.27E-07 | 1.14E-08 | -7.14191 | 9.68275 | -2.400679 | hsa_circRNA_102728 | AATGGAAGATGGTGGTTTTTAGATCGTATGGCTGATGACGACTGGT |
| ASCRP001799 | 1.33E-07 | 1.22E-08 | -7.12059 | 9.6151 | -2.050048 | hsa_circRNA_101440 | ATTGGAACAAGTGGTGATGGCACTTCTCAAATACTGGCCAAAGACT |
| ASCRP003063 | 1.34E-07 | 1.24E-08 | -7.11508 | 9.59764 | -1.358019 | hsa_circRNA_102751 | TGTGCAGGAGCTGGCCCGGATTGCGGACAGTAAGGATCATGTGTTT |
| ASCRP000535 | 2.00E-07 | 2.07E-08 | -6.95563 | 9.09093 | -1.643736 | hsa_circRNA_100146 | TAGTGCCAAGGAAAGCAGCTGGCCCTTCTCAAGACCAATTCGGCTG |
| ASCRP002787 | 2.08E-07 | 2.22E-08 | -6.93414 | 9.02253 | -2.024462 | hsa_circRNA_102470 | GGCTGTGAGGGGTTTCTATGTTGCACTGAGACTGGTGGCCTGTGCA |
| ASCRP001596 | 2.37E-07 | 2.67E-08 | -6.87765 | 8.84265 | -1.1935 | hsa_circRNA_101233 | CCTTACGAACCGCACTAACTCTCCAACAATAAATACATTTGATAAG |
| ASCRP003680 | 2.47E-07 | 2.80E-08 | -6.86254 | 8.79449 | -1.482754 | hsa_circRNA_103382 | TCAGCTGCCTAGTGCTGGGGAGTCAGGGAAGAGCACCATCGTCAAG |
| ASCRP004952 | 2.63E-07 | 3.01E-08 | -6.84074 | 8.72501 | -2.897545 | hsa_circRNA_104700 | GGAAAAAGAGGAAAGATTTCTGCCCAGCAGACCGGGTTATCCCAGT |
| ASCRP001005 | 3.64E-07 | 4.34E-08 | -6.72683 | 8.36166 | -2.111706 | hsa_circRNA_100631 | TCTTAGACAGGCAGCGGAGGAGGAAATGGCAGGTCCTAATCAACTC |
| ASCRP004768 | 3.64E-07 | 4.40E-08 | -6.72245 | 8.34769 | -1.766895 | hsa_circRNA_104514 | AGGGCATGGAGTACCTGCAAGGTGTGGAGTTACAGTCCGAGACAGT |
| ASCRP000286 | 3.64E-07 | 4.43E-08 | -6.72063 | 8.34186 | -1.568061 | hsa_circRNA_001506 | CGTCGTGATGGTAGTCTCCCACTATTTTATATCTTTGTTACTTCAA |
| ASCRP004682 | 3.89E-07 | 4.82E-08 | -6.69486 | 8.25959 | -2.425894 | hsa_circRNA_104426 | ACTTTTAAGTGGAGTGGTGGATCAGACCAAAGATGGGTGGCTTTAA |
| ASCRP004893 | 4.66E-07 | 5.89E-08 | -6.63241 | 8.06011 | -1.391962 | hsa_circRNA_104640 | TGAAATGGTGTCTCCCAGTGCTCCAGACAATGAAACATCCAAATCT |
| ASCRP001006 | 5.93E-07 | 7.88E-08 | -6.54269 | 7.77333 | -1.928798 | hsa_circRNA_100632 | TACTGGAAAGGCAGCGGAGGAGGAAATGGCAGGTCCTAATCAACTC |
| ASCRP002376 | 7.35E-07 | 1.01E-07 | -6.46544 | 7.52625 | -2.336495 | hsa_circRNA_102041 | GCGAAAACACAGATGGTGATGGAATTTCCTGATAATGTGTTAAATC |
| ASCRP001407 | 9.67E-07 | 1.37E-07 | -6.37234 | 7.22837 | -1.181296 | hsa_circRNA_101037 | TCCATGATGAGGTGGCTGGGATCGTTGGCAGAGCGGACGTGTTAGC |
| ASCRP004971 | 1.26E-06 | 1.87E-07 | -6.27705 | 6.92336 | -1.237218 | hsa_circRNA_104720 | ACATGGCCCAGTTGCTGAACCTGTTTGCATGAGTTGCTCCTGACGG |
| ASCRP000189 | 1.88E-06 | 2.96E-07 | -6.13491 | 6.46841 | -1.032047 | hsa_circRNA_001059 | ATTCTGGGTAAAAGGACAGGGGGATGGCGTCTCCCACAGACGGTAA |
| ASCRP004228 | 1.88E-06 | 2.97E-07 | -6.13389 | 6.46513 | -1.031327 | hsa_circRNA_103948 | TGTTGGCTCTCCTTAAACAGGATATACACATGACACCATCCACTGA |
| ASCRP000389 | 2.21E-06 | 3.63E-07 | -6.07226 | 6.26792 | -1.905329 | hsa_circRNA_002172 | TTGGAACAGACTCACGGCCAGCGAAGTGAGTTCCCAGAGAACGGGG |
| ASCRP002944 | 2.21E-06 | 3.63E-07 | -6.07223 | 6.26784 | -2.53302 | hsa_circRNA_102631 | AAGATCAGTGTGTGGAAATCAGCCTAGAGGCAACCAAAAACATGGT |
| ASCRP000071 | 2.37E-06 | 3.95E-07 | -6.046 | 6.1839 | -1.845721 | hsa_circRNA_000585 | AACAGACTCACGGCCAGCGAACTGAGTGCGTCCTGTCACTCCACTC |
| ASCRP002734 | 2.38E-06 | 4.01E-07 | -6.04207 | 6.17132 | -1.081499 | hsa_circRNA_102415 | CTACATGGCTGTTCTCCGGGGCTTGGCGTACCTCCGAGAGAAGCAC |
| ASCRP003252 | 2.67E-06 | 4.58E-07 | -6.00069 | 6.03898 | -1.377152 | hsa_circRNA_102945 | TTGCCCTGGGGAAGAGAGGATGGTGATGCACCGGTTACAAAAGATG |
| ASCRP002567 | 2.93E-06 | 5.12E-07 | -5.96652 | 5.9297 | -1.11878 | hsa_circRNA_102239 | ACTACAGGACTGCGGACAAGGGCTGGCAGGTGCACATTCAGGTTCC |
| ASCRP002074 | 3.18E-06 | 5.67E-07 | -5.93502 | 5.82901 | -1.084797 | hsa_circRNA_101727 | TATTCAGAGACTGAGAAGGAGGTGGCTGGCCGTGCGGCTGGAGTGT |
| ASCRP005030 | 3.32E-06 | 5.93E-07 | -5.92125 | 5.78498 | -1.729729 | hsa_circRNA_104780 | AAGTGAAGAACTGTGAGGTGGAGCAGTGGGACTCGGATGAGCCCAT |
| ASCRP005393 | 3.82E-06 | 7.20E-07 | -5.86177 | 5.59495 | -1.252007 | hsa_circRNA_400100 | GGGCGGGCTCACATGGCTGGGTACGGAAAGGAGAGAGCAAACACTC |
| ASCRP000248 | 4.03E-06 | 7.70E-07 | -5.8411 | 5.52893 | -1.065441 | hsa_circRNA_001302 | CGGGTAGGTGGTGCCCTTGCTGACCTGGGTGATGGCCTTCTCCCCG |
| ASCRP000285 | 4.16E-06 | 8.02E-07 | -5.8286 | 5.489 | -1.644012 | hsa_circRNA_001503 | AGGAGGCTGCAGGTGGAGTGTTATAGTTTTGCCGCTGGACTCTTCC |
| ASCRP001855 | 4.53E-06 | 8.84E-07 | -5.79852 | 5.39297 | -1.102139 | hsa_circRNA_101504 | AGGACTGCTGGTGTGGACACTGCAAGAGACTTGCACCTGAGTATGA |
| ASCRP000752 | 4.61E-06 | 9.01E-07 | -5.79248 | 5.37369 | -1.238319 | hsa_circRNA_100367 | GCCCAGCGTCTCCTTCAGTGAATCTACAGACCTATTTTCTCAGGAG |
| ASCRP003902 | 5.27E-06 | 1.05E-06 | -5.74507 | 5.22245 | -1.371399 | hsa_circRNA_103607 | TGGGACTCCTGGTGACTGCTGAGGTTGGAAAGCTCTTGGGTGAAGA |
| ASCRP000210 | 5.58E-06 | 1.13E-06 | -5.72405 | 5.15543 | -1.915324 | hsa_circRNA_001143 | TGAAAAATAAGTGAGTGTGGCTTACACATGTATTTCCTGATCATTT |
| ASCRP000689 | 5.91E-06 | 1.20E-06 | -5.70325 | 5.08911 | -2.104069 | hsa_circRNA_100302 | ATGAGCGGTGGTGGCACTGGTACCCGGGTGATTTGCTCAGATTGTT |
| ASCRP005129 | 7.34E-06 | 1.58E-06 | -5.61947 | 4.82227 | -1.259848 | hsa_circRNA_104882 | TATGGCCTAAAACAGGAGGCTGGCAGGATGTACAGTTTTTATCACA |
| ASCRP002552 | 8.56E-06 | 1.87E-06 | -5.56767 | 4.65752 | -1.072282 | hsa_circRNA_102224 | CTGATGTGTTTTATAAGGTTGGCTGGATATTTACAGACCTCGTCTC |
| ASCRP000562 | 9.41E-06 | 2.07E-06 | -5.53682 | 4.55949 | -1.327438 | hsa_circRNA_100173 | AATATCGATGGGAGGTGGTTGACTCAATGGTTCAGCATTTTAAAGT |
| ASCRP001256 | 1.09E-05 | 2.41E-06 | -5.48872 | 4.40674 | -2.428807 | hsa_circRNA_100883 | CATTATGAAGTGTGTGGACCAGTTGACAAAGATCCAAACTGAATTA |
| ASCRP005154 | 1.28E-05 | 2.89E-06 | -5.43275 | 4.22926 | -1.989256 | hsa_circRNA_104907 | AAGGATGGAGGTGTGTTCCAGAGAACAAGCCTTCAGACATTTGCTA |
| ASCRP003719 | 1.46E-05 | 3.33E-06 | -5.3895 | 4.09229 | -1.018895 | hsa_circRNA_103421 | AGTCAGCAGAACTGAAATAGGTGATGGATGTGGACACACTGTACTA |
| ASCRP002554 | 1.46E-05 | 3.34E-06 | -5.38814 | 4.08799 | -1.040938 | hsa_circRNA_102226 | AAGTTTGTTACTGCAGTGGCTACAGGTTGGCTGGATATTTACAGAC |
| ASCRP004162 | 1.47E-05 | 3.36E-06 | -5.38597 | 4.08111 | -1.462226 | hsa_circRNA_103881 | ATTTCATGACTAACCAGTGATGGTGATTGCTCACACCAAAGCCTTG |
| ASCRP003726 | 1.53E-05 | 3.51E-06 | -5.37251 | 4.03851 | -1.475576 | hsa_circRNA_103428 | TCAAATCTGTGTGGAGCAGGTGCCTTAGCTGGACCAATTATTGTGG |
| ASCRP003437 | 1.53E-05 | 3.53E-06 | -5.37134 | 4.03481 | -2.259061 | hsa_circRNA_103134 | ATGGTGATCGGTGGTATGTTTTTAGCCACAGGTAGTACTGATCATG |
| ASCRP003968 | 1.53E-05 | 3.53E-06 | -5.37112 | 4.03413 | -2.046672 | hsa_circRNA_103674 | AAAGTGGAGTGTGCCCGGGATGAGACTGTAGTCCGAGTCCCTGGAA |
| ASCRP000354 | 1.55E-05 | 3.58E-06 | -5.36671 | 4.02018 | -1.328312 | hsa_circRNA_001950 | ATGGGGCCTGGCGGGCTTTGGGCTTTACTATATATGGATGGATCCA |
| ASCRP000182 | 1.63E-05 | 3.80E-06 | -5.34858 | 3.96285 | -1.410179 | hsa_circRNA_001040 | AGAGCTGCAGGGGCCCGTTTGTGTTGTGGAGCAAGGTTCTCAAAAG |
| ASCRP003718 | 1.72E-05 | 4.05E-06 | -5.32832 | 3.89881 | -1.049864 | hsa_circRNA_103420 | CTGTTATAGATAAACAAGGTGATGGATGTGGACACACTGTACTAGG |
| ASCRP005274 | 1.79E-05 | 4.24E-06 | -5.31405 | 3.85371 | -1.046412 | hsa_circRNA_105027 | AAAGATGCAGGTGCTCTCCCGTTCTCTGCTGGCTCCCTTCTTGTTT |
| ASCRP001615 | 1.80E-05 | 4.25E-06 | -5.31324 | 3.85116 | -1.223986 | hsa_circRNA_101252 | TAGTGAGCAGAATTCAATTCGTCATAATCTGTCCCTACACAGCAAG |
| ASCRP002913 | 2.21E-05 | 5.39E-06 | -5.23989 | 3.6198 | -2.103009 | hsa_circRNA_102600 | AACTGGCCCGGAAACAGGAGGACGTGTGGATGTGGAAGCAGCCAAG |
| ASCRP001967 | 3.21E-05 | 8.31E-06 | -5.10491 | 3.19556 | -1.782803 | hsa_circRNA_101618 | GATGTGCAGGGTGTGCAACTGAGGAACATGGCTCAAGAAACTAATC |
| ASCRP001647 | 3.34E-05 | 8.70E-06 | -5.09076 | 3.15121 | -1.177853 | hsa_circRNA_101287 | CAAGATACAAGATCTCCCCAATAATATGATTTACCAAGTCGCCATT |
| ASCRP003059 | 3.39E-05 | 8.84E-06 | -5.08564 | 3.13517 | -1.257028 | hsa_circRNA_102747 | GAAAAACTTTCTGATCTTATGGTTGGTGATGAGGCAAGTGAATTAC |
| ASCRP004178 | 3.40E-05 | 8.90E-06 | -5.08359 | 3.12875 | -1.494956 | hsa_circRNA_103898 | GATTAATGTCAATGATTTGCATCTGCTGGTGATGATGGAATTGTAG |
| ASCRP005260 | 4.17E-05 | 1.11E-05 | -5.01489 | 2.91387 | -2.164293 | hsa_circRNA_105013 | TGAAAGTTCTTCCAGGTGATGGTGAGGTAGTAGAACAGGTAATTGG |
| ASCRP000881 | 4.36E-05 | 1.17E-05 | -4.99867 | 2.86324 | -1.129109 | hsa_circRNA_100499 | AACCACTCTGGAATACTTGCTGGCTGGGGCAGATATCATTGAAACA |
| ASCRP001588 | 4.79E-05 | 1.30E-05 | -4.96633 | 2.7624 | -1.44442 | hsa_circRNA_101225 | AATACAGGAGGCTGCAAGGTGTGACTGTTGTAAATCTCAAGGAACT |
| ASCRP002286 | 5.04E-05 | 1.39E-05 | -4.94519 | 2.69657 | -1.313488 | hsa_circRNA_101945 | TTGGCTTTGCTGGCAGTGTATACGGTAAAATATGCCTTGATCAGTG |
| ASCRP002105 | 5.20E-05 | 1.45E-05 | -4.93152 | 2.65402 | -1.415554 | hsa_circRNA_101759 | AAGGTGGATGTGATTGCCAAGCGTGTGCTGGGACTGCACCTCCAGC |
| ASCRP004014 | 5.53E-05 | 1.55E-05 | -4.91065 | 2.58912 | -1.811619 | hsa_circRNA_103723 | TTCACAGCAGGTTTTGCGTGTGAAGCTGTGTGGAAATGTGAAATAC |
| ASCRP004642 | 5.74E-05 | 1.61E-05 | -4.89769 | 2.54887 | -1.268128 | hsa_circRNA_104381 | TCATGACTGAACAGTGCTCCGTATGTGGATGTGATCTGTTTGAACA |
| ASCRP001646 | 6.19E-05 | 1.76E-05 | -4.87062 | 2.46483 | -1.00414 | hsa_circRNA_101286 | CTTGATGAAGCAGAAGATGATTTTAAAAAAGTGATGGTGACCCTGA |
| ASCRP001530 | 7.15E-05 | 2.08E-05 | -4.81711 | 2.29911 | -1.175794 | hsa_circRNA_101167 | AAGGAAGCAAGATATACATCAGGGGATGTGAGAGTGTGGGACACCC |
| ASCRP002053 | 9.19E-05 | 2.78E-05 | -4.7263 | 2.01893 | -2.074783 | hsa_circRNA_101706 | GAAACTGTTATGATTGCTCATTTAGAAAGTGGATGTGTGGGATAAC |
| ASCRP001655 | 9.57E-05 | 2.92E-05 | -4.71062 | 1.97073 | -1.192056 | hsa_circRNA_101295 | TTCTTGGTACAGTGGTGGAAGTTGATGGGTCGAAACTAAATGTGAC |
| ASCRP004599 | 1.31E-04 | 4.10E-05 | -4.60296 | 1.64083 | -1.083302 | hsa_circRNA_104336 | GTTTCTGGTGGTAGTATCTTCCCCAGCAGATGTTGCTGAAAAAGCT |
| ASCRP004660 | 1.36E-04 | 4.25E-05 | -4.59106 | 1.6045 | -2.159949 | hsa_circRNA_104400 | CCAGCAGAAGGTGGTCGTGTGATGGTAACAGATGCTGACAGGTCAA |
| ASCRP004473 | 1.58E-04 | 5.00E-05 | -4.5394 | 1.44717 | -1.782267 | hsa_circRNA_104200 | TGGAGCTTCTTGATATTGCGGTGGTGGAGATGAGCGATGCCTTCCG |
| ASCRP004689 | 1.66E-04 | 5.28E-05 | -4.52235 | 1.39535 | -1.04011 | hsa_circRNA_104433 | TCAAGTGTCGAGATTGCCATCTGCCCCAACAACCATGAGGTGCATA |
| ASCRP003232 | 1.72E-04 | 5.50E-05 | -4.50906 | 1.355 | -1.05572 | hsa_circRNA_102924 | TTCCGGTGTGGTCGTCCTTGGTGGAAGGAACCATGAACTGGCATCT |
| ASCRP004209 | 1.92E-04 | 6.14E-05 | -4.47383 | 1.24826 | -1.005472 | hsa_circRNA_103929 | AGTGTGGCTGTCACAAAGTTATTACGACCTATTGCCCTGCTAACAA |
| ASCRP000302 | 2.51E-04 | 8.33E-05 | -4.37589 | 0.95295 | -1.972801 | hsa_circRNA_001587 | TTTGCAATGGTGGGGTGGTGAGGGATGCGGGCTGCAGGCAAGTATG |
| ASCRP001224 | 2.70E-04 | 9.06E-05 | -4.34868 | 0.87132 | -2.022209 | hsa_circRNA_100850 | CCATCGTGGGGTGGTGAAGGTGGGTCTGGTTGAAGACTCTCCCTCC |
| ASCRP002148 | 2.72E-04 | 9.15E-05 | -4.34551 | 0.86181 | -1.314033 | hsa_circRNA_101803 | TTTGTGGAGCTGTTGTTAATAACAGTACTGGTAGTGGAAGGGATTG |
| ASCRP005364 | 3.30E-04 | 1.16E-04 | -4.26915 | 0.63373 | -1.184709 | hsa_circRNA_400070 | TGTCCAAGAACTCCCATTGTCCCAGTCCCTGGCTCGGAGCTCTGTG |
| ASCRP003925 | 3.30E-04 | 1.16E-04 | -4.26882 | 0.63276 | -1.027822 | hsa_circRNA_103630 | TAATTTAAGCAAAATCAGGTTGGAGTTGCTGTGGGTGAGCTGCTGT |
| ASCRP004318 | 3.34E-04 | 1.18E-04 | -4.26415 | 0.61885 | -1.366883 | hsa_circRNA_104044 | AACTGAAGTGGCTATGTGGTTGATGTTGCAGAATGATGAGCCGGAG |
| ASCRP003285 | 3.97E-04 | 1.41E-04 | -4.2051 | 0.44357 | -1.352861 | hsa_circRNA_102978 | ATGCTGGAAGGCTGTGGGTGAGCGTGGAGGATGCTCAGATGCACAC |
| ASCRP002204 | 4.00E-04 | 1.43E-04 | -4.20132 | 0.4324 | -1.129292 | hsa_circRNA_101861 | AGGTCTGGCTGTTTATTGATGAGATATGTGTTGAGACGGATTCTCC |
| ASCRP000263 | 4.55E-04 | 1.63E-04 | -4.15843 | 0.30571 | -1.73781 | hsa_circRNA_001389 | AGGTGAGGAGGCATGTGTAGCTGTGTAAAGGTGAGGCATGCAAGTG |
| ASCRP000253 | 5.15E-04 | 1.87E-04 | -4.11299 | 0.17207 | -1.976079 | hsa_circRNA_001350 | CTTAGGTGGTGTGGGAAGATCTGTCTGAGAAGAAACAAGAAAGCAA |
| ASCRP003085 | 5.35E-04 | 1.96E-04 | -4.09819 | 0.12868 | -1.819405 | hsa_circRNA_102774 | ATCTGGAAGGAACATCTTCGCCTGGTGTGGTGGAAAGTCCAACATC |
| ASCRP004661 | 5.47E-04 | 2.02E-04 | -4.08837 | 0.09993 | -2.005394 | hsa_circRNA_104401 | ACTATTGAAGGTGGTCGTGTGATGGTAACAGATGCTGACAGGTCAA |
| ASCRP002380 | 6.61E-04 | 2.47E-04 | -4.0208 | -0.09722 | -1.68964 | hsa_circRNA_102045 | TGAGATTGAGAAACATGGTGGTGGCTTTGAAGGAGCTGTCTATTCG |
| ASCRP000153 | 6.62E-04 | 2.49E-04 | -4.01935 | -0.10144 | -1.145375 | hsa_circRNA_000926 | GGGTGATGTGGAAAAACTTTCTGCCTTATTTTTTGAACAATTGAGC |
| ASCRP005323 | 6.71E-04 | 2.52E-04 | -4.01419 | -0.11644 | -2.044425 | hsa_circRNA_400027 | TGGATCGATGCATTTGCAGAAACAAAGATTGTGTGTGGATCGATGA |
| ASCRP004383 | 7.45E-04 | 2.87E-04 | -3.97197 | -0.23883 | -1.119677 | hsa_circRNA_104110 | ATTTGCCAAGTGTGTAAATATGTTGCTGTGGAGCTGAAGTCAGCCT |
| ASCRP003146 | 7.45E-04 | 2.87E-04 | -3.97144 | -0.24037 | -1.493293 | hsa_circRNA_102838 | GAAGAAGAAGGTAACGGCGACTGTGACTGTGGTGAATGTGTGTGCA |
| ASCRP004372 | 8.01E-04 | 3.12E-04 | -3.94437 | -0.31853 | -1.942593 | hsa_circRNA_104099 | ACATGCATCGATGTGGATGAGTGTGTGGAGGGGACTGACAACTGCC |
| ASCRP002693 | 8.82E-04 | 3.44E-04 | -3.91178 | -0.41234 | -1.514355 | hsa_circRNA_102374 | ATTAAACAAGGTGCATTGGATCGTTGTGTGATGGGGATAACAGCAG |
| ASCRP000140 | 1.07E-03 | 4.25E-04 | -3.84018 | -0.61719 | -1.992672 | hsa_circRNA_000864 | CAGCAGTGTGGACTGTTGGCTGGTAAAGTGTGTGCAATGTGAGGAA |
| ASCRP000333 | 1.17E-03 | 4.73E-04 | -3.80417 | -0.71957 | -1.140602 | hsa_circRNA_001800 | GCTAACTCAGGGTGGGTTATGTGGAGCTGTTCATACTGCAGATATG |
| ASCRP000007 | 1.43E-03 | 5.88E-04 | -3.73058 | -0.92739 | -1.548592 | hsa_circRNA_000046 | GTCTCTTGAGGTGAGGGTGTGCAGCTTGGTAGGGATTGGGGTCCCT |
| ASCRP001639 | 1.67E-03 | 6.96E-04 | -3.67365 | -1.08683 | -1.415715 | hsa_circRNA_101278 | TTGGAAGTTTTTCTGATCTGTGGTTGTGGTTCCGGAGAATCTGGTT |
| ASCRP004574 | 1.86E-03 | 7.84E-04 | -3.63245 | -1.20143 | -1.855988 | hsa_circRNA_104310 | TTATGGACACGGTCTTTCTTATTCAGGTGTGTGTAACTGGTGTGTG |
| ASCRP001592 | 1.88E-03 | 7.98E-04 | -3.62666 | -1.21748 | -1.252717 | hsa_circRNA_101229 | CTGTGCACAGAATCGCATGTGTGAGTGCCCCTAGTGTTTACCAGAA |
| ASCRP003286 | 2.07E-03 | 8.83E-04 | -3.5917 | -1.31417 | -1.151086 | hsa_circRNA_102979 | GATGCTGAAGGCTGTGGGTGAGCGTGGAGGATGCTCAGATGCACAC |
| ASCRP001093 | 2.15E-03 | 9.27E-04 | -3.57506 | -1.36 | -1.534988 | hsa_circRNA_100719 | GGTGGTGAAGTAGTGAAGCTTTTTATAACTATGATGCCAGAGGAGC |
| ASCRP001568 | 2.20E-03 | 9.50E-04 | -3.56655 | -1.38339 | -1.439707 | hsa_circRNA_101205 | TTCCGCTGGGGGTGTGCTGGACCGGTTTTCTCAAATTCAGCCAAAG |
| ASCRP001122 | 2.20E-03 | 9.52E-04 | -3.56567 | -1.38583 | -1.213309 | hsa_circRNA_100748 | TGTGGATGTGGAAGAAAGTGATGAGAGTTTTGCCGAATTGACAAGC |
| ASCRP002099 | 2.40E-03 | 1.05E-03 | -3.53303 | -1.47532 | -1.614561 | hsa_circRNA_101753 | GTGGAAAACGTGGTATGTGTGCAACAGAGAGAAATTATGCGAATCA |
| ASCRP001771 | 2.41E-03 | 1.05E-03 | -3.5313 | -1.48004 | -1.128259 | hsa_circRNA_101412 | CTTTGTCTAGCCCCAGTGCAGTGAAACCAGCAGTGTGTGTAGCAGC |
| ASCRP002381 | 2.54E-03 | 1.12E-03 | -3.50963 | -1.53921 | -1.443908 | hsa_circRNA_102046 | CGTATATGAAAGAGGAAGTGGATAGAAACATGGTGGTGGCTTTGAA |
| ASCRP000267 | 2.59E-03 | 1.15E-03 | -3.50101 | -1.56268 | -1.680979 | hsa_circRNA_001405 | TGAATGGATGGTAGATGGAGGCAGGTGCATGTGTGATGGGAAGTGT |
| ASCRP001732 | 2.63E-03 | 1.17E-03 | -3.49474 | -1.57974 | -1.517077 | hsa_circRNA_101373 | AGACTAAAAGGAAAATGCTGTCCAGTGGGGTGTGTACATCAACTGT |
| ASCRP000462 | 2.66E-03 | 1.18E-03 | -3.49076 | -1.59056 | -1.232781 | hsa_circRNA_100072 | CCGGCACAGGGTGTGTAGCTGGGACGGTGCTGGTCTGAGCTGGACC |
| ASCRP000026 | 2.75E-03 | 1.23E-03 | -3.47759 | -1.6263 | -1.0538 | hsa_circRNA_000250 | ATGTGGGGGTGGAGGCTTAATTAACGTTTGTAAATCCCTCTTGAGA |
| ASCRP000022 | 2.85E-03 | 1.28E-03 | -3.46324 | -1.6652 | -1.418339 | hsa_circRNA_000200 | GTGGTTAAGACAGTGTTGTGTTGAAGGGACTGTTTATAATTTTCAC |
| ASCRP001502 | 2.95E-03 | 1.33E-03 | -3.44924 | -1.70305 | -1.201961 | hsa_circRNA_101136 | AAACAAAGTCGTGTGCATGGCGTCCTTGGAGGATGAGCTGTGTGGC |
| ASCRP001434 | 3.19E-03 | 1.45E-03 | -3.41865 | -1.78543 | -1.032793 | hsa_circRNA_101066 | TGGTAGTCATCCTCTCCATTGTGGGTGGTGGTGAATGCCTTGGTGG |
| ASCRP000017 | 3.23E-03 | 1.48E-03 | -3.4127 | -1.80143 | -1.1883 | hsa_circRNA_000166 | CGAAGTGAGTTCAATGGCTGAGGTGAGGTGAGTTCCCAGAGAACGG |
| ASCRP004805 | 3.32E-03 | 1.52E-03 | -3.40194 | -1.83029 | -1.26645 | hsa_circRNA_104551 | GTGTGCATTGGTTTTGGAAGGAAGGATGTTGTAGAACACTTACTAC |
| ASCRP000116 | 3.41E-03 | 1.57E-03 | -3.39023 | -1.86165 | -1.134723 | hsa_circRNA_000780 | CTTAGGAAACCTGCTGTGGAGTGGGATGTCCTCTGTGTGCATCATG |
| ASCRP002452 | 3.43E-03 | 1.58E-03 | -3.38858 | -1.86606 | -1.003133 | hsa_circRNA_102120 | CGCTTAGCATTGTGCAAGTGGAGCTGTGCTTTGCTTACAACCAGAG |
| ASCRP000027 | 3.43E-03 | 1.59E-03 | -3.38751 | -1.86891 | -1.209434 | hsa_circRNA_000274 | GGGTAGGGAGGTGAGGGCCTGGTGTGGGGTTTCCCAAGGAGAGCGC |
| ASCRP001643 | 3.50E-03 | 1.62E-03 | -3.38014 | -1.88862 | -1.407571 | hsa_circRNA_101282 | AAACAGCAAAACAGACATTGCTACAAGTGGTTGGTGTGGTCTCTGT |
| ASCRP004040 | 3.54E-03 | 1.64E-03 | -3.37479 | -1.90289 | -1.417719 | hsa_circRNA_103749 | ATGGAAAGCAGGTGAGAGTTACGTGTGTGCATCCAATGAACCATTT |
| ASCRP001942 | 3.78E-03 | 1.77E-03 | -3.34883 | -1.97202 | -1.207373 | hsa_circRNA_101592 | TCTTGTAGCAGCGCCTCCTCGATGCATCACTGTAAGCGATACCGCT |
| ASCRP004324 | 3.90E-03 | 1.83E-03 | -3.337 | -2.00343 | -1.213387 | hsa_circRNA_104050 | TATGCTTTACTGGGATCTTGTGTGTGATAATGCCTGGAAGGTCCAT |
| ASCRP000172 | 4.14E-03 | 1.95E-03 | -3.31351 | -2.06559 | -1.704088 | hsa_circRNA_000993 | TGTTAATTCTGGGATCAAGGTGGTCGGACATTGTGTGGTGTAATGA |
| ASCRP002284 | 4.27E-03 | 2.02E-03 | -3.30083 | -2.09902 | -1.348997 | hsa_circRNA_101943 | CTCGGATAAGTGTTGCTGTGGATGTATGGTCGACGCTGGCTGATTT |
| ASCRP000244 | 4.32E-03 | 2.06E-03 | -3.29411 | -2.11674 | -1.048073 | hsa_circRNA_001288 | ATAAAGGATACTTTCTGGAGGTTGGTGGTCCAGTAGCACAGATAAG |
| ASCRP005342 | 5.14E-03 | 2.51E-03 | -3.22398 | -2.3002 | -1.140314 | hsa_circRNA_400046 | CACTGGGGATGCACAGACTTACAGGGGTGTGTATGCATGCACCTGT |
| ASCRP004466 | 6.17E-03 | 3.09E-03 | -3.1483 | -2.49556 | -1.099762 | hsa_circRNA_104193 | GATTTCCATGTTGCAGATAATAATGAAGATGTTGATGGTGATGGTG |
| ASCRP003509 | 6.76E-03 | 3.43E-03 | -3.1094 | -2.59492 | -1.504908 | hsa_circRNA_103211 | AGACCTCGCCATTGAGTATGAGGTGGTGTGGACCCTGCAAGATCCT |
| ASCRP002545 | 7.43E-03 | 3.82E-03 | -3.07001 | -2.69472 | -1.018714 | hsa_circRNA_102217 | AACTCCCTCATCGGAGCTGGTGGTGGCTCTGAGTCATCTTGTGGTT |
| ASCRP000158 | 7.45E-03 | 3.83E-03 | -3.06875 | -2.69791 | -1.04571 | hsa_circRNA_000942 | ATGACAGAGGCTGGGGTGTTGGGAGATCAGACTTGGATTTGTGAAT |
| ASCRP004605 | 8.50E-03 | 4.44E-03 | -3.01383 | -2.83573 | -1.463526 | hsa_circRNA_104342 | GGTGGTGTAAAAGTGTGAAAGAAAATGTCTTTATTTAAAGCCCGTG |
| ASCRP001611 | 8.81E-03 | 4.65E-03 | -2.9969 | -2.8779 | -1.2748 | hsa_circRNA_101248 | CAACTGTCTGCTGGTTGGTGGAGAATTTGACTTGGAGATGAACTTT |
| ASCRP003053 | 1.04E-02 | 5.56E-03 | -2.92969 | -3.04381 | -1.269647 | hsa_circRNA_102741 | AAGACTAGAGCTCCTTTTTGGTTCAGGTGTGCTGGTGTCTCTAAGC |
| ASCRP001939 | 1.05E-02 | 5.66E-03 | -2.92305 | -3.06008 | -1.335674 | hsa_circRNA_101589 | TAGAGTATCAAGCAGAGTCAATGCATGTGTTGATGTGGTGCTCTCA |
| ASCRP000012 | 1.14E-02 | 6.22E-03 | -2.88727 | -3.14729 | -1.138264 | hsa_circRNA_000104 | AAGAACCAAGAATGCAGAAGTCTGGGGGTGGTGGAGAGCCAGGCCA |
| ASCRP000310 | 1.18E-02 | 6.50E-03 | -2.87054 | -3.18783 | -1.474289 | hsa_circRNA_001653 | ACTTGTGTGTGGGTGACTAAGTGGATGCATGTGTGCCCCTGCACTC |
| ASCRP005329 | 1.22E-02 | 6.74E-03 | -2.85664 | -3.22139 | -1.414536 | hsa_circRNA_400033 | ACCGATTTCTAGTGTGTGTTGGTGATGGGGCCGCAGCTGGTTTACC |
| ASCRP003742 | 1.49E-02 | 8.38E-03 | -2.7725 | -3.42224 | -1.228269 | hsa_circRNA_103444 | ACCACTCAAGATGTATGGTCTGCTGGCTGTGTGTTGGCTGAGCTGT |
| ASCRP004767 | 1.69E-02 | 9.70E-03 | -2.71554 | -3.55589 | -1.170783 | hsa_circRNA_104513 | ACTCTGTACAATGCCGGTGGTGGTGTGCACATTGAGCCCCGGTATA |
| ASCRP000167 | 1.70E-02 | 9.76E-03 | -2.71299 | -3.56182 | -1.429968 | hsa_circRNA_000963 | TGGGTGGTGTCGGGTAGGCCTCGCAATGTATTCAAGTTACTCCAGG |
| ASCRP002915 | 1.80E-02 | 1.04E-02 | -2.68825 | -3.61923 | -1.142887 | hsa_circRNA_102602 | AGAGGAATTGGTTGTTGGTGTGGATCAGAAGATGAGGAGGCACCTT |
| ASCRP002823 | 2.13E-02 | 1.24E-02 | -2.61707 | -3.78239 | -1.296548 | hsa_circRNA_102509 | GGTGCTGTTGGTGTTGTTCGATCCTTTGGTACAGAAGACAGACCGA |
| ASCRP004399 | 2.51E-02 | 1.49E-02 | -2.54324 | -3.94835 | -1.317202 | hsa_circRNA_104126 | ATCATCAATGGGAAGGTGGCAATGCAGTGGTGGATGGTTGTGGCAA |
| ASCRP002417 | 2.65E-02 | 1.58E-02 | -2.51882 | -4.00246 | -1.100145 | hsa_circRNA_102082 | GGCTTCCATGATGCTGAGTTTGTGTGTGAACGGACACTGAAATATT |
| ASCRP000150 | 2.87E-02 | 1.74E-02 | -2.48118 | -4.08517 | -1.14929 | hsa_circRNA_000911 | AAACATCACTATGGCTGGTGAACGTGTGTGCTGGTGTCCCTGGGCA |
| ASCRP005052 | 4.38E-02 | 2.79E-02 | -2.28125 | -4.50882 | -1.003351 | hsa_circRNA_104803 | AAATTTGGTGCCTGGAGTAGGAGTTTGGTGGTAATCCATTTGCTTC |
| ASCRP001034 | 4.59E-02 | 2.93E-02 | -2.25977 | -4.55273 | -1.203492 | hsa_circRNA_100660 | TCTACAAGAGATTGTGGTGGTGCCATGCAGCAAGAATCAGAGAGAT |
| ASCRP002764 | 4.97E-02 | 3.19E-02 | -2.22252 | -4.62809 | -1.169538 | hsa_circRNA_102445 | TCAAGGACAAGATGAAGATGTGTGTGTCTTTAAGTGCTCAGTGTCC |
