# Supplementary material for: circRNA circ_102049 Implicates in Pancreatic Ductal Adenocarcinoma Progression through Activating CD80 by Targeting miR-455-3p
Source: Mediators Inflamm. 2021 Jan 7;2021:8819990. doi: 10.1155/2021/8819990 (PMC7811564; doi:10.1155/2021/8819990)
Supplement: Supplementary 5 — Table S3 The target miRNAs of overlapped DECs according to the CircInteractome database. [file 8819990.f5.doc]

**Supplementary Table S3: The target miRNAs of overlapped DECs according to the CircInteractome database**

| Predicted target genes for results   # Column A: miRNA, the name of the miRNA. # Column B: circRNA_ID, The ID of circRNA. # Column C Type, the type of the circRNA. # Column D Diagrams, the diagrams illustrate key features for miRNA binding. See Ref [1,2] for details. # Column E: Total, the total number of the binding sites on the targets. # Column F: Context+, the sum of the context+ scores used in TargetScan after version 6.0. More negative is better. See Ref [3,4] for details. # Column G: Context, the sum of the context scores used in TargetScan before version 5.x. More negative is better. See Ref [1] for details. # Column H Structure, the sum of the structure scores used in miRanda (Ref [5]). The higher the better. # Column I: Energy, the sum of the free energy predicted by miRanda (Ref [5]). More negative is better. # Column J ~ Column U: counting numbers for different seed match types. # Column V: UTR Length, the length of UTR sequence  # Note: Currently, there is not enough data for constructing phylogenetic tree of different species for LncRNAs. The "Conservation" section is meaningless for LncRNA. All sites on LncRNA are treated as non-conserved ones. Column A: The red marked miRNAs represent a poor prognosis with highly expressed in Kaplan-Meier plotter (http://kmplot.com/analysis/), the green marked miRNAs represent a poor prognosis with lowly expressed, and the gray red marked miRNAs with no statistical significance  Column B: The red marked represent upregulated circRNAs and green marked represent downregulated circRNAs. # Reference: [1] Grimson A., et al. MicroRNA Targeting Specificity in Mammals: Determinants beyond Seed Pairing. Molecular Cell, Volume 27, Issue 1, 91-105, 6 July 2007. [2] Amy E. Pasquinelli. MicroRNAs and their targets: recognition, regulation and an emerging reciprocal relationship. Nature Reviews Genetics, Volume 13, 271-282, 1 April 2012. [3] Robin C Friedman, et al. Most Mammalian mRNAs Are Conserved Targets of MicroRNAs. Genome Research, Volume 19, 92-105, 2009. [4] David M Garcia., et al. Weak Seed-Pairing Stability and High Target-Site Abundance Decrease the Proficiency of lsy-6 and Other miRNAs. Nat. Struct. Mol. Biol., Volume 18, 1139-1146, 2011. [5] Enright AJ., el al. miRanda algorithm: MicroRNA targets in Drosophila. Genome Biology, Volume 5, R1, 2003. | | | | | | | | | | | | | | | | | | |
| --- | --- | --- | --- | --- | --- | --- | --- | --- | --- | --- | --- | --- | --- | --- | --- | --- | --- | --- |
|
|
|
|
|
|
|
|
|
|
|
|
|
|
|
|
|
|
|
|
|
|
| **miRNA** | **Target** | **TargetScan** | | **miRanda** | | **Conserved Sites** | | | | | | **Poorly Conserved Sites** | | | | | | **Annotations** |
| **miRNA** | **circRNA_ID** | **Context+** | **Context** | **Structure** | **Energy** | **8mer** | **7mer-m8** | **7mer-A1** | **6mer** | **Offset 6mer** | **Imperfect** | **8mer** | **7mer-m8** | **7mer-A1** | **6mer** | **Offset 6mer** | **Imperfect** | **UTR Length** |
| hsa-miR-326 | hsa_circRNA_000274 | -0.331 | -0.247 | 150 | -19.39 | 0 | 0 | 0 | 0 | 0 | 0 | 1 | 0 | 0 | 0 | 0 | 0 | 254 |
| hsa-miR-3940-3p | hsa_circRNA_000274 | -0.323 | -0.191 | 161 | -24.92 | 0 | 0 | 0 | 0 | 0 | 0 | 0 | 1 | 0 | 0 | 0 | 0 | 254 |
| hsa-miR-4313 | hsa_circRNA_000274 | -0.321 | -0.228 | 166 | -26.05 | 0 | 0 | 0 | 0 | 0 | 0 | 0 | 1 | 0 | 0 | 0 | 0 | 254 |
| hsa-miR-4652-5p | hsa_circRNA_000274 | -0.406 | -0.237 | 153 | -16.68 | 0 | 0 | 0 | 0 | 0 | 0 | 1 | 0 | 0 | 0 | 0 | 0 | 254 |
| hsa-miR-4667-5p | hsa_circRNA_000274 | -0.382 | -0.249 | 153 | -21.86 | 0 | 0 | 0 | 0 | 0 | 0 | 1 | 0 | 0 | 0 | 0 | 0 | 254 |
| hsa-miR-4700-5p | hsa_circRNA_000274 | -0.35 | -0.243 | 147 | -23.73 | 0 | 0 | 0 | 0 | 0 | 0 | 1 | 0 | 0 | 0 | 0 | 0 | 254 |
| hsa-miR-4708-3p | hsa_circRNA_000274 | -0.351 | -0.219 | 146 | -18.38 | 0 | 0 | 0 | 0 | 0 | 0 | 1 | 0 | 0 | 0 | 0 | 0 | 254 |
| hsa-miR-4750-3p | hsa_circRNA_000274 | -0.33 | -0.216 | 140 | -16.68 | 0 | 0 | 0 | 0 | 0 | 0 | 1 | 0 | 0 | 0 | 0 | 0 | 254 |
| hsa-miR-6805-5p | hsa_circRNA_000274 | -0.355 | -0.267 | 150 | -22.09 | 0 | 0 | 0 | 0 | 0 | 0 | 0 | 1 | 0 | 0 | 0 | 0 | 254 |
| hsa-miR-6846-5p | hsa_circRNA_000274 | -0.308 | -0.212 | 143 | -19.47 | 0 | 0 | 0 | 0 | 0 | 0 | 0 | 1 | 0 | 0 | 0 | 0 | 254 |
| hsa-miR-6848-5p | hsa_circRNA_000274 | -0.317 | -0.228 | 142 | -21.8 | 0 | 0 | 0 | 0 | 0 | 0 | 0 | 1 | 0 | 0 | 0 | 0 | 254 |
| hsa-miR-764 | hsa_circRNA_000274 | -0.355 | -0.283 | 147 | -16.27 | 0 | 0 | 0 | 0 | 0 | 0 | 1 | 0 | 0 | 0 | 0 | 0 | 254 |
| hsa-miR-8089 | hsa_circRNA_000274 | -0.329 | -0.239 | 145 | -20.34 | 0 | 0 | 0 | 0 | 0 | 0 | 1 | 0 | 0 | 0 | 0 | 0 | 254 |
| hsa-miR-1255a | hsa_circRNA_100117 | -0.327 | -0.317 | 164 | -15.25 | 0 | 0 | 0 | 0 | 0 | 0 | 1 | 0 | 0 | 0 | 0 | 0 | 429 |
| hsa-miR-1255b-5p | hsa_circRNA_100117 | -0.316 | -0.315 | 160 | -15.94 | 0 | 0 | 0 | 0 | 0 | 0 | 1 | 0 | 0 | 0 | 0 | 0 | 429 |
| hsa-miR-1294 | hsa_circRNA_100117 | -0.52 | -0.499 | 154 | -20.19 | 0 | 0 | 0 | 0 | 0 | 0 | 1 | 0 | 0 | 0 | 0 | 0 | 429 |
| hsa-miR-134-5p | hsa_circRNA_100117 | -0.408 | -0.421 | 154 | -21.66 | 0 | 0 | 0 | 0 | 0 | 0 | 1 | 0 | 0 | 0 | 0 | 0 | 429 |
| hsa-miR-142-3p | hsa_circRNA_100117 | -0.303 | -0.263 | 153 | -13.54 | 0 | 0 | 0 | 0 | 0 | 0 | 1 | 0 | 0 | 0 | 0 | 0 | 429 |
| hsa-miR-202-3p | hsa_circRNA_100117 | -0.405 | -0.434 | 156 | -19.58 | 0 | 0 | 0 | 0 | 0 | 0 | 1 | 0 | 0 | 0 | 0 | 0 | 429 |
| hsa-miR-296-3p | hsa_circRNA_100117 | -0.394 | -0.35 | 151 | -19.77 | 0 | 0 | 0 | 0 | 0 | 0 | 1 | 0 | 0 | 0 | 0 | 0 | 429 |
| hsa-miR-3118 | hsa_circRNA_100117 | -0.408 | -0.421 | 156 | -17.38 | 0 | 0 | 0 | 0 | 0 | 0 | 1 | 0 | 0 | 0 | 0 | 0 | 429 |
| hsa-miR-3653-5p | hsa_circRNA_100117 | -0.34 | -0.378 | 153 | -16.85 | 0 | 0 | 0 | 0 | 0 | 0 | 1 | 0 | 0 | 0 | 0 | 0 | 429 |
| hsa-miR-4490 | hsa_circRNA_100117 | -0.312 | -0.368 | 141 | -15.6 | 0 | 0 | 0 | 0 | 0 | 0 | 1 | 0 | 0 | 0 | 0 | 0 | 429 |
| hsa-miR-4501 | hsa_circRNA_100117 | -0.382 | -0.436 | 148 | -15.22 | 0 | 0 | 0 | 0 | 0 | 0 | 1 | 0 | 0 | 0 | 0 | 0 | 429 |
| hsa-miR-4747-5p | hsa_circRNA_100117 | -0.394 | -0.41 | 147 | -24.52 | 0 | 0 | 0 | 0 | 0 | 0 | 1 | 0 | 0 | 0 | 0 | 0 | 429 |
| hsa-miR-4751 | hsa_circRNA_100117 | -0.535 | -0.487 | 148 | -15.33 | 0 | 0 | 0 | 0 | 0 | 0 | 1 | 0 | 0 | 0 | 0 | 0 | 429 |
| hsa-miR-4774-5p | hsa_circRNA_100117 | -0.335 | -0.337 | 153 | -18.19 | 0 | 0 | 0 | 0 | 0 | 0 | 1 | 0 | 0 | 0 | 0 | 0 | 429 |
| hsa-miR-5196-5p | hsa_circRNA_100117 | -0.405 | -0.412 | 158 | -27.34 | 0 | 0 | 0 | 0 | 0 | 0 | 1 | 0 | 0 | 0 | 0 | 0 | 429 |
| hsa-miR-5681b | hsa_circRNA_100117 | -0.301 | -0.334 | 149 | -13.9 | 0 | 0 | 0 | 0 | 0 | 0 | 1 | 0 | 0 | 0 | 0 | 0 | 429 |
| hsa-miR-5691 | hsa_circRNA_100117 | -0.305 | -0.321 | 140 | -14.76 | 0 | 0 | 0 | 0 | 0 | 0 | 1 | 0 | 0 | 0 | 0 | 0 | 429 |
| hsa-miR-627-5p | hsa_circRNA_100117 | -0.58 | -0.528 | 291 | -27.39 | 0 | 0 | 0 | 0 | 0 | 0 | 2 | 0 | 0 | 0 | 0 | 0 | 429 |
| hsa-miR-653-3p | hsa_circRNA_100117 | -0.423 | -0.427 | 145 | -15.54 | 0 | 0 | 0 | 0 | 0 | 0 | 1 | 0 | 0 | 0 | 0 | 0 | 429 |
| hsa-miR-6719-3p | hsa_circRNA_100117 | -0.342 | -0.471 | 299 | -29.18 | 0 | 0 | 0 | 0 | 0 | 0 | 1 | 1 | 0 | 0 | 0 | 0 | 429 |
| hsa-miR-6780a-3p | hsa_circRNA_100117 | -0.323 | -0.389 | 140 | -11.1 | 0 | 0 | 0 | 0 | 0 | 0 | 1 | 0 | 0 | 0 | 0 | 0 | 429 |
| hsa-miR-6805-3p | hsa_circRNA_100117 | -0.305 | -0.321 | 142 | -12.62 | 0 | 0 | 0 | 0 | 0 | 0 | 1 | 0 | 0 | 0 | 0 | 0 | 429 |
| hsa-miR-1256 | hsa_circRNA_100302 | -0.409 | -0.416 | 150 | -11.42 | 0 | 0 | 0 | 0 | 0 | 0 | 1 | 0 | 0 | 0 | 0 | 0 | 291 |
| hsa-miR-3155a | hsa_circRNA_100302 | -0.43 | -0.35 | 153 | -24.13 | 0 | 0 | 0 | 0 | 0 | 0 | 1 | 0 | 0 | 0 | 0 | 0 | 291 |
| hsa-miR-3155b | hsa_circRNA_100302 | -0.399 | -0.343 | 151 | -19.86 | 0 | 0 | 0 | 0 | 0 | 0 | 1 | 0 | 0 | 0 | 0 | 0 | 291 |
| hsa-miR-3619-3p | hsa_circRNA_100302 | -0.385 | -0.31 | 143 | -18.91 | 0 | 0 | 0 | 0 | 0 | 0 | 1 | 0 | 0 | 0 | 0 | 0 | 291 |
| hsa-miR-4254 | hsa_circRNA_100302 | -0.394 | -0.37 | 141 | -15.64 | 0 | 0 | 0 | 0 | 0 | 0 | 1 | 0 | 0 | 0 | 0 | 0 | 291 |
| hsa-miR-4436b-3p | hsa_circRNA_100302 | -0.389 | -0.352 | 142 | -16.69 | 0 | 0 | 0 | 0 | 0 | 0 | 1 | 0 | 0 | 0 | 0 | 0 | 291 |
| hsa-miR-4520-3p | hsa_circRNA_100302 | -0.399 | -0.368 | 288 | -33.19 | 0 | 0 | 0 | 0 | 0 | 0 | 0 | 2 | 0 | 0 | 0 | 0 | 291 |
| hsa-miR-4632-5p | hsa_circRNA_100302 | -0.41 | -0.356 | 145 | -24.2 | 0 | 0 | 0 | 0 | 0 | 0 | 1 | 0 | 0 | 0 | 0 | 0 | 291 |
| hsa-miR-4776-5p | hsa_circRNA_100302 | -0.48 | -0.373 | 153 | -23.82 | 0 | 0 | 0 | 0 | 0 | 0 | 1 | 0 | 0 | 0 | 0 | 0 | 291 |
| hsa-miR-484 | hsa_circRNA_100302 | -0.378 | -0.339 | 140 | -15.9 | 0 | 0 | 0 | 0 | 0 | 0 | 1 | 0 | 0 | 0 | 0 | 0 | 291 |
| hsa-miR-485-5p | hsa_circRNA_100302 | -0.353 | -0.305 | 156 | -22.04 | 0 | 0 | 0 | 0 | 0 | 0 | 1 | 0 | 0 | 0 | 0 | 0 | 291 |
| hsa-miR-5193 | hsa_circRNA_100302 | -0.372 | -0.348 | 140 | -15.45 | 0 | 0 | 0 | 0 | 0 | 0 | 1 | 0 | 0 | 0 | 0 | 0 | 291 |
| hsa-miR-6735-5p | hsa_circRNA_100302 | -0.4 | -0.354 | 288 | -42.99 | 0 | 0 | 0 | 0 | 0 | 0 | 1 | 0 | 0 | 0 | 0 | 1 | 291 |
| hsa-miR-6825-5p | hsa_circRNA_100302 | -0.458 | -0.395 | 149 | -22.02 | 0 | 0 | 0 | 0 | 0 | 0 | 1 | 0 | 0 | 0 | 0 | 0 | 291 |
| hsa-miR-6852-5p | hsa_circRNA_100302 | -0.435 | -0.388 | 140 | -18.51 | 0 | 0 | 0 | 0 | 0 | 0 | 1 | 0 | 0 | 0 | 0 | 0 | 291 |
| hsa-miR-6879-5p | hsa_circRNA_100302 | -0.431 | -0.36 | 146 | -23.85 | 0 | 0 | 0 | 0 | 0 | 0 | 1 | 0 | 0 | 0 | 0 | 0 | 291 |
| hsa-miR-6884-5p | hsa_circRNA_100302 | -0.364 | -0.307 | 154 | -21.31 | 0 | 0 | 0 | 0 | 0 | 0 | 1 | 0 | 0 | 0 | 0 | 0 | 291 |
| hsa-miR-7843-5p | hsa_circRNA_100302 | -0.41 | -0.356 | 145 | -21.9 | 0 | 0 | 0 | 0 | 0 | 0 | 1 | 0 | 0 | 0 | 0 | 0 | 291 |
| hsa-miR-1273g-3p | hsa_circRNA_100790 | -0.456 | -0.473 | 140 | -15.06 | 0 | 0 | 0 | 0 | 0 | 0 | 1 | 0 | 0 | 0 | 0 | 0 | 261 |
| hsa-miR-218-1-3p | hsa_circRNA_100790 | -0.424 | -0.423 | 147 | -12.59 | 0 | 0 | 0 | 0 | 0 | 0 | 1 | 0 | 0 | 0 | 0 | 0 | 261 |
| hsa-miR-3137 | hsa_circRNA_100790 | -0.406 | -0.355 | 165 | -26.26 | 0 | 0 | 0 | 0 | 0 | 0 | 1 | 0 | 0 | 0 | 0 | 0 | 261 |
| hsa-miR-3663-5p | hsa_circRNA_100790 | -0.336 | -0.347 | 148 | -16.36 | 0 | 0 | 0 | 0 | 0 | 0 | 1 | 0 | 0 | 0 | 0 | 0 | 261 |
| hsa-miR-4423-5p | hsa_circRNA_100790 | -0.412 | -0.387 | 147 | -13.07 | 0 | 0 | 0 | 0 | 0 | 0 | 1 | 0 | 0 | 0 | 0 | 0 | 261 |
| hsa-miR-4433b-5p | hsa_circRNA_100790 | -0.339 | -0.259 | 147 | -14.86 | 0 | 0 | 0 | 0 | 0 | 0 | 1 | 0 | 0 | 0 | 0 | 0 | 261 |
| hsa-miR-4708-5p | hsa_circRNA_100790 | -0.371 | -0.355 | 149 | -19.24 | 0 | 0 | 0 | 0 | 0 | 0 | 1 | 0 | 0 | 0 | 0 | 0 | 261 |
| hsa-miR-485-5p | hsa_circRNA_100790 | -0.367 | -0.359 | 149 | -17.48 | 0 | 0 | 0 | 0 | 0 | 0 | 1 | 0 | 0 | 0 | 0 | 0 | 261 |
| hsa-miR-5691 | hsa_circRNA_100790 | -0.382 | -0.351 | 156 | -23.28 | 0 | 0 | 0 | 0 | 0 | 0 | 1 | 0 | 0 | 0 | 0 | 0 | 261 |
| hsa-miR-6509-3p | hsa_circRNA_100790 | -0.374 | -0.381 | 147 | -15.68 | 0 | 0 | 0 | 0 | 0 | 0 | 1 | 0 | 0 | 0 | 0 | 0 | 261 |
| hsa-miR-6753-3p | hsa_circRNA_100790 | -0.411 | -0.421 | 145 | -15.39 | 0 | 0 | 0 | 0 | 0 | 0 | 1 | 0 | 0 | 0 | 0 | 0 | 261 |
| hsa-miR-6805-3p | hsa_circRNA_100790 | -0.319 | -0.339 | 140 | -13.5 | 0 | 0 | 0 | 0 | 0 | 0 | 1 | 0 | 0 | 0 | 0 | 0 | 261 |
| hsa-miR-6884-5p | hsa_circRNA_100790 | -0.388 | -0.363 | 157 | -20.32 | 0 | 0 | 0 | 0 | 0 | 0 | 1 | 0 | 0 | 0 | 0 | 0 | 261 |
| hsa-miR-7107-3p | hsa_circRNA_100790 | -0.442 | -0.427 | 142 | -14.06 | 0 | 0 | 0 | 0 | 0 | 0 | 1 | 0 | 0 | 0 | 0 | 0 | 261 |
| hsa-miR-744-3p | hsa_circRNA_100790 | -0.433 | -0.43 | 146 | -10.03 | 0 | 0 | 0 | 0 | 0 | 0 | 1 | 0 | 0 | 0 | 0 | 0 | 261 |
| hsa-miR-148a-5p | hsa_circRNA_100904 | -0.341 | -0.45 | 147 | -12.69 | 0 | 0 | 0 | 0 | 0 | 0 | 1 | 0 | 0 | 0 | 0 | 0 | 409 |
| hsa-miR-181a-3p | hsa_circRNA_100904 | -0.559 | -0.435 | 142 | -14.81 | 0 | 0 | 0 | 0 | 0 | 0 | 1 | 0 | 0 | 0 | 0 | 0 | 409 |
| hsa-miR-1915-3p | hsa_circRNA_100904 | -0.491 | -0.443 | 146 | -23.34 | 0 | 0 | 0 | 0 | 0 | 0 | 1 | 0 | 0 | 0 | 0 | 0 | 409 |
| hsa-miR-221-3p | hsa_circRNA_100904 | -0.309 | -0.333 | 145 | -11.55 | 0 | 0 | 0 | 0 | 0 | 0 | 1 | 0 | 0 | 0 | 0 | 0 | 409 |
| hsa-miR-222-3p | hsa_circRNA_100904 | -0.309 | -0.333 | 147 | -11.82 | 0 | 0 | 0 | 0 | 0 | 0 | 1 | 0 | 0 | 0 | 0 | 0 | 409 |
| hsa-miR-29b-1-5p | hsa_circRNA_100904 | -0.32 | -0.411 | 151 | -19.61 | 0 | 0 | 0 | 0 | 0 | 0 | 1 | 0 | 0 | 0 | 0 | 0 | 409 |
| hsa-miR-3119 | hsa_circRNA_100904 | -0.314 | -0.423 | 145 | -11 | 0 | 0 | 0 | 0 | 0 | 0 | 1 | 0 | 0 | 0 | 0 | 0 | 409 |
| hsa-miR-320e | hsa_circRNA_100904 | -0.347 | -0.368 | 143 | -15.76 | 0 | 0 | 0 | 0 | 0 | 0 | 1 | 0 | 0 | 0 | 0 | 0 | 409 |
| hsa-miR-4448 | hsa_circRNA_100904 | -0.322 | -0.337 | 140 | -14.18 | 0 | 0 | 0 | 0 | 0 | 0 | 1 | 0 | 0 | 0 | 0 | 0 | 409 |
| hsa-miR-517a-3p | hsa_circRNA_100904 | -0.378 | -0.299 | 144 | -18.2 | 0 | 0 | 0 | 0 | 0 | 0 | 0 | 1 | 0 | 0 | 0 | 0 | 409 |
| hsa-miR-517b-3p | hsa_circRNA_100904 | -0.378 | -0.299 | 144 | -18.2 | 0 | 0 | 0 | 0 | 0 | 0 | 0 | 1 | 0 | 0 | 0 | 0 | 409 |
| hsa-miR-517c-3p | hsa_circRNA_100904 | -0.378 | -0.299 | 144 | -18.2 | 0 | 0 | 0 | 0 | 0 | 0 | 0 | 1 | 0 | 0 | 0 | 0 | 409 |
| hsa-miR-5192 | hsa_circRNA_100904 | -0.415 | -0.422 | 158 | -24.74 | 0 | 0 | 0 | 0 | 0 | 0 | 1 | 0 | 0 | 0 | 0 | 0 | 409 |
| hsa-miR-532-5p | hsa_circRNA_100904 | -0.343 | -0.397 | 146 | -10.96 | 0 | 0 | 0 | 0 | 0 | 0 | 1 | 0 | 0 | 0 | 0 | 0 | 409 |
| hsa-miR-6516-5p | hsa_circRNA_100904 | -0.32 | -0.369 | 156 | -16.32 | 0 | 0 | 0 | 0 | 0 | 0 | 1 | 0 | 0 | 0 | 0 | 0 | 409 |
| hsa-miR-6764-5p | hsa_circRNA_100904 | -0.48 | -0.441 | 140 | -21.21 | 0 | 0 | 0 | 0 | 0 | 0 | 1 | 0 | 0 | 0 | 0 | 0 | 409 |
| hsa-miR-1294 | hsa_circRNA_101226 | -0.433 | -0.438 | 156 | -16.73 | 0 | 0 | 0 | 0 | 0 | 0 | 1 | 0 | 0 | 0 | 0 | 0 | 246 |
| hsa-miR-22-5p | hsa_circRNA_101226 | -0.311 | -0.441 | 145 | -11.29 | 0 | 0 | 0 | 0 | 0 | 0 | 1 | 0 | 0 | 0 | 0 | 0 | 246 |
| hsa-miR-3120-5p | hsa_circRNA_101226 | -0.335 | -0.408 | 140 | -12.07 | 0 | 0 | 0 | 0 | 0 | 0 | 1 | 0 | 0 | 0 | 0 | 0 | 246 |
| hsa-miR-4527 | hsa_circRNA_101226 | -0.531 | -0.45 | 289 | -37.38 | 0 | 0 | 0 | 0 | 0 | 0 | 1 | 0 | 0 | 0 | 0 | 1 | 246 |
| hsa-miR-518a-5p | hsa_circRNA_101226 | -0.316 | -0.445 | 146 | -12.44 | 0 | 0 | 0 | 0 | 0 | 0 | 1 | 0 | 0 | 0 | 0 | 0 | 246 |
| hsa-miR-527 | hsa_circRNA_101226 | -0.316 | -0.445 | 146 | -12.44 | 0 | 0 | 0 | 0 | 0 | 0 | 1 | 0 | 0 | 0 | 0 | 0 | 246 |
| hsa-miR-562 | hsa_circRNA_101226 | -0.409 | -0.448 | 152 | -12.67 | 0 | 0 | 0 | 0 | 0 | 0 | 1 | 0 | 0 | 0 | 0 | 0 | 246 |
| hsa-miR-616-3p | hsa_circRNA_101226 | -0.445 | -0.502 | 164 | -20.47 | 0 | 0 | 0 | 0 | 0 | 0 | 1 | 0 | 0 | 0 | 0 | 0 | 246 |
| hsa-miR-6503-5p | hsa_circRNA_101226 | -0.51 | -0.446 | 150 | -15.64 | 0 | 0 | 0 | 0 | 0 | 0 | 1 | 0 | 0 | 0 | 0 | 0 | 246 |
| hsa-miR-6768-5p | hsa_circRNA_101226 | -0.312 | -0.321 | 156 | -22.72 | 0 | 0 | 0 | 0 | 0 | 0 | 1 | 0 | 0 | 0 | 0 | 0 | 246 |
| hsa-miR-1180-3p | hsa_circRNA_101656 | -0.558 | -0.373 | 148 | -20.77 | 0 | 0 | 0 | 0 | 0 | 0 | 1 | 0 | 0 | 0 | 0 | 0 | 538 |
| hsa-miR-3126-5p | hsa_circRNA_101656 | -0.438 | -0.316 | 152 | -23.44 | 0 | 0 | 0 | 0 | 0 | 0 | 1 | 0 | 0 | 0 | 0 | 0 | 538 |
| hsa-miR-4456 | hsa_circRNA_101656 | -0.395 | -0.359 | 140 | -13.19 | 0 | 0 | 0 | 0 | 0 | 0 | 1 | 0 | 0 | 0 | 0 | 0 | 538 |
| hsa-miR-4701-5p | hsa_circRNA_101656 | -0.387 | -0.322 | 145 | -19.71 | 0 | 0 | 0 | 0 | 0 | 0 | 1 | 0 | 0 | 0 | 0 | 0 | 538 |
| hsa-miR-4755-3p | hsa_circRNA_101656 | -0.445 | -0.384 | 142 | -14.85 | 0 | 0 | 0 | 0 | 0 | 0 | 1 | 0 | 0 | 0 | 0 | 0 | 538 |
| hsa-miR-4763-5p | hsa_circRNA_101656 | -0.525 | -0.37 | 144 | -16.95 | 0 | 0 | 0 | 0 | 0 | 0 | 1 | 0 | 0 | 0 | 0 | 0 | 538 |
| hsa-miR-588 | hsa_circRNA_101656 | -0.408 | -0.326 | 148 | -22.38 | 0 | 0 | 0 | 0 | 0 | 0 | 1 | 0 | 0 | 0 | 0 | 0 | 538 |
| hsa-miR-593-5p | hsa_circRNA_101656 | -0.372 | -0.181 | 153 | -25.33 | 0 | 0 | 0 | 0 | 0 | 0 | 1 | 0 | 0 | 0 | 0 | 0 | 538 |
| hsa-miR-6875-5p | hsa_circRNA_101656 | -0.406 | -0.31 | 158 | -26.96 | 0 | 0 | 0 | 0 | 0 | 0 | 1 | 0 | 0 | 0 | 0 | 0 | 538 |
| hsa-miR-6880-5p | hsa_circRNA_101656 | -0.455 | -0.347 | 154 | -27.6 | 0 | 0 | 0 | 0 | 0 | 0 | 1 | 0 | 0 | 0 | 0 | 0 | 538 |
| hsa-miR-891a-3p | hsa_circRNA_101656 | -0.338 | -0.225 | 149 | -19.76 | 0 | 0 | 0 | 0 | 0 | 0 | 1 | 0 | 0 | 0 | 0 | 0 | 538 |
| hsa-miR-1207-3p | hsa_circRNA_101798 | -0.359 | -0.334 | 140 | -15.15 | 0 | 0 | 0 | 0 | 0 | 0 | 1 | 0 | 0 | 0 | 0 | 0 | 313 |
| hsa-miR-1237-3p | hsa_circRNA_101798 | -0.37 | -0.412 | 149 | -22.04 | 0 | 0 | 0 | 0 | 0 | 0 | 1 | 0 | 0 | 0 | 0 | 0 | 313 |
| hsa-miR-138-5p | hsa_circRNA_101798 | -0.447 | -0.345 | 149 | -18.71 | 0 | 0 | 0 | 0 | 0 | 0 | 1 | 0 | 0 | 0 | 0 | 0 | 313 |
| hsa-miR-185-5p | hsa_circRNA_101798 | -0.334 | -0.3 | 158 | -20.14 | 0 | 0 | 0 | 0 | 0 | 0 | 1 | 0 | 0 | 0 | 0 | 0 | 313 |
| hsa-miR-3689a-5p | hsa_circRNA_101798 | -0.358 | -0.451 | 155 | -18.5 | 0 | 0 | 0 | 0 | 0 | 0 | 1 | 0 | 0 | 0 | 0 | 0 | 313 |
| hsa-miR-3689b-5p | hsa_circRNA_101798 | -0.358 | -0.451 | 155 | -18.5 | 0 | 0 | 0 | 0 | 0 | 0 | 1 | 0 | 0 | 0 | 0 | 0 | 313 |
| hsa-miR-3689e | hsa_circRNA_101798 | -0.358 | -0.451 | 155 | -18.5 | 0 | 0 | 0 | 0 | 0 | 0 | 1 | 0 | 0 | 0 | 0 | 0 | 313 |
| hsa-miR-3689f | hsa_circRNA_101798 | -0.348 | -0.449 | 151 | -15.37 | 0 | 0 | 0 | 0 | 0 | 0 | 1 | 0 | 0 | 0 | 0 | 0 | 313 |
| hsa-miR-4306 | hsa_circRNA_101798 | -0.334 | -0.3 | 158 | -19.97 | 0 | 0 | 0 | 0 | 0 | 0 | 1 | 0 | 0 | 0 | 0 | 0 | 313 |
| hsa-miR-4469 | hsa_circRNA_101798 | -0.352 | -0.271 | 153 | -25.84 | 0 | 0 | 0 | 0 | 0 | 0 | 1 | 0 | 0 | 0 | 0 | 0 | 313 |
| hsa-miR-4525 | hsa_circRNA_101798 | -0.373 | -0.263 | 153 | -20.02 | 0 | 0 | 0 | 0 | 0 | 0 | 1 | 0 | 0 | 0 | 0 | 0 | 313 |
| hsa-miR-4529-5p | hsa_circRNA_101798 | -0.352 | -0.197 | 155 | -17.09 | 0 | 0 | 0 | 0 | 0 | 0 | 1 | 0 | 0 | 0 | 0 | 0 | 313 |
| hsa-miR-4644 | hsa_circRNA_101798 | -0.324 | -0.298 | 144 | -21.34 | 0 | 0 | 0 | 0 | 0 | 0 | 1 | 0 | 0 | 0 | 0 | 0 | 313 |
| hsa-miR-4667-3p | hsa_circRNA_101798 | -0.316 | -0.235 | 140 | -24.55 | 0 | 0 | 0 | 0 | 0 | 0 | 1 | 0 | 0 | 0 | 0 | 0 | 313 |
| hsa-miR-4764-3p | hsa_circRNA_101798 | -0.415 | -0.474 | 152 | -14.43 | 0 | 0 | 0 | 0 | 0 | 0 | 1 | 0 | 0 | 0 | 0 | 0 | 313 |
| hsa-miR-5010-5p | hsa_circRNA_101798 | -0.394 | -0.267 | 158 | -21.38 | 0 | 0 | 0 | 0 | 0 | 0 | 1 | 0 | 0 | 0 | 0 | 0 | 313 |
| hsa-miR-6868-3p | hsa_circRNA_101798 | -0.531 | -0.658 | 312 | -37.92 | 0 | 0 | 0 | 0 | 0 | 0 | 1 | 1 | 0 | 0 | 0 | 0 | 313 |
| hsa-miR-7152-3p | hsa_circRNA_101798 | -0.354 | -0.211 | 149 | -22.39 | 0 | 0 | 0 | 0 | 0 | 0 | 1 | 0 | 0 | 0 | 0 | 0 | 313 |
| hsa-miR-214-3p | hsa_circRNA_102049 | -0.402 | -0.351 | 151 | -23.09 | 0 | 0 | 0 | 0 | 0 | 0 | 1 | 0 | 0 | 0 | 0 | 0 | 250 |
| hsa-miR-342-3p | hsa_circRNA_102049 | -0.315 | -0.357 | 140 | -15.04 | 0 | 0 | 0 | 0 | 0 | 0 | 1 | 0 | 0 | 0 | 0 | 0 | 250 |
| hsa-miR-3619-5p | hsa_circRNA_102049 | -0.391 | -0.349 | 148 | -23.08 | 0 | 0 | 0 | 0 | 0 | 0 | 1 | 0 | 0 | 0 | 0 | 0 | 250 |
| hsa-miR-455-3p | hsa_circRNA_102049 | -0.364 | -0.32 | 297 | -36.6 | 0 | 0 | 0 | 0 | 0 | 0 | 0 | 2 | 0 | 0 | 0 | 0 | 250 |
| hsa-miR-4722-3p | hsa_circRNA_102049 | -0.429 | -0.422 | 141 | -19.06 | 0 | 0 | 0 | 0 | 0 | 0 | 1 | 0 | 0 | 0 | 0 | 0 | 250 |
| hsa-miR-516a-3p | hsa_circRNA_102049 | -0.346 | -0.344 | 146 | -14.72 | 0 | 0 | 0 | 0 | 0 | 0 | 1 | 0 | 0 | 0 | 0 | 0 | 250 |
| hsa-miR-516b-3p | hsa_circRNA_102049 | -0.346 | -0.344 | 146 | -14.72 | 0 | 0 | 0 | 0 | 0 | 0 | 1 | 0 | 0 | 0 | 0 | 0 | 250 |
| hsa-miR-6508-3p | hsa_circRNA_102049 | -0.357 | -0.336 | 153 | -18.64 | 0 | 0 | 0 | 0 | 0 | 0 | 0 | 1 | 0 | 0 | 0 | 0 | 250 |
| hsa-miR-6727-3p | hsa_circRNA_102049 | -0.44 | -0.424 | 142 | -21.34 | 0 | 0 | 0 | 0 | 0 | 0 | 1 | 0 | 0 | 0 | 0 | 0 | 250 |
| hsa-miR-6780a-3p | hsa_circRNA_102049 | -0.364 | -0.329 | 159 | -15.6 | 0 | 0 | 0 | 0 | 0 | 0 | 1 | 0 | 0 | 0 | 0 | 0 | 250 |
| hsa-miR-6884-3p | hsa_circRNA_102049 | -0.359 | -0.346 | 144 | -13.06 | 0 | 0 | 0 | 0 | 0 | 0 | 1 | 0 | 0 | 0 | 0 | 0 | 250 |
| hsa-miR-6895-5p | hsa_circRNA_102049 | -0.31 | -0.232 | 140 | -15.56 | 0 | 0 | 0 | 0 | 0 | 0 | 0 | 1 | 0 | 0 | 0 | 0 | 250 |
| hsa-miR-7162-5p | hsa_circRNA_102049 | -0.346 | -0.344 | 147 | -16.15 | 0 | 0 | 0 | 0 | 0 | 0 | 1 | 0 | 0 | 0 | 0 | 0 | 250 |
| hsa-miR-761 | hsa_circRNA_102049 | -0.37 | -0.345 | 163 | -21.75 | 0 | 0 | 0 | 0 | 0 | 0 | 1 | 0 | 0 | 0 | 0 | 0 | 250 |
| hsa-miR-15a-5p | hsa_circRNA_102359 | -0.326 | -0.288 | 142 | -14.63 | 0 | 0 | 0 | 0 | 0 | 0 | 1 | 0 | 0 | 0 | 0 | 0 | 178 |
| hsa-miR-15b-5p | hsa_circRNA_102359 | -0.347 | -0.292 | 148 | -15.11 | 0 | 0 | 0 | 0 | 0 | 0 | 1 | 0 | 0 | 0 | 0 | 0 | 178 |
| hsa-miR-16-5p | hsa_circRNA_102359 | -0.337 | -0.29 | 146 | -16.4 | 0 | 0 | 0 | 0 | 0 | 0 | 1 | 0 | 0 | 0 | 0 | 0 | 178 |
| hsa-miR-195-5p | hsa_circRNA_102359 | -0.326 | -0.288 | 141 | -15.7 | 0 | 0 | 0 | 0 | 0 | 0 | 1 | 0 | 0 | 0 | 0 | 0 | 178 |
| hsa-miR-221-5p | hsa_circRNA_102359 | -0.315 | -0.297 | 146 | -15.45 | 0 | 0 | 0 | 0 | 0 | 0 | 0 | 1 | 0 | 0 | 0 | 0 | 178 |
| hsa-miR-424-5p | hsa_circRNA_102359 | -0.316 | -0.286 | 141 | -14.35 | 0 | 0 | 0 | 0 | 0 | 0 | 1 | 0 | 0 | 0 | 0 | 0 | 178 |
| hsa-miR-4502 | hsa_circRNA_102359 | -0.318 | -0.362 | 151 | -16.41 | 0 | 0 | 0 | 0 | 0 | 0 | 1 | 0 | 0 | 0 | 0 | 0 | 178 |
| hsa-miR-497-5p | hsa_circRNA_102359 | -0.305 | -0.284 | 140 | -13.21 | 0 | 0 | 0 | 0 | 0 | 0 | 1 | 0 | 0 | 0 | 0 | 0 | 178 |
| hsa-miR-6838-5p | hsa_circRNA_102359 | -0.358 | -0.294 | 155 | -16.46 | 0 | 0 | 0 | 0 | 0 | 0 | 1 | 0 | 0 | 0 | 0 | 0 | 178 |
| hsa-miR-3121-5p | hsa_circRNA_102465 | -0.336 | -0.34 | 145 | -15.59 | 0 | 0 | 0 | 0 | 0 | 0 | 1 | 0 | 0 | 0 | 0 | 0 | 126 |
| hsa-miR-6784-5p | hsa_circRNA_102465 | -0.634 | -0.367 | 157 | -27.53 | 0 | 0 | 0 | 0 | 0 | 0 | 1 | 0 | 0 | 0 | 0 | 0 | 126 |
| hsa-miR-4677-3p | hsa_circRNA_102741 | -0.367 | -0.407 | 149 | -17.52 | 0 | 0 | 0 | 0 | 0 | 0 | 1 | 0 | 0 | 0 | 0 | 0 | 441 |
| hsa-miR-6075 | hsa_circRNA_102741 | -0.305 | -0.148 | 159 | -27.41 | 0 | 0 | 0 | 0 | 0 | 0 | 0 | 1 | 0 | 0 | 0 | 0 | 441 |
| hsa-miR-6781-5p | hsa_circRNA_102741 | -0.673 | -0.436 | 141 | -18.92 | 0 | 0 | 0 | 0 | 0 | 0 | 1 | 0 | 0 | 0 | 0 | 0 | 441 |
| hsa-miR-760 | hsa_circRNA_102741 | -0.414 | -0.374 | 142 | -17.75 | 0 | 0 | 0 | 0 | 0 | 0 | 1 | 0 | 0 | 0 | 0 | 0 | 441 |
| hsa-miR-766-3p | hsa_circRNA_102741 | -0.343 | -0.315 | 140 | -21.1 | 0 | 0 | 0 | 0 | 0 | 0 | 1 | 0 | 0 | 0 | 0 | 0 | 441 |
| hsa-miR-8057 | hsa_circRNA_102741 | -0.356 | -0.3 | 150 | -17.62 | 0 | 0 | 0 | 0 | 0 | 0 | 1 | 0 | 0 | 0 | 0 | 0 | 441 |
| hsa-miR-152-5p | hsa_circRNA_102984 | -0.465 | -0.477 | 294 | -35.04 | 0 | 0 | 0 | 0 | 0 | 0 | 1 | 1 | 0 | 0 | 0 | 0 | 421 |
| hsa-miR-155-3p | hsa_circRNA_102984 | -0.34 | -0.365 | 142 | -11.78 | 0 | 0 | 0 | 0 | 0 | 0 | 1 | 0 | 0 | 0 | 0 | 0 | 421 |
| hsa-miR-1587 | hsa_circRNA_102984 | -0.502 | -0.449 | 147 | -25.03 | 0 | 0 | 0 | 0 | 0 | 0 | 1 | 0 | 0 | 0 | 0 | 0 | 421 |
| hsa-miR-1915-3p | hsa_circRNA_102984 | -0.404 | -0.322 | 140 | -18.05 | 0 | 0 | 0 | 0 | 0 | 0 | 1 | 0 | 0 | 0 | 0 | 0 | 421 |
| hsa-miR-26b-3p | hsa_circRNA_102984 | -0.307 | -0.362 | 288 | -23.4 | 0 | 0 | 0 | 0 | 0 | 0 | 1 | 1 | 0 | 0 | 0 | 0 | 421 |
| hsa-miR-3620-5p | hsa_circRNA_102984 | -0.492 | -0.447 | 148 | -25.76 | 0 | 0 | 0 | 0 | 0 | 0 | 1 | 0 | 0 | 0 | 0 | 0 | 421 |
| hsa-miR-4536-5p | hsa_circRNA_102984 | -0.386 | -0.326 | 148 | -18.64 | 0 | 0 | 0 | 0 | 0 | 0 | 1 | 0 | 0 | 0 | 0 | 0 | 421 |
| hsa-miR-4656 | hsa_circRNA_102984 | -0.469 | -0.432 | 151 | -25.31 | 0 | 0 | 0 | 0 | 0 | 0 | 1 | 0 | 0 | 0 | 0 | 0 | 421 |
| hsa-miR-4746-3p | hsa_circRNA_102984 | -0.557 | -0.312 | 145 | -17.49 | 0 | 0 | 0 | 0 | 0 | 0 | 1 | 0 | 0 | 0 | 0 | 0 | 421 |
| hsa-miR-4757-5p | hsa_circRNA_102984 | -0.46 | -0.445 | 140 | -18.25 | 0 | 0 | 0 | 0 | 0 | 0 | 1 | 0 | 0 | 0 | 0 | 0 | 421 |
| hsa-miR-4799-3p | hsa_circRNA_102984 | -0.308 | -0.249 | 151 | -15.47 | 0 | 0 | 0 | 0 | 0 | 0 | 1 | 0 | 0 | 0 | 0 | 0 | 421 |
| hsa-miR-5588-5p | hsa_circRNA_102984 | -0.308 | -0.249 | 160 | -19.02 | 0 | 0 | 0 | 0 | 0 | 0 | 1 | 0 | 0 | 0 | 0 | 0 | 421 |
| hsa-miR-582-3p | hsa_circRNA_102984 | -0.496 | -0.553 | 293 | -28.19 | 0 | 0 | 0 | 0 | 0 | 0 | 1 | 1 | 0 | 0 | 0 | 0 | 421 |
| hsa-miR-642a-3p | hsa_circRNA_102984 | -0.306 | -0.351 | 148 | -12.52 | 0 | 0 | 0 | 0 | 0 | 0 | 1 | 0 | 0 | 0 | 0 | 0 | 421 |
| hsa-miR-642b-3p | hsa_circRNA_102984 | -0.306 | -0.351 | 148 | -13.41 | 0 | 0 | 0 | 0 | 0 | 0 | 1 | 0 | 0 | 0 | 0 | 0 | 421 |
| hsa-miR-671-5p | hsa_circRNA_102984 | -0.408 | -0.365 | 143 | -18.51 | 0 | 0 | 0 | 0 | 0 | 0 | 1 | 0 | 0 | 0 | 0 | 0 | 421 |
| hsa-miR-6744-3p | hsa_circRNA_102984 | -0.481 | -0.449 | 149 | -18.66 | 0 | 0 | 0 | 0 | 0 | 0 | 1 | 0 | 0 | 0 | 0 | 0 | 421 |
| hsa-miR-6764-5p | hsa_circRNA_102984 | -0.435 | -0.329 | 160 | -25.36 | 0 | 0 | 0 | 0 | 0 | 0 | 1 | 0 | 0 | 0 | 0 | 0 | 421 |
| hsa-miR-6826-3p | hsa_circRNA_102984 | -0.421 | -0.26 | 159 | -25.66 | 0 | 0 | 0 | 0 | 0 | 0 | 1 | 0 | 0 | 0 | 0 | 0 | 421 |
| hsa-miR-6878-3p | hsa_circRNA_102984 | -0.556 | -0.47 | 170 | -22.41 | 0 | 0 | 0 | 0 | 0 | 0 | 1 | 0 | 0 | 0 | 0 | 0 | 421 |
| hsa-miR-6887-3p | hsa_circRNA_102984 | -0.411 | -0.286 | 140 | -19.42 | 0 | 0 | 0 | 0 | 0 | 0 | 1 | 0 | 0 | 0 | 0 | 0 | 421 |
| hsa-miR-1972 | hsa_circRNA_103076 | -0.476 | -0.407 | 144 | -21.93 | 0 | 0 | 0 | 0 | 0 | 0 | 1 | 0 | 0 | 0 | 0 | 0 | 339 |
| hsa-miR-378g | hsa_circRNA_103076 | -0.381 | -0.299 | 144 | -16.64 | 0 | 0 | 0 | 0 | 0 | 0 | 1 | 0 | 0 | 0 | 0 | 0 | 339 |
| hsa-miR-455-3p | hsa_circRNA_103076 | -0.459 | -0.49 | 147 | -16.11 | 0 | 0 | 0 | 0 | 0 | 0 | 1 | 0 | 0 | 0 | 0 | 0 | 339 |
| hsa-miR-4718 | hsa_circRNA_103076 | -0.348 | -0.314 | 140 | -13.75 | 0 | 0 | 0 | 0 | 0 | 0 | 1 | 0 | 0 | 0 | 0 | 0 | 339 |
| hsa-miR-5691 | hsa_circRNA_103076 | -0.314 | -0.304 | 158 | -19.25 | 0 | 0 | 0 | 0 | 0 | 0 | 1 | 0 | 0 | 0 | 0 | 0 | 339 |
| hsa-miR-5707 | hsa_circRNA_103076 | -0.414 | -0.397 | 148 | -11.41 | 0 | 0 | 0 | 0 | 0 | 0 | 1 | 0 | 0 | 0 | 0 | 0 | 339 |
| hsa-miR-588 | hsa_circRNA_103076 | -0.305 | -0.28 | 147 | -17.87 | 0 | 0 | 0 | 0 | 0 | 0 | 0 | 1 | 0 | 0 | 0 | 0 | 339 |
| hsa-miR-6777-3p | hsa_circRNA_103076 | -0.365 | -0.337 | 142 | -16.96 | 0 | 0 | 0 | 0 | 0 | 0 | 1 | 0 | 0 | 0 | 0 | 0 | 339 |
| hsa-miR-6829-5p | hsa_circRNA_103076 | -0.525 | -0.412 | 158 | -25.96 | 0 | 0 | 0 | 0 | 0 | 0 | 1 | 0 | 0 | 0 | 0 | 0 | 339 |
| hsa-miR-7112-3p | hsa_circRNA_103076 | -0.453 | -0.474 | 143 | -14.94 | 0 | 0 | 0 | 0 | 0 | 0 | 1 | 0 | 0 | 0 | 0 | 0 | 339 |
| hsa-miR-7114-5p | hsa_circRNA_103076 | -0.381 | -0.437 | 141 | -19.3 | 0 | 0 | 0 | 0 | 0 | 0 | 1 | 0 | 0 | 0 | 0 | 0 | 339 |
| hsa-miR-3663-5p | hsa_circRNA_103089 | -0.318 | -0.254 | 146 | -26.97 | 0 | 0 | 0 | 0 | 0 | 0 | 1 | 0 | 0 | 0 | 0 | 0 | 259 |
| hsa-miR-425-3p | hsa_circRNA_103089 | -0.512 | -0.362 | 144 | -13.85 | 0 | 0 | 0 | 0 | 0 | 0 | 1 | 0 | 0 | 0 | 0 | 0 | 259 |
| hsa-miR-744-5p | hsa_circRNA_103089 | -0.525 | -0.189 | 155 | -29.45 | 0 | 0 | 0 | 0 | 0 | 0 | 1 | 0 | 0 | 0 | 0 | 0 | 259 |
| hsa-miR-17-3p | hsa_circRNA_103285 | -0.379 | -0.382 | 156 | -23.24 | 0 | 0 | 0 | 0 | 0 | 0 | 1 | 0 | 0 | 0 | 0 | 0 | 196 |
| hsa-miR-335-5p | hsa_circRNA_103285 | -0.361 | -0.394 | 147 | -14.59 | 0 | 0 | 0 | 0 | 0 | 0 | 1 | 0 | 0 | 0 | 0 | 0 | 196 |
| hsa-miR-4694-5p | hsa_circRNA_103285 | -0.374 | -0.4 | 149 | -14.4 | 0 | 0 | 0 | 0 | 0 | 0 | 1 | 0 | 0 | 0 | 0 | 0 | 196 |
| hsa-miR-708-5p | hsa_circRNA_103285 | -0.308 | -0.318 | 159 | -21.07 | 0 | 0 | 0 | 0 | 0 | 0 | 0 | 1 | 0 | 0 | 0 | 0 | 196 |
| hsa-let-7a-5p | hsa_circRNA_103390 | -0.365 | -0.344 | 150 | -20.36 | 0 | 0 | 0 | 0 | 0 | 0 | 1 | 0 | 0 | 0 | 0 | 0 | 234 |
| hsa-let-7b-5p | hsa_circRNA_103390 | -0.365 | -0.344 | 154 | -18.82 | 0 | 0 | 0 | 0 | 0 | 0 | 1 | 0 | 0 | 0 | 0 | 0 | 234 |
| hsa-let-7c-5p | hsa_circRNA_103390 | -0.365 | -0.344 | 150 | -18.34 | 0 | 0 | 0 | 0 | 0 | 0 | 1 | 0 | 0 | 0 | 0 | 0 | 234 |
| hsa-let-7d-5p | hsa_circRNA_103390 | -0.376 | -0.346 | 151 | -18.48 | 0 | 0 | 0 | 0 | 0 | 0 | 1 | 0 | 0 | 0 | 0 | 0 | 234 |
| hsa-let-7e-5p | hsa_circRNA_103390 | -0.355 | -0.342 | 142 | -19.95 | 0 | 0 | 0 | 0 | 0 | 0 | 1 | 0 | 0 | 0 | 0 | 0 | 234 |
| hsa-let-7f-5p | hsa_circRNA_103390 | -0.386 | -0.348 | 149 | -20.13 | 0 | 0 | 0 | 0 | 0 | 0 | 1 | 0 | 0 | 0 | 0 | 0 | 234 |
| hsa-let-7g-5p | hsa_circRNA_103390 | -0.365 | -0.344 | 155 | -21.29 | 0 | 0 | 0 | 0 | 0 | 0 | 1 | 0 | 0 | 0 | 0 | 0 | 234 |
| hsa-let-7i-5p | hsa_circRNA_103390 | -0.365 | -0.344 | 150 | -19.55 | 0 | 0 | 0 | 0 | 0 | 0 | 1 | 0 | 0 | 0 | 0 | 0 | 234 |
| hsa-miR-1229-5p | hsa_circRNA_103390 | -0.35 | -0.182 | 159 | -26.75 | 0 | 0 | 0 | 0 | 0 | 0 | 1 | 0 | 0 | 0 | 0 | 0 | 234 |
| hsa-miR-1273g-3p | hsa_circRNA_103390 | -0.354 | -0.303 | 150 | -17.28 | 0 | 0 | 0 | 0 | 0 | 0 | 1 | 0 | 0 | 0 | 0 | 0 | 234 |
| hsa-miR-15a-3p | hsa_circRNA_103390 | -0.372 | -0.307 | 145 | -16.19 | 0 | 0 | 0 | 0 | 0 | 0 | 1 | 0 | 0 | 0 | 0 | 0 | 234 |
| hsa-miR-328-5p | hsa_circRNA_103390 | -0.305 | -0.147 | 147 | -25.91 | 0 | 0 | 0 | 0 | 0 | 0 | 0 | 1 | 0 | 0 | 0 | 0 | 234 |
| hsa-miR-4458 | hsa_circRNA_103390 | -0.355 | -0.342 | 140 | -13.39 | 0 | 0 | 0 | 0 | 0 | 0 | 1 | 0 | 0 | 0 | 0 | 0 | 234 |
| hsa-miR-4500 | hsa_circRNA_103390 | -0.365 | -0.344 | 149 | -17.79 | 0 | 0 | 0 | 0 | 0 | 0 | 1 | 0 | 0 | 0 | 0 | 0 | 234 |
| hsa-miR-4690-3p | hsa_circRNA_103390 | -0.39 | -0.283 | 161 | -25.99 | 0 | 0 | 0 | 0 | 0 | 0 | 1 | 0 | 0 | 0 | 0 | 0 | 234 |
| hsa-miR-5685 | hsa_circRNA_103390 | -0.369 | -0.279 | 147 | -24.08 | 0 | 0 | 0 | 0 | 0 | 0 | 1 | 0 | 0 | 0 | 0 | 0 | 234 |
| hsa-miR-597-3p | hsa_circRNA_103390 | -0.318 | -0.265 | 153 | -22.42 | 0 | 0 | 0 | 0 | 0 | 0 | 1 | 0 | 0 | 0 | 0 | 0 | 234 |
| hsa-miR-6885-5p | hsa_circRNA_103390 | -0.305 | -0.147 | 148 | -23.65 | 0 | 0 | 0 | 0 | 0 | 0 | 0 | 1 | 0 | 0 | 0 | 0 | 234 |
| hsa-miR-98-5p | hsa_circRNA_103390 | -0.365 | -0.344 | 154 | -18.23 | 0 | 0 | 0 | 0 | 0 | 0 | 1 | 0 | 0 | 0 | 0 | 0 | 234 |
| hsa-miR-1231 | hsa_circRNA_103655 | -0.433 | -0.465 | 141 | -15.88 | 0 | 0 | 0 | 0 | 0 | 0 | 1 | 0 | 0 | 0 | 0 | 0 | 1634 |
| hsa-miR-1287-5p | hsa_circRNA_103655 | -0.377 | -0.427 | 433 | -57.72 | 0 | 0 | 0 | 0 | 0 | 0 | 1 | 0 | 0 | 0 | 2 | 0 | 1634 |
| hsa-miR-1288-3p | hsa_circRNA_103655 | -0.607 | -0.505 | 438 | -54.54 | 0 | 0 | 0 | 0 | 0 | 0 | 1 | 1 | 0 | 0 | 0 | 1 | 1634 |
| hsa-miR-1294 | hsa_circRNA_103655 | -0.327 | -0.296 | 147 | -16.18 | 0 | 0 | 0 | 0 | 0 | 0 | 1 | 0 | 0 | 0 | 0 | 0 | 1634 |
| hsa-miR-143-3p | hsa_circRNA_103655 | -0.343 | -0.412 | 305 | -41.29 | 0 | 0 | 0 | 0 | 0 | 0 | 1 | 0 | 0 | 0 | 1 | 0 | 1634 |
| hsa-miR-15a-5p | hsa_circRNA_103655 | -0.502 | -0.449 | 298 | -38.57 | 0 | 0 | 0 | 0 | 0 | 0 | 1 | 1 | 0 | 0 | 0 | 0 | 1634 |
| hsa-miR-15b-5p | hsa_circRNA_103655 | -0.502 | -0.449 | 296 | -30.46 | 0 | 0 | 0 | 0 | 0 | 0 | 1 | 1 | 0 | 0 | 0 | 0 | 1634 |
| hsa-miR-16-5p | hsa_circRNA_103655 | -0.477 | -0.485 | 302 | -34.05 | 0 | 0 | 0 | 0 | 0 | 0 | 1 | 1 | 0 | 0 | 0 | 0 | 1634 |
| hsa-miR-182-5p | hsa_circRNA_103655 | -0.379 | -0.475 | 145 | -11.63 | 0 | 0 | 0 | 0 | 0 | 0 | 1 | 0 | 0 | 0 | 0 | 0 | 1634 |
| hsa-miR-184 | hsa_circRNA_103655 | -0.452 | -0.256 | 146 | -23.26 | 0 | 0 | 0 | 0 | 0 | 0 | 1 | 0 | 0 | 0 | 0 | 0 | 1634 |
| hsa-miR-1911-3p | hsa_circRNA_103655 | -0.576 | -0.428 | 309 | -34.49 | 0 | 0 | 0 | 0 | 0 | 0 | 1 | 1 | 0 | 0 | 0 | 0 | 1634 |
| hsa-miR-195-5p | hsa_circRNA_103655 | -0.457 | -0.467 | 290 | -26.92 | 0 | 0 | 0 | 0 | 0 | 0 | 1 | 1 | 0 | 0 | 0 | 0 | 1634 |
| hsa-miR-298 | hsa_circRNA_103655 | -0.515 | -0.62 | 464 | -64.05 | 0 | 0 | 0 | 0 | 0 | 0 | 1 | 1 | 0 | 0 | 1 | 0 | 1634 |
| hsa-miR-3147 | hsa_circRNA_103655 | -0.415 | -0.359 | 436 | -76.14 | 0 | 0 | 0 | 0 | 0 | 0 | 1 | 0 | 0 | 0 | 0 | 2 | 1634 |
| hsa-miR-3184-5p | hsa_circRNA_103655 | -0.396 | -0.311 | 281 | -43.24 | 0 | 0 | 0 | 0 | 0 | 0 | 1 | 0 | 0 | 0 | 0 | 1 | 1634 |
| hsa-miR-323a-5p | hsa_circRNA_103655 | -0.392 | -0.336 | 293 | -40.32 | 0 | 0 | 0 | 0 | 0 | 0 | 0 | 2 | 0 | 0 | 0 | 0 | 1634 |
| hsa-miR-330-3p | hsa_circRNA_103655 | -0.408 | -0.657 | 284 | -32.13 | 0 | 0 | 0 | 0 | 0 | 0 | 1 | 1 | 0 | 0 | 0 | 0 | 1634 |
| hsa-miR-3692-5p | hsa_circRNA_103655 | -0.35 | -0.322 | 149 | -19.87 | 0 | 0 | 0 | 0 | 0 | 0 | 1 | 0 | 0 | 0 | 0 | 0 | 1634 |
| hsa-miR-423-5p | hsa_circRNA_103655 | -0.428 | -0.317 | 153 | -23.75 | 0 | 0 | 0 | 0 | 0 | 0 | 1 | 0 | 0 | 0 | 0 | 0 | 1634 |
| hsa-miR-424-5p | hsa_circRNA_103655 | -0.43 | -0.421 | 293 | -30.4 | 0 | 0 | 0 | 0 | 0 | 0 | 1 | 1 | 0 | 0 | 0 | 0 | 1634 |
| hsa-miR-4269 | hsa_circRNA_103655 | -0.329 | -0.306 | 306 | -42.98 | 0 | 0 | 0 | 0 | 0 | 0 | 0 | 2 | 0 | 0 | 0 | 0 | 1634 |
| hsa-miR-4316 | hsa_circRNA_103655 | -0.321 | -0.282 | 150 | -20.94 | 0 | 0 | 0 | 0 | 0 | 0 | 1 | 0 | 0 | 0 | 0 | 0 | 1634 |
| hsa-miR-4324 | hsa_circRNA_103655 | -0.387 | -0.482 | 434 | -42.54 | 0 | 0 | 0 | 0 | 0 | 0 | 1 | 2 | 0 | 0 | 0 | 0 | 1634 |
| hsa-miR-4446-3p | hsa_circRNA_103655 | -0.573 | -0.501 | 421 | -60.14 | 0 | 0 | 0 | 0 | 0 | 0 | 1 | 1 | 0 | 0 | 1 | 0 | 1634 |
| hsa-miR-4456 | hsa_circRNA_103655 | -0.407 | -0.368 | 433 | -44.86 | 0 | 0 | 0 | 0 | 0 | 0 | 0 | 3 | 0 | 0 | 0 | 0 | 1634 |
| hsa-miR-4468 | hsa_circRNA_103655 | -0.444 | -0.448 | 299 | -28.42 | 0 | 0 | 0 | 0 | 0 | 0 | 1 | 1 | 0 | 0 | 0 | 0 | 1634 |
| hsa-miR-4487 | hsa_circRNA_103655 | -0.347 | -0.305 | 156 | -20.23 | 0 | 0 | 0 | 0 | 0 | 0 | 1 | 0 | 0 | 0 | 0 | 0 | 1634 |
| hsa-miR-4494 | hsa_circRNA_103655 | -0.416 | -0.458 | 287 | -36.36 | 0 | 0 | 0 | 0 | 0 | 0 | 1 | 0 | 0 | 0 | 0 | 1 | 1634 |
| hsa-miR-4505 | hsa_circRNA_103655 | -0.325 | -0.174 | 300 | -46.78 | 0 | 0 | 0 | 0 | 0 | 0 | 1 | 1 | 0 | 0 | 0 | 0 | 1634 |
| hsa-miR-4524a-5p | hsa_circRNA_103655 | -0.373 | -0.34 | 146 | -12.03 | 0 | 0 | 0 | 0 | 0 | 0 | 1 | 0 | 0 | 0 | 0 | 0 | 1634 |
| hsa-miR-4524b-5p | hsa_circRNA_103655 | -0.373 | -0.34 | 145 | -10.53 | 0 | 0 | 0 | 0 | 0 | 0 | 1 | 0 | 0 | 0 | 0 | 0 | 1634 |
| hsa-miR-4675 | hsa_circRNA_103655 | -0.427 | -0.383 | 295 | -35.69 | 0 | 0 | 0 | 0 | 0 | 0 | 0 | 2 | 0 | 0 | 0 | 0 | 1634 |
| hsa-miR-4741 | hsa_circRNA_103655 | -0.446 | -0.414 | 287 | -37.33 | 0 | 0 | 0 | 0 | 0 | 0 | 0 | 2 | 0 | 0 | 0 | 0 | 1634 |
| hsa-miR-4770 | hsa_circRNA_103655 | -0.307 | -0.374 | 156 | -16.18 | 0 | 0 | 0 | 0 | 0 | 0 | 1 | 0 | 0 | 0 | 0 | 0 | 1634 |
| hsa-miR-486-3p | hsa_circRNA_103655 | -0.576 | -0.453 | 286 | -33.18 | 0 | 0 | 0 | 0 | 0 | 0 | 1 | 1 | 0 | 0 | 0 | 0 | 1634 |
| hsa-miR-497-5p | hsa_circRNA_103655 | -0.514 | -0.437 | 288 | -31.19 | 0 | 0 | 0 | 0 | 0 | 0 | 1 | 1 | 0 | 0 | 0 | 0 | 1634 |
| hsa-miR-548au-3p | hsa_circRNA_103655 | -0.373 | -0.351 | 280 | -25.25 | 0 | 0 | 0 | 0 | 0 | 0 | 1 | 1 | 0 | 0 | 0 | 0 | 1634 |
| hsa-miR-5787 | hsa_circRNA_103655 | -0.324 | -0.188 | 291 | -45.76 | 0 | 0 | 0 | 0 | 0 | 0 | 1 | 1 | 0 | 0 | 0 | 0 | 1634 |
| hsa-miR-601 | hsa_circRNA_103655 | -0.4 | -0.365 | 307 | -43.99 | 0 | 0 | 0 | 0 | 0 | 0 | 0 | 1 | 1 | 0 | 0 | 0 | 1634 |
| hsa-miR-6505-5p | hsa_circRNA_103655 | -0.311 | -0.438 | 147 | -16.15 | 0 | 0 | 0 | 0 | 0 | 0 | 1 | 0 | 0 | 0 | 0 | 0 | 1634 |
| hsa-miR-6510-5p | hsa_circRNA_103655 | -0.316 | -0.275 | 145 | -16.88 | 0 | 0 | 0 | 0 | 0 | 0 | 1 | 0 | 0 | 0 | 0 | 0 | 1634 |
| hsa-miR-6511b-5p | hsa_circRNA_103655 | -0.481 | -0.385 | 170 | -31.4 | 0 | 0 | 0 | 0 | 0 | 0 | 1 | 0 | 0 | 0 | 0 | 0 | 1634 |
| hsa-miR-653-3p | hsa_circRNA_103655 | -0.355 | -0.375 | 292 | -27.43 | 0 | 0 | 0 | 0 | 0 | 0 | 1 | 1 | 0 | 0 | 0 | 0 | 1634 |
| hsa-miR-6715b-5p | hsa_circRNA_103655 | -0.311 | -0.275 | 296 | -39.52 | 0 | 0 | 0 | 0 | 0 | 0 | 0 | 2 | 0 | 0 | 0 | 0 | 1634 |
| hsa-miR-6805-5p | hsa_circRNA_103655 | -0.318 | -0.224 | 145 | -25.13 | 0 | 0 | 0 | 0 | 0 | 0 | 0 | 1 | 0 | 0 | 0 | 0 | 1634 |
| hsa-miR-6811-5p | hsa_circRNA_103655 | -0.45 | -0.379 | 148 | -18.3 | 0 | 0 | 0 | 0 | 0 | 0 | 1 | 0 | 0 | 0 | 0 | 0 | 1634 |
| hsa-miR-6838-5p | hsa_circRNA_103655 | -0.518 | -0.506 | 311 | -37.64 | 0 | 0 | 0 | 0 | 0 | 0 | 1 | 1 | 0 | 0 | 0 | 0 | 1634 |
| hsa-miR-6857-3p | hsa_circRNA_103655 | -0.369 | -0.408 | 142 | -16.69 | 0 | 0 | 0 | 0 | 0 | 0 | 1 | 0 | 0 | 0 | 0 | 0 | 1634 |
| hsa-miR-7160-3p | hsa_circRNA_103655 | -0.352 | -0.262 | 141 | -17.7 | 0 | 0 | 0 | 0 | 0 | 0 | 0 | 1 | 0 | 0 | 0 | 0 | 1634 |
| hsa-miR-7160-5p | hsa_circRNA_103655 | -0.409 | -0.432 | 140 | -17.1 | 0 | 0 | 0 | 0 | 0 | 0 | 1 | 0 | 0 | 0 | 0 | 0 | 1634 |
| hsa-miR-765 | hsa_circRNA_103655 | -0.304 | -0.291 | 287 | -40.81 | 0 | 0 | 0 | 0 | 0 | 0 | 1 | 0 | 0 | 0 | 0 | 1 | 1634 |
| hsa-miR-7847-3p | hsa_circRNA_103655 | -0.348 | -0.326 | 152 | -19.41 | 0 | 0 | 0 | 0 | 0 | 0 | 1 | 0 | 0 | 0 | 0 | 0 | 1634 |
| hsa-miR-7978 | hsa_circRNA_103655 | -0.405 | -0.415 | 144 | -13.9 | 0 | 0 | 0 | 0 | 0 | 0 | 1 | 0 | 0 | 0 | 0 | 0 | 1634 |
| hsa-miR-8071 | hsa_circRNA_103655 | -0.615 | -0.472 | 436 | -59.99 | 0 | 0 | 0 | 0 | 0 | 0 | 2 | 0 | 0 | 0 | 0 | 1 | 1634 |
| hsa-miR-8075 | hsa_circRNA_103655 | -0.636 | -0.661 | 581 | -74.61 | 0 | 0 | 0 | 0 | 0 | 0 | 1 | 2 | 0 | 0 | 0 | 1 | 1634 |
| hsa-miR-128-1-5p | hsa_circRNA_104003 | -0.457 | -0.189 | 149 | -24.89 | 0 | 0 | 0 | 0 | 0 | 0 | 1 | 0 | 0 | 0 | 0 | 0 | 372 |
| hsa-miR-128-2-5p | hsa_circRNA_104003 | -0.415 | -0.181 | 140 | -20.27 | 0 | 0 | 0 | 0 | 0 | 0 | 1 | 0 | 0 | 0 | 0 | 0 | 372 |
| hsa-miR-1291 | hsa_circRNA_104003 | -0.413 | -0.226 | 149 | -26.25 | 0 | 0 | 0 | 0 | 0 | 0 | 1 | 0 | 0 | 0 | 0 | 0 | 372 |
| hsa-miR-1825 | hsa_circRNA_104003 | -0.318 | -0.206 | 145 | -17.59 | 0 | 0 | 0 | 0 | 0 | 0 | 1 | 0 | 0 | 0 | 0 | 0 | 372 |
| hsa-miR-25-5p | hsa_circRNA_104003 | -0.422 | -0.184 | 295 | -41.56 | 0 | 0 | 0 | 0 | 0 | 0 | 1 | 0 | 0 | 0 | 0 | 1 | 372 |
| hsa-miR-296-5p | hsa_circRNA_104003 | -0.504 | -0.238 | 141 | -18.35 | 0 | 0 | 0 | 0 | 0 | 0 | 1 | 0 | 0 | 0 | 0 | 0 | 372 |
| hsa-miR-4492 | hsa_circRNA_104003 | -0.415 | -0.303 | 144 | -19.5 | 0 | 0 | 0 | 0 | 0 | 0 | 1 | 0 | 0 | 0 | 0 | 0 | 372 |
| hsa-miR-4498 | hsa_circRNA_104003 | -0.425 | -0.305 | 143 | -23.59 | 0 | 0 | 0 | 0 | 0 | 0 | 1 | 0 | 0 | 0 | 0 | 0 | 372 |
| hsa-miR-4527 | hsa_circRNA_104003 | -0.419 | -0.277 | 150 | -20.17 | 0 | 0 | 0 | 0 | 0 | 0 | 1 | 0 | 0 | 0 | 0 | 0 | 372 |
| hsa-miR-4697-3p | hsa_circRNA_104003 | -0.327 | -0.219 | 159 | -24.26 | 0 | 0 | 0 | 0 | 0 | 0 | 1 | 0 | 0 | 0 | 0 | 0 | 372 |
| hsa-miR-4730 | hsa_circRNA_104003 | -0.494 | -0.152 | 179 | -33.08 | 0 | 0 | 0 | 0 | 0 | 0 | 1 | 0 | 0 | 0 | 0 | 0 | 372 |
| hsa-miR-5001-5p | hsa_circRNA_104003 | -0.446 | -0.309 | 156 | -24.08 | 0 | 0 | 0 | 0 | 0 | 0 | 1 | 0 | 0 | 0 | 0 | 0 | 372 |
| hsa-miR-541-3p | hsa_circRNA_104003 | -0.529 | -0.237 | 296 | -43.77 | 0 | 0 | 0 | 0 | 0 | 0 | 1 | 1 | 0 | 0 | 0 | 0 | 372 |
| hsa-miR-5693 | hsa_circRNA_104003 | -0.311 | -0.231 | 149 | -18.1 | 0 | 0 | 0 | 0 | 0 | 0 | 1 | 0 | 0 | 0 | 0 | 0 | 372 |
| hsa-miR-6503-5p | hsa_circRNA_104003 | -0.409 | -0.275 | 149 | -14.82 | 0 | 0 | 0 | 0 | 0 | 0 | 1 | 0 | 0 | 0 | 0 | 0 | 372 |
| hsa-miR-654-5p | hsa_circRNA_104003 | -0.537 | -0.266 | 299 | -43.76 | 0 | 0 | 0 | 0 | 0 | 0 | 1 | 1 | 0 | 0 | 0 | 0 | 372 |
| hsa-miR-6775-3p | hsa_circRNA_104003 | -0.413 | -0.226 | 281 | -42.91 | 0 | 0 | 0 | 0 | 0 | 0 | 1 | 0 | 0 | 0 | 0 | 1 | 372 |
| hsa-miR-6814-3p | hsa_circRNA_104003 | -0.399 | -0.203 | 146 | -20.18 | 0 | 0 | 0 | 0 | 0 | 0 | 1 | 0 | 0 | 0 | 0 | 0 | 372 |
| hsa-miR-6872-5p | hsa_circRNA_104003 | -0.43 | -0.209 | 158 | -27.3 | 0 | 0 | 0 | 0 | 0 | 0 | 1 | 0 | 0 | 0 | 0 | 0 | 372 |
| hsa-miR-762 | hsa_circRNA_104003 | -0.436 | -0.307 | 145 | -29.59 | 0 | 0 | 0 | 0 | 0 | 0 | 1 | 0 | 0 | 0 | 0 | 0 | 372 |
| hsa-miR-7704 | hsa_circRNA_104003 | -0.375 | -0.177 | 289 | -51.68 | 0 | 0 | 0 | 0 | 0 | 0 | 1 | 0 | 0 | 0 | 0 | 1 | 372 |
| hsa-miR-3907 | hsa_circRNA_104310 | -0.476 | -0.337 | 151 | -20.02 | 0 | 0 | 0 | 0 | 0 | 0 | 1 | 0 | 0 | 0 | 0 | 0 | 245 |
| hsa-miR-3913-3p | hsa_circRNA_104310 | -0.3 | -0.29 | 151 | -15.29 | 0 | 0 | 0 | 0 | 0 | 0 | 1 | 0 | 0 | 0 | 0 | 0 | 245 |
| hsa-miR-3974 | hsa_circRNA_104310 | -0.324 | -0.372 | 305 | -34.08 | 0 | 0 | 0 | 0 | 0 | 0 | 0 | 2 | 0 | 0 | 0 | 0 | 245 |
| hsa-miR-4800-5p | hsa_circRNA_104310 | -0.332 | -0.287 | 158 | -25.72 | 0 | 0 | 0 | 0 | 0 | 0 | 0 | 1 | 0 | 0 | 0 | 0 | 245 |
| hsa-miR-1228-3p | hsa_circRNA_104313 | -0.336 | -0.34 | 147 | -16.91 | 0 | 0 | 0 | 0 | 0 | 0 | 1 | 0 | 0 | 0 | 0 | 0 | 291 |
| hsa-miR-3925-3p | hsa_circRNA_104313 | -0.334 | -0.36 | 148 | -20.01 | 0 | 0 | 0 | 0 | 0 | 0 | 1 | 0 | 0 | 0 | 0 | 0 | 291 |
| hsa-miR-4277 | hsa_circRNA_104313 | -0.308 | -0.439 | 143 | -13.44 | 0 | 0 | 0 | 0 | 0 | 0 | 1 | 0 | 0 | 0 | 0 | 0 | 291 |
| hsa-miR-623 | hsa_circRNA_104313 | -0.32 | -0.315 | 157 | -22.25 | 0 | 0 | 0 | 0 | 0 | 0 | 1 | 0 | 0 | 0 | 0 | 0 | 291 |
| hsa-miR-670-3p | hsa_circRNA_104313 | -0.432 | -0.52 | 295 | -25.72 | 0 | 0 | 0 | 0 | 0 | 0 | 1 | 1 | 0 | 0 | 0 | 0 | 291 |
| hsa-miR-7110-3p | hsa_circRNA_104313 | -0.368 | -0.422 | 141 | -19.38 | 0 | 0 | 0 | 0 | 0 | 0 | 1 | 0 | 0 | 0 | 0 | 0 | 291 |
| hsa-miR-7151-5p | hsa_circRNA_104313 | -0.372 | -0.32 | 166 | -24.11 | 0 | 0 | 0 | 0 | 0 | 0 | 1 | 0 | 0 | 0 | 0 | 0 | 291 |
| hsa-miR-1251-3p | hsa_circRNA_104759 | -0.31 | -0.299 | 159 | -19.12 | 0 | 0 | 0 | 0 | 0 | 0 | 1 | 0 | 0 | 0 | 0 | 0 | 472 |
| hsa-miR-135b-3p | hsa_circRNA_104759 | -0.415 | -0.37 | 140 | -12.07 | 0 | 0 | 0 | 0 | 0 | 0 | 1 | 0 | 0 | 0 | 0 | 0 | 472 |
| hsa-miR-3135b | hsa_circRNA_104759 | -0.395 | -0.314 | 163 | -30.47 | 0 | 0 | 0 | 0 | 0 | 0 | 1 | 0 | 0 | 0 | 0 | 0 | 472 |
| hsa-miR-3136-3p | hsa_circRNA_104759 | -0.441 | -0.337 | 149 | -19.19 | 0 | 0 | 0 | 0 | 0 | 0 | 1 | 0 | 0 | 0 | 0 | 0 | 472 |
| hsa-miR-3150b-3p | hsa_circRNA_104759 | -0.306 | -0.271 | 148 | -21.66 | 0 | 0 | 0 | 0 | 0 | 0 | 1 | 0 | 0 | 0 | 0 | 0 | 472 |
| hsa-miR-3188 | hsa_circRNA_104759 | -0.32 | -0.27 | 162 | -28.31 | 0 | 0 | 0 | 0 | 0 | 0 | 1 | 0 | 0 | 0 | 0 | 0 | 472 |
| hsa-miR-3192-5p | hsa_circRNA_104759 | -0.346 | -0.323 | 149 | -19.79 | 0 | 0 | 0 | 0 | 0 | 0 | 1 | 0 | 0 | 0 | 0 | 0 | 472 |
| hsa-miR-3692-5p | hsa_circRNA_104759 | -0.395 | -0.36 | 153 | -21.84 | 0 | 0 | 0 | 0 | 0 | 0 | 1 | 0 | 0 | 0 | 0 | 0 | 472 |
| hsa-miR-3928-3p | hsa_circRNA_104759 | -0.331 | -0.309 | 150 | -26.2 | 0 | 0 | 0 | 0 | 0 | 0 | 1 | 0 | 0 | 0 | 0 | 0 | 472 |
| hsa-miR-4300 | hsa_circRNA_104759 | -0.395 | -0.286 | 150 | -22.67 | 0 | 0 | 0 | 0 | 0 | 0 | 1 | 0 | 0 | 0 | 0 | 0 | 472 |
| hsa-miR-4425 | hsa_circRNA_104759 | -0.382 | -0.316 | 155 | -20.9 | 0 | 0 | 0 | 0 | 0 | 0 | 1 | 0 | 0 | 0 | 0 | 0 | 472 |
| hsa-miR-4649-3p | hsa_circRNA_104759 | -0.398 | -0.373 | 156 | -17.06 | 0 | 0 | 0 | 0 | 0 | 0 | 1 | 0 | 0 | 0 | 0 | 0 | 472 |
| hsa-miR-4651 | hsa_circRNA_104759 | -0.471 | -0.372 | 148 | -22.42 | 0 | 0 | 0 | 0 | 0 | 0 | 1 | 0 | 0 | 0 | 0 | 0 | 472 |
| hsa-miR-4776-3p | hsa_circRNA_104759 | -0.326 | -0.372 | 145 | -15.3 | 0 | 0 | 0 | 0 | 0 | 0 | 1 | 0 | 0 | 0 | 0 | 0 | 472 |
| hsa-miR-4784 | hsa_circRNA_104759 | -0.306 | -0.271 | 146 | -19.95 | 0 | 0 | 0 | 0 | 0 | 0 | 1 | 0 | 0 | 0 | 0 | 0 | 472 |
| hsa-miR-548s | hsa_circRNA_104759 | -0.31 | -0.265 | 301 | -35.74 | 0 | 0 | 0 | 0 | 0 | 0 | 1 | 0 | 0 | 0 | 0 | 1 | 472 |
| hsa-miR-5591-5p | hsa_circRNA_104759 | -0.426 | -0.292 | 142 | -16.42 | 0 | 0 | 0 | 0 | 0 | 0 | 1 | 0 | 0 | 0 | 0 | 0 | 472 |
| hsa-miR-6071 | hsa_circRNA_104759 | -0.487 | -0.512 | 280 | -29.45 | 0 | 0 | 0 | 0 | 0 | 0 | 1 | 1 | 0 | 0 | 0 | 0 | 472 |
| hsa-miR-608 | hsa_circRNA_104759 | -0.471 | -0.372 | 165 | -27.67 | 0 | 0 | 0 | 0 | 0 | 0 | 1 | 0 | 0 | 0 | 0 | 0 | 472 |
| hsa-miR-6081 | hsa_circRNA_104759 | -0.422 | -0.363 | 294 | -47.64 | 0 | 0 | 0 | 0 | 0 | 0 | 1 | 0 | 0 | 0 | 0 | 1 | 472 |
| hsa-miR-6726-5p | hsa_circRNA_104759 | -0.405 | -0.288 | 155 | -24.93 | 0 | 0 | 0 | 0 | 0 | 0 | 1 | 0 | 0 | 0 | 0 | 0 | 472 |
| hsa-miR-6737-5p | hsa_circRNA_104759 | -0.405 | -0.29 | 158 | -29.87 | 0 | 0 | 0 | 0 | 0 | 0 | 1 | 0 | 0 | 0 | 0 | 0 | 472 |
| hsa-miR-6812-5p | hsa_circRNA_104759 | -0.374 | -0.284 | 146 | -21.72 | 0 | 0 | 0 | 0 | 0 | 0 | 1 | 0 | 0 | 0 | 0 | 0 | 472 |
| hsa-miR-6815-3p | hsa_circRNA_104759 | -0.444 | -0.417 | 287 | -29.92 | 0 | 0 | 0 | 0 | 0 | 0 | 1 | 1 | 0 | 0 | 0 | 0 | 472 |
| hsa-miR-6819-5p | hsa_circRNA_104759 | -0.374 | -0.284 | 152 | -24.45 | 0 | 0 | 0 | 0 | 0 | 0 | 1 | 0 | 0 | 0 | 0 | 0 | 472 |
| hsa-miR-6846-5p | hsa_circRNA_104759 | -0.51 | -0.356 | 149 | -27.82 | 0 | 0 | 0 | 0 | 0 | 0 | 1 | 0 | 0 | 0 | 0 | 0 | 472 |
| hsa-miR-6848-5p | hsa_circRNA_104759 | -0.521 | -0.358 | 305 | -63.32 | 0 | 0 | 0 | 0 | 0 | 0 | 1 | 0 | 0 | 0 | 0 | 1 | 472 |
| hsa-miR-7155-3p | hsa_circRNA_104759 | -0.452 | -0.339 | 150 | -19.05 | 0 | 0 | 0 | 0 | 0 | 0 | 1 | 0 | 0 | 0 | 0 | 0 | 472 |
| hsa-miR-920 | hsa_circRNA_104759 | -0.395 | -0.286 | 151 | -19.68 | 0 | 0 | 0 | 0 | 0 | 0 | 1 | 0 | 0 | 0 | 0 | 0 | 472 |
| hsa-miR-1197 | hsa_circRNA_400027 | -0.302 | -0.282 | 153 | -21.75 | 0 | 0 | 0 | 0 | 0 | 0 | 1 | 0 | 0 | 0 | 0 | 0 | 1180 |
| hsa-miR-1295b-5p | hsa_circRNA_400027 | -0.559 | -0.469 | 456 | -47.72 | 0 | 0 | 0 | 0 | 0 | 0 | 1 | 2 | 0 | 0 | 0 | 0 | 1180 |
| hsa-miR-1912 | hsa_circRNA_400027 | -0.539 | -0.451 | 466 | -60.75 | 0 | 0 | 0 | 0 | 0 | 0 | 1 | 2 | 0 | 0 | 0 | 0 | 1180 |
| hsa-miR-1914-5p | hsa_circRNA_400027 | -0.371 | -0.335 | 147 | -23.09 | 0 | 0 | 0 | 0 | 0 | 0 | 1 | 0 | 0 | 0 | 0 | 0 | 1180 |
| hsa-miR-214-5p | hsa_circRNA_400027 | -0.392 | -0.35 | 150 | -20.01 | 0 | 0 | 0 | 0 | 0 | 0 | 1 | 0 | 0 | 0 | 0 | 0 | 1180 |
| hsa-miR-2467-3p | hsa_circRNA_400027 | -0.448 | -0.399 | 160 | -21.82 | 0 | 0 | 0 | 0 | 0 | 0 | 1 | 0 | 0 | 0 | 0 | 0 | 1180 |
| hsa-miR-328-3p | hsa_circRNA_400027 | -0.463 | -0.288 | 168 | -29.38 | 0 | 0 | 0 | 0 | 0 | 0 | 1 | 0 | 0 | 0 | 0 | 0 | 1180 |
| hsa-miR-3922-5p | hsa_circRNA_400027 | -0.329 | -0.273 | 148 | -22.6 | 0 | 0 | 0 | 0 | 0 | 0 | 1 | 0 | 0 | 0 | 0 | 0 | 1180 |
| hsa-miR-4462 | hsa_circRNA_400027 | -0.47 | -0.311 | 152 | -22.05 | 0 | 0 | 0 | 0 | 0 | 0 | 1 | 0 | 0 | 0 | 0 | 0 | 1180 |
| hsa-miR-4529-5p | hsa_circRNA_400027 | -0.383 | -0.304 | 153 | -18.66 | 0 | 0 | 0 | 0 | 0 | 0 | 1 | 0 | 0 | 0 | 0 | 0 | 1180 |
| hsa-miR-4685-5p | hsa_circRNA_400027 | -0.468 | -0.344 | 317 | -63.07 | 0 | 0 | 0 | 0 | 0 | 0 | 1 | 0 | 0 | 0 | 0 | 1 | 1180 |
| hsa-miR-4722-3p | hsa_circRNA_400027 | -0.374 | -0.317 | 149 | -20.47 | 0 | 0 | 0 | 0 | 0 | 0 | 1 | 0 | 0 | 0 | 0 | 0 | 1180 |
| hsa-miR-4769-3p | hsa_circRNA_400027 | -0.43 | -0.443 | 303 | -43.8 | 0 | 0 | 0 | 0 | 0 | 0 | 1 | 1 | 0 | 0 | 0 | 0 | 1180 |
| hsa-miR-5586-5p | hsa_circRNA_400027 | -0.309 | -0.248 | 145 | -15.31 | 0 | 0 | 0 | 0 | 0 | 0 | 1 | 0 | 0 | 0 | 0 | 0 | 1180 |
| hsa-miR-637 | hsa_circRNA_400027 | -0.398 | -0.266 | 168 | -32.1 | 0 | 0 | 0 | 0 | 0 | 0 | 1 | 0 | 0 | 0 | 0 | 0 | 1180 |
| hsa-miR-665 | hsa_circRNA_400027 | -0.338 | -0.313 | 147 | -21.11 | 0 | 0 | 0 | 0 | 0 | 0 | 1 | 0 | 0 | 0 | 0 | 0 | 1180 |
| hsa-miR-671-5p | hsa_circRNA_400027 | -0.336 | -0.293 | 293 | -43.84 | 0 | 0 | 0 | 0 | 0 | 0 | 0 | 2 | 0 | 0 | 0 | 0 | 1180 |
| hsa-miR-6727-3p | hsa_circRNA_400027 | -0.395 | -0.321 | 157 | -22.57 | 0 | 0 | 0 | 0 | 0 | 0 | 1 | 0 | 0 | 0 | 0 | 0 | 1180 |
| hsa-miR-6769a-3p | hsa_circRNA_400027 | -0.415 | -0.272 | 143 | -15.15 | 0 | 0 | 0 | 0 | 0 | 0 | 1 | 0 | 0 | 0 | 0 | 0 | 1180 |
| hsa-miR-6780b-3p | hsa_circRNA_400027 | -0.516 | -0.524 | 294 | -36.42 | 0 | 0 | 0 | 0 | 0 | 0 | 1 | 1 | 0 | 0 | 0 | 0 | 1180 |
| hsa-miR-6817-5p | hsa_circRNA_400027 | -0.381 | -0.392 | 280 | -35.33 | 0 | 0 | 0 | 0 | 0 | 0 | 1 | 1 | 0 | 0 | 0 | 0 | 1180 |
| hsa-miR-6837-5p | hsa_circRNA_400027 | -0.426 | -0.336 | 140 | -17.79 | 0 | 0 | 0 | 0 | 0 | 0 | 1 | 0 | 0 | 0 | 0 | 0 | 1180 |
| hsa-miR-6861-3p | hsa_circRNA_400027 | -0.464 | -0.334 | 141 | -15.24 | 0 | 0 | 0 | 0 | 0 | 0 | 1 | 0 | 0 | 0 | 0 | 0 | 1180 |
| hsa-miR-8076 | hsa_circRNA_400027 | -0.326 | -0.427 | 151 | -12.7 | 0 | 0 | 0 | 0 | 0 | 0 | 1 | 0 | 0 | 0 | 0 | 0 | 1180 |
| hsa-miR-1203 | hsa_circRNA_400068 | -0.356 | -0.227 | 153 | -23.11 | 0 | 0 | 0 | 0 | 0 | 0 | 0 | 1 | 0 | 0 | 0 | 0 | 340 |
| hsa-miR-1226-3p | hsa_circRNA_400068 | -0.428 | -0.355 | 142 | -17.42 | 0 | 0 | 0 | 0 | 0 | 0 | 1 | 0 | 0 | 0 | 0 | 0 | 340 |
| hsa-miR-210-5p | hsa_circRNA_400068 | -0.338 | -0.139 | 303 | -49.08 | 0 | 0 | 0 | 0 | 0 | 0 | 0 | 2 | 0 | 0 | 0 | 0 | 340 |
| hsa-miR-2861 | hsa_circRNA_400068 | -0.437 | -0.279 | 145 | -19.34 | 0 | 0 | 0 | 0 | 0 | 0 | 1 | 0 | 0 | 0 | 0 | 0 | 340 |
| hsa-miR-3184-5p | hsa_circRNA_400068 | -0.376 | -0.173 | 160 | -31.07 | 0 | 0 | 0 | 0 | 0 | 0 | 1 | 0 | 0 | 0 | 0 | 0 | 340 |
| hsa-miR-3194-5p | hsa_circRNA_400068 | -0.488 | -0.313 | 169 | -35.25 | 0 | 0 | 0 | 0 | 0 | 0 | 1 | 0 | 0 | 0 | 0 | 0 | 340 |
| hsa-miR-423-5p | hsa_circRNA_400068 | -0.345 | -0.167 | 140 | -23.4 | 0 | 0 | 0 | 0 | 0 | 0 | 1 | 0 | 0 | 0 | 0 | 0 | 340 |
| hsa-miR-874-5p | hsa_circRNA_400068 | -0.456 | -0.191 | 157 | -24.04 | 0 | 0 | 0 | 0 | 0 | 0 | 1 | 0 | 0 | 0 | 0 | 0 | 340 |
| hsa-miR-1207-5p | hsa_circRNA_400091 | -0.463 | -0.379 | 150 | -22.54 | 0 | 0 | 0 | 0 | 0 | 0 | 1 | 0 | 0 | 0 | 0 | 0 | 200 |
| hsa-miR-1295b-3p | hsa_circRNA_400091 | -0.354 | -0.215 | 154 | -20.55 | 0 | 0 | 0 | 0 | 0 | 0 | 1 | 0 | 0 | 0 | 0 | 0 | 200 |
| hsa-miR-1301-3p | hsa_circRNA_400091 | -0.354 | -0.311 | 154 | -22.49 | 0 | 0 | 0 | 0 | 0 | 0 | 1 | 0 | 0 | 0 | 0 | 0 | 200 |
| hsa-miR-221-5p | hsa_circRNA_400091 | -0.373 | -0.285 | 152 | -14.97 | 0 | 0 | 0 | 0 | 0 | 0 | 1 | 0 | 0 | 0 | 0 | 0 | 200 |
| hsa-miR-4763-3p | hsa_circRNA_400091 | -0.453 | -0.405 | 290 | -49.59 | 0 | 0 | 0 | 0 | 0 | 0 | 1 | 0 | 0 | 1 | 0 | 0 | 200 |
| hsa-miR-486-3p | hsa_circRNA_400091 | -0.454 | -0.342 | 144 | -18.05 | 0 | 0 | 0 | 0 | 0 | 0 | 1 | 0 | 0 | 0 | 0 | 0 | 200 |
| hsa-miR-5001-3p | hsa_circRNA_400091 | -0.308 | -0.27 | 147 | -16.39 | 0 | 0 | 0 | 0 | 0 | 0 | 1 | 0 | 0 | 0 | 0 | 0 | 200 |
| hsa-miR-5047 | hsa_circRNA_400091 | -0.386 | -0.317 | 152 | -20.63 | 0 | 0 | 0 | 0 | 0 | 0 | 1 | 0 | 0 | 0 | 0 | 0 | 200 |
| hsa-miR-663b | hsa_circRNA_400091 | -0.565 | -0.349 | 150 | -28.01 | 0 | 0 | 0 | 0 | 0 | 0 | 1 | 0 | 0 | 0 | 0 | 0 | 200 |
| hsa-miR-6738-3p | hsa_circRNA_400091 | -0.317 | -0.298 | 150 | -15.62 | 0 | 0 | 0 | 0 | 0 | 0 | 1 | 0 | 0 | 0 | 0 | 0 | 200 |
| hsa-miR-6811-3p | hsa_circRNA_400091 | -0.396 | -0.347 | 144 | -12.63 | 0 | 0 | 0 | 0 | 0 | 0 | 1 | 0 | 0 | 0 | 0 | 0 | 200 |
| hsa-miR-8073 | hsa_circRNA_400091 | -0.373 | -0.285 | 153 | -24.17 | 0 | 0 | 0 | 0 | 0 | 0 | 1 | 0 | 0 | 0 | 0 | 0 | 200 |
