# Supplementary material for: circRNA circ_102049 Implicates in Pancreatic Ductal Adenocarcinoma Progression through Activating CD80 by Targeting miR-455-3p
Source: Mediators Inflamm. 2021 Jan 7;2021:8819990. doi: 10.1155/2021/8819990 (PMC7811564; doi:10.1155/2021/8819990)
Supplement: Supplementary 6 — Table S4 The target miRNAs of overlapped DECs according to the KM plotter analysis. [file 8819990.f6.doc]

**Supplementary Table S4: The target miRNAs of overlapped DECs according to the KM plotter analysis**

| **Predicted target genes for results**  # Column A: miRNA, the name of the miRNA. # Column B: circRNA_ID, The ID of circRNA. # Column C Type, the type of the circRNA. # Column D Diagrams, the diagrams illustrate key features for miRNA binding. See Ref [1,2] for details. # Column E: Total, the total number of the binding sites on the targets. # Column F: Context+, the sum of the context+ scores used in TargetScan after version 6.0. More negative is better. See Ref [3,4] for details. # Column G: Context, the sum of the context scores used in TargetScan before version 5.x. More negative is better. See Ref [1] for details. # Column H Structure, the sum of the structure scores used in miRanda (Ref [5]). The higher the better. # Column I: Energy, the sum of the free energy predicted by miRanda (Ref [5]). More negative is better. # Column J ~ Column U: counting numbers for different seed match types. # Column V: UTR Length, the length of UTR sequence  # Note: Currently, there is not enough data for constructing phylogenetic tree of different species for LncRNAs. The "Conservation" section is meaningless for LncRNA. All sites on LncRNA are treated as non-conserved ones. Column A: The red marked miRNAs represent a poor prognosis with highly expressed in Kaplan-Meier plotter (http://kmplot.com/analysis/), and the green marked miRNAs represent a poor prognosis with lowly expressed. Column B: The red marked represent upregulated circRNAs and green marked represent downregulated circRNAs.  # Reference: [1] Grimson A., et al. MicroRNA Targeting Specificity in Mammals: Determinants beyond Seed Pairing. Molecular Cell, Volume 27, Issue 1, 91-105, 6 July 2007. [2] Amy E. Pasquinelli. MicroRNAs and their targets: recognition, regulation and an emerging reciprocal relationship. Nature Reviews Genetics, Volume 13, 271-282, 1 April 2012. [3] Robin C Friedman, et al. Most Mammalian mRNAs Are Conserved Targets of MicroRNAs. Genome Research, Volume 19, 92-105, 2009. [4] David M Garcia., et al. Weak Seed-Pairing Stability and High Target-Site Abundance Decrease the Proficiency of lsy-6 and Other miRNAs. Nat. Struct. Mol. Biol., Volume 18, 1139-1146, 2011. [5] Enright AJ., el al. miRanda algorithm: MicroRNA targets in Drosophila. Genome Biology, Volume 5, R1, 2003. | | | | | | | | | | | | | | | | | | |
| --- | --- | --- | --- | --- | --- | --- | --- | --- | --- | --- | --- | --- | --- | --- | --- | --- | --- | --- |
|
|
|
|
|
|
|
|
|
|
|
|
|
|
|
|
|
|
|
|
|
|
|
|
| **miRNA** | **Target** | **TargetScan** | | **miRanda** | | **Conserved Sites** | | | | | | **Poorly Conserved Sites** | | | | | | **Annotations** |
| **miRNA** | **circRNA_ID** | **Context+** | **Context** | **Structure** | **Energy** | **8mer** | **7mer-m8** | **7mer-A1** | **6mer** | **Offset 6mer** | **Imperfect** | **8mer** | **7mer-m8** | **7mer-A1** | **6mer** | **Offset 6mer** | **Imperfect** | **UTR Length** |
| hsa-miR-4313 | hsa_circRNA_000274 | -0.321 | -0.228 | 166 | -26.05 | 0 | 0 | 0 | 0 | 0 | 0 | 0 | 1 | 0 | 0 | 0 | 0 | 254 |
| hsa-miR-4652-5p | hsa_circRNA_000274 | -0.406 | -0.237 | 153 | -16.68 | 0 | 0 | 0 | 0 | 0 | 0 | 1 | 0 | 0 | 0 | 0 | 0 | 254 |
| hsa-miR-4667-5p | hsa_circRNA_000274 | -0.382 | -0.249 | 153 | -21.86 | 0 | 0 | 0 | 0 | 0 | 0 | 1 | 0 | 0 | 0 | 0 | 0 | 254 |
| hsa-miR-4700-5p | hsa_circRNA_000274 | -0.35 | -0.243 | 147 | -23.73 | 0 | 0 | 0 | 0 | 0 | 0 | 1 | 0 | 0 | 0 | 0 | 0 | 254 |
| hsa-miR-4708-3p | hsa_circRNA_000274 | -0.351 | -0.219 | 146 | -18.38 | 0 | 0 | 0 | 0 | 0 | 0 | 1 | 0 | 0 | 0 | 0 | 0 | 254 |
| hsa-miR-4750-3p | hsa_circRNA_000274 | -0.33 | -0.216 | 140 | -16.68 | 0 | 0 | 0 | 0 | 0 | 0 | 1 | 0 | 0 | 0 | 0 | 0 | 254 |
| hsa-miR-6805-5p | hsa_circRNA_000274 | -0.355 | -0.267 | 150 | -22.09 | 0 | 0 | 0 | 0 | 0 | 0 | 0 | 1 | 0 | 0 | 0 | 0 | 254 |
| hsa-miR-6846-5p | hsa_circRNA_000274 | -0.308 | -0.212 | 143 | -19.47 | 0 | 0 | 0 | 0 | 0 | 0 | 0 | 1 | 0 | 0 | 0 | 0 | 254 |
| hsa-miR-6848-5p | hsa_circRNA_000274 | -0.317 | -0.228 | 142 | -21.8 | 0 | 0 | 0 | 0 | 0 | 0 | 0 | 1 | 0 | 0 | 0 | 0 | 254 |
| hsa-miR-764 | hsa_circRNA_000274 | -0.355 | -0.283 | 147 | -16.27 | 0 | 0 | 0 | 0 | 0 | 0 | 1 | 0 | 0 | 0 | 0 | 0 | 254 |
| hsa-miR-8089 | hsa_circRNA_000274 | -0.329 | -0.239 | 145 | -20.34 | 0 | 0 | 0 | 0 | 0 | 0 | 1 | 0 | 0 | 0 | 0 | 0 | 254 |
| hsa-miR-296-3p | hsa_circRNA_100117 | -0.394 | -0.35 | 151 | -19.77 | 0 | 0 | 0 | 0 | 0 | 0 | 1 | 0 | 0 | 0 | 0 | 0 | 429 |
| hsa-miR-4501 | hsa_circRNA_100117 | -0.382 | -0.436 | 148 | -15.22 | 0 | 0 | 0 | 0 | 0 | 0 | 1 | 0 | 0 | 0 | 0 | 0 | 429 |
| hsa-miR-627-5p | hsa_circRNA_100117 | -0.58 | -0.528 | 291 | -27.39 | 0 | 0 | 0 | 0 | 0 | 0 | 2 | 0 | 0 | 0 | 0 | 0 | 429 |
| hsa-miR-653-3p | hsa_circRNA_100117 | -0.423 | -0.427 | 145 | -15.54 | 0 | 0 | 0 | 0 | 0 | 0 | 1 | 0 | 0 | 0 | 0 | 0 | 429 |
| hsa-miR-1256 | hsa_circRNA_100302 | -0.409 | -0.416 | 150 | -11.42 | 0 | 0 | 0 | 0 | 0 | 0 | 1 | 0 | 0 | 0 | 0 | 0 | 291 |
| hsa-miR-3155a | hsa_circRNA_100302 | -0.43 | -0.35 | 153 | -24.13 | 0 | 0 | 0 | 0 | 0 | 0 | 1 | 0 | 0 | 0 | 0 | 0 | 291 |
| hsa-miR-3155b | hsa_circRNA_100302 | -0.399 | -0.343 | 151 | -19.86 | 0 | 0 | 0 | 0 | 0 | 0 | 1 | 0 | 0 | 0 | 0 | 0 | 291 |
| hsa-miR-3619-3p | hsa_circRNA_100302 | -0.385 | -0.31 | 143 | -18.91 | 0 | 0 | 0 | 0 | 0 | 0 | 1 | 0 | 0 | 0 | 0 | 0 | 291 |
| hsa-miR-4254 | hsa_circRNA_100302 | -0.394 | -0.37 | 141 | -15.64 | 0 | 0 | 0 | 0 | 0 | 0 | 1 | 0 | 0 | 0 | 0 | 0 | 291 |
| hsa-miR-4436b-3p | hsa_circRNA_100302 | -0.389 | -0.352 | 142 | -16.69 | 0 | 0 | 0 | 0 | 0 | 0 | 1 | 0 | 0 | 0 | 0 | 0 | 291 |
| hsa-miR-4520-3p | hsa_circRNA_100302 | -0.399 | -0.368 | 288 | -33.19 | 0 | 0 | 0 | 0 | 0 | 0 | 0 | 2 | 0 | 0 | 0 | 0 | 291 |
| hsa-miR-4632-5p | hsa_circRNA_100302 | -0.41 | -0.356 | 145 | -24.2 | 0 | 0 | 0 | 0 | 0 | 0 | 1 | 0 | 0 | 0 | 0 | 0 | 291 |
| hsa-miR-4776-5p | hsa_circRNA_100302 | -0.48 | -0.373 | 153 | -23.82 | 0 | 0 | 0 | 0 | 0 | 0 | 1 | 0 | 0 | 0 | 0 | 0 | 291 |
| hsa-miR-5193 | hsa_circRNA_100302 | -0.372 | -0.348 | 140 | -15.45 | 0 | 0 | 0 | 0 | 0 | 0 | 1 | 0 | 0 | 0 | 0 | 0 | 291 |
| hsa-miR-6735-5p | hsa_circRNA_100302 | -0.4 | -0.354 | 288 | -42.99 | 0 | 0 | 0 | 0 | 0 | 0 | 1 | 0 | 0 | 0 | 0 | 1 | 291 |
| hsa-miR-6825-5p | hsa_circRNA_100302 | -0.458 | -0.395 | 149 | -22.02 | 0 | 0 | 0 | 0 | 0 | 0 | 1 | 0 | 0 | 0 | 0 | 0 | 291 |
| hsa-miR-6852-5p | hsa_circRNA_100302 | -0.435 | -0.388 | 140 | -18.51 | 0 | 0 | 0 | 0 | 0 | 0 | 1 | 0 | 0 | 0 | 0 | 0 | 291 |
| hsa-miR-6879-5p | hsa_circRNA_100302 | -0.431 | -0.36 | 146 | -23.85 | 0 | 0 | 0 | 0 | 0 | 0 | 1 | 0 | 0 | 0 | 0 | 0 | 291 |
| hsa-miR-6884-5p | hsa_circRNA_100302 | -0.364 | -0.307 | 154 | -21.31 | 0 | 0 | 0 | 0 | 0 | 0 | 1 | 0 | 0 | 0 | 0 | 0 | 291 |
| hsa-miR-7843-5p | hsa_circRNA_100302 | -0.41 | -0.356 | 145 | -21.9 | 0 | 0 | 0 | 0 | 0 | 0 | 1 | 0 | 0 | 0 | 0 | 0 | 291 |
| hsa-miR-218-1-3p | hsa_circRNA_100790 | -0.424 | -0.423 | 147 | -12.59 | 0 | 0 | 0 | 0 | 0 | 0 | 1 | 0 | 0 | 0 | 0 | 0 | 261 |
| hsa-miR-4423-5p | hsa_circRNA_100790 | -0.412 | -0.387 | 147 | -13.07 | 0 | 0 | 0 | 0 | 0 | 0 | 1 | 0 | 0 | 0 | 0 | 0 | 261 |
| hsa-miR-485-5p | hsa_circRNA_100790 | -0.367 | -0.359 | 149 | -17.48 | 0 | 0 | 0 | 0 | 0 | 0 | 1 | 0 | 0 | 0 | 0 | 0 | 261 |
| hsa-miR-744-3p | hsa_circRNA_100790 | -0.433 | -0.43 | 146 | -10.03 | 0 | 0 | 0 | 0 | 0 | 0 | 1 | 0 | 0 | 0 | 0 | 0 | 261 |
| hsa-miR-148a-5p | hsa_circRNA_100904 | -0.341 | -0.45 | 147 | -12.69 | 0 | 0 | 0 | 0 | 0 | 0 | 1 | 0 | 0 | 0 | 0 | 0 | 409 |
| hsa-miR-29b-1-5p | hsa_circRNA_100904 | -0.32 | -0.411 | 151 | -19.61 | 0 | 0 | 0 | 0 | 0 | 0 | 1 | 0 | 0 | 0 | 0 | 0 | 409 |
| hsa-miR-532-5p | hsa_circRNA_100904 | -0.343 | -0.397 | 146 | -10.96 | 0 | 0 | 0 | 0 | 0 | 0 | 1 | 0 | 0 | 0 | 0 | 0 | 409 |
| hsa-miR-1180-3p | hsa_circRNA_101656 | -0.558 | -0.373 | 148 | -20.77 | 0 | 0 | 0 | 0 | 0 | 0 | 1 | 0 | 0 | 0 | 0 | 0 | 538 |
| hsa-miR-185-5p | hsa_circRNA_101798 | -0.334 | -0.3 | 158 | -20.14 | 0 | 0 | 0 | 0 | 0 | 0 | 1 | 0 | 0 | 0 | 0 | 0 | 313 |
| hsa-miR-5010-5p | hsa_circRNA_101798 | -0.394 | -0.267 | 158 | -21.38 | 0 | 0 | 0 | 0 | 0 | 0 | 1 | 0 | 0 | 0 | 0 | 0 | 313 |
| hsa-miR-6868-3p | hsa_circRNA_101798 | -0.531 | -0.658 | 312 | -37.92 | 0 | 0 | 0 | 0 | 0 | 0 | 1 | 1 | 0 | 0 | 0 | 0 | 313 |
| hsa-miR-342-3p | hsa_circRNA_102049 | -0.315 | -0.357 | 140 | -15.04 | 0 | 0 | 0 | 0 | 0 | 0 | 1 | 0 | 0 | 0 | 0 | 0 | 250 |
| hsa-miR-455-3p | hsa_circRNA_102049 | -0.364 | -0.32 | 297 | -36.6 | 0 | 0 | 0 | 0 | 0 | 0 | 0 | 2 | 0 | 0 | 0 | 0 | 250 |
| hsa-miR-15a-5p | hsa_circRNA_102359 | -0.326 | -0.288 | 142 | -14.63 | 0 | 0 | 0 | 0 | 0 | 0 | 1 | 0 | 0 | 0 | 0 | 0 | 178 |
| hsa-miR-16-5p | hsa_circRNA_102359 | -0.337 | -0.29 | 146 | -16.4 | 0 | 0 | 0 | 0 | 0 | 0 | 1 | 0 | 0 | 0 | 0 | 0 | 178 |
| hsa-miR-4677-3p | hsa_circRNA_102741 | -0.367 | -0.407 | 149 | -17.52 | 0 | 0 | 0 | 0 | 0 | 0 | 1 | 0 | 0 | 0 | 0 | 0 | 441 |
| hsa-miR-6075 | hsa_circRNA_102741 | -0.305 | -0.148 | 159 | -27.41 | 0 | 0 | 0 | 0 | 0 | 0 | 0 | 1 | 0 | 0 | 0 | 0 | 441 |
| hsa-miR-6781-5p | hsa_circRNA_102741 | -0.673 | -0.436 | 141 | -18.92 | 0 | 0 | 0 | 0 | 0 | 0 | 1 | 0 | 0 | 0 | 0 | 0 | 441 |
| hsa-miR-8057 | hsa_circRNA_102741 | -0.356 | -0.3 | 150 | -17.62 | 0 | 0 | 0 | 0 | 0 | 0 | 1 | 0 | 0 | 0 | 0 | 0 | 441 |
| hsa-miR-26b-3p | hsa_circRNA_102984 | -0.307 | -0.362 | 288 | -23.4 | 0 | 0 | 0 | 0 | 0 | 0 | 1 | 1 | 0 | 0 | 0 | 0 | 421 |
| hsa-miR-642a-3p | hsa_circRNA_102984 | -0.306 | -0.351 | 148 | -12.52 | 0 | 0 | 0 | 0 | 0 | 0 | 1 | 0 | 0 | 0 | 0 | 0 | 421 |
| hsa-miR-671-5p | hsa_circRNA_102984 | -0.408 | -0.365 | 143 | -18.51 | 0 | 0 | 0 | 0 | 0 | 0 | 1 | 0 | 0 | 0 | 0 | 0 | 421 |
| hsa-miR-6764-5p | hsa_circRNA_102984 | -0.435 | -0.329 | 160 | -25.36 | 0 | 0 | 0 | 0 | 0 | 0 | 1 | 0 | 0 | 0 | 0 | 0 | 421 |
| hsa-miR-455-3p | hsa_circRNA_103076 | -0.459 | -0.49 | 147 | -16.11 | 0 | 0 | 0 | 0 | 0 | 0 | 1 | 0 | 0 | 0 | 0 | 0 | 339 |
| hsa-miR-744-5p | hsa_circRNA_103089 | -0.525 | -0.189 | 155 | -29.45 | 0 | 0 | 0 | 0 | 0 | 0 | 1 | 0 | 0 | 0 | 0 | 0 | 259 |
| hsa-miR-335-5p | hsa_circRNA_103285 | -0.361 | -0.394 | 147 | -14.59 | 0 | 0 | 0 | 0 | 0 | 0 | 1 | 0 | 0 | 0 | 0 | 0 | 196 |
| hsa-let-7d-5p | hsa_circRNA_103390 | -0.376 | -0.346 | 151 | -18.48 | 0 | 0 | 0 | 0 | 0 | 0 | 1 | 0 | 0 | 0 | 0 | 0 | 234 |
| hsa-let-7g-5p | hsa_circRNA_103390 | -0.365 | -0.344 | 155 | -21.29 | 0 | 0 | 0 | 0 | 0 | 0 | 1 | 0 | 0 | 0 | 0 | 0 | 234 |
| hsa-miR-15a-3p | hsa_circRNA_103390 | -0.372 | -0.307 | 145 | -16.19 | 0 | 0 | 0 | 0 | 0 | 0 | 1 | 0 | 0 | 0 | 0 | 0 | 234 |
| hsa-miR-328-5p | hsa_circRNA_103390 | -0.305 | -0.147 | 147 | -25.91 | 0 | 0 | 0 | 0 | 0 | 0 | 0 | 1 | 0 | 0 | 0 | 0 | 234 |
| hsa-miR-98-5p | hsa_circRNA_103390 | -0.365 | -0.344 | 154 | -18.23 | 0 | 0 | 0 | 0 | 0 | 0 | 1 | 0 | 0 | 0 | 0 | 0 | 234 |
| hsa-miR-15a-5p | hsa_circRNA_103655 | -0.502 | -0.449 | 298 | -38.57 | 0 | 0 | 0 | 0 | 0 | 0 | 1 | 1 | 0 | 0 | 0 | 0 | 1634 |
| hsa-miR-16-5p | hsa_circRNA_103655 | -0.477 | -0.485 | 302 | -34.05 | 0 | 0 | 0 | 0 | 0 | 0 | 1 | 1 | 0 | 0 | 0 | 0 | 1634 |
| hsa-miR-486-3p | hsa_circRNA_103655 | -0.576 | -0.453 | 286 | -33.18 | 0 | 0 | 0 | 0 | 0 | 0 | 1 | 1 | 0 | 0 | 0 | 0 | 1634 |
| hsa-miR-653-3p | hsa_circRNA_103655 | -0.355 | -0.375 | 292 | -27.43 | 0 | 0 | 0 | 0 | 0 | 0 | 1 | 1 | 0 | 0 | 0 | 0 | 1634 |
| hsa-miR-128-1-5p | hsa_circRNA_104003 | -0.457 | -0.189 | 149 | -24.89 | 0 | 0 | 0 | 0 | 0 | 0 | 1 | 0 | 0 | 0 | 0 | 0 | 372 |
| hsa-miR-128-2-5p | hsa_circRNA_104003 | -0.415 | -0.181 | 140 | -20.27 | 0 | 0 | 0 | 0 | 0 | 0 | 1 | 0 | 0 | 0 | 0 | 0 | 372 |
| hsa-miR-1291 | hsa_circRNA_104003 | -0.413 | -0.226 | 149 | -26.25 | 0 | 0 | 0 | 0 | 0 | 0 | 1 | 0 | 0 | 0 | 0 | 0 | 372 |
| hsa-miR-296-5p | hsa_circRNA_104003 | -0.504 | -0.238 | 141 | -18.35 | 0 | 0 | 0 | 0 | 0 | 0 | 1 | 0 | 0 | 0 | 0 | 0 | 372 |
| hsa-miR-3907 | hsa_circRNA_104310 | -0.476 | -0.337 | 151 | -20.02 | 0 | 0 | 0 | 0 | 0 | 0 | 1 | 0 | 0 | 0 | 0 | 0 | 245 |
| hsa-miR-3913-3p | hsa_circRNA_104310 | -0.3 | -0.29 | 151 | -15.29 | 0 | 0 | 0 | 0 | 0 | 0 | 1 | 0 | 0 | 0 | 0 | 0 | 245 |
| hsa-miR-3974 | hsa_circRNA_104310 | -0.324 | -0.372 | 305 | -34.08 | 0 | 0 | 0 | 0 | 0 | 0 | 0 | 2 | 0 | 0 | 0 | 0 | 245 |
| hsa-miR-4800-5p | hsa_circRNA_104310 | -0.332 | -0.287 | 158 | -25.72 | 0 | 0 | 0 | 0 | 0 | 0 | 0 | 1 | 0 | 0 | 0 | 0 | 245 |
| hsa-miR-670-3p | hsa_circRNA_104313 | -0.432 | -0.52 | 295 | -25.72 | 0 | 0 | 0 | 0 | 0 | 0 | 1 | 1 | 0 | 0 | 0 | 0 | 291 |
| hsa-miR-1295b-5p | hsa_circRNA_400027 | -0.559 | -0.469 | 456 | -47.72 | 0 | 0 | 0 | 0 | 0 | 0 | 1 | 2 | 0 | 0 | 0 | 0 | 1180 |
| hsa-miR-1912 | hsa_circRNA_400027 | -0.539 | -0.451 | 466 | -60.75 | 0 | 0 | 0 | 0 | 0 | 0 | 1 | 2 | 0 | 0 | 0 | 0 | 1180 |
| hsa-miR-1914-5p | hsa_circRNA_400027 | -0.371 | -0.335 | 147 | -23.09 | 0 | 0 | 0 | 0 | 0 | 0 | 1 | 0 | 0 | 0 | 0 | 0 | 1180 |
| hsa-miR-2467-3p | hsa_circRNA_400027 | -0.448 | -0.399 | 160 | -21.82 | 0 | 0 | 0 | 0 | 0 | 0 | 1 | 0 | 0 | 0 | 0 | 0 | 1180 |
| hsa-miR-3922-5p | hsa_circRNA_400027 | -0.329 | -0.273 | 148 | -22.6 | 0 | 0 | 0 | 0 | 0 | 0 | 1 | 0 | 0 | 0 | 0 | 0 | 1180 |
| hsa-miR-4462 | hsa_circRNA_400027 | -0.47 | -0.311 | 152 | -22.05 | 0 | 0 | 0 | 0 | 0 | 0 | 1 | 0 | 0 | 0 | 0 | 0 | 1180 |
| hsa-miR-4529-5p | hsa_circRNA_400027 | -0.383 | -0.304 | 153 | -18.66 | 0 | 0 | 0 | 0 | 0 | 0 | 1 | 0 | 0 | 0 | 0 | 0 | 1180 |
| hsa-miR-4685-5p | hsa_circRNA_400027 | -0.468 | -0.344 | 317 | -63.07 | 0 | 0 | 0 | 0 | 0 | 0 | 1 | 0 | 0 | 0 | 0 | 1 | 1180 |
| hsa-miR-4722-3p | hsa_circRNA_400027 | -0.374 | -0.317 | 149 | -20.47 | 0 | 0 | 0 | 0 | 0 | 0 | 1 | 0 | 0 | 0 | 0 | 0 | 1180 |
| hsa-miR-4769-3p | hsa_circRNA_400027 | -0.43 | -0.443 | 303 | -43.8 | 0 | 0 | 0 | 0 | 0 | 0 | 1 | 1 | 0 | 0 | 0 | 0 | 1180 |
| hsa-miR-637 | hsa_circRNA_400027 | -0.398 | -0.266 | 168 | -32.1 | 0 | 0 | 0 | 0 | 0 | 0 | 1 | 0 | 0 | 0 | 0 | 0 | 1180 |
| hsa-miR-665 | hsa_circRNA_400027 | -0.338 | -0.313 | 147 | -21.11 | 0 | 0 | 0 | 0 | 0 | 0 | 1 | 0 | 0 | 0 | 0 | 0 | 1180 |
| hsa-miR-6727-3p | hsa_circRNA_400027 | -0.395 | -0.321 | 157 | -22.57 | 0 | 0 | 0 | 0 | 0 | 0 | 1 | 0 | 0 | 0 | 0 | 0 | 1180 |
| hsa-miR-6769a-3p | hsa_circRNA_400027 | -0.415 | -0.272 | 143 | -15.15 | 0 | 0 | 0 | 0 | 0 | 0 | 1 | 0 | 0 | 0 | 0 | 0 | 1180 |
| hsa-miR-6780b-3p | hsa_circRNA_400027 | -0.516 | -0.524 | 294 | -36.42 | 0 | 0 | 0 | 0 | 0 | 0 | 1 | 1 | 0 | 0 | 0 | 0 | 1180 |
| hsa-miR-6817-5p | hsa_circRNA_400027 | -0.381 | -0.392 | 280 | -35.33 | 0 | 0 | 0 | 0 | 0 | 0 | 1 | 1 | 0 | 0 | 0 | 0 | 1180 |
| hsa-miR-6861-3p | hsa_circRNA_400027 | -0.464 | -0.334 | 141 | -15.24 | 0 | 0 | 0 | 0 | 0 | 0 | 1 | 0 | 0 | 0 | 0 | 0 | 1180 |
| hsa-miR-8076 | hsa_circRNA_400027 | -0.326 | -0.427 | 151 | -12.7 | 0 | 0 | 0 | 0 | 0 | 0 | 1 | 0 | 0 | 0 | 0 | 0 | 1180 |
| hsa-miR-874-5p | hsa_circRNA_400068 | -0.456 | -0.191 | 157 | -24.04 | 0 | 0 | 0 | 0 | 0 | 0 | 1 | 0 | 0 | 0 | 0 | 0 | 340 |
| hsa-miR-1301-3p | hsa_circRNA_400091 | -0.354 | -0.311 | 154 | -22.49 | 0 | 0 | 0 | 0 | 0 | 0 | 1 | 0 | 0 | 0 | 0 | 0 | 200 |
| hsa-miR-486-3p | hsa_circRNA_400091 | -0.454 | -0.342 | 144 | -18.05 | 0 | 0 | 0 | 0 | 0 | 0 | 1 | 0 | 0 | 0 | 0 | 0 | 200 |
