# Supplementary material for: circRNA circ_102049 Implicates in Pancreatic Ductal Adenocarcinoma Progression through Activating CD80 by Targeting miR-455-3p
Source: Mediators Inflamm. 2021 Jan 7;2021:8819990. doi: 10.1155/2021/8819990 (PMC7811564; doi:10.1155/2021/8819990)
Supplement: Supplementary 7 — Table S5 The potential target genes of 53 highly expressed miRNAs with poor survival of PDAC patients. [file 8819990.f7.doc]

**Supplementary Table S5: The potential target genes of 53 highly expressed miRNAs with poor survival of PDAC patients**

| **miRNA** | **Target name** | **targetScan7.1** | **mirdbV5** | **NumSum** | **miRTarBase(Y/N)** |
| --- | --- | --- | --- | --- | --- |
| hsa-miR-4313 | LITAF | 1 | 1 | 2 | N |
| hsa-miR-4313 | NSMF | 1 | 1 | 2 | N |
| hsa-miR-4313 | SCN1B | 1 | 1 | 2 | N |
| hsa-miR-4313 | XPO7 | 1 | 1 | 2 | N |
| hsa-miR-4313 | E2F4 | 1 | 1 | 2 | N |
| hsa-miR-4313 | DHCR24 | 1 | 1 | 2 | N |
| hsa-miR-4313 | SLC25A27 | 1 | 1 | 2 | N |
| hsa-miR-4313 | HEXIM1 | 1 | 1 | 2 | N |
| hsa-miR-4313 | TMEM164 | 1 | 1 | 2 | N |
| hsa-miR-4313 | KCNJ12 | 1 | 1 | 2 | N |
| hsa-miR-4313 | CLCA1 | 1 | 1 | 2 | N |
| hsa-miR-4313 | TBX15 | 1 | 1 | 2 | N |
| hsa-miR-4313 | ZNF362 | 1 | 1 | 2 | N |
| hsa-miR-4313 | SHISA3 | 1 | 1 | 2 | N |
| hsa-miR-4313 | ADARB2 | 1 | 1 | 2 | N |
| hsa-miR-4313 | RAB1B | 1 | 1 | 2 | N |
| hsa-miR-4313 | CYP26B1 | 1 | 1 | 2 | N |
| hsa-miR-4313 | DACH1 | 1 | 1 | 2 | N |
| hsa-miR-4313 | TNFRSF12A | 1 | 1 | 2 | N |
| hsa-miR-4313 | KLHDC7A | 1 | 1 | 2 | N |
| hsa-miR-4313 | CACNG7 | 1 | 1 | 2 | N |
| hsa-miR-4313 | PHF12 | 1 | 1 | 2 | N |
| hsa-miR-4313 | SBK1 | 1 | 1 | 2 | N |
| hsa-miR-4313 | SGCB | 1 | 1 | 2 | N |
| hsa-miR-4313 | KCNE4 | 1 | 1 | 2 | N |
| hsa-miR-4313 | ZDHHC8 | 1 | 1 | 2 | Y |
| hsa-miR-4313 | PITPNM3 | 1 | 1 | 2 | N |
| hsa-miR-4313 | NDFIP1 | 1 | 1 | 2 | N |
| hsa-miR-4313 | PLK3 | 1 | 1 | 2 | N |
| hsa-miR-4313 | FAM89B | 1 | 1 | 2 | N |
| hsa-miR-4313 | LONRF1 | 1 | 1 | 2 | N |
| hsa-miR-4313 | GAS1 | 1 | 1 | 2 | N |
| hsa-miR-4313 | LPIN1 | 1 | 1 | 2 | N |
| hsa-miR-4313 | BAHD1 | 1 | 1 | 2 | N |
| hsa-miR-4313 | DDX4 | 1 | 1 | 2 | N |
| hsa-miR-4313 | MID1IP1 | 1 | 1 | 2 | N |
| hsa-miR-4313 | NR4A2 | 1 | 1 | 2 | N |
| hsa-miR-4313 | B4GALT2 | 1 | 1 | 2 | N |
| hsa-miR-4313 | MBD6 | 1 | 1 | 2 | N |
| hsa-miR-4313 | CDK15 | 1 | 1 | 2 | N |
| hsa-miR-4652-5p | PRCP | 1 | 1 | 2 | N |
| hsa-miR-4652-5p | ARNTL | 1 | 1 | 2 | N |
| hsa-miR-4652-5p | SIDT1 | 1 | 1 | 2 | N |
| hsa-miR-4652-5p | SERPINA10 | 1 | 1 | 2 | N |
| hsa-miR-4652-5p | FAM98C | 1 | 1 | 2 | N |
| hsa-miR-4652-5p | TMEM110 | 1 | 1 | 2 | N |
| hsa-miR-4652-5p | RNF150 | 1 | 1 | 2 | N |
| hsa-miR-4652-5p | ARL6IP1 | 1 | 1 | 2 | N |
| hsa-miR-4652-5p | GJB1 | 1 | 1 | 2 | N |
| hsa-miR-4652-5p | M6PR | 1 | 1 | 2 | N |
| hsa-miR-4652-5p | PACSIN1 | 1 | 1 | 2 | N |
| hsa-miR-4652-5p | PPP1R16B | 1 | 1 | 2 | N |
| hsa-miR-4652-5p | RASSF4 | 1 | 1 | 2 | N |
| hsa-miR-4652-5p | ZNF285 | 1 | 1 | 2 | N |
| hsa-miR-4652-5p | ZMYND15 | 1 | 1 | 2 | N |
| hsa-miR-4652-5p | RND1 | 1 | 1 | 2 | N |
| hsa-miR-4652-5p | ZNF664 | 1 | 1 | 2 | N |
| hsa-miR-4652-5p | KIAA1161 | 1 | 1 | 2 | N |
| hsa-miR-4652-5p | PSMD5 | 1 | 1 | 2 | N |
| hsa-miR-4652-5p | PDF | 1 | 1 | 2 | N |
| hsa-miR-4652-5p | FAM217A | 1 | 1 | 2 | N |
| hsa-miR-4652-5p | F2R | 1 | 1 | 2 | N |
| hsa-miR-4652-5p | SLC35F1 | 1 | 1 | 2 | N |
| hsa-miR-4652-5p | RET | 1 | 1 | 2 | N |
| hsa-miR-4652-5p | KIAA0040 | 1 | 1 | 2 | N |
| hsa-miR-4652-5p | C20orf27 | 1 | 1 | 2 | N |
| hsa-miR-4652-5p | CLIP3 | 1 | 1 | 2 | N |
| hsa-miR-4652-5p | TMEM67 | 1 | 1 | 2 | N |
| hsa-miR-4652-5p | VTI1A | 1 | 1 | 2 | N |
| hsa-miR-4652-5p | KCTD15 | 1 | 1 | 2 | N |
| hsa-miR-4652-5p | FURIN | 1 | 1 | 2 | N |
| hsa-miR-4667-5p | SLC16A2 | 1 | 1 | 2 | N |
| hsa-miR-4667-5p | SH2D3C | 1 | 1 | 2 | N |
| hsa-miR-4667-5p | RNASE13 | 1 | 1 | 2 | N |
| hsa-miR-4667-5p | DUSP3 | 1 | 1 | 2 | N |
| hsa-miR-4667-5p | LOXHD1 | 1 | 1 | 2 | N |
| hsa-miR-4667-5p | SAMD12 | 1 | 1 | 2 | N |
| hsa-miR-4667-5p | POPDC3 | 1 | 1 | 2 | N |
| hsa-miR-4667-5p | PCGF2 | 1 | 1 | 2 | N |
| hsa-miR-4667-5p | SPATA2L | 1 | 1 | 2 | N |
| hsa-miR-4667-5p | PSMB11 | 1 | 1 | 2 | N |
| hsa-miR-4667-5p | BCL2L1 | 1 | 1 | 2 | N |
| hsa-miR-4667-5p | NEK9 | 1 | 1 | 2 | N |
| hsa-miR-4667-5p | PCDHGA12 | 1 | 1 | 2 | N |
| hsa-miR-4667-5p | MUT | 1 | 1 | 2 | N |
| hsa-miR-4667-5p | RBM14 | 1 | 1 | 2 | N |
| hsa-miR-4667-5p | PVRL4 | 1 | 1 | 2 | N |
| hsa-miR-4667-5p | PPP1R9B | 1 | 1 | 2 | Y |
| hsa-miR-4667-5p | RASSF4 | 1 | 1 | 2 | N |
| hsa-miR-4667-5p | ERGIC1 | 1 | 1 | 2 | N |
| hsa-miR-4667-5p | ICK | 1 | 1 | 2 | N |
| hsa-miR-4667-5p | SEPN1 | 1 | 1 | 2 | N |
| hsa-miR-4667-5p | PRKCG | 1 | 1 | 2 | N |
| hsa-miR-4667-5p | SPR | 1 | 1 | 2 | N |
| hsa-miR-4667-5p | MS4A15 | 1 | 1 | 2 | N |
| hsa-miR-4667-5p | FOXRED1 | 1 | 1 | 2 | N |
| hsa-miR-4667-5p | SPIB | 1 | 1 | 2 | N |
| hsa-miR-4667-5p | CARTPT | 1 | 1 | 2 | N |
| hsa-miR-4667-5p | SYT12 | 1 | 1 | 2 | N |
| hsa-miR-4667-5p | DAGLA | 1 | 1 | 2 | N |
| hsa-miR-4667-5p | SLC38A7 | 1 | 1 | 2 | N |
| hsa-miR-4667-5p | ELMOD1 | 1 | 1 | 2 | N |
| hsa-miR-4667-5p | PPP1R1B | 1 | 1 | 2 | N |
| hsa-miR-4667-5p | CCL2 | 1 | 1 | 2 | N |
| hsa-miR-4667-5p | HSDL1 | 1 | 1 | 2 | N |
| hsa-miR-4667-5p | PDPK1 | 1 | 1 | 2 | N |
| hsa-miR-4667-5p | PCDHGC5 | 1 | 1 | 2 | N |
| hsa-miR-4667-5p | RGSL1 | 1 | 1 | 2 | N |
| hsa-miR-4667-5p | CAPN12 | 1 | 1 | 2 | N |
| hsa-miR-4667-5p | RBBP5 | 1 | 1 | 2 | N |
| hsa-miR-4667-5p | ITGA10 | 1 | 1 | 2 | N |
| hsa-miR-4667-5p | PCDHGA2 | 1 | 1 | 2 | N |
| hsa-miR-4667-5p | NCDN | 1 | 1 | 2 | N |
| hsa-miR-4667-5p | TSC22D4 | 1 | 1 | 2 | N |
| hsa-miR-4667-5p | PCDHGA7 | 1 | 1 | 2 | N |
| hsa-miR-4667-5p | OSBPL7 | 1 | 1 | 2 | N |
| hsa-miR-4667-5p | ZNF548 | 1 | 1 | 2 | N |
| hsa-miR-4667-5p | ARID3B | 1 | 1 | 2 | N |
| hsa-miR-4667-5p | ZNF687 | 1 | 1 | 2 | N |
| hsa-miR-4667-5p | RNF170 | 1 | 1 | 2 | N |
| hsa-miR-4667-5p | FGF18 | 1 | 1 | 2 | N |
| hsa-miR-4667-5p | PSMD5 | 1 | 1 | 2 | N |
| hsa-miR-4667-5p | GPR64 | 1 | 1 | 2 | N |
| hsa-miR-4667-5p | GDNF | 1 | 1 | 2 | N |
| hsa-miR-4667-5p | UBE2L6 | 1 | 1 | 2 | N |
| hsa-miR-4667-5p | DMRTA2 | 1 | 1 | 2 | N |
| hsa-miR-4667-5p | PCDHGA11 | 1 | 1 | 2 | N |
| hsa-miR-4667-5p | TBC1D13 | 1 | 1 | 2 | Y |
| hsa-miR-4667-5p | P2RY2 | 1 | 1 | 2 | N |
| hsa-miR-4667-5p | TSPAN11 | 1 | 1 | 2 | N |
| hsa-miR-4667-5p | ARFIP1 | 1 | 1 | 2 | N |
| hsa-miR-4667-5p | PPP1R10 | 1 | 1 | 2 | N |
| hsa-miR-4667-5p | RRN3 | 1 | 1 | 2 | N |
| hsa-miR-4667-5p | MRO | 1 | 1 | 2 | N |
| hsa-miR-4667-5p | LZTS1 | 1 | 1 | 2 | N |
| hsa-miR-4667-5p | ZBTB39 | 1 | 1 | 2 | N |
| hsa-miR-4667-5p | C17orf107 | 1 | 1 | 2 | N |
| hsa-miR-4667-5p | TMEM63B | 1 | 1 | 2 | N |
| hsa-miR-4667-5p | SSX2 | 1 | 1 | 2 | N |
| hsa-miR-4667-5p | TSPAN18 | 1 | 1 | 2 | N |
| hsa-miR-4667-5p | SSX7 | 1 | 1 | 2 | N |
| hsa-miR-4667-5p | KIF24 | 1 | 1 | 2 | N |
| hsa-miR-4667-5p | NUMA1 | 1 | 1 | 2 | N |
| hsa-miR-4667-5p | CACNB1 | 1 | 1 | 2 | N |
| hsa-miR-4667-5p | SLC25A42 | 1 | 1 | 2 | N |
| hsa-miR-4667-5p | GAB2 | 1 | 1 | 2 | N |
| hsa-miR-4667-5p | CABP4 | 1 | 1 | 2 | N |
| hsa-miR-4667-5p | SYT2 | 1 | 1 | 2 | N |
| hsa-miR-4667-5p | PCDHGB3 | 1 | 1 | 2 | N |
| hsa-miR-4667-5p | NR6A1 | 1 | 1 | 2 | N |
| hsa-miR-4667-5p | KCNK3 | 1 | 1 | 2 | N |
| hsa-miR-4667-5p | NCEH1 | 1 | 1 | 2 | Y |
| hsa-miR-4667-5p | VTI1A | 1 | 1 | 2 | N |
| hsa-miR-4667-5p | PXN | 1 | 1 | 2 | N |
| hsa-miR-4667-5p | KDM8 | 1 | 1 | 2 | N |
| hsa-miR-4667-5p | TTF2 | 1 | 1 | 2 | N |
| hsa-miR-4667-5p | FAM228B | 1 | 1 | 2 | N |
| hsa-miR-4667-5p | THAP11 | 1 | 1 | 2 | N |
| hsa-miR-4667-5p | SSX2B | 1 | 1 | 2 | N |
| hsa-miR-4667-5p | UBE2L3 | 1 | 1 | 2 | N |
| hsa-miR-4667-5p | CELF6 | 1 | 1 | 2 | N |
| hsa-miR-4667-5p | TP53INP2 | 1 | 1 | 2 | N |
| hsa-miR-4667-5p | NHLH1 | 1 | 1 | 2 | N |
| hsa-miR-4667-5p | DRG2 | 1 | 1 | 2 | N |
| hsa-miR-4667-5p | GLIPR1 | 1 | 1 | 2 | N |
| hsa-miR-4667-5p | PCDHGA1 | 1 | 1 | 2 | N |
| hsa-miR-4667-5p | SLC45A3 | 1 | 1 | 2 | N |
| hsa-miR-4667-5p | BAK1 | 1 | 1 | 2 | N |
| hsa-miR-4667-5p | ATF6B | 1 | 1 | 2 | N |
| hsa-miR-4667-5p | CIC | 1 | 1 | 2 | N |
| hsa-miR-4667-5p | TMEM127 | 1 | 1 | 2 | N |
| hsa-miR-4667-5p | CYBRD1 | 1 | 1 | 2 | Y |
| hsa-miR-4667-5p | C17orf103 | 1 | 1 | 2 | N |
| hsa-miR-4667-5p | PTMS | 1 | 1 | 2 | N |
| hsa-miR-4667-5p | MAT2A | 1 | 1 | 2 | N |
| hsa-miR-4667-5p | PCDHGA5 | 1 | 1 | 2 | N |
| hsa-miR-4667-5p | HCCS | 1 | 1 | 2 | N |
| hsa-miR-4667-5p | CELSR2 | 1 | 1 | 2 | N |
| hsa-miR-4667-5p | XPO7 | 1 | 1 | 2 | N |
| hsa-miR-4667-5p | C4orf46 | 1 | 1 | 2 | N |
| hsa-miR-4667-5p | FBLN1 | 1 | 1 | 2 | N |
| hsa-miR-4667-5p | SCAMP4 | 1 | 1 | 2 | N |
| hsa-miR-4667-5p | NAA40 | 1 | 1 | 2 | N |
| hsa-miR-4667-5p | CTAGE1 | 1 | 1 | 2 | N |
| hsa-miR-4667-5p | IQSEC3 | 1 | 1 | 2 | N |
| hsa-miR-4667-5p | PCDHGB7 | 1 | 1 | 2 | N |
| hsa-miR-4667-5p | DHRS3 | 1 | 1 | 2 | N |
| hsa-miR-4667-5p | CDR2L | 1 | 1 | 2 | N |
| hsa-miR-4667-5p | NR1I3 | 1 | 1 | 2 | N |
| hsa-miR-4667-5p | SYPL2 | 1 | 1 | 2 | N |
| hsa-miR-4667-5p | PCDHGA10 | 1 | 1 | 2 | N |
| hsa-miR-4667-5p | PCDHGC3 | 1 | 1 | 2 | N |
| hsa-miR-4667-5p | ABCC10 | 1 | 1 | 2 | N |
| hsa-miR-4667-5p | RGL3 | 1 | 1 | 2 | N |
| hsa-miR-4667-5p | PCDHGA9 | 1 | 1 | 2 | N |
| hsa-miR-4667-5p | THRA | 1 | 1 | 2 | N |
| hsa-miR-4667-5p | PFKFB4 | 1 | 1 | 2 | N |
| hsa-miR-4667-5p | IVL | 1 | 1 | 2 | N |
| hsa-miR-4667-5p | KDELR1 | 1 | 1 | 2 | N |
| hsa-miR-4667-5p | SELM | 1 | 1 | 2 | N |
| hsa-miR-4667-5p | ARHGEF6 | 1 | 1 | 2 | N |
| hsa-miR-4667-5p | ITPRIP | 1 | 1 | 2 | N |
| hsa-miR-4667-5p | EGLN3 | 1 | 1 | 2 | N |
| hsa-miR-4667-5p | ATXN1L | 1 | 1 | 2 | N |
| hsa-miR-4667-5p | ARHGAP36 | 1 | 1 | 2 | N |
| hsa-miR-4667-5p | ABCG4 | 1 | 1 | 2 | N |
| hsa-miR-4667-5p | VAT1 | 1 | 1 | 2 | N |
| hsa-miR-4667-5p | ADPGK | 1 | 1 | 2 | N |
| hsa-miR-4667-5p | TCF7 | 1 | 1 | 2 | N |
| hsa-miR-4667-5p | FBXO41 | 1 | 1 | 2 | N |
| hsa-miR-4667-5p | LRRC16B | 1 | 1 | 2 | N |
| hsa-miR-4667-5p | ZNF831 | 1 | 1 | 2 | N |
| hsa-miR-4667-5p | MECR | 1 | 1 | 2 | N |
| hsa-miR-4667-5p | GPD1 | 1 | 1 | 2 | N |
| hsa-miR-4667-5p | SNX12 | 1 | 1 | 2 | N |
| hsa-miR-4667-5p | TM9SF4 | 1 | 1 | 2 | N |
| hsa-miR-4667-5p | SLC6A9 | 1 | 1 | 2 | N |
| hsa-miR-4667-5p | IGFBP5 | 1 | 1 | 2 | N |
| hsa-miR-4667-5p | CCDC178 | 1 | 1 | 2 | N |
| hsa-miR-4667-5p | FUT11 | 1 | 1 | 2 | N |
| hsa-miR-4667-5p | SLC25A32 | 1 | 1 | 2 | N |
| hsa-miR-4667-5p | KIAA0319 | 1 | 1 | 2 | N |
| hsa-miR-4667-5p | SSX4 | 1 | 1 | 2 | N |
| hsa-miR-4667-5p | VSTM4 | 1 | 1 | 2 | N |
| hsa-miR-4667-5p | PCDHGA8 | 1 | 1 | 2 | N |
| hsa-miR-4667-5p | PTPLAD1 | 1 | 1 | 2 | N |
| hsa-miR-4667-5p | STX17 | 1 | 1 | 2 | N |
| hsa-miR-4667-5p | SUSD2 | 1 | 1 | 2 | N |
| hsa-miR-4667-5p | C15orf56 | 1 | 1 | 2 | N |
| hsa-miR-4667-5p | FAIM2 | 1 | 1 | 2 | N |
| hsa-miR-4667-5p | FGR | 1 | 1 | 2 | N |
| hsa-miR-4667-5p | SCAMP5 | 1 | 1 | 2 | N |
| hsa-miR-4667-5p | UNC5B | 1 | 1 | 2 | Y |
| hsa-miR-4667-5p | UNC13B | 1 | 1 | 2 | N |
| hsa-miR-4667-5p | CPLX2 | 1 | 1 | 2 | N |
| hsa-miR-4667-5p | SLC7A2 | 1 | 1 | 2 | N |
| hsa-miR-4667-5p | MBD6 | 1 | 1 | 2 | N |
| hsa-miR-4667-5p | RARB | 1 | 1 | 2 | N |
| hsa-miR-4667-5p | CABP5 | 1 | 1 | 2 | N |
| hsa-miR-4667-5p | LASP1 | 1 | 1 | 2 | N |
| hsa-miR-4667-5p | VAMP2 | 1 | 1 | 2 | N |
| hsa-miR-4667-5p | SSX4B | 1 | 1 | 2 | N |
| hsa-miR-4667-5p | CD207 | 1 | 1 | 2 | N |
| hsa-miR-4667-5p | PCDHGA3 | 1 | 1 | 2 | N |
| hsa-miR-4667-5p | TOM1L2 | 1 | 1 | 2 | N |
| hsa-miR-4667-5p | SALL1 | 1 | 1 | 2 | N |
| hsa-miR-4667-5p | ZNF302 | 1 | 1 | 2 | N |
| hsa-miR-4667-5p | MAPK1 | 1 | 1 | 2 | N |
| hsa-miR-4667-5p | NAGA | 1 | 1 | 2 | N |
| hsa-miR-4667-5p | LYPD6 | 1 | 1 | 2 | N |
| hsa-miR-4667-5p | TSPYL5 | 1 | 1 | 2 | N |
| hsa-miR-4667-5p | CALN1 | 1 | 1 | 2 | N |
| hsa-miR-4667-5p | ATP8B2 | 1 | 1 | 2 | N |
| hsa-miR-4667-5p | KCNJ10 | 1 | 1 | 2 | N |
| hsa-miR-4667-5p | ORMDL3 | 1 | 1 | 2 | N |
| hsa-miR-4667-5p | GLP1R | 1 | 1 | 2 | N |
| hsa-miR-4700-5p | ABCC10 | 1 | 1 | 2 | N |
| hsa-miR-4700-5p | TSPAN11 | 1 | 1 | 2 | N |
| hsa-miR-4700-5p | PTPLAD1 | 1 | 1 | 2 | N |
| hsa-miR-4700-5p | DUSP3 | 1 | 1 | 2 | N |
| hsa-miR-4700-5p | PCDHGA8 | 1 | 1 | 2 | N |
| hsa-miR-4700-5p | HAPLN4 | 1 | 1 | 2 | N |
| hsa-miR-4700-5p | SEPN1 | 1 | 1 | 2 | N |
| hsa-miR-4700-5p | C17orf107 | 1 | 1 | 2 | N |
| hsa-miR-4700-5p | IVL | 1 | 1 | 2 | N |
| hsa-miR-4700-5p | PCDHGA5 | 1 | 1 | 2 | N |
| hsa-miR-4700-5p | ERGIC1 | 1 | 1 | 2 | N |
| hsa-miR-4700-5p | SLC7A2 | 1 | 1 | 2 | N |
| hsa-miR-4700-5p | MS4A15 | 1 | 1 | 2 | N |
| hsa-miR-4700-5p | MECR | 1 | 1 | 2 | N |
| hsa-miR-4700-5p | SNX12 | 1 | 1 | 2 | N |
| hsa-miR-4700-5p | LASP1 | 1 | 1 | 2 | N |
| hsa-miR-4700-5p | NCDN | 1 | 1 | 2 | N |
| hsa-miR-4700-5p | KCNJ10 | 1 | 1 | 2 | N |
| hsa-miR-4700-5p | NEK9 | 1 | 1 | 2 | N |
| hsa-miR-4700-5p | UBE2L3 | 1 | 1 | 2 | N |
| hsa-miR-4700-5p | TMEM127 | 1 | 1 | 2 | N |
| hsa-miR-4700-5p | SAMD12 | 1 | 1 | 2 | N |
| hsa-miR-4700-5p | IGFBP5 | 1 | 1 | 2 | N |
| hsa-miR-4700-5p | NR6A1 | 1 | 1 | 2 | N |
| hsa-miR-4700-5p | SYT2 | 1 | 1 | 2 | N |
| hsa-miR-4700-5p | TSPAN18 | 1 | 1 | 2 | N |
| hsa-miR-4700-5p | PCDHGA10 | 1 | 1 | 2 | N |
| hsa-miR-4700-5p | VAT1 | 1 | 1 | 2 | N |
| hsa-miR-4700-5p | TTF2 | 1 | 1 | 2 | N |
| hsa-miR-4700-5p | GLP1R | 1 | 1 | 2 | N |
| hsa-miR-4700-5p | PDPK1 | 1 | 1 | 2 | N |
| hsa-miR-4700-5p | CARTPT | 1 | 1 | 2 | N |
| hsa-miR-4700-5p | PCDHGA1 | 1 | 1 | 2 | N |
| hsa-miR-4700-5p | PCDHGB7 | 1 | 1 | 2 | N |
| hsa-miR-4700-5p | KCNK3 | 1 | 1 | 2 | N |
| hsa-miR-4700-5p | FBLN1 | 1 | 1 | 2 | N |
| hsa-miR-4700-5p | UNC13B | 1 | 1 | 2 | N |
| hsa-miR-4700-5p | SLC36A3 | 1 | 1 | 2 | N |
| hsa-miR-4700-5p | CABP4 | 1 | 1 | 2 | N |
| hsa-miR-4700-5p | ARHGAP36 | 1 | 1 | 2 | N |
| hsa-miR-4700-5p | PCDHGC5 | 1 | 1 | 2 | N |
| hsa-miR-4700-5p | PRKCG | 1 | 1 | 2 | N |
| hsa-miR-4700-5p | POPDC3 | 1 | 1 | 2 | N |
| hsa-miR-4700-5p | PFKFB4 | 1 | 1 | 2 | N |
| hsa-miR-4700-5p | ABCG4 | 1 | 1 | 2 | N |
| hsa-miR-4700-5p | PCDHGB3 | 1 | 1 | 2 | N |
| hsa-miR-4700-5p | UNC5B | 1 | 1 | 2 | Y |
| hsa-miR-4700-5p | NHLH1 | 1 | 1 | 2 | N |
| hsa-miR-4700-5p | SPATA2L | 1 | 1 | 2 | N |
| hsa-miR-4700-5p | RGL3 | 1 | 1 | 2 | N |
| hsa-miR-4700-5p | RASSF4 | 1 | 1 | 2 | N |
| hsa-miR-4700-5p | XPO7 | 1 | 1 | 2 | N |
| hsa-miR-4700-5p | LOXHD1 | 1 | 1 | 2 | N |
| hsa-miR-4700-5p | PCDHGA7 | 1 | 1 | 2 | N |
| hsa-miR-4700-5p | KDELR1 | 1 | 1 | 2 | N |
| hsa-miR-4700-5p | UBE2L6 | 1 | 1 | 2 | N |
| hsa-miR-4700-5p | RRN3 | 1 | 1 | 2 | N |
| hsa-miR-4700-5p | HCCS | 1 | 1 | 2 | N |
| hsa-miR-4700-5p | SYT12 | 1 | 1 | 2 | N |
| hsa-miR-4700-5p | IQSEC3 | 1 | 1 | 2 | N |
| hsa-miR-4700-5p | SLC16A2 | 1 | 1 | 2 | N |
| hsa-miR-4700-5p | MAPK1 | 1 | 1 | 2 | N |
| hsa-miR-4700-5p | SPATA33 | 1 | 1 | 2 | N |
| hsa-miR-4700-5p | KIAA0319 | 1 | 1 | 2 | N |
| hsa-miR-4700-5p | GLIPR1 | 1 | 1 | 2 | N |
| hsa-miR-4700-5p | CDR2L | 1 | 1 | 2 | N |
| hsa-miR-4700-5p | CAPN12 | 1 | 1 | 2 | N |
| hsa-miR-4700-5p | SSX1 | 1 | 1 | 2 | N |
| hsa-miR-4700-5p | PXN | 1 | 1 | 2 | N |
| hsa-miR-4700-5p | C15orf56 | 1 | 1 | 2 | N |
| hsa-miR-4700-5p | NAA40 | 1 | 1 | 2 | N |
| hsa-miR-4700-5p | ATP8B2 | 1 | 1 | 2 | N |
| hsa-miR-4700-5p | GDNF | 1 | 1 | 2 | N |
| hsa-miR-4700-5p | FAM127B | 1 | 1 | 2 | N |
| hsa-miR-4700-5p | CPLX2 | 1 | 1 | 2 | N |
| hsa-miR-4700-5p | RGSL1 | 1 | 1 | 2 | N |
| hsa-miR-4700-5p | NAGA | 1 | 1 | 2 | N |
| hsa-miR-4700-5p | SLC25A32 | 1 | 1 | 2 | N |
| hsa-miR-4700-5p | SH2D3C | 1 | 1 | 2 | N |
| hsa-miR-4700-5p | BAK1 | 1 | 1 | 2 | N |
| hsa-miR-4700-5p | FOXRED1 | 1 | 1 | 2 | N |
| hsa-miR-4700-5p | TOM1L2 | 1 | 1 | 2 | N |
| hsa-miR-4700-5p | ARFIP1 | 1 | 1 | 2 | N |
| hsa-miR-4700-5p | RBM14 | 1 | 1 | 2 | N |
| hsa-miR-4700-5p | TSPYL5 | 1 | 1 | 2 | N |
| hsa-miR-4700-5p | GAB2 | 1 | 1 | 2 | N |
| hsa-miR-4700-5p | CABP5 | 1 | 1 | 2 | N |
| hsa-miR-4700-5p | TP53INP2 | 1 | 1 | 2 | N |
| hsa-miR-4700-5p | SLC38A7 | 1 | 1 | 2 | N |
| hsa-miR-4700-5p | CD207 | 1 | 1 | 2 | N |
| hsa-miR-4700-5p | CCL2 | 1 | 1 | 2 | N |
| hsa-miR-4700-5p | NUMA1 | 1 | 1 | 2 | N |
| hsa-miR-4700-5p | PCDHGC3 | 1 | 1 | 2 | N |
| hsa-miR-4700-5p | SPIB | 1 | 1 | 2 | N |
| hsa-miR-4700-5p | HSF2BP | 1 | 1 | 2 | N |
| hsa-miR-4700-5p | DAGLA | 1 | 1 | 2 | N |
| hsa-miR-4700-5p | GPD1 | 1 | 1 | 2 | N |
| hsa-miR-4700-5p | MAT2A | 1 | 1 | 2 | N |
| hsa-miR-4700-5p | FUT11 | 1 | 1 | 2 | N |
| hsa-miR-4700-5p | DHRS3 | 1 | 1 | 2 | N |
| hsa-miR-4700-5p | SLC6A9 | 1 | 1 | 2 | N |
| hsa-miR-4700-5p | RARG | 1 | 1 | 2 | N |
| hsa-miR-4700-5p | MUT | 1 | 1 | 2 | N |
| hsa-miR-4700-5p | RARB | 1 | 1 | 2 | N |
| hsa-miR-4700-5p | MRO | 1 | 1 | 2 | N |
| hsa-miR-4700-5p | OSBPL7 | 1 | 1 | 2 | N |
| hsa-miR-4700-5p | ATXN1L | 1 | 1 | 2 | N |
| hsa-miR-4700-5p | PCDHGA3 | 1 | 1 | 2 | N |
| hsa-miR-4700-5p | NRIP2 | 1 | 1 | 2 | N |
| hsa-miR-4700-5p | ICK | 1 | 1 | 2 | N |
| hsa-miR-4700-5p | SDC3 | 1 | 1 | 2 | N |
| hsa-miR-4700-5p | LYPD6 | 1 | 1 | 2 | N |
| hsa-miR-4700-5p | PCDHGA11 | 1 | 1 | 2 | N |
| hsa-miR-4700-5p | NR1I3 | 1 | 1 | 2 | N |
| hsa-miR-4700-5p | ATN1 | 1 | 1 | 2 | N |
| hsa-miR-4700-5p | ZBTB39 | 1 | 1 | 2 | N |
| hsa-miR-4700-5p | PPP1R1B | 1 | 1 | 2 | N |
| hsa-miR-4700-5p | CIC | 1 | 1 | 2 | N |
| hsa-miR-4700-5p | SCAMP4 | 1 | 1 | 2 | N |
| hsa-miR-4700-5p | FBXO41 | 1 | 1 | 2 | N |
| hsa-miR-4700-5p | TM9SF4 | 1 | 1 | 2 | N |
| hsa-miR-4700-5p | ADPGK | 1 | 1 | 2 | N |
| hsa-miR-4700-5p | SPERT | 1 | 1 | 2 | N |
| hsa-miR-4700-5p | RNASE13 | 1 | 1 | 2 | N |
| hsa-miR-4700-5p | KDM8 | 1 | 1 | 2 | N |
| hsa-miR-4700-5p | VTI1A | 1 | 1 | 2 | N |
| hsa-miR-4700-5p | LRRC16B | 1 | 1 | 2 | N |
| hsa-miR-4700-5p | BCL2L1 | 1 | 1 | 2 | N |
| hsa-miR-4700-5p | ARHGEF6 | 1 | 1 | 2 | N |
| hsa-miR-4700-5p | C4orf46 | 1 | 1 | 2 | N |
| hsa-miR-4700-5p | SYPL2 | 1 | 1 | 2 | N |
| hsa-miR-4700-5p | P2RY2 | 1 | 1 | 2 | N |
| hsa-miR-4700-5p | ZNF831 | 1 | 1 | 2 | N |
| hsa-miR-4700-5p | ZNF548 | 1 | 1 | 2 | N |
| hsa-miR-4700-5p | PCDHGA12 | 1 | 1 | 2 | N |
| hsa-miR-4700-5p | LZTS1 | 1 | 1 | 2 | N |
| hsa-miR-4700-5p | PSMD5 | 1 | 1 | 2 | N |
| hsa-miR-4700-5p | THAP11 | 1 | 1 | 2 | N |
| hsa-miR-4700-5p | FAIM2 | 1 | 1 | 2 | N |
| hsa-miR-4700-5p | ZNF302 | 1 | 1 | 2 | N |
| hsa-miR-4700-5p | ITGA10 | 1 | 1 | 2 | N |
| hsa-miR-4700-5p | CTAGE1 | 1 | 1 | 2 | N |
| hsa-miR-4700-5p | PSMB11 | 1 | 1 | 2 | N |
| hsa-miR-4700-5p | THRA | 1 | 1 | 2 | N |
| hsa-miR-4700-5p | SELM | 1 | 1 | 2 | N |
| hsa-miR-4700-5p | ITPRIP | 1 | 1 | 2 | N |
| hsa-miR-4700-5p | MBD6 | 1 | 1 | 2 | N |
| hsa-miR-4700-5p | PPP1R10 | 1 | 1 | 2 | N |
| hsa-miR-4700-5p | ARID3B | 1 | 1 | 2 | N |
| hsa-miR-4700-5p | TSC22D4 | 1 | 1 | 2 | N |
| hsa-miR-4700-5p | TCF7 | 1 | 1 | 2 | N |
| hsa-miR-4700-5p | NGB | 1 | 1 | 2 | N |
| hsa-miR-4700-5p | SLC25A42 | 1 | 1 | 2 | N |
| hsa-miR-4700-5p | EGLN3 | 1 | 1 | 2 | N |
| hsa-miR-4700-5p | SUSD2 | 1 | 1 | 2 | N |
| hsa-miR-4700-5p | PCDHGA2 | 1 | 1 | 2 | N |
| hsa-miR-4700-5p | FAM228B | 1 | 1 | 2 | N |
| hsa-miR-4700-5p | TBC1D13 | 1 | 1 | 2 | Y |
| hsa-miR-4700-5p | ATF6B | 1 | 1 | 2 | N |
| hsa-miR-4700-5p | STX17 | 1 | 1 | 2 | N |
| hsa-miR-4700-5p | SALL1 | 1 | 1 | 2 | N |
| hsa-miR-4700-5p | DRG2 | 1 | 1 | 2 | N |
| hsa-miR-4700-5p | FGF18 | 1 | 1 | 2 | N |
| hsa-miR-4700-5p | PCDHGA9 | 1 | 1 | 2 | N |
| hsa-miR-4700-5p | CELF6 | 1 | 1 | 2 | N |
| hsa-miR-4700-5p | CACNB1 | 1 | 1 | 2 | N |
| hsa-miR-4700-5p | TMEM63B | 1 | 1 | 2 | N |
| hsa-miR-4700-5p | CALN1 | 1 | 1 | 2 | N |
| hsa-miR-4700-5p | HSDL1 | 1 | 1 | 2 | N |
| hsa-miR-4700-5p | RNF170 | 1 | 1 | 2 | N |
| hsa-miR-4700-5p | CCDC178 | 1 | 1 | 2 | N |
| hsa-miR-4700-5p | SPR | 1 | 1 | 2 | N |
| hsa-miR-4700-5p | ELMOD1 | 1 | 1 | 2 | N |
| hsa-miR-4700-5p | KIF24 | 1 | 1 | 2 | N |
| hsa-miR-4700-5p | NCEH1 | 1 | 1 | 2 | Y |
| hsa-miR-4700-5p | VAMP2 | 1 | 1 | 2 | N |
| hsa-miR-4708-3p | CCR10 | 1 | 1 | 2 | N |
| hsa-miR-4708-3p | FAM181A | 1 | 1 | 2 | N |
| hsa-miR-4708-3p | LUC7L3 | 1 | 1 | 2 | N |
| hsa-miR-4708-3p | NFIA | 1 | 1 | 2 | N |
| hsa-miR-4708-3p | NTM | 1 | 1 | 2 | N |
| hsa-miR-4708-3p | C19orf82 | 1 | 1 | 2 | N |
| hsa-miR-4708-3p | PRKG1 | 1 | 1 | 2 | N |
| hsa-miR-4708-3p | GCNT3 | 1 | 1 | 2 | N |
| hsa-miR-4708-3p | PALM2 | 1 | 1 | 2 | N |
| hsa-miR-4708-3p | CPN2 | 1 | 1 | 2 | N |
| hsa-miR-4708-3p | CCDC103 | 1 | 1 | 2 | N |
| hsa-miR-4708-3p | ASB7 | 1 | 1 | 2 | N |
| hsa-miR-4708-3p | AKAP6 | 1 | 1 | 2 | N |
| hsa-miR-4708-3p | TCF21 | 1 | 1 | 2 | N |
| hsa-miR-4708-3p | DYNLL1 | 1 | 1 | 2 | N |
| hsa-miR-4708-3p | RALGAPB | 1 | 1 | 2 | N |
| hsa-miR-4708-3p | APLF | 1 | 1 | 2 | Y |
| hsa-miR-4708-3p | PCDH8 | 1 | 1 | 2 | N |
| hsa-miR-4708-3p | LINGO2 | 1 | 1 | 2 | N |
| hsa-miR-4708-3p | FGFR1OP | 1 | 1 | 2 | N |
| hsa-miR-4708-3p | USP38 | 1 | 1 | 2 | N |
| hsa-miR-4708-3p | PRDM5 | 1 | 1 | 2 | N |
| hsa-miR-4708-3p | LTBP2 | 1 | 1 | 2 | Y |
| hsa-miR-4708-3p | TP53INP1 | 1 | 1 | 2 | N |
| hsa-miR-4708-3p | FAM65B | 1 | 1 | 2 | N |
| hsa-miR-4708-3p | UBE2O | 1 | 1 | 2 | N |
| hsa-miR-4708-3p | SLC9A6 | 1 | 1 | 2 | N |
| hsa-miR-4708-3p | NECAP1 | 1 | 1 | 2 | N |
| hsa-miR-4708-3p | MCU | 1 | 1 | 2 | N |
| hsa-miR-4708-3p | RNF169 | 1 | 1 | 2 | N |
| hsa-miR-4708-3p | CTNNB1 | 1 | 1 | 2 | N |
| hsa-miR-4708-3p | SIDT2 | 1 | 1 | 2 | N |
| hsa-miR-4708-3p | GRB2 | 1 | 1 | 2 | N |
| hsa-miR-4708-3p | SRSF2 | 1 | 1 | 2 | Y |
| hsa-miR-4708-3p | UBTD1 | 1 | 1 | 2 | N |
| hsa-miR-4708-3p | SLC22A3 | 1 | 1 | 2 | N |
| hsa-miR-4708-3p | NOTCH3 | 1 | 1 | 2 | N |
| hsa-miR-4708-3p | CHIC2 | 1 | 1 | 2 | N |
| hsa-miR-4708-3p | LGALS8 | 1 | 1 | 2 | N |
| hsa-miR-4708-3p | KIAA1024 | 1 | 1 | 2 | N |
| hsa-miR-4708-3p | MYRIP | 1 | 1 | 2 | N |
| hsa-miR-4708-3p | CASP2 | 1 | 1 | 2 | N |
| hsa-miR-4708-3p | ZNF275 | 1 | 1 | 2 | N |
| hsa-miR-4708-3p | SPCS3 | 1 | 1 | 2 | N |
| hsa-miR-4708-3p | ITGB5 | 1 | 1 | 2 | N |
| hsa-miR-4708-3p | CAPRIN1 | 1 | 1 | 2 | N |
| hsa-miR-4708-3p | SPOCK3 | 1 | 1 | 2 | N |
| hsa-miR-4708-3p | C10orf128 | 1 | 1 | 2 | N |
| hsa-miR-4708-3p | SERP1 | 1 | 1 | 2 | N |
| hsa-miR-4708-3p | SMAD1 | 1 | 1 | 2 | N |
| hsa-miR-4708-3p | LPHN2 | 1 | 1 | 2 | N |
| hsa-miR-4708-3p | DAAM1 | 1 | 1 | 2 | N |
| hsa-miR-4708-3p | TMEFF2 | 1 | 1 | 2 | N |
| hsa-miR-4708-3p | SPRR1B | 1 | 1 | 2 | N |
| hsa-miR-4708-3p | SYNCRIP | 1 | 1 | 2 | N |
| hsa-miR-4708-3p | SETD7 | 1 | 1 | 2 | N |
| hsa-miR-4708-3p | DUOXA1 | 1 | 1 | 2 | N |
| hsa-miR-4708-3p | UXS1 | 1 | 1 | 2 | N |
| hsa-miR-4708-3p | HNRNPLL | 1 | 1 | 2 | N |
| hsa-miR-4708-3p | FRMD4B | 1 | 1 | 2 | N |
| hsa-miR-4708-3p | NEMF | 1 | 1 | 2 | N |
| hsa-miR-4708-3p | DOLPP1 | 1 | 1 | 2 | N |
| hsa-miR-4708-3p | CETN2 | 1 | 1 | 2 | N |
| hsa-miR-4708-3p | ZNF521 | 1 | 1 | 2 | N |
| hsa-miR-4708-3p | MATR3 | 1 | 1 | 2 | Y |
| hsa-miR-4708-3p | CCNE2 | 1 | 1 | 2 | N |
| hsa-miR-4708-3p | HOGA1 | 1 | 1 | 2 | N |
| hsa-miR-4708-3p | SMC1B | 1 | 1 | 2 | N |
| hsa-miR-4708-3p | BEAN1 | 1 | 1 | 2 | N |
| hsa-miR-4708-3p | PUM2 | 1 | 1 | 2 | N |
| hsa-miR-4708-3p | RBCK1 | 1 | 1 | 2 | N |
| hsa-miR-4708-3p | PRKD1 | 1 | 1 | 2 | N |
| hsa-miR-4708-3p | OPRK1 | 1 | 1 | 2 | N |
| hsa-miR-4708-3p | NUP210 | 1 | 1 | 2 | N |
| hsa-miR-4708-3p | PTK2B | 1 | 1 | 2 | N |
| hsa-miR-4708-3p | SLC25A13 | 1 | 1 | 2 | N |
| hsa-miR-4750-3p | WBP1L | 1 | 1 | 2 | N |
| hsa-miR-4750-3p | SYNPO2L | 1 | 1 | 2 | N |
| hsa-miR-4750-3p | KLF3 | 1 | 1 | 2 | N |
| hsa-miR-4750-3p | SNX8 | 1 | 1 | 2 | N |
| hsa-miR-4750-3p | TMEM154 | 1 | 1 | 2 | N |
| hsa-miR-4750-3p | RHEB | 1 | 1 | 2 | N |
| hsa-miR-4750-3p | COPS7B | 1 | 1 | 2 | N |
| hsa-miR-4750-3p | C9orf43 | 1 | 1 | 2 | N |
| hsa-miR-4750-3p | CYP2C18 | 1 | 1 | 2 | N |
| hsa-miR-4750-3p | INSM2 | 1 | 1 | 2 | N |
| hsa-miR-4750-3p | RIC3 | 1 | 1 | 2 | N |
| hsa-miR-4750-3p | ZNF189 | 1 | 1 | 2 | N |
| hsa-miR-4750-3p | HS6ST1 | 1 | 1 | 2 | N |
| hsa-miR-4750-3p | LSM14B | 1 | 1 | 2 | N |
| hsa-miR-4750-3p | PAK6 | 1 | 1 | 2 | N |
| hsa-miR-4750-3p | PPTC7 | 1 | 1 | 2 | N |
| hsa-miR-4750-3p | TUFT1 | 1 | 1 | 2 | N |
| hsa-miR-4750-3p | MTF1 | 1 | 1 | 2 | N |
| hsa-miR-4750-3p | NEDD9 | 1 | 1 | 2 | N |
| hsa-miR-4750-3p | FLRT1 | 1 | 1 | 2 | N |
| hsa-miR-4750-3p | YIPF4 | 1 | 1 | 2 | N |
| hsa-miR-4750-3p | RFX2 | 1 | 1 | 2 | N |
| hsa-miR-4750-3p | HEATR5B | 1 | 1 | 2 | N |
| hsa-miR-4750-3p | RHOQ | 1 | 1 | 2 | N |
| hsa-miR-4750-3p | RTKN2 | 1 | 1 | 2 | N |
| hsa-miR-4750-3p | RGP1 | 1 | 1 | 2 | N |
| hsa-miR-4750-3p | NPNT | 1 | 1 | 2 | N |
| hsa-miR-4750-3p | TRAF1 | 1 | 1 | 2 | N |
| hsa-miR-4750-3p | RNF44 | 1 | 1 | 2 | N |
| hsa-miR-4750-3p | NPAS3 | 1 | 1 | 2 | N |
| hsa-miR-6805-5p | KMT2D | 1 | 1 | 2 | N |
| hsa-miR-6805-5p | EGFL8 | 1 | 1 | 2 | N |
| hsa-miR-6805-5p | TSC22D4 | 1 | 1 | 2 | N |
| hsa-miR-6805-5p | UNC5B | 1 | 1 | 2 | N |
| hsa-miR-6805-5p | PVRL1 | 1 | 1 | 2 | N |
| hsa-miR-6805-5p | MAP7D1 | 1 | 1 | 2 | N |
| hsa-miR-6805-5p | METAP1 | 1 | 1 | 2 | N |
| hsa-miR-6805-5p | ESRRA | 1 | 1 | 2 | N |
| hsa-miR-6805-5p | PTP4A2 | 1 | 1 | 2 | N |
| hsa-miR-6805-5p | AHDC1 | 1 | 1 | 2 | Y |
| hsa-miR-6805-5p | BHLHE40 | 1 | 1 | 2 | N |
| hsa-miR-6805-5p | T | 1 | 1 | 2 | N |
| hsa-miR-6805-5p | CDKN1A | 1 | 1 | 2 | Y |
| hsa-miR-6805-5p | FZD9 | 1 | 1 | 2 | N |
| hsa-miR-6805-5p | FAM163A | 1 | 1 | 2 | N |
| hsa-miR-6805-5p | KCNC3 | 1 | 1 | 2 | N |
| hsa-miR-6805-5p | WFIKKN2 | 1 | 1 | 2 | N |
| hsa-miR-6805-5p | MANSC1 | 1 | 1 | 2 | N |
| hsa-miR-6805-5p | SVOP | 1 | 1 | 2 | N |
| hsa-miR-6805-5p | RAPGEF1 | 1 | 1 | 2 | Y |
| hsa-miR-6805-5p | SIPA1L1 | 1 | 1 | 2 | N |
| hsa-miR-6805-5p | CHD5 | 1 | 1 | 2 | N |
| hsa-miR-6805-5p | NIPAL3 | 1 | 1 | 2 | N |
| hsa-miR-6805-5p | SBK1 | 1 | 1 | 2 | N |
| hsa-miR-6805-5p | KIAA0195 | 1 | 1 | 2 | N |
| hsa-miR-6805-5p | SOX12 | 1 | 1 | 2 | Y |
| hsa-miR-6805-5p | DPYSL2 | 1 | 1 | 2 | N |
| hsa-miR-6805-5p | KLK4 | 1 | 1 | 2 | N |
| hsa-miR-6805-5p | SLC6A17 | 1 | 1 | 2 | N |
| hsa-miR-6805-5p | ATXN2L | 1 | 1 | 2 | N |
| hsa-miR-6805-5p | BAIAP2L2 | 1 | 1 | 2 | N |
| hsa-miR-6805-5p | KCNA3 | 1 | 1 | 2 | N |
| hsa-miR-6805-5p | TRABD2B | 1 | 1 | 2 | N |
| hsa-miR-6805-5p | SLC25A23 | 1 | 1 | 2 | N |
| hsa-miR-6805-5p | DAZL | 1 | 1 | 2 | N |
| hsa-miR-6805-5p | DACT3 | 1 | 1 | 2 | N |
| hsa-miR-6805-5p | MLLT6 | 1 | 1 | 2 | N |
| hsa-miR-6805-5p | DMBX1 | 1 | 1 | 2 | N |
| hsa-miR-6805-5p | NCS1 | 1 | 1 | 2 | Y |
| hsa-miR-6805-5p | SPRY4 | 1 | 1 | 2 | N |
| hsa-miR-6805-5p | QRICH1 | 1 | 1 | 2 | N |
| hsa-miR-6805-5p | KIAA1644 | 1 | 1 | 2 | N |
| hsa-miR-6805-5p | R3HDM4 | 1 | 1 | 2 | N |
| hsa-miR-6805-5p | SCNN1A | 1 | 1 | 2 | N |
| hsa-miR-6805-5p | GANAB | 1 | 1 | 2 | N |
| hsa-miR-6805-5p | EFNA3 | 1 | 1 | 2 | N |
| hsa-miR-6805-5p | NEDD8 | 1 | 1 | 2 | N |
| hsa-miR-6805-5p | PHKA2 | 1 | 1 | 2 | N |
| hsa-miR-6805-5p | TP53INP2 | 1 | 1 | 2 | N |
| hsa-miR-6805-5p | TWSG1 | 1 | 1 | 2 | N |
| hsa-miR-6805-5p | ZNF385A | 1 | 1 | 2 | Y |
| hsa-miR-6805-5p | MINOS1 | 1 | 1 | 2 | N |
| hsa-miR-6805-5p | FOXN1 | 1 | 1 | 2 | N |
| hsa-miR-6805-5p | RHOC | 1 | 1 | 2 | N |
| hsa-miR-6805-5p | NLGN2 | 1 | 1 | 2 | N |
| hsa-miR-6805-5p | MARK2 | 1 | 1 | 2 | Y |
| hsa-miR-6805-5p | CNPY3 | 1 | 1 | 2 | N |
| hsa-miR-6805-5p | MAF | 1 | 1 | 2 | N |
| hsa-miR-6805-5p | SOX10 | 1 | 1 | 2 | N |
| hsa-miR-6805-5p | TSPAN11 | 1 | 1 | 2 | N |
| hsa-miR-6805-5p | DAGLA | 1 | 1 | 2 | N |
| hsa-miR-6805-5p | ZKSCAN2 | 1 | 1 | 2 | N |
| hsa-miR-6805-5p | DBF4B | 1 | 1 | 2 | N |
| hsa-miR-6805-5p | ASL | 1 | 1 | 2 | N |
| hsa-miR-6805-5p | PDAP1 | 1 | 1 | 2 | N |
| hsa-miR-6805-5p | DGCR8 | 1 | 1 | 2 | N |
| hsa-miR-6805-5p | H1F0 | 1 | 1 | 2 | N |
| hsa-miR-6805-5p | BHLHE41 | 1 | 1 | 2 | N |
| hsa-miR-6805-5p | ZNRF3 | 1 | 1 | 2 | N |
| hsa-miR-6805-5p | LPHN1 | 1 | 1 | 2 | N |
| hsa-miR-6805-5p | RNF141 | 1 | 1 | 2 | N |
| hsa-miR-6805-5p | B4GALT2 | 1 | 1 | 2 | N |
| hsa-miR-6805-5p | ISL1 | 1 | 1 | 2 | N |
| hsa-miR-6805-5p | NFIC | 1 | 1 | 2 | Y |
| hsa-miR-6805-5p | MRS2 | 1 | 1 | 2 | N |
| hsa-miR-6805-5p | PPP2R4 | 1 | 1 | 2 | N |
| hsa-miR-6805-5p | AR | 1 | 1 | 2 | N |
| hsa-miR-6805-5p | TMEM127 | 1 | 1 | 2 | N |
| hsa-miR-6805-5p | ATP2A3 | 1 | 1 | 2 | N |
| hsa-miR-6805-5p | TMEM235 | 1 | 1 | 2 | N |
| hsa-miR-6805-5p | CD93 | 1 | 1 | 2 | N |
| hsa-miR-6805-5p | CSRNP1 | 1 | 1 | 2 | N |
| hsa-miR-6805-5p | CLK2 | 1 | 1 | 2 | N |
| hsa-miR-6805-5p | TMCC2 | 1 | 1 | 2 | N |
| hsa-miR-6805-5p | SERPINA1 | 1 | 1 | 2 | N |
| hsa-miR-6805-5p | VSTM2L | 1 | 1 | 2 | N |
| hsa-miR-6805-5p | PMF1 | 1 | 1 | 2 | N |
| hsa-miR-6805-5p | VPS37C | 1 | 1 | 2 | Y |
| hsa-miR-6805-5p | PPP2R2A | 1 | 1 | 2 | Y |
| hsa-miR-6805-5p | TNRC6B | 1 | 1 | 2 | N |
| hsa-miR-6805-5p | NR1D1 | 1 | 1 | 2 | N |
| hsa-miR-6805-5p | MEGF11 | 1 | 1 | 2 | N |
| hsa-miR-6805-5p | SRR | 1 | 1 | 2 | N |
| hsa-miR-6805-5p | FAM131A | 1 | 1 | 2 | N |
| hsa-miR-6805-5p | TATDN2 | 1 | 1 | 2 | N |
| hsa-miR-6846-5p | RUSC2 | 1 | 1 | 2 | N |
| hsa-miR-6846-5p | DNM1 | 1 | 1 | 2 | N |
| hsa-miR-6846-5p | SPRN | 1 | 1 | 2 | N |
| hsa-miR-6846-5p | KCNQ4 | 1 | 1 | 2 | N |
| hsa-miR-6846-5p | BHLHE40 | 1 | 1 | 2 | N |
| hsa-miR-6846-5p | PDE4A | 1 | 1 | 2 | Y |
| hsa-miR-6846-5p | CTIF | 1 | 1 | 2 | N |
| hsa-miR-6846-5p | CACNB3 | 1 | 1 | 2 | N |
| hsa-miR-6846-5p | GSC | 1 | 1 | 2 | N |
| hsa-miR-6846-5p | AQP3 | 1 | 1 | 2 | N |
| hsa-miR-6846-5p | COMMD7 | 1 | 1 | 2 | N |
| hsa-miR-6846-5p | GEMIN8 | 1 | 1 | 2 | N |
| hsa-miR-6846-5p | ARG1 | 1 | 1 | 2 | N |
| hsa-miR-6846-5p | GPD2 | 1 | 1 | 2 | N |
| hsa-miR-6846-5p | PPP1R9B | 1 | 1 | 2 | N |
| hsa-miR-6846-5p | BRD2 | 1 | 1 | 2 | N |
| hsa-miR-6846-5p | MISP | 1 | 1 | 2 | N |
| hsa-miR-6846-5p | DCAF12 | 1 | 1 | 2 | N |
| hsa-miR-6846-5p | NTSR1 | 1 | 1 | 2 | N |
| hsa-miR-6846-5p | TNF | 1 | 1 | 2 | N |
| hsa-miR-6846-5p | FOSB | 1 | 1 | 2 | N |
| hsa-miR-6846-5p | DDX39B | 1 | 1 | 2 | Y |
| hsa-miR-6846-5p | NTNG1 | 1 | 1 | 2 | N |
| hsa-miR-6846-5p | NAPA | 1 | 1 | 2 | N |
| hsa-miR-6846-5p | NID1 | 1 | 1 | 2 | N |
| hsa-miR-6846-5p | CPLX1 | 1 | 1 | 2 | N |
| hsa-miR-6846-5p | SBF1 | 1 | 1 | 2 | Y |
| hsa-miR-6846-5p | ABTB2 | 1 | 1 | 2 | N |
| hsa-miR-6846-5p | ANKRD13B | 1 | 1 | 2 | N |
| hsa-miR-6846-5p | CRHR1 | 1 | 1 | 2 | N |
| hsa-miR-6846-5p | SRD5A1 | 1 | 1 | 2 | N |
| hsa-miR-6846-5p | CHKB | 1 | 1 | 2 | N |
| hsa-miR-6846-5p | ALDH1B1 | 1 | 1 | 2 | N |
| hsa-miR-6846-5p | SCNN1A | 1 | 1 | 2 | N |
| hsa-miR-6846-5p | UNC5B | 1 | 1 | 2 | N |
| hsa-miR-6846-5p | MLST8 | 1 | 1 | 2 | N |
| hsa-miR-6846-5p | CNN1 | 1 | 1 | 2 | N |
| hsa-miR-6846-5p | IGF2 | 1 | 1 | 2 | N |
| hsa-miR-6846-5p | KMT2D | 1 | 1 | 2 | N |
| hsa-miR-6846-5p | FAM212B | 1 | 1 | 2 | N |
| hsa-miR-6846-5p | CCDC97 | 1 | 1 | 2 | N |
| hsa-miR-6846-5p | LSG1 | 1 | 1 | 2 | N |
| hsa-miR-6846-5p | NDEL1 | 1 | 1 | 2 | N |
| hsa-miR-6846-5p | SCAMP5 | 1 | 1 | 2 | N |
| hsa-miR-6846-5p | DYNC1I1 | 1 | 1 | 2 | N |
| hsa-miR-6846-5p | CABIN1 | 1 | 1 | 2 | N |
| hsa-miR-6846-5p | PXN | 1 | 1 | 2 | N |
| hsa-miR-6846-5p | SCUBE3 | 1 | 1 | 2 | N |
| hsa-miR-6846-5p | B3GNT7 | 1 | 1 | 2 | N |
| hsa-miR-6846-5p | PRELP | 1 | 1 | 2 | Y |
| hsa-miR-6846-5p | FOXK1 | 1 | 1 | 2 | N |
| hsa-miR-6846-5p | HNF4A | 1 | 1 | 2 | N |
| hsa-miR-6846-5p | C16orf45 | 1 | 1 | 2 | N |
| hsa-miR-6846-5p | RAI1 | 1 | 1 | 2 | N |
| hsa-miR-6846-5p | UBE3A | 1 | 1 | 2 | N |
| hsa-miR-6846-5p | NECAP2 | 1 | 1 | 2 | N |
| hsa-miR-6846-5p | SOX13 | 1 | 1 | 2 | N |
| hsa-miR-6846-5p | TPSB2 | 1 | 1 | 2 | N |
| hsa-miR-6846-5p | TP53INP2 | 1 | 1 | 2 | N |
| hsa-miR-6846-5p | SPRED3 | 1 | 1 | 2 | N |
| hsa-miR-6846-5p | SIT1 | 1 | 1 | 2 | N |
| hsa-miR-6846-5p | SEPT5 | 1 | 1 | 2 | N |
| hsa-miR-6846-5p | SLC45A3 | 1 | 1 | 2 | N |
| hsa-miR-6846-5p | USP21 | 1 | 1 | 2 | N |
| hsa-miR-6846-5p | ITPK1 | 1 | 1 | 2 | N |
| hsa-miR-6846-5p | SEC11A | 1 | 1 | 2 | N |
| hsa-miR-6846-5p | VAMP2 | 1 | 1 | 2 | N |
| hsa-miR-6846-5p | MYRF | 1 | 1 | 2 | N |
| hsa-miR-6846-5p | CDK16 | 1 | 1 | 2 | N |
| hsa-miR-6846-5p | HPCAL4 | 1 | 1 | 2 | N |
| hsa-miR-6846-5p | FLNC | 1 | 1 | 2 | N |
| hsa-miR-6846-5p | KIAA1161 | 1 | 1 | 2 | N |
| hsa-miR-6846-5p | B3GNT3 | 1 | 1 | 2 | N |
| hsa-miR-6846-5p | METAP1 | 1 | 1 | 2 | N |
| hsa-miR-6846-5p | OR51E2 | 1 | 1 | 2 | N |
| hsa-miR-6846-5p | TNFRSF10B | 1 | 1 | 2 | N |
| hsa-miR-6846-5p | NCKAP5L | 1 | 1 | 2 | Y |
| hsa-miR-6846-5p | SHB | 1 | 1 | 2 | N |
| hsa-miR-6846-5p | GALNT6 | 1 | 1 | 2 | N |
| hsa-miR-6846-5p | ELMOD3 | 1 | 1 | 2 | N |
| hsa-miR-6846-5p | THY1 | 1 | 1 | 2 | N |
| hsa-miR-6846-5p | RGMA | 1 | 1 | 2 | N |
| hsa-miR-6846-5p | TSPO | 1 | 1 | 2 | N |
| hsa-miR-6846-5p | ACKR2 | 1 | 1 | 2 | N |
| hsa-miR-6846-5p | MPP2 | 1 | 1 | 2 | N |
| hsa-miR-6846-5p | PRKACA | 1 | 1 | 2 | N |
| hsa-miR-6846-5p | NCDN | 1 | 1 | 2 | N |
| hsa-miR-6846-5p | NFIC | 1 | 1 | 2 | Y |
| hsa-miR-6846-5p | PRKCA | 1 | 1 | 2 | N |
| hsa-miR-6846-5p | NACC1 | 1 | 1 | 2 | Y |
| hsa-miR-6846-5p | CPLX2 | 1 | 1 | 2 | N |
| hsa-miR-6846-5p | TTYH3 | 1 | 1 | 2 | N |
| hsa-miR-6846-5p | TSPAN11 | 1 | 1 | 2 | N |
| hsa-miR-6846-5p | KCNJ10 | 1 | 1 | 2 | N |
| hsa-miR-6846-5p | C2orf43 | 1 | 1 | 2 | N |
| hsa-miR-6846-5p | NAT8L | 1 | 1 | 2 | N |
| hsa-miR-6846-5p | TCEANC2 | 1 | 1 | 2 | N |
| hsa-miR-6846-5p | FAM53B | 1 | 1 | 2 | N |
| hsa-miR-6846-5p | CUX1 | 1 | 1 | 2 | N |
| hsa-miR-6846-5p | RAVER1 | 1 | 1 | 2 | N |
| **miRNA** |  | **targetScan7.1** | **mirdbV5** | **NumSum** | **miRTarBase(Y/N)** |
| hsa-miR-6848-5p | PXN | 1 | 1 | 2 | N |
| hsa-miR-6848-5p | AQP3 | 1 | 1 | 2 | N |
| hsa-miR-6848-5p | CPLX1 | 1 | 1 | 2 | N |
| hsa-miR-6848-5p | TTYH3 | 1 | 1 | 2 | N |
| hsa-miR-6848-5p | DCAF12 | 1 | 1 | 2 | N |
| hsa-miR-6848-5p | NAPA | 1 | 1 | 2 | N |
| hsa-miR-6848-5p | PPP1R9B | 1 | 1 | 2 | N |
| hsa-miR-6848-5p | TCEANC2 | 1 | 1 | 2 | N |
| hsa-miR-6848-5p | FAM212B | 1 | 1 | 2 | N |
| hsa-miR-6848-5p | CTIF | 1 | 1 | 2 | N |
| hsa-miR-6848-5p | NECAP2 | 1 | 1 | 2 | N |
| hsa-miR-6848-5p | ELMOD3 | 1 | 1 | 2 | N |
| hsa-miR-6848-5p | NFIC | 1 | 1 | 2 | Y |
| hsa-miR-6848-5p | TP53INP2 | 1 | 1 | 2 | N |
| hsa-miR-6848-5p | NAT8L | 1 | 1 | 2 | N |
| hsa-miR-6848-5p | KIAA1161 | 1 | 1 | 2 | N |
| hsa-miR-6848-5p | FAM53B | 1 | 1 | 2 | N |
| hsa-miR-6848-5p | DNM1 | 1 | 1 | 2 | N |
| hsa-miR-6848-5p | BRD2 | 1 | 1 | 2 | N |
| hsa-miR-6848-5p | PRKCA | 1 | 1 | 2 | N |
| hsa-miR-6848-5p | TPSB2 | 1 | 1 | 2 | N |
| hsa-miR-6848-5p | NCKAP5L | 1 | 1 | 2 | Y |
| hsa-miR-6848-5p | MPP2 | 1 | 1 | 2 | N |
| hsa-miR-6848-5p | FOSB | 1 | 1 | 2 | N |
| hsa-miR-6848-5p | GEMIN8 | 1 | 1 | 2 | N |
| hsa-miR-6848-5p | SEC11A | 1 | 1 | 2 | N |
| hsa-miR-6848-5p | NDEL1 | 1 | 1 | 2 | N |
| hsa-miR-6848-5p | CRHR1 | 1 | 1 | 2 | N |
| hsa-miR-6848-5p | GALNT6 | 1 | 1 | 2 | N |
| hsa-miR-6848-5p | BHLHE40 | 1 | 1 | 2 | N |
| hsa-miR-6848-5p | CPLX2 | 1 | 1 | 2 | N |
| hsa-miR-6848-5p | IGF2 | 1 | 1 | 2 | N |
| hsa-miR-6848-5p | SIT1 | 1 | 1 | 2 | N |
| hsa-miR-6848-5p | SPRN | 1 | 1 | 2 | N |
| hsa-miR-6848-5p | TSPAN11 | 1 | 1 | 2 | N |
| hsa-miR-6848-5p | RGMA | 1 | 1 | 2 | N |
| hsa-miR-6848-5p | CHKB | 1 | 1 | 2 | N |
| hsa-miR-6848-5p | TNF | 1 | 1 | 2 | N |
| hsa-miR-6848-5p | PRELP | 1 | 1 | 2 | Y |
| hsa-miR-6848-5p | RAI1 | 1 | 1 | 2 | N |
| hsa-miR-6848-5p | UNC5B | 1 | 1 | 2 | N |
| hsa-miR-6848-5p | SBF1 | 1 | 1 | 2 | Y |
| hsa-miR-6848-5p | USP21 | 1 | 1 | 2 | N |
| hsa-miR-6848-5p | SEMA4G | 1 | 1 | 2 | N |
| hsa-miR-6848-5p | ITPK1 | 1 | 1 | 2 | N |
| hsa-miR-6848-5p | COMMD7 | 1 | 1 | 2 | N |
| hsa-miR-6848-5p | ZNF362 | 1 | 1 | 2 | N |
| hsa-miR-6848-5p | SOX13 | 1 | 1 | 2 | N |
| hsa-miR-6848-5p | MISP | 1 | 1 | 2 | N |
| hsa-miR-6848-5p | NTSR1 | 1 | 1 | 2 | N |
| hsa-miR-6848-5p | C2orf43 | 1 | 1 | 2 | N |
| hsa-miR-6848-5p | GSC | 1 | 1 | 2 | N |
| hsa-miR-6848-5p | B3GNT3 | 1 | 1 | 2 | N |
| hsa-miR-6848-5p | RUSC2 | 1 | 1 | 2 | N |
| hsa-miR-6848-5p | FOXK1 | 1 | 1 | 2 | N |
| hsa-miR-6848-5p | OR51E2 | 1 | 1 | 2 | N |
| hsa-miR-6848-5p | CACNB3 | 1 | 1 | 2 | N |
| hsa-miR-6848-5p | SPRED3 | 1 | 1 | 2 | N |
| hsa-miR-6848-5p | VAMP2 | 1 | 1 | 2 | N |
| hsa-miR-6848-5p | LSG1 | 1 | 1 | 2 | N |
| hsa-miR-6848-5p | MYRF | 1 | 1 | 2 | N |
| hsa-miR-6848-5p | KMT2D | 1 | 1 | 2 | N |
| hsa-miR-6848-5p | SHB | 1 | 1 | 2 | N |
| hsa-miR-6848-5p | THY1 | 1 | 1 | 2 | N |
| hsa-miR-6848-5p | HPCAL4 | 1 | 1 | 2 | N |
| hsa-miR-6848-5p | PRKACA | 1 | 1 | 2 | N |
| hsa-miR-6848-5p | CDX1 | 1 | 1 | 2 | N |
| hsa-miR-6848-5p | SCNN1A | 1 | 1 | 2 | N |
| hsa-miR-6848-5p | HEG1 | 1 | 1 | 2 | N |
| hsa-miR-6848-5p | MLST8 | 1 | 1 | 2 | N |
| hsa-miR-6848-5p | ANKRD13B | 1 | 1 | 2 | N |
| hsa-miR-6848-5p | HNF4A | 1 | 1 | 2 | N |
| hsa-miR-6848-5p | C16orf45 | 1 | 1 | 2 | N |
| hsa-miR-6848-5p | ACKR2 | 1 | 1 | 2 | N |
| hsa-miR-6848-5p | PDE4A | 1 | 1 | 2 | Y |
| hsa-miR-6848-5p | KCNQ4 | 1 | 1 | 2 | N |
| hsa-miR-6848-5p | TNFRSF10B | 1 | 1 | 2 | N |
| hsa-miR-6848-5p | B3GNT7 | 1 | 1 | 2 | N |
| hsa-miR-6848-5p | METAP1 | 1 | 1 | 2 | N |
| hsa-miR-6848-5p | ABTB2 | 1 | 1 | 2 | N |
| hsa-miR-6848-5p | SCAMP5 | 1 | 1 | 2 | N |
| hsa-miR-6848-5p | NID1 | 1 | 1 | 2 | N |
| hsa-miR-6848-5p | SLC45A3 | 1 | 1 | 2 | N |
| hsa-miR-6848-5p | ARG1 | 1 | 1 | 2 | N |
| hsa-miR-6848-5p | SEPT5 | 1 | 1 | 2 | N |
| hsa-miR-6848-5p | CBX6 | 1 | 1 | 2 | Y |
| hsa-miR-6848-5p | NTNG1 | 1 | 1 | 2 | N |
| hsa-miR-6848-5p | KCNJ10 | 1 | 1 | 2 | N |
| hsa-miR-6848-5p | SCUBE3 | 1 | 1 | 2 | N |
| hsa-miR-6848-5p | NCDN | 1 | 1 | 2 | N |
| hsa-miR-6848-5p | GPD2 | 1 | 1 | 2 | N |
| hsa-miR-6848-5p | NACC1 | 1 | 1 | 2 | Y |
| hsa-miR-6848-5p | CUX1 | 1 | 1 | 2 | N |
| hsa-miR-6848-5p | CDK16 | 1 | 1 | 2 | N |
| hsa-miR-6848-5p | UBE3A | 1 | 1 | 2 | N |
| hsa-miR-6848-5p | SRD5A1 | 1 | 1 | 2 | N |
| hsa-miR-764 | DNPEP | 1 | 1 | 2 | N |
| hsa-miR-764 | TMEM106A | 1 | 1 | 2 | N |
| hsa-miR-764 | ZBED6CL | 1 | 1 | 2 | N |
| hsa-miR-764 | KCNJ1 | 1 | 1 | 2 | N |
| hsa-miR-764 | CCR3 | 1 | 1 | 2 | N |
| hsa-miR-764 | CCL25 | 1 | 1 | 2 | N |
| hsa-miR-764 | SLC35D3 | 1 | 1 | 2 | N |
| hsa-miR-764 | SMIM12 | 1 | 1 | 2 | N |
| hsa-miR-764 | TRRAP | 1 | 1 | 2 | N |
| hsa-miR-764 | MANF | 1 | 1 | 2 | N |
| hsa-miR-764 | PTK6 | 1 | 1 | 2 | N |
| hsa-miR-764 | DNAH17 | 1 | 1 | 2 | N |
| hsa-miR-764 | SPCS1 | 1 | 1 | 2 | N |
| hsa-miR-764 | YBX1 | 1 | 1 | 2 | N |
| hsa-miR-764 | JMJD7-PLA2G4B | 1 | 1 | 2 | N |
| hsa-miR-764 | RAF1 | 1 | 1 | 2 | N |
| hsa-miR-764 | CHIC1 | 1 | 1 | 2 | N |
| hsa-miR-764 | DUSP22 | 1 | 1 | 2 | N |
| hsa-miR-764 | EIF4EBP2 | 1 | 1 | 2 | N |
| hsa-miR-8089 | PCDHGC5 | 1 | 1 | 2 | N |
| hsa-miR-8089 | LRRC16B | 1 | 1 | 2 | N |
| hsa-miR-8089 | NAGA | 1 | 1 | 2 | N |
| hsa-miR-8089 | TBC1D13 | 1 | 1 | 2 | Y |
| hsa-miR-8089 | SLC38A7 | 1 | 1 | 2 | N |
| hsa-miR-8089 | NCEH1 | 1 | 1 | 2 | Y |
| hsa-miR-8089 | PCDHGA12 | 1 | 1 | 2 | N |
| hsa-miR-8089 | SPIB | 1 | 1 | 2 | N |
| hsa-miR-8089 | HCCS | 1 | 1 | 2 | N |
| hsa-miR-8089 | GDNF | 1 | 1 | 2 | N |
| hsa-miR-8089 | OSBPL7 | 1 | 1 | 2 | N |
| hsa-miR-8089 | ARFIP1 | 1 | 1 | 2 | N |
| hsa-miR-8089 | DUSP3 | 1 | 1 | 2 | N |
| hsa-miR-8089 | TTF2 | 1 | 1 | 2 | N |
| hsa-miR-8089 | P2RY2 | 1 | 1 | 2 | N |
| hsa-miR-8089 | NEK9 | 1 | 1 | 2 | N |
| hsa-miR-8089 | CARTPT | 1 | 1 | 2 | N |
| hsa-miR-8089 | LOXHD1 | 1 | 1 | 2 | N |
| hsa-miR-8089 | BAK1 | 1 | 1 | 2 | N |
| hsa-miR-8089 | SNX12 | 1 | 1 | 2 | N |
| hsa-miR-8089 | KLK4 | 1 | 1 | 2 | N |
| hsa-miR-8089 | LYPD6 | 1 | 1 | 2 | N |
| hsa-miR-8089 | PFKFB4 | 1 | 1 | 2 | N |
| hsa-miR-8089 | SLC25A42 | 1 | 1 | 2 | N |
| hsa-miR-8089 | NUMA1 | 1 | 1 | 2 | N |
| hsa-miR-8089 | KIF24 | 1 | 1 | 2 | N |
| hsa-miR-8089 | PCDHGA1 | 1 | 1 | 2 | N |
| hsa-miR-8089 | NHLH1 | 1 | 1 | 2 | N |
| hsa-miR-8089 | FOXRED1 | 1 | 1 | 2 | N |
| hsa-miR-8089 | SLC7A2 | 1 | 1 | 2 | N |
| hsa-miR-8089 | MEF2D | 1 | 1 | 2 | N |
| hsa-miR-8089 | GLP1R | 1 | 1 | 2 | N |
| hsa-miR-8089 | CDR2L | 1 | 1 | 2 | N |
| hsa-miR-8089 | XPO7 | 1 | 1 | 2 | N |
| hsa-miR-8089 | FBLN1 | 1 | 1 | 2 | N |
| hsa-miR-8089 | PPP1R9B | 1 | 1 | 2 | Y |
| hsa-miR-8089 | RASSF4 | 1 | 1 | 2 | N |
| hsa-miR-8089 | CCDC178 | 1 | 1 | 2 | N |
| hsa-miR-8089 | MAPK1 | 1 | 1 | 2 | N |
| hsa-miR-8089 | MUT | 1 | 1 | 2 | N |
| hsa-miR-8089 | KDM8 | 1 | 1 | 2 | N |
| hsa-miR-8089 | RARB | 1 | 1 | 2 | N |
| hsa-miR-8089 | ATF6B | 1 | 1 | 2 | N |
| hsa-miR-8089 | PCDHGA5 | 1 | 1 | 2 | N |
| hsa-miR-8089 | CCL2 | 1 | 1 | 2 | N |
| hsa-miR-8089 | LASP1 | 1 | 1 | 2 | N |
| hsa-miR-8089 | DHRS3 | 1 | 1 | 2 | N |
| hsa-miR-8089 | SAMD12 | 1 | 1 | 2 | N |
| hsa-miR-8089 | TMEM63B | 1 | 1 | 2 | N |
| hsa-miR-8089 | KIAA1755 | 1 | 1 | 2 | N |
| hsa-miR-8089 | SALL1 | 1 | 1 | 2 | N |
| hsa-miR-8089 | CTAGE1 | 1 | 1 | 2 | N |
| hsa-miR-8089 | FUT11 | 1 | 1 | 2 | N |
| hsa-miR-8089 | MECR | 1 | 1 | 2 | N |
| hsa-miR-8089 | ELMOD1 | 1 | 1 | 2 | N |
| hsa-miR-8089 | SYT2 | 1 | 1 | 2 | N |
| hsa-miR-8089 | TSPYL5 | 1 | 1 | 2 | N |
| hsa-miR-8089 | NR1I3 | 1 | 1 | 2 | N |
| hsa-miR-8089 | MS4A15 | 1 | 1 | 2 | N |
| hsa-miR-8089 | IVL | 1 | 1 | 2 | N |
| hsa-miR-8089 | NR6A1 | 1 | 1 | 2 | N |
| hsa-miR-8089 | ITPRIP | 1 | 1 | 2 | N |
| hsa-miR-8089 | SYPL2 | 1 | 1 | 2 | N |
| hsa-miR-8089 | CPLX2 | 1 | 1 | 2 | N |
| hsa-miR-8089 | RBM14 | 1 | 1 | 2 | N |
| hsa-miR-8089 | PCGF1 | 1 | 1 | 2 | N |
| hsa-miR-8089 | ZCCHC24 | 1 | 1 | 2 | N |
| hsa-miR-8089 | SLC25A32 | 1 | 1 | 2 | N |
| hsa-miR-8089 | FAM228B | 1 | 1 | 2 | N |
| hsa-miR-8089 | ARID3B | 1 | 1 | 2 | N |
| hsa-miR-8089 | PSMD5 | 1 | 1 | 2 | N |
| hsa-miR-8089 | SCAMP4 | 1 | 1 | 2 | N |
| hsa-miR-8089 | PCDHGB3 | 1 | 1 | 2 | N |
| hsa-miR-8089 | HSDL1 | 1 | 1 | 2 | N |
| hsa-miR-8089 | PTPLAD1 | 1 | 1 | 2 | N |
| hsa-miR-8089 | ATXN1L | 1 | 1 | 2 | N |
| hsa-miR-8089 | PCDHGA9 | 1 | 1 | 2 | N |
| hsa-miR-8089 | IQSEC3 | 1 | 1 | 2 | N |
| hsa-miR-8089 | ARHGEF6 | 1 | 1 | 2 | N |
| hsa-miR-8089 | RNASE13 | 1 | 1 | 2 | N |
| hsa-miR-8089 | NGB | 1 | 1 | 2 | N |
| hsa-miR-8089 | CABP5 | 1 | 1 | 2 | N |
| hsa-miR-8089 | IGFBP5 | 1 | 1 | 2 | N |
| hsa-miR-8089 | GPD1 | 1 | 1 | 2 | N |
| hsa-miR-8089 | SLC2A4 | 1 | 1 | 2 | N |
| hsa-miR-8089 | SLC16A2 | 1 | 1 | 2 | N |
| hsa-miR-8089 | TSPAN11 | 1 | 1 | 2 | N |
| hsa-miR-8089 | RNF170 | 1 | 1 | 2 | N |
| hsa-miR-8089 | THAP11 | 1 | 1 | 2 | N |
| hsa-miR-8089 | CELF6 | 1 | 1 | 2 | N |
| hsa-miR-8089 | PCDHGB7 | 1 | 1 | 2 | N |
| hsa-miR-8089 | BCL2L1 | 1 | 1 | 2 | N |
| hsa-miR-8089 | RRN3 | 1 | 1 | 2 | N |
| hsa-miR-8089 | DAGLA | 1 | 1 | 2 | N |
| hsa-miR-8089 | CAPN12 | 1 | 1 | 2 | N |
| hsa-miR-8089 | SLC6A9 | 1 | 1 | 2 | N |
| hsa-miR-8089 | ZNF831 | 1 | 1 | 2 | N |
| hsa-miR-8089 | LYPD3 | 1 | 1 | 2 | N |
| hsa-miR-8089 | UNC13B | 1 | 1 | 2 | N |
| hsa-miR-8089 | FAIM2 | 1 | 1 | 2 | N |
| hsa-miR-8089 | ZNF548 | 1 | 1 | 2 | N |
| hsa-miR-8089 | PCDHGA10 | 1 | 1 | 2 | N |
| hsa-miR-8089 | PCDHGC3 | 1 | 1 | 2 | N |
| hsa-miR-8089 | CACNB1 | 1 | 1 | 2 | N |
| hsa-miR-8089 | STX17 | 1 | 1 | 2 | N |
| hsa-miR-8089 | TP53INP2 | 1 | 1 | 2 | N |
| hsa-miR-8089 | TCF7 | 1 | 1 | 2 | N |
| hsa-miR-8089 | PCDHGA3 | 1 | 1 | 2 | N |
| hsa-miR-8089 | ABCG4 | 1 | 1 | 2 | N |
| hsa-miR-8089 | MBD6 | 1 | 1 | 2 | N |
| hsa-miR-8089 | SH2D3C | 1 | 1 | 2 | N |
| hsa-miR-8089 | TOM1L2 | 1 | 1 | 2 | N |
| hsa-miR-8089 | PSMB11 | 1 | 1 | 2 | N |
| hsa-miR-8089 | B4GALT1 | 1 | 1 | 2 | N |
| hsa-miR-8089 | ARHGAP36 | 1 | 1 | 2 | N |
| hsa-miR-8089 | SFRP5 | 1 | 1 | 2 | N |
| hsa-miR-8089 | MAT2A | 1 | 1 | 2 | N |
| hsa-miR-8089 | PDPK1 | 1 | 1 | 2 | N |
| hsa-miR-8089 | PCDHGA8 | 1 | 1 | 2 | N |
| hsa-miR-8089 | GLIPR1 | 1 | 1 | 2 | N |
| hsa-miR-8089 | SELM | 1 | 1 | 2 | N |
| hsa-miR-8089 | ICK | 1 | 1 | 2 | N |
| hsa-miR-8089 | UBE2L3 | 1 | 1 | 2 | N |
| hsa-miR-8089 | PPP1R1B | 1 | 1 | 2 | N |
| hsa-miR-8089 | CD207 | 1 | 1 | 2 | N |
| hsa-miR-8089 | RRAGA | 1 | 1 | 2 | N |
| hsa-miR-8089 | C17orf107 | 1 | 1 | 2 | N |
| hsa-miR-8089 | PPP1R10 | 1 | 1 | 2 | N |
| hsa-miR-8089 | CD22 | 1 | 1 | 2 | N |
| hsa-miR-8089 | ATP8B2 | 1 | 1 | 2 | N |
| hsa-miR-8089 | C15orf56 | 1 | 1 | 2 | N |
| hsa-miR-8089 | TM9SF4 | 1 | 1 | 2 | N |
| hsa-miR-8089 | CABP4 | 1 | 1 | 2 | N |
| hsa-miR-8089 | CIC | 1 | 1 | 2 | N |
| hsa-miR-8089 | SPATA2L | 1 | 1 | 2 | N |
| hsa-miR-8089 | SPR | 1 | 1 | 2 | N |
| hsa-miR-8089 | POPDC3 | 1 | 1 | 2 | N |
| hsa-miR-8089 | EGLN3 | 1 | 1 | 2 | N |
| hsa-miR-8089 | ADPGK | 1 | 1 | 2 | N |
| hsa-miR-8089 | MRO | 1 | 1 | 2 | N |
| hsa-miR-8089 | SYT12 | 1 | 1 | 2 | N |
| hsa-miR-8089 | ERGIC1 | 1 | 1 | 2 | N |
| hsa-miR-8089 | KCNK3 | 1 | 1 | 2 | N |
| hsa-miR-8089 | FGF18 | 1 | 1 | 2 | N |
| hsa-miR-8089 | NCDN | 1 | 1 | 2 | N |
| hsa-miR-8089 | C4orf46 | 1 | 1 | 2 | N |
| hsa-miR-8089 | PCDHGA2 | 1 | 1 | 2 | N |
| hsa-miR-8089 | NAA40 | 1 | 1 | 2 | N |
| hsa-miR-8089 | GAB2 | 1 | 1 | 2 | N |
| hsa-miR-8089 | PCDHGA11 | 1 | 1 | 2 | N |
| hsa-miR-8089 | IGF2 | 1 | 1 | 2 | N |
| hsa-miR-8089 | THRA | 1 | 1 | 2 | N |
| hsa-miR-8089 | VAMP2 | 1 | 1 | 2 | N |
| hsa-miR-8089 | ABCC10 | 1 | 1 | 2 | N |
| hsa-miR-8089 | TMEM127 | 1 | 1 | 2 | N |
| hsa-miR-8089 | KCNJ10 | 1 | 1 | 2 | N |
| hsa-miR-8089 | CLIP3 | 1 | 1 | 2 | N |
| hsa-miR-8089 | FBXO41 | 1 | 1 | 2 | N |
| hsa-miR-8089 | LZTS1 | 1 | 1 | 2 | N |
| hsa-miR-8089 | PRKCG | 1 | 1 | 2 | N |
| hsa-miR-8089 | PCDHGA7 | 1 | 1 | 2 | N |
| hsa-miR-8089 | TSPAN18 | 1 | 1 | 2 | N |
| hsa-miR-8089 | CALN1 | 1 | 1 | 2 | N |
| hsa-miR-8089 | SYN1 | 1 | 1 | 2 | N |
| hsa-miR-8089 | SEPN1 | 1 | 1 | 2 | N |
| hsa-miR-8089 | RGSL1 | 1 | 1 | 2 | N |
| hsa-miR-8089 | UNC5B | 1 | 1 | 2 | Y |
| hsa-miR-8089 | RGL3 | 1 | 1 | 2 | N |
| hsa-miR-8089 | KIAA0319 | 1 | 1 | 2 | N |
| hsa-miR-8089 | UBE2L6 | 1 | 1 | 2 | N |
| hsa-miR-8089 | DRG2 | 1 | 1 | 2 | N |
| hsa-miR-8089 | VTI1A | 1 | 1 | 2 | N |
| hsa-miR-8089 | PXN | 1 | 1 | 2 | N |
| hsa-miR-8089 | VAT1 | 1 | 1 | 2 | N |
| hsa-miR-8089 | SUSD2 | 1 | 1 | 2 | N |
| hsa-miR-8089 | KDELR1 | 1 | 1 | 2 | N |
| hsa-miR-8089 | ZBTB39 | 1 | 1 | 2 | N |
| hsa-miR-8089 | ZNF302 | 1 | 1 | 2 | N |
| hsa-miR-1256 | FAM222B | 1 | 1 | 2 | N |
| hsa-miR-1256 | NR2E1 | 1 | 1 | 2 | N |
| hsa-miR-1256 | RAP1B | 1 | 1 | 2 | N |
| hsa-miR-1256 | RALGPS1 | 1 | 1 | 2 | N |
| hsa-miR-1256 | RAC1 | 1 | 1 | 2 | N |
| hsa-miR-1256 | CT62 | 1 | 1 | 2 | N |
| hsa-miR-1256 | NUTF2 | 1 | 1 | 2 | N |
| hsa-miR-1256 | VAPB | 1 | 1 | 2 | N |
| hsa-miR-1256 | KDM1B | 1 | 1 | 2 | N |
| hsa-miR-1256 | LRRC18 | 1 | 1 | 2 | N |
| hsa-miR-1256 | PDZRN4 | 1 | 1 | 2 | N |
| hsa-miR-1256 | KDELC2 | 1 | 1 | 2 | N |
| hsa-miR-1256 | AFF4 | 1 | 1 | 2 | N |
| hsa-miR-1256 | RFXAP | 1 | 1 | 2 | N |
| hsa-miR-1256 | MBD2 | 1 | 1 | 2 | N |
| hsa-miR-1256 | TBC1D9B | 1 | 1 | 2 | N |
| hsa-miR-1256 | RAP2C | 1 | 1 | 2 | N |
| hsa-miR-1256 | EYA4 | 1 | 1 | 2 | N |
| hsa-miR-1256 | C12orf42 | 1 | 1 | 2 | N |
| hsa-miR-1256 | TMEM196 | 1 | 1 | 2 | N |
| hsa-miR-1256 | MARCH4 | 1 | 1 | 2 | N |
| hsa-miR-1256 | TGFA | 1 | 1 | 2 | N |
| hsa-miR-1256 | SLC23A3 | 1 | 1 | 2 | N |
| hsa-miR-1256 | CNEP1R1 | 1 | 1 | 2 | Y |
| hsa-miR-1256 | HADHB | 1 | 1 | 2 | N |
| hsa-miR-1256 | ZNF474 | 1 | 1 | 2 | N |
| hsa-miR-1256 | LDOC1L | 1 | 1 | 2 | N |
| hsa-miR-1256 | AKIRIN2 | 1 | 1 | 2 | N |
| hsa-miR-1256 | RER1 | 1 | 1 | 2 | Y |
| hsa-miR-3155a | GRAMD1C | 1 | 1 | 2 | N |
| hsa-miR-3155a | FGF1 | 1 | 1 | 2 | N |
| hsa-miR-3155a | WFS1 | 1 | 1 | 2 | N |
| hsa-miR-3155a | THBD | 1 | 1 | 2 | N |
| hsa-miR-3155a | FOXP3 | 1 | 1 | 2 | N |
| hsa-miR-3155a | HOXA5 | 1 | 1 | 2 | N |
| hsa-miR-3155a | CPLX3 | 1 | 1 | 2 | N |
| hsa-miR-3155a | EMC6 | 1 | 1 | 2 | N |
| hsa-miR-3155a | B3GNT9 | 1 | 1 | 2 | N |
| hsa-miR-3155a | C22orf26 | 1 | 1 | 2 | N |
| hsa-miR-3155a | ABLIM2 | 1 | 1 | 2 | N |
| hsa-miR-3155a | LAMB3 | 1 | 1 | 2 | N |
| hsa-miR-3155a | KCNJ14 | 1 | 1 | 2 | N |
| hsa-miR-3155a | MED8 | 1 | 1 | 2 | N |
| hsa-miR-3155a | SNN | 1 | 1 | 2 | N |
| hsa-miR-3155a | IGBP1 | 1 | 1 | 2 | N |
| hsa-miR-3155a | RHOBTB1 | 1 | 1 | 2 | N |
| hsa-miR-3155a | STEAP3 | 1 | 1 | 2 | N |
| hsa-miR-3155a | HLA-DOB | 1 | 1 | 2 | N |
| hsa-miR-3155a | PLEKHF1 | 1 | 1 | 2 | N |
| hsa-miR-3155a | ACVR1B | 1 | 1 | 2 | N |
| hsa-miR-3155a | MYCBP2 | 1 | 1 | 2 | N |
| hsa-miR-3155a | CHMP6 | 1 | 1 | 2 | N |
| hsa-miR-3155a | HIVEP2 | 1 | 1 | 2 | N |
| hsa-miR-3155a | MGAT5 | 1 | 1 | 2 | Y |
| hsa-miR-3155a | EPX | 1 | 1 | 2 | N |
| hsa-miR-3155a | TOX2 | 1 | 1 | 2 | N |
| hsa-miR-3155a | HOXA11 | 1 | 1 | 2 | N |
| hsa-miR-3155a | NUP54 | 1 | 1 | 2 | N |
| hsa-miR-3155a | TLCD1 | 1 | 1 | 2 | N |
| hsa-miR-3155a | PEA15 | 1 | 1 | 2 | N |
| hsa-miR-3155a | IFNAR1 | 1 | 1 | 2 | N |
| hsa-miR-3155a | SORBS1 | 1 | 1 | 2 | N |
| hsa-miR-3155a | SERTAD1 | 1 | 1 | 2 | N |
| hsa-miR-3155a | IL21R | 1 | 1 | 2 | N |
| hsa-miR-3155a | PTGER4 | 1 | 1 | 2 | N |
| hsa-miR-3155a | AVL9 | 1 | 1 | 2 | N |
| hsa-miR-3155a | C10orf2 | 1 | 1 | 2 | N |
| hsa-miR-3155a | NDOR1 | 1 | 1 | 2 | N |
| hsa-miR-3155a | HNF1A | 1 | 1 | 2 | N |
| hsa-miR-3155a | CST3 | 1 | 1 | 2 | N |
| hsa-miR-3155a | ANAPC7 | 1 | 1 | 2 | Y |
| hsa-miR-3155a | HN1 | 1 | 1 | 2 | N |
| hsa-miR-3155a | YTHDF3 | 1 | 1 | 2 | N |
| hsa-miR-3155a | TNFSF9 | 1 | 1 | 2 | N |
| hsa-miR-3155a | SLC17A9 | 1 | 1 | 2 | N |
| hsa-miR-3155b | HOXA5 | 1 | 1 | 2 | N |
| hsa-miR-3155b | HIVEP2 | 1 | 1 | 2 | N |
| hsa-miR-3155b | THBD | 1 | 1 | 2 | N |
| hsa-miR-3155b | GRAMD1C | 1 | 1 | 2 | N |
| hsa-miR-3155b | SORBS1 | 1 | 1 | 2 | N |
| hsa-miR-3155b | CHMP6 | 1 | 1 | 2 | N |
| hsa-miR-3155b | CST3 | 1 | 1 | 2 | N |
| hsa-miR-3155b | MED8 | 1 | 1 | 2 | N |
| hsa-miR-3155b | SLC17A9 | 1 | 1 | 2 | N |
| hsa-miR-3155b | IGBP1 | 1 | 1 | 2 | N |
| hsa-miR-3155b | IL21R | 1 | 1 | 2 | N |
| hsa-miR-3155b | NDOR1 | 1 | 1 | 2 | N |
| hsa-miR-3155b | WFS1 | 1 | 1 | 2 | N |
| hsa-miR-3155b | IFNAR1 | 1 | 1 | 2 | N |
| hsa-miR-3155b | EPX | 1 | 1 | 2 | N |
| hsa-miR-3155b | RHOBTB1 | 1 | 1 | 2 | N |
| hsa-miR-3155b | ABLIM2 | 1 | 1 | 2 | N |
| hsa-miR-3155b | ANAPC7 | 1 | 1 | 2 | Y |
| hsa-miR-3155b | TNFSF9 | 1 | 1 | 2 | N |
| hsa-miR-3155b | PTGER4 | 1 | 1 | 2 | N |
| hsa-miR-3155b | PLEKHF1 | 1 | 1 | 2 | N |
| hsa-miR-3155b | CPLX3 | 1 | 1 | 2 | N |
| hsa-miR-3155b | HN1 | 1 | 1 | 2 | N |
| hsa-miR-3155b | AVL9 | 1 | 1 | 2 | N |
| hsa-miR-3155b | SNN | 1 | 1 | 2 | N |
| hsa-miR-3155b | EMC6 | 1 | 1 | 2 | N |
| hsa-miR-3155b | PEA15 | 1 | 1 | 2 | N |
| hsa-miR-3155b | MYCBP2 | 1 | 1 | 2 | N |
| hsa-miR-3155b | KCNJ14 | 1 | 1 | 2 | N |
| hsa-miR-3155b | YTHDF3 | 1 | 1 | 2 | N |
| hsa-miR-3155b | C22orf26 | 1 | 1 | 2 | N |
| hsa-miR-3155b | TOX2 | 1 | 1 | 2 | N |
| hsa-miR-3155b | HNF1A | 1 | 1 | 2 | N |
| hsa-miR-3155b | ACVR1B | 1 | 1 | 2 | N |
| hsa-miR-3155b | B3GNT9 | 1 | 1 | 2 | N |
| hsa-miR-3155b | LAMB3 | 1 | 1 | 2 | N |
| hsa-miR-3155b | FOXP3 | 1 | 1 | 2 | N |
| hsa-miR-3155b | HOXA11 | 1 | 1 | 2 | N |
| hsa-miR-3155b | NUP54 | 1 | 1 | 2 | N |
| hsa-miR-3155b | HLA-DOB | 1 | 1 | 2 | N |
| hsa-miR-3155b | FGF1 | 1 | 1 | 2 | N |
| hsa-miR-3155b | C10orf2 | 1 | 1 | 2 | N |
| hsa-miR-3155b | TLCD1 | 1 | 1 | 2 | N |
| hsa-miR-3155b | MGAT5 | 1 | 1 | 2 | Y |
| hsa-miR-3155b | STEAP3 | 1 | 1 | 2 | N |
| hsa-miR-3155b | SERTAD1 | 1 | 1 | 2 | N |
| hsa-miR-3619-3p | VPS36 | 1 | 1 | 2 | N |
| hsa-miR-3619-3p | GXYLT1 | 1 | 1 | 2 | N |
| hsa-miR-3619-3p | SMC2 | 1 | 1 | 2 | N |
| hsa-miR-3619-3p | SYT9 | 1 | 1 | 2 | N |
| hsa-miR-3619-3p | SMCO4 | 1 | 1 | 2 | N |
| hsa-miR-3619-3p | ARHGAP29 | 1 | 1 | 2 | N |
| hsa-miR-3619-3p | GNGT2 | 1 | 1 | 2 | N |
| hsa-miR-3619-3p | COL4A1 | 1 | 1 | 2 | N |
| hsa-miR-3619-3p | LYSMD3 | 1 | 1 | 2 | N |
| hsa-miR-3619-3p | ARHGAP5 | 1 | 1 | 2 | N |
| hsa-miR-3619-3p | KRT19 | 1 | 1 | 2 | N |
| hsa-miR-3619-3p | CTNNB1 | 1 | 1 | 2 | N |
| hsa-miR-3619-3p | OCIAD1 | 1 | 1 | 2 | N |
| hsa-miR-3619-3p | CYP4F3 | 1 | 1 | 2 | N |
| hsa-miR-4254 | ARHGAP25 | 1 | 1 | 2 | N |
| hsa-miR-4254 | SLAMF8 | 1 | 1 | 2 | N |
| hsa-miR-4254 | ULK2 | 1 | 1 | 2 | N |
| hsa-miR-4254 | CLLU1 | 1 | 1 | 2 | N |
| hsa-miR-4254 | CASP7 | 1 | 1 | 2 | N |
| hsa-miR-4254 | RPIA | 1 | 1 | 2 | N |
| hsa-miR-4254 | GPSM1 | 1 | 1 | 2 | N |
| hsa-miR-4254 | HBEGF | 1 | 1 | 2 | Y |
| hsa-miR-4254 | FARS2 | 1 | 1 | 2 | N |
| hsa-miR-4254 | VAMP7 | 1 | 1 | 2 | N |
| hsa-miR-4254 | SAMD11 | 1 | 1 | 2 | N |
| hsa-miR-4254 | KLRF1 | 1 | 1 | 2 | N |
| hsa-miR-4254 | SLC2A1 | 1 | 1 | 2 | N |
| hsa-miR-4254 | FSCN3 | 1 | 1 | 2 | N |
| hsa-miR-4254 | CSRNP2 | 1 | 1 | 2 | N |
| hsa-miR-4254 | PPP1R14C | 1 | 1 | 2 | N |
| hsa-miR-4254 | PKIA | 1 | 1 | 2 | N |
| hsa-miR-4254 | DNER | 1 | 1 | 2 | N |
| hsa-miR-4254 | PRUNE | 1 | 1 | 2 | N |
| hsa-miR-4254 | TBC1D13 | 1 | 1 | 2 | N |
| hsa-miR-4254 | GABARAPL2 | 1 | 1 | 2 | N |
| hsa-miR-4254 | RASL12 | 1 | 1 | 2 | N |
| hsa-miR-4254 | GSTP1 | 1 | 1 | 2 | N |
| hsa-miR-4254 | C9orf114 | 1 | 1 | 2 | N |
| hsa-miR-4254 | GPD1 | 1 | 1 | 2 | N |
| hsa-miR-4254 | C19orf43 | 1 | 1 | 2 | N |
| hsa-miR-4254 | CDK2AP2 | 1 | 1 | 2 | N |
| hsa-miR-4254 | LY6H | 1 | 1 | 2 | N |
| hsa-miR-4254 | SIPA1L3 | 1 | 1 | 2 | N |
| hsa-miR-4254 | URM1 | 1 | 1 | 2 | N |
| hsa-miR-4254 | TCERG1L | 1 | 1 | 2 | N |
| hsa-miR-4254 | ZNF512B | 1 | 1 | 2 | Y |
| hsa-miR-4436b-3p | STAB2 | 1 | 1 | 2 | N |
| hsa-miR-4436b-3p | LIN28A | 1 | 1 | 2 | N |
| hsa-miR-4436b-3p | SCML1 | 1 | 1 | 2 | N |
| hsa-miR-4436b-3p | TTC39A | 1 | 1 | 2 | N |
| hsa-miR-4436b-3p | TMEM110 | 1 | 1 | 2 | N |
| hsa-miR-4436b-3p | KIAA0247 | 1 | 1 | 2 | N |
| hsa-miR-4436b-3p | TSPAN9 | 1 | 1 | 2 | N |
| hsa-miR-4436b-3p | LSM12 | 1 | 1 | 2 | N |
| hsa-miR-4436b-3p | TLL2 | 1 | 1 | 2 | N |
| hsa-miR-4436b-3p | CX3CL1 | 1 | 1 | 2 | N |
| hsa-miR-4436b-3p | RHBDF2 | 1 | 1 | 2 | Y |
| hsa-miR-4436b-3p | ETV4 | 1 | 1 | 2 | N |
| hsa-miR-4436b-3p | CACFD1 | 1 | 1 | 2 | N |
| hsa-miR-4436b-3p | FHL1 | 1 | 1 | 2 | N |
| hsa-miR-4436b-3p | SDC3 | 1 | 1 | 2 | N |
| hsa-miR-4436b-3p | NEURL1B | 1 | 1 | 2 | N |
| hsa-miR-4436b-3p | GABRA4 | 1 | 1 | 2 | N |
| hsa-miR-4436b-3p | FNDC3B | 1 | 1 | 2 | N |
| hsa-miR-4436b-3p | ATG9A | 1 | 1 | 2 | N |
| hsa-miR-4436b-3p | FBXO41 | 1 | 1 | 2 | Y |
| hsa-miR-4436b-3p | VPS39 | 1 | 1 | 2 | N |
| hsa-miR-4436b-3p | WIPF3 | 1 | 1 | 2 | N |
| hsa-miR-4436b-3p | ZIC3 | 1 | 1 | 2 | N |
| hsa-miR-4436b-3p | FGF23 | 1 | 1 | 2 | N |
| hsa-miR-4436b-3p | SH3GL2 | 1 | 1 | 2 | N |
| hsa-miR-4436b-3p | GRM2 | 1 | 1 | 2 | N |
| hsa-miR-4436b-3p | FAM214B | 1 | 1 | 2 | N |
| hsa-miR-4436b-3p | MAPKAPK2 | 1 | 1 | 2 | N |
| hsa-miR-4436b-3p | C14orf1 | 1 | 1 | 2 | N |
| hsa-miR-4436b-3p | LDLRAP1 | 1 | 1 | 2 | N |
| hsa-miR-4436b-3p | NAMPT | 1 | 1 | 2 | N |
| hsa-miR-4436b-3p | MPZ | 1 | 1 | 2 | N |
| hsa-miR-4436b-3p | TRIM26 | 1 | 1 | 2 | N |
| hsa-miR-4436b-3p | CCDC114 | 1 | 1 | 2 | N |
| hsa-miR-4436b-3p | AGAP3 | 1 | 1 | 2 | N |
| hsa-miR-4436b-3p | GDNF | 1 | 1 | 2 | N |
| hsa-miR-4436b-3p | SLC6A9 | 1 | 1 | 2 | Y |
| hsa-miR-4436b-3p | KCNIP1 | 1 | 1 | 2 | N |
| hsa-miR-4436b-3p | WASF2 | 1 | 1 | 2 | N |
| hsa-miR-4436b-3p | NRIP2 | 1 | 1 | 2 | N |
| hsa-miR-4436b-3p | HAP1 | 1 | 1 | 2 | N |
| hsa-miR-4436b-3p | HJURP | 1 | 1 | 2 | N |
| hsa-miR-4436b-3p | HLA-DOB | 1 | 1 | 2 | N |
| hsa-miR-4436b-3p | PIGY | 1 | 1 | 2 | N |
| hsa-miR-4436b-3p | SOX13 | 1 | 1 | 2 | N |
| hsa-miR-4436b-3p | CYBRD1 | 1 | 1 | 2 | N |
| hsa-miR-4436b-3p | SLC6A17 | 1 | 1 | 2 | N |
| hsa-miR-4436b-3p | ANKFY1 | 1 | 1 | 2 | N |
| hsa-miR-4436b-3p | CDC42SE1 | 1 | 1 | 2 | Y |
| hsa-miR-4436b-3p | RELT | 1 | 1 | 2 | N |
| hsa-miR-4436b-3p | TEX264 | 1 | 1 | 2 | N |
| hsa-miR-4436b-3p | C17orf67 | 1 | 1 | 2 | N |
| hsa-miR-4436b-3p | USP7 | 1 | 1 | 2 | N |
| hsa-miR-4436b-3p | SOWAHC | 1 | 1 | 2 | Y |
| hsa-miR-4436b-3p | CDIP1 | 1 | 1 | 2 | N |
| hsa-miR-4436b-3p | HMX2 | 1 | 1 | 2 | N |
| hsa-miR-4436b-3p | TBC1D9B | 1 | 1 | 2 | N |
| hsa-miR-4436b-3p | SOX10 | 1 | 1 | 2 | N |
| hsa-miR-4436b-3p | CACNA2D2 | 1 | 1 | 2 | N |
| hsa-miR-4436b-3p | CLSTN2 | 1 | 1 | 2 | N |
| hsa-miR-4436b-3p | SMARCD1 | 1 | 1 | 2 | N |
| hsa-miR-4436b-3p | SH2D5 | 1 | 1 | 2 | N |
| hsa-miR-4436b-3p | NPLOC4 | 1 | 1 | 2 | Y |
| hsa-miR-4436b-3p | C11orf44 | 1 | 1 | 2 | N |
| hsa-miR-4436b-3p | SRRD | 1 | 1 | 2 | N |
| hsa-miR-4436b-3p | FAM86A | 1 | 1 | 2 | N |
| hsa-miR-4436b-3p | TRIM35 | 1 | 1 | 2 | N |
| hsa-miR-4436b-3p | SEPT3 | 1 | 1 | 2 | N |
| hsa-miR-4436b-3p | TMEM154 | 1 | 1 | 2 | N |
| hsa-miR-4436b-3p | TRIM9 | 1 | 1 | 2 | N |
| hsa-miR-4436b-3p | PARP6 | 1 | 1 | 2 | N |
| hsa-miR-4436b-3p | SLC37A3 | 1 | 1 | 2 | N |
| hsa-miR-4436b-3p | STK35 | 1 | 1 | 2 | N |
| hsa-miR-4436b-3p | RASSF2 | 1 | 1 | 2 | N |
| hsa-miR-4436b-3p | KCNK3 | 1 | 1 | 2 | N |
| hsa-miR-4436b-3p | AIF1L | 1 | 1 | 2 | N |
| hsa-miR-4436b-3p | CHAF1A | 1 | 1 | 2 | N |
| hsa-miR-4436b-3p | C11orf86 | 1 | 1 | 2 | N |
| hsa-miR-4436b-3p | TMEM127 | 1 | 1 | 2 | N |
| hsa-miR-4436b-3p | KIRREL | 1 | 1 | 2 | N |
| hsa-miR-4436b-3p | PUM2 | 1 | 1 | 2 | Y |
| hsa-miR-4436b-3p | SSR1 | 1 | 1 | 2 | N |
| hsa-miR-4436b-3p | PPP1R9B | 1 | 1 | 2 | N |
| hsa-miR-4436b-3p | FAM53C | 1 | 1 | 2 | N |
| hsa-miR-4436b-3p | SV2A | 1 | 1 | 2 | N |
| hsa-miR-4436b-3p | RASSF4 | 1 | 1 | 2 | N |
| hsa-miR-4436b-3p | GPR85 | 1 | 1 | 2 | N |
| hsa-miR-4436b-3p | TRIM4 | 1 | 1 | 2 | N |
| hsa-miR-4436b-3p | COL5A3 | 1 | 1 | 2 | N |
| hsa-miR-4436b-3p | FAM155B | 1 | 1 | 2 | N |
| hsa-miR-4436b-3p | ITCH | 1 | 1 | 2 | N |
| hsa-miR-4436b-3p | LUZP1 | 1 | 1 | 2 | Y |
| hsa-miR-4436b-3p | PIANP | 1 | 1 | 2 | N |
| hsa-miR-4436b-3p | ABHD12 | 1 | 1 | 2 | N |
| hsa-miR-4436b-3p | ARL4C | 1 | 1 | 2 | N |
| hsa-miR-4436b-3p | ARL6IP1 | 1 | 1 | 2 | Y |
| hsa-miR-4436b-3p | HNRNPR | 1 | 1 | 2 | N |
| hsa-miR-4436b-3p | PPP6C | 1 | 1 | 2 | N |
| hsa-miR-4436b-3p | CIC | 1 | 1 | 2 | N |
| hsa-miR-4436b-3p | ADIPOR2 | 1 | 1 | 2 | N |
| hsa-miR-4436b-3p | RALYL | 1 | 1 | 2 | N |
| hsa-miR-4436b-3p | LINGO1 | 1 | 1 | 2 | N |
| hsa-miR-4436b-3p | AURKA | 1 | 1 | 2 | Y |
| hsa-miR-4436b-3p | ORAI3 | 1 | 1 | 2 | N |
| hsa-miR-4436b-3p | LBH | 1 | 1 | 2 | N |
| hsa-miR-4436b-3p | EFNB3 | 1 | 1 | 2 | N |
| hsa-miR-4436b-3p | RGMB | 1 | 1 | 2 | Y |
| hsa-miR-4436b-3p | AK3 | 1 | 1 | 2 | N |
| hsa-miR-4436b-3p | TRIM46 | 1 | 1 | 2 | N |
| hsa-miR-4436b-3p | PATZ1 | 1 | 1 | 2 | N |
| hsa-miR-4436b-3p | NAB2 | 1 | 1 | 2 | N |
| hsa-miR-4436b-3p | CSF1R | 1 | 1 | 2 | N |
| hsa-miR-4436b-3p | WDTC1 | 1 | 1 | 2 | N |
| hsa-miR-4436b-3p | PGS1 | 1 | 1 | 2 | N |
| hsa-miR-4436b-3p | DCP1A | 1 | 1 | 2 | N |
| hsa-miR-4436b-3p | STK19 | 1 | 1 | 2 | N |
| hsa-miR-4436b-3p | GLIS2 | 1 | 1 | 2 | N |
| hsa-miR-4436b-3p | XYLT1 | 1 | 1 | 2 | N |
| hsa-miR-4436b-3p | ST3GAL5 | 1 | 1 | 2 | N |
| hsa-miR-4436b-3p | C9orf84 | 1 | 1 | 2 | N |
| hsa-miR-4436b-3p | HMG20A | 1 | 1 | 2 | N |
| hsa-miR-4436b-3p | SLC31A2 | 1 | 1 | 2 | N |
| hsa-miR-4436b-3p | RTKN | 1 | 1 | 2 | N |
| hsa-miR-4436b-3p | TBC1D13 | 1 | 1 | 2 | N |
| hsa-miR-4436b-3p | HTRA3 | 1 | 1 | 2 | N |
| hsa-miR-4436b-3p | NOTCH2 | 1 | 1 | 2 | N |
| hsa-miR-4436b-3p | RD3 | 1 | 1 | 2 | N |
| hsa-miR-4436b-3p | MAML1 | 1 | 1 | 2 | N |
| hsa-miR-4436b-3p | KIF21B | 1 | 1 | 2 | N |
| hsa-miR-4436b-3p | ANKRA2 | 1 | 1 | 2 | N |
| hsa-miR-4436b-3p | ADAM10 | 1 | 1 | 2 | N |
| hsa-miR-4436b-3p | ZNF862 | 1 | 1 | 2 | N |
| hsa-miR-4436b-3p | DDI2 | 1 | 1 | 2 | N |
| hsa-miR-4436b-3p | SLC37A1 | 1 | 1 | 2 | N |
| hsa-miR-4436b-3p | RAB1B | 1 | 1 | 2 | Y |
| hsa-miR-4436b-3p | ARHGEF6 | 1 | 1 | 2 | N |
| hsa-miR-4436b-3p | ADAMTS4 | 1 | 1 | 2 | N |
| hsa-miR-4436b-3p | SOCS4 | 1 | 1 | 2 | N |
| hsa-miR-4436b-3p | TNFAIP8L3 | 1 | 1 | 2 | N |
| hsa-miR-4436b-3p | TMEM25 | 1 | 1 | 2 | N |
| hsa-miR-4436b-3p | PAPD7 | 1 | 1 | 2 | N |
| hsa-miR-4436b-3p | CXCR5 | 1 | 1 | 2 | N |
| hsa-miR-4436b-3p | TCEA2 | 1 | 1 | 2 | N |
| hsa-miR-4436b-3p | NAT16 | 1 | 1 | 2 | N |
| hsa-miR-4436b-3p | NEUROD4 | 1 | 1 | 2 | N |
| hsa-miR-4436b-3p | DHDDS | 1 | 1 | 2 | N |
| hsa-miR-4436b-3p | AGO1 | 1 | 1 | 2 | Y |
| hsa-miR-4436b-3p | SYT7 | 1 | 1 | 2 | Y |
| hsa-miR-4436b-3p | C17orf85 | 1 | 1 | 2 | N |
| hsa-miR-4436b-3p | NCKAP5L | 1 | 1 | 2 | N |
| hsa-miR-4436b-3p | MAFG | 1 | 1 | 2 | N |
| hsa-miR-4436b-3p | PVRL1 | 1 | 1 | 2 | N |
| hsa-miR-4436b-3p | CBX7 | 1 | 1 | 2 | N |
| hsa-miR-4436b-3p | ULK3 | 1 | 1 | 2 | N |
| hsa-miR-4436b-3p | ZSCAN31 | 1 | 1 | 2 | N |
| hsa-miR-4520-3p | VIPAS39 | 1 | 1 | 2 | N |
| hsa-miR-4520-3p | RAB10 | 1 | 1 | 2 | Y |
| hsa-miR-4520-3p | SYPL2 | 1 | 1 | 2 | N |
| hsa-miR-4520-3p | MAPKAPK2 | 1 | 1 | 2 | N |
| hsa-miR-4520-3p | GNG12 | 1 | 1 | 2 | N |
| hsa-miR-4520-3p | PGBD5 | 1 | 1 | 2 | N |
| hsa-miR-4520-3p | EVPL | 1 | 1 | 2 | N |
| hsa-miR-4520-3p | HSPH1 | 1 | 1 | 2 | N |
| hsa-miR-4520-3p | SEPT3 | 1 | 1 | 2 | N |
| hsa-miR-4520-3p | NDRG3 | 1 | 1 | 2 | N |
| hsa-miR-4520-3p | COL4A1 | 1 | 1 | 2 | N |
| hsa-miR-4520-3p | GSPT2 | 1 | 1 | 2 | N |
| hsa-miR-4520-3p | GRB2 | 1 | 1 | 2 | N |
| hsa-miR-4520-3p | NHLH1 | 1 | 1 | 2 | N |
| hsa-miR-4520-3p | KIAA1644 | 1 | 1 | 2 | N |
| hsa-miR-4520-3p | PPP1R3D | 1 | 1 | 2 | N |
| hsa-miR-4520-3p | SDHD | 1 | 1 | 2 | N |
| hsa-miR-4520-3p | PODXL | 1 | 1 | 2 | N |
| hsa-miR-4520-3p | PRKX | 1 | 1 | 2 | Y |
| hsa-miR-4520-3p | POLR2C | 1 | 1 | 2 | N |
| hsa-miR-4520-3p | LHFPL3 | 1 | 1 | 2 | N |
| hsa-miR-4520-3p | RNF24 | 1 | 1 | 2 | N |
| hsa-miR-4520-3p | PIM3 | 1 | 1 | 2 | N |
| hsa-miR-4520-3p | SND1 | 1 | 1 | 2 | N |
| hsa-miR-4520-3p | SLC25A15 | 1 | 1 | 2 | N |
| hsa-miR-4520-3p | LRRTM1 | 1 | 1 | 2 | N |
| hsa-miR-4520-3p | PSMA3 | 1 | 1 | 2 | N |
| hsa-miR-4520-3p | RAB25 | 1 | 1 | 2 | Y |
| hsa-miR-4520-3p | RHEB | 1 | 1 | 2 | N |
| hsa-miR-4520-3p | CENPB | 1 | 1 | 2 | N |
| hsa-miR-4520-3p | MLEC | 1 | 1 | 2 | N |
| hsa-miR-4520-3p | PSMA4 | 1 | 1 | 2 | N |
| hsa-miR-4520-3p | NDUFA8 | 1 | 1 | 2 | N |
| hsa-miR-4520-3p | YWHAG | 1 | 1 | 2 | N |
| hsa-miR-4520-3p | FRS3 | 1 | 1 | 2 | N |
| hsa-miR-4520-3p | HIST2H2BE | 1 | 1 | 2 | N |
| hsa-miR-4520-3p | CADM3 | 1 | 1 | 2 | N |
| hsa-miR-4520-3p | SIKE1 | 1 | 1 | 2 | N |
| hsa-miR-4520-3p | FOXN2 | 1 | 1 | 2 | N |
| hsa-miR-4520-3p | TRIM41 | 1 | 1 | 2 | N |
| hsa-miR-4520-3p | PTPN5 | 1 | 1 | 2 | N |
| hsa-miR-4520-3p | ACVR1B | 1 | 1 | 2 | N |
| hsa-miR-4632-5p | VPS39 | 1 | 1 | 2 | N |
| hsa-miR-4632-5p | NRIP2 | 1 | 1 | 2 | N |
| hsa-miR-4632-5p | GPR85 | 1 | 1 | 2 | N |
| hsa-miR-4632-5p | HECTD3 | 1 | 1 | 2 | N |
| hsa-miR-4632-5p | LUZP1 | 1 | 1 | 2 | Y |
| hsa-miR-4632-5p | HTRA3 | 1 | 1 | 2 | N |
| hsa-miR-4632-5p | SMARCD1 | 1 | 1 | 2 | N |
| hsa-miR-4632-5p | RTKN | 1 | 1 | 2 | N |
| hsa-miR-4632-5p | TNFAIP8L3 | 1 | 1 | 2 | N |
| hsa-miR-4632-5p | RASSF4 | 1 | 1 | 2 | N |
| hsa-miR-4632-5p | WDTC1 | 1 | 1 | 2 | N |
| hsa-miR-4632-5p | COL5A3 | 1 | 1 | 2 | N |
| hsa-miR-4632-5p | WASF2 | 1 | 1 | 2 | N |
| hsa-miR-4632-5p | PIGY | 1 | 1 | 2 | N |
| hsa-miR-4632-5p | SH2D5 | 1 | 1 | 2 | N |
| hsa-miR-4632-5p | RELT | 1 | 1 | 2 | N |
| hsa-miR-4632-5p | GABRA4 | 1 | 1 | 2 | N |
| hsa-miR-4632-5p | KCNK3 | 1 | 1 | 2 | N |
| hsa-miR-4632-5p | ATP9B | 1 | 1 | 2 | N |
| hsa-miR-4632-5p | MAPKAPK2 | 1 | 1 | 2 | N |
| hsa-miR-4632-5p | CBX6 | 1 | 1 | 2 | Y |
| hsa-miR-4632-5p | CSF1R | 1 | 1 | 2 | N |
| hsa-miR-4632-5p | PATZ1 | 1 | 1 | 2 | N |
| hsa-miR-4632-5p | GRM2 | 1 | 1 | 2 | N |
| hsa-miR-4632-5p | PAPD7 | 1 | 1 | 2 | N |
| hsa-miR-4632-5p | FAM214B | 1 | 1 | 2 | N |
| hsa-miR-4632-5p | MGLL | 1 | 1 | 2 | N |
| hsa-miR-4632-5p | GLIS2 | 1 | 1 | 2 | N |
| hsa-miR-4632-5p | LINGO1 | 1 | 1 | 2 | N |
| hsa-miR-4632-5p | CACNA2D2 | 1 | 1 | 2 | N |
| hsa-miR-4632-5p | MPZ | 1 | 1 | 2 | N |
| hsa-miR-4632-5p | SOCS4 | 1 | 1 | 2 | N |
| hsa-miR-4632-5p | AK3 | 1 | 1 | 2 | N |
| hsa-miR-4632-5p | TRIM26 | 1 | 1 | 2 | N |
| hsa-miR-4632-5p | NOTCH2 | 1 | 1 | 2 | N |
| hsa-miR-4632-5p | STAB2 | 1 | 1 | 2 | N |
| hsa-miR-4632-5p | SLC31A2 | 1 | 1 | 2 | N |
| hsa-miR-4632-5p | ORAI3 | 1 | 1 | 2 | N |
| hsa-miR-4632-5p | ULK3 | 1 | 1 | 2 | N |
| hsa-miR-4632-5p | KCNIP1 | 1 | 1 | 2 | N |
| hsa-miR-4632-5p | RHBDF2 | 1 | 1 | 2 | Y |
| hsa-miR-4632-5p | ADAM10 | 1 | 1 | 2 | N |
| hsa-miR-4632-5p | ZNF862 | 1 | 1 | 2 | N |
| hsa-miR-4632-5p | CILP | 1 | 1 | 2 | N |
| hsa-miR-4632-5p | CACFD1 | 1 | 1 | 2 | N |
| hsa-miR-4632-5p | RAB1B | 1 | 1 | 2 | N |
| hsa-miR-4632-5p | AIF1L | 1 | 1 | 2 | N |
| hsa-miR-4632-5p | PPP6C | 1 | 1 | 2 | N |
| hsa-miR-4632-5p | NPLOC4 | 1 | 1 | 2 | Y |
| hsa-miR-4632-5p | DAGLA | 1 | 1 | 2 | N |
| hsa-miR-4632-5p | LSM12 | 1 | 1 | 2 | N |
| hsa-miR-4632-5p | KIRREL | 1 | 1 | 2 | N |
| hsa-miR-4632-5p | AGAP3 | 1 | 1 | 2 | N |
| hsa-miR-4632-5p | SEPT3 | 1 | 1 | 2 | N |
| hsa-miR-4632-5p | ZIC3 | 1 | 1 | 2 | N |
| hsa-miR-4632-5p | SV2A | 1 | 1 | 2 | N |
| hsa-miR-4632-5p | C14orf1 | 1 | 1 | 2 | N |
| hsa-miR-4632-5p | PGS1 | 1 | 1 | 2 | N |
| hsa-miR-4632-5p | CBX7 | 1 | 1 | 2 | N |
| hsa-miR-4632-5p | NAB2 | 1 | 1 | 2 | N |
| hsa-miR-4632-5p | NAT16 | 1 | 1 | 2 | N |
| hsa-miR-4632-5p | ETV4 | 1 | 1 | 2 | N |
| hsa-miR-4632-5p | KMT2D | 1 | 1 | 2 | Y |
| hsa-miR-4632-5p | SLC37A1 | 1 | 1 | 2 | N |
| hsa-miR-4632-5p | SSR1 | 1 | 1 | 2 | N |
| hsa-miR-4632-5p | TRIM9 | 1 | 1 | 2 | N |
| hsa-miR-4632-5p | FAM19A3 | 1 | 1 | 2 | N |
| hsa-miR-4632-5p | CCDC114 | 1 | 1 | 2 | N |
| hsa-miR-4632-5p | CHAF1A | 1 | 1 | 2 | N |
| hsa-miR-4632-5p | CIC | 1 | 1 | 2 | N |
| hsa-miR-4632-5p | HAP1 | 1 | 1 | 2 | N |
| hsa-miR-4632-5p | SLC6A9 | 1 | 1 | 2 | Y |
| hsa-miR-4632-5p | RALYL | 1 | 1 | 2 | N |
| hsa-miR-4632-5p | DDI2 | 1 | 1 | 2 | N |
| hsa-miR-4632-5p | LASP1 | 1 | 1 | 2 | N |
| hsa-miR-4632-5p | XYLT1 | 1 | 1 | 2 | N |
| hsa-miR-4632-5p | KIAA0247 | 1 | 1 | 2 | N |
| hsa-miR-4632-5p | TMEM154 | 1 | 1 | 2 | N |
| hsa-miR-4632-5p | C17orf67 | 1 | 1 | 2 | N |
| hsa-miR-4632-5p | ARL4C | 1 | 1 | 2 | N |
| hsa-miR-4632-5p | ZSCAN31 | 1 | 1 | 2 | N |
| hsa-miR-4632-5p | AGO1 | 1 | 1 | 2 | Y |
| hsa-miR-4632-5p | NAA30 | 1 | 1 | 2 | N |
| hsa-miR-4632-5p | FNDC3B | 1 | 1 | 2 | N |
| hsa-miR-4632-5p | STK35 | 1 | 1 | 2 | N |
| hsa-miR-4632-5p | CNIH2 | 1 | 1 | 2 | N |
| hsa-miR-4632-5p | FHL1 | 1 | 1 | 2 | N |
| hsa-miR-4632-5p | CX3CL1 | 1 | 1 | 2 | N |
| hsa-miR-4632-5p | PUM2 | 1 | 1 | 2 | Y |
| hsa-miR-4632-5p | HLA-DOB | 1 | 1 | 2 | N |
| hsa-miR-4632-5p | CDC42SE1 | 1 | 1 | 2 | Y |
| hsa-miR-4632-5p | DCP1A | 1 | 1 | 2 | N |
| hsa-miR-4632-5p | TLL2 | 1 | 1 | 2 | N |
| hsa-miR-4632-5p | WIPF3 | 1 | 1 | 2 | N |
| hsa-miR-4632-5p | TRIM35 | 1 | 1 | 2 | N |
| hsa-miR-4632-5p | SDC3 | 1 | 1 | 2 | N |
| hsa-miR-4632-5p | THEM6 | 1 | 1 | 2 | N |
| hsa-miR-4632-5p | TCEA2 | 1 | 1 | 2 | N |
| hsa-miR-4632-5p | PARP6 | 1 | 1 | 2 | N |
| hsa-miR-4632-5p | SLC26A9 | 1 | 1 | 2 | N |
| hsa-miR-4632-5p | ANKFY1 | 1 | 1 | 2 | N |
| hsa-miR-4632-5p | KIF21B | 1 | 1 | 2 | N |
| hsa-miR-4632-5p | SOX10 | 1 | 1 | 2 | N |
| hsa-miR-4632-5p | FAM86A | 1 | 1 | 2 | N |
| hsa-miR-4632-5p | ARL6IP1 | 1 | 1 | 2 | Y |
| hsa-miR-4632-5p | CTNND1 | 1 | 1 | 2 | N |
| hsa-miR-4632-5p | ANKRA2 | 1 | 1 | 2 | N |
| hsa-miR-4632-5p | TMEM110 | 1 | 1 | 2 | N |
| hsa-miR-4632-5p | NEUROD4 | 1 | 1 | 2 | N |
| hsa-miR-4632-5p | TMEM127 | 1 | 1 | 2 | N |
| hsa-miR-4632-5p | RASSF2 | 1 | 1 | 2 | N |
| hsa-miR-4632-5p | CTDNEP1 | 1 | 1 | 2 | Y |
| hsa-miR-4632-5p | CLSTN2 | 1 | 1 | 2 | N |
| hsa-miR-4632-5p | TTC39A | 1 | 1 | 2 | N |
| hsa-miR-4632-5p | ADAMTS4 | 1 | 1 | 2 | N |
| hsa-miR-4632-5p | CYBRD1 | 1 | 1 | 2 | N |
| hsa-miR-4632-5p | HMG20A | 1 | 1 | 2 | N |
| hsa-miR-4632-5p | REG4 | 1 | 1 | 2 | N |
| hsa-miR-4632-5p | ADIPOR2 | 1 | 1 | 2 | N |
| hsa-miR-4632-5p | LIN28A | 1 | 1 | 2 | N |
| hsa-miR-4632-5p | SYT7 | 1 | 1 | 2 | Y |
| hsa-miR-4632-5p | ABHD12 | 1 | 1 | 2 | N |
| hsa-miR-4632-5p | HJURP | 1 | 1 | 2 | N |
| hsa-miR-4632-5p | ARHGEF6 | 1 | 1 | 2 | N |
| hsa-miR-4632-5p | ATG9A | 1 | 1 | 2 | N |
| hsa-miR-4632-5p | NEU1 | 1 | 1 | 2 | N |
| hsa-miR-4632-5p | GDNF | 1 | 1 | 2 | N |
| hsa-miR-4632-5p | FAM195B | 1 | 1 | 2 | N |
| hsa-miR-4632-5p | PIANP | 1 | 1 | 2 | N |
| hsa-miR-4632-5p | MISP | 1 | 1 | 2 | N |
| hsa-miR-4632-5p | ITCH | 1 | 1 | 2 | N |
| hsa-miR-4632-5p | CDIP1 | 1 | 1 | 2 | N |
| hsa-miR-4632-5p | USP7 | 1 | 1 | 2 | N |
| hsa-miR-4632-5p | KCNE1 | 1 | 1 | 2 | N |
| hsa-miR-4632-5p | RGMB | 1 | 1 | 2 | Y |
| hsa-miR-4632-5p | SOWAHC | 1 | 1 | 2 | Y |
| hsa-miR-4632-5p | FBXO41 | 1 | 1 | 2 | Y |
| hsa-miR-4632-5p | PVRL1 | 1 | 1 | 2 | N |
| hsa-miR-4632-5p | SCML1 | 1 | 1 | 2 | N |
| hsa-miR-4632-5p | C17orf85 | 1 | 1 | 2 | N |
| hsa-miR-4632-5p | EFNB3 | 1 | 1 | 2 | N |
| hsa-miR-4632-5p | FAM53C | 1 | 1 | 2 | N |
| hsa-miR-4632-5p | TRIM4 | 1 | 1 | 2 | N |
| hsa-miR-4632-5p | TMEM25 | 1 | 1 | 2 | N |
| hsa-miR-4632-5p | TSPAN9 | 1 | 1 | 2 | N |
| hsa-miR-4632-5p | HMX2 | 1 | 1 | 2 | N |
| hsa-miR-4632-5p | ST3GAL5 | 1 | 1 | 2 | N |
| hsa-miR-4632-5p | MARVELD1 | 1 | 1 | 2 | N |
| hsa-miR-4632-5p | C11orf86 | 1 | 1 | 2 | N |
| hsa-miR-4632-5p | AURKA | 1 | 1 | 2 | Y |
| hsa-miR-4632-5p | LBH | 1 | 1 | 2 | N |
| hsa-miR-4632-5p | TRIM46 | 1 | 1 | 2 | N |
| hsa-miR-4632-5p | SLC37A3 | 1 | 1 | 2 | N |
| hsa-miR-4632-5p | NAMPT | 1 | 1 | 2 | N |
| hsa-miR-4632-5p | MAFG | 1 | 1 | 2 | N |
| hsa-miR-4632-5p | SLC6A17 | 1 | 1 | 2 | N |
| hsa-miR-4632-5p | TBC1D9B | 1 | 1 | 2 | N |
| hsa-miR-4632-5p | PPP1R9B | 1 | 1 | 2 | N |
| hsa-miR-4632-5p | SOX13 | 1 | 1 | 2 | N |
| hsa-miR-4632-5p | SRRD | 1 | 1 | 2 | N |
| hsa-miR-4632-5p | HNRNPR | 1 | 1 | 2 | N |
| hsa-miR-4632-5p | TBC1D13 | 1 | 1 | 2 | N |
| hsa-miR-4632-5p | LDLRAP1 | 1 | 1 | 2 | N |
| hsa-miR-4632-5p | SH3GL2 | 1 | 1 | 2 | N |
| hsa-miR-4632-5p | C9orf84 | 1 | 1 | 2 | N |
| hsa-miR-4632-5p | FAM155B | 1 | 1 | 2 | N |
| hsa-miR-4632-5p | STK19 | 1 | 1 | 2 | N |
| hsa-miR-4776-5p | SLC38A2 | 1 | 1 | 2 | N |
| hsa-miR-4776-5p | FOXO1 | 1 | 1 | 2 | N |
| hsa-miR-4776-5p | NXT2 | 1 | 1 | 2 | N |
| hsa-miR-4776-5p | OCIAD1 | 1 | 1 | 2 | N |
| hsa-miR-4776-5p | GLRX3 | 1 | 1 | 2 | N |
| hsa-miR-4776-5p | IKBIP | 1 | 1 | 2 | N |
| hsa-miR-4776-5p | RGS2 | 1 | 1 | 2 | N |
| hsa-miR-4776-5p | TNFSF10 | 1 | 1 | 2 | N |
| hsa-miR-4776-5p | COL4A1 | 1 | 1 | 2 | N |
| hsa-miR-4776-5p | CRYGS | 1 | 1 | 2 | N |
| hsa-miR-4776-5p | ARHGAP29 | 1 | 1 | 2 | N |
| hsa-miR-4776-5p | NMT1 | 1 | 1 | 2 | N |
| hsa-miR-4776-5p | ASB7 | 1 | 1 | 2 | N |
| hsa-miR-4776-5p | FAM198B | 1 | 1 | 2 | N |
| hsa-miR-4776-5p | SMC2 | 1 | 1 | 2 | N |
| hsa-miR-4776-5p | MAML2 | 1 | 1 | 2 | N |
| hsa-miR-4776-5p | TFCP2L1 | 1 | 1 | 2 | N |
| hsa-miR-4776-5p | KRT8 | 1 | 1 | 2 | Y |
| hsa-miR-4776-5p | TCF21 | 1 | 1 | 2 | N |
| hsa-miR-4776-5p | TMPRSS5 | 1 | 1 | 2 | N |
| hsa-miR-4776-5p | TSPAN7 | 1 | 1 | 2 | N |
| hsa-miR-4776-5p | GPR173 | 1 | 1 | 2 | N |
| hsa-miR-4776-5p | DSTN | 1 | 1 | 2 | N |
| hsa-miR-4776-5p | TOP1 | 1 | 1 | 2 | N |
| hsa-miR-4776-5p | KCNK9 | 1 | 1 | 2 | N |
| hsa-miR-4776-5p | RBM14-RBM4 | 1 | 1 | 2 | N |
| hsa-miR-5193 | MRPS35 | 1 | 1 | 2 | N |
| hsa-miR-5193 | NAV1 | 1 | 1 | 2 | Y |
| hsa-miR-5193 | NAA50 | 1 | 1 | 2 | N |
| hsa-miR-5193 | ZNF114 | 1 | 1 | 2 | N |
| hsa-miR-5193 | PALD1 | 1 | 1 | 2 | Y |
| hsa-miR-5193 | CCK | 1 | 1 | 2 | N |
| hsa-miR-5193 | PNMA3 | 1 | 1 | 2 | N |
| hsa-miR-5193 | CCNF | 1 | 1 | 2 | N |
| hsa-miR-5193 | TLCD2 | 1 | 1 | 2 | Y |
| hsa-miR-5193 | FAM167B | 1 | 1 | 2 | N |
| hsa-miR-5193 | DAAM2 | 1 | 1 | 2 | N |
| hsa-miR-5193 | FAM212B | 1 | 1 | 2 | N |
| hsa-miR-5193 | SNX30 | 1 | 1 | 2 | N |
| hsa-miR-5193 | PRMT3 | 1 | 1 | 2 | N |
| hsa-miR-5193 | ANKRD54 | 1 | 1 | 2 | N |
| hsa-miR-5193 | GSG1L | 1 | 1 | 2 | N |
| hsa-miR-5193 | ING1 | 1 | 1 | 2 | Y |
| hsa-miR-5193 | C16orf90 | 1 | 1 | 2 | N |
| hsa-miR-5193 | SNPH | 1 | 1 | 2 | N |
| hsa-miR-5193 | SFMBT2 | 1 | 1 | 2 | N |
| hsa-miR-5193 | SCN4A | 1 | 1 | 2 | N |
| hsa-miR-5193 | ZNF24 | 1 | 1 | 2 | N |
| hsa-miR-5193 | LPHN1 | 1 | 1 | 2 | N |
| hsa-miR-5193 | DMBX1 | 1 | 1 | 2 | N |
| hsa-miR-5193 | DLG3 | 1 | 1 | 2 | N |
| hsa-miR-5193 | PEA15 | 1 | 1 | 2 | Y |
| hsa-miR-5193 | GLB1L2 | 1 | 1 | 2 | N |
| hsa-miR-5193 | TREML2 | 1 | 1 | 2 | N |
| hsa-miR-5193 | NYAP1 | 1 | 1 | 2 | N |
| hsa-miR-5193 | MICAL3 | 1 | 1 | 2 | N |
| hsa-miR-5193 | SP6 | 1 | 1 | 2 | N |
| hsa-miR-5193 | RASSF1 | 1 | 1 | 2 | N |
| hsa-miR-5193 | GJB1 | 1 | 1 | 2 | N |
| hsa-miR-5193 | IL25 | 1 | 1 | 2 | N |
| hsa-miR-5193 | LAMP5 | 1 | 1 | 2 | N |
| hsa-miR-5193 | SNX8 | 1 | 1 | 2 | N |
| hsa-miR-5193 | EID1 | 1 | 1 | 2 | N |
| hsa-miR-5193 | MGAT5 | 1 | 1 | 2 | N |
| hsa-miR-5193 | SNX18 | 1 | 1 | 2 | N |
| hsa-miR-5193 | PPP1R17 | 1 | 1 | 2 | N |
| hsa-miR-5193 | DST | 1 | 1 | 2 | N |
| hsa-miR-5193 | RAP1GAP2 | 1 | 1 | 2 | N |
| hsa-miR-5193 | SYT11 | 1 | 1 | 2 | N |
| hsa-miR-5193 | RHOBTB2 | 1 | 1 | 2 | N |
| hsa-miR-5193 | NFAM1 | 1 | 1 | 2 | Y |
| hsa-miR-5193 | SLC22A6 | 1 | 1 | 2 | Y |
| hsa-miR-5193 | PIGR | 1 | 1 | 2 | N |
| hsa-miR-5193 | KIAA0513 | 1 | 1 | 2 | N |
| hsa-miR-5193 | TNFRSF19 | 1 | 1 | 2 | N |
| hsa-miR-5193 | CNBP | 1 | 1 | 2 | N |
| hsa-miR-5193 | AP1G1 | 1 | 1 | 2 | N |
| hsa-miR-5193 | SEMA3G | 1 | 1 | 2 | N |
| hsa-miR-5193 | HAS3 | 1 | 1 | 2 | N |
| hsa-miR-5193 | MIEF2 | 1 | 1 | 2 | N |
| hsa-miR-5193 | JDP2 | 1 | 1 | 2 | N |
| hsa-miR-5193 | PRKAG1 | 1 | 1 | 2 | Y |
| hsa-miR-5193 | PAFAH1B2 | 1 | 1 | 2 | N |
| hsa-miR-5193 | EMP1 | 1 | 1 | 2 | N |
| hsa-miR-5193 | NAA11 | 1 | 1 | 2 | N |
| hsa-miR-5193 | FAM20B | 1 | 1 | 2 | Y |
| hsa-miR-5193 | THSD1 | 1 | 1 | 2 | N |
| hsa-miR-5193 | CABLES1 | 1 | 1 | 2 | N |
| hsa-miR-5193 | CAMK2N1 | 1 | 1 | 2 | N |
| hsa-miR-5193 | KCNJ9 | 1 | 1 | 2 | N |
| hsa-miR-5193 | ARRDC3 | 1 | 1 | 2 | Y |
| hsa-miR-5193 | ATP2B4 | 1 | 1 | 2 | N |
| hsa-miR-5193 | SLC38A5 | 1 | 1 | 2 | Y |
| hsa-miR-5193 | EHD3 | 1 | 1 | 2 | N |
| hsa-miR-5193 | LIF | 1 | 1 | 2 | Y |
| hsa-miR-5193 | FADS1 | 1 | 1 | 2 | N |
| hsa-miR-5193 | JOSD1 | 1 | 1 | 2 | N |
| hsa-miR-5193 | DCAF7 | 1 | 1 | 2 | N |
| hsa-miR-5193 | MKLN1 | 1 | 1 | 2 | N |
| hsa-miR-5193 | AGPAT1 | 1 | 1 | 2 | N |
| hsa-miR-5193 | DPP8 | 1 | 1 | 2 | N |
| hsa-miR-5193 | SOX4 | 1 | 1 | 2 | N |
| hsa-miR-5193 | HDAC7 | 1 | 1 | 2 | Y |
| hsa-miR-5193 | SNW1 | 1 | 1 | 2 | Y |
| hsa-miR-5193 | HOMER1 | 1 | 1 | 2 | N |
| hsa-miR-5193 | SOX10 | 1 | 1 | 2 | N |
| hsa-miR-5193 | GLP1R | 1 | 1 | 2 | N |
| hsa-miR-5193 | CHD6 | 1 | 1 | 2 | N |
| hsa-miR-5193 | HOXA9 | 1 | 1 | 2 | N |
| hsa-miR-5193 | PLCG1 | 1 | 1 | 2 | N |
| hsa-miR-5193 | ZBTB37 | 1 | 1 | 2 | N |
| hsa-miR-5193 | PLCXD2 | 1 | 1 | 2 | N |
| hsa-miR-6735-5p | STK19 | 1 | 1 | 2 | N |
| hsa-miR-6735-5p | STAB2 | 1 | 1 | 2 | N |
| hsa-miR-6735-5p | CX3CL1 | 1 | 1 | 2 | N |
| hsa-miR-6735-5p | LIN28A | 1 | 1 | 2 | N |
| hsa-miR-6735-5p | PATZ1 | 1 | 1 | 2 | N |
| hsa-miR-6735-5p | HJURP | 1 | 1 | 2 | N |
| hsa-miR-6735-5p | CACFD1 | 1 | 1 | 2 | N |
| hsa-miR-6735-5p | TNFAIP8L3 | 1 | 1 | 2 | N |
| hsa-miR-6735-5p | PPP6C | 1 | 1 | 2 | N |
| hsa-miR-6735-5p | FAM86A | 1 | 1 | 2 | N |
| hsa-miR-6735-5p | HNRNPR | 1 | 1 | 2 | N |
| hsa-miR-6735-5p | PGS1 | 1 | 1 | 2 | N |
| hsa-miR-6735-5p | IDUA | 1 | 1 | 2 | N |
| hsa-miR-6735-5p | KCNIP1 | 1 | 1 | 2 | N |
| hsa-miR-6735-5p | LDLRAP1 | 1 | 1 | 2 | N |
| hsa-miR-6735-5p | ADAMTS4 | 1 | 1 | 2 | N |
| hsa-miR-6735-5p | SLC6A17 | 1 | 1 | 2 | N |
| hsa-miR-6735-5p | ANKRA2 | 1 | 1 | 2 | N |
| hsa-miR-6735-5p | AIF1L | 1 | 1 | 2 | N |
| hsa-miR-6735-5p | ARL4C | 1 | 1 | 2 | N |
| hsa-miR-6735-5p | FAM155B | 1 | 1 | 2 | N |
| hsa-miR-6735-5p | ST3GAL5 | 1 | 1 | 2 | N |
| hsa-miR-6735-5p | RTKN | 1 | 1 | 2 | N |
| hsa-miR-6735-5p | SLC31A2 | 1 | 1 | 2 | N |
| hsa-miR-6735-5p | LSM12 | 1 | 1 | 2 | N |
| hsa-miR-6735-5p | ZNF862 | 1 | 1 | 2 | N |
| hsa-miR-6735-5p | ZIC3 | 1 | 1 | 2 | N |
| hsa-miR-6735-5p | PIANP | 1 | 1 | 2 | N |
| hsa-miR-6735-5p | CYBRD1 | 1 | 1 | 2 | N |
| hsa-miR-6735-5p | CCDC114 | 1 | 1 | 2 | N |
| hsa-miR-6735-5p | KCNK3 | 1 | 1 | 2 | N |
| hsa-miR-6735-5p | WASF2 | 1 | 1 | 2 | N |
| hsa-miR-6735-5p | SLC37A3 | 1 | 1 | 2 | N |
| hsa-miR-6735-5p | NOTCH2 | 1 | 1 | 2 | N |
| hsa-miR-6735-5p | PRMT2 | 1 | 1 | 2 | N |
| hsa-miR-6735-5p | CSF1R | 1 | 1 | 2 | N |
| hsa-miR-6735-5p | C14orf1 | 1 | 1 | 2 | N |
| hsa-miR-6735-5p | KIAA0247 | 1 | 1 | 2 | N |
| hsa-miR-6735-5p | FHL1 | 1 | 1 | 2 | N |
| hsa-miR-6735-5p | EFNB3 | 1 | 1 | 2 | N |
| hsa-miR-6735-5p | CHAF1A | 1 | 1 | 2 | N |
| hsa-miR-6735-5p | AK3 | 1 | 1 | 2 | N |
| hsa-miR-6735-5p | WDTC1 | 1 | 1 | 2 | N |
| hsa-miR-6735-5p | AGO1 | 1 | 1 | 2 | Y |
| hsa-miR-6735-5p | SCML1 | 1 | 1 | 2 | N |
| hsa-miR-6735-5p | HTRA3 | 1 | 1 | 2 | N |
| hsa-miR-6735-5p | ZSCAN31 | 1 | 1 | 2 | N |
| hsa-miR-6735-5p | ARL6IP1 | 1 | 1 | 2 | Y |
| hsa-miR-6735-5p | SRRD | 1 | 1 | 2 | N |
| hsa-miR-6735-5p | SH3GL2 | 1 | 1 | 2 | N |
| hsa-miR-6735-5p | ARHGEF6 | 1 | 1 | 2 | N |
| hsa-miR-6735-5p | AGAP3 | 1 | 1 | 2 | N |
| hsa-miR-6735-5p | SMARCD1 | 1 | 1 | 2 | N |
| hsa-miR-6735-5p | ADAM10 | 1 | 1 | 2 | N |
| hsa-miR-6735-5p | CLSTN2 | 1 | 1 | 2 | N |
| hsa-miR-6735-5p | RELT | 1 | 1 | 2 | N |
| hsa-miR-6735-5p | ATG9A | 1 | 1 | 2 | N |
| hsa-miR-6735-5p | SSR1 | 1 | 1 | 2 | N |
| hsa-miR-6735-5p | FKBP8 | 1 | 1 | 2 | Y |
| hsa-miR-6735-5p | NRIP2 | 1 | 1 | 2 | N |
| hsa-miR-6735-5p | TCEA2 | 1 | 1 | 2 | N |
| hsa-miR-6735-5p | ULK3 | 1 | 1 | 2 | N |
| hsa-miR-6735-5p | CIC | 1 | 1 | 2 | N |
| hsa-miR-6735-5p | SLC6A9 | 1 | 1 | 2 | Y |
| hsa-miR-6735-5p | COL5A3 | 1 | 1 | 2 | N |
| hsa-miR-6735-5p | NAB2 | 1 | 1 | 2 | N |
| hsa-miR-6735-5p | FAM53C | 1 | 1 | 2 | N |
| hsa-miR-6735-5p | RASSF4 | 1 | 1 | 2 | N |
| hsa-miR-6735-5p | PVRL1 | 1 | 1 | 2 | N |
| hsa-miR-6735-5p | VPS39 | 1 | 1 | 2 | N |
| hsa-miR-6735-5p | GRM2 | 1 | 1 | 2 | N |
| hsa-miR-6735-5p | GABRA4 | 1 | 1 | 2 | N |
| hsa-miR-6735-5p | LBH | 1 | 1 | 2 | N |
| hsa-miR-6735-5p | HMX2 | 1 | 1 | 2 | N |
| hsa-miR-6735-5p | KIRREL | 1 | 1 | 2 | N |
| hsa-miR-6735-5p | GDNF | 1 | 1 | 2 | N |
| hsa-miR-6735-5p | NEUROD4 | 1 | 1 | 2 | N |
| hsa-miR-6735-5p | RAB1B | 1 | 1 | 2 | N |
| hsa-miR-6735-5p | HLA-DOB | 1 | 1 | 2 | N |
| hsa-miR-6735-5p | DDIT4L | 1 | 1 | 2 | N |
| hsa-miR-6735-5p | SH2D5 | 1 | 1 | 2 | N |
| hsa-miR-6735-5p | C17orf85 | 1 | 1 | 2 | N |
| hsa-miR-6735-5p | PIGY | 1 | 1 | 2 | N |
| hsa-miR-6735-5p | TSPAN9 | 1 | 1 | 2 | N |
| hsa-miR-6735-5p | TRIM46 | 1 | 1 | 2 | N |
| hsa-miR-6735-5p | SOWAHC | 1 | 1 | 2 | Y |
| hsa-miR-6735-5p | RAB3B | 1 | 1 | 2 | N |
| hsa-miR-6735-5p | PPM1M | 1 | 1 | 2 | N |
| hsa-miR-6735-5p | ITCH | 1 | 1 | 2 | N |
| hsa-miR-6735-5p | SOX13 | 1 | 1 | 2 | N |
| hsa-miR-6735-5p | AURKA | 1 | 1 | 2 | Y |
| hsa-miR-6735-5p | DCP1A | 1 | 1 | 2 | N |
| hsa-miR-6735-5p | MPZ | 1 | 1 | 2 | N |
| hsa-miR-6735-5p | DDI2 | 1 | 1 | 2 | N |
| hsa-miR-6735-5p | SYNGR1 | 1 | 1 | 2 | N |
| hsa-miR-6735-5p | CACNA2D2 | 1 | 1 | 2 | N |
| hsa-miR-6735-5p | CCNL2 | 1 | 1 | 2 | N |
| hsa-miR-6735-5p | ANKFY1 | 1 | 1 | 2 | N |
| hsa-miR-6735-5p | TMEM127 | 1 | 1 | 2 | N |
| hsa-miR-6735-5p | HMG20A | 1 | 1 | 2 | N |
| hsa-miR-6735-5p | LFNG | 1 | 1 | 2 | N |
| hsa-miR-6735-5p | ABHD12 | 1 | 1 | 2 | N |
| hsa-miR-6735-5p | TBC1D13 | 1 | 1 | 2 | N |
| hsa-miR-6735-5p | ETV4 | 1 | 1 | 2 | N |
| hsa-miR-6735-5p | C9orf84 | 1 | 1 | 2 | N |
| hsa-miR-6735-5p | FAM214B | 1 | 1 | 2 | N |
| hsa-miR-6735-5p | CDC42SE1 | 1 | 1 | 2 | Y |
| hsa-miR-6735-5p | TRIM9 | 1 | 1 | 2 | N |
| hsa-miR-6735-5p | RHBDF2 | 1 | 1 | 2 | Y |
| hsa-miR-6735-5p | ANKRD23 | 1 | 1 | 2 | N |
| hsa-miR-6735-5p | TTC39A | 1 | 1 | 2 | N |
| hsa-miR-6735-5p | TRIM26 | 1 | 1 | 2 | N |
| hsa-miR-6735-5p | KIF21B | 1 | 1 | 2 | N |
| hsa-miR-6735-5p | XYLT1 | 1 | 1 | 2 | N |
| hsa-miR-6735-5p | TLL2 | 1 | 1 | 2 | N |
| hsa-miR-6735-5p | SDC3 | 1 | 1 | 2 | N |
| hsa-miR-6735-5p | PARP6 | 1 | 1 | 2 | N |
| hsa-miR-6735-5p | C11orf86 | 1 | 1 | 2 | N |
| hsa-miR-6735-5p | SLC37A1 | 1 | 1 | 2 | N |
| hsa-miR-6735-5p | PAPD7 | 1 | 1 | 2 | N |
| hsa-miR-6735-5p | RALYL | 1 | 1 | 2 | N |
| hsa-miR-6735-5p | RASSF2 | 1 | 1 | 2 | N |
| hsa-miR-6735-5p | TMEM154 | 1 | 1 | 2 | N |
| hsa-miR-6735-5p | STK35 | 1 | 1 | 2 | N |
| hsa-miR-6735-5p | LINGO1 | 1 | 1 | 2 | N |
| hsa-miR-6735-5p | CASS4 | 1 | 1 | 2 | N |
| hsa-miR-6735-5p | TRIM4 | 1 | 1 | 2 | N |
| hsa-miR-6735-5p | RGMB | 1 | 1 | 2 | Y |
| hsa-miR-6735-5p | PUM2 | 1 | 1 | 2 | Y |
| hsa-miR-6735-5p | TRAPPC3 | 1 | 1 | 2 | N |
| hsa-miR-6735-5p | TMEM110 | 1 | 1 | 2 | N |
| hsa-miR-6735-5p | SOCS4 | 1 | 1 | 2 | N |
| hsa-miR-6735-5p | LUZP1 | 1 | 1 | 2 | Y |
| hsa-miR-6735-5p | ADIPOR2 | 1 | 1 | 2 | N |
| hsa-miR-6735-5p | TMEM25 | 1 | 1 | 2 | N |
| hsa-miR-6735-5p | C17orf67 | 1 | 1 | 2 | N |
| hsa-miR-6735-5p | PPP1R9B | 1 | 1 | 2 | N |
| hsa-miR-6735-5p | FBXO41 | 1 | 1 | 2 | Y |
| hsa-miR-6735-5p | TRIM35 | 1 | 1 | 2 | N |
| hsa-miR-6735-5p | CBX7 | 1 | 1 | 2 | N |
| hsa-miR-6735-5p | SEPT3 | 1 | 1 | 2 | N |
| hsa-miR-6735-5p | NAT16 | 1 | 1 | 2 | N |
| hsa-miR-6735-5p | FNDC3B | 1 | 1 | 2 | N |
| hsa-miR-6735-5p | SOX10 | 1 | 1 | 2 | N |
| hsa-miR-6735-5p | CDIP1 | 1 | 1 | 2 | N |
| hsa-miR-6735-5p | HAP1 | 1 | 1 | 2 | N |
| hsa-miR-6735-5p | MAFG | 1 | 1 | 2 | N |
| hsa-miR-6735-5p | NPLOC4 | 1 | 1 | 2 | Y |
| hsa-miR-6735-5p | ORAI3 | 1 | 1 | 2 | N |
| hsa-miR-6735-5p | NAMPT | 1 | 1 | 2 | N |
| hsa-miR-6735-5p | GPR85 | 1 | 1 | 2 | N |
| hsa-miR-6735-5p | TBC1D9B | 1 | 1 | 2 | N |
| hsa-miR-6735-5p | ARHGEF4 | 1 | 1 | 2 | N |
| hsa-miR-6735-5p | SV2A | 1 | 1 | 2 | N |
| hsa-miR-6735-5p | WIPF3 | 1 | 1 | 2 | N |
| hsa-miR-6735-5p | SYT7 | 1 | 1 | 2 | Y |
| hsa-miR-6735-5p | USP7 | 1 | 1 | 2 | N |
| hsa-miR-6825-5p | MGAT5B | 1 | 1 | 2 | Y |
| hsa-miR-6825-5p | CAPN15 | 1 | 1 | 2 | N |
| hsa-miR-6825-5p | SAP18 | 1 | 1 | 2 | Y |
| hsa-miR-6825-5p | THY1 | 1 | 1 | 2 | Y |
| hsa-miR-6825-5p | ADCYAP1R1 | 1 | 1 | 2 | N |
| hsa-miR-6825-5p | STAT2 | 1 | 1 | 2 | N |
| hsa-miR-6825-5p | ITGA3 | 1 | 1 | 2 | Y |
| hsa-miR-6825-5p | COL8A2 | 1 | 1 | 2 | N |
| hsa-miR-6825-5p | NACC1 | 1 | 1 | 2 | Y |
| hsa-miR-6825-5p | PRKCG | 1 | 1 | 2 | N |
| hsa-miR-6825-5p | PHYHIP | 1 | 1 | 2 | Y |
| hsa-miR-6825-5p | RBM28 | 1 | 1 | 2 | N |
| hsa-miR-6825-5p | C1orf21 | 1 | 1 | 2 | N |
| hsa-miR-6825-5p | OTX1 | 1 | 1 | 2 | N |
| hsa-miR-6825-5p | ZNF703 | 1 | 1 | 2 | N |
| hsa-miR-6825-5p | IRF2BPL | 1 | 1 | 2 | N |
| hsa-miR-6825-5p | SYNDIG1L | 1 | 1 | 2 | N |
| hsa-miR-6825-5p | SGSM1 | 1 | 1 | 2 | N |
| hsa-miR-6825-5p | NRGN | 1 | 1 | 2 | Y |
| hsa-miR-6825-5p | LASP1 | 1 | 1 | 2 | N |
| hsa-miR-6825-5p | B3GNT7 | 1 | 1 | 2 | N |
| hsa-miR-6825-5p | FOXP4 | 1 | 1 | 2 | N |
| hsa-miR-6825-5p | PHOX2A | 1 | 1 | 2 | N |
| hsa-miR-6825-5p | EPHB3 | 1 | 1 | 2 | N |
| hsa-miR-6825-5p | MAP1A | 1 | 1 | 2 | N |
| hsa-miR-6825-5p | PRR12 | 1 | 1 | 2 | Y |
| hsa-miR-6825-5p | FAM53C | 1 | 1 | 2 | Y |
| hsa-miR-6825-5p | AFAP1 | 1 | 1 | 2 | N |
| hsa-miR-6825-5p | ERF | 1 | 1 | 2 | N |
| hsa-miR-6825-5p | SYN1 | 1 | 1 | 2 | N |
| hsa-miR-6825-5p | NR6A1 | 1 | 1 | 2 | N |
| hsa-miR-6825-5p | SPATA2L | 1 | 1 | 2 | N |
| hsa-miR-6825-5p | SNX33 | 1 | 1 | 2 | N |
| hsa-miR-6825-5p | CS | 1 | 1 | 2 | N |
| hsa-miR-6825-5p | COPS7A | 1 | 1 | 2 | N |
| hsa-miR-6825-5p | NR1D1 | 1 | 1 | 2 | N |
| hsa-miR-6825-5p | SUV420H2 | 1 | 1 | 2 | N |
| hsa-miR-6825-5p | GNB1 | 1 | 1 | 2 | N |
| hsa-miR-6825-5p | ZC3H3 | 1 | 1 | 2 | N |
| hsa-miR-6825-5p | MVB12B | 1 | 1 | 2 | N |
| hsa-miR-6825-5p | PDE4A | 1 | 1 | 2 | Y |
| hsa-miR-6825-5p | PCDHGA7 | 1 | 1 | 2 | N |
| hsa-miR-6825-5p | TOMM40 | 1 | 1 | 2 | N |
| hsa-miR-6825-5p | ABCG4 | 1 | 1 | 2 | N |
| hsa-miR-6825-5p | SNAI3 | 1 | 1 | 2 | N |
| hsa-miR-6825-5p | SPRED2 | 1 | 1 | 2 | N |
| hsa-miR-6825-5p | RARG | 1 | 1 | 2 | N |
| hsa-miR-6825-5p | DACT2 | 1 | 1 | 2 | N |
| hsa-miR-6825-5p | PCDHGA3 | 1 | 1 | 2 | N |
| hsa-miR-6825-5p | SF3A2 | 1 | 1 | 2 | N |
| hsa-miR-6825-5p | PLP2 | 1 | 1 | 2 | N |
| hsa-miR-6825-5p | FAM131A | 1 | 1 | 2 | N |
| hsa-miR-6825-5p | SLC7A8 | 1 | 1 | 2 | N |
| hsa-miR-6825-5p | SPRYD3 | 1 | 1 | 2 | N |
| hsa-miR-6825-5p | SZRD1 | 1 | 1 | 2 | N |
| hsa-miR-6825-5p | BBS1 | 1 | 1 | 2 | N |
| hsa-miR-6825-5p | ZFP91 | 1 | 1 | 2 | N |
| hsa-miR-6825-5p | SMAP2 | 1 | 1 | 2 | N |
| hsa-miR-6825-5p | TBL1X | 1 | 1 | 2 | N |
| hsa-miR-6825-5p | MYO1C | 1 | 1 | 2 | Y |
| hsa-miR-6825-5p | BSN | 1 | 1 | 2 | N |
| hsa-miR-6825-5p | EPHA8 | 1 | 1 | 2 | N |
| hsa-miR-6825-5p | C11orf87 | 1 | 1 | 2 | N |
| hsa-miR-6825-5p | LYSMD1 | 1 | 1 | 2 | N |
| hsa-miR-6825-5p | SMARCC2 | 1 | 1 | 2 | N |
| hsa-miR-6825-5p | KLHDC3 | 1 | 1 | 2 | N |
| hsa-miR-6825-5p | DBNDD2 | 1 | 1 | 2 | N |
| hsa-miR-6825-5p | MATN1 | 1 | 1 | 2 | N |
| hsa-miR-6825-5p | CLSTN1 | 1 | 1 | 2 | Y |
| hsa-miR-6825-5p | YWHAZ | 1 | 1 | 2 | Y |
| hsa-miR-6825-5p | E2F3 | 1 | 1 | 2 | N |
| hsa-miR-6825-5p | PEAR1 | 1 | 1 | 2 | N |
| hsa-miR-6825-5p | SCN4B | 1 | 1 | 2 | N |
| hsa-miR-6825-5p | SLC2A12 | 1 | 1 | 2 | N |
| hsa-miR-6825-5p | RPP25 | 1 | 1 | 2 | Y |
| hsa-miR-6825-5p | PSD | 1 | 1 | 2 | N |
| hsa-miR-6825-5p | SEPT3 | 1 | 1 | 2 | N |
| hsa-miR-6825-5p | TMEM222 | 1 | 1 | 2 | N |
| hsa-miR-6825-5p | MCRS1 | 1 | 1 | 2 | N |
| hsa-miR-6825-5p | STAT3 | 1 | 1 | 2 | N |
| hsa-miR-6825-5p | ZNF385A | 1 | 1 | 2 | N |
| hsa-miR-6825-5p | CARM1 | 1 | 1 | 2 | N |
| hsa-miR-6825-5p | CLINT1 | 1 | 1 | 2 | N |
| hsa-miR-6825-5p | CDK5R2 | 1 | 1 | 2 | N |
| hsa-miR-6825-5p | PHF19 | 1 | 1 | 2 | N |
| hsa-miR-6825-5p | SLC29A4 | 1 | 1 | 2 | N |
| hsa-miR-6825-5p | ZCCHC24 | 1 | 1 | 2 | N |
| hsa-miR-6825-5p | PTPRA | 1 | 1 | 2 | N |
| hsa-miR-6825-5p | RNF112 | 1 | 1 | 2 | N |
| hsa-miR-6825-5p | KRTAP10-11 | 1 | 1 | 2 | N |
| hsa-miR-6825-5p | PCDHGC3 | 1 | 1 | 2 | N |
| hsa-miR-6825-5p | SLC9A3R2 | 1 | 1 | 2 | N |
| hsa-miR-6825-5p | SERTM1 | 1 | 1 | 2 | N |
| hsa-miR-6825-5p | LGALS12 | 1 | 1 | 2 | N |
| hsa-miR-6825-5p | HDAC3 | 1 | 1 | 2 | N |
| hsa-miR-6825-5p | TP53INP2 | 1 | 1 | 2 | N |
| hsa-miR-6825-5p | H2AFX | 1 | 1 | 2 | N |
| hsa-miR-6825-5p | SAMD13 | 1 | 1 | 2 | N |
| hsa-miR-6825-5p | SHISA6 | 1 | 1 | 2 | Y |
| hsa-miR-6825-5p | UBE2M | 1 | 1 | 2 | N |
| hsa-miR-6825-5p | MOB3A | 1 | 1 | 2 | Y |
| hsa-miR-6825-5p | FAM102A | 1 | 1 | 2 | N |
| hsa-miR-6825-5p | KCND1 | 1 | 1 | 2 | Y |
| hsa-miR-6825-5p | KIRREL3 | 1 | 1 | 2 | N |
| hsa-miR-6825-5p | CABP2 | 1 | 1 | 2 | N |
| hsa-miR-6825-5p | CDC42SE1 | 1 | 1 | 2 | N |
| hsa-miR-6825-5p | UBE2Q1 | 1 | 1 | 2 | N |
| hsa-miR-6825-5p | TEAD2 | 1 | 1 | 2 | N |
| hsa-miR-6825-5p | ZNF579 | 1 | 1 | 2 | N |
| hsa-miR-6825-5p | PRELP | 1 | 1 | 2 | Y |
| hsa-miR-6825-5p | PCDHGB7 | 1 | 1 | 2 | N |
| hsa-miR-6825-5p | GTSF1 | 1 | 1 | 2 | N |
| hsa-miR-6825-5p | SLC12A5 | 1 | 1 | 2 | N |
| hsa-miR-6825-5p | PCDHGB3 | 1 | 1 | 2 | N |
| hsa-miR-6825-5p | C6orf89 | 1 | 1 | 2 | N |
| hsa-miR-6825-5p | SLC4A2 | 1 | 1 | 2 | Y |
| hsa-miR-6825-5p | ASIC1 | 1 | 1 | 2 | N |
| hsa-miR-6825-5p | TRIM66 | 1 | 1 | 2 | N |
| hsa-miR-6825-5p | HP1BP3 | 1 | 1 | 2 | N |
| hsa-miR-6825-5p | FAM83H | 1 | 1 | 2 | Y |
| hsa-miR-6825-5p | ZC3H18 | 1 | 1 | 2 | N |
| hsa-miR-6825-5p | SMYD5 | 1 | 1 | 2 | N |
| hsa-miR-6825-5p | GSK3A | 1 | 1 | 2 | N |
| hsa-miR-6825-5p | HS3ST4 | 1 | 1 | 2 | N |
| hsa-miR-6825-5p | LANCL2 | 1 | 1 | 2 | N |
| hsa-miR-6825-5p | IQSEC2 | 1 | 1 | 2 | N |
| hsa-miR-6825-5p | MIP | 1 | 1 | 2 | N |
| hsa-miR-6825-5p | YWHAE | 1 | 1 | 2 | Y |
| hsa-miR-6825-5p | URM1 | 1 | 1 | 2 | Y |
| hsa-miR-6825-5p | SLC38A5 | 1 | 1 | 2 | N |
| hsa-miR-6825-5p | PHLPP1 | 1 | 1 | 2 | N |
| hsa-miR-6825-5p | EPHB4 | 1 | 1 | 2 | N |
| hsa-miR-6825-5p | SCAI | 1 | 1 | 2 | N |
| hsa-miR-6825-5p | TMEM63B | 1 | 1 | 2 | N |
| hsa-miR-6825-5p | AP3S2 | 1 | 1 | 2 | N |
| hsa-miR-6825-5p | SLC2A4 | 1 | 1 | 2 | N |
| hsa-miR-6825-5p | ALOXE3 | 1 | 1 | 2 | N |
| hsa-miR-6825-5p | CCND2 | 1 | 1 | 2 | N |
| hsa-miR-6825-5p | LBH | 1 | 1 | 2 | N |
| hsa-miR-6825-5p | SMUG1 | 1 | 1 | 2 | N |
| hsa-miR-6825-5p | UBE2Z | 1 | 1 | 2 | N |
| hsa-miR-6825-5p | DES | 1 | 1 | 2 | N |
| hsa-miR-6825-5p | MAML3 | 1 | 1 | 2 | N |
| hsa-miR-6825-5p | SLC6A6 | 1 | 1 | 2 | N |
| hsa-miR-6825-5p | TBX6 | 1 | 1 | 2 | N |
| hsa-miR-6825-5p | PDXP | 1 | 1 | 2 | N |
| hsa-miR-6825-5p | GMEB2 | 1 | 1 | 2 | N |
| hsa-miR-6825-5p | MARK2 | 1 | 1 | 2 | Y |
| hsa-miR-6825-5p | RIMS4 | 1 | 1 | 2 | N |
| hsa-miR-6825-5p | ARID3B | 1 | 1 | 2 | N |
| hsa-miR-6825-5p | TMUB2 | 1 | 1 | 2 | N |
| hsa-miR-6825-5p | MBD6 | 1 | 1 | 2 | N |
| hsa-miR-6825-5p | ADM | 1 | 1 | 2 | N |
| hsa-miR-6825-5p | ATP6V0E2 | 1 | 1 | 2 | N |
| hsa-miR-6825-5p | PPP5C | 1 | 1 | 2 | N |
| hsa-miR-6825-5p | C1orf198 | 1 | 1 | 2 | N |
| hsa-miR-6825-5p | KCNJ12 | 1 | 1 | 2 | N |
| hsa-miR-6825-5p | C2CD2L | 1 | 1 | 2 | N |
| hsa-miR-6825-5p | ANKRD52 | 1 | 1 | 2 | Y |
| hsa-miR-6825-5p | C11orf84 | 1 | 1 | 2 | N |
| hsa-miR-6825-5p | CRABP2 | 1 | 1 | 2 | N |
| hsa-miR-6825-5p | SRF | 1 | 1 | 2 | N |
| hsa-miR-6825-5p | SLITRK5 | 1 | 1 | 2 | Y |
| hsa-miR-6825-5p | FAM127C | 1 | 1 | 2 | N |
| hsa-miR-6825-5p | AMOTL1 | 1 | 1 | 2 | Y |
| hsa-miR-6825-5p | RAB5C | 1 | 1 | 2 | Y |
| hsa-miR-6825-5p | CD3EAP | 1 | 1 | 2 | N |
| hsa-miR-6825-5p | AQP2 | 1 | 1 | 2 | N |
| hsa-miR-6825-5p | BTRC | 1 | 1 | 2 | N |
| hsa-miR-6825-5p | CENPT | 1 | 1 | 2 | N |
| hsa-miR-6825-5p | BTNL8 | 1 | 1 | 2 | N |
| hsa-miR-6825-5p | DAB2IP | 1 | 1 | 2 | N |
| hsa-miR-6825-5p | DDX51 | 1 | 1 | 2 | N |
| hsa-miR-6825-5p | POU2F2 | 1 | 1 | 2 | N |
| hsa-miR-6825-5p | KIAA1671 | 1 | 1 | 2 | N |
| hsa-miR-6825-5p | HECTD4 | 1 | 1 | 2 | N |
| hsa-miR-6825-5p | ELK1 | 1 | 1 | 2 | N |
| hsa-miR-6825-5p | PACS1 | 1 | 1 | 2 | N |
| hsa-miR-6825-5p | SMARCA2 | 1 | 1 | 2 | N |
| hsa-miR-6825-5p | FXYD6 | 1 | 1 | 2 | N |
| hsa-miR-6825-5p | HGSNAT | 1 | 1 | 2 | N |
| hsa-miR-6825-5p | NCDN | 1 | 1 | 2 | N |
| hsa-miR-6825-5p | THAP11 | 1 | 1 | 2 | N |
| hsa-miR-6825-5p | PTPRU | 1 | 1 | 2 | N |
| hsa-miR-6825-5p | LRRN2 | 1 | 1 | 2 | N |
| hsa-miR-6825-5p | LRRC28 | 1 | 1 | 2 | N |
| hsa-miR-6825-5p | WDTC1 | 1 | 1 | 2 | N |
| hsa-miR-6825-5p | KIF21B | 1 | 1 | 2 | N |
| hsa-miR-6825-5p | PCDHGC5 | 1 | 1 | 2 | N |
| hsa-miR-6825-5p | SCRT1 | 1 | 1 | 2 | N |
| hsa-miR-6825-5p | BRPF1 | 1 | 1 | 2 | N |
| hsa-miR-6825-5p | VGLL1 | 1 | 1 | 2 | N |
| hsa-miR-6825-5p | PRKAR1B | 1 | 1 | 2 | N |
| hsa-miR-6825-5p | BOK | 1 | 1 | 2 | N |
| hsa-miR-6825-5p | PCDHGA1 | 1 | 1 | 2 | N |
| hsa-miR-6825-5p | DOLPP1 | 1 | 1 | 2 | N |
| hsa-miR-6825-5p | CBX8 | 1 | 1 | 2 | Y |
| hsa-miR-6825-5p | PNPLA2 | 1 | 1 | 2 | Y |
| hsa-miR-6825-5p | ELN | 1 | 1 | 2 | N |
| hsa-miR-6825-5p | OGDH | 1 | 1 | 2 | N |
| hsa-miR-6825-5p | KIAA0754 | 1 | 1 | 2 | N |
| hsa-miR-6825-5p | PPP2R1A | 1 | 1 | 2 | N |
| hsa-miR-6825-5p | TULP1 | 1 | 1 | 2 | Y |
| hsa-miR-6825-5p | PCDHGA2 | 1 | 1 | 2 | N |
| hsa-miR-6825-5p | KCNJ11 | 1 | 1 | 2 | N |
| hsa-miR-6825-5p | PVRL1 | 1 | 1 | 2 | N |
| hsa-miR-6825-5p | ATP2A3 | 1 | 1 | 2 | N |
| hsa-miR-6825-5p | MINK1 | 1 | 1 | 2 | N |
| hsa-miR-6825-5p | RASSF2 | 1 | 1 | 2 | N |
| hsa-miR-6825-5p | TNS1 | 1 | 1 | 2 | N |
| hsa-miR-6825-5p | ATP5G2 | 1 | 1 | 2 | N |
| hsa-miR-6825-5p | R3HDM4 | 1 | 1 | 2 | N |
| hsa-miR-6825-5p | EPAS1 | 1 | 1 | 2 | N |
| hsa-miR-6825-5p | XPNPEP1 | 1 | 1 | 2 | N |
| hsa-miR-6825-5p | ASB8 | 1 | 1 | 2 | N |
| hsa-miR-6825-5p | RPH3AL | 1 | 1 | 2 | N |
| hsa-miR-6825-5p | DCAKD | 1 | 1 | 2 | Y |
| hsa-miR-6825-5p | DAO | 1 | 1 | 2 | N |
| hsa-miR-6825-5p | SCAMP4 | 1 | 1 | 2 | Y |
| hsa-miR-6825-5p | BRK1 | 1 | 1 | 2 | N |
| hsa-miR-6825-5p | PREB | 1 | 1 | 2 | N |
| hsa-miR-6825-5p | SLC9A1 | 1 | 1 | 2 | N |
| hsa-miR-6825-5p | NFIC | 1 | 1 | 2 | N |
| hsa-miR-6825-5p | ST3GAL1 | 1 | 1 | 2 | N |
| hsa-miR-6825-5p | NPTXR | 1 | 1 | 2 | N |
| hsa-miR-6825-5p | CNTNAP5 | 1 | 1 | 2 | N |
| hsa-miR-6825-5p | MEST | 1 | 1 | 2 | N |
| hsa-miR-6825-5p | NFASC | 1 | 1 | 2 | Y |
| hsa-miR-6825-5p | CYP21A2 | 1 | 1 | 2 | N |
| hsa-miR-6825-5p | DAGLA | 1 | 1 | 2 | N |
| hsa-miR-6825-5p | NECAP1 | 1 | 1 | 2 | N |
| hsa-miR-6825-5p | NOTCH2 | 1 | 1 | 2 | N |
| hsa-miR-6825-5p | B3GAT1 | 1 | 1 | 2 | N |
| hsa-miR-6825-5p | SHISA9 | 1 | 1 | 2 | N |
| hsa-miR-6825-5p | EFNB3 | 1 | 1 | 2 | N |
| hsa-miR-6825-5p | KDM5C | 1 | 1 | 2 | N |
| hsa-miR-6825-5p | CENPB | 1 | 1 | 2 | N |
| hsa-miR-6825-5p | DIRAS1 | 1 | 1 | 2 | N |
| hsa-miR-6825-5p | NAA15 | 1 | 1 | 2 | N |
| hsa-miR-6825-5p | TAOK3 | 1 | 1 | 2 | N |
| hsa-miR-6825-5p | AP1M1 | 1 | 1 | 2 | N |
| hsa-miR-6825-5p | NHLH1 | 1 | 1 | 2 | N |
| hsa-miR-6825-5p | ACBD4 | 1 | 1 | 2 | N |
| hsa-miR-6825-5p | ZFP3 | 1 | 1 | 2 | N |
| hsa-miR-6825-5p | SMEK1 | 1 | 1 | 2 | N |
| hsa-miR-6825-5p | NDRG1 | 1 | 1 | 2 | N |
| hsa-miR-6825-5p | KLHDC8A | 1 | 1 | 2 | N |
| hsa-miR-6825-5p | NDUFA4L2 | 1 | 1 | 2 | N |
| hsa-miR-6825-5p | CBS | 1 | 1 | 2 | Y |
| hsa-miR-6825-5p | GTPBP1 | 1 | 1 | 2 | Y |
| hsa-miR-6825-5p | CBX6 | 1 | 1 | 2 | Y |
| hsa-miR-6825-5p | COL5A3 | 1 | 1 | 2 | N |
| hsa-miR-6825-5p | EIF5A | 1 | 1 | 2 | Y |
| hsa-miR-6825-5p | IGF2 | 1 | 1 | 2 | N |
| hsa-miR-6825-5p | UBALD1 | 1 | 1 | 2 | N |
| hsa-miR-6825-5p | NGFR | 1 | 1 | 2 | Y |
| hsa-miR-6825-5p | HSPB7 | 1 | 1 | 2 | N |
| hsa-miR-6825-5p | CREB3L2 | 1 | 1 | 2 | N |
| hsa-miR-6825-5p | AGO1 | 1 | 1 | 2 | N |
| hsa-miR-6825-5p | VDAC1 | 1 | 1 | 2 | N |
| hsa-miR-6825-5p | SLC7A1 | 1 | 1 | 2 | N |
| hsa-miR-6825-5p | USF2 | 1 | 1 | 2 | N |
| hsa-miR-6825-5p | TMEM104 | 1 | 1 | 2 | N |
| hsa-miR-6825-5p | CAPN5 | 1 | 1 | 2 | N |
| hsa-miR-6825-5p | NXN | 1 | 1 | 2 | Y |
| hsa-miR-6825-5p | LBX1 | 1 | 1 | 2 | Y |
| hsa-miR-6825-5p | PSAP | 1 | 1 | 2 | Y |
| hsa-miR-6825-5p | SOX12 | 1 | 1 | 2 | N |
| hsa-miR-6825-5p | BEGAIN | 1 | 1 | 2 | N |
| hsa-miR-6825-5p | ZSCAN5A | 1 | 1 | 2 | N |
| hsa-miR-6825-5p | NTSR1 | 1 | 1 | 2 | Y |
| hsa-miR-6825-5p | MYH9 | 1 | 1 | 2 | N |
| hsa-miR-6825-5p | SEMA4G | 1 | 1 | 2 | N |
| hsa-miR-6825-5p | NFYC | 1 | 1 | 2 | N |
| hsa-miR-6825-5p | MLLT6 | 1 | 1 | 2 | N |
| hsa-miR-6825-5p | SIRPA | 1 | 1 | 2 | N |
| hsa-miR-6825-5p | SLC17A9 | 1 | 1 | 2 | N |
| hsa-miR-6825-5p | ATN1 | 1 | 1 | 2 | N |
| hsa-miR-6825-5p | VDR | 1 | 1 | 2 | N |
| hsa-miR-6825-5p | FAM127B | 1 | 1 | 2 | N |
| hsa-miR-6825-5p | DPP9 | 1 | 1 | 2 | N |
| hsa-miR-6825-5p | FBXL16 | 1 | 1 | 2 | N |
| hsa-miR-6825-5p | NUDC | 1 | 1 | 2 | N |
| hsa-miR-6825-5p | FOSL2 | 1 | 1 | 2 | N |
| hsa-miR-6825-5p | YBX2 | 1 | 1 | 2 | N |
| hsa-miR-6825-5p | LMNA | 1 | 1 | 2 | N |
| hsa-miR-6825-5p | NAT8L | 1 | 1 | 2 | N |
| hsa-miR-6825-5p | FAM131B | 1 | 1 | 2 | N |
| hsa-miR-6825-5p | RAB5B | 1 | 1 | 2 | N |
| hsa-miR-6825-5p | GPR173 | 1 | 1 | 2 | Y |
| hsa-miR-6825-5p | IDS | 1 | 1 | 2 | Y |
| hsa-miR-6825-5p | GZF1 | 1 | 1 | 2 | N |
| hsa-miR-6825-5p | PACSIN1 | 1 | 1 | 2 | Y |
| hsa-miR-6825-5p | PFN1 | 1 | 1 | 2 | Y |
| hsa-miR-6825-5p | GIT1 | 1 | 1 | 2 | N |
| hsa-miR-6825-5p | ABHD12 | 1 | 1 | 2 | Y |
| hsa-miR-6825-5p | CELF3 | 1 | 1 | 2 | N |
| hsa-miR-6825-5p | SPSB4 | 1 | 1 | 2 | N |
| hsa-miR-6825-5p | NRARP | 1 | 1 | 2 | N |
| hsa-miR-6825-5p | FAIM2 | 1 | 1 | 2 | N |
| hsa-miR-6825-5p | PNOC | 1 | 1 | 2 | N |
| hsa-miR-6825-5p | TMEM151A | 1 | 1 | 2 | N |
| hsa-miR-6825-5p | MESDC1 | 1 | 1 | 2 | N |
| hsa-miR-6825-5p | CDR2L | 1 | 1 | 2 | N |
| hsa-miR-6825-5p | EFNA4 | 1 | 1 | 2 | N |
| hsa-miR-6825-5p | PCDHGA10 | 1 | 1 | 2 | N |
| hsa-miR-6825-5p | AMHR2 | 1 | 1 | 2 | N |
| hsa-miR-6825-5p | LIX1L | 1 | 1 | 2 | N |
| hsa-miR-6825-5p | RPH3A | 1 | 1 | 2 | N |
| hsa-miR-6825-5p | HIVEP3 | 1 | 1 | 2 | N |
| hsa-miR-6825-5p | SEMA6B | 1 | 1 | 2 | N |
| hsa-miR-6825-5p | CNPY3 | 1 | 1 | 2 | N |
| hsa-miR-6825-5p | TMEM86A | 1 | 1 | 2 | N |
| hsa-miR-6825-5p | B3GNT5 | 1 | 1 | 2 | N |
| hsa-miR-6825-5p | ALPP | 1 | 1 | 2 | N |
| hsa-miR-6825-5p | TSPAN15 | 1 | 1 | 2 | N |
| hsa-miR-6825-5p | CDC42BPA | 1 | 1 | 2 | N |
| hsa-miR-6825-5p | APOBEC3D | 1 | 1 | 2 | N |
| hsa-miR-6825-5p | CDKN1A | 1 | 1 | 2 | Y |
| hsa-miR-6825-5p | BSDC1 | 1 | 1 | 2 | N |
| hsa-miR-6825-5p | PAX9 | 1 | 1 | 2 | N |
| hsa-miR-6825-5p | N4BP1 | 1 | 1 | 2 | N |
| hsa-miR-6825-5p | WIPF2 | 1 | 1 | 2 | N |
| hsa-miR-6825-5p | GNB3 | 1 | 1 | 2 | N |
| hsa-miR-6825-5p | PPP2R4 | 1 | 1 | 2 | N |
| hsa-miR-6825-5p | ELMSAN1 | 1 | 1 | 2 | N |
| hsa-miR-6825-5p | NAV2 | 1 | 1 | 2 | Y |
| hsa-miR-6825-5p | ATF7IP2 | 1 | 1 | 2 | N |
| hsa-miR-6825-5p | SLC8A2 | 1 | 1 | 2 | N |
| hsa-miR-6825-5p | MAPK3 | 1 | 1 | 2 | N |
| hsa-miR-6825-5p | PCDHGA12 | 1 | 1 | 2 | N |
| hsa-miR-6825-5p | TAB1 | 1 | 1 | 2 | N |
| hsa-miR-6825-5p | CAPN11 | 1 | 1 | 2 | N |
| hsa-miR-6825-5p | SYCP2L | 1 | 1 | 2 | N |
| hsa-miR-6825-5p | MGAT3 | 1 | 1 | 2 | N |
| hsa-miR-6825-5p | PCDHGA5 | 1 | 1 | 2 | N |
| hsa-miR-6825-5p | IGLON5 | 1 | 1 | 2 | N |
| hsa-miR-6825-5p | COL6A1 | 1 | 1 | 2 | N |
| hsa-miR-6825-5p | ZNF609 | 1 | 1 | 2 | N |
| hsa-miR-6825-5p | COPZ1 | 1 | 1 | 2 | N |
| hsa-miR-6825-5p | PCDHGA9 | 1 | 1 | 2 | N |
| hsa-miR-6825-5p | CBX7 | 1 | 1 | 2 | N |
| hsa-miR-6825-5p | AES | 1 | 1 | 2 | N |
| hsa-miR-6825-5p | IFT140 | 1 | 1 | 2 | N |
| hsa-miR-6825-5p | RNF26 | 1 | 1 | 2 | N |
| hsa-miR-6825-5p | PIP4K2B | 1 | 1 | 2 | N |
| hsa-miR-6825-5p | VAMP2 | 1 | 1 | 2 | N |
| hsa-miR-6825-5p | ARHGAP9 | 1 | 1 | 2 | N |
| hsa-miR-6825-5p | BRPF3 | 1 | 1 | 2 | N |
| hsa-miR-6825-5p | MAPT | 1 | 1 | 2 | N |
| hsa-miR-6825-5p | TIMP4 | 1 | 1 | 2 | N |
| hsa-miR-6825-5p | SPTBN4 | 1 | 1 | 2 | N |
| hsa-miR-6825-5p | BANF1 | 1 | 1 | 2 | N |
| hsa-miR-6825-5p | GPSM1 | 1 | 1 | 2 | N |
| hsa-miR-6825-5p | MPP2 | 1 | 1 | 2 | N |
| hsa-miR-6825-5p | BAAT | 1 | 1 | 2 | N |
| hsa-miR-6825-5p | PPFIA3 | 1 | 1 | 2 | N |
| hsa-miR-6825-5p | GINS1 | 1 | 1 | 2 | N |
| hsa-miR-6825-5p | RPS6KA2 | 1 | 1 | 2 | Y |
| hsa-miR-6825-5p | PTMS | 1 | 1 | 2 | N |
| hsa-miR-6825-5p | DNM1 | 1 | 1 | 2 | N |
| hsa-miR-6825-5p | ADCY9 | 1 | 1 | 2 | Y |
| hsa-miR-6825-5p | BOLL | 1 | 1 | 2 | N |
| hsa-miR-6825-5p | TMEM184B | 1 | 1 | 2 | N |
| hsa-miR-6825-5p | MINOS1-NBL1 | 1 | 1 | 2 | Y |
| hsa-miR-6825-5p | HSPB6 | 1 | 1 | 2 | N |
| hsa-miR-6825-5p | SAMD4B | 1 | 1 | 2 | N |
| hsa-miR-6825-5p | ING4 | 1 | 1 | 2 | Y |
| hsa-miR-6825-5p | HIF3A | 1 | 1 | 2 | N |
| hsa-miR-6825-5p | UROC1 | 1 | 1 | 2 | N |
| hsa-miR-6825-5p | ZBTB4 | 1 | 1 | 2 | N |
| hsa-miR-6825-5p | SORCS2 | 1 | 1 | 2 | Y |
| hsa-miR-6825-5p | HOXB6 | 1 | 1 | 2 | Y |
| hsa-miR-6825-5p | SIDT2 | 1 | 1 | 2 | N |
| hsa-miR-6825-5p | MEIS2 | 1 | 1 | 2 | N |
| hsa-miR-6825-5p | NEUROG2 | 1 | 1 | 2 | N |
| hsa-miR-6825-5p | PXN | 1 | 1 | 2 | N |
| hsa-miR-6825-5p | GPR132 | 1 | 1 | 2 | N |
| hsa-miR-6825-5p | SGSM2 | 1 | 1 | 2 | N |
| hsa-miR-6825-5p | APLNR | 1 | 1 | 2 | N |
| hsa-miR-6825-5p | RAMP2 | 1 | 1 | 2 | N |
| hsa-miR-6825-5p | KCNQ4 | 1 | 1 | 2 | N |
| hsa-miR-6825-5p | ARHGEF6 | 1 | 1 | 2 | N |
| hsa-miR-6825-5p | DTX4 | 1 | 1 | 2 | N |
| hsa-miR-6825-5p | DENND1A | 1 | 1 | 2 | N |
| hsa-miR-6825-5p | TCF7 | 1 | 1 | 2 | N |
| hsa-miR-6825-5p | SLCO2B1 | 1 | 1 | 2 | N |
| hsa-miR-6825-5p | BTG2 | 1 | 1 | 2 | Y |
| hsa-miR-6825-5p | PLXNA4 | 1 | 1 | 2 | N |
| hsa-miR-6825-5p | LENG8 | 1 | 1 | 2 | Y |
| hsa-miR-6825-5p | UNK | 1 | 1 | 2 | Y |
| hsa-miR-6825-5p | KCTD21 | 1 | 1 | 2 | N |
| hsa-miR-6825-5p | ADAMTSL1 | 1 | 1 | 2 | N |
| hsa-miR-6825-5p | HMGA1 | 1 | 1 | 2 | N |
| hsa-miR-6825-5p | MXD3 | 1 | 1 | 2 | N |
| hsa-miR-6825-5p | ZSCAN31 | 1 | 1 | 2 | N |
| hsa-miR-6825-5p | DPYSL4 | 1 | 1 | 2 | N |
| hsa-miR-6825-5p | PCDHGA11 | 1 | 1 | 2 | N |
| hsa-miR-6825-5p | DHRS11 | 1 | 1 | 2 | N |
| hsa-miR-6825-5p | AP2A1 | 1 | 1 | 2 | N |
| hsa-miR-6825-5p | LHPP | 1 | 1 | 2 | N |
| hsa-miR-6825-5p | MTSS1L | 1 | 1 | 2 | N |
| hsa-miR-6825-5p | WDR5B | 1 | 1 | 2 | N |
| hsa-miR-6825-5p | ATP2B2 | 1 | 1 | 2 | N |
| hsa-miR-6825-5p | SPTB | 1 | 1 | 2 | N |
| hsa-miR-6825-5p | SLC16A9 | 1 | 1 | 2 | N |
| hsa-miR-6825-5p | KDM6B | 1 | 1 | 2 | Y |
| hsa-miR-6825-5p | DDAH1 | 1 | 1 | 2 | N |
| hsa-miR-6825-5p | TNFAIP1 | 1 | 1 | 2 | N |
| hsa-miR-6825-5p | CSDC2 | 1 | 1 | 2 | N |
| hsa-miR-6825-5p | COPS7B | 1 | 1 | 2 | Y |
| hsa-miR-6825-5p | NEUROD2 | 1 | 1 | 2 | Y |
| hsa-miR-6825-5p | ILK | 1 | 1 | 2 | N |
| hsa-miR-6825-5p | MTUS1 | 1 | 1 | 2 | N |
| hsa-miR-6825-5p | DBNDD1 | 1 | 1 | 2 | N |
| hsa-miR-6825-5p | ZBTB7A | 1 | 1 | 2 | Y |
| hsa-miR-6825-5p | MEX3A | 1 | 1 | 2 | Y |
| hsa-miR-6825-5p | LFNG | 1 | 1 | 2 | N |
| hsa-miR-6825-5p | TSPYL5 | 1 | 1 | 2 | N |
| hsa-miR-6825-5p | TOR2A | 1 | 1 | 2 | Y |
| hsa-miR-6825-5p | SLC18A1 | 1 | 1 | 2 | N |
| hsa-miR-6825-5p | PDE1B | 1 | 1 | 2 | N |
| hsa-miR-6825-5p | MIS18A | 1 | 1 | 2 | N |
| hsa-miR-6825-5p | KIAA0247 | 1 | 1 | 2 | N |
| hsa-miR-6825-5p | JPH4 | 1 | 1 | 2 | N |
| hsa-miR-6825-5p | SP6 | 1 | 1 | 2 | N |
| hsa-miR-6825-5p | NOVA2 | 1 | 1 | 2 | N |
| hsa-miR-6825-5p | C17orf103 | 1 | 1 | 2 | N |
| hsa-miR-6825-5p | TUBB3 | 1 | 1 | 2 | N |
| hsa-miR-6825-5p | RAB35 | 1 | 1 | 2 | N |
| hsa-miR-6825-5p | DYRK1B | 1 | 1 | 2 | Y |
| hsa-miR-6825-5p | ZFP36L1 | 1 | 1 | 2 | N |
| hsa-miR-6825-5p | BACH2 | 1 | 1 | 2 | N |
| hsa-miR-6825-5p | NEK7 | 1 | 1 | 2 | N |
| hsa-miR-6825-5p | DDB1 | 1 | 1 | 2 | N |
| hsa-miR-6825-5p | PRLHR | 1 | 1 | 2 | N |
| hsa-miR-6825-5p | PLEKHH1 | 1 | 1 | 2 | N |
| hsa-miR-6825-5p | GATA4 | 1 | 1 | 2 | N |
| hsa-miR-6825-5p | TEX261 | 1 | 1 | 2 | N |
| hsa-miR-6825-5p | AGPAT1 | 1 | 1 | 2 | N |
| hsa-miR-6825-5p | IER5 | 1 | 1 | 2 | Y |
| hsa-miR-6825-5p | GRIK3 | 1 | 1 | 2 | N |
| hsa-miR-6825-5p | SRRM4 | 1 | 1 | 2 | N |
| hsa-miR-6825-5p | HCFC1 | 1 | 1 | 2 | Y |
| hsa-miR-6825-5p | EAF1 | 1 | 1 | 2 | N |
| hsa-miR-6825-5p | NLGN2 | 1 | 1 | 2 | Y |
| hsa-miR-6825-5p | CHAF1A | 1 | 1 | 2 | N |
| hsa-miR-6825-5p | MRO | 1 | 1 | 2 | N |
| hsa-miR-6825-5p | SPRY4 | 1 | 1 | 2 | N |
| hsa-miR-6825-5p | MYLK2 | 1 | 1 | 2 | N |
| hsa-miR-6825-5p | ATXN1L | 1 | 1 | 2 | N |
| hsa-miR-6825-5p | MYRF | 1 | 1 | 2 | N |
| hsa-miR-6825-5p | NAB2 | 1 | 1 | 2 | N |
| hsa-miR-6825-5p | MBD1 | 1 | 1 | 2 | N |
| hsa-miR-6825-5p | ELF4 | 1 | 1 | 2 | N |
| hsa-miR-6825-5p | CDK18 | 1 | 1 | 2 | N |
| hsa-miR-6825-5p | POU3F1 | 1 | 1 | 2 | N |
| hsa-miR-6825-5p | GOLT1A | 1 | 1 | 2 | N |
| hsa-miR-6825-5p | VASH1 | 1 | 1 | 2 | N |
| hsa-miR-6825-5p | CNIH2 | 1 | 1 | 2 | N |
| hsa-miR-6825-5p | STXBP1 | 1 | 1 | 2 | N |
| hsa-miR-6825-5p | DUSP3 | 1 | 1 | 2 | N |
| hsa-miR-6825-5p | FAM127A | 1 | 1 | 2 | N |
| hsa-miR-6825-5p | CBL | 1 | 1 | 2 | N |
| hsa-miR-6825-5p | PHF8 | 1 | 1 | 2 | Y |
| hsa-miR-6825-5p | PDX1 | 1 | 1 | 2 | N |
| hsa-miR-6825-5p | PPARD | 1 | 1 | 2 | N |
| hsa-miR-6825-5p | NFAM1 | 1 | 1 | 2 | N |
| hsa-miR-6825-5p | PPP1R9B | 1 | 1 | 2 | Y |
| hsa-miR-6825-5p | SORBS3 | 1 | 1 | 2 | N |
| hsa-miR-6825-5p | EPB41L1 | 1 | 1 | 2 | Y |
| hsa-miR-6825-5p | PIP4K2C | 1 | 1 | 2 | N |
| hsa-miR-6825-5p | PKLR | 1 | 1 | 2 | N |
| hsa-miR-6825-5p | SREBF2 | 1 | 1 | 2 | Y |
| hsa-miR-6825-5p | PHF23 | 1 | 1 | 2 | N |
| hsa-miR-6825-5p | LCE1E | 1 | 1 | 2 | N |
| hsa-miR-6825-5p | CYP26B1 | 1 | 1 | 2 | N |
| hsa-miR-6825-5p | CTDSP1 | 1 | 1 | 2 | N |
| hsa-miR-6825-5p | OAF | 1 | 1 | 2 | N |
| hsa-miR-6825-5p | TRIM3 | 1 | 1 | 2 | N |
| hsa-miR-6825-5p | KBTBD4 | 1 | 1 | 2 | N |
| hsa-miR-6825-5p | OLFML2A | 1 | 1 | 2 | N |
| hsa-miR-6825-5p | MKNK2 | 1 | 1 | 2 | Y |
| hsa-miR-6825-5p | TTYH3 | 1 | 1 | 2 | N |
| hsa-miR-6825-5p | ARF5 | 1 | 1 | 2 | N |
| hsa-miR-6825-5p | STX11 | 1 | 1 | 2 | N |
| hsa-miR-6825-5p | SLIT3 | 1 | 1 | 2 | N |
| hsa-miR-6825-5p | DAAM2 | 1 | 1 | 2 | N |
| hsa-miR-6825-5p | KIF5A | 1 | 1 | 2 | N |
| hsa-miR-6825-5p | PILRB | 1 | 1 | 2 | N |
| hsa-miR-6825-5p | ZBTB46 | 1 | 1 | 2 | N |
| hsa-miR-6825-5p | PCDHGA8 | 1 | 1 | 2 | N |
| hsa-miR-6825-5p | SIRPB1 | 1 | 1 | 2 | N |
| hsa-miR-6825-5p | CLIP2 | 1 | 1 | 2 | N |
| hsa-miR-6825-5p | RAC1 | 1 | 1 | 2 | Y |
| hsa-miR-6825-5p | HPCAL1 | 1 | 1 | 2 | N |
| hsa-miR-6825-5p | LDLRAP1 | 1 | 1 | 2 | N |
| hsa-miR-6825-5p | TGIF1 | 1 | 1 | 2 | Y |
| hsa-miR-6825-5p | FA2H | 1 | 1 | 2 | N |
| hsa-miR-6825-5p | TMEM63C | 1 | 1 | 2 | N |
| hsa-miR-6825-5p | MYPOP | 1 | 1 | 2 | N |
| hsa-miR-6825-5p | CACNA1H | 1 | 1 | 2 | N |
| hsa-miR-6825-5p | SYNGAP1 | 1 | 1 | 2 | N |
| hsa-miR-6825-5p | TP63 | 1 | 1 | 2 | N |
| hsa-miR-6825-5p | PRSS53 | 1 | 1 | 2 | N |
| hsa-miR-6825-5p | CTDSPL | 1 | 1 | 2 | N |
| hsa-miR-6825-5p | NRBP1 | 1 | 1 | 2 | Y |
| hsa-miR-6825-5p | PML | 1 | 1 | 2 | N |
| hsa-miR-6825-5p | ARRB1 | 1 | 1 | 2 | Y |
| hsa-miR-6825-5p | KCNS1 | 1 | 1 | 2 | N |
| hsa-miR-6825-5p | DDX17 | 1 | 1 | 2 | N |
| hsa-miR-6825-5p | LY6H | 1 | 1 | 2 | N |
| hsa-miR-6825-5p | PPT2 | 1 | 1 | 2 | N |
| hsa-miR-6825-5p | PRSS27 | 1 | 1 | 2 | N |
| hsa-miR-6825-5p | ARHGDIA | 1 | 1 | 2 | N |
| hsa-miR-6825-5p | GPD1 | 1 | 1 | 2 | N |
| hsa-miR-6825-5p | EFNB1 | 1 | 1 | 2 | Y |
| hsa-miR-6825-5p | TNRC6A | 1 | 1 | 2 | N |
| hsa-miR-6825-5p | PTRF | 1 | 1 | 2 | N |
| hsa-miR-6825-5p | RIMS3 | 1 | 1 | 2 | N |
| hsa-miR-6825-5p | FOXO4 | 1 | 1 | 2 | N |
| hsa-miR-6825-5p | VAT1 | 1 | 1 | 2 | N |
| hsa-miR-6825-5p | SHISA7 | 1 | 1 | 2 | N |
| hsa-miR-6825-5p | HOXB7 | 1 | 1 | 2 | N |
| hsa-miR-6825-5p | TP73 | 1 | 1 | 2 | N |
| hsa-miR-6825-5p | SCN4A | 1 | 1 | 2 | N |
| hsa-miR-6825-5p | SOX13 | 1 | 1 | 2 | N |
| hsa-miR-6825-5p | YPEL4 | 1 | 1 | 2 | N |
| hsa-miR-6852-5p | PTPRT | 1 | 1 | 2 | N |
| hsa-miR-6852-5p | CNPY3 | 1 | 1 | 2 | N |
| hsa-miR-6852-5p | PSCA | 1 | 1 | 2 | N |
| hsa-miR-6852-5p | CLDN9 | 1 | 1 | 2 | N |
| hsa-miR-6852-5p | LRRC15 | 1 | 1 | 2 | N |
| hsa-miR-6852-5p | ULK2 | 1 | 1 | 2 | N |
| hsa-miR-6852-5p | CCDC97 | 1 | 1 | 2 | N |
| hsa-miR-6852-5p | TNRC18 | 1 | 1 | 2 | N |
| hsa-miR-6852-5p | SLC6A17 | 1 | 1 | 2 | N |
| hsa-miR-6852-5p | DNPH1 | 1 | 1 | 2 | N |
| hsa-miR-6852-5p | COPZ2 | 1 | 1 | 2 | N |
| hsa-miR-6852-5p | CPLX2 | 1 | 1 | 2 | N |
| hsa-miR-6852-5p | FAM107A | 1 | 1 | 2 | N |
| hsa-miR-6852-5p | ARNT2 | 1 | 1 | 2 | N |
| hsa-miR-6852-5p | ARID3B | 1 | 1 | 2 | N |
| hsa-miR-6852-5p | HOXB5 | 1 | 1 | 2 | Y |
| hsa-miR-6852-5p | TEPP | 1 | 1 | 2 | N |
| hsa-miR-6852-5p | PACSIN1 | 1 | 1 | 2 | N |
| hsa-miR-6852-5p | SCAMP4 | 1 | 1 | 2 | Y |
| hsa-miR-6852-5p | SPATA3 | 1 | 1 | 2 | N |
| hsa-miR-6852-5p | CDV3 | 1 | 1 | 2 | N |
| hsa-miR-6852-5p | B3GNT7 | 1 | 1 | 2 | N |
| hsa-miR-6852-5p | PEX14 | 1 | 1 | 2 | N |
| hsa-miR-6852-5p | NTSR1 | 1 | 1 | 2 | N |
| hsa-miR-6852-5p | SNX32 | 1 | 1 | 2 | N |
| hsa-miR-6852-5p | UBE2Z | 1 | 1 | 2 | N |
| hsa-miR-6852-5p | AMOTL2 | 1 | 1 | 2 | Y |
| hsa-miR-6852-5p | FBXO41 | 1 | 1 | 2 | N |
| hsa-miR-6852-5p | ZC3H7B | 1 | 1 | 2 | N |
| hsa-miR-6852-5p | SPRY4 | 1 | 1 | 2 | N |
| hsa-miR-6852-5p | SLC16A2 | 1 | 1 | 2 | N |
| hsa-miR-6852-5p | ZNF282 | 1 | 1 | 2 | N |
| hsa-miR-6852-5p | SFTPA2 | 1 | 1 | 2 | N |
| hsa-miR-6852-5p | CC2D1A | 1 | 1 | 2 | N |
| hsa-miR-6852-5p | SORBS3 | 1 | 1 | 2 | N |
| hsa-miR-6852-5p | BTNL8 | 1 | 1 | 2 | N |
| hsa-miR-6852-5p | CBX2 | 1 | 1 | 2 | N |
| hsa-miR-6852-5p | MIEF2 | 1 | 1 | 2 | N |
| hsa-miR-6852-5p | NFIC | 1 | 1 | 2 | Y |
| hsa-miR-6852-5p | AGK | 1 | 1 | 2 | N |
| hsa-miR-6852-5p | ANKRD13A | 1 | 1 | 2 | N |
| hsa-miR-6852-5p | ATG9A | 1 | 1 | 2 | N |
| hsa-miR-6852-5p | TEF | 1 | 1 | 2 | N |
| hsa-miR-6852-5p | BPIFA3 | 1 | 1 | 2 | N |
| hsa-miR-6852-5p | RIN3 | 1 | 1 | 2 | N |
| hsa-miR-6852-5p | TNFAIP8L1 | 1 | 1 | 2 | N |
| hsa-miR-6852-5p | ITGA10 | 1 | 1 | 2 | N |
| hsa-miR-6852-5p | TP73 | 1 | 1 | 2 | N |
| hsa-miR-6852-5p | DCTN5 | 1 | 1 | 2 | N |
| hsa-miR-6852-5p | LHX3 | 1 | 1 | 2 | N |
| hsa-miR-6852-5p | USH1G | 1 | 1 | 2 | N |
| hsa-miR-6852-5p | DOT1L | 1 | 1 | 2 | N |
| hsa-miR-6852-5p | NLGN3 | 1 | 1 | 2 | N |
| hsa-miR-6852-5p | TCTA | 1 | 1 | 2 | N |
| hsa-miR-6852-5p | BTN2A1 | 1 | 1 | 2 | N |
| hsa-miR-6852-5p | C1QTNF6 | 1 | 1 | 2 | N |
| hsa-miR-6852-5p | BPIFC | 1 | 1 | 2 | N |
| hsa-miR-6852-5p | ABCC1 | 1 | 1 | 2 | N |
| hsa-miR-6852-5p | LIMK2 | 1 | 1 | 2 | N |
| hsa-miR-6852-5p | ETV3L | 1 | 1 | 2 | N |
| hsa-miR-6852-5p | MMP24 | 1 | 1 | 2 | N |
| hsa-miR-6852-5p | ATXN7L3 | 1 | 1 | 2 | Y |
| hsa-miR-6852-5p | DKK3 | 1 | 1 | 2 | N |
| hsa-miR-6852-5p | BTG2 | 1 | 1 | 2 | N |
| hsa-miR-6852-5p | TIMP4 | 1 | 1 | 2 | N |
| hsa-miR-6852-5p | NFYC | 1 | 1 | 2 | N |
| hsa-miR-6852-5p | FATE1 | 1 | 1 | 2 | N |
| hsa-miR-6852-5p | NRIP2 | 1 | 1 | 2 | N |
| hsa-miR-6852-5p | MAFK | 1 | 1 | 2 | N |
| hsa-miR-6852-5p | NR2C2AP | 1 | 1 | 2 | N |
| hsa-miR-6852-5p | RAVER1 | 1 | 1 | 2 | N |
| hsa-miR-6852-5p | AMMECR1 | 1 | 1 | 2 | N |
| hsa-miR-6852-5p | PPARD | 1 | 1 | 2 | N |
| hsa-miR-6852-5p | IMPDH1 | 1 | 1 | 2 | N |
| hsa-miR-6852-5p | S1PR3 | 1 | 1 | 2 | N |
| hsa-miR-6852-5p | C14orf28 | 1 | 1 | 2 | N |
| hsa-miR-6852-5p | BTN2A2 | 1 | 1 | 2 | N |
| hsa-miR-6852-5p | ATG7 | 1 | 1 | 2 | N |
| hsa-miR-6852-5p | TCF19 | 1 | 1 | 2 | N |
| hsa-miR-6852-5p | MICALL1 | 1 | 1 | 2 | N |
| hsa-miR-6852-5p | RPS6KA1 | 1 | 1 | 2 | N |
| hsa-miR-6852-5p | PPP1R17 | 1 | 1 | 2 | N |
| hsa-miR-6852-5p | FCER2 | 1 | 1 | 2 | N |
| hsa-miR-6852-5p | CACNB1 | 1 | 1 | 2 | N |
| hsa-miR-6852-5p | APBA1 | 1 | 1 | 2 | N |
| hsa-miR-6852-5p | RAD9A | 1 | 1 | 2 | N |
| hsa-miR-6852-5p | TM9SF4 | 1 | 1 | 2 | N |
| hsa-miR-6852-5p | WDR55 | 1 | 1 | 2 | N |
| hsa-miR-6852-5p | TMEM194A | 1 | 1 | 2 | N |
| hsa-miR-6852-5p | RALY | 1 | 1 | 2 | N |
| hsa-miR-6852-5p | CHST3 | 1 | 1 | 2 | N |
| hsa-miR-6852-5p | LDB1 | 1 | 1 | 2 | N |
| hsa-miR-6852-5p | VEGFA | 1 | 1 | 2 | N |
| hsa-miR-6852-5p | ARRB1 | 1 | 1 | 2 | N |
| hsa-miR-6852-5p | TCF7 | 1 | 1 | 2 | N |
| hsa-miR-6852-5p | RNF40 | 1 | 1 | 2 | N |
| hsa-miR-6852-5p | TBL2 | 1 | 1 | 2 | N |
| hsa-miR-6852-5p | C7orf50 | 1 | 1 | 2 | N |
| hsa-miR-6852-5p | LPHN1 | 1 | 1 | 2 | N |
| hsa-miR-6852-5p | STAC | 1 | 1 | 2 | N |
| hsa-miR-6852-5p | BMF | 1 | 1 | 2 | N |
| hsa-miR-6852-5p | STX1A | 1 | 1 | 2 | N |
| hsa-miR-6852-5p | SLC25A42 | 1 | 1 | 2 | N |
| hsa-miR-6852-5p | KCNH4 | 1 | 1 | 2 | N |
| hsa-miR-6852-5p | SPR | 1 | 1 | 2 | N |
| hsa-miR-6852-5p | PCBP3 | 1 | 1 | 2 | N |
| hsa-miR-6852-5p | GRINA | 1 | 1 | 2 | N |
| hsa-miR-6852-5p | SOX10 | 1 | 1 | 2 | N |
| hsa-miR-6852-5p | SESN1 | 1 | 1 | 2 | N |
| hsa-miR-6852-5p | CPEB4 | 1 | 1 | 2 | N |
| hsa-miR-6852-5p | PDGFRB | 1 | 1 | 2 | N |
| hsa-miR-6852-5p | ADORA1 | 1 | 1 | 2 | N |
| hsa-miR-6852-5p | CALN1 | 1 | 1 | 2 | N |
| hsa-miR-6852-5p | PVRL1 | 1 | 1 | 2 | N |
| hsa-miR-6852-5p | SUSD2 | 1 | 1 | 2 | N |
| hsa-miR-6852-5p | CCDC64B | 1 | 1 | 2 | N |
| hsa-miR-6852-5p | HDAC1 | 1 | 1 | 2 | N |
| hsa-miR-6852-5p | SNPH | 1 | 1 | 2 | N |
| hsa-miR-6852-5p | ZBTB7A | 1 | 1 | 2 | N |
| hsa-miR-6852-5p | C14orf180 | 1 | 1 | 2 | N |
| hsa-miR-6852-5p | SHISA7 | 1 | 1 | 2 | N |
| hsa-miR-6852-5p | NPTX1 | 1 | 1 | 2 | N |
| hsa-miR-6852-5p | LZTS1 | 1 | 1 | 2 | N |
| hsa-miR-6852-5p | MECR | 1 | 1 | 2 | N |
| hsa-miR-6852-5p | TNKS1BP1 | 1 | 1 | 2 | N |
| hsa-miR-6852-5p | VSTM4 | 1 | 1 | 2 | N |
| hsa-miR-6852-5p | MBD6 | 1 | 1 | 2 | N |
| hsa-miR-6852-5p | THBS3 | 1 | 1 | 2 | N |
| hsa-miR-6852-5p | TRADD | 1 | 1 | 2 | N |
| hsa-miR-6852-5p | ITPRIP | 1 | 1 | 2 | N |
| hsa-miR-6852-5p | GLIS2 | 1 | 1 | 2 | N |
| hsa-miR-6852-5p | PACS1 | 1 | 1 | 2 | N |
| hsa-miR-6852-5p | IGFBP5 | 1 | 1 | 2 | N |
| hsa-miR-6852-5p | SCN3B | 1 | 1 | 2 | N |
| hsa-miR-6852-5p | NCDN | 1 | 1 | 2 | N |
| hsa-miR-6852-5p | TNFAIP2 | 1 | 1 | 2 | N |
| hsa-miR-6852-5p | EPHA10 | 1 | 1 | 2 | N |
| hsa-miR-6852-5p | HMGA1 | 1 | 1 | 2 | N |
| hsa-miR-6852-5p | PHF2 | 1 | 1 | 2 | N |
| hsa-miR-6852-5p | DNAL4 | 1 | 1 | 2 | N |
| hsa-miR-6852-5p | FXYD3 | 1 | 1 | 2 | N |
| hsa-miR-6852-5p | NR2F6 | 1 | 1 | 2 | N |
| hsa-miR-6852-5p | ATF6B | 1 | 1 | 2 | N |
| hsa-miR-6852-5p | ENC1 | 1 | 1 | 2 | N |
| hsa-miR-6852-5p | BOK | 1 | 1 | 2 | N |
| hsa-miR-6852-5p | ENTHD2 | 1 | 1 | 2 | N |
| hsa-miR-6852-5p | BBS1 | 1 | 1 | 2 | N |
| hsa-miR-6852-5p | SSBP4 | 1 | 1 | 2 | N |
| hsa-miR-6852-5p | BARHL1 | 1 | 1 | 2 | N |
| hsa-miR-6852-5p | TMEM63C | 1 | 1 | 2 | N |
| hsa-miR-6852-5p | HAUS5 | 1 | 1 | 2 | N |
| hsa-miR-6852-5p | MMP15 | 1 | 1 | 2 | N |
| hsa-miR-6852-5p | SLC39A2 | 1 | 1 | 2 | N |
| hsa-miR-6852-5p | ANP32B | 1 | 1 | 2 | Y |
| hsa-miR-6852-5p | WWP2 | 1 | 1 | 2 | N |
| hsa-miR-6852-5p | STC1 | 1 | 1 | 2 | N |
| hsa-miR-6852-5p | NUBP2 | 1 | 1 | 2 | N |
| hsa-miR-6852-5p | TMEM127 | 1 | 1 | 2 | N |
| hsa-miR-6852-5p | STRN4 | 1 | 1 | 2 | N |
| hsa-miR-6852-5p | GPD1 | 1 | 1 | 2 | N |
| hsa-miR-6852-5p | FXYD6 | 1 | 1 | 2 | N |
| hsa-miR-6852-5p | ALX4 | 1 | 1 | 2 | N |
| hsa-miR-6852-5p | ZNF496 | 1 | 1 | 2 | N |
| hsa-miR-6852-5p | DRG2 | 1 | 1 | 2 | N |
| hsa-miR-6852-5p | RRP7A | 1 | 1 | 2 | N |
| hsa-miR-6852-5p | PLEKHO2 | 1 | 1 | 2 | N |
| hsa-miR-6852-5p | ARHGAP19 | 1 | 1 | 2 | N |
| hsa-miR-6852-5p | RNF165 | 1 | 1 | 2 | N |
| hsa-miR-6852-5p | HAP1 | 1 | 1 | 2 | N |
| hsa-miR-6852-5p | NACC1 | 1 | 1 | 2 | N |
| hsa-miR-6852-5p | AKNA | 1 | 1 | 2 | N |
| hsa-miR-6852-5p | SNURF | 1 | 1 | 2 | N |
| hsa-miR-6852-5p | PRIMA1 | 1 | 1 | 2 | N |
| hsa-miR-6852-5p | PARP6 | 1 | 1 | 2 | N |
| hsa-miR-6852-5p | FOXO4 | 1 | 1 | 2 | N |
| hsa-miR-6852-5p | TSPAN18 | 1 | 1 | 2 | N |
| hsa-miR-6852-5p | FAM131B | 1 | 1 | 2 | N |
| hsa-miR-6852-5p | WSCD2 | 1 | 1 | 2 | N |
| hsa-miR-6852-5p | FAM53C | 1 | 1 | 2 | Y |
| hsa-miR-6852-5p | GPKOW | 1 | 1 | 2 | N |
| hsa-miR-6852-5p | ICOSLG | 1 | 1 | 2 | N |
| hsa-miR-6852-5p | TTYH3 | 1 | 1 | 2 | N |
| hsa-miR-6852-5p | SLC25A22 | 1 | 1 | 2 | N |
| hsa-miR-6852-5p | LRRC25 | 1 | 1 | 2 | N |
| hsa-miR-6852-5p | DAGLA | 1 | 1 | 2 | N |
| hsa-miR-6852-5p | STK4 | 1 | 1 | 2 | N |
| hsa-miR-6852-5p | LRRC16B | 1 | 1 | 2 | N |
| hsa-miR-6852-5p | SLC6A3 | 1 | 1 | 2 | N |
| hsa-miR-6852-5p | RBM14 | 1 | 1 | 2 | N |
| hsa-miR-6879-5p | TTC39A | 1 | 1 | 2 | N |
| hsa-miR-6879-5p | CLSTN2 | 1 | 1 | 2 | N |
| hsa-miR-6879-5p | SLC6A9 | 1 | 1 | 2 | Y |
| hsa-miR-6879-5p | ZIC3 | 1 | 1 | 2 | N |
| hsa-miR-6879-5p | WIPF3 | 1 | 1 | 2 | N |
| hsa-miR-6879-5p | TMEM25 | 1 | 1 | 2 | N |
| hsa-miR-6879-5p | TMEM110 | 1 | 1 | 2 | N |
| hsa-miR-6879-5p | HJURP | 1 | 1 | 2 | N |
| hsa-miR-6879-5p | FHL1 | 1 | 1 | 2 | N |
| hsa-miR-6879-5p | LASP1 | 1 | 1 | 2 | N |
| hsa-miR-6879-5p | KCNK3 | 1 | 1 | 2 | N |
| hsa-miR-6879-5p | KIF21B | 1 | 1 | 2 | N |
| hsa-miR-6879-5p | FAM214B | 1 | 1 | 2 | N |
| hsa-miR-6879-5p | CX3CL1 | 1 | 1 | 2 | N |
| hsa-miR-6879-5p | SLC26A9 | 1 | 1 | 2 | N |
| hsa-miR-6879-5p | TRIM9 | 1 | 1 | 2 | N |
| hsa-miR-6879-5p | ETV4 | 1 | 1 | 2 | N |
| hsa-miR-6879-5p | RMND5B | 1 | 1 | 2 | N |
| hsa-miR-6879-5p | LDLRAP1 | 1 | 1 | 2 | N |
| hsa-miR-6879-5p | ITCH | 1 | 1 | 2 | N |
| hsa-miR-6879-5p | HMX2 | 1 | 1 | 2 | N |
| hsa-miR-6879-5p | ULK3 | 1 | 1 | 2 | N |
| hsa-miR-6879-5p | SLC37A3 | 1 | 1 | 2 | N |
| hsa-miR-6879-5p | CTNND1 | 1 | 1 | 2 | N |
| hsa-miR-6879-5p | FAM155B | 1 | 1 | 2 | N |
| hsa-miR-6879-5p | HLA-DOB | 1 | 1 | 2 | N |
| hsa-miR-6879-5p | CBX6 | 1 | 1 | 2 | Y |
| hsa-miR-6879-5p | CYBRD1 | 1 | 1 | 2 | N |
| hsa-miR-6879-5p | PVRL1 | 1 | 1 | 2 | N |
| hsa-miR-6879-5p | C17orf67 | 1 | 1 | 2 | N |
| hsa-miR-6879-5p | FAM53A | 1 | 1 | 2 | N |
| hsa-miR-6879-5p | SSR1 | 1 | 1 | 2 | N |
| hsa-miR-6879-5p | ADIPOR2 | 1 | 1 | 2 | N |
| hsa-miR-6879-5p | MPZ | 1 | 1 | 2 | N |
| hsa-miR-6879-5p | SV2A | 1 | 1 | 2 | N |
| hsa-miR-6879-5p | RGS7BP | 1 | 1 | 2 | N |
| hsa-miR-6879-5p | PPP1R9B | 1 | 1 | 2 | N |
| hsa-miR-6879-5p | GABRA4 | 1 | 1 | 2 | N |
| hsa-miR-6879-5p | TSPAN9 | 1 | 1 | 2 | N |
| hsa-miR-6879-5p | ZSCAN31 | 1 | 1 | 2 | N |
| hsa-miR-6879-5p | SLC31A2 | 1 | 1 | 2 | N |
| hsa-miR-6879-5p | SOX13 | 1 | 1 | 2 | N |
| hsa-miR-6879-5p | SYT7 | 1 | 1 | 2 | Y |
| hsa-miR-6879-5p | DCP1A | 1 | 1 | 2 | N |
| hsa-miR-6879-5p | TNFAIP8L3 | 1 | 1 | 2 | N |
| hsa-miR-6879-5p | PUM2 | 1 | 1 | 2 | Y |
| hsa-miR-6879-5p | RHBDF2 | 1 | 1 | 2 | Y |
| hsa-miR-6879-5p | USP7 | 1 | 1 | 2 | N |
| hsa-miR-6879-5p | SOX10 | 1 | 1 | 2 | N |
| hsa-miR-6879-5p | KIAA0247 | 1 | 1 | 2 | N |
| hsa-miR-6879-5p | ATP9B | 1 | 1 | 2 | N |
| hsa-miR-6879-5p | TRIM35 | 1 | 1 | 2 | N |
| hsa-miR-6879-5p | NOTCH2 | 1 | 1 | 2 | N |
| hsa-miR-6879-5p | SEPT3 | 1 | 1 | 2 | N |
| hsa-miR-6879-5p | HMG20A | 1 | 1 | 2 | N |
| hsa-miR-6879-5p | FNDC3B | 1 | 1 | 2 | N |
| hsa-miR-6879-5p | HTRA3 | 1 | 1 | 2 | N |
| hsa-miR-6879-5p | PPP6C | 1 | 1 | 2 | N |
| hsa-miR-6879-5p | TMEM127 | 1 | 1 | 2 | N |
| hsa-miR-6879-5p | ARHGEF6 | 1 | 1 | 2 | N |
| hsa-miR-6879-5p | NAT16 | 1 | 1 | 2 | N |
| hsa-miR-6879-5p | VPS39 | 1 | 1 | 2 | N |
| hsa-miR-6879-5p | MAFG | 1 | 1 | 2 | N |
| hsa-miR-6879-5p | TEX261 | 1 | 1 | 2 | N |
| hsa-miR-6879-5p | C17orf85 | 1 | 1 | 2 | N |
| hsa-miR-6879-5p | TRIM46 | 1 | 1 | 2 | N |
| hsa-miR-6879-5p | RTKN | 1 | 1 | 2 | N |
| hsa-miR-6879-5p | KIRREL | 1 | 1 | 2 | N |
| hsa-miR-6879-5p | ZNF34 | 1 | 1 | 2 | N |
| hsa-miR-6879-5p | PIGY | 1 | 1 | 2 | N |
| hsa-miR-6879-5p | NAMPT | 1 | 1 | 2 | N |
| hsa-miR-6879-5p | CACNA2D2 | 1 | 1 | 2 | N |
| hsa-miR-6879-5p | RALYL | 1 | 1 | 2 | N |
| hsa-miR-6879-5p | TLL2 | 1 | 1 | 2 | N |
| hsa-miR-6879-5p | HAP1 | 1 | 1 | 2 | N |
| hsa-miR-6879-5p | PATZ1 | 1 | 1 | 2 | N |
| hsa-miR-6879-5p | STK19 | 1 | 1 | 2 | N |
| hsa-miR-6879-5p | SH2D5 | 1 | 1 | 2 | N |
| hsa-miR-6879-5p | AK3 | 1 | 1 | 2 | N |
| hsa-miR-6879-5p | NCKAP5L | 1 | 1 | 2 | N |
| hsa-miR-6879-5p | CSF1R | 1 | 1 | 2 | N |
| hsa-miR-6879-5p | RASSF4 | 1 | 1 | 2 | N |
| hsa-miR-6879-5p | TBX15 | 1 | 1 | 2 | N |
| hsa-miR-6879-5p | LINGO1 | 1 | 1 | 2 | N |
| hsa-miR-6879-5p | CTDNEP1 | 1 | 1 | 2 | Y |
| hsa-miR-6879-5p | KCNQ3 | 1 | 1 | 2 | N |
| hsa-miR-6879-5p | MAPKAPK2 | 1 | 1 | 2 | N |
| hsa-miR-6879-5p | AGAP3 | 1 | 1 | 2 | N |
| hsa-miR-6879-5p | NAB2 | 1 | 1 | 2 | N |
| hsa-miR-6879-5p | CCDC114 | 1 | 1 | 2 | N |
| hsa-miR-6879-5p | RAB1B | 1 | 1 | 2 | N |
| hsa-miR-6879-5p | DMTN | 1 | 1 | 2 | N |
| hsa-miR-6879-5p | WDTC1 | 1 | 1 | 2 | N |
| hsa-miR-6879-5p | FBXO41 | 1 | 1 | 2 | Y |
| hsa-miR-6879-5p | SOWAHC | 1 | 1 | 2 | Y |
| hsa-miR-6879-5p | CDC42SE1 | 1 | 1 | 2 | Y |
| hsa-miR-6879-5p | SLC37A1 | 1 | 1 | 2 | N |
| hsa-miR-6879-5p | PAPD7 | 1 | 1 | 2 | N |
| hsa-miR-6879-5p | DDI2 | 1 | 1 | 2 | N |
| hsa-miR-6879-5p | WASF2 | 1 | 1 | 2 | N |
| hsa-miR-6879-5p | SLC6A17 | 1 | 1 | 2 | N |
| hsa-miR-6879-5p | TBC1D13 | 1 | 1 | 2 | N |
| hsa-miR-6879-5p | NEU1 | 1 | 1 | 2 | N |
| hsa-miR-6879-5p | CHAF1A | 1 | 1 | 2 | N |
| hsa-miR-6879-5p | EFNB3 | 1 | 1 | 2 | N |
| hsa-miR-6879-5p | FAM86A | 1 | 1 | 2 | N |
| hsa-miR-6879-5p | SDC3 | 1 | 1 | 2 | N |
| hsa-miR-6879-5p | DDX17 | 1 | 1 | 2 | N |
| hsa-miR-6879-5p | ADAM10 | 1 | 1 | 2 | N |
| hsa-miR-6879-5p | COL5A3 | 1 | 1 | 2 | N |
| hsa-miR-6879-5p | TRIM4 | 1 | 1 | 2 | N |
| hsa-miR-6879-5p | AIF1L | 1 | 1 | 2 | N |
| hsa-miR-6879-5p | NEUROD4 | 1 | 1 | 2 | N |
| hsa-miR-6879-5p | C6orf89 | 1 | 1 | 2 | N |
| hsa-miR-6879-5p | RELT | 1 | 1 | 2 | N |
| hsa-miR-6879-5p | CACFD1 | 1 | 1 | 2 | N |
| hsa-miR-6879-5p | GRM2 | 1 | 1 | 2 | N |
| hsa-miR-6879-5p | XYLT1 | 1 | 1 | 2 | N |
| hsa-miR-6879-5p | ORAI3 | 1 | 1 | 2 | N |
| hsa-miR-6879-5p | C11orf86 | 1 | 1 | 2 | N |
| hsa-miR-6879-5p | MRPL4 | 1 | 1 | 2 | N |
| hsa-miR-6879-5p | ARL4C | 1 | 1 | 2 | N |
| hsa-miR-6879-5p | LUZP1 | 1 | 1 | 2 | Y |
| hsa-miR-6879-5p | SH3GL2 | 1 | 1 | 2 | N |
| hsa-miR-6879-5p | GPR85 | 1 | 1 | 2 | N |
| hsa-miR-6879-5p | AURKA | 1 | 1 | 2 | Y |
| hsa-miR-6879-5p | TBC1D9B | 1 | 1 | 2 | N |
| hsa-miR-6879-5p | PARP6 | 1 | 1 | 2 | N |
| hsa-miR-6879-5p | LIN28A | 1 | 1 | 2 | N |
| hsa-miR-6879-5p | COPZ1 | 1 | 1 | 2 | N |
| hsa-miR-6879-5p | ABHD12 | 1 | 1 | 2 | N |
| hsa-miR-6879-5p | PIANP | 1 | 1 | 2 | N |
| hsa-miR-6879-5p | C9orf84 | 1 | 1 | 2 | N |
| hsa-miR-6879-5p | STAB2 | 1 | 1 | 2 | N |
| hsa-miR-6879-5p | RGMB | 1 | 1 | 2 | Y |
| hsa-miR-6879-5p | ZNF862 | 1 | 1 | 2 | N |
| hsa-miR-6879-5p | NPLOC4 | 1 | 1 | 2 | Y |
| hsa-miR-6879-5p | RASSF2 | 1 | 1 | 2 | N |
| hsa-miR-6879-5p | TRIM26 | 1 | 1 | 2 | N |
| hsa-miR-6879-5p | AGO1 | 1 | 1 | 2 | Y |
| hsa-miR-6879-5p | LSM12 | 1 | 1 | 2 | N |
| hsa-miR-6879-5p | FAM53C | 1 | 1 | 2 | N |
| hsa-miR-6879-5p | PGS1 | 1 | 1 | 2 | N |
| hsa-miR-6879-5p | GDNF | 1 | 1 | 2 | N |
| hsa-miR-6879-5p | NRIP2 | 1 | 1 | 2 | N |
| hsa-miR-6879-5p | SCML1 | 1 | 1 | 2 | N |
| hsa-miR-6879-5p | HNRNPR | 1 | 1 | 2 | N |
| hsa-miR-6879-5p | CIC | 1 | 1 | 2 | N |
| hsa-miR-6879-5p | SMARCD1 | 1 | 1 | 2 | N |
| hsa-miR-6879-5p | TCEA2 | 1 | 1 | 2 | N |
| hsa-miR-6879-5p | ADAMTS4 | 1 | 1 | 2 | N |
| hsa-miR-6879-5p | ANKFY1 | 1 | 1 | 2 | N |
| hsa-miR-6879-5p | HECTD3 | 1 | 1 | 2 | N |
| hsa-miR-6879-5p | KMT2D | 1 | 1 | 2 | Y |
| hsa-miR-6879-5p | CBX7 | 1 | 1 | 2 | N |
| hsa-miR-6879-5p | SOCS4 | 1 | 1 | 2 | N |
| hsa-miR-6879-5p | C14orf1 | 1 | 1 | 2 | N |
| hsa-miR-6879-5p | STK35 | 1 | 1 | 2 | N |
| hsa-miR-6879-5p | CDIP1 | 1 | 1 | 2 | N |
| hsa-miR-6879-5p | ST3GAL5 | 1 | 1 | 2 | N |
| hsa-miR-6879-5p | ARL6IP1 | 1 | 1 | 2 | Y |
| hsa-miR-6879-5p | ANKRA2 | 1 | 1 | 2 | N |
| hsa-miR-6879-5p | FAM195B | 1 | 1 | 2 | N |
| hsa-miR-6879-5p | SRRD | 1 | 1 | 2 | N |
| hsa-miR-6879-5p | KCNIP1 | 1 | 1 | 2 | N |
| hsa-miR-6879-5p | ATG9A | 1 | 1 | 2 | N |
| hsa-miR-6879-5p | LBH | 1 | 1 | 2 | N |
| hsa-miR-6879-5p | TMEM154 | 1 | 1 | 2 | N |
| hsa-miR-6884-5p | TMEM151B | 1 | 1 | 2 | N |
| hsa-miR-6884-5p | ARL2BP | 1 | 1 | 2 | N |
| hsa-miR-6884-5p | ADIPOR2 | 1 | 1 | 2 | N |
| hsa-miR-6884-5p | ZNF384 | 1 | 1 | 2 | N |
| hsa-miR-6884-5p | PARP11 | 1 | 1 | 2 | N |
| hsa-miR-6884-5p | ALX4 | 1 | 1 | 2 | N |
| hsa-miR-6884-5p | RAB8B | 1 | 1 | 2 | N |
| hsa-miR-6884-5p | PAK1 | 1 | 1 | 2 | N |
| hsa-miR-6884-5p | PPAN-P2RY11 | 1 | 1 | 2 | N |
| hsa-miR-6884-5p | ST3GAL1 | 1 | 1 | 2 | N |
| hsa-miR-6884-5p | DNAJC5G | 1 | 1 | 2 | N |
| hsa-miR-6884-5p | EFNA1 | 1 | 1 | 2 | N |
| hsa-miR-6884-5p | SLC16A2 | 1 | 1 | 2 | N |
| hsa-miR-6884-5p | PTMS | 1 | 1 | 2 | N |
| hsa-miR-6884-5p | MGST3 | 1 | 1 | 2 | N |
| hsa-miR-6884-5p | ZBTB39 | 1 | 1 | 2 | N |
| hsa-miR-6884-5p | MGAT5B | 1 | 1 | 2 | N |
| hsa-miR-6884-5p | CKS1B | 1 | 1 | 2 | Y |
| hsa-miR-6884-5p | SDC3 | 1 | 1 | 2 | N |
| hsa-miR-6884-5p | CTDNEP1 | 1 | 1 | 2 | N |
| hsa-miR-6884-5p | GAB2 | 1 | 1 | 2 | N |
| hsa-miR-6884-5p | HIF3A | 1 | 1 | 2 | N |
| hsa-miR-6884-5p | DAND5 | 1 | 1 | 2 | N |
| hsa-miR-6884-5p | RBPMS | 1 | 1 | 2 | N |
| hsa-miR-6884-5p | VPS26A | 1 | 1 | 2 | N |
| hsa-miR-6884-5p | ETNK2 | 1 | 1 | 2 | N |
| hsa-miR-6884-5p | ACTR3 | 1 | 1 | 2 | N |
| hsa-miR-6884-5p | DCAF7 | 1 | 1 | 2 | N |
| hsa-miR-6884-5p | SLC36A3 | 1 | 1 | 2 | N |
| hsa-miR-6884-5p | FRMPD3 | 1 | 1 | 2 | N |
| hsa-miR-6884-5p | TMEM104 | 1 | 1 | 2 | N |
| hsa-miR-7843-5p | XYLT1 | 1 | 1 | 2 | N |
| hsa-miR-7843-5p | ABHD12 | 1 | 1 | 2 | N |
| hsa-miR-7843-5p | VPS39 | 1 | 1 | 2 | N |
| hsa-miR-7843-5p | ADAM10 | 1 | 1 | 2 | N |
| hsa-miR-7843-5p | SOX10 | 1 | 1 | 2 | N |
| hsa-miR-7843-5p | C9orf84 | 1 | 1 | 2 | N |
| hsa-miR-7843-5p | TMEM154 | 1 | 1 | 2 | N |
| hsa-miR-7843-5p | RGMB | 1 | 1 | 2 | Y |
| hsa-miR-7843-5p | C17orf85 | 1 | 1 | 2 | N |
| hsa-miR-7843-5p | LIN28A | 1 | 1 | 2 | N |
| hsa-miR-7843-5p | MAFG | 1 | 1 | 2 | N |
| hsa-miR-7843-5p | GPR85 | 1 | 1 | 2 | N |
| hsa-miR-7843-5p | AWAT2 | 1 | 1 | 2 | N |
| hsa-miR-7843-5p | CIC | 1 | 1 | 2 | N |
| hsa-miR-7843-5p | ORAI3 | 1 | 1 | 2 | N |
| hsa-miR-7843-5p | CACNA2D2 | 1 | 1 | 2 | N |
| hsa-miR-7843-5p | NAMPT | 1 | 1 | 2 | N |
| hsa-miR-7843-5p | SOX13 | 1 | 1 | 2 | N |
| hsa-miR-7843-5p | CDC42SE1 | 1 | 1 | 2 | Y |
| hsa-miR-7843-5p | CBX7 | 1 | 1 | 2 | N |
| hsa-miR-7843-5p | ZSCAN31 | 1 | 1 | 2 | N |
| hsa-miR-7843-5p | PARP6 | 1 | 1 | 2 | N |
| hsa-miR-7843-5p | NPLOC4 | 1 | 1 | 2 | Y |
| hsa-miR-7843-5p | C14orf1 | 1 | 1 | 2 | N |
| hsa-miR-7843-5p | LDLRAP1 | 1 | 1 | 2 | N |
| hsa-miR-7843-5p | ARL6IP1 | 1 | 1 | 2 | Y |
| hsa-miR-7843-5p | DCP1A | 1 | 1 | 2 | N |
| hsa-miR-7843-5p | KCNK3 | 1 | 1 | 2 | N |
| hsa-miR-7843-5p | TTC39A | 1 | 1 | 2 | N |
| hsa-miR-7843-5p | CX3CL1 | 1 | 1 | 2 | N |
| hsa-miR-7843-5p | CACFD1 | 1 | 1 | 2 | N |
| hsa-miR-7843-5p | SOWAHC | 1 | 1 | 2 | Y |
| hsa-miR-7843-5p | ITCH | 1 | 1 | 2 | N |
| hsa-miR-7843-5p | TRIM46 | 1 | 1 | 2 | N |
| hsa-miR-7843-5p | TRIM35 | 1 | 1 | 2 | N |
| hsa-miR-7843-5p | SH3GL2 | 1 | 1 | 2 | N |
| hsa-miR-7843-5p | ANKFY1 | 1 | 1 | 2 | N |
| hsa-miR-7843-5p | HMG20A | 1 | 1 | 2 | N |
| hsa-miR-7843-5p | WIPF3 | 1 | 1 | 2 | N |
| hsa-miR-7843-5p | PVRL1 | 1 | 1 | 2 | N |
| hsa-miR-7843-5p | ST3GAL5 | 1 | 1 | 2 | N |
| hsa-miR-7843-5p | TNFAIP8L3 | 1 | 1 | 2 | N |
| hsa-miR-7843-5p | TRIM4 | 1 | 1 | 2 | N |
| hsa-miR-7843-5p | RELT | 1 | 1 | 2 | N |
| hsa-miR-7843-5p | SDC3 | 1 | 1 | 2 | N |
| hsa-miR-7843-5p | AGAP3 | 1 | 1 | 2 | N |
| hsa-miR-7843-5p | STX1A | 1 | 1 | 2 | N |
| hsa-miR-7843-5p | TLL2 | 1 | 1 | 2 | N |
| hsa-miR-7843-5p | RASSF2 | 1 | 1 | 2 | N |
| hsa-miR-7843-5p | RHBDF2 | 1 | 1 | 2 | Y |
| hsa-miR-7843-5p | AURKA | 1 | 1 | 2 | Y |
| hsa-miR-7843-5p | PIANP | 1 | 1 | 2 | N |
| hsa-miR-7843-5p | CSF1R | 1 | 1 | 2 | N |
| hsa-miR-7843-5p | ZNF862 | 1 | 1 | 2 | N |
| hsa-miR-7843-5p | HJURP | 1 | 1 | 2 | N |
| hsa-miR-7843-5p | AGO1 | 1 | 1 | 2 | Y |
| hsa-miR-7843-5p | FBXO41 | 1 | 1 | 2 | Y |
| hsa-miR-7843-5p | NRIP2 | 1 | 1 | 2 | N |
| hsa-miR-7843-5p | CHAF1A | 1 | 1 | 2 | N |
| hsa-miR-7843-5p | RALYL | 1 | 1 | 2 | N |
| hsa-miR-7843-5p | RTKN | 1 | 1 | 2 | N |
| hsa-miR-7843-5p | C17orf67 | 1 | 1 | 2 | N |
| hsa-miR-7843-5p | LUZP1 | 1 | 1 | 2 | Y |
| hsa-miR-7843-5p | PAPD7 | 1 | 1 | 2 | N |
| hsa-miR-7843-5p | TMEM110 | 1 | 1 | 2 | N |
| hsa-miR-7843-5p | SMARCD1 | 1 | 1 | 2 | N |
| hsa-miR-7843-5p | FAM155B | 1 | 1 | 2 | N |
| hsa-miR-7843-5p | ADIPOR2 | 1 | 1 | 2 | N |
| hsa-miR-7843-5p | EFNB3 | 1 | 1 | 2 | N |
| hsa-miR-7843-5p | SV2A | 1 | 1 | 2 | N |
| hsa-miR-7843-5p | NAT16 | 1 | 1 | 2 | N |
| hsa-miR-7843-5p | PUM2 | 1 | 1 | 2 | Y |
| hsa-miR-7843-5p | ATG9A | 1 | 1 | 2 | N |
| hsa-miR-7843-5p | ETV4 | 1 | 1 | 2 | N |
| hsa-miR-7843-5p | NOTCH2 | 1 | 1 | 2 | N |
| hsa-miR-7843-5p | CLSTN2 | 1 | 1 | 2 | N |
| hsa-miR-7843-5p | TSPAN9 | 1 | 1 | 2 | N |
| hsa-miR-7843-5p | HNRNPR | 1 | 1 | 2 | N |
| hsa-miR-7843-5p | ZIC3 | 1 | 1 | 2 | N |
| hsa-miR-7843-5p | LSM12 | 1 | 1 | 2 | N |
| hsa-miR-7843-5p | PATZ1 | 1 | 1 | 2 | N |
| hsa-miR-7843-5p | STK19 | 1 | 1 | 2 | N |
| hsa-miR-7843-5p | SLC6A9 | 1 | 1 | 2 | Y |
| hsa-miR-7843-5p | USP7 | 1 | 1 | 2 | N |
| hsa-miR-7843-5p | HAP1 | 1 | 1 | 2 | N |
| hsa-miR-7843-5p | SEPT3 | 1 | 1 | 2 | N |
| hsa-miR-7843-5p | CYBRD1 | 1 | 1 | 2 | N |
| hsa-miR-7843-5p | AK3 | 1 | 1 | 2 | N |
| hsa-miR-7843-5p | FHL1 | 1 | 1 | 2 | N |
| hsa-miR-7843-5p | WASF2 | 1 | 1 | 2 | N |
| hsa-miR-7843-5p | STAB2 | 1 | 1 | 2 | N |
| hsa-miR-7843-5p | STK35 | 1 | 1 | 2 | N |
| hsa-miR-7843-5p | RASSF4 | 1 | 1 | 2 | N |
| hsa-miR-7843-5p | HLA-DOB | 1 | 1 | 2 | N |
| hsa-miR-7843-5p | GDNF | 1 | 1 | 2 | N |
| hsa-miR-7843-5p | HECTD3 | 1 | 1 | 2 | N |
| hsa-miR-7843-5p | ARHGEF6 | 1 | 1 | 2 | N |
| hsa-miR-7843-5p | LINGO1 | 1 | 1 | 2 | N |
| hsa-miR-7843-5p | SRRD | 1 | 1 | 2 | N |
| hsa-miR-7843-5p | KIRREL | 1 | 1 | 2 | N |
| hsa-miR-7843-5p | COL5A3 | 1 | 1 | 2 | N |
| hsa-miR-7843-5p | WDTC1 | 1 | 1 | 2 | N |
| hsa-miR-7843-5p | SLC37A1 | 1 | 1 | 2 | N |
| hsa-miR-7843-5p | MPZ | 1 | 1 | 2 | N |
| hsa-miR-7843-5p | ARL4C | 1 | 1 | 2 | N |
| hsa-miR-7843-5p | FNDC3B | 1 | 1 | 2 | N |
| hsa-miR-7843-5p | CCDC114 | 1 | 1 | 2 | N |
| hsa-miR-7843-5p | PGS1 | 1 | 1 | 2 | N |
| hsa-miR-7843-5p | FAM214B | 1 | 1 | 2 | N |
| hsa-miR-7843-5p | GRM2 | 1 | 1 | 2 | N |
| hsa-miR-7843-5p | ULK3 | 1 | 1 | 2 | N |
| hsa-miR-7843-5p | TBC1D13 | 1 | 1 | 2 | N |
| hsa-miR-7843-5p | MAPKAPK2 | 1 | 1 | 2 | N |
| hsa-miR-7843-5p | SLC31A2 | 1 | 1 | 2 | N |
| hsa-miR-7843-5p | PPP1R9B | 1 | 1 | 2 | N |
| hsa-miR-7843-5p | PIGY | 1 | 1 | 2 | N |
| hsa-miR-7843-5p | ANKRA2 | 1 | 1 | 2 | N |
| hsa-miR-7843-5p | C11orf86 | 1 | 1 | 2 | N |
| hsa-miR-7843-5p | KIAA0247 | 1 | 1 | 2 | N |
| hsa-miR-7843-5p | SSR1 | 1 | 1 | 2 | N |
| hsa-miR-7843-5p | CDIP1 | 1 | 1 | 2 | N |
| hsa-miR-7843-5p | SCML1 | 1 | 1 | 2 | N |
| hsa-miR-7843-5p | AIF1L | 1 | 1 | 2 | N |
| hsa-miR-7843-5p | NAB2 | 1 | 1 | 2 | N |
| hsa-miR-7843-5p | CST9 | 1 | 1 | 2 | N |
| hsa-miR-7843-5p | TRIM9 | 1 | 1 | 2 | N |
| hsa-miR-7843-5p | DDI2 | 1 | 1 | 2 | N |
| hsa-miR-7843-5p | RAB1B | 1 | 1 | 2 | N |
| hsa-miR-7843-5p | KIF21B | 1 | 1 | 2 | N |
| hsa-miR-7843-5p | ADAMTS4 | 1 | 1 | 2 | N |
| hsa-miR-7843-5p | TRIM26 | 1 | 1 | 2 | N |
| hsa-miR-7843-5p | TMEM25 | 1 | 1 | 2 | N |
| hsa-miR-7843-5p | SYT7 | 1 | 1 | 2 | Y |
| hsa-miR-7843-5p | FAM86A | 1 | 1 | 2 | N |
| hsa-miR-7843-5p | TBC1D9B | 1 | 1 | 2 | N |
| hsa-miR-7843-5p | SH2D5 | 1 | 1 | 2 | N |
| hsa-miR-7843-5p | SLC37A3 | 1 | 1 | 2 | N |
| hsa-miR-7843-5p | HMX2 | 1 | 1 | 2 | N |
| hsa-miR-7843-5p | HTRA3 | 1 | 1 | 2 | N |
| hsa-miR-7843-5p | SOCS4 | 1 | 1 | 2 | N |
| hsa-miR-7843-5p | SLC6A17 | 1 | 1 | 2 | N |
| hsa-miR-7843-5p | FAM53C | 1 | 1 | 2 | N |
| hsa-miR-7843-5p | GABRA4 | 1 | 1 | 2 | N |
| hsa-miR-7843-5p | NEUROD4 | 1 | 1 | 2 | N |
| hsa-miR-7843-5p | KCNIP1 | 1 | 1 | 2 | N |
| hsa-miR-7843-5p | TCEA2 | 1 | 1 | 2 | N |
| hsa-miR-7843-5p | PPP6C | 1 | 1 | 2 | N |
| hsa-miR-7843-5p | TMEM127 | 1 | 1 | 2 | N |
| hsa-miR-7843-5p | LBH | 1 | 1 | 2 | N |
| hsa-miR-4677-3p | MSX2 | 1 | 1 | 2 | N |
| hsa-miR-4677-3p | GOLGA8J | 1 | 1 | 2 | N |
| hsa-miR-4677-3p | GOLGA8M | 1 | 1 | 2 | N |
| hsa-miR-4677-3p | LCLAT1 | 1 | 1 | 2 | N |
| hsa-miR-4677-3p | PCDHB14 | 1 | 1 | 2 | N |
| hsa-miR-4677-3p | LPPR5 | 1 | 1 | 2 | N |
| hsa-miR-4677-3p | PLS1 | 1 | 1 | 2 | N |
| hsa-miR-4677-3p | ZNF586 | 1 | 1 | 2 | N |
| hsa-miR-4677-3p | DCAF12 | 1 | 1 | 2 | N |
| hsa-miR-4677-3p | C8orf58 | 1 | 1 | 2 | N |
| hsa-miR-4677-3p | C1orf52 | 1 | 1 | 2 | N |
| hsa-miR-4677-3p | CD8B | 1 | 1 | 2 | N |
| hsa-miR-4677-3p | TNFSF4 | 1 | 1 | 2 | N |
| hsa-miR-4677-3p | RAB1A | 1 | 1 | 2 | N |
| hsa-miR-4677-3p | CPA3 | 1 | 1 | 2 | N |
| hsa-miR-4677-3p | JPH3 | 1 | 1 | 2 | N |
| hsa-miR-4677-3p | GOLGA8K | 1 | 1 | 2 | N |
| hsa-miR-4677-3p | CXXC5 | 1 | 1 | 2 | Y |
| hsa-miR-4677-3p | RAET1E | 1 | 1 | 2 | N |
| hsa-miR-4677-3p | CXCL14 | 1 | 1 | 2 | N |
| hsa-miR-4677-3p | CHRDL1 | 1 | 1 | 2 | Y |
| hsa-miR-4677-3p | CD300A | 1 | 1 | 2 | N |
| hsa-miR-4677-3p | PPM1N | 1 | 1 | 2 | Y |
| hsa-miR-4677-3p | PALM | 1 | 1 | 2 | N |
| hsa-miR-4677-3p | KLF7 | 1 | 1 | 2 | N |
| hsa-miR-4677-3p | CASP14 | 1 | 1 | 2 | N |
| hsa-miR-4677-3p | CYP7B1 | 1 | 1 | 2 | N |
| hsa-miR-4677-3p | HEBP1 | 1 | 1 | 2 | N |
| hsa-miR-4677-3p | FLI1 | 1 | 1 | 2 | N |
| hsa-miR-4677-3p | TRIP13 | 1 | 1 | 2 | N |
| hsa-miR-4677-3p | UBAC1 | 1 | 1 | 2 | N |
| hsa-miR-4677-3p | PEX11B | 1 | 1 | 2 | N |
| hsa-miR-4677-3p | SAYSD1 | 1 | 1 | 2 | N |
| hsa-miR-4677-3p | FCRLB | 1 | 1 | 2 | N |
| hsa-miR-4677-3p | RHOV | 1 | 1 | 2 | N |
| hsa-miR-4677-3p | GOLGA8H | 1 | 1 | 2 | N |
| hsa-miR-4677-3p | ABHD14A | 1 | 1 | 2 | N |
| hsa-miR-6075 | FOXP1 | 1 | 1 | 2 | N |
| hsa-miR-6075 | CREB3L1 | 1 | 1 | 2 | N |
| hsa-miR-6075 | OVOL1 | 1 | 1 | 2 | N |
| hsa-miR-6075 | PRIMA1 | 1 | 1 | 2 | N |
| hsa-miR-6075 | KLHDC3 | 1 | 1 | 2 | N |
| hsa-miR-6075 | KIF2C | 1 | 1 | 2 | Y |
| hsa-miR-6781-5p | MPRIP | 1 | 1 | 2 | Y |
| hsa-miR-6781-5p | SCGB3A1 | 1 | 1 | 2 | N |
| hsa-miR-6781-5p | MIEF2 | 1 | 1 | 2 | N |
| hsa-miR-6781-5p | SBF1 | 1 | 1 | 2 | N |
| hsa-miR-6781-5p | ZMIZ1 | 1 | 1 | 2 | N |
| hsa-miR-6781-5p | GNG13 | 1 | 1 | 2 | N |
| hsa-miR-6781-5p | AKT1S1 | 1 | 1 | 2 | N |
| hsa-miR-6781-5p | KMT2D | 1 | 1 | 2 | N |
| hsa-miR-6781-5p | DLX1 | 1 | 1 | 2 | N |
| hsa-miR-6781-5p | SIX5 | 1 | 1 | 2 | N |
| hsa-miR-6781-5p | HIC1 | 1 | 1 | 2 | N |
| hsa-miR-8057 | FAF2 | 1 | 1 | 2 | N |
| hsa-miR-8057 | GCSAM | 1 | 1 | 2 | N |
| hsa-miR-8057 | SCYL1 | 1 | 1 | 2 | N |
| hsa-miR-8057 | C4orf19 | 1 | 1 | 2 | N |
| hsa-miR-8057 | E2F4 | 1 | 1 | 2 | N |
| hsa-miR-8057 | RAB33B | 1 | 1 | 2 | N |
| hsa-miR-8057 | HRK | 1 | 1 | 2 | N |
| hsa-miR-8057 | TCAIM | 1 | 1 | 2 | N |
| hsa-miR-8057 | CLASP1 | 1 | 1 | 2 | N |
| hsa-miR-8057 | SYCP1 | 1 | 1 | 2 | N |
| hsa-miR-8057 | TNIP2 | 1 | 1 | 2 | N |
| hsa-miR-8057 | RICTOR | 1 | 1 | 2 | N |
| hsa-miR-8057 | UBE2L6 | 1 | 1 | 2 | N |
| hsa-miR-8057 | ATF3 | 1 | 1 | 2 | N |
| hsa-miR-8057 | GALK2 | 1 | 1 | 2 | N |
| hsa-miR-8057 | CRYBG3 | 1 | 1 | 2 | N |
| hsa-miR-8057 | CDH10 | 1 | 1 | 2 | N |
| hsa-miR-8057 | ELOVL6 | 1 | 1 | 2 | N |
| hsa-miR-8057 | MTUS2 | 1 | 1 | 2 | N |
| hsa-miR-8057 | ARL13B | 1 | 1 | 2 | N |
| hsa-miR-8057 | ANP32B | 1 | 1 | 2 | Y |
| hsa-miR-8057 | SAV1 | 1 | 1 | 2 | N |
| hsa-miR-8057 | HIBADH | 1 | 1 | 2 | N |
| hsa-miR-8057 | KLK9 | 1 | 1 | 2 | N |
| hsa-miR-8057 | SLC2A5 | 1 | 1 | 2 | N |
| hsa-miR-8057 | RSPO3 | 1 | 1 | 2 | N |
| hsa-miR-8057 | ATXN1L | 1 | 1 | 2 | N |
| hsa-miR-8057 | SERPINB1 | 1 | 1 | 2 | N |
| hsa-miR-8057 | C10orf118 | 1 | 1 | 2 | N |
| hsa-miR-3907 | COL5A2 | 1 | 1 | 2 | N |
| hsa-miR-3907 | AIG1 | 1 | 1 | 2 | N |
| hsa-miR-3907 | UBE2Z | 1 | 1 | 2 | N |
| hsa-miR-3907 | LEP | 1 | 1 | 2 | N |
| hsa-miR-3907 | SLC2A3 | 1 | 1 | 2 | Y |
| hsa-miR-3907 | DUSP3 | 1 | 1 | 2 | N |
| hsa-miR-3907 | SMC6 | 1 | 1 | 2 | N |
| hsa-miR-3907 | ZNF526 | 1 | 1 | 2 | N |
| hsa-miR-3907 | HLF | 1 | 1 | 2 | N |
| hsa-miR-3907 | REEP1 | 1 | 1 | 2 | N |
| hsa-miR-3907 | TEAD1 | 1 | 1 | 2 | N |
| hsa-miR-3907 | SLC2A14 | 1 | 1 | 2 | N |
| hsa-miR-3907 | RNF2 | 1 | 1 | 2 | N |
| hsa-miR-3907 | ANXA9 | 1 | 1 | 2 | N |
| hsa-miR-3907 | ZFAND2A | 1 | 1 | 2 | N |
| hsa-miR-3907 | PURA | 1 | 1 | 2 | N |
| hsa-miR-3907 | RPUSD1 | 1 | 1 | 2 | N |
| hsa-miR-3907 | PCSK9 | 1 | 1 | 2 | N |
| hsa-miR-3907 | SLC1A3 | 1 | 1 | 2 | N |
| hsa-miR-3907 | ITGB3BP | 1 | 1 | 2 | N |
| hsa-miR-3907 | MORC4 | 1 | 1 | 2 | N |
| hsa-miR-3907 | E2F2 | 1 | 1 | 2 | N |
| hsa-miR-3907 | WDR44 | 1 | 1 | 2 | N |
| hsa-miR-3907 | COLGALT1 | 1 | 1 | 2 | N |
| hsa-miR-3907 | LGI3 | 1 | 1 | 2 | N |
| hsa-miR-3907 | LRCH1 | 1 | 1 | 2 | N |
| hsa-miR-3907 | C10orf118 | 1 | 1 | 2 | N |
| hsa-miR-3907 | PLEKHA6 | 1 | 1 | 2 | N |
| hsa-miR-3907 | TMEM213 | 1 | 1 | 2 | N |
| hsa-miR-3907 | SP100 | 1 | 1 | 2 | Y |
| hsa-miR-3907 | LENG8 | 1 | 1 | 2 | N |
| hsa-miR-3907 | CTDSP1 | 1 | 1 | 2 | N |
| hsa-miR-3907 | ZCCHC13 | 1 | 1 | 2 | N |
| hsa-miR-3907 | FKRP | 1 | 1 | 2 | N |
| hsa-miR-3907 | THRSP | 1 | 1 | 2 | N |
| hsa-miR-3907 | ENSA | 1 | 1 | 2 | N |
| hsa-miR-3907 | GCNT4 | 1 | 1 | 2 | N |
| hsa-miR-3907 | LYPD5 | 1 | 1 | 2 | N |
| hsa-miR-3907 | TBPL1 | 1 | 1 | 2 | Y |
| hsa-miR-3907 | ABLIM3 | 1 | 1 | 2 | N |
| hsa-miR-3913-3p | SLC35A5 | 1 | 1 | 2 | N |
| hsa-miR-3913-3p | EFCAB11 | 1 | 1 | 2 | N |
| hsa-miR-3913-3p | UPF3B | 1 | 1 | 2 | N |
| hsa-miR-3913-3p | AKIRIN2 | 1 | 1 | 2 | N |
| hsa-miR-3913-3p | NRIP1 | 1 | 1 | 2 | N |
| hsa-miR-3913-3p | ZNF12 | 1 | 1 | 2 | N |
| hsa-miR-3913-3p | RAB3C | 1 | 1 | 2 | N |
| hsa-miR-3913-3p | EMC7 | 1 | 1 | 2 | N |
| hsa-miR-3913-3p | PARM1 | 1 | 1 | 2 | N |
| hsa-miR-3913-3p | CMTM6 | 1 | 1 | 2 | Y |
| hsa-miR-3913-3p | SPTLC1 | 1 | 1 | 2 | N |
| hsa-miR-3913-3p | ANKRD49 | 1 | 1 | 2 | N |
| hsa-miR-3913-3p | ATP1B3 | 1 | 1 | 2 | N |
| hsa-miR-3913-3p | GINS4 | 1 | 1 | 2 | N |
| hsa-miR-3913-3p | SNX3 | 1 | 1 | 2 | N |
| hsa-miR-3913-3p | G3BP2 | 1 | 1 | 2 | N |
| hsa-miR-3913-3p | B4GALT3 | 1 | 1 | 2 | N |
| hsa-miR-3913-3p | VMA21 | 1 | 1 | 2 | N |
| hsa-miR-3913-3p | PPARGC1B | 1 | 1 | 2 | N |
| hsa-miR-3913-3p | PRRC2C | 1 | 1 | 2 | N |
| hsa-miR-3913-3p | UBE2I | 1 | 1 | 2 | N |
| hsa-miR-3913-3p | SHISA7 | 1 | 1 | 2 | N |
| hsa-miR-3913-3p | RIPK4 | 1 | 1 | 2 | N |
| hsa-miR-3913-3p | SKA2 | 1 | 1 | 2 | N |
| hsa-miR-3913-3p | NFIL3 | 1 | 1 | 2 | N |
| hsa-miR-3913-3p | CDK1 | 1 | 1 | 2 | N |
| hsa-miR-3913-3p | PNISR | 1 | 1 | 2 | N |
| hsa-miR-3913-3p | PPP1CB | 1 | 1 | 2 | N |
| hsa-miR-3913-3p | RAP1GDS1 | 1 | 1 | 2 | N |
| hsa-miR-3913-3p | MXI1 | 1 | 1 | 2 | N |
| hsa-miR-3913-3p | ANKRD28 | 1 | 1 | 2 | N |
| hsa-miR-3913-3p | FAM117B | 1 | 1 | 2 | Y |
| hsa-miR-3913-3p | VWA2 | 1 | 1 | 2 | N |
| hsa-miR-3913-3p | SRSF1 | 1 | 1 | 2 | N |
| hsa-miR-3913-3p | EMG1 | 1 | 1 | 2 | N |
| hsa-miR-3913-3p | ZNF790 | 1 | 1 | 2 | N |
| hsa-miR-3913-3p | KIAA1715 | 1 | 1 | 2 | N |
| hsa-miR-3913-3p | TMEM117 | 1 | 1 | 2 | N |
| hsa-miR-3913-3p | RGS18 | 1 | 1 | 2 | N |
| hsa-miR-3913-3p | RAN | 1 | 1 | 2 | N |
| hsa-miR-3913-3p | MRPL1 | 1 | 1 | 2 | N |
| hsa-miR-3913-3p | SUZ12 | 1 | 1 | 2 | N |
| hsa-miR-3974 | CYP26B1 | 1 | 1 | 2 | N |
| hsa-miR-3974 | DPH6 | 1 | 1 | 2 | N |
| hsa-miR-3974 | KLHL15 | 1 | 1 | 2 | N |
| hsa-miR-3974 | ADH6 | 1 | 1 | 2 | N |
| hsa-miR-3974 | NTF3 | 1 | 1 | 2 | N |
| hsa-miR-3974 | UBE2L3 | 1 | 1 | 2 | N |
| hsa-miR-3974 | LST1 | 1 | 1 | 2 | N |
| hsa-miR-3974 | CLDN1 | 1 | 1 | 2 | N |
| hsa-miR-3974 | WIF1 | 1 | 1 | 2 | N |
| hsa-miR-3974 | C4orf19 | 1 | 1 | 2 | N |
| hsa-miR-3974 | NCAM2 | 1 | 1 | 2 | N |
| hsa-miR-3974 | SMOC2 | 1 | 1 | 2 | N |
| hsa-miR-3974 | KLHL34 | 1 | 1 | 2 | N |
| hsa-miR-3974 | LIN52 | 1 | 1 | 2 | N |
| hsa-miR-3974 | PTBP1 | 1 | 1 | 2 | N |
| hsa-miR-3974 | NHLH2 | 1 | 1 | 2 | N |
| hsa-miR-3974 | LMAN2 | 1 | 1 | 2 | N |
| hsa-miR-3974 | SAMD13 | 1 | 1 | 2 | N |
| hsa-miR-3974 | CIAO1 | 1 | 1 | 2 | N |
| hsa-miR-3974 | IL13RA1 | 1 | 1 | 2 | N |
| hsa-miR-3974 | COPS4 | 1 | 1 | 2 | N |
| hsa-miR-3974 | USP1 | 1 | 1 | 2 | N |
| hsa-miR-3974 | HIBADH | 1 | 1 | 2 | N |
| hsa-miR-3974 | PTP4A1 | 1 | 1 | 2 | N |
| hsa-miR-3974 | TFRC | 1 | 1 | 2 | N |
| hsa-miR-3974 | ZMAT3 | 1 | 1 | 2 | N |
| hsa-miR-3974 | CWC27 | 1 | 1 | 2 | N |
| hsa-miR-3974 | C16orf72 | 1 | 1 | 2 | N |
| hsa-miR-3974 | BMP4 | 1 | 1 | 2 | N |
| hsa-miR-3974 | NDRG2 | 1 | 1 | 2 | N |
| hsa-miR-3974 | KCNE4 | 1 | 1 | 2 | N |
| hsa-miR-3974 | REPS2 | 1 | 1 | 2 | N |
| hsa-miR-3974 | SRSF1 | 1 | 1 | 2 | N |
| hsa-miR-3974 | RAB22A | 1 | 1 | 2 | N |
| hsa-miR-3974 | CBLL1 | 1 | 1 | 2 | N |
| hsa-miR-3974 | RND3 | 1 | 1 | 2 | N |
| hsa-miR-3974 | MBTPS2 | 1 | 1 | 2 | N |
| hsa-miR-3974 | MPV17 | 1 | 1 | 2 | N |
| hsa-miR-3974 | GPR68 | 1 | 1 | 2 | N |
| hsa-miR-3974 | POU3F2 | 1 | 1 | 2 | N |
| hsa-miR-3974 | PTEN | 1 | 1 | 2 | N |
| hsa-miR-4800-5p | INA | 1 | 1 | 2 | N |
| hsa-miR-4800-5p | ATP10B | 1 | 1 | 2 | N |
| hsa-miR-4800-5p | SECISBP2L | 1 | 1 | 2 | N |
| hsa-miR-4800-5p | WNT3 | 1 | 1 | 2 | N |
| hsa-miR-4800-5p | LPO | 1 | 1 | 2 | N |
| hsa-miR-4800-5p | PSMA8 | 1 | 1 | 2 | N |
| hsa-miR-4800-5p | PCDH19 | 1 | 1 | 2 | N |
| hsa-miR-4800-5p | SSB | 1 | 1 | 2 | N |
| hsa-miR-4800-5p | HTR2A | 1 | 1 | 2 | N |
| hsa-miR-4800-5p | ZNF732 | 1 | 1 | 2 | N |
| hsa-miR-4800-5p | FMR1 | 1 | 1 | 2 | N |
| hsa-miR-4800-5p | ARMC1 | 1 | 1 | 2 | N |
| hsa-miR-4800-5p | SMCO4 | 1 | 1 | 2 | N |
| hsa-miR-4800-5p | LDHAL6A | 1 | 1 | 2 | N |
| hsa-miR-4800-5p | GLRX3 | 1 | 1 | 2 | N |
| hsa-miR-4800-5p | MIS12 | 1 | 1 | 2 | N |
| hsa-miR-4800-5p | ZBTB20 | 1 | 1 | 2 | N |
| hsa-miR-1295b-5p | TGFBR2 | 1 | 1 | 2 | N |
| hsa-miR-1295b-5p | GSTCD | 1 | 1 | 2 | N |
| hsa-miR-1295b-5p | DFNA5 | 1 | 1 | 2 | N |
| hsa-miR-1295b-5p | RB1 | 1 | 1 | 2 | N |
| hsa-miR-1295b-5p | ZNF484 | 1 | 1 | 2 | N |
| hsa-miR-1295b-5p | AGFG1 | 1 | 1 | 2 | N |
| hsa-miR-1295b-5p | DDX17 | 1 | 1 | 2 | N |
| hsa-miR-1295b-5p | TFEC | 1 | 1 | 2 | N |
| hsa-miR-1295b-5p | NUS1 | 1 | 1 | 2 | N |
| hsa-miR-1295b-5p | PLA2G3 | 1 | 1 | 2 | N |
| hsa-miR-1295b-5p | SPCS2 | 1 | 1 | 2 | N |
| hsa-miR-1295b-5p | SIRPA | 1 | 1 | 2 | N |
| hsa-miR-1295b-5p | CLNS1A | 1 | 1 | 2 | N |
| hsa-miR-1295b-5p | DIXDC1 | 1 | 1 | 2 | N |
| hsa-miR-1295b-5p | SEPT6 | 1 | 1 | 2 | N |
| hsa-miR-1295b-5p | SLITRK2 | 1 | 1 | 2 | N |
| hsa-miR-1295b-5p | TMEM194A | 1 | 1 | 2 | N |
| hsa-miR-1295b-5p | ZNF621 | 1 | 1 | 2 | N |
| hsa-miR-1295b-5p | CMTR1 | 1 | 1 | 2 | N |
| hsa-miR-1295b-5p | PBX1 | 1 | 1 | 2 | N |
| hsa-miR-1295b-5p | UPF1 | 1 | 1 | 2 | N |
| hsa-miR-1295b-5p | KIAA0247 | 1 | 1 | 2 | N |
| hsa-miR-1295b-5p | FGF1 | 1 | 1 | 2 | Y |
| hsa-miR-1295b-5p | LYPD1 | 1 | 1 | 2 | N |
| hsa-miR-1295b-5p | AKIRIN1 | 1 | 1 | 2 | N |
| hsa-miR-1295b-5p | PCSK9 | 1 | 1 | 2 | Y |
| hsa-miR-1295b-5p | SLC46A2 | 1 | 1 | 2 | N |
| hsa-miR-1295b-5p | TMED2 | 1 | 1 | 2 | N |
| hsa-miR-1295b-5p | SCEL | 1 | 1 | 2 | N |
| hsa-miR-1295b-5p | PCDHB12 | 1 | 1 | 2 | N |
| hsa-miR-1295b-5p | TEX261 | 1 | 1 | 2 | N |
| hsa-miR-1295b-5p | JPH3 | 1 | 1 | 2 | N |
| hsa-miR-1295b-5p | SERAC1 | 1 | 1 | 2 | N |
| hsa-miR-1295b-5p | MRPL33 | 1 | 1 | 2 | N |
| hsa-miR-1295b-5p | FOXL1 | 1 | 1 | 2 | N |
| hsa-miR-1295b-5p | RBMS1 | 1 | 1 | 2 | N |
| hsa-miR-1295b-5p | TM9SF3 | 1 | 1 | 2 | N |
| hsa-miR-1295b-5p | TMEM64 | 1 | 1 | 2 | N |
| hsa-miR-1295b-5p | ATOH7 | 1 | 1 | 2 | N |
| hsa-miR-1295b-5p | TMEM9B | 1 | 1 | 2 | N |
| hsa-miR-1295b-5p | ORMDL3 | 1 | 1 | 2 | N |
| hsa-miR-1295b-5p | SH3BP5L | 1 | 1 | 2 | N |
| hsa-miR-1295b-5p | MTHFSD | 1 | 1 | 2 | N |
| hsa-miR-1295b-5p | NLGN3 | 1 | 1 | 2 | N |
| hsa-miR-1295b-5p | MKX | 1 | 1 | 2 | N |
| hsa-miR-1295b-5p | SMAP2 | 1 | 1 | 2 | N |
| hsa-miR-1295b-5p | DYM | 1 | 1 | 2 | N |
| hsa-miR-1295b-5p | TGFBR1 | 1 | 1 | 2 | N |
| hsa-miR-1295b-5p | WRNIP1 | 1 | 1 | 2 | N |
| hsa-miR-1295b-5p | ADCYAP1R1 | 1 | 1 | 2 | N |
| hsa-miR-1912 | ZNF484 | 1 | 1 | 2 | N |
| hsa-miR-1912 | NUS1 | 1 | 1 | 2 | N |
| hsa-miR-1912 | MTHFSD | 1 | 1 | 2 | N |
| hsa-miR-1912 | FGF1 | 1 | 1 | 2 | Y |
| hsa-miR-1912 | SCEL | 1 | 1 | 2 | N |
| hsa-miR-1912 | RNF32 | 1 | 1 | 2 | N |
| hsa-miR-1912 | MRPL33 | 1 | 1 | 2 | N |
| hsa-miR-1912 | SH3BP5L | 1 | 1 | 2 | N |
| hsa-miR-1912 | ORMDL3 | 1 | 1 | 2 | N |
| hsa-miR-1912 | GSTCD | 1 | 1 | 2 | N |
| hsa-miR-1912 | CLNS1A | 1 | 1 | 2 | N |
| hsa-miR-1912 | SLC46A2 | 1 | 1 | 2 | N |
| hsa-miR-1912 | DDX17 | 1 | 1 | 2 | N |
| hsa-miR-1912 | RB1 | 1 | 1 | 2 | N |
| hsa-miR-1912 | RBMS1 | 1 | 1 | 2 | N |
| hsa-miR-1912 | TMEM64 | 1 | 1 | 2 | N |
| hsa-miR-1912 | MKX | 1 | 1 | 2 | N |
| hsa-miR-1912 | SMAP2 | 1 | 1 | 2 | N |
| hsa-miR-1912 | TM9SF3 | 1 | 1 | 2 | N |
| hsa-miR-1912 | SLITRK2 | 1 | 1 | 2 | N |
| hsa-miR-1912 | KIAA0247 | 1 | 1 | 2 | N |
| hsa-miR-1912 | PLA2G3 | 1 | 1 | 2 | N |
| hsa-miR-1912 | CMTR1 | 1 | 1 | 2 | N |
| hsa-miR-1912 | KRT85 | 1 | 1 | 2 | N |
| hsa-miR-1912 | DIXDC1 | 1 | 1 | 2 | N |
| hsa-miR-1912 | TEX261 | 1 | 1 | 2 | N |
| hsa-miR-1912 | ZNF621 | 1 | 1 | 2 | N |
| hsa-miR-1912 | TMEM194A | 1 | 1 | 2 | N |
| hsa-miR-1912 | PBX1 | 1 | 1 | 2 | N |
| hsa-miR-1912 | FOXL1 | 1 | 1 | 2 | N |
| hsa-miR-1912 | NLGN3 | 1 | 1 | 2 | N |
| hsa-miR-1912 | DFNA5 | 1 | 1 | 2 | N |
| hsa-miR-1912 | SEPT6 | 1 | 1 | 2 | N |
| hsa-miR-1912 | TMEM9B | 1 | 1 | 2 | N |
| hsa-miR-1912 | SPCS2 | 1 | 1 | 2 | N |
| hsa-miR-1912 | JPH3 | 1 | 1 | 2 | N |
| hsa-miR-1912 | PCSK9 | 1 | 1 | 2 | Y |
| hsa-miR-1912 | LYPD1 | 1 | 1 | 2 | N |
| hsa-miR-1912 | GABARAPL2 | 1 | 1 | 2 | N |
| hsa-miR-1912 | WRNIP1 | 1 | 1 | 2 | N |
| hsa-miR-1912 | TGFBR2 | 1 | 1 | 2 | N |
| hsa-miR-1912 | ATOH7 | 1 | 1 | 2 | N |
| hsa-miR-1912 | PCDHB12 | 1 | 1 | 2 | N |
| hsa-miR-1912 | DYM | 1 | 1 | 2 | N |
| hsa-miR-1912 | ADCYAP1R1 | 1 | 1 | 2 | N |
| hsa-miR-1912 | TFEC | 1 | 1 | 2 | N |
| hsa-miR-1912 | TGFBR1 | 1 | 1 | 2 | N |
| hsa-miR-1912 | SIRPA | 1 | 1 | 2 | N |
| hsa-miR-1912 | AKIRIN1 | 1 | 1 | 2 | N |
| hsa-miR-1912 | TMED2 | 1 | 1 | 2 | N |
| hsa-miR-1912 | AGFG1 | 1 | 1 | 2 | N |
| hsa-miR-1912 | SERAC1 | 1 | 1 | 2 | N |
| hsa-miR-1912 | UPF1 | 1 | 1 | 2 | N |
| hsa-miR-1914-5p | KIAA1644 | 1 | 1 | 2 | N |
| hsa-miR-1914-5p | ZADH2 | 1 | 1 | 2 | N |
| hsa-miR-1914-5p | MYO16 | 1 | 1 | 2 | N |
| hsa-miR-1914-5p | CNGA3 | 1 | 1 | 2 | N |
| hsa-miR-1914-5p | ZBTB10 | 1 | 1 | 2 | N |
| hsa-miR-1914-5p | ATP2C1 | 1 | 1 | 2 | N |
| hsa-miR-1914-5p | GSK3B | 1 | 1 | 2 | N |
| hsa-miR-1914-5p | ZNF43 | 1 | 1 | 2 | N |
| hsa-miR-1914-5p | PIPOX | 1 | 1 | 2 | N |
| hsa-miR-1914-5p | TOR1AIP2 | 1 | 1 | 2 | N |
| hsa-miR-1914-5p | SLC33A1 | 1 | 1 | 2 | N |
| hsa-miR-1914-5p | KCNC4 | 1 | 1 | 2 | N |
| hsa-miR-1914-5p | KCNQ4 | 1 | 1 | 2 | N |
| hsa-miR-1914-5p | TGM2 | 1 | 1 | 2 | N |
| hsa-miR-1914-5p | AGGF1 | 1 | 1 | 2 | N |
| hsa-miR-1914-5p | TSPY4 | 1 | 1 | 2 | N |
| hsa-miR-1914-5p | GPR39 | 1 | 1 | 2 | N |
| hsa-miR-1914-5p | EXOC5 | 1 | 1 | 2 | N |
| hsa-miR-1914-5p | ST18 | 1 | 1 | 2 | N |
| hsa-miR-1914-5p | MEA1 | 1 | 1 | 2 | N |
| hsa-miR-1914-5p | DESI2 | 1 | 1 | 2 | N |
| hsa-miR-1914-5p | RAP2A | 1 | 1 | 2 | N |
| hsa-miR-1914-5p | ZNF98 | 1 | 1 | 2 | N |
| hsa-miR-1914-5p | KLHL29 | 1 | 1 | 2 | N |
| hsa-miR-1914-5p | STK10 | 1 | 1 | 2 | N |
| hsa-miR-1914-5p | TSPY3 | 1 | 1 | 2 | N |
| hsa-miR-1914-5p | SFT2D2 | 1 | 1 | 2 | N |
| hsa-miR-1914-5p | RGS20 | 1 | 1 | 2 | N |
| hsa-miR-1914-5p | EMX2 | 1 | 1 | 2 | N |
| hsa-miR-1914-5p | ZNF430 | 1 | 1 | 2 | N |
| hsa-miR-1914-5p | ZNF681 | 1 | 1 | 2 | N |
| hsa-miR-1914-5p | TSPY8 | 1 | 1 | 2 | N |
| hsa-miR-1914-5p | CBX7 | 1 | 1 | 2 | N |
| hsa-miR-1914-5p | TSPY1 | 1 | 1 | 2 | N |
| hsa-miR-1914-5p | ZNF675 | 1 | 1 | 2 | N |
| hsa-miR-1914-5p | CD96 | 1 | 1 | 2 | N |
| hsa-miR-1914-5p | KLHDC8A | 1 | 1 | 2 | N |
| hsa-miR-1914-5p | MBTD1 | 1 | 1 | 2 | N |
| hsa-miR-1914-5p | SPIN1 | 1 | 1 | 2 | N |
| hsa-miR-1914-5p | SKIDA1 | 1 | 1 | 2 | N |
| hsa-miR-1914-5p | FOXP2 | 1 | 1 | 2 | N |
| hsa-miR-1914-5p | FAM169A | 1 | 1 | 2 | N |
| hsa-miR-1914-5p | ZNF492 | 1 | 1 | 2 | N |
| hsa-miR-1914-5p | SENP8 | 1 | 1 | 2 | N |
| hsa-miR-1914-5p | MYCT1 | 1 | 1 | 2 | N |
| hsa-miR-1914-5p | MAP3K11 | 1 | 1 | 2 | N |
| hsa-miR-1914-5p | UBE2Z | 1 | 1 | 2 | N |
| hsa-miR-1914-5p | ZNF92 | 1 | 1 | 2 | N |
| hsa-miR-1914-5p | PODXL | 1 | 1 | 2 | N |
| hsa-miR-1914-5p | ZNF716 | 1 | 1 | 2 | N |
| hsa-miR-1914-5p | ZNF112 | 1 | 1 | 2 | N |
| hsa-miR-1914-5p | SET | 1 | 1 | 2 | N |
| hsa-miR-1914-5p | DDX19B | 1 | 1 | 2 | N |
| hsa-miR-1914-5p | SPSB1 | 1 | 1 | 2 | N |
| hsa-miR-1914-5p | TSPY2 | 1 | 1 | 2 | N |
| hsa-miR-1914-5p | ZNF254 | 1 | 1 | 2 | N |
| hsa-miR-1914-5p | EXT2 | 1 | 1 | 2 | N |
| hsa-miR-2467-3p | NR5A1 | 1 | 1 | 2 | N |
| hsa-miR-2467-3p | SC5D | 1 | 1 | 2 | N |
| hsa-miR-2467-3p | KLHL21 | 1 | 1 | 2 | N |
| hsa-miR-2467-3p | ARL17A | 1 | 1 | 2 | N |
| hsa-miR-2467-3p | KCNJ10 | 1 | 1 | 2 | N |
| hsa-miR-2467-3p | AGO1 | 1 | 1 | 2 | Y |
| hsa-miR-2467-3p | CECR6 | 1 | 1 | 2 | N |
| hsa-miR-2467-3p | GCSAM | 1 | 1 | 2 | N |
| hsa-miR-2467-3p | FXYD5 | 1 | 1 | 2 | N |
| hsa-miR-2467-3p | TEAD2 | 1 | 1 | 2 | N |
| hsa-miR-2467-3p | LIMD2 | 1 | 1 | 2 | N |
| hsa-miR-2467-3p | FABP3 | 1 | 1 | 2 | Y |
| hsa-miR-2467-3p | UBL4A | 1 | 1 | 2 | N |
| hsa-miR-2467-3p | LHPP | 1 | 1 | 2 | N |
| hsa-miR-2467-3p | MPDU1 | 1 | 1 | 2 | Y |
| hsa-miR-2467-3p | WDR4 | 1 | 1 | 2 | N |
| hsa-miR-2467-3p | FAM134C | 1 | 1 | 2 | N |
| hsa-miR-2467-3p | KIAA1644 | 1 | 1 | 2 | N |
| hsa-miR-2467-3p | MOB3C | 1 | 1 | 2 | N |
| hsa-miR-2467-3p | WASF2 | 1 | 1 | 2 | N |
| hsa-miR-2467-3p | NCS1 | 1 | 1 | 2 | N |
| hsa-miR-2467-3p | SSX2B | 1 | 1 | 2 | N |
| hsa-miR-2467-3p | FOXP2 | 1 | 1 | 2 | Y |
| hsa-miR-2467-3p | GPATCH8 | 1 | 1 | 2 | N |
| hsa-miR-2467-3p | KPNA6 | 1 | 1 | 2 | N |
| hsa-miR-2467-3p | ZNF793 | 1 | 1 | 2 | N |
| hsa-miR-2467-3p | ZFYVE28 | 1 | 1 | 2 | N |
| hsa-miR-2467-3p | EYA1 | 1 | 1 | 2 | N |
| hsa-miR-2467-3p | EPS15L1 | 1 | 1 | 2 | Y |
| hsa-miR-2467-3p | FAM132B | 1 | 1 | 2 | N |
| hsa-miR-2467-3p | TMEM110 | 1 | 1 | 2 | N |
| hsa-miR-2467-3p | GSPT2 | 1 | 1 | 2 | N |
| hsa-miR-2467-3p | PFDN1 | 1 | 1 | 2 | N |
| hsa-miR-2467-3p | SRXN1 | 1 | 1 | 2 | N |
| hsa-miR-2467-3p | BAP1 | 1 | 1 | 2 | N |
| hsa-miR-2467-3p | DMTN | 1 | 1 | 2 | N |
| hsa-miR-2467-3p | NRBP1 | 1 | 1 | 2 | Y |
| hsa-miR-2467-3p | SDHD | 1 | 1 | 2 | N |
| hsa-miR-2467-3p | MOB3B | 1 | 1 | 2 | N |
| hsa-miR-2467-3p | TRIM29 | 1 | 1 | 2 | N |
| hsa-miR-2467-3p | HRH3 | 1 | 1 | 2 | N |
| hsa-miR-2467-3p | NFAM1 | 1 | 1 | 2 | N |
| hsa-miR-2467-3p | H3F3B | 1 | 1 | 2 | N |
| hsa-miR-2467-3p | TTC4 | 1 | 1 | 2 | N |
| hsa-miR-2467-3p | TPCN1 | 1 | 1 | 2 | N |
| hsa-miR-2467-3p | CYB561 | 1 | 1 | 2 | N |
| hsa-miR-2467-3p | EFHD1 | 1 | 1 | 2 | N |
| hsa-miR-2467-3p | ICK | 1 | 1 | 2 | N |
| hsa-miR-2467-3p | FRS3 | 1 | 1 | 2 | N |
| hsa-miR-2467-3p | ADAM19 | 1 | 1 | 2 | N |
| hsa-miR-2467-3p | ARL3 | 1 | 1 | 2 | N |
| hsa-miR-2467-3p | IL1RN | 1 | 1 | 2 | N |
| hsa-miR-2467-3p | SSX4B | 1 | 1 | 2 | N |
| hsa-miR-2467-3p | FAM102A | 1 | 1 | 2 | Y |
| hsa-miR-2467-3p | CYP1A1 | 1 | 1 | 2 | N |
| hsa-miR-2467-3p | SSX4 | 1 | 1 | 2 | N |
| hsa-miR-2467-3p | GBF1 | 1 | 1 | 2 | N |
| hsa-miR-2467-3p | KRTAP3-1 | 1 | 1 | 2 | N |
| hsa-miR-2467-3p | PHF19 | 1 | 1 | 2 | N |
| hsa-miR-2467-3p | VOPP1 | 1 | 1 | 2 | N |
| hsa-miR-2467-3p | SLC2A4 | 1 | 1 | 2 | N |
| hsa-miR-2467-3p | IGDCC4 | 1 | 1 | 2 | Y |
| hsa-miR-2467-3p | HEY2 | 1 | 1 | 2 | N |
| hsa-miR-2467-3p | SSX1 | 1 | 1 | 2 | N |
| hsa-miR-2467-3p | TUFT1 | 1 | 1 | 2 | N |
| hsa-miR-2467-3p | SGSM1 | 1 | 1 | 2 | N |
| hsa-miR-2467-3p | OPCML | 1 | 1 | 2 | N |
| hsa-miR-2467-3p | HIF1AN | 1 | 1 | 2 | N |
| hsa-miR-2467-3p | ATXN1L | 1 | 1 | 2 | Y |
| hsa-miR-2467-3p | ONECUT2 | 1 | 1 | 2 | N |
| hsa-miR-2467-3p | MORF4L2 | 1 | 1 | 2 | Y |
| hsa-miR-2467-3p | CX3CL1 | 1 | 1 | 2 | N |
| hsa-miR-2467-3p | SLC41A1 | 1 | 1 | 2 | N |
| hsa-miR-2467-3p | WDR19 | 1 | 1 | 2 | N |
| hsa-miR-2467-3p | ASCL1 | 1 | 1 | 2 | N |
| hsa-miR-2467-3p | KLF12 | 1 | 1 | 2 | N |
| hsa-miR-2467-3p | SEC22C | 1 | 1 | 2 | N |
| hsa-miR-2467-3p | HIF3A | 1 | 1 | 2 | N |
| hsa-miR-2467-3p | CHAD | 1 | 1 | 2 | N |
| hsa-miR-2467-3p | AR | 1 | 1 | 2 | Y |
| hsa-miR-2467-3p | TNNI1 | 1 | 1 | 2 | N |
| hsa-miR-2467-3p | KLC4 | 1 | 1 | 2 | N |
| hsa-miR-2467-3p | SSX2 | 1 | 1 | 2 | N |
| hsa-miR-2467-3p | DCAF7 | 1 | 1 | 2 | N |
| hsa-miR-2467-3p | CDK14 | 1 | 1 | 2 | N |
| hsa-miR-2467-3p | SLC25A42 | 1 | 1 | 2 | N |
| hsa-miR-2467-3p | TMEM127 | 1 | 1 | 2 | Y |
| hsa-miR-2467-3p | FKBP1A | 1 | 1 | 2 | N |
| hsa-miR-2467-3p | FOXP4 | 1 | 1 | 2 | N |
| hsa-miR-2467-3p | NR1H2 | 1 | 1 | 2 | N |
| hsa-miR-2467-3p | RAB11B | 1 | 1 | 2 | N |
| hsa-miR-2467-3p | MAP7D1 | 1 | 1 | 2 | N |
| hsa-miR-2467-3p | PRG2 | 1 | 1 | 2 | N |
| hsa-miR-2467-3p | DAD1 | 1 | 1 | 2 | N |
| hsa-miR-2467-3p | TMEM198 | 1 | 1 | 2 | N |
| hsa-miR-2467-3p | NEK9 | 1 | 1 | 2 | N |
| hsa-miR-2467-3p | BRSK2 | 1 | 1 | 2 | N |
| hsa-miR-2467-3p | GABRA1 | 1 | 1 | 2 | N |
| hsa-miR-2467-3p | TEF | 1 | 1 | 2 | N |
| hsa-miR-2467-3p | KCNQ4 | 1 | 1 | 2 | N |
| hsa-miR-2467-3p | PLEKHA6 | 1 | 1 | 2 | N |
| hsa-miR-2467-3p | SENP8 | 1 | 1 | 2 | N |
| hsa-miR-2467-3p | C20orf27 | 1 | 1 | 2 | Y |
| hsa-miR-2467-3p | KIAA0513 | 1 | 1 | 2 | N |
| hsa-miR-2467-3p | AGPAT4 | 1 | 1 | 2 | N |
| hsa-miR-2467-3p | SSX3 | 1 | 1 | 2 | N |
| hsa-miR-2467-3p | RASD2 | 1 | 1 | 2 | N |
| hsa-miR-2467-3p | NFATC4 | 1 | 1 | 2 | N |
| hsa-miR-2467-3p | CTSD | 1 | 1 | 2 | N |
| hsa-miR-2467-3p | PVRL3 | 1 | 1 | 2 | N |
| hsa-miR-2467-3p | ST3GAL1 | 1 | 1 | 2 | N |
| hsa-miR-2467-3p | SNX27 | 1 | 1 | 2 | Y |
| hsa-miR-2467-3p | CCDC97 | 1 | 1 | 2 | N |
| hsa-miR-2467-3p | C12orf5 | 1 | 1 | 2 | N |
| hsa-miR-2467-3p | ARL8B | 1 | 1 | 2 | N |
| hsa-miR-2467-3p | FAM189A2 | 1 | 1 | 2 | N |
| hsa-miR-2467-3p | TWIST1 | 1 | 1 | 2 | Y |
| hsa-miR-2467-3p | C18orf25 | 1 | 1 | 2 | N |
| hsa-miR-2467-3p | PGAM5 | 1 | 1 | 2 | Y |
| hsa-miR-2467-3p | HSD11B2 | 1 | 1 | 2 | N |
| hsa-miR-2467-3p | ATXN7L3 | 1 | 1 | 2 | N |
| hsa-miR-2467-3p | ARHGEF4 | 1 | 1 | 2 | N |
| hsa-miR-2467-3p | NRSN2 | 1 | 1 | 2 | N |
| hsa-miR-2467-3p | AP1S1 | 1 | 1 | 2 | N |
| hsa-miR-2467-3p | BTRC | 1 | 1 | 2 | N |
| hsa-miR-2467-3p | GPR137 | 1 | 1 | 2 | N |
| hsa-miR-2467-3p | NPLOC4 | 1 | 1 | 2 | N |
| hsa-miR-2467-3p | TCF7L1 | 1 | 1 | 2 | N |
| hsa-miR-2467-3p | DPF1 | 1 | 1 | 2 | N |
| hsa-miR-2467-3p | KSR1 | 1 | 1 | 2 | N |
| hsa-miR-2467-3p | CYTH4 | 1 | 1 | 2 | N |
| hsa-miR-2467-3p | KY | 1 | 1 | 2 | N |
| hsa-miR-2467-3p | ZMAT3 | 1 | 1 | 2 | N |
| hsa-miR-2467-3p | N4BP1 | 1 | 1 | 2 | N |
| hsa-miR-2467-3p | RHOXF1 | 1 | 1 | 2 | N |
| hsa-miR-2467-3p | CD209 | 1 | 1 | 2 | N |
| hsa-miR-2467-3p | KCNAB2 | 1 | 1 | 2 | N |
| hsa-miR-3922-5p | TERF2IP | 1 | 1 | 2 | N |
| hsa-miR-3922-5p | RNF214 | 1 | 1 | 2 | N |
| hsa-miR-3922-5p | KLHL34 | 1 | 1 | 2 | N |
| hsa-miR-3922-5p | MARCH8 | 1 | 1 | 2 | N |
| hsa-miR-3922-5p | TAB2 | 1 | 1 | 2 | N |
| hsa-miR-3922-5p | MAPK13 | 1 | 1 | 2 | N |
| hsa-miR-3922-5p | KCTD15 | 1 | 1 | 2 | N |
| hsa-miR-3922-5p | SLC45A1 | 1 | 1 | 2 | N |
| hsa-miR-3922-5p | RNF13 | 1 | 1 | 2 | N |
| hsa-miR-3922-5p | TBX22 | 1 | 1 | 2 | N |
| hsa-miR-3922-5p | C10orf105 | 1 | 1 | 2 | N |
| hsa-miR-3922-5p | STX2 | 1 | 1 | 2 | N |
| hsa-miR-3922-5p | ZNF195 | 1 | 1 | 2 | N |
| hsa-miR-3922-5p | PIK3R6 | 1 | 1 | 2 | N |
| hsa-miR-3922-5p | RXRB | 1 | 1 | 2 | N |
| hsa-miR-3922-5p | PRKAR2B | 1 | 1 | 2 | N |
| hsa-miR-3922-5p | TNPO1 | 1 | 1 | 2 | N |
| hsa-miR-3922-5p | FKBP3 | 1 | 1 | 2 | N |
| hsa-miR-3922-5p | ERG | 1 | 1 | 2 | N |
| hsa-miR-3922-5p | B3GNT1 | 1 | 1 | 2 | N |
| hsa-miR-3922-5p | BARX2 | 1 | 1 | 2 | N |
| hsa-miR-3922-5p | GATC | 1 | 1 | 2 | N |
| hsa-miR-3922-5p | TP53 | 1 | 1 | 2 | N |
| hsa-miR-3922-5p | NIP7 | 1 | 1 | 2 | N |
| hsa-miR-3922-5p | PPM1D | 1 | 1 | 2 | N |
| hsa-miR-3922-5p | ZFP3 | 1 | 1 | 2 | N |
| hsa-miR-3922-5p | NAP1L1 | 1 | 1 | 2 | N |
| hsa-miR-3922-5p | GNA12 | 1 | 1 | 2 | N |
| hsa-miR-3922-5p | SHC1 | 1 | 1 | 2 | N |
| hsa-miR-3922-5p | ARSJ | 1 | 1 | 2 | N |
| hsa-miR-3922-5p | TES | 1 | 1 | 2 | N |
| hsa-miR-3922-5p | ZFX | 1 | 1 | 2 | N |
| hsa-miR-3922-5p | CYP4F11 | 1 | 1 | 2 | N |
| hsa-miR-3922-5p | SSPN | 1 | 1 | 2 | N |
| hsa-miR-3922-5p | RIC3 | 1 | 1 | 2 | N |
| hsa-miR-3922-5p | ABHD4 | 1 | 1 | 2 | N |
| hsa-miR-3922-5p | TLCD2 | 1 | 1 | 2 | N |
| hsa-miR-3922-5p | ESYT2 | 1 | 1 | 2 | N |
| hsa-miR-3922-5p | MSRB3 | 1 | 1 | 2 | N |
| hsa-miR-3922-5p | OTOA | 1 | 1 | 2 | N |
| hsa-miR-3922-5p | SCN4B | 1 | 1 | 2 | N |
| hsa-miR-3922-5p | BUB1B | 1 | 1 | 2 | N |
| hsa-miR-3922-5p | ABL1 | 1 | 1 | 2 | N |
| hsa-miR-3922-5p | NAGS | 1 | 1 | 2 | N |
| hsa-miR-3922-5p | FLI1 | 1 | 1 | 2 | N |
| hsa-miR-3922-5p | IL3 | 1 | 1 | 2 | N |
| hsa-miR-3922-5p | CD47 | 1 | 1 | 2 | N |
| hsa-miR-3922-5p | SLC5A9 | 1 | 1 | 2 | N |
| hsa-miR-3922-5p | PIK3IP1 | 1 | 1 | 2 | N |
| hsa-miR-3922-5p | INTS2 | 1 | 1 | 2 | Y |
| hsa-miR-3922-5p | CHRNA1 | 1 | 1 | 2 | N |
| hsa-miR-4462 | KIAA1644 | 1 | 1 | 2 | N |
| hsa-miR-4462 | DNAJC5B | 1 | 1 | 2 | N |
| hsa-miR-4462 | DTX1 | 1 | 1 | 2 | N |
| hsa-miR-4462 | ZNF597 | 1 | 1 | 2 | N |
| hsa-miR-4462 | AIDA | 1 | 1 | 2 | N |
| hsa-miR-4462 | ATP2A1 | 1 | 1 | 2 | N |
| hsa-miR-4462 | HABP4 | 1 | 1 | 2 | N |
| hsa-miR-4462 | GAREML | 1 | 1 | 2 | N |
| hsa-miR-4529-5p | PARS2 | 1 | 1 | 2 | N |
| hsa-miR-4529-5p | PLIN1 | 1 | 1 | 2 | N |
| hsa-miR-4529-5p | CNNM3 | 1 | 1 | 2 | N |
| hsa-miR-4529-5p | LONRF1 | 1 | 1 | 2 | N |
| hsa-miR-4529-5p | DIO1 | 1 | 1 | 2 | N |
| hsa-miR-4529-5p | BRMS1L | 1 | 1 | 2 | N |
| hsa-miR-4529-5p | CTBP2 | 1 | 1 | 2 | N |
| hsa-miR-4529-5p | H3F3B | 1 | 1 | 2 | N |
| hsa-miR-4529-5p | FIGNL1 | 1 | 1 | 2 | Y |
| hsa-miR-4529-5p | FAM208A | 1 | 1 | 2 | N |
| hsa-miR-4529-5p | PIP4K2C | 1 | 1 | 2 | N |
| hsa-miR-4529-5p | LDB3 | 1 | 1 | 2 | N |
| hsa-miR-4529-5p | AQP2 | 1 | 1 | 2 | N |
| hsa-miR-4529-5p | ADAM12 | 1 | 1 | 2 | N |
| hsa-miR-4529-5p | UNC5D | 1 | 1 | 2 | N |
| hsa-miR-4529-5p | RND1 | 1 | 1 | 2 | N |
| hsa-miR-4529-5p | MEX3C | 1 | 1 | 2 | N |
| hsa-miR-4529-5p | LMNB1 | 1 | 1 | 2 | Y |
| hsa-miR-4529-5p | SHISA9 | 1 | 1 | 2 | Y |
| hsa-miR-4529-5p | ALG10 | 1 | 1 | 2 | N |
| hsa-miR-4529-5p | PER2 | 1 | 1 | 2 | N |
| hsa-miR-4529-5p | CSDE1 | 1 | 1 | 2 | N |
| hsa-miR-4529-5p | EIF4G1 | 1 | 1 | 2 | N |
| hsa-miR-4529-5p | HSPA12A | 1 | 1 | 2 | N |
| hsa-miR-4529-5p | DNAJC14 | 1 | 1 | 2 | N |
| hsa-miR-4529-5p | FNBP4 | 1 | 1 | 2 | N |
| hsa-miR-4529-5p | POLR1E | 1 | 1 | 2 | N |
| hsa-miR-4529-5p | TRIM44 | 1 | 1 | 2 | N |
| hsa-miR-4529-5p | HEATR5B | 1 | 1 | 2 | N |
| hsa-miR-4529-5p | PSTPIP2 | 1 | 1 | 2 | N |
| hsa-miR-4529-5p | UNC119B | 1 | 1 | 2 | N |
| hsa-miR-4685-5p | HEMK1 | 1 | 1 | 2 | N |
| hsa-miR-4685-5p | ZDHHC5 | 1 | 1 | 2 | Y |
| hsa-miR-4685-5p | KLHDC8A | 1 | 1 | 2 | N |
| hsa-miR-4685-5p | HMGCL | 1 | 1 | 2 | N |
| hsa-miR-4685-5p | RAPGEF5 | 1 | 1 | 2 | N |
| hsa-miR-4685-5p | KLHL21 | 1 | 1 | 2 | Y |
| hsa-miR-4685-5p | SPNS2 | 1 | 1 | 2 | Y |
| hsa-miR-4685-5p | KIAA0247 | 1 | 1 | 2 | N |
| hsa-miR-4685-5p | NFAM1 | 1 | 1 | 2 | N |
| hsa-miR-4685-5p | USP42 | 1 | 1 | 2 | N |
| hsa-miR-4685-5p | CSF1 | 1 | 1 | 2 | N |
| hsa-miR-4685-5p | LRRC20 | 1 | 1 | 2 | N |
| hsa-miR-4685-5p | CALCOCO1 | 1 | 1 | 2 | N |
| hsa-miR-4685-5p | RXRB | 1 | 1 | 2 | N |
| hsa-miR-4685-5p | PITPNM3 | 1 | 1 | 2 | N |
| hsa-miR-4685-5p | FLRT1 | 1 | 1 | 2 | N |
| hsa-miR-4685-5p | CA7 | 1 | 1 | 2 | N |
| hsa-miR-4685-5p | RXRG | 1 | 1 | 2 | N |
| hsa-miR-4685-5p | UNC13D | 1 | 1 | 2 | N |
| hsa-miR-4685-5p | FOXN4 | 1 | 1 | 2 | N |
| hsa-miR-4685-5p | EAF1 | 1 | 1 | 2 | N |
| hsa-miR-4685-5p | GRIK3 | 1 | 1 | 2 | N |
| hsa-miR-4685-5p | SUSD5 | 1 | 1 | 2 | N |
| hsa-miR-4685-5p | TACC1 | 1 | 1 | 2 | N |
| hsa-miR-4685-5p | SLC23A3 | 1 | 1 | 2 | N |
| hsa-miR-4685-5p | DGKQ | 1 | 1 | 2 | N |
| hsa-miR-4685-5p | RPL13A | 1 | 1 | 2 | Y |
| hsa-miR-4685-5p | B3GNT7 | 1 | 1 | 2 | N |
| hsa-miR-4685-5p | NHLRC3 | 1 | 1 | 2 | N |
| hsa-miR-4685-5p | CYTH1 | 1 | 1 | 2 | N |
| hsa-miR-4685-5p | CCDC114 | 1 | 1 | 2 | N |
| hsa-miR-4685-5p | PSMA3 | 1 | 1 | 2 | N |
| hsa-miR-4685-5p | NPLOC4 | 1 | 1 | 2 | N |
| hsa-miR-4685-5p | MOB3A | 1 | 1 | 2 | N |
| hsa-miR-4685-5p | ADAMTS10 | 1 | 1 | 2 | N |
| hsa-miR-4685-5p | PLEKHA6 | 1 | 1 | 2 | N |
| hsa-miR-4685-5p | DEXI | 1 | 1 | 2 | N |
| hsa-miR-4685-5p | THPO | 1 | 1 | 2 | N |
| hsa-miR-4685-5p | BTG2 | 1 | 1 | 2 | Y |
| hsa-miR-4685-5p | MDGA1 | 1 | 1 | 2 | N |
| hsa-miR-4685-5p | RAB38 | 1 | 1 | 2 | N |
| hsa-miR-4685-5p | ZNF512B | 1 | 1 | 2 | N |
| hsa-miR-4685-5p | MS4A7 | 1 | 1 | 2 | N |
| hsa-miR-4685-5p | BTBD9 | 1 | 1 | 2 | N |
| hsa-miR-4685-5p | ARMC7 | 1 | 1 | 2 | N |
| hsa-miR-4685-5p | ACTR1A | 1 | 1 | 2 | N |
| hsa-miR-4685-5p | ORAI2 | 1 | 1 | 2 | N |
| hsa-miR-4685-5p | ZNF609 | 1 | 1 | 2 | N |
| hsa-miR-4685-5p | HAPLN4 | 1 | 1 | 2 | N |
| hsa-miR-4685-5p | C17orf103 | 1 | 1 | 2 | N |
| hsa-miR-4685-5p | GAS7 | 1 | 1 | 2 | N |
| hsa-miR-4685-5p | KRI1 | 1 | 1 | 2 | N |
| hsa-miR-4685-5p | PDAP1 | 1 | 1 | 2 | N |
| hsa-miR-4685-5p | CLIC5 | 1 | 1 | 2 | N |
| hsa-miR-4685-5p | KPNA6 | 1 | 1 | 2 | N |
| hsa-miR-4685-5p | TBC1D22A | 1 | 1 | 2 | N |
| hsa-miR-4685-5p | PTPN12 | 1 | 1 | 2 | N |
| hsa-miR-4685-5p | NAA60 | 1 | 1 | 2 | N |
| hsa-miR-4685-5p | CHGA | 1 | 1 | 2 | N |
| hsa-miR-4685-5p | STIM1 | 1 | 1 | 2 | N |
| hsa-miR-4685-5p | CYB561D1 | 1 | 1 | 2 | N |
| hsa-miR-4685-5p | OPRL1 | 1 | 1 | 2 | N |
| hsa-miR-4685-5p | CISH | 1 | 1 | 2 | N |
| hsa-miR-4685-5p | SLC25A1 | 1 | 1 | 2 | N |
| hsa-miR-4685-5p | BBS4 | 1 | 1 | 2 | N |
| hsa-miR-4685-5p | GPR173 | 1 | 1 | 2 | N |
| hsa-miR-4685-5p | RAD9A | 1 | 1 | 2 | N |
| hsa-miR-4685-5p | MEGF11 | 1 | 1 | 2 | N |
| hsa-miR-4685-5p | SMARCD2 | 1 | 1 | 2 | N |
| hsa-miR-4685-5p | FMOD | 1 | 1 | 2 | N |
| hsa-miR-4685-5p | DEDD2 | 1 | 1 | 2 | N |
| hsa-miR-4685-5p | OLFML2A | 1 | 1 | 2 | N |
| hsa-miR-4685-5p | MGAT5B | 1 | 1 | 2 | N |
| hsa-miR-4685-5p | LRRC15 | 1 | 1 | 2 | N |
| hsa-miR-4685-5p | PSMD9 | 1 | 1 | 2 | N |
| hsa-miR-4685-5p | ANKRD54 | 1 | 1 | 2 | N |
| hsa-miR-4685-5p | APH1A | 1 | 1 | 2 | N |
| hsa-miR-4685-5p | VPS39 | 1 | 1 | 2 | N |
| hsa-miR-4685-5p | TNS1 | 1 | 1 | 2 | N |
| hsa-miR-4685-5p | NDOR1 | 1 | 1 | 2 | N |
| hsa-miR-4685-5p | WDTC1 | 1 | 1 | 2 | N |
| hsa-miR-4685-5p | BIN3 | 1 | 1 | 2 | N |
| hsa-miR-4685-5p | DAB2IP | 1 | 1 | 2 | N |
| hsa-miR-4685-5p | KCNJ10 | 1 | 1 | 2 | N |
| hsa-miR-4685-5p | C9orf3 | 1 | 1 | 2 | N |
| hsa-miR-4685-5p | SYT9 | 1 | 1 | 2 | N |
| hsa-miR-4685-5p | TPPP | 1 | 1 | 2 | N |
| hsa-miR-4685-5p | FAM102A | 1 | 1 | 2 | N |
| hsa-miR-4685-5p | EPHB6 | 1 | 1 | 2 | N |
| hsa-miR-4685-5p | ALS2CL | 1 | 1 | 2 | N |
| hsa-miR-4685-5p | RAB5B | 1 | 1 | 2 | N |
| hsa-miR-4685-5p | ASIC1 | 1 | 1 | 2 | N |
| hsa-miR-4685-5p | CCDC13 | 1 | 1 | 2 | N |
| hsa-miR-4685-5p | TTPAL | 1 | 1 | 2 | N |
| hsa-miR-4685-5p | RHOJ | 1 | 1 | 2 | N |
| hsa-miR-4685-5p | DPCR1 | 1 | 1 | 2 | N |
| hsa-miR-4685-5p | LPCAT1 | 1 | 1 | 2 | N |
| hsa-miR-4685-5p | DPH1 | 1 | 1 | 2 | N |
| hsa-miR-4685-5p | CST9 | 1 | 1 | 2 | N |
| hsa-miR-4685-5p | CHAC1 | 1 | 1 | 2 | N |
| hsa-miR-4685-5p | TMEM222 | 1 | 1 | 2 | N |
| hsa-miR-4685-5p | PNMAL2 | 1 | 1 | 2 | N |
| hsa-miR-4685-5p | MVB12B | 1 | 1 | 2 | N |
| hsa-miR-4685-5p | CAPN13 | 1 | 1 | 2 | N |
| hsa-miR-4685-5p | PVRL1 | 1 | 1 | 2 | N |
| hsa-miR-4685-5p | CDK18 | 1 | 1 | 2 | N |
| hsa-miR-4685-5p | SOCS7 | 1 | 1 | 2 | N |
| hsa-miR-4685-5p | GCHFR | 1 | 1 | 2 | N |
| hsa-miR-4685-5p | BOLA1 | 1 | 1 | 2 | N |
| hsa-miR-4685-5p | PLAUR | 1 | 1 | 2 | N |
| hsa-miR-4685-5p | NCKAP5L | 1 | 1 | 2 | N |
| hsa-miR-4685-5p | TTYH3 | 1 | 1 | 2 | Y |
| hsa-miR-4685-5p | SURF4 | 1 | 1 | 2 | Y |
| hsa-miR-4685-5p | UBALD1 | 1 | 1 | 2 | N |
| hsa-miR-4685-5p | VDR | 1 | 1 | 2 | N |
| hsa-miR-4685-5p | TAL1 | 1 | 1 | 2 | N |
| hsa-miR-4685-5p | TMEM127 | 1 | 1 | 2 | N |
| hsa-miR-4685-5p | TNS3 | 1 | 1 | 2 | N |
| hsa-miR-4685-5p | RAB11FIP5 | 1 | 1 | 2 | N |
| hsa-miR-4685-5p | KCTD20 | 1 | 1 | 2 | N |
| hsa-miR-4685-5p | SEZ6 | 1 | 1 | 2 | N |
| hsa-miR-4685-5p | GRB10 | 1 | 1 | 2 | N |
| hsa-miR-4685-5p | TUSC5 | 1 | 1 | 2 | N |
| hsa-miR-4685-5p | SEZ6L2 | 1 | 1 | 2 | N |
| hsa-miR-4685-5p | FRMD4B | 1 | 1 | 2 | N |
| hsa-miR-4685-5p | PPP2R5D | 1 | 1 | 2 | N |
| hsa-miR-4685-5p | CLEC2L | 1 | 1 | 2 | N |
| hsa-miR-4685-5p | DCHS1 | 1 | 1 | 2 | N |
| hsa-miR-4685-5p | ATP2B1 | 1 | 1 | 2 | N |
| hsa-miR-4685-5p | DENND4B | 1 | 1 | 2 | N |
| hsa-miR-4685-5p | CHCHD4 | 1 | 1 | 2 | N |
| hsa-miR-4685-5p | PPP3CB | 1 | 1 | 2 | N |
| hsa-miR-4685-5p | DHRS13 | 1 | 1 | 2 | N |
| hsa-miR-4685-5p | SLC43A2 | 1 | 1 | 2 | N |
| hsa-miR-4685-5p | GATC | 1 | 1 | 2 | N |
| hsa-miR-4685-5p | REPIN1 | 1 | 1 | 2 | N |
| hsa-miR-4685-5p | CDIP1 | 1 | 1 | 2 | N |
| hsa-miR-4685-5p | PPFIA4 | 1 | 1 | 2 | N |
| hsa-miR-4685-5p | KIAA1045 | 1 | 1 | 2 | N |
| hsa-miR-4685-5p | TBC1D13 | 1 | 1 | 2 | N |
| hsa-miR-4685-5p | ABLIM2 | 1 | 1 | 2 | N |
| hsa-miR-4685-5p | TMEM155 | 1 | 1 | 2 | N |
| hsa-miR-4685-5p | ITCH | 1 | 1 | 2 | N |
| hsa-miR-4685-5p | ERF | 1 | 1 | 2 | N |
| hsa-miR-4685-5p | CYB5R3 | 1 | 1 | 2 | N |
| hsa-miR-4685-5p | WBP1L | 1 | 1 | 2 | N |
| hsa-miR-4685-5p | SELV | 1 | 1 | 2 | N |
| hsa-miR-4685-5p | GTF3C1 | 1 | 1 | 2 | N |
| hsa-miR-4685-5p | DEGS2 | 1 | 1 | 2 | N |
| hsa-miR-4685-5p | SOX7 | 1 | 1 | 2 | N |
| hsa-miR-4685-5p | KCNJ5 | 1 | 1 | 2 | N |
| hsa-miR-4685-5p | LRRC8E | 1 | 1 | 2 | N |
| hsa-miR-4685-5p | ESR1 | 1 | 1 | 2 | N |
| hsa-miR-4685-5p | KCNQ4 | 1 | 1 | 2 | N |
| hsa-miR-4685-5p | SBK1 | 1 | 1 | 2 | Y |
| hsa-miR-4685-5p | PEX14 | 1 | 1 | 2 | N |
| hsa-miR-4685-5p | IL1RN | 1 | 1 | 2 | N |
| hsa-miR-4685-5p | FAM53A | 1 | 1 | 2 | N |
| hsa-miR-4685-5p | ANKRD34A | 1 | 1 | 2 | N |
| hsa-miR-4685-5p | MIEF2 | 1 | 1 | 2 | N |
| hsa-miR-4685-5p | PGF | 1 | 1 | 2 | N |
| hsa-miR-4685-5p | SRSF5 | 1 | 1 | 2 | N |
| hsa-miR-4685-5p | CPXM2 | 1 | 1 | 2 | N |
| hsa-miR-4685-5p | ETV4 | 1 | 1 | 2 | N |
| hsa-miR-4685-5p | FOXP4 | 1 | 1 | 2 | N |
| hsa-miR-4685-5p | SOWAHC | 1 | 1 | 2 | Y |
| hsa-miR-4685-5p | MINOS1 | 1 | 1 | 2 | N |
| hsa-miR-4685-5p | CTSH | 1 | 1 | 2 | N |
| hsa-miR-4722-3p | CRTC1 | 1 | 1 | 2 | N |
| hsa-miR-4722-3p | MAP3K13 | 1 | 1 | 2 | N |
| hsa-miR-4722-3p | CDIP1 | 1 | 1 | 2 | N |
| hsa-miR-4722-3p | LEPR | 1 | 1 | 2 | N |
| hsa-miR-4722-3p | NAT8L | 1 | 1 | 2 | N |
| hsa-miR-4722-3p | NEURL1B | 1 | 1 | 2 | N |
| hsa-miR-4722-3p | PSG1 | 1 | 1 | 2 | N |
| hsa-miR-4722-3p | ITFG2 | 1 | 1 | 2 | N |
| hsa-miR-4722-3p | ACRC | 1 | 1 | 2 | N |
| hsa-miR-4722-3p | SLC25A42 | 1 | 1 | 2 | N |
| hsa-miR-4722-3p | SCN4B | 1 | 1 | 2 | N |
| hsa-miR-4722-3p | AADAT | 1 | 1 | 2 | N |
| hsa-miR-4722-3p | PDIK1L | 1 | 1 | 2 | N |
| hsa-miR-4722-3p | FAM189A2 | 1 | 1 | 2 | N |
| hsa-miR-4722-3p | CMTR1 | 1 | 1 | 2 | N |
| hsa-miR-4722-3p | AKTIP | 1 | 1 | 2 | N |
| hsa-miR-4722-3p | MARCH1 | 1 | 1 | 2 | N |
| hsa-miR-4722-3p | PKNOX2 | 1 | 1 | 2 | N |
| hsa-miR-4722-3p | VPS53 | 1 | 1 | 2 | N |
| hsa-miR-4722-3p | N4BP3 | 1 | 1 | 2 | N |
| hsa-miR-4722-3p | ANKRD20A3 | 1 | 1 | 2 | N |
| hsa-miR-4722-3p | CHP1 | 1 | 1 | 2 | N |
| hsa-miR-4722-3p | PBX2 | 1 | 1 | 2 | N |
| hsa-miR-4722-3p | SERP2 | 1 | 1 | 2 | N |
| hsa-miR-4722-3p | MFSD6 | 1 | 1 | 2 | Y |
| hsa-miR-4722-3p | NUP214 | 1 | 1 | 2 | N |
| hsa-miR-4722-3p | PRR15L | 1 | 1 | 2 | N |
| hsa-miR-4722-3p | PSG8 | 1 | 1 | 2 | N |
| hsa-miR-4722-3p | STX3 | 1 | 1 | 2 | N |
| hsa-miR-4722-3p | GALK2 | 1 | 1 | 2 | N |
| hsa-miR-4722-3p | HSDL1 | 1 | 1 | 2 | N |
| hsa-miR-4722-3p | ANKMY2 | 1 | 1 | 2 | N |
| hsa-miR-4722-3p | CAPZA1 | 1 | 1 | 2 | Y |
| hsa-miR-4722-3p | PBX1 | 1 | 1 | 2 | Y |
| hsa-miR-4722-3p | PLEKHG7 | 1 | 1 | 2 | N |
| hsa-miR-4722-3p | DCAF10 | 1 | 1 | 2 | N |
| hsa-miR-4722-3p | MLEC | 1 | 1 | 2 | N |
| hsa-miR-4722-3p | PSG4 | 1 | 1 | 2 | N |
| hsa-miR-4722-3p | BSN | 1 | 1 | 2 | N |
| hsa-miR-4722-3p | SLC25A30 | 1 | 1 | 2 | N |
| hsa-miR-4722-3p | RNF165 | 1 | 1 | 2 | Y |
| hsa-miR-4722-3p | NTMT1 | 1 | 1 | 2 | N |
| hsa-miR-4722-3p | C15orf62 | 1 | 1 | 2 | N |
| hsa-miR-4722-3p | KAT6B | 1 | 1 | 2 | N |
| hsa-miR-4722-3p | TDRKH | 1 | 1 | 2 | N |
| hsa-miR-4722-3p | DTWD2 | 1 | 1 | 2 | N |
| hsa-miR-4769-3p | IP6K3 | 1 | 1 | 2 | N |
| hsa-miR-4769-3p | TM9SF2 | 1 | 1 | 2 | N |
| hsa-miR-4769-3p | ARL4A | 1 | 1 | 2 | N |
| hsa-miR-4769-3p | NEU3 | 1 | 1 | 2 | N |
| hsa-miR-4769-3p | ZNF423 | 1 | 1 | 2 | N |
| hsa-miR-4769-3p | PRIM1 | 1 | 1 | 2 | N |
| hsa-miR-4769-3p | ZNF384 | 1 | 1 | 2 | N |
| hsa-miR-4769-3p | COMMD8 | 1 | 1 | 2 | N |
| hsa-miR-4769-3p | APPL1 | 1 | 1 | 2 | N |
| hsa-miR-4769-3p | POU4F2 | 1 | 1 | 2 | N |
| hsa-miR-4769-3p | COPS2 | 1 | 1 | 2 | N |
| hsa-miR-4769-3p | WIPI2 | 1 | 1 | 2 | N |
| hsa-miR-4769-3p | TMEM133 | 1 | 1 | 2 | Y |
| hsa-miR-4769-3p | HRASLS | 1 | 1 | 2 | N |
| hsa-miR-4769-3p | BCL6 | 1 | 1 | 2 | N |
| hsa-miR-4769-3p | CRHBP | 1 | 1 | 2 | N |
| hsa-miR-4769-3p | FAM3C | 1 | 1 | 2 | N |
| hsa-miR-4769-3p | DAB1 | 1 | 1 | 2 | N |
| hsa-miR-4769-3p | SEC22C | 1 | 1 | 2 | N |
| hsa-miR-4769-3p | PAK2 | 1 | 1 | 2 | N |
| hsa-miR-4769-3p | SHOC2 | 1 | 1 | 2 | N |
| hsa-miR-4769-3p | CSNK1G1 | 1 | 1 | 2 | N |
| hsa-miR-4769-3p | SYT9 | 1 | 1 | 2 | N |
| hsa-miR-4769-3p | SEMA6A | 1 | 1 | 2 | N |
| hsa-miR-4769-3p | CSTF3 | 1 | 1 | 2 | N |
| hsa-miR-4769-3p | SIGLEC6 | 1 | 1 | 2 | N |
| hsa-miR-4769-3p | ATF1 | 1 | 1 | 2 | N |
| hsa-miR-4769-3p | AKTIP | 1 | 1 | 2 | N |
| hsa-miR-4769-3p | RADIL | 1 | 1 | 2 | N |
| hsa-miR-4769-3p | ETV1 | 1 | 1 | 2 | N |
| hsa-miR-4769-3p | SMIM14 | 1 | 1 | 2 | N |
| hsa-miR-4769-3p | ADAM9 | 1 | 1 | 2 | N |
| hsa-miR-4769-3p | CLIC4 | 1 | 1 | 2 | N |
| hsa-miR-4769-3p | TMEM254 | 1 | 1 | 2 | N |
| hsa-miR-4769-3p | ZNF843 | 1 | 1 | 2 | N |
| hsa-miR-4769-3p | GABRA1 | 1 | 1 | 2 | N |
| hsa-miR-4769-3p | IL36RN | 1 | 1 | 2 | N |
| hsa-miR-4769-3p | CCDC126 | 1 | 1 | 2 | N |
| hsa-miR-4769-3p | WDR3 | 1 | 1 | 2 | N |
| hsa-miR-4769-3p | MEOX2 | 1 | 1 | 2 | N |
| hsa-miR-637 | TSPAN11 | 1 | 1 | 2 | N |
| hsa-miR-637 | GPSM1 | 1 | 1 | 2 | N |
| hsa-miR-637 | SYK | 1 | 1 | 2 | N |
| hsa-miR-637 | RNASE13 | 1 | 1 | 2 | N |
| hsa-miR-637 | SMYD5 | 1 | 1 | 2 | N |
| hsa-miR-637 | NECAB3 | 1 | 1 | 2 | N |
| hsa-miR-637 | SLC25A36 | 1 | 1 | 2 | N |
| hsa-miR-637 | FAF2 | 1 | 1 | 2 | N |
| hsa-miR-637 | PVRL1 | 1 | 1 | 2 | N |
| hsa-miR-637 | LRRC15 | 1 | 1 | 2 | N |
| hsa-miR-637 | UBAP1 | 1 | 1 | 2 | N |
| hsa-miR-637 | CUX1 | 1 | 1 | 2 | N |
| hsa-miR-637 | PIP4K2C | 1 | 1 | 2 | N |
| hsa-miR-637 | CNIH2 | 1 | 1 | 2 | N |
| hsa-miR-637 | LASP1 | 1 | 1 | 2 | N |
| hsa-miR-637 | RERE | 1 | 1 | 2 | N |
| hsa-miR-637 | HSD11B2 | 1 | 1 | 2 | N |
| hsa-miR-637 | TNRC18 | 1 | 1 | 2 | N |
| hsa-miR-637 | HNF4A | 1 | 1 | 2 | N |
| hsa-miR-637 | GBGT1 | 1 | 1 | 2 | N |
| hsa-miR-637 | PANX2 | 1 | 1 | 2 | N |
| hsa-miR-637 | CTDSP1 | 1 | 1 | 2 | N |
| hsa-miR-637 | FCHSD1 | 1 | 1 | 2 | N |
| hsa-miR-637 | TSSK1B | 1 | 1 | 2 | N |
| hsa-miR-637 | HIF3A | 1 | 1 | 2 | N |
| hsa-miR-637 | MOB3A | 1 | 1 | 2 | N |
| hsa-miR-637 | SLC8A2 | 1 | 1 | 2 | N |
| hsa-miR-637 | SPRY4 | 1 | 1 | 2 | N |
| hsa-miR-637 | ABTB2 | 1 | 1 | 2 | N |
| hsa-miR-637 | PCDHGA10 | 1 | 1 | 2 | N |
| hsa-miR-637 | AFAP1 | 1 | 1 | 2 | N |
| hsa-miR-637 | RXRB | 1 | 1 | 2 | N |
| hsa-miR-637 | C20orf112 | 1 | 1 | 2 | N |
| hsa-miR-637 | DMWD | 1 | 1 | 2 | N |
| hsa-miR-637 | PECR | 1 | 1 | 2 | N |
| hsa-miR-637 | POLR2M | 1 | 1 | 2 | N |
| hsa-miR-637 | GIGYF1 | 1 | 1 | 2 | N |
| hsa-miR-637 | PCDHGA11 | 1 | 1 | 2 | N |
| hsa-miR-637 | DNTTIP1 | 1 | 1 | 2 | N |
| hsa-miR-637 | CNTNAP1 | 1 | 1 | 2 | N |
| hsa-miR-637 | PCDHGA9 | 1 | 1 | 2 | N |
| hsa-miR-637 | NFIX | 1 | 1 | 2 | N |
| hsa-miR-637 | STRADA | 1 | 1 | 2 | N |
| hsa-miR-637 | KCTD12 | 1 | 1 | 2 | N |
| hsa-miR-637 | CACNA2D2 | 1 | 1 | 2 | N |
| hsa-miR-637 | SOX13 | 1 | 1 | 2 | N |
| hsa-miR-637 | FOXA1 | 1 | 1 | 2 | N |
| hsa-miR-637 | MAP1A | 1 | 1 | 2 | N |
| hsa-miR-637 | NCDN | 1 | 1 | 2 | N |
| hsa-miR-637 | SCUBE3 | 1 | 1 | 2 | N |
| hsa-miR-637 | SPRED3 | 1 | 1 | 2 | N |
| hsa-miR-637 | C1orf106 | 1 | 1 | 2 | N |
| hsa-miR-637 | PCDHGB3 | 1 | 1 | 2 | N |
| hsa-miR-637 | CPLX2 | 1 | 1 | 2 | N |
| hsa-miR-637 | AKR1E2 | 1 | 1 | 2 | N |
| hsa-miR-637 | MAFG | 1 | 1 | 2 | N |
| hsa-miR-637 | SOX10 | 1 | 1 | 2 | N |
| hsa-miR-637 | ZBTB47 | 1 | 1 | 2 | N |
| hsa-miR-637 | CRYZ | 1 | 1 | 2 | N |
| hsa-miR-637 | FAM212B | 1 | 1 | 2 | N |
| hsa-miR-637 | DUSP9 | 1 | 1 | 2 | N |
| hsa-miR-637 | SEPT3 | 1 | 1 | 2 | N |
| hsa-miR-637 | RNF141 | 1 | 1 | 2 | N |
| hsa-miR-637 | PABPC4 | 1 | 1 | 2 | N |
| hsa-miR-637 | NFIC | 1 | 1 | 2 | N |
| hsa-miR-637 | SRCIN1 | 1 | 1 | 2 | N |
| hsa-miR-637 | IQSEC2 | 1 | 1 | 2 | N |
| hsa-miR-637 | PADI1 | 1 | 1 | 2 | N |
| hsa-miR-637 | S1PR3 | 1 | 1 | 2 | N |
| hsa-miR-637 | CRISP2 | 1 | 1 | 2 | N |
| hsa-miR-637 | ITGA3 | 1 | 1 | 2 | N |
| hsa-miR-637 | EPSTI1 | 1 | 1 | 2 | N |
| hsa-miR-637 | LDLRAD3 | 1 | 1 | 2 | N |
| hsa-miR-637 | SLC43A2 | 1 | 1 | 2 | N |
| hsa-miR-637 | PCDHGA1 | 1 | 1 | 2 | N |
| hsa-miR-637 | USF2 | 1 | 1 | 2 | N |
| hsa-miR-637 | NR4A1 | 1 | 1 | 2 | N |
| hsa-miR-637 | LMX1B | 1 | 1 | 2 | N |
| hsa-miR-637 | PMEL | 1 | 1 | 2 | N |
| hsa-miR-637 | NAPA | 1 | 1 | 2 | N |
| hsa-miR-637 | ADD2 | 1 | 1 | 2 | N |
| hsa-miR-637 | DYRK1B | 1 | 1 | 2 | N |
| hsa-miR-637 | ZNF853 | 1 | 1 | 2 | N |
| hsa-miR-637 | MYO6 | 1 | 1 | 2 | N |
| hsa-miR-637 | EPHB3 | 1 | 1 | 2 | N |
| hsa-miR-637 | C1orf43 | 1 | 1 | 2 | N |
| hsa-miR-637 | SEPT7 | 1 | 1 | 2 | N |
| hsa-miR-637 | RING1 | 1 | 1 | 2 | N |
| hsa-miR-637 | FAM131B | 1 | 1 | 2 | N |
| hsa-miR-637 | PLCD3 | 1 | 1 | 2 | N |
| hsa-miR-637 | TEAD3 | 1 | 1 | 2 | N |
| hsa-miR-637 | HEYL | 1 | 1 | 2 | N |
| hsa-miR-637 | FOSB | 1 | 1 | 2 | N |
| hsa-miR-637 | KRTAP10-10 | 1 | 1 | 2 | N |
| hsa-miR-637 | SGTA | 1 | 1 | 2 | N |
| hsa-miR-637 | EEFSEC | 1 | 1 | 2 | N |
| hsa-miR-637 | PCDHGA3 | 1 | 1 | 2 | N |
| hsa-miR-637 | PACSIN1 | 1 | 1 | 2 | N |
| hsa-miR-637 | SYT4 | 1 | 1 | 2 | N |
| hsa-miR-637 | DTNB | 1 | 1 | 2 | N |
| hsa-miR-637 | NLGN3 | 1 | 1 | 2 | N |
| hsa-miR-637 | PCDHGA12 | 1 | 1 | 2 | N |
| hsa-miR-637 | ADAMTS10 | 1 | 1 | 2 | N |
| hsa-miR-637 | CLDN2 | 1 | 1 | 2 | N |
| hsa-miR-637 | PAX8 | 1 | 1 | 2 | N |
| hsa-miR-637 | LY6E | 1 | 1 | 2 | N |
| hsa-miR-637 | KCNMB1 | 1 | 1 | 2 | N |
| hsa-miR-637 | ZNF524 | 1 | 1 | 2 | N |
| hsa-miR-637 | RAB35 | 1 | 1 | 2 | N |
| hsa-miR-637 | DOK7 | 1 | 1 | 2 | N |
| hsa-miR-637 | ZNF395 | 1 | 1 | 2 | N |
| hsa-miR-637 | PPP1R9B | 1 | 1 | 2 | Y |
| hsa-miR-637 | CDK18 | 1 | 1 | 2 | N |
| hsa-miR-637 | KCNK3 | 1 | 1 | 2 | N |
| hsa-miR-637 | MNT | 1 | 1 | 2 | N |
| hsa-miR-637 | DGKG | 1 | 1 | 2 | N |
| hsa-miR-637 | MYO3B | 1 | 1 | 2 | N |
| hsa-miR-637 | LZTS3 | 1 | 1 | 2 | N |
| hsa-miR-637 | TBC1D13 | 1 | 1 | 2 | N |
| hsa-miR-637 | SHISA9 | 1 | 1 | 2 | N |
| hsa-miR-637 | KRT1 | 1 | 1 | 2 | N |
| hsa-miR-637 | PCDHGB7 | 1 | 1 | 2 | N |
| hsa-miR-637 | SLC25A22 | 1 | 1 | 2 | N |
| hsa-miR-637 | ARHGEF4 | 1 | 1 | 2 | N |
| hsa-miR-637 | CISH | 1 | 1 | 2 | N |
| hsa-miR-637 | RARA | 1 | 1 | 2 | N |
| hsa-miR-637 | PCDHGA2 | 1 | 1 | 2 | N |
| hsa-miR-637 | PCDHGC3 | 1 | 1 | 2 | N |
| hsa-miR-637 | COL4A1 | 1 | 1 | 2 | Y |
| hsa-miR-637 | SULF2 | 1 | 1 | 2 | N |
| hsa-miR-637 | MARVELD1 | 1 | 1 | 2 | N |
| hsa-miR-637 | SLC25A42 | 1 | 1 | 2 | N |
| hsa-miR-637 | DAGLA | 1 | 1 | 2 | N |
| hsa-miR-637 | DAB2IP | 1 | 1 | 2 | Y |
| hsa-miR-637 | TAGLN | 1 | 1 | 2 | N |
| hsa-miR-637 | DCP1A | 1 | 1 | 2 | N |
| hsa-miR-637 | USP21 | 1 | 1 | 2 | N |
| hsa-miR-637 | TMEM151A | 1 | 1 | 2 | N |
| hsa-miR-637 | GSK3A | 1 | 1 | 2 | N |
| hsa-miR-637 | S1PR2 | 1 | 1 | 2 | N |
| hsa-miR-637 | GATS | 1 | 1 | 2 | N |
| hsa-miR-637 | FOXN1 | 1 | 1 | 2 | N |
| hsa-miR-637 | SLC6A17 | 1 | 1 | 2 | N |
| hsa-miR-637 | HMG20B | 1 | 1 | 2 | N |
| hsa-miR-637 | FAM163A | 1 | 1 | 2 | N |
| hsa-miR-637 | MPZ | 1 | 1 | 2 | N |
| hsa-miR-637 | VAMP2 | 1 | 1 | 2 | N |
| hsa-miR-637 | CDR2L | 1 | 1 | 2 | N |
| hsa-miR-637 | PCDHGA5 | 1 | 1 | 2 | N |
| hsa-miR-637 | TCEB3 | 1 | 1 | 2 | N |
| hsa-miR-637 | SGCD | 1 | 1 | 2 | N |
| hsa-miR-637 | FOXO4 | 1 | 1 | 2 | N |
| hsa-miR-637 | CPSF7 | 1 | 1 | 2 | N |
| hsa-miR-637 | FOXP4 | 1 | 1 | 2 | N |
| hsa-miR-637 | RRAS | 1 | 1 | 2 | N |
| hsa-miR-637 | DNAJC30 | 1 | 1 | 2 | N |
| hsa-miR-637 | GAS7 | 1 | 1 | 2 | N |
| hsa-miR-637 | PDE4A | 1 | 1 | 2 | N |
| hsa-miR-637 | RFX4 | 1 | 1 | 2 | N |
| hsa-miR-637 | PCDHGA8 | 1 | 1 | 2 | N |
| hsa-miR-637 | TMEM106A | 1 | 1 | 2 | N |
| hsa-miR-637 | HS1BP3 | 1 | 1 | 2 | N |
| hsa-miR-637 | PLA2G2F | 1 | 1 | 2 | N |
| hsa-miR-637 | URM1 | 1 | 1 | 2 | N |
| hsa-miR-637 | HS6ST1 | 1 | 1 | 2 | Y |
| hsa-miR-637 | LRRC38 | 1 | 1 | 2 | N |
| hsa-miR-637 | THY1 | 1 | 1 | 2 | N |
| hsa-miR-637 | GGA1 | 1 | 1 | 2 | N |
| hsa-miR-637 | PCDHGA7 | 1 | 1 | 2 | N |
| hsa-miR-637 | GRM3 | 1 | 1 | 2 | N |
| hsa-miR-637 | EMC10 | 1 | 1 | 2 | N |
| hsa-miR-637 | PPIL1 | 1 | 1 | 2 | N |
| hsa-miR-637 | WNT7A | 1 | 1 | 2 | N |
| hsa-miR-637 | CNN1 | 1 | 1 | 2 | N |
| hsa-miR-637 | RGS11 | 1 | 1 | 2 | N |
| hsa-miR-637 | KDM8 | 1 | 1 | 2 | N |
| hsa-miR-637 | PHLPP1 | 1 | 1 | 2 | N |
| hsa-miR-665 | PSMF1 | 1 | 1 | 2 | Y |
| hsa-miR-665 | PRSS8 | 1 | 1 | 2 | Y |
| hsa-miR-665 | DPF2 | 1 | 1 | 2 | N |
| hsa-miR-665 | RASGEF1B | 1 | 1 | 2 | N |
| hsa-miR-665 | KCNN3 | 1 | 1 | 2 | N |
| hsa-miR-665 | HABP4 | 1 | 1 | 2 | N |
| hsa-miR-665 | PGPEP1 | 1 | 1 | 2 | Y |
| hsa-miR-665 | TRIM8 | 1 | 1 | 2 | N |
| hsa-miR-665 | KREMEN1 | 1 | 1 | 2 | Y |
| hsa-miR-665 | SEMA3G | 1 | 1 | 2 | N |
| hsa-miR-665 | ZNF710 | 1 | 1 | 2 | N |
| hsa-miR-665 | HOXB5 | 1 | 1 | 2 | N |
| hsa-miR-665 | SUMO1 | 1 | 1 | 2 | N |
| hsa-miR-665 | GATS | 1 | 1 | 2 | N |
| hsa-miR-665 | CARHSP1 | 1 | 1 | 2 | N |
| hsa-miR-665 | EMC8 | 1 | 1 | 2 | N |
| hsa-miR-665 | ANKRD13C | 1 | 1 | 2 | N |
| hsa-miR-665 | MGAT1 | 1 | 1 | 2 | N |
| hsa-miR-665 | COPS7B | 1 | 1 | 2 | N |
| hsa-miR-665 | IGFN1 | 1 | 1 | 2 | N |
| hsa-miR-665 | KIF21B | 1 | 1 | 2 | Y |
| hsa-miR-665 | MCRS1 | 1 | 1 | 2 | N |
| hsa-miR-665 | CDC25A | 1 | 1 | 2 | N |
| hsa-miR-665 | ENO2 | 1 | 1 | 2 | N |
| hsa-miR-665 | CHST3 | 1 | 1 | 2 | N |
| hsa-miR-665 | PRR14L | 1 | 1 | 2 | N |
| hsa-miR-665 | STX3 | 1 | 1 | 2 | N |
| hsa-miR-665 | SDC1 | 1 | 1 | 2 | N |
| hsa-miR-665 | HEYL | 1 | 1 | 2 | Y |
| hsa-miR-665 | CHKB | 1 | 1 | 2 | N |
| hsa-miR-665 | TIMP3 | 1 | 1 | 2 | N |
| hsa-miR-665 | LUZP1 | 1 | 1 | 2 | N |
| hsa-miR-665 | TXNL1 | 1 | 1 | 2 | N |
| hsa-miR-665 | OCSTAMP | 1 | 1 | 2 | N |
| hsa-miR-665 | EXT1 | 1 | 1 | 2 | N |
| hsa-miR-665 | CYTH3 | 1 | 1 | 2 | N |
| hsa-miR-6727-3p | N4BP3 | 1 | 1 | 2 | N |
| hsa-miR-6727-3p | DCAF10 | 1 | 1 | 2 | N |
| hsa-miR-6727-3p | CAPZA1 | 1 | 1 | 2 | Y |
| hsa-miR-6727-3p | MFSD6 | 1 | 1 | 2 | Y |
| hsa-miR-6727-3p | HSDL1 | 1 | 1 | 2 | N |
| hsa-miR-6727-3p | ANKMY2 | 1 | 1 | 2 | N |
| hsa-miR-6727-3p | PKNOX2 | 1 | 1 | 2 | N |
| hsa-miR-6727-3p | PDIK1L | 1 | 1 | 2 | N |
| hsa-miR-6727-3p | SLC25A42 | 1 | 1 | 2 | N |
| hsa-miR-6727-3p | PSG4 | 1 | 1 | 2 | N |
| hsa-miR-6727-3p | VPS53 | 1 | 1 | 2 | N |
| hsa-miR-6727-3p | DTWD2 | 1 | 1 | 2 | N |
| hsa-miR-6727-3p | AADAT | 1 | 1 | 2 | N |
| hsa-miR-6727-3p | PLEKHG7 | 1 | 1 | 2 | N |
| hsa-miR-6727-3p | CHP1 | 1 | 1 | 2 | N |
| hsa-miR-6727-3p | PSG1 | 1 | 1 | 2 | N |
| hsa-miR-6727-3p | C15orf62 | 1 | 1 | 2 | N |
| hsa-miR-6727-3p | CD247 | 1 | 1 | 2 | N |
| hsa-miR-6727-3p | PBX1 | 1 | 1 | 2 | Y |
| hsa-miR-6727-3p | NUP214 | 1 | 1 | 2 | N |
| hsa-miR-6727-3p | BSN | 1 | 1 | 2 | N |
| hsa-miR-6727-3p | KAT6B | 1 | 1 | 2 | N |
| hsa-miR-6727-3p | ACRC | 1 | 1 | 2 | N |
| hsa-miR-6727-3p | SCN4B | 1 | 1 | 2 | N |
| hsa-miR-6727-3p | TDRKH | 1 | 1 | 2 | N |
| hsa-miR-6727-3p | CRTC1 | 1 | 1 | 2 | N |
| hsa-miR-6727-3p | SLC25A30 | 1 | 1 | 2 | N |
| hsa-miR-6727-3p | CMTR1 | 1 | 1 | 2 | N |
| hsa-miR-6727-3p | SERP2 | 1 | 1 | 2 | N |
| hsa-miR-6727-3p | LEPR | 1 | 1 | 2 | N |
| hsa-miR-6727-3p | GALK2 | 1 | 1 | 2 | N |
| hsa-miR-6727-3p | ITFG2 | 1 | 1 | 2 | N |
| hsa-miR-6727-3p | PSG8 | 1 | 1 | 2 | N |
| hsa-miR-6727-3p | ANKRD20A3 | 1 | 1 | 2 | N |
| hsa-miR-6727-3p | AKTIP | 1 | 1 | 2 | N |
| hsa-miR-6727-3p | RNF165 | 1 | 1 | 2 | Y |
| hsa-miR-6727-3p | PBX2 | 1 | 1 | 2 | N |
| hsa-miR-6727-3p | STX3 | 1 | 1 | 2 | N |
| hsa-miR-6727-3p | MARCH1 | 1 | 1 | 2 | N |
| hsa-miR-6727-3p | CDIP1 | 1 | 1 | 2 | N |
| hsa-miR-6727-3p | FAM189A2 | 1 | 1 | 2 | N |
| hsa-miR-6727-3p | NEURL1B | 1 | 1 | 2 | N |
| hsa-miR-6727-3p | NAT8L | 1 | 1 | 2 | N |
| hsa-miR-6727-3p | MAP3K13 | 1 | 1 | 2 | N |
| hsa-miR-6727-3p | NTMT1 | 1 | 1 | 2 | N |
| hsa-miR-6727-3p | PRR15L | 1 | 1 | 2 | N |
| hsa-miR-6769a-3p | PTN | 1 | 1 | 2 | N |
| hsa-miR-6769a-3p | FAM104A | 1 | 1 | 2 | N |
| hsa-miR-6769a-3p | AMPD3 | 1 | 1 | 2 | N |
| hsa-miR-6769a-3p | EREG | 1 | 1 | 2 | N |
| hsa-miR-6769a-3p | LYPLA2 | 1 | 1 | 2 | N |
| hsa-miR-6769a-3p | GRIPAP1 | 1 | 1 | 2 | N |
| hsa-miR-6769a-3p | NRIP3 | 1 | 1 | 2 | N |
| hsa-miR-6769a-3p | ALKBH5 | 1 | 1 | 2 | N |
| hsa-miR-6769a-3p | PPP2R4 | 1 | 1 | 2 | N |
| hsa-miR-6769a-3p | RDH12 | 1 | 1 | 2 | N |
| hsa-miR-6769a-3p | PKP1 | 1 | 1 | 2 | N |
| hsa-miR-6769a-3p | C21orf62 | 1 | 1 | 2 | N |
| hsa-miR-6769a-3p | KCTD4 | 1 | 1 | 2 | N |
| hsa-miR-6769a-3p | ZNF706 | 1 | 1 | 2 | N |
| hsa-miR-6769a-3p | SLC7A14 | 1 | 1 | 2 | N |
| hsa-miR-6769a-3p | STMN1 | 1 | 1 | 2 | Y |
| hsa-miR-6769a-3p | DBI | 1 | 1 | 2 | N |
| hsa-miR-6769a-3p | ARGLU1 | 1 | 1 | 2 | Y |
| hsa-miR-6780b-3p | ZNF12 | 1 | 1 | 2 | N |
| hsa-miR-6780b-3p | TXLNG | 1 | 1 | 2 | N |
| hsa-miR-6780b-3p | FOXN2 | 1 | 1 | 2 | N |
| hsa-miR-6780b-3p | PUS7L | 1 | 1 | 2 | N |
| hsa-miR-6780b-3p | CERS6 | 1 | 1 | 2 | N |
| hsa-miR-6780b-3p | APPL1 | 1 | 1 | 2 | N |
| hsa-miR-6780b-3p | DHRS7B | 1 | 1 | 2 | N |
| hsa-miR-6780b-3p | RNF157 | 1 | 1 | 2 | N |
| hsa-miR-6780b-3p | EBF1 | 1 | 1 | 2 | N |
| hsa-miR-6780b-3p | MMP13 | 1 | 1 | 2 | N |
| hsa-miR-6780b-3p | BNIP2 | 1 | 1 | 2 | N |
| hsa-miR-6780b-3p | TMEM213 | 1 | 1 | 2 | N |
| hsa-miR-6780b-3p | TMEM200A | 1 | 1 | 2 | N |
| hsa-miR-6780b-3p | GPBP1L1 | 1 | 1 | 2 | N |
| hsa-miR-6780b-3p | AGPAT1 | 1 | 1 | 2 | N |
| hsa-miR-6780b-3p | TRIM43 | 1 | 1 | 2 | N |
| hsa-miR-6780b-3p | XRCC5 | 1 | 1 | 2 | N |
| hsa-miR-6780b-3p | BIRC2 | 1 | 1 | 2 | N |
| hsa-miR-6780b-3p | MID1 | 1 | 1 | 2 | N |
| hsa-miR-6780b-3p | ITIH6 | 1 | 1 | 2 | N |
| hsa-miR-6780b-3p | IRF2BP2 | 1 | 1 | 2 | Y |
| hsa-miR-6780b-3p | FAM216B | 1 | 1 | 2 | N |
| hsa-miR-6780b-3p | DNAJC27 | 1 | 1 | 2 | N |
| hsa-miR-6780b-3p | FAM210A | 1 | 1 | 2 | N |
| hsa-miR-6780b-3p | RANBP1 | 1 | 1 | 2 | Y |
| hsa-miR-6780b-3p | PCDH20 | 1 | 1 | 2 | N |
| hsa-miR-6780b-3p | KLF3 | 1 | 1 | 2 | N |
| hsa-miR-6780b-3p | VAPB | 1 | 1 | 2 | N |
| hsa-miR-6817-5p | WIPI2 | 1 | 1 | 2 | N |
| hsa-miR-6817-5p | SEC22C | 1 | 1 | 2 | N |
| hsa-miR-6817-5p | TM9SF2 | 1 | 1 | 2 | N |
| hsa-miR-6817-5p | FAM3C | 1 | 1 | 2 | N |
| hsa-miR-6817-5p | COMMD8 | 1 | 1 | 2 | N |
| hsa-miR-6817-5p | TMEM133 | 1 | 1 | 2 | Y |
| hsa-miR-6817-5p | POU4F2 | 1 | 1 | 2 | N |
| hsa-miR-6817-5p | GABRA1 | 1 | 1 | 2 | N |
| hsa-miR-6817-5p | AKTIP | 1 | 1 | 2 | N |
| hsa-miR-6817-5p | CRHBP | 1 | 1 | 2 | N |
| hsa-miR-6817-5p | IL36RN | 1 | 1 | 2 | N |
| hsa-miR-6817-5p | CSTF3 | 1 | 1 | 2 | N |
| hsa-miR-6817-5p | RADIL | 1 | 1 | 2 | N |
| hsa-miR-6817-5p | MEOX2 | 1 | 1 | 2 | N |
| hsa-miR-6817-5p | ETV1 | 1 | 1 | 2 | N |
| hsa-miR-6817-5p | CSNK1G1 | 1 | 1 | 2 | N |
| hsa-miR-6817-5p | ZNF423 | 1 | 1 | 2 | N |
| hsa-miR-6817-5p | ARL4A | 1 | 1 | 2 | N |
| hsa-miR-6817-5p | CCDC126 | 1 | 1 | 2 | N |
| hsa-miR-6817-5p | BCL6 | 1 | 1 | 2 | N |
| hsa-miR-6817-5p | DAB1 | 1 | 1 | 2 | N |
| hsa-miR-6817-5p | WDR3 | 1 | 1 | 2 | N |
| hsa-miR-6817-5p | TMEM254 | 1 | 1 | 2 | N |
| hsa-miR-6817-5p | ZNF384 | 1 | 1 | 2 | N |
| hsa-miR-6817-5p | CLIC4 | 1 | 1 | 2 | N |
| hsa-miR-6817-5p | ZNF843 | 1 | 1 | 2 | N |
| hsa-miR-6817-5p | PAK2 | 1 | 1 | 2 | N |
| hsa-miR-6817-5p | SIGLEC6 | 1 | 1 | 2 | N |
| hsa-miR-6817-5p | ATF1 | 1 | 1 | 2 | N |
| hsa-miR-6817-5p | PANK1 | 1 | 1 | 2 | N |
| hsa-miR-6817-5p | SYT9 | 1 | 1 | 2 | N |
| hsa-miR-6817-5p | SEMA6A | 1 | 1 | 2 | N |
| hsa-miR-6817-5p | NEU3 | 1 | 1 | 2 | N |
| hsa-miR-6817-5p | COPS2 | 1 | 1 | 2 | N |
| hsa-miR-6817-5p | SHOC2 | 1 | 1 | 2 | N |
| hsa-miR-6817-5p | LRRFIP1 | 1 | 1 | 2 | N |
| hsa-miR-6817-5p | IP6K3 | 1 | 1 | 2 | N |
| hsa-miR-6817-5p | HRASLS | 1 | 1 | 2 | N |
| hsa-miR-6817-5p | PRIM1 | 1 | 1 | 2 | N |
| hsa-miR-6817-5p | SMIM14 | 1 | 1 | 2 | N |
| hsa-miR-6817-5p | C11orf21 | 1 | 1 | 2 | N |
| hsa-miR-6817-5p | ADAM9 | 1 | 1 | 2 | N |
| hsa-miR-6817-5p | APPL1 | 1 | 1 | 2 | N |
| hsa-miR-6861-3p | SHISA7 | 1 | 1 | 2 | N |
| hsa-miR-6861-3p | SVOP | 1 | 1 | 2 | N |
| hsa-miR-6861-3p | ASB7 | 1 | 1 | 2 | N |
| hsa-miR-6861-3p | MAFB | 1 | 1 | 2 | N |
| hsa-miR-6861-3p | GABRA1 | 1 | 1 | 2 | N |
| hsa-miR-6861-3p | CNOT2 | 1 | 1 | 2 | N |
| hsa-miR-6861-3p | PIGM | 1 | 1 | 2 | N |
| hsa-miR-6861-3p | STEAP3 | 1 | 1 | 2 | N |
| hsa-miR-6861-3p | ETF1 | 1 | 1 | 2 | N |
| hsa-miR-6861-3p | KIAA0040 | 1 | 1 | 2 | N |
| hsa-miR-6861-3p | GPC1 | 1 | 1 | 2 | N |
| hsa-miR-6861-3p | IFNAR1 | 1 | 1 | 2 | N |
| hsa-miR-6861-3p | AKIRIN2 | 1 | 1 | 2 | N |
| hsa-miR-6861-3p | NUDT15 | 1 | 1 | 2 | N |
| hsa-miR-6861-3p | DCHS1 | 1 | 1 | 2 | N |
| hsa-miR-6861-3p | TMEM50A | 1 | 1 | 2 | N |
| hsa-miR-6861-3p | ABHD3 | 1 | 1 | 2 | N |
| hsa-miR-6861-3p | ZNF211 | 1 | 1 | 2 | N |
| hsa-miR-6861-3p | MTFR1L | 1 | 1 | 2 | N |
| hsa-miR-6861-3p | ZBTB20 | 1 | 1 | 2 | N |
| hsa-miR-6861-3p | GRM6 | 1 | 1 | 2 | N |
| hsa-miR-6861-3p | CBFA2T3 | 1 | 1 | 2 | N |
| hsa-miR-6861-3p | AKAP12 | 1 | 1 | 2 | N |
| hsa-miR-6861-3p | SYS1 | 1 | 1 | 2 | N |
| hsa-miR-8076 | SGPP1 | 1 | 1 | 2 | N |
| hsa-miR-8076 | HAND2 | 1 | 1 | 2 | N |
| hsa-miR-8076 | C2orf66 | 1 | 1 | 2 | N |
| hsa-miR-8076 | TRIM50 | 1 | 1 | 2 | N |
| hsa-miR-8076 | CDK1 | 1 | 1 | 2 | N |
| hsa-miR-8076 | POC5 | 1 | 1 | 2 | N |
| hsa-miR-8076 | BOLA3 | 1 | 1 | 2 | Y |
| hsa-miR-8076 | NUDCD2 | 1 | 1 | 2 | N |
| hsa-miR-8076 | LRRTM2 | 1 | 1 | 2 | N |
| hsa-miR-8076 | DSE | 1 | 1 | 2 | N |
| hsa-miR-8076 | ATP6V1G1 | 1 | 1 | 2 | N |
| hsa-miR-8076 | MEIS1 | 1 | 1 | 2 | N |
| hsa-miR-8076 | PAQR3 | 1 | 1 | 2 | N |
| hsa-miR-8076 | EPC2 | 1 | 1 | 2 | N |
| hsa-miR-8076 | CSF2 | 1 | 1 | 2 | N |
| hsa-miR-8076 | SEPT14 | 1 | 1 | 2 | N |
| hsa-miR-8076 | GALR1 | 1 | 1 | 2 | N |
| hsa-miR-8076 | LIX1 | 1 | 1 | 2 | N |
| hsa-miR-8076 | PURG | 1 | 1 | 2 | N |
| hsa-miR-8076 | SEPT7 | 1 | 1 | 2 | N |
| hsa-miR-8076 | ZNF664 | 1 | 1 | 2 | N |
| hsa-miR-8076 | SENP2 | 1 | 1 | 2 | N |
| hsa-miR-8076 | PLAG1 | 1 | 1 | 2 | N |
| hsa-miR-8076 | PPP1CC | 1 | 1 | 2 | N |
| hsa-miR-8076 | SLC12A2 | 1 | 1 | 2 | N |
| hsa-miR-8076 | IRF2BP2 | 1 | 1 | 2 | Y |
| hsa-miR-8076 | C8orf22 | 1 | 1 | 2 | N |
| hsa-miR-8076 | BVES | 1 | 1 | 2 | Y |
| hsa-miR-8076 | SBSPON | 1 | 1 | 2 | N |
| hsa-miR-8076 | AK3 | 1 | 1 | 2 | N |
| hsa-miR-8076 | GABPA | 1 | 1 | 2 | N |
| hsa-miR-8076 | GNB5 | 1 | 1 | 2 | N |
| hsa-miR-8076 | RQCD1 | 1 | 1 | 2 | N |
| hsa-miR-8076 | PCSK5 | 1 | 1 | 2 | N |
| hsa-miR-8076 | GRIA2 | 1 | 1 | 2 | N |
| hsa-miR-8076 | UBL3 | 1 | 1 | 2 | N |
| hsa-miR-8076 | PAPD5 | 1 | 1 | 2 | N |
| hsa-miR-8076 | POLR2K | 1 | 1 | 2 | N |
| hsa-miR-8076 | CRADD | 1 | 1 | 2 | Y |
| hsa-miR-8076 | MPLKIP | 1 | 1 | 2 | N |
| hsa-miR-8076 | SMARCAD1 | 1 | 1 | 2 | N |
| hsa-miR-8076 | ANXA4 | 1 | 1 | 2 | N |
| hsa-miR-8076 | FMR1 | 1 | 1 | 2 | N |
| hsa-miR-8076 | GNAQ | 1 | 1 | 2 | N |
| hsa-miR-8076 | EWSR1 | 1 | 1 | 2 | N |
| hsa-miR-8076 | CCDC82 | 1 | 1 | 2 | N |
| hsa-miR-8076 | RBM45 | 1 | 1 | 2 | N |
| hsa-miR-8076 | UBE2D1 | 1 | 1 | 2 | N |
| hsa-miR-8076 | FAM13B | 1 | 1 | 2 | N |
