# Supplementary material for: circRNA circ_102049 Implicates in Pancreatic Ductal Adenocarcinoma Progression through Activating CD80 by Targeting miR-455-3p
Source: Mediators Inflamm. 2021 Jan 7;2021:8819990. doi: 10.1155/2021/8819990 (PMC7811564; doi:10.1155/2021/8819990)
Supplement: Supplementary 8 — Table S6 The potential target genes of 38 lowly expressed miRNAs with poor survival of PDAC patients. [file 8819990.f8.doc]

**Supplementary Table S6: The potential target genes of 38 lowly expressed miRNA with poor survival of PDAC patients.**

| **miRNA** | **Target name** | **targetScan7.1** | **mirdbV5** | **NumSum** | **miRTarBase(Y/N)** |
| --- | --- | --- | --- | --- | --- |
| hsa-miR-296-3p | STRN3 | 1 | 1 | 2 | N |
| hsa-miR-296-3p | ZBTB20 | 1 | 1 | 2 | N |
| hsa-miR-296-3p | SOCS6 | 1 | 1 | 2 | N |
| hsa-miR-4501 | ZNF655 | 1 | 1 | 2 | N |
| hsa-miR-4501 | C2orf73 | 1 | 1 | 2 | N |
| hsa-miR-4501 | LRRC2 | 1 | 1 | 2 | N |
| hsa-miR-4501 | PAQR9 | 1 | 1 | 2 | N |
| hsa-miR-4501 | ZFAND1 | 1 | 1 | 2 | N |
| hsa-miR-4501 | FBXO6 | 1 | 1 | 2 | N |
| hsa-miR-4501 | ANGPTL3 | 1 | 1 | 2 | N |
| hsa-miR-4501 | RAB21 | 1 | 1 | 2 | N |
| hsa-miR-4501 | RIT1 | 1 | 1 | 2 | N |
| hsa-miR-4501 | CA10 | 1 | 1 | 2 | N |
| hsa-miR-4501 | DDAH1 | 1 | 1 | 2 | N |
| hsa-miR-4501 | DEFB118 | 1 | 1 | 2 | N |
| hsa-miR-4501 | ITGB1 | 1 | 1 | 2 | N |
| hsa-miR-4501 | NKX3-1 | 1 | 1 | 2 | N |
| hsa-miR-4501 | SYT17 | 1 | 1 | 2 | N |
| hsa-miR-4501 | KIAA0040 | 1 | 1 | 2 | N |
| hsa-miR-4501 | NUDT4 | 1 | 1 | 2 | N |
| hsa-miR-4501 | CCPG1 | 1 | 1 | 2 | N |
| hsa-miR-4501 | AGTR1 | 1 | 1 | 2 | N |
| hsa-miR-4501 | OSMR | 1 | 1 | 2 | N |
| hsa-miR-4501 | STAT1 | 1 | 1 | 2 | N |
| hsa-miR-4501 | MBNL1 | 1 | 1 | 2 | N |
| hsa-miR-4501 | GK | 1 | 1 | 2 | N |
| hsa-miR-4501 | VAPB | 1 | 1 | 2 | N |
| hsa-miR-4501 | TKTL1 | 1 | 1 | 2 | N |
| hsa-miR-4501 | FSD1L | 1 | 1 | 2 | N |
| hsa-miR-4501 | MCIDAS | 1 | 1 | 2 | N |
| hsa-miR-627-5p | COMT | 1 | 1 | 2 | N |
| hsa-miR-627-5p | SKIL | 1 | 1 | 2 | N |
| hsa-miR-627-5p | SCN3B | 1 | 1 | 2 | N |
| hsa-miR-627-5p | CNOT2 | 1 | 1 | 2 | N |
| hsa-miR-627-5p | CA7 | 1 | 1 | 2 | N |
| hsa-miR-627-5p | FAM204A | 1 | 1 | 2 | N |
| hsa-miR-627-5p | HIRIP3 | 1 | 1 | 2 | N |
| hsa-miR-627-5p | MAP2K4 | 1 | 1 | 2 | N |
| hsa-miR-627-5p | HTR2A | 1 | 1 | 2 | N |
| hsa-miR-627-5p | SLC25A31 | 1 | 1 | 2 | N |
| hsa-miR-627-5p | CTSH | 1 | 1 | 2 | N |
| hsa-miR-627-5p | FAM78A | 1 | 1 | 2 | N |
| hsa-miR-627-5p | ROM1 | 1 | 1 | 2 | N |
| hsa-miR-627-5p | IFNG | 1 | 1 | 2 | N |
| hsa-miR-653-3p | BRD8 | 1 | 1 | 2 | N |
| hsa-miR-653-3p | OMG | 1 | 1 | 2 | N |
| hsa-miR-653-3p | EYA1 | 1 | 1 | 2 | N |
| hsa-miR-653-3p | YIPF6 | 1 | 1 | 2 | N |
| hsa-miR-653-3p | DYNLL1 | 1 | 1 | 2 | N |
| hsa-miR-653-3p | STMN4 | 1 | 1 | 2 | N |
| hsa-miR-653-3p | PARP14 | 1 | 1 | 2 | N |
| hsa-miR-653-3p | ACADM | 1 | 1 | 2 | N |
| hsa-miR-653-3p | PTPN20A | 1 | 1 | 2 | N |
| hsa-miR-653-3p | ZDHHC17 | 1 | 1 | 2 | N |
| hsa-miR-653-3p | IRF1 | 1 | 1 | 2 | N |
| hsa-miR-653-3p | PPP1R1A | 1 | 1 | 2 | N |
| hsa-miR-653-3p | VIP | 1 | 1 | 2 | N |
| hsa-miR-653-3p | EZH2 | 1 | 1 | 2 | N |
| hsa-miR-653-3p | PER3 | 1 | 1 | 2 | N |
| hsa-miR-653-3p | LACC1 | 1 | 1 | 2 | N |
| hsa-miR-653-3p | CNKSR2 | 1 | 1 | 2 | N |
| hsa-miR-653-3p | CDC42SE2 | 1 | 1 | 2 | N |
| hsa-miR-653-3p | RNF6 | 1 | 1 | 2 | N |
| hsa-miR-653-3p | MAML1 | 1 | 1 | 2 | N |
| hsa-miR-653-3p | CCDC28A | 1 | 1 | 2 | N |
| hsa-miR-653-3p | GPR137C | 1 | 1 | 2 | N |
| hsa-miR-653-3p | MAP1B | 1 | 1 | 2 | N |
| hsa-miR-653-3p | PFKFB1 | 1 | 1 | 2 | N |
| hsa-miR-653-3p | RPE | 1 | 1 | 2 | N |
| hsa-miR-653-3p | HRH4 | 1 | 1 | 2 | N |
| hsa-miR-653-3p | YWHAG | 1 | 1 | 2 | N |
| hsa-miR-653-3p | UBTD2 | 1 | 1 | 2 | N |
| hsa-miR-653-3p | WDR26 | 1 | 1 | 2 | N |
| hsa-miR-653-3p | SNX31 | 1 | 1 | 2 | N |
| hsa-miR-653-3p | GABRA5 | 1 | 1 | 2 | N |
| hsa-miR-653-3p | SELP | 1 | 1 | 2 | N |
| hsa-miR-653-3p | AGXT2 | 1 | 1 | 2 | N |
| hsa-miR-653-3p | C11orf44 | 1 | 1 | 2 | N |
| hsa-miR-653-3p | TOMM20 | 1 | 1 | 2 | N |
| hsa-miR-653-3p | PRKCH | 1 | 1 | 2 | N |
| hsa-miR-653-3p | YIPF4 | 1 | 1 | 2 | N |
| hsa-miR-653-3p | GNG5 | 1 | 1 | 2 | N |
| hsa-miR-653-3p | PEF1 | 1 | 1 | 2 | N |
| hsa-miR-653-3p | CHST4 | 1 | 1 | 2 | N |
| hsa-miR-653-3p | POPDC3 | 1 | 1 | 2 | N |
| hsa-miR-653-3p | SSFA2 | 1 | 1 | 2 | N |
| hsa-miR-653-3p | CTDSPL2 | 1 | 1 | 2 | N |
| hsa-miR-653-3p | TSPAN5 | 1 | 1 | 2 | N |
| hsa-miR-653-3p | CCT6A | 1 | 1 | 2 | N |
| hsa-miR-653-3p | AMMECR1L | 1 | 1 | 2 | N |
| hsa-miR-653-3p | SMARCAD1 | 1 | 1 | 2 | N |
| hsa-miR-653-3p | PVRL3 | 1 | 1 | 2 | N |
| hsa-miR-653-3p | DNAJC6 | 1 | 1 | 2 | N |
| hsa-miR-653-3p | CD53 | 1 | 1 | 2 | N |
| hsa-miR-653-3p | FBXL5 | 1 | 1 | 2 | N |
| hsa-miR-653-3p | PTPN20B | 1 | 1 | 2 | N |
| hsa-miR-653-3p | FAM117B | 1 | 1 | 2 | N |
| hsa-miR-653-3p | ANGPTL7 | 1 | 1 | 2 | N |
| hsa-miR-653-3p | ZNF329 | 1 | 1 | 2 | N |
| hsa-miR-218-1-3p | CPNE4 | 1 | 1 | 2 | N |
| hsa-miR-218-1-3p | SOCS1 | 1 | 1 | 2 | N |
| hsa-miR-218-1-3p | IRS2 | 1 | 1 | 2 | N |
| hsa-miR-218-1-3p | LDLRAP1 | 1 | 1 | 2 | N |
| hsa-miR-218-1-3p | MAFG | 1 | 1 | 2 | N |
| hsa-miR-218-1-3p | ADAM12 | 1 | 1 | 2 | N |
| hsa-miR-218-1-3p | KBTBD3 | 1 | 1 | 2 | N |
| hsa-miR-218-1-3p | MAPK9 | 1 | 1 | 2 | N |
| hsa-miR-218-1-3p | PANK3 | 1 | 1 | 2 | N |
| hsa-miR-218-1-3p | RFXANK | 1 | 1 | 2 | N |
| hsa-miR-218-1-3p | CD8B | 1 | 1 | 2 | N |
| hsa-miR-218-1-3p | LSM14B | 1 | 1 | 2 | N |
| hsa-miR-218-1-3p | NRBF2 | 1 | 1 | 2 | N |
| hsa-miR-218-1-3p | AHNAK2 | 1 | 1 | 2 | N |
| hsa-miR-218-1-3p | GALP | 1 | 1 | 2 | N |
| hsa-miR-218-1-3p | GSTO2 | 1 | 1 | 2 | N |
| hsa-miR-218-1-3p | HOXA11 | 1 | 1 | 2 | N |
| hsa-miR-218-1-3p | C10orf12 | 1 | 1 | 2 | N |
| hsa-miR-218-1-3p | ATF3 | 1 | 1 | 2 | N |
| hsa-miR-218-1-3p | MID1IP1 | 1 | 1 | 2 | N |
| hsa-miR-218-1-3p | MYO10 | 1 | 1 | 2 | Y |
| hsa-miR-218-1-3p | SMARCC1 | 1 | 1 | 2 | N |
| hsa-miR-218-1-3p | RFX2 | 1 | 1 | 2 | N |
| hsa-miR-218-1-3p | GRAMD2 | 1 | 1 | 2 | N |
| hsa-miR-218-1-3p | MKL2 | 1 | 1 | 2 | N |
| hsa-miR-218-1-3p | ITM2C | 1 | 1 | 2 | N |
| hsa-miR-218-1-3p | BTG2 | 1 | 1 | 2 | Y |
| hsa-miR-218-1-3p | ZNF362 | 1 | 1 | 2 | N |
| hsa-miR-218-1-3p | PIRT | 1 | 1 | 2 | N |
| hsa-miR-218-1-3p | INHBB | 1 | 1 | 2 | N |
| hsa-miR-218-1-3p | C7orf49 | 1 | 1 | 2 | N |
| hsa-miR-218-1-3p | UBE2E2 | 1 | 1 | 2 | N |
| hsa-miR-218-1-3p | TP53INP2 | 1 | 1 | 2 | N |
| hsa-miR-218-1-3p | MANBA | 1 | 1 | 2 | N |
| hsa-miR-218-1-3p | PPP3CB | 1 | 1 | 2 | N |
| hsa-miR-218-1-3p | MFAP3L | 1 | 1 | 2 | N |
| hsa-miR-218-1-3p | RNF10 | 1 | 1 | 2 | N |
| hsa-miR-218-1-3p | DOCK7 | 1 | 1 | 2 | N |
| hsa-miR-218-1-3p | DAZAP2 | 1 | 1 | 2 | N |
| hsa-miR-218-1-3p | APOL6 | 1 | 1 | 2 | N |
| hsa-miR-218-1-3p | FAM63B | 1 | 1 | 2 | N |
| hsa-miR-218-1-3p | WNT5A | 1 | 1 | 2 | N |
| hsa-miR-218-1-3p | HIBADH | 1 | 1 | 2 | N |
| hsa-miR-218-1-3p | C21orf62 | 1 | 1 | 2 | N |
| hsa-miR-218-1-3p | C7 | 1 | 1 | 2 | N |
| hsa-miR-218-1-3p | CLN5 | 1 | 1 | 2 | N |
| hsa-miR-218-1-3p | ZNF471 | 1 | 1 | 2 | N |
| hsa-miR-218-1-3p | SRPK2 | 1 | 1 | 2 | N |
| hsa-miR-218-1-3p | RTKN | 1 | 1 | 2 | N |
| hsa-miR-4423-5p | FOPNL | 1 | 1 | 2 | N |
| hsa-miR-4423-5p | ANAPC16 | 1 | 1 | 2 | N |
| hsa-miR-4423-5p | SH3BGRL3 | 1 | 1 | 2 | N |
| hsa-miR-4423-5p | FGF9 | 1 | 1 | 2 | N |
| hsa-miR-4423-5p | VTCN1 | 1 | 1 | 2 | N |
| hsa-miR-4423-5p | TBC1D23 | 1 | 1 | 2 | N |
| hsa-miR-4423-5p | MCM9 | 1 | 1 | 2 | N |
| hsa-miR-4423-5p | KCTD4 | 1 | 1 | 2 | N |
| hsa-miR-4423-5p | VIP | 1 | 1 | 2 | N |
| hsa-miR-4423-5p | CACUL1 | 1 | 1 | 2 | N |
| hsa-miR-4423-5p | COPS2 | 1 | 1 | 2 | N |
| hsa-miR-4423-5p | HNRNPUL1 | 1 | 1 | 2 | N |
| hsa-miR-4423-5p | RND2 | 1 | 1 | 2 | N |
| hsa-miR-4423-5p | COQ10B | 1 | 1 | 2 | N |
| hsa-miR-4423-5p | KCTD18 | 1 | 1 | 2 | N |
| hsa-miR-485-5p | RAB8B | 1 | 1 | 2 | N |
| hsa-miR-485-5p | MGAT5B | 1 | 1 | 2 | N |
| hsa-miR-485-5p | TMEM104 | 1 | 1 | 2 | N |
| hsa-miR-485-5p | ALX4 | 1 | 1 | 2 | N |
| hsa-miR-485-5p | SDC3 | 1 | 1 | 2 | N |
| hsa-miR-485-5p | SLC36A3 | 1 | 1 | 2 | N |
| hsa-miR-485-5p | FRMPD3 | 1 | 1 | 2 | N |
| hsa-miR-485-5p | CTDNEP1 | 1 | 1 | 2 | N |
| hsa-miR-485-5p | MGST3 | 1 | 1 | 2 | N |
| hsa-miR-485-5p | HIF3A | 1 | 1 | 2 | Y |
| hsa-miR-485-5p | ETNK2 | 1 | 1 | 2 | N |
| hsa-miR-485-5p | RBPMS | 1 | 1 | 2 | N |
| hsa-miR-485-5p | EFNA1 | 1 | 1 | 2 | N |
| hsa-miR-485-5p | DNAJC5G | 1 | 1 | 2 | N |
| hsa-miR-485-5p | ST3GAL1 | 1 | 1 | 2 | N |
| hsa-miR-485-5p | GAB2 | 1 | 1 | 2 | N |
| hsa-miR-485-5p | ADIPOR2 | 1 | 1 | 2 | N |
| hsa-miR-485-5p | DCAF7 | 1 | 1 | 2 | N |
| hsa-miR-485-5p | PTMS | 1 | 1 | 2 | N |
| hsa-miR-485-5p | ZBTB39 | 1 | 1 | 2 | N |
| hsa-miR-485-5p | SLC16A2 | 1 | 1 | 2 | N |
| hsa-miR-485-5p | CKS1B | 1 | 1 | 2 | Y |
| hsa-miR-485-5p | TMEM151B | 1 | 1 | 2 | N |
| hsa-miR-485-5p | PAK1 | 1 | 1 | 2 | N |
| hsa-miR-485-5p | ARL2BP | 1 | 1 | 2 | N |
| hsa-miR-485-5p | DAND5 | 1 | 1 | 2 | N |
| hsa-miR-485-5p | ZNF384 | 1 | 1 | 2 | N |
| hsa-miR-485-5p | PARP11 | 1 | 1 | 2 | N |
| hsa-miR-485-5p | VPS26A | 1 | 1 | 2 | N |
| hsa-miR-485-5p | ACTR3 | 1 | 1 | 2 | N |
| hsa-miR-744-3p | AZIN1 | 1 | 1 | 2 | Y |
| hsa-miR-744-3p | URI1 | 1 | 1 | 2 | N |
| hsa-miR-744-3p | FAM47B | 1 | 1 | 2 | N |
| hsa-miR-744-3p | LRIF1 | 1 | 1 | 2 | N |
| hsa-miR-744-3p | KCTD4 | 1 | 1 | 2 | N |
| hsa-miR-744-3p | ANAPC16 | 1 | 1 | 2 | N |
| hsa-miR-744-3p | MTMR9 | 1 | 1 | 2 | N |
| hsa-miR-744-3p | EPS15 | 1 | 1 | 2 | N |
| hsa-miR-744-3p | FAM109A | 1 | 1 | 2 | N |
| hsa-miR-744-3p | ESCO1 | 1 | 1 | 2 | N |
| hsa-miR-744-3p | RBM11 | 1 | 1 | 2 | N |
| hsa-miR-744-3p | COPS2 | 1 | 1 | 2 | N |
| hsa-miR-744-3p | NDST3 | 1 | 1 | 2 | N |
| hsa-miR-744-3p | FAM47A | 1 | 1 | 2 | N |
| hsa-miR-744-3p | TMED7 | 1 | 1 | 2 | N |
| hsa-miR-744-3p | ABCC4 | 1 | 1 | 2 | N |
| hsa-miR-744-3p | RNPS1 | 1 | 1 | 2 | N |
| hsa-miR-744-3p | ARIH2 | 1 | 1 | 2 | N |
| hsa-miR-744-3p | ARHGAP23 | 1 | 1 | 2 | N |
| hsa-miR-744-3p | SLC25A44 | 1 | 1 | 2 | N |
| hsa-miR-744-3p | FMR1 | 1 | 1 | 2 | N |
| hsa-miR-744-3p | FAM177B | 1 | 1 | 2 | N |
| hsa-miR-744-3p | HNRNPUL1 | 1 | 1 | 2 | N |
| hsa-miR-744-3p | CD302 | 1 | 1 | 2 | N |
| hsa-miR-744-3p | KPNA3 | 1 | 1 | 2 | N |
| hsa-miR-744-3p | FGF9 | 1 | 1 | 2 | N |
| hsa-miR-744-3p | STK39 | 1 | 1 | 2 | N |
| hsa-miR-744-3p | EDN3 | 1 | 1 | 2 | N |
| hsa-miR-744-3p | GPR85 | 1 | 1 | 2 | N |
| hsa-miR-744-3p | PPP2R1B | 1 | 1 | 2 | N |
| hsa-miR-744-3p | PHF2 | 1 | 1 | 2 | N |
| hsa-miR-744-3p | ZNF594 | 1 | 1 | 2 | N |
| hsa-miR-744-3p | CYBRD1 | 1 | 1 | 2 | N |
| hsa-miR-744-3p | SH3BGRL3 | 1 | 1 | 2 | N |
| hsa-miR-744-3p | TMEM140 | 1 | 1 | 2 | N |
| hsa-miR-744-3p | ZNF800 | 1 | 1 | 2 | N |
| hsa-miR-148a-5p | NUP50 | 1 | 1 | 2 | N |
| hsa-miR-148a-5p | ABRACL | 1 | 1 | 2 | N |
| hsa-miR-148a-5p | SPOCK3 | 1 | 1 | 2 | N |
| hsa-miR-148a-5p | PHTF2 | 1 | 1 | 2 | N |
| hsa-miR-148a-5p | THUMPD3 | 1 | 1 | 2 | N |
| hsa-miR-148a-5p | TXN2 | 1 | 1 | 2 | N |
| hsa-miR-148a-5p | HSPA5 | 1 | 1 | 2 | N |
| hsa-miR-148a-5p | CSNK1A1 | 1 | 1 | 2 | N |
| hsa-miR-148a-5p | FAM169A | 1 | 1 | 2 | N |
| hsa-miR-148a-5p | SIX4 | 1 | 1 | 2 | N |
| hsa-miR-148a-5p | STIL | 1 | 1 | 2 | N |
| hsa-miR-148a-5p | GSR | 1 | 1 | 2 | N |
| hsa-miR-148a-5p | UBE2G1 | 1 | 1 | 2 | N |
| hsa-miR-148a-5p | TCEAL1 | 1 | 1 | 2 | N |
| hsa-miR-148a-5p | DNASE2 | 1 | 1 | 2 | N |
| hsa-miR-148a-5p | MBLAC2 | 1 | 1 | 2 | N |
| hsa-miR-148a-5p | ZFP42 | 1 | 1 | 2 | N |
| hsa-miR-148a-5p | PIGC | 1 | 1 | 2 | N |
| hsa-miR-148a-5p | USP3 | 1 | 1 | 2 | N |
| hsa-miR-148a-5p | RAB8B | 1 | 1 | 2 | N |
| hsa-miR-148a-5p | NIN | 1 | 1 | 2 | N |
| hsa-miR-148a-5p | C21orf59 | 1 | 1 | 2 | N |
| hsa-miR-148a-5p | TMEM212 | 1 | 1 | 2 | N |
| hsa-miR-148a-5p | HIST1H2BD | 1 | 1 | 2 | N |
| hsa-miR-148a-5p | TMED7 | 1 | 1 | 2 | N |
| hsa-miR-148a-5p | AGL | 1 | 1 | 2 | N |
| hsa-miR-148a-5p | TMEM199 | 1 | 1 | 2 | N |
| hsa-miR-148a-5p | CYorf17 | 1 | 1 | 2 | N |
| hsa-miR-148a-5p | COL10A1 | 1 | 1 | 2 | N |
| hsa-miR-29b-1-5p | PROS1 | 1 | 1 | 2 | N |
| hsa-miR-29b-1-5p | CLEC2B | 1 | 1 | 2 | N |
| hsa-miR-29b-1-5p | REM1 | 1 | 1 | 2 | N |
| hsa-miR-29b-1-5p | NEUROD1 | 1 | 1 | 2 | Y |
| hsa-miR-29b-1-5p | EP300 | 1 | 1 | 2 | N |
| hsa-miR-29b-1-5p | PARP2 | 1 | 1 | 2 | N |
| hsa-miR-29b-1-5p | PWWP2A | 1 | 1 | 2 | N |
| hsa-miR-29b-1-5p | AGAP3 | 1 | 1 | 2 | N |
| hsa-miR-29b-1-5p | SLMO2 | 1 | 1 | 2 | N |
| hsa-miR-29b-1-5p | CNEP1R1 | 1 | 1 | 2 | N |
| hsa-miR-29b-1-5p | VSIG1 | 1 | 1 | 2 | N |
| hsa-miR-29b-1-5p | RUNDC3A | 1 | 1 | 2 | N |
| hsa-miR-29b-1-5p | NSFL1C | 1 | 1 | 2 | N |
| hsa-miR-29b-1-5p | FGB | 1 | 1 | 2 | N |
| hsa-miR-29b-1-5p | POU2F2 | 1 | 1 | 2 | N |
| hsa-miR-29b-1-5p | LYRM1 | 1 | 1 | 2 | N |
| hsa-miR-29b-1-5p | BPNT1 | 1 | 1 | 2 | N |
| hsa-miR-29b-1-5p | DIXDC1 | 1 | 1 | 2 | N |
| hsa-miR-29b-1-5p | DDIT4L | 1 | 1 | 2 | N |
| hsa-miR-29b-1-5p | TNPO3 | 1 | 1 | 2 | N |
| hsa-miR-29b-1-5p | MLANA | 1 | 1 | 2 | N |
| hsa-miR-29b-1-5p | ACTC1 | 1 | 1 | 2 | Y |
| hsa-miR-29b-1-5p | MMD | 1 | 1 | 2 | N |
| hsa-miR-29b-1-5p | WFDC10B | 1 | 1 | 2 | N |
| hsa-miR-29b-1-5p | NPR2 | 1 | 1 | 2 | N |
| hsa-miR-29b-1-5p | EWSR1 | 1 | 1 | 2 | N |
| hsa-miR-29b-1-5p | RGS22 | 1 | 1 | 2 | N |
| hsa-miR-29b-1-5p | FOLR1 | 1 | 1 | 2 | Y |
| hsa-miR-29b-1-5p | C1orf43 | 1 | 1 | 2 | N |
| hsa-miR-29b-1-5p | PDYN | 1 | 1 | 2 | N |
| hsa-miR-29b-1-5p | ZDHHC5 | 1 | 1 | 2 | Y |
| hsa-miR-29b-1-5p | ASF1A | 1 | 1 | 2 | N |
| hsa-miR-29b-1-5p | STC1 | 1 | 1 | 2 | N |
| hsa-miR-29b-1-5p | EXT1 | 1 | 1 | 2 | N |
| hsa-miR-29b-1-5p | CA13 | 1 | 1 | 2 | N |
| hsa-miR-29b-1-5p | FIGN | 1 | 1 | 2 | N |
| hsa-miR-29b-1-5p | LHFPL2 | 1 | 1 | 2 | N |
| hsa-miR-29b-1-5p | WFDC10A | 1 | 1 | 2 | N |
| hsa-miR-29b-1-5p | FA2H | 1 | 1 | 2 | N |
| hsa-miR-532-5p | RCOR1 | 1 | 1 | 2 | N |
| hsa-miR-532-5p | CXCL2 | 1 | 1 | 2 | Y |
| hsa-miR-532-5p | STC2 | 1 | 1 | 2 | N |
| hsa-miR-532-5p | CCDC64 | 1 | 1 | 2 | N |
| hsa-miR-532-5p | GBP3 | 1 | 1 | 2 | N |
| hsa-miR-532-5p | DENND6A | 1 | 1 | 2 | N |
| hsa-miR-532-5p | RASSF5 | 1 | 1 | 2 | N |
| hsa-miR-532-5p | CCNG1 | 1 | 1 | 2 | N |
| hsa-miR-532-5p | CXCL1 | 1 | 1 | 2 | N |
| hsa-miR-532-5p | RAB11A | 1 | 1 | 2 | N |
| hsa-miR-532-5p | SESTD1 | 1 | 1 | 2 | N |
| hsa-miR-532-5p | LINGO2 | 1 | 1 | 2 | N |
| hsa-miR-532-5p | C11orf87 | 1 | 1 | 2 | N |
| hsa-miR-532-5p | TAP1 | 1 | 1 | 2 | N |
| hsa-miR-532-5p | CPEB3 | 1 | 1 | 2 | N |
| hsa-miR-532-5p | NFATC2IP | 1 | 1 | 2 | N |
| hsa-miR-532-5p | KRAS | 1 | 1 | 2 | N |
| hsa-miR-532-5p | SLC39A8 | 1 | 1 | 2 | N |
| hsa-miR-1180-3p | ETS1 | 1 | 1 | 2 | N |
| hsa-miR-1180-3p | MKNK2 | 1 | 1 | 2 | Y |
| hsa-miR-1180-3p | PRIMA1 | 1 | 1 | 2 | N |
| hsa-miR-1180-3p | ZSWIM6 | 1 | 1 | 2 | Y |
| hsa-miR-1180-3p | TEF | 1 | 1 | 2 | N |
| hsa-miR-1180-3p | KANSL1 | 1 | 1 | 2 | Y |
| hsa-miR-185-5p | IKZF4 | 1 | 1 | 2 | N |
| hsa-miR-185-5p | DNM1 | 1 | 1 | 2 | N |
| hsa-miR-185-5p | CCND2 | 1 | 1 | 2 | Y |
| hsa-miR-185-5p | KIF3C | 1 | 1 | 2 | N |
| hsa-miR-185-5p | PCDHA1 | 1 | 1 | 2 | N |
| hsa-miR-185-5p | PCDHA10 | 1 | 1 | 2 | N |
| hsa-miR-185-5p | PCDHA9 | 1 | 1 | 2 | N |
| hsa-miR-185-5p | TRIM44 | 1 | 1 | 2 | N |
| hsa-miR-185-5p | TEAD1 | 1 | 1 | 2 | N |
| hsa-miR-185-5p | SPATA2 | 1 | 1 | 2 | Y |
| hsa-miR-185-5p | PCDHA6 | 1 | 1 | 2 | N |
| hsa-miR-185-5p | TOX3 | 1 | 1 | 2 | N |
| hsa-miR-185-5p | PAK6 | 1 | 1 | 2 | N |
| hsa-miR-185-5p | NMNAT2 | 1 | 1 | 2 | N |
| hsa-miR-185-5p | SLC16A2 | 1 | 1 | 2 | N |
| hsa-miR-185-5p | ATP6V1F | 1 | 1 | 2 | N |
| hsa-miR-185-5p | PCDHA12 | 1 | 1 | 2 | N |
| hsa-miR-185-5p | SF1 | 1 | 1 | 2 | Y |
| hsa-miR-185-5p | PCDHA5 | 1 | 1 | 2 | N |
| hsa-miR-185-5p | GPR26 | 1 | 1 | 2 | N |
| hsa-miR-185-5p | EIF5A | 1 | 1 | 2 | N |
| hsa-miR-185-5p | PCDHA11 | 1 | 1 | 2 | N |
| hsa-miR-185-5p | SGMS1 | 1 | 1 | 2 | N |
| hsa-miR-185-5p | SLC39A14 | 1 | 1 | 2 | N |
| hsa-miR-185-5p | PCDHAC1 | 1 | 1 | 2 | N |
| hsa-miR-185-5p | PCDHA8 | 1 | 1 | 2 | N |
| hsa-miR-185-5p | NFATC3 | 1 | 1 | 2 | Y |
| hsa-miR-185-5p | SLC25A15 | 1 | 1 | 2 | N |
| hsa-miR-185-5p | PBX2 | 1 | 1 | 2 | Y |
| hsa-miR-185-5p | PCDHA4 | 1 | 1 | 2 | N |
| hsa-miR-185-5p | XYLT1 | 1 | 1 | 2 | N |
| hsa-miR-185-5p | ZC3H11A | 1 | 1 | 2 | Y |
| hsa-miR-185-5p | CDC42 | 1 | 1 | 2 | Y |
| hsa-miR-185-5p | PCDHA3 | 1 | 1 | 2 | N |
| hsa-miR-185-5p | CAPZB | 1 | 1 | 2 | Y |
| hsa-miR-185-5p | GPR61 | 1 | 1 | 2 | N |
| hsa-miR-185-5p | RAB35 | 1 | 1 | 2 | N |
| hsa-miR-185-5p | PCDHAC2 | 1 | 1 | 2 | N |
| hsa-miR-185-5p | THRA | 1 | 1 | 2 | N |
| hsa-miR-185-5p | CAMK2D | 1 | 1 | 2 | Y |
| hsa-miR-185-5p | SNX30 | 1 | 1 | 2 | N |
| hsa-miR-185-5p | PCDHA2 | 1 | 1 | 2 | N |
| hsa-miR-185-5p | PHF7 | 1 | 1 | 2 | N |
| hsa-miR-185-5p | SIX3 | 1 | 1 | 2 | N |
| hsa-miR-185-5p | TANC2 | 1 | 1 | 2 | N |
| hsa-miR-185-5p | FAM76A | 1 | 1 | 2 | N |
| hsa-miR-185-5p | BSN | 1 | 1 | 2 | N |
| hsa-miR-185-5p | SOX13 | 1 | 1 | 2 | N |
| hsa-miR-185-5p | DLG2 | 1 | 1 | 2 | N |
| hsa-miR-185-5p | LCOR | 1 | 1 | 2 | N |
| hsa-miR-185-5p | PCDHA13 | 1 | 1 | 2 | N |
| hsa-miR-185-5p | SMG7 | 1 | 1 | 2 | Y |
| hsa-miR-185-5p | ATP1A3 | 1 | 1 | 2 | N |
| hsa-miR-185-5p | CA10 | 1 | 1 | 2 | N |
| hsa-miR-185-5p | CDH4 | 1 | 1 | 2 | N |
| hsa-miR-185-5p | SLC25A53 | 1 | 1 | 2 | N |
| hsa-miR-185-5p | PCDHA7 | 1 | 1 | 2 | N |
| hsa-miR-5010-5p | HOXB3 | 1 | 1 | 2 | N |
| hsa-miR-5010-5p | FNDC7 | 1 | 1 | 2 | N |
| hsa-miR-5010-5p | GUCA1B | 1 | 1 | 2 | N |
| hsa-miR-5010-5p | RNF216 | 1 | 1 | 2 | N |
| hsa-miR-5010-5p | RNF125 | 1 | 1 | 2 | N |
| hsa-miR-5010-5p | NUDT15 | 1 | 1 | 2 | N |
| hsa-miR-5010-5p | CUX1 | 1 | 1 | 2 | N |
| hsa-miR-5010-5p | PLK3 | 1 | 1 | 2 | N |
| hsa-miR-5010-5p | SLC5A8 | 1 | 1 | 2 | N |
| hsa-miR-5010-5p | NAPG | 1 | 1 | 2 | N |
| hsa-miR-5010-5p | FILIP1L | 1 | 1 | 2 | N |
| hsa-miR-5010-5p | KDELR3 | 1 | 1 | 2 | N |
| hsa-miR-5010-5p | PSENEN | 1 | 1 | 2 | N |
| hsa-miR-5010-5p | MSS51 | 1 | 1 | 2 | N |
| hsa-miR-5010-5p | NCAN | 1 | 1 | 2 | N |
| hsa-miR-5010-5p | SHISA7 | 1 | 1 | 2 | N |
| hsa-miR-5010-5p | STC1 | 1 | 1 | 2 | N |
| hsa-miR-5010-5p | ARHGEF10 | 1 | 1 | 2 | N |
| hsa-miR-5010-5p | TACR1 | 1 | 1 | 2 | N |
| hsa-miR-5010-5p | GNG7 | 1 | 1 | 2 | N |
| hsa-miR-5010-5p | RAB11FIP5 | 1 | 1 | 2 | N |
| hsa-miR-5010-5p | PPT2 | 1 | 1 | 2 | N |
| hsa-miR-5010-5p | SLC35A4 | 1 | 1 | 2 | N |
| hsa-miR-5010-5p | ENTPD2 | 1 | 1 | 2 | N |
| hsa-miR-5010-5p | TSPYL2 | 1 | 1 | 2 | N |
| hsa-miR-5010-5p | STIM2 | 1 | 1 | 2 | N |
| hsa-miR-5010-5p | C1orf106 | 1 | 1 | 2 | N |
| hsa-miR-5010-5p | COPG1 | 1 | 1 | 2 | N |
| hsa-miR-5010-5p | STMN2 | 1 | 1 | 2 | N |
| hsa-miR-5010-5p | PTPRR | 1 | 1 | 2 | N |
| hsa-miR-5010-5p | NAB2 | 1 | 1 | 2 | N |
| hsa-miR-6868-3p | SYCE1 | 1 | 1 | 2 | N |
| hsa-miR-6868-3p | MARCKS | 1 | 1 | 2 | N |
| hsa-miR-6868-3p | DNAH5 | 1 | 1 | 2 | N |
| hsa-miR-6868-3p | EIF4E | 1 | 1 | 2 | N |
| hsa-miR-6868-3p | RAB11FIP2 | 1 | 1 | 2 | N |
| hsa-miR-6868-3p | EVA1C | 1 | 1 | 2 | N |
| hsa-miR-6868-3p | GTF2E2 | 1 | 1 | 2 | N |
| hsa-miR-6868-3p | SOX4 | 1 | 1 | 2 | N |
| hsa-miR-6868-3p | TMEM138 | 1 | 1 | 2 | Y |
| hsa-miR-6868-3p | GADD45A | 1 | 1 | 2 | N |
| hsa-miR-6868-3p | PZP | 1 | 1 | 2 | N |
| hsa-miR-6868-3p | EHF | 1 | 1 | 2 | N |
| hsa-miR-6868-3p | PRDM1 | 1 | 1 | 2 | N |
| hsa-miR-6868-3p | NEGR1 | 1 | 1 | 2 | Y |
| hsa-miR-6868-3p | DPYSL3 | 1 | 1 | 2 | N |
| hsa-miR-6868-3p | OTX2 | 1 | 1 | 2 | N |
| hsa-miR-6868-3p | KY | 1 | 1 | 2 | N |
| hsa-miR-6868-3p | SETD7 | 1 | 1 | 2 | N |
| hsa-miR-6868-3p | DTWD2 | 1 | 1 | 2 | N |
| hsa-miR-6868-3p | TAF4B | 1 | 1 | 2 | N |
| hsa-miR-6868-3p | DECR2 | 1 | 1 | 2 | N |
| hsa-miR-6868-3p | GNG10 | 1 | 1 | 2 | N |
| hsa-miR-6868-3p | TXLNB | 1 | 1 | 2 | N |
| hsa-miR-6868-3p | C10orf128 | 1 | 1 | 2 | N |
| hsa-miR-6868-3p | SPTY2D1 | 1 | 1 | 2 | N |
| hsa-miR-6868-3p | C19orf43 | 1 | 1 | 2 | N |
| hsa-miR-6868-3p | C1orf115 | 1 | 1 | 2 | N |
| hsa-miR-6868-3p | RSRC2 | 1 | 1 | 2 | Y |
| hsa-miR-6868-3p | GXYLT1 | 1 | 1 | 2 | N |
| hsa-miR-6868-3p | SPG20 | 1 | 1 | 2 | N |
| hsa-miR-6868-3p | KLHL23 | 1 | 1 | 2 | N |
| hsa-miR-6868-3p | EPDR1 | 1 | 1 | 2 | N |
| hsa-miR-6868-3p | TSHR | 1 | 1 | 2 | N |
| hsa-miR-6868-3p | ARRDC3 | 1 | 1 | 2 | N |
| hsa-miR-6868-3p | SYT1 | 1 | 1 | 2 | N |
| hsa-miR-6868-3p | NOTCH2NL | 1 | 1 | 2 | N |
| hsa-miR-6868-3p | ZHX1 | 1 | 1 | 2 | N |
| hsa-miR-6868-3p | EMX2 | 1 | 1 | 2 | Y |
| hsa-miR-6868-3p | WDR89 | 1 | 1 | 2 | N |
| hsa-miR-6868-3p | KLRK1 | 1 | 1 | 2 | N |
| hsa-miR-6868-3p | TRIM2 | 1 | 1 | 2 | N |
| hsa-miR-6868-3p | ZNF287 | 1 | 1 | 2 | N |
| hsa-miR-6868-3p | C8orf86 | 1 | 1 | 2 | N |
| hsa-miR-6868-3p | DNAJC25-GNG10 | 1 | 1 | 2 | N |
| hsa-miR-6868-3p | RUNDC3B | 1 | 1 | 2 | N |
| hsa-miR-6868-3p | GATA2 | 1 | 1 | 2 | N |
| hsa-miR-6868-3p | FBXO32 | 1 | 1 | 2 | N |
| hsa-miR-6868-3p | DIO2 | 1 | 1 | 2 | N |
| hsa-miR-6868-3p | LYN | 1 | 1 | 2 | N |
| hsa-miR-6868-3p | RND1 | 1 | 1 | 2 | N |
| hsa-miR-6868-3p | RGS1 | 1 | 1 | 2 | N |
| hsa-miR-342-3p | KCNA4 | 1 | 1 | 2 | N |
| hsa-miR-342-3p | SYNPO2L | 1 | 1 | 2 | Y |
| hsa-miR-342-3p | KDM6B | 1 | 1 | 2 | N |
| hsa-miR-342-3p | EPC1 | 1 | 1 | 2 | N |
| hsa-miR-342-3p | UBE2D2 | 1 | 1 | 2 | N |
| hsa-miR-342-3p | FUT8 | 1 | 1 | 2 | N |
| hsa-miR-342-3p | PKDCC | 1 | 1 | 2 | N |
| hsa-miR-342-3p | MRFAP1 | 1 | 1 | 2 | N |
| hsa-miR-342-3p | FAM53C | 1 | 1 | 2 | N |
| hsa-miR-342-3p | MATN1 | 1 | 1 | 2 | N |
| hsa-miR-342-3p | DTNBP1 | 1 | 1 | 2 | N |
| hsa-miR-342-3p | RGS4 | 1 | 1 | 2 | Y |
| hsa-miR-342-3p | OSER1 | 1 | 1 | 2 | N |
| hsa-miR-342-3p | ID4 | 1 | 1 | 2 | Y |
| hsa-miR-342-3p | GXYLT1 | 1 | 1 | 2 | N |
| hsa-miR-342-3p | FOSB | 1 | 1 | 2 | N |
| hsa-miR-342-3p | FAM208A | 1 | 1 | 2 | N |
| hsa-miR-342-3p | MMS19 | 1 | 1 | 2 | N |
| hsa-miR-455-3p | TMEFF1 | 1 | 1 | 2 | N |
| hsa-miR-455-3p | COLEC12 | 1 | 1 | 2 | N |
| hsa-miR-455-3p | ESCO1 | 1 | 1 | 2 | N |
| hsa-miR-455-3p | PRKAB2 | 1 | 1 | 2 | N |
| hsa-miR-455-3p | CUL3 | 1 | 1 | 2 | N |
| hsa-miR-455-3p | ARFGAP2 | 1 | 1 | 2 | N |
| hsa-miR-455-3p | UBE2Q2 | 1 | 1 | 2 | N |
| hsa-miR-455-3p | GABARAPL2 | 1 | 1 | 2 | N |
| hsa-miR-455-3p | ACAN | 1 | 1 | 2 | N |
| hsa-miR-455-3p | CD80 | 1 | 1 | 2 | N |
| hsa-miR-455-3p | ELF3 | 1 | 1 | 2 | N |
| hsa-miR-455-3p | HOXC4 | 1 | 1 | 2 | N |
| hsa-miR-455-3p | NLN | 1 | 1 | 2 | N |
| hsa-miR-455-3p | SLC25A3 | 1 | 1 | 2 | N |
| hsa-miR-455-3p | ZBTB18 | 1 | 1 | 2 | N |
| hsa-miR-455-3p | RTN4 | 1 | 1 | 2 | N |
| hsa-miR-455-3p | TTK | 1 | 1 | 2 | N |
| hsa-miR-455-3p | AGO4 | 1 | 1 | 2 | N |
| hsa-miR-455-3p | HM13 | 1 | 1 | 2 | N |
| hsa-miR-455-3p | STK17B | 1 | 1 | 2 | N |
| hsa-miR-455-3p | LHX2 | 1 | 1 | 2 | N |
| hsa-miR-455-3p | MSANTD3-TMEFF1 | 1 | 1 | 2 | N |
| hsa-miR-15a-5p | ARMCX2 | 1 | 1 | 2 | N |
| hsa-miR-15a-5p | INSR | 1 | 1 | 2 | N |
| hsa-miR-15a-5p | UBFD1 | 1 | 1 | 2 | N |
| hsa-miR-15a-5p | ZC2HC1A | 1 | 1 | 2 | N |
| hsa-miR-15a-5p | RAP2C | 1 | 1 | 2 | Y |
| hsa-miR-15a-5p | PCDHA11 | 1 | 1 | 2 | N |
| hsa-miR-15a-5p | HELZ | 1 | 1 | 2 | N |
| hsa-miR-15a-5p | CPD | 1 | 1 | 2 | N |
| hsa-miR-15a-5p | GABARAPL1 | 1 | 1 | 2 | Y |
| hsa-miR-15a-5p | KIF23 | 1 | 1 | 2 | Y |
| hsa-miR-15a-5p | IARS | 1 | 1 | 2 | N |
| hsa-miR-15a-5p | SIRT4 | 1 | 1 | 2 | Y |
| hsa-miR-15a-5p | TTC14 | 1 | 1 | 2 | N |
| hsa-miR-15a-5p | ADRB2 | 1 | 1 | 2 | N |
| hsa-miR-15a-5p | CCND1 | 1 | 1 | 2 | Y |
| hsa-miR-15a-5p | PDIK1L | 1 | 1 | 2 | Y |
| hsa-miR-15a-5p | PTPN3 | 1 | 1 | 2 | N |
| hsa-miR-15a-5p | RNF217 | 1 | 1 | 2 | N |
| hsa-miR-15a-5p | SUMO3 | 1 | 1 | 2 | N |
| hsa-miR-15a-5p | SPRED1 | 1 | 1 | 2 | Y |
| hsa-miR-15a-5p | CDCA4 | 1 | 1 | 2 | Y |
| hsa-miR-15a-5p | KCNJ2 | 1 | 1 | 2 | N |
| hsa-miR-15a-5p | ZBTB39 | 1 | 1 | 2 | N |
| hsa-miR-15a-5p | ARHGDIA | 1 | 1 | 2 | Y |
| hsa-miR-15a-5p | PAFAH1B1 | 1 | 1 | 2 | Y |
| hsa-miR-15a-5p | TNFSF13B | 1 | 1 | 2 | N |
| hsa-miR-15a-5p | HPCAL4 | 1 | 1 | 2 | N |
| hsa-miR-15a-5p | RNF125 | 1 | 1 | 2 | N |
| hsa-miR-15a-5p | BTRC | 1 | 1 | 2 | Y |
| hsa-miR-15a-5p | RNF144B | 1 | 1 | 2 | N |
| hsa-miR-15a-5p | N4BP1 | 1 | 1 | 2 | Y |
| hsa-miR-15a-5p | FERMT2 | 1 | 1 | 2 | N |
| hsa-miR-15a-5p | ZHX1 | 1 | 1 | 2 | N |
| hsa-miR-15a-5p | ANO3 | 1 | 1 | 2 | N |
| hsa-miR-15a-5p | CCDC19 | 1 | 1 | 2 | N |
| hsa-miR-15a-5p | NXPH1 | 1 | 1 | 2 | N |
| hsa-miR-15a-5p | PCDHA4 | 1 | 1 | 2 | N |
| hsa-miR-15a-5p | PCDHA7 | 1 | 1 | 2 | N |
| hsa-miR-15a-5p | AGO4 | 1 | 1 | 2 | Y |
| hsa-miR-15a-5p | LATS2 | 1 | 1 | 2 | N |
| hsa-miR-15a-5p | COPS2 | 1 | 1 | 2 | N |
| hsa-miR-15a-5p | ATG9A | 1 | 1 | 2 | Y |
| hsa-miR-15a-5p | FAM91A1 | 1 | 1 | 2 | N |
| hsa-miR-15a-5p | SLC4A4 | 1 | 1 | 2 | N |
| hsa-miR-15a-5p | BCL7A | 1 | 1 | 2 | Y |
| hsa-miR-15a-5p | SOCS6 | 1 | 1 | 2 | N |
| hsa-miR-15a-5p | LUZP1 | 1 | 1 | 2 | Y |
| hsa-miR-15a-5p | PLEKHA5 | 1 | 1 | 2 | N |
| hsa-miR-15a-5p | AMER1 | 1 | 1 | 2 | Y |
| hsa-miR-15a-5p | GSTCD | 1 | 1 | 2 | N |
| hsa-miR-15a-5p | KIF5A | 1 | 1 | 2 | N |
| hsa-miR-15a-5p | TBPL1 | 1 | 1 | 2 | Y |
| hsa-miR-15a-5p | PTH | 1 | 1 | 2 | N |
| hsa-miR-15a-5p | NUP50 | 1 | 1 | 2 | Y |
| hsa-miR-15a-5p | ATXN2 | 1 | 1 | 2 | N |
| hsa-miR-15a-5p | ZMAT3 | 1 | 1 | 2 | Y |
| hsa-miR-15a-5p | RAB9A | 1 | 1 | 2 | N |
| hsa-miR-15a-5p | PVRL1 | 1 | 1 | 2 | N |
| hsa-miR-15a-5p | ATXN7L3 | 1 | 1 | 2 | N |
| hsa-miR-15a-5p | POLR3F | 1 | 1 | 2 | N |
| hsa-miR-15a-5p | SLC9A6 | 1 | 1 | 2 | Y |
| hsa-miR-15a-5p | TSPYL2 | 1 | 1 | 2 | Y |
| hsa-miR-15a-5p | BTAF1 | 1 | 1 | 2 | N |
| hsa-miR-15a-5p | FSD1 | 1 | 1 | 2 | N |
| hsa-miR-15a-5p | UBE2Q1 | 1 | 1 | 2 | Y |
| hsa-miR-15a-5p | KLHL18 | 1 | 1 | 2 | N |
| hsa-miR-15a-5p | PCDHAC1 | 1 | 1 | 2 | N |
| hsa-miR-15a-5p | CRKL | 1 | 1 | 2 | Y |
| hsa-miR-15a-5p | ZCCHC3 | 1 | 1 | 2 | Y |
| hsa-miR-15a-5p | IPO7 | 1 | 1 | 2 | N |
| hsa-miR-15a-5p | SLC41A2 | 1 | 1 | 2 | N |
| hsa-miR-15a-5p | DLL1 | 1 | 1 | 2 | N |
| hsa-miR-15a-5p | MOB3B | 1 | 1 | 2 | N |
| hsa-miR-15a-5p | CCNT2 | 1 | 1 | 2 | Y |
| hsa-miR-15a-5p | ZBTB44 | 1 | 1 | 2 | N |
| hsa-miR-15a-5p | XPO7 | 1 | 1 | 2 | N |
| hsa-miR-15a-5p | PURA | 1 | 1 | 2 | Y |
| hsa-miR-15a-5p | TAB3 | 1 | 1 | 2 | N |
| hsa-miR-15a-5p | ARL3 | 1 | 1 | 2 | N |
| hsa-miR-15a-5p | KDSR | 1 | 1 | 2 | N |
| hsa-miR-15a-5p | CLDN2 | 1 | 1 | 2 | N |
| hsa-miR-15a-5p | SNX16 | 1 | 1 | 2 | Y |
| hsa-miR-15a-5p | TMEM178B | 1 | 1 | 2 | N |
| hsa-miR-15a-5p | PISD | 1 | 1 | 2 | Y |
| hsa-miR-15a-5p | TRANK1 | 1 | 1 | 2 | N |
| hsa-miR-15a-5p | FAM110C | 1 | 1 | 2 | N |
| hsa-miR-15a-5p | KIF1B | 1 | 1 | 2 | N |
| hsa-miR-15a-5p | VEGFA | 1 | 1 | 2 | Y |
| hsa-miR-15a-5p | CLCN4 | 1 | 1 | 2 | N |
| hsa-miR-15a-5p | PRDM4 | 1 | 1 | 2 | Y |
| hsa-miR-15a-5p | TMEM255A | 1 | 1 | 2 | N |
| hsa-miR-15a-5p | MAMSTR | 1 | 1 | 2 | N |
| hsa-miR-15a-5p | HIGD1A | 1 | 1 | 2 | Y |
| hsa-miR-15a-5p | GRM7 | 1 | 1 | 2 | N |
| hsa-miR-15a-5p | ENTPD7 | 1 | 1 | 2 | Y |
| hsa-miR-15a-5p | RASGEF1B | 1 | 1 | 2 | N |
| hsa-miR-15a-5p | PCDHA12 | 1 | 1 | 2 | N |
| hsa-miR-15a-5p | MAP7 | 1 | 1 | 2 | N |
| hsa-miR-15a-5p | SHOC2 | 1 | 1 | 2 | Y |
| hsa-miR-15a-5p | USP42 | 1 | 1 | 2 | Y |
| hsa-miR-15a-5p | GNAI3 | 1 | 1 | 2 | N |
| hsa-miR-15a-5p | TENM2 | 1 | 1 | 2 | N |
| hsa-miR-15a-5p | FGF2 | 1 | 1 | 2 | Y |
| hsa-miR-15a-5p | HSPA4L | 1 | 1 | 2 | Y |
| hsa-miR-15a-5p | LSM11 | 1 | 1 | 2 | Y |
| hsa-miR-15a-5p | PCDHA5 | 1 | 1 | 2 | N |
| hsa-miR-15a-5p | CACUL1 | 1 | 1 | 2 | Y |
| hsa-miR-15a-5p | WEE1 | 1 | 1 | 2 | Y |
| hsa-miR-15a-5p | RECK | 1 | 1 | 2 | Y |
| hsa-miR-15a-5p | CPEB3 | 1 | 1 | 2 | Y |
| hsa-miR-15a-5p | ATXN1L | 1 | 1 | 2 | N |
| hsa-miR-15a-5p | CDC25A | 1 | 1 | 2 | Y |
| hsa-miR-15a-5p | C2orf42 | 1 | 1 | 2 | Y |
| hsa-miR-15a-5p | ZBTB46 | 1 | 1 | 2 | N |
| hsa-miR-15a-5p | DCP1A | 1 | 1 | 2 | N |
| hsa-miR-15a-5p | DENND6A | 1 | 1 | 2 | Y |
| hsa-miR-15a-5p | DOLPP1 | 1 | 1 | 2 | N |
| hsa-miR-15a-5p | RAD23B | 1 | 1 | 2 | Y |
| hsa-miR-15a-5p | KCNN4 | 1 | 1 | 2 | N |
| hsa-miR-15a-5p | SYNJ1 | 1 | 1 | 2 | N |
| hsa-miR-15a-5p | SLIT2 | 1 | 1 | 2 | N |
| hsa-miR-15a-5p | SCOC | 1 | 1 | 2 | N |
| hsa-miR-15a-5p | TLK1 | 1 | 1 | 2 | Y |
| hsa-miR-15a-5p | CYP26B1 | 1 | 1 | 2 | Y |
| hsa-miR-15a-5p | CC2D1B | 1 | 1 | 2 | N |
| hsa-miR-15a-5p | RAB9B | 1 | 1 | 2 | Y |
| hsa-miR-15a-5p | SESN1 | 1 | 1 | 2 | N |
| hsa-miR-15a-5p | FASN | 1 | 1 | 2 | Y |
| hsa-miR-15a-5p | TUBA1A | 1 | 1 | 2 | N |
| hsa-miR-15a-5p | EGLN1 | 1 | 1 | 2 | N |
| hsa-miR-15a-5p | FLT3 | 1 | 1 | 2 | N |
| hsa-miR-15a-5p | JARID2 | 1 | 1 | 2 | Y |
| hsa-miR-15a-5p | PLEKHA1 | 1 | 1 | 2 | Y |
| hsa-miR-15a-5p | APLN | 1 | 1 | 2 | N |
| hsa-miR-15a-5p | CAPRIN1 | 1 | 1 | 2 | N |
| hsa-miR-15a-5p | SMAD7 | 1 | 1 | 2 | Y |
| hsa-miR-15a-5p | SLC35G1 | 1 | 1 | 2 | N |
| hsa-miR-15a-5p | BTLA | 1 | 1 | 2 | N |
| hsa-miR-15a-5p | SEH1L | 1 | 1 | 2 | N |
| hsa-miR-15a-5p | CBX4 | 1 | 1 | 2 | Y |
| hsa-miR-15a-5p | PCDHA9 | 1 | 1 | 2 | N |
| hsa-miR-15a-5p | CREBRF | 1 | 1 | 2 | Y |
| hsa-miR-15a-5p | SCN8A | 1 | 1 | 2 | N |
| hsa-miR-15a-5p | OMG | 1 | 1 | 2 | N |
| hsa-miR-15a-5p | CDC37L1 | 1 | 1 | 2 | Y |
| hsa-miR-15a-5p | TPD52L3 | 1 | 1 | 2 | N |
| hsa-miR-15a-5p | BFAR | 1 | 1 | 2 | N |
| hsa-miR-15a-5p | ARL2 | 1 | 1 | 2 | N |
| hsa-miR-15a-5p | VPS4A | 1 | 1 | 2 | Y |
| hsa-miR-15a-5p | LCOR | 1 | 1 | 2 | N |
| hsa-miR-15a-5p | STRADB | 1 | 1 | 2 | Y |
| hsa-miR-15a-5p | HTR2A | 1 | 1 | 2 | N |
| hsa-miR-15a-5p | MOB4 | 1 | 1 | 2 | Y |
| hsa-miR-15a-5p | TACC1 | 1 | 1 | 2 | N |
| hsa-miR-15a-5p | C20orf112 | 1 | 1 | 2 | N |
| hsa-miR-15a-5p | IKBKB | 1 | 1 | 2 | N |
| hsa-miR-15a-5p | CSRNP1 | 1 | 1 | 2 | N |
| hsa-miR-15a-5p | UNC80 | 1 | 1 | 2 | N |
| hsa-miR-15a-5p | C16orf72 | 1 | 1 | 2 | Y |
| hsa-miR-15a-5p | IHH | 1 | 1 | 2 | N |
| hsa-miR-15a-5p | RASEF | 1 | 1 | 2 | Y |
| hsa-miR-15a-5p | RELN | 1 | 1 | 2 | N |
| hsa-miR-15a-5p | NISCH | 1 | 1 | 2 | N |
| hsa-miR-15a-5p | C8orf58 | 1 | 1 | 2 | N |
| hsa-miR-15a-5p | BCL2L2 | 1 | 1 | 2 | N |
| hsa-miR-15a-5p | SPSB4 | 1 | 1 | 2 | N |
| hsa-miR-15a-5p | CPEB2 | 1 | 1 | 2 | Y |
| hsa-miR-15a-5p | SALL4 | 1 | 1 | 2 | N |
| hsa-miR-15a-5p | SYDE2 | 1 | 1 | 2 | N |
| hsa-miR-15a-5p | CHEK1 | 1 | 1 | 2 | Y |
| hsa-miR-15a-5p | ANKS1A | 1 | 1 | 2 | N |
| hsa-miR-15a-5p | SYT4 | 1 | 1 | 2 | N |
| hsa-miR-15a-5p | GPN1 | 1 | 1 | 2 | N |
| hsa-miR-15a-5p | CDC42SE2 | 1 | 1 | 2 | Y |
| hsa-miR-15a-5p | PRR15L | 1 | 1 | 2 | N |
| hsa-miR-15a-5p | RAB11FIP2 | 1 | 1 | 2 | Y |
| hsa-miR-15a-5p | ARFGAP2 | 1 | 1 | 2 | N |
| hsa-miR-15a-5p | RSBN1 | 1 | 1 | 2 | N |
| hsa-miR-15a-5p | ZNF449 | 1 | 1 | 2 | Y |
| hsa-miR-15a-5p | PCDHA2 | 1 | 1 | 2 | N |
| hsa-miR-15a-5p | DNAJB4 | 1 | 1 | 2 | N |
| hsa-miR-15a-5p | IRAK2 | 1 | 1 | 2 | N |
| hsa-miR-15a-5p | MAP3K9 | 1 | 1 | 2 | N |
| hsa-miR-15a-5p | RNF24 | 1 | 1 | 2 | N |
| hsa-miR-15a-5p | CASK | 1 | 1 | 2 | Y |
| hsa-miR-15a-5p | DMTF1 | 1 | 1 | 2 | Y |
| hsa-miR-15a-5p | PTPRR | 1 | 1 | 2 | N |
| hsa-miR-15a-5p | SLC15A4 | 1 | 1 | 2 | N |
| hsa-miR-15a-5p | CAPZA2 | 1 | 1 | 2 | Y |
| hsa-miR-15a-5p | TSPAN5 | 1 | 1 | 2 | N |
| hsa-miR-15a-5p | KIF21A | 1 | 1 | 2 | N |
| hsa-miR-15a-5p | PCDHA13 | 1 | 1 | 2 | N |
| hsa-miR-15a-5p | PAPPA | 1 | 1 | 2 | N |
| hsa-miR-15a-5p | PDK4 | 1 | 1 | 2 | N |
| hsa-miR-15a-5p | SEPT2 | 1 | 1 | 2 | Y |
| hsa-miR-15a-5p | PHF19 | 1 | 1 | 2 | Y |
| hsa-miR-15a-5p | IST1 | 1 | 1 | 2 | N |
| hsa-miR-15a-5p | RSPO3 | 1 | 1 | 2 | N |
| hsa-miR-15a-5p | USP3 | 1 | 1 | 2 | Y |
| hsa-miR-15a-5p | MAP2K1 | 1 | 1 | 2 | N |
| hsa-miR-15a-5p | DYRK1B | 1 | 1 | 2 | N |
| hsa-miR-15a-5p | STK33 | 1 | 1 | 2 | N |
| hsa-miR-15a-5p | WIPI2 | 1 | 1 | 2 | Y |
| hsa-miR-15a-5p | LITAF | 1 | 1 | 2 | Y |
| hsa-miR-15a-5p | ELMSAN1 | 1 | 1 | 2 | N |
| hsa-miR-15a-5p | SLC9A8 | 1 | 1 | 2 | N |
| hsa-miR-15a-5p | G0S2 | 1 | 1 | 2 | N |
| hsa-miR-15a-5p | PPM1E | 1 | 1 | 2 | N |
| hsa-miR-15a-5p | CDK5R1 | 1 | 1 | 2 | N |
| hsa-miR-15a-5p | E2F7 | 1 | 1 | 2 | Y |
| hsa-miR-15a-5p | ASH1L | 1 | 1 | 2 | Y |
| hsa-miR-15a-5p | FAM133B | 1 | 1 | 2 | N |
| hsa-miR-15a-5p | ABL2 | 1 | 1 | 2 | Y |
| hsa-miR-15a-5p | MYT1L | 1 | 1 | 2 | N |
| hsa-miR-15a-5p | UBE4B | 1 | 1 | 2 | N |
| hsa-miR-15a-5p | RFWD2 | 1 | 1 | 2 | Y |
| hsa-miR-15a-5p | TCAIM | 1 | 1 | 2 | N |
| hsa-miR-15a-5p | PCDHA1 | 1 | 1 | 2 | N |
| hsa-miR-15a-5p | PCDHA6 | 1 | 1 | 2 | N |
| hsa-miR-15a-5p | MYLK | 1 | 1 | 2 | N |
| hsa-miR-15a-5p | ARIH1 | 1 | 1 | 2 | Y |
| hsa-miR-15a-5p | FBXO21 | 1 | 1 | 2 | N |
| hsa-miR-15a-5p | MED26 | 1 | 1 | 2 | N |
| hsa-miR-15a-5p | CDK17 | 1 | 1 | 2 | Y |
| hsa-miR-15a-5p | YWHAH | 1 | 1 | 2 | Y |
| hsa-miR-15a-5p | PPAP2B | 1 | 1 | 2 | N |
| hsa-miR-15a-5p | HPSE2 | 1 | 1 | 2 | N |
| hsa-miR-15a-5p | PLAG1 | 1 | 1 | 2 | Y |
| hsa-miR-15a-5p | PLSCR4 | 1 | 1 | 2 | N |
| hsa-miR-15a-5p | RARB | 1 | 1 | 2 | Y |
| hsa-miR-15a-5p | UBE2V1 | 1 | 1 | 2 | Y |
| hsa-miR-15a-5p | C1orf21 | 1 | 1 | 2 | Y |
| hsa-miR-15a-5p | PTPN4 | 1 | 1 | 2 | N |
| hsa-miR-15a-5p | SGK1 | 1 | 1 | 2 | N |
| hsa-miR-15a-5p | AREL1 | 1 | 1 | 2 | N |
| hsa-miR-15a-5p | ZNF622 | 1 | 1 | 2 | Y |
| hsa-miR-15a-5p | SLC36A1 | 1 | 1 | 2 | N |
| hsa-miR-15a-5p | GATAD2A | 1 | 1 | 2 | Y |
| hsa-miR-15a-5p | ZNF275 | 1 | 1 | 2 | Y |
| hsa-miR-15a-5p | MKNK1 | 1 | 1 | 2 | N |
| hsa-miR-15a-5p | CCNE1 | 1 | 1 | 2 | Y |
| hsa-miR-15a-5p | PTHLH | 1 | 1 | 2 | N |
| hsa-miR-15a-5p | PEX13 | 1 | 1 | 2 | Y |
| hsa-miR-15a-5p | GPR63 | 1 | 1 | 2 | N |
| hsa-miR-15a-5p | C1QL3 | 1 | 1 | 2 | N |
| hsa-miR-15a-5p | PCDHA3 | 1 | 1 | 2 | N |
| hsa-miR-15a-5p | FBXW7 | 1 | 1 | 2 | N |
| hsa-miR-15a-5p | FGFR1 | 1 | 1 | 2 | N |
| hsa-miR-15a-5p | BTG2 | 1 | 1 | 2 | Y |
| hsa-miR-15a-5p | WNT7A | 1 | 1 | 2 | N |
| hsa-miR-15a-5p | RFX3 | 1 | 1 | 2 | N |
| hsa-miR-15a-5p | ST8SIA3 | 1 | 1 | 2 | N |
| hsa-miR-15a-5p | ISLR | 1 | 1 | 2 | N |
| hsa-miR-15a-5p | E2F3 | 1 | 1 | 2 | Y |
| hsa-miR-15a-5p | CLDN12 | 1 | 1 | 2 | N |
| hsa-miR-15a-5p | AQP11 | 1 | 1 | 2 | N |
| hsa-miR-15a-5p | NRBP1 | 1 | 1 | 2 | N |
| hsa-miR-15a-5p | KIF5C | 1 | 1 | 2 | N |
| hsa-miR-15a-5p | MIPOL1 | 1 | 1 | 2 | N |
| hsa-miR-15a-5p | CLSPN | 1 | 1 | 2 | Y |
| hsa-miR-15a-5p | AEBP2 | 1 | 1 | 2 | N |
| hsa-miR-15a-5p | PRKAR2A | 1 | 1 | 2 | Y |
| hsa-miR-15a-5p | YWHAQ | 1 | 1 | 2 | Y |
| hsa-miR-15a-5p | PID1 | 1 | 1 | 2 | N |
| hsa-miR-15a-5p | ZMYM2 | 1 | 1 | 2 | N |
| hsa-miR-15a-5p | GAREM | 1 | 1 | 2 | N |
| hsa-miR-15a-5p | WBP11 | 1 | 1 | 2 | N |
| hsa-miR-15a-5p | RBM6 | 1 | 1 | 2 | N |
| hsa-miR-15a-5p | PDIA6 | 1 | 1 | 2 | Y |
| hsa-miR-15a-5p | MAPK8 | 1 | 1 | 2 | N |
| hsa-miR-15a-5p | SEMA6D | 1 | 1 | 2 | N |
| hsa-miR-15a-5p | TMEM100 | 1 | 1 | 2 | Y |
| hsa-miR-15a-5p | KIAA1432 | 1 | 1 | 2 | N |
| hsa-miR-15a-5p | TNRC6B | 1 | 1 | 2 | Y |
| hsa-miR-15a-5p | AKT3 | 1 | 1 | 2 | Y |
| hsa-miR-15a-5p | STX17 | 1 | 1 | 2 | Y |
| hsa-miR-15a-5p | PAQR3 | 1 | 1 | 2 | N |
| hsa-miR-15a-5p | GORASP2 | 1 | 1 | 2 | N |
| hsa-miR-15a-5p | MEOX2 | 1 | 1 | 2 | N |
| hsa-miR-15a-5p | VAPB | 1 | 1 | 2 | N |
| hsa-miR-15a-5p | PCMT1 | 1 | 1 | 2 | Y |
| hsa-miR-15a-5p | CARM1 | 1 | 1 | 2 | Y |
| hsa-miR-15a-5p | CD28 | 1 | 1 | 2 | N |
| hsa-miR-15a-5p | PLXNC1 | 1 | 1 | 2 | N |
| hsa-miR-15a-5p | USP25 | 1 | 1 | 2 | N |
| hsa-miR-15a-5p | PPM1D | 1 | 1 | 2 | N |
| hsa-miR-15a-5p | RASSF8 | 1 | 1 | 2 | N |
| hsa-miR-15a-5p | SETD3 | 1 | 1 | 2 | N |
| hsa-miR-15a-5p | ZSWIM3 | 1 | 1 | 2 | N |
| hsa-miR-15a-5p | CUL2 | 1 | 1 | 2 | Y |
| hsa-miR-15a-5p | WNT3A | 1 | 1 | 2 | Y |
| hsa-miR-15a-5p | AXIN2 | 1 | 1 | 2 | Y |
| hsa-miR-15a-5p | DPY19L4 | 1 | 1 | 2 | N |
| hsa-miR-15a-5p | YTHDC1 | 1 | 1 | 2 | Y |
| hsa-miR-15a-5p | SMPD1 | 1 | 1 | 2 | N |
| hsa-miR-15a-5p | ACTR2 | 1 | 1 | 2 | Y |
| hsa-miR-15a-5p | SPRYD3 | 1 | 1 | 2 | N |
| hsa-miR-15a-5p | SRPR | 1 | 1 | 2 | N |
| hsa-miR-15a-5p | RBBP6 | 1 | 1 | 2 | Y |
| hsa-miR-15a-5p | GPATCH8 | 1 | 1 | 2 | Y |
| hsa-miR-15a-5p | RNF138 | 1 | 1 | 2 | Y |
| hsa-miR-15a-5p | TMEM55A | 1 | 1 | 2 | N |
| hsa-miR-15a-5p | DYNC1I1 | 1 | 1 | 2 | N |
| hsa-miR-15a-5p | TMEM161B | 1 | 1 | 2 | Y |
| hsa-miR-15a-5p | DDX3Y | 1 | 1 | 2 | Y |
| hsa-miR-15a-5p | CNOT6L | 1 | 1 | 2 | N |
| hsa-miR-15a-5p | HOXA10 | 1 | 1 | 2 | Y |
| hsa-miR-15a-5p | ZBTB34 | 1 | 1 | 2 | Y |
| hsa-miR-15a-5p | SNCG | 1 | 1 | 2 | Y |
| hsa-miR-15a-5p | LURAP1L | 1 | 1 | 2 | Y |
| hsa-miR-15a-5p | PCDHAC2 | 1 | 1 | 2 | N |
| hsa-miR-15a-5p | ELL | 1 | 1 | 2 | N |
| hsa-miR-16-5p | RFWD2 | 1 | 1 | 2 | Y |
| hsa-miR-16-5p | RBBP6 | 1 | 1 | 2 | Y |
| hsa-miR-16-5p | CYP26B1 | 1 | 1 | 2 | Y |
| hsa-miR-16-5p | SPRYD3 | 1 | 1 | 2 | Y |
| hsa-miR-16-5p | CD28 | 1 | 1 | 2 | N |
| hsa-miR-16-5p | CDCA4 | 1 | 1 | 2 | Y |
| hsa-miR-16-5p | SCOC | 1 | 1 | 2 | N |
| hsa-miR-16-5p | DYNC1I1 | 1 | 1 | 2 | N |
| hsa-miR-16-5p | CPD | 1 | 1 | 2 | N |
| hsa-miR-16-5p | FBXO21 | 1 | 1 | 2 | N |
| hsa-miR-16-5p | PEX13 | 1 | 1 | 2 | Y |
| hsa-miR-16-5p | SCN8A | 1 | 1 | 2 | N |
| hsa-miR-16-5p | CHEK1 | 1 | 1 | 2 | Y |
| hsa-miR-16-5p | CRKL | 1 | 1 | 2 | Y |
| hsa-miR-16-5p | PTPN4 | 1 | 1 | 2 | N |
| hsa-miR-16-5p | MED26 | 1 | 1 | 2 | N |
| hsa-miR-16-5p | PCDHAC1 | 1 | 1 | 2 | N |
| hsa-miR-16-5p | MYT1L | 1 | 1 | 2 | N |
| hsa-miR-16-5p | SRPR | 1 | 1 | 2 | N |
| hsa-miR-16-5p | ZNF449 | 1 | 1 | 2 | Y |
| hsa-miR-16-5p | KDSR | 1 | 1 | 2 | Y |
| hsa-miR-16-5p | KIAA1432 | 1 | 1 | 2 | N |
| hsa-miR-16-5p | PCDHA6 | 1 | 1 | 2 | N |
| hsa-miR-16-5p | BCL2L2 | 1 | 1 | 2 | N |
| hsa-miR-16-5p | PDK4 | 1 | 1 | 2 | Y |
| hsa-miR-16-5p | RFX3 | 1 | 1 | 2 | N |
| hsa-miR-16-5p | ST8SIA3 | 1 | 1 | 2 | N |
| hsa-miR-16-5p | UBFD1 | 1 | 1 | 2 | Y |
| hsa-miR-16-5p | RAB9A | 1 | 1 | 2 | N |
| hsa-miR-16-5p | ZBTB39 | 1 | 1 | 2 | N |
| hsa-miR-16-5p | NISCH | 1 | 1 | 2 | Y |
| hsa-miR-16-5p | KIF21A | 1 | 1 | 2 | N |
| hsa-miR-16-5p | PRKAR2A | 1 | 1 | 2 | Y |
| hsa-miR-16-5p | GPN1 | 1 | 1 | 2 | N |
| hsa-miR-16-5p | SETD3 | 1 | 1 | 2 | N |
| hsa-miR-16-5p | NRBP1 | 1 | 1 | 2 | N |
| hsa-miR-16-5p | SEH1L | 1 | 1 | 2 | Y |
| hsa-miR-16-5p | ATG9A | 1 | 1 | 2 | Y |
| hsa-miR-16-5p | CREBRF | 1 | 1 | 2 | Y |
| hsa-miR-16-5p | PCDHA12 | 1 | 1 | 2 | N |
| hsa-miR-16-5p | AXIN2 | 1 | 1 | 2 | Y |
| hsa-miR-16-5p | AMER1 | 1 | 1 | 2 | Y |
| hsa-miR-16-5p | PRDM4 | 1 | 1 | 2 | Y |
| hsa-miR-16-5p | DOLPP1 | 1 | 1 | 2 | N |
| hsa-miR-16-5p | SPRED1 | 1 | 1 | 2 | Y |
| hsa-miR-16-5p | VPS4A | 1 | 1 | 2 | Y |
| hsa-miR-16-5p | PCMT1 | 1 | 1 | 2 | Y |
| hsa-miR-16-5p | C16orf72 | 1 | 1 | 2 | Y |
| hsa-miR-16-5p | CPEB2 | 1 | 1 | 2 | Y |
| hsa-miR-16-5p | ISLR | 1 | 1 | 2 | N |
| hsa-miR-16-5p | SIRT4 | 1 | 1 | 2 | Y |
| hsa-miR-16-5p | HELZ | 1 | 1 | 2 | Y |
| hsa-miR-16-5p | CCDC19 | 1 | 1 | 2 | N |
| hsa-miR-16-5p | CBX4 | 1 | 1 | 2 | Y |
| hsa-miR-16-5p | C1QL3 | 1 | 1 | 2 | N |
| hsa-miR-16-5p | YTHDC1 | 1 | 1 | 2 | Y |
| hsa-miR-16-5p | C20orf112 | 1 | 1 | 2 | N |
| hsa-miR-16-5p | C2orf42 | 1 | 1 | 2 | Y |
| hsa-miR-16-5p | RELN | 1 | 1 | 2 | N |
| hsa-miR-16-5p | KLHL18 | 1 | 1 | 2 | N |
| hsa-miR-16-5p | STRADB | 1 | 1 | 2 | Y |
| hsa-miR-16-5p | COPS2 | 1 | 1 | 2 | N |
| hsa-miR-16-5p | UBE4B | 1 | 1 | 2 | N |
| hsa-miR-16-5p | INSR | 1 | 1 | 2 | N |
| hsa-miR-16-5p | TSPYL2 | 1 | 1 | 2 | N |
| hsa-miR-16-5p | CNOT6L | 1 | 1 | 2 | N |
| hsa-miR-16-5p | RBM6 | 1 | 1 | 2 | Y |
| hsa-miR-16-5p | SNCG | 1 | 1 | 2 | Y |
| hsa-miR-16-5p | TMEM55A | 1 | 1 | 2 | N |
| hsa-miR-16-5p | PCDHA2 | 1 | 1 | 2 | N |
| hsa-miR-16-5p | PCDHA5 | 1 | 1 | 2 | N |
| hsa-miR-16-5p | ZHX1 | 1 | 1 | 2 | N |
| hsa-miR-16-5p | HPCAL4 | 1 | 1 | 2 | N |
| hsa-miR-16-5p | WNT3A | 1 | 1 | 2 | Y |
| hsa-miR-16-5p | FASN | 1 | 1 | 2 | Y |
| hsa-miR-16-5p | CC2D1B | 1 | 1 | 2 | N |
| hsa-miR-16-5p | HPSE2 | 1 | 1 | 2 | N |
| hsa-miR-16-5p | LITAF | 1 | 1 | 2 | Y |
| hsa-miR-16-5p | RNF217 | 1 | 1 | 2 | Y |
| hsa-miR-16-5p | MYLK | 1 | 1 | 2 | N |
| hsa-miR-16-5p | AGO4 | 1 | 1 | 2 | Y |
| hsa-miR-16-5p | DYRK1B | 1 | 1 | 2 | N |
| hsa-miR-16-5p | SHOC2 | 1 | 1 | 2 | Y |
| hsa-miR-16-5p | PTHLH | 1 | 1 | 2 | N |
| hsa-miR-16-5p | SLIT2 | 1 | 1 | 2 | N |
| hsa-miR-16-5p | RAB11FIP2 | 1 | 1 | 2 | Y |
| hsa-miR-16-5p | SALL4 | 1 | 1 | 2 | N |
| hsa-miR-16-5p | RAB9B | 1 | 1 | 2 | Y |
| hsa-miR-16-5p | ZNF622 | 1 | 1 | 2 | Y |
| hsa-miR-16-5p | PVRL1 | 1 | 1 | 2 | N |
| hsa-miR-16-5p | IRAK2 | 1 | 1 | 2 | N |
| hsa-miR-16-5p | ARFGAP2 | 1 | 1 | 2 | N |
| hsa-miR-16-5p | HSPA4L | 1 | 1 | 2 | Y |
| hsa-miR-16-5p | TRANK1 | 1 | 1 | 2 | N |
| hsa-miR-16-5p | MOB4 | 1 | 1 | 2 | Y |
| hsa-miR-16-5p | ARIH1 | 1 | 1 | 2 | Y |
| hsa-miR-16-5p | SLC9A6 | 1 | 1 | 2 | Y |
| hsa-miR-16-5p | FERMT2 | 1 | 1 | 2 | N |
| hsa-miR-16-5p | GAREM | 1 | 1 | 2 | N |
| hsa-miR-16-5p | CARM1 | 1 | 1 | 2 | Y |
| hsa-miR-16-5p | TAB3 | 1 | 1 | 2 | N |
| hsa-miR-16-5p | FSD1 | 1 | 1 | 2 | N |
| hsa-miR-16-5p | SESN1 | 1 | 1 | 2 | N |
| hsa-miR-16-5p | AREL1 | 1 | 1 | 2 | N |
| hsa-miR-16-5p | ACTR2 | 1 | 1 | 2 | Y |
| hsa-miR-16-5p | PTPN3 | 1 | 1 | 2 | Y |
| hsa-miR-16-5p | PTH | 1 | 1 | 2 | N |
| hsa-miR-16-5p | CLSPN | 1 | 1 | 2 | Y |
| hsa-miR-16-5p | ZSWIM3 | 1 | 1 | 2 | N |
| hsa-miR-16-5p | STK33 | 1 | 1 | 2 | Y |
| hsa-miR-16-5p | TUBA1A | 1 | 1 | 2 | Y |
| hsa-miR-16-5p | SNX16 | 1 | 1 | 2 | Y |
| hsa-miR-16-5p | WIPI2 | 1 | 1 | 2 | Y |
| hsa-miR-16-5p | ANO3 | 1 | 1 | 2 | N |
| hsa-miR-16-5p | FAM110C | 1 | 1 | 2 | N |
| hsa-miR-16-5p | CAPZA2 | 1 | 1 | 2 | Y |
| hsa-miR-16-5p | CCNE1 | 1 | 1 | 2 | Y |
| hsa-miR-16-5p | ZMAT3 | 1 | 1 | 2 | Y |
| hsa-miR-16-5p | PCDHA13 | 1 | 1 | 2 | N |
| hsa-miR-16-5p | USP3 | 1 | 1 | 2 | Y |
| hsa-miR-16-5p | PCDHA7 | 1 | 1 | 2 | N |
| hsa-miR-16-5p | SEPT2 | 1 | 1 | 2 | Y |
| hsa-miR-16-5p | SMAD7 | 1 | 1 | 2 | Y |
| hsa-miR-16-5p | YWHAH | 1 | 1 | 2 | Y |
| hsa-miR-16-5p | PDIK1L | 1 | 1 | 2 | Y |
| hsa-miR-16-5p | WEE1 | 1 | 1 | 2 | Y |
| hsa-miR-16-5p | MKNK1 | 1 | 1 | 2 | N |
| hsa-miR-16-5p | SLC36A1 | 1 | 1 | 2 | N |
| hsa-miR-16-5p | SOCS6 | 1 | 1 | 2 | N |
| hsa-miR-16-5p | PLEKHA5 | 1 | 1 | 2 | N |
| hsa-miR-16-5p | KIF5A | 1 | 1 | 2 | Y |
| hsa-miR-16-5p | ZBTB44 | 1 | 1 | 2 | N |
| hsa-miR-16-5p | SLC4A4 | 1 | 1 | 2 | N |
| hsa-miR-16-5p | MAFK | 1 | 1 | 2 | Y |
| hsa-miR-16-5p | PID1 | 1 | 1 | 2 | N |
| hsa-miR-16-5p | CLCN4 | 1 | 1 | 2 | N |
| hsa-miR-16-5p | ZCCHC3 | 1 | 1 | 2 | Y |
| hsa-miR-16-5p | PHF19 | 1 | 1 | 2 | Y |
| hsa-miR-16-5p | CLDN2 | 1 | 1 | 2 | Y |
| hsa-miR-16-5p | FGF2 | 1 | 1 | 2 | Y |
| hsa-miR-16-5p | NUP50 | 1 | 1 | 2 | Y |
| hsa-miR-16-5p | RAP2C | 1 | 1 | 2 | Y |
| hsa-miR-16-5p | CDC25A | 1 | 1 | 2 | Y |
| hsa-miR-16-5p | HOXA10 | 1 | 1 | 2 | Y |
| hsa-miR-16-5p | N4BP1 | 1 | 1 | 2 | Y |
| hsa-miR-16-5p | CPEB3 | 1 | 1 | 2 | Y |
| hsa-miR-16-5p | UNC80 | 1 | 1 | 2 | N |
| hsa-miR-16-5p | IPO7 | 1 | 1 | 2 | Y |
| hsa-miR-16-5p | KIF23 | 1 | 1 | 2 | Y |
| hsa-miR-16-5p | DNAJB4 | 1 | 1 | 2 | Y |
| hsa-miR-16-5p | PRR15L | 1 | 1 | 2 | N |
| hsa-miR-16-5p | ARMCX2 | 1 | 1 | 2 | Y |
| hsa-miR-16-5p | RSBN1 | 1 | 1 | 2 | N |
| hsa-miR-16-5p | MAP3K9 | 1 | 1 | 2 | N |
| hsa-miR-16-5p | NXPH1 | 1 | 1 | 2 | N |
| hsa-miR-16-5p | IST1 | 1 | 1 | 2 | N |
| hsa-miR-16-5p | CCND1 | 1 | 1 | 2 | Y |
| hsa-miR-16-5p | PCDHA3 | 1 | 1 | 2 | N |
| hsa-miR-16-5p | CASK | 1 | 1 | 2 | Y |
| hsa-miR-16-5p | ELMSAN1 | 1 | 1 | 2 | N |
| hsa-miR-16-5p | OMG | 1 | 1 | 2 | N |
| hsa-miR-16-5p | SMPD1 | 1 | 1 | 2 | N |
| hsa-miR-16-5p | MAP7 | 1 | 1 | 2 | Y |
| hsa-miR-16-5p | PCDHA4 | 1 | 1 | 2 | N |
| hsa-miR-16-5p | PCDHA9 | 1 | 1 | 2 | N |
| hsa-miR-16-5p | BFAR | 1 | 1 | 2 | Y |
| hsa-miR-16-5p | RNF138 | 1 | 1 | 2 | Y |
| hsa-miR-16-5p | SLC41A2 | 1 | 1 | 2 | N |
| hsa-miR-16-5p | ELL | 1 | 1 | 2 | N |
| hsa-miR-16-5p | FLT3 | 1 | 1 | 2 | N |
| hsa-miR-16-5p | G0S2 | 1 | 1 | 2 | N |
| hsa-miR-16-5p | PTPRR | 1 | 1 | 2 | N |
| hsa-miR-16-5p | AQP11 | 1 | 1 | 2 | N |
| hsa-miR-16-5p | TTC14 | 1 | 1 | 2 | N |
| hsa-miR-16-5p | BTG2 | 1 | 1 | 2 | Y |
| hsa-miR-16-5p | SUMO3 | 1 | 1 | 2 | N |
| hsa-miR-16-5p | KIF5C | 1 | 1 | 2 | N |
| hsa-miR-16-5p | DDX3Y | 1 | 1 | 2 | Y |
| hsa-miR-16-5p | EGLN1 | 1 | 1 | 2 | N |
| hsa-miR-16-5p | ENTPD7 | 1 | 1 | 2 | Y |
| hsa-miR-16-5p | CDC42SE2 | 1 | 1 | 2 | Y |
| hsa-miR-16-5p | VEGFA | 1 | 1 | 2 | Y |
| hsa-miR-16-5p | TENM2 | 1 | 1 | 2 | N |
| hsa-miR-16-5p | MOB3B | 1 | 1 | 2 | N |
| hsa-miR-16-5p | CDK17 | 1 | 1 | 2 | Y |
| hsa-miR-16-5p | BTAF1 | 1 | 1 | 2 | Y |
| hsa-miR-16-5p | RASEF | 1 | 1 | 2 | Y |
| hsa-miR-16-5p | GNAI3 | 1 | 1 | 2 | N |
| hsa-miR-16-5p | GABARAPL1 | 1 | 1 | 2 | Y |
| hsa-miR-16-5p | SYT4 | 1 | 1 | 2 | N |
| hsa-miR-16-5p | STX17 | 1 | 1 | 2 | Y |
| hsa-miR-16-5p | PLSCR4 | 1 | 1 | 2 | Y |
| hsa-miR-16-5p | AEBP2 | 1 | 1 | 2 | N |
| hsa-miR-16-5p | SYDE2 | 1 | 1 | 2 | N |
| hsa-miR-16-5p | ATXN1L | 1 | 1 | 2 | N |
| hsa-miR-16-5p | RSPO3 | 1 | 1 | 2 | N |
| hsa-miR-16-5p | PURA | 1 | 1 | 2 | Y |
| hsa-miR-16-5p | C1orf21 | 1 | 1 | 2 | Y |
| hsa-miR-16-5p | HTR2A | 1 | 1 | 2 | N |
| hsa-miR-16-5p | TSPAN5 | 1 | 1 | 2 | N |
| hsa-miR-16-5p | IKBKB | 1 | 1 | 2 | N |
| hsa-miR-16-5p | E2F7 | 1 | 1 | 2 | Y |
| hsa-miR-16-5p | UBE2V1 | 1 | 1 | 2 | Y |
| hsa-miR-16-5p | GPATCH8 | 1 | 1 | 2 | Y |
| hsa-miR-16-5p | DMTF1 | 1 | 1 | 2 | Y |
| hsa-miR-16-5p | EIF4B | 1 | 1 | 2 | Y |
| hsa-miR-16-5p | ABL2 | 1 | 1 | 2 | Y |
| hsa-miR-16-5p | CACUL1 | 1 | 1 | 2 | Y |
| hsa-miR-16-5p | PPM1D | 1 | 1 | 2 | Y |
| hsa-miR-16-5p | FAM133B | 1 | 1 | 2 | N |
| hsa-miR-16-5p | USP42 | 1 | 1 | 2 | Y |
| hsa-miR-16-5p | PDIA6 | 1 | 1 | 2 | Y |
| hsa-miR-16-5p | RASSF8 | 1 | 1 | 2 | N |
| hsa-miR-16-5p | TMEM178B | 1 | 1 | 2 | N |
| hsa-miR-16-5p | KCNJ2 | 1 | 1 | 2 | N |
| hsa-miR-16-5p | PAFAH1B1 | 1 | 1 | 2 | Y |
| hsa-miR-16-5p | FBXW7 | 1 | 1 | 2 | Y |
| hsa-miR-16-5p | SCN3A | 1 | 1 | 2 | N |
| hsa-miR-16-5p | RNF144B | 1 | 1 | 2 | Y |
| hsa-miR-16-5p | CSRNP1 | 1 | 1 | 2 | N |
| hsa-miR-16-5p | LCOR | 1 | 1 | 2 | N |
| hsa-miR-16-5p | C8orf58 | 1 | 1 | 2 | N |
| hsa-miR-16-5p | RAD23B | 1 | 1 | 2 | Y |
| hsa-miR-16-5p | TMEM161B | 1 | 1 | 2 | Y |
| hsa-miR-16-5p | UBE2Q1 | 1 | 1 | 2 | Y |
| hsa-miR-16-5p | TMCC1 | 1 | 1 | 2 | Y |
| hsa-miR-16-5p | CUL2 | 1 | 1 | 2 | Y |
| hsa-miR-16-5p | FAM91A1 | 1 | 1 | 2 | N |
| hsa-miR-16-5p | MAMSTR | 1 | 1 | 2 | N |
| hsa-miR-16-5p | JARID2 | 1 | 1 | 2 | Y |
| hsa-miR-16-5p | ARL2 | 1 | 1 | 2 | Y |
| hsa-miR-16-5p | GRM7 | 1 | 1 | 2 | N |
| hsa-miR-16-5p | ZC2HC1A | 1 | 1 | 2 | N |
| hsa-miR-16-5p | PLEKHA1 | 1 | 1 | 2 | Y |
| hsa-miR-16-5p | DPY19L4 | 1 | 1 | 2 | N |
| hsa-miR-16-5p | RNF24 | 1 | 1 | 2 | N |
| hsa-miR-16-5p | BTLA | 1 | 1 | 2 | N |
| hsa-miR-16-5p | ASH1L | 1 | 1 | 2 | Y |
| hsa-miR-16-5p | GATAD2A | 1 | 1 | 2 | Y |
| hsa-miR-16-5p | PPM1E | 1 | 1 | 2 | N |
| hsa-miR-16-5p | MAP2K1 | 1 | 1 | 2 | N |
| hsa-miR-16-5p | TMEM255A | 1 | 1 | 2 | Y |
| hsa-miR-16-5p | ATXN2 | 1 | 1 | 2 | N |
| hsa-miR-16-5p | PCDHA11 | 1 | 1 | 2 | N |
| hsa-miR-16-5p | IHH | 1 | 1 | 2 | N |
| hsa-miR-16-5p | GSTCD | 1 | 1 | 2 | N |
| hsa-miR-16-5p | CCNT2 | 1 | 1 | 2 | Y |
| hsa-miR-16-5p | TNFSF13B | 1 | 1 | 2 | N |
| hsa-miR-16-5p | IARS | 1 | 1 | 2 | Y |
| hsa-miR-16-5p | ARHGDIA | 1 | 1 | 2 | Y |
| hsa-miR-16-5p | BTRC | 1 | 1 | 2 | Y |
| hsa-miR-16-5p | TNRC6B | 1 | 1 | 2 | Y |
| hsa-miR-16-5p | PISD | 1 | 1 | 2 | Y |
| hsa-miR-16-5p | ANKS1A | 1 | 1 | 2 | N |
| hsa-miR-16-5p | ZNF275 | 1 | 1 | 2 | Y |
| hsa-miR-16-5p | ATXN7L3 | 1 | 1 | 2 | Y |
| hsa-miR-16-5p | SGK1 | 1 | 1 | 2 | N |
| hsa-miR-16-5p | POLR3F | 1 | 1 | 2 | N |
| hsa-miR-16-5p | DLL1 | 1 | 1 | 2 | N |
| hsa-miR-16-5p | WNT7A | 1 | 1 | 2 | N |
| hsa-miR-16-5p | VAPB | 1 | 1 | 2 | N |
| hsa-miR-16-5p | FGFR1 | 1 | 1 | 2 | Y |
| hsa-miR-16-5p | LSM11 | 1 | 1 | 2 | Y |
| hsa-miR-16-5p | YWHAQ | 1 | 1 | 2 | Y |
| hsa-miR-16-5p | GPR63 | 1 | 1 | 2 | N |
| hsa-miR-16-5p | RASGEF1B | 1 | 1 | 2 | N |
| hsa-miR-16-5p | MAPK8 | 1 | 1 | 2 | N |
| hsa-miR-16-5p | WBP11 | 1 | 1 | 2 | Y |
| hsa-miR-16-5p | FAM73A | 1 | 1 | 2 | N |
| hsa-miR-16-5p | PAPPA | 1 | 1 | 2 | N |
| hsa-miR-16-5p | USP25 | 1 | 1 | 2 | N |
| hsa-miR-16-5p | PPAP2B | 1 | 1 | 2 | N |
| hsa-miR-16-5p | LUZP1 | 1 | 1 | 2 | Y |
| hsa-miR-16-5p | ADRB2 | 1 | 1 | 2 | N |
| hsa-miR-16-5p | SLC35G1 | 1 | 1 | 2 | N |
| hsa-miR-16-5p | SEMA6D | 1 | 1 | 2 | N |
| hsa-miR-16-5p | CAPRIN1 | 1 | 1 | 2 | Y |
| hsa-miR-16-5p | CDC37L1 | 1 | 1 | 2 | Y |
| hsa-miR-16-5p | LURAP1L | 1 | 1 | 2 | Y |
| hsa-miR-16-5p | APLN | 1 | 1 | 2 | N |
| hsa-miR-16-5p | CLDN12 | 1 | 1 | 2 | N |
| hsa-miR-16-5p | KIF1B | 1 | 1 | 2 | Y |
| hsa-miR-16-5p | TACC1 | 1 | 1 | 2 | N |
| hsa-miR-16-5p | PAQR3 | 1 | 1 | 2 | Y |
| hsa-miR-16-5p | RARB | 1 | 1 | 2 | Y |
| hsa-miR-16-5p | SPSB4 | 1 | 1 | 2 | N |
| hsa-miR-16-5p | PCDHA1 | 1 | 1 | 2 | N |
| hsa-miR-16-5p | SLC15A4 | 1 | 1 | 2 | N |
| hsa-miR-16-5p | TBPL1 | 1 | 1 | 2 | Y |
| hsa-miR-16-5p | TCAIM | 1 | 1 | 2 | N |
| hsa-miR-16-5p | PLAG1 | 1 | 1 | 2 | Y |
| hsa-miR-16-5p | TMEM100 | 1 | 1 | 2 | Y |
| hsa-miR-16-5p | MEOX2 | 1 | 1 | 2 | N |
| hsa-miR-16-5p | TPD52L3 | 1 | 1 | 2 | N |
| hsa-miR-16-5p | DENND6A | 1 | 1 | 2 | Y |
| hsa-miR-16-5p | GORASP2 | 1 | 1 | 2 | N |
| hsa-miR-16-5p | SLC9A8 | 1 | 1 | 2 | N |
| hsa-miR-16-5p | RECK | 1 | 1 | 2 | Y |
| hsa-miR-16-5p | TLK1 | 1 | 1 | 2 | Y |
| hsa-miR-16-5p | PCDHAC2 | 1 | 1 | 2 | N |
| hsa-miR-16-5p | SYNJ1 | 1 | 1 | 2 | Y |
| hsa-miR-16-5p | LATS2 | 1 | 1 | 2 | N |
| hsa-miR-16-5p | ARL3 | 1 | 1 | 2 | Y |
| hsa-miR-16-5p | AKT3 | 1 | 1 | 2 | Y |
| hsa-miR-16-5p | RNF125 | 1 | 1 | 2 | N |
| hsa-miR-16-5p | KCNN4 | 1 | 1 | 2 | Y |
| hsa-miR-16-5p | DCP1A | 1 | 1 | 2 | N |
| hsa-miR-16-5p | XPO7 | 1 | 1 | 2 | Y |
| hsa-miR-16-5p | ZMYM2 | 1 | 1 | 2 | N |
| hsa-miR-16-5p | ZBTB34 | 1 | 1 | 2 | Y |
| hsa-miR-16-5p | PLXNC1 | 1 | 1 | 2 | N |
| hsa-miR-16-5p | ZBTB46 | 1 | 1 | 2 | N |
| hsa-miR-26b-3p | FGF1 | 1 | 1 | 2 | N |
| hsa-miR-26b-3p | C10orf118 | 1 | 1 | 2 | N |
| hsa-miR-26b-3p | PRRX1 | 1 | 1 | 2 | N |
| hsa-miR-26b-3p | UBE2D3 | 1 | 1 | 2 | N |
| hsa-miR-26b-3p | SLC18A2 | 1 | 1 | 2 | N |
| hsa-miR-26b-3p | PIK3CA | 1 | 1 | 2 | N |
| hsa-miR-26b-3p | RSPO2 | 1 | 1 | 2 | N |
| hsa-miR-26b-3p | LMBR1 | 1 | 1 | 2 | N |
| hsa-miR-26b-3p | SDCBP2 | 1 | 1 | 2 | N |
| hsa-miR-26b-3p | ZMAT4 | 1 | 1 | 2 | N |
| hsa-miR-26b-3p | STYK1 | 1 | 1 | 2 | N |
| hsa-miR-26b-3p | CEP135 | 1 | 1 | 2 | N |
| hsa-miR-26b-3p | SLC6A16 | 1 | 1 | 2 | N |
| hsa-miR-26b-3p | PSMF1 | 1 | 1 | 2 | N |
| hsa-miR-26b-3p | ZNF148 | 1 | 1 | 2 | N |
| hsa-miR-26b-3p | GLO1 | 1 | 1 | 2 | N |
| hsa-miR-26b-3p | BCL2L2 | 1 | 1 | 2 | N |
| hsa-miR-26b-3p | PIK3R4 | 1 | 1 | 2 | N |
| hsa-miR-26b-3p | CDK5R1 | 1 | 1 | 2 | N |
| hsa-miR-26b-3p | GPX8 | 1 | 1 | 2 | N |
| hsa-miR-26b-3p | TMEM38B | 1 | 1 | 2 | N |
| hsa-miR-26b-3p | SLIT2 | 1 | 1 | 2 | N |
| hsa-miR-26b-3p | OGFRL1 | 1 | 1 | 2 | N |
| hsa-miR-26b-3p | TRDN | 1 | 1 | 2 | N |
| hsa-miR-26b-3p | CTDSPL | 1 | 1 | 2 | N |
| hsa-miR-26b-3p | ZBTB8OS | 1 | 1 | 2 | N |
| hsa-miR-26b-3p | NXPH2 | 1 | 1 | 2 | N |
| hsa-miR-642a-3p | BCL2L2-PABPN1 | 1 | 1 | 2 | N |
| hsa-miR-642a-3p | DIP2C | 1 | 1 | 2 | N |
| hsa-miR-642a-3p | PDCD10 | 1 | 1 | 2 | N |
| hsa-miR-642a-3p | RIT2 | 1 | 1 | 2 | N |
| hsa-miR-642a-3p | NSG1 | 1 | 1 | 2 | N |
| hsa-miR-642a-3p | NXPH1 | 1 | 1 | 2 | N |
| hsa-miR-642a-3p | RAB28 | 1 | 1 | 2 | N |
| hsa-miR-642a-3p | SLC30A3 | 1 | 1 | 2 | N |
| hsa-miR-642a-3p | VGLL3 | 1 | 1 | 2 | N |
| hsa-miR-642a-3p | EML6 | 1 | 1 | 2 | N |
| hsa-miR-642a-3p | PDE5A | 1 | 1 | 2 | N |
| hsa-miR-642a-3p | EDAR | 1 | 1 | 2 | N |
| hsa-miR-642a-3p | AP1S3 | 1 | 1 | 2 | N |
| hsa-miR-642a-3p | ZNF177 | 1 | 1 | 2 | N |
| hsa-miR-642a-3p | ANO5 | 1 | 1 | 2 | N |
| hsa-miR-642a-3p | ADORA2B | 1 | 1 | 2 | N |
| hsa-miR-642a-3p | IFNG | 1 | 1 | 2 | N |
| hsa-miR-642a-3p | ARL6 | 1 | 1 | 2 | N |
| hsa-miR-642a-3p | ZNF449 | 1 | 1 | 2 | N |
| hsa-miR-642a-3p | FAM169A | 1 | 1 | 2 | N |
| hsa-miR-642a-3p | SERINC1 | 1 | 1 | 2 | N |
| hsa-miR-642a-3p | NNAT | 1 | 1 | 2 | N |
| hsa-miR-642a-3p | GCSAM | 1 | 1 | 2 | N |
| hsa-miR-642a-3p | SYT17 | 1 | 1 | 2 | N |
| hsa-miR-642a-3p | EDDM3A | 1 | 1 | 2 | N |
| hsa-miR-642a-3p | ELOVL6 | 1 | 1 | 2 | N |
| hsa-miR-642a-3p | DHX32 | 1 | 1 | 2 | N |
| hsa-miR-642a-3p | SPESP1 | 1 | 1 | 2 | N |
| hsa-miR-642a-3p | RPE | 1 | 1 | 2 | N |
| hsa-miR-642a-3p | RNF11 | 1 | 1 | 2 | N |
| hsa-miR-642a-3p | PABPN1 | 1 | 1 | 2 | N |
| hsa-miR-642a-3p | SPRY1 | 1 | 1 | 2 | N |
| hsa-miR-642a-3p | AMHR2 | 1 | 1 | 2 | N |
| hsa-miR-642a-3p | BCOR | 1 | 1 | 2 | N |
| hsa-miR-642a-3p | USP27X | 1 | 1 | 2 | N |
| hsa-miR-642a-3p | NCOA7 | 1 | 1 | 2 | N |
| hsa-miR-671-5p | YPEL2 | 1 | 1 | 2 | Y |
| hsa-miR-671-5p | CDK2AP2 | 1 | 1 | 2 | Y |
| hsa-miR-671-5p | CAPN3 | 1 | 1 | 2 | N |
| hsa-miR-671-5p | PASD1 | 1 | 1 | 2 | N |
| hsa-miR-671-5p | SYT9 | 1 | 1 | 2 | N |
| hsa-miR-671-5p | DHCR24 | 1 | 1 | 2 | N |
| hsa-miR-671-5p | BCR | 1 | 1 | 2 | N |
| hsa-miR-671-5p | VPS45 | 1 | 1 | 2 | N |
| hsa-miR-671-5p | GOLIM4 | 1 | 1 | 2 | N |
| hsa-miR-671-5p | ADAMTSL5 | 1 | 1 | 2 | N |
| hsa-miR-671-5p | SGK2 | 1 | 1 | 2 | N |
| hsa-miR-671-5p | PIK3IP1 | 1 | 1 | 2 | N |
| hsa-miR-671-5p | LRRC59 | 1 | 1 | 2 | N |
| hsa-miR-671-5p | THBS1 | 1 | 1 | 2 | N |
| hsa-miR-671-5p | CA7 | 1 | 1 | 2 | N |
| hsa-miR-671-5p | SYNPR | 1 | 1 | 2 | N |
| hsa-miR-671-5p | GPR107 | 1 | 1 | 2 | N |
| hsa-miR-671-5p | HIVEP3 | 1 | 1 | 2 | N |
| hsa-miR-671-5p | SASH3 | 1 | 1 | 2 | N |
| hsa-miR-671-5p | SYPL2 | 1 | 1 | 2 | N |
| hsa-miR-671-5p | NFYA | 1 | 1 | 2 | N |
| hsa-miR-671-5p | KCNA6 | 1 | 1 | 2 | N |
| hsa-miR-671-5p | CRTC3 | 1 | 1 | 2 | Y |
| hsa-miR-671-5p | CD209 | 1 | 1 | 2 | N |
| hsa-miR-671-5p | USP46 | 1 | 1 | 2 | N |
| hsa-miR-671-5p | RAG1 | 1 | 1 | 2 | N |
| hsa-miR-671-5p | ATOX1 | 1 | 1 | 2 | N |
| hsa-miR-671-5p | VPS52 | 1 | 1 | 2 | N |
| hsa-miR-671-5p | VPS26A | 1 | 1 | 2 | N |
| hsa-miR-671-5p | TET3 | 1 | 1 | 2 | N |
| hsa-miR-671-5p | VSNL1 | 1 | 1 | 2 | N |
| hsa-miR-671-5p | C1QA | 1 | 1 | 2 | N |
| hsa-miR-671-5p | CDR2L | 1 | 1 | 2 | N |
| hsa-miR-671-5p | TAF8 | 1 | 1 | 2 | Y |
| hsa-miR-671-5p | FGFR2 | 1 | 1 | 2 | N |
| hsa-miR-671-5p | CAMKK2 | 1 | 1 | 2 | N |
| hsa-miR-671-5p | C16orf72 | 1 | 1 | 2 | N |
| hsa-miR-671-5p | FAM83F | 1 | 1 | 2 | N |
| hsa-miR-671-5p | DDX39B | 1 | 1 | 2 | Y |
| hsa-miR-671-5p | SHISA5 | 1 | 1 | 2 | N |
| hsa-miR-671-5p | CACNG1 | 1 | 1 | 2 | N |
| hsa-miR-671-5p | KRT38 | 1 | 1 | 2 | N |
| hsa-miR-671-5p | RAB6B | 1 | 1 | 2 | N |
| hsa-miR-671-5p | SMCO3 | 1 | 1 | 2 | N |
| hsa-miR-671-5p | HNRNPUL1 | 1 | 1 | 2 | Y |
| hsa-miR-671-5p | FAM110B | 1 | 1 | 2 | N |
| hsa-miR-671-5p | ACBD6 | 1 | 1 | 2 | N |
| hsa-miR-671-5p | TBC1D24 | 1 | 1 | 2 | N |
| hsa-miR-671-5p | HLA-DRB1 | 1 | 1 | 2 | N |
| hsa-miR-671-5p | TBC1D13 | 1 | 1 | 2 | N |
| hsa-miR-671-5p | PRR5L | 1 | 1 | 2 | N |
| hsa-miR-671-5p | PTPN20A | 1 | 1 | 2 | N |
| hsa-miR-671-5p | STEAP2 | 1 | 1 | 2 | N |
| hsa-miR-671-5p | CREB3L2 | 1 | 1 | 2 | N |
| hsa-miR-671-5p | KRT9 | 1 | 1 | 2 | N |
| hsa-miR-671-5p | ST8SIA5 | 1 | 1 | 2 | N |
| hsa-miR-671-5p | RNF38 | 1 | 1 | 2 | N |
| hsa-miR-671-5p | GALNT10 | 1 | 1 | 2 | N |
| hsa-miR-671-5p | KIT | 1 | 1 | 2 | N |
| hsa-miR-671-5p | CAPN15 | 1 | 1 | 2 | Y |
| hsa-miR-671-5p | EMC10 | 1 | 1 | 2 | N |
| hsa-miR-671-5p | C10orf25 | 1 | 1 | 2 | N |
| hsa-miR-671-5p | WFIKKN2 | 1 | 1 | 2 | N |
| hsa-miR-671-5p | LEP | 1 | 1 | 2 | N |
| hsa-miR-671-5p | USP36 | 1 | 1 | 2 | N |
| hsa-miR-671-5p | CALN1 | 1 | 1 | 2 | N |
| hsa-miR-671-5p | C12orf77 | 1 | 1 | 2 | N |
| hsa-miR-671-5p | EDN1 | 1 | 1 | 2 | N |
| hsa-miR-671-5p | GATS | 1 | 1 | 2 | N |
| hsa-miR-671-5p | SLC30A6 | 1 | 1 | 2 | N |
| hsa-miR-671-5p | CHMP1A | 1 | 1 | 2 | N |
| hsa-miR-671-5p | TRIM67 | 1 | 1 | 2 | N |
| hsa-miR-671-5p | PTPN20B | 1 | 1 | 2 | N |
| hsa-miR-671-5p | NFKB1 | 1 | 1 | 2 | N |
| hsa-miR-671-5p | ACTR2 | 1 | 1 | 2 | Y |
| hsa-miR-671-5p | MDM4 | 1 | 1 | 2 | N |
| hsa-miR-671-5p | LGALS3BP | 1 | 1 | 2 | Y |
| hsa-miR-671-5p | TNFRSF1B | 1 | 1 | 2 | N |
| hsa-miR-671-5p | CSNK2A2 | 1 | 1 | 2 | N |
| hsa-miR-671-5p | SATB2 | 1 | 1 | 2 | Y |
| hsa-miR-671-5p | PRC1 | 1 | 1 | 2 | N |
| hsa-miR-671-5p | CPNE2 | 1 | 1 | 2 | Y |
| hsa-miR-671-5p | C14orf180 | 1 | 1 | 2 | N |
| hsa-miR-671-5p | SPTBN2 | 1 | 1 | 2 | N |
| hsa-miR-671-5p | PI4K2A | 1 | 1 | 2 | N |
| hsa-miR-671-5p | EHD3 | 1 | 1 | 2 | N |
| hsa-miR-671-5p | ACY1 | 1 | 1 | 2 | Y |
| hsa-miR-6764-5p | CDIPT | 1 | 1 | 2 | N |
| hsa-miR-6764-5p | MINK1 | 1 | 1 | 2 | N |
| hsa-miR-6764-5p | C9orf69 | 1 | 1 | 2 | N |
| hsa-miR-6764-5p | HDAC7 | 1 | 1 | 2 | N |
| hsa-miR-6764-5p | WSCD1 | 1 | 1 | 2 | N |
| hsa-miR-6764-5p | IKBKE | 1 | 1 | 2 | N |
| hsa-miR-6764-5p | C17orf103 | 1 | 1 | 2 | N |
| hsa-miR-6764-5p | SMCR8 | 1 | 1 | 2 | N |
| hsa-miR-6764-5p | RPH3A | 1 | 1 | 2 | N |
| hsa-miR-6764-5p | PDGFRB | 1 | 1 | 2 | N |
| hsa-miR-6764-5p | SLC7A8 | 1 | 1 | 2 | N |
| hsa-miR-6764-5p | FAM107A | 1 | 1 | 2 | N |
| hsa-miR-6764-5p | PRRT4 | 1 | 1 | 2 | N |
| hsa-miR-6764-5p | YPEL4 | 1 | 1 | 2 | N |
| hsa-miR-6764-5p | SLC18A1 | 1 | 1 | 2 | N |
| hsa-miR-6764-5p | ETNK2 | 1 | 1 | 2 | N |
| hsa-miR-6764-5p | GJC3 | 1 | 1 | 2 | N |
| hsa-miR-6764-5p | DIO3 | 1 | 1 | 2 | N |
| hsa-miR-6764-5p | LRRC20 | 1 | 1 | 2 | N |
| hsa-miR-6764-5p | PRRT1 | 1 | 1 | 2 | N |
| hsa-miR-6764-5p | FBXL18 | 1 | 1 | 2 | N |
| hsa-miR-6764-5p | ZFYVE27 | 1 | 1 | 2 | N |
| hsa-miR-6764-5p | ALX4 | 1 | 1 | 2 | N |
| hsa-miR-6764-5p | SGK223 | 1 | 1 | 2 | N |
| hsa-miR-6764-5p | ICOSLG | 1 | 1 | 2 | N |
| hsa-miR-6764-5p | FAIM2 | 1 | 1 | 2 | N |
| hsa-miR-6764-5p | CD79A | 1 | 1 | 2 | N |
| hsa-miR-6764-5p | LRSAM1 | 1 | 1 | 2 | N |
| hsa-miR-6764-5p | CA7 | 1 | 1 | 2 | N |
| hsa-miR-6764-5p | C19orf25 | 1 | 1 | 2 | N |
| hsa-miR-6764-5p | TGIF2 | 1 | 1 | 2 | N |
| hsa-miR-6764-5p | NF2 | 1 | 1 | 2 | N |
| hsa-miR-6764-5p | GPSM3 | 1 | 1 | 2 | N |
| hsa-miR-6764-5p | PTGES2 | 1 | 1 | 2 | Y |
| hsa-miR-6764-5p | NABP2 | 1 | 1 | 2 | N |
| hsa-miR-6764-5p | PNMAL2 | 1 | 1 | 2 | N |
| hsa-miR-6764-5p | TAB1 | 1 | 1 | 2 | Y |
| hsa-miR-6764-5p | CXXC1 | 1 | 1 | 2 | N |
| hsa-miR-6764-5p | STIM1 | 1 | 1 | 2 | N |
| hsa-miR-6764-5p | TRPM2 | 1 | 1 | 2 | N |
| hsa-miR-6764-5p | SOX13 | 1 | 1 | 2 | N |
| hsa-miR-6764-5p | SURF4 | 1 | 1 | 2 | N |
| hsa-miR-6764-5p | LRRC15 | 1 | 1 | 2 | N |
| hsa-miR-6764-5p | MAP4 | 1 | 1 | 2 | N |
| hsa-miR-6764-5p | WNT3A | 1 | 1 | 2 | N |
| hsa-miR-6764-5p | MAFF | 1 | 1 | 2 | N |
| hsa-miR-6764-5p | CHTF8 | 1 | 1 | 2 | N |
| hsa-miR-6764-5p | SPRR2F | 1 | 1 | 2 | N |
| hsa-miR-6764-5p | PTCHD1 | 1 | 1 | 2 | Y |
| hsa-miR-6764-5p | RAD9A | 1 | 1 | 2 | N |
| hsa-miR-6764-5p | ANKRD34C | 1 | 1 | 2 | N |
| hsa-miR-6764-5p | HEYL | 1 | 1 | 2 | Y |
| hsa-miR-6764-5p | REXO4 | 1 | 1 | 2 | N |
| hsa-miR-6764-5p | C12orf49 | 1 | 1 | 2 | N |
| hsa-miR-6764-5p | ZDHHC18 | 1 | 1 | 2 | N |
| hsa-miR-6764-5p | PABPC1 | 1 | 1 | 2 | N |
| hsa-miR-6764-5p | TFE3 | 1 | 1 | 2 | N |
| hsa-miR-6764-5p | PAX7 | 1 | 1 | 2 | N |
| hsa-miR-6764-5p | NFAM1 | 1 | 1 | 2 | N |
| hsa-miR-6764-5p | NDOR1 | 1 | 1 | 2 | N |
| hsa-miR-6764-5p | SPSB1 | 1 | 1 | 2 | N |
| hsa-miR-6764-5p | ZMYND12 | 1 | 1 | 2 | N |
| hsa-miR-6764-5p | PPP2R5D | 1 | 1 | 2 | N |
| hsa-miR-6764-5p | SRC | 1 | 1 | 2 | N |
| hsa-miR-6764-5p | ADCYAP1R1 | 1 | 1 | 2 | N |
| hsa-miR-6764-5p | SYCP2L | 1 | 1 | 2 | N |
| hsa-miR-6764-5p | HHIPL2 | 1 | 1 | 2 | N |
| hsa-miR-6764-5p | BTD | 1 | 1 | 2 | N |
| hsa-miR-6764-5p | SLC9A5 | 1 | 1 | 2 | N |
| hsa-miR-6764-5p | PNLIPRP3 | 1 | 1 | 2 | N |
| hsa-miR-6764-5p | SMPD1 | 1 | 1 | 2 | N |
| hsa-miR-6764-5p | LDOC1L | 1 | 1 | 2 | N |
| hsa-miR-6764-5p | PPP2R4 | 1 | 1 | 2 | N |
| hsa-miR-6764-5p | STAC2 | 1 | 1 | 2 | N |
| hsa-miR-6764-5p | DCHS1 | 1 | 1 | 2 | N |
| hsa-miR-6764-5p | GMEB2 | 1 | 1 | 2 | N |
| hsa-miR-6764-5p | TMEM222 | 1 | 1 | 2 | N |
| hsa-miR-6764-5p | KCNC2 | 1 | 1 | 2 | N |
| hsa-miR-6764-5p | GRK6 | 1 | 1 | 2 | N |
| hsa-miR-6764-5p | OR2H1 | 1 | 1 | 2 | N |
| hsa-miR-6764-5p | LDLRAP1 | 1 | 1 | 2 | N |
| hsa-miR-6764-5p | TRPC4AP | 1 | 1 | 2 | N |
| hsa-miR-6764-5p | EPN1 | 1 | 1 | 2 | N |
| hsa-miR-6764-5p | RIMS4 | 1 | 1 | 2 | N |
| hsa-miR-6764-5p | GCK | 1 | 1 | 2 | N |
| hsa-miR-6764-5p | NHSL1 | 1 | 1 | 2 | N |
| hsa-miR-6764-5p | PRKCQ | 1 | 1 | 2 | N |
| hsa-miR-6764-5p | CTDNEP1 | 1 | 1 | 2 | N |
| hsa-miR-6764-5p | CNTN2 | 1 | 1 | 2 | N |
| hsa-miR-6764-5p | CTSD | 1 | 1 | 2 | N |
| hsa-miR-6764-5p | SORCS2 | 1 | 1 | 2 | N |
| hsa-miR-6764-5p | MISP | 1 | 1 | 2 | N |
| hsa-miR-6764-5p | ZNF474 | 1 | 1 | 2 | N |
| hsa-miR-6764-5p | SIGIRR | 1 | 1 | 2 | N |
| hsa-miR-6764-5p | ZBTB2 | 1 | 1 | 2 | N |
| hsa-miR-6764-5p | FRMD8 | 1 | 1 | 2 | N |
| hsa-miR-6764-5p | OLFML2A | 1 | 1 | 2 | Y |
| hsa-miR-6764-5p | ORAI2 | 1 | 1 | 2 | N |
| hsa-miR-6764-5p | HNF1A | 1 | 1 | 2 | N |
| hsa-miR-6764-5p | PBXIP1 | 1 | 1 | 2 | N |
| hsa-miR-6764-5p | VOPP1 | 1 | 1 | 2 | N |
| hsa-miR-6764-5p | DGCR2 | 1 | 1 | 2 | N |
| hsa-miR-6764-5p | IL18RAP | 1 | 1 | 2 | N |
| hsa-miR-6764-5p | ITPK1 | 1 | 1 | 2 | N |
| hsa-miR-6764-5p | TAGLN2 | 1 | 1 | 2 | N |
| hsa-miR-6764-5p | SRF | 1 | 1 | 2 | N |
| hsa-miR-6764-5p | BLOC1S3 | 1 | 1 | 2 | N |
| hsa-miR-6764-5p | TMEM47 | 1 | 1 | 2 | N |
| hsa-miR-6764-5p | NHLRC3 | 1 | 1 | 2 | N |
| hsa-miR-6764-5p | LIN28A | 1 | 1 | 2 | N |
| hsa-miR-6764-5p | CLCN6 | 1 | 1 | 2 | N |
| hsa-miR-6764-5p | FAM53A | 1 | 1 | 2 | N |
| hsa-miR-6764-5p | CDIP1 | 1 | 1 | 2 | N |
| hsa-miR-6764-5p | KIAA0319L | 1 | 1 | 2 | N |
| hsa-miR-6764-5p | GDF5 | 1 | 1 | 2 | N |
| hsa-miR-6764-5p | BET1L | 1 | 1 | 2 | N |
| hsa-miR-6764-5p | SET | 1 | 1 | 2 | N |
| hsa-miR-6764-5p | CPXM2 | 1 | 1 | 2 | N |
| hsa-miR-6764-5p | PBX1 | 1 | 1 | 2 | N |
| hsa-miR-6764-5p | RELT | 1 | 1 | 2 | N |
| hsa-miR-6764-5p | C2orf72 | 1 | 1 | 2 | N |
| hsa-miR-6764-5p | RHOF | 1 | 1 | 2 | N |
| hsa-miR-6764-5p | NPLOC4 | 1 | 1 | 2 | N |
| hsa-miR-6764-5p | ZFP36L1 | 1 | 1 | 2 | N |
| hsa-miR-6764-5p | TSKU | 1 | 1 | 2 | N |
| hsa-miR-6764-5p | ITFG3 | 1 | 1 | 2 | N |
| hsa-miR-6764-5p | SYNC | 1 | 1 | 2 | N |
| hsa-miR-6764-5p | TSTA3 | 1 | 1 | 2 | N |
| hsa-miR-6764-5p | DHX36 | 1 | 1 | 2 | N |
| hsa-miR-6764-5p | SREBF2 | 1 | 1 | 2 | N |
| hsa-miR-6764-5p | MAPKAPK2 | 1 | 1 | 2 | N |
| hsa-miR-6764-5p | POU6F1 | 1 | 1 | 2 | N |
| hsa-miR-6764-5p | PLA2G4E | 1 | 1 | 2 | N |
| hsa-miR-6764-5p | MXI1 | 1 | 1 | 2 | N |
| hsa-miR-6764-5p | DEAF1 | 1 | 1 | 2 | N |
| hsa-miR-6764-5p | MB | 1 | 1 | 2 | N |
| hsa-miR-6764-5p | TMEM184B | 1 | 1 | 2 | Y |
| hsa-miR-6764-5p | SNX33 | 1 | 1 | 2 | N |
| hsa-miR-6764-5p | OVOL1 | 1 | 1 | 2 | N |
| hsa-miR-6764-5p | XRCC3 | 1 | 1 | 2 | N |
| hsa-miR-6764-5p | OCM | 1 | 1 | 2 | N |
| hsa-miR-6764-5p | PDE1B | 1 | 1 | 2 | N |
| hsa-miR-6764-5p | LSS | 1 | 1 | 2 | N |
| hsa-miR-6764-5p | MGAT3 | 1 | 1 | 2 | N |
| hsa-miR-6764-5p | TMEM229B | 1 | 1 | 2 | N |
| hsa-miR-6764-5p | KLHL30 | 1 | 1 | 2 | N |
| hsa-miR-6764-5p | HSPB7 | 1 | 1 | 2 | N |
| hsa-miR-6764-5p | PBX2 | 1 | 1 | 2 | N |
| hsa-miR-6764-5p | ZNF609 | 1 | 1 | 2 | N |
| hsa-miR-6764-5p | ADRA2B | 1 | 1 | 2 | N |
| hsa-miR-6764-5p | ALAD | 1 | 1 | 2 | N |
| hsa-miR-6764-5p | PLXNA2 | 1 | 1 | 2 | N |
| hsa-miR-6764-5p | DUSP13 | 1 | 1 | 2 | N |
| hsa-miR-6764-5p | EXOC3L2 | 1 | 1 | 2 | N |
| hsa-miR-6764-5p | C10orf105 | 1 | 1 | 2 | N |
| hsa-miR-6764-5p | PCDHGA7 | 1 | 1 | 2 | N |
| hsa-miR-455-3p | TMEFF1 | 1 | 1 | 2 | N |
| hsa-miR-455-3p | COLEC12 | 1 | 1 | 2 | N |
| hsa-miR-455-3p | ESCO1 | 1 | 1 | 2 | N |
| hsa-miR-455-3p | PRKAB2 | 1 | 1 | 2 | N |
| hsa-miR-455-3p | CUL3 | 1 | 1 | 2 | N |
| hsa-miR-455-3p | ARFGAP2 | 1 | 1 | 2 | N |
| hsa-miR-455-3p | UBE2Q2 | 1 | 1 | 2 | N |
| hsa-miR-455-3p | GABARAPL2 | 1 | 1 | 2 | N |
| hsa-miR-455-3p | ACAN | 1 | 1 | 2 | N |
| hsa-miR-455-3p | CD80 | 1 | 1 | 2 | N |
| hsa-miR-455-3p | ELF3 | 1 | 1 | 2 | N |
| hsa-miR-455-3p | HOXC4 | 1 | 1 | 2 | N |
| hsa-miR-455-3p | NLN | 1 | 1 | 2 | N |
| hsa-miR-455-3p | SLC25A3 | 1 | 1 | 2 | N |
| hsa-miR-455-3p | ZBTB18 | 1 | 1 | 2 | N |
| hsa-miR-455-3p | RTN4 | 1 | 1 | 2 | N |
| hsa-miR-455-3p | TTK | 1 | 1 | 2 | N |
| hsa-miR-455-3p | AGO4 | 1 | 1 | 2 | N |
| hsa-miR-455-3p | HM13 | 1 | 1 | 2 | N |
| hsa-miR-455-3p | STK17B | 1 | 1 | 2 | N |
| hsa-miR-455-3p | LHX2 | 1 | 1 | 2 | N |
| hsa-miR-455-3p | MSANTD3-TMEFF1 | 1 | 1 | 2 | N |
| hsa-miR-744-5p | SH3BGRL3 | 1 | 1 | 2 | Y |
| hsa-miR-744-5p | SCRT1 | 1 | 1 | 2 | N |
| hsa-miR-744-5p | TMEM253 | 1 | 1 | 2 | N |
| hsa-miR-744-5p | PPP5C | 1 | 1 | 2 | N |
| hsa-miR-335-5p | CALU | 1 | 1 | 2 | N |
| hsa-miR-335-5p | SEPHS1 | 1 | 1 | 2 | N |
| hsa-miR-335-5p | PRKAA2 | 1 | 1 | 2 | N |
| hsa-miR-335-5p | SORCS1 | 1 | 1 | 2 | N |
| hsa-miR-335-5p | SREK1IP1 | 1 | 1 | 2 | N |
| hsa-miR-335-5p | NXPH2 | 1 | 1 | 2 | N |
| hsa-miR-335-5p | ZMPSTE24 | 1 | 1 | 2 | N |
| hsa-miR-335-5p | HAND1 | 1 | 1 | 2 | N |
| hsa-miR-335-5p | KDSR | 1 | 1 | 2 | N |
| hsa-miR-335-5p | SMARCA2 | 1 | 1 | 2 | N |
| hsa-miR-335-5p | CASP7 | 1 | 1 | 2 | N |
| hsa-let-7d-5p | RAB11FIP4 | 1 | 1 | 2 | Y |
| hsa-let-7d-5p | FRMD4B | 1 | 1 | 2 | N |
| hsa-let-7d-5p | GALNT1 | 1 | 1 | 2 | N |
| hsa-let-7d-5p | FNIP1 | 1 | 1 | 2 | N |
| hsa-let-7d-5p | PCGF3 | 1 | 1 | 2 | Y |
| hsa-let-7d-5p | PARD6B | 1 | 1 | 2 | N |
| hsa-let-7d-5p | FNDC3A | 1 | 1 | 2 | Y |
| hsa-let-7d-5p | AMT | 1 | 1 | 2 | N |
| hsa-let-7d-5p | C15orf41 | 1 | 1 | 2 | N |
| hsa-let-7d-5p | OSBPL3 | 1 | 1 | 2 | N |
| hsa-let-7d-5p | SLC5A9 | 1 | 1 | 2 | N |
| hsa-let-7d-5p | PPAPDC2 | 1 | 1 | 2 | N |
| hsa-let-7d-5p | RGS16 | 1 | 1 | 2 | N |
| hsa-let-7d-5p | CCL7 | 1 | 1 | 2 | N |
| hsa-let-7d-5p | ARRDC4 | 1 | 1 | 2 | Y |
| hsa-let-7d-5p | BZW1 | 1 | 1 | 2 | Y |
| hsa-let-7d-5p | ZFYVE26 | 1 | 1 | 2 | N |
| hsa-let-7d-5p | KLHL23 | 1 | 1 | 2 | N |
| hsa-let-7d-5p | TMPPE | 1 | 1 | 2 | N |
| hsa-let-7d-5p | SCD | 1 | 1 | 2 | N |
| hsa-let-7d-5p | ZBTB8B | 1 | 1 | 2 | N |
| hsa-let-7d-5p | CDC34 | 1 | 1 | 2 | N |
| hsa-let-7d-5p | MAP4K3 | 1 | 1 | 2 | N |
| hsa-let-7d-5p | CLDN12 | 1 | 1 | 2 | Y |
| hsa-let-7d-5p | ZNF512B | 1 | 1 | 2 | N |
| hsa-let-7d-5p | MED8 | 1 | 1 | 2 | N |
| hsa-let-7d-5p | CEP135 | 1 | 1 | 2 | Y |
| hsa-let-7d-5p | PAPPA | 1 | 1 | 2 | N |
| hsa-let-7d-5p | TARBP2 | 1 | 1 | 2 | N |
| hsa-let-7d-5p | DDX19A | 1 | 1 | 2 | N |
| hsa-let-7d-5p | USP38 | 1 | 1 | 2 | Y |
| hsa-let-7d-5p | TGFBR3 | 1 | 1 | 2 | Y |
| hsa-let-7d-5p | PGRMC1 | 1 | 1 | 2 | Y |
| hsa-let-7d-5p | RNF20 | 1 | 1 | 2 | N |
| hsa-let-7d-5p | C15orf39 | 1 | 1 | 2 | N |
| hsa-let-7d-5p | TMEM110 | 1 | 1 | 2 | N |
| hsa-let-7d-5p | PALD1 | 1 | 1 | 2 | N |
| hsa-let-7d-5p | ACVR1C | 1 | 1 | 2 | N |
| hsa-let-7d-5p | PLXNC1 | 1 | 1 | 2 | N |
| hsa-let-7d-5p | SLC25A27 | 1 | 1 | 2 | N |
| hsa-let-7d-5p | RALB | 1 | 1 | 2 | N |
| hsa-let-7d-5p | FZD3 | 1 | 1 | 2 | N |
| hsa-let-7d-5p | ADRB2 | 1 | 1 | 2 | N |
| hsa-let-7d-5p | NYNRIN | 1 | 1 | 2 | N |
| hsa-let-7d-5p | ZNF275 | 1 | 1 | 2 | N |
| hsa-let-7d-5p | LRIG3 | 1 | 1 | 2 | Y |
| hsa-let-7d-5p | ADAMTS8 | 1 | 1 | 2 | N |
| hsa-let-7d-5p | NR6A1 | 1 | 1 | 2 | Y |
| hsa-let-7d-5p | MAPK6 | 1 | 1 | 2 | Y |
| hsa-let-7d-5p | SLC20A1 | 1 | 1 | 2 | Y |
| hsa-let-7d-5p | RSPO2 | 1 | 1 | 2 | N |
| hsa-let-7d-5p | APBB3 | 1 | 1 | 2 | N |
| hsa-let-7d-5p | LIPT2 | 1 | 1 | 2 | N |
| hsa-let-7d-5p | ATP8B4 | 1 | 1 | 2 | N |
| hsa-let-7d-5p | PLEKHA8 | 1 | 1 | 2 | N |
| hsa-let-7d-5p | STX3 | 1 | 1 | 2 | Y |
| hsa-let-7d-5p | GNG5 | 1 | 1 | 2 | Y |
| hsa-let-7d-5p | DDX26B | 1 | 1 | 2 | N |
| hsa-let-7d-5p | KCTD21 | 1 | 1 | 2 | Y |
| hsa-let-7d-5p | THRSP | 1 | 1 | 2 | N |
| hsa-let-7d-5p | ARG2 | 1 | 1 | 2 | N |
| hsa-let-7d-5p | GAS7 | 1 | 1 | 2 | N |
| hsa-let-7d-5p | UTRN | 1 | 1 | 2 | N |
| hsa-let-7d-5p | BEGAIN | 1 | 1 | 2 | N |
| hsa-let-7d-5p | GXYLT1 | 1 | 1 | 2 | N |
| hsa-let-7d-5p | MFSD4 | 1 | 1 | 2 | N |
| hsa-let-7d-5p | SLC25A18 | 1 | 1 | 2 | N |
| hsa-let-7d-5p | SKIL | 1 | 1 | 2 | N |
| hsa-let-7d-5p | ADAMTS15 | 1 | 1 | 2 | N |
| hsa-let-7d-5p | NRAS | 1 | 1 | 2 | N |
| hsa-let-7d-5p | IGDCC3 | 1 | 1 | 2 | N |
| hsa-let-7d-5p | UHRF2 | 1 | 1 | 2 | N |
| hsa-let-7d-5p | COL24A1 | 1 | 1 | 2 | N |
| hsa-let-7d-5p | RDX | 1 | 1 | 2 | Y |
| hsa-let-7d-5p | ADRB3 | 1 | 1 | 2 | N |
| hsa-let-7d-5p | AP1S1 | 1 | 1 | 2 | Y |
| hsa-let-7d-5p | RRM2 | 1 | 1 | 2 | Y |
| hsa-let-7d-5p | NGF | 1 | 1 | 2 | N |
| hsa-let-7d-5p | TRIM71 | 1 | 1 | 2 | Y |
| hsa-let-7d-5p | FIGN | 1 | 1 | 2 | Y |
| hsa-let-7d-5p | GALC | 1 | 1 | 2 | N |
| hsa-let-7d-5p | TET3 | 1 | 1 | 2 | N |
| hsa-let-7d-5p | AKAP6 | 1 | 1 | 2 | N |
| hsa-let-7d-5p | GNPTAB | 1 | 1 | 2 | N |
| hsa-let-7d-5p | THAP9 | 1 | 1 | 2 | N |
| hsa-let-7d-5p | LRIG2 | 1 | 1 | 2 | N |
| hsa-let-7d-5p | ULK2 | 1 | 1 | 2 | N |
| hsa-let-7d-5p | PXT1 | 1 | 1 | 2 | N |
| hsa-let-7d-5p | CLP1 | 1 | 1 | 2 | N |
| hsa-let-7d-5p | YOD1 | 1 | 1 | 2 | Y |
| hsa-let-7d-5p | CCR7 | 1 | 1 | 2 | N |
| hsa-let-7d-5p | RASGRP1 | 1 | 1 | 2 | N |
| hsa-let-7d-5p | AHCTF1 | 1 | 1 | 2 | N |
| hsa-let-7d-5p | E2F5 | 1 | 1 | 2 | N |
| hsa-let-7d-5p | TRIM41 | 1 | 1 | 2 | N |
| hsa-let-7d-5p | SMC1A | 1 | 1 | 2 | Y |
| hsa-let-7d-5p | SLC5A6 | 1 | 1 | 2 | Y |
| hsa-let-7d-5p | RPUSD2 | 1 | 1 | 2 | Y |
| hsa-let-7d-5p | BZW2 | 1 | 1 | 2 | N |
| hsa-let-7d-5p | XKR8 | 1 | 1 | 2 | N |
| hsa-let-7d-5p | STK40 | 1 | 1 | 2 | Y |
| hsa-let-7d-5p | NDST2 | 1 | 1 | 2 | N |
| hsa-let-7d-5p | DLST | 1 | 1 | 2 | N |
| hsa-let-7d-5p | SMIM3 | 1 | 1 | 2 | N |
| hsa-let-7d-5p | IQCB1 | 1 | 1 | 2 | N |
| hsa-let-7d-5p | NME6 | 1 | 1 | 2 | N |
| hsa-let-7d-5p | B3GNT7 | 1 | 1 | 2 | N |
| hsa-let-7d-5p | COL4A6 | 1 | 1 | 2 | N |
| hsa-let-7d-5p | C5orf51 | 1 | 1 | 2 | Y |
| hsa-let-7d-5p | NXT2 | 1 | 1 | 2 | N |
| hsa-let-7d-5p | PRPF38B | 1 | 1 | 2 | N |
| hsa-let-7d-5p | NAA30 | 1 | 1 | 2 | Y |
| hsa-let-7d-5p | PRRX1 | 1 | 1 | 2 | N |
| hsa-let-7d-5p | IGF2BP3 | 1 | 1 | 2 | Y |
| hsa-let-7d-5p | TMEM2 | 1 | 1 | 2 | Y |
| hsa-let-7d-5p | LIN28B | 1 | 1 | 2 | Y |
| hsa-let-7d-5p | LBR | 1 | 1 | 2 | Y |
| hsa-let-7d-5p | ACER2 | 1 | 1 | 2 | Y |
| hsa-let-7d-5p | DDI2 | 1 | 1 | 2 | N |
| hsa-let-7d-5p | ESPL1 | 1 | 1 | 2 | Y |
| hsa-let-7d-5p | PBX1 | 1 | 1 | 2 | N |
| hsa-let-7d-5p | GRPEL2 | 1 | 1 | 2 | Y |
| hsa-let-7d-5p | COIL | 1 | 1 | 2 | Y |
| hsa-let-7d-5p | GPR26 | 1 | 1 | 2 | N |
| hsa-let-7d-5p | SMARCAD1 | 1 | 1 | 2 | Y |
| hsa-let-7d-5p | ARHGAP28 | 1 | 1 | 2 | N |
| hsa-let-7d-5p | PDPR | 1 | 1 | 2 | N |
| hsa-let-7d-5p | C8orf58 | 1 | 1 | 2 | N |
| hsa-let-7d-5p | GATM | 1 | 1 | 2 | Y |
| hsa-let-7d-5p | GLRX | 1 | 1 | 2 | N |
| hsa-let-7d-5p | IGF1R | 1 | 1 | 2 | Y |
| hsa-let-7d-5p | ZNF583 | 1 | 1 | 2 | N |
| hsa-let-7d-5p | TGFBR1 | 1 | 1 | 2 | Y |
| hsa-let-7d-5p | PPP1R15B | 1 | 1 | 2 | Y |
| hsa-let-7d-5p | GCNT4 | 1 | 1 | 2 | N |
| hsa-let-7d-5p | BIN3 | 1 | 1 | 2 | Y |
| hsa-let-7d-5p | FOXP2 | 1 | 1 | 2 | N |
| hsa-let-7d-5p | CASP3 | 1 | 1 | 2 | N |
| hsa-let-7d-5p | EIF4G2 | 1 | 1 | 2 | Y |
| hsa-let-7d-5p | NHLRC3 | 1 | 1 | 2 | Y |
| hsa-let-7d-5p | BACH1 | 1 | 1 | 2 | Y |
| hsa-let-7d-5p | HMGA1 | 1 | 1 | 2 | Y |
| hsa-let-7d-5p | MTUS1 | 1 | 1 | 2 | Y |
| hsa-let-7d-5p | SCN4B | 1 | 1 | 2 | N |
| hsa-let-7d-5p | CPEB1 | 1 | 1 | 2 | N |
| hsa-let-7d-5p | E2F6 | 1 | 1 | 2 | Y |
| hsa-let-7d-5p | SPRYD4 | 1 | 1 | 2 | Y |
| hsa-let-7d-5p | PBX3 | 1 | 1 | 2 | N |
| hsa-let-7d-5p | RIOK3 | 1 | 1 | 2 | N |
| hsa-let-7d-5p | KLHL31 | 1 | 1 | 2 | N |
| hsa-let-7d-5p | DDX19B | 1 | 1 | 2 | N |
| hsa-let-7d-5p | CPA4 | 1 | 1 | 2 | Y |
| hsa-let-7d-5p | MXD1 | 1 | 1 | 2 | Y |
| hsa-let-7d-5p | CCNJ | 1 | 1 | 2 | N |
| hsa-let-7d-5p | HIC2 | 1 | 1 | 2 | N |
| hsa-let-7d-5p | HMGA2 | 1 | 1 | 2 | Y |
| hsa-let-7d-5p | EDN1 | 1 | 1 | 2 | Y |
| hsa-let-7d-5p | KLHDC8B | 1 | 1 | 2 | Y |
| hsa-let-7d-5p | LIMD2 | 1 | 1 | 2 | Y |
| hsa-let-7d-5p | TTLL4 | 1 | 1 | 2 | N |
| hsa-let-7d-5p | HAND1 | 1 | 1 | 2 | Y |
| hsa-let-7g-5p | CLP1 | 1 | 1 | 2 | N |
| hsa-let-7g-5p | OSBPL3 | 1 | 1 | 2 | N |
| hsa-let-7g-5p | MTUS1 | 1 | 1 | 2 | Y |
| hsa-let-7g-5p | STX3 | 1 | 1 | 2 | Y |
| hsa-let-7g-5p | EDN1 | 1 | 1 | 2 | Y |
| hsa-let-7g-5p | SLC5A6 | 1 | 1 | 2 | Y |
| hsa-let-7g-5p | PDPR | 1 | 1 | 2 | N |
| hsa-let-7g-5p | KCTD21 | 1 | 1 | 2 | Y |
| hsa-let-7g-5p | ACVR1C | 1 | 1 | 2 | N |
| hsa-let-7g-5p | COL4A6 | 1 | 1 | 2 | N |
| hsa-let-7g-5p | LRIG3 | 1 | 1 | 2 | Y |
| hsa-let-7g-5p | C5orf51 | 1 | 1 | 2 | Y |
| hsa-let-7g-5p | HMGA2 | 1 | 1 | 2 | Y |
| hsa-let-7g-5p | MAPK6 | 1 | 1 | 2 | Y |
| hsa-let-7g-5p | RIOK3 | 1 | 1 | 2 | N |
| hsa-let-7g-5p | ADAMTS8 | 1 | 1 | 2 | N |
| hsa-let-7g-5p | FRMD4B | 1 | 1 | 2 | N |
| hsa-let-7g-5p | PLXNC1 | 1 | 1 | 2 | N |
| hsa-let-7g-5p | DPF2 | 1 | 1 | 2 | N |
| hsa-let-7g-5p | SLC25A18 | 1 | 1 | 2 | N |
| hsa-let-7g-5p | LIMD2 | 1 | 1 | 2 | Y |
| hsa-let-7g-5p | DDI2 | 1 | 1 | 2 | N |
| hsa-let-7g-5p | PPP1R15B | 1 | 1 | 2 | Y |
| hsa-let-7g-5p | NRAS | 1 | 1 | 2 | N |
| hsa-let-7g-5p | HIC2 | 1 | 1 | 2 | N |
| hsa-let-7g-5p | PGRMC1 | 1 | 1 | 2 | Y |
| hsa-let-7g-5p | NME6 | 1 | 1 | 2 | N |
| hsa-let-7g-5p | PLEKHA8 | 1 | 1 | 2 | N |
| hsa-let-7g-5p | TMEM2 | 1 | 1 | 2 | N |
| hsa-let-7g-5p | SLC5A9 | 1 | 1 | 2 | N |
| hsa-let-7g-5p | KLHL31 | 1 | 1 | 2 | N |
| hsa-let-7g-5p | ARG2 | 1 | 1 | 2 | N |
| hsa-let-7g-5p | LIN28B | 1 | 1 | 2 | N |
| hsa-let-7g-5p | PAPPA | 1 | 1 | 2 | N |
| hsa-let-7g-5p | CEP135 | 1 | 1 | 2 | Y |
| hsa-let-7g-5p | PCGF3 | 1 | 1 | 2 | Y |
| hsa-let-7g-5p | FOXP2 | 1 | 1 | 2 | N |
| hsa-let-7g-5p | THAP9 | 1 | 1 | 2 | N |
| hsa-let-7g-5p | HAND1 | 1 | 1 | 2 | Y |
| hsa-let-7g-5p | ZNF275 | 1 | 1 | 2 | N |
| hsa-let-7g-5p | TMPPE | 1 | 1 | 2 | N |
| hsa-let-7g-5p | TGFBR1 | 1 | 1 | 2 | Y |
| hsa-let-7g-5p | MAP4K3 | 1 | 1 | 2 | N |
| hsa-let-7g-5p | TRIM41 | 1 | 1 | 2 | N |
| hsa-let-7g-5p | GATM | 1 | 1 | 2 | Y |
| hsa-let-7g-5p | GRPEL2 | 1 | 1 | 2 | Y |
| hsa-let-7g-5p | SCN4B | 1 | 1 | 2 | N |
| hsa-let-7g-5p | KLHDC8B | 1 | 1 | 2 | Y |
| hsa-let-7g-5p | EIF4G2 | 1 | 1 | 2 | Y |
| hsa-let-7g-5p | AMT | 1 | 1 | 2 | N |
| hsa-let-7g-5p | LIPH | 1 | 1 | 2 | N |
| hsa-let-7g-5p | RNF20 | 1 | 1 | 2 | N |
| hsa-let-7g-5p | SKIL | 1 | 1 | 2 | N |
| hsa-let-7g-5p | C15orf41 | 1 | 1 | 2 | N |
| hsa-let-7g-5p | SLC20A1 | 1 | 1 | 2 | Y |
| hsa-let-7g-5p | PALD1 | 1 | 1 | 2 | N |
| hsa-let-7g-5p | LBR | 1 | 1 | 2 | N |
| hsa-let-7g-5p | GCNT4 | 1 | 1 | 2 | N |
| hsa-let-7g-5p | GALC | 1 | 1 | 2 | N |
| hsa-let-7g-5p | AHCTF1 | 1 | 1 | 2 | N |
| hsa-let-7g-5p | HMGA1 | 1 | 1 | 2 | Y |
| hsa-let-7g-5p | RSPO2 | 1 | 1 | 2 | N |
| hsa-let-7g-5p | PBX3 | 1 | 1 | 2 | N |
| hsa-let-7g-5p | CCL7 | 1 | 1 | 2 | N |
| hsa-let-7g-5p | BEGAIN | 1 | 1 | 2 | N |
| hsa-let-7g-5p | RAB11FIP4 | 1 | 1 | 2 | Y |
| hsa-let-7g-5p | APBB3 | 1 | 1 | 2 | N |
| hsa-let-7g-5p | ZBTB8B | 1 | 1 | 2 | N |
| hsa-let-7g-5p | LIPT2 | 1 | 1 | 2 | N |
| hsa-let-7g-5p | RALB | 1 | 1 | 2 | N |
| hsa-let-7g-5p | RGS16 | 1 | 1 | 2 | N |
| hsa-let-7g-5p | GPR26 | 1 | 1 | 2 | N |
| hsa-let-7g-5p | NGF | 1 | 1 | 2 | N |
| hsa-let-7g-5p | PRPF38B | 1 | 1 | 2 | N |
| hsa-let-7g-5p | AKAP6 | 1 | 1 | 2 | N |
| hsa-let-7g-5p | COL24A1 | 1 | 1 | 2 | N |
| hsa-let-7g-5p | E2F6 | 1 | 1 | 2 | Y |
| hsa-let-7g-5p | RDX | 1 | 1 | 2 | Y |
| hsa-let-7g-5p | GAS7 | 1 | 1 | 2 | N |
| hsa-let-7g-5p | ADRB3 | 1 | 1 | 2 | N |
| hsa-let-7g-5p | BZW2 | 1 | 1 | 2 | N |
| hsa-let-7g-5p | YOD1 | 1 | 1 | 2 | Y |
| hsa-let-7g-5p | IGF1R | 1 | 1 | 2 | Y |
| hsa-let-7g-5p | CDC34 | 1 | 1 | 2 | N |
| hsa-let-7g-5p | GNPTAB | 1 | 1 | 2 | N |
| hsa-let-7g-5p | TTLL4 | 1 | 1 | 2 | N |
| hsa-let-7g-5p | ULK2 | 1 | 1 | 2 | N |
| hsa-let-7g-5p | KLHL23 | 1 | 1 | 2 | N |
| hsa-let-7g-5p | CCR7 | 1 | 1 | 2 | N |
| hsa-let-7g-5p | TMEM110 | 1 | 1 | 2 | N |
| hsa-let-7g-5p | USP38 | 1 | 1 | 2 | Y |
| hsa-let-7g-5p | RPUSD2 | 1 | 1 | 2 | N |
| hsa-let-7g-5p | PPAPDC2 | 1 | 1 | 2 | N |
| hsa-let-7g-5p | NAA30 | 1 | 1 | 2 | Y |
| hsa-let-7g-5p | ZFYVE26 | 1 | 1 | 2 | N |
| hsa-let-7g-5p | STK40 | 1 | 1 | 2 | N |
| hsa-let-7g-5p | NXT2 | 1 | 1 | 2 | N |
| hsa-let-7g-5p | TET3 | 1 | 1 | 2 | N |
| hsa-let-7g-5p | ARHGAP28 | 1 | 1 | 2 | N |
| hsa-let-7g-5p | DDX19A | 1 | 1 | 2 | N |
| hsa-let-7g-5p | ACER2 | 1 | 1 | 2 | Y |
| hsa-let-7g-5p | DLST | 1 | 1 | 2 | N |
| hsa-let-7g-5p | ZNF512B | 1 | 1 | 2 | N |
| hsa-let-7g-5p | MXD1 | 1 | 1 | 2 | Y |
| hsa-let-7g-5p | B3GNT7 | 1 | 1 | 2 | N |
| hsa-let-7g-5p | PARD6B | 1 | 1 | 2 | N |
| hsa-let-7g-5p | SMARCAD1 | 1 | 1 | 2 | Y |
| hsa-let-7g-5p | IGF2BP3 | 1 | 1 | 2 | Y |
| hsa-let-7g-5p | GALNT1 | 1 | 1 | 2 | N |
| hsa-let-7g-5p | NDST2 | 1 | 1 | 2 | N |
| hsa-let-7g-5p | CLDN12 | 1 | 1 | 2 | Y |
| hsa-let-7g-5p | FNIP1 | 1 | 1 | 2 | N |
| hsa-let-7g-5p | CCNJ | 1 | 1 | 2 | N |
| hsa-let-7g-5p | ADAMTS15 | 1 | 1 | 2 | N |
| hsa-let-7g-5p | IGDCC3 | 1 | 1 | 2 | N |
| hsa-let-7g-5p | ADRB2 | 1 | 1 | 2 | N |
| hsa-let-7g-5p | ESPL1 | 1 | 1 | 2 | Y |
| hsa-let-7g-5p | GLRX | 1 | 1 | 2 | N |
| hsa-let-7g-5p | NHLRC3 | 1 | 1 | 2 | Y |
| hsa-let-7g-5p | ARRDC4 | 1 | 1 | 2 | N |
| hsa-let-7g-5p | CASP3 | 1 | 1 | 2 | Y |
| hsa-let-7g-5p | RASGRP1 | 1 | 1 | 2 | N |
| hsa-let-7g-5p | GXYLT1 | 1 | 1 | 2 | N |
| hsa-let-7g-5p | BACH1 | 1 | 1 | 2 | Y |
| hsa-let-7g-5p | ATP8B4 | 1 | 1 | 2 | N |
| hsa-let-7g-5p | THRSP | 1 | 1 | 2 | N |
| hsa-let-7g-5p | PBX1 | 1 | 1 | 2 | N |
| hsa-let-7g-5p | E2F5 | 1 | 1 | 2 | N |
| hsa-let-7g-5p | GNG5 | 1 | 1 | 2 | Y |
| hsa-let-7g-5p | FNDC3A | 1 | 1 | 2 | Y |
| hsa-let-7g-5p | MED8 | 1 | 1 | 2 | N |
| hsa-let-7g-5p | ZNF583 | 1 | 1 | 2 | N |
| hsa-let-7g-5p | LRIG2 | 1 | 1 | 2 | N |
| hsa-let-7g-5p | MFSD4 | 1 | 1 | 2 | N |
| hsa-let-7g-5p | TGFBR3 | 1 | 1 | 2 | Y |
| hsa-let-7g-5p | BZW1 | 1 | 1 | 2 | Y |
| hsa-let-7g-5p | NYNRIN | 1 | 1 | 2 | N |
| hsa-let-7g-5p | CPA4 | 1 | 1 | 2 | Y |
| hsa-let-7g-5p | BIN3 | 1 | 1 | 2 | N |
| hsa-let-7g-5p | SMIM3 | 1 | 1 | 2 | N |
| hsa-let-7g-5p | C15orf39 | 1 | 1 | 2 | N |
| hsa-let-7g-5p | PRRX1 | 1 | 1 | 2 | N |
| hsa-let-7g-5p | TRIM71 | 1 | 1 | 2 | Y |
| hsa-let-7g-5p | UHRF2 | 1 | 1 | 2 | N |
| hsa-let-7g-5p | RRM2 | 1 | 1 | 2 | Y |
| hsa-let-7g-5p | XKR8 | 1 | 1 | 2 | N |
| hsa-let-7g-5p | SLC25A27 | 1 | 1 | 2 | N |
| hsa-let-7g-5p | DDX19B | 1 | 1 | 2 | N |
| hsa-let-7g-5p | COIL | 1 | 1 | 2 | Y |
| hsa-let-7g-5p | CPEB1 | 1 | 1 | 2 | N |
| hsa-let-7g-5p | DDX26B | 1 | 1 | 2 | N |
| hsa-let-7g-5p | SCD | 1 | 1 | 2 | N |
| hsa-let-7g-5p | UTRN | 1 | 1 | 2 | N |
| hsa-let-7g-5p | PXT1 | 1 | 1 | 2 | N |
| hsa-let-7g-5p | IQCB1 | 1 | 1 | 2 | N |
| hsa-let-7g-5p | FIGN | 1 | 1 | 2 | Y |
| hsa-let-7g-5p | FZD3 | 1 | 1 | 2 | N |
| hsa-let-7g-5p | AP1S1 | 1 | 1 | 2 | Y |
| hsa-let-7g-5p | TARBP2 | 1 | 1 | 2 | N |
| hsa-let-7g-5p | C8orf58 | 1 | 1 | 2 | N |
| hsa-let-7g-5p | SPRYD4 | 1 | 1 | 2 | N |
| hsa-let-7g-5p | NR6A1 | 1 | 1 | 2 | Y |
| hsa-miR-15a-3p | RAB8B | 1 | 1 | 2 | N |
| hsa-miR-15a-3p | STRN3 | 1 | 1 | 2 | N |
| hsa-miR-15a-3p | PAQR5 | 1 | 1 | 2 | N |
| hsa-miR-15a-3p | GID8 | 1 | 1 | 2 | N |
| hsa-miR-15a-3p | NBN | 1 | 1 | 2 | N |
| hsa-miR-15a-3p | CTBP2 | 1 | 1 | 2 | N |
| hsa-miR-15a-3p | PNRC2 | 1 | 1 | 2 | N |
| hsa-miR-15a-3p | RAB31 | 1 | 1 | 2 | N |
| hsa-miR-15a-3p | NPNT | 1 | 1 | 2 | N |
| hsa-miR-15a-3p | PLA2G2F | 1 | 1 | 2 | N |
| hsa-miR-15a-3p | KLHDC2 | 1 | 1 | 2 | N |
| hsa-miR-15a-3p | ARHGAP26 | 1 | 1 | 2 | N |
| hsa-miR-15a-3p | KCNA1 | 1 | 1 | 2 | N |
| hsa-miR-15a-3p | TPD52 | 1 | 1 | 2 | N |
| hsa-miR-15a-3p | ZBTB4 | 1 | 1 | 2 | N |
| hsa-miR-15a-3p | TP53TG3D | 1 | 1 | 2 | N |
| hsa-miR-15a-3p | GLCCI1 | 1 | 1 | 2 | N |
| hsa-miR-15a-3p | ARMCX2 | 1 | 1 | 2 | N |
| hsa-miR-15a-3p | C17orf97 | 1 | 1 | 2 | N |
| hsa-miR-15a-3p | BNC2 | 1 | 1 | 2 | N |
| hsa-miR-15a-3p | ARHGEF3 | 1 | 1 | 2 | N |
| hsa-miR-15a-3p | ADAMDEC1 | 1 | 1 | 2 | N |
| hsa-miR-15a-3p | SETD8 | 1 | 1 | 2 | N |
| hsa-miR-15a-3p | DHX15 | 1 | 1 | 2 | N |
| hsa-miR-15a-3p | CPEB2 | 1 | 1 | 2 | N |
| hsa-miR-15a-3p | RAVER2 | 1 | 1 | 2 | N |
| hsa-miR-15a-3p | SLC41A2 | 1 | 1 | 2 | N |
| hsa-miR-15a-3p | SSPN | 1 | 1 | 2 | N |
| hsa-miR-15a-3p | CYP19A1 | 1 | 1 | 2 | N |
| hsa-miR-15a-3p | RAB21 | 1 | 1 | 2 | N |
| hsa-miR-15a-3p | C3orf70 | 1 | 1 | 2 | N |
| hsa-miR-15a-3p | LMNB1 | 1 | 1 | 2 | Y |
| hsa-miR-15a-3p | CREBZF | 1 | 1 | 2 | N |
| hsa-miR-15a-3p | PARP16 | 1 | 1 | 2 | N |
| hsa-miR-15a-3p | DEFB118 | 1 | 1 | 2 | N |
| hsa-miR-328-5p | KHSRP | 1 | 1 | 2 | Y |
| hsa-miR-328-5p | DNM1 | 1 | 1 | 2 | N |
| hsa-miR-328-5p | MEIS1 | 1 | 1 | 2 | N |
| hsa-miR-328-5p | FURIN | 1 | 1 | 2 | Y |
| hsa-miR-328-5p | WDR52 | 1 | 1 | 2 | N |
| hsa-miR-328-5p | RAMP1 | 1 | 1 | 2 | N |
| hsa-miR-328-5p | PXN | 1 | 1 | 2 | N |
| hsa-miR-328-5p | CPLX2 | 1 | 1 | 2 | N |
| hsa-miR-328-5p | CXCL14 | 1 | 1 | 2 | N |
| hsa-miR-328-5p | BPIFB4 | 1 | 1 | 2 | N |
| hsa-miR-328-5p | EMP3 | 1 | 1 | 2 | N |
| hsa-miR-328-5p | RARA | 1 | 1 | 2 | N |
| hsa-miR-328-5p | SALL1 | 1 | 1 | 2 | N |
| hsa-miR-328-5p | MYOM3 | 1 | 1 | 2 | N |
| hsa-miR-328-5p | IL27 | 1 | 1 | 2 | N |
| hsa-miR-328-5p | DYNLRB1 | 1 | 1 | 2 | N |
| hsa-miR-328-5p | PVRL1 | 1 | 1 | 2 | N |
| hsa-miR-328-5p | PRKACA | 1 | 1 | 2 | N |
| hsa-miR-328-5p | VAMP2 | 1 | 1 | 2 | N |
| hsa-miR-328-5p | DNAJB2 | 1 | 1 | 2 | N |
| hsa-miR-328-5p | COL5A3 | 1 | 1 | 2 | N |
| hsa-miR-328-5p | C12orf66 | 1 | 1 | 2 | N |
| hsa-miR-328-5p | CA10 | 1 | 1 | 2 | N |
| hsa-miR-328-5p | CHI3L1 | 1 | 1 | 2 | N |
| hsa-miR-328-5p | SRCIN1 | 1 | 1 | 2 | N |
| hsa-miR-328-5p | LHX4 | 1 | 1 | 2 | N |
| hsa-miR-328-5p | FOXJ1 | 1 | 1 | 2 | N |
| hsa-miR-328-5p | VSTM2L | 1 | 1 | 2 | N |
| hsa-miR-328-5p | SOD3 | 1 | 1 | 2 | N |
| hsa-miR-328-5p | LYPLA2 | 1 | 1 | 2 | N |
| hsa-miR-328-5p | DAB2IP | 1 | 1 | 2 | N |
| hsa-miR-328-5p | CD7 | 1 | 1 | 2 | N |
| hsa-miR-328-5p | SLC19A1 | 1 | 1 | 2 | N |
| hsa-miR-328-5p | WWP2 | 1 | 1 | 2 | Y |
| hsa-miR-328-5p | NEFH | 1 | 1 | 2 | N |
| hsa-miR-328-5p | PDE4A | 1 | 1 | 2 | N |
| hsa-miR-328-5p | SERTAD4 | 1 | 1 | 2 | N |
| hsa-miR-328-5p | ZHX2 | 1 | 1 | 2 | N |
| hsa-miR-328-5p | SPATA20 | 1 | 1 | 2 | N |
| hsa-miR-328-5p | ATXN7L3 | 1 | 1 | 2 | N |
| hsa-miR-328-5p | KDSR | 1 | 1 | 2 | N |
| hsa-miR-328-5p | ACTL6B | 1 | 1 | 2 | N |
| hsa-miR-328-5p | CECR6 | 1 | 1 | 2 | N |
| hsa-miR-328-5p | KCND1 | 1 | 1 | 2 | N |
| hsa-miR-328-5p | SYDE1 | 1 | 1 | 2 | N |
| hsa-miR-328-5p | LENG8 | 1 | 1 | 2 | N |
| hsa-miR-328-5p | GRIN3A | 1 | 1 | 2 | N |
| hsa-miR-328-5p | PPM1H | 1 | 1 | 2 | N |
| hsa-miR-328-5p | FBN3 | 1 | 1 | 2 | N |
| hsa-miR-328-5p | ZNF385B | 1 | 1 | 2 | N |
| hsa-miR-328-5p | TUBB4A | 1 | 1 | 2 | N |
| hsa-miR-328-5p | NGFR | 1 | 1 | 2 | N |
| hsa-miR-328-5p | TENC1 | 1 | 1 | 2 | N |
| hsa-miR-328-5p | TCF7L2 | 1 | 1 | 2 | N |
| hsa-miR-328-5p | HIC1 | 1 | 1 | 2 | N |
| hsa-miR-328-5p | ANKRD40 | 1 | 1 | 2 | N |
| hsa-miR-328-5p | ATP1A3 | 1 | 1 | 2 | N |
| hsa-miR-328-5p | MINOS1-NBL1 | 1 | 1 | 2 | N |
| hsa-miR-328-5p | ZCCHC24 | 1 | 1 | 2 | N |
| hsa-miR-328-5p | CACNB3 | 1 | 1 | 2 | N |
| hsa-miR-328-5p | CENPB | 1 | 1 | 2 | N |
| hsa-miR-328-5p | ISYNA1 | 1 | 1 | 2 | N |
| hsa-miR-328-5p | GM2A | 1 | 1 | 2 | N |
| hsa-miR-328-5p | GIGYF1 | 1 | 1 | 2 | Y |
| hsa-miR-328-5p | ELK1 | 1 | 1 | 2 | N |
| hsa-miR-328-5p | DNMBP | 1 | 1 | 2 | N |
| hsa-miR-328-5p | PPP1R14B | 1 | 1 | 2 | N |
| hsa-miR-328-5p | CACNG1 | 1 | 1 | 2 | N |
| hsa-miR-328-5p | ARL4C | 1 | 1 | 2 | Y |
| hsa-miR-328-5p | FCRLA | 1 | 1 | 2 | N |
| hsa-miR-328-5p | NFIC | 1 | 1 | 2 | N |
| hsa-miR-328-5p | KLRD1 | 1 | 1 | 2 | N |
| hsa-miR-328-5p | RPRD1B | 1 | 1 | 2 | N |
| hsa-miR-328-5p | ZMAT5 | 1 | 1 | 2 | Y |
| hsa-miR-328-5p | C1orf106 | 1 | 1 | 2 | N |
| hsa-miR-328-5p | ZNF646 | 1 | 1 | 2 | N |
| hsa-miR-328-5p | BAIAP2 | 1 | 1 | 2 | N |
| hsa-miR-328-5p | E2F2 | 1 | 1 | 2 | N |
| hsa-miR-328-5p | ANGPT4 | 1 | 1 | 2 | N |
| hsa-miR-328-5p | C9orf72 | 1 | 1 | 2 | N |
| hsa-miR-328-5p | WDTC1 | 1 | 1 | 2 | N |
| hsa-miR-328-5p | BCORL1 | 1 | 1 | 2 | N |
| hsa-miR-328-5p | MPP2 | 1 | 1 | 2 | N |
| hsa-miR-328-5p | JUNB | 1 | 1 | 2 | N |
| hsa-miR-328-5p | CAPN15 | 1 | 1 | 2 | N |
| hsa-miR-328-5p | EFNB3 | 1 | 1 | 2 | N |
| hsa-miR-328-5p | KLF16 | 1 | 1 | 2 | N |
| hsa-miR-328-5p | NCDN | 1 | 1 | 2 | N |
| hsa-miR-328-5p | RAB3A | 1 | 1 | 2 | N |
| hsa-miR-328-5p | BRSK1 | 1 | 1 | 2 | N |
| hsa-miR-328-5p | ARL8A | 1 | 1 | 2 | N |
| hsa-miR-328-5p | CLSTN3 | 1 | 1 | 2 | N |
| hsa-miR-328-5p | DTX3 | 1 | 1 | 2 | N |
| hsa-miR-328-5p | CDKN1A | 1 | 1 | 2 | Y |
| hsa-miR-328-5p | MYOD1 | 1 | 1 | 2 | N |
| hsa-miR-328-5p | SMARCD2 | 1 | 1 | 2 | N |
| hsa-miR-328-5p | ZNF609 | 1 | 1 | 2 | N |
| hsa-miR-328-5p | OTOGL | 1 | 1 | 2 | N |
| hsa-miR-328-5p | ANKRD66 | 1 | 1 | 2 | N |
| hsa-miR-328-5p | HMGA1 | 1 | 1 | 2 | N |
| hsa-miR-328-5p | EPHB3 | 1 | 1 | 2 | N |
| hsa-miR-328-5p | SYNGAP1 | 1 | 1 | 2 | N |
| hsa-miR-328-5p | ZBTB46 | 1 | 1 | 2 | Y |
| hsa-miR-328-5p | TPSB2 | 1 | 1 | 2 | N |
| hsa-miR-328-5p | RRP1B | 1 | 1 | 2 | N |
| hsa-miR-328-5p | RAB22A | 1 | 1 | 2 | N |
| hsa-miR-328-5p | CTIF | 1 | 1 | 2 | N |
| hsa-miR-328-5p | TTYH3 | 1 | 1 | 2 | N |
| hsa-miR-328-5p | PAFAH1B2 | 1 | 1 | 2 | N |
| hsa-miR-328-5p | TRPC5 | 1 | 1 | 2 | N |
| hsa-miR-328-5p | IQSEC2 | 1 | 1 | 2 | N |
| hsa-miR-328-5p | FOXA1 | 1 | 1 | 2 | N |
| hsa-miR-328-5p | KIAA1644 | 1 | 1 | 2 | N |
| hsa-miR-328-5p | GNAI2 | 1 | 1 | 2 | N |
| hsa-miR-328-5p | LGI3 | 1 | 1 | 2 | N |
| hsa-miR-328-5p | DGKG | 1 | 1 | 2 | N |
| hsa-miR-328-5p | ERI3 | 1 | 1 | 2 | N |
| hsa-miR-98-5p | EIF4G2 | 1 | 1 | 2 | Y |
| hsa-miR-98-5p | RDX | 1 | 1 | 2 | Y |
| hsa-miR-98-5p | LIN28B | 1 | 1 | 2 | N |
| hsa-miR-98-5p | MTUS1 | 1 | 1 | 2 | Y |
| hsa-miR-98-5p | IQCB1 | 1 | 1 | 2 | Y |
| hsa-miR-98-5p | KLHL23 | 1 | 1 | 2 | N |
| hsa-miR-98-5p | LBR | 1 | 1 | 2 | N |
| hsa-miR-98-5p | HAND1 | 1 | 1 | 2 | Y |
| hsa-miR-98-5p | AMT | 1 | 1 | 2 | N |
| hsa-miR-98-5p | STK40 | 1 | 1 | 2 | N |
| hsa-miR-98-5p | RGS16 | 1 | 1 | 2 | Y |
| hsa-miR-98-5p | RASGRP1 | 1 | 1 | 2 | N |
| hsa-miR-98-5p | SKIL | 1 | 1 | 2 | N |
| hsa-miR-98-5p | CLP1 | 1 | 1 | 2 | Y |
| hsa-miR-98-5p | NME6 | 1 | 1 | 2 | Y |
| hsa-miR-98-5p | PALD1 | 1 | 1 | 2 | N |
| hsa-miR-98-5p | DDX19B | 1 | 1 | 2 | N |
| hsa-miR-98-5p | IGF2BP3 | 1 | 1 | 2 | Y |
| hsa-miR-98-5p | COL4A6 | 1 | 1 | 2 | N |
| hsa-miR-98-5p | MAPK6 | 1 | 1 | 2 | Y |
| hsa-miR-98-5p | B3GNT7 | 1 | 1 | 2 | N |
| hsa-miR-98-5p | ADAMTS8 | 1 | 1 | 2 | N |
| hsa-miR-98-5p | HMGA2 | 1 | 1 | 2 | Y |
| hsa-miR-98-5p | ESPL1 | 1 | 1 | 2 | Y |
| hsa-miR-98-5p | AP1S1 | 1 | 1 | 2 | Y |
| hsa-miR-98-5p | RRM2 | 1 | 1 | 2 | Y |
| hsa-miR-98-5p | FNDC3A | 1 | 1 | 2 | Y |
| hsa-miR-98-5p | COL24A1 | 1 | 1 | 2 | N |
| hsa-miR-98-5p | C5orf51 | 1 | 1 | 2 | Y |
| hsa-miR-98-5p | LIMD2 | 1 | 1 | 2 | Y |
| hsa-miR-98-5p | CPEB1 | 1 | 1 | 2 | N |
| hsa-miR-98-5p | CLDN12 | 1 | 1 | 2 | Y |
| hsa-miR-98-5p | IGF1R | 1 | 1 | 2 | Y |
| hsa-miR-98-5p | SMARCAD1 | 1 | 1 | 2 | Y |
| hsa-miR-98-5p | PRRX1 | 1 | 1 | 2 | N |
| hsa-miR-98-5p | RALB | 1 | 1 | 2 | N |
| hsa-miR-98-5p | SLC20A1 | 1 | 1 | 2 | Y |
| hsa-miR-98-5p | TET3 | 1 | 1 | 2 | Y |
| hsa-miR-98-5p | RNF20 | 1 | 1 | 2 | N |
| hsa-miR-98-5p | E2F6 | 1 | 1 | 2 | Y |
| hsa-miR-98-5p | PDPR | 1 | 1 | 2 | N |
| hsa-miR-98-5p | THRSP | 1 | 1 | 2 | N |
| hsa-miR-98-5p | SCN4B | 1 | 1 | 2 | N |
| hsa-miR-98-5p | GAS7 | 1 | 1 | 2 | N |
| hsa-miR-98-5p | EDN1 | 1 | 1 | 2 | Y |
| hsa-miR-98-5p | CEP135 | 1 | 1 | 2 | Y |
| hsa-miR-98-5p | RIOK3 | 1 | 1 | 2 | N |
| hsa-miR-98-5p | PBX1 | 1 | 1 | 2 | N |
| hsa-miR-98-5p | ZNF512B | 1 | 1 | 2 | N |
| hsa-miR-98-5p | CASP3 | 1 | 1 | 2 | Y |
| hsa-miR-98-5p | FNIP1 | 1 | 1 | 2 | N |
| hsa-miR-98-5p | PPP1R15B | 1 | 1 | 2 | Y |
| hsa-miR-98-5p | THAP9 | 1 | 1 | 2 | N |
| hsa-miR-98-5p | OSBPL3 | 1 | 1 | 2 | N |
| hsa-miR-98-5p | ARRDC4 | 1 | 1 | 2 | N |
| hsa-miR-98-5p | FRMD4B | 1 | 1 | 2 | Y |
| hsa-miR-98-5p | FZD3 | 1 | 1 | 2 | Y |
| hsa-miR-98-5p | ZBTB8B | 1 | 1 | 2 | N |
| hsa-miR-98-5p | FIGN | 1 | 1 | 2 | Y |
| hsa-miR-98-5p | FOXP2 | 1 | 1 | 2 | N |
| hsa-miR-98-5p | NDST2 | 1 | 1 | 2 | N |
| hsa-miR-98-5p | GALC | 1 | 1 | 2 | N |
| hsa-miR-98-5p | KLHL31 | 1 | 1 | 2 | N |
| hsa-miR-98-5p | HIC2 | 1 | 1 | 2 | N |
| hsa-miR-98-5p | APBB3 | 1 | 1 | 2 | N |
| hsa-miR-98-5p | CCL7 | 1 | 1 | 2 | N |
| hsa-miR-98-5p | LRIG3 | 1 | 1 | 2 | Y |
| hsa-miR-98-5p | TARBP2 | 1 | 1 | 2 | Y |
| hsa-miR-98-5p | COIL | 1 | 1 | 2 | Y |
| hsa-miR-98-5p | E2F5 | 1 | 1 | 2 | Y |
| hsa-miR-98-5p | BZW2 | 1 | 1 | 2 | N |
| hsa-miR-98-5p | MXD1 | 1 | 1 | 2 | Y |
| hsa-miR-98-5p | ULK2 | 1 | 1 | 2 | N |
| hsa-miR-98-5p | PCGF3 | 1 | 1 | 2 | Y |
| hsa-miR-98-5p | NR6A1 | 1 | 1 | 2 | Y |
| hsa-miR-98-5p | AHCTF1 | 1 | 1 | 2 | N |
| hsa-miR-98-5p | RSPO2 | 1 | 1 | 2 | N |
| hsa-miR-98-5p | ZNF583 | 1 | 1 | 2 | N |
| hsa-miR-98-5p | RPUSD2 | 1 | 1 | 2 | N |
| hsa-miR-98-5p | AKAP6 | 1 | 1 | 2 | N |
| hsa-miR-98-5p | TGFBR1 | 1 | 1 | 2 | Y |
| hsa-miR-98-5p | MED8 | 1 | 1 | 2 | Y |
| hsa-miR-98-5p | C15orf41 | 1 | 1 | 2 | N |
| hsa-miR-98-5p | TRIM71 | 1 | 1 | 2 | Y |
| hsa-miR-98-5p | UHRF2 | 1 | 1 | 2 | N |
| hsa-miR-98-5p | TRIM41 | 1 | 1 | 2 | N |
| hsa-miR-98-5p | SMIM3 | 1 | 1 | 2 | N |
| hsa-miR-98-5p | IGDCC3 | 1 | 1 | 2 | N |
| hsa-miR-98-5p | LRIG2 | 1 | 1 | 2 | N |
| hsa-miR-98-5p | NYNRIN | 1 | 1 | 2 | N |
| hsa-miR-98-5p | BIN3 | 1 | 1 | 2 | Y |
| hsa-miR-98-5p | TMEM2 | 1 | 1 | 2 | Y |
| hsa-miR-98-5p | GNPTAB | 1 | 1 | 2 | Y |
| hsa-miR-98-5p | PAPPA | 1 | 1 | 2 | N |
| hsa-miR-98-5p | GCNT4 | 1 | 1 | 2 | N |
| hsa-miR-98-5p | PPAPDC2 | 1 | 1 | 2 | N |
| hsa-miR-98-5p | PXT1 | 1 | 1 | 2 | N |
| hsa-miR-98-5p | SLC5A9 | 1 | 1 | 2 | N |
| hsa-miR-98-5p | GRPEL2 | 1 | 1 | 2 | Y |
| hsa-miR-98-5p | TMEM110 | 1 | 1 | 2 | N |
| hsa-miR-98-5p | CDC34 | 1 | 1 | 2 | Y |
| hsa-miR-98-5p | GPR26 | 1 | 1 | 2 | N |
| hsa-miR-98-5p | SLC25A27 | 1 | 1 | 2 | N |
| hsa-miR-98-5p | TGFBR3 | 1 | 1 | 2 | Y |
| hsa-miR-98-5p | NGF | 1 | 1 | 2 | N |
| hsa-miR-98-5p | CCR7 | 1 | 1 | 2 | N |
| hsa-miR-98-5p | RAB11FIP4 | 1 | 1 | 2 | Y |
| hsa-miR-98-5p | SLC25A18 | 1 | 1 | 2 | N |
| hsa-miR-98-5p | ATP8B4 | 1 | 1 | 2 | N |
| hsa-miR-98-5p | PLXNC1 | 1 | 1 | 2 | N |
| hsa-miR-98-5p | USP38 | 1 | 1 | 2 | Y |
| hsa-miR-98-5p | GLRX | 1 | 1 | 2 | N |
| hsa-miR-98-5p | SLC5A6 | 1 | 1 | 2 | Y |
| hsa-miR-98-5p | DLST | 1 | 1 | 2 | N |
| hsa-miR-98-5p | DDI2 | 1 | 1 | 2 | N |
| hsa-miR-98-5p | ADRB2 | 1 | 1 | 2 | N |
| hsa-miR-98-5p | BZW1 | 1 | 1 | 2 | Y |
| hsa-miR-98-5p | KLHDC8B | 1 | 1 | 2 | Y |
| hsa-miR-98-5p | KCTD21 | 1 | 1 | 2 | Y |
| hsa-miR-98-5p | ADRB3 | 1 | 1 | 2 | N |
| hsa-miR-98-5p | ZFYVE26 | 1 | 1 | 2 | N |
| hsa-miR-98-5p | ARG2 | 1 | 1 | 2 | N |
| hsa-miR-98-5p | PGRMC1 | 1 | 1 | 2 | Y |
| hsa-miR-98-5p | PRPF38B | 1 | 1 | 2 | N |
| hsa-miR-98-5p | ACVR1C | 1 | 1 | 2 | N |
| hsa-miR-98-5p | CCNJ | 1 | 1 | 2 | Y |
| hsa-miR-98-5p | STX3 | 1 | 1 | 2 | Y |
| hsa-miR-98-5p | TTLL4 | 1 | 1 | 2 | Y |
| hsa-miR-98-5p | YOD1 | 1 | 1 | 2 | Y |
| hsa-miR-98-5p | ARHGAP28 | 1 | 1 | 2 | Y |
| hsa-miR-98-5p | PARD6B | 1 | 1 | 2 | N |
| hsa-miR-98-5p | XKR8 | 1 | 1 | 2 | N |
| hsa-miR-98-5p | TMPPE | 1 | 1 | 2 | N |
| hsa-miR-98-5p | NXT2 | 1 | 1 | 2 | N |
| hsa-miR-98-5p | SPRYD4 | 1 | 1 | 2 | N |
| hsa-miR-98-5p | SCD | 1 | 1 | 2 | N |
| hsa-miR-98-5p | HMGA1 | 1 | 1 | 2 | Y |
| hsa-miR-98-5p | DDX19A | 1 | 1 | 2 | N |
| hsa-miR-98-5p | CPA4 | 1 | 1 | 2 | Y |
| hsa-miR-98-5p | UTRN | 1 | 1 | 2 | N |
| hsa-miR-98-5p | ADAMTS15 | 1 | 1 | 2 | N |
| hsa-miR-98-5p | PLEKHA8 | 1 | 1 | 2 | N |
| hsa-miR-98-5p | ACER2 | 1 | 1 | 2 | Y |
| hsa-miR-98-5p | GXYLT1 | 1 | 1 | 2 | N |
| hsa-miR-98-5p | NHLRC3 | 1 | 1 | 2 | Y |
| hsa-miR-98-5p | MAP4K3 | 1 | 1 | 2 | N |
| hsa-miR-98-5p | BACH1 | 1 | 1 | 2 | Y |
| hsa-miR-98-5p | GALNT1 | 1 | 1 | 2 | N |
| hsa-miR-98-5p | ZNF275 | 1 | 1 | 2 | N |
| hsa-miR-98-5p | GATM | 1 | 1 | 2 | Y |
| hsa-miR-98-5p | GNG5 | 1 | 1 | 2 | Y |
| hsa-miR-98-5p | PBX3 | 1 | 1 | 2 | Y |
| hsa-miR-98-5p | C15orf39 | 1 | 1 | 2 | N |
| hsa-miR-98-5p | NRAS | 1 | 1 | 2 | Y |
| hsa-miR-98-5p | NAA30 | 1 | 1 | 2 | Y |
| hsa-miR-98-5p | MFSD4 | 1 | 1 | 2 | N |
| hsa-miR-98-5p | DDX26B | 1 | 1 | 2 | N |
| hsa-miR-98-5p | BEGAIN | 1 | 1 | 2 | N |
| hsa-miR-98-5p | LIPT2 | 1 | 1 | 2 | N |
| hsa-miR-98-5p | C8orf58 | 1 | 1 | 2 | N |
| hsa-miR-15a-5p | ARMCX2 | 1 | 1 | 2 | N |
| hsa-miR-15a-5p | INSR | 1 | 1 | 2 | N |
| hsa-miR-15a-5p | UBFD1 | 1 | 1 | 2 | N |
| hsa-miR-15a-5p | ZC2HC1A | 1 | 1 | 2 | N |
| hsa-miR-15a-5p | RAP2C | 1 | 1 | 2 | Y |
| hsa-miR-15a-5p | PCDHA11 | 1 | 1 | 2 | N |
| hsa-miR-15a-5p | HELZ | 1 | 1 | 2 | N |
| hsa-miR-15a-5p | CPD | 1 | 1 | 2 | N |
| hsa-miR-15a-5p | GABARAPL1 | 1 | 1 | 2 | Y |
| hsa-miR-15a-5p | KIF23 | 1 | 1 | 2 | Y |
| hsa-miR-15a-5p | IARS | 1 | 1 | 2 | N |
| hsa-miR-15a-5p | SIRT4 | 1 | 1 | 2 | Y |
| hsa-miR-15a-5p | TTC14 | 1 | 1 | 2 | N |
| hsa-miR-15a-5p | ADRB2 | 1 | 1 | 2 | N |
| hsa-miR-15a-5p | CCND1 | 1 | 1 | 2 | Y |
| hsa-miR-15a-5p | PDIK1L | 1 | 1 | 2 | Y |
| hsa-miR-15a-5p | PTPN3 | 1 | 1 | 2 | N |
| hsa-miR-15a-5p | RNF217 | 1 | 1 | 2 | N |
| hsa-miR-15a-5p | SUMO3 | 1 | 1 | 2 | N |
| hsa-miR-15a-5p | SPRED1 | 1 | 1 | 2 | Y |
| hsa-miR-15a-5p | CDCA4 | 1 | 1 | 2 | Y |
| hsa-miR-15a-5p | KCNJ2 | 1 | 1 | 2 | N |
| hsa-miR-15a-5p | ZBTB39 | 1 | 1 | 2 | N |
| hsa-miR-15a-5p | ARHGDIA | 1 | 1 | 2 | Y |
| hsa-miR-15a-5p | PAFAH1B1 | 1 | 1 | 2 | Y |
| hsa-miR-15a-5p | TNFSF13B | 1 | 1 | 2 | N |
| hsa-miR-15a-5p | HPCAL4 | 1 | 1 | 2 | N |
| hsa-miR-15a-5p | RNF125 | 1 | 1 | 2 | N |
| hsa-miR-15a-5p | BTRC | 1 | 1 | 2 | Y |
| hsa-miR-15a-5p | RNF144B | 1 | 1 | 2 | N |
| hsa-miR-15a-5p | N4BP1 | 1 | 1 | 2 | Y |
| hsa-miR-15a-5p | FERMT2 | 1 | 1 | 2 | N |
| hsa-miR-15a-5p | ZHX1 | 1 | 1 | 2 | N |
| hsa-miR-15a-5p | ANO3 | 1 | 1 | 2 | N |
| hsa-miR-15a-5p | CCDC19 | 1 | 1 | 2 | N |
| hsa-miR-15a-5p | NXPH1 | 1 | 1 | 2 | N |
| hsa-miR-15a-5p | PCDHA4 | 1 | 1 | 2 | N |
| hsa-miR-15a-5p | PCDHA7 | 1 | 1 | 2 | N |
| hsa-miR-15a-5p | AGO4 | 1 | 1 | 2 | Y |
| hsa-miR-15a-5p | LATS2 | 1 | 1 | 2 | N |
| hsa-miR-15a-5p | COPS2 | 1 | 1 | 2 | N |
| hsa-miR-15a-5p | ATG9A | 1 | 1 | 2 | Y |
| hsa-miR-15a-5p | FAM91A1 | 1 | 1 | 2 | N |
| hsa-miR-15a-5p | SLC4A4 | 1 | 1 | 2 | N |
| hsa-miR-15a-5p | BCL7A | 1 | 1 | 2 | Y |
| hsa-miR-15a-5p | SOCS6 | 1 | 1 | 2 | N |
| hsa-miR-15a-5p | LUZP1 | 1 | 1 | 2 | Y |
| hsa-miR-15a-5p | PLEKHA5 | 1 | 1 | 2 | N |
| hsa-miR-15a-5p | AMER1 | 1 | 1 | 2 | Y |
| hsa-miR-15a-5p | GSTCD | 1 | 1 | 2 | N |
| hsa-miR-15a-5p | KIF5A | 1 | 1 | 2 | N |
| hsa-miR-15a-5p | TBPL1 | 1 | 1 | 2 | Y |
| hsa-miR-15a-5p | PTH | 1 | 1 | 2 | N |
| hsa-miR-15a-5p | NUP50 | 1 | 1 | 2 | Y |
| hsa-miR-15a-5p | ATXN2 | 1 | 1 | 2 | N |
| hsa-miR-15a-5p | ZMAT3 | 1 | 1 | 2 | Y |
| hsa-miR-15a-5p | RAB9A | 1 | 1 | 2 | N |
| hsa-miR-15a-5p | PVRL1 | 1 | 1 | 2 | N |
| hsa-miR-15a-5p | ATXN7L3 | 1 | 1 | 2 | N |
| hsa-miR-15a-5p | POLR3F | 1 | 1 | 2 | N |
| hsa-miR-15a-5p | SLC9A6 | 1 | 1 | 2 | Y |
| hsa-miR-15a-5p | TSPYL2 | 1 | 1 | 2 | Y |
| hsa-miR-15a-5p | BTAF1 | 1 | 1 | 2 | N |
| hsa-miR-15a-5p | FSD1 | 1 | 1 | 2 | N |
| hsa-miR-15a-5p | UBE2Q1 | 1 | 1 | 2 | Y |
| hsa-miR-15a-5p | KLHL18 | 1 | 1 | 2 | N |
| hsa-miR-15a-5p | PCDHAC1 | 1 | 1 | 2 | N |
| hsa-miR-15a-5p | CRKL | 1 | 1 | 2 | Y |
| hsa-miR-15a-5p | ZCCHC3 | 1 | 1 | 2 | Y |
| hsa-miR-15a-5p | IPO7 | 1 | 1 | 2 | N |
| hsa-miR-15a-5p | SLC41A2 | 1 | 1 | 2 | N |
| hsa-miR-15a-5p | DLL1 | 1 | 1 | 2 | N |
| hsa-miR-15a-5p | MOB3B | 1 | 1 | 2 | N |
| hsa-miR-15a-5p | CCNT2 | 1 | 1 | 2 | Y |
| hsa-miR-15a-5p | ZBTB44 | 1 | 1 | 2 | N |
| hsa-miR-15a-5p | XPO7 | 1 | 1 | 2 | N |
| hsa-miR-15a-5p | PURA | 1 | 1 | 2 | Y |
| hsa-miR-15a-5p | TAB3 | 1 | 1 | 2 | N |
| hsa-miR-15a-5p | ARL3 | 1 | 1 | 2 | N |
| hsa-miR-15a-5p | KDSR | 1 | 1 | 2 | N |
| hsa-miR-15a-5p | CLDN2 | 1 | 1 | 2 | N |
| hsa-miR-15a-5p | SNX16 | 1 | 1 | 2 | Y |
| hsa-miR-15a-5p | TMEM178B | 1 | 1 | 2 | N |
| hsa-miR-15a-5p | PISD | 1 | 1 | 2 | Y |
| hsa-miR-15a-5p | TRANK1 | 1 | 1 | 2 | N |
| hsa-miR-15a-5p | FAM110C | 1 | 1 | 2 | N |
| hsa-miR-15a-5p | KIF1B | 1 | 1 | 2 | N |
| hsa-miR-15a-5p | VEGFA | 1 | 1 | 2 | Y |
| hsa-miR-15a-5p | CLCN4 | 1 | 1 | 2 | N |
| hsa-miR-15a-5p | PRDM4 | 1 | 1 | 2 | Y |
| hsa-miR-15a-5p | TMEM255A | 1 | 1 | 2 | N |
| hsa-miR-15a-5p | MAMSTR | 1 | 1 | 2 | N |
| hsa-miR-15a-5p | HIGD1A | 1 | 1 | 2 | Y |
| hsa-miR-15a-5p | GRM7 | 1 | 1 | 2 | N |
| hsa-miR-15a-5p | ENTPD7 | 1 | 1 | 2 | Y |
| hsa-miR-15a-5p | RASGEF1B | 1 | 1 | 2 | N |
| hsa-miR-15a-5p | PCDHA12 | 1 | 1 | 2 | N |
| hsa-miR-15a-5p | MAP7 | 1 | 1 | 2 | N |
| hsa-miR-15a-5p | SHOC2 | 1 | 1 | 2 | Y |
| hsa-miR-15a-5p | USP42 | 1 | 1 | 2 | Y |
| hsa-miR-15a-5p | GNAI3 | 1 | 1 | 2 | N |
| hsa-miR-15a-5p | TENM2 | 1 | 1 | 2 | N |
| hsa-miR-15a-5p | FGF2 | 1 | 1 | 2 | Y |
| hsa-miR-15a-5p | HSPA4L | 1 | 1 | 2 | Y |
| hsa-miR-15a-5p | LSM11 | 1 | 1 | 2 | Y |
| hsa-miR-15a-5p | PCDHA5 | 1 | 1 | 2 | N |
| hsa-miR-15a-5p | CACUL1 | 1 | 1 | 2 | Y |
| hsa-miR-15a-5p | WEE1 | 1 | 1 | 2 | Y |
| hsa-miR-15a-5p | RECK | 1 | 1 | 2 | Y |
| hsa-miR-15a-5p | CPEB3 | 1 | 1 | 2 | Y |
| hsa-miR-15a-5p | ATXN1L | 1 | 1 | 2 | N |
| hsa-miR-15a-5p | CDC25A | 1 | 1 | 2 | Y |
| hsa-miR-15a-5p | C2orf42 | 1 | 1 | 2 | Y |
| hsa-miR-15a-5p | ZBTB46 | 1 | 1 | 2 | N |
| hsa-miR-15a-5p | DCP1A | 1 | 1 | 2 | N |
| hsa-miR-15a-5p | DENND6A | 1 | 1 | 2 | Y |
| hsa-miR-15a-5p | DOLPP1 | 1 | 1 | 2 | N |
| hsa-miR-15a-5p | RAD23B | 1 | 1 | 2 | Y |
| hsa-miR-15a-5p | KCNN4 | 1 | 1 | 2 | N |
| hsa-miR-15a-5p | SYNJ1 | 1 | 1 | 2 | N |
| hsa-miR-15a-5p | SLIT2 | 1 | 1 | 2 | N |
| hsa-miR-15a-5p | SCOC | 1 | 1 | 2 | N |
| hsa-miR-15a-5p | TLK1 | 1 | 1 | 2 | Y |
| hsa-miR-15a-5p | CYP26B1 | 1 | 1 | 2 | Y |
| hsa-miR-15a-5p | CC2D1B | 1 | 1 | 2 | N |
| hsa-miR-15a-5p | RAB9B | 1 | 1 | 2 | Y |
| hsa-miR-15a-5p | SESN1 | 1 | 1 | 2 | N |
| hsa-miR-15a-5p | FASN | 1 | 1 | 2 | Y |
| hsa-miR-15a-5p | TUBA1A | 1 | 1 | 2 | N |
| hsa-miR-15a-5p | EGLN1 | 1 | 1 | 2 | N |
| hsa-miR-15a-5p | FLT3 | 1 | 1 | 2 | N |
| hsa-miR-15a-5p | JARID2 | 1 | 1 | 2 | Y |
| hsa-miR-15a-5p | PLEKHA1 | 1 | 1 | 2 | Y |
| hsa-miR-15a-5p | APLN | 1 | 1 | 2 | N |
| hsa-miR-15a-5p | CAPRIN1 | 1 | 1 | 2 | N |
| hsa-miR-15a-5p | SMAD7 | 1 | 1 | 2 | Y |
| hsa-miR-15a-5p | SLC35G1 | 1 | 1 | 2 | N |
| hsa-miR-15a-5p | BTLA | 1 | 1 | 2 | N |
| hsa-miR-15a-5p | SEH1L | 1 | 1 | 2 | N |
| hsa-miR-15a-5p | CBX4 | 1 | 1 | 2 | Y |
| hsa-miR-15a-5p | PCDHA9 | 1 | 1 | 2 | N |
| hsa-miR-15a-5p | CREBRF | 1 | 1 | 2 | Y |
| hsa-miR-15a-5p | SCN8A | 1 | 1 | 2 | N |
| hsa-miR-15a-5p | OMG | 1 | 1 | 2 | N |
| hsa-miR-15a-5p | CDC37L1 | 1 | 1 | 2 | Y |
| hsa-miR-15a-5p | TPD52L3 | 1 | 1 | 2 | N |
| hsa-miR-15a-5p | BFAR | 1 | 1 | 2 | N |
| hsa-miR-15a-5p | ARL2 | 1 | 1 | 2 | N |
| hsa-miR-15a-5p | VPS4A | 1 | 1 | 2 | Y |
| hsa-miR-15a-5p | LCOR | 1 | 1 | 2 | N |
| hsa-miR-15a-5p | STRADB | 1 | 1 | 2 | Y |
| hsa-miR-15a-5p | HTR2A | 1 | 1 | 2 | N |
| hsa-miR-15a-5p | MOB4 | 1 | 1 | 2 | Y |
| hsa-miR-15a-5p | TACC1 | 1 | 1 | 2 | N |
| hsa-miR-15a-5p | C20orf112 | 1 | 1 | 2 | N |
| hsa-miR-15a-5p | IKBKB | 1 | 1 | 2 | N |
| hsa-miR-15a-5p | CSRNP1 | 1 | 1 | 2 | N |
| hsa-miR-15a-5p | UNC80 | 1 | 1 | 2 | N |
| hsa-miR-15a-5p | C16orf72 | 1 | 1 | 2 | Y |
| hsa-miR-15a-5p | IHH | 1 | 1 | 2 | N |
| hsa-miR-15a-5p | RASEF | 1 | 1 | 2 | Y |
| hsa-miR-15a-5p | RELN | 1 | 1 | 2 | N |
| hsa-miR-15a-5p | NISCH | 1 | 1 | 2 | N |
| hsa-miR-15a-5p | C8orf58 | 1 | 1 | 2 | N |
| hsa-miR-15a-5p | BCL2L2 | 1 | 1 | 2 | N |
| hsa-miR-15a-5p | SPSB4 | 1 | 1 | 2 | N |
| hsa-miR-15a-5p | CPEB2 | 1 | 1 | 2 | Y |
| hsa-miR-15a-5p | SALL4 | 1 | 1 | 2 | N |
| hsa-miR-15a-5p | SYDE2 | 1 | 1 | 2 | N |
| hsa-miR-15a-5p | CHEK1 | 1 | 1 | 2 | Y |
| hsa-miR-15a-5p | ANKS1A | 1 | 1 | 2 | N |
| hsa-miR-15a-5p | SYT4 | 1 | 1 | 2 | N |
| hsa-miR-15a-5p | GPN1 | 1 | 1 | 2 | N |
| hsa-miR-15a-5p | CDC42SE2 | 1 | 1 | 2 | Y |
| hsa-miR-15a-5p | PRR15L | 1 | 1 | 2 | N |
| hsa-miR-15a-5p | RAB11FIP2 | 1 | 1 | 2 | Y |
| hsa-miR-15a-5p | ARFGAP2 | 1 | 1 | 2 | N |
| hsa-miR-15a-5p | RSBN1 | 1 | 1 | 2 | N |
| hsa-miR-15a-5p | ZNF449 | 1 | 1 | 2 | Y |
| hsa-miR-15a-5p | PCDHA2 | 1 | 1 | 2 | N |
| hsa-miR-15a-5p | DNAJB4 | 1 | 1 | 2 | N |
| hsa-miR-15a-5p | IRAK2 | 1 | 1 | 2 | N |
| hsa-miR-15a-5p | MAP3K9 | 1 | 1 | 2 | N |
| hsa-miR-15a-5p | RNF24 | 1 | 1 | 2 | N |
| hsa-miR-15a-5p | CASK | 1 | 1 | 2 | Y |
| hsa-miR-15a-5p | DMTF1 | 1 | 1 | 2 | Y |
| hsa-miR-15a-5p | PTPRR | 1 | 1 | 2 | N |
| hsa-miR-15a-5p | SLC15A4 | 1 | 1 | 2 | N |
| hsa-miR-15a-5p | CAPZA2 | 1 | 1 | 2 | Y |
| hsa-miR-15a-5p | TSPAN5 | 1 | 1 | 2 | N |
| hsa-miR-15a-5p | KIF21A | 1 | 1 | 2 | N |
| hsa-miR-15a-5p | PCDHA13 | 1 | 1 | 2 | N |
| hsa-miR-15a-5p | PAPPA | 1 | 1 | 2 | N |
| hsa-miR-15a-5p | PDK4 | 1 | 1 | 2 | N |
| hsa-miR-15a-5p | SEPT2 | 1 | 1 | 2 | Y |
| hsa-miR-15a-5p | PHF19 | 1 | 1 | 2 | Y |
| hsa-miR-15a-5p | IST1 | 1 | 1 | 2 | N |
| hsa-miR-15a-5p | RSPO3 | 1 | 1 | 2 | N |
| hsa-miR-15a-5p | USP3 | 1 | 1 | 2 | Y |
| hsa-miR-15a-5p | MAP2K1 | 1 | 1 | 2 | N |
| hsa-miR-15a-5p | DYRK1B | 1 | 1 | 2 | N |
| hsa-miR-15a-5p | STK33 | 1 | 1 | 2 | N |
| hsa-miR-15a-5p | WIPI2 | 1 | 1 | 2 | Y |
| hsa-miR-15a-5p | LITAF | 1 | 1 | 2 | Y |
| hsa-miR-15a-5p | ELMSAN1 | 1 | 1 | 2 | N |
| hsa-miR-15a-5p | SLC9A8 | 1 | 1 | 2 | N |
| hsa-miR-15a-5p | G0S2 | 1 | 1 | 2 | N |
| hsa-miR-15a-5p | PPM1E | 1 | 1 | 2 | N |
| hsa-miR-15a-5p | CDK5R1 | 1 | 1 | 2 | N |
| hsa-miR-15a-5p | E2F7 | 1 | 1 | 2 | Y |
| hsa-miR-15a-5p | ASH1L | 1 | 1 | 2 | Y |
| hsa-miR-15a-5p | FAM133B | 1 | 1 | 2 | N |
| hsa-miR-15a-5p | ABL2 | 1 | 1 | 2 | Y |
| hsa-miR-15a-5p | MYT1L | 1 | 1 | 2 | N |
| hsa-miR-15a-5p | UBE4B | 1 | 1 | 2 | N |
| hsa-miR-15a-5p | RFWD2 | 1 | 1 | 2 | Y |
| hsa-miR-15a-5p | TCAIM | 1 | 1 | 2 | N |
| hsa-miR-15a-5p | PCDHA1 | 1 | 1 | 2 | N |
| hsa-miR-15a-5p | PCDHA6 | 1 | 1 | 2 | N |
| hsa-miR-15a-5p | MYLK | 1 | 1 | 2 | N |
| hsa-miR-15a-5p | ARIH1 | 1 | 1 | 2 | Y |
| hsa-miR-15a-5p | FBXO21 | 1 | 1 | 2 | N |
| hsa-miR-15a-5p | MED26 | 1 | 1 | 2 | N |
| hsa-miR-15a-5p | CDK17 | 1 | 1 | 2 | Y |
| hsa-miR-15a-5p | YWHAH | 1 | 1 | 2 | Y |
| hsa-miR-15a-5p | PPAP2B | 1 | 1 | 2 | N |
| hsa-miR-15a-5p | HPSE2 | 1 | 1 | 2 | N |
| hsa-miR-15a-5p | PLAG1 | 1 | 1 | 2 | Y |
| hsa-miR-15a-5p | PLSCR4 | 1 | 1 | 2 | N |
| hsa-miR-15a-5p | RARB | 1 | 1 | 2 | Y |
| hsa-miR-15a-5p | UBE2V1 | 1 | 1 | 2 | Y |
| hsa-miR-15a-5p | C1orf21 | 1 | 1 | 2 | Y |
| hsa-miR-15a-5p | PTPN4 | 1 | 1 | 2 | N |
| hsa-miR-15a-5p | SGK1 | 1 | 1 | 2 | N |
| hsa-miR-15a-5p | AREL1 | 1 | 1 | 2 | N |
| hsa-miR-15a-5p | ZNF622 | 1 | 1 | 2 | Y |
| hsa-miR-15a-5p | SLC36A1 | 1 | 1 | 2 | N |
| hsa-miR-15a-5p | GATAD2A | 1 | 1 | 2 | Y |
| hsa-miR-15a-5p | ZNF275 | 1 | 1 | 2 | Y |
| hsa-miR-15a-5p | MKNK1 | 1 | 1 | 2 | N |
| hsa-miR-15a-5p | CCNE1 | 1 | 1 | 2 | Y |
| hsa-miR-15a-5p | PTHLH | 1 | 1 | 2 | N |
| hsa-miR-15a-5p | PEX13 | 1 | 1 | 2 | Y |
| hsa-miR-15a-5p | GPR63 | 1 | 1 | 2 | N |
| hsa-miR-15a-5p | C1QL3 | 1 | 1 | 2 | N |
| hsa-miR-15a-5p | PCDHA3 | 1 | 1 | 2 | N |
| hsa-miR-15a-5p | FBXW7 | 1 | 1 | 2 | N |
| hsa-miR-15a-5p | FGFR1 | 1 | 1 | 2 | N |
| hsa-miR-15a-5p | BTG2 | 1 | 1 | 2 | Y |
| hsa-miR-15a-5p | WNT7A | 1 | 1 | 2 | N |
| hsa-miR-15a-5p | RFX3 | 1 | 1 | 2 | N |
| hsa-miR-15a-5p | ST8SIA3 | 1 | 1 | 2 | N |
| hsa-miR-15a-5p | ISLR | 1 | 1 | 2 | N |
| hsa-miR-15a-5p | E2F3 | 1 | 1 | 2 | Y |
| hsa-miR-15a-5p | CLDN12 | 1 | 1 | 2 | N |
| hsa-miR-15a-5p | AQP11 | 1 | 1 | 2 | N |
| hsa-miR-15a-5p | NRBP1 | 1 | 1 | 2 | N |
| hsa-miR-15a-5p | KIF5C | 1 | 1 | 2 | N |
| hsa-miR-15a-5p | MIPOL1 | 1 | 1 | 2 | N |
| hsa-miR-15a-5p | CLSPN | 1 | 1 | 2 | Y |
| hsa-miR-15a-5p | AEBP2 | 1 | 1 | 2 | N |
| hsa-miR-15a-5p | PRKAR2A | 1 | 1 | 2 | Y |
| hsa-miR-15a-5p | YWHAQ | 1 | 1 | 2 | Y |
| hsa-miR-15a-5p | PID1 | 1 | 1 | 2 | N |
| hsa-miR-15a-5p | ZMYM2 | 1 | 1 | 2 | N |
| hsa-miR-15a-5p | GAREM | 1 | 1 | 2 | N |
| hsa-miR-15a-5p | WBP11 | 1 | 1 | 2 | N |
| hsa-miR-15a-5p | RBM6 | 1 | 1 | 2 | N |
| hsa-miR-15a-5p | PDIA6 | 1 | 1 | 2 | Y |
| hsa-miR-15a-5p | MAPK8 | 1 | 1 | 2 | N |
| hsa-miR-15a-5p | SEMA6D | 1 | 1 | 2 | N |
| hsa-miR-15a-5p | TMEM100 | 1 | 1 | 2 | Y |
| hsa-miR-15a-5p | KIAA1432 | 1 | 1 | 2 | N |
| hsa-miR-15a-5p | TNRC6B | 1 | 1 | 2 | Y |
| hsa-miR-15a-5p | AKT3 | 1 | 1 | 2 | Y |
| hsa-miR-15a-5p | STX17 | 1 | 1 | 2 | Y |
| hsa-miR-15a-5p | PAQR3 | 1 | 1 | 2 | N |
| hsa-miR-15a-5p | GORASP2 | 1 | 1 | 2 | N |
| hsa-miR-15a-5p | MEOX2 | 1 | 1 | 2 | N |
| hsa-miR-15a-5p | VAPB | 1 | 1 | 2 | N |
| hsa-miR-15a-5p | PCMT1 | 1 | 1 | 2 | Y |
| hsa-miR-15a-5p | CARM1 | 1 | 1 | 2 | Y |
| hsa-miR-15a-5p | CD28 | 1 | 1 | 2 | N |
| hsa-miR-15a-5p | PLXNC1 | 1 | 1 | 2 | N |
| hsa-miR-15a-5p | USP25 | 1 | 1 | 2 | N |
| hsa-miR-15a-5p | PPM1D | 1 | 1 | 2 | N |
| hsa-miR-15a-5p | RASSF8 | 1 | 1 | 2 | N |
| hsa-miR-15a-5p | SETD3 | 1 | 1 | 2 | N |
| hsa-miR-15a-5p | ZSWIM3 | 1 | 1 | 2 | N |
| hsa-miR-15a-5p | CUL2 | 1 | 1 | 2 | Y |
| hsa-miR-15a-5p | WNT3A | 1 | 1 | 2 | Y |
| hsa-miR-15a-5p | AXIN2 | 1 | 1 | 2 | Y |
| hsa-miR-15a-5p | DPY19L4 | 1 | 1 | 2 | N |
| hsa-miR-15a-5p | YTHDC1 | 1 | 1 | 2 | Y |
| hsa-miR-15a-5p | SMPD1 | 1 | 1 | 2 | N |
| hsa-miR-15a-5p | ACTR2 | 1 | 1 | 2 | Y |
| hsa-miR-15a-5p | SPRYD3 | 1 | 1 | 2 | N |
| hsa-miR-15a-5p | SRPR | 1 | 1 | 2 | N |
| hsa-miR-15a-5p | RBBP6 | 1 | 1 | 2 | Y |
| hsa-miR-15a-5p | GPATCH8 | 1 | 1 | 2 | Y |
| hsa-miR-15a-5p | RNF138 | 1 | 1 | 2 | Y |
| hsa-miR-15a-5p | TMEM55A | 1 | 1 | 2 | N |
| hsa-miR-15a-5p | DYNC1I1 | 1 | 1 | 2 | N |
| hsa-miR-15a-5p | TMEM161B | 1 | 1 | 2 | Y |
| hsa-miR-15a-5p | DDX3Y | 1 | 1 | 2 | Y |
| hsa-miR-15a-5p | CNOT6L | 1 | 1 | 2 | N |
| hsa-miR-15a-5p | HOXA10 | 1 | 1 | 2 | Y |
| hsa-miR-15a-5p | ZBTB34 | 1 | 1 | 2 | Y |
| hsa-miR-15a-5p | SNCG | 1 | 1 | 2 | Y |
| hsa-miR-15a-5p | LURAP1L | 1 | 1 | 2 | Y |
| hsa-miR-15a-5p | PCDHAC2 | 1 | 1 | 2 | N |
| hsa-miR-15a-5p | ELL | 1 | 1 | 2 | N |
| hsa-miR-16-5p | RFWD2 | 1 | 1 | 2 | Y |
| hsa-miR-16-5p | RBBP6 | 1 | 1 | 2 | Y |
| hsa-miR-16-5p | CYP26B1 | 1 | 1 | 2 | Y |
| hsa-miR-16-5p | SPRYD3 | 1 | 1 | 2 | Y |
| hsa-miR-16-5p | CD28 | 1 | 1 | 2 | N |
| hsa-miR-16-5p | CDCA4 | 1 | 1 | 2 | Y |
| hsa-miR-16-5p | SCOC | 1 | 1 | 2 | N |
| hsa-miR-16-5p | DYNC1I1 | 1 | 1 | 2 | N |
| hsa-miR-16-5p | CPD | 1 | 1 | 2 | N |
| hsa-miR-16-5p | FBXO21 | 1 | 1 | 2 | N |
| hsa-miR-16-5p | PEX13 | 1 | 1 | 2 | Y |
| hsa-miR-16-5p | SCN8A | 1 | 1 | 2 | N |
| hsa-miR-16-5p | CHEK1 | 1 | 1 | 2 | Y |
| hsa-miR-16-5p | CRKL | 1 | 1 | 2 | Y |
| hsa-miR-16-5p | PTPN4 | 1 | 1 | 2 | N |
| hsa-miR-16-5p | MED26 | 1 | 1 | 2 | N |
| hsa-miR-16-5p | PCDHAC1 | 1 | 1 | 2 | N |
| hsa-miR-16-5p | MYT1L | 1 | 1 | 2 | N |
| hsa-miR-16-5p | SRPR | 1 | 1 | 2 | N |
| hsa-miR-16-5p | ZNF449 | 1 | 1 | 2 | Y |
| hsa-miR-16-5p | KDSR | 1 | 1 | 2 | Y |
| hsa-miR-16-5p | KIAA1432 | 1 | 1 | 2 | N |
| hsa-miR-16-5p | PCDHA6 | 1 | 1 | 2 | N |
| hsa-miR-16-5p | BCL2L2 | 1 | 1 | 2 | N |
| hsa-miR-16-5p | PDK4 | 1 | 1 | 2 | Y |
| hsa-miR-16-5p | RFX3 | 1 | 1 | 2 | N |
| hsa-miR-16-5p | ST8SIA3 | 1 | 1 | 2 | N |
| hsa-miR-16-5p | UBFD1 | 1 | 1 | 2 | Y |
| hsa-miR-16-5p | RAB9A | 1 | 1 | 2 | N |
| hsa-miR-16-5p | ZBTB39 | 1 | 1 | 2 | N |
| hsa-miR-16-5p | NISCH | 1 | 1 | 2 | Y |
| hsa-miR-16-5p | KIF21A | 1 | 1 | 2 | N |
| hsa-miR-16-5p | PRKAR2A | 1 | 1 | 2 | Y |
| hsa-miR-16-5p | GPN1 | 1 | 1 | 2 | N |
| hsa-miR-16-5p | SETD3 | 1 | 1 | 2 | N |
| hsa-miR-16-5p | NRBP1 | 1 | 1 | 2 | N |
| hsa-miR-16-5p | SEH1L | 1 | 1 | 2 | Y |
| hsa-miR-16-5p | ATG9A | 1 | 1 | 2 | Y |
| hsa-miR-16-5p | CREBRF | 1 | 1 | 2 | Y |
| hsa-miR-16-5p | PCDHA12 | 1 | 1 | 2 | N |
| hsa-miR-16-5p | AXIN2 | 1 | 1 | 2 | Y |
| hsa-miR-16-5p | AMER1 | 1 | 1 | 2 | Y |
| hsa-miR-16-5p | PRDM4 | 1 | 1 | 2 | Y |
| hsa-miR-16-5p | DOLPP1 | 1 | 1 | 2 | N |
| hsa-miR-16-5p | SPRED1 | 1 | 1 | 2 | Y |
| hsa-miR-16-5p | VPS4A | 1 | 1 | 2 | Y |
| hsa-miR-16-5p | PCMT1 | 1 | 1 | 2 | Y |
| hsa-miR-16-5p | C16orf72 | 1 | 1 | 2 | Y |
| hsa-miR-16-5p | CPEB2 | 1 | 1 | 2 | Y |
| hsa-miR-16-5p | ISLR | 1 | 1 | 2 | N |
| hsa-miR-16-5p | SIRT4 | 1 | 1 | 2 | Y |
| hsa-miR-16-5p | HELZ | 1 | 1 | 2 | Y |
| hsa-miR-16-5p | CCDC19 | 1 | 1 | 2 | N |
| hsa-miR-16-5p | CBX4 | 1 | 1 | 2 | Y |
| hsa-miR-16-5p | C1QL3 | 1 | 1 | 2 | N |
| hsa-miR-16-5p | YTHDC1 | 1 | 1 | 2 | Y |
| hsa-miR-16-5p | C20orf112 | 1 | 1 | 2 | N |
| hsa-miR-16-5p | C2orf42 | 1 | 1 | 2 | Y |
| hsa-miR-16-5p | RELN | 1 | 1 | 2 | N |
| hsa-miR-16-5p | KLHL18 | 1 | 1 | 2 | N |
| hsa-miR-16-5p | STRADB | 1 | 1 | 2 | Y |
| hsa-miR-16-5p | COPS2 | 1 | 1 | 2 | N |
| hsa-miR-16-5p | UBE4B | 1 | 1 | 2 | N |
| hsa-miR-16-5p | INSR | 1 | 1 | 2 | N |
| hsa-miR-16-5p | TSPYL2 | 1 | 1 | 2 | N |
| hsa-miR-16-5p | CNOT6L | 1 | 1 | 2 | N |
| hsa-miR-16-5p | RBM6 | 1 | 1 | 2 | Y |
| hsa-miR-16-5p | SNCG | 1 | 1 | 2 | Y |
| hsa-miR-16-5p | TMEM55A | 1 | 1 | 2 | N |
| hsa-miR-16-5p | PCDHA2 | 1 | 1 | 2 | N |
| hsa-miR-16-5p | PCDHA5 | 1 | 1 | 2 | N |
| hsa-miR-16-5p | ZHX1 | 1 | 1 | 2 | N |
| hsa-miR-16-5p | HPCAL4 | 1 | 1 | 2 | N |
| hsa-miR-16-5p | WNT3A | 1 | 1 | 2 | Y |
| hsa-miR-16-5p | FASN | 1 | 1 | 2 | Y |
| hsa-miR-16-5p | CC2D1B | 1 | 1 | 2 | N |
| hsa-miR-16-5p | HPSE2 | 1 | 1 | 2 | N |
| hsa-miR-16-5p | LITAF | 1 | 1 | 2 | Y |
| hsa-miR-16-5p | RNF217 | 1 | 1 | 2 | Y |
| hsa-miR-16-5p | MYLK | 1 | 1 | 2 | N |
| hsa-miR-16-5p | AGO4 | 1 | 1 | 2 | Y |
| hsa-miR-16-5p | DYRK1B | 1 | 1 | 2 | N |
| hsa-miR-16-5p | SHOC2 | 1 | 1 | 2 | Y |
| hsa-miR-16-5p | PTHLH | 1 | 1 | 2 | N |
| hsa-miR-16-5p | SLIT2 | 1 | 1 | 2 | N |
| hsa-miR-16-5p | RAB11FIP2 | 1 | 1 | 2 | Y |
| hsa-miR-16-5p | SALL4 | 1 | 1 | 2 | N |
| hsa-miR-16-5p | RAB9B | 1 | 1 | 2 | Y |
| hsa-miR-16-5p | ZNF622 | 1 | 1 | 2 | Y |
| hsa-miR-16-5p | PVRL1 | 1 | 1 | 2 | N |
| hsa-miR-16-5p | IRAK2 | 1 | 1 | 2 | N |
| hsa-miR-16-5p | ARFGAP2 | 1 | 1 | 2 | N |
| hsa-miR-16-5p | HSPA4L | 1 | 1 | 2 | Y |
| hsa-miR-16-5p | TRANK1 | 1 | 1 | 2 | N |
| hsa-miR-16-5p | MOB4 | 1 | 1 | 2 | Y |
| hsa-miR-16-5p | ARIH1 | 1 | 1 | 2 | Y |
| hsa-miR-16-5p | SLC9A6 | 1 | 1 | 2 | Y |
| hsa-miR-16-5p | FERMT2 | 1 | 1 | 2 | N |
| hsa-miR-16-5p | GAREM | 1 | 1 | 2 | N |
| hsa-miR-16-5p | CARM1 | 1 | 1 | 2 | Y |
| hsa-miR-16-5p | TAB3 | 1 | 1 | 2 | N |
| hsa-miR-16-5p | FSD1 | 1 | 1 | 2 | N |
| hsa-miR-16-5p | SESN1 | 1 | 1 | 2 | N |
| hsa-miR-16-5p | AREL1 | 1 | 1 | 2 | N |
| hsa-miR-16-5p | ACTR2 | 1 | 1 | 2 | Y |
| hsa-miR-16-5p | PTPN3 | 1 | 1 | 2 | Y |
| hsa-miR-16-5p | PTH | 1 | 1 | 2 | N |
| hsa-miR-16-5p | CLSPN | 1 | 1 | 2 | Y |
| hsa-miR-16-5p | ZSWIM3 | 1 | 1 | 2 | N |
| hsa-miR-16-5p | STK33 | 1 | 1 | 2 | Y |
| hsa-miR-16-5p | TUBA1A | 1 | 1 | 2 | Y |
| hsa-miR-16-5p | SNX16 | 1 | 1 | 2 | Y |
| hsa-miR-16-5p | WIPI2 | 1 | 1 | 2 | Y |
| hsa-miR-16-5p | ANO3 | 1 | 1 | 2 | N |
| hsa-miR-16-5p | FAM110C | 1 | 1 | 2 | N |
| hsa-miR-16-5p | CAPZA2 | 1 | 1 | 2 | Y |
| hsa-miR-16-5p | CCNE1 | 1 | 1 | 2 | Y |
| hsa-miR-16-5p | ZMAT3 | 1 | 1 | 2 | Y |
| hsa-miR-16-5p | PCDHA13 | 1 | 1 | 2 | N |
| hsa-miR-16-5p | USP3 | 1 | 1 | 2 | Y |
| hsa-miR-16-5p | PCDHA7 | 1 | 1 | 2 | N |
| hsa-miR-16-5p | SEPT2 | 1 | 1 | 2 | Y |
| hsa-miR-16-5p | SMAD7 | 1 | 1 | 2 | Y |
| hsa-miR-16-5p | YWHAH | 1 | 1 | 2 | Y |
| hsa-miR-16-5p | PDIK1L | 1 | 1 | 2 | Y |
| hsa-miR-16-5p | WEE1 | 1 | 1 | 2 | Y |
| hsa-miR-16-5p | MKNK1 | 1 | 1 | 2 | N |
| hsa-miR-16-5p | SLC36A1 | 1 | 1 | 2 | N |
| hsa-miR-16-5p | SOCS6 | 1 | 1 | 2 | N |
| hsa-miR-16-5p | PLEKHA5 | 1 | 1 | 2 | N |
| hsa-miR-16-5p | KIF5A | 1 | 1 | 2 | Y |
| hsa-miR-16-5p | ZBTB44 | 1 | 1 | 2 | N |
| hsa-miR-16-5p | SLC4A4 | 1 | 1 | 2 | N |
| hsa-miR-16-5p | MAFK | 1 | 1 | 2 | Y |
| hsa-miR-16-5p | PID1 | 1 | 1 | 2 | N |
| hsa-miR-16-5p | CLCN4 | 1 | 1 | 2 | N |
| hsa-miR-16-5p | ZCCHC3 | 1 | 1 | 2 | Y |
| hsa-miR-16-5p | PHF19 | 1 | 1 | 2 | Y |
| hsa-miR-16-5p | CLDN2 | 1 | 1 | 2 | Y |
| hsa-miR-16-5p | FGF2 | 1 | 1 | 2 | Y |
| hsa-miR-16-5p | NUP50 | 1 | 1 | 2 | Y |
| hsa-miR-16-5p | RAP2C | 1 | 1 | 2 | Y |
| hsa-miR-16-5p | CDC25A | 1 | 1 | 2 | Y |
| hsa-miR-16-5p | HOXA10 | 1 | 1 | 2 | Y |
| hsa-miR-16-5p | N4BP1 | 1 | 1 | 2 | Y |
| hsa-miR-16-5p | CPEB3 | 1 | 1 | 2 | Y |
| hsa-miR-16-5p | UNC80 | 1 | 1 | 2 | N |
| hsa-miR-16-5p | IPO7 | 1 | 1 | 2 | Y |
| hsa-miR-16-5p | KIF23 | 1 | 1 | 2 | Y |
| hsa-miR-16-5p | DNAJB4 | 1 | 1 | 2 | Y |
| hsa-miR-16-5p | PRR15L | 1 | 1 | 2 | N |
| hsa-miR-16-5p | ARMCX2 | 1 | 1 | 2 | Y |
| hsa-miR-16-5p | RSBN1 | 1 | 1 | 2 | N |
| hsa-miR-16-5p | MAP3K9 | 1 | 1 | 2 | N |
| hsa-miR-16-5p | NXPH1 | 1 | 1 | 2 | N |
| hsa-miR-16-5p | IST1 | 1 | 1 | 2 | N |
| hsa-miR-16-5p | CCND1 | 1 | 1 | 2 | Y |
| hsa-miR-16-5p | PCDHA3 | 1 | 1 | 2 | N |
| hsa-miR-16-5p | CASK | 1 | 1 | 2 | Y |
| hsa-miR-16-5p | ELMSAN1 | 1 | 1 | 2 | N |
| hsa-miR-16-5p | OMG | 1 | 1 | 2 | N |
| hsa-miR-16-5p | SMPD1 | 1 | 1 | 2 | N |
| hsa-miR-16-5p | MAP7 | 1 | 1 | 2 | Y |
| hsa-miR-16-5p | PCDHA4 | 1 | 1 | 2 | N |
| hsa-miR-16-5p | PCDHA9 | 1 | 1 | 2 | N |
| hsa-miR-16-5p | BFAR | 1 | 1 | 2 | Y |
| hsa-miR-16-5p | RNF138 | 1 | 1 | 2 | Y |
| hsa-miR-16-5p | SLC41A2 | 1 | 1 | 2 | N |
| hsa-miR-16-5p | ELL | 1 | 1 | 2 | N |
| hsa-miR-16-5p | FLT3 | 1 | 1 | 2 | N |
| hsa-miR-16-5p | G0S2 | 1 | 1 | 2 | N |
| hsa-miR-16-5p | PTPRR | 1 | 1 | 2 | N |
| hsa-miR-16-5p | AQP11 | 1 | 1 | 2 | N |
| hsa-miR-16-5p | TTC14 | 1 | 1 | 2 | N |
| hsa-miR-16-5p | BTG2 | 1 | 1 | 2 | Y |
| hsa-miR-16-5p | SUMO3 | 1 | 1 | 2 | N |
| hsa-miR-16-5p | KIF5C | 1 | 1 | 2 | N |
| hsa-miR-16-5p | DDX3Y | 1 | 1 | 2 | Y |
| hsa-miR-16-5p | EGLN1 | 1 | 1 | 2 | N |
| hsa-miR-16-5p | ENTPD7 | 1 | 1 | 2 | Y |
| hsa-miR-16-5p | CDC42SE2 | 1 | 1 | 2 | Y |
| hsa-miR-16-5p | VEGFA | 1 | 1 | 2 | Y |
| hsa-miR-16-5p | TENM2 | 1 | 1 | 2 | N |
| hsa-miR-16-5p | MOB3B | 1 | 1 | 2 | N |
| hsa-miR-16-5p | CDK17 | 1 | 1 | 2 | Y |
| hsa-miR-16-5p | BTAF1 | 1 | 1 | 2 | Y |
| hsa-miR-16-5p | RASEF | 1 | 1 | 2 | Y |
| hsa-miR-16-5p | GNAI3 | 1 | 1 | 2 | N |
| hsa-miR-16-5p | GABARAPL1 | 1 | 1 | 2 | Y |
| hsa-miR-16-5p | SYT4 | 1 | 1 | 2 | N |
| hsa-miR-16-5p | STX17 | 1 | 1 | 2 | Y |
| hsa-miR-16-5p | PLSCR4 | 1 | 1 | 2 | Y |
| hsa-miR-16-5p | AEBP2 | 1 | 1 | 2 | N |
| hsa-miR-16-5p | SYDE2 | 1 | 1 | 2 | N |
| hsa-miR-16-5p | ATXN1L | 1 | 1 | 2 | N |
| hsa-miR-16-5p | RSPO3 | 1 | 1 | 2 | N |
| hsa-miR-16-5p | PURA | 1 | 1 | 2 | Y |
| hsa-miR-16-5p | C1orf21 | 1 | 1 | 2 | Y |
| hsa-miR-16-5p | HTR2A | 1 | 1 | 2 | N |
| hsa-miR-16-5p | TSPAN5 | 1 | 1 | 2 | N |
| hsa-miR-16-5p | IKBKB | 1 | 1 | 2 | N |
| hsa-miR-16-5p | E2F7 | 1 | 1 | 2 | Y |
| hsa-miR-16-5p | UBE2V1 | 1 | 1 | 2 | Y |
| hsa-miR-16-5p | GPATCH8 | 1 | 1 | 2 | Y |
| hsa-miR-16-5p | DMTF1 | 1 | 1 | 2 | Y |
| hsa-miR-16-5p | EIF4B | 1 | 1 | 2 | Y |
| hsa-miR-16-5p | ABL2 | 1 | 1 | 2 | Y |
| hsa-miR-16-5p | CACUL1 | 1 | 1 | 2 | Y |
| hsa-miR-16-5p | PPM1D | 1 | 1 | 2 | Y |
| hsa-miR-16-5p | FAM133B | 1 | 1 | 2 | N |
| hsa-miR-16-5p | USP42 | 1 | 1 | 2 | Y |
| hsa-miR-16-5p | PDIA6 | 1 | 1 | 2 | Y |
| hsa-miR-16-5p | RASSF8 | 1 | 1 | 2 | N |
| hsa-miR-16-5p | TMEM178B | 1 | 1 | 2 | N |
| hsa-miR-16-5p | KCNJ2 | 1 | 1 | 2 | N |
| hsa-miR-16-5p | PAFAH1B1 | 1 | 1 | 2 | Y |
| hsa-miR-16-5p | FBXW7 | 1 | 1 | 2 | Y |
| hsa-miR-16-5p | SCN3A | 1 | 1 | 2 | N |
| hsa-miR-16-5p | RNF144B | 1 | 1 | 2 | Y |
| hsa-miR-16-5p | CSRNP1 | 1 | 1 | 2 | N |
| hsa-miR-16-5p | LCOR | 1 | 1 | 2 | N |
| hsa-miR-16-5p | C8orf58 | 1 | 1 | 2 | N |
| hsa-miR-16-5p | RAD23B | 1 | 1 | 2 | Y |
| hsa-miR-16-5p | TMEM161B | 1 | 1 | 2 | Y |
| hsa-miR-16-5p | UBE2Q1 | 1 | 1 | 2 | Y |
| hsa-miR-16-5p | TMCC1 | 1 | 1 | 2 | Y |
| hsa-miR-16-5p | CUL2 | 1 | 1 | 2 | Y |
| hsa-miR-16-5p | FAM91A1 | 1 | 1 | 2 | N |
| hsa-miR-16-5p | MAMSTR | 1 | 1 | 2 | N |
| hsa-miR-16-5p | JARID2 | 1 | 1 | 2 | Y |
| hsa-miR-16-5p | ARL2 | 1 | 1 | 2 | Y |
| hsa-miR-16-5p | GRM7 | 1 | 1 | 2 | N |
| hsa-miR-16-5p | ZC2HC1A | 1 | 1 | 2 | N |
| hsa-miR-16-5p | PLEKHA1 | 1 | 1 | 2 | Y |
| hsa-miR-16-5p | DPY19L4 | 1 | 1 | 2 | N |
| hsa-miR-16-5p | RNF24 | 1 | 1 | 2 | N |
| hsa-miR-16-5p | BTLA | 1 | 1 | 2 | N |
| hsa-miR-16-5p | ASH1L | 1 | 1 | 2 | Y |
| hsa-miR-16-5p | GATAD2A | 1 | 1 | 2 | Y |
| hsa-miR-16-5p | PPM1E | 1 | 1 | 2 | N |
| hsa-miR-16-5p | MAP2K1 | 1 | 1 | 2 | N |
| hsa-miR-16-5p | TMEM255A | 1 | 1 | 2 | Y |
| hsa-miR-16-5p | ATXN2 | 1 | 1 | 2 | N |
| hsa-miR-16-5p | PCDHA11 | 1 | 1 | 2 | N |
| hsa-miR-16-5p | IHH | 1 | 1 | 2 | N |
| hsa-miR-16-5p | GSTCD | 1 | 1 | 2 | N |
| hsa-miR-16-5p | CCNT2 | 1 | 1 | 2 | Y |
| hsa-miR-16-5p | TNFSF13B | 1 | 1 | 2 | N |
| hsa-miR-16-5p | IARS | 1 | 1 | 2 | Y |
| hsa-miR-16-5p | ARHGDIA | 1 | 1 | 2 | Y |
| hsa-miR-16-5p | BTRC | 1 | 1 | 2 | Y |
| hsa-miR-16-5p | TNRC6B | 1 | 1 | 2 | Y |
| hsa-miR-16-5p | PISD | 1 | 1 | 2 | Y |
| hsa-miR-16-5p | ANKS1A | 1 | 1 | 2 | N |
| hsa-miR-16-5p | ZNF275 | 1 | 1 | 2 | Y |
| hsa-miR-16-5p | ATXN7L3 | 1 | 1 | 2 | Y |
| hsa-miR-16-5p | SGK1 | 1 | 1 | 2 | N |
| hsa-miR-16-5p | POLR3F | 1 | 1 | 2 | N |
| hsa-miR-16-5p | DLL1 | 1 | 1 | 2 | N |
| hsa-miR-16-5p | WNT7A | 1 | 1 | 2 | N |
| hsa-miR-16-5p | VAPB | 1 | 1 | 2 | N |
| hsa-miR-16-5p | FGFR1 | 1 | 1 | 2 | Y |
| hsa-miR-16-5p | LSM11 | 1 | 1 | 2 | Y |
| hsa-miR-16-5p | YWHAQ | 1 | 1 | 2 | Y |
| hsa-miR-16-5p | GPR63 | 1 | 1 | 2 | N |
| hsa-miR-16-5p | RASGEF1B | 1 | 1 | 2 | N |
| hsa-miR-16-5p | MAPK8 | 1 | 1 | 2 | N |
| hsa-miR-16-5p | WBP11 | 1 | 1 | 2 | Y |
| hsa-miR-16-5p | FAM73A | 1 | 1 | 2 | N |
| hsa-miR-16-5p | PAPPA | 1 | 1 | 2 | N |
| hsa-miR-16-5p | USP25 | 1 | 1 | 2 | N |
| hsa-miR-16-5p | PPAP2B | 1 | 1 | 2 | N |
| hsa-miR-16-5p | LUZP1 | 1 | 1 | 2 | Y |
| hsa-miR-16-5p | ADRB2 | 1 | 1 | 2 | N |
| hsa-miR-16-5p | SLC35G1 | 1 | 1 | 2 | N |
| hsa-miR-16-5p | SEMA6D | 1 | 1 | 2 | N |
| hsa-miR-16-5p | CAPRIN1 | 1 | 1 | 2 | Y |
| hsa-miR-16-5p | CDC37L1 | 1 | 1 | 2 | Y |
| hsa-miR-16-5p | LURAP1L | 1 | 1 | 2 | Y |
| hsa-miR-16-5p | APLN | 1 | 1 | 2 | N |
| hsa-miR-16-5p | CLDN12 | 1 | 1 | 2 | N |
| hsa-miR-16-5p | KIF1B | 1 | 1 | 2 | Y |
| hsa-miR-16-5p | TACC1 | 1 | 1 | 2 | N |
| hsa-miR-16-5p | PAQR3 | 1 | 1 | 2 | Y |
| hsa-miR-16-5p | RARB | 1 | 1 | 2 | Y |
| hsa-miR-16-5p | SPSB4 | 1 | 1 | 2 | N |
| hsa-miR-16-5p | PCDHA1 | 1 | 1 | 2 | N |
| hsa-miR-16-5p | SLC15A4 | 1 | 1 | 2 | N |
| hsa-miR-16-5p | TBPL1 | 1 | 1 | 2 | Y |
| hsa-miR-16-5p | TCAIM | 1 | 1 | 2 | N |
| hsa-miR-16-5p | PLAG1 | 1 | 1 | 2 | Y |
| hsa-miR-16-5p | TMEM100 | 1 | 1 | 2 | Y |
| hsa-miR-16-5p | MEOX2 | 1 | 1 | 2 | N |
| hsa-miR-16-5p | TPD52L3 | 1 | 1 | 2 | N |
| hsa-miR-16-5p | DENND6A | 1 | 1 | 2 | Y |
| hsa-miR-16-5p | GORASP2 | 1 | 1 | 2 | N |
| hsa-miR-16-5p | SLC9A8 | 1 | 1 | 2 | N |
| hsa-miR-16-5p | RECK | 1 | 1 | 2 | Y |
| hsa-miR-16-5p | TLK1 | 1 | 1 | 2 | Y |
| hsa-miR-16-5p | PCDHAC2 | 1 | 1 | 2 | N |
| hsa-miR-16-5p | SYNJ1 | 1 | 1 | 2 | Y |
| hsa-miR-16-5p | LATS2 | 1 | 1 | 2 | N |
| hsa-miR-16-5p | ARL3 | 1 | 1 | 2 | Y |
| hsa-miR-16-5p | AKT3 | 1 | 1 | 2 | Y |
| hsa-miR-16-5p | RNF125 | 1 | 1 | 2 | N |
| hsa-miR-16-5p | KCNN4 | 1 | 1 | 2 | Y |
| hsa-miR-16-5p | DCP1A | 1 | 1 | 2 | N |
| hsa-miR-16-5p | XPO7 | 1 | 1 | 2 | Y |
| hsa-miR-16-5p | ZMYM2 | 1 | 1 | 2 | N |
| hsa-miR-16-5p | ZBTB34 | 1 | 1 | 2 | Y |
| hsa-miR-16-5p | PLXNC1 | 1 | 1 | 2 | N |
| hsa-miR-16-5p | ZBTB46 | 1 | 1 | 2 | N |
| hsa-miR-486-3p | PPARD | 1 | 1 | 2 | N |
| hsa-miR-486-3p | CLIP2 | 1 | 1 | 2 | N |
| hsa-miR-486-3p | C17orf62 | 1 | 1 | 2 | N |
| hsa-miR-486-3p | GPSM1 | 1 | 1 | 2 | N |
| hsa-miR-486-3p | SEPT3 | 1 | 1 | 2 | N |
| hsa-miR-486-3p | GBAS | 1 | 1 | 2 | N |
| hsa-miR-486-3p | CD209 | 1 | 1 | 2 | N |
| hsa-miR-486-3p | RNF41 | 1 | 1 | 2 | Y |
| hsa-miR-486-3p | ZDHHC8 | 1 | 1 | 2 | N |
| hsa-miR-486-3p | SPDEF | 1 | 1 | 2 | N |
| hsa-miR-486-3p | MICALL1 | 1 | 1 | 2 | N |
| hsa-miR-486-3p | SPIB | 1 | 1 | 2 | N |
| hsa-miR-486-3p | WNT4 | 1 | 1 | 2 | N |
| hsa-miR-486-3p | CCND3 | 1 | 1 | 2 | N |
| hsa-miR-486-3p | SORT1 | 1 | 1 | 2 | N |
| hsa-miR-486-3p | GLIS2 | 1 | 1 | 2 | N |
| hsa-miR-486-3p | PPP1R14B | 1 | 1 | 2 | N |
| hsa-miR-486-3p | LGI3 | 1 | 1 | 2 | N |
| hsa-miR-486-3p | CPLX2 | 1 | 1 | 2 | N |
| hsa-miR-486-3p | KXD1 | 1 | 1 | 2 | N |
| hsa-miR-486-3p | MAP3K11 | 1 | 1 | 2 | N |
| hsa-miR-486-3p | NPTX1 | 1 | 1 | 2 | N |
| hsa-miR-486-3p | POGK | 1 | 1 | 2 | N |
| hsa-miR-486-3p | AGO1 | 1 | 1 | 2 | N |
| hsa-miR-486-3p | IQSEC2 | 1 | 1 | 2 | N |
| hsa-miR-486-3p | SRCIN1 | 1 | 1 | 2 | Y |
| hsa-miR-486-3p | SZRD1 | 1 | 1 | 2 | Y |
| hsa-miR-486-3p | FAM101B | 1 | 1 | 2 | N |
| hsa-miR-486-3p | TRAF3 | 1 | 1 | 2 | N |
| hsa-miR-486-3p | USF1 | 1 | 1 | 2 | N |
| hsa-miR-486-3p | CTDSP2 | 1 | 1 | 2 | N |
| hsa-miR-486-3p | FLNC | 1 | 1 | 2 | N |
| hsa-miR-486-3p | KCNAB2 | 1 | 1 | 2 | N |
| hsa-miR-486-3p | C16orf70 | 1 | 1 | 2 | N |
| hsa-miR-486-3p | PDE2A | 1 | 1 | 2 | N |
| hsa-miR-486-3p | KCNQ4 | 1 | 1 | 2 | N |
| hsa-miR-486-3p | MRVI1 | 1 | 1 | 2 | N |
| hsa-miR-486-3p | SLAMF8 | 1 | 1 | 2 | N |
| hsa-miR-486-3p | ZNF512B | 1 | 1 | 2 | N |
| hsa-miR-486-3p | CTSB | 1 | 1 | 2 | N |
| hsa-miR-486-3p | HS6ST1 | 1 | 1 | 2 | N |
| hsa-miR-486-3p | TSPAN11 | 1 | 1 | 2 | N |
| hsa-miR-486-3p | PHOX2A | 1 | 1 | 2 | N |
| hsa-miR-486-3p | RNF4 | 1 | 1 | 2 | N |
| hsa-miR-486-3p | CNTNAP1 | 1 | 1 | 2 | N |
| hsa-miR-486-3p | KMT2D | 1 | 1 | 2 | Y |
| hsa-miR-486-3p | KDELR1 | 1 | 1 | 2 | N |
| hsa-miR-486-3p | ZC3H12A | 1 | 1 | 2 | N |
| hsa-miR-486-3p | SLC6A8 | 1 | 1 | 2 | N |
| hsa-miR-486-3p | MARK2 | 1 | 1 | 2 | N |
| hsa-miR-486-3p | KIF21B | 1 | 1 | 2 | N |
| hsa-miR-486-3p | MDGA1 | 1 | 1 | 2 | N |
| hsa-miR-486-3p | CACNA2D2 | 1 | 1 | 2 | Y |
| hsa-miR-486-3p | CYFIP2 | 1 | 1 | 2 | N |
| hsa-miR-486-3p | DIRAS1 | 1 | 1 | 2 | N |
| hsa-miR-486-3p | CASKIN1 | 1 | 1 | 2 | Y |
| hsa-miR-486-3p | SLC48A1 | 1 | 1 | 2 | N |
| hsa-miR-486-3p | SH3KBP1 | 1 | 1 | 2 | N |
| hsa-miR-486-3p | PLEKHO2 | 1 | 1 | 2 | N |
| hsa-miR-486-3p | CHST1 | 1 | 1 | 2 | N |
| hsa-miR-486-3p | NEFH | 1 | 1 | 2 | N |
| hsa-miR-486-3p | RIMS4 | 1 | 1 | 2 | N |
| hsa-miR-486-3p | PRRT2 | 1 | 1 | 2 | N |
| hsa-miR-486-3p | NDOR1 | 1 | 1 | 2 | N |
| hsa-miR-486-3p | PLEK | 1 | 1 | 2 | N |
| hsa-miR-486-3p | GATAD2B | 1 | 1 | 2 | N |
| hsa-miR-486-3p | TMEM104 | 1 | 1 | 2 | N |
| hsa-miR-486-3p | LRP1 | 1 | 1 | 2 | N |
| hsa-miR-486-3p | LPHN1 | 1 | 1 | 2 | N |
| hsa-miR-486-3p | NFASC | 1 | 1 | 2 | N |
| hsa-miR-486-3p | KLF12 | 1 | 1 | 2 | N |
| hsa-miR-486-3p | SNX32 | 1 | 1 | 2 | N |
| hsa-miR-486-3p | GRIN1 | 1 | 1 | 2 | N |
| hsa-miR-486-3p | TMEM178A | 1 | 1 | 2 | N |
| hsa-miR-486-3p | TP53INP2 | 1 | 1 | 2 | N |
| hsa-miR-486-3p | DMBX1 | 1 | 1 | 2 | N |
| hsa-miR-486-3p | TMEM234 | 1 | 1 | 2 | N |
| hsa-miR-486-3p | CHIT1 | 1 | 1 | 2 | N |
| hsa-miR-486-3p | JAKMIP3 | 1 | 1 | 2 | N |
| hsa-miR-486-3p | BCL7B | 1 | 1 | 2 | N |
| hsa-miR-486-3p | HRH3 | 1 | 1 | 2 | N |
| hsa-miR-486-3p | C8orf46 | 1 | 1 | 2 | N |
| hsa-miR-486-3p | KIRREL | 1 | 1 | 2 | N |
| hsa-miR-486-3p | STK10 | 1 | 1 | 2 | N |
| hsa-miR-486-3p | UPB1 | 1 | 1 | 2 | N |
| hsa-miR-486-3p | TSPAN9 | 1 | 1 | 2 | N |
| hsa-miR-486-3p | IFITM5 | 1 | 1 | 2 | N |
| hsa-miR-486-3p | PEX26 | 1 | 1 | 2 | N |
| hsa-miR-486-3p | TESK2 | 1 | 1 | 2 | N |
| hsa-miR-486-3p | ARHGAP1 | 1 | 1 | 2 | N |
| hsa-miR-486-3p | CLCF1 | 1 | 1 | 2 | N |
| hsa-miR-486-3p | FMR1 | 1 | 1 | 2 | N |
| hsa-miR-486-3p | DBF4B | 1 | 1 | 2 | N |
| hsa-miR-486-3p | TMEM164 | 1 | 1 | 2 | N |
| hsa-miR-486-3p | RNF165 | 1 | 1 | 2 | N |
| hsa-miR-486-3p | WFIKKN2 | 1 | 1 | 2 | N |
| hsa-miR-486-3p | PEMT | 1 | 1 | 2 | N |
| hsa-miR-486-3p | RPL3L | 1 | 1 | 2 | N |
| hsa-miR-486-3p | SNW1 | 1 | 1 | 2 | N |
| hsa-miR-486-3p | CBFA2T3 | 1 | 1 | 2 | N |
| hsa-miR-486-3p | GM2A | 1 | 1 | 2 | N |
| hsa-miR-486-3p | SCN2B | 1 | 1 | 2 | N |
| hsa-miR-486-3p | CTDSP1 | 1 | 1 | 2 | N |
| hsa-miR-486-3p | KLHDC7B | 1 | 1 | 2 | N |
| hsa-miR-486-3p | FAM155B | 1 | 1 | 2 | N |
| hsa-miR-486-3p | WNT5B | 1 | 1 | 2 | N |
| hsa-miR-486-3p | DAB2IP | 1 | 1 | 2 | N |
| hsa-miR-486-3p | NKD1 | 1 | 1 | 2 | N |
| hsa-miR-486-3p | PAFAH1B2 | 1 | 1 | 2 | N |
| hsa-miR-486-3p | ABCG4 | 1 | 1 | 2 | N |
| hsa-miR-486-3p | SLC9A8 | 1 | 1 | 2 | N |
| hsa-miR-486-3p | NSD1 | 1 | 1 | 2 | Y |
| hsa-miR-486-3p | IL2RB | 1 | 1 | 2 | N |
| hsa-miR-486-3p | NCKAP5L | 1 | 1 | 2 | N |
| hsa-miR-486-3p | CCND2 | 1 | 1 | 2 | N |
| hsa-miR-486-3p | SH3GLB2 | 1 | 1 | 2 | N |
| hsa-miR-486-3p | FBXO41 | 1 | 1 | 2 | N |
| hsa-miR-486-3p | CLIP3 | 1 | 1 | 2 | N |
| hsa-miR-486-3p | VSX1 | 1 | 1 | 2 | N |
| hsa-miR-486-3p | EN1 | 1 | 1 | 2 | N |
| hsa-miR-486-3p | CTNNBIP1 | 1 | 1 | 2 | N |
| hsa-miR-486-3p | GDI1 | 1 | 1 | 2 | N |
| hsa-miR-486-3p | NOTUM | 1 | 1 | 2 | N |
| hsa-miR-486-3p | VAT1 | 1 | 1 | 2 | N |
| hsa-miR-486-3p | RAP1GAP2 | 1 | 1 | 2 | N |
| hsa-miR-486-3p | ZFHX3 | 1 | 1 | 2 | Y |
| hsa-miR-486-3p | PNKD | 1 | 1 | 2 | N |
| hsa-miR-486-3p | ANKRD54 | 1 | 1 | 2 | N |
| hsa-miR-486-3p | IGSF9 | 1 | 1 | 2 | N |
| hsa-miR-486-3p | RAI14 | 1 | 1 | 2 | N |
| hsa-miR-486-3p | SLC41A1 | 1 | 1 | 2 | N |
| hsa-miR-486-3p | SNAI3 | 1 | 1 | 2 | N |
| hsa-miR-486-3p | PALM | 1 | 1 | 2 | N |
| hsa-miR-486-3p | TIE1 | 1 | 1 | 2 | N |
| hsa-miR-486-3p | ADAM19 | 1 | 1 | 2 | N |
| hsa-miR-486-3p | MED22 | 1 | 1 | 2 | N |
| hsa-miR-486-3p | TMEM235 | 1 | 1 | 2 | N |
| hsa-miR-486-3p | TTYH3 | 1 | 1 | 2 | N |
| hsa-miR-486-3p | VPREB3 | 1 | 1 | 2 | N |
| hsa-miR-486-3p | MAPK15 | 1 | 1 | 2 | N |
| hsa-miR-486-3p | SRC | 1 | 1 | 2 | N |
| hsa-miR-486-3p | TMEM229B | 1 | 1 | 2 | N |
| hsa-miR-486-3p | AGPAT1 | 1 | 1 | 2 | N |
| hsa-miR-486-3p | FAM212B | 1 | 1 | 2 | N |
| hsa-miR-486-3p | DNMBP | 1 | 1 | 2 | N |
| hsa-miR-486-3p | ERGIC1 | 1 | 1 | 2 | N |
| hsa-miR-486-3p | NPTXR | 1 | 1 | 2 | Y |
| hsa-miR-486-3p | SOX10 | 1 | 1 | 2 | N |
| hsa-miR-486-3p | VPS9D1 | 1 | 1 | 2 | N |
| hsa-miR-486-3p | HSPG2 | 1 | 1 | 2 | N |
| hsa-miR-486-3p | SLC6A9 | 1 | 1 | 2 | N |
| hsa-miR-486-3p | HIF1AN | 1 | 1 | 2 | N |
| hsa-miR-486-3p | RELT | 1 | 1 | 2 | N |
| hsa-miR-486-3p | MESDC1 | 1 | 1 | 2 | N |
| hsa-miR-486-3p | TRPC4AP | 1 | 1 | 2 | N |
| hsa-miR-486-3p | CD276 | 1 | 1 | 2 | Y |
| hsa-miR-486-3p | CDC42SE1 | 1 | 1 | 2 | N |
| hsa-miR-486-3p | NFIC | 1 | 1 | 2 | Y |
| hsa-miR-486-3p | PLCB1 | 1 | 1 | 2 | N |
| hsa-miR-486-3p | MKNK2 | 1 | 1 | 2 | Y |
| hsa-miR-486-3p | DPF2 | 1 | 1 | 2 | N |
| hsa-miR-486-3p | ELAC2 | 1 | 1 | 2 | N |
| hsa-miR-486-3p | POU2F2 | 1 | 1 | 2 | N |
| hsa-miR-486-3p | AP1B1 | 1 | 1 | 2 | N |
| hsa-miR-486-3p | NEDD8 | 1 | 1 | 2 | N |
| hsa-miR-486-3p | RGAG4 | 1 | 1 | 2 | N |
| hsa-miR-486-3p | TRAFD1 | 1 | 1 | 2 | N |
| hsa-miR-486-3p | CECR6 | 1 | 1 | 2 | N |
| hsa-miR-486-3p | E2F4 | 1 | 1 | 2 | N |
| hsa-miR-486-3p | TBC1D22B | 1 | 1 | 2 | N |
| hsa-miR-486-3p | SLC7A8 | 1 | 1 | 2 | N |
| hsa-miR-486-3p | HSPB6 | 1 | 1 | 2 | N |
| hsa-miR-486-3p | RANGAP1 | 1 | 1 | 2 | N |
| hsa-miR-486-3p | RHOF | 1 | 1 | 2 | N |
| hsa-miR-486-3p | MARCH1 | 1 | 1 | 2 | N |
| hsa-miR-486-3p | LRRC61 | 1 | 1 | 2 | N |
| hsa-miR-486-3p | CDC25B | 1 | 1 | 2 | N |
| hsa-miR-486-3p | NHLH1 | 1 | 1 | 2 | N |
| hsa-miR-486-3p | ITGA11 | 1 | 1 | 2 | N |
| hsa-miR-486-3p | NAA60 | 1 | 1 | 2 | N |
| hsa-miR-486-3p | TRIOBP | 1 | 1 | 2 | N |
| hsa-miR-486-3p | RELA | 1 | 1 | 2 | N |
| hsa-miR-486-3p | DBNDD1 | 1 | 1 | 2 | N |
| hsa-miR-486-3p | TMEM132E | 1 | 1 | 2 | N |
| hsa-miR-486-3p | ARF3 | 1 | 1 | 2 | N |
| hsa-miR-486-3p | GPC6 | 1 | 1 | 2 | N |
| hsa-miR-486-3p | STIM1 | 1 | 1 | 2 | N |
| hsa-miR-486-3p | RHOG | 1 | 1 | 2 | N |
| hsa-miR-486-3p | FXYD6 | 1 | 1 | 2 | N |
| hsa-miR-486-3p | LUZP1 | 1 | 1 | 2 | Y |
| hsa-miR-486-3p | PAK6 | 1 | 1 | 2 | N |
| hsa-miR-486-3p | PRX | 1 | 1 | 2 | N |
| hsa-miR-486-3p | TUBB | 1 | 1 | 2 | Y |
| hsa-miR-486-3p | PTGFRN | 1 | 1 | 2 | N |
| hsa-miR-486-3p | B3GALT5 | 1 | 1 | 2 | N |
| hsa-miR-486-3p | FRS3 | 1 | 1 | 2 | N |
| hsa-miR-486-3p | VASH1 | 1 | 1 | 2 | N |
| hsa-miR-486-3p | C17orf97 | 1 | 1 | 2 | N |
| hsa-miR-486-3p | MAPK3 | 1 | 1 | 2 | N |
| hsa-miR-486-3p | TMEM63C | 1 | 1 | 2 | N |
| hsa-miR-486-3p | MDM4 | 1 | 1 | 2 | Y |
| hsa-miR-486-3p | GATA4 | 1 | 1 | 2 | N |
| hsa-miR-486-3p | DLL4 | 1 | 1 | 2 | N |
| hsa-miR-486-3p | KLHDC3 | 1 | 1 | 2 | N |
| hsa-miR-486-3p | R3HDM4 | 1 | 1 | 2 | N |
| hsa-miR-486-3p | SYT2 | 1 | 1 | 2 | N |
| hsa-miR-486-3p | DLGAP4 | 1 | 1 | 2 | Y |
| hsa-miR-486-3p | DTX3 | 1 | 1 | 2 | N |
| hsa-miR-486-3p | MEP1A | 1 | 1 | 2 | N |
| hsa-miR-486-3p | VAMP2 | 1 | 1 | 2 | N |
| hsa-miR-486-3p | CALN1 | 1 | 1 | 2 | N |
| hsa-miR-486-3p | TSPAN5 | 1 | 1 | 2 | N |
| hsa-miR-486-3p | PXN | 1 | 1 | 2 | N |
| hsa-miR-486-3p | H6PD | 1 | 1 | 2 | N |
| hsa-miR-486-3p | TM9SF4 | 1 | 1 | 2 | N |
| hsa-miR-486-3p | SF3A1 | 1 | 1 | 2 | N |
| hsa-miR-486-3p | DIEXF | 1 | 1 | 2 | N |
| hsa-miR-486-3p | ULK1 | 1 | 1 | 2 | N |
| hsa-miR-486-3p | LRRC55 | 1 | 1 | 2 | N |
| hsa-miR-486-3p | DLL3 | 1 | 1 | 2 | N |
| hsa-miR-486-3p | TRIM62 | 1 | 1 | 2 | N |
| hsa-miR-486-3p | ADH6 | 1 | 1 | 2 | N |
| hsa-miR-486-3p | ATF3 | 1 | 1 | 2 | N |
| hsa-miR-486-3p | MAP3K10 | 1 | 1 | 2 | N |
| hsa-miR-486-3p | FLOT2 | 1 | 1 | 2 | N |
| hsa-miR-486-3p | FAM131C | 1 | 1 | 2 | N |
| hsa-miR-486-3p | ARRB2 | 1 | 1 | 2 | N |
| hsa-miR-486-3p | FAM102A | 1 | 1 | 2 | N |
| hsa-miR-486-3p | NGFR | 1 | 1 | 2 | N |
| hsa-miR-486-3p | SCRIB | 1 | 1 | 2 | N |
| hsa-miR-486-3p | HOMER2 | 1 | 1 | 2 | N |
| hsa-miR-486-3p | ANP32A | 1 | 1 | 2 | N |
| hsa-miR-486-3p | TXLNA | 1 | 1 | 2 | Y |
| hsa-miR-486-3p | PTMS | 1 | 1 | 2 | Y |
| hsa-miR-486-3p | PACS2 | 1 | 1 | 2 | N |
| hsa-miR-486-3p | GRM4 | 1 | 1 | 2 | N |
| hsa-miR-486-3p | PIK3R2 | 1 | 1 | 2 | N |
| hsa-miR-486-3p | ECM1 | 1 | 1 | 2 | Y |
| hsa-miR-486-3p | ENTPD2 | 1 | 1 | 2 | N |
| hsa-miR-486-3p | VOPP1 | 1 | 1 | 2 | N |
| hsa-miR-486-3p | SLC39A13 | 1 | 1 | 2 | N |
| hsa-miR-486-3p | ALDH3B2 | 1 | 1 | 2 | N |
| hsa-miR-486-3p | RAB2B | 1 | 1 | 2 | N |
| hsa-miR-486-3p | ITGB2 | 1 | 1 | 2 | N |
| hsa-miR-486-3p | IMPDH1 | 1 | 1 | 2 | N |
| hsa-miR-486-3p | CELSR3 | 1 | 1 | 2 | N |
| hsa-miR-486-3p | UAP1L1 | 1 | 1 | 2 | N |
| hsa-miR-486-3p | STK32B | 1 | 1 | 2 | N |
| hsa-miR-486-3p | GIMAP4 | 1 | 1 | 2 | N |
| hsa-miR-486-3p | S100A10 | 1 | 1 | 2 | N |
| hsa-miR-486-3p | PDGFRB | 1 | 1 | 2 | N |
| hsa-miR-486-3p | AATK | 1 | 1 | 2 | N |
| hsa-miR-486-3p | SNPH | 1 | 1 | 2 | N |
| hsa-miR-486-3p | HES7 | 1 | 1 | 2 | N |
| hsa-miR-486-3p | SURF4 | 1 | 1 | 2 | N |
| hsa-miR-486-3p | TRABD2B | 1 | 1 | 2 | N |
| hsa-miR-486-3p | MCM5 | 1 | 1 | 2 | N |
| hsa-miR-486-3p | SLC43A2 | 1 | 1 | 2 | N |
| hsa-miR-486-3p | ZCCHC13 | 1 | 1 | 2 | N |
| hsa-miR-486-3p | SBK1 | 1 | 1 | 2 | Y |
| hsa-miR-486-3p | CNIH2 | 1 | 1 | 2 | N |
| hsa-miR-486-3p | BRF1 | 1 | 1 | 2 | N |
| hsa-miR-486-3p | SCN1B | 1 | 1 | 2 | N |
| hsa-miR-486-3p | DCAF7 | 1 | 1 | 2 | N |
| hsa-miR-486-3p | CSRNP1 | 1 | 1 | 2 | N |
| hsa-miR-486-3p | GRM2 | 1 | 1 | 2 | N |
| hsa-miR-486-3p | TBC1D13 | 1 | 1 | 2 | N |
| hsa-miR-486-3p | IGSF5 | 1 | 1 | 2 | N |
| hsa-miR-486-3p | DGAT1 | 1 | 1 | 2 | N |
| hsa-miR-486-3p | NACC2 | 1 | 1 | 2 | Y |
| hsa-miR-486-3p | MAP1LC3A | 1 | 1 | 2 | N |
| hsa-miR-486-3p | TP73 | 1 | 1 | 2 | N |
| hsa-miR-486-3p | SPTBN4 | 1 | 1 | 2 | N |
| hsa-miR-486-3p | PVRL1 | 1 | 1 | 2 | N |
| hsa-miR-486-3p | SDC3 | 1 | 1 | 2 | N |
| hsa-miR-486-3p | CBX6 | 1 | 1 | 2 | N |
| hsa-miR-486-3p | ARID1B | 1 | 1 | 2 | N |
| hsa-miR-486-3p | KCNJ3 | 1 | 1 | 2 | N |
| hsa-miR-486-3p | ZNF710 | 1 | 1 | 2 | N |
| hsa-miR-486-3p | FOSL2 | 1 | 1 | 2 | N |
| hsa-miR-486-3p | CCDC97 | 1 | 1 | 2 | N |
| hsa-miR-486-3p | TFE3 | 1 | 1 | 2 | N |
| hsa-miR-486-3p | PTK2B | 1 | 1 | 2 | N |
| hsa-miR-486-3p | CRTC3 | 1 | 1 | 2 | N |
| hsa-miR-486-3p | ATXN7L3 | 1 | 1 | 2 | Y |
| hsa-miR-486-3p | ZNF772 | 1 | 1 | 2 | N |
| hsa-miR-486-3p | HCN3 | 1 | 1 | 2 | N |
| hsa-miR-653-3p | BRD8 | 1 | 1 | 2 | N |
| hsa-miR-653-3p | OMG | 1 | 1 | 2 | N |
| hsa-miR-653-3p | EYA1 | 1 | 1 | 2 | N |
| hsa-miR-653-3p | YIPF6 | 1 | 1 | 2 | N |
| hsa-miR-653-3p | DYNLL1 | 1 | 1 | 2 | N |
| hsa-miR-653-3p | STMN4 | 1 | 1 | 2 | N |
| hsa-miR-653-3p | PARP14 | 1 | 1 | 2 | N |
| hsa-miR-653-3p | ACADM | 1 | 1 | 2 | N |
| hsa-miR-653-3p | PTPN20A | 1 | 1 | 2 | N |
| hsa-miR-653-3p | ZDHHC17 | 1 | 1 | 2 | N |
| hsa-miR-653-3p | IRF1 | 1 | 1 | 2 | N |
| hsa-miR-653-3p | PPP1R1A | 1 | 1 | 2 | N |
| hsa-miR-653-3p | VIP | 1 | 1 | 2 | N |
| hsa-miR-653-3p | EZH2 | 1 | 1 | 2 | N |
| hsa-miR-653-3p | PER3 | 1 | 1 | 2 | N |
| hsa-miR-653-3p | LACC1 | 1 | 1 | 2 | N |
| hsa-miR-653-3p | CNKSR2 | 1 | 1 | 2 | N |
| hsa-miR-653-3p | CDC42SE2 | 1 | 1 | 2 | N |
| hsa-miR-653-3p | RNF6 | 1 | 1 | 2 | N |
| hsa-miR-653-3p | MAML1 | 1 | 1 | 2 | N |
| hsa-miR-653-3p | CCDC28A | 1 | 1 | 2 | N |
| hsa-miR-653-3p | GPR137C | 1 | 1 | 2 | N |
| hsa-miR-653-3p | MAP1B | 1 | 1 | 2 | N |
| hsa-miR-653-3p | PFKFB1 | 1 | 1 | 2 | N |
| hsa-miR-653-3p | RPE | 1 | 1 | 2 | N |
| hsa-miR-653-3p | HRH4 | 1 | 1 | 2 | N |
| hsa-miR-653-3p | YWHAG | 1 | 1 | 2 | N |
| hsa-miR-653-3p | UBTD2 | 1 | 1 | 2 | N |
| hsa-miR-653-3p | WDR26 | 1 | 1 | 2 | N |
| hsa-miR-653-3p | SNX31 | 1 | 1 | 2 | N |
| hsa-miR-653-3p | GABRA5 | 1 | 1 | 2 | N |
| hsa-miR-653-3p | SELP | 1 | 1 | 2 | N |
| hsa-miR-653-3p | AGXT2 | 1 | 1 | 2 | N |
| hsa-miR-653-3p | C11orf44 | 1 | 1 | 2 | N |
| hsa-miR-653-3p | TOMM20 | 1 | 1 | 2 | N |
| hsa-miR-653-3p | PRKCH | 1 | 1 | 2 | N |
| hsa-miR-653-3p | YIPF4 | 1 | 1 | 2 | N |
| hsa-miR-653-3p | GNG5 | 1 | 1 | 2 | N |
| hsa-miR-653-3p | PEF1 | 1 | 1 | 2 | N |
| hsa-miR-653-3p | CHST4 | 1 | 1 | 2 | N |
| hsa-miR-653-3p | POPDC3 | 1 | 1 | 2 | N |
| hsa-miR-653-3p | SSFA2 | 1 | 1 | 2 | N |
| hsa-miR-653-3p | CTDSPL2 | 1 | 1 | 2 | N |
| hsa-miR-653-3p | TSPAN5 | 1 | 1 | 2 | N |
| hsa-miR-653-3p | CCT6A | 1 | 1 | 2 | N |
| hsa-miR-653-3p | AMMECR1L | 1 | 1 | 2 | N |
| hsa-miR-653-3p | SMARCAD1 | 1 | 1 | 2 | N |
| hsa-miR-653-3p | PVRL3 | 1 | 1 | 2 | N |
| hsa-miR-653-3p | DNAJC6 | 1 | 1 | 2 | N |
| hsa-miR-653-3p | CD53 | 1 | 1 | 2 | N |
| hsa-miR-653-3p | FBXL5 | 1 | 1 | 2 | N |
| hsa-miR-653-3p | PTPN20B | 1 | 1 | 2 | N |
| hsa-miR-653-3p | FAM117B | 1 | 1 | 2 | N |
| hsa-miR-653-3p | ANGPTL7 | 1 | 1 | 2 | N |
| hsa-miR-653-3p | ZNF329 | 1 | 1 | 2 | N |
| hsa-miR-128-1-5p | SNCG | 1 | 1 | 2 | N |
| hsa-miR-128-1-5p | ALDH1B1 | 1 | 1 | 2 | N |
| hsa-miR-128-1-5p | PRRT1 | 1 | 1 | 2 | N |
| hsa-miR-128-1-5p | CBFA2T3 | 1 | 1 | 2 | N |
| hsa-miR-128-1-5p | DEGS2 | 1 | 1 | 2 | N |
| hsa-miR-128-1-5p | INPP5A | 1 | 1 | 2 | N |
| hsa-miR-128-1-5p | TBC1D25 | 1 | 1 | 2 | N |
| hsa-miR-128-1-5p | UNC5A | 1 | 1 | 2 | N |
| hsa-miR-128-1-5p | GNG13 | 1 | 1 | 2 | N |
| hsa-miR-128-1-5p | STAU1 | 1 | 1 | 2 | N |
| hsa-miR-128-1-5p | CDH15 | 1 | 1 | 2 | N |
| hsa-miR-128-2-5p | SNCG | 1 | 1 | 2 | N |
| hsa-miR-128-2-5p | DEGS2 | 1 | 1 | 2 | N |
| hsa-miR-128-2-5p | ALDH1B1 | 1 | 1 | 2 | N |
| hsa-miR-128-2-5p | CDH15 | 1 | 1 | 2 | N |
| hsa-miR-128-2-5p | STAU1 | 1 | 1 | 2 | N |
| hsa-miR-128-2-5p | TBC1D25 | 1 | 1 | 2 | N |
| hsa-miR-128-2-5p | PRRT1 | 1 | 1 | 2 | N |
| hsa-miR-128-2-5p | CBFA2T3 | 1 | 1 | 2 | N |
| hsa-miR-128-2-5p | GNG13 | 1 | 1 | 2 | N |
| hsa-miR-128-2-5p | UNC5A | 1 | 1 | 2 | N |
| hsa-miR-128-2-5p | INPP5A | 1 | 1 | 2 | N |
| hsa-miR-1291 | ENTPD6 | 1 | 1 | 2 | N |
| hsa-miR-1291 | SLC9A5 | 1 | 1 | 2 | N |
| hsa-miR-1291 | DYNC1I1 | 1 | 1 | 2 | N |
| hsa-miR-1291 | PACSIN1 | 1 | 1 | 2 | N |
| hsa-miR-1291 | UNC13B | 1 | 1 | 2 | N |
| hsa-miR-1291 | KCNQ5 | 1 | 1 | 2 | N |
| hsa-miR-1291 | PPP2R4 | 1 | 1 | 2 | N |
| hsa-miR-1291 | MED22 | 1 | 1 | 2 | N |
| hsa-miR-1291 | HPCAL4 | 1 | 1 | 2 | N |
| hsa-miR-1291 | CYP2C18 | 1 | 1 | 2 | N |
| hsa-miR-1291 | RNF125 | 1 | 1 | 2 | N |
| hsa-miR-1291 | MESDC1 | 1 | 1 | 2 | N |
| hsa-miR-1291 | USP37 | 1 | 1 | 2 | N |
| hsa-miR-1291 | TGM4 | 1 | 1 | 2 | N |
| hsa-miR-1291 | FAM160A2 | 1 | 1 | 2 | N |
| hsa-miR-1291 | PAK2 | 1 | 1 | 2 | N |
| hsa-miR-1291 | MGAT5 | 1 | 1 | 2 | N |
| hsa-miR-1291 | TAB1 | 1 | 1 | 2 | N |
| hsa-miR-1291 | AK3 | 1 | 1 | 2 | N |
| hsa-miR-1291 | PPM1F | 1 | 1 | 2 | Y |
| hsa-miR-1291 | LNX2 | 1 | 1 | 2 | N |
| hsa-miR-1291 | C1orf172 | 1 | 1 | 2 | N |
| hsa-miR-1291 | PHF19 | 1 | 1 | 2 | N |
| hsa-miR-1291 | IQCE | 1 | 1 | 2 | N |
| hsa-miR-1291 | KCNK5 | 1 | 1 | 2 | N |
| hsa-miR-1291 | CIB1 | 1 | 1 | 2 | N |
| hsa-miR-1291 | AGPAT1 | 1 | 1 | 2 | N |
| hsa-miR-1291 | RGS9BP | 1 | 1 | 2 | N |
| hsa-miR-1291 | KLHDC7A | 1 | 1 | 2 | N |
| hsa-miR-1291 | LOXL4 | 1 | 1 | 2 | N |
| hsa-miR-1291 | LRRC32 | 1 | 1 | 2 | N |
| hsa-miR-1291 | IL1B | 1 | 1 | 2 | N |
| hsa-miR-1291 | DERL3 | 1 | 1 | 2 | N |
| hsa-miR-1291 | CYTH1 | 1 | 1 | 2 | N |
| hsa-miR-1291 | RELT | 1 | 1 | 2 | N |
| hsa-miR-1291 | KCNH4 | 1 | 1 | 2 | N |
| hsa-miR-1291 | SLC6A20 | 1 | 1 | 2 | N |
| hsa-miR-1291 | CABP7 | 1 | 1 | 2 | N |
| hsa-miR-1291 | FGFR3 | 1 | 1 | 2 | N |
| hsa-miR-1291 | KLHDC10 | 1 | 1 | 2 | N |
| hsa-miR-1291 | VSIG4 | 1 | 1 | 2 | N |
| hsa-miR-1291 | TBC1D5 | 1 | 1 | 2 | N |
| hsa-miR-1291 | GM2A | 1 | 1 | 2 | N |
| hsa-miR-1291 | HIC1 | 1 | 1 | 2 | N |
| hsa-miR-1291 | HES5 | 1 | 1 | 2 | N |
| hsa-miR-1291 | APBA1 | 1 | 1 | 2 | N |
| hsa-miR-1291 | VASH1 | 1 | 1 | 2 | N |
| hsa-miR-1291 | ATG4D | 1 | 1 | 2 | N |
| hsa-miR-1291 | SYTL1 | 1 | 1 | 2 | N |
| hsa-miR-1291 | HIATL1 | 1 | 1 | 2 | N |
| hsa-miR-1291 | NF2 | 1 | 1 | 2 | N |
| hsa-miR-1291 | AQP1 | 1 | 1 | 2 | N |
| hsa-miR-1291 | ABCC1 | 1 | 1 | 2 | Y |
| hsa-miR-1291 | TIMM22 | 1 | 1 | 2 | N |
| hsa-miR-1291 | SHKBP1 | 1 | 1 | 2 | N |
| hsa-miR-1291 | FAM189A1 | 1 | 1 | 2 | N |
| hsa-miR-1291 | FBXO10 | 1 | 1 | 2 | N |
| hsa-miR-1291 | ADORA2A | 1 | 1 | 2 | N |
| hsa-miR-1291 | C2orf72 | 1 | 1 | 2 | N |
| hsa-miR-1291 | SPSB4 | 1 | 1 | 2 | N |
| hsa-miR-1291 | WNT11 | 1 | 1 | 2 | N |
| hsa-miR-1291 | RASSF1 | 1 | 1 | 2 | N |
| hsa-miR-1291 | SNX19 | 1 | 1 | 2 | N |
| hsa-miR-1291 | SH3GL1 | 1 | 1 | 2 | N |
| hsa-miR-1291 | C11orf21 | 1 | 1 | 2 | N |
| hsa-miR-1291 | RPP14 | 1 | 1 | 2 | N |
| hsa-miR-1291 | UBE2Z | 1 | 1 | 2 | N |
| hsa-miR-1291 | SLC6A7 | 1 | 1 | 2 | N |
| hsa-miR-1291 | CD74 | 1 | 1 | 2 | N |
| hsa-miR-1291 | CCDC97 | 1 | 1 | 2 | N |
| hsa-miR-1291 | ARHGDIB | 1 | 1 | 2 | N |
| hsa-miR-1291 | IMPA2 | 1 | 1 | 2 | N |
| hsa-miR-1291 | GLYCTK | 1 | 1 | 2 | N |
| hsa-miR-1291 | XKR7 | 1 | 1 | 2 | N |
| hsa-miR-1291 | ARL4C | 1 | 1 | 2 | N |
| hsa-miR-1291 | CYB561A3 | 1 | 1 | 2 | N |
| hsa-miR-1291 | PGAP3 | 1 | 1 | 2 | N |
| hsa-miR-1291 | GPSM1 | 1 | 1 | 2 | N |
| hsa-miR-1291 | ESYT1 | 1 | 1 | 2 | N |
| hsa-miR-1291 | PHYHIP | 1 | 1 | 2 | N |
| hsa-miR-1291 | RAF1 | 1 | 1 | 2 | N |
| hsa-miR-1291 | GALNT13 | 1 | 1 | 2 | N |
| hsa-miR-1291 | SIRT3 | 1 | 1 | 2 | N |
| hsa-miR-1291 | PGM5 | 1 | 1 | 2 | N |
| hsa-miR-1291 | ARID3B | 1 | 1 | 2 | N |
| hsa-miR-1291 | ST6GALNAC6 | 1 | 1 | 2 | N |
| hsa-miR-1291 | PHOX2A | 1 | 1 | 2 | N |
| hsa-miR-1291 | KIAA0319L | 1 | 1 | 2 | N |
| hsa-miR-1291 | CD79A | 1 | 1 | 2 | N |
| hsa-miR-1291 | CC2D1A | 1 | 1 | 2 | N |
| hsa-miR-1291 | USP45 | 1 | 1 | 2 | N |
| hsa-miR-1291 | RALGDS | 1 | 1 | 2 | N |
| hsa-miR-1291 | CAMKK1 | 1 | 1 | 2 | N |
| hsa-miR-1291 | SRSF9 | 1 | 1 | 2 | N |
| hsa-miR-1291 | CUX1 | 1 | 1 | 2 | N |
| hsa-miR-1291 | NPTXR | 1 | 1 | 2 | N |
| hsa-miR-1291 | RNF123 | 1 | 1 | 2 | N |
| hsa-miR-1291 | CDC42EP1 | 1 | 1 | 2 | N |
| hsa-miR-1291 | LGR4 | 1 | 1 | 2 | N |
| hsa-miR-1291 | TMEM151A | 1 | 1 | 2 | N |
| hsa-miR-1291 | FAIM2 | 1 | 1 | 2 | N |
| hsa-miR-1291 | SLC12A7 | 1 | 1 | 2 | Y |
| hsa-miR-1291 | IQSEC3 | 1 | 1 | 2 | Y |
| hsa-miR-1291 | DNAJB5 | 1 | 1 | 2 | N |
| hsa-miR-1291 | LRRC48 | 1 | 1 | 2 | N |
| hsa-miR-1291 | TMEM254 | 1 | 1 | 2 | N |
| hsa-miR-1291 | PML | 1 | 1 | 2 | N |
| hsa-miR-1291 | FAM153A | 1 | 1 | 2 | N |
| hsa-miR-1291 | KREMEN1 | 1 | 1 | 2 | N |
| hsa-miR-1291 | SAMD14 | 1 | 1 | 2 | N |
| hsa-miR-1291 | LMOD1 | 1 | 1 | 2 | N |
| hsa-miR-1291 | VWA5A | 1 | 1 | 2 | Y |
| hsa-miR-1291 | RS1 | 1 | 1 | 2 | N |
| hsa-miR-296-5p | CAD | 1 | 1 | 2 | N |
| hsa-miR-296-5p | SOX12 | 1 | 1 | 2 | Y |
| hsa-miR-296-5p | IPO11 | 1 | 1 | 2 | N |
| hsa-miR-296-5p | SMG7 | 1 | 1 | 2 | N |
| hsa-miR-296-5p | FBRS | 1 | 1 | 2 | N |
| hsa-miR-296-5p | GAB2 | 1 | 1 | 2 | N |
| hsa-miR-296-5p | ADAMTS10 | 1 | 1 | 2 | N |
| hsa-miR-296-5p | DIRAS1 | 1 | 1 | 2 | N |
| hsa-miR-296-5p | DYRK1B | 1 | 1 | 2 | N |
| hsa-miR-296-5p | ACTR1A | 1 | 1 | 2 | N |
| hsa-miR-296-5p | LYPLA2 | 1 | 1 | 2 | N |
| hsa-miR-296-5p | RTN2 | 1 | 1 | 2 | N |
| hsa-miR-296-5p | NFIC | 1 | 1 | 2 | Y |
| hsa-miR-296-5p | AMMECR1L | 1 | 1 | 2 | N |
| hsa-miR-296-5p | SLC30A3 | 1 | 1 | 2 | N |
| hsa-miR-296-5p | VKORC1L1 | 1 | 1 | 2 | N |
| hsa-miR-296-5p | RNF44 | 1 | 1 | 2 | Y |
| hsa-miR-296-5p | SLC25A22 | 1 | 1 | 2 | N |
| hsa-miR-296-5p | KCTD15 | 1 | 1 | 2 | N |
| hsa-miR-296-5p | EPN1 | 1 | 1 | 2 | N |
| hsa-miR-296-5p | HIPK1 | 1 | 1 | 2 | N |
| hsa-miR-296-5p | PPP2R5B | 1 | 1 | 2 | N |
| hsa-miR-296-5p | SRF | 1 | 1 | 2 | N |
| hsa-miR-296-5p | NUMBL | 1 | 1 | 2 | N |
| hsa-miR-296-5p | RAB37 | 1 | 1 | 2 | N |
| hsa-miR-296-5p | NYNRIN | 1 | 1 | 2 | N |
| hsa-miR-296-5p | HMGA1 | 1 | 1 | 2 | Y |
| hsa-miR-296-5p | HM13 | 1 | 1 | 2 | N |
| hsa-miR-296-5p | CBX6 | 1 | 1 | 2 | N |
| hsa-miR-296-5p | BAHD1 | 1 | 1 | 2 | N |
| hsa-miR-670-3p | DONSON | 1 | 1 | 2 | N |
| hsa-miR-670-3p | RBM15 | 1 | 1 | 2 | N |
| hsa-miR-670-3p | OMA1 | 1 | 1 | 2 | N |
| hsa-miR-670-3p | YTHDF2 | 1 | 1 | 2 | N |
| hsa-miR-670-3p | CCR7 | 1 | 1 | 2 | N |
| hsa-miR-670-3p | ID1 | 1 | 1 | 2 | N |
| hsa-miR-670-3p | GSPT1 | 1 | 1 | 2 | N |
| hsa-miR-670-3p | PEX2 | 1 | 1 | 2 | N |
| hsa-miR-670-3p | ZKSCAN1 | 1 | 1 | 2 | N |
| hsa-miR-670-3p | IGFBP5 | 1 | 1 | 2 | N |
| hsa-miR-670-3p | HMG20A | 1 | 1 | 2 | N |
| hsa-miR-670-3p | ENPP2 | 1 | 1 | 2 | N |
| hsa-miR-670-3p | TBPL1 | 1 | 1 | 2 | N |
| hsa-miR-670-3p | MPST | 1 | 1 | 2 | N |
| hsa-miR-670-3p | HOXC4 | 1 | 1 | 2 | N |
| hsa-miR-670-3p | CCNB2 | 1 | 1 | 2 | N |
| hsa-miR-670-3p | RTN4 | 1 | 1 | 2 | N |
| hsa-miR-670-3p | PKP4 | 1 | 1 | 2 | N |
| hsa-miR-670-3p | KCNK1 | 1 | 1 | 2 | N |
| hsa-miR-670-3p | LDLRAP1 | 1 | 1 | 2 | N |
| hsa-miR-670-3p | MBNL1 | 1 | 1 | 2 | Y |
| hsa-miR-874-5p | TMEM127 | 1 | 1 | 2 | N |
| hsa-miR-874-5p | LRRC56 | 1 | 1 | 2 | N |
| hsa-miR-874-5p | GRAP | 1 | 1 | 2 | N |
| hsa-miR-874-5p | MAGEB6 | 1 | 1 | 2 | N |
| hsa-miR-874-5p | BFSP1 | 1 | 1 | 2 | N |
| hsa-miR-874-5p | TM9SF4 | 1 | 1 | 2 | N |
| hsa-miR-874-5p | TTYH3 | 1 | 1 | 2 | N |
| hsa-miR-874-5p | RAB4B | 1 | 1 | 2 | N |
| hsa-miR-874-5p | CHMP1A | 1 | 1 | 2 | N |
| hsa-miR-874-5p | PEF1 | 1 | 1 | 2 | N |
| hsa-miR-874-5p | TNPO1 | 1 | 1 | 2 | N |
| hsa-miR-874-5p | FZD7 | 1 | 1 | 2 | N |
| hsa-miR-874-5p | MPI | 1 | 1 | 2 | N |
| hsa-miR-874-5p | ACTR1A | 1 | 1 | 2 | N |
| hsa-miR-874-5p | TMEM121 | 1 | 1 | 2 | N |
| hsa-miR-874-5p | PREX1 | 1 | 1 | 2 | N |
| hsa-miR-874-5p | RAB43 | 1 | 1 | 2 | N |
| hsa-miR-874-5p | KRT80 | 1 | 1 | 2 | N |
| hsa-miR-874-5p | SYPL2 | 1 | 1 | 2 | N |
| hsa-miR-874-5p | CEP85 | 1 | 1 | 2 | N |
| hsa-miR-874-5p | ITGA5 | 1 | 1 | 2 | N |
| hsa-miR-874-5p | SLC35C2 | 1 | 1 | 2 | N |
| hsa-miR-874-5p | CSF2RA | 1 | 1 | 2 | N |
| hsa-miR-874-5p | KDM4A | 1 | 1 | 2 | N |
| hsa-miR-874-5p | RNF26 | 1 | 1 | 2 | N |
| hsa-miR-874-5p | EPHB3 | 1 | 1 | 2 | N |
| hsa-miR-874-5p | ACOT12 | 1 | 1 | 2 | N |
| hsa-miR-874-5p | NPTX1 | 1 | 1 | 2 | N |
| hsa-miR-874-5p | ARHGAP19 | 1 | 1 | 2 | N |
| hsa-miR-874-5p | NCBP2 | 1 | 1 | 2 | N |
| hsa-miR-874-5p | CHCHD4 | 1 | 1 | 2 | Y |
| hsa-miR-874-5p | TSPAN9 | 1 | 1 | 2 | N |
| hsa-miR-874-5p | EFCAB4B | 1 | 1 | 2 | N |
| hsa-miR-874-5p | SGSM1 | 1 | 1 | 2 | N |
| hsa-miR-874-5p | VASH1 | 1 | 1 | 2 | N |
| hsa-miR-874-5p | LPCAT3 | 1 | 1 | 2 | Y |
| hsa-miR-874-5p | TBX6 | 1 | 1 | 2 | N |
| hsa-miR-874-5p | ARID3B | 1 | 1 | 2 | N |
| hsa-miR-874-5p | ZSWIM8 | 1 | 1 | 2 | N |
| hsa-miR-874-5p | MED18 | 1 | 1 | 2 | N |
| hsa-miR-874-5p | CPSF7 | 1 | 1 | 2 | N |
| hsa-miR-874-5p | TRPC4AP | 1 | 1 | 2 | N |
| hsa-miR-874-5p | GRHL2 | 1 | 1 | 2 | N |
| hsa-miR-874-5p | VAT1 | 1 | 1 | 2 | N |
| hsa-miR-874-5p | WDR26 | 1 | 1 | 2 | N |
| hsa-miR-874-5p | HMBS | 1 | 1 | 2 | N |
| hsa-miR-874-5p | RNF44 | 1 | 1 | 2 | N |
| hsa-miR-874-5p | CPLX3 | 1 | 1 | 2 | N |
| hsa-miR-874-5p | SPIB | 1 | 1 | 2 | N |
| hsa-miR-874-5p | IL17REL | 1 | 1 | 2 | N |
| hsa-miR-874-5p | ICAM1 | 1 | 1 | 2 | N |
| hsa-miR-874-5p | BARX1 | 1 | 1 | 2 | N |
| hsa-miR-874-5p | EPN1 | 1 | 1 | 2 | N |
| hsa-miR-874-5p | RUSC2 | 1 | 1 | 2 | N |
| hsa-miR-874-5p | PKP1 | 1 | 1 | 2 | N |
| hsa-miR-874-5p | PI4KB | 1 | 1 | 2 | N |
| hsa-miR-874-5p | CYB5R3 | 1 | 1 | 2 | N |
| hsa-miR-874-5p | TMEM79 | 1 | 1 | 2 | N |
| hsa-miR-874-5p | DBN1 | 1 | 1 | 2 | N |
| hsa-miR-874-5p | ZC3H7B | 1 | 1 | 2 | N |
| hsa-miR-874-5p | GPX3 | 1 | 1 | 2 | N |
| hsa-miR-874-5p | SNPH | 1 | 1 | 2 | N |
| hsa-miR-874-5p | DNAJC24 | 1 | 1 | 2 | N |
| hsa-miR-874-5p | PDPK1 | 1 | 1 | 2 | N |
| hsa-miR-874-5p | LMO3 | 1 | 1 | 2 | N |
| hsa-miR-874-5p | FBRS | 1 | 1 | 2 | N |
| hsa-miR-874-5p | MX2 | 1 | 1 | 2 | N |
| hsa-miR-874-5p | DYRK1B | 1 | 1 | 2 | N |
| hsa-miR-874-5p | CLP1 | 1 | 1 | 2 | N |
| hsa-miR-874-5p | CD34 | 1 | 1 | 2 | N |
| hsa-miR-874-5p | MLEC | 1 | 1 | 2 | N |
| hsa-miR-874-5p | WDR82 | 1 | 1 | 2 | N |
| hsa-miR-874-5p | GABARAPL1 | 1 | 1 | 2 | N |
| hsa-miR-874-5p | HMGA1 | 1 | 1 | 2 | N |
| hsa-miR-874-5p | BMP7 | 1 | 1 | 2 | N |
| hsa-miR-874-5p | FYCO1 | 1 | 1 | 2 | N |
| hsa-miR-874-5p | ZNF512B | 1 | 1 | 2 | N |
| hsa-miR-874-5p | GPR173 | 1 | 1 | 2 | N |
| hsa-miR-874-5p | RAD23B | 1 | 1 | 2 | N |
| hsa-miR-874-5p | GNB2 | 1 | 1 | 2 | N |
| hsa-miR-874-5p | STK40 | 1 | 1 | 2 | N |
| hsa-miR-874-5p | TMEM104 | 1 | 1 | 2 | N |
| hsa-miR-874-5p | CBX1 | 1 | 1 | 2 | N |
| hsa-miR-874-5p | GRAMD2 | 1 | 1 | 2 | N |
| hsa-miR-874-5p | GZF1 | 1 | 1 | 2 | N |
| hsa-miR-874-5p | MAP3K11 | 1 | 1 | 2 | N |
| hsa-miR-874-5p | BRPF1 | 1 | 1 | 2 | N |
| hsa-miR-874-5p | PGPEP1 | 1 | 1 | 2 | N |
| hsa-miR-1301-3p | TMEM200B | 1 | 1 | 2 | N |
| hsa-miR-1301-3p | ZBTB18 | 1 | 1 | 2 | Y |
| hsa-miR-1301-3p | UBQLN3 | 1 | 1 | 2 | N |
| hsa-miR-1301-3p | KLHL31 | 1 | 1 | 2 | N |
| hsa-miR-1301-3p | FDX1L | 1 | 1 | 2 | N |
| hsa-miR-1301-3p | FAM160B1 | 1 | 1 | 2 | N |
| hsa-miR-1301-3p | NPTXR | 1 | 1 | 2 | Y |
| hsa-miR-1301-3p | PTRF | 1 | 1 | 2 | N |
| hsa-miR-1301-3p | C1QTNF9 | 1 | 1 | 2 | N |
| hsa-miR-1301-3p | FBXO11 | 1 | 1 | 2 | N |
| hsa-miR-1301-3p | SYT11 | 1 | 1 | 2 | N |
| hsa-miR-1301-3p | CRELD2 | 1 | 1 | 2 | N |
| hsa-miR-1301-3p | SLITRK4 | 1 | 1 | 2 | N |
| hsa-miR-1301-3p | ATP2B1 | 1 | 1 | 2 | N |
| hsa-miR-1301-3p | SIRPB2 | 1 | 1 | 2 | N |
| hsa-miR-1301-3p | MYBL1 | 1 | 1 | 2 | N |
| hsa-miR-1301-3p | HOXC4 | 1 | 1 | 2 | N |
| hsa-miR-1301-3p | SASH3 | 1 | 1 | 2 | N |
| hsa-miR-1301-3p | IL7 | 1 | 1 | 2 | N |
| hsa-miR-1301-3p | B3GAT1 | 1 | 1 | 2 | N |
| hsa-miR-1301-3p | SRXN1 | 1 | 1 | 2 | N |
| hsa-miR-1301-3p | SULT1E1 | 1 | 1 | 2 | N |
| hsa-miR-1301-3p | NXT2 | 1 | 1 | 2 | N |
| hsa-miR-1301-3p | RNF222 | 1 | 1 | 2 | N |
| hsa-miR-1301-3p | NPPA | 1 | 1 | 2 | N |
| hsa-miR-1301-3p | ELMOD1 | 1 | 1 | 2 | N |
| hsa-miR-1301-3p | DDX47 | 1 | 1 | 2 | N |
| hsa-miR-1301-3p | LIMA1 | 1 | 1 | 2 | N |
| hsa-miR-1301-3p | IGDCC4 | 1 | 1 | 2 | N |
| hsa-miR-1301-3p | G3BP2 | 1 | 1 | 2 | N |
| hsa-miR-1301-3p | TEDDM1 | 1 | 1 | 2 | N |
| hsa-miR-1301-3p | ANAPC11 | 1 | 1 | 2 | N |
| hsa-miR-1301-3p | MAP3K4 | 1 | 1 | 2 | N |
| hsa-miR-1301-3p | IL6ST | 1 | 1 | 2 | N |
| hsa-miR-1301-3p | KRT23 | 1 | 1 | 2 | N |
| hsa-miR-1301-3p | PHACTR3 | 1 | 1 | 2 | N |
| hsa-miR-1301-3p | ARL5A | 1 | 1 | 2 | N |
| hsa-miR-1301-3p | SKIL | 1 | 1 | 2 | Y |
| hsa-miR-1301-3p | PHF19 | 1 | 1 | 2 | Y |
| hsa-miR-1301-3p | ZNF333 | 1 | 1 | 2 | Y |
| hsa-miR-1301-3p | MBNL1 | 1 | 1 | 2 | Y |
| hsa-miR-1301-3p | RHEB | 1 | 1 | 2 | N |
| hsa-miR-1301-3p | LDLRAD2 | 1 | 1 | 2 | N |
| hsa-miR-1301-3p | C1orf65 | 1 | 1 | 2 | N |
| hsa-miR-1301-3p | DNAJA3 | 1 | 1 | 2 | N |
| hsa-miR-1301-3p | A4GNT | 1 | 1 | 2 | N |
| hsa-miR-1301-3p | RNF38 | 1 | 1 | 2 | N |
| hsa-miR-1301-3p | TUBB4A | 1 | 1 | 2 | N |
| hsa-miR-1301-3p | CDK17 | 1 | 1 | 2 | N |
| hsa-miR-1301-3p | EWSR1 | 1 | 1 | 2 | N |
| hsa-miR-1301-3p | EZH2 | 1 | 1 | 2 | N |
| hsa-miR-1301-3p | HOPX | 1 | 1 | 2 | N |
| hsa-miR-1301-3p | DNAJC27 | 1 | 1 | 2 | N |
| hsa-miR-1301-3p | RSAD2 | 1 | 1 | 2 | N |
| hsa-miR-1301-3p | NAA60 | 1 | 1 | 2 | N |
| hsa-miR-1301-3p | DNAJB11 | 1 | 1 | 2 | N |
| hsa-miR-1301-3p | DCX | 1 | 1 | 2 | N |
| hsa-miR-1301-3p | AP1S3 | 1 | 1 | 2 | N |
| hsa-miR-1301-3p | MINOS1-NBL1 | 1 | 1 | 2 | Y |
| hsa-miR-1301-3p | EYA3 | 1 | 1 | 2 | N |
| hsa-miR-1301-3p | MARCKSL1 | 1 | 1 | 2 | N |
| hsa-miR-1301-3p | RAB1A | 1 | 1 | 2 | N |
| hsa-miR-1301-3p | KMT2D | 1 | 1 | 2 | Y |
| hsa-miR-1301-3p | KIF23 | 1 | 1 | 2 | Y |
| hsa-miR-486-3p | PPARD | 1 | 1 | 2 | N |
| hsa-miR-486-3p | CLIP2 | 1 | 1 | 2 | N |
| hsa-miR-486-3p | C17orf62 | 1 | 1 | 2 | N |
| hsa-miR-486-3p | GPSM1 | 1 | 1 | 2 | N |
| hsa-miR-486-3p | SEPT3 | 1 | 1 | 2 | N |
| hsa-miR-486-3p | GBAS | 1 | 1 | 2 | N |
| hsa-miR-486-3p | CD209 | 1 | 1 | 2 | N |
| hsa-miR-486-3p | RNF41 | 1 | 1 | 2 | Y |
| hsa-miR-486-3p | ZDHHC8 | 1 | 1 | 2 | N |
| hsa-miR-486-3p | SPDEF | 1 | 1 | 2 | N |
| hsa-miR-486-3p | MICALL1 | 1 | 1 | 2 | N |
| hsa-miR-486-3p | SPIB | 1 | 1 | 2 | N |
| hsa-miR-486-3p | WNT4 | 1 | 1 | 2 | N |
| hsa-miR-486-3p | CCND3 | 1 | 1 | 2 | N |
| hsa-miR-486-3p | SORT1 | 1 | 1 | 2 | N |
| hsa-miR-486-3p | GLIS2 | 1 | 1 | 2 | N |
| hsa-miR-486-3p | PPP1R14B | 1 | 1 | 2 | N |
| hsa-miR-486-3p | LGI3 | 1 | 1 | 2 | N |
| hsa-miR-486-3p | CPLX2 | 1 | 1 | 2 | N |
| hsa-miR-486-3p | KXD1 | 1 | 1 | 2 | N |
| hsa-miR-486-3p | MAP3K11 | 1 | 1 | 2 | N |
| hsa-miR-486-3p | NPTX1 | 1 | 1 | 2 | N |
| hsa-miR-486-3p | POGK | 1 | 1 | 2 | N |
| hsa-miR-486-3p | AGO1 | 1 | 1 | 2 | N |
| hsa-miR-486-3p | IQSEC2 | 1 | 1 | 2 | N |
| hsa-miR-486-3p | SRCIN1 | 1 | 1 | 2 | Y |
| hsa-miR-486-3p | SZRD1 | 1 | 1 | 2 | Y |
| hsa-miR-486-3p | FAM101B | 1 | 1 | 2 | N |
| hsa-miR-486-3p | TRAF3 | 1 | 1 | 2 | N |
| hsa-miR-486-3p | USF1 | 1 | 1 | 2 | N |
| hsa-miR-486-3p | CTDSP2 | 1 | 1 | 2 | N |
| hsa-miR-486-3p | FLNC | 1 | 1 | 2 | N |
| hsa-miR-486-3p | KCNAB2 | 1 | 1 | 2 | N |
| hsa-miR-486-3p | C16orf70 | 1 | 1 | 2 | N |
| hsa-miR-486-3p | PDE2A | 1 | 1 | 2 | N |
| hsa-miR-486-3p | KCNQ4 | 1 | 1 | 2 | N |
| hsa-miR-486-3p | MRVI1 | 1 | 1 | 2 | N |
| hsa-miR-486-3p | SLAMF8 | 1 | 1 | 2 | N |
| hsa-miR-486-3p | ZNF512B | 1 | 1 | 2 | N |
| hsa-miR-486-3p | CTSB | 1 | 1 | 2 | N |
| hsa-miR-486-3p | HS6ST1 | 1 | 1 | 2 | N |
| hsa-miR-486-3p | TSPAN11 | 1 | 1 | 2 | N |
| hsa-miR-486-3p | PHOX2A | 1 | 1 | 2 | N |
| hsa-miR-486-3p | RNF4 | 1 | 1 | 2 | N |
| hsa-miR-486-3p | CNTNAP1 | 1 | 1 | 2 | N |
| hsa-miR-486-3p | KMT2D | 1 | 1 | 2 | Y |
| hsa-miR-486-3p | KDELR1 | 1 | 1 | 2 | N |
| hsa-miR-486-3p | ZC3H12A | 1 | 1 | 2 | N |
| hsa-miR-486-3p | SLC6A8 | 1 | 1 | 2 | N |
| hsa-miR-486-3p | MARK2 | 1 | 1 | 2 | N |
| hsa-miR-486-3p | KIF21B | 1 | 1 | 2 | N |
| hsa-miR-486-3p | MDGA1 | 1 | 1 | 2 | N |
| hsa-miR-486-3p | CACNA2D2 | 1 | 1 | 2 | Y |
| hsa-miR-486-3p | CYFIP2 | 1 | 1 | 2 | N |
| hsa-miR-486-3p | DIRAS1 | 1 | 1 | 2 | N |
| hsa-miR-486-3p | CASKIN1 | 1 | 1 | 2 | Y |
| hsa-miR-486-3p | SLC48A1 | 1 | 1 | 2 | N |
| hsa-miR-486-3p | SH3KBP1 | 1 | 1 | 2 | N |
| hsa-miR-486-3p | PLEKHO2 | 1 | 1 | 2 | N |
| hsa-miR-486-3p | CHST1 | 1 | 1 | 2 | N |
| hsa-miR-486-3p | NEFH | 1 | 1 | 2 | N |
| hsa-miR-486-3p | RIMS4 | 1 | 1 | 2 | N |
| hsa-miR-486-3p | PRRT2 | 1 | 1 | 2 | N |
| hsa-miR-486-3p | NDOR1 | 1 | 1 | 2 | N |
| hsa-miR-486-3p | PLEK | 1 | 1 | 2 | N |
| hsa-miR-486-3p | GATAD2B | 1 | 1 | 2 | N |
| hsa-miR-486-3p | TMEM104 | 1 | 1 | 2 | N |
| hsa-miR-486-3p | LRP1 | 1 | 1 | 2 | N |
| hsa-miR-486-3p | LPHN1 | 1 | 1 | 2 | N |
| hsa-miR-486-3p | NFASC | 1 | 1 | 2 | N |
| hsa-miR-486-3p | KLF12 | 1 | 1 | 2 | N |
| hsa-miR-486-3p | SNX32 | 1 | 1 | 2 | N |
| hsa-miR-486-3p | GRIN1 | 1 | 1 | 2 | N |
| hsa-miR-486-3p | TMEM178A | 1 | 1 | 2 | N |
| hsa-miR-486-3p | TP53INP2 | 1 | 1 | 2 | N |
| hsa-miR-486-3p | DMBX1 | 1 | 1 | 2 | N |
| hsa-miR-486-3p | TMEM234 | 1 | 1 | 2 | N |
| hsa-miR-486-3p | CHIT1 | 1 | 1 | 2 | N |
| hsa-miR-486-3p | JAKMIP3 | 1 | 1 | 2 | N |
| hsa-miR-486-3p | BCL7B | 1 | 1 | 2 | N |
| hsa-miR-486-3p | HRH3 | 1 | 1 | 2 | N |
| hsa-miR-486-3p | C8orf46 | 1 | 1 | 2 | N |
| hsa-miR-486-3p | KIRREL | 1 | 1 | 2 | N |
| hsa-miR-486-3p | STK10 | 1 | 1 | 2 | N |
| hsa-miR-486-3p | UPB1 | 1 | 1 | 2 | N |
| hsa-miR-486-3p | TSPAN9 | 1 | 1 | 2 | N |
| hsa-miR-486-3p | IFITM5 | 1 | 1 | 2 | N |
| hsa-miR-486-3p | PEX26 | 1 | 1 | 2 | N |
| hsa-miR-486-3p | TESK2 | 1 | 1 | 2 | N |
| hsa-miR-486-3p | ARHGAP1 | 1 | 1 | 2 | N |
| hsa-miR-486-3p | CLCF1 | 1 | 1 | 2 | N |
| hsa-miR-486-3p | FMR1 | 1 | 1 | 2 | N |
| hsa-miR-486-3p | DBF4B | 1 | 1 | 2 | N |
| hsa-miR-486-3p | TMEM164 | 1 | 1 | 2 | N |
| hsa-miR-486-3p | RNF165 | 1 | 1 | 2 | N |
| hsa-miR-486-3p | WFIKKN2 | 1 | 1 | 2 | N |
| hsa-miR-486-3p | PEMT | 1 | 1 | 2 | N |
| hsa-miR-486-3p | RPL3L | 1 | 1 | 2 | N |
| hsa-miR-486-3p | SNW1 | 1 | 1 | 2 | N |
| hsa-miR-486-3p | CBFA2T3 | 1 | 1 | 2 | N |
| hsa-miR-486-3p | GM2A | 1 | 1 | 2 | N |
| hsa-miR-486-3p | SCN2B | 1 | 1 | 2 | N |
| hsa-miR-486-3p | CTDSP1 | 1 | 1 | 2 | N |
| hsa-miR-486-3p | KLHDC7B | 1 | 1 | 2 | N |
| hsa-miR-486-3p | FAM155B | 1 | 1 | 2 | N |
| hsa-miR-486-3p | WNT5B | 1 | 1 | 2 | N |
| hsa-miR-486-3p | DAB2IP | 1 | 1 | 2 | N |
| hsa-miR-486-3p | NKD1 | 1 | 1 | 2 | N |
| hsa-miR-486-3p | PAFAH1B2 | 1 | 1 | 2 | N |
| hsa-miR-486-3p | ABCG4 | 1 | 1 | 2 | N |
| hsa-miR-486-3p | SLC9A8 | 1 | 1 | 2 | N |
| hsa-miR-486-3p | NSD1 | 1 | 1 | 2 | Y |
| hsa-miR-486-3p | IL2RB | 1 | 1 | 2 | N |
| hsa-miR-486-3p | NCKAP5L | 1 | 1 | 2 | N |
| hsa-miR-486-3p | CCND2 | 1 | 1 | 2 | N |
| hsa-miR-486-3p | SH3GLB2 | 1 | 1 | 2 | N |
| hsa-miR-486-3p | FBXO41 | 1 | 1 | 2 | N |
| hsa-miR-486-3p | CLIP3 | 1 | 1 | 2 | N |
| hsa-miR-486-3p | VSX1 | 1 | 1 | 2 | N |
| hsa-miR-486-3p | EN1 | 1 | 1 | 2 | N |
| hsa-miR-486-3p | CTNNBIP1 | 1 | 1 | 2 | N |
| hsa-miR-486-3p | GDI1 | 1 | 1 | 2 | N |
| hsa-miR-486-3p | NOTUM | 1 | 1 | 2 | N |
| hsa-miR-486-3p | VAT1 | 1 | 1 | 2 | N |
| hsa-miR-486-3p | RAP1GAP2 | 1 | 1 | 2 | N |
| hsa-miR-486-3p | ZFHX3 | 1 | 1 | 2 | Y |
| hsa-miR-486-3p | PNKD | 1 | 1 | 2 | N |
| hsa-miR-486-3p | ANKRD54 | 1 | 1 | 2 | N |
| hsa-miR-486-3p | IGSF9 | 1 | 1 | 2 | N |
| hsa-miR-486-3p | RAI14 | 1 | 1 | 2 | N |
| hsa-miR-486-3p | SLC41A1 | 1 | 1 | 2 | N |
| hsa-miR-486-3p | SNAI3 | 1 | 1 | 2 | N |
| hsa-miR-486-3p | PALM | 1 | 1 | 2 | N |
| hsa-miR-486-3p | TIE1 | 1 | 1 | 2 | N |
| hsa-miR-486-3p | ADAM19 | 1 | 1 | 2 | N |
| hsa-miR-486-3p | MED22 | 1 | 1 | 2 | N |
| hsa-miR-486-3p | TMEM235 | 1 | 1 | 2 | N |
| hsa-miR-486-3p | TTYH3 | 1 | 1 | 2 | N |
| hsa-miR-486-3p | VPREB3 | 1 | 1 | 2 | N |
| hsa-miR-486-3p | MAPK15 | 1 | 1 | 2 | N |
| hsa-miR-486-3p | SRC | 1 | 1 | 2 | N |
| hsa-miR-486-3p | TMEM229B | 1 | 1 | 2 | N |
| hsa-miR-486-3p | AGPAT1 | 1 | 1 | 2 | N |
| hsa-miR-486-3p | FAM212B | 1 | 1 | 2 | N |
| hsa-miR-486-3p | DNMBP | 1 | 1 | 2 | N |
| hsa-miR-486-3p | ERGIC1 | 1 | 1 | 2 | N |
| hsa-miR-486-3p | NPTXR | 1 | 1 | 2 | Y |
| hsa-miR-486-3p | SOX10 | 1 | 1 | 2 | N |
| hsa-miR-486-3p | VPS9D1 | 1 | 1 | 2 | N |
| hsa-miR-486-3p | HSPG2 | 1 | 1 | 2 | N |
| hsa-miR-486-3p | SLC6A9 | 1 | 1 | 2 | N |
| hsa-miR-486-3p | HIF1AN | 1 | 1 | 2 | N |
| hsa-miR-486-3p | RELT | 1 | 1 | 2 | N |
| hsa-miR-486-3p | MESDC1 | 1 | 1 | 2 | N |
| hsa-miR-486-3p | TRPC4AP | 1 | 1 | 2 | N |
| hsa-miR-486-3p | CD276 | 1 | 1 | 2 | Y |
| hsa-miR-486-3p | CDC42SE1 | 1 | 1 | 2 | N |
| hsa-miR-486-3p | NFIC | 1 | 1 | 2 | Y |
| hsa-miR-486-3p | PLCB1 | 1 | 1 | 2 | N |
| hsa-miR-486-3p | MKNK2 | 1 | 1 | 2 | Y |
| hsa-miR-486-3p | DPF2 | 1 | 1 | 2 | N |
| hsa-miR-486-3p | ELAC2 | 1 | 1 | 2 | N |
| hsa-miR-486-3p | POU2F2 | 1 | 1 | 2 | N |
| hsa-miR-486-3p | AP1B1 | 1 | 1 | 2 | N |
| hsa-miR-486-3p | NEDD8 | 1 | 1 | 2 | N |
| hsa-miR-486-3p | RGAG4 | 1 | 1 | 2 | N |
| hsa-miR-486-3p | TRAFD1 | 1 | 1 | 2 | N |
| hsa-miR-486-3p | CECR6 | 1 | 1 | 2 | N |
| hsa-miR-486-3p | E2F4 | 1 | 1 | 2 | N |
| hsa-miR-486-3p | TBC1D22B | 1 | 1 | 2 | N |
| hsa-miR-486-3p | SLC7A8 | 1 | 1 | 2 | N |
| hsa-miR-486-3p | HSPB6 | 1 | 1 | 2 | N |
| hsa-miR-486-3p | RANGAP1 | 1 | 1 | 2 | N |
| hsa-miR-486-3p | RHOF | 1 | 1 | 2 | N |
| hsa-miR-486-3p | MARCH1 | 1 | 1 | 2 | N |
| hsa-miR-486-3p | LRRC61 | 1 | 1 | 2 | N |
| hsa-miR-486-3p | CDC25B | 1 | 1 | 2 | N |
| hsa-miR-486-3p | NHLH1 | 1 | 1 | 2 | N |
| hsa-miR-486-3p | ITGA11 | 1 | 1 | 2 | N |
| hsa-miR-486-3p | NAA60 | 1 | 1 | 2 | N |
| hsa-miR-486-3p | TRIOBP | 1 | 1 | 2 | N |
| hsa-miR-486-3p | RELA | 1 | 1 | 2 | N |
| hsa-miR-486-3p | DBNDD1 | 1 | 1 | 2 | N |
| hsa-miR-486-3p | TMEM132E | 1 | 1 | 2 | N |
| hsa-miR-486-3p | ARF3 | 1 | 1 | 2 | N |
| hsa-miR-486-3p | GPC6 | 1 | 1 | 2 | N |
| hsa-miR-486-3p | STIM1 | 1 | 1 | 2 | N |
| hsa-miR-486-3p | RHOG | 1 | 1 | 2 | N |
| hsa-miR-486-3p | FXYD6 | 1 | 1 | 2 | N |
| hsa-miR-486-3p | LUZP1 | 1 | 1 | 2 | Y |
| hsa-miR-486-3p | PAK6 | 1 | 1 | 2 | N |
| hsa-miR-486-3p | PRX | 1 | 1 | 2 | N |
| hsa-miR-486-3p | TUBB | 1 | 1 | 2 | Y |
| hsa-miR-486-3p | PTGFRN | 1 | 1 | 2 | N |
| hsa-miR-486-3p | B3GALT5 | 1 | 1 | 2 | N |
| hsa-miR-486-3p | FRS3 | 1 | 1 | 2 | N |
| hsa-miR-486-3p | VASH1 | 1 | 1 | 2 | N |
| hsa-miR-486-3p | C17orf97 | 1 | 1 | 2 | N |
| hsa-miR-486-3p | MAPK3 | 1 | 1 | 2 | N |
| hsa-miR-486-3p | TMEM63C | 1 | 1 | 2 | N |
| hsa-miR-486-3p | MDM4 | 1 | 1 | 2 | Y |
| hsa-miR-486-3p | GATA4 | 1 | 1 | 2 | N |
| hsa-miR-486-3p | DLL4 | 1 | 1 | 2 | N |
| hsa-miR-486-3p | KLHDC3 | 1 | 1 | 2 | N |
| hsa-miR-486-3p | R3HDM4 | 1 | 1 | 2 | N |
| hsa-miR-486-3p | SYT2 | 1 | 1 | 2 | N |
| hsa-miR-486-3p | DLGAP4 | 1 | 1 | 2 | Y |
| hsa-miR-486-3p | DTX3 | 1 | 1 | 2 | N |
| hsa-miR-486-3p | MEP1A | 1 | 1 | 2 | N |
| hsa-miR-486-3p | VAMP2 | 1 | 1 | 2 | N |
| hsa-miR-486-3p | CALN1 | 1 | 1 | 2 | N |
| hsa-miR-486-3p | TSPAN5 | 1 | 1 | 2 | N |
| hsa-miR-486-3p | PXN | 1 | 1 | 2 | N |
| hsa-miR-486-3p | H6PD | 1 | 1 | 2 | N |
| hsa-miR-486-3p | TM9SF4 | 1 | 1 | 2 | N |
| hsa-miR-486-3p | SF3A1 | 1 | 1 | 2 | N |
| hsa-miR-486-3p | DIEXF | 1 | 1 | 2 | N |
| hsa-miR-486-3p | ULK1 | 1 | 1 | 2 | N |
| hsa-miR-486-3p | LRRC55 | 1 | 1 | 2 | N |
| hsa-miR-486-3p | DLL3 | 1 | 1 | 2 | N |
| hsa-miR-486-3p | TRIM62 | 1 | 1 | 2 | N |
| hsa-miR-486-3p | ADH6 | 1 | 1 | 2 | N |
| hsa-miR-486-3p | ATF3 | 1 | 1 | 2 | N |
| hsa-miR-486-3p | MAP3K10 | 1 | 1 | 2 | N |
| hsa-miR-486-3p | FLOT2 | 1 | 1 | 2 | N |
| hsa-miR-486-3p | FAM131C | 1 | 1 | 2 | N |
| hsa-miR-486-3p | ARRB2 | 1 | 1 | 2 | N |
| hsa-miR-486-3p | FAM102A | 1 | 1 | 2 | N |
| hsa-miR-486-3p | NGFR | 1 | 1 | 2 | N |
| hsa-miR-486-3p | SCRIB | 1 | 1 | 2 | N |
| hsa-miR-486-3p | HOMER2 | 1 | 1 | 2 | N |
| hsa-miR-486-3p | ANP32A | 1 | 1 | 2 | N |
| hsa-miR-486-3p | TXLNA | 1 | 1 | 2 | Y |
| hsa-miR-486-3p | PTMS | 1 | 1 | 2 | Y |
| hsa-miR-486-3p | PACS2 | 1 | 1 | 2 | N |
| hsa-miR-486-3p | GRM4 | 1 | 1 | 2 | N |
| hsa-miR-486-3p | PIK3R2 | 1 | 1 | 2 | N |
| hsa-miR-486-3p | ECM1 | 1 | 1 | 2 | Y |
| hsa-miR-486-3p | ENTPD2 | 1 | 1 | 2 | N |
| hsa-miR-486-3p | VOPP1 | 1 | 1 | 2 | N |
| hsa-miR-486-3p | SLC39A13 | 1 | 1 | 2 | N |
| hsa-miR-486-3p | ALDH3B2 | 1 | 1 | 2 | N |
| hsa-miR-486-3p | RAB2B | 1 | 1 | 2 | N |
| hsa-miR-486-3p | ITGB2 | 1 | 1 | 2 | N |
| hsa-miR-486-3p | IMPDH1 | 1 | 1 | 2 | N |
| hsa-miR-486-3p | CELSR3 | 1 | 1 | 2 | N |
| hsa-miR-486-3p | UAP1L1 | 1 | 1 | 2 | N |
| hsa-miR-486-3p | STK32B | 1 | 1 | 2 | N |
| hsa-miR-486-3p | GIMAP4 | 1 | 1 | 2 | N |
| hsa-miR-486-3p | S100A10 | 1 | 1 | 2 | N |
| hsa-miR-486-3p | PDGFRB | 1 | 1 | 2 | N |
| hsa-miR-486-3p | AATK | 1 | 1 | 2 | N |
| hsa-miR-486-3p | SNPH | 1 | 1 | 2 | N |
| hsa-miR-486-3p | HES7 | 1 | 1 | 2 | N |
| hsa-miR-486-3p | SURF4 | 1 | 1 | 2 | N |
| hsa-miR-486-3p | TRABD2B | 1 | 1 | 2 | N |
| hsa-miR-486-3p | MCM5 | 1 | 1 | 2 | N |
| hsa-miR-486-3p | SLC43A2 | 1 | 1 | 2 | N |
| hsa-miR-486-3p | ZCCHC13 | 1 | 1 | 2 | N |
| hsa-miR-486-3p | SBK1 | 1 | 1 | 2 | Y |
| hsa-miR-486-3p | CNIH2 | 1 | 1 | 2 | N |
| hsa-miR-486-3p | BRF1 | 1 | 1 | 2 | N |
| hsa-miR-486-3p | SCN1B | 1 | 1 | 2 | N |
| hsa-miR-486-3p | DCAF7 | 1 | 1 | 2 | N |
| hsa-miR-486-3p | CSRNP1 | 1 | 1 | 2 | N |
| hsa-miR-486-3p | GRM2 | 1 | 1 | 2 | N |
| hsa-miR-486-3p | TBC1D13 | 1 | 1 | 2 | N |
| hsa-miR-486-3p | IGSF5 | 1 | 1 | 2 | N |
| hsa-miR-486-3p | DGAT1 | 1 | 1 | 2 | N |
| hsa-miR-486-3p | NACC2 | 1 | 1 | 2 | Y |
| hsa-miR-486-3p | MAP1LC3A | 1 | 1 | 2 | N |
| hsa-miR-486-3p | TP73 | 1 | 1 | 2 | N |
| hsa-miR-486-3p | SPTBN4 | 1 | 1 | 2 | N |
| hsa-miR-486-3p | PVRL1 | 1 | 1 | 2 | N |
| hsa-miR-486-3p | SDC3 | 1 | 1 | 2 | N |
| hsa-miR-486-3p | CBX6 | 1 | 1 | 2 | N |
| hsa-miR-486-3p | ARID1B | 1 | 1 | 2 | N |
| hsa-miR-486-3p | KCNJ3 | 1 | 1 | 2 | N |
| hsa-miR-486-3p | ZNF710 | 1 | 1 | 2 | N |
| hsa-miR-486-3p | FOSL2 | 1 | 1 | 2 | N |
| hsa-miR-486-3p | CCDC97 | 1 | 1 | 2 | N |
| hsa-miR-486-3p | TFE3 | 1 | 1 | 2 | N |
| hsa-miR-486-3p | PTK2B | 1 | 1 | 2 | N |
| hsa-miR-486-3p | CRTC3 | 1 | 1 | 2 | N |
| hsa-miR-486-3p | ATXN7L3 | 1 | 1 | 2 | Y |
| hsa-miR-486-3p | ZNF772 | 1 | 1 | 2 | N |
| hsa-miR-486-3p | HCN3 | 1 | 1 | 2 | N |
