# Supplementary material for: circRNA circ_102049 Implicates in Pancreatic Ductal Adenocarcinoma Progression through Activating CD80 by Targeting miR-455-3p
Source: Mediators Inflamm. 2021 Jan 7;2021:8819990. doi: 10.1155/2021/8819990 (PMC7811564; doi:10.1155/2021/8819990)
Supplement: Supplementary 11 — Table S9 The inflammation-associated genes in the GeneCards database. [file 8819990.f11.doc]

**Supplementary Table S9: The inflammation-associated genes in the Genecards database.**

| Gene Symbol | Description | Relevance score |
| --- | --- | --- |
| IL6 | Interleukin 6 | 91.93 |
| TNF | Tumor Necrosis Factor | 88.14 |
| IL10 | Interleukin 10 | 79.85 |
| TLR4 | Toll Like Receptor 4 | 70.91 |
| NLRP3 | NLR Family Pyrin Domain Containing 3 | 63.56 |
| NOD2 | Nucleotide Binding Oligomerization Domain Containing 2 | 58.79 |
| TLR2 | Toll Like Receptor 2 | 54.44 |
| HLA-DRB1 | Major Histocompatibility Complex, Class II, DR Beta 1 | 54.42 |
| IL1RN | Interleukin 1 Receptor Antagonist | 53.38 |
| CRP | C-Reactive Protein | 50 |
| PRTN3 | Proteinase 3 | 47.62 |
| IL13 | Interleukin 13 | 47.09 |
| HLA-B | Major Histocompatibility Complex, Class I, B | 46.92 |
| TNFRSF1A | TNF Receptor Superfamily Member 1A | 46.24 |
| CXCL8 | C-X-C Motif Chemokine Ligand 8 | 46.11 |
| MEFV | MEFV Innate Immuity Regulator, Pyrin | 45.81 |
| ELANE | Elastase, Neutrophil Expressed | 45.53 |
| IL1B | Interleukin 1 Beta | 45.16 |
| CTLA4 | Cytotoxic T-Lymphocyte Associated Protein 4 | 44.73 |
| FOXP3 | Forkhead Box P3 | 43.67 |
| IL23R | Interleukin 23 Receptor | 40.4 |
| FAS | Fas Cell Surface Death Receptor | 39.31 |
| TGFB1 | Transforming Growth Factor Beta 1 | 38.7 |
| PTPN22 | Protein Tyrosine Phosphatase Non-Receptor Type 22 | 37.39 |
| TLR3 | Toll Like Receptor 3 | 37.22 |
| IL10RA | Interleukin 10 Receptor Subunit Alpha | 36.82 |
| STAT1 | Signal Transducer And Activator Of Transcription 1 | 36.26 |
| IL17A | Interleukin 17A | 35.58 |
| CCR5 | C-C Motif Chemokine Receptor 5 | 35.53 |
| STAT3 | Signal Transducer And Activator Of Transcription 3 | 34.83 |
| IFNG | Interferon Gamma | 34.59 |
| CD40LG | CD40 Ligand | 34.57 |
| CCL11 | C-C Motif Chemokine Ligand 11 | 34.22 |
| FASLG | Fas Ligand | 34.15 |
| CCL2 | C-C Motif Chemokine Ligand 2 | 33.25 |
| HLA-DQB1 | Major Histocompatibility Complex, Class II, DQ Beta 1 | 33.13 |
| HLA-DPB1 | Major Histocompatibility Complex, Class II, DP Beta 1 | 32.61 |
| IRF5 | Interferon Regulatory Factor 5 | 32.4 |
| NLRP1 | NLR Family Pyrin Domain Containing 1 | 32.29 |
| IL4 | Interleukin 4 | 31.73 |
| MBL2 | Mannose Binding Lectin 2 | 31.69 |
| HLA-A | Major Histocompatibility Complex, Class I, A | 31.29 |
| ALB | Albumin | 31.18 |
| LTA | Lymphotoxin Alpha | 31.09 |
| CCR6 | C-C Motif Chemokine Receptor 6 | 30.82 |
| ABCB1 | ATP Binding Cassette Subfamily B Member 1 | 30.4 |
| STAT4 | Signal Transducer And Activator Of Transcription 4 | 30.17 |
| SERPINA1 | Serpin Family A Member 1 | 30.1 |
| PSTPIP1 | Proline-Serine-Threonine Phosphatase Interacting Protein 1 | 30.07 |
| TNFAIP3 | TNF Alpha Induced Protein 3 | 29.94 |
| ITGAM | Integrin Subunit Alpha M | 29.92 |
| ICAM1 | Intercellular Adhesion Molecule 1 | 29.86 |
| IL10RB | Interleukin 10 Receptor Subunit Beta | 29.74 |
| MPO | Myeloperoxidase | 29.68 |
| TP53 | Tumor Protein P53 | 29.42 |
| CIITA | Class II Major Histocompatibility Complex Transactivator | 29.36 |
| FCGR2A | Fc Fragment Of IgG Receptor IIa | 28.91 |
| MMP1 | Matrix Metallopeptidase 1 | 28.88 |
| IL18 | Interleukin 18 | 28.57 |
| HLA-DQA1 | Major Histocompatibility Complex, Class II, DQ Alpha 1 | 28.5 |
| LACC1 | Laccase Domain Containing 1 | 28.29 |
| ITGB2 | Integrin Subunit Beta 2 | 28.27 |
| MVK | Mevalonate Kinase | 28.2 |
| IRGM | Immunity Related GTPase M | 27.85 |
| MMP9 | Matrix Metallopeptidase 9 | 27.35 |
| MIR155 | MicroRNA 155 | 27.34 |
| PTGS2 | Prostaglandin-Endoperoxide Synthase 2 | 27.24 |
| CCR1 | C-C Motif Chemokine Receptor 1 | 27.21 |
| HLA-DPA1 | Major Histocompatibility Complex, Class II, DP Alpha 1 | 27.06 |
| PLG | Plasminogen | 26.91 |
| IL12RB1 | Interleukin 12 Receptor Subunit Beta 1 | 26.87 |
| ERAP1 | Endoplasmic Reticulum Aminopeptidase 1 | 26.87 |
| CFTR | CF Transmembrane Conductance Regulator | 26.85 |
| IL1A | Interleukin 1 Alpha | 26.82 |
| NLRC4 | NLR Family CARD Domain Containing 4 | 26.75 |
| IL5 | Interleukin 5 | 26.66 |
| MIR21 | MicroRNA 21 | 26.63 |
| IL2 | Interleukin 2 | 26.55 |
| VCAM1 | Vascular Cell Adhesion Molecule 1 | 26.42 |
| ATG16L1 | Autophagy Related 16 Like 1 | 26.19 |
| MYD88 | MYD88 Innate Immune Signal Transduction Adaptor | 26.16 |
| MIF | Macrophage Migration Inhibitory Factor | 26 |
| SELE | Selectin E | 25.56 |
| SPP1 | Secreted Phosphoprotein 1 | 25.56 |
| NLRP12 | NLR Family Pyrin Domain Containing 12 | 25.33 |
| TNFRSF1B | TNF Receptor Superfamily Member 1B | 25.07 |
| ADA2 | Adenosine Deaminase 2 | 25.07 |
| IFIH1 | Interferon Induced With Helicase C Domain 1 | 24.95 |
| CCL5 | C-C Motif Chemokine Ligand 5 | 24.93 |
| TNFSF15 | TNF Superfamily Member 15 | 24.46 |
| HMGB1 | High Mobility Group Box 1 | 24.44 |
| FCGR3B | Fc Fragment Of IgG Receptor IIIb | 24.35 |
| C4A | Complement C4A (Rodgers Blood Group) | 24.29 |
| CXCL10 | C-X-C Motif Chemokine Ligand 10 | 24.28 |
| EPX | Eosinophil Peroxidase | 24.26 |
| BTNL2 | Butyrophilin Like 2 | 24.12 |
| CD4 | CD4 Molecule | 24 |
| FLG | Filaggrin | 23.99 |
| S100A9 | S100 Calcium Binding Protein A9 | 23.93 |
| CD19 | CD19 Molecule | 23.51 |
| RIPK1 | Receptor Interacting Serine/Threonine Kinase 1 | 23.45 |
| VEGFA | Vascular Endothelial Growth Factor A | 23.37 |
| IL2RA | Interleukin 2 Receptor Subunit Alpha | 23.17 |
| MUC1 | Mucin 1, Cell Surface Associated | 23.16 |
| HMOX1 | Heme Oxygenase 1 | 23.13 |
| IL21 | Interleukin 21 | 23.13 |
| DNASE1 | Deoxyribonuclease 1 | 23.12 |
| IL12A | Interleukin 12A | 22.79 |
| IL1R1 | Interleukin 1 Receptor Type 1 | 22.71 |
| RNASE3 | Ribonuclease A Family Member 3 | 22.42 |
| TLR9 | Toll Like Receptor 9 | 22.42 |
| ALOX5 | Arachidonate 5-Lipoxygenase | 22.4 |
| IRF1 | Interferon Regulatory Factor 1 | 22.24 |
| NFKB1 | Nuclear Factor Kappa B Subunit 1 | 22.19 |
| IL12B | Interleukin 12B | 22.02 |
| TBX21 | T-Box Transcription Factor 21 | 21.93 |
| AIRE | Autoimmune Regulator | 21.82 |
| C3 | Complement C3 | 21.64 |
| F2 | Coagulation Factor II, Thrombin | 21.59 |
| IKBKB | Inhibitor Of Nuclear Factor Kappa B Kinase Subunit Beta | 21.56 |
| CSF2 | Colony Stimulating Factor 2 | 21.3 |
| SLC22A4 | Solute Carrier Family 22 Member 4 | 21.02 |
| APOE | Apolipoprotein E | 20.99 |
| PADI4 | Peptidyl Arginine Deiminase 4 | 20.95 |
| MIR146A | MicroRNA 146a | 20.81 |
| SMAD3 | SMAD Family Member 3 | 20.74 |
| CD79A | CD79a Molecule | 20.74 |
| INAVA | Innate Immunity Activator | 20.74 |
| CTNNB1 | Catenin Beta 1 | 20.65 |
| DNASE1L3 | Deoxyribonuclease 1 Like 3 | 20.57 |
| CCL3 | C-C Motif Chemokine Ligand 3 | 20.52 |
| TTR | Transthyretin | 20.34 |
| SPINK5 | Serine Peptidase Inhibitor Kazal Type 5 | 20.23 |
| NOS2 | Nitric Oxide Synthase 2 | 20.18 |
| S100A8 | S100 Calcium Binding Protein A8 | 20.14 |
| INS | Insulin | 19.98 |
| FCGR2B | Fc Fragment Of IgG Receptor IIb | 19.77 |
| CD55 | CD55 Molecule (Cromer Blood Group) | 19.76 |
| OTULIN | OTU Deubiquitinase With Linear Linkage Specificity | 19.62 |
| TGFB2 | Transforming Growth Factor Beta 2 | 19.6 |
| PDCD1 | Programmed Cell Death 1 | 19.49 |
| SELP | Selectin P | 19.43 |
| ICOSLG | Inducible T Cell Costimulator Ligand | 19.39 |
| HAVCR2 | Hepatitis A Virus Cellular Receptor 2 | 19.23 |
| MUC5B | Mucin 5B, Oligomeric Mucus/Gel-Forming | 19.14 |
| CASP1 | Caspase 1 | 19.13 |
| FOXJ1 | Forkhead Box J1 | 19.02 |
| S100A12 | S100 Calcium Binding Protein A12 | 19.02 |
| CXCL1 | C-X-C Motif Chemokine Ligand 1 | 18.96 |
| SAA1 | Serum Amyloid A1 | 18.9 |
| MMP2 | Matrix Metallopeptidase 2 | 18.88 |
| RASGRP1 | RAS Guanyl Releasing Protein 1 | 18.65 |
| MST1 | Macrophage Stimulating 1 | 18.64 |
| TREX1 | Three Prime Repair Exonuclease 1 | 18.62 |
| SERPINA3 | Serpin Family A Member 3 | 18.62 |
| CXCR3 | C-X-C Motif Chemokine Receptor 3 | 18.59 |
| BDNF | Brain Derived Neurotrophic Factor | 18.59 |
| PIK3CA | Phosphatidylinositol-4,5-Bisphosphate 3-Kinase Catalytic Subunit Alpha | 18.44 |
| CTSG | Cathepsin G | 18.39 |
| CCL4 | C-C Motif Chemokine Ligand 4 | 18.36 |
| IFNA1 | Interferon Alpha 1 | 18.33 |
| WAS | WASP Actin Nucleation Promoting Factor | 18.33 |
| TLR5 | Toll Like Receptor 5 | 18.27 |
| ADIPOQ | Adiponectin, C1Q And Collagen Domain Containing | 18.13 |
| CAT | Catalase | 17.95 |
| C1S | Complement C1s | 17.95 |
| IL2RB | Interleukin 2 Receptor Subunit Beta | 17.93 |
| BACH2 | BTB Domain And CNC Homolog 2 | 17.92 |
| SELL | Selectin L | 17.88 |
| IKBKG | Inhibitor Of Nuclear Factor Kappa B Kinase Regulatory Subunit Gamma | 17.87 |
| STING1 | Stimulator Of Interferon Response CGAMP Interactor 1 | 17.77 |
| CD8A | CD8a Molecule | 17.76 |
| TIMP1 | TIMP Metallopeptidase Inhibitor 1 | 17.71 |
| HSPD1 | Heat Shock Protein Family D (Hsp60) Member 1 | 17.68 |
| MIR126 | MicroRNA 126 | 17.68 |
| IL36RN | Interleukin 36 Receptor Antagonist | 17.66 |
| MMP3 | Matrix Metallopeptidase 3 | 17.65 |
| IL33 | Interleukin 33 | 17.57 |
| TNIP1 | TNFAIP3 Interacting Protein 1 | 17.57 |
| CD40 | CD40 Molecule | 17.54 |
| CSF3 | Colony Stimulating Factor 3 | 17.47 |
| PPARG | Peroxisome Proliferator Activated Receptor Gamma | 17.47 |
| TNFRSF13C | TNF Receptor Superfamily Member 13C | 17.42 |
| AGER | Advanced Glycosylation End-Product Specific Receptor | 17.42 |
| SPINK1 | Serine Peptidase Inhibitor Kazal Type 1 | 17.42 |
| CYBA | Cytochrome B-245 Alpha Chain | 17.42 |
| CHI3L1 | Chitinase 3 Like 1 | 17.42 |
| NCF4 | Neutrophil Cytosolic Factor 4 | 17.36 |
| CCR3 | C-C Motif Chemokine Receptor 3 | 17.36 |
| HLA-G | Major Histocompatibility Complex, Class I, G | 17.33 |
| IRAK1 | Interleukin 1 Receptor Associated Kinase 1 | 17.26 |
| CD28 | CD28 Molecule | 17.15 |
| IL23A | Interleukin 23 Subunit Alpha | 17.12 |
| MIR145 | MicroRNA 145 | 17.07 |
| TSLP | Thymic Stromal Lymphopoietin | 17.06 |
| CD80 | CD80 Molecule | 17.06 |
| AKT1 | AKT Serine/Threonine Kinase 1 | 16.96 |
| ACE | Angiotensin I Converting Enzyme | 16.95 |
| TBK1 | TANK Binding Kinase 1 | 16.94 |
| CR2 | Complement C3d Receptor 2 | 16.94 |
| PTGS1 | Prostaglandin-Endoperoxide Synthase 1 | 16.81 |
| LTF | Lactotransferrin | 16.8 |
| CYBB | Cytochrome B-245 Beta Chain | 16.79 |
| IFNB1 | Interferon Beta 1 | 16.77 |
| DEFB4A | Defensin Beta 4A | 16.72 |
| IL17F | Interleukin 17F | 16.7 |
| GPT | Glutamic--Pyruvic Transaminase | 16.62 |
| PTX3 | Pentraxin 3 | 16.61 |
| KNG1 | Kininogen 1 | 16.59 |
| TERT | Telomerase Reverse Transcriptase | 16.58 |
| ANXA1 | Annexin A1 | 16.55 |
| IL4R | Interleukin 4 Receptor | 16.53 |
| KLRC4 | Killer Cell Lectin Like Receptor C4 | 16.45 |
| IL12A-AS1 | IL12A Antisense RNA 1 | 16.45 |
| LPL | Lipoprotein Lipase | 16.45 |
| ETS1 | ETS Proto-Oncogene 1, Transcription Factor | 16.45 |
| UBAC2 | UBA Domain Containing 2 | 16.38 |
| MBTPS2 | Membrane Bound Transcription Factor Peptidase, Site 2 | 16.36 |
| IL1RAPL2 | Interleukin 1 Receptor Accessory Protein Like 2 | 16.32 |
| CXCR4 | C-X-C Motif Chemokine Receptor 4 | 16.29 |
| IL15 | Interleukin 15 | 16.29 |
| HP | Haptoglobin | 16.28 |
| KRAS | KRAS Proto-Oncogene, GTPase | 16.28 |
| MIR122 | MicroRNA 122 | 16.13 |
| LEP | Leptin | 16.11 |
| CXCL2 | C-X-C Motif Chemokine Ligand 2 | 16.11 |
| C4B | Complement C4B (Chido Blood Group) | 16.1 |
| TLR6 | Toll Like Receptor 6 | 16.06 |
| LCN2 | Lipocalin 2 | 16.01 |
| IL7R | Interleukin 7 Receptor | 15.98 |
| ADA | Adenosine Deaminase | 15.97 |
| SERPINE1 | Serpin Family E Member 1 | 15.92 |
| SH2D1A | SH2 Domain Containing 1A | 15.88 |
| MIR150 | MicroRNA 150 | 15.87 |
| SAMHD1 | SAM And HD Domain Containing Deoxynucleoside Triphosphate Triphosphohydrolase 1 | 15.77 |
| TNFSF11 | TNF Superfamily Member 11 | 15.68 |
| RELA | RELA Proto-Oncogene, NF-KB Subunit | 15.65 |
| TEK | TEK Receptor Tyrosine Kinase | 15.61 |
| NR3C1 | Nuclear Receptor Subfamily 3 Group C Member 1 | 15.61 |
| TNFRSF13B | TNF Receptor Superfamily Member 13B | 15.55 |
| NCF1 | Neutrophil Cytosolic Factor 1 | 15.51 |
| RBCK1 | RANBP2-Type And C3HC4-Type Zinc Finger Containing 1 | 15.51 |
| PRSS1 | Serine Protease 1 | 15.48 |
| TNFSF12 | TNF Superfamily Member 12 | 15.35 |
| CXCL9 | C-X-C Motif Chemokine Ligand 9 | 15.3 |
| APOH | Apolipoprotein H | 15.28 |
| CXCL12 | C-X-C Motif Chemokine Ligand 12 | 15.27 |
| PSMB8 | Proteasome 20S Subunit Beta 8 | 15.26 |
| IL9 | Interleukin 9 | 15.22 |
| B2M | Beta-2-Microglobulin | 15.19 |
| ADRB2 | Adrenoceptor Beta 2 | 15.18 |
| POLA1 | DNA Polymerase Alpha 1, Catalytic Subunit | 15.18 |
| NOD1 | Nucleotide Binding Oligomerization Domain Containing 1 | 15.15 |
| NCF2 | Neutrophil Cytosolic Factor 2 | 15.12 |
| SFTPC | Surfactant Protein C | 15.09 |
| TNFSF13B | TNF Superfamily Member 13b | 15.01 |
| SLC9A3 | Solute Carrier Family 9 Member A3 | 14.98 |
| TCF4 | Transcription Factor 4 | 14.97 |
| FERMT1 | Fermitin Family Member 1 | 14.95 |
| CASR | Calcium Sensing Receptor | 14.94 |
| ADAR | Adenosine Deaminase RNA Specific | 14.91 |
| JAK2 | Janus Kinase 2 | 14.8 |
| CASP10 | Caspase 10 | 14.73 |
| MPZ | Myelin Protein Zero | 14.71 |
| PRKCD | Protein Kinase C Delta | 14.7 |
| RETN | Resistin | 14.67 |
| FLNA | Filamin A | 14.63 |
| CP | Ceruloplasmin | 14.63 |
| THBD | Thrombomodulin | 14.62 |
| SERPINB1 | Serpin Family B Member 1 | 14.61 |
| TGFBR1 | Transforming Growth Factor Beta Receptor 1 | 14.55 |
| PTPRC | Protein Tyrosine Phosphatase Receptor Type C | 14.55 |
| CASP3 | Caspase 3 | 14.53 |
| FGFR2 | Fibroblast Growth Factor Receptor 2 | 14.48 |
| CD44 | CD44 Molecule (Indian Blood Group) | 14.44 |
| ABCB4 | ATP Binding Cassette Subfamily B Member 4 | 14.38 |
| PLA2G2A | Phospholipase A2 Group IIA | 14.27 |
| DNAL1 | Dynein Axonemal Light Chain 1 | 14.26 |
| TLR7 | Toll Like Receptor 7 | 14.25 |
| SFTPA2 | Surfactant Protein A2 | 14.25 |
| IGHE | Immunoglobulin Heavy Constant Epsilon | 14.24 |
| MMEL1 | Membrane Metalloendopeptidase Like 1 | 14.19 |
| CD86 | CD86 Molecule | 14.16 |
| F2RL1 | F2R Like Trypsin Receptor 1 | 14.15 |
| GZMB | Granzyme B | 14.14 |
| SCGB1A1 | Secretoglobin Family 1A Member 1 | 14.14 |
| PPARA | Peroxisome Proliferator Activated Receptor Alpha | 14.13 |
| EPCAM | Epithelial Cell Adhesion Molecule | 14.11 |
| TGFBR2 | Transforming Growth Factor Beta Receptor 2 | 14.07 |
| ITGA4 | Integrin Subunit Alpha 4 | 14.06 |
| GDNF | Glial Cell Derived Neurotrophic Factor | 14.05 |
| EGFR | Epidermal Growth Factor Receptor | 14.02 |
| ICOS | Inducible T Cell Costimulator | 14.01 |
| NFKBIA | NFKB Inhibitor Alpha | 13.98 |
| RET | Ret Proto-Oncogene | 13.94 |
| CD244 | CD244 Molecule | 13.93 |
| IL22 | Interleukin 22 | 13.85 |
| NR1H3 | Nuclear Receptor Subfamily 1 Group H Member 3 | 13.84 |
| IL37 | Interleukin 37 | 13.83 |
| CALCA | Calcitonin Related Polypeptide Alpha | 13.83 |
| IFNGR1 | Interferon Gamma Receptor 1 | 13.8 |
| RSPH4A | Radial Spoke Head Component 4A | 13.71 |
| RSPH9 | Radial Spoke Head Component 9 | 13.71 |
| PTGDR2 | Prostaglandin D2 Receptor 2 | 13.7 |
| UBE2L3 | Ubiquitin Conjugating Enzyme E2 L3 | 13.69 |
| GPR35 | G Protein-Coupled Receptor 35 | 13.67 |
| MIR222 | MicroRNA 222 | 13.66 |
| NFKB2 | Nuclear Factor Kappa B Subunit 2 | 13.66 |
| PIK3CG | Phosphatidylinositol-4,5-Bisphosphate 3-Kinase Catalytic Subunit Gamma | 13.65 |
| TAC1 | Tachykinin Precursor 1 | 13.64 |
| CXCR2 | C-X-C Motif Chemokine Receptor 2 | 13.62 |
| MAPK8 | Mitogen-Activated Protein Kinase 8 | 13.59 |
| MIR34A | MicroRNA 34a | 13.58 |
| MAPK14 | Mitogen-Activated Protein Kinase 14 | 13.56 |
| SOCS3 | Suppressor Of Cytokine Signaling 3 | 13.55 |
| FCGR3A | Fc Fragment Of IgG Receptor IIIa | 13.55 |
| F5 | Coagulation Factor V | 13.54 |
| TLR1 | Toll Like Receptor 1 | 13.48 |
| ITGB4 | Integrin Subunit Beta 4 | 13.46 |
| PLA2G7 | Phospholipase A2 Group VII | 13.46 |
| NR1H4 | Nuclear Receptor Subfamily 1 Group H Member 4 | 13.42 |
| CSF1 | Colony Stimulating Factor 1 | 13.33 |
| SLPI | Secretory Leukocyte Peptidase Inhibitor | 13.32 |
| JUN | Jun Proto-Oncogene, AP-1 Transcription Factor Subunit | 13.31 |
| CD14 | CD14 Molecule | 13.3 |
| BPI | Bactericidal Permeability Increasing Protein | 13.3 |
| SFTPD | Surfactant Protein D | 13.29 |
| RNASEH2B | Ribonuclease H2 Subunit B | 13.29 |
| PTPN2 | Protein Tyrosine Phosphatase Non-Receptor Type 2 | 13.29 |
| ADAM17 | ADAM Metallopeptidase Domain 17 | 13.25 |
| PSMB9 | Proteasome 20S Subunit Beta 9 | 13.24 |
| COL7A1 | Collagen Type VII Alpha 1 Chain | 13.21 |
| ITGAL | Integrin Subunit Alpha L | 13.2 |
| IFNA2 | Interferon Alpha 2 | 13.2 |
| MMP8 | Matrix Metallopeptidase 8 | 13.2 |
| GGT1 | Gamma-Glutamyltransferase 1 | 13.2 |
| MRAP | Melanocortin 2 Receptor Accessory Protein | 13.19 |
| ACKR2 | Atypical Chemokine Receptor 2 | 13.15 |
| GJB2 | Gap Junction Protein Beta 2 | 13.14 |
| TNFRSF11B | TNF Receptor Superfamily Member 11b | 13.12 |
| SERPINC1 | Serpin Family C Member 1 | 13.1 |
| VWF | Von Willebrand Factor | 13.1 |
| CCR7 | C-C Motif Chemokine Receptor 7 | 13.07 |
| PRSS2 | Serine Protease 2 | 13.07 |
| F3 | Coagulation Factor III, Tissue Factor | 13.06 |
| CYBC1 | Cytochrome B-245 Chaperone 1 | 13.02 |
| HLA-C | Major Histocompatibility Complex, Class I, C | 13.02 |
| PYCARD | PYD And CARD Domain Containing | 12.99 |
| C5 | Complement C5 | 12.98 |
| DNAAF2 | Dynein Axonemal Assembly Factor 2 | 12.95 |
| IRAK4 | Interleukin 1 Receptor Associated Kinase 4 | 12.95 |
| CFI | Complement Factor I | 12.92 |
| ENG | Endoglin | 12.91 |
| KRT18 | Keratin 18 | 12.89 |
| SFTPA1 | Surfactant Protein A1 | 12.88 |
| RNF31 | Ring Finger Protein 31 | 12.87 |
| DDX58 | DExD/H-Box Helicase 58 | 12.86 |
| BLK | BLK Proto-Oncogene, Src Family Tyrosine Kinase | 12.82 |
| FGFR3 | Fibroblast Growth Factor Receptor 3 | 12.82 |
| IL16 | Interleukin 16 | 12.81 |
| CASP8 | Caspase 8 | 12.81 |
| TNFSF4 | TNF Superfamily Member 4 | 12.79 |
| LMNA | Lamin A/C | 12.78 |
| C1R | Complement C1r | 12.77 |
| ABCG2 | ATP Binding Cassette Subfamily G Member 2 (Junior Blood Group) | 12.74 |
| MIR200C | MicroRNA 200c | 12.72 |
| RTEL1 | Regulator Of Telomere Elongation Helicase 1 | 12.72 |
| XIAP | X-Linked Inhibitor Of Apoptosis | 12.7 |
| APOA1 | Apolipoprotein A1 | 12.67 |
| COL17A1 | Collagen Type XVII Alpha 1 Chain | 12.66 |
| CARD14 | Caspase Recruitment Domain Family Member 14 | 12.59 |
| IL3 | Interleukin 3 | 12.58 |
| GATA3 | GATA Binding Protein 3 | 12.58 |
| CX3CL1 | C-X3-C Motif Chemokine Ligand 1 | 12.55 |
| LPIN2 | Lipin 2 | 12.52 |
| PLEC | Plectin | 12.49 |
| CD163 | CD163 Molecule | 12.49 |
| CCL20 | C-C Motif Chemokine Ligand 20 | 12.48 |
| CDH1 | Cadherin 1 | 12.48 |
| ABCB11 | ATP Binding Cassette Subfamily B Member 11 | 12.46 |
| BGLAP | Bone Gamma-Carboxyglutamate Protein | 12.46 |
| CD274 | CD274 Molecule | 12.45 |
| IL17RA | Interleukin 17 Receptor A | 12.38 |
| MIR132 | MicroRNA 132 | 12.38 |
| CCR4 | C-C Motif Chemokine Receptor 4 | 12.37 |
| CCR2 | C-C Motif Chemokine Receptor 2 | 12.34 |
| CAMP | Cathelicidin Antimicrobial Peptide | 12.33 |
| TLR8 | Toll Like Receptor 8 | 12.32 |
| RSPH1 | Radial Spoke Head Component 1 | 12.31 |
| CTRC | Chymotrypsin C | 12.31 |
| IL6R | Interleukin 6 Receptor | 12.28 |
| NFE2L2 | Nuclear Factor, Erythroid 2 Like 2 | 12.24 |
| RNASEH2A | Ribonuclease H2 Subunit A | 12.24 |
| RNASEH2C | Ribonuclease H2 Subunit C | 12.24 |
| CD81 | CD81 Molecule | 12.24 |
| BTK | Bruton Tyrosine Kinase | 12.23 |
| VDR | Vitamin D Receptor | 12.19 |
| IRF3 | Interferon Regulatory Factor 3 | 12.18 |
| COL1A1 | Collagen Type I Alpha 1 Chain | 12.18 |
| MIR221 | MicroRNA 221 | 12.14 |
| MMP12 | Matrix Metallopeptidase 12 | 12.1 |
| CD69 | CD69 Molecule | 12.07 |
| FLNC | Filamin C | 12.07 |
| TF | Transferrin | 12.06 |
| APC | APC Regulator Of WNT Signaling Pathway | 12.05 |
| MTHFR | Methylenetetrahydrofolate Reductase | 12.02 |
| NGF | Nerve Growth Factor | 12.01 |
| MIR15B | MicroRNA 15b | 12 |
| REL | REL Proto-Oncogene, NF-KB Subunit | 12 |
| TLR10 | Toll Like Receptor 10 | 11.91 |
| TICAM1 | Toll Like Receptor Adaptor Molecule 1 | 11.87 |
| MAPK1 | Mitogen-Activated Protein Kinase 1 | 11.86 |
| H2AC18 | H2A Clustered Histone 18 | 11.83 |
| CCL17 | C-C Motif Chemokine Ligand 17 | 11.81 |
| CLEC7A | C-Type Lectin Domain Containing 7A | 11.8 |
| AQP4 | Aquaporin 4 | 11.79 |
| EDN1 | Endothelin 1 | 11.77 |
| DNAI2 | Dynein Axonemal Intermediate Chain 2 | 11.77 |
| MIR31 | MicroRNA 31 | 11.75 |
| MUC5AC | Mucin 5AC, Oligomeric Mucus/Gel-Forming | 11.75 |
| IL7 | Interleukin 7 | 11.74 |
| GHRL | Ghrelin And Obestatin Prepropeptide | 11.73 |
| ELN | Elastin | 11.73 |
| ENO1 | Enolase 1 | 11.73 |
| TERC | Telomerase RNA Component | 11.72 |
| DUOX2 | Dual Oxidase 2 | 11.7 |
| HYDIN | HYDIN Axonemal Central Pair Apparatus Protein | 11.67 |
| CXCL13 | C-X-C Motif Chemokine Ligand 13 | 11.67 |
| VIP | Vasoactive Intestinal Peptide | 11.62 |
| NME8 | NME/NM23 Family Member 8 | 11.6 |
| CCDC40 | Coiled-Coil Domain Containing 40 | 11.6 |
| DRC1 | Dynein Regulatory Complex Subunit 1 | 11.6 |
| F13A1 | Coagulation Factor XIII A Chain | 11.55 |
| CXCL5 | C-X-C Motif Chemokine Ligand 5 | 11.52 |
| PMM2 | Phosphomannomutase 2 | 11.51 |
| CX3CR1 | C-X3-C Motif Chemokine Receptor 1 | 11.51 |
| CD36 | CD36 Molecule | 11.5 |
| GJB6 | Gap Junction Protein Beta 6 | 11.47 |
| PRF1 | Perforin 1 | 11.44 |
| TTC7A | Tetratricopeptide Repeat Domain 7A | 11.41 |
| NPPB | Natriuretic Peptide B | 11.37 |
| IDO1 | Indoleamine 2,3-Dioxygenase 1 | 11.35 |
| MIR20A | MicroRNA 20a | 11.33 |
| TFRC | Transferrin Receptor | 11.32 |
| WG | Wegener Granulomatosis | 11.31 |
| CHUK | Component Of Inhibitor Of Nuclear Factor Kappa B Kinase Complex | 11.3 |
| CCL26 | C-C Motif Chemokine Ligand 26 | 11.3 |
| TGFB3 | Transforming Growth Factor Beta 3 | 11.27 |
| SLC11A1 | Solute Carrier Family 11 Member 1 | 11.26 |
| CXCR1 | C-X-C Motif Chemokine Receptor 1 | 11.26 |
| MIR223 | MicroRNA 223 | 11.24 |
| LAMC2 | Laminin Subunit Gamma 2 | 11.21 |
| PON1 | Paraoxonase 1 | 11.2 |
| LBP | Lipopolysaccharide Binding Protein | 11.18 |
| POMC | Proopiomelanocortin | 11.18 |
| LRBA | LPS Responsive Beige-Like Anchor Protein | 11.18 |
| C5AR1 | Complement C5a Receptor 1 | 11.14 |
| JAK3 | Janus Kinase 3 | 11.12 |
| HPS1 | HPS1 Biogenesis Of Lysosomal Organelles Complex 3 Subunit 1 | 11.12 |
| IGF1 | Insulin Like Growth Factor 1 | 11.11 |
| MX1 | MX Dynamin Like GTPase 1 | 11.1 |
| DNAAF1 | Dynein Axonemal Assembly Factor 1 | 11.09 |
| FGF2 | Fibroblast Growth Factor 2 | 11.07 |
| FOS | Fos Proto-Oncogene, AP-1 Transcription Factor Subunit | 11.07 |
| ABCA3 | ATP Binding Cassette Subfamily A Member 3 | 11.05 |
| SELPLG | Selectin P Ligand | 11.03 |
| CCL7 | C-C Motif Chemokine Ligand 7 | 10.98 |
| DMD | Dystrophin | 10.96 |
| DNAI1 | Dynein Axonemal Intermediate Chain 1 | 10.96 |
| CCDC103 | Coiled-Coil Domain Containing 103 | 10.96 |
| ITGA6 | Integrin Subunit Alpha 6 | 10.95 |
| CD247 | CD247 Molecule | 10.94 |
| JAK1 | Janus Kinase 1 | 10.91 |
| DNAH5 | Dynein Axonemal Heavy Chain 5 | 10.87 |
| DNAAF5 | Dynein Axonemal Assembly Factor 5 | 10.87 |
| CAPN5 | Calpain 5 | 10.84 |
| MIR142 | MicroRNA 142 | 10.84 |
| MIR192 | MicroRNA 192 | 10.84 |
| ZAP70 | Zeta Chain Of T Cell Receptor Associated Protein Kinase 70 | 10.83 |
| MECP2 | Methyl-CpG Binding Protein 2 | 10.81 |
| IL1RL1 | Interleukin 1 Receptor Like 1 | 10.81 |
| DNAH11 | Dynein Axonemal Heavy Chain 11 | 10.8 |
| CCDC114 | Coiled-Coil Domain Containing 114 | 10.8 |
| DNAAF3 | Dynein Axonemal Assembly Factor 3 | 10.8 |
| CCDC39 | Coiled-Coil Domain Containing 39 | 10.8 |
| MCIDAS | Multiciliate Differentiation And DNA Synthesis Associated Cell Cycle Protein | 10.8 |
| COMP | Cartilage Oligomeric Matrix Protein | 10.77 |
| SOD1 | Superoxide Dismutase 1 | 10.74 |
| MBP | Myelin Basic Protein | 10.73 |
| ITGAX | Integrin Subunit Alpha X | 10.73 |
| PSMB4 | Proteasome 20S Subunit Beta 4 | 10.72 |
| WIPF1 | WAS/WASL Interacting Protein Family Member 1 | 10.7 |
| PGM3 | Phosphoglucomutase 3 | 10.68 |
| TNFAIP6 | TNF Alpha Induced Protein 6 | 10.68 |
| TRPV1 | Transient Receptor Potential Cation Channel Subfamily V Member 1 | 10.66 |
| MIR143 | MicroRNA 143 | 10.63 |
| CCL13 | C-C Motif Chemokine Ligand 13 | 10.62 |
| XDH | Xanthine Dehydrogenase | 10.61 |
| IRF8 | Interferon Regulatory Factor 8 | 10.59 |
| CCL24 | C-C Motif Chemokine Ligand 24 | 10.56 |
| MOG | Myelin Oligodendrocyte Glycoprotein | 10.47 |
| SIRT1 | Sirtuin 1 | 10.43 |
| UNC93B1 | Unc-93 Homolog B1, TLR Signaling Regulator | 10.41 |
| IL11 | Interleukin 11 | 10.41 |
| APOL1 | Apolipoprotein L1 | 10.41 |
| SAA4 | Serum Amyloid A4, Constitutive | 10.41 |
| IL21R | Interleukin 21 Receptor | 10.39 |
| MMP13 | Matrix Metallopeptidase 13 | 10.38 |
| CALR | Calreticulin | 10.37 |
| SLC17A5 | Solute Carrier Family 17 Member 5 | 10.37 |
| HFE | Homeostatic Iron Regulator | 10.35 |
| TYR | Tyrosinase | 10.3 |
| LAMB3 | Laminin Subunit Beta 3 | 10.28 |
| MIR148A | MicroRNA 148a | 10.28 |
| SYK | Spleen Associated Tyrosine Kinase | 10.24 |
| CCN2 | Cellular Communication Network Factor 2 | 10.23 |
| NLRP6 | NLR Family Pyrin Domain Containing 6 | 10.22 |
| GCG | Glucagon | 10.21 |
| REG3A | Regenerating Family Member 3 Alpha | 10.16 |
| IL12RB2 | Interleukin 12 Receptor Subunit Beta 2 | 10.15 |
| MIR199A1 | MicroRNA 199a-1 | 10.14 |
| SAG | S-Antigen Visual Arrestin | 10.1 |
| NOX1 | NADPH Oxidase 1 | 10.1 |
| ATP12A | ATPase H+/K+ Transporting Non-Gastric Alpha2 Subunit | 10.1 |
| STAT6 | Signal Transducer And Activator Of Transcription 6 | 10.09 |
| HAMP | Hepcidin Antimicrobial Peptide | 10.09 |
| CDKN1A | Cyclin Dependent Kinase Inhibitor 1A | 10.09 |
| ARMC4 | Armadillo Repeat Containing 4 | 10.09 |
| ALOX15 | Arachidonate 15-Lipoxygenase | 10.07 |
| PLCG2 | Phospholipase C Gamma 2 | 10.05 |
| CPA1 | Carboxypeptidase A1 | 10.04 |
| FBN1 | Fibrillin 1 | 10.04 |
| NCSTN | Nicastrin | 10.03 |
| TNFRSF10A | TNF Receptor Superfamily Member 10a | 10.03 |
| PTH | Parathyroid Hormone | 10.03 |
| NOS3 | Nitric Oxide Synthase 3 | 10.02 |
| TACR1 | Tachykinin Receptor 1 | 10.02 |
| DNAH1 | Dynein Axonemal Heavy Chain 1 | 10.01 |
| RBP4 | Retinol Binding Protein 4 | 9.99 |
| RBP3 | Retinol Binding Protein 3 | 9.99 |
| CD27 | CD27 Molecule | 9.98 |
| MIR125A | MicroRNA 125a | 9.97 |
| PECAM1 | Platelet And Endothelial Cell Adhesion Molecule 1 | 9.95 |
| HIF1A | Hypoxia Inducible Factor 1 Subunit Alpha | 9.94 |
| CARMIL2 | Capping Protein Regulator And Myosin 1 Linker 2 | 9.93 |
| FGF23 | Fibroblast Growth Factor 23 | 9.9 |
| TRAF3 | TNF Receptor Associated Factor 3 | 9.89 |
| BCL2 | BCL2 Apoptosis Regulator | 9.87 |
| ANXA5 | Annexin A5 | 9.85 |
| CCNO | Cyclin O | 9.84 |
| DNAH9 | Dynein Axonemal Heavy Chain 9 | 9.84 |
| SPAG1 | Sperm Associated Antigen 1 | 9.84 |
| ZMYND10 | Zinc Finger MYND-Type Containing 10 | 9.84 |
| LRRC6 | Leucine Rich Repeat Containing 6 | 9.84 |
| CCDC151 | Coiled-Coil Domain Containing 151 | 9.84 |
| RSPH3 | Radial Spoke Head 3 | 9.84 |
| CFAP298 | Cilia And Flagella Associated Protein 298 | 9.84 |
| A2ML1 | Alpha-2-Macroglobulin Like 1 | 9.84 |
| SAMD9 | Sterile Alpha Motif Domain Containing 9 | 9.84 |
| LAMA3 | Laminin Subunit Alpha 3 | 9.84 |
| ERBB2 | Erb-B2 Receptor Tyrosine Kinase 2 | 9.83 |
| SOCS1 | Suppressor Of Cytokine Signaling 1 | 9.79 |
| FGF10 | Fibroblast Growth Factor 10 | 9.78 |
| TYK2 | Tyrosine Kinase 2 | 9.78 |
| GAPDH | Glyceraldehyde-3-Phosphate Dehydrogenase | 9.78 |
| USB1 | U6 SnRNA Biogenesis Phosphodiesterase 1 | 9.78 |
| HGF | Hepatocyte Growth Factor | 9.77 |
| TRIM21 | Tripartite Motif Containing 21 | 9.76 |
| DPP4 | Dipeptidyl Peptidase 4 | 9.75 |
| RELN | Reelin | 9.75 |
| TRAF3IP2 | TRAF3 Interacting Protein 2 | 9.75 |
| LRP1 | LDL Receptor Related Protein 1 | 9.75 |
| EGF | Epidermal Growth Factor | 9.72 |
| TGM2 | Transglutaminase 2 | 9.71 |
| AP1S3 | Adaptor Related Protein Complex 1 Subunit Sigma 3 | 9.68 |
| PDE4A | Phosphodiesterase 4A | 9.67 |
| CFHR2 | Complement Factor H Related 2 | 9.66 |
| ESR1 | Estrogen Receptor 1 | 9.66 |
| DSP | Desmoplakin | 9.66 |
| TAP1 | Transporter 1, ATP Binding Cassette Subfamily B Member | 9.66 |
| TPO | Thyroid Peroxidase | 9.64 |
| MTOR | Mechanistic Target Of Rapamycin Kinase | 9.64 |
| ATP4A | ATPase H+/K+ Transporting Subunit Alpha | 9.62 |
| MIR17 | MicroRNA 17 | 9.62 |
| HTR1A | 5-Hydroxytryptamine Receptor 1A | 9.62 |
| DSG1 | Desmoglein 1 | 9.6 |
| SST | Somatostatin | 9.6 |
| FAM13A | Family With Sequence Similarity 13 Member A | 9.57 |
| IL18R1 | Interleukin 18 Receptor 1 | 9.56 |
| MS4A2 | Membrane Spanning 4-Domains A2 | 9.56 |
| BANK1 | B Cell Scaffold Protein With Ankyrin Repeats 1 | 9.56 |
| PTGER2 | Prostaglandin E Receptor 2 | 9.55 |
| TPMT | Thiopurine S-Methyltransferase | 9.54 |
| IL27 | Interleukin 27 | 9.53 |
| HAVCR1 | Hepatitis A Virus Cellular Receptor 1 | 9.51 |
| TREM1 | Triggering Receptor Expressed On Myeloid Cells 1 | 9.5 |
| SOD2 | Superoxide Dismutase 2 | 9.49 |
| MIR93 | MicroRNA 93 | 9.49 |
| SERPINA6 | Serpin Family A Member 6 | 9.46 |
| POU2AF1 | POU Class 2 Homeobox Associating Factor 1 | 9.46 |
| PTEN | Phosphatase And Tensin Homolog | 9.45 |
| CCK | Cholecystokinin | 9.43 |
| BRCA2 | BRCA2 DNA Repair Associated | 9.41 |
| MICA | MHC Class I Polypeptide-Related Sequence A | 9.41 |
| GJA1 | Gap Junction Protein Alpha 1 | 9.41 |
| TJP1 | Tight Junction Protein 1 | 9.4 |
| MALAT1 | Metastasis Associated Lung Adenocarcinoma Transcript 1 | 9.37 |
| GFAP | Glial Fibrillary Acidic Protein | 9.37 |
| MIR141 | MicroRNA 141 | 9.37 |
| PSEN1 | Presenilin 1 | 9.36 |
| RIPK2 | Receptor Interacting Serine/Threonine Kinase 2 | 9.35 |
| HSPA4 | Heat Shock Protein Family A (Hsp70) Member 4 | 9.35 |
| FCGR1A | Fc Fragment Of IgG Receptor Ia | 9.34 |
| BCL6 | BCL6 Transcription Repressor | 9.33 |
| ARG1 | Arginase 1 | 9.32 |
| AFP | Alpha Fetoprotein | 9.31 |
| KRT8 | Keratin 8 | 9.3 |
| TAP2 | Transporter 2, ATP Binding Cassette Subfamily B Member | 9.3 |
| PI3 | Peptidase Inhibitor 3 | 9.3 |
| HRH1 | Histamine Receptor H1 | 9.29 |
| IL32 | Interleukin 32 | 9.28 |
| RAC1 | Rac Family Small GTPase 1 | 9.28 |
| BMP6 | Bone Morphogenetic Protein 6 | 9.28 |
| SHARPIN | SHANK Associated RH Domain Interactor | 9.25 |
| CTSC | Cathepsin C | 9.25 |
| SMAD4 | SMAD Family Member 4 | 9.24 |
| CCL22 | C-C Motif Chemokine Ligand 22 | 9.23 |
| EPRS1 | Glutamyl-Prolyl-TRNA Synthetase 1 | 9.19 |
| KLRK1 | Killer Cell Lectin Like Receptor K1 | 9.16 |
| CCR9 | C-C Motif Chemokine Receptor 9 | 9.15 |
| CXCL11 | C-X-C Motif Chemokine Ligand 11 | 9.15 |
| CTSB | Cathepsin B | 9.15 |
| CFH | Complement Factor H | 9.14 |
| DOCK8 | Dedicator Of Cytokinesis 8 | 9.14 |
| LACTB | Lactamase Beta | 9.13 |
| JAZF1 | JAZF Zinc Finger 1 | 9.12 |
| CREB1 | CAMP Responsive Element Binding Protein 1 | 9.09 |
| PRKCQ | Protein Kinase C Theta | 9.08 |
| IFNAR1 | Interferon Alpha And Beta Receptor Subunit 1 | 9.03 |
| MADCAM1 | Mucosal Vascular Addressin Cell Adhesion Molecule 1 | 9.03 |
| KRT19 | Keratin 19 | 9.02 |
| AIM2 | Absent In Melanoma 2 | 9.02 |
| FN1 | Fibronectin 1 | 9.02 |
| DPYD | Dihydropyrimidine Dehydrogenase | 9.02 |
| KIAA0319L | KIAA0319 Like | 9.02 |
| PXK | PX Domain Containing Serine/Threonine Kinase Like | 9.02 |
| CELIAC2 | Celiac Disease 2 | 9.02 |
| SLEB3 | Systemic Lupus Erythematosus Susceptibility 3 | 9.02 |
| CELIAC10 | Celiac Disease, Susceptibility To, 10 | 9.02 |
| CELIAC11 | Celiac Disease, Susceptibility To, 11 | 9.02 |
| CELIAC12 | Celiac Disease, Susceptibility To, 12 | 9.02 |
| CELIAC13 | Celiac Disease, Susceptibility To, 13 | 9.02 |
| CELIAC5 | Celiac Disease, Susceptibility To, 5 | 9.02 |
| CELIAC6 | Celiac Disease, Susceptibility To, 6 | 9.02 |
| CELIAC7 | Celiac Disease, Susceptibility To, 7 | 9.02 |
| CELIAC8 | Celiac Disease, Susceptibility To, 8 | 9.02 |
| CELIAC9 | Celiac Disease, Susceptibility To, 9 | 9.02 |
| SLEB12 | Systemic Lupus Erythematosus, Susceptibility To, 12 | 9.02 |
| SLEB13 | Systemic Lupus Erythematosus, Susceptibility To, 13 | 9.02 |
| SLEB14 | Systemic Lupus Erythematosus, Susceptiblity To, 14 | 9.02 |
| SLEB15 | Systemic Lupus Erthematosus, Susceptibility To, 15 | 9.02 |
| SLEB4 | Systemic Lupus Erythematosus, Susceptibility To, 4 | 9.02 |
| SLEB5 | Systemic Lupus Erythematosus, Susceptibility To, 5 | 9.02 |
| SLEB7 | Systemic Lupus Erythematosus, Susceptibility To, 7 | 9.02 |
| SLEB8 | Systemic Lupus Erythematosus, Susceptibility To, 8 | 9.02 |
| SULT1A3 | Sulfotransferase Family 1A Member 3 | 9.01 |
| CR1 | Complement C3b/C4b Receptor 1 (Knops Blood Group) | 9 |
| UMOD | Uromodulin | 9 |
| LCK | LCK Proto-Oncogene, Src Family Tyrosine Kinase | 8.99 |
| DEFB1 | Defensin Beta 1 | 8.98 |
| COL2A1 | Collagen Type II Alpha 1 Chain | 8.97 |
| MUC2 | Mucin 2, Oligomeric Mucus/Gel-Forming | 8.97 |
| GNE | Glucosamine (UDP-N-Acetyl)-2-Epimerase/N-Acetylmannosamine Kinase | 8.97 |
| TNFRSF4 | TNF Receptor Superfamily Member 4 | 8.96 |
| HMGCR | 3-Hydroxy-3-Methylglutaryl-CoA Reductase | 8.94 |
| DDX41 | DEAD-Box Helicase 41 | 8.92 |
| GSTM1 | Glutathione S-Transferase Mu 1 | 8.92 |
| CD46 | CD46 Molecule | 8.9 |
| GUSB | Glucuronidase Beta | 8.88 |
| ACVR1 | Activin A Receptor Type 1 | 8.87 |
| PRG2 | Proteoglycan 2, Pro Eosinophil Major Basic Protein | 8.86 |
| P2RX7 | Purinergic Receptor P2X 7 | 8.85 |
| HNMT | Histamine N-Methyltransferase | 8.85 |
| SMAD7 | SMAD Family Member 7 | 8.83 |
| LGALS1 | Galectin 1 | 8.82 |
| KRT10 | Keratin 10 | 8.81 |
| HSD3B7 | Hydroxy-Delta-5-Steroid Dehydrogenase, 3 Beta- And Steroid Delta-Isomerase 7 | 8.8 |
| CCL21 | C-C Motif Chemokine Ligand 21 | 8.79 |
| CYSLTR1 | Cysteinyl Leukotriene Receptor 1 | 8.79 |
| NFKBIL1 | NFKB Inhibitor Like 1 | 8.77 |
| GAS8 | Growth Arrest Specific 8 | 8.76 |
| TTC25 | Tetratricopeptide Repeat Domain 25 | 8.76 |
| DNAAF4 | Dynein Axonemal Assembly Factor 4 | 8.76 |
| MME | Membrane Metalloendopeptidase | 8.76 |
| KDR | Kinase Insert Domain Receptor | 8.76 |
| CFB | Complement Factor B | 8.74 |
| CARD9 | Caspase Recruitment Domain Family Member 9 | 8.71 |
| ACAN | Aggrecan | 8.71 |
| RARRES2 | Retinoic Acid Receptor Responder 2 | 8.7 |
| PROCR | Protein C Receptor | 8.69 |
| CXCR5 | C-X-C Motif Chemokine Receptor 5 | 8.67 |
| MUC16 | Mucin 16, Cell Surface Associated | 8.67 |
| CLC | Charcot-Leyden Crystal Galectin | 8.67 |
| CRLF1 | Cytokine Receptor Like Factor 1 | 8.66 |
| CST3 | Cystatin C | 8.64 |
| S100B | S100 Calcium Binding Protein B | 8.64 |
| CCL18 | C-C Motif Chemokine Ligand 18 | 8.63 |
| HSP90AA1 | Heat Shock Protein 90 Alpha Family Class A Member 1 | 8.63 |
| ACP5 | Acid Phosphatase 5, Tartrate Resistant | 8.63 |
| PLAT | Plasminogen Activator, Tissue Type | 8.62 |
| ARPC1B | Actin Related Protein 2/3 Complex Subunit 1B | 8.62 |
| TG | Thyroglobulin | 8.6 |
| CCDC65 | Coiled-Coil Domain Containing 65 | 8.6 |
| LRRC56 | Leucine Rich Repeat Containing 56 | 8.6 |
| DNAJB13 | DnaJ Heat Shock Protein Family (Hsp40) Member B13 | 8.6 |
| GAS2L2 | Growth Arrest Specific 2 Like 2 | 8.6 |
| DNAAF6 | Dynein Axonemal Assembly Factor 6 | 8.6 |
| CFAP300 | Cilia And Flagella Associated Protein 300 | 8.6 |
| PPBP | Pro-Platelet Basic Protein | 8.59 |
| NCAM1 | Neural Cell Adhesion Molecule 1 | 8.59 |
| BCL2L1 | BCL2 Like 1 | 8.58 |
| CYCS | Cytochrome C, Somatic | 8.57 |
| MIR27A | MicroRNA 27a | 8.56 |
| SKIV2L | Ski2 Like RNA Helicase | 8.55 |
| CAV1 | Caveolin 1 | 8.55 |
| POSTN | Periostin | 8.55 |
| C1QA | Complement C1q A Chain | 8.54 |
| SLC22A5 | Solute Carrier Family 22 Member 5 | 8.54 |
| CCL27 | C-C Motif Chemokine Ligand 27 | 8.52 |
| ANKH | ANKH Inorganic Pyrophosphate Transport Regulator | 8.5 |
| MIR10B | MicroRNA 10b | 8.5 |
| MIR148B | MicroRNA 148b | 8.5 |
| UCN | Urocortin | 8.49 |
| SLC39A4 | Solute Carrier Family 39 Member 4 | 8.48 |
| HLA-DOA | Major Histocompatibility Complex, Class II, DO Alpha | 8.47 |
| RSAD2 | Radical S-Adenosyl Methionine Domain Containing 2 | 8.46 |
| COMT | Catechol-O-Methyltransferase | 8.46 |
| CAPN3 | Calpain 3 | 8.45 |
| APOB | Apolipoprotein B | 8.44 |
| TIMP2 | TIMP Metallopeptidase Inhibitor 2 | 8.44 |
| CYLD | CYLD Lysine 63 Deubiquitinase | 8.42 |
| NAMPT | Nicotinamide Phosphoribosyltransferase | 8.42 |
| TNFRSF18 | TNF Receptor Superfamily Member 18 | 8.41 |
| HLA-DQA2 | Major Histocompatibility Complex, Class II, DQ Alpha 2 | 8.4 |
| ADM | Adrenomedullin | 8.39 |
| PYY | Peptide YY | 8.39 |
| SERPIND1 | Serpin Family D Member 1 | 8.39 |
| GFI1 | Growth Factor Independent 1 Transcriptional Repressor | 8.37 |
| CHIT1 | Chitinase 1 | 8.36 |
| PLA2G1B | Phospholipase A2 Group IB | 8.36 |
| F2R | Coagulation Factor II Thrombin Receptor | 8.35 |
| KITLG | KIT Ligand | 8.32 |
| AREG | Amphiregulin | 8.31 |
| KRT17 | Keratin 17 | 8.3 |
| BRINP3 | BMP/Retinoic Acid Inducible Neural Specific 3 | 8.3 |
| FCAR | Fc Fragment Of IgA Receptor | 8.29 |
| F13B | Coagulation Factor XIII B Chain | 8.28 |
| IL18BP | Interleukin 18 Binding Protein | 8.28 |
| MLX | MAX Dimerization Protein MLX | 8.27 |
| HSPG2 | Heparan Sulfate Proteoglycan 2 | 8.26 |
| DSG2 | Desmoglein 2 | 8.25 |
| MIR30A | MicroRNA 30a | 8.25 |
| MYC | MYC Proto-Oncogene, BHLH Transcription Factor | 8.25 |
| KRT14 | Keratin 14 | 8.24 |
| SPIB | Spi-B Transcription Factor | 8.23 |
| TNPO3 | Transportin 3 | 8.23 |
| CD1D | CD1d Molecule | 8.22 |
| PTGDR | Prostaglandin D2 Receptor | 8.21 |
| COL11A2 | Collagen Type XI Alpha 2 Chain | 8.21 |
| MET | MET Proto-Oncogene, Receptor Tyrosine Kinase | 8.21 |
| TAPBP | TAP Binding Protein | 8.2 |
| PIK3C2A | Phosphatidylinositol-4-Phosphate 3-Kinase Catalytic Subunit Type 2 Alpha | 8.2 |
| NR1H2 | Nuclear Receptor Subfamily 1 Group H Member 2 | 8.18 |
| GC | GC Vitamin D Binding Protein | 8.18 |
| CLDN2 | Claudin 2 | 8.18 |
| ANKRD55 | Ankyrin Repeat Domain 55 | 8.15 |
| CD59 | CD59 Molecule (CD59 Blood Group) | 8.14 |
| TRAF6 | TNF Receptor Associated Factor 6 | 8.14 |
| MAP3K7 | Mitogen-Activated Protein Kinase Kinase Kinase 7 | 8.12 |
| SFTPB | Surfactant Protein B | 8.11 |
| SIGLEC5 | Sialic Acid Binding Ig Like Lectin 5 | 8.1 |
| GAST | Gastrin | 8.1 |
| TARS1 | Threonyl-TRNA Synthetase 1 | 8.1 |
| IRF7 | Interferon Regulatory Factor 7 | 8.09 |
| PIK3R1 | Phosphoinositide-3-Kinase Regulatory Subunit 1 | 8.09 |
| COG6 | Component Of Oligomeric Golgi Complex 6 | 8.09 |
| H19 | H19 Imprinted Maternally Expressed Transcript | 8.09 |
| ORM1 | Orosomucoid 1 | 8.08 |
| LTB4R | Leukotriene B4 Receptor | 8.05 |
| AMACR | Alpha-Methylacyl-CoA Racemase | 8.05 |
| FLT1 | Fms Related Receptor Tyrosine Kinase 1 | 8.04 |
| TTC37 | Tetratricopeptide Repeat Domain 37 | 8.04 |
| GSN | Gelsolin | 8.01 |
| DDX3X | DEAD-Box Helicase 3 X-Linked | 7.99 |
| PARN | Poly(A)-Specific Ribonuclease | 7.99 |
| LTA4H | Leukotriene A4 Hydrolase | 7.99 |
| WDR19 | WD Repeat Domain 19 | 7.98 |
| KRT7 | Keratin 7 | 7.98 |
| APP | Amyloid Beta Precursor Protein | 7.98 |
| LGALS3 | Galectin 3 | 7.98 |
| IL18RAP | Interleukin 18 Receptor Accessory Protein | 7.97 |
| RAG2 | Recombination Activating 2 | 7.97 |
| MASP2 | Mannan Binding Lectin Serine Peptidase 2 | 7.97 |
| DARS2 | Aspartyl-TRNA Synthetase 2, Mitochondrial | 7.95 |
| TMEM67 | Transmembrane Protein 67 | 7.95 |
| SS3 | Sarcoidosis, Susceptibility To, 3 | 7.95 |
| CYSLTR2 | Cysteinyl Leukotriene Receptor 2 | 7.95 |
| MICB | MHC Class I Polypeptide-Related Sequence B | 7.95 |
| CDX2 | Caudal Type Homeobox 2 | 7.94 |
| ADAMTS13 | ADAM Metallopeptidase With Thrombospondin Type 1 Motif 13 | 7.94 |
| NR1I2 | Nuclear Receptor Subfamily 1 Group I Member 2 | 7.91 |
| VTN | Vitronectin | 7.91 |
| FGF7 | Fibroblast Growth Factor 7 | 7.9 |
| ANGPT1 | Angiopoietin 1 | 7.89 |
| PF4 | Platelet Factor 4 | 7.88 |
| C1QC | Complement C1q C Chain | 7.86 |
| IGES | Immunoglobulin E Concentration, Serum | 7.85 |
| CYP2C19 | Cytochrome P450 Family 2 Subfamily C Member 19 | 7.84 |
| KMT2A | Lysine Methyltransferase 2A | 7.84 |
| RFX5 | Regulatory Factor X5 | 7.83 |
| C1QB | Complement C1q B Chain | 7.83 |
| EDNRB | Endothelin Receptor Type B | 7.83 |
| HPSE | Heparanase | 7.81 |
| HECTD4 | HECT Domain E3 Ubiquitin Protein Ligase 4 | 7.81 |
| FIP1L1 | Factor Interacting With PAPOLA And CPSF1 | 7.81 |
| ICAM3 | Intercellular Adhesion Molecule 3 | 7.8 |
| IL6ST | Interleukin 6 Signal Transducer | 7.8 |
| CYP27B1 | Cytochrome P450 Family 27 Subfamily B Member 1 | 7.8 |
| ITK | IL2 Inducible T Cell Kinase | 7.79 |
| LBR | Lamin B Receptor | 7.78 |
| MALT1 | MALT1 Paracaspase | 7.77 |
| IL26 | Interleukin 26 | 7.77 |
| CYP3A4 | Cytochrome P450 Family 3 Subfamily A Member 4 | 7.77 |
| HLA-DRA | Major Histocompatibility Complex, Class II, DR Alpha | 7.76 |
| ALPL | Alkaline Phosphatase, Biomineralization Associated | 7.76 |
| HPGD | 15-Hydroxyprostaglandin Dehydrogenase | 7.76 |
| SAT1 | Spermidine/Spermine N1-Acetyltransferase 1 | 7.73 |
| MIR326 | MicroRNA 326 | 7.73 |
| KL | Klotho | 7.73 |
| PLA2G6 | Phospholipase A2 Group VI | 7.69 |
| COPA | COPI Coat Complex Subunit Alpha | 7.68 |
| RXRB | Retinoid X Receptor Beta | 7.68 |
| AOC3 | Amine Oxidase Copper Containing 3 | 7.67 |
| ADAM33 | ADAM Metallopeptidase Domain 33 | 7.66 |
| CYP7A1 | Cytochrome P450 Family 7 Subfamily A Member 1 | 7.65 |
| CFHR5 | Complement Factor H Related 5 | 7.65 |
| RELB | RELB Proto-Oncogene, NF-KB Subunit | 7.65 |
| COL6A2 | Collagen Type VI Alpha 2 Chain | 7.65 |
| PAX6 | Paired Box 6 | 7.65 |
| NPHP1 | Nephrocystin 1 | 7.64 |
| JAGN1 | Jagunal Homolog 1 | 7.64 |
| FCER2 | Fc Fragment Of IgE Receptor II | 7.64 |
| AGTR1 | Angiotensin II Receptor Type 1 | 7.64 |
| CFP | Complement Factor Properdin | 7.63 |
| PRL | Prolactin | 7.62 |
| CMA1 | Chymase 1 | 7.62 |
| ARG2 | Arginase 2 | 7.62 |
| GPIHBP1 | Glycosylphosphatidylinositol Anchored High Density Lipoprotein Binding Protein 1 | 7.61 |
| MIR196B | MicroRNA 196b | 7.6 |
| RFXANK | Regulatory Factor X Associated Ankyrin Containing Protein | 7.57 |
| RFXAP | Regulatory Factor X Associated Protein | 7.57 |
| NPHP4 | Nephrocystin 4 | 7.57 |
| CD209 | CD209 Molecule | 7.56 |
| MLH1 | MutL Homolog 1 | 7.54 |
| TNFRSF25 | TNF Receptor Superfamily Member 25 | 7.54 |
| CCR8 | C-C Motif Chemokine Receptor 8 | 7.53 |
| MUC7 | Mucin 7, Secreted | 7.53 |
| CDKN2A | Cyclin Dependent Kinase Inhibitor 2A | 7.52 |
| APOC2 | Apolipoprotein C2 | 7.52 |
| ERCC6 | ERCC Excision Repair 6, Chromatin Remodeling Factor | 7.49 |
| MIR210 | MicroRNA 210 | 7.49 |
| CPN1 | Carboxypeptidase N Subunit 1 | 7.48 |
| MYO9B | Myosin IXB | 7.48 |
| SCT | Secretin | 7.48 |
| GRN | Granulin Precursor | 7.47 |
| CYP2E1 | Cytochrome P450 Family 2 Subfamily E Member 1 | 7.44 |
| G6PD | Glucose-6-Phosphate Dehydrogenase | 7.44 |
| GUCY2C | Guanylate Cyclase 2C | 7.43 |
| ATOD3 | Dermatitis, Atopic, 3 | 7.43 |
| NOS1 | Nitric Oxide Synthase 1 | 7.43 |
| CRYAA | Crystallin Alpha A | 7.43 |
| RORC | RAR Related Orphan Receptor C | 7.42 |
| VEGFC | Vascular Endothelial Growth Factor C | 7.41 |
| FGA | Fibrinogen Alpha Chain | 7.41 |
| IL31 | Interleukin 31 | 7.4 |
| MIR214 | MicroRNA 214 | 7.4 |
| IL15RA | Interleukin 15 Receptor Subunit Alpha | 7.4 |
| IKZF1 | IKAROS Family Zinc Finger 1 | 7.38 |
| EPO | Erythropoietin | 7.38 |
| CRH | Corticotropin Releasing Hormone | 7.38 |
| PRG4 | Proteoglycan 4 | 7.37 |
| GAS6 | Growth Arrest Specific 6 | 7.37 |
| ATOD1 | Dermatitis, Atopic | 7.36 |
| ATOD5 | Dermatitis, Atopic, 5 | 7.36 |
| ATOD6 | Dermatitis, Atopic, 6 | 7.36 |
| EOE1 | Esophagitis, Eosinophilic | 7.36 |
| EOE2 | Esophagitis, Eosinophilic, 2 | 7.36 |
| AQP5 | Aquaporin 5 | 7.35 |
| FCRL3 | Fc Receptor Like 3 | 7.35 |
| KRT16 | Keratin 16 | 7.34 |
| MB | Myoglobin | 7.33 |
| CSN1S1 | Casein Alpha S1 | 7.33 |
| CLCA1 | Chloride Channel Accessory 1 | 7.33 |
| SDC1 | Syndecan 1 | 7.32 |
| TNFRSF11A | TNF Receptor Superfamily Member 11a | 7.32 |
| IFNAR2 | Interferon Alpha And Beta Receptor Subunit 2 | 7.31 |
| GSTP1 | Glutathione S-Transferase Pi 1 | 7.3 |
| INVS | Inversin | 7.28 |
| MIRLET7E | MicroRNA Let-7e | 7.27 |
| MAVS | Mitochondrial Antiviral Signaling Protein | 7.26 |
| RTN4 | Reticulon 4 | 7.26 |
| OSM | Oncostatin M | 7.26 |
| HDAC2 | Histone Deacetylase 2 | 7.25 |
| BRAF | B-Raf Proto-Oncogene, Serine/Threonine Kinase | 7.25 |
| FABP4 | Fatty Acid Binding Protein 4 | 7.25 |
| TNFSF10 | TNF Superfamily Member 10 | 7.25 |
| AZU1 | Azurocidin 1 | 7.25 |
| SLC37A4 | Solute Carrier Family 37 Member 4 | 7.24 |
| XBP1 | X-Box Binding Protein 1 | 7.24 |
| TRAF1 | TNF Receptor Associated Factor 1 | 7.21 |
| MIR125B1 | MicroRNA 125b-1 | 7.21 |
| CD58 | CD58 Molecule | 7.2 |
| FUT2 | Fucosyltransferase 2 | 7.2 |
| ALOX5AP | Arachidonate 5-Lipoxygenase Activating Protein | 7.2 |
| TXN | Thioredoxin | 7.19 |
| ANGPT2 | Angiopoietin 2 | 7.19 |
| CXCL6 | C-X-C Motif Chemokine Ligand 6 | 7.18 |
| MUC4 | Mucin 4, Cell Surface Associated | 7.16 |
| CCL8 | C-C Motif Chemokine Ligand 8 | 7.16 |
| FCER1A | Fc Fragment Of IgE Receptor Ia | 7.16 |
| MAPK3 | Mitogen-Activated Protein Kinase 3 | 7.16 |
| CLU | Clusterin | 7.15 |
| TGM1 | Transglutaminase 1 | 7.14 |
| MIR195 | MicroRNA 195 | 7.14 |
| CD68 | CD68 Molecule | 7.13 |
| STAT5A | Signal Transducer And Activator Of Transcription 5A | 7.12 |
| SLC6A4 | Solute Carrier Family 6 Member 4 | 7.12 |
| GAD2 | Glutamate Decarboxylase 2 | 7.12 |
| MIR203A | MicroRNA 203a | 7.12 |
| HCRT | Hypocretin Neuropeptide Precursor | 7.11 |
| CAMK4 | Calcium/Calmodulin Dependent Protein Kinase IV | 7.1 |
| IRF4 | Interferon Regulatory Factor 4 | 7.09 |
| CLDN1 | Claudin 1 | 7.09 |
| RAG1 | Recombination Activating 1 | 7.09 |
| MIR140 | MicroRNA 140 | 7.09 |
| TNFSF13 | TNF Superfamily Member 13 | 7.09 |
| CEACAM5 | CEA Cell Adhesion Molecule 5 | 7.08 |
| PARP1 | Poly(ADP-Ribose) Polymerase 1 | 7.08 |
| RO60 | Ro60, Y RNA Binding Protein | 7.07 |
| CSF3R | Colony Stimulating Factor 3 Receptor | 7.06 |
| DLG5 | Discs Large MAGUK Scaffold Protein 5 | 7.06 |
| MIR451A | MicroRNA 451a | 7.05 |
| LGI1 | Leucine Rich Glioma Inactivated 1 | 7.05 |
| TP63 | Tumor Protein P63 | 7.04 |
| PLA2G2D | Phospholipase A2 Group IID | 7.04 |
| SERPINF2 | Serpin Family F Member 2 | 7.03 |
| FPR1 | Formyl Peptide Receptor 1 | 7.02 |
| CDKN1B | Cyclin Dependent Kinase Inhibitor 1B | 7.02 |
| CXCL16 | C-X-C Motif Chemokine Ligand 16 | 7.02 |
| AGT | Angiotensinogen | 7.02 |
| CCND1 | Cyclin D1 | 7.01 |
| CAST | Calpastatin | 7.01 |
| LMX1B | LIM Homeobox Transcription Factor 1 Beta | 7 |
| FLT4 | Fms Related Receptor Tyrosine Kinase 4 | 6.98 |
| MIR146B | MicroRNA 146b | 6.98 |
| IL5RA | Interleukin 5 Receptor Subunit Alpha | 6.98 |
| TNNI3 | Troponin I3, Cardiac Type | 6.97 |
| MC3R | Melanocortin 3 Receptor | 6.97 |
| LIF | LIF Interleukin 6 Family Cytokine | 6.97 |
| PNPLA3 | Patatin Like Phospholipase Domain Containing 3 | 6.96 |
| GSR | Glutathione-Disulfide Reductase | 6.96 |
| FCN2 | Ficolin 2 | 6.96 |
| PCCA | Propionyl-CoA Carboxylase Subunit Alpha | 6.95 |
| IFNL3 | Interferon Lambda 3 | 6.94 |
| F8 | Coagulation Factor VIII | 6.94 |
| AHSG | Alpha 2-HS Glycoprotein | 6.93 |
| SEPSECS | Sep (O-Phosphoserine) TRNA:Sec (Selenocysteine) TRNA Synthase | 6.93 |
| DCDC2 | Doublecortin Domain Containing 2 | 6.93 |
| ISG15 | ISG15 Ubiquitin Like Modifier | 6.92 |
| CLEC16A | C-Type Lectin Domain Containing 16A | 6.92 |
| ADSL | Adenylosuccinate Lyase | 6.91 |
| CCL19 | C-C Motif Chemokine Ligand 19 | 6.91 |
| PLVAP | Plasmalemma Vesicle Associated Protein | 6.89 |
| PTGES | Prostaglandin E Synthase | 6.89 |
| BDKRB1 | Bradykinin Receptor B1 | 6.88 |
| MEN1 | Menin 1 | 6.88 |
| SLC39A8 | Solute Carrier Family 39 Member 8 | 6.87 |
| NPHP3 | Nephrocystin 3 | 6.86 |
| NKX2-3 | NK2 Homeobox 3 | 6.86 |
| IRAK3 | Interleukin 1 Receptor Associated Kinase 3 | 6.86 |
| HLA-DRB4 | Major Histocompatibility Complex, Class II, DR Beta 4 | 6.86 |
| HRH2 | Histamine Receptor H2 | 6.84 |
| NPY | Neuropeptide Y | 6.84 |
| STUB1 | STIP1 Homology And U-Box Containing Protein 1 | 6.84 |
| IGFBP3 | Insulin Like Growth Factor Binding Protein 3 | 6.84 |
| ORMDL3 | ORMDL Sphingolipid Biosynthesis Regulator 3 | 6.84 |
| NAT2 | N-Acetyltransferase 2 | 6.83 |
| RHO | Rhodopsin | 6.82 |
| MIR452 | MicroRNA 452 | 6.82 |
| IL2RG | Interleukin 2 Receptor Subunit Gamma | 6.81 |
| PIK3CD | Phosphatidylinositol-4,5-Bisphosphate 3-Kinase Catalytic Subunit Delta | 6.81 |
| ITCH | Itchy E3 Ubiquitin Protein Ligase | 6.81 |
| G6PC | Glucose-6-Phosphatase Catalytic Subunit | 6.79 |
| SCN9A | Sodium Voltage-Gated Channel Alpha Subunit 9 | 6.79 |
| OPRM1 | Opioid Receptor Mu 1 | 6.79 |
| PLAU | Plasminogen Activator, Urokinase | 6.79 |
| ITPKC | Inositol-Trisphosphate 3-Kinase C | 6.79 |
| IFI16 | Interferon Gamma Inducible Protein 16 | 6.79 |
| ADGRE5 | Adhesion G Protein-Coupled Receptor E5 | 6.77 |
| SRC | SRC Proto-Oncogene, Non-Receptor Tyrosine Kinase | 6.75 |
| COL3A1 | Collagen Type III Alpha 1 Chain | 6.75 |
| IGHM | Immunoglobulin Heavy Constant Mu | 6.75 |
| PPARGC1A | PPARG Coactivator 1 Alpha | 6.74 |
| SNCA | Synuclein Alpha | 6.74 |
| LEPR | Leptin Receptor | 6.73 |
| TCIRG1 | T Cell Immune Regulator 1, ATPase H+ Transporting V0 Subunit A3 | 6.73 |
| CEP290 | Centrosomal Protein 290 | 6.72 |
| PSC | Cholangitis, Primary Sclerosing | 6.72 |
| IBD11 | Inflammatory Bowel Disease 11 | 6.72 |
| HPRT1 | Hypoxanthine Phosphoribosyltransferase 1 | 6.71 |
| HRG | Histidine Rich Glycoprotein | 6.71 |
| BCL10 | BCL10 Immune Signaling Adaptor | 6.7 |
| MIR197 | MicroRNA 197 | 6.67 |
| MMP7 | Matrix Metallopeptidase 7 | 6.67 |
| ITGB1 | Integrin Subunit Beta 1 | 6.67 |
| TAGAP | T Cell Activation RhoGTPase Activating Protein | 6.65 |
| F2RL3 | F2R Like Thrombin Or Trypsin Receptor 3 | 6.64 |
| DPEP1 | Dipeptidase 1 | 6.64 |
| THBS1 | Thrombospondin 1 | 6.64 |
| CHIA | Chitinase Acidic | 6.64 |
| HNF4A | Hepatocyte Nuclear Factor 4 Alpha | 6.64 |
| MACIR | Macrophage Immunometabolism Regulator | 6.62 |
| MEP1B | Meprin A Subunit Beta | 6.61 |
| MIR381 | MicroRNA 381 | 6.6 |
| ERVW-1 | Endogenous Retrovirus Group W Member 1, Envelope | 6.58 |
| PRSS3 | Serine Protease 3 | 6.57 |
| DCN | Decorin | 6.56 |
| CCN6 | Cellular Communication Network Factor 6 | 6.55 |
| LDLR | Low Density Lipoprotein Receptor | 6.55 |
| IL17C | Interleukin 17C | 6.54 |
| NAT9 | N-Acetyltransferase 9 (Putative) | 6.53 |
| MIR99A | MicroRNA 99a | 6.53 |
| LY96 | Lymphocyte Antigen 96 | 6.52 |
| PRKCA | Protein Kinase C Alpha | 6.52 |
| CNR1 | Cannabinoid Receptor 1 | 6.52 |
| CDH23 | Cadherin Related 23 | 6.52 |
| UCP2 | Uncoupling Protein 2 | 6.51 |
| ITGAE | Integrin Subunit Alpha E | 6.51 |
| ENO2 | Enolase 2 | 6.51 |
| ILRUN | Inflammation And Lipid Regulator With UBA-Like And NBR1-Like Domains | 6.51 |
| ENTPD1 | Ectonucleoside Triphosphate Diphosphohydrolase 1 | 6.5 |
| DNMT1 | DNA Methyltransferase 1 | 6.49 |
| CCR10 | C-C Motif Chemokine Receptor 10 | 6.49 |
| MERTK | MER Proto-Oncogene, Tyrosine Kinase | 6.49 |
| MLXIPL | MLX Interacting Protein Like | 6.49 |
| CHRM3 | Cholinergic Receptor Muscarinic 3 | 6.49 |
| GLUL | Glutamate-Ammonia Ligase | 6.48 |
| NPSR1 | Neuropeptide S Receptor 1 | 6.48 |
| DLAT | Dihydrolipoamide S-Acetyltransferase | 6.47 |
| LMBRD1 | LMBR1 Domain Containing 1 | 6.47 |
| CLEC4C | C-Type Lectin Domain Family 4 Member C | 6.47 |
| MAPT | Microtubule Associated Protein Tau | 6.47 |
| SCGB3A2 | Secretoglobin Family 3A Member 2 | 6.45 |
| SPN | Sialophorin | 6.45 |
| MIR423 | MicroRNA 423 | 6.45 |
| IFNG-AS1 | IFNG Antisense RNA 1 | 6.44 |
| MT-ND1 | Mitochondrially Encoded NADH:Ubiquinone Oxidoreductase Core Subunit 1 | 6.44 |
| COL5A1 | Collagen Type V Alpha 1 Chain | 6.44 |
| MIR144 | MicroRNA 144 | 6.42 |
| LALBA | Lactalbumin Alpha | 6.42 |
| SDCCAG8 | SHH Signaling And Ciliogenesis Regulator SDCCAG8 | 6.41 |
| HBB | Hemoglobin Subunit Beta | 6.4 |
| MMUT | Methylmalonyl-CoA Mutase | 6.4 |
| SSB | Small RNA Binding Exonuclease Protection Factor La | 6.4 |
| CST6 | Cystatin E/M | 6.4 |
| PSENEN | Presenilin Enhancer, Gamma-Secretase Subunit | 6.39 |
| MIR22 | MicroRNA 22 | 6.39 |
| FOXC2 | Forkhead Box C2 | 6.39 |
| TOR1A | Torsin Family 1 Member A | 6.39 |
| SCNN1A | Sodium Channel Epithelial 1 Subunit Alpha | 6.39 |
| MIR483 | MicroRNA 483 | 6.38 |
| HBG2 | Hemoglobin Subunit Gamma 2 | 6.38 |
| SNRPB | Small Nuclear Ribonucleoprotein Polypeptides B And B1 | 6.38 |
| ALPK1 | Alpha Kinase 1 | 6.37 |
| GAL | Galanin And GMAP Prepropeptide | 6.37 |
| MSN | Moesin | 6.37 |
| TSPO | Translocator Protein | 6.37 |
| SPTB | Spectrin Beta, Erythrocytic | 6.37 |
| CBLIF | Cobalamin Binding Intrinsic Factor | 6.36 |
| GGTLC3 | Gamma-Glutamyltransferase Light Chain Family Member 3 | 6.36 |
| OCLN | Occludin | 6.35 |
| TGFA | Transforming Growth Factor Alpha | 6.35 |
| HRH4 | Histamine Receptor H4 | 6.34 |
| REG4 | Regenerating Family Member 4 | 6.34 |
| CDC73 | Cell Division Cycle 73 | 6.34 |
| MT-TF | Mitochondrially Encoded TRNA-Phe (UUU/C) | 6.34 |
| RPGR | Retinitis Pigmentosa GTPase Regulator | 6.33 |
| ERAP2 | Endoplasmic Reticulum Aminopeptidase 2 | 6.32 |
| PTGDS | Prostaglandin D2 Synthase | 6.32 |
| ESR2 | Estrogen Receptor 2 | 6.32 |
| BTLA | B And T Lymphocyte Associated | 6.32 |
| HLA-DRB5 | Major Histocompatibility Complex, Class II, DR Beta 5 | 6.3 |
| SERPINH1 | Serpin Family H Member 1 | 6.3 |
| MT-CYB | Mitochondrially Encoded Cytochrome B | 6.29 |
| ABCC6 | ATP Binding Cassette Subfamily C Member 6 | 6.29 |
| SRP54 | Signal Recognition Particle 54 | 6.28 |
| TPT1 | Tumor Protein, Translationally-Controlled 1 | 6.28 |
| ICAM2 | Intercellular Adhesion Molecule 2 | 6.27 |
| JAG1 | Jagged Canonical Notch Ligand 1 | 6.26 |
| CCL1 | C-C Motif Chemokine Ligand 1 | 6.26 |
| CELA3B | Chymotrypsin Like Elastase 3B | 6.25 |
| AHR | Aryl Hydrocarbon Receptor | 6.24 |
| COX5A | Cytochrome C Oxidase Subunit 5A | 6.24 |
| PAPPA | Pappalysin 1 | 6.24 |
| AQP1 | Aquaporin 1 (Colton Blood Group) | 6.24 |
| PGF | Placental Growth Factor | 6.23 |
| C4B_2 | Complement Component 4B (Chido Blood Group), Copy 2 | 6.23 |
| ALDH2 | Aldehyde Dehydrogenase 2 Family Member | 6.23 |
| ATN1 | Atrophin 1 | 6.23 |
| PIP | Prolactin Induced Protein | 6.23 |
| IL1R2 | Interleukin 1 Receptor Type 2 | 6.21 |
| GNAS | GNAS Complex Locus | 6.21 |
| IL19 | Interleukin 19 | 6.21 |
| RAC2 | Rac Family Small GTPase 2 | 6.2 |
| ABCC2 | ATP Binding Cassette Subfamily C Member 2 | 6.2 |
| DEFA5 | Defensin Alpha 5 | 6.2 |
| MIR494 | MicroRNA 494 | 6.2 |
| PIPOX | Pipecolic Acid And Sarcosine Oxidase | 6.2 |
| PLAUR | Plasminogen Activator, Urokinase Receptor | 6.19 |
| KIR3DL1 | Killer Cell Immunoglobulin Like Receptor, Three Ig Domains And Long Cytoplasmic Tail 1 | 6.19 |
| LRRK2 | Leucine Rich Repeat Kinase 2 | 6.19 |
| REN | Renin | 6.19 |
| MIR152 | MicroRNA 152 | 6.18 |
| LTC4S | Leukotriene C4 Synthase | 6.18 |
| IVL | Involucrin | 6.18 |
| RUNX2 | RUNX Family Transcription Factor 2 | 6.18 |
| MGP | Matrix Gla Protein | 6.17 |
| GNAQ | G Protein Subunit Alpha Q | 6.17 |
| PRDM1 | PR/SET Domain 1 | 6.17 |
| IRS1 | Insulin Receptor Substrate 1 | 6.17 |
| ALPP | Alkaline Phosphatase, Placental | 6.17 |
| EPHX1 | Epoxide Hydrolase 1 | 6.17 |
| SREBF1 | Sterol Regulatory Element Binding Transcription Factor 1 | 6.17 |
| LYN | LYN Proto-Oncogene, Src Family Tyrosine Kinase | 6.16 |
| MUC6 | Mucin 6, Oligomeric Mucus/Gel-Forming | 6.16 |
| MAP3K5 | Mitogen-Activated Protein Kinase Kinase Kinase 5 | 6.16 |
| CSTA | Cystatin A | 6.15 |
| MSH2 | MutS Homolog 2 | 6.15 |
| MIR106B | MicroRNA 106b | 6.15 |
| SOD3 | Superoxide Dismutase 3 | 6.15 |
| CHGA | Chromogranin A | 6.15 |
| FABP5 | Fatty Acid Binding Protein 5 | 6.14 |
| IKBKE | Inhibitor Of Nuclear Factor Kappa B Kinase Subunit Epsilon | 6.14 |
| BMP2 | Bone Morphogenetic Protein 2 | 6.13 |
| RNASEL | Ribonuclease L | 6.13 |
| MS4A1 | Membrane Spanning 4-Domains A1 | 6.13 |
| CDH26 | Cadherin 26 | 6.12 |
| SLC10A1 | Solute Carrier Family 10 Member 1 | 6.11 |
| IFITM3 | Interferon Induced Transmembrane Protein 3 | 6.11 |
| ERCC2 | ERCC Excision Repair 2, TFIIH Core Complex Helicase Subunit | 6.1 |
| SMAD2 | SMAD Family Member 2 | 6.1 |
| ATP8B1 | ATPase Phospholipid Transporting 8B1 | 6.1 |
| CD38 | CD38 Molecule | 6.09 |
| SCD | Stearoyl-CoA Desaturase | 6.09 |
| TSHR | Thyroid Stimulating Hormone Receptor | 6.08 |
| SCNN1B | Sodium Channel Epithelial 1 Subunit Beta | 6.08 |
| HRAS | HRas Proto-Oncogene, GTPase | 6.08 |
| CYP7B1 | Cytochrome P450 Family 7 Subfamily B Member 1 | 6.08 |
| FABP2 | Fatty Acid Binding Protein 2 | 6.07 |
| PIGN | Phosphatidylinositol Glycan Anchor Biosynthesis Class N | 6.07 |
| EIF2AK2 | Eukaryotic Translation Initiation Factor 2 Alpha Kinase 2 | 6.07 |
| CNTNAP2 | Contactin Associated Protein 2 | 6.06 |
| DPYSL5 | Dihydropyrimidinase Like 5 | 6.06 |
| PTGER4 | Prostaglandin E Receptor 4 | 6.06 |
| H3C14 | H3 Clustered Histone 14 | 6.06 |
| NGFR | Nerve Growth Factor Receptor | 6.06 |
| SETBP1 | SET Binding Protein 1 | 6.06 |
| TRAF3IP1 | TRAF3 Interacting Protein 1 | 6.06 |
| CEACAM6 | CEA Cell Adhesion Molecule 6 | 6.05 |
| LPO | Lactoperoxidase | 6.05 |
| HDAC9 | Histone Deacetylase 9 | 6.05 |
| OLR1 | Oxidized Low Density Lipoprotein Receptor 1 | 6.05 |
| PNP | Purine Nucleoside Phosphorylase | 6.04 |
| MRC1 | Mannose Receptor C-Type 1 | 6.04 |
| ATG5 | Autophagy Related 5 | 6.03 |
| IFNGR2 | Interferon Gamma Receptor 2 | 6.03 |
| ATM | ATM Serine/Threonine Kinase | 6.02 |
| BTD | Biotinidase | 6.02 |
| CRLF2 | Cytokine Receptor Like Factor 2 | 6.01 |
| ADAMTS5 | ADAM Metallopeptidase With Thrombospondin Type 1 Motif 5 | 6.01 |
| AMPH | Amphiphysin | 6.01 |
| PES1 | Pescadillo Ribosomal Biogenesis Factor 1 | 6.01 |
| CENPB | Centromere Protein B | 6.01 |
| RHNO1 | RAD9-HUS1-RAD1 Interacting Nuclear Orphan 1 | 6.01 |
| MUCL3 | Mucin Like 3 | 6.01 |
| ATOD7 | Dermatitis, Atopic, Susceptibility To, 7 | 6.01 |
| ATOD8 | Dermatitis, Atopic, 8 | 6.01 |
| ATOD9 | Dermatitis, Atopic, 9 | 6.01 |
| ASRT3 | Asthma-Related Traits, Susceptibility To, 3 | 6.01 |
| ASRT4 | Asthma-Related Traits, Susceptibility To, 4 | 6.01 |
| ASRT6 | Asthma-Related Traits, Susceptibility To, 6 | 6.01 |
| ASRT8 | Asthma-Related Traits, Susceptibility To, 8 | 6.01 |
| COPD | Pulmonary Disease, Chronic Obstructive, Severe Early-Onset | 6.01 |
| IBD25 | Inflammatory Bowel Disease-25 | 6.01 |
| CD34 | CD34 Molecule | 6 |
| TXK | TXK Tyrosine Kinase | 5.99 |
| IFIT1 | Interferon Induced Protein With Tetratricopeptide Repeats 1 | 5.99 |
| SLC2A10 | Solute Carrier Family 2 Member 10 | 5.99 |
| SAA2 | Serum Amyloid A2 | 5.99 |
| PTPN11 | Protein Tyrosine Phosphatase Non-Receptor Type 11 | 5.98 |
| CRHR1 | Corticotropin Releasing Hormone Receptor 1 | 5.98 |
| IFT172 | Intraflagellar Transport 172 | 5.98 |
| CLEC1A | C-Type Lectin Domain Family 1 Member A | 5.97 |
| ZC3H12A | Zinc Finger CCCH-Type Containing 12A | 5.97 |
| GSK3B | Glycogen Synthase Kinase 3 Beta | 5.96 |
| CETP | Cholesteryl Ester Transfer Protein | 5.94 |
| ST14 | ST14 Transmembrane Serine Protease Matriptase | 5.93 |
| SERPINF1 | Serpin Family F Member 1 | 5.93 |
| S100A7 | S100 Calcium Binding Protein A7 | 5.92 |
| EBI3 | Epstein-Barr Virus Induced 3 | 5.9 |
| TBX4 | T-Box Transcription Factor 4 | 5.89 |
| CLEC4M | C-Type Lectin Domain Family 4 Member M | 5.89 |
| SP140 | SP140 Nuclear Body Protein | 5.89 |
| NEAT1 | Nuclear Paraspeckle Assembly Transcript 1 | 5.89 |
| CCRL2 | C-C Motif Chemokine Receptor Like 2 | 5.88 |
| ENPP1 | Ectonucleotide Pyrophosphatase/Phosphodiesterase 1 | 5.87 |
| DCTD | DCMP Deaminase | 5.87 |
| IBSP | Integrin Binding Sialoprotein | 5.86 |
| PRKACA | Protein Kinase CAMP-Activated Catalytic Subunit Alpha | 5.85 |
| SLC10A2 | Solute Carrier Family 10 Member 2 | 5.85 |
| PNPLA2 | Patatin Like Phospholipase Domain Containing 2 | 5.85 |
| CARD11 | Caspase Recruitment Domain Family Member 11 | 5.84 |
| C3AR1 | Complement C3a Receptor 1 | 5.84 |
| GDF15 | Growth Differentiation Factor 15 | 5.84 |
| RAP1A | RAP1A, Member Of RAS Oncogene Family | 5.84 |
| PLA2G4A | Phospholipase A2 Group IVA | 5.84 |
| DSG3 | Desmoglein 3 | 5.83 |
| HSPA5 | Heat Shock Protein Family A (Hsp70) Member 5 | 5.83 |
| LCE3C | Late Cornified Envelope 3C | 5.83 |
| LCE3B | Late Cornified Envelope 3B | 5.83 |
| PRKAA1 | Protein Kinase AMP-Activated Catalytic Subunit Alpha 1 | 5.82 |
| DKC1 | Dyskerin Pseudouridine Synthase 1 | 5.82 |
| LRP2 | LDL Receptor Related Protein 2 | 5.82 |
| HLA-DMA | Major Histocompatibility Complex, Class II, DM Alpha | 5.82 |
| TFF2 | Trefoil Factor 2 | 5.82 |
| DSC2 | Desmocollin 2 | 5.82 |
| C2 | Complement C2 | 5.81 |
| GPI | Glucose-6-Phosphate Isomerase | 5.81 |
| PREP | Prolyl Endopeptidase | 5.8 |
| DYSF | Dysferlin | 5.8 |
| MIR324 | MicroRNA 324 | 5.8 |
| HSPA1L | Heat Shock Protein Family A (Hsp70) Member 1 Like | 5.79 |
| GPBAR1 | G Protein-Coupled Bile Acid Receptor 1 | 5.79 |
| CARD8 | Caspase Recruitment Domain Family Member 8 | 5.79 |
| CYP2D6 | Cytochrome P450 Family 2 Subfamily D Member 6 | 5.79 |
| PNLIP | Pancreatic Lipase | 5.76 |
| MEG3 | Maternally Expressed 3 | 5.76 |
| LYZ | Lysozyme | 5.75 |
| ZMPSTE24 | Zinc Metallopeptidase STE24 | 5.75 |
| PLP1 | Proteolipid Protein 1 | 5.75 |
| CNTF | Ciliary Neurotrophic Factor | 5.75 |
| FCN3 | Ficolin 3 | 5.75 |
| LAP3 | Leucine Aminopeptidase 3 | 5.75 |
| MIR328 | MicroRNA 328 | 5.75 |
| CLDN18 | Claudin 18 | 5.74 |
| OFD1 | OFD1 Centriole And Centriolar Satellite Protein | 5.74 |
| NR0B2 | Nuclear Receptor Subfamily 0 Group B Member 2 | 5.74 |
| ODC1 | Ornithine Decarboxylase 1 | 5.74 |
| PML | PML Nuclear Body Scaffold | 5.73 |
| SH2B3 | SH2B Adaptor Protein 3 | 5.73 |
| MIR15A | MicroRNA 15a | 5.73 |
| SPINK13 | Serine Peptidase Inhibitor Kazal Type 13 | 5.72 |
| LGALS8 | Galectin 8 | 5.72 |
| CXCR6 | C-X-C Motif Chemokine Receptor 6 | 5.71 |
| TNC | Tenascin C | 5.71 |
| GRB2 | Growth Factor Receptor Bound Protein 2 | 5.71 |
| HTRA1 | HtrA Serine Peptidase 1 | 5.71 |
| SCARB2 | Scavenger Receptor Class B Member 2 | 5.71 |
| KRT6A | Keratin 6A | 5.7 |
| COBL | Cordon-Bleu WH2 Repeat Protein | 5.69 |
| MIR23A | MicroRNA 23a | 5.69 |
| MIR29C | MicroRNA 29c | 5.69 |
| ADAMTS4 | ADAM Metallopeptidase With Thrombospondin Type 1 Motif 4 | 5.69 |
| FERMT3 | Fermitin Family Member 3 | 5.69 |
| CSF1R | Colony Stimulating Factor 1 Receptor | 5.69 |
| STAT2 | Signal Transducer And Activator Of Transcription 2 | 5.69 |
| TBXA2R | Thromboxane A2 Receptor | 5.69 |
| ITGB3 | Integrin Subunit Beta 3 | 5.68 |
| CTSL | Cathepsin L | 5.68 |
| KLK3 | Kallikrein Related Peptidase 3 | 5.68 |
| SHBG | Sex Hormone Binding Globulin | 5.68 |
| DDIT3 | DNA Damage Inducible Transcript 3 | 5.67 |
| IL25 | Interleukin 25 | 5.67 |
| APOC3 | Apolipoprotein C3 | 5.67 |
| ALMS1 | ALMS1 Centrosome And Basal Body Associated Protein | 5.67 |
| NR4A2 | Nuclear Receptor Subfamily 4 Group A Member 2 | 5.67 |
| IL17D | Interleukin 17D | 5.67 |
| MCL1 | MCL1 Apoptosis Regulator, BCL2 Family Member | 5.66 |
| GRIN2A | Glutamate Ionotropic Receptor NMDA Type Subunit 2A | 5.66 |
| TKT | Transketolase | 5.65 |
| KIT | KIT Proto-Oncogene, Receptor Tyrosine Kinase | 5.65 |
| HSPA2 | Heat Shock Protein Family A (Hsp70) Member 2 | 5.64 |
| MIR9-1 | MicroRNA 9-1 | 5.63 |
| DEFB103B | Defensin Beta 103B | 5.63 |
| MYH9 | Myosin Heavy Chain 9 | 5.63 |
| SCARB1 | Scavenger Receptor Class B Member 1 | 5.62 |
| OTC | Ornithine Carbamoyltransferase | 5.62 |
| DCXR | Dicarbonyl And L-Xylulose Reductase | 5.62 |
| SPG7 | SPG7 Matrix AAA Peptidase Subunit, Paraplegin | 5.62 |
| RING1 | Ring Finger Protein 1 | 5.62 |
| HSD17B8 | Hydroxysteroid 17-Beta Dehydrogenase 8 | 5.62 |
| SEMA6A | Semaphorin 6A | 5.62 |
| ZNF354A | Zinc Finger Protein 354A | 5.62 |
| LIN54 | Lin-54 DREAM MuvB Core Complex Component | 5.62 |
| WSCD1 | WSC Domain Containing 1 | 5.62 |
| CCDC86 | Coiled-Coil Domain Containing 86 | 5.62 |
| OR1E1 | Olfactory Receptor Family 1 Subfamily E Member 1 | 5.62 |
| PLGLB1 | Plasminogen Like B1 | 5.62 |
| USP50 | Ubiquitin Specific Peptidase 50 | 5.62 |
| FRG2C | FSHD Region Gene 2 Family Member C | 5.62 |
| HLA-DPB2 | Major Histocompatibility Complex, Class II, DP Beta 2 (Pseudogene) | 5.62 |
| MIR30D | MicroRNA 30d | 5.62 |
| MIR198 | MicroRNA 198 | 5.62 |
| MIR486-1 | MicroRNA 486-1 | 5.62 |
| RMRP | RNA Component Of Mitochondrial RNA Processing Endoribonuclease | 5.62 |
| ACKR1 | Atypical Chemokine Receptor 1 (Duffy Blood Group) | 5.62 |
| ADAMTS2 | ADAM Metallopeptidase With Thrombospondin Type 1 Motif 2 | 5.61 |
| KARS1 | Lysyl-TRNA Synthetase 1 | 5.61 |
| HLA-E | Major Histocompatibility Complex, Class I, E | 5.6 |
| NHP2 | NHP2 Ribonucleoprotein | 5.59 |
| NOP10 | NOP10 Ribonucleoprotein | 5.59 |
| HTR3A | 5-Hydroxytryptamine Receptor 3A | 5.59 |
| NLRP7 | NLR Family Pyrin Domain Containing 7 | 5.59 |
| CCN1 | Cellular Communication Network Factor 1 | 5.59 |
| CDKN2B | Cyclin Dependent Kinase Inhibitor 2B | 5.59 |
| TNFRSF8 | TNF Receptor Superfamily Member 8 | 5.58 |
| SLC29A3 | Solute Carrier Family 29 Member 3 | 5.58 |
| NQO1 | NAD(P)H Quinone Dehydrogenase 1 | 5.58 |
| DPP9 | Dipeptidyl Peptidase 9 | 5.58 |
| MIR184 | MicroRNA 184 | 5.57 |
| PANX1 | Pannexin 1 | 5.57 |
| FSTL1 | Follistatin Like 1 | 5.57 |
| MIR181A1 | MicroRNA 181a-1 | 5.56 |
| OPLAH | 5-Oxoprolinase, ATP-Hydrolysing | 5.55 |
| GH1 | Growth Hormone 1 | 5.55 |
| TNFRSF17 | TNF Receptor Superfamily Member 17 | 5.55 |
| GDF5 | Growth Differentiation Factor 5 | 5.55 |
| HLCS | Holocarboxylase Synthetase | 5.54 |
| CST9 | Cystatin 9 | 5.54 |
| PTPRN | Protein Tyrosine Phosphatase Receptor Type N | 5.54 |
| POLG | DNA Polymerase Gamma, Catalytic Subunit | 5.54 |
| MAZ | MYC Associated Zinc Finger Protein | 5.54 |
| SQSTM1 | Sequestosome 1 | 5.53 |
| SLAMF7 | SLAM Family Member 7 | 5.52 |
| TNFRSF12A | TNF Receptor Superfamily Member 12A | 5.52 |
| MT-ND6 | Mitochondrially Encoded NADH:Ubiquinone Oxidoreductase Core Subunit 6 | 5.52 |
| FASN | Fatty Acid Synthase | 5.52 |
| HOTAIR | HOX Transcript Antisense RNA | 5.52 |
| AIP | Aryl Hydrocarbon Receptor Interacting Protein | 5.52 |
| MIR149 | MicroRNA 149 | 5.52 |
| HBEGF | Heparin Binding EGF Like Growth Factor | 5.51 |
| PLA2G10 | Phospholipase A2 Group X | 5.51 |
| PRPS1 | Phosphoribosyl Pyrophosphate Synthetase 1 | 5.51 |
| CGAS | Cyclic GMP-AMP Synthase | 5.51 |
| WT1 | WT1 Transcription Factor | 5.5 |
| IQCB1 | IQ Motif Containing B1 | 5.5 |
| CEP164 | Centrosomal Protein 164 | 5.5 |
| MMP14 | Matrix Metallopeptidase 14 | 5.49 |
| NELFCD | Negative Elongation Factor Complex Member C/D | 5.49 |
| GGT2 | Gamma-Glutamyltransferase 2 | 5.49 |
| GPHN | Gephyrin | 5.49 |
| ITGA2 | Integrin Subunit Alpha 2 | 5.48 |
| DMBT1 | Deleted In Malignant Brain Tumors 1 | 5.48 |
| DST | Dystonin | 5.48 |
| IBD5 | Inflammatory Bowel Disease 5 | 5.48 |
| NTRK1 | Neurotrophic Receptor Tyrosine Kinase 1 | 5.48 |
| INSR | Insulin Receptor | 5.47 |
| AR | Androgen Receptor | 5.47 |
| NT5E | 5'-Nucleotidase Ecto | 5.47 |
| SERPINI2 | Serpin Family I Member 2 | 5.47 |
| OPTN | Optineurin | 5.47 |
| LIPC | Lipase C, Hepatic Type | 5.47 |
| MIR130A | MicroRNA 130a | 5.47 |
| MIR345 | MicroRNA 345 | 5.46 |
| MT-ND4 | Mitochondrially Encoded NADH:Ubiquinone Oxidoreductase Core Subunit 4 | 5.45 |
| IRF9 | Interferon Regulatory Factor 9 | 5.44 |
| DUOX1 | Dual Oxidase 1 | 5.43 |
| GSDMD | Gasdermin D | 5.43 |
| ITLN1 | Intelectin 1 | 5.43 |
| ADORA2B | Adenosine A2b Receptor | 5.43 |
| EGR1 | Early Growth Response 1 | 5.43 |
| LPAR1 | Lysophosphatidic Acid Receptor 1 | 5.42 |
| LORICRIN | Loricrin Cornified Envelope Precursor Protein | 5.42 |
| CYP21A2 | Cytochrome P450 Family 21 Subfamily A Member 2 | 5.42 |
| HLA-DMB | Major Histocompatibility Complex, Class II, DM Beta | 5.42 |
| TFF3 | Trefoil Factor 3 | 5.41 |
| CSK | C-Terminal Src Kinase | 5.41 |
| GP1BA | Glycoprotein Ib Platelet Subunit Alpha | 5.41 |
| CPA3 | Carboxypeptidase A3 | 5.4 |
| HLA-DRB3 | Major Histocompatibility Complex, Class II, DR Beta 3 | 5.39 |
| RNASE2 | Ribonuclease A Family Member 2 | 5.39 |
| CFLAR | CASP8 And FADD Like Apoptosis Regulator | 5.39 |
| SCNN1G | Sodium Channel Epithelial 1 Subunit Gamma | 5.39 |
| IL36G | Interleukin 36 Gamma | 5.38 |
| PROC | Protein C, Inactivator Of Coagulation Factors Va And VIIIa | 5.38 |
| COL4A3 | Collagen Type IV Alpha 3 Chain | 5.37 |
| FGF19 | Fibroblast Growth Factor 19 | 5.36 |
| GNA11 | G Protein Subunit Alpha 11 | 5.36 |
| MIR590 | MicroRNA 590 | 5.36 |
| AMBP | Alpha-1-Microglobulin/Bikunin Precursor | 5.36 |
| SLC30A8 | Solute Carrier Family 30 Member 8 | 5.35 |
| DEK | DEK Proto-Oncogene | 5.35 |
| CNTNAP1 | Contactin Associated Protein 1 | 5.35 |
| KRT1 | Keratin 1 | 5.34 |
| CYP2C9 | Cytochrome P450 Family 2 Subfamily C Member 9 | 5.34 |
| TRPA1 | Transient Receptor Potential Cation Channel Subfamily A Member 1 | 5.33 |
| KLK7 | Kallikrein Related Peptidase 7 | 5.33 |
| LCN1 | Lipocalin 1 | 5.32 |
| MIR124-1 | MicroRNA 124-1 | 5.32 |
| TINF2 | TERF1 Interacting Nuclear Factor 2 | 5.31 |
| WRAP53 | WD Repeat Containing Antisense To TP53 | 5.31 |
| TIA1 | TIA1 Cytotoxic Granule Associated RNA Binding Protein | 5.31 |
| DDB2 | Damage Specific DNA Binding Protein 2 | 5.31 |
| ASGR1 | Asialoglycoprotein Receptor 1 | 5.3 |
| MIR25 | MicroRNA 25 | 5.3 |
| IBD3 | Inflammatory Bowel Disease 3 | 5.3 |
| IBD7 | Inflammatory Bowel Disease 7 | 5.3 |
| CASP4 | Caspase 4 | 5.3 |
| EREG | Epiregulin | 5.3 |
| CHEK2 | Checkpoint Kinase 2 | 5.3 |
| ADCY10 | Adenylate Cyclase 10 | 5.3 |
| HPX | Hemopexin | 5.3 |
| VIM | Vimentin | 5.29 |
| SPARC | Secreted Protein Acidic And Cysteine Rich | 5.29 |
| MIRLET7A1 | MicroRNA Let-7a-1 | 5.29 |
| MIR424 | MicroRNA 424 | 5.29 |
| ADORA2A | Adenosine A2a Receptor | 5.29 |
| CD1A | CD1a Molecule | 5.28 |
| CD22 | CD22 Molecule | 5.28 |
| MIR346 | MicroRNA 346 | 5.28 |
| STX1A | Syntaxin 1A | 5.28 |
| CNTN2 | Contactin 2 | 5.28 |
| CNGA3 | Cyclic Nucleotide Gated Channel Subunit Alpha 3 | 5.28 |
| IBD2 | Inflammatory Bowel Disease 2 | 5.28 |
| IBD8 | Inflammatory Bowel Disease 8 | 5.28 |
| IFI27 | Interferon Alpha Inducible Protein 27 | 5.27 |
| GIMAP5 | GTPase, IMAP Family Member 5 | 5.27 |
| PHEX | Phosphate Regulating Endopeptidase Homolog X-Linked | 5.27 |
| ADRB3 | Adrenoceptor Beta 3 | 5.27 |
| RPL7 | Ribosomal Protein L7 | 5.26 |
| CD70 | CD70 Molecule | 5.26 |
| CD5 | CD5 Molecule | 5.25 |
| SREBF2 | Sterol Regulatory Element Binding Transcription Factor 2 | 5.24 |
| ACTN4 | Actinin Alpha 4 | 5.24 |
| TRIM39 | Tripartite Motif Containing 39 | 5.23 |
| ASPRV1 | Aspartic Peptidase Retroviral Like 1 | 5.23 |
| MTUS2 | Microtubule Associated Scaffold Protein 2 | 5.23 |
| MIR199B | MicroRNA 199b | 5.23 |
| CNR2 | Cannabinoid Receptor 2 | 5.22 |
| NOTCH1 | Notch Receptor 1 | 5.22 |
| ATP1A3 | ATPase Na+/K+ Transporting Subunit Alpha 3 | 5.21 |
| INPP5E | Inositol Polyphosphate-5-Phosphatase E | 5.21 |
| BLOC1S6 | Biogenesis Of Lysosomal Organelles Complex 1 Subunit 6 | 5.21 |
| SRSF6 | Serine And Arginine Rich Splicing Factor 6 | 5.21 |
| LCOR | Ligand Dependent Nuclear Receptor Corepressor | 5.21 |
| CEP43 | Centrosomal Protein 43 | 5.21 |
| MIR296 | MicroRNA 296 | 5.21 |
| MIR500A | MicroRNA 500a | 5.21 |
| IBD4 | Inflammatory Bowel Disease 4 | 5.21 |
| IBD6 | Inflammatory Bowel Disease 6 | 5.21 |
| IBD9 | Inflammatory Bowel Disease 9 | 5.21 |
| MS2 | Multiple Sclerosis, Susceptibility To, 2 | 5.21 |
| MS3 | Multiple Sclerosis, Susceptibility To, 3 | 5.21 |
| MS4 | Multiple Sclerosis, Susceptiblity To, 4 | 5.21 |
| IBD12 | Inflammatory Bowel Disease 12 | 5.21 |
| IBD15 | Inflammatory Bowel Disease-15 | 5.21 |
| IBD16 | Inflammatory Bowel Disease-16 | 5.21 |
| IBD18 | Inflammatory Bowel Disease-18 | 5.21 |
| IBD20 | Inflammatory Bowel Disease-20 | 5.21 |
| IBD21 | Inflammatory Bowel Disease-21 | 5.21 |
| IBD22 | Inflammatory Bowel Disease-22 | 5.21 |
| IBD23 | Inflammatory Bowel Disease-23 | 5.21 |
| IBD24 | Inflammatory Bowel Disease-24 | 5.21 |
| IBD26 | Inflammatory Bowel Disease-26 | 5.21 |
| IBD27 | Inflammatory Bowel Disease-27 | 5.21 |
| THY1 | Thy-1 Cell Surface Antigen | 5.2 |
| MIR185 | MicroRNA 185 | 5.19 |
| GHR | Growth Hormone Receptor | 5.19 |
| ACTB | Actin Beta | 5.19 |
| MIR181B1 | MicroRNA 181b-1 | 5.19 |
| BMP4 | Bone Morphogenetic Protein 4 | 5.19 |
| MSMO1 | Methylsterol Monooxygenase 1 | 5.19 |
| ABCG8 | ATP Binding Cassette Subfamily G Member 8 | 5.18 |
| SP1 | Sp1 Transcription Factor | 5.18 |
| IGFBP1 | Insulin Like Growth Factor Binding Protein 1 | 5.17 |
| PGR | Progesterone Receptor | 5.17 |
| MT-CO1 | Mitochondrially Encoded Cytochrome C Oxidase I | 5.17 |
| CPT2 | Carnitine Palmitoyltransferase 2 | 5.16 |
| IL13RA2 | Interleukin 13 Receptor Subunit Alpha 2 | 5.16 |
| C1orf141 | Chromosome 1 Open Reading Frame 141 | 5.16 |
| CYP4F22 | Cytochrome P450 Family 4 Subfamily F Member 22 | 5.15 |
| HGD | Homogentisate 1,2-Dioxygenase | 5.14 |
| S1PR1 | Sphingosine-1-Phosphate Receptor 1 | 5.14 |
| FGF21 | Fibroblast Growth Factor 21 | 5.14 |
| CXCL3 | C-X-C Motif Chemokine Ligand 3 | 5.14 |
| KLRD1 | Killer Cell Lectin Like Receptor D1 | 5.14 |
| STAT5B | Signal Transducer And Activator Of Transcription 5B | 5.14 |
| ENPP2 | Ectonucleotide Pyrophosphatase/Phosphodiesterase 2 | 5.13 |
| FZD4 | Frizzled Class Receptor 4 | 5.13 |
| AKR1B1 | Aldo-Keto Reductase Family 1 Member B | 5.12 |
| CCL25 | C-C Motif Chemokine Ligand 25 | 5.12 |
| C9orf72 | C9orf72-SMCR8 Complex Subunit | 5.12 |
| SLAMF1 | Signaling Lymphocytic Activation Molecule Family Member 1 | 5.12 |
| BRCA1 | BRCA1 DNA Repair Associated | 5.12 |
| MIR130B | MicroRNA 130b | 5.12 |
| OXT | Oxytocin/Neurophysin I Prepropeptide | 5.12 |
| CERS3 | Ceramide Synthase 3 | 5.12 |
| APEX1 | Apurinic/Apyrimidinic Endodeoxyribonuclease 1 | 5.11 |
| CYP1A1 | Cytochrome P450 Family 1 Subfamily A Member 1 | 5.11 |
| HOXA1 | Homeobox A1 | 5.11 |
| KIR2DL1 | Killer Cell Immunoglobulin Like Receptor, Two Ig Domains And Long Cytoplasmic Tail 1 | 5.11 |
| THRIL | TNF And HNRNPL Related Immunoregulatory Long Non-Coding RNA | 5.11 |
| CA2 | Carbonic Anhydrase 2 | 5.1 |
| HSD11B1 | Hydroxysteroid 11-Beta Dehydrogenase 1 | 5.1 |
| BSCL2 | BSCL2 Lipid Droplet Biogenesis Associated, Seipin | 5.1 |
| CIDEC | Cell Death Inducing DFFA Like Effector C | 5.09 |
| LIPN | Lipase Family Member N | 5.08 |
| CCKAR | Cholecystokinin A Receptor | 5.08 |
| SUMO4 | Small Ubiquitin Like Modifier 4 | 5.08 |
| JRKL | JRK Like | 5.08 |
| LYST | Lysosomal Trafficking Regulator | 5.08 |
| ANXA2 | Annexin A2 | 5.07 |
| MSTN | Myostatin | 5.06 |
| FGF1 | Fibroblast Growth Factor 1 | 5.06 |
| MSH6 | MutS Homolog 6 | 5.05 |
| OAS1 | 2'-5'-Oligoadenylate Synthetase 1 | 5.05 |
| MAN2A1 | Mannosidase Alpha Class 2A Member 1 | 5.05 |
| DYNLT1 | Dynein Light Chain Tctex-Type 1 | 5.05 |
| ANKRD20A1 | Ankyrin Repeat Domain 20 Family Member A1 | 5.05 |
| PDE4D | Phosphodiesterase 4D | 5.05 |
| SLC40A1 | Solute Carrier Family 40 Member 1 | 5.05 |
| TTN | Titin | 5.05 |
| NDUFA13 | NADH:Ubiquinone Oxidoreductase Subunit A13 | 5.05 |
| PDGFB | Platelet Derived Growth Factor Subunit B | 5.04 |
| MIR320A | MicroRNA 320a | 5.04 |
| CYP1A2 | Cytochrome P450 Family 1 Subfamily A Member 2 | 5.03 |
| MYLK | Myosin Light Chain Kinase | 5.03 |
| CLEC4E | C-Type Lectin Domain Family 4 Member E | 5.03 |
| PPIG | Peptidylprolyl Isomerase G | 5.03 |
| LPP | LIM Domain Containing Preferred Translocation Partner In Lipoma | 5.02 |
| DSC1 | Desmocollin 1 | 5.02 |
| PPP2CA | Protein Phosphatase 2 Catalytic Subunit Alpha | 5.02 |
| GATA1 | GATA Binding Protein 1 | 5.02 |
| GPS2 | G Protein Pathway Suppressor 2 | 5.02 |
| KDM6A | Lysine Demethylase 6A | 5.01 |
| ACE2 | Angiotensin I Converting Enzyme 2 | 5 |
| MIRLET7B | MicroRNA Let-7b | 5 |
| BLOC1S1 | Biogenesis Of Lysosomal Organelles Complex 1 Subunit 1 | 5 |
| SLC5A2 | Solute Carrier Family 5 Member 2 | 4.99 |
| MTTP | Microsomal Triglyceride Transfer Protein | 4.99 |
| SNRPE | Small Nuclear Ribonucleoprotein Polypeptide E | 4.98 |
| SNRPA | Small Nuclear Ribonucleoprotein Polypeptide A | 4.98 |
| STX11 | Syntaxin 11 | 4.98 |
| SNRPD1 | Small Nuclear Ribonucleoprotein D1 Polypeptide | 4.98 |
| GIMAP4 | GTPase, IMAP Family Member 4 | 4.98 |
| PNMA2 | PNMA Family Member 2 | 4.98 |
| ZFP90 | ZFP90 Zinc Finger Protein | 4.98 |
| GIMAP2 | GTPase, IMAP Family Member 2 | 4.98 |
| CCDC180 | Coiled-Coil Domain Containing 180 | 4.98 |
| MUC12 | Mucin 12, Cell Surface Associated | 4.98 |
| MIR642A | MicroRNA 642a | 4.98 |
| ENSG00000266919 |  | 4.98 |
| ENSG00000278769 |  | 4.98 |
| ADCYAP1 | Adenylate Cyclase Activating Polypeptide 1 | 4.98 |
| NOX5 | NADPH Oxidase 5 | 4.98 |
| CBS | Cystathionine Beta-Synthase | 4.98 |
| MAP3K8 | Mitogen-Activated Protein Kinase Kinase Kinase 8 | 4.97 |
| FPR2 | Formyl Peptide Receptor 2 | 4.96 |
| CD83 | CD83 Molecule | 4.96 |
| PROS1 | Protein S | 4.96 |
| IFIT3 | Interferon Induced Protein With Tetratricopeptide Repeats 3 | 4.95 |
| PPARD | Peroxisome Proliferator Activated Receptor Delta | 4.94 |
| TOLLIP | Toll Interacting Protein | 4.94 |
| AEBP1 | AE Binding Protein 1 | 4.94 |
| NTF3 | Neurotrophin 3 | 4.94 |
| STEAP4 | STEAP4 Metalloreductase | 4.93 |
| KLRC2 | Killer Cell Lectin Like Receptor C2 | 4.93 |
| FMR1 | FMRP Translational Regulator 1 | 4.92 |
| OCRL | OCRL Inositol Polyphosphate-5-Phosphatase | 4.92 |
| MPLKIP | M-Phase Specific PLK1 Interacting Protein | 4.92 |
| PDPN | Podoplanin | 4.92 |
| KLRC1 | Killer Cell Lectin Like Receptor C1 | 4.91 |
| CLEC11A | C-Type Lectin Domain Containing 11A | 4.91 |
| ANPEP | Alanyl Aminopeptidase, Membrane | 4.91 |
| NOX4 | NADPH Oxidase 4 | 4.91 |
| ITGAV | Integrin Subunit Alpha V | 4.9 |
| OTUD7B | OTU Deubiquitinase 7B | 4.9 |
| NKX2-1 | NK2 Homeobox 1 | 4.9 |
| PSMD4 | Proteasome 26S Subunit, Non-ATPase 4 | 4.9 |
| HMGB2 | High Mobility Group Box 2 | 4.9 |
| GNRH1 | Gonadotropin Releasing Hormone 1 | 4.9 |
| HJV | Hemojuvelin BMP Co-Receptor | 4.9 |
| ANGPTL4 | Angiopoietin Like 4 | 4.9 |
| RNPC3 | RNA Binding Region (RNP1, RRM) Containing 3 | 4.89 |
| TMPO | Thymopoietin | 4.89 |
| TGM3 | Transglutaminase 3 | 4.89 |
| TMEM79 | Transmembrane Protein 79 | 4.89 |
| XPO1 | Exportin 1 | 4.89 |
| MIR182 | MicroRNA 182 | 4.89 |
| BIRC5 | Baculoviral IAP Repeat Containing 5 | 4.88 |
| NUP210 | Nucleoporin 210 | 4.88 |
| ATF4 | Activating Transcription Factor 4 | 4.88 |
| CD276 | CD276 Molecule | 4.87 |
| ADIPOR1 | Adiponectin Receptor 1 | 4.87 |
| LAT | Linker For Activation Of T Cells | 4.87 |
| ASGR2 | Asialoglycoprotein Receptor 2 | 4.87 |
| COL5A2 | Collagen Type V Alpha 2 Chain | 4.87 |
| S100A7A | S100 Calcium Binding Protein A7A | 4.87 |
| VDAC1 | Voltage Dependent Anion Channel 1 | 4.86 |
| CREM | CAMP Responsive Element Modulator | 4.85 |
| PHF11 | PHD Finger Protein 11 | 4.85 |
| CYP3A5 | Cytochrome P450 Family 3 Subfamily A Member 5 | 4.84 |
| CYP4F3 | Cytochrome P450 Family 4 Subfamily F Member 3 | 4.84 |
| RB1 | RB Transcriptional Corepressor 1 | 4.84 |
| FRZB | Frizzled Related Protein | 4.83 |
| CELA1 | Chymotrypsin Like Elastase 1 | 4.82 |
| MIR24-1 | MicroRNA 24-1 | 4.82 |
| DDX1 | DEAD-Box Helicase 1 | 4.82 |
| SLAMF6 | SLAM Family Member 6 | 4.82 |
| KIAA1109 | KIAA1109 | 4.82 |
| CORO1A | Coronin 1A | 4.82 |
| ADAMTSL1 | ADAMTS Like 1 | 4.82 |
| SLC5A4 | Solute Carrier Family 5 Member 4 | 4.82 |
| ADIPOR2 | Adiponectin Receptor 2 | 4.82 |
| TIRAP | TIR Domain Containing Adaptor Protein | 4.82 |
| CYP19A1 | Cytochrome P450 Family 19 Subfamily A Member 1 | 4.81 |
| LPA | Lipoprotein(A) | 4.81 |
| WNT4 | Wnt Family Member 4 | 4.81 |
| SHH | Sonic Hedgehog Signaling Molecule | 4.8 |
| TMPRSS15 | Transmembrane Serine Protease 15 | 4.8 |
| IFNL4 | Interferon Lambda 4 (Gene/Pseudogene) | 4.8 |
| FGFR1 | Fibroblast Growth Factor Receptor 1 | 4.8 |
| VPS37A | VPS37A Subunit Of ESCRT-I | 4.8 |
| SLC25A13 | Solute Carrier Family 25 Member 13 | 4.8 |
| GAS5 | Growth Arrest Specific 5 | 4.8 |
| TFF1 | Trefoil Factor 1 | 4.79 |
| CTSK | Cathepsin K | 4.79 |
| AGPAT2 | 1-Acylglycerol-3-Phosphate O-Acyltransferase 2 | 4.79 |
| ORM2 | Orosomucoid 2 | 4.79 |
| KNTC1 | Kinetochore Associated 1 | 4.79 |
| CTC1 | CST Telomere Replication Complex Component 1 | 4.78 |
| PRDM16 | PR/SET Domain 16 | 4.78 |
| SNRPN | Small Nuclear Ribonucleoprotein Polypeptide N | 4.78 |
| RGS1 | Regulator Of G Protein Signaling 1 | 4.78 |
| CD79B | CD79b Molecule | 4.78 |
| LTB | Lymphotoxin Beta | 4.78 |
| TNNT2 | Troponin T2, Cardiac Type | 4.77 |
| P2RY12 | Purinergic Receptor P2Y12 | 4.77 |
| SLC1A2 | Solute Carrier Family 1 Member 2 | 4.77 |
| IL9R | Interleukin 9 Receptor | 4.77 |
| SERPINA7 | Serpin Family A Member 7 | 4.76 |
| MECOM | MDS1 And EVI1 Complex Locus | 4.75 |
| ALG3 | ALG3 Alpha-1,3- Mannosyltransferase | 4.75 |
| NAGS | N-Acetylglutamate Synthase | 4.75 |
| LMO2 | LIM Domain Only 2 | 4.75 |
| LY9 | Lymphocyte Antigen 9 | 4.75 |
| CD7 | CD7 Molecule | 4.75 |
| PRRT2 | Proline Rich Transmembrane Protein 2 | 4.75 |
| UBE4A | Ubiquitination Factor E4A | 4.75 |
| SNRNP70 | Small Nuclear Ribonucleoprotein U1 Subunit 70 | 4.75 |
| CFHR4 | Complement Factor H Related 4 | 4.75 |
| IGSF6 | Immunoglobulin Superfamily Member 6 | 4.75 |
| NUDT10 | Nudix Hydrolase 10 | 4.75 |
| CDR1 | Cerebellar Degeneration Related Protein 1 | 4.75 |
| MIR196A1 | MicroRNA 196a-1 | 4.75 |
| MIR409 | MicroRNA 409 | 4.75 |
| MIR518B | MicroRNA 518b | 4.75 |
| MIR622 | MicroRNA 622 | 4.75 |
| NCF4-AS1 | NCF4 Antisense RNA 1 | 4.75 |
| LOC106029312 | Williams-Beuren Syndrome Medial Block B Recombination Region | 4.75 |
| DECR1 | 2,4-Dienoyl-CoA Reductase 1 | 4.75 |
| CD72 | CD72 Molecule | 4.75 |
| EPSTI1 | Epithelial Stromal Interaction 1 | 4.75 |
| H2BC21 | H2B Clustered Histone 21 | 4.75 |
| P2RY2 | Purinergic Receptor P2Y2 | 4.74 |
| PPM1L | Protein Phosphatase, Mg2+/Mn2+ Dependent 1L | 4.74 |
| BIRC3 | Baculoviral IAP Repeat Containing 3 | 4.73 |
| SDHB | Succinate Dehydrogenase Complex Iron Sulfur Subunit B | 4.73 |
| UFSP2 | UFM1 Specific Peptidase 2 | 4.73 |
| LAMA2 | Laminin Subunit Alpha 2 | 4.73 |
| DEFB4B | Defensin Beta 4B | 4.73 |
| CLEC12A | C-Type Lectin Domain Family 12 Member A | 4.73 |
| CDKN2B-AS1 | CDKN2B Antisense RNA 1 | 4.72 |
| TRPV4 | Transient Receptor Potential Cation Channel Subfamily V Member 4 | 4.72 |
| METTL9 | Methyltransferase Like 9 | 4.72 |
| P2RX3 | Purinergic Receptor P2X 3 | 4.71 |
| COL4A4 | Collagen Type IV Alpha 4 Chain | 4.71 |
| RAF1 | Raf-1 Proto-Oncogene, Serine/Threonine Kinase | 4.7 |
| OTOP1 | Otopetrin 1 | 4.7 |
| HMGCL | 3-Hydroxy-3-Methylglutaryl-CoA Lyase | 4.69 |
| COL4A5 | Collagen Type IV Alpha 5 Chain | 4.69 |
| IRF2BP2 | Interferon Regulatory Factor 2 Binding Protein 2 | 4.69 |
| MDM2 | MDM2 Proto-Oncogene | 4.68 |
| IL1RL2 | Interleukin 1 Receptor Like 2 | 4.68 |
| SIGLEC8 | Sialic Acid Binding Ig Like Lectin 8 | 4.68 |
| ERF | ETS2 Repressor Factor | 4.68 |
| SLC15A4 | Solute Carrier Family 15 Member 4 | 4.68 |
| MIR134 | MicroRNA 134 | 4.68 |
| MIR638 | MicroRNA 638 | 4.68 |
| MIR657 | MicroRNA 657 | 4.68 |
| MIR637 | MicroRNA 637 | 4.68 |
| MIR92B | MicroRNA 92b | 4.68 |
| MIR663A | MicroRNA 663a | 4.68 |
| MLN | Motilin | 4.68 |
| H4-16 | H4 Histone 16 | 4.68 |
| CASP9 | Caspase 9 | 4.68 |
| GATA6 | GATA Binding Protein 6 | 4.67 |
| GZMA | Granzyme A | 4.67 |
| PRKAB1 | Protein Kinase AMP-Activated Non-Catalytic Subunit Beta 1 | 4.66 |
| NLRP13 | NLR Family Pyrin Domain Containing 13 | 4.66 |
| EDA | Ectodysplasin A | 4.66 |
| VCP | Valosin Containing Protein | 4.65 |
| CHKA | Choline Kinase Alpha | 4.65 |
| RPLP0 | Ribosomal Protein Lateral Stalk Subunit P0 | 4.65 |
| BLNK | B Cell Linker | 4.65 |
| IGLL1 | Immunoglobulin Lambda Like Polypeptide 1 | 4.65 |
| MDK | Midkine | 4.65 |
| BAX | BCL2 Associated X, Apoptosis Regulator | 4.64 |
| MSR1 | Macrophage Scavenger Receptor 1 | 4.64 |
| SPTA1 | Spectrin Alpha, Erythrocytic 1 | 4.64 |
| LAMA4 | Laminin Subunit Alpha 4 | 4.64 |
| FOXP1 | Forkhead Box P1 | 4.63 |
| EZH2 | Enhancer Of Zeste 2 Polycomb Repressive Complex 2 Subunit | 4.63 |
| GSDMB | Gasdermin B | 4.63 |
| XRCC6 | X-Ray Repair Cross Complementing 6 | 4.63 |
| XRCC5 | X-Ray Repair Cross Complementing 5 | 4.63 |
| TM6SF2 | Transmembrane 6 Superfamily Member 2 | 4.63 |
| CD2 | CD2 Molecule | 4.63 |
| DCLRE1C | DNA Cross-Link Repair 1C | 4.62 |
| ADORA1 | Adenosine A1 Receptor | 4.62 |
| CEACAM8 | CEA Cell Adhesion Molecule 8 | 4.62 |
| CPB2 | Carboxypeptidase B2 | 4.62 |
| CASP7 | Caspase 7 | 4.61 |
| TCF3 | Transcription Factor 3 | 4.61 |
| NLRP9 | NLR Family Pyrin Domain Containing 9 | 4.61 |
| CD24 | CD24 Molecule | 4.61 |
| SLC5A11 | Solute Carrier Family 5 Member 11 | 4.61 |
| FOXD2-AS1 | FOXD2 Adjacent Opposite Strand RNA 1 | 4.61 |
| EIF2S1 | Eukaryotic Translation Initiation Factor 2 Subunit Alpha | 4.61 |
| IL22RA1 | Interleukin 22 Receptor Subunit Alpha 1 | 4.6 |
| EXT1 | Exostosin Glycosyltransferase 1 | 4.6 |
| DBT | Dihydrolipoamide Branched Chain Transacylase E2 | 4.6 |
| EDNRA | Endothelin Receptor Type A | 4.6 |
| RYR1 | Ryanodine Receptor 1 | 4.6 |
| GCH1 | GTP Cyclohydrolase 1 | 4.59 |
| TYRP1 | Tyrosinase Related Protein 1 | 4.59 |
| NPPA | Natriuretic Peptide A | 4.58 |
| PPP1CA | Protein Phosphatase 1 Catalytic Subunit Alpha | 4.58 |
| DEFB103A | Defensin Beta 103A | 4.58 |
| LACRT | Lacritin | 4.58 |
| MROCKI | MARCKS Cis Regulating LncRNA Promoter Of Cytokines And Inflammation | 4.58 |
| LTK | Leukocyte Receptor Tyrosine Kinase | 4.58 |
| TRIM5 | Tripartite Motif Containing 5 | 4.58 |
| TGM6 | Transglutaminase 6 | 4.58 |
| SLC51A | Solute Carrier Family 51 Subunit Alpha | 4.58 |
| MTUS1 | Microtubule Associated Scaffold Protein 1 | 4.58 |
| WDFY4 | WDFY Family Member 4 | 4.58 |
| PRDM10 | PR/SET Domain 10 | 4.58 |
| H2AC20 | H2A Clustered Histone 20 | 4.58 |
| MIR96 | MicroRNA 96 | 4.58 |
| MIR208A | MicroRNA 208a | 4.58 |
| MIR629 | MicroRNA 629 | 4.58 |
| MIR769 | MicroRNA 769 | 4.58 |
| IRS2 | Insulin Receptor Substrate 2 | 4.57 |
| NTS | Neurotensin | 4.57 |
| SLC25A15 | Solute Carrier Family 25 Member 15 | 4.57 |
| PKP1 | Plakophilin 1 | 4.57 |
| FAAH | Fatty Acid Amide Hydrolase | 4.56 |
| ATP7A | ATPase Copper Transporting Alpha | 4.56 |
| MLKL | Mixed Lineage Kinase Domain Like Pseudokinase | 4.56 |
| SLC26A3 | Solute Carrier Family 26 Member 3 | 4.56 |
| IGF2 | Insulin Like Growth Factor 2 | 4.56 |
| SERPINA4 | Serpin Family A Member 4 | 4.56 |
| VEGFD | Vascular Endothelial Growth Factor D | 4.55 |
| AKR1D1 | Aldo-Keto Reductase Family 1 Member D1 | 4.54 |
| KLK5 | Kallikrein Related Peptidase 5 | 4.54 |
| TYMS | Thymidylate Synthetase | 4.54 |
| MC1R | Melanocortin 1 Receptor | 4.54 |
| SLC2A4 | Solute Carrier Family 2 Member 4 | 4.54 |
| CYP1B1 | Cytochrome P450 Family 1 Subfamily B Member 1 | 4.53 |
| MC4R | Melanocortin 4 Receptor | 4.53 |
| ABCA1 | ATP Binding Cassette Subfamily A Member 1 | 4.53 |
| MMP10 | Matrix Metallopeptidase 10 | 4.52 |
| BACE1 | Beta-Secretase 1 | 4.52 |
| TBX1 | T-Box Transcription Factor 1 | 4.51 |
| IL13RA1 | Interleukin 13 Receptor Subunit Alpha 1 | 4.51 |
| LRRC8A | Leucine Rich Repeat Containing 8 VRAC Subunit A | 4.51 |
| TPI1 | Triosephosphate Isomerase 1 | 4.51 |
| GOT2 | Glutamic-Oxaloacetic Transaminase 2 | 4.51 |
| LCT | Lactase | 4.51 |
| SLC30A10 | Solute Carrier Family 30 Member 10 | 4.51 |
| KEL | Kell Metallo-Endopeptidase (Kell Blood Group) | 4.51 |
| RASGRP3 | RAS Guanyl Releasing Protein 3 | 4.51 |
| HLA-DOB | Major Histocompatibility Complex, Class II, DO Beta | 4.51 |
| SSRP1 | Structure Specific Recognition Protein 1 | 4.51 |
| XK | X-Linked Kx Blood Group | 4.51 |
| UBASH3A | Ubiquitin Associated And SH3 Domain Containing A | 4.51 |
| TOR1B | Torsin Family 1 Member B | 4.51 |
| LAPTM4A | Lysosomal Protein Transmembrane 4 Alpha | 4.51 |
| ALYREF | Aly/REF Export Factor | 4.51 |
| SNRPB2 | Small Nuclear Ribonucleoprotein Polypeptide B2 | 4.51 |
| TRIM68 | Tripartite Motif Containing 68 | 4.51 |
| ADAD1 | Adenosine Deaminase Domain Containing 1 | 4.51 |
| PPP1R12C | Protein Phosphatase 1 Regulatory Subunit 12C | 4.51 |
| SNRPD3 | Small Nuclear Ribonucleoprotein D3 Polypeptide | 4.51 |
| LAX1 | Lymphocyte Transmembrane Adaptor 1 | 4.51 |
| TIMD4 | T Cell Immunoglobulin And Mucin Domain Containing 4 | 4.51 |
| SCHIP1 | Schwannomin Interacting Protein 1 | 4.51 |
| PHRF1 | PHD And Ring Finger Domains 1 | 4.51 |
| PUS10 | Pseudouridine Synthase 10 | 4.51 |
| TMEM201 | Transmembrane Protein 201 | 4.51 |
| NPS | Neuropeptide S | 4.51 |
| CXorf21 | Chromosome X Open Reading Frame 21 | 4.51 |
| PEDS1 | Plasmanylethanolamine Desaturase 1 | 4.51 |
| NCF1C | Neutrophil Cytosolic Factor 1C Pseudogene | 4.51 |
| MIR433 | MicroRNA 433 | 4.51 |
| MIR615 | MicroRNA 615 | 4.51 |
| NCF1B | Neutrophil Cytosolic Factor 1B Pseudogene | 4.51 |
| MIR154 | MicroRNA 154 | 4.51 |
| MIR484 | MicroRNA 484 | 4.51 |
| MIR654 | MicroRNA 654 | 4.51 |
| MIR612 | MicroRNA 612 | 4.51 |
| MIR608 | MicroRNA 608 | 4.51 |
| MIR557 | MicroRNA 557 | 4.51 |
| MIR575 | MicroRNA 575 | 4.51 |
| MIR601 | MicroRNA 601 | 4.51 |
| MIR583 | MicroRNA 583 | 4.51 |
| MIR662 | MicroRNA 662 | 4.51 |
| MIR600 | MicroRNA 600 | 4.51 |
| MIR658 | MicroRNA 658 | 4.51 |
| MIR611 | MicroRNA 611 | 4.51 |
| MIR325 | MicroRNA 325 | 4.51 |
| MIR596 | MicroRNA 596 | 4.51 |
| MIR602 | MicroRNA 602 | 4.51 |
| LINC01193 | Long Intergenic Non-Protein Coding RNA 1193 | 4.51 |
| ENSG00000207300 |  | 4.51 |
| SLEN3 | Systemic Lupus Erythematosus With Nephritis 3 | 4.51 |
| SLEH1 | Systemic Lupus Erythematosus With Hemolytic Anemia 1 | 4.51 |
| SLEN1 | Systemic Lupus Erythematosus With Nephritis 1 | 4.51 |
| SLEN2 | Systemic Lupus Erythematosus With Nephritis 2 | 4.51 |
| CLEC5A | C-Type Lectin Domain Containing 5A | 4.5 |
| TSPAN12 | Tetraspanin 12 | 4.49 |
| APOBEC3G | Apolipoprotein B MRNA Editing Enzyme Catalytic Subunit 3G | 4.49 |
| KDM4C | Lysine Demethylase 4C | 4.49 |
| ALOX12 | Arachidonate 12-Lipoxygenase, 12S Type | 4.49 |
| MAG | Myelin Associated Glycoprotein | 4.48 |
| NFATC1 | Nuclear Factor Of Activated T Cells 1 | 4.47 |
| GBA | Glucosylceramidase Beta | 4.47 |
| RNF125 | Ring Finger Protein 125 | 4.47 |
| PDE5A | Phosphodiesterase 5A | 4.46 |
| FOXP2 | Forkhead Box P2 | 4.45 |
| BAP1 | BRCA1 Associated Protein 1 | 4.44 |
| RREB1 | Ras Responsive Element Binding Protein 1 | 4.44 |
| FOXE3 | Forkhead Box E3 | 4.44 |
| MIR335 | MicroRNA 335 | 4.44 |
| AIF1 | Allograft Inflammatory Factor 1 | 4.44 |
| ADAM8 | ADAM Metallopeptidase Domain 8 | 4.43 |
| GREM1 | Gremlin 1, DAN Family BMP Antagonist | 4.43 |
| ADRB1 | Adrenoceptor Beta 1 | 4.42 |
| HAPLN1 | Hyaluronan And Proteoglycan Link Protein 1 | 4.41 |
| F11R | F11 Receptor | 4.41 |
| MIR205 | MicroRNA 205 | 4.4 |
| PLIN1 | Perilipin 1 | 4.4 |
| GRP | Gastrin Releasing Peptide | 4.39 |
| ITGA1 | Integrin Subunit Alpha 1 | 4.39 |
| ENPP3 | Ectonucleotide Pyrophosphatase/Phosphodiesterase 3 | 4.39 |
| RICTOR | RPTOR Independent Companion Of MTOR Complex 2 | 4.38 |
| JAM3 | Junctional Adhesion Molecule 3 | 4.38 |
| KRT20 | Keratin 20 | 4.37 |
| SERPINB4 | Serpin Family B Member 4 | 4.37 |
| MIR455 | MicroRNA 455 | 4.37 |
| LRG1 | Leucine Rich Alpha-2-Glycoprotein 1 | 4.36 |
| KRT5 | Keratin 5 | 4.36 |
| NR5A2 | Nuclear Receptor Subfamily 5 Group A Member 2 | 4.36 |
| PKD1 | Polycystin 1, Transient Receptor Potential Channel Interacting | 4.36 |
| S100A4 | S100 Calcium Binding Protein A4 | 4.35 |
| RHOA | Ras Homolog Family Member A | 4.35 |
| GPX4 | Glutathione Peroxidase 4 | 4.35 |
| CEBPB | CCAAT Enhancer Binding Protein Beta | 4.34 |
| MIR200B | MicroRNA 200b | 4.33 |
| CYP4F2 | Cytochrome P450 Family 4 Subfamily F Member 2 | 4.33 |
| COL9A1 | Collagen Type IX Alpha 1 Chain | 4.33 |
| GAD1 | Glutamate Decarboxylase 1 | 4.32 |
| IGF1R | Insulin Like Growth Factor 1 Receptor | 4.32 |
| NDP | Norrin Cystine Knot Growth Factor NDP | 4.32 |
| SP100 | SP100 Nuclear Antigen | 4.32 |
| TTC21B | Tetratricopeptide Repeat Domain 21B | 4.32 |
| MIR215 | MicroRNA 215 | 4.32 |
| ADORA3 | Adenosine A3 Receptor | 4.32 |
| AGA | Aspartylglucosaminidase | 4.31 |
| HSPA1A | Heat Shock Protein Family A (Hsp70) Member 1A | 4.31 |
| DDX39B | DExD-Box Helicase 39B | 4.31 |
| MAN2B1 | Mannosidase Alpha Class 2B Member 1 | 4.31 |
| TUG1 | Taurine Up-Regulated 1 | 4.29 |
| DIO2 | Iodothyronine Deiodinase 2 | 4.29 |
| ADGRE3 | Adhesion G Protein-Coupled Receptor E3 | 4.29 |
| GBP5 | Guanylate Binding Protein 5 | 4.28 |
| AP3B1 | Adaptor Related Protein Complex 3 Subunit Beta 1 | 4.28 |
| GLRA1 | Glycine Receptor Alpha 1 | 4.28 |
| AQP3 | Aquaporin 3 (Gill Blood Group) | 4.28 |
| FKBP5 | FKBP Prolyl Isomerase 5 | 4.28 |
| CXADR | CXADR Ig-Like Cell Adhesion Molecule | 4.27 |
| PCNA | Proliferating Cell Nuclear Antigen | 4.27 |
| DGKE | Diacylglycerol Kinase Epsilon | 4.27 |
| ECE1 | Endothelin Converting Enzyme 1 | 4.27 |
| PDGFRA | Platelet Derived Growth Factor Receptor Alpha | 4.27 |
| HM13 | Histocompatibility Minor 13 | 4.27 |
| PMEL | Premelanosome Protein | 4.27 |
| SIGIRR | Single Ig And TIR Domain Containing | 4.27 |
| F9 | Coagulation Factor IX | 4.26 |
| RPL11 | Ribosomal Protein L11 | 4.25 |
| CD151 | CD151 Molecule (Raph Blood Group) | 4.25 |
| COL11A1 | Collagen Type XI Alpha 1 Chain | 4.25 |
| MMAB | Metabolism Of Cobalamin Associated B | 4.25 |
| RDH12 | Retinol Dehydrogenase 12 | 4.25 |
| A4GALT | Alpha 1,4-Galactosyltransferase (P Blood Group) | 4.25 |
| MMACHC | Metabolism Of Cobalamin Associated C | 4.25 |
| ATP11A | ATPase Phospholipid Transporting 11A | 4.25 |
| GLIS2 | GLIS Family Zinc Finger 2 | 4.25 |
| HPS5 | HPS5 Biogenesis Of Lysosomal Organelles Complex 2 Subunit 2 | 4.25 |
| PLA2G4F | Phospholipase A2 Group IVF | 4.25 |
| HVCN1 | Hydrogen Voltage Gated Channel 1 | 4.25 |
| SPCS1 | Signal Peptidase Complex Subunit 1 | 4.25 |
| PTPRQ | Protein Tyrosine Phosphatase Receptor Type Q | 4.25 |
| STN1 | STN1 Subunit Of CST Complex | 4.25 |
| MIR200A | MicroRNA 200a | 4.25 |
| MIR30E | MicroRNA 30e | 4.25 |
| MIRLET7D | MicroRNA Let-7d | 4.25 |
| MIR28 | MicroRNA 28 | 4.25 |
| AIS2 | Autoimmune Susceptibility 2 | 4.25 |
| AIS3 | Autoimmune Susceptibility 3 (Vitiligo Specific) | 4.25 |
| HT | Hashimoto Thyroiditis | 4.25 |
| AIS4 | Autoimmune Disease, Susceptibility To, 4 | 4.25 |
| GRD1 | Graves Disease, Susceptiblity To, 1 | 4.25 |
| GRD2 | Graves Disease, Susceptibility To, 2 | 4.25 |
| ENDO1 | Endometriosis, Susceptibility To, 1 | 4.25 |
| SM2 | Hepatic Fibrosis Susceptibility Due To Schistosoma Mansoni Infection | 4.25 |
| NAFLD1 | Fatty Liver Disease 1, Susceptiblity To | 4.25 |
| NAFLD2 | Fatty Liver Disease, Nonalcoholic, Susceptibility To, 2 | 4.25 |
| PBC2 | Biliary Cirrhosis, Primary, 2 | 4.25 |
| PBC3 | Biliary Cirrhosis, Primary, 3 | 4.25 |
| PBC4 | Biliary Cirrhosis, Primary, 4 | 4.25 |
| PBC5 | Biliary Cirrhosis, Primary, 5 | 4.25 |
| PGA3 | Pepsinogen A3 | 4.25 |
| PGA5 | Pepsinogen A5 | 4.25 |
| FHIT | Fragile Histidine Triad Diadenosine Triphosphatase | 4.25 |
| CDSN | Corneodesmosin | 4.25 |
| MIR27B | MicroRNA 27b | 4.25 |
| TNFAIP8L2 | TNF Alpha Induced Protein 8 Like 2 | 4.24 |
| E2F1 | E2F Transcription Factor 1 | 4.24 |
| TYMP | Thymidine Phosphorylase | 4.23 |
| NF2 | Neurofibromin 2 | 4.23 |
| MIR199A2 | MicroRNA 199a-2 | 4.23 |
| SLC6A5 | Solute Carrier Family 6 Member 5 | 4.23 |
| MIR135A1 | MicroRNA 135a-1 | 4.23 |
| HNF1B | HNF1 Homeobox B | 4.22 |
| EPC2 | Enhancer Of Polycomb Homolog 2 | 4.22 |
| PGA4 | Pepsinogen A4 | 4.22 |
| APRT | Adenine Phosphoribosyltransferase | 4.22 |
| DHX58 | DExH-Box Helicase 58 | 4.22 |
| CST4 | Cystatin S | 4.22 |
| NARS1 | Asparaginyl-TRNA Synthetase 1 | 4.22 |
| NR1I3 | Nuclear Receptor Subfamily 1 Group I Member 3 | 4.22 |
| XPNPEP3 | X-Prolyl Aminopeptidase 3 | 4.21 |
| MT-ND5 | Mitochondrially Encoded NADH:Ubiquinone Oxidoreductase Core Subunit 5 | 4.21 |
| UBD | Ubiquitin D | 4.21 |
| RPS27A | Ribosomal Protein S27a | 4.21 |
| ACACA | Acetyl-CoA Carboxylase Alpha | 4.21 |
| SLC15A1 | Solute Carrier Family 15 Member 1 | 4.21 |
| USP18 | Ubiquitin Specific Peptidase 18 | 4.2 |
| MIR125B2 | MicroRNA 125b-2 | 4.2 |
| TRH | Thyrotropin Releasing Hormone | 4.2 |
| TACR2 | Tachykinin Receptor 2 | 4.19 |
| ZEB2 | Zinc Finger E-Box Binding Homeobox 2 | 4.18 |
| PDGFRB | Platelet Derived Growth Factor Receptor Beta | 4.18 |
| ECM1 | Extracellular Matrix Protein 1 | 4.18 |
| ABCC1 | ATP Binding Cassette Subfamily C Member 1 | 4.18 |
| TRIM33 | Tripartite Motif Containing 33 | 4.17 |
| PITX2 | Paired Like Homeodomain 2 | 4.17 |
| TPSAB1 | Tryptase Alpha/Beta 1 | 4.17 |
| RXRA | Retinoid X Receptor Alpha | 4.16 |
| RIPK3 | Receptor Interacting Serine/Threonine Kinase 3 | 4.16 |
| MRGPRX2 | MAS Related GPR Family Member X2 | 4.16 |
| NOTCH2 | Notch Receptor 2 | 4.16 |
| FBXL19 | F-Box And Leucine Rich Repeat Protein 19 | 4.16 |
| STXBP1 | Syntaxin Binding Protein 1 | 4.16 |
| PEPD | Peptidase D | 4.15 |
| THBS4 | Thrombospondin 4 | 4.15 |
| DNMT3B | DNA Methyltransferase 3 Beta | 4.15 |
| G6PC3 | Glucose-6-Phosphatase Catalytic Subunit 3 | 4.15 |
| GARS1 | Glycyl-TRNA Synthetase 1 | 4.15 |
| HARS1 | Histidyl-TRNA Synthetase 1 | 4.15 |
| MIR186 | MicroRNA 186 | 4.15 |
| BANCR | BRAF-Activated Non-Protein Coding RNA | 4.15 |
| MAF | MAF BZIP Transcription Factor | 4.15 |
| MIR19B1 | MicroRNA 19b-1 | 4.14 |
| CKLF | Chemokine Like Factor | 4.14 |
| MC2R | Melanocortin 2 Receptor | 4.14 |
| APOA5 | Apolipoprotein A5 | 4.14 |
| ERCC3 | ERCC Excision Repair 3, TFIIH Core Complex Helicase Subunit | 4.13 |
| ARRB2 | Arrestin Beta 2 | 4.13 |
| SERPINB2 | Serpin Family B Member 2 | 4.13 |
| JUP | Junction Plakoglobin | 4.12 |
| MIR29B1 | MicroRNA 29b-1 | 4.12 |
| FYN | FYN Proto-Oncogene, Src Family Tyrosine Kinase | 4.12 |
| TGIF1 | TGFB Induced Factor Homeobox 1 | 4.12 |
| CCKBR | Cholecystokinin B Receptor | 4.12 |
| AXIN1 | Axin 1 | 4.11 |
| PDZK1 | PDZ Domain Containing 1 | 4.11 |
| GLRB | Glycine Receptor Beta | 4.11 |
| TINCR | TINCR Ubiquitin Domain Containing | 4.1 |
| MIR7-3 | MicroRNA 7-3 | 4.1 |
| TH | Tyrosine Hydroxylase | 4.1 |
| CASP14 | Caspase 14 | 4.1 |
| REG1A | Regenerating Family Member 1 Alpha | 4.1 |
| AMH | Anti-Mullerian Hormone | 4.1 |
| NRAS | NRAS Proto-Oncogene, GTPase | 4.1 |
| MIR127 | MicroRNA 127 | 4.1 |
| LIPG | Lipase G, Endothelial Type | 4.1 |
| SLCO1B1 | Solute Carrier Organic Anion Transporter Family Member 1B1 | 4.09 |
| CDKN2C | Cyclin Dependent Kinase Inhibitor 2C | 4.08 |
| EOLA1 | Endothelium And Lymphocyte Associated ASCH Domain 1 | 4.08 |
| UNC13D | Unc-13 Homolog D | 4.07 |
| CSN3 | Casein Kappa | 4.07 |
| DAG1 | Dystroglycan 1 | 4.07 |
| MIR20B | MicroRNA 20b | 4.07 |
| LIPE | Lipase E, Hormone Sensitive Type | 4.07 |
| AGTR2 | Angiotensin II Receptor Type 2 | 4.07 |
| BDKRB2 | Bradykinin Receptor B2 | 4.06 |
| RAPGEF3 | Rap Guanine Nucleotide Exchange Factor 3 | 4.05 |
| PHGDH | Phosphoglycerate Dehydrogenase | 4.05 |
| BST2 | Bone Marrow Stromal Cell Antigen 2 | 4.05 |
| FSCN1 | Fascin Actin-Bundling Protein 1 | 4.05 |
| RPE65 | Retinoid Isomerohydrolase RPE65 | 4.05 |
| CARD6 | Caspase Recruitment Domain Family Member 6 | 4.05 |
| DBH | Dopamine Beta-Hydroxylase | 4.04 |
| PCCB | Propionyl-CoA Carboxylase Subunit Beta | 4.04 |
| THPO | Thrombopoietin | 4.04 |
| RC3H1 | Ring Finger And CCCH-Type Domains 1 | 4.04 |
| SCN11A | Sodium Voltage-Gated Channel Alpha Subunit 11 | 4.03 |
| SPTAN1 | Spectrin Alpha, Non-Erythrocytic 1 | 4.03 |
| MATN1 | Matrilin 1 | 4.03 |
| SLC35C1 | Solute Carrier Family 35 Member C1 | 4.03 |
| POMP | Proteasome Maturation Protein | 4.02 |
| CTSD | Cathepsin D | 4.01 |
| FKRP | Fukutin Related Protein | 4.01 |
| GOLM1 | Golgi Membrane Protein 1 | 4.01 |
| CALCOCO2 | Calcium Binding And Coiled-Coil Domain 2 | 4.01 |
| KLF6 | Kruppel Like Factor 6 | 4.01 |
| ITIH4 | Inter-Alpha-Trypsin Inhibitor Heavy Chain 4 | 4 |
| SLC2A9 | Solute Carrier Family 2 Member 9 | 4 |
| CAPN14 | Calpain 14 | 3.99 |
| CHST4 | Carbohydrate Sulfotransferase 4 | 3.99 |
| GSTT1 | Glutathione S-Transferase Theta 1 | 3.99 |
| MIR193A | MicroRNA 193a | 3.99 |
| PSPH | Phosphoserine Phosphatase | 3.98 |
| ATAD1 | ATPase Family AAA Domain Containing 1 | 3.98 |
| TRAPPC11 | Trafficking Protein Particle Complex 11 | 3.98 |
| TIMP3 | TIMP Metallopeptidase Inhibitor 3 | 3.98 |
| PRKN | Parkin RBR E3 Ubiquitin Protein Ligase | 3.98 |
| PDGFA | Platelet Derived Growth Factor Subunit A | 3.98 |
| PLD3 | Phospholipase D Family Member 3 | 3.98 |
| CYP8B1 | Cytochrome P450 Family 8 Subfamily B Member 1 | 3.98 |
| EPC1 | Enhancer Of Polycomb Homolog 1 | 3.98 |
| NT5C1A | 5'-Nucleotidase, Cytosolic IA | 3.98 |
| SLC17A1 | Solute Carrier Family 17 Member 1 | 3.98 |
| DMAP1 | DNA Methyltransferase 1 Associated Protein 1 | 3.98 |
| CFDP1 | Craniofacial Development Protein 1 | 3.98 |
| SH2D3A | SH2 Domain Containing 3A | 3.98 |
| OLAH | Oleoyl-ACP Hydrolase | 3.98 |
| SRCIN1 | SRC Kinase Signaling Inhibitor 1 | 3.98 |
| SDAD1 | SDA1 Domain Containing 1 | 3.98 |
| OR5V1 | Olfactory Receptor Family 5 Subfamily V Member 1 | 3.98 |
| CYREN | Cell Cycle Regulator Of NHEJ | 3.98 |
| UCA1 | Urothelial Cancer Associated 1 | 3.98 |
| MIR342 | MicroRNA 342 | 3.98 |
| FENDRR | FOXF1 Adjacent Non-Coding Developmental Regulatory RNA | 3.98 |
| FLG-AS1 | FLG Antisense RNA 1 | 3.98 |
| MIR3936 | MicroRNA 3936 | 3.98 |
| IGSF6-DREV1 | Region Containing Immunoglobulin Superfamily, Member 6 And DREV1 | 3.98 |
| FCGRT | Fc Fragment Of IgG Receptor And Transporter | 3.98 |
| RARB | Retinoic Acid Receptor Beta | 3.97 |
| H6PD | Hexose-6-Phosphate Dehydrogenase/Glucose 1-Dehydrogenase | 3.97 |
| COL1A2 | Collagen Type I Alpha 2 Chain | 3.97 |
| MIR378A | MicroRNA 378a | 3.96 |
| LYVE1 | Lymphatic Vessel Endothelial Hyaluronan Receptor 1 | 3.95 |
| OMP | Olfactory Marker Protein | 3.95 |
| MIR34C | MicroRNA 34c | 3.95 |
| SBDS | SBDS Ribosome Maturation Factor | 3.94 |
| EPB42 | Erythrocyte Membrane Protein Band 4.2 | 3.94 |
| EDARADD | EDAR Associated Death Domain | 3.94 |
| ALPI | Alkaline Phosphatase, Intestinal | 3.94 |
| SCN1A | Sodium Voltage-Gated Channel Alpha Subunit 1 | 3.94 |
| LXN | Latexin | 3.94 |
| DRD2 | Dopamine Receptor D2 | 3.93 |
| HYOU1 | Hypoxia Up-Regulated 1 | 3.93 |
| PRODH | Proline Dehydrogenase 1 | 3.92 |
| SLC22A12 | Solute Carrier Family 22 Member 12 | 3.92 |
| SLC22A11 | Solute Carrier Family 22 Member 11 | 3.92 |
| STIM2 | Stromal Interaction Molecule 2 | 3.92 |
| FBXL7 | F-Box And Leucine Rich Repeat Protein 7 | 3.92 |
| ACP1 | Acid Phosphatase 1 | 3.92 |
| ADAMTS3 | ADAM Metallopeptidase With Thrombospondin Type 1 Motif 3 | 3.92 |
| KRT13 | Keratin 13 | 3.92 |
| SLC19A1 | Solute Carrier Family 19 Member 1 | 3.92 |
| OXGR1 | Oxoglutarate Receptor 1 | 3.92 |
| MIR23B | MicroRNA 23b | 3.92 |
| KLF2 | Kruppel Like Factor 2 | 3.91 |
| PCSK9 | Proprotein Convertase Subtilisin/Kexin Type 9 | 3.91 |
| MUTYH | MutY DNA Glycosylase | 3.91 |
| HSPB1 | Heat Shock Protein Family B (Small) Member 1 | 3.9 |
| HAS1 | Hyaluronan Synthase 1 | 3.89 |
| EIF4E | Eukaryotic Translation Initiation Factor 4E | 3.89 |
| CRHR2 | Corticotropin Releasing Hormone Receptor 2 | 3.89 |
| TNFRSF6B | TNF Receptor Superfamily Member 6b | 3.89 |
| ELF3 | E74 Like ETS Transcription Factor 3 | 3.89 |
| NR4A3 | Nuclear Receptor Subfamily 4 Group A Member 3 | 3.89 |
| HNF1A | HNF1 Homeobox A | 3.88 |
| AKT2 | AKT Serine/Threonine Kinase 2 | 3.88 |
| PRRC2A | Proline Rich Coiled-Coil 2A | 3.88 |
| RANBP2 | RAN Binding Protein 2 | 3.87 |
| GP6 | Glycoprotein VI Platelet | 3.87 |
| ACTA2 | Actin Alpha 2, Smooth Muscle | 3.87 |
| RHOD | Ras Homolog Family Member D | 3.86 |
| PRSS8 | Serine Protease 8 | 3.86 |
| LMOD1 | Leiomodin 1 | 3.86 |
| COL8A2 | Collagen Type VIII Alpha 2 Chain | 3.86 |
| TARDBP | TAR DNA Binding Protein | 3.85 |
| DHODH | Dihydroorotate Dehydrogenase (Quinone) | 3.85 |
| NEK9 | NIMA Related Kinase 9 | 3.85 |
| SLC30A2 | Solute Carrier Family 30 Member 2 | 3.85 |
| WDR35 | WD Repeat Domain 35 | 3.85 |
| ZFAT | Zinc Finger And AT-Hook Domain Containing | 3.85 |
| ZNF750 | Zinc Finger Protein 750 | 3.85 |
| NSMCE3 | NSE3 Homolog, SMC5-SMC6 Complex Component | 3.85 |
| RNU4ATAC | RNA, U4atac Small Nuclear (U12-Dependent Splicing) | 3.85 |
| ABCC3 | ATP Binding Cassette Subfamily C Member 3 | 3.85 |
| SLC22A2 | Solute Carrier Family 22 Member 2 | 3.84 |
| TCN2 | Transcobalamin 2 | 3.84 |
| UROS | Uroporphyrinogen III Synthase | 3.84 |
| CPA6 | Carboxypeptidase A6 | 3.83 |
| MIR103A1 | MicroRNA 103a-1 | 3.83 |
| LOX | Lysyl Oxidase | 3.83 |
| CFHR1 | Complement Factor H Related 1 | 3.83 |
| FGL2 | Fibrinogen Like 2 | 3.83 |
| PKD2 | Polycystin 2, Transient Receptor Potential Cation Channel | 3.82 |
| SP110 | SP110 Nuclear Body Protein | 3.82 |
| DDC | Dopa Decarboxylase | 3.82 |
| GZMM | Granzyme M | 3.82 |
| C1QBP | Complement C1q Binding Protein | 3.82 |
| NPHS1 | NPHS1 Adhesion Molecule, Nephrin | 3.82 |
| SLC26A4 | Solute Carrier Family 26 Member 4 | 3.82 |
| TPH1 | Tryptophan Hydroxylase 1 | 3.82 |
| MAFB | MAF BZIP Transcription Factor B | 3.82 |
| LCAT | Lecithin-Cholesterol Acyltransferase | 3.82 |
| ADCY7 | Adenylate Cyclase 7 | 3.82 |
| ESM1 | Endothelial Cell Specific Molecule 1 | 3.81 |
| DMP1 | Dentin Matrix Acidic Phosphoprotein 1 | 3.81 |
| ABCC4 | ATP Binding Cassette Subfamily C Member 4 | 3.81 |
| ABCD1 | ATP Binding Cassette Subfamily D Member 1 | 3.81 |
| MIR338 | MicroRNA 338 | 3.8 |
| CD2AP | CD2 Associated Protein | 3.8 |
| KIR2DL3 | Killer Cell Immunoglobulin Like Receptor, Two Ig Domains And Long Cytoplasmic Tail 3 | 3.8 |
| IL17RC | Interleukin 17 Receptor C | 3.8 |
| IREB2 | Iron Responsive Element Binding Protein 2 | 3.79 |
| RTN4R | Reticulon 4 Receptor | 3.79 |
| MUC3A | Mucin 3A, Cell Surface Associated | 3.78 |
| CPB1 | Carboxypeptidase B1 | 3.78 |
| CLDN6 | Claudin 6 | 3.78 |
| KIF21B | Kinesin Family Member 21B | 3.78 |
| SPATA2 | Spermatogenesis Associated 2 | 3.78 |
| PGC | Progastricsin | 3.78 |
| WRN | WRN RecQ Like Helicase | 3.77 |
| CHRNA5 | Cholinergic Receptor Nicotinic Alpha 5 Subunit | 3.77 |
| AGR2 | Anterior Gradient 2, Protein Disulphide Isomerase Family Member | 3.77 |
| JMJD1C | Jumonji Domain Containing 1C | 3.77 |
| DNTT | DNA Nucleotidylexotransferase | 3.76 |
| MIR98 | MicroRNA 98 | 3.76 |
| MYH6 | Myosin Heavy Chain 6 | 3.76 |
| CD3E | CD3e Molecule | 3.75 |
| LST1 | Leukocyte Specific Transcript 1 | 3.75 |
| RPSA | Ribosomal Protein SA | 3.75 |
| FBL | Fibrillarin | 3.75 |
| MYO7A | Myosin VIIA | 3.75 |
| SUCLG2 | Succinate-CoA Ligase GDP-Forming Subunit Beta | 3.75 |
| CLDN11 | Claudin 11 | 3.75 |
| MYOM2 | Myomesin 2 | 3.75 |
| CLDN9 | Claudin 9 | 3.75 |
| ELMOD2 | ELMO Domain Containing 2 | 3.75 |
| EPPIN | Epididymal Peptidase Inhibitor | 3.75 |
| COL26A1 | Collagen Type XXVI Alpha 1 Chain | 3.75 |
| EMSY | EMSY Transcriptional Repressor, BRCA2 Interacting | 3.75 |
| GP2 | Glycoprotein 2 | 3.75 |
| ANTXR1 | ANTXR Cell Adhesion Molecule 1 | 3.75 |
| SLC5A1 | Solute Carrier Family 5 Member 1 | 3.75 |
| MMP19 | Matrix Metallopeptidase 19 | 3.74 |
| CNP | 2',3'-Cyclic Nucleotide 3' Phosphodiesterase | 3.74 |
| VIPR1 | Vasoactive Intestinal Peptide Receptor 1 | 3.74 |
| FFAR4 | Free Fatty Acid Receptor 4 | 3.74 |
| PADI2 | Peptidyl Arginine Deiminase 2 | 3.73 |
| MIR26A1 | MicroRNA 26a-1 | 3.73 |
| CALB2 | Calbindin 2 | 3.73 |
| PIGR | Polymeric Immunoglobulin Receptor | 3.73 |
| MIR497 | MicroRNA 497 | 3.73 |
| UBC | Ubiquitin C | 3.72 |
| FLT3 | Fms Related Receptor Tyrosine Kinase 3 | 3.72 |
| F7 | Coagulation Factor VII | 3.72 |
| FERMT2 | Fermitin Family Member 2 | 3.72 |
| MTHFD1 | Methylenetetrahydrofolate Dehydrogenase, Cyclohydrolase And Formyltetrahydrofolate Synthetase 1 | 3.71 |
| EZR | Ezrin | 3.71 |
| ARRB1 | Arrestin Beta 1 | 3.71 |
| SPINT2 | Serine Peptidase Inhibitor, Kunitz Type 2 | 3.7 |
| SERPING1 | Serpin Family G Member 1 | 3.7 |
| SGK1 | Serum/Glucocorticoid Regulated Kinase 1 | 3.7 |
| DHFR | Dihydrofolate Reductase | 3.69 |
| MIR16-1 | MicroRNA 16-1 | 3.69 |
| SNAI1 | Snail Family Transcriptional Repressor 1 | 3.68 |
| GRIN2B | Glutamate Ionotropic Receptor NMDA Type Subunit 2B | 3.68 |
| NGLY1 | N-Glycanase 1 | 3.68 |
| SLC22A6 | Solute Carrier Family 22 Member 6 | 3.68 |
| CPA4 | Carboxypeptidase A4 | 3.68 |
| SLC22A8 | Solute Carrier Family 22 Member 8 | 3.68 |
| SLC17A3 | Solute Carrier Family 17 Member 3 | 3.68 |
| SLC16A9 | Solute Carrier Family 16 Member 9 | 3.68 |
| CD1E | CD1e Molecule | 3.68 |
| TEF | TEF Transcription Factor, PAR BZIP Family Member | 3.68 |
| CLEC4G | C-Type Lectin Domain Family 4 Member G | 3.68 |
| SIAE | Sialic Acid Acetylesterase | 3.68 |
| SLC22A13 | Solute Carrier Family 22 Member 13 | 3.68 |
| ILVBL | IlvB Acetolactate Synthase Like | 3.68 |
| VMP1 | Vacuole Membrane Protein 1 | 3.68 |
| ALDH16A1 | Aldehyde Dehydrogenase 16 Family Member A1 | 3.68 |
| MORC3 | MORC Family CW-Type Zinc Finger 3 | 3.68 |
| UACA | Uveal Autoantigen With Coiled-Coil Domains And Ankyrin Repeats | 3.68 |
| DENND1B | DENN Domain Containing 1B | 3.68 |
| ZNF622 | Zinc Finger Protein 622 | 3.68 |
| SPPL2C | Signal Peptide Peptidase Like 2C | 3.68 |
| CARMIL1 | Capping Protein Regulator And Myosin 1 Linker 1 | 3.68 |
| TRIM52 | Tripartite Motif Containing 52 | 3.68 |
| OTULINL | OTU Deubiquitinase With Linear Linkage Specificity Like | 3.68 |
| MARCHF11 | Membrane Associated Ring-CH-Type Finger 11 | 3.68 |
| MEG8 | Maternally Expressed 8, Small Nucleolar RNA Host Gene | 3.68 |
| MIR151A | MicroRNA 151a | 3.68 |
| CBSL | Cystathionine Beta-Synthase Like | 3.68 |
| RN7SL1 | RNA Component Of Signal Recognition Particle 7SL1 | 3.68 |
| NRIR | Negative Regulator Of Interferon Response | 3.68 |
| SOCS5 | Suppressor Of Cytokine Signaling 5 | 3.67 |
| UGT1A1 | UDP Glucuronosyltransferase Family 1 Member A1 | 3.67 |
| SHC1 | SHC Adaptor Protein 1 | 3.67 |
| KDM6B | Lysine Demethylase 6B | 3.66 |
| RYR2 | Ryanodine Receptor 2 | 3.65 |
| SULT2A1 | Sulfotransferase Family 2A Member 1 | 3.65 |
| HAX1 | HCLS1 Associated Protein X-1 | 3.65 |
| MMRN1 | Multimerin 1 | 3.65 |
| HAPLN3 | Hyaluronan And Proteoglycan Link Protein 3 | 3.65 |
| CISH | Cytokine Inducible SH2 Containing Protein | 3.65 |
| SIGLEC1 | Sialic Acid Binding Ig Like Lectin 1 | 3.65 |
| NUP107 | Nucleoporin 107 | 3.65 |
| CEBPA | CCAAT Enhancer Binding Protein Alpha | 3.65 |
| CPD | Carboxypeptidase D | 3.65 |
| MIR551B | MicroRNA 551b | 3.65 |
| WNT5A | Wnt Family Member 5A | 3.65 |
| TNMD | Tenomodulin | 3.65 |
| LGALS3BP | Galectin 3 Binding Protein | 3.65 |
| CPQ | Carboxypeptidase Q | 3.64 |
| SRF | Serum Response Factor | 3.64 |
| LGALS4 | Galectin 4 | 3.64 |
| MCAM | Melanoma Cell Adhesion Molecule | 3.64 |
| CRNDE | Colorectal Neoplasia Differentially Expressed | 3.63 |
| CHRNA3 | Cholinergic Receptor Nicotinic Alpha 3 Subunit | 3.63 |
| MYOC | Myocilin | 3.63 |
| CABIN1 | Calcineurin Binding Protein 1 | 3.63 |
| SCTR | Secretin Receptor | 3.63 |
| SEMA3C | Semaphorin 3C | 3.63 |
| GRIN1 | Glutamate Ionotropic Receptor NMDA Type Subunit 1 | 3.63 |
| MIR29A | MicroRNA 29a | 3.63 |
| VEGFB | Vascular Endothelial Growth Factor B | 3.62 |
| PROX1 | Prospero Homeobox 1 | 3.62 |
| SOX9 | SRY-Box Transcription Factor 9 | 3.62 |
| DCT | Dopachrome Tautomerase | 3.62 |
| SLC26A2 | Solute Carrier Family 26 Member 2 | 3.61 |
| KIF7 | Kinesin Family Member 7 | 3.61 |
| NOG | Noggin | 3.61 |
| MIR495 | MicroRNA 495 | 3.61 |
| ZC3H12D | Zinc Finger CCCH-Type Containing 12D | 3.61 |
| NTRK2 | Neurotrophic Receptor Tyrosine Kinase 2 | 3.6 |
| KIDINS220 | Kinase D Interacting Substrate 220 | 3.6 |
| WLS | Wnt Ligand Secretion Mediator | 3.6 |
| CDHR3 | Cadherin Related Family Member 3 | 3.6 |
| PRR4 | Proline Rich 4 | 3.6 |
| MIR361 | MicroRNA 361 | 3.6 |
| MIR10A | MicroRNA 10a | 3.6 |
| RPS6KB1 | Ribosomal Protein S6 Kinase B1 | 3.6 |
| CD6 | CD6 Molecule | 3.6 |
| SYVN1 | Synoviolin 1 | 3.6 |
| HSP90AB1 | Heat Shock Protein 90 Alpha Family Class B Member 1 | 3.6 |
| TREM2 | Triggering Receptor Expressed On Myeloid Cells 2 | 3.59 |
| MT-CO3 | Mitochondrially Encoded Cytochrome C Oxidase III | 3.59 |
| MT-TH | Mitochondrially Encoded TRNA-His (CAU/C) | 3.59 |
| GLA | Galactosidase Alpha | 3.58 |
| HOXA13 | Homeobox A13 | 3.58 |
| POLR3A | RNA Polymerase III Subunit A | 3.58 |
| LMNB2 | Lamin B2 | 3.58 |
| ZNF341 | Zinc Finger Protein 341 | 3.58 |
| PTK2B | Protein Tyrosine Kinase 2 Beta | 3.56 |
| KLF4 | Kruppel Like Factor 4 | 3.56 |
| OGG1 | 8-Oxoguanine DNA Glycosylase | 3.56 |
| ITGA3 | Integrin Subunit Alpha 3 | 3.56 |
| SLC6A19 | Solute Carrier Family 6 Member 19 | 3.55 |
| ERCC4 | ERCC Excision Repair 4, Endonuclease Catalytic Subunit | 3.55 |
| ERCC5 | ERCC Excision Repair 5, Endonuclease | 3.55 |
| CD200R1 | CD200 Receptor 1 | 3.55 |
| GATA4 | GATA Binding Protein 4 | 3.55 |
| FURIN | Furin, Paired Basic Amino Acid Cleaving Enzyme | 3.55 |
| NEU1 | Neuraminidase 1 | 3.55 |
| CLEC4A | C-Type Lectin Domain Family 4 Member A | 3.55 |
| SCN10A | Sodium Voltage-Gated Channel Alpha Subunit 10 | 3.54 |
| SMARCA2 | SWI/SNF Related, Matrix Associated, Actin Dependent Regulator Of Chromatin, Subfamily A, Member 2 | 3.54 |
| SYP | Synaptophysin | 3.54 |
| ABCG5 | ATP Binding Cassette Subfamily G Member 5 | 3.54 |
| AICDA | Activation Induced Cytidine Deaminase | 3.54 |
| ISG20 | Interferon Stimulated Exonuclease Gene 20 | 3.54 |
| AIMP1 | Aminoacyl TRNA Synthetase Complex Interacting Multifunctional Protein 1 | 3.53 |
| TMEM203 | Transmembrane Protein 203 | 3.53 |
| MIR99B | MicroRNA 99b | 3.53 |
| TSC2 | TSC Complex Subunit 2 | 3.53 |
| MGMT | O-6-Methylguanine-DNA Methyltransferase | 3.53 |
| TYROBP | Transmembrane Immune Signaling Adaptor TYROBP | 3.52 |
| HSPA1B | Heat Shock Protein Family A (Hsp70) Member 1B | 3.52 |
| MIR708 | MicroRNA 708 | 3.52 |
| CD5L | CD5 Molecule Like | 3.52 |
| XPA | XPA, DNA Damage Recognition And Repair Factor | 3.51 |
| A2M | Alpha-2-Macroglobulin | 3.51 |
| HUWE1 | HECT, UBA And WWE Domain Containing E3 Ubiquitin Protein Ligase 1 | 3.5 |
| CD1B | CD1b Molecule | 3.5 |
| SERPINB3 | Serpin Family B Member 3 | 3.5 |
| RETNLB | Resistin Like Beta | 3.5 |
| SIRT6 | Sirtuin 6 | 3.5 |
| LIG4 | DNA Ligase 4 | 3.5 |
| TNFSF18 | TNF Superfamily Member 18 | 3.49 |
| PDP1 | Pyruvate Dehyrogenase Phosphatase Catalytic Subunit 1 | 3.49 |
| RNF113A | Ring Finger Protein 113A | 3.49 |
| CARD16 | Caspase Recruitment Domain Family Member 16 | 3.49 |
| HTR2A | 5-Hydroxytryptamine Receptor 2A | 3.48 |
| PTMA | Prothymosin Alpha | 3.48 |
| JMJD6 | Jumonji Domain Containing 6, Arginine Demethylase And Lysine Hydroxylase | 3.47 |
| HDAC11 | Histone Deacetylase 11 | 3.47 |
| PTPRJ | Protein Tyrosine Phosphatase Receptor Type J | 3.47 |
| GRK5 | G Protein-Coupled Receptor Kinase 5 | 3.47 |
| ABCB7 | ATP Binding Cassette Subfamily B Member 7 | 3.47 |
| SUMO1 | Small Ubiquitin Like Modifier 1 | 3.47 |
| ZEB1 | Zinc Finger E-Box Binding Homeobox 1 | 3.46 |
| DNAJB1 | DnaJ Heat Shock Protein Family (Hsp40) Member B1 | 3.46 |
| ADH7 | Alcohol Dehydrogenase 7 (Class IV), Mu Or Sigma Polypeptide | 3.46 |
| LIG3 | DNA Ligase 3 | 3.46 |
| PSMA7 | Proteasome 20S Subunit Alpha 7 | 3.46 |
| PSMD2 | Proteasome 26S Subunit, Non-ATPase 2 | 3.46 |
| NFYB | Nuclear Transcription Factor Y Subunit Beta | 3.46 |
| MLIP | Muscular LMNA Interacting Protein | 3.46 |
| SAGE1 | Sarcoma Antigen 1 | 3.46 |
| MIR139 | MicroRNA 139 | 3.46 |
| RASSF1 | Ras Association Domain Family Member 1 | 3.46 |
| GTF2IRD1 | GTF2I Repeat Domain Containing 1 | 3.45 |
| DEFA1 | Defensin Alpha 1 | 3.45 |
| DPP10 | Dipeptidyl Peptidase Like 10 | 3.45 |
| PAK1 | P21 (RAC1) Activated Kinase 1 | 3.45 |
| NTF4 | Neurotrophin 4 | 3.45 |
| RPL13A | Ribosomal Protein L13a | 3.44 |
| ACHE | Acetylcholinesterase (Cartwright Blood Group) | 3.44 |
| GPR151 | G Protein-Coupled Receptor 151 | 3.44 |
| MIR212 | MicroRNA 212 | 3.44 |
| MITF | Melanocyte Inducing Transcription Factor | 3.44 |
| FCER1G | Fc Fragment Of IgE Receptor Ig | 3.44 |
| KRT3 | Keratin 3 | 3.43 |
| HOXA-AS2 | HOXA Cluster Antisense RNA 2 | 3.43 |
| CACNA1A | Calcium Voltage-Gated Channel Subunit Alpha1 A | 3.43 |
| OAT | Ornithine Aminotransferase | 3.43 |
| CRB1 | Crumbs Cell Polarity Complex Component 1 | 3.43 |
| PVT1 | Pvt1 Oncogene | 3.43 |
| MIR33B | MicroRNA 33b | 3.43 |
| MIR675 | MicroRNA 675 | 3.43 |
| CBR3-AS1 | CBR3 Antisense RNA 1 | 3.43 |
| MIR383 | MicroRNA 383 | 3.43 |
| SDHA | Succinate Dehydrogenase Complex Flavoprotein Subunit A | 3.43 |
| MIR30B | MicroRNA 30b | 3.43 |
| FOLR2 | Folate Receptor Beta | 3.42 |
| MKI67 | Marker Of Proliferation Ki-67 | 3.42 |
| GTF2E2 | General Transcription Factor IIE Subunit 2 | 3.42 |
| GTF2H5 | General Transcription Factor IIH Subunit 5 | 3.42 |
| CRYAB | Crystallin Alpha B | 3.41 |
| SEMA7A | Semaphorin 7A (John Milton Hagen Blood Group) | 3.41 |
| DICER1 | Dicer 1, Ribonuclease III | 3.4 |
| ACVRL1 | Activin A Receptor Like Type 1 | 3.4 |
| KMT2D | Lysine Methyltransferase 2D | 3.4 |
| DSPP | Dentin Sialophosphoprotein | 3.39 |
| STK36 | Serine/Threonine Kinase 36 | 3.39 |
| ADAMTS1 | ADAM Metallopeptidase With Thrombospondin Type 1 Motif 1 | 3.39 |
| KAT5 | Lysine Acetyltransferase 5 | 3.39 |
| MLANA | Melan-A | 3.39 |
| KIF3A | Kinesin Family Member 3A | 3.39 |
| ALK | ALK Receptor Tyrosine Kinase | 3.38 |
| IGFBP5 | Insulin Like Growth Factor Binding Protein 5 | 3.38 |
| KRT15 | Keratin 15 | 3.38 |
| NR3C2 | Nuclear Receptor Subfamily 3 Group C Member 2 | 3.38 |
| GPR1 | G Protein-Coupled Receptor 1 | 3.38 |
| GLP1R | Glucagon Like Peptide 1 Receptor | 3.38 |
| C1QTNF3 | C1q And TNF Related 3 | 3.37 |
| BSG | Basigin (Ok Blood Group) | 3.37 |
| PON2 | Paraoxonase 2 | 3.37 |
| DDR1 | Discoidin Domain Receptor Tyrosine Kinase 1 | 3.36 |
| FUT3 | Fucosyltransferase 3 (Lewis Blood Group) | 3.36 |
| EDN3 | Endothelin 3 | 3.36 |
| RUNX3 | RUNX Family Transcription Factor 3 | 3.36 |
| MFN2 | Mitofusin 2 | 3.36 |
| ALDH7A1 | Aldehyde Dehydrogenase 7 Family Member A1 | 3.36 |
| NT5C2 | 5'-Nucleotidase, Cytosolic II | 3.36 |
| NDUFS1 | NADH:Ubiquinone Oxidoreductase Core Subunit S1 | 3.36 |
| ADCY9 | Adenylate Cyclase 9 | 3.36 |
| MYO5B | Myosin VB | 3.36 |
| ADARB1 | Adenosine Deaminase RNA Specific B1 | 3.36 |
| SLC26A5 | Solute Carrier Family 26 Member 5 | 3.36 |
| CDC42BPB | CDC42 Binding Protein Kinase Beta | 3.36 |
| RPL34 | Ribosomal Protein L34 | 3.36 |
| CILP | Cartilage Intermediate Layer Protein | 3.36 |
| MSLN | Mesothelin | 3.36 |
| DSG4 | Desmoglein 4 | 3.36 |
| NT5C3A | 5'-Nucleotidase, Cytosolic IIIA | 3.36 |
| ATAD2 | ATPase Family AAA Domain Containing 2 | 3.36 |
| MEST | Mesoderm Specific Transcript | 3.36 |
| LMF1 | Lipase Maturation Factor 1 | 3.36 |
| DCAF8 | DDB1 And CUL4 Associated Factor 8 | 3.36 |
| GHITM | Growth Hormone Inducible Transmembrane Protein | 3.36 |
| MMP27 | Matrix Metallopeptidase 27 | 3.36 |
| ADAM30 | ADAM Metallopeptidase Domain 30 | 3.36 |
| GXYLT2 | Glucoside Xylosyltransferase 2 | 3.36 |
| SPAG6 | Sperm Associated Antigen 6 | 3.36 |
| PAM16 | Presequence Translocase Associated Motor 16 | 3.36 |
| MAS1L | MAS1 Proto-Oncogene Like, G Protein-Coupled Receptor | 3.36 |
| EVPL | Envoplakin | 3.36 |
| VWA2 | Von Willebrand Factor A Domain Containing 2 | 3.36 |
| MYRF | Myelin Regulatory Factor | 3.36 |
| APOLD1 | Apolipoprotein L Domain Containing 1 | 3.36 |
| KRTDAP | Keratinocyte Differentiation Associated Protein | 3.36 |
| WRAP73 | WD Repeat Containing, Antisense To TP73 | 3.36 |
| KRTAP9-2 | Keratin Associated Protein 9-2 | 3.36 |
| PRNT | Prion Locus LncRNA, Testis Expressed | 3.36 |
| MIR128-2 | MicroRNA 128-2 | 3.36 |
| MIR339 | MicroRNA 339 | 3.36 |
| SNHG5 | Small Nucleolar RNA Host Gene 5 | 3.36 |
| MIR340 | MicroRNA 340 | 3.36 |
| ZFAS1 | ZNFX1 Antisense RNA 1 | 3.36 |
| MIR299 | MicroRNA 299 | 3.36 |
| IBD19 | Inflammatory Bowel Disease 19 | 3.36 |
| NPM1 | Nucleophosmin 1 | 3.36 |
| ALOX12B | Arachidonate 12-Lipoxygenase, 12R Type | 3.36 |
| FST | Follistatin | 3.36 |
| HSPA8 | Heat Shock Protein Family A (Hsp70) Member 8 | 3.35 |
| ACTC1 | Actin Alpha Cardiac Muscle 1 | 3.35 |
| PZP | PZP Alpha-2-Macroglobulin Like | 3.35 |
| MGST2 | Microsomal Glutathione S-Transferase 2 | 3.35 |
| ADAMTS10 | ADAM Metallopeptidase With Thrombospondin Type 1 Motif 10 | 3.35 |
| MIR100 | MicroRNA 100 | 3.34 |
| GCKR | Glucokinase Regulator | 3.34 |
| PRKCZ | Protein Kinase C Zeta | 3.34 |
| HSD3B1 | Hydroxy-Delta-5-Steroid Dehydrogenase, 3 Beta- And Steroid Delta-Isomerase 1 | 3.34 |
| FGF3 | Fibroblast Growth Factor 3 | 3.33 |
| SMARCD2 | SWI/SNF Related, Matrix Associated, Actin Dependent Regulator Of Chromatin, Subfamily D, Member 2 | 3.33 |
| IFN1@ | Interferon, Type 1, Cluster | 3.33 |
| SPEF2 | Sperm Flagellar 2 | 3.32 |
| CFAP221 | Cilia And Flagella Associated Protein 221 | 3.32 |
| CTNNA1 | Catenin Alpha 1 | 3.32 |
| IL20 | Interleukin 20 | 3.32 |
| HPSE2 | Heparanase 2 (Inactive) | 3.32 |
| XPC | XPC Complex Subunit, DNA Damage Recognition And Repair Factor | 3.32 |
| ALOXE3 | Arachidonate Lipoxygenase 3 | 3.32 |
| FGF9 | Fibroblast Growth Factor 9 | 3.32 |
| MAPK10 | Mitogen-Activated Protein Kinase 10 | 3.31 |
| EIF2AK3 | Eukaryotic Translation Initiation Factor 2 Alpha Kinase 3 | 3.31 |
| PLCG1 | Phospholipase C Gamma 1 | 3.31 |
| KEAP1 | Kelch Like ECH Associated Protein 1 | 3.3 |
| NRP1 | Neuropilin 1 | 3.3 |
| TST | Thiosulfate Sulfurtransferase | 3.3 |
| KLK4 | Kallikrein Related Peptidase 4 | 3.3 |
| MIR204 | MicroRNA 204 | 3.3 |
| MIR183 | MicroRNA 183 | 3.3 |
| ATP7B | ATPase Copper Transporting Beta | 3.3 |
| DDR2 | Discoidin Domain Receptor Tyrosine Kinase 2 | 3.29 |
| PRKD1 | Protein Kinase D1 | 3.29 |
| NIPAL4 | NIPA Like Domain Containing 4 | 3.29 |
| TNFRSF9 | TNF Receptor Superfamily Member 9 | 3.29 |
| GLUD1 | Glutamate Dehydrogenase 1 | 3.29 |
| BMPR2 | Bone Morphogenetic Protein Receptor Type 2 | 3.28 |
| PKP2 | Plakophilin 2 | 3.28 |
| SLC22A3 | Solute Carrier Family 22 Member 3 | 3.28 |
| CNDP2 | Carnosine Dipeptidase 2 | 3.28 |
| PMP22 | Peripheral Myelin Protein 22 | 3.28 |
| GDF7 | Growth Differentiation Factor 7 | 3.28 |
| CTNNA3 | Catenin Alpha 3 | 3.28 |
| ACR | Acrosin | 3.28 |
| DCAF1 | DDB1 And CUL4 Associated Factor 1 | 3.28 |
| YAP1 | Yes1 Associated Transcriptional Regulator | 3.26 |
| PTGER3 | Prostaglandin E Receptor 3 | 3.26 |
| NLRP8 | NLR Family Pyrin Domain Containing 8 | 3.26 |
| FOXG1 | Forkhead Box G1 | 3.25 |
| EXO1 | Exonuclease 1 | 3.25 |
| TPR | Translocated Promoter Region, Nuclear Basket Protein | 3.25 |
| HHIP | Hedgehog Interacting Protein | 3.25 |
| KRT6B | Keratin 6B | 3.25 |
| GP5 | Glycoprotein V Platelet | 3.25 |
| LRIG2 | Leucine Rich Repeats And Immunoglobulin Like Domains 2 | 3.25 |
| PLA2G4B | Phospholipase A2 Group IVB | 3.25 |
| FGFBP2 | Fibroblast Growth Factor Binding Protein 2 | 3.25 |
| OGA | O-GlcNAcase | 3.25 |
| NPSR1-AS1 | NPSR1 Antisense RNA 1 | 3.25 |
| AAA1 | Aortic Aneurysm, Familial Abdominal 1 | 3.25 |
| CLCN5 | Chloride Voltage-Gated Channel 5 | 3.25 |
| WNT3A | Wnt Family Member 3A | 3.25 |
| FUT7 | Fucosyltransferase 7 | 3.24 |
| NOTCH3 | Notch Receptor 3 | 3.24 |
| ADAM10 | ADAM Metallopeptidase Domain 10 | 3.24 |
| MTDH | Metadherin | 3.24 |
| PAH | Phenylalanine Hydroxylase | 3.23 |
| MATN3 | Matrilin 3 | 3.23 |
| BCKDHB | Branched Chain Keto Acid Dehydrogenase E1 Subunit Beta | 3.23 |
| AP2S1 | Adaptor Related Protein Complex 2 Subunit Sigma 1 | 3.23 |
| GJB4 | Gap Junction Protein Beta 4 | 3.23 |
| FOXD3 | Forkhead Box D3 | 3.23 |
| TBX19 | T-Box Transcription Factor 19 | 3.23 |
| GLIS3 | GLIS Family Zinc Finger 3 | 3.23 |
| ASPN | Asporin | 3.23 |
| TRAPPC2 | Trafficking Protein Particle Complex 2 | 3.23 |
| CAVIN1 | Caveolae Associated Protein 1 | 3.23 |
| CGA | Glycoprotein Hormones, Alpha Polypeptide | 3.23 |
| S100A11 | S100 Calcium Binding Protein A11 | 3.23 |
| SLCO2A1 | Solute Carrier Organic Anion Transporter Family Member 2A1 | 3.23 |
| NR2F2 | Nuclear Receptor Subfamily 2 Group F Member 2 | 3.22 |
| ABCA12 | ATP Binding Cassette Subfamily A Member 12 | 3.22 |
| FADD | Fas Associated Via Death Domain | 3.21 |
| GP1BB | Glycoprotein Ib Platelet Subunit Beta | 3.21 |
| SHANK3 | SH3 And Multiple Ankyrin Repeat Domains 3 | 3.21 |
| TWIST1 | Twist Family BHLH Transcription Factor 1 | 3.2 |
| PLD1 | Phospholipase D1 | 3.2 |
| DKK1 | Dickkopf WNT Signaling Pathway Inhibitor 1 | 3.2 |
| DDB1 | Damage Specific DNA Binding Protein 1 | 3.19 |
| MIR301A | MicroRNA 301a | 3.19 |
| CEACAM1 | CEA Cell Adhesion Molecule 1 | 3.19 |
| HK1 | Hexokinase 1 | 3.19 |
| FBLN5 | Fibulin 5 | 3.19 |
| SELENOS | Selenoprotein S | 3.19 |
| MFHAS1 | Malignant Fibrous Histiocytoma Amplified Sequence 1 | 3.19 |
| SLC7A7 | Solute Carrier Family 7 Member 7 | 3.18 |
| NIPBL | NIPBL Cohesin Loading Factor | 3.18 |
| BLVRB | Biliverdin Reductase B | 3.18 |
| CSGALNACT1 | Chondroitin Sulfate N-Acetylgalactosaminyltransferase 1 | 3.18 |
| SRPX2 | Sushi Repeat Containing Protein X-Linked 2 | 3.18 |
| LAT2 | Linker For Activation Of T Cells Family Member 2 | 3.18 |
| RSF1 | Remodeling And Spacing Factor 1 | 3.18 |
| ADRA1B | Adrenoceptor Alpha 1B | 3.18 |
| CHRM1 | Cholinergic Receptor Muscarinic 1 | 3.18 |
| GRIA1 | Glutamate Ionotropic Receptor AMPA Type Subunit 1 | 3.18 |
| CCL23 | C-C Motif Chemokine Ligand 23 | 3.18 |
| GLP2R | Glucagon Like Peptide 2 Receptor | 3.17 |
| RPS6KA5 | Ribosomal Protein S6 Kinase A5 | 3.17 |
| CHD4 | Chromodomain Helicase DNA Binding Protein 4 | 3.17 |
| SLC30A4 | Solute Carrier Family 30 Member 4 | 3.17 |
| MAP2K1 | Mitogen-Activated Protein Kinase Kinase 1 | 3.17 |
| RDX | Radixin | 3.17 |
| NLRP10 | NLR Family Pyrin Domain Containing 10 | 3.17 |
| PSIP1 | PC4 And SFRS1 Interacting Protein 1 | 3.16 |
| MIRLET7C | MicroRNA Let-7c | 3.16 |
| CD160 | CD160 Molecule | 3.15 |
| PRMT5 | Protein Arginine Methyltransferase 5 | 3.15 |
| NR1D1 | Nuclear Receptor Subfamily 1 Group D Member 1 | 3.15 |
| SDC2 | Syndecan 2 | 3.15 |
| TYRO3 | TYRO3 Protein Tyrosine Kinase | 3.15 |
| MTRR | 5-Methyltetrahydrofolate-Homocysteine Methyltransferase Reductase | 3.15 |
| BST1 | Bone Marrow Stromal Cell Antigen 1 | 3.15 |
| RNF168 | Ring Finger Protein 168 | 3.14 |
| CDKN3 | Cyclin Dependent Kinase Inhibitor 3 | 3.14 |
| TAF1 | TATA-Box Binding Protein Associated Factor 1 | 3.13 |
| CCN4 | Cellular Communication Network Factor 4 | 3.13 |
| TRPM8 | Transient Receptor Potential Cation Channel Subfamily M Member 8 | 3.13 |
| SAE1 | SUMO1 Activating Enzyme Subunit 1 | 3.13 |
| NDUFB8 | NADH:Ubiquinone Oxidoreductase Subunit B8 | 3.13 |
| C7 | Complement C7 | 3.13 |
| FBLIM1 | Filamin Binding LIM Protein 1 | 3.13 |
| SOST | Sclerostin | 3.13 |
| NLRP4 | NLR Family Pyrin Domain Containing 4 | 3.12 |
| KLKB1 | Kallikrein B1 | 3.12 |
| DAPK1 | Death Associated Protein Kinase 1 | 3.11 |
| COG2 | Component Of Oligomeric Golgi Complex 2 | 3.11 |
| LEF1 | Lymphoid Enhancer Binding Factor 1 | 3.1 |
| NEFL | Neurofilament Light | 3.1 |
| ATIC | 5-Aminoimidazole-4-Carboxamide Ribonucleotide Formyltransferase/IMP Cyclohydrolase | 3.1 |
| STK24 | Serine/Threonine Kinase 24 | 3.1 |
| RNASET2 | Ribonuclease T2 | 3.1 |
| FTCD | Formimidoyltransferase Cyclodeaminase | 3.1 |
| SURF1 | SURF1 Cytochrome C Oxidase Assembly Factor | 3.1 |
| CLIC1 | Chloride Intracellular Channel 1 | 3.1 |
| GLCCI1 | Glucocorticoid Induced 1 | 3.1 |
| LAD1 | Ladinin 1 | 3.1 |
| CFAP43 | Cilia And Flagella Associated Protein 43 | 3.1 |
| PIK3CB | Phosphatidylinositol-4,5-Bisphosphate 3-Kinase Catalytic Subunit Beta | 3.1 |
| IL24 | Interleukin 24 | 3.1 |
| NLRP14 | NLR Family Pyrin Domain Containing 14 | 3.1 |
| RAB27A | RAB27A, Member RAS Oncogene Family | 3.1 |
| CLDN4 | Claudin 4 | 3.1 |
| TSC1 | TSC Complex Subunit 1 | 3.09 |
| PROM1 | Prominin 1 | 3.09 |
| CD207 | CD207 Molecule | 3.09 |
| OXA1L | OXA1L Mitochondrial Inner Membrane Protein | 3.08 |
| FLNB | Filamin B | 3.08 |
| TREML1 | Triggering Receptor Expressed On Myeloid Cells Like 1 | 3.08 |
| SCN5A | Sodium Voltage-Gated Channel Alpha Subunit 5 | 3.08 |
| PCK1 | Phosphoenolpyruvate Carboxykinase 1 | 3.08 |
| KCNJ11 | Potassium Inwardly Rectifying Channel Subfamily J Member 11 | 3.08 |
| GALNT3 | Polypeptide N-Acetylgalactosaminyltransferase 3 | 3.08 |
| CHKB | Choline Kinase Beta | 3.08 |
| ETFDH | Electron Transfer Flavoprotein Dehydrogenase | 3.08 |
| RLBP1 | Retinaldehyde Binding Protein 1 | 3.08 |
| GYPC | Glycophorin C (Gerbich Blood Group) | 3.08 |
| CTSE | Cathepsin E | 3.08 |
| PPL | Periplakin | 3.08 |
| NRTN | Neurturin | 3.08 |
| AZIN1 | Antizyme Inhibitor 1 | 3.08 |
| MFF | Mitochondrial Fission Factor | 3.08 |
| IFITM2 | Interferon Induced Transmembrane Protein 2 | 3.08 |
| AARS1 | Alanyl-TRNA Synthetase 1 | 3.08 |
| LAMTOR5 | Late Endosomal/Lysosomal Adaptor, MAPK And MTOR Activator 5 | 3.08 |
| TREX2 | Three Prime Repair Exonuclease 2 | 3.08 |
| SUPT20H | SPT20 Homolog, SAGA Complex Component | 3.08 |
| IFI44L | Interferon Induced Protein 44 Like | 3.08 |
| MIR574 | MicroRNA 574 | 3.08 |
| MIR224 | MicroRNA 224 | 3.08 |
| PSORS4 | Psoriasis Susceptibility 4 | 3.08 |
| COL18A1 | Collagen Type XVIII Alpha 1 Chain | 3.07 |
| ATF3 | Activating Transcription Factor 3 | 3.07 |
| CACNA1C | Calcium Voltage-Gated Channel Subunit Alpha1 C | 3.06 |
| CYP24A1 | Cytochrome P450 Family 24 Subfamily A Member 1 | 3.06 |
| PRKG1 | Protein Kinase CGMP-Dependent 1 | 3.06 |
| CTSS | Cathepsin S | 3.05 |
| ADH1C | Alcohol Dehydrogenase 1C (Class I), Gamma Polypeptide | 3.05 |
| PLXND1 | Plexin D1 | 3.05 |
| ASAH1 | N-Acylsphingosine Amidohydrolase 1 | 3.05 |
| IGFBP2 | Insulin Like Growth Factor Binding Protein 2 | 3.05 |
| LY86 | Lymphocyte Antigen 86 | 3.05 |
| HBA2 | Hemoglobin Subunit Alpha 2 | 3.05 |
| NR1D2 | Nuclear Receptor Subfamily 1 Group D Member 2 | 3.05 |
| GBE1 | 1,4-Alpha-Glucan Branching Enzyme 1 | 3.05 |
| SRY | Sex Determining Region Y | 3.05 |
| VASP | Vasodilator Stimulated Phosphoprotein | 3.04 |
| CD3D | CD3d Molecule | 3.04 |
| SLIT2 | Slit Guidance Ligand 2 | 3.04 |
| TRIB2 | Tribbles Pseudokinase 2 | 3.04 |
| EGLN3 | Egl-9 Family Hypoxia Inducible Factor 3 | 3.03 |
| F2RL2 | Coagulation Factor II Thrombin Receptor Like 2 | 3.03 |
| HTR7 | 5-Hydroxytryptamine Receptor 7 | 3.03 |
| ITGB6 | Integrin Subunit Beta 6 | 3.03 |
| PSMD1 | Proteasome 26S Subunit, Non-ATPase 1 | 3.03 |
| ABO | ABO, Alpha 1-3-N-Acetylgalactosaminyltransferase And Alpha 1-3-Galactosyltransferase | 3.03 |
| CDK2 | Cyclin Dependent Kinase 2 | 3.02 |
| TSC22D3 | TSC22 Domain Family Member 3 | 3.02 |
| BCHE | Butyrylcholinesterase | 3.02 |
| TRG | T Cell Receptor Gamma Locus | 3.02 |
| BGN | Biglycan | 3.02 |
| IL31RA | Interleukin 31 Receptor A | 3.01 |
| EBAG9 | Estrogen Receptor Binding Site Associated Antigen 9 | 3.01 |
| CDK4 | Cyclin Dependent Kinase 4 | 3.01 |
| IGSF3 | Immunoglobulin Superfamily Member 3 | 3.01 |
| UFD1 | Ubiquitin Recognition Factor In ER Associated Degradation 1 | 3.01 |
| ACADM | Acyl-CoA Dehydrogenase Medium Chain | 3.01 |
| ITPA | Inosine Triphosphatase | 3.01 |
| PPP1CB | Protein Phosphatase 1 Catalytic Subunit Beta | 3.01 |
| PPP1CC | Protein Phosphatase 1 Catalytic Subunit Gamma | 3.01 |
| SOX10 | SRY-Box Transcription Factor 10 | 3.01 |
| SMAD9 | SMAD Family Member 9 | 3.01 |
| RSPO1 | R-Spondin 1 | 3.01 |
| OCA2 | OCA2 Melanosomal Transmembrane Protein | 3.01 |
| PMPCA | Peptidase, Mitochondrial Processing Subunit Alpha | 3.01 |
| PADI3 | Peptidyl Arginine Deiminase 3 | 3.01 |
| SLC22A18 | Solute Carrier Family 22 Member 18 | 3.01 |
| B3GALNT1 | Beta-1,3-N-Acetylgalactosaminyltransferase 1 (Globoside Blood Group) | 3.01 |
| ATRIP | ATR Interacting Protein | 3.01 |
| AHI1 | Abelson Helper Integration Site 1 | 3.01 |
| MPV17 | Mitochondrial Inner Membrane Protein MPV17 | 3.01 |
| NEB | Nebulin | 3.01 |
| SETX | Senataxin | 3.01 |
| BACE2 | Beta-Secretase 2 | 3.01 |
| B4GALNT2 | Beta-1,4-N-Acetyl-Galactosaminyltransferase 2 | 3.01 |
| ADARB2 | Adenosine Deaminase RNA Specific B2 (Inactive) | 3.01 |
| HPS3 | HPS3 Biogenesis Of Lysosomal Organelles Complex 2 Subunit 1 | 3.01 |
| MMAA | Metabolism Of Cobalamin Associated A | 3.01 |
| DHX36 | DEAH-Box Helicase 36 | 3.01 |
| SH2D2A | SH2 Domain Containing 2A | 3.01 |
| SH3BP5 | SH3 Domain Binding Protein 5 | 3.01 |
| PPP3R2 | Protein Phosphatase 3 Regulatory Subunit B, Beta | 3.01 |
| TMEM43 | Transmembrane Protein 43 | 3.01 |
| CNGB3 | Cyclic Nucleotide Gated Channel Subunit Beta 3 | 3.01 |
| AKTIP | AKT Interacting Protein | 3.01 |
| ETV5 | ETS Variant Transcription Factor 5 | 3.01 |
| HPS6 | HPS6 Biogenesis Of Lysosomal Organelles Complex 2 Subunit 3 | 3.01 |
| DHRS2 | Dehydrogenase/Reductase 2 | 3.01 |
| DCHS1 | Dachsous Cadherin-Related 1 | 3.01 |
| HOGA1 | 4-Hydroxy-2-Oxoglutarate Aldolase 1 | 3.01 |
| SEMA3D | Semaphorin 3D | 3.01 |
| RASGRP4 | RAS Guanyl Releasing Protein 4 | 3.01 |
| TRMU | TRNA 5-Methylaminomethyl-2-Thiouridylate Methyltransferase | 3.01 |
| DCTN4 | Dynactin Subunit 4 | 3.01 |
| DNASE2 | Deoxyribonuclease 2, Lysosomal | 3.01 |
| KCNK6 | Potassium Two Pore Domain Channel Subfamily K Member 6 | 3.01 |
| USP53 | Ubiquitin Specific Peptidase 53 | 3.01 |
| VPS13B | Vacuolar Protein Sorting 13 Homolog B | 3.01 |
| EVC | EvC Ciliary Complex Subunit 1 | 3.01 |
| CLCA4 | Chloride Channel Accessory 4 | 3.01 |
| IMPG2 | Interphotoreceptor Matrix Proteoglycan 2 | 3.01 |
| TTC12 | Tetratricopeptide Repeat Domain 12 | 3.01 |
| ARMC5 | Armadillo Repeat Containing 5 | 3.01 |
| CDK5RAP1 | CDK5 Regulatory Subunit Associated Protein 1 | 3.01 |
| MRI1 | Methylthioribose-1-Phosphate Isomerase 1 | 3.01 |
| EHD4 | EH Domain Containing 4 | 3.01 |
| UPK1A | Uroplakin 1A | 3.01 |
| MAPK8IP2 | Mitogen-Activated Protein Kinase 8 Interacting Protein 2 | 3.01 |
| CLDND1 | Claudin Domain Containing 1 | 3.01 |
| NEK10 | NIMA Related Kinase 10 | 3.01 |
| PLB1 | Phospholipase B1 | 3.01 |
| LECT2 | Leukocyte Cell Derived Chemotaxin 2 | 3.01 |
| PSTPIP2 | Proline-Serine-Threonine Phosphatase Interacting Protein 2 | 3.01 |
| FAM107B | Family With Sequence Similarity 107 Member B | 3.01 |
| CRISP1 | Cysteine Rich Secretory Protein 1 | 3.01 |
| SHF | Src Homology 2 Domain Containing F | 3.01 |
| TXLNB | Taxilin Beta | 3.01 |
| PGBD1 | PiggyBac Transposable Element Derived 1 | 3.01 |
| USP40 | Ubiquitin Specific Peptidase 40 | 3.01 |
| ZNF334 | Zinc Finger Protein 334 | 3.01 |
| XIRP1 | Xin Actin Binding Repeat Containing 1 | 3.01 |
| TRIM56 | Tripartite Motif Containing 56 | 3.01 |
| ATP5PO | ATP Synthase Peripheral Stalk Subunit OSCP | 3.01 |
| TBATA | Thymus, Brain And Testes Associated | 3.01 |
| P3H3 | Prolyl 3-Hydroxylase 3 | 3.01 |
| P3H4 | Prolyl 3-Hydroxylase Family Member 4 (Inactive) | 3.01 |
| PRSS57 | Serine Protease 57 | 3.01 |
| PATE1 | Prostate And Testis Expressed 1 | 3.01 |
| SCIMP | SLP Adaptor And CSK Interacting Membrane Protein | 3.01 |
| VWA8 | Von Willebrand Factor A Domain Containing 8 | 3.01 |
| TLDC2 | TBC/LysM-Associated Domain Containing 2 | 3.01 |
| CASTOR3 | CASTOR Family Member 3 | 3.01 |
| MRGPRG-AS1 | MRGPRG Antisense RNA 1 | 3.01 |
| FBXL19-AS1 | FBXL19 Antisense RNA 1 | 3.01 |
| MIR491 | MicroRNA 491 | 3.01 |
| SCARNA5 | Small Cajal Body-Specific RNA 5 | 3.01 |
| SCARNA6 | Small Cajal Body-Specific RNA 6 | 3.01 |
| SNHG28 | Small Nucleolar RNA Host Gene 28 | 3.01 |
| SNHG29 | Small Nucleolar RNA Host Gene 29 | 3.01 |
| CYTOR | Cytoskeleton Regulator RNA | 3.01 |
| SNORA54 | Small Nucleolar RNA, H/ACA Box 54 | 3.01 |
| SNORD35A | Small Nucleolar RNA, C/D Box 35A | 3.01 |
| LINC00229 | Long Intergenic Non-Protein Coding RNA 229 | 3.01 |
| SNORA59A | Small Nucleolar RNA, H/ACA Box 59A | 3.01 |
| MIR376A1 | MicroRNA 376a-1 | 3.01 |
| LINC01191 | Long Intergenic Non-Protein Coding RNA 1191 | 3.01 |
| RNASEH2B-AS1 | RNASEH2B Antisense RNA 1 | 3.01 |
| SMIM35 | Small Integral Membrane Protein 35 | 3.01 |
| MT-TG | Mitochondrially Encoded TRNA-Gly (GGN) | 3.01 |
| ENSG00000260743 |  | 3.01 |
| PSORS5 | Psoriasis Susceptibility 5 | 3.01 |
| ATRIP-TREX1 | ATRIP-TREX1 Readthrough | 3.01 |
| PSORS6 | Psoriasis Susceptibility 6 | 3.01 |
| PSORS9 | Psoriasis Susceptibility 9 | 3.01 |
| PSORS10 | Psoriasis Susceptibility 10 | 3.01 |
| PSORS3 | Psoriasis Susceptibility 3 | 3.01 |
| PSORS7 | Psoriasis Susceptibility 7 | 3.01 |
| PSORS8 | Psoriasis Susceptibility 8 | 3.01 |
| GBD2 | Gallbladder Disease 2 | 3.01 |
| GBD3 | Gallbladder Disease 3 | 3.01 |
| LPRS | Leprosy, Susceptibility To 1 | 3.01 |
| LPRS6 | Leprosy, Susceptiblity To, 6 | 3.01 |
| AOMS1 | Abdominal Obesity-Metabolic Syndrome QTL1 | 3.01 |
| AOMS2 | Abdominal Obesity-Metabolic Syndrome QTL2 | 3.01 |
| AGSPX | Angio Serpiginosum | 3.01 |
| MBNP | ?Membranous Nephropathy, Susceptibility To | 3.01 |
| CTEPH1 | Pulmonary Hypertension, Chronic Thromboembolic, Without Deep Vein Thrombosis, Susceptibility To | 3.01 |
| DUPC1 | Dupuytren Contracture 1 | 3.01 |
| DEL11P13 | Wilms Tumor, Aniridia, Genitourinary Anomalies And Mental Retardation Syndrome | 3.01 |
| PDON2 | Periodontitis, Localized Aggressive | 3.01 |
| SLSN3 | Senior-Loken Syndrome 3 | 3.01 |
| HSCR5 | Hirschsprung Disease, Susceptibility To, 5 | 3.01 |
| HSCR6 | Hirschsprung Disease, Short-Segment, 2 | 3.01 |
| HSCR7 | Hirschsprung Disease, Short-Segment, 3 | 3.01 |
| HSCR8 | Hirschsprung Disease Modifier 2 | 3.01 |
| HSCR9 | Hirschsprung Disease, Susceptibility To, 9 | 3.01 |
| PSORS11 | Psoriasis Susceptibility 11 | 3.01 |
| LOC117038795 | CRISPRi-FlowFISH-Validated PRDX2 Regulatory Element | 3.01 |
| LAMP1 | Lysosomal Associated Membrane Protein 1 | 3 |
| TNFRSF10C | TNF Receptor Superfamily Member 10c | 3 |
| GPX1 | Glutathione Peroxidase 1 | 3 |
| PSMB3 | Proteasome 20S Subunit Beta 3 | 3 |
| GHRH | Growth Hormone Releasing Hormone | 3 |
| KIF11 | Kinesin Family Member 11 | 2.99 |
| PNPLA1 | Patatin Like Phospholipase Domain Containing 1 | 2.99 |
| APPL1 | Adaptor Protein, Phosphotyrosine Interacting With PH Domain And Leucine Zipper 1 | 2.99 |
| FOXC1 | Forkhead Box C1 | 2.99 |
| MIR18A | MicroRNA 18a | 2.99 |
| VCAN | Versican | 2.99 |
| ERG | ETS Transcription Factor ERG | 2.99 |
| AQP9 | Aquaporin 9 | 2.98 |
| NCR3 | Natural Cytotoxicity Triggering Receptor 3 | 2.98 |
| ID1 | Inhibitor Of DNA Binding 1, HLH Protein | 2.98 |
| RHBDF2 | Rhomboid 5 Homolog 2 | 2.98 |
| DEFA3 | Defensin Alpha 3 | 2.98 |
| CDC42 | Cell Division Cycle 42 | 2.98 |
| IFT122 | Intraflagellar Transport 122 | 2.97 |
| C2CD3 | C2 Domain Containing 3 Centriole Elongation Regulator | 2.97 |
| NDUFB11 | NADH:Ubiquinone Oxidoreductase Subunit B11 | 2.97 |
| PKHD1 | PKHD1 Ciliary IPT Domain Containing Fibrocystin/Polyductin | 2.97 |
| CHRNA7 | Cholinergic Receptor Nicotinic Alpha 7 Subunit | 2.96 |
| TIMP4 | TIMP Metallopeptidase Inhibitor 4 | 2.96 |
| FZD8 | Frizzled Class Receptor 8 | 2.96 |
| XRCC1 | X-Ray Repair Cross Complementing 1 | 2.96 |
| NDFIP1 | Nedd4 Family Interacting Protein 1 | 2.96 |
| AURKA | Aurora Kinase A | 2.95 |
| ASS1 | Argininosuccinate Synthase 1 | 2.95 |
| CD52 | CD52 Molecule | 2.95 |
| SMAD1 | SMAD Family Member 1 | 2.94 |
| MPP1 | Membrane Palmitoylated Protein 1 | 2.94 |
| FCN1 | Ficolin 1 | 2.94 |
| CLCN7 | Chloride Voltage-Gated Channel 7 | 2.94 |
| SEC24C | SEC24 Homolog C, COPII Coat Complex Component | 2.94 |
| HIRA | Histone Cell Cycle Regulator | 2.94 |
| IARS2 | Isoleucyl-TRNA Synthetase 2, Mitochondrial | 2.94 |
| MAB21L1 | Mab-21 Like 1 | 2.94 |
| ARVCF | ARVCF Delta Catenin Family Member | 2.94 |
| TSHB | Thyroid Stimulating Hormone Subunit Beta | 2.94 |
| NLRP11 | NLR Family Pyrin Domain Containing 11 | 2.94 |
| PRXL2A | Peroxiredoxin Like 2A | 2.94 |
| CES1 | Carboxylesterase 1 | 2.93 |
| TGFBI | Transforming Growth Factor Beta Induced | 2.93 |
| CKM | Creatine Kinase, M-Type | 2.93 |
| EPAS1 | Endothelial PAS Domain Protein 1 | 2.93 |
| RASGRP2 | RAS Guanyl Releasing Protein 2 | 2.92 |
| SLC2A1 | Solute Carrier Family 2 Member 1 | 2.92 |
| RHBDD3 | Rhomboid Domain Containing 3 | 2.92 |
| CDKAL1 | CDK5 Regulatory Subunit Associated Protein 1 Like 1 | 2.92 |
| CD1C | CD1c Molecule | 2.92 |
| CD200 | CD200 Molecule | 2.91 |
| AXL | AXL Receptor Tyrosine Kinase | 2.91 |
| ZNF580 | Zinc Finger Protein 580 | 2.91 |
| DEFB126 | Defensin Beta 126 | 2.91 |
| MIR382 | MicroRNA 382 | 2.9 |
| ERBB3 | Erb-B2 Receptor Tyrosine Kinase 3 | 2.9 |
| MDH2 | Malate Dehydrogenase 2 | 2.9 |
| SENP8 | SUMO Peptidase Family Member, NEDD8 Specific | 2.9 |
| GNLY | Granulysin | 2.9 |
| MIR107 | MicroRNA 107 | 2.9 |
| FBP1 | Fructose-Bisphosphatase 1 | 2.89 |
| CPS1 | Carbamoyl-Phosphate Synthase 1 | 2.89 |
| POLD1 | DNA Polymerase Delta 1, Catalytic Subunit | 2.89 |
| B9D2 | B9 Domain Containing 2 | 2.89 |
| PPP1R14A | Protein Phosphatase 1 Regulatory Inhibitor Subunit 14A | 2.89 |
| MED30 | Mediator Complex Subunit 30 | 2.89 |
| CCP110 | Centriolar Coiled-Coil Protein 110 | 2.89 |
| CTNND1 | Catenin Delta 1 | 2.89 |
| PPIA | Peptidylprolyl Isomerase A | 2.89 |
| SH3KBP1 | SH3 Domain Containing Kinase Binding Protein 1 | 2.89 |
| TRAF2 | TNF Receptor Associated Factor 2 | 2.88 |
| NR5A1 | Nuclear Receptor Subfamily 5 Group A Member 1 | 2.88 |
| LRP5 | LDL Receptor Related Protein 5 | 2.88 |
| CDH3 | Cadherin 3 | 2.88 |
| LMNB1 | Lamin B1 | 2.88 |
| SLCO1B3 | Solute Carrier Organic Anion Transporter Family Member 1B3 | 2.88 |
| BANF1 | BAF Nuclear Assembly Factor 1 | 2.88 |
| HEY2 | Hes Related Family BHLH Transcription Factor With YRPW Motif 2 | 2.88 |
| PKP3 | Plakophilin 3 | 2.88 |
| FYB2 | FYN Binding Protein 2 | 2.88 |
| IL17RB | Interleukin 17 Receptor B | 2.87 |
| PRKCB | Protein Kinase C Beta | 2.87 |
| IL36A | Interleukin 36 Alpha | 2.87 |
| BPIFA1 | BPI Fold Containing Family A Member 1 | 2.87 |
| FBXW4 | F-Box And WD Repeat Domain Containing 4 | 2.87 |
| MIR490 | MicroRNA 490 | 2.87 |
| GPX3 | Glutathione Peroxidase 3 | 2.86 |
| RORA | RAR Related Orphan Receptor A | 2.86 |
| IL1F10 | Interleukin 1 Family Member 10 | 2.86 |
| S1PR3 | Sphingosine-1-Phosphate Receptor 3 | 2.85 |
| DES | Desmin | 2.85 |
| ANGPTL3 | Angiopoietin Like 3 | 2.85 |
| CCS | Copper Chaperone For Superoxide Dismutase | 2.85 |
| DSC3 | Desmocollin 3 | 2.85 |
| U2AF1 | U2 Small Nuclear RNA Auxiliary Factor 1 | 2.85 |
| NEUROG3 | Neurogenin 3 | 2.85 |
| ELAVL3 | ELAV Like RNA Binding Protein 3 | 2.85 |
| LVRN | Laeverin | 2.85 |
| CCL4L1 | C-C Motif Chemokine Ligand 4 Like 1 | 2.85 |
| NRON | Non-Coding Repressor Of NFAT | 2.85 |
| WNT10B | Wnt Family Member 10B | 2.84 |
| MYH2 | Myosin Heavy Chain 2 | 2.84 |
| BMP8B | Bone Morphogenetic Protein 8b | 2.84 |
| SUN2 | Sad1 And UNC84 Domain Containing 2 | 2.84 |
| MIR133A1 | MicroRNA 133a-1 | 2.84 |
| NLRX1 | NLR Family Member X1 | 2.83 |
| CHD7 | Chromodomain Helicase DNA Binding Protein 7 | 2.83 |
| ITGA2B | Integrin Subunit Alpha 2b | 2.83 |
| SIK3 | SIK Family Kinase 3 | 2.83 |
| AREL1 | Apoptosis Resistant E3 Ubiquitin Protein Ligase 1 | 2.82 |
| MFGE8 | Milk Fat Globule EGF And Factor V/VIII Domain Containing | 2.82 |
| MGST1 | Microsomal Glutathione S-Transferase 1 | 2.82 |
| TAC3 | Tachykinin Precursor 3 | 2.81 |
| ITGAD | Integrin Subunit Alpha D | 2.81 |
| MAOA | Monoamine Oxidase A | 2.81 |
| CERK | Ceramide Kinase | 2.8 |
| CLDN7 | Claudin 7 | 2.8 |
| IL17B | Interleukin 17B | 2.8 |
| MASP1 | Mannan Binding Lectin Serine Peptidase 1 | 2.79 |
| IL36B | Interleukin 36 Beta | 2.79 |
| SULT2B1 | Sulfotransferase Family 2B Member 1 | 2.79 |
| CREBBP | CREB Binding Protein | 2.78 |
| HDAC4 | Histone Deacetylase 4 | 2.78 |
| PYDC1 | Pyrin Domain Containing 1 | 2.78 |
| TET2 | Tet Methylcytosine Dioxygenase 2 | 2.78 |
| BMPR1A | Bone Morphogenetic Protein Receptor Type 1A | 2.78 |
| FHL2 | Four And A Half LIM Domains 2 | 2.78 |
| IGH | Immunoglobulin Heavy Locus | 2.78 |
| CXCL14 | C-X-C Motif Chemokine Ligand 14 | 2.78 |
| IFT88 | Intraflagellar Transport 88 | 2.78 |
| MIR32 | MicroRNA 32 | 2.78 |
| CSPG4 | Chondroitin Sulfate Proteoglycan 4 | 2.77 |
| CLDN5 | Claudin 5 | 2.77 |
| ZBP1 | Z-DNA Binding Protein 1 | 2.77 |
| BMPR1B | Bone Morphogenetic Protein Receptor Type 1B | 2.77 |
| MIR19A | MicroRNA 19a | 2.76 |
| LGR5 | Leucine Rich Repeat Containing G Protein-Coupled Receptor 5 | 2.76 |
| CCAT1 | Colon Cancer Associated Transcript 1 | 2.76 |
| LEPQTL1 | Leptin, Serum Levels Of | 2.76 |
| ENPP7 | Ectonucleotide Pyrophosphatase/Phosphodiesterase 7 | 2.75 |
| NF1 | Neurofibromin 1 | 2.75 |
| LINC01194 | Long Intergenic Non-Protein Coding RNA 1194 | 2.75 |
| FAP | Fibroblast Activation Protein Alpha | 2.75 |
| BAD | BCL2 Associated Agonist Of Cell Death | 2.75 |
| NOTCH4 | Notch Receptor 4 | 2.75 |
| PTCH1 | Patched 1 | 2.74 |
| MTR | 5-Methyltetrahydrofolate-Homocysteine Methyltransferase | 2.74 |
| PSORS1C1 | Psoriasis Susceptibility 1 Candidate 1 | 2.74 |
| PKM | Pyruvate Kinase M1/2 | 2.74 |
| ACTA1 | Actin Alpha 1, Skeletal Muscle | 2.74 |
| EDN2 | Endothelin 2 | 2.74 |
| HDAC8 | Histone Deacetylase 8 | 2.74 |
| DUSP1 | Dual Specificity Phosphatase 1 | 2.73 |
| MT-CO2 | Mitochondrially Encoded Cytochrome C Oxidase II | 2.73 |
| NKX3-1 | NK3 Homeobox 1 | 2.73 |
| GRIA3 | Glutamate Ionotropic Receptor AMPA Type Subunit 3 | 2.72 |
| NME1 | NME/NM23 Nucleoside Diphosphate Kinase 1 | 2.72 |
| HNRNPA1 | Heterogeneous Nuclear Ribonucleoprotein A1 | 2.72 |
| SETD2 | SET Domain Containing 2, Histone Lysine Methyltransferase | 2.72 |
| GPX2 | Glutathione Peroxidase 2 | 2.72 |
| SLC46A1 | Solute Carrier Family 46 Member 1 | 2.72 |
| MYSM1 | Myb Like, SWIRM And MPN Domains 1 | 2.72 |
| TMPRSS2 | Transmembrane Serine Protease 2 | 2.72 |
| TMSB4X | Thymosin Beta 4 X-Linked | 2.72 |
| NBN | Nibrin | 2.72 |
| PLCD1 | Phospholipase C Delta 1 | 2.71 |
| SLC4A1 | Solute Carrier Family 4 Member 1 (Diego Blood Group) | 2.71 |
| POLH | DNA Polymerase Eta | 2.71 |
| SLC12A3 | Solute Carrier Family 12 Member 3 | 2.71 |
| AP1B1 | Adaptor Related Protein Complex 1 Subunit Beta 1 | 2.71 |
| CLCNKB | Chloride Voltage-Gated Channel Kb | 2.71 |
| SEMA3A | Semaphorin 3A | 2.71 |
| APOD | Apolipoprotein D | 2.71 |
| CEP83 | Centrosomal Protein 83 | 2.7 |
| TFR2 | Transferrin Receptor 2 | 2.7 |
| CYP17A1 | Cytochrome P450 Family 17 Subfamily A Member 1 | 2.7 |
| TAZ | Tafazzin | 2.7 |
| BEST1 | Bestrophin 1 | 2.7 |
| FBXO11 | F-Box Protein 11 | 2.7 |
| GLS2 | Glutaminase 2 | 2.7 |
| TBX18 | T-Box Transcription Factor 18 | 2.7 |
| CSN2 | Casein Beta | 2.7 |
| NECTIN2 | Nectin Cell Adhesion Molecule 2 | 2.7 |
| CNMD | Chondromodulin | 2.7 |
| MMP25 | Matrix Metallopeptidase 25 | 2.7 |
| STS | Steroid Sulfatase | 2.7 |
| SLC37A1 | Solute Carrier Family 37 Member 1 | 2.69 |
| TASP1 | Taspase 1 | 2.69 |
| GPR84 | G Protein-Coupled Receptor 84 | 2.69 |
| CYP2B6 | Cytochrome P450 Family 2 Subfamily B Member 6 | 2.68 |
| CDK9 | Cyclin Dependent Kinase 9 | 2.68 |
| TAT | Tyrosine Aminotransferase | 2.68 |
| BID | BH3 Interacting Domain Death Agonist | 2.67 |
| DNM1 | Dynamin 1 | 2.67 |
| THRB | Thyroid Hormone Receptor Beta | 2.67 |
| RPS6KB2 | Ribosomal Protein S6 Kinase B2 | 2.67 |
| L1CAM | L1 Cell Adhesion Molecule | 2.67 |
| ATP2C1 | ATPase Secretory Pathway Ca2+ Transporting 1 | 2.67 |
| FGF4 | Fibroblast Growth Factor 4 | 2.67 |
| BTC | Betacellulin | 2.67 |
| PSMA3 | Proteasome 20S Subunit Alpha 3 | 2.67 |
| EYA4 | EYA Transcriptional Coactivator And Phosphatase 4 | 2.67 |
| LRPAP1 | LDL Receptor Related Protein Associated Protein 1 | 2.67 |
| CYFIP2 | Cytoplasmic FMR1 Interacting Protein 2 | 2.67 |
| BBS4 | Bardet-Biedl Syndrome 4 | 2.67 |
| CHD3 | Chromodomain Helicase DNA Binding Protein 3 | 2.67 |
| IFITM1 | Interferon Induced Transmembrane Protein 1 | 2.67 |
| PRDM2 | PR/SET Domain 2 | 2.67 |
| IVNS1ABP | Influenza Virus NS1A Binding Protein | 2.67 |
| CKAP4 | Cytoskeleton Associated Protein 4 | 2.67 |
| UPK3A | Uroplakin 3A | 2.67 |
| MOBP | Myelin Associated Oligodendrocyte Basic Protein | 2.67 |
| REG3G | Regenerating Family Member 3 Gamma | 2.67 |
| SLC51B | Solute Carrier Family 51 Subunit Beta | 2.67 |
| POLR1H | RNA Polymerase I Subunit H | 2.67 |
| ARHGAP45 | Rho GTPase Activating Protein 45 | 2.67 |
| MIR133B | MicroRNA 133b | 2.67 |
| LINC-ROR | Long Intergenic Non-Protein Coding RNA, Regulator Of Reprogramming | 2.67 |
| LINC00426 | Long Intergenic Non-Protein Coding RNA 426 | 2.67 |
| ALRH | Allergic Rhinitis | 2.67 |
| AGGF1 | Angiogenic Factor With G-Patch And FHA Domains 1 | 2.67 |
| PHKA2 | Phosphorylase Kinase Regulatory Subunit Alpha 2 | 2.66 |
| NMT1 | N-Myristoyltransferase 1 | 2.66 |
| GLMN | Glomulin, FKBP Associated Protein | 2.66 |
| ARMC10 | Armadillo Repeat Containing 10 | 2.66 |
| MAP3K14 | Mitogen-Activated Protein Kinase Kinase Kinase 14 | 2.66 |
| DAB2IP | DAB2 Interacting Protein | 2.66 |
| TCF7L2 | Transcription Factor 7 Like 2 | 2.66 |
| SMARCA4 | SWI/SNF Related, Matrix Associated, Actin Dependent Regulator Of Chromatin, Subfamily A, Member 4 | 2.66 |
| FOXM1 | Forkhead Box M1 | 2.65 |
| PLK1 | Polo Like Kinase 1 | 2.65 |
| SCG2 | Secretogranin II | 2.65 |
| GLI1 | GLI Family Zinc Finger 1 | 2.65 |
| SLC39A14 | Solute Carrier Family 39 Member 14 | 2.65 |
| CBL | Cbl Proto-Oncogene | 2.64 |
| PINK1 | PTEN Induced Kinase 1 | 2.64 |
| HAS2 | Hyaluronan Synthase 2 | 2.63 |
| VAV1 | Vav Guanine Nucleotide Exchange Factor 1 | 2.63 |
| TNFAIP2 | TNF Alpha Induced Protein 2 | 2.62 |
| SIRT3 | Sirtuin 3 | 2.61 |
| C1QTNF12 | C1q And TNF Related 12 | 2.61 |
| MMP26 | Matrix Metallopeptidase 26 | 2.6 |
| PAX3 | Paired Box 3 | 2.6 |
| PC | Pyruvate Carboxylase | 2.6 |
| TALDO1 | Transaldolase 1 | 2.6 |
| CACNA1E | Calcium Voltage-Gated Channel Subunit Alpha1 E | 2.6 |
| ACY1 | Aminoacylase 1 | 2.6 |
| ACADS | Acyl-CoA Dehydrogenase Short Chain | 2.6 |
| GPT2 | Glutamic--Pyruvic Transaminase 2 | 2.6 |
| SUCLA2 | Succinate-CoA Ligase ADP-Forming Subunit Beta | 2.6 |
| CACNA1F | Calcium Voltage-Gated Channel Subunit Alpha1 F | 2.6 |
| HADHA | Hydroxyacyl-CoA Dehydrogenase Trifunctional Multienzyme Complex Subunit Alpha | 2.6 |
| MRAS | Muscle RAS Oncogene Homolog | 2.6 |
| EMD | Emerin | 2.6 |
| FARSB | Phenylalanyl-TRNA Synthetase Subunit Beta | 2.6 |
| ABCA4 | ATP Binding Cassette Subfamily A Member 4 | 2.6 |
| PABPN1 | Poly(A) Binding Protein Nuclear 1 | 2.6 |
| SCN1B | Sodium Voltage-Gated Channel Beta Subunit 1 | 2.6 |
| ABCD4 | ATP Binding Cassette Subfamily D Member 4 | 2.6 |
| DPP6 | Dipeptidyl Peptidase Like 6 | 2.6 |
| SLC6A13 | Solute Carrier Family 6 Member 13 | 2.6 |
| SLC25A6 | Solute Carrier Family 25 Member 6 | 2.6 |
| CENPF | Centromere Protein F | 2.6 |
| POLR2B | RNA Polymerase II Subunit B | 2.6 |
| NUP98 | Nucleoporin 98 And 96 Precursor | 2.6 |
| XPNPEP1 | X-Prolyl Aminopeptidase 1 | 2.6 |
| NPHS2 | NPHS2 Stomatin Family Member, Podocin | 2.6 |
| RPS9 | Ribosomal Protein S9 | 2.6 |
| SLC30A5 | Solute Carrier Family 30 Member 5 | 2.6 |
| SLC34A3 | Solute Carrier Family 34 Member 3 | 2.6 |
| SIL1 | SIL1 Nucleotide Exchange Factor | 2.6 |
| PHOX2A | Paired Like Homeobox 2A | 2.6 |
| POLI | DNA Polymerase Iota | 2.6 |
| RS1 | Retinoschisin 1 | 2.6 |
| SRP72 | Signal Recognition Particle 72 | 2.6 |
| TPPP | Tubulin Polymerization Promoting Protein | 2.6 |
| SDSL | Serine Dehydratase Like | 2.6 |
| ARMC9 | Armadillo Repeat Containing 9 | 2.6 |
| LY6E | Lymphocyte Antigen 6 Family Member E | 2.6 |
| DECR2 | 2,4-Dienoyl-CoA Reductase 2 | 2.6 |
| PSCA | Prostate Stem Cell Antigen | 2.6 |
| SH2D3C | SH2 Domain Containing 3C | 2.6 |
| SLC30A7 | Solute Carrier Family 30 Member 7 | 2.6 |
| LILRB3 | Leukocyte Immunoglobulin Like Receptor B3 | 2.6 |
| TBCB | Tubulin Folding Cofactor B | 2.6 |
| DHX37 | DEAH-Box Helicase 37 | 2.6 |
| SLC52A1 | Solute Carrier Family 52 Member 1 | 2.6 |
| PSMD5 | Proteasome 26S Subunit, Non-ATPase 5 | 2.6 |
| RCE1 | Ras Converting CAAX Endopeptidase 1 | 2.6 |
| ASZ1 | Ankyrin Repeat, SAM And Basic Leucine Zipper Domain Containing 1 | 2.6 |
| CHIC2 | Cysteine Rich Hydrophobic Domain 2 | 2.6 |
| SLC25A16 | Solute Carrier Family 25 Member 16 | 2.6 |
| NUDT15 | Nudix Hydrolase 15 | 2.6 |
| LIN9 | Lin-9 DREAM MuvB Core Complex Component | 2.6 |
| KIR3DL2 | Killer Cell Immunoglobulin Like Receptor, Three Ig Domains And Long Cytoplasmic Tail 2 | 2.6 |
| KCMF1 | Potassium Channel Modulatory Factor 1 | 2.6 |
| INTU | Inturned Planar Cell Polarity Protein | 2.6 |
| ZWINT | ZW10 Interacting Kinetochore Protein | 2.6 |
| FKBPL | FKBP Prolyl Isomerase Like | 2.6 |
| MRPL49 | Mitochondrial Ribosomal Protein L49 | 2.6 |
| CPLX3 | Complexin 3 | 2.6 |
| IRGC | Immunity Related GTPase Cinema | 2.6 |
| KIR3DL3 | Killer Cell Immunoglobulin Like Receptor, Three Ig Domains And Long Cytoplasmic Tail 3 | 2.6 |
| TTLL1 | Tubulin Tyrosine Ligase Like 1 | 2.6 |
| P3H2 | Prolyl 3-Hydroxylase 2 | 2.6 |
| ZBTB8OS | Zinc Finger And BTB Domain Containing 8 Opposite Strand | 2.6 |
| CAGE1 | Cancer Antigen 1 | 2.6 |
| MEPE | Matrix Extracellular Phosphoglycoprotein | 2.6 |
| EFHC2 | EF-Hand Domain Containing 2 | 2.6 |
| NXPH4 | Neurexophilin 4 | 2.6 |
| ISLR2 | Immunoglobulin Superfamily Containing Leucine Rich Repeat 2 | 2.6 |
| MED8 | Mediator Complex Subunit 8 | 2.6 |
| C18orf25 | Chromosome 18 Open Reading Frame 25 | 2.6 |
| OPALIN | Oligodendrocytic Myelin Paranodal And Inner Loop Protein | 2.6 |
| SCRN3 | Secernin 3 | 2.6 |
| POC5 | POC5 Centriolar Protein | 2.6 |
| OBP2A | Odorant Binding Protein 2A | 2.6 |
| ITLN2 | Intelectin 2 | 2.6 |
| CLASRP | CLK4 Associating Serine/Arginine Rich Protein | 2.6 |
| SPESP1 | Sperm Equatorial Segment Protein 1 | 2.6 |
| PPP1R26 | Protein Phosphatase 1 Regulatory Subunit 26 | 2.6 |
| TMEM134 | Transmembrane Protein 134 | 2.6 |
| STH | Saitohin | 2.6 |
| ERVMER34-1 | Endogenous Retrovirus Group MER34 Member 1, Envelope | 2.6 |
| ZNRD2 | Zinc Ribbon Domain Containing 2 | 2.6 |
| PCA3 | Prostate Cancer Associated 3 | 2.6 |
| MIR369 | MicroRNA 369 | 2.6 |
| BDNF-AS | BDNF Antisense RNA | 2.6 |
| MIR1275 | MicroRNA 1275 | 2.6 |
| MIR376C | MicroRNA 376c | 2.6 |
| AFAP1-AS1 | AFAP1 Antisense RNA 1 | 2.6 |
| MIR584 | MicroRNA 584 | 2.6 |
| MIR487B | MicroRNA 487b | 2.6 |
| MIR422A | MicroRNA 422a | 2.6 |
| MIR379 | MicroRNA 379 | 2.6 |
| MIR501 | MicroRNA 501 | 2.6 |
| SNORA40 | Small Nucleolar RNA, H/ACA Box 40 | 2.6 |
| MIR18B | MicroRNA 18b | 2.6 |
| SNORD50A | Small Nucleolar RNA, C/D Box 50A | 2.6 |
| MIR362 | MicroRNA 362 | 2.6 |
| MIR664A | MicroRNA 664a | 2.6 |
| MIR599 | MicroRNA 599 | 2.6 |
| CXCR2P1 | C-X-C Motif Chemokine Receptor 2 Pseudogene 1 | 2.6 |
| NPTN-IT1 | NPTN Intronic Transcript 1 | 2.6 |
| SNORD79 | Small Nucleolar RNA, C/D Box 79 | 2.6 |
| LINC02384 | Long Intergenic Non-Protein Coding RNA 2384 | 2.6 |
| VIS1 | Viral Integration Site 1 | 2.6 |
| ENSG00000280878 |  | 2.6 |
| GCASPC | Gall Bladder Cancer Associated Suppressor Of Pyruvate Carboxylase LncRNA | 2.6 |
| MS | Multiple Sclerosis | 2.6 |
| DIP | Interstitial Pneumonitis, Desquamative, Familial | 2.6 |
| AKR1A1 | Aldo-Keto Reductase Family 1 Member A1 | 2.6 |
| FLI1 | Fli-1 Proto-Oncogene, ETS Transcription Factor | 2.6 |
| STAR | Steroidogenic Acute Regulatory Protein | 2.6 |
| GPR32 | G Protein-Coupled Receptor 32 | 2.6 |
| SYNCRIP | Synaptotagmin Binding Cytoplasmic RNA Interacting Protein | 2.6 |
| PNOC | Prepronociceptin | 2.6 |
| CCR5AS | CCR5 Antisense RNA | 2.59 |
| NAGLU | N-Acetyl-Alpha-Glucosaminidase | 2.59 |
| UNC119 | Unc-119 Lipid Binding Chaperone | 2.59 |
| CDK6 | Cyclin Dependent Kinase 6 | 2.59 |
| S100A1 | S100 Calcium Binding Protein A1 | 2.59 |
| TNIP2 | TNFAIP3 Interacting Protein 2 | 2.59 |
| DLL4 | Delta Like Canonical Notch Ligand 4 | 2.59 |
| CLEC4D | C-Type Lectin Domain Family 4 Member D | 2.59 |
| CD48 | CD48 Molecule | 2.58 |
| CDR1-AS | CDR1 Antisense RNA | 2.58 |
| EPHA3 | EPH Receptor A3 | 2.58 |
| HOXA9 | Homeobox A9 | 2.58 |
| SRD5A1 | Steroid 5 Alpha-Reductase 1 | 2.57 |
| HABP2 | Hyaluronan Binding Protein 2 | 2.57 |
| SMAD6 | SMAD Family Member 6 | 2.57 |
| OCM2 | Oncomodulin 2 | 2.57 |
| HNRNPA2B1 | Heterogeneous Nuclear Ribonucleoprotein A2/B1 | 2.57 |
| IDUA | Alpha-L-Iduronidase | 2.57 |
| EIF4EBP1 | Eukaryotic Translation Initiation Factor 4E Binding Protein 1 | 2.57 |
| UPF1 | UPF1 RNA Helicase And ATPase | 2.57 |
| SLC39A2 | Solute Carrier Family 39 Member 2 | 2.57 |
| NUMA1 | Nuclear Mitotic Apparatus Protein 1 | 2.56 |
| TRPC6 | Transient Receptor Potential Cation Channel Subfamily C Member 6 | 2.56 |
| HSD3B2 | Hydroxy-Delta-5-Steroid Dehydrogenase, 3 Beta- And Steroid Delta-Isomerase 2 | 2.56 |
| PDGFC | Platelet Derived Growth Factor C | 2.55 |
| ERN1 | Endoplasmic Reticulum To Nucleus Signaling 1 | 2.55 |
| SEMA4D | Semaphorin 4D | 2.55 |
| KLK6 | Kallikrein Related Peptidase 6 | 2.54 |
| MRE11 | MRE11 Homolog, Double Strand Break Repair Nuclease | 2.54 |
| COX4I2 | Cytochrome C Oxidase Subunit 4I2 | 2.54 |
| TANK | TRAF Family Member Associated NFKB Activator | 2.54 |
| ATG7 | Autophagy Related 7 | 2.54 |
| SMPD1 | Sphingomyelin Phosphodiesterase 1 | 2.54 |
| BECN1 | Beclin 1 | 2.54 |
| FGB | Fibrinogen Beta Chain | 2.53 |
| IRF2 | Interferon Regulatory Factor 2 | 2.53 |
| CASP5 | Caspase 5 | 2.53 |
| FFAR2 | Free Fatty Acid Receptor 2 | 2.53 |
| REG1B | Regenerating Family Member 1 Beta | 2.53 |
| MIR92A1 | MicroRNA 92a-1 | 2.53 |
| HMOX2 | Heme Oxygenase 2 | 2.53 |
| BCL2L11 | BCL2 Like 11 | 2.53 |
| ADCY5 | Adenylate Cyclase 5 | 2.52 |
| CLEC2A | C-Type Lectin Domain Family 2 Member A | 2.52 |
| UCHL1 | Ubiquitin C-Terminal Hydrolase L1 | 2.52 |
| PIM2 | Pim-2 Proto-Oncogene, Serine/Threonine Kinase | 2.52 |
| ATF6 | Activating Transcription Factor 6 | 2.52 |
| SGCB | Sarcoglycan Beta | 2.52 |
| ANK1 | Ankyrin 1 | 2.51 |
| DAB2 | DAB Adaptor Protein 2 | 2.51 |
| USP8 | Ubiquitin Specific Peptidase 8 | 2.51 |
| CEL | Carboxyl Ester Lipase | 2.51 |
| PRKCG | Protein Kinase C Gamma | 2.51 |
| SFRP4 | Secreted Frizzled Related Protein 4 | 2.51 |
| GPR65 | G Protein-Coupled Receptor 65 | 2.5 |
| CLDN3 | Claudin 3 | 2.5 |
| VIL1 | Villin 1 | 2.5 |
| ACVR2A | Activin A Receptor Type 2A | 2.5 |
| ADD1 | Adducin 1 | 2.5 |
| ERBB4 | Erb-B2 Receptor Tyrosine Kinase 4 | 2.49 |
| EFNB2 | Ephrin B2 | 2.49 |
| PPP3CA | Protein Phosphatase 3 Catalytic Subunit Alpha | 2.49 |
| CDH2 | Cadherin 2 | 2.49 |
| MAPK8IP1 | Mitogen-Activated Protein Kinase 8 Interacting Protein 1 | 2.49 |
| BMP1 | Bone Morphogenetic Protein 1 | 2.49 |
| TJP2 | Tight Junction Protein 2 | 2.49 |
| ELOVL6 | ELOVL Fatty Acid Elongase 6 | 2.49 |
| LY75 | Lymphocyte Antigen 75 | 2.49 |
| RNASE7 | Ribonuclease A Family Member 7 | 2.49 |
| ARMS2 | Age-Related Maculopathy Susceptibility 2 | 2.49 |
| MIR106A | MicroRNA 106a | 2.49 |
| MGAT5 | Alpha-1,6-Mannosylglycoprotein 6-Beta-N-Acetylglucosaminyltransferase | 2.49 |
| A4GNT | Alpha-1,4-N-Acetylglucosaminyltransferase | 2.49 |
| HIVEP3 | HIVEP Zinc Finger 3 | 2.49 |
| MIR373 | MicroRNA 373 | 2.48 |
| PROK2 | Prokineticin 2 | 2.48 |
| ADH1B | Alcohol Dehydrogenase 1B (Class I), Beta Polypeptide | 2.48 |
| LPIN1 | Lipin 1 | 2.48 |
| MSX2 | Msh Homeobox 2 | 2.47 |
| COCH | Cochlin | 2.47 |
| PSEN2 | Presenilin 2 | 2.47 |
| CYP27A1 | Cytochrome P450 Family 27 Subfamily A Member 1 | 2.47 |
| SLC39A1 | Solute Carrier Family 39 Member 1 | 2.47 |
| MIR625 | MicroRNA 625 | 2.47 |
| KRT9 | Keratin 9 | 2.47 |
| CD96 | CD96 Molecule | 2.47 |
| APIP | APAF1 Interacting Protein | 2.47 |
| ZDHHC19 | Zinc Finger DHHC-Type Palmitoyltransferase 19 | 2.47 |
| BRI3 | Brain Protein I3 | 2.47 |
| JAG2 | Jagged Canonical Notch Ligand 2 | 2.46 |
| CDH5 | Cadherin 5 | 2.46 |
| APOA2 | Apolipoprotein A2 | 2.46 |
| GATA2 | GATA Binding Protein 2 | 2.46 |
| RAB5A | RAB5A, Member RAS Oncogene Family | 2.46 |
| CADM1 | Cell Adhesion Molecule 1 | 2.46 |
| YBX3 | Y-Box Binding Protein 3 | 2.46 |
| MIR33A | MicroRNA 33a | 2.46 |
| MYH7 | Myosin Heavy Chain 7 | 2.45 |
| SNX10 | Sorting Nexin 10 | 2.45 |
| LSP1 | Lymphocyte Specific Protein 1 | 2.44 |
| SMC1A | Structural Maintenance Of Chromosomes 1A | 2.44 |
| SMC3 | Structural Maintenance Of Chromosomes 3 | 2.44 |
| RAD21 | RAD21 Cohesin Complex Component | 2.44 |
| REV3L | REV3 Like, DNA Directed Polymerase Zeta Catalytic Subunit | 2.44 |
| CLTCL1 | Clathrin Heavy Chain Like 1 | 2.44 |
| GPR101 | G Protein-Coupled Receptor 101 | 2.44 |
| SETD5 | SET Domain Containing 5 | 2.44 |
| DNAJC21 | DnaJ Heat Shock Protein Family (Hsp40) Member C21 | 2.44 |
| EFL1 | Elongation Factor Like GTPase 1 | 2.44 |
| CEBPE | CCAAT Enhancer Binding Protein Epsilon | 2.44 |
| PCSK1 | Proprotein Convertase Subtilisin/Kexin Type 1 | 2.44 |
| ID3 | Inhibitor Of DNA Binding 3, HLH Protein | 2.44 |
| KRIT1 | KRIT1 Ankyrin Repeat Containing | 2.44 |
| APBB1IP | Amyloid Beta Precursor Protein Binding Family B Member 1 Interacting Protein | 2.44 |
| FREM2 | FRAS1 Related Extracellular Matrix 2 | 2.44 |
| NCOA4 | Nuclear Receptor Coactivator 4 | 2.44 |
| DYNC2H1 | Dynein Cytoplasmic 2 Heavy Chain 1 | 2.44 |
| FADS3 | Fatty Acid Desaturase 3 | 2.44 |
| HLA-F | Major Histocompatibility Complex, Class I, F | 2.44 |
| PLPP1 | Phospholipid Phosphatase 1 | 2.44 |
| CARD17 | Caspase Recruitment Domain Family Member 17 | 2.44 |
| HTR1B | 5-Hydroxytryptamine Receptor 1B | 2.43 |
| GALNS | Galactosamine (N-Acetyl)-6-Sulfatase | 2.43 |
| KDM1A | Lysine Demethylase 1A | 2.43 |
| PTHLH | Parathyroid Hormone Like Hormone | 2.43 |
| MAOB | Monoamine Oxidase B | 2.43 |
| MEP1A | Meprin A Subunit Alpha | 2.43 |
| MARCO | Macrophage Receptor With Collagenous Structure | 2.43 |
| UCMA | Upper Zone Of Growth Plate And Cartilage Matrix Associated | 2.43 |
| MIR7-1 | MicroRNA 7-1 | 2.43 |
| CAV3 | Caveolin 3 | 2.42 |
| RAI1 | Retinoic Acid Induced 1 | 2.42 |
| FGFR4 | Fibroblast Growth Factor Receptor 4 | 2.42 |
| GPD2 | Glycerol-3-Phosphate Dehydrogenase 2 | 2.42 |
| FAM20C | FAM20C Golgi Associated Secretory Pathway Kinase | 2.42 |
| ANO1 | Anoctamin 1 | 2.42 |
| RTN3 | Reticulon 3 | 2.42 |
| IFRD1 | Interferon Related Developmental Regulator 1 | 2.42 |
| RAB3D | RAB3D, Member RAS Oncogene Family | 2.42 |
| KRT12 | Keratin 12 | 2.42 |
| AFM | Afamin | 2.42 |
| C1GALT1C1 | C1GALT1 Specific Chaperone 1 | 2.42 |
| UTP6 | UTP6 Small Subunit Processome Component | 2.42 |
| TXLNA | Taxilin Alpha | 2.42 |
| GPR183 | G Protein-Coupled Receptor 183 | 2.42 |
| FZD5 | Frizzled Class Receptor 5 | 2.41 |
| PGRMC2 | Progesterone Receptor Membrane Component 2 | 2.41 |
| GPR83 | G Protein-Coupled Receptor 83 | 2.41 |
| ORAI1 | ORAI Calcium Release-Activated Calcium Modulator 1 | 2.4 |
| KCNJ5 | Potassium Inwardly Rectifying Channel Subfamily J Member 5 | 2.4 |
| NPR1 | Natriuretic Peptide Receptor 1 | 2.4 |
| MIR499A | MicroRNA 499a | 2.4 |
| CD47 | CD47 Molecule | 2.4 |
| CYP11A1 | Cytochrome P450 Family 11 Subfamily A Member 1 | 2.4 |
| ADH5 | Alcohol Dehydrogenase 5 (Class III), Chi Polypeptide | 2.4 |
| GMDS | GDP-Mannose 4,6-Dehydratase | 2.4 |
| STXBP2 | Syntaxin Binding Protein 2 | 2.4 |
| SLC4A2 | Solute Carrier Family 4 Member 2 | 2.4 |
| TFAM | Transcription Factor A, Mitochondrial | 2.4 |
| KPNA1 | Karyopherin Subunit Alpha 1 | 2.4 |
| TLN1 | Talin 1 | 2.4 |
| FGF20 | Fibroblast Growth Factor 20 | 2.4 |
| PENK | Proenkephalin | 2.4 |
| FSIP1 | Fibrous Sheath Interacting Protein 1 | 2.4 |
| IFNA4 | Interferon Alpha 4 | 2.4 |
| CD63 | CD63 Molecule | 2.4 |
| HCAR2 | Hydroxycarboxylic Acid Receptor 2 | 2.39 |
| KLRG1 | Killer Cell Lectin Like Receptor G1 | 2.39 |
| FANCC | FA Complementation Group C | 2.39 |
| FUCA1 | Alpha-L-Fucosidase 1 | 2.39 |
| FUS | FUS RNA Binding Protein | 2.39 |
| SRD5A2 | Steroid 5 Alpha-Reductase 2 | 2.39 |
| SUCNR1 | Succinate Receptor 1 | 2.39 |
| CD248 | CD248 Molecule | 2.39 |
| EGR3 | Early Growth Response 3 | 2.39 |
| HBE1 | Hemoglobin Subunit Epsilon 1 | 2.39 |
| PSAP | Prosaposin | 2.38 |
| BPGM | Bisphosphoglycerate Mutase | 2.38 |
| PLN | Phospholamban | 2.37 |
| OPRL1 | Opioid Related Nociceptin Receptor 1 | 2.37 |
| MIR138-1 | MicroRNA 138-1 | 2.37 |
| CA8 | Carbonic Anhydrase 8 | 2.37 |
| ARHGEF1 | Rho Guanine Nucleotide Exchange Factor 1 | 2.37 |
| E2F3 | E2F Transcription Factor 3 | 2.37 |
| DHX9 | DExH-Box Helicase 9 | 2.37 |
| OAS3 | 2'-5'-Oligoadenylate Synthetase 3 | 2.37 |
| SLC30A1 | Solute Carrier Family 30 Member 1 | 2.37 |
| BBS5 | Bardet-Biedl Syndrome 5 | 2.37 |
| CC2D2A | Coiled-Coil And C2 Domain Containing 2A | 2.37 |
| IMMT | Inner Membrane Mitochondrial Protein | 2.37 |
| SLC30A6 | Solute Carrier Family 30 Member 6 | 2.37 |
| TCFL5 | Transcription Factor Like 5 | 2.37 |
| ALLC | Allantoicase | 2.37 |
| H3-3A | H3.3 Histone A | 2.37 |
| TRIM67 | Tripartite Motif Containing 67 | 2.37 |
| WASHC5 | WASH Complex Subunit 5 | 2.37 |
| FAM155A | Family With Sequence Similarity 155 Member A | 2.37 |
| WASHC1 | WASH Complex Subunit 1 | 2.37 |
| SFRP1 | Secreted Frizzled Related Protein 1 | 2.37 |
| RARS1 | Arginyl-TRNA Synthetase 1 | 2.36 |
| F10 | Coagulation Factor X | 2.36 |
| CD9 | CD9 Molecule | 2.36 |
| SDHD | Succinate Dehydrogenase Complex Subunit D | 2.35 |
| C8B | Complement C8 Beta Chain | 2.35 |
| TIMM8A | Translocase Of Inner Mitochondrial Membrane 8A | 2.35 |
| SGCG | Sarcoglycan Gamma | 2.35 |
| AFF4 | AF4/FMR2 Family Member 4 | 2.35 |
| C8A | Complement C8 Alpha Chain | 2.35 |
| HCCS | Holocytochrome C Synthase | 2.35 |
| PSMD12 | Proteasome 26S Subunit, Non-ATPase 12 | 2.35 |
| NHLRC1 | NHL Repeat Containing E3 Ubiquitin Protein Ligase 1 | 2.35 |
| CSPP1 | Centrosome And Spindle Pole Associated Protein 1 | 2.35 |
| FAN1 | FANCD2 And FANCI Associated Nuclease 1 | 2.35 |
| NELFA | Negative Elongation Factor Complex Member A | 2.35 |
| KIAA0586 | KIAA0586 | 2.35 |
| CFAP410 | Cilia And Flagella Associated Protein 410 | 2.35 |
| MAGI2 | Membrane Associated Guanylate Kinase, WW And PDZ Domain Containing 2 | 2.35 |
| CHST2 | Carbohydrate Sulfotransferase 2 | 2.35 |
| VLDLR | Very Low Density Lipoprotein Receptor | 2.34 |
| FFAR3 | Free Fatty Acid Receptor 3 | 2.34 |
| LDHA | Lactate Dehydrogenase A | 2.34 |
| MIR218-1 | MicroRNA 218-1 | 2.34 |
| THNSL2 | Threonine Synthase Like 2 | 2.33 |
| NSMCE2 | NSE2 (MMS21) Homolog, SMC5-SMC6 Complex SUMO Ligase | 2.33 |
| FZD1 | Frizzled Class Receptor 1 | 2.33 |
| TBX3 | T-Box Transcription Factor 3 | 2.33 |
| ZFHX3 | Zinc Finger Homeobox 3 | 2.33 |
| MIR103A2 | MicroRNA 103a-2 | 2.33 |
| PSG2 | Pregnancy Specific Beta-1-Glycoprotein 2 | 2.32 |
| BICC1 | BicC Family RNA Binding Protein 1 | 2.32 |
| TOP1 | DNA Topoisomerase I | 2.32 |
| COL10A1 | Collagen Type X Alpha 1 Chain | 2.32 |
| PLIN2 | Perilipin 2 | 2.32 |
| FXN | Frataxin | 2.32 |
| RHBDF1 | Rhomboid 5 Homolog 1 | 2.32 |
| RC3H2 | Ring Finger And CCCH-Type Domains 2 | 2.32 |
| ACP3 | Acid Phosphatase 3 | 2.31 |
| MAPK11 | Mitogen-Activated Protein Kinase 11 | 2.3 |
| TBP | TATA-Box Binding Protein | 2.3 |
| DNM1L | Dynamin 1 Like | 2.3 |
| MFAP5 | Microfibril Associated Protein 5 | 2.3 |
| SNX9 | Sorting Nexin 9 | 2.3 |
| ITGB8 | Integrin Subunit Beta 8 | 2.3 |
| COIL | Coilin | 2.3 |
| SERPINB13 | Serpin Family B Member 13 | 2.3 |
| TSG101 | Tumor Susceptibility 101 | 2.3 |
| AMY2A | Amylase Alpha 2A | 2.3 |
| CH25H | Cholesterol 25-Hydroxylase | 2.3 |
| SEPTIN4 | Septin 4 | 2.3 |
| MIR519D | MicroRNA 519d | 2.3 |
| LOXL2 | Lysyl Oxidase Like 2 | 2.3 |
| TRIM22 | Tripartite Motif Containing 22 | 2.3 |
| CHST1 | Carbohydrate Sulfotransferase 1 | 2.29 |
| MZB1 | Marginal Zone B And B1 Cell Specific Protein | 2.29 |
| TNFSF14 | TNF Superfamily Member 14 | 2.29 |
| COX4I1 | Cytochrome C Oxidase Subunit 4I1 | 2.29 |
| PVR | PVR Cell Adhesion Molecule | 2.29 |
| CDX1 | Caudal Type Homeobox 1 | 2.29 |
| KCNMA1 | Potassium Calcium-Activated Channel Subfamily M Alpha 1 | 2.29 |
| SLC9A3R1 | SLC9A3 Regulator 1 | 2.29 |
| MPG | N-Methylpurine DNA Glycosylase | 2.29 |
| MIR135B | MicroRNA 135b | 2.29 |
| TGFB2-OT1 | TGFB2 Overlapping Transcript 1 | 2.29 |
| PLCB4 | Phospholipase C Beta 4 | 2.28 |
| MIR101-1 | MicroRNA 101-1 | 2.28 |
| TXNIP | Thioredoxin Interacting Protein | 2.28 |
| IGF2R | Insulin Like Growth Factor 2 Receptor | 2.28 |
| WNT2 | Wnt Family Member 2 | 2.27 |
| CLCN3 | Chloride Voltage-Gated Channel 3 | 2.27 |
| PERP | P53 Apoptosis Effector Related To PMP22 | 2.27 |
| ABCD2 | ATP Binding Cassette Subfamily D Member 2 | 2.27 |
| MIR30C1 | MicroRNA 30c-1 | 2.27 |
| HTR4 | 5-Hydroxytryptamine Receptor 4 | 2.27 |
| SALL1 | Spalt Like Transcription Factor 1 | 2.27 |
| KPNB1 | Karyopherin Subunit Beta 1 | 2.27 |
| FABP6 | Fatty Acid Binding Protein 6 | 2.27 |
| NID1 | Nidogen 1 | 2.27 |
| PPY | Pancreatic Polypeptide | 2.27 |
| SLC9A3R2 | SLC9A3 Regulator 2 | 2.27 |
| STAP2 | Signal Transducing Adaptor Family Member 2 | 2.26 |
| NUPR1 | Nuclear Protein 1, Transcriptional Regulator | 2.26 |
| IGFLR1 | IGF Like Family Receptor 1 | 2.26 |
| NOCT | Nocturnin | 2.26 |
| PDGFRL | Platelet Derived Growth Factor Receptor Like | 2.25 |
| CANX | Calnexin | 2.25 |
| MMP17 | Matrix Metallopeptidase 17 | 2.25 |
| ROM1 | Retinal Outer Segment Membrane Protein 1 | 2.25 |
| IKZF3 | IKAROS Family Zinc Finger 3 | 2.25 |
| RGS6 | Regulator Of G Protein Signaling 6 | 2.25 |
| BCL7B | BAF Chromatin Remodeling Complex Subunit BCL7B | 2.25 |
| ALPG | Alkaline Phosphatase, Germ Cell | 2.25 |
| MIR31HG | MIR31 Host Gene | 2.25 |
| CCNE1 | Cyclin E1 | 2.24 |
| NCK1 | NCK Adaptor Protein 1 | 2.24 |
| GRB7 | Growth Factor Receptor Bound Protein 7 | 2.24 |
| SKP2 | S-Phase Kinase Associated Protein 2 | 2.24 |
| CTRL | Chymotrypsin Like | 2.24 |
| NAV2 | Neuron Navigator 2 | 2.24 |
| MIR503 | MicroRNA 503 | 2.24 |
| APLN | Apelin | 2.24 |
| CASP12 | Caspase 12 (Gene/Pseudogene) | 2.24 |
| PIN1 | Peptidylprolyl Cis/Trans Isomerase, NIMA-Interacting 1 | 2.23 |
| ATP2A2 | ATPase Sarcoplasmic/Endoplasmic Reticulum Ca2+ Transporting 2 | 2.22 |
| GAA | Glucosidase Alpha, Acid | 2.22 |
| FGF8 | Fibroblast Growth Factor 8 | 2.22 |
| IHH | Indian Hedgehog Signaling Molecule | 2.22 |
| FKBP1A | FKBP Prolyl Isomerase 1A | 2.22 |
| GYPA | Glycophorin A (MNS Blood Group) | 2.22 |
| CKB | Creatine Kinase B | 2.22 |
| STMN1 | Stathmin 1 | 2.22 |
| LTBP2 | Latent Transforming Growth Factor Beta Binding Protein 2 | 2.22 |
| CAV2 | Caveolin 2 | 2.22 |
| SMAD5 | SMAD Family Member 5 | 2.22 |
| SLC39A7 | Solute Carrier Family 39 Member 7 | 2.22 |
| KLK8 | Kallikrein Related Peptidase 8 | 2.22 |
| C1GALT1 | Core 1 Synthase, Glycoprotein-N-Acetylgalactosamine 3-Beta-Galactosyltransferase 1 | 2.22 |
| TRIP10 | Thyroid Hormone Receptor Interactor 10 | 2.22 |
| ATOH1 | Atonal BHLH Transcription Factor 1 | 2.22 |
| EDC4 | Enhancer Of MRNA Decapping 4 | 2.22 |
| PTPMT1 | Protein Tyrosine Phosphatase Mitochondrial 1 | 2.22 |
| CHORDC1 | Cysteine And Histidine Rich Domain Containing 1 | 2.22 |
| YIPF7 | Yip1 Domain Family Member 7 | 2.22 |
| MIR193B | MicroRNA 193b | 2.22 |
| STX8 | Syntaxin 8 | 2.22 |
| GKN2 | Gastrokine 2 | 2.22 |
| MIR3936HG | MIR3936 Host Gene | 2.22 |
| HBA1 | Hemoglobin Subunit Alpha 1 | 2.2 |
| KYNU | Kynureninase | 2.2 |
| DLD | Dihydrolipoamide Dehydrogenase | 2.2 |
| GALK1 | Galactokinase 1 | 2.2 |
| PAX8 | Paired Box 8 | 2.2 |
| OGDH | Oxoglutarate Dehydrogenase | 2.2 |
| SLC36A2 | Solute Carrier Family 36 Member 2 | 2.2 |
| WNT2B | Wnt Family Member 2B | 2.2 |
| CRX | Cone-Rod Homeobox | 2.2 |
| SLC6A12 | Solute Carrier Family 6 Member 12 | 2.2 |
| UBR1 | Ubiquitin Protein Ligase E3 Component N-Recognin 1 | 2.2 |
| CCM2 | CCM2 Scaffold Protein | 2.2 |
| MAP4K3 | Mitogen-Activated Protein Kinase Kinase Kinase Kinase 3 | 2.2 |
| DYNLL1 | Dynein Light Chain LC8-Type 1 | 2.2 |
| BBS2 | Bardet-Biedl Syndrome 2 | 2.2 |
| GART | Phosphoribosylglycinamide Formyltransferase, Phosphoribosylglycinamide Synthetase, Phosphoribosylaminoimidazole Synthetase | 2.2 |
| ARR3 | Arrestin 3 | 2.2 |
| ADAM22 | ADAM Metallopeptidase Domain 22 | 2.2 |
| ST8SIA2 | ST8 Alpha-N-Acetyl-Neuraminide Alpha-2,8-Sialyltransferase 2 | 2.2 |
| WNT9A | Wnt Family Member 9A | 2.2 |
| CLASP1 | Cytoplasmic Linker Associated Protein 1 | 2.2 |
| PLS1 | Plastin 1 | 2.2 |
| PTF1A | Pancreas Associated Transcription Factor 1a | 2.2 |
| TCN1 | Transcobalamin 1 | 2.2 |
| PRSS21 | Serine Protease 21 | 2.2 |
| CFHR3 | Complement Factor H Related 3 | 2.2 |
| SHOX2 | Short Stature Homeobox 2 | 2.2 |
| SLC15A2 | Solute Carrier Family 15 Member 2 | 2.2 |
| KPNA5 | Karyopherin Subunit Alpha 5 | 2.2 |
| NCAPG | Non-SMC Condensin I Complex Subunit G | 2.2 |
| NAA15 | N-Alpha-Acetyltransferase 15, NatA Auxiliary Subunit | 2.2 |
| FAF2 | Fas Associated Factor Family Member 2 | 2.2 |
| SLC41A1 | Solute Carrier Family 41 Member 1 | 2.2 |
| RASSF6 | Ras Association Domain Family Member 6 | 2.2 |
| TRAT1 | T Cell Receptor Associated Transmembrane Adaptor 1 | 2.2 |
| STX2 | Syntaxin 2 | 2.2 |
| ZNF408 | Zinc Finger Protein 408 | 2.2 |
| CCDC80 | Coiled-Coil Domain Containing 80 | 2.2 |
| CST9L | Cystatin 9 Like | 2.2 |
| HTR3E | 5-Hydroxytryptamine Receptor 3E | 2.2 |
| WDR46 | WD Repeat Domain 46 | 2.2 |
| YIPF1 | Yip1 Domain Family Member 1 | 2.2 |
| FNDC4 | Fibronectin Type III Domain Containing 4 | 2.2 |
| CLNK | Cytokine Dependent Hematopoietic Cell Linker | 2.2 |
| SERPINA9 | Serpin Family A Member 9 | 2.2 |
| ZPBP2 | Zona Pellucida Binding Protein 2 | 2.2 |
| LIME1 | Lck Interacting Transmembrane Adaptor 1 | 2.2 |
| STATH | Statherin | 2.2 |
| COL6A5 | Collagen Type VI Alpha 5 Chain | 2.2 |
| MT-ND2 | Mitochondrially Encoded NADH:Ubiquinone Oxidoreductase Core Subunit 2 | 2.2 |
| CGB3 | Chorionic Gonadotropin Subunit Beta 3 | 2.2 |
| MT-ATP6 | Mitochondrially Encoded ATP Synthase Membrane Subunit 6 | 2.2 |
| DEFB118 | Defensin Beta 118 | 2.2 |
| OTOL1 | Otolin 1 | 2.2 |
| HCP5 | HLA Complex P5 | 2.2 |
| CEMIP2 | Cell Migration Inducing Hyaluronidase 2 | 2.2 |
| MIR429 | MicroRNA 429 | 2.2 |
| MIR187 | MicroRNA 187 | 2.2 |
| MT-TK | Mitochondrially Encoded TRNA-Lys (AAA/G) | 2.2 |
| LIVAR | Liver Cell Viability Associated LncRNA | 2.2 |
| ANG | Angiogenin | 2.19 |
| JPH2 | Junctophilin 2 | 2.19 |
| ARID5A | AT-Rich Interaction Domain 5A | 2.19 |
| HHLA2 | HERV-H LTR-Associating 2 | 2.19 |
| LRRC19 | Leucine Rich Repeat Containing 19 | 2.19 |
| SOX12 | SRY-Box Transcription Factor 12 | 2.19 |
| LCE3A | Late Cornified Envelope 3A | 2.19 |
| PTPN6 | Protein Tyrosine Phosphatase Non-Receptor Type 6 | 2.19 |
| BAK1 | BCL2 Antagonist/Killer 1 | 2.18 |
| HDC | Histidine Decarboxylase | 2.18 |
| DHCR7 | 7-Dehydrocholesterol Reductase | 2.18 |
| MT-TL1 | Mitochondrially Encoded TRNA-Leu (UUA/G) 1 | 2.16 |
| ANGPTL2 | Angiopoietin Like 2 | 2.16 |
| CYP11B1 | Cytochrome P450 Family 11 Subfamily B Member 1 | 2.16 |
| GPLD1 | Glycosylphosphatidylinositol Specific Phospholipase D1 | 2.16 |
| BIRC2 | Baculoviral IAP Repeat Containing 2 | 2.15 |
| PRKAR1A | Protein Kinase CAMP-Dependent Type I Regulatory Subunit Alpha | 2.15 |
| CD3G | CD3g Molecule | 2.15 |
| EXTL3 | Exostosin Like Glycosyltransferase 3 | 2.15 |
| BCOR | BCL6 Corepressor | 2.15 |
| VPS33B | VPS33B Late Endosome And Lysosome Associated | 2.15 |
| SOX18 | SRY-Box Transcription Factor 18 | 2.15 |
| PIEZO1 | Piezo Type Mechanosensitive Ion Channel Component 1 | 2.15 |
| E2F5 | E2F Transcription Factor 5 | 2.15 |
| MID1 | Midline 1 | 2.14 |
| MIRLET7I | MicroRNA Let-7i | 2.14 |
| ATP8A1 | ATPase Phospholipid Transporting 8A1 | 2.13 |
| CACNA1S | Calcium Voltage-Gated Channel Subunit Alpha1 S | 2.13 |
| RAB7A | RAB7A, Member RAS Oncogene Family | 2.13 |
| AVPR2 | Arginine Vasopressin Receptor 2 | 2.13 |
| PDHX | Pyruvate Dehydrogenase Complex Component X | 2.13 |
| PDX1 | Pancreatic And Duodenal Homeobox 1 | 2.13 |
| SCN8A | Sodium Voltage-Gated Channel Alpha Subunit 8 | 2.13 |
| SCP2 | Sterol Carrier Protein 2 | 2.13 |
| USP9X | Ubiquitin Specific Peptidase 9 X-Linked | 2.13 |
| MUSK | Muscle Associated Receptor Tyrosine Kinase | 2.13 |
| NR0B1 | Nuclear Receptor Subfamily 0 Group B Member 1 | 2.13 |
| SLC20A2 | Solute Carrier Family 20 Member 2 | 2.13 |
| ZIC3 | Zic Family Member 3 | 2.13 |
| FGF17 | Fibroblast Growth Factor 17 | 2.13 |
| GPC3 | Glypican 3 | 2.13 |
| AGL | Amylo-Alpha-1, 6-Glucosidase, 4-Alpha-Glucanotransferase | 2.13 |
| HINT1 | Histidine Triad Nucleotide Binding Protein 1 | 2.13 |
| SUCLG1 | Succinate-CoA Ligase GDP/ADP-Forming Subunit Alpha | 2.13 |
| VAPB | VAMP Associated Protein B And C | 2.13 |
| CARTPT | CART Prepropeptide | 2.13 |
| PEX1 | Peroxisomal Biogenesis Factor 1 | 2.13 |
| PDE6D | Phosphodiesterase 6D | 2.13 |
| GUCY2D | Guanylate Cyclase 2D, Retinal | 2.13 |
| PEX19 | Peroxisomal Biogenesis Factor 19 | 2.13 |
| PEX2 | Peroxisomal Biogenesis Factor 2 | 2.13 |
| SLC27A5 | Solute Carrier Family 27 Member 5 | 2.13 |
| SLC39A13 | Solute Carrier Family 39 Member 13 | 2.13 |
| PSMD7 | Proteasome 26S Subunit, Non-ATPase 7 | 2.13 |
| WASF2 | WASP Family Member 2 | 2.13 |
| VAPA | VAMP Associated Protein A | 2.13 |
| WASF1 | WASP Family Member 1 | 2.13 |
| GMPPA | GDP-Mannose Pyrophosphorylase A | 2.13 |
| AIPL1 | Aryl Hydrocarbon Receptor Interacting Protein Like 1 | 2.13 |
| AK5 | Adenylate Kinase 5 | 2.13 |
| E2F2 | E2F Transcription Factor 2 | 2.13 |
| RPS17 | Ribosomal Protein S17 | 2.13 |
| PNKD | PNKD Metallo-Beta-Lactamase Domain Containing | 2.13 |
| PIGV | Phosphatidylinositol Glycan Anchor Biosynthesis Class V | 2.13 |
| PDCD10 | Programmed Cell Death 10 | 2.13 |
| FLCN | Folliculin | 2.13 |
| BRSK2 | BR Serine/Threonine Kinase 2 | 2.13 |
| FGF16 | Fibroblast Growth Factor 16 | 2.13 |
| ATG12 | Autophagy Related 12 | 2.13 |
| FGF18 | Fibroblast Growth Factor 18 | 2.13 |
| FBN2 | Fibrillin 2 | 2.13 |
| BMP15 | Bone Morphogenetic Protein 15 | 2.13 |
| MATR3 | Matrin 3 | 2.13 |
| FOXF1 | Forkhead Box F1 | 2.13 |
| CYB5B | Cytochrome B5 Type B | 2.13 |
| CDIPT | CDP-Diacylglycerol--Inositol 3-Phosphatidyltransferase | 2.13 |
| NEK3 | NIMA Related Kinase 3 | 2.13 |
| NEK8 | NIMA Related Kinase 8 | 2.13 |
| PEMT | Phosphatidylethanolamine N-Methyltransferase | 2.13 |
| PTPN4 | Protein Tyrosine Phosphatase Non-Receptor Type 4 | 2.13 |
| WNT8B | Wnt Family Member 8B | 2.13 |
| WNT6 | Wnt Family Member 6 | 2.13 |
| ATP6V1D | ATPase H+ Transporting V1 Subunit D | 2.13 |
| ARL2 | ADP Ribosylation Factor Like GTPase 2 | 2.13 |
| MAGT1 | Magnesium Transporter 1 | 2.13 |
| ADAMTS17 | ADAM Metallopeptidase With Thrombospondin Type 1 Motif 17 | 2.13 |
| FMN2 | Formin 2 | 2.13 |
| CEP250 | Centrosomal Protein 250 | 2.13 |
| CNOT4 | CCR4-NOT Transcription Complex Subunit 4 | 2.13 |
| COTL1 | Coactosin Like F-Actin Binding Protein 1 | 2.13 |
| CKAP5 | Cytoskeleton Associated Protein 5 | 2.13 |
| MKS1 | MKS Transition Zone Complex Subunit 1 | 2.13 |
| RPGRIP1 | RPGR Interacting Protein 1 | 2.13 |
| NR2F6 | Nuclear Receptor Subfamily 2 Group F Member 6 | 2.13 |
| SLC45A2 | Solute Carrier Family 45 Member 2 | 2.13 |
| OPN4 | Opsin 4 | 2.13 |
| SLC39A10 | Solute Carrier Family 39 Member 10 | 2.13 |
| SLC39A5 | Solute Carrier Family 39 Member 5 | 2.13 |
| SLC39A6 | Solute Carrier Family 39 Member 6 | 2.13 |
| NUDT5 | Nudix Hydrolase 5 | 2.13 |
| TCTN3 | Tectonic Family Member 3 | 2.13 |
| USH1G | USH1 Protein Network Component Sans | 2.13 |
| RASSF5 | Ras Association Domain Family Member 5 | 2.13 |
| KAT8 | Lysine Acetyltransferase 8 | 2.13 |
| VPS45 | Vacuolar Protein Sorting 45 Homolog | 2.13 |
| VSX2 | Visual System Homeobox 2 | 2.13 |
| GGCT | Gamma-Glutamylcyclotransferase | 2.13 |
| GEN1 | GEN1 Holliday Junction 5' Flap Endonuclease | 2.13 |
| MAPKBP1 | Mitogen-Activated Protein Kinase Binding Protein 1 | 2.13 |
| ANXA9 | Annexin A9 | 2.13 |
| FGF11 | Fibroblast Growth Factor 11 | 2.13 |
| ACIN1 | Apoptotic Chromatin Condensation Inducer 1 | 2.13 |
| FSCN2 | Fascin Actin-Bundling Protein 2, Retinal | 2.13 |
| FRAS1 | Fraser Extracellular Matrix Complex Subunit 1 | 2.13 |
| CTPS2 | CTP Synthase 2 | 2.13 |
| HFM1 | Helicase For Meiosis 1 | 2.13 |
| MKRN1 | Makorin Ring Finger Protein 1 | 2.13 |
| DR1 | Down-Regulator Of Transcription 1 | 2.13 |
| RPGRIP1L | RPGRIP1 Like | 2.13 |
| ELP4 | Elongator Acetyltransferase Complex Subunit 4 | 2.13 |
| SON | SON DNA And RNA Binding Protein | 2.13 |
| SPOCK1 | SPARC (Osteonectin), Cwcv And Kazal Like Domains Proteoglycan 1 | 2.13 |
| PPP1R9B | Protein Phosphatase 1 Regulatory Subunit 9B | 2.13 |
| IFT140 | Intraflagellar Transport 140 | 2.13 |
| IFT57 | Intraflagellar Transport 57 | 2.13 |
| SP2 | Sp2 Transcription Factor | 2.13 |
| SNRPC | Small Nuclear Ribonucleoprotein Polypeptide C | 2.13 |
| SLC43A2 | Solute Carrier Family 43 Member 2 | 2.13 |
| RPS4X | Ribosomal Protein S4 X-Linked | 2.13 |
| TINAG | Tubulointerstitial Nephritis Antigen | 2.13 |
| TCTN2 | Tectonic Family Member 2 | 2.13 |
| SYMPK | Symplekin | 2.13 |
| PRPH2 | Peripherin 2 | 2.13 |
| BBS1 | Bardet-Biedl Syndrome 1 | 2.13 |
| ARHGAP21 | Rho GTPase Activating Protein 21 | 2.13 |
| FGF22 | Fibroblast Growth Factor 22 | 2.13 |
| ANKS6 | Ankyrin Repeat And Sterile Alpha Motif Domain Containing 6 | 2.13 |
| ESCO1 | Establishment Of Sister Chromatid Cohesion N-Acetyltransferase 1 | 2.13 |
| GTPBP1 | GTP Binding Protein 1 | 2.13 |
| IER3IP1 | Immediate Early Response 3 Interacting Protein 1 | 2.13 |
| PPP1R7 | Protein Phosphatase 1 Regulatory Subunit 7 | 2.13 |
| PPME1 | Protein Phosphatase Methylesterase 1 | 2.13 |
| SLC39A12 | Solute Carrier Family 39 Member 12 | 2.13 |
| SHOX | Short Stature Homeobox | 2.13 |
| PHC3 | Polyhomeotic Homolog 3 | 2.13 |
| L3MBTL1 | L3MBTL Histone Methyl-Lysine Binding Protein 1 | 2.13 |
| USH2A | Usherin | 2.13 |
| KLHL12 | Kelch Like Family Member 12 | 2.13 |
| SCYL3 | SCY1 Like Pseudokinase 3 | 2.13 |
| GABRQ | Gamma-Aminobutyric Acid Type A Receptor Subunit Theta | 2.13 |
| ARL13B | ADP Ribosylation Factor Like GTPase 13B | 2.13 |
| RNASE6 | Ribonuclease A Family Member K6 | 2.13 |
| RNH1 | Ribonuclease/Angiogenin Inhibitor 1 | 2.13 |
| PPFIBP2 | PPFIA Binding Protein 2 | 2.13 |
| IFT80 | Intraflagellar Transport 80 | 2.13 |
| POC1B | POC1 Centriolar Protein B | 2.13 |
| PLP2 | Proteolipid Protein 2 | 2.13 |
| TCTN1 | Tectonic Family Member 1 | 2.13 |
| TRAPPC10 | Trafficking Protein Particle Complex 10 | 2.13 |
| TXNDC15 | Thioredoxin Domain Containing 15 | 2.13 |
| TCF25 | Transcription Factor 25 | 2.13 |
| SUPV3L1 | Suv3 Like RNA Helicase | 2.13 |
| TMEM231 | Transmembrane Protein 231 | 2.13 |
| INSM1 | INSM Transcriptional Repressor 1 | 2.13 |
| VSX1 | Visual System Homeobox 1 | 2.13 |
| LIPI | Lipase I | 2.13 |
| LUC7L2 | LUC7 Like 2, Pre-MRNA Splicing Factor | 2.13 |
| B9D1 | B9 Domain Containing 1 | 2.13 |
| MAP3K15 | Mitogen-Activated Protein Kinase Kinase Kinase 15 | 2.13 |
| ANKRD30A | Ankyrin Repeat Domain 30A | 2.13 |
| ADSS1 | Adenylosuccinate Synthase 1 | 2.13 |
| FNIP1 | Folliculin Interacting Protein 1 | 2.13 |
| FNIP2 | Folliculin Interacting Protein 2 | 2.13 |
| HRC | Histidine Rich Calcium Binding Protein | 2.13 |
| EXOC1 | Exocyst Complex Component 1 | 2.13 |
| HACD1 | 3-Hydroxyacyl-CoA Dehydratase 1 | 2.13 |
| CNTLN | Centlein | 2.13 |
| IFT20 | Intraflagellar Transport 20 | 2.13 |
| IFT52 | Intraflagellar Transport 52 | 2.13 |
| RRP8 | Ribosomal RNA Processing 8 | 2.13 |
| SLC37A3 | Solute Carrier Family 37 Member 3 | 2.13 |
| TMEM216 | Transmembrane Protein 216 | 2.13 |
| TMEM138 | Transmembrane Protein 138 | 2.13 |
| MAGEE1 | MAGE Family Member E1 | 2.13 |
| SPNS1 | Sphingolipid Transporter 1 (Putative) | 2.13 |
| LCORL | Ligand Dependent Nuclear Receptor Corepressor Like | 2.13 |
| LAMB4 | Laminin Subunit Beta 4 | 2.13 |
| UGT1A8 | UDP Glucuronosyltransferase Family 1 Member A8 | 2.13 |
| LETM2 | Leucine Zipper And EF-Hand Containing Transmembrane Protein 2 | 2.13 |
| UNK | Unk Zinc Finger | 2.13 |
| ZER1 | Zyg-11 Related Cell Cycle Regulator | 2.13 |
| EPGN | Epithelial Mitogen | 2.13 |
| CEP97 | Centrosomal Protein 97 | 2.13 |
| SPAG7 | Sperm Associated Antigen 7 | 2.13 |
| GET4 | Guided Entry Of Tail-Anchored Proteins Factor 4 | 2.13 |
| CBWD1 | COBW Domain Containing 1 | 2.13 |
| GMEB2 | Glucocorticoid Modulatory Element Binding Protein 2 | 2.13 |
| CCDC85B | Coiled-Coil Domain Containing 85B | 2.13 |
| CTAG2 | Cancer/Testis Antigen 2 | 2.13 |
| CTAGE1 | Cutaneous T Cell Lymphoma-Associated Antigen 1 | 2.13 |
| RHOXF1 | Rhox Homeobox Family Member 1 | 2.13 |
| PAGR1 | PAXIP1 Associated Glutamate Rich Protein 1 | 2.13 |
| SDR39U1 | Short Chain Dehydrogenase/Reductase Family 39U Member 1 | 2.13 |
| RPS4Y1 | Ribosomal Protein S4 Y-Linked 1 | 2.13 |
| TWNK | Twinkle MtDNA Helicase | 2.13 |
| TSPY1 | Testis Specific Protein Y-Linked 1 | 2.13 |
| SYNPO2L | Synaptopodin 2 Like | 2.13 |
| TANC2 | Tetratricopeptide Repeat, Ankyrin Repeat And Coiled-Coil Containing 2 | 2.13 |
| WDR13 | WD Repeat Domain 13 | 2.13 |
| ZNF17 | Zinc Finger Protein 17 | 2.13 |
| ASB17 | Ankyrin Repeat And SOCS Box Containing 17 | 2.13 |
| ASCL3 | Achaete-Scute Family BHLH Transcription Factor 3 | 2.13 |
| BPIFA2 | BPI Fold Containing Family A Member 2 | 2.13 |
| FRMD1 | FERM Domain Containing 1 | 2.13 |
| CMTR2 | Cap Methyltransferase 2 | 2.13 |
| SPAG8 | Sperm Associated Antigen 8 | 2.13 |
| PARPBP | PARP1 Binding Protein | 2.13 |
| ODF3 | Outer Dense Fiber Of Sperm Tails 3 | 2.13 |
| ZAN | Zonadhesin | 2.13 |
| LLPH | LLP Homolog, Long-Term Synaptic Facilitation Factor | 2.13 |
| ARL16 | ADP Ribosylation Factor Like GTPase 16 | 2.13 |
| CSTL1 | Cystatin Like 1 | 2.13 |
| MT-ND3 | Mitochondrially Encoded NADH:Ubiquinone Oxidoreductase Core Subunit 3 | 2.13 |
| PSMG4 | Proteasome Assembly Chaperone 4 | 2.13 |
| PSORS1C2 | Psoriasis Susceptibility 1 Candidate 2 | 2.13 |
| SSC5D | Scavenger Receptor Cysteine Rich Family Member With 5 Domains | 2.13 |
| STPG1 | Sperm Tail PG-Rich Repeat Containing 1 | 2.13 |
| MROH7 | Maestro Heat Like Repeat Family Member 7 | 2.13 |
| VWA7 | Von Willebrand Factor A Domain Containing 7 | 2.13 |
| GCNT7 | Glucosaminyl (N-Acetyl) Transferase Family Member 7 | 2.13 |
| C1orf210 | Chromosome 1 Open Reading Frame 210 | 2.13 |
| RNASE10 | Ribonuclease A Family Member 10 (Inactive) | 2.13 |
| CLRN2 | Clarin 2 | 2.13 |
| ZNF425 | Zinc Finger Protein 425 | 2.13 |
| DAW1 | Dynein Assembly Factor With WD Repeats 1 | 2.13 |
| SMIM20 | Small Integral Membrane Protein 20 | 2.13 |
| KRTAP4-4 | Keratin Associated Protein 4-4 | 2.13 |
| MT-ND4L | Mitochondrially Encoded NADH:Ubiquinone Oxidoreductase Core Subunit 4L | 2.13 |
| KAAG1 | Kidney Associated Antigen 1 | 2.13 |
| KRTAP10-8 | Keratin Associated Protein 10-8 | 2.13 |
| FAM184B | Family With Sequence Similarity 184 Member B | 2.13 |
| CEP20 | Centrosomal Protein 20 | 2.13 |
| KRTAP4-11 | Keratin Associated Protein 4-11 | 2.13 |
| SCX | Scleraxis BHLH Transcription Factor | 2.13 |
| NCBP3 | Nuclear Cap Binding Subunit 3 | 2.13 |
| KCNJ18 | Potassium Inwardly Rectifying Channel Subfamily J Member 18 | 2.13 |
| KRTAP12-3 | Keratin Associated Protein 12-3 | 2.13 |
| VHLL | VHL Like | 2.13 |
| CBWD3 | COBW Domain Containing 3 | 2.13 |
| KRTAP10-7 | Keratin Associated Protein 10-7 | 2.13 |
| KRTAP9-8 | Keratin Associated Protein 9-8 | 2.13 |
| HOTTIP | HOXA Distal Transcript Antisense RNA | 2.13 |
| PHGR1 | Proline, Histidine And Glycine Rich 1 | 2.13 |
| GFY | Golgi Associated Olfactory Signaling Regulator | 2.13 |
| MIR191 | MicroRNA 191 | 2.13 |
| PTENP1 | Phosphatase And Tensin Homolog Pseudogene 1 | 2.13 |
| APELA | Apelin Receptor Early Endogenous Ligand | 2.13 |
| FAM30A | Family With Sequence Similarity 30 Member A | 2.13 |
| MIR330 | MicroRNA 330 | 2.13 |
| MIR331 | MicroRNA 331 | 2.13 |
| PCGEM1 | PCGEM1 Prostate-Specific Transcript | 2.13 |
| MIR129-1 | MicroRNA 129-1 | 2.13 |
| MIR582 | MicroRNA 582 | 2.13 |
| SNORA75 | Small Nucleolar RNA, H/ACA Box 75 | 2.13 |
| PCAT1 | Prostate Cancer Associated Transcript 1 | 2.13 |
| SNORA73B | Small Nucleolar RNA, H/ACA Box 73B | 2.13 |
| RNU11 | RNA, U11 Small Nuclear | 2.13 |
| SNORD13 | Small Nucleolar RNA, C/D Box 13 | 2.13 |
| MIR95 | MicroRNA 95 | 2.13 |
| ZFAT-AS1 | ZFAT Antisense RNA 1 | 2.13 |
| LINC00922 | Long Intergenic Non-Protein Coding RNA 922 | 2.13 |
| MIR411 | MicroRNA 411 | 2.13 |
| PRNCR1 | Prostate Cancer Associated Non-Coding RNA 1 | 2.13 |
| LINC01133 | Long Intergenic Non-Protein Coding RNA 1133 | 2.13 |
| BACE1-AS | BACE1 Antisense RNA | 2.13 |
| SNORD29 | Small Nucleolar RNA, C/D Box 29 | 2.13 |
| LINC01589 | Long Intergenic Non-Protein Coding RNA 1589 | 2.13 |
| MINCR | MYC-Induced Long Non-Coding RNA | 2.13 |
| MIR663AHG | MIR663A Host Gene | 2.13 |
| MIR1288 | MicroRNA 1288 | 2.13 |
| MT-TR | Mitochondrially Encoded TRNA-Arg (CGN) | 2.13 |
| MIR1206 | MicroRNA 1206 | 2.13 |
| FLNC-AS1 | FLNC Antisense RNA 1 | 2.13 |
| LNCARSR | LncRNA Regulator Of Akt Signaling Associated With HCC And RCC | 2.13 |
| MIR1279 | MicroRNA 1279 | 2.13 |
| NMRAL2P | NmrA Like Redox Sensor 2, Pseudogene | 2.13 |
| ENSG00000262202 |  | 2.13 |
| RNU12-2P | RNA, U12 Small Nuclear 2, Pseudogene | 2.13 |
| HVBS7 | Hepatitis B Virus Integration Site 7 | 2.13 |
| HVBS8 | Hepatitis B Virus Integration Site 8 | 2.13 |
| GRDX | Graves Disease, Susceptibility To, X-Linked | 2.13 |
| OMS | Otitis Media, Susceptibility To | 2.13 |
| LOC106014249 | PAX6 Upstream Regulatory Region | 2.13 |
| CANT1 | Calcium Activated Nucleotidase 1 | 2.12 |
| MAPKAPK2 | MAPK Activated Protein Kinase 2 | 2.12 |
| SLC16A1 | Solute Carrier Family 16 Member 1 | 2.11 |
| ERLIN1 | ER Lipid Raft Associated 1 | 2.11 |
| ZFP36 | ZFP36 Ring Finger Protein | 2.11 |
| MTSS1 | MTSS I-BAR Domain Containing 1 | 2.09 |
| ACTG2 | Actin Gamma 2, Smooth Muscle | 2.09 |
| MT-TS1 | Mitochondrially Encoded TRNA-Ser (UCN) 1 | 2.09 |
| MT-TS2 | Mitochondrially Encoded TRNA-Ser (AGU/C) 2 | 2.09 |
| MT-TW | Mitochondrially Encoded TRNA-Trp (UGA/G) | 2.09 |
| MT-TQ | Mitochondrially Encoded TRNA-Gln (CAA/G) | 2.09 |
| NTRK3 | Neurotrophic Receptor Tyrosine Kinase 3 | 2.09 |
| PROKR1 | Prokineticin Receptor 1 | 2.09 |
| CLEC2B | C-Type Lectin Domain Family 2 Member B | 2.09 |
| GADD45A | Growth Arrest And DNA Damage Inducible Alpha | 2.08 |
| CASP2 | Caspase 2 | 2.08 |
| AK2 | Adenylate Kinase 2 | 2.08 |
| ACADVL | Acyl-CoA Dehydrogenase Very Long Chain | 2.08 |
| GANAB | Glucosidase II Alpha Subunit | 2.08 |
| PRMT7 | Protein Arginine Methyltransferase 7 | 2.08 |
| BCS1L | BCS1 Homolog, Ubiquinol-Cytochrome C Reductase Complex Chaperone | 2.08 |
| EMP2 | Epithelial Membrane Protein 2 | 2.08 |
| STX16 | Syntaxin 16 | 2.08 |
| GJC2 | Gap Junction Protein Gamma 2 | 2.08 |
| TPP2 | Tripeptidyl Peptidase 2 | 2.08 |
| VPS33A | VPS33A Core Subunit Of CORVET And HOPS Complexes | 2.08 |
| COG8 | Component Of Oligomeric Golgi Complex 8 | 2.08 |
| KCTD1 | Potassium Channel Tetramerization Domain Containing 1 | 2.08 |
| ATOH7 | Atonal BHLH Transcription Factor 7 | 2.08 |
| BNC2 | Basonuclin 2 | 2.08 |
| SDR9C7 | Short Chain Dehydrogenase/Reductase Family 9C Member 7 | 2.08 |
| NADK2 | NAD Kinase 2, Mitochondrial | 2.08 |
| VIPAS39 | VPS33B Interacting Protein, Apical-Basolateral Polarity Regulator, Spe-39 Homolog | 2.08 |
| LAGE3 | L Antigen Family Member 3 | 2.08 |
| KDF1 | Keratinocyte Differentiation Factor 1 | 2.08 |
| NLRC5 | NLR Family CARD Domain Containing 5 | 2.08 |
| CDK5 | Cyclin Dependent Kinase 5 | 2.07 |
| INHBA | Inhibin Subunit Beta A | 2.07 |
| RARA | Retinoic Acid Receptor Alpha | 2.07 |
| PNPO | Pyridoxamine 5'-Phosphate Oxidase | 2.07 |
| WNT11 | Wnt Family Member 11 | 2.07 |
| ASH1L | ASH1 Like Histone Lysine Methyltransferase | 2.07 |
| RECQL4 | RecQ Like Helicase 4 | 2.07 |
| SLC26A9 | Solute Carrier Family 26 Member 9 | 2.07 |
| SPDEF | SAM Pointed Domain Containing ETS Transcription Factor | 2.07 |
| HCAR1 | Hydroxycarboxylic Acid Receptor 1 | 2.07 |
| STAU1 | Staufen Double-Stranded RNA Binding Protein 1 | 2.07 |
| PRKCE | Protein Kinase C Epsilon | 2.07 |
| PTK2 | Protein Tyrosine Kinase 2 | 2.07 |
| SERPINB5 | Serpin Family B Member 5 | 2.07 |
| TRB | T Cell Receptor Beta Locus | 2.06 |
| FOXO3 | Forkhead Box O3 | 2.06 |
| IL1RAP | Interleukin 1 Receptor Accessory Protein | 2.05 |
| RHOC | Ras Homolog Family Member C | 2.05 |
| AKR1C2 | Aldo-Keto Reductase Family 1 Member C2 | 2.04 |
| DVL1 | Dishevelled Segment Polarity Protein 1 | 2.04 |
| RNF5 | Ring Finger Protein 5 | 2.04 |
| WNT16 | Wnt Family Member 16 | 2.04 |
| CAPZB | Capping Actin Protein Of Muscle Z-Line Subunit Beta | 2.04 |
| ABCF2 | ATP Binding Cassette Subfamily F Member 2 | 2.04 |
| KIF21A | Kinesin Family Member 21A | 2.04 |
| WDR62 | WD Repeat Domain 62 | 2.04 |
| TRPC4AP | Transient Receptor Potential Cation Channel Subfamily C Member 4 Associated Protein | 2.04 |
| WDR20 | WD Repeat Domain 20 | 2.04 |
| WDTC1 | WD And Tetratricopeptide Repeats 1 | 2.04 |
| CIB1 | Calcium And Integrin Binding 1 | 2.04 |
| CMKLR1 | Chemerin Chemokine-Like Receptor 1 | 2.04 |
| RHOH | Ras Homolog Family Member H | 2.04 |
| TP73 | Tumor Protein P73 | 2.04 |
| FPR3 | Formyl Peptide Receptor 3 | 2.04 |
| PLEK | Pleckstrin | 2.03 |
| MC5R | Melanocortin 5 Receptor | 2.02 |
| LIMK1 | LIM Domain Kinase 1 | 2.02 |
| PRKDC | Protein Kinase, DNA-Activated, Catalytic Subunit | 2.02 |
| POR | Cytochrome P450 Oxidoreductase | 2.02 |
| ACTG1 | Actin Gamma 1 | 2.02 |
| TUBA1B | Tubulin Alpha 1b | 2.02 |
| AGPAT1 | 1-Acylglycerol-3-Phosphate O-Acyltransferase 1 | 2.02 |
| FETUB | Fetuin B | 2.02 |
| MYH11 | Myosin Heavy Chain 11 | 2.01 |
| SERPINA12 | Serpin Family A Member 12 | 2.01 |
| GNAI1 | G Protein Subunit Alpha I1 | 2.01 |
| KPNA2 | Karyopherin Subunit Alpha 2 | 2.01 |
| NRG1 | Neuregulin 1 | 2 |
| TBL1XR1 | TBL1X Receptor 1 | 2 |
| TMC6 | Transmembrane Channel Like 6 | 2 |
| WNT1 | Wnt Family Member 1 | 1.99 |
| NPC2 | NPC Intracellular Cholesterol Transporter 2 | 1.99 |
| ACTN1 | Actinin Alpha 1 | 1.99 |
| DNAH8 | Dynein Axonemal Heavy Chain 8 | 1.99 |
| SPHK1 | Sphingosine Kinase 1 | 1.99 |
| CTTN | Cortactin | 1.98 |
| MGAM | Maltase-Glucoamylase | 1.98 |
| BRD4 | Bromodomain Containing 4 | 1.98 |
| S100A6 | S100 Calcium Binding Protein A6 | 1.97 |
| NMUR1 | Neuromedin U Receptor 1 | 1.97 |
| SLC27A2 | Solute Carrier Family 27 Member 2 | 1.97 |
| NPTN | Neuroplastin | 1.97 |
| SLC25A17 | Solute Carrier Family 25 Member 17 | 1.97 |
| SHE | Src Homology 2 Domain Containing E | 1.97 |
| FECH | Ferrochelatase | 1.97 |
| KDSR | 3-Ketodihydrosphingosine Reductase | 1.97 |
| AUTS2 | Activator Of Transcription And Developmental Regulator AUTS2 | 1.97 |
| ABCF1 | ATP Binding Cassette Subfamily F Member 1 | 1.96 |
| FCSK | Fucose Kinase | 1.96 |
| NCOA3 | Nuclear Receptor Coactivator 3 | 1.96 |
| PSMA6 | Proteasome 20S Subunit Alpha 6 | 1.95 |
| GABBR1 | Gamma-Aminobutyric Acid Type B Receptor Subunit 1 | 1.94 |
| SNAI2 | Snail Family Transcriptional Repressor 2 | 1.94 |
| IFNL1 | Interferon Lambda 1 | 1.94 |
| PRKAA2 | Protein Kinase AMP-Activated Catalytic Subunit Alpha 2 | 1.94 |
| LGALS9 | Galectin 9 | 1.94 |
| ITGA5 | Integrin Subunit Alpha 5 | 1.94 |
| MIA2 | MIA SH3 Domain ER Export Factor 2 | 1.94 |
| SLC8A1 | Solute Carrier Family 8 Member A1 | 1.93 |
| KMO | Kynurenine 3-Monooxygenase | 1.93 |
| CALCRL | Calcitonin Receptor Like Receptor | 1.93 |
| DCLK1 | Doublecortin Like Kinase 1 | 1.93 |
| AHNAK | AHNAK Nucleoprotein | 1.93 |
| CASP6 | Caspase 6 | 1.93 |
| SIRPA | Signal Regulatory Protein Alpha | 1.93 |
| OASL | 2'-5'-Oligoadenylate Synthetase Like | 1.92 |
| YWHAZ | Tyrosine 3-Monooxygenase/Tryptophan 5-Monooxygenase Activation Protein Zeta | 1.92 |
| S100A10 | S100 Calcium Binding Protein A10 | 1.92 |
| IFI35 | Interferon Induced Protein 35 | 1.92 |
| PTPN3 | Protein Tyrosine Phosphatase Non-Receptor Type 3 | 1.92 |
| IL3RA | Interleukin 3 Receptor Subunit Alpha | 1.92 |
| CPOX | Coproporphyrinogen Oxidase | 1.91 |
| PRNP | Prion Protein | 1.9 |
| TRIM25 | Tripartite Motif Containing 25 | 1.9 |
| CARD10 | Caspase Recruitment Domain Family Member 10 | 1.9 |
| SCRIB | Scribble Planar Cell Polarity Protein | 1.9 |
| NUP85 | Nucleoporin 85 | 1.9 |
| ZBTB16 | Zinc Finger And BTB Domain Containing 16 | 1.9 |
| SEC61A1 | SEC61 Translocon Subunit Alpha 1 | 1.9 |
| VHL | Von Hippel-Lindau Tumor Suppressor | 1.9 |
| YWHAB | Tyrosine 3-Monooxygenase/Tryptophan 5-Monooxygenase Activation Protein Beta | 1.89 |
| NR4A1 | Nuclear Receptor Subfamily 4 Group A Member 1 | 1.89 |
| MAP2K7 | Mitogen-Activated Protein Kinase Kinase 7 | 1.89 |
| AOC1 | Amine Oxidase Copper Containing 1 | 1.89 |
| POMGNT2 | Protein O-Linked Mannose N-Acetylglucosaminyltransferase 2 (Beta 1,4-) | 1.89 |
| LRP6 | LDL Receptor Related Protein 6 | 1.88 |
| S100P | S100 Calcium Binding Protein P | 1.87 |
| CCND2 | Cyclin D2 | 1.87 |
| RXFP1 | Relaxin Family Peptide Receptor 1 | 1.87 |
| CRBN | Cereblon | 1.87 |
| EFNA1 | Ephrin A1 | 1.87 |
| CD82 | CD82 Molecule | 1.87 |
| USP2 | Ubiquitin Specific Peptidase 2 | 1.87 |
| ACTR3 | Actin Related Protein 3 | 1.87 |
| HPR | Haptoglobin-Related Protein | 1.87 |
| CHL1 | Cell Adhesion Molecule L1 Like | 1.87 |
| CNTN5 | Contactin 5 | 1.87 |
| SPRR1B | Small Proline Rich Protein 1B | 1.86 |
| WARS1 | Tryptophanyl-TRNA Synthetase 1 | 1.86 |
| LHCGR | Luteinizing Hormone/Choriogonadotropin Receptor | 1.85 |
| SMARCC2 | SWI/SNF Related, Matrix Associated, Actin Dependent Regulator Of Chromatin Subfamily C Member 2 | 1.85 |
| ULK1 | Unc-51 Like Autophagy Activating Kinase 1 | 1.85 |
| MAP1LC3B | Microtubule Associated Protein 1 Light Chain 3 Beta | 1.85 |
| SP3 | Sp3 Transcription Factor | 1.85 |
| PSMC6 | Proteasome 26S Subunit, ATPase 6 | 1.85 |
| AHSP | Alpha Hemoglobin Stabilizing Protein | 1.85 |
| HULC | Hepatocellular Carcinoma Up-Regulated Long Non-Coding RNA | 1.85 |
| SLC7A5 | Solute Carrier Family 7 Member 5 | 1.85 |
| GNA14 | G Protein Subunit Alpha 14 | 1.85 |
| CUX1 | Cut Like Homeobox 1 | 1.85 |
| PPP1R12A | Protein Phosphatase 1 Regulatory Subunit 12A | 1.85 |
| UFM1 | Ubiquitin Fold Modifier 1 | 1.85 |
| PI4K2A | Phosphatidylinositol 4-Kinase Type 2 Alpha | 1.85 |
| CLDN8 | Claudin 8 | 1.85 |
| TRIM40 | Tripartite Motif Containing 40 | 1.84 |
| NLRP2 | NLR Family Pyrin Domain Containing 2 | 1.84 |
| STK10 | Serine/Threonine Kinase 10 | 1.84 |
| CDC25C | Cell Division Cycle 25C | 1.83 |
| ADRA1A | Adrenoceptor Alpha 1A | 1.83 |
| PTS | 6-Pyruvoyltetrahydropterin Synthase | 1.83 |
| CACNA2D1 | Calcium Voltage-Gated Channel Auxiliary Subunit Alpha2delta 1 | 1.83 |
| TRPM4 | Transient Receptor Potential Cation Channel Subfamily M Member 4 | 1.83 |
| NDUFS4 | NADH:Ubiquinone Oxidoreductase Subunit S4 | 1.83 |
| LAMP2 | Lysosomal Associated Membrane Protein 2 | 1.83 |
| BHLHE40 | Basic Helix-Loop-Helix Family Member E40 | 1.83 |
| TOMM40 | Translocase Of Outer Mitochondrial Membrane 40 | 1.83 |
| NDUFS3 | NADH:Ubiquinone Oxidoreductase Core Subunit S3 | 1.83 |
| SF3B1 | Splicing Factor 3b Subunit 1 | 1.83 |
| BCL11B | BAF Chromatin Remodeling Complex Subunit BCL11B | 1.83 |
| TNFRSF21 | TNF Receptor Superfamily Member 21 | 1.82 |
| MGST3 | Microsomal Glutathione S-Transferase 3 | 1.82 |
| ABHD12 | Abhydrolase Domain Containing 12, Lysophospholipase | 1.82 |
| TOP2A | DNA Topoisomerase II Alpha | 1.82 |
| SLC6A3 | Solute Carrier Family 6 Member 3 | 1.82 |
| CDO1 | Cysteine Dioxygenase Type 1 | 1.82 |
| PKP4 | Plakophilin 4 | 1.82 |
| PLA2R1 | Phospholipase A2 Receptor 1 | 1.82 |
| IK | IK Cytokine | 1.82 |
| RHBDL2 | Rhomboid Like 2 | 1.82 |
| MIR374A | MicroRNA 374a | 1.82 |
| KCNH2 | Potassium Voltage-Gated Channel Subfamily H Member 2 | 1.82 |
| KCNQ1 | Potassium Voltage-Gated Channel Subfamily Q Member 1 | 1.82 |
| GLB1 | Galactosidase Beta 1 | 1.82 |
| DGAT1 | Diacylglycerol O-Acyltransferase 1 | 1.82 |
| AQP2 | Aquaporin 2 | 1.82 |
| ASCL1 | Achaete-Scute Family BHLH Transcription Factor 1 | 1.82 |
| WNT5B | Wnt Family Member 5B | 1.82 |
| TNXB | Tenascin XB | 1.82 |
| CD84 | CD84 Molecule | 1.82 |
| TTPA | Alpha Tocopherol Transfer Protein | 1.82 |
| CTNNAL1 | Catenin Alpha Like 1 | 1.82 |
| LGR4 | Leucine Rich Repeat Containing G Protein-Coupled Receptor 4 | 1.82 |
| IL20RA | Interleukin 20 Receptor Subunit Alpha | 1.82 |
| DCD | Dermcidin | 1.82 |
| MIAT | Myocardial Infarction Associated Transcript | 1.82 |
| CSNK1A1 | Casein Kinase 1 Alpha 1 | 1.8 |
| GLI3 | GLI Family Zinc Finger 3 | 1.8 |
| GNAO1 | G Protein Subunit Alpha O1 | 1.8 |
| GRM5 | Glutamate Metabotropic Receptor 5 | 1.8 |
| SOS1 | SOS Ras/Rac Guanine Nucleotide Exchange Factor 1 | 1.8 |
| UBE2C | Ubiquitin Conjugating Enzyme E2 C | 1.8 |
| TXNRD2 | Thioredoxin Reductase 2 | 1.8 |
| SERPINI1 | Serpin Family I Member 1 | 1.8 |
| ARSB | Arylsulfatase B | 1.8 |
| FZD7 | Frizzled Class Receptor 7 | 1.8 |
| DRD3 | Dopamine Receptor D3 | 1.8 |
| DGKA | Diacylglycerol Kinase Alpha | 1.8 |
| KCNN3 | Potassium Calcium-Activated Channel Subfamily N Member 3 | 1.8 |
| FBLN1 | Fibulin 1 | 1.8 |
| EFEMP2 | EGF Containing Fibulin Extracellular Matrix Protein 2 | 1.8 |
| EMX2 | Empty Spiracles Homeobox 2 | 1.8 |
| CACNA2D2 | Calcium Voltage-Gated Channel Auxiliary Subunit Alpha2delta 2 | 1.8 |
| CISD2 | CDGSH Iron Sulfur Domain 2 | 1.8 |
| PSMD3 | Proteasome 26S Subunit, Non-ATPase 3 | 1.8 |
| PAEP | Progestagen Associated Endometrial Protein | 1.8 |
| CKMT1A | Creatine Kinase, Mitochondrial 1A | 1.8 |
| CHPF | Chondroitin Polymerizing Factor | 1.8 |
| NAA50 | N-Alpha-Acetyltransferase 50, NatE Catalytic Subunit | 1.8 |
| SPON1 | Spondin 1 | 1.8 |
| PROK1 | Prokineticin 1 | 1.8 |
| TNRC6B | Trinucleotide Repeat Containing Adaptor 6B | 1.8 |
| INTS7 | Integrator Complex Subunit 7 | 1.8 |
| KRT79 | Keratin 79 | 1.8 |
| NUP43 | Nucleoporin 43 | 1.8 |
| SNAP47 | Synaptosome Associated Protein 47 | 1.8 |
| RIOX2 | Ribosomal Oxygenase 2 | 1.8 |
| LYPD8 | LY6/PLAUR Domain Containing 8 | 1.8 |
| ZEB1-AS1 | ZEB1 Antisense RNA 1 | 1.8 |
| MIR744 | MicroRNA 744 | 1.8 |
| PFKM | Phosphofructokinase, Muscle | 1.8 |
| CACNA1G | Calcium Voltage-Gated Channel Subunit Alpha1 G | 1.8 |
| BLM | BLM RecQ Like Helicase | 1.8 |
| EXT2 | Exostosin Glycosyltransferase 2 | 1.8 |
| TRPS1 | Transcriptional Repressor GATA Binding 1 | 1.8 |
| PAPSS2 | 3'-Phosphoadenosine 5'-Phosphosulfate Synthase 2 | 1.8 |
| PIGA | Phosphatidylinositol Glycan Anchor Biosynthesis Class A | 1.8 |
| TRPM1 | Transient Receptor Potential Cation Channel Subfamily M Member 1 | 1.8 |
| COL9A3 | Collagen Type IX Alpha 3 Chain | 1.8 |
| DSE | Dermatan Sulfate Epimerase | 1.8 |
| MPDU1 | Mannose-P-Dolichol Utilization Defect 1 | 1.8 |
| LEMD3 | LEM Domain Containing 3 | 1.8 |
| HOXD10 | Homeobox D10 | 1.8 |
| CCBE1 | Collagen And Calcium Binding EGF Domains 1 | 1.8 |
| VPS13A | Vacuolar Protein Sorting 13 Homolog A | 1.8 |
| GINS1 | GINS Complex Subunit 1 | 1.8 |
| CLEC10A | C-Type Lectin Domain Containing 10A | 1.79 |
| HS6ST3 | Heparan Sulfate 6-O-Sulfotransferase 3 | 1.79 |
| PPIB | Peptidylprolyl Isomerase B | 1.78 |
| ALDH1A2 | Aldehyde Dehydrogenase 1 Family Member A2 | 1.78 |
| SOX2 | SRY-Box Transcription Factor 2 | 1.78 |
| MATK | Megakaryocyte-Associated Tyrosine Kinase | 1.78 |
| AGXT | Alanine--Glyoxylate And Serine--Pyruvate Aminotransferase | 1.78 |
| LAMA1 | Laminin Subunit Alpha 1 | 1.78 |
| PSMA4 | Proteasome 20S Subunit Alpha 4 | 1.78 |
| UGT2B7 | UDP Glucuronosyltransferase Family 2 Member B7 | 1.78 |
| ARPC2 | Actin Related Protein 2/3 Complex Subunit 2 | 1.78 |
| SNCB | Synuclein Beta | 1.78 |
| KPNA3 | Karyopherin Subunit Alpha 3 | 1.78 |
| ADH1A | Alcohol Dehydrogenase 1A (Class I), Alpha Polypeptide | 1.78 |
| TERF2IP | TERF2 Interacting Protein | 1.78 |
| NRG3 | Neuregulin 3 | 1.78 |
| PROKR2 | Prokineticin Receptor 2 | 1.78 |
| LIN28B | Lin-28 Homolog B | 1.78 |
| SP7 | Sp7 Transcription Factor | 1.78 |
| GPC5 | Glypican 5 | 1.78 |
| DKK3 | Dickkopf WNT Signaling Pathway Inhibitor 3 | 1.78 |
| NXF1 | Nuclear RNA Export Factor 1 | 1.78 |
| BICD1 | BICD Cargo Adaptor 1 | 1.78 |
| ELAVL4 | ELAV Like RNA Binding Protein 4 | 1.78 |
| PPP1R12B | Protein Phosphatase 1 Regulatory Subunit 12B | 1.78 |
| CCHCR1 | Coiled-Coil Alpha-Helical Rod Protein 1 | 1.78 |
| MUL1 | Mitochondrial E3 Ubiquitin Protein Ligase 1 | 1.78 |
| EXOSC1 | Exosome Component 1 | 1.78 |
| NAV1 | Neuron Navigator 1 | 1.78 |
| LGALS7 | Galectin 7 | 1.78 |
| BAHD1 | Bromo Adjacent Homology Domain Containing 1 | 1.78 |
| COMMD9 | COMM Domain Containing 9 | 1.78 |
| MMD | Monocyte To Macrophage Differentiation Associated | 1.78 |
| IFNA8 | Interferon Alpha 8 | 1.78 |
| SRL | Sarcalumenin | 1.78 |
| CCDC88B | Coiled-Coil Domain Containing 88B | 1.78 |
| MIR425 | MicroRNA 425 | 1.78 |
| MIR92A2 | MicroRNA 92a-2 | 1.78 |
| LTB4R2 | Leukotriene B4 Receptor 2 | 1.77 |
| NFKBIB | NFKB Inhibitor Beta | 1.77 |
| MYDGF | Myeloid Derived Growth Factor | 1.77 |
| FGG | Fibrinogen Gamma Chain | 1.77 |
| PLTP | Phospholipid Transfer Protein | 1.76 |
| CEBPD | CCAAT Enhancer Binding Protein Delta | 1.76 |
| NTN1 | Netrin 1 | 1.76 |
| KIR2DL4 | Killer Cell Immunoglobulin Like Receptor, Two Ig Domains And Long Cytoplasmic Tail 4 | 1.75 |
| DPP8 | Dipeptidyl Peptidase 8 | 1.75 |
| MAGEA3 | MAGE Family Member A3 | 1.75 |
| MIR24-2 | MicroRNA 24-2 | 1.75 |
| FSHR | Follicle Stimulating Hormone Receptor | 1.75 |
| CACNB2 | Calcium Voltage-Gated Channel Auxiliary Subunit Beta 2 | 1.75 |
| ACTN2 | Actinin Alpha 2 | 1.75 |
| CA4 | Carbonic Anhydrase 4 | 1.75 |
| SPTLC1 | Serine Palmitoyltransferase Long Chain Base Subunit 1 | 1.75 |
| BLVRA | Biliverdin Reductase A | 1.75 |
| HPD | 4-Hydroxyphenylpyruvate Dioxygenase | 1.75 |
| SGCD | Sarcoglycan Delta | 1.75 |
| UMPS | Uridine Monophosphate Synthetase | 1.75 |
| GFRA1 | GDNF Family Receptor Alpha 1 | 1.75 |
| ABCC9 | ATP Binding Cassette Subfamily C Member 9 | 1.75 |
| DRD1 | Dopamine Receptor D1 | 1.75 |
| KIF5A | Kinesin Family Member 5A | 1.75 |
| PSMB5 | Proteasome 20S Subunit Beta 5 | 1.75 |
| GPC6 | Glypican 6 | 1.75 |
| MCFD2 | Multiple Coagulation Factor Deficiency 2, ER Cargo Receptor Complex Subunit | 1.75 |
| PIK3R3 | Phosphoinositide-3-Kinase Regulatory Subunit 3 | 1.75 |
| PSMB1 | Proteasome 20S Subunit Beta 1 | 1.75 |
| LOXL4 | Lysyl Oxidase Like 4 | 1.75 |
| ATXN10 | Ataxin 10 | 1.75 |
| IQSEC1 | IQ Motif And Sec7 Domain ArfGEF 1 | 1.75 |
| CTHRC1 | Collagen Triple Helix Repeat Containing 1 | 1.75 |
| CHST11 | Carbohydrate Sulfotransferase 11 | 1.75 |
| DHH | Desert Hedgehog Signaling Molecule | 1.75 |
| PCYT1B | Phosphate Cytidylyltransferase 1, Choline, Beta | 1.75 |
| PKD2L1 | Polycystin 2 Like 1, Transient Receptor Potential Cation Channel | 1.75 |
| NR2E3 | Nuclear Receptor Subfamily 2 Group E Member 3 | 1.75 |
| EBF1 | EBF Transcription Factor 1 | 1.75 |
| FUCA2 | Alpha-L-Fucosidase 2 | 1.75 |
| DMRT1 | Doublesex And Mab-3 Related Transcription Factor 1 | 1.75 |
| DSCAM | DS Cell Adhesion Molecule | 1.75 |
| DTX1 | Deltex E3 Ubiquitin Ligase 1 | 1.75 |
| ECT2 | Epithelial Cell Transforming 2 | 1.75 |
| PDCL | Phosducin Like | 1.75 |
| USP6 | Ubiquitin Specific Peptidase 6 | 1.75 |
| USP6NL | USP6 N-Terminal Like | 1.75 |
| ZNF423 | Zinc Finger Protein 423 | 1.75 |
| KRT2 | Keratin 2 | 1.75 |
| FIS1 | Fission, Mitochondrial 1 | 1.75 |
| TOMM20 | Translocase Of Outer Mitochondrial Membrane 20 | 1.75 |
| TRAPPC9 | Trafficking Protein Particle Complex 9 | 1.75 |
| EYA2 | EYA Transcriptional Coactivator And Phosphatase 2 | 1.75 |
| FLG2 | Filaggrin Family Member 2 | 1.75 |
| LRFN2 | Leucine Rich Repeat And Fibronectin Type III Domain Containing 2 | 1.75 |
| AEBP2 | AE Binding Protein 2 | 1.75 |
| NXT1 | Nuclear Transport Factor 2 Like Export Factor 1 | 1.75 |
| WDR11 | WD Repeat Domain 11 | 1.75 |
| ASB3 | Ankyrin Repeat And SOCS Box Containing 3 | 1.75 |
| GTDC1 | Glycosyltransferase Like Domain Containing 1 | 1.75 |
| EMCN | Endomucin | 1.75 |
| PKNOX2 | PBX/Knotted 1 Homeobox 2 | 1.75 |
| SP8 | Sp8 Transcription Factor | 1.75 |
| CLDN23 | Claudin 23 | 1.75 |
| SEC14L3 | SEC14 Like Lipid Binding 3 | 1.75 |
| DENND3 | DENN Domain Containing 3 | 1.75 |
| NREP | Neuronal Regeneration Related Protein | 1.75 |
| PNRC2 | Proline Rich Nuclear Receptor Coactivator 2 | 1.75 |
| RASGEF1A | RasGEF Domain Family Member 1A | 1.75 |
| GPHA2 | Glycoprotein Hormone Subunit Alpha 2 | 1.75 |
| SPECC1 | Sperm Antigen With Calponin Homology And Coiled-Coil Domains 1 | 1.75 |
| GPAT3 | Glycerol-3-Phosphate Acyltransferase 3 | 1.75 |
| IFNA7 | Interferon Alpha 7 | 1.75 |
| SFTA3 | Surfactant Associated 3 | 1.75 |
| PRM1 | Protamine 1 | 1.75 |
| SQOR | Sulfide Quinone Oxidoreductase | 1.75 |
| DCDC2C | Doublecortin Domain Containing 2C | 1.75 |
| DEFB128 | Defensin Beta 128 | 1.75 |
| HDAC1 | Histone Deacetylase 1 | 1.73 |
| EPHX2 | Epoxide Hydrolase 2 | 1.73 |
| APOM | Apolipoprotein M | 1.73 |
| PGM1 | Phosphoglucomutase 1 | 1.73 |
| POLE | DNA Polymerase Epsilon, Catalytic Subunit | 1.73 |
| HSPA9 | Heat Shock Protein Family A (Hsp70) Member 9 | 1.73 |
| ALDH3A2 | Aldehyde Dehydrogenase 3 Family Member A2 | 1.73 |
| MYH14 | Myosin Heavy Chain 14 | 1.73 |
| SMARCAD1 | SWI/SNF-Related, Matrix-Associated Actin-Dependent Regulator Of Chromatin, Subfamily A, Containing DEAD/H Box 1 | 1.73 |
| UBE2A | Ubiquitin Conjugating Enzyme E2 A | 1.73 |
| TBX2 | T-Box Transcription Factor 2 | 1.73 |
| SUOX | Sulfite Oxidase | 1.73 |
| GJB3 | Gap Junction Protein Beta 3 | 1.73 |
| MCCC2 | Methylcrotonoyl-CoA Carboxylase 2 | 1.73 |
| BCKDHA | Branched Chain Keto Acid Dehydrogenase E1 Subunit Alpha | 1.73 |
| CDK10 | Cyclin Dependent Kinase 10 | 1.73 |
| SDHC | Succinate Dehydrogenase Complex Subunit C | 1.73 |
| EBP | EBP Cholestenol Delta-Isomerase | 1.73 |
| SEC23B | SEC23 Homolog B, COPII Coat Complex Component | 1.73 |
| KIF22 | Kinesin Family Member 22 | 1.73 |
| SRD5A3 | Steroid 5 Alpha-Reductase 3 | 1.73 |
| XYLT1 | Xylosyltransferase 1 | 1.73 |
| MEIS2 | Meis Homeobox 2 | 1.73 |
| COL9A2 | Collagen Type IX Alpha 2 Chain | 1.73 |
| NSUN2 | NOP2/Sun RNA Methyltransferase 2 | 1.73 |
| B4GALT7 | Beta-1,4-Galactosyltransferase 7 | 1.73 |
| CHST14 | Carbohydrate Sulfotransferase 14 | 1.73 |
| MNX1 | Motor Neuron And Pancreas Homeobox 1 | 1.73 |
| SHOC2 | SHOC2 Leucine Rich Repeat Scaffold Protein | 1.73 |
| EDAR | Ectodysplasin A Receptor | 1.73 |
| PPA2 | Inorganic Pyrophosphatase 2 | 1.73 |
| TGM5 | Transglutaminase 5 | 1.73 |
| FAT4 | FAT Atypical Cadherin 4 | 1.73 |
| DNAJB11 | DnaJ Heat Shock Protein Family (Hsp40) Member B11 | 1.73 |
| MORC2 | MORC Family CW-Type Zinc Finger 2 | 1.73 |
| TBCK | TBC1 Domain Containing Kinase | 1.73 |
| RBM8A | RNA Binding Motif Protein 8A | 1.73 |
| TMC8 | Transmembrane Channel Like 8 | 1.73 |
| KANSL1 | KAT8 Regulatory NSL Complex Subunit 1 | 1.73 |
| SH3PXD2B | SH3 And PX Domains 2B | 1.73 |
| SHPK | Sedoheptulokinase | 1.73 |
| ZNF687 | Zinc Finger Protein 687 | 1.73 |
| FAM111B | Family With Sequence Similarity 111 Member B | 1.73 |
| EPG5 | Ectopic P-Granules Autophagy Protein 5 Homolog | 1.73 |
| RTTN | Rotatin | 1.73 |
| NABP1 | Nucleic Acid Binding Protein 1 | 1.73 |
| NAXD | NAD(P)HX Dehydratase | 1.73 |
| GGCX | Gamma-Glutamyl Carboxylase | 1.72 |
| NSMAF | Neutral Sphingomyelinase Activation Associated Factor | 1.72 |
| ADAM32 | ADAM Metallopeptidase Domain 32 | 1.72 |
| TECPR2 | Tectonin Beta-Propeller Repeat Containing 2 | 1.72 |
| CLEC14A | C-Type Lectin Domain Containing 14A | 1.72 |
| MAP2K4 | Mitogen-Activated Protein Kinase Kinase 4 | 1.72 |
| SOAT1 | Sterol O-Acyltransferase 1 | 1.71 |
| F11 | Coagulation Factor XI | 1.71 |
| UCN2 | Urocortin 2 | 1.71 |
| FMO3 | Flavin Containing Dimethylaniline Monoxygenase 3 | 1.7 |
| P2RY6 | Pyrimidinergic Receptor P2Y6 | 1.7 |
| BCAR1 | BCAR1 Scaffold Protein, Cas Family Member | 1.7 |
| MSMB | Microseminoprotein Beta | 1.7 |
| SFRP2 | Secreted Frizzled Related Protein 2 | 1.69 |
| PIM1 | Pim-1 Proto-Oncogene, Serine/Threonine Kinase | 1.69 |
| PTPRO | Protein Tyrosine Phosphatase Receptor Type O | 1.69 |
| KLRB1 | Killer Cell Lectin Like Receptor B1 | 1.68 |
| ENO3 | Enolase 3 | 1.68 |
| HADHB | Hydroxyacyl-CoA Dehydrogenase Trifunctional Multienzyme Complex Subunit Beta | 1.68 |
| ETV6 | ETS Variant Transcription Factor 6 | 1.68 |
| FOLH1 | Folate Hydrolase 1 | 1.68 |
| RASA1 | RAS P21 Protein Activator 1 | 1.68 |
| FAF1 | Fas Associated Factor 1 | 1.68 |
| GRIK3 | Glutamate Ionotropic Receptor Kainate Type Subunit 3 | 1.68 |
| LEFTY2 | Left-Right Determination Factor 2 | 1.68 |
| STX3 | Syntaxin 3 | 1.68 |
| GNA15 | G Protein Subunit Alpha 15 | 1.68 |
| ARHGAP4 | Rho GTPase Activating Protein 4 | 1.68 |
| CA3 | Carbonic Anhydrase 3 | 1.68 |
| ACTN3 | Actinin Alpha 3 | 1.68 |
| HOXB5 | Homeobox B5 | 1.68 |
| NPL | N-Acetylneuraminate Pyruvate Lyase | 1.68 |
| EML4 | EMAP Like 4 | 1.68 |
| TPPP3 | Tubulin Polymerization Promoting Protein Family Member 3 | 1.68 |
| RAB32 | RAB32, Member RAS Oncogene Family | 1.68 |
| SLC9A5 | Solute Carrier Family 9 Member A5 | 1.68 |
| CD300E | CD300e Molecule | 1.68 |
| BATF2 | Basic Leucine Zipper ATF-Like Transcription Factor 2 | 1.68 |
| PMS2P2 | PMS1 Homolog 2, Mismatch Repair System Component Pseudogene 2 | 1.68 |
| PANDAR | Promoter Of CDKN1A Antisense DNA Damage Activated RNA | 1.68 |
| AKR1C3 | Aldo-Keto Reductase Family 1 Member C3 | 1.67 |
| ITGB5 | Integrin Subunit Beta 5 | 1.67 |
| SCN4A | Sodium Voltage-Gated Channel Alpha Subunit 4 | 1.67 |
| MSH3 | MutS Homolog 3 | 1.67 |
| UGT1A6 | UDP Glucuronosyltransferase Family 1 Member A6 | 1.67 |
| MAPK7 | Mitogen-Activated Protein Kinase 7 | 1.67 |
| CD74 | CD74 Molecule | 1.66 |
| FCRL6 | Fc Receptor Like 6 | 1.66 |
| HELLS | Helicase, Lymphoid Specific | 1.66 |
| DLL1 | Delta Like Canonical Notch Ligand 1 | 1.66 |
| BMI1 | BMI1 Proto-Oncogene, Polycomb Ring Finger | 1.66 |
| NRP2 | Neuropilin 2 | 1.66 |
| VTI1B | Vesicle Transport Through Interaction With T-SNAREs 1B | 1.66 |
| ARTN | Artemin | 1.66 |
| GIP | Gastric Inhibitory Polypeptide | 1.66 |
| ELAVL1 | ELAV Like RNA Binding Protein 1 | 1.65 |
| F12 | Coagulation Factor XII | 1.65 |
| PRKAG2 | Protein Kinase AMP-Activated Non-Catalytic Subunit Gamma 2 | 1.64 |
| HSD17B4 | Hydroxysteroid 17-Beta Dehydrogenase 4 | 1.64 |
| AVP | Arginine Vasopressin | 1.64 |
| AMFR | Autocrine Motility Factor Receptor | 1.64 |
| SI | Sucrase-Isomaltase | 1.64 |
| SERPINE2 | Serpin Family E Member 2 | 1.64 |
| S100A2 | S100 Calcium Binding Protein A2 | 1.64 |
| CDK2AP1 | Cyclin Dependent Kinase 2 Associated Protein 1 | 1.64 |
| MIR671 | MicroRNA 671 | 1.64 |
| PTGER1 | Prostaglandin E Receptor 1 | 1.64 |
| MYO9A | Myosin IXA | 1.63 |
| RAB11A | RAB11A, Member RAS Oncogene Family | 1.62 |
| CTSA | Cathepsin A | 1.62 |
| EYA1 | EYA Transcriptional Coactivator And Phosphatase 1 | 1.62 |
| GSTM3 | Glutathione S-Transferase Mu 3 | 1.62 |
| CDK1 | Cyclin Dependent Kinase 1 | 1.62 |
| PHOX2B | Paired Like Homeobox 2B | 1.62 |
| HOXA10 | Homeobox A10 | 1.62 |
| COL14A1 | Collagen Type XIV Alpha 1 Chain | 1.62 |
| ALDH9A1 | Aldehyde Dehydrogenase 9 Family Member A1 | 1.62 |
| ADD2 | Adducin 2 | 1.62 |
| FOXL2 | Forkhead Box L2 | 1.62 |
| SCUBE2 | Signal Peptide, CUB Domain And EGF Like Domain Containing 2 | 1.62 |
| GULP1 | GULP PTB Domain Containing Engulfment Adaptor 1 | 1.62 |
| TAS2R38 | Taste 2 Receptor Member 38 | 1.62 |
| OCM | Oncomodulin | 1.62 |
| MIR375 | MicroRNA 375 | 1.62 |
| VTRNA2-1 | Vault RNA 2-1 | 1.62 |
| TAB1 | TGF-Beta Activated Kinase 1 (MAP3K7) Binding Protein 1 | 1.62 |
| AGK | Acylglycerol Kinase | 1.62 |
| EP300 | E1A Binding Protein P300 | 1.62 |
| DEAF1 | DEAF1 Transcription Factor | 1.61 |
| BMP7 | Bone Morphogenetic Protein 7 | 1.6 |
| RRM2B | Ribonucleotide Reductase Regulatory TP53 Inducible Subunit M2B | 1.6 |
| AXIN2 | Axin 2 | 1.6 |
| PLCB1 | Phospholipase C Beta 1 | 1.6 |
| SPTLC2 | Serine Palmitoyltransferase Long Chain Base Subunit 2 | 1.6 |
| CCNB1 | Cyclin B1 | 1.6 |
| GOT1 | Glutamic-Oxaloacetic Transaminase 1 | 1.6 |
| ERCC1 | ERCC Excision Repair 1, Endonuclease Non-Catalytic Subunit | 1.6 |
| E2F4 | E2F Transcription Factor 4 | 1.6 |
| CALM1 | Calmodulin 1 | 1.6 |
| RALA | RAS Like Proto-Oncogene A | 1.6 |
| ARID1A | AT-Rich Interaction Domain 1A | 1.6 |
| ADH4 | Alcohol Dehydrogenase 4 (Class II), Pi Polypeptide | 1.6 |
| ACTR2 | Actin Related Protein 2 | 1.6 |
| FMO1 | Flavin Containing Dimethylaniline Monoxygenase 1 | 1.6 |
| MGAT1 | Alpha-1,3-Mannosyl-Glycoprotein 2-Beta-N-Acetylglucosaminyltransferase | 1.6 |
| CASQ1 | Calsequestrin 1 | 1.6 |
| LNX1 | Ligand Of Numb-Protein X 1 | 1.6 |
| NDUFA9 | NADH:Ubiquinone Oxidoreductase Subunit A9 | 1.6 |
| SH3GL1 | SH3 Domain Containing GRB2 Like 1, Endophilin A2 | 1.6 |
| SLC7A1 | Solute Carrier Family 7 Member 1 | 1.6 |
| IQGAP1 | IQ Motif Containing GTPase Activating Protein 1 | 1.6 |
| CCNA1 | Cyclin A1 | 1.6 |
| LCP2 | Lymphocyte Cytosolic Protein 2 | 1.6 |
| SULT1A2 | Sulfotransferase Family 1A Member 2 | 1.6 |
| ANK2 | Ankyrin 2 | 1.6 |
| BMP5 | Bone Morphogenetic Protein 5 | 1.6 |
| CAPNS1 | Calpain Small Subunit 1 | 1.6 |
| PSMC5 | Proteasome 26S Subunit, ATPase 5 | 1.6 |
| UGT2B4 | UDP Glucuronosyltransferase Family 2 Member B4 | 1.6 |
| KALRN | Kalirin RhoGEF Kinase | 1.6 |
| APOBEC3A | Apolipoprotein B MRNA Editing Enzyme Catalytic Subunit 3A | 1.6 |
| AP3D1 | Adaptor Related Protein Complex 3 Subunit Delta 1 | 1.6 |
| NDUFB4 | NADH:Ubiquinone Oxidoreductase Subunit B4 | 1.6 |
| TERF2 | Telomeric Repeat Binding Factor 2 | 1.6 |
| FGD3 | FYVE, RhoGEF And PH Domain Containing 3 | 1.6 |
| AGFG1 | ArfGAP With FG Repeats 1 | 1.6 |
| CST5 | Cystatin D | 1.6 |
| RGL2 | Ral Guanine Nucleotide Dissociation Stimulator Like 2 | 1.6 |
| FGD5 | FYVE, RhoGEF And PH Domain Containing 5 | 1.6 |
| ANO5 | Anoctamin 5 | 1.6 |
| RCVRN | Recoverin | 1.6 |
| MAGEA1 | MAGE Family Member A1 | 1.6 |
| ADAMTS9 | ADAM Metallopeptidase With Thrombospondin Type 1 Motif 9 | 1.6 |
| DNAJC1 | DnaJ Heat Shock Protein Family (Hsp40) Member C1 | 1.6 |
| SPIN1 | Spindlin 1 | 1.6 |
| NUDT6 | Nudix Hydrolase 6 | 1.6 |
| SNAPIN | SNAP Associated Protein | 1.6 |
| GSDMA | Gasdermin A | 1.6 |
| PHF5A | PHD Finger Protein 5A | 1.6 |
| IPO8 | Importin 8 | 1.6 |
| UBAC1 | UBA Domain Containing 1 | 1.6 |
| WRNIP1 | WRN Helicase Interacting Protein 1 | 1.6 |
| NAV3 | Neuron Navigator 3 | 1.6 |
| DEFB104A | Defensin Beta 104A | 1.6 |
| TPRA1 | Transmembrane Protein Adipocyte Associated 1 | 1.6 |
| DUX4 | Double Homeobox 4 | 1.6 |
| PLAAT4 | Phospholipase A And Acyltransferase 4 | 1.6 |
| SERPINA2 | Serpin Family A Member 2 (Gene/Pseudogene) | 1.6 |
| KCNQ1OT1 | KCNQ1 Opposite Strand/Antisense Transcript 1 | 1.6 |
| DANCR | Differentiation Antagonizing Non-Protein Coding RNA | 1.6 |
| MIR485 | MicroRNA 485 | 1.6 |
| VNN1 | Vanin 1 | 1.6 |
| WNT7A | Wnt Family Member 7A | 1.57 |
| ATP2A1 | ATPase Sarcoplasmic/Endoplasmic Reticulum Ca2+ Transporting 1 | 1.57 |
| HTR2C | 5-Hydroxytryptamine Receptor 2C | 1.57 |
| AKR1C4 | Aldo-Keto Reductase Family 1 Member C4 | 1.57 |
| GABBR2 | Gamma-Aminobutyric Acid Type B Receptor Subunit 2 | 1.57 |
| MCM4 | Minichromosome Maintenance Complex Component 4 | 1.57 |
| GNRHR | Gonadotropin Releasing Hormone Receptor | 1.57 |
| GK | Glycerol Kinase | 1.57 |
| CACNA1D | Calcium Voltage-Gated Channel Subunit Alpha1 D | 1.57 |
| POLB | DNA Polymerase Beta | 1.57 |
| VKORC1 | Vitamin K Epoxide Reductase Complex Subunit 1 | 1.57 |
| GNAI3 | G Protein Subunit Alpha I3 | 1.57 |
| PAX2 | Paired Box 2 | 1.57 |
| SLC12A1 | Solute Carrier Family 12 Member 1 | 1.57 |
| GJA5 | Gap Junction Protein Alpha 5 | 1.57 |
| GPC4 | Glypican 4 | 1.57 |
| ADRA1D | Adrenoceptor Alpha 1D | 1.57 |
| ABAT | 4-Aminobutyrate Aminotransferase | 1.57 |
| GABRA2 | Gamma-Aminobutyric Acid Type A Receptor Subunit Alpha2 | 1.57 |
| CYP2R1 | Cytochrome P450 Family 2 Subfamily R Member 1 | 1.57 |
| HNRNPK | Heterogeneous Nuclear Ribonucleoprotein K | 1.57 |
| SOS2 | SOS Ras/Rho Guanine Nucleotide Exchange Factor 2 | 1.57 |
| SMARCB1 | SWI/SNF Related, Matrix Associated, Actin Dependent Regulator Of Chromatin, Subfamily B, Member 1 | 1.57 |
| PITX1 | Paired Like Homeodomain 1 | 1.57 |
| CHD1 | Chromodomain Helicase DNA Binding Protein 1 | 1.57 |
| OTX2 | Orthodenticle Homeobox 2 | 1.57 |
| WFS1 | Wolframin ER Transmembrane Glycoprotein | 1.57 |
| GCNT2 | Glucosaminyl (N-Acetyl) Transferase 2 (I Blood Group) | 1.57 |
| CAMKK1 | Calcium/Calmodulin Dependent Protein Kinase Kinase 1 | 1.57 |
| CCNA2 | Cyclin A2 | 1.57 |
| ASPA | Aspartoacylase | 1.57 |
| FKBP4 | FKBP Prolyl Isomerase 4 | 1.57 |
| ABCD3 | ATP Binding Cassette Subfamily D Member 3 | 1.57 |
| CTPS1 | CTP Synthase 1 | 1.57 |
| CPT1B | Carnitine Palmitoyltransferase 1B | 1.57 |
| SLC6A6 | Solute Carrier Family 6 Member 6 | 1.57 |
| NPR3 | Natriuretic Peptide Receptor 3 | 1.57 |
| LINGO1 | Leucine Rich Repeat And Ig Domain Containing 1 | 1.57 |
| AKAP9 | A-Kinase Anchoring Protein 9 | 1.57 |
| GNAZ | G Protein Subunit Alpha Z | 1.57 |
| FANCG | FA Complementation Group G | 1.57 |
| AGPS | Alkylglycerone Phosphate Synthase | 1.57 |
| FSHB | Follicle Stimulating Hormone Subunit Beta | 1.57 |
| CRYZ | Crystallin Zeta | 1.57 |
| CLCN4 | Chloride Voltage-Gated Channel 4 | 1.57 |
| DNAJC5 | DnaJ Heat Shock Protein Family (Hsp40) Member C5 | 1.57 |
| NME3 | NME/NM23 Nucleoside Diphosphate Kinase 3 | 1.57 |
| SLC12A4 | Solute Carrier Family 12 Member 4 | 1.57 |
| PTPN5 | Protein Tyrosine Phosphatase Non-Receptor Type 5 | 1.57 |
| UBE2T | Ubiquitin Conjugating Enzyme E2 T | 1.57 |
| KLK2 | Kallikrein Related Peptidase 2 | 1.57 |
| HSPH1 | Heat Shock Protein Family H (Hsp110) Member 1 | 1.57 |
| WNT7B | Wnt Family Member 7B | 1.57 |
| GNB2 | G Protein Subunit Beta 2 | 1.57 |
| AP2B1 | Adaptor Related Protein Complex 2 Subunit Beta 1 | 1.57 |
| ANKRD1 | Ankyrin Repeat Domain 1 | 1.57 |
| MCF2L | MCF.2 Cell Line Derived Transforming Sequence Like | 1.57 |
| GDAP1 | Ganglioside Induced Differentiation Associated Protein 1 | 1.57 |
| PSMA2 | Proteasome 20S Subunit Alpha 2 | 1.57 |
| SLC5A8 | Solute Carrier Family 5 Member 8 | 1.57 |
| ADD3 | Adducin 3 | 1.57 |
| MINK1 | Misshapen Like Kinase 1 | 1.57 |
| LRIG1 | Leucine Rich Repeats And Immunoglobulin Like Domains 1 | 1.57 |
| AP3B2 | Adaptor Related Protein Complex 3 Subunit Beta 2 | 1.57 |
| BHMT | Betaine--Homocysteine S-Methyltransferase | 1.57 |
| HOXD13 | Homeobox D13 | 1.57 |
| CCNE2 | Cyclin E2 | 1.57 |
| DHPS | Deoxyhypusine Synthase | 1.57 |
| MTA3 | Metastasis Associated 1 Family Member 3 | 1.57 |
| DTNBP1 | Dystrobrevin Binding Protein 1 | 1.57 |
| SGCA | Sarcoglycan Alpha | 1.57 |
| SCNN1D | Sodium Channel Epithelial 1 Subunit Delta | 1.57 |
| PODXL | Podocalyxin Like | 1.57 |
| PLIN3 | Perilipin 3 | 1.57 |
| TCF7L1 | Transcription Factor 7 Like 1 | 1.57 |
| PTPRU | Protein Tyrosine Phosphatase Receptor Type U | 1.57 |
| CACNA2D3 | Calcium Voltage-Gated Channel Auxiliary Subunit Alpha2delta 3 | 1.57 |
| B3GNT2 | UDP-GlcNAc:BetaGal Beta-1,3-N-Acetylglucosaminyltransferase 2 | 1.57 |
| CRYGD | Crystallin Gamma D | 1.57 |
| DCBLD2 | Discoidin, CUB And LCCL Domain Containing 2 | 1.57 |
| DPP7 | Dipeptidyl Peptidase 7 | 1.57 |
| PSMC2 | Proteasome 26S Subunit, ATPase 2 | 1.57 |
| INCENP | Inner Centromere Protein | 1.57 |
| PSMC4 | Proteasome 26S Subunit, ATPase 4 | 1.57 |
| TIAL1 | TIA1 Cytotoxic Granule Associated RNA Binding Protein Like 1 | 1.57 |
| GGA3 | Golgi Associated, Gamma Adaptin Ear Containing, ARF Binding Protein 3 | 1.57 |
| MBNL1 | Muscleblind Like Splicing Regulator 1 | 1.57 |
| FNBP1 | Formin Binding Protein 1 | 1.57 |
| HPS4 | HPS4 Biogenesis Of Lysosomal Organelles Complex 3 Subunit 2 | 1.57 |
| COG5 | Component Of Oligomeric Golgi Complex 5 | 1.57 |
| DYM | Dymeclin | 1.57 |
| DDX42 | DEAD-Box Helicase 42 | 1.57 |
| ICAM5 | Intercellular Adhesion Molecule 5 | 1.57 |
| IFT81 | Intraflagellar Transport 81 | 1.57 |
| OSBP | Oxysterol Binding Protein | 1.57 |
| TNS1 | Tensin 1 | 1.57 |
| LILRA2 | Leukocyte Immunoglobulin Like Receptor A2 | 1.57 |
| TMEFF2 | Transmembrane Protein With EGF Like And Two Follistatin Like Domains 2 | 1.57 |
| RCN1 | Reticulocalbin 1 | 1.57 |
| GKN1 | Gastrokine 1 | 1.57 |
| COL15A1 | Collagen Type XV Alpha 1 Chain | 1.57 |
| RIOK3 | RIO Kinase 3 | 1.57 |
| MX2 | MX Dynamin Like GTPase 2 | 1.57 |
| TMEM106B | Transmembrane Protein 106B | 1.57 |
| LRIG3 | Leucine Rich Repeats And Immunoglobulin Like Domains 3 | 1.57 |
| ERN2 | Endoplasmic Reticulum To Nucleus Signaling 2 | 1.57 |
| GUCA2A | Guanylate Cyclase Activator 2A | 1.57 |
| E2F7 | E2F Transcription Factor 7 | 1.57 |
| NCKIPSD | NCK Interacting Protein With SH3 Domain | 1.57 |
| PDC | Phosducin | 1.57 |
| NT5C1B | 5'-Nucleotidase, Cytosolic IB | 1.57 |
| LCMT2 | Leucine Carboxyl Methyltransferase 2 | 1.57 |
| KLK14 | Kallikrein Related Peptidase 14 | 1.57 |
| RAB3IL1 | RAB3A Interacting Protein Like 1 | 1.57 |
| ZC3HC1 | Zinc Finger C3HC-Type Containing 1 | 1.57 |
| ANOS1 | Anosmin 1 | 1.57 |
| TTC39B | Tetratricopeptide Repeat Domain 39B | 1.57 |
| KRT75 | Keratin 75 | 1.57 |
| BBS12 | Bardet-Biedl Syndrome 12 | 1.57 |
| AHRR | Aryl-Hydrocarbon Receptor Repressor | 1.57 |
| ANKRD44 | Ankyrin Repeat Domain 44 | 1.57 |
| CMIP | C-Maf Inducing Protein | 1.57 |
| THSD7A | Thrombospondin Type 1 Domain Containing 7A | 1.57 |
| XRN1 | 5'-3' Exoribonuclease 1 | 1.57 |
| TNP1 | Transition Protein 1 | 1.57 |
| SYNRG | Synergin Gamma | 1.57 |
| DMBX1 | Diencephalon/Mesencephalon Homeobox 1 | 1.57 |
| CABS1 | Calcium Binding Protein, Spermatid Associated 1 | 1.57 |
| RETREG1 | Reticulophagy Regulator 1 | 1.57 |
| DEFB127 | Defensin Beta 127 | 1.57 |
| MT-ATP8 | Mitochondrially Encoded ATP Synthase Membrane Subunit 8 | 1.57 |
| GET1 | Guided Entry Of Tail-Anchored Proteins Factor 1 | 1.57 |
| SOX2-OT | SOX2 Overlapping Transcript | 1.57 |
| LINC01554 | Long Intergenic Non-Protein Coding RNA 1554 | 1.57 |
| MIRLET7G | MicroRNA Let-7g | 1.57 |
| HNF1A-AS1 | HNF1A Antisense RNA 1 | 1.57 |
| LINC00261 | Long Intergenic Non-Protein Coding RNA 261 | 1.57 |
| MIR4435-2HG | MIR4435-2 Host Gene | 1.57 |
| HAGLR | HOXD Antisense Growth-Associated Long Non-Coding RNA | 1.57 |
| MIR505 | MicroRNA 505 | 1.57 |
| DUX4L1 | Double Homeobox 4 Like 1 (Pseudogene) | 1.57 |
| PACERR | PTGS2 Antisense NFKB1 Complex-Mediated Expression Regulator RNA | 1.57 |
| PRINS | Psoriasis Associated Non-Protein Coding RNA Induced By Stress | 1.57 |
| LOC100505549 | Uncharacterized LOC100505549 | 1.57 |
| LOC111674463 | CFTR Promoter Region | 1.57 |
| PIAS1 | Protein Inhibitor Of Activated STAT 1 | 1.57 |
| MKKS | McKusick-Kaufman Syndrome | 1.56 |
| PRDX2 | Peroxiredoxin 2 | 1.56 |
| PLA2G5 | Phospholipase A2 Group V | 1.54 |
| PDYN | Prodynorphin | 1.54 |
| OSMR | Oncostatin M Receptor | 1.54 |
| SFRP5 | Secreted Frizzled Related Protein 5 | 1.54 |
| TFPI | Tissue Factor Pathway Inhibitor | 1.53 |
| TFAP2A | Transcription Factor AP-2 Alpha | 1.53 |
| TNFRSF10B | TNF Receptor Superfamily Member 10b | 1.53 |
| PAFAH1B1 | Platelet Activating Factor Acetylhydrolase 1b Regulatory Subunit 1 | 1.52 |
| UBB | Ubiquitin B | 1.52 |
| GRK2 | G Protein-Coupled Receptor Kinase 2 | 1.51 |
| CCN3 | Cellular Communication Network Factor 3 | 1.51 |
| PTPRF | Protein Tyrosine Phosphatase Receptor Type F | 1.5 |
| MYL2 | Myosin Light Chain 2 | 1.5 |
| CASK | Calcium/Calmodulin Dependent Serine Protein Kinase | 1.5 |
| FZD2 | Frizzled Class Receptor 2 | 1.5 |
| IMPDH1 | Inosine Monophosphate Dehydrogenase 1 | 1.5 |
| PIP5K1C | Phosphatidylinositol-4-Phosphate 5-Kinase Type 1 Gamma | 1.5 |
| VRK1 | VRK Serine/Threonine Kinase 1 | 1.5 |
| ANTXR2 | ANTXR Cell Adhesion Molecule 2 | 1.5 |
| FAH | Fumarylacetoacetate Hydrolase | 1.5 |
| ADCY3 | Adenylate Cyclase 3 | 1.5 |
| HCN4 | Hyperpolarization Activated Cyclic Nucleotide Gated Potassium Channel 4 | 1.5 |
| SCN3A | Sodium Voltage-Gated Channel Alpha Subunit 3 | 1.5 |
| NUMB | NUMB Endocytic Adaptor Protein | 1.5 |
| KCNQ2 | Potassium Voltage-Gated Channel Subfamily Q Member 2 | 1.5 |
| KCNJ1 | Potassium Inwardly Rectifying Channel Subfamily J Member 1 | 1.5 |
| MAN1B1 | Mannosidase Alpha Class 1B Member 1 | 1.5 |
| ARF1 | ADP Ribosylation Factor 1 | 1.5 |
| ALDH6A1 | Aldehyde Dehydrogenase 6 Family Member A1 | 1.5 |
| ABCB6 | ATP Binding Cassette Subfamily B Member 6 (Langereis Blood Group) | 1.5 |
| GABRG2 | Gamma-Aminobutyric Acid Type A Receptor Subunit Gamma2 | 1.5 |
| CHN1 | Chimerin 1 | 1.5 |
| GRHPR | Glyoxylate And Hydroxypyruvate Reductase | 1.5 |
| DDX5 | DEAD-Box Helicase 5 | 1.5 |
| PRKAR1B | Protein Kinase CAMP-Dependent Type I Regulatory Subunit Beta | 1.5 |
| WNT3 | Wnt Family Member 3 | 1.5 |
| WNK1 | WNK Lysine Deficient Protein Kinase 1 | 1.5 |
| WEE1 | WEE1 G2 Checkpoint Kinase | 1.5 |
| GJA8 | Gap Junction Protein Alpha 8 | 1.5 |
| CACNB4 | Calcium Voltage-Gated Channel Auxiliary Subunit Beta 4 | 1.5 |
| ACOX1 | Acyl-CoA Oxidase 1 | 1.5 |
| CLCN2 | Chloride Voltage-Gated Channel 2 | 1.5 |
| DPYS | Dihydropyrimidinase | 1.5 |
| RPL10 | Ribosomal Protein L10 | 1.5 |
| NEFH | Neurofilament Heavy | 1.5 |
| PISD | Phosphatidylserine Decarboxylase | 1.5 |
| PDE6B | Phosphodiesterase 6B | 1.5 |
| PRKG2 | Protein Kinase CGMP-Dependent 2 | 1.5 |
| PLOD3 | Procollagen-Lysine,2-Oxoglutarate 5-Dioxygenase 3 | 1.5 |
| SLC16A2 | Solute Carrier Family 16 Member 2 | 1.5 |
| KCNQ3 | Potassium Voltage-Gated Channel Subfamily Q Member 3 | 1.5 |
| SYNJ1 | Synaptojanin 1 | 1.5 |
| IVD | Isovaleryl-CoA Dehydrogenase | 1.5 |
| ZIC1 | Zic Family Member 1 | 1.5 |
| WNT10A | Wnt Family Member 10A | 1.5 |
| AFG3L2 | AFG3 Like Matrix AAA Peptidase Subunit 2 | 1.5 |
| BAAT | Bile Acid-CoA:Amino Acid N-Acyltransferase | 1.5 |
| ARL3 | ADP Ribosylation Factor Like GTPase 3 | 1.5 |
| MGAT2 | Alpha-1,6-Mannosyl-Glycoprotein 2-Beta-N-Acetylglucosaminyltransferase | 1.5 |
| GSTA3 | Glutathione S-Transferase Alpha 3 | 1.5 |
| CHMP2B | Charged Multivesicular Body Protein 2B | 1.5 |
| DGUOK | Deoxyguanosine Kinase | 1.5 |
| RPL15 | Ribosomal Protein L15 | 1.5 |
| EHHADH | Enoyl-CoA Hydratase And 3-Hydroxyacyl CoA Dehydrogenase | 1.5 |
| PSMA1 | Proteasome 20S Subunit Alpha 1 | 1.5 |
| SGSH | N-Sulfoglucosamine Sulfohydrolase | 1.5 |
| OPA1 | OPA1 Mitochondrial Dynamin Like GTPase | 1.5 |
| SLC34A1 | Solute Carrier Family 34 Member 1 | 1.5 |
| STAG2 | Stromal Antigen 2 | 1.5 |
| KCNE1 | Potassium Voltage-Gated Channel Subfamily E Regulatory Subunit 1 | 1.5 |
| PRKAR2B | Protein Kinase CAMP-Dependent Type II Regulatory Subunit Beta | 1.5 |
| PDE6G | Phosphodiesterase 6G | 1.5 |
| NDUFA1 | NADH:Ubiquinone Oxidoreductase Subunit A1 | 1.5 |
| KCNJ10 | Potassium Inwardly Rectifying Channel Subfamily J Member 10 | 1.5 |
| XPNPEP2 | X-Prolyl Aminopeptidase 2 | 1.5 |
| ACSF3 | Acyl-CoA Synthetase Family Member 3 | 1.5 |
| GFRA2 | GDNF Family Receptor Alpha 2 | 1.5 |
| C9 | Complement C9 | 1.5 |
| BDH1 | 3-Hydroxybutyrate Dehydrogenase 1 | 1.5 |
| BAIAP2 | BAR/IMD Domain Containing Adaptor Protein 2 | 1.5 |
| GPC1 | Glypican 1 | 1.5 |
| ARHGEF10 | Rho Guanine Nucleotide Exchange Factor 10 | 1.5 |
| MCEE | Methylmalonyl-CoA Epimerase | 1.5 |
| BCL11A | BAF Chromatin Remodeling Complex Subunit BCL11A | 1.5 |
| LOXL1 | Lysyl Oxidase Like 1 | 1.5 |
| CTNNA2 | Catenin Alpha 2 | 1.5 |
| HOXB1 | Homeobox B1 | 1.5 |
| CDKL5 | Cyclin Dependent Kinase Like 5 | 1.5 |
| CLPB | Caseinolytic Mitochondrial Matrix Peptidase Chaperone Subunit B | 1.5 |
| CKMT2 | Creatine Kinase, Mitochondrial 2 | 1.5 |
| COLEC11 | Collectin Subfamily Member 11 | 1.5 |
| PEX7 | Peroxisomal Biogenesis Factor 7 | 1.5 |
| PEX14 | Peroxisomal Biogenesis Factor 14 | 1.5 |
| PSMA5 | Proteasome 20S Subunit Alpha 5 | 1.5 |
| SH2B1 | SH2B Adaptor Protein 1 | 1.5 |
| PPM1G | Protein Phosphatase, Mg2+/Mn2+ Dependent 1G | 1.5 |
| SNX5 | Sorting Nexin 5 | 1.5 |
| RPS2 | Ribosomal Protein S2 | 1.5 |
| HSD17B1 | Hydroxysteroid 17-Beta Dehydrogenase 1 | 1.5 |
| PLCZ1 | Phospholipase C Zeta 1 | 1.5 |
| SLC44A1 | Solute Carrier Family 44 Member 1 | 1.5 |
| PSMD14 | Proteasome 26S Subunit, Non-ATPase 14 | 1.5 |
| PSME2 | Proteasome Activator Subunit 2 | 1.5 |
| TPP1 | Tripeptidyl Peptidase 1 | 1.5 |
| UBA5 | Ubiquitin Like Modifier Activating Enzyme 5 | 1.5 |
| TRHR | Thyrotropin Releasing Hormone Receptor | 1.5 |
| TRIM24 | Tripartite Motif Containing 24 | 1.5 |
| TTBK2 | Tau Tubulin Kinase 2 | 1.5 |
| PEX6 | Peroxisomal Biogenesis Factor 6 | 1.5 |
| TXNL4A | Thioredoxin Like 4A | 1.5 |
| HAND2 | Heart And Neural Crest Derivatives Expressed 2 | 1.5 |
| SULT1A1 | Sulfotransferase Family 1A Member 1 | 1.5 |
| CACNB1 | Calcium Voltage-Gated Channel Auxiliary Subunit Beta 1 | 1.5 |
| ARFGAP1 | ADP Ribosylation Factor GTPase Activating Protein 1 | 1.5 |
| ACP2 | Acid Phosphatase 2, Lysosomal | 1.5 |
| C6 | Complement C6 | 1.5 |
| CAPN9 | Calpain 9 | 1.5 |
| GUCA1A | Guanylate Cyclase Activator 1A | 1.5 |
| MMADHC | Metabolism Of Cobalamin Associated D | 1.5 |
| PEX3 | Peroxisomal Biogenesis Factor 3 | 1.5 |
| PEX5 | Peroxisomal Biogenesis Factor 5 | 1.5 |
| PRPF31 | Pre-MRNA Processing Factor 31 | 1.5 |
| S1PR4 | Sphingosine-1-Phosphate Receptor 4 | 1.5 |
| NFYC | Nuclear Transcription Factor Y Subunit Gamma | 1.5 |
| RPL7A | Ribosomal Protein L7a | 1.5 |
| SLC35A3 | Solute Carrier Family 35 Member A3 | 1.5 |
| PIGQ | Phosphatidylinositol Glycan Anchor Biosynthesis Class Q | 1.5 |
| SRGAP1 | SLIT-ROBO Rho GTPase Activating Protein 1 | 1.5 |
| TRIP11 | Thyroid Hormone Receptor Interactor 11 | 1.5 |
| LDHC | Lactate Dehydrogenase C | 1.5 |
| RAB11B | RAB11B, Member RAS Oncogene Family | 1.5 |
| TULP1 | TUB Like Protein 1 | 1.5 |
| EFNB3 | Ephrin B3 | 1.5 |
| ITGA8 | Integrin Subunit Alpha 8 | 1.5 |
| SSR4 | Signal Sequence Receptor Subunit 4 | 1.5 |
| YME1L1 | YME1 Like 1 ATPase | 1.5 |
| GCM2 | Glial Cells Missing Transcription Factor 2 | 1.5 |
| GOPC | Golgi Associated PDZ And Coiled-Coil Motif Containing | 1.5 |
| CCT3 | Chaperonin Containing TCP1 Subunit 3 | 1.5 |
| CD164 | CD164 Molecule | 1.5 |
| GLYAT | Glycine-N-Acyltransferase | 1.5 |
| ATG3 | Autophagy Related 3 | 1.5 |
| ATP8A2 | ATPase Phospholipid Transporting 8A2 | 1.5 |
| FBXO7 | F-Box Protein 7 | 1.5 |
| ASCC1 | Activating Signal Cointegrator 1 Complex Subunit 1 | 1.5 |
| ANGPTL1 | Angiopoietin Like 1 | 1.5 |
| BCAP31 | B Cell Receptor Associated Protein 31 | 1.5 |
| GALK2 | Galactokinase 2 | 1.5 |
| MFN1 | Mitofusin 1 | 1.5 |
| HPCA | Hippocalcin | 1.5 |
| CNNM2 | Cyclin And CBS Domain Divalent Metal Cation Transport Mediator 2 | 1.5 |
| HHAT | Hedgehog Acyltransferase | 1.5 |
| CLDN16 | Claudin 16 | 1.5 |
| MN1 | MN1 Proto-Oncogene, Transcriptional Regulator | 1.5 |
| DNAL4 | Dynein Axonemal Light Chain 4 | 1.5 |
| MYPN | Myopalladin | 1.5 |
| NDEL1 | NudE Neurodevelopment Protein 1 Like 1 | 1.5 |
| NDUFA5 | NADH:Ubiquinone Oxidoreductase Subunit A5 | 1.5 |
| ELOVL1 | ELOVL Fatty Acid Elongase 1 | 1.5 |
| CDH17 | Cadherin 17 | 1.5 |
| PEX10 | Peroxisomal Biogenesis Factor 10 | 1.5 |
| PEX26 | Peroxisomal Biogenesis Factor 26 | 1.5 |
| SPEG | Striated Muscle Enriched Protein Kinase | 1.5 |
| NRL | Neural Retina Leucine Zipper | 1.5 |
| PARL | Presenilin Associated Rhomboid Like | 1.5 |
| RPN2 | Ribophorin II | 1.5 |
| SLC6A11 | Solute Carrier Family 6 Member 11 | 1.5 |
| SLC6A20 | Solute Carrier Family 6 Member 20 | 1.5 |
| PIWIL1 | Piwi Like RNA-Mediated Gene Silencing 1 | 1.5 |
| PLEKHA1 | Pleckstrin Homology Domain Containing A1 | 1.5 |
| SLC47A1 | Solute Carrier Family 47 Member 1 | 1.5 |
| NTNG1 | Netrin G1 | 1.5 |
| NT5C | 5', 3'-Nucleotidase, Cytosolic | 1.5 |
| RPS3A | Ribosomal Protein S3A | 1.5 |
| PSMD11 | Proteasome 26S Subunit, Non-ATPase 11 | 1.5 |
| PSMC3 | Proteasome 26S Subunit, ATPase 3 | 1.5 |
| PIGL | Phosphatidylinositol Glycan Anchor Biosynthesis Class L | 1.5 |
| TPX2 | TPX2 Microtubule Nucleation Factor | 1.5 |
| UBA52 | Ubiquitin A-52 Residue Ribosomal Protein Fusion Product 1 | 1.5 |
| TES | Testin LIM Domain Protein | 1.5 |
| SULT4A1 | Sulfotransferase Family 4A Member 1 | 1.5 |
| RBL1 | RB Transcriptional Corepressor Like 1 | 1.5 |
| LDB3 | LIM Domain Binding 3 | 1.5 |
| SDS | Serine Dehydratase | 1.5 |
| MSH5 | MutS Homolog 5 | 1.5 |
| POLG2 | DNA Polymerase Gamma 2, Accessory Subunit | 1.5 |
| PPIH | Peptidylprolyl Isomerase H | 1.5 |
| PPIL2 | Peptidylprolyl Isomerase Like 2 | 1.5 |
| PSMB2 | Proteasome 20S Subunit Beta 2 | 1.5 |
| USP22 | Ubiquitin Specific Peptidase 22 | 1.5 |
| WNT8A | Wnt Family Member 8A | 1.5 |
| WASL | WASP Like Actin Nucleation Promoting Factor | 1.5 |
| WNT9B | Wnt Family Member 9B | 1.5 |
| CCT7 | Chaperonin Containing TCP1 Subunit 7 | 1.5 |
| BBS10 | Bardet-Biedl Syndrome 10 | 1.5 |
| EXOSC8 | Exosome Component 8 | 1.5 |
| EXOSC9 | Exosome Component 9 | 1.5 |
| AP3S1 | Adaptor Related Protein Complex 3 Subunit Sigma 1 | 1.5 |
| ARFGAP3 | ADP Ribosylation Factor GTPase Activating Protein 3 | 1.5 |
| ARHGAP15 | Rho GTPase Activating Protein 15 | 1.5 |
| ERLIN2 | ER Lipid Raft Associated 2 | 1.5 |
| FKBP14 | FKBP Prolyl Isomerase 14 | 1.5 |
| MBD1 | Methyl-CpG Binding Domain Protein 1 | 1.5 |
| ADAMTSL2 | ADAMTS Like 2 | 1.5 |
| ACSBG1 | Acyl-CoA Synthetase Bubblegum Family Member 1 | 1.5 |
| BVES | Blood Vessel Epicardial Substance | 1.5 |
| CRYGC | Crystallin Gamma C | 1.5 |
| CRYGS | Crystallin Gamma S | 1.5 |
| CRIPT | CXXC Repeat Containing Interactor Of PDZ3 Domain | 1.5 |
| CTDSPL | CTD Small Phosphatase Like | 1.5 |
| CPSF4 | Cleavage And Polyadenylation Specific Factor 4 | 1.5 |
| CLIC2 | Chloride Intracellular Channel 2 | 1.5 |
| CDK12 | Cyclin Dependent Kinase 12 | 1.5 |
| CLPS | Colipase | 1.5 |
| COG4 | Component Of Oligomeric Golgi Complex 4 | 1.5 |
| CHST15 | Carbohydrate Sulfotransferase 15 | 1.5 |
| CER1 | Cerberus 1, DAN Family BMP Antagonist | 1.5 |
| DNAJC3 | DnaJ Heat Shock Protein Family (Hsp40) Member C3 | 1.5 |
| E2F6 | E2F Transcription Factor 6 | 1.5 |
| CDCA7L | Cell Division Cycle Associated 7 Like | 1.5 |
| CD320 | CD320 Molecule | 1.5 |
| GREM2 | Gremlin 2, DAN Family BMP Antagonist | 1.5 |
| DEF6 | DEF6 Guanine Nucleotide Exchange Factor | 1.5 |
| COL27A1 | Collagen Type XXVII Alpha 1 Chain | 1.5 |
| PEX13 | Peroxisomal Biogenesis Factor 13 | 1.5 |
| SIM1 | SIM BHLH Transcription Factor 1 | 1.5 |
| IDI2 | Isopentenyl-Diphosphate Delta Isomerase 2 | 1.5 |
| PROP1 | PROP Paired-Like Homeobox 1 | 1.5 |
| SLCO1C1 | Solute Carrier Organic Anion Transporter Family Member 1C1 | 1.5 |
| POLR2F | RNA Polymerase II, I And III Subunit F | 1.5 |
| SLC35A2 | Solute Carrier Family 35 Member A2 | 1.5 |
| SLC35D1 | Solute Carrier Family 35 Member D1 | 1.5 |
| OPN1SW | Opsin 1, Short Wave Sensitive | 1.5 |
| SLC4A11 | Solute Carrier Family 4 Member 11 | 1.5 |
| SLC38A4 | Solute Carrier Family 38 Member 4 | 1.5 |
| NUP133 | Nucleoporin 133 | 1.5 |
| NTNG2 | Netrin G2 | 1.5 |
| TULP3 | TUB Like Protein 3 | 1.5 |
| UBQLN4 | Ubiquilin 4 | 1.5 |
| TSHZ1 | Teashirt Zinc Finger Homeobox 1 | 1.5 |
| TACC1 | Transforming Acidic Coiled-Coil Containing Protein 1 | 1.5 |
| RAB38 | RAB38, Member RAS Oncogene Family | 1.5 |
| KIF13A | Kinesin Family Member 13A | 1.5 |
| STX5 | Syntaxin 5 | 1.5 |
| RAB8A | RAB8A, Member RAS Oncogene Family | 1.5 |
| RBPMS | RNA Binding Protein, MRNA Processing Factor | 1.5 |
| SSR2 | Signal Sequence Receptor Subunit 2 | 1.5 |
| ZC3H14 | Zinc Finger CCCH-Type Containing 14 | 1.5 |
| WASF3 | WASP Family Member 3 | 1.5 |
| VPS53 | VPS53 Subunit Of GARP Complex | 1.5 |
| CACYBP | Calcyclin Binding Protein | 1.5 |
| MAN1C1 | Mannosidase Alpha Class 1C Member 1 | 1.5 |
| EXOSC10 | Exosome Component 10 | 1.5 |
| FEZF1 | FEZ Family Zinc Finger 1 | 1.5 |
| LZTFL1 | Leucine Zipper Transcription Factor Like 1 | 1.5 |
| AHSA1 | Activator Of HSP90 ATPase Activity 1 | 1.5 |
| BFSP2 | Beaded Filament Structural Protein 2 | 1.5 |
| AASDHPPT | Aminoadipate-Semialdehyde Dehydrogenase-Phosphopantetheinyl Transferase | 1.5 |
| FRAT1 | FRAT Regulator Of WNT Signaling Pathway 1 | 1.5 |
| ATP8B2 | ATPase Phospholipid Transporting 8B2 | 1.5 |
| EXTL1 | Exostosin Like Glycosyltransferase 1 | 1.5 |
| CEP41 | Centrosomal Protein 41 | 1.5 |
| GOLGA5 | Golgin A5 | 1.5 |
| CNKSR2 | Connector Enhancer Of Kinase Suppressor Of Ras 2 | 1.5 |
| CNNM4 | Cyclin And CBS Domain Divalent Metal Cation Transport Mediator 4 | 1.5 |
| HERC5 | HECT And RLD Domain Containing E3 Ubiquitin Protein Ligase 5 | 1.5 |
| COG7 | Component Of Oligomeric Golgi Complex 7 | 1.5 |
| RIMS2 | Regulating Synaptic Membrane Exocytosis 2 | 1.5 |
| COLQ | Collagen Like Tail Subunit Of Asymmetric Acetylcholinesterase | 1.5 |
| CHGB | Chromogranin B | 1.5 |
| CHST5 | Carbohydrate Sulfotransferase 5 | 1.5 |
| CHMP2A | Charged Multivesicular Body Protein 2A | 1.5 |
| MLLT1 | MLLT1 Super Elongation Complex Subunit | 1.5 |
| MYBBP1A | MYB Binding Protein 1a | 1.5 |
| CDAN1 | Codanin 1 | 1.5 |
| CDCA2 | Cell Division Cycle Associated 2 | 1.5 |
| CDC5L | Cell Division Cycle 5 Like | 1.5 |
| EIF3I | Eukaryotic Translation Initiation Factor 3 Subunit I | 1.5 |
| RIT2 | Ras Like Without CAAX 2 | 1.5 |
| PEX12 | Peroxisomal Biogenesis Factor 12 | 1.5 |
| NPTX2 | Neuronal Pentraxin 2 | 1.5 |
| NPTXR | Neuronal Pentraxin Receptor | 1.5 |
| PAPOLG | Poly(A) Polymerase Gamma | 1.5 |
| PLCL1 | Phospholipase C Like 1 (Inactive) | 1.5 |
| SNX2 | Sorting Nexin 2 | 1.5 |
| PCDH8 | Protocadherin 8 | 1.5 |
| OPA3 | Outer Mitochondrial Membrane Lipid Metabolism Regulator OPA3 | 1.5 |
| SMTN | Smoothelin | 1.5 |
| SMNDC1 | Survival Motor Neuron Domain Containing 1 | 1.5 |
| SLC27A3 | Solute Carrier Family 27 Member 3 | 1.5 |
| OAS2 | 2'-5'-Oligoadenylate Synthetase 2 | 1.5 |
| SLC25A46 | Solute Carrier Family 25 Member 46 | 1.5 |
| NUP160 | Nucleoporin 160 | 1.5 |
| SLC52A3 | Solute Carrier Family 52 Member 3 | 1.5 |
| PSMC3IP | PSMC3 Interacting Protein | 1.5 |
| PIGC | Phosphatidylinositol Glycan Anchor Biosynthesis Class C | 1.5 |
| SRSF7 | Serine And Arginine Rich Splicing Factor 7 | 1.5 |
| PSMD8 | Proteasome 26S Subunit, Non-ATPase 8 | 1.5 |
| PHOSPHO1 | Phosphoethanolamine/Phosphocholine Phosphatase 1 | 1.5 |
| LCA5 | Lebercilin LCA5 | 1.5 |
| UGT3A1 | UDP Glycosyltransferase Family 3 Member A1 | 1.5 |
| LDHAL6B | Lactate Dehydrogenase A Like 6B | 1.5 |
| TTC3 | Tetratricopeptide Repeat Domain 3 | 1.5 |
| STRADB | STE20 Related Adaptor Beta | 1.5 |
| RCBTB1 | RCC1 And BTB Domain Containing Protein 1 | 1.5 |
| KCNIP1 | Potassium Voltage-Gated Channel Interacting Protein 1 | 1.5 |
| TLL2 | Tolloid Like 2 | 1.5 |
| TMEM126A | Transmembrane Protein 126A | 1.5 |
| PSPN | Persephin | 1.5 |
| DTNB | Dystrobrevin Beta | 1.5 |
| HAP1 | Huntingtin Associated Protein 1 | 1.5 |
| NEXN | Nexilin F-Actin Binding Protein | 1.5 |
| STX12 | Syntaxin 12 | 1.5 |
| PPP1R8 | Protein Phosphatase 1 Regulatory Subunit 8 | 1.5 |
| RASAL1 | RAS Protein Activator Like 1 | 1.5 |
| RBM28 | RNA Binding Motif Protein 28 | 1.5 |
| VPS28 | VPS28 Subunit Of ESCRT-I | 1.5 |
| WDR36 | WD Repeat Domain 36 | 1.5 |
| BBS7 | Bardet-Biedl Syndrome 7 | 1.5 |
| AP3M1 | Adaptor Related Protein Complex 3 Subunit Mu 1 | 1.5 |
| LRRK1 | Leucine Rich Repeat Kinase 1 | 1.5 |
| ACER3 | Alkaline Ceramidase 3 | 1.5 |
| FMN1 | Formin 1 | 1.5 |
| MAN2A2 | Mannosidase Alpha Class 2A Member 2 | 1.5 |
| RHOJ | Ras Homolog Family Member J | 1.5 |
| CMAS | Cytidine Monophosphate N-Acetylneuraminic Acid Synthetase | 1.5 |
| HMCN1 | Hemicentin 1 | 1.5 |
| RGMB | Repulsive Guidance Molecule BMP Co-Receptor B | 1.5 |
| HBD | Hemoglobin Subunit Delta | 1.5 |
| DNALI1 | Dynein Axonemal Light Intermediate Chain 1 | 1.5 |
| MYOM1 | Myomesin 1 | 1.5 |
| RPH3AL | Rabphilin 3A Like (Without C2 Domains) | 1.5 |
| CSPG5 | Chondroitin Sulfate Proteoglycan 5 | 1.5 |
| PEX16 | Peroxisomal Biogenesis Factor 16 | 1.5 |
| SGTA | Small Glutamine Rich Tetratricopeptide Repeat Containing Alpha | 1.5 |
| POU4F1 | POU Class 4 Homeobox 1 | 1.5 |
| POU4F2 | POU Class 4 Homeobox 2 | 1.5 |
| SLC7A3 | Solute Carrier Family 7 Member 3 | 1.5 |
| SMG7 | SMG7 Nonsense Mediated MRNA Decay Factor | 1.5 |
| SLC12A8 | Solute Carrier Family 12 Member 8 | 1.5 |
| DYNC1I2 | Dynein Cytoplasmic 1 Intermediate Chain 2 | 1.5 |
| PSMA8 | Proteasome 20S Subunit Alpha 8 | 1.5 |
| KIF17 | Kinesin Family Member 17 | 1.5 |
| FRMD4A | FERM Domain Containing 4A | 1.5 |
| CALR3 | Calreticulin 3 | 1.5 |
| CABP4 | Calcium Binding Protein 4 | 1.5 |
| ARFRP1 | ADP Ribosylation Factor Related Protein 1 | 1.5 |
| FCHSD1 | FCH And Double SH3 Domains 1 | 1.5 |
| ANKK1 | Ankyrin Repeat And Kinase Domain Containing 1 | 1.5 |
| AGR3 | Anterior Gradient 3, Protein Disulphide Isomerase Family Member | 1.5 |
| ADAMTS14 | ADAM Metallopeptidase With Thrombospondin Type 1 Motif 14 | 1.5 |
| CRNN | Cornulin | 1.5 |
| CREB3L4 | CAMP Responsive Element Binding Protein 3 Like 4 | 1.5 |
| CDR2 | Cerebellar Degeneration Related Protein 2 | 1.5 |
| CNNM3 | Cyclin And CBS Domain Divalent Metal Cation Transport Mediator 3 | 1.5 |
| CMTM7 | CKLF Like MARVEL Transmembrane Domain Containing 7 | 1.5 |
| CLN6 | CLN6 Transmembrane ER Protein | 1.5 |
| HES7 | Hes Family BHLH Transcription Factor 7 | 1.5 |
| CHD6 | Chromodomain Helicase DNA Binding Protein 6 | 1.5 |
| DNAJC13 | DnaJ Heat Shock Protein Family (Hsp40) Member C13 | 1.5 |
| DENR | Density Regulated Re-Initiation And Release Factor | 1.5 |
| DERL2 | Derlin 2 | 1.5 |
| DOCK11 | Dedicator Of Cytokinesis 11 | 1.5 |
| DDRGK1 | DDRGK Domain Containing 1 | 1.5 |
| RNF185 | Ring Finger Protein 185 | 1.5 |
| MPPED2 | Metallophosphoesterase Domain Containing 2 | 1.5 |
| CSMD2 | CUB And Sushi Multiple Domains 2 | 1.5 |
| NDFIP2 | Nedd4 Family Interacting Protein 2 | 1.5 |
| NDUFB1 | NADH:Ubiquinone Oxidoreductase Subunit B1 | 1.5 |
| PRUNE2 | Prune Homolog 2 With BCH Domain | 1.5 |
| PRPF19 | Pre-MRNA Processing Factor 19 | 1.5 |
| PDZD11 | PDZ Domain Containing 11 | 1.5 |
| SPATA7 | Spermatogenesis Associated 7 | 1.5 |
| SFMBT1 | Scm Like With Four Mbt Domains 1 | 1.5 |
| IFT27 | Intraflagellar Transport 27 | 1.5 |
| IFT43 | Intraflagellar Transport 43 | 1.5 |
| SOX8 | SRY-Box Transcription Factor 8 | 1.5 |
| SAMD12 | Sterile Alpha Motif Domain Containing 12 | 1.5 |
| SDR16C5 | Short Chain Dehydrogenase/Reductase Family 16C Member 5 | 1.5 |
| ENC1 | Ectodermal-Neural Cortex 1 | 1.5 |
| PLRG1 | Pleiotropic Regulator 1 | 1.5 |
| OMA1 | OMA1 Zinc Metallopeptidase | 1.5 |
| SLC52A2 | Solute Carrier Family 52 Member 2 | 1.5 |
| SLC5A12 | Solute Carrier Family 5 Member 12 | 1.5 |
| SLC17A2 | Solute Carrier Family 17 Member 2 | 1.5 |
| PHF10 | PHD Finger Protein 10 | 1.5 |
| UFC1 | Ubiquitin-Fold Modifier Conjugating Enzyme 1 | 1.5 |
| LIN7C | Lin-7 Homolog C, Crumbs Cell Polarity Complex Component | 1.5 |
| KHDRBS3 | KH RNA Binding Domain Containing, Signal Transduction Associated 3 | 1.5 |
| TMEM30A | Transmembrane Protein 30A | 1.5 |
| TAF1C | TATA-Box Binding Protein Associated Factor, RNA Polymerase I Subunit C | 1.5 |
| SPA17 | Sperm Autoantigenic Protein 17 | 1.5 |
| SNX32 | Sorting Nexin 32 | 1.5 |
| TRAPPC6A | Trafficking Protein Particle Complex 6A | 1.5 |
| NAA30 | N-Alpha-Acetyltransferase 30, NatC Catalytic Subunit | 1.5 |
| NHS | NHS Actin Remodeling Regulator | 1.5 |
| RASA4 | RAS P21 Protein Activator 4 | 1.5 |
| RAB3IP | RAB3A Interacting Protein | 1.5 |
| STX18 | Syntaxin 18 | 1.5 |
| PACRG | Parkin Coregulated | 1.5 |
| ZHX2 | Zinc Fingers And Homeoboxes 2 | 1.5 |
| ZKSCAN1 | Zinc Finger With KRAB And SCAN Domains 1 | 1.5 |
| ZIC4 | Zic Family Member 4 | 1.5 |
| VPREB1 | V-Set Pre-B Cell Surrogate Light Chain 1 | 1.5 |
| ZFHX4 | Zinc Finger Homeobox 4 | 1.5 |
| GATB | Glutamyl-TRNA Amidotransferase Subunit B | 1.5 |
| GBGT1 | Globoside Alpha-1,3-N-Acetylgalactosaminyltransferase 1 (FORS Blood Group) | 1.5 |
| CCNB3 | Cyclin B3 | 1.5 |
| GINS2 | GINS Complex Subunit 2 | 1.5 |
| GPC2 | Glypican 2 | 1.5 |
| AQP10 | Aquaporin 10 | 1.5 |
| MANEA | Mannosidase Endo-Alpha | 1.5 |
| LRCH1 | Leucine Rich Repeats And Calponin Homology Domain Containing 1 | 1.5 |
| ARFGAP2 | ADP Ribosylation Factor GTPase Activating Protein 2 | 1.5 |
| ARHGEF25 | Rho Guanine Nucleotide Exchange Factor 25 | 1.5 |
| ATP11C | ATPase Phospholipid Transporting 11C | 1.5 |
| FBXW2 | F-Box And WD Repeat Domain Containing 2 | 1.5 |
| LRRC1 | Leucine Rich Repeat Containing 1 | 1.5 |
| AMELX | Amelogenin X-Linked | 1.5 |
| AHCTF1 | AT-Hook Containing Transcription Factor 1 | 1.5 |
| ANKRD49 | Ankyrin Repeat Domain 49 | 1.5 |
| BOLL | Boule Homolog, RNA Binding Protein | 1.5 |
| MCU | Mitochondrial Calcium Uniporter | 1.5 |
| ACAD11 | Acyl-CoA Dehydrogenase Family Member 11 | 1.5 |
| BUD13 | BUD13 Homolog | 1.5 |
| C12orf65 | Chromosome 12 Open Reading Frame 65 | 1.5 |
| C19orf12 | Chromosome 19 Open Reading Frame 12 | 1.5 |
| ANKRD2 | Ankyrin Repeat Domain 2 | 1.5 |
| CEP104 | Centrosomal Protein 104 | 1.5 |
| CCNDBP1 | Cyclin D1 Binding Protein 1 | 1.5 |
| CNNM1 | Cyclin And CBS Domain Divalent Metal Cation Transport Mediator 1 | 1.5 |
| CLUAP1 | Clusterin Associated Protein 1 | 1.5 |
| COG1 | Component Of Oligomeric Golgi Complex 1 | 1.5 |
| COLGALT1 | Collagen Beta(1-O)Galactosyltransferase 1 | 1.5 |
| CHSY3 | Chondroitin Sulfate Synthase 3 | 1.5 |
| MKX | Mohawk Homeobox | 1.5 |
| CHPF2 | Chondroitin Polymerizing Factor 2 | 1.5 |
| CEP89 | Centrosomal Protein 89 | 1.5 |
| DERL1 | Derlin 1 | 1.5 |
| DMRT2 | Doublesex And Mab-3 Related Transcription Factor 2 | 1.5 |
| DMTF1 | Cyclin D Binding Myb Like Transcription Factor 1 | 1.5 |
| CDCA4 | Cell Division Cycle Associated 4 | 1.5 |
| MON1A | MON1 Homolog A, Secretory Trafficking Associated | 1.5 |
| DAND5 | DAN Domain BMP Antagonist Family Member 5 | 1.5 |
| NDUFC1 | NADH:Ubiquinone Oxidoreductase Subunit C1 | 1.5 |
| NDUFAF5 | NADH:Ubiquinone Oxidoreductase Complex Assembly Factor 5 | 1.5 |
| DPPA2 | Developmental Pluripotency Associated 2 | 1.5 |
| HS1BP3 | HCLS1 Binding Protein 3 | 1.5 |
| SERAC1 | Serine Active Site Containing 1 | 1.5 |
| NOTUM | Notum, Palmitoleoyl-Protein Carboxylesterase | 1.5 |
| SHKBP1 | SH3KBP1 Binding Protein 1 | 1.5 |
| PAF1 | PAF1 Homolog, Paf1/RNA Polymerase II Complex Component | 1.5 |
| SNTG2 | Syntrophin Gamma 2 | 1.5 |
| SCARA3 | Scavenger Receptor Class A Member 3 | 1.5 |
| SMU1 | SMU1 DNA Replication Regulator And Spliceosomal Factor | 1.5 |
| SPSB2 | SplA/Ryanodine Receptor Domain And SOCS Box Containing 2 | 1.5 |
| IMMP1L | Inner Mitochondrial Membrane Peptidase Subunit 1 | 1.5 |
| TXNDC17 | Thioredoxin Domain Containing 17 | 1.5 |
| TTC14 | Tetratricopeptide Repeat Domain 14 | 1.5 |
| JMY | Junction Mediating And Regulatory Protein, P53 Cofactor | 1.5 |
| JRK | Jrk Helix-Turn-Helix Protein | 1.5 |
| TCF21 | Transcription Factor 21 | 1.5 |
| STOML1 | Stomatin Like 1 | 1.5 |
| SUGCT | Succinyl-CoA:Glutarate-CoA Transferase | 1.5 |
| TLX2 | T Cell Leukemia Homeobox 2 | 1.5 |
| TM9SF3 | Transmembrane 9 Superfamily Member 3 | 1.5 |
| KXD1 | KxDL Motif Containing 1 | 1.5 |
| TMEM237 | Transmembrane Protein 237 | 1.5 |
| TMEM107 | Transmembrane Protein 107 | 1.5 |
| DNAH17 | Dynein Axonemal Heavy Chain 17 | 1.5 |
| DNAH7 | Dynein Axonemal Heavy Chain 7 | 1.5 |
| NAA20 | N-Alpha-Acetyltransferase 20, NatB Catalytic Subunit | 1.5 |
| HAPLN2 | Hyaluronan And Proteoglycan Link Protein 2 | 1.5 |
| HAPLN4 | Hyaluronan And Proteoglycan Link Protein 4 | 1.5 |
| SAMD9L | Sterile Alpha Motif Domain Containing 9 Like | 1.5 |
| DLEC1 | DLEC1 Cilia And Flagella Associated Protein | 1.5 |
| RND2 | Rho Family GTPase 2 | 1.5 |
| SNAPC4 | Small Nuclear RNA Activating Complex Polypeptide 4 | 1.5 |
| SYCP1 | Synaptonemal Complex Protein 1 | 1.5 |
| IFI44 | Interferon Induced Protein 44 | 1.5 |
| PPP6R3 | Protein Phosphatase 6 Regulatory Subunit 3 | 1.5 |
| KIF18A | Kinesin Family Member 18A | 1.5 |
| WBP11 | WW Domain Binding Protein 11 | 1.5 |
| ZNF346 | Zinc Finger Protein 346 | 1.5 |
| VPS37C | VPS37C Subunit Of ESCRT-I | 1.5 |
| UNC119B | Unc-119 Lipid Binding Chaperone B | 1.5 |
| VIT | Vitrin | 1.5 |
| BLOC1S2 | Biogenesis Of Lysosomal Organelles Complex 1 Subunit 2 | 1.5 |
| BLOC1S3 | Biogenesis Of Lysosomal Organelles Complex 1 Subunit 3 | 1.5 |
| FRA10AC1 | FRA10A Associated CGG Repeat 1 | 1.5 |
| GNAT3 | G Protein Subunit Alpha Transducin 3 | 1.5 |
| CCDC136 | Coiled-Coil Domain Containing 136 | 1.5 |
| FAM161A | FAM161 Centrosomal Protein A | 1.5 |
| FAM163A | Family With Sequence Similarity 163 Member A | 1.5 |
| FAT3 | FAT Atypical Cadherin 3 | 1.5 |
| BAIAP2L2 | BAR/IMD Domain Containing Adaptor Protein 2 Like 2 | 1.5 |
| FAM76B | Family With Sequence Similarity 76 Member B | 1.5 |
| FAM83D | Family With Sequence Similarity 83 Member D | 1.5 |
| ANKS3 | Ankyrin Repeat And Sterile Alpha Motif Domain Containing 3 | 1.5 |
| BORA | BORA Aurora Kinase A Activator | 1.5 |
| C21orf91 | Chromosome 21 Open Reading Frame 91 | 1.5 |
| MALL | Mal, T Cell Differentiation Protein Like | 1.5 |
| CRYZL1 | Crystallin Zeta Like 1 | 1.5 |
| CMTR1 | Cap Methyltransferase 1 | 1.5 |
| DNAJC17 | DnaJ Heat Shock Protein Family (Hsp40) Member C17 | 1.5 |
| DNAJC24 | DnaJ Heat Shock Protein Family (Hsp40) Member C24 | 1.5 |
| DESI2 | Desumoylating Isopeptidase 2 | 1.5 |
| DYDC1 | DPY30 Domain Containing 1 | 1.5 |
| CCDC92 | Coiled-Coil Domain Containing 92 | 1.5 |
| DDX53 | DEAD-Box Helicase 53 | 1.5 |
| MRAP2 | Melanocortin 2 Receptor Accessory Protein 2 | 1.5 |
| NCLN | Nicalin | 1.5 |
| NEUROD4 | Neuronal Differentiation 4 | 1.5 |
| SPIRE2 | Spire Type Actin Nucleation Factor 2 | 1.5 |
| SIDT1 | SID1 Transmembrane Family Member 1 | 1.5 |
| IDNK | IDNK Gluconokinase | 1.5 |
| PPP1R3F | Protein Phosphatase 1 Regulatory Subunit 3F | 1.5 |
| PCDHA12 | Protocadherin Alpha 12 | 1.5 |
| RRP1B | Ribosomal RNA Processing 1B | 1.5 |
| EMP1 | Epithelial Membrane Protein 1 | 1.5 |
| NFATC2IP | Nuclear Factor Of Activated T Cells 2 Interacting Protein | 1.5 |
| NXT2 | Nuclear Transport Factor 2 Like Export Factor 2 | 1.5 |
| SLC37A2 | Solute Carrier Family 37 Member 2 | 1.5 |
| NT5DC2 | 5'-Nucleotidase Domain Containing 2 | 1.5 |
| SIKE1 | Suppressor Of IKBKE 1 | 1.5 |
| PID1 | Phosphotyrosine Interaction Domain Containing 1 | 1.5 |
| UFL1 | UFM1 Specific Ligase 1 | 1.5 |
| LGALSL | Galectin Like | 1.5 |
| TUT1 | Terminal Uridylyl Transferase 1, U6 SnRNA-Specific | 1.5 |
| LDHAL6A | Lactate Dehydrogenase A Like 6A | 1.5 |
| TSKU | Tsukushi, Small Leucine Rich Proteoglycan | 1.5 |
| TTC38 | Tetratricopeptide Repeat Domain 38 | 1.5 |
| RAX2 | Retina And Anterior Neural Fold Homeobox 2 | 1.5 |
| KCTD13 | Potassium Channel Tetramerization Domain Containing 13 | 1.5 |
| RBM20 | RNA Binding Motif Protein 20 | 1.5 |
| KIF24 | Kinesin Family Member 24 | 1.5 |
| SUGP2 | SURP And G-Patch Domain Containing 2 | 1.5 |
| TMEM214 | Transmembrane Protein 214 | 1.5 |
| E2F8 | E2F Transcription Factor 8 | 1.5 |
| EFR3A | EFR3 Homolog A | 1.5 |
| STX10 | Syntaxin 10 | 1.5 |
| RAB20 | RAB20, Member RAS Oncogene Family | 1.5 |
| PCDHA9 | Protocadherin Alpha 9 | 1.5 |
| WDR6 | WD Repeat Domain 6 | 1.5 |
| ZNF14 | Zinc Finger Protein 14 | 1.5 |
| ZNF467 | Zinc Finger Protein 467 | 1.5 |
| VILL | Villin Like | 1.5 |
| ZNRF3 | Zinc And Ring Finger 3 | 1.5 |
| ZBTB14 | Zinc Finger And BTB Domain Containing 14 | 1.5 |
| BLOC1S5 | Biogenesis Of Lysosomal Organelles Complex 1 Subunit 5 | 1.5 |
| FRY | FRY Microtubule Binding Protein | 1.5 |
| GTSF1 | Gametocyte Specific Factor 1 | 1.5 |
| RGCC | Regulator Of Cell Cycle | 1.5 |
| DET1 | DET1 Partner Of COP1 E3 Ubiquitin Ligase | 1.5 |
| NACA2 | Nascent Polypeptide Associated Complex Subunit Alpha 2 | 1.5 |
| RNF112 | Ring Finger Protein 112 | 1.5 |
| PRM2 | Protamine 2 | 1.5 |
| PCDHAC2 | Protocadherin Alpha Subfamily C, 2 | 1.5 |
| PCDHA10 | Protocadherin Alpha 10 | 1.5 |
| SLC35F3 | Solute Carrier Family 35 Member F3 | 1.5 |
| URGCP | Upregulator Of Cell Proliferation | 1.5 |
| TTC30B | Tetratricopeptide Repeat Domain 30B | 1.5 |
| TCHH | Trichohyalin | 1.5 |
| TCHP | Trichoplein Keratin Filament Binding | 1.5 |
| KIF26A | Kinesin Family Member 26A | 1.5 |
| TMEM159 | Transmembrane Protein 159 | 1.5 |
| PDCD11 | Programmed Cell Death 11 | 1.5 |
| RUFY3 | RUN And FYVE Domain Containing 3 | 1.5 |
| KRT28 | Keratin 28 | 1.5 |
| WHAMM | WASP Homolog Associated With Actin, Golgi Membranes And Microtubules | 1.5 |
| ZBED1 | Zinc Finger BED-Type Containing 1 | 1.5 |
| GFRA4 | GDNF Family Receptor Alpha 4 | 1.5 |
| CAMTA2 | Calmodulin Binding Transcription Activator 2 | 1.5 |
| MIEF2 | Mitochondrial Elongation Factor 2 | 1.5 |
| MARVELD1 | MARVEL Domain Containing 1 | 1.5 |
| FAM186B | Family With Sequence Similarity 186 Member B | 1.5 |
| ARHGAP36 | Rho GTPase Activating Protein 36 | 1.5 |
| LYNX1 | Ly6/Neurotoxin 1 | 1.5 |
| FBF1 | Fas Binding Factor 1 | 1.5 |
| ASTL | Astacin Like Metalloendopeptidase | 1.5 |
| FAM81B | Family With Sequence Similarity 81 Member B | 1.5 |
| FAM83G | Family With Sequence Similarity 83 Member G | 1.5 |
| ALG10B | ALG10 Alpha-1,2-Glucosyltransferase B | 1.5 |
| FLYWCH1 | FLYWCH-Type Zinc Finger 1 | 1.5 |
| GIN1 | Gypsy Retrotransposon Integrase 1 | 1.5 |
| H3-3B | H3.3 Histone B | 1.5 |
| MTMR10 | Myotubularin Related Protein 10 | 1.5 |
| DMRT3 | Doublesex And Mab-3 Related Transcription Factor 3 | 1.5 |
| DYDC2 | DPY30 Domain Containing 2 | 1.5 |
| CDC42SE1 | CDC42 Small Effector 1 | 1.5 |
| MPPED1 | Metallophosphoesterase Domain Containing 1 | 1.5 |
| RNF103 | Ring Finger Protein 103 | 1.5 |
| PRRG4 | Proline Rich And Gla Domain 4 | 1.5 |
| PCDHA11 | Protocadherin Alpha 11 | 1.5 |
| NT5DC3 | 5'-Nucleotidase Domain Containing 3 | 1.5 |
| LCN9 | Lipocalin 9 | 1.5 |
| TNP2 | Transition Protein 2 | 1.5 |
| URB2 | URB2 Ribosome Biogenesis Homolog | 1.5 |
| TBRG1 | Transforming Growth Factor Beta Regulator 1 | 1.5 |
| RBM48 | RNA Binding Motif Protein 48 | 1.5 |
| REPIN1 | Replication Initiator 1 | 1.5 |
| TMEM219 | Transmembrane Protein 219 | 1.5 |
| SCLT1 | Sodium Channel And Clathrin Linker 1 | 1.5 |
| ZCWPW1 | Zinc Finger CW-Type And PWWP Domain Containing 1 | 1.5 |
| ADIRF | Adipogenesis Regulatory Factor | 1.5 |
| BLOC1S4 | Biogenesis Of Lysosomal Organelles Complex 1 Subunit 4 | 1.5 |
| CC2D1B | Coiled-Coil And C2 Domain Containing 1B | 1.5 |
| GPAT4 | Glycerol-3-Phosphate Acyltransferase 4 | 1.5 |
| CCZ1 | CCZ1 Homolog, Vacuolar Protein Trafficking And Biogenesis Associated | 1.5 |
| CCDC60 | Coiled-Coil Domain Containing 60 | 1.5 |
| CCDC61 | Coiled-Coil Domain Containing 61 | 1.5 |
| MIEF1 | Mitochondrial Elongation Factor 1 | 1.5 |
| ESX1 | ESX Homeobox 1 | 1.5 |
| ARL14EP | ADP Ribosylation Factor Like GTPase 14 Effector Protein | 1.5 |
| BPIFB3 | BPI Fold Containing Family B Member 3 | 1.5 |
| BPIFB6 | BPI Fold Containing Family B Member 6 | 1.5 |
| C7orf50 | Chromosome 7 Open Reading Frame 50 | 1.5 |
| C1orf56 | Chromosome 1 Open Reading Frame 56 | 1.5 |
| C12orf29 | Chromosome 12 Open Reading Frame 29 | 1.5 |
| CYLC1 | Cylicin 1 | 1.5 |
| HEPACAM2 | HEPACAM Family Member 2 | 1.5 |
| HMGXB3 | HMG-Box Containing 3 | 1.5 |
| CLUH | Clustered Mitochondria Homolog | 1.5 |
| DEFB125 | Defensin Beta 125 | 1.5 |
| DCDC1 | Doublecortin Domain Containing 1 | 1.5 |
| CNST | Consortin, Connexin Sorting Protein | 1.5 |
| PCDHA13 | Protocadherin Alpha 13 | 1.5 |
| OR1F1 | Olfactory Receptor Family 1 Subfamily F Member 1 | 1.5 |
| SMDT1 | Single-Pass Membrane Protein With Aspartate Rich Tail 1 | 1.5 |
| OR4D10 | Olfactory Receptor Family 4 Subfamily D Member 10 | 1.5 |
| SEM1 | SEM1 26S Proteasome Complex Subunit | 1.5 |
| PHACTR4 | Phosphatase And Actin Regulator 4 | 1.5 |
| UFSP1 | UFM1 Specific Peptidase 1 (Inactive) | 1.5 |
| LGALS7B | Galectin 7B | 1.5 |
| PCDHAC1 | Protocadherin Alpha Subfamily C, 1 | 1.5 |
| TAAR9 | Trace Amine Associated Receptor 9 | 1.5 |
| ZIK1 | Zinc Finger Protein Interacting With K Protein 1 | 1.5 |
| ZMAT1 | Zinc Finger Matrin-Type 1 | 1.5 |
| ZNF609 | Zinc Finger Protein 609 | 1.5 |
| ZNF606 | Zinc Finger Protein 606 | 1.5 |
| ZNF740 | Zinc Finger Protein 740 | 1.5 |
| MARCHF5 | Membrane Associated Ring-CH-Type Finger 5 | 1.5 |
| CCDC122 | Coiled-Coil Domain Containing 122 | 1.5 |
| GLT6D1 | Glycosyltransferase 6 Domain Containing 1 | 1.5 |
| FAM162B | Family With Sequence Similarity 162 Member B | 1.5 |
| ARL13A | ADP Ribosylation Factor Like GTPase 13A | 1.5 |
| FREM3 | FRAS1 Related Extracellular Matrix 3 | 1.5 |
| CDR2L | Cerebellar Degeneration Related Protein 2 Like | 1.5 |
| MRPL58 | Mitochondrial Ribosomal Protein L58 | 1.5 |
| DYNC2I1 | Dynein 2 Intermediate Chain 1 | 1.5 |
| MYZAP | Myocardial Zonula Adherens Protein | 1.5 |
| CCDC34 | Coiled-Coil Domain Containing 34 | 1.5 |
| DEFB121 | Defensin Beta 121 | 1.5 |
| LNP1 | Leukemia NUP98 Fusion Partner 1 | 1.5 |
| PRRG1 | Proline Rich And Gla Domain 1 | 1.5 |
| PRRG2 | Proline Rich And Gla Domain 2 | 1.5 |
| SEC22C | SEC22 Homolog C, Vesicle Trafficking Protein | 1.5 |
| PALD1 | Phosphatase Domain Containing Paladin 1 | 1.5 |
| OR1D5 | Olfactory Receptor Family 1 Subfamily D Member 5 | 1.5 |
| OR10S1 | Olfactory Receptor Family 10 Subfamily S Member 1 | 1.5 |
| PNMA3 | PNMA Family Member 3 | 1.5 |
| URB1 | URB1 Ribosome Biogenesis Homolog | 1.5 |
| KIFBP | Kinesin Family Binding Protein | 1.5 |
| TMEM17 | Transmembrane Protein 17 | 1.5 |
| NPB | Neuropeptide B | 1.5 |
| TIAF1 | TGFB1-Induced Anti-Apoptotic Factor 1 | 1.5 |
| TIGD1 | Tigger Transposable Element Derived 1 | 1.5 |
| ZNF492 | Zinc Finger Protein 492 | 1.5 |
| ZNF99 | Zinc Finger Protein 99 | 1.5 |
| CC2D2B | Coiled-Coil And C2 Domain Containing 2B | 1.5 |
| MAP10 | Microtubule Associated Protein 10 | 1.5 |
| C12orf60 | Chromosome 12 Open Reading Frame 60 | 1.5 |
| CRPPA | CDP-L-Ribitol Pyrophosphorylase A | 1.5 |
| RTP2 | Receptor Transporter Protein 2 | 1.5 |
| TENT4A | Terminal Nucleotidyltransferase 4A | 1.5 |
| TEN1 | TEN1 Subunit Of CST Complex | 1.5 |
| SEPTIN3 | Septin 3 | 1.5 |
| PCDHA8 | Protocadherin Alpha 8 | 1.5 |
| WASHC4 | WASH Complex Subunit 4 | 1.5 |
| MARCHF6 | Membrane Associated Ring-CH-Type Finger 6 | 1.5 |
| GDPGP1 | GDP-D-Glucose Phosphorylase 1 | 1.5 |
| CCZ1B | CCZ1 Homolog B, Vacuolar Protein Trafficking And Biogenesis Associated | 1.5 |
| AWAT1 | Acyl-CoA Wax Alcohol Acyltransferase 1 | 1.5 |
| FAM216A | Family With Sequence Similarity 216 Member A | 1.5 |
| C11orf95 | Chromosome 11 Open Reading Frame 95 | 1.5 |
| DEFB124 | Defensin Beta 124 | 1.5 |
| SPINK9 | Serine Peptidase Inhibitor Kazal Type 9 | 1.5 |
| SPAG11B | Sperm Associated Antigen 11B | 1.5 |
| NOTO | Notochord Homeobox | 1.5 |
| SPX | Spexin Hormone | 1.5 |
| TMEM167B | Transmembrane Protein 167B | 1.5 |
| DLEU1 | Deleted In Lymphocytic Leukemia 1 | 1.5 |
| AKR7L | Aldo-Keto Reductase Family 7 Like (Gene/Pseudogene) | 1.5 |
| LTO1 | LTO1 Maturation Factor Of ABCE1 | 1.5 |
| LRRC70 | Leucine Rich Repeat Containing 70 | 1.5 |
| C10orf55 | Chromosome 10 Open Reading Frame 55 (Putative) | 1.5 |
| CPLANE1 | Ciliogenesis And Planar Polarity Effector 1 | 1.5 |
| DEFB104B | Defensin Beta 104B | 1.5 |
| DEFB132 | Defensin Beta 132 | 1.5 |
| DELE1 | DAP3 Binding Cell Death Enhancer 1 | 1.5 |
| RTRAF | RNA Transcription, Translation And Transport Factor | 1.5 |
| SMIM24 | Small Integral Membrane Protein 24 | 1.5 |
| ONECUT3 | One Cut Homeobox 3 | 1.5 |
| NSG2 | Neuronal Vesicle Trafficking Associated 2 | 1.5 |
| PXT1 | Peroxisomal Testis Enriched Protein 1 | 1.5 |
| DENND2B | DENN Domain Containing 2B | 1.5 |
| PIFO | Primary Cilia Formation | 1.5 |
| WDR87 | WD Repeat Domain 87 | 1.5 |
| VPS50 | VPS50 Subunit Of EARP/GARPII Complex | 1.5 |
| MINDY1 | MINDY Lysine 48 Deubiquitinase 1 | 1.5 |
| ASAH2B | N-Acylsphingosine Amidohydrolase 2B | 1.5 |
| HMSD | Histocompatibility Minor Serpin Domain Containing | 1.5 |
| CFAP74 | Cilia And Flagella Associated Protein 74 | 1.5 |
| CIAO3 | Cytosolic Iron-Sulfur Assembly Component 3 | 1.5 |
| LCE1B | Late Cornified Envelope 1B | 1.5 |
| RELCH | RAB11 Binding And LisH Domain, Coiled-Coil And HEAT Repeat Containing | 1.5 |
| NEXMIF | Neurite Extension And Migration Factor | 1.5 |
| C16orf95 | Chromosome 16 Open Reading Frame 95 | 1.5 |
| OR8K3 | Olfactory Receptor Family 8 Subfamily K Member 3 (Gene/Pseudogene) | 1.5 |
| TRMT9B | TRNA Methyltransferase 9B (Putative) | 1.5 |
| CARMIL3 | Capping Protein Regulator And Myosin 1 Linker 3 | 1.5 |
| CASC2 | Cancer Susceptibility 2 | 1.5 |
| CBARP | CACN Subunit Beta Associated Regulatory Protein | 1.5 |
| BICDL1 | BICD Family Like Cargo Adaptor 1 | 1.5 |
| C4orf47 | Chromosome 4 Open Reading Frame 47 | 1.5 |
| FRG2 | FSHD Region Gene 2 | 1.5 |
| RAMAC | RNA Guanine-7 Methyltransferase Activating Subunit | 1.5 |
| RSPH10B2 | Radial Spoke Head 10 Homolog B2 | 1.5 |
| WT1-AS | WT1 Antisense RNA | 1.5 |
| IGF2-AS | IGF2 Antisense RNA | 1.5 |
| SNHG32 | Small Nucleolar RNA Host Gene 32 | 1.5 |
| RCC1L | RCC1 Like | 1.5 |
| XIST | X Inactive Specific Transcript | 1.5 |
| ARMH3 | Armadillo Like Helical Domain Containing 3 | 1.5 |
| FAM187A | Family With Sequence Similarity 187 Member A | 1.5 |
| ITPRID1 | ITPR Interacting Domain Containing 1 | 1.5 |
| FER1L4 | Fer-1 Like Family Member 4 (Pseudogene) | 1.5 |
| SPEM2 | SPEM Family Member 2 | 1.5 |
| SNHG12 | Small Nucleolar RNA Host Gene 12 | 1.5 |
| TDRG1 | Testis Development Related 1 | 1.5 |
| TMEM121B | Transmembrane Protein 121B | 1.5 |
| ADM5 | Adrenomedullin 5 (Putative) | 1.5 |
| ANXA2P2 | Annexin A2 Pseudogene 2 | 1.5 |
| SNHG1 | Small Nucleolar RNA Host Gene 1 | 1.5 |
| PSORS1C3 | Psoriasis Susceptibility 1 Candidate 3 | 1.5 |
| MT1DP | Metallothionein 1D, Pseudogene | 1.5 |
| LINC01006 | Long Intergenic Non-Protein Coding RNA 1006 | 1.5 |
| HOXA11-AS | HOXA11 Antisense RNA | 1.5 |
| SNHG7 | Small Nucleolar RNA Host Gene 7 | 1.5 |
| LINC00052 | Long Intergenic Non-Protein Coding RNA 52 | 1.5 |
| MTRNR2L5 | MT-RNR2 Like 5 | 1.5 |
| EGOT | Eosinophil Granule Ontogeny Transcript | 1.5 |
| WASH3P | WASP Family Homolog 3, Pseudogene | 1.5 |
| LINC00473 | Long Intergenic Non-Protein Coding RNA 473 | 1.5 |
| RN7SK | RNA Component Of 7SK Nuclear Ribonucleoprotein | 1.5 |
| OR3A4P | Olfactory Receptor Family 3 Subfamily A Member 4 Pseudogene | 1.5 |
| TSNAX-DISC1 | TSNAX-DISC1 Readthrough (NMD Candidate) | 1.5 |
| MTRNR2L6 | MT-RNR2 Like 6 | 1.5 |
| CASC15 | Cancer Susceptibility 15 | 1.5 |
| GGT3P | Gamma-Glutamyltransferase 3 Pseudogene | 1.5 |
| MIR1224 | MicroRNA 1224 | 1.5 |
| RNU6-1 | RNA, U6 Small Nuclear 1 | 1.5 |
| MIR454 | MicroRNA 454 | 1.5 |
| PAPPA-AS1 | PAPPA Antisense RNA 1 | 1.5 |
| SNHG8 | Small Nucleolar RNA Host Gene 8 | 1.5 |
| SNHG15 | Small Nucleolar RNA Host Gene 15 | 1.5 |
| TRAPPC2B | Trafficking Protein Particle Complex 2B | 1.5 |
| PWRN1 | Prader-Willi Region Non-Protein Coding RNA 1 | 1.5 |
| KRT18P55 | Keratin 18 Pseudogene 55 | 1.5 |
| ERVH48-1 | Endogenous Retrovirus Group 48 Member 1 | 1.5 |
| AFDN-DT | AFDN Divergent Transcript | 1.5 |
| BCAR4 | Breast Cancer Anti-Estrogen Resistance 4 | 1.5 |
| MIR598 | MicroRNA 598 | 1.5 |
| RNU6ATAC | RNA, U6atac Small Nuclear (U12-Dependent Splicing) | 1.5 |
| SNORD63 | Small Nucleolar RNA, C/D Box 63 | 1.5 |
| PRDM16-DT | PRDM16 Divergent Transcript | 1.5 |
| SNHG16 | Small Nucleolar RNA Host Gene 16 | 1.5 |
| SNHG20 | Small Nucleolar RNA Host Gene 20 | 1.5 |
| KANTR | KDM5C Adjacent Transcript | 1.5 |
| RUNX1-IT1 | RUNX1 Intronic Transcript 1 | 1.5 |
| SNORA70 | Small Nucleolar RNA, H/ACA Box 70 | 1.5 |
| WASH6P | WASP Family Homolog 6, Pseudogene | 1.5 |
| LINC00668 | Long Intergenic Non-Protein Coding RNA 668 | 1.5 |
| ABHD11-AS1 | ABHD11 Antisense RNA 1 (Tail To Tail) | 1.5 |
| MIR518A1 | MicroRNA 518a-1 | 1.5 |
| MIR532 | MicroRNA 532 | 1.5 |
| CECR3 | Cat Eye Syndrome Chromosome Region, Candidate 3 | 1.5 |
| DUX4L8 | Double Homeobox 4 Like 8 (Pseudogene) | 1.5 |
| MIR512-1 | MicroRNA 512-1 | 1.5 |
| RNU5A-1 | RNA, U5A Small Nuclear 1 | 1.5 |
| RPL34-DT | RPL34 Divergent Transcript | 1.5 |
| SFTA1P | Surfactant Associated 1, LncRNA | 1.5 |
| SNORD42B | Small Nucleolar RNA, C/D Box 42B | 1.5 |
| SNORD61 | Small Nucleolar RNA, C/D Box 61 | 1.5 |
| PCDHA@ | Protocadherin Alpha Cluster, Complex Locus | 1.5 |
| SNHG6 | Small Nucleolar RNA Host Gene 6 | 1.5 |
| TUSC7 | Tumor Suppressor Candidate 7 | 1.5 |
| MYLK-AS1 | MYLK Antisense RNA 1 | 1.5 |
| HIF1A-AS2 | HIF1A Antisense RNA 2 | 1.5 |
| ZFHX4-AS1 | ZFHX4 Antisense RNA 1 | 1.5 |
| LINC00673 | Long Intergenic Non-Protein Coding RNA 673 | 1.5 |
| GATA6-AS1 | GATA6 Antisense RNA 1 (Head To Head) | 1.5 |
| FEZF1-AS1 | FEZF1 Antisense RNA 1 | 1.5 |
| MACC1-AS1 | MACC1 Antisense RNA 1 | 1.5 |
| ENTPD1-AS1 | ENTPD1 Antisense RNA 1 | 1.5 |
| DPYD-AS1 | DPYD Antisense RNA 1 | 1.5 |
| DUX4L7 | Double Homeobox 4 Like 7 (Pseudogene) | 1.5 |
| MIR509-1 | MicroRNA 509-1 | 1.5 |
| HNRNPUL2-BSCL2 | HNRNPUL2-BSCL2 Readthrough (NMD Candidate) | 1.5 |
| SNORD69 | Small Nucleolar RNA, C/D Box 69 | 1.5 |
| SNORD96A | Small Nucleolar RNA, C/D Box 96A | 1.5 |
| SNHG14 | Small Nucleolar RNA Host Gene 14 | 1.5 |
| INHBA-AS1 | INHBA Antisense RNA 1 | 1.5 |
| ZNF667-AS1 | ZNF667 Antisense RNA 1 (Head To Head) | 1.5 |
| LINC01234 | Long Intergenic Non-Protein Coding RNA 1234 | 1.5 |
| GACAT2 | Gastric Cancer Associated Transcript 2 | 1.5 |
| GACAT3 | Gastric Cancer Associated Transcript 3 | 1.5 |
| GAPLINC | Gastric Adenocarcinoma Associated, Positive CD44 Regulator, Long Intergenic Non-Coding RNA | 1.5 |
| MIR1183 | MicroRNA 1183 | 1.5 |
| BLACAT1 | Bladder Cancer Associated Transcript 1 | 1.5 |
| CASC9 | Cancer Susceptibility 9 | 1.5 |
| MIR578 | MicroRNA 578 | 1.5 |
| CEBPA-DT | CEBPA Divergent Transcript | 1.5 |
| HAGLROS | HAGLR Opposite Strand LncRNA | 1.5 |
| IL21-AS1 | IL21 Antisense RNA 1 | 1.5 |
| SLC7A11-AS1 | SLC7A11 Antisense RNA 1 | 1.5 |
| SNORA2C | Small Nucleolar RNA, H/ACA Box 2C | 1.5 |
| SNORA31 | Small Nucleolar RNA, H/ACA Box 31 | 1.5 |
| SPRY4-IT1 | SPRY4 Intronic Transcript 1 | 1.5 |
| TMEM238L | Transmembrane Protein 238 Like | 1.5 |
| SAMMSON | Survival Associated Mitochondrial Melanoma Specific Oncogenic Non-Coding RNA | 1.5 |
| SNORA3A | Small Nucleolar RNA, H/ACA Box 3A | 1.5 |
| LINC00901 | Long Intergenic Non-Protein Coding RNA 901 | 1.5 |
| LINC00941 | Long Intergenic Non-Protein Coding RNA 941 | 1.5 |
| LINC00963 | Long Intergenic Non-Protein Coding RNA 963 | 1.5 |
| VPS9D1-AS1 | VPS9D1 Antisense RNA 1 | 1.5 |
| LINC00540 | Long Intergenic Non-Protein Coding RNA 540 | 1.5 |
| LINC00572 | Long Intergenic Non-Protein Coding RNA 572 | 1.5 |
| LINC01150 | Long Intergenic Non-Protein Coding RNA 1150 | 1.5 |
| MIR1299 | MicroRNA 1299 | 1.5 |
| MAP3K20-AS1 | MAP3K20 Antisense RNA 1 | 1.5 |
| AGAP2-AS1 | AGAP2 Antisense RNA 1 | 1.5 |
| MDC1-AS1 | MDC1 Antisense RNA 1 | 1.5 |
| A2M-AS1 | A2M Antisense RNA 1 | 1.5 |
| CCAT2 | Colon Cancer Associated Transcript 2 | 1.5 |
| MIR518D | MicroRNA 518d | 1.5 |
| CFAP298-TCP10L | CFAP298-TCP10L Readthrough | 1.5 |
| DPYD-AS2 | DPYD Antisense RNA 2 | 1.5 |
| MSTO2P | Misato Family Member 2, Pseudogene | 1.5 |
| NPHP3-AS1 | NPHP3 Antisense RNA 1 | 1.5 |
| SH3RF3-AS1 | SH3RF3 Antisense RNA 1 | 1.5 |
| PANTR1 | POU3F3 Adjacent Non-Coding Transcript 1 | 1.5 |
| EGFLAM-AS1 | EGFLAM Antisense RNA 1 | 1.5 |
| SLURP2 | Secreted LY6/PLAUR Domain Containing 2 | 1.5 |
| SMIM31 | Small Integral Membrane Protein 31 | 1.5 |
| TRERNA1 | Translation Regulatory Long Non-Coding RNA 1 | 1.5 |
| KCNK15-AS1 | KCNK15 And WISP2 Antisense RNA 1 | 1.5 |
| SUCLG2-AS1 | SUCLG2 Antisense RNA 1 (Head To Head) | 1.5 |
| TMPO-AS1 | TMPO Antisense RNA 1 | 1.5 |
| SCHLAP1 | SWI/SNF Complex Antagonist Associated With Prostate Cancer 1 | 1.5 |
| EML2-AS1 | EML2 Antisense RNA 1 | 1.5 |
| ZNF571-AS1 | ZNF571 Antisense RNA 1 | 1.5 |
| LINC01094 | Long Intergenic Non-Protein Coding RNA 1094 | 1.5 |
| LINC00628 | Long Intergenic Non-Protein Coding RNA 628 | 1.5 |
| BNC2-AS1 | BNC2 Antisense RNA 1 | 1.5 |
| FRGCA | FOXM1-Regulated, Gastric Cancer Associated | 1.5 |
| CTBP1-AS | CTBP1 Antisense RNA | 1.5 |
| NCRUPAR | Non-Protein Coding RNA, Upstream Of F2R/PAR1 | 1.5 |
| NPHP3-ACAD11 | NPHP3-ACAD11 Readthrough (NMD Candidate) | 1.5 |
| SNORD76 | Small Nucleolar RNA, C/D Box 76 | 1.5 |
| SILC1 | Sciatic Injury Induced LincRNA Upregulator Of SOX11 | 1.5 |
| TTC21B-AS1 | TTC21B Antisense RNA 1 | 1.5 |
| MTRNR2L12 | MT-RNR2 Like 12 | 1.5 |
| TGFB2-AS1 | TGFB2 Antisense RNA 1 (Head To Head) | 1.5 |
| LINC01844 | Long Intergenic Non-Protein Coding RNA 1844 | 1.5 |
| LINC01307 | Long Intergenic Non-Protein Coding RNA 1307 | 1.5 |
| WASF4P | WASP Family Member 4, Pseudogene | 1.5 |
| MIR4274 | MicroRNA 4274 | 1.5 |
| MIR548D1 | MicroRNA 548d-1 | 1.5 |
| SUMO1P3 | SUMO1 Pseudogene 3 | 1.5 |
| ITGA6-AS1 | ITGA6 Antisense RNA 1 | 1.5 |
| LINC01097 | Long Intergenic Non-Protein Coding RNA 1097 | 1.5 |
| GACAT1 | Gastric Cancer Associated Transcript 1 | 1.5 |
| MHRT | Myosin Heavy Chain Associated RNA Transcript | 1.5 |
| GHET1 | Gastric Carcinoma Proliferation Enhancing Transcript 1 | 1.5 |
| MIR6090 | MicroRNA 6090 | 1.5 |
| MMADHC-DT | MMADHC Divergent Transcript | 1.5 |
| DPYD-IT1 | DPYD Intronic Transcript 1 | 1.5 |
| MT-TC | Mitochondrially Encoded TRNA-Cys (UGU/C) | 1.5 |
| PCNA-AS1 | PCNA Antisense RNA 1 | 1.5 |
| LOC102723566 | Uncharacterized LOC102723566 | 1.5 |
| SLC5A4-AS1 | SLC5A4 Antisense RNA 1 | 1.5 |
| SLC9A3-AS1 | SLC9A3 Antisense RNA 1 | 1.5 |
| TNFRSF10A-AS1 | TNFRSF10A Antisense RNA 1 | 1.5 |
| THBS4-AS1 | THBS4 Antisense RNA 1 | 1.5 |
| KRT7-AS | KRT7 Antisense RNA 1 | 1.5 |
| LINC01772 | Long Intergenic Non-Protein Coding RNA 1772 | 1.5 |
| LINC01612 | Long Intergenic Non-Protein Coding RNA 1612 | 1.5 |
| CFTR-AS1 | CFTR Antisense RNA 1 | 1.5 |
| DUXAP8 | Double Homeobox A Pseudogene 8 | 1.5 |
| SKAP1-AS1 | SKAP1 Antisense RNA 1 | 1.5 |
| ENSG00000229717 |  | 1.5 |
| WASF5P | WASP Family Member 5, Pseudogene | 1.5 |
| LINC02258 | Long Intergenic Non-Protein Coding RNA 2258 | 1.5 |
| LINC02461 | Long Intergenic Non-Protein Coding RNA 2461 | 1.5 |
| LINC01856 | Long Intergenic Non-Protein Coding RNA 1856 | 1.5 |
| RN7SL7P | RNA, 7SL, Cytoplasmic 7, Pseudogene | 1.5 |
| DUXAP9 | Double Homeobox A Pseudogene 9 | 1.5 |
| ENSG00000246528 |  | 1.5 |
| TP53COR1 | Tumor Protein P53 Pathway Corepressor 1 | 1.5 |
| LOC101448202 | Uncharacterized LOC101448202 | 1.5 |
| SNORD116@ | Small Nucleolar RNA, C/D Box 116 Cluster | 1.5 |
| ENSG00000253389 |  | 1.5 |
| ABCD1P4 | ATP Binding Cassette Subfamily D Member 1 Pseudogene 4 | 1.5 |
| ENSG00000226277 |  | 1.5 |
| ENSG00000232406 |  | 1.5 |
| ENSG00000249201 |  | 1.5 |
| ENSG00000277200 |  | 1.5 |
| ABCD1P2 | ATP Binding Cassette Subfamily D Member 1 Pseudogene 2 | 1.5 |
| ABCD1P3 | ATP Binding Cassette Subfamily D Member 1 Pseudogene 3 | 1.5 |
| GCRG224 | Gastric Cancer-Related Gene GCRG224 | 1.5 |
| LOC101927278 | Uncharacterized LOC101927278 | 1.5 |
| ENSG00000259039 |  | 1.5 |
| ENSG00000250406 |  | 1.5 |
| ENSG00000266990 |  | 1.5 |
| ENSG00000285159 |  | 1.5 |
| PRS | Prieto X-Linked Mental Retardation Syndrome | 1.5 |
| PCDHACT | Protocadherin Alpha Constant | 1.5 |
| ENSG00000278998 |  | 1.5 |
| ABCD1P1 | ATP Binding Cassette Subfamily D Member 1 Pseudogene 1 | 1.5 |
| LNCRNA-ATB | Long Noncoding RNA Activated By TGF-Beta | 1.5 |
| CTD | Coats Disease | 1.5 |
| LOC105372446 | Uncharacterized LOC105372446 | 1.5 |
| PRD | Primary Retinal Dysplasia | 1.5 |
| CFM1 | Cystic Fibrosis Modifier 1 | 1.5 |
| VDI | Vesicular Stomatitis Virus Defective Interfering Particle Suppression | 1.5 |
| MSD | Microcephaly With Spastic Diplegia (Paine Syndrome) | 1.5 |
| LOC100500719 | HPS1, Biogenesis Of Lysosomal Organelles Complex 3 Subunit 1 Pseudogene | 1.5 |
| MYP1 | Myopia 1 (X-Linked) | 1.5 |
| TCONS_00068220 | Uncharacterized TCONS_00068220 | 1.5 |
| BED | Bornholm Eye Disease | 1.5 |
| MCS+9.7 | RET Intron 1 Enhancer | 1.5 |
| MRXSBWB | Brooks-Wisniewski-Brown Syndrome | 1.5 |
| ENSG00000188078 |  | 1.5 |
| LOC113939944 | Sharpr-MPRA Regulatory Region 9539 | 1.5 |
| LOC113523647 | CFTR +21.5 Kb DNase I Hypersensitive Site | 1.5 |
| LOC113604967 | DNase I Hypersensitive Site 83.7 Kb Downstream Of CFTR | 1.5 |
| LOC113633875 | CFTR Intron 18a DNase I Hypersensitive Site | 1.5 |
| LOC113633876 | CFTR Intron 18b DNase I Hypersensitive Site | 1.5 |
| LOC113633877 | CFTR Intron 19 DNase I Hypersensitive Site | 1.5 |
| LOC113664106 | CFTR Intron 2 DNase I Hypersensitive Site | 1.5 |
| LOC113664107 | CFTR Intron 3 DNase I Hypersensitive Site | 1.5 |
| LOC111674464 | CFTR Intron 1 Enhancer | 1.5 |
| LOC111674465 | DNase I Hypersensitive Site -80 Kb Upstream Of CFTR | 1.5 |
| LOC111674466 | DNase I Hypersensitive Site -20.9 Kb Upstream Of CFTR | 1.5 |
| LOC111674467 | CFTR Intron 20 Enhancer | 1.5 |
| LOC111674470 | DNase I Hypersensitive Region 15.6 Kb Downstream Of CFTR | 1.5 |
| LOC111674471 | DNase I Hypersensitive Region 5.4 To 7.4 Kb Downstream Of CFTR | 1.5 |
| LOC111674472 | DNase I Hypersensitive Sites In Introns 16 And 17a Of CFTR | 1.5 |
| LOC111674473 | DNase I Hypersensitive Sites In Intron 10ab Of CFTR | 1.5 |
| LOC111674474 | DNase I Hypersensitive Site In Intron 10c Of CFTR | 1.5 |
| LOC111674475 | CFTR Intron 11 Enhancer | 1.5 |
| LOC111674476 | CFTR -35 Kb Upstream Enhancer | 1.5 |
| LOC111674477 | CFTR Intron 23 Enhancer | 1.5 |
| LOC111674478 | CFTR -44 Kb Upstream Enhancer | 1.5 |
| LOC111674479 | CFTR +36.6 Kb Downstream Enhancer | 1.5 |
| LOC110121502 | VISTA Enhancer Hs2326 | 1.5 |
| LOC107988032 | Xq28 Proximal FLNA-EMD Recombination Region | 1.5 |
| LOC108491823 | CTTNBP2 Intron Enhancer-Blocking Element 7-1-1 | 1.5 |
| LOC108281186 | AJAP1-NPHP4 Intergenic Nontranscribed DNase I Hypersensitive Site-Defined Enhancer | 1.5 |
| LOC109461476 | GDF5 Promoter Region | 1.5 |
| LOC107457585 | Meiotic Recombination Hotspot J | 1.5 |
| LOC107982234 | WT1/WT1-AS Bi-Directional Promoter Region | 1.5 |
| LOC106736614 | RET 5' Regulatory Region | 1.5 |
| TAB2 | TGF-Beta Activated Kinase 1 (MAP3K7) Binding Protein 2 | 1.5 |
| HDAC6 | Histone Deacetylase 6 | 1.49 |
| PTAFR | Platelet Activating Factor Receptor | 1.49 |
| FOXO1 | Forkhead Box O1 | 1.48 |
| GNPTAB | N-Acetylglucosamine-1-Phosphate Transferase Subunits Alpha And Beta | 1.47 |
| ZBTB24 | Zinc Finger And BTB Domain Containing 24 | 1.47 |
| FTL | Ferritin Light Chain | 1.47 |
| IL22RA2 | Interleukin 22 Receptor Subunit Alpha 2 | 1.46 |
| MAPK9 | Mitogen-Activated Protein Kinase 9 | 1.45 |
| ABCG1 | ATP Binding Cassette Subfamily G Member 1 | 1.45 |
| FTH1 | Ferritin Heavy Chain 1 | 1.45 |
| GABPA | GA Binding Protein Transcription Factor Subunit Alpha | 1.45 |
| ACKR3 | Atypical Chemokine Receptor 3 | 1.45 |
| PDE7A | Phosphodiesterase 7A | 1.45 |
| PLAA | Phospholipase A2 Activating Protein | 1.45 |
| SLC35A1 | Solute Carrier Family 35 Member A1 | 1.45 |
| POU5F1 | POU Class 5 Homeobox 1 | 1.44 |
| PDE4B | Phosphodiesterase 4B | 1.44 |
| PTPN1 | Protein Tyrosine Phosphatase Non-Receptor Type 1 | 1.44 |
| EDIL3 | EGF Like Repeats And Discoidin Domains 3 | 1.43 |
| KCNA2 | Potassium Voltage-Gated Channel Subfamily A Member 2 | 1.43 |
| MAP2K3 | Mitogen-Activated Protein Kinase Kinase 3 | 1.42 |
| S1PR2 | Sphingosine-1-Phosphate Receptor 2 | 1.42 |
| OPRD1 | Opioid Receptor Delta 1 | 1.42 |
| CYP2J2 | Cytochrome P450 Family 2 Subfamily J Member 2 | 1.42 |
| CD93 | CD93 Molecule | 1.4 |
| CXCL17 | C-X-C Motif Chemokine Ligand 17 | 1.4 |
| SIK2 | Salt Inducible Kinase 2 | 1.4 |
| FADS2 | Fatty Acid Desaturase 2 | 1.4 |
| TBXT | T-Box Transcription Factor T | 1.39 |
| KIF15 | Kinesin Family Member 15 | 1.39 |
| UCN3 | Urocortin 3 | 1.39 |
| PHB | Prohibitin | 1.39 |
| NPPC | Natriuretic Peptide C | 1.39 |
| NFKBIE | NFKB Inhibitor Epsilon | 1.38 |
| ROCK1 | Rho Associated Coiled-Coil Containing Protein Kinase 1 | 1.37 |
| SOCS2 | Suppressor Of Cytokine Signaling 2 | 1.37 |
| MAP3K3 | Mitogen-Activated Protein Kinase Kinase Kinase 3 | 1.37 |
| APOC1 | Apolipoprotein C1 | 1.37 |
| GPER1 | G Protein-Coupled Estrogen Receptor 1 | 1.37 |
| PLCE1 | Phospholipase C Epsilon 1 | 1.36 |
| PXN | Paxillin | 1.36 |
| SDC4 | Syndecan 4 | 1.36 |
| TNFRSF14 | TNF Receptor Superfamily Member 14 | 1.36 |
| APCS | Amyloid P Component, Serum | 1.36 |
| TCF7 | Transcription Factor 7 | 1.35 |
| HMMR | Hyaluronan Mediated Motility Receptor | 1.35 |
| CDH11 | Cadherin 11 | 1.35 |
| MAP3K1 | Mitogen-Activated Protein Kinase Kinase Kinase 1 | 1.35 |
| TRPM2 | Transient Receptor Potential Cation Channel Subfamily M Member 2 | 1.33 |
| COPS5 | COP9 Signalosome Subunit 5 | 1.33 |
| FTO | FTO Alpha-Ketoglutarate Dependent Dioxygenase | 1.33 |
| CSF2RA | Colony Stimulating Factor 2 Receptor Subunit Alpha | 1.33 |
| MYOD1 | Myogenic Differentiation 1 | 1.32 |
| MST1R | Macrophage Stimulating 1 Receptor | 1.32 |
| FGF14 | Fibroblast Growth Factor 14 | 1.32 |
| TRADD | TNFRSF1A Associated Via Death Domain | 1.32 |
| LTBR | Lymphotoxin Beta Receptor | 1.32 |
| MEF2A | Myocyte Enhancer Factor 2A | 1.31 |
| PTGFR | Prostaglandin F Receptor | 1.31 |
| NFAT5 | Nuclear Factor Of Activated T Cells 5 | 1.31 |
| MIR206 | MicroRNA 206 | 1.3 |
| PTN | Pleiotrophin | 1.3 |
| PON3 | Paraoxonase 3 | 1.3 |
| IL34 | Interleukin 34 | 1.3 |
| YBX1 | Y-Box Binding Protein 1 | 1.29 |
| MAP2K6 | Mitogen-Activated Protein Kinase Kinase 6 | 1.29 |
| PPM1D | Protein Phosphatase, Mg2+/Mn2+ Dependent 1D | 1.29 |
| GRHL3 | Grainyhead Like Transcription Factor 3 | 1.27 |
| WDR26 | WD Repeat Domain 26 | 1.27 |
| IRF6 | Interferon Regulatory Factor 6 | 1.27 |
| CIRBP | Cold Inducible RNA Binding Protein | 1.26 |
| HDAC3 | Histone Deacetylase 3 | 1.26 |
| TAB3 | TGF-Beta Activated Kinase 1 (MAP3K7) Binding Protein 3 | 1.26 |
| TNFSF9 | TNF Superfamily Member 9 | 1.26 |
| P2RX4 | Purinergic Receptor P2X 4 | 1.26 |
| TRIB3 | Tribbles Pseudokinase 3 | 1.25 |
| MYOG | Myogenin | 1.25 |
| HSD11B2 | Hydroxysteroid 11-Beta Dehydrogenase 2 | 1.25 |
| FABP12 | Fatty Acid Binding Protein 12 | 1.24 |
| ARSA | Arylsulfatase A | 1.24 |
| BCL3 | BCL3 Transcription Coactivator | 1.24 |
| ESRRB | Estrogen Related Receptor Beta | 1.24 |
| CD226 | CD226 Molecule | 1.24 |
| RNASE1 | Ribonuclease A Family Member 1, Pancreatic | 1.24 |
| PF4V1 | Platelet Factor 4 Variant 1 | 1.24 |
| MRTFA | Myocardin Related Transcription Factor A | 1.24 |
| BTG2 | BTG Anti-Proliferation Factor 2 | 1.24 |
| PRDX5 | Peroxiredoxin 5 | 1.24 |
| LIPA | Lipase A, Lysosomal Acid Type | 1.23 |
| ADAM15 | ADAM Metallopeptidase Domain 15 | 1.23 |
| CRHBP | Corticotropin Releasing Hormone Binding Protein | 1.23 |
| CTF1 | Cardiotrophin 1 | 1.23 |
| RALBP1 | RalA Binding Protein 1 | 1.23 |
| TINAGL1 | Tubulointerstitial Nephritis Antigen Like 1 | 1.23 |
| SIGLEC9 | Sialic Acid Binding Ig Like Lectin 9 | 1.23 |
| FADS1 | Fatty Acid Desaturase 1 | 1.23 |
| WWTR1 | WW Domain Containing Transcription Regulator 1 | 1.22 |
| THBS2 | Thrombospondin 2 | 1.22 |
| ITGB7 | Integrin Subunit Beta 7 | 1.22 |
| APOA4 | Apolipoprotein A4 | 1.21 |
| STK39 | Serine/Threonine Kinase 39 | 1.21 |
| TACR3 | Tachykinin Receptor 3 | 1.21 |
| FLT3LG | Fms Related Receptor Tyrosine Kinase 3 Ligand | 1.21 |
| SAA3P | Serum Amyloid A3, Pseudogene | 1.21 |
| TK2 | Thymidine Kinase 2 | 1.2 |
| NAA10 | N-Alpha-Acetyltransferase 10, NatA Catalytic Subunit | 1.2 |
| ATP6AP2 | ATPase H+ Transporting Accessory Protein 2 | 1.2 |
| OXTR | Oxytocin Receptor | 1.2 |
| NCOR2 | Nuclear Receptor Corepressor 2 | 1.2 |
| FOSL2 | FOS Like 2, AP-1 Transcription Factor Subunit | 1.2 |
| TFPI2 | Tissue Factor Pathway Inhibitor 2 | 1.2 |
| CRISP3 | Cysteine Rich Secretory Protein 3 | 1.2 |
| PPARGC1B | PPARG Coactivator 1 Beta | 1.2 |
| HYAL1 | Hyaluronidase 1 | 1.19 |
| NPC1 | NPC Intracellular Cholesterol Transporter 1 | 1.19 |
| LGALS2 | Galectin 2 | 1.19 |
| ABL1 | ABL Proto-Oncogene 1, Non-Receptor Tyrosine Kinase | 1.19 |
| RPS19 | Ribosomal Protein S19 | 1.18 |
| DOCK2 | Dedicator Of Cytokinesis 2 | 1.18 |
| CSF2RB | Colony Stimulating Factor 2 Receptor Subunit Beta | 1.18 |
| TFEB | Transcription Factor EB | 1.17 |
| AGO2 | Argonaute RISC Catalytic Component 2 | 1.17 |
| NBR1 | NBR1 Autophagy Cargo Receptor | 1.17 |
| LRRFIP1 | LRR Binding FLII Interacting Protein 1 | 1.17 |
| PDE3B | Phosphodiesterase 3B | 1.17 |
| KLK1 | Kallikrein 1 | 1.17 |
| DUSP2 | Dual Specificity Phosphatase 2 | 1.17 |
| FLII | FLII Actin Remodeling Protein | 1.16 |
| NTSR1 | Neurotensin Receptor 1 | 1.16 |
| TAGLN | Transgelin | 1.16 |
| NLRC3 | NLR Family CARD Domain Containing 3 | 1.16 |
| UBE2N | Ubiquitin Conjugating Enzyme E2 N | 1.16 |
| CPT1A | Carnitine Palmitoyltransferase 1A | 1.15 |
| TTF2 | Transcription Termination Factor 2 | 1.15 |
| SCAP | SREBF Chaperone | 1.15 |
| PRDX6 | Peroxiredoxin 6 | 1.15 |
| GAP43 | Growth Associated Protein 43 | 1.15 |
| MIR181A2 | MicroRNA 181a-2 | 1.15 |
| INPP5D | Inositol Polyphosphate-5-Phosphatase D | 1.15 |
| PLOD1 | Procollagen-Lysine,2-Oxoglutarate 5-Dioxygenase 1 | 1.14 |
| STK4 | Serine/Threonine Kinase 4 | 1.14 |
| CUL4A | Cullin 4A | 1.14 |
| CORT | Cortistatin | 1.14 |
| ST2 | Suppression Of Tumorigenicity 2 | 1.14 |
| NDRG1 | N-Myc Downstream Regulated 1 | 1.14 |
| PDIA3 | Protein Disulfide Isomerase Family A Member 3 | 1.14 |
| TOR2A | Torsin Family 2 Member A | 1.14 |
| PIAS2 | Protein Inhibitor Of Activated STAT 2 | 1.13 |
| VTCN1 | V-Set Domain Containing T Cell Activation Inhibitor 1 | 1.13 |
| SPI1 | Spi-1 Proto-Oncogene | 1.13 |
| ARNT | Aryl Hydrocarbon Receptor Nuclear Translocator | 1.13 |
| PTGES2 | Prostaglandin E Synthase 2 | 1.13 |
| ROR2 | Receptor Tyrosine Kinase Like Orphan Receptor 2 | 1.12 |
| GTF2I | General Transcription Factor IIi | 1.12 |
| GSK3A | Glycogen Synthase Kinase 3 Alpha | 1.12 |
| SLC25A1 | Solute Carrier Family 25 Member 1 | 1.12 |
| ANXA4 | Annexin A4 | 1.12 |
| GRIP1 | Glutamate Receptor Interacting Protein 1 | 1.12 |
| MRGPRX1 | MAS Related GPR Family Member X1 | 1.12 |
| ALOX15B | Arachidonate 15-Lipoxygenase Type B | 1.12 |
| ALDOA | Aldolase, Fructose-Bisphosphate A | 1.12 |
| GDF2 | Growth Differentiation Factor 2 | 1.12 |
| TNNT1 | Troponin T1, Slow Skeletal Type | 1.12 |
| ROBO4 | Roundabout Guidance Receptor 4 | 1.12 |
| MBOAT7 | Membrane Bound O-Acyltransferase Domain Containing 7 | 1.12 |
| BCL2L12 | BCL2 Like 12 | 1.12 |
| GZMK | Granzyme K | 1.12 |
| BBC3 | BCL2 Binding Component 3 | 1.12 |
| MYLK3 | Myosin Light Chain Kinase 3 | 1.12 |
| NFATC2 | Nuclear Factor Of Activated T Cells 2 | 1.11 |
| ETS2 | ETS Proto-Oncogene 2, Transcription Factor | 1.11 |
| USP10 | Ubiquitin Specific Peptidase 10 | 1.11 |
| FNDC5 | Fibronectin Type III Domain Containing 5 | 1.11 |
| FOSL1 | FOS Like 1, AP-1 Transcription Factor Subunit | 1.1 |
| TPM1 | Tropomyosin 1 | 1.1 |
| DLK1 | Delta Like Non-Canonical Notch Ligand 1 | 1.1 |
| CUL4B | Cullin 4B | 1.1 |
| STIM1 | Stromal Interaction Molecule 1 | 1.1 |
| KLF5 | Kruppel Like Factor 5 | 1.1 |
| FMOD | Fibromodulin | 1.1 |
| GRIK2 | Glutamate Ionotropic Receptor Kainate Type Subunit 2 | 1.09 |
| OPRK1 | Opioid Receptor Kappa 1 | 1.09 |
| IPMK | Inositol Polyphosphate Multikinase | 1.09 |
| MIR181C | MicroRNA 181c | 1.09 |
| TBL2 | Transducin Beta Like 2 | 1.09 |
| GORAB | Golgin, RAB6 Interacting | 1.09 |
| RCAN1 | Regulator Of Calcineurin 1 | 1.09 |
| UBE2I | Ubiquitin Conjugating Enzyme E2 I | 1.09 |
| PDCD4 | Programmed Cell Death 4 | 1.09 |
| TRIB1 | Tribbles Pseudokinase 1 | 1.09 |
| UBE2D2 | Ubiquitin Conjugating Enzyme E2 D2 | 1.08 |
| DIO3 | Iodothyronine Deiodinase 3 | 1.08 |
| RNF20 | Ring Finger Protein 20 | 1.08 |
| TNFSF8 | TNF Superfamily Member 8 | 1.08 |
| SLC12A2 | Solute Carrier Family 12 Member 2 | 1.08 |
| C1D | C1D Nuclear Receptor Corepressor | 1.08 |
| COL4A1 | Collagen Type IV Alpha 1 Chain | 1.08 |
| CFD | Complement Factor D | 1.08 |
| EPHB4 | EPH Receptor B4 | 1.08 |
| P2RY11 | Purinergic Receptor P2Y11 | 1.08 |
| FABP1 | Fatty Acid Binding Protein 1 | 1.08 |
| UNC5B | Unc-5 Netrin Receptor B | 1.08 |
| OGN | Osteoglycin | 1.07 |
| CFL1 | Cofilin 1 | 1.07 |
| IRAK2 | Interleukin 1 Receptor Associated Kinase 2 | 1.07 |
| SETD7 | SET Domain Containing 7, Histone Lysine Methyltransferase | 1.07 |
| MNDA | Myeloid Cell Nuclear Differentiation Antigen | 1.07 |
| ASH2L | ASH2 Like, Histone Lysine Methyltransferase Complex Subunit | 1.07 |
| NEUROG1 | Neurogenin 1 | 1.07 |
| ENTPD2 | Ectonucleoside Triphosphate Diphosphohydrolase 2 | 1.07 |
| FBXO3 | F-Box Protein 3 | 1.07 |
| MSBP1 | Minisatellite Binding Protein 1 | 1.07 |
| CD2-LCR | CD2 Locus Control Region | 1.07 |
| IAPP | Islet Amyloid Polypeptide | 1.07 |
| GALR1 | Galanin Receptor 1 | 1.06 |
| TRAF5 | TNF Receptor Associated Factor 5 | 1.06 |
| HYAL2 | Hyaluronidase 2 | 1.06 |
| SLC3A2 | Solute Carrier Family 3 Member 2 | 1.06 |
| SRGN | Serglycin | 1.05 |
| PRDX1 | Peroxiredoxin 1 | 1.05 |
| CGB5 | Chorionic Gonadotropin Subunit Beta 5 | 1.05 |
| RUNX1 | RUNX Family Transcription Factor 1 | 1.04 |
| PELI1 | Pellino E3 Ubiquitin Protein Ligase 1 | 1.04 |
| HSP90B1 | Heat Shock Protein 90 Beta Family Member 1 | 1.04 |
| RECK | Reversion Inducing Cysteine Rich Protein With Kazal Motifs | 1.04 |
| PARK7 | Parkinsonism Associated Deglycase | 1.04 |
| UTS2 | Urotensin 2 | 1.04 |
| DEPTOR | DEP Domain Containing MTOR Interacting Protein | 1.04 |
| TRIM63 | Tripartite Motif Containing 63 | 1.04 |
| NTAN1 | N-Terminal Asparagine Amidase | 1.04 |
| XCL1 | X-C Motif Chemokine Ligand 1 | 1.04 |
| CCL16 | C-C Motif Chemokine Ligand 16 | 1.03 |
| PCK2 | Phosphoenolpyruvate Carboxykinase 2, Mitochondrial | 1.03 |
| CCL28 | C-C Motif Chemokine Ligand 28 | 1.03 |
| BTRC | Beta-Transducin Repeat Containing E3 Ubiquitin Protein Ligase | 1.03 |
| MT2A | Metallothionein 2A | 1.02 |
| APAF1 | Apoptotic Peptidase Activating Factor 1 | 1.01 |
| PTGIR | Prostaglandin I2 Receptor | 1.01 |
| LILRB1 | Leukocyte Immunoglobulin Like Receptor B1 | 1.01 |
| SUV39H1 | Suppressor Of Variegation 3-9 Homolog 1 | 1.01 |
| PRPF8 | Pre-MRNA Processing Factor 8 | 1.01 |
| SMOX | Spermine Oxidase | 1.01 |
| BCR | BCR Activator Of RhoGEF And GTPase | 1.01 |
| RBX1 | Ring-Box 1 | 1.01 |
| USP17L2 | Ubiquitin Specific Peptidase 17 Like Family Member 2 | 1.01 |
| USP14 | Ubiquitin Specific Peptidase 14 | 1.01 |
| CASZ1 | Castor Zinc Finger 1 | 1.01 |
| ALCAM | Activated Leukocyte Cell Adhesion Molecule | 1 |
| GPR55 | G Protein-Coupled Receptor 55 | 1 |
| SERPINB9 | Serpin Family B Member 9 | 1 |
| LITAF | Lipopolysaccharide Induced TNF Factor | 1 |
| SKP1 | S-Phase Kinase Associated Protein 1 | 0.99 |
| SMPD3 | Sphingomyelin Phosphodiesterase 3 | 0.99 |
| KLF15 | Kruppel Like Factor 15 | 0.99 |
| LIN28A | Lin-28 Homolog A | 0.99 |
| H2BC3 | H2B Clustered Histone 3 | 0.99 |
| CYP11B2 | Cytochrome P450 Family 11 Subfamily B Member 2 | 0.99 |
| FABP3 | Fatty Acid Binding Protein 3 | 0.99 |
| USF1 | Upstream Transcription Factor 1 | 0.99 |
| COMMD1 | Copper Metabolism Domain Containing 1 | 0.99 |
| NUCB2 | Nucleobindin 2 | 0.99 |
| NAIP | NLR Family Apoptosis Inhibitory Protein | 0.99 |
| PIBF1 | Progesterone Immunomodulatory Binding Factor 1 | 0.99 |
| RHOB | Ras Homolog Family Member B | 0.98 |
| TIE1 | Tyrosine Kinase With Immunoglobulin Like And EGF Like Domains 1 | 0.98 |
| EEF2K | Eukaryotic Elongation Factor 2 Kinase | 0.98 |
| DROSHA | Drosha Ribonuclease III | 0.98 |
| RBBP5 | RB Binding Protein 5, Histone Lysine Methyltransferase Complex Subunit | 0.98 |
| RPS6KA3 | Ribosomal Protein S6 Kinase A3 | 0.98 |
| PKLR | Pyruvate Kinase L/R | 0.98 |
| TBX5 | T-Box Transcription Factor 5 | 0.97 |
| SLC7A11 | Solute Carrier Family 7 Member 11 | 0.97 |
| CUL1 | Cullin 1 | 0.97 |
| HTT | Huntingtin | 0.97 |
| FFAR1 | Free Fatty Acid Receptor 1 | 0.97 |
| CAMK2G | Calcium/Calmodulin Dependent Protein Kinase II Gamma | 0.97 |
| MAP2K2 | Mitogen-Activated Protein Kinase Kinase 2 | 0.97 |
| STK11 | Serine/Threonine Kinase 11 | 0.97 |
| PPP2CB | Protein Phosphatase 2 Catalytic Subunit Beta | 0.96 |
| TRPC5 | Transient Receptor Potential Cation Channel Subfamily C Member 5 | 0.96 |
| NEIL2 | Nei Like DNA Glycosylase 2 | 0.96 |
| PRKCI | Protein Kinase C Iota | 0.96 |
| YY1 | YY1 Transcription Factor | 0.96 |
| SLC29A2 | Solute Carrier Family 29 Member 2 | 0.96 |
| ELK1 | ETS Transcription Factor ELK1 | 0.96 |
| B3GAT1 | Beta-1,3-Glucuronyltransferase 1 | 0.96 |
| PTGES3 | Prostaglandin E Synthase 3 | 0.96 |
| JAM2 | Junctional Adhesion Molecule 2 | 0.96 |
| CEACAM4 | CEA Cell Adhesion Molecule 4 | 0.96 |
| MAP3K11 | Mitogen-Activated Protein Kinase Kinase Kinase 11 | 0.96 |
| SLC23A1 | Solute Carrier Family 23 Member 1 | 0.96 |
| FBXO32 | F-Box Protein 32 | 0.96 |
| RACK1 | Receptor For Activated C Kinase 1 | 0.96 |
| DLG1 | Discs Large MAGUK Scaffold Protein 1 | 0.95 |
| LTBP3 | Latent Transforming Growth Factor Beta Binding Protein 3 | 0.95 |
| DPF2 | Double PHD Fingers 2 | 0.95 |
| IDE | Insulin Degrading Enzyme | 0.95 |
| PTGIS | Prostaglandin I2 Synthase | 0.95 |
| ASIC1 | Acid Sensing Ion Channel Subunit 1 | 0.95 |
| UCP3 | Uncoupling Protein 3 | 0.95 |
| WWOX | WW Domain Containing Oxidoreductase | 0.94 |
| YWHAQ | Tyrosine 3-Monooxygenase/Tryptophan 5-Monooxygenase Activation Protein Theta | 0.94 |
| HSPA6 | Heat Shock Protein Family A (Hsp70) Member 6 | 0.94 |
| PGRMC1 | Progesterone Receptor Membrane Component 1 | 0.94 |
| RNF2 | Ring Finger Protein 2 | 0.94 |
| EHF | ETS Homologous Factor | 0.94 |
| BMPER | BMP Binding Endothelial Regulator | 0.94 |
| CHID1 | Chitinase Domain Containing 1 | 0.94 |
| SPRY2 | Sprouty RTK Signaling Antagonist 2 | 0.94 |
| RPS6 | Ribosomal Protein S6 | 0.94 |
| ADPRH | ADP-Ribosylarginine Hydrolase | 0.94 |
| CCN5 | Cellular Communication Network Factor 5 | 0.94 |
| EPHB2 | EPH Receptor B2 | 0.93 |
| NEDD4 | NEDD4 E3 Ubiquitin Protein Ligase | 0.93 |
| NAT1 | N-Acetyltransferase 1 | 0.93 |
| LTBP1 | Latent Transforming Growth Factor Beta Binding Protein 1 | 0.93 |
| HSPA14 | Heat Shock Protein Family A (Hsp70) Member 14 | 0.93 |
| PTCRA | Pre T Cell Antigen Receptor Alpha | 0.93 |
| HMBOX1 | Homeobox Containing 1 | 0.93 |
| SIRT2 | Sirtuin 2 | 0.93 |
| TIAM1 | TIAM Rac1 Associated GEF 1 | 0.93 |
| HAS3 | Hyaluronan Synthase 3 | 0.93 |
| CCL15 | C-C Motif Chemokine Ligand 15 | 0.93 |
| PDCD1LG2 | Programmed Cell Death 1 Ligand 2 | 0.92 |
| IDS | Iduronate 2-Sulfatase | 0.92 |
| UNG | Uracil DNA Glycosylase | 0.92 |
| MSX1 | Msh Homeobox 1 | 0.92 |
| ARHGAP29 | Rho GTPase Activating Protein 29 | 0.92 |
| DPH1 | Diphthamide Biosynthesis 1 | 0.92 |
| OSTM1 | Osteoclastogenesis Associated Transmembrane Protein 1 | 0.92 |
| CLIP2 | CAP-Gly Domain Containing Linker Protein 2 | 0.92 |
| P4HTM | Prolyl 4-Hydroxylase, Transmembrane | 0.92 |
| SELENON | Selenoprotein N | 0.92 |
| HTR1D | 5-Hydroxytryptamine Receptor 1D | 0.91 |
| GSTA1 | Glutathione S-Transferase Alpha 1 | 0.91 |
| DNMT3A | DNA Methyltransferase 3 Alpha | 0.91 |
| NEDD9 | Neural Precursor Cell Expressed, Developmentally Down-Regulated 9 | 0.91 |
| PLA2G3 | Phospholipase A2 Group III | 0.91 |
| GPSM1 | G Protein Signaling Modulator 1 | 0.91 |
| CCAR2 | Cell Cycle And Apoptosis Regulator 2 | 0.91 |
| SELENOP | Selenoprotein P | 0.91 |
| ATP1A1 | ATPase Na+/K+ Transporting Subunit Alpha 1 | 0.91 |
| MEF2C | Myocyte Enhancer Factor 2C | 0.91 |
| DUSP6 | Dual Specificity Phosphatase 6 | 0.91 |
| COL4A2 | Collagen Type IV Alpha 2 Chain | 0.91 |
| CNTFR | Ciliary Neurotrophic Factor Receptor | 0.91 |
| ST6GAL1 | ST6 Beta-Galactoside Alpha-2,6-Sialyltransferase 1 | 0.91 |
| CBY1 | Chibby Family Member 1, Beta Catenin Antagonist | 0.91 |
| HNRNPL | Heterogeneous Nuclear Ribonucleoprotein L | 0.91 |
| DUSP22 | Dual Specificity Phosphatase 22 | 0.91 |
| LILRB4 | Leukocyte Immunoglobulin Like Receptor B4 | 0.91 |
| SWAP70 | Switching B Cell Complex Subunit SWAP70 | 0.91 |
| CLCF1 | Cardiotrophin Like Cytokine Factor 1 | 0.91 |
| TREML2 | Triggering Receptor Expressed On Myeloid Cells Like 2 | 0.91 |
| GJB1 | Gap Junction Protein Beta 1 | 0.9 |
| LPAR3 | Lysophosphatidic Acid Receptor 3 | 0.9 |
| RIPK4 | Receptor Interacting Serine/Threonine Kinase 4 | 0.9 |
| C4BPB | Complement Component 4 Binding Protein Beta | 0.9 |
| NEO1 | Neogenin 1 | 0.9 |
| SPINT1 | Serine Peptidase Inhibitor, Kunitz Type 1 | 0.9 |
| PTGR1 | Prostaglandin Reductase 1 | 0.9 |
| ADGRE2 | Adhesion G Protein-Coupled Receptor E2 | 0.9 |
| MIRLET7A2 | MicroRNA Let-7a-2 | 0.9 |
| MIRLET7A3 | MicroRNA Let-7a-3 | 0.9 |
| TMPRSS6 | Transmembrane Serine Protease 6 | 0.9 |
| PLD2 | Phospholipase D2 | 0.89 |
| HDAC7 | Histone Deacetylase 7 | 0.89 |
| PIK3C3 | Phosphatidylinositol 3-Kinase Catalytic Subunit Type 3 | 0.89 |
| GAB1 | GRB2 Associated Binding Protein 1 | 0.89 |
| TRIM28 | Tripartite Motif Containing 28 | 0.89 |
| USP4 | Ubiquitin Specific Peptidase 4 | 0.89 |
| CDH13 | Cadherin 13 | 0.89 |
| UBASH3B | Ubiquitin Associated And SH3 Domain Containing B | 0.89 |
| ADAMTS12 | ADAM Metallopeptidase With Thrombospondin Type 1 Motif 12 | 0.89 |
| NES | Nestin | 0.89 |
| CALCB | Calcitonin Related Polypeptide Beta | 0.89 |
| MIR34B | MicroRNA 34b | 0.89 |
| MYF5 | Myogenic Factor 5 | 0.89 |
| ZBTB33 | Zinc Finger And BTB Domain Containing 33 | 0.89 |
| MAS1 | MAS1 Proto-Oncogene, G Protein-Coupled Receptor | 0.89 |
| ATRX | ATRX Chromatin Remodeler | 0.88 |
| L2HGDH | L-2-Hydroxyglutarate Dehydrogenase | 0.88 |
| TMEM70 | Transmembrane Protein 70 | 0.88 |
| SEMA4A | Semaphorin 4A | 0.88 |
| NM | Neutrophil Migration | 0.88 |
| KCNK3 | Potassium Two Pore Domain Channel Subfamily K Member 3 | 0.87 |
| MKNK1 | MAPK Interacting Serine/Threonine Kinase 1 | 0.87 |
| GRIK1 | Glutamate Ionotropic Receptor Kainate Type Subunit 1 | 0.87 |
| GRK6 | G Protein-Coupled Receptor Kinase 6 | 0.87 |
| ATP6V0A2 | ATPase H+ Transporting V0 Subunit A2 | 0.87 |
| IL11RA | Interleukin 11 Receptor Subunit Alpha | 0.87 |
| NRIP1 | Nuclear Receptor Interacting Protein 1 | 0.87 |
| INHBB | Inhibin Subunit Beta B | 0.87 |
| USP25 | Ubiquitin Specific Peptidase 25 | 0.87 |
| POLR2L | RNA Polymerase II, I And III Subunit L | 0.87 |
| KLF7 | Kruppel Like Factor 7 | 0.87 |
| LIPF | Lipase F, Gastric Type | 0.87 |
| CD300LF | CD300 Molecule Like Family Member F | 0.87 |
| PARG | Poly(ADP-Ribose) Glycohydrolase | 0.87 |
| IER3 | Immediate Early Response 3 | 0.87 |
| COMMD6 | COMM Domain Containing 6 | 0.87 |
| MIRLET7F1 | MicroRNA Let-7f-1 | 0.87 |
| MIR124-3 | MicroRNA 124-3 | 0.87 |
| MSBP2 | Minisatellite Binding Protein 2 | 0.87 |
| GRIA2 | Glutamate Ionotropic Receptor AMPA Type Subunit 2 | 0.87 |
| ACKR4 | Atypical Chemokine Receptor 4 | 0.87 |
| MIR216A | MicroRNA 216a | 0.87 |
| MCM2 | Minichromosome Maintenance Complex Component 2 | 0.86 |
| NINJ1 | Ninjurin 1 | 0.86 |
| GSS | Glutathione Synthetase | 0.86 |
| GNMT | Glycine N-Methyltransferase | 0.86 |
| CSTB | Cystatin B | 0.86 |
| GPD1 | Glycerol-3-Phosphate Dehydrogenase 1 | 0.86 |
| ARF6 | ADP Ribosylation Factor 6 | 0.86 |
| ACSL1 | Acyl-CoA Synthetase Long Chain Family Member 1 | 0.86 |
| EOMES | Eomesodermin | 0.86 |
| BNIP3 | BCL2 Interacting Protein 3 | 0.86 |
| IL17RD | Interleukin 17 Receptor D | 0.86 |
| MAFK | MAF BZIP Transcription Factor K | 0.86 |
| RAI14 | Retinoic Acid Induced 14 | 0.86 |
| NAXE | NAD(P)HX Epimerase | 0.86 |
| VSIR | V-Set Immunoregulatory Receptor | 0.86 |
| RARG | Retinoic Acid Receptor Gamma | 0.85 |
| PBK | PDZ Binding Kinase | 0.85 |
| PRMT6 | Protein Arginine Methyltransferase 6 | 0.85 |
| PCYT1A | Phosphate Cytidylyltransferase 1, Choline, Alpha | 0.85 |
| KCNJ6 | Potassium Inwardly Rectifying Channel Subfamily J Member 6 | 0.85 |
| TLK2 | Tousled Like Kinase 2 | 0.85 |
| CDC45 | Cell Division Cycle 45 | 0.85 |
| RFC2 | Replication Factor C Subunit 2 | 0.85 |
| EPM2A | EPM2A Glucan Phosphatase, Laforin | 0.85 |
| NDE1 | NudE Neurodevelopment Protein 1 | 0.85 |
| PANK2 | Pantothenate Kinase 2 | 0.85 |
| SLC25A24 | Solute Carrier Family 25 Member 24 | 0.85 |
| NSD1 | Nuclear Receptor Binding SET Domain Protein 1 | 0.85 |
| VANGL1 | VANGL Planar Cell Polarity Protein 1 | 0.85 |
| NFIX | Nuclear Factor I X | 0.85 |
| COQ2 | Coenzyme Q2, Polyprenyltransferase | 0.85 |
| SLC30A9 | Solute Carrier Family 30 Member 9 | 0.85 |
| NXN | Nucleoredoxin | 0.85 |
| LETM1 | Leucine Zipper And EF-Hand Containing Transmembrane Protein 1 | 0.85 |
| TBCD | Tubulin Folding Cofactor D | 0.85 |
| BAZ1B | Bromodomain Adjacent To Zinc Finger Domain 1B | 0.85 |
| BPTF | Bromodomain PHD Finger Transcription Factor | 0.85 |
| BSND | Barttin CLCNK Type Accessory Subunit Beta | 0.85 |
| COX7B | Cytochrome C Oxidase Subunit 7B | 0.85 |
| PDSS2 | Decaprenyl Diphosphate Synthase Subunit 2 | 0.85 |
| ORC6 | Origin Recognition Complex Subunit 6 | 0.85 |
| SRCAP | Snf2 Related CREBBP Activator Protein | 0.85 |
| MED25 | Mediator Complex Subunit 25 | 0.85 |
| CRELD1 | Cysteine Rich With EGF Like Domains 1 | 0.85 |
| DLX4 | Distal-Less Homeobox 4 | 0.85 |
| TBC1D24 | TBC1 Domain Family Member 24 | 0.85 |
| ANKRD11 | Ankyrin Repeat Domain 11 | 0.85 |
| NECTIN1 | Nectin Cell Adhesion Molecule 1 | 0.85 |
| IQSEC2 | IQ Motif And Sec7 Domain ArfGEF 2 | 0.85 |
| PUS3 | Pseudouridine Synthase 3 | 0.85 |
| KPTN | Kaptin, Actin Binding Protein | 0.85 |
| TGDS | TDP-Glucose 4,6-Dehydratase | 0.85 |
| ADAT3 | Adenosine Deaminase TRNA Specific 3 | 0.85 |
| CCDC47 | Coiled-Coil Domain Containing 47 | 0.85 |
| NSD2 | Nuclear Receptor Binding SET Domain Protein 2 | 0.85 |
| PRKCH | Protein Kinase C Eta | 0.84 |
| FDFT1 | Farnesyl-Diphosphate Farnesyltransferase 1 | 0.84 |
| HOXA11 | Homeobox A11 | 0.84 |
| ADAMTS7 | ADAM Metallopeptidase With Thrombospondin Type 1 Motif 7 | 0.84 |
| HGFAC | HGF Activator | 0.84 |
| SAPCD2 | Suppressor APC Domain Containing 2 | 0.84 |
| MIR302B | MicroRNA 302b | 0.84 |
| PHACTR1 | Phosphatase And Actin Regulator 1 | 0.84 |
| RGMA | Repulsive Guidance Molecule BMP Co-Receptor A | 0.84 |
| SET | SET Nuclear Proto-Oncogene | 0.84 |
| PRKACB | Protein Kinase CAMP-Activated Catalytic Subunit Beta | 0.84 |
| UBE2D3 | Ubiquitin Conjugating Enzyme E2 D3 | 0.84 |
| COL4A6 | Collagen Type IV Alpha 6 Chain | 0.84 |
| RPS3 | Ribosomal Protein S3 | 0.84 |
| NR2E1 | Nuclear Receptor Subfamily 2 Group E Member 1 | 0.84 |
| TRPV3 | Transient Receptor Potential Cation Channel Subfamily V Member 3 | 0.84 |
| STK38 | Serine/Threonine Kinase 38 | 0.84 |
| KSR1 | Kinase Suppressor Of Ras 1 | 0.84 |
| UTS2R | Urotensin 2 Receptor | 0.84 |
| RGS16 | Regulator Of G Protein Signaling 16 | 0.84 |
| AHCYL2 | Adenosylhomocysteinase Like 2 | 0.84 |
| DOCK4 | Dedicator Of Cytokinesis 4 | 0.84 |
| STK40 | Serine/Threonine Kinase 40 | 0.84 |
| CLEC6A | C-Type Lectin Domain Containing 6A | 0.84 |
| ICAM4 | Intercellular Adhesion Molecule 4 (Landsteiner-Wiener Blood Group) | 0.84 |
| C2CD4C | C2 Calcium Dependent Domain Containing 4C | 0.84 |
| MIR449A | MicroRNA 449a | 0.84 |
| MIR194-1 | MicroRNA 194-1 | 0.84 |
| MUC8 | Mucin 8 | 0.84 |
| CSNK2A2 | Casein Kinase 2 Alpha 2 | 0.84 |
| PRMT1 | Protein Arginine Methyltransferase 1 | 0.83 |
| MARCKS | Myristoylated Alanine Rich Protein Kinase C Substrate | 0.83 |
| NCEH1 | Neutral Cholesterol Ester Hydrolase 1 | 0.83 |
| C1QTNF6 | C1q And TNF Related 6 | 0.83 |
| MIR370 | MicroRNA 370 | 0.83 |
| MIR544A | MicroRNA 544a | 0.83 |
| NKILA | NF-KappaB Interacting LncRNA | 0.83 |
| SLC7A2 | Solute Carrier Family 7 Member 2 | 0.82 |
| AGRP | Agouti Related Neuropeptide | 0.81 |
| MEF2D | Myocyte Enhancer Factor 2D | 0.81 |
| ARNTL | Aryl Hydrocarbon Receptor Nuclear Translocator Like | 0.81 |
| SMG1 | SMG1 Nonsense Mediated MRNA Decay Associated PI3K Related Kinase | 0.81 |
| KAT6B | Lysine Acetyltransferase 6B | 0.81 |
| TRIM27 | Tripartite Motif Containing 27 | 0.81 |
| HIF3A | Hypoxia Inducible Factor 3 Subunit Alpha | 0.81 |
| ITIH2 | Inter-Alpha-Trypsin Inhibitor Heavy Chain 2 | 0.81 |
| ENTPD7 | Ectonucleoside Triphosphate Diphosphohydrolase 7 | 0.81 |
| S100A14 | S100 Calcium Binding Protein A14 | 0.81 |
| CIP2A | Cellular Inhibitor Of PP2A | 0.81 |
| DEFB109B | Defensin Beta 109B | 0.81 |
| MAP3K4 | Mitogen-Activated Protein Kinase Kinase Kinase 4 | 0.81 |
| IGFBP4 | Insulin Like Growth Factor Binding Protein 4 | 0.81 |
| QKI | QKI, KH Domain Containing RNA Binding | 0.81 |
| PARD3 | Par-3 Family Cell Polarity Regulator | 0.81 |
| CPEB1 | Cytoplasmic Polyadenylation Element Binding Protein 1 | 0.81 |
| ERRFI1 | ERBB Receptor Feedback Inhibitor 1 | 0.81 |
| NXPH1 | Neurexophilin 1 | 0.81 |
| LRRC25 | Leucine Rich Repeat Containing 25 | 0.81 |
| C2CD4B | C2 Calcium Dependent Domain Containing 4B | 0.81 |
| C2CD4A | C2 Calcium Dependent Domain Containing 4A | 0.81 |
| DEFB114 | Defensin Beta 114 | 0.81 |
| MIR194-2 | MicroRNA 194-2 | 0.81 |
| MIR219A2 | MicroRNA 219a-2 | 0.81 |
| TRD | T Cell Receptor Delta Locus | 0.81 |
| GRM1 | Glutamate Metabotropic Receptor 1 | 0.8 |
| EPHA1 | EPH Receptor A1 | 0.8 |
| PRCP | Prolylcarboxypeptidase | 0.8 |
| PIAS4 | Protein Inhibitor Of Activated STAT 4 | 0.8 |
| NODAL | Nodal Growth Differentiation Factor | 0.8 |
| KLF10 | Kruppel Like Factor 10 | 0.8 |
| CDK20 | Cyclin Dependent Kinase 20 | 0.8 |
| C1QTNF1 | C1q And TNF Related 1 | 0.8 |
| DDT | D-Dopachrome Tautomerase | 0.8 |
| WFDC2 | WAP Four-Disulfide Core Domain 2 | 0.8 |
| PMCH | Pro-Melanin Concentrating Hormone | 0.8 |
| C1QTNF9 | C1q And TNF Related 9 | 0.8 |
| ECRG4 | ECRG4 Augurin Precursor | 0.8 |
| MAP3K12 | Mitogen-Activated Protein Kinase Kinase Kinase 12 | 0.78 |
| PSMB7 | Proteasome 20S Subunit Beta 7 | 0.78 |
| GNG2 | G Protein Subunit Gamma 2 | 0.78 |
| LONP1 | Lon Peptidase 1, Mitochondrial | 0.78 |
| FUT8 | Fucosyltransferase 8 | 0.78 |
| NRF1 | Nuclear Respiratory Factor 1 | 0.78 |
| ZBTB17 | Zinc Finger And BTB Domain Containing 17 | 0.78 |
| SLC16A4 | Solute Carrier Family 16 Member 4 | 0.78 |
| VSIG4 | V-Set And Immunoglobulin Domain Containing 4 | 0.78 |
| ARSH | Arylsulfatase Family Member H | 0.78 |
| ERVK-6 | Endogenous Retrovirus Group K Member 6, Envelope | 0.78 |
| RAD51 | RAD51 Recombinase | 0.78 |
| FEN1 | Flap Structure-Specific Endonuclease 1 | 0.78 |
| DHCR24 | 24-Dehydrocholesterol Reductase | 0.78 |
| NDST1 | N-Deacetylase And N-Sulfotransferase 1 | 0.78 |
| MARK2 | Microtubule Affinity Regulating Kinase 2 | 0.78 |
| BRD2 | Bromodomain Containing 2 | 0.78 |
| AKR1C1 | Aldo-Keto Reductase Family 1 Member C1 | 0.78 |
| UBR5 | Ubiquitin Protein Ligase E3 Component N-Recognin 5 | 0.78 |
| SND1 | Staphylococcal Nuclease And Tudor Domain Containing 1 | 0.78 |
| GIGYF2 | GRB10 Interacting GYF Protein 2 | 0.78 |
| EGFL7 | EGF Like Domain Multiple 7 | 0.78 |
| AP2A1 | Adaptor Related Protein Complex 2 Subunit Alpha 1 | 0.78 |
| AOAH | Acyloxyacyl Hydrolase | 0.78 |
| SERPINB10 | Serpin Family B Member 10 | 0.78 |
| TNFAIP8 | TNF Alpha Induced Protein 8 | 0.78 |
| PI16 | Peptidase Inhibitor 16 | 0.78 |
| NKRF | NFKB Repressing Factor | 0.78 |
| NOL6 | Nucleolar Protein 6 | 0.78 |
| CEMIP | Cell Migration Inducing Hyaluronidase 1 | 0.78 |
| ROMO1 | Reactive Oxygen Species Modulator 1 | 0.78 |
| NOC4L | Nucleolar Complex Associated 4 Homolog | 0.78 |
| MARCHF7 | Membrane Associated Ring-CH-Type Finger 7 | 0.78 |
| IGKV2D-29 | Immunoglobulin Kappa Variable 2D-29 | 0.78 |
| MIR302E | MicroRNA 302e | 0.78 |
| DEFA6 | Defensin Alpha 6 | 0.78 |
| MAFG | MAF BZIP Transcription Factor G | 0.77 |
| HSF1 | Heat Shock Transcription Factor 1 | 0.76 |
| CTBP1 | C-Terminal Binding Protein 1 | 0.76 |
| TEAD1 | TEA Domain Transcription Factor 1 | 0.76 |
| HCN2 | Hyperpolarization Activated Cyclic Nucleotide Gated Potassium And Sodium Channel 2 | 0.76 |
| SKI | SKI Proto-Oncogene | 0.76 |
| UGDH | UDP-Glucose 6-Dehydrogenase | 0.76 |
| TDO2 | Tryptophan 2,3-Dioxygenase | 0.76 |
| MAP3K6 | Mitogen-Activated Protein Kinase Kinase Kinase 6 | 0.76 |
| CRAT | Carnitine O-Acetyltransferase | 0.76 |
| EPHB3 | EPH Receptor B3 | 0.76 |
| WDR5 | WD Repeat Domain 5 | 0.76 |
| M6PR | Mannose-6-Phosphate Receptor, Cation Dependent | 0.76 |
| ACSS2 | Acyl-CoA Synthetase Short Chain Family Member 2 | 0.76 |
| HCRTR1 | Hypocretin Receptor 1 | 0.76 |
| SELENBP1 | Selenium Binding Protein 1 | 0.76 |
| PSMD9 | Proteasome 26S Subunit, Non-ATPase 9 | 0.76 |
| TAOK1 | TAO Kinase 1 | 0.76 |
| FANCM | FA Complementation Group M | 0.76 |
| GSTM2 | Glutathione S-Transferase Mu 2 | 0.76 |
| CADM3 | Cell Adhesion Molecule 3 | 0.76 |
| SIX2 | SIX Homeobox 2 | 0.76 |
| PA2G4 | Proliferation-Associated 2G4 | 0.76 |
| BDH2 | 3-Hydroxybutyrate Dehydrogenase 2 | 0.76 |
| CD101 | CD101 Molecule | 0.76 |
| ANGPTL7 | Angiopoietin Like 7 | 0.76 |
| CHI3L2 | Chitinase 3 Like 2 | 0.76 |
| SIGLEC7 | Sialic Acid Binding Ig Like Lectin 7 | 0.76 |
| KMT2B | Lysine Methyltransferase 2B | 0.76 |
| SPAM1 | Sperm Adhesion Molecule 1 | 0.76 |
| BCL2L14 | BCL2 Like 14 | 0.76 |
| SIN3B | SIN3 Transcription Regulator Family Member B | 0.76 |
| RND1 | Rho Family GTPase 1 | 0.76 |
| NINJ2 | Ninjurin 2 | 0.76 |
| ORMDL1 | ORMDL Sphingolipid Biosynthesis Regulator 1 | 0.76 |
| IRF1-AS1 | IRF1 Antisense RNA 1 | 0.76 |
| MIR128-1 | MicroRNA 128-1 | 0.76 |
| MIR124-2 | MicroRNA 124-2 | 0.76 |
| MIR377 | MicroRNA 377 | 0.76 |
| CARM1 | Coactivator Associated Arginine Methyltransferase 1 | 0.74 |
| ALDH1A1 | Aldehyde Dehydrogenase 1 Family Member A1 | 0.74 |
| PRKACG | Protein Kinase CAMP-Activated Catalytic Subunit Gamma | 0.74 |
| PIKFYVE | Phosphoinositide Kinase, FYVE-Type Zinc Finger Containing | 0.74 |
| DAO | D-Amino Acid Oxidase | 0.74 |
| ATP6V1E1 | ATPase H+ Transporting V1 Subunit E1 | 0.74 |
| BCL2L2 | BCL2 Like 2 | 0.74 |
| BMX | BMX Non-Receptor Tyrosine Kinase | 0.74 |
| SIAH2 | Siah E3 Ubiquitin Protein Ligase 2 | 0.74 |
| SIX1 | SIX Homeobox 1 | 0.74 |
| LIAS | Lipoic Acid Synthetase | 0.74 |
| INHA | Inhibin Subunit Alpha | 0.74 |
| TRAP1 | TNF Receptor Associated Protein 1 | 0.74 |
| ABI1 | Abl Interactor 1 | 0.74 |
| CYP3A43 | Cytochrome P450 Family 3 Subfamily A Member 43 | 0.74 |
| MCM3AP | Minichromosome Maintenance Complex Component 3 Associated Protein | 0.74 |
| CALU | Calumenin | 0.74 |
| B3GALT5 | Beta-1,3-Galactosyltransferase 5 | 0.74 |
| ATG4B | Autophagy Related 4B Cysteine Peptidase | 0.74 |
| GADD45G | Growth Arrest And DNA Damage Inducible Gamma | 0.74 |
| PHLPP1 | PH Domain And Leucine Rich Repeat Protein Phosphatase 1 | 0.74 |
| PUM2 | Pumilio RNA Binding Family Member 2 | 0.74 |
| CELSR3 | Cadherin EGF LAG Seven-Pass G-Type Receptor 3 | 0.74 |
| MVP | Major Vault Protein | 0.74 |
| SNIP1 | Smad Nuclear Interacting Protein 1 | 0.74 |
| KCNN1 | Potassium Calcium-Activated Channel Subfamily N Member 1 | 0.74 |
| KLHL2 | Kelch Like Family Member 2 | 0.74 |
| NAPRT | Nicotinate Phosphoribosyltransferase | 0.74 |
| YTHDF2 | YTH N6-Methyladenosine RNA Binding Protein 2 | 0.74 |
| MIB2 | Mindbomb E3 Ubiquitin Protein Ligase 2 | 0.74 |
| LRFN5 | Leucine Rich Repeat And Fibronectin Type III Domain Containing 5 | 0.74 |
| SCRN1 | Secernin 1 | 0.74 |
| PGGT1B | Protein Geranylgeranyltransferase Type I Subunit Beta | 0.74 |
| TACC2 | Transforming Acidic Coiled-Coil Containing Protein 2 | 0.74 |
| RAB37 | RAB37, Member RAS Oncogene Family | 0.74 |
| ZFPM1 | Zinc Finger Protein, FOG Family Member 1 | 0.74 |
| ADAMDEC1 | ADAM Like Decysin 1 | 0.74 |
| RWDD3 | RWD Domain Containing 3 | 0.74 |
| ZC3H12C | Zinc Finger CCCH-Type Containing 12C | 0.74 |
| MAP3K19 | Mitogen-Activated Protein Kinase Kinase Kinase 19 | 0.74 |
| BUD23 | BUD23 RRNA Methyltransferase And Ribosome Maturation Factor | 0.74 |
| DEXI | Dexi Homolog | 0.74 |
| H2BS1 | H2B.S Histone 1 | 0.74 |
| LINC00305 | Long Intergenic Non-Protein Coding RNA 305 | 0.74 |
| MIR147B | MicroRNA 147b | 0.74 |
| MIR1908 | MicroRNA 1908 | 0.74 |
| MIR7-2 | MicroRNA 7-2 | 0.74 |
| MIR2355 | MicroRNA 2355 | 0.74 |
| MIR665 | MicroRNA 665 | 0.74 |
| HBB-LCR | Beta-Globin Locus Control Region | 0.74 |
| C5AR2 | Complement Component 5a Receptor 2 | 0.73 |
| LIFR | LIF Receptor Subunit Alpha | 0.72 |
| PGAM5 | PGAM Family Member 5, Mitochondrial Serine/Threonine Protein Phosphatase | 0.71 |
| ABHD15-AS1 | ABHD15 Antisense RNA 1 | 0.71 |
| LINC01230 | Long Intergenic Non-Protein Coding RNA 1230 | 0.71 |
| MAPKAPK3 | MAPK Activated Protein Kinase 3 | 0.71 |
| PI4KA | Phosphatidylinositol 4-Kinase Alpha | 0.71 |
| PIK3R4 | Phosphoinositide-3-Kinase Regulatory Subunit 4 | 0.71 |
| SLC12A5 | Solute Carrier Family 12 Member 5 | 0.71 |
| PYGM | Glycogen Phosphorylase, Muscle Associated | 0.71 |
| AMPD1 | Adenosine Monophosphate Deaminase 1 | 0.71 |
| GFER | Growth Factor, Augmenter Of Liver Regeneration | 0.71 |
| CHRNA1 | Cholinergic Receptor Nicotinic Alpha 1 Subunit | 0.71 |
| GRM3 | Glutamate Metabotropic Receptor 3 | 0.71 |
| DDOST | Dolichyl-Diphosphooligosaccharide--Protein Glycosyltransferase Non-Catalytic Subunit | 0.71 |
| CACNG2 | Calcium Voltage-Gated Channel Auxiliary Subunit Gamma 2 | 0.71 |
| FDXR | Ferredoxin Reductase | 0.71 |
| CHRM5 | Cholinergic Receptor Muscarinic 5 | 0.71 |
| GRM2 | Glutamate Metabotropic Receptor 2 | 0.71 |
| IGFALS | Insulin Like Growth Factor Binding Protein Acid Labile Subunit | 0.71 |
| POU2F2 | POU Class 2 Homeobox 2 | 0.71 |
| SUMF1 | Sulfatase Modifying Factor 1 | 0.71 |
| PLXNA1 | Plexin A1 | 0.71 |
| TLE4 | TLE Family Member 4, Transcriptional Corepressor | 0.71 |
| GPD1L | Glycerol-3-Phosphate Dehydrogenase 1 Like | 0.71 |
| RNF8 | Ring Finger Protein 8 | 0.71 |
| TNR | Tenascin R | 0.71 |
| ZFPM2 | Zinc Finger Protein, FOG Family Member 2 | 0.71 |
| B3GNT3 | UDP-GlcNAc:BetaGal Beta-1,3-N-Acetylglucosaminyltransferase 3 | 0.71 |
| CNPY3 | Canopy FGF Signaling Regulator 3 | 0.71 |
| DGCR8 | DGCR8 Microprocessor Complex Subunit | 0.71 |
| PADI1 | Peptidyl Arginine Deiminase 1 | 0.71 |
| B4GALT5 | Beta-1,4-Galactosyltransferase 5 | 0.71 |
| AMBRA1 | Autophagy And Beclin 1 Regulator 1 | 0.71 |
| RGN | Regucalcin | 0.71 |
| KSR2 | Kinase Suppressor Of Ras 2 | 0.71 |
| PDXP | Pyridoxal Phosphatase | 0.71 |
| IFI30 | IFI30 Lysosomal Thiol Reductase | 0.71 |
| TGIF2 | TGFB Induced Factor Homeobox 2 | 0.71 |
| USP19 | Ubiquitin Specific Peptidase 19 | 0.71 |
| HEXIM1 | HEXIM P-TEFb Complex Subunit 1 | 0.71 |
| SRSF5 | Serine And Arginine Rich Splicing Factor 5 | 0.71 |
| SLU7 | SLU7 Homolog, Splicing Factor | 0.71 |
| SEMA3G | Semaphorin 3G | 0.71 |
| ATG14 | Autophagy Related 14 | 0.71 |
| CLEC2D | C-Type Lectin Domain Family 2 Member D | 0.71 |
| HLA-DQB2 | Major Histocompatibility Complex, Class II, DQ Beta 2 | 0.71 |
| TRIM3 | Tripartite Motif Containing 3 | 0.71 |
| BEST3 | Bestrophin 3 | 0.71 |
| PLA2G2F | Phospholipase A2 Group IIF | 0.71 |
| TRIM58 | Tripartite Motif Containing 58 | 0.71 |
| TNFAIP8L3 | TNF Alpha Induced Protein 8 Like 3 | 0.71 |
| TNFAIP8L1 | TNF Alpha Induced Protein 8 Like 1 | 0.71 |
| KLF14 | Kruppel Like Factor 14 | 0.71 |
| NAT16 | N-Acetyltransferase 16 (Putative) | 0.71 |
| MIR30C2 | MicroRNA 30c-2 | 0.71 |
| MIR219A1 | MicroRNA 219a-1 | 0.71 |
| MIR802 | MicroRNA 802 | 0.71 |
| MIR650 | MicroRNA 650 | 0.71 |
| CKBE | Creatine Kinase, Ectopic Expression | 0.71 |
| LOC111099027 | Transmembrane Protease Serine 2 Breakpoint Cluster Recombination Region | 0.71 |
| LOC111099028 | ERG, ETS Transcription Factor Breakpoint Cluster Recombination Region | 0.71 |
| KLK11 | Kallikrein Related Peptidase 11 | 0.71 |
| ATF2 | Activating Transcription Factor 2 | 0.7 |
| STAB1 | Stabilin 1 | 0.7 |
| MAPK13 | Mitogen-Activated Protein Kinase 13 | 0.69 |
| LOC111365141 | NOS2 5' Regulatory Region | 0.68 |
| CACNA1B | Calcium Voltage-Gated Channel Subunit Alpha1 B | 0.68 |
| MPL | MPL Proto-Oncogene, Thrombopoietin Receptor | 0.68 |
| CYP2A6 | Cytochrome P450 Family 2 Subfamily A Member 6 | 0.68 |
| MAPK12 | Mitogen-Activated Protein Kinase 12 | 0.67 |
| ADK | Adenosine Kinase | 0.67 |
| AGRN | Agrin | 0.67 |
| UCP1 | Uncoupling Protein 1 | 0.65 |
| CSNK2B | Casein Kinase 2 Beta | 0.64 |
| NDUFV1 | NADH:Ubiquinone Oxidoreductase Core Subunit V1 | 0.64 |
| PDE10A | Phosphodiesterase 10A | 0.64 |
| POMGNT1 | Protein O-Linked Mannose N-Acetylglucosaminyltransferase 1 (Beta 1,2-) | 0.64 |
| POMT1 | Protein O-Mannosyltransferase 1 | 0.64 |
| CCT5 | Chaperonin Containing TCP1 Subunit 5 | 0.64 |
| MLYCD | Malonyl-CoA Decarboxylase | 0.64 |
| NLK | Nemo Like Kinase | 0.64 |
| PMP2 | Peripheral Myelin Protein 2 | 0.64 |
| RNF216 | Ring Finger Protein 216 | 0.64 |
| SEC31A | SEC31 Homolog A, COPII Coat Complex Component | 0.64 |
| SMOC2 | SPARC Related Modular Calcium Binding 2 | 0.64 |
| FAU | FAU Ubiquitin Like And Ribosomal Protein S30 Fusion | 0.64 |
| CPN2 | Carboxypeptidase N Subunit 2 | 0.64 |
| PAFAH2 | Platelet Activating Factor Acetylhydrolase 2 | 0.64 |
| OTUB1 | OTU Deubiquitinase, Ubiquitin Aldehyde Binding 1 | 0.64 |
| LRRC32 | Leucine Rich Repeat Containing 32 | 0.64 |
| ACSM2A | Acyl-CoA Synthetase Medium Chain Family Member 2A | 0.64 |
| NLRP5 | NLR Family Pyrin Domain Containing 5 | 0.64 |
| ZDHHC6 | Zinc Finger DHHC-Type Palmitoyltransferase 6 | 0.64 |
| CCNG2 | Cyclin G2 | 0.64 |
| TPSG1 | Tryptase Gamma 1 | 0.64 |
| MOK | MOK Protein Kinase | 0.64 |
| FOXQ1 | Forkhead Box Q1 | 0.64 |
| NNAT | Neuronatin | 0.64 |
| LARGE1 | LARGE Xylosyl- And Glucuronyltransferase 1 | 0.64 |
| VOPP1 | VOPP1 WW Domain Binding Protein | 0.64 |
| CCDC3 | Coiled-Coil Domain Containing 3 | 0.64 |
| FAM47E | Family With Sequence Similarity 47 Member E | 0.64 |
| XCL2 | X-C Motif Chemokine Ligand 2 | 0.64 |
| DTHD1 | Death Domain Containing 1 | 0.64 |
| DNM3OS | DNM3 Opposite Strand/Antisense RNA | 0.64 |
| MGAT3-AS1 | MGAT3 Antisense RNA 1 | 0.64 |
| NLRP2B | NLR Family Pyrin Domain Containing 2B | 0.64 |
| NLRP3P1 | NLR Family Pyrin Domain Containing 3 Pseudogene 1 | 0.64 |
| NLRP9P1 | NLR Family Pyrin Domain Containing 9 Pseudogene 1 | 0.64 |
| LOC111162620 | TAp63 Promoter Of Tumor Protein P63 | 0.64 |
| SLC2A2 | Solute Carrier Family 2 Member 2 | 0.64 |
| MFSD2A | Major Facilitator Superfamily Domain Containing 2A | 0.64 |
| ELOB | Elongin B | 0.64 |
| KAT2B | Lysine Acetyltransferase 2B | 0.64 |
| TAX1BP1 | Tax1 Binding Protein 1 | 0.64 |
| ADRA2A | Adrenoceptor Alpha 2A | 0.63 |
| SFN | Stratifin | 0.63 |
| INPP4A | Inositol Polyphosphate-4-Phosphatase Type I A | 0.63 |
| CRABP1 | Cellular Retinoic Acid Binding Protein 1 | 0.63 |
| SSH1 | Slingshot Protein Phosphatase 1 | 0.63 |
| RBP2 | Retinol Binding Protein 2 | 0.63 |
| CREB3 | CAMP Responsive Element Binding Protein 3 | 0.63 |
| MIR511 | MicroRNA 511 | 0.63 |
| HNRNPD | Heterogeneous Nuclear Ribonucleoprotein D | 0.62 |
| GBP1 | Guanylate Binding Protein 1 | 0.62 |
| KCNK9 | Potassium Two Pore Domain Channel Subfamily K Member 9 | 0.61 |
| FBXW11 | F-Box And WD Repeat Domain Containing 11 | 0.61 |
| MBD2 | Methyl-CpG Binding Domain Protein 2 | 0.61 |
| NFE2 | Nuclear Factor, Erythroid 2 | 0.61 |
| CWF19L1 | CWF19 Like Cell Cycle Control Factor 1 | 0.61 |
| ZNF281 | Zinc Finger Protein 281 | 0.61 |
| PHF12 | PHD Finger Protein 12 | 0.61 |
| TMEM258 | Transmembrane Protein 258 | 0.61 |
| NEURL3 | Neuralized E3 Ubiquitin Protein Ligase 3 | 0.61 |
| DUSP19 | Dual Specificity Phosphatase 19 | 0.6 |
| LAMP3 | Lysosomal Associated Membrane Protein 3 | 0.6 |
| ABL2 | ABL Proto-Oncogene 2, Non-Receptor Tyrosine Kinase | 0.59 |
| HIPK2 | Homeodomain Interacting Protein Kinase 2 | 0.59 |
| ROBO1 | Roundabout Guidance Receptor 1 | 0.59 |
| ID2 | Inhibitor Of DNA Binding 2 | 0.59 |
| FOXO4 | Forkhead Box O4 | 0.59 |
| MKNK2 | MAPK Interacting Serine/Threonine Kinase 2 | 0.59 |
| MUS81 | MUS81 Structure-Specific Endonuclease Subunit | 0.59 |
| MAP4 | Microtubule Associated Protein 4 | 0.59 |
| AAAS | Aladin WD Repeat Nucleoporin | 0.59 |
| NUAK2 | NUAK Family Kinase 2 | 0.59 |
| FASTK | Fas Activated Serine/Threonine Kinase | 0.59 |
| NRBP1 | Nuclear Receptor Binding Protein 1 | 0.59 |
| GADD45GIP1 | GADD45G Interacting Protein 1 | 0.59 |
| FBXO17 | F-Box Protein 17 | 0.59 |
| CARD19 | Caspase Recruitment Domain Family Member 19 | 0.59 |
| HOXA-AS3 | HOXA Cluster Antisense RNA 3 | 0.59 |
| CTH | Cystathionine Gamma-Lyase | 0.59 |
| SPHK2 | Sphingosine Kinase 2 | 0.59 |
| C4BPA | Complement Component 4 Binding Protein Alpha | 0.59 |
| STC1 | Stanniocalcin 1 | 0.59 |
| GHSR | Growth Hormone Secretagogue Receptor | 0.58 |
| YWHAG | Tyrosine 3-Monooxygenase/Tryptophan 5-Monooxygenase Activation Protein Gamma | 0.57 |
| CDC25A | Cell Division Cycle 25A | 0.57 |
| DYRK1B | Dual Specificity Tyrosine Phosphorylation Regulated Kinase 1B | 0.57 |
| CTBP2 | C-Terminal Binding Protein 2 | 0.57 |
| USP1 | Ubiquitin Specific Peptidase 1 | 0.57 |
| SLK | STE20 Like Kinase | 0.57 |
| KRT4 | Keratin 4 | 0.57 |
| ENTPD3 | Ectonucleoside Triphosphate Diphosphohydrolase 3 | 0.57 |
| NCAN | Neurocan | 0.57 |
| IQGAP2 | IQ Motif Containing GTPase Activating Protein 2 | 0.57 |
| SUZ12 | SUZ12 Polycomb Repressive Complex 2 Subunit | 0.57 |
| BCAM | Basal Cell Adhesion Molecule (Lutheran Blood Group) | 0.57 |
| UBE3C | Ubiquitin Protein Ligase E3C | 0.57 |
| USP20 | Ubiquitin Specific Peptidase 20 | 0.57 |
| TMC1 | Transmembrane Channel Like 1 | 0.57 |
| ZDHHC8 | Zinc Finger DHHC-Type Palmitoyltransferase 8 | 0.57 |
| CDC23 | Cell Division Cycle 23 | 0.57 |
| DDX39A | DExD-Box Helicase 39A | 0.57 |
| NOP58 | NOP58 Ribonucleoprotein | 0.57 |
| SNTB2 | Syntrophin Beta 2 | 0.57 |
| AQP8 | Aquaporin 8 | 0.57 |
| DHDH | Dihydrodiol Dehydrogenase | 0.57 |
| SLCO4C1 | Solute Carrier Organic Anion Transporter Family Member 4C1 | 0.57 |
| PLXDC2 | Plexin Domain Containing 2 | 0.57 |
| STARD7 | StAR Related Lipid Transfer Domain Containing 7 | 0.57 |
| GGT6 | Gamma-Glutamyltransferase 6 | 0.57 |
| HIC2 | HIC ZBTB Transcriptional Repressor 2 | 0.57 |
| OSGIN1 | Oxidative Stress Induced Growth Inhibitor 1 | 0.57 |
| RTP3 | Receptor Transporter Protein 3 | 0.57 |
| UBAP2 | Ubiquitin Associated Protein 2 | 0.57 |
| DEFA1B | Defensin Alpha 1B | 0.57 |
| PGAP6 | Post-Glycosylphosphatidylinositol Attachment To Proteins 6 | 0.57 |
| PRAC2 | PRAC2 Small Nuclear Protein | 0.57 |
| MIR3178 | MicroRNA 3178 | 0.57 |
| CHAT | Choline O-Acetyltransferase | 0.57 |
| NFKBIZ | NFKB Inhibitor Zeta | 0.57 |
| MYB | MYB Proto-Oncogene, Transcription Factor | 0.56 |
| FBXW7 | F-Box And WD Repeat Domain Containing 7 | 0.56 |
| AZGP1 | Alpha-2-Glycoprotein 1, Zinc-Binding | 0.56 |
| TEC | Tec Protein Tyrosine Kinase | 0.55 |
| BAMBI | BMP And Activin Membrane Bound Inhibitor | 0.55 |
| CD300A | CD300a Molecule | 0.54 |
| CTSH | Cathepsin H | 0.54 |
| GLO1 | Glyoxalase I | 0.54 |
| NCOR1 | Nuclear Receptor Corepressor 1 | 0.54 |
| BCL2A1 | BCL2 Related Protein A1 | 0.54 |
| ATP1B1 | ATPase Na+/K+ Transporting Subunit Beta 1 | 0.54 |
| PFN1 | Profilin 1 | 0.54 |
| PDK3 | Pyruvate Dehydrogenase Kinase 3 | 0.54 |
| YWHAH | Tyrosine 3-Monooxygenase/Tryptophan 5-Monooxygenase Activation Protein Eta | 0.54 |
| SIN3A | SIN3 Transcription Regulator Family Member A | 0.54 |
| HSD17B10 | Hydroxysteroid 17-Beta Dehydrogenase 10 | 0.54 |
| IMPA2 | Inositol Monophosphatase 2 | 0.54 |
| ALS2 | Alsin Rho Guanine Nucleotide Exchange Factor ALS2 | 0.54 |
| EWSR1 | EWS RNA Binding Protein 1 | 0.54 |
| RGS14 | Regulator Of G Protein Signaling 14 | 0.54 |
| EIF5A | Eukaryotic Translation Initiation Factor 5A | 0.54 |
| PAM | Peptidylglycine Alpha-Amidating Monooxygenase | 0.54 |
| PPP2R5C | Protein Phosphatase 2 Regulatory Subunit B'Gamma | 0.54 |
| SOX17 | SRY-Box Transcription Factor 17 | 0.54 |
| VPS35 | VPS35 Retromer Complex Component | 0.54 |
| CRTC1 | CREB Regulated Transcription Coactivator 1 | 0.54 |
| PAICS | Phosphoribosylaminoimidazole Carboxylase And Phosphoribosylaminoimidazolesuccinocarboxamide Synthase | 0.54 |
| TLK1 | Tousled Like Kinase 1 | 0.54 |
| SEC13 | SEC13 Homolog, Nuclear Pore And COPII Coat Complex Component | 0.54 |
| STAM | Signal Transducing Adaptor Molecule | 0.54 |
| DNA2 | DNA Replication Helicase/Nuclease 2 | 0.54 |
| CAPZA2 | Capping Actin Protein Of Muscle Z-Line Subunit Alpha 2 | 0.54 |
| CDCA8 | Cell Division Cycle Associated 8 | 0.54 |
| TLE2 | TLE Family Member 2, Transcriptional Corepressor | 0.54 |
| IGBP1 | Immunoglobulin Binding Protein 1 | 0.54 |
| DOCK7 | Dedicator Of Cytokinesis 7 | 0.54 |
| GNL3 | G Protein Nucleolar 3 | 0.54 |
| CBX1 | Chromobox 1 | 0.54 |
| MICAL1 | Microtubule Associated Monooxygenase, Calponin And LIM Domain Containing 1 | 0.54 |
| HIC1 | HIC ZBTB Transcriptional Repressor 1 | 0.54 |
| CLK2 | CDC Like Kinase 2 | 0.54 |
| DDX18 | DEAD-Box Helicase 18 | 0.54 |
| NDST2 | N-Deacetylase And N-Sulfotransferase 2 | 0.54 |
| SETD1A | SET Domain Containing 1A, Histone Lysine Methyltransferase | 0.54 |
| POLA2 | DNA Polymerase Alpha 2, Accessory Subunit | 0.54 |
| GGA1 | Golgi Associated, Gamma Adaptin Ear Containing, ARF Binding Protein 1 | 0.54 |
| MCOLN3 | Mucolipin 3 | 0.54 |
| MED15 | Mediator Complex Subunit 15 | 0.54 |
| CRLS1 | Cardiolipin Synthase 1 | 0.54 |
| CDCP1 | CUB Domain Containing Protein 1 | 0.54 |
| ELL | Elongation Factor For RNA Polymerase II | 0.54 |
| NUBPL | Nucleotide Binding Protein Like | 0.54 |
| TECTA | Tectorin Alpha | 0.54 |
| REEP1 | Receptor Accessory Protein 1 | 0.54 |
| ZBTB18 | Zinc Finger And BTB Domain Containing 18 | 0.54 |
| LZTS1 | Leucine Zipper Tumor Suppressor 1 | 0.54 |
| KRR1 | KRR1 Small Subunit Processome Component Homolog | 0.54 |
| USP34 | Ubiquitin Specific Peptidase 34 | 0.54 |
| WDR48 | WD Repeat Domain 48 | 0.54 |
| GNL2 | G Protein Nucleolar 2 | 0.54 |
| GOLGB1 | Golgin B1 | 0.54 |
| FBXW5 | F-Box And WD Repeat Domain Containing 5 | 0.54 |
| COPS6 | COP9 Signalosome Subunit 6 | 0.54 |
| MTMR12 | Myotubularin Related Protein 12 | 0.54 |
| DUSP14 | Dual Specificity Phosphatase 14 | 0.54 |
| DUSP15 | Dual Specificity Phosphatase 15 | 0.54 |
| ELK4 | ETS Transcription Factor ELK4 | 0.54 |
| PARP12 | Poly(ADP-Ribose) Polymerase Family Member 12 | 0.54 |
| RUSC2 | RUN And SH3 Domain Containing 2 | 0.54 |
| SLC43A3 | Solute Carrier Family 43 Member 3 | 0.54 |
| USP12 | Ubiquitin Specific Peptidase 12 | 0.54 |
| PPIL3 | Peptidylprolyl Isomerase Like 3 | 0.54 |
| ZFP64 | ZFP64 Zinc Finger Protein | 0.54 |
| FBXW8 | F-Box And WD Repeat Domain Containing 8 | 0.54 |
| CRIP1 | Cysteine Rich Protein 1 | 0.54 |
| CRIP2 | Cysteine Rich Protein 2 | 0.54 |
| MMS19 | MMS19 Homolog, Cytosolic Iron-Sulfur Assembly Component | 0.54 |
| DARS1 | Aspartyl-TRNA Synthetase 1 | 0.54 |
| ST7 | Suppression Of Tumorigenicity 7 | 0.54 |
| TTC4 | Tetratricopeptide Repeat Domain 4 | 0.54 |
| IFNL2 | Interferon Lambda 2 | 0.54 |
| WDHD1 | WD Repeat And HMG-Box DNA Binding Protein 1 | 0.54 |
| VBP1 | VHL Binding Protein 1 | 0.54 |
| ATG2A | Autophagy Related 2A | 0.54 |
| BCL7C | BAF Chromatin Remodeling Complex Subunit BCL7C | 0.54 |
| GAB3 | GRB2 Associated Binding Protein 3 | 0.54 |
| LRRTM2 | Leucine Rich Repeat Transmembrane Neuronal 2 | 0.54 |
| SETD1B | SET Domain Containing 1B, Histone Lysine Methyltransferase | 0.54 |
| SEC22B | SEC22 Homolog B, Vesicle Trafficking Protein | 0.54 |
| TTBK1 | Tau Tubulin Kinase 1 | 0.54 |
| KIFC2 | Kinesin Family Member C2 | 0.54 |
| PPP6R1 | Protein Phosphatase 6 Regulatory Subunit 1 | 0.54 |
| VPS16 | VPS16 Core Subunit Of CORVET And HOPS Complexes | 0.54 |
| LMTK3 | Lemur Tyrosine Kinase 3 | 0.54 |
| GOLIM4 | Golgi Integral Membrane Protein 4 | 0.54 |
| TENM2 | Teneurin Transmembrane Protein 2 | 0.54 |
| JKAMP | JNK1/MAPK8 Associated Membrane Protein | 0.54 |
| WDR75 | WD Repeat Domain 75 | 0.54 |
| GPANK1 | G-Patch Domain And Ankyrin Repeats 1 | 0.54 |
| CCDC120 | Coiled-Coil Domain Containing 120 | 0.54 |
| ATAD5 | ATPase Family AAA Domain Containing 5 | 0.54 |
| FAM126B | Family With Sequence Similarity 126 Member B | 0.54 |
| DCAF5 | DDB1 And CUL4 Associated Factor 5 | 0.54 |
| SPHKAP | SPHK1 Interactor, AKAP Domain Containing | 0.54 |
| LSM12 | LSM12 Homolog | 0.54 |
| GRWD1 | Glutamate Rich WD Repeat Containing 1 | 0.54 |
| DCAF4 | DDB1 And CUL4 Associated Factor 4 | 0.54 |
| DCAF11 | DDB1 And CUL4 Associated Factor 11 | 0.54 |
| WDR54 | WD Repeat Domain 54 | 0.54 |
| WDR5B | WD Repeat Domain 5B | 0.54 |
| DYNC2I2 | Dynein 2 Intermediate Chain 2 | 0.54 |
| DCAF16 | DDB1 And CUL4 Associated Factor 16 | 0.54 |
| IGLL5 | Immunoglobulin Lambda Like Polypeptide 5 | 0.54 |
| ZBTB47 | Zinc Finger And BTB Domain Containing 47 | 0.54 |
| PRR14L | Proline Rich 14 Like | 0.54 |
| PJVK | Pejvakin | 0.54 |
| TMSB4XP8 | TMSB4X Pseudogene 8 | 0.54 |
| CSNK2A1 | Casein Kinase 2 Alpha 1 | 0.53 |
| PRDX4 | Peroxiredoxin 4 | 0.53 |
| P2RX1 | Purinergic Receptor P2X 1 | 0.53 |
| HCK | HCK Proto-Oncogene, Src Family Tyrosine Kinase | 0.53 |
| CYP2C8 | Cytochrome P450 Family 2 Subfamily C Member 8 | 0.53 |
| NR2C2 | Nuclear Receptor Subfamily 2 Group C Member 2 | 0.53 |
| JUND | JunD Proto-Oncogene, AP-1 Transcription Factor Subunit | 0.53 |
| ASIC3 | Acid Sensing Ion Channel Subunit 3 | 0.53 |
| LOC110806262 | Solute Carrier Family 6 Member 4 Gene Promoter | 0.53 |
| VCL | Vinculin | 0.53 |
| PDPK1 | 3-Phosphoinositide Dependent Protein Kinase 1 | 0.53 |
| CAPN1 | Calpain 1 | 0.52 |
| FUT4 | Fucosyltransferase 4 | 0.52 |
| RAMP1 | Receptor Activity Modifying Protein 1 | 0.52 |
| TRA | T Cell Receptor Alpha Locus | 0.52 |
| HK2 | Hexokinase 2 | 0.52 |
| KCNN4 | Potassium Calcium-Activated Channel Subfamily N Member 4 | 0.51 |
| GCLC | Glutamate-Cysteine Ligase Catalytic Subunit | 0.5 |
| AIFM1 | Apoptosis Inducing Factor Mitochondria Associated 1 | 0.5 |
| DDAH2 | Dimethylarginine Dimethylaminohydrolase 2 | 0.5 |
| JUNB | JunB Proto-Oncogene, AP-1 Transcription Factor Subunit | 0.5 |
| P2RY1 | Purinergic Receptor P2Y1 | 0.5 |
| GNB3 | G Protein Subunit Beta 3 | 0.49 |
| TNIP3 | TNFAIP3 Interacting Protein 3 | 0.49 |
| ROCK2 | Rho Associated Coiled-Coil Containing Protein Kinase 2 | 0.49 |
| TNFRSF10D | TNF Receptor Superfamily Member 10d | 0.49 |
| HSPB2 | Heat Shock Protein Family B (Small) Member 2 | 0.49 |
| CST1 | Cystatin SN | 0.49 |
| GPR15 | G Protein-Coupled Receptor 15 | 0.49 |
| BAIAP2L1 | BAR/IMD Domain Containing Adaptor Protein 2 Like 1 | 0.49 |
| DUSP10 | Dual Specificity Phosphatase 10 | 0.48 |
| HSPE1 | Heat Shock Protein Family E (Hsp10) Member 1 | 0.48 |
| SLC9A1 | Solute Carrier Family 9 Member A1 | 0.47 |
| SLC11A2 | Solute Carrier Family 11 Member 2 | 0.47 |
| SYT1 | Synaptotagmin 1 | 0.47 |
| SGPL1 | Sphingosine-1-Phosphate Lyase 1 | 0.47 |
| NEDD8 | NEDD8 Ubiquitin Like Modifier | 0.47 |
| KHSRP | KH-Type Splicing Regulatory Protein | 0.47 |
| RBM4 | RNA Binding Motif Protein 4 | 0.47 |
| TRIM8 | Tripartite Motif Containing 8 | 0.47 |
| GHRHR | Growth Hormone Releasing Hormone Receptor | 0.47 |
| CYB561 | Cytochrome B561 | 0.47 |
| GOLGA2 | Golgin A2 | 0.47 |
| NCAPD2 | Non-SMC Condensin I Complex Subunit D2 | 0.47 |
| NCAPD3 | Non-SMC Condensin II Complex Subunit D3 | 0.47 |
| GH2 | Growth Hormone 2 | 0.47 |
| EMB | Embigin | 0.47 |
| PLXNB2 | Plexin B2 | 0.47 |
| UBE2Q1 | Ubiquitin Conjugating Enzyme E2 Q1 | 0.47 |
| OTUB2 | OTU Deubiquitinase, Ubiquitin Aldehyde Binding 2 | 0.47 |
| TRIM62 | Tripartite Motif Containing 62 | 0.47 |
| ZBTB12 | Zinc Finger And BTB Domain Containing 12 | 0.47 |
| DCUN1D5 | Defective In Cullin Neddylation 1 Domain Containing 5 | 0.47 |
| TDRD10 | Tudor Domain Containing 10 | 0.47 |
| WTAPP1 | Wilms Tumor 1 Associated Protein Pseudogene 1 | 0.47 |
| MTCO2P12 | MT-CO2 Pseudogene 12 | 0.47 |
| UTRN | Utrophin | 0.47 |
| EPHA2 | EPH Receptor A2 | 0.47 |
| ATR | ATR Serine/Threonine Kinase | 0.47 |
| ELOC | Elongin C | 0.47 |
| OSCAR | Osteoclast Associated Ig-Like Receptor | 0.46 |
| CAMK2B | Calcium/Calmodulin Dependent Protein Kinase II Beta | 0.46 |
| TUBB | Tubulin Beta Class I | 0.46 |
| CHRM2 | Cholinergic Receptor Muscarinic 2 | 0.46 |
| TRPC3 | Transient Receptor Potential Cation Channel Subfamily C Member 3 | 0.46 |
| COL6A3 | Collagen Type VI Alpha 3 Chain | 0.46 |
| VIPR2 | Vasoactive Intestinal Peptide Receptor 2 | 0.46 |
| ANXA6 | Annexin A6 | 0.46 |
| BRAP | BRCA1 Associated Protein | 0.46 |
| GPRC5B | G Protein-Coupled Receptor Class C Group 5 Member B | 0.46 |
| CHRFAM7A | CHRNA7 (Exons 5-10) And FAM7A (Exons A-E) Fusion | 0.46 |
| TBXAS1 | Thromboxane A Synthase 1 | 0.46 |
| PFKFB3 | 6-Phosphofructo-2-Kinase/Fructose-2,6-Biphosphatase 3 | 0.46 |
| HEXB | Hexosaminidase Subunit Beta | 0.44 |
| GLS | Glutaminase | 0.44 |
| BATF | Basic Leucine Zipper ATF-Like Transcription Factor | 0.44 |
| SORT1 | Sortilin 1 | 0.44 |
| MIP | Major Intrinsic Protein Of Lens Fiber | 0.44 |
| LAIR1 | Leukocyte Associated Immunoglobulin Like Receptor 1 | 0.44 |
| TAGLN2 | Transgelin 2 | 0.44 |
| IGKC | Immunoglobulin Kappa Constant | 0.44 |
| B4GALT1 | Beta-1,4-Galactosyltransferase 1 | 0.44 |
| CBLB | Cbl Proto-Oncogene B | 0.44 |
| PEBP1 | Phosphatidylethanolamine Binding Protein 1 | 0.44 |
| HES1 | Hes Family BHLH Transcription Factor 1 | 0.44 |
| PGLYRP3 | Peptidoglycan Recognition Protein 3 | 0.44 |
| LAYN | Layilin | 0.44 |
| CHEK1 | Checkpoint Kinase 1 | 0.43 |
| HDAC5 | Histone Deacetylase 5 | 0.43 |
| ADAM12 | ADAM Metallopeptidase Domain 12 | 0.43 |
| CA9 | Carbonic Anhydrase 9 | 0.43 |
| ACO1 | Aconitase 1 | 0.43 |
| GPNMB | Glycoprotein Nmb | 0.43 |
| DDIT4 | DNA Damage Inducible Transcript 4 | 0.43 |
| MAP1LC3A | Microtubule Associated Protein 1 Light Chain 3 Alpha | 0.43 |
| PHB2 | Prohibitin 2 | 0.43 |
| CREG1 | Cellular Repressor Of E1A Stimulated Genes 1 | 0.43 |
| TIFA | TRAF Interacting Protein With Forkhead Associated Domain | 0.43 |
| SCGB2A1 | Secretoglobin Family 2A Member 1 | 0.43 |
| PANX3 | Pannexin 3 | 0.43 |
| DNM2 | Dynamin 2 | 0.42 |
| PRLR | Prolactin Receptor | 0.42 |
| DIABLO | Diablo IAP-Binding Mitochondrial Protein | 0.42 |
| LMAN1 | Lectin, Mannose Binding 1 | 0.42 |
| CD177 | CD177 Molecule | 0.42 |
| SLC28A3 | Solute Carrier Family 28 Member 3 | 0.42 |
| LPCAT3 | Lysophosphatidylcholine Acyltransferase 3 | 0.42 |
| PYDC2 | Pyrin Domain Containing 2 | 0.42 |
| MGLL | Monoglyceride Lipase | 0.42 |
| GLRX | Glutaredoxin | 0.42 |
| MBTPS1 | Membrane Bound Transcription Factor Peptidase, Site 1 | 0.42 |
| FOXA1 | Forkhead Box A1 | 0.42 |
| GPR68 | G Protein-Coupled Receptor 68 | 0.42 |
| ATOX1 | Antioxidant 1 Copper Chaperone | 0.42 |
| PCSK6 | Proprotein Convertase Subtilisin/Kexin Type 6 | 0.42 |
| SMPD2 | Sphingomyelin Phosphodiesterase 2 | 0.42 |
| ASIC2 | Acid Sensing Ion Channel Subunit 2 | 0.42 |
| RNLS | Renalase, FAD Dependent Amine Oxidase | 0.42 |
| APOL3 | Apolipoprotein L3 | 0.42 |
| CRISPLD2 | Cysteine Rich Secretory Protein LCCL Domain Containing 2 | 0.42 |
| PJA2 | Praja Ring Finger Ubiquitin Ligase 2 | 0.42 |
| JCHAIN | Joining Chain Of Multimeric IgA And IgM | 0.42 |
| EFS | Embryonal Fyn-Associated Substrate | 0.42 |
| IGHG1 | Immunoglobulin Heavy Constant Gamma 1 (G1m Marker) | 0.42 |
| IL27RA | Interleukin 27 Receptor Subunit Alpha | 0.42 |
| LAG3 | Lymphocyte Activating 3 | 0.42 |
| CEACAM3 | CEA Cell Adhesion Molecule 3 | 0.41 |
| EPOR | Erythropoietin Receptor | 0.4 |
| ADAM9 | ADAM Metallopeptidase Domain 9 | 0.4 |
| TXNRD1 | Thioredoxin Reductase 1 | 0.4 |
| USP7 | Ubiquitin Specific Peptidase 7 | 0.4 |
| CUL3 | Cullin 3 | 0.4 |
| DNM3 | Dynamin 3 | 0.4 |
| FOSB | FosB Proto-Oncogene, AP-1 Transcription Factor Subunit | 0.4 |
| CNOT3 | CCR4-NOT Transcription Complex Subunit 3 | 0.4 |
| CDC37 | Cell Division Cycle 37, HSP90 Cochaperone | 0.4 |
| CPVL | Carboxypeptidase Vitellogenic Like | 0.4 |
| SIRT4 | Sirtuin 4 | 0.4 |
| NCR2 | Natural Cytotoxicity Triggering Receptor 2 | 0.4 |
| OXER1 | Oxoeicosanoid Receptor 1 | 0.4 |
| SCG5 | Secretogranin V | 0.4 |
| CUEDC2 | CUE Domain Containing 2 | 0.4 |
| MOXD1 | Monooxygenase DBH Like 1 | 0.4 |
| HERC4 | HECT And RLD Domain Containing E3 Ubiquitin Protein Ligase 4 | 0.4 |
| MUC17 | Mucin 17, Cell Surface Associated | 0.4 |
| RPS6KA1 | Ribosomal Protein S6 Kinase A1 | 0.4 |
| LAMA5 | Laminin Subunit Alpha 5 | 0.4 |
| CTCFL | CCCTC-Binding Factor Like | 0.4 |
| MBOAT1 | Membrane Bound O-Acyltransferase Domain Containing 1 | 0.4 |
| ILK | Integrin Linked Kinase | 0.4 |
| FOXA2 | Forkhead Box A2 | 0.4 |
| TRPV2 | Transient Receptor Potential Cation Channel Subfamily V Member 2 | 0.4 |
| EGLN1 | Egl-9 Family Hypoxia Inducible Factor 1 | 0.39 |
| SLC3A1 | Solute Carrier Family 3 Member 1 | 0.39 |
| CAMKK2 | Calcium/Calmodulin Dependent Protein Kinase Kinase 2 | 0.39 |
| MMP11 | Matrix Metallopeptidase 11 | 0.39 |
| GRAP2 | GRB2 Related Adaptor Protein 2 | 0.39 |
| TRAF4 | TNF Receptor Associated Factor 4 | 0.39 |
| HRH3 | Histamine Receptor H3 | 0.39 |
| TRPC1 | Transient Receptor Potential Cation Channel Subfamily C Member 1 | 0.39 |
| SDCBP | Syndecan Binding Protein | 0.39 |
| SSTR4 | Somatostatin Receptor 4 | 0.39 |
| MYOCD | Myocardin | 0.39 |
| NR6A1 | Nuclear Receptor Subfamily 6 Group A Member 1 | 0.39 |
| MARVELD2 | MARVEL Domain Containing 2 | 0.39 |
| GPR4 | G Protein-Coupled Receptor 4 | 0.39 |
| PFDN4 | Prefoldin Subunit 4 | 0.39 |
| MKRN2 | Makorin Ring Finger Protein 2 | 0.39 |
| RFC3 | Replication Factor C Subunit 3 | 0.39 |
| CCDC50 | Coiled-Coil Domain Containing 50 | 0.39 |
| SPOCK3 | SPARC (Osteonectin), Cwcv And Kazal Like Domains Proteoglycan 3 | 0.39 |
| PPRC1 | PPARG Related Coactivator 1 | 0.39 |
| APOC4 | Apolipoprotein C4 | 0.39 |
| SPNS2 | Sphingolipid Transporter 2 | 0.39 |
| ADSS2 | Adenylosuccinate Synthase 2 | 0.39 |
| CEP85 | Centrosomal Protein 85 | 0.39 |
| COMMD10 | COMM Domain Containing 10 | 0.39 |
| RADIL | Rap Associating With DIL Domain | 0.39 |
| IGHA1 | Immunoglobulin Heavy Constant Alpha 1 | 0.39 |
| UMAD1 | UBAP1-MVB12-Associated (UMA) Domain Containing 1 | 0.39 |
| AKR1B10 | Aldo-Keto Reductase Family 1 Member B10 | 0.38 |
| GPRC6A | G Protein-Coupled Receptor Class C Group 6 Member A | 0.38 |
| H2AX | H2A.X Variant Histone | 0.38 |
| SLC29A1 | Solute Carrier Family 29 Member 1 (Augustine Blood Group) | 0.37 |
| MAP4K4 | Mitogen-Activated Protein Kinase Kinase Kinase Kinase 4 | 0.37 |
| NMI | N-Myc And STAT Interactor | 0.37 |
| NOXO1 | NADPH Oxidase Organizer 1 | 0.37 |
| OLFM4 | Olfactomedin 4 | 0.37 |
| MBL3P | Mannose-Binding Lectin Family Member 3, Pseudogene | 0.37 |
| ITPR1 | Inositol 1,4,5-Trisphosphate Receptor Type 1 | 0.37 |
| NFIA | Nuclear Factor I A | 0.37 |
| EGR2 | Early Growth Response 2 | 0.37 |
| TRPM3 | Transient Receptor Potential Cation Channel Subfamily M Member 3 | 0.37 |
| SESN2 | Sestrin 2 | 0.37 |
| PARP14 | Poly(ADP-Ribose) Polymerase Family Member 14 | 0.37 |
| TMSB10 | Thymosin Beta 10 | 0.37 |
| SIGLEC14 | Sialic Acid Binding Ig Like Lectin 14 | 0.37 |
| CACNA1H | Calcium Voltage-Gated Channel Subunit Alpha1 H | 0.37 |
| NEDD4L | NEDD4 Like E3 Ubiquitin Protein Ligase | 0.37 |
| ACLY | ATP Citrate Lyase | 0.37 |
| ACACB | Acetyl-CoA Carboxylase Beta | 0.37 |
| CDC6 | Cell Division Cycle 6 | 0.37 |
| EHMT2 | Euchromatic Histone Lysine Methyltransferase 2 | 0.37 |
| PPT1 | Palmitoyl-Protein Thioesterase 1 | 0.37 |
| CBX5 | Chromobox 5 | 0.37 |
| CRADD | CASP2 And RIPK1 Domain Containing Adaptor With Death Domain | 0.37 |
| CITED2 | Cbp/P300 Interacting Transactivator With Glu/Asp Rich Carboxy-Terminal Domain 2 | 0.37 |
| TRPC4 | Transient Receptor Potential Cation Channel Subfamily C Member 4 | 0.37 |
| MAP4K1 | Mitogen-Activated Protein Kinase Kinase Kinase Kinase 1 | 0.37 |
| MAPKAP1 | MAPK Associated Protein 1 | 0.37 |
| EEF1A1 | Eukaryotic Translation Elongation Factor 1 Alpha 1 | 0.37 |
| ELOVL5 | ELOVL Fatty Acid Elongase 5 | 0.37 |
| PRDX3 | Peroxiredoxin 3 | 0.37 |
| ITGA9 | Integrin Subunit Alpha 9 | 0.37 |
| TFE3 | Transcription Factor Binding To IGHM Enhancer 3 | 0.37 |
| AIMP2 | Aminoacyl TRNA Synthetase Complex Interacting Multifunctional Protein 2 | 0.37 |
| DAPK2 | Death Associated Protein Kinase 2 | 0.37 |
| PAWR | Pro-Apoptotic WT1 Regulator | 0.37 |
| TRIM2 | Tripartite Motif Containing 2 | 0.37 |
| RAP2A | RAP2A, Member Of RAS Oncogene Family | 0.37 |
| POP1 | POP1 Homolog, Ribonuclease P/MRP Subunit | 0.37 |
| METTL3 | Methyltransferase Like 3 | 0.37 |
| CD53 | CD53 Molecule | 0.37 |
| USF2 | Upstream Transcription Factor 2, C-Fos Interacting | 0.37 |
| TET1 | Tet Methylcytosine Dioxygenase 1 | 0.37 |
| MAL | Mal, T Cell Differentiation Protein | 0.37 |
| CREB3L3 | CAMP Responsive Element Binding Protein 3 Like 3 | 0.37 |
| PIEZO2 | Piezo Type Mechanosensitive Ion Channel Component 2 | 0.37 |
| CYP4F8 | Cytochrome P450 Family 4 Subfamily F Member 8 | 0.37 |
| ZNF598 | Zinc Finger Protein 598, E3 Ubiquitin Ligase | 0.37 |
| H3C1 | H3 Clustered Histone 1 | 0.37 |
| OR10J1 | Olfactory Receptor Family 10 Subfamily J Member 1 | 0.37 |
| CCL3L1 | C-C Motif Chemokine Ligand 3 Like 1 | 0.37 |
| TGFBR3 | Transforming Growth Factor Beta Receptor 3 | 0.36 |
| MT1A | Metallothionein 1A | 0.36 |
| AVEN | Apoptosis And Caspase Activation Inhibitor | 0.36 |
| INPPL1 | Inositol Polyphosphate Phosphatase Like 1 | 0.35 |
| DIAPH1 | Diaphanous Related Formin 1 | 0.35 |
| SLC1A1 | Solute Carrier Family 1 Member 1 | 0.35 |
| HMGA1 | High Mobility Group AT-Hook 1 | 0.35 |
| POU2F1 | POU Class 2 Homeobox 1 | 0.35 |
| SNAP23 | Synaptosome Associated Protein 23 | 0.35 |
| PSMB10 | Proteasome 20S Subunit Beta 10 | 0.35 |
| UCHL3 | Ubiquitin C-Terminal Hydrolase L3 | 0.35 |
| HS3ST1 | Heparan Sulfate-Glucosamine 3-Sulfotransferase 1 | 0.35 |
| HPGDS | Hematopoietic Prostaglandin D Synthase | 0.35 |
| CD99 | CD99 Molecule (Xg Blood Group) | 0.35 |
| PITRM1 | Pitrilysin Metallopeptidase 1 | 0.35 |
| RFX1 | Regulatory Factor X1 | 0.35 |
| ATP5F1A | ATP Synthase F1 Subunit Alpha | 0.35 |
| JPH4 | Junctophilin 4 | 0.35 |
| ATP5F1B | ATP Synthase F1 Subunit Beta | 0.35 |
| MIR372 | MicroRNA 372 | 0.35 |
| FGR | FGR Proto-Oncogene, Src Family Tyrosine Kinase | 0.34 |
| KCNJ2 | Potassium Inwardly Rectifying Channel Subfamily J Member 2 | 0.34 |
| UBE3A | Ubiquitin Protein Ligase E3A | 0.34 |
| PDK1 | Pyruvate Dehydrogenase Kinase 1 | 0.34 |
| PLCB2 | Phospholipase C Beta 2 | 0.34 |
| PARP2 | Poly(ADP-Ribose) Polymerase 2 | 0.34 |
| GNB1 | G Protein Subunit Beta 1 | 0.34 |
| ARHGEF2 | Rho/Rac Guanine Nucleotide Exchange Factor 2 | 0.34 |
| SIAH1 | Siah E3 Ubiquitin Protein Ligase 1 | 0.34 |
| CBR3 | Carbonyl Reductase 3 | 0.34 |
| PPP2R2A | Protein Phosphatase 2 Regulatory Subunit Balpha | 0.34 |
| EIF2AK4 | Eukaryotic Translation Initiation Factor 2 Alpha Kinase 4 | 0.34 |
| CD33 | CD33 Molecule | 0.34 |
| SH3GL2 | SH3 Domain Containing GRB2 Like 2, Endophilin A1 | 0.34 |
| SLIT1 | Slit Guidance Ligand 1 | 0.34 |
| SLC2A5 | Solute Carrier Family 2 Member 5 | 0.34 |
| SLC18A3 | Solute Carrier Family 18 Member A3 | 0.34 |
| P2RY4 | Pyrimidinergic Receptor P2Y4 | 0.34 |
| WWP2 | WW Domain Containing E3 Ubiquitin Protein Ligase 2 | 0.34 |
| ARHGDIB | Rho GDP Dissociation Inhibitor Beta | 0.34 |
| PARD6A | Par-6 Family Cell Polarity Regulator Alpha | 0.34 |
| PRKRA | Protein Activator Of Interferon Induced Protein Kinase EIF2AK2 | 0.34 |
| CTNND2 | Catenin Delta 2 | 0.34 |
| MLC1 | Modulator Of VRAC Current 1 | 0.34 |
| EFNA2 | Ephrin A2 | 0.34 |
| SRSF1 | Serine And Arginine Rich Splicing Factor 1 | 0.34 |
| USP5 | Ubiquitin Specific Peptidase 5 | 0.34 |
| CSAD | Cysteine Sulfinic Acid Decarboxylase | 0.34 |
| PGLYRP1 | Peptidoglycan Recognition Protein 1 | 0.34 |
| ACMSD | Aminocarboxymuconate Semialdehyde Decarboxylase | 0.34 |
| SMC4 | Structural Maintenance Of Chromosomes 4 | 0.34 |
| TNPO1 | Transportin 1 | 0.34 |
| AGTRAP | Angiotensin II Receptor Associated Protein | 0.34 |
| CYGB | Cytoglobin | 0.34 |
| TSPAN5 | Tetraspanin 5 | 0.34 |
| MPRIP | Myosin Phosphatase Rho Interacting Protein | 0.34 |
| RAD51B | RAD51 Paralog B | 0.34 |
| SPINK7 | Serine Peptidase Inhibitor Kazal Type 7 | 0.34 |
| SPARCL1 | SPARC Like 1 | 0.34 |
| PGLYRP2 | Peptidoglycan Recognition Protein 2 | 0.34 |
| COQ10A | Coenzyme Q10A | 0.34 |
| PLEKHO1 | Pleckstrin Homology Domain Containing O1 | 0.34 |
| FCGR2C | Fc Fragment Of IgG Receptor IIc (Gene/Pseudogene) | 0.34 |
| ADM2 | Adrenomedullin 2 | 0.34 |
| C12orf43 | Chromosome 12 Open Reading Frame 43 | 0.34 |
| OR10J5 | Olfactory Receptor Family 10 Subfamily J Member 5 | 0.34 |
| CCL3L3 | C-C Motif Chemokine Ligand 3 Like 3 | 0.34 |
| PATJ | PATJ Crumbs Cell Polarity Complex Component | 0.34 |
| MIR155HG | MIR155 Host Gene | 0.34 |
| HMGB1P1 | High Mobility Group Box 1 Pseudogene 1 | 0.34 |
| LINC00917 | Long Intergenic Non-Protein Coding RNA 917 | 0.34 |
| MIR1246 | MicroRNA 1246 | 0.34 |
| LOC401864 | Chloride Intracellular Channel 1 Pseudogene | 0.34 |
| LOC107832851 | SIRT1 Promoter Region | 0.34 |
| GCK | Glucokinase | 0.33 |
| DYRK1A | Dual Specificity Tyrosine Phosphorylation Regulated Kinase 1A | 0.33 |
| NEK2 | NIMA Related Kinase 2 | 0.33 |
| ATF1 | Activating Transcription Factor 1 | 0.33 |
| CUBN | Cubilin | 0.33 |
| HEXA | Hexosaminidase Subunit Alpha | 0.33 |
| PLOD2 | Procollagen-Lysine,2-Oxoglutarate 5-Dioxygenase 2 | 0.33 |
| CPE | Carboxypeptidase E | 0.33 |
| NFE2L1 | Nuclear Factor, Erythroid 2 Like 1 | 0.33 |
| EFNA5 | Ephrin A5 | 0.33 |
| ANXA3 | Annexin A3 | 0.33 |
| MED1 | Mediator Complex Subunit 1 | 0.33 |
| SLURP1 | Secreted LY6/PLAUR Domain Containing 1 | 0.33 |
| PTP4A1 | Protein Tyrosine Phosphatase 4A1 | 0.33 |
| ST3GAL4 | ST3 Beta-Galactoside Alpha-2,3-Sialyltransferase 4 | 0.33 |
| TOB1 | Transducer Of ERBB2, 1 | 0.33 |
| PCSK5 | Proprotein Convertase Subtilisin/Kexin Type 5 | 0.33 |
| KHDRBS1 | KH RNA Binding Domain Containing, Signal Transduction Associated 1 | 0.33 |
| RNF114 | Ring Finger Protein 114 | 0.33 |
| SULF2 | Sulfatase 2 | 0.33 |
| AKAP5 | A-Kinase Anchoring Protein 5 | 0.33 |
| FAT1 | FAT Atypical Cadherin 1 | 0.33 |
| IL17RE | Interleukin 17 Receptor E | 0.33 |
| PPP1R13L | Protein Phosphatase 1 Regulatory Subunit 13 Like | 0.33 |
| HSPB6 | Heat Shock Protein Family B (Small) Member 6 | 0.33 |
| AMOTL1 | Angiomotin Like 1 | 0.33 |
| CSMD1 | CUB And Sushi Multiple Domains 1 | 0.33 |
| AZIN2 | Antizyme Inhibitor 2 | 0.33 |
| GTF3A | General Transcription Factor IIIA | 0.33 |
| NAALADL2 | N-Acetylated Alpha-Linked Acidic Dipeptidase Like 2 | 0.33 |
| TIGIT | T Cell Immunoreceptor With Ig And ITIM Domains | 0.33 |
| MACROD2 | Mono-ADP Ribosylhydrolase 2 | 0.33 |
| PTPA | Protein Phosphatase 2 Phosphatase Activator | 0.33 |
| HSH2D | Hematopoietic SH2 Domain Containing | 0.33 |
| CEP131 | Centrosomal Protein 131 | 0.33 |
| LILRA3 | Leukocyte Immunoglobulin Like Receptor A3 | 0.33 |
| ENTR1 | Endosome Associated Trafficking Regulator 1 | 0.33 |
| ANGPTL8 | Angiopoietin Like 8 | 0.33 |
| DEFB106A | Defensin Beta 106A | 0.33 |
| MINDY3 | MINDY Lysine 48 Deubiquitinase 3 | 0.33 |
| MIR217 | MicroRNA 217 | 0.33 |
| MIR9-2 | MicroRNA 9-2 | 0.33 |
| PAK4 | P21 (RAC1) Activated Kinase 4 | 0.32 |
| SIK1 | Salt Inducible Kinase 1 | 0.32 |
| ROS1 | ROS Proto-Oncogene 1, Receptor Tyrosine Kinase | 0.32 |
| RPA1 | Replication Protein A1 | 0.32 |
| SHMT1 | Serine Hydroxymethyltransferase 1 | 0.32 |
| PAK6 | P21 (RAC1) Activated Kinase 6 | 0.32 |
| GRID2 | Glutamate Ionotropic Receptor Delta Type Subunit 2 | 0.32 |
| NONO | Non-POU Domain Containing Octamer Binding | 0.32 |
| RPTOR | Regulatory Associated Protein Of MTOR Complex 1 | 0.32 |
| KCNA3 | Potassium Voltage-Gated Channel Subfamily A Member 3 | 0.32 |
| CDH15 | Cadherin 15 | 0.32 |
| SRR | Serine Racemase | 0.32 |
| WWP1 | WW Domain Containing E3 Ubiquitin Protein Ligase 1 | 0.32 |
| GPR132 | G Protein-Coupled Receptor 132 | 0.32 |
| ECSIT | ECSIT Signaling Integrator | 0.32 |
| KCTD15 | Potassium Channel Tetramerization Domain Containing 15 | 0.32 |
| BCO2 | Beta-Carotene Oxygenase 2 | 0.32 |
| MMP28 | Matrix Metallopeptidase 28 | 0.32 |
| GRIN3A | Glutamate Ionotropic Receptor NMDA Type Subunit 3A | 0.32 |
| OLFML3 | Olfactomedin Like 3 | 0.32 |
| SRA1 | Steroid Receptor RNA Activator 1 | 0.32 |
| CDNF | Cerebral Dopamine Neurotrophic Factor | 0.32 |
| RHBDD2 | Rhomboid Domain Containing 2 | 0.32 |
| RNF11 | Ring Finger Protein 11 | 0.32 |
| SIGLEC11 | Sialic Acid Binding Ig Like Lectin 11 | 0.32 |
| PAK5 | P21 (RAC1) Activated Kinase 5 | 0.32 |
| FYB1 | FYN Binding Protein 1 | 0.32 |
| RHBDD1 | Rhomboid Domain Containing 1 | 0.32 |
| RHBDL1 | Rhomboid Like 1 | 0.32 |
| RHBDL3 | Rhomboid Like 3 | 0.32 |
| HSPA12B | Heat Shock Protein Family A (Hsp70) Member 12B | 0.32 |
| TFPT | TCF3 Fusion Partner | 0.32 |
| ADGRB1 | Adhesion G Protein-Coupled Receptor B1 | 0.32 |
| NCR3LG1 | Natural Killer Cell Cytotoxicity Receptor 3 Ligand 1 | 0.32 |
| NIBAN1 | Niban Apoptosis Regulator 1 | 0.32 |
| MIR136 | MicroRNA 136 | 0.32 |
| MIR210HG | MIR210 Host Gene | 0.32 |
| BUB1B-PAK6 | BUB1B-PAK6 Readthrough | 0.32 |
| LINC00887 | Long Intergenic Non-Protein Coding RNA 887 | 0.32 |
| LINC01629 | Long Intergenic Non-Protein Coding RNA 1629 | 0.32 |
| FRAXA | Fragile Site, Folic Acid Type, Rare, Fra(X)(Q27.3) A (Macroorchidism, Mental Retardation) | 0.32 |
| CSNK1D | Casein Kinase 1 Delta | 0.31 |
| FES | FES Proto-Oncogene, Tyrosine Kinase | 0.31 |
| CALCR | Calcitonin Receptor | 0.31 |
| CAPN2 | Calpain 2 | 0.31 |
| FOLR1 | Folate Receptor Alpha | 0.31 |
| PLCB3 | Phospholipase C Beta 3 | 0.31 |
| DCC | DCC Netrin 1 Receptor | 0.31 |
| COASY | Coenzyme A Synthase | 0.31 |
| DBI | Diazepam Binding Inhibitor, Acyl-CoA Binding Protein | 0.31 |
| PKN2 | Protein Kinase N2 | 0.31 |
| RAN | RAN, Member RAS Oncogene Family | 0.31 |
| AQP7 | Aquaporin 7 | 0.31 |
| PARVA | Parvin Alpha | 0.31 |
| PAFAH1B2 | Platelet Activating Factor Acetylhydrolase 1b Catalytic Subunit 2 | 0.31 |
| EGLN2 | Egl-9 Family Hypoxia Inducible Factor 2 | 0.31 |
| PTPRD | Protein Tyrosine Phosphatase Receptor Type D | 0.31 |
| CUL5 | Cullin 5 | 0.31 |
| GDI1 | GDP Dissociation Inhibitor 1 | 0.31 |
| GJA4 | Gap Junction Protein Alpha 4 | 0.31 |
| GALC | Galactosylceramidase | 0.31 |
| CYP4A11 | Cytochrome P450 Family 4 Subfamily A Member 11 | 0.31 |
| DISC1 | DISC1 Scaffold Protein | 0.31 |
| UBE2V2 | Ubiquitin Conjugating Enzyme E2 V2 | 0.31 |
| DDAH1 | Dimethylarginine Dimethylaminohydrolase 1 | 0.31 |
| PLA2G4C | Phospholipase A2 Group IVC | 0.31 |
| ROBO2 | Roundabout Guidance Receptor 2 | 0.31 |
| EIF4G2 | Eukaryotic Translation Initiation Factor 4 Gamma 2 | 0.31 |
| UBE2V1 | Ubiquitin Conjugating Enzyme E2 V1 | 0.31 |
| STX4 | Syntaxin 4 | 0.31 |
| CUL2 | Cullin 2 | 0.31 |
| ADAM19 | ADAM Metallopeptidase Domain 19 | 0.31 |
| FUT9 | Fucosyltransferase 9 | 0.31 |
| RFC4 | Replication Factor C Subunit 4 | 0.31 |
| RND3 | Rho Family GTPase 3 | 0.31 |
| SLIT3 | Slit Guidance Ligand 3 | 0.31 |
| CYP2A13 | Cytochrome P450 Family 2 Subfamily A Member 13 | 0.31 |
| AMOT | Angiomotin | 0.31 |
| AGXT2 | Alanine--Glyoxylate Aminotransferase 2 | 0.31 |
| G3BP1 | G3BP Stress Granule Assembly Factor 1 | 0.31 |
| CDK19 | Cyclin Dependent Kinase 19 | 0.31 |
| MRC2 | Mannose Receptor C Type 2 | 0.31 |
| EIF6 | Eukaryotic Translation Initiation Factor 6 | 0.31 |
| PSMD10 | Proteasome 26S Subunit, Non-ATPase 10 | 0.31 |
| WSB1 | WD Repeat And SOCS Box Containing 1 | 0.31 |
| BAG4 | BAG Cochaperone 4 | 0.31 |
| ADAM28 | ADAM Metallopeptidase Domain 28 | 0.31 |
| NIPSNAP1 | Nipsnap Homolog 1 | 0.31 |
| SLC9A8 | Solute Carrier Family 9 Member A8 | 0.31 |
| TNRC6A | Trinucleotide Repeat Containing Adaptor 6A | 0.31 |
| TRIP6 | Thyroid Hormone Receptor Interactor 6 | 0.31 |
| RCC2 | Regulator Of Chromosome Condensation 2 | 0.31 |
| SH3RF1 | SH3 Domain Containing Ring Finger 1 | 0.31 |
| SAFB | Scaffold Attachment Factor B | 0.31 |
| TRO | Trophinin | 0.31 |
| KLHL6 | Kelch Like Family Member 6 | 0.31 |
| S100A13 | S100 Calcium Binding Protein A13 | 0.31 |
| VASH1 | Vasohibin 1 | 0.31 |
| DUOXA2 | Dual Oxidase Maturation Factor 2 | 0.31 |
| CST2 | Cystatin SA | 0.31 |
| TONSL | Tonsoku Like, DNA Repair Protein | 0.31 |
| SYTL1 | Synaptotagmin Like 1 | 0.31 |
| HSPA13 | Heat Shock Protein Family A (Hsp70) Member 13 | 0.31 |
| METRNL | Meteorin Like, Glial Cell Differentiation Regulator | 0.31 |
| CCDC14 | Coiled-Coil Domain Containing 14 | 0.31 |
| HECTD2 | HECT Domain E3 Ubiquitin Protein Ligase 2 | 0.31 |
| SCGB3A1 | Secretoglobin Family 3A Member 1 | 0.31 |
| C1QTNF4 | C1q And TNF Related 4 | 0.31 |
| ADGRE1 | Adhesion G Protein-Coupled Receptor E1 | 0.31 |
| SOCS7 | Suppressor Of Cytokine Signaling 7 | 0.31 |
| PODXL2 | Podocalyxin Like 2 | 0.31 |
| TAC4 | Tachykinin Precursor 4 | 0.31 |
| MPIG6B | Megakaryocyte And Platelet Inhibitory Receptor G6b | 0.31 |
| CNOT9 | CCR4-NOT Transcription Complex Subunit 9 | 0.31 |
| SCGB1D1 | Secretoglobin Family 1D Member 1 | 0.31 |
| NUP42 | Nucleoporin 42 | 0.31 |
| H3C15 | H3 Clustered Histone 15 | 0.31 |
| TCIM | Transcriptional And Immune Response Regulator | 0.31 |
| TSBP1 | Testis Expressed Basic Protein 1 | 0.31 |
| FAM215A | Family With Sequence Similarity 215 Member A | 0.31 |
| MIR135A2 | MicroRNA 135a-2 | 0.31 |
| MIR942 | MicroRNA 942 | 0.31 |
| CRPP1 | C-Reactive Protein Pseudogene 1 | 0.31 |
| MAP2K5 | Mitogen-Activated Protein Kinase Kinase 5 | 0.3 |
| SLC1A3 | Solute Carrier Family 1 Member 3 | 0.29 |
| YWHAE | Tyrosine 3-Monooxygenase/Tryptophan 5-Monooxygenase Activation Protein Epsilon | 0.29 |
| CHRNA4 | Cholinergic Receptor Nicotinic Alpha 4 Subunit | 0.29 |
| PRKAG1 | Protein Kinase AMP-Activated Non-Catalytic Subunit Gamma 1 | 0.29 |
| ADCY6 | Adenylate Cyclase 6 | 0.29 |
| CCND3 | Cyclin D3 | 0.29 |
| SSTR2 | Somatostatin Receptor 2 | 0.29 |
| RBPJ | Recombination Signal Binding Protein For Immunoglobulin Kappa J Region | 0.29 |
| CDK8 | Cyclin Dependent Kinase 8 | 0.29 |
| MIB1 | Mindbomb E3 Ubiquitin Protein Ligase 1 | 0.29 |
| EPHA5 | EPH Receptor A5 | 0.29 |
| ABCC8 | ATP Binding Cassette Subfamily C Member 8 | 0.29 |
| ACAD8 | Acyl-CoA Dehydrogenase Family Member 8 | 0.29 |
| EIF4G1 | Eukaryotic Translation Initiation Factor 4 Gamma 1 | 0.29 |
| IGFBP7 | Insulin Like Growth Factor Binding Protein 7 | 0.29 |
| TUBA4A | Tubulin Alpha 4a | 0.29 |
| LPAR2 | Lysophosphatidic Acid Receptor 2 | 0.29 |
| EPS8 | Epidermal Growth Factor Receptor Pathway Substrate 8 | 0.29 |
| MMP16 | Matrix Metallopeptidase 16 | 0.29 |
| DLC1 | DLC1 Rho GTPase Activating Protein | 0.29 |
| EFEMP1 | EGF Containing Fibulin Extracellular Matrix Protein 1 | 0.29 |
| PPP2R5D | Protein Phosphatase 2 Regulatory Subunit B'Delta | 0.29 |
| NLGN1 | Neuroligin 1 | 0.29 |
| PTPRN2 | Protein Tyrosine Phosphatase Receptor Type N2 | 0.29 |
| GIT1 | GIT ArfGAP 1 | 0.29 |
| GAB2 | GRB2 Associated Binding Protein 2 | 0.29 |
| GSTO1 | Glutathione S-Transferase Omega 1 | 0.29 |
| RHOQ | Ras Homolog Family Member Q | 0.29 |
| RGS4 | Regulator Of G Protein Signaling 4 | 0.29 |
| RGS10 | Regulator Of G Protein Signaling 10 | 0.29 |
| MLF1 | Myeloid Leukemia Factor 1 | 0.29 |
| HPN | Hepsin | 0.29 |
| CRY1 | Cryptochrome Circadian Regulator 1 | 0.29 |
| PER3 | Period Circadian Regulator 3 | 0.29 |
| SERPINA5 | Serpin Family A Member 5 | 0.29 |
| UBQLN1 | Ubiquilin 1 | 0.29 |
| KIF2C | Kinesin Family Member 2C | 0.29 |
| POLR1A | RNA Polymerase I Subunit A | 0.29 |
| AKR7A2 | Aldo-Keto Reductase Family 7 Member A2 | 0.29 |
| CALML3 | Calmodulin Like 3 | 0.29 |
| BMP3 | Bone Morphogenetic Protein 3 | 0.29 |
| PRPF4 | Pre-MRNA Processing Factor 4 | 0.29 |
| SENP1 | SUMO Specific Peptidase 1 | 0.29 |
| DTNA | Dystrobrevin Alpha | 0.29 |
| GTF2B | General Transcription Factor IIB | 0.29 |
| GLRA3 | Glycine Receptor Alpha 3 | 0.29 |
| FLOT2 | Flotillin 2 | 0.29 |
| GALNT1 | Polypeptide N-Acetylgalactosaminyltransferase 1 | 0.29 |
| GSTA2 | Glutathione S-Transferase Alpha 2 | 0.29 |
| HAGH | Hydroxyacylglutathione Hydrolase | 0.29 |
| DUSP5 | Dual Specificity Phosphatase 5 | 0.29 |
| CYTH2 | Cytohesin 2 | 0.29 |
| NCS1 | Neuronal Calcium Sensor 1 | 0.29 |
| RPL4 | Ribosomal Protein L4 | 0.29 |
| PIAS3 | Protein Inhibitor Of Activated STAT 3 | 0.29 |
| PICK1 | Protein Interacting With PRKCA 1 | 0.29 |
| KDM2B | Lysine Demethylase 2B | 0.29 |
| SOX11 | SRY-Box Transcription Factor 11 | 0.29 |
| PCSK2 | Proprotein Convertase Subtilisin/Kexin Type 2 | 0.29 |
| OXSR1 | Oxidative Stress Responsive Kinase 1 | 0.29 |
| PRIM1 | DNA Primase Subunit 1 | 0.29 |
| RANGAP1 | Ran GTPase Activating Protein 1 | 0.29 |
| FGL1 | Fibrinogen Like 1 | 0.29 |
| CLEC1B | C-Type Lectin Domain Family 1 Member B | 0.29 |
| CBFA2T3 | CBFA2/RUNX1 Partner Transcriptional Co-Repressor 3 | 0.29 |
| CNOT1 | CCR4-NOT Transcription Complex Subunit 1 | 0.29 |
| HDGF | Heparin Binding Growth Factor | 0.29 |
| SMOC1 | SPARC Related Modular Calcium Binding 1 | 0.29 |
| IQGAP3 | IQ Motif Containing GTPase Activating Protein 3 | 0.29 |
| TPM4 | Tropomyosin 4 | 0.29 |
| RARRES1 | Retinoic Acid Receptor Responder 1 | 0.29 |
| KIF5C | Kinesin Family Member 5C | 0.29 |
| TELO2 | Telomere Maintenance 2 | 0.29 |
| THOC2 | THO Complex 2 | 0.29 |
| ZNF148 | Zinc Finger Protein 148 | 0.29 |
| ULK3 | Unc-51 Like Kinase 3 | 0.29 |
| GOLPH3 | Golgi Phosphoprotein 3 | 0.29 |
| MANF | Mesencephalic Astrocyte Derived Neurotrophic Factor | 0.29 |
| ANGPTL6 | Angiopoietin Like 6 | 0.29 |
| BLZF1 | Basic Leucine Zipper Nuclear Factor 1 | 0.29 |
| FOXA3 | Forkhead Box A3 | 0.29 |
| GADD45B | Growth Arrest And DNA Damage Inducible Beta | 0.29 |
| COLEC12 | Collectin Subfamily Member 12 | 0.29 |
| CHST12 | Carbohydrate Sulfotransferase 12 | 0.29 |
| CETN3 | Centrin 3 | 0.29 |
| DNER | Delta/Notch Like EGF Repeat Containing | 0.29 |
| MSTO1 | Misato Mitochondrial Distribution And Morphology Regulator 1 | 0.29 |
| MPP5 | Membrane Palmitoylated Protein 5 | 0.29 |
| RNF41 | Ring Finger Protein 41 | 0.29 |
| EIF3C | Eukaryotic Translation Initiation Factor 3 Subunit C | 0.29 |
| IKZF2 | IKAROS Family Zinc Finger 2 | 0.29 |
| PDLIM5 | PDZ And LIM Domain 5 | 0.29 |
| PFDN5 | Prefoldin Subunit 5 | 0.29 |
| SNRK | SNF Related Kinase | 0.29 |
| SDCBP2 | Syndecan Binding Protein 2 | 0.29 |
| SEMA4G | Semaphorin 4G | 0.29 |
| PTBP2 | Polypyrimidine Tract Binding Protein 2 | 0.29 |
| ULBP3 | UL16 Binding Protein 3 | 0.29 |
| STARD13 | StAR Related Lipid Transfer Domain Containing 13 | 0.29 |
| KIF13B | Kinesin Family Member 13B | 0.29 |
| KIF14 | Kinesin Family Member 14 | 0.29 |
| REEP5 | Receptor Accessory Protein 5 | 0.29 |
| RAB9A | RAB9A, Member RAS Oncogene Family | 0.29 |
| STK26 | Serine/Threonine Kinase 26 | 0.29 |
| CD180 | CD180 Molecule | 0.29 |
| NPAS3 | Neuronal PAS Domain Protein 3 | 0.29 |
| ZNF217 | Zinc Finger Protein 217 | 0.29 |
| FARP1 | FERM, ARH/RhoGEF And Pleckstrin Domain Protein 1 | 0.29 |
| ATG13 | Autophagy Related 13 | 0.29 |
| ANAPC7 | Anaphase Promoting Complex Subunit 7 | 0.29 |
| CEP135 | Centrosomal Protein 135 | 0.29 |
| DCP1A | Decapping MRNA 1A | 0.29 |
| SCARF1 | Scavenger Receptor Class F Member 1 | 0.29 |
| OBSL1 | Obscurin Like Cytoskeletal Adaptor 1 | 0.29 |
| ILF3 | Interleukin Enhancer Binding Factor 3 | 0.29 |
| ITSN2 | Intersectin 2 | 0.29 |
| EIF3K | Eukaryotic Translation Initiation Factor 3 Subunit K | 0.29 |
| TICAM2 | Toll Like Receptor Adaptor Molecule 2 | 0.29 |
| AZI2 | 5-Azacytidine Induced 2 | 0.29 |
| AP2A2 | Adaptor Related Protein Complex 2 Subunit Alpha 2 | 0.29 |
| FAM83H | Family With Sequence Similarity 83 Member H | 0.29 |
| HEATR1 | HEAT Repeat Containing 1 | 0.29 |
| DNAJB4 | DnaJ Heat Shock Protein Family (Hsp40) Member B4 | 0.29 |
| DENND5A | DENN Domain Containing 5A | 0.29 |
| MYO1B | Myosin IB | 0.29 |
| LNX2 | Ligand Of Numb-Protein X 2 | 0.29 |
| RNF144B | Ring Finger Protein 144B | 0.29 |
| PDLIM2 | PDZ And LIM Domain 2 | 0.29 |
| SKA1 | Spindle And Kinetochore Associated Complex Subunit 1 | 0.29 |
| SCPEP1 | Serine Carboxypeptidase 1 | 0.29 |
| SASS6 | SAS-6 Centriolar Assembly Protein | 0.29 |
| NID2 | Nidogen 2 | 0.29 |
| NTPCR | Nucleoside-Triphosphatase, Cancer-Related | 0.29 |
| SRP19 | Signal Recognition Particle 19 | 0.29 |
| STAU2 | Staufen Double-Stranded RNA Binding Protein 2 | 0.29 |
| NCAPG2 | Non-SMC Condensin II Complex Subunit G2 | 0.29 |
| IL4I1 | Interleukin 4 Induced 1 | 0.29 |
| YOD1 | YOD1 Deubiquitinase | 0.29 |
| EPB41L4B | Erythrocyte Membrane Protein Band 4.1 Like 4B | 0.29 |
| FUBP3 | Far Upstream Element Binding Protein 3 | 0.29 |
| PELI3 | Pellino E3 Ubiquitin Protein Ligase Family Member 3 | 0.29 |
| SLC46A3 | Solute Carrier Family 46 Member 3 | 0.29 |
| SLC22A23 | Solute Carrier Family 22 Member 23 | 0.29 |
| TENM3 | Teneurin Transmembrane Protein 3 | 0.29 |
| CYP20A1 | Cytochrome P450 Family 20 Subfamily A Member 1 | 0.29 |
| OSR1 | Odd-Skipped Related Transcription Factor 1 | 0.29 |
| ZFAND6 | Zinc Finger AN1-Type Containing 6 | 0.29 |
| YARS1 | Tyrosyl-TRNA Synthetase 1 | 0.29 |
| YTHDF1 | YTH N6-Methyladenosine RNA Binding Protein 1 | 0.29 |
| YTHDF3 | YTH N6-Methyladenosine RNA Binding Protein 3 | 0.29 |
| UBXN1 | UBX Domain Protein 1 | 0.29 |
| GOLGA3 | Golgin A3 | 0.29 |
| FAM135B | Family With Sequence Similarity 135 Member B | 0.29 |
| MAGI3 | Membrane Associated Guanylate Kinase, WW And PDZ Domain Containing 3 | 0.29 |
| ERO1A | Endoplasmic Reticulum Oxidoreductase 1 Alpha | 0.29 |
| GSTT2 | Glutathione S-Transferase Theta 2 (Gene/Pseudogene) | 0.29 |
| CKAP2 | Cytoskeleton Associated Protein 2 | 0.29 |
| RNF115 | Ring Finger Protein 115 | 0.29 |
| EMILIN2 | Elastin Microfibril Interfacer 2 | 0.29 |
| PCOLCE2 | Procollagen C-Endopeptidase Enhancer 2 | 0.29 |
| PCP4 | Purkinje Cell Protein 4 | 0.29 |
| SDK2 | Sidekick Cell Adhesion Molecule 2 | 0.29 |
| RRP9 | Ribosomal RNA Processing 9, U3 Small Nucleolar RNA Binding Protein | 0.29 |
| PLEKHB2 | Pleckstrin Homology Domain Containing B2 | 0.29 |
| PLBD1 | Phospholipase B Domain Containing 1 | 0.29 |
| OIT3 | Oncoprotein Induced Transcript 3 | 0.29 |
| SSNA1 | SS Nuclear Autoantigen 1 | 0.29 |
| UEVLD | UEV And Lactate/Malate Dehyrogenase Domains | 0.29 |
| TROAP | Trophinin Associated Protein | 0.29 |
| KRT80 | Keratin 80 | 0.29 |
| USP54 | Ubiquitin Specific Peptidase 54 | 0.29 |
| CAPN15 | Calpain 15 | 0.29 |
| MPHOSPH9 | M-Phase Phosphoprotein 9 | 0.29 |
| IFNA10 | Interferon Alpha 10 | 0.29 |
| TRMT6 | TRNA Methyltransferase 6 | 0.29 |
| DUS3L | Dihydrouridine Synthase 3 Like | 0.29 |
| ZFAND2B | Zinc Finger AN1-Type Containing 2B | 0.29 |
| CCDC77 | Coiled-Coil Domain Containing 77 | 0.29 |
| MELTF | Melanotransferrin | 0.29 |
| CNOT10 | CCR4-NOT Transcription Complex Subunit 10 | 0.29 |
| DDX60L | DExD/H-Box 60 Like | 0.29 |
| NDNF | Neuron Derived Neurotrophic Factor | 0.29 |
| TXLNG | Taxilin Gamma | 0.29 |
| LEPROT | Leptin Receptor Overlapping Transcript | 0.29 |
| KHNYN | KH And NYN Domain Containing | 0.29 |
| THAP4 | THAP Domain Containing 4 | 0.29 |
| YLPM1 | YLP Motif Containing 1 | 0.29 |
| CLEC9A | C-Type Lectin Domain Containing 9A | 0.29 |
| CCDC112 | Coiled-Coil Domain Containing 112 | 0.29 |
| SPATA2L | Spermatogenesis Associated 2 Like | 0.29 |
| MYCBPAP | MYCBP Associated Protein | 0.29 |
| ZCCHC3 | Zinc Finger CCHC-Type Containing 3 | 0.29 |
| ZNF395 | Zinc Finger Protein 395 | 0.29 |
| VPS37D | VPS37D Subunit Of ESCRT-I | 0.29 |
| CCDC138 | Coiled-Coil Domain Containing 138 | 0.29 |
| GOLGA7B | Golgin A7 Family Member B | 0.29 |
| H4C1 | H4 Clustered Histone 1 | 0.29 |
| NCKAP5L | NCK Associated Protein 5 Like | 0.29 |
| TBC1D31 | TBC1 Domain Family Member 31 | 0.29 |
| DRGX | Dorsal Root Ganglia Homeobox | 0.29 |
| ATP5IF1 | ATP Synthase Inhibitory Factor Subunit 1 | 0.29 |
| CEP95 | Centrosomal Protein 95 | 0.29 |
| KIAA1671 | KIAA1671 | 0.29 |
| C1orf122 | Chromosome 1 Open Reading Frame 122 | 0.29 |
| LRRC69 | Leucine Rich Repeat Containing 69 | 0.29 |
| IGHG2 | Immunoglobulin Heavy Constant Gamma 2 (G2m Marker) | 0.29 |
| DIPK2A | Divergent Protein Kinase Domain 2A | 0.29 |
| MEAK7 | MTOR Associated Protein, Eak-7 Homolog | 0.29 |
| ACOD1 | Aconitate Decarboxylase 1 | 0.29 |
| H2BU1 | H2B.U Histone 1 | 0.29 |
| RTL6 | Retrotransposon Gag Like 6 | 0.29 |
| TEDC1 | Tubulin Epsilon And Delta Complex 1 | 0.29 |
| TMEM178B | Transmembrane Protein 178B | 0.29 |
| TNXA | Tenascin XA (Pseudogene) | 0.29 |
| IGHA2 | Immunoglobulin Heavy Constant Alpha 2 (A2m Marker) | 0.29 |
| MIR218-2 | MicroRNA 218-2 | 0.29 |
| MIR520C | MicroRNA 520c | 0.29 |
| MIR492 | MicroRNA 492 | 0.29 |
| HNRNPCL2 | Heterogeneous Nuclear Ribonucleoprotein C Like 2 | 0.29 |
| MIR873 | MicroRNA 873 | 0.29 |
| LINC01548 | Long Intergenic Non-Protein Coding RNA 1548 | 0.29 |
| MKRN4P | Makorin Ring Finger Protein 4, Pseudogene | 0.29 |
| MIR522 | MicroRNA 522 | 0.29 |
| LOC100506403 | Uncharacterized LOC100506403 | 0.29 |
| GOT2P1 | GOT2 Pseudogene 1 | 0.29 |
| LINC01826 | Long Intergenic Non-Protein Coding RNA 1826 | 0.29 |
| BDP1P | B Double Prime 1 Pseudogene | 0.29 |
| CAMK2A | Calcium/Calmodulin Dependent Protein Kinase II Alpha | 0.27 |
| P4HB | Prolyl 4-Hydroxylase Subunit Beta | 0.27 |
| CAMK2D | Calcium/Calmodulin Dependent Protein Kinase II Delta | 0.27 |
| PSAT1 | Phosphoserine Aminotransferase 1 | 0.27 |
| PGK1 | Phosphoglycerate Kinase 1 | 0.27 |
| FER | FER Tyrosine Kinase | 0.27 |
| DLG4 | Discs Large MAGUK Scaffold Protein 4 | 0.27 |
| CDC34 | Cell Division Cycle 34, Ubiqiutin Conjugating Enzyme | 0.27 |
| HTRA2 | HtrA Serine Peptidase 2 | 0.27 |
| PIK3C2B | Phosphatidylinositol-4-Phosphate 3-Kinase Catalytic Subunit Type 2 Beta | 0.27 |
| LAMB1 | Laminin Subunit Beta 1 | 0.27 |
| MTNR1B | Melatonin Receptor 1B | 0.27 |
| MYH10 | Myosin Heavy Chain 10 | 0.27 |
| PAX5 | Paired Box 5 | 0.27 |
| ALAS2 | 5'-Aminolevulinate Synthase 2 | 0.27 |
| FGF5 | Fibroblast Growth Factor 5 | 0.27 |
| DFFA | DNA Fragmentation Factor Subunit Alpha | 0.27 |
| PDK4 | Pyruvate Dehydrogenase Kinase 4 | 0.27 |
| SCO2 | Synthesis Of Cytochrome C Oxidase 2 | 0.27 |
| PTPN12 | Protein Tyrosine Phosphatase Non-Receptor Type 12 | 0.27 |
| MCM5 | Minichromosome Maintenance Complex Component 5 | 0.27 |
| GNA13 | G Protein Subunit Alpha 13 | 0.27 |
| MAPRE1 | Microtubule Associated Protein RP/EB Family Member 1 | 0.27 |
| ATP2A3 | ATPase Sarcoplasmic/Endoplasmic Reticulum Ca2+ Transporting 3 | 0.27 |
| DAXX | Death Domain Associated Protein | 0.27 |
| SHMT2 | Serine Hydroxymethyltransferase 2 | 0.27 |
| PPM1B | Protein Phosphatase, Mg2+/Mn2+ Dependent 1B | 0.27 |
| IGF2BP2 | Insulin Like Growth Factor 2 MRNA Binding Protein 2 | 0.27 |
| NFATC4 | Nuclear Factor Of Activated T Cells 4 | 0.27 |
| SLC5A6 | Solute Carrier Family 5 Member 6 | 0.27 |
| HSPB8 | Heat Shock Protein Family B (Small) Member 8 | 0.27 |
| POLR2A | RNA Polymerase II Subunit A | 0.27 |
| RAB3A | RAB3A, Member RAS Oncogene Family | 0.27 |
| MAP3K10 | Mitogen-Activated Protein Kinase Kinase Kinase 10 | 0.27 |
| FBLN2 | Fibulin 2 | 0.27 |
| BLMH | Bleomycin Hydrolase | 0.27 |
| ABCA7 | ATP Binding Cassette Subfamily A Member 7 | 0.27 |
| CYP3A7 | Cytochrome P450 Family 3 Subfamily A Member 7 | 0.27 |
| RHOT1 | Ras Homolog Family Member T1 | 0.27 |
| DFFB | DNA Fragmentation Factor Subunit Beta | 0.27 |
| DLGAP1 | DLG Associated Protein 1 | 0.27 |
| DUSP4 | Dual Specificity Phosphatase 4 | 0.27 |
| COL6A1 | Collagen Type VI Alpha 1 Chain | 0.27 |
| RNGTT | RNA Guanylyltransferase And 5'-Phosphatase | 0.27 |
| DAB1 | DAB Adaptor Protein 1 | 0.27 |
| HTR2B | 5-Hydroxytryptamine Receptor 2B | 0.27 |
| ENDOG | Endonuclease G | 0.27 |
| HSD17B7 | Hydroxysteroid 17-Beta Dehydrogenase 7 | 0.27 |
| SSTR1 | Somatostatin Receptor 1 | 0.27 |
| P2RX2 | Purinergic Receptor P2X 2 | 0.27 |
| POLL | DNA Polymerase Lambda | 0.27 |
| PARP4 | Poly(ADP-Ribose) Polymerase Family Member 4 | 0.27 |
| BNIP3L | BCL2 Interacting Protein 3 Like | 0.27 |
| GGH | Gamma-Glutamyl Hydrolase | 0.27 |
| SLC28A2 | Solute Carrier Family 28 Member 2 | 0.27 |
| SLC25A10 | Solute Carrier Family 25 Member 10 | 0.27 |
| RAD23A | RAD23 Homolog A, Nucleotide Excision Repair Protein | 0.27 |
| LGMN | Legumain | 0.27 |
| P2RY14 | Purinergic Receptor P2Y14 | 0.27 |
| CAMK1D | Calcium/Calmodulin Dependent Protein Kinase ID | 0.27 |
| GBA2 | Glucosylceramidase Beta 2 | 0.27 |
| GALR2 | Galanin Receptor 2 | 0.27 |
| ARHGEF3 | Rho Guanine Nucleotide Exchange Factor 3 | 0.27 |
| FGF13 | Fibroblast Growth Factor 13 | 0.27 |
| ATL1 | Atlastin GTPase 1 | 0.27 |
| FLOT1 | Flotillin 1 | 0.27 |
| ABCC11 | ATP Binding Cassette Subfamily C Member 11 | 0.27 |
| CHN2 | Chimerin 2 | 0.27 |
| MTF1 | Metal Regulatory Transcription Factor 1 | 0.27 |
| DGKQ | Diacylglycerol Kinase Theta | 0.27 |
| NDUFA8 | NADH:Ubiquinone Oxidoreductase Subunit A8 | 0.27 |
| PABPC1 | Poly(A) Binding Protein Cytoplasmic 1 | 0.27 |
| RXRG | Retinoid X Receptor Gamma | 0.27 |
| NFASC | Neurofascin | 0.27 |
| NMNAT2 | Nicotinamide Nucleotide Adenylyltransferase 2 | 0.27 |
| RPLP2 | Ribosomal Protein Lateral Stalk Subunit P2 | 0.27 |
| NISCH | Nischarin | 0.27 |
| SMYD3 | SET And MYND Domain Containing 3 | 0.27 |
| SLC39A3 | Solute Carrier Family 39 Member 3 | 0.27 |
| SEMA5A | Semaphorin 5A | 0.27 |
| PHLPP2 | PH Domain And Leucine Rich Repeat Protein Phosphatase 2 | 0.27 |
| TOM1 | Target Of Myb1 Membrane Trafficking Protein | 0.27 |
| RANBP9 | RAN Binding Protein 9 | 0.27 |
| JDP2 | Jun Dimerization Protein 2 | 0.27 |
| KCNE2 | Potassium Voltage-Gated Channel Subfamily E Regulatory Subunit 2 | 0.27 |
| XRCC3 | X-Ray Repair Cross Complementing 3 | 0.27 |
| ABLIM1 | Actin Binding LIM Protein 1 | 0.27 |
| ARHGEF4 | Rho Guanine Nucleotide Exchange Factor 4 | 0.27 |
| LTBP4 | Latent Transforming Growth Factor Beta Binding Protein 4 | 0.27 |
| ACTR3B | Actin Related Protein 3B | 0.27 |
| CEP55 | Centrosomal Protein 55 | 0.27 |
| CELSR2 | Cadherin EGF LAG Seven-Pass G-Type Receptor 2 | 0.27 |
| CNTN6 | Contactin 6 | 0.27 |
| DLG2 | Discs Large MAGUK Scaffold Protein 2 | 0.27 |
| MYH1 | Myosin Heavy Chain 1 | 0.27 |
| ICA1 | Islet Cell Autoantigen 1 | 0.27 |
| PMS1 | PMS1 Homolog 1, Mismatch Repair System Component | 0.27 |
| NFIC | Nuclear Factor I C | 0.27 |
| SLC9A2 | Solute Carrier Family 9 Member A2 | 0.27 |
| SENP3 | SUMO Specific Peptidase 3 | 0.27 |
| TRAIP | TRAF Interacting Protein | 0.27 |
| LILRB2 | Leukocyte Immunoglobulin Like Receptor B2 | 0.27 |
| UNC5C | Unc-5 Netrin Receptor C | 0.27 |
| RAB18 | RAB18, Member RAS Oncogene Family | 0.27 |
| SUMO2 | Small Ubiquitin Like Modifier 2 | 0.27 |
| RAB6A | RAB6A, Member RAS Oncogene Family | 0.27 |
| STK16 | Serine/Threonine Kinase 16 | 0.27 |
| LIMS1 | LIM Zinc Finger Domain Containing 1 | 0.27 |
| STAMBPL1 | STAM Binding Protein Like 1 | 0.27 |
| VNN2 | Vanin 2 | 0.27 |
| GDF9 | Growth Differentiation Factor 9 | 0.27 |
| CCDC22 | Coiled-Coil Domain Containing 22 | 0.27 |
| ARHGEF16 | Rho Guanine Nucleotide Exchange Factor 16 | 0.27 |
| ASAP1 | ArfGAP With SH3 Domain, Ankyrin Repeat And PH Domain 1 | 0.27 |
| AFAP1 | Actin Filament Associated Protein 1 | 0.27 |
| BHLHE41 | Basic Helix-Loop-Helix Family Member E41 | 0.27 |
| COL8A1 | Collagen Type VIII Alpha 1 Chain | 0.27 |
| DSTN | Destrin, Actin Depolymerizing Factor | 0.27 |
| NANOG | Nanog Homeobox | 0.27 |
| GPR119 | G Protein-Coupled Receptor 119 | 0.27 |
| DBNL | Drebrin Like | 0.27 |
| NELL1 | Neural EGFL Like 1 | 0.27 |
| CRTC3 | CREB Regulated Transcription Coactivator 3 | 0.27 |
| SLC9A9 | Solute Carrier Family 9 Member A9 | 0.27 |
| SLCO1A2 | Solute Carrier Organic Anion Transporter Family Member 1A2 | 0.27 |
| SUPT5H | SPT5 Homolog, DSIF Elongation Factor Subunit | 0.27 |
| RAB4B | RAB4B, Member RAS Oncogene Family | 0.27 |
| TMPRSS11D | Transmembrane Serine Protease 11D | 0.27 |
| EIF2A | Eukaryotic Translation Initiation Factor 2A | 0.27 |
| MSI1 | Musashi RNA Binding Protein 1 | 0.27 |
| KCNIP4 | Potassium Voltage-Gated Channel Interacting Protein 4 | 0.27 |
| DDX21 | DExD-Box Helicase 21 | 0.27 |
| NFE2L3 | Nuclear Factor, Erythroid 2 Like 3 | 0.27 |
| TJP3 | Tight Junction Protein 3 | 0.27 |
| IFNK | Interferon Kappa | 0.27 |
| VGF | VGF Nerve Growth Factor Inducible | 0.27 |
| AUP1 | AUP1 Lipid Droplet Regulating VLDL Assembly Factor | 0.27 |
| APOF | Apolipoprotein F | 0.27 |
| BET1 | Bet1 Golgi Vesicular Membrane Trafficking Protein | 0.27 |
| BCAS1 | Brain Enriched Myelin Associated Protein 1 | 0.27 |
| MBNL2 | Muscleblind Like Splicing Regulator 2 | 0.27 |
| ADGRL2 | Adhesion G Protein-Coupled Receptor L2 | 0.27 |
| RLN2 | Relaxin 2 | 0.27 |
| HOPX | HOP Homeobox | 0.27 |
| CPEB4 | Cytoplasmic Polyadenylation Element Binding Protein 4 | 0.27 |
| CD99L2 | CD99 Molecule Like 2 | 0.27 |
| SIGLEC10 | Sialic Acid Binding Ig Like Lectin 10 | 0.27 |
| SARM1 | Sterile Alpha And TIR Motif Containing 1 | 0.27 |
| ST18 | ST18 C2H2C-Type Zinc Finger Transcription Factor | 0.27 |
| RBFOX1 | RNA Binding Fox-1 Homolog 1 | 0.27 |
| KIF6 | Kinesin Family Member 6 | 0.27 |
| INSL5 | Insulin Like 5 | 0.27 |
| PLA2G4D | Phospholipase A2 Group IVD | 0.27 |
| ZNF7 | Zinc Finger Protein 7 | 0.27 |
| ARHGEF10L | Rho Guanine Nucleotide Exchange Factor 10 Like | 0.27 |
| ANKS1A | Ankyrin Repeat And Sterile Alpha Motif Domain Containing 1A | 0.27 |
| COMMD7 | COMM Domain Containing 7 | 0.27 |
| EFHD1 | EF-Hand Domain Family Member D1 | 0.27 |
| DCP1B | Decapping MRNA 1B | 0.27 |
| SETD6 | SET Domain Containing 6, Protein Lysine Methyltransferase | 0.27 |
| TBL1X | Transducin Beta Like 1 X-Linked | 0.27 |
| WDPCP | WD Repeat Containing Planar Cell Polarity Effector | 0.27 |
| ZNF536 | Zinc Finger Protein 536 | 0.27 |
| GRXCR1 | Glutaredoxin And Cysteine Rich Domain Containing 1 | 0.27 |
| SGCZ | Sarcoglycan Zeta | 0.27 |
| ITGBL1 | Integrin Subunit Beta Like 1 | 0.27 |
| TTLL7 | Tubulin Tyrosine Ligase Like 7 | 0.27 |
| FRMD4B | FERM Domain Containing 4B | 0.27 |
| HECW1 | HECT, C2 And WW Domain Containing E3 Ubiquitin Protein Ligase 1 | 0.27 |
| PAAF1 | Proteasomal ATPase Associated Factor 1 | 0.27 |
| ITIH5 | Inter-Alpha-Trypsin Inhibitor Heavy Chain 5 | 0.27 |
| TRIM47 | Tripartite Motif Containing 47 | 0.27 |
| CCDC69 | Coiled-Coil Domain Containing 69 | 0.27 |
| ADGRG3 | Adhesion G Protein-Coupled Receptor G3 | 0.27 |
| C6orf89 | Chromosome 6 Open Reading Frame 89 | 0.27 |
| DCHS2 | Dachsous Cadherin-Related 2 | 0.27 |
| CYTL1 | Cytokine Like 1 | 0.27 |
| IFIT5 | Interferon Induced Protein With Tetratricopeptide Repeats 5 | 0.27 |
| UBXN7 | UBX Domain Protein 7 | 0.27 |
| TSPAN17 | Tetraspanin 17 | 0.27 |
| TMEM100 | Transmembrane Protein 100 | 0.27 |
| COMMD5 | COMM Domain Containing 5 | 0.27 |
| H3-4 | H3.4 Histone | 0.27 |
| EID1 | EP300 Interacting Inhibitor Of Differentiation 1 | 0.27 |
| KLRF1 | Killer Cell Lectin Like Receptor F1 | 0.27 |
| KLHL29 | Kelch Like Family Member 29 | 0.27 |
| H1-0 | H1.0 Linker Histone | 0.27 |
| CPTP | Ceramide-1-Phosphate Transfer Protein | 0.27 |
| CREBRF | CREB3 Regulatory Factor | 0.27 |
| OR10J3 | Olfactory Receptor Family 10 Subfamily J Member 3 | 0.27 |
| CACTIN | Cactin, Spliceosome C Complex Subunit | 0.27 |
| GATD3A | Glutamine Amidotransferase Like Class 1 Domain Containing 3A | 0.27 |
| IGHG3 | Immunoglobulin Heavy Constant Gamma 3 (G3m Marker) | 0.27 |
| MIR302A | MicroRNA 302a | 0.27 |
| BAIAP2-DT | BAIAP2 Divergent Transcript | 0.27 |
| LOC154449 | Uncharacterized LOC154449 | 0.27 |
| WAKMAR2 | Wound And Keratinocyte Migration Associated LncRNA 2 | 0.27 |
| LINC01725 | Long Intergenic Non-Protein Coding RNA 1725 | 0.27 |
| ECRP | Ribonuclease A Family Member 2 Pseudogene | 0.27 |
| ACTBP8 | ACTB Pseudogene 8 | 0.27 |
| LINC01228 | Long Intergenic Non-Protein Coding RNA 1228 | 0.27 |
| EEF1A1P27 | Eukaryotic Translation Elongation Factor 1 Alpha 1 Pseudogene 27 | 0.27 |
| FTLP17 | Ferritin Light Chain Pseudogene 17 | 0.27 |
| HDLC3 | High Density Lipoprotein Cholesterol, Low Serum, 3 | 0.27 |
| PARK12 | Parkinson Disease 12 (Susceptibility) | 0.27 |
| LOC117307477 | CD209 Promoter Region | 0.27 |
| NSF | N-Ethylmaleimide Sensitive Factor, Vesicle Fusing ATPase | 0.26 |
| TRIM32 | Tripartite Motif Containing 32 | 0.25 |
| GCLM | Glutamate-Cysteine Ligase Modifier Subunit | 0.25 |
| PPIC | Peptidylprolyl Isomerase C | 0.25 |
| MAPK8IP3 | Mitogen-Activated Protein Kinase 8 Interacting Protein 3 | 0.25 |
| CNOT8 | CCR4-NOT Transcription Complex Subunit 8 | 0.25 |
| CHRDL1 | Chordin Like 1 | 0.25 |
| ADGRG1 | Adhesion G Protein-Coupled Receptor G1 | 0.25 |
| SIGLEC12 | Sialic Acid Binding Ig Like Lectin 12 | 0.25 |
| TSTD1 | Thiosulfate Sulfurtransferase Like Domain Containing 1 | 0.25 |
| MIR138-2 | MicroRNA 138-2 | 0.25 |
| NFIA-AS1 | NFIA Antisense RNA 1 | 0.25 |
| MAX | MYC Associated Factor X | 0.24 |
| ADCY1 | Adenylate Cyclase 1 | 0.24 |
| RHEB | Ras Homolog, MTORC1 Binding | 0.24 |
| PDHA1 | Pyruvate Dehydrogenase E1 Subunit Alpha 1 | 0.24 |
| GCDH | Glutaryl-CoA Dehydrogenase | 0.24 |
| GABRB2 | Gamma-Aminobutyric Acid Type A Receptor Subunit Beta2 | 0.24 |
| KCNB1 | Potassium Voltage-Gated Channel Subfamily B Member 1 | 0.24 |
| BCAT1 | Branched Chain Amino Acid Transaminase 1 | 0.24 |
| GP9 | Glycoprotein IX Platelet | 0.24 |
| SLC9A6 | Solute Carrier Family 9 Member A6 | 0.24 |
| SLC19A3 | Solute Carrier Family 19 Member 3 | 0.24 |
| SIGMAR1 | Sigma Non-Opioid Intracellular Receptor 1 | 0.24 |
| KCNH1 | Potassium Voltage-Gated Channel Subfamily H Member 1 | 0.24 |
| SOX5 | SRY-Box Transcription Factor 5 | 0.24 |
| ADCYAP1R1 | ADCYAP Receptor Type I | 0.24 |
| ATP2B2 | ATPase Plasma Membrane Ca2+ Transporting 2 | 0.24 |
| ARAF | A-Raf Proto-Oncogene, Serine/Threonine Kinase | 0.24 |
| DVL2 | Dishevelled Segment Polarity Protein 2 | 0.24 |
| EHMT1 | Euchromatic Histone Lysine Methyltransferase 1 | 0.24 |
| NPY1R | Neuropeptide Y Receptor Y1 | 0.24 |
| PPP2R2B | Protein Phosphatase 2 Regulatory Subunit Bbeta | 0.24 |
| OXCT1 | 3-Oxoacid CoA-Transferase 1 | 0.24 |
| PPM1A | Protein Phosphatase, Mg2+/Mn2+ Dependent 1A | 0.24 |
| PCBD1 | Pterin-4 Alpha-Carbinolamine Dehydratase 1 | 0.24 |
| SLC5A5 | Solute Carrier Family 5 Member 5 | 0.24 |
| TOP2B | DNA Topoisomerase II Beta | 0.24 |
| STEAP3 | STEAP3 Metalloreductase | 0.24 |
| RALB | RAS Like Proto-Oncogene B | 0.24 |
| PRKD2 | Protein Kinase D2 | 0.24 |
| STAMBP | STAM Binding Protein | 0.24 |
| PIP4K2A | Phosphatidylinositol-5-Phosphate 4-Kinase Type 2 Alpha | 0.24 |
| ATXN3 | Ataxin 3 | 0.24 |
| MCM7 | Minichromosome Maintenance Complex Component 7 | 0.24 |
| AASS | Aminoadipate-Semialdehyde Synthase | 0.24 |
| GABRB1 | Gamma-Aminobutyric Acid Type A Receptor Subunit Beta1 | 0.24 |
| CYP2C18 | Cytochrome P450 Family 2 Subfamily C Member 18 | 0.24 |
| CHRNB2 | Cholinergic Receptor Nicotinic Beta 2 Subunit | 0.24 |
| DYNC1H1 | Dynein Cytoplasmic 1 Heavy Chain 1 | 0.24 |
| DAPK3 | Death Associated Protein Kinase 3 | 0.24 |
| NPC1L1 | NPC1 Like Intracellular Cholesterol Transporter 1 | 0.24 |
| PAX7 | Paired Box 7 | 0.24 |
| PLXNB1 | Plexin B1 | 0.24 |
| LAMB2 | Laminin Subunit Beta 2 | 0.24 |
| KHK | Ketohexokinase | 0.24 |
| RAD23B | RAD23 Homolog B, Nucleotide Excision Repair Protein | 0.24 |
| PYCR2 | Pyrroline-5-Carboxylate Reductase 2 | 0.24 |
| KISS1R | KISS1 Receptor | 0.24 |
| PTDSS1 | Phosphatidylserine Synthase 1 | 0.24 |
| CFL2 | Cofilin 2 | 0.24 |
| TFDP1 | Transcription Factor Dp-1 | 0.24 |
| CAMK1 | Calcium/Calmodulin Dependent Protein Kinase I | 0.24 |
| CBX3 | Chromobox 3 | 0.24 |
| ACD | ACD Shelterin Complex Subunit And Telomerase Recruitment Factor | 0.24 |
| FPGS | Folylpolyglutamate Synthase | 0.24 |
| FABP7 | Fatty Acid Binding Protein 7 | 0.24 |
| DGAT2 | Diacylglycerol O-Acyltransferase 2 | 0.24 |
| GRB14 | Growth Factor Receptor Bound Protein 14 | 0.24 |
| HTR6 | 5-Hydroxytryptamine Receptor 6 | 0.24 |
| POU3F2 | POU Class 3 Homeobox 2 | 0.24 |
| SLC1A5 | Solute Carrier Family 1 Member 5 | 0.24 |
| KCNN2 | Potassium Calcium-Activated Channel Subfamily N Member 2 | 0.24 |
| TECR | Trans-2,3-Enoyl-CoA Reductase | 0.24 |
| TLL1 | Tolloid Like 1 | 0.24 |
| ZFP36L1 | ZFP36 Ring Finger Protein Like 1 | 0.24 |
| VAV3 | Vav Guanine Nucleotide Exchange Factor 3 | 0.24 |
| ABCB8 | ATP Binding Cassette Subfamily B Member 8 | 0.24 |
| GJC1 | Gap Junction Protein Gamma 1 | 0.24 |
| ABCB5 | ATP Binding Cassette Subfamily B Member 5 | 0.24 |
| FOXE1 | Forkhead Box E1 | 0.24 |
| FGFRL1 | Fibroblast Growth Factor Receptor Like 1 | 0.24 |
| HR | HR Lysine Demethylase And Nuclear Receptor Corepressor | 0.24 |
| CDK5RAP2 | CDK5 Regulatory Subunit Associated Protein 2 | 0.24 |
| COX15 | Cytochrome C Oxidase Assembly Homolog COX15 | 0.24 |
| MTMR3 | Myotubularin Related Protein 3 | 0.24 |
| MLST8 | MTOR Associated Protein, LST8 Homolog | 0.24 |
| IGFBP6 | Insulin Like Growth Factor Binding Protein 6 | 0.24 |
| SPRY1 | Sprouty RTK Signaling Antagonist 1 | 0.24 |
| RARS2 | Arginyl-TRNA Synthetase 2, Mitochondrial | 0.24 |
| RBMX | RNA Binding Motif Protein X-Linked | 0.24 |
| KIF1C | Kinesin Family Member 1C | 0.24 |
| GNA12 | G Protein Subunit Alpha 12 | 0.24 |
| MAST1 | Microtubule Associated Serine/Threonine Kinase 1 | 0.24 |
| AP4B1 | Adaptor Related Protein Complex 4 Subunit Beta 1 | 0.24 |
| LPCAT2 | Lysophosphatidylcholine Acyltransferase 2 | 0.24 |
| EPB41L3 | Erythrocyte Membrane Protein Band 4.1 Like 3 | 0.24 |
| EPHA6 | EPH Receptor A6 | 0.24 |
| GSTM4 | Glutathione S-Transferase Mu 4 | 0.24 |
| GUCY1A2 | Guanylate Cyclase 1 Soluble Subunit Alpha 2 | 0.24 |
| CELF2 | CUGBP Elav-Like Family Member 2 | 0.24 |
| CLTB | Clathrin Light Chain B | 0.24 |
| CLCA2 | Chloride Channel Accessory 2 | 0.24 |
| RIN2 | Ras And Rab Interactor 2 | 0.24 |
| MSRB2 | Methionine Sulfoxide Reductase B2 | 0.24 |
| MRPS22 | Mitochondrial Ribosomal Protein S22 | 0.24 |
| MYO3A | Myosin IIIA | 0.24 |
| CDH4 | Cadherin 4 | 0.24 |
| CYTH1 | Cytohesin 1 | 0.24 |
| SHANK2 | SH3 And Multiple Ankyrin Repeat Domains 2 | 0.24 |
| ENSA | Endosulfine Alpha | 0.24 |
| ENAH | ENAH Actin Regulator | 0.24 |
| SLC2A6 | Solute Carrier Family 2 Member 6 | 0.24 |
| NTM | Neurotrimin | 0.24 |
| SLC22A7 | Solute Carrier Family 22 Member 7 | 0.24 |
| PTBP1 | Polypyrimidine Tract Binding Protein 1 | 0.24 |
| RAB28 | RAB28, Member RAS Oncogene Family | 0.24 |
| KISS1 | KiSS-1 Metastasis Suppressor | 0.24 |
| SORBS1 | Sorbin And SH3 Domain Containing 1 | 0.24 |
| DSTYK | Dual Serine/Threonine And Tyrosine Protein Kinase | 0.24 |
| ANAPC1 | Anaphase Promoting Complex Subunit 1 | 0.24 |
| GALNT13 | Polypeptide N-Acetylgalactosaminyltransferase 13 | 0.24 |
| GLRX5 | Glutaredoxin 5 | 0.24 |
| APEH | Acylaminoacyl-Peptide Hydrolase | 0.24 |
| FARSA | Phenylalanyl-TRNA Synthetase Subunit Alpha | 0.24 |
| AKAP12 | A-Kinase Anchoring Protein 12 | 0.24 |
| EPN2 | Epsin 2 | 0.24 |
| ANO10 | Anoctamin 10 | 0.24 |
| MAPK15 | Mitogen-Activated Protein Kinase 15 | 0.24 |
| AATF | Apoptosis Antagonizing Transcription Factor | 0.24 |
| CWC27 | CWC27 Spliceosome Associated Cyclophilin | 0.24 |
| CAMLG | Calcium Modulating Ligand | 0.24 |
| AP1M2 | Adaptor Related Protein Complex 1 Subunit Mu 2 | 0.24 |
| RGS5 | Regulator Of G Protein Signaling 5 | 0.24 |
| RGS7 | Regulator Of G Protein Signaling 7 | 0.24 |
| CLN5 | CLN5 Intracellular Trafficking Protein | 0.24 |
| HLX | H2.0 Like Homeobox | 0.24 |
| DIO1 | Iodothyronine Deiodinase 1 | 0.24 |
| NAAA | N-Acylethanolamine Acid Amidase | 0.24 |
| ECI2 | Enoyl-CoA Delta Isomerase 2 | 0.24 |
| MYO3B | Myosin IIIB | 0.24 |
| RFC5 | Replication Factor C Subunit 5 | 0.24 |
| CPLX2 | Complexin 2 | 0.24 |
| SPAST | Spastin | 0.24 |
| SGPP1 | Sphingosine-1-Phosphate Phosphatase 1 | 0.24 |
| PANK4 | Pantothenate Kinase 4 (Inactive) | 0.24 |
| PCMT1 | Protein-L-Isoaspartate (D-Aspartate) O-Methyltransferase | 0.24 |
| SOSTDC1 | Sclerostin Domain Containing 1 | 0.24 |
| RSU1 | Ras Suppressor Protein 1 | 0.24 |
| SLCO3A1 | Solute Carrier Organic Anion Transporter Family Member 3A1 | 0.24 |
| OLFM1 | Olfactomedin 1 | 0.24 |
| ONECUT1 | One Cut Homeobox 1 | 0.24 |
| NUP153 | Nucleoporin 153 | 0.24 |
| SS18 | SS18 Subunit Of BAF Chromatin Remodeling Complex | 0.24 |
| TRAF7 | TNF Receptor Associated Factor 7 | 0.24 |
| KCNV2 | Potassium Voltage-Gated Channel Modifier Subfamily V Member 2 | 0.24 |
| SUPT3H | SPT3 Homolog, SAGA And STAGA Complex Component | 0.24 |
| SORBS3 | Sorbin And SH3 Domain Containing 3 | 0.24 |
| TTC8 | Tetratricopeptide Repeat Domain 8 | 0.24 |
| DYNC1I1 | Dynein Cytoplasmic 1 Intermediate Chain 1 | 0.24 |
| NACC1 | Nucleus Accumbens Associated 1 | 0.24 |
| HTATIP2 | HIV-1 Tat Interactive Protein 2 | 0.24 |
| PPA1 | Inorganic Pyrophosphatase 1 | 0.24 |
| PI4K2B | Phosphatidylinositol 4-Kinase Type 2 Beta | 0.24 |
| UNC13A | Unc-13 Homolog A | 0.24 |
| ZBTB7B | Zinc Finger And BTB Domain Containing 7B | 0.24 |
| C1QTNF5 | C1q And TNF Related 5 | 0.24 |
| CD109 | CD109 Molecule | 0.24 |
| EXOC6 | Exocyst Complex Component 6 | 0.24 |
| APOBEC1 | Apolipoprotein B MRNA Editing Enzyme Catalytic Subunit 1 | 0.24 |
| ARHGAP24 | Rho GTPase Activating Protein 24 | 0.24 |
| AK8 | Adenylate Kinase 8 | 0.24 |
| ANKS1B | Ankyrin Repeat And Sterile Alpha Motif Domain Containing 1B | 0.24 |
| ATP8B3 | ATPase Phospholipid Transporting 8B3 | 0.24 |
| CDK5R2 | Cyclin Dependent Kinase 5 Regulatory Subunit 2 | 0.24 |
| CEP152 | Centrosomal Protein 152 | 0.24 |
| RHPN2 | Rhophilin Rho GTPase Binding Protein 2 | 0.24 |
| COQ4 | Coenzyme Q4 | 0.24 |
| CLYBL | Citramalyl-CoA Lyase | 0.24 |
| MRPL13 | Mitochondrial Ribosomal Protein L13 | 0.24 |
| NAP1L1 | Nucleosome Assembly Protein 1 Like 1 | 0.24 |
| CYTH3 | Cytohesin 3 | 0.24 |
| PSD3 | Pleckstrin And Sec7 Domain Containing 3 | 0.24 |
| PELP1 | Proline, Glutamate And Leucine Rich Protein 1 | 0.24 |
| IGSF8 | Immunoglobulin Superfamily Member 8 | 0.24 |
| IFIT2 | Interferon Induced Protein With Tetratricopeptide Repeats 2 | 0.24 |
| PCDH7 | Protocadherin 7 | 0.24 |
| RPP30 | Ribonuclease P/MRP Subunit P30 | 0.24 |
| PKN3 | Protein Kinase N3 | 0.24 |
| TUBGCP6 | Tubulin Gamma Complex Associated Protein 6 | 0.24 |
| TXNDC5 | Thioredoxin Domain Containing 5 | 0.24 |
| LEMD2 | LEM Domain Nuclear Envelope Protein 2 | 0.24 |
| UBR2 | Ubiquitin Protein Ligase E3 Component N-Recognin 2 | 0.24 |
| LHPP | Phospholysine Phosphohistidine Inorganic Pyrophosphate Phosphatase | 0.24 |
| TSEN34 | TRNA Splicing Endonuclease Subunit 34 | 0.24 |
| PVALB | Parvalbumin | 0.24 |
| RASAL2 | RAS Protein Activator Like 2 | 0.24 |
| SYTL2 | Synaptotagmin Like 2 | 0.24 |
| KPNA6 | Karyopherin Subunit Alpha 6 | 0.24 |
| KLHL1 | Kelch Like Family Member 1 | 0.24 |
| RAB17 | RAB17, Member RAS Oncogene Family | 0.24 |
| KANK2 | KN Motif And Ankyrin Repeat Domains 2 | 0.24 |
| ID4 | Inhibitor Of DNA Binding 4, HLH Protein | 0.24 |
| MYO10 | Myosin X | 0.24 |
| DUSP13 | Dual Specificity Phosphatase 13 | 0.24 |
| MT1F | Metallothionein 1F | 0.24 |
| PRDM5 | PR/SET Domain 5 | 0.24 |
| RBBP6 | RB Binding Protein 6, Ubiquitin Ligase | 0.24 |
| KRT81 | Keratin 81 | 0.24 |
| KRT86 | Keratin 86 | 0.24 |
| USP47 | Ubiquitin Specific Peptidase 47 | 0.24 |
| LLGL2 | LLGL Scribble Cell Polarity Complex Component 2 | 0.24 |
| GEM | GTP Binding Protein Overexpressed In Skeletal Muscle | 0.24 |
| LRRFIP2 | LRR Binding FLII Interacting Protein 2 | 0.24 |
| LRRC7 | Leucine Rich Repeat Containing 7 | 0.24 |
| BRAT1 | BRCA1 Associated ATM Activator 1 | 0.24 |
| AATK | Apoptosis Associated Tyrosine Kinase | 0.24 |
| MAEA | Macrophage Erythroblast Attacher, E3 Ubiquitin Ligase | 0.24 |
| CRYL1 | Crystallin Lambda 1 | 0.24 |
| GTF2E1 | General Transcription Factor IIE Subunit 1 | 0.24 |
| DUSP23 | Dual Specificity Phosphatase 23 | 0.24 |
| NDRG2 | NDRG Family Member 2 | 0.24 |
| SH3TC2 | SH3 Domain And Tetratricopeptide Repeats 2 | 0.24 |
| SLMAP | Sarcolemma Associated Protein | 0.24 |
| PLXNA4 | Plexin A4 | 0.24 |
| SRP14 | Signal Recognition Particle 14 | 0.24 |
| ST13 | ST13 Hsp70 Interacting Protein | 0.24 |
| KCNK15 | Potassium Two Pore Domain Channel Subfamily K Member 15 | 0.24 |
| KLF12 | Kruppel Like Factor 12 | 0.24 |
| SUMF2 | Sulfatase Modifying Factor 2 | 0.24 |
| HMGCLL1 | 3-Hydroxymethyl-3-Methylglutaryl-CoA Lyase Like 1 | 0.24 |
| HIGD1A | HIG1 Hypoxia Inducible Domain Family Member 1A | 0.24 |
| SPPL2A | Signal Peptide Peptidase Like 2A | 0.24 |
| KRT83 | Keratin 83 | 0.24 |
| CALML5 | Calmodulin Like 5 | 0.24 |
| GBP4 | Guanylate Binding Protein 4 | 0.24 |
| MID1IP1 | MID1 Interacting Protein 1 | 0.24 |
| FGGY | FGGY Carbohydrate Kinase Domain Containing | 0.24 |
| ANXA10 | Annexin A10 | 0.24 |
| ARHGAP12 | Rho GTPase Activating Protein 12 | 0.24 |
| FEM1A | Fem-1 Homolog A | 0.24 |
| BDP1 | B Double Prime 1, Subunit Of RNA Polymerase III Transcription Initiation Factor IIIB | 0.24 |
| MCRS1 | Microspherule Protein 1 | 0.24 |
| ABHD2 | Abhydrolase Domain Containing 2, Acylglycerol Lipase | 0.24 |
| GAL3ST1 | Galactose-3-O-Sulfotransferase 1 | 0.24 |
| FHOD3 | Formin Homology 2 Domain Containing 3 | 0.24 |
| CUTC | CutC Copper Transporter | 0.24 |
| CPPED1 | Calcineurin Like Phosphoesterase Domain Containing 1 | 0.24 |
| RMI1 | RecQ Mediated Genome Instability 1 | 0.24 |
| HEPHL1 | Hephaestin Like 1 | 0.24 |
| FOXL1 | Forkhead Box L1 | 0.24 |
| ANO2 | Anoctamin 2 | 0.24 |
| COBLL1 | Cordon-Bleu WH2 Repeat Protein Like 1 | 0.24 |
| DPY30 | Dpy-30 Histone Methyltransferase Complex Regulatory Subunit | 0.24 |
| MYO18A | Myosin XVIIIA | 0.24 |
| MOV10L1 | Mov10 Like RISC Complex RNA Helicase 1 | 0.24 |
| CDH18 | Cadherin 18 | 0.24 |
| SPAG16 | Sperm Associated Antigen 16 | 0.24 |
| PDZD2 | PDZ Domain Containing 2 | 0.24 |
| NOS1AP | Nitric Oxide Synthase 1 Adaptor Protein | 0.24 |
| SIPA1L1 | Signal Induced Proliferation Associated 1 Like 1 | 0.24 |
| NPM2 | Nucleophosmin/Nucleoplasmin 2 | 0.24 |
| SEZ6L2 | Seizure Related 6 Homolog Like 2 | 0.24 |
| IFNA5 | Interferon Alpha 5 | 0.24 |
| PAXIP1 | PAX Interacting Protein 1 | 0.24 |
| PCDH18 | Protocadherin 18 | 0.24 |
| PMEPA1 | Prostate Transmembrane Protein, Androgen Induced 1 | 0.24 |
| NUDT7 | Nudix Hydrolase 7 | 0.24 |
| TRAPPC12 | Trafficking Protein Particle Complex 12 | 0.24 |
| TUBD1 | Tubulin Delta 1 | 0.24 |
| UBXN11 | UBX Domain Protein 11 | 0.24 |
| TAF5 | TATA-Box Binding Protein Associated Factor 5 | 0.24 |
| RBM38 | RNA Binding Motif Protein 38 | 0.24 |
| KCNH4 | Potassium Voltage-Gated Channel Subfamily H Member 4 | 0.24 |
| TM9SF2 | Transmembrane 9 Superfamily Member 2 | 0.24 |
| TMOD1 | Tropomodulin 1 | 0.24 |
| TMPRSS11A | Transmembrane Serine Protease 11A | 0.24 |
| COL19A1 | Collagen Type XIX Alpha 1 Chain | 0.24 |
| SH3BGRL | SH3 Domain Binding Glutamate Rich Protein Like | 0.24 |
| CUL9 | Cullin 9 | 0.24 |
| ORC3 | Origin Recognition Complex Subunit 3 | 0.24 |
| HYAL3 | Hyaluronidase 3 | 0.24 |
| KCNK5 | Potassium Two Pore Domain Channel Subfamily K Member 5 | 0.24 |
| TIMM8B | Translocase Of Inner Mitochondrial Membrane 8 Homolog B | 0.24 |
| WDR59 | WD Repeat Domain 59 | 0.24 |
| ZFP36L2 | ZFP36 Ring Finger Protein Like 2 | 0.24 |
| ZC3HAV1 | Zinc Finger CCCH-Type Containing, Antiviral 1 | 0.24 |
| AKAP8L | A-Kinase Anchoring Protein 8 Like | 0.24 |
| ADAMTS19 | ADAM Metallopeptidase With Thrombospondin Type 1 Motif 19 | 0.24 |
| ADAMTS20 | ADAM Metallopeptidase With Thrombospondin Type 1 Motif 20 | 0.24 |
| FXYD5 | FXYD Domain Containing Ion Transport Regulator 5 | 0.24 |
| FXYD7 | FXYD Domain Containing Ion Transport Regulator 7 | 0.24 |
| GLB1L3 | Galactosidase Beta 1 Like 3 | 0.24 |
| B3GAT2 | Beta-1,3-Glucuronyltransferase 2 | 0.24 |
| ATG16L2 | Autophagy Related 16 Like 2 | 0.24 |
| ARAP2 | ArfGAP With RhoGAP Domain, Ankyrin Repeat And PH Domain 2 | 0.24 |
| LPCAT1 | Lysophosphatidylcholine Acyltransferase 1 | 0.24 |
| ARHGAP11A | Rho GTPase Activating Protein 11A | 0.24 |
| ARHGAP28 | Rho GTPase Activating Protein 28 | 0.24 |
| ASTN2 | Astrotactin 2 | 0.24 |
| BCAS3 | BCAS3 Microtubule Associated Cell Migration Factor | 0.24 |
| MDGA2 | MAM Domain Containing Glycosylphosphatidylinositol Anchor 2 | 0.24 |
| ADAMTS15 | ADAM Metallopeptidase With Thrombospondin Type 1 Motif 15 | 0.24 |
| AAMP | Angio Associated Migratory Cell Protein | 0.24 |
| BTBD3 | BTB Domain Containing 3 | 0.24 |
| HS3ST4 | Heparan Sulfate-Glucosamine 3-Sulfotransferase 4 | 0.24 |
| RLN3 | Relaxin 3 | 0.24 |
| CPSF2 | Cleavage And Polyadenylation Specific Factor 2 | 0.24 |
| CEP72 | Centrosomal Protein 72 | 0.24 |
| CORO2A | Coronin 2A | 0.24 |
| HERPUD2 | HERPUD Family Member 2 | 0.24 |
| CLMN | Calmin | 0.24 |
| CIDEB | Cell Death Inducing DFFA Like Effector B | 0.24 |
| DHX57 | DExH-Box Helicase 57 | 0.24 |
| MRPS24 | Mitochondrial Ribosomal Protein S24 | 0.24 |
| MYADM | Myeloid Associated Differentiation Marker | 0.24 |
| RP1L1 | RP1 Like 1 | 0.24 |
| GREB1 | Growth Regulating Estrogen Receptor Binding 1 | 0.24 |
| CDH20 | Cadherin 20 | 0.24 |
| RNF32 | Ring Finger Protein 32 | 0.24 |
| MPP7 | Membrane Palmitoylated Protein 7 | 0.24 |
| COL23A1 | Collagen Type XXIII Alpha 1 Chain | 0.24 |
| NCKAP1L | NCK Associated Protein 1 Like | 0.24 |
| IGSF11 | Immunoglobulin Superfamily Member 11 | 0.24 |
| IFNA6 | Interferon Alpha 6 | 0.24 |
| PPM1H | Protein Phosphatase, Mg2+/Mn2+ Dependent 1H | 0.24 |
| PPP1R11 | Protein Phosphatase 1 Regulatory Inhibitor Subunit 11 | 0.24 |
| SORCS2 | Sortilin Related VPS10 Domain Containing Receptor 2 | 0.24 |
| PCF11 | PCF11 Cleavage And Polyadenylation Factor Subunit | 0.24 |
| SAMD4A | Sterile Alpha Motif Domain Containing 4A | 0.24 |
| SDK1 | Sidekick Cell Adhesion Molecule 1 | 0.24 |
| PLEKHA6 | Pleckstrin Homology Domain Containing A6 | 0.24 |
| INSC | INSC Spindle Orientation Adaptor Protein | 0.24 |
| SRSF10 | Serine And Arginine Rich Splicing Factor 10 | 0.24 |
| TOM1L1 | Target Of Myb1 Like 1 Membrane Trafficking Protein | 0.24 |
| TSPAN33 | Tetraspanin 33 | 0.24 |
| SYT6 | Synaptotagmin 6 | 0.24 |
| ITFG1 | Integrin Alpha FG-GAP Repeat Containing 1 | 0.24 |
| SUGT1 | SGT1 Homolog, MIS12 Kinetochore Complex Assembly Cochaperone | 0.24 |
| TMEM18 | Transmembrane Protein 18 | 0.24 |
| TMEM132D | Transmembrane Protein 132D | 0.24 |
| PSRC1 | Proline And Serine Rich Coiled-Coil 1 | 0.24 |
| MYO15A | Myosin XVA | 0.24 |
| SCAMP5 | Secretory Carrier Membrane Protein 5 | 0.24 |
| KRT31 | Keratin 31 | 0.24 |
| KRT33B | Keratin 33B | 0.24 |
| KRT35 | Keratin 35 | 0.24 |
| KRT85 | Keratin 85 | 0.24 |
| YTHDC1 | YTH Domain Containing 1 | 0.24 |
| LINGO2 | Leucine Rich Repeat And Ig Domain Containing 2 | 0.24 |
| GFOD1 | Glucose-Fructose Oxidoreductase Domain Containing 1 | 0.24 |
| MEGF9 | Multiple EGF Like Domains 9 | 0.24 |
| CCDC91 | Coiled-Coil Domain Containing 91 | 0.24 |
| ATG2B | Autophagy Related 2B | 0.24 |
| ARMC1 | Armadillo Repeat Containing 1 | 0.24 |
| FBXL5 | F-Box And Leucine Rich Repeat Protein 5 | 0.24 |
| MAL2 | Mal, T Cell Differentiation Protein 2 | 0.24 |
| LZTS2 | Leucine Zipper Tumor Suppressor 2 | 0.24 |
| MACROD1 | Mono-ADP Ribosylhydrolase 1 | 0.24 |
| MACROH2A1 | MacroH2A.1 Histone | 0.24 |
| AFAP1L1 | Actin Filament Associated Protein 1 Like 1 | 0.24 |
| ADGRV1 | Adhesion G Protein-Coupled Receptor V1 | 0.24 |
| C8orf37 | Chromosome 8 Open Reading Frame 37 | 0.24 |
| FIBCD1 | Fibrinogen C Domain Containing 1 | 0.24 |
| CPNE9 | Copine Family Member 9 | 0.24 |
| COMMD3 | COMM Domain Containing 3 | 0.24 |
| COMMD8 | COMM Domain Containing 8 | 0.24 |
| MRPL48 | Mitochondrial Ribosomal Protein L48 | 0.24 |
| EDEM3 | ER Degradation Enhancing Alpha-Mannosidase Like Protein 3 | 0.24 |
| ECD | Ecdysoneless Cell Cycle Regulator | 0.24 |
| MYO16 | Myosin XVI | 0.24 |
| MOSPD2 | Motile Sperm Domain Containing 2 | 0.24 |
| MPHOSPH6 | M-Phase Phosphoprotein 6 | 0.24 |
| RNF123 | Ring Finger Protein 123 | 0.24 |
| RNFT1 | Ring Finger Protein, Transmembrane 1 | 0.24 |
| NCDN | Neurochondrin | 0.24 |
| IFNA21 | Interferon Alpha 21 | 0.24 |
| PLEKHG6 | Pleckstrin Homology And RhoGEF Domain Containing G6 | 0.24 |
| IFNA13 | Interferon Alpha 13 | 0.24 |
| PBX4 | PBX Homeobox 4 | 0.24 |
| NIPSNAP3B | Nipsnap Homolog 3B | 0.24 |
| SLBP | Stem-Loop Binding Protein | 0.24 |
| PLPP3 | Phospholipid Phosphatase 3 | 0.24 |
| PLD5 | Phospholipase D Family Member 5 | 0.24 |
| SLC36A4 | Solute Carrier Family 36 Member 4 | 0.24 |
| PHACTR2 | Phosphatase And Actin Regulator 2 | 0.24 |
| PHTF1 | Putative Homeodomain Transcription Factor 1 | 0.24 |
| PHTF2 | Putative Homeodomain Transcription Factor 2 | 0.24 |
| U2AF1L4 | U2 Small Nuclear RNA Auxiliary Factor 1 Like 4 | 0.24 |
| TSPAN14 | Tetraspanin 14 | 0.24 |
| KDM8 | Lysine Demethylase 8 | 0.24 |
| SUSD1 | Sushi Domain Containing 1 | 0.24 |
| TMEM30B | Transmembrane Protein 30B | 0.24 |
| TMED9 | Transmembrane P24 Trafficking Protein 9 | 0.24 |
| TRAPPC5 | Trafficking Protein Particle Complex 5 | 0.24 |
| DND1 | DND MicroRNA-Mediated Repression Inhibitor 1 | 0.24 |
| CNTNAP5 | Contactin Associated Protein Family Member 5 | 0.24 |
| SAMD4B | Sterile Alpha Motif Domain Containing 4B | 0.24 |
| KRT34 | Keratin 34 | 0.24 |
| ZPR1 | ZPR1 Zinc Finger | 0.24 |
| ZBTB46 | Zinc Finger And BTB Domain Containing 46 | 0.24 |
| ZNF558 | Zinc Finger Protein 558 | 0.24 |
| AKIRIN1 | Akirin 1 | 0.24 |
| AKIRIN2 | Akirin 2 | 0.24 |
| ACBD7 | Acyl-CoA Binding Domain Containing 7 | 0.24 |
| CASP8AP2 | Caspase 8 Associated Protein 2 | 0.24 |
| FBXL12 | F-Box And Leucine Rich Repeat Protein 12 | 0.24 |
| FAM53B | Family With Sequence Similarity 53 Member B | 0.24 |
| APOO | Apolipoprotein O | 0.24 |
| ADGRB3 | Adhesion G Protein-Coupled Receptor B3 | 0.24 |
| FOXD2 | Forkhead Box D2 | 0.24 |
| FSCB | Fibrous Sheath CABYR Binding Protein | 0.24 |
| ARL4C | ADP Ribosylation Factor Like GTPase 4C | 0.24 |
| RIMS4 | Regulating Synaptic Membrane Exocytosis 4 | 0.24 |
| COMMD4 | COMM Domain Containing 4 | 0.24 |
| MTERF1 | Mitochondrial Transcription Termination Factor 1 | 0.24 |
| MXD3 | MAX Dimerization Protein 3 | 0.24 |
| EEPD1 | Endonuclease/Exonuclease/Phosphatase Family Domain Containing 1 | 0.24 |
| NBEAL1 | Neurobeachin Like 1 | 0.24 |
| CCDC33 | Coiled-Coil Domain Containing 33 | 0.24 |
| GPR141 | G Protein-Coupled Receptor 141 | 0.24 |
| SPAG17 | Sperm Associated Antigen 17 | 0.24 |
| PRAM1 | PML-RARA Regulated Adaptor Molecule 1 | 0.24 |
| OR2F2 | Olfactory Receptor Family 2 Subfamily F Member 2 | 0.24 |
| PKHD1L1 | PKHD1 Like 1 | 0.24 |
| OLFML2B | Olfactomedin Like 2B | 0.24 |
| SLC25A2 | Solute Carrier Family 25 Member 2 | 0.24 |
| IQCA1 | IQ Motif Containing With AAA Domain 1 | 0.24 |
| SRRM4 | Serine/Arginine Repetitive Matrix 4 | 0.24 |
| TIPARP | TCDD Inducible Poly(ADP-Ribose) Polymerase | 0.24 |
| LGSN | Lengsin, Lens Protein With Glutamine Synthetase Domain | 0.24 |
| RASEF | RAS And EF-Hand Domain Containing | 0.24 |
| TENM4 | Teneurin Transmembrane Protein 4 | 0.24 |
| STON2 | Stonin 2 | 0.24 |
| KBTBD11 | Kelch Repeat And BTB Domain Containing 11 | 0.24 |
| SPP2 | Secreted Phosphoprotein 2 | 0.24 |
| TGFBRAP1 | Transforming Growth Factor Beta Receptor Associated Protein 1 | 0.24 |
| ZNF804A | Zinc Finger Protein 804A | 0.24 |
| VWA5A | Von Willebrand Factor A Domain Containing 5A | 0.24 |
| METTL15 | Methyltransferase Like 15 | 0.24 |
| FNDC1 | Fibronectin Type III Domain Containing 1 | 0.24 |
| GLIS1 | GLIS Family Zinc Finger 1 | 0.24 |
| B3GALT1 | Beta-1,3-Galactosyltransferase 1 | 0.24 |
| FHDC1 | FH2 Domain Containing 1 | 0.24 |
| LRIF1 | Ligand Dependent Nuclear Receptor Interacting Factor 1 | 0.24 |
| EVA1A | Eva-1 Homolog A, Regulator Of Programmed Cell Death | 0.24 |
| EVI5L | Ecotropic Viral Integration Site 5 Like | 0.24 |
| MAGEB4 | MAGE Family Member B4 | 0.24 |
| ERBIN | Erbb2 Interacting Protein | 0.24 |
| ALKBH5 | AlkB Homolog 5, RNA Demethylase | 0.24 |
| AGMO | Alkylglycerol Monooxygenase | 0.24 |
| BCL2L15 | BCL2 Like 15 | 0.24 |
| BIVM | Basic, Immunoglobulin-Like Variable Motif Containing | 0.24 |
| ACTL8 | Actin Like 8 | 0.24 |
| C2orf42 | Chromosome 2 Open Reading Frame 42 | 0.24 |
| FICD | FIC Domain Protein Adenylyltransferase | 0.24 |
| FERD3L | Fer3 Like BHLH Transcription Factor | 0.24 |
| CYLC2 | Cylicin 2 | 0.24 |
| RMDN3 | Regulator Of Microtubule Dynamics 3 | 0.24 |
| ESF1 | ESF1 Nucleolar Pre-RRNA Processing Protein Homolog | 0.24 |
| DMRTB1 | DMRT Like Family B With Proline Rich C-Terminal 1 | 0.24 |
| MVB12B | Multivesicular Body Subunit 12B | 0.24 |
| GPSM3 | G Protein Signaling Modulator 3 | 0.24 |
| DAOA | D-Amino Acid Oxidase Activator | 0.24 |
| DAPL1 | Death Associated Protein Like 1 | 0.24 |
| RNFT2 | Ring Finger Protein, Transmembrane 2 | 0.24 |
| SPNS3 | Sphingolipid Transporter 3 (Putative) | 0.24 |
| SPATA8 | Spermatogenesis Associated 8 | 0.24 |
| SPC24 | SPC24 Component Of NDC80 Kinetochore Complex | 0.24 |
| PRTFDC1 | Phosphoribosyl Transferase Domain Containing 1 | 0.24 |
| SHISA6 | Shisa Family Member 6 | 0.24 |
| SNRNP48 | Small Nuclear Ribonucleoprotein U11/U12 Subunit 48 | 0.24 |
| SNX29 | Sorting Nexin 29 | 0.24 |
| RSBN1 | Round Spermatid Basic Protein 1 | 0.24 |
| RSBN1L | Round Spermatid Basic Protein 1 Like | 0.24 |
| PLEKHG1 | Pleckstrin Homology And RhoGEF Domain Containing G1 | 0.24 |
| TPGS2 | Tubulin Polyglutamylase Complex Subunit 2 | 0.24 |
| TPRN | Taperin | 0.24 |
| UBXN2A | UBX Domain Protein 2A | 0.24 |
| UBTD1 | Ubiquitin Domain Containing 1 | 0.24 |
| TRUB2 | TruB Pseudouridine Synthase Family Member 2 | 0.24 |
| UMODL1 | Uromodulin Like 1 | 0.24 |
| KLHL31 | Kelch Like Family Member 31 | 0.24 |
| TEX12 | Testis Expressed 12 | 0.24 |
| PYROXD2 | Pyridine Nucleotide-Disulphide Oxidoreductase Domain 2 | 0.24 |
| TMC2 | Transmembrane Channel Like 2 | 0.24 |
| TMEM45B | Transmembrane Protein 45B | 0.24 |
| IFNA17 | Interferon Alpha 17 | 0.24 |
| TXNDC11 | Thioredoxin Domain Containing 11 | 0.24 |
| PILRB | Paired Immunoglobin Like Type 2 Receptor Beta | 0.24 |
| KRT33A | Keratin 33A | 0.24 |
| ZG16B | Zymogen Granule Protein 16B | 0.24 |
| ZC3H4 | Zinc Finger CCCH-Type Containing 4 | 0.24 |
| ZNF470 | Zinc Finger Protein 470 | 0.24 |
| ZNF446 | Zinc Finger Protein 446 | 0.24 |
| ZNF649 | Zinc Finger Protein 649 | 0.24 |
| VGLL3 | Vestigial Like Family Member 3 | 0.24 |
| CARD18 | Caspase Recruitment Domain Family Member 18 | 0.24 |
| CCDC13 | Coiled-Coil Domain Containing 13 | 0.24 |
| ASB18 | Ankyrin Repeat And SOCS Box Containing 18 | 0.24 |
| ATXN7L1 | Ataxin 7 Like 1 | 0.24 |
| FAM110A | Family With Sequence Similarity 110 Member A | 0.24 |
| LYSMD3 | LysM Domain Containing 3 | 0.24 |
| ATAD2B | ATPase Family AAA Domain Containing 2B | 0.24 |
| ANKAR | Ankyrin And Armadillo Repeat Containing | 0.24 |
| BTBD8 | BTB Domain Containing 8 | 0.24 |
| C8orf34 | Chromosome 8 Open Reading Frame 34 | 0.24 |
| ANKRD16 | Ankyrin Repeat Domain 16 | 0.24 |
[truncated: 123,674 more chars]
