# Supplementary material for: circRNA circ_102049 Implicates in Pancreatic Ductal Adenocarcinoma Progression through Activating CD80 by Targeting miR-455-3p
Source: Mediators Inflamm. 2021 Jan 7;2021:8819990. doi: 10.1155/2021/8819990 (PMC7811564; doi:10.1155/2021/8819990)
Supplement: Supplementary 9 — Table S7 The differentially methylated genes in the GSE14245 dataset. [file 8819990.f9.pdf]

Supplementary Table S7 The differentially methylated genes in the GSE14245 dataset

ID	adj.P.Val	P.Value	t	B	logFC	RANGE_START	RANGE_END	RANGE_GB
cg22674699	4.79E-52	1.19E-57	2.31E+01	120.7358545	3.92E-01	176987918	176988041	NC_000002.11
cg16732616	1.60E-49	1.20E-54	2.20E+01	113.8727168	4.04E-01	50886782	50886905	NC_000001.10
cg24221648	2.96E-48	3.68E-53	2.14E+01	110.4694261	3.66E-01	79170283	79170406	NC_000013.10
cg14473102	1.11E-47	1.66E-52	2.12E+01	108.9708643	3.58E-01	176994448	176994571	NC_000002.11
cg01059818	1.69E-47	2.93E-52	2.11E+01	108.4057534	2.48E-01	26370690	26370813	NC_000017.10
cg06304097	1.01E-46	2.01E-51	2.08E+01	106.4944987	4.06E-01	133109867	133109990	NC_000010.10
cg14451382	7.18E-45	2.32E-49	2.01E+01	101.7751627	2.96E-01	1876397	1876520	NC_000005.9
cg18375860	1.71E-41	1.65E-45	1.87E+01	92.9595531	3.20E-01	237205409	237205532	NC_000001.10
cg27423760	1.71E-41	1.69E-45	1.87E+01	92.9310716	3.38E-01	51186463	51186586	NC_000016.9
cg02835462	1.71E-41	1.70E-45	1.87E+01	92.9250063	2.31E-01	46391567	46391690	NC_000001.10
cg12526197	2.27E-41	2.37E-45	1.87E+01	92.5982992	2.16E-01	230416938	230417061	NC_000001.10
cg23992403	3.16E-41	3.62E-45	1.86E+01	92.1767336	2.90E-01	226012347	226012470	NC_000001.10
cg03306374	3.45E-41	4.12E-45	1.86E+01	92.047072	4.69E-01	23847325	23847448	NC_000016.9
cg03156893	4.08E-41	5.07E-45	1.86E+01	91.841312	3.43E-01	23847675	23847798	NC_000016.9
cg05732750	9.52E-41	1.23E-44	1.84E+01	90.9598158	3.05E-01	6704045	6704168	NC_000007.13
cg18074820	1.21E-40	1.63E-44	1.84E+01	90.6822949	2.05E-01	99203739	99203862	NC_000015.9
cg11030420	3.94E-40	5.88E-44	1.82E+01	89.4040994	1.83E-01	136758308	136758431	NC_000006.11
cg17495912	7.00E-40	1.06E-43	1.81E+01	88.816848	3.45E-01	37006340	37006463	NC_000013.10
cg14759565	1.77E-39	2.86E-43	1.80E+01	87.8304741	2.43E-01	65360123	65360246	NC_000011.9
cg13438893	7.21E-39	1.25E-42	1.78E+01	86.3607392	3.39E-01	79170303	79170426	NC_000013.10
cg18952796	8.39E-39	1.50E-42	1.77E+01	86.181958	3.67E-01	98246633	98246756	NC_000007.13
cg13755795	1.29E-38	2.40E-42	1.77E+01	85.716485	3.31E-01	51185772	51185895	NC_000016.9
cg11552694	1.81E-38	3.53E-42	1.76E+01	85.3300147	3.98E-01	2752149	2752272	NC_000005.9

cg11595545	2.64E-38	5.48E-42	1.75E+01	84.8946456	4.22E-01	111217497	111217620	NC_000001.10
cg12918457	3.17E-38	6.78E-42	1.75E+01	84.6824463	3.53E-01	115630748	115630871	NC_000011.9
cg03758150	5.40E-38	1.23E-41	1.74E+01	84.086968	3.67E-01	30866192	30866315	NC_000019.9
cg01464835	9.87E-38	2.45E-41	1.73E+01	83.405803	3.49E-01	30016147	30016270	NC_000019.9
cg17954142	1.11E-37	2.80E-41	1.73E+01	83.2698631	2.13E-01	230417096	230417219	NC_000001.10
cg16675507	1.21E-37	3.18E-41	1.73E+01	83.144288	3.15E-01	155108371	155108494	NC_000005.9
cg06647382	1.37E-37	3.69E-41	1.73E+01	82.9973561	1.69E-01	48175734	48175857	NC_000017.10
cg19384289	1.69E-37	4.65E-41	1.72E+01	82.7659913	3.20E-01	176994365	176994488	NC_000002.11
cg03109827	2.19E-37	6.08E-41	1.72E+01	82.4997155	3.40E-01	133110349	133110472	NC_000010.10
cg07011231	3.75E-37	1.06E-40	1.71E+01	81.9467385	1.65E-01	73329813	73329936	NC_000002.11
cg26504274	5.83E-37	1.71E-40	1.70E+01	81.4713915	1.83E-01	2485409	2485532	NC_000011.9
cg03337430	6.76E-37	2.05E-40	1.70E+01	81.2932649	2.27E-01	226012507	226012630	NC_000001.10
cg10158541	6.76E-37	2.05E-40	1.70E+01	81.2920935	3.03E-01	37006265	37006388	NC_000013.10
cg02609724	8.00E-37	2.50E-40	1.70E+01	81.0921083	2.06E-01	139506447	139506570	NC_000005.9
cg06177254	1.23E-36	3.97E-40	1.69E+01	80.6350671	2.33E-01	77303975	77304098	NC_000011.9
cg09161419	1.46E-36	4.76E-40	1.69E+01	80.4527223	2.09E-01	119593593	119593716	NC_000011.9
cg25096740	1.47E-36	4.81E-40	1.69E+01	80.4434868	2.14E-01	20140828	20140951	NC_000001.10
cg20885063	1.75E-36	5.90E-40	1.69E+01	80.2403957	2.04E-01	17939419	17939542	NC_000017.10
cg15617814	1.80E-36	6.12E-40	1.69E+01	80.2038668	3.93E-01	131780492	131780615	NC_000011.9
cg23331421	2.42E-36	8.59E-40	1.68E+01	79.8664908	3.15E-01	30866202	30866325	NC_000019.9
cg11041817	2.51E-36	8.99E-40	1.68E+01	79.8205216	2.05E-01	46685327	46685450	NC_000017.10
cg03493668	3.52E-36	1.32E-39	1.67E+01	79.4392749	1.64E-01	77776607	77776730	NC_000011.9
cg11546137	4.26E-36	1.63E-39	1.67E+01	79.2303307	3.01E-01	176972563	176972686	NC_000002.11
cg04215359	5.07E-36	1.98E-39	1.67E+01	79.0378307	1.96E-01	153381623	153381746	NC_000002.11
cg17468616	7.15E-36	2.86E-39	1.66E+01	78.669991	2.58E-01	226013212	226013335	NC_000001.10

cg06724588	7.91E-36	3.22E-39	1.66E+01	78.5514885	2.94E-01	51185459	51185582	NC_000016.9
cg08204280	8.26E-36	3.40E-39	1.66E+01	78.4992413	3.09E-01	2751839	2751962	NC_000005.9
cg24416513	8.26E-36	3.41E-39	1.66E+01	78.4959408	4.10E-01	176994764	176994887	NC_000002.11
cg24472231	1.95E-35	8.95E-39	1.65E+01	77.5352147	3.10E-01	105471979	105472102	NC_000002.11
cg03928367	2.27E-35	1.06E-38	1.64E+01	77.367466	2.52E-01	34186995	34187118	NC_000020.10
cg11950982	2.45E-35	1.15E-38	1.64E+01	77.2851732	1.69E-01	46269126	46269249	NC_000021.8
cg27019093	2.49E-35	1.18E-38	1.64E+01	77.2588372	2.15E-01	4235082	4235205	NC_000017.10
cg14666369	2.95E-35	1.42E-38	1.64E+01	77.0761708	3.11E-01	121164371	121164494	NC_000012.11
cg27393010	3.31E-35	1.61E-38	1.64E+01	76.950595	3.38E-01	121940216	121940339	NC_000007.13
cg18716164	3.53E-35	1.74E-38	1.64E+01	76.8753153	3.40E-01	30019647	30019770	NC_000019.9
cg02371631	3.61E-35	1.78E-38	1.64E+01	76.8499172	2.48E-01	202748718	202748841	NC_000002.11
cg18466162	3.94E-35	1.97E-38	1.64E+01	76.7519356	2.57E-01	114792873	114792996	NC_000005.9
cg20302133	5.18E-35	2.66E-38	1.63E+01	76.4507597	3.70E-01	111217194	111217317	NC_000001.10
cg23619365	5.86E-35	3.07E-38	1.63E+01	76.3085298	3.09E-01	112712009	112712132	NC_000013.10
cg23130131	6.75E-35	3.61E-38	1.63E+01	76.1491143	2.67E-01	111544299	111544422	NC_000004.11
cg12040830	8.98E-35	4.95E-38	1.62E+01	75.8330271	4.17E-01	112833773	112833896	NC_000011.9
cg19674091	9.92E-35	5.50E-38	1.62E+01	75.7298024	3.08E-01	90643766	90643889	NC_000015.9
cg04711162	1.07E-34	6.05E-38	1.62E+01	75.6338337	2.92E-01	30017511	30017634	NC_000019.9
cg16046493	1.08E-34	6.10E-38	1.62E+01	75.6263696	2.48E-01	58551954	58552077	NC_000003.11
cg24079790	1.24E-34	7.08E-38	1.62E+01	75.4783847	2.39E-01	107993467	107993590	NC_000011.9
cg22153181	1.43E-34	8.34E-38	1.61E+01	75.314793	2.49E-01	172734431	172734554	NC_000004.11
cg19196335	1.53E-34	9.08E-38	1.61E+01	75.2305879	2.28E-01	27184094	27184217	NC_000007.13
cg07082358	1.99E-34	1.22E-37	1.61E+01	74.9347162	2.21E-01	102981127	102981250	NC_000007.13
cg07113642	2.02E-34	1.25E-37	1.61E+01	74.914747	3.92E-01	2781262	2781385	NC_000020.10
cg24613080	2.05E-34	1.27E-37	1.61E+01	74.9005293	2.87E-01	32484035	32484158	NC_000017.10

cg20927661	2.46E-34	1.54E-37	1.61E+01	74.7033843	3.77E-01	5837002	5837125	NC_000002.11
cg10837685	2.54E-34	1.60E-37	1.61E+01	74.6695729	2.13E-01	22968612	22968735	NC_000015.9
cg17509561	2.57E-34	1.62E-37	1.60E+01	74.6528707	2.00E-01	19419126	19419249	NC_000008.10
cg23731805	2.64E-34	1.69E-37	1.60E+01	74.6153139	1.68E-01	56479243	56479366	NC_000012.11
cg25683325	2.87E-34	1.86E-37	1.60E+01	74.5154017	3.94E-01	158151058	158151181	NC_000001.10
cg09709600	2.94E-34	1.91E-37	1.60E+01	74.4888403	1.99E-01	65359521	65359644	NC_000011.9
cg06711560	3.79E-34	2.59E-37	1.60E+01	74.1875895	2.34E-01	139746365	139746488	NC_000009.11
cg08475953	3.92E-34	2.69E-37	1.60E+01	74.1504664	3.41E-01	178004091	178004214	NC_000005.9
cg14952488	3.99E-34	2.74E-37	1.60E+01	74.1306784	2.08E-01	53821365	53821488	NC_000005.9
cg08568670	5.04E-34	3.53E-37	1.59E+01	73.8789361	1.80E-01	48593395	48593518	NC_000003.11
cg15442800	5.18E-34	3.65E-37	1.59E+01	73.845367	2.42E-01	56755990	56756113	NC_000020.10
cg03502002	5.78E-34	4.10E-37	1.59E+01	73.7315239	3.76E-01	74962133	74962256	NC_000018.9
cg26521404	7.23E-34	5.33E-37	1.59E+01	73.4695597	2.95E-01	27204981	27205104	NC_000007.13
cg18885162	1.04E-33	8.02E-37	1.58E+01	73.0634917	2.30E-01	41529269	41529392	NC_000006.11
cg09874822	1.09E-33	8.46E-37	1.58E+01	73.0103831	3.58E-01	1203997	1204120	NC_000016.9
cg16997642	1.10E-33	8.63E-37	1.58E+01	72.9904306	2.48E-01	27184159	27184282	NC_000007.13
cg10936057	1.21E-33	9.57E-37	1.58E+01	72.8882737	2.95E-01	708425	708548	NC_000010.10
cg09880291	1.24E-33	9.80E-37	1.58E+01	72.8644828	2.63E-01	27184109	27184232	NC_000007.13
cg27360326	1.26E-33	1.00E-36	1.58E+01	72.8411454	3.30E-01	53422691	53422814	NC_000013.10
cg17098850	1.28E-33	1.02E-36	1.58E+01	72.8255641	2.08E-01	22968689	22968812	NC_000015.9
cg16784745	1.45E-33	1.17E-36	1.58E+01	72.6904412	2.40E-01	54731086	54731209	NC_000012.11
cg15808943	1.60E-33	1.29E-36	1.57E+01	72.5885812	3.50E-01	176994665	176994788	NC_000002.11
cg17369513	1.63E-33	1.33E-36	1.57E+01	72.5630253	1.61E-01	98121165	98121288	NC_000005.9
cg01447112	2.02E-33	1.66E-36	1.57E+01	72.3405871	3.43E-01	6703803	6703926	NC_000007.13
cg09671258	2.14E-33	1.76E-36	1.57E+01	72.280455	3.91E-01	180202530	180202653	NC_000001.10

cg02456226	2.51E-33	2.10E-36	1.57E+01	72.1066813	3.35E-01	18429703	18429826	NC_000010.10
cg08823554	2.69E-33	2.25E-36	1.57E+01	72.0364981	3.12E-01	46269191	46269314	NC_000021.8
cg04330449	2.98E-33	2.52E-36	1.57E+01	71.9244872	3.02E-01	134871166	134871289	NC_000005.9
cg14754787	3.01E-33	2.56E-36	1.57E+01	71.9088984	2.89E-01	35299600	35299723	NC_000017.10
cg10327976	3.03E-33	2.58E-36	1.56E+01	71.8995327	1.92E-01	42843303	42843426	NC_000015.9
cg02391433	3.52E-33	3.03E-36	1.56E+01	71.7399883	1.07E-01	37574923	37575046	NC_000009.11
cg12985235	4.24E-33	3.72E-36	1.56E+01	71.5374603	2.61E-01	4345965	4346088	NC_000019.9
cg27200446	4.24E-33	3.72E-36	1.56E+01	71.536893	3.70E-01	41606439	41606562	NC_000006.11
cg25611369	4.75E-33	4.19E-36	1.56E+01	71.4186962	2.92E-01	69460638	69460761	NC_000011.9
cg06991565	5.36E-33	4.81E-36	1.56E+01	71.2817707	1.55E-01	31733799	31733922	NC_000006.11
cg25495534	5.81E-33	5.26E-36	1.55E+01	71.1924572	2.19E-01	65431222	65431345	NC_000010.10
cg05554151	6.83E-33	6.31E-36	1.55E+01	71.0116779	1.79E-01	56700796	56700919	NC_000016.9
cg06068085	7.91E-33	7.45E-36	1.55E+01	70.8469683	2.28E-01	127871927	127872050	NC_000005.9
cg06295294	8.21E-33	7.79E-36	1.55E+01	70.801718	1.70E-01	159956323	159956446	NC_000004.11
cg08526074	8.66E-33	8.26E-36	1.55E+01	70.7439125	3.58E-01	51184562	51184685	NC_000016.9
cg08235864	8.82E-33	8.44E-36	1.55E+01	70.7230078	3.08E-01	2751822	2751945	NC_000005.9
cg26013553	1.05E-32	1.01E-35	1.55E+01	70.5427589	3.21E-01	111217406	111217529	NC_000001.10
cg13267264	1.39E-32	1.36E-35	1.54E+01	70.2473646	3.72E-01	70983600	70983723	NC_000008.10
cg08533364	1.55E-32	1.55E-35	1.54E+01	70.1200986	1.38E-01	3147191	3147314	NC_000020.10
cg12281517	1.64E-32	1.65E-35	1.54E+01	70.0583382	1.94E-01	153576804	153576927	NC_000004.11
cg08315202	1.67E-32	1.69E-35	1.54E+01	70.0318981	3.31E-01	98246006	98246129	NC_000007.13
cg01789499	2.09E-32	2.16E-35	1.53E+01	69.7880294	2.21E-01	81109888	81110011	NC_000004.11
cg25331919	2.30E-32	2.39E-35	1.53E+01	69.6868411	2.24E-01	47092023	47092146	NC_000018.9
cg21436938	2.34E-32	2.46E-35	1.53E+01	69.6586432	1.79E-01	116061416	116061539	NC_000009.11
cg18961681	2.40E-32	2.54E-35	1.53E+01	69.6271005	2.91E-01	32467657	32467780	NC_000007.13

cg23291301	2.56E-32	2.72E-35	1.53E+01	69.5567489	2.90E-01	105472469	105472592	NC_000002.11
cg03585823	2.70E-32	2.89E-35	1.53E+01	69.4996004	2.30E-01	56693070	56693193	NC_000016.9
cg23642130	3.51E-32	3.81E-35	1.53E+01	69.2245893	2.98E-01	30866365	30866488	NC_000019.9
cg04222358	3.74E-32	4.09E-35	1.53E+01	69.1516749	2.60E-01	2140256	2140379	NC_000003.11
cg03287574	4.53E-32	5.06E-35	1.52E+01	68.9412257	2.83E-01	1886956	1887079	NC_000005.9
cg18348647	4.64E-32	5.22E-35	1.52E+01	68.910357	3.74E-01	37006107	37006230	NC_000013.10
cg15412087	4.64E-32	5.22E-35	1.52E+01	68.9099452	2.06E-01	120085145	120085268	NC_000011.9
cg06262497	5.24E-32	5.95E-35	1.52E+01	68.7798271	2.34E-01	3564676	3564799	NC_000001.10
cg14195103	5.38E-32	6.13E-35	1.52E+01	68.7508999	2.33E-01	73283583	73283706	NC_000017.10
cg25181651	6.52E-32	7.62E-35	1.52E+01	68.5345088	3.19E-01	1748132	1748255	NC_000002.11
cg03769116	8.46E-32	1.01E-34	1.51E+01	68.2522346	2.62E-01	34833674	34833797	NC_000019.9
cg26187962	9.14E-32	1.10E-34	1.51E+01	68.1656062	1.76E-01	226011953	226012076	NC_000001.10
cg21408624	9.46E-32	1.15E-34	1.51E+01	68.1230063	1.87E-01	50800055	50800178	NC_000007.13
cg03544320	1.14E-31	1.43E-34	1.51E+01	67.9097931	2.81E-01	5894691	5894814	NC_000004.11
cg14252236	1.16E-31	1.45E-34	1.51E+01	67.8911947	1.39E-01	143784002	143784125	NC_000002.11
cg09016242	1.16E-31	1.47E-34	1.51E+01	67.8802289	3.61E-01	51185110	51185233	NC_000016.9
cg08272731	1.22E-31	1.55E-34	1.51E+01	67.8294945	3.23E-01	75602167	75602290	NC_000001.10
cg25090514	1.29E-31	1.64E-34	1.51E+01	67.7688134	2.46E-01	2038743	2038866	NC_000005.9
cg27058257	1.29E-31	1.65E-34	1.51E+01	67.7667428	4.14E-01	30019529	30019652	NC_000019.9
cg00939495	1.37E-31	1.76E-34	1.50E+01	67.7010194	2.88E-01	9783398	9783521	NC_000004.11
cg02306798	1.44E-31	1.86E-34	1.50E+01	67.6473453	2.08E-01	14245929	14246052	NC_000005.9
cg24102242	1.49E-31	1.93E-34	1.50E+01	67.6104323	1.87E-01	3327319	3327442	NC_000006.11
cg08152546	1.62E-31	2.13E-34	1.50E+01	67.5114668	2.80E-01	112717207	112717330	NC_000013.10
cg02864757	1.64E-31	2.16E-34	1.50E+01	67.4989088	2.78E-01	51184583	51184706	NC_000016.9
cg23632875	1.74E-31	2.31E-34	1.50E+01	67.4303254	3.92E-01	133110244	133110367	NC_000010.10

cg26132774	1.80E-31	2.40E-34	1.50E+01	67.3934756	3.52E-01	79170146	79170269	NC_000013.10
cg23906738	1.83E-31	2.44E-34	1.50E+01	67.3770034	3.47E-01	36987301	36987424	NC_000014.8
cg20146541	1.89E-31	2.53E-34	1.50E+01	67.3415624	3.17E-01	248020697	248020820	NC_000001.10
cg23586440	1.90E-31	2.55E-34	1.50E+01	67.3334588	2.32E-01	31067009	31067132	NC_000020.10
cg04347874	2.03E-31	2.74E-34	1.50E+01	67.2613401	3.81E-01	36987408	36987531	NC_000014.8
cg00384539	2.07E-31	2.79E-34	1.50E+01	67.2416515	2.87E-01	70983567	70983690	NC_000008.10
cg09907509	2.36E-31	3.25E-34	1.50E+01	67.0917372	2.44E-01	37248244	37248367	NC_000013.10
cg24389585	2.38E-31	3.28E-34	1.50E+01	67.0816679	2.06E-01	27184125	27184248	NC_000007.13
cg12630983	2.48E-31	3.43E-34	1.50E+01	67.0376501	1.47E-01	127872200	127872323	NC_000005.9
cg22784954	2.64E-31	3.67E-34	1.49E+01	66.969209	3.15E-01	5140646	5140769	NC_000005.9
cg05977021	2.88E-31	4.04E-34	1.49E+01	66.8736918	1.95E-01	92242370	92242493	NC_000007.13
cg06528737	2.90E-31	4.07E-34	1.49E+01	66.866706	1.90E-01	11574303	11574426	NC_000010.10
cg15811515	2.91E-31	4.10E-34	1.49E+01	66.8606484	3.40E-01	31580989	31581112	NC_000016.9
cg25397945	2.92E-31	4.11E-34	1.49E+01	66.8566393	3.24E-01	37096148	37096271	NC_000019.9
cg06429887	3.23E-31	4.60E-34	1.49E+01	66.7447556	2.80E-01	24600500	24600623	NC_000014.8
cg14001664	3.42E-31	4.89E-34	1.49E+01	66.6843338	2.45E-01	132088801	132088924	NC_000002.11
cg18897632	3.67E-31	5.26E-34	1.49E+01	66.6121707	3.87E-01	5836231	5836354	NC_000002.11
cg25955565	3.69E-31	5.30E-34	1.49E+01	66.6044742	1.43E-01	71863756	71863879	NC_000014.8
cg06813419	3.85E-31	5.58E-34	1.49E+01	66.552857	2.08E-01	211502171	211502294	NC_000001.10
cg21253742	3.91E-31	5.68E-34	1.49E+01	66.5360427	2.27E-01	85305079	85305202	NC_000015.9
cg21082141	3.91E-31	5.69E-34	1.49E+01	66.5343329	1.80E-01	81111985	81112108	NC_000004.11
cg17241776	4.47E-31	6.58E-34	1.49E+01	66.3893787	2.83E-01	51417469	51417592	NC_000013.10
cg15653173	4.79E-31	7.08E-34	1.48E+01	66.3170108	2.90E-01	112723034	112723157	NC_000013.10
cg11679455	4.98E-31	7.39E-34	1.48E+01	66.2741736	2.38E-01	8100761	8100884	NC_000010.10
cg14954154	5.34E-31	8.01E-34	1.48E+01	66.194612	1.34E-01	90853443	90853566	NC_000014.8

cg06367286	5.60E-31	8.44E-34	1.48E+01	66.1425419	2.30E-01	2887873	2887996	NC_000007.13
cg12338417	5.72E-31	8.65E-34	1.48E+01	66.1174012	2.75E-01	32860178	32860301	NC_000003.11
cg13580196	6.74E-31	1.03E-33	1.48E+01	65.9405085	2.23E-01	85529414	85529537	NC_000015.9
cg09835225	7.44E-31	1.15E-33	1.48E+01	65.8342658	3.31E-01	18429643	18429766	NC_000010.10
cg03078269	8.39E-31	1.31E-33	1.48E+01	65.7084596	3.38E-01	54354974	54355097	NC_000012.11
cg01392544	9.52E-31	1.49E-33	1.47E+01	65.5768328	3.69E-01	18429760	18429883	NC_000010.10
cg02674925	9.70E-31	1.52E-33	1.47E+01	65.5546773	8.91E-02	233474079	233474202	NC_000001.10
cg02478448	9.96E-31	1.57E-33	1.47E+01	65.524073	3.59E-01	37006063	37006186	NC_000013.10
cg16412082	1.03E-30	1.64E-33	1.47E+01	65.48384	2.03E-01	114610635	114610758	NC_000010.10
cg10617909	1.05E-30	1.67E-33	1.47E+01	65.4648594	3.79E-01	180527697	180527820	NC_000005.9
cg01805540	1.20E-30	1.91E-33	1.47E+01	65.3288548	3.60E-01	18429749	18429872	NC_000010.10
cg13408027	1.20E-30	1.93E-33	1.47E+01	65.3218209	1.95E-01	116133216	116133339	NC_000009.11
cg27561818	1.22E-30	1.95E-33	1.47E+01	65.3066272	2.95E-01	57832760	57832883	NC_000016.9
cg24144083	1.30E-30	2.11E-33	1.47E+01	65.2304045	3.18E-01	36805814	36805937	NC_000003.11
cg26921611	1.32E-30	2.15E-33	1.47E+01	65.211497	2.05E-01	37917868	37917991	NC_000008.10
cg06923233	1.44E-30	2.36E-33	1.47E+01	65.1183356	1.25E-01	2899061	2899184	NC_000016.9
cg02012703	1.44E-30	2.37E-33	1.47E+01	65.117239	3.08E-01	30016478	30016601	NC_000019.9
cg06521544	1.62E-30	2.68E-33	1.47E+01	64.991875	1.02E-01	57114657	57114780	NC_000003.11
cg25100011	1.65E-30	2.74E-33	1.47E+01	64.9701333	1.01E-01	33146725	33146848	NC_000001.10
cg23121993	2.07E-30	3.48E-33	1.46E+01	64.7335513	3.75E-01	1883954	1884077	NC_000005.9
cg11601252	2.12E-30	3.56E-33	1.46E+01	64.7100983	3.38E-01	68122139	68122262	NC_000015.9
cg20539752	2.13E-30	3.58E-33	1.46E+01	64.7035679	2.57E-01	176994305	176994428	NC_000002.11
cg01423964	2.20E-30	3.72E-33	1.46E+01	64.6671156	3.47E-01	111217575	111217698	NC_000001.10
cg14217074	2.41E-30	4.10E-33	1.46E+01	64.5689623	1.56E-01	187528979	187529102	NC_000003.11
cg08795964	2.42E-30	4.13E-33	1.46E+01	64.5628236	2.66E-01	72468987	72469110	NC_000008.10

cg25720804	2.45E-30	4.19E-33	1.46E+01	64.5484508	2.52E-01	170736389	170736512	NC_000005.9
cg08860498	2.46E-30	4.20E-33	1.46E+01	64.5449256	1.10E-01	40838861	40838984	NC_000017.10
cg19339146	2.62E-30	4.52E-33	1.46E+01	64.4737179	2.23E-01	62397911	62398034	NC_000004.11
cg18862481	2.67E-30	4.61E-33	1.46E+01	64.4532783	2.83E-01	129693370	129693493	NC_000003.11
cg21545862	2.84E-30	4.92E-33	1.46E+01	64.3885791	3.24E-01	174159294	174159417	NC_000005.9
cg08466792	2.89E-30	5.02E-33	1.46E+01	64.3677285	3.40E-01	3603227	3603350	NC_000005.9
cg05964918	2.89E-30	5.03E-33	1.46E+01	64.3669103	2.02E-01	96953953	96954076	NC_000010.10
cg04353438	2.95E-30	5.15E-33	1.46E+01	64.3429959	2.66E-01	5621182	5621305	NC_000019.9
cg18150339	3.06E-30	5.39E-33	1.46E+01	64.2981656	1.05E-01	10374150	10374273	NC_000005.9
cg10632765	3.12E-30	5.49E-33	1.46E+01	64.2800814	2.30E-01	6534916	6535039	NC_000019.9
cg06000994	3.16E-30	5.58E-33	1.46E+01	64.2633235	2.60E-01	30866194	30866317	NC_000019.9
cg18948722	3.36E-30	5.94E-33	1.45E+01	64.2018504	3.06E-01	37006116	37006239	NC_000013.10
cg15821562	3.38E-30	5.99E-33	1.45E+01	64.193805	2.39E-01	90083275	90083398	NC_000014.8
cg25185173	3.43E-30	6.08E-33	1.45E+01	64.1774455	3.33E-01	154712580	154712703	NC_000004.11
cg19715094	3.60E-30	6.43E-33	1.45E+01	64.1231931	2.55E-01	118402540	118402663	NC_000011.9
cg07421737	3.81E-30	6.84E-33	1.45E+01	64.0616765	1.98E-01	67811641	67811764	NC_000011.9
cg25191628	3.90E-30	7.02E-33	1.45E+01	64.0350012	2.84E-01	50886949	50887072	NC_000001.10
cg18515031	3.95E-30	7.12E-33	1.45E+01	64.0211509	2.39E-01	73988469	73988592	NC_000010.10
cg10266904	4.33E-30	7.84E-33	1.45E+01	63.9255501	2.07E-01	18570097	18570220	NC_000016.9
cg24633978	4.47E-30	8.11E-33	1.45E+01	63.8914734	2.40E-01	176972581	176972704	NC_000002.11
cg04950627	4.66E-30	8.50E-33	1.45E+01	63.845306	1.10E-01	39758157	39758280	NC_000002.11
cg07252731	4.72E-30	8.62E-33	1.45E+01	63.8308863	3.31E-01	88799999	88800122	NC_000015.9
cg00025795	4.74E-30	8.67E-33	1.45E+01	63.8255948	7.55E-02	77983156	77983279	NC_000017.10
cg05579037	4.92E-30	9.05E-33	1.45E+01	63.7825201	2.22E-01	27184853	27184976	NC_000007.13
cg12164282	5.16E-30	9.54E-33	1.45E+01	63.7296981	2.77E-01	1748112	1748235	NC_000002.11

cg13796823	5.17E-30	9.58E-33	1.45E+01	63.7255807	2.94E-01	130686592	130686715	NC_000006.11
cg00492784	5.34E-30	9.90E-33	1.45E+01	63.6930208	1.05E-01	52681818	52681941	NC_000012.11
cg27618240	5.46E-30	1.02E-32	1.45E+01	63.6674467	3.45E-01	20824770	20824893	NC_000007.13
cg16876236	5.78E-30	1.08E-32	1.45E+01	63.6063696	1.92E-01	17679640	17679763	NC_000020.10
cg10163955	5.98E-30	1.12E-32	1.45E+01	63.5696684	1.54E-01	8101402	8101525	NC_000010.10
cg09772661	6.06E-30	1.14E-32	1.45E+01	63.5514311	2.94E-01	7794952	7795075	NC_000019.9
cg02547394	6.13E-30	1.16E-32	1.44E+01	63.5371885	2.36E-01	112721789	112721912	NC_000013.10
cg23071286	6.43E-30	1.22E-32	1.44E+01	63.4844873	1.99E-01	91360201	91360324	NC_000015.9
cg19712603	6.49E-30	1.23E-32	1.44E+01	63.4744469	3.06E-01	53422777	53422900	NC_000013.10
cg17317266	6.55E-30	1.25E-32	1.44E+01	63.4636189	1.79E-01	11495261	11495384	NC_000019.9
cg21185289	6.61E-30	1.27E-32	1.44E+01	63.4473662	3.43E-01	74743437	74743560	NC_000002.11
cg08039116	6.66E-30	1.28E-32	1.44E+01	63.4374916	2.27E-01	43152254	43152377	NC_000007.13
cg12230883	6.73E-30	1.30E-32	1.44E+01	63.4253271	2.34E-01	184035527	184035650	NC_000003.11
cg03045635	6.73E-30	1.30E-32	1.44E+01	63.4239172	2.54E-01	9783198	9783321	NC_000004.11
cg19870512	6.77E-30	1.31E-32	1.44E+01	63.4162802	3.57E-01	4919081	4919204	NC_000012.11
cg25019378	6.77E-30	1.31E-32	1.44E+01	63.4146127	2.35E-01	158749367	158749490	NC_000006.11
cg02776080	6.97E-30	1.35E-32	1.44E+01	63.3834056	1.30E-01	29999615	29999738	NC_000006.11
cg26524347	7.00E-30	1.36E-32	1.44E+01	63.3764095	1.88E-01	121738449	121738572	NC_000012.11
cg25307168	7.85E-30	1.53E-32	1.44E+01	63.2580497	2.71E-01	37353035	37353158	NC_000020.10
cg19919217	7.90E-30	1.54E-32	1.44E+01	63.2512326	2.03E-01	38248105	38248228	NC_000017.10
cg19764418	8.00E-30	1.57E-32	1.44E+01	63.2369086	2.32E-01	237205098	237205221	NC_000001.10
cg07143083	8.01E-30	1.57E-32	1.44E+01	63.2345345	2.41E-01	70597921	70598044	NC_000007.13
cg02711479	8.04E-30	1.58E-32	1.44E+01	63.2294312	2.49E-01	36849024	36849147	NC_000001.10
cg10960709	8.43E-30	1.67E-32	1.44E+01	63.1720847	1.87E-01	140117543	140117666	NC_000007.13
cg06469345	8.67E-30	1.73E-32	1.44E+01	63.1402005	2.22E-01	9783246	9783369	NC_000004.11

cg27501612	9.72E-30	1.94E-32	1.44E+01	63.0232625	1.42E-01	56977363	56977486	NC_000016.9
cg13572771	1.01E-29	2.02E-32	1.44E+01	62.9841242	1.71E-01	68871428	68871551	NC_000014.8
cg12085698	1.01E-29	2.04E-32	1.44E+01	62.9754153	2.36E-01	13983784	13983907	NC_000011.9
cg05057720	1.29E-29	2.65E-32	1.43E+01	62.7136815	3.28E-01	38724675	38724798	NC_000014.8
cg01381846	1.44E-29	2.97E-32	1.43E+01	62.6000635	2.96E-01	27204785	27204908	NC_000007.13
cg07314409	1.47E-29	3.04E-32	1.43E+01	62.5769356	1.46E-01	193532456	193532579	NC_000003.11
cg16326743	1.49E-29	3.08E-32	1.43E+01	62.5632831	1.23E-01	176522599	176522722	NC_000005.9
cg18757468	1.51E-29	3.13E-32	1.43E+01	62.5497252	2.66E-01	65352168	65352291	NC_000011.9
cg16354688	1.55E-29	3.22E-32	1.43E+01	62.5193346	2.48E-01	16370265	16370388	NC_000001.10
cg16550651	1.60E-29	3.34E-32	1.43E+01	62.4829745	1.68E-01	40268381	40268504	NC_000017.10
cg05835105	1.75E-29	3.68E-32	1.43E+01	62.3886291	3.31E-01	111543401	111543524	NC_000004.11
cg16827475	1.77E-29	3.72E-32	1.43E+01	62.3759057	2.87E-01	131767526	131767649	NC_000010.10
cg09524907	1.77E-29	3.73E-32	1.43E+01	62.3728572	3.40E-01	56014406	56014529	NC_000008.10
cg02423579	1.96E-29	4.15E-32	1.43E+01	62.2671752	1.90E-01	2872169	2872292	NC_000007.13
cg13699355	1.97E-29	4.18E-32	1.43E+01	62.2602244	4.17E-01	468179	468302	NC_000002.11
cg18652900	2.02E-29	4.32E-32	1.43E+01	62.2288406	3.49E-01	15580445	15580568	NC_000019.9
cg13973641	2.04E-29	4.35E-32	1.43E+01	62.2213279	1.45E-01	109691025	109691148	NC_000009.11
cg00310215	2.49E-29	5.40E-32	1.42E+01	62.0067299	3.06E-01	51185346	51185469	NC_000016.9
cg07565505	2.51E-29	5.44E-32	1.42E+01	61.9993303	3.10E-01	1887300	1887423	NC_000005.9
cg06440348	2.52E-29	5.47E-32	1.42E+01	61.9934135	1.82E-01	81111527	81111650	NC_000004.11
cg11543229	2.53E-29	5.50E-32	1.42E+01	61.9875471	1.47E-01	74382996	74383119	NC_000017.10
cg00575672	2.56E-29	5.58E-32	1.42E+01	61.9741482	2.01E-01	100644171	100644294	NC_000004.11
cg23806621	2.57E-29	5.61E-32	1.42E+01	61.9684172	2.30E-01	61638501	61638624	NC_000020.10
cg22953960	2.59E-29	5.65E-32	1.42E+01	61.9616974	1.98E-01	22766081	22766204	NC_000010.10
cg14060496	2.71E-29	5.91E-32	1.42E+01	61.9157	3.05E-01	61638518	61638641	NC_000020.10

cg20377305	2.75E-29	6.02E-32	1.42E+01	61.8979461	3.11E-01	96650668	96650791	NC_000007.13
cg25188395	2.77E-29	6.08E-32	1.42E+01	61.8881858	3.05E-01	27204052	27204175	NC_000007.13
cg17839237	2.81E-29	6.20E-32	1.42E+01	61.8681753	3.87E-01	19157193	19157316	NC_000007.13
cg22491927	2.86E-29	6.32E-32	1.42E+01	61.8497145	3.39E-01	13617091	13617214	NC_000019.9
cg20185017	2.96E-29	6.59E-32	1.42E+01	61.8085853	3.35E-01	18429661	18429784	NC_000010.10
cg09118932	3.49E-29	7.85E-32	1.42E+01	61.6340447	3.17E-01	1748337	1748460	NC_000002.11
cg06097580	3.53E-29	7.96E-32	1.42E+01	61.6207043	2.14E-01	56456934	56457057	NC_000016.9
cg15396686	3.57E-29	8.06E-32	1.42E+01	61.6081965	2.96E-01	124897048	124897171	NC_000010.10
cg26520908	3.67E-29	8.32E-32	1.42E+01	61.5766969	1.81E-01	3191876	3191999	NC_000001.10
cg20750832	3.73E-29	8.46E-32	1.42E+01	61.5598127	2.47E-01	170742345	170742468	NC_000005.9
cg17078686	3.80E-29	8.64E-32	1.42E+01	61.5388371	2.76E-01	105471965	105472088	NC_000002.11
cg07821629	3.91E-29	8.93E-32	1.42E+01	61.5062905	3.21E-01	38670840	38670963	NC_000007.13
cg00582971	4.02E-29	9.23E-32	1.42E+01	61.4728703	3.26E-01	178422128	178422251	NC_000005.9
cg00378228	4.08E-29	9.37E-32	1.42E+01	61.4577095	1.26E-01	10122153	10122276	NC_000004.11
cg05446471	4.17E-29	9.60E-32	1.41E+01	61.4341457	2.68E-01	13522740	13522863	NC_000003.11
cg10548254	4.26E-29	9.80E-32	1.41E+01	61.4131119	1.81E-01	50656772	50656895	NC_000022.10
cg05895956	4.36E-29	1.01E-31	1.41E+01	61.3875083	1.67E-01	42216981	42217104	NC_000020.10
cg10853431	4.46E-29	1.04E-31	1.41E+01	61.3588196	1.89E-01	11574300	11574423	NC_000010.10
cg00747890	4.46E-29	1.04E-31	1.41E+01	61.3585057	2.25E-01	90854534	90854657	NC_000014.8
cg16924061	4.46E-29	1.04E-31	1.41E+01	61.3584684	2.12E-01	71955528	71955651	NC_000018.9
cg18206027	4.56E-29	1.06E-31	1.41E+01	61.3369962	2.90E-01	49813486	49813609	NC_000007.13
cg07996393	4.63E-29	1.08E-31	1.41E+01	61.3195759	1.96E-01	43792951	43793074	NC_000006.11
cg19875547	4.77E-29	1.11E-31	1.41E+01	61.2853955	3.45E-01	38670804	38670927	NC_000007.13
cg21759280	5.03E-29	1.18E-31	1.41E+01	61.2251218	2.15E-01	6534936	6535059	NC_000019.9
cg16308533	5.09E-29	1.20E-31	1.41E+01	61.2127237	1.96E-01	40838983	40839106	NC_000017.10

cg06598836	5.21E-29	1.23E-31	1.41E+01	61.1863661	3.28E-01	2140699	2140822	NC_000003.11
cg17868307	5.71E-29	1.36E-31	1.41E+01	61.0886748	2.60E-01	73597649	73597772	NC_000007.13
cg23937608	6.02E-29	1.44E-31	1.41E+01	61.0342711	1.04E-01	49317231	49317354	NC_000012.11
cg15358549	6.08E-29	1.45E-31	1.41E+01	61.0237756	2.34E-01	127872074	127872197	NC_000005.9
cg10303487	6.52E-29	1.56E-31	1.41E+01	60.9517288	2.86E-01	105479058	105479181	NC_000008.10
cg15975865	6.87E-29	1.65E-31	1.41E+01	60.8925077	3.68E-01	127413831	127413954	NC_000002.11
cg14715696	7.04E-29	1.70E-31	1.41E+01	60.8675919	2.98E-01	176945122	176945245	NC_000002.11
cg16627346	7.10E-29	1.71E-31	1.41E+01	60.8581894	2.44E-01	96057706	96057829	NC_000002.11
cg01699217	7.13E-29	1.72E-31	1.41E+01	60.8516487	3.11E-01	61638488	61638611	NC_000020.10
cg02950701	7.77E-29	1.89E-31	1.41E+01	60.7607312	1.94E-01	35656925	35657048	NC_000010.10
cg12349580	8.74E-29	2.14E-31	1.40E+01	60.6362596	1.50E-01	94681045	94681168	NC_000012.11
cg15689969	9.27E-29	2.29E-31	1.40E+01	60.5714922	2.06E-01	14374397	14374520	NC_000005.9
cg09412882	9.43E-29	2.33E-31	1.40E+01	60.5522953	2.28E-01	12271701	12271824	NC_000011.9
cg19852958	1.09E-28	2.71E-31	1.40E+01	60.4014216	3.39E-01	13545760	13545883	NC_000004.11
cg12435059	1.10E-28	2.72E-31	1.40E+01	60.3979399	1.30E-01	37291314	37291437	NC_000003.11
cg07460095	1.15E-28	2.87E-31	1.40E+01	60.3464056	8.03E-02	132373398	132373521	NC_000003.11
cg19854437	1.24E-28	3.11E-31	1.40E+01	60.2641243	2.15E-01	99891629	99891752	NC_000014.8
cg02300154	1.26E-28	3.19E-31	1.40E+01	60.2383605	3.53E-01	70597058	70597181	NC_000007.13
cg09374673	1.27E-28	3.21E-31	1.40E+01	60.2332231	1.51E-01	144986488	144986611	NC_000008.10
cg15556990	1.28E-28	3.24E-31	1.40E+01	60.2255885	1.65E-01	80544479	80544602	NC_000007.13
cg17214479	1.30E-28	3.30E-31	1.40E+01	60.206437	1.97E-01	32560449	32560572	NC_000013.10
cg21370856	1.35E-28	3.46E-31	1.40E+01	60.1600405	3.29E-01	23848003	23848126	NC_000016.9
cg09238180	1.39E-28	3.57E-31	1.40E+01	60.1274241	3.29E-01	176993632	176993755	NC_000002.11
cg02745847	1.40E-28	3.59E-31	1.40E+01	60.1216396	2.36E-01	47075880	47076003	NC_000017.10
cg19806642	1.42E-28	3.66E-31	1.40E+01	60.1045748	2.77E-01	178422140	178422263	NC_000005.9

cg01820007	1.45E-28	3.73E-31	1.40E+01	60.0847143	1.64E-01	52858131	52858254	NC_000005.9
cg03449456	1.46E-28	3.76E-31	1.40E+01	60.0768035	1.75E-01	3191219	3191342	NC_000001.10
cg26115633	1.49E-28	3.84E-31	1.39E+01	60.0543275	3.22E-01	129535893	129536016	NC_000010.10
cg07272353	1.54E-28	4.00E-31	1.39E+01	60.0161201	1.69E-01	241631239	241631362	NC_000002.11
cg03935183	1.58E-28	4.10E-31	1.39E+01	59.9892351	1.60E-01	8100563	8100686	NC_000010.10
cg21093166	1.81E-28	4.73E-31	1.39E+01	59.8474203	2.40E-01	2752145	2752268	NC_000005.9
cg15834072	1.82E-28	4.76E-31	1.39E+01	59.8412935	2.44E-01	155411603	155411726	NC_000004.11
cg09813525	1.86E-28	4.87E-31	1.39E+01	59.819059	3.48E-01	53420386	53420509	NC_000013.10
cg10603275	1.88E-28	4.96E-31	1.39E+01	59.8019806	3.30E-01	239665	239788	NC_000003.11
cg25502818	1.89E-28	4.97E-31	1.39E+01	59.7988098	1.69E-01	225410509	225410632	NC_000002.11
cg11940285	2.12E-28	5.64E-31	1.39E+01	59.6730754	2.98E-01	129693385	129693508	NC_000003.11
cg04525408	2.23E-28	5.95E-31	1.39E+01	59.6207265	1.90E-01	74383274	74383397	NC_000017.10
cg17316316	2.25E-28	6.00E-31	1.39E+01	59.6115508	2.69E-01	180525108	180525231	NC_000005.9
cg03422911	2.27E-28	6.05E-31	1.39E+01	59.6033627	2.53E-01	237205295	237205418	NC_000001.10
cg02250259	2.29E-28	6.12E-31	1.39E+01	59.5922166	8.78E-02	27273366	27273489	NC_000007.13
cg12180703	2.45E-28	6.59E-31	1.39E+01	59.5178956	2.41E-01	37353037	37353160	NC_000020.10
cg24547992	2.56E-28	6.92E-31	1.39E+01	59.4702053	1.87E-01	62557944	62558067	NC_000011.9
cg09823859	2.66E-28	7.21E-31	1.39E+01	59.4296862	2.06E-01	88328294	88328417	NC_000013.10
cg09491120	2.74E-28	7.45E-31	1.39E+01	59.3966584	2.29E-01	1394986	1395109	NC_000006.11
cg16494192	2.85E-28	7.77E-31	1.38E+01	59.3546715	2.55E-01	50818652	50818775	NC_000010.10
cg03044249	2.98E-28	8.12E-31	1.38E+01	59.3114535	3.37E-01	70597065	70597188	NC_000007.13
cg23668184	3.01E-28	8.21E-31	1.38E+01	59.2999975	2.55E-01	5836713	5836836	NC_000002.11
cg05141602	3.02E-28	8.26E-31	1.38E+01	59.294298	1.35E-01	234626084	234626207	NC_000002.11
cg13609939	3.08E-28	8.43E-31	1.38E+01	59.2738061	2.32E-01	126105160	126105283	NC_000010.10
cg27462475	3.15E-28	8.64E-31	1.38E+01	59.2486362	2.24E-01	99736304	99736427	NC_000013.10

cg14063129	3.15E-28	8.65E-31	1.38E+01	59.2483537	1.57E-01	105802387	105802510	NC_000014.8
cg01898698	3.24E-28	8.92E-31	1.38E+01	59.2178714	1.85E-01	105472759	105472882	NC_000002.11
cg14037652	3.31E-28	9.14E-31	1.38E+01	59.192935	2.03E-01	93390387	93390510	NC_000010.10
cg06135139	3.33E-28	9.21E-31	1.38E+01	59.1855952	2.77E-01	61637856	61637979	NC_000020.10
cg00969405	3.67E-28	1.02E-30	1.38E+01	59.0847933	2.60E-01	27184441	27184564	NC_000007.13
cg15512534	3.69E-28	1.03E-30	1.38E+01	59.0785726	2.01E-01	205271231	205271354	NC_000001.10
cg24657817	3.70E-28	1.03E-30	1.38E+01	59.0736275	3.25E-01	42153708	42153831	NC_000004.11
cg10556005	3.70E-28	1.03E-30	1.38E+01	59.0733334	2.54E-01	233467801	233467924	NC_000001.10
cg27028923	3.75E-28	1.05E-30	1.38E+01	59.05659	2.83E-01	1765764	1765887	NC_000019.9
cg19769715	3.83E-28	1.07E-30	1.38E+01	59.0350614	2.03E-01	149636363	149636486	NC_000005.9
cg06577205	3.85E-28	1.08E-30	1.38E+01	59.0306677	2.87E-01	15500714	15500837	NC_000005.9
cg06551540	3.86E-28	1.08E-30	1.38E+01	59.0266288	1.95E-01	45617878	45618001	NC_000012.11
cg06215569	4.22E-28	1.19E-30	1.38E+01	58.9308341	3.53E-01	110611465	110611588	NC_000001.10
cg04513777	4.27E-28	1.21E-30	1.38E+01	58.9176241	2.28E-01	15493409	15493532	NC_000016.9
cg08396193	4.38E-28	1.24E-30	1.38E+01	58.8875081	2.03E-01	27193709	27193832	NC_000007.13
cg18533201	4.61E-28	1.31E-30	1.38E+01	58.8350446	1.98E-01	97157453	97157576	NC_000008.10
cg23316253	4.71E-28	1.34E-30	1.38E+01	58.8103631	2.45E-01	8076277	8076400	NC_000010.10
cg22182451	5.03E-28	1.44E-30	1.38E+01	58.7408064	1.20E-01	72486158	72486281	NC_000015.9
cg11629889	5.34E-28	1.54E-30	1.38E+01	58.6772971	1.64E-01	40839022	40839145	NC_000017.10
cg11439596	5.69E-28	1.65E-30	1.37E+01	58.6063237	3.16E-01	1931376	1931499	NC_000005.9
cg24809973	5.69E-28	1.65E-30	1.37E+01	58.6051115	2.60E-01	72468820	72468943	NC_000008.10
cg05736768	5.74E-28	1.67E-30	1.37E+01	58.5968257	2.50E-01	105479248	105479371	NC_000008.10
cg19515186	5.85E-28	1.70E-30	1.37E+01	58.5743308	1.97E-01	73478549	73478672	NC_000002.11
cg22869726	6.00E-28	1.75E-30	1.37E+01	58.5478701	3.05E-01	34398265	34398388	NC_000021.8
cg20391432	6.71E-28	1.97E-30	1.37E+01	58.4313333	1.31E-01	36667594	36667717	NC_000002.11

cg14520423	6.78E-28	1.99E-30	1.37E+01	58.4184809	1.99E-01	131681980	131682103	NC_000009.11
cg18690385	7.09E-28	2.09E-30	1.37E+01	58.3714122	2.13E-01	47078966	47079089	NC_000001.10
cg07266046	7.28E-28	2.15E-30	1.37E+01	58.3429409	2.17E-01	202804175	202804298	NC_000002.11
cg18279094	7.33E-28	2.17E-30	1.37E+01	58.3348917	2.26E-01	63790044	63790167	NC_000001.10
cg27065717	7.58E-28	2.25E-30	1.37E+01	58.2975999	2.12E-01	85608934	85609057	NC_000016.9
cg25189564	8.37E-28	2.50E-30	1.37E+01	58.1923347	3.61E-01	158938051	158938174	NC_000007.13
cg05053327	8.71E-28	2.61E-30	1.37E+01	58.1501036	1.55E-01	40837383	40837506	NC_000017.10
cg13314145	9.08E-28	2.73E-30	1.37E+01	58.1045508	3.08E-01	98246001	98246124	NC_000007.13
cg09969277	9.28E-28	2.80E-30	1.37E+01	58.0815805	2.77E-01	89922328	89922451	NC_000015.9
cg21357629	9.99E-28	3.03E-30	1.37E+01	58.0003381	3.85E-01	47063665	47063788	NC_000021.8
cg16073378	1.03E-27	3.13E-30	1.37E+01	57.9706357	2.56E-01	468413	468536	NC_000002.11
cg20782724	1.08E-27	3.30E-30	1.36E+01	57.9173611	1.73E-01	47518657	47518780	NC_000007.13
cg17376609	1.13E-27	3.46E-30	1.36E+01	57.8697977	1.86E-01	88328813	88328936	NC_000013.10
cg24925945	1.17E-27	3.59E-30	1.36E+01	57.8332171	2.01E-01	20055434	20055557	NC_000011.9
cg10224098	1.19E-27	3.63E-30	1.36E+01	57.821263	3.08E-01	44873229	44873352	NC_000001.10
cg01498700	1.20E-27	3.68E-30	1.36E+01	57.8088102	1.59E-01	75241064	75241187	NC_000016.9
cg08349400	1.20E-27	3.69E-30	1.36E+01	57.8056022	1.27E-01	53396138	53396261	NC_000006.11
cg08672919	1.25E-27	3.84E-30	1.36E+01	57.7666918	1.14E-01	169338788	169338911	NC_000006.11
cg04093645	1.26E-27	3.89E-30	1.36E+01	57.7543251	2.21E-01	127872037	127872160	NC_000005.9
cg12757676	1.31E-27	4.05E-30	1.36E+01	57.7120737	1.89E-01	100799558	100799681	NC_000002.11
cg06962177	1.37E-27	4.25E-30	1.36E+01	57.6651531	3.53E-01	63785946	63786069	NC_000001.10
cg03207666	1.38E-27	4.28E-30	1.36E+01	57.6584805	2.13E-01	27183950	27184073	NC_000007.13
cg07318128	1.38E-27	4.29E-30	1.36E+01	57.6568512	2.00E-01	46683806	46683929	NC_000017.10
cg17961200	1.40E-27	4.37E-30	1.36E+01	57.638292	2.82E-01	135937572	135937695	NC_000009.11
cg25007447	1.40E-27	4.37E-30	1.36E+01	57.6381519	1.65E-01	93521330	93521453	NC_000012.11

cg24496475	1.43E-27	4.48E-30	1.36E+01	57.6136046	2.41E-01	1400189	1400312	NC_000004.11
cg15316843	1.45E-27	4.54E-30	1.36E+01	57.5998573	3.15E-01	65282946	65283069	NC_000008.10
cg09491542	1.53E-27	4.82E-30	1.36E+01	57.5402414	1.68E-01	154835773	154835896	NC_000006.11
cg11211486	1.65E-27	5.22E-30	1.36E+01	57.4598225	1.37E-01	46033800	46033923	NC_000003.11
cg06060135	1.79E-27	5.69E-30	1.36E+01	57.3749228	3.61E-01	3596704	3596827	NC_000005.9
cg14384532	1.81E-27	5.75E-30	1.36E+01	57.3646208	2.73E-01	88800624	88800747	NC_000015.9
cg02087954	1.87E-27	5.99E-30	1.36E+01	57.324427	3.07E-01	100609534	100609657	NC_000007.13
cg14658493	1.90E-27	6.08E-30	1.36E+01	57.309701	2.49E-01	27184077	27184200	NC_000007.13
cg26595278	1.93E-27	6.18E-30	1.36E+01	57.2934321	2.95E-01	32009163	32009286	NC_000011.9
cg26504021	1.95E-27	6.25E-30	1.36E+01	57.2822009	2.57E-01	2752882	2753005	NC_000005.9
cg13300273	1.99E-27	6.38E-30	1.35E+01	57.2615529	2.80E-01	200842890	200843013	NC_000001.10
cg02574509	2.05E-27	6.58E-30	1.35E+01	57.2298412	2.97E-01	240255377	240255500	NC_000001.10
cg16783279	2.21E-27	7.14E-30	1.35E+01	57.1492358	3.15E-01	125425625	125425748	NC_000010.10
cg18247055	2.22E-27	7.18E-30	1.35E+01	57.1439731	2.21E-01	22634226	22634349	NC_000010.10
cg10433549	2.22E-27	7.20E-30	1.35E+01	57.1407651	1.54E-01	41472748	41472871	NC_000017.10
cg14587524	2.31E-27	7.53E-30	1.35E+01	57.0957961	3.36E-01	38183262	38183385	NC_000019.9
cg05137358	2.32E-27	7.58E-30	1.35E+01	57.0898964	3.29E-01	37006127	37006250	NC_000013.10
cg11750165	2.36E-27	7.73E-30	1.35E+01	57.0701312	2.29E-01	112722233	112722356	NC_000013.10
cg04475027	2.39E-27	7.85E-30	1.35E+01	57.0553597	3.18E-01	128752040	128752163	NC_000012.11
cg14051544	2.43E-27	7.99E-30	1.35E+01	57.037707	2.70E-01	170303286	170303409	NC_000003.11
cg23301563	2.45E-27	8.08E-30	1.35E+01	57.0263081	1.56E-01	101770526	101770649	NC_000002.11
cg06183338	2.46E-27	8.12E-30	1.35E+01	57.0207903	3.24E-01	178004204	178004327	NC_000005.9
cg19697232	2.47E-27	8.15E-30	1.35E+01	57.0177368	1.25E-01	16854566	16854689	NC_000005.9
cg20764887	2.47E-27	8.17E-30	1.35E+01	57.0156907	2.96E-01	178004012	178004135	NC_000005.9
cg08569979	2.60E-27	8.60E-30	1.35E+01	56.9647096	1.27E-01	41053715	41053838	NC_000013.10

cg00022235	2.60E-27	8.62E-30	1.35E+01	56.9614513	1.78E-01	11764847	11764970	NC_000016.9
cg11028624	2.62E-27	8.69E-30	1.35E+01	56.9536192	1.96E-01	13983793	13983916	NC_000011.9
cg14500070	2.62E-27	8.70E-30	1.35E+01	56.9523895	2.14E-01	141119541	141119664	NC_000003.11
cg01217876	2.73E-27	9.08E-30	1.35E+01	56.9101218	1.64E-01	74498163	74498286	NC_000017.10
cg14441976	2.75E-27	9.15E-30	1.35E+01	56.9027342	3.24E-01	128301083	128301206	NC_000005.9
cg02394395	2.82E-27	9.43E-30	1.35E+01	56.8728957	1.24E-01	94825257	94825380	NC_000010.10
cg12744820	2.82E-27	9.45E-30	1.35E+01	56.870238	2.89E-01	137814960	137815083	NC_000006.11
cg14353137	2.87E-27	9.66E-30	1.35E+01	56.8490888	2.98E-01	67873799	67873922	NC_000008.10
cg03804213	2.89E-27	9.72E-30	1.35E+01	56.8424233	2.82E-01	37248078	37248201	NC_000013.10
cg12577942	2.98E-27	1.01E-29	1.35E+01	56.8081832	2.08E-01	90168963	90169086	NC_000004.11
cg01796166	3.11E-27	1.05E-29	1.35E+01	56.7633428	2.70E-01	95239586	95239709	NC_000014.8
cg05506365	3.32E-27	1.13E-29	1.35E+01	56.6944416	2.39E-01	105472193	105472316	NC_000002.11
cg07770968	3.35E-27	1.14E-29	1.35E+01	56.6851856	3.48E-01	53852422	53852545	NC_000008.10
cg27619367	3.38E-27	1.15E-29	1.35E+01	56.674404	1.36E-01	13150556	13150679	NC_000012.11
cg00502618	3.72E-27	1.27E-29	1.35E+01	56.5764353	1.69E-01	26952697	26952820	NC_000022.10
cg23216434	3.89E-27	1.33E-29	1.34E+01	56.5300227	1.47E-01	67871362	67871485	NC_000001.10
cg05294276	3.89E-27	1.33E-29	1.34E+01	56.5284549	2.15E-01	112618918	112619041	NC_000010.10
cg17774559	3.89E-27	1.33E-29	1.34E+01	56.5275236	3.02E-01	1879698	1879821	NC_000005.9
cg01075002	3.98E-27	1.37E-29	1.34E+01	56.5028097	1.65E-01	155450882	155451005	NC_000006.11
cg14015441	4.11E-27	1.41E-29	1.34E+01	56.4711491	2.14E-01	105479318	105479441	NC_000008.10
cg27388763	4.35E-27	1.50E-29	1.34E+01	56.4111191	1.27E-01	44648385	44648508	NC_000017.10
cg21573200	4.38E-27	1.51E-29	1.34E+01	56.4020242	2.75E-01	2739602	2739725	NC_000005.9
cg21932672	4.43E-27	1.53E-29	1.34E+01	56.3890413	1.62E-01	95584445	95584568	NC_000001.10
cg14647620	4.58E-27	1.59E-29	1.34E+01	56.352271	1.47E-01	100462742	100462865	NC_000009.11
cg02194398	4.59E-27	1.60E-29	1.34E+01	56.3491359	7.93E-02	142556368	142556491	NC_000004.11

cg26392545	4.61E-27	1.60E-29	1.34E+01	56.3457415	1.58E-01	28187379	28187502	NC_000013.10
cg04557544	4.74E-27	1.65E-29	1.34E+01	56.315748	3.50E-01	12996046	12996169	NC_000016.9
cg26393977	5.08E-27	1.78E-29	1.34E+01	56.243203	1.98E-01	37612713	37612836	NC_000008.10
cg09142313	5.12E-27	1.79E-29	1.34E+01	56.2336865	3.08E-01	72677859	72677982	NC_000005.9
cg09493505	5.13E-27	1.80E-29	1.34E+01	56.2293231	3.63E-01	49813111	49813234	NC_000007.13
cg24401487	5.27E-27	1.85E-29	1.34E+01	56.2019108	1.88E-01	230416880	230417003	NC_000001.10
cg08722660	5.34E-27	1.88E-29	1.34E+01	56.1868851	1.19E-01	201099406	201099529	NC_000001.10
cg02482218	5.39E-27	1.90E-29	1.34E+01	56.1772551	2.61E-01	12995568	12995691	NC_000016.9
cg21464220	5.45E-27	1.92E-29	1.34E+01	56.1634969	2.56E-01	242242798	242242921	NC_000002.11
cg09474331	5.49E-27	1.94E-29	1.34E+01	56.1568195	3.95E-01	54926805	54926928	NC_000019.9
cg24288527	5.66E-27	2.00E-29	1.34E+01	56.1246248	2.24E-01	93390347	93390470	NC_000010.10
cg25260137	5.75E-27	2.04E-29	1.34E+01	56.1067549	2.71E-01	65282185	65282308	NC_000008.10
cg08209711	5.94E-27	2.11E-29	1.34E+01	56.0722748	1.70E-01	32825499	32825622	NC_000006.11
cg11667258	6.21E-27	2.21E-29	1.34E+01	56.026597	2.70E-01	155107529	155107652	NC_000005.9
cg03530754	6.31E-27	2.25E-29	1.34E+01	56.0087921	3.09E-01	128751460	128751583	NC_000012.11
cg01660911	6.32E-27	2.25E-29	1.34E+01	56.0068485	3.13E-01	17082772	17082895	NC_000022.10
cg07846220	6.38E-27	2.28E-29	1.34E+01	55.9961015	3.06E-01	7117680	7117803	NC_000018.9
cg17429382	6.75E-27	2.43E-29	1.34E+01	55.9332855	3.11E-01	3786246	3786369	NC_000019.9
cg13468680	6.83E-27	2.46E-29	1.34E+01	55.9185239	1.67E-01	139393050	139393173	NC_000009.11
cg12781700	7.06E-27	2.56E-29	1.34E+01	55.8815055	3.24E-01	42733729	42733852	NC_000017.10
cg02732915	7.18E-27	2.60E-29	1.33E+01	55.8643346	1.48E-01	81120670	81120793	NC_000004.11
cg04597433	7.19E-27	2.60E-29	1.33E+01	55.8625217	2.77E-01	9783206	9783329	NC_000004.11
cg12756396	7.39E-27	2.69E-29	1.33E+01	55.8323714	2.24E-01	50886969	50887092	NC_000001.10
cg26450044	7.50E-27	2.73E-29	1.33E+01	55.815759	8.83E-02	21927746	21927869	NC_000016.9
cg23128056	7.83E-27	2.86E-29	1.33E+01	55.7688117	1.02E-01	39674626	39674749	NC_000017.10

cg26523264	8.07E-27	2.95E-29	1.33E+01	55.737481	1.33E-01	8649844	8649967	NC_000019.9
cg27616541	8.26E-27	3.03E-29	1.33E+01	55.7120048	1.73E-01	40837288	40837411	NC_000017.10
cg05151154	8.36E-27	3.08E-29	1.33E+01	55.6974328	3.82E-01	51184786	51184909	NC_000016.9
cg00552235	8.37E-27	3.08E-29	1.33E+01	55.6956697	3.18E-01	100943163	100943286	NC_000007.13
cg09614415	8.39E-27	3.09E-29	1.33E+01	55.6927169	2.46E-01	129535509	129535632	NC_000010.10
cg11178863	8.47E-27	3.12E-29	1.33E+01	55.6832455	3.49E-01	13839785	13839908	NC_000001.10
cg24089600	8.51E-27	3.14E-29	1.33E+01	55.6771657	2.91E-01	110960177	110960300	NC_000013.10
cg16348962	8.57E-27	3.17E-29	1.33E+01	55.6686182	1.28E-01	70491417	70491540	NC_000016.9
cg18082337	8.75E-27	3.24E-29	1.33E+01	55.6461207	2.95E-01	147113726	147113849	NC_000003.11
cg20884887	8.77E-27	3.25E-29	1.33E+01	55.6424445	3.69E-01	27208285	27208408	NC_000007.13
cg22910769	9.01E-27	3.35E-29	1.33E+01	55.6131806	1.87E-01	72651035	72651158	NC_000010.10
cg07926618	9.12E-27	3.39E-29	1.33E+01	55.6005367	2.29E-01	17247876	17247999	NC_000001.10
cg00177388	9.15E-27	3.41E-29	1.33E+01	55.5961379	2.58E-01	72677886	72678009	NC_000005.9
cg07533148	9.66E-27	3.61E-29	1.33E+01	55.5384876	2.90E-01	248020812	248020935	NC_000001.10
cg09997062	9.79E-27	3.66E-29	1.33E+01	55.5247945	1.31E-01	39708372	39708495	NC_000017.10
cg03609960	1.04E-26	3.91E-29	1.33E+01	55.4585059	2.90E-01	99288805	99288928	NC_000012.11
cg07847863	1.04E-26	3.93E-29	1.33E+01	55.4536118	2.73E-01	53419963	53420086	NC_000013.10
cg05648614	1.07E-26	4.03E-29	1.33E+01	55.4295058	2.22E-01	5180520	5180643	NC_000003.11
cg04992127	1.09E-26	4.11E-29	1.33E+01	55.408766	2.72E-01	2751854	2751977	NC_000005.9
cg16517298	1.14E-26	4.32E-29	1.33E+01	55.3590005	1.98E-01	230413174	230413297	NC_000001.10
cg09873510	1.26E-26	4.79E-29	1.33E+01	55.2564251	8.88E-02	65016588	65016711	NC_000005.9
cg02976898	1.27E-26	4.86E-29	1.33E+01	55.2421821	1.56E-01	76742856	76742979	NC_000010.10
cg10778841	1.30E-26	4.99E-29	1.33E+01	55.215479	3.03E-01	106401319	106401442	NC_000010.10
cg13563298	1.31E-26	5.01E-29	1.33E+01	55.2117345	3.05E-01	95948059	95948182	NC_000009.11
cg14596987	1.32E-26	5.09E-29	1.33E+01	55.1957019	1.69E-01	98249189	98249312	NC_000009.11

cg12296772	1.40E-26	5.39E-29	1.32E+01	55.1390505	2.99E-01	17271067	17271190	NC_000008.10
cg26866142	1.40E-26	5.43E-29	1.32E+01	55.1319075	1.73E-01	205779678	205779801	NC_000001.10
cg02467990	1.43E-26	5.53E-29	1.32E+01	55.1136121	3.86E-01	49813102	49813225	NC_000007.13
cg18483908	1.43E-26	5.54E-29	1.32E+01	55.111664	2.82E-01	19017420	19017543	NC_000022.10
cg05343808	1.43E-26	5.55E-29	1.32E+01	55.1108856	2.14E-01	50857635	50857758	NC_000007.13
cg03442254	1.50E-26	5.85E-29	1.32E+01	55.058987	1.80E-01	1849073	1849196	NC_000001.10
cg10671668	1.52E-26	5.92E-29	1.32E+01	55.0456218	2.96E-01	4919230	4919353	NC_000012.11
cg10004079	1.53E-26	5.97E-29	1.32E+01	55.0375483	2.08E-01	26372530	26372653	NC_000017.10
cg16855929	1.53E-26	5.99E-29	1.32E+01	55.0346077	2.36E-01	231561414	231561537	NC_000001.10
cg03727333	1.54E-26	6.02E-29	1.32E+01	55.0292309	1.51E-01	52379467	52379590	NC_000010.10
cg11431723	1.56E-26	6.11E-29	1.32E+01	55.0150502	1.68E-01	149126842	149126965	NC_000003.11
cg05475485	1.57E-26	6.15E-29	1.32E+01	55.0086512	1.70E-01	67810027	67810150	NC_000011.9
cg08832906	1.59E-26	6.25E-29	1.32E+01	54.9929282	3.10E-01	139208852	139208975	NC_000007.13
cg24914884	1.60E-26	6.31E-29	1.32E+01	54.9837013	1.83E-01	64588128	64588251	NC_000002.11
cg20405017	1.60E-26	6.31E-29	1.32E+01	54.9833678	2.23E-01	50235393	50235516	NC_000017.10
cg25691167	1.64E-26	6.48E-29	1.32E+01	54.9566959	3.15E-01	19184961	19185084	NC_000007.13
cg23259001	1.64E-26	6.48E-29	1.32E+01	54.9559453	1.78E-01	105800169	105800292	NC_000014.8
cg14699728	1.66E-26	6.58E-29	1.32E+01	54.9410141	3.12E-01	66188522	66188645	NC_000011.9
cg25308508	1.69E-26	6.67E-29	1.32E+01	54.9270761	2.53E-01	3592638	3592761	NC_000005.9
cg22797031	1.86E-26	7.41E-29	1.32E+01	54.822563	3.14E-01	170630070	170630193	NC_000001.10
cg24304309	1.90E-26	7.60E-29	1.32E+01	54.7977261	1.86E-01	154577895	154578018	NC_000001.10
cg16461861	1.92E-26	7.70E-29	1.32E+01	54.7847834	1.27E-01	43180148	43180271	NC_000005.9
cg23190190	1.93E-26	7.73E-29	1.32E+01	54.780937	1.14E-01	70031725	70031848	NC_000011.9
cg06782035	1.93E-26	7.74E-29	1.32E+01	54.7793566	3.58E-01	16179135	16179258	NC_000005.9
cg17373343	1.95E-26	7.82E-29	1.32E+01	54.7700901	2.18E-01	6026554	6026677	NC_000019.9

cg25208017	1.95E-26	7.85E-29	1.32E+01	54.7657116	3.81E-01	240255486	240255609	NC_000001.10
cg07106737	2.00E-26	8.04E-29	1.32E+01	54.741678	1.34E-01	88599035	88599158	NC_000016.9
cg07195057	2.04E-26	8.25E-29	1.32E+01	54.7167291	1.16E-01	907118	907241	NC_000001.10
cg06779449	2.05E-26	8.29E-29	1.32E+01	54.7121113	3.09E-01	42544663	42544786	NC_000020.10
cg07860213	2.06E-26	8.32E-29	1.32E+01	54.7083506	2.54E-01	70982039	70982162	NC_000008.10
cg00826626	2.07E-26	8.37E-29	1.32E+01	54.7026076	1.51E-01	19360878	19361001	NC_000014.8
cg01289769	2.08E-26	8.43E-29	1.32E+01	54.695534	2.37E-01	125077434	125077557	NC_000003.11
cg07049592	2.09E-26	8.45E-29	1.32E+01	54.6923057	1.99E-01	27184450	27184573	NC_000007.13
cg04210254	2.17E-26	8.81E-29	1.32E+01	54.650635	2.13E-01	22327148	22327271	NC_000001.10
cg19074340	2.22E-26	9.05E-29	1.32E+01	54.6247226	3.96E-01	32456912	32457035	NC_000011.9
cg19352726	2.23E-26	9.07E-29	1.32E+01	54.6222023	1.95E-01	42757805	42757928	NC_000006.11
cg13056222	2.24E-26	9.13E-29	1.32E+01	54.6161036	1.76E-01	204219199	204219322	NC_000001.10
cg00352652	2.31E-26	9.42E-29	1.32E+01	54.5846543	2.19E-01	88547533	88547656	NC_000016.9
cg18384778	2.37E-26	9.73E-29	1.32E+01	54.5519403	2.83E-01	57618136	57618259	NC_000019.9
cg01826337	2.39E-26	9.80E-29	1.32E+01	54.544876	1.42E-01	1846001	1846124	NC_000001.10
cg14235574	2.47E-26	1.02E-28	1.32E+01	54.5075243	2.15E-01	112080226	112080349	NC_000009.11
cg18009496	2.51E-26	1.03E-28	1.32E+01	54.4929139	3.20E-01	1883940	1884063	NC_000005.9
cg20465933	2.53E-26	1.04E-28	1.32E+01	54.4815738	1.42E-01	152376761	152376884	NC_000004.11
cg22770911	2.63E-26	1.09E-28	1.31E+01	54.4432722	1.83E-01	8101307	8101430	NC_000010.10
cg25750507	2.68E-26	1.11E-28	1.31E+01	54.4232579	1.99E-01	46684004	46684127	NC_000017.10
cg12560987	2.68E-26	1.11E-28	1.31E+01	54.42219	2.55E-01	1287065	1287188	NC_000007.13
cg04456238	2.72E-26	1.13E-28	1.31E+01	54.4052551	2.36E-01	32450104	32450227	NC_000011.9
cg14535746	2.72E-26	1.13E-28	1.31E+01	54.4047568	1.67E-01	96652787	96652910	NC_000012.11
cg12027899	2.81E-26	1.17E-28	1.31E+01	54.3693278	1.77E-01	58385947	58386070	NC_000011.9
cg06117133	2.85E-26	1.19E-28	1.31E+01	54.3540519	1.32E-01	67966621	67966744	NC_000016.9

cg00559444	2.85E-26	1.19E-28	1.31E+01	54.353588	1.69E-01	22028743	22028866	NC_000008.10
cg01870247	2.85E-26	1.19E-28	1.31E+01	54.3497844	1.27E-01	31354127	31354250	NC_000020.10
cg05261299	2.96E-26	1.24E-28	1.31E+01	54.3114615	1.98E-01	52437299	52437422	NC_000012.11
cg05522011	3.00E-26	1.26E-28	1.31E+01	54.2987983	2.05E-01	81122726	81122849	NC_000004.11
cg11075099	3.04E-26	1.27E-28	1.31E+01	54.284252	1.74E-01	27829087	27829210	NC_000013.10
cg07068756	3.06E-26	1.29E-28	1.31E+01	54.275551	3.53E-01	41258910	41259033	NC_000004.11
cg15384598	3.18E-26	1.34E-28	1.31E+01	54.2358679	3.03E-01	24045549	24045672	NC_000014.8
cg25974626	3.28E-26	1.38E-28	1.31E+01	54.2018915	1.14E-01	32977142	32977265	NC_000007.13
cg14416371	3.28E-26	1.39E-28	1.31E+01	54.2009176	3.70E-01	43602847	43602970	NC_000011.9
cg13794530	3.33E-26	1.41E-28	1.31E+01	54.1858396	3.14E-01	158937494	158937617	NC_000007.13
cg00688962	3.38E-26	1.43E-28	1.31E+01	54.1706777	2.14E-01	21950567	21950690	NC_000004.11
cg02721665	3.47E-26	1.47E-28	1.31E+01	54.1403963	2.29E-01	125425583	125425706	NC_000010.10
cg08305436	3.62E-26	1.54E-28	1.31E+01	54.0949976	2.32E-01	22806184	22806307	NC_000019.9
cg20989443	3.65E-26	1.56E-28	1.31E+01	54.0839105	2.06E-01	230301268	230301391	NC_000001.10
cg27659787	3.93E-26	1.68E-28	1.31E+01	54.0067608	2.62E-01	111544291	111544414	NC_000004.11
cg24626752	3.97E-26	1.70E-28	1.31E+01	53.9962998	1.91E-01	88328274	88328397	NC_000013.10
cg14030258	4.08E-26	1.76E-28	1.31E+01	53.9641631	1.70E-01	29591301	29591424	NC_000001.10
cg19694519	4.17E-26	1.80E-28	1.31E+01	53.9419813	1.66E-01	22389206	22389329	NC_000008.10
cg11232245	4.21E-26	1.82E-28	1.31E+01	53.9307438	2.05E-01	101284535	101284658	NC_000010.10
cg04138185	4.29E-26	1.85E-28	1.31E+01	53.9123198	3.60E-01	84322584	84322707	NC_000015.9
cg09793121	4.36E-26	1.89E-28	1.31E+01	53.8949207	2.50E-01	34398263	34398386	NC_000021.8
cg02642962	4.80E-26	2.09E-28	1.31E+01	53.7906003	1.72E-01	124610404	124610527	NC_000010.10
cg09595050	4.82E-26	2.10E-28	1.31E+01	53.7858274	2.11E-01	81110205	81110328	NC_000004.11
cg20817131	4.97E-26	2.17E-28	1.30E+01	53.7534231	2.35E-01	27184167	27184290	NC_000007.13
cg14624314	5.10E-26	2.23E-28	1.30E+01	53.7272673	2.46E-01	145328877	145329000	NC_000005.9

cg25517810	5.13E-26	2.25E-28	1.30E+01	53.7189078	2.86E-01	105474385	105474508	NC_000002.11
cg01819995	5.25E-26	2.30E-28	1.30E+01	53.6960043	1.66E-01	30537979	30538102	NC_000020.10
cg27416372	5.34E-26	2.34E-28	1.30E+01	53.6780516	2.32E-01	147110229	147110352	NC_000003.11
cg02091605	5.37E-26	2.36E-28	1.30E+01	53.669648	2.56E-01	1776102	1776225	NC_000019.9
cg14698646	5.47E-26	2.41E-28	1.30E+01	53.6510029	2.42E-01	46684750	46684873	NC_000017.10
cg20754261	5.49E-26	2.42E-28	1.30E+01	53.6466173	2.92E-01	468330	468453	NC_000002.11
cg26050906	5.52E-26	2.44E-28	1.30E+01	53.6397203	1.53E-01	50800202	50800325	NC_000007.13
cg09388605	6.09E-26	2.70E-28	1.30E+01	53.5375158	2.64E-01	56013800	56013923	NC_000008.10
cg16052972	6.11E-26	2.71E-28	1.30E+01	53.5330983	1.35E-01	12184833	12184956	NC_000003.11
cg09521703	6.21E-26	2.76E-28	1.30E+01	53.5155911	2.11E-01	55944864	55944987	NC_000019.9
cg01485075	6.40E-26	2.86E-28	1.30E+01	53.4823359	3.47E-01	22817371	22817494	NC_000019.9
cg10366867	6.45E-26	2.88E-28	1.30E+01	53.472575	1.28E-01	88804589	88804712	NC_000016.9
cg08767286	6.56E-26	2.94E-28	1.30E+01	53.4545772	2.15E-01	237077265	237077388	NC_000002.11
cg23847712	6.62E-26	2.97E-28	1.30E+01	53.444571	2.64E-01	9783196	9783319	NC_000004.11
cg15239304	6.72E-26	3.01E-28	1.30E+01	53.4282063	1.28E-01	224810907	224811030	NC_000002.11
cg02628107	6.93E-26	3.11E-28	1.30E+01	53.3962419	1.03E-01	10279354	10279477	NC_000005.9
cg07033372	6.98E-26	3.14E-28	1.30E+01	53.3878416	2.57E-01	37353032	37353155	NC_000020.10
cg18578405	7.13E-26	3.21E-28	1.30E+01	53.3653732	2.80E-01	12996057	12996180	NC_000016.9
cg20522046	7.13E-26	3.21E-28	1.30E+01	53.3643474	1.66E-01	52024792	52024915	NC_000013.10
cg19937061	7.51E-26	3.40E-28	1.30E+01	53.3097038	2.93E-01	25320133	25320256	NC_000013.10
cg21678445	7.54E-26	3.41E-28	1.30E+01	53.3048235	3.80E-01	22930283	22930406	NC_000018.9
cg25415246	7.58E-26	3.43E-28	1.30E+01	53.299088	2.13E-01	1762710	1762833	NC_000019.9
cg05012672	7.65E-26	3.47E-28	1.30E+01	53.2880054	1.89E-01	3189830	3189953	NC_000019.9
cg22504528	7.75E-26	3.52E-28	1.30E+01	53.2745308	2.35E-01	26951369	26951492	NC_000022.10
cg09061993	7.80E-26	3.55E-28	1.30E+01	53.2668933	2.26E-01	211499620	211499743	NC_000001.10

cg01535698	7.81E-26	3.55E-28	1.30E+01	53.2652853	3.20E-01	240255136	240255259	NC_000001.10
cg16352072	7.82E-26	3.56E-28	1.30E+01	53.2627174	1.54E-01	910223	910346	NC_000001.10
cg11946583	7.82E-26	3.56E-28	1.30E+01	53.2623703	2.12E-01	75674098	75674221	NC_000007.13
cg03171770	7.86E-26	3.58E-28	1.30E+01	53.2561403	2.72E-01	43393728	43393851	NC_000010.10
cg05206884	8.00E-26	3.66E-28	1.30E+01	53.2359281	3.24E-01	33454324	33454447	NC_000022.10
cg25999442	8.04E-26	3.68E-28	1.30E+01	53.2303754	3.55E-01	34113398	34113521	NC_000019.9
cg03187713	8.31E-26	3.81E-28	1.30E+01	53.1963268	1.55E-01	51330698	51330821	NC_000007.13
cg18372607	8.36E-26	3.83E-28	1.30E+01	53.1896586	2.91E-01	31827084	31827207	NC_000011.9
cg16962557	8.41E-26	3.86E-28	1.30E+01	53.1837772	1.52E-01	153587387	153587510	NC_000004.11
cg06062984	8.52E-26	3.92E-28	1.30E+01	53.1678037	2.68E-01	22413960	22414083	NC_000003.11
cg02393617	8.52E-26	3.92E-28	1.30E+01	53.1675704	1.21E-01	20087765	20087888	NC_000011.9
cg17963548	8.65E-26	3.98E-28	1.30E+01	53.1510776	1.31E-01	224824723	224824846	NC_000002.11
cg03221715	8.69E-26	4.01E-28	1.30E+01	53.1455475	1.61E-01	129148362	129148485	NC_000003.11
cg01283246	8.70E-26	4.01E-28	1.30E+01	53.1442064	3.20E-01	135266135	135266258	NC_000005.9
cg03952724	8.77E-26	4.05E-28	1.30E+01	53.1347826	1.10E-01	30333365	30333488	NC_000008.10
cg22167515	8.82E-26	4.07E-28	1.30E+01	53.1294436	2.70E-01	91192466	91192589	NC_000001.10
cg15874092	8.82E-26	4.08E-28	1.30E+01	53.1281281	2.75E-01	59827921	59828044	NC_000020.10
cg08092050	8.83E-26	4.08E-28	1.30E+01	53.1270978	1.70E-01	78734858	78734981	NC_000015.9
cg00940260	8.93E-26	4.13E-28	1.30E+01	53.1142112	1.94E-01	47064750	47064873	NC_000001.10
cg20049415	8.93E-26	4.14E-28	1.30E+01	53.113777	2.18E-01	21377671	21377794	NC_000020.10
cg27225130	8.96E-26	4.15E-28	1.30E+01	53.1096345	1.32E-01	208218009	208218132	NC_000001.10
cg07125166	9.06E-26	4.21E-28	1.30E+01	53.097201	1.94E-01	48413613	48413736	NC_000003.11
cg24266670	9.37E-26	4.36E-28	1.29E+01	53.0610418	3.00E-01	115630629	115630752	NC_000011.9
cg02983163	9.39E-26	4.37E-28	1.29E+01	53.0588185	2.06E-01	47010461	47010584	NC_000021.8
cg08802358	9.39E-26	4.38E-28	1.29E+01	53.0580741	2.01E-01	176524797	176524920	NC_000005.9

cg16179589	9.41E-26	4.39E-28	1.29E+01	53.055086	2.34E-01	36107292	36107415	NC_000017.10
cg06271327	9.56E-26	4.46E-28	1.29E+01	53.0391459	6.72E-02	8062512	8062635	NC_000017.10
cg27649239	9.92E-26	4.64E-28	1.29E+01	53.0006023	1.99E-01	68120393	68120516	NC_000015.9
cg11399546	9.92E-26	4.64E-28	1.29E+01	53.0005375	2.38E-01	226730561	226730684	NC_000001.10
cg14465148	1.03E-25	4.80E-28	1.29E+01	52.9650706	1.30E-01	3329791	3329914	NC_000006.11
cg10293925	1.03E-25	4.81E-28	1.29E+01	52.9635053	3.20E-01	38670985	38671108	NC_000007.13
cg26862964	1.03E-25	4.83E-28	1.29E+01	52.9602485	1.62E-01	114878283	114878406	NC_000004.11
cg12201110	1.05E-25	4.96E-28	1.29E+01	52.934099	1.49E-01	95321963	95322086	NC_000005.9
cg00625351	1.06E-25	5.01E-28	1.29E+01	52.9242912	1.33E-01	34048130	34048253	NC_000003.11
cg14684068	1.06E-25	5.01E-28	1.29E+01	52.9238583	1.70E-01	63894400	63894523	NC_000011.9
cg22795586	1.07E-25	5.06E-28	1.29E+01	52.9132906	2.25E-01	170629555	170629678	NC_000001.10
cg12017745	1.09E-25	5.15E-28	1.29E+01	52.8963707	2.29E-01	65352327	65352450	NC_000011.9
cg13530474	1.09E-25	5.17E-28	1.29E+01	52.892209	1.85E-01	97339939	97340062	NC_000008.10
cg05300538	1.09E-25	5.17E-28	1.29E+01	52.8916622	9.33E-02	64898766	64898889	NC_000011.9
cg06535102	1.14E-25	5.40E-28	1.29E+01	52.8489295	1.63E-01	95101861	95101984	NC_000001.10
cg19774425	1.14E-25	5.42E-28	1.29E+01	52.8445373	1.36E-01	51327477	51327600	NC_000019.9
cg09632858	1.15E-25	5.48E-28	1.29E+01	52.8347975	1.88E-01	27219548	27219671	NC_000016.9
cg18657094	1.16E-25	5.53E-28	1.29E+01	52.8252341	2.91E-01	2140030	2140153	NC_000003.11
cg16989443	1.17E-25	5.57E-28	1.29E+01	52.8188674	1.98E-01	1849195	1849318	NC_000001.10
cg05468917	1.20E-25	5.72E-28	1.29E+01	52.7915235	1.33E-01	2882180	2882303	NC_000016.9
cg09469566	1.20E-25	5.74E-28	1.29E+01	52.788148	2.14E-01	112717244	112717367	NC_000013.10
cg24031355	1.25E-25	6.01E-28	1.29E+01	52.7429803	3.07E-01	22634439	22634562	NC_000010.10
cg10089865	1.26E-25	6.03E-28	1.29E+01	52.7389695	1.38E-01	8100286	8100409	NC_000010.10
cg00872726	1.26E-25	6.05E-28	1.29E+01	52.736686	2.33E-01	97499908	97500031	NC_000014.8
cg21885046	1.26E-25	6.06E-28	1.29E+01	52.7337596	3.08E-01	130647004	130647127	NC_000012.11

cg24792365	1.27E-25	6.09E-28	1.29E+01	52.7295842	1.01E-01	188869178	188869301	NC_000003.11
cg04136610	1.27E-25	6.10E-28	1.29E+01	52.7283066	3.40E-01	5139878	5140001	NC_000005.9
cg09907936	1.29E-25	6.21E-28	1.29E+01	52.7092165	3.35E-01	58570466	58570589	NC_000019.9
cg15382538	1.31E-25	6.32E-28	1.29E+01	52.693261	2.33E-01	72678193	72678316	NC_000005.9
cg06353485	1.33E-25	6.41E-28	1.29E+01	52.6790254	1.88E-01	3409927	3410050	NC_000004.11
cg16631083	1.33E-25	6.43E-28	1.29E+01	52.6750514	2.55E-01	33250039	33250162	NC_000021.8
cg06891164	1.34E-25	6.46E-28	1.29E+01	52.6702815	1.39E-01	105451249	105451372	NC_000014.8
cg24663256	1.43E-25	6.97E-28	1.29E+01	52.5948464	2.86E-01	21950307	21950430	NC_000004.11
cg14373380	1.44E-25	7.04E-28	1.29E+01	52.5850387	1.55E-01	16268457	16268580	NC_000001.10
cg12159189	1.48E-25	7.24E-28	1.29E+01	52.5579306	2.79E-01	17850521	17850644	NC_000022.10
cg11281005	1.48E-25	7.24E-28	1.29E+01	52.5576818	1.74E-01	113396948	113397071	NC_000013.10
cg06159486	1.48E-25	7.26E-28	1.29E+01	52.554653	2.57E-01	18429691	18429814	NC_000010.10
cg05333149	1.49E-25	7.28E-28	1.29E+01	52.5512687	1.78E-01	29660173	29660296	NC_000022.10
cg12477716	1.55E-25	7.60E-28	1.29E+01	52.5093661	3.92E-01	47073436	47073559	NC_000017.10
cg06625077	1.60E-25	7.86E-28	1.29E+01	52.476322	1.61E-01	67963638	67963761	NC_000016.9
cg03190219	1.63E-25	8.04E-28	1.29E+01	52.4528014	2.19E-01	25898688	25898811	NC_000007.13
cg14197071	1.65E-25	8.13E-28	1.29E+01	52.4417359	2.49E-01	81110459	81110582	NC_000004.11
cg20143970	1.66E-25	8.19E-28	1.29E+01	52.4345752	1.53E-01	323570	323693	NC_000006.11
cg12178147	1.70E-25	8.42E-28	1.29E+01	52.4073689	1.95E-01	109576929	109577052	NC_000012.11
cg01874697	1.70E-25	8.43E-28	1.29E+01	52.4067302	2.45E-01	106401517	106401640	NC_000010.10
cg11097433	1.73E-25	8.56E-28	1.29E+01	52.3908062	2.83E-01	108924557	108924680	NC_000010.10
cg03181248	1.76E-25	8.72E-28	1.28E+01	52.3722328	2.54E-01	175199670	175199793	NC_000002.11
cg12599168	1.77E-25	8.76E-28	1.28E+01	52.3677347	2.23E-01	142554539	142554662	NC_000007.13
cg00461236	1.77E-25	8.79E-28	1.28E+01	52.3647476	1.84E-01	86704016	86704139	NC_000016.9
cg01804278	1.81E-25	8.97E-28	1.28E+01	52.3449854	1.88E-01	63894802	63894925	NC_000011.9

cg03939132	1.81E-25	8.99E-28	1.28E+01	52.3426853	1.76E-01	19316305	19316428	NC_000001.10
cg08858649	1.83E-25	9.09E-28	1.28E+01	52.3309038	2.45E-01	30139903	30140026	NC_000006.11
cg06601579	1.86E-25	9.24E-28	1.28E+01	52.3147313	2.01E-01	142966	143089	NC_000007.13
cg21183256	1.96E-25	9.77E-28	1.28E+01	52.2590232	2.75E-01	73163868	73163991	NC_000008.10
cg11507187	1.99E-25	9.97E-28	1.28E+01	52.2393955	1.28E-01	149636432	149636555	NC_000005.9
cg21899596	1.99E-25	9.98E-28	1.28E+01	52.2388078	3.56E-01	57522493	57522616	NC_000004.11
cg23243867	2.00E-25	1.00E-27	1.28E+01	52.2352402	3.16E-01	77334045	77334168	NC_000001.10
cg11911286	2.02E-25	1.01E-27	1.28E+01	52.2251741	2.44E-01	109177239	109177362	NC_000002.11
cg01024458	2.03E-25	1.02E-27	1.28E+01	52.2155227	2.86E-01	8703816	8703939	NC_000001.10
cg10177528	2.10E-25	1.06E-27	1.28E+01	52.1808197	1.82E-01	211499638	211499761	NC_000001.10
cg14123923	2.14E-25	1.08E-27	1.28E+01	52.1605119	3.07E-01	79176572	79176695	NC_000013.10
cg26313188	2.16E-25	1.09E-27	1.28E+01	52.1499655	1.73E-01	9225416	9225539	NC_000017.10
cg19679633	2.17E-25	1.09E-27	1.28E+01	52.1478437	3.28E-01	2752278	2752401	NC_000005.9
cg07906527	2.17E-25	1.10E-27	1.28E+01	52.1439071	1.61E-01	204116483	204116606	NC_000001.10
cg00801823	2.27E-25	1.15E-27	1.28E+01	52.1012073	1.30E-01	64061354	64061477	NC_000005.9
cg26328893	2.31E-25	1.17E-27	1.28E+01	52.0778082	1.47E-01	105747540	105747663	NC_000014.8
cg25016420	2.36E-25	1.20E-27	1.28E+01	52.0559283	2.06E-01	12989053	12989176	NC_000020.10
cg21135135	2.43E-25	1.23E-27	1.28E+01	52.0277263	2.73E-01	70597687	70597810	NC_000007.13
cg10164264	2.50E-25	1.27E-27	1.28E+01	51.998698	1.79E-01	1373477	1373600	NC_000004.11
cg17643662	2.50E-25	1.27E-27	1.28E+01	51.9952949	1.55E-01	30407052	30407175	NC_000020.10
cg21054521	2.60E-25	1.33E-27	1.28E+01	51.9530524	2.51E-01	112759386	112759509	NC_000013.10
cg21538631	2.62E-25	1.34E-27	1.28E+01	51.9442496	2.44E-01	53391662	53391785	NC_000001.10
cg12006284	2.63E-25	1.35E-27	1.28E+01	51.9403932	2.14E-01	32449638	32449761	NC_000011.9
cg20062116	2.69E-25	1.38E-27	1.28E+01	51.9187635	1.73E-01	98780453	98780576	NC_000010.10
cg02123767	2.70E-25	1.38E-27	1.28E+01	51.9140252	2.41E-01	71295007	71295130	NC_000017.10

cg26389281	2.79E-25	1.44E-27	1.28E+01	51.8771873	1.31E-01	915978	916101	NC_000017.10
cg14556188	2.81E-25	1.45E-27	1.28E+01	51.867256	1.16E-01	144523541	144523664	NC_000008.10
cg12729166	2.83E-25	1.46E-27	1.28E+01	51.8603983	1.47E-01	100110390	100110513	NC_000001.10
cg07547765	2.85E-25	1.47E-27	1.28E+01	51.8552168	2.42E-01	46685448	46685571	NC_000017.10
cg10093934	2.87E-25	1.48E-27	1.28E+01	51.8445869	1.51E-01	173423557	173423680	NC_000002.11
cg10991081	2.95E-25	1.53E-27	1.28E+01	51.8154891	9.56E-02	201960135	201960258	NC_000001.10
cg14397309	2.96E-25	1.53E-27	1.28E+01	51.8112926	1.27E-01	48437909	48438032	NC_000012.11
cg11452043	3.01E-25	1.56E-27	1.28E+01	51.7938863	2.00E-01	131814000	131814123	NC_000002.11
cg00228689	3.02E-25	1.57E-27	1.28E+01	51.7906638	1.16E-01	53742112	53742235	NC_000001.10
cg20311863	3.03E-25	1.58E-27	1.28E+01	51.7838529	2.34E-01	91184126	91184249	NC_000001.10
cg23479922	3.04E-25	1.58E-27	1.28E+01	51.7824521	2.13E-01	16179633	16179756	NC_000005.9
cg08963581	3.11E-25	1.62E-27	1.28E+01	51.7586808	1.40E-01	72863301	72863424	NC_000016.9
cg22763718	3.19E-25	1.66E-27	1.28E+01	51.7330029	2.89E-01	53422780	53422903	NC_000013.10
cg22996718	3.20E-25	1.67E-27	1.28E+01	51.7293113	9.14E-02	26138213	26138336	NC_000022.10
cg18273421	3.23E-25	1.69E-27	1.28E+01	51.7138444	1.25E-01	31734554	31734677	NC_000006.11
cg04117356	3.25E-25	1.70E-27	1.28E+01	51.7074799	2.24E-01	4406024	4406147	NC_000017.10
cg24008395	3.29E-25	1.72E-27	1.28E+01	51.6958631	9.87E-02	56069567	56069690	NC_000017.10
cg00127167	3.34E-25	1.75E-27	1.27E+01	51.6807941	2.46E-01	5140029	5140152	NC_000005.9
cg03693099	3.38E-25	1.77E-27	1.27E+01	51.6677728	2.06E-01	135936901	135937024	NC_000009.11
cg00315395	3.46E-25	1.82E-27	1.27E+01	51.6432858	2.46E-01	129145951	129146074	NC_000011.9
cg22757824	3.51E-25	1.85E-27	1.27E+01	51.6260669	3.13E-01	110960186	110960309	NC_000013.10
cg12440350	3.55E-25	1.87E-27	1.27E+01	51.612936	1.41E-01	17084335	17084458	NC_000001.10
cg20673829	3.58E-25	1.89E-27	1.27E+01	51.6054472	2.91E-01	158938060	158938183	NC_000007.13
cg03291548	3.61E-25	1.91E-27	1.27E+01	51.5943198	1.52E-01	18266804	18266927	NC_000022.10
cg13458645	3.61E-25	1.91E-27	1.27E+01	51.5942367	2.79E-01	111544271	111544394	NC_000004.11

cg02927058	3.63E-25	1.92E-27	1.27E+01	51.5896417	1.75E-01	10116780	10116903	NC_000001.10
cg01093311	3.63E-25	1.92E-27	1.27E+01	51.5870593	1.42E-01	31491009	31491132	NC_000014.8
cg22304612	3.65E-25	1.93E-27	1.27E+01	51.5822882	2.67E-01	2781241	2781364	NC_000020.10
cg01856162	3.80E-25	2.02E-27	1.27E+01	51.5386669	1.89E-01	233350940	233351063	NC_000002.11
cg24804436	3.84E-25	2.04E-27	1.27E+01	51.528813	8.19E-02	42955017	42955140	NC_000006.11
cg23042510	3.89E-25	2.07E-27	1.27E+01	51.5143618	2.02E-01	131757695	131757818	NC_000010.10
cg03692651	3.91E-25	2.08E-27	1.27E+01	51.5088576	3.23E-01	22444593	22444716	NC_000019.9
cg15446391	3.97E-25	2.12E-27	1.27E+01	51.4910434	1.83E-01	32452370	32452493	NC_000011.9
cg06382344	4.02E-25	2.15E-27	1.27E+01	51.4775452	2.88E-01	162280519	162280642	NC_000002.11
cg16508480	4.08E-25	2.18E-27	1.27E+01	51.4610551	3.35E-01	5139874	5139997	NC_000005.9
cg01713086	4.10E-25	2.19E-27	1.27E+01	51.4551183	6.10E-02	28268413	28268536	NC_000008.10
cg01645753	4.16E-25	2.23E-27	1.27E+01	51.4380756	3.04E-01	83316734	83316857	NC_000015.9
cg26814276	4.24E-25	2.28E-27	1.27E+01	51.4169395	3.11E-01	172734266	172734389	NC_000004.11
cg07014673	4.28E-25	2.30E-27	1.27E+01	51.4071625	3.21E-01	237078733	237078856	NC_000002.11
cg17445490	4.32E-25	2.33E-27	1.27E+01	51.3962504	1.87E-01	21642517	21642640	NC_000001.10
cg14658067	4.46E-25	2.41E-27	1.27E+01	51.3635993	2.50E-01	1273394	1273517	NC_000007.13
cg16239296	4.52E-25	2.44E-27	1.27E+01	51.3496902	1.13E-01	83736925	83737048	NC_000015.9
cg04159077	4.55E-25	2.46E-27	1.27E+01	51.3425896	1.42E-01	81110820	81110943	NC_000010.10
cg05937737	4.65E-25	2.52E-27	1.27E+01	51.3173447	2.28E-01	170303540	170303663	NC_000003.11
cg24621437	4.69E-25	2.54E-27	1.27E+01	51.3092218	3.04E-01	108924560	108924683	NC_000010.10
cg16709232	4.74E-25	2.57E-27	1.27E+01	51.2975776	2.76E-01	72322154	72322277	NC_000017.10
cg03560996	4.77E-25	2.59E-27	1.27E+01	51.2912384	1.31E-01	31733908	31734031	NC_000006.11
cg06528306	4.81E-25	2.61E-27	1.27E+01	51.2815428	3.57E-01	53852661	53852784	NC_000008.10
cg13944175	4.87E-25	2.65E-27	1.27E+01	51.2688412	2.40E-01	52222698	52222821	NC_000019.9
cg18777119	4.90E-25	2.67E-27	1.27E+01	51.2609275	2.99E-01	30017829	30017952	NC_000019.9

cg09767822	4.90E-25	2.67E-27	1.27E+01	51.2608241	1.80E-01	20178040	20178163	NC_000011.9
cg19922137	4.94E-25	2.69E-27	1.27E+01	51.2529572	2.35E-01	210111561	210111684	NC_000001.10
cg14058551	5.07E-25	2.76E-27	1.27E+01	51.226796	1.96E-01	115313103	115313226	NC_000009.11
cg08759276	5.13E-25	2.80E-27	1.27E+01	51.2125313	1.62E-01	86644262	86644385	NC_000016.9
cg24604013	5.17E-25	2.83E-27	1.27E+01	51.202594	2.45E-01	112721325	112721448	NC_000013.10
cg11071231	5.22E-25	2.86E-27	1.27E+01	51.1936863	2.95E-01	57069907	57070030	NC_000008.10
cg19126300	5.33E-25	2.92E-27	1.27E+01	51.1710217	3.08E-01	32457162	32457285	NC_000011.9
cg21265287	5.35E-25	2.94E-27	1.27E+01	51.165017	1.09E-01	1228383	1228506	NC_000001.10
cg19761848	5.38E-25	2.95E-27	1.27E+01	51.1602407	2.77E-01	237076815	237076938	NC_000002.11
cg15054725	5.42E-25	2.98E-27	1.27E+01	51.1526634	2.97E-01	223176606	223176729	NC_000002.11
cg06932616	5.53E-25	3.05E-27	1.27E+01	51.1289175	1.31E-01	48908335	48908458	NC_000019.9
cg09627302	5.55E-25	3.06E-27	1.27E+01	51.1249992	1.35E-01	31432787	31432910	NC_000003.11
cg16175941	5.68E-25	3.14E-27	1.27E+01	51.0993599	2.50E-01	154779103	154779226	NC_000006.11
cg24101039	5.69E-25	3.14E-27	1.27E+01	51.0976547	2.55E-01	21470054	21470177	NC_000007.13
cg12480375	5.83E-25	3.23E-27	1.27E+01	51.0698188	1.40E-01	30135523	30135646	NC_000006.11
cg10453719	5.86E-25	3.25E-27	1.27E+01	51.0641762	2.42E-01	165305050	165305173	NC_000004.11
cg13075537	6.15E-25	3.42E-27	1.27E+01	51.0129326	2.68E-01	25810696	25810819	NC_000001.10
cg13914024	6.24E-25	3.48E-27	1.27E+01	50.9969114	1.76E-01	161009644	161009767	NC_000001.10
cg27356162	6.33E-25	3.54E-27	1.26E+01	50.9809573	1.49E-01	65347283	65347406	NC_000015.9
cg23658709	6.39E-25	3.57E-27	1.26E+01	50.9711375	1.70E-01	38120185	38120308	NC_000006.11
cg02415779	6.43E-25	3.60E-27	1.26E+01	50.9629114	2.68E-01	18527778	18527901	NC_000022.10
cg04534765	6.46E-25	3.62E-27	1.26E+01	50.9590607	2.93E-01	74962369	74962492	NC_000018.9
cg05447556	6.49E-25	3.64E-27	1.26E+01	50.9523585	1.16E-01	38377633	38377756	NC_000022.10
cg11958675	6.50E-25	3.65E-27	1.26E+01	50.950921	1.40E-01	35618813	35618936	NC_000010.10
cg24681438	6.51E-25	3.65E-27	1.26E+01	50.949103	1.59E-01	241627226	241627349	NC_000002.11

cg24708471	6.51E-25	3.66E-27	1.26E+01	50.9481804	3.58E-01	30719437	30719560	NC_000019.9
cg21597025	6.59E-25	3.71E-27	1.26E+01	50.9346126	1.61E-01	142837564	142837687	NC_000003.11
cg14514120	6.80E-25	3.83E-27	1.26E+01	50.9012188	1.62E-01	64801624	64801747	NC_000012.11
cg01047586	6.80E-25	3.83E-27	1.26E+01	50.9011982	3.24E-01	176933407	176933530	NC_000002.11
cg08146323	6.86E-25	3.87E-27	1.26E+01	50.8915323	2.56E-01	57183118	57183241	NC_000019.9
cg11342234	7.28E-25	4.12E-27	1.26E+01	50.8292497	1.58E-01	2710838	2710961	NC_000011.9
cg26709300	7.33E-25	4.15E-27	1.26E+01	50.8225103	2.25E-01	30106682	30106805	NC_000016.9
cg12740910	7.37E-25	4.18E-27	1.26E+01	50.815778	1.85E-01	147233887	147234010	NC_000001.10
cg16763574	7.45E-25	4.22E-27	1.26E+01	50.8045327	1.81E-01	111937052	111937175	NC_000013.10
cg03217795	7.45E-25	4.23E-27	1.26E+01	50.8031524	3.30E-01	23847556	23847679	NC_000016.9
cg00540067	7.45E-25	4.23E-27	1.26E+01	50.8028567	2.15E-01	126074344	126074467	NC_000003.11
cg10266211	7.54E-25	4.28E-27	1.26E+01	50.7903426	1.94E-01	4860082	4860205	NC_000004.11
cg27473355	7.64E-25	4.35E-27	1.26E+01	50.7744706	1.20E-01	28915234	28915357	NC_000016.9
cg16729415	7.67E-25	4.37E-27	1.26E+01	50.7710462	3.60E-01	35047203	35047326	NC_000015.9
cg02259384	7.80E-25	4.45E-27	1.26E+01	50.7529059	1.82E-01	58523373	58523496	NC_000003.11
cg17214456	7.85E-25	4.49E-27	1.26E+01	50.7445958	1.06E-01	102216984	102217107	NC_000012.11
cg02421985	7.89E-25	4.51E-27	1.26E+01	50.7393352	2.56E-01	180527547	180527670	NC_000005.9
cg05167251	8.04E-25	4.60E-27	1.26E+01	50.7188318	2.53E-01	176987465	176987588	NC_000002.11
cg14487131	8.12E-25	4.65E-27	1.26E+01	50.7090254	3.76E-01	79633737	79633860	NC_000009.11
cg11665583	8.23E-25	4.72E-27	1.26E+01	50.6947898	1.24E-01	26893201	26893324	NC_000022.10
cg11728747	8.53E-25	4.91E-27	1.26E+01	50.6549922	2.47E-01	29037910	29038033	NC_000007.13
cg05797615	8.62E-25	4.97E-27	1.26E+01	50.6428751	1.74E-01	220925515	220925638	NC_000001.10
cg21680980	8.82E-25	5.09E-27	1.26E+01	50.618556	1.28E-01	221069572	221069695	NC_000001.10
cg18127659	9.03E-25	5.22E-27	1.26E+01	50.5936343	1.38E-01	170617566	170617689	NC_000006.11
cg10811502	9.19E-25	5.32E-27	1.26E+01	50.5755304	1.54E-01	30407370	30407493	NC_000020.10

cg07963234	9.23E-25	5.34E-27	1.26E+01	50.5709487	2.64E-01	5894923	5895046	NC_000004.11
cg09591329	9.44E-25	5.48E-27	1.26E+01	50.5459396	1.67E-01	102388664	102388787	NC_000002.11
cg21641458	9.49E-25	5.51E-27	1.26E+01	50.5397031	2.18E-01	27185136	27185259	NC_000007.13
cg04698114	9.94E-25	5.79E-27	1.26E+01	50.491325	2.58E-01	51184379	51184502	NC_000016.9
cg19101893	1.00E-24	5.85E-27	1.26E+01	50.4801462	1.91E-01	43283862	43283985	NC_000001.10
cg01709312	1.01E-24	5.87E-27	1.26E+01	50.477104	2.11E-01	112630835	112630958	NC_000010.10
cg18627235	1.01E-24	5.92E-27	1.26E+01	50.4687399	2.84E-01	96342747	96342870	NC_000014.8
cg02387639	1.02E-24	6.00E-27	1.26E+01	50.4564541	1.12E-01	179384293	179384416	NC_000003.11
cg25612297	1.03E-24	6.04E-27	1.26E+01	50.4494044	1.58E-01	64046701	64046824	NC_000002.11
cg07502439	1.04E-24	6.11E-27	1.26E+01	50.4376285	2.24E-01	51185461	51185584	NC_000016.9
cg15499701	1.07E-24	6.29E-27	1.26E+01	50.4090089	9.29E-02	33208306	33208429	NC_000019.9
cg04413148	1.07E-24	6.31E-27	1.26E+01	50.4059359	1.95E-01	67965867	67965990	NC_000016.9
cg13495205	1.07E-24	6.31E-27	1.26E+01	50.4055966	3.27E-01	4714033	4714156	NC_000001.10
cg20266316	1.08E-24	6.33E-27	1.26E+01	50.4017268	2.81E-01	98247587	98247710	NC_000007.13
cg07022279	1.08E-24	6.38E-27	1.26E+01	50.39538	1.79E-01	64703637	64703760	NC_000011.9
cg04671611	1.08E-24	6.38E-27	1.26E+01	50.3952683	2.77E-01	6623787	6623910	NC_000016.9
cg26047066	1.08E-24	6.38E-27	1.26E+01	50.3949559	2.83E-01	221069136	221069259	NC_000001.10
cg21606866	1.10E-24	6.48E-27	1.26E+01	50.3797247	1.92E-01	43735174	43735297	NC_000001.10
cg19296501	1.10E-24	6.51E-27	1.26E+01	50.3751568	1.12E-01	1371045	1371168	NC_000004.11
cg03422651	1.10E-24	6.51E-27	1.26E+01	50.3742656	1.47E-01	2542324	2542447	NC_000016.9
cg26920808	1.11E-24	6.55E-27	1.26E+01	50.368019	1.75E-01	72237548	72237671	NC_000012.11
cg00421335	1.13E-24	6.68E-27	1.26E+01	50.3492238	1.63E-01	71206528	71206651	NC_000002.11
cg13264399	1.17E-24	6.93E-27	1.26E+01	50.3118248	1.65E-01	176944987	176945110	NC_000002.11
cg23348372	1.17E-24	6.94E-27	1.26E+01	50.3115553	1.93E-01	86282139	86282262	NC_000002.11
cg06610464	1.17E-24	6.96E-27	1.26E+01	50.308095	1.65E-01	40243608	40243731	NC_000020.10

cg27324804	1.18E-24	7.01E-27	1.26E+01	50.3013678	1.54E-01	101509161	101509284	NC_000007.13
cg12456714	1.20E-24	7.16E-27	1.25E+01	50.2799027	3.02E-01	16180393	16180516	NC_000005.9
cg18422423	1.21E-24	7.19E-27	1.25E+01	50.2754127	2.14E-01	51576922	51577045	NC_000003.11
cg08569799	1.21E-24	7.23E-27	1.25E+01	50.2702181	3.14E-01	1886828	1886951	NC_000005.9
cg06003834	1.22E-24	7.27E-27	1.25E+01	50.2647371	1.28E-01	66354967	66355090	NC_000005.9
cg00001747	1.22E-24	7.30E-27	1.25E+01	50.2603537	3.18E-01	105460263	105460386	NC_000002.11
cg00458564	1.24E-24	7.41E-27	1.25E+01	50.245627	1.51E-01	114847039	114847162	NC_000013.10
cg20427198	1.24E-24	7.41E-27	1.25E+01	50.2455647	2.16E-01	46969267	46969390	NC_000022.10
cg05745622	1.27E-24	7.62E-27	1.25E+01	50.2176262	9.31E-02	80510469	80510592	NC_000017.10
cg10985281	1.29E-24	7.76E-27	1.25E+01	50.1994195	1.34E-01	194573301	194573424	NC_000003.11
cg05178576	1.30E-24	7.82E-27	1.25E+01	50.1918768	2.72E-01	77468486	77468609	NC_000016.9
cg10356210	1.31E-24	7.88E-27	1.25E+01	50.1852473	1.83E-01	26946568	26946691	NC_000002.11
cg25527090	1.32E-24	7.92E-27	1.25E+01	50.1796384	2.44E-01	118894180	118894303	NC_000010.10
cg19923650	1.33E-24	7.97E-27	1.25E+01	50.1730043	2.66E-01	172659730	172659853	NC_000005.9
cg14482712	1.33E-24	7.97E-27	1.25E+01	50.172897	1.90E-01	40348303	40348426	NC_000015.9
cg25324105	1.33E-24	8.04E-27	1.25E+01	50.1648225	3.11E-01	38183259	38183382	NC_000019.9
cg25411239	1.33E-24	8.04E-27	1.25E+01	50.1643584	1.57E-01	3094859	3094982	NC_000001.10
cg20205704	1.33E-24	8.05E-27	1.25E+01	50.1636181	1.72E-01	2213196	2213319	NC_000017.10
cg20443254	1.35E-24	8.17E-27	1.25E+01	50.1492545	3.10E-01	108169020	108169143	NC_000012.11
cg27376182	1.36E-24	8.22E-27	1.25E+01	50.1428958	3.17E-01	3233068	3233191	NC_000016.9
cg03943218	1.36E-24	8.22E-27	1.25E+01	50.1426166	2.90E-01	102419600	102419723	NC_000010.10
cg14564616	1.38E-24	8.34E-27	1.25E+01	50.1286427	3.10E-01	1876337	1876460	NC_000005.9
cg03238797	1.38E-24	8.38E-27	1.25E+01	50.1238769	2.81E-01	77468893	77469016	NC_000016.9
cg17509967	1.42E-24	8.64E-27	1.25E+01	50.0938258	2.63E-01	13617094	13617217	NC_000019.9
cg26417985	1.43E-24	8.68E-27	1.25E+01	50.089072	1.81E-01	17633832	17633955	NC_000020.10

cg06688293	1.45E-24	8.82E-27	1.25E+01	50.0724153	1.64E-01	16469294	16469417	NC_000005.9
cg24556441	1.46E-24	8.91E-27	1.25E+01	50.0626536	2.75E-01	33453994	33454117	NC_000022.10
cg09978395	1.48E-24	9.04E-27	1.25E+01	50.0488268	1.81E-01	37092121	37092244	NC_000021.8
cg05037270	1.51E-24	9.20E-27	1.25E+01	50.0305933	1.90E-01	21504121	21504244	NC_000001.10
cg17541528	1.58E-24	9.67E-27	1.25E+01	49.9813277	3.18E-01	110959650	110959773	NC_000013.10
cg00718444	1.58E-24	9.68E-27	1.25E+01	49.9807041	1.90E-01	145685564	145685687	NC_000008.10
cg06425919	1.59E-24	9.75E-27	1.25E+01	49.9726649	2.51E-01	17741925	17742048	NC_000011.9
cg27578963	1.60E-24	9.78E-27	1.25E+01	49.9699349	1.32E-01	88803282	88803405	NC_000016.9
cg10966580	1.60E-24	9.83E-27	1.25E+01	49.9645698	2.36E-01	6223973	6224096	NC_000004.11
cg25486824	1.61E-24	9.89E-27	1.25E+01	49.9592766	2.02E-01	13983790	13983913	NC_000011.9
cg05305413	1.62E-24	9.93E-27	1.25E+01	49.954808	1.92E-01	22327119	22327242	NC_000001.10
cg01878308	1.63E-24	1.00E-26	1.25E+01	49.9436689	2.06E-01	86719502	86719625	NC_000002.11
cg19824388	1.64E-24	1.01E-26	1.25E+01	49.9406141	1.29E-01	50192542	50192665	NC_000019.9
cg05185738	1.71E-24	1.05E-26	1.25E+01	49.8953287	2.40E-01	70371992	70372115	NC_000015.9
cg25722041	1.72E-24	1.06E-26	1.25E+01	49.8907918	1.69E-01	8623473	8623596	NC_000001.10
cg18347642	1.82E-24	1.13E-26	1.25E+01	49.8294717	2.21E-01	21950766	21950889	NC_000004.11
cg03419646	1.82E-24	1.13E-26	1.25E+01	49.8286516	1.96E-01	47297268	47297391	NC_000017.10
cg20291049	1.83E-24	1.13E-26	1.25E+01	49.8229626	2.17E-01	105472217	105472340	NC_000002.11
cg08070327	1.84E-24	1.14E-26	1.25E+01	49.8141392	2.05E-01	27184059	27184182	NC_000007.13
cg03894103	1.88E-24	1.17E-26	1.25E+01	49.7922774	2.06E-01	44586901	44587024	NC_000002.11
cg20505375	1.88E-24	1.17E-26	1.25E+01	49.7922147	1.61E-01	143374469	143374592	NC_000008.10
cg15108047	1.88E-24	1.17E-26	1.25E+01	49.7894715	1.76E-01	19742828	19742951	NC_000020.10
cg20773956	1.90E-24	1.19E-26	1.25E+01	49.7788025	1.63E-01	155582325	155582448	NC_000001.10
cg02287325	1.91E-24	1.19E-26	1.25E+01	49.7755417	1.73E-01	75252355	75252478	NC_000016.9
cg03794214	1.94E-24	1.22E-26	1.25E+01	49.7542795	2.39E-01	42912017	42912140	NC_000022.10

cg06947913	1.95E-24	1.22E-26	1.25E+01	49.7537402	3.54E-01	50297793	50297916	NC_000012.11
cg11599526	1.95E-24	1.22E-26	1.25E+01	49.7507796	1.76E-01	150449509	150449632	NC_000005.9
cg08139729	1.96E-24	1.23E-26	1.25E+01	49.7459529	1.89E-01	41984243	41984366	NC_000001.10
cg14866863	2.00E-24	1.25E-26	1.25E+01	49.7227993	2.49E-01	58332116	58332239	NC_000014.8
cg17332245	2.00E-24	1.26E-26	1.25E+01	49.7222628	1.61E-01	20406947	20407070	NC_000006.11
cg16086620	2.01E-24	1.26E-26	1.25E+01	49.7201837	3.19E-01	93389557	93389680	NC_000014.8
cg01452847	2.03E-24	1.28E-26	1.25E+01	49.7059404	2.42E-01	46711341	46711464	NC_000017.10
cg01688536	2.05E-24	1.29E-26	1.25E+01	49.6953173	3.29E-01	35296968	35297091	NC_000007.13
cg23891360	2.09E-24	1.32E-26	1.25E+01	49.6746439	2.92E-01	130388234	130388357	NC_000012.11
cg27376707	2.09E-24	1.32E-26	1.25E+01	49.6712237	2.62E-01	6533187	6533310	NC_000016.9
cg07793163	2.12E-24	1.34E-26	1.25E+01	49.6569913	2.01E-01	12680897	12681020	NC_000012.11
cg05526731	2.14E-24	1.36E-26	1.25E+01	49.6460021	2.11E-01	185263	185386	NC_000017.10
cg04834608	2.19E-24	1.39E-26	1.25E+01	49.620145	2.44E-01	3706634	3706757	NC_000016.9
cg23180938	2.20E-24	1.39E-26	1.25E+01	49.6176923	3.18E-01	115152485	115152608	NC_000005.9
cg05099508	2.23E-24	1.41E-26	1.25E+01	49.6036443	3.00E-01	22634432	22634555	NC_000010.10
cg21277995	2.29E-24	1.45E-26	1.24E+01	49.5761957	3.77E-01	393239	393362	NC_000006.11
cg25317585	2.29E-24	1.46E-26	1.24E+01	49.5728966	3.47E-01	102568886	102569009	NC_000013.10
cg24454144	2.30E-24	1.46E-26	1.24E+01	49.5692152	3.10E-01	37353126	37353249	NC_000020.10
cg01200640	2.30E-24	1.46E-26	1.24E+01	49.569125	2.83E-01	687384	687507	NC_000008.10
cg07337025	2.31E-24	1.47E-26	1.24E+01	49.5620226	1.61E-01	135996211	135996334	NC_000003.11
cg02623991	2.37E-24	1.51E-26	1.24E+01	49.5362999	3.21E-01	54926431	54926554	NC_000019.9
cg15415452	2.44E-24	1.56E-26	1.24E+01	49.5065077	2.97E-01	112759355	112759478	NC_000013.10
cg27015161	2.47E-24	1.58E-26	1.24E+01	49.4925632	1.70E-01	24816070	24816193	NC_000002.11
cg12252039	2.49E-24	1.59E-26	1.24E+01	49.4847701	1.78E-01	51087971	51088094	NC_000007.13
cg25181284	2.49E-24	1.60E-26	1.24E+01	49.4836308	7.54E-02	50497037	50497160	NC_000012.11

cg05141333	2.53E-24	1.62E-26	1.24E+01	49.4677309	2.17E-01	145048363	145048486	NC_000008.10
cg26503073	2.54E-24	1.63E-26	1.24E+01	49.4616706	2.44E-01	6704074	6704197	NC_000007.13
cg00058923	2.58E-24	1.66E-26	1.24E+01	49.4442622	1.89E-01	161069167	161069290	NC_000001.10
cg15556502	2.58E-24	1.66E-26	1.24E+01	49.4435708	3.09E-01	43602845	43602968	NC_000011.9
cg26296488	2.59E-24	1.67E-26	1.24E+01	49.440336	3.54E-01	9783192	9783315	NC_000004.11
cg00316151	2.61E-24	1.68E-26	1.24E+01	49.4315376	1.57E-01	14576504	14576627	NC_000016.9
cg20899584	2.62E-24	1.68E-26	1.24E+01	49.4295565	6.90E-02	33396983	33397106	NC_000011.9
cg00154727	2.63E-24	1.70E-26	1.24E+01	49.4236014	1.96E-01	50863804	50863927	NC_000007.13
cg14724925	2.67E-24	1.73E-26	1.24E+01	49.405839	1.57E-01	134675438	134675561	NC_000010.10
cg12405139	2.70E-24	1.75E-26	1.24E+01	49.3942094	2.18E-01	8106035	8106158	NC_000010.10
cg00177451	2.70E-24	1.75E-26	1.24E+01	49.3940057	3.63E-01	51186266	51186389	NC_000016.9
cg16803678	2.71E-24	1.75E-26	1.24E+01	49.3915815	1.47E-01	133941748	133941871	NC_000003.11
cg22954449	2.73E-24	1.76E-26	1.24E+01	49.3840137	3.65E-01	5140001	5140124	NC_000005.9
cg23839136	2.74E-24	1.77E-26	1.24E+01	49.381304	2.58E-01	70597599	70597722	NC_000007.13
cg01500945	2.74E-24	1.77E-26	1.24E+01	49.3788041	3.09E-01	51185672	51185795	NC_000016.9
cg02200717	2.80E-24	1.81E-26	1.24E+01	49.3579282	2.44E-01	21511676	21511799	NC_000014.8
cg20718350	2.80E-24	1.81E-26	1.24E+01	49.3575566	3.36E-01	103352294	103352417	NC_000012.11
cg04904318	2.80E-24	1.82E-26	1.24E+01	49.3553075	2.42E-01	46607828	46607951	NC_000017.10
cg03409338	2.82E-24	1.83E-26	1.24E+01	49.3485526	1.25E-01	591353	591476	NC_000016.9
cg26247373	2.85E-24	1.85E-26	1.24E+01	49.3370554	1.31E-01	57655696	57655819	NC_000012.11
cg26844246	2.90E-24	1.89E-26	1.24E+01	49.3170375	1.87E-01	170736277	170736400	NC_000005.9
cg16908948	2.92E-24	1.90E-26	1.24E+01	49.3093399	1.76E-01	45500256	45500379	NC_000010.10
cg25946538	2.93E-24	1.91E-26	1.24E+01	49.3067071	1.45E-01	2482531	2482654	NC_000011.9
cg11224582	2.97E-24	1.93E-26	1.24E+01	49.2921555	3.04E-01	4919138	4919261	NC_000012.11
cg16123202	2.98E-24	1.94E-26	1.24E+01	49.2876553	2.29E-01	1287145	1287268	NC_000007.13

cg12859429	3.04E-24	1.99E-26	1.24E+01	49.2666352	1.79E-01	20046011	20046134	NC_000011.9
cg06876872	3.04E-24	1.99E-26	1.24E+01	49.2649795	1.81E-01	202110290	202110413	NC_000001.10
cg05221264	3.04E-24	1.99E-26	1.24E+01	49.2647448	1.58E-01	15783513	15783636	NC_000001.10
cg08572278	3.04E-24	1.99E-26	1.24E+01	49.2631017	1.07E-01	62467955	62468078	NC_000004.11
cg09499844	3.05E-24	2.00E-26	1.24E+01	49.2592192	1.92E-01	129045722	129045845	NC_000008.10
cg02652344	3.13E-24	2.06E-26	1.24E+01	49.2319546	2.69E-01	123553595	123553718	NC_000009.11
cg04100397	3.15E-24	2.07E-26	1.24E+01	49.2259332	8.89E-02	110875551	110875674	NC_000002.11
cg08566455	3.22E-24	2.12E-26	1.24E+01	49.2010158	2.54E-01	130971164	130971287	NC_000002.11
cg22992279	3.25E-24	2.14E-26	1.24E+01	49.1912578	2.25E-01	26414901	26415024	NC_000004.11
cg24607283	3.27E-24	2.16E-26	1.24E+01	49.1846439	1.69E-01	11302249	11302372	NC_000003.11
cg01618923	3.41E-24	2.26E-26	1.24E+01	49.1378305	2.01E-01	49883335	49883458	NC_000013.10
cg25916711	3.42E-24	2.27E-26	1.24E+01	49.1349558	2.28E-01	172660080	172660203	NC_000005.9
cg19539519	3.44E-24	2.28E-26	1.24E+01	49.1289306	1.50E-01	26553687	26553810	NC_000017.10
cg22978087	3.49E-24	2.32E-26	1.24E+01	49.1132362	2.93E-01	24814126	24814249	NC_000008.10
cg03603866	3.53E-24	2.34E-26	1.24E+01	49.1011505	1.17E-01	107027625	107027748	NC_000013.10
cg20988960	3.56E-24	2.37E-26	1.24E+01	49.0924547	1.67E-01	3191815	3191938	NC_000001.10
cg03964958	3.63E-24	2.42E-26	1.24E+01	49.0688985	2.54E-01	176964720	176964843	NC_000002.11
cg22489321	3.64E-24	2.43E-26	1.24E+01	49.0664135	2.23E-01	100643388	100643511	NC_000013.10
cg23290344	3.65E-24	2.43E-26	1.24E+01	49.0638886	2.55E-01	24771466	24771589	NC_000008.10
cg22125805	3.66E-24	2.44E-26	1.24E+01	49.0608031	2.86E-01	119212248	119212371	NC_000012.11
cg27003782	3.74E-24	2.50E-26	1.24E+01	49.037045	2.47E-01	125651391	125651514	NC_000010.10
cg17236169	3.80E-24	2.54E-26	1.24E+01	49.0214374	2.02E-01	64480683	64480806	NC_000011.9
cg19506628	3.83E-24	2.56E-26	1.24E+01	49.012114	1.15E-01	653442	653565	NC_000005.9
cg12347480	3.90E-24	2.62E-26	1.24E+01	48.9924448	1.31E-01	27497024	27497147	NC_000017.10
cg14532519	4.03E-24	2.71E-26	1.24E+01	48.958259	1.26E-01	42612642	42612765	NC_000022.10

cg13974192	4.05E-24	2.72E-26	1.24E+01	48.9537283	2.54E-01	53775410	53775533	NC_000013.10
cg09641127	4.09E-24	2.75E-26	1.24E+01	48.9428342	2.54E-01	10193895	10194018	NC_000002.11
cg06392169	4.10E-24	2.76E-26	1.24E+01	48.9409092	3.42E-01	391936	392059	NC_000006.11
cg25390165	4.14E-24	2.78E-26	1.24E+01	48.9310999	1.97E-01	27184188	27184311	NC_000007.13
cg14723977	4.21E-24	2.83E-26	1.24E+01	48.9134481	1.13E-01	15532806	15532929	NC_000019.9
cg16670307	4.21E-24	2.83E-26	1.24E+01	48.9127214	1.92E-01	131720640	131720763	NC_000011.9
cg17107017	4.21E-24	2.84E-26	1.24E+01	48.9111731	2.03E-01	116925613	116925736	NC_000001.10
cg06163274	4.21E-24	2.84E-26	1.24E+01	48.9109327	2.13E-01	52627619	52627742	NC_000018.9
cg15405084	4.29E-24	2.90E-26	1.23E+01	48.8907322	1.09E-01	187864009	187864132	NC_000004.11
cg15788059	4.33E-24	2.92E-26	1.23E+01	48.8825552	2.07E-01	26961237	26961360	NC_000022.10
cg01893212	4.33E-24	2.93E-26	1.23E+01	48.8813928	3.81E-01	49813088	49813211	NC_000007.13
cg09860921	4.36E-24	2.95E-26	1.23E+01	48.8741676	1.39E-01	45116241	45116364	NC_000001.10
cg15629893	4.36E-24	2.95E-26	1.23E+01	48.8740096	1.68E-01	22327131	22327254	NC_000001.10
cg26682580	4.38E-24	2.97E-26	1.23E+01	48.8673694	2.77E-01	128752058	128752181	NC_000012.11
cg21680729	4.39E-24	2.97E-26	1.23E+01	48.8651032	1.49E-01	17426422	17426545	NC_000017.10
cg15684162	4.42E-24	3.00E-26	1.23E+01	48.8566854	1.60E-01	61712639	61712762	NC_000017.10
cg17302155	4.44E-24	3.01E-26	1.23E+01	48.8523656	3.02E-01	100061111	100061234	NC_000006.11
cg02366961	4.48E-24	3.04E-26	1.23E+01	48.8430335	1.49E-01	19095211	19095334	NC_000011.9
cg09426834	4.57E-24	3.11E-26	1.23E+01	48.8212571	3.26E-01	946648	946771	NC_000002.11
cg22759823	4.57E-24	3.11E-26	1.23E+01	48.8211376	3.25E-01	57610870	57610993	NC_000019.9
cg18516557	4.65E-24	3.16E-26	1.23E+01	48.8035298	2.13E-01	177004015	177004138	NC_000002.11
cg15332951	4.65E-24	3.17E-26	1.23E+01	48.8023633	2.05E-01	21194739	21194862	NC_000017.10
cg23054189	4.70E-24	3.20E-26	1.23E+01	48.7907699	3.04E-01	248020692	248020815	NC_000001.10
cg02172150	4.76E-24	3.24E-26	1.23E+01	48.7787887	2.71E-01	70597774	70597897	NC_000007.13
cg12393318	4.85E-24	3.32E-26	1.23E+01	48.7562629	2.41E-01	87981068	87981191	NC_000005.9

cg12799689	4.85E-24	3.32E-26	1.23E+01	48.7558748	3.26E-01	67067893	67068016	NC_000018.9
cg05148385	4.85E-24	3.32E-26	1.23E+01	48.7543407	9.51E-02	213950646	213950769	NC_000002.11
cg09194815	5.00E-24	3.43E-26	1.23E+01	48.7224617	2.05E-01	100845274	100845397	NC_000013.10
cg06646174	5.05E-24	3.47E-26	1.23E+01	48.7130492	1.51E-01	116948652	116948775	NC_000011.9
cg13532185	5.09E-24	3.50E-26	1.23E+01	48.7036726	1.57E-01	130078849	130078972	NC_000011.9
cg00947023	5.19E-24	3.57E-26	1.23E+01	48.6841429	1.71E-01	11953402	11953525	NC_000017.10
cg24150172	5.22E-24	3.59E-26	1.23E+01	48.6780053	3.33E-01	55372111	55372234	NC_000008.10
cg07536910	5.25E-24	3.62E-26	1.23E+01	48.6702144	2.61E-01	176948105	176948228	NC_000002.11
cg15048991	5.25E-24	3.62E-26	1.23E+01	48.6697597	3.32E-01	5139866	5139989	NC_000005.9
cg16619425	5.38E-24	3.71E-26	1.23E+01	48.6452063	2.96E-01	59828528	59828651	NC_000020.10
cg03984502	5.63E-24	3.90E-26	1.23E+01	48.5957768	1.23E-01	151805661	151805784	NC_000001.10
cg26207909	5.63E-24	3.90E-26	1.23E+01	48.5950922	2.34E-01	103986467	103986590	NC_000014.8
cg11473001	5.71E-24	3.96E-26	1.23E+01	48.5794237	1.92E-01	131780499	131780622	NC_000011.9
cg17902007	5.86E-24	4.07E-26	1.23E+01	48.5529064	1.63E-01	101184847	101184970	NC_000013.10
cg00132509	5.92E-24	4.12E-26	1.23E+01	48.5414022	2.36E-01	3329936	3330059	NC_000006.11
cg09936426	5.97E-24	4.16E-26	1.23E+01	48.5325593	2.38E-01	13839913	13840036	NC_000001.10
cg03462380	6.09E-24	4.24E-26	1.23E+01	48.5128384	2.60E-01	27112902	27113025	NC_000015.9
cg05534710	6.15E-24	4.28E-26	1.23E+01	48.5025645	2.93E-01	3595728	3595851	NC_000005.9
cg03190911	6.18E-24	4.31E-26	1.23E+01	48.4964083	1.35E-01	33273011	33273134	NC_000006.11
cg06999084	6.28E-24	4.39E-26	1.23E+01	48.4791141	1.35E-01	88873729	88873852	NC_000016.9
cg00781795	6.29E-24	4.39E-26	1.23E+01	48.4772482	1.48E-01	55815677	55815800	NC_000018.9
cg04812556	6.38E-24	4.46E-26	1.23E+01	48.4614991	2.92E-01	9112715	9112838	NC_000011.9
cg26947626	6.39E-24	4.47E-26	1.23E+01	48.460684	2.84E-01	72469430	72469553	NC_000008.10
cg16236960	6.41E-24	4.49E-26	1.23E+01	48.4557597	1.92E-01	135011937	135012060	NC_000002.11
cg17860133	6.43E-24	4.51E-26	1.23E+01	48.4522043	1.84E-01	117468345	117468468	NC_000012.11

cg27234067	6.48E-24	4.54E-26	1.23E+01	48.4435912	2.01E-01	32460799	32460922	NC_000011.9
cg22962425	6.56E-24	4.60E-26	1.23E+01	48.4312235	1.09E-01	58891595	58891718	NC_000014.8
cg15833565	6.61E-24	4.64E-26	1.23E+01	48.4227423	1.70E-01	44741190	44741313	NC_000001.10
cg20866932	6.63E-24	4.66E-26	1.23E+01	48.418661	1.79E-01	150034315	150034438	NC_000007.13
cg00190206	6.65E-24	4.68E-26	1.23E+01	48.4152644	1.71E-01	176999353	176999476	NC_000002.11
cg05807722	6.71E-24	4.72E-26	1.23E+01	48.4064341	1.44E-01	67697020	67697143	NC_000016.9
cg06186808	6.71E-24	4.72E-26	1.23E+01	48.405183	1.45E-01	24600901	24601024	NC_000014.8
cg10595952	6.83E-24	4.81E-26	1.23E+01	48.3867586	2.56E-01	2097052	2097175	NC_000005.9
cg11823511	6.86E-24	4.83E-26	1.23E+01	48.3821159	2.61E-01	91183697	91183820	NC_000001.10
cg23644589	6.88E-24	4.85E-26	1.23E+01	48.3788916	1.61E-01	228404256	228404379	NC_000001.10
cg21223771	6.90E-24	4.87E-26	1.23E+01	48.3748087	2.27E-01	95945201	95945324	NC_000009.11
cg25821781	6.93E-24	4.90E-26	1.23E+01	48.3692208	1.75E-01	2325143	2325266	NC_000017.10
cg20979852	6.95E-24	4.92E-26	1.23E+01	48.3657817	3.12E-01	140770683	140770806	NC_000003.11
cg22959260	7.16E-24	5.06E-26	1.23E+01	48.3363389	1.97E-01	40382862	40382985	NC_000015.9
cg01382137	7.19E-24	5.09E-26	1.23E+01	48.3305652	3.11E-01	53423078	53423201	NC_000013.10
cg12485556	7.23E-24	5.12E-26	1.23E+01	48.3246338	1.69E-01	105774617	105774740	NC_000006.11
cg04184836	7.27E-24	5.15E-26	1.23E+01	48.3193381	3.89E-01	83316640	83316763	NC_000015.9
cg10500503	7.35E-24	5.21E-26	1.23E+01	48.3074371	1.96E-01	57011246	57011369	NC_000001.10
cg20312205	7.45E-24	5.29E-26	1.23E+01	48.2936667	2.88E-01	84456308	84456431	NC_000013.10
cg06338150	7.50E-24	5.33E-26	1.23E+01	48.2852729	1.45E-01	139392895	139393018	NC_000009.11
cg16137772	7.52E-24	5.35E-26	1.23E+01	48.2813767	2.00E-01	43937721	43937844	NC_000020.10
cg07969308	7.62E-24	5.42E-26	1.23E+01	48.2681285	9.77E-02	460746	460869	NC_000016.9
cg14446951	7.62E-24	5.43E-26	1.23E+01	48.2670393	1.10E-01	36605829	36605952	NC_000002.11
cg25623768	7.62E-24	5.43E-26	1.23E+01	48.2663605	3.03E-01	45241008	45241131	NC_000002.11
cg00762738	7.68E-24	5.48E-26	1.23E+01	48.2582773	1.95E-01	205780033	205780156	NC_000001.10

cg06410537	7.70E-24	5.50E-26	1.23E+01	48.2542603	1.90E-01	170736572	170736695	NC_000005.9
cg26990587	7.72E-24	5.52E-26	1.23E+01	48.251047	2.57E-01	100253901	100254024	NC_000007.13
cg17105834	7.77E-24	5.55E-26	1.23E+01	48.2443355	3.10E-01	177133566	177133689	NC_000001.10
cg07870999	7.83E-24	5.61E-26	1.23E+01	48.2352116	2.36E-01	155260895	155261018	NC_000007.13
cg05778278	7.86E-24	5.63E-26	1.23E+01	48.2312866	1.41E-01	54505303	54505426	NC_000008.10
cg23244488	7.88E-24	5.65E-26	1.23E+01	48.2279952	2.77E-01	19146032	19146155	NC_000007.13
cg25958283	7.91E-24	5.67E-26	1.23E+01	48.2246388	3.39E-01	11752089	11752212	NC_000018.9
cg03304610	7.95E-24	5.70E-26	1.23E+01	48.2185738	3.23E-01	172734276	172734399	NC_000004.11
cg22620221	8.01E-24	5.74E-26	1.22E+01	48.2109492	2.58E-01	153584416	153584539	NC_000007.13
cg08268892	8.05E-24	5.77E-26	1.22E+01	48.2059115	9.91E-02	157131429	157131552	NC_000006.11
cg16638580	8.07E-24	5.79E-26	1.22E+01	48.2026516	1.24E-01	39890911	39891034	NC_000019.9
cg23106559	8.14E-24	5.85E-26	1.22E+01	48.1929798	1.44E-01	58167483	58167606	NC_000012.11
cg05888894	8.23E-24	5.92E-26	1.22E+01	48.1815765	2.21E-01	119107030	119107153	NC_000008.10
cg22457637	8.27E-24	5.95E-26	1.22E+01	48.1767453	1.66E-01	142142872	142142995	NC_000008.10
cg15638709	8.36E-24	6.02E-26	1.22E+01	48.164083	2.58E-01	110226387	110226510	NC_000010.10
cg23934404	8.48E-24	6.11E-26	1.22E+01	48.1493791	3.12E-01	112758491	112758614	NC_000013.10
cg22789318	8.71E-24	6.28E-26	1.22E+01	48.1219864	1.59E-01	981643	981766	NC_000017.10
cg25247290	8.77E-24	6.33E-26	1.22E+01	48.1138524	2.40E-01	32461212	32461335	NC_000011.9
cg02155398	8.81E-24	6.36E-26	1.22E+01	48.1089657	2.76E-01	45160490	45160613	NC_000002.11
cg14657517	8.89E-24	6.43E-26	1.22E+01	48.0981159	2.87E-01	32456910	32457033	NC_000011.9
cg04489066	8.89E-24	6.44E-26	1.22E+01	48.097961	1.99E-01	42211509	42211632	NC_000015.9
cg22754569	8.92E-24	6.46E-26	1.22E+01	48.0947458	1.72E-01	59564444	59564567	NC_000008.10
cg07271473	9.13E-24	6.62E-26	1.22E+01	48.0691906	1.43E-01	40124792	40124915	NC_000021.8
cg04227922	9.21E-24	6.69E-26	1.22E+01	48.059979	2.26E-01	119615496	119615619	NC_000002.11
cg08747377	9.21E-24	6.69E-26	1.22E+01	48.0588682	3.43E-01	82660670	82660793	NC_000016.9

cg21039708	9.26E-24	6.74E-26	1.22E+01	48.0527116	3.44E-01	57278729	57278852	NC_000014.8
cg25732961	9.32E-24	6.78E-26	1.22E+01	48.045796	2.17E-01	7755877	7756000	NC_000017.10
cg15929797	9.35E-24	6.81E-26	1.22E+01	48.0418505	2.32E-01	172660796	172660919	NC_000005.9
cg18887759	9.43E-24	6.88E-26	1.22E+01	48.0320423	1.72E-01	50324282	50324405	NC_000019.9
cg19760241	9.59E-24	7.01E-26	1.22E+01	48.0135437	2.35E-01	35300010	35300133	NC_000017.10
cg06750832	9.59E-24	7.01E-26	1.22E+01	48.0134164	3.19E-01	111217691	111217814	NC_000001.10
cg13912196	9.78E-24	7.15E-26	1.22E+01	47.9932883	2.14E-01	70372127	70372250	NC_000015.9
cg24968692	9.96E-24	7.30E-26	1.22E+01	47.9731725	1.45E-01	131032645	131032768	NC_000007.13
cg18092028	9.98E-24	7.31E-26	1.22E+01	47.9709529	2.95E-01	59827212	59827335	NC_000020.10
cg03867465	1.02E-23	7.48E-26	1.22E+01	47.9483352	1.93E-01	183062301	183062424	NC_000004.11
cg13829550	1.02E-23	7.51E-26	1.22E+01	47.9441512	2.64E-01	74893112	74893235	NC_000014.8
cg04917226	1.03E-23	7.56E-26	1.22E+01	47.938457	2.74E-01	19157839	19157962	NC_000007.13
cg27230984	1.04E-23	7.61E-26	1.22E+01	47.9317776	1.43E-01	916362	916485	NC_000017.10
cg12325536	1.05E-23	7.76E-26	1.22E+01	47.9118009	1.97E-01	87968658	87968781	NC_000005.9
cg21625737	1.06E-23	7.81E-26	1.22E+01	47.9056626	1.99E-01	1011049	1011172	NC_000011.9
cg11344566	1.08E-23	7.94E-26	1.22E+01	47.8895805	1.73E-01	124782885	124783008	NC_000002.11
cg07926691	1.08E-23	7.95E-26	1.22E+01	47.8886318	3.06E-01	38670987	38671110	NC_000007.13
cg15132565	1.08E-23	7.96E-26	1.22E+01	47.8872081	3.17E-01	72740154	72740277	NC_000005.9
cg00295794	1.08E-23	7.97E-26	1.22E+01	47.8858431	2.87E-01	100641409	100641532	NC_000013.10
cg04865220	1.09E-23	8.06E-26	1.22E+01	47.8741234	2.00E-01	73886532	73886655	NC_000015.9
cg23322933	1.09E-23	8.09E-26	1.22E+01	47.8706417	2.68E-01	61109207	61109330	NC_000014.8
cg23858040	1.10E-23	8.13E-26	1.22E+01	47.8653828	3.85E-01	11059577	11059700	NC_000010.10
cg17832688	1.11E-23	8.19E-26	1.22E+01	47.8585172	9.59E-02	44017421	44017544	NC_000001.10
cg18316735	1.11E-23	8.23E-26	1.22E+01	47.8542307	1.30E-01	204339486	204339609	NC_000001.10
cg19508622	1.11E-23	8.24E-26	1.22E+01	47.8527833	2.45E-01	129103558	129103681	NC_000008.10

cg25339054	1.13E-23	8.37E-26	1.22E+01	47.8368978	3.00E-01	50673808	50673931	NC_000005.9
cg03444587	1.14E-23	8.47E-26	1.22E+01	47.8253826	1.67E-01	2054271	2054394	NC_000016.9
cg13265572	1.15E-23	8.54E-26	1.22E+01	47.8172057	1.58E-01	182694617	182694740	NC_000003.11
cg00905220	1.16E-23	8.59E-26	1.22E+01	47.8111458	1.70E-01	982154	982277	NC_000017.10
cg21631754	1.17E-23	8.73E-26	1.22E+01	47.7946637	1.91E-01	45737485	45737608	NC_000019.9
cg09531492	1.18E-23	8.75E-26	1.22E+01	47.7925758	1.57E-01	50632560	50632683	NC_000022.10
cg14696396	1.18E-23	8.81E-26	1.22E+01	47.7855696	2.96E-01	83776446	83776569	NC_000015.9
cg23723818	1.18E-23	8.82E-26	1.22E+01	47.7843085	1.29E-01	57015477	57015600	NC_000001.10
cg05903444	1.18E-23	8.83E-26	1.22E+01	47.7841276	1.85E-01	2751041	2751164	NC_000005.9
cg17391928	1.19E-23	8.88E-26	1.22E+01	47.777727	2.54E-01	128301230	128301353	NC_000005.9
cg25539045	1.19E-23	8.90E-26	1.22E+01	47.7755135	3.22E-01	129535186	129535309	NC_000010.10
cg20054111	1.21E-23	9.02E-26	1.22E+01	47.7629532	1.76E-01	140829046	140829169	NC_000004.11
cg19256368	1.21E-23	9.03E-26	1.22E+01	47.7613167	1.96E-01	83619037	83619160	NC_000015.9
cg05452645	1.22E-23	9.15E-26	1.22E+01	47.7488572	1.98E-01	81117647	81117770	NC_000004.11
cg10312981	1.25E-23	9.36E-26	1.22E+01	47.7254327	1.52E-01	87577304	87577427	NC_000016.9
cg12048508	1.28E-23	9.59E-26	1.22E+01	47.7014641	8.98E-02	65418238	65418361	NC_000012.11
cg20441606	1.28E-23	9.60E-26	1.22E+01	47.700887	1.53E-01	79084557	79084680	NC_000017.10
cg09138865	1.29E-23	9.67E-26	1.22E+01	47.6939124	1.72E-01	88427435	88427558	NC_000010.10
cg08186575	1.30E-23	9.74E-26	1.22E+01	47.6858286	1.26E-01	4371730	4371853	NC_000019.9
cg05295006	1.30E-23	9.76E-26	1.22E+01	47.6842065	2.45E-01	172734347	172734470	NC_000004.11
cg03900143	1.31E-23	9.88E-26	1.22E+01	47.6717195	2.73E-01	147111660	147111783	NC_000003.11
cg16697214	1.32E-23	9.90E-26	1.22E+01	47.670191	3.03E-01	50343361	50343484	NC_000007.13
cg05376374	1.32E-23	9.90E-26	1.22E+01	47.669867	3.16E-01	43602920	43603043	NC_000011.9
cg12483476	1.32E-23	9.95E-26	1.22E+01	47.664655	2.65E-01	178421711	178421834	NC_000005.9
cg10370850	1.32E-23	9.98E-26	1.22E+01	47.6618655	1.15E-01	70027093	70027216	NC_000011.9

cg26476852	1.35E-23	1.02E-25	1.22E+01	47.6404922	2.45E-01	27204728	27204851	NC_000007.13
cg24687655	1.37E-23	1.03E-25	1.22E+01	47.6269554	1.58E-01	110398293	110398416	NC_000013.10
cg04543008	1.37E-23	1.04E-25	1.22E+01	47.6250775	2.98E-01	71955332	71955455	NC_000011.9
cg02128191	1.37E-23	1.04E-25	1.22E+01	47.6230683	7.72E-02	669285	669408	NC_000010.10
cg18770350	1.43E-23	1.08E-25	1.22E+01	47.5802265	3.15E-01	236849966	236850089	NC_000001.10
cg12122146	1.48E-23	1.13E-25	1.22E+01	47.5421367	3.23E-01	130388216	130388339	NC_000012.11
cg05463136	1.48E-23	1.13E-25	1.22E+01	47.5401038	1.01E-01	16286371	16286494	NC_000003.11
cg19736009	1.49E-23	1.13E-25	1.22E+01	47.5381119	1.67E-01	73994187	73994310	NC_000010.10
cg02672378	1.49E-23	1.14E-25	1.22E+01	47.5338939	1.58E-01	41506029	41506152	NC_000006.11
cg02091786	1.50E-23	1.14E-25	1.22E+01	47.5296212	1.17E-01	62415525	62415648	NC_000011.9
cg14073722	1.53E-23	1.16E-25	1.21E+01	47.5093767	2.82E-01	50235737	50235860	NC_000017.10
cg19497031	1.53E-23	1.17E-25	1.21E+01	47.5069217	2.72E-01	79176272	79176395	NC_000013.10
cg26813646	1.55E-23	1.19E-25	1.21E+01	47.491136	2.25E-01	50817651	50817774	NC_000010.10
cg20277356	1.56E-23	1.19E-25	1.21E+01	47.4845101	1.65E-01	123353800	123353923	NC_000010.10
cg11173579	1.62E-23	1.25E-25	1.21E+01	47.4412632	2.62E-01	33247885	33248008	NC_000021.8
cg18486102	1.64E-23	1.26E-25	1.21E+01	47.4338352	3.61E-01	50297777	50297900	NC_000012.11
cg25746778	1.68E-23	1.30E-25	1.21E+01	47.4032336	2.44E-01	131758331	131758454	NC_000010.10
cg22729543	1.77E-23	1.37E-25	1.21E+01	47.3475068	2.28E-01	98991418	98991541	NC_000007.13
cg00637144	1.78E-23	1.37E-25	1.21E+01	47.3453989	1.70E-01	52360268	52360391	NC_000013.10
cg02219383	1.78E-23	1.38E-25	1.21E+01	47.3417164	1.37E-01	43385111	43385234	NC_000003.11
cg27317541	1.83E-23	1.42E-25	1.21E+01	47.3142966	9.18E-02	17454056	17454179	NC_000017.10
cg04180086	1.85E-23	1.43E-25	1.21E+01	47.3019588	2.52E-01	1878499	1878622	NC_000005.9
cg11953609	1.86E-23	1.44E-25	1.21E+01	47.2965503	1.17E-01	170629048	170629171	NC_000001.10
cg02457680	1.86E-23	1.44E-25	1.21E+01	47.2960128	2.19E-01	154475139	154475262	NC_000001.10
cg03873105	1.86E-23	1.45E-25	1.21E+01	47.2942578	1.53E-01	76096456	76096579	NC_000011.9

cg16664405	1.90E-23	1.48E-25	1.21E+01	47.2719568	2.66E-01	111216962	111217085	NC_000010.10
cg08498138	1.91E-23	1.48E-25	1.21E+01	47.2682684	1.21E-01	14576651	14576774	NC_000016.9
cg03568039	1.92E-23	1.50E-25	1.21E+01	47.2605546	1.53E-01	52561293	52561416	NC_000020.10
cg26794346	1.92E-23	1.50E-25	1.21E+01	47.2586284	1.09E-01	16472305	16472428	NC_000006.11
cg19640789	1.93E-23	1.51E-25	1.21E+01	47.252484	7.22E-02	8650112	8650235	NC_000019.9
cg05757474	1.95E-23	1.52E-25	1.21E+01	47.2438125	2.05E-01	63894504	63894627	NC_000011.9
cg13985639	1.96E-23	1.53E-25	1.21E+01	47.2364589	2.28E-01	99344969	99345092	NC_000010.10
cg00145904	1.98E-23	1.55E-25	1.21E+01	47.2278115	1.27E-01	10697703	10697826	NC_000001.10
cg06974135	2.04E-23	1.60E-25	1.21E+01	47.1954395	9.50E-02	58824741	58824864	NC_000005.9
cg11512704	2.07E-23	1.62E-25	1.21E+01	47.181923	1.81E-01	2057358	2057481	NC_000012.11
cg13358636	2.07E-23	1.62E-25	1.21E+01	47.1800935	2.57E-01	124782263	124782386	NC_000002.11
cg04206025	2.10E-23	1.65E-25	1.21E+01	47.1635917	1.98E-01	16975443	16975566	NC_000001.10
cg25015733	2.13E-23	1.68E-25	1.21E+01	47.1475595	2.62E-01	99342986	99343109	NC_000002.11
cg24888814	2.16E-23	1.70E-25	1.21E+01	47.1327329	1.35E-01	54723931	54724054	NC_000012.11
cg17863912	2.18E-23	1.71E-25	1.21E+01	47.1250253	2.25E-01	176994362	176994485	NC_000002.11
cg07018577	2.20E-23	1.73E-25	1.21E+01	47.1144019	2.26E-01	180831396	180831519	NC_000001.10
cg23029851	2.20E-23	1.74E-25	1.21E+01	47.1120418	1.84E-01	293878	294001	NC_000011.9
cg03361789	2.28E-23	1.80E-25	1.21E+01	47.076324	1.38E-01	74005651	74005774	NC_000017.10
cg17217443	2.28E-23	1.81E-25	1.21E+01	47.0730661	1.08E-01	36511291	36511414	NC_000017.10
cg26674966	2.28E-23	1.81E-25	1.21E+01	47.0724919	1.99E-01	22929993	22930116	NC_000015.9
cg26667091	2.28E-23	1.81E-25	1.21E+01	47.0723311	1.73E-01	65352232	65352355	NC_000011.9
cg17160666	2.30E-23	1.82E-25	1.21E+01	47.0665701	1.89E-01	8545003	8545126	NC_000010.10
cg05753794	2.31E-23	1.83E-25	1.21E+01	47.0578301	1.52E-01	150781341	150781464	NC_000007.13
cg00767886	2.33E-23	1.84E-25	1.21E+01	47.0524232	1.55E-01	42193112	42193235	NC_000015.9
cg16146718	2.33E-23	1.85E-25	1.21E+01	47.0511389	1.91E-01	145537915	145538038	NC_000008.10

cg18229521	2.33E-23	1.85E-25	1.21E+01	47.049392	2.98E-01	92790285	92790408	NC_000014.8
cg15007156	2.34E-23	1.86E-25	1.21E+01	47.0444073	2.57E-01	14925135	14925258	NC_000001.10
cg18354248	2.34E-23	1.86E-25	1.21E+01	47.0437712	1.68E-01	93390311	93390434	NC_000010.10
cg08036188	2.39E-23	1.89E-25	1.21E+01	47.0256521	9.17E-02	46522560	46522683	NC_000017.10
cg08460580	2.40E-23	1.90E-25	1.21E+01	47.020356	9.39E-02	689187	689310	NC_000006.11
cg08182501	2.44E-23	1.94E-25	1.21E+01	47.0021468	1.64E-01	177003406	177003529	NC_000002.11
cg01939477	2.44E-23	1.94E-25	1.21E+01	47.0019154	3.36E-01	43602879	43603002	NC_000011.9
cg12764034	2.46E-23	1.95E-25	1.21E+01	46.9943146	2.20E-01	75602846	75602969	NC_000001.10
cg19329160	2.48E-23	1.98E-25	1.21E+01	46.9835596	2.38E-01	79182095	79182218	NC_000013.10
cg07922933	2.50E-23	2.00E-25	1.21E+01	46.9737034	1.95E-01	228548842	228548965	NC_000001.10
cg20735050	2.51E-23	2.00E-25	1.21E+01	46.9690571	2.45E-01	94521117	94521240	NC_000011.9
cg27276079	2.52E-23	2.01E-25	1.21E+01	46.9659693	1.93E-01	42211520	42211643	NC_000015.9
cg09980058	2.53E-23	2.02E-25	1.21E+01	46.9635239	2.46E-01	18898325	18898448	NC_000019.9
cg19288904	2.53E-23	2.02E-25	1.21E+01	46.9623538	3.00E-01	98836248	98836371	NC_000015.9
cg10516993	2.55E-23	2.04E-25	1.21E+01	46.9514058	2.56E-01	158147446	158147569	NC_000001.10
cg21908235	2.64E-23	2.11E-25	1.21E+01	46.9189588	2.83E-01	146551744	146551867	NC_000001.10
cg03599078	2.67E-23	2.13E-25	1.21E+01	46.9065726	1.95E-01	8078314	8078437	NC_000010.10
cg14078335	2.68E-23	2.15E-25	1.21E+01	46.8999232	2.48E-01	71760291	71760414	NC_000011.9
cg15989068	2.79E-23	2.24E-25	1.21E+01	46.857323	1.93E-01	5837057	5837180	NC_000002.11
cg18988498	2.80E-23	2.25E-25	1.21E+01	46.8542374	1.86E-01	31824262	31824385	NC_000011.9
cg06818435	2.84E-23	2.28E-25	1.21E+01	46.8404936	1.55E-01	33279680	33279803	NC_000001.10
cg03858756	2.87E-23	2.31E-25	1.20E+01	46.8272682	2.95E-01	176994637	176994760	NC_000002.11
cg15139644	2.90E-23	2.34E-25	1.20E+01	46.8159945	2.80E-01	35065386	35065509	NC_000018.9
cg22541735	2.91E-23	2.35E-25	1.20E+01	46.8121235	3.23E-01	176988203	176988326	NC_000002.11
cg24760961	2.93E-23	2.36E-25	1.20E+01	46.8075616	1.41E-01	130782216	130782339	NC_000011.9

cg20066716	2.93E-23	2.36E-25	1.20E+01	46.8070156	2.92E-01	49815181	49815304	NC_000007.13
cg05627639	2.95E-23	2.38E-25	1.20E+01	46.7970737	3.21E-01	34113449	34113572	NC_000019.9
cg19259125	2.97E-23	2.40E-25	1.20E+01	46.7884755	1.65E-01	113396924	113397047	NC_000013.10
cg02022519	2.98E-23	2.41E-25	1.20E+01	46.7872504	1.27E-01	115830067	115830190	NC_000011.9
cg26134895	2.98E-23	2.41E-25	1.20E+01	46.7851959	3.75E-01	15500270	15500393	NC_000005.9
cg03730428	2.99E-23	2.42E-25	1.20E+01	46.781706	2.23E-01	32009004	32009127	NC_000011.9
cg19206040	3.00E-23	2.43E-25	1.20E+01	46.776472	2.99E-01	37500441	37500564	NC_000001.10
cg07917052	3.00E-23	2.43E-25	1.20E+01	46.776311	1.10E-01	51110982	51111105	NC_000013.10
cg13234587	3.02E-23	2.45E-25	1.20E+01	46.7689493	7.03E-02	189148133	189148256	NC_000003.11
cg04064644	3.07E-23	2.49E-25	1.20E+01	46.7523281	2.33E-01	1876719	1876842	NC_000005.9
cg24135615	3.07E-23	2.49E-25	1.20E+01	46.7521187	1.26E-01	37042201	37042324	NC_000017.10
cg07007754	3.09E-23	2.51E-25	1.20E+01	46.7470354	2.05E-01	114847329	114847452	NC_000013.10
cg04423294	3.09E-23	2.51E-25	1.20E+01	46.7453486	1.44E-01	188479059	188479182	NC_000003.11
cg07898582	3.13E-23	2.54E-25	1.20E+01	46.7324872	1.79E-01	2953930	2954053	NC_000007.13
cg01366419	3.15E-23	2.56E-25	1.20E+01	46.7260393	2.46E-01	70597091	70597214	NC_000007.13
cg25116216	3.16E-23	2.57E-25	1.20E+01	46.7228988	3.19E-01	10101581	10101704	NC_000017.10
cg00619126	3.19E-23	2.60E-25	1.20E+01	46.7101638	2.43E-01	105459775	105459898	NC_000002.11
cg18380175	3.20E-23	2.61E-25	1.20E+01	46.7067712	2.58E-01	77334070	77334193	NC_000001.10
cg00911881	3.21E-23	2.62E-25	1.20E+01	46.7049661	1.52E-01	11605722	11605845	NC_000008.10
cg03993087	3.21E-23	2.62E-25	1.20E+01	46.7023469	1.44E-01	80499721	80499844	NC_000010.10
cg00377332	3.25E-23	2.65E-25	1.20E+01	46.6903718	1.92E-01	45761250	45761373	NC_000003.11
cg05185021	3.25E-23	2.65E-25	1.20E+01	46.6903131	2.14E-01	2324794	2324917	NC_000017.10
cg09685182	3.26E-23	2.66E-25	1.20E+01	46.6871819	2.18E-01	130236037	130236160	NC_000003.11
cg04999691	3.26E-23	2.67E-25	1.20E+01	46.6862726	1.62E-01	150027050	150027173	NC_000007.13
cg01130127	3.27E-23	2.68E-25	1.20E+01	46.6816191	3.16E-01	51228821	51228944	NC_000019.9

cg27460824	3.28E-23	2.68E-25	1.20E+01	46.6800384	2.03E-01	24750164	24750287	NC_000013.10
cg11657808	3.29E-23	2.70E-25	1.20E+01	46.6750659	3.07E-01	237205950	237206073	NC_000001.10
cg10179300	3.30E-23	2.70E-25	1.20E+01	46.6737882	1.61E-01	14147618	14147741	NC_000005.9
cg04991747	3.43E-23	2.82E-25	1.20E+01	46.6304043	1.56E-01	230207211	230207334	NC_000001.10
cg26650638	3.48E-23	2.85E-25	1.20E+01	46.6180395	3.13E-01	86320857	86320980	NC_000016.9
cg05501996	3.52E-23	2.89E-25	1.20E+01	46.6044058	2.25E-01	50673730	50673853	NC_000005.9
cg04234070	3.53E-23	2.91E-25	1.20E+01	46.6001498	1.38E-01	95275575	95275698	NC_000008.10
cg02753742	3.53E-23	2.91E-25	1.20E+01	46.5994056	1.06E-01	159956264	159956387	NC_000004.11
cg26122980	3.61E-23	2.98E-25	1.20E+01	46.5766321	3.51E-01	38671001	38671124	NC_000007.13
cg22564695	3.65E-23	3.01E-25	1.20E+01	46.5652165	1.45E-01	13080436	13080559	NC_000019.9
cg20872937	3.68E-23	3.04E-25	1.20E+01	46.5556055	3.23E-01	74961968	74962091	NC_000018.9
cg19047707	3.68E-23	3.04E-25	1.20E+01	46.5555517	2.46E-01	233351525	233351648	NC_000002.11
cg12923203	3.69E-23	3.05E-25	1.20E+01	46.5519123	1.64E-01	90771952	90772075	NC_000015.9
cg26804891	3.72E-23	3.08E-25	1.20E+01	46.5432216	1.42E-01	2055242	2055365	NC_000016.9
cg08961621	3.79E-23	3.14E-25	1.20E+01	46.5222615	1.67E-01	39644244	39644367	NC_000021.8
cg20162240	3.80E-23	3.15E-25	1.20E+01	46.5192291	1.43E-01	129733373	129733496	NC_000010.10
cg03574723	3.87E-23	3.22E-25	1.20E+01	46.499523	2.18E-01	125425994	125426117	NC_000010.10
cg10042132	3.91E-23	3.25E-25	1.20E+01	46.4895506	7.95E-02	81938575	81938698	NC_000014.8
cg18348836	3.96E-23	3.29E-25	1.20E+01	46.4756587	2.85E-01	60282400	60282523	NC_000001.10
cg01281157	4.01E-23	3.34E-25	1.20E+01	46.4624718	2.77E-01	178422260	178422383	NC_000005.9
cg27593537	4.05E-23	3.37E-25	1.20E+01	46.4543885	1.37E-01	102810606	102810729	NC_000010.10
cg18411108	4.11E-23	3.42E-25	1.20E+01	46.4379857	2.72E-01	63955727	63955850	NC_000003.11
cg14160480	4.15E-23	3.46E-25	1.20E+01	46.4263297	2.17E-01	15697398	15697521	NC_000001.10
cg18556676	4.15E-23	3.46E-25	1.20E+01	46.4259759	2.21E-01	22095111	22095234	NC_000012.11
cg20569034	4.17E-23	3.48E-25	1.20E+01	46.4206019	2.01E-01	58121550	58121673	NC_000012.11

cg14297867	4.18E-23	3.49E-25	1.20E+01	46.417726	1.63E-01	27526172	27526295	NC_000009.11
cg12475759	4.20E-23	3.50E-25	1.20E+01	46.4143895	2.56E-01	4714672	4714795	NC_000001.10
cg19589652	4.22E-23	3.52E-25	1.20E+01	46.4087282	1.75E-01	56000809	56000932	NC_000019.9
cg19400113	4.22E-23	3.53E-25	1.20E+01	46.407887	1.33E-01	189546778	189546901	NC_000003.11
cg10250663	4.22E-23	3.53E-25	1.20E+01	46.4076211	3.09E-01	55358458	55358581	NC_000016.9
cg13074532	4.30E-23	3.60E-25	1.20E+01	46.3873155	9.30E-02	120156359	120156482	NC_000012.11
cg06641279	4.35E-23	3.64E-25	1.20E+01	46.3762155	1.31E-01	22307561	22307684	NC_000001.10
cg19513834	4.36E-23	3.65E-25	1.20E+01	46.3737621	3.42E-01	105470561	105470684	NC_000002.11
cg05426006	4.36E-23	3.66E-25	1.20E+01	46.3720955	2.41E-01	45781603	45781726	NC_000013.10
cg09634469	4.38E-23	3.67E-25	1.20E+01	46.367738	1.80E-01	21128411	21128534	NC_000022.10
cg03807298	4.50E-23	3.78E-25	1.20E+01	46.3397639	3.80E-01	174159039	174159162	NC_000005.9
cg21550372	4.54E-23	3.82E-25	1.20E+01	46.3297318	1.58E-01	100908908	100909031	NC_000014.8
cg25024074	4.56E-23	3.84E-25	1.20E+01	46.3235581	3.18E-01	182322501	182322624	NC_000002.11
cg05708656	4.59E-23	3.86E-25	1.20E+01	46.3184057	1.74E-01	71792305	71792428	NC_000016.9
cg04880278	4.60E-23	3.87E-25	1.20E+01	46.3157254	1.37E-01	11597172	11597295	NC_000008.10
cg07882671	4.64E-23	3.91E-25	1.20E+01	46.3056837	3.85E-01	1883005	1883128	NC_000005.9
cg22812005	4.68E-23	3.95E-25	1.20E+01	46.2956094	1.30E-01	244216231	244216354	NC_000001.10
cg21802055	4.70E-23	3.97E-25	1.20E+01	46.2914459	2.64E-01	4868794	4868917	NC_000004.11
cg04475936	4.77E-23	4.03E-25	1.20E+01	46.2743049	1.64E-01	2112184	2112307	NC_000005.9
cg23204968	4.78E-23	4.04E-25	1.20E+01	46.2735468	2.21E-01	27183816	27183939	NC_000007.13
cg27631389	4.78E-23	4.04E-25	1.20E+01	46.2721444	1.81E-01	12263892	12264015	NC_000011.9
cg07723431	4.83E-23	4.08E-25	1.20E+01	46.2624453	9.10E-02	2044540	2044663	NC_000019.9
cg16583088	4.92E-23	4.16E-25	1.20E+01	46.2431007	2.01E-01	197395440	197395563	NC_000003.11
cg02505749	4.96E-23	4.20E-25	1.20E+01	46.2339105	2.80E-01	140715735	140715858	NC_000008.10
cg02700891	5.01E-23	4.25E-25	1.20E+01	46.2229488	2.92E-01	129693586	129693709	NC_000003.11

cg27172557	5.07E-23	4.32E-25	1.20E+01	46.2073472	1.92E-01	91012362	91012485	NC_000014.8
cg05500840	5.15E-23	4.39E-25	1.20E+01	46.1915691	2.26E-01	176972068	176972191	NC_000002.11
cg05999324	5.16E-23	4.40E-25	1.20E+01	46.1879517	2.02E-01	112655570	112655693	NC_000010.10
cg00274203	5.17E-23	4.41E-25	1.20E+01	46.1870543	1.88E-01	56661537	56661660	NC_000012.11
cg06965409	5.17E-23	4.41E-25	1.20E+01	46.1858647	7.61E-02	1611973	1612096	NC_000016.9
cg08485158	5.17E-23	4.42E-25	1.20E+01	46.1846369	1.85E-01	35766021	35766144	NC_000006.11
cg04585435	5.17E-23	4.42E-25	1.20E+01	46.184536	1.78E-01	4211943	4212066	NC_000017.10
cg06585708	5.26E-23	4.49E-25	1.20E+01	46.1678394	2.30E-01	3602413	3602536	NC_000005.9
cg03112869	5.29E-23	4.52E-25	1.20E+01	46.1612787	1.87E-01	48413769	48413892	NC_000003.11
cg14517454	5.34E-23	4.58E-25	1.20E+01	46.1490432	8.90E-02	26671918	26672041	NC_000001.10
cg22490780	5.35E-23	4.58E-25	1.20E+01	46.1479839	2.43E-01	16867064	16867187	NC_000005.9
cg08806408	5.39E-23	4.62E-25	1.20E+01	46.1405106	2.92E-01	51185001	51185124	NC_000016.9
cg20810478	5.39E-23	4.62E-25	1.19E+01	46.1386808	2.74E-01	248020632	248020755	NC_000001.10
cg25076197	5.45E-23	4.68E-25	1.19E+01	46.1269606	1.03E-01	104681440	104681563	NC_000007.13
cg10659886	5.53E-23	4.75E-25	1.19E+01	46.1119514	1.89E-01	58629719	58629842	NC_000019.9
cg04021697	5.53E-23	4.76E-25	1.19E+01	46.1110684	3.09E-01	3567303	3567426	NC_000001.10
cg14507560	5.55E-23	4.77E-25	1.19E+01	46.108235	3.49E-01	1882885	1883008	NC_000005.9
cg01992684	5.57E-23	4.79E-25	1.19E+01	46.1044925	1.22E-01	154147579	154147702	NC_000005.9
cg02345417	5.57E-23	4.79E-25	1.19E+01	46.1043092	1.78E-01	111631732	111631855	NC_000011.9
cg10564464	5.70E-23	4.91E-25	1.19E+01	46.0800284	1.78E-01	134963133	134963256	NC_000002.11
cg23358612	5.73E-23	4.93E-25	1.19E+01	46.0751694	2.36E-01	1275258	1275381	NC_000007.13
cg18022344	5.86E-23	5.05E-25	1.19E+01	46.050902	1.63E-01	21665238	21665361	NC_000016.9
cg24398479	5.89E-23	5.09E-25	1.19E+01	46.0442298	2.73E-01	27192061	27192184	NC_000007.13
cg02857643	5.90E-23	5.09E-25	1.19E+01	46.0435033	1.30E-01	48696108	48696231	NC_000017.10
cg18326021	5.94E-23	5.13E-25	1.19E+01	46.036166	2.59E-01	106401479	106401602	NC_000010.10

cg19211295	6.03E-23	5.21E-25	1.19E+01	46.0204231	1.36E-01	6328369	6328492	NC_000004.11
cg00483562	6.24E-23	5.40E-25	1.19E+01	45.9846817	2.86E-01	1931781	1931904	NC_000005.9
cg02334126	6.28E-23	5.44E-25	1.19E+01	45.9768675	1.36E-01	121017209	121017332	NC_000012.11
cg02135861	6.29E-23	5.45E-25	1.19E+01	45.9754177	1.88E-01	2752139	2752262	NC_000005.9
cg13536060	6.35E-23	5.50E-25	1.19E+01	45.9666779	1.63E-01	51189671	51189794	NC_000019.9
cg01126567	6.40E-23	5.55E-25	1.19E+01	45.9581626	2.63E-01	20085047	20085170	NC_000016.9
cg09017434	6.41E-23	5.56E-25	1.19E+01	45.9561295	1.83E-01	16179660	16179783	NC_000005.9
cg26010655	6.43E-23	5.58E-25	1.19E+01	45.9529937	1.69E-01	181911749	181911872	NC_000003.11
cg26978776	6.44E-23	5.59E-25	1.19E+01	45.9508704	1.83E-01	124605370	124605493	NC_000010.10
cg20815339	6.50E-23	5.65E-25	1.19E+01	45.9399087	1.43E-01	7435357	7435480	NC_000004.11
cg06653699	6.55E-23	5.70E-25	1.19E+01	45.9316294	2.82E-01	51185082	51185205	NC_000016.9
cg08238265	6.56E-23	5.70E-25	1.19E+01	45.9310988	1.91E-01	64682912	64683035	NC_000014.8
cg23994802	6.59E-23	5.73E-25	1.19E+01	45.926347	1.23E-01	241621882	241622005	NC_000002.11
cg26333652	6.64E-23	5.78E-25	1.19E+01	45.9178763	1.82E-01	2750758	2750881	NC_000005.9
cg16110314	6.67E-23	5.81E-25	1.19E+01	45.9125038	1.99E-01	246203683	246203806	NC_000001.10
cg15520279	6.75E-23	5.88E-25	1.19E+01	45.9004719	2.39E-01	176995088	176995211	NC_000002.11
cg05267269	6.79E-23	5.92E-25	1.19E+01	45.8935447	1.57E-01	101461653	101461776	NC_000002.11
cg11878045	6.81E-23	5.94E-25	1.19E+01	45.8902971	1.61E-01	2482536	2482659	NC_000011.9
cg26675654	6.89E-23	6.02E-25	1.19E+01	45.8763619	2.18E-01	1269693	1269816	NC_000007.13
cg12426612	6.89E-23	6.02E-25	1.19E+01	45.8760145	2.06E-01	25905125	25905248	NC_000017.10
cg26693553	6.96E-23	6.08E-25	1.19E+01	45.8663804	7.03E-02	3147056	3147179	NC_000010.10
cg15427886	6.98E-23	6.10E-25	1.19E+01	45.8633609	2.41E-01	55379663	55379786	NC_000008.10
cg24691891	7.02E-23	6.14E-25	1.19E+01	45.8568505	2.25E-01	33829934	33830057	NC_000001.10
cg11159090	7.08E-23	6.21E-25	1.19E+01	45.8464278	7.06E-02	72633627	72633750	NC_000005.9
cg13214504	7.15E-23	6.27E-25	1.19E+01	45.8360436	1.62E-01	240251238	240251361	NC_000002.11

cg12714007	7.23E-23	6.34E-25	1.19E+01	45.8247447	1.87E-01	119233234	119233357	NC_000011.9
cg12973941	7.35E-23	6.46E-25	1.19E+01	45.8074285	2.57E-01	13545639	13545762	NC_000004.11
cg23624016	7.36E-23	6.47E-25	1.19E+01	45.8049692	2.06E-01	43968501	43968624	NC_000007.13
cg08497311	7.39E-23	6.50E-25	1.19E+01	45.8000256	1.46E-01	88806610	88806733	NC_000016.9
cg07915921	7.39E-23	6.50E-25	1.19E+01	45.8000202	2.95E-01	54321502	54321625	NC_000012.11
cg17548735	7.46E-23	6.56E-25	1.19E+01	45.7912369	2.43E-01	41020969	41021092	NC_000006.11
cg11651237	7.51E-23	6.62E-25	1.19E+01	45.7830482	2.31E-01	27216529	27216652	NC_000015.9
cg17741689	7.52E-23	6.62E-25	1.19E+01	45.781927	2.60E-01	4714574	4714697	NC_000001.10
cg00582524	7.80E-23	6.88E-25	1.19E+01	45.7434524	2.31E-01	51184392	51184515	NC_000016.9
cg08290240	8.06E-23	7.13E-25	1.19E+01	45.7092976	2.87E-01	59827195	59827318	NC_000020.10
cg06679720	8.08E-23	7.15E-25	1.19E+01	45.7060097	2.46E-01	51417686	51417809	NC_000013.10
cg04560767	8.29E-23	7.34E-25	1.19E+01	45.6800557	8.31E-02	19107491	19107614	NC_000022.10
cg27269917	8.30E-23	7.35E-25	1.19E+01	45.678339	2.05E-01	18853737	18853860	NC_000017.10
cg19814981	8.32E-23	7.38E-25	1.19E+01	45.6747264	2.34E-01	3592435	3592558	NC_000005.9
cg20800509	8.34E-23	7.40E-25	1.19E+01	45.6723998	3.60E-01	104513083	104513206	NC_000008.10
cg21371645	8.48E-23	7.52E-25	1.19E+01	45.6553603	1.20E-01	7147256	7147379	NC_000017.10
cg18973238	8.50E-23	7.55E-25	1.19E+01	45.6517098	1.66E-01	64169293	64169416	NC_000001.10
cg21433558	8.54E-23	7.59E-25	1.19E+01	45.6466398	1.78E-01	40837037	40837160	NC_000017.10
cg09663584	8.65E-23	7.69E-25	1.19E+01	45.6342023	1.03E-01	77347405	77347528	NC_000015.9
cg26820209	8.68E-23	7.72E-25	1.19E+01	45.629613	2.03E-01	22303326	22303449	NC_000001.10
cg16976499	8.72E-23	7.76E-25	1.19E+01	45.6244199	1.55E-01	76330505	76330628	NC_000006.11
cg18456140	8.78E-23	7.82E-25	1.19E+01	45.6168669	2.16E-01	67810268	67810391	NC_000011.9
cg11823159	8.91E-23	7.94E-25	1.19E+01	45.601366	1.52E-01	128611859	128611982	NC_000002.11
cg12921171	8.98E-23	8.02E-25	1.19E+01	45.5918508	1.47E-01	63204710	63204833	NC_000015.9
cg26194092	9.09E-23	8.13E-25	1.19E+01	45.5782364	1.69E-01	15819330	15819453	NC_000001.10

cg10631097	9.26E-23	8.30E-25	1.19E+01	45.5579405	1.60E-01	10144309	10144432	NC_000002.11
cg24604988	9.43E-23	8.46E-25	1.19E+01	45.5393841	2.15E-01	32421988	32422111	NC_000020.10
cg17987112	9.46E-23	8.49E-25	1.19E+01	45.5357735	3.50E-01	174158654	174158777	NC_000005.9
cg10013501	9.47E-23	8.49E-25	1.19E+01	45.5351595	1.57E-01	25257813	25257936	NC_000001.10
cg00786909	9.52E-23	8.54E-25	1.19E+01	45.5295171	1.95E-01	227163427	227163550	NC_000001.10
cg17113147	9.55E-23	8.57E-25	1.19E+01	45.5257385	1.91E-01	753545	753668	NC_000017.10
cg06962944	9.59E-23	8.62E-25	1.19E+01	45.5200783	3.17E-01	42544728	42544851	NC_000020.10
cg24456002	9.66E-23	8.69E-25	1.19E+01	45.5122451	2.18E-01	96911165	96911288	NC_000015.9
cg10158997	9.67E-23	8.70E-25	1.19E+01	45.5112607	1.59E-01	31734106	31734229	NC_000006.11
cg24186506	9.85E-23	8.87E-25	1.19E+01	45.4921166	1.66E-01	125780810	125780933	NC_000010.10
cg12111714	9.89E-23	8.91E-25	1.19E+01	45.4871247	2.24E-01	26043472	26043595	NC_000013.10
cg14854850	1.00E-22	9.03E-25	1.19E+01	45.474041	1.08E-01	48237752	48237875	NC_000012.11
cg05783449	1.03E-22	9.29E-25	1.18E+01	45.4458825	1.79E-01	64868710	64868833	NC_000002.11
cg24109980	1.04E-22	9.44E-25	1.18E+01	45.430381	2.94E-01	50818707	50818830	NC_000010.10
cg07918545	1.05E-22	9.46E-25	1.18E+01	45.4273562	3.15E-01	129535669	129535792	NC_000010.10
cg03531687	1.06E-22	9.56E-25	1.18E+01	45.4176008	1.15E-01	25638551	25638674	NC_000003.11
cg01851378	1.07E-22	9.70E-25	1.18E+01	45.403032	2.66E-01	51186925	51187048	NC_000016.9
cg22997113	1.07E-22	9.74E-25	1.18E+01	45.3987092	2.24E-01	27170241	27170364	NC_000007.13
cg16538471	1.07E-22	9.74E-25	1.18E+01	45.3987086	1.21E-01	20153546	20153669	NC_000007.13
cg17432857	1.09E-22	9.89E-25	1.18E+01	45.3839732	2.42E-01	27184438	27184561	NC_000007.13
cg19255084	1.10E-22	9.97E-25	1.18E+01	45.3761857	9.95E-02	113412723	113412846	NC_000003.11
cg24794906	1.10E-22	9.97E-25	1.18E+01	45.3752609	1.05E-01	37836827	37836950	NC_000021.8
cg07442479	1.12E-22	1.02E-24	1.18E+01	45.3513489	2.11E-01	37839769	37839892	NC_000005.9
cg16126178	1.13E-22	1.03E-24	1.18E+01	45.3432782	1.36E-01	105239857	105239980	NC_000014.8
cg24847829	1.16E-22	1.05E-24	1.18E+01	45.3197468	3.26E-01	136834464	136834587	NC_000005.9

cg05115106	1.16E-22	1.06E-24	1.18E+01	45.3178332	6.28E-02	75250344	75250467	NC_000017.10
cg05528516	1.16E-22	1.06E-24	1.18E+01	45.3148829	9.99E-02	1070040	1070163	NC_000001.10
cg07838483	1.18E-22	1.08E-24	1.18E+01	45.2957494	2.13E-01	30202712	30202835	NC_000019.9
cg06214745	1.20E-22	1.10E-24	1.18E+01	45.2823974	1.07E-01	1051817	1051940	NC_000017.10
cg11843333	1.20E-22	1.10E-24	1.18E+01	45.2823934	1.34E-01	15719138	15719261	NC_000004.11
cg15937832	1.21E-22	1.11E-24	1.18E+01	45.2678132	7.05E-02	26368795	26368918	NC_000008.10
cg17716724	1.25E-22	1.14E-24	1.18E+01	45.2416244	9.92E-02	200118677	200118800	NC_000001.10
cg21216220	1.25E-22	1.14E-24	1.18E+01	45.238746	1.37E-01	35732945	35733068	NC_000017.10
cg03611809	1.25E-22	1.14E-24	1.18E+01	45.2385915	1.69E-01	43709209	43709332	NC_000001.10
cg22288657	1.26E-22	1.16E-24	1.18E+01	45.2280564	1.58E-01	688002	688125	NC_000017.10
cg21929943	1.27E-22	1.16E-24	1.18E+01	45.2211083	2.62E-01	152622815	152622938	NC_000007.13
cg09086835	1.27E-22	1.17E-24	1.18E+01	45.2206891	1.85E-01	89922345	89922468	NC_000015.9
cg22424284	1.28E-22	1.17E-24	1.18E+01	45.2130381	2.58E-01	118166402	118166525	NC_000001.10
cg02430183	1.28E-22	1.18E-24	1.18E+01	45.2111098	2.12E-01	95947766	95947889	NC_000009.11
cg18763223	1.28E-22	1.18E-24	1.18E+01	45.2109054	1.09E-01	74334701	74334824	NC_000015.9
cg00982535	1.29E-22	1.19E-24	1.18E+01	45.2007295	1.88E-01	118166013	118166136	NC_000001.10
cg14058329	1.31E-22	1.20E-24	1.18E+01	45.1886717	2.03E-01	27183946	27184069	NC_000007.13
cg14014225	1.31E-22	1.20E-24	1.18E+01	45.1885856	1.44E-01	38479236	38479359	NC_000022.10
cg19593767	1.33E-22	1.22E-24	1.18E+01	45.1736915	2.30E-01	42544666	42544789	NC_000020.10
cg05335030	1.33E-22	1.22E-24	1.18E+01	45.172064	1.72E-01	50632819	50632942	NC_000022.10
cg23811117	1.33E-22	1.23E-24	1.18E+01	45.1706263	8.61E-02	158155310	158155433	NC_000002.11
cg12981843	1.36E-22	1.26E-24	1.18E+01	45.1465496	1.40E-01	779218	779341	NC_000016.9
cg10386483	1.36E-22	1.26E-24	1.18E+01	45.143096	3.04E-01	35047189	35047312	NC_000015.9
cg16749093	1.40E-22	1.30E-24	1.18E+01	45.1148287	1.65E-01	65352605	65352728	NC_000011.9
cg09923593	1.40E-22	1.30E-24	1.18E+01	45.1104057	1.51E-01	65287548	65287671	NC_000002.11

cg23942526	1.42E-22	1.32E-24	1.18E+01	45.0996231	1.62E-01	27882598	27882721	NC_000007.13
cg01614020	1.44E-22	1.33E-24	1.18E+01	45.0866445	3.20E-01	24814137	24814260	NC_000008.10
cg03283421	1.44E-22	1.34E-24	1.18E+01	45.0842301	2.46E-01	124610984	124611107	NC_000010.10
cg21908828	1.45E-22	1.34E-24	1.18E+01	45.0790121	1.37E-01	154386136	154386259	NC_000004.11
cg05578673	1.47E-22	1.37E-24	1.18E+01	45.0608695	2.15E-01	90307339	90307462	NC_000001.10
cg23065561	1.48E-22	1.37E-24	1.18E+01	45.0571452	1.83E-01	53522876	53522999	NC_000004.11
cg13938785	1.48E-22	1.38E-24	1.18E+01	45.0556237	2.22E-01	140471399	140471522	NC_000009.11
cg26997966	1.50E-22	1.40E-24	1.18E+01	45.0397409	1.25E-01	117155868	117155991	NC_000011.9
cg19219655	1.54E-22	1.43E-24	1.18E+01	45.0142036	1.27E-01	10187086	10187209	NC_000002.11
cg16523655	1.54E-22	1.44E-24	1.18E+01	45.0118567	1.26E-01	30103301	30103424	NC_000006.11
cg10155522	1.54E-22	1.44E-24	1.18E+01	45.0103481	2.07E-01	94868555	94868678	NC_000011.9
cg05369857	1.55E-22	1.45E-24	1.18E+01	45.0040952	2.90E-01	101923702	101923825	NC_000014.8
cg14697605	1.55E-22	1.45E-24	1.18E+01	45.0030829	1.01E-01	22240140	22240263	NC_000008.10
cg14882265	1.56E-22	1.45E-24	1.18E+01	45.0007984	2.01E-01	27184375	27184498	NC_000007.13
cg18405631	1.56E-22	1.46E-24	1.18E+01	45.0001315	1.28E-01	171155686	171155809	NC_000003.11
cg18524262	1.56E-22	1.46E-24	1.18E+01	44.9990664	1.95E-01	55862111	55862234	NC_000018.9
cg20482698	1.57E-22	1.47E-24	1.18E+01	44.9933291	2.71E-01	236849994	236850117	NC_000001.10
cg21865150	1.58E-22	1.48E-24	1.18E+01	44.9817499	2.79E-01	46804239	46804362	NC_000017.10
cg07532965	1.59E-22	1.49E-24	1.18E+01	44.9790414	1.58E-01	24667149	24667272	NC_000014.8
cg13784195	1.59E-22	1.49E-24	1.18E+01	44.9780041	2.06E-01	27104564	27104687	NC_000021.8
cg18256498	1.59E-22	1.49E-24	1.18E+01	44.9758491	1.61E-01	173125575	173125698	NC_000005.9
cg25113237	1.59E-22	1.49E-24	1.18E+01	44.9742513	3.34E-01	2756113	2756236	NC_000005.9
cg13053408	1.62E-22	1.52E-24	1.18E+01	44.9580524	1.07E-01	10146330	10146453	NC_000003.11
cg04494197	1.62E-22	1.52E-24	1.18E+01	44.9572702	1.93E-01	1011415	1011538	NC_000011.9
cg07985164	1.62E-22	1.52E-24	1.18E+01	44.9555358	1.72E-01	93196523	93196646	NC_000015.9

cg07099331	1.64E-22	1.54E-24	1.18E+01	44.9446961	2.50E-01	102419617	102419740	NC_000010.10
cg08572611	1.65E-22	1.55E-24	1.18E+01	44.9356165	2.60E-01	100253914	100254037	NC_000007.13
cg03893271	1.65E-22	1.55E-24	1.18E+01	44.9354116	2.72E-01	70597458	70597581	NC_000007.13
cg14013695	1.67E-22	1.58E-24	1.18E+01	44.9204854	2.39E-01	27184176	27184299	NC_000007.13
cg16457842	1.71E-22	1.61E-24	1.18E+01	44.8967015	1.57E-01	84417445	84417568	NC_000006.11
cg24980653	1.72E-22	1.63E-24	1.18E+01	44.8885779	3.23E-01	1399235	1399358	NC_000004.11
cg05162256	1.72E-22	1.63E-24	1.18E+01	44.8877502	1.30E-01	53522945	53523068	NC_000004.11
cg07060551	1.74E-22	1.65E-24	1.18E+01	44.875085	1.81E-01	51198381	51198504	NC_000019.9
cg05938267	1.75E-22	1.66E-24	1.18E+01	44.8714962	7.75E-02	77228897	77229020	NC_000016.9
cg21198455	1.77E-22	1.68E-24	1.18E+01	44.8577229	1.91E-01	131679993	131680116	NC_000009.11
cg04457114	1.84E-22	1.74E-24	1.18E+01	44.8207422	2.02E-01	174159705	174159828	NC_000005.9
cg24200426	1.84E-22	1.75E-24	1.18E+01	44.8187173	1.08E-01	50184585	50184708	NC_000019.9
cg27353143	1.84E-22	1.75E-24	1.18E+01	44.8162719	2.07E-01	38308057	38308180	NC_000019.9
cg18501001	1.86E-22	1.77E-24	1.18E+01	44.8044852	1.82E-01	76497386	76497509	NC_000017.10
cg26400421	1.87E-22	1.78E-24	1.18E+01	44.7982391	1.38E-01	33334443	33334566	NC_000011.9
cg03507412	1.88E-22	1.79E-24	1.18E+01	44.792833	6.14E-02	6491411	6491534	NC_000011.9
cg19263228	1.90E-22	1.81E-24	1.18E+01	44.7827733	1.48E-01	3191660	3191783	NC_000001.10
cg00646347	1.91E-22	1.82E-24	1.18E+01	44.7777616	1.93E-01	135044856	135044979	NC_000010.10
cg14834653	1.96E-22	1.87E-24	1.17E+01	44.7504692	1.56E-01	123353791	123353914	NC_000010.10
cg16679302	1.98E-22	1.90E-24	1.17E+01	44.737076	1.14E-01	85622276	85622399	NC_000016.9
cg23342718	2.01E-22	1.93E-24	1.17E+01	44.7217627	2.77E-01	115135576	115135699	NC_000012.11
cg15868425	2.02E-22	1.94E-24	1.17E+01	44.7155576	1.77E-01	105643522	105643645	NC_000014.8
cg24902995	2.06E-22	1.97E-24	1.17E+01	44.6982058	1.91E-01	922165	922288	NC_000007.13
cg19333201	2.11E-22	2.03E-24	1.17E+01	44.6705936	1.57E-01	53137547	53137670	NC_000010.10
cg07790615	2.11E-22	2.03E-24	1.17E+01	44.6701741	3.27E-01	237205174	237205297	NC_000001.10

cg04295172	2.12E-22	2.04E-24	1.17E+01	44.6667362	1.38E-01	108365051	108365174	NC_000011.9
cg09467345	2.17E-22	2.09E-24	1.17E+01	44.6416059	1.49E-01	30407202	30407325	NC_000020.10
cg11075451	2.20E-22	2.12E-24	1.17E+01	44.6252425	1.49E-01	89600119	89600242	NC_000001.10
cg03744763	2.21E-22	2.13E-24	1.17E+01	44.6226696	2.14E-01	27184737	27184860	NC_000007.13
cg02582387	2.22E-22	2.15E-24	1.17E+01	44.6136157	2.68E-01	20879813	20879936	NC_000001.10
cg23850283	2.22E-22	2.15E-24	1.17E+01	44.6132696	1.86E-01	114261369	114261492	NC_000002.11
cg12786452	2.23E-22	2.15E-24	1.17E+01	44.6127067	1.69E-01	62309293	62309416	NC_000017.10
cg24856726	2.25E-22	2.17E-24	1.17E+01	44.6027054	2.94E-01	108169333	108169456	NC_000012.11
cg03650119	2.25E-22	2.17E-24	1.17E+01	44.6020229	1.98E-01	228346014	228346137	NC_000001.10
cg25403721	2.25E-22	2.18E-24	1.17E+01	44.6005461	2.63E-01	114885372	114885495	NC_000012.11
cg02930721	2.25E-22	2.18E-24	1.17E+01	44.6003419	1.81E-01	89375113	89375236	NC_000016.9
cg23415434	2.26E-22	2.19E-24	1.17E+01	44.595847	2.14E-01	131798869	131798992	NC_000002.11
cg22236375	2.26E-22	2.19E-24	1.17E+01	44.5946509	8.01E-02	100089996	100090119	NC_000002.11
cg12801067	2.27E-22	2.20E-24	1.17E+01	44.5896848	1.20E-01	89380779	89380902	NC_000012.11
cg04252204	2.29E-22	2.21E-24	1.17E+01	44.583097	1.51E-01	80401500	80401623	NC_000017.10
cg07160746	2.29E-22	2.22E-24	1.17E+01	44.5788778	2.03E-01	43726765	43726888	NC_000020.10
cg01088387	2.29E-22	2.22E-24	1.17E+01	44.5787522	8.51E-02	78215445	78215568	NC_000011.9
cg16404157	2.31E-22	2.24E-24	1.17E+01	44.5736006	2.56E-01	38724648	38724771	NC_000014.8
cg03840594	2.37E-22	2.30E-24	1.17E+01	44.5449817	1.47E-01	99283553	99283676	NC_000006.11
cg27606499	2.37E-22	2.31E-24	1.17E+01	44.5421799	2.47E-01	147111120	147111243	NC_000003.11
cg18920088	2.38E-22	2.31E-24	1.17E+01	44.5408885	2.10E-01	151346409	151346532	NC_000006.11
cg18987410	2.38E-22	2.31E-24	1.17E+01	44.5397605	2.43E-01	124791197	124791320	NC_000011.9
cg06488443	2.40E-22	2.34E-24	1.17E+01	44.5284964	1.67E-01	162280341	162280464	NC_000002.11
cg06009783	2.42E-22	2.35E-24	1.17E+01	44.5228081	1.71E-01	64588440	64588563	NC_000002.11
cg24709001	2.46E-22	2.39E-24	1.17E+01	44.5069817	1.29E-01	3235319	3235442	NC_000004.11

cg10536898	2.47E-22	2.40E-24	1.17E+01	44.5017751	2.04E-01	50673227	50673350	NC_000005.9
cg05966235	2.48E-22	2.42E-24	1.17E+01	44.4963883	1.60E-01	28915196	28915319	NC_000016.9
cg06158650	2.50E-22	2.44E-24	1.17E+01	44.4881979	2.57E-01	119522637	119522760	NC_000001.10
cg09574499	2.50E-22	2.44E-24	1.17E+01	44.4863932	2.04E-01	27168962	27169085	NC_000007.13
cg08313939	2.50E-22	2.44E-24	1.17E+01	44.4857642	2.43E-01	37353117	37353240	NC_000020.10
cg26693451	2.51E-22	2.44E-24	1.17E+01	44.4847795	8.59E-02	78163892	78164015	NC_000017.10
cg04510788	2.51E-22	2.45E-24	1.17E+01	44.481536	2.05E-01	37498900	37499023	NC_000001.10
cg21300373	2.51E-22	2.45E-24	1.17E+01	44.4808037	2.09E-01	165304540	165304663	NC_000004.11
cg18267047	2.53E-22	2.47E-24	1.17E+01	44.4743569	1.69E-01	30121774	30121897	NC_000006.11
cg04170948	2.55E-22	2.49E-24	1.17E+01	44.4667526	1.48E-01	56979519	56979642	NC_000016.9
cg13284646	2.59E-22	2.54E-24	1.17E+01	44.4484314	1.49E-01	32505474	32505597	NC_000017.10
cg24937685	2.63E-22	2.57E-24	1.17E+01	44.4350672	1.57E-01	129806002	129806125	NC_000011.9
cg20227326	2.65E-22	2.60E-24	1.17E+01	44.4255615	1.42E-01	25227128	25227251	NC_000008.10
cg13306921	2.67E-22	2.62E-24	1.17E+01	44.4179614	1.59E-01	54863529	54863652	NC_000001.10
cg14565725	2.71E-22	2.65E-24	1.17E+01	44.4030517	1.96E-01	119532044	119532167	NC_000001.10
cg04904331	2.72E-22	2.67E-24	1.17E+01	44.3974927	3.62E-01	49813033	49813156	NC_000007.13
cg11932644	2.73E-22	2.68E-24	1.17E+01	44.3932561	1.80E-01	129230657	129230780	NC_000004.11
cg01281038	2.74E-22	2.69E-24	1.17E+01	44.3917156	1.58E-01	67966256	67966379	NC_000016.9
cg09302895	2.74E-22	2.69E-24	1.17E+01	44.3908012	1.99E-01	237077311	237077434	NC_000002.11
cg21875330	2.78E-22	2.73E-24	1.17E+01	44.3740644	1.92E-01	37127874	37127997	NC_000014.8
cg14636164	2.79E-22	2.74E-24	1.17E+01	44.372203	1.09E-01	127121878	127122001	NC_000009.11
cg20704654	2.79E-22	2.74E-24	1.17E+01	44.3708989	2.06E-01	30072118	30072241	NC_000020.10
cg12863545	2.79E-22	2.75E-24	1.17E+01	44.368754	3.22E-01	8869136	8869259	NC_000017.10
cg27578811	2.81E-22	2.76E-24	1.17E+01	44.3628069	2.22E-01	83316223	83316346	NC_000015.9
cg20309677	2.81E-22	2.77E-24	1.17E+01	44.3606481	1.50E-01	116925753	116925876	NC_000001.10

cg17873048	2.86E-22	2.82E-24	1.17E+01	44.3439959	2.50E-01	46697700	46697823	NC_000017.10
cg22508930	2.89E-22	2.85E-24	1.17E+01	44.3317735	1.41E-01	69016159	69016282	NC_000012.11
cg01237333	2.91E-22	2.88E-24	1.17E+01	44.3226763	1.51E-01	113530	113653	NC_000009.11
cg17116895	2.93E-22	2.89E-24	1.17E+01	44.3171538	8.18E-02	112133054	112133177	NC_000006.11
cg14096889	2.94E-22	2.91E-24	1.17E+01	44.3125145	2.46E-01	84322591	84322714	NC_000015.9
cg01983105	2.95E-22	2.92E-24	1.17E+01	44.309416	1.78E-01	103421224	103421347	NC_000004.11
cg22911054	2.95E-22	2.92E-24	1.17E+01	44.3075049	2.36E-01	68603379	68603502	NC_000011.9
cg13678973	2.95E-22	2.92E-24	1.17E+01	44.3071108	2.85E-01	50513870	50513993	NC_000001.10
cg12754421	2.99E-22	2.96E-24	1.17E+01	44.2940258	2.68E-01	105480790	105480913	NC_000011.9
cg00676269	2.99E-22	2.96E-24	1.17E+01	44.2935041	1.35E-01	46429970	46430093	NC_000022.10
cg02742906	2.99E-22	2.96E-24	1.17E+01	44.2933142	2.86E-01	112758625	112758748	NC_000013.10
cg25447202	3.01E-22	2.98E-24	1.17E+01	44.2867522	9.89E-02	3409885	3410008	NC_000004.11
cg13857947	3.01E-22	2.99E-24	1.17E+01	44.2864565	1.80E-01	240318818	240318941	NC_000002.11
cg24693478	3.01E-22	2.99E-24	1.17E+01	44.2862118	1.08E-01	208218000	208218123	NC_000001.10
cg27343663	3.02E-22	3.00E-24	1.17E+01	44.2819715	6.00E-02	79082811	79082934	NC_000017.10
cg14888296	3.02E-22	3.00E-24	1.17E+01	44.2804617	1.66E-01	48413707	48413830	NC_000003.11
cg25104105	3.03E-22	3.01E-24	1.17E+01	44.2786136	3.23E-01	14925198	14925321	NC_000001.10
cg18940047	3.05E-22	3.04E-24	1.17E+01	44.2699658	2.19E-01	153749736	153749859	NC_000007.13
cg00790847	3.09E-22	3.08E-24	1.17E+01	44.2553081	1.49E-01	40497575	40497698	NC_000012.11
cg16021909	3.10E-22	3.09E-24	1.17E+01	44.2506295	2.63E-01	248021091	248021214	NC_000001.10
cg14048837	3.12E-22	3.11E-24	1.17E+01	44.2449663	1.98E-01	109658527	109658650	NC_000009.11
cg18597315	3.16E-22	3.15E-24	1.17E+01	44.232378	1.78E-01	40549191	40549314	NC_000015.9
cg25575115	3.19E-22	3.19E-24	1.17E+01	44.2209887	1.01E-01	101175912	101176035	NC_000013.10
cg19912142	3.20E-22	3.20E-24	1.17E+01	44.2183837	2.87E-01	119494671	119494794	NC_000010.10
cg23572908	3.20E-22	3.20E-24	1.17E+01	44.2170605	3.42E-01	158937969	158938092	NC_000007.13

cg08965374	3.22E-22	3.22E-24	1.17E+01	44.2124418	1.26E-01	124370088	124370211	NC_000005.9
cg27151303	3.25E-22	3.25E-24	1.17E+01	44.2008048	2.26E-01	27184821	27184944	NC_000007.13
cg05058846	3.26E-22	3.26E-24	1.17E+01	44.1978045	1.40E-01	79270707	79270830	NC_000010.10
cg04213746	3.27E-22	3.28E-24	1.17E+01	44.1929877	2.30E-01	8106003	8106126	NC_000010.10
cg27391357	3.31E-22	3.32E-24	1.17E+01	44.1810851	1.92E-01	107017800	107017923	NC_000004.11
cg10952925	3.31E-22	3.32E-24	1.17E+01	44.1807577	1.97E-01	22327803	22327926	NC_000001.10
cg08842021	3.31E-22	3.32E-24	1.17E+01	44.1807364	9.44E-02	45481731	45481854	NC_000006.11
cg10613332	3.32E-22	3.33E-24	1.17E+01	44.1777093	1.33E-01	13368845	13368968	NC_000019.9
cg27201802	3.33E-22	3.34E-24	1.17E+01	44.173601	1.49E-01	30138225	30138348	NC_000006.11
cg02451516	3.34E-22	3.35E-24	1.17E+01	44.1715417	1.63E-01	74657147	74657270	NC_000016.9
cg24049451	3.36E-22	3.37E-24	1.17E+01	44.1657228	1.01E-01	37826163	37826286	NC_000017.10
cg00760272	3.36E-22	3.38E-24	1.17E+01	44.1632963	6.96E-02	44067042	44067165	NC_000002.11
cg06180630	3.37E-22	3.39E-24	1.17E+01	44.1599376	8.72E-02	3401607	3401730	NC_000001.10
cg09578475	3.40E-22	3.42E-24	1.17E+01	44.1513193	2.86E-01	9473688	9473811	NC_000019.9
cg26514080	3.41E-22	3.44E-24	1.17E+01	44.146924	1.14E-01	33239538	33239661	NC_000001.10
cg18946437	3.43E-22	3.46E-24	1.17E+01	44.1409009	1.61E-01	25335346	25335469	NC_000022.10
cg24892571	3.45E-22	3.48E-24	1.17E+01	44.1353957	5.68E-02	66284828	66284951	NC_000012.11
cg06504162	3.49E-22	3.52E-24	1.17E+01	44.1233615	1.77E-01	11559800	11559923	NC_000011.9
cg17433998	3.52E-22	3.55E-24	1.17E+01	44.1142243	1.83E-01	130025478	130025601	NC_000007.13
cg06616014	3.52E-22	3.56E-24	1.17E+01	44.1128167	1.95E-01	48413738	48413861	NC_000003.11
cg18786873	3.56E-22	3.59E-24	1.17E+01	44.1034913	2.76E-01	110610899	110611022	NC_000001.10
cg04082793	3.56E-22	3.60E-24	1.17E+01	44.1018637	1.15E-01	11513849	11513972	NC_000004.11
cg27378890	3.57E-22	3.60E-24	1.17E+01	44.1000716	1.31E-01	10834591	10834714	NC_000016.9
cg12999453	3.59E-22	3.62E-24	1.17E+01	44.0944666	1.86E-01	59482319	59482442	NC_000017.10
cg00912625	3.60E-22	3.63E-24	1.17E+01	44.0909748	2.70E-01	2140977	2141100	NC_000003.11

cg19719409	3.60E-22	3.65E-24	1.17E+01	44.0879599	1.50E-01	27130324	27130447	NC_000007.13
cg12964697	3.61E-22	3.65E-24	1.17E+01	44.0858975	1.69E-01	2140231	2140354	NC_000007.13
cg26277754	3.62E-22	3.67E-24	1.17E+01	44.0811925	2.17E-01	50673033	50673156	NC_000005.9
cg17806437	3.62E-22	3.67E-24	1.17E+01	44.0811129	2.13E-01	42211581	42211704	NC_000015.9
cg10666081	3.64E-22	3.69E-24	1.17E+01	44.0757615	1.01E-01	105985002	105985125	NC_000002.11
cg00334063	3.64E-22	3.69E-24	1.17E+01	44.0754509	2.46E-01	147106010	147106133	NC_000003.11
cg13707793	3.66E-22	3.71E-24	1.16E+01	44.070745	2.34E-01	139045301	139045424	NC_000005.9
cg25710107	3.66E-22	3.71E-24	1.16E+01	44.0696009	2.18E-01	101187390	101187513	NC_000013.10
cg03264601	3.69E-22	3.74E-24	1.16E+01	44.0617231	3.06E-01	55500670	55500793	NC_000020.10
cg14045872	3.70E-22	3.76E-24	1.16E+01	44.0564732	2.97E-01	49813065	49813188	NC_000007.13
cg26328180	3.71E-22	3.77E-24	1.16E+01	44.0536794	1.34E-01	91874214	91874337	NC_000002.11
cg27243166	3.74E-22	3.80E-24	1.16E+01	44.0471149	1.59E-01	64479496	64479619	NC_000011.9
cg16472542	3.79E-22	3.85E-24	1.16E+01	44.0328378	1.10E-01	20020575	20020698	NC_000022.10
cg24123634	3.83E-22	3.90E-24	1.16E+01	44.020403	1.20E-01	52008360	52008483	NC_000010.10
cg14113442	3.83E-22	3.91E-24	1.16E+01	44.0190567	7.01E-02	44078112	44078235	NC_000021.8
cg23246769	3.87E-22	3.95E-24	1.16E+01	44.0081421	1.50E-01	124461296	124461419	NC_000012.11
cg19267252	3.88E-22	3.96E-24	1.16E+01	44.0063084	2.46E-01	105459317	105459440	NC_000002.11
cg22175781	3.89E-22	3.97E-24	1.16E+01	44.0044892	1.09E-01	101175948	101176071	NC_000013.10
cg24536114	3.89E-22	3.97E-24	1.16E+01	44.0030295	1.22E-01	89448405	89448528	NC_000001.10
cg15966151	3.95E-22	4.03E-24	1.16E+01	43.987231	1.78E-01	27238472	27238595	NC_000001.10
cg03735012	3.95E-22	4.04E-24	1.16E+01	43.9868493	2.64E-01	55964092	55964215	NC_000019.9
cg24948406	3.98E-22	4.07E-24	1.16E+01	43.9783272	2.10E-01	46608268	46608391	NC_000017.10
cg15705563	4.01E-22	4.10E-24	1.16E+01	43.9720642	1.09E-01	33273014	33273137	NC_000006.11
cg01192746	4.04E-22	4.13E-24	1.16E+01	43.9640572	2.09E-01	179717478	179717601	NC_000005.9
cg05184377	4.06E-22	4.16E-24	1.16E+01	43.9574932	1.73E-01	50976646	50976769	NC_000010.10

cg21049762	4.07E-22	4.16E-24	1.16E+01	43.9560259	1.68E-01	67805456	67805579	NC_000011.9
cg04860563	4.11E-22	4.21E-24	1.16E+01	43.9443458	1.10E-01	189524470	189524593	NC_000003.11
cg01835580	4.14E-22	4.25E-24	1.16E+01	43.9361909	2.06E-01	21511501	21511624	NC_000014.8
cg26770907	4.17E-22	4.27E-24	1.16E+01	43.9298447	1.95E-01	48844808	48844931	NC_000016.9
cg18829521	4.18E-22	4.30E-24	1.16E+01	43.925126	1.09E-01	24496598	24496721	NC_000010.10
cg21836358	4.20E-22	4.32E-24	1.16E+01	43.9198346	2.41E-01	63790202	63790325	NC_000001.10
cg04127342	4.28E-22	4.41E-24	1.16E+01	43.8999545	2.58E-01	57358130	57358253	NC_000008.10
cg18259189	4.29E-22	4.41E-24	1.16E+01	43.8987407	5.59E-02	70020941	70021064	NC_000011.9
cg07562888	4.29E-22	4.42E-24	1.16E+01	43.8967526	2.19E-01	80883720	80883843	NC_000010.10
cg03311556	4.33E-22	4.46E-24	1.16E+01	43.887562	1.90E-01	81108070	81108193	NC_000004.11
cg22795470	4.33E-22	4.46E-24	1.16E+01	43.8868783	2.42E-01	43152542	43152665	NC_000007.13
cg21510284	4.35E-22	4.48E-24	1.16E+01	43.8835164	1.68E-01	103854927	103855050	NC_000014.8
cg15805451	4.42E-22	4.57E-24	1.16E+01	43.8644071	1.68E-01	51135775	51135898	NC_000007.13
cg06582663	4.45E-22	4.60E-24	1.16E+01	43.8571366	1.95E-01	79043264	79043387	NC_000017.10
cg07800670	4.49E-22	4.64E-24	1.16E+01	43.8482706	1.32E-01	56765087	56765210	NC_000006.11
cg01242602	4.49E-22	4.64E-24	1.16E+01	43.8477454	6.40E-02	99871510	99871633	NC_000006.11
cg12595013	4.51E-22	4.67E-24	1.16E+01	43.8429728	1.96E-01	147128123	147128246	NC_000003.11
cg01581084	4.52E-22	4.68E-24	1.16E+01	43.8402374	2.55E-01	99961874	99961997	NC_000008.10
cg20230732	4.59E-22	4.76E-24	1.16E+01	43.8240351	1.50E-01	128721448	128721571	NC_000003.11
cg05520712	4.61E-22	4.77E-24	1.16E+01	43.8205934	1.11E-01	53079912	53080035	NC_000005.9
cg17934871	4.67E-22	4.84E-24	1.16E+01	43.8064247	2.70E-01	37248423	37248546	NC_000013.10
cg12685548	4.69E-22	4.87E-24	1.16E+01	43.800722	1.56E-01	180644451	180644574	NC_000003.11
cg23244289	4.70E-22	4.87E-24	1.16E+01	43.7996204	2.85E-01	79331135	79331258	NC_000005.9
cg15067606	4.70E-22	4.88E-24	1.16E+01	43.7993275	1.36E-01	113458144	113458267	NC_000003.11
cg18731024	4.80E-22	4.98E-24	1.16E+01	43.7775419	2.29E-01	2998264	2998387	NC_000005.9

cg08643997	4.80E-22	4.99E-24	1.16E+01	43.7771959	1.09E-01	224136349	224136472	NC_000001.10
cg01354473	4.81E-22	5.00E-24	1.16E+01	43.7743167	1.87E-01	27204803	27204926	NC_000007.13
cg14836602	4.82E-22	5.01E-24	1.16E+01	43.7715787	2.64E-01	3592718	3592841	NC_000005.9
cg10216615	4.86E-22	5.06E-24	1.16E+01	43.7626012	2.78E-01	37353158	37353281	NC_000020.10
cg04150488	4.88E-22	5.08E-24	1.16E+01	43.7575263	1.25E-01	58799794	58799917	NC_000012.11
cg22702772	4.88E-22	5.09E-24	1.16E+01	43.75676	2.98E-01	48699012	48699135	NC_000003.11
cg01814898	4.92E-22	5.14E-24	1.16E+01	43.7474727	2.20E-01	110530427	110530550	NC_000001.10
cg24415208	4.93E-22	5.14E-24	1.16E+01	43.747145	2.88E-01	54466441	54466564	NC_000019.9
cg16768018	4.97E-22	5.18E-24	1.16E+01	43.7385651	2.46E-01	147108843	147108966	NC_000003.11
cg11389172	4.98E-22	5.19E-24	1.16E+01	43.7362901	2.97E-01	50818299	50818422	NC_000010.10
cg23666536	5.01E-22	5.24E-24	1.16E+01	43.7280997	1.41E-01	114240177	114240300	NC_000001.10
cg27501362	5.05E-22	5.28E-24	1.16E+01	43.7208106	1.38E-01	24796628	24796751	NC_000022.10
cg09124223	5.05E-22	5.28E-24	1.16E+01	43.7207823	2.28E-01	153749738	153749861	NC_000007.13
cg01939453	5.09E-22	5.32E-24	1.16E+01	43.7120933	2.68E-01	166077483	166077606	NC_000006.11
cg27241576	5.10E-22	5.34E-24	1.16E+01	43.7088443	7.12E-02	81663253	81663376	NC_000016.9
cg07792478	5.11E-22	5.36E-24	1.16E+01	43.7061263	3.07E-01	65290484	65290607	NC_000008.10
cg23495279	5.12E-22	5.37E-24	1.16E+01	43.7042003	2.56E-01	4849827	4849950	NC_000008.10
cg15373592	5.14E-22	5.39E-24	1.16E+01	43.6998924	2.26E-01	56405167	56405290	NC_000006.11
cg15443067	5.14E-22	5.39E-24	1.16E+01	43.6992501	5.67E-02	564731	564854	NC_000012.11
cg05260031	5.15E-22	5.40E-24	1.16E+01	43.6973678	2.02E-01	145677584	145677707	NC_000008.10
cg13751267	5.17E-22	5.42E-24	1.16E+01	43.6939195	1.26E-01	178416402	178416525	NC_000002.11
cg02473540	5.17E-22	5.42E-24	1.16E+01	43.6937755	2.34E-01	58570454	58570577	NC_000019.9
cg07823492	5.18E-22	5.44E-24	1.16E+01	43.6910558	2.41E-01	46608099	46608222	NC_000017.10
cg03790250	5.21E-22	5.47E-24	1.16E+01	43.6850273	2.38E-01	20256326	20256449	NC_000004.11
cg18065874	5.23E-22	5.49E-24	1.16E+01	43.6807528	1.47E-01	37244283	37244406	NC_000008.10

cg17972789	5.23E-22	5.50E-24	1.16E+01	43.6800175	2.25E-01	70372105	70372228	NC_000015.9
cg14362113	5.26E-22	5.53E-24	1.16E+01	43.6736036	1.64E-01	22956527	22956650	NC_000006.11
cg17326855	5.28E-22	5.56E-24	1.16E+01	43.669615	1.96E-01	118435653	118435776	NC_000001.10
cg11106836	5.31E-22	5.59E-24	1.16E+01	43.6637748	1.37E-01	117852822	117852945	NC_000011.9
cg04183158	5.37E-22	5.66E-24	1.16E+01	43.6515928	1.98E-01	1010983	1011106	NC_000011.9
cg15228928	5.38E-22	5.66E-24	1.16E+01	43.6506864	3.05E-01	112727521	112727644	NC_000013.10
cg26069037	5.44E-22	5.73E-24	1.16E+01	43.6389998	1.04E-01	38479240	38479363	NC_000022.10
cg25424419	5.44E-22	5.73E-24	1.16E+01	43.6389861	1.70E-01	26284491	26284614	NC_000017.10
cg01962750	5.46E-22	5.76E-24	1.16E+01	43.6335034	1.29E-01	8298983	8299106	NC_000002.11
cg04290346	5.47E-22	5.77E-24	1.16E+01	43.63177	3.00E-01	20177852	20177975	NC_000011.9
cg22879689	5.49E-22	5.79E-24	1.16E+01	43.6284275	1.59E-01	925143	925266	NC_000007.13
cg16065021	5.49E-22	5.79E-24	1.16E+01	43.6283995	2.44E-01	127940654	127940777	NC_000012.11
cg06122635	5.53E-22	5.84E-24	1.16E+01	43.6199852	3.01E-01	105461368	105461491	NC_000002.11
cg14841514	5.63E-22	5.96E-24	1.16E+01	43.6005144	1.51E-01	80866619	80866742	NC_000010.10
cg25139636	5.64E-22	5.96E-24	1.16E+01	43.5990168	1.30E-01	69084888	69085011	NC_000015.9
cg26807475	5.69E-22	6.02E-24	1.16E+01	43.5897082	1.08E-01	16671081	16671204	NC_000005.9
cg22918700	5.70E-22	6.04E-24	1.16E+01	43.5868697	1.25E-01	24600067	24600190	NC_000014.8
cg21350575	5.74E-22	6.08E-24	1.16E+01	43.5797718	3.12E-01	19617439	19617562	NC_000021.8
cg06616729	5.76E-22	6.11E-24	1.16E+01	43.575925	3.13E-01	55379248	55379371	NC_000008.10
cg04784672	5.78E-22	6.12E-24	1.16E+01	43.5733794	2.83E-01	42077674	42077797	NC_000014.8
cg02914667	5.83E-22	6.19E-24	1.16E+01	43.5629114	1.62E-01	63894453	63894576	NC_000011.9
cg11328149	5.97E-22	6.34E-24	1.16E+01	43.5386628	7.79E-02	31689459	31689582	NC_000006.11
cg23901212	6.00E-22	6.38E-24	1.16E+01	43.5326154	1.42E-01	228632807	228632930	NC_000002.11
cg06503286	6.09E-22	6.47E-24	1.16E+01	43.5178609	1.64E-01	241626691	241626814	NC_000002.11
cg23220071	6.11E-22	6.50E-24	1.16E+01	43.5133109	1.45E-01	227061009	227061132	NC_000001.10

cg27173965	6.12E-22	6.51E-24	1.16E+01	43.5125537	1.79E-01	68841715	68841838	NC_000001.10
cg10295552	6.21E-22	6.61E-24	1.16E+01	43.4971106	2.12E-01	35464574	35464697	NC_000017.10
cg12127472	6.26E-22	6.67E-24	1.16E+01	43.4876886	2.58E-01	42733724	42733847	NC_000017.10
cg15591616	6.29E-22	6.71E-24	1.16E+01	43.4825073	1.61E-01	8024678	8024801	NC_000001.10
cg24101492	6.29E-22	6.72E-24	1.16E+01	43.4811407	2.74E-01	96343119	96343242	NC_000014.8
cg05839533	6.40E-22	6.84E-24	1.16E+01	43.4631022	1.11E-01	39642907	39643030	NC_000017.10
cg22851691	6.57E-22	7.02E-24	1.16E+01	43.4368763	2.81E-01	46711446	46711569	NC_000017.10
cg20803857	6.57E-22	7.03E-24	1.16E+01	43.4357492	2.87E-01	107502615	107502738	NC_000002.11
cg24721899	6.72E-22	7.19E-24	1.16E+01	43.4128514	2.28E-01	13543985	13544108	NC_000004.11
cg22008075	6.74E-22	7.22E-24	1.16E+01	43.4097055	1.77E-01	118215567	118215690	NC_000001.10
cg17509220	6.77E-22	7.26E-24	1.16E+01	43.4043832	2.78E-01	13617012	13617135	NC_000019.9
cg08812189	6.78E-22	7.27E-24	1.16E+01	43.4026208	2.89E-01	147110367	147110490	NC_000003.11
cg07830644	6.79E-22	7.29E-24	1.16E+01	43.4002276	1.32E-01	172409790	172409913	NC_000005.9
cg12551813	6.81E-22	7.31E-24	1.16E+01	43.3975147	1.03E-01	200781121	200781244	NC_000001.10
cg04908960	6.82E-22	7.32E-24	1.16E+01	43.3952129	2.45E-01	30425679	30425802	NC_000016.9
cg14676407	6.83E-22	7.33E-24	1.16E+01	43.3937875	1.93E-01	125651370	125651493	NC_000010.10
cg01477835	6.86E-22	7.37E-24	1.16E+01	43.3890856	1.87E-01	87989696	87989819	NC_000004.11
cg02347113	6.98E-22	7.50E-24	1.15E+01	43.3712393	1.66E-01	101752032	101752155	NC_000002.11
cg18197377	6.98E-22	7.50E-24	1.15E+01	43.3709446	2.60E-01	115134688	115134811	NC_000012.11
cg14029170	7.06E-22	7.59E-24	1.15E+01	43.359243	1.55E-01	53388842	53388965	NC_000006.11
cg09524455	7.09E-22	7.62E-24	1.15E+01	43.3554197	2.65E-01	2751872	2751995	NC_000005.9
cg07710971	7.25E-22	7.82E-24	1.15E+01	43.3305134	1.62E-01	46920836	46920959	NC_000016.9
cg04788956	7.26E-22	7.82E-24	1.15E+01	43.3299286	1.37E-01	67696138	67696261	NC_000016.9
cg11036833	7.36E-22	7.93E-24	1.15E+01	43.3159584	2.55E-01	115152494	115152617	NC_000005.9
cg17193062	7.53E-22	8.13E-24	1.15E+01	43.2915816	9.91E-02	32545455	32545578	NC_000003.11

cg01385836	7.67E-22	8.29E-24	1.15E+01	43.2724247	1.41E-01	190644623	190644746	NC_000002.11
cg22501243	7.68E-22	8.30E-24	1.15E+01	43.2705202	8.27E-02	122611269	122611392	NC_000012.11
cg08447992	7.76E-22	8.40E-24	1.15E+01	43.2589728	1.72E-01	93389086	93389209	NC_000010.10
cg10877130	7.76E-22	8.40E-24	1.15E+01	43.2589108	9.15E-02	69285772	69285895	NC_000011.9
cg07453407	7.94E-22	8.60E-24	1.15E+01	43.2359432	2.30E-01	46404031	46404154	NC_000019.9
cg15034345	7.94E-22	8.60E-24	1.15E+01	43.235213	3.36E-01	5836111	5836234	NC_000002.11
cg27287438	8.04E-22	8.72E-24	1.15E+01	43.2218538	1.75E-01	1066396	1066519	NC_000007.13
cg21289124	8.12E-22	8.81E-24	1.15E+01	43.2118158	1.37E-01	26951249	26951372	NC_000022.10
cg02237470	8.12E-22	8.82E-24	1.15E+01	43.211052	2.98E-01	53852443	53852566	NC_000008.10
cg06818851	8.24E-22	8.96E-24	1.15E+01	43.1949332	1.03E-01	907708	907831	NC_000001.10
cg12717584	8.42E-22	9.16E-24	1.15E+01	43.1732859	1.20E-01	23122112	23122235	NC_000001.10
cg06824581	8.51E-22	9.27E-24	1.15E+01	43.1616439	8.55E-02	20515978	20516101	NC_000001.10
cg00457495	8.52E-22	9.27E-24	1.15E+01	43.1608879	1.33E-01	49883384	49883507	NC_000022.10
cg05901579	8.59E-22	9.36E-24	1.15E+01	43.1512186	3.54E-01	88800567	88800690	NC_000015.9
cg24800630	8.65E-22	9.43E-24	1.15E+01	43.1438753	9.99E-02	156747654	156747777	NC_000006.11
cg06507244	8.71E-22	9.49E-24	1.15E+01	43.1376048	1.81E-01	127569494	127569617	NC_000010.10
cg14669274	8.76E-22	9.55E-24	1.15E+01	43.1312229	3.12E-01	143592375	143592498	NC_000008.10
cg03963198	8.80E-22	9.61E-24	1.15E+01	43.1256704	2.60E-01	1882871	1882994	NC_000005.9
cg05222243	8.82E-22	9.63E-24	1.15E+01	43.1232491	1.42E-01	71672822	71672945	NC_000010.10
cg15092343	8.87E-22	9.69E-24	1.15E+01	43.1175801	2.91E-01	4860061	4860184	NC_000004.11
cg25492363	9.09E-22	9.93E-24	1.15E+01	43.0925018	2.18E-01	57319872	57319995	NC_000011.9
cg20340508	9.09E-22	9.93E-24	1.15E+01	43.0924432	3.57E-01	34442377	34442500	NC_000021.8
cg05526341	9.10E-22	9.96E-24	1.15E+01	43.0903378	1.28E-01	66489362	66489485	NC_000003.11
cg16738453	9.11E-22	9.97E-24	1.15E+01	43.0888985	2.13E-01	57029014	57029137	NC_000001.10
cg17608570	9.13E-22	1.00E-23	1.15E+01	43.0864243	1.81E-01	130228545	130228668	NC_000010.10

cg24430140	9.18E-22	1.01E-23	1.15E+01	43.08092	2.56E-01	57276642	57276765	NC_000019.9
cg08513123	9.27E-22	1.02E-23	1.15E+01	43.0707601	1.51E-01	30121930	30122053	NC_000006.11
cg02004979	9.28E-22	1.02E-23	1.15E+01	43.0693821	2.49E-01	141043480	141043603	NC_000003.11
cg09013267	9.35E-22	1.03E-23	1.15E+01	43.0605522	8.62E-02	176762943	176763066	NC_000005.9
cg17239236	9.47E-22	1.04E-23	1.15E+01	43.0475757	3.70E-01	71802184	71802307	NC_000007.13
cg05221057	9.47E-22	1.04E-23	1.15E+01	43.0471868	2.80E-01	112711587	112711710	NC_000013.10
cg20793665	9.53E-22	1.05E-23	1.15E+01	43.0410043	1.84E-01	232549224	232549347	NC_000002.11
cg13445796	9.60E-22	1.06E-23	1.15E+01	43.0326865	2.99E-01	20181725	20181848	NC_000011.9
cg02731193	9.62E-22	1.06E-23	1.15E+01	43.0307432	1.11E-01	53407186	53407309	NC_000006.11
cg16510099	9.71E-22	1.07E-23	1.15E+01	43.0199459	1.43E-01	63851177	63851300	NC_000011.9
cg17050632	9.79E-22	1.08E-23	1.15E+01	43.0125031	1.17E-01	141577526	141577649	NC_000008.10
cg21472165	9.79E-22	1.08E-23	1.15E+01	43.0118371	1.37E-01	82749904	82750027	NC_000006.11
cg14289542	9.80E-22	1.08E-23	1.15E+01	43.0105152	2.26E-01	112726662	112726785	NC_000013.10
cg22887845	9.82E-22	1.08E-23	1.15E+01	43.0081462	2.42E-01	41544539	41544662	NC_000006.11
cg17592667	9.82E-22	1.08E-23	1.15E+01	43.0077429	1.91E-01	1848649	1848772	NC_000001.10
cg13512268	9.93E-22	1.10E-23	1.15E+01	42.9945472	2.22E-01	27183973	27184096	NC_000007.13
cg16856049	9.97E-22	1.10E-23	1.15E+01	42.9905447	2.39E-01	115134709	115134832	NC_000012.11
cg02242894	1.00E-21	1.11E-23	1.15E+01	42.9832235	5.78E-02	31494799	31494922	NC_000016.9
cg22312354	1.01E-21	1.12E-23	1.15E+01	42.9746898	1.80E-01	56158714	56158837	NC_000019.9
cg23444251	1.02E-21	1.12E-23	1.15E+01	42.9697527	1.52E-01	123353298	123353421	NC_000010.10
cg08347047	1.02E-21	1.13E-23	1.15E+01	42.9689398	2.39E-01	21680426	21680549	NC_000012.11
cg17555373	1.02E-21	1.13E-23	1.15E+01	42.9687015	1.95E-01	53775108	53775231	NC_000013.10
cg09619146	1.02E-21	1.13E-23	1.15E+01	42.966836	1.98E-01	125651034	125651157	NC_000010.10
cg07557903	1.02E-21	1.13E-23	1.15E+01	42.9665776	1.78E-01	144600645	144600768	NC_000008.10
cg08067346	1.02E-21	1.13E-23	1.15E+01	42.9646634	2.44E-01	25011481	25011604	NC_000016.9

cg07179981	1.02E-21	1.13E-23	1.15E+01	42.9630247	2.02E-01	922051	922174	NC_000007.13
cg08641514	1.02E-21	1.13E-23	1.15E+01	42.9620652	2.36E-01	25987606	25987729	NC_000007.13
cg08448701	1.03E-21	1.14E-23	1.15E+01	42.9576567	2.69E-01	21686282	21686405	NC_000020.10
cg01350077	1.04E-21	1.16E-23	1.15E+01	42.9419983	2.95E-01	41606317	41606440	NC_000006.11
cg26319015	1.04E-21	1.16E-23	1.15E+01	42.941631	1.43E-01	5647145	5647268	NC_000007.13
cg06373870	1.04E-21	1.16E-23	1.15E+01	42.94007	1.96E-01	81117853	81117976	NC_000004.11
cg10481072	1.05E-21	1.17E-23	1.15E+01	42.9342179	1.54E-01	92459517	92459640	NC_000007.13
cg13461482	1.08E-21	1.20E-23	1.15E+01	42.9069472	1.54E-01	67809271	67809394	NC_000011.9
cg05661282	1.09E-21	1.21E-23	1.15E+01	42.8999351	3.20E-01	58220370	58220493	NC_000019.9
cg11867552	1.10E-21	1.23E-23	1.15E+01	42.8839364	1.73E-01	50801000	50801123	NC_000007.13
cg10420161	1.11E-21	1.24E-23	1.15E+01	42.8750252	1.49E-01	102899983	102900106	NC_000010.10
cg17525406	1.12E-21	1.24E-23	1.15E+01	42.8684602	2.71E-01	4715520	4715643	NC_000001.10
cg09923953	1.12E-21	1.25E-23	1.15E+01	42.8659028	2.06E-01	51525638	51525761	NC_000019.9
cg09754845	1.12E-21	1.25E-23	1.15E+01	42.862153	2.75E-01	1408818	1408941	NC_000007.13
cg18343437	1.13E-21	1.26E-23	1.15E+01	42.8599472	2.45E-01	142528415	142528538	NC_000008.10
cg04812726	1.13E-21	1.26E-23	1.15E+01	42.8594637	1.64E-01	171875049	171875172	NC_000005.9
cg26775079	1.13E-21	1.26E-23	1.15E+01	42.858678	1.70E-01	56158830	56158953	NC_000019.9
cg16930572	1.13E-21	1.27E-23	1.15E+01	42.8524153	1.49E-01	16761118	16761241	NC_000012.11
cg14043702	1.14E-21	1.27E-23	1.15E+01	42.8488809	1.77E-01	101136251	101136374	NC_000001.10
cg05336395	1.14E-21	1.27E-23	1.15E+01	42.8457629	1.71E-01	53421688	53421811	NC_000013.10
cg03469054	1.15E-21	1.28E-23	1.15E+01	42.8411085	2.56E-01	130387861	130387984	NC_000012.11
cg22682201	1.17E-21	1.30E-23	1.15E+01	42.8219775	2.04E-01	41020847	41020970	NC_000006.11
cg13569987	1.17E-21	1.31E-23	1.15E+01	42.8145815	1.62E-01	26975979	26976102	NC_000017.10
cg04813119	1.19E-21	1.34E-23	1.15E+01	42.7972421	1.72E-01	75181254	75181377	NC_000017.10
cg14503395	1.22E-21	1.37E-23	1.15E+01	42.7765945	2.01E-01	126128745	126128868	NC_000009.11

cg12776966	1.23E-21	1.38E-23	1.15E+01	42.7689851	2.65E-01	30021276	30021399	NC_000019.9
cg21485062	1.23E-21	1.38E-23	1.15E+01	42.7664072	1.27E-01	42949773	42949896	NC_000007.13
cg05869585	1.24E-21	1.39E-23	1.15E+01	42.758379	1.26E-01	8890233	8890356	NC_000016.9
cg06906472	1.25E-21	1.40E-23	1.15E+01	42.752389	2.78E-01	107502801	107502924	NC_000002.11
cg08006727	1.25E-21	1.40E-23	1.15E+01	42.7496377	1.61E-01	116061614	116061737	NC_000009.11
cg20391793	1.27E-21	1.42E-23	1.15E+01	42.7354318	7.85E-02	106135414	106135537	NC_000002.11
cg06707356	1.28E-21	1.44E-23	1.15E+01	42.7268784	1.48E-01	11832612	11832735	NC_000016.9
cg11401159	1.28E-21	1.44E-23	1.15E+01	42.7264163	1.83E-01	6759129	6759252	NC_000005.9
cg27493243	1.28E-21	1.44E-23	1.15E+01	42.7259798	1.24E-01	233366070	233366193	NC_000002.11
cg09975561	1.28E-21	1.44E-23	1.15E+01	42.7214568	9.71E-02	71187554	71187677	NC_000016.9
cg22282131	1.28E-21	1.44E-23	1.15E+01	42.7210282	1.38E-01	127439670	127439793	NC_000007.13
cg15273575	1.29E-21	1.46E-23	1.15E+01	42.7132442	1.71E-01	145090803	145090926	NC_000006.11
cg11307865	1.29E-21	1.46E-23	1.15E+01	42.7114339	8.29E-02	27587175	27587298	NC_000012.11
cg02135728	1.29E-21	1.46E-23	1.15E+01	42.7112757	1.45E-01	97486613	97486736	NC_000003.11
cg17438849	1.30E-21	1.46E-23	1.15E+01	42.7091264	2.93E-01	43152468	43152591	NC_000007.13
cg11793269	1.30E-21	1.47E-23	1.15E+01	42.7052112	2.49E-01	2752545	2752668	NC_000005.9
cg07268058	1.30E-21	1.47E-23	1.15E+01	42.7047886	2.49E-01	76630260	76630383	NC_000015.9
cg21694591	1.31E-21	1.48E-23	1.14E+01	42.6961359	1.62E-01	118165133	118165256	NC_000001.10
cg03881775	1.32E-21	1.49E-23	1.14E+01	42.6877953	1.86E-01	147111135	147111258	NC_000003.11
cg24403845	1.33E-21	1.50E-23	1.14E+01	42.6829137	3.56E-01	108924366	108924489	NC_000010.10
cg14928149	1.33E-21	1.50E-23	1.14E+01	42.6819335	1.64E-01	155485385	155485508	NC_000007.13
cg12374721	1.33E-21	1.51E-23	1.14E+01	42.6781199	3.02E-01	46799640	46799763	NC_000017.10
cg10201616	1.34E-21	1.52E-23	1.14E+01	42.6733245	2.32E-01	11198556	11198679	NC_000019.9
cg07631435	1.37E-21	1.55E-23	1.14E+01	42.6503651	1.99E-01	114043903	114044026	NC_000011.9
cg18926450	1.38E-21	1.56E-23	1.14E+01	42.6416691	2.23E-01	110368234	110368357	NC_000012.11

cg04205744	1.39E-21	1.57E-23	1.14E+01	42.6350611	2.05E-01	86606367	86606490	NC_000016.9
cg17221945	1.40E-21	1.59E-23	1.14E+01	42.6283968	1.67E-01	39953603	39953726	NC_000001.10
cg07081061	1.40E-21	1.59E-23	1.14E+01	42.6276712	1.23E-01	3350901	3351024	NC_000006.11
cg10358533	1.41E-21	1.60E-23	1.14E+01	42.6181938	2.97E-01	34114209	34114332	NC_000019.9
cg24004943	1.41E-21	1.61E-23	1.14E+01	42.6148644	1.64E-01	127569905	127570028	NC_000010.10
cg24937747	1.42E-21	1.62E-23	1.14E+01	42.609749	2.34E-01	1882902	1883025	NC_000005.9
cg25923577	1.42E-21	1.62E-23	1.14E+01	42.6094462	3.83E-01	61276447	61276570	NC_000011.9
cg12989041	1.42E-21	1.62E-23	1.14E+01	42.6050303	1.64E-01	159049761	159049884	NC_000006.11
cg02906874	1.44E-21	1.64E-23	1.14E+01	42.5939475	1.65E-01	125767965	125768088	NC_000010.10
cg01802258	1.45E-21	1.66E-23	1.14E+01	42.5839151	3.26E-01	92790097	92790220	NC_000014.8
cg22609785	1.45E-21	1.66E-23	1.14E+01	42.5836161	1.69E-01	80416672	80416795	NC_000010.10
cg08751950	1.45E-21	1.66E-23	1.14E+01	42.5834139	2.04E-01	2416396	2416519	NC_000007.13
cg19791143	1.47E-21	1.68E-23	1.14E+01	42.5701396	3.95E-02	29778848	29778971	NC_000008.10
cg07316978	1.48E-21	1.69E-23	1.14E+01	42.5644237	1.41E-01	157529107	157529230	NC_000006.11
cg16396488	1.48E-21	1.70E-23	1.14E+01	42.5612137	2.03E-01	120766223	120766346	NC_000012.11
cg22526226	1.51E-21	1.73E-23	1.14E+01	42.5438885	9.95E-02	46169000	46169123	NC_000012.11
cg03368099	1.51E-21	1.73E-23	1.14E+01	42.543564	1.90E-01	27184521	27184644	NC_000007.13
cg27552378	1.55E-21	1.78E-23	1.14E+01	42.5142494	1.93E-01	3702776	3702899	NC_000016.9
cg21664351	1.55E-21	1.78E-23	1.14E+01	42.5121866	2.01E-01	19841423	19841546	NC_000011.9
cg01530101	1.56E-21	1.78E-23	1.14E+01	42.5112508	1.72E-01	2891098	2891221	NC_000011.9
cg03101522	1.56E-21	1.79E-23	1.14E+01	42.505155	1.29E-01	127897503	127897626	NC_000006.11
cg02126424	1.58E-21	1.81E-23	1.14E+01	42.4961931	1.91E-01	67965099	67965222	NC_000016.9
cg26059468	1.58E-21	1.82E-23	1.14E+01	42.4909154	2.74E-01	55370579	55370702	NC_000008.10
cg21393713	1.60E-21	1.84E-23	1.14E+01	42.4811287	3.42E-01	69064801	69064924	NC_000007.13
cg06365177	1.61E-21	1.86E-23	1.14E+01	42.470572	1.57E-01	30798589	30798712	NC_000020.10

cg03532926	1.62E-21	1.86E-23	1.14E+01	42.4700535	2.40E-01	153584839	153584962	NC_000007.13
cg05011468	1.63E-21	1.87E-23	1.14E+01	42.4632135	1.22E-01	96997582	96997705	NC_000002.11
cg01204060	1.65E-21	1.90E-23	1.14E+01	42.4495815	1.25E-01	4227342	4227465	NC_000020.10
cg14378259	1.65E-21	1.90E-23	1.14E+01	42.4494065	6.71E-02	37765272	37765395	NC_000022.10
cg14215786	1.66E-21	1.92E-23	1.14E+01	42.4378754	1.48E-01	100857629	100857752	NC_000014.8
cg17012160	1.68E-21	1.94E-23	1.14E+01	42.4263937	2.12E-01	240255691	240255814	NC_000001.10
cg04322298	1.69E-21	1.95E-23	1.14E+01	42.4202817	1.30E-01	190635899	190636022	NC_000002.11
cg04849541	1.70E-21	1.96E-23	1.14E+01	42.4170359	3.24E-01	1931620	1931743	NC_000005.9
cg16244648	1.72E-21	1.99E-23	1.14E+01	42.4031962	9.65E-02	141555043	141555166	NC_000005.9
cg14184817	1.72E-21	1.99E-23	1.14E+01	42.4028885	1.94E-01	45737483	45737606	NC_000019.9
cg10521808	1.72E-21	1.99E-23	1.14E+01	42.4018757	5.98E-02	7313750	7313873	NC_000001.10
cg04332526	1.72E-21	2.00E-23	1.14E+01	42.3996247	2.00E-01	3707039	3707162	NC_000016.9
cg09067029	1.72E-21	2.00E-23	1.14E+01	42.3992345	1.53E-01	186954259	186954382	NC_000001.10
cg12307484	1.74E-21	2.01E-23	1.14E+01	42.3917253	2.41E-01	19158721	19158844	NC_000007.13
cg13987572	1.74E-21	2.02E-23	1.14E+01	42.3865267	1.36E-01	140477134	140477257	NC_000009.11
cg04908382	1.75E-21	2.03E-23	1.14E+01	42.3843905	1.03E-01	105415856	105415979	NC_000014.8
cg19412007	1.75E-21	2.03E-23	1.14E+01	42.383246	9.40E-02	32825056	32825179	NC_000006.11
cg07206256	1.75E-21	2.03E-23	1.14E+01	42.3817086	8.81E-02	194573323	194573446	NC_000003.11
cg09971314	1.75E-21	2.04E-23	1.14E+01	42.379555	2.83E-01	28415023	28415146	NC_000006.11
cg06302806	1.75E-21	2.04E-23	1.14E+01	42.3794845	1.74E-01	43939970	43940093	NC_000020.10
cg17133446	1.77E-21	2.06E-23	1.14E+01	42.368263	1.87E-01	26961427	26961550	NC_000022.10
cg19505194	1.79E-21	2.08E-23	1.14E+01	42.3597255	1.64E-01	15801943	15802066	NC_000001.10
cg27088639	1.85E-21	2.15E-23	1.14E+01	42.3253514	1.37E-01	64682573	64682696	NC_000014.8
cg13435326	1.85E-21	2.15E-23	1.14E+01	42.3246869	1.82E-01	178907655	178907778	NC_000003.11
cg13434308	1.85E-21	2.15E-23	1.14E+01	42.3236924	2.27E-01	29936486	29936609	NC_000012.11

cg05064936	1.85E-21	2.16E-23	1.14E+01	42.3232031	1.60E-01	45764416	45764539	NC_000017.10
cg03184368	1.86E-21	2.17E-23	1.14E+01	42.3162103	1.89E-01	40555722	40555845	NC_000004.11
cg05364072	1.86E-21	2.17E-23	1.14E+01	42.3160165	1.68E-01	36643981	36644104	NC_000001.10
cg10126205	1.87E-21	2.17E-23	1.14E+01	42.3147136	2.89E-01	19158664	19158787	NC_000007.13
cg26206456	1.88E-21	2.19E-23	1.14E+01	42.3062732	2.09E-01	992484	992607	NC_000008.10
cg06868026	1.89E-21	2.21E-23	1.14E+01	42.3005043	2.04E-01	46787813	46787936	NC_000021.8
cg03700449	1.89E-21	2.21E-23	1.14E+01	42.2995476	2.77E-01	103352326	103352449	NC_000012.11
cg11416620	1.90E-21	2.22E-23	1.14E+01	42.2953499	1.34E-01	129072147	129072270	NC_000002.11
cg11081251	1.91E-21	2.23E-23	1.14E+01	42.2892636	1.32E-01	65375977	65376100	NC_000015.9
cg14958141	1.91E-21	2.23E-23	1.14E+01	42.2884551	1.73E-01	38394114	38394237	NC_000019.9
cg21174375	1.92E-21	2.25E-23	1.14E+01	42.282	2.23E-01	64681225	64681348	NC_000014.8
cg17352462	1.93E-21	2.25E-23	1.14E+01	42.2799441	8.16E-02	134364082	134364205	NC_000010.10
cg24046689	1.94E-21	2.27E-23	1.14E+01	42.2716676	1.77E-01	922235	922358	NC_000007.13
cg01429449	1.94E-21	2.28E-23	1.14E+01	42.2695441	1.96E-01	176936397	176936520	NC_000002.11
cg07524000	1.95E-21	2.28E-23	1.14E+01	42.2673194	1.67E-01	816020	816143	NC_000016.9
cg19142026	1.97E-21	2.30E-23	1.14E+01	42.2572171	2.90E-01	27170394	27170517	NC_000007.13
cg03256198	1.97E-21	2.30E-23	1.14E+01	42.2569265	2.32E-01	230283231	230283354	NC_000001.10
cg13839457	1.97E-21	2.31E-23	1.14E+01	42.2565397	3.47E-01	115630889	115631012	NC_000011.9
cg11294513	1.97E-21	2.31E-23	1.14E+01	42.2539387	2.97E-01	58220295	58220418	NC_000019.9
cg22867629	2.01E-21	2.36E-23	1.14E+01	42.2319247	1.31E-01	96699068	96699191	NC_000014.8
cg07120346	2.02E-21	2.37E-23	1.14E+01	42.2310099	2.68E-01	58141890	58142013	NC_000006.11
cg04990472	2.02E-21	2.37E-23	1.14E+01	42.2270607	1.62E-01	69950043	69950166	NC_000014.8
cg17130486	2.04E-21	2.39E-23	1.14E+01	42.2197867	1.60E-01	52437360	52437483	NC_000012.11
cg21880222	2.04E-21	2.39E-23	1.14E+01	42.2190398	1.66E-01	103327770	103327893	NC_000002.11
cg26923490	2.06E-21	2.42E-23	1.14E+01	42.2093362	2.45E-01	49575475	49575598	NC_000019.9

cg24013351	2.07E-21	2.43E-23	1.14E+01	42.2023173	1.02E-01	37670820	37670943	NC_000014.8
cg24319902	2.08E-21	2.45E-23	1.14E+01	42.1948849	2.21E-01	41166990	41167113	NC_000008.10
cg04140596	2.12E-21	2.49E-23	1.14E+01	42.179562	1.74E-01	228404239	228404362	NC_000001.10
cg04819959	2.12E-21	2.49E-23	1.14E+01	42.1787161	1.54E-01	130135065	130135188	NC_000007.13
cg03654783	2.14E-21	2.52E-23	1.14E+01	42.1683748	1.41E-01	156634034	156634157	NC_000001.10
cg09917805	2.14E-21	2.52E-23	1.14E+01	42.1679028	1.48E-01	4763596	4763719	NC_000004.11
cg02133510	2.14E-21	2.53E-23	1.14E+01	42.1660567	1.05E-01	32044959	32045082	NC_000006.11
cg05863502	2.17E-21	2.56E-23	1.14E+01	42.1540821	2.29E-01	140771990	140772113	NC_000009.11
cg19131667	2.17E-21	2.56E-23	1.14E+01	42.1536032	1.98E-01	17236940	17237063	NC_000003.11
cg12015737	2.17E-21	2.56E-23	1.14E+01	42.1520356	2.45E-01	27184030	27184153	NC_000007.13
cg06675190	2.17E-21	2.57E-23	1.14E+01	42.1487244	2.29E-01	89346205	89346328	NC_000015.9
cg00862117	2.20E-21	2.60E-23	1.14E+01	42.136074	1.89E-01	176111	176234	NC_000012.11
cg10169070	2.23E-21	2.64E-23	1.14E+01	42.120718	1.36E-01	114098428	114098551	NC_000006.11
cg20148575	2.24E-21	2.65E-23	1.14E+01	42.1190629	3.79E-01	132182671	132182794	NC_000002.11
cg13962186	2.27E-21	2.69E-23	1.14E+01	42.1028923	2.41E-01	79633650	79633773	NC_000009.11
cg01417692	2.28E-21	2.70E-23	1.14E+01	42.0993724	2.22E-01	47078581	47078704	NC_000001.10
cg23024254	2.29E-21	2.71E-23	1.14E+01	42.0945988	7.00E-02	33433579	33433702	NC_000001.10
cg14416248	2.29E-21	2.72E-23	1.14E+01	42.0934287	1.62E-01	3170095	3170218	NC_000004.11
cg13786429	2.30E-21	2.73E-23	1.14E+01	42.0889079	1.07E-01	35210094	35210217	NC_000021.8
cg14415914	2.31E-21	2.73E-23	1.14E+01	42.0876413	1.28E-01	36537273	36537396	NC_000022.10
cg04629133	2.32E-21	2.75E-23	1.14E+01	42.0829589	1.71E-01	30791409	30791532	NC_000020.10
cg14024461	2.37E-21	2.81E-23	1.14E+01	42.0605548	2.25E-01	228646047	228646170	NC_000001.10
cg18002447	2.38E-21	2.83E-23	1.14E+01	42.0535378	1.84E-01	59482956	59483079	NC_000017.10
cg08662665	2.41E-21	2.87E-23	1.14E+01	42.0405788	1.78E-01	30865823	30865946	NC_000019.9
cg16405026	2.41E-21	2.87E-23	1.14E+01	42.0402804	3.29E-01	145281942	145282065	NC_000002.11

cg24364723	2.42E-21	2.87E-23	1.14E+01	42.0381554	1.04E-01	22838414	22838537	NC_000001.10
cg06763054	2.43E-21	2.88E-23	1.14E+01	42.0342796	1.71E-01	17270834	17270957	NC_000008.10
cg12763813	2.43E-21	2.90E-23	1.14E+01	42.0303253	8.91E-02	123278445	123278568	NC_000010.10
cg06925438	2.47E-21	2.94E-23	1.14E+01	42.0156654	1.48E-01	480121	480244	NC_000005.9
cg04819499	2.47E-21	2.94E-23	1.13E+01	42.0136653	2.49E-01	43045014	43045137	NC_000017.10
cg09268963	2.47E-21	2.95E-23	1.13E+01	42.0134105	2.19E-01	64692302	64692425	NC_000014.8
cg18944047	2.48E-21	2.95E-23	1.13E+01	42.0107621	2.57E-01	72322143	72322266	NC_000017.10
cg22108469	2.51E-21	2.99E-23	1.13E+01	41.9985167	2.88E-01	174158984	174159107	NC_000005.9
cg11199046	2.52E-21	3.00E-23	1.13E+01	41.9943094	1.90E-01	115136308	115136431	NC_000012.11
cg11888359	2.53E-21	3.02E-23	1.13E+01	41.9901406	1.78E-01	4936127	4936250	NC_000019.9
cg27635470	2.55E-21	3.04E-23	1.13E+01	41.98105	1.97E-01	75241125	75241248	NC_000016.9
cg18596689	2.60E-21	3.10E-23	1.13E+01	41.9619714	1.80E-01	35766051	35766174	NC_000006.11
cg17038626	2.62E-21	3.14E-23	1.13E+01	41.9508917	2.52E-01	2435931	2436054	NC_000012.11
cg09762115	2.63E-21	3.14E-23	1.13E+01	41.9492071	1.86E-01	86095308	86095431	NC_000002.11
cg07787771	2.63E-21	3.15E-23	1.13E+01	41.9481531	1.81E-01	104341826	104341949	NC_000010.10
cg17087479	2.64E-21	3.16E-23	1.13E+01	41.9441501	3.17E-01	214156255	214156378	NC_000001.10
cg02973693	2.66E-21	3.19E-23	1.13E+01	41.9344141	1.79E-01	73085092	73085215	NC_000016.9
cg18579862	2.67E-21	3.20E-23	1.13E+01	41.932355	3.46E-01	58095595	58095718	NC_000019.9
cg23855818	2.67E-21	3.20E-23	1.13E+01	41.9318151	1.89E-01	105323396	105323519	NC_000012.11
cg13767223	2.67E-21	3.20E-23	1.13E+01	41.9316713	6.12E-02	96009863	96009986	NC_000009.11
cg19456540	2.68E-21	3.22E-23	1.13E+01	41.9261295	2.89E-01	60976285	60976408	NC_000014.8
cg23895963	2.72E-21	3.27E-23	1.13E+01	41.9105618	1.54E-01	117471115	117471238	NC_000012.11
cg14062050	2.73E-21	3.28E-23	1.13E+01	41.906441	2.99E-01	1886994	1887117	NC_000005.9
cg08249988	2.75E-21	3.30E-23	1.13E+01	41.8992508	3.13E-01	19322818	19322941	NC_000019.9
cg11611505	2.75E-21	3.31E-23	1.13E+01	41.8979795	9.86E-02	3373478	3373601	NC_000010.10

cg14156751	2.77E-21	3.32E-23	1.13E+01	41.8931019	1.99E-01	50976571	50976694	NC_000010.10
cg08720517	2.77E-21	3.34E-23	1.13E+01	41.8899004	2.60E-01	138729919	138730042	NC_000005.9
cg13100190	2.83E-21	3.41E-23	1.13E+01	41.8671246	2.10E-01	114053961	114054084	NC_000006.11
cg25069596	2.84E-21	3.42E-23	1.13E+01	41.8656024	1.35E-01	916509	916632	NC_000017.10
cg11659652	2.86E-21	3.45E-23	1.13E+01	41.8577481	1.61E-01	129797840	129797963	NC_000010.10
cg06419180	2.90E-21	3.49E-23	1.13E+01	41.8434952	1.37E-01	19262414	19262537	NC_000008.10
cg20575761	2.92E-21	3.52E-23	1.13E+01	41.8368809	2.04E-01	57275166	57275289	NC_000014.8
cg01068023	2.93E-21	3.54E-23	1.13E+01	41.8299028	1.37E-01	761370	761493	NC_000001.10
cg18734095	2.96E-21	3.57E-23	1.13E+01	41.821204	1.59E-01	50062005	50062128	NC_000019.9
cg23651826	3.02E-21	3.65E-23	1.13E+01	41.799502	2.45E-01	115630531	115630654	NC_000011.9
cg16002355	3.03E-21	3.67E-23	1.13E+01	41.7958532	3.20E-01	111544387	111544510	NC_000004.11
cg12799677	3.07E-21	3.72E-23	1.13E+01	41.7810726	1.14E-01	165631596	165631719	NC_000001.10
cg01990572	3.10E-21	3.76E-23	1.13E+01	41.7719915	1.05E-01	8555659	8555782	NC_000010.10
cg17504999	3.14E-21	3.80E-23	1.13E+01	41.7594203	2.39E-01	134072723	134072846	NC_000004.11
cg05260714	3.23E-21	3.93E-23	1.13E+01	41.7282472	1.62E-01	25344500	25344623	NC_000022.10
cg16792230	3.25E-21	3.95E-23	1.13E+01	41.7225403	1.69E-01	27629665	27629788	NC_000016.9
cg00903932	3.26E-21	3.97E-23	1.13E+01	41.7175427	1.74E-01	101258727	101258850	NC_000001.10
cg27480241	3.31E-21	4.03E-23	1.13E+01	41.7027936	1.63E-01	179310165	179310288	NC_000002.11
cg15534461	3.31E-21	4.03E-23	1.13E+01	41.7010829	2.11E-01	118575033	118575156	NC_000012.11
cg16700920	3.31E-21	4.04E-23	1.13E+01	41.7008067	1.73E-01	133810060	133810183	NC_000010.10
cg13824665	3.33E-21	4.05E-23	1.13E+01	41.6967318	1.05E-01	76183993	76184116	NC_000014.8
cg27288226	3.35E-21	4.08E-23	1.13E+01	41.68905	2.43E-01	57618436	57618559	NC_000019.9
cg13227624	3.36E-21	4.09E-23	1.13E+01	41.6869362	8.26E-02	104352735	104352858	NC_000010.10
cg26681523	3.36E-21	4.09E-23	1.13E+01	41.6863923	1.59E-01	22854408	22854531	NC_000001.10
cg26337821	3.39E-21	4.14E-23	1.13E+01	41.6754869	1.55E-01	123364675	123364798	NC_000007.13

cg00157668	3.40E-21	4.15E-23	1.13E+01	41.6726737	2.09E-01	109674526	109674649	NC_000010.10
cg18066946	3.40E-21	4.16E-23	1.13E+01	41.6703633	1.19E-01	126803477	126803600	NC_000010.10
cg26034658	3.41E-21	4.17E-23	1.13E+01	41.6688111	1.92E-01	65836743	65836866	NC_000011.9
cg26120073	3.42E-21	4.18E-23	1.13E+01	41.6658719	1.74E-01	91841955	91842078	NC_000014.8
cg11293312	3.44E-21	4.21E-23	1.13E+01	41.6585957	1.61E-01	49252020	49252143	NC_000019.9
cg03653317	3.50E-21	4.29E-23	1.13E+01	41.6399976	1.98E-01	118165598	118165721	NC_000001.10
cg19238875	3.50E-21	4.29E-23	1.13E+01	41.6392044	1.37E-01	61612218	61612341	NC_000003.11
cg09624466	3.51E-21	4.30E-23	1.13E+01	41.63767	2.88E-01	57278710	57278833	NC_000014.8
cg10300684	3.51E-21	4.30E-23	1.13E+01	41.6376353	2.99E-01	29236323	29236446	NC_000014.8
cg16181903	3.51E-21	4.30E-23	1.13E+01	41.6368591	1.22E-01	134828123	134828246	NC_000010.10
cg02468316	3.52E-21	4.32E-23	1.13E+01	41.6326058	1.35E-01	50801032	50801155	NC_000007.13
cg25019707	3.53E-21	4.33E-23	1.13E+01	41.6306914	1.02E-01	69994152	69994275	NC_000011.9
cg20002958	3.55E-21	4.36E-23	1.13E+01	41.6245352	1.82E-01	221282600	221282723	NC_000002.11
cg26295057	3.56E-21	4.37E-23	1.13E+01	41.621367	3.55E-01	37839829	37839952	NC_000005.9
cg11464895	3.58E-21	4.40E-23	1.13E+01	41.6150933	2.16E-01	135044789	135044912	NC_000010.10
cg02149136	3.59E-21	4.41E-23	1.13E+01	41.6121892	1.17E-01	85995039	85995162	NC_000011.9
cg21410980	3.61E-21	4.44E-23	1.13E+01	41.6066306	1.38E-01	40121831	40121954	NC_000004.11
cg26003814	3.61E-21	4.45E-23	1.13E+01	41.604597	8.21E-02	16444599	16444722	NC_000001.10
cg12845177	3.62E-21	4.46E-23	1.13E+01	41.6020789	1.89E-01	8650523	8650646	NC_000019.9
cg25635000	3.64E-21	4.48E-23	1.13E+01	41.5969655	1.75E-01	26952678	26952801	NC_000022.10
cg00474798	3.65E-21	4.50E-23	1.13E+01	41.592479	1.64E-01	84684489	84684612	NC_000002.11
cg10402471	3.67E-21	4.52E-23	1.13E+01	41.5885865	7.72E-02	200377740	200377863	NC_000001.10
cg07712904	3.69E-21	4.55E-23	1.13E+01	41.580995	1.51E-01	40389891	40390014	NC_000015.9
cg08210637	3.70E-21	4.56E-23	1.13E+01	41.578381	2.31E-01	58160654	58160777	NC_000016.9
cg10656845	3.71E-21	4.57E-23	1.13E+01	41.577172	1.80E-01	44159780	44159903	NC_000007.13

cg04785284	3.72E-21	4.60E-23	1.13E+01	41.5715012	6.70E-02	86702451	86702574	NC_000016.9
cg19864468	3.73E-21	4.60E-23	1.13E+01	41.570061	1.98E-01	113346566	113346689	NC_000002.11
cg18270675	3.74E-21	4.62E-23	1.13E+01	41.5658371	1.55E-01	85422118	85422241	NC_000011.9
cg08516516	3.75E-21	4.63E-23	1.13E+01	41.564139	2.81E-01	115152492	115152615	NC_000005.9
cg05941108	3.77E-21	4.66E-23	1.13E+01	41.5573782	2.86E-01	1884212	1884335	NC_000005.9
cg13245817	3.79E-21	4.69E-23	1.13E+01	41.5524814	6.96E-02	47481224	47481347	NC_000007.13
cg05339076	3.82E-21	4.73E-23	1.13E+01	41.5441101	1.80E-01	47513051	47513174	NC_000022.10
cg26053840	3.85E-21	4.76E-23	1.13E+01	41.5358625	2.08E-01	38644662	38644785	NC_000006.11
cg22966391	3.90E-21	4.82E-23	1.13E+01	41.5233436	1.66E-01	2416675	2416798	NC_000007.13
cg21179088	3.92E-21	4.86E-23	1.13E+01	41.5156004	2.81E-01	54612333	54612456	NC_000007.13
cg14570652	3.96E-21	4.90E-23	1.13E+01	41.5072687	2.00E-01	139393736	139393859	NC_000009.11
cg04454951	4.02E-21	4.99E-23	1.13E+01	41.49089	3.26E-01	49813031	49813154	NC_000007.13
cg20748132	4.04E-21	5.01E-23	1.13E+01	41.4856238	2.16E-01	125217707	125217830	NC_000012.11
cg13398440	4.04E-21	5.02E-23	1.13E+01	41.4838044	1.95E-01	178688308	178688431	NC_000005.9
cg25664813	4.11E-21	5.11E-23	1.13E+01	41.4665126	2.45E-01	1123123	1123246	NC_000005.9
cg25966682	4.15E-21	5.17E-23	1.13E+01	41.455437	1.02E-01	57577597	57577720	NC_000012.11
cg14091497	4.16E-21	5.17E-23	1.13E+01	41.4540124	8.83E-02	139430748	139430871	NC_000009.11
cg02198895	4.17E-21	5.20E-23	1.13E+01	41.4499168	1.31E-01	110759424	110759547	NC_000013.10
cg10179363	4.23E-21	5.26E-23	1.13E+01	41.4367276	1.47E-01	90352112	90352235	NC_000015.9
cg13912599	4.26E-21	5.30E-23	1.13E+01	41.429876	1.02E-01	150959380	150959503	NC_000001.10
cg13175830	4.29E-21	5.34E-23	1.13E+01	41.4223592	1.71E-01	123353956	123354079	NC_000010.10
cg15944367	4.30E-21	5.36E-23	1.13E+01	41.4188167	1.65E-01	36897829	36897952	NC_000017.10
cg09487611	4.31E-21	5.37E-23	1.13E+01	41.4164496	2.50E-01	5019311	5019434	NC_000012.11
cg22286978	4.33E-21	5.40E-23	1.13E+01	41.410717	3.33E-01	58858806	58858929	NC_000019.9
cg16939364	4.38E-21	5.47E-23	1.13E+01	41.3983037	2.19E-01	56015750	56015873	NC_000008.10

cg17403609	4.40E-21	5.49E-23	1.13E+01	41.3944032	2.20E-01	180075862	180075985	NC_000005.9
cg21158528	4.48E-21	5.60E-23	1.13E+01	41.3748955	2.15E-01	211501629	211501752	NC_000001.10
cg09053680	4.50E-21	5.62E-23	1.13E+01	41.371633	3.55E-01	135044114	135044237	NC_000010.10
cg16107331	4.50E-21	5.62E-23	1.13E+01	41.3712164	7.82E-02	73623052	73623175	NC_000015.9
cg19570244	4.51E-21	5.64E-23	1.13E+01	41.3675659	3.23E-01	32457158	32457281	NC_000011.9
cg06659380	4.55E-21	5.69E-23	1.13E+01	41.3590437	1.61E-01	52162604	52162727	NC_000013.10
cg19625388	4.55E-21	5.70E-23	1.13E+01	41.3586773	1.11E-01	113977418	113977541	NC_000013.10
cg23332582	4.57E-21	5.73E-23	1.13E+01	41.3533569	3.16E-01	140770308	140770431	NC_000003.11
cg13544006	4.63E-21	5.80E-23	1.13E+01	41.3408658	3.16E-01	34189411	34189534	NC_000020.10
cg26594445	4.63E-21	5.80E-23	1.13E+01	41.3406925	1.14E-01	64888605	64888728	NC_000011.9
cg11308643	4.71E-21	5.91E-23	1.12E+01	41.3222069	2.22E-01	105480788	105480911	NC_000011.9
cg08508724	4.73E-21	5.94E-23	1.12E+01	41.3168541	7.45E-02	30122045	30122168	NC_000006.11
cg04051396	4.76E-21	5.99E-23	1.12E+01	41.308643	2.09E-01	93130178	93130301	NC_000012.11
cg02030452	4.83E-21	6.08E-23	1.12E+01	41.294082	1.68E-01	65589615	65589738	NC_000011.9
cg17181598	4.84E-21	6.09E-23	1.12E+01	41.2924448	2.15E-01	118165662	118165785	NC_000001.10
cg10812717	4.86E-21	6.11E-23	1.12E+01	41.2883055	9.49E-02	158183846	158183969	NC_000002.11
cg20398486	4.87E-21	6.13E-23	1.12E+01	41.2858271	2.64E-01	176944442	176944565	NC_000002.11
cg18149207	4.87E-21	6.13E-23	1.12E+01	41.2855318	1.91E-01	151805634	151805757	NC_000001.10
cg22526990	4.87E-21	6.14E-23	1.12E+01	41.2844659	1.90E-01	33788511	33788634	NC_000019.9
cg14694622	4.90E-21	6.18E-23	1.12E+01	41.278255	1.40E-01	171836486	171836609	NC_000003.11
cg00385596	4.90E-21	6.18E-23	1.12E+01	41.278162	1.36E-01	76088726	76088849	NC_000011.9
cg05185038	4.91E-21	6.20E-23	1.12E+01	41.2750295	1.56E-01	105945685	105945808	NC_000014.8
cg09395612	4.93E-21	6.21E-23	1.12E+01	41.2723125	1.98E-01	86018936	86019059	NC_000016.9
cg00539976	4.94E-21	6.23E-23	1.12E+01	41.2691225	1.80E-01	1878508	1878631	NC_000005.9
cg04217218	4.97E-21	6.27E-23	1.12E+01	41.2630286	1.57E-01	14693888	14694011	NC_000009.11

cg21426003	4.97E-21	6.27E-23	1.12E+01	41.2626418	2.79E-01	237076811	237076934	NC_000002.11
cg18036653	4.98E-21	6.29E-23	1.12E+01	41.2597421	1.33E-01	39484787	39484910	NC_000017.10
cg25906620	5.08E-21	6.43E-23	1.12E+01	41.2387782	1.81E-01	11985726	11985849	NC_000018.9
cg14101442	5.11E-21	6.46E-23	1.12E+01	41.2329728	9.54E-02	130742236	130742359	NC_000011.9
cg16012489	5.12E-21	6.49E-23	1.12E+01	41.229192	1.33E-01	11489852	11489975	NC_000008.10
cg21151122	5.13E-21	6.50E-23	1.12E+01	41.2267445	1.06E-01	241568880	241569003	NC_000002.11
cg09427374	5.19E-21	6.58E-23	1.12E+01	41.2159331	1.91E-01	86095320	86095443	NC_000002.11
cg10659805	5.27E-21	6.69E-23	1.12E+01	41.1983531	2.65E-01	96631680	96631803	NC_000007.13
cg03276408	5.27E-21	6.69E-23	1.12E+01	41.1982942	2.77E-01	112712475	112712598	NC_000013.10
cg04682580	5.31E-21	6.74E-23	1.12E+01	41.1919455	1.30E-01	53016396	53016519	NC_000001.10
cg20129213	5.35E-21	6.79E-23	1.12E+01	41.184113	3.66E-01	104512317	104512440	NC_000008.10
cg14035202	5.36E-21	6.82E-23	1.12E+01	41.1801354	1.10E-01	136338301	136338424	NC_000009.11
cg15985191	5.36E-21	6.82E-23	1.12E+01	41.1800866	2.29E-01	18429357	18429480	NC_000010.10
cg16790374	5.43E-21	6.91E-23	1.12E+01	41.166168	1.88E-01	57212793	57212916	NC_000008.10
cg01454947	5.44E-21	6.93E-23	1.12E+01	41.1644389	2.18E-01	99223011	99223134	NC_000002.11
cg01023808	5.54E-21	7.06E-23	1.12E+01	41.1452337	1.84E-01	97857492	97857615	NC_000007.13
cg26802026	5.55E-21	7.07E-23	1.12E+01	41.1440381	2.47E-01	127872329	127872452	NC_000005.9
cg07806674	5.56E-21	7.08E-23	1.12E+01	41.1421605	1.73E-01	27994978	27995101	NC_000001.10
cg23536473	5.57E-21	7.10E-23	1.12E+01	41.140135	2.83E-01	19184555	19184678	NC_000007.13
cg07728793	5.59E-21	7.13E-23	1.12E+01	41.1353365	5.02E-02	238771724	238771847	NC_000002.11
cg05098381	5.59E-21	7.14E-23	1.12E+01	41.134881	1.81E-01	71301786	71301909	NC_000003.11
cg07213616	5.59E-21	7.14E-23	1.12E+01	41.1343857	1.06E-01	60725347	60725470	NC_000005.9
cg24372485	5.62E-21	7.18E-23	1.12E+01	41.1292538	1.61E-01	2078331	2078454	NC_000019.9
cg15446073	5.63E-21	7.19E-23	1.12E+01	41.1273418	9.11E-02	62339136	62339259	NC_000011.9
cg08918120	5.63E-21	7.20E-23	1.12E+01	41.12656	6.57E-02	88294021	88294144	NC_000016.9

cg07850418	5.67E-21	7.24E-23	1.12E+01	41.1201974	2.53E-01	147110378	147110501	NC_000003.11
cg05413282	5.71E-21	7.30E-23	1.12E+01	41.1118353	2.41E-01	33796088	33796211	NC_000009.11
cg26452868	5.81E-21	7.44E-23	1.12E+01	41.0933436	3.12E-01	57387715	57387838	NC_000010.10
cg18172877	5.82E-21	7.45E-23	1.12E+01	41.0915421	2.33E-01	1878672	1878795	NC_000005.9
cg07177011	5.83E-21	7.47E-23	1.12E+01	41.0889591	1.89E-01	1849305	1849428	NC_000001.10
cg02657828	5.85E-21	7.50E-23	1.12E+01	41.0848159	1.55E-01	86703935	86704058	NC_000016.9
cg07103493	5.86E-21	7.51E-23	1.12E+01	41.084124	2.15E-01	128301232	128301355	NC_000005.9
cg12505170	5.89E-21	7.56E-23	1.12E+01	41.0781543	2.88E-01	72595685	72595808	NC_000005.9
cg04336624	5.90E-21	7.57E-23	1.12E+01	41.0755774	8.83E-02	29167281	29167404	NC_000016.9
cg00733324	5.91E-21	7.58E-23	1.12E+01	41.0748239	1.69E-01	67845244	67845367	NC_000014.8
cg22568703	5.91E-21	7.58E-23	1.12E+01	41.0746229	9.65E-02	129733117	129733240	NC_000010.10
cg04704053	5.91E-21	7.59E-23	1.12E+01	41.0737374	2.58E-01	15090275	15090398	NC_000019.9
cg07147449	5.92E-21	7.60E-23	1.12E+01	41.0725506	2.96E-01	115136072	115136195	NC_000012.11
cg09492893	5.97E-21	7.67E-23	1.12E+01	41.0635522	8.96E-02	149939853	149939976	NC_000005.9
cg07382920	6.05E-21	7.78E-23	1.12E+01	41.049572	2.15E-01	3567646	3567769	NC_000001.10
cg03659519	6.06E-21	7.79E-23	1.12E+01	41.0473973	2.63E-01	74961966	74962089	NC_000018.9
cg24840099	6.06E-21	7.79E-23	1.12E+01	41.0473254	1.97E-01	4864430	4864553	NC_000004.11
cg05371578	6.06E-21	7.80E-23	1.12E+01	41.046968	1.80E-01	147128157	147128280	NC_000003.11
cg16027761	6.08E-21	7.82E-23	1.12E+01	41.0436377	3.07E-01	53422871	53422994	NC_000013.10
cg24258347	6.10E-21	7.84E-23	1.12E+01	41.0410801	2.33E-01	13983851	13983974	NC_000011.9
cg16752592	6.10E-21	7.85E-23	1.12E+01	41.0399936	2.20E-01	145537709	145537832	NC_000008.10
cg01033419	6.12E-21	7.88E-23	1.12E+01	41.0368212	1.74E-01	56387378	56387501	NC_000006.11
cg00410895	6.14E-21	7.90E-23	1.12E+01	41.0334405	1.87E-01	43283884	43284007	NC_000001.10
cg21104155	6.21E-21	8.01E-23	1.12E+01	41.0207491	1.55E-01	68040530	68040653	NC_000014.8
cg08172947	6.23E-21	8.04E-23	1.12E+01	41.0168542	2.16E-01	41868550	41868673	NC_000022.10

cg06712089	6.24E-21	8.05E-23	1.12E+01	41.015728	5.03E-02	139128651	139128774	NC_000009.11
cg20449685	6.27E-21	8.09E-23	1.12E+01	41.0103267	2.34E-01	58545728	58545851	NC_000019.9
cg24695614	6.29E-21	8.12E-23	1.12E+01	41.0065906	5.43E-02	3143611	3143734	NC_000010.10
cg10739556	6.33E-21	8.17E-23	1.12E+01	40.9999549	2.79E-01	27192056	27192179	NC_000007.13
cg11215976	6.33E-21	8.18E-23	1.12E+01	40.9995273	2.54E-01	236849942	236850065	NC_000001.10
cg26517714	6.34E-21	8.18E-23	1.12E+01	40.998778	2.42E-01	170736245	170736368	NC_000005.9
cg01642521	6.35E-21	8.20E-23	1.12E+01	40.9963387	3.22E-01	20618250	20618373	NC_000011.9
cg16597537	6.42E-21	8.30E-23	1.12E+01	40.985029	9.07E-02	20252617	20252740	NC_000022.10
cg04273148	6.47E-21	8.38E-23	1.12E+01	40.9758603	2.22E-01	57808038	57808161	NC_000016.9
cg00748072	6.49E-21	8.40E-23	1.12E+01	40.9733722	1.70E-01	174862824	174862947	NC_000002.11
cg22674789	6.51E-21	8.43E-23	1.12E+01	40.9693481	1.66E-01	81740565	81740688	NC_000010.10
cg24368408	6.56E-21	8.50E-23	1.12E+01	40.9609782	1.53E-01	27361313	27361436	NC_000002.11
cg14958635	6.68E-21	8.66E-23	1.12E+01	40.9425734	2.22E-01	134870977	134871100	NC_000005.9
cg26454350	6.68E-21	8.66E-23	1.12E+01	40.942487	9.27E-02	27493204	27493327	NC_000017.10
cg26876703	6.72E-21	8.71E-23	1.12E+01	40.9365389	1.99E-01	48540366	48540489	NC_000017.10
cg02285003	6.72E-21	8.72E-23	1.12E+01	40.9362785	1.09E-01	32296777	32296900	NC_000005.9
cg12377139	6.72E-21	8.72E-23	1.12E+01	40.9359922	1.82E-01	22634218	22634341	NC_000010.10
cg03345668	6.76E-21	8.77E-23	1.12E+01	40.9297986	1.52E-01	47081524	47081647	NC_000001.10
cg15409013	6.80E-21	8.83E-23	1.12E+01	40.9237657	2.86E-01	5139855	5139978	NC_000005.9
cg10172887	6.81E-21	8.85E-23	1.12E+01	40.9213991	1.85E-01	170578592	170578715	NC_000006.11
cg26252958	6.81E-21	8.85E-23	1.12E+01	40.921151	1.99E-01	105847870	105847993	NC_000006.11
cg00741333	6.85E-21	8.90E-23	1.12E+01	40.9157406	1.81E-01	43197675	43197798	NC_000006.11
cg08044097	6.85E-21	8.90E-23	1.12E+01	40.9156246	2.41E-01	55371013	55371136	NC_000008.10
cg24224020	6.85E-21	8.90E-23	1.12E+01	40.9152051	2.57E-01	111544443	111544566	NC_000004.11
cg12797017	6.94E-21	9.03E-23	1.12E+01	40.9006285	1.16E-01	89059249	89059372	NC_000014.8

cg08813062	7.01E-21	9.13E-23	1.12E+01	40.8904815	1.91E-01	175199576	175199699	NC_000002.11
cg15043723	7.03E-21	9.16E-23	1.12E+01	40.8865836	1.73E-01	8106006	8106129	NC_000010.10
cg18347903	7.05E-21	9.19E-23	1.12E+01	40.8841853	8.76E-02	427580	427703	NC_000002.11
cg02621287	7.07E-21	9.22E-23	1.12E+01	40.880968	2.46E-01	11653278	11653401	NC_000012.11
cg18722841	7.10E-21	9.26E-23	1.12E+01	40.8765497	2.76E-01	71954982	71955105	NC_000011.9
cg09804711	7.13E-21	9.30E-23	1.12E+01	40.8715065	1.25E-01	61403556	61403679	NC_000020.10
cg01878345	7.13E-21	9.31E-23	1.12E+01	40.8708834	2.70E-01	105471960	105472083	NC_000002.11
cg25379036	7.13E-21	9.32E-23	1.12E+01	40.8702899	1.25E-01	125544500	125544623	NC_000006.11
cg19651694	7.13E-21	9.32E-23	1.12E+01	40.8702871	2.24E-01	71955164	71955287	NC_000011.9
cg15107670	7.17E-21	9.37E-23	1.12E+01	40.8648985	2.42E-01	32456759	32456882	NC_000011.9
cg25409308	7.17E-21	9.37E-23	1.12E+01	40.8646193	1.33E-01	111602911	111603034	NC_000011.9
cg18450832	7.18E-21	9.38E-23	1.12E+01	40.8632371	1.19E-01	37250092	37250215	NC_000005.9
cg07573209	7.20E-21	9.41E-23	1.12E+01	40.8604385	2.92E-01	76630095	76630218	NC_000015.9
cg07149609	7.21E-21	9.43E-23	1.12E+01	40.8584404	2.51E-01	170630734	170630857	NC_000001.10
cg11345432	7.26E-21	9.50E-23	1.12E+01	40.8510005	1.20E-01	125767838	125767961	NC_000010.10
cg03109914	7.27E-21	9.51E-23	1.12E+01	40.8501871	1.48E-01	823348	823471	NC_000011.9
cg09656848	7.56E-21	9.90E-23	1.12E+01	40.8102377	3.25E-01	61276678	61276801	NC_000011.9
cg07700514	7.56E-21	9.91E-23	1.12E+01	40.8090386	3.77E-01	56228467	56228590	NC_000016.9
cg15070710	7.58E-21	9.94E-23	1.12E+01	40.8061267	2.15E-01	19532345	19532468	NC_000001.10
cg02478836	7.80E-21	1.02E-22	1.12E+01	40.775859	1.30E-01	47510037	47510160	NC_000022.10
cg01285862	7.89E-21	1.04E-22	1.12E+01	40.7647678	1.63E-01	153414365	153414488	NC_000005.9
cg08623156	7.90E-21	1.04E-22	1.12E+01	40.7622953	1.08E-01	113577046	113577169	NC_000012.11
cg16246661	7.91E-21	1.04E-22	1.12E+01	40.7609393	2.04E-01	230272611	230272734	NC_000001.10
cg25444386	7.97E-21	1.05E-22	1.12E+01	40.7528664	2.75E-01	55990944	55991067	NC_000004.11
cg02660165	8.04E-21	1.06E-22	1.12E+01	40.7438114	1.37E-01	160391109	160391232	NC_000001.10

cg24498500	8.06E-21	1.06E-22	1.12E+01	40.7411313	1.77E-01	118166247	118166370	NC_000001.10
cg09915729	8.08E-21	1.06E-22	1.12E+01	40.7383123	2.07E-01	121513707	121513830	NC_000010.10
cg04402007	8.23E-21	1.09E-22	1.12E+01	40.7178105	2.69E-01	35093901	35094024	NC_000008.10
cg20987924	8.25E-21	1.09E-22	1.12E+01	40.7152583	2.45E-01	60296981	60297104	NC_000015.9
cg25555505	8.25E-21	1.09E-22	1.12E+01	40.7150514	1.26E-01	4791054	4791177	NC_000007.13
cg22455914	8.32E-21	1.10E-22	1.12E+01	40.706861	3.92E-01	7116977	7117100	NC_000018.9
cg16407471	8.34E-21	1.10E-22	1.12E+01	40.7029287	2.93E-01	43602914	43603037	NC_000011.9
cg05787035	8.38E-21	1.11E-22	1.12E+01	40.6982522	1.28E-01	202171635	202171758	NC_000001.10
cg14893932	8.39E-21	1.11E-22	1.12E+01	40.6975674	1.28E-01	66584493	66584616	NC_000015.9
cg20115910	8.60E-21	1.14E-22	1.12E+01	40.6720229	9.38E-02	49934906	49935029	NC_000012.11
cg26756625	8.62E-21	1.14E-22	1.12E+01	40.6695156	2.14E-01	126779875	126779998	NC_000009.11
cg08810842	8.62E-21	1.14E-22	1.12E+01	40.6692617	2.39E-01	22548546	22548669	NC_000008.10
cg02172312	8.62E-21	1.14E-22	1.12E+01	40.6682884	2.45E-01	62069337	62069460	NC_000016.9
cg20664238	8.65E-21	1.15E-22	1.12E+01	40.66531	2.82E-01	88798877	88799000	NC_000015.9
cg10909506	8.74E-21	1.16E-22	1.12E+01	40.6543414	1.59E-01	38081995	38082118	NC_000017.10
cg01226231	8.75E-21	1.16E-22	1.12E+01	40.6530973	1.12E-01	139935196	139935319	NC_000005.9
cg20707409	8.81E-21	1.17E-22	1.11E+01	40.6454619	1.58E-01	124605957	124606080	NC_000010.10
cg07135701	8.87E-21	1.18E-22	1.11E+01	40.6391863	1.15E-01	23519441	23519564	NC_000016.9
cg05774699	8.87E-21	1.18E-22	1.11E+01	40.6381621	2.06E-01	27184316	27184439	NC_000007.13
cg17924901	8.91E-21	1.18E-22	1.11E+01	40.633395	1.49E-01	3475233	3475356	NC_000004.11
cg15639951	8.94E-21	1.19E-22	1.11E+01	40.6300311	1.73E-01	43367609	43367732	NC_000017.10
cg08065231	8.97E-21	1.19E-22	1.11E+01	40.626679	2.98E-01	9473684	9473807	NC_000019.9
cg17141972	9.05E-21	1.20E-22	1.11E+01	40.6171706	1.60E-01	77547510	77547633	NC_000006.11
cg13465477	9.13E-21	1.21E-22	1.11E+01	40.6075323	2.40E-01	133534961	133535084	NC_000009.11
cg15031661	9.18E-21	1.22E-22	1.11E+01	40.6012331	2.88E-01	240256603	240256726	NC_000001.10

cg17927493	9.21E-21	1.22E-22	1.11E+01	40.5987869	1.64E-01	81997731	81997854	NC_000014.8
cg22762091	9.23E-21	1.23E-22	1.11E+01	40.5955828	1.97E-01	132052843	132052966	NC_000008.10
cg15585794	9.27E-21	1.23E-22	1.11E+01	40.5918543	2.85E-01	131780329	131780452	NC_000011.9
cg09868598	9.29E-21	1.24E-22	1.11E+01	40.5890126	2.18E-01	50674015	50674138	NC_000005.9
cg18493314	9.30E-21	1.24E-22	1.11E+01	40.5882477	1.44E-01	21899075	21899198	NC_000014.8
cg22620090	9.34E-21	1.24E-22	1.11E+01	40.5826185	2.96E-01	105400993	105401116	NC_000006.11
cg23881278	9.35E-21	1.25E-22	1.11E+01	40.5813992	2.86E-01	113346299	113346422	NC_000011.9
cg26675485	9.47E-21	1.26E-22	1.11E+01	40.5679666	1.57E-01	67968879	67969002	NC_000016.9
cg13151751	9.51E-21	1.27E-22	1.11E+01	40.5637661	1.20E-01	89993815	89993938	NC_000001.10
cg03243768	9.51E-21	1.27E-22	1.11E+01	40.5631117	2.10E-01	9115107	9115230	NC_000011.9
cg20100801	9.57E-21	1.28E-22	1.11E+01	40.5561937	1.84E-01	95987654	95987777	NC_000012.11
cg25475443	9.57E-21	1.28E-22	1.11E+01	40.5559717	9.20E-02	125806388	125806511	NC_000010.10
cg16626318	9.72E-21	1.30E-22	1.11E+01	40.5395832	1.01E-01	37288164	37288287	NC_000003.11
cg23476745	9.81E-21	1.31E-22	1.11E+01	40.5304522	1.24E-01	79076552	79076675	NC_000017.10
cg08042322	9.82E-21	1.31E-22	1.11E+01	40.5298429	1.65E-01	104233595	104233718	NC_000012.11
cg03957649	9.94E-21	1.33E-22	1.11E+01	40.5165203	8.96E-02	30121853	30121976	NC_000006.11
cg24705818	9.98E-21	1.34E-22	1.11E+01	40.5123559	1.68E-01	18964128	18964251	NC_000010.10
cg07575617	1.00E-20	1.34E-22	1.11E+01	40.5074772	1.09E-01	218460685	218460808	NC_000001.10
cg08165221	1.00E-20	1.34E-22	1.11E+01	40.5073023	2.17E-01	5833017	5833140	NC_000002.11
cg20168230	1.01E-20	1.35E-22	1.11E+01	40.5011693	2.05E-01	37499649	37499772	NC_000001.10
cg03013699	1.01E-20	1.36E-22	1.11E+01	40.4979949	5.76E-02	50772477	50772600	NC_000007.13
cg09004195	1.02E-20	1.37E-22	1.11E+01	40.4903947	1.04E-01	222323493	222323616	NC_000002.11
cg18863595	1.02E-20	1.37E-22	1.11E+01	40.4868464	2.63E-01	6902845	6902968	NC_000003.11
cg07707505	1.02E-20	1.37E-22	1.11E+01	40.4867475	1.68E-01	12185435	12185558	NC_000001.10
cg22295334	1.03E-20	1.38E-22	1.11E+01	40.4817507	1.22E-01	135835943	135836066	NC_000008.10

cg02148562	1.03E-20	1.38E-22	1.11E+01	40.4805936	2.19E-01	128766988	128767111	NC_000008.10
cg16142306	1.04E-20	1.39E-22	1.11E+01	40.4712466	2.24E-01	41258935	41259058	NC_000004.11
cg25342872	1.04E-20	1.40E-22	1.11E+01	40.4633682	1.32E-01	79175132	79175255	NC_000017.10
cg01570424	1.05E-20	1.41E-22	1.11E+01	40.4600183	3.29E-01	2038922	2039045	NC_000005.9
cg08487909	1.05E-20	1.41E-22	1.11E+01	40.4576359	1.22E-01	30904981	30905104	NC_000006.11
cg19596468	1.05E-20	1.41E-22	1.11E+01	40.4571881	1.78E-01	4864110	4864233	NC_000004.11
cg00298405	1.05E-20	1.41E-22	1.11E+01	40.455413	1.21E-01	46835192	46835315	NC_000022.10
cg10888790	1.05E-20	1.42E-22	1.11E+01	40.4512216	1.60E-01	131766874	131766997	NC_000010.10
cg27277463	1.06E-20	1.42E-22	1.11E+01	40.44907	3.22E-01	62585031	62585154	NC_000012.11
cg17538572	1.06E-20	1.43E-22	1.11E+01	40.4462906	3.17E-01	94834763	94834886	NC_000010.10
cg26524638	1.06E-20	1.43E-22	1.11E+01	40.4461865	1.33E-01	2481449	2481572	NC_000011.9
cg07600374	1.06E-20	1.43E-22	1.11E+01	40.4415188	1.19E-01	134278787	134278910	NC_000006.11
cg12656740	1.07E-20	1.44E-22	1.11E+01	40.4391877	1.10E-01	205422437	205422560	NC_000001.10
cg08013557	1.07E-20	1.44E-22	1.11E+01	40.4353415	2.38E-01	147115043	147115166	NC_000003.11
cg08094235	1.07E-20	1.45E-22	1.11E+01	40.4339502	1.49E-01	3463558	3463681	NC_000002.11
cg12177001	1.08E-20	1.46E-22	1.11E+01	40.4244026	1.22E-01	94576148	94576271	NC_000014.8
cg18507379	1.09E-20	1.47E-22	1.11E+01	40.4188191	2.99E-01	140787507	140787630	NC_000005.9
cg08829841	1.09E-20	1.47E-22	1.11E+01	40.4184102	2.16E-01	129535348	129535471	NC_000010.10
cg13067139	1.09E-20	1.47E-22	1.11E+01	40.4182822	8.90E-02	155221277	155221400	NC_000001.10
cg24342610	1.09E-20	1.48E-22	1.11E+01	40.4123145	1.19E-01	50331002	50331125	NC_000003.11
cg08080923	1.10E-20	1.49E-22	1.11E+01	40.4056263	1.33E-01	47763943	47764066	NC_000019.9
cg08893692	1.10E-20	1.49E-22	1.11E+01	40.4039482	2.80E-01	72595741	72595864	NC_000005.9
cg22795216	1.10E-20	1.49E-22	1.11E+01	40.4030687	1.87E-01	47078649	47078772	NC_000001.10
cg07737063	1.10E-20	1.50E-22	1.11E+01	40.4000521	2.23E-01	1486341	1486464	NC_000011.9
cg08296263	1.11E-20	1.51E-22	1.11E+01	40.391815	1.51E-01	28938433	28938556	NC_000008.10

cg08158952	1.12E-20	1.51E-22	1.11E+01	40.3882531	2.87E-01	57183016	57183139	NC_000019.9
cg01691522	1.12E-20	1.52E-22	1.11E+01	40.38483	1.20E-01	42998521	42998644	NC_000005.9
cg02852421	1.12E-20	1.52E-22	1.11E+01	40.3844186	2.53E-01	70372614	70372737	NC_000015.9
cg02119792	1.12E-20	1.52E-22	1.11E+01	40.3832292	3.00E-01	47010818	47010941	NC_000021.8
cg05543520	1.12E-20	1.52E-22	1.11E+01	40.3831329	2.20E-01	66500703	66500826	NC_000003.11
cg04562755	1.12E-20	1.52E-22	1.11E+01	40.3823968	1.67E-01	64090011	64090134	NC_000001.10
cg06431953	1.12E-20	1.52E-22	1.11E+01	40.3814788	1.65E-01	112630726	112630849	NC_000010.10
cg18794282	1.13E-20	1.54E-22	1.11E+01	40.3720011	1.44E-01	48253633	48253756	NC_000008.10
cg08627010	1.13E-20	1.54E-22	1.11E+01	40.3705716	1.67E-01	125651697	125651820	NC_000010.10
cg08776356	1.14E-20	1.55E-22	1.11E+01	40.3669146	2.66E-01	51186260	51186383	NC_000016.9
cg17176911	1.15E-20	1.56E-22	1.11E+01	40.3564782	1.58E-01	150261107	150261230	NC_000003.11
cg06762395	1.18E-20	1.60E-22	1.11E+01	40.3328114	1.23E-01	75263297	75263420	NC_000016.9
cg18725867	1.18E-20	1.60E-22	1.11E+01	40.332026	2.84E-01	37834958	37835081	NC_000005.9
cg25033990	1.19E-20	1.61E-22	1.11E+01	40.3240736	2.22E-01	32484014	32484137	NC_000017.10
cg00495201	1.19E-20	1.62E-22	1.11E+01	40.3229315	1.99E-01	137825623	137825746	NC_000005.9
cg06741803	1.19E-20	1.62E-22	1.11E+01	40.3187004	1.43E-01	46807522	46807645	NC_000001.10
cg13808641	1.20E-20	1.63E-22	1.11E+01	40.3124836	9.81E-02	96006533	96006656	NC_000009.11
cg25059165	1.20E-20	1.63E-22	1.11E+01	40.3124249	1.05E-01	33171467	33171590	NC_000006.11
cg05239811	1.20E-20	1.63E-22	1.11E+01	40.3118879	1.49E-01	31734147	31734270	NC_000006.11
cg22112000	1.20E-20	1.64E-22	1.11E+01	40.3084406	1.56E-01	5647182	5647305	NC_000007.13
cg22978326	1.21E-20	1.66E-22	1.11E+01	40.2970065	1.39E-01	171761287	171761410	NC_000003.11
cg07828377	1.24E-20	1.70E-22	1.11E+01	40.2730558	1.16E-01	109496	109619	NC_000017.10
cg26956874	1.24E-20	1.70E-22	1.11E+01	40.2722549	2.66E-01	237206643	237206766	NC_000001.10
cg05977017	1.25E-20	1.71E-22	1.11E+01	40.2691315	1.11E-01	126856298	126856421	NC_000005.9
cg15467759	1.25E-20	1.71E-22	1.11E+01	40.2668511	7.55E-02	149900327	149900450	NC_000005.9

cg17800654	1.25E-20	1.71E-22	1.11E+01	40.26489	2.39E-01	50888590	50888713	NC_000001.10
cg05560435	1.26E-20	1.72E-22	1.11E+01	40.2618986	2.66E-01	172671526	172671649	NC_000005.9
cg09850764	1.26E-20	1.73E-22	1.11E+01	40.2580896	1.49E-01	1406322	1406445	NC_000016.9
cg21584979	1.26E-20	1.73E-22	1.11E+01	40.254212	1.77E-01	235878176	235878299	NC_000002.11
cg26509731	1.27E-20	1.74E-22	1.11E+01	40.2513166	2.06E-01	80578590	80578713	NC_000006.11
cg26284985	1.29E-20	1.77E-22	1.11E+01	40.2353159	1.48E-01	110426718	110426841	NC_000006.11
cg05237641	1.30E-20	1.79E-22	1.11E+01	40.22088	1.75E-01	128077307	128077430	NC_000010.10
cg24872782	1.31E-20	1.79E-22	1.11E+01	40.2203168	2.87E-01	15500249	15500372	NC_000005.9
cg17397631	1.31E-20	1.80E-22	1.11E+01	40.2175411	2.78E-01	779880	780003	NC_000004.11
cg18755296	1.31E-20	1.81E-22	1.11E+01	40.2128967	2.77E-01	53852446	53852569	NC_000008.10
cg09321400	1.32E-20	1.81E-22	1.11E+01	40.2102075	2.73E-01	55690443	55690566	NC_000016.9
cg04224064	1.32E-20	1.82E-22	1.11E+01	40.2049732	2.37E-01	36992233	36992356	NC_000014.8
cg17035132	1.33E-20	1.83E-22	1.11E+01	40.200572	1.33E-01	2504160	2504283	NC_000011.9
cg20319604	1.33E-20	1.83E-22	1.11E+01	40.1996602	1.69E-01	44428936	44429059	NC_000002.11
cg27062795	1.38E-20	1.90E-22	1.11E+01	40.1621188	2.74E-01	56879613	56879736	NC_000019.9
cg04958794	1.38E-20	1.91E-22	1.11E+01	40.1586328	2.60E-01	97685274	97685397	NC_000014.8
cg15459774	1.39E-20	1.91E-22	1.11E+01	40.1560494	1.45E-01	8331529	8331652	NC_000010.10
cg15111065	1.40E-20	1.93E-22	1.11E+01	40.1475219	1.92E-01	129491725	129491848	NC_000007.13
cg10550033	1.42E-20	1.96E-22	1.11E+01	40.1323737	1.89E-01	59983940	59984063	NC_000013.10
cg05223720	1.42E-20	1.97E-22	1.11E+01	40.1290465	3.05E-01	71801793	71801916	NC_000007.13
cg16767916	1.43E-20	1.97E-22	1.11E+01	40.1247424	1.72E-01	105473128	105473251	NC_000002.11
cg01310768	1.44E-20	1.99E-22	1.11E+01	40.1177594	1.71E-01	89823130	89823253	NC_000005.9
cg00446413	1.44E-20	1.99E-22	1.11E+01	40.1145176	2.03E-01	153749206	153749329	NC_000007.13
cg17226356	1.44E-20	2.00E-22	1.11E+01	40.1133754	1.12E-01	48600317	48600440	NC_000003.11
cg08289056	1.45E-20	2.01E-22	1.11E+01	40.1057421	1.59E-01	6233666	6233789	NC_000010.10

cg02825211	1.46E-20	2.02E-22	1.11E+01	40.104195	1.88E-01	8808663	8808786	NC_000019.9
cg08351085	1.46E-20	2.02E-22	1.11E+01	40.1031493	1.59E-01	113980035	113980158	NC_000013.10
cg03044684	1.47E-20	2.04E-22	1.11E+01	40.0935966	2.66E-01	33248032	33248155	NC_000021.8
cg22821931	1.47E-20	2.04E-22	1.11E+01	40.0914134	1.55E-01	50800837	50800960	NC_000007.13
cg23665381	1.49E-20	2.07E-22	1.11E+01	40.079915	2.60E-01	5710098	5710221	NC_000004.11
cg02807786	1.50E-20	2.07E-22	1.11E+01	40.0754494	1.36E-01	125141606	125141729	NC_000012.11
cg15079455	1.52E-20	2.10E-22	1.11E+01	40.0621498	1.12E-01	127989828	127989951	NC_000003.11
cg11696200	1.54E-20	2.14E-22	1.11E+01	40.0432065	2.50E-01	33768013	33768136	NC_000013.10
cg06146977	1.59E-20	2.21E-22	1.11E+01	40.0144473	1.75E-01	43469464	43469587	NC_000013.10
cg15854333	1.59E-20	2.21E-22	1.11E+01	40.0138063	8.91E-02	33171765	33171888	NC_000006.11
cg08065657	1.65E-20	2.31E-22	1.11E+01	39.970653	2.27E-01	52223099	52223222	NC_000019.9
cg27031484	1.66E-20	2.31E-22	1.11E+01	39.9685675	1.78E-01	129502834	129502957	NC_000007.13
cg01295203	1.66E-20	2.32E-22	1.10E+01	39.9641195	2.28E-01	70984199	70984322	NC_000008.10
cg24656388	1.71E-20	2.38E-22	1.10E+01	39.9373903	1.43E-01	8085918	8086041	NC_000006.11
cg25799348	1.73E-20	2.42E-22	1.10E+01	39.9236644	1.26E-01	4729417	4729540	NC_000007.13
cg24345261	1.74E-20	2.44E-22	1.10E+01	39.9156524	7.85E-02	154196770	154196893	NC_000004.11
cg24733475	1.75E-20	2.45E-22	1.10E+01	39.9108575	7.11E-02	64694047	64694170	NC_000014.8
cg10477621	1.75E-20	2.46E-22	1.10E+01	39.9074417	2.22E-01	19184950	19185073	NC_000007.13
cg00431835	1.77E-20	2.47E-22	1.10E+01	39.9003493	1.76E-01	86796158	86796281	NC_000016.9
cg24469464	1.77E-20	2.48E-22	1.10E+01	39.8992625	2.03E-01	4860157	4860280	NC_000004.11
cg09753632	1.79E-20	2.52E-22	1.10E+01	39.8829454	8.34E-02	95413861	95413984	NC_000005.9
cg03059131	1.81E-20	2.53E-22	1.10E+01	39.8769145	3.10E-01	60296996	60297119	NC_000015.9
cg00128386	1.81E-20	2.53E-22	1.10E+01	39.8766157	1.80E-01	85665539	85665662	NC_000016.9
cg01610605	1.82E-20	2.55E-22	1.10E+01	39.8701597	2.11E-01	6069399	6069522	NC_000016.9
cg05083202	1.82E-20	2.56E-22	1.10E+01	39.8668155	1.69E-01	40146149	40146272	NC_000021.8

cg24032666	1.83E-20	2.57E-22	1.10E+01	39.8635085	2.89E-01	24814035	24814158	NC_000008.10
cg24284973	1.83E-20	2.57E-22	1.10E+01	39.8627918	2.74E-01	131780357	131780480	NC_000011.9
cg01693350	1.83E-20	2.58E-22	1.10E+01	39.8598677	1.81E-01	32452187	32452310	NC_000011.9
cg12894814	1.85E-20	2.61E-22	1.10E+01	39.8470852	1.66E-01	22232412	22232535	NC_000007.13
cg11829371	1.85E-20	2.61E-22	1.10E+01	39.8468739	1.04E-01	981724	981847	NC_000017.10
cg00418282	1.89E-20	2.66E-22	1.10E+01	39.8290654	2.73E-01	2740132	2740255	NC_000005.9
cg24297348	1.91E-20	2.69E-22	1.10E+01	39.8159256	8.74E-02	112706316	112706439	NC_000003.11
cg27532754	1.93E-20	2.72E-22	1.10E+01	39.8062371	1.72E-01	134905361	134905484	NC_000010.10
cg15310162	1.93E-20	2.73E-22	1.10E+01	39.8043927	2.20E-01	37852822	37852945	NC_000021.8
cg08575330	1.93E-20	2.73E-22	1.10E+01	39.8042258	2.79E-01	175199828	175199951	NC_000002.11
cg18180407	1.95E-20	2.75E-22	1.10E+01	39.7947007	2.06E-01	55047736	55047859	NC_000001.10
cg15040499	1.95E-20	2.76E-22	1.10E+01	39.7908469	1.48E-01	35861263	35861386	NC_000017.10
cg08407408	1.96E-20	2.77E-22	1.10E+01	39.7896535	1.77E-01	123007555	123007678	NC_000012.11
cg00812833	1.97E-20	2.79E-22	1.10E+01	39.7801456	2.82E-01	242687412	242687535	NC_000001.10
cg16428857	1.98E-20	2.80E-22	1.10E+01	39.7773507	1.18E-01	33248616	33248739	NC_000006.11
cg01150683	2.02E-20	2.86E-22	1.10E+01	39.7581658	1.91E-01	111216646	111216769	NC_000010.10
cg04775304	2.03E-20	2.88E-22	1.10E+01	39.7516126	7.32E-02	75266396	75266519	NC_000016.9
cg26710891	2.04E-20	2.88E-22	1.10E+01	39.7486763	1.84E-01	2324923	2325046	NC_000017.10
cg02743222	2.04E-20	2.88E-22	1.10E+01	39.7486751	2.15E-01	170736335	170736458	NC_000005.9
cg06482428	2.04E-20	2.89E-22	1.10E+01	39.748103	2.12E-01	21950173	21950296	NC_000004.11
cg20883194	2.04E-20	2.89E-22	1.10E+01	39.7455716	1.48E-01	45801769	45801892	NC_000012.11
cg20170533	2.05E-20	2.91E-22	1.10E+01	39.7408128	2.22E-01	51417846	51417969	NC_000013.10
cg26769186	2.06E-20	2.93E-22	1.10E+01	39.7337578	8.46E-02	196010417	196010540	NC_000003.11
cg10153349	2.10E-20	2.98E-22	1.10E+01	39.7170908	1.74E-01	13983893	13984016	NC_000011.9
cg15928093	2.13E-20	3.02E-22	1.10E+01	39.7025997	2.59E-01	130647213	130647336	NC_000012.11

cg18795809	2.14E-20	3.04E-22	1.10E+01	39.6978721	3.10E-01	10458531	10458654	NC_000004.11
cg18255813	2.14E-20	3.04E-22	1.10E+01	39.6970848	2.42E-01	7195966	7196089	NC_000006.11
cg07471614	2.15E-20	3.07E-22	1.10E+01	39.688029	2.08E-01	125855152	125855275	NC_000008.10
cg16823083	2.16E-20	3.07E-22	1.10E+01	39.6871186	2.08E-01	3567412	3567535	NC_000001.10
cg12974388	2.16E-20	3.07E-22	1.10E+01	39.6853543	2.86E-01	237072998	237073121	NC_000002.11
cg23495748	2.17E-20	3.10E-22	1.10E+01	39.6783979	3.04E-01	175223636	175223759	NC_000005.9
cg18129971	2.19E-20	3.13E-22	1.10E+01	39.6687028	1.04E-01	9884912	9885035	NC_000018.9
cg13138089	2.19E-20	3.13E-22	1.10E+01	39.6683133	1.32E-01	233251770	233251893	NC_000002.11
cg12212311	2.20E-20	3.14E-22	1.10E+01	39.6643238	2.51E-01	77558916	77559039	NC_000018.9
cg06223767	2.22E-20	3.16E-22	1.10E+01	39.6571221	3.14E-01	393616	393739	NC_000006.11
cg20634442	2.23E-20	3.19E-22	1.10E+01	39.6493346	2.33E-01	75524696	75524819	NC_000017.10
cg07281879	2.24E-20	3.19E-22	1.10E+01	39.6485955	1.89E-01	32459626	32459749	NC_000011.9
cg04304509	2.25E-20	3.21E-22	1.10E+01	39.6430506	1.48E-01	230215193	230215316	NC_000001.10
cg19755314	2.26E-20	3.22E-22	1.10E+01	39.63764	1.90E-01	143220	143343	NC_000007.13
cg10530883	2.28E-20	3.26E-22	1.10E+01	39.6278884	2.37E-01	3596207	3596330	NC_000005.9
cg19267861	2.31E-20	3.30E-22	1.10E+01	39.6145859	2.23E-01	61809724	61809847	NC_000020.10
cg24370974	2.32E-20	3.32E-22	1.10E+01	39.6095121	1.64E-01	31721356	31721479	NC_000006.11
cg26509915	2.33E-20	3.34E-22	1.10E+01	39.6039268	1.14E-01	69285633	69285756	NC_000011.9
cg16475811	2.34E-20	3.35E-22	1.10E+01	39.6013003	1.39E-01	69308726	69308849	NC_000011.9
cg19936016	2.34E-20	3.35E-22	1.10E+01	39.5997938	1.60E-01	1206045	1206168	NC_000004.11
cg11667086	2.34E-20	3.35E-22	1.10E+01	39.599768	1.90E-01	31848554	31848677	NC_000011.9
cg06360427	2.34E-20	3.35E-22	1.10E+01	39.5989158	2.23E-01	74962672	74962795	NC_000018.9
cg13686789	2.35E-20	3.37E-22	1.10E+01	39.5934196	1.04E-01	72655401	72655524	NC_000009.11
cg16332256	2.36E-20	3.38E-22	1.10E+01	39.5923217	2.93E-01	9534388	9534511	NC_000004.11
cg03926852	2.36E-20	3.38E-22	1.10E+01	39.5915816	9.43E-02	31681562	31681685	NC_000006.11

cg06593118	2.39E-20	3.42E-22	1.10E+01	39.5793042	2.28E-01	117711896	117712019	NC_000011.9
cg11254700	2.42E-20	3.48E-22	1.10E+01	39.5618735	2.81E-01	53561386	53561509	NC_000019.9
cg00922781	2.43E-20	3.49E-22	1.10E+01	39.5582001	2.02E-01	128077080	128077203	NC_000010.10
cg01851970	2.44E-20	3.50E-22	1.10E+01	39.5562164	9.62E-02	73579244	73579367	NC_000005.9
cg13023623	2.44E-20	3.51E-22	1.10E+01	39.5536253	2.28E-01	57276257	57276380	NC_000014.8
cg25953239	2.45E-20	3.53E-22	1.10E+01	39.5487268	2.41E-01	176982342	176982465	NC_000002.11
cg27428744	2.46E-20	3.54E-22	1.10E+01	39.5461001	1.68E-01	65508251	65508374	NC_000016.9
cg17529832	2.48E-20	3.56E-22	1.10E+01	39.5387289	2.78E-01	105461096	105461219	NC_000002.11
cg19949955	2.48E-20	3.57E-22	1.10E+01	39.5354836	2.31E-01	33402443	33402566	NC_000014.8
cg11776930	2.49E-20	3.59E-22	1.10E+01	39.5313123	1.43E-01	88427400	88427523	NC_000010.10
cg15362957	2.50E-20	3.60E-22	1.10E+01	39.5285183	1.55E-01	97684772	97684895	NC_000014.8
cg18856388	2.53E-20	3.64E-22	1.10E+01	39.5167535	3.08E-01	1875886	1876009	NC_000005.9
cg12751432	2.53E-20	3.65E-22	1.10E+01	39.5156921	3.06E-01	56015066	56015189	NC_000008.10
cg03680996	2.54E-20	3.65E-22	1.10E+01	39.5139258	1.62E-01	10725534	10725657	NC_000006.11
cg23151413	2.60E-20	3.75E-22	1.10E+01	39.4887846	1.88E-01	79350405	79350528	NC_000002.11
cg23169059	2.63E-20	3.79E-22	1.10E+01	39.477334	8.76E-02	167761204	167761327	NC_000006.11
cg22865128	2.64E-20	3.82E-22	1.10E+01	39.4699762	1.56E-01	241627555	241627678	NC_000002.11
cg26492368	2.65E-20	3.82E-22	1.10E+01	39.4690007	2.09E-01	22634733	22634856	NC_000010.10
cg22860848	2.66E-20	3.84E-22	1.10E+01	39.4653576	1.31E-01	241631345	241631468	NC_000002.11
cg14991487	2.66E-20	3.85E-22	1.10E+01	39.4619661	2.60E-01	176987404	176987527	NC_000002.11
cg13277254	2.66E-20	3.85E-22	1.10E+01	39.4618966	1.46E-01	171333801	171333924	NC_000005.9
cg06613775	2.67E-20	3.86E-22	1.10E+01	39.4585291	1.58E-01	88897441	88897564	NC_000012.11
cg20979061	2.68E-20	3.87E-22	1.10E+01	39.4570732	1.57E-01	49939949	49940072	NC_000019.9
cg19653979	2.76E-20	4.00E-22	1.10E+01	39.4245551	8.48E-02	11881462	11881585	NC_000002.11
cg27642784	2.81E-20	4.07E-22	1.10E+01	39.4059955	1.91E-01	157813670	157813793	NC_000003.11

cg06518628	2.81E-20	4.08E-22	1.10E+01	39.4044685	2.48E-01	104513704	104513827	NC_000008.10
cg10696504	2.85E-20	4.13E-22	1.10E+01	39.3916161	1.31E-01	25201541	25201664	NC_000013.10
cg13478737	2.95E-20	4.29E-22	1.10E+01	39.353695	1.46E-01	133807443	133807566	NC_000009.11
cg07401230	2.99E-20	4.35E-22	1.10E+01	39.3407607	3.18E-01	9763877	9764000	NC_000008.10
cg14038391	3.00E-20	4.37E-22	1.10E+01	39.3353977	2.87E-01	102900130	102900253	NC_000010.10
cg03229780	3.01E-20	4.38E-22	1.10E+01	39.3339784	2.70E-01	108238372	108238495	NC_000012.11
cg01815671	3.05E-20	4.44E-22	1.10E+01	39.3212229	2.77E-01	126113663	126113786	NC_000003.11
cg26246807	3.07E-20	4.47E-22	1.10E+01	39.3144161	3.44E-01	58095659	58095782	NC_000019.9
cg19528797	3.07E-20	4.47E-22	1.10E+01	39.3140913	1.74E-01	117468110	117468233	NC_000012.11
cg19326874	3.07E-20	4.47E-22	1.10E+01	39.3129065	1.50E-01	37903243	37903366	NC_000004.11
cg15985184	3.07E-20	4.48E-22	1.10E+01	39.3119226	2.37E-01	51830311	51830434	NC_000019.9
cg16975225	3.13E-20	4.56E-22	1.10E+01	39.2937281	1.60E-01	52437342	52437465	NC_000012.11
cg07527426	3.14E-20	4.58E-22	1.10E+01	39.2901612	8.42E-02	44125473	44125596	NC_000006.11
cg07575466	3.14E-20	4.59E-22	1.10E+01	39.2881957	1.56E-01	40769708	40769831	NC_000001.10
cg04437648	3.16E-20	4.61E-22	1.09E+01	39.2836604	9.55E-02	234627450	234627573	NC_000002.11
cg08621473	3.16E-20	4.61E-22	1.09E+01	39.2833151	2.34E-01	23734512	23734635	NC_000013.10
cg06356454	3.17E-20	4.62E-22	1.09E+01	39.2799571	1.67E-01	241631498	241631621	NC_000002.11
cg21298249	3.18E-20	4.65E-22	1.09E+01	39.2751897	1.49E-01	77734286	77734409	NC_000015.9
cg15047344	3.19E-20	4.66E-22	1.09E+01	39.2730942	1.16E-01	42997034	42997157	NC_000006.11
cg19909349	3.19E-20	4.66E-22	1.09E+01	39.2726604	2.94E-01	7118092	7118215	NC_000018.9
cg11814826	3.20E-20	4.68E-22	1.09E+01	39.2683915	9.74E-02	26666694	26666817	NC_000017.10
cg21049487	3.23E-20	4.73E-22	1.09E+01	39.2578315	1.50E-01	104328065	104328188	NC_000012.11
cg17594242	3.24E-20	4.74E-22	1.09E+01	39.2546656	1.34E-01	115654782	115654905	NC_000001.10
cg26034341	3.26E-20	4.78E-22	1.09E+01	39.2467297	2.15E-01	477314	477437	NC_000017.10
cg08333333	3.28E-20	4.81E-22	1.09E+01	39.2415343	2.12E-01	57182844	57182967	NC_000019.9

cg24555562	3.30E-20	4.83E-22	1.09E+01	39.2360918	1.57E-01	242827096	242827219	NC_000002.11
cg00503690	3.31E-20	4.85E-22	1.09E+01	39.2317008	1.15E-01	66431055	66431178	NC_000003.11
cg03124514	3.31E-20	4.86E-22	1.09E+01	39.2313076	1.38E-01	42943857	42943980	NC_000005.9
cg10534873	3.32E-20	4.87E-22	1.09E+01	39.2294932	1.84E-01	56591902	56592025	NC_000014.8
cg13723983	3.32E-20	4.87E-22	1.09E+01	39.2292697	1.08E-01	132383362	132383485	NC_000009.11
cg12012932	3.32E-20	4.87E-22	1.09E+01	39.22788	2.78E-01	30484033	30484156	NC_000015.9
cg13057167	3.36E-20	4.93E-22	1.09E+01	39.2168773	8.77E-02	103750565	103750688	NC_000014.8
cg06407390	3.36E-20	4.93E-22	1.09E+01	39.2164784	1.49E-01	134723491	134723614	NC_000010.10
cg24824686	3.36E-20	4.94E-22	1.09E+01	39.2150374	1.66E-01	129797777	129797900	NC_000010.10
cg05977002	3.37E-20	4.95E-22	1.09E+01	39.2122447	2.04E-01	92908051	92908174	NC_000005.9
cg01856023	3.38E-20	4.96E-22	1.09E+01	39.209537	1.34E-01	81969883	81970006	NC_000016.9
cg09048251	3.40E-20	5.00E-22	1.09E+01	39.2030432	2.18E-01	77253974	77254097	NC_000005.9
cg14364472	3.42E-20	5.02E-22	1.09E+01	39.1976101	2.15E-01	139394549	139394672	NC_000009.11
cg04653800	3.43E-20	5.04E-22	1.09E+01	39.1938951	2.16E-01	7910535	7910658	NC_000002.11
cg23089825	3.47E-20	5.11E-22	1.09E+01	39.1807409	2.73E-01	170630558	170630681	NC_000001.10
cg06548118	3.48E-20	5.13E-22	1.09E+01	39.1775634	1.53E-01	38812043	38812166	NC_000002.11
cg12559685	3.49E-20	5.14E-22	1.09E+01	39.1755462	8.48E-02	40839469	40839592	NC_000017.10
cg15107132	3.50E-20	5.15E-22	1.09E+01	39.1737505	1.35E-01	63991130	63991253	NC_000011.9
cg07065111	3.50E-20	5.15E-22	1.09E+01	39.1727977	2.50E-01	5831213	5831336	NC_000002.11
cg19672997	3.52E-20	5.19E-22	1.09E+01	39.1652005	2.02E-01	48144369	48144492	NC_000014.8
cg05181041	3.53E-20	5.20E-22	1.09E+01	39.163857	2.75E-01	128076941	128077064	NC_000010.10
cg17766651	3.53E-20	5.20E-22	1.09E+01	39.1628218	2.00E-01	76303552	76303675	NC_000012.11
cg17132535	3.55E-20	5.24E-22	1.09E+01	39.1564755	1.76E-01	47133191	47133314	NC_000002.11
cg17429000	3.60E-20	5.31E-22	1.09E+01	39.1426381	2.30E-01	76936636	76936759	NC_000005.9
cg01783070	3.61E-20	5.32E-22	1.09E+01	39.1399818	2.93E-01	21686293	21686416	NC_000020.10

cg18088415	3.61E-20	5.33E-22	1.09E+01	39.1396927	1.72E-01	31770979	31771102	NC_000006.11
cg05374412	3.61E-20	5.33E-22	1.09E+01	39.1395723	3.00E-01	82660727	82660850	NC_000016.9
cg07317062	3.61E-20	5.34E-22	1.09E+01	39.1376706	2.45E-01	27170388	27170511	NC_000007.13
cg25221239	3.63E-20	5.37E-22	1.09E+01	39.1323851	2.11E-01	131764419	131764542	NC_000010.10
cg09800974	3.64E-20	5.37E-22	1.09E+01	39.1310162	2.21E-01	178688272	178688395	NC_000005.9
cg06120492	3.64E-20	5.38E-22	1.09E+01	39.1306005	1.20E-01	38529102	38529225	NC_000003.11
cg27391267	3.65E-20	5.40E-22	1.09E+01	39.1262455	1.93E-01	58545333	58545456	NC_000019.9
cg04319844	3.66E-20	5.41E-22	1.09E+01	39.123957	9.60E-02	41519026	41519149	NC_000008.10
cg07266350	3.71E-20	5.49E-22	1.09E+01	39.109526	2.38E-01	49460521	49460644	NC_000003.11
cg13876553	3.72E-20	5.51E-22	1.09E+01	39.106588	1.94E-01	426894	427017	NC_000009.11
cg08701621	3.72E-20	5.51E-22	1.09E+01	39.1061298	3.33E-01	58570419	58570542	NC_000019.9
cg22562383	3.73E-20	5.52E-22	1.09E+01	39.1046271	2.41E-01	1123154	1123277	NC_000005.9
cg11510891	3.73E-20	5.53E-22	1.09E+01	39.1025641	1.51E-01	41053747	41053870	NC_000013.10
cg14780068	3.76E-20	5.57E-22	1.09E+01	39.0961262	1.48E-01	201751846	201751969	NC_000001.10
cg07111441	3.76E-20	5.57E-22	1.09E+01	39.0948258	9.54E-02	47130423	47130546	NC_000001.10
cg21997746	3.76E-20	5.58E-22	1.09E+01	39.0937806	2.07E-01	22718216	22718339	NC_000006.11
cg09422450	3.78E-20	5.61E-22	1.09E+01	39.088288	2.18E-01	79623618	79623741	NC_000016.9
cg01729827	3.79E-20	5.62E-22	1.09E+01	39.086207	2.50E-01	54579084	54579207	NC_000020.10
cg09505233	3.80E-20	5.64E-22	1.09E+01	39.0825633	1.61E-01	133810087	133810210	NC_000010.10
cg14072515	3.89E-20	5.77E-22	1.09E+01	39.0605028	3.17E-01	125732604	125732727	NC_000010.10
cg00695558	3.96E-20	5.88E-22	1.09E+01	39.0415826	1.19E-01	42992137	42992260	NC_000008.10
cg02879159	3.96E-20	5.89E-22	1.09E+01	39.0396391	1.55E-01	30135449	30135572	NC_000006.11
cg24301352	3.96E-20	5.89E-22	1.09E+01	39.0395132	1.74E-01	3189957	3190080	NC_000019.9
cg14267754	4.00E-20	5.96E-22	1.09E+01	39.0288182	2.48E-01	99424203	99424326	NC_000007.13
cg05044994	4.02E-20	5.98E-22	1.09E+01	39.024804	1.13E-01	187896605	187896728	NC_000003.11

cg23231692	4.03E-20	6.00E-22	1.09E+01	39.0218604	9.29E-02	636167	636290	NC_000011.9
cg14944647	4.03E-20	6.00E-22	1.09E+01	39.0207251	2.54E-01	43602857	43602980	NC_000011.9
cg20721467	4.04E-20	6.02E-22	1.09E+01	39.018999	1.74E-01	76597927	76598050	NC_000010.10
cg25023379	4.04E-20	6.03E-22	1.09E+01	39.0169746	1.17E-01	70138712	70138835	NC_000014.8
cg02633363	4.07E-20	6.07E-22	1.09E+01	39.0097437	1.35E-01	149906179	149906302	NC_000005.9
cg05184456	4.08E-20	6.09E-22	1.09E+01	39.0066273	1.85E-01	145557639	145557762	NC_000008.10
cg02213971	4.11E-20	6.13E-22	1.09E+01	38.9995937	1.53E-01	69994230	69994353	NC_000011.9
cg13257146	4.12E-20	6.15E-22	1.09E+01	38.9974403	5.89E-02	42076143	42076266	NC_000003.11
cg09887059	4.16E-20	6.22E-22	1.09E+01	38.985994	2.58E-01	113916664	113916787	NC_000012.11
cg21178568	4.16E-20	6.22E-22	1.09E+01	38.9849828	1.06E-01	97390853	97390976	NC_000009.11
cg23877720	4.17E-20	6.24E-22	1.09E+01	38.9818876	2.38E-01	49815117	49815240	NC_000007.13
cg24425021	4.18E-20	6.26E-22	1.09E+01	38.9800876	2.69E-01	79177520	79177643	NC_000013.10
cg08146945	4.19E-20	6.28E-22	1.09E+01	38.9766683	1.76E-01	86538307	86538430	NC_000016.9
cg20597139	4.20E-20	6.29E-22	1.09E+01	38.9748905	8.41E-02	64451491	64451614	NC_000015.9
cg05916700	4.20E-20	6.29E-22	1.09E+01	38.9739426	1.39E-01	93130344	93130467	NC_000012.11
cg15672437	4.21E-20	6.31E-22	1.09E+01	38.971952	3.02E-01	113764555	113764678	NC_000013.10
cg24190603	4.21E-20	6.31E-22	1.09E+01	38.9712418	2.95E-01	84418433	84418556	NC_000006.11
cg07489048	4.24E-20	6.36E-22	1.09E+01	38.9641387	1.86E-01	29236898	29237021	NC_000014.8
cg06147743	4.25E-20	6.37E-22	1.09E+01	38.9613922	8.58E-02	42835588	42835711	NC_000020.10
cg23209575	4.26E-20	6.39E-22	1.09E+01	38.9585035	1.64E-01	77436344	77436467	NC_000017.10
cg12363472	4.27E-20	6.41E-22	1.09E+01	38.9554135	1.80E-01	166218867	166218990	NC_000006.11
cg17780246	4.31E-20	6.48E-22	1.09E+01	38.9454749	1.84E-01	54481467	54481590	NC_000019.9
cg12411994	4.31E-20	6.48E-22	1.09E+01	38.9451058	5.31E-02	150017070	150017193	NC_000007.13
cg13545633	4.32E-20	6.49E-22	1.09E+01	38.9435202	1.18E-01	75524350	75524473	NC_000017.10
cg01860957	4.32E-20	6.49E-22	1.09E+01	38.9435148	8.46E-02	78163790	78163913	NC_000017.10

cg04011497	4.32E-20	6.50E-22	1.09E+01	38.9420431	1.74E-01	78338052	78338175	NC_000017.10
cg25354617	4.33E-20	6.51E-22	1.09E+01	38.9402484	1.95E-01	3241813	3241936	NC_000004.11
cg12904880	4.39E-20	6.60E-22	1.09E+01	38.9269167	2.36E-01	30139832	30139955	NC_000006.11
cg18343862	4.39E-20	6.61E-22	1.09E+01	38.9248661	9.21E-02	10590003	10590126	NC_000011.9
cg25967031	4.48E-20	6.75E-22	1.09E+01	38.9038855	2.24E-01	27171203	27171326	NC_000007.13
cg10298052	4.48E-20	6.76E-22	1.09E+01	38.903243	2.16E-01	57391055	57391178	NC_000010.10
cg01472043	4.49E-20	6.77E-22	1.09E+01	38.9012643	1.45E-01	11868183	11868306	NC_000010.10
cg04619046	4.50E-20	6.79E-22	1.09E+01	38.8992274	1.77E-01	64541730	64541853	NC_000011.9
cg25219728	4.54E-20	6.85E-22	1.09E+01	38.8896282	1.37E-01	75895858	75895981	NC_000004.11
cg12072964	4.55E-20	6.86E-22	1.09E+01	38.8885953	3.22E-01	7117321	7117444	NC_000018.9
cg01323381	4.58E-20	6.91E-22	1.09E+01	38.8816555	2.54E-01	27184264	27184387	NC_000007.13
cg10144751	4.63E-20	6.99E-22	1.09E+01	38.870217	1.23E-01	1618846	1618969	NC_000016.9
cg04607024	4.66E-20	7.05E-22	1.09E+01	38.8615748	1.27E-01	67696204	67696327	NC_000016.9
cg24575234	4.68E-20	7.07E-22	1.09E+01	38.8580675	2.36E-01	136553884	136554007	NC_000007.13
cg06024951	4.69E-20	7.10E-22	1.09E+01	38.8547813	1.00E-01	74670386	74670509	NC_000015.9
cg06305026	4.71E-20	7.12E-22	1.09E+01	38.8518313	5.91E-02	155432132	155432255	NC_000006.11
cg09191036	4.71E-20	7.12E-22	1.09E+01	38.8512678	1.81E-01	9783363	9783486	NC_000004.11
cg16863382	4.72E-20	7.14E-22	1.09E+01	38.8488697	1.76E-01	75252695	75252818	NC_000016.9
cg13364311	4.75E-20	7.19E-22	1.09E+01	38.8423934	1.43E-01	222333289	222333412	NC_000002.11
cg22828305	4.78E-20	7.24E-22	1.09E+01	38.8347906	1.14E-01	124722991	124723114	NC_000003.11
cg14668632	4.78E-20	7.25E-22	1.09E+01	38.83413	1.19E-01	2872130	2872253	NC_000007.13
cg26212135	4.79E-20	7.25E-22	1.09E+01	38.833639	1.15E-01	3456947	3457070	NC_000010.10
cg27260891	4.83E-20	7.33E-22	1.09E+01	38.8225727	1.15E-01	70028721	70028844	NC_000011.9
cg10307632	4.86E-20	7.37E-22	1.09E+01	38.8179093	1.16E-01	206621802	206621925	NC_000002.11
cg01410878	4.88E-20	7.40E-22	1.09E+01	38.8135492	2.68E-01	32908285	32908408	NC_000017.10

cg24499462	4.88E-20	7.40E-22	1.09E+01	38.8133652	8.56E-02	37095576	37095699	NC_000015.9
cg26894523	4.89E-20	7.42E-22	1.09E+01	38.8110285	1.99E-01	107725	107848	NC_000004.11
cg09963962	4.90E-20	7.44E-22	1.09E+01	38.8077153	5.90E-02	64812414	64812537	NC_000011.9
cg20763327	4.93E-20	7.48E-22	1.09E+01	38.8027161	1.59E-01	16667384	16667507	NC_000005.9
cg18480906	5.00E-20	7.59E-22	1.09E+01	38.7879796	9.44E-02	14014169	14014292	NC_000005.9
cg13912117	5.02E-20	7.63E-22	1.09E+01	38.7830913	2.95E-01	132054555	132054678	NC_000008.10
cg21676440	5.04E-20	7.67E-22	1.09E+01	38.7782421	2.49E-01	134493324	134493447	NC_000006.11
cg11220663	5.05E-20	7.68E-22	1.09E+01	38.7764123	2.33E-01	70994863	70994986	NC_000002.11
cg12824952	5.10E-20	7.76E-22	1.09E+01	38.7656892	1.08E-01	74042596	74042719	NC_000011.9
cg00111823	5.23E-20	7.96E-22	1.09E+01	38.7409774	1.07E-01	38479215	38479338	NC_000022.10
cg08141862	5.28E-20	8.06E-22	1.09E+01	38.7291481	1.23E-01	177458423	177458546	NC_000003.11
cg08217024	5.33E-20	8.13E-22	1.09E+01	38.7203417	3.13E-01	48145108	48145231	NC_000014.8
cg00163006	5.41E-20	8.26E-22	1.09E+01	38.7045903	1.17E-01	4417124	4417247	NC_000017.10
cg20496928	5.41E-20	8.26E-22	1.09E+01	38.7037997	9.66E-02	80179899	80180022	NC_000017.10
cg11381539	5.44E-20	8.31E-22	1.09E+01	38.6981615	2.69E-01	71801535	71801658	NC_000007.13
cg17423650	5.46E-20	8.35E-22	1.09E+01	38.6938563	1.12E-01	185218725	185218848	NC_000001.10
cg16475423	5.53E-20	8.45E-22	1.09E+01	38.6818572	1.04E-01	145106459	145106582	NC_000002.11
cg17374034	5.55E-20	8.48E-22	1.09E+01	38.6781024	5.63E-02	39000613	39000736	NC_000015.9
cg06813782	5.57E-20	8.53E-22	1.09E+01	38.6726925	1.51E-01	31046836	31046959	NC_000020.10
cg17509681	5.58E-20	8.54E-22	1.09E+01	38.6717293	1.68E-01	37930596	37930719	NC_000008.10
cg18403551	5.61E-20	8.58E-22	1.09E+01	38.6660451	2.99E-01	140770306	140770429	NC_000003.11
cg01331275	5.61E-20	8.59E-22	1.09E+01	38.6651878	1.98E-01	9908576	9908699	NC_000002.11
cg22702328	5.66E-20	8.68E-22	1.09E+01	38.6555645	2.29E-01	50235965	50236088	NC_000017.10
cg04492228	5.66E-20	8.68E-22	1.09E+01	38.655301	2.02E-01	8101513	8101636	NC_000010.10
cg02392025	5.69E-20	8.71E-22	1.09E+01	38.6510789	1.44E-01	56658502	56658625	NC_000019.9

cg23398109	5.79E-20	8.88E-22	1.09E+01	38.6322159	1.57E-01	26658767	26658890	NC_000017.10
cg04415798	5.81E-20	8.92E-22	1.09E+01	38.6279026	2.71E-01	37128586	37128709	NC_000014.8
cg21946698	5.81E-20	8.92E-22	1.09E+01	38.6277479	1.94E-01	326615	326738	NC_000016.9
cg14875171	5.84E-20	8.96E-22	1.09E+01	38.6232687	2.27E-01	50574196	50574319	NC_000002.11
cg25056332	5.91E-20	9.08E-22	1.08E+01	38.6099449	1.39E-01	64682466	64682589	NC_000014.8
cg25520960	5.93E-20	9.12E-22	1.08E+01	38.6058232	1.36E-01	219863984	219864107	NC_000002.11
cg06454760	5.97E-20	9.18E-22	1.08E+01	38.5993837	3.10E-01	58570427	58570550	NC_000019.9
cg23457506	5.98E-20	9.20E-22	1.08E+01	38.5974015	1.19E-01	30687453	30687576	NC_000002.11
cg11994674	5.98E-20	9.20E-22	1.08E+01	38.597279	2.42E-01	47547767	47547890	NC_000017.10
cg27528510	5.99E-20	9.21E-22	1.08E+01	38.5957935	2.68E-01	101006058	101006181	NC_000007.13
cg21790570	6.03E-20	9.29E-22	1.08E+01	38.5881974	6.05E-02	38133323	38133446	NC_000017.10
cg08330031	6.03E-20	9.30E-22	1.08E+01	38.5869374	2.15E-01	104623503	104623626	NC_000010.10
cg06462796	6.04E-20	9.30E-22	1.08E+01	38.5861852	1.54E-01	79634861	79634984	NC_000009.11
cg15365218	6.04E-20	9.32E-22	1.08E+01	38.584963	8.09E-02	33396859	33396982	NC_000011.9
cg23092421	6.05E-20	9.33E-22	1.08E+01	38.5836728	2.00E-01	49253689	49253812	NC_000019.9
cg20852557	6.06E-20	9.35E-22	1.08E+01	38.5814027	1.69E-01	184035519	184035642	NC_000003.11
cg00287312	6.08E-20	9.38E-22	1.08E+01	38.5785498	3.27E-01	53420425	53420548	NC_000013.10
cg03357798	6.11E-20	9.43E-22	1.08E+01	38.5726452	2.77E-01	69243285	69243408	NC_000008.10
cg04279973	6.11E-20	9.43E-22	1.08E+01	38.5724764	2.59E-01	23846968	23847091	NC_000016.9
cg22550847	6.14E-20	9.49E-22	1.08E+01	38.5665065	1.03E-01	79240082	79240205	NC_000004.11
cg07584981	6.17E-20	9.54E-22	1.08E+01	38.5616424	1.39E-01	38172854	38172977	NC_000017.10
cg01927745	6.19E-20	9.57E-22	1.08E+01	38.5580636	1.73E-01	72677723	72677846	NC_000005.9
cg26109568	6.20E-20	9.59E-22	1.08E+01	38.5562279	1.81E-01	105697685	105697808	NC_000002.11
cg24852548	6.22E-20	9.62E-22	1.08E+01	38.5528803	3.49E-01	57522632	57522755	NC_000004.11
cg05494604	6.23E-20	9.64E-22	1.08E+01	38.5505257	3.05E-01	23733862	23733985	NC_000013.10

cg00210133	6.24E-20	9.66E-22	1.08E+01	38.5493859	1.53E-01	50632714	50632837	NC_000022.10
cg19885761	6.27E-20	9.71E-22	1.08E+01	38.5440294	2.49E-01	175223646	175223769	NC_000005.9
cg12733154	6.30E-20	9.75E-22	1.08E+01	38.5397684	1.52E-01	69801321	69801444	NC_000011.9
cg21553980	6.34E-20	9.82E-22	1.08E+01	38.5328446	1.58E-01	109444	109567	NC_000017.10
cg27528817	6.34E-20	9.83E-22	1.08E+01	38.5320293	1.43E-01	72860419	72860542	NC_000003.11
cg03224572	6.39E-20	9.90E-22	1.08E+01	38.5242413	2.58E-01	97172012	97172135	NC_000008.10
cg06991300	6.41E-20	9.94E-22	1.08E+01	38.520644	2.44E-01	122301724	122301847	NC_000004.11
cg09916030	6.41E-20	9.95E-22	1.08E+01	38.5199347	5.86E-02	37949197	37949320	NC_000021.8
cg01003961	6.42E-20	9.97E-22	1.08E+01	38.5180277	1.67E-01	20824706	20824829	NC_000007.13
cg18225577	6.43E-20	9.97E-22	1.08E+01	38.5176374	1.81E-01	49713901	49714024	NC_000019.9
cg06785999	6.43E-20	9.98E-22	1.08E+01	38.5161081	2.94E-01	60975964	60976087	NC_000014.8
cg08087868	6.51E-20	1.01E-21	1.08E+01	38.5033147	1.86E-01	51188129	51188252	NC_000016.9
cg03704308	6.56E-20	1.02E-21	1.08E+01	38.4966053	2.86E-01	42533414	42533537	NC_000007.13
cg23121787	6.61E-20	1.03E-21	1.08E+01	38.4876818	1.92E-01	42211437	42211560	NC_000015.9
cg18473234	6.61E-20	1.03E-21	1.08E+01	38.4872197	1.19E-01	5097819	5097942	NC_000019.9
cg15618758	6.61E-20	1.03E-21	1.08E+01	38.4871764	1.63E-01	157168834	157168957	NC_000006.11
cg01540023	6.62E-20	1.03E-21	1.08E+01	38.485547	7.34E-02	98950565	98950688	NC_000013.10
cg17087899	6.73E-20	1.05E-21	1.08E+01	38.4686041	1.45E-01	151038698	151038821	NC_000007.13
cg03786842	6.81E-20	1.06E-21	1.08E+01	38.4555055	1.34E-01	127569751	127569874	NC_000010.10
cg09554406	6.83E-20	1.06E-21	1.08E+01	38.4531136	1.54E-01	49714044	49714167	NC_000019.9
cg06241792	6.86E-20	1.07E-21	1.08E+01	38.4492544	3.13E-01	21680669	21680792	NC_000012.11
cg16992627	6.90E-20	1.08E-21	1.08E+01	38.4419527	9.74E-02	32081610	32081733	NC_000006.11
cg16719582	6.91E-20	1.08E-21	1.08E+01	38.4402293	1.20E-01	133090971	133091094	NC_000012.11
cg20847228	6.93E-20	1.08E-21	1.08E+01	38.4366656	1.82E-01	50297087	50297210	NC_000003.11
cg09748975	6.95E-20	1.08E-21	1.08E+01	38.4350104	1.77E-01	4864532	4864655	NC_000004.11

cg04550052	6.96E-20	1.09E-21	1.08E+01	38.4320384	2.28E-01	51184355	51184478	NC_000016.9
cg04200485	7.00E-20	1.09E-21	1.08E+01	38.426255	1.26E-01	95900374	95900497	NC_000011.9
cg08146483	7.02E-20	1.10E-21	1.08E+01	38.4230691	2.74E-01	75602412	75602535	NC_000001.10
cg12982322	7.02E-20	1.10E-21	1.08E+01	38.4230531	1.74E-01	32449821	32449944	NC_000011.9
cg07302069	7.03E-20	1.10E-21	1.08E+01	38.4213232	3.71E-01	27196286	27196409	NC_000007.13
cg13791971	7.03E-20	1.10E-21	1.08E+01	38.420942	2.01E-01	97945584	97945707	NC_000009.11
cg10152131	7.04E-20	1.10E-21	1.08E+01	38.4205027	3.00E-01	51111677	51111800	NC_000019.9
cg06719900	7.06E-20	1.10E-21	1.08E+01	38.4161464	2.12E-01	109292894	109293017	NC_000011.9
cg14791193	7.08E-20	1.11E-21	1.08E+01	38.4139933	1.41E-01	16702476	16702599	NC_000001.10
cg05026135	7.10E-20	1.11E-21	1.08E+01	38.41087	2.73E-01	30713427	30713550	NC_000019.9
cg00021933	7.14E-20	1.12E-21	1.08E+01	38.4049145	1.16E-01	86082316	86082439	NC_000001.10
cg06727067	7.24E-20	1.13E-21	1.08E+01	38.3898008	1.85E-01	92582155	92582278	NC_000005.9
cg20310894	7.35E-20	1.15E-21	1.08E+01	38.3741148	1.22E-01	100125898	100126021	NC_000014.8
cg21578219	7.40E-20	1.16E-21	1.08E+01	38.367556	2.55E-01	18434542	18434665	NC_000001.10
cg16005494	7.48E-20	1.17E-21	1.08E+01	38.3554589	1.89E-01	70597713	70597836	NC_000007.13
cg03591376	7.52E-20	1.18E-21	1.08E+01	38.3492248	8.21E-02	45998201	45998324	NC_000019.9
cg22250605	7.57E-20	1.19E-21	1.08E+01	38.3422086	1.49E-01	189804187	189804310	NC_000003.11
cg13057576	7.57E-20	1.19E-21	1.08E+01	38.3420795	2.07E-01	64692154	64692277	NC_000014.8
cg12552402	7.65E-20	1.20E-21	1.08E+01	38.331885	1.37E-01	72083379	72083502	NC_000012.11
cg00573165	7.68E-20	1.21E-21	1.08E+01	38.327147	1.28E-01	20250849	20250972	NC_000001.10
cg04905644	7.68E-20	1.21E-21	1.08E+01	38.3271117	1.99E-01	28938493	28938616	NC_000008.10
cg18374393	7.71E-20	1.21E-21	1.08E+01	38.3231412	1.82E-01	105737848	105737971	NC_000014.8
cg11326311	7.73E-20	1.22E-21	1.08E+01	38.3199872	1.61E-01	58400854	58400977	NC_000011.9
cg12091642	7.75E-20	1.22E-21	1.08E+01	38.3177965	3.08E-01	61104566	61104689	NC_000014.8
cg05142982	7.81E-20	1.23E-21	1.08E+01	38.3097354	3.12E-01	495736	495859	NC_000008.10

cg18098286	7.81E-20	1.23E-21	1.08E+01	38.3088159	8.53E-02	234544271	234544394	NC_000002.11
cg23154526	7.83E-20	1.23E-21	1.08E+01	38.3065775	2.40E-01	60297323	60297446	NC_000015.9
cg14098847	8.02E-20	1.27E-21	1.08E+01	38.2810028	2.47E-01	101923218	101923341	NC_000014.8
cg00874589	8.04E-20	1.27E-21	1.08E+01	38.2784885	1.08E-01	112405614	112405737	NC_000003.11
cg27445278	8.09E-20	1.28E-21	1.08E+01	38.2723044	1.48E-01	26961465	26961588	NC_000022.10
cg06193628	8.09E-20	1.28E-21	1.08E+01	38.2718185	1.93E-01	228526145	228526268	NC_000002.11
cg23484951	8.09E-20	1.28E-21	1.08E+01	38.271295	2.24E-01	144241407	144241530	NC_000008.10
cg02230017	8.12E-20	1.28E-21	1.08E+01	38.2674966	2.89E-01	6069019	6069142	NC_000016.9
cg00851532	8.15E-20	1.29E-21	1.08E+01	38.2642061	1.93E-01	64680245	64680368	NC_000014.8
cg21287017	8.17E-20	1.29E-21	1.08E+01	38.2611507	8.53E-02	9498109	9498232	NC_000020.10
cg05404010	8.23E-20	1.30E-21	1.08E+01	38.2528407	2.55E-01	51186211	51186334	NC_000016.9
cg15437741	8.27E-20	1.31E-21	1.08E+01	38.2482249	1.58E-01	80194576	80194699	NC_000017.10
cg26552361	8.32E-20	1.32E-21	1.08E+01	38.2411988	9.31E-02	172293806	172293929	NC_000005.9
cg11049828	8.33E-20	1.32E-21	1.08E+01	38.239912	6.18E-02	69290831	69290954	NC_000011.9
cg11300341	8.47E-20	1.34E-21	1.08E+01	38.2226195	1.48E-01	17689562	17689685	NC_000017.10
cg03058660	8.48E-20	1.34E-21	1.08E+01	38.2206856	2.24E-01	48919198	48919321	NC_000019.9
cg14603098	8.50E-20	1.35E-21	1.08E+01	38.2184134	2.97E-01	32484259	32484382	NC_000017.10
cg16670554	8.53E-20	1.35E-21	1.08E+01	38.2150902	2.19E-01	68118985	68119108	NC_000015.9
cg18919209	8.54E-20	1.35E-21	1.08E+01	38.2138034	1.14E-01	96012039	96012162	NC_000011.9
cg10168149	8.65E-20	1.37E-21	1.08E+01	38.2000859	2.62E-01	200335558	200335681	NC_000002.11
cg21154627	8.69E-20	1.38E-21	1.08E+01	38.194589	2.56E-01	170303190	170303313	NC_000003.11
cg25136495	8.74E-20	1.39E-21	1.08E+01	38.1887318	2.28E-01	6729718	6729841	NC_000012.11
cg03426602	8.78E-20	1.39E-21	1.08E+01	38.1847725	1.79E-01	227312417	227312540	NC_000002.11
cg16417374	8.85E-20	1.41E-21	1.08E+01	38.1762427	1.89E-01	26098310	26098433	NC_000001.10
cg27111250	8.90E-20	1.42E-21	1.08E+01	38.1695454	2.14E-01	81111177	81111300	NC_000004.11

cg14874750	8.93E-20	1.42E-21	1.08E+01	38.1660796	3.05E-01	101006063	101006186	NC_000007.13
cg09686443	8.96E-20	1.42E-21	1.08E+01	38.163237	2.40E-01	126113658	126113781	NC_000003.11
cg20629316	8.96E-20	1.43E-21	1.08E+01	38.163052	2.44E-01	64413782	64413905	NC_000001.10
cg06178310	8.97E-20	1.43E-21	1.08E+01	38.1613182	5.41E-02	190105653	190105776	NC_000003.11
cg05749855	8.97E-20	1.43E-21	1.08E+01	38.1607649	2.21E-01	89472771	89472894	NC_000016.9
cg02187214	8.97E-20	1.43E-21	1.08E+01	38.1606774	1.85E-01	86599468	86599591	NC_000016.9
cg06051411	9.06E-20	1.44E-21	1.08E+01	38.1516336	2.06E-01	73044991	73045114	NC_000017.10
cg14667871	9.11E-20	1.45E-21	1.08E+01	38.1452436	2.54E-01	15500833	15500956	NC_000005.9
cg11847933	9.12E-20	1.45E-21	1.08E+01	38.1436046	1.60E-01	227130394	227130517	NC_000001.10
cg03209854	9.15E-20	1.46E-21	1.08E+01	38.140256	2.36E-01	5140003	5140126	NC_000005.9
cg05030680	9.16E-20	1.46E-21	1.08E+01	38.1394659	2.63E-01	111542366	111542489	NC_000004.11
cg16508028	9.17E-20	1.46E-21	1.08E+01	38.1380837	1.60E-01	145678763	145678886	NC_000008.10
cg00761700	9.19E-20	1.47E-21	1.08E+01	38.1349957	1.63E-01	95727673	95727796	NC_000014.8
cg22686881	9.25E-20	1.48E-21	1.08E+01	38.1288077	1.79E-01	170737255	170737378	NC_000005.9
cg08167286	9.25E-20	1.48E-21	1.08E+01	38.1284095	8.58E-02	31779529	31779652	NC_000006.11
cg17436134	9.25E-20	1.48E-21	1.08E+01	38.1280607	2.68E-01	119536288	119536411	NC_000001.10
cg03413355	9.30E-20	1.48E-21	1.08E+01	38.1232367	1.50E-01	56537579	56537702	NC_000015.9
cg19443257	9.31E-20	1.49E-21	1.08E+01	38.1214364	3.23E-01	140770549	140770672	NC_000003.11
cg16787600	9.45E-20	1.51E-21	1.08E+01	38.1062559	3.17E-01	106400880	106401003	NC_000010.10
cg23356505	9.49E-20	1.52E-21	1.08E+01	38.1014387	9.84E-02	144613012	144613135	NC_000006.11
cg00379720	9.51E-20	1.52E-21	1.08E+01	38.0989523	2.27E-01	23185968	23186091	NC_000019.9
cg03929977	9.60E-20	1.54E-21	1.08E+01	38.0893436	2.57E-01	5019017	5019140	NC_000012.11
cg22791678	9.60E-20	1.54E-21	1.08E+01	38.0887516	1.99E-01	41552580	41552703	NC_000006.11
cg25762262	9.62E-20	1.54E-21	1.08E+01	38.0869783	1.18E-01	194056482	194056605	NC_000003.11
cg23208176	9.67E-20	1.55E-21	1.08E+01	38.0809474	2.82E-01	1876168	1876291	NC_000005.9

cg01259220	9.72E-20	1.56E-21	1.08E+01	38.0764036	1.61E-01	914964	915087	NC_000007.13
cg17040483	9.84E-20	1.58E-21	1.08E+01	38.0624994	1.92E-01	131221478	131221601	NC_000007.13
cg06162185	9.88E-20	1.58E-21	1.08E+01	38.0589121	1.70E-01	8546551	8546674	NC_000010.10
cg12727138	9.97E-20	1.60E-21	1.08E+01	38.0495674	1.88E-01	162102947	162103070	NC_000002.11
cg23901592	9.97E-20	1.60E-21	1.08E+01	38.0491049	1.97E-01	90126322	90126445	NC_000014.8
cg26931862	9.98E-20	1.60E-21	1.08E+01	38.0478912	1.95E-01	54349169	54349292	NC_000012.11
cg18063733	1.00E-19	1.61E-21	1.08E+01	38.0441902	2.92E-01	50265443	50265566	NC_000005.9
cg03385262	1.00E-19	1.61E-21	1.08E+01	38.0419661	1.17E-01	128890086	128890209	NC_000004.11
cg25684999	1.00E-19	1.61E-21	1.08E+01	38.0405035	2.55E-01	23480803	23480926	NC_000010.10
cg20333001	1.01E-19	1.63E-21	1.08E+01	38.0306507	1.62E-01	242212896	242213019	NC_000002.11
cg21647227	1.02E-19	1.64E-21	1.08E+01	38.0266167	2.22E-01	119527111	119527234	NC_000001.10
cg17789809	1.03E-19	1.66E-21	1.08E+01	38.0094326	2.00E-01	181343322	181343445	NC_000003.11
cg00688802	1.04E-19	1.66E-21	1.08E+01	38.008849	1.18E-01	227861715	227861838	NC_000002.11
cg15652169	1.04E-19	1.67E-21	1.08E+01	38.0056447	1.05E-01	141864511	141864634	NC_000005.9
cg27017251	1.05E-19	1.68E-21	1.08E+01	37.9979156	1.24E-01	22334553	22334676	NC_000022.10
cg02703822	1.07E-19	1.72E-21	1.08E+01	37.9780899	1.54E-01	81680007	81680130	NC_000008.10
cg00818229	1.07E-19	1.73E-21	1.08E+01	37.9729622	8.12E-02	40815902	40816025	NC_000022.10
cg12072376	1.08E-19	1.73E-21	1.08E+01	37.9684139	1.17E-01	76000521	76000644	NC_000017.10
cg11643022	1.08E-19	1.74E-21	1.08E+01	37.9627897	4.69E-02	141589945	141590068	NC_000005.9
cg14282050	1.09E-19	1.76E-21	1.08E+01	37.9546738	1.74E-01	20353615	20353738	NC_000009.11
cg27595860	1.10E-19	1.77E-21	1.08E+01	37.9466685	1.98E-01	145557636	145557759	NC_000008.10
cg01440556	1.10E-19	1.77E-21	1.08E+01	37.9460293	1.12E-01	242165309	242165432	NC_000002.11
cg13637871	1.10E-19	1.78E-21	1.08E+01	37.9423131	1.60E-01	130924728	130924851	NC_000009.11
cg23883696	1.13E-19	1.82E-21	1.07E+01	37.9213341	2.12E-01	70534298	70534421	NC_000018.9
cg02432280	1.13E-19	1.83E-21	1.07E+01	37.9147954	3.09E-01	152622368	152622491	NC_000007.13

cg10373086	1.14E-19	1.84E-21	1.07E+01	37.9083605	1.85E-01	111544286	111544409	NC_000004.11
cg07169046	1.14E-19	1.84E-21	1.07E+01	37.9071136	1.42E-01	39120381	39120504	NC_000015.9
cg01603847	1.14E-19	1.85E-21	1.07E+01	37.9057069	2.96E-01	6449461	6449584	NC_000005.9
cg22586653	1.14E-19	1.85E-21	1.07E+01	37.9043098	2.44E-01	115136098	115136221	NC_000012.11
cg04428163	1.15E-19	1.87E-21	1.07E+01	37.8951153	1.51E-01	1294021	1294144	NC_000020.10
cg23736843	1.15E-19	1.87E-21	1.07E+01	37.8948749	2.75E-01	113541915	113542038	NC_000012.11
cg26015019	1.16E-19	1.87E-21	1.07E+01	37.8920082	1.25E-01	11631138	11631261	NC_000003.11
cg00800512	1.16E-19	1.88E-21	1.07E+01	37.8875244	2.49E-01	58095518	58095641	NC_000019.9
cg01869554	1.16E-19	1.88E-21	1.07E+01	37.8862291	1.18E-01	90772557	90772680	NC_000015.9
cg24129977	1.17E-19	1.89E-21	1.07E+01	37.8807923	2.57E-01	1748872	1748995	NC_000002.11
cg06246941	1.18E-19	1.92E-21	1.07E+01	37.8688753	2.00E-01	45671708	45671831	NC_000015.9
cg26687072	1.19E-19	1.93E-21	1.07E+01	37.8618449	1.86E-01	46608345	46608468	NC_000017.10
cg02587316	1.19E-19	1.93E-21	1.07E+01	37.860338	3.10E-01	37096321	37096444	NC_000019.9
cg04577162	1.21E-19	1.96E-21	1.07E+01	37.8484036	1.81E-01	73667397	73667520	NC_000007.13
cg12824796	1.21E-19	1.96E-21	1.07E+01	37.8457841	2.84E-01	131768928	131769051	NC_000010.10
cg04276723	1.21E-19	1.97E-21	1.07E+01	37.8442356	2.10E-01	38670597	38670720	NC_000007.13
cg07187971	1.22E-19	1.98E-21	1.07E+01	37.8375765	1.54E-01	60481018	60481141	NC_000018.9
cg27009703	1.23E-19	2.00E-21	1.07E+01	37.8258969	1.52E-01	27204894	27205017	NC_000007.13
cg08295111	1.23E-19	2.00E-21	1.07E+01	37.8254969	1.58E-01	133866097	133866220	NC_000005.9
cg04389897	1.23E-19	2.01E-21	1.07E+01	37.8236199	3.22E-01	10398601	10398724	NC_000006.11
cg19094872	1.24E-19	2.02E-21	1.07E+01	37.8170574	1.66E-01	242065898	242066021	NC_000002.11
cg04923773	1.24E-19	2.02E-21	1.07E+01	37.8158214	7.58E-02	96161502	96161625	NC_000012.11
cg09618102	1.25E-19	2.03E-21	1.07E+01	37.8103899	2.69E-01	1747364	1747487	NC_000002.11
cg14023291	1.25E-19	2.03E-21	1.07E+01	37.8097166	1.68E-01	124615180	124615303	NC_000009.11
cg24334259	1.26E-19	2.04E-21	1.07E+01	37.8053365	1.01E-01	138311416	138311539	NC_000003.11

cg23007540	1.26E-19	2.06E-21	1.07E+01	37.7992377	1.71E-01	142179688	142179811	NC_000008.10
cg14730445	1.26E-19	2.06E-21	1.07E+01	37.7989128	2.55E-01	101603581	101603704	NC_000012.11
cg17575394	1.27E-19	2.06E-21	1.07E+01	37.7955201	1.12E-01	49248930	49249053	NC_000012.11
cg07780543	1.27E-19	2.07E-21	1.07E+01	37.7932457	2.30E-01	22094750	22094873	NC_000012.11
cg17007640	1.27E-19	2.07E-21	1.07E+01	37.7930154	1.95E-01	29243504	29243627	NC_000014.8
cg09647110	1.27E-19	2.07E-21	1.07E+01	37.7908967	1.55E-01	44772303	44772426	NC_000002.11
cg27254482	1.27E-19	2.08E-21	1.07E+01	37.7894295	2.26E-01	34398085	34398208	NC_000021.8
cg22466784	1.28E-19	2.09E-21	1.07E+01	37.7825957	1.21E-01	540176	540299	NC_000007.13
cg01405040	1.29E-19	2.10E-21	1.07E+01	37.7767368	2.56E-01	176964137	176964260	NC_000002.11
cg06870213	1.29E-19	2.11E-21	1.07E+01	37.7759751	2.06E-01	55444335	55444458	NC_000006.11
cg02799090	1.29E-19	2.11E-21	1.07E+01	37.7734497	1.54E-01	130508166	130508289	NC_000010.10
cg15817769	1.29E-19	2.11E-21	1.07E+01	37.7731228	2.03E-01	230272542	230272665	NC_000001.10
cg12159995	1.30E-19	2.11E-21	1.07E+01	37.7719736	1.79E-01	70398671	70398794	NC_000011.9
cg06746009	1.30E-19	2.13E-21	1.07E+01	37.7659315	2.05E-01	101896452	101896575	NC_000007.13
cg06496010	1.31E-19	2.13E-21	1.07E+01	37.7635396	1.18E-01	97201196	97201319	NC_000002.11
cg24727311	1.31E-19	2.14E-21	1.07E+01	37.7607472	2.76E-01	13424269	13424392	NC_000008.10
cg17526573	1.31E-19	2.14E-21	1.07E+01	37.7599909	3.10E-01	50574708	50574831	NC_000002.11
cg01532168	1.31E-19	2.14E-21	1.07E+01	37.7598141	2.47E-01	140715557	140715680	NC_000008.10
cg02347074	1.31E-19	2.15E-21	1.07E+01	37.7548995	1.98E-01	223155925	223156048	NC_000002.11
cg02812891	1.34E-19	2.19E-21	1.07E+01	37.7368198	1.52E-01	233251773	233251896	NC_000002.11
cg22724228	1.34E-19	2.20E-21	1.07E+01	37.7320043	1.14E-01	154826964	154827087	NC_000006.11
cg21517947	1.34E-19	2.20E-21	1.07E+01	37.7317333	2.93E-01	41167107	41167230	NC_000008.10
cg11539055	1.35E-19	2.21E-21	1.07E+01	37.7289339	1.07E-01	80886264	80886387	NC_000004.11
cg05375744	1.35E-19	2.21E-21	1.07E+01	37.7273791	1.36E-01	54634871	54634994	NC_000019.9
cg03063639	1.36E-19	2.22E-21	1.07E+01	37.7222922	3.36E-01	83776422	83776545	NC_000015.9

cg18324707	1.36E-19	2.23E-21	1.07E+01	37.7191574	2.57E-01	137531801	137531924	NC_000007.13
cg09897064	1.38E-19	2.26E-21	1.07E+01	37.7051206	1.02E-01	56188065	56188188	NC_000020.10
cg16144331	1.38E-19	2.27E-21	1.07E+01	37.7031788	1.19E-01	142251375	142251498	NC_000008.10
cg09051560	1.40E-19	2.29E-21	1.07E+01	37.6916925	6.61E-02	35118158	35118281	NC_000006.11
cg23003783	1.41E-19	2.32E-21	1.07E+01	37.6803994	2.13E-01	178422071	178422194	NC_000005.9
cg17663418	1.41E-19	2.32E-21	1.07E+01	37.679628	1.90E-01	184013877	184014000	NC_000003.11
cg07704235	1.46E-19	2.40E-21	1.07E+01	37.6461857	9.32E-02	46501202	46501325	NC_000018.9
cg00282426	1.46E-19	2.41E-21	1.07E+01	37.6435141	1.87E-01	136843224	136843347	NC_000009.11
cg17353412	1.46E-19	2.41E-21	1.07E+01	37.6413312	1.94E-01	27141774	27141897	NC_000007.13
cg17499719	1.47E-19	2.41E-21	1.07E+01	37.6401947	1.32E-01	17588410	17588533	NC_000020.10
cg05446629	1.47E-19	2.43E-21	1.07E+01	37.635232	2.88E-01	134281742	134281865	NC_000011.9
cg18767920	1.47E-19	2.43E-21	1.07E+01	37.6340064	1.49E-01	33048493	33048616	NC_000018.9
cg21097881	1.48E-19	2.43E-21	1.07E+01	37.6319767	3.31E-01	24323939	24324062	NC_000007.13
cg02596427	1.48E-19	2.44E-21	1.07E+01	37.6298087	9.42E-02	47295058	47295181	NC_000002.11
cg23663774	1.49E-19	2.45E-21	1.07E+01	37.6245354	3.11E-01	111555440	111555563	NC_000004.11
cg12001304	1.50E-19	2.48E-21	1.07E+01	37.6152871	2.94E-01	229045020	229045143	NC_000002.11
cg19826115	1.52E-19	2.50E-21	1.07E+01	37.6043037	8.37E-02	143673332	143673455	NC_000002.11
cg10550245	1.52E-19	2.51E-21	1.07E+01	37.6017576	1.58E-01	48141749	48141872	NC_000007.13
cg06159352	1.52E-19	2.52E-21	1.07E+01	37.5983032	3.53E-01	37487613	37487736	NC_000007.13
cg00562553	1.53E-19	2.52E-21	1.07E+01	37.5959166	1.81E-01	27169740	27169863	NC_000007.13
cg20672930	1.53E-19	2.53E-21	1.07E+01	37.5923363	1.39E-01	259396	259519	NC_000012.11
cg19054524	1.53E-19	2.53E-21	1.07E+01	37.5919758	2.47E-01	21686273	21686396	NC_000020.10
cg23783862	1.54E-19	2.55E-21	1.07E+01	37.5855972	1.58E-01	82658145	82658268	NC_000008.10
cg21548038	1.54E-19	2.55E-21	1.07E+01	37.5849706	5.03E-02	50517157	50517280	NC_000014.8
cg11251614	1.54E-19	2.55E-21	1.07E+01	37.5849301	1.03E-01	36839846	36839969	NC_000006.11

cg24061141	1.55E-19	2.56E-21	1.07E+01	37.5832225	2.44E-01	35293869	35293992	NC_000007.13
cg25001102	1.56E-19	2.57E-21	1.07E+01	37.5763654	2.20E-01	221068935	221069058	NC_000001.10
cg03335246	1.56E-19	2.58E-21	1.07E+01	37.57379	1.90E-01	4860431	4860554	NC_000004.11
cg27050153	1.56E-19	2.59E-21	1.07E+01	37.5695537	1.95E-01	102030110	102030233	NC_000014.8
cg13173552	1.57E-19	2.61E-21	1.07E+01	37.5642237	1.44E-01	16091599	16091722	NC_000001.10
cg14317001	1.58E-19	2.61E-21	1.07E+01	37.5621995	6.44E-02	144462776	144462899	NC_000008.10
cg02500960	1.58E-19	2.62E-21	1.07E+01	37.5580743	1.40E-01	8122233	8122356	NC_000004.11
cg17498296	1.58E-19	2.62E-21	1.07E+01	37.5575418	3.12E-01	124907540	124907663	NC_000010.10
cg05874561	1.58E-19	2.63E-21	1.07E+01	37.5565563	3.05E-01	154709828	154709951	NC_000004.11
cg13085403	1.59E-19	2.63E-21	1.07E+01	37.5545893	1.97E-01	232901128	232901251	NC_000001.10
cg10799492	1.61E-19	2.67E-21	1.07E+01	37.5398714	9.01E-02	42591850	42591973	NC_000008.10
cg02675546	1.61E-19	2.68E-21	1.07E+01	37.5365333	1.60E-01	29292730	29292853	NC_000008.10
cg02383130	1.62E-19	2.68E-21	1.07E+01	37.534831	3.02E-01	38670957	38671080	NC_000007.13
cg02197303	1.62E-19	2.68E-21	1.07E+01	37.5346244	1.43E-01	3240621	3240744	NC_000004.11
cg22846776	1.62E-19	2.69E-21	1.07E+01	37.5331483	1.72E-01	2324861	2324984	NC_000017.10
cg06597095	1.63E-19	2.70E-21	1.07E+01	37.5281722	1.44E-01	43480361	43480484	NC_000005.9
cg12627299	1.63E-19	2.72E-21	1.07E+01	37.5228558	1.32E-01	66444235	66444358	NC_000011.9
cg18275316	1.64E-19	2.74E-21	1.07E+01	37.5158107	1.89E-01	3607063	3607186	NC_000005.9
cg04430996	1.65E-19	2.75E-21	1.07E+01	37.510573	7.45E-02	591437	591560	NC_000016.9
cg13694927	1.65E-19	2.75E-21	1.07E+01	37.5105318	1.80E-01	27184712	27184835	NC_000007.13
cg02809746	1.65E-19	2.75E-21	1.07E+01	37.5101223	2.48E-01	49312033	49312156	NC_000016.9
cg03112087	1.65E-19	2.75E-21	1.07E+01	37.5100645	3.23E-01	142053720	142053843	NC_000004.11
cg20826024	1.66E-19	2.76E-21	1.07E+01	37.5079341	1.08E-01	31529154	31529277	NC_000006.11
cg09797337	1.66E-19	2.76E-21	1.07E+01	37.507259	2.19E-01	112709538	112709661	NC_000013.10
cg00845900	1.68E-19	2.80E-21	1.07E+01	37.4945318	1.50E-01	129931729	129931852	NC_000007.13

cg19505136	1.68E-19	2.80E-21	1.07E+01	37.4943592	2.50E-01	239506	239629	NC_000003.11
cg11183632	1.68E-19	2.81E-21	1.07E+01	37.4906092	2.68E-01	21503152	21503275	NC_000020.10
cg19304150	1.69E-19	2.83E-21	1.07E+01	37.48297	2.28E-01	114696350	114696473	NC_000001.10
cg24401026	1.69E-19	2.83E-21	1.07E+01	37.4823289	1.47E-01	136732287	136732410	NC_000006.11
cg19777853	1.70E-19	2.85E-21	1.07E+01	37.477015	8.43E-02	26643109	26643232	NC_000001.10
cg14938313	1.71E-19	2.85E-21	1.07E+01	37.4742746	9.66E-02	70028676	70028799	NC_000011.9
cg03146949	1.71E-19	2.86E-21	1.07E+01	37.470843	2.92E-01	56879662	56879785	NC_000019.9
cg21610839	1.72E-19	2.88E-21	1.07E+01	37.4665949	1.63E-01	23861312	23861435	NC_000003.11
cg25339566	1.72E-19	2.88E-21	1.07E+01	37.4655001	2.56E-01	67218165	67218288	NC_000001.10
cg01693830	1.73E-19	2.89E-21	1.07E+01	37.462072	1.48E-01	127790462	127790585	NC_000003.11
cg16850173	1.73E-19	2.90E-21	1.07E+01	37.457472	2.75E-01	140033611	140033734	NC_000009.11
cg11626496	1.74E-19	2.91E-21	1.07E+01	37.4552597	2.31E-01	25320388	25320511	NC_000013.10
cg15603568	1.74E-19	2.92E-21	1.07E+01	37.4518868	3.25E-01	105481283	105481406	NC_000011.9
cg01243371	1.75E-19	2.92E-21	1.07E+01	37.4503069	2.20E-01	86547386	86547509	NC_000016.9
cg08594218	1.76E-19	2.94E-21	1.07E+01	37.4449798	2.96E-01	46803008	46803131	NC_000017.10
cg18105665	1.77E-19	2.97E-21	1.07E+01	37.4353026	1.84E-01	93257417	93257540	NC_000014.8
cg04865659	1.77E-19	2.97E-21	1.07E+01	37.4352196	1.08E-01	90771840	90771963	NC_000015.9
cg17713613	1.77E-19	2.97E-21	1.07E+01	37.4339527	1.93E-01	21541765	21541888	NC_000019.9
cg05686497	1.78E-19	2.98E-21	1.07E+01	37.4309371	2.63E-01	127874587	127874710	NC_000005.9
cg02276973	1.79E-19	3.00E-21	1.07E+01	37.4239274	1.20E-01	90610486	90610609	NC_000015.9
cg13092806	1.82E-19	3.05E-21	1.07E+01	37.4095555	3.11E-01	177043255	177043378	NC_000002.11
cg13555335	1.82E-19	3.05E-21	1.07E+01	37.4091362	5.62E-02	140583757	140583880	NC_000009.11
cg27655671	1.82E-19	3.05E-21	1.07E+01	37.4074578	1.59E-01	38207741	38207864	NC_000006.11
cg08430009	1.82E-19	3.06E-21	1.07E+01	37.405251	2.95E-01	19138124	19138247	NC_000022.10
cg11334771	1.83E-19	3.06E-21	1.07E+01	37.4039386	2.63E-01	71955337	71955460	NC_000011.9

cg16509851	1.83E-19	3.06E-21	1.07E+01	37.4033106	2.14E-01	174430614	174430737	NC_000004.11
cg00073837	1.83E-19	3.07E-21	1.07E+01	37.40241	2.16E-01	223177008	223177131	NC_000002.11
cg00608779	1.83E-19	3.07E-21	1.07E+01	37.4015797	1.31E-01	132436490	132436613	NC_000009.11
cg21231400	1.83E-19	3.07E-21	1.07E+01	37.400808	2.23E-01	155108085	155108208	NC_000005.9
cg25522738	1.83E-19	3.08E-21	1.07E+01	37.3980939	1.68E-01	2416486	2416609	NC_000007.13
cg26814650	1.84E-19	3.09E-21	1.07E+01	37.3965171	1.47E-01	1269399	1269522	NC_000001.10
cg11662315	1.84E-19	3.10E-21	1.07E+01	37.3934274	5.46E-02	43717310	43717433	NC_000021.8
cg09825670	1.85E-19	3.10E-21	1.07E+01	37.3906917	1.50E-01	1611999	1612122	NC_000001.10
cg20195319	1.87E-19	3.14E-21	1.07E+01	37.3794212	8.96E-02	47568297	47568420	NC_000007.13
cg15549927	1.87E-19	3.15E-21	1.07E+01	37.3761384	1.73E-01	153749756	153749879	NC_000007.13
cg27165867	1.87E-19	3.15E-21	1.07E+01	37.3750912	6.08E-02	105738592	105738715	NC_000014.8
cg04254769	1.91E-19	3.21E-21	1.07E+01	37.357464	1.60E-01	141674673	141674796	NC_000005.9
cg10187707	1.91E-19	3.22E-21	1.07E+01	37.3553754	8.00E-02	49626842	49626965	NC_000020.10
cg27260772	1.91E-19	3.22E-21	1.07E+01	37.3529624	3.13E-01	50791202	50791325	NC_000006.11
cg26423824	1.92E-19	3.25E-21	1.07E+01	37.3463149	9.93E-02	25898825	25898948	NC_000017.10
cg12646649	1.93E-19	3.26E-21	1.07E+01	37.3408	2.83E-01	102987257	102987380	NC_000010.10
cg25923609	1.96E-19	3.31E-21	1.07E+01	37.3279999	2.92E-01	3607092	3607215	NC_000005.9
cg22294804	1.96E-19	3.31E-21	1.07E+01	37.3254984	1.05E-01	26484149	26484272	NC_000008.10
cg24219974	1.98E-19	3.36E-21	1.07E+01	37.3135354	1.66E-01	14729722	14729845	NC_000006.11
cg09013789	2.00E-19	3.38E-21	1.07E+01	37.3053243	8.04E-02	138776683	138776806	NC_000007.13
cg24637364	2.02E-19	3.42E-21	1.07E+01	37.2952666	2.62E-01	66259084	66259207	NC_000001.10
cg02215776	2.02E-19	3.43E-21	1.07E+01	37.2918667	1.05E-01	3116792	3116915	NC_000010.10
cg26549595	2.04E-19	3.45E-21	1.07E+01	37.2857757	1.58E-01	145048235	145048358	NC_000008.10
cg13251292	2.06E-19	3.50E-21	1.07E+01	37.2724216	1.21E-01	94790112	94790235	NC_000014.8
cg15880211	2.06E-19	3.50E-21	1.07E+01	37.2720764	1.64E-01	50250494	50250617	NC_000022.10

cg11073558	2.06E-19	3.50E-21	1.07E+01	37.2706512	2.46E-01	97171901	97172024	NC_000008.10
cg13615983	2.06E-19	3.50E-21	1.07E+01	37.2705906	1.27E-01	139746152	139746275	NC_000009.11
cg01561200	2.07E-19	3.52E-21	1.07E+01	37.2655134	1.43E-01	93176520	93176643	NC_000001.10
cg20302957	2.09E-19	3.55E-21	1.06E+01	37.2580576	1.02E-01	58870494	58870617	NC_000011.9
cg24248680	2.10E-19	3.56E-21	1.06E+01	37.2538355	1.75E-01	124610364	124610487	NC_000010.10
cg27058882	2.12E-19	3.60E-21	1.06E+01	37.2449999	1.30E-01	27187966	27188089	NC_000017.10
cg17647091	2.12E-19	3.60E-21	1.06E+01	37.2429313	1.46E-01	54474036	54474159	NC_000012.11
cg10538440	2.13E-19	3.63E-21	1.06E+01	37.2354393	1.31E-01	38117682	38117805	NC_000021.8
cg15711902	2.14E-19	3.63E-21	1.06E+01	37.2346327	3.01E-01	170630457	170630580	NC_000001.10
cg06659727	2.14E-19	3.64E-21	1.06E+01	37.2330144	2.08E-01	110959428	110959551	NC_000013.10
cg01845451	2.14E-19	3.65E-21	1.06E+01	37.2305519	1.47E-01	67805250	67805373	NC_000011.9
cg19064258	2.15E-19	3.66E-21	1.06E+01	37.2280921	2.32E-01	22826117	22826240	NC_000016.9
cg24239808	2.15E-19	3.66E-21	1.06E+01	37.2278287	6.71E-02	15641908	15642031	NC_000003.11
cg24053163	2.15E-19	3.66E-21	1.06E+01	37.2264285	1.83E-01	92503425	92503548	NC_000014.8
cg19098763	2.16E-19	3.68E-21	1.06E+01	37.2213253	2.80E-01	50513661	50513784	NC_000001.10
cg00955686	2.17E-19	3.69E-21	1.06E+01	37.2191448	5.79E-02	36620450	36620573	NC_000001.10
cg08189989	2.17E-19	3.70E-21	1.06E+01	37.2166785	2.83E-01	105459164	105459287	NC_000002.11
cg14848450	2.18E-19	3.71E-21	1.06E+01	37.2148022	2.28E-01	28590760	28590883	NC_000010.10
cg14987002	2.18E-19	3.72E-21	1.06E+01	37.2119151	9.03E-02	151496830	151496953	NC_000004.11
cg19379102	2.18E-19	3.72E-21	1.06E+01	37.2103701	1.09E-01	37576698	37576821	NC_000022.10
cg01009664	2.19E-19	3.73E-21	1.06E+01	37.2096028	2.79E-01	129693613	129693736	NC_000003.11
cg27219961	2.19E-19	3.74E-21	1.06E+01	37.2070511	8.04E-02	1692107	1692230	NC_000008.10
cg09236176	2.19E-19	3.74E-21	1.06E+01	37.2066853	2.89E-01	77179455	77179578	NC_000017.10
cg16707405	2.20E-19	3.75E-21	1.06E+01	37.2040623	2.65E-01	115152413	115152536	NC_000005.9
cg15885337	2.20E-19	3.75E-21	1.06E+01	37.2031709	2.81E-01	132813819	132813942	NC_000011.9

cg04228709	2.21E-19	3.76E-21	1.06E+01	37.1995486	1.35E-01	155537930	155538053	NC_000006.11
cg11965370	2.22E-19	3.78E-21	1.06E+01	37.194188	2.35E-01	131780946	131781069	NC_000011.9
cg02572033	2.23E-19	3.81E-21	1.06E+01	37.1871075	1.60E-01	217444	217567	NC_000017.10
cg15073906	2.24E-19	3.83E-21	1.06E+01	37.1814831	2.26E-01	178887722	178887845	NC_000001.10
cg22961814	2.26E-19	3.86E-21	1.06E+01	37.1747076	9.95E-02	112670508	112670631	NC_000010.10
cg06306260	2.26E-19	3.87E-21	1.06E+01	37.1732074	1.75E-01	99330962	99331085	NC_000007.13
cg02279670	2.31E-19	3.94E-21	1.06E+01	37.1530771	3.09E-01	79177195	79177318	NC_000013.10
cg17391741	2.31E-19	3.95E-21	1.06E+01	37.1515938	1.69E-01	141071381	141071504	NC_000005.9
cg12355110	2.31E-19	3.96E-21	1.06E+01	37.150367	1.96E-01	57617754	57617877	NC_000019.9
cg21516162	2.31E-19	3.96E-21	1.06E+01	37.1491001	1.97E-01	2953889	2954012	NC_000007.13
cg10185489	2.32E-19	3.98E-21	1.06E+01	37.1448248	9.48E-02	31079555	31079678	NC_000006.11
cg21660452	2.35E-19	4.02E-21	1.06E+01	37.134404	1.80E-01	64397807	64397930	NC_000011.9
cg12306195	2.35E-19	4.02E-21	1.06E+01	37.1333978	2.12E-01	5892350	5892473	NC_000004.11
cg16767801	2.35E-19	4.02E-21	1.06E+01	37.1329694	2.40E-01	21686548	21686671	NC_000020.10
cg20148389	2.35E-19	4.03E-21	1.06E+01	37.1310747	5.60E-02	41053543	41053666	NC_000013.10
cg14477205	2.36E-19	4.04E-21	1.06E+01	37.1281108	1.39E-01	20817076	20817199	NC_000001.10
cg05923687	2.41E-19	4.14E-21	1.06E+01	37.1047095	2.30E-01	63795711	63795834	NC_000001.10
cg19350360	2.43E-19	4.17E-21	1.06E+01	37.0967872	2.21E-01	100913948	100914071	NC_000015.9
cg09997532	2.43E-19	4.17E-21	1.06E+01	37.0966808	7.33E-02	11403999	11404122	NC_000004.11
cg14024321	2.43E-19	4.18E-21	1.06E+01	37.0954886	8.95E-02	95286432	95286555	NC_000009.11
cg23876072	2.43E-19	4.18E-21	1.06E+01	37.0944944	1.35E-01	69931921	69932044	NC_000011.9
cg14784253	2.46E-19	4.23E-21	1.06E+01	37.084473	1.34E-01	30473983	30474106	NC_000007.13
cg07198552	2.46E-19	4.24E-21	1.06E+01	37.0820669	2.30E-01	59828977	59829100	NC_000020.10
cg01419831	2.47E-19	4.25E-21	1.06E+01	37.0800647	3.10E-01	162283705	162283828	NC_000002.11
cg10681992	2.47E-19	4.25E-21	1.06E+01	37.0788125	1.01E-01	6964527	6964650	NC_000004.11

cg25144207	2.47E-19	4.26E-21	1.06E+01	37.0767874	1.73E-01	4864302	4864425	NC_000004.11
cg01615424	2.49E-19	4.29E-21	1.06E+01	37.0707631	2.48E-01	32639059	32639182	NC_000015.9
cg01718742	2.50E-19	4.31E-21	1.06E+01	37.0649158	2.27E-01	62070184	62070307	NC_000016.9
cg07645809	2.55E-19	4.39E-21	1.06E+01	37.0469483	7.21E-02	168677970	168678093	NC_000006.11
cg25575937	2.55E-19	4.40E-21	1.06E+01	37.0437025	1.59E-01	48414383	48414506	NC_000003.11
cg08053523	2.56E-19	4.41E-21	1.06E+01	37.0419432	1.25E-01	220026863	220026986	NC_000002.11
cg08629895	2.57E-19	4.44E-21	1.06E+01	37.0356357	1.34E-01	41787856	41787979	NC_000017.10
cg04433909	2.58E-19	4.45E-21	1.06E+01	37.0339452	1.06E-01	38368572	38368695	NC_000015.9
cg16656078	2.62E-19	4.53E-21	1.06E+01	37.0147653	1.85E-01	95278638	95278761	NC_000005.9
cg01246835	2.63E-19	4.55E-21	1.06E+01	37.0106904	2.27E-01	6703708	6703831	NC_000007.13
cg08091192	2.64E-19	4.56E-21	1.06E+01	37.008695	2.50E-01	147114986	147115109	NC_000003.11
cg05667838	2.64E-19	4.57E-21	1.06E+01	37.0079336	8.85E-02	31015282	31015405	NC_000007.13
cg26809635	2.66E-19	4.61E-21	1.06E+01	36.9989652	3.47E-01	54355087	54355210	NC_000012.11
cg26460092	2.69E-19	4.66E-21	1.06E+01	36.9874041	3.34E-01	83776420	83776543	NC_000015.9
cg03966669	2.69E-19	4.67E-21	1.06E+01	36.9865163	1.56E-01	4763799	4763922	NC_000016.9
cg25203481	2.70E-19	4.67E-21	1.06E+01	36.9860948	1.78E-01	2891082	2891205	NC_000011.9
cg01184975	2.70E-19	4.68E-21	1.06E+01	36.9840161	2.77E-01	114852091	114852214	NC_000012.11
cg15827843	2.70E-19	4.68E-21	1.06E+01	36.9829581	1.66E-01	1123765	1123888	NC_000005.9
cg17117243	2.71E-19	4.70E-21	1.06E+01	36.9795198	1.36E-01	109341365	109341488	NC_000006.11
cg11014373	2.76E-19	4.79E-21	1.06E+01	36.961117	3.12E-01	105479054	105479177	NC_000002.11
cg14043816	2.78E-19	4.83E-21	1.06E+01	36.9528781	1.60E-01	229166684	229166807	NC_000001.10
cg21385480	2.79E-19	4.84E-21	1.06E+01	36.9499397	1.76E-01	66454763	66454886	NC_000003.11
cg24202221	2.79E-19	4.85E-21	1.06E+01	36.9488858	1.33E-01	38694132	38694255	NC_000002.11
cg21681320	2.80E-19	4.86E-21	1.06E+01	36.9455662	1.80E-01	3011044	3011167	NC_000001.10
cg05311626	2.83E-19	4.93E-21	1.06E+01	36.9327637	1.17E-01	1320114	1320237	NC_000011.9

cg00473624	2.85E-19	4.96E-21	1.06E+01	36.9251811	2.34E-01	62139710	62139833	NC_000011.9
cg07914084	2.86E-19	4.97E-21	1.06E+01	36.9228631	3.23E-01	237205999	237206122	NC_000001.10
cg15841063	2.87E-19	5.00E-21	1.06E+01	36.9183045	2.56E-01	63789500	63789623	NC_000001.10
cg09268125	2.92E-19	5.08E-21	1.06E+01	36.901718	1.45E-01	67695975	67696098	NC_000016.9
cg24561661	2.93E-19	5.11E-21	1.06E+01	36.8971021	2.59E-01	176972812	176972935	NC_000002.11
cg10689404	2.94E-19	5.12E-21	1.06E+01	36.8941696	1.82E-01	35300645	35300768	NC_000017.10
cg15045356	2.95E-19	5.14E-21	1.06E+01	36.8901734	8.65E-02	11969590	11969713	NC_000011.9
cg24923694	2.95E-19	5.15E-21	1.06E+01	36.8887231	1.99E-01	25905747	25905870	NC_000008.10
cg20981086	2.96E-19	5.15E-21	1.06E+01	36.8882985	2.34E-01	101923256	101923379	NC_000014.8
cg00324719	2.96E-19	5.15E-21	1.06E+01	36.8875302	1.36E-01	1155602	1155725	NC_000008.10
cg08862035	2.96E-19	5.16E-21	1.06E+01	36.8871634	2.49E-01	2617432	2617555	NC_000002.11
cg14904079	2.97E-19	5.18E-21	1.06E+01	36.8819925	1.48E-01	103514655	103514778	NC_000013.10
cg17611647	2.99E-19	5.22E-21	1.06E+01	36.8756836	1.07E-01	75260857	75260980	NC_000017.10
cg06392753	2.99E-19	5.22E-21	1.06E+01	36.8744374	2.43E-01	102783282	102783405	NC_000002.11
cg14345012	3.04E-19	5.30E-21	1.06E+01	36.8593134	1.47E-01	26189240	26189363	NC_000020.10
cg16759221	3.04E-19	5.31E-21	1.06E+01	36.858211	1.64E-01	45003025	45003148	NC_000017.10
cg07220650	3.05E-19	5.33E-21	1.06E+01	36.8546557	1.29E-01	2336575	2336698	NC_000001.10
cg15393937	3.10E-19	5.42E-21	1.06E+01	36.8375161	2.26E-01	2161383	2161506	NC_000011.9
cg13982529	3.11E-19	5.44E-21	1.06E+01	36.8339612	2.47E-01	87974398	87974521	NC_000005.9
cg14252519	3.16E-19	5.52E-21	1.06E+01	36.818936	3.24E-01	137533851	137533974	NC_000009.11
cg20546778	3.17E-19	5.55E-21	1.06E+01	36.8134643	2.13E-01	75263599	75263722	NC_000008.10
cg14470895	3.18E-19	5.56E-21	1.06E+01	36.8116146	2.68E-01	115152431	115152554	NC_000005.9
cg17787134	3.19E-19	5.59E-21	1.06E+01	36.8070613	2.67E-01	47073522	47073645	NC_000017.10
cg22562942	3.20E-19	5.61E-21	1.06E+01	36.8027316	1.87E-01	24772309	24772432	NC_000008.10
cg25506432	3.22E-19	5.65E-21	1.06E+01	36.7973179	1.61E-01	27184065	27184188	NC_000007.13

cg00182639	3.22E-19	5.66E-21	1.06E+01	36.7952782	1.74E-01	114841671	114841794	NC_000012.11
cg03354113	3.23E-19	5.66E-21	1.06E+01	36.7949132	3.34E-01	79396793	79396916	NC_000010.10
cg05152503	3.23E-19	5.67E-21	1.06E+01	36.7939055	1.43E-01	30407098	30407221	NC_000020.10
cg17194668	3.27E-19	5.74E-21	1.06E+01	36.7815678	1.48E-01	85186517	85186640	NC_000015.9
cg15122439	3.27E-19	5.74E-21	1.06E+01	36.7805347	2.30E-01	200469026	200469149	NC_000002.11
cg12377641	3.27E-19	5.76E-21	1.06E+01	36.7781595	1.50E-01	11868179	11868302	NC_000010.10
cg00252733	3.28E-19	5.76E-21	1.06E+01	36.7777779	7.59E-02	102727239	102727362	NC_000010.10
cg13293266	3.28E-19	5.77E-21	1.06E+01	36.7755532	8.94E-02	118591684	118591807	NC_000002.11
cg16940012	3.34E-19	5.87E-21	1.06E+01	36.758451	2.52E-01	174159564	174159687	NC_000005.9
cg12647587	3.37E-19	5.94E-21	1.06E+01	36.7474789	1.48E-01	96954252	96954375	NC_000010.10
cg02767771	3.40E-19	5.98E-21	1.06E+01	36.7401274	2.96E-01	135043492	135043615	NC_000010.10
cg14568830	3.40E-19	5.99E-21	1.06E+01	36.738309	2.29E-01	23463243	23463366	NC_000010.10
cg13803688	3.41E-19	6.01E-21	1.06E+01	36.7359275	1.70E-01	110610452	110610575	NC_000001.10
cg16630482	3.44E-19	6.06E-21	1.06E+01	36.7274287	2.79E-01	133657751	133657874	NC_000011.9
cg11834658	3.44E-19	6.06E-21	1.06E+01	36.7270112	1.44E-01	21611224	21611347	NC_000001.10
cg17192315	3.47E-19	6.12E-21	1.06E+01	36.7179924	1.13E-01	61375622	61375745	NC_000018.9
cg01262036	3.49E-19	6.16E-21	1.06E+01	36.7109489	7.06E-02	105513535	105513658	NC_000014.8
cg18630667	3.49E-19	6.16E-21	1.06E+01	36.7108012	3.07E-01	37096323	37096446	NC_000019.9
cg08612137	3.50E-19	6.18E-21	1.06E+01	36.708032	2.54E-01	42533206	42533329	NC_000007.13
cg10719970	3.52E-19	6.23E-21	1.06E+01	36.700325	1.63E-01	41529932	41530055	NC_000006.11
cg04326540	3.54E-19	6.25E-21	1.06E+01	36.6963551	1.53E-01	105747951	105748074	NC_000014.8
cg04415176	3.55E-19	6.27E-21	1.06E+01	36.6930799	2.57E-01	176957842	176957965	NC_000002.11
cg04022561	3.55E-19	6.28E-21	1.06E+01	36.6924207	2.29E-01	50673245	50673368	NC_000005.9
cg00806490	3.57E-19	6.31E-21	1.06E+01	36.6870491	2.27E-01	82660873	82660996	NC_000016.9
cg11878331	3.58E-19	6.34E-21	1.06E+01	36.6829814	2.65E-01	72469507	72469630	NC_000008.10

cg07849944	3.62E-19	6.41E-21	1.06E+01	36.6709868	2.46E-01	6623738	6623861	NC_000016.9
cg26332560	3.64E-19	6.44E-21	1.06E+01	36.6665789	2.23E-01	132052887	132053010	NC_000008.10
cg19045729	3.70E-19	6.55E-21	1.06E+01	36.6496776	1.02E-01	104252630	104252753	NC_000010.10
cg25677548	3.70E-19	6.57E-21	1.06E+01	36.6472234	8.25E-02	41247912	41248035	NC_000015.9
cg08143349	3.72E-19	6.60E-21	1.06E+01	36.6423195	1.16E-01	27180888	27181011	NC_000007.13
cg23758305	3.72E-19	6.61E-21	1.06E+01	36.6408353	2.51E-01	70982867	70982990	NC_000008.10
cg19689151	3.72E-19	6.61E-21	1.06E+01	36.6407493	1.66E-01	105778041	105778164	NC_000014.8
cg20019985	3.73E-19	6.62E-21	1.06E+01	36.6390737	1.85E-01	32008961	32009084	NC_000011.9
cg07537750	3.74E-19	6.63E-21	1.06E+01	36.6374487	1.50E-01	64703510	64703633	NC_000011.9
cg01705052	3.75E-19	6.66E-21	1.06E+01	36.634091	3.07E-01	12996234	12996357	NC_000016.9
cg23312743	3.75E-19	6.67E-21	1.06E+01	36.632628	1.03E-01	64877311	64877434	NC_000011.9
cg02474076	3.78E-19	6.72E-21	1.06E+01	36.624882	1.30E-01	127223255	127223378	NC_000007.13
cg22747746	3.80E-19	6.75E-21	1.06E+01	36.6198468	2.34E-01	23564193	23564316	NC_000008.10
cg04718883	3.81E-19	6.77E-21	1.06E+01	36.6172677	1.08E-01	151346358	151346481	NC_000006.11
cg04020999	3.81E-19	6.77E-21	1.06E+01	36.6172324	1.02E-01	110352548	110352671	NC_000012.11
cg24013378	3.81E-19	6.77E-21	1.06E+01	36.6166911	7.45E-02	69480958	69481081	NC_000011.9
cg22001496	3.82E-19	6.79E-21	1.06E+01	36.6138658	3.25E-01	69243486	69243609	NC_000008.10
cg03696599	3.83E-19	6.83E-21	1.06E+01	36.6090284	2.02E-01	124782261	124782384	NC_000002.11
cg10055580	3.84E-19	6.84E-21	1.06E+01	36.60688	1.85E-01	46707781	46707904	NC_000017.10
cg01743841	3.85E-19	6.85E-21	1.06E+01	36.6049867	2.92E-01	61992494	61992617	NC_000020.10
cg26554592	3.88E-19	6.92E-21	1.06E+01	36.5953035	2.68E-01	108924398	108924521	NC_000010.10
cg02201132	3.89E-19	6.93E-21	1.06E+01	36.5946809	1.52E-01	242212884	242213007	NC_000002.11
cg05962092	3.94E-19	7.03E-21	1.05E+01	36.5799697	1.69E-01	49575664	49575787	NC_000019.9
cg26353287	3.94E-19	7.04E-21	1.05E+01	36.5786295	1.94E-01	42954090	42954213	NC_000005.9
cg13752649	3.94E-19	7.04E-21	1.05E+01	36.578446	1.38E-01	46608559	46608682	NC_000017.10

cg22818319	3.95E-19	7.05E-21	1.05E+01	36.5773241	1.53E-01	118372162	118372285	NC_000010.10
cg02510924	4.02E-19	7.19E-21	1.05E+01	36.5580456	6.06E-02	127802658	127802781	NC_000003.11
cg08712082	4.08E-19	7.30E-21	1.05E+01	36.5422675	1.66E-01	30673506	30673629	NC_000007.13
cg00816224	4.09E-19	7.32E-21	1.05E+01	36.5395649	2.12E-01	17083482	17083605	NC_000022.10
cg27317647	4.16E-19	7.44E-21	1.05E+01	36.5234082	1.70E-01	145562760	145562883	NC_000008.10
cg04674315	4.17E-19	7.46E-21	1.05E+01	36.5207823	1.79E-01	25897907	25898030	NC_000007.13
cg13301003	4.19E-19	7.50E-21	1.05E+01	36.5158346	1.93E-01	32449254	32449377	NC_000011.9
cg26477488	4.22E-19	7.56E-21	1.05E+01	36.507507	2.86E-01	153858936	153859059	NC_000005.9
cg06928993	4.23E-19	7.58E-21	1.05E+01	36.5045629	2.22E-01	49313421	49313544	NC_000019.9
cg00549566	4.28E-19	7.67E-21	1.05E+01	36.4927376	3.46E-01	37488253	37488376	NC_000007.13
cg07319253	4.29E-19	7.70E-21	1.05E+01	36.4898905	1.70E-01	17249877	17250000	NC_000001.10
cg27237300	4.29E-19	7.71E-21	1.05E+01	36.4880014	2.77E-01	34442292	34442415	NC_000021.8
cg26767593	4.31E-19	7.74E-21	1.05E+01	36.484046	1.63E-01	30015647	30015770	NC_000008.10
cg14486338	4.35E-19	7.82E-21	1.05E+01	36.4743731	1.92E-01	99440279	99440402	NC_000008.10
cg11501438	4.36E-19	7.83E-21	1.05E+01	36.473443	2.32E-01	20755949	20756072	NC_000017.10
cg17489908	4.36E-19	7.83E-21	1.05E+01	36.4728354	2.01E-01	8101566	8101689	NC_000010.10
cg18734428	4.38E-19	7.86E-21	1.05E+01	36.4685655	2.59E-01	84322277	84322400	NC_000015.9
cg06243400	4.40E-19	7.91E-21	1.05E+01	36.4632751	2.22E-01	19157813	19157936	NC_000007.13
cg02400787	4.46E-19	8.03E-21	1.05E+01	36.448273	5.98E-02	6399178	6399301	NC_000012.11
cg08383929	4.48E-19	8.06E-21	1.05E+01	36.443852	1.17E-01	180646754	180646877	NC_000005.9
cg11574745	4.50E-19	8.10E-21	1.05E+01	36.4387362	1.86E-01	5087031	5087154	NC_000006.11
cg23705060	4.51E-19	8.12E-21	1.05E+01	36.4367311	1.34E-01	132443404	132443527	NC_000012.11
cg10564112	4.56E-19	8.21E-21	1.05E+01	36.4260959	1.04E-01	91842149	91842272	NC_000014.8
cg04270085	4.59E-19	8.27E-21	1.05E+01	36.4180591	1.28E-01	12223636	12223759	NC_000002.11
cg22142922	4.60E-19	8.30E-21	1.05E+01	36.4155216	1.68E-01	69969430	69969553	NC_000011.9

cg04541474	4.61E-19	8.32E-21	1.05E+01	36.41304	3.03E-01	44873592	44873715	NC_000001.10
cg11462939	4.63E-19	8.35E-21	1.05E+01	36.4092608	6.70E-02	99606117	99606240	NC_000013.10
cg20501882	4.69E-19	8.47E-21	1.05E+01	36.3949754	1.60E-01	107542178	107542301	NC_000007.13
cg09557313	4.71E-19	8.49E-21	1.05E+01	36.3920213	2.54E-01	53313174	53313297	NC_000013.10
cg21236845	4.76E-19	8.60E-21	1.05E+01	36.3796737	2.34E-01	98115329	98115452	NC_000005.9
cg16591875	4.87E-19	8.81E-21	1.05E+01	36.3556126	1.28E-01	23932864	23932987	NC_000015.9
cg18298050	4.87E-19	8.81E-21	1.05E+01	36.3555091	1.25E-01	26829938	26830061	NC_000022.10
cg01449136	4.87E-19	8.81E-21	1.05E+01	36.355355	2.20E-01	61109317	61109440	NC_000014.8
cg04853843	4.88E-19	8.82E-21	1.05E+01	36.3542423	2.30E-01	55358461	55358584	NC_000016.9
cg19016714	4.90E-19	8.86E-21	1.05E+01	36.3501087	5.54E-02	80806029	80806152	NC_000010.10
cg25904659	4.90E-19	8.87E-21	1.05E+01	36.3490223	1.54E-01	916272	916395	NC_000017.10
cg13613439	4.94E-19	8.94E-21	1.05E+01	36.3416208	9.00E-02	131683312	131683435	NC_000009.11
cg05038396	4.95E-19	8.97E-21	1.05E+01	36.3376479	1.39E-01	86363010	86363133	NC_000002.11
cg10241684	4.96E-19	8.98E-21	1.05E+01	36.3367439	7.76E-02	14998547	14998670	NC_000003.11
cg01730970	4.99E-19	9.04E-21	1.05E+01	36.3308318	2.36E-01	27687074	27687197	NC_000001.10
cg17245290	4.99E-19	9.05E-21	1.05E+01	36.3293852	1.26E-01	35504020	35504143	NC_000010.10
cg20064693	5.03E-19	9.13E-21	1.05E+01	36.3202637	6.60E-02	228601611	228601734	NC_000001.10
cg26485937	5.04E-19	9.14E-21	1.05E+01	36.3191684	1.78E-01	140802432	140802555	NC_000005.9
cg08045301	5.04E-19	9.15E-21	1.05E+01	36.3181997	2.38E-01	71887487	71887610	NC_000016.9
cg00953825	5.06E-19	9.18E-21	1.05E+01	36.3148176	7.20E-02	60757134	60757257	NC_000017.10
cg06942814	5.08E-19	9.23E-21	1.05E+01	36.3098219	1.41E-01	27170819	27170942	NC_000007.13
cg16111364	5.09E-19	9.25E-21	1.05E+01	36.3079943	1.07E-01	122891696	122891819	NC_000012.11
cg00063369	5.10E-19	9.26E-21	1.05E+01	36.3069839	1.40E-01	213811478	213811601	NC_000002.11
cg25157999	5.13E-19	9.31E-21	1.05E+01	36.3009667	1.03E-01	657046	657169	NC_000012.11
cg13516768	5.14E-19	9.34E-21	1.05E+01	36.2979741	1.00E-01	61599125	61599248	NC_000020.10

cg12667031	5.16E-19	9.38E-21	1.05E+01	36.2936925	7.64E-02	169878852	169878975	NC_000001.10
cg02595832	5.16E-19	9.38E-21	1.05E+01	36.2932033	2.89E-01	129535786	129535909	NC_000010.10
cg05917460	5.18E-19	9.41E-21	1.05E+01	36.2901698	3.05E-01	37126902	37127025	NC_000014.8
cg26768816	5.21E-19	9.47E-21	1.05E+01	36.2841038	2.99E-02	30302793	30302916	NC_000019.9
cg24613957	5.24E-19	9.53E-21	1.05E+01	36.2776905	1.19E-01	20250684	20250807	NC_000001.10
cg24904818	5.26E-19	9.57E-21	1.05E+01	36.2734183	9.12E-02	49713323	49713446	NC_000019.9
cg04865180	5.32E-19	9.69E-21	1.05E+01	36.2614478	2.83E-01	23734385	23734508	NC_000013.10
cg09737668	5.36E-19	9.77E-21	1.05E+01	36.2535848	9.05E-02	159923525	159923648	NC_000001.10
cg19722706	5.39E-19	9.82E-21	1.05E+01	36.2484199	1.28E-01	83732977	83733100	NC_000015.9
cg03813164	5.39E-19	9.82E-21	1.05E+01	36.2478996	3.45E-01	11059725	11059848	NC_000010.10
cg26150514	5.42E-19	9.87E-21	1.05E+01	36.2428629	1.69E-01	96907209	96907332	NC_000015.9
cg11147786	5.43E-19	9.91E-21	1.05E+01	36.2393916	1.79E-01	19157739	19157862	NC_000007.13
cg11379081	5.46E-19	9.96E-21	1.05E+01	36.2343453	1.98E-01	209405050	209405173	NC_000001.10
cg18878432	5.50E-19	1.00E-20	1.05E+01	36.2263449	2.08E-01	46683409	46683532	NC_000017.10
cg08733739	5.61E-19	1.02E-20	1.05E+01	36.2061705	7.75E-02	13438879	13439002	NC_000006.11
cg07821427	5.61E-19	1.03E-20	1.05E+01	36.2053079	3.35E-01	77822419	77822542	NC_000016.9
cg03704667	5.63E-19	1.03E-20	1.05E+01	36.2026268	1.34E-01	69152610	69152733	NC_000016.9
cg01341355	5.64E-19	1.03E-20	1.05E+01	36.1993695	1.34E-01	31505493	31505616	NC_000006.11
cg07592513	5.66E-19	1.04E-20	1.05E+01	36.195713	1.20E-01	49994239	49994362	NC_000019.9
cg12853539	5.68E-19	1.04E-20	1.05E+01	36.1925586	1.37E-01	125623465	125623588	NC_000006.11
cg09979356	5.70E-19	1.04E-20	1.05E+01	36.1889658	9.83E-02	23725375	23725498	NC_000016.9
cg22289434	5.70E-19	1.04E-20	1.05E+01	36.1876105	2.44E-01	34188940	34189063	NC_000020.10
cg16521917	5.78E-19	1.06E-20	1.05E+01	36.1735726	2.38E-01	145813946	145814069	NC_000007.13
cg02583418	5.86E-19	1.07E-20	1.05E+01	36.1591547	2.27E-01	105473340	105473463	NC_000002.11
cg26998044	5.91E-19	1.08E-20	1.05E+01	36.1513796	3.13E-01	8869155	8869278	NC_000017.10

cg07017374	5.98E-19	1.10E-20	1.05E+01	36.1393199	3.47E-01	28674451	28674574	NC_000013.10
cg20423781	5.99E-19	1.10E-20	1.05E+01	36.1379052	1.53E-01	31031734	31031857	NC_000002.11
cg17075865	6.00E-19	1.10E-20	1.05E+01	36.1363945	8.89E-02	121287467	121287590	NC_000010.10
cg23836168	6.08E-19	1.12E-20	1.05E+01	36.1219406	8.12E-02	77084618	77084741	NC_000017.10
cg15443767	6.09E-19	1.12E-20	1.05E+01	36.1197561	1.11E-01	157943975	157944098	NC_000002.11
cg16197088	6.16E-19	1.13E-20	1.05E+01	36.1074466	1.69E-01	823500	823623	NC_000011.9
cg16733813	6.17E-19	1.13E-20	1.05E+01	36.1061189	1.29E-01	228295568	228295691	NC_000001.10
cg02825373	6.17E-19	1.13E-20	1.05E+01	36.1053017	2.90E-01	55501101	55501224	NC_000020.10
cg27543385	6.20E-19	1.14E-20	1.05E+01	36.1004157	1.71E-01	71800219	71800342	NC_000017.10
cg00248856	6.23E-19	1.15E-20	1.05E+01	36.0950671	1.11E-01	69464272	69464395	NC_000011.9
cg03058444	6.25E-19	1.15E-20	1.05E+01	36.0919662	1.53E-01	1873540	1873663	NC_000006.11
cg08676975	6.26E-19	1.15E-20	1.05E+01	36.0903599	2.98E-01	37005582	37005705	NC_000013.10
cg10297617	6.26E-19	1.15E-20	1.05E+01	36.0893994	1.89E-01	91610405	91610528	NC_000014.8
cg25383605	6.26E-19	1.15E-20	1.05E+01	36.0889833	1.19E-01	155538047	155538170	NC_000006.11
cg20959189	6.28E-19	1.16E-20	1.05E+01	36.0858732	1.42E-01	141122632	141122755	NC_000003.11
cg05663453	6.32E-19	1.16E-20	1.05E+01	36.0792249	1.61E-01	22782452	22782575	NC_000001.10
cg12198813	6.35E-19	1.17E-20	1.05E+01	36.0750033	1.67E-01	2891077	2891200	NC_000011.9
cg05940984	6.36E-19	1.17E-20	1.05E+01	36.0722727	2.45E-01	32448678	32448801	NC_000011.9
cg04221877	6.39E-19	1.18E-20	1.05E+01	36.0673472	1.57E-01	25257515	25257638	NC_000001.10
cg07016605	6.40E-19	1.18E-20	1.05E+01	36.0655177	1.55E-01	80194706	80194829	NC_000017.10
cg24880892	6.42E-19	1.18E-20	1.05E+01	36.0631255	5.89E-02	39034493	39034616	NC_000017.10
cg22372285	6.42E-19	1.18E-20	1.05E+01	36.062841	1.13E-01	125951005	125951128	NC_000011.9
cg26268287	6.42E-19	1.19E-20	1.05E+01	36.0617404	9.02E-02	114957848	114957971	NC_000005.9
cg23040011	6.45E-19	1.19E-20	1.05E+01	36.0572505	1.46E-01	56594902	56595025	NC_000014.8
cg16334617	6.46E-19	1.19E-20	1.05E+01	36.0551941	6.76E-02	126306823	126306946	NC_000011.9

cg22940988	6.47E-19	1.20E-20	1.05E+01	36.0533158	7.10E-02	67538446	67538569	NC_000015.9
cg08819431	6.48E-19	1.20E-20	1.05E+01	36.0514601	2.13E-01	139067293	139067416	NC_000005.9
cg14981055	6.49E-19	1.20E-20	1.05E+01	36.0493622	1.14E-01	56658336	56658459	NC_000019.9
cg26692294	6.51E-19	1.20E-20	1.05E+01	36.0470297	2.78E-01	6449001	6449124	NC_000005.9
cg05762309	6.58E-19	1.22E-20	1.05E+01	36.0355806	8.41E-02	30121843	30121966	NC_000006.11
cg27648808	6.63E-19	1.23E-20	1.05E+01	36.0285681	6.60E-02	168439973	168440096	NC_000005.9
cg02455820	6.64E-19	1.23E-20	1.05E+01	36.0266967	2.72E-01	51147769	51147892	NC_000016.9
cg27515369	6.64E-19	1.23E-20	1.05E+01	36.0265036	3.72E-01	140770599	140770722	NC_000003.11
cg10741025	6.65E-19	1.23E-20	1.05E+01	36.0238977	2.64E-01	37132665	37132788	NC_000014.8
cg10869641	6.66E-19	1.23E-20	1.05E+01	36.0231159	5.78E-02	170184833	170184956	NC_000006.11
cg00895997	6.66E-19	1.23E-20	1.05E+01	36.0228006	2.05E-01	122840109	122840232	NC_000007.13
cg27403628	6.67E-19	1.23E-20	1.05E+01	36.0217505	2.57E-01	17679263	17679386	NC_000020.10
cg06383163	6.68E-19	1.24E-20	1.05E+01	36.0192273	1.77E-01	139509073	139509196	NC_000008.10
cg08222702	6.72E-19	1.24E-20	1.05E+01	36.0139732	1.05E-01	122897859	122897982	NC_000010.10
cg14323675	6.81E-19	1.26E-20	1.05E+01	36.0000217	1.97E-01	17907018	17907141	NC_000009.11
cg14285308	6.81E-19	1.26E-20	1.05E+01	35.9995908	6.37E-02	20517512	20517635	NC_000001.10
cg13459498	6.83E-19	1.27E-20	1.05E+01	35.9957109	2.68E-01	42544792	42544915	NC_000020.10
cg19372975	6.85E-19	1.27E-20	1.05E+01	35.9930499	1.92E-01	242212901	242213024	NC_000002.11
cg06904649	6.91E-19	1.28E-20	1.05E+01	35.9840287	7.92E-02	64291323	64291446	NC_000015.9
cg16896847	6.92E-19	1.28E-20	1.05E+01	35.9816745	3.03E-01	144512042	144512165	NC_000008.10
cg01058717	6.96E-19	1.29E-20	1.05E+01	35.9754134	1.42E-01	1487068	1487191	NC_000001.10
cg03142393	6.97E-19	1.30E-20	1.05E+01	35.9738103	6.90E-02	96327674	96327797	NC_000002.11
cg05020604	6.99E-19	1.30E-20	1.05E+01	35.9708754	3.05E-01	37096329	37096452	NC_000019.9
cg06584784	6.99E-19	1.30E-20	1.05E+01	35.9707186	1.19E-01	86606923	86607046	NC_000016.9
cg21538208	7.02E-19	1.30E-20	1.05E+01	35.9668086	2.17E-01	4864488	4864611	NC_000004.11

cg17045801	7.08E-19	1.32E-20	1.05E+01	35.9575484	2.92E-01	43153223	43153346	NC_000007.13
cg19037143	7.11E-19	1.32E-20	1.05E+01	35.9528805	8.09E-02	187524750	187524873	NC_000004.11
cg20535067	7.12E-19	1.32E-20	1.05E+01	35.9518435	1.00E-01	134794320	134794443	NC_000010.10
cg17241310	7.13E-19	1.33E-20	1.05E+01	35.9496146	2.01E-01	91182856	91182979	NC_000001.10
cg06355272	7.20E-19	1.34E-20	1.05E+01	35.9400836	6.85E-02	3576929	3577052	NC_000010.10
cg02952703	7.23E-19	1.35E-20	1.05E+01	35.9349898	1.47E-01	14584130	14584253	NC_000019.9
cg13071306	7.25E-19	1.35E-20	1.05E+01	35.9323544	1.19E-01	6314113	6314236	NC_000001.10
cg18901940	7.27E-19	1.35E-20	1.05E+01	35.9305682	1.41E-01	69869364	69869487	NC_000010.10
cg09025327	7.39E-19	1.38E-20	1.04E+01	35.9128301	1.24E-01	9309004	9309127	NC_000001.10
cg26145461	7.42E-19	1.38E-20	1.04E+01	35.9083777	9.03E-02	2510637	2510760	NC_000006.11
cg08393317	7.55E-19	1.41E-20	1.04E+01	35.8913825	2.94E-01	54445463	54445586	NC_000019.9
cg05046306	7.56E-19	1.41E-20	1.04E+01	35.8890517	1.90E-01	46698273	46698396	NC_000017.10
cg08857144	7.57E-19	1.41E-20	1.04E+01	35.8879044	2.64E-01	70995351	70995474	NC_000002.11
cg20277905	7.60E-19	1.42E-20	1.04E+01	35.8837842	2.02E-01	61809716	61809839	NC_000020.10
cg00152008	7.63E-19	1.42E-20	1.04E+01	35.8798305	2.46E-01	50889225	50889348	NC_000001.10
cg03475671	7.64E-19	1.43E-20	1.04E+01	35.8783477	8.96E-02	90317562	90317685	NC_000015.9
cg24469013	7.66E-19	1.43E-20	1.04E+01	35.8755239	1.52E-01	72946534	72946657	NC_000011.9
cg23383871	7.68E-19	1.43E-20	1.04E+01	35.8732298	2.35E-01	47934987	47935110	NC_000020.10
cg02438781	7.72E-19	1.44E-20	1.04E+01	35.8667169	1.89E-01	22469533	22469656	NC_000019.9
cg01889899	7.81E-19	1.46E-20	1.04E+01	35.855502	1.07E-01	101922827	101922950	NC_000015.9
cg17219656	7.83E-19	1.46E-20	1.04E+01	35.8525946	1.36E-01	236924443	236924566	NC_000001.10
cg22628240	7.88E-19	1.47E-20	1.04E+01	35.8453619	1.79E-01	235107837	235107960	NC_000001.10
cg13060997	7.98E-19	1.49E-20	1.04E+01	35.8321195	2.94E-01	37006611	37006734	NC_000013.10
cg15219163	8.01E-19	1.50E-20	1.04E+01	35.8278984	6.79E-02	70842128	70842251	NC_000011.9
cg27127645	8.02E-19	1.50E-20	1.04E+01	35.8275987	1.32E-01	55220956	55221079	NC_000014.8

cg11021995	8.04E-19	1.51E-20	1.04E+01	35.8243482	2.90E-01	37488577	37488700	NC_000007.13
cg02027945	8.07E-19	1.51E-20	1.04E+01	35.8208367	3.53E-01	20618230	20618353	NC_000011.9
cg14324370	8.12E-19	1.52E-20	1.04E+01	35.8140511	2.76E-01	177042789	177042912	NC_000002.11
cg04207632	8.13E-19	1.52E-20	1.04E+01	35.8118762	1.75E-01	6986609	6986732	NC_000004.11
cg10383568	8.20E-19	1.54E-20	1.04E+01	35.8039166	1.85E-01	6589761	6589884	NC_000011.9
cg27425459	8.20E-19	1.54E-20	1.04E+01	35.8032755	1.59E-01	105697589	105697712	NC_000002.11
cg19909787	8.21E-19	1.54E-20	1.04E+01	35.801357	2.44E-01	124907708	124907831	NC_000010.10
cg07714085	8.22E-19	1.54E-20	1.04E+01	35.8002863	1.36E-01	46424852	46424975	NC_000002.11
cg16294838	8.22E-19	1.54E-20	1.04E+01	35.8001247	2.17E-01	169195305	169195428	NC_000005.9
cg22805485	8.34E-19	1.57E-20	1.04E+01	35.7853729	2.10E-01	13983818	13983941	NC_000011.9
cg09179999	8.35E-19	1.57E-20	1.04E+01	35.7836259	4.76E-02	77811681	77811804	NC_000015.9
cg03143486	8.37E-19	1.57E-20	1.04E+01	35.7809736	1.60E-01	811491	811614	NC_000007.13
cg19643053	8.41E-19	1.58E-20	1.04E+01	35.7759616	2.44E-01	27184271	27184394	NC_000007.13
cg12331980	8.44E-19	1.59E-20	1.04E+01	35.7728797	1.52E-01	179749654	179749777	NC_000002.11
cg17869851	8.52E-19	1.60E-20	1.04E+01	35.7627366	1.04E-01	230976414	230976537	NC_000001.10
cg01672943	8.56E-19	1.61E-20	1.04E+01	35.7572289	3.12E-01	37125292	37125415	NC_000014.8
cg16370720	8.58E-19	1.61E-20	1.04E+01	35.7552469	4.87E-02	108907710	108907833	NC_000006.11
cg00209786	8.62E-19	1.62E-20	1.04E+01	35.7495145	1.20E-01	56752779	56752902	NC_000020.10
cg05747555	8.66E-19	1.63E-20	1.04E+01	35.7447538	1.30E-01	60778881	60779004	NC_000017.10
cg18907180	8.68E-19	1.63E-20	1.04E+01	35.7428858	2.41E-01	97499987	97500110	NC_000014.8
cg19591056	8.76E-19	1.65E-20	1.04E+01	35.7329216	2.84E-01	240254988	240255111	NC_000001.10
cg15979173	8.76E-19	1.65E-20	1.04E+01	35.7324266	1.67E-01	91182128	91182251	NC_000001.10
cg23712458	8.86E-19	1.67E-20	1.04E+01	35.7208373	1.77E-01	189002	189125	NC_000017.10
cg06873316	8.91E-19	1.68E-20	1.04E+01	35.7146951	2.57E-01	20690682	20690805	NC_000011.9
cg26789779	8.96E-19	1.69E-20	1.04E+01	35.70857	2.43E-01	37839943	37840066	NC_000005.9

cg15266705	8.99E-19	1.70E-20	1.04E+01	35.7051881	2.69E-01	115136096	115136219	NC_000012.11
cg03262554	8.99E-19	1.70E-20	1.04E+01	35.7049261	2.48E-01	136474735	136474858	NC_000009.11
cg16700555	9.13E-19	1.73E-20	1.04E+01	35.6891754	1.80E-01	29700860	29700983	NC_000019.9
cg20018563	9.19E-19	1.74E-20	1.04E+01	35.6818579	1.65E-01	92690264	92690387	NC_000015.9
cg10901633	9.27E-19	1.75E-20	1.04E+01	35.6737912	1.77E-01	4814459	4814582	NC_000003.11
cg04946749	9.28E-19	1.76E-20	1.04E+01	35.6715442	3.04E-01	74865838	74865961	NC_000017.10
cg20713968	9.29E-19	1.76E-20	1.04E+01	35.6711753	2.32E-01	1737564	1737687	NC_000008.10
cg24656269	9.30E-19	1.76E-20	1.04E+01	35.6696873	2.45E-01	132271581	132271704	NC_000011.9
cg14776201	9.31E-19	1.76E-20	1.04E+01	35.6686743	3.23E-01	5836256	5836379	NC_000002.11
cg06424065	9.36E-19	1.77E-20	1.04E+01	35.6632003	2.32E-01	6247640	6247763	NC_000004.11
cg13291694	9.37E-19	1.77E-20	1.04E+01	35.6616125	9.53E-02	136584563	136584686	NC_000006.11
cg00028935	9.39E-19	1.78E-20	1.04E+01	35.6593513	2.33E-01	147114406	147114529	NC_000003.11
cg20522483	9.45E-19	1.79E-20	1.04E+01	35.6524388	1.42E-01	119659495	119659618	NC_000004.11
cg19560588	9.53E-19	1.81E-20	1.04E+01	35.6436164	2.55E-01	25892614	25892737	NC_000007.13
cg10959353	9.62E-19	1.82E-20	1.04E+01	35.6339951	2.35E-01	229046515	229046638	NC_000002.11
cg16499947	9.62E-19	1.83E-20	1.04E+01	35.6335075	7.97E-02	123329113	123329236	NC_000010.10
cg04220930	9.63E-19	1.83E-20	1.04E+01	35.6327944	1.67E-01	52814681	52814804	NC_000008.10
cg17086686	9.64E-19	1.83E-20	1.04E+01	35.6307673	1.39E-01	134578213	134578336	NC_000008.10
cg01578017	9.79E-19	1.86E-20	1.04E+01	35.6151187	3.23E-01	84814683	84814806	NC_000007.13
cg16587616	9.80E-19	1.86E-20	1.04E+01	35.6143397	2.27E-01	62996022	62996145	NC_000006.11
cg10038009	9.89E-19	1.88E-20	1.04E+01	35.6035638	2.96E-01	31228202	31228325	NC_000016.9
cg18127648	9.90E-19	1.88E-20	1.04E+01	35.6023979	1.09E-01	79652632	79652755	NC_000010.10
cg23722790	9.91E-19	1.88E-20	1.04E+01	35.6017508	1.79E-01	67467602	67467725	NC_000001.10
cg16166796	1.00E-18	1.90E-20	1.04E+01	35.5924379	2.97E-01	220174201	220174324	NC_000002.11
cg17796333	1.00E-18	1.91E-20	1.04E+01	35.5899227	7.97E-02	52511342	52511465	NC_000006.11

cg06809252	1.00E-18	1.91E-20	1.04E+01	35.5882046	2.21E-01	110612044	110612167	NC_000001.10
cg12071328	1.00E-18	1.91E-20	1.04E+01	35.588129	3.04E-01	20690930	20691053	NC_000011.9
cg01269620	1.00E-18	1.91E-20	1.04E+01	35.5870985	3.06E-01	166074191	166074314	NC_000006.11
cg16389924	1.01E-18	1.91E-20	1.04E+01	35.5862597	8.76E-02	79298604	79298727	NC_000015.9
cg19910231	1.01E-18	1.92E-20	1.04E+01	35.5853271	1.12E-01	3167371	3167494	NC_000010.10
cg08939095	1.01E-18	1.92E-20	1.04E+01	35.5836499	2.48E-01	83953068	83953191	NC_000015.9
cg10786098	1.04E-18	1.99E-20	1.04E+01	35.5471468	1.41E-01	117468289	117468412	NC_000012.11
cg03416228	1.04E-18	1.99E-20	1.04E+01	35.5462916	1.32E-01	65360321	65360444	NC_000011.9
cg08586541	1.05E-18	2.01E-20	1.04E+01	35.5388059	2.41E-01	51198888	51199011	NC_000019.9
cg13772815	1.05E-18	2.01E-20	1.04E+01	35.5384763	1.11E-01	20492631	20492754	NC_000017.10
cg14690510	1.05E-18	2.01E-20	1.04E+01	35.5366676	1.09E-01	60757589	60757712	NC_000017.10
cg14678084	1.06E-18	2.03E-20	1.04E+01	35.5301698	1.81E-01	127627251	127627374	NC_000007.13
cg12919006	1.07E-18	2.04E-20	1.04E+01	35.5249724	2.70E-01	124782713	124782836	NC_000002.11
cg03711485	1.07E-18	2.04E-20	1.04E+01	35.5233461	2.31E-01	101296036	101296159	NC_000010.10
cg01105418	1.07E-18	2.04E-20	1.04E+01	35.5233421	2.36E-01	244214593	244214716	NC_000001.10
cg08241225	1.07E-18	2.04E-20	1.04E+01	35.5231034	9.32E-02	36041510	36041633	NC_000019.9
cg27603283	1.07E-18	2.05E-20	1.04E+01	35.519094	2.11E-01	4998649	4998772	NC_000007.13
cg15487498	1.08E-18	2.06E-20	1.04E+01	35.5127457	1.30E-01	230372938	230373061	NC_000001.10
cg00864684	1.08E-18	2.07E-20	1.04E+01	35.5064766	1.27E-01	125825166	125825289	NC_000010.10
cg10257471	1.09E-18	2.08E-20	1.04E+01	35.5042659	1.70E-01	83933961	83934084	NC_000016.9
cg18485844	1.09E-18	2.09E-20	1.04E+01	35.5003745	3.29E-01	2291652	2291775	NC_000011.9
cg24060451	1.10E-18	2.11E-20	1.04E+01	35.4912082	2.76E-01	33454209	33454332	NC_000022.10
cg06469895	1.10E-18	2.11E-20	1.04E+01	35.4891359	1.38E-01	69418206	69418329	NC_000016.9
cg03603062	1.11E-18	2.14E-20	1.04E+01	35.477247	1.98E-01	95224434	95224557	NC_000007.13
cg24885417	1.12E-18	2.14E-20	1.04E+01	35.4751981	2.86E-01	24323764	24323887	NC_000007.13

cg23852994	1.12E-18	2.15E-20	1.04E+01	35.4695198	1.01E-01	67076568	67076691	NC_000015.9
cg23277659	1.13E-18	2.17E-20	1.04E+01	35.4625338	1.48E-01	47066498	47066621	NC_000001.10
cg08798685	1.13E-18	2.17E-20	1.04E+01	35.4614283	1.76E-01	27730294	27730417	NC_000006.11
cg05181279	1.14E-18	2.19E-20	1.04E+01	35.4544287	1.30E-01	11982794	11982917	NC_000011.9
cg06639332	1.14E-18	2.19E-20	1.04E+01	35.4506253	2.76E-01	37248305	37248428	NC_000013.10
cg24192845	1.14E-18	2.20E-20	1.04E+01	35.4500665	9.32E-02	15497029	15497152	NC_000006.11
cg08708684	1.15E-18	2.21E-20	1.04E+01	35.4450821	3.05E-01	135043506	135043629	NC_000010.10
cg25211622	1.15E-18	2.21E-20	1.04E+01	35.4440942	2.04E-01	794443	794566	NC_000011.9
cg10050410	1.16E-18	2.23E-20	1.04E+01	35.4357404	1.02E-01	4035293	4035416	NC_000010.10
cg13469286	1.17E-18	2.25E-20	1.04E+01	35.4274475	1.29E-01	134680756	134680879	NC_000010.10
cg20627646	1.17E-18	2.25E-20	1.04E+01	35.4253156	1.63E-01	103943308	103943431	NC_000008.10
cg16571983	1.17E-18	2.25E-20	1.04E+01	35.4240171	2.18E-01	78492922	78493045	NC_000013.10
cg15826444	1.17E-18	2.26E-20	1.04E+01	35.4231265	9.52E-02	20767574	20767697	NC_000002.11
cg10655629	1.17E-18	2.26E-20	1.04E+01	35.4227692	1.33E-01	480253	480376	NC_000005.9
cg26949037	1.17E-18	2.26E-20	1.04E+01	35.4217342	5.52E-02	31003936	31004059	NC_000016.9
cg05106231	1.18E-18	2.27E-20	1.04E+01	35.4192725	1.09E-01	7338152	7338275	NC_000017.10
cg11668749	1.18E-18	2.28E-20	1.04E+01	35.4140283	1.68E-01	229568360	229568483	NC_000001.10
cg26078977	1.20E-18	2.32E-20	1.04E+01	35.3960052	1.93E-01	59189467	59189590	NC_000005.9
cg05523056	1.20E-18	2.32E-20	1.04E+01	35.3948165	2.20E-01	77468458	77468581	NC_000016.9
cg19947104	1.21E-18	2.32E-20	1.04E+01	35.3935528	2.10E-01	17757342	17757465	NC_000011.9
cg16535788	1.21E-18	2.33E-20	1.04E+01	35.3926607	1.63E-01	80395405	80395528	NC_000017.10
cg24183575	1.21E-18	2.33E-20	1.04E+01	35.3905717	1.93E-01	49497929	49498052	NC_000003.11
cg00890054	1.21E-18	2.34E-20	1.04E+01	35.3863304	1.40E-01	9405823	9405946	NC_000001.10
cg00542638	1.21E-18	2.34E-20	1.04E+01	35.3860637	1.50E-01	20007500	20007623	NC_000022.10
cg22036988	1.23E-18	2.38E-20	1.04E+01	35.3705514	2.99E-01	140770345	140770468	NC_000003.11

cg01096617	1.23E-18	2.38E-20	1.04E+01	35.3689756	2.11E-01	58569180	58569303	NC_000016.9
cg03140968	1.24E-18	2.39E-20	1.04E+01	35.3647715	2.18E-01	21686264	21686387	NC_000020.10
cg05678749	1.24E-18	2.39E-20	1.04E+01	35.3643836	1.82E-01	27216819	27216942	NC_000015.9
cg16787483	1.25E-18	2.42E-20	1.04E+01	35.3541499	1.91E-01	88328251	88328374	NC_000013.10
cg25426892	1.26E-18	2.43E-20	1.04E+01	35.349572	1.74E-01	100942959	100943082	NC_000007.13
cg16409261	1.26E-18	2.44E-20	1.04E+01	35.3458738	2.11E-01	61703735	61703858	NC_000020.10
cg07804728	1.27E-18	2.45E-20	1.04E+01	35.3425292	2.01E-01	129284050	129284173	NC_000010.10
cg24127719	1.27E-18	2.45E-20	1.04E+01	35.3404491	2.26E-01	38068660	38068783	NC_000021.8
cg05574357	1.27E-18	2.46E-20	1.04E+01	35.3365125	2.10E-01	115134362	115134485	NC_000012.11
cg24930713	1.27E-18	2.46E-20	1.04E+01	35.3358305	1.17E-01	47048981	47049104	NC_000017.10
cg12932195	1.28E-18	2.47E-20	1.04E+01	35.3317049	1.61E-01	50313343	50313466	NC_000022.10
cg16051656	1.28E-18	2.48E-20	1.04E+01	35.331112	1.65E-01	41162610	41162733	NC_000003.11
cg13718664	1.28E-18	2.49E-20	1.04E+01	35.3267749	1.70E-01	3854593	3854716	NC_000005.9
cg05809668	1.29E-18	2.49E-20	1.04E+01	35.3263053	2.97E-01	35301188	35301311	NC_000007.13
cg24792289	1.29E-18	2.50E-20	1.04E+01	35.3205448	2.26E-01	134825895	134826018	NC_000005.9
cg06434428	1.30E-18	2.51E-20	1.04E+01	35.3184941	1.09E-01	83016630	83016753	NC_000005.9
cg00761344	1.31E-18	2.53E-20	1.04E+01	35.3095723	1.10E-01	40103693	40103816	NC_000001.10
cg03261682	1.32E-18	2.56E-20	1.04E+01	35.2998688	1.68E-01	6233757	6233880	NC_000010.10
cg12907379	1.32E-18	2.56E-20	1.04E+01	35.2993815	2.52E-01	100624130	100624253	NC_000013.10
cg14209299	1.32E-18	2.56E-20	1.04E+01	35.2964292	2.31E-01	107502793	107502916	NC_000002.11
cg13703871	1.32E-18	2.57E-20	1.04E+01	35.2946086	2.85E-01	9473674	9473797	NC_000019.9
cg26351229	1.33E-18	2.58E-20	1.04E+01	35.2921411	1.94E-01	132053780	132053903	NC_000008.10
cg13007988	1.34E-18	2.59E-20	1.04E+01	35.2862066	1.78E-01	49449136	49449259	NC_000012.11
cg12537619	1.34E-18	2.60E-20	1.04E+01	35.282457	1.15E-01	3187923	3188046	NC_000011.9
cg00660765	1.34E-18	2.61E-20	1.04E+01	35.2801504	8.52E-02	69371349	69371472	NC_000003.11

cg07356488	1.34E-18	2.61E-20	1.04E+01	35.2786304	1.51E-01	121477737	121477860	NC_000011.9
cg15186181	1.35E-18	2.61E-20	1.04E+01	35.2779678	2.72E-01	55370434	55370557	NC_000008.10
cg09137301	1.35E-18	2.61E-20	1.04E+01	35.2772627	1.54E-01	150026998	150027121	NC_000007.13
cg25423888	1.35E-18	2.62E-20	1.04E+01	35.2734115	4.80E-02	102286179	102286302	NC_000010.10
cg07883457	1.36E-18	2.63E-20	1.04E+01	35.269451	1.33E-01	58147897	58148020	NC_000006.11
cg04738965	1.36E-18	2.65E-20	1.04E+01	35.2636145	1.82E-01	147127662	147127785	NC_000003.11
cg22036165	1.37E-18	2.65E-20	1.04E+01	35.2618735	7.85E-02	101871315	101871438	NC_000008.10
cg18854045	1.37E-18	2.67E-20	1.04E+01	35.2562596	5.95E-02	11271359	11271482	NC_000002.11
cg24903893	1.38E-18	2.68E-20	1.04E+01	35.2516516	1.69E-01	158522515	158522638	NC_000005.9
cg23797411	1.42E-18	2.77E-20	1.03E+01	35.2194172	2.85E-01	106399877	106400000	NC_000010.10
cg02390606	1.43E-18	2.78E-20	1.03E+01	35.2164899	4.79E-02	42979979	42980102	NC_000017.10
cg13977835	1.44E-18	2.80E-20	1.03E+01	35.2089327	1.39E-01	23883338	23883461	NC_000001.10
cg13292542	1.44E-18	2.81E-20	1.03E+01	35.2061371	2.15E-01	80394588	80394711	NC_000017.10
cg14564076	1.44E-18	2.81E-20	1.03E+01	35.2046949	2.55E-01	153584873	153584996	NC_000007.13
cg24436462	1.45E-18	2.82E-20	1.03E+01	35.2029953	2.03E-01	183595745	183595868	NC_000001.10
cg07052063	1.46E-18	2.85E-20	1.03E+01	35.1914271	1.32E-01	99255236	99255359	NC_000010.10
cg12334871	1.47E-18	2.87E-20	1.03E+01	35.1833669	2.43E-01	149993971	149994094	NC_000005.9
cg13847853	1.48E-18	2.88E-20	1.03E+01	35.1796642	5.85E-02	39696336	39696459	NC_000017.10
cg21995919	1.48E-18	2.88E-20	1.03E+01	35.1794845	3.09E-01	182322279	182322402	NC_000002.11
cg09797577	1.49E-18	2.90E-20	1.03E+01	35.1736998	2.52E-01	28778226	28778349	NC_000006.11
cg26162582	1.50E-18	2.93E-20	1.03E+01	35.1656697	3.27E-01	4918391	4918514	NC_000012.11
cg25973534	1.50E-18	2.93E-20	1.03E+01	35.1625504	2.92E-01	5139853	5139976	NC_000005.9
cg07881405	1.51E-18	2.95E-20	1.03E+01	35.1582071	2.78E-01	3597487	3597610	NC_000005.9
cg21145524	1.51E-18	2.95E-20	1.03E+01	35.1571837	2.97E-01	239207	239330	NC_000003.11
cg24039697	1.52E-18	2.97E-20	1.03E+01	35.1505875	2.69E-01	8094534	8094657	NC_000010.10

cg21459340	1.52E-18	2.97E-20	1.03E+01	35.149285	2.64E-01	2781324	2781447	NC_000020.10
cg10507231	1.52E-18	2.97E-20	1.03E+01	35.149044	9.29E-02	204459638	204459761	NC_000001.10
cg01361263	1.52E-18	2.98E-20	1.03E+01	35.1480365	1.89E-01	58051763	58051886	NC_000003.11
cg02642565	1.52E-18	2.98E-20	1.03E+01	35.1463824	1.43E-01	4579402	4579525	NC_000016.9
cg10364040	1.53E-18	2.99E-20	1.03E+01	35.144979	2.74E-01	176981064	176981187	NC_000002.11
cg05134019	1.53E-18	2.99E-20	1.03E+01	35.1440238	2.07E-01	227059463	227059586	NC_000001.10
cg24347663	1.53E-18	2.99E-20	1.03E+01	35.1433256	2.68E-01	70995349	70995472	NC_000002.11
cg07157230	1.53E-18	3.00E-20	1.03E+01	35.1415405	1.78E-01	105363943	105364066	NC_000014.8
cg27029821	1.55E-18	3.03E-20	1.03E+01	35.1294328	1.35E-01	70983741	70983864	NC_000008.10
cg19727439	1.55E-18	3.05E-20	1.03E+01	35.1247247	2.59E-01	37500508	37500631	NC_000001.10
cg14642432	1.56E-18	3.07E-20	1.03E+01	35.1173518	2.45E-01	6533163	6533286	NC_000016.9
cg18267049	1.57E-18	3.08E-20	1.03E+01	35.1154981	2.43E-01	101875028	101875151	NC_000010.10
cg27413385	1.57E-18	3.08E-20	1.03E+01	35.1149175	1.45E-01	7515425	7515548	NC_000017.10
cg13073589	1.57E-18	3.08E-20	1.03E+01	35.1146655	1.33E-01	172517922	172518045	NC_000001.10
cg16797818	1.57E-18	3.08E-20	1.03E+01	35.1130461	1.20E-01	70463309	70463432	NC_000012.11
cg02128244	1.58E-18	3.10E-20	1.03E+01	35.1071648	2.03E-01	31848008	31848131	NC_000019.9
cg23010538	1.59E-18	3.13E-20	1.03E+01	35.0978676	3.46E-01	190447290	190447413	NC_000001.10
cg10437806	1.60E-18	3.15E-20	1.03E+01	35.0935336	2.54E-01	21680508	21680631	NC_000012.11
cg11254527	1.60E-18	3.15E-20	1.03E+01	35.0911454	1.45E-01	35873695	35873818	NC_000001.10
cg05743346	1.61E-18	3.16E-20	1.03E+01	35.0879216	3.01E-01	71800987	71801110	NC_000007.13
cg13587564	1.61E-18	3.17E-20	1.03E+01	35.0844836	1.68E-01	137003752	137003875	NC_000009.11
cg02765177	1.61E-18	3.18E-20	1.03E+01	35.0838946	1.86E-01	124605963	124606086	NC_000010.10
cg18340983	1.62E-18	3.18E-20	1.03E+01	35.0831374	6.56E-02	72941691	72941814	NC_000011.9
cg16422814	1.62E-18	3.18E-20	1.03E+01	35.0830988	2.07E-01	123691602	123691725	NC_000010.10
cg07152216	1.62E-18	3.19E-20	1.03E+01	35.0786705	2.50E-01	147141219	147141342	NC_000003.11

cg27408345	1.64E-18	3.22E-20	1.03E+01	35.0690813	1.73E-01	112630738	112630861	NC_000010.10
cg13019491	1.64E-18	3.23E-20	1.03E+01	35.0670467	2.80E-01	60977856	60977979	NC_000014.8
cg05646686	1.64E-18	3.23E-20	1.03E+01	35.0668741	2.13E-01	33402368	33402491	NC_000014.8
cg09409845	1.65E-18	3.26E-20	1.03E+01	35.0593784	1.28E-01	128657791	128657914	NC_000008.10
cg03781266	1.66E-18	3.27E-20	1.03E+01	35.0557037	2.65E-01	43153023	43153146	NC_000007.13
cg09639151	1.66E-18	3.28E-20	1.03E+01	35.0515417	2.09E-01	140810920	140811043	NC_000005.9
cg26634219	1.67E-18	3.29E-20	1.03E+01	35.0501108	2.22E-01	46608277	46608400	NC_000017.10
cg01017689	1.67E-18	3.29E-20	1.03E+01	35.0498908	8.05E-02	76376266	76376389	NC_000005.9
cg27190946	1.67E-18	3.29E-20	1.03E+01	35.0483127	2.12E-01	32639011	32639134	NC_000015.9
cg20676716	1.68E-18	3.31E-20	1.03E+01	35.0421516	2.04E-01	177053568	177053691	NC_000002.11
cg16463460	1.68E-18	3.31E-20	1.03E+01	35.0418144	1.74E-01	32454718	32454841	NC_000011.9
cg02801786	1.68E-18	3.32E-20	1.03E+01	35.0412794	2.68E-01	113914222	113914345	NC_000012.11
cg04914996	1.68E-18	3.32E-20	1.03E+01	35.0399424	1.28E-01	186915259	186915382	NC_000003.11
cg01519253	1.69E-18	3.34E-20	1.03E+01	35.0353888	2.44E-01	114886463	114886586	NC_000012.11
cg03886699	1.70E-18	3.35E-20	1.03E+01	35.0316591	9.33E-02	212698918	212699041	NC_000001.10
cg16711792	1.72E-18	3.39E-20	1.03E+01	35.0192093	2.30E-01	32930054	32930177	NC_000021.8
cg13754948	1.72E-18	3.40E-20	1.03E+01	35.0173042	1.41E-01	155679340	155679463	NC_000007.13
cg14230666	1.72E-18	3.41E-20	1.03E+01	35.0143071	2.58E-01	84322946	84323069	NC_000015.9
cg24815973	1.73E-18	3.41E-20	1.03E+01	35.0132466	1.34E-01	3140901	3141024	NC_000006.11
cg04944853	1.73E-18	3.43E-20	1.03E+01	35.0091752	1.99E-01	139252106	139252229	NC_000003.11
cg21239439	1.74E-18	3.44E-20	1.03E+01	35.0050461	1.20E-01	170038665	170038788	NC_000006.11
cg05398903	1.75E-18	3.45E-20	1.03E+01	35.001058	2.63E-01	115135045	115135168	NC_000012.11
cg18371475	1.75E-18	3.47E-20	1.03E+01	34.9977057	2.05E-01	2751550	2751673	NC_000005.9
cg24280540	1.76E-18	3.47E-20	1.03E+01	34.9952555	2.72E-01	69243510	69243633	NC_000008.10
cg03637815	1.78E-18	3.52E-20	1.03E+01	34.982665	2.51E-01	95359718	95359841	NC_000013.10

cg05042123	1.80E-18	3.56E-20	1.03E+01	34.9702351	8.91E-02	2590002	2590125	NC_000004.11
cg14055887	1.80E-18	3.57E-20	1.03E+01	34.967487	1.53E-01	136236665	136236788	NC_000009.11
cg20139706	1.83E-18	3.64E-20	1.03E+01	34.9498717	1.86E-01	237068750	237068873	NC_000002.11
cg09799983	1.84E-18	3.64E-20	1.03E+01	34.9477763	2.37E-01	38301756	38301879	NC_000002.11
cg21115346	1.85E-18	3.68E-20	1.03E+01	34.9386363	2.05E-01	130021544	130021667	NC_000007.13
cg03843000	1.86E-18	3.68E-20	1.03E+01	34.9377157	2.87E-01	114846968	114847091	NC_000012.11
cg18060030	1.86E-18	3.69E-20	1.03E+01	34.9360065	1.15E-01	168967238	168967361	NC_000003.11
cg22518433	1.87E-18	3.70E-20	1.03E+01	34.9322289	2.38E-01	49253931	49254054	NC_000019.9
cg00252282	1.87E-18	3.71E-20	1.03E+01	34.9307808	2.76E-01	5542736	5542859	NC_000012.11
cg01566592	1.87E-18	3.71E-20	1.03E+01	34.9289234	3.53E-01	104512858	104512981	NC_000008.10
cg14833621	1.87E-18	3.72E-20	1.03E+01	34.9261933	1.78E-01	47801438	47801561	NC_000012.11
cg13046832	1.88E-18	3.73E-20	1.03E+01	34.9246778	2.61E-01	29254680	29254803	NC_000014.8
cg00963169	1.88E-18	3.74E-20	1.03E+01	34.921303	2.54E-01	50513927	50514050	NC_000001.10
cg20464143	1.89E-18	3.75E-20	1.03E+01	34.9199316	2.28E-01	16728219	16728342	NC_000005.9
cg24909309	1.89E-18	3.76E-20	1.03E+01	34.9178549	1.94E-01	38387291	38387414	NC_000008.10
cg01627823	1.89E-18	3.76E-20	1.03E+01	34.9177532	2.76E-01	37125531	37125654	NC_000014.8
cg20993754	1.89E-18	3.76E-20	1.03E+01	34.9171432	1.22E-01	55226987	55227110	NC_000002.11
cg05713794	1.89E-18	3.76E-20	1.03E+01	34.9156528	5.68E-02	194119861	194119984	NC_000003.11
cg02523640	1.89E-18	3.77E-20	1.03E+01	34.9146262	2.43E-01	129534613	129534736	NC_000010.10
cg14872657	1.90E-18	3.79E-20	1.03E+01	34.9097337	2.57E-01	43153216	43153339	NC_000007.13
cg24680586	1.90E-18	3.79E-20	1.03E+01	34.9094127	3.15E-01	105036727	105036850	NC_000010.10
cg20611911	1.91E-18	3.81E-20	1.03E+01	34.9045004	2.35E-01	3567930	3568053	NC_000001.10
cg17279592	1.92E-18	3.83E-20	1.03E+01	34.8994324	1.21E-01	170038733	170038856	NC_000006.11
cg04802221	1.93E-18	3.84E-20	1.03E+01	34.895043	9.15E-02	67223955	67224078	NC_000016.9
cg02912476	1.96E-18	3.90E-20	1.03E+01	34.8800877	1.15E-01	2035087	2035210	NC_000001.10

cg09010671	1.96E-18	3.91E-20	1.03E+01	34.8768553	3.05E-01	79177763	79177886	NC_000013.10
cg13884295	1.96E-18	3.92E-20	1.03E+01	34.876414	1.68E-01	50618355	50618478	NC_000003.11
cg12189382	1.96E-18	3.92E-20	1.03E+01	34.8760644	5.76E-02	79077463	79077586	NC_000017.10
cg23287992	1.97E-18	3.92E-20	1.03E+01	34.8743678	1.29E-01	31734390	31734513	NC_000006.11
cg18120446	1.98E-18	3.94E-20	1.03E+01	34.8694611	1.87E-01	13531076	13531199	NC_000004.11
cg17278793	1.98E-18	3.95E-20	1.03E+01	34.8673353	9.40E-02	149767909	149768032	NC_000001.10
cg26750489	2.00E-18	3.99E-20	1.03E+01	34.8583879	6.33E-02	33171106	33171229	NC_000006.11
cg12536527	2.00E-18	4.00E-20	1.03E+01	34.8559542	1.88E-01	81637024	81637147	NC_000007.13
cg23205387	2.01E-18	4.02E-20	1.03E+01	34.8507932	2.04E-01	152623043	152623166	NC_000007.13
cg15478767	2.02E-18	4.03E-20	1.03E+01	34.8486799	1.23E-01	178039086	178039209	NC_000003.11
cg21220462	2.02E-18	4.03E-20	1.03E+01	34.8478209	1.74E-01	92005563	92005686	NC_000009.11
cg02792792	2.03E-18	4.06E-20	1.03E+01	34.8413435	2.29E-01	115152420	115152543	NC_000005.9
cg18819620	2.05E-18	4.10E-20	1.03E+01	34.8298745	6.28E-02	155450816	155450939	NC_000006.11
cg18609306	2.06E-18	4.11E-20	1.03E+01	34.827393	1.56E-01	81015941	81016064	NC_000017.10
cg19570924	2.06E-18	4.13E-20	1.03E+01	34.8236718	2.27E-01	3457015	3457138	NC_000010.10
cg16982087	2.07E-18	4.14E-20	1.03E+01	34.8221617	1.90E-01	924964	925087	NC_000007.13
cg16241878	2.07E-18	4.14E-20	1.03E+01	34.8216985	8.52E-02	44328648	44328771	NC_000015.9
cg15920654	2.07E-18	4.14E-20	1.03E+01	34.8216779	2.15E-01	4869091	4869214	NC_000004.11
cg18529845	2.08E-18	4.16E-20	1.03E+01	34.8162862	3.21E-01	31806042	31806165	NC_000002.11
cg24946597	2.09E-18	4.19E-20	1.03E+01	34.8100004	2.97E-01	31497464	31497587	NC_000008.10
cg07048527	2.10E-18	4.21E-20	1.03E+01	34.8055753	2.13E-01	46700449	46700572	NC_000017.10
cg25479320	2.11E-18	4.24E-20	1.03E+01	34.7985848	8.98E-02	30127152	30127275	NC_000006.11
cg07572028	2.11E-18	4.24E-20	1.03E+01	34.7966878	1.38E-01	3695198	3695321	NC_000001.10
cg01370449	2.11E-18	4.24E-20	1.03E+01	34.7966408	1.89E-01	27183369	27183492	NC_000007.13
cg25727671	2.12E-18	4.26E-20	1.03E+01	34.7927648	2.05E-01	27193351	27193474	NC_000007.13

cg11668923	2.13E-18	4.27E-20	1.03E+01	34.7910437	2.55E-01	128076936	128077059	NC_000010.10
cg10722226	2.13E-18	4.27E-20	1.03E+01	34.7907054	2.17E-01	214156203	214156326	NC_000001.10
cg19958511	2.14E-18	4.29E-20	1.03E+01	34.7856496	1.02E-01	134821825	134821948	NC_000010.10
cg08199563	2.14E-18	4.30E-20	1.03E+01	34.7829082	1.17E-01	55816212	55816335	NC_000019.9
cg00812438	2.18E-18	4.37E-20	1.03E+01	34.7667155	2.48E-01	127940086	127940209	NC_000012.11
cg14141102	2.18E-18	4.38E-20	1.03E+01	34.7663914	9.84E-02	107974557	107974680	NC_000012.11
cg13525804	2.21E-18	4.44E-20	1.03E+01	34.7511819	1.43E-01	33857698	33857821	NC_000009.11
cg26163578	2.22E-18	4.46E-20	1.03E+01	34.747609	2.25E-01	57154589	57154712	NC_000019.9
cg01614102	2.23E-18	4.48E-20	1.03E+01	34.743013	2.21E-01	115134918	115135041	NC_000012.11
cg20427331	2.23E-18	4.49E-20	1.03E+01	34.7408585	1.59E-01	52000062	52000185	NC_000013.10
cg01261206	2.25E-18	4.53E-20	1.03E+01	34.7330035	2.18E-01	1011281	1011404	NC_000011.9
cg00778995	2.25E-18	4.54E-20	1.03E+01	34.730705	2.69E-01	105470558	105470681	NC_000002.11
cg00181125	2.27E-18	4.57E-20	1.03E+01	34.7226685	2.32E-01	22095182	22095305	NC_000012.11
cg26049726	2.27E-18	4.59E-20	1.03E+01	34.7200464	2.02E-01	73518702	73518825	NC_000002.11
cg08208595	2.30E-18	4.63E-20	1.03E+01	34.7093561	9.74E-02	105793554	105793677	NC_000014.8
cg14345213	2.30E-18	4.63E-20	1.03E+01	34.7093526	1.60E-01	100176407	100176530	NC_000009.11
cg12164321	2.31E-18	4.66E-20	1.03E+01	34.7046226	2.34E-01	125651085	125651208	NC_000010.10
cg16942595	2.31E-18	4.66E-20	1.03E+01	34.7030948	1.46E-01	149375316	149375439	NC_000006.11
cg17297362	2.33E-18	4.70E-20	1.03E+01	34.6946777	1.79E-01	233641507	233641630	NC_000002.11
cg22014983	2.36E-18	4.78E-20	1.03E+01	34.6795234	7.97E-02	39637465	39637588	NC_000002.11
cg26074603	2.37E-18	4.80E-20	1.03E+01	34.6739777	2.85E-01	75603068	75603191	NC_000012.11
cg02842158	2.37E-18	4.81E-20	1.03E+01	34.6735279	1.43E-01	50943720	50943843	NC_000022.10
cg02986692	2.38E-18	4.81E-20	1.03E+01	34.6716662	5.07E-02	156030348	156030471	NC_000006.11
cg03522150	2.39E-18	4.83E-20	1.03E+01	34.6683025	2.43E-01	46969673	46969796	NC_000011.9
cg27097660	2.39E-18	4.84E-20	1.03E+01	34.6673338	1.53E-01	75241082	75241205	NC_000016.9

cg22356339	2.42E-18	4.90E-20	1.03E+01	34.6539533	2.54E-01	103352454	103352577	NC_000012.11
cg08074851	2.44E-18	4.95E-20	1.03E+01	34.644672	2.20E-01	47797590	47797713	NC_000002.11
cg03487983	2.44E-18	4.95E-20	1.03E+01	34.643949	1.37E-01	67729501	67729624	NC_000014.8
cg16987305	2.46E-18	4.98E-20	1.03E+01	34.6372038	2.94E-01	65991596	65991719	NC_000001.10
cg21649258	2.48E-18	5.03E-20	1.03E+01	34.6280057	2.72E-01	107503672	107503795	NC_000002.11
cg08567279	2.49E-18	5.05E-20	1.03E+01	34.6240209	2.70E-01	53313529	53313652	NC_000013.10
cg14354398	2.49E-18	5.06E-20	1.03E+01	34.6223814	8.10E-02	113539759	113539882	NC_000013.10
cg13628577	2.50E-18	5.07E-20	1.03E+01	34.620657	2.04E-01	122431121	122431244	NC_000005.9
cg16973406	2.50E-18	5.07E-20	1.03E+01	34.6195266	1.05E-01	43265991	43266114	NC_000022.10
cg14211735	2.51E-18	5.10E-20	1.03E+01	34.6153647	1.64E-01	78837981	78838104	NC_000015.9
cg14755690	2.51E-18	5.10E-20	1.03E+01	34.6135752	2.25E-01	128868265	128868388	NC_000007.13
cg03096785	2.52E-18	5.11E-20	1.03E+01	34.6116343	2.01E-01	15764869	15764992	NC_000001.10
cg19088962	2.52E-18	5.13E-20	1.03E+01	34.6081369	6.53E-02	54576274	54576397	NC_000012.11
cg26091021	2.53E-18	5.14E-20	1.03E+01	34.6070134	2.63E-01	177021826	177021949	NC_000002.11
cg04991805	2.55E-18	5.20E-20	1.03E+01	34.595367	2.44E-01	99790910	99791033	NC_000010.10
cg18002116	2.56E-18	5.20E-20	1.03E+01	34.5945376	2.18E-01	53419596	53419719	NC_000013.10
cg13784966	2.56E-18	5.21E-20	1.03E+01	34.5924504	1.36E-01	3158710	3158833	NC_000010.10
cg25261694	2.58E-18	5.25E-20	1.03E+01	34.5866231	1.80E-01	88559053	88559176	NC_000010.10
cg14436491	2.62E-18	5.33E-20	1.02E+01	34.5705961	1.16E-01	176893	177016	NC_000009.11
cg07926644	2.62E-18	5.34E-20	1.02E+01	34.5682162	1.45E-01	4011511	4011634	NC_000019.9
cg10784386	2.63E-18	5.37E-20	1.02E+01	34.564065	2.53E-01	79330943	79331066	NC_000005.9
cg23454797	2.63E-18	5.37E-20	1.02E+01	34.5627203	1.93E-01	27183990	27184113	NC_000007.13
cg00992417	2.63E-18	5.37E-20	1.02E+01	34.5626928	1.61E-01	917891	918014	NC_000005.9
cg16564132	2.65E-18	5.40E-20	1.02E+01	34.5576269	1.35E-01	47193871	47193994	NC_000007.13
cg01724683	2.65E-18	5.42E-20	1.02E+01	34.5546329	6.77E-02	75180961	75181084	NC_000017.10

cg12526923	2.65E-18	5.42E-20	1.02E+01	34.5541282	2.33E-01	17439208	17439331	NC_000019.9
cg12600530	2.66E-18	5.43E-20	1.02E+01	34.5523551	1.81E-01	86687001	86687124	NC_000005.9
cg11644515	2.66E-18	5.44E-20	1.02E+01	34.5506778	1.30E-01	35827824	35827947	NC_000021.8
cg13329346	2.66E-18	5.44E-20	1.02E+01	34.5505204	1.20E-01	172750611	172750734	NC_000005.9
cg06232807	2.66E-18	5.44E-20	1.02E+01	34.5496839	2.00E-01	51185132	51185255	NC_000016.9
cg07811198	2.70E-18	5.53E-20	1.02E+01	34.5348918	2.05E-01	153585368	153585491	NC_000007.13
cg16519587	2.73E-18	5.58E-20	1.02E+01	34.5259501	2.77E-01	26614649	26614772	NC_000006.11
cg02289739	2.75E-18	5.62E-20	1.02E+01	34.5177942	1.21E-01	131156554	131156677	NC_000007.13
cg06266582	2.76E-18	5.65E-20	1.02E+01	34.5134801	2.24E-01	64871778	64871901	NC_000011.9
cg10981651	2.77E-18	5.66E-20	1.02E+01	34.5103598	1.98E-01	153853545	153853668	NC_000005.9
cg09123188	2.77E-18	5.67E-20	1.02E+01	34.5091384	2.57E-01	100913934	100914057	NC_000015.9
cg24848035	2.77E-18	5.67E-20	1.02E+01	34.5087949	2.58E-01	101118525	101118648	NC_000008.10
cg06560026	2.77E-18	5.68E-20	1.02E+01	34.507678	1.24E-01	6624242	6624365	NC_000016.9
cg12768681	2.80E-18	5.73E-20	1.02E+01	34.4990673	2.85E-01	20879547	20879670	NC_000001.10
cg26795540	2.81E-18	5.76E-20	1.02E+01	34.4937118	1.12E-01	35082070	35082193	NC_000010.10
cg17517480	2.81E-18	5.76E-20	1.02E+01	34.4931903	1.62E-01	22303542	22303665	NC_000001.10
cg13732302	2.81E-18	5.77E-20	1.02E+01	34.4922304	7.94E-02	44222207	44222330	NC_000017.10
cg20779964	2.82E-18	5.78E-20	1.02E+01	34.489707	1.90E-01	54580070	54580193	NC_000020.10
cg12432010	2.83E-18	5.81E-20	1.02E+01	34.484726	1.74E-01	176081	176204	NC_000012.11
cg22381068	2.84E-18	5.83E-20	1.02E+01	34.4812784	1.87E-01	906363	906486	NC_000001.10
cg19047292	2.85E-18	5.84E-20	1.02E+01	34.4794229	3.00E-01	56228442	56228565	NC_000016.9
cg10721834	2.87E-18	5.89E-20	1.02E+01	34.4723347	1.88E-01	22990066	22990189	NC_000019.9
cg02081266	2.88E-18	5.92E-20	1.02E+01	34.4664128	2.82E-01	59529618	59529741	NC_000017.10
cg12465501	2.90E-18	5.96E-20	1.02E+01	34.4607246	1.53E-01	211518776	211518899	NC_000001.10
cg10717869	2.91E-18	5.98E-20	1.02E+01	34.4566626	1.98E-01	205780912	205781035	NC_000001.10

cg15416279	2.92E-18	6.00E-20	1.02E+01	34.4541461	2.14E-01	3606795	3606918	NC_000005.9
cg10586087	2.92E-18	6.01E-20	1.02E+01	34.4521037	7.12E-02	148376773	148376896	NC_000005.9
cg14114931	2.92E-18	6.01E-20	1.02E+01	34.4510065	1.25E-01	50943910	50944033	NC_000022.10
cg13805533	2.93E-18	6.03E-20	1.02E+01	34.4476159	1.51E-01	19674482	19674605	NC_000020.10
cg27142536	2.94E-18	6.05E-20	1.02E+01	34.4449702	2.42E-01	20618198	20618321	NC_000011.9
cg26344513	2.94E-18	6.05E-20	1.02E+01	34.4447704	1.60E-01	22930613	22930736	NC_000015.9
cg26777883	2.96E-18	6.09E-20	1.02E+01	34.4388253	2.87E-01	688417	688540	NC_000008.10
cg15448975	2.96E-18	6.10E-20	1.02E+01	34.4373603	1.66E-01	5894930	5895053	NC_000004.11
cg20707345	2.98E-18	6.14E-20	1.02E+01	34.4305719	1.07E-01	67695867	67695990	NC_000016.9
cg13449606	3.00E-18	6.18E-20	1.02E+01	34.4248386	5.73E-02	27686328	27686451	NC_000001.10
cg12299795	3.00E-18	6.19E-20	1.02E+01	34.4221897	2.29E-01	64982093	64982216	NC_000001.10
cg07929264	3.01E-18	6.20E-20	1.02E+01	34.4213779	1.47E-01	89064822	89064945	NC_000002.11
cg13748640	3.01E-18	6.20E-20	1.02E+01	34.4204962	1.34E-01	102858493	102858616	NC_000009.11
cg02253760	3.01E-18	6.21E-20	1.02E+01	34.4187894	2.98E-01	147077616	147077739	NC_000003.11
cg21286173	3.02E-18	6.23E-20	1.02E+01	34.4161664	7.32E-02	38121490	38121613	NC_000008.10
cg15577706	3.02E-18	6.23E-20	1.02E+01	34.4161137	8.76E-02	33763575	33763698	NC_000017.10
cg06749715	3.02E-18	6.23E-20	1.02E+01	34.4156858	2.61E-01	37132291	37132414	NC_000014.8
cg12933903	3.04E-18	6.28E-20	1.02E+01	34.4086889	1.50E-01	20223923	20224046	NC_000003.11
cg11171221	3.05E-18	6.29E-20	1.02E+01	34.4066327	2.30E-01	124902844	124902967	NC_000010.10
cg26330304	3.05E-18	6.30E-20	1.02E+01	34.4057217	1.92E-01	90569017	90569140	NC_000015.9
cg22603247	3.08E-18	6.35E-20	1.02E+01	34.3968302	3.26E-01	124907553	124907676	NC_000010.10
cg15260552	3.08E-18	6.36E-20	1.02E+01	34.3960322	9.66E-02	64338402	64338525	NC_000012.11
cg24773418	3.09E-18	6.39E-20	1.02E+01	34.3905446	2.83E-01	33402512	33402635	NC_000014.8
cg04784475	3.10E-18	6.41E-20	1.02E+01	34.3882382	2.45E-01	38183130	38183253	NC_000019.9
cg25051341	3.10E-18	6.41E-20	1.02E+01	34.3878368	3.09E-01	100061307	100061430	NC_000006.11

cg09507526	3.10E-18	6.41E-20	1.02E+01	34.3877413	2.80E-01	23847816	23847939	NC_000016.9
cg18331515	3.10E-18	6.41E-20	1.02E+01	34.3874002	2.93E-01	178772385	178772508	NC_000005.9
cg06055845	3.10E-18	6.42E-20	1.02E+01	34.385907	1.54E-01	40788787	40788910	NC_000019.9
cg13063344	3.12E-18	6.47E-20	1.02E+01	34.378967	2.40E-01	132906443	132906566	NC_000012.11
cg22115076	3.13E-18	6.47E-20	1.02E+01	34.3779008	2.31E-01	50673588	50673711	NC_000005.9
cg12489915	3.14E-18	6.50E-20	1.02E+01	34.373405	1.66E-01	49194795	49194918	NC_000017.10
cg25681339	3.16E-18	6.54E-20	1.02E+01	34.368409	2.14E-01	1174148	1174271	NC_000017.10
cg09359114	3.16E-18	6.55E-20	1.02E+01	34.36665	2.40E-01	96650096	96650219	NC_000007.13
cg19107469	3.17E-18	6.57E-20	1.02E+01	34.3639382	1.93E-01	55200445	55200568	NC_000020.10
cg07598407	3.17E-18	6.57E-20	1.02E+01	34.3630471	1.31E-01	145048767	145048890	NC_000008.10
cg11403907	3.19E-18	6.62E-20	1.02E+01	34.3565168	1.86E-01	98569645	98569768	NC_000001.10
cg25943821	3.19E-18	6.62E-20	1.02E+01	34.3558641	1.22E-01	32283464	32283587	NC_000003.11
cg05976714	3.20E-18	6.63E-20	1.02E+01	34.3541513	2.62E-01	180076481	180076604	NC_000005.9
cg00663972	3.21E-18	6.65E-20	1.02E+01	34.351129	2.17E-01	112721725	112721848	NC_000013.10
cg04342955	3.21E-18	6.66E-20	1.02E+01	34.3504156	3.17E-01	12995296	12995419	NC_000016.9
cg03011535	3.22E-18	6.69E-20	1.02E+01	34.3451389	2.87E-01	42544794	42544917	NC_000020.10
cg09234616	3.24E-18	6.73E-20	1.02E+01	34.3401065	2.03E-01	32452592	32452715	NC_000011.9
cg18468354	3.26E-18	6.78E-20	1.02E+01	34.3318206	2.36E-01	36993517	36993640	NC_000014.8
cg07912789	3.26E-18	6.78E-20	1.02E+01	34.3317217	2.45E-01	3600273	3600396	NC_000012.11
cg19651132	3.29E-18	6.84E-20	1.02E+01	34.323773	3.00E-01	5018805	5018928	NC_000012.11
cg00209893	3.32E-18	6.90E-20	1.02E+01	34.3145222	7.90E-02	220097009	220097132	NC_000002.11
cg08526991	3.34E-18	6.96E-20	1.02E+01	34.3059898	2.09E-01	5831394	5831517	NC_000002.11
cg01889143	3.35E-18	6.98E-20	1.02E+01	34.3039798	2.72E-01	134825791	134825914	NC_000005.9
cg00966405	3.39E-18	7.06E-20	1.02E+01	34.2924162	1.38E-01	5535748	5535871	NC_000007.13
cg15607538	3.39E-18	7.06E-20	1.02E+01	34.2915077	3.13E-01	133484853	133484976	NC_000012.11

cg02012771	3.39E-18	7.07E-20	1.02E+01	34.2911491	9.15E-02	75646783	75646906	NC_000015.9
cg16638540	3.40E-18	7.09E-20	1.02E+01	34.2879775	2.20E-01	58570468	58570591	NC_000019.9
cg26886462	3.42E-18	7.13E-20	1.02E+01	34.2823184	1.24E-01	155108449	155108572	NC_000005.9
cg08790440	3.42E-18	7.13E-20	1.02E+01	34.2817986	1.69E-01	124782831	124782954	NC_000002.11
cg14610335	3.42E-18	7.14E-20	1.02E+01	34.2813804	1.42E-01	117012651	117012774	NC_000009.11
cg09937500	3.42E-18	7.14E-20	1.02E+01	34.2805565	1.47E-01	208175271	208175394	NC_000002.11
cg01080927	3.43E-18	7.15E-20	1.02E+01	34.2795289	1.77E-01	125341187	125341310	NC_000006.11
cg00557947	3.43E-18	7.16E-20	1.02E+01	34.2780276	2.96E-01	102419245	102419368	NC_000010.10
cg02350039	3.45E-18	7.21E-20	1.02E+01	34.271247	1.64E-01	138813263	138813386	NC_000006.11
cg26737985	3.45E-18	7.21E-20	1.02E+01	34.2711632	2.05E-01	170263544	170263667	NC_000004.11
cg08182446	3.47E-18	7.24E-20	1.02E+01	34.2671558	2.65E-01	27216012	27216135	NC_000015.9
cg00346208	3.48E-18	7.27E-20	1.02E+01	34.2628235	1.73E-01	20669905	20670028	NC_000001.10
cg00808403	3.50E-18	7.32E-20	1.02E+01	34.2568089	1.02E-01	47052636	47052759	NC_000003.11
cg25712695	3.51E-18	7.34E-20	1.02E+01	34.2532951	8.77E-02	120741653	120741776	NC_000012.11
cg20649017	3.53E-18	7.39E-20	1.02E+01	34.2469435	2.46E-01	176980837	176980960	NC_000002.11
cg08355659	3.55E-18	7.42E-20	1.02E+01	34.2430536	1.48E-01	54399190	54399313	NC_000012.11
cg23690166	3.57E-18	7.46E-20	1.02E+01	34.2370503	2.31E-01	46711017	46711140	NC_000017.10
cg27038101	3.57E-18	7.47E-20	1.02E+01	34.2367274	1.53E-01	67965804	67965927	NC_000016.9
cg23938834	3.57E-18	7.48E-20	1.02E+01	34.2350741	1.28E-01	101303628	101303751	NC_000008.10
cg10406295	3.58E-18	7.49E-20	1.02E+01	34.232955	2.65E-01	41167113	41167236	NC_000008.10
cg27125965	3.60E-18	7.53E-20	1.02E+01	34.2280068	1.00E-01	11707246	11707369	NC_000019.9
cg12513880	3.60E-18	7.54E-20	1.02E+01	34.2265113	2.38E-01	115136298	115136421	NC_000012.11
cg26664161	3.60E-18	7.54E-20	1.02E+01	34.2263125	2.14E-01	135265937	135266060	NC_000005.9
cg26306435	3.60E-18	7.55E-20	1.02E+01	34.225748	1.40E-01	125805783	125805906	NC_000010.10
cg14125530	3.62E-18	7.59E-20	1.02E+01	34.2207576	1.16E-01	70329240	70329363	NC_000012.11

cg09266229	3.62E-18	7.59E-20	1.02E+01	34.2204876	1.16E-01	111697455	111697578	NC_000013.10
cg18017383	3.64E-18	7.62E-20	1.02E+01	34.2162604	1.11E-01	51379874	51379997	NC_000003.11
cg01054478	3.64E-18	7.63E-20	1.02E+01	34.2154646	2.37E-01	180100963	180101086	NC_000005.9
cg17092583	3.66E-18	7.67E-20	1.02E+01	34.2094258	7.09E-02	735575	735698	NC_000011.9
cg00004996	3.66E-18	7.69E-20	1.02E+01	34.2078586	1.17E-01	17271535	17271658	NC_000008.10
cg23714705	3.68E-18	7.72E-20	1.02E+01	34.2035862	1.48E-01	134360912	134361035	NC_000010.10
cg20978694	3.69E-18	7.75E-20	1.02E+01	34.1994349	2.75E-01	2061804	2061927	NC_000004.11
cg14352682	3.71E-18	7.78E-20	1.02E+01	34.1952709	1.35E-01	30614168	30614291	NC_000006.11
cg00463011	3.73E-18	7.83E-20	1.02E+01	34.189236	1.37E-01	30550239	30550362	NC_000016.9
cg21758446	3.74E-18	7.85E-20	1.02E+01	34.1873502	1.32E-01	71518879	71519002	NC_000012.11
cg06034255	3.77E-18	7.93E-20	1.02E+01	34.1772354	1.29E-01	125806958	125807081	NC_000010.10
cg07568194	3.78E-18	7.94E-20	1.02E+01	34.1761675	2.27E-01	77468850	77468973	NC_000016.9
cg04086977	3.78E-18	7.94E-20	1.02E+01	34.1758766	1.10E-01	134829832	134829955	NC_000010.10
cg27009556	3.79E-18	7.96E-20	1.02E+01	34.1725851	1.40E-01	81813969	81814092	NC_000016.9
cg12284836	3.80E-18	7.98E-20	1.02E+01	34.1702762	1.08E-01	36481121	36481244	NC_000007.13
cg14908122	3.80E-18	7.99E-20	1.02E+01	34.1687522	3.98E-02	120971358	120971481	NC_000012.11
cg19685567	3.82E-18	8.03E-20	1.02E+01	34.1640953	7.83E-02	155639944	155640067	NC_000007.13
cg12356890	3.82E-18	8.04E-20	1.02E+01	34.1626622	3.99E-01	11059727	11059850	NC_000010.10
cg21629500	3.83E-18	8.06E-20	1.02E+01	34.1603811	2.87E-01	2754753	2754876	NC_000005.9
cg24035107	3.84E-18	8.07E-20	1.02E+01	34.1594073	2.34E-01	2394399	2394522	NC_000017.10
cg19014214	3.84E-18	8.08E-20	1.02E+01	34.1581664	1.48E-01	122230364	122230487	NC_000011.9
cg05409693	3.87E-18	8.15E-20	1.02E+01	34.1491556	1.60E-01	78680140	78680263	NC_000012.11
cg12887317	3.88E-18	8.17E-20	1.02E+01	34.1475449	1.06E-01	42921456	42921579	NC_000005.9
cg23204113	3.90E-18	8.21E-20	1.02E+01	34.1423266	1.49E-01	91408027	91408150	NC_000010.10
cg21643731	3.90E-18	8.23E-20	1.02E+01	34.1402045	8.03E-02	133545053	133545176	NC_000007.13

cg06341513	3.91E-18	8.23E-20	1.02E+01	34.1398496	3.45E-01	61277017	61277140	NC_000011.9
cg04507915	3.93E-18	8.28E-20	1.02E+01	34.1343208	1.55E-01	32861407	32861530	NC_000003.11
cg23786625	3.93E-18	8.29E-20	1.02E+01	34.1327605	2.64E-01	106402741	106402864	NC_000010.10
cg02657836	3.93E-18	8.30E-20	1.02E+01	34.1320411	1.94E-01	155109126	155109249	NC_000005.9
cg23151051	3.93E-18	8.30E-20	1.02E+01	34.1319799	1.19E-01	75789908	75790031	NC_000002.11
cg27223047	3.96E-18	8.37E-20	1.02E+01	34.1236076	2.41E-01	127874825	127874948	NC_000005.9
cg04658707	3.98E-18	8.40E-20	1.02E+01	34.1199465	1.59E-01	47453889	47454012	NC_000021.8
cg18533546	3.99E-18	8.42E-20	1.02E+01	34.1177915	1.05E-01	1845250	1845373	NC_000008.10
cg00745725	3.99E-18	8.43E-20	1.02E+01	34.1165346	2.18E-01	27216288	27216411	NC_000015.9
cg00551679	4.00E-18	8.45E-20	1.02E+01	34.1142756	2.19E-01	86547530	86547653	NC_000016.9
cg20422099	4.00E-18	8.45E-20	1.02E+01	34.1139347	2.86E-01	178771393	178771516	NC_000005.9
cg06496344	4.03E-18	8.51E-20	1.02E+01	34.1069105	2.51E-01	23253834	23253957	NC_000019.9
cg17749384	4.04E-18	8.54E-20	1.02E+01	34.1029655	1.87E-01	28571186	28571309	NC_000010.10
cg24313571	4.05E-18	8.55E-20	1.02E+01	34.1016362	1.60E-01	823352	823475	NC_000011.9
cg01495122	4.08E-18	8.63E-20	1.02E+01	34.0925085	2.09E-01	41167087	41167210	NC_000008.10
cg08140236	4.09E-18	8.66E-20	1.02E+01	34.0897439	8.50E-02	337132	337255	NC_000016.9
cg16218477	4.10E-18	8.67E-20	1.02E+01	34.087978	1.59E-01	1066167	1066290	NC_000007.13
cg16642284	4.11E-18	8.69E-20	1.02E+01	34.0862828	3.51E-01	129535378	129535501	NC_000010.10
cg21363377	4.11E-18	8.70E-20	1.02E+01	34.0852368	1.49E-01	127651533	127651656	NC_000007.13
cg18716083	4.14E-18	8.76E-20	1.02E+01	34.0778288	1.26E-01	111506418	111506541	NC_000005.9
cg09894698	4.18E-18	8.86E-20	1.02E+01	34.066886	2.78E-01	2291347	2291470	NC_000011.9
cg12213850	4.18E-18	8.86E-20	1.02E+01	34.0666275	5.87E-02	71157272	71157395	NC_000010.10
cg14526718	4.23E-18	8.97E-20	1.02E+01	34.0546312	2.26E-01	176932840	176932963	NC_000002.11
cg23944586	4.27E-18	9.06E-20	1.02E+01	34.0449415	7.49E-02	19598794	19598917	NC_000011.9
cg18202449	4.28E-18	9.07E-20	1.02E+01	34.0431642	2.72E-01	50513654	50513777	NC_000001.10

cg14238959	4.28E-18	9.08E-20	1.02E+01	34.0425543	2.54E-01	114885270	114885393	NC_000012.11
cg10339775	4.28E-18	9.09E-20	1.02E+01	34.0416435	7.78E-02	111493027	111493150	NC_000007.13
cg23235241	4.28E-18	9.10E-20	1.02E+01	34.0406623	3.16E-01	25704505	25704628	NC_000016.9
cg15501942	4.31E-18	9.16E-20	1.02E+01	34.0342456	1.53E-01	2482442	2482565	NC_000011.9
cg13070156	4.33E-18	9.20E-20	1.02E+01	34.029351	1.00E-01	134997000	134997123	NC_000010.10
cg19998400	4.35E-18	9.25E-20	1.02E+01	34.0238432	8.79E-02	235875195	235875318	NC_000002.11
cg02344784	4.36E-18	9.27E-20	1.02E+01	34.0215205	2.04E-01	104178138	104178261	NC_000012.11
cg20604446	4.37E-18	9.29E-20	1.02E+01	34.0203718	1.47E-01	175292778	175292901	NC_000002.11
cg05576262	4.43E-18	9.42E-20	1.02E+01	34.0056832	2.49E-01	3234344	3234467	NC_000016.9
cg15921239	4.44E-18	9.45E-20	1.02E+01	34.0025331	1.59E-01	35765215	35765338	NC_000006.11
cg15393022	4.44E-18	9.45E-20	1.02E+01	34.0025101	1.38E-01	54775837	54775960	NC_000001.10
cg03574652	4.46E-18	9.49E-20	1.02E+01	33.9988894	1.20E-01	187100815	187100938	NC_000003.11
cg15484532	4.49E-18	9.57E-20	1.02E+01	33.990651	2.03E-01	4714171	4714294	NC_000001.10
cg07362258	4.51E-18	9.61E-20	1.02E+01	33.9865603	1.08E-01	41109707	41109830	NC_000013.10
cg11763094	4.53E-18	9.65E-20	1.02E+01	33.9820008	2.40E-01	84322323	84322446	NC_000015.9
cg24042452	4.58E-18	9.77E-20	1.02E+01	33.9703384	1.59E-01	156305664	156305787	NC_000001.10
cg18368845	4.62E-18	9.84E-20	1.02E+01	33.9624929	1.81E-01	41173440	41173563	NC_000013.10
cg25942450	4.65E-18	9.93E-20	1.02E+01	33.9535733	1.85E-01	170736251	170736374	NC_000005.9
cg18465962	4.66E-18	9.94E-20	1.02E+01	33.9527113	9.28E-02	100767262	100767385	NC_000007.13
cg08190858	4.68E-18	9.99E-20	1.02E+01	33.9478417	2.74E-01	54321346	54321469	NC_000012.11
cg08445263	4.69E-18	1.00E-19	1.02E+01	33.9454197	2.22E-01	27216277	27216400	NC_000015.9
cg16504626	4.70E-18	1.00E-19	1.02E+01	33.9423966	3.04E-01	57070013	57070136	NC_000008.10
cg25690589	4.74E-18	1.01E-19	1.02E+01	33.9344695	1.31E-01	60285478	60285601	NC_000015.9
cg16206659	4.80E-18	1.03E-19	1.02E+01	33.9206025	2.15E-01	189203762	189203885	NC_000004.11
cg16752876	4.82E-18	1.03E-19	1.02E+01	33.9150806	2.19E-01	19553237	19553360	NC_000002.11

cg10039444	4.82E-18	1.03E-19	1.02E+01	33.9147185	8.77E-02	56233676	56233799	NC_000014.8
cg19019537	4.83E-18	1.03E-19	1.02E+01	33.9129653	3.63E-01	495817	495940	NC_000008.10
cg08086720	4.84E-18	1.04E-19	1.02E+01	33.9117858	2.65E-01	115135003	115135126	NC_000012.11
cg21389753	4.86E-18	1.04E-19	1.01E+01	33.9059492	2.39E-01	59480674	59480797	NC_000017.10
cg24457118	4.90E-18	1.05E-19	1.01E+01	33.898183	1.81E-01	155167337	155167460	NC_000001.10
cg15736783	4.92E-18	1.05E-19	1.01E+01	33.8944939	8.45E-02	80178612	80178735	NC_000006.11
cg21210370	4.93E-18	1.06E-19	1.01E+01	33.8926423	1.15E-01	143609637	143609760	NC_000008.10
cg19951107	4.94E-18	1.06E-19	1.01E+01	33.8886776	1.37E-01	111760098	111760221	NC_000003.11
cg05936441	4.96E-18	1.06E-19	1.01E+01	33.8857795	1.81E-01	37356844	37356967	NC_000020.10
cg17352975	4.98E-18	1.07E-19	1.01E+01	33.8808939	2.56E-01	5297429	5297552	NC_000020.10
cg15563974	4.99E-18	1.07E-19	1.01E+01	33.8798982	8.23E-02	38864256	38864379	NC_000019.9
cg08809260	5.00E-18	1.07E-19	1.01E+01	33.8758595	1.95E-01	177054140	177054263	NC_000002.11
cg07136554	5.04E-18	1.08E-19	1.01E+01	33.868567	1.12E-01	52542167	52542290	NC_000014.8
cg02213822	5.07E-18	1.09E-19	1.01E+01	33.8620644	1.29E-01	128985576	128985699	NC_000009.11
cg06651311	5.09E-18	1.09E-19	1.01E+01	33.858613	2.90E-01	37246230	37246353	NC_000004.11
cg14377687	5.10E-18	1.10E-19	1.01E+01	33.8560341	1.24E-01	1220228	1220351	NC_000011.9
cg09171111	5.14E-18	1.11E-19	1.01E+01	33.8475421	1.56E-01	100506730	100506853	NC_000001.10
cg06226516	5.14E-18	1.11E-19	1.01E+01	33.8474101	1.37E-01	13072314	13072437	NC_000007.13
cg03299269	5.15E-18	1.11E-19	1.01E+01	33.8448338	1.16E-01	250003	250126	NC_000002.11
cg05132851	5.16E-18	1.11E-19	1.01E+01	33.8430005	1.35E-01	134828962	134829085	NC_000010.10
cg01408881	5.16E-18	1.11E-19	1.01E+01	33.8427405	9.81E-02	65566249	65566372	NC_000012.11
cg05573844	5.17E-18	1.11E-19	1.01E+01	33.8410269	2.12E-01	54354590	54354713	NC_000012.11
cg22192275	5.20E-18	1.12E-19	1.01E+01	33.8352129	1.48E-01	217528557	217528680	NC_000002.11
cg20517050	5.20E-18	1.12E-19	1.01E+01	33.8341729	2.42E-01	27183806	27183929	NC_000007.13
cg09537620	5.21E-18	1.12E-19	1.01E+01	33.8334245	2.29E-01	31826574	31826697	NC_000011.9

cg18318715	5.21E-18	1.12E-19	1.01E+01	33.8327545	5.23E-02	181068076	181068199	NC_000001.10
cg11323198	5.25E-18	1.13E-19	1.01E+01	33.8246364	2.32E-01	62069806	62069929	NC_000016.9
cg23349790	5.26E-18	1.13E-19	1.01E+01	33.8231092	2.92E-01	18434576	18434699	NC_000001.10
cg22758454	5.27E-18	1.13E-19	1.01E+01	33.8217238	2.45E-01	16570483	16570606	NC_000017.10
cg26168118	5.32E-18	1.15E-19	1.01E+01	33.8119724	1.60E-01	105751711	105751834	NC_000014.8
cg22173510	5.32E-18	1.15E-19	1.01E+01	33.811203	1.60E-01	753400	753523	NC_000017.10
cg12612118	5.37E-18	1.16E-19	1.01E+01	33.8012498	2.83E-01	392555	392678	NC_000006.11
cg01501009	5.45E-18	1.18E-19	1.01E+01	33.7860657	2.13E-01	57183268	57183391	NC_000019.9
cg09286367	5.47E-18	1.18E-19	1.01E+01	33.7833439	1.47E-01	5535692	5535815	NC_000007.13
cg21200408	5.47E-18	1.18E-19	1.01E+01	33.7832034	2.51E-01	21378211	21378334	NC_000020.10
cg02307288	5.50E-18	1.19E-19	1.01E+01	33.7764494	1.17E-01	135549111	135549234	NC_000005.9
cg27518272	5.52E-18	1.19E-19	1.01E+01	33.7724978	8.97E-02	63918395	63918518	NC_000003.11
cg08286181	5.54E-18	1.20E-19	1.01E+01	33.7690794	2.33E-01	115134991	115135114	NC_000012.11
cg18749629	5.57E-18	1.20E-19	1.01E+01	33.7632284	7.74E-02	144566769	144566892	NC_000008.10
cg18401406	5.57E-18	1.20E-19	1.01E+01	33.7627614	9.13E-02	37247537	37247660	NC_000013.10
cg12498326	5.60E-18	1.21E-19	1.01E+01	33.7572641	9.01E-02	130599682	130599805	NC_000012.11
cg12258042	5.61E-18	1.21E-19	1.01E+01	33.7558987	2.45E-01	4852036	4852159	NC_000008.10
cg20670075	5.68E-18	1.23E-19	1.01E+01	33.7422751	2.27E-01	113909388	113909511	NC_000012.11
cg26328510	5.69E-18	1.23E-19	1.01E+01	33.7414547	3.29E-01	11059718	11059841	NC_000010.10
cg15294361	5.70E-18	1.23E-19	1.01E+01	33.738579	4.38E-02	675222	675345	NC_000012.11
cg02203336	5.70E-18	1.23E-19	1.01E+01	33.7379907	1.54E-01	31031755	31031878	NC_000002.11
cg09237849	5.84E-18	1.26E-19	1.01E+01	33.7139391	9.03E-02	22974668	22974791	NC_000008.10
cg15724256	5.84E-18	1.27E-19	1.01E+01	33.7137608	2.19E-01	119602857	119602980	NC_000002.11
cg15251319	5.87E-18	1.27E-19	1.01E+01	33.7088922	8.06E-02	194836302	194836425	NC_000003.11
cg27205902	5.88E-18	1.27E-19	1.01E+01	33.7066417	1.52E-01	1006985	1007108	NC_000011.9

cg25574603	5.89E-18	1.28E-19	1.01E+01	33.704232	1.33E-01	13256766	13256889	NC_000012.11
cg15767218	5.91E-18	1.28E-19	1.01E+01	33.7013491	1.55E-01	69949353	69949476	NC_000011.9
cg14386867	5.93E-18	1.29E-19	1.01E+01	33.6966168	7.82E-02	140086051	140086174	NC_000009.11
cg15993401	5.94E-18	1.29E-19	1.01E+01	33.6958275	1.17E-01	31079306	31079429	NC_000006.11
cg27357571	5.94E-18	1.29E-19	1.01E+01	33.6948307	2.60E-01	34398226	34398349	NC_000021.8
cg19026124	5.95E-18	1.29E-19	1.01E+01	33.6940641	1.46E-01	6056784	6056907	NC_000020.10
cg15625675	5.98E-18	1.30E-19	1.01E+01	33.689014	1.22E-01	114957464	114957587	NC_000005.9
cg13044838	5.99E-18	1.30E-19	1.01E+01	33.6859939	6.17E-02	61594176	61594299	NC_000020.10
cg10511902	6.00E-18	1.30E-19	1.01E+01	33.6848798	1.52E-01	91842949	91843072	NC_000014.8
cg05647602	6.04E-18	1.31E-19	1.01E+01	33.6783114	1.05E-01	18258664	18258787	NC_000022.10
cg26937772	6.05E-18	1.31E-19	1.01E+01	33.6765133	1.27E-01	56704905	56705028	NC_000016.9
cg23316360	6.06E-18	1.32E-19	1.01E+01	33.6743501	2.24E-01	78492916	78493039	NC_000013.10
cg12716639	6.08E-18	1.32E-19	1.01E+01	33.6699727	1.70E-01	119234198	119234321	NC_000011.9
cg22599115	6.10E-18	1.33E-19	1.01E+01	33.6674442	1.06E-01	1223262	1223385	NC_000005.9
cg09590897	6.14E-18	1.34E-19	1.01E+01	33.6595395	1.47E-01	3961702	3961825	NC_000019.9
cg17160984	6.17E-18	1.34E-19	1.01E+01	33.655785	1.50E-01	131765778	131765901	NC_000010.10
cg16449219	6.17E-18	1.34E-19	1.01E+01	33.6544349	2.36E-01	51290169	51290292	NC_000014.8
cg09410234	6.17E-18	1.34E-19	1.01E+01	33.6542588	2.79E-01	111217715	111217838	NC_000001.10
cg16927040	6.18E-18	1.34E-19	1.01E+01	33.6537115	2.10E-01	187388128	187388251	NC_000003.11
cg22512256	6.21E-18	1.35E-19	1.01E+01	33.6479453	1.15E-01	8221132	8221255	NC_000004.11
cg17101296	6.21E-18	1.35E-19	1.01E+01	33.6478502	2.66E-01	145925708	145925831	NC_000008.10
cg26988853	6.21E-18	1.35E-19	1.01E+01	33.6478171	1.20E-01	67695978	67696101	NC_000016.9
cg14229207	6.22E-18	1.35E-19	1.01E+01	33.6460861	2.51E-01	135929747	135929870	NC_000009.11
cg00329272	6.23E-18	1.36E-19	1.01E+01	33.64459	1.13E-01	3191481	3191604	NC_000001.10
cg27284034	6.25E-18	1.36E-19	1.01E+01	33.6404223	1.36E-01	75252211	75252334	NC_000016.9

cg12351768	6.26E-18	1.36E-19	1.01E+01	33.639431	1.89E-01	113296192	113296315	NC_000013.10
cg00316508	6.26E-18	1.36E-19	1.01E+01	33.6392065	9.86E-02	146828698	146828821	NC_000004.11
cg13488201	6.27E-18	1.37E-19	1.01E+01	33.6363661	1.86E-01	128077323	128077446	NC_000010.10
cg23245711	6.31E-18	1.38E-19	1.01E+01	33.6296198	1.50E-01	48413678	48413801	NC_000003.11
cg13476204	6.35E-18	1.39E-19	1.01E+01	33.6226351	1.86E-01	75241216	75241339	NC_000016.9
cg20337445	6.37E-18	1.39E-19	1.01E+01	33.6205467	1.04E-01	35947818	35947941	NC_000022.10
cg16964348	6.39E-18	1.39E-19	1.01E+01	33.6169495	3.65E-01	24323799	24323922	NC_000007.13
cg04704085	6.44E-18	1.41E-19	1.01E+01	33.6082794	1.23E-01	47513235	47513358	NC_000022.10
cg04669574	6.46E-18	1.41E-19	1.01E+01	33.604787	2.26E-01	50297945	50298068	NC_000012.11
cg14885748	6.46E-18	1.41E-19	1.01E+01	33.6047816	2.20E-01	127211262	127211385	NC_000012.11
cg03897125	6.51E-18	1.42E-19	1.01E+01	33.596921	6.62E-02	73767359	73767482	NC_000010.10
cg25976755	6.57E-18	1.44E-19	1.01E+01	33.5861778	1.11E-01	2481495	2481618	NC_000011.9
cg22843912	6.59E-18	1.44E-19	1.01E+01	33.5832137	1.14E-01	121274655	121274778	NC_000012.11
cg08453021	6.60E-18	1.44E-19	1.01E+01	33.5826017	2.72E-01	37488575	37488698	NC_000007.13
cg10432569	6.63E-18	1.45E-19	1.01E+01	33.5769924	2.41E-01	46709858	46709981	NC_000017.10
cg17294725	6.65E-18	1.46E-19	1.01E+01	33.5741277	1.68E-01	1272559	1272682	NC_000007.13
cg09799658	6.66E-18	1.46E-19	1.01E+01	33.5718879	2.55E-01	19157304	19157427	NC_000007.13
cg00712762	6.67E-18	1.46E-19	1.01E+01	33.5702466	9.45E-02	36351470	36351593	NC_000001.10
cg25574024	6.72E-18	1.47E-19	1.01E+01	33.5624937	2.51E-01	2161894	2162017	NC_000011.9
cg10629020	6.75E-18	1.48E-19	1.01E+01	33.5585805	5.80E-02	981730	981853	NC_000017.10
cg05713044	6.80E-18	1.49E-19	1.01E+01	33.5506301	1.88E-01	16671496	16671619	NC_000005.9
cg23791824	6.80E-18	1.49E-19	1.01E+01	33.5498438	1.40E-01	56722243	56722366	NC_000020.10
cg25409794	6.80E-18	1.49E-19	1.01E+01	33.549783	3.07E-02	14020824	14020947	NC_000019.9
cg27088038	6.81E-18	1.49E-19	1.01E+01	33.5491149	1.16E-01	92959927	92960050	NC_000014.8
cg11548404	6.84E-18	1.50E-19	1.01E+01	33.5437748	1.11E-01	133076622	133076745	NC_000012.11

cg03511513	6.87E-18	1.51E-19	1.01E+01	33.5384231	1.49E-01	90472777	90472900	NC_000012.11
cg03315215	6.89E-18	1.51E-19	1.01E+01	33.5354438	2.26E-01	53422847	53422970	NC_000013.10
cg00017221	6.93E-18	1.52E-19	1.01E+01	33.5296454	2.36E-01	149719536	149719659	NC_000001.10
cg25015867	6.96E-18	1.53E-19	1.01E+01	33.5249472	2.43E-01	22929808	22929931	NC_000018.9
cg26329756	6.96E-18	1.53E-19	1.01E+01	33.5243928	7.16E-02	99250482	99250605	NC_000015.9
cg22746058	6.99E-18	1.54E-19	1.01E+01	33.5209086	2.13E-01	23481176	23481299	NC_000010.10
cg08031769	7.00E-18	1.54E-19	1.01E+01	33.5194069	1.56E-01	41042969	41043092	NC_000003.11
cg01674009	7.02E-18	1.54E-19	1.01E+01	33.5157371	1.21E-01	45655294	45655417	NC_000019.9
cg04617948	7.03E-18	1.55E-19	1.01E+01	33.5137694	2.33E-01	129535138	129535261	NC_000010.10
cg16935065	7.05E-18	1.55E-19	1.01E+01	33.511597	3.09E-01	107503233	107503356	NC_000002.11
cg02035348	7.05E-18	1.55E-19	1.01E+01	33.5109185	8.41E-02	69624697	69624820	NC_000011.9
cg12637676	7.08E-18	1.56E-19	1.01E+01	33.5063674	1.47E-01	11587161	11587284	NC_000008.10
cg25724283	7.13E-18	1.57E-19	1.01E+01	33.4993843	2.60E-01	36705475	36705598	NC_000013.10
cg17535595	7.22E-18	1.59E-19	1.01E+01	33.4859429	3.34E-01	53422808	53422931	NC_000013.10
cg00318436	7.23E-18	1.59E-19	1.01E+01	33.484212	1.62E-01	67696181	67696304	NC_000016.9
cg00100012	7.23E-18	1.59E-19	1.01E+01	33.4841972	1.97E-01	37302983	37303106	NC_000020.10
cg02245533	7.23E-18	1.60E-19	1.01E+01	33.4832912	1.04E-01	735913	736036	NC_000011.9
cg12868970	7.24E-18	1.60E-19	1.01E+01	33.4826501	1.75E-01	189569600	189569723	NC_000003.11
cg10856812	7.24E-18	1.60E-19	1.01E+01	33.4818499	1.93E-01	117523153	117523276	NC_000012.11
cg15566448	7.28E-18	1.61E-19	1.01E+01	33.4765979	9.57E-02	11808230	11808353	NC_000001.10
cg05864326	7.31E-18	1.62E-19	1.01E+01	33.4710321	2.38E-01	177030150	177030273	NC_000002.11
cg11047382	7.31E-18	1.62E-19	1.01E+01	33.4708153	3.96E-02	1812614	1812737	NC_000008.10
cg14859460	7.39E-18	1.63E-19	1.01E+01	33.4605442	2.32E-01	178422244	178422367	NC_000005.9
cg13747435	7.47E-18	1.65E-19	1.01E+01	33.449031	4.73E-02	130631782	130631905	NC_000009.11
cg18051552	7.52E-18	1.66E-19	1.01E+01	33.4418505	1.40E-01	71548732	71548855	NC_000006.11

cg16856286	7.56E-18	1.67E-19	1.01E+01	33.4365669	2.50E-01	54332992	54333115	NC_000012.11
cg24158307	7.57E-18	1.67E-19	1.01E+01	33.4356516	4.21E-02	95599667	95599790	NC_000014.8
cg22655696	7.58E-18	1.68E-19	1.01E+01	33.4345089	1.65E-01	131757191	131757314	NC_000010.10
cg18297437	7.59E-18	1.68E-19	1.01E+01	33.4325554	1.93E-01	147131332	147131455	NC_000003.11
cg17911318	7.60E-18	1.68E-19	1.01E+01	33.4311489	2.68E-01	74962000	74962123	NC_000018.9
cg10594506	7.68E-18	1.70E-19	1.01E+01	33.4197909	7.96E-02	155166889	155167012	NC_000001.10
cg09204108	7.75E-18	1.72E-19	1.01E+01	33.410063	1.49E-01	42211679	42211802	NC_000015.9
cg21851672	7.81E-18	1.73E-19	1.01E+01	33.4016473	1.80E-01	200007863	200007986	NC_000001.10
cg06748955	7.86E-18	1.75E-19	1.01E+01	33.3949614	1.07E-01	3094696	3094819	NC_000001.10
cg08540745	7.88E-18	1.75E-19	1.01E+01	33.3930557	1.75E-01	25867637	25867760	NC_000001.10
cg00014998	7.93E-18	1.76E-19	1.01E+01	33.3863138	2.13E-01	177014962	177015085	NC_000002.11
cg04249642	7.98E-18	1.77E-19	1.01E+01	33.3798317	1.12E-01	8117354	8117477	NC_000010.10
cg08684959	7.98E-18	1.77E-19	1.01E+01	33.3793754	8.66E-02	70031709	70031832	NC_000011.9
cg06121800	7.99E-18	1.77E-19	1.01E+01	33.3783707	7.20E-02	134996984	134997107	NC_000010.10
cg25400358	7.99E-18	1.77E-19	1.01E+01	33.3781413	1.37E-01	64054159	64054282	NC_000011.9
cg05658487	8.01E-18	1.78E-19	1.01E+01	33.3747956	1.91E-01	1887583	1887706	NC_000005.9
cg18664856	8.01E-18	1.78E-19	1.01E+01	33.3746229	9.33E-02	105240263	105240386	NC_000014.8
cg20505728	8.05E-18	1.79E-19	1.01E+01	33.3694756	1.23E-01	43800538	43800661	NC_000003.11
cg09717809	8.06E-18	1.79E-19	1.01E+01	33.3684674	1.99E-01	74375930	74376053	NC_000002.11
cg01790274	8.07E-18	1.79E-19	1.01E+01	33.3678395	1.23E-01	136241020	136241143	NC_000009.11
cg23508908	8.09E-18	1.80E-19	1.01E+01	33.3650634	1.15E-01	84771783	84771906	NC_000006.11
cg16890121	8.09E-18	1.80E-19	1.01E+01	33.364567	2.00E-01	28198579	28198702	NC_000013.10
cg22434409	8.10E-18	1.80E-19	1.01E+01	33.3633691	2.78E-01	21950722	21950845	NC_000004.11
cg26831119	8.11E-18	1.80E-19	1.01E+01	33.3624007	2.54E-01	111550830	111550953	NC_000004.11
cg09905605	8.13E-18	1.81E-19	1.01E+01	33.3591728	1.57E-01	31053323	31053446	NC_000016.9

cg17550175	8.23E-18	1.83E-19	1.01E+01	33.3473328	1.05E-01	95322270	95322393	NC_000010.10
cg13939859	8.27E-18	1.84E-19	1.01E+01	33.3410414	2.43E-01	91183519	91183642	NC_000001.10
cg04944090	8.30E-18	1.85E-19	1.01E+01	33.3379559	1.61E-01	85635486	85635609	NC_000016.9
cg14905600	8.32E-18	1.85E-19	1.01E+01	33.3351978	1.94E-01	134226361	134226484	NC_000010.10
cg23295683	8.32E-18	1.85E-19	1.01E+01	33.3350266	1.68E-01	81998796	81998919	NC_000014.8
cg24309428	8.32E-18	1.85E-19	1.01E+01	33.3348141	2.14E-01	89224381	89224504	NC_000011.9
cg03679565	8.41E-18	1.88E-19	1.01E+01	33.3235186	2.95E-01	1882775	1882898	NC_000005.9
cg26348803	8.41E-18	1.88E-19	1.01E+01	33.3225426	1.58E-01	202331299	202331422	NC_000001.10
cg27638288	8.44E-18	1.88E-19	1.01E+01	33.3191663	1.32E-01	12071946	12072069	NC_000011.9
cg05869438	8.46E-18	1.89E-19	1.01E+01	33.316585	9.98E-02	1112006	1112129	NC_000011.9
cg19962424	8.48E-18	1.89E-19	1.01E+01	33.3142527	2.15E-01	35110045	35110168	NC_000011.9
cg05590233	8.56E-18	1.91E-19	1.01E+01	33.3042416	1.08E-01	109545	109668	NC_000017.10
cg15890103	8.60E-18	1.92E-19	1.01E+01	33.2997716	7.54E-02	86663160	86663283	NC_000011.9
cg27398263	8.61E-18	1.92E-19	1.01E+01	33.2985412	3.17E-01	79177700	79177823	NC_000013.10
cg21787249	8.63E-18	1.93E-19	1.01E+01	33.2957283	1.58E-01	39230144	39230267	NC_000003.11
cg14024893	8.65E-18	1.93E-19	1.01E+01	33.2927871	1.39E-01	139943146	139943269	NC_000009.11
cg05815404	8.74E-18	1.95E-19	1.01E+01	33.2825207	1.89E-01	78825501	78825624	NC_000017.10
cg18113295	8.75E-18	1.96E-19	1.01E+01	33.2813472	1.51E-01	232135730	232135853	NC_000002.11
cg06758591	8.75E-18	1.96E-19	1.01E+01	33.2807092	1.34E-01	177140203	177140326	NC_000001.10
cg14391419	8.76E-18	1.96E-19	1.01E+01	33.2798081	2.48E-01	19158647	19158770	NC_000007.13
cg14168530	8.77E-18	1.96E-19	1.01E+01	33.2789399	3.23E-01	45155991	45156114	NC_000002.11
cg23204707	8.80E-18	1.97E-19	1.01E+01	33.2754761	8.00E-02	234726683	234726806	NC_000002.11
cg20792062	8.90E-18	1.99E-19	1.01E+01	33.2632955	1.87E-01	5153288	5153411	NC_000012.11
cg12766736	8.90E-18	1.99E-19	1.01E+01	33.2628921	1.37E-01	32460656	32460779	NC_000011.9
cg14394550	8.93E-18	2.00E-19	1.01E+01	33.2602035	2.59E-01	22548400	22548523	NC_000008.10

cg02316283	8.94E-18	2.00E-19	1.01E+01	33.2584449	5.07E-02	23313866	23313989	NC_000014.8
cg00388812	8.95E-18	2.01E-19	1.01E+01	33.2569646	1.63E-01	99961376	99961499	NC_000008.10
cg09888049	8.96E-18	2.01E-19	1.01E+01	33.2564513	1.68E-01	114848083	114848206	NC_000013.10
cg02892577	8.96E-18	2.01E-19	1.01E+01	33.2553917	1.95E-01	121181759	121181882	NC_000010.10
cg09878914	8.98E-18	2.01E-19	1.01E+01	33.2528638	1.25E-01	31507316	31507439	NC_000006.11
cg15318546	9.01E-18	2.02E-19	1.01E+01	33.2501899	2.16E-01	133069091	133069214	NC_000012.11
cg22117637	9.01E-18	2.02E-19	1.01E+01	33.2493852	1.03E-01	223285495	223285618	NC_000002.11
cg10595406	9.05E-18	2.03E-19	1.00E+01	33.2453965	1.44E-01	2586303	2586426	NC_000017.10
cg22344581	9.06E-18	2.03E-19	1.00E+01	33.2438468	7.97E-02	30650712	30650835	NC_000017.10
cg25915838	9.06E-18	2.03E-19	1.00E+01	33.2434513	2.53E-01	100999077	100999200	NC_000011.9
cg06952671	9.06E-18	2.03E-19	1.00E+01	33.2431401	3.28E-01	182322268	182322391	NC_000002.11
cg02039267	9.08E-18	2.04E-19	1.00E+01	33.241361	1.88E-01	118894069	118894192	NC_000010.10
cg06427772	9.12E-18	2.05E-19	1.00E+01	33.2371671	7.40E-02	134230646	134230769	NC_000010.10
cg25631414	9.17E-18	2.06E-19	1.00E+01	33.2312408	8.61E-02	169641941	169642064	NC_000006.11
cg12396325	9.17E-18	2.06E-19	1.00E+01	33.2310775	2.28E-01	105235606	105235729	NC_000008.10
cg06200670	9.18E-18	2.06E-19	1.00E+01	33.2301981	1.37E-01	226730581	226730704	NC_000001.10
cg08845329	9.19E-18	2.06E-19	1.00E+01	33.2281446	9.50E-02	80932127	80932250	NC_000017.10
cg26468007	9.28E-18	2.09E-19	1.00E+01	33.2179795	2.95E-01	4998714	4998837	NC_000007.13
cg00682096	9.32E-18	2.10E-19	1.00E+01	33.2133903	2.03E-01	46672924	46673047	NC_000017.10
cg00747187	9.33E-18	2.10E-19	1.00E+01	33.2129096	1.29E-01	97800068	97800191	NC_000007.13
cg09746044	9.36E-18	2.10E-19	1.00E+01	33.2093291	1.13E-01	44221832	44221955	NC_000015.9
cg15954353	9.52E-18	2.14E-19	1.00E+01	33.1913241	1.99E-01	5403337	5403460	NC_000017.10
cg11033014	9.52E-18	2.14E-19	1.00E+01	33.1909323	9.67E-02	46805673	46805796	NC_000022.10
cg10381113	9.52E-18	2.14E-19	1.00E+01	33.1908256	1.75E-01	24797136	24797259	NC_000013.10
cg20728791	9.57E-18	2.16E-19	1.00E+01	33.1854785	1.74E-01	112675455	112675578	NC_000010.10

cg21874706	9.62E-18	2.17E-19	1.00E+01	33.1798359	1.04E-01	39744055	39744178	NC_000017.10
cg06213876	9.79E-18	2.21E-19	1.00E+01	33.1617852	1.55E-01	16357187	16357310	NC_000016.9
cg14654886	9.81E-18	2.21E-19	1.00E+01	33.1597363	2.75E-01	5710319	5710442	NC_000004.11
cg13586987	9.85E-18	2.22E-19	1.00E+01	33.1549463	5.00E-02	16282230	16282353	NC_000006.11
cg00123055	9.90E-18	2.24E-19	1.00E+01	33.1493064	1.76E-01	55370951	55371074	NC_000008.10
cg13641903	9.90E-18	2.24E-19	1.00E+01	33.1492812	1.18E-01	32452608	32452731	NC_000011.9
cg07072722	9.91E-18	2.24E-19	1.00E+01	33.1486954	1.90E-01	131781246	131781369	NC_000011.9
cg05317455	9.92E-18	2.24E-19	1.00E+01	33.1468724	6.52E-02	69811162	69811285	NC_000011.9
cg01624637	9.92E-18	2.24E-19	1.00E+01	33.1466975	1.51E-01	87739377	87739500	NC_000016.9
cg15921518	9.93E-18	2.24E-19	1.00E+01	33.146278	1.15E-01	180664022	180664145	NC_000005.9
cg01436998	9.94E-18	2.25E-19	1.00E+01	33.1450541	2.49E-01	38308263	38308386	NC_000019.9
cg01762091	9.95E-18	2.25E-19	1.00E+01	33.1441762	2.04E-01	7197775	7197898	NC_000017.10
cg22679474	1.00E-17	2.27E-19	1.00E+01	33.1353862	6.37E-02	63929152	63929275	NC_000003.11
cg01487892	1.00E-17	2.27E-19	1.00E+01	33.1338807	9.74E-02	46895183	46895306	NC_000021.8
cg26430027	1.01E-17	2.28E-19	1.00E+01	33.1304228	1.55E-01	137393288	137393411	NC_000009.11
cg13963807	1.01E-17	2.28E-19	1.00E+01	33.1284622	7.14E-02	67165707	67165830	NC_000011.9
cg27052183	1.01E-17	2.29E-19	1.00E+01	33.1246448	1.18E-01	66411986	66412109	NC_000011.9
cg19594626	1.02E-17	2.30E-19	1.00E+01	33.122339	1.35E-01	133077819	133077942	NC_000006.11
cg14505585	1.03E-17	2.32E-19	1.00E+01	33.1110548	2.34E-01	17082770	17082893	NC_000022.10
cg04031454	1.03E-17	2.33E-19	1.00E+01	33.1070051	7.89E-02	71028968	71029091	NC_000010.10
cg05262829	1.03E-17	2.34E-19	1.00E+01	33.1052433	2.13E-01	226921576	226921699	NC_000001.10
cg10734448	1.04E-17	2.35E-19	1.00E+01	33.0999864	1.23E-01	32052373	32052496	NC_000006.11
cg00765233	1.04E-17	2.35E-19	1.00E+01	33.0981392	1.48E-01	235877260	235877383	NC_000002.11
cg25157472	1.05E-17	2.38E-19	1.00E+01	33.0876206	1.53E-01	12996362	12996485	NC_000003.11
cg11167100	1.05E-17	2.38E-19	1.00E+01	33.0866452	3.33E-01	83776269	83776392	NC_000015.9

cg18739887	1.05E-17	2.38E-19	1.00E+01	33.0865129	1.76E-01	109675284	109675407	NC_000010.10
cg14062468	1.06E-17	2.40E-19	1.00E+01	33.0796242	2.46E-01	33453893	33454016	NC_000022.10
cg22041073	1.06E-17	2.41E-19	1.00E+01	33.0757568	9.95E-02	29604114	29604237	NC_000006.11
cg10333808	1.07E-17	2.42E-19	1.00E+01	33.0693739	2.47E-01	22487459	22487582	NC_000012.11
cg18236477	1.07E-17	2.42E-19	1.00E+01	33.06893	2.02E-01	26043066	26043189	NC_000013.10
cg05337753	1.07E-17	2.44E-19	1.00E+01	33.0640674	1.93E-01	75373407	75373530	NC_000017.10
cg19058765	1.08E-17	2.45E-19	1.00E+01	33.0585563	1.43E-01	64226103	64226226	NC_000017.10
cg24748769	1.08E-17	2.46E-19	1.00E+01	33.0536575	2.22E-01	57271015	57271138	NC_000014.8
cg08417728	1.09E-17	2.47E-19	1.00E+01	33.0524438	2.73E-01	17850647	17850770	NC_000022.10
cg19153228	1.09E-17	2.47E-19	1.00E+01	33.0522895	1.66E-01	173902435	173902558	NC_000002.11
cg04609801	1.09E-17	2.47E-19	1.00E+01	33.0505573	1.68E-01	28609176	28609299	NC_000016.9
cg03950807	1.09E-17	2.48E-19	1.00E+01	33.0447675	1.01E-01	81082931	81083054	NC_000015.9
cg20945566	1.10E-17	2.49E-19	1.00E+01	33.0422206	2.70E-01	46800020	46800143	NC_000017.10
cg08239858	1.10E-17	2.51E-19	1.00E+01	33.0347157	1.89E-01	2739131	2739254	NC_000005.9
cg26588943	1.10E-17	2.51E-19	1.00E+01	33.0344647	1.94E-01	172734718	172734841	NC_000004.11
cg19866325	1.11E-17	2.52E-19	1.00E+01	33.0297082	1.79E-01	47078752	47078875	NC_000001.10
cg01975714	1.11E-17	2.52E-19	1.00E+01	33.0289501	1.07E-01	18981736	18981859	NC_000022.10
cg02773698	1.11E-17	2.53E-19	1.00E+01	33.0268065	1.45E-01	72682460	72682583	NC_000011.9
cg17174566	1.11E-17	2.54E-19	1.00E+01	33.02463	1.12E-01	148635022	148635145	NC_000002.11
cg13209481	1.11E-17	2.54E-19	1.00E+01	33.0241395	1.92E-01	36973483	36973606	NC_000014.8
cg16190732	1.11E-17	2.54E-19	1.00E+01	33.0236557	3.22E-01	34113010	34113133	NC_000019.9
cg01727145	1.12E-17	2.54E-19	1.00E+01	33.0218357	1.63E-01	220313422	220313545	NC_000002.11
cg05241461	1.13E-17	2.57E-19	1.00E+01	33.0124458	2.57E-01	22816980	22817103	NC_000019.9
cg25211875	1.13E-17	2.57E-19	1.00E+01	33.0095514	8.39E-02	55944483	55944606	NC_000015.9
cg03703637	1.13E-17	2.58E-19	1.00E+01	33.0087119	3.09E-01	178771776	178771899	NC_000005.9

cg05499134	1.14E-17	2.59E-19	1.00E+01	33.0031354	5.36E-02	88294692	88294815	NC_000016.9
cg19098710	1.14E-17	2.61E-19	1.00E+01	32.9956717	2.49E-01	113541698	113541821	NC_000012.11
cg13606990	1.15E-17	2.62E-19	1.00E+01	32.9927967	1.65E-01	32848016	32848139	NC_000018.9
cg24154951	1.15E-17	2.62E-19	1.00E+01	32.9920732	1.92E-01	177001426	177001549	NC_000002.11
cg07703530	1.15E-17	2.62E-19	1.00E+01	32.9909099	1.74E-01	81422474	81422597	NC_000008.10
cg20902425	1.15E-17	2.63E-19	1.00E+01	32.9874856	6.23E-02	46805678	46805801	NC_000022.10
cg04682699	1.16E-17	2.65E-19	1.00E+01	32.9815095	1.96E-01	50248845	50248968	NC_000003.11
cg17607298	1.16E-17	2.66E-19	1.00E+01	32.9769758	2.00E-01	112727981	112728104	NC_000013.10
cg02919422	1.18E-17	2.69E-19	1.00E+01	32.9671134	1.74E-01	55370544	55370667	NC_000008.10
cg10422777	1.18E-17	2.69E-19	1.00E+01	32.9667026	2.31E-01	79631227	79631350	NC_000009.11
cg12775934	1.18E-17	2.69E-19	1.00E+01	32.9648183	3.67E-02	62603273	62603396	NC_000011.9
cg23551917	1.19E-17	2.72E-19	1.00E+01	32.9547162	1.26E-01	45077070	45077193	NC_000003.11
cg11067712	1.20E-17	2.75E-19	1.00E+01	32.9452287	1.22E-01	105850644	105850767	NC_000011.9
cg12175729	1.20E-17	2.75E-19	1.00E+01	32.9451128	2.70E-01	140715540	140715663	NC_000008.10
cg13601435	1.20E-17	2.76E-19	1.00E+01	32.9415561	3.12E-01	120507431	120507554	NC_000009.11
cg23486580	1.21E-17	2.77E-19	1.00E+01	32.9360275	1.10E-01	105688375	105688498	NC_000014.8
cg06494105	1.21E-17	2.78E-19	1.00E+01	32.9350202	9.39E-02	3961553	3961676	NC_000019.9
cg21251230	1.22E-17	2.78E-19	1.00E+01	32.9319218	2.00E-01	129566665	129566788	NC_000009.11
cg09578028	1.23E-17	2.81E-19	1.00E+01	32.9225995	2.33E-01	176987411	176987534	NC_000002.11
cg07312654	1.23E-17	2.81E-19	1.00E+01	32.9224873	2.10E-01	132053773	132053896	NC_000008.10
cg13375463	1.23E-17	2.82E-19	1.00E+01	32.9193899	1.45E-01	27899025	27899148	NC_000001.10
cg19915413	1.23E-17	2.83E-19	1.00E+01	32.9156803	7.86E-02	79977640	79977763	NC_000017.10
cg02327997	1.24E-17	2.85E-19	1.00E+01	32.9091802	1.71E-01	128077318	128077441	NC_000010.10
cg09830022	1.24E-17	2.85E-19	1.00E+01	32.9086406	4.19E-02	104862488	104862611	NC_000010.10
cg03284640	1.24E-17	2.85E-19	1.00E+01	32.9081176	1.23E-01	125804060	125804183	NC_000010.10

cg17078116	1.24E-17	2.85E-19	1.00E+01	32.9074818	1.89E-01	24772344	24772467	NC_000008.10
cg01542143	1.25E-17	2.87E-19	1.00E+01	32.9029112	1.65E-01	3240754	3240877	NC_000004.11
cg24512644	1.25E-17	2.87E-19	1.00E+01	32.9012509	1.76E-01	41248902	41249025	NC_000022.10
cg10703826	1.25E-17	2.88E-19	1.00E+01	32.8992906	2.39E-01	119532116	119532239	NC_000001.10
cg26984343	1.26E-17	2.89E-19	1.00E+01	32.8960171	1.98E-01	37006133	37006256	NC_000013.10
cg15260428	1.26E-17	2.89E-19	1.00E+01	32.894025	2.13E-01	132381902	132382025	NC_000012.11
cg23699196	1.27E-17	2.91E-19	1.00E+01	32.8871294	2.41E-01	131780334	131780457	NC_000011.9
cg23559689	1.27E-17	2.92E-19	1.00E+01	32.884886	3.04E-01	105481292	105481415	NC_000011.9
cg16476975	1.28E-17	2.95E-19	9.99	32.8740927	2.95E-01	155164995	155165118	NC_000007.13
cg09562938	1.29E-17	2.97E-19	9.99	32.8685017	9.17E-02	62307787	62307910	NC_000017.10
cg14369938	1.30E-17	2.99E-19	9.99	32.8624717	2.80E-01	32338150	32338273	NC_000007.13
cg11289296	1.30E-17	2.99E-19	9.99	32.8606236	8.96E-02	74383131	74383254	NC_000017.10
cg24880701	1.30E-17	3.00E-19	9.99	32.8593393	1.62E-01	68121563	68121686	NC_000015.9
cg03401357	1.30E-17	3.00E-19	9.99	32.8577778	2.95E-01	46951477	46951600	NC_000001.10
cg00320094	1.31E-17	3.02E-19	9.99	32.8511663	2.12E-01	68695183	68695306	NC_000001.10
cg12238343	1.32E-17	3.05E-19	9.99	32.8425171	2.68E-01	33936402	33936525	NC_000005.9
cg13707645	1.32E-17	3.05E-19	9.99	32.8420808	1.12E-01	131682162	131682285	NC_000009.11
cg04913118	1.33E-17	3.06E-19	9.99	32.838545	1.46E-01	32826171	32826294	NC_000006.11
cg00400832	1.33E-17	3.07E-19	9.99	32.8342458	2.17E-01	96650323	96650446	NC_000007.13
cg15790820	1.34E-17	3.08E-19	9.99	32.8303174	2.70E-01	2292510	2292633	NC_000011.9
cg12724826	1.34E-17	3.09E-19	9.99	32.8277388	1.05E-01	31630051	31630174	NC_000006.11
cg20334627	1.34E-17	3.09E-19	9.99	32.8275034	2.99E-01	130686697	130686820	NC_000006.11
cg13070738	1.35E-17	3.10E-19	9.99	32.8245378	1.39E-01	144054288	144054411	NC_000007.13
cg07540084	1.35E-17	3.11E-19	9.99	32.8206183	2.08E-01	2537210	2537333	NC_000005.9
cg08470764	1.36E-17	3.13E-19	9.99	32.8154256	1.66E-01	100609792	100609915	NC_000007.13

cg19414741	1.36E-17	3.14E-19	9.98	32.8128246	2.06E-01	57358240	57358363	NC_000008.10
cg07758738	1.37E-17	3.17E-19	9.98	32.8034866	1.72E-01	12911528	12911651	NC_000018.9
cg08318726	1.37E-17	3.17E-19	9.98	32.8027896	2.25E-01	114844265	114844388	NC_000012.11
cg18764577	1.37E-17	3.17E-19	9.98	32.8021058	2.30E-01	7117790	7117913	NC_000018.9
cg10987503	1.38E-17	3.20E-19	9.98	32.7953209	1.15E-01	922825	922948	NC_000007.13
cg26381364	1.39E-17	3.21E-19	9.98	32.7921162	2.66E-01	103889772	103889895	NC_000012.11
cg18705773	1.40E-17	3.23E-19	9.98	32.7855648	2.79E-01	170743564	170743687	NC_000005.9
cg09492451	1.40E-17	3.23E-19	9.98	32.784311	2.56E-01	54967389	54967512	NC_000016.9
cg15001431	1.40E-17	3.24E-19	9.98	32.7818013	1.70E-01	108538614	108538737	NC_000011.9
cg05729352	1.40E-17	3.25E-19	9.98	32.7800689	1.19E-01	109524367	109524490	NC_000002.11
cg27338807	1.41E-17	3.26E-19	9.98	32.7743652	1.48E-01	87739385	87739508	NC_000016.9
cg09534159	1.42E-17	3.29E-19	9.98	32.7669481	2.87E-01	10458918	10459041	NC_000004.11
cg08737543	1.44E-17	3.33E-19	9.98	32.7539155	1.21E-01	10118766	10118889	NC_000001.10
cg22633932	1.44E-17	3.35E-19	9.97	32.7491067	1.23E-01	140472802	140472925	NC_000004.11
cg01146232	1.44E-17	3.35E-19	9.97	32.7490499	2.53E-01	51185147	51185270	NC_000016.9
cg14007067	1.45E-17	3.37E-19	9.97	32.7430756	2.43E-01	76740258	76740381	NC_000018.9
cg11337945	1.47E-17	3.41E-19	9.97	32.7299039	2.72E-01	2755158	2755281	NC_000005.9
cg20303815	1.48E-17	3.44E-19	9.97	32.7216978	1.16E-01	69706981	69707104	NC_000011.9
cg23263937	1.49E-17	3.47E-19	9.97	32.7141261	2.73E-01	27192656	27192779	NC_000007.13
cg26371320	1.50E-17	3.47E-19	9.97	32.7125225	2.35E-01	28778091	28778214	NC_000006.11
cg21078322	1.50E-17	3.48E-19	9.97	32.7121887	2.14E-01	134491163	134491286	NC_000006.11
cg11293190	1.50E-17	3.48E-19	9.97	32.709819	1.50E-01	1523856	1523979	NC_000019.9
cg11375458	1.50E-17	3.49E-19	9.97	32.7083168	1.88E-01	177003996	177004119	NC_000002.11
cg26992415	1.50E-17	3.49E-19	9.97	32.7068483	1.49E-01	94533087	94533210	NC_000011.9
cg18319533	1.51E-17	3.50E-19	9.97	32.7046878	2.28E-01	1011315	1011438	NC_000011.9

cg10297491	1.51E-17	3.52E-19	9.97	32.6996366	3.27E-01	11059677	11059800	NC_000010.10
cg23935522	1.52E-17	3.53E-19	9.97	32.696793	1.16E-01	77162317	77162440	NC_000012.11
cg18800739	1.53E-17	3.55E-19	9.97	32.6897365	1.13E-01	144626962	144627085	NC_000006.11
cg20498685	1.53E-17	3.57E-19	9.97	32.6860271	2.68E-01	19156086	19156209	NC_000007.13
cg06495961	1.53E-17	3.57E-19	9.97	32.6845821	2.39E-01	153584582	153584705	NC_000007.13
cg02167020	1.55E-17	3.60E-19	9.96	32.6759021	2.12E-01	102899949	102900072	NC_000010.10
cg03764518	1.55E-17	3.61E-19	9.96	32.6751608	2.15E-01	57388402	57388525	NC_000010.10
cg08676249	1.56E-17	3.63E-19	9.96	32.6693844	2.39E-01	69064868	69064991	NC_000007.13
cg11562901	1.56E-17	3.63E-19	9.96	32.668892	1.66E-01	56318135	56318258	NC_000012.11
cg11868921	1.56E-17	3.65E-19	9.96	32.6640326	1.38E-01	154812487	154812610	NC_000006.11
cg06313718	1.56E-17	3.65E-19	9.96	32.6636408	1.06E-01	49714199	49714322	NC_000003.11
cg23294090	1.57E-17	3.65E-19	9.96	32.6622448	2.46E-01	139509672	139509795	NC_000008.10
cg10516089	1.57E-17	3.67E-19	9.96	32.6588245	5.75E-02	50800620	50800743	NC_000007.13
cg26624744	1.57E-17	3.67E-19	9.96	32.6581238	1.73E-01	156692507	156692630	NC_000005.9
cg13078140	1.58E-17	3.69E-19	9.96	32.6518261	2.85E-01	20255082	20255205	NC_000004.11
cg18675128	1.59E-17	3.72E-19	9.96	32.6457689	1.04E-01	71177172	71177295	NC_000010.10
cg21694941	1.60E-17	3.74E-19	9.96	32.6402865	1.64E-01	57358590	57358713	NC_000008.10
cg00235367	1.62E-17	3.78E-19	9.96	32.6282183	2.09E-01	147110322	147110445	NC_000003.11
cg04620418	1.63E-17	3.80E-19	9.96	32.6233245	1.93E-01	65282285	65282408	NC_000008.10
cg04248279	1.64E-17	3.82E-19	9.96	32.6174709	1.72E-01	184833	184956	NC_000017.10
cg00046625	1.64E-17	3.83E-19	9.95	32.6165778	2.47E-01	66258760	66258883	NC_000001.10
cg00733001	1.64E-17	3.83E-19	9.95	32.6154624	1.25E-01	679432	679555	NC_000008.10
cg17499173	1.64E-17	3.85E-19	9.95	32.6119222	8.06E-02	736285	736408	NC_000019.9
cg27639662	1.65E-17	3.85E-19	9.95	32.6108198	1.68E-01	81111393	81111516	NC_000004.11
cg27313012	1.65E-17	3.87E-19	9.95	32.6066482	1.13E-01	233497542	233497665	NC_000001.10

cg21012296	1.67E-17	3.91E-19	9.95	32.5963607	2.62E-01	21502728	21502851	NC_000020.10
cg02520593	1.67E-17	3.92E-19	9.95	32.5923569	1.49E-01	109015999	109016122	NC_000012.11
cg02511231	1.68E-17	3.93E-19	9.95	32.5899693	3.52E-01	170738274	170738397	NC_000005.9
cg17016394	1.68E-17	3.94E-19	9.95	32.5886154	3.41E-01	34442360	34442483	NC_000021.8
cg10601582	1.69E-17	3.95E-19	9.95	32.5857224	2.61E-01	22605234	22605357	NC_000019.9
cg26530319	1.69E-17	3.95E-19	9.95	32.5844965	1.50E-01	221060923	221061046	NC_000001.10
cg02164046	1.69E-17	3.96E-19	9.95	32.5823648	2.21E-01	187388148	187388271	NC_000003.11
cg11559967	1.69E-17	3.97E-19	9.95	32.5797527	5.54E-02	38363872	38363995	NC_000022.10
cg06766541	1.70E-17	3.98E-19	9.95	32.5785445	1.11E-01	128556540	128556663	NC_000007.13
cg01560873	1.71E-17	4.00E-19	9.95	32.5725113	2.35E-01	3116792	3116915	NC_000005.9
cg14042889	1.71E-17	4.01E-19	9.95	32.5691724	1.93E-01	27169208	27169331	NC_000007.13
cg04370314	1.71E-17	4.02E-19	9.95	32.5689878	2.45E-01	100643312	100643435	NC_000013.10
cg22519702	1.71E-17	4.02E-19	9.95	32.5679171	1.39E-01	147204189	147204312	NC_000005.9
cg02668075	1.71E-17	4.02E-19	9.95	32.5671801	8.54E-02	109558595	109558718	NC_000002.11
cg23302682	1.72E-17	4.04E-19	9.95	32.564015	2.90E-01	166580952	166581075	NC_000006.11
cg06908618	1.72E-17	4.04E-19	9.95	32.5634723	2.09E-01	87739453	87739576	NC_000016.9
cg15597540	1.72E-17	4.04E-19	9.95	32.5620217	7.35E-02	50387202	50387325	NC_000003.11
cg18307838	1.72E-17	4.04E-19	9.95	32.5617454	1.14E-01	228380005	228380128	NC_000002.11
cg00286701	1.72E-17	4.05E-19	9.95	32.5610338	9.81E-02	2583402	2583525	NC_000007.13
cg14532376	1.73E-17	4.06E-19	9.95	32.5582833	1.45E-01	133799686	133799809	NC_000009.11
cg10099056	1.73E-17	4.07E-19	9.95	32.5557284	1.04E-01	227131420	227131543	NC_000001.10
cg14249876	1.74E-17	4.09E-19	9.95	32.5511468	3.65E-01	44143993	44144116	NC_000007.13
cg07737566	1.75E-17	4.11E-19	9.94	32.5464038	1.80E-01	50847263	50847386	NC_000007.13
cg17553300	1.75E-17	4.11E-19	9.94	32.5452414	1.83E-01	55508460	55508583	NC_000003.11
cg13803632	1.75E-17	4.12E-19	9.94	32.5437969	6.20E-02	81968855	81968978	NC_000001.10

cg15344640	1.76E-17	4.14E-19	9.94	32.5385778	9.82E-02	176774729	176774852	NC_000005.9
cg22043936	1.76E-17	4.15E-19	9.94	32.5368168	9.70E-02	28504564	28504687	NC_000001.10
cg09968620	1.78E-17	4.18E-19	9.94	32.527987	2.55E-01	176987383	176987506	NC_000002.11
cg09287629	1.78E-17	4.19E-19	9.94	32.5273825	1.55E-01	203036208	203036331	NC_000002.11
cg10329579	1.78E-17	4.19E-19	9.94	32.5265596	2.32E-01	17754830	17754953	NC_000001.10
cg16311306	1.79E-17	4.21E-19	9.94	32.5228456	1.01E-01	203244158	203244281	NC_000001.10
cg09815977	1.79E-17	4.22E-19	9.94	32.519838	1.32E-01	7021987	7022110	NC_000012.11
cg18406033	1.80E-17	4.24E-19	9.94	32.5152851	2.38E-01	223159869	223159992	NC_000002.11
cg07297322	1.80E-17	4.24E-19	9.94	32.5143627	1.59E-01	74661825	74661948	NC_000016.9
cg27058486	1.82E-17	4.28E-19	9.94	32.5059846	2.71E-01	34112683	34112806	NC_000019.9
cg07788092	1.82E-17	4.30E-19	9.94	32.5017409	2.14E-01	9473691	9473814	NC_000019.9
cg12271981	1.83E-17	4.31E-19	9.94	32.4998507	2.28E-01	88326752	88326875	NC_000013.10
cg04548856	1.83E-17	4.31E-19	9.94	32.4994929	2.39E-01	57284528	57284651	NC_000014.8
cg03656099	1.83E-17	4.31E-19	9.94	32.4988406	1.63E-01	59481954	59482077	NC_000017.10
cg01342411	1.83E-17	4.33E-19	9.94	32.4953322	2.19E-01	75263551	75263674	NC_000016.9
cg14627862	1.84E-17	4.34E-19	9.94	32.4926426	1.44E-01	169641232	169641355	NC_000006.11
cg15848561	1.84E-17	4.35E-19	9.94	32.4896507	1.27E-01	101924186	101924309	NC_000014.8
cg18437365	1.85E-17	4.37E-19	9.94	32.4861911	2.14E-01	112717484	112717607	NC_000013.10
cg01748268	1.85E-17	4.37E-19	9.94	32.4849752	8.90E-02	30122143	30122266	NC_000006.11
cg03579904	1.86E-17	4.38E-19	9.93	32.4828395	1.92E-01	87739430	87739553	NC_000016.9
cg14802771	1.86E-17	4.39E-19	9.93	32.4816038	8.60E-02	232903780	232903903	NC_000002.11
cg02036494	1.87E-17	4.41E-19	9.93	32.4753311	1.40E-01	130467986	130468109	NC_000009.11
cg08319991	1.87E-17	4.42E-19	9.93	32.4743111	3.00E-01	41259283	41259406	NC_000004.11
cg11868485	1.87E-17	4.42E-19	9.93	32.4729952	1.92E-01	140782938	140783061	NC_000004.11
cg04819539	1.87E-17	4.43E-19	9.93	32.4715964	6.48E-02	58167550	58167673	NC_000012.11

cg15439725	1.88E-17	4.44E-19	9.93	32.4702591	2.07E-01	299311	299434	NC_000004.11
cg20398120	1.88E-17	4.45E-19	9.93	32.4676437	7.15E-02	219696690	219696813	NC_000002.11
cg18724565	1.89E-17	4.46E-19	9.93	32.4655224	1.80E-01	5633068	5633191	NC_000007.13
cg19861741	1.90E-17	4.49E-19	9.93	32.4574587	2.25E-01	170742608	170742731	NC_000005.9
cg00343633	1.90E-17	4.50E-19	9.93	32.4566515	3.39E-01	105481306	105481429	NC_000011.9
cg24446548	1.90E-17	4.50E-19	9.93	32.4564676	3.07E-01	19157263	19157386	NC_000007.13
cg09225840	1.91E-17	4.52E-19	9.93	32.4524587	1.08E-01	12248163	12248286	NC_000001.10
cg12610471	1.91E-17	4.52E-19	9.93	32.4511759	1.64E-01	22634199	22634322	NC_000010.10
cg22720790	1.92E-17	4.56E-19	9.93	32.4430617	1.79E-01	58716032	58716155	NC_000019.9
cg17048000	1.93E-17	4.56E-19	9.93	32.4421512	1.17E-01	235921243	235921366	NC_000002.11
cg12503717	1.93E-17	4.57E-19	9.93	32.4414477	9.86E-02	59664229	59664352	NC_000011.9
cg10888854	1.93E-17	4.57E-19	9.93	32.4407179	7.99E-02	56644191	56644314	NC_000014.8
cg11081894	1.93E-17	4.57E-19	9.93	32.4397631	1.63E-01	101283847	101283970	NC_000010.10
cg08630080	1.93E-17	4.58E-19	9.93	32.4396271	5.26E-02	69605276	69605399	NC_000015.9
cg20881910	1.94E-17	4.60E-19	9.93	32.4350735	2.42E-01	131780332	131780455	NC_000011.9
cg07557260	1.94E-17	4.61E-19	9.93	32.4322747	2.24E-01	132052870	132052993	NC_000008.10
cg20313677	1.95E-17	4.62E-19	9.93	32.4303137	1.03E-01	3738610	3738733	NC_000001.10
cg05196969	1.95E-17	4.62E-19	9.93	32.4294876	2.34E-01	76627594	76627717	NC_000015.9
cg04635983	1.96E-17	4.64E-19	9.93	32.425292	1.49E-01	3602558	3602681	NC_000005.9
cg22403344	1.97E-17	4.67E-19	9.92	32.4185844	2.56E-01	95691500	95691623	NC_000002.11
cg11382529	1.97E-17	4.68E-19	9.92	32.417855	1.82E-01	65490613	65490736	NC_000008.10
cg18034737	1.98E-17	4.70E-19	9.92	32.4139688	2.65E-01	188916865	188916988	NC_000004.11
cg08573038	1.98E-17	4.71E-19	9.92	32.4105459	1.32E-01	110399302	110399425	NC_000012.11
cg11469391	1.98E-17	4.71E-19	9.92	32.4098277	2.18E-01	155242445	155242568	NC_000007.13
cg16677112	1.99E-17	4.72E-19	9.92	32.408291	2.34E-01	155103905	155104028	NC_000001.10

cg27373390	2.00E-17	4.75E-19	9.92	32.402594	1.75E-01	37095036	37095159	NC_000003.11
cg18266783	2.00E-17	4.75E-19	9.92	32.4016259	1.91E-01	156466729	156466852	NC_000001.10
cg04111071	2.02E-17	4.79E-19	9.92	32.3933981	2.24E-01	19813299	19813422	NC_000007.13
cg21245492	2.02E-17	4.81E-19	9.92	32.39017	1.86E-01	99250440	99250563	NC_000015.9
cg08059402	2.03E-17	4.84E-19	9.92	32.3844844	8.78E-02	194188646	194188769	NC_000003.11
cg23134259	2.03E-17	4.84E-19	9.92	32.3842308	1.78E-01	173039661	173039784	NC_000005.9
cg08364561	2.04E-17	4.86E-19	9.92	32.3807194	2.36E-01	31846844	31846967	NC_000011.9
cg10826733	2.05E-17	4.88E-19	9.92	32.3754141	1.51E-01	128482561	128482684	NC_000007.13
cg12085155	2.06E-17	4.91E-19	9.92	32.3697528	1.05E-01	37769679	37769802	NC_000022.10
cg01800148	2.06E-17	4.91E-19	9.92	32.3692886	2.05E-01	9130886	9131009	NC_000001.10
cg01972751	2.06E-17	4.92E-19	9.92	32.3676577	2.58E-01	137814728	137814851	NC_000006.11
cg13502382	2.07E-17	4.94E-19	9.92	32.3642728	1.69E-01	167192488	167192611	NC_000006.11
cg07983905	2.07E-17	4.95E-19	9.92	32.3622392	2.24E-01	3073561	3073684	NC_000020.10
cg02430935	2.08E-17	4.95E-19	9.92	32.3615028	2.12E-01	124909028	124909151	NC_000010.10
cg15547669	2.08E-17	4.96E-19	9.92	32.3586828	2.70E-01	2781122	2781245	NC_000020.10
cg21107103	2.09E-17	4.98E-19	9.92	32.3563477	1.64E-01	18630052	18630175	NC_000011.9
cg00489401	2.09E-17	4.99E-19	9.92	32.3542325	2.15E-01	180075875	180075998	NC_000005.9
cg15718581	2.10E-17	5.00E-19	9.91	32.3511938	2.66E-01	188916581	188916704	NC_000004.11
cg21606928	2.13E-17	5.08E-19	9.91	32.336351	2.24E-01	23501122	23501245	NC_000013.10
cg07377907	2.13E-17	5.08E-19	9.91	32.3361389	9.66E-02	69150318	69150441	NC_000016.9
cg08528626	2.16E-17	5.15E-19	9.91	32.322105	2.46E-01	107503728	107503851	NC_000002.11
cg25751484	2.16E-17	5.15E-19	9.91	32.3220514	1.38E-01	13915817	13915940	NC_000019.9
cg25809841	2.16E-17	5.17E-19	9.91	32.3185811	2.22E-01	51162684	51162807	NC_000019.9
cg06558014	2.17E-17	5.20E-19	9.91	32.312948	2.64E-01	100051116	100051239	NC_000006.11
cg24671810	2.18E-17	5.22E-19	9.91	32.3088635	1.36E-01	1026978	1027101	NC_000017.10

cg05498785	2.19E-17	5.23E-19	9.91	32.3079838	8.86E-02	85990170	85990293	NC_000016.9
cg01991967	2.20E-17	5.25E-19	9.91	32.3024629	1.44E-01	113884734	113884857	NC_000002.11
cg02315940	2.21E-17	5.29E-19	9.91	32.2952586	3.17E-01	139653872	139653995	NC_000003.11
cg02783918	2.22E-17	5.32E-19	9.91	32.2908235	1.70E-01	86547544	86547667	NC_000016.9
cg04927004	2.23E-17	5.33E-19	9.91	32.2880948	1.31E-01	61809348	61809471	NC_000020.10
cg13197551	2.23E-17	5.33E-19	9.91	32.2873759	1.06E-01	60709957	60710080	NC_000020.10
cg25602684	2.24E-17	5.36E-19	9.9	32.2834269	2.40E-01	37839918	37840041	NC_000005.9
cg24896053	2.24E-17	5.37E-19	9.9	32.2817244	1.65E-01	8553099	8553222	NC_000010.10
cg15466862	2.24E-17	5.37E-19	9.9	32.2801249	1.92E-01	112722333	112722456	NC_000013.10
cg19191600	2.25E-17	5.38E-19	9.9	32.2787425	3.96E-02	30613506	30613629	NC_000006.11
cg13797914	2.25E-17	5.40E-19	9.9	32.2748794	8.77E-02	42192995	42193118	NC_000015.9
cg23068913	2.27E-17	5.44E-19	9.9	32.2671799	2.57E-01	23463377	23463500	NC_000010.10
cg03936663	2.29E-17	5.50E-19	9.9	32.2574329	1.97E-01	218098422	218098545	NC_000001.10
cg06893273	2.29E-17	5.51E-19	9.9	32.2562531	2.01E-01	46604774	46604897	NC_000017.10
cg03276484	2.30E-17	5.51E-19	9.9	32.2550331	7.27E-02	194803749	194803872	NC_000003.11
cg24414344	2.31E-17	5.54E-19	9.9	32.2494465	1.38E-01	39019556	39019679	NC_000017.10
cg08151857	2.31E-17	5.55E-19	9.9	32.2480582	3.20E-01	46691820	46691943	NC_000017.10
cg06720544	2.31E-17	5.55E-19	9.9	32.2473172	1.48E-01	65641018	65641141	NC_000002.11
cg01150105	2.32E-17	5.56E-19	9.9	32.2464864	1.70E-01	175139160	175139283	NC_000003.11
cg01777643	2.34E-17	5.61E-19	9.9	32.237562	2.46E-01	224805287	224805410	NC_000001.10
cg01315916	2.34E-17	5.63E-19	9.9	32.2341283	1.85E-01	3591609	3591732	NC_000005.9
cg10743390	2.34E-17	5.63E-19	9.9	32.2337772	1.60E-01	30020728	30020851	NC_000019.9
cg26791307	2.35E-17	5.65E-19	9.9	32.23087	1.87E-01	97629235	97629358	NC_000015.9
cg12667732	2.39E-17	5.74E-19	9.89	32.2145127	1.51E-01	92673594	92673717	NC_000015.9
cg06164660	2.39E-17	5.75E-19	9.89	32.2123712	2.26E-01	128751582	128751705	NC_000012.11

cg08076955	2.39E-17	5.75E-19	9.89	32.2122821	1.13E-01	46608462	46608585	NC_000017.10
cg02250846	2.41E-17	5.79E-19	9.89	32.2066093	2.69E-01	23258484	23258607	NC_000019.9
cg04209913	2.41E-17	5.79E-19	9.89	32.2063412	2.25E-01	37836246	37836369	NC_000005.9
cg04155485	2.41E-17	5.81E-19	9.89	32.2034006	1.22E-01	5535934	5536057	NC_000007.13
cg23413567	2.42E-17	5.82E-19	9.89	32.2019692	1.49E-01	41223175	41223298	NC_000013.10
cg27269962	2.42E-17	5.84E-19	9.89	32.1980188	1.58E-01	127540997	127541120	NC_000007.13
cg04175111	2.43E-17	5.84E-19	9.89	32.1971335	2.03E-01	2334971	2335094	NC_000005.9
cg14089067	2.43E-17	5.84E-19	9.89	32.1970157	1.50E-01	55442785	55442908	NC_000001.10
cg22660578	2.44E-17	5.88E-19	9.89	32.1907688	2.30E-01	35294029	35294152	NC_000017.10
cg18773844	2.44E-17	5.89E-19	9.89	32.1895172	9.65E-02	69463247	69463370	NC_000011.9
cg00228281	2.44E-17	5.89E-19	9.89	32.1891434	1.33E-01	50800963	50801086	NC_000007.13
cg00792424	2.46E-17	5.92E-19	9.89	32.1846187	2.55E-01	10386160	10386283	NC_000006.11
cg23740135	2.46E-17	5.93E-19	9.89	32.1817811	1.58E-01	41621208	41621331	NC_000006.11
cg12520549	2.47E-17	5.95E-19	9.89	32.178781	1.54E-01	162280741	162280864	NC_000002.11
cg19031475	2.48E-17	5.98E-19	9.89	32.1747058	1.02E-01	134659481	134659604	NC_000010.10
cg05924485	2.50E-17	6.02E-19	9.89	32.1669143	1.85E-01	22990378	22990501	NC_000019.9
cg13724788	2.50E-17	6.03E-19	9.89	32.1654272	2.79E-01	19322778	19322901	NC_000019.9
cg10514097	2.50E-17	6.04E-19	9.89	32.1647428	2.17E-01	86544787	86544910	NC_000016.9
cg17751591	2.52E-17	6.07E-19	9.89	32.1587237	1.10E-01	80139098	80139221	NC_000017.10
cg26565021	2.52E-17	6.08E-19	9.89	32.1581784	2.86E-01	34112825	34112948	NC_000019.9
cg01790144	2.52E-17	6.08E-19	9.89	32.157124	9.42E-02	84476098	84476221	NC_000016.9
cg23266594	2.52E-17	6.09E-19	9.89	32.1555228	1.68E-01	149546691	149546814	NC_000005.9
cg04362839	2.53E-17	6.12E-19	9.88	32.1511925	9.56E-02	105688421	105688544	NC_000014.8
cg07208172	2.53E-17	6.12E-19	9.88	32.1507368	1.19E-01	32818353	32818476	NC_000001.10
cg18740861	2.54E-17	6.14E-19	9.88	32.147892	8.61E-02	134998487	134998610	NC_000010.10

cg26858704	2.55E-17	6.16E-19	9.88	32.144063	2.71E-01	4918848	4918971	NC_000012.11
cg26685294	2.56E-17	6.20E-19	9.88	32.1385503	7.48E-02	67696047	67696170	NC_000016.9
cg14497054	2.57E-17	6.21E-19	9.88	32.1364441	1.53E-01	34261116	34261239	NC_000012.11
cg19859290	2.58E-17	6.24E-19	9.88	32.1325157	3.07E-01	20618399	20618522	NC_000011.9
cg03032025	2.58E-17	6.24E-19	9.88	32.1318862	1.83E-01	173316748	173316871	NC_000005.9
cg04802545	2.59E-17	6.25E-19	9.88	32.1296936	1.13E-01	2423231	2423354	NC_000011.9
cg02332982	2.61E-17	6.31E-19	9.88	32.1217297	2.60E-01	45444420	45444543	NC_000012.11
cg22670033	2.61E-17	6.32E-19	9.88	32.1188467	1.42E-01	180646608	180646731	NC_000005.9
cg23030040	2.64E-17	6.39E-19	9.88	32.1087504	4.83E-02	3414302	3414425	NC_000004.11
cg17816908	2.66E-17	6.43E-19	9.88	32.1016989	2.16E-01	41167109	41167232	NC_000008.10
cg08885198	2.67E-17	6.46E-19	9.88	32.0976009	6.66E-02	79051613	79051736	NC_000017.10
cg13865360	2.67E-17	6.47E-19	9.88	32.0967637	1.06E-01	15476258	15476381	NC_000001.10
cg00831710	2.68E-17	6.51E-19	9.88	32.0903842	1.73E-01	144240945	144241068	NC_000008.10
cg11664818	2.69E-17	6.53E-19	9.87	32.087132	1.66E-01	37852665	37852788	NC_000021.8
cg18065686	2.71E-17	6.58E-19	9.87	32.0790164	1.20E-01	23411171	23411294	NC_000008.10
cg05593641	2.71E-17	6.59E-19	9.87	32.0785816	2.70E-01	106398816	106398939	NC_000010.10
cg03570636	2.74E-17	6.65E-19	9.87	32.0683924	1.77E-01	2753375	2753498	NC_000005.9
cg27292667	2.75E-17	6.67E-19	9.87	32.0667699	9.56E-02	1049628	1049751	NC_000016.9
cg05542338	2.77E-17	6.73E-19	9.87	32.0571851	2.63E-01	5710407	5710530	NC_000004.11
cg13596497	2.78E-17	6.75E-19	9.87	32.053748	2.43E-01	110959104	110959227	NC_000013.10
cg15916399	2.78E-17	6.75E-19	9.87	32.0535913	2.12E-01	67068457	67068580	NC_000018.9
cg10633658	2.78E-17	6.77E-19	9.87	32.0519416	1.89E-01	58147525	58147648	NC_000006.11
cg18173058	2.80E-17	6.80E-19	9.87	32.0471073	2.05E-01	114838359	114838482	NC_000012.11
cg19385090	2.80E-17	6.81E-19	9.87	32.0452854	2.04E-01	27292614	27292737	NC_000007.13
cg08442064	2.80E-17	6.81E-19	9.87	32.0448402	1.45E-01	139394779	139394902	NC_000003.11

cg07766263	2.82E-17	6.85E-19	9.87	32.0390972	1.73E-01	170303045	170303168	NC_000003.11
cg04247508	2.83E-17	6.87E-19	9.87	32.0361899	5.91E-02	48557420	48557543	NC_000008.10
cg18590299	2.86E-17	6.95E-19	9.87	32.0253739	1.15E-01	3975803	3975926	NC_000017.10
cg22729726	2.86E-17	6.96E-19	9.87	32.0241525	1.79E-01	3123854	3123977	NC_000001.10
cg08945642	2.86E-17	6.96E-19	9.87	32.0236333	8.47E-02	205863	205986	NC_000005.9
cg05571970	2.88E-17	7.02E-19	9.86	32.0148128	1.86E-01	166422182	166422305	NC_000006.11
cg27179533	2.89E-17	7.03E-19	9.86	32.0135883	2.44E-01	101006052	101006175	NC_000007.13
cg01522592	2.89E-17	7.04E-19	9.86	32.0127358	1.63E-01	75235311	75235434	NC_000011.9
cg00067600	2.89E-17	7.05E-19	9.86	32.0115764	1.61E-01	34109141	34109264	NC_000003.11
cg22163834	2.90E-17	7.07E-19	9.86	32.0081018	9.70E-02	32025965	32026088	NC_000006.11
cg13840252	2.90E-17	7.07E-19	9.86	32.0078613	1.05E-01	10732744	10732867	NC_000001.10
cg03331387	2.91E-17	7.08E-19	9.86	32.0066473	9.88E-02	127114962	127115085	NC_000003.11
cg15720516	2.91E-17	7.09E-19	9.86	32.0055636	1.02E-01	12907933	12908056	NC_000019.9
cg27313941	2.91E-17	7.10E-19	9.86	32.0042833	1.78E-01	27186554	27186677	NC_000007.13
cg10188823	2.91E-17	7.11E-19	9.86	32.0030814	2.91E-01	29876206	29876329	NC_000022.10
cg03091551	2.92E-17	7.12E-19	9.86	32.0015466	1.98E-01	57275967	57276090	NC_000014.8
cg12169568	2.92E-17	7.12E-19	9.86	32.0011351	7.99E-02	89708532	89708655	NC_000014.8
cg20289688	2.92E-17	7.13E-19	9.86	31.9995896	2.09E-01	17742502	17742625	NC_000011.9
cg04861929	2.93E-17	7.15E-19	9.86	31.9973289	2.32E-01	109293070	109293193	NC_000011.9
cg05208056	2.94E-17	7.18E-19	9.86	31.9931518	1.85E-01	106502915	106503038	NC_000002.11
cg02311364	2.94E-17	7.18E-19	9.86	31.9930959	1.29E-01	13001256	13001379	NC_000003.11
cg00332937	2.96E-17	7.24E-19	9.86	31.98463	2.18E-01	72740460	72740583	NC_000005.9
cg09268961	2.99E-17	7.31E-19	9.86	31.9747026	1.70E-01	14400943	14401066	NC_000016.9
cg08755736	3.00E-17	7.35E-19	9.86	31.9702143	1.69E-01	95957928	95958051	NC_000008.10
cg02291403	3.01E-17	7.36E-19	9.86	31.9678832	2.39E-01	130646461	130646584	NC_000012.11

cg22079195	3.01E-17	7.37E-19	9.86	31.9677939	1.08E-01	100799756	100799879	NC_000002.11
cg16733714	3.02E-17	7.40E-19	9.86	31.9634275	4.67E-02	54318953	54319076	NC_000001.10
cg13747145	3.04E-17	7.43E-19	9.86	31.95895	9.55E-02	33261000	33261123	NC_000003.11
cg10631546	3.05E-17	7.47E-19	9.85	31.9536003	9.97E-02	114903101	114903224	NC_000004.11
cg27420520	3.06E-17	7.50E-19	9.85	31.9504643	2.43E-01	103352267	103352390	NC_000012.11
cg09497124	3.10E-17	7.59E-19	9.85	31.9380409	6.33E-02	43883469	43883592	NC_000015.9
cg08549332	3.11E-17	7.61E-19	9.85	31.9352143	1.17E-01	101558461	101558584	NC_000007.13
cg15960924	3.12E-17	7.65E-19	9.85	31.9300941	1.74E-01	32151282	32151405	NC_000003.11
cg13929328	3.13E-17	7.66E-19	9.85	31.9287522	1.94E-01	129535898	129536021	NC_000010.10
cg21229268	3.15E-17	7.73E-19	9.85	31.9200883	2.02E-01	34442350	34442473	NC_000021.8
cg15262242	3.15E-17	7.73E-19	9.85	31.9193508	3.10E-01	47301741	47301864	NC_000017.10
cg00515457	3.17E-17	7.78E-19	9.85	31.9138605	2.50E-01	35396203	35396326	NC_000019.9
cg27248474	3.17E-17	7.78E-19	9.85	31.9135386	1.77E-01	88991238	88991361	NC_000016.9
cg23551605	3.18E-17	7.80E-19	9.85	31.9105353	1.28E-01	236442942	236443065	NC_000002.11
cg07062388	3.19E-17	7.82E-19	9.85	31.9081264	7.76E-02	70507787	70507910	NC_000006.11
cg23663318	3.20E-17	7.84E-19	9.85	31.9055417	1.08E-01	89448407	89448530	NC_000001.10
cg04124457	3.20E-17	7.84E-19	9.85	31.9052542	1.32E-01	56036455	56036578	NC_000015.9
cg03724721	3.21E-17	7.89E-19	9.85	31.899673	1.45E-01	19939061	19939184	NC_000022.10
cg23251122	3.22E-17	7.90E-19	9.85	31.8984254	1.14E-01	35947995	35948118	NC_000022.10
cg10164795	3.22E-17	7.90E-19	9.85	31.898114	1.41E-01	44181838	44181961	NC_000010.10
cg09743650	3.23E-17	7.94E-19	9.85	31.8930431	1.37E-01	85675160	85675283	NC_000016.9
cg19760441	3.24E-17	7.96E-19	9.84	31.8907766	5.94E-02	32825139	32825262	NC_000006.11
cg25902889	3.26E-17	8.01E-19	9.84	31.8844755	1.96E-01	4305090	4305213	NC_000019.9
cg27621340	3.27E-17	8.02E-19	9.84	31.8829092	2.68E-01	89346269	89346392	NC_000015.9
cg16754788	3.28E-17	8.06E-19	9.84	31.8779586	2.05E-01	97803770	97803893	NC_000008.10

cg02661431	3.30E-17	8.11E-19	9.84	31.8725315	9.47E-02	45010881	45011004	NC_000001.10
cg19182986	3.30E-17	8.12E-19	9.84	31.8709723	1.20E-01	124613500	124613623	NC_000011.9
cg11028052	3.32E-17	8.16E-19	9.84	31.8665058	1.04E-01	81154387	81154510	NC_000015.9
cg23003534	3.33E-17	8.19E-19	9.84	31.8630886	2.61E-01	105036747	105036870	NC_000010.10
cg21915998	3.33E-17	8.19E-19	9.84	31.8627534	1.51E-01	67060872	67060995	NC_000002.11
cg16342949	3.33E-17	8.19E-19	9.84	31.8622095	1.10E-01	63883584	63883707	NC_000011.9
cg23329015	3.34E-17	8.20E-19	9.84	31.8608561	1.14E-01	1027680	1027803	NC_000017.10
cg07444414	3.38E-17	8.32E-19	9.84	31.8472945	1.27E-01	32117377	32117500	NC_000006.11
cg14394692	3.40E-17	8.37E-19	9.84	31.8406342	2.36E-01	30517589	30517712	NC_000015.9
cg00028844	3.40E-17	8.38E-19	9.84	31.8401605	1.38E-01	43938184	43938307	NC_000020.10
cg16501028	3.40E-17	8.38E-19	9.84	31.8397204	1.56E-01	32450000	32450123	NC_000011.9
cg22539706	3.42E-17	8.43E-19	9.84	31.8340567	9.94E-02	24646727	24646850	NC_000010.10
cg00916179	3.42E-17	8.44E-19	9.84	31.83325	1.15E-01	43992805	43992928	NC_000002.11
cg06874656	3.45E-17	8.51E-19	9.83	31.824909	2.23E-01	131780353	131780476	NC_000011.9
cg10023272	3.47E-17	8.55E-19	9.83	31.820371	1.94E-01	110611823	110611946	NC_000001.10
cg26133372	3.47E-17	8.55E-19	9.83	31.8203533	3.42E-02	1537930	1538053	NC_000005.9
cg17686487	3.47E-17	8.55E-19	9.83	31.8194773	2.11E-01	50686281	50686404	NC_000005.9
cg11058904	3.48E-17	8.57E-19	9.83	31.8173252	1.01E-01	69987299	69987422	NC_000011.9
cg18409985	3.50E-17	8.63E-19	9.83	31.811174	1.38E-01	113513520	113513643	NC_000013.10
cg07598357	3.51E-17	8.66E-19	9.83	31.8069208	1.91E-01	170595195	170595318	NC_000006.11
cg05132905	3.52E-17	8.69E-19	9.83	31.8040426	8.30E-02	38600367	38600490	NC_000002.11
cg19913496	3.52E-17	8.69E-19	9.83	31.8039778	1.51E-01	116694158	116694281	NC_000001.10
cg11155547	3.55E-17	8.77E-19	9.83	31.7945313	6.34E-02	42856236	42856359	NC_000006.11
cg06660530	3.58E-17	8.84E-19	9.83	31.7866188	2.09E-01	61809911	61810034	NC_000020.10
cg24739457	3.59E-17	8.88E-19	9.83	31.7821331	2.12E-01	205821442	205821565	NC_000001.10

cg25652781	3.63E-17	8.97E-19	9.83	31.7723433	6.02E-02	153586344	153586467	NC_000004.11
cg18489289	3.64E-17	8.99E-19	9.83	31.7701363	1.92E-01	44876292	44876415	NC_000010.10
cg04023150	3.66E-17	9.05E-19	9.83	31.7639916	3.11E-01	44873064	44873187	NC_000001.10
cg18407189	3.68E-17	9.10E-19	9.82	31.7578083	8.89E-02	67810421	67810544	NC_000011.9
cg16437211	3.70E-17	9.15E-19	9.82	31.753078	1.28E-01	142844692	142844815	NC_000008.10
cg18818210	3.73E-17	9.22E-19	9.82	31.7450581	1.26E-01	147286378	147286501	NC_000005.9
cg12682684	3.73E-17	9.25E-19	9.82	31.7425231	2.24E-01	66535403	66535526	NC_000004.11
cg13473356	3.79E-17	9.39E-19	9.82	31.7271128	2.29E-01	179754613	179754736	NC_000003.11
cg11903177	3.79E-17	9.39E-19	9.82	31.7270765	1.69E-01	17379358	17379481	NC_000017.10
cg00783000	3.80E-17	9.43E-19	9.82	31.7232976	1.38E-01	101007650	101007773	NC_000014.8
cg24997888	3.81E-17	9.44E-19	9.82	31.7216248	1.23E-01	179042747	179042870	NC_000001.10
cg00661970	3.81E-17	9.45E-19	9.82	31.7211033	2.62E-01	115135027	115135150	NC_000012.11
cg14137354	3.81E-17	9.45E-19	9.82	31.7208844	1.84E-01	95993155	95993278	NC_000009.11
cg00811065	3.83E-17	9.50E-19	9.82	31.71598	1.97E-01	1287159	1287282	NC_000007.13
cg02172669	3.83E-17	9.50E-19	9.82	31.7150978	1.51E-01	149410459	149410582	NC_000007.13
cg19736604	3.84E-17	9.54E-19	9.82	31.7113101	9.14E-02	32052289	32052412	NC_000006.11
cg13302823	3.84E-17	9.54E-19	9.82	31.7111456	2.04E-01	145559792	145559915	NC_000008.10
cg02567323	3.85E-17	9.55E-19	9.82	31.710026	1.67E-01	48671699	48671822	NC_000002.11
cg22290744	3.85E-17	9.57E-19	9.82	31.7087204	2.53E-01	5710016	5710139	NC_000004.11
cg04606397	3.87E-17	9.61E-19	9.82	31.7040406	1.63E-01	157129618	157129741	NC_000006.11
cg13660679	3.88E-17	9.64E-19	9.82	31.7013697	9.21E-02	133570890	133571013	NC_000009.11
cg25884711	3.89E-17	9.66E-19	9.82	31.6988052	3.39E-01	24323840	24323963	NC_000007.13
cg02400740	3.90E-17	9.69E-19	9.82	31.6955187	2.20E-01	19157938	19158061	NC_000007.13
cg15111708	3.90E-17	9.70E-19	9.82	31.6949916	9.50E-02	154146853	154146976	NC_000005.9
cg00348762	3.93E-17	9.77E-19	9.81	31.6873891	2.01E-01	56228114	56228237	NC_000016.9

cg25994871	3.95E-17	9.82E-19	9.81	31.6832145	1.47E-01	107967614	107967737	NC_000007.13
cg21972431	3.95E-17	9.82E-19	9.81	31.6828314	1.67E-01	17812356	17812479	NC_000007.13
cg04773818	3.95E-17	9.83E-19	9.81	31.6821774	2.47E-01	31496966	31497089	NC_000008.10
cg05210258	3.97E-17	9.89E-19	9.81	31.6760634	2.60E-01	102569538	102569661	NC_000013.10
cg26581504	3.98E-17	9.91E-19	9.81	31.6741796	1.41E-01	112046963	112047086	NC_000011.9
cg27032146	3.98E-17	9.92E-19	9.81	31.6730603	2.12E-01	96652115	96652238	NC_000007.13
cg20591728	4.00E-17	9.97E-19	9.81	31.6679571	1.80E-01	46683606	46683729	NC_000017.10
cg16197186	4.01E-17	9.98E-19	9.81	31.667108	1.01E-01	12413887	12414010	NC_000003.11
cg17910121	4.02E-17	1.00E-18	9.81	31.6623288	2.03E-01	28034669	28034792	NC_000010.10
cg01287000	4.03E-17	1.00E-18	9.81	31.6612908	1.49E-01	145938564	145938687	NC_000008.10
cg16720807	4.03E-17	1.00E-18	9.81	31.6605116	1.68E-01	75786714	75786837	NC_000002.11
cg12500879	4.03E-17	1.00E-18	9.81	31.6603782	2.76E-01	31848649	31848772	NC_000011.9
cg04023204	4.05E-17	1.01E-18	9.81	31.6557285	9.80E-02	68281979	68282102	NC_000016.9
cg11611932	4.05E-17	1.01E-18	9.81	31.6546142	8.44E-02	46073483	46073606	NC_000007.13
cg18007757	4.06E-17	1.01E-18	9.81	31.6526163	4.19E-02	110358928	110359051	NC_000013.10
cg22694818	4.08E-17	1.02E-18	9.81	31.6487318	1.86E-01	75595970	75596093	NC_000001.10
cg10334767	4.08E-17	1.02E-18	9.81	31.6480731	2.77E-01	10459128	10459251	NC_000004.11
cg26290155	4.08E-17	1.02E-18	9.81	31.6471824	1.89E-01	69449994	69450117	NC_000014.8
cg07747616	4.09E-17	1.02E-18	9.81	31.6448014	1.37E-01	7729794	7729917	NC_000006.11
cg10276506	4.13E-17	1.03E-18	9.81	31.6356302	9.17E-02	31690170	31690293	NC_000006.11
cg17545805	4.13E-17	1.03E-18	9.81	31.6347524	4.05E-02	134551796	134551919	NC_000010.10
cg08463758	4.14E-17	1.03E-18	9.81	31.6329578	1.96E-01	39995649	39995772	NC_000020.10
cg26998274	4.14E-17	1.03E-18	9.81	31.6325915	2.73E-01	84456258	84456381	NC_000013.10
cg20829347	4.14E-17	1.03E-18	9.81	31.6316761	1.60E-01	112720927	112721050	NC_000013.10
cg25970454	4.15E-17	1.04E-18	9.81	31.6302201	1.46E-01	76957699	76957822	NC_000012.11

cg23845153	4.17E-17	1.04E-18	9.8	31.6242307	9.97E-02	7750448	7750571	NC_000004.11
cg09387587	4.18E-17	1.04E-18	9.8	31.6226798	5.69E-02	90000322	90000445	NC_000016.9
cg11097541	4.18E-17	1.04E-18	9.8	31.6223672	1.84E-01	27208188	27208311	NC_000007.13
cg06522679	4.20E-17	1.05E-18	9.8	31.6176025	8.15E-02	18287231	18287354	NC_000011.9
cg21356750	4.20E-17	1.05E-18	9.8	31.6171905	9.53E-02	31864236	31864359	NC_000006.11
cg25643223	4.22E-17	1.06E-18	9.8	31.6106647	1.89E-01	24787195	24787318	NC_000013.10
cg22557091	4.25E-17	1.06E-18	9.8	31.6051884	2.35E-01	31827920	31828043	NC_000011.9
cg16648062	4.27E-17	1.07E-18	9.8	31.5984519	2.53E-01	104512168	104512291	NC_000008.10
cg15187276	4.28E-17	1.07E-18	9.8	31.5973992	9.30E-02	62531608	62531731	NC_000011.9
cg02866106	4.29E-17	1.07E-18	9.8	31.5952299	1.85E-01	136553110	136553233	NC_000007.13
cg09825082	4.29E-17	1.07E-18	9.8	31.5941077	2.08E-01	2567907	2568030	NC_000002.11
cg02253499	4.29E-17	1.08E-18	9.8	31.5929588	1.40E-01	12602046	12602169	NC_000003.11
cg00495860	4.31E-17	1.08E-18	9.8	31.5882528	2.32E-01	38065524	38065647	NC_000021.8
cg17079833	4.32E-17	1.08E-18	9.8	31.5877628	3.31E-01	22487847	22487970	NC_000012.11
cg14645650	4.32E-17	1.08E-18	9.8	31.5875318	7.00E-02	25228801	25228924	NC_000001.10
cg13519035	4.32E-17	1.08E-18	9.8	31.5873215	2.06E-01	34261006	34261129	NC_000012.11
cg25463470	4.38E-17	1.10E-18	9.8	31.5714514	1.99E-01	112721712	112721835	NC_000013.10
cg02864844	4.38E-17	1.10E-18	9.8	31.5713999	3.02E-01	149917263	149917386	NC_000007.13
cg03737442	4.39E-17	1.10E-18	9.8	31.5697246	1.09E-01	11177295	11177418	NC_000003.11
cg26310913	4.39E-17	1.10E-18	9.8	31.5693923	1.18E-01	239554854	239554977	NC_000001.10
cg00902516	4.39E-17	1.10E-18	9.8	31.5685325	1.72E-01	6233774	6233897	NC_000010.10
cg02150536	4.42E-17	1.11E-18	9.8	31.5625614	4.33E-02	11302179	11302302	NC_000003.11
cg21966860	4.43E-17	1.11E-18	9.79	31.5585269	1.62E-01	145538097	145538220	NC_000008.10
cg21779611	4.48E-17	1.12E-18	9.79	31.5483044	2.57E-01	76738999	76739122	NC_000018.9
cg09714184	4.49E-17	1.13E-18	9.79	31.5458767	1.08E-01	15740113	15740236	NC_000016.9

cg27553667	4.50E-17	1.13E-18	9.79	31.5417848	2.34E-01	27216083	27216206	NC_000015.9
cg14027297	4.51E-17	1.13E-18	9.79	31.540829	8.64E-02	26911633	26911756	NC_000022.10
cg15934634	4.51E-17	1.13E-18	9.79	31.5404537	1.73E-01	68920095	68920218	NC_000011.9
cg18605137	4.52E-17	1.14E-18	9.79	31.5376398	1.12E-01	80915044	80915167	NC_000017.10
cg04219410	4.59E-17	1.16E-18	9.79	31.5216663	6.78E-02	21106687	21106810	NC_000020.10
cg18101138	4.61E-17	1.16E-18	9.79	31.5163684	1.83E-01	4942469	4942592	NC_000005.9
cg18603028	4.72E-17	1.19E-18	9.78	31.4934106	2.18E-01	136834392	136834515	NC_000005.9
cg08886973	4.75E-17	1.20E-18	9.78	31.4873955	2.35E-01	166402416	166402539	NC_000006.11
cg03278146	4.75E-17	1.20E-18	9.78	31.4862548	2.93E-01	5197327	5197450	NC_000018.9
cg15798050	4.76E-17	1.20E-18	9.78	31.4849518	9.85E-02	47513521	47513644	NC_000022.10
cg14461586	4.76E-17	1.20E-18	9.78	31.4839879	7.26E-02	122707223	122707346	NC_000010.10
cg11021317	4.78E-17	1.21E-18	9.78	31.4795379	9.40E-02	11471730	11471853	NC_000004.11
cg23288059	4.78E-17	1.21E-18	9.78	31.4794853	2.48E-01	38724945	38725068	NC_000014.8
cg16794506	4.81E-17	1.21E-18	9.78	31.4736919	2.30E-01	119600002	119600125	NC_000002.11
cg25060525	4.81E-17	1.21E-18	9.78	31.4724628	6.46E-02	3235295	3235418	NC_000004.11
cg11162839	4.83E-17	1.22E-18	9.78	31.4689686	1.25E-01	135688971	135689094	NC_000003.11
cg11401784	4.85E-17	1.22E-18	9.78	31.4642931	1.55E-01	16092264	16092387	NC_000001.10
cg02947450	4.87E-17	1.23E-18	9.78	31.4595265	9.53E-02	22766861	22766984	NC_000010.10
cg24952102	4.87E-17	1.23E-18	9.78	31.4590985	8.26E-02	93062752	93062875	NC_000011.9
cg27054655	4.88E-17	1.23E-18	9.78	31.457829	1.49E-01	178772969	178773092	NC_000005.9
cg11976616	4.89E-17	1.24E-18	9.78	31.4551361	2.27E-01	41213172	41213295	NC_000005.9
cg16794682	4.92E-17	1.24E-18	9.78	31.4489437	9.70E-02	69460951	69461074	NC_000011.9
cg17178489	4.93E-17	1.25E-18	9.78	31.4468241	1.27E-01	22896140	22896263	NC_000015.9
cg01008267	4.94E-17	1.25E-18	9.78	31.4448057	8.50E-02	47515249	47515372	NC_000022.10
cg19600023	4.96E-17	1.25E-18	9.78	31.4402914	1.25E-01	150525304	150525427	NC_000006.11

cg04360793	4.97E-17	1.26E-18	9.78	31.4378781	2.37E-01	79472361	79472484	NC_000001.10
cg20510724	4.97E-17	1.26E-18	9.78	31.4370812	2.48E-01	89346795	89346918	NC_000015.9
cg14099664	5.00E-17	1.26E-18	9.78	31.4321754	1.38E-01	32545349	32545472	NC_000003.11
cg02656891	5.03E-17	1.27E-18	9.77	31.4264112	2.23E-01	34642519	34642642	NC_000001.10
cg22484980	5.07E-17	1.28E-18	9.77	31.4178392	1.15E-01	68815449	68815572	NC_000011.9
cg06805834	5.08E-17	1.29E-18	9.77	31.415865	9.82E-02	45081274	45081397	NC_000003.11
cg18811550	5.09E-17	1.29E-18	9.77	31.4140661	1.66E-01	27198311	27198434	NC_000007.13
cg23253569	5.09E-17	1.29E-18	9.77	31.4123371	2.77E-01	34398222	34398345	NC_000021.8
cg20661138	5.10E-17	1.29E-18	9.77	31.4100693	2.25E-01	145925314	145925437	NC_000008.10
cg19304273	5.11E-17	1.30E-18	9.77	31.4084392	1.42E-01	49070231	49070354	NC_000011.9
cg01219205	5.12E-17	1.30E-18	9.77	31.4056662	1.54E-01	33937433	33937556	NC_000001.10
cg07139350	5.15E-17	1.31E-18	9.77	31.4001529	1.82E-01	12416368	12416491	NC_000001.10
cg07193766	5.18E-17	1.31E-18	9.77	31.3952101	3.23E-01	32457127	32457250	NC_000011.9
cg14626309	5.19E-17	1.32E-18	9.77	31.392778	2.38E-01	299051	299174	NC_000004.11
cg14879760	5.23E-17	1.33E-18	9.77	31.3850657	2.28E-01	50673965	50674088	NC_000005.9
cg24988036	5.24E-17	1.33E-18	9.77	31.3816499	1.64E-01	32260699	32260822	NC_000020.10
cg02434880	5.26E-17	1.34E-18	9.77	31.377389	1.08E-01	30620732	30620855	NC_000006.11
cg22409854	5.30E-17	1.35E-18	9.77	31.3702576	2.51E-01	88877301	88877424	NC_000006.11
cg08722638	5.31E-17	1.35E-18	9.77	31.3685395	1.44E-01	43035419	43035542	NC_000002.11
cg05766140	5.32E-17	1.35E-18	9.77	31.3668976	2.98E-01	391743	391866	NC_000006.11
cg00061039	5.33E-17	1.36E-18	9.76	31.3629573	2.14E-01	187186953	187187076	NC_000004.11
cg17120366	5.35E-17	1.36E-18	9.76	31.3593939	1.56E-01	28571322	28571445	NC_000010.10
cg18126097	5.35E-17	1.36E-18	9.76	31.3588737	2.38E-01	200014673	200014796	NC_000001.10
cg14492800	5.37E-17	1.36E-18	9.76	31.3565962	2.53E-01	37839923	37840046	NC_000005.9
cg12491966	5.38E-17	1.37E-18	9.76	31.354595	1.29E-01	46925242	46925365	NC_000021.8

cg10824063	5.39E-17	1.37E-18	9.76	31.3516259	2.39E-01	20690934	20691057	NC_000011.9
cg08274637	5.43E-17	1.38E-18	9.76	31.3439372	1.43E-01	51417923	51418046	NC_000013.10
cg09830455	5.46E-17	1.39E-18	9.76	31.3380643	1.56E-01	70886197	70886320	NC_000011.9
cg01670608	5.50E-17	1.40E-18	9.76	31.3298445	1.66E-01	35765093	35765216	NC_000006.11
cg13692446	5.52E-17	1.41E-18	9.76	31.3270312	2.51E-01	112759719	112759842	NC_000013.10
cg01670896	5.53E-17	1.41E-18	9.76	31.3250512	1.70E-01	1933522	1933645	NC_000008.10
cg04772968	5.53E-17	1.41E-18	9.76	31.3249404	1.87E-01	12404112	12404235	NC_000019.9
cg05843106	5.53E-17	1.41E-18	9.76	31.3247103	1.88E-01	186030462	186030585	NC_000004.11
cg03976877	5.58E-17	1.42E-18	9.76	31.3160904	3.17E-01	158937610	158937733	NC_000007.13
cg07121488	5.58E-17	1.42E-18	9.76	31.3159345	6.91E-02	40581105	40581228	NC_000015.9
cg23528705	5.62E-17	1.43E-18	9.76	31.3080889	2.08E-01	1275252	1275375	NC_000007.13
cg04207385	5.67E-17	1.45E-18	9.75	31.2978272	2.63E-01	88877064	88877187	NC_000006.11
cg08498254	5.68E-17	1.45E-18	9.75	31.2968136	2.20E-01	155109505	155109628	NC_000005.9
cg13791254	5.70E-17	1.46E-18	9.75	31.2923309	2.20E-01	100616607	100616730	NC_000009.11
cg16334314	5.71E-17	1.46E-18	9.75	31.2912043	2.58E-01	84418789	84418912	NC_000006.11
cg04559779	5.75E-17	1.47E-18	9.75	31.2837378	2.50E-01	65291730	65291853	NC_000008.10
cg05161082	5.77E-17	1.47E-18	9.75	31.2798556	2.74E-01	21680409	21680532	NC_000012.11
cg21234123	5.77E-17	1.47E-18	9.75	31.2797975	7.89E-02	139435339	139435462	NC_000009.11
cg08323651	5.78E-17	1.48E-18	9.75	31.2782112	2.12E-01	136553855	136553978	NC_000007.13
cg21033440	5.80E-17	1.48E-18	9.75	31.2738472	2.01E-01	65409861	65409984	NC_000011.9
cg27032232	5.81E-17	1.48E-18	9.75	31.2732401	2.40E-01	153584609	153584732	NC_000007.13
cg12419135	5.82E-17	1.49E-18	9.75	31.2715822	2.92E-01	29719023	29719146	NC_000017.10
cg22425711	5.85E-17	1.50E-18	9.75	31.2662897	9.52E-02	38200251	38200374	NC_000001.10
cg12566756	5.86E-17	1.50E-18	9.75	31.2636967	2.40E-01	106975149	106975272	NC_000012.11
cg00729039	5.86E-17	1.50E-18	9.75	31.2630394	1.14E-01	148722798	148722921	NC_000007.13

cg22226398	5.87E-17	1.50E-18	9.75	31.2612494	1.04E-01	235295459	235295582	NC_000001.10
cg15196806	5.90E-17	1.51E-18	9.75	31.2561838	1.79E-01	27171213	27171336	NC_000007.13
cg18394552	5.90E-17	1.51E-18	9.75	31.2558834	1.87E-01	159428643	159428766	NC_000005.9
cg21369812	5.91E-17	1.51E-18	9.75	31.2535178	1.17E-01	73471699	73471822	NC_000010.10
cg06239131	5.92E-17	1.52E-18	9.75	31.2519856	1.02E-01	2483786	2483909	NC_000001.10
cg04839289	5.93E-17	1.52E-18	9.75	31.249845	1.33E-01	24045274	24045397	NC_000014.8
cg24624901	5.93E-17	1.52E-18	9.75	31.249711	2.71E-01	19184273	19184396	NC_000007.13
cg16282242	5.93E-17	1.52E-18	9.75	31.2496484	4.05E-02	94975980	94976103	NC_000012.11
cg07944936	5.94E-17	1.52E-18	9.75	31.2481177	1.81E-01	107950685	107950808	NC_000006.11
cg25113462	5.99E-17	1.54E-18	9.75	31.2386121	1.45E-01	239299293	239299416	NC_000002.11
cg17284804	6.02E-17	1.54E-18	9.75	31.2342797	2.28E-01	67218135	67218258	NC_000001.10
cg25012961	6.03E-17	1.55E-18	9.74	31.2322063	2.04E-01	124034288	124034411	NC_000005.9
cg15837333	6.07E-17	1.56E-18	9.74	31.2241578	1.22E-01	64519179	64519302	NC_000012.11
cg00332021	6.08E-17	1.56E-18	9.74	31.2223856	1.96E-01	17083412	17083535	NC_000022.10
cg18566177	6.14E-17	1.58E-18	9.74	31.2137911	2.36E-01	134491483	134491606	NC_000006.11
cg23411518	6.14E-17	1.58E-18	9.74	31.2128527	1.53E-01	57680479	57680602	NC_000015.9
cg22660196	6.14E-17	1.58E-18	9.74	31.2127178	9.31E-02	143932803	143932926	NC_000006.11
cg12537437	6.18E-17	1.59E-18	9.74	31.206067	1.80E-01	74871202	74871325	NC_000011.9
cg24860020	6.21E-17	1.60E-18	9.74	31.2002754	4.24E-02	122515725	122515848	NC_000008.10
cg15814504	6.24E-17	1.60E-18	9.74	31.1965548	2.66E-01	50694985	50695108	NC_000005.9
cg16638920	6.27E-17	1.61E-18	9.74	31.1904635	1.85E-01	87974012	87974135	NC_000005.9
cg27364012	6.35E-17	1.63E-18	9.74	31.1781638	1.43E-01	109322605	109322728	NC_000006.11
cg02585142	6.38E-17	1.64E-18	9.74	31.1735692	1.45E-01	89434967	89435090	NC_000015.9
cg02428119	6.38E-17	1.64E-18	9.74	31.1726131	2.72E-01	58520874	58520997	NC_000019.9
cg00097146	6.38E-17	1.64E-18	9.74	31.1725674	2.21E-01	45171818	45171941	NC_000002.11

cg13048147	6.39E-17	1.65E-18	9.74	31.1705643	1.57E-01	73044542	73044665	NC_000017.10
cg22735650	6.40E-17	1.65E-18	9.74	31.1700099	6.19E-02	71504159	71504282	NC_000015.9
cg08211028	6.40E-17	1.65E-18	9.74	31.1690685	2.44E-01	109674963	109675086	NC_000010.10
cg03200786	6.45E-17	1.66E-18	9.73	31.1618312	1.41E-01	2483916	2484039	NC_000004.11
cg01118679	6.48E-17	1.67E-18	9.73	31.1566658	6.20E-02	87018135	87018258	NC_000001.10
cg08620154	6.48E-17	1.67E-18	9.73	31.1565581	2.78E-01	69244553	69244676	NC_000008.10
cg05206661	6.49E-17	1.67E-18	9.73	31.1549167	5.91E-02	11271531	11271654	NC_000002.11
cg14413262	6.50E-17	1.68E-18	9.73	31.1529427	9.07E-02	8384046	8384169	NC_000001.10
cg02412684	6.55E-17	1.69E-18	9.73	31.1455628	1.22E-01	123354194	123354317	NC_000010.10
cg05085500	6.55E-17	1.69E-18	9.73	31.145502	2.24E-01	19692172	19692295	NC_000006.11
cg10640181	6.57E-17	1.69E-18	9.73	31.1426174	5.46E-02	16704094	16704217	NC_000005.9
cg02217367	6.58E-17	1.70E-18	9.73	31.1405041	5.50E-02	926438	926561	NC_000017.10
cg24895178	6.58E-17	1.70E-18	9.73	31.1396816	1.32E-01	14207927	14208050	NC_000017.10
cg02533364	6.62E-17	1.71E-18	9.73	31.1338475	1.10E-01	79076083	79076206	NC_000017.10
cg04835383	6.63E-17	1.71E-18	9.73	31.1326545	1.51E-01	28888029	28888152	NC_000006.11
cg18932798	6.65E-17	1.72E-18	9.73	31.1294722	2.72E-01	105037503	105037626	NC_000010.10
cg06372151	6.65E-17	1.72E-18	9.73	31.1282439	1.66E-01	49340730	49340853	NC_000012.11
cg17744883	6.69E-17	1.73E-18	9.73	31.1224346	1.97E-01	2279386	2279509	NC_000005.9
cg24876960	6.72E-17	1.74E-18	9.73	31.1184552	2.71E-01	1883214	1883337	NC_000005.9
cg21003878	6.73E-17	1.74E-18	9.73	31.1156725	1.14E-01	24872323	24872446	NC_000014.8
cg26272270	6.75E-17	1.74E-18	9.73	31.1135953	2.54E-01	18430144	18430267	NC_000010.10
cg24375626	6.88E-17	1.78E-18	9.72	31.0936284	2.04E-01	182098716	182098839	NC_000001.10
cg08787083	6.96E-17	1.80E-18	9.72	31.0816842	1.19E-01	129868324	129868447	NC_000010.10
cg06320418	6.98E-17	1.81E-18	9.72	31.0783976	1.36E-01	766888	767011	NC_000016.9
cg17390350	6.99E-17	1.81E-18	9.72	31.077517	2.67E-01	42267747	42267870	NC_000007.13

cg06334484	7.02E-17	1.82E-18	9.72	31.0723289	1.22E-01	35948145	35948268	NC_000007.13
cg23207990	7.04E-17	1.82E-18	9.72	31.0692174	2.32E-01	154710535	154710658	NC_000004.11
cg01367097	7.05E-17	1.83E-18	9.72	31.0677948	1.55E-01	163037784	163037907	NC_000001.10
cg27337857	7.05E-17	1.83E-18	9.72	31.0673879	1.24E-01	18628714	18628837	NC_000007.13
cg09617993	7.08E-17	1.84E-18	9.72	31.0630294	2.18E-01	3239294	3239417	NC_000016.9
cg01790438	7.12E-17	1.85E-18	9.72	31.0576162	1.46E-01	10332117	10332240	NC_000005.9
cg01760189	7.18E-17	1.86E-18	9.72	31.0483405	1.42E-01	35656896	35657019	NC_000017.10
cg22694087	7.26E-17	1.88E-18	9.71	31.0370811	6.93E-02	128273476	128273599	NC_000003.11
cg23081855	7.29E-17	1.89E-18	9.71	31.0322039	2.09E-01	134889789	134889912	NC_000007.13
cg03816707	7.30E-17	1.90E-18	9.71	31.0305625	1.78E-01	43726544	43726667	NC_000020.10
cg16498194	7.31E-17	1.90E-18	9.71	31.0292099	1.60E-01	130647874	130647997	NC_000012.11
cg09257635	7.33E-17	1.90E-18	9.71	31.0268495	1.95E-01	228346181	228346304	NC_000001.10
cg02318926	7.33E-17	1.90E-18	9.71	31.0264438	2.38E-01	83952345	83952468	NC_000015.9
cg02880877	7.33E-17	1.90E-18	9.71	31.0262456	1.61E-01	125623484	125623607	NC_000006.11
cg00429509	7.38E-17	1.92E-18	9.71	31.019109	1.27E-01	365132	365255	NC_000002.11
cg26993198	7.40E-17	1.92E-18	9.71	31.0166236	2.12E-01	153859740	153859863	NC_000005.9
cg03968618	7.43E-17	1.93E-18	9.71	31.0120202	1.96E-01	186758663	186758786	NC_000004.11
cg10845893	7.45E-17	1.94E-18	9.71	31.0103674	1.61E-01	139420170	139420293	NC_000005.9
cg07514158	7.47E-17	1.94E-18	9.71	31.0072643	2.43E-01	24267526	24267649	NC_000016.9
cg27316626	7.48E-17	1.95E-18	9.71	31.0056435	1.75E-01	73450762	73450885	NC_000008.10
cg17744110	7.48E-17	1.95E-18	9.71	31.0048696	1.53E-01	47107173	47107296	NC_000019.9
cg14359292	7.49E-17	1.95E-18	9.71	31.0041492	2.02E-01	27170892	27171015	NC_000007.13
cg05213609	7.52E-17	1.96E-18	9.71	30.9997481	2.30E-01	51185407	51185530	NC_000016.9
cg26390050	7.59E-17	1.98E-18	9.71	30.9895795	9.66E-02	144621170	144621293	NC_000008.10
cg11338420	7.61E-17	1.98E-18	9.71	30.9879639	9.94E-02	144621219	144621342	NC_000008.10

cg11414560	7.61E-17	1.98E-18	9.71	30.9877644	2.73E-01	58179996	58180119	NC_000020.10
cg00573504	7.64E-17	1.99E-18	9.71	30.9831944	1.80E-01	1123809	1123932	NC_000005.9
cg22872857	7.66E-17	1.99E-18	9.71	30.9805294	1.87E-01	21794985	21795108	NC_000008.10
cg20844851	7.67E-17	2.00E-18	9.71	30.9787953	2.50E-01	117591827	117591950	NC_000006.11
cg17857070	7.67E-17	2.00E-18	9.71	30.9786026	2.39E-01	14320475	14320598	NC_000019.9
cg14548038	7.69E-17	2.00E-18	9.71	30.9766696	8.62E-02	140178418	140178541	NC_000009.11
cg23774356	7.69E-17	2.00E-18	9.71	30.9763645	3.05E-01	19137874	19137997	NC_000022.10
cg08448668	7.76E-17	2.02E-18	9.7	30.9670952	8.90E-02	245852157	245852280	NC_000001.10
cg17245135	7.77E-17	2.02E-18	9.7	30.9660278	1.22E-01	88018524	88018647	NC_000005.9
cg26500398	7.80E-17	2.03E-18	9.7	30.9612873	7.06E-02	22974616	22974739	NC_000008.10
cg06369992	7.84E-17	2.04E-18	9.7	30.9566171	2.51E-01	119494494	119494617	NC_000010.10
cg10888399	7.84E-17	2.05E-18	9.7	30.9559351	9.21E-02	18061368	18061491	NC_000022.10
cg05546264	7.90E-17	2.06E-18	9.7	30.9487318	1.44E-01	187507476	187507599	NC_000004.11
cg06685737	7.91E-17	2.06E-18	9.7	30.9472919	2.17E-01	35301730	35301853	NC_000007.13
cg22346124	7.93E-17	2.07E-18	9.7	30.9446349	2.97E-01	2291677	2291800	NC_000011.9
cg20380465	7.93E-17	2.07E-18	9.7	30.9446029	8.03E-02	13919497	13919620	NC_000005.9
cg17804850	7.95E-17	2.08E-18	9.7	30.9408007	1.56E-01	60052386	60052509	NC_000018.9
cg05546878	8.00E-17	2.09E-18	9.7	30.9351952	8.48E-02	219696695	219696818	NC_000002.11
cg20470857	8.05E-17	2.10E-18	9.7	30.928509	1.60E-01	119915783	119915906	NC_000002.11
cg08097359	8.10E-17	2.12E-18	9.7	30.9225135	1.53E-01	50064054	50064177	NC_000019.9
cg08389151	8.10E-17	2.12E-18	9.7	30.9224335	1.25E-01	58862087	58862210	NC_000019.9
cg03496114	8.11E-17	2.12E-18	9.7	30.9206002	2.05E-01	80329459	80329582	NC_000017.10
cg08133669	8.12E-17	2.12E-18	9.7	30.9195387	1.56E-01	222790218	222790341	NC_000001.10
cg19788941	8.12E-17	2.12E-18	9.7	30.9191006	1.73E-01	23907989	23908112	NC_000002.11
cg00956199	8.13E-17	2.13E-18	9.7	30.9170979	2.13E-01	8078325	8078448	NC_000010.10

cg03230154	8.16E-17	2.14E-18	9.7	30.9130383	2.52E-01	22817176	22817299	NC_000019.9
cg24468199	8.16E-17	2.14E-18	9.7	30.9130266	1.21E-01	65410028	65410151	NC_000011.9
cg16195091	8.16E-17	2.14E-18	9.7	30.9128207	2.22E-01	76227996	76228119	NC_000017.10
cg26304088	8.17E-17	2.14E-18	9.7	30.9126623	1.38E-01	51462100	51462223	NC_000019.9
cg23999224	8.17E-17	2.14E-18	9.7	30.9114994	9.08E-02	39836003	39836126	NC_000019.9
cg10569606	8.25E-17	2.16E-18	9.69	30.9017113	2.37E-01	5019004	5019127	NC_000012.11
cg26422458	8.28E-17	2.17E-18	9.69	30.898462	2.25E-01	79472452	79472575	NC_000001.10
cg26705960	8.31E-17	2.18E-18	9.69	30.8945962	2.42E-01	100999009	100999132	NC_000011.9
cg24011260	8.35E-17	2.19E-18	9.69	30.8891074	2.61E-01	153584748	153584871	NC_000007.13
cg04123776	8.36E-17	2.19E-18	9.69	30.8876357	2.77E-01	170630602	170630725	NC_000001.10
cg10273340	8.42E-17	2.21E-18	9.69	30.8804716	2.96E-01	56224793	56224916	NC_000016.9
cg04834436	8.49E-17	2.23E-18	9.69	30.8715572	1.79E-01	55362754	55362877	NC_000016.9
cg13567542	8.54E-17	2.24E-18	9.69	30.8664183	2.25E-01	30140018	30140141	NC_000006.11
cg07462756	8.54E-17	2.24E-18	9.69	30.8660317	1.99E-01	4859985	4860108	NC_000004.11
cg14611258	8.55E-17	2.24E-18	9.69	30.8643119	1.56E-01	17946468	17946591	NC_000017.10
cg14763548	8.67E-17	2.28E-18	9.69	30.8494045	3.30E-01	25062447	25062570	NC_000020.10
cg26878836	8.68E-17	2.28E-18	9.69	30.8482997	1.10E-01	68183902	68184025	NC_000011.9
cg18587340	8.74E-17	2.29E-18	9.69	30.8420716	1.74E-01	39995654	39995777	NC_000020.10
cg08066943	8.74E-17	2.30E-18	9.69	30.8411567	1.99E-01	147136922	147137045	NC_000003.11
cg01940460	8.75E-17	2.30E-18	9.68	30.839889	7.91E-02	191207242	191207365	NC_000002.11
cg24870688	8.78E-17	2.31E-18	9.68	30.8371403	1.22E-01	168399841	168399964	NC_000006.11
cg02246645	8.81E-17	2.32E-18	9.68	30.8331231	2.48E-01	103352235	103352358	NC_000012.11
cg16519433	8.82E-17	2.32E-18	9.68	30.8317645	4.62E-02	3143596	3143719	NC_000010.10
cg06636427	8.88E-17	2.33E-18	9.68	30.8246835	2.00E-01	1273200	1273323	NC_000007.13
cg19124242	8.93E-17	2.35E-18	9.68	30.8181192	1.74E-01	105747184	105747307	NC_000012.11

cg27567561	8.99E-17	2.37E-18	9.68	30.811829	1.32E-01	107974733	107974856	NC_000012.11
cg09776094	9.00E-17	2.37E-18	9.68	30.810808	8.16E-02	89357477	89357600	NC_000016.9
cg20449659	9.00E-17	2.37E-18	9.68	30.8107658	1.60E-01	32421752	32421875	NC_000011.9
cg05821789	9.03E-17	2.38E-18	9.68	30.8066467	2.75E-01	122848098	122848221	NC_000011.9
cg04983128	9.06E-17	2.39E-18	9.68	30.8031116	4.56E-02	10654462	10654585	NC_000001.10
cg02775230	9.07E-17	2.39E-18	9.68	30.8024603	1.72E-01	75263800	75263923	NC_000016.9
cg03289872	9.15E-17	2.41E-18	9.68	30.7935426	2.50E-01	56989102	56989225	NC_000019.9
cg00185829	9.17E-17	2.41E-18	9.68	30.7913152	3.06E-01	50064374	50064497	NC_000022.10
cg27347269	9.20E-17	2.43E-18	9.68	30.7870142	3.10E-01	67067867	67067990	NC_000018.9
cg17305436	9.27E-17	2.44E-18	9.68	30.7792422	2.38E-01	19837319	19837442	NC_000006.11
cg15950068	9.32E-17	2.46E-18	9.67	30.7732629	2.28E-01	84418659	84418782	NC_000006.11
cg01375869	9.33E-17	2.46E-18	9.67	30.7728516	1.21E-01	134828713	134828836	NC_000010.10
cg07514159	9.35E-17	2.47E-18	9.67	30.7700797	1.04E-01	80982257	80982380	NC_000010.10
cg18276638	9.37E-17	2.47E-18	9.67	30.7681562	2.56E-01	214360619	214360742	NC_000001.10
cg04243901	9.38E-17	2.48E-18	9.67	30.766406	8.11E-02	125441307	125441430	NC_000012.11
cg14239494	9.38E-17	2.48E-18	9.67	30.7660921	8.26E-02	40245598	40245721	NC_000020.10
cg22643811	9.41E-17	2.48E-18	9.67	30.7633487	2.29E-01	48885446	48885569	NC_000022.10
cg17394126	9.43E-17	2.49E-18	9.67	30.7609186	5.53E-02	137787060	137787183	NC_000003.11
cg13415242	9.45E-17	2.49E-18	9.67	30.7590488	7.69E-02	69568346	69568469	NC_000010.10
cg05157140	9.47E-17	2.50E-18	9.67	30.7569392	2.69E-01	1272545	1272668	NC_000007.13
cg18147485	9.49E-17	2.51E-18	9.67	30.7540234	3.68E-01	110986685	110986808	NC_000008.10
cg07104660	9.54E-17	2.52E-18	9.67	30.7486142	2.47E-01	67873511	67873634	NC_000008.10
cg21834684	9.59E-17	2.53E-18	9.67	30.7432644	1.58E-01	81797427	81797550	NC_000014.8
cg22717014	9.61E-17	2.54E-18	9.67	30.7404121	2.48E-01	111542473	111542596	NC_000004.11
cg02747390	9.62E-17	2.55E-18	9.67	30.7391868	9.93E-02	104939819	104939942	NC_000007.13

cg09530108	9.68E-17	2.56E-18	9.67	30.7332801	1.44E-01	36586710	36586833	NC_000002.11
cg11661914	9.68E-17	2.56E-18	9.67	30.732249	1.95E-01	49180849	49180972	NC_000012.11
cg10836101	9.68E-17	2.56E-18	9.67	30.7322487	1.80E-01	30020886	30021009	NC_000019.9
cg10319073	9.69E-17	2.56E-18	9.67	30.7319577	1.01E-01	123351548	123351671	NC_000012.11
cg00228796	9.69E-17	2.57E-18	9.67	30.731113	1.59E-01	21670736	21670859	NC_000001.10
cg24837370	9.70E-17	2.57E-18	9.67	30.7303367	2.23E-01	5833774	5833897	NC_000002.11
cg04007726	9.74E-17	2.58E-18	9.67	30.7253667	1.66E-01	80981129	80981252	NC_000010.10
cg02708922	9.75E-17	2.58E-18	9.67	30.7244733	1.43E-01	3502790	3502913	NC_000002.11
cg09569989	9.84E-17	2.61E-18	9.67	30.7151269	1.08E-01	38053419	38053542	NC_000004.11
cg02417576	9.86E-17	2.61E-18	9.67	30.7133204	9.21E-02	46846389	46846512	NC_000021.8
cg18583931	9.87E-17	2.62E-18	9.67	30.7123428	1.22E-01	71378283	71378406	NC_000015.9
cg16595750	9.89E-17	2.62E-18	9.67	30.7100899	1.18E-01	123338597	123338720	NC_000010.10
cg27580905	9.89E-17	2.62E-18	9.67	30.7100019	1.45E-01	39335085	39335208	NC_000002.11
cg25094569	9.93E-17	2.63E-18	9.66	30.7056562	2.22E-01	32448769	32448892	NC_000011.9
cg10898258	1.01E-16	2.67E-18	9.66	30.6929236	6.07E-02	104192495	104192618	NC_000014.8
cg18349138	1.01E-16	2.68E-18	9.66	30.6892445	2.61E-01	19617138	19617261	NC_000021.8
cg19764973	1.01E-16	2.68E-18	9.66	30.6868205	6.28E-02	59525433	59525556	NC_000011.9
cg08248516	1.01E-16	2.69E-18	9.66	30.6857331	2.34E-01	27194745	27194868	NC_000007.13
cg09851951	1.01E-16	2.69E-18	9.66	30.6843502	2.22E-01	54323498	54323621	NC_000016.9
cg22328746	1.02E-16	2.70E-18	9.66	30.6816438	1.14E-01	186578455	186578578	NC_000004.11
cg24774396	1.02E-16	2.70E-18	9.66	30.6809082	1.65E-01	706005	706128	NC_000017.10
cg05275605	1.02E-16	2.71E-18	9.66	30.6771869	1.13E-01	46844883	46845006	NC_000021.8
cg05835726	1.03E-16	2.74E-18	9.66	30.6673099	2.12E-01	27183861	27183984	NC_000007.13
cg04308769	1.03E-16	2.74E-18	9.66	30.665181	2.01E-01	200007623	200007746	NC_000001.10
cg18617005	1.03E-16	2.75E-18	9.66	30.6642102	2.77E-01	140787504	140787627	NC_000005.9

cg25855768	1.03E-16	2.75E-18	9.66	30.6621462	1.36E-01	127472121	127472244	NC_000007.13
cg20585500	1.04E-16	2.77E-18	9.66	30.6567122	1.52E-01	64703192	64703315	NC_000011.9
cg09587487	1.04E-16	2.77E-18	9.66	30.6560064	1.13E-01	178993784	178993907	NC_000001.10
cg01477546	1.05E-16	2.78E-18	9.66	30.6516555	7.46E-02	86096098	86096221	NC_000002.11
cg22816059	1.05E-16	2.79E-18	9.66	30.6489829	8.55E-02	422114	422237	NC_000005.9
cg25053002	1.05E-16	2.80E-18	9.65	30.6436106	6.99E-02	3142765	3142888	NC_000010.10
cg26963839	1.05E-16	2.81E-18	9.65	30.6421693	5.34E-02	134230196	134230319	NC_000010.10
cg05015495	1.06E-16	2.81E-18	9.65	30.6418536	8.45E-02	93957782	93957905	NC_000005.9
cg27120816	1.06E-16	2.82E-18	9.65	30.6391098	2.20E-01	62070164	62070287	NC_000016.9
cg19961522	1.06E-16	2.82E-18	9.65	30.6381372	1.62E-01	30407388	30407511	NC_000020.10
cg10266455	1.06E-16	2.82E-18	9.65	30.6379073	1.02E-01	174411207	174411330	NC_000002.11
cg15708153	1.06E-16	2.83E-18	9.65	30.6356299	3.24E-01	56879554	56879677	NC_000019.9
cg21790626	1.06E-16	2.83E-18	9.65	30.6330083	2.76E-01	58220494	58220617	NC_000019.9
cg12133498	1.07E-16	2.85E-18	9.65	30.625914	1.82E-01	148248073	148248196	NC_000001.10
cg16640855	1.08E-16	2.87E-18	9.65	30.6192073	2.54E-01	182543233	182543356	NC_000002.11
cg26769435	1.08E-16	2.88E-18	9.65	30.6157547	8.52E-02	53397932	53398055	NC_000008.10
cg20239391	1.08E-16	2.88E-18	9.65	30.6151418	1.59E-01	45030732	45030855	NC_000020.10
cg05267893	1.09E-16	2.90E-18	9.65	30.6112877	5.51E-02	156213621	156213744	NC_000001.10
cg20434239	1.09E-16	2.90E-18	9.65	30.6089136	4.09E-02	43028046	43028169	NC_000017.10
cg16897114	1.10E-16	2.92E-18	9.65	30.6026293	2.54E-01	5153174	5153297	NC_000012.11
cg03199646	1.10E-16	2.92E-18	9.65	30.6025019	9.25E-02	105408428	105408551	NC_000007.13
cg25897161	1.10E-16	2.94E-18	9.65	30.5964878	1.34E-01	64367485	64367608	NC_000015.9
cg16400238	1.10E-16	2.95E-18	9.65	30.5934897	6.19E-02	27237364	27237487	NC_000017.10
cg01069965	1.11E-16	2.95E-18	9.65	30.5927354	1.60E-01	48331548	48331671	NC_000020.10
cg20985450	1.11E-16	2.95E-18	9.65	30.5927088	2.91E-01	100621382	100621505	NC_000013.10

cg17290701	1.11E-16	2.96E-18	9.65	30.5905252	3.02E-01	11059714	11059837	NC_000010.10
cg10888878	1.11E-16	2.96E-18	9.65	30.5889939	6.64E-02	48459961	48460084	NC_000001.10
cg23369632	1.11E-16	2.97E-18	9.65	30.5852996	2.06E-01	56438637	56438760	NC_000006.11
cg14189141	1.12E-16	2.98E-18	9.65	30.5814275	2.27E-01	1042605	1042728	NC_000009.11
cg00578032	1.12E-16	2.99E-18	9.65	30.5812143	1.71E-01	38308069	38308192	NC_000019.9
cg13427828	1.12E-16	2.99E-18	9.65	30.5800865	1.03E-01	42301921	42302044	NC_000003.11
cg27318539	1.12E-16	2.99E-18	9.65	30.5799248	7.67E-02	10220494	10220617	NC_000019.9
cg26102342	1.12E-16	2.99E-18	9.64	30.5782626	1.11E-01	98878655	98878778	NC_000012.11
cg24394172	1.12E-16	3.01E-18	9.64	30.5739401	1.83E-01	46995203	46995326	NC_000004.11
cg07349208	1.13E-16	3.02E-18	9.64	30.5709119	2.11E-01	111532679	111532802	NC_000004.11
cg08621277	1.13E-16	3.02E-18	9.64	30.570611	7.06E-02	68271518	68271641	NC_000011.9
cg24148757	1.13E-16	3.03E-18	9.64	30.5669586	8.32E-02	112942995	112943118	NC_000001.10
cg05754861	1.13E-16	3.04E-18	9.64	30.5646836	1.47E-01	99961768	99961891	NC_000008.10
cg03714676	1.14E-16	3.05E-18	9.64	30.5615307	1.84E-01	48174649	48174772	NC_000008.10
cg18391899	1.14E-16	3.06E-18	9.64	30.5568983	2.27E-01	23462616	23462739	NC_000010.10
cg19938826	1.14E-16	3.06E-18	9.64	30.5554036	1.46E-01	145218181	145218304	NC_000005.9
cg12453014	1.15E-16	3.08E-18	9.64	30.5518386	2.54E-01	119801142	119801265	NC_000005.9
cg06645033	1.15E-16	3.08E-18	9.64	30.5513689	1.56E-01	50890678	50890801	NC_000001.10
cg24596008	1.15E-16	3.08E-18	9.64	30.5503592	6.05E-02	242741273	242741396	NC_000002.11
cg07857469	1.15E-16	3.09E-18	9.64	30.5484199	1.58E-01	81107185	81107308	NC_000004.11
cg04613834	1.15E-16	3.09E-18	9.64	30.5457699	1.70E-01	12996419	12996542	NC_000016.9
cg17103088	1.16E-16	3.10E-18	9.64	30.5444362	1.44E-01	3600595	3600718	NC_000019.9
cg23303685	1.16E-16	3.10E-18	9.64	30.5433276	1.56E-01	157521999	157522122	NC_000006.11
cg03413465	1.16E-16	3.10E-18	9.64	30.5432946	2.18E-01	172734776	172734899	NC_000004.11
cg03975694	1.16E-16	3.12E-18	9.64	30.5381884	2.06E-01	38042472	38042595	NC_000019.9

cg00525392	1.17E-16	3.14E-18	9.64	30.531941	1.46E-01	19744123	19744246	NC_000018.9
cg00529958	1.17E-16	3.15E-18	9.64	30.5284976	1.84E-01	100624288	100624411	NC_000013.10
cg00295485	1.18E-16	3.16E-18	9.64	30.5248991	1.53E-01	106755721	106755844	NC_000002.11
cg18249173	1.19E-16	3.19E-18	9.64	30.5144502	2.70E-01	35293245	35293368	NC_000007.13
cg05107500	1.19E-16	3.20E-18	9.63	30.513007	1.67E-01	6070292	6070415	NC_000016.9
cg09936609	1.19E-16	3.20E-18	9.63	30.5126784	1.16E-01	232283700	232283823	NC_000002.11
cg14244674	1.19E-16	3.21E-18	9.63	30.509378	1.06E-01	140148456	140148579	NC_000009.11
cg26053480	1.20E-16	3.22E-18	9.63	30.5051264	1.88E-01	25898758	25898881	NC_000007.13
cg25952663	1.20E-16	3.24E-18	9.63	30.5003683	1.00E-01	54450826	54450949	NC_000012.11
cg24699097	1.20E-16	3.24E-18	9.63	30.5001872	1.09E-01	29795469	29795592	NC_000017.10
cg17047566	1.21E-16	3.25E-18	9.63	30.4982609	6.99E-02	38120258	38120381	NC_000006.11
cg15378605	1.21E-16	3.26E-18	9.63	30.4948937	1.30E-01	125805592	125805715	NC_000010.10
cg19864758	1.22E-16	3.29E-18	9.63	30.4847317	2.54E-01	17206720	17206843	NC_000020.10
cg18643383	1.22E-16	3.29E-18	9.63	30.4845893	8.26E-02	69864882	69865005	NC_000002.11
cg14750277	1.22E-16	3.30E-18	9.63	30.482922	2.54E-01	73164024	73164147	NC_000008.10
cg18918418	1.23E-16	3.30E-18	9.63	30.4821802	8.61E-02	216745386	216745509	NC_000002.11
cg14738806	1.23E-16	3.32E-18	9.63	30.4756328	2.24E-01	115102713	115102836	NC_000012.11
cg03981213	1.23E-16	3.32E-18	9.63	30.4750034	1.73E-01	60766554	60766677	NC_000002.11
cg12118269	1.24E-16	3.35E-18	9.63	30.4667787	2.35E-01	102893980	102894103	NC_000010.10
cg04490714	1.25E-16	3.36E-18	9.63	30.4647319	2.03E-01	55690564	55690687	NC_000016.9
cg10004365	1.25E-16	3.37E-18	9.63	30.4599754	1.32E-01	134657833	134657956	NC_000010.10
cg11718868	1.26E-16	3.40E-18	9.63	30.4521031	1.53E-01	94487237	94487360	NC_000010.10
cg03916756	1.27E-16	3.42E-18	9.62	30.446883	6.81E-02	79032487	79032610	NC_000017.10
cg11804381	1.27E-16	3.43E-18	9.62	30.4426274	5.92E-02	36662414	36662537	NC_000022.10
cg26293019	1.28E-16	3.45E-18	9.62	30.4379013	2.36E-01	50688913	50689036	NC_000005.9

cg07027430	1.28E-16	3.46E-18	9.62	30.4363173	1.96E-01	7354685	7354808	NC_000016.9
cg09430118	1.29E-16	3.47E-18	9.62	30.4313843	2.11E-01	37095681	37095804	NC_000019.9
cg13879483	1.29E-16	3.47E-18	9.62	30.4307869	3.35E-01	95942907	95943030	NC_000012.11
cg04672979	1.29E-16	3.49E-18	9.62	30.4272059	1.38E-01	13369111	13369234	NC_000012.11
cg04549162	1.30E-16	3.50E-18	9.62	30.424526	1.92E-01	125425690	125425813	NC_000010.10
cg00187692	1.30E-16	3.50E-18	9.62	30.4228936	2.25E-01	52199954	52200077	NC_000013.10
cg02874376	1.30E-16	3.51E-18	9.62	30.4219826	2.42E-01	101193397	101193520	NC_000014.8
cg09821185	1.30E-16	3.51E-18	9.62	30.421542	2.41E-01	101923366	101923489	NC_000014.8
cg17976839	1.30E-16	3.51E-18	9.62	30.4211397	1.15E-01	50923823	50923946	NC_000013.10
cg17283453	1.30E-16	3.52E-18	9.62	30.4172153	1.82E-01	9473880	9474003	NC_000019.9
cg10289790	1.30E-16	3.53E-18	9.62	30.4165181	1.60E-01	105359915	105360038	NC_000014.8
cg23401445	1.30E-16	3.53E-18	9.62	30.4163277	1.80E-01	113442419	113442542	NC_000004.11
cg03431585	1.31E-16	3.55E-18	9.62	30.4108276	9.54E-02	44995633	44995756	NC_000020.10
cg13012916	1.31E-16	3.55E-18	9.62	30.4090897	2.34E-01	36973691	36973814	NC_000014.8
cg25451660	1.32E-16	3.56E-18	9.62	30.4074731	1.43E-01	11641324	11641447	NC_000011.9
cg10767192	1.32E-16	3.56E-18	9.62	30.4064208	2.00E-01	115135606	115135729	NC_000012.11
cg05984554	1.32E-16	3.57E-18	9.62	30.4048347	2.17E-01	30019369	30019492	NC_000019.9
cg20401252	1.33E-16	3.60E-18	9.62	30.3956805	1.84E-01	5834062	5834185	NC_000002.11
cg20265470	1.34E-16	3.63E-18	9.62	30.3881081	6.32E-02	20503098	20503221	NC_000001.10
cg19339311	1.34E-16	3.63E-18	9.62	30.3868873	7.83E-02	42213292	42213415	NC_000020.10
cg17076890	1.34E-16	3.63E-18	9.62	30.3864585	2.08E-01	23481421	23481544	NC_000010.10
cg08855249	1.36E-16	3.67E-18	9.61	30.3758114	5.42E-02	207975363	207975486	NC_000001.10
cg14649650	1.37E-16	3.71E-18	9.61	30.3659812	3.17E-01	13933996	13934119	NC_000010.10
cg09888336	1.37E-16	3.71E-18	9.61	30.3648061	8.73E-02	57180335	57180458	NC_000004.11
cg04556126	1.37E-16	3.72E-18	9.61	30.3628913	2.35E-01	147106561	147106684	NC_000003.11

cg03602280	1.38E-16	3.74E-18	9.61	30.3580534	2.67E-01	11148914	11149037	NC_000018.9
cg23222169	1.38E-16	3.74E-18	9.61	30.3573911	2.07E-01	47710949	47711072	NC_000013.10
cg26877508	1.38E-16	3.74E-18	9.61	30.3571378	1.86E-01	71495324	71495447	NC_000015.9
cg07050692	1.38E-16	3.75E-18	9.61	30.355298	2.40E-01	61664695	61664818	NC_000008.10
cg16497340	1.39E-16	3.76E-18	9.61	30.3524409	1.64E-01	180601183	180601306	NC_000005.9
cg26590537	1.39E-16	3.76E-18	9.61	30.3521824	2.50E-01	5020202	5020325	NC_000012.11
cg19157034	1.39E-16	3.77E-18	9.61	30.3504504	2.27E-01	89905761	89905884	NC_000015.9
cg09643136	1.40E-16	3.80E-18	9.61	30.3434528	1.72E-01	223176683	223176806	NC_000002.11
cg24767148	1.40E-16	3.80E-18	9.61	30.3430477	2.53E-01	23481786	23481909	NC_000010.10
cg12381873	1.40E-16	3.80E-18	9.61	30.3427865	1.30E-01	72270059	72270182	NC_000017.10
cg12860686	1.41E-16	3.82E-18	9.61	30.3363319	2.20E-01	2753876	2753999	NC_000005.9
cg16294325	1.41E-16	3.84E-18	9.61	30.3320217	1.18E-01	42929934	42930057	NC_000006.11
cg14717906	1.41E-16	3.84E-18	9.61	30.3306324	1.28E-01	138641864	138641987	NC_000005.9
cg05349077	1.42E-16	3.86E-18	9.61	30.3279372	1.83E-01	630159	630282	NC_000016.9
cg21830368	1.42E-16	3.86E-18	9.61	30.3262303	2.18E-01	75373327	75373450	NC_000017.10
cg25606066	1.43E-16	3.88E-18	9.61	30.3205358	1.50E-01	245844087	245844210	NC_000001.10
cg07829842	1.44E-16	3.92E-18	9.6	30.3108063	2.73E-01	56228495	56228618	NC_000016.9
cg23987444	1.45E-16	3.93E-18	9.6	30.3080125	3.15E-01	50817810	50817933	NC_000010.10
cg20190649	1.45E-16	3.94E-18	9.6	30.3064826	1.70E-01	46255433	46255556	NC_000017.10
cg20696432	1.46E-16	3.96E-18	9.6	30.3011909	1.29E-01	18881907	18882030	NC_000017.10
cg14750948	1.46E-16	3.96E-18	9.6	30.3003517	2.09E-01	147130477	147130600	NC_000003.11
cg16234978	1.46E-16	3.97E-18	9.6	30.2997219	2.06E-01	1704095	1704218	NC_000007.13
cg06041278	1.46E-16	3.97E-18	9.6	30.2996621	1.62E-01	43937467	43937590	NC_000020.10
cg03518469	1.46E-16	3.97E-18	9.6	30.298333	6.55E-02	88791646	88791769	NC_000016.9
cg19673115	1.46E-16	3.98E-18	9.6	30.2960549	6.70E-02	3105482	3105605	NC_000010.10

cg04171235	1.47E-16	4.01E-18	9.6	30.2894765	1.58E-01	46604393	46604516	NC_000017.10
cg10628201	1.48E-16	4.03E-18	9.6	30.2852276	9.97E-02	21977597	21977720	NC_000022.10
cg01307939	1.48E-16	4.03E-18	9.6	30.2845656	2.43E-01	98467571	98467694	NC_000007.13
cg19125081	1.48E-16	4.03E-18	9.6	30.284096	7.22E-02	242219370	242219493	NC_000002.11
cg20354141	1.50E-16	4.09E-18	9.6	30.2684914	1.99E-01	100609766	100609889	NC_000007.13
cg14447193	1.50E-16	4.10E-18	9.6	30.265756	1.34E-01	87433864	87433987	NC_000009.11
cg14522302	1.52E-16	4.15E-18	9.6	30.2556544	6.47E-02	226300601	226300724	NC_000001.10
cg06585566	1.53E-16	4.17E-18	9.59	30.2502246	2.52E-01	57276794	57276917	NC_000019.9
cg04739647	1.53E-16	4.17E-18	9.59	30.2495803	1.97E-01	176987462	176987585	NC_000002.11
cg06610849	1.53E-16	4.18E-18	9.59	30.2486218	2.59E-01	58520847	58520970	NC_000019.9
cg18633154	1.54E-16	4.21E-18	9.59	30.2418609	1.99E-01	77296234	77296357	NC_000014.8
cg21305068	1.55E-16	4.25E-18	9.59	30.2316467	1.08E-01	45622219	45622342	NC_000021.8
cg11787626	1.56E-16	4.25E-18	9.59	30.2310294	3.81E-02	147233738	147233861	NC_000001.10
cg21627409	1.56E-16	4.26E-18	9.59	30.2283286	2.35E-01	140810106	140810229	NC_000005.9
cg25120992	1.56E-16	4.27E-18	9.59	30.2257077	7.57E-02	31735435	31735558	NC_000006.11
cg24509810	1.57E-16	4.28E-18	9.59	30.2237699	2.00E-01	80549634	80549757	NC_000002.11
cg21816308	1.57E-16	4.30E-18	9.59	30.2207533	2.31E-01	39044140	39044263	NC_000001.10
cg24574863	1.58E-16	4.31E-18	9.59	30.2166103	1.05E-01	42954752	42954875	NC_000005.9
cg12740087	1.58E-16	4.32E-18	9.59	30.2153461	6.81E-02	224445738	224445861	NC_000001.10
cg14317414	1.58E-16	4.32E-18	9.59	30.2153231	1.68E-01	109991376	109991499	NC_000009.11
cg26751356	1.59E-16	4.35E-18	9.59	30.2085135	2.24E-01	112712635	112712758	NC_000013.10
cg01371799	1.59E-16	4.36E-18	9.59	30.2052145	1.65E-01	1264049	1264172	NC_000007.13
cg27649806	1.60E-16	4.37E-18	9.59	30.203969	9.38E-02	35101217	35101340	NC_000009.11
cg14562712	1.60E-16	4.38E-18	9.59	30.2017402	2.81E-01	100823377	100823500	NC_000007.13
cg14983606	1.60E-16	4.38E-18	9.59	30.2015282	2.46E-01	65493706	65493829	NC_000008.10

cg07671291	1.60E-16	4.40E-18	9.59	30.1978178	9.81E-02	67523109	67523232	NC_000017.10
cg19980199	1.61E-16	4.40E-18	9.59	30.197001	1.04E-01	480033	480156	NC_000005.9
cg23746628	1.61E-16	4.41E-18	9.59	30.1939184	1.61E-01	74439996	74440119	NC_000004.11
cg02944637	1.63E-16	4.46E-18	9.58	30.1832695	1.30E-01	19733345	19733468	NC_000016.9
cg12121983	1.63E-16	4.47E-18	9.58	30.180614	1.04E-01	1387582	1387705	NC_000017.10
cg00765783	1.64E-16	4.49E-18	9.58	30.1764117	2.46E-01	12996587	12996710	NC_000016.9
cg14518868	1.64E-16	4.50E-18	9.58	30.1754364	2.18E-02	69864352	69864475	NC_000012.11
cg11831238	1.65E-16	4.52E-18	9.58	30.1696891	1.92E-01	20182021	20182144	NC_000011.9
cg03566787	1.65E-16	4.53E-18	9.58	30.1685289	1.13E-01	47517381	47517504	NC_000011.9
cg04928096	1.65E-16	4.53E-18	9.58	30.1675582	7.93E-02	338079	338202	NC_000016.9
cg15554678	1.65E-16	4.54E-18	9.58	30.1669237	2.22E-01	17083563	17083686	NC_000022.10
cg20776543	1.66E-16	4.55E-18	9.58	30.1641901	1.56E-01	921156	921279	NC_000007.13
cg10658542	1.66E-16	4.55E-18	9.58	30.1628239	2.84E-01	33592642	33592765	NC_000012.11
cg21856645	1.67E-16	4.59E-18	9.58	30.1560122	6.30E-02	23517243	23517366	NC_000014.8
cg04514249	1.67E-16	4.59E-18	9.58	30.1543116	2.82E-01	144621385	144621508	NC_000004.11
cg26587870	1.68E-16	4.62E-18	9.58	30.1481427	1.62E-01	27730563	27730686	NC_000006.11
cg26204042	1.69E-16	4.65E-18	9.58	30.1424695	1.74E-01	110163765	110163888	NC_000011.9
cg24154839	1.70E-16	4.66E-18	9.58	30.1396186	1.95E-01	46995741	46995864	NC_000004.11
cg17629929	1.70E-16	4.68E-18	9.58	30.1367766	2.41E-01	1875548	1875671	NC_000005.9
cg11099899	1.70E-16	4.68E-18	9.58	30.1357315	1.87E-01	37852334	37852457	NC_000021.8
cg11361442	1.71E-16	4.70E-18	9.58	30.1315309	1.51E-01	68841024	68841147	NC_000011.9
cg05327509	1.71E-16	4.72E-18	9.58	30.1281217	8.73E-02	101175894	101176017	NC_000013.10
cg01069451	1.72E-16	4.73E-18	9.58	30.1263989	1.13E-01	3210387	3210510	NC_000020.10
cg06722633	1.72E-16	4.74E-18	9.58	30.1241097	1.93E-01	37499309	37499432	NC_000001.10
cg06668065	1.72E-16	4.75E-18	9.58	30.1217171	2.07E-01	92909069	92909192	NC_000005.9

cg08324703	1.73E-16	4.76E-18	9.57	30.1185731	1.86E-01	679514	679637	NC_000010.10
cg25496678	1.73E-16	4.77E-18	9.57	30.1170465	2.47E-01	118899407	118899530	NC_000010.10
cg00986824	1.74E-16	4.78E-18	9.57	30.1151919	1.97E-01	75601465	75601588	NC_000012.11
cg17606491	1.74E-16	4.78E-18	9.57	30.1151781	9.70E-02	125806390	125806513	NC_000010.10
cg08101174	1.74E-16	4.80E-18	9.57	30.111157	9.33E-02	104196541	104196664	NC_000010.10
cg05245094	1.75E-16	4.82E-18	9.57	30.1070604	1.42E-01	85669572	85669695	NC_000016.9
cg06077738	1.76E-16	4.85E-18	9.57	30.0996981	9.75E-02	63872429	63872552	NC_000003.11
cg10710457	1.77E-16	4.87E-18	9.57	30.0964854	4.58E-02	3143236	3143359	NC_000010.10
cg22715788	1.77E-16	4.88E-18	9.57	30.0948613	1.25E-01	2624148	2624271	NC_000007.13
cg13557668	1.77E-16	4.88E-18	9.57	30.0946959	2.39E-01	135265931	135266054	NC_000005.9
cg13982505	1.78E-16	4.90E-18	9.57	30.0895176	1.60E-01	233642094	233642217	NC_000002.11
cg16538343	1.78E-16	4.92E-18	9.57	30.0861038	7.76E-02	68772961	68773084	NC_000011.9
cg07920503	1.80E-16	4.96E-18	9.57	30.0784833	2.99E-01	25745406	25745529	NC_000013.10
cg08701047	1.80E-16	4.96E-18	9.57	30.0783386	2.16E-01	130085199	130085322	NC_000010.10
cg04943927	1.80E-16	4.98E-18	9.57	30.0752501	9.82E-02	12937632	12937755	NC_000012.11
cg25984671	1.80E-16	4.98E-18	9.57	30.0751449	2.27E-01	591083	591206	NC_000020.10
cg19353431	1.81E-16	4.99E-18	9.57	30.0731764	1.89E-01	170578785	170578908	NC_000006.11
cg13021333	1.81E-16	4.99E-18	9.57	30.0730764	2.65E-01	6004207	6004330	NC_000006.11
cg04332534	1.81E-16	5.00E-18	9.57	30.070945	2.43E-01	37096487	37096610	NC_000019.9
cg04541368	1.82E-16	5.02E-18	9.57	30.0671659	2.21E-01	92909429	92909552	NC_000005.9
cg14308254	1.82E-16	5.04E-18	9.57	30.0623283	1.34E-01	124219699	124219822	NC_000008.10
cg05237374	1.83E-16	5.06E-18	9.57	30.059534	2.05E-01	66980127	66980250	NC_000014.8
cg06204922	1.83E-16	5.06E-18	9.57	30.0576659	2.90E-01	5019933	5020056	NC_000012.11
cg00663077	1.84E-16	5.08E-18	9.56	30.0554008	2.41E-01	188916709	188916832	NC_000004.11
cg18077971	1.84E-16	5.08E-18	9.56	30.0553387	2.55E-01	223164867	223164990	NC_000002.11

cg26950756	1.84E-16	5.08E-18	9.56	30.0548746	9.59E-02	78707683	78707806	NC_000015.9
cg21219666	1.84E-16	5.09E-18	9.56	30.0529876	7.58E-02	40166108	40166231	NC_000017.10
cg13432737	1.85E-16	5.11E-18	9.56	30.047928	8.91E-02	37922420	37922543	NC_000017.10
cg01397513	1.86E-16	5.15E-18	9.56	30.0409755	1.42E-01	85670135	85670258	NC_000016.9
cg03122264	1.87E-16	5.17E-18	9.56	30.0374015	8.85E-02	127797208	127797331	NC_000006.11
cg06816350	1.87E-16	5.17E-18	9.56	30.0372827	2.22E-01	2278885	2279008	NC_000005.9
cg21883754	1.87E-16	5.17E-18	9.56	30.036899	2.07E-01	54926437	54926560	NC_000019.9
cg03111576	1.87E-16	5.18E-18	9.56	30.0359188	5.03E-02	11268374	11268497	NC_000016.9
cg09257178	1.87E-16	5.18E-18	9.56	30.0353854	9.33E-02	114197839	114197962	NC_000002.11
cg09252766	1.88E-16	5.22E-18	9.56	30.0278146	7.90E-02	134722243	134722366	NC_000010.10
cg21479226	1.89E-16	5.23E-18	9.56	30.0260198	3.37E-01	69244510	69244633	NC_000008.10
cg11372090	1.89E-16	5.23E-18	9.56	30.0249843	3.01E-01	37488660	37488783	NC_000007.13
cg04494873	1.89E-16	5.24E-18	9.56	30.0244355	1.65E-01	72677287	72677410	NC_000005.9
cg00260012	1.89E-16	5.24E-18	9.56	30.0237858	1.45E-01	31031702	31031825	NC_000002.11
cg03751030	1.90E-16	5.28E-18	9.56	30.0169205	2.12E-01	56015576	56015699	NC_000008.10
cg13976988	1.91E-16	5.28E-18	9.56	30.0158087	1.54E-01	38607402	38607525	NC_000005.9
cg02757432	1.91E-16	5.29E-18	9.56	30.0154146	1.63E-01	125426048	125426171	NC_000010.10
cg27296759	1.91E-16	5.30E-18	9.56	30.0136134	1.51E-01	71503901	71504024	NC_000005.9
cg08236767	1.91E-16	5.30E-18	9.56	30.0121717	2.87E-01	107503662	107503785	NC_000002.11
cg26195356	1.91E-16	5.31E-18	9.56	30.0109771	2.11E-01	2743601	2743724	NC_000005.9
cg23051392	1.92E-16	5.32E-18	9.56	30.0090991	2.21E-01	34043481	34043604	NC_000005.9
cg26053291	1.92E-16	5.32E-18	9.56	30.0085613	1.52E-01	89064631	89064754	NC_000002.11
cg06094615	1.93E-16	5.35E-18	9.56	30.0031072	2.23E-01	50887578	50887701	NC_000010.10
cg06401021	1.94E-16	5.39E-18	9.56	29.9965086	3.01E-01	55443868	55443991	NC_000006.11
cg18239942	1.94E-16	5.39E-18	9.56	29.9952946	1.10E-01	105793879	105794002	NC_000014.8

cg04816311	1.95E-16	5.41E-18	9.56	29.9929754	1.78E-01	1066650	1066773	NC_000007.13
cg23528400	1.95E-16	5.41E-18	9.56	29.9924573	2.93E-01	32859438	32859561	NC_000003.11
cg22145666	1.95E-16	5.41E-18	9.56	29.9917206	8.32E-02	234663900	234664023	NC_000002.11
cg10806483	1.95E-16	5.41E-18	9.56	29.9916438	8.91E-02	102459329	102459452	NC_000012.11
cg13594465	1.95E-16	5.42E-18	9.55	29.9902027	1.16E-01	71491485	71491608	NC_000015.9
cg10034890	1.95E-16	5.43E-18	9.55	29.9888008	2.90E-01	230578216	230578339	NC_000002.11
cg16504397	1.96E-16	5.44E-18	9.55	29.9860058	1.29E-01	27523945	27524068	NC_000011.9
cg02066441	1.97E-16	5.46E-18	9.55	29.982398	1.63E-01	17858588	17858711	NC_000001.10
cg14712186	1.97E-16	5.48E-18	9.55	29.9797039	2.05E-01	91184413	91184536	NC_000001.10
cg19741167	1.99E-16	5.52E-18	9.55	29.9719113	2.54E-01	86263270	86263393	NC_000002.11
cg02499453	1.99E-16	5.53E-18	9.55	29.9713455	1.06E-01	32572722	32572845	NC_000022.10
cg11075176	1.99E-16	5.53E-18	9.55	29.9702631	1.49E-01	155476729	155476852	NC_000007.13
cg08361180	2.00E-16	5.58E-18	9.55	29.9623711	7.08E-02	103432427	103432550	NC_000010.10
cg06272038	2.01E-16	5.59E-18	9.55	29.960539	2.55E-01	2292512	2292635	NC_000011.9
cg01610488	2.02E-16	5.63E-18	9.55	29.9537155	2.35E-01	72987870	72987993	NC_000008.10
cg04720592	2.02E-16	5.63E-18	9.55	29.952107	1.59E-01	118165724	118165847	NC_000001.10
cg18757267	2.03E-16	5.64E-18	9.55	29.9512206	7.05E-02	164704545	164704668	NC_000001.10
cg02281208	2.04E-16	5.68E-18	9.55	29.9448614	2.28E-01	27216008	27216131	NC_000015.9
cg19894253	2.04E-16	5.69E-18	9.55	29.9424831	1.38E-01	124261709	124261832	NC_000008.10
cg18034637	2.04E-16	5.70E-18	9.55	29.9413759	1.66E-01	111846087	111846210	NC_000011.9
cg27163412	2.05E-16	5.73E-18	9.55	29.9362029	8.58E-02	137358801	137358924	NC_000009.11
cg02282631	2.06E-16	5.74E-18	9.55	29.9334718	2.20E-01	42953543	42953666	NC_000005.9
cg27296293	2.06E-16	5.75E-18	9.55	29.9324286	2.86E-01	101454680	101454803	NC_000011.9
cg07641160	2.07E-16	5.77E-18	9.55	29.927693	2.97E-01	117086850	117086973	NC_000006.11
cg07273003	2.07E-16	5.78E-18	9.55	29.9260432	2.19E-01	16955416	16955539	NC_000011.9

cg25605754	2.08E-16	5.79E-18	9.54	29.9249577	1.20E-01	116595196	116595319	NC_000007.13
cg04575395	2.08E-16	5.80E-18	9.54	29.9232066	2.53E-01	5892072	5892195	NC_000004.11
cg13918042	2.08E-16	5.82E-18	9.54	29.9208111	2.16E-01	98596430	98596553	NC_000010.10
cg16509158	2.09E-16	5.82E-18	9.54	29.9198256	9.46E-02	95894040	95894163	NC_000011.9
cg22122013	2.09E-16	5.82E-18	9.54	29.9191679	8.40E-02	97564501	97564624	NC_000002.11
cg18957463	2.09E-16	5.85E-18	9.54	29.9154107	1.44E-01	47597617	47597740	NC_000004.11
cg08499046	2.10E-16	5.85E-18	9.54	29.9142152	2.05E-01	31824327	31824450	NC_000011.9
cg03122532	2.10E-16	5.87E-18	9.54	29.9117213	1.69E-01	39644216	39644339	NC_000021.8
cg05552490	2.11E-16	5.90E-18	9.54	29.9064082	1.45E-01	43952511	43952634	NC_000006.11
cg26402660	2.12E-16	5.94E-18	9.54	29.9006101	2.17E-01	27179268	27179391	NC_000007.13
cg00526336	2.13E-16	5.96E-18	9.54	29.8963432	7.90E-02	230415902	230416025	NC_000001.10
cg19905504	2.13E-16	5.96E-18	9.54	29.8962819	8.61E-02	242165248	242165371	NC_000002.11
cg10178917	2.14E-16	5.99E-18	9.54	29.8918928	7.30E-02	99665210	99665333	NC_000014.8
cg03346706	2.14E-16	5.99E-18	9.54	29.8915732	1.64E-01	3162988	3163111	NC_000019.9
cg06375328	2.15E-16	6.00E-18	9.54	29.890317	5.27E-02	69308768	69308891	NC_000011.9
cg07295918	2.15E-16	6.01E-18	9.54	29.8886298	9.91E-02	69463131	69463254	NC_000011.9
cg02511172	2.15E-16	6.01E-18	9.54	29.8885609	6.50E-02	233085209	233085332	NC_000001.10
cg10132208	2.15E-16	6.02E-18	9.54	29.886168	1.64E-01	58545542	58545665	NC_000019.9
cg07204550	2.17E-16	6.07E-18	9.54	29.8788712	1.88E-01	21950155	21950278	NC_000004.11
cg18527574	2.17E-16	6.09E-18	9.54	29.875616	1.66E-01	222434303	222434426	NC_000002.11
cg25875213	2.18E-16	6.10E-18	9.54	29.8742649	2.76E-01	38183055	38183178	NC_000019.9
cg14950829	2.18E-16	6.11E-18	9.54	29.8723707	2.00E-01	53419617	53419740	NC_000013.10
cg15150652	2.19E-16	6.14E-18	9.54	29.866982	1.51E-01	82637778	82637901	NC_000005.9
cg09434624	2.19E-16	6.15E-18	9.54	29.865459	1.36E-01	8010881	8011004	NC_000011.9
cg02630214	2.20E-16	6.16E-18	9.54	29.8636213	2.01E-01	147098585	147098708	NC_000003.11

cg24911113	2.21E-16	6.19E-18	9.53	29.8595405	2.07E-01	23185714	23185837	NC_000019.9
cg13079837	2.21E-16	6.19E-18	9.53	29.8586127	1.35E-01	1104452	1104575	NC_000012.11
cg20948997	2.22E-16	6.23E-18	9.53	29.853044	1.91E-01	56014433	56014556	NC_000008.10
cg25641533	2.23E-16	6.25E-18	9.53	29.8499091	1.25E-01	31734580	31734703	NC_000006.11
cg13272644	2.23E-16	6.25E-18	9.53	29.8493901	2.22E-01	37499476	37499599	NC_000001.10
cg08105748	2.23E-16	6.26E-18	9.53	29.8478984	2.04E-02	57086875	57086998	NC_000006.11
cg18685561	2.24E-16	6.29E-18	9.53	29.8431367	2.16E-01	165305022	165305145	NC_000004.11
cg10362542	2.24E-16	6.30E-18	9.53	29.8421246	3.35E-01	77179709	77179832	NC_000017.10
cg26407316	2.25E-16	6.31E-18	9.53	29.8398333	1.25E-01	50801745	50801868	NC_000014.8
cg18543991	2.25E-16	6.33E-18	9.53	29.8365191	1.87E-01	145871392	145871515	NC_000003.11
cg02842496	2.26E-16	6.36E-18	9.53	29.831887	2.45E-01	132053262	132053385	NC_000008.10
cg16848154	2.26E-16	6.36E-18	9.53	29.8318418	1.40E-01	2785183	2785306	NC_000020.10
cg25657222	2.27E-16	6.39E-18	9.53	29.8277225	1.45E-01	6556270	6556393	NC_000017.10
cg07496545	2.27E-16	6.39E-18	9.53	29.8274498	1.77E-01	140683737	140683860	NC_000005.9
cg18668753	2.28E-16	6.41E-18	9.53	29.8236339	1.17E-01	57352657	57352780	NC_000019.9
cg09142135	2.28E-16	6.42E-18	9.53	29.823479	1.42E-01	67809980	67810103	NC_000011.9
cg23989821	2.28E-16	6.42E-18	9.53	29.823227	2.77E-01	60952405	60952528	NC_000014.8
cg03904711	2.28E-16	6.42E-18	9.53	29.8224687	1.21E-01	118865255	118865378	NC_000003.11
cg07177123	2.33E-16	6.56E-18	9.53	29.8008025	6.26E-02	30909162	30909285	NC_000007.13
cg14621053	2.34E-16	6.58E-18	9.53	29.7986235	1.65E-01	128077315	128077438	NC_000010.10
cg21096141	2.34E-16	6.60E-18	9.53	29.7959087	1.71E-01	83627633	83627756	NC_000004.11
cg13750802	2.36E-16	6.64E-18	9.52	29.7900763	1.03E-01	41055185	41055308	NC_000013.10
cg07148651	2.37E-16	6.68E-18	9.52	29.7839203	1.52E-01	1155617	1155740	NC_000008.10
cg23390118	2.37E-16	6.68E-18	9.52	29.7837331	1.41E-01	2339513	2339636	NC_000012.11
cg21864868	2.37E-16	6.69E-18	9.52	29.7824305	1.95E-01	46673002	46673125	NC_000017.10

cg04894619	2.37E-16	6.69E-18	9.52	29.7815102	2.11E-01	179754615	179754738	NC_000003.11
cg17098147	2.40E-16	6.76E-18	9.52	29.7717331	1.68E-01	22634142	22634265	NC_000010.10
cg01316476	2.42E-16	6.82E-18	9.52	29.7632306	1.56E-01	25899194	25899317	NC_000007.13
cg22506453	2.42E-16	6.83E-18	9.52	29.7615645	1.71E-01	39122753	39122876	NC_000017.10
cg19593741	2.42E-16	6.84E-18	9.52	29.7603275	1.30E-01	31734217	31734340	NC_000006.11
cg02739346	2.44E-16	6.89E-18	9.52	29.7529387	1.81E-01	119600519	119600642	NC_000002.11
cg23476802	2.45E-16	6.90E-18	9.52	29.7508449	1.50E-01	30407261	30407384	NC_000020.10
cg14978741	2.45E-16	6.91E-18	9.52	29.750504	7.24E-02	21460005	21460128	NC_000010.10
cg01182048	2.46E-16	6.93E-18	9.52	29.7465092	1.17E-01	71005638	71005761	NC_000010.10
cg10870587	2.46E-16	6.93E-18	9.52	29.746454	9.80E-02	95249627	95249750	NC_000010.10
cg10552126	2.47E-16	6.96E-18	9.52	29.7427475	1.71E-01	154002064	154002187	NC_000007.13
cg27659049	2.48E-16	7.01E-18	9.52	29.7353375	2.17E-01	115173713	115173836	NC_000012.11
cg08701998	2.49E-16	7.03E-18	9.52	29.7334912	1.11E-01	31430863	31430986	NC_000013.10
cg05892568	2.50E-16	7.06E-18	9.51	29.728427	2.14E-01	30015782	30015905	NC_000019.9
cg16130238	2.53E-16	7.15E-18	9.51	29.715888	8.86E-02	22958402	22958525	NC_000014.8
cg13142700	2.53E-16	7.16E-18	9.51	29.7149783	2.76E-01	11034501	11034624	NC_000003.11
cg18254364	2.54E-16	7.19E-18	9.51	29.7106567	9.98E-02	24741821	24741944	NC_000014.8
cg15797110	2.56E-16	7.23E-18	9.51	29.7048578	2.64E-01	27196314	27196437	NC_000007.13
cg10676084	2.56E-16	7.24E-18	9.51	29.7033253	1.99E-01	27017560	27017683	NC_000015.9
cg11717507	2.58E-16	7.30E-18	9.51	29.6953406	2.60E-01	114838825	114838948	NC_000012.11
cg01952234	2.58E-16	7.30E-18	9.51	29.6949535	2.76E-01	32457130	32457253	NC_000011.9
cg11751117	2.59E-16	7.32E-18	9.51	29.6929113	1.84E-01	56663411	56663534	NC_000014.8
cg15873149	2.59E-16	7.33E-18	9.51	29.6920821	2.40E-01	147137180	147137303	NC_000003.11
cg26782150	2.61E-16	7.38E-18	9.51	29.6852075	1.60E-01	664719	664842	NC_000010.10
cg04071270	2.62E-16	7.40E-18	9.51	29.6815895	1.62E-01	140457553	140457676	NC_000005.9

cg03699469	2.62E-16	7.42E-18	9.51	29.680136	2.34E-01	144242029	144242152	NC_000008.10
cg26333393	2.63E-16	7.46E-18	9.51	29.6747179	1.39E-01	125651726	125651849	NC_000010.10
cg00076307	2.64E-16	7.47E-18	9.51	29.673121	1.96E-01	128300967	128301090	NC_000005.9
cg11965976	2.66E-16	7.53E-18	9.5	29.6653698	3.08E-01	127874463	127874586	NC_000005.9
cg25403974	2.67E-16	7.55E-18	9.5	29.6620284	7.58E-02	150105495	150105618	NC_000007.13
cg20959866	2.67E-16	7.55E-18	9.5	29.6619607	1.90E-01	4714008	4714131	NC_000001.10
cg00459623	2.67E-16	7.56E-18	9.5	29.6607726	3.05E-01	176936581	176936704	NC_000002.11
cg00824018	2.67E-16	7.56E-18	9.5	29.660632	1.90E-01	105037661	105037784	NC_000010.10
cg06671614	2.68E-16	7.60E-18	9.5	29.6554145	1.41E-01	44876162	44876285	NC_000010.10
cg12737285	2.68E-16	7.60E-18	9.5	29.6550895	1.09E-01	192362155	192362278	NC_000002.11
cg15302311	2.68E-16	7.61E-18	9.5	29.6542624	1.23E-01	100584395	100584518	NC_000006.11
cg08159291	2.70E-16	7.65E-18	9.5	29.6494197	1.99E-01	4715615	4715738	NC_000001.10
cg00188627	2.70E-16	7.67E-18	9.5	29.6467005	1.76E-01	159557779	159557902	NC_000003.11
cg10502121	2.71E-16	7.69E-18	9.5	29.6445886	1.47E-01	53286906	53287029	NC_000007.13
cg23443847	2.71E-16	7.69E-18	9.5	29.6436939	8.17E-02	126170483	126170606	NC_000003.11
cg25798792	2.73E-16	7.74E-18	9.5	29.6372252	2.25E-01	66535351	66535474	NC_000004.11
cg03810326	2.73E-16	7.75E-18	9.5	29.6364797	6.45E-02	43230937	43231060	NC_000006.11
cg12057127	2.76E-16	7.85E-18	9.5	29.6240829	1.83E-01	46699155	46699278	NC_000017.10
cg06282952	2.77E-16	7.86E-18	9.5	29.6230245	1.60E-01	148859147	148859270	NC_000006.11
cg10730712	2.77E-16	7.87E-18	9.5	29.620745	2.91E-01	178017827	178017950	NC_000005.9
cg17481117	2.84E-16	8.08E-18	9.49	29.5956706	1.66E-01	27113459	27113582	NC_000015.9
cg03908391	2.85E-16	8.10E-18	9.49	29.5929894	1.49E-01	57427170	57427293	NC_000020.10
cg21699044	2.86E-16	8.13E-18	9.49	29.5890268	2.14E-01	65281689	65281812	NC_000008.10
cg01572468	2.86E-16	8.14E-18	9.49	29.5875932	5.83E-02	75225065	75225188	NC_000011.9
cg10883258	2.87E-16	8.16E-18	9.49	29.5851826	9.22E-02	179239447	179239570	NC_000005.9

cg09775628	2.87E-16	8.18E-18	9.49	29.5833324	3.23E-02	1479573	1479696	NC_000019.9
cg16557964	2.89E-16	8.21E-18	9.49	29.5791109	1.30E-01	100253838	100253961	NC_000003.11
cg11566394	2.90E-16	8.26E-18	9.49	29.573245	2.24E-01	4856709	4856832	NC_000004.11
cg04112058	2.90E-16	8.26E-18	9.49	29.5727183	1.00E-01	46648124	46648247	NC_000016.9
cg08658787	2.91E-16	8.29E-18	9.49	29.5701855	1.56E-01	113916646	113916769	NC_000012.11
cg11859489	2.91E-16	8.29E-18	9.49	29.5701085	1.79E-01	92459058	92459181	NC_000015.9
cg21331088	2.92E-16	8.32E-18	9.49	29.5663053	2.54E-01	908290	908413	NC_000018.9
cg23242052	2.92E-16	8.32E-18	9.49	29.5660361	2.32E-01	176947786	176947909	NC_000002.11
cg07544045	2.94E-16	8.37E-18	9.49	29.5602381	8.00E-02	158592420	158592543	NC_000006.11
cg22082456	2.94E-16	8.38E-18	9.49	29.5591915	1.54E-01	53392538	53392661	NC_000001.10
cg21356962	2.95E-16	8.41E-18	9.49	29.5549787	7.52E-02	243035414	243035537	NC_000002.11
cg16922279	2.96E-16	8.43E-18	9.49	29.5532253	2.03E-01	71955439	71955562	NC_000011.9
cg04525757	2.96E-16	8.45E-18	9.49	29.5506238	2.00E-01	29235973	29236096	NC_000014.8
cg00853742	2.98E-16	8.49E-18	9.49	29.5459638	1.53E-01	39990972	39991095	NC_000020.10
cg01046104	2.98E-16	8.50E-18	9.49	29.5449083	2.81E-01	58095588	58095711	NC_000019.9
cg14823825	2.98E-16	8.52E-18	9.49	29.5429038	1.55E-01	26170211	26170334	NC_000008.10
cg15241920	2.99E-16	8.54E-18	9.49	29.5402205	3.05E-01	54926514	54926637	NC_000019.9
cg06124883	3.00E-16	8.56E-18	9.49	29.5384222	1.62E-01	15525193	15525316	NC_000001.10
cg00397673	3.00E-16	8.58E-18	9.49	29.5360186	1.57E-01	56940418	56940541	NC_000018.9
cg20954484	3.01E-16	8.61E-18	9.48	29.5325686	1.16E-01	42727014	42727137	NC_000003.11
cg10254035	3.03E-16	8.64E-18	9.48	29.5283545	1.57E-01	21741635	21741758	NC_000006.11
cg18081940	3.04E-16	8.70E-18	9.48	29.521903	1.98E-01	154475173	154475296	NC_000001.10
cg16673508	3.05E-16	8.71E-18	9.48	29.5211111	9.67E-02	1056794	1056917	NC_000016.9
cg05168977	3.06E-16	8.75E-18	9.48	29.5158554	1.45E-01	98247142	98247265	NC_000007.13
cg17971129	3.07E-16	8.77E-18	9.48	29.5136964	1.45E-01	3866461	3866584	NC_000006.11

cg24530480	3.08E-16	8.80E-18	9.48	29.5105202	1.56E-01	1008155	1008278	NC_000008.10
cg15617155	3.08E-16	8.82E-18	9.48	29.5087555	2.47E-01	63785321	63785444	NC_000001.10
cg04569897	3.08E-16	8.82E-18	9.48	29.5079212	1.16E-01	45609407	45609530	NC_000006.11
cg19257274	3.10E-16	8.86E-18	9.48	29.503382	1.87E-01	207990250	207990373	NC_000001.10
cg13172905	3.10E-16	8.87E-18	9.48	29.5027733	1.87E-01	30341765	30341888	NC_000008.10
cg17104824	3.10E-16	8.88E-18	9.48	29.5021288	2.00E-01	177014959	177015082	NC_000002.11
cg02396253	3.10E-16	8.88E-18	9.48	29.502089	1.16E-01	135271056	135271179	NC_000006.11
cg26090619	3.10E-16	8.88E-18	9.48	29.5017458	1.23E-01	80330167	80330290	NC_000017.10
cg03876413	3.10E-16	8.88E-18	9.48	29.5011993	2.46E-01	32338240	32338363	NC_000007.13
cg10327980	3.11E-16	8.91E-18	9.48	29.4986151	1.52E-01	24601001	24601124	NC_000014.8
cg25209842	3.12E-16	8.93E-18	9.48	29.4965864	2.18E-01	103536342	103536465	NC_000010.10
cg10677144	3.13E-16	8.97E-18	9.48	29.4913883	1.63E-01	3220106	3220229	NC_000018.9
cg00355281	3.14E-16	8.98E-18	9.48	29.4901416	2.78E-01	24323767	24323890	NC_000007.13
cg13895004	3.14E-16	9.00E-18	9.48	29.4878161	9.95E-02	112217129	112217252	NC_000009.11
cg13486833	3.15E-16	9.01E-18	9.48	29.4867218	1.23E-01	71507300	71507423	NC_000015.9
cg15995935	3.16E-16	9.06E-18	9.48	29.4816294	1.04E-01	111871839	111871962	NC_000002.11
cg24782949	3.17E-16	9.08E-18	9.48	29.4796088	1.60E-01	179749875	179749998	NC_000002.11
cg08320122	3.17E-16	9.10E-18	9.48	29.4773989	1.19E-01	62090588	62090711	NC_000004.11
cg14054928	3.20E-16	9.16E-18	9.47	29.4705334	2.17E-01	50235328	50235451	NC_000017.10
cg10211414	3.20E-16	9.17E-18	9.47	29.4694187	1.69E-01	8875053	8875176	NC_000001.10
cg06943925	3.20E-16	9.19E-18	9.47	29.4674558	1.74E-01	132145434	132145557	NC_000005.9
cg22752533	3.23E-16	9.26E-18	9.47	29.4599065	1.68E-01	44657948	44658071	NC_000020.10
cg14710529	3.25E-16	9.31E-18	9.47	29.4543544	1.32E-01	9387472	9387595	NC_000002.11
cg22321267	3.25E-16	9.33E-18	9.47	29.4529922	9.92E-02	5647332	5647455	NC_000007.13
cg08455781	3.25E-16	9.33E-18	9.47	29.4528803	1.22E-01	85669875	85669998	NC_000016.9

cg13353160	3.25E-16	9.33E-18	9.47	29.4524839	9.01E-02	153567375	153567498	NC_000004.11
cg10311602	3.25E-16	9.33E-18	9.47	29.4524084	7.22E-02	53739778	53739901	NC_000001.10
cg27217474	3.27E-16	9.38E-18	9.47	29.4472416	2.01E-01	46697543	46697666	NC_000017.10
cg13846270	3.28E-16	9.41E-18	9.47	29.4442608	1.54E-01	51417929	51418052	NC_000013.10
cg03243226	3.28E-16	9.42E-18	9.47	29.4432755	2.52E-01	105480979	105481102	NC_000011.9
cg01806590	3.30E-16	9.48E-18	9.47	29.4364022	1.40E-01	23066421	23066544	NC_000014.8
cg10356613	3.30E-16	9.50E-18	9.47	29.435214	3.25E-01	35294491	35294614	NC_000017.10
cg26309134	3.31E-16	9.50E-18	9.47	29.4344181	3.04E-01	56879571	56879694	NC_000019.9
cg18130905	3.31E-16	9.52E-18	9.47	29.4330397	1.56E-01	30517666	30517789	NC_000018.9
cg01309744	3.31E-16	9.52E-18	9.47	29.4327013	1.99E-01	155167401	155167524	NC_000007.13
cg02914422	3.32E-16	9.55E-18	9.47	29.4300514	2.26E-01	32110145	32110268	NC_000007.13
cg24925839	3.33E-16	9.57E-18	9.47	29.4275709	5.36E-02	698480	698603	NC_000004.11
cg17868687	3.34E-16	9.60E-18	9.47	29.4247507	6.23E-02	242197976	242198099	NC_000002.11
cg10985145	3.35E-16	9.64E-18	9.47	29.4206094	1.78E-01	135227049	135227172	NC_000005.9
cg02093732	3.35E-16	9.64E-18	9.47	29.4204997	1.48E-01	30517601	30517724	NC_000015.9
cg08047969	3.35E-16	9.64E-18	9.47	29.41981	1.04E-01	29266384	29266507	NC_000016.9
cg00866976	3.38E-16	9.72E-18	9.47	29.4116877	2.76E-01	56224782	56224905	NC_000016.9
cg21475402	3.38E-16	9.73E-18	9.47	29.4113388	1.79E-01	156612140	156612263	NC_000001.10
cg17256609	3.38E-16	9.74E-18	9.47	29.4105378	1.28E-01	41643375	41643498	NC_000008.10
cg12557626	3.38E-16	9.74E-18	9.47	29.4096213	1.14E-01	113977332	113977455	NC_000013.10
cg00240432	3.39E-16	9.77E-18	9.47	29.4072581	2.75E-01	19157296	19157419	NC_000007.13
cg05409131	3.41E-16	9.84E-18	9.46	29.4000412	1.11E-01	132070595	132070718	NC_000003.11
cg15772157	3.43E-16	9.88E-18	9.46	29.3961483	1.51E-01	22766320	22766443	NC_000010.10
cg15022051	3.43E-16	9.90E-18	9.46	29.393629	2.02E-01	170396116	170396239	NC_000006.11
cg18078087	3.44E-16	9.91E-18	9.46	29.3924517	7.49E-02	15005463	15005586	NC_000004.11

cg17691543	3.44E-16	9.92E-18	9.46	29.3921015	1.52E-01	49759312	49759435	NC_000003.11
cg10469980	3.44E-16	9.93E-18	9.46	29.3908791	1.95E-01	85660101	85660224	NC_000016.9
cg06443644	3.45E-16	9.97E-18	9.46	29.3872037	1.35E-01	1846046	1846169	NC_000001.10
cg07104706	3.47E-16	1.00E-17	9.46	29.3829748	2.52E-01	84456127	84456250	NC_000013.10
cg06717850	3.48E-16	1.00E-17	9.46	29.3792075	1.78E-01	57617602	57617725	NC_000019.9
cg16646879	3.49E-16	1.01E-17	9.46	29.3769285	9.73E-02	21844541	21844664	NC_000016.9
cg08431563	3.49E-16	1.01E-17	9.46	29.3766785	2.66E-01	77334207	77334330	NC_000001.10
cg16307965	3.49E-16	1.01E-17	9.46	29.3756475	1.26E-01	143593367	143593490	NC_000002.11
cg17320707	3.49E-16	1.01E-17	9.46	29.374774	2.45E-01	119307931	119308054	NC_000010.10
cg26925408	3.49E-16	1.01E-17	9.46	29.3743292	1.53E-01	147212748	147212871	NC_000005.9
cg12354056	3.50E-16	1.01E-17	9.46	29.3739433	1.88E-01	186136503	186136626	NC_000003.11
cg19594305	3.51E-16	1.01E-17	9.46	29.3702918	2.38E-01	34112991	34113114	NC_000019.9
cg02687288	3.52E-16	1.02E-17	9.46	29.365136	2.01E-01	97839561	97839684	NC_000005.9
cg09554951	3.53E-16	1.02E-17	9.46	29.3626896	1.68E-01	2890725	2890848	NC_000011.9
cg06528626	3.54E-16	1.02E-17	9.46	29.3609377	1.36E-01	16761002	16761125	NC_000012.11
cg22050611	3.54E-16	1.02E-17	9.46	29.3608254	1.75E-01	1321954	1322077	NC_000011.9
cg09936561	3.54E-16	1.02E-17	9.46	29.3603168	1.46E-01	9783623	9783746	NC_000004.11
cg25436126	3.54E-16	1.03E-17	9.46	29.35908	7.00E-02	10942676	10942799	NC_000002.11
cg01165177	3.55E-16	1.03E-17	9.46	29.3585922	4.43E-02	68772710	68772833	NC_000011.9
cg11472279	3.59E-16	1.04E-17	9.46	29.3461763	3.38E-01	11059733	11059856	NC_000010.10
cg20457796	3.59E-16	1.04E-17	9.46	29.3443656	1.74E-01	50819500	50819623	NC_000010.10
cg16991765	3.60E-16	1.04E-17	9.46	29.343796	1.72E-01	178594097	178594220	NC_000005.9
cg01228134	3.60E-16	1.04E-17	9.46	29.3434078	1.49E-01	233351921	233352044	NC_000002.11
cg00674365	3.62E-16	1.05E-17	9.45	29.3372124	3.21E-01	57019069	57019192	NC_000019.9
cg15339231	3.62E-16	1.05E-17	9.45	29.3363363	2.20E-01	96650407	96650530	NC_000007.13

cg02497428	3.62E-16	1.05E-17	9.45	29.3355351	1.45E-01	21665138	21665261	NC_000016.9
cg16234490	3.62E-16	1.05E-17	9.45	29.3354319	1.46E-01	77138082	77138205	NC_000004.11
cg06472636	3.63E-16	1.05E-17	9.45	29.3331237	9.43E-02	168174824	168174947	NC_000005.9
cg21593606	3.65E-16	1.06E-17	9.45	29.3286834	1.10E-01	98676195	98676318	NC_000007.13
cg14524704	3.67E-16	1.06E-17	9.45	29.3236398	1.42E-01	25899168	25899291	NC_000007.13
cg10409860	3.67E-16	1.06E-17	9.45	29.3233948	7.46E-02	81969992	81970115	NC_000016.9
cg02431089	3.67E-16	1.06E-17	9.45	29.3233759	5.78E-02	4884396	4884519	NC_000006.11
cg20357228	3.67E-16	1.06E-17	9.45	29.3223166	5.42E-02	32817977	32818100	NC_000006.11
cg07066163	3.69E-16	1.07E-17	9.45	29.3174007	1.15E-01	85635689	85635812	NC_000016.9
cg12070646	3.69E-16	1.07E-17	9.45	29.3152732	9.46E-02	71149811	71149934	NC_000002.11
cg07742235	3.71E-16	1.08E-17	9.45	29.3106918	5.92E-02	69308752	69308875	NC_000011.9
cg14230810	3.71E-16	1.08E-17	9.45	29.3099812	6.15E-02	74024488	74024611	NC_000017.10
cg01799115	3.73E-16	1.08E-17	9.45	29.305291	1.99E-01	135827395	135827518	NC_000008.10
cg02173969	3.74E-16	1.08E-17	9.45	29.3034166	1.34E-01	28882863	28882986	NC_000006.11
cg07811634	3.74E-16	1.09E-17	9.45	29.3018847	4.16E-02	97857989	97858112	NC_000007.13
cg06365535	3.75E-16	1.09E-17	9.45	29.299513	1.54E-01	59534102	59534225	NC_000017.10
cg21051580	3.75E-16	1.09E-17	9.45	29.2988588	1.91E-01	4998415	4998538	NC_000007.13
cg18988856	3.76E-16	1.09E-17	9.45	29.2959225	1.44E-01	99425577	99425700	NC_000007.13
cg02423064	3.76E-16	1.09E-17	9.45	29.2955093	1.87E-01	32484008	32484131	NC_000017.10
cg27652869	3.77E-16	1.10E-17	9.45	29.2933742	1.07E-01	172216846	172216969	NC_000005.9
cg23465650	3.77E-16	1.10E-17	9.45	29.293234	1.30E-01	158522899	158523022	NC_000005.9
cg01631277	3.79E-16	1.10E-17	9.45	29.2873784	1.93E-01	53203309	53203432	NC_000014.8
cg12069267	3.80E-16	1.10E-17	9.45	29.2864075	1.41E-01	1407858	1407981	NC_000004.11
cg21169914	3.82E-16	1.11E-17	9.45	29.2792841	2.08E-01	165304488	165304611	NC_000004.11
cg08885409	3.82E-16	1.11E-17	9.45	29.2787314	1.06E-01	55969571	55969694	NC_000017.10

cg23664385	3.85E-16	1.12E-17	9.44	29.2720966	1.63E-01	187467427	187467550	NC_000003.11
cg24283289	3.86E-16	1.12E-17	9.44	29.2695992	1.25E-01	19016892	19017015	NC_000022.10
cg19674032	3.88E-16	1.13E-17	9.44	29.2641658	1.05E-01	111682796	111682919	NC_000013.10
cg12940822	3.88E-16	1.13E-17	9.44	29.2635551	2.58E-01	127873397	127873520	NC_000005.9
cg02897005	3.88E-16	1.13E-17	9.44	29.2622379	2.12E-01	47064129	47064252	NC_000021.8
cg00175709	3.88E-16	1.13E-17	9.44	29.26216	2.07E-01	207670014	207670137	NC_000001.10
cg24871414	3.91E-16	1.14E-17	9.44	29.2542751	2.12E-01	95663987	95664110	NC_000002.11
cg20113824	3.92E-16	1.14E-17	9.44	29.2525639	2.08E-01	70946946	70947069	NC_000008.10
cg14373923	3.92E-16	1.14E-17	9.44	29.2521743	2.24E-01	61638433	61638556	NC_000020.10
cg17669365	3.94E-16	1.15E-17	9.44	29.247776	7.40E-02	71163953	71164076	NC_000002.11
cg14354327	3.94E-16	1.15E-17	9.44	29.2455677	1.42E-01	131763676	131763799	NC_000010.10
cg19821713	3.98E-16	1.16E-17	9.44	29.2351424	1.24E-01	96909816	96909939	NC_000015.9
cg22457238	4.00E-16	1.17E-17	9.44	29.2307869	2.07E-01	58570995	58571118	NC_000019.9
cg17031944	4.00E-16	1.17E-17	9.44	29.2300943	6.96E-02	21614078	21614201	NC_000001.10
cg03275201	4.00E-16	1.17E-17	9.44	29.2298128	1.48E-01	80602016	80602139	NC_000017.10
cg11557382	4.01E-16	1.17E-17	9.44	29.2290493	2.13E-01	153446256	153446379	NC_000007.13
cg21367811	4.01E-16	1.17E-17	9.44	29.2288212	2.07E-01	27169136	27169259	NC_000007.13
cg24818418	4.04E-16	1.18E-17	9.44	29.2191664	1.48E-01	110834590	110834713	NC_000004.11
cg08870743	4.05E-16	1.18E-17	9.44	29.2168194	2.74E-01	34398199	34398322	NC_000021.8
cg24136205	4.06E-16	1.19E-17	9.44	29.2150336	1.52E-01	100624293	100624416	NC_000013.10
cg21484228	4.07E-16	1.19E-17	9.44	29.2124367	2.29E-01	32859417	32859540	NC_000003.11
cg22154659	4.08E-16	1.19E-17	9.43	29.2085498	1.86E-01	27134369	27134492	NC_000007.13
cg04847275	4.10E-16	1.20E-17	9.43	29.2054282	2.09E-01	95693828	95693951	NC_000014.8
cg00775939	4.12E-16	1.21E-17	9.43	29.1984444	1.51E-01	118301448	118301571	NC_000010.10
cg11716272	4.13E-16	1.21E-17	9.43	29.197004	2.10E-01	5837348	5837471	NC_000002.11

cg00906476	4.13E-16	1.21E-17	9.43	29.1966291	9.87E-02	139046200	139046323	NC_000005.9
cg06584433	4.15E-16	1.21E-17	9.43	29.1918976	1.88E-01	124605905	124606028	NC_000010.10
cg04353095	4.15E-16	1.21E-17	9.43	29.1915509	2.95E-01	140770588	140770711	NC_000003.11
cg05232118	4.17E-16	1.22E-17	9.43	29.1855826	1.04E-01	216660598	216660721	NC_000002.11
cg12682032	4.22E-16	1.24E-17	9.43	29.1748175	1.83E-01	83617937	83618060	NC_000015.9
cg25866143	4.22E-16	1.24E-17	9.43	29.1747437	1.69E-01	27183262	27183385	NC_000007.13
cg14830367	4.23E-16	1.24E-17	9.43	29.1717691	1.03E-01	178459176	178459299	NC_000001.10
cg00632019	4.23E-16	1.24E-17	9.43	29.17152	1.01E-01	27217587	27217710	NC_000016.9
cg09054633	4.23E-16	1.24E-17	9.43	29.1713816	1.92E-01	136834294	136834417	NC_000005.9
cg17642941	4.24E-16	1.24E-17	9.43	29.1689178	1.80E-01	27198429	27198552	NC_000007.13
cg01662158	4.25E-16	1.25E-17	9.43	29.1663742	4.44E-02	156030265	156030388	NC_000006.11
cg23619399	4.27E-16	1.25E-17	9.43	29.1626453	2.28E-01	79169753	79169876	NC_000013.10
cg05233288	4.28E-16	1.25E-17	9.43	29.1603574	1.31E-01	10534164	10534287	NC_000001.10
cg24019371	4.29E-16	1.26E-17	9.43	29.1558794	2.47E-01	4716554	4716677	NC_000001.10
cg06685968	4.29E-16	1.26E-17	9.43	29.1558526	3.05E-01	27192246	27192369	NC_000007.13
cg27512659	4.31E-16	1.26E-17	9.43	29.152959	1.03E-01	4697405	4697528	NC_000010.10
cg03894892	4.33E-16	1.27E-17	9.43	29.1464216	1.68E-01	153445197	153445320	NC_000007.13
cg19630629	4.34E-16	1.27E-17	9.42	29.1454366	2.27E-01	136556193	136556316	NC_000007.13
cg19156875	4.36E-16	1.28E-17	9.42	29.1396882	2.69E-01	43974446	43974569	NC_000017.10
cg03092399	4.36E-16	1.28E-17	9.42	29.1396575	1.02E-01	108149307	108149430	NC_000011.9
cg10495997	4.36E-16	1.28E-17	9.42	29.1393583	9.47E-02	72438402	72438525	NC_000011.9
cg00441573	4.37E-16	1.28E-17	9.42	29.1378159	1.71E-01	43428471	43428594	NC_000010.10
cg04074001	4.40E-16	1.29E-17	9.42	29.1312999	1.05E-01	148861409	148861532	NC_000004.11
cg00305996	4.42E-16	1.30E-17	9.42	29.1256656	1.70E-01	227655319	227655442	NC_000002.11
cg05740668	4.43E-16	1.30E-17	9.42	29.1237274	6.52E-02	132203203	132203326	NC_000002.11

cg01811674	4.44E-16	1.30E-17	9.42	29.1214092	1.73E-01	150697210	150697333	NC_000006.11
cg09735896	4.45E-16	1.31E-17	9.42	29.1192498	8.67E-02	39019978	39020101	NC_000017.10
cg03164275	4.45E-16	1.31E-17	9.42	29.1175036	2.67E-01	219826805	219826928	NC_000002.11
cg16277843	4.48E-16	1.32E-17	9.42	29.1124748	1.10E-01	169671468	169671591	NC_000006.11
cg04223553	4.48E-16	1.32E-17	9.42	29.1117457	1.80E-01	153852934	153853057	NC_000005.9
cg10996039	4.48E-16	1.32E-17	9.42	29.1115814	2.04E-01	27178861	27178984	NC_000007.13
cg15922693	4.48E-16	1.32E-17	9.42	29.1107619	1.64E-01	51610688	51610811	NC_000012.11
cg25616547	4.49E-16	1.32E-17	9.42	29.108136	2.14E-01	115134344	115134467	NC_000012.11
cg21512644	4.50E-16	1.32E-17	9.42	29.1056739	2.21E-01	156130016	156130139	NC_000004.11
cg17228900	4.52E-16	1.33E-17	9.42	29.1021159	2.81E-01	391764	391887	NC_000006.11
cg21092551	4.52E-16	1.33E-17	9.42	29.1016747	2.19E-01	29993498	29993621	NC_000015.9
cg05513616	4.52E-16	1.33E-17	9.42	29.101172	3.28E-02	1538510	1538633	NC_000005.9
cg13618880	4.53E-16	1.33E-17	9.42	29.099705	1.76E-01	170579474	170579597	NC_000006.11
cg02475871	4.54E-16	1.34E-17	9.42	29.0966779	3.37E-02	5684969	5685092	NC_000019.9
cg01955203	4.54E-16	1.34E-17	9.42	29.0964898	1.34E-01	124515502	124515625	NC_000008.10
cg04168670	4.56E-16	1.34E-17	9.42	29.0920726	1.62E-01	124782817	124782940	NC_000002.11
cg01351783	4.58E-16	1.35E-17	9.42	29.0885779	8.74E-02	7220085	7220208	NC_000006.11
cg20655099	4.58E-16	1.35E-17	9.42	29.0885559	1.35E-01	5394889	5395012	NC_000007.13
cg08452658	4.58E-16	1.35E-17	9.42	29.0881556	3.01E-01	83776271	83776394	NC_000015.9
cg26464586	4.59E-16	1.35E-17	9.42	29.0843436	1.44E-01	43921712	43921835	NC_000006.11
cg16347811	4.61E-16	1.36E-17	9.42	29.0812515	1.25E-01	29322804	29322927	NC_000007.13
cg12880658	4.62E-16	1.36E-17	9.41	29.0784686	2.38E-01	115152386	115152509	NC_000005.9
cg10659480	4.64E-16	1.37E-17	9.41	29.0733679	6.44E-02	34055800	34055923	NC_000003.11
cg09967440	4.65E-16	1.37E-17	9.41	29.0722792	1.93E-01	127444969	127445092	NC_000010.10
cg21945949	4.67E-16	1.38E-17	9.41	29.0682433	1.48E-01	64683610	64683733	NC_000002.11

cg10512951	4.72E-16	1.39E-17	9.41	29.0566228	2.03E-01	70500634	70500757	NC_000006.11
cg05348776	4.75E-16	1.40E-17	9.41	29.0493792	2.19E-01	95693784	95693907	NC_000014.8
cg24000873	4.75E-16	1.40E-17	9.41	29.0484774	2.14E-01	24813653	24813776	NC_000008.10
cg23863891	4.76E-16	1.41E-17	9.41	29.0472588	1.75E-01	155363328	155363451	NC_000007.13
cg06545143	4.77E-16	1.41E-17	9.41	29.0451843	1.87E-01	166402638	166402761	NC_000006.11
cg15415136	4.78E-16	1.41E-17	9.41	29.0426156	1.81E-01	38042418	38042541	NC_000019.9
cg23715830	4.79E-16	1.42E-17	9.41	29.0400758	2.60E-01	13974614	13974737	NC_000003.11
cg15889057	4.81E-16	1.42E-17	9.41	29.0368176	1.49E-01	53602179	53602302	NC_000012.11
cg27345762	4.82E-16	1.42E-17	9.41	29.0335226	1.81E-01	30734041	30734164	NC_000007.13
cg10767336	4.83E-16	1.43E-17	9.41	29.0318345	1.88E-01	5019066	5019189	NC_000012.11
cg19095000	4.83E-16	1.43E-17	9.41	29.0306945	1.39E-01	11711141	11711264	NC_000001.10
cg01696114	4.84E-16	1.43E-17	9.41	29.0300617	1.22E-01	1616448	1616571	NC_000004.11
cg01566199	4.84E-16	1.43E-17	9.41	29.0298025	1.53E-01	26197239	26197362	NC_000001.10
cg04245645	4.85E-16	1.44E-17	9.41	29.0262134	2.59E-01	96343016	96343139	NC_000014.8
cg07978472	4.86E-16	1.44E-17	9.41	29.0254716	2.59E-01	122686493	122686616	NC_000004.11
cg02463418	4.86E-16	1.44E-17	9.41	29.0245668	2.33E-01	66535575	66535698	NC_000004.11
cg22117468	4.89E-16	1.45E-17	9.41	29.0188984	1.11E-01	70176293	70176416	NC_000002.11
cg06069030	4.89E-16	1.45E-17	9.41	29.0186203	8.92E-02	89920590	89920713	NC_000016.9
cg15201291	4.89E-16	1.45E-17	9.41	29.0185541	1.45E-01	96830102	96830225	NC_000010.10
cg12083852	4.90E-16	1.45E-17	9.4	29.0158092	1.37E-01	151282686	151282809	NC_000001.10
cg12689506	4.91E-16	1.45E-17	9.4	29.0140404	1.43E-01	100607835	100607958	NC_000007.13
cg20270188	4.93E-16	1.46E-17	9.4	29.0099876	1.67E-01	156611963	156612086	NC_000001.10
cg22514987	4.97E-16	1.47E-17	9.4	29.0017148	8.59E-02	52271182	52271305	NC_000006.11
cg04971534	4.98E-16	1.47E-17	9.4	28.9992559	2.16E-01	27333432	27333555	NC_000017.10
cg18473544	5.00E-16	1.48E-17	9.4	28.9950334	1.23E-01	14409599	14409722	NC_000003.11

cg22379472	5.00E-16	1.48E-17	9.4	28.9936911	2.48E-01	47413617	47413740	NC_000007.13
cg24089936	5.01E-16	1.48E-17	9.4	28.992671	1.28E-01	49341594	49341717	NC_000001.10
cg11002404	5.02E-16	1.49E-17	9.4	28.991127	2.67E-01	11060038	11060161	NC_000010.10
cg18773260	5.02E-16	1.49E-17	9.4	28.9902292	1.17E-01	46685292	46685415	NC_000017.10
cg15445725	5.02E-16	1.49E-17	9.4	28.9898512	1.53E-01	194469106	194469229	NC_000003.11
cg06274159	5.05E-16	1.50E-17	9.4	28.9846168	2.68E-01	188916867	188916990	NC_000004.11
cg26128092	5.05E-16	1.50E-17	9.4	28.9845597	1.99E-01	3567550	3567673	NC_000001.10
cg01168289	5.07E-16	1.51E-17	9.4	28.9786366	1.62E-01	32421514	32421637	NC_000011.9
cg10365563	5.08E-16	1.51E-17	9.4	28.9773608	1.99E-01	21469772	21469895	NC_000007.13
cg12927617	5.08E-16	1.51E-17	9.4	28.9769386	1.41E-01	117084602	117084725	NC_000009.11
cg08585669	5.09E-16	1.51E-17	9.4	28.9755369	5.85E-02	37853032	37853155	NC_000017.10
cg02677947	5.13E-16	1.52E-17	9.4	28.9680264	1.32E-01	739463	739586	NC_000016.9
cg04068980	5.14E-16	1.53E-17	9.4	28.9656218	2.47E-01	50674203	50674326	NC_000005.9
cg22067676	5.15E-16	1.53E-17	9.4	28.9639897	2.03E-01	126281331	126281454	NC_000006.11
cg19329644	5.16E-16	1.53E-17	9.4	28.9608936	8.95E-02	135010873	135010996	NC_000010.10
cg19963602	5.17E-16	1.53E-17	9.4	28.9596914	3.60E-02	94975601	94975724	NC_000012.11
cg01969701	5.19E-16	1.54E-17	9.4	28.9542024	2.21E-01	69286145	69286268	NC_000011.9
cg19125370	5.20E-16	1.55E-17	9.4	28.9524795	1.74E-01	10390532	10390655	NC_000006.11
cg15948088	5.21E-16	1.55E-17	9.39	28.9503074	2.13E-01	109293068	109293191	NC_000011.9
cg12547839	5.21E-16	1.55E-17	9.39	28.9499542	7.54E-02	74416551	74416674	NC_000017.10
cg18753541	5.22E-16	1.55E-17	9.39	28.9482103	1.45E-01	121841743	121841866	NC_000004.11
cg10320659	5.24E-16	1.56E-17	9.39	28.9445819	1.74E-01	62959262	62959385	NC_000020.10
cg04360049	5.26E-16	1.56E-17	9.39	28.9407927	2.95E-01	158150797	158150920	NC_000001.10
cg12478381	5.26E-16	1.56E-17	9.39	28.9406206	2.59E-01	26565249	26565372	NC_000022.10
cg21384402	5.26E-16	1.56E-17	9.39	28.940537	2.54E-01	105036701	105036824	NC_000010.10

cg05294930	5.26E-16	1.57E-17	9.39	28.9398218	7.33E-02	72220937	72221060	NC_000014.8
cg08406370	5.28E-16	1.57E-17	9.39	28.9372209	2.72E-01	23846893	23847016	NC_000016.9
cg03363161	5.28E-16	1.57E-17	9.39	28.9358089	2.08E-01	147139184	147139307	NC_000003.11
cg07137606	5.29E-16	1.57E-17	9.39	28.9345117	2.10E-01	242687018	242687141	NC_000001.10
cg03265074	5.33E-16	1.59E-17	9.39	28.9264948	1.36E-01	52603300	52603423	NC_000012.11
cg26382551	5.33E-16	1.59E-17	9.39	28.9260319	2.02E-01	11047284	11047407	NC_000010.10
cg12648074	5.33E-16	1.59E-17	9.39	28.9256028	1.89E-01	140864474	140864597	NC_000005.9
cg14972654	5.34E-16	1.59E-17	9.39	28.9248917	1.33E-01	111771263	111771386	NC_000010.10
cg26846409	5.34E-16	1.59E-17	9.39	28.9248489	1.41E-01	21640304	21640427	NC_000008.10
cg09605164	5.35E-16	1.59E-17	9.39	28.9227688	2.59E-01	152622787	152622910	NC_000007.13
cg05158165	5.37E-16	1.60E-17	9.39	28.9186827	4.16E-02	108965753	108965876	NC_000006.11
cg03907016	5.38E-16	1.60E-17	9.39	28.9172003	1.83E-01	6534760	6534883	NC_000019.9
cg22191326	5.39E-16	1.61E-17	9.39	28.9136523	2.42E-01	98836379	98836502	NC_000015.9
cg22737473	5.41E-16	1.61E-17	9.39	28.9106411	1.43E-01	200823609	200823732	NC_000002.11
cg27645498	5.41E-16	1.61E-17	9.39	28.9098173	1.49E-01	125145553	125145676	NC_000012.11
cg01728554	5.44E-16	1.62E-17	9.39	28.9047924	1.80E-01	155107661	155107784	NC_000005.9
cg25529304	5.47E-16	1.63E-17	9.39	28.8983046	1.11E-01	43034529	43034652	NC_000005.9
cg13842237	5.49E-16	1.64E-17	9.39	28.894964	2.03E-01	47064079	47064202	NC_000021.8
cg02466815	5.51E-16	1.65E-17	9.39	28.890242	2.47E-01	177054573	177054696	NC_000002.11
cg23625660	5.56E-16	1.66E-17	9.38	28.8813519	1.92E-01	135266516	135266639	NC_000005.9
cg13702005	5.58E-16	1.67E-17	9.38	28.8774384	2.86E-01	103889789	103889912	NC_000012.11
cg23207077	5.60E-16	1.67E-17	9.38	28.8741446	1.63E-01	905988	906111	NC_000001.10
cg11732321	5.70E-16	1.70E-17	9.38	28.856361	4.37E-02	99841169	99841292	NC_000008.10
cg04207084	5.71E-16	1.71E-17	9.38	28.8536886	1.67E-01	157477846	157477969	NC_000007.13
cg10242602	5.72E-16	1.71E-17	9.38	28.851745	2.09E-01	188916875	188916998	NC_000004.11

cg05080154	5.75E-16	1.72E-17	9.38	28.8459179	1.92E-01	76739409	76739532	NC_000018.9
cg16662785	5.78E-16	1.73E-17	9.38	28.8415006	1.81E-01	36246115	36246238	NC_000022.10
cg01115565	5.80E-16	1.74E-17	9.38	28.8375831	8.03E-02	29584222	29584345	NC_000001.10
cg10715092	5.80E-16	1.74E-17	9.38	28.8369887	2.18E-01	26190354	26190477	NC_000020.10
cg08322102	5.83E-16	1.75E-17	9.38	28.8316139	3.03E-01	77595321	77595444	NC_000008.10
cg10363246	5.84E-16	1.75E-17	9.38	28.8303952	2.78E-01	5019482	5019605	NC_000012.11
cg19147218	5.85E-16	1.75E-17	9.38	28.8291617	2.53E-01	27197555	27197678	NC_000007.13
cg27240402	5.87E-16	1.76E-17	9.38	28.8254031	7.57E-02	1364170	1364293	NC_000004.11
cg07664198	5.90E-16	1.77E-17	9.37	28.8189695	1.93E-01	136553882	136554005	NC_000007.13
cg22397673	5.92E-16	1.77E-17	9.37	28.8161796	5.94E-02	31851106	31851229	NC_000006.11
cg25612428	5.92E-16	1.78E-17	9.37	28.8150478	1.08E-01	10649867	10649990	NC_000005.9
cg19942495	5.93E-16	1.78E-17	9.37	28.8143921	1.68E-01	32484027	32484150	NC_000017.10
cg19706534	5.93E-16	1.78E-17	9.37	28.8134087	1.23E-01	120197644	120197767	NC_000002.11
cg07609844	5.97E-16	1.79E-17	9.37	28.8069683	1.26E-01	151389355	151389478	NC_000002.11
cg12741420	5.98E-16	1.79E-17	9.37	28.8056658	2.40E-01	392131	392254	NC_000006.11
cg26257969	5.99E-16	1.80E-17	9.37	28.80379	9.09E-02	48722474	48722597	NC_000012.11
cg20439473	5.99E-16	1.80E-17	9.37	28.8023871	9.96E-02	56061070	56061193	NC_000017.10
cg21003981	5.99E-16	1.80E-17	9.37	28.8021841	1.79E-02	9649299	9649422	NC_000001.10
cg03168029	6.01E-16	1.80E-17	9.37	28.7999439	1.27E-01	1221398	1221521	NC_000011.9
cg13654525	6.04E-16	1.82E-17	9.37	28.7934224	1.57E-01	65360509	65360632	NC_000011.9
cg09370594	6.05E-16	1.82E-17	9.37	28.7929632	1.20E-01	2291872	2291995	NC_000019.9
cg03226000	6.07E-16	1.82E-17	9.37	28.7895316	2.47E-01	55690378	55690501	NC_000016.9
cg05209584	6.07E-16	1.82E-17	9.37	28.7894965	2.55E-01	127873283	127873406	NC_000005.9
cg20841748	6.09E-16	1.83E-17	9.37	28.784884	1.40E-01	40838078	40838201	NC_000017.10
cg05888551	6.09E-16	1.83E-17	9.37	28.7846403	6.67E-02	56750788	56750911	NC_000020.10

cg17694795	6.10E-16	1.83E-17	9.37	28.7833201	1.99E-01	67874366	67874489	NC_000008.10
cg00260235	6.12E-16	1.84E-17	9.37	28.7800909	3.92E-02	69931262	69931385	NC_000011.9
cg26925231	6.12E-16	1.84E-17	9.37	28.7792381	1.96E-01	15094380	15094503	NC_000008.10
cg24772525	6.13E-16	1.84E-17	9.37	28.7790018	1.56E-01	172014861	172014984	NC_000002.11
cg08536805	6.16E-16	1.85E-17	9.37	28.7734961	7.18E-02	107748222	107748345	NC_000003.11
cg03970036	6.19E-16	1.86E-17	9.37	28.7682432	2.48E-01	220174232	220174355	NC_000002.11
cg25307665	6.20E-16	1.87E-17	9.37	28.765584	1.82E-01	27183694	27183817	NC_000007.13
cg12206317	6.20E-16	1.87E-17	9.37	28.7651887	1.12E-01	224026237	224026360	NC_000001.10
cg03376794	6.21E-16	1.87E-17	9.37	28.7641293	2.95E-01	25704507	25704630	NC_000016.9
cg09543255	6.24E-16	1.88E-17	9.37	28.7596077	2.36E-01	170302640	170302763	NC_000003.11
cg16945378	6.24E-16	1.88E-17	9.37	28.7592873	6.06E-02	37451213	37451336	NC_000022.10
cg00486340	6.24E-16	1.88E-17	9.37	28.7590857	2.09E-01	4860026	4860149	NC_000004.11
cg22205276	6.26E-16	1.89E-17	9.36	28.7544942	1.56E-01	60052372	60052495	NC_000018.9
cg22959054	6.32E-16	1.90E-17	9.36	28.7459432	1.27E-01	16761114	16761237	NC_000012.11
cg25555059	6.33E-16	1.91E-17	9.36	28.7441068	1.98E-01	128301488	128301611	NC_000005.9
cg21477176	6.33E-16	1.91E-17	9.36	28.7428742	2.02E-01	54445888	54446011	NC_000019.9
cg16848624	6.35E-16	1.92E-17	9.36	28.7395097	2.26E-01	45614290	45614413	NC_000007.13
cg02878244	6.38E-16	1.92E-17	9.36	28.7359956	2.01E-01	20181911	20182034	NC_000011.9
cg02397552	6.39E-16	1.93E-17	9.36	28.7341406	1.20E-01	77576078	77576201	NC_000013.10
cg02611934	6.40E-16	1.93E-17	9.36	28.7316492	1.53E-01	88329407	88329530	NC_000013.10
cg07899681	6.40E-16	1.93E-17	9.36	28.7314682	1.69E-01	22444714	22444837	NC_000019.9
cg18103859	6.43E-16	1.94E-17	9.36	28.7266741	3.34E-01	61110002	61110125	NC_000014.8
cg13919438	6.43E-16	1.94E-17	9.36	28.7263849	2.14E-01	39995546	39995669	NC_000020.10
cg01482645	6.45E-16	1.95E-17	9.36	28.7239036	2.29E-01	104513024	104513147	NC_000008.10
cg04227961	6.46E-16	1.95E-17	9.36	28.7221921	2.31E-01	113541993	113542116	NC_000012.11

cg14835443	6.46E-16	1.95E-17	9.36	28.7221042	2.09E-01	47225543	47225666	NC_000012.11
cg23641560	6.47E-16	1.96E-17	9.36	28.7200017	1.11E-01	182755044	182755167	NC_000002.11
cg09972259	6.47E-16	1.96E-17	9.36	28.7192091	2.15E-01	112711854	112711977	NC_000013.10
cg04109655	6.48E-16	1.96E-17	9.36	28.7181632	9.11E-02	2114887	2115010	NC_000007.13
cg07612562	6.55E-16	1.98E-17	9.36	28.7065439	2.41E-01	145813915	145814038	NC_000007.13
cg16204414	6.57E-16	1.99E-17	9.36	28.7041455	1.93E-01	10325494	10325617	NC_000003.11
cg07808555	6.58E-16	1.99E-17	9.36	28.7022253	2.70E-01	111217712	111217835	NC_000001.10
cg09022422	6.58E-16	1.99E-17	9.36	28.7019698	2.32E-01	10857798	10857921	NC_000003.11
cg18343881	6.61E-16	2.00E-17	9.36	28.6968621	1.57E-01	83016717	83016840	NC_000005.9
cg13362636	6.66E-16	2.02E-17	9.35	28.6888622	1.82E-01	82289309	82289432	NC_000002.11
cg26835683	6.68E-16	2.02E-17	9.35	28.686121	1.13E-01	160180434	160180557	NC_000001.10
cg15032142	6.71E-16	2.03E-17	9.35	28.6812893	2.11E-01	28414780	28414903	NC_000006.11
cg13680388	6.72E-16	2.04E-17	9.35	28.6797715	2.00E-01	57471844	57471967	NC_000020.10
cg04611203	6.75E-16	2.05E-17	9.35	28.6744019	1.94E-01	2781438	2781561	NC_000020.10
cg21045464	6.78E-16	2.05E-17	9.35	28.670859	2.45E-01	115135057	115135180	NC_000012.11
cg19839281	6.79E-16	2.06E-17	9.35	28.6689721	1.34E-01	238362492	238362615	NC_000002.11
cg10244666	6.81E-16	2.06E-17	9.35	28.6660662	2.44E-01	32421808	32421931	NC_000011.9
cg14123942	6.85E-16	2.08E-17	9.35	28.6592489	2.48E-01	104500322	104500445	NC_000009.11
cg11893763	6.85E-16	2.08E-17	9.35	28.6590205	1.91E-01	125425688	125425811	NC_000010.10
cg27444994	6.85E-16	2.08E-17	9.35	28.6589023	2.51E-01	62070072	62070195	NC_000016.9
cg06829686	6.88E-16	2.09E-17	9.35	28.6553759	2.69E-01	47910843	47910966	NC_000001.10
cg00154357	6.89E-16	2.09E-17	9.35	28.6532488	1.23E-01	147111280	147111403	NC_000003.11
cg12060744	6.92E-16	2.10E-17	9.35	28.6488164	2.57E-01	58095581	58095704	NC_000019.9
cg00788025	6.94E-16	2.11E-17	9.35	28.6454995	9.76E-02	962749	962872	NC_000004.11
cg14672084	6.95E-16	2.11E-17	9.35	28.6443284	2.00E-01	140857941	140858064	NC_000005.9

cg25718223	6.97E-16	2.12E-17	9.35	28.6402803	1.21E-01	13915877	13916000	NC_000019.9
cg11124652	6.98E-16	2.12E-17	9.35	28.6395621	1.25E-01	62462782	62462905	NC_000002.11
cg21683069	6.98E-16	2.12E-17	9.35	28.6387939	1.78E-01	147781600	147781723	NC_000001.10
cg11323255	7.00E-16	2.13E-17	9.35	28.6357597	1.49E-01	1493168	1493291	NC_000019.9
cg16256390	7.00E-16	2.13E-17	9.35	28.635739	2.35E-01	99712336	99712459	NC_000014.8
cg27174778	7.01E-16	2.13E-17	9.35	28.6351537	9.90E-02	44186519	44186642	NC_000007.13
cg05883442	7.01E-16	2.13E-17	9.35	28.63432	1.26E-01	67476137	67476260	NC_000016.9
cg17722002	7.06E-16	2.15E-17	9.34	28.6267592	1.85E-01	121940381	121940504	NC_000007.13
cg09127607	7.07E-16	2.15E-17	9.34	28.624983	2.12E-01	69914213	69914336	NC_000010.10
cg01348055	7.08E-16	2.16E-17	9.34	28.6235543	1.30E-01	10272788	10272911	NC_000016.9
cg07372195	7.13E-16	2.17E-17	9.34	28.6160886	1.38E-01	67310842	67310965	NC_000017.10
cg13672348	7.20E-16	2.19E-17	9.34	28.6069377	5.88E-02	140588475	140588598	NC_000009.11
cg05783915	7.22E-16	2.20E-17	9.34	28.604593	2.47E-01	119302062	119302185	NC_000010.10
cg09567180	7.23E-16	2.20E-17	9.34	28.6030419	1.45E-01	124609416	124609539	NC_000010.10
cg25730685	7.24E-16	2.21E-17	9.34	28.6009992	2.73E-01	2375010	2375133	NC_000001.10
cg03882967	7.25E-16	2.21E-17	9.34	28.6001475	1.00E-01	182117858	182117981	NC_000003.11
cg14852384	7.25E-16	2.21E-17	9.34	28.598988	1.66E-01	229569877	229570000	NC_000001.10
cg10581811	7.28E-16	2.22E-17	9.34	28.5954006	3.37E-02	58257267	58257390	NC_000012.11
cg05087577	7.29E-16	2.22E-17	9.34	28.5927098	5.19E-02	43532611	43532734	NC_000001.10
cg16935061	7.31E-16	2.23E-17	9.34	28.590217	1.86E-01	1395278	1395401	NC_000006.11
cg20293594	7.32E-16	2.23E-17	9.34	28.5889573	2.66E-01	27196296	27196419	NC_000007.13
cg05495949	7.39E-16	2.25E-17	9.34	28.5789947	2.77E-01	4923140	4923263	NC_000007.13
cg08802533	7.40E-16	2.26E-17	9.34	28.5771148	1.27E-01	27073253	27073376	NC_000002.11
cg24932457	7.41E-16	2.26E-17	9.34	28.5758757	1.99E-01	175568559	175568682	NC_000001.10
cg12924936	7.42E-16	2.27E-17	9.34	28.5742364	2.51E-01	2279098	2279221	NC_000005.9

cg05471509	7.49E-16	2.29E-17	9.34	28.5657877	1.10E-01	114109420	114109543	NC_000013.10
cg01947130	7.49E-16	2.29E-17	9.34	28.565495	2.34E-01	60287196	60287319	NC_000015.9
cg22560214	7.49E-16	2.29E-17	9.34	28.5643722	1.47E-01	13531213	13531336	NC_000004.11
cg23383138	7.50E-16	2.29E-17	9.34	28.5636556	1.38E-01	30885012	30885135	NC_000022.10
cg16685313	7.51E-16	2.30E-17	9.33	28.561229	1.63E-01	233036012	233036135	NC_000001.10
cg15839448	7.53E-16	2.30E-17	9.33	28.5596427	1.92E-01	41166530	41166653	NC_000008.10
cg14800197	7.62E-16	2.33E-17	9.33	28.5471599	1.35E-01	121735681	121735804	NC_000002.11
cg26913576	7.62E-16	2.33E-17	9.33	28.5466352	9.46E-02	2151698	2151821	NC_000011.9
cg04572969	7.62E-16	2.33E-17	9.33	28.5465318	8.71E-02	3503480	3503603	NC_000001.10
cg14335816	7.64E-16	2.34E-17	9.33	28.543352	1.05E-01	1069505	1069628	NC_000017.10
cg12451155	7.73E-16	2.37E-17	9.33	28.531361	1.45E-01	29065427	29065550	NC_000001.10
cg21452281	7.74E-16	2.37E-17	9.33	28.5293928	1.76E-01	189203965	189204088	NC_000004.11
cg25632105	7.75E-16	2.37E-17	9.33	28.5287355	1.85E-01	136553728	136553851	NC_000007.13
cg08112737	7.76E-16	2.38E-17	9.33	28.5274832	9.99E-02	57178051	57178174	NC_000004.11
cg14155184	7.77E-16	2.38E-17	9.33	28.5258312	8.54E-02	44120425	44120548	NC_000006.11
cg10357909	7.77E-16	2.38E-17	9.33	28.5252686	6.87E-02	479994	480117	NC_000016.9
cg19656282	7.77E-16	2.38E-17	9.33	28.525073	2.01E-01	74742786	74742909	NC_000002.11
cg16472050	7.78E-16	2.38E-17	9.33	28.5245787	1.75E-01	172945698	172945821	NC_000002.11
cg05525743	7.79E-16	2.39E-17	9.33	28.5222868	2.58E-01	30716524	30716647	NC_000019.9
cg24953703	7.82E-16	2.39E-17	9.33	28.5194722	1.63E-01	41236975	41237098	NC_000015.9
cg03149432	7.82E-16	2.40E-17	9.33	28.5188098	1.85E-01	37498721	37498844	NC_000001.10
cg18788664	7.84E-16	2.40E-17	9.33	28.5165283	2.36E-01	221068171	221068294	NC_000001.10
cg16730825	7.86E-16	2.41E-17	9.33	28.5129323	1.71E-01	928439	928562	NC_000011.9
cg12288551	7.87E-16	2.41E-17	9.33	28.512378	1.24E-01	50801220	50801343	NC_000007.13
cg05991454	7.87E-16	2.41E-17	9.33	28.5115825	2.12E-01	147558435	147558558	NC_000004.11

cg05732394	7.90E-16	2.42E-17	9.33	28.5081056	1.04E-01	44255249	44255372	NC_000007.13
cg03242458	7.92E-16	2.43E-17	9.33	28.5052643	9.68E-02	30135644	30135767	NC_000006.11
cg05011265	8.00E-16	2.46E-17	9.32	28.4942382	8.01E-02	22958399	22958522	NC_000014.8
cg13191508	8.01E-16	2.46E-17	9.32	28.4934853	1.37E-01	144600686	144600809	NC_000008.10
cg21100077	8.03E-16	2.47E-17	9.32	28.4901043	1.66E-01	100627366	100627489	NC_000013.10
cg11709905	8.05E-16	2.47E-17	9.32	28.487292	9.70E-02	736287	736410	NC_000011.9
cg06946708	8.05E-16	2.47E-17	9.32	28.4872669	2.12E-01	125732373	125732496	NC_000010.10
cg21097354	8.07E-16	2.48E-17	9.32	28.4854572	1.93E-01	27179161	27179284	NC_000007.13
cg06170425	8.09E-16	2.49E-17	9.32	28.4823683	1.05E-01	4164087	4164210	NC_000016.9
cg14427009	8.13E-16	2.50E-17	9.32	28.4776409	1.63E-01	58207058	58207181	NC_000013.10
cg16518590	8.19E-16	2.52E-17	9.32	28.4699313	4.76E-02	51104801	51104924	NC_000007.13
cg02885007	8.23E-16	2.53E-17	9.32	28.4652229	1.42E-01	176987605	176987728	NC_000002.11
cg01002253	8.23E-16	2.53E-17	9.32	28.4647687	2.33E-01	164264914	164265037	NC_000004.11
cg00652274	8.23E-16	2.53E-17	9.32	28.4646903	1.67E-01	115576803	115576926	NC_000003.11
cg02728050	8.24E-16	2.53E-17	9.32	28.4638597	2.13E-01	50818153	50818276	NC_000010.10
cg06311863	8.25E-16	2.54E-17	9.32	28.4616163	2.13E-01	55508686	55508809	NC_000003.11
cg26848718	8.26E-16	2.54E-17	9.32	28.4603561	1.82E-01	32454975	32455098	NC_000011.9
cg16347256	8.27E-16	2.54E-17	9.32	28.4599046	1.57E-01	57285160	57285283	NC_000001.10
cg16730509	8.31E-16	2.56E-17	9.32	28.4542981	1.27E-01	102787588	102787711	NC_000014.8
cg19265000	8.32E-16	2.56E-17	9.32	28.4532708	1.10E-01	49460706	49460829	NC_000012.11
cg05924171	8.32E-16	2.56E-17	9.32	28.4530025	6.12E-02	67471737	67471860	NC_000011.9
cg06898293	8.33E-16	2.56E-17	9.32	28.451656	2.03E-01	114750703	114750826	NC_000007.13
cg04317399	8.36E-16	2.58E-17	9.32	28.447389	1.94E-01	27170313	27170436	NC_000007.13
cg13352750	8.37E-16	2.58E-17	9.32	28.4459248	2.41E-01	27225123	27225246	NC_000007.13
cg14386999	8.40E-16	2.59E-17	9.32	28.4420974	7.41E-02	44906672	44906795	NC_000021.8

cg25593565	8.41E-16	2.59E-17	9.32	28.4419659	1.00E-01	5646827	5646950	NC_000007.13
cg24145007	8.44E-16	2.60E-17	9.32	28.437773	8.50E-02	245869090	245869213	NC_000001.10
cg10410852	8.47E-16	2.61E-17	9.31	28.4332919	1.15E-01	74714308	74714431	NC_000010.10
cg11348701	8.50E-16	2.62E-17	9.31	28.4304772	9.80E-02	171704223	171704346	NC_000002.11
cg03184290	8.53E-16	2.63E-17	9.31	28.4265314	2.33E-01	111550666	111550789	NC_000004.11
cg05018513	8.54E-16	2.63E-17	9.31	28.4253251	1.37E-01	105173980	105174103	NC_000014.8
cg04404540	8.54E-16	2.63E-17	9.31	28.4251919	2.19E-01	72270444	72270567	NC_000017.10
cg15925365	8.57E-16	2.64E-17	9.31	28.4219686	2.10E-01	52858998	52859121	NC_000006.11
cg02232208	8.57E-16	2.64E-17	9.31	28.4211377	2.21E-01	147137147	147137270	NC_000003.11
cg04269530	8.60E-16	2.65E-17	9.31	28.4185692	1.49E-01	48919401	48919524	NC_000019.9
cg20392607	8.60E-16	2.65E-17	9.31	28.4184807	2.77E-01	5018798	5018921	NC_000012.11
cg18434588	8.65E-16	2.67E-17	9.31	28.4117199	1.13E-01	80095132	80095255	NC_000005.9
cg04279449	8.68E-16	2.68E-17	9.31	28.4078375	1.19E-01	703918	704041	NC_000011.9
cg18336674	8.70E-16	2.69E-17	9.31	28.4059225	1.41E-01	170735264	170735387	NC_000005.9
cg17252960	8.71E-16	2.69E-17	9.31	28.4052647	2.37E-01	19837350	19837473	NC_000006.11
cg06070644	8.72E-16	2.69E-17	9.31	28.4032201	8.67E-02	25677759	25677882	NC_000004.11
cg14930075	8.79E-16	2.72E-17	9.31	28.3949301	2.79E-01	140715018	140715141	NC_000008.10
cg06538872	8.80E-16	2.72E-17	9.31	28.3939434	8.29E-02	947343	947466	NC_000001.10
cg21546184	8.83E-16	2.73E-17	9.31	28.3898705	2.40E-01	65283649	65283772	NC_000008.10
cg03646189	8.84E-16	2.73E-17	9.31	28.3893287	2.46E-01	2756101	2756224	NC_000005.9
cg10731700	8.87E-16	2.74E-17	9.31	28.3858655	2.65E-01	1760259	1760382	NC_000019.9
cg26432350	8.89E-16	2.75E-17	9.31	28.3828117	1.36E-01	47040357	47040480	NC_000003.11
cg18252309	8.89E-16	2.75E-17	9.31	28.3827387	2.28E-01	153583867	153583990	NC_000007.13
cg01520105	8.89E-16	2.75E-17	9.31	28.3827284	1.06E-01	111887243	111887366	NC_000006.11
cg06735554	8.92E-16	2.76E-17	9.31	28.379153	1.19E-01	1221726	1221849	NC_000011.9

cg23484785	8.93E-16	2.76E-17	9.31	28.3780072	1.26E-01	105343352	105343475	NC_000004.11
cg12395726	8.93E-16	2.76E-17	9.31	28.3772318	5.63E-02	33248687	33248810	NC_000006.11
cg24812837	8.98E-16	2.78E-17	9.31	28.3721546	2.53E-01	102894120	102894243	NC_000010.10
cg06616093	8.99E-16	2.78E-17	9.31	28.3703832	6.63E-02	134828082	134828205	NC_000010.10
cg06737561	9.05E-16	2.80E-17	9.3	28.3634675	1.85E-01	50821518	50821641	NC_000010.10
cg24890294	9.08E-16	2.81E-17	9.3	28.3597868	7.22E-02	31617322	31617445	NC_000006.11
cg05314639	9.11E-16	2.82E-17	9.3	28.3572024	1.21E-01	114109729	114109852	NC_000013.10
cg04168853	9.12E-16	2.83E-17	9.3	28.3554285	2.20E-01	38346023	38346146	NC_000019.9
cg15265853	9.13E-16	2.83E-17	9.3	28.3542027	5.25E-02	29401371	29401494	NC_000017.10
cg07618409	9.18E-16	2.85E-17	9.3	28.3482271	1.01E-01	55742491	55742614	NC_000017.10
cg12304520	9.21E-16	2.85E-17	9.3	28.3455833	2.25E-01	140810123	140810246	NC_000005.9
cg07642170	9.23E-16	2.86E-17	9.3	28.3430635	5.77E-02	44218746	44218869	NC_000019.9
cg23921338	9.25E-16	2.87E-17	9.3	28.3408338	7.83E-02	70206922	70207045	NC_000002.11
cg19253850	9.31E-16	2.89E-17	9.3	28.3344777	1.29E-01	129796229	129796352	NC_000010.10
cg08492619	9.32E-16	2.89E-17	9.3	28.3334741	2.72E-01	88324193	88324316	NC_000013.10
cg09221867	9.40E-16	2.92E-17	9.3	28.3244407	2.54E-01	134071663	134071786	NC_000004.11
cg11630404	9.41E-16	2.92E-17	9.3	28.3221938	2.63E-01	113442346	113442469	NC_000004.11
cg24333629	9.43E-16	2.93E-17	9.3	28.320208	2.32E-01	229045958	229046081	NC_000002.11
cg24317217	9.43E-16	2.93E-17	9.3	28.3201026	1.90E-01	70231495	70231618	NC_000003.11
cg07180307	9.44E-16	2.93E-17	9.3	28.3194934	2.53E-01	109294168	109294291	NC_000011.9
cg25950235	9.52E-16	2.96E-17	9.3	28.3110067	2.63E-01	89910753	89910876	NC_000015.9
cg23266743	9.53E-16	2.96E-17	9.3	28.309802	2.18E-01	130388198	130388321	NC_000012.11
cg09417809	9.53E-16	2.96E-17	9.3	28.309119	1.92E-01	115131223	115131346	NC_000012.11
cg24389054	9.54E-16	2.96E-17	9.3	28.3084065	1.65E-01	27194013	27194136	NC_000007.13
cg03679755	9.55E-16	2.97E-17	9.3	28.3074492	2.12E-01	101875035	101875158	NC_000010.10

cg13950829	9.55E-16	2.97E-17	9.3	28.306717	2.87E-01	11060652	11060775	NC_000010.10
cg10124710	9.58E-16	2.98E-17	9.29	28.3036039	1.63E-01	146544267	146544390	NC_000001.10
cg23683588	9.71E-16	3.02E-17	9.29	28.2902833	2.04E-01	37136283	37136406	NC_000014.8
cg18722086	9.73E-16	3.03E-17	9.29	28.2879729	1.48E-01	113380072	113380195	NC_000003.11
cg08097882	9.73E-16	3.03E-17	9.29	28.2878898	2.35E-01	79177516	79177639	NC_000013.10
cg06745684	9.75E-16	3.03E-17	9.29	28.2860526	9.02E-02	37949239	37949362	NC_000021.8
cg12924956	9.75E-16	3.03E-17	9.29	28.2859063	1.61E-01	24600569	24600692	NC_000014.8
cg20100910	9.75E-16	3.03E-17	9.29	28.285119	2.44E-01	102475429	102475552	NC_000010.10
cg10615414	9.78E-16	3.04E-17	9.29	28.2824554	2.36E-01	37353096	37353219	NC_000020.10
cg17840132	9.79E-16	3.05E-17	9.29	28.2808808	3.69E-02	39380842	39380965	NC_000005.9
cg13407456	9.84E-16	3.06E-17	9.29	28.2756806	2.68E-01	69243398	69243521	NC_000008.10
cg09765089	9.87E-16	3.07E-17	9.29	28.2729387	2.95E-01	27291346	27291469	NC_000007.13
cg07659054	9.88E-16	3.07E-17	9.29	28.2720322	2.14E-01	27134225	27134348	NC_000007.13
cg26730369	1.01E-15	3.15E-17	9.29	28.2490267	2.54E-01	26724001	26724124	NC_000008.10
cg16590466	1.01E-15	3.15E-17	9.29	28.2483966	7.19E-02	30795267	30795390	NC_000016.9
cg25693769	1.02E-15	3.17E-17	9.28	28.2406103	1.49E-01	33261112	33261235	NC_000003.11
cg19877064	1.02E-15	3.18E-17	9.28	28.2375272	5.90E-02	30435467	30435590	NC_000003.11
cg05837769	1.02E-15	3.19E-17	9.28	28.2369544	1.44E-01	65358078	65358201	NC_000011.9
cg04304705	1.02E-15	3.19E-17	9.28	28.2362122	1.88E-01	48884960	48885083	NC_000022.10
cg02765731	1.02E-15	3.20E-17	9.28	28.233842	1.77E-01	83436797	83436920	NC_000012.11
cg03888064	1.03E-15	3.20E-17	9.28	28.233003	1.68E-01	156305656	156305779	NC_000001.10
cg01142635	1.03E-15	3.20E-17	9.28	28.2319377	1.87E-01	6224117	6224240	NC_000004.11
cg16690757	1.03E-15	3.21E-17	9.28	28.2305213	1.86E-01	26680932	26681055	NC_000010.10
cg20276585	1.03E-15	3.21E-17	9.28	28.2281191	2.84E-01	81952330	81952453	NC_000004.11
cg12431879	1.03E-15	3.21E-17	9.28	28.2279043	1.73E-01	17082787	17082910	NC_000022.10

cg01995743	1.03E-15	3.22E-17	9.28	28.2277348	2.43E-01	118229732	118229855	NC_000006.11
cg05159909	1.03E-15	3.22E-17	9.28	28.2256422	6.77E-02	56515094	56515217	NC_000012.11
cg18349911	1.04E-15	3.24E-17	9.28	28.22051	2.15E-01	1877135	1877258	NC_000005.9
cg26916764	1.04E-15	3.24E-17	9.28	28.2189597	5.24E-02	115268937	115269060	NC_000001.10
cg26433102	1.04E-15	3.25E-17	9.28	28.2177317	2.42E-01	391441	391564	NC_000006.11
cg07492143	1.04E-15	3.25E-17	9.28	28.2172845	1.12E-01	67695812	67695935	NC_000016.9
cg04842426	1.04E-15	3.26E-17	9.28	28.2142324	2.09E-01	136555777	136555900	NC_000007.13
cg00321644	1.04E-15	3.26E-17	9.28	28.213613	6.51E-02	977110	977233	NC_000012.11
cg20035362	1.04E-15	3.26E-17	9.28	28.2134768	9.98E-02	10161366	10161489	NC_000003.11
cg08026818	1.05E-15	3.27E-17	9.28	28.2115872	2.65E-01	77823221	77823344	NC_000016.9
cg09131151	1.05E-15	3.30E-17	9.28	28.2031089	1.35E-01	133782453	133782576	NC_000011.9
cg14338246	1.06E-15	3.31E-17	9.28	28.1991902	7.39E-02	75960767	75960890	NC_000017.10
cg06073141	1.07E-15	3.35E-17	9.28	28.1877213	4.56E-02	190105762	190105885	NC_000003.11
cg05697849	1.07E-15	3.35E-17	9.28	28.1870681	1.72E-01	50513766	50513889	NC_000001.10
cg05932360	1.07E-15	3.35E-17	9.28	28.1859183	1.14E-01	15313285	15313408	NC_000006.11
cg00597931	1.08E-15	3.37E-17	9.28	28.1804736	1.49E-01	142179697	142179820	NC_000008.10
cg20936854	1.08E-15	3.38E-17	9.28	28.1792687	5.79E-02	44527408	44527531	NC_000020.10
cg09806671	1.08E-15	3.38E-17	9.28	28.1790579	1.24E-01	165324679	165324802	NC_000001.10
cg16781502	1.08E-15	3.38E-17	9.28	28.1777009	1.62E-01	134774214	134774337	NC_000010.10
cg11544647	1.08E-15	3.40E-17	9.27	28.1737616	9.07E-02	6659957	6660080	NC_000019.9
cg00929376	1.08E-15	3.40E-17	9.27	28.1735742	1.57E-01	25258236	25258359	NC_000001.10
cg16440561	1.09E-15	3.40E-17	9.27	28.1726748	2.49E-01	220312854	220312977	NC_000002.11
cg13928102	1.09E-15	3.40E-17	9.27	28.1710351	1.65E-01	76360050	76360173	NC_000005.9
cg16945186	1.09E-15	3.41E-17	9.27	28.1686798	2.09E-01	115103960	115104083	NC_000012.11
cg16150752	1.09E-15	3.42E-17	9.27	28.1660718	2.74E-01	16180072	16180195	NC_000005.9

cg27464184	1.09E-15	3.42E-17	9.27	28.1659312	2.87E-01	114075881	114076004	NC_000012.11
cg23474501	1.09E-15	3.43E-17	9.27	28.164177	2.14E-01	134902168	134902291	NC_000010.10
cg22488256	1.10E-15	3.46E-17	9.27	28.1557625	2.35E-01	66258046	66258169	NC_000001.10
cg13588946	1.10E-15	3.46E-17	9.27	28.1554067	1.46E-01	69678511	69678634	NC_000014.8
cg16509192	1.11E-15	3.47E-17	9.27	28.1515931	1.25E-01	122107024	122107147	NC_000003.11
cg18750960	1.11E-15	3.47E-17	9.27	28.1514596	2.07E-01	177016417	177016540	NC_000002.11
cg01382489	1.11E-15	3.48E-17	9.27	28.1497622	5.73E-02	44949625	44949748	NC_000021.8
cg17966167	1.11E-15	3.48E-17	9.27	28.1484143	2.69E-01	37488564	37488687	NC_000007.13
cg06894723	1.12E-15	3.51E-17	9.27	28.1408615	1.07E-01	110398142	110398265	NC_000013.10
cg18430128	1.12E-15	3.51E-17	9.27	28.1400777	2.33E-01	58570491	58570614	NC_000019.9
cg22206608	1.12E-15	3.51E-17	9.27	28.1399521	2.11E-01	125732632	125732755	NC_000010.10
cg27041794	1.13E-15	3.55E-17	9.27	28.1309002	2.70E-01	11059708	11059831	NC_000010.10
cg11838639	1.13E-15	3.55E-17	9.27	28.1283184	6.94E-02	3512917	3513040	NC_000010.10
cg14212180	1.14E-15	3.57E-17	9.27	28.1239393	4.01E-02	3365201	3365324	NC_000019.9
cg09582952	1.14E-15	3.57E-17	9.27	28.1231864	2.39E-01	29033187	29033310	NC_000017.10
cg13686795	1.14E-15	3.57E-17	9.27	28.1229571	7.67E-02	151522573	151522696	NC_000007.13
cg10615842	1.14E-15	3.59E-17	9.27	28.118046	2.45E-01	20619158	20619281	NC_000011.9
cg11342468	1.15E-15	3.60E-17	9.27	28.114929	1.95E-01	103889784	103889907	NC_000012.11
cg08705882	1.15E-15	3.60E-17	9.27	28.1148844	6.62E-02	94337576	94337699	NC_000010.10
cg13323416	1.15E-15	3.61E-17	9.27	28.1128208	1.31E-01	45609367	45609490	NC_000006.11
cg27340506	1.15E-15	3.61E-17	9.27	28.1125965	1.31E-01	87082045	87082168	NC_000008.10
cg11861308	1.15E-15	3.61E-17	9.27	28.1121982	5.85E-02	17002043	17002166	NC_000022.10
cg12226006	1.15E-15	3.61E-17	9.27	28.1119528	9.19E-02	123350909	123351032	NC_000010.10
cg22374057	1.16E-15	3.64E-17	9.26	28.1046423	1.72E-01	11148682	11148805	NC_000018.9
cg05446615	1.16E-15	3.64E-17	9.26	28.1043142	6.44E-02	88547861	88547984	NC_000016.9

cg21053982	1.16E-15	3.64E-17	9.26	28.1035917	3.33E-02	90578774	90578897	NC_000005.9
cg06837746	1.16E-15	3.66E-17	9.26	28.0992034	7.42E-02	8774972	8775095	NC_000003.11
cg02814289	1.16E-15	3.66E-17	9.26	28.0984508	5.42E-02	717213	717336	NC_000016.9
cg05655106	1.17E-15	3.67E-17	9.26	28.0978165	1.25E-01	353905	354028	NC_000005.9
cg13344169	1.17E-15	3.70E-17	9.26	28.0899234	2.27E-01	71161178	71161301	NC_000017.10
cg23610736	1.18E-15	3.70E-17	9.26	28.088312	1.18E-01	186957729	186957852	NC_000001.10
cg13096407	1.18E-15	3.73E-17	9.26	28.080916	7.18E-02	32885502	32885625	NC_000013.10
cg25654952	1.19E-15	3.76E-17	9.26	28.0719033	1.12E-01	7861342	7861465	NC_000007.13
cg02182795	1.19E-15	3.76E-17	9.26	28.0718767	2.53E-01	57069688	57069811	NC_000008.10
cg22167267	1.20E-15	3.78E-17	9.26	28.0665289	1.14E-01	80132832	80132955	NC_000017.10
cg10575652	1.20E-15	3.78E-17	9.26	28.0664661	7.97E-02	123351652	123351775	NC_000012.11
cg01033865	1.20E-15	3.79E-17	9.26	28.0655622	2.35E-01	115630896	115631019	NC_000011.9
cg14485004	1.20E-15	3.79E-17	9.26	28.0638846	2.39E-01	75524913	75525036	NC_000017.10
cg06857316	1.20E-15	3.79E-17	9.26	28.0638739	8.16E-02	97846357	97846480	NC_000007.13
cg00810939	1.20E-15	3.80E-17	9.26	28.0633845	1.79E-01	24463749	24463872	NC_000001.10
cg24378227	1.21E-15	3.81E-17	9.26	28.0603707	1.63E-01	1396771	1396894	NC_000006.11
cg05502103	1.21E-15	3.82E-17	9.26	28.0571531	1.09E-01	588936	589059	NC_000007.13
cg16801887	1.21E-15	3.83E-17	9.26	28.0554489	2.80E-01	86541480	86541603	NC_000016.9
cg09729848	1.22E-15	3.83E-17	9.26	28.0536504	2.55E-01	178770998	178771121	NC_000005.9
cg15858959	1.22E-15	3.85E-17	9.26	28.0487279	1.23E-01	71863687	71863810	NC_000014.8
cg04194457	1.23E-15	3.88E-17	9.25	28.0414552	8.65E-02	52493238	52493361	NC_000020.10
cg08439930	1.23E-15	3.89E-17	9.25	28.0392432	2.92E-01	51185060	51185183	NC_000016.9
cg07684152	1.23E-15	3.89E-17	9.25	28.0380399	4.51E-02	130670760	130670883	NC_000012.11
cg15646778	1.23E-15	3.90E-17	9.25	28.0370448	1.21E-01	33376062	33376185	NC_000006.11
cg07042546	1.24E-15	3.90E-17	9.25	28.0355631	1.49E-01	117104996	117105119	NC_000011.9

cg19161330	1.24E-15	3.91E-17	9.25	28.0335991	8.43E-02	184290643	184290766	NC_000003.11
cg18676967	1.24E-15	3.92E-17	9.25	28.0312508	1.38E-01	233217476	233217599	NC_000002.11
cg24928181	1.25E-15	3.94E-17	9.25	28.0252942	1.04E-01	151424835	151424958	NC_000002.11
cg02970836	1.25E-15	3.95E-17	9.25	28.0240904	2.54E-01	3237991	3238114	NC_000016.9
cg06533244	1.25E-15	3.96E-17	9.25	28.0213174	1.75E-01	27258460	27258583	NC_000006.11
cg16886487	1.26E-15	3.97E-17	9.25	28.0180853	1.13E-01	50944575	50944698	NC_000022.10
cg14236662	1.26E-15	3.98E-17	9.25	28.0166733	2.53E-01	176988284	176988407	NC_000002.11
cg01323104	1.26E-15	4.00E-17	9.25	28.0117324	1.58E-01	87958903	87959026	NC_000004.11
cg20691140	1.27E-15	4.00E-17	9.25	28.0106398	2.18E-01	137522667	137522790	NC_000002.11
cg12836959	1.27E-15	4.01E-17	9.25	28.0091942	2.47E-01	147098568	147098691	NC_000003.11
cg26462212	1.27E-15	4.01E-17	9.25	28.0079649	3.43E-02	339544	339667	NC_000016.9
cg27564715	1.27E-15	4.02E-17	9.25	28.0073939	1.17E-01	1773758	1773881	NC_000008.10
cg12487153	1.27E-15	4.03E-17	9.25	28.0033571	8.98E-02	13560875	13560998	NC_000006.11
cg24865955	1.28E-15	4.04E-17	9.25	28.0023444	6.12E-02	141576963	141577086	NC_000005.9
cg08471734	1.28E-15	4.05E-17	9.25	27.9995938	8.73E-02	15808817	15808940	NC_000001.10
cg12854979	1.28E-15	4.05E-17	9.25	27.9994721	1.03E-01	78134143	78134266	NC_000017.10
cg02781618	1.28E-15	4.06E-17	9.25	27.9958693	2.04E-01	42153695	42153818	NC_000004.11
cg20462512	1.28E-15	4.07E-17	9.25	27.9954858	1.85E-01	5543155	5543278	NC_000012.11
cg16960943	1.28E-15	4.07E-17	9.25	27.9954378	8.86E-02	236417185	236417308	NC_000002.11
cg13088368	1.28E-15	4.07E-17	9.25	27.9950863	1.90E-01	42544716	42544839	NC_000020.10
cg23290121	1.28E-15	4.07E-17	9.25	27.9946679	1.18E-01	241612069	241612192	NC_000002.11
cg26385172	1.29E-15	4.09E-17	9.25	27.9886027	2.36E-01	115135169	115135292	NC_000012.11
cg06325515	1.29E-15	4.09E-17	9.25	27.9884003	5.76E-02	75610567	75610690	NC_000016.9
cg23317501	1.29E-15	4.09E-17	9.25	27.988313	1.94E-01	35991215	35991338	NC_000005.9
cg03577655	1.30E-15	4.11E-17	9.25	27.9858611	2.61E-01	49815365	49815488	NC_000007.13

cg02116996	1.30E-15	4.12E-17	9.24	27.9813996	3.36E-02	30126364	30126487	NC_000006.11
cg03445516	1.30E-15	4.13E-17	9.24	27.978919	1.58E-01	95664233	95664356	NC_000002.11
cg15207742	1.30E-15	4.13E-17	9.24	27.9789168	2.50E-01	43438809	43438932	NC_000020.10
cg17463655	1.30E-15	4.14E-17	9.24	27.9781171	1.41E-01	97846790	97846913	NC_000007.13
cg06994787	1.31E-15	4.14E-17	9.24	27.977848	1.90E-01	11834960	11835083	NC_000016.9
cg14780632	1.31E-15	4.14E-17	9.24	27.9764317	2.54E-01	65816521	65816644	NC_000011.9
cg22521696	1.31E-15	4.15E-17	9.24	27.9761047	1.96E-01	38183210	38183333	NC_000019.9
cg00461022	1.32E-15	4.18E-17	9.24	27.9690652	1.21E-01	16618052	16618175	NC_000005.9
cg23771603	1.32E-15	4.18E-17	9.24	27.9688825	2.53E-01	26223310	26223433	NC_000010.10
cg00897799	1.32E-15	4.18E-17	9.24	27.9681262	9.84E-02	47367897	47368020	NC_000013.10
cg13542964	1.32E-15	4.18E-17	9.24	27.9675744	2.06E-01	10458667	10458790	NC_000004.11
cg14633892	1.32E-15	4.19E-17	9.24	27.9663825	1.08E-01	56810749	56810872	NC_000003.11
cg22546168	1.32E-15	4.19E-17	9.24	27.9652352	1.94E-01	135050326	135050449	NC_000010.10
cg25685456	1.33E-15	4.21E-17	9.24	27.9612483	8.78E-02	143269881	143270004	NC_000006.11
cg09083627	1.33E-15	4.21E-17	9.24	27.9608012	2.76E-01	88324597	88324720	NC_000013.10
cg09159223	1.33E-15	4.22E-17	9.24	27.9595272	8.03E-02	36018688	36018811	NC_000007.13
cg03483150	1.33E-15	4.22E-17	9.24	27.959358	2.01E-01	57358651	57358774	NC_000008.10
cg25886621	1.33E-15	4.22E-17	9.24	27.9581726	1.66E-01	93130251	93130374	NC_000012.11
cg16635592	1.33E-15	4.23E-17	9.24	27.9561562	1.36E-01	66870371	66870494	NC_000003.11
cg02391713	1.34E-15	4.25E-17	9.24	27.950565	2.61E-01	105400985	105401108	NC_000006.11
cg24979233	1.34E-15	4.26E-17	9.24	27.949684	1.30E-01	1800284	1800407	NC_000004.11
cg24703706	1.35E-15	4.28E-17	9.24	27.9455598	1.13E-01	47408658	47408781	NC_000007.13
cg17697739	1.35E-15	4.28E-17	9.24	27.9453501	1.26E-01	1873340	1873463	NC_000006.11
cg05131623	1.35E-15	4.28E-17	9.24	27.9451254	2.72E-01	179755285	179755408	NC_000003.11
cg24610236	1.35E-15	4.28E-17	9.24	27.943838	3.41E-01	30722114	30722237	NC_000007.13

cg21511365	1.35E-15	4.29E-17	9.24	27.9419275	2.36E-01	9763739	9763862	NC_000008.10
cg08753373	1.35E-15	4.30E-17	9.24	27.940464	2.60E-01	134000124	134000247	NC_000010.10
cg26790818	1.35E-15	4.30E-17	9.24	27.9394488	1.21E-01	187786456	187786579	NC_000003.11
cg17653824	1.35E-15	4.30E-17	9.24	27.9390907	1.64E-01	5153054	5153177	NC_000012.11
cg04596004	1.36E-15	4.31E-17	9.24	27.936874	1.81E-01	112710155	112710278	NC_000013.10
cg15554023	1.36E-15	4.33E-17	9.24	27.9322332	1.63E-01	144008937	144009060	NC_000001.10
cg11137321	1.36E-15	4.34E-17	9.24	27.9308853	6.90E-02	148010015	148010138	NC_000001.10
cg09971562	1.37E-15	4.35E-17	9.24	27.9296054	1.33E-01	128694679	128694802	NC_000011.9
cg05718036	1.37E-15	4.35E-17	9.24	27.929019	2.68E-01	81952593	81952716	NC_000004.11
cg13814720	1.37E-15	4.36E-17	9.24	27.9267231	6.64E-02	91193175	91193298	NC_000009.11
cg12658374	1.37E-15	4.36E-17	9.24	27.92654	1.23E-01	37323563	37323686	NC_000017.10
cg11438428	1.37E-15	4.36E-17	9.24	27.9258834	1.90E-01	23481594	23481717	NC_000010.10
cg14601251	1.37E-15	4.36E-17	9.24	27.9258734	6.35E-02	65913984	65914107	NC_000015.9
cg15732768	1.37E-15	4.37E-17	9.24	27.9249612	1.97E-01	96651537	96651660	NC_000007.13
cg18785679	1.37E-15	4.37E-17	9.24	27.9243831	1.58E-01	135032504	135032627	NC_000010.10
cg04733537	1.37E-15	4.38E-17	9.24	27.9226502	2.15E-01	130388748	130388871	NC_000012.11
cg26123532	1.37E-15	4.38E-17	9.24	27.922286	8.64E-02	158027027	158027150	NC_000002.11
cg01993818	1.38E-15	4.38E-17	9.24	27.9217115	1.27E-01	34270437	34270560	NC_000014.8
cg00886598	1.38E-15	4.38E-17	9.24	27.9209165	1.09E-01	42812844	42812967	NC_000005.9
cg11302943	1.38E-15	4.40E-17	9.23	27.9179678	2.33E-01	99790596	99790719	NC_000010.10
cg04039957	1.38E-15	4.40E-17	9.23	27.9176871	1.08E-01	143013207	143013330	NC_000007.13
cg03708680	1.38E-15	4.40E-17	9.23	27.9163029	1.08E-01	134829025	134829148	NC_000010.10
cg25981315	1.39E-15	4.44E-17	9.23	27.908727	1.62E-01	39806701	39806824	NC_000008.10
cg17397592	1.39E-15	4.44E-17	9.23	27.9082969	2.14E-01	237416338	237416461	NC_000002.11
cg26789064	1.39E-15	4.44E-17	9.23	27.9073227	2.19E-01	4866130	4866253	NC_000004.11

cg16054280	1.39E-15	4.45E-17	9.23	27.9066789	1.41E-01	122517974	122518097	NC_000003.11
cg16151651	1.40E-15	4.46E-17	9.23	27.9047453	1.56E-01	1609119	1609242	NC_000002.11
cg27180492	1.40E-15	4.47E-17	9.23	27.9022137	1.73E-01	27442106	27442229	NC_000006.11
cg08223984	1.40E-15	4.47E-17	9.23	27.9020054	1.09E-01	195476016	195476139	NC_000003.11
cg27516568	1.40E-15	4.47E-17	9.23	27.9014563	1.64E-01	122686667	122686790	NC_000004.11
cg18812579	1.40E-15	4.47E-17	9.23	27.9012386	9.04E-02	85906252	85906375	NC_000011.9
cg06755297	1.40E-15	4.48E-17	9.23	27.8989974	3.78E-02	220441097	220441220	NC_000001.10
cg09639715	1.41E-15	4.48E-17	9.23	27.8984106	1.03E-01	42994008	42994131	NC_000017.10
cg18249634	1.41E-15	4.49E-17	9.23	27.8979954	2.43E-01	32860467	32860590	NC_000003.11
cg23619431	1.41E-15	4.52E-17	9.23	27.891479	8.67E-02	74536068	74536191	NC_000017.10
cg01525538	1.42E-15	4.54E-17	9.23	27.8865702	1.71E-01	228785987	228786110	NC_000001.10
cg03323462	1.42E-15	4.54E-17	9.23	27.886233	1.95E-01	35300677	35300800	NC_000017.10
cg21585100	1.42E-15	4.55E-17	9.23	27.8844075	1.31E-01	55922747	55922870	NC_000018.9
cg07603511	1.42E-15	4.55E-17	9.23	27.8842813	1.69E-01	174160335	174160458	NC_000005.9
cg25445101	1.44E-15	4.59E-17	9.23	27.8752941	8.02E-02	26878566	26878689	NC_000017.10
cg21883598	1.44E-15	4.59E-17	9.23	27.8742873	1.91E-01	45404157	45404280	NC_000015.9
cg16171849	1.44E-15	4.60E-17	9.23	27.8739587	1.56E-01	139254260	139254383	NC_000003.11
cg24872610	1.44E-15	4.61E-17	9.23	27.8721276	1.57E-01	3158994	3159117	NC_000010.10
cg20977794	1.44E-15	4.61E-17	9.23	27.8713136	2.03E-01	51560748	51560871	NC_000014.8
cg02941135	1.45E-15	4.64E-17	9.23	27.8647114	1.16E-01	81122314	81122437	NC_000004.11
cg14110073	1.45E-15	4.64E-17	9.23	27.8638343	2.99E-01	51071328	51071451	NC_000019.9
cg07605229	1.45E-15	4.65E-17	9.23	27.8629644	1.56E-01	210467524	210467647	NC_000002.11
cg27140914	1.45E-15	4.65E-17	9.23	27.8628732	1.67E-01	39095348	39095471	NC_000003.11
cg02011374	1.46E-15	4.68E-17	9.23	27.8552144	1.95E-01	1761780	1761903	NC_000019.9
cg00503840	1.47E-15	4.69E-17	9.22	27.8531244	1.55E-01	96650509	96650632	NC_000007.13

cg17108209	1.47E-15	4.70E-17	9.22	27.8527625	9.68E-02	117484306	117484429	NC_000012.11
cg05028087	1.47E-15	4.70E-17	9.22	27.8516328	2.77E-01	2757221	2757344	NC_000005.9
cg12485185	1.47E-15	4.72E-17	9.22	27.8486849	2.25E-01	34782820	34782943	NC_000015.9
cg03075662	1.47E-15	4.72E-17	9.22	27.846794	5.47E-02	146213648	146213771	NC_000003.11
cg21036840	1.49E-15	4.77E-17	9.22	27.8382866	7.37E-02	169400357	169400480	NC_000006.11
cg05829782	1.49E-15	4.77E-17	9.22	27.8373002	3.02E-01	74707911	74708034	NC_000014.8
cg14093715	1.49E-15	4.78E-17	9.22	27.8349552	2.43E-01	126776180	126776303	NC_000009.11
cg07880943	1.49E-15	4.78E-17	9.22	27.834793	1.46E-01	46744500	46744623	NC_000013.10
cg17537177	1.49E-15	4.79E-17	9.22	27.8329354	2.46E-01	130646129	130646252	NC_000012.11
cg16206460	1.49E-15	4.79E-17	9.22	27.8326783	2.97E-01	15580700	15580823	NC_000019.9
cg04474190	1.50E-15	4.83E-17	9.22	27.8255583	1.39E-01	188868788	188868911	NC_000003.11
cg26709285	1.51E-15	4.84E-17	9.22	27.8228612	1.75E-01	48099146	48099269	NC_000020.10
cg05770240	1.51E-15	4.85E-17	9.22	27.8213745	9.64E-02	124502244	124502367	NC_000011.9
cg16348470	1.51E-15	4.86E-17	9.22	27.8189288	2.31E-01	34642609	34642732	NC_000001.10
cg04672706	1.52E-15	4.88E-17	9.22	27.8150781	2.14E-01	55370423	55370546	NC_000008.10
cg05037662	1.52E-15	4.88E-17	9.22	27.8147055	2.15E-01	25704296	25704419	NC_000016.9
cg22868282	1.53E-15	4.90E-17	9.22	27.8107381	2.37E-01	47224954	47225077	NC_000012.11
cg24073466	1.53E-15	4.90E-17	9.22	27.8098483	2.50E-01	60937031	60937154	NC_000010.10
cg26149275	1.53E-15	4.91E-17	9.22	27.8092072	1.94E-01	176950007	176950130	NC_000002.11
cg21719361	1.53E-15	4.92E-17	9.22	27.8070409	1.51E-01	50944355	50944478	NC_000022.10
cg18239431	1.53E-15	4.92E-17	9.22	27.8058668	1.60E-01	25898220	25898343	NC_000008.10
cg14600384	1.53E-15	4.93E-17	9.22	27.805036	1.11E-01	131769364	131769487	NC_000012.11
cg22879667	1.54E-15	4.94E-17	9.22	27.802323	1.86E-01	4868441	4868564	NC_000004.11
cg18771839	1.55E-15	4.97E-17	9.22	27.7965297	5.36E-02	69931206	69931329	NC_000011.9
cg06972213	1.55E-15	4.98E-17	9.22	27.7955302	1.12E-01	39643395	39643518	NC_000017.10

cg16015712	1.55E-15	4.99E-17	9.22	27.7917654	2.38E-01	53308597	53308720	NC_000001.10
cg05659097	1.56E-15	5.02E-17	9.21	27.7869394	2.76E-01	2757244	2757367	NC_000005.9
cg16197717	1.57E-15	5.05E-17	9.21	27.7805398	1.89E-01	125650957	125651080	NC_000010.10
cg16437087	1.58E-15	5.10E-17	9.21	27.7719749	7.82E-02	193919045	193919168	NC_000003.11
cg05350411	1.58E-15	5.10E-17	9.21	27.7716787	2.00E-01	569174	569297	NC_000004.11
cg11121935	1.59E-15	5.11E-17	9.21	27.7700197	1.15E-01	105359964	105360087	NC_000014.8
cg09409539	1.59E-15	5.11E-17	9.21	27.7687971	1.88E-01	95664243	95664366	NC_000002.11
cg20230721	1.59E-15	5.13E-17	9.21	27.7647916	3.32E-01	93519855	93519978	NC_000007.13
cg20073893	1.60E-15	5.14E-17	9.21	27.7627709	1.51E-01	22945457	22945580	NC_000014.8
cg20121142	1.61E-15	5.18E-17	9.21	27.7563242	2.21E-01	19158001	19158124	NC_000007.13
cg25778535	1.61E-15	5.18E-17	9.21	27.7561988	2.69E-01	8190572	8190695	NC_000011.9
cg25656283	1.61E-15	5.20E-17	9.21	27.7512774	1.23E-01	50733196	50733319	NC_000010.10
cg02111741	1.61E-15	5.20E-17	9.21	27.751204	1.10E-01	26683554	26683677	NC_000017.10
cg24526587	1.62E-15	5.22E-17	9.21	27.748237	9.50E-02	164587676	164587799	NC_000001.10
cg11739399	1.62E-15	5.22E-17	9.21	27.7480422	1.53E-01	206669785	206669908	NC_000001.10
cg14923370	1.62E-15	5.22E-17	9.21	27.7479398	1.25E-01	134622540	134622663	NC_000010.10
cg09774787	1.62E-15	5.23E-17	9.21	27.746831	2.76E-01	55690381	55690504	NC_000016.9
cg06455422	1.63E-15	5.25E-17	9.21	27.7423393	1.88E-01	98875360	98875483	NC_000013.10
cg04246113	1.63E-15	5.26E-17	9.21	27.7413088	1.80E-01	120316152	120316275	NC_000001.10
cg05959346	1.65E-15	5.31E-17	9.21	27.7309543	1.31E-01	40102679	40102802	NC_000001.10
cg11233163	1.66E-15	5.34E-17	9.2	27.7253714	1.89E-01	23955549	23955672	NC_000003.11
cg11898646	1.68E-15	5.41E-17	9.2	27.7126888	1.62E-01	158522427	158522550	NC_000005.9
cg27456185	1.68E-15	5.42E-17	9.2	27.7117296	1.77E-01	93196011	93196134	NC_000015.9
cg00082664	1.68E-15	5.42E-17	9.2	27.7099529	2.78E-01	154710796	154710919	NC_000004.11
cg20044410	1.68E-15	5.43E-17	9.2	27.7094694	2.14E-01	10463350	10463473	NC_000004.11

cg18322569	1.69E-15	5.45E-17	9.2	27.7053896	2.43E-01	91182777	91182900	NC_000001.10
cg14384093	1.69E-15	5.45E-17	9.2	27.7046062	1.79E-01	111878565	111878688	NC_000009.11
cg27546237	1.69E-15	5.47E-17	9.2	27.7012103	2.47E-01	110960452	110960575	NC_000013.10
cg26354128	1.69E-15	5.48E-17	9.2	27.7006815	2.58E-01	93897195	93897318	NC_000014.8
cg21852408	1.70E-15	5.48E-17	9.2	27.7001764	2.26E-01	109294190	109294313	NC_000011.9
cg16006349	1.70E-15	5.48E-17	9.2	27.6993945	1.94E-01	54360460	54360583	NC_000012.11
cg25001845	1.70E-15	5.50E-17	9.2	27.6965704	6.21E-02	75201709	75201832	NC_000014.8
cg21236414	1.71E-15	5.52E-17	9.2	27.6932122	1.19E-01	894185	894308	NC_000004.11
cg24471254	1.72E-15	5.58E-17	9.2	27.6819918	1.87E-01	100253792	100253915	NC_000007.13
cg25789533	1.73E-15	5.59E-17	9.2	27.6807449	9.04E-02	47104483	47104606	NC_000019.9
cg17326994	1.73E-15	5.60E-17	9.2	27.6789864	6.78E-02	32511779	32511902	NC_000017.10
cg27504802	1.73E-15	5.61E-17	9.2	27.6773545	2.70E-01	231298651	231298774	NC_000001.10
cg01627492	1.73E-15	5.61E-17	9.2	27.6770942	6.95E-02	18916400	18916523	NC_000017.10
cg15861694	1.73E-15	5.61E-17	9.2	27.677067	7.10E-02	2706745	2706868	NC_000011.9
cg05292954	1.73E-15	5.61E-17	9.2	27.6764356	2.94E-01	57050620	57050743	NC_000019.9
cg13894719	1.73E-15	5.62E-17	9.2	27.675552	1.09E-01	56534223	56534346	NC_000004.11
cg22164539	1.74E-15	5.64E-17	9.2	27.6709439	9.31E-02	32081613	32081736	NC_000006.11
cg10377644	1.76E-15	5.68E-17	9.2	27.6636635	3.35E-02	590582	590705	NC_000016.9
cg20051292	1.76E-15	5.70E-17	9.2	27.6617273	2.17E-01	101118512	101118635	NC_000008.10
cg03025148	1.77E-15	5.72E-17	9.19	27.6576424	5.40E-02	26936997	26937120	NC_000022.10
cg19725343	1.77E-15	5.74E-17	9.19	27.653587	2.85E-01	51830960	51831083	NC_000019.9
cg14820653	1.78E-15	5.76E-17	9.19	27.6505256	6.02E-02	173046018	173046141	NC_000002.11
cg24954895	1.79E-15	5.79E-17	9.19	27.6454812	2.24E-01	140800761	140800884	NC_000005.9
cg06239593	1.79E-15	5.82E-17	9.19	27.6402007	7.00E-02	130748639	130748762	NC_000003.11
cg05587627	1.80E-15	5.83E-17	9.19	27.6390849	1.79E-01	118657363	118657486	NC_000011.9

cg09441069	1.80E-15	5.84E-17	9.19	27.6367976	1.25E-01	47569479	47569602	NC_000021.8
cg07336617	1.80E-15	5.85E-17	9.19	27.6347658	1.77E-01	76935080	76935203	NC_000005.9
cg27635976	1.82E-15	5.91E-17	9.19	27.6245406	1.55E-01	88984067	88984190	NC_000016.9
cg23202496	1.83E-15	5.93E-17	9.19	27.6219918	6.41E-02	35488318	35488441	NC_000020.10
cg09017619	1.83E-15	5.95E-17	9.19	27.6187756	2.49E-01	27196320	27196443	NC_000007.13
cg04658772	1.83E-15	5.96E-17	9.19	27.6172531	2.13E-01	77822433	77822556	NC_000016.9
cg18490614	1.84E-15	5.97E-17	9.19	27.6147348	1.65E-01	27213362	27213485	NC_000015.9
cg14804903	1.84E-15	5.99E-17	9.19	27.6122288	1.24E-01	86418754	86418877	NC_000002.11
cg14936938	1.84E-15	5.99E-17	9.19	27.6114991	1.91E-01	122118171	122118294	NC_000008.10
cg09693765	1.85E-15	6.00E-17	9.19	27.61014	1.12E-01	45075639	45075762	NC_000021.8
cg06998224	1.85E-15	6.03E-17	9.19	27.6060052	1.29E-01	128113907	128114030	NC_000005.9
cg15992648	1.86E-15	6.06E-17	9.19	27.6004045	1.05E-01	155640784	155640907	NC_000007.13
cg07803375	1.88E-15	6.12E-17	9.18	27.5907936	2.49E-01	811206	811329	NC_000007.13
cg01559597	1.88E-15	6.12E-17	9.18	27.5905636	1.32E-01	14716193	14716316	NC_000006.11
cg07831164	1.88E-15	6.13E-17	9.18	27.5884957	1.51E-01	10836225	10836348	NC_000016.9
cg11947981	1.88E-15	6.14E-17	9.18	27.5880775	2.77E-01	182322749	182322872	NC_000002.11
cg09252495	1.89E-15	6.14E-17	9.18	27.5870646	2.52E-01	21503898	21504021	NC_000020.10
cg10527010	1.91E-15	6.22E-17	9.18	27.5751907	2.21E-01	10857795	10857918	NC_000003.11
cg09193347	1.92E-15	6.25E-17	9.18	27.5703832	1.96E-01	79381867	79381990	NC_000015.9
cg27096416	1.92E-15	6.26E-17	9.18	27.5688437	6.97E-02	140599947	140600070	NC_000007.13
cg04107939	1.92E-15	6.27E-17	9.18	27.5664759	2.06E-01	171520494	171520617	NC_000003.11
cg13989664	1.93E-15	6.28E-17	9.18	27.5644432	1.86E-01	126374295	126374418	NC_000012.11
cg20812722	1.93E-15	6.29E-17	9.18	27.5633392	1.05E-01	25525951	25526074	NC_000001.10
cg27065876	1.93E-15	6.29E-17	9.18	27.5627616	1.01E-01	1840421	1840544	NC_000016.9
cg25356621	1.93E-15	6.31E-17	9.18	27.5610307	8.06E-02	74686282	74686405	NC_000012.11

cg08063125	1.94E-15	6.32E-17	9.18	27.5592721	2.89E-01	56989543	56989666	NC_000019.9
cg00293996	1.94E-15	6.32E-17	9.18	27.5583334	1.07E-01	37915391	37915514	NC_000021.8
cg07040894	1.94E-15	6.33E-17	9.18	27.55779	1.02E-01	128291985	128292108	NC_000002.11
cg04194947	1.95E-15	6.37E-17	9.18	27.5511702	1.81E-01	177012191	177012314	NC_000002.11
cg22931725	1.95E-15	6.37E-17	9.18	27.5503213	1.65E-01	240255627	240255750	NC_000001.10
cg26437826	1.96E-15	6.39E-17	9.18	27.5482555	1.81E-01	76936361	76936484	NC_000005.9
cg02459519	1.96E-15	6.39E-17	9.18	27.5481499	8.27E-02	62401855	62401978	NC_000017.10
cg08214995	1.96E-15	6.41E-17	9.18	27.545271	1.75E-01	22826243	22826366	NC_000016.9
cg20463526	1.96E-15	6.41E-17	9.18	27.5449955	2.85E-01	91191001	91191124	NC_000001.10
cg03834919	1.97E-15	6.43E-17	9.18	27.5422987	1.58E-01	862427	862550	NC_000007.13
cg04522596	1.97E-15	6.43E-17	9.18	27.5413714	1.64E-01	98878060	98878183	NC_000012.11
cg05201777	1.97E-15	6.44E-17	9.18	27.5409455	1.31E-01	112598561	112598684	NC_000010.10
cg02387745	1.97E-15	6.44E-17	9.18	27.5406994	1.45E-01	74317457	74317580	NC_000004.11
cg27630192	1.97E-15	6.44E-17	9.18	27.5404777	1.48E-01	108213750	108213873	NC_000007.13
cg06399302	1.98E-15	6.46E-17	9.18	27.5375917	2.46E-01	34113214	34113337	NC_000019.9
cg23491743	1.98E-15	6.46E-17	9.18	27.5364982	1.55E-01	241989271	241989394	NC_000002.11
cg04870949	1.98E-15	6.47E-17	9.18	27.536308	1.24E-01	114109857	114109980	NC_000013.10
cg17074989	1.99E-15	6.50E-17	9.17	27.5305631	1.51E-01	58880752	58880875	NC_000015.9
cg04900080	2.00E-15	6.53E-17	9.17	27.5263948	1.79E-01	229570665	229570788	NC_000001.10
cg15418593	2.00E-15	6.53E-17	9.17	27.5257292	1.70E-01	97492133	97492256	NC_000014.8
cg04342092	2.00E-15	6.54E-17	9.17	27.5256451	2.11E-01	58095468	58095591	NC_000019.9
cg14801038	2.00E-15	6.54E-17	9.17	27.5252746	1.67E-01	134214307	134214430	NC_000006.11
cg18877506	2.00E-15	6.54E-17	9.17	27.5250897	2.18E-01	13910555	13910678	NC_000001.10
cg27557796	2.01E-15	6.57E-17	9.17	27.5199657	7.98E-02	31688702	31688825	NC_000006.11
cg21126583	2.01E-15	6.57E-17	9.17	27.5199398	2.90E-01	145813421	145813544	NC_000007.13

cg03225817	2.01E-15	6.59E-17	9.17	27.5174894	2.52E-01	105481317	105481440	NC_000011.9
cg08291024	2.02E-15	6.60E-17	9.17	27.5154003	2.26E-01	165324411	165324534	NC_000001.10
cg25372296	2.02E-15	6.60E-17	9.17	27.5152947	2.04E-01	98510328	98510451	NC_000001.10
cg06012215	2.02E-15	6.62E-17	9.17	27.5122285	1.12E-01	111216867	111216990	NC_000010.10
cg14838600	2.03E-15	6.66E-17	9.17	27.5069998	1.32E-01	151442967	151443090	NC_000007.13
cg22190437	2.04E-15	6.68E-17	9.17	27.5038491	1.84E-01	229045417	229045540	NC_000002.11
cg18790597	2.04E-15	6.70E-17	9.17	27.501416	2.44E-01	17783331	17783454	NC_000004.11
cg27369542	2.05E-15	6.70E-17	9.17	27.5004579	9.38E-02	14207890	14208013	NC_000017.10
cg01468220	2.05E-15	6.71E-17	9.17	27.5002939	1.40E-01	779026	779149	NC_000016.9
cg23291280	2.05E-15	6.71E-17	9.17	27.4996426	2.15E-01	31847970	31848093	NC_000019.9
cg08720786	2.05E-15	6.72E-17	9.17	27.497723	1.06E-01	48981581	48981704	NC_000010.10
cg16887264	2.05E-15	6.72E-17	9.17	27.4975467	2.28E-01	147561775	147561898	NC_000004.11
cg17029354	2.06E-15	6.77E-17	9.17	27.4911248	2.07E-01	115134904	115135027	NC_000012.11
cg05415871	2.06E-15	6.77E-17	9.17	27.4900424	1.76E-01	1008188	1008311	NC_000008.10
cg10640072	2.07E-15	6.78E-17	9.17	27.4896238	1.74E-01	99985888	99986011	NC_000008.10
cg02765535	2.07E-15	6.78E-17	9.17	27.4895987	1.43E-01	96149803	96149926	NC_000012.11
cg21782511	2.07E-15	6.79E-17	9.17	27.4882582	6.67E-02	1808376	1808499	NC_000004.11
cg00160886	2.07E-15	6.80E-17	9.17	27.4864565	1.34E-01	157216105	157216228	NC_000003.11
cg00017437	2.08E-15	6.84E-17	9.17	27.4811533	2.00E-01	2706335	2706458	NC_000001.10
cg00853103	2.08E-15	6.84E-17	9.17	27.4804578	2.17E-01	6533789	6533912	NC_000016.9
cg01970904	2.09E-15	6.86E-17	9.17	27.4778377	1.35E-01	172014013	172014136	NC_000002.11
cg01826138	2.09E-15	6.87E-17	9.17	27.4767368	4.39E-02	181843183	181843306	NC_000002.11
cg04618897	2.10E-15	6.90E-17	9.17	27.4724284	6.91E-02	4829256	4829379	NC_000007.13
cg13211324	2.11E-15	6.95E-17	9.16	27.4648405	1.37E-01	113373736	113373859	NC_000003.11
cg06896909	2.11E-15	6.95E-17	9.16	27.4646483	1.64E-01	19813297	19813420	NC_000007.13

cg16782974	2.12E-15	6.95E-17	9.16	27.464247	1.35E-01	98290350	98290473	NC_000010.10
cg03018771	2.12E-15	6.96E-17	9.16	27.4638811	1.56E-01	27940619	27940742	NC_000001.10
cg14477581	2.12E-15	6.97E-17	9.16	27.4618641	1.25E-01	72899321	72899444	NC_000009.11
cg18670076	2.12E-15	6.97E-17	9.16	27.4616426	1.12E-01	189675162	189675285	NC_000003.11
cg05656251	2.13E-15	6.99E-17	9.16	27.4588322	6.86E-02	3125906	3126029	NC_000001.10
cg18729001	2.13E-15	7.02E-17	9.16	27.4546645	1.99E-01	45443808	45443931	NC_000012.11
cg12062936	2.14E-15	7.04E-17	9.16	27.4516867	1.24E-01	18801087	18801210	NC_000007.13
cg23208513	2.14E-15	7.05E-17	9.16	27.4508878	2.18E-01	101118377	101118500	NC_000008.10
cg26024843	2.14E-15	7.06E-17	9.16	27.4498672	2.30E-01	137533949	137534072	NC_000009.11
cg06674731	2.15E-15	7.06E-17	9.16	27.4488508	1.83E-01	154335014	154335137	NC_000002.11
cg17762554	2.15E-15	7.07E-17	9.16	27.4483235	1.54E-01	112435953	112436076	NC_000002.11
cg02497558	2.15E-15	7.07E-17	9.16	27.4474411	1.27E-01	46608570	46608693	NC_000017.10
cg23254031	2.16E-15	7.10E-17	9.16	27.4436506	1.94E-01	48144765	48144888	NC_000014.8
cg26831087	2.16E-15	7.12E-17	9.16	27.4408388	1.23E-01	1259070	1259193	NC_000001.10
cg17951138	2.16E-15	7.12E-17	9.16	27.4401955	2.03E-01	92908392	92908515	NC_000005.9
cg24741563	2.16E-15	7.13E-17	9.16	27.439679	9.21E-02	58545335	58545458	NC_000017.10
cg09537792	2.17E-15	7.15E-17	9.16	27.4372509	1.32E-01	89438892	89439015	NC_000016.9
cg14446622	2.19E-15	7.21E-17	9.16	27.4288643	8.07E-02	100894501	100894624	NC_000009.11
cg02390588	2.19E-15	7.22E-17	9.16	27.4272792	9.62E-02	53722253	53722376	NC_000012.11
cg15867939	2.21E-15	7.27E-17	9.16	27.4195989	1.97E-01	140770523	140770646	NC_000003.11
cg09619271	2.21E-15	7.28E-17	9.16	27.4185619	2.32E-01	45961289	45961412	NC_000007.13
cg15965716	2.21E-15	7.29E-17	9.16	27.4173823	1.62E-01	164688315	164688438	NC_000001.10
cg25020820	2.23E-15	7.34E-17	9.16	27.4100996	1.00E-01	43230751	43230874	NC_000006.11
cg06915270	2.23E-15	7.36E-17	9.16	27.4087113	7.04E-02	183891360	183891483	NC_000001.10
cg08530237	2.26E-15	7.45E-17	9.15	27.3957946	2.45E-01	22094563	22094686	NC_000012.11

cg14685796	2.27E-15	7.49E-17	9.15	27.3911406	1.94E-01	50265660	50265783	NC_000005.9
cg02438611	2.27E-15	7.49E-17	9.15	27.3904954	1.77E-01	55828107	55828230	NC_000017.10
cg07990546	2.27E-15	7.51E-17	9.15	27.3885544	2.30E-01	74742309	74742432	NC_000002.11
cg17486097	2.28E-15	7.53E-17	9.15	27.3851515	1.87E-01	35093411	35093534	NC_000008.10
cg04618528	2.28E-15	7.54E-17	9.15	27.3842788	5.31E-02	39645876	39645999	NC_000017.10
cg06310816	2.28E-15	7.54E-17	9.15	27.3838879	2.63E-01	42267719	42267842	NC_000007.13
cg03714619	2.28E-15	7.54E-17	9.15	27.3837825	2.61E-01	45160445	45160568	NC_000002.11
cg00246932	2.28E-15	7.54E-17	9.15	27.3836219	2.78E-01	56228498	56228621	NC_000016.9
cg00581154	2.28E-15	7.55E-17	9.15	27.3828187	1.47E-01	3123695	3123818	NC_000001.10
cg24873562	2.29E-15	7.55E-17	9.15	27.3822022	2.50E-01	124902349	124902472	NC_000010.10
cg14078092	2.29E-15	7.58E-17	9.15	27.3792369	9.49E-02	123463530	123463653	NC_000012.11
cg07583894	2.29E-15	7.58E-17	9.15	27.3792178	2.58E-01	12936512	12936635	NC_000019.9
cg25573386	2.29E-15	7.58E-17	9.15	27.3782902	1.47E-01	18890579	18890702	NC_000012.11
cg16242629	2.30E-15	7.60E-17	9.15	27.3760017	2.05E-01	30354246	30354369	NC_000012.11
cg22177840	2.30E-15	7.61E-17	9.15	27.3750726	1.05E-01	90139296	90139419	NC_000003.11
cg07960450	2.30E-15	7.61E-17	9.15	27.3747568	1.91E-01	45614300	45614423	NC_000007.13
cg00848392	2.32E-15	7.66E-17	9.15	27.3683786	1.27E-01	31734401	31734524	NC_000006.11
cg01360618	2.32E-15	7.66E-17	9.15	27.3682066	2.89E-01	69591659	69591782	NC_000003.11
cg07132449	2.32E-15	7.69E-17	9.15	27.3650496	4.41E-02	189432110	189432233	NC_000003.11
cg01359200	2.33E-15	7.71E-17	9.15	27.3621086	7.87E-02	194763212	194763335	NC_000003.11
cg08358166	2.33E-15	7.72E-17	9.15	27.3609146	2.37E-01	18538271	18538394	NC_000017.10
cg10827094	2.33E-15	7.73E-17	9.15	27.3597128	1.37E-01	117468061	117468184	NC_000012.11
cg26483578	2.34E-15	7.76E-17	9.15	27.3558749	2.80E-01	130686708	130686831	NC_000006.11
cg14135814	2.35E-15	7.78E-17	9.15	27.3535805	2.36E-01	119419329	119419452	NC_000012.11
cg15440688	2.35E-15	7.79E-17	9.15	27.3517029	1.55E-01	95237637	95237760	NC_000014.8

cg21528112	2.36E-15	7.81E-17	9.15	27.3492384	2.29E-01	11034978	11035101	NC_000003.11
cg09243147	2.36E-15	7.82E-17	9.15	27.3476898	8.34E-02	101399998	101400121	NC_000003.11
cg11877129	2.37E-15	7.86E-17	9.15	27.3427321	2.32E-01	128752356	128752479	NC_000012.11
cg15383705	2.37E-15	7.87E-17	9.15	27.3419653	1.17E-01	114699120	114699243	NC_000010.10
cg04223420	2.38E-15	7.87E-17	9.15	27.3412862	1.98E-01	134363877	134364000	NC_000005.9
cg01382072	2.38E-15	7.89E-17	9.14	27.3394066	1.23E-01	86787277	86787400	NC_000016.9
cg26452056	2.38E-15	7.90E-17	9.14	27.338196	2.04E-01	131770909	131771032	NC_000010.10
cg07502829	2.39E-15	7.94E-17	9.14	27.333583	1.66E-01	112708288	112708411	NC_000013.10
cg02667667	2.41E-15	8.00E-17	9.14	27.3257518	2.67E-01	56228505	56228628	NC_000016.9
cg27615388	2.42E-15	8.02E-17	9.14	27.323328	2.02E-01	63257092	63257215	NC_000005.9
cg21488279	2.42E-15	8.02E-17	9.14	27.3228154	1.13E-01	122434178	122434301	NC_000005.9
cg05471296	2.42E-15	8.03E-17	9.14	27.3216856	2.62E-01	27196302	27196425	NC_000007.13
cg00840332	2.43E-15	8.05E-17	9.14	27.3190753	1.85E-01	127881269	127881392	NC_000007.13
cg01085837	2.44E-15	8.09E-17	9.14	27.314703	2.37E-01	22094461	22094584	NC_000012.11
cg26940910	2.44E-15	8.09E-17	9.14	27.314678	5.47E-02	206627837	206627960	NC_000001.10
cg17847723	2.44E-15	8.09E-17	9.14	27.3144127	1.95E-01	114075981	114076104	NC_000012.11
cg10379472	2.44E-15	8.10E-17	9.14	27.3134339	1.23E-01	71475452	71475575	NC_000016.9
cg05048377	2.44E-15	8.12E-17	9.14	27.3113464	2.06E-01	3237964	3238087	NC_000016.9
cg03356595	2.45E-15	8.14E-17	9.14	27.3086101	2.36E-01	51187807	51187930	NC_000016.9
cg20021204	2.46E-15	8.16E-17	9.14	27.3065006	1.08E-01	77413730	77413853	NC_000015.9
cg14650610	2.47E-15	8.19E-17	9.14	27.3019212	3.28E-01	136834492	136834615	NC_000005.9
cg07167946	2.47E-15	8.21E-17	9.14	27.3000711	1.89E-01	1878205	1878328	NC_000005.9
cg09683350	2.49E-15	8.27E-17	9.14	27.2925873	2.33E-01	89914466	89914589	NC_000015.9
cg14216068	2.49E-15	8.27E-17	9.14	27.2921921	1.85E-01	27146445	27146568	NC_000007.13
cg00393837	2.50E-15	8.31E-17	9.14	27.2883793	2.20E-01	105470050	105470173	NC_000002.11

cg26429655	2.50E-15	8.32E-17	9.14	27.2865562	2.79E-01	178772390	178772513	NC_000005.9
cg02264079	2.51E-15	8.35E-17	9.14	27.283111	1.54E-01	31053489	31053612	NC_000016.9
cg06898168	2.52E-15	8.39E-17	9.14	27.2779956	1.31E-01	5447353	5447476	NC_000007.13
cg09685096	2.53E-15	8.42E-17	9.13	27.2746275	1.25E-01	86157111	86157234	NC_000001.10
cg06410849	2.53E-15	8.43E-17	9.13	27.2741035	1.20E-01	66955988	66956111	NC_000016.9
cg22898797	2.54E-15	8.46E-17	9.13	27.2699747	2.86E-01	102484232	102484355	NC_000010.10
cg16577707	2.54E-15	8.47E-17	9.13	27.269603	6.08E-02	154181866	154181989	NC_000005.9
cg04134748	2.55E-15	8.48E-17	9.13	27.2678174	1.81E-01	3052175	3052298	NC_000001.10
cg19884262	2.57E-15	8.58E-17	9.13	27.2567831	1.97E-01	129535968	129536091	NC_000010.10
cg06888121	2.62E-15	8.72E-17	9.13	27.2407384	1.62E-01	27169068	27169191	NC_000007.13
cg23596808	2.63E-15	8.76E-17	9.13	27.2352737	5.92E-02	45349963	45350086	NC_000006.11
cg01206259	2.63E-15	8.77E-17	9.13	27.2348014	1.03E-01	121167974	121168097	NC_000010.10
cg03250019	2.64E-15	8.80E-17	9.13	27.2312351	2.14E-01	5197066	5197189	NC_000018.9
cg06488505	2.64E-15	8.80E-17	9.13	27.2310205	7.37E-02	28934075	28934198	NC_000017.10
cg13010161	2.64E-15	8.82E-17	9.13	27.2294284	1.42E-01	121488902	121489025	NC_000002.11
cg12131827	2.65E-15	8.82E-17	9.13	27.2290018	1.34E-01	21640270	21640393	NC_000008.10
cg22830113	2.66E-15	8.86E-17	9.13	27.2249179	3.44E-01	127783168	127783291	NC_000002.11
cg07745624	2.66E-15	8.88E-17	9.13	27.2219622	1.51E-01	21950952	21951075	NC_000004.11
cg23965109	2.66E-15	8.89E-17	9.13	27.2214861	8.80E-02	186739029	186739152	NC_000003.11
cg14306683	2.67E-15	8.89E-17	9.13	27.2208674	4.68E-02	43300632	43300755	NC_000022.10
cg01517185	2.67E-15	8.90E-17	9.13	27.2202472	5.95E-02	358490	358613	NC_000016.9
cg09522056	2.69E-15	8.99E-17	9.12	27.2105823	1.72E-01	31830979	31831102	NC_000016.9
cg01110440	2.69E-15	8.99E-17	9.12	27.2103821	1.35E-01	110707714	110707837	NC_000006.11
cg20457275	2.71E-15	9.05E-17	9.12	27.2031628	1.43E-01	1394071	1394194	NC_000006.11
cg13930388	2.71E-15	9.05E-17	9.12	27.2030858	1.34E-01	133799399	133799522	NC_000009.11

cg22717227	2.71E-15	9.06E-17	9.12	27.2028077	1.89E-01	50887568	50887691	NC_000010.10
cg17591595	2.72E-15	9.09E-17	9.12	27.19883	1.62E-01	27171051	27171174	NC_000007.13
cg23596233	2.72E-15	9.10E-17	9.12	27.1982385	2.80E-01	134281663	134281786	NC_000011.9
cg02854229	2.73E-15	9.11E-17	9.12	27.1969437	1.29E-01	101962994	101963117	NC_000014.8
cg04837847	2.74E-15	9.15E-17	9.12	27.1923868	1.14E-01	178482132	178482255	NC_000001.10
cg07713705	2.74E-15	9.17E-17	9.12	27.1906268	1.45E-01	128712399	128712522	NC_000011.9
cg06697380	2.75E-15	9.19E-17	9.12	27.1882982	1.09E-01	126634803	126634926	NC_000005.9
cg07876051	2.75E-15	9.21E-17	9.12	27.1867028	1.47E-01	3159145	3159268	NC_000010.10
cg06913501	2.77E-15	9.25E-17	9.12	27.1824084	2.10E-01	180101143	180101266	NC_000005.9
cg15230781	2.77E-15	9.26E-17	9.12	27.1811525	2.29E-01	37353700	37353823	NC_000020.10
cg11230940	2.77E-15	9.27E-17	9.12	27.179347	1.72E-01	3103774	3103897	NC_000005.9
cg07653901	2.78E-15	9.30E-17	9.12	27.1765527	1.11E-01	50250238	50250361	NC_000022.10
cg21313071	2.78E-15	9.31E-17	9.12	27.175247	1.38E-01	49640589	49640712	NC_000020.10
cg01718447	2.79E-15	9.32E-17	9.12	27.1741977	2.39E-01	30722327	30722450	NC_000007.13
cg19601666	2.79E-15	9.34E-17	9.12	27.1725697	1.04E-01	71172089	71172212	NC_000001.10
cg21399832	2.81E-15	9.39E-17	9.12	27.1670609	1.70E-01	117847410	117847533	NC_000004.11
cg12407978	2.81E-15	9.41E-17	9.12	27.1649711	1.31E-01	10590159	10590282	NC_000011.9
cg17279551	2.82E-15	9.44E-17	9.12	27.1621081	9.22E-02	139970459	139970582	NC_000005.9
cg11384848	2.82E-15	9.45E-17	9.12	27.1602104	2.45E-01	140770864	140770987	NC_000003.11
cg09914742	2.83E-15	9.49E-17	9.12	27.1561554	2.08E-01	5296943	5297066	NC_000020.10
cg03132773	2.83E-15	9.50E-17	9.12	27.1556269	2.47E-01	22824877	22825000	NC_000016.9
cg01708273	2.84E-15	9.50E-17	9.12	27.1551314	2.02E-01	176974060	176974183	NC_000002.11
cg22445447	2.84E-15	9.51E-17	9.12	27.1547186	7.48E-02	41712591	41712714	NC_000015.9
cg22097425	2.84E-15	9.52E-17	9.12	27.1530921	2.50E-01	125774311	125774434	NC_000011.9
cg16615154	2.85E-15	9.55E-17	9.12	27.1501734	1.44E-01	40567553	40567676	NC_000006.11

cg17199800	2.86E-15	9.60E-17	9.11	27.1455952	2.21E-01	96632777	96632900	NC_000007.13
cg07730329	2.87E-15	9.63E-17	9.11	27.1424158	1.93E-01	140810137	140810260	NC_000005.9
cg20014988	2.88E-15	9.65E-17	9.11	27.1403398	1.79E-01	52363132	52363255	NC_000010.10
cg08837627	2.90E-15	9.74E-17	9.11	27.1307129	1.68E-01	22715300	22715423	NC_000019.9
cg25636182	2.91E-15	9.75E-17	9.11	27.1296736	1.32E-01	8122559	8122682	NC_000004.11
cg02746725	2.91E-15	9.75E-17	9.11	27.1295031	2.12E-01	177054306	177054429	NC_000002.11
cg18264383	2.92E-15	9.80E-17	9.11	27.1245341	3.00E-02	45730917	45731040	NC_000003.11
cg06099465	2.93E-15	9.84E-17	9.11	27.1210062	1.34E-01	1631034	1631157	NC_000017.10
cg12394567	2.94E-15	9.88E-17	9.11	27.1169357	3.67E-02	3143783	3143906	NC_000010.10
cg15413269	2.94E-15	9.89E-17	9.11	27.1161554	1.20E-01	106784011	106784134	NC_000008.10
cg15173079	2.95E-15	9.91E-17	9.11	27.1132661	8.53E-02	3154078	3154201	NC_000006.11
cg13453747	2.95E-15	9.93E-17	9.11	27.1121801	1.28E-01	137038185	137038308	NC_000009.11
cg19849478	2.97E-15	9.99E-17	9.11	27.1052726	1.41E-01	122592277	122592400	NC_000012.11
cg24899806	2.98E-15	1.00E-16	9.11	27.1040584	1.44E-01	119914282	119914405	NC_000007.13
cg00099240	2.98E-15	1.00E-16	9.11	27.1036429	1.41E-01	33454632	33454755	NC_000022.10
cg14768164	2.98E-15	1.00E-16	9.11	27.1017848	2.00E-01	145025049	145025172	NC_000002.11
cg00809969	2.98E-15	1.00E-16	9.11	27.1015257	1.42E-01	27181067	27181190	NC_000007.13
cg20058043	2.99E-15	1.01E-16	9.11	27.0986617	2.68E-01	130646370	130646493	NC_000012.11
cg15778457	3.00E-15	1.01E-16	9.11	27.0965123	2.48E-01	155165295	155165418	NC_000007.13
cg13320436	3.00E-15	1.01E-16	9.11	27.0951954	1.64E-01	55138014	55138137	NC_000008.10
cg04774711	3.01E-15	1.01E-16	9.11	27.0920314	2.16E-01	21684138	21684261	NC_000020.10
cg20014398	3.02E-15	1.01E-16	9.11	27.0902147	2.37E-01	31825792	31825915	NC_000011.9
cg23712342	3.03E-15	1.02E-16	9.11	27.0857961	2.02E-01	60936794	60936917	NC_000010.10
cg00955035	3.03E-15	1.02E-16	9.11	27.0846202	1.34E-01	233253378	233253501	NC_000002.11
cg15541630	3.03E-15	1.02E-16	9.11	27.0844844	1.62E-01	87081770	87081893	NC_000008.10

cg26960012	3.04E-15	1.02E-16	9.1	27.0833159	8.63E-02	2889915	2890038	NC_000016.9
cg21085022	3.04E-15	1.02E-16	9.1	27.0831817	1.62E-01	34648170	34648293	NC_000006.11
cg03585720	3.04E-15	1.02E-16	9.1	27.0803693	1.64E-01	235048762	235048885	NC_000001.10
cg00804525	3.05E-15	1.03E-16	9.1	27.0783196	1.31E-01	68943500	68943623	NC_000016.9
cg22990888	3.05E-15	1.03E-16	9.1	27.0780083	9.80E-02	30585904	30586027	NC_000008.10
cg03698009	3.06E-15	1.03E-16	9.1	27.0764815	2.35E-01	27204349	27204472	NC_000007.13
cg09061207	3.07E-15	1.04E-16	9.1	27.0703931	1.51E-01	31504181	31504304	NC_000006.11
cg25039722	3.07E-15	1.04E-16	9.1	27.0702618	2.45E-01	50236344	50236467	NC_000017.10
cg06580943	3.07E-15	1.04E-16	9.1	27.0701115	1.02E-01	168405834	168405957	NC_000006.11
cg18194945	3.08E-15	1.04E-16	9.1	27.0688314	2.02E-01	157820591	157820714	NC_000003.11
cg10067970	3.08E-15	1.04E-16	9.1	27.0678963	2.69E-01	95359666	95359789	NC_000013.10
cg09059319	3.09E-15	1.04E-16	9.1	27.0642032	1.68E-01	99986175	99986298	NC_000008.10
cg09726866	3.09E-15	1.04E-16	9.1	27.0641005	1.06E-01	79083545	79083668	NC_000004.11
cg06018240	3.10E-15	1.05E-16	9.1	27.0595597	6.76E-02	150768388	150768511	NC_000007.13
cg09088437	3.12E-15	1.05E-16	9.1	27.0539898	9.14E-02	109559012	109559135	NC_000002.11
cg00803758	3.12E-15	1.05E-16	9.1	27.0534687	1.64E-01	184539762	184539885	NC_000004.11
cg06638723	3.14E-15	1.06E-16	9.1	27.0481712	1.05E-01	186447018	186447141	NC_000001.10
cg20826165	3.14E-15	1.06E-16	9.1	27.0470311	1.86E-01	64491841	64491964	NC_000012.11
cg25817701	3.14E-15	1.06E-16	9.1	27.0463585	1.70E-01	2140143	2140266	NC_000007.13
cg09996597	3.15E-15	1.06E-16	9.1	27.0444136	1.76E-01	1844927	1845050	NC_000016.9
cg00371453	3.15E-15	1.06E-16	9.1	27.0428929	2.21E-01	7854482	7854605	NC_000019.9
cg14935646	3.19E-15	1.08E-16	9.1	27.0324286	1.51E-01	27215464	27215587	NC_000007.13
cg04922810	3.19E-15	1.08E-16	9.1	27.0323793	2.24E-01	30721888	30722011	NC_000007.13
cg06266962	3.19E-15	1.08E-16	9.1	27.0316364	9.94E-02	20871424	20871547	NC_000016.9
cg01597641	3.22E-15	1.09E-16	9.1	27.0226039	2.49E-01	155241970	155242093	NC_000007.13

cg03387135	3.22E-15	1.09E-16	9.1	27.0221767	2.57E-01	61809827	61809950	NC_000020.10
cg01534316	3.22E-15	1.09E-16	9.1	27.0208299	1.38E-01	112061221	112061344	NC_000001.10
cg04667180	3.22E-15	1.09E-16	9.09	27.0199222	1.08E-01	150389906	150390029	NC_000007.13
cg11426800	3.23E-15	1.09E-16	9.09	27.018653	1.43E-01	23710000	23710123	NC_000002.11
cg25818697	3.23E-15	1.09E-16	9.09	27.0178285	1.43E-01	69934795	69934918	NC_000011.9
cg06546806	3.23E-15	1.09E-16	9.09	27.0176171	2.73E-01	237078704	237078827	NC_000002.11
cg22721334	3.25E-15	1.10E-16	9.09	27.0108275	2.96E-01	58609618	58609741	NC_000019.9
cg26698580	3.25E-15	1.10E-16	9.09	27.0107291	8.11E-02	67695984	67696107	NC_000016.9
cg07402669	3.27E-15	1.11E-16	9.09	27.004572	2.17E-01	57090015	57090138	NC_000020.10
cg19343464	3.28E-15	1.11E-16	9.09	27.0017322	2.62E-01	105481509	105481632	NC_000011.9
cg08095196	3.28E-15	1.11E-16	9.09	27.0009232	2.41E-01	35047023	35047146	NC_000015.9
cg11267630	3.28E-15	1.11E-16	9.09	27.0000599	2.46E-01	46811527	46811650	NC_000017.10
cg16594299	3.29E-15	1.11E-16	9.09	26.998453	4.81E-02	40324054	40324177	NC_000001.10
cg26254045	3.29E-15	1.11E-16	9.09	26.9982555	1.73E-01	38886658	38886781	NC_000019.9
cg13721404	3.30E-15	1.12E-16	9.09	26.9953299	2.26E-01	31739202	31739325	NC_000018.9
cg09633129	3.30E-15	1.12E-16	9.09	26.9949675	5.04E-02	2936284	2936407	NC_000004.11
cg00571033	3.30E-15	1.12E-16	9.09	26.9945609	1.53E-01	9760957	9761080	NC_000008.10
cg15104644	3.30E-15	1.12E-16	9.09	26.9930639	2.30E-01	231297103	231297226	NC_000001.10
cg09427718	3.31E-15	1.12E-16	9.09	26.9923438	1.29E-01	23819929	23820052	NC_000004.11
cg09941601	3.31E-15	1.12E-16	9.09	26.9909427	1.72E-01	86606899	86607022	NC_000016.9
cg23086243	3.32E-15	1.12E-16	9.09	26.9885367	7.82E-02	34954931	34955054	NC_000017.10
cg23645199	3.33E-15	1.13E-16	9.09	26.9836713	1.83E-01	233498625	233498748	NC_000002.11
cg16422098	3.34E-15	1.13E-16	9.09	26.9823582	2.69E-01	15761881	15762004	NC_000010.10
cg23034757	3.35E-15	1.13E-16	9.09	26.9799742	1.99E-01	140787864	140787987	NC_000005.9
cg04902729	3.35E-15	1.14E-16	9.09	26.9780611	2.66E-01	181452707	181452830	NC_000001.10

cg02640173	3.36E-15	1.14E-16	9.09	26.9749037	9.75E-02	121605707	121605830	NC_000002.11
cg02119363	3.37E-15	1.14E-16	9.09	26.9726579	2.16E-01	179754603	179754726	NC_000003.11
cg10269365	3.37E-15	1.14E-16	9.09	26.9709181	1.76E-01	223166989	223167112	NC_000002.11
cg07350078	3.38E-15	1.15E-16	9.09	26.9678332	5.58E-02	206637459	206637582	NC_000001.10
cg14038731	3.40E-15	1.15E-16	9.09	26.9636984	1.36E-01	110732536	110732659	NC_000006.11
cg03072621	3.40E-15	1.15E-16	9.09	26.9632504	2.16E-01	6069929	6070052	NC_000016.9
cg11502555	3.41E-15	1.16E-16	9.09	26.961086	1.86E-01	100627341	100627464	NC_000013.10
cg00903371	3.41E-15	1.16E-16	9.09	26.9593621	1.39E-01	238360134	238360257	NC_000002.11
cg11324219	3.41E-15	1.16E-16	9.09	26.958628	7.22E-02	97857356	97857479	NC_000007.13
cg22428147	3.42E-15	1.16E-16	9.09	26.9566351	2.22E-01	137531791	137531914	NC_000007.13
cg17948913	3.42E-15	1.16E-16	9.09	26.9565462	1.33E-01	572064	572187	NC_000005.9
cg03940684	3.42E-15	1.16E-16	9.08	26.9559127	2.28E-01	239733	239856	NC_000003.11
cg15463538	3.44E-15	1.17E-16	9.08	26.9493338	5.10E-02	13315209	13315332	NC_000019.9
cg18449120	3.46E-15	1.17E-16	9.08	26.9460948	1.61E-01	5832882	5833005	NC_000002.11
cg02807849	3.46E-15	1.17E-16	9.08	26.9455313	1.22E-01	48908102	48908225	NC_000019.9
cg00950846	3.46E-15	1.18E-16	9.08	26.9430903	2.21E-01	62069317	62069440	NC_000016.9
cg12215739	3.47E-15	1.18E-16	9.08	26.9428614	1.94E-01	177014966	177015089	NC_000002.11
cg20317521	3.49E-15	1.19E-16	9.08	26.9356769	8.88E-02	79026312	79026435	NC_000017.10
cg23806894	3.51E-15	1.19E-16	9.08	26.9303944	2.39E-01	111535528	111535651	NC_000004.11
cg00411072	3.53E-15	1.20E-16	9.08	26.9241617	1.31E-01	46660940	46661063	NC_000017.10
cg07703790	3.55E-15	1.21E-16	9.08	26.9187755	1.51E-01	28897214	28897337	NC_000018.9
cg19019849	3.56E-15	1.21E-16	9.08	26.9148568	1.73E-01	13533145	13533268	NC_000004.11
cg27446570	3.60E-15	1.22E-16	9.08	26.9047584	8.45E-02	3809532	3809655	NC_000001.10
cg26656287	3.60E-15	1.23E-16	9.08	26.9031077	8.51E-02	1120889	1121012	NC_000011.9
cg04487296	3.61E-15	1.23E-16	9.08	26.9003361	1.73E-01	149389669	149389792	NC_000007.13

cg04303439	3.62E-15	1.23E-16	9.08	26.8990582	1.21E-01	1124847	1124970	NC_000005.9
cg01691827	3.62E-15	1.23E-16	9.08	26.8983583	1.37E-01	86599189	86599312	NC_000016.9
cg14186641	3.63E-15	1.24E-16	9.08	26.8945997	3.30E-01	88876741	88876864	NC_000006.11
cg11526778	3.64E-15	1.24E-16	9.07	26.8919516	1.21E-01	28115783	28115906	NC_000007.13
cg09938049	3.64E-15	1.24E-16	9.07	26.8910639	1.33E-01	54747206	54747329	NC_000012.11
cg18435449	3.65E-15	1.24E-16	9.07	26.8894123	2.58E-01	58095445	58095568	NC_000019.9
cg15264904	3.66E-15	1.25E-16	9.07	26.8864157	8.81E-02	180218509	180218632	NC_000005.9
cg08001256	3.67E-15	1.25E-16	9.07	26.8842556	7.52E-02	90611340	90611463	NC_000015.9
cg18792031	3.71E-15	1.27E-16	9.07	26.870605	1.43E-01	94574750	94574873	NC_000008.10
cg11387088	3.74E-15	1.28E-16	9.07	26.8640011	6.73E-02	55962574	55962697	NC_000017.10
cg04787888	3.74E-15	1.28E-16	9.07	26.863838	2.03E-01	86547322	86547445	NC_000016.9
cg10232395	3.75E-15	1.28E-16	9.07	26.8608934	1.69E-01	61613721	61613844	NC_000008.10
cg26267340	3.75E-15	1.28E-16	9.07	26.8594611	1.80E-01	31497829	31497952	NC_000008.10
cg20248516	3.76E-15	1.28E-16	9.07	26.8589341	1.77E-01	119602901	119603024	NC_000002.11
cg08251037	3.77E-15	1.29E-16	9.07	26.8555725	1.75E-01	70597778	70597901	NC_000007.13
cg08481873	3.78E-15	1.29E-16	9.07	26.8530057	1.49E-01	241653301	241653424	NC_000002.11
cg19237294	3.78E-15	1.29E-16	9.07	26.8514091	1.81E-01	35065236	35065359	NC_000018.9
cg01541645	3.84E-15	1.31E-16	9.07	26.8356244	1.99E-01	61104329	61104452	NC_000014.8
cg01813393	3.86E-15	1.32E-16	9.07	26.8308379	1.40E-01	62729423	62729546	NC_000001.10
cg16418810	3.86E-15	1.32E-16	9.07	26.8289403	2.46E-01	5713100	5713223	NC_000004.11
cg00179921	3.88E-15	1.33E-16	9.06	26.8252608	1.10E-01	39927437	39927560	NC_000022.10
cg14458834	3.88E-15	1.33E-16	9.06	26.8251287	3.53E-01	46655394	46655517	NC_000017.10
cg13767135	3.88E-15	1.33E-16	9.06	26.8247005	1.09E-01	69949459	69949582	NC_000011.9
cg15466177	3.89E-15	1.33E-16	9.06	26.8225125	6.01E-02	102675179	102675302	NC_000014.8
cg05342467	3.89E-15	1.33E-16	9.06	26.8221193	4.74E-02	2029732	2029855	NC_000007.13

cg16360372	3.89E-15	1.33E-16	9.06	26.8205472	8.86E-02	147212728	147212851	NC_000005.9
cg06568323	3.90E-15	1.34E-16	9.06	26.8176549	1.05E-01	100745507	100745630	NC_000013.10
cg09528825	3.91E-15	1.34E-16	9.06	26.8172713	2.96E-01	28074388	28074511	NC_000016.9
cg06233731	3.92E-15	1.34E-16	9.06	26.8145387	1.08E-01	18890872	18890995	NC_000012.11
cg20762480	3.94E-15	1.35E-16	9.06	26.8075403	1.48E-01	39538854	39538977	NC_000012.11
cg16399049	3.94E-15	1.35E-16	9.06	26.8071782	1.72E-01	22825843	22825966	NC_000016.9
cg17840719	3.95E-15	1.35E-16	9.06	26.8063912	2.57E-01	178368253	178368376	NC_000005.9
cg08267591	3.97E-15	1.36E-16	9.06	26.8007936	1.58E-01	165693745	165693868	NC_000002.11
cg04850284	3.97E-15	1.36E-16	9.06	26.7997562	1.44E-01	15707786	15707909	NC_000001.10
cg13201808	3.98E-15	1.36E-16	9.06	26.7969014	2.81E-01	43569269	43569392	NC_000011.9
cg00614182	3.98E-15	1.37E-16	9.06	26.7965049	2.19E-01	31498006	31498129	NC_000008.10
cg13090007	3.99E-15	1.37E-16	9.06	26.7946318	1.22E-01	142981593	142981716	NC_000008.10
cg19301960	4.01E-15	1.38E-16	9.06	26.7891688	1.47E-01	1271312	1271435	NC_000019.9
cg13313226	4.01E-15	1.38E-16	9.06	26.7889417	1.27E-01	124665869	124665992	NC_000011.9
cg21857375	4.02E-15	1.38E-16	9.06	26.7881711	4.52E-02	182781107	182781230	NC_000002.11
cg02610222	4.03E-15	1.38E-16	9.06	26.7846497	1.86E-01	147560282	147560405	NC_000004.11
cg17054060	4.03E-15	1.38E-16	9.06	26.784146	1.77E-01	3606081	3606204	NC_000005.9
cg22802813	4.04E-15	1.39E-16	9.06	26.7819248	2.30E-01	95942761	95942884	NC_000012.11
cg14640592	4.05E-15	1.39E-16	9.06	26.7794521	1.52E-01	203044726	203044849	NC_000001.10
cg22227034	4.06E-15	1.39E-16	9.06	26.7773642	1.33E-01	12252976	12253099	NC_000001.10
cg07190914	4.07E-15	1.39E-16	9.06	26.7754479	1.60E-01	4500072	4500195	NC_000010.10
cg16826777	4.07E-15	1.40E-16	9.06	26.774432	1.56E-01	43376882	43377005	NC_000017.10
cg09159022	4.07E-15	1.40E-16	9.06	26.7742473	2.37E-01	4849522	4849645	NC_000008.10
cg10453425	4.08E-15	1.40E-16	9.06	26.772175	2.35E-01	87636594	87636717	NC_000016.9
cg23842255	4.08E-15	1.40E-16	9.06	26.7721456	1.58E-01	29876363	29876486	NC_000022.10

cg20406460	4.09E-15	1.41E-16	9.06	26.7677011	2.24E-01	3606489	3606612	NC_000005.9
cg08136772	4.10E-15	1.41E-16	9.06	26.7670592	1.56E-01	53420933	53421056	NC_000013.10
cg14309093	4.10E-15	1.41E-16	9.06	26.7654747	7.43E-02	133529722	133529845	NC_000010.10
cg11143671	4.12E-15	1.42E-16	9.05	26.7612763	1.22E-01	180904018	180904141	NC_000001.10
cg11248413	4.12E-15	1.42E-16	9.05	26.7603038	2.35E-01	134871658	134871781	NC_000005.9
cg08529882	4.12E-15	1.42E-16	9.05	26.7598797	1.97E-01	124902456	124902579	NC_000010.10
cg12357484	4.13E-15	1.42E-16	9.05	26.7594912	1.23E-01	69987414	69987537	NC_000011.9
cg15672768	4.14E-15	1.42E-16	9.05	26.7563567	1.53E-01	140741220	140741343	NC_000005.9
cg09385093	4.14E-15	1.42E-16	9.05	26.7558263	2.71E-01	119607885	119608008	NC_000002.11
cg09598437	4.14E-15	1.42E-16	9.05	26.7551324	1.60E-01	52643118	52643241	NC_000012.11
cg05532446	4.15E-15	1.43E-16	9.05	26.7522479	8.31E-02	47173093	47173216	NC_000016.9
cg04778012	4.17E-15	1.43E-16	9.05	26.7485835	1.60E-01	99775508	99775631	NC_000007.13
cg19502744	4.17E-15	1.43E-16	9.05	26.748475	1.92E-01	31805915	31806038	NC_000002.11
cg08423575	4.17E-15	1.43E-16	9.05	26.7479465	1.51E-01	22826800	22826923	NC_000001.10
cg10526277	4.17E-15	1.43E-16	9.05	26.7474575	2.37E-01	157813363	157813486	NC_000003.11
cg15170605	4.17E-15	1.44E-16	9.05	26.7473786	3.49E-01	70995459	70995582	NC_000002.11
cg00578304	4.17E-15	1.44E-16	9.05	26.7473334	1.21E-01	2968627	2968750	NC_000007.13
cg08401657	4.17E-15	1.44E-16	9.05	26.746908	1.12E-01	10399046	10399169	NC_000006.11
cg06563713	4.18E-15	1.44E-16	9.05	26.7455826	1.22E-01	11950917	11951040	NC_000018.9
cg17257947	4.18E-15	1.44E-16	9.05	26.7455514	4.54E-02	22541476	22541599	NC_000008.10
cg11930477	4.18E-15	1.44E-16	9.05	26.7447637	1.60E-01	31841980	31842103	NC_000011.9
cg16757724	4.18E-15	1.44E-16	9.05	26.7440369	1.49E-01	43935528	43935651	NC_000020.10
cg18894648	4.19E-15	1.44E-16	9.05	26.7428794	2.80E-01	87974446	87974569	NC_000005.9
cg13077930	4.20E-15	1.44E-16	9.05	26.7412416	1.51E-01	74269525	74269648	NC_000004.11
cg20684197	4.20E-15	1.45E-16	9.05	26.7404058	1.19E-01	142066938	142067061	NC_000005.9

cg03755123	4.20E-15	1.45E-16	9.05	26.7397057	1.84E-01	135043508	135043631	NC_000010.10
cg23544007	4.21E-15	1.45E-16	9.05	26.7385144	1.46E-01	88913736	88913859	NC_000002.11
cg22512438	4.21E-15	1.45E-16	9.05	26.7380926	2.36E-01	129693489	129693612	NC_000003.11
cg08611331	4.21E-15	1.45E-16	9.05	26.7370288	6.91E-02	30853476	30853599	NC_000016.9
cg04088969	4.21E-15	1.45E-16	9.05	26.7367394	2.84E-01	11060048	11060171	NC_000010.10
cg14757311	4.22E-15	1.45E-16	9.05	26.7350343	1.69E-01	962818	962941	NC_000016.9
cg19274703	4.22E-15	1.45E-16	9.05	26.7344063	1.73E-01	41806451	41806574	NC_000022.10
cg23938553	4.22E-15	1.46E-16	9.05	26.7336564	4.22E-02	48591529	48591652	NC_000017.10
cg20637609	4.23E-15	1.46E-16	9.05	26.7320302	1.72E-01	46825011	46825134	NC_000017.10
cg00003298	4.24E-15	1.46E-16	9.05	26.7308054	2.70E-01	113901210	113901333	NC_000012.11
cg20585038	4.26E-15	1.47E-16	9.05	26.7258069	1.66E-01	89148933	89149056	NC_000015.9
cg23603789	4.26E-15	1.47E-16	9.05	26.7252098	3.44E-02	242572545	242572668	NC_000002.11
cg19407095	4.26E-15	1.47E-16	9.05	26.7251567	1.54E-01	112721778	112721901	NC_000013.10
cg13950603	4.26E-15	1.47E-16	9.05	26.7236339	2.55E-01	7795619	7795742	NC_000019.9
cg06050631	4.28E-15	1.47E-16	9.05	26.7206176	1.68E-01	140098850	140098973	NC_000006.11
cg10964388	4.28E-15	1.48E-16	9.05	26.7193851	1.36E-01	96069024	96069147	NC_000012.11
cg11743349	4.28E-15	1.48E-16	9.05	26.7188018	1.78E-01	21950864	21950987	NC_000004.11
cg04425920	4.29E-15	1.48E-16	9.05	26.7175458	2.30E-01	50604518	50604641	NC_000010.10
cg06675478	4.30E-15	1.48E-16	9.05	26.7153257	1.29E-01	112721786	112721909	NC_000013.10
cg20132590	4.30E-15	1.48E-16	9.05	26.7143485	1.70E-01	73673893	73674016	NC_000003.11
cg14270687	4.31E-15	1.49E-16	9.05	26.7120063	1.77E-01	32953574	32953697	NC_000017.10
cg17254405	4.31E-15	1.49E-16	9.05	26.7114062	5.85E-02	68514089	68514212	NC_000011.9
cg04285318	4.32E-15	1.49E-16	9.05	26.710679	7.91E-02	192016481	192016604	NC_000002.11
cg15221604	4.33E-15	1.49E-16	9.05	26.7078119	2.58E-01	124738735	124738858	NC_000011.9
cg01080368	4.41E-15	1.52E-16	9.04	26.6881716	1.29E-01	135480456	135480579	NC_000002.11

cg15569451	4.42E-15	1.53E-16	9.04	26.686326	5.16E-02	44041324	44041447	NC_000020.10
cg10325088	4.44E-15	1.53E-16	9.04	26.6815707	4.05E-02	217724849	217724972	NC_000001.10
cg08404546	4.45E-15	1.54E-16	9.04	26.6785915	1.25E-01	37910993	37911116	NC_000022.10
cg10512745	4.45E-15	1.54E-16	9.04	26.6780265	2.97E-01	50884480	50884603	NC_000001.10
cg07850527	4.46E-15	1.54E-16	9.04	26.6761108	3.07E-01	89268040	89268163	NC_000016.9
cg27116912	4.50E-15	1.55E-16	9.04	26.6685465	2.30E-01	176947940	176948063	NC_000002.11
cg00287737	4.50E-15	1.56E-16	9.04	26.6671134	1.34E-01	894070	894193	NC_000004.11
cg22561647	4.53E-15	1.57E-16	9.04	26.6611666	1.57E-01	60747596	60747719	NC_000017.10
cg08492173	4.53E-15	1.57E-16	9.04	26.6598875	1.91E-01	3597311	3597434	NC_000005.9
cg04480285	4.54E-15	1.57E-16	9.04	26.6587984	7.94E-02	61097029	61097152	NC_000011.9
cg14091493	4.55E-15	1.57E-16	9.04	26.6559919	8.67E-02	136796914	136797037	NC_000009.11
cg08475379	4.57E-15	1.58E-16	9.04	26.6522753	1.59E-01	37302989	37303112	NC_000020.10
cg12563372	4.57E-15	1.58E-16	9.04	26.6514643	2.04E-01	25908503	25908626	NC_000008.10
cg08941080	4.58E-15	1.58E-16	9.04	26.6495666	5.01E-02	66494112	66494235	NC_000005.9
cg01052879	4.60E-15	1.59E-16	9.04	26.6452898	2.26E-01	61809830	61809953	NC_000020.10
cg19030579	4.60E-15	1.59E-16	9.04	26.645135	2.63E-01	50888710	50888833	NC_000001.10
cg16668896	4.60E-15	1.59E-16	9.04	26.6446503	1.46E-01	54900801	54900924	NC_000007.13
cg11935896	4.60E-15	1.59E-16	9.04	26.6438476	6.43E-02	148409702	148409825	NC_000005.9
cg25952964	4.62E-15	1.60E-16	9.04	26.6403976	1.31E-01	109607272	109607395	NC_000002.11
cg02856026	4.62E-15	1.60E-16	9.04	26.6403058	1.37E-01	22328339	22328462	NC_000001.10
cg15203618	4.62E-15	1.60E-16	9.04	26.6389138	7.36E-02	85646125	85646248	NC_000011.9
cg23954541	4.63E-15	1.60E-16	9.04	26.6378812	7.50E-02	16969609	16969732	NC_000001.10
cg04852339	4.63E-15	1.60E-16	9.04	26.6376069	1.14E-01	159841571	159841694	NC_000002.11
cg05065879	4.64E-15	1.61E-16	9.03	26.6358317	1.71E-01	123229471	123229594	NC_000011.9
cg00629117	4.64E-15	1.61E-16	9.03	26.6348847	1.98E-01	1844932	1845055	NC_000016.9

cg21266559	4.64E-15	1.61E-16	9.03	26.6347275	2.22E-01	34114285	34114408	NC_000019.9
cg02332936	4.65E-15	1.61E-16	9.03	26.6338048	1.93E-01	115140584	115140707	NC_000009.11
cg25081106	4.67E-15	1.62E-16	9.03	26.629498	3.00E-01	88324169	88324292	NC_000013.10
cg16907558	4.68E-15	1.62E-16	9.03	26.6267956	1.88E-01	71017637	71017760	NC_000002.11
cg07371029	4.68E-15	1.62E-16	9.03	26.6256279	1.22E-01	134743011	134743134	NC_000010.10
cg07440398	4.68E-15	1.62E-16	9.03	26.6250562	1.54E-01	99712966	99713089	NC_000014.8
cg21245816	4.69E-15	1.63E-16	9.03	26.6232837	1.42E-01	43044675	43044798	NC_000005.9
cg03205584	4.72E-15	1.64E-16	9.03	26.6173481	3.00E-01	79258407	79258530	NC_000012.11
cg12591770	4.76E-15	1.65E-16	9.03	26.6095843	1.63E-01	100913571	100913694	NC_000015.9
cg14891410	4.76E-15	1.65E-16	9.03	26.6080945	1.59E-01	32458769	32458892	NC_000011.9
cg16018002	4.77E-15	1.65E-16	9.03	26.6068136	1.11E-01	11251104	11251227	NC_000006.11
cg14810242	4.78E-15	1.66E-16	9.03	26.6030225	7.44E-02	102087008	102087131	NC_000002.11
cg23318063	4.78E-15	1.66E-16	9.03	26.6030179	2.41E-01	58332751	58332874	NC_000014.8
cg11794956	4.80E-15	1.67E-16	9.03	26.5990543	2.10E-01	92443321	92443444	NC_000005.9
cg13680844	4.80E-15	1.67E-16	9.03	26.5988585	5.51E-02	52255405	52255528	NC_000003.11
cg15710961	4.82E-15	1.67E-16	9.03	26.5954955	4.63E-02	56572591	56572714	NC_000006.11
cg04521004	4.83E-15	1.68E-16	9.03	26.5939057	1.90E-01	157821262	157821385	NC_000003.11
cg21093192	4.85E-15	1.68E-16	9.03	26.5891332	2.60E-01	54412993	54413116	NC_000019.9
cg15959715	4.86E-15	1.69E-16	9.03	26.5862199	2.53E-01	26506713	26506836	NC_000010.10
cg01178680	4.87E-15	1.69E-16	9.03	26.584336	1.23E-01	74425552	74425675	NC_000015.9
cg12554268	4.89E-15	1.70E-16	9.03	26.5814049	1.01E-01	27338633	27338756	NC_000007.13
cg02052905	4.89E-15	1.70E-16	9.03	26.5802719	1.38E-01	88796355	88796478	NC_000011.9
cg23398332	4.90E-15	1.70E-16	9.03	26.5789759	7.90E-02	128485943	128486066	NC_000008.10
cg11144641	4.91E-15	1.71E-16	9.03	26.5764841	1.92E-01	134871566	134871689	NC_000005.9
cg00470641	4.91E-15	1.71E-16	9.03	26.5751791	1.36E-01	3096219	3096342	NC_000010.10

cg08036309	4.92E-15	1.71E-16	9.03	26.5738855	1.85E-01	13536991	13537114	NC_000004.11
cg27639104	4.92E-15	1.71E-16	9.02	26.5728474	2.92E-02	2482929	2483052	NC_000011.9
cg22460443	4.93E-15	1.71E-16	9.02	26.5721028	8.51E-02	90610983	90611106	NC_000015.9
cg27512716	4.95E-15	1.72E-16	9.02	26.5663168	1.41E-01	44219675	44219798	NC_000019.9
cg15267232	4.97E-15	1.73E-16	9.02	26.5620242	2.43E-01	8097689	8097812	NC_000010.10
cg04652943	4.98E-15	1.73E-16	9.02	26.5612717	1.22E-01	3750565	3750688	NC_000002.11
cg25640822	4.98E-15	1.73E-16	9.02	26.5600885	2.76E-01	134871645	134871768	NC_000005.9
cg00910518	4.98E-15	1.73E-16	9.02	26.5598491	1.62E-01	91195213	91195336	NC_000001.10
cg07681938	5.00E-15	1.74E-16	9.02	26.5562757	1.95E-01	62070234	62070357	NC_000016.9
cg20023231	5.01E-15	1.74E-16	9.02	26.5549243	1.97E-01	22825282	22825405	NC_000016.9
cg22661893	5.01E-15	1.75E-16	9.02	26.5535551	2.00E-01	89224559	89224682	NC_000011.9
cg14855519	5.02E-15	1.75E-16	9.02	26.5521082	1.63E-01	25898191	25898314	NC_000008.10
cg17665601	5.04E-15	1.76E-16	9.02	26.5474784	4.15E-02	131162292	131162415	NC_000008.10
cg12651914	5.05E-15	1.76E-16	9.02	26.5463861	1.96E-01	130642779	130642902	NC_000012.11
cg20567847	5.06E-15	1.76E-16	9.02	26.5435889	2.84E-01	155167038	155167161	NC_000007.13
cg27530352	5.09E-15	1.78E-16	9.02	26.5364548	9.03E-02	11294188	11294311	NC_000003.11
cg04096767	5.12E-15	1.78E-16	9.02	26.5321599	1.51E-01	32449450	32449573	NC_000011.9
cg08688393	5.12E-15	1.79E-16	9.02	26.5310857	1.52E-01	70371326	70371449	NC_000015.9
cg01240823	5.14E-15	1.79E-16	9.02	26.5275711	1.04E-01	92667819	92667942	NC_000015.9
cg14916150	5.16E-15	1.80E-16	9.02	26.5233307	2.12E-01	4868983	4869106	NC_000004.11
cg11416384	5.20E-15	1.82E-16	9.02	26.5148708	3.08E-01	37246827	37246950	NC_000004.11
cg12728623	5.21E-15	1.82E-16	9.02	26.5141047	1.27E-01	19938992	19939115	NC_000022.10
cg06794441	5.21E-15	1.82E-16	9.02	26.5138851	1.18E-01	67520733	67520856	NC_000001.10
cg19202601	5.22E-15	1.82E-16	9.02	26.5120433	7.81E-02	30070168	30070291	NC_000022.10
cg23669876	5.22E-15	1.82E-16	9.02	26.5114873	1.12E-01	36489276	36489399	NC_000001.10

cg21986027	5.22E-15	1.82E-16	9.02	26.5107097	1.83E-01	169238138	169238261	NC_000006.11
cg25644556	5.24E-15	1.83E-16	9.01	26.5065483	2.68E-01	27209582	27209705	NC_000007.13
cg17482963	5.28E-15	1.84E-16	9.01	26.4998773	1.58E-01	27591911	27592034	NC_000008.10
cg26344747	5.30E-15	1.85E-16	9.01	26.496355	1.46E-01	21193332	21193455	NC_000017.10
cg02906863	5.30E-15	1.85E-16	9.01	26.4954487	1.39E-01	22275386	22275509	NC_000022.10
cg24176563	5.30E-15	1.85E-16	9.01	26.4954171	2.20E-01	133562776	133562899	NC_000006.11
cg20731875	5.33E-15	1.86E-16	9.01	26.4905757	1.48E-01	14207701	14207824	NC_000017.10
cg18710784	5.33E-15	1.86E-16	9.01	26.4885159	2.50E-01	132813563	132813686	NC_000011.9
cg08490115	5.34E-15	1.87E-16	9.01	26.487242	2.41E-01	30038675	30038798	NC_000011.9
cg02909298	5.34E-15	1.87E-16	9.01	26.4867735	1.09E-01	56207327	56207450	NC_000014.8
cg02597493	5.37E-15	1.88E-16	9.01	26.4818071	8.29E-02	79298637	79298760	NC_000015.9
cg17669581	5.38E-15	1.88E-16	9.01	26.479898	1.67E-01	54968251	54968374	NC_000004.11
cg25841625	5.38E-15	1.88E-16	9.01	26.4791671	1.79E-01	77119588	77119711	NC_000004.11
cg26813561	5.39E-15	1.89E-16	9.01	26.4773733	7.26E-02	112171802	112171925	NC_000012.11
cg08901867	5.39E-15	1.89E-16	9.01	26.4771151	1.21E-01	46871042	46871165	NC_000017.10
cg04201744	5.40E-15	1.89E-16	9.01	26.4761547	6.63E-02	178976295	178976418	NC_000002.11
cg00153106	5.42E-15	1.89E-16	9.01	26.472526	2.21E-01	172671561	172671684	NC_000005.9
cg11530678	5.42E-15	1.89E-16	9.01	26.4723896	6.93E-02	67081012	67081135	NC_000015.9
cg02773399	5.44E-15	1.90E-16	9.01	26.4673458	4.74E-02	110226215	110226338	NC_000009.11
cg00661855	5.45E-15	1.91E-16	9.01	26.4647789	1.19E-01	3152428	3152551	NC_000006.11
cg15768103	5.47E-15	1.92E-16	9.01	26.4618491	2.58E-01	127413936	127414059	NC_000002.11
cg19993589	5.48E-15	1.92E-16	9.01	26.4597011	2.07E-01	177140510	177140633	NC_000001.10
cg27434509	5.51E-15	1.93E-16	9.01	26.4540342	2.12E-01	5710337	5710460	NC_000004.11
cg11214140	5.55E-15	1.94E-16	9.01	26.447605	1.82E-01	55370429	55370552	NC_000008.10
cg04036366	5.55E-15	1.95E-16	9.01	26.4464139	1.17E-01	79976900	79977023	NC_000017.10

cg21463349	5.56E-15	1.95E-16	9	26.4453416	1.64E-01	119600948	119601071	NC_000002.11
cg18035229	5.56E-15	1.95E-16	9	26.4447483	2.21E-01	70984270	70984393	NC_000008.10
cg09403666	5.57E-15	1.95E-16	9	26.443334	1.92E-01	23461493	23461616	NC_000010.10
cg09548799	5.60E-15	1.96E-16	9	26.4367315	1.00E-01	67839134	67839257	NC_000008.10
cg10523671	5.60E-15	1.96E-16	9	26.4366782	1.46E-01	121612956	121613079	NC_000003.11
cg05965387	5.62E-15	1.97E-16	9	26.4330542	1.47E-01	51418053	51418176	NC_000013.10
cg14523847	5.64E-15	1.98E-16	9	26.4301832	2.25E-01	153584597	153584720	NC_000007.13
cg27157482	5.64E-15	1.98E-16	9	26.4301455	1.86E-01	27215606	27215729	NC_000007.13
cg24626674	5.65E-15	1.98E-16	9	26.428524	7.96E-02	57958333	57958456	NC_000011.9
cg13506166	5.66E-15	1.99E-16	9	26.4263661	3.71E-02	81404956	81405079	NC_000014.8
cg02814641	5.70E-15	2.00E-16	9	26.4185587	1.68E-01	167832664	167832787	NC_000006.11
cg02519879	5.70E-15	2.00E-16	9	26.4184488	1.32E-01	122286677	122286800	NC_000003.11
cg23939866	5.75E-15	2.02E-16	9	26.410164	6.27E-02	154181852	154181975	NC_000005.9
cg21095561	5.76E-15	2.02E-16	9	26.4075831	2.19E-01	42733994	42734117	NC_000017.10
cg20725704	5.79E-15	2.03E-16	9	26.40281	7.54E-02	10950238	10950361	NC_000002.11
cg24632241	5.79E-15	2.03E-16	9	26.4027117	2.75E-01	80530431	80530554	NC_000002.11
cg19144684	5.79E-15	2.03E-16	9	26.4023884	2.10E-01	137522960	137523083	NC_000002.11
cg13596833	5.80E-15	2.04E-16	9	26.400134	2.45E-01	31827899	31828022	NC_000011.9
cg16536329	5.82E-15	2.05E-16	9	26.3961277	2.12E-01	178368185	178368308	NC_000005.9
cg23623622	5.84E-15	2.05E-16	9	26.3940098	1.94E-01	30139880	30140003	NC_000006.11
cg11722553	5.86E-15	2.06E-16	9	26.3905484	5.90E-02	10829162	10829285	NC_000012.11
cg05740045	5.86E-15	2.06E-16	9	26.3901286	1.75E-01	65499926	65500049	NC_000008.10
cg26889572	5.86E-15	2.06E-16	9	26.3895435	1.08E-01	106126690	106126813	NC_000002.11
cg21230493	5.88E-15	2.07E-16	9	26.3864299	2.02E-01	26722496	26722619	NC_000008.10
cg13885535	5.88E-15	2.07E-16	9	26.3862082	1.23E-01	124315614	124315737	NC_000009.11

cg17892178	5.89E-15	2.07E-16	9	26.3838212	2.24E-01	6902824	6902947	NC_000003.11
cg22290117	5.90E-15	2.08E-16	8.99	26.3821067	1.18E-01	57427173	57427296	NC_000020.10
cg06838985	5.92E-15	2.08E-16	8.99	26.3786776	1.72E-01	101453871	101453994	NC_000011.9
cg21581504	5.93E-15	2.09E-16	8.99	26.3765491	1.53E-01	68545960	68546083	NC_000002.11
cg10726463	5.95E-15	2.09E-16	8.99	26.3736959	1.06E-01	38243770	38243893	NC_000022.10
cg13540960	5.99E-15	2.11E-16	8.99	26.3662208	2.07E-01	32450244	32450367	NC_000011.9
cg08534653	6.01E-15	2.12E-16	8.99	26.3625834	2.17E-01	1747700	1747823	NC_000002.11
cg23692680	6.02E-15	2.12E-16	8.99	26.3606984	1.90E-01	101445897	101446020	NC_000008.10
cg04317962	6.03E-15	2.12E-16	8.99	26.3603459	1.99E-01	79623625	79623748	NC_000016.9
cg27334919	6.03E-15	2.12E-16	8.99	26.3592204	1.95E-01	19158378	19158501	NC_000007.13
cg25963041	6.05E-15	2.13E-16	8.99	26.3564572	3.27E-01	57049777	57049900	NC_000019.9
cg15991405	6.05E-15	2.13E-16	8.99	26.3558978	1.89E-01	176988480	176988603	NC_000002.11
cg03092191	6.06E-15	2.13E-16	8.99	26.3548071	2.29E-01	1272710	1272833	NC_000007.13
cg14008883	6.08E-15	2.14E-16	8.99	26.351086	1.54E-01	50818911	50819034	NC_000010.10
cg08273957	6.08E-15	2.14E-16	8.99	26.3502848	2.22E-01	29227941	29228064	NC_000014.8
cg10321393	6.09E-15	2.15E-16	8.99	26.3492076	9.76E-02	15905387	15905510	NC_000001.10
cg26224652	6.09E-15	2.15E-16	8.99	26.3490077	5.60E-02	57181252	57181375	NC_000004.11
cg16064793	6.09E-15	2.15E-16	8.99	26.3486607	1.69E-01	157021142	157021265	NC_000005.9
cg21101386	6.12E-15	2.16E-16	8.99	26.3439519	1.31E-01	35125857	35125980	NC_000020.10
cg09227138	6.12E-15	2.16E-16	8.99	26.3437298	2.07E-01	84419244	84419367	NC_000006.11
cg21502786	6.14E-15	2.17E-16	8.99	26.3398691	2.39E-01	56234476	56234599	NC_000017.10
cg13381984	6.15E-15	2.17E-16	8.99	26.3388757	1.50E-01	127881344	127881467	NC_000007.13
cg04059773	6.15E-15	2.17E-16	8.99	26.3387682	2.46E-01	53561563	53561686	NC_000019.9
cg04495995	6.16E-15	2.17E-16	8.99	26.3366256	2.90E-01	19147157	19147280	NC_000007.13
cg06704093	6.16E-15	2.18E-16	8.99	26.3359005	2.13E-01	110226132	110226255	NC_000010.10

cg09679642	6.17E-15	2.18E-16	8.99	26.3352311	6.95E-02	79805210	79805333	NC_000017.10
cg20244327	6.17E-15	2.18E-16	8.99	26.3349119	1.51E-01	3283946	3284069	NC_000010.10
cg11363444	6.18E-15	2.18E-16	8.99	26.3324416	1.16E-01	153079618	153079741	NC_000002.11
cg07080358	6.19E-15	2.19E-16	8.99	26.3312456	2.53E-01	68546507	68546630	NC_000002.11
cg18729086	6.19E-15	2.19E-16	8.99	26.3303034	9.75E-02	101187440	101187563	NC_000010.10
cg17415381	6.20E-15	2.19E-16	8.99	26.3297242	1.37E-01	61514690	61514813	NC_000017.10
cg25209111	6.25E-15	2.21E-16	8.99	26.3208118	1.43E-01	104009148	104009271	NC_000010.10
cg15718811	6.25E-15	2.21E-16	8.99	26.3203694	1.53E-01	120427810	120427933	NC_000008.10
cg21214293	6.26E-15	2.21E-16	8.99	26.3189411	1.55E-01	145713882	145714005	NC_000005.9
cg24847685	6.28E-15	2.22E-16	8.98	26.3154837	2.59E-01	147087653	147087776	NC_000003.11
cg04949206	6.30E-15	2.23E-16	8.98	26.3128156	1.95E-01	176971971	176972094	NC_000002.11
cg13874817	6.33E-15	2.24E-16	8.98	26.3078152	2.27E-01	140770751	140770874	NC_000003.11
cg01556677	6.34E-15	2.24E-16	8.98	26.3050142	7.17E-02	80534871	80534994	NC_000007.13
cg06002288	6.38E-15	2.26E-16	8.98	26.2988425	1.94E-01	44500261	44500384	NC_000018.9
cg05333146	6.41E-15	2.27E-16	8.98	26.2939427	1.47E-01	67183725	67183848	NC_000016.9
cg23305365	6.43E-15	2.27E-16	8.98	26.2916761	1.09E-01	105379392	105379515	NC_000008.10
cg26512459	6.43E-15	2.28E-16	8.98	26.2906108	6.71E-02	3435203	3435326	NC_000011.9
cg18106668	6.45E-15	2.28E-16	8.98	26.2886331	2.15E-01	24323261	24323384	NC_000007.13
cg01024444	6.46E-15	2.29E-16	8.98	26.2861904	1.96E-01	52222446	52222569	NC_000019.9
cg08149459	6.49E-15	2.30E-16	8.98	26.2818876	1.13E-01	77304195	77304318	NC_000014.8
cg09825979	6.49E-15	2.30E-16	8.98	26.281802	1.61E-01	27179432	27179555	NC_000007.13
cg23615741	6.50E-15	2.30E-16	8.98	26.280049	2.02E-01	101297642	101297765	NC_000010.10
cg15670863	6.50E-15	2.30E-16	8.98	26.2798886	8.92E-02	49110292	49110415	NC_000019.9
cg07865267	6.52E-15	2.31E-16	8.98	26.2763522	1.29E-01	1421517	1421640	NC_000017.10
cg14802355	6.53E-15	2.31E-16	8.98	26.2755384	1.58E-01	103683140	103683263	NC_000004.11

cg19814174	6.54E-15	2.32E-16	8.98	26.2740963	1.50E-01	213704987	213705110	NC_000001.10
cg14732324	6.58E-15	2.33E-16	8.98	26.2665881	2.91E-01	528621	528744	NC_000005.9
cg05258261	6.59E-15	2.34E-16	8.98	26.2652252	2.37E-01	140770608	140770731	NC_000003.11
cg04876124	6.60E-15	2.34E-16	8.98	26.2633651	1.31E-01	3160911	3161034	NC_000001.10
cg10314760	6.60E-15	2.34E-16	8.98	26.263147	1.35E-01	123353654	123353777	NC_000010.10
cg21871232	6.61E-15	2.34E-16	8.98	26.2625665	1.88E-01	95693881	95694004	NC_000014.8
cg19003326	6.61E-15	2.35E-16	8.98	26.2613737	1.30E-01	60720320	60720443	NC_000005.9
cg25312935	6.62E-15	2.35E-16	8.98	26.2598372	5.45E-02	211860642	211860765	NC_000001.10
cg09788586	6.63E-15	2.35E-16	8.98	26.2596173	1.75E-01	114337676	114337799	NC_000012.11
cg00413617	6.63E-15	2.35E-16	8.98	26.2591411	1.36E-01	17757415	17757538	NC_000011.9
cg15092219	6.63E-15	2.35E-16	8.98	26.2588604	1.56E-01	247511364	247511487	NC_000001.10
cg07746943	6.63E-15	2.35E-16	8.98	26.2585816	2.82E-01	238615	238738	NC_000003.11
cg13475333	6.63E-15	2.35E-16	8.98	26.258176	1.96E-01	139585616	139585739	NC_000009.11
cg26079753	6.64E-15	2.36E-16	8.98	26.2571976	2.51E-01	54355528	54355651	NC_000012.11
cg10537450	6.65E-15	2.36E-16	8.98	26.2557617	1.34E-01	147789973	147790096	NC_000001.10
cg22660933	6.65E-15	2.36E-16	8.97	26.2547495	1.03E-01	46608355	46608478	NC_000017.10
cg01394052	6.71E-15	2.38E-16	8.97	26.2460253	3.79E-02	27684997	27685120	NC_000001.10
cg18836576	6.72E-15	2.39E-16	8.97	26.2444856	5.85E-02	33677751	33677874	NC_000006.11
cg00962755	6.72E-15	2.39E-16	8.97	26.2430684	1.49E-01	201439001	201439124	NC_000001.10
cg21161253	6.73E-15	2.39E-16	8.97	26.2417833	2.55E-01	61638149	61638272	NC_000020.10
cg09444060	6.84E-15	2.43E-16	8.97	26.2257712	5.58E-02	28887940	28888063	NC_000006.11
cg12072560	6.85E-15	2.43E-16	8.97	26.2245799	2.59E-01	12254173	12254296	NC_000018.9
cg22242857	6.90E-15	2.45E-16	8.97	26.2163926	1.85E-01	76751952	76752075	NC_000018.9
cg12166610	6.93E-15	2.46E-16	8.97	26.2127148	1.91E-01	31498256	31498379	NC_000008.10
cg18855674	6.93E-15	2.47E-16	8.97	26.2118207	1.92E-01	72469553	72469676	NC_000008.10

cg18451814	6.97E-15	2.48E-16	8.97	26.2063377	1.98E-01	57278188	57278311	NC_000014.8
cg16620392	7.00E-15	2.49E-16	8.97	26.2023041	1.24E-01	131765379	131765502	NC_000010.10
cg21999939	7.03E-15	2.50E-16	8.97	26.1975167	2.73E-01	2755004	2755127	NC_000005.9
cg12500949	7.03E-15	2.50E-16	8.97	26.1972854	9.95E-02	88357920	88358043	NC_000002.11
cg11468819	7.04E-15	2.51E-16	8.97	26.1961003	1.72E-01	4942612	4942735	NC_000005.9
cg18837713	7.04E-15	2.51E-16	8.97	26.1949235	1.21E-01	77159365	77159488	NC_000012.11
cg18408326	7.05E-15	2.51E-16	8.97	26.194599	2.35E-01	18429037	18429160	NC_000010.10
cg01191815	7.07E-15	2.52E-16	8.97	26.1917943	7.01E-02	63084959	63085082	NC_000015.9
cg04763554	7.09E-15	2.52E-16	8.96	26.1886312	2.76E-01	98511258	98511381	NC_000001.10
cg25756435	7.12E-15	2.54E-16	8.96	26.183234	2.10E-01	12952129	12952252	NC_000019.9
cg24535046	7.14E-15	2.54E-16	8.96	26.1811553	5.87E-02	21730858	21730981	NC_000017.10
cg18098400	7.15E-15	2.55E-16	8.96	26.1798193	2.29E-01	25621328	25621451	NC_000013.10
cg17156983	7.15E-15	2.55E-16	8.96	26.1791674	1.69E-01	18951159	18951282	NC_000010.10
cg08629267	7.18E-15	2.56E-16	8.96	26.1749548	1.33E-01	239014798	239014921	NC_000002.11
cg09873584	7.18E-15	2.56E-16	8.96	26.1749334	1.41E-01	60348937	60349060	NC_000010.10
cg10310457	7.23E-15	2.58E-16	8.96	26.1684718	1.75E-01	5217523	5217646	NC_000016.9
cg14880894	7.24E-15	2.58E-16	8.96	26.1668655	1.88E-01	78697859	78697982	NC_000004.11
cg25975712	7.25E-15	2.59E-16	8.96	26.1643638	2.74E-01	48971051	48971174	NC_000022.10
cg23487201	7.29E-15	2.60E-16	8.96	26.1598547	1.87E-01	57090122	57090245	NC_000020.10
cg11747897	7.29E-15	2.60E-16	8.96	26.1598141	6.23E-02	46860105	46860228	NC_000022.10
cg12154110	7.30E-15	2.60E-16	8.96	26.158055	1.14E-01	49254220	49254343	NC_000019.9
cg03275648	7.36E-15	2.63E-16	8.96	26.1488356	9.60E-02	96940848	96940971	NC_000009.11
cg05994320	7.37E-15	2.63E-16	8.96	26.1482446	1.74E-01	113727753	113727876	NC_000007.13
cg24380053	7.37E-15	2.63E-16	8.96	26.1474448	1.62E-01	13028632	13028755	NC_000003.11
cg06001519	7.39E-15	2.64E-16	8.96	26.1454745	2.13E-01	23500838	23500961	NC_000013.10

cg04331756	7.43E-15	2.65E-16	8.96	26.1394621	1.18E-01	8546532	8546655	NC_000010.10
cg23230158	7.45E-15	2.66E-16	8.96	26.1372488	1.44E-01	230260837	230260960	NC_000001.10
cg22356083	7.47E-15	2.67E-16	8.96	26.1346361	8.84E-02	55861964	55862087	NC_000019.9
cg03002848	7.47E-15	2.67E-16	8.96	26.1340355	2.10E-01	135050343	135050466	NC_000010.10
cg18857369	7.49E-15	2.67E-16	8.96	26.1319397	1.24E-01	105518248	105518371	NC_000014.8
cg12250896	7.49E-15	2.68E-16	8.96	26.1306925	2.63E-01	134001112	134001235	NC_000010.10
cg17712694	7.54E-15	2.69E-16	8.95	26.1241321	2.56E-01	16180068	16180191	NC_000005.9
cg17598190	7.55E-15	2.70E-16	8.95	26.1227996	4.28E-02	3162054	3162177	NC_000010.10
cg07124126	7.56E-15	2.70E-16	8.95	26.1221959	7.70E-02	81927346	81927469	NC_000016.9
cg01340163	7.57E-15	2.70E-16	8.95	26.1208251	1.56E-01	55990582	55990705	NC_000004.11
cg21615397	7.57E-15	2.71E-16	8.95	26.1199068	1.85E-01	55714393	55714516	NC_000018.9
cg12425321	7.59E-15	2.71E-16	8.95	26.1180394	9.73E-02	31733914	31734037	NC_000006.11
cg05203809	7.59E-15	2.71E-16	8.95	26.1169217	1.56E-01	12281858	12281981	NC_000016.9
cg05079049	7.60E-15	2.72E-16	8.95	26.1165876	1.47E-01	55364843	55364966	NC_000016.9
cg19369022	7.60E-15	2.72E-16	8.95	26.1159697	2.33E-01	51111388	51111511	NC_000019.9
cg21282630	7.62E-15	2.72E-16	8.95	26.1138221	2.60E-01	27945125	27945248	NC_000021.8
cg10410142	7.62E-15	2.72E-16	8.95	26.1130821	2.45E-01	158141542	158141665	NC_000004.11
cg12746120	7.63E-15	2.73E-16	8.95	26.1115311	8.96E-02	110398188	110398311	NC_000013.10
cg07972135	7.63E-15	2.73E-16	8.95	26.111067	2.47E-01	105481322	105481445	NC_000011.9
cg13962153	7.64E-15	2.73E-16	8.95	26.1104339	1.58E-01	36644157	36644280	NC_000001.10
cg09323727	7.69E-15	2.75E-16	8.95	26.1034996	2.60E-01	176936436	176936559	NC_000002.11
cg09637449	7.77E-15	2.78E-16	8.95	26.0929074	6.40E-02	73479754	73479877	NC_000010.10
cg09042277	7.79E-15	2.79E-16	8.95	26.0901398	2.13E-01	114840854	114840977	NC_000012.11
cg13983640	7.80E-15	2.79E-16	8.95	26.0895024	1.61E-01	41010223	41010346	NC_000006.11
cg26282731	7.80E-15	2.79E-16	8.95	26.0890132	1.90E-01	148939288	148939411	NC_000004.11

cg04390523	7.81E-15	2.80E-16	8.95	26.0875048	2.33E-01	78493100	78493223	NC_000013.10
cg19580344	7.82E-15	2.80E-16	8.95	26.0862444	1.23E-01	16669915	16670038	NC_000005.9
cg09767658	7.84E-15	2.81E-16	8.95	26.0833274	1.30E-01	8433089	8433212	NC_000001.10
cg09576978	7.84E-15	2.81E-16	8.95	26.0830386	9.65E-02	41554080	41554203	NC_000008.10
cg26589665	7.87E-15	2.82E-16	8.95	26.0798344	1.10E-01	71623290	71623413	NC_000015.9
cg07495363	7.88E-15	2.82E-16	8.95	26.0783317	2.83E-01	198651076	198651199	NC_000002.11
cg02810149	7.88E-15	2.82E-16	8.95	26.078032	1.13E-01	45608938	45609061	NC_000006.11
cg14323928	7.90E-15	2.83E-16	8.95	26.0760152	6.21E-02	31393845	31393968	NC_000015.9
cg12379145	7.90E-15	2.83E-16	8.95	26.0748812	1.31E-01	14030611	14030734	NC_000001.10
cg07704151	7.90E-15	2.83E-16	8.95	26.0746516	1.13E-01	100327897	100328020	NC_000003.11
cg06563089	7.91E-15	2.83E-16	8.95	26.07416	1.66E-01	75601157	75601280	NC_000012.11
cg01995480	7.92E-15	2.84E-16	8.95	26.0720469	2.68E-01	45405827	45405950	NC_000022.10
cg18805066	7.94E-15	2.85E-16	8.95	26.069768	2.14E-01	27134259	27134382	NC_000007.13
cg01687878	7.94E-15	2.85E-16	8.95	26.0694261	1.64E-01	123616817	123616940	NC_000012.11
cg24636944	7.95E-15	2.85E-16	8.95	26.0678132	1.25E-01	92615296	92615419	NC_000005.9
cg00281664	7.96E-15	2.85E-16	8.95	26.0673803	1.31E-01	113384048	113384171	NC_000013.10
cg26707887	7.98E-15	2.86E-16	8.94	26.064042	1.29E-01	67027311	67027434	NC_000005.9
cg22815766	7.98E-15	2.86E-16	8.94	26.064037	1.08E-01	134022784	134022907	NC_000011.9
cg14223293	8.00E-15	2.87E-16	8.94	26.0618413	2.13E-01	3602272	3602395	NC_000005.9
cg14995475	8.04E-15	2.88E-16	8.94	26.0568969	1.80E-01	6534774	6534897	NC_000019.9
cg21464565	8.05E-15	2.89E-16	8.94	26.0548351	1.88E-01	27141139	27141262	NC_000007.13
cg19952303	8.06E-15	2.89E-16	8.94	26.054047	1.93E-01	85306323	85306446	NC_000012.11
cg16009129	8.07E-15	2.90E-16	8.94	26.0521132	4.72E-02	6106379	6106502	NC_000019.9
cg05076914	8.08E-15	2.90E-16	8.94	26.0506316	1.69E-01	168842302	168842425	NC_000006.11
cg26348902	8.11E-15	2.91E-16	8.94	26.0466159	1.90E-01	1399938	1400061	NC_000004.11

cg03951219	8.13E-15	2.92E-16	8.94	26.0447512	2.94E-01	159141440	159141563	NC_000001.10
cg18356785	8.15E-15	2.93E-16	8.94	26.0418973	1.76E-01	47611780	47611903	NC_000011.9
cg10980774	8.17E-15	2.93E-16	8.94	26.0396276	2.81E-01	112832547	112832670	NC_000011.9
cg17477818	8.18E-15	2.94E-16	8.94	26.0381691	1.17E-01	10203128	10203251	NC_000019.9
cg26838023	8.21E-15	2.95E-16	8.94	26.0345688	1.04E-01	30072314	30072437	NC_000020.10
cg07520506	8.22E-15	2.95E-16	8.94	26.0328981	1.81E-01	7593007	7593130	NC_000012.11
cg22946150	8.29E-15	2.98E-16	8.94	26.0244717	2.61E-01	84116107	84116230	NC_000015.9
cg26094789	8.32E-15	2.99E-16	8.94	26.0208436	1.62E-01	37135586	37135709	NC_000014.8
cg23933759	8.34E-15	3.00E-16	8.94	26.0172449	1.19E-01	37836671	37836794	NC_000021.8
cg04250451	8.36E-15	3.01E-16	8.94	26.0147477	1.18E-01	25257784	25257907	NC_000001.10
cg09249675	8.37E-15	3.01E-16	8.94	26.0135838	1.54E-01	3401572	3401695	NC_000019.9
cg16134430	8.37E-15	3.01E-16	8.94	26.0133922	4.74E-02	34636155	34636278	NC_000021.8
cg07047831	8.37E-15	3.01E-16	8.94	26.0132652	8.59E-02	186918647	186918770	NC_000003.11
cg13188098	8.38E-15	3.02E-16	8.94	26.0127646	1.42E-01	4918335	4918458	NC_000012.11
cg09304047	8.38E-15	3.02E-16	8.94	26.0126035	1.76E-01	53747541	53747664	NC_000002.11
cg19955823	8.38E-15	3.02E-16	8.94	26.0124949	3.94E-02	90428806	90428929	NC_000014.8
cg19227031	8.38E-15	3.02E-16	8.94	26.0122146	1.13E-01	32118315	32118438	NC_000006.11
cg00516513	8.39E-15	3.02E-16	8.94	26.0105069	2.69E-01	41753803	41753926	NC_000004.11
cg15630907	8.39E-15	3.02E-16	8.94	26.0103981	1.88E-01	115630921	115631044	NC_000011.9
cg19988235	8.41E-15	3.03E-16	8.94	26.0086131	1.24E-01	9945589	9945712	NC_000002.11
cg13262687	8.41E-15	3.03E-16	8.94	26.008009	2.40E-01	147559579	147559702	NC_000004.11
cg00153110	8.41E-15	3.03E-16	8.94	26.0078483	2.23E-01	175201062	175201185	NC_000002.11
cg03983645	8.42E-15	3.03E-16	8.94	26.0072222	2.07E-01	20181887	20182010	NC_000011.9
cg04656171	8.45E-15	3.05E-16	8.94	26.0030367	1.36E-01	42211689	42211812	NC_000015.9
cg16635010	8.47E-15	3.05E-16	8.94	26.000938	5.89E-02	179563497	179563620	NC_000005.9

cg00711088	8.47E-15	3.05E-16	8.93	26.0007867	2.08E-01	1401409	1401532	NC_000004.11
cg15800722	8.47E-15	3.05E-16	8.93	26.000311	1.88E-01	42144929	42145052	NC_000017.10
cg14447703	8.48E-15	3.06E-16	8.93	25.9986222	9.35E-02	30846517	30846640	NC_000006.11
cg16089796	8.55E-15	3.08E-16	8.93	25.9903874	9.08E-02	128382383	128382506	NC_000002.11
cg01613249	8.59E-15	3.10E-16	8.93	25.9861935	1.23E-01	54727293	54727416	NC_000002.11
cg05593336	8.59E-15	3.10E-16	8.93	25.9853788	1.56E-01	85361633	85361756	NC_000006.11
cg10101109	8.62E-15	3.11E-16	8.93	25.9818907	1.28E-01	112455255	112455378	NC_000012.11
cg16664996	8.63E-15	3.11E-16	8.93	25.9812022	9.62E-02	88385249	88385372	NC_000016.9
cg12172720	8.65E-15	3.12E-16	8.93	25.9778863	1.82E-01	150019603	150019726	NC_000005.9
cg20723705	8.66E-15	3.13E-16	8.93	25.9774745	1.94E-01	122118676	122118799	NC_000011.9
cg01010285	8.66E-15	3.13E-16	8.93	25.9767195	7.49E-02	70176103	70176226	NC_000002.11
cg26068180	8.66E-15	3.13E-16	8.93	25.976684	8.02E-02	46053650	46053773	NC_000001.10
cg10528576	8.72E-15	3.15E-16	8.93	25.9703934	2.32E-01	101192913	101193036	NC_000014.8
cg04726439	8.77E-15	3.17E-16	8.93	25.9641324	9.99E-02	18898394	18898517	NC_000019.9
cg18185987	8.78E-15	3.17E-16	8.93	25.9630397	1.34E-01	88477518	88477641	NC_000014.8
cg26191586	8.80E-15	3.18E-16	8.93	25.9603233	8.65E-02	53794760	53794883	NC_000001.10
cg03885098	8.87E-15	3.21E-16	8.93	25.9519063	1.55E-01	207991780	207991903	NC_000001.10
cg06507987	8.88E-15	3.21E-16	8.93	25.9512117	1.38E-01	27686768	27686891	NC_000001.10
cg23816347	8.88E-15	3.21E-16	8.93	25.9503874	2.20E-01	126113640	126113763	NC_000003.11
cg13932450	8.91E-15	3.22E-16	8.93	25.9475153	1.83E-01	43879598	43879721	NC_000006.11
cg06859084	8.92E-15	3.22E-16	8.93	25.9464331	2.05E-01	137523042	137523165	NC_000002.11
cg24531255	8.98E-15	3.25E-16	8.93	25.9388228	2.96E-01	93520012	93520135	NC_000007.13
cg05872540	9.04E-15	3.27E-16	8.92	25.9327602	4.63E-02	17673325	17673448	NC_000020.10
cg07179825	9.05E-15	3.27E-16	8.92	25.9315667	1.84E-01	119635157	119635280	NC_000001.10
cg14741366	9.05E-15	3.27E-16	8.92	25.9314387	5.68E-02	49571568	49571691	NC_000020.10

cg04845915	9.05E-15	3.28E-16	8.92	25.9311428	1.42E-01	140802804	140802927	NC_000005.9
cg14772925	9.05E-15	3.28E-16	8.92	25.9305029	1.50E-01	100998943	100999066	NC_000011.9
cg17555825	9.10E-15	3.29E-16	8.92	25.925862	1.65E-01	76924190	76924313	NC_000005.9
cg19656577	9.10E-15	3.29E-16	8.92	25.9253596	2.55E-01	3600229	3600352	NC_000012.11
cg19742492	9.12E-15	3.30E-16	8.92	25.9227721	9.12E-02	69937541	69937664	NC_000011.9
cg18016181	9.12E-15	3.30E-16	8.92	25.9223838	2.13E-01	117608	117731	NC_000009.11
cg14056306	9.13E-15	3.31E-16	8.92	25.9220672	3.50E-02	73997820	73997943	NC_000017.10
cg25377605	9.14E-15	3.31E-16	8.92	25.9208699	8.73E-02	10513717	10513840	NC_000008.10
cg14153069	9.20E-15	3.33E-16	8.92	25.9139667	1.02E-01	131683257	131683380	NC_000009.11
cg14910055	9.21E-15	3.34E-16	8.92	25.9129823	9.77E-02	43266228	43266351	NC_000022.10
cg27291304	9.27E-15	3.36E-16	8.92	25.9053785	2.02E-01	22193479	22193602	NC_000019.9
cg11894856	9.30E-15	3.37E-16	8.92	25.9024437	3.09E-02	93327173	93327296	NC_000012.11
cg19543317	9.30E-15	3.37E-16	8.92	25.9023084	1.13E-01	75918155	75918278	NC_000014.8
cg13324546	9.32E-15	3.38E-16	8.92	25.9001082	1.94E-01	23564031	23564154	NC_000008.10
cg01834022	9.32E-15	3.38E-16	8.92	25.8991983	2.40E-01	5710411	5710534	NC_000004.11
cg23093589	9.34E-15	3.39E-16	8.92	25.896932	1.87E-01	20824932	20825055	NC_000007.13
cg12523526	9.38E-15	3.41E-16	8.92	25.8925954	2.67E-01	2756005	2756128	NC_000005.9
cg24517501	9.39E-15	3.41E-16	8.92	25.8913669	1.36E-01	92952702	92952825	NC_000001.10
cg08085772	9.39E-15	3.41E-16	8.92	25.8913107	3.97E-02	12218160	12218283	NC_000003.11
cg08228703	9.40E-15	3.41E-16	8.92	25.8908318	2.49E-01	74893097	74893220	NC_000014.8
cg22418909	9.44E-15	3.43E-16	8.92	25.8859812	1.87E-01	41166738	41166861	NC_000008.10
cg12693436	9.46E-15	3.43E-16	8.92	25.8840733	8.27E-02	47130971	47131094	NC_000010.10
cg17805540	9.47E-15	3.44E-16	8.92	25.8835334	1.82E-01	13543730	13543853	NC_000004.11
cg00497967	9.47E-15	3.44E-16	8.92	25.8828971	1.89E-01	77822438	77822561	NC_000016.9
cg17046577	9.47E-15	3.44E-16	8.92	25.8825336	2.04E-01	84815361	84815484	NC_000007.13

cg22570288	9.49E-15	3.45E-16	8.92	25.8808617	5.17E-02	15970272	15970395	NC_000004.11
cg01721450	9.51E-15	3.45E-16	8.92	25.8784223	8.75E-02	127788457	127788580	NC_000003.11
cg23813156	9.53E-15	3.46E-16	8.92	25.8760349	2.26E-01	47935243	47935366	NC_000020.10
cg04986934	9.56E-15	3.47E-16	8.91	25.8731975	5.61E-02	39019908	39020031	NC_000017.10
cg11184748	9.57E-15	3.48E-16	8.91	25.8717661	1.57E-01	36806179	36806302	NC_000003.11
cg25340966	9.57E-15	3.48E-16	8.91	25.8717094	2.21E-01	119532195	119532318	NC_000001.10
cg10441628	9.59E-15	3.48E-16	8.91	25.870145	1.58E-01	159399432	159399555	NC_000005.9
cg03951603	9.59E-15	3.48E-16	8.91	25.8699859	2.01E-01	89903565	89903688	NC_000015.9
cg01346596	9.59E-15	3.48E-16	8.91	25.8699275	1.98E-01	11868146	11868269	NC_000010.10
cg02320543	9.61E-15	3.49E-16	8.91	25.8673923	2.14E-01	61109545	61109668	NC_000014.8
cg09616494	9.65E-15	3.51E-16	8.91	25.8627311	6.43E-02	36333625	36333748	NC_000004.11
cg21437208	9.66E-15	3.51E-16	8.91	25.8618261	2.38E-02	79935213	79935336	NC_000017.10
cg01959730	9.67E-15	3.52E-16	8.91	25.8607191	2.18E-01	119548825	119548948	NC_000001.10
cg27060240	9.70E-15	3.53E-16	8.91	25.8572556	9.59E-02	62412324	62412447	NC_000002.11
cg10297792	9.76E-15	3.55E-16	8.91	25.8511331	1.77E-01	115129446	115129569	NC_000012.11
cg09175749	9.78E-15	3.56E-16	8.91	25.8494184	2.07E-01	4715467	4715590	NC_000001.10
cg01997272	9.78E-15	3.56E-16	8.91	25.8491282	1.68E-01	103740476	103740599	NC_000014.8
cg11277087	9.81E-15	3.57E-16	8.91	25.8454733	1.96E-01	23258096	23258219	NC_000019.9
cg17498773	9.81E-15	3.57E-16	8.91	25.8453643	2.48E-01	107799532	107799655	NC_000011.9
cg21521683	9.83E-15	3.58E-16	8.91	25.8437411	3.20E-01	22487682	22487805	NC_000012.11
cg10741153	9.86E-15	3.59E-16	8.91	25.8406245	1.88E-01	29284353	29284476	NC_000019.9
cg21068030	9.87E-15	3.59E-16	8.91	25.8390326	1.05E-01	7762413	7762536	NC_000017.10
cg11793699	9.89E-15	3.60E-16	8.91	25.8373701	1.69E-01	156814901	156815024	NC_000001.10
cg12446446	9.91E-15	3.61E-16	8.91	25.8347709	1.30E-01	161865479	161865602	NC_000006.11
cg09822423	9.92E-15	3.61E-16	8.91	25.8342799	7.03E-02	192509700	192509823	NC_000001.10

cg07884222	9.95E-15	3.63E-16	8.91	25.8302979	1.56E-01	60302404	60302527	NC_000015.9
cg00433861	9.97E-15	3.63E-16	8.91	25.8282189	1.41E-01	67070170	67070293	NC_000018.9
cg00344801	9.98E-15	3.64E-16	8.91	25.8275077	1.01E-01	46685728	46685851	NC_000022.10
cg00159100	9.98E-15	3.64E-16	8.91	25.827089	3.09E-01	37487633	37487756	NC_000007.13
cg25439758	9.99E-15	3.64E-16	8.91	25.8260869	9.44E-02	67817606	67817729	NC_000011.9
cg07445112	1.00E-14	3.66E-16	8.91	25.8222678	3.69E-02	45628839	45628962	NC_000022.10
cg21507078	1.00E-14	3.66E-16	8.91	25.821036	1.97E-01	138444145	138444268	NC_000005.9
cg03547757	1.01E-14	3.67E-16	8.91	25.8198492	1.10E-01	57425515	57425638	NC_000020.10
cg24715245	1.01E-14	3.68E-16	8.91	25.8170721	1.67E-01	41258794	41258917	NC_000004.11
cg17349389	1.01E-14	3.68E-16	8.91	25.8161262	2.26E-01	80530770	80530893	NC_000002.11
cg21997986	1.01E-14	3.70E-16	8.9	25.8102237	1.10E-01	102745414	102745537	NC_000004.11
cg24524352	1.02E-14	3.71E-16	8.9	25.8078431	2.58E-01	95522876	95522999	NC_000002.11
cg08893109	1.02E-14	3.71E-16	8.9	25.8069984	1.97E-01	6313050	6313173	NC_000007.13
cg05667379	1.02E-14	3.73E-16	8.9	25.8017922	2.12E-01	115920764	115920887	NC_000002.11
cg04748834	1.03E-14	3.75E-16	8.9	25.7982102	2.53E-01	239229	239352	NC_000003.11
cg09009536	1.03E-14	3.75E-16	8.9	25.7974167	1.95E-01	66535383	66535506	NC_000004.11
cg26772847	1.03E-14	3.75E-16	8.9	25.7968381	1.48E-01	62589487	62589610	NC_000015.9
cg22983083	1.03E-14	3.76E-16	8.9	25.7952737	2.14E-01	8656852	8656975	NC_000019.9
cg25553279	1.03E-14	3.76E-16	8.9	25.7951287	1.20E-01	29203014	29203137	NC_000002.11
cg22771960	1.03E-14	3.77E-16	8.9	25.7912301	1.31E-01	3961667	3961790	NC_000019.9
cg15562912	1.04E-14	3.79E-16	8.9	25.7877023	2.17E-01	140770617	140770740	NC_000003.11
cg05784193	1.04E-14	3.79E-16	8.9	25.7871544	1.83E-01	192847589	192847712	NC_000001.10
cg19234613	1.04E-14	3.79E-16	8.9	25.7857565	8.02E-02	107832502	107832625	NC_000012.11
cg05667348	1.04E-14	3.79E-16	8.9	25.7855058	2.46E-01	118892581	118892704	NC_000010.10
cg26659079	1.04E-14	3.80E-16	8.9	25.7842497	1.88E-01	5836181	5836304	NC_000002.11

cg14271531	1.04E-14	3.81E-16	8.9	25.7815798	2.19E-01	19837622	19837745	NC_000006.11
cg06794543	1.05E-14	3.85E-16	8.9	25.7713728	1.62E-01	42749748	42749871	NC_000015.9
cg25024920	1.05E-14	3.85E-16	8.9	25.7700857	5.91E-02	60783915	60784038	NC_000017.10
cg19203065	1.06E-14	3.89E-16	8.9	25.7613982	5.98E-02	11598968	11599091	NC_000002.11
cg24805411	1.07E-14	3.90E-16	8.9	25.7592921	1.76E-01	56158851	56158974	NC_000019.9
cg12989085	1.07E-14	3.90E-16	8.9	25.7592377	5.61E-02	1367637	1367760	NC_000017.10
cg10613684	1.07E-14	3.92E-16	8.9	25.7539115	1.01E-01	245849102	245849225	NC_000001.10
cg19836431	1.08E-14	3.94E-16	8.9	25.7487377	9.33E-02	66620018	66620141	NC_000011.9
cg15762032	1.08E-14	3.95E-16	8.89	25.7454698	2.03E-01	26506933	26507056	NC_000010.10
cg01748892	1.08E-14	3.96E-16	8.89	25.7423449	1.34E-01	27184667	27184790	NC_000007.13
cg22622477	1.09E-14	3.99E-16	8.89	25.7356461	1.44E-01	46685442	46685565	NC_000017.10
cg14932016	1.09E-14	3.99E-16	8.89	25.7355986	2.81E-01	236849653	236849776	NC_000001.10
cg20980783	1.09E-14	4.00E-16	8.89	25.7328669	2.25E-01	67874178	67874301	NC_000008.10
cg17030173	1.09E-14	4.00E-16	8.89	25.7324076	2.54E-01	16180062	16180185	NC_000005.9
cg23093116	1.10E-14	4.03E-16	8.89	25.7266555	6.23E-02	117489843	117489966	NC_000001.10
cg26053547	1.10E-14	4.04E-16	8.89	25.7241972	6.78E-02	77549871	77549994	NC_000018.9
cg22834052	1.10E-14	4.04E-16	8.89	25.7241436	1.30E-01	86362424	86362547	NC_000002.11
cg17059658	1.10E-14	4.04E-16	8.89	25.7239761	2.60E-01	905101	905224	NC_000018.9
cg17763260	1.10E-14	4.04E-16	8.89	25.7233509	6.97E-02	12969611	12969734	NC_000019.9
cg01910938	1.11E-14	4.06E-16	8.89	25.7195756	3.40E-02	136729091	136729214	NC_000003.11
cg13831540	1.11E-14	4.06E-16	8.89	25.7195066	1.30E-01	130128420	130128543	NC_000007.13
cg05341781	1.11E-14	4.06E-16	8.89	25.7185853	1.87E-01	164723567	164723690	NC_000001.10
cg21743182	1.11E-14	4.07E-16	8.89	25.7156317	1.92E-01	90339132	90339255	NC_000007.13
cg24219966	1.11E-14	4.08E-16	8.89	25.7146191	9.85E-02	224950887	224951010	NC_000002.11
cg08511818	1.11E-14	4.08E-16	8.89	25.7129929	1.45E-01	30104637	30104760	NC_000020.10

cg06310927	1.11E-14	4.08E-16	8.89	25.71279	1.47E-01	114292220	114292343	NC_000013.10
cg20219457	1.11E-14	4.09E-16	8.89	25.7120728	3.23E-01	36705446	36705569	NC_000013.10
cg22448090	1.12E-14	4.09E-16	8.89	25.7107807	7.07E-02	200978598	200978721	NC_000001.10
cg13521620	1.12E-14	4.10E-16	8.89	25.708901	1.89E-01	57410687	57410810	NC_000017.10
cg11644424	1.12E-14	4.10E-16	8.89	25.7088186	8.78E-02	157258856	157258979	NC_000006.11
cg14074285	1.12E-14	4.11E-16	8.89	25.7063338	2.19E-01	4868908	4869031	NC_000004.11
cg03013554	1.13E-14	4.14E-16	8.89	25.6982498	1.02E-01	46320816	46320939	NC_000021.8
cg02570644	1.13E-14	4.17E-16	8.89	25.6931743	1.08E-01	68123248	68123371	NC_000016.9
cg05522774	1.14E-14	4.17E-16	8.89	25.6917696	2.22E-01	34443443	34443566	NC_000021.8
cg26643142	1.14E-14	4.17E-16	8.89	25.6917457	1.75E-01	54399494	54399617	NC_000012.11
cg02743070	1.14E-14	4.18E-16	8.89	25.6889278	1.63E-01	80834309	80834432	NC_000010.10
cg27502580	1.14E-14	4.19E-16	8.89	25.6879698	7.98E-02	169247421	169247544	NC_000003.11
cg25241823	1.14E-14	4.19E-16	8.89	25.6872913	1.27E-01	18983753	18983876	NC_000019.9
cg14078730	1.14E-14	4.20E-16	8.89	25.6839655	1.54E-01	63896557	63896680	NC_000011.9
cg21113776	1.15E-14	4.21E-16	8.88	25.6816071	1.01E-01	2499915	2500038	NC_000007.13
cg00340850	1.15E-14	4.22E-16	8.88	25.6809899	1.88E-01	55500523	55500646	NC_000020.10
cg06572465	1.15E-14	4.22E-16	8.88	25.6803243	2.32E-01	140800929	140801052	NC_000005.9
cg05496377	1.15E-14	4.22E-16	8.88	25.6802825	1.86E-01	1776225	1776348	NC_000019.9
cg22284043	1.15E-14	4.22E-16	8.88	25.6799901	2.25E-01	92051576	92051699	NC_000013.10
cg18127922	1.15E-14	4.23E-16	8.88	25.678933	1.42E-01	46672255	46672378	NC_000017.10
cg25305887	1.15E-14	4.23E-16	8.88	25.6773567	6.73E-02	8555579	8555702	NC_000010.10
cg02653557	1.16E-14	4.25E-16	8.88	25.6741182	2.54E-01	58095424	58095547	NC_000019.9
cg17997363	1.16E-14	4.25E-16	8.88	25.6733006	9.36E-02	123353337	123353460	NC_000010.10
cg02508229	1.16E-14	4.25E-16	8.88	25.6728509	1.78E-01	27336989	27337112	NC_000001.10
cg17103314	1.16E-14	4.26E-16	8.88	25.671509	8.05E-02	51487968	51488091	NC_000019.9

cg22598744	1.16E-14	4.26E-16	8.88	25.6714339	1.17E-01	5890866	5890989	NC_000009.11
cg00120810	1.16E-14	4.29E-16	8.88	25.6651412	9.79E-02	11195317	11195440	NC_000003.11
cg11114242	1.17E-14	4.30E-16	8.88	25.6608769	1.64E-01	136756541	136756664	NC_000006.11
cg05224741	1.17E-14	4.32E-16	8.88	25.6563266	2.70E-01	10422322	10422445	NC_000006.11
cg20889445	1.18E-14	4.33E-16	8.88	25.6557976	1.90E-01	111029566	111029689	NC_000012.11
cg23147373	1.18E-14	4.33E-16	8.88	25.6553157	1.02E-01	64954212	64954335	NC_000014.8
cg02205073	1.18E-14	4.33E-16	8.88	25.6551777	1.81E-01	19175822	19175945	NC_000016.9
cg01583131	1.18E-14	4.34E-16	8.88	25.6531849	1.91E-01	27744675	27744798	NC_000011.9
cg07200060	1.18E-14	4.34E-16	8.88	25.6520977	1.76E-01	78483672	78483795	NC_000010.10
cg08966413	1.18E-14	4.35E-16	8.88	25.651389	6.37E-02	23154937	23155060	NC_000016.9
cg10084644	1.18E-14	4.35E-16	8.88	25.6502062	1.93E-01	99775521	99775644	NC_000007.13
cg05620165	1.18E-14	4.36E-16	8.88	25.6472061	9.06E-02	105583493	105583616	NC_000010.10
cg09147777	1.19E-14	4.37E-16	8.88	25.6459836	1.88E-01	105480771	105480894	NC_000011.9
cg11015251	1.19E-14	4.37E-16	8.88	25.645714	1.40E-01	27170554	27170677	NC_000007.13
cg19358877	1.19E-14	4.40E-16	8.88	25.6397802	2.84E-01	57019279	57019402	NC_000019.9
cg25264948	1.19E-14	4.40E-16	8.88	25.6397036	1.29E-01	31734232	31734355	NC_000006.11
cg19628456	1.19E-14	4.40E-16	8.88	25.6387638	8.17E-02	31779381	31779504	NC_000006.11
cg05262549	1.19E-14	4.40E-16	8.88	25.6381785	1.51E-01	183256588	183256711	NC_000001.10
cg21768835	1.20E-14	4.41E-16	8.88	25.6369239	1.16E-01	97857938	97858061	NC_000007.13
cg12050054	1.20E-14	4.41E-16	8.88	25.6367393	6.79E-02	105963656	105963779	NC_000014.8
cg12369869	1.20E-14	4.41E-16	8.88	25.6367146	1.68E-01	184035508	184035631	NC_000003.11
cg06307046	1.20E-14	4.41E-16	8.88	25.6359286	7.39E-02	16685776	16685899	NC_000007.13
cg01856455	1.20E-14	4.42E-16	8.88	25.635456	6.63E-02	22289520	22289643	NC_000022.10
cg15402529	1.20E-14	4.45E-16	8.88	25.628808	1.82E-01	115135333	115135456	NC_000012.11
cg12493761	1.21E-14	4.45E-16	8.88	25.6283256	1.62E-01	43253257	43253380	NC_000012.11

cg15029248	1.21E-14	4.46E-16	8.88	25.6260712	8.50E-02	2379937	2380060	NC_000001.10
cg05663573	1.21E-14	4.46E-16	8.88	25.6258779	2.46E-01	170302890	170303013	NC_000003.11
cg13716840	1.21E-14	4.46E-16	8.88	25.6247869	1.10E-01	956576	956699	NC_000005.9
cg21145136	1.21E-14	4.47E-16	8.88	25.6227184	2.84E-01	84116115	84116238	NC_000015.9
cg10784341	1.21E-14	4.48E-16	8.88	25.622332	1.22E-01	143929300	143929423	NC_000006.11
cg18954144	1.21E-14	4.48E-16	8.88	25.6211233	1.68E-01	5896473	5896596	NC_000002.11
cg00039480	1.21E-14	4.48E-16	8.88	25.6205115	7.85E-02	19620267	19620390	NC_000017.10
cg19076587	1.22E-14	4.49E-16	8.87	25.6182069	9.62E-02	46079554	46079677	NC_000011.9
cg11484576	1.22E-14	4.50E-16	8.87	25.6159499	1.89E-01	63272554	63272677	NC_000011.9
cg07875993	1.22E-14	4.51E-16	8.87	25.6137435	1.19E-01	79096535	79096658	NC_000004.11
cg09034695	1.22E-14	4.52E-16	8.87	25.6127097	9.92E-02	4272875	4272998	NC_000017.10
cg04467618	1.23E-14	4.53E-16	8.87	25.609462	1.78E-01	134210946	134211069	NC_000006.11
cg00006626	1.23E-14	4.55E-16	8.87	25.6069208	1.02E-01	154127462	154127585	NC_000001.10
cg13965272	1.23E-14	4.55E-16	8.87	25.6060919	1.12E-01	117993982	117994105	NC_000009.11
cg16854606	1.23E-14	4.55E-16	8.87	25.6053576	1.38E-01	13080743	13080866	NC_000019.9
cg26279070	1.24E-14	4.60E-16	8.87	25.5956423	1.52E-01	130971343	130971466	NC_000002.11
cg19610529	1.24E-14	4.60E-16	8.87	25.5955862	2.02E-01	61108807	61108930	NC_000014.8
cg07244354	1.25E-14	4.61E-16	8.87	25.5932639	1.25E-01	131757801	131757924	NC_000010.10
cg19449377	1.25E-14	4.61E-16	8.87	25.5927923	1.07E-01	39041199	39041322	NC_000017.10
cg13219127	1.25E-14	4.62E-16	8.87	25.5912875	2.13E-01	1322131	1322254	NC_000011.9
cg02362439	1.25E-14	4.62E-16	8.87	25.5904606	6.66E-02	33171324	33171447	NC_000006.11
cg27376072	1.25E-14	4.63E-16	8.87	25.5886375	5.13E-02	18101567	18101690	NC_000017.10
cg03267742	1.26E-14	4.65E-16	8.87	25.5844408	1.46E-01	686545	686668	NC_000017.10
cg25482786	1.26E-14	4.65E-16	8.87	25.5835445	2.29E-01	238496	238619	NC_000003.11
cg18445088	1.26E-14	4.66E-16	8.87	25.5827963	1.41E-01	40081812	40081935	NC_000022.10

cg13492692	1.26E-14	4.67E-16	8.87	25.5809551	1.79E-01	126777673	126777796	NC_000009.11
cg25368116	1.26E-14	4.67E-16	8.87	25.5800355	1.26E-01	87064843	87064966	NC_000011.9
cg27301032	1.26E-14	4.68E-16	8.87	25.5789083	2.48E-01	112721425	112721548	NC_000013.10
cg07246273	1.26E-14	4.68E-16	8.87	25.5788492	2.57E-01	119212313	119212436	NC_000012.11
cg12973118	1.27E-14	4.69E-16	8.87	25.5749723	1.73E-01	223168941	223169064	NC_000002.11
cg09062462	1.27E-14	4.71E-16	8.87	25.5720054	7.00E-02	85220242	85220365	NC_000001.10
cg03064067	1.27E-14	4.71E-16	8.87	25.5716029	2.47E-01	85306916	85307039	NC_000012.11
cg01381795	1.27E-14	4.72E-16	8.87	25.5700122	1.15E-01	54496611	54496734	NC_000019.9
cg09890775	1.28E-14	4.73E-16	8.87	25.5670278	1.54E-01	131758694	131758817	NC_000010.10
cg00024280	1.29E-14	4.77E-16	8.87	25.5599663	1.87E-01	17501257	17501380	NC_000019.9
cg01148073	1.29E-14	4.78E-16	8.87	25.5574091	1.30E-01	82126267	82126390	NC_000016.9
cg16230141	1.30E-14	4.81E-16	8.86	25.5501967	2.46E-01	37488162	37488285	NC_000007.13
cg10823473	1.30E-14	4.82E-16	8.86	25.5488299	1.48E-01	76630514	76630637	NC_000015.9
cg10989897	1.30E-14	4.83E-16	8.86	25.5468851	2.30E-01	50513645	50513768	NC_000001.10
cg14151976	1.31E-14	4.85E-16	8.86	25.5435572	1.17E-01	37565559	37565682	NC_000009.11
cg21982950	1.31E-14	4.85E-16	8.86	25.5424911	1.86E-01	1876200	1876323	NC_000005.9
cg14062537	1.31E-14	4.87E-16	8.86	25.5387712	1.55E-01	93272333	93272456	NC_000010.10
cg07698121	1.31E-14	4.87E-16	8.86	25.538176	1.84E-01	85659946	85660069	NC_000016.9
cg05111081	1.31E-14	4.88E-16	8.86	25.537073	1.20E-01	92425496	92425619	NC_000015.9
cg12075445	1.32E-14	4.89E-16	8.86	25.5343803	2.84E-01	132906010	132906133	NC_000012.11
cg12524640	1.32E-14	4.90E-16	8.86	25.5329727	1.47E-01	2983056	2983179	NC_000001.10
cg15829622	1.32E-14	4.91E-16	8.86	25.5313535	4.81E-02	45730731	45730854	NC_000021.8
cg21100518	1.32E-14	4.91E-16	8.86	25.5305704	1.29E-01	29595002	29595125	NC_000006.11
cg03512426	1.32E-14	4.92E-16	8.86	25.5289599	1.36E-01	131763683	131763806	NC_000010.10
cg04431946	1.33E-14	4.93E-16	8.86	25.5262005	2.67E-01	47797569	47797692	NC_000002.11

cg12935350	1.33E-14	4.96E-16	8.86	25.5203264	1.81E-01	105697777	105697900	NC_000002.11
cg09792881	1.33E-14	4.96E-16	8.86	25.5201473	2.29E-01	50884544	50884667	NC_000001.10
cg20808078	1.33E-14	4.96E-16	8.86	25.5200168	6.61E-02	68515209	68515332	NC_000001.10
cg25482091	1.34E-14	4.99E-16	8.86	25.515621	8.18E-02	143478766	143478889	NC_000002.11
cg13208922	1.34E-14	4.99E-16	8.86	25.5142578	1.87E-01	299022	299145	NC_000004.11
cg08963972	1.34E-14	5.00E-16	8.86	25.5136725	8.75E-02	5021694	5021817	NC_000004.11
cg26092180	1.35E-14	5.01E-16	8.86	25.5106426	4.30E-02	108310912	108311035	NC_000003.11
cg21907850	1.35E-14	5.02E-16	8.86	25.5095781	1.44E-01	186130024	186130147	NC_000004.11
cg19836595	1.35E-14	5.02E-16	8.86	25.5083865	8.53E-02	115395513	115395636	NC_000003.11
cg15680755	1.35E-14	5.03E-16	8.86	25.507187	2.25E-01	54411668	54411791	NC_000019.9
cg20332645	1.35E-14	5.03E-16	8.86	25.5059882	1.71E-01	1400869	1400992	NC_000004.11
cg18406934	1.35E-14	5.04E-16	8.86	25.505178	1.41E-01	23183238	23183361	NC_000008.10
cg07370496	1.35E-14	5.04E-16	8.86	25.5051509	1.13E-01	36806213	36806336	NC_000003.11
cg00991875	1.35E-14	5.04E-16	8.86	25.5050868	2.09E-01	161100376	161100499	NC_000006.11
cg13609167	1.35E-14	5.04E-16	8.86	25.5044463	7.55E-02	2827061	2827184	NC_000004.11
cg22070991	1.36E-14	5.05E-16	8.86	25.503075	1.89E-01	4856904	4857027	NC_000004.11
cg27454136	1.36E-14	5.05E-16	8.86	25.5026237	2.60E-01	5889876	5889999	NC_000004.11
cg04598121	1.36E-14	5.06E-16	8.86	25.5017636	1.78E-01	57358505	57358628	NC_000008.10
cg08862890	1.36E-14	5.06E-16	8.86	25.5005751	3.42E-01	169064451	169064574	NC_000005.9
cg11197258	1.36E-14	5.07E-16	8.86	25.4997347	2.26E-01	124845001	124845124	NC_000012.11
cg23597629	1.37E-14	5.10E-16	8.85	25.4930874	1.71E-01	229569670	229569793	NC_000001.10
cg21565421	1.37E-14	5.10E-16	8.85	25.4927275	1.36E-01	74592665	74592788	NC_000015.9
cg16357388	1.37E-14	5.10E-16	8.85	25.4923514	2.11E-01	5892083	5892206	NC_000004.11
cg08441170	1.37E-14	5.11E-16	8.85	25.49099	2.72E-01	26223100	26223223	NC_000010.10
cg05058758	1.37E-14	5.12E-16	8.85	25.4896475	1.78E-01	143784246	143784369	NC_000002.11

cg24996709	1.38E-14	5.16E-16	8.85	25.4807102	1.28E-01	33170838	33170961	NC_000006.11
cg21889448	1.39E-14	5.17E-16	8.85	25.4799929	1.53E-01	62637815	62637938	NC_000004.11
cg17307442	1.39E-14	5.17E-16	8.85	25.4796138	2.38E-01	119302053	119302176	NC_000010.10
cg01240056	1.39E-14	5.20E-16	8.85	25.4734035	2.45E-01	1263927	1264050	NC_000007.13
cg07676859	1.39E-14	5.20E-16	8.85	25.4729999	2.61E-01	23015932	23016055	NC_000020.10
cg14662355	1.40E-14	5.21E-16	8.85	25.4716573	2.29E-01	165324810	165324933	NC_000001.10
cg20746830	1.40E-14	5.22E-16	8.85	25.4692731	8.41E-02	94586670	94586793	NC_000001.10
cg07022343	1.40E-14	5.23E-16	8.85	25.4684505	1.57E-01	230208170	230208293	NC_000001.10
cg21301879	1.41E-14	5.25E-16	8.85	25.4652076	6.13E-02	128148379	128148502	NC_000002.11
cg10799327	1.41E-14	5.25E-16	8.85	25.4644487	1.30E-01	170484840	170484963	NC_000003.11
cg02760031	1.41E-14	5.25E-16	8.85	25.4639206	2.13E-01	591151	591274	NC_000020.10
cg13316720	1.41E-14	5.27E-16	8.85	25.4613441	1.29E-01	25840984	25841107	NC_000017.10
cg04961466	1.41E-14	5.27E-16	8.85	25.4610718	2.00E-01	164264985	164265108	NC_000004.11
cg19971655	1.41E-14	5.27E-16	8.85	25.4608194	6.74E-02	55464927	55465050	NC_000001.10
cg02915259	1.42E-14	5.29E-16	8.85	25.4575461	5.57E-02	60224185	60224308	NC_000001.10
cg01860778	1.42E-14	5.30E-16	8.85	25.4558301	9.62E-02	26189510	26189633	NC_000020.10
cg05203875	1.42E-14	5.30E-16	8.85	25.4545349	1.20E-01	1801294	1801417	NC_000004.11
cg03057083	1.42E-14	5.31E-16	8.85	25.4535063	2.78E-01	12995300	12995423	NC_000016.9
cg00589850	1.42E-14	5.31E-16	8.85	25.4528225	1.72E-01	140857995	140858118	NC_000005.9
cg00247557	1.42E-14	5.32E-16	8.85	25.4512881	1.86E-01	5197071	5197194	NC_000018.9
cg25773223	1.42E-14	5.32E-16	8.85	25.4505707	9.32E-02	3680061	3680184	NC_000010.10
cg14741726	1.43E-14	5.34E-16	8.85	25.4482662	1.08E-01	3475262	3475385	NC_000004.11
cg03936436	1.43E-14	5.34E-16	8.85	25.447426	7.52E-02	67962191	67962314	NC_000016.9
cg18328792	1.44E-14	5.39E-16	8.85	25.4387424	1.10E-01	90417102	90417225	NC_000014.8
cg26562772	1.44E-14	5.40E-16	8.85	25.4369644	2.11E-01	221890431	221890554	NC_000001.10

cg04418162	1.44E-14	5.40E-16	8.85	25.4367687	1.41E-01	125687269	125687392	NC_000003.11
cg10277836	1.45E-14	5.42E-16	8.85	25.4329377	1.47E-01	32446172	32446295	NC_000002.11
cg00295382	1.46E-14	5.45E-16	8.84	25.427753	1.81E-01	40364870	40364993	NC_000001.10
cg11711057	1.46E-14	5.48E-16	8.84	25.4228652	1.66E-01	107974444	107974567	NC_000012.11
cg12636882	1.46E-14	5.48E-16	8.84	25.4226727	1.56E-01	3180930	3181053	NC_000004.11
cg17336584	1.47E-14	5.49E-16	8.84	25.4206739	1.95E-01	12750256	12750379	NC_000006.11
cg12926104	1.47E-14	5.51E-16	8.84	25.4160665	2.40E-01	19157339	19157462	NC_000007.13
cg00130866	1.47E-14	5.52E-16	8.84	25.4140461	7.73E-02	124052565	124052688	NC_000008.10
cg14543200	1.48E-14	5.54E-16	8.84	25.4111419	1.30E-01	61977951	61978074	NC_000014.8
cg23650423	1.48E-14	5.54E-16	8.84	25.4106311	2.53E-01	101454676	101454799	NC_000011.9
cg04569082	1.48E-14	5.55E-16	8.84	25.4099364	2.50E-01	127940323	127940446	NC_000012.11
cg05619892	1.49E-14	5.58E-16	8.84	25.4046225	2.46E-01	40755187	40755310	NC_000008.10
cg18034859	1.51E-14	5.67E-16	8.84	25.3881891	6.44E-02	30406688	30406811	NC_000020.10
cg04574459	1.52E-14	5.69E-16	8.84	25.3850283	2.63E-01	47010768	47010891	NC_000021.8
cg14619806	1.53E-14	5.73E-16	8.84	25.3779648	1.07E-01	125768098	125768221	NC_000010.10
cg02798576	1.53E-14	5.73E-16	8.84	25.377711	2.07E-01	101290168	101290291	NC_000010.10
cg15543616	1.53E-14	5.75E-16	8.84	25.3751055	2.13E-01	5019024	5019147	NC_000012.11
cg03603018	1.53E-14	5.76E-16	8.84	25.3726963	8.68E-02	25298714	25298837	NC_000001.10
cg06243393	1.54E-14	5.79E-16	8.84	25.3683104	4.47E-02	9885015	9885138	NC_000018.9
cg08732352	1.54E-14	5.79E-16	8.84	25.3675209	2.35E-01	35298391	35298514	NC_000007.13
cg06520279	1.55E-14	5.83E-16	8.83	25.3609368	9.79E-02	44236918	44237041	NC_000018.9
cg12463703	1.55E-14	5.83E-16	8.83	25.3603701	9.03E-02	127007401	127007524	NC_000003.11
cg11596779	1.55E-14	5.84E-16	8.83	25.3593938	8.14E-02	77188947	77189070	NC_000005.9
cg17165841	1.56E-14	5.85E-16	8.83	25.3568016	2.10E-01	207507001	207507124	NC_000002.11
cg07161721	1.56E-14	5.88E-16	8.83	25.3519674	2.50E-01	72459953	72460076	NC_000008.10

cg16679297	1.56E-14	5.89E-16	8.83	25.3507931	1.76E-01	168534069	168534192	NC_000005.9
cg11957331	1.57E-14	5.90E-16	8.83	25.3493008	2.67E-01	42828156	42828279	NC_000019.9
cg17066932	1.57E-14	5.91E-16	8.83	25.347777	1.07E-01	4312184	4312307	NC_000010.10
cg03572760	1.57E-14	5.91E-16	8.83	25.3472326	9.93E-02	42287595	42287718	NC_000017.10
cg15479964	1.57E-14	5.91E-16	8.83	25.3467302	4.65E-02	99341525	99341648	NC_000010.10
cg24481035	1.57E-14	5.92E-16	8.83	25.3463414	6.15E-02	54388422	54388545	NC_000019.9
cg01105140	1.58E-14	5.94E-16	8.83	25.341509	1.77E-01	149137869	149137992	NC_000001.10
cg12306833	1.58E-14	5.96E-16	8.83	25.3395948	7.86E-02	50227389	50227512	NC_000003.11
cg23036678	1.58E-14	5.97E-16	8.83	25.3381621	1.12E-01	70002831	70002954	NC_000011.9
cg05748163	1.58E-14	5.97E-16	8.83	25.3375808	1.62E-01	25897763	25897886	NC_000008.10
cg15291740	1.59E-14	6.00E-16	8.83	25.3331575	1.79E-01	48478766	48478889	NC_000017.10
cg11213369	1.59E-14	6.00E-16	8.83	25.332614	1.96E-01	1270925	1271048	NC_000007.13
cg25560398	1.59E-14	6.00E-16	8.83	25.3318686	8.42E-02	233252170	233252293	NC_000002.11
cg26764362	1.60E-14	6.03E-16	8.83	25.3281032	7.91E-02	45451585	45451708	NC_000006.11
cg16101806	1.61E-14	6.06E-16	8.83	25.323123	4.24E-02	10701439	10701562	NC_000005.9
cg07936950	1.61E-14	6.06E-16	8.83	25.322763	2.33E-01	172953134	172953257	NC_000002.11
cg24129390	1.61E-14	6.07E-16	8.83	25.3201071	1.74E-01	33780205	33780328	NC_000013.10
cg03984866	1.62E-14	6.12E-16	8.83	25.3127494	1.80E-01	8161776	8161899	NC_000004.11
cg27600205	1.63E-14	6.14E-16	8.83	25.3089615	2.24E-01	134070416	134070539	NC_000004.11
cg16599703	1.63E-14	6.15E-16	8.83	25.3078919	1.96E-01	37500358	37500481	NC_000001.10
cg05356754	1.63E-14	6.16E-16	8.83	25.306771	9.37E-02	24685985	24686108	NC_000014.8
cg10366093	1.65E-14	6.21E-16	8.82	25.2977498	2.10E-01	57414457	57414580	NC_000011.9
cg01823510	1.65E-14	6.22E-16	8.82	25.2971372	2.01E-01	242776354	242776477	NC_000002.11
cg23037403	1.65E-14	6.22E-16	8.82	25.2969968	2.03E-01	178368183	178368306	NC_000005.9
cg02402744	1.65E-14	6.22E-16	8.82	25.2959919	1.31E-01	88261612	88261735	NC_000016.9

cg17478228	1.65E-14	6.23E-16	8.82	25.2946332	1.32E-01	11591593	11591716	NC_000019.9
cg07034660	1.65E-14	6.24E-16	8.82	25.2929911	2.55E-01	38670412	38670535	NC_000007.13
cg13422257	1.67E-14	6.30E-16	8.82	25.2839324	4.85E-02	125801889	125802012	NC_000010.10
cg20870512	1.67E-14	6.32E-16	8.82	25.2804385	2.76E-01	1272515	1272638	NC_000007.13
cg08123207	1.68E-14	6.35E-16	8.82	25.2769164	1.16E-01	32118312	32118435	NC_000006.11
cg13693652	1.68E-14	6.35E-16	8.82	25.2768048	4.84E-02	86888629	86888752	NC_000001.10
cg21786381	1.68E-14	6.35E-16	8.82	25.2759526	1.34E-01	75234078	75234201	NC_000011.9
cg03261948	1.68E-14	6.37E-16	8.82	25.2735227	1.10E-01	30140417	30140540	NC_000006.11
cg09233651	1.69E-14	6.39E-16	8.82	25.2696881	2.06E-01	114838567	114838690	NC_000012.11
cg15082473	1.69E-14	6.40E-16	8.82	25.269063	5.24E-02	114801587	114801710	NC_000013.10
cg12052661	1.70E-14	6.42E-16	8.82	25.2652027	1.30E-01	140772545	140772668	NC_000009.11
cg23833588	1.70E-14	6.44E-16	8.82	25.2616495	3.05E-01	46961583	46961706	NC_000013.10
cg14392740	1.70E-14	6.45E-16	8.82	25.2605139	4.93E-02	44832616	44832739	NC_000015.9
cg15627072	1.71E-14	6.47E-16	8.82	25.257434	1.04E-01	2432621	2432744	NC_000001.10
cg12032648	1.71E-14	6.48E-16	8.82	25.2569439	1.97E-01	166676897	166677020	NC_000002.11
cg01401365	1.71E-14	6.49E-16	8.82	25.2541781	5.31E-02	75314761	75314884	NC_000015.9
cg06439005	1.72E-14	6.51E-16	8.82	25.2518647	1.48E-01	172754848	172754971	NC_000005.9
cg15904939	1.72E-14	6.51E-16	8.82	25.2512951	9.30E-02	1828124	1828247	NC_000019.9
cg15953602	1.73E-14	6.57E-16	8.82	25.2426012	2.04E-01	75896720	75896843	NC_000008.10
cg06833732	1.73E-14	6.57E-16	8.82	25.242041	2.51E-01	175299035	175299158	NC_000005.9
cg08432727	1.73E-14	6.58E-16	8.82	25.2411355	1.86E-01	5831722	5831845	NC_000002.11
cg13519194	1.74E-14	6.59E-16	8.81	25.2394177	1.06E-01	43539572	43539695	NC_000010.10
cg01223193	1.74E-14	6.61E-16	8.81	25.2360625	7.14E-02	89002822	89002945	NC_000015.9
cg15961092	1.74E-14	6.62E-16	8.81	25.2350486	4.49E-02	45681130	45681253	NC_000014.8
cg15353031	1.75E-14	6.63E-16	8.81	25.2338352	2.00E-01	50887632	50887755	NC_000010.10

cg16605327	1.75E-14	6.63E-16	8.81	25.2337787	2.28E-01	114846849	114846972	NC_000012.11
cg24971209	1.75E-14	6.63E-16	8.81	25.2333897	2.32E-01	144242106	144242229	NC_000008.10
cg26349474	1.75E-14	6.65E-16	8.81	25.2314234	1.78E-01	46705637	46705760	NC_000017.10
cg11017065	1.75E-14	6.66E-16	8.81	25.2290393	3.62E-01	128564874	128564997	NC_000011.9
cg12799666	1.76E-14	6.68E-16	8.81	25.2260567	1.23E-01	44066402	44066525	NC_000015.9
cg05583014	1.76E-14	6.69E-16	8.81	25.224735	1.08E-01	48509019	48509142	NC_000003.11
cg15961473	1.76E-14	6.70E-16	8.81	25.2231054	8.05E-02	36646362	36646485	NC_000017.10
cg11994115	1.77E-14	6.71E-16	8.81	25.2217172	9.60E-02	40360856	40360979	NC_000019.9
cg18425434	1.77E-14	6.72E-16	8.81	25.2198609	1.85E-01	118429648	118429771	NC_000010.10
cg06935856	1.77E-14	6.73E-16	8.81	25.218971	7.02E-02	7701546	7701669	NC_000017.10
cg04027805	1.78E-14	6.76E-16	8.81	25.2149359	1.50E-01	4715479	4715602	NC_000001.10
cg02941385	1.78E-14	6.76E-16	8.81	25.2140226	9.51E-02	69284017	69284140	NC_000014.8
cg14970666	1.78E-14	6.76E-16	8.81	25.2139759	2.03E-01	2038989	2039112	NC_000005.9
cg09743950	1.78E-14	6.78E-16	8.81	25.2113855	1.84E-01	31341875	31341998	NC_000016.9
cg09969797	1.79E-14	6.80E-16	8.81	25.2086502	8.86E-02	150543360	150543483	NC_000007.13
cg11664500	1.79E-14	6.80E-16	8.81	25.208441	2.63E-01	133562479	133562602	NC_000006.11
cg14037665	1.79E-14	6.82E-16	8.81	25.2054491	2.24E-01	1748617	1748740	NC_000002.11
cg04539515	1.80E-14	6.84E-16	8.81	25.2033061	1.62E-01	221068794	221068917	NC_000001.10
cg04642105	1.80E-14	6.85E-16	8.81	25.2013149	1.54E-01	1239119	1239242	NC_000004.11
cg20704028	1.81E-14	6.87E-16	8.81	25.1982977	7.03E-02	121493706	121493829	NC_000010.10
cg04622125	1.81E-14	6.87E-16	8.81	25.1980018	9.38E-02	46876658	46876781	NC_000021.8
cg06536868	1.81E-14	6.88E-16	8.81	25.19739	1.58E-01	27530884	27531007	NC_000002.11
cg01486814	1.81E-14	6.88E-16	8.81	25.1970167	1.71E-01	88799186	88799309	NC_000011.9
cg26055812	1.81E-14	6.88E-16	8.81	25.1965872	1.35E-01	52198580	52198703	NC_000001.10
cg07466788	1.81E-14	6.89E-16	8.81	25.1949503	1.27E-01	80194552	80194675	NC_000017.10

cg13677149	1.81E-14	6.90E-16	8.81	25.1936859	1.92E-01	27284789	27284912	NC_000007.13
cg03734874	1.82E-14	6.93E-16	8.81	25.1903602	2.06E-01	105071382	105071505	NC_000014.8
cg11561737	1.82E-14	6.95E-16	8.81	25.186676	1.91E-01	46796908	46797031	NC_000017.10
cg02145319	1.83E-14	6.99E-16	8.81	25.1807989	4.94E-02	339492	339615	NC_000016.9
cg13281139	1.84E-14	7.02E-16	8.8	25.1776957	2.55E-01	20255061	20255184	NC_000004.11
cg18553305	1.84E-14	7.02E-16	8.8	25.1771105	5.36E-02	3112665	3112788	NC_000012.11
cg27049766	1.84E-14	7.02E-16	8.8	25.176512	2.72E-01	58220516	58220639	NC_000019.9
cg07212778	1.85E-14	7.05E-16	8.8	25.1730193	2.05E-01	119916510	119916633	NC_000002.11
cg14491284	1.85E-14	7.06E-16	8.8	25.1711064	1.11E-01	74846046	74846169	NC_000004.11
cg25473201	1.87E-14	7.12E-16	8.8	25.1634683	7.13E-02	168677942	168678065	NC_000006.11
cg13577778	1.87E-14	7.13E-16	8.8	25.1620424	6.93E-02	69481517	69481640	NC_000014.8
cg06940614	1.87E-14	7.13E-16	8.8	25.1613345	2.43E-01	38182773	38182896	NC_000019.9
cg06566087	1.87E-14	7.14E-16	8.8	25.1609847	9.89E-02	234655709	234655832	NC_000002.11
cg15377283	1.87E-14	7.15E-16	8.8	25.159257	2.11E-01	55371369	55371492	NC_000008.10
cg21853871	1.87E-14	7.15E-16	8.8	25.1584843	1.60E-01	46628717	46628840	NC_000017.10
cg04319462	1.87E-14	7.16E-16	8.8	25.1581793	1.45E-01	33284965	33285088	NC_000006.11
cg19161124	1.88E-14	7.18E-16	8.8	25.154683	1.43E-01	153583563	153583686	NC_000007.13
cg02216394	1.88E-14	7.19E-16	8.8	25.1539157	9.06E-02	30178715	30178838	NC_000006.11
cg18141888	1.89E-14	7.22E-16	8.8	25.148956	1.72E-01	21487412	21487535	NC_000020.10
cg07052737	1.89E-14	7.22E-16	8.8	25.1488755	1.31E-01	26224428	26224551	NC_000002.11
cg21817858	1.89E-14	7.23E-16	8.8	25.1481902	2.00E-01	141133337	141133460	NC_000005.9
cg12599275	1.89E-14	7.23E-16	8.8	25.1479421	9.89E-02	3011274	3011397	NC_000001.10
cg24830559	1.90E-14	7.24E-16	8.8	25.1462063	7.48E-02	7048926	7049049	NC_000004.11
cg08709276	1.90E-14	7.26E-16	8.8	25.1433465	1.15E-01	41020450	41020573	NC_000006.11
cg05533326	1.91E-14	7.28E-16	8.8	25.1406543	3.12E-02	29128686	29128809	NC_000016.9

cg24622589	1.91E-14	7.29E-16	8.8	25.1402613	1.68E-01	58613446	58613569	NC_000003.11
cg06445348	1.91E-14	7.29E-16	8.8	25.1399088	3.01E-01	166917009	166917132	NC_000001.10
cg19380001	1.91E-14	7.30E-16	8.8	25.1389425	2.09E-01	50889427	50889550	NC_000001.10
cg16445423	1.91E-14	7.31E-16	8.8	25.1376306	1.49E-01	98609870	98609993	NC_000002.11
cg15231989	1.91E-14	7.31E-16	8.8	25.1372305	1.59E-01	2742433	2742556	NC_000007.13
cg15044957	1.92E-14	7.32E-16	8.8	25.1352952	2.25E-01	30038672	30038795	NC_000011.9
cg13481132	1.92E-14	7.32E-16	8.8	25.135249	2.13E-01	138606811	138606934	NC_000009.11
cg06745378	1.92E-14	7.32E-16	8.8	25.1351759	1.25E-01	55841452	55841575	NC_000002.11
cg23448584	1.92E-14	7.34E-16	8.8	25.1335077	2.66E-01	2781316	2781439	NC_000020.10
cg01070209	1.92E-14	7.35E-16	8.8	25.132159	2.35E-01	119419331	119419454	NC_000012.11
cg12236088	1.92E-14	7.35E-16	8.8	25.1311953	1.20E-01	105275812	105275935	NC_000002.11
cg18487598	1.93E-14	7.38E-16	8.8	25.1279793	1.19E-01	178735372	178735495	NC_000005.9
cg21858380	1.93E-14	7.38E-16	8.8	25.1275219	2.08E-01	119916486	119916609	NC_000002.11
cg09232478	1.93E-14	7.39E-16	8.8	25.1263975	2.09E-01	119548852	119548975	NC_000001.10
cg18237607	1.93E-14	7.40E-16	8.8	25.1245761	2.64E-01	37840075	37840198	NC_000005.9
cg24692310	1.94E-14	7.42E-16	8.8	25.1229466	1.57E-01	60915630	60915753	NC_000011.9
cg00382227	1.94E-14	7.43E-16	8.8	25.1216224	1.49E-01	334557	334680	NC_000016.9
cg00091285	1.95E-14	7.45E-16	8.8	25.1180719	2.00E-01	98511031	98511154	NC_000001.10
cg21921016	1.95E-14	7.48E-16	8.79	25.1145624	1.35E-01	42330638	42330761	NC_000008.10
cg19753174	1.95E-14	7.48E-16	8.79	25.1142061	1.32E-01	219351218	219351341	NC_000001.10
cg03251349	1.95E-14	7.49E-16	8.79	25.1133453	6.20E-02	30104916	30105039	NC_000006.11
cg16764637	1.96E-14	7.50E-16	8.79	25.1123553	1.52E-01	27194731	27194854	NC_000007.13
cg27444913	1.96E-14	7.50E-16	8.79	25.1118669	1.21E-01	230244525	230244648	NC_000001.10
cg26149682	1.97E-14	7.54E-16	8.79	25.1059899	1.41E-01	10417663	10417786	NC_000006.11
cg12114834	1.97E-14	7.57E-16	8.79	25.1031032	2.27E-01	122848518	122848641	NC_000011.9

cg04598517	1.97E-14	7.57E-16	8.79	25.1022099	2.57E-01	1445542	1445665	NC_000005.9
cg15362695	1.98E-14	7.58E-16	8.79	25.1009476	6.52E-02	156954183	156954306	NC_000006.11
cg12071888	1.98E-14	7.58E-16	8.79	25.1008872	2.07E-01	91195159	91195282	NC_000001.10
cg15798385	1.98E-14	7.61E-16	8.79	25.0968499	1.78E-01	27281569	27281692	NC_000007.13
cg14961476	1.99E-14	7.64E-16	8.79	25.0931279	2.11E-01	9130738	9130861	NC_000001.10
cg20279673	2.00E-14	7.66E-16	8.79	25.0904023	2.29E-01	17850431	17850554	NC_000022.10
cg03190944	2.01E-14	7.71E-16	8.79	25.0839634	9.14E-02	88388231	88388354	NC_000016.9
cg19897071	2.01E-14	7.72E-16	8.79	25.0831122	1.57E-01	35991382	35991505	NC_000005.9
cg16182986	2.02E-14	7.74E-16	8.79	25.0810721	1.95E-01	16180419	16180542	NC_000005.9
cg17029062	2.02E-14	7.75E-16	8.79	25.0798776	2.21E-01	32859445	32859568	NC_000003.11
cg19025113	2.02E-14	7.75E-16	8.79	25.0787664	1.87E-01	45155201	45155324	NC_000002.11
cg05756220	2.02E-14	7.76E-16	8.79	25.0785909	2.06E-01	30038685	30038808	NC_000011.9
cg02640041	2.02E-14	7.77E-16	8.79	25.0767424	2.07E-01	33936520	33936643	NC_000005.9
cg27348223	2.03E-14	7.81E-16	8.79	25.0719036	2.07E-01	1445354	1445477	NC_000005.9
cg01569660	2.04E-14	7.82E-16	8.79	25.0703798	8.39E-02	65369213	65369336	NC_000015.9
cg27337021	2.04E-14	7.85E-16	8.79	25.0661744	4.83E-02	139947627	139947750	NC_000005.9
cg19127283	2.05E-14	7.86E-16	8.79	25.0652371	2.51E-01	32859407	32859530	NC_000003.11
cg15149095	2.05E-14	7.86E-16	8.79	25.0650301	1.96E-01	210111672	210111795	NC_000001.10
cg07239452	2.05E-14	7.87E-16	8.79	25.0646067	3.55E-02	1864422	1864545	NC_000008.10
cg05507514	2.05E-14	7.88E-16	8.79	25.062455	1.07E-01	153842846	153842969	NC_000003.11
cg18274480	2.06E-14	7.91E-16	8.79	25.0593898	1.74E-01	145713863	145713986	NC_000005.9
cg08951901	2.07E-14	7.95E-16	8.79	25.0535352	6.92E-02	32052293	32052416	NC_000006.11
cg21244177	2.07E-14	7.97E-16	8.79	25.0522633	1.56E-01	63795848	63795971	NC_000001.10
cg17341170	2.07E-14	7.98E-16	8.78	25.0501078	9.11E-02	2481545	2481668	NC_000011.9
cg15044699	2.08E-14	8.01E-16	8.78	25.0472537	5.94E-02	100081382	100081505	NC_000010.10

cg26530498	2.08E-14	8.01E-16	8.78	25.0464349	2.07E-01	119916431	119916554	NC_000002.11
cg04453180	2.10E-14	8.06E-16	8.78	25.0401524	2.09E-01	140856330	140856453	NC_000005.9
cg20495476	2.10E-14	8.08E-16	8.78	25.0374894	9.36E-02	32506497	32506620	NC_000022.10
cg19285478	2.10E-14	8.09E-16	8.78	25.0364207	1.57E-01	63849412	63849535	NC_000011.9
cg08934976	2.10E-14	8.10E-16	8.78	25.0357692	6.07E-02	1264529	1264652	NC_000005.9
cg02051616	2.11E-14	8.13E-16	8.78	25.0316662	1.56E-01	172661132	172661255	NC_000005.9
cg24543937	2.13E-14	8.20E-16	8.78	25.0238372	1.35E-01	71282102	71282225	NC_000017.10
cg10822665	2.13E-14	8.21E-16	8.78	25.0224026	1.08E-01	1028394	1028517	NC_000017.10
cg05313771	2.14E-14	8.23E-16	8.78	25.0201208	1.87E-01	1762435	1762558	NC_000019.9
cg15699267	2.14E-14	8.25E-16	8.78	25.0180021	2.44E-01	61809557	61809680	NC_000020.10
cg08579468	2.14E-14	8.25E-16	8.78	25.0173229	8.71E-02	81324215	81324338	NC_000016.9
cg22129809	2.14E-14	8.25E-16	8.78	25.0169831	8.25E-02	139260099	139260222	NC_000003.11
cg15712559	2.15E-14	8.29E-16	8.78	25.0130372	2.66E-01	58180616	58180739	NC_000020.10
cg02256631	2.15E-14	8.29E-16	8.78	25.0122322	2.80E-01	31342952	31343075	NC_000016.9
cg01574663	2.15E-14	8.30E-16	8.78	25.01131	1.51E-01	22825769	22825892	NC_000016.9
cg24249925	2.16E-14	8.31E-16	8.78	25.0104094	1.51E-01	162702571	162702694	NC_000001.10
cg05392323	2.16E-14	8.32E-16	8.78	25.0097019	1.47E-01	23698143	23698266	NC_000001.10
cg00469207	2.16E-14	8.32E-16	8.78	25.0087283	2.76E-01	152622455	152622578	NC_000007.13
cg24292761	2.16E-14	8.33E-16	8.78	25.0074332	1.71E-01	117584665	117584788	NC_000006.11
cg10443049	2.17E-14	8.35E-16	8.78	25.0053391	2.58E-01	8481537	8481660	NC_000007.13
cg13445199	2.17E-14	8.36E-16	8.78	25.0049889	2.63E-01	10459132	10459255	NC_000004.11
cg20985014	2.17E-14	8.38E-16	8.78	25.0022931	6.03E-02	112374151	112374274	NC_000006.11
cg06845853	2.17E-14	8.38E-16	8.78	25.0021113	1.90E-01	32461240	32461363	NC_000011.9
cg17284168	2.18E-14	8.42E-16	8.78	24.9979155	2.19E-01	134491531	134491654	NC_000006.11
cg11390378	2.18E-14	8.42E-16	8.78	24.9971862	2.35E-01	147576227	147576350	NC_000004.11

cg23918610	2.19E-14	8.47E-16	8.78	24.9917523	3.50E-02	119018141	119018264	NC_000008.10
cg26018544	2.19E-14	8.47E-16	8.78	24.9912286	6.03E-02	25230915	25231038	NC_000001.10
cg23054883	2.20E-14	8.49E-16	8.78	24.9897107	1.84E-01	130647580	130647703	NC_000012.11
cg17839324	2.20E-14	8.51E-16	8.77	24.987154	1.22E-01	3123500	3123623	NC_000001.10
cg16712637	2.22E-14	8.57E-16	8.77	24.9794099	1.78E-01	103352000	103352123	NC_000012.11
cg23901967	2.23E-14	8.61E-16	8.77	24.9753761	1.26E-01	36890321	36890444	NC_000017.10
cg13163930	2.24E-14	8.65E-16	8.77	24.9707969	2.58E-01	27647896	27648019	NC_000006.11
cg14328457	2.24E-14	8.66E-16	8.77	24.9692619	2.14E-01	37381475	37381598	NC_000017.10
cg03707749	2.25E-14	8.69E-16	8.77	24.9655808	1.85E-01	86240461	86240584	NC_000012.11
cg19524723	2.25E-14	8.70E-16	8.77	24.964551	1.29E-01	60052048	60052171	NC_000018.9
cg03874199	2.25E-14	8.71E-16	8.77	24.9642631	1.91E-01	176964456	176964579	NC_000002.11
cg07716052	2.26E-14	8.73E-16	8.77	24.9616045	1.43E-01	32454748	32454871	NC_000011.9
cg23075337	2.27E-14	8.76E-16	8.77	24.9582884	2.19E-01	8481460	8481583	NC_000007.13
cg09088193	2.27E-14	8.78E-16	8.77	24.9557284	9.96E-02	39122765	39122888	NC_000017.10
cg02722434	2.28E-14	8.82E-16	8.77	24.9519745	8.35E-02	91362469	91362592	NC_000015.9
cg17224937	2.29E-14	8.86E-16	8.77	24.9464897	1.51E-01	125055879	125056002	NC_000005.9
cg16007456	2.30E-14	8.90E-16	8.77	24.9426798	2.92E-01	101004638	101004761	NC_000001.10
cg20535425	2.30E-14	8.91E-16	8.77	24.9418926	1.08E-01	57458955	57459078	NC_000004.11
cg07125412	2.31E-14	8.93E-16	8.77	24.9387818	1.33E-01	60770205	60770328	NC_000014.8
cg00378510	2.31E-14	8.96E-16	8.77	24.9358271	1.90E-01	2291020	2291143	NC_000019.9
cg16656895	2.32E-14	8.98E-16	8.77	24.9342124	1.43E-01	119305317	119305440	NC_000010.10
cg21715599	2.32E-14	9.00E-16	8.77	24.9319538	1.04E-01	92463554	92463677	NC_000015.9
cg27513573	2.33E-14	9.01E-16	8.77	24.9307934	2.21E-01	131768098	131768221	NC_000010.10
cg02084912	2.33E-14	9.01E-16	8.77	24.9302687	4.48E-02	10325711	10325834	NC_000003.11
cg25037595	2.33E-14	9.02E-16	8.77	24.9289204	1.51E-01	2756320	2756443	NC_000005.9

cg03934713	2.33E-14	9.03E-16	8.77	24.9287069	1.55E-01	6069198	6069321	NC_000016.9
cg17352045	2.33E-14	9.03E-16	8.77	24.9284909	1.25E-01	32847702	32847825	NC_000006.11
cg27479396	2.34E-14	9.07E-16	8.76	24.9241281	1.13E-01	56215568	56215691	NC_000019.9
cg21170752	2.34E-14	9.08E-16	8.76	24.9222027	3.86E-02	148291514	148291637	NC_000005.9
cg07087457	2.35E-14	9.10E-16	8.76	24.9207248	1.57E-01	135032461	135032584	NC_000010.10
cg07380026	2.35E-14	9.11E-16	8.76	24.9191359	1.39E-01	1296007	1296130	NC_000005.9
cg09899185	2.35E-14	9.13E-16	8.76	24.9178353	2.16E-01	50695720	50695843	NC_000005.9
cg27578381	2.36E-14	9.14E-16	8.76	24.916476	1.55E-01	76814686	76814809	NC_000017.10
cg01572434	2.36E-14	9.15E-16	8.76	24.915087	1.33E-01	130219926	130220049	NC_000005.9
cg27227281	2.36E-14	9.15E-16	8.76	24.9148646	1.13E-01	105026171	105026294	NC_000007.13
cg11418477	2.36E-14	9.17E-16	8.76	24.9129119	1.53E-01	175199741	175199864	NC_000002.11
cg19709146	2.37E-14	9.18E-16	8.76	24.9122603	1.22E-01	47745115	47745238	NC_000011.9
cg05095591	2.37E-14	9.19E-16	8.76	24.9111348	2.39E-01	147129184	147129307	NC_000003.11
cg26937267	2.37E-14	9.20E-16	8.76	24.9096914	5.96E-02	1315171	1315294	NC_000011.9
cg13246235	2.38E-14	9.22E-16	8.76	24.907768	2.17E-01	12749978	12750101	NC_000006.11
cg27510182	2.38E-14	9.22E-16	8.76	24.9076221	2.51E-01	58715553	58715676	NC_000001.10
cg06010032	2.38E-14	9.25E-16	8.76	24.904681	1.86E-01	139508813	139508936	NC_000008.10
cg18836626	2.39E-14	9.29E-16	8.76	24.9005168	2.02E-01	26042746	26042869	NC_000013.10
cg08619712	2.39E-14	9.29E-16	8.76	24.9004074	9.43E-02	2288051	2288174	NC_000011.9
cg15006869	2.40E-14	9.31E-16	8.76	24.8976683	1.07E-01	49141173	49141296	NC_000022.10
cg03985727	2.40E-14	9.32E-16	8.76	24.8968781	2.32E-01	15727525	15727648	NC_000007.13
cg20239565	2.41E-14	9.35E-16	8.76	24.8942864	1.20E-01	80394416	80394539	NC_000017.10
cg27247697	2.41E-14	9.35E-16	8.76	24.8940519	2.28E-01	87018054	87018177	NC_000002.11
cg05309948	2.41E-14	9.35E-16	8.76	24.8933278	2.50E-01	2292447	2292570	NC_000011.9
cg14380840	2.41E-14	9.36E-16	8.76	24.8929551	1.01E-01	3600710	3600833	NC_000010.10

cg00803910	2.41E-14	9.38E-16	8.76	24.8904953	1.45E-01	197896800	197896923	NC_000003.11
cg06496885	2.43E-14	9.43E-16	8.76	24.8848814	7.47E-02	3354972	3355095	NC_000010.10
cg15128876	2.43E-14	9.45E-16	8.76	24.8833077	1.19E-01	161146073	161146196	NC_000001.10
cg16696270	2.44E-14	9.48E-16	8.76	24.8801857	2.01E-01	132052934	132053057	NC_000008.10
cg03328107	2.44E-14	9.49E-16	8.76	24.8790762	8.67E-02	3413994	3414117	NC_000001.10
cg03888033	2.44E-14	9.50E-16	8.76	24.8782854	1.75E-01	156818905	156819028	NC_000001.10
cg19633390	2.45E-14	9.53E-16	8.76	24.8748628	1.74E-01	110381307	110381430	NC_000013.10
cg27535885	2.47E-14	9.59E-16	8.76	24.8684526	8.86E-02	47070807	47070930	NC_000001.10
cg05926586	2.48E-14	9.64E-16	8.76	24.8634408	1.61E-01	74592786	74592909	NC_000015.9
cg24863175	2.48E-14	9.64E-16	8.76	24.8633443	1.25E-01	186840433	186840556	NC_000001.10
cg09979478	2.50E-14	9.71E-16	8.75	24.8559906	2.47E-01	145925997	145926120	NC_000008.10
cg11631547	2.50E-14	9.74E-16	8.75	24.8534671	1.49E-01	113669901	113670024	NC_000011.9
cg19718563	2.51E-14	9.77E-16	8.75	24.8502442	1.47E-01	37266748	37266871	NC_000019.9
cg26516080	2.51E-14	9.78E-16	8.75	24.8497254	7.14E-02	124776158	124776281	NC_000012.11
cg10741478	2.52E-14	9.82E-16	8.75	24.8450057	2.03E-01	35687300	35687423	NC_000007.13
cg05570831	2.52E-14	9.83E-16	8.75	24.8440212	2.61E-02	75513561	75513684	NC_000016.9
cg11540358	2.53E-14	9.84E-16	8.75	24.8431517	3.58E-02	133003684	133003807	NC_000006.11
cg19517718	2.53E-14	9.85E-16	8.75	24.8424507	1.67E-01	1747082	1747205	NC_000002.11
cg17082347	2.54E-14	9.90E-16	8.75	24.8372577	2.14E-01	68118006	68118129	NC_000015.9
cg21366688	2.55E-14	9.95E-16	8.75	24.831984	2.22E-01	134491421	134491544	NC_000006.11
cg21355828	2.57E-14	1.00E-15	8.75	24.8259222	1.65E-01	149389814	149389937	NC_000007.13
cg11708136	2.58E-14	1.01E-15	8.75	24.8197491	1.63E-01	88799191	88799314	NC_000011.9
cg20204986	2.60E-14	1.01E-15	8.75	24.8152809	1.84E-01	32448067	32448190	NC_000011.9
cg07877229	2.60E-14	1.02E-15	8.75	24.8124782	1.12E-01	142554307	142554430	NC_000007.13
cg05784532	2.62E-14	1.02E-15	8.75	24.8049385	1.48E-01	230284198	230284321	NC_000001.10

cg24377495	2.62E-14	1.02E-15	8.75	24.8046551	1.47E-01	6660075	6660198	NC_000019.9
cg27313147	2.63E-14	1.03E-15	8.75	24.8010094	1.20E-01	155679151	155679274	NC_000007.13
cg02730156	2.63E-14	1.03E-15	8.75	24.7999145	1.98E-01	38069950	38070073	NC_000021.8
cg06243084	2.63E-14	1.03E-15	8.74	24.7998344	1.78E-01	63990629	63990752	NC_000011.9
cg01543173	2.64E-14	1.03E-15	8.74	24.7992	2.78E-01	34442534	34442657	NC_000021.8
cg11229862	2.64E-14	1.03E-15	8.74	24.7969076	1.76E-01	126113682	126113805	NC_000003.11
cg22882665	2.65E-14	1.03E-15	8.74	24.7954216	2.60E-01	45241110	45241233	NC_000002.11
cg00022911	2.65E-14	1.04E-15	8.74	24.793157	2.45E-01	19372392	19372515	NC_000019.9
cg01610632	2.66E-14	1.04E-15	8.74	24.7906593	1.86E-01	147127579	147127702	NC_000003.11
cg12544557	2.66E-14	1.04E-15	8.74	24.7886225	1.26E-01	39019545	39019668	NC_000017.10
cg02798181	2.67E-14	1.04E-15	8.74	24.7872428	9.73E-02	72820074	72820197	NC_000010.10
cg04638468	2.67E-14	1.04E-15	8.74	24.7858488	2.79E-01	122686453	122686576	NC_000004.11
cg06596654	2.70E-14	1.05E-15	8.74	24.774898	1.39E-01	86527923	86528046	NC_000016.9
cg12821278	2.70E-14	1.05E-15	8.74	24.7744164	2.84E-01	122131895	122132018	NC_000009.11
cg16206678	2.70E-14	1.06E-15	8.74	24.7741652	1.25E-01	30122152	30122275	NC_000006.11
cg01500055	2.70E-14	1.06E-15	8.74	24.7739916	1.33E-01	46143709	46143832	NC_000017.10
cg00298065	2.71E-14	1.06E-15	8.74	24.7690042	1.57E-01	58715577	58715700	NC_000019.9
cg10922935	2.71E-14	1.06E-15	8.74	24.7684099	2.82E-01	78452048	78452171	NC_000017.10
cg20925841	2.73E-14	1.07E-15	8.74	24.7625513	3.06E-01	121104400	121104523	NC_000002.11
cg25951981	2.73E-14	1.07E-15	8.74	24.7613198	1.77E-01	46995743	46995866	NC_000004.11
cg18925664	2.75E-14	1.07E-15	8.74	24.7558558	1.07E-01	95785126	95785249	NC_000011.9
cg19443075	2.75E-14	1.08E-15	8.74	24.7547811	1.70E-01	2161403	2161526	NC_000011.9
cg26381514	2.75E-14	1.08E-15	8.74	24.7538227	1.59E-01	26963489	26963612	NC_000012.11
cg11199713	2.75E-14	1.08E-15	8.74	24.7523721	1.55E-01	112721773	112721896	NC_000013.10
cg15648239	2.75E-14	1.08E-15	8.74	24.7523265	9.69E-02	119248136	119248259	NC_000011.9

cg05824757	2.76E-14	1.08E-15	8.74	24.7492807	9.86E-02	72574568	72574691	NC_000010.10
cg12247536	2.77E-14	1.08E-15	8.74	24.7469753	8.35E-02	113428107	113428230	NC_000012.11
cg04220881	2.79E-14	1.09E-15	8.74	24.7387592	1.74E-01	152524814	152524937	NC_000007.13
cg09655666	2.79E-14	1.10E-15	8.74	24.7375644	1.68E-01	34820190	34820313	NC_000017.10
cg18177414	2.80E-14	1.10E-15	8.73	24.7365596	2.24E-01	149389929	149390052	NC_000007.13
cg19368911	2.80E-14	1.10E-15	8.73	24.7363987	1.28E-01	245541456	245541579	NC_000001.10
cg16704073	2.81E-14	1.10E-15	8.73	24.7315331	1.22E-01	64702921	64703044	NC_000011.9
cg11992015	2.81E-14	1.10E-15	8.73	24.7301056	4.29E-02	110876674	110876797	NC_000002.11
cg23921871	2.81E-14	1.10E-15	8.73	24.7300539	1.10E-01	109577383	109577506	NC_000012.11
cg22910462	2.81E-14	1.10E-15	8.73	24.7298742	1.36E-01	17680578	17680701	NC_000022.10
cg11209521	2.82E-14	1.10E-15	8.73	24.7290037	1.65E-01	68209155	68209278	NC_000001.10
cg07090539	2.85E-14	1.12E-15	8.73	24.71874	1.16E-01	70619853	70619976	NC_000017.10
cg19965031	2.85E-14	1.12E-15	8.73	24.7175121	1.14E-01	100038389	100038512	NC_000014.8
cg11727383	2.85E-14	1.12E-15	8.73	24.715798	1.92E-01	102988389	102988512	NC_000010.10
cg06256858	2.85E-14	1.12E-15	8.73	24.7157325	1.66E-01	5890289	5890412	NC_000004.11
cg07462540	2.85E-14	1.12E-15	8.73	24.7155413	2.58E-01	8473279	8473402	NC_000007.13
cg06668555	2.86E-14	1.12E-15	8.73	24.7141544	1.87E-01	139509129	139509252	NC_000008.10
cg14752336	2.86E-14	1.12E-15	8.73	24.7125831	1.63E-01	106400454	106400577	NC_000010.10
cg23180235	2.88E-14	1.13E-15	8.73	24.7056524	8.89E-02	8577752	8577875	NC_000003.11
cg22486834	2.88E-14	1.13E-15	8.73	24.7047135	2.13E-01	52859107	52859230	NC_000006.11
cg10120807	2.89E-14	1.14E-15	8.73	24.7020813	1.82E-01	69350190	69350313	NC_000016.9
cg01935405	2.89E-14	1.14E-15	8.73	24.7020081	1.45E-01	88569083	88569206	NC_000016.9
cg16887170	2.90E-14	1.14E-15	8.73	24.6989043	7.96E-02	19451180	19451303	NC_000017.10
cg01466121	2.90E-14	1.14E-15	8.73	24.6986749	1.00E-01	202136483	202136606	NC_000002.11
cg27160395	2.91E-14	1.14E-15	8.73	24.6960174	1.86E-01	177014949	177015072	NC_000002.11

cg16332701	2.91E-14	1.14E-15	8.73	24.6959771	6.37E-02	3502815	3502938	NC_000002.11
cg03024760	2.91E-14	1.14E-15	8.73	24.6959383	2.36E-01	27215997	27216120	NC_000015.9
cg00892368	2.91E-14	1.14E-15	8.73	24.6953656	1.18E-01	32847704	32847827	NC_000006.11
cg07315755	2.93E-14	1.15E-15	8.73	24.6859309	7.88E-02	90611414	90611537	NC_000015.9
cg21106486	2.95E-14	1.16E-15	8.73	24.6787345	1.71E-01	207843084	207843207	NC_000001.10
cg24896649	2.96E-14	1.16E-15	8.73	24.6781645	2.02E-01	188916726	188916849	NC_000004.11
cg14410016	2.96E-14	1.16E-15	8.73	24.6772644	1.39E-01	176986659	176986782	NC_000002.11
cg26142412	2.97E-14	1.17E-15	8.72	24.6721515	2.44E-01	20185077	20185200	NC_000011.9
cg20900557	2.97E-14	1.17E-15	8.72	24.6716498	9.66E-02	232283631	232283754	NC_000002.11
cg26656135	2.98E-14	1.17E-15	8.72	24.6701313	2.44E-01	133562774	133562897	NC_000006.11
cg01099448	2.99E-14	1.18E-15	8.72	24.6677953	7.76E-02	214182538	214182661	NC_000002.11
cg07552182	2.99E-14	1.18E-15	8.72	24.667001	1.16E-01	89458394	89458517	NC_000005.9
cg03762760	3.00E-14	1.18E-15	8.72	24.661699	1.01E-01	33031216	33031339	NC_000012.11
cg03391684	3.01E-14	1.18E-15	8.72	24.6609003	2.19E-01	14925524	14925647	NC_000001.10
cg06344553	3.01E-14	1.18E-15	8.72	24.66069	1.20E-01	131684893	131685016	NC_000010.10
cg21415530	3.01E-14	1.19E-15	8.72	24.6593998	1.63E-01	140715802	140715925	NC_000008.10
cg13496660	3.01E-14	1.19E-15	8.72	24.6582551	1.38E-01	2424182	2424305	NC_000019.9
cg00824767	3.02E-14	1.19E-15	8.72	24.6570992	2.30E-01	23488272	23488395	NC_000010.10
cg12907598	3.02E-14	1.19E-15	8.72	24.6563231	7.71E-02	6676673	6676796	NC_000001.10
cg11466131	3.02E-14	1.19E-15	8.72	24.6539288	1.46E-01	37954778	37954901	NC_000022.10
cg01670677	3.03E-14	1.19E-15	8.72	24.6534674	2.48E-01	71955322	71955445	NC_000011.9
cg09310891	3.03E-14	1.19E-15	8.72	24.6528365	2.55E-01	7854403	7854526	NC_000019.9
cg14888916	3.04E-14	1.20E-15	8.72	24.6493879	2.16E-01	99951420	99951543	NC_000008.10
cg06954368	3.04E-14	1.20E-15	8.72	24.6475968	1.29E-01	38930080	38930203	NC_000005.9
cg06161299	3.04E-14	1.20E-15	8.72	24.6475074	1.06E-01	74633150	74633273	NC_000018.9

cg23346462	3.05E-14	1.20E-15	8.72	24.6448211	2.51E-01	179754533	179754656	NC_000003.11
cg04281464	3.05E-14	1.20E-15	8.72	24.6446515	2.71E-01	70014873	70014996	NC_000014.8
cg11523712	3.06E-14	1.21E-15	8.72	24.6427408	2.15E-01	176957055	176957178	NC_000002.11
cg07211140	3.07E-14	1.21E-15	8.72	24.6390394	1.66E-01	32455025	32455148	NC_000011.9
cg06602871	3.11E-14	1.23E-15	8.72	24.6251714	1.09E-01	66466186	66466309	NC_000016.9
cg01795199	3.12E-14	1.23E-15	8.72	24.6216525	4.97E-02	1808807	1808930	NC_000004.11
cg24125828	3.14E-14	1.24E-15	8.72	24.6147961	1.69E-01	32117049	32117172	NC_000006.11
cg16330450	3.15E-14	1.24E-15	8.71	24.6108965	2.55E-01	53852030	53852153	NC_000008.10
cg21487550	3.15E-14	1.25E-15	8.71	24.6105278	1.34E-01	127056955	127057078	NC_000003.11
cg12594635	3.16E-14	1.25E-15	8.71	24.6087198	1.77E-01	54129457	54129580	NC_000012.11
cg00421139	3.17E-14	1.25E-15	8.71	24.6046906	2.91E-01	97172961	97173084	NC_000008.10
cg02510267	3.17E-14	1.25E-15	8.71	24.6040259	2.31E-01	20690807	20690930	NC_000011.9
cg03442425	3.17E-14	1.25E-15	8.71	24.6035772	2.00E-01	32907639	32907762	NC_000017.10
cg26444528	3.18E-14	1.25E-15	8.71	24.6028729	1.52E-01	58207859	58207982	NC_000013.10
cg20834681	3.18E-14	1.26E-15	8.71	24.6021592	1.32E-01	5153947	5154070	NC_000012.11
cg04973669	3.18E-14	1.26E-15	8.71	24.6014054	8.89E-02	33272928	33273051	NC_000006.11
cg11081638	3.18E-14	1.26E-15	8.71	24.5998973	6.03E-02	58619867	58619990	NC_000003.11
cg15748490	3.19E-14	1.26E-15	8.71	24.5996345	2.34E-01	240254960	240255083	NC_000001.10
cg05687174	3.19E-14	1.26E-15	8.71	24.5986127	1.60E-01	31382507	31382630	NC_000005.9
cg03959937	3.20E-14	1.26E-15	8.71	24.5959404	9.88E-02	10910819	10910942	NC_000016.9
cg26644049	3.20E-14	1.27E-15	8.71	24.5945225	1.31E-01	57276917	57277040	NC_000019.9
cg07103129	3.20E-14	1.27E-15	8.71	24.5937207	1.88E-01	50787964	50788087	NC_000006.11
cg24637252	3.22E-14	1.27E-15	8.71	24.5899999	9.82E-02	71402902	71403025	NC_000015.9
cg03238075	3.22E-14	1.27E-15	8.71	24.5891269	2.25E-01	21113363	21113486	NC_000017.10
cg09500565	3.22E-14	1.27E-15	8.71	24.587451	3.74E-02	100061911	100062034	NC_000002.11

cg03078363	3.23E-14	1.28E-15	8.71	24.5860418	2.24E-01	54408664	54408787	NC_000012.11
cg14658804	3.24E-14	1.28E-15	8.71	24.5824069	2.89E-01	168728213	168728336	NC_000005.9
cg09610766	3.24E-14	1.28E-15	8.71	24.5813686	1.38E-01	105166570	105166693	NC_000003.11
cg15778745	3.25E-14	1.29E-15	8.71	24.5787581	2.86E-01	88324570	88324693	NC_000013.10
cg03062549	3.26E-14	1.29E-15	8.71	24.5754193	2.34E-01	34786594	34786717	NC_000015.9
cg14002960	3.26E-14	1.29E-15	8.71	24.5741322	1.91E-01	31739423	31739546	NC_000018.9
cg18291939	3.29E-14	1.30E-15	8.71	24.5668055	7.61E-02	1219407	1219530	NC_000011.9
cg17153133	3.29E-14	1.30E-15	8.71	24.5667097	1.27E-01	134482010	134482133	NC_000006.11
cg08980987	3.29E-14	1.30E-15	8.71	24.5663369	1.28E-01	25054905	25055028	NC_000012.11
cg00530925	3.29E-14	1.30E-15	8.71	24.5648331	2.54E-01	23463094	23463217	NC_000010.10
cg01703291	3.30E-14	1.31E-15	8.71	24.5634168	1.50E-01	85574661	85574784	NC_000015.9
cg13413955	3.30E-14	1.31E-15	8.71	24.5626679	1.73E-01	842914	843037	NC_000009.11
cg12616421	3.30E-14	1.31E-15	8.71	24.5620307	6.13E-02	32017224	32017347	NC_000006.11
cg17958132	3.30E-14	1.31E-15	8.71	24.5618885	9.98E-02	915739	915862	NC_000017.10
cg11606826	3.31E-14	1.31E-15	8.71	24.5607928	1.61E-01	30540994	30541117	NC_000006.11
cg18197808	3.32E-14	1.31E-15	8.71	24.5572266	1.73E-01	46776356	46776479	NC_000012.11
cg02643054	3.32E-14	1.31E-15	8.71	24.5570719	2.38E-01	27206544	27206667	NC_000007.13
cg23655210	3.32E-14	1.32E-15	8.71	24.5561905	1.55E-01	14129214	14129337	NC_000018.9
cg08620267	3.33E-14	1.32E-15	8.71	24.5547986	7.01E-02	85126437	85126560	NC_000016.9
cg20732478	3.33E-14	1.32E-15	8.71	24.5522445	1.51E-01	176993643	176993766	NC_000002.11
cg26630231	3.33E-14	1.32E-15	8.71	24.5521519	2.13E-01	112726482	112726605	NC_000013.10
cg09450097	3.33E-14	1.32E-15	8.71	24.5517202	9.94E-02	62194059	62194182	NC_000004.11
cg07959138	3.35E-14	1.33E-15	8.7	24.5474305	1.00E-01	87601970	87602093	NC_000001.10
cg18627360	3.35E-14	1.33E-15	8.7	24.5468177	2.50E-01	61809834	61809957	NC_000020.10
cg25570913	3.37E-14	1.33E-15	8.7	24.542065	2.79E-01	112759893	112760016	NC_000013.10

cg18213472	3.37E-14	1.33E-15	8.7	24.5417864	2.52E-01	84419189	84419312	NC_000006.11
cg23876292	3.37E-14	1.34E-15	8.7	24.5416591	1.56E-01	57024517	57024640	NC_000018.9
cg18326819	3.37E-14	1.34E-15	8.7	24.541492	1.24E-01	46801257	46801380	NC_000021.8
cg22591040	3.38E-14	1.34E-15	8.7	24.5366039	9.05E-02	58106772	58106895	NC_000003.11
cg17875845	3.39E-14	1.35E-15	8.7	24.5340804	1.47E-01	87932378	87932501	NC_000004.11
cg08383315	3.39E-14	1.35E-15	8.7	24.5326625	2.48E-01	8190565	8190688	NC_000011.9
cg25457200	3.41E-14	1.35E-15	8.7	24.5279299	1.26E-01	65811116	65811239	NC_000015.9
cg06830971	3.41E-14	1.36E-15	8.7	24.5267334	6.54E-02	76890776	76890899	NC_000017.10
cg03557916	3.42E-14	1.36E-15	8.7	24.5256575	1.14E-01	114109949	114110072	NC_000013.10
cg21077559	3.44E-14	1.36E-15	8.7	24.5200635	2.02E-01	122686319	122686442	NC_000004.11
cg02467024	3.45E-14	1.37E-15	8.7	24.5144154	1.17E-01	8187972	8188095	NC_000012.11
cg05113373	3.47E-14	1.38E-15	8.7	24.5090614	1.06E-01	962823	962946	NC_000016.9
cg06889975	3.47E-14	1.38E-15	8.7	24.5084364	8.59E-02	22558722	22558845	NC_000008.10
cg03532040	3.48E-14	1.38E-15	8.7	24.5072017	1.15E-01	51301449	51301572	NC_000019.9
cg23191956	3.48E-14	1.38E-15	8.7	24.5063493	1.86E-01	29235123	29235246	NC_000014.8
cg14225678	3.48E-14	1.38E-15	8.7	24.5060531	2.95E-01	54612325	54612448	NC_000007.13
cg21707187	3.48E-14	1.39E-15	8.7	24.5052175	2.45E-01	54345862	54345985	NC_000012.11
cg13882284	3.50E-14	1.39E-15	8.7	24.5012883	1.45E-01	4859996	4860119	NC_000004.11
cg06324719	3.50E-14	1.39E-15	8.7	24.4989652	1.05E-01	70118762	70118885	NC_000015.9
cg10841552	3.52E-14	1.40E-15	8.7	24.4948185	1.65E-01	20622931	20623054	NC_000011.9
cg09591830	3.54E-14	1.41E-15	8.7	24.4893394	9.25E-02	74975236	74975359	NC_000002.11
cg02522041	3.54E-14	1.41E-15	8.7	24.4885576	9.19E-02	1249008	1249131	NC_000011.9
cg25146557	3.54E-14	1.41E-15	8.7	24.4879713	1.77E-01	207507421	207507544	NC_000002.11
cg23531049	3.55E-14	1.41E-15	8.69	24.4855167	7.40E-02	42075277	42075400	NC_000015.9
cg16805094	3.56E-14	1.42E-15	8.69	24.4824301	1.43E-01	169629875	169629998	NC_000006.11

cg27507261	3.57E-14	1.42E-15	8.69	24.4808203	1.67E-01	5403053	5403176	NC_000017.10
cg12573849	3.57E-14	1.42E-15	8.69	24.4797093	1.69E-01	105470711	105470834	NC_000002.11
cg26827852	3.58E-14	1.43E-15	8.69	24.4766899	4.26E-02	89887630	89887753	NC_000001.10
cg10134425	3.59E-14	1.43E-15	8.69	24.4745992	1.10E-01	153934955	153935078	NC_000004.11
cg08911275	3.59E-14	1.43E-15	8.69	24.4744545	3.04E-01	176957462	176957585	NC_000002.11
cg12890291	3.60E-14	1.43E-15	8.69	24.472061	9.44E-02	22234163	22234286	NC_000007.13
cg20684251	3.60E-14	1.43E-15	8.69	24.4718679	4.07E-02	51840554	51840677	NC_000019.9
cg23989963	3.60E-14	1.43E-15	8.69	24.4705949	1.96E-01	83952420	83952543	NC_000015.9
cg10448808	3.61E-14	1.44E-15	8.69	24.4691385	2.44E-01	86599745	86599868	NC_000016.9
cg01455981	3.62E-14	1.44E-15	8.69	24.4662406	9.87E-02	20511095	20511218	NC_000001.10
cg09642925	3.62E-14	1.44E-15	8.69	24.4661321	1.72E-01	114261793	114261916	NC_000002.11
cg02597299	3.64E-14	1.45E-15	8.69	24.4604245	1.92E-01	151304559	151304682	NC_000005.9
cg24181605	3.64E-14	1.45E-15	8.69	24.4602916	1.56E-01	62458847	62458970	NC_000015.9
cg06020207	3.64E-14	1.45E-15	8.69	24.4599275	1.54E-01	131766412	131766535	NC_000010.10
cg23311929	3.64E-14	1.45E-15	8.69	24.4595903	7.31E-02	126698647	126698770	NC_000010.10
cg07439546	3.64E-14	1.45E-15	8.69	24.4592814	1.71E-01	8813357	8813480	NC_000016.9
cg07609788	3.65E-14	1.45E-15	8.69	24.4574324	1.86E-01	39995652	39995775	NC_000020.10
cg18991611	3.65E-14	1.46E-15	8.69	24.4549036	3.32E-01	49468828	49468951	NC_000008.10
cg26879349	3.66E-14	1.46E-15	8.69	24.4526709	8.35E-02	26109215	26109338	NC_000015.9
cg02582957	3.66E-14	1.46E-15	8.69	24.4523839	1.87E-01	182547955	182548078	NC_000002.11
cg10127275	3.67E-14	1.46E-15	8.69	24.4511325	1.93E-01	165304496	165304619	NC_000004.11
cg26406401	3.67E-14	1.46E-15	8.69	24.4506181	5.98E-02	3499759	3499882	NC_000001.10
cg21776417	3.69E-14	1.47E-15	8.69	24.4456179	2.23E-01	147142415	147142538	NC_000003.11
cg24793584	3.69E-14	1.47E-15	8.69	24.4455686	5.39E-02	80557532	80557655	NC_000017.10
cg05347845	3.69E-14	1.47E-15	8.69	24.4440664	3.36E-01	229569892	229570015	NC_000001.10

cg18680788	3.70E-14	1.48E-15	8.69	24.4414134	2.95E-01	172754896	172755019	NC_000005.9
cg19699140	3.70E-14	1.48E-15	8.69	24.4409352	9.14E-02	53302900	53303023	NC_000013.10
cg26834713	3.71E-14	1.48E-15	8.69	24.4395642	6.17E-02	241567666	241567789	NC_000002.11
cg14638883	3.71E-14	1.48E-15	8.69	24.4387101	2.85E-01	166582201	166582324	NC_000006.11
cg08845023	3.74E-14	1.49E-15	8.69	24.4307614	5.93E-02	125767421	125767544	NC_000010.10
cg08206156	3.75E-14	1.50E-15	8.69	24.4282158	6.61E-02	240658646	240658769	NC_000002.11
cg24211976	3.75E-14	1.50E-15	8.69	24.4280312	1.70E-01	60285744	60285867	NC_000015.9
cg24326021	3.76E-14	1.50E-15	8.69	24.4249291	1.56E-01	56188459	56188582	NC_000019.9
cg21761355	3.76E-14	1.50E-15	8.69	24.4234635	1.15E-01	131930674	131930797	NC_000012.11
cg18849102	3.77E-14	1.51E-15	8.68	24.4204911	1.56E-01	4859935	4860058	NC_000004.11
cg20099968	3.78E-14	1.51E-15	8.68	24.4200171	9.27E-02	1364147	1364270	NC_000004.11
cg18956933	3.78E-14	1.51E-15	8.68	24.4195084	2.28E-01	147136904	147137027	NC_000003.11
cg14629665	3.79E-14	1.52E-15	8.68	24.415815	8.28E-02	136819477	136819600	NC_000009.11
cg23460843	3.79E-14	1.52E-15	8.68	24.4157319	3.02E-01	127644075	127644198	NC_000002.11
cg03281661	3.79E-14	1.52E-15	8.68	24.4148053	2.04E-01	3239458	3239581	NC_000016.9
cg25666233	3.81E-14	1.52E-15	8.68	24.4110223	1.68E-01	74424975	74425098	NC_000015.9
cg02402214	3.82E-14	1.53E-15	8.68	24.4064284	9.21E-02	32046950	32047073	NC_000006.11
cg09463220	3.83E-14	1.53E-15	8.68	24.4051091	1.21E-01	129349316	129349439	NC_000003.11
cg10317138	3.83E-14	1.53E-15	8.68	24.4048972	2.96E-01	128075923	128076046	NC_000010.10
cg15442037	3.83E-14	1.53E-15	8.68	24.4043967	1.51E-01	69286293	69286416	NC_000011.9
cg18514065	3.84E-14	1.54E-15	8.68	24.4019374	1.13E-01	104165052	104165175	NC_000014.8
cg13450437	3.85E-14	1.54E-15	8.68	24.3999449	9.47E-02	133797391	133797514	NC_000009.11
cg20847039	3.85E-14	1.54E-15	8.68	24.3992471	1.42E-01	48388426	48388549	NC_000013.10
cg27392850	3.85E-14	1.54E-15	8.68	24.3990842	2.68E-01	9764073	9764196	NC_000008.10
cg03338754	3.85E-14	1.54E-15	8.68	24.3981222	1.88E-01	113765397	113765520	NC_000013.10

cg03463411	3.86E-14	1.54E-15	8.68	24.3973934	1.68E-01	81118188	81118311	NC_000004.11
cg15452970	3.87E-14	1.55E-15	8.68	24.3941795	1.24E-01	91190620	91190743	NC_000001.10
cg17233506	3.87E-14	1.55E-15	8.68	24.3937851	1.68E-01	46608293	46608416	NC_000017.10
cg01943414	3.88E-14	1.55E-15	8.68	24.3915149	4.88E-02	60757632	60757755	NC_000017.10
cg10633051	3.88E-14	1.55E-15	8.68	24.3913122	1.30E-01	168042982	168043105	NC_000002.11
cg10958086	3.88E-14	1.56E-15	8.68	24.3904373	2.08E-02	952903	953026	NC_000017.10
cg19244312	3.90E-14	1.56E-15	8.68	24.3859931	1.02E-01	171964315	171964438	NC_000003.11
cg23022999	3.90E-14	1.56E-15	8.68	24.3852145	1.30E-01	39368345	39368468	NC_000019.9
cg24688502	3.91E-14	1.57E-15	8.68	24.3811943	1.06E-01	62159885	62160008	NC_000004.11
cg27226147	3.92E-14	1.57E-15	8.68	24.380991	1.28E-01	27447492	27447615	NC_000006.11
cg27192990	3.92E-14	1.57E-15	8.68	24.3793395	5.18E-02	129479024	129479147	NC_000006.11
cg08482436	3.92E-14	1.57E-15	8.68	24.3790802	2.32E-01	96647888	96648011	NC_000007.13
cg11256132	3.92E-14	1.57E-15	8.68	24.37826	7.04E-02	244515893	244516016	NC_000001.10
cg10849498	3.93E-14	1.58E-15	8.68	24.3768857	1.11E-01	31829800	31829923	NC_000006.11
cg07755245	3.93E-14	1.58E-15	8.68	24.3767728	1.13E-01	4891238	4891361	NC_000019.9
cg08948225	3.93E-14	1.58E-15	8.68	24.3759181	4.39E-02	27288300	27288423	NC_000008.10
cg16072304	3.94E-14	1.58E-15	8.68	24.3744645	1.78E-01	153859646	153859769	NC_000005.9
cg00970313	3.96E-14	1.59E-15	8.68	24.3684907	2.30E-01	37125914	37126037	NC_000014.8
cg15313740	3.97E-14	1.60E-15	8.68	24.3652556	1.51E-01	171625337	171625460	NC_000002.11
cg20989480	3.99E-14	1.60E-15	8.68	24.3613908	9.46E-02	32451461	32451584	NC_000011.9
cg12476242	4.01E-14	1.61E-15	8.67	24.3558642	1.38E-01	127813395	127813518	NC_000003.11
cg18318649	4.02E-14	1.61E-15	8.67	24.3537814	2.52E-01	87974369	87974492	NC_000005.9
cg11797430	4.02E-14	1.62E-15	8.67	24.3528668	1.00E-01	2761364	2761487	NC_000012.11
cg15819352	4.05E-14	1.63E-15	8.67	24.3450398	8.71E-02	1845940	1846063	NC_000001.10
cg08553437	4.06E-14	1.63E-15	8.67	24.3429727	2.48E-01	122686456	122686579	NC_000004.11

cg25898111	4.06E-14	1.63E-15	8.67	24.34149	9.79E-02	3316292	3316415	NC_000010.10
cg22214414	4.07E-14	1.64E-15	8.67	24.3399753	9.98E-02	58507232	58507355	NC_000020.10
cg20764212	4.07E-14	1.64E-15	8.67	24.3397802	8.77E-02	6331035	6331158	NC_000004.11
cg13843613	4.07E-14	1.64E-15	8.67	24.3395974	2.16E-01	177140126	177140249	NC_000001.10
cg17816173	4.08E-14	1.64E-15	8.67	24.3373659	1.49E-01	158377440	158377563	NC_000007.13
cg09615505	4.08E-14	1.64E-15	8.67	24.3370448	2.00E-01	176977285	176977408	NC_000002.11
cg24483767	4.09E-14	1.64E-15	8.67	24.3360416	1.46E-01	46850840	46850963	NC_000021.8
cg00090261	4.10E-14	1.65E-15	8.67	24.3327972	2.16E-01	101118548	101118671	NC_000008.10
cg02828785	4.11E-14	1.66E-15	8.67	24.3286223	1.41E-01	12846546	12846669	NC_000012.11
cg03487040	4.13E-14	1.66E-15	8.67	24.3257004	1.00E-01	55511435	55511558	NC_000002.11
cg12314713	4.13E-14	1.66E-15	8.67	24.324747	2.63E-01	47074576	47074699	NC_000017.10
cg22916722	4.13E-14	1.66E-15	8.67	24.3240154	2.37E-01	76605056	76605179	NC_000014.8
cg15084543	4.13E-14	1.66E-15	8.67	24.3238343	2.01E-01	79472408	79472531	NC_000001.10
cg07096883	4.13E-14	1.66E-15	8.67	24.3238093	1.75E-01	149979234	149979357	NC_000007.13
cg05331731	4.14E-14	1.67E-15	8.67	24.3223507	1.49E-01	11147146	11147269	NC_000018.9
cg23540210	4.14E-14	1.67E-15	8.67	24.3212406	1.39E-01	180332054	180332177	NC_000003.11
cg26727693	4.15E-14	1.67E-15	8.67	24.3198467	1.80E-01	63544175	63544298	NC_000012.11
cg02234120	4.15E-14	1.67E-15	8.67	24.3190326	8.33E-02	234626351	234626474	NC_000002.11
cg14408465	4.15E-14	1.67E-15	8.67	24.3187969	2.31E-01	48143434	48143557	NC_000014.8
cg25836061	4.15E-14	1.67E-15	8.67	24.3182584	1.17E-01	19939028	19939151	NC_000022.10
cg21124530	4.16E-14	1.68E-15	8.67	24.3172079	4.20E-02	17689373	17689496	NC_000007.13
cg04386759	4.20E-14	1.69E-15	8.67	24.3067306	2.18E-01	170554795	170554918	NC_000006.11
cg07834249	4.22E-14	1.70E-15	8.67	24.3012018	7.34E-02	495271	495394	NC_000005.9
cg06338562	4.23E-14	1.71E-15	8.67	24.2988039	2.06E-01	37488645	37488768	NC_000007.13
cg00423153	4.24E-14	1.71E-15	8.66	24.2961595	2.22E-01	229046785	229046908	NC_000002.11

cg14273116	4.24E-14	1.71E-15	8.66	24.2960471	1.75E-01	107897	108020	NC_000004.11
cg19115882	4.25E-14	1.72E-15	8.66	24.2934183	1.45E-01	26919145	26919268	NC_000002.11
cg01664666	4.26E-14	1.72E-15	8.66	24.2904805	2.56E-01	242687465	242687588	NC_000001.10
cg06686995	4.27E-14	1.72E-15	8.66	24.289162	8.73E-02	179228489	179228612	NC_000005.9
cg10239098	4.28E-14	1.73E-15	8.66	24.2868498	2.41E-01	176995556	176995679	NC_000002.11
cg09740313	4.28E-14	1.73E-15	8.66	24.2862671	7.83E-02	115540541	115540664	NC_000010.10
cg19305604	4.28E-14	1.73E-15	8.66	24.285509	8.22E-02	132958038	132958161	NC_000012.11
cg18238908	4.29E-14	1.73E-15	8.66	24.2850107	1.37E-01	33893978	33894101	NC_000001.10
cg11064738	4.29E-14	1.73E-15	8.66	24.2848513	1.22E-01	68985557	68985680	NC_000014.8
cg11866396	4.29E-14	1.73E-15	8.66	24.2834324	7.34E-02	5322940	5323063	NC_000017.10
cg13801656	4.30E-14	1.74E-15	8.66	24.2819968	6.94E-02	4685060	4685183	NC_000009.11
cg22715837	4.32E-14	1.75E-15	8.66	24.2757846	2.37E-01	113723162	113723285	NC_000007.13
cg25316120	4.35E-14	1.76E-15	8.66	24.2704397	1.01E-01	106986197	106986320	NC_000002.11
cg04344565	4.38E-14	1.77E-15	8.66	24.2636705	2.11E-01	140715646	140715769	NC_000008.10
cg23156916	4.38E-14	1.77E-15	8.66	24.261611	1.96E-01	688132	688255	NC_000008.10
cg04670857	4.39E-14	1.77E-15	8.66	24.2610741	2.23E-01	79740208	79740331	NC_000002.11
cg08198773	4.39E-14	1.77E-15	8.66	24.2604194	1.05E-01	1697536	1697659	NC_000008.10
cg22624278	4.39E-14	1.78E-15	8.66	24.25945	1.07E-01	131237708	131237831	NC_000012.11
cg00998387	4.40E-14	1.78E-15	8.66	24.2584163	2.28E-01	65281572	65281695	NC_000008.10
cg10967350	4.40E-14	1.78E-15	8.66	24.2572397	1.72E-01	2141304	2141427	NC_000003.11
cg00031013	4.40E-14	1.78E-15	8.66	24.2566041	1.03E-01	7757933	7758056	NC_000017.10
cg13960084	4.41E-14	1.78E-15	8.66	24.2554164	2.01E-01	140773147	140773270	NC_000009.11
cg09364590	4.41E-14	1.78E-15	8.66	24.2553496	1.07E-01	155537901	155538024	NC_000006.11
cg09834794	4.43E-14	1.79E-15	8.66	24.2496737	2.15E-01	147087702	147087825	NC_000003.11
cg13669804	4.43E-14	1.79E-15	8.66	24.2494421	4.72E-02	148712659	148712782	NC_000003.11

cg19046904	4.45E-14	1.80E-15	8.66	24.2444344	1.09E-01	46426201	46426324	NC_000022.10
cg01636354	4.47E-14	1.81E-15	8.66	24.2406663	7.36E-02	134078647	134078770	NC_000003.11
cg13222752	4.47E-14	1.81E-15	8.66	24.2403067	2.31E-01	74892569	74892692	NC_000014.8
cg11573679	4.48E-14	1.81E-15	8.66	24.2385339	2.80E-01	68546467	68546590	NC_000002.11
cg07522372	4.52E-14	1.83E-15	8.65	24.2299367	1.01E-01	13390440	13390563	NC_000018.9
cg17018096	4.52E-14	1.83E-15	8.65	24.2291013	1.51E-01	8657692	8657815	NC_000019.9
cg06969129	4.52E-14	1.83E-15	8.65	24.2286857	8.88E-02	5645984	5646107	NC_000007.13
cg05107535	4.52E-14	1.83E-15	8.65	24.2278002	1.71E-01	3242850	3242973	NC_000016.9
cg21701379	4.53E-14	1.84E-15	8.65	24.2259893	1.98E-01	31079694	31079817	NC_000012.11
cg17269633	4.53E-14	1.84E-15	8.65	24.2255158	1.73E-01	42733600	42733723	NC_000017.10
cg24004139	4.54E-14	1.84E-15	8.65	24.223198	7.31E-02	26670703	26670826	NC_000001.10
cg08535373	4.55E-14	1.84E-15	8.65	24.2225363	2.37E-01	37352612	37352735	NC_000020.10
cg27272235	4.55E-14	1.85E-15	8.65	24.2208668	2.03E-01	221061070	221061193	NC_000001.10
cg06525347	4.56E-14	1.85E-15	8.65	24.2191437	1.86E-01	133561630	133561753	NC_000006.11
cg03380645	4.56E-14	1.85E-15	8.65	24.2184931	1.39E-01	85895048	85895171	NC_000002.11
cg15674181	4.57E-14	1.85E-15	8.65	24.2183631	7.37E-02	3158688	3158811	NC_000010.10
cg22187630	4.57E-14	1.85E-15	8.65	24.2181974	1.95E-01	13616871	13616994	NC_000019.9
cg27477373	4.57E-14	1.85E-15	8.65	24.2174515	2.93E-01	56879645	56879768	NC_000019.9
cg20098875	4.57E-14	1.86E-15	8.65	24.2160441	1.17E-01	5093370	5093493	NC_000019.9
cg00375589	4.58E-14	1.86E-15	8.65	24.2148714	8.70E-02	15898759	15898882	NC_000003.11
cg13457172	4.60E-14	1.87E-15	8.65	24.2111952	2.43E-01	118897847	118897970	NC_000010.10
cg12700904	4.60E-14	1.87E-15	8.65	24.2111485	2.25E-01	13590720	13590843	NC_000003.11
cg06536988	4.60E-14	1.87E-15	8.65	24.2096337	7.77E-02	153586440	153586563	NC_000004.11
cg02878544	4.61E-14	1.87E-15	8.65	24.2071759	1.18E-01	2559709	2559832	NC_000016.9
cg15590989	4.61E-14	1.87E-15	8.65	24.2068812	1.91E-01	183062460	183062583	NC_000004.11

cg13172637	4.62E-14	1.88E-15	8.65	24.2050895	2.64E-01	97685823	97685946	NC_000014.8
cg06432036	4.64E-14	1.88E-15	8.65	24.2016441	1.73E-01	170595385	170595508	NC_000006.11
cg03699182	4.67E-14	1.90E-15	8.65	24.1945747	2.61E-01	121104187	121104310	NC_000002.11
cg27485730	4.68E-14	1.90E-15	8.65	24.1916267	7.60E-02	46857675	46857798	NC_000022.10
cg06239923	4.69E-14	1.91E-15	8.65	24.1899212	1.05E-01	55923774	55923897	NC_000020.10
cg20891301	4.69E-14	1.91E-15	8.65	24.1896182	1.22E-01	4864711	4864834	NC_000004.11
cg25865542	4.70E-14	1.91E-15	8.65	24.1881049	7.98E-02	32495794	32495917	NC_000003.11
cg11799589	4.70E-14	1.91E-15	8.65	24.1877702	1.28E-01	44443270	44443393	NC_000001.10
cg06672560	4.71E-14	1.92E-15	8.65	24.1849585	1.92E-01	54580175	54580298	NC_000020.10
cg01202666	4.72E-14	1.92E-15	8.65	24.1829093	1.72E-01	19157650	19157773	NC_000007.13
cg24232092	4.73E-14	1.93E-15	8.65	24.1796718	1.05E-01	1364344	1364467	NC_000004.11
cg02013838	4.75E-14	1.93E-15	8.65	24.1769156	2.40E-01	105460715	105460838	NC_000002.11
cg17134770	4.75E-14	1.93E-15	8.65	24.1760378	1.19E-01	183390353	183390476	NC_000001.10
cg07931411	4.78E-14	1.94E-15	8.64	24.170223	2.60E-01	15580721	15580844	NC_000019.9
cg24519147	4.78E-14	1.94E-15	8.64	24.1701651	1.43E-01	188916496	188916619	NC_000004.11
cg02769743	4.78E-14	1.94E-15	8.64	24.1699968	1.19E-01	9668402	9668525	NC_000001.10
cg00589371	4.80E-14	1.95E-15	8.64	24.1659308	2.03E-01	63417783	63417906	NC_000018.9
cg17621718	4.80E-14	1.95E-15	8.64	24.1658621	2.23E-01	42306737	42306860	NC_000003.11
cg08279008	4.80E-14	1.95E-15	8.64	24.1647831	2.43E-01	86263224	86263347	NC_000002.11
cg13186590	4.80E-14	1.96E-15	8.64	24.1642411	1.91E-01	5928013	5928136	NC_000004.11
cg03217253	4.82E-14	1.96E-15	8.64	24.1612137	2.30E-01	53758055	53758178	NC_000019.9
cg10498703	4.83E-14	1.97E-15	8.64	24.1578628	1.48E-01	64680996	64681119	NC_000014.8
cg26228014	4.84E-14	1.97E-15	8.64	24.1573752	1.36E-01	47073138	47073261	NC_000001.10
cg18537027	4.84E-14	1.97E-15	8.64	24.1565943	1.28E-01	64601816	64601939	NC_000005.9
cg19212391	4.85E-14	1.97E-15	8.64	24.1548391	2.21E-01	72432790	72432913	NC_000017.10

cg19408626	4.86E-14	1.98E-15	8.64	24.1528619	1.51E-01	12189773	12189896	NC_000001.10
cg23824713	4.87E-14	1.98E-15	8.64	24.1503891	1.20E-01	61157738	61157861	NC_000018.9
cg15310492	4.89E-14	1.99E-15	8.64	24.1451505	2.01E-01	30038677	30038800	NC_000011.9
cg06581175	4.89E-14	1.99E-15	8.64	24.1449325	2.12E-01	96632635	96632758	NC_000007.13
cg17802600	4.90E-14	2.00E-15	8.64	24.1432986	2.32E-01	30717501	30717624	NC_000019.9
cg08407434	4.91E-14	2.00E-15	8.64	24.1420951	8.44E-02	43001691	43001814	NC_000020.10
cg16412609	4.91E-14	2.00E-15	8.64	24.140939	1.12E-01	31579805	31579928	NC_000016.9
cg13188011	4.91E-14	2.00E-15	8.64	24.140243	1.88E-01	39319326	39319449	NC_000020.10
cg02302165	4.92E-14	2.01E-15	8.64	24.1391239	7.57E-02	178751288	178751411	NC_000001.10
cg23719508	4.93E-14	2.01E-15	8.64	24.1371903	1.40E-01	2623999	2624122	NC_000007.13
cg23217946	4.99E-14	2.04E-15	8.64	24.1248488	2.29E-01	22817039	22817162	NC_000019.9
cg08629884	5.01E-14	2.05E-15	8.64	24.1200957	1.95E-01	1719983	1720106	NC_000004.11
cg25331550	5.02E-14	2.05E-15	8.64	24.1185557	1.24E-01	3471397	3471520	NC_000004.11
cg27227797	5.04E-14	2.06E-15	8.64	24.1132547	1.28E-01	375831	375954	NC_000019.9
cg00741609	5.05E-14	2.06E-15	8.64	24.1116407	1.81E-01	102893925	102894048	NC_000010.10
cg08004425	5.09E-14	2.08E-15	8.63	24.1038804	2.35E-01	37499547	37499670	NC_000001.10
cg02920216	5.10E-14	2.08E-15	8.63	24.1011661	1.94E-01	19616939	19617062	NC_000021.8
cg00745735	5.11E-14	2.09E-15	8.63	24.0989372	9.80E-02	69868860	69868983	NC_000010.10
cg14510812	5.11E-14	2.09E-15	8.63	24.0986983	2.17E-01	85306797	85306920	NC_000012.11
cg16851046	5.12E-14	2.09E-15	8.63	24.0974764	1.68E-01	21291173	21291296	NC_000013.10
cg15000279	5.13E-14	2.09E-15	8.63	24.0964676	2.11E-01	29285009	29285132	NC_000019.9
cg26850144	5.13E-14	2.09E-15	8.63	24.0963727	1.05E-01	69635508	69635631	NC_000011.9
cg19847945	5.13E-14	2.10E-15	8.63	24.0950159	1.71E-01	60783784	60783907	NC_000017.10
cg13597544	5.16E-14	2.11E-15	8.63	24.0900739	1.12E-01	79365635	79365758	NC_000017.10
cg18309817	5.16E-14	2.11E-15	8.63	24.0896802	1.72E-01	12254153	12254276	NC_000018.9

cg02550218	5.17E-14	2.11E-15	8.63	24.0869247	1.49E-01	89767709	89767832	NC_000005.9
cg15950251	5.17E-14	2.12E-15	8.63	24.0863766	8.45E-02	139658345	139658468	NC_000007.13
cg13909534	5.21E-14	2.13E-15	8.63	24.0800778	2.13E-01	178772372	178772495	NC_000005.9
cg22574825	5.21E-14	2.13E-15	8.63	24.0789	2.47E-01	29068987	29069110	NC_000013.10
cg21042456	5.22E-14	2.13E-15	8.63	24.077623	2.43E-01	1270558	1270681	NC_000007.13
cg08668790	5.22E-14	2.14E-15	8.63	24.077475	2.65E-01	58220662	58220785	NC_000019.9
cg24863335	5.24E-14	2.14E-15	8.63	24.0734636	2.33E-01	70535406	70535529	NC_000018.9
cg15012678	5.25E-14	2.15E-15	8.63	24.07069	1.29E-01	197285336	197285459	NC_000003.11
cg00132208	5.26E-14	2.15E-15	8.63	24.069711	5.90E-02	10120780	10120903	NC_000004.11
cg03307465	5.26E-14	2.15E-15	8.63	24.0689293	2.93E-01	40679519	40679642	NC_000002.11
cg26248537	5.26E-14	2.15E-15	8.63	24.0689119	4.73E-02	8376216	8376339	NC_000001.10
cg26698460	5.27E-14	2.16E-15	8.63	24.0661271	1.69E-01	58716004	58716127	NC_000019.9
cg08845721	5.28E-14	2.16E-15	8.63	24.0644864	1.81E-01	142780693	142780816	NC_000005.9
cg24408656	5.28E-14	2.16E-15	8.63	24.0643736	2.31E-01	21503861	21503984	NC_000020.10
cg06990571	5.31E-14	2.17E-15	8.63	24.0599206	1.76E-01	132986509	132986632	NC_000008.10
cg12860391	5.31E-14	2.18E-15	8.63	24.0589307	2.16E-01	33602821	33602944	NC_000015.9
cg18415687	5.33E-14	2.18E-15	8.63	24.0555208	2.57E-01	35047411	35047534	NC_000015.9
cg12149795	5.34E-14	2.19E-15	8.63	24.053757	1.79E-01	47882121	47882244	NC_000021.8
cg13881299	5.34E-14	2.19E-15	8.63	24.0535435	4.21E-02	122684632	122684755	NC_000012.11
cg01192077	5.35E-14	2.19E-15	8.63	24.0518391	1.46E-01	158523507	158523630	NC_000005.9
cg26170604	5.37E-14	2.20E-15	8.62	24.0467077	2.68E-01	8482614	8482737	NC_000007.13
cg13572145	5.38E-14	2.20E-15	8.62	24.0462896	1.77E-01	80529621	80529744	NC_000002.11
cg26578726	5.39E-14	2.21E-15	8.62	24.0440859	1.42E-01	56289611	56289734	NC_000014.8
cg13068215	5.39E-14	2.21E-15	8.62	24.0429061	2.10E-01	240286481	240286604	NC_000001.10
cg17437939	5.39E-14	2.21E-15	8.62	24.042682	2.64E-01	97361252	97361375	NC_000007.13

cg00795341	5.39E-14	2.21E-15	8.62	24.0425616	2.27E-01	79330929	79331052	NC_000005.9
cg10601026	5.42E-14	2.22E-15	8.62	24.0383307	1.25E-01	64579140	64579263	NC_000010.10
cg23979876	5.43E-14	2.23E-15	8.62	24.0352632	1.11E-01	141299001	141299124	NC_000004.11
cg09553380	5.44E-14	2.23E-15	8.62	24.0336832	2.71E-01	207308829	207308952	NC_000002.11
cg14287112	5.44E-14	2.23E-15	8.62	24.0333904	2.59E-01	133562485	133562608	NC_000006.11
cg23967544	5.44E-14	2.23E-15	8.62	24.0327084	1.59E-01	172672684	172672807	NC_000005.9
cg17003736	5.45E-14	2.24E-15	8.62	24.0318211	1.63E-01	147111308	147111431	NC_000003.11
cg14212402	5.45E-14	2.24E-15	8.62	24.030185	8.89E-02	236300937	236301060	NC_000002.11
cg05349039	5.46E-14	2.24E-15	8.62	24.0292489	7.48E-02	46845110	46845233	NC_000021.8
cg25990363	5.48E-14	2.25E-15	8.62	24.0258231	1.22E-01	4144783	4144906	NC_000004.11
cg20263853	5.50E-14	2.26E-15	8.62	24.0214228	1.37E-01	8824177	8824300	NC_000001.10
cg05089090	5.51E-14	2.26E-15	8.62	24.0196595	2.82E-01	37005570	37005693	NC_000013.10
cg21613389	5.51E-14	2.27E-15	8.62	24.0190321	1.57E-01	2807593	2807716	NC_000017.10
cg05214685	5.52E-14	2.27E-15	8.62	24.0186055	1.72E-01	223177493	223177616	NC_000002.11
cg00951108	5.53E-14	2.27E-15	8.62	24.0149537	4.64E-02	110416982	110417105	NC_000001.10
cg16635314	5.55E-14	2.28E-15	8.62	24.0126924	2.09E-01	31580299	31580422	NC_000016.9
cg06052479	5.55E-14	2.28E-15	8.62	24.0125704	8.65E-02	1050258	1050381	NC_000016.9
cg21930842	5.56E-14	2.29E-15	8.62	24.009329	1.44E-01	241631663	241631786	NC_000002.11
cg21745612	5.59E-14	2.30E-15	8.62	24.0038026	1.91E-01	32907636	32907759	NC_000017.10
cg12460765	5.60E-14	2.30E-15	8.62	24.0026119	2.65E-02	2415105	2415228	NC_000007.13
cg13462129	5.61E-14	2.31E-15	8.62	24.0000526	2.05E-01	96651281	96651404	NC_000007.13
cg16381449	5.62E-14	2.31E-15	8.62	23.9980081	4.38E-02	44281505	44281628	NC_000015.9
cg00920668	5.63E-14	2.32E-15	8.62	23.9967292	1.80E-01	207991122	207991245	NC_000001.10
cg14693804	5.63E-14	2.32E-15	8.62	23.9966607	7.41E-02	92757970	92758093	NC_000005.9
cg06260709	5.63E-14	2.32E-15	8.62	23.9965757	1.17E-01	48626621	48626744	NC_000020.10

cg04131898	5.65E-14	2.32E-15	8.62	23.9937342	1.93E-01	32459428	32459551	NC_000011.9
cg07992340	5.65E-14	2.32E-15	8.62	23.993521	9.86E-02	70320238	70320361	NC_000016.9
cg12124922	5.66E-14	2.33E-15	8.62	23.9913807	2.92E-01	158150649	158150772	NC_000001.10
cg03386971	5.66E-14	2.33E-15	8.62	23.990733	1.09E-01	46328304	46328427	NC_000022.10
cg02808379	5.66E-14	2.33E-15	8.62	23.9902448	8.28E-02	3502739	3502862	NC_000002.11
cg01944655	5.68E-14	2.34E-15	8.62	23.9877276	2.00E-01	119418804	119418927	NC_000012.11
cg23632985	5.70E-14	2.35E-15	8.61	23.9838451	2.36E-01	20184637	20184760	NC_000011.9
cg13490403	5.70E-14	2.35E-15	8.61	23.9831309	2.08E-01	124982413	124982536	NC_000009.11
cg01419567	5.70E-14	2.35E-15	8.61	23.9828706	2.40E-01	77594595	77594718	NC_000008.10
cg16079541	5.70E-14	2.35E-15	8.61	23.9825998	7.54E-02	30848846	30848969	NC_000006.11
cg27111463	5.71E-14	2.35E-15	8.61	23.9823334	2.21E-01	88751405	88751528	NC_000002.11
cg20331241	5.71E-14	2.35E-15	8.61	23.9810867	4.81E-02	32052409	32052532	NC_000006.11
cg05238769	5.71E-14	2.35E-15	8.61	23.9807285	2.03E-01	34399260	34399383	NC_000021.8
cg23865240	5.73E-14	2.36E-15	8.61	23.9771848	1.84E-01	27134109	27134232	NC_000007.13
cg10887069	5.73E-14	2.36E-15	8.61	23.9769064	1.28E-01	178993698	178993821	NC_000001.10
cg06463958	5.75E-14	2.37E-15	8.61	23.9746695	3.08E-01	166582393	166582516	NC_000006.11
cg04702989	5.79E-14	2.39E-15	8.61	23.9672241	1.08E-01	31053962	31054085	NC_000016.9
cg17946668	5.79E-14	2.39E-15	8.61	23.966609	3.69E-02	231006796	231006919	NC_000001.10
cg09684066	5.80E-14	2.39E-15	8.61	23.9657857	1.17E-01	183891565	183891688	NC_000001.10
cg17014436	5.80E-14	2.39E-15	8.61	23.9650142	6.42E-02	45206948	45207071	NC_000021.8
cg09813248	5.80E-14	2.39E-15	8.61	23.9641744	1.73E-01	121164194	121164317	NC_000012.11
cg23971170	5.81E-14	2.40E-15	8.61	23.9630888	2.09E-01	165326269	165326392	NC_000001.10
cg24736989	5.82E-14	2.40E-15	8.61	23.9607813	2.13E-01	60973904	60974027	NC_000014.8
cg03994318	5.86E-14	2.42E-15	8.61	23.954737	2.66E-01	109147921	109148044	NC_000013.10
cg08704302	5.87E-14	2.42E-15	8.61	23.9527172	1.24E-01	65373833	65373956	NC_000015.9

cg14772639	5.89E-14	2.43E-15	8.61	23.9482553	1.36E-01	128711564	128711687	NC_000002.11
cg03847373	5.90E-14	2.44E-15	8.61	23.9465053	2.39E-01	50265767	50265890	NC_000005.9
cg10457689	5.91E-14	2.44E-15	8.61	23.9442265	1.27E-01	66621696	66621819	NC_000008.10
cg10056132	5.92E-14	2.45E-15	8.61	23.9432733	1.66E-01	50887731	50887854	NC_000010.10
cg07922843	5.94E-14	2.46E-15	8.61	23.9393374	1.69E-01	92083173	92083296	NC_000001.10
cg23052568	5.95E-14	2.46E-15	8.61	23.9382153	2.86E-01	42995527	42995650	NC_000005.9
cg16706905	5.96E-14	2.46E-15	8.61	23.9364601	4.59E-02	2650027	2650150	NC_000007.13
cg16460383	5.97E-14	2.47E-15	8.61	23.9346586	1.76E-01	818890	819013	NC_000017.10
cg00699993	6.02E-14	2.49E-15	8.61	23.9258881	2.05E-01	158141570	158141693	NC_000004.11
cg16851417	6.02E-14	2.49E-15	8.61	23.9248976	2.03E-01	38077042	38077165	NC_000021.8
cg10859327	6.02E-14	2.49E-15	8.61	23.9244794	1.45E-01	101704782	101704905	NC_000010.10
cg13638420	6.04E-14	2.50E-15	8.61	23.9223918	1.83E-01	32448293	32448416	NC_000011.9
cg13976823	6.04E-14	2.50E-15	8.61	23.9221985	8.39E-02	137396107	137396230	NC_000009.11
cg02333960	6.05E-14	2.51E-15	8.6	23.919018	1.33E-01	1012935	1013058	NC_000007.13
cg13561879	6.09E-14	2.52E-15	8.6	23.9134283	1.76E-01	35092687	35092810	NC_000008.10
cg19810598	6.10E-14	2.53E-15	8.6	23.9117039	2.13E-01	50892674	50892797	NC_000001.10
cg19472098	6.11E-14	2.53E-15	8.6	23.9093034	1.97E-01	175809	175932	NC_000012.11
cg21546671	6.11E-14	2.53E-15	8.6	23.9088592	2.91E-01	46655387	46655510	NC_000017.10
cg05109569	6.12E-14	2.54E-15	8.6	23.9073392	1.57E-01	27146430	27146553	NC_000007.13
cg02960016	6.14E-14	2.54E-15	8.6	23.9046824	8.97E-02	11192067	11192190	NC_000003.11
cg18808076	6.15E-14	2.55E-15	8.6	23.9032533	4.83E-02	3074184	3074307	NC_000011.9
cg16471110	6.15E-14	2.55E-15	8.6	23.9024281	3.47E-02	77133818	77133941	NC_000018.9
cg00933415	6.16E-14	2.56E-15	8.6	23.8999309	6.56E-02	33135453	33135576	NC_000006.11
cg18520696	6.19E-14	2.57E-15	8.6	23.8947944	1.51E-01	4631822	4631945	NC_000017.10
cg00288463	6.20E-14	2.57E-15	8.6	23.8945715	1.21E-01	223382426	223382549	NC_000002.11

cg19047670	6.20E-14	2.57E-15	8.6	23.8936078	7.10E-02	69461451	69461574	NC_000011.9
cg04553307	6.21E-14	2.58E-15	8.6	23.8920757	1.26E-01	922775	922898	NC_000007.13
cg15980539	6.21E-14	2.58E-15	8.6	23.8914987	1.96E-01	152128865	152128988	NC_000006.11
cg09096555	6.22E-14	2.58E-15	8.6	23.8909015	1.33E-01	72848358	72848481	NC_000017.10
cg20631104	6.22E-14	2.58E-15	8.6	23.8904787	2.44E-01	81951446	81951569	NC_000004.11
cg21220614	6.23E-14	2.59E-15	8.6	23.8885508	9.59E-02	43266108	43266231	NC_000022.10
cg07879474	6.24E-14	2.59E-15	8.6	23.8867352	8.26E-02	139064216	139064339	NC_000005.9
cg25741259	6.24E-14	2.59E-15	8.6	23.8864993	1.11E-01	6022752	6022875	NC_000019.9
cg24957532	6.25E-14	2.59E-15	8.6	23.8856608	8.94E-02	118730026	118730149	NC_000005.9
cg01216877	6.26E-14	2.60E-15	8.6	23.8831949	1.37E-01	24869928	24870051	NC_000003.11
cg07969619	6.26E-14	2.60E-15	8.6	23.8831005	2.09E-01	115136279	115136402	NC_000012.11
cg07722657	6.27E-14	2.60E-15	8.6	23.88206	1.64E-01	221060360	221060483	NC_000001.10
cg03159124	6.30E-14	2.62E-15	8.6	23.8767552	2.51E-01	7118077	7118200	NC_000018.9
cg14817655	6.30E-14	2.62E-15	8.6	23.8765628	2.02E-01	188916724	188916847	NC_000004.11
cg03001098	6.31E-14	2.62E-15	8.6	23.8747423	1.22E-01	55712097	55712220	NC_000007.13
cg07126525	6.33E-14	2.63E-15	8.6	23.8716527	2.35E-01	166218519	166218642	NC_000006.11
cg09538603	6.35E-14	2.64E-15	8.6	23.8685335	5.81E-02	11219986	11220109	NC_000016.9
cg26608174	6.35E-14	2.64E-15	8.6	23.8681837	1.63E-01	46711035	46711158	NC_000017.10
cg17816394	6.36E-14	2.64E-15	8.6	23.8667229	2.40E-01	235814009	235814132	NC_000001.10
cg18110483	6.37E-14	2.65E-15	8.6	23.8642013	2.00E-01	79330959	79331082	NC_000005.9
cg03995968	6.38E-14	2.65E-15	8.6	23.8632268	2.08E-01	65953812	65953935	NC_000016.9
cg01307461	6.38E-14	2.65E-15	8.6	23.8629958	5.57E-02	177963966	177964089	NC_000003.11
cg06730250	6.38E-14	2.65E-15	8.6	23.8623411	9.65E-02	177821870	177821993	NC_000005.9
cg03314029	6.38E-14	2.65E-15	8.6	23.8621789	2.25E-01	88496788	88496911	NC_000016.9
cg11649268	6.40E-14	2.66E-15	8.59	23.8589089	5.90E-02	2322610	2322733	NC_000017.10

cg07993372	6.41E-14	2.67E-15	8.59	23.85816	2.92E-02	427084	427207	NC_000016.9
cg07255841	6.43E-14	2.67E-15	8.59	23.8548093	1.66E-01	531305	531428	NC_000005.9
cg01186062	6.44E-14	2.68E-15	8.59	23.8533177	4.42E-02	66420823	66420946	NC_000007.13
cg15715477	6.45E-14	2.68E-15	8.59	23.8512899	2.41E-01	65283042	65283165	NC_000008.10
cg21019662	6.45E-14	2.68E-15	8.59	23.8512825	1.20E-01	107176420	107176543	NC_000012.11
cg08865099	6.47E-14	2.69E-15	8.59	23.848356	1.54E-01	27281581	27281704	NC_000007.13
cg21711132	6.47E-14	2.69E-15	8.59	23.8481618	2.00E-01	27215952	27216075	NC_000015.9
cg08560111	6.48E-14	2.70E-15	8.59	23.8455199	2.23E-01	19147487	19147610	NC_000007.13
cg25903143	6.50E-14	2.71E-15	8.59	23.8434976	1.41E-01	68920466	68920589	NC_000011.9
cg20498414	6.50E-14	2.71E-15	8.59	23.8426104	2.89E-01	43439400	43439523	NC_000020.10
cg18675097	6.51E-14	2.71E-15	8.59	23.8412599	1.81E-01	28227127	28227250	NC_000006.11
cg14789818	6.52E-14	2.72E-15	8.59	23.8388775	1.50E-01	227748712	227748835	NC_000001.10
cg26555310	6.55E-14	2.73E-15	8.59	23.834318	1.19E-01	68914717	68914840	NC_000001.10
cg17214381	6.57E-14	2.74E-15	8.59	23.8319145	2.14E-01	188916668	188916791	NC_000004.11
cg15843567	6.57E-14	2.74E-15	8.59	23.8312371	2.59E-01	26223206	26223329	NC_000010.10
cg24529858	6.58E-14	2.74E-15	8.59	23.8292252	1.87E-01	187186949	187187072	NC_000004.11
cg06018296	6.61E-14	2.76E-15	8.59	23.8248517	1.10E-01	52707138	52707261	NC_000013.10
cg12120861	6.61E-14	2.76E-15	8.59	23.8245323	1.21E-01	61815378	61815501	NC_000017.10
cg23640615	6.61E-14	2.76E-15	8.59	23.8244518	1.60E-01	40034517	40034640	NC_000001.10
cg01972418	6.63E-14	2.76E-15	8.59	23.8226469	2.14E-01	37125430	37125553	NC_000014.8
cg11282657	6.63E-14	2.76E-15	8.59	23.8222636	1.50E-01	79615101	79615224	NC_000017.10
cg24736380	6.64E-14	2.77E-15	8.59	23.8198481	5.53E-02	3144101	3144224	NC_000010.10
cg11312896	6.66E-14	2.78E-15	8.59	23.816481	2.60E-01	58545122	58545245	NC_000019.9
cg00467553	6.68E-14	2.79E-15	8.59	23.8143143	1.11E-01	38391432	38391555	NC_000009.11
cg26300652	6.68E-14	2.79E-15	8.59	23.8141702	2.41E-01	33593004	33593127	NC_000012.11

cg27170782	6.70E-14	2.80E-15	8.59	23.8113092	2.27E-01	46800028	46800151	NC_000017.10
cg01757312	6.74E-14	2.81E-15	8.59	23.805171	1.73E-01	112720565	112720688	NC_000013.10
cg21128568	6.75E-14	2.82E-15	8.59	23.8038173	1.32E-01	216411327	216411450	NC_000002.11
cg20743280	6.77E-14	2.83E-15	8.59	23.8002805	2.43E-01	22930884	22931007	NC_000018.9
cg14055896	6.77E-14	2.83E-15	8.59	23.7999226	2.44E-01	54481859	54481982	NC_000019.9
cg08421685	6.78E-14	2.83E-15	8.59	23.7987199	2.28E-01	238611	238734	NC_000003.11
cg18799217	6.78E-14	2.83E-15	8.59	23.7979852	1.53E-01	87985589	87985712	NC_000005.9
cg26198463	6.80E-14	2.84E-15	8.58	23.7959732	1.69E-01	72987744	72987867	NC_000008.10
cg05184882	6.83E-14	2.85E-15	8.58	23.7913032	7.64E-02	55509593	55509716	NC_000017.10
cg02323356	6.86E-14	2.87E-15	8.58	23.7868226	1.26E-01	220313153	220313276	NC_000002.11
cg00836482	6.86E-14	2.87E-15	8.58	23.7861285	2.59E-01	119528956	119529079	NC_000003.11
cg22871668	6.87E-14	2.87E-15	8.58	23.7850332	2.66E-01	133562492	133562615	NC_000006.11
cg16393742	6.89E-14	2.88E-15	8.58	23.7824707	1.62E-01	132922227	132922350	NC_000012.11
cg10813720	6.89E-14	2.88E-15	8.58	23.781833	6.62E-02	26670374	26670497	NC_000001.10
cg04141813	6.89E-14	2.88E-15	8.58	23.7814066	2.30E-01	216450	216573	NC_000016.9
cg08958294	6.90E-14	2.88E-15	8.58	23.7805809	2.44E-01	146350131	146350254	NC_000006.11
cg15941948	6.90E-14	2.88E-15	8.58	23.7803556	1.67E-01	2751552	2751675	NC_000005.9
cg19676553	6.91E-14	2.89E-15	8.58	23.7786836	1.48E-01	105751629	105751752	NC_000014.8
cg09853371	6.91E-14	2.89E-15	8.58	23.7781202	2.78E-01	57522145	57522268	NC_000004.11
cg13371883	6.93E-14	2.90E-15	8.58	23.7750172	1.55E-01	101511926	101512049	NC_000002.11
cg02240013	6.94E-14	2.90E-15	8.58	23.7739456	5.23E-02	48680392	48680515	NC_000003.11
cg26526374	6.96E-14	2.91E-15	8.58	23.7712579	2.39E-01	109674854	109674977	NC_000010.10
cg05934012	7.05E-14	2.95E-15	8.58	23.7579053	1.91E-01	1533852	1533975	NC_000001.10
cg01197831	7.06E-14	2.96E-15	8.58	23.7560951	1.19E-01	97355941	97356064	NC_000009.11
cg08195247	7.07E-14	2.96E-15	8.58	23.7552013	2.18E-01	176933286	176933409	NC_000002.11

cg21830459	7.07E-14	2.96E-15	8.58	23.7543765	1.47E-01	17680563	17680686	NC_000022.10
cg02052915	7.10E-14	2.97E-15	8.58	23.750782	1.42E-01	46797568	46797691	NC_000017.10
cg25326048	7.15E-14	2.99E-15	8.58	23.7432968	1.85E-01	153446106	153446229	NC_000007.13
cg13210467	7.15E-14	3.00E-15	8.58	23.7428073	1.32E-01	99775443	99775566	NC_000007.13
cg26051135	7.15E-14	3.00E-15	8.58	23.7424583	6.51E-02	47087133	47087256	NC_000010.10
cg14608424	7.16E-14	3.00E-15	8.58	23.740847	5.27E-02	1079641	1079764	NC_000017.10
cg11273250	7.18E-14	3.01E-15	8.58	23.7387473	6.63E-02	42716703	42716826	NC_000006.11
cg17922359	7.18E-14	3.01E-15	8.58	23.7383252	1.77E-01	13530923	13531046	NC_000004.11
cg12877723	7.18E-14	3.01E-15	8.58	23.7381149	2.03E-01	57358312	57358435	NC_000008.10
cg20527270	7.19E-14	3.02E-15	8.58	23.7364134	2.78E-01	49468688	49468811	NC_000008.10
cg17338208	7.21E-14	3.02E-15	8.57	23.7340519	2.90E-01	93520024	93520147	NC_000007.13
cg15973818	7.21E-14	3.02E-15	8.57	23.733548	1.22E-01	53623995	53624118	NC_000008.10
cg18552380	7.21E-14	3.03E-15	8.57	23.7329635	2.28E-01	97685945	97686068	NC_000014.8
cg25038926	7.24E-14	3.04E-15	8.57	23.7291559	6.86E-02	111556206	111556329	NC_000002.11
cg10531355	7.26E-14	3.05E-15	8.57	23.7266726	1.54E-01	79489249	79489372	NC_000005.9
cg15347189	7.27E-14	3.05E-15	8.57	23.7251714	1.88E-01	187387999	187388122	NC_000003.11
cg01342746	7.27E-14	3.05E-15	8.57	23.7243975	1.52E-01	44449571	44449694	NC_000004.11
cg16791016	7.28E-14	3.05E-15	8.57	23.7238832	4.49E-02	85354618	85354741	NC_000016.9
cg16614288	7.30E-14	3.06E-15	8.57	23.720936	1.05E-01	17608110	17608233	NC_000019.9
cg14367229	7.31E-14	3.07E-15	8.57	23.7193131	1.78E-01	28367741	28367864	NC_000013.10
cg12411964	7.31E-14	3.07E-15	8.57	23.7186484	6.08E-02	66818958	66819081	NC_000001.10
cg18689332	7.33E-14	3.08E-15	8.57	23.7164421	1.70E-01	114837666	114837789	NC_000012.11
cg04452534	7.34E-14	3.08E-15	8.57	23.7147241	1.40E-01	56133370	56133493	NC_000005.9
cg22665696	7.34E-14	3.08E-15	8.57	23.7139173	2.17E-01	131768755	131768878	NC_000010.10
cg09740598	7.35E-14	3.09E-15	8.57	23.7132957	1.30E-01	26808425	26808548	NC_000017.10

cg21959090	7.38E-14	3.10E-15	8.57	23.7091133	7.10E-02	45744435	45744558	NC_000019.9
cg10427452	7.38E-14	3.10E-15	8.57	23.708901	2.63E-01	3122030	3122153	NC_000005.9
cg12882697	7.39E-14	3.11E-15	8.57	23.7074054	1.82E-01	1445549	1445672	NC_000005.9
cg04575427	7.40E-14	3.11E-15	8.57	23.7059628	1.19E-01	44144711	44144834	NC_000020.10
cg20894457	7.44E-14	3.12E-15	8.57	23.7010988	8.80E-02	24584366	24584489	NC_000022.10
cg09527126	7.47E-14	3.14E-15	8.57	23.6961729	2.67E-01	128675283	128675406	NC_000012.11
cg25198120	7.52E-14	3.16E-15	8.57	23.6902577	1.31E-01	121708646	121708769	NC_000003.11
cg14604066	7.52E-14	3.16E-15	8.57	23.6891346	1.56E-01	139590572	139590695	NC_000009.11
cg11413576	7.55E-14	3.18E-15	8.57	23.6853116	1.04E-01	59650783	59650906	NC_000008.10
cg16884902	7.57E-14	3.18E-15	8.57	23.6831405	1.90E-01	20617974	20618097	NC_000011.9
cg18564075	7.58E-14	3.19E-15	8.57	23.6818616	8.57E-02	116705063	116705186	NC_000012.11
cg10770742	7.58E-14	3.19E-15	8.57	23.6818378	2.65E-01	151107285	151107408	NC_000007.13
cg23279152	7.61E-14	3.20E-15	8.57	23.6772794	8.49E-02	75856823	75856946	NC_000004.11
cg07166291	7.62E-14	3.21E-15	8.57	23.6752128	1.50E-01	95971994	95972117	NC_000002.11
cg16136290	7.63E-14	3.21E-15	8.57	23.6738448	7.81E-02	112280233	112280356	NC_000001.10
cg13450005	7.63E-14	3.21E-15	8.57	23.6732975	2.28E-01	32457257	32457380	NC_000011.9
cg07447431	7.63E-14	3.21E-15	8.57	23.6732939	4.39E-02	27795808	27795931	NC_000010.10
cg01019587	7.64E-14	3.21E-15	8.57	23.6730903	2.04E-01	53286921	53287044	NC_000007.13
cg04712635	7.69E-14	3.24E-15	8.56	23.6653249	9.54E-02	141203013	141203136	NC_000005.9
cg25993718	7.71E-14	3.25E-15	8.56	23.66293	1.77E-01	54580196	54580319	NC_000020.10
cg11129341	7.72E-14	3.25E-15	8.56	23.6621072	1.29E-01	19870761	19870884	NC_000011.9
cg04935449	7.72E-14	3.25E-15	8.56	23.6613061	1.77E-01	139208871	139208994	NC_000007.13
cg20823772	7.73E-14	3.26E-15	8.56	23.6605168	1.77E-01	99962013	99962136	NC_000008.10
cg14832406	7.73E-14	3.26E-15	8.56	23.6597638	1.61E-01	75602451	75602574	NC_000001.10
cg20435370	7.73E-14	3.26E-15	8.56	23.6594914	2.00E-01	80646370	80646493	NC_000009.11

cg02145310	7.76E-14	3.27E-15	8.56	23.6560184	1.21E-01	80844809	80844932	NC_000010.10
cg20677171	7.77E-14	3.28E-15	8.56	23.6539207	1.03E-01	56608297	56608420	NC_000019.9
cg14978637	7.78E-14	3.28E-15	8.56	23.6532021	1.31E-01	41950817	41950940	NC_000001.10
cg09628488	7.78E-14	3.28E-15	8.56	23.6527291	1.11E-01	122598941	122599064	NC_000008.10
cg26171271	7.79E-14	3.28E-15	8.56	23.6521081	8.42E-02	36518358	36518481	NC_000006.11
cg20617977	7.79E-14	3.29E-15	8.56	23.651359	9.44E-02	38053457	38053580	NC_000004.11
cg26736812	7.79E-14	3.29E-15	8.56	23.6511529	2.02E-01	48042353	48042476	NC_000017.10
cg09935495	7.79E-14	3.29E-15	8.56	23.6507491	9.27E-02	31690157	31690280	NC_000006.11
cg06295238	7.80E-14	3.29E-15	8.56	23.650004	6.16E-02	1205447	1205570	NC_000004.11
cg19134568	7.80E-14	3.29E-15	8.56	23.6493355	1.42E-01	147561339	147561462	NC_000004.11
cg10943359	7.81E-14	3.30E-15	8.56	23.6484726	1.64E-01	165304531	165304654	NC_000004.11
cg04115680	7.82E-14	3.30E-15	8.56	23.6473708	2.50E-01	75889229	75889352	NC_000007.13
cg08195921	7.82E-14	3.30E-15	8.56	23.6468276	9.79E-02	131701622	131701745	NC_000012.11
cg16616521	7.85E-14	3.31E-15	8.56	23.643902	2.40E-01	31821388	31821511	NC_000011.9
cg02252323	7.88E-14	3.33E-15	8.56	23.6388452	1.76E-01	22414339	22414462	NC_000003.11
cg10193069	7.89E-14	3.33E-15	8.56	23.6380295	1.57E-01	50058971	50059094	NC_000019.9
cg12679749	7.95E-14	3.36E-15	8.56	23.6306704	9.55E-02	81101251	81101374	NC_000004.11
cg27621129	7.96E-14	3.36E-15	8.56	23.6291751	1.68E-01	19152121	19152244	NC_000007.13
cg19724555	7.97E-14	3.37E-15	8.56	23.6272498	1.96E-01	132906439	132906562	NC_000012.11
cg09403956	7.97E-14	3.37E-15	8.56	23.6268176	1.02E-01	31324774	31324897	NC_000010.10
cg05327864	7.98E-14	3.37E-15	8.56	23.6261092	2.45E-01	136554352	136554475	NC_000007.13
cg20182232	8.00E-14	3.38E-15	8.56	23.6240681	8.69E-02	118865365	118865488	NC_000003.11
cg25729445	8.06E-14	3.41E-15	8.56	23.6156058	1.83E-01	29595347	29595470	NC_000006.11
cg20080983	8.07E-14	3.41E-15	8.56	23.6144842	8.10E-02	29430271	29430394	NC_000022.10
cg09137718	8.09E-14	3.42E-15	8.56	23.6116582	1.15E-01	140239682	140239805	NC_000007.13

cg03790422	8.09E-14	3.42E-15	8.56	23.6114792	3.77E-02	39552818	39552941	NC_000017.10
cg15237494	8.10E-14	3.43E-15	8.55	23.6101049	2.21E-01	28339334	28339457	NC_000021.8
cg00920572	8.11E-14	3.43E-15	8.55	23.6094412	1.29E-01	54349349	54349472	NC_000012.11
cg13764991	8.13E-14	3.44E-15	8.55	23.6065938	2.19E-01	1052046	1052169	NC_000009.11
cg23508264	8.16E-14	3.45E-15	8.55	23.6025874	1.73E-01	62415024	62415147	NC_000011.9
cg24658517	8.17E-14	3.45E-15	8.55	23.6018875	1.65E-01	221065322	221065445	NC_000001.10
cg13791131	8.21E-14	3.47E-15	8.55	23.5966953	2.35E-01	2161892	2162015	NC_000011.9
cg14016236	8.23E-14	3.48E-15	8.55	23.593749	2.19E-01	171792545	171792668	NC_000003.11
cg08468187	8.24E-14	3.49E-15	8.55	23.5930987	1.30E-01	54963323	54963446	NC_000019.9
cg17424999	8.28E-14	3.50E-15	8.55	23.5881064	1.85E-01	20622988	20623111	NC_000011.9
cg07139668	8.29E-14	3.51E-15	8.55	23.5858535	3.35E-02	46686806	46686929	NC_000001.10
cg05783139	8.30E-14	3.52E-15	8.55	23.5847574	2.22E-01	198650985	198651108	NC_000002.11
cg01022121	8.31E-14	3.52E-15	8.55	23.583067	1.07E-01	2410885	2411008	NC_000001.10
cg10755973	8.32E-14	3.52E-15	8.55	23.582475	2.65E-01	126773879	126774002	NC_000009.11
cg04339613	8.32E-14	3.53E-15	8.55	23.5820188	1.28E-01	131757303	131757426	NC_000010.10
cg06219206	8.33E-14	3.53E-15	8.55	23.5807246	1.26E-01	24383177	24383300	NC_000012.11
cg08363794	8.34E-14	3.54E-15	8.55	23.5791284	1.69E-01	176986460	176986583	NC_000002.11
cg02244431	8.35E-14	3.54E-15	8.55	23.5789623	1.80E-01	236588226	236588349	NC_000002.11
cg00408285	8.35E-14	3.54E-15	8.55	23.5784006	1.51E-01	85670262	85670385	NC_000016.9
cg06879152	8.39E-14	3.56E-15	8.55	23.5734585	1.62E-01	60937041	60937164	NC_000010.10
cg26188621	8.39E-14	3.56E-15	8.55	23.5727993	1.41E-01	76983413	76983536	NC_000010.10
cg11925066	8.40E-14	3.56E-15	8.55	23.5717962	1.24E-01	132182304	132182427	NC_000002.11
cg25321212	8.43E-14	3.57E-15	8.55	23.5684978	9.25E-02	128993187	128993310	NC_000010.10
cg25953146	8.43E-14	3.57E-15	8.55	23.5683005	6.13E-02	132558939	132559062	NC_000002.11
cg18417954	8.45E-14	3.59E-15	8.55	23.565359	1.52E-01	55672513	55672636	NC_000019.9

cg16172329	8.48E-14	3.60E-15	8.55	23.5615339	1.71E-01	162907368	162907491	NC_000002.11
cg07483638	8.49E-14	3.60E-15	8.55	23.5604991	5.95E-02	155674621	155674744	NC_000007.13
cg20631014	8.51E-14	3.61E-15	8.55	23.5582548	2.05E-01	84419242	84419365	NC_000006.11
cg12600556	8.51E-14	3.61E-15	8.55	23.5578521	8.96E-02	94504969	94505092	NC_000001.10
cg06598987	8.51E-14	3.61E-15	8.55	23.5578416	6.26E-02	25846548	25846671	NC_000017.10
cg13382504	8.54E-14	3.62E-15	8.55	23.5544344	4.43E-02	112170902	112171025	NC_000006.11
cg00122851	8.55E-14	3.63E-15	8.55	23.5537211	6.13E-02	1413159	1413282	NC_000016.9
cg21539229	8.57E-14	3.64E-15	8.55	23.5506408	1.30E-01	48413146	48413269	NC_000003.11
cg11791452	8.61E-14	3.66E-15	8.54	23.5460394	1.14E-01	121663137	121663260	NC_000002.11
cg04154911	8.62E-14	3.66E-15	8.54	23.5443729	8.21E-02	36725928	36726051	NC_000002.11
cg23262897	8.64E-14	3.67E-15	8.54	23.5419037	1.52E-01	140718655	140718778	NC_000005.9
cg19673418	8.67E-14	3.68E-15	8.54	23.5386448	4.54E-02	31600630	31600753	NC_000006.11
cg18777299	8.67E-14	3.68E-15	8.54	23.5385682	1.52E-01	26994740	26994863	NC_000004.11
cg02837536	8.67E-14	3.68E-15	8.54	23.5383178	2.12E-01	4771682	4771805	NC_000001.10
cg04188273	8.67E-14	3.69E-15	8.54	23.5378895	2.47E-01	110223830	110223953	NC_000004.11
cg08415508	8.69E-14	3.69E-15	8.54	23.5357612	9.81E-02	144303645	144303768	NC_000008.10
cg10586423	8.73E-14	3.71E-15	8.54	23.5318726	8.79E-02	111015513	111015636	NC_000001.10
cg02829688	8.73E-14	3.71E-15	8.54	23.5308273	1.65E-01	119527008	119527131	NC_000001.10
cg23427269	8.75E-14	3.72E-15	8.54	23.5290285	2.75E-01	70995444	70995567	NC_000002.11
cg04481096	8.76E-14	3.72E-15	8.54	23.5277597	2.26E-01	27142100	27142223	NC_000007.13
cg05527869	8.78E-14	3.73E-15	8.54	23.52523	3.17E-01	35294476	35294599	NC_000017.10
cg27582240	8.81E-14	3.75E-15	8.54	23.5216518	1.30E-01	75380514	75380637	NC_000018.9
cg14700684	8.82E-14	3.75E-15	8.54	23.52087	1.11E-01	22086413	22086536	NC_000008.10
cg11410718	8.85E-14	3.77E-15	8.54	23.5168117	1.93E-01	27170412	27170535	NC_000007.13
cg06734979	8.85E-14	3.77E-15	8.54	23.5164794	7.62E-02	88457445	88457568	NC_000016.9

cg24408200	8.88E-14	3.78E-15	8.54	23.5128245	1.10E-01	80914695	80914818	NC_000017.10
cg26186727	8.89E-14	3.79E-15	8.54	23.5117021	1.92E-01	70534534	70534657	NC_000018.9
cg03718025	8.93E-14	3.80E-15	8.54	23.5075193	1.45E-01	12038128	12038251	NC_000018.9
cg08478121	8.94E-14	3.80E-15	8.54	23.5066547	1.31E-01	88545571	88545694	NC_000016.9
cg15990629	8.94E-14	3.81E-15	8.54	23.5062988	1.09E-01	25054873	25054996	NC_000012.11
cg02191909	8.96E-14	3.81E-15	8.54	23.5045452	7.70E-02	33273716	33273839	NC_000006.11
cg14907769	8.98E-14	3.82E-15	8.54	23.5021745	2.30E-01	49468941	49469064	NC_000008.10
cg24835238	9.03E-14	3.85E-15	8.54	23.4958453	1.38E-01	34872733	34872856	NC_000015.9
cg05726756	9.04E-14	3.85E-15	8.54	23.4952204	1.86E-01	46608288	46608411	NC_000017.10
cg21942576	9.04E-14	3.85E-15	8.54	23.4950527	9.97E-02	46126885	46127008	NC_000001.10
cg02745211	9.04E-14	3.85E-15	8.54	23.4945845	2.67E-01	132757106	132757229	NC_000003.11
cg07102450	9.05E-14	3.86E-15	8.54	23.493627	8.99E-02	4355464	4355587	NC_000016.9
cg09573795	9.05E-14	3.86E-15	8.54	23.4934297	1.63E-01	4863874	4863997	NC_000004.11
cg24928938	9.07E-14	3.86E-15	8.54	23.4915928	6.61E-02	4371934	4372057	NC_000019.9
cg10539928	9.07E-14	3.87E-15	8.54	23.4909841	4.80E-02	164693462	164693585	NC_000001.10
cg03437186	9.09E-14	3.87E-15	8.54	23.4891772	2.32E-01	45614848	45614971	NC_000007.13
cg01998213	9.10E-14	3.88E-15	8.54	23.4877929	1.96E-01	19149989	19150112	NC_000007.13
cg13589108	9.11E-14	3.88E-15	8.54	23.4864957	2.39E-01	177140680	177140803	NC_000001.10
cg05524354	9.12E-14	3.89E-15	8.53	23.4850282	1.55E-01	129797760	129797883	NC_000010.10
cg10157936	9.17E-14	3.91E-15	8.53	23.4798249	1.01E-01	105220767	105220890	NC_000014.8
cg24216506	9.18E-14	3.91E-15	8.53	23.4785737	8.66E-02	3572257	3572380	NC_000010.10
cg10985956	9.18E-14	3.92E-15	8.53	23.4783326	6.94E-02	87774698	87774821	NC_000001.10
cg12300724	9.19E-14	3.92E-15	8.53	23.4774768	2.37E-01	72460135	72460258	NC_000008.10
cg05787507	9.19E-14	3.92E-15	8.53	23.4772191	4.74E-02	32751472	32751595	NC_000001.10
cg04249522	9.19E-14	3.92E-15	8.53	23.4771361	2.09E-01	166218512	166218635	NC_000006.11

cg15725639	9.20E-14	3.92E-15	8.53	23.4762204	1.62E-01	47856924	47857047	NC_000019.9
cg17612681	9.21E-14	3.93E-15	8.53	23.474699	1.36E-01	66298197	66298320	NC_000002.11
cg02463137	9.23E-14	3.94E-15	8.53	23.4720142	7.74E-02	125778039	125778162	NC_000010.10
cg08732466	9.25E-14	3.95E-15	8.53	23.4699347	1.44E-01	2982592	2982715	NC_000001.10
cg08337773	9.25E-14	3.95E-15	8.53	23.4697916	7.58E-02	72100575	72100698	NC_000015.9
cg14695492	9.28E-14	3.96E-15	8.53	23.4660971	1.51E-01	143545574	143545697	NC_000008.10
cg17208528	9.31E-14	3.97E-15	8.53	23.4637843	2.54E-01	134211114	134211237	NC_000006.11
cg06720014	9.31E-14	3.98E-15	8.53	23.4630928	7.46E-02	19390728	19390851	NC_000001.10
cg23646375	9.33E-14	3.98E-15	8.53	23.4612431	1.62E-01	209847618	209847741	NC_000001.10
cg18539122	9.34E-14	3.99E-15	8.53	23.4599997	2.00E-01	79169872	79169995	NC_000013.10
cg23538468	9.34E-14	3.99E-15	8.53	23.4599071	9.76E-02	140588019	140588142	NC_000009.11
cg25938646	9.35E-14	3.99E-15	8.53	23.4587059	1.94E-01	84456681	84456804	NC_000013.10
cg06337342	9.37E-14	4.00E-15	8.53	23.4565273	7.55E-02	27963567	27963690	NC_000007.13
cg11724970	9.40E-14	4.02E-15	8.53	23.4528918	1.51E-01	27182493	27182616	NC_000007.13
cg02662828	9.41E-14	4.02E-15	8.53	23.4521115	1.13E-01	48492848	48492971	NC_000004.11
cg26101942	9.44E-14	4.04E-15	8.53	23.4480403	7.10E-02	28232176	28232299	NC_000008.10
cg12689205	9.44E-14	4.04E-15	8.53	23.44778	1.30E-01	110321674	110321797	NC_000013.10
cg22769941	9.46E-14	4.04E-15	8.53	23.4463515	2.42E-01	20690628	20690751	NC_000011.9
cg17322443	9.47E-14	4.05E-15	8.53	23.4453842	1.76E-01	152133420	152133543	NC_000007.13
cg12143789	9.49E-14	4.06E-15	8.53	23.4433179	2.26E-01	72594461	72594584	NC_000005.9
cg10931190	9.52E-14	4.07E-15	8.53	23.4396485	1.91E-01	110406506	110406629	NC_000005.9
cg09316122	9.52E-14	4.07E-15	8.53	23.439088	2.31E-01	228195522	228195645	NC_000001.10
cg17039236	9.54E-14	4.08E-15	8.53	23.4369768	2.12E-01	77548049	77548172	NC_000018.9
cg12467749	9.55E-14	4.08E-15	8.53	23.4364402	2.12E-01	35294430	35294553	NC_000007.13
cg10984178	9.56E-14	4.09E-15	8.53	23.4349596	1.15E-01	946099	946222	NC_000001.10

cg13914354	9.56E-14	4.09E-15	8.53	23.4348173	1.02E-01	139118767	139118890	NC_000009.11
cg10602135	9.57E-14	4.09E-15	8.53	23.4341294	4.94E-02	167645394	167645517	NC_000005.9
cg14473327	9.57E-14	4.10E-15	8.53	23.4339526	2.40E-01	976634	976757	NC_000009.11
cg22157015	9.58E-14	4.10E-15	8.53	23.4324812	4.29E-02	38120891	38121014	NC_000006.11
cg15465878	9.59E-14	4.11E-15	8.53	23.4310911	4.26E-02	45183988	45184111	NC_000001.10
cg13929627	9.60E-14	4.11E-15	8.53	23.4308308	1.71E-01	79635445	79635568	NC_000009.11
cg19815574	9.60E-14	4.11E-15	8.53	23.4302119	1.23E-01	47152384	47152507	NC_000019.9
cg19861858	9.61E-14	4.11E-15	8.53	23.4292941	1.25E-01	1019352	1019475	NC_000008.10
cg03882167	9.61E-14	4.12E-15	8.53	23.4291218	2.28E-01	131514163	131514286	NC_000002.11
cg24228819	9.61E-14	4.12E-15	8.53	23.4289788	2.34E-01	136553868	136553991	NC_000007.13
cg14361001	9.64E-14	4.13E-15	8.53	23.4257011	1.21E-01	83989198	83989321	NC_000009.11
cg09234518	9.66E-14	4.14E-15	8.53	23.4238542	1.98E-01	24771114	24771237	NC_000008.10
cg16654801	9.73E-14	4.17E-15	8.52	23.4162403	1.75E-01	119301763	119301886	NC_000010.10
cg01637244	9.74E-14	4.18E-15	8.52	23.4146351	1.54E-01	25833304	25833427	NC_000003.11
cg02437766	9.75E-14	4.18E-15	8.52	23.4132255	9.76E-02	53483018	53483141	NC_000005.9
cg15123428	9.78E-14	4.19E-15	8.52	23.4108969	1.88E-01	10186139	10186262	NC_000002.11
cg03526459	9.79E-14	4.20E-15	8.52	23.4093985	1.95E-01	146549940	146550063	NC_000001.10
cg13023870	9.80E-14	4.21E-15	8.52	23.4076362	2.26E-01	37124018	37124141	NC_000014.8
cg13808012	9.80E-14	4.21E-15	8.52	23.4075574	1.41E-01	244094935	244095058	NC_000001.10
cg03323696	9.81E-14	4.21E-15	8.52	23.4073785	1.86E-01	59189120	59189243	NC_000005.9
cg05134015	9.81E-14	4.21E-15	8.52	23.4072464	1.80E-01	36973365	36973488	NC_000014.8
cg14172137	9.84E-14	4.22E-15	8.52	23.4041026	9.95E-02	114725242	114725365	NC_000006.11
cg11642106	9.85E-14	4.23E-15	8.52	23.4026425	2.08E-01	27772805	27772928	NC_000003.11
cg12877251	9.85E-14	4.23E-15	8.52	23.4022773	2.72E-01	9760880	9761003	NC_000008.10
cg06723694	9.85E-14	4.23E-15	8.52	23.402136	6.01E-02	1034059	1034182	NC_000016.9

cg04439561	9.87E-14	4.24E-15	8.52	23.4002489	1.72E-01	5541403	5541526	NC_000012.11
cg03485909	9.89E-14	4.25E-15	8.52	23.3983199	1.33E-01	121033957	121034080	NC_000007.13
cg11549166	9.89E-14	4.25E-15	8.52	23.3975881	6.14E-02	1846098	1846221	NC_000001.10
cg15839088	9.90E-14	4.25E-15	8.52	23.3967025	1.46E-01	71316797	71316920	NC_000005.9
cg11940973	9.92E-14	4.26E-15	8.52	23.3947396	1.19E-01	145939975	145940098	NC_000005.9
cg06817534	9.93E-14	4.27E-15	8.52	23.3937573	5.17E-02	12025211	12025334	NC_000006.11
cg10857427	9.93E-14	4.27E-15	8.52	23.3928631	1.65E-01	15091492	15091615	NC_000012.11
cg04543289	9.94E-14	4.27E-15	8.52	23.392601	1.78E-01	24267559	24267682	NC_000016.9
cg24437715	9.95E-14	4.28E-15	8.52	23.3914125	2.05E-01	237072391	237072514	NC_000002.11
cg10522607	9.95E-14	4.28E-15	8.52	23.3908325	1.65E-01	1595933	1596056	NC_000002.11
cg02706881	9.95E-14	4.28E-15	8.52	23.3906084	8.34E-02	46845775	46845898	NC_000021.8
cg03782202	9.97E-14	4.28E-15	8.52	23.3894174	1.43E-01	176971820	176971943	NC_000002.11
cg01638592	1.00E-13	4.31E-15	8.52	23.3844949	3.01E-01	111472183	111472306	NC_000012.11
cg19068510	1.00E-13	4.31E-15	8.52	23.3842683	2.44E-01	100642106	100642229	NC_000013.10
cg08095183	1.00E-13	4.31E-15	8.52	23.3825088	9.09E-02	69947048	69947171	NC_000011.9
cg02071005	1.00E-13	4.32E-15	8.52	23.3805622	2.17E-01	175200844	175200967	NC_000002.11
cg23008562	1.01E-13	4.33E-15	8.52	23.3790905	9.45E-02	2891065	2891188	NC_000011.9
cg25050447	1.01E-13	4.33E-15	8.52	23.3782394	1.04E-01	78165522	78165645	NC_000017.10
cg25256538	1.01E-13	4.34E-15	8.52	23.3764888	6.39E-02	159343074	159343197	NC_000006.11
cg15067806	1.01E-13	4.34E-15	8.52	23.3759067	2.10E-01	149146748	149146871	NC_000001.10
cg20984812	1.01E-13	4.35E-15	8.52	23.3737618	4.87E-02	171765992	171766115	NC_000001.10
cg15191648	1.01E-13	4.36E-15	8.52	23.3723654	1.71E-01	76740262	76740385	NC_000018.9
cg03433597	1.01E-13	4.36E-15	8.52	23.3720754	1.40E-01	22806448	22806571	NC_000019.9
cg09083359	1.02E-13	4.37E-15	8.52	23.3695161	1.79E-01	3854537	3854660	NC_000005.9
cg13491296	1.03E-13	4.42E-15	8.51	23.3587746	1.96E-01	156390613	156390736	NC_000001.10

cg08602190	1.03E-13	4.43E-15	8.51	23.35657	2.34E-01	128076260	128076383	NC_000010.10
cg18443359	1.03E-13	4.43E-15	8.51	23.3559092	1.81E-01	134374693	134374816	NC_000005.9
cg02181287	1.03E-13	4.43E-15	8.51	23.355712	1.13E-01	77785784	77785907	NC_000014.8
cg13807341	1.03E-13	4.44E-15	8.51	23.3549918	9.16E-02	118445984	118446107	NC_000011.9
cg14488962	1.03E-13	4.45E-15	8.51	23.350854	1.24E-01	131510278	131510401	NC_000011.9
cg27375286	1.04E-13	4.47E-15	8.51	23.3477374	1.36E-01	11518493	11518616	NC_000002.11
cg23936031	1.05E-13	4.51E-15	8.51	23.3376049	1.56E-01	27183133	27183256	NC_000007.13
cg23727983	1.05E-13	4.53E-15	8.51	23.3345292	2.60E-01	125774082	125774205	NC_000011.9
cg06687091	1.05E-13	4.53E-15	8.51	23.3342412	1.92E-01	43912991	43913114	NC_000018.9
cg27034819	1.05E-13	4.53E-15	8.51	23.3334205	1.92E-01	88799526	88799649	NC_000015.9
cg10344832	1.05E-13	4.54E-15	8.51	23.3327189	1.57E-01	68119609	68119732	NC_000015.9
cg06186330	1.05E-13	4.54E-15	8.51	23.3323329	7.67E-02	46522513	46522636	NC_000017.10
cg22849427	1.05E-13	4.54E-15	8.51	23.3320662	2.24E-01	155108695	155108818	NC_000005.9
cg26325430	1.05E-13	4.55E-15	8.51	23.3301034	9.17E-02	33639059	33639182	NC_000013.10
cg25961952	1.06E-13	4.55E-15	8.51	23.3290452	1.53E-01	134523470	134523593	NC_000005.9
cg01610231	1.06E-13	4.56E-15	8.51	23.3278063	2.00E-01	111555314	111555437	NC_000004.11
cg19514381	1.06E-13	4.57E-15	8.51	23.3263508	1.63E-01	25899118	25899241	NC_000007.13
cg13537774	1.06E-13	4.57E-15	8.51	23.3254283	1.47E-01	84088338	84088461	NC_000009.11
cg24693189	1.06E-13	4.57E-15	8.51	23.3252005	6.58E-02	33165288	33165411	NC_000001.10
cg11115235	1.06E-13	4.57E-15	8.51	23.3247785	2.38E-01	6247215	6247338	NC_000004.11
cg13062406	1.06E-13	4.58E-15	8.51	23.3243889	2.63E-01	18813544	18813667	NC_000011.9
cg00705730	1.06E-13	4.59E-15	8.51	23.3204177	1.22E-01	106438120	106438243	NC_000002.11
cg14708905	1.06E-13	4.59E-15	8.51	23.3203205	1.24E-01	30113074	30113197	NC_000006.11
cg02371121	1.07E-13	4.61E-15	8.51	23.3162704	1.15E-01	1050484	1050607	NC_000016.9
cg08145949	1.07E-13	4.61E-15	8.51	23.3160698	8.43E-02	1798209	1798332	NC_000004.11

cg13248581	1.07E-13	4.64E-15	8.51	23.3111132	1.44E-01	17680545	17680668	NC_000022.10
cg07857243	1.08E-13	4.65E-15	8.51	23.308345	2.28E-01	77822441	77822564	NC_000016.9
cg22728186	1.08E-13	4.66E-15	8.51	23.3061682	8.26E-02	10712967	10713090	NC_000001.10
cg19035993	1.08E-13	4.66E-15	8.51	23.3052681	6.09E-02	32108701	32108824	NC_000022.10
cg07035165	1.08E-13	4.67E-15	8.51	23.3046337	2.00E-01	113698506	113698629	NC_000005.9
cg03772567	1.09E-13	4.69E-15	8.51	23.2997086	1.90E-01	55676815	55676938	NC_000001.10
cg08426444	1.09E-13	4.70E-15	8.5	23.2969695	1.88E-01	73164019	73164142	NC_000008.10
cg13143349	1.09E-13	4.71E-15	8.5	23.2965414	1.91E-01	1931544	1931667	NC_000005.9
cg20673721	1.09E-13	4.71E-15	8.5	23.2958917	1.31E-01	25859382	25859505	NC_000017.10
cg11511443	1.09E-13	4.71E-15	8.5	23.2958241	1.40E-01	16761406	16761529	NC_000012.11
cg24592546	1.09E-13	4.71E-15	8.5	23.2957301	1.30E-01	30136790	30136913	NC_000006.11
cg15638239	1.09E-13	4.73E-15	8.5	23.2908347	8.50E-02	3025736	3025859	NC_000007.13
cg21059392	1.10E-13	4.74E-15	8.5	23.2895788	9.11E-02	101922750	101922873	NC_000014.8
cg01879723	1.10E-13	4.74E-15	8.5	23.2883957	1.56E-01	141119262	141119385	NC_000003.11
cg16079312	1.10E-13	4.76E-15	8.5	23.2854202	1.22E-01	3016476	3016599	NC_000011.9
cg14534277	1.10E-13	4.77E-15	8.5	23.284229	1.35E-01	28623874	28623997	NC_000018.9
cg12947035	1.10E-13	4.77E-15	8.5	23.2841197	9.98E-02	41644902	41645025	NC_000019.9
cg23178364	1.10E-13	4.77E-15	8.5	23.2833129	1.45E-01	165425105	165425228	NC_000002.11
cg13319711	1.10E-13	4.77E-15	8.5	23.283169	1.66E-01	39015544	39015667	NC_000007.13
cg02955098	1.10E-13	4.77E-15	8.5	23.2825821	1.42E-01	3473665	3473788	NC_000001.10
cg23578346	1.11E-13	4.79E-15	8.5	23.2797961	1.69E-01	3750973	3751096	NC_000002.11
cg04699462	1.11E-13	4.79E-15	8.5	23.2780883	7.34E-02	101328918	101329041	NC_000007.13
cg19595835	1.12E-13	4.83E-15	8.5	23.2707683	2.03E-01	158150945	158151068	NC_000001.10
cg04181542	1.12E-13	4.85E-15	8.5	23.2665716	9.66E-02	100584390	100584513	NC_000006.11
cg09371439	1.12E-13	4.85E-15	8.5	23.2662156	1.60E-01	84322594	84322717	NC_000015.9

cg22711009	1.12E-13	4.86E-15	8.5	23.2642129	8.67E-02	118576132	118576255	NC_000010.10
cg10795666	1.13E-13	4.88E-15	8.5	23.2608619	2.30E-01	49794635	49794758	NC_000013.10
cg01927921	1.13E-13	4.91E-15	8.5	23.2546796	1.23E-01	3787906	3788029	NC_000005.9
cg14507146	1.14E-13	4.92E-15	8.5	23.2520295	1.80E-01	34043333	34043456	NC_000005.9
cg09366312	1.14E-13	4.94E-15	8.5	23.248637	2.16E-01	5891001	5891124	NC_000018.9
cg02119348	1.14E-13	4.94E-15	8.5	23.2476448	1.39E-01	6069731	6069854	NC_000016.9
cg13922494	1.14E-13	4.96E-15	8.5	23.2443708	8.43E-02	106890760	106890883	NC_000005.9
cg15922080	1.15E-13	5.00E-15	8.5	23.2370987	8.69E-02	32046906	32047029	NC_000006.11
cg18421360	1.15E-13	5.00E-15	8.49	23.2368899	5.58E-02	90415181	90415304	NC_000005.9
cg27471124	1.16E-13	5.02E-15	8.49	23.2327486	1.21E-01	109292789	109292912	NC_000011.9
cg00182202	1.16E-13	5.02E-15	8.49	23.2321463	4.28E-02	4257902	4258025	NC_000016.9
cg07303275	1.16E-13	5.03E-15	8.49	23.2301007	8.26E-02	75499416	75499539	NC_000016.9
cg09464883	1.16E-13	5.03E-15	8.49	23.2300177	1.09E-01	79689621	79689744	NC_000004.11
cg24151755	1.16E-13	5.04E-15	8.49	23.2289706	1.13E-01	230238754	230238877	NC_000001.10
cg08125821	1.16E-13	5.05E-15	8.49	23.2275333	2.15E-01	114445779	114445902	NC_000008.10
cg25677092	1.17E-13	5.07E-15	8.49	23.22367	1.26E-01	120398684	120398807	NC_000003.11
cg20588069	1.17E-13	5.07E-15	8.49	23.2223299	1.82E-01	4860439	4860562	NC_000004.11
cg04856896	1.17E-13	5.09E-15	8.49	23.2194661	1.58E-01	32665450	32665573	NC_000001.10
cg21697851	1.18E-13	5.11E-15	8.49	23.2152022	2.00E-01	38076869	38076992	NC_000021.8
cg23077820	1.18E-13	5.12E-15	8.49	23.2124182	1.25E-01	223154176	223154299	NC_000002.11
cg27344859	1.18E-13	5.13E-15	8.49	23.2114085	1.90E-01	61808313	61808436	NC_000020.10
cg12827405	1.18E-13	5.14E-15	8.49	23.2099332	1.17E-01	10431475	10431598	NC_000006.11
cg10445315	1.18E-13	5.14E-15	8.49	23.2095828	2.49E-01	38066047	38066170	NC_000021.8
cg03496122	1.18E-13	5.14E-15	8.49	23.2092824	2.15E-01	26664404	26664527	NC_000003.11
cg01967586	1.19E-13	5.15E-15	8.49	23.2075876	9.06E-02	228894546	228894669	NC_000001.10

cg14265823	1.19E-13	5.15E-15	8.49	23.2065588	2.07E-01	223163326	223163449	NC_000002.11
cg11946503	1.19E-13	5.19E-15	8.49	23.1991549	1.62E-01	134870514	134870637	NC_000005.9
cg07049167	1.20E-13	5.22E-15	8.49	23.1949681	8.82E-02	166818506	166818629	NC_000001.10
cg13546308	1.20E-13	5.22E-15	8.49	23.1947038	1.15E-01	153366155	153366278	NC_000003.11
cg19746565	1.20E-13	5.23E-15	8.49	23.1928134	1.07E-01	11742488	11742611	NC_000008.10
cg03595755	1.21E-13	5.26E-15	8.49	23.1858733	2.12E-01	1931251	1931374	NC_000005.9
cg25577842	1.21E-13	5.27E-15	8.49	23.1840136	1.70E-01	32459760	32459883	NC_000011.9
cg06950634	1.21E-13	5.27E-15	8.49	23.1839957	1.28E-01	304351	304474	NC_000011.9
cg02128112	1.21E-13	5.28E-15	8.49	23.1822608	1.42E-01	48844757	48844880	NC_000016.9
cg00298357	1.22E-13	5.30E-15	8.49	23.1797773	6.55E-02	148758791	148758914	NC_000005.9
cg02938172	1.22E-13	5.31E-15	8.49	23.1772964	1.82E-01	185152	185275	NC_000017.10
cg21150271	1.22E-13	5.32E-15	8.49	23.1759904	1.33E-01	21553912	21554035	NC_000008.10
cg12173558	1.23E-13	5.34E-15	8.48	23.172119	1.65E-01	69244734	69244857	NC_000008.10
cg23584308	1.23E-13	5.35E-15	8.48	23.1700282	8.46E-02	105352991	105353114	NC_000014.8
cg27338202	1.23E-13	5.37E-15	8.48	23.1662607	1.41E-01	187481967	187482090	NC_000003.11
cg15131808	1.24E-13	5.38E-15	8.48	23.1634266	2.75E-01	528580	528703	NC_000005.9
cg06873498	1.24E-13	5.39E-15	8.48	23.1618055	1.32E-01	85652969	85653092	NC_000016.9
cg23239647	1.24E-13	5.39E-15	8.48	23.1617828	9.10E-02	105273411	105273534	NC_000002.11
cg03448619	1.24E-13	5.39E-15	8.48	23.1617795	5.37E-02	45840784	45840907	NC_000003.11
cg16317291	1.24E-13	5.40E-15	8.48	23.1604922	9.85E-02	7076865	7076988	NC_000012.11
cg17633576	1.24E-13	5.40E-15	8.48	23.1602326	1.68E-01	16726789	16726912	NC_000012.11
cg04742515	1.24E-13	5.42E-15	8.48	23.1566248	1.31E-01	239949301	239949424	NC_000002.11
cg24228040	1.25E-13	5.44E-15	8.48	23.1531698	2.00E-01	50496857	50496980	NC_000022.10
cg20574415	1.26E-13	5.47E-15	8.48	23.1474207	1.86E-01	79382807	79382930	NC_000015.9
cg01175812	1.26E-13	5.48E-15	8.48	23.1469932	1.58E-01	4919001	4919124	NC_000012.11

cg23318156	1.26E-13	5.49E-15	8.48	23.1445226	7.27E-02	35392306	35392429	NC_000006.11
cg15643703	1.26E-13	5.49E-15	8.48	23.1445036	1.06E-01	46722806	46722929	NC_000021.8
cg13298841	1.27E-13	5.54E-15	8.48	23.1352671	2.34E-01	43152021	43152144	NC_000007.13
cg10761315	1.28E-13	5.57E-15	8.48	23.1297453	1.66E-01	104552034	104552157	NC_000014.8
cg26272220	1.28E-13	5.59E-15	8.48	23.1272887	2.73E-01	81471884	81472007	NC_000012.11
cg04084026	1.28E-13	5.59E-15	8.48	23.1268918	1.97E-01	57832309	57832432	NC_000016.9
cg04460747	1.29E-13	5.63E-15	8.48	23.1202856	7.63E-02	52469005	52469128	NC_000006.11
cg21307628	1.29E-13	5.63E-15	8.48	23.1190666	1.34E-01	112360952	112361075	NC_000003.11
cg12688965	1.29E-13	5.64E-15	8.48	23.1174449	1.31E-01	144582077	144582200	NC_000008.10
cg24328816	1.29E-13	5.64E-15	8.48	23.1174281	6.04E-02	73096502	73096625	NC_000017.10
cg02875118	1.29E-13	5.65E-15	8.48	23.1164557	2.09E-01	112833009	112833132	NC_000011.9
cg06052090	1.30E-13	5.66E-15	8.48	23.1147481	8.45E-02	55100608	55100731	NC_000007.13
cg25772365	1.30E-13	5.66E-15	8.48	23.1138528	1.48E-01	140762224	140762347	NC_000006.11
cg01604051	1.30E-13	5.67E-15	8.47	23.1122224	1.02E-01	50851503	50851626	NC_000007.13
cg23372684	1.30E-13	5.68E-15	8.47	23.1106665	1.25E-01	178004685	178004808	NC_000005.9
cg19416570	1.31E-13	5.70E-15	8.47	23.106726	1.49E-01	58715677	58715800	NC_000019.9
cg26281476	1.31E-13	5.71E-15	8.47	23.1062237	8.93E-02	62719007	62719130	NC_000004.11
cg02347249	1.31E-13	5.74E-15	8.47	23.1009907	1.05E-01	90268539	90268662	NC_000012.11
cg16413030	1.32E-13	5.76E-15	8.47	23.0964087	1.60E-01	176937704	176937827	NC_000002.11
cg09056876	1.33E-13	5.82E-15	8.47	23.086136	1.31E-01	156718398	156718521	NC_000006.11
cg00963378	1.33E-13	5.83E-15	8.47	23.085102	2.06E-01	20716638	20716761	NC_000013.10
cg17754510	1.34E-13	5.86E-15	8.47	23.0790589	2.78E-01	10391412	10391535	NC_000006.11
cg03352106	1.34E-13	5.87E-15	8.47	23.0781476	2.29E-01	21926476	21926599	NC_000012.11
cg19665362	1.34E-13	5.87E-15	8.47	23.0779686	2.58E-01	83316838	83316961	NC_000015.9
cg06570224	1.34E-13	5.88E-15	8.47	23.0766257	1.44E-01	157812475	157812598	NC_000003.11

cg05235540	1.34E-13	5.88E-15	8.47	23.0763926	1.06E-01	30582174	30582297	NC_000006.11
cg26046467	1.35E-13	5.91E-15	8.47	23.0709001	1.44E-01	59000498	59000621	NC_000018.9
cg23971440	1.35E-13	5.92E-15	8.47	23.0701266	1.05E-01	73007729	73007852	NC_000011.9
cg02828023	1.35E-13	5.93E-15	8.47	23.0685337	2.44E-01	107503834	107503957	NC_000002.11
cg18010718	1.35E-13	5.93E-15	8.47	23.0682165	2.25E-01	74075195	74075318	NC_000017.10
cg04209911	1.35E-13	5.93E-15	8.47	23.0680412	2.00E-01	177140145	177140268	NC_000001.10
cg15200412	1.36E-13	5.94E-15	8.47	23.0667854	8.98E-02	990294	990417	NC_000019.9
cg22333868	1.36E-13	5.95E-15	8.47	23.0642353	1.60E-01	79611355	79611478	NC_000012.11
cg00458754	1.36E-13	5.98E-15	8.47	23.0605175	1.26E-01	88427813	88427936	NC_000010.10
cg06046431	1.36E-13	5.98E-15	8.47	23.0604931	1.75E-01	27744490	27744613	NC_000011.9
cg19946641	1.37E-13	5.99E-15	8.47	23.058915	8.50E-02	130909093	130909216	NC_000012.11
cg11166453	1.37E-13	5.99E-15	8.47	23.0576296	1.42E-01	247681781	247681904	NC_000001.10
cg12544191	1.37E-13	6.01E-15	8.47	23.0543074	1.53E-01	172671754	172671877	NC_000005.9
cg13984173	1.37E-13	6.03E-15	8.47	23.0519748	1.66E-01	40674438	40674561	NC_000015.9
cg03537802	1.37E-13	6.03E-15	8.47	23.0518924	7.86E-02	101868388	101868511	NC_000011.9
cg18023041	1.37E-13	6.03E-15	8.47	23.0516662	1.04E-01	19831490	19831613	NC_000011.9
cg25940202	1.38E-13	6.04E-15	8.46	23.0503855	4.67E-02	207984794	207984917	NC_000001.10
cg05520988	1.38E-13	6.04E-15	8.46	23.0501398	1.67E-01	31506568	31506691	NC_000006.11
cg24177393	1.38E-13	6.05E-15	8.46	23.0483702	1.66E-01	43037517	43037640	NC_000005.9
cg02384251	1.39E-13	6.08E-15	8.46	23.0429483	1.18E-01	1205914	1206037	NC_000004.11
cg13069211	1.39E-13	6.09E-15	8.46	23.0424248	8.01E-02	134878340	134878463	NC_000010.10
cg02483701	1.39E-13	6.11E-15	8.46	23.0384659	1.64E-01	27215610	27215733	NC_000007.13
cg02900183	1.39E-13	6.11E-15	8.46	23.0383464	1.09E-01	16284421	16284544	NC_000019.9
cg20778451	1.40E-13	6.15E-15	8.46	23.0326734	1.86E-01	178368205	178368328	NC_000005.9
cg01301138	1.40E-13	6.15E-15	8.46	23.0322494	1.44E-01	82660630	82660753	NC_000016.9

cg01106989	1.40E-13	6.15E-15	8.46	23.0320018	1.28E-01	52858459	52858582	NC_000006.11
cg23683501	1.40E-13	6.17E-15	8.46	23.0296873	8.01E-02	52270108	52270231	NC_000006.11
cg01795955	1.40E-13	6.17E-15	8.46	23.0290856	1.47E-01	200468626	200468749	NC_000002.11
cg05490233	1.41E-13	6.19E-15	8.46	23.0252922	1.26E-01	129321847	129321970	NC_000003.11
cg26836233	1.41E-13	6.19E-15	8.46	23.0252504	2.04E-01	10857800	10857923	NC_000003.11
cg10821845	1.41E-13	6.20E-15	8.46	23.0244235	2.42E-01	144241104	144241227	NC_000008.10
cg25078444	1.41E-13	6.20E-15	8.46	23.0243876	2.29E-01	29235193	29235316	NC_000014.8
cg12810096	1.41E-13	6.21E-15	8.46	23.0231597	2.08E-01	32484112	32484235	NC_000017.10
cg25078150	1.42E-13	6.23E-15	8.46	23.0187588	2.68E-01	118922841	118922964	NC_000010.10
cg16086559	1.42E-13	6.24E-15	8.46	23.0184839	1.81E-01	96911531	96911654	NC_000015.9
cg11704005	1.42E-13	6.25E-15	8.46	23.015583	1.69E-01	19002948	19003071	NC_000001.10
cg01791587	1.43E-13	6.28E-15	8.46	23.0120421	1.67E-01	110693985	110694108	NC_000001.10
cg04338871	1.43E-13	6.28E-15	8.46	23.0110834	1.39E-01	70002333	70002456	NC_000011.9
cg09974780	1.43E-13	6.31E-15	8.46	23.0065238	1.29E-01	61601128	61601251	NC_000005.9
cg21290313	1.44E-13	6.32E-15	8.46	23.0053209	1.05E-01	20158232	20158355	NC_000007.13
cg03882242	1.44E-13	6.34E-15	8.46	23.0016323	2.38E-01	103889798	103889921	NC_000012.11
cg19701087	1.45E-13	6.37E-15	8.46	22.9978545	1.92E-01	52859008	52859131	NC_000006.11
cg15575538	1.45E-13	6.38E-15	8.46	22.9965081	1.57E-01	25894005	25894128	NC_000007.13
cg18049569	1.45E-13	6.38E-15	8.46	22.9955396	5.21E-02	3154105	3154228	NC_000006.11
cg03191794	1.45E-13	6.39E-15	8.46	22.9950964	1.23E-01	83102075	83102198	NC_000012.11
cg12181459	1.45E-13	6.40E-15	8.46	22.992517	1.37E-01	8098328	8098451	NC_000010.10
cg02538579	1.46E-13	6.42E-15	8.46	22.9901597	4.77E-02	43513399	43513522	NC_000005.9
cg02190597	1.46E-13	6.43E-15	8.46	22.9888166	5.29E-02	88821234	88821357	NC_000016.9
cg21170658	1.46E-13	6.43E-15	8.45	22.9879093	1.46E-01	137515695	137515818	NC_000009.11
cg22638505	1.46E-13	6.43E-15	8.45	22.9874985	1.23E-01	21541772	21541895	NC_000019.9

cg19006302	1.46E-13	6.45E-15	8.45	22.9852594	1.06E-01	54058767	54058890	NC_000019.9
cg15775138	1.46E-13	6.45E-15	8.45	22.9849619	2.61E-01	127744389	127744512	NC_000007.13
cg24369185	1.46E-13	6.45E-15	8.45	22.9844908	2.32E-01	41341601	41341724	NC_000006.11
cg24768365	1.47E-13	6.49E-15	8.45	22.9784064	1.34E-01	43944652	43944775	NC_000012.11
cg18301583	1.47E-13	6.50E-15	8.45	22.9779873	2.42E-01	53635967	53636090	NC_000019.9
cg24845274	1.47E-13	6.50E-15	8.45	22.9772526	1.71E-01	136555697	136555820	NC_000007.13
cg11308277	1.47E-13	6.50E-15	8.45	22.9772143	1.80E-01	75603343	75603466	NC_000012.11
cg12793525	1.48E-13	6.51E-15	8.45	22.976695	1.43E-01	44875989	44876112	NC_000010.10
cg12492087	1.48E-13	6.51E-15	8.45	22.9765987	1.50E-01	42749885	42750008	NC_000015.9
cg05214690	1.48E-13	6.51E-15	8.45	22.9762147	2.70E-01	178772203	178772326	NC_000005.9
cg17419731	1.48E-13	6.51E-15	8.45	22.9759481	1.37E-01	5535731	5535854	NC_000007.13
cg19261306	1.48E-13	6.54E-15	8.45	22.9717657	1.44E-01	59234911	59235034	NC_000001.10
cg08758850	1.49E-13	6.55E-15	8.45	22.9695217	1.10E-01	127533554	127533677	NC_000009.11
cg16935734	1.49E-13	6.55E-15	8.45	22.96947	1.28E-01	77901368	77901491	NC_000018.9
cg25431366	1.49E-13	6.55E-15	8.45	22.9694009	1.09E-01	238887209	238887332	NC_000002.11
cg08964780	1.49E-13	6.56E-15	8.45	22.9684567	2.16E-01	27209463	27209586	NC_000007.13
cg15736524	1.50E-13	6.60E-15	8.45	22.9624958	1.82E-01	43568922	43569045	NC_000011.9
cg08521987	1.50E-13	6.61E-15	8.45	22.9613286	3.05E-01	119000927	119001050	NC_000010.10
cg08832227	1.50E-13	6.62E-15	8.45	22.9595285	1.85E-01	5020693	5020816	NC_000012.11
cg12193277	1.50E-13	6.62E-15	8.45	22.9588546	1.16E-01	39547181	39547304	NC_000022.10
cg20523169	1.50E-13	6.62E-15	8.45	22.9587914	1.55E-01	75880269	75880392	NC_000017.10
cg27211576	1.50E-13	6.63E-15	8.45	22.9584301	1.07E-01	75093165	75093288	NC_000015.9
cg13080379	1.51E-13	6.65E-15	8.45	22.9549776	1.77E-01	119527156	119527279	NC_000001.10
cg10939004	1.51E-13	6.65E-15	8.45	22.9542548	1.06E-01	194072233	194072356	NC_000003.11
cg00444151	1.51E-13	6.67E-15	8.45	22.9514361	1.48E-01	33823619	33823742	NC_000017.10

cg26860935	1.51E-13	6.69E-15	8.45	22.9494802	2.11E-01	1407989	1408112	NC_000004.11
cg12285988	1.52E-13	6.72E-15	8.45	22.9441028	1.63E-01	50890701	50890824	NC_000001.10
cg16553435	1.52E-13	6.73E-15	8.45	22.9432888	2.08E-01	89267943	89268066	NC_000016.9
cg12972705	1.53E-13	6.75E-15	8.45	22.9397849	1.87E-01	1915076	1915199	NC_000008.10
cg13532463	1.53E-13	6.75E-15	8.45	22.9395601	1.53E-01	101126107	101126230	NC_000009.11
cg23048481	1.53E-13	6.76E-15	8.45	22.9383406	2.29E-01	39044196	39044319	NC_000001.10
cg26557834	1.54E-13	6.79E-15	8.45	22.9343679	1.48E-01	134570471	134570594	NC_000006.11
cg01261798	1.54E-13	6.80E-15	8.45	22.9332094	2.56E-01	134000034	134000157	NC_000010.10
cg02452944	1.54E-13	6.81E-15	8.45	22.9312737	1.92E-01	140810109	140810232	NC_000005.9
cg13589559	1.54E-13	6.81E-15	8.45	22.9309422	2.86E-02	111786616	111786739	NC_000009.11
cg08881785	1.54E-13	6.82E-15	8.45	22.9304109	1.17E-01	155465076	155465199	NC_000003.11
cg25426743	1.55E-13	6.84E-15	8.45	22.9268579	9.71E-02	27166103	27166226	NC_000007.13
cg01831743	1.55E-13	6.88E-15	8.44	22.9220263	1.18E-01	110352941	110353064	NC_000012.11
cg20712489	1.55E-13	6.88E-15	8.44	22.9220187	1.10E-01	228475395	228475518	NC_000002.11
cg15427906	1.55E-13	6.88E-15	8.44	22.9217237	2.56E-01	20825216	20825339	NC_000007.13
cg13930892	1.55E-13	6.88E-15	8.44	22.9208777	2.68E-01	2292000	2292123	NC_000011.9
cg23388392	1.56E-13	6.89E-15	8.44	22.9200906	1.14E-01	39577117	39577240	NC_000017.10
cg17542408	1.56E-13	6.90E-15	8.44	22.9180042	1.93E-01	78673100	78673223	NC_000011.9
cg04549333	1.56E-13	6.91E-15	8.44	22.9175038	2.02E-01	44325823	44325946	NC_000011.9
cg05085230	1.56E-13	6.92E-15	8.44	22.9153408	2.15E-01	133562461	133562584	NC_000006.11
cg23289079	1.56E-13	6.92E-15	8.44	22.9150643	1.89E-01	122430476	122430599	NC_000005.9
cg08491681	1.57E-13	6.96E-15	8.44	22.9099472	1.56E-01	33781288	33781411	NC_000013.10
cg19761272	1.58E-13	6.99E-15	8.44	22.9049978	2.21E-01	76605176	76605299	NC_000014.8
cg27279301	1.59E-13	7.03E-15	8.44	22.8995975	1.28E-01	105945287	105945410	NC_000014.8
cg26396370	1.59E-13	7.04E-15	8.44	22.8987727	9.22E-02	10194752	10194875	NC_000002.11

cg23051261	1.59E-13	7.06E-15	8.44	22.8962551	5.86E-02	10799025	10799148	NC_000001.10
cg08620044	1.59E-13	7.07E-15	8.44	22.894815	2.06E-01	87980976	87981099	NC_000005.9
cg08282037	1.60E-13	7.08E-15	8.44	22.8932164	8.51E-02	55595978	55596101	NC_000019.9
cg09884341	1.60E-13	7.09E-15	8.44	22.892055	2.24E-01	87974464	87974587	NC_000005.9
cg12300353	1.60E-13	7.09E-15	8.44	22.8914225	2.20E-01	44450358	44450481	NC_000004.11
cg11197630	1.60E-13	7.10E-15	8.44	22.8897442	1.36E-01	56712591	56712714	NC_000005.9
cg21328033	1.60E-13	7.11E-15	8.44	22.8891344	1.83E-01	50236765	50236888	NC_000017.10
cg02039271	1.61E-13	7.12E-15	8.44	22.8872006	4.50E-02	156008127	156008250	NC_000003.11
cg18692678	1.61E-13	7.14E-15	8.44	22.884761	1.84E-01	114838772	114838895	NC_000012.11
cg01586208	1.61E-13	7.16E-15	8.44	22.8822933	1.20E-01	22806647	22806770	NC_000019.9
cg23335460	1.61E-13	7.16E-15	8.44	22.8814245	2.08E-01	99790593	99790716	NC_000010.10
cg12668482	1.61E-13	7.17E-15	8.44	22.8811014	1.98E-01	72987762	72987885	NC_000008.10
cg21748836	1.62E-13	7.17E-15	8.44	22.8807664	6.99E-02	239313057	239313180	NC_000002.11
cg17494585	1.62E-13	7.18E-15	8.44	22.8787161	1.62E-01	117705743	117705866	NC_000001.10
cg25317941	1.63E-13	7.24E-15	8.44	22.8711661	1.44E-01	233351153	233351276	NC_000002.11
cg24002183	1.63E-13	7.25E-15	8.44	22.8702757	1.16E-01	15568574	15568697	NC_000019.9
cg05887092	1.64E-13	7.27E-15	8.44	22.866619	1.21E-01	76393375	76393498	NC_000017.10
cg13876809	1.64E-13	7.27E-15	8.44	22.8665859	7.82E-02	101005971	101006094	NC_000001.10
cg06167664	1.64E-13	7.28E-15	8.44	22.8659619	2.42E-01	27072564	27072687	NC_000002.11
cg17775899	1.64E-13	7.28E-15	8.44	22.8659077	1.38E-01	27179426	27179549	NC_000007.13
cg04562217	1.64E-13	7.30E-15	8.43	22.8630908	1.87E-01	79815718	79815841	NC_000003.11
cg01731023	1.65E-13	7.35E-15	8.43	22.8564915	8.45E-02	1363486	1363609	NC_000004.11
cg02266732	1.65E-13	7.35E-15	8.43	22.8564681	1.87E-01	63257710	63257833	NC_000005.9
cg16918989	1.65E-13	7.35E-15	8.43	22.8562783	2.65E-01	8869213	8869336	NC_000017.10
cg04063345	1.66E-13	7.36E-15	8.43	22.8547102	1.87E-01	152130058	152130181	NC_000006.11

cg07013734	1.66E-13	7.36E-15	8.43	22.8545265	2.49E-01	101454683	101454806	NC_000011.9
cg07861603	1.66E-13	7.37E-15	8.43	22.8536893	1.46E-01	3085246	3085369	NC_000016.9
cg26259537	1.66E-13	7.40E-15	8.43	22.8500114	2.40E-01	27209247	27209370	NC_000007.13
cg01489926	1.66E-13	7.40E-15	8.43	22.8489613	9.05E-02	84423960	84424083	NC_000016.9
cg22668218	1.67E-13	7.43E-15	8.43	22.846045	1.85E-01	161100017	161100140	NC_000006.11
cg03202545	1.67E-13	7.43E-15	8.43	22.8455149	8.00E-02	139359312	139359435	NC_000007.13
cg23689712	1.67E-13	7.45E-15	8.43	22.8423756	1.98E-01	165205314	165205437	NC_000001.10
cg18921980	1.68E-13	7.46E-15	8.43	22.8420519	2.14E-01	175594943	175595066	NC_000002.11
cg10173932	1.68E-13	7.46E-15	8.43	22.8414675	1.36E-01	130950761	130950884	NC_000012.11
cg04261138	1.68E-13	7.48E-15	8.43	22.8392016	1.85E-01	87968636	87968759	NC_000005.9
cg07397481	1.68E-13	7.48E-15	8.43	22.8389106	8.21E-02	81927366	81927489	NC_000016.9
cg08931404	1.68E-13	7.50E-15	8.43	22.8367638	4.18E-02	29227083	29227206	NC_000013.10
cg07175883	1.69E-13	7.54E-15	8.43	22.8315159	2.24E-01	176957908	176958031	NC_000002.11
cg10543634	1.69E-13	7.54E-15	8.43	22.8302572	1.84E-01	67651491	67651614	NC_000015.9
cg02970384	1.70E-13	7.56E-15	8.43	22.8282062	1.64E-01	229567560	229567683	NC_000001.10
cg19051015	1.70E-13	7.57E-15	8.43	22.8264249	1.54E-01	46697414	46697537	NC_000017.10
cg07379434	1.70E-13	7.58E-15	8.43	22.8259663	2.08E-01	49814597	49814720	NC_000007.13
cg17957186	1.70E-13	7.58E-15	8.43	22.8250817	2.08E-01	79177895	79178018	NC_000013.10
cg08223003	1.70E-13	7.59E-15	8.43	22.824967	1.14E-01	134285320	134285443	NC_000008.10
cg05797224	1.71E-13	7.63E-15	8.43	22.8197698	6.84E-02	94277532	94277655	NC_000001.10
cg14590098	1.71E-13	7.64E-15	8.43	22.8184518	1.96E-01	65290848	65290971	NC_000008.10
cg25191725	1.71E-13	7.64E-15	8.43	22.8178243	1.31E-01	26554221	26554344	NC_000017.10
cg08644980	1.71E-13	7.64E-15	8.43	22.8176956	9.34E-02	1265487	1265610	NC_000016.9
cg18815647	1.72E-13	7.64E-15	8.43	22.8176001	1.84E-01	25744676	25744799	NC_000013.10
cg07706352	1.72E-13	7.64E-15	8.43	22.8172498	1.31E-01	10077245	10077368	NC_000019.9

cg20263686	1.72E-13	7.64E-15	8.43	22.8172401	8.15E-02	41643429	41643552	NC_000008.10
cg13364624	1.72E-13	7.66E-15	8.43	22.8147691	1.10E-01	111250761	111250884	NC_000013.10
cg11795276	1.73E-13	7.70E-15	8.43	22.8094887	1.45E-01	54454252	54454375	NC_000012.11
cg13917578	1.73E-13	7.72E-15	8.43	22.8080864	1.16E-01	143547850	143547973	NC_000005.9
cg02833919	1.73E-13	7.72E-15	8.43	22.8076682	8.24E-02	143210645	143210768	NC_000008.10
cg21670003	1.73E-13	7.73E-15	8.43	22.8066576	1.18E-01	68867965	68868088	NC_000011.9
cg12865888	1.73E-13	7.74E-15	8.43	22.8053634	2.29E-01	141220177	141220300	NC_000008.10
cg24838010	1.73E-13	7.74E-15	8.43	22.8052801	1.94E-01	78194695	78194818	NC_000017.10
cg15533075	1.74E-13	7.74E-15	8.43	22.8047611	1.52E-01	172734843	172734966	NC_000004.11
cg19024969	1.74E-13	7.77E-15	8.42	22.8013098	2.34E-01	32905966	32906089	NC_000017.10
cg16907527	1.75E-13	7.79E-15	8.42	22.7989899	1.67E-01	43744388	43744511	NC_000006.11
cg09463204	1.75E-13	7.80E-15	8.42	22.7978888	1.14E-01	33177867	33177990	NC_000015.9
cg21956337	1.75E-13	7.80E-15	8.42	22.7975491	2.46E-01	88799707	88799830	NC_000015.9
cg00400448	1.75E-13	7.80E-15	8.42	22.7969231	1.59E-01	60285691	60285814	NC_000015.9
cg17508941	1.75E-13	7.81E-15	8.42	22.7965986	1.64E-01	19183280	19183403	NC_000007.13
cg27023727	1.76E-13	7.84E-15	8.42	22.7925755	1.29E-01	71687739	71687862	NC_000014.8
cg06543087	1.76E-13	7.84E-15	8.42	22.7925227	2.26E-01	108508548	108508671	NC_000001.10
cg08383399	1.76E-13	7.87E-15	8.42	22.7882831	1.90E-01	4852488	4852611	NC_000008.10
cg02866628	1.76E-13	7.88E-15	8.42	22.7878268	2.02E-01	54612475	54612598	NC_000007.13
cg14302308	1.77E-13	7.90E-15	8.42	22.7842332	1.34E-01	38694110	38694233	NC_000009.11
cg11950383	1.78E-13	7.95E-15	8.42	22.7791268	1.69E-01	34400072	34400195	NC_000021.8
cg24935332	1.78E-13	7.95E-15	8.42	22.7781786	1.41E-01	31846819	31846942	NC_000011.9
cg00444360	1.78E-13	7.96E-15	8.42	22.7770021	1.19E-01	88270612	88270735	NC_000011.9
cg10575261	1.78E-13	7.97E-15	8.42	22.7765574	2.07E-01	178368160	178368283	NC_000005.9
cg13685951	1.79E-13	7.98E-15	8.42	22.7743649	1.91E-01	177140322	177140445	NC_000001.10

cg14546497	1.79E-13	8.00E-15	8.42	22.77265	9.07E-02	119538353	119538476	NC_000011.9
cg14409941	1.79E-13	8.02E-15	8.42	22.7695717	2.56E-01	178771781	178771904	NC_000005.9
cg12836011	1.80E-13	8.03E-15	8.42	22.7681848	2.19E-01	166795260	166795383	NC_000004.11
cg16804284	1.80E-13	8.05E-15	8.42	22.7658898	2.22E-01	29237480	29237603	NC_000014.8
cg27555582	1.81E-13	8.10E-15	8.42	22.7597004	2.44E-01	22094009	22094132	NC_000012.11
cg25013052	1.82E-13	8.13E-15	8.42	22.756882	7.10E-02	31838774	31838897	NC_000006.11
cg03938516	1.82E-13	8.16E-15	8.42	22.7532523	3.19E-02	53731693	53731816	NC_000001.10
cg11457640	1.82E-13	8.16E-15	8.42	22.7522119	1.59E-01	40567376	40567499	NC_000006.11
cg21769619	1.83E-13	8.18E-15	8.42	22.7506904	1.29E-01	32454768	32454891	NC_000011.9
cg00681365	1.83E-13	8.19E-15	8.42	22.7487323	5.49E-02	62387003	62387126	NC_000004.11
cg09733312	1.84E-13	8.24E-15	8.42	22.7427371	4.06E-02	63863556	63863679	NC_000010.10
cg15874877	1.84E-13	8.25E-15	8.42	22.7424975	1.44E-01	140799572	140799695	NC_000005.9
cg02630854	1.84E-13	8.26E-15	8.41	22.7411915	1.08E-01	177652347	177652470	NC_000003.11
cg08712932	1.85E-13	8.27E-15	8.41	22.7394289	2.16E-01	133562463	133562586	NC_000006.11
cg23660949	1.85E-13	8.29E-15	8.41	22.7377185	1.06E-01	79072761	79072884	NC_000017.10
cg16857825	1.85E-13	8.29E-15	8.41	22.7370506	1.30E-01	35994825	35994948	NC_000006.11
cg23513966	1.85E-13	8.29E-15	8.41	22.7366694	1.10E-01	76630209	76630332	NC_000015.9
cg25164624	1.85E-13	8.30E-15	8.41	22.7356815	2.63E-01	3641641	3641764	NC_000020.10
cg08000065	1.86E-13	8.34E-15	8.41	22.7312418	2.08E-01	35092823	35092946	NC_000008.10
cg19446165	1.86E-13	8.36E-15	8.41	22.7292605	1.40E-01	185756	185879	NC_000017.10
cg19224201	1.87E-13	8.36E-15	8.41	22.72901	1.66E-01	119615015	119615138	NC_000002.11
cg24558112	1.87E-13	8.40E-15	8.41	22.7238747	8.37E-02	26364710	26364833	NC_000008.10
cg09477453	1.87E-13	8.40E-15	8.41	22.723844	1.68E-01	1276617	1276740	NC_000007.13
cg08229320	1.88E-13	8.41E-15	8.41	22.7228609	1.48E-01	45941351	45941474	NC_000010.10
cg25066972	1.88E-13	8.43E-15	8.41	22.721112	1.87E-01	82002568	82002691	NC_000014.8

cg19545040	1.88E-13	8.44E-15	8.41	22.7198974	5.35E-02	103700513	103700636	NC_000010.10
cg10169241	1.88E-13	8.45E-15	8.41	22.7182628	1.45E-01	1467032	1467155	NC_000019.9
cg23514417	1.89E-13	8.46E-15	8.41	22.7172022	1.27E-01	31938533	31938656	NC_000006.11
cg04568492	1.89E-13	8.46E-15	8.41	22.7170981	2.44E-01	229568252	229568375	NC_000001.10
cg06132727	1.89E-13	8.48E-15	8.41	22.714948	1.45E-01	174422191	174422314	NC_000004.11
cg05994094	1.89E-13	8.49E-15	8.41	22.7134724	2.32E-01	31020806	31020929	NC_000018.9
cg22641350	1.89E-13	8.50E-15	8.41	22.7129138	1.60E-01	1008191	1008314	NC_000008.10
cg10663408	1.90E-13	8.51E-15	8.41	22.7116721	1.92E-01	130021651	130021774	NC_000007.13
cg20076842	1.90E-13	8.51E-15	8.41	22.7112403	1.75E-01	28617743	28617866	NC_000003.11
cg04920358	1.90E-13	8.51E-15	8.41	22.7108266	2.37E-01	49468800	49468923	NC_000008.10
cg22136329	1.90E-13	8.53E-15	8.41	22.7088596	3.14E-02	27296033	27296156	NC_000007.13
cg11229101	1.90E-13	8.54E-15	8.41	22.7078656	1.14E-01	134230885	134231008	NC_000010.10
cg08698580	1.91E-13	8.57E-15	8.41	22.7047935	9.07E-02	233708989	233709112	NC_000002.11
cg06787004	1.91E-13	8.57E-15	8.41	22.7042973	1.14E-01	19938981	19939104	NC_000022.10
cg02978421	1.91E-13	8.59E-15	8.41	22.7016976	2.42E-01	27135780	27135903	NC_000007.13
cg04330683	1.92E-13	8.62E-15	8.41	22.6989982	1.55E-01	22715410	22715533	NC_000019.9
cg23530707	1.92E-13	8.62E-15	8.41	22.6988451	7.40E-02	72983995	72984118	NC_000007.13
cg15447540	1.92E-13	8.64E-15	8.41	22.6961819	1.83E-01	132054713	132054836	NC_000008.10
cg13350783	1.92E-13	8.64E-15	8.41	22.6959462	5.63E-02	39661354	39661477	NC_000017.10
cg07125905	1.93E-13	8.65E-15	8.41	22.6955633	1.55E-01	140767364	140767487	NC_000005.9
cg14777244	1.93E-13	8.66E-15	8.41	22.6938166	7.62E-02	49110198	49110321	NC_000019.9
cg14972743	1.94E-13	8.69E-15	8.41	22.6901343	2.23E-01	58609960	58610083	NC_000019.9
cg08171336	1.94E-13	8.70E-15	8.41	22.6894839	1.19E-01	144682400	144682523	NC_000008.10
cg10787911	1.94E-13	8.74E-15	8.41	22.6854743	9.06E-02	142368316	142368439	NC_000008.10
cg16674020	1.95E-13	8.77E-15	8.41	22.6819549	1.58E-01	4174482	4174605	NC_000007.13

cg12699647	1.96E-13	8.81E-15	8.4	22.6767254	1.68E-01	80401857	80401980	NC_000017.10
cg19728382	1.97E-13	8.84E-15	8.4	22.6736975	1.45E-01	172757276	172757399	NC_000005.9
cg15252243	1.97E-13	8.84E-15	8.4	22.6735613	1.75E-01	90031538	90031661	NC_000004.11
cg18770669	1.99E-13	8.93E-15	8.4	22.6638153	1.30E-01	5541220	5541343	NC_000012.11
cg24104241	1.99E-13	8.94E-15	8.4	22.66249	2.13E-01	229567626	229567749	NC_000001.10
cg23102483	1.99E-13	8.94E-15	8.4	22.6623837	2.88E-02	47267132	47267255	NC_000011.9
cg06092815	1.99E-13	8.94E-15	8.4	22.6622223	1.79E-01	229046406	229046529	NC_000002.11
cg22571664	1.99E-13	8.96E-15	8.4	22.6608351	1.82E-01	32713112	32713235	NC_000005.9
cg12782992	2.00E-13	8.99E-15	8.4	22.6568099	2.20E-01	9026055	9026178	NC_000011.9
cg13921308	2.00E-13	8.99E-15	8.4	22.6567165	8.25E-02	214166160	214166283	NC_000001.10
cg17462107	2.00E-13	9.00E-15	8.4	22.6555273	1.11E-01	66199867	66199990	NC_000005.9
cg18708502	2.00E-13	9.02E-15	8.4	22.654046	1.32E-01	21588555	21588678	NC_000013.10
cg13454407	2.01E-13	9.03E-15	8.4	22.652934	2.04E-01	110986982	110987105	NC_000008.10
cg14156314	2.01E-13	9.05E-15	8.4	22.6507428	7.16E-02	139959160	139959283	NC_000009.11
cg14094447	2.01E-13	9.06E-15	8.4	22.6496839	2.51E-02	74383399	74383522	NC_000009.11
cg08640634	2.01E-13	9.06E-15	8.4	22.6494116	2.52E-01	108486450	108486573	NC_000006.11
cg16348254	2.03E-13	9.12E-15	8.4	22.6427044	6.52E-02	43837992	43838115	NC_000010.10
cg13525837	2.03E-13	9.13E-15	8.4	22.6414675	2.22E-01	110986371	110986494	NC_000008.10
cg19388032	2.03E-13	9.17E-15	8.4	22.6378881	1.16E-01	33171491	33171614	NC_000006.11
cg10362591	2.04E-13	9.17E-15	8.4	22.6370791	2.21E-01	55689865	55689988	NC_000016.9
cg08116263	2.04E-13	9.18E-15	8.4	22.6369667	4.88E-02	132837117	132837240	NC_000006.11
cg11671688	2.04E-13	9.18E-15	8.4	22.6360933	1.62E-01	110301075	110301198	NC_000006.11
cg23374312	2.05E-13	9.22E-15	8.4	22.6317458	9.51E-02	21565335	21565458	NC_000014.8
cg05895545	2.05E-13	9.24E-15	8.4	22.6305075	1.50E-01	50695337	50695460	NC_000005.9
cg03942855	2.06E-13	9.27E-15	8.4	22.6264702	2.09E-01	110210925	110211048	NC_000001.10

cg05037024	2.06E-13	9.31E-15	8.4	22.6226221	8.34E-02	155848920	155849043	NC_000005.9
cg03981954	2.07E-13	9.32E-15	8.4	22.6213041	2.10E-01	121942010	121942133	NC_000007.13
cg10148473	2.07E-13	9.33E-15	8.4	22.6208315	2.59E-01	75601824	75601947	NC_000012.11
cg14928719	2.07E-13	9.34E-15	8.4	22.6193945	9.41E-02	139079940	139080063	NC_000002.11
cg10332723	2.07E-13	9.35E-15	8.4	22.6181304	6.19E-02	84600152	84600275	NC_000016.9
cg27111150	2.08E-13	9.37E-15	8.39	22.6157525	1.40E-01	97183248	97183371	NC_000010.10
cg04021502	2.08E-13	9.38E-15	8.39	22.6153059	7.99E-02	1270598	1270721	NC_000005.9
cg27016494	2.08E-13	9.39E-15	8.39	22.613796	1.75E-01	96651586	96651709	NC_000007.13
cg20286200	2.08E-13	9.39E-15	8.39	22.6137415	2.57E-01	133562267	133562390	NC_000006.11
cg00625939	2.08E-13	9.40E-15	8.39	22.6131908	9.60E-02	189028032	189028155	NC_000004.11
cg04340928	2.08E-13	9.41E-15	8.39	22.61231	1.29E-01	55067613	55067736	NC_000016.9
cg03983008	2.09E-13	9.42E-15	8.39	22.611018	1.04E-01	179002268	179002391	NC_000005.9
cg22559002	2.09E-13	9.44E-15	8.39	22.6086377	9.33E-02	63898392	63898515	NC_000017.10
cg16696002	2.09E-13	9.44E-15	8.39	22.60849	1.60E-01	172189728	172189851	NC_000001.10
cg14914238	2.09E-13	9.45E-15	8.39	22.6077997	2.02E-01	83620321	83620444	NC_000015.9
cg02442509	2.10E-13	9.47E-15	8.39	22.6055265	1.93E-01	45156207	45156330	NC_000002.11
cg05043349	2.10E-13	9.49E-15	8.39	22.6034303	2.41E-01	65290113	65290236	NC_000008.10
cg13620526	2.11E-13	9.52E-15	8.39	22.6001102	4.97E-02	24629560	24629683	NC_000014.8
cg00104348	2.11E-13	9.54E-15	8.39	22.5989947	7.52E-02	10772424	10772547	NC_000012.11
cg15146859	2.11E-13	9.54E-15	8.39	22.5980602	2.59E-01	74961737	74961860	NC_000018.9
cg00569418	2.11E-13	9.55E-15	8.39	22.5978738	5.47E-02	50192100	50192223	NC_000019.9
cg00290506	2.11E-13	9.55E-15	8.39	22.597816	2.52E-01	224804226	224804349	NC_000001.10
cg15069295	2.11E-13	9.55E-15	8.39	22.5975448	1.82E-01	137522461	137522584	NC_000002.11
cg13255208	2.12E-13	9.57E-15	8.39	22.595533	1.19E-01	104165183	104165306	NC_000014.8
cg22200649	2.12E-13	9.59E-15	8.39	22.5931792	5.52E-02	65961644	65961767	NC_000012.11

cg09570682	2.12E-13	9.60E-15	8.39	22.592768	2.46E-01	106429443	106429566	NC_000006.11
cg13614229	2.14E-13	9.68E-15	8.39	22.5845728	1.49E-01	131217218	131217341	NC_000012.11
cg02317400	2.14E-13	9.68E-15	8.39	22.5845587	1.86E-01	135050345	135050468	NC_000010.10
cg15952725	2.14E-13	9.70E-15	8.39	22.582376	1.64E-01	15783190	15783313	NC_000001.10
cg24335984	2.15E-13	9.70E-15	8.39	22.5817227	8.12E-02	50361554	50361677	NC_000003.11
cg22447539	2.15E-13	9.73E-15	8.39	22.5786104	1.76E-01	5633086	5633209	NC_000007.13
cg16793187	2.16E-13	9.79E-15	8.39	22.5725311	1.53E-01	115530431	115530554	NC_000011.9
cg17131044	2.18E-13	9.85E-15	8.39	22.5672799	1.83E-01	20619012	20619135	NC_000011.9
cg24022656	2.19E-13	9.90E-15	8.39	22.5623926	1.87E-01	157291349	157291472	NC_000007.13
cg04689058	2.20E-13	9.95E-15	8.39	22.5567588	1.36E-01	66953825	66953948	NC_000016.9
cg07552868	2.20E-13	9.98E-15	8.38	22.5536547	8.38E-02	144994826	144994949	NC_000001.10
cg13493652	2.21E-13	1.00E-14	8.38	22.5523444	8.85E-02	6385721	6385844	NC_000007.13
cg12967666	2.22E-13	1.00E-14	8.38	22.548621	9.26E-02	53745145	53745268	NC_000014.8
cg26344227	2.22E-13	1.00E-14	8.38	22.5479259	2.10E-01	53287126	53287249	NC_000007.13
cg01800521	2.22E-13	1.01E-14	8.38	22.5446188	2.17E-01	21503456	21503579	NC_000020.10
cg18710412	2.23E-13	1.01E-14	8.38	22.5435328	1.06E-01	90654191	90654314	NC_000005.9
cg26983710	2.23E-13	1.01E-14	8.38	22.5432009	2.30E-01	154728202	154728325	NC_000002.11
cg14428048	2.23E-13	1.01E-14	8.38	22.5426047	1.75E-01	121940344	121940467	NC_000007.13
cg00333226	2.23E-13	1.01E-14	8.38	22.5409975	1.70E-01	49813763	49813886	NC_000007.13
cg13927454	2.23E-13	1.01E-14	8.38	22.5396028	1.33E-01	90798982	90799105	NC_000009.11
cg08876932	2.24E-13	1.01E-14	8.38	22.5373442	2.10E-01	71955547	71955670	NC_000011.9
cg27655158	2.25E-13	1.02E-14	8.38	22.5342281	2.64E-01	1396593	1396716	NC_000004.11
cg09662162	2.25E-13	1.02E-14	8.38	22.5306055	7.26E-02	1212457	1212580	NC_000016.9
cg26808747	2.26E-13	1.03E-14	8.38	22.525728	1.13E-01	128077376	128077499	NC_000010.10
cg26720125	2.27E-13	1.03E-14	8.38	22.5228264	2.23E-01	38077673	38077796	NC_000021.8

cg24632756	2.27E-13	1.03E-14	8.38	22.5216786	1.83E-01	33603026	33603149	NC_000015.9
cg22455694	2.28E-13	1.03E-14	8.38	22.5205888	1.44E-01	176974001	176974124	NC_000002.11
cg17631357	2.28E-13	1.03E-14	8.38	22.518661	5.80E-02	79298234	79298357	NC_000015.9
cg23069057	2.29E-13	1.04E-14	8.38	22.5139969	1.27E-01	14867316	14867439	NC_000004.11
cg09661632	2.29E-13	1.04E-14	8.38	22.5127084	6.43E-02	59525666	59525789	NC_000017.10
cg27219874	2.29E-13	1.04E-14	8.38	22.5116182	6.88E-02	67696095	67696218	NC_000016.9
cg24463471	2.30E-13	1.04E-14	8.38	22.5112983	1.55E-01	25257978	25258101	NC_000001.10
cg10024876	2.30E-13	1.04E-14	8.38	22.5112445	1.95E-01	69592049	69592172	NC_000003.11
cg15824707	2.30E-13	1.05E-14	8.38	22.5073763	1.02E-01	45403733	45403856	NC_000015.9
cg05141924	2.31E-13	1.05E-14	8.38	22.5031541	5.48E-02	28669866	28669989	NC_000002.11
cg18279134	2.31E-13	1.05E-14	8.38	22.5022595	1.13E-01	16394568	16394691	NC_000001.10
cg14507868	2.32E-13	1.06E-14	8.38	22.4983671	2.33E-01	107503819	107503942	NC_000002.11
cg17287155	2.33E-13	1.06E-14	8.38	22.4964895	9.20E-02	393347	393470	NC_000005.9
cg10763247	2.33E-13	1.06E-14	8.38	22.4954056	7.76E-02	61066614	61066737	NC_000003.11
cg24334591	2.33E-13	1.06E-14	8.37	22.4940144	2.53E-01	37005562	37005685	NC_000013.10
cg23016129	2.34E-13	1.06E-14	8.37	22.4917508	1.30E-01	22634224	22634347	NC_000010.10
cg09780241	2.34E-13	1.06E-14	8.37	22.4907859	2.49E-01	111535597	111535720	NC_000004.11
cg05250768	2.35E-13	1.07E-14	8.37	22.4880827	2.67E-01	27209270	27209393	NC_000007.13
cg17727929	2.35E-13	1.07E-14	8.37	22.4878667	2.24E-01	38069338	38069461	NC_000021.8
cg02803819	2.35E-13	1.07E-14	8.37	22.4861083	2.09E-01	27141067	27141190	NC_000007.13
cg10307076	2.36E-13	1.07E-14	8.37	22.4834688	2.78E-02	3572221	3572344	NC_000010.10
cg18813020	2.36E-13	1.07E-14	8.37	22.4808896	1.67E-01	122434472	122434595	NC_000005.9
cg06916740	2.37E-13	1.08E-14	8.37	22.4786982	9.90E-02	91188911	91189034	NC_000006.11
cg06448705	2.37E-13	1.08E-14	8.37	22.478484	1.84E-01	135701094	135701217	NC_000005.9
cg16014492	2.37E-13	1.08E-14	8.37	22.4756644	6.98E-02	88018953	88019076	NC_000016.9

cg06228507	2.37E-13	1.08E-14	8.37	22.4748343	1.67E-01	61637360	61637483	NC_000020.10
cg18731327	2.39E-13	1.09E-14	8.37	22.4700119	1.40E-01	147114352	147114475	NC_000003.11
cg24346905	2.39E-13	1.09E-14	8.37	22.4698053	1.91E-01	108023249	108023372	NC_000001.10
cg11419814	2.39E-13	1.09E-14	8.37	22.4680543	1.63E-01	22056533	22056656	NC_000013.10
cg05854217	2.39E-13	1.09E-14	8.37	22.4674538	1.64E-01	1595819	1595942	NC_000002.11
cg14522150	2.39E-13	1.09E-14	8.37	22.4668819	1.17E-01	50616478	50616601	NC_000010.10
cg22677260	2.40E-13	1.09E-14	8.37	22.4657074	7.31E-02	55814218	55814341	NC_000019.9
cg05791938	2.40E-13	1.09E-14	8.37	22.4645296	5.98E-02	97229682	97229805	NC_000010.10
cg05845376	2.40E-13	1.10E-14	8.37	22.4616102	1.67E-01	140683632	140683755	NC_000005.9
cg22974462	2.41E-13	1.10E-14	8.37	22.4601194	5.54E-02	29177553	29177676	NC_000008.10
cg13924554	2.42E-13	1.10E-14	8.37	22.4562793	5.33E-02	50710952	50711075	NC_000013.10
cg15278646	2.42E-13	1.10E-14	8.37	22.4546144	2.09E-01	28341980	28342103	NC_000015.9
cg16896079	2.42E-13	1.10E-14	8.37	22.4533839	2.65E-01	41582362	41582485	NC_000012.11
cg06117072	2.43E-13	1.11E-14	8.37	22.4510654	1.66E-01	50791385	50791508	NC_000006.11
cg12848065	2.43E-13	1.11E-14	8.37	22.4485473	1.89E-01	207507528	207507651	NC_000002.11
cg08954761	2.44E-13	1.11E-14	8.37	22.4480006	6.20E-02	88294101	88294224	NC_000016.9
cg24362812	2.44E-13	1.11E-14	8.37	22.4467672	2.61E-01	11148769	11148892	NC_000018.9
cg05743885	2.44E-13	1.11E-14	8.37	22.4455827	1.94E-01	27196365	27196488	NC_000007.13
cg21859781	2.44E-13	1.11E-14	8.37	22.4454789	2.49E-01	97173034	97173157	NC_000008.10
cg01881265	2.44E-13	1.12E-14	8.37	22.4439651	9.52E-02	934534	934657	NC_000017.10
cg21938148	2.45E-13	1.12E-14	8.37	22.4433538	2.89E-01	110958977	110959100	NC_000013.10
cg01769468	2.45E-13	1.12E-14	8.37	22.4431408	9.85E-02	86888585	86888708	NC_000001.10
cg21523719	2.45E-13	1.12E-14	8.37	22.440571	1.16E-01	24128335	24128458	NC_000001.10
cg25056510	2.46E-13	1.12E-14	8.37	22.4376545	9.74E-02	32729745	32729868	NC_000015.9
cg06089606	2.46E-13	1.12E-14	8.37	22.4374592	9.97E-02	89909180	89909303	NC_000003.11

cg11100481	2.46E-13	1.12E-14	8.37	22.4365951	1.27E-01	149867577	149867700	NC_000005.9
cg20052718	2.46E-13	1.12E-14	8.37	22.4363024	1.69E-01	19156902	19157025	NC_000007.13
cg09890213	2.47E-13	1.13E-14	8.36	22.4321002	1.12E-01	52409033	52409156	NC_000012.11
cg26156448	2.47E-13	1.13E-14	8.36	22.4318046	1.75E-01	21378200	21378323	NC_000020.10
cg19787644	2.48E-13	1.13E-14	8.36	22.430763	1.02E-01	94532246	94532369	NC_000012.11
cg02344820	2.48E-13	1.13E-14	8.36	22.4301445	1.40E-01	155167511	155167634	NC_000007.13
cg01283863	2.48E-13	1.13E-14	8.36	22.4300447	4.85E-02	33863759	33863882	NC_000020.10
cg26355072	2.48E-13	1.13E-14	8.36	22.4271979	5.34E-02	141674679	141674802	NC_000005.9
cg20269729	2.48E-13	1.14E-14	8.36	22.4265555	4.58E-02	33152008	33152131	NC_000006.11
cg27569174	2.49E-13	1.14E-14	8.36	22.4262287	7.44E-02	177952602	177952725	NC_000001.10
cg05187193	2.50E-13	1.14E-14	8.36	22.4211203	8.80E-02	168397037	168397160	NC_000006.11
cg21176263	2.50E-13	1.14E-14	8.36	22.4206735	6.13E-02	129455497	129455620	NC_000009.11
cg06714088	2.51E-13	1.15E-14	8.36	22.4166172	7.76E-02	167093800	167093923	NC_000001.10
cg13551330	2.51E-13	1.15E-14	8.36	22.4157962	5.34E-02	32388149	32388272	NC_000009.11
cg22108374	2.51E-13	1.15E-14	8.36	22.4154952	1.46E-01	74557527	74557650	NC_000015.9
cg26118906	2.52E-13	1.15E-14	8.36	22.41332	1.92E-01	58332488	58332611	NC_000014.8
cg04523867	2.52E-13	1.15E-14	8.36	22.413313	9.75E-02	56667262	56667385	NC_000016.9
cg25622154	2.52E-13	1.15E-14	8.36	22.4096566	1.84E-01	228657536	228657659	NC_000001.10
cg05720454	2.53E-13	1.16E-14	8.36	22.4080348	2.13E-01	34442511	34442634	NC_000021.8
cg22868951	2.53E-13	1.16E-14	8.36	22.4077854	1.54E-01	129767588	129767711	NC_000004.11
cg13717541	2.53E-13	1.16E-14	8.36	22.4055599	2.15E-01	95727643	95727766	NC_000014.8
cg16945312	2.54E-13	1.16E-14	8.36	22.4038572	2.09E-01	1393028	1393151	NC_000006.11
cg18266257	2.54E-13	1.16E-14	8.36	22.4017222	6.79E-02	32803444	32803567	NC_000006.11
cg16903885	2.55E-13	1.17E-14	8.36	22.4006395	1.06E-01	134795548	134795671	NC_000010.10
cg07904028	2.55E-13	1.17E-14	8.36	22.3983216	9.81E-02	6328508	6328631	NC_000004.11

cg24502901	2.55E-13	1.17E-14	8.36	22.3979287	6.15E-02	24086220	24086343	NC_000001.10
cg22171758	2.56E-13	1.17E-14	8.36	22.3968348	8.74E-02	54146133	54146256	NC_000012.11
cg19109431	2.56E-13	1.17E-14	8.36	22.3939204	1.26E-01	44234511	44234634	NC_000006.11
cg25970377	2.57E-13	1.18E-14	8.36	22.3925931	1.70E-01	57089933	57090056	NC_000020.10
cg07517893	2.58E-13	1.18E-14	8.36	22.3881714	1.98E-01	45889164	45889287	NC_000019.9
cg07647164	2.58E-13	1.18E-14	8.36	22.3874676	1.98E-01	214360690	214360813	NC_000001.10
cg09188385	2.60E-13	1.19E-14	8.36	22.3784701	1.06E-01	5645728	5645851	NC_000007.13
cg04345475	2.60E-13	1.19E-14	8.36	22.3782768	1.08E-01	6501591	6501714	NC_000011.9
cg00012762	2.60E-13	1.19E-14	8.36	22.3779107	2.83E-02	72744649	72744772	NC_000017.10
cg22855968	2.60E-13	1.19E-14	8.36	22.3776087	9.93E-02	134673162	134673285	NC_000006.11
cg10755235	2.61E-13	1.20E-14	8.36	22.3743351	2.13E-01	60974039	60974162	NC_000014.8
cg26274166	2.63E-13	1.21E-14	8.35	22.3661192	1.50E-01	50060174	50060297	NC_000019.9
cg17403512	2.63E-13	1.21E-14	8.35	22.3658963	9.65E-02	28700396	28700519	NC_000015.9
cg13852234	2.64E-13	1.21E-14	8.35	22.3624877	8.67E-02	7952192	7952315	NC_000017.10
cg07996594	2.64E-13	1.21E-14	8.35	22.3608968	1.49E-01	25257505	25257628	NC_000001.10
cg14045327	2.65E-13	1.21E-14	8.35	22.3602142	1.38E-01	387968	388091	NC_000019.9
cg07274716	2.66E-13	1.22E-14	8.35	22.3567732	1.73E-01	134363973	134364096	NC_000005.9
cg23245942	2.66E-13	1.22E-14	8.35	22.3543095	1.78E-01	46698973	46699096	NC_000017.10
cg07800665	2.67E-13	1.23E-14	8.35	22.3507533	2.55E-01	21657926	21658049	NC_000019.9
cg05586304	2.68E-13	1.23E-14	8.35	22.3485729	2.14E-01	75629039	75629162	NC_000003.11
cg07799386	2.68E-13	1.23E-14	8.35	22.3471399	1.34E-01	122430821	122430944	NC_000005.9
cg12892506	2.70E-13	1.24E-14	8.35	22.3402569	2.26E-01	147113918	147114041	NC_000003.11
cg24892510	2.70E-13	1.24E-14	8.35	22.3400164	2.12E-01	53636309	53636432	NC_000019.9
cg03231647	2.70E-13	1.24E-14	8.35	22.3398264	1.64E-01	237081650	237081773	NC_000002.11
cg19202813	2.70E-13	1.24E-14	8.35	22.3394105	1.33E-01	189216616	189216739	NC_000002.11

cg22916586	2.70E-13	1.24E-14	8.35	22.337132	1.53E-01	12686957	12687080	NC_000012.11
cg04293733	2.71E-13	1.25E-14	8.35	22.3348769	2.39E-01	98511629	98511752	NC_000001.10
cg18723560	2.71E-13	1.25E-14	8.35	22.3344541	2.19E-01	51228831	51228954	NC_000019.9
cg03717503	2.72E-13	1.25E-14	8.35	22.3321362	1.15E-01	86734176	86734299	NC_000016.9
cg09606807	2.73E-13	1.25E-14	8.35	22.327971	1.69E-01	96579908	96580031	NC_000015.9
cg17382918	2.73E-13	1.26E-14	8.35	22.3274865	1.23E-01	57042544	57042667	NC_000020.10
cg10702770	2.73E-13	1.26E-14	8.35	22.3268912	1.30E-01	39283780	39283903	NC_000001.10
cg12055515	2.73E-13	1.26E-14	8.35	22.3259999	1.66E-01	66735203	66735326	NC_000002.11
cg07150062	2.74E-13	1.26E-14	8.35	22.324462	2.10E-01	104552032	104552155	NC_000014.8
cg18639233	2.74E-13	1.26E-14	8.35	22.3222621	2.41E-01	60975846	60975969	NC_000014.8
cg00936187	2.75E-13	1.26E-14	8.35	22.3212149	9.41E-02	88801685	88801808	NC_000016.9
cg14146657	2.75E-13	1.27E-14	8.35	22.3192328	1.35E-01	128412220	128412343	NC_000009.11
cg22138430	2.75E-13	1.27E-14	8.35	22.31857	2.08E-01	92789500	92789623	NC_000014.8
cg02705681	2.76E-13	1.27E-14	8.35	22.3155859	9.89E-02	102118300	102118423	NC_000007.13
cg11873482	2.76E-13	1.27E-14	8.35	22.3149871	2.60E-01	97361244	97361367	NC_000007.13
cg18740429	2.77E-13	1.28E-14	8.35	22.3118013	1.81E-01	134844716	134844839	NC_000010.10
cg26564815	2.77E-13	1.28E-14	8.35	22.3107271	9.68E-02	74350933	74351056	NC_000011.9
cg18828619	2.77E-13	1.28E-14	8.35	22.3103818	9.09E-02	56615077	56615200	NC_000014.8
cg03365311	2.78E-13	1.28E-14	8.35	22.3098223	2.14E-01	43602965	43603088	NC_000011.9
cg20644626	2.78E-13	1.28E-14	8.34	22.3072174	1.01E-01	74274353	74274476	NC_000004.11
cg11990902	2.79E-13	1.29E-14	8.34	22.3031803	4.28E-02	44196412	44196535	NC_000006.11
cg04846243	2.81E-13	1.29E-14	8.34	22.297854	2.61E-01	47074883	47075006	NC_000017.10
cg13718100	2.81E-13	1.30E-14	8.34	22.2962562	1.04E-01	133796449	133796572	NC_000009.11
cg02628840	2.82E-13	1.30E-14	8.34	22.2951495	1.04E-01	46860579	46860702	NC_000021.8
cg27601873	2.82E-13	1.30E-14	8.34	22.2947866	5.73E-02	2163879	2164002	NC_000016.9

cg19623360	2.82E-13	1.30E-14	8.34	22.2939454	1.58E-01	27191914	27192037	NC_000007.13
cg25655854	2.82E-13	1.30E-14	8.34	22.2931418	1.13E-01	99772732	99772855	NC_000004.11
cg06107104	2.82E-13	1.30E-14	8.34	22.2927409	1.29E-01	73617762	73617885	NC_000017.10
cg14802400	2.83E-13	1.30E-14	8.34	22.2901592	9.77E-02	104608583	104608706	NC_000012.11
cg04807108	2.83E-13	1.31E-14	8.34	22.2881239	1.13E-01	78697933	78698056	NC_000015.9
cg02970297	2.84E-13	1.31E-14	8.34	22.2868948	1.95E-01	102920962	102921085	NC_000007.13
cg19115393	2.84E-13	1.31E-14	8.34	22.286314	2.31E-01	59829060	59829183	NC_000020.10
cg05640128	2.84E-13	1.31E-14	8.34	22.2860995	1.95E-01	154002270	154002393	NC_000007.13
cg06568783	2.84E-13	1.31E-14	8.34	22.286042	8.04E-02	45388128	45388251	NC_000017.10
cg22547775	2.84E-13	1.31E-14	8.34	22.2846482	1.24E-01	2537634	2537757	NC_000005.9
cg23698950	2.85E-13	1.31E-14	8.34	22.2818331	1.06E-01	30110123	30110246	NC_000006.11
cg20443451	2.85E-13	1.32E-14	8.34	22.2808984	1.41E-01	31497233	31497356	NC_000006.11
cg08580032	2.86E-13	1.32E-14	8.34	22.2797045	1.01E-01	77562500	77562623	NC_000018.9
cg14127567	2.87E-13	1.33E-14	8.34	22.2734562	5.44E-02	60817041	60817164	NC_000017.10
cg26465206	2.87E-13	1.33E-14	8.34	22.2734292	1.40E-01	13670022	13670145	NC_000010.10
cg16138150	2.88E-13	1.33E-14	8.34	22.2717131	2.43E-01	37839684	37839807	NC_000005.9
cg03610729	2.88E-13	1.33E-14	8.34	22.271428	9.78E-02	168199897	168200020	NC_000005.9
cg24080247	2.88E-13	1.33E-14	8.34	22.2704707	2.24E-01	38082613	38082736	NC_000021.8
cg20890210	2.88E-13	1.33E-14	8.34	22.2703352	2.06E-01	73450181	73450304	NC_000008.10
cg07831417	2.88E-13	1.33E-14	8.34	22.2703058	2.23E-01	122848204	122848327	NC_000011.9
cg07188591	2.89E-13	1.33E-14	8.34	22.2678675	2.50E-01	148854315	148854438	NC_000001.10
cg04465731	2.90E-13	1.34E-14	8.34	22.2628326	6.43E-02	85398200	85398323	NC_000011.9
cg16284437	2.91E-13	1.34E-14	8.34	22.2600061	1.20E-01	76751616	76751739	NC_000018.9
cg08652487	2.93E-13	1.36E-14	8.34	22.2518512	1.64E-01	229569795	229569918	NC_000001.10
cg04072323	2.93E-13	1.36E-14	8.34	22.2516888	2.53E-01	229046325	229046448	NC_000002.11

cg00985983	2.94E-13	1.36E-14	8.33	22.2475506	1.39E-01	30728510	30728633	NC_000022.10
cg24315340	2.95E-13	1.36E-14	8.33	22.2458644	1.41E-01	146058215	146058338	NC_000006.11
cg02311794	2.95E-13	1.37E-14	8.33	22.2443128	1.20E-01	86302971	86303094	NC_000015.9
cg16484042	2.96E-13	1.37E-14	8.33	22.240371	1.39E-01	14403405	14403528	NC_000016.9
cg00704780	2.97E-13	1.37E-14	8.33	22.237654	1.26E-01	242207920	242208043	NC_000002.11
cg06493207	2.97E-13	1.38E-14	8.33	22.2372877	1.08E-01	174160825	174160948	NC_000005.9
cg15173134	2.98E-13	1.38E-14	8.33	22.2347868	2.90E-01	119000638	119000761	NC_000010.10
cg10808783	2.98E-13	1.38E-14	8.33	22.2337198	2.18E-01	1467921	1468044	NC_000019.9
cg08285415	2.99E-13	1.38E-14	8.33	22.2316959	5.57E-02	78283681	78283804	NC_000015.9
cg16428251	3.00E-13	1.39E-14	8.33	22.2289877	2.17E-01	137483479	137483602	NC_000003.11
cg09473315	3.00E-13	1.39E-14	8.33	22.2276572	1.42E-01	76228233	76228356	NC_000017.10
cg19389397	3.01E-13	1.39E-14	8.33	22.2256585	1.15E-01	42973877	42974000	NC_000022.10
cg13356896	3.01E-13	1.39E-14	8.33	22.223322	2.60E-01	198650987	198651110	NC_000002.11
cg23620184	3.01E-13	1.39E-14	8.33	22.2232949	1.41E-01	23772616	23772739	NC_000014.8
cg14628071	3.02E-13	1.40E-14	8.33	22.2199369	5.30E-02	138670633	138670756	NC_000009.11
cg14639185	3.02E-13	1.40E-14	8.33	22.2196064	8.83E-02	108634147	108634270	NC_000012.11
cg13924432	3.03E-13	1.40E-14	8.33	22.2186007	1.55E-01	220223283	220223406	NC_000002.11
cg18112235	3.03E-13	1.40E-14	8.33	22.2176446	8.87E-02	196451226	196451349	NC_000002.11
cg23963517	3.03E-13	1.41E-14	8.33	22.2159276	1.61E-01	60473872	60473995	NC_000010.10
cg19075081	3.04E-13	1.41E-14	8.33	22.2148873	8.94E-02	70714378	70714501	NC_000016.9
cg25262548	3.05E-13	1.41E-14	8.33	22.2116007	1.39E-01	158982975	158983098	NC_000006.11
cg20556402	3.05E-13	1.41E-14	8.33	22.2115342	1.28E-01	41616858	41616981	NC_000006.11
cg11774346	3.06E-13	1.42E-14	8.33	22.2066878	1.57E-01	46797899	46798022	NC_000021.8
cg07549278	3.06E-13	1.42E-14	8.33	22.2062981	1.61E-01	1204244	1204367	NC_000016.9
cg18391611	3.06E-13	1.42E-14	8.33	22.2062284	8.43E-02	56042991	56043114	NC_000019.9

cg19317211	3.06E-13	1.42E-14	8.33	22.2061604	2.47E-01	2801711	2801834	NC_000020.10
cg02761480	3.07E-13	1.42E-14	8.33	22.2036274	2.41E-01	77822771	77822894	NC_000016.9
cg05622465	3.07E-13	1.42E-14	8.33	22.2023228	2.11E-01	110613072	110613195	NC_000001.10
cg15175377	3.08E-13	1.43E-14	8.33	22.200008	1.48E-01	68920434	68920557	NC_000011.9
cg05738240	3.08E-13	1.43E-14	8.33	22.1985653	1.79E-01	114337927	114338050	NC_000012.11
cg16631199	3.09E-13	1.44E-14	8.33	22.1951913	1.64E-01	101841253	101841376	NC_000010.10
cg12615535	3.10E-13	1.44E-14	8.33	22.1939249	1.95E-01	139257713	139257836	NC_000003.11
cg08405910	3.10E-13	1.44E-14	8.33	22.1938117	9.96E-02	106501075	106501198	NC_000002.11
cg01646268	3.10E-13	1.44E-14	8.33	22.1932373	1.46E-01	14367071	14367194	NC_000011.9
cg24174232	3.11E-13	1.44E-14	8.33	22.1914364	1.17E-01	29818175	29818298	NC_000017.10
cg16459364	3.11E-13	1.44E-14	8.33	22.1899519	1.73E-01	24771125	24771248	NC_000008.10
cg10390058	3.13E-13	1.45E-14	8.32	22.1846501	2.26E-01	74962794	74962917	NC_000018.9
cg24675150	3.13E-13	1.46E-14	8.32	22.1815484	2.57E-01	119212160	119212283	NC_000012.11
cg18787401	3.14E-13	1.46E-14	8.32	22.1809304	2.42E-01	56879559	56879682	NC_000019.9
cg01787574	3.14E-13	1.46E-14	8.32	22.1798808	1.87E-01	16758057	16758180	NC_000012.11
cg22937632	3.14E-13	1.46E-14	8.32	22.1784111	8.40E-02	33563946	33564069	NC_000011.9
cg16294566	3.14E-13	1.46E-14	8.32	22.1781613	2.20E-01	32458656	32458779	NC_000011.9
cg13235366	3.15E-13	1.46E-14	8.32	22.1756621	8.58E-02	44032202	44032325	NC_000001.10
cg01307130	3.16E-13	1.47E-14	8.32	22.1746642	2.57E-01	71329040	71329163	NC_000010.10
cg13464448	3.16E-13	1.47E-14	8.32	22.1741583	2.03E-01	130297513	130297636	NC_000011.9
cg18026588	3.16E-13	1.47E-14	8.32	22.1734249	1.99E-01	27018037	27018160	NC_000015.9
cg11710887	3.16E-13	1.47E-14	8.32	22.1720374	8.03E-02	158748803	158748926	NC_000007.13
cg20429172	3.16E-13	1.47E-14	8.32	22.1714938	2.00E-01	248020436	248020559	NC_000001.10
cg09914773	3.17E-13	1.47E-14	8.32	22.1700845	9.11E-02	99383224	99383347	NC_000007.13
cg16364121	3.18E-13	1.48E-14	8.32	22.1677984	2.93E-01	35291924	35292047	NC_000017.10

cg19574193	3.18E-13	1.48E-14	8.32	22.1672525	9.68E-02	20136824	20136947	NC_000014.8
cg17052466	3.18E-13	1.48E-14	8.32	22.166598	8.36E-02	2423382	2423505	NC_000011.9
cg03144922	3.18E-13	1.48E-14	8.32	22.1663811	1.78E-01	111540992	111541115	NC_000004.11
cg00168118	3.18E-13	1.48E-14	8.32	22.1655669	1.53E-01	85669675	85669798	NC_000016.9
cg17082640	3.18E-13	1.48E-14	8.32	22.1649437	1.11E-01	57205380	57205503	NC_000008.10
cg17827020	3.19E-13	1.48E-14	8.32	22.1622567	1.94E-01	180202784	180202907	NC_000001.10
cg01661742	3.20E-13	1.49E-14	8.32	22.1607367	1.07E-01	142141192	142141315	NC_000004.11
cg13422870	3.21E-13	1.49E-14	8.32	22.1559954	8.29E-02	102671668	102671791	NC_000009.11
cg09856107	3.21E-13	1.50E-14	8.32	22.1547917	9.64E-02	12701776	12701899	NC_000003.11
cg17924936	3.22E-13	1.50E-14	8.32	22.1532712	2.24E-01	1763800	1763923	NC_000019.9
cg09862721	3.22E-13	1.50E-14	8.32	22.1516499	4.06E-02	152155003	152155126	NC_000002.11
cg03605361	3.22E-13	1.50E-14	8.32	22.1512189	6.45E-02	179715673	179715796	NC_000005.9
cg16300838	3.23E-13	1.50E-14	8.32	22.151044	6.06E-02	135109211	135109334	NC_000010.10
cg12971867	3.23E-13	1.50E-14	8.32	22.1483869	8.70E-02	50528564	50528687	NC_000022.10
cg13890379	3.23E-13	1.51E-14	8.32	22.1480217	1.42E-01	67070324	67070447	NC_000018.9
cg25089587	3.24E-13	1.51E-14	8.32	22.1476695	9.00E-02	104184551	104184674	NC_000014.8
cg08587321	3.24E-13	1.51E-14	8.32	22.145488	7.60E-02	31473811	31473934	NC_000016.9
cg19925849	3.25E-13	1.51E-14	8.32	22.1427969	1.67E-01	54445377	54445500	NC_000019.9
cg16921310	3.25E-13	1.51E-14	8.32	22.1418792	2.20E-01	103351987	103352110	NC_000012.11
cg01209655	3.26E-13	1.52E-14	8.32	22.1412107	4.13E-02	30041081	30041204	NC_000006.11
cg06388730	3.27E-13	1.52E-14	8.32	22.1382482	1.62E-01	35297699	35297822	NC_000007.13
cg23055707	3.28E-13	1.53E-14	8.32	22.1350719	1.27E-01	1681153	1681276	NC_000002.11
cg17095728	3.28E-13	1.53E-14	8.32	22.1345392	2.34E-01	231297516	231297639	NC_000001.10
cg02000882	3.28E-13	1.53E-14	8.32	22.1329044	2.29E-01	86542113	86542236	NC_000016.9
cg10831607	3.29E-13	1.53E-14	8.32	22.1317783	2.26E-01	115134374	115134497	NC_000012.11

cg04434339	3.29E-13	1.53E-14	8.32	22.1313458	2.40E-01	107503547	107503670	NC_000002.11
cg09151131	3.29E-13	1.53E-14	8.32	22.1305918	1.38E-01	48552276	48552399	NC_000017.10
cg06825039	3.29E-13	1.53E-14	8.32	22.129405	1.81E-01	20824905	20825028	NC_000007.13
cg16686174	3.31E-13	1.54E-14	8.32	22.1246611	1.25E-01	75373081	75373204	NC_000017.10
cg00364956	3.31E-13	1.54E-14	8.31	22.1232996	1.16E-01	33777219	33777342	NC_000017.10
cg05433657	3.32E-13	1.55E-14	8.31	22.1210259	5.10E-02	71006703	71006826	NC_000010.10
cg18239481	3.32E-13	1.55E-14	8.31	22.1208222	4.98E-02	5600805	5600928	NC_000010.10
cg03237868	3.33E-13	1.55E-14	8.31	22.1187187	7.72E-02	52919914	52920037	NC_000004.11
cg17460447	3.35E-13	1.56E-14	8.31	22.1109748	2.98E-01	5712937	5713060	NC_000004.11
cg08040824	3.35E-13	1.56E-14	8.31	22.1103248	7.09E-02	189420438	189420561	NC_000003.11
cg22481263	3.35E-13	1.56E-14	8.31	22.1102832	1.03E-01	906493	906616	NC_000001.10
cg03705299	3.36E-13	1.57E-14	8.31	22.1094844	6.08E-02	8721021	8721144	NC_000011.9
cg24921186	3.36E-13	1.57E-14	8.31	22.1089335	7.64E-02	43045001	43045124	NC_000005.9
cg05732832	3.36E-13	1.57E-14	8.31	22.1075895	2.11E-01	4868723	4868846	NC_000004.11
cg13458210	3.37E-13	1.57E-14	8.31	22.105465	9.46E-02	161010071	161010194	NC_000001.10
cg00052482	3.37E-13	1.57E-14	8.31	22.1035012	1.47E-01	151226139	151226262	NC_000001.10
cg07095230	3.37E-13	1.58E-14	8.31	22.1032969	2.20E-01	59480730	59480853	NC_000017.10
cg13566648	3.38E-13	1.58E-14	8.31	22.1030235	1.78E-01	12750030	12750153	NC_000006.11
cg03515663	3.39E-13	1.58E-14	8.31	22.0984981	1.70E-01	157478510	157478633	NC_000007.13
cg22111442	3.40E-13	1.59E-14	8.31	22.0967798	1.17E-01	31441196	31441319	NC_000001.10
cg10732215	3.40E-13	1.59E-14	8.31	22.0954702	2.85E-01	22625465	22625588	NC_000010.10
cg25961733	3.41E-13	1.59E-14	8.31	22.0927398	3.96E-02	134228239	134228362	NC_000010.10
cg21306006	3.41E-13	1.59E-14	8.31	22.0917876	1.92E-01	166402081	166402204	NC_000006.11
cg01369750	3.41E-13	1.59E-14	8.31	22.0917506	2.05E-01	688018	688141	NC_000008.10
cg27361370	3.42E-13	1.60E-14	8.31	22.0901339	1.95E-01	157813327	157813450	NC_000003.11

cg00926400	3.42E-13	1.60E-14	8.31	22.0891846	2.21E-01	198651498	198651621	NC_000002.11
cg15013830	3.44E-13	1.61E-14	8.31	22.0824272	5.43E-02	24581791	24581914	NC_000022.10
cg08985063	3.45E-13	1.61E-14	8.31	22.0802311	1.60E-01	4866800	4866923	NC_000004.11
cg23038560	3.45E-13	1.61E-14	8.31	22.0792454	6.81E-02	94582513	94582636	NC_000014.8
cg02990869	3.46E-13	1.62E-14	8.31	22.0781234	1.95E-01	165304842	165304965	NC_000004.11
cg19049194	3.46E-13	1.62E-14	8.31	22.0767336	1.81E-01	175193754	175193877	NC_000002.11
cg04868962	3.47E-13	1.62E-14	8.31	22.0744227	1.77E-01	180427177	180427300	NC_000002.11
cg04623131	3.47E-13	1.62E-14	8.31	22.0730319	1.14E-01	97564564	97564687	NC_000002.11
cg18354370	3.48E-13	1.63E-14	8.31	22.0702539	9.65E-02	50393044	50393167	NC_000003.11
cg14552982	3.49E-13	1.63E-14	8.31	22.0689135	1.07E-01	133568226	133568349	NC_000009.11
cg25393009	3.49E-13	1.63E-14	8.31	22.0678636	1.62E-01	14691641	14691764	NC_000011.9
cg08705958	3.49E-13	1.63E-14	8.31	22.0674675	6.90E-02	113497324	113497447	NC_000013.10
cg25944168	3.50E-13	1.64E-14	8.31	22.0657373	1.05E-01	88862221	88862344	NC_000002.11
cg19794395	3.51E-13	1.64E-14	8.3	22.0627426	6.75E-02	58158293	58158416	NC_000012.11
cg26184391	3.51E-13	1.64E-14	8.3	22.0627401	1.07E-01	24004643	24004766	NC_000004.11
cg12461677	3.52E-13	1.65E-14	8.3	22.0595111	1.34E-01	58862089	58862212	NC_000019.9
cg26584983	3.52E-13	1.65E-14	8.3	22.0589425	1.36E-01	93798975	93799098	NC_000012.11
cg25426203	3.52E-13	1.65E-14	8.3	22.0581137	1.22E-01	88600793	88600916	NC_000010.10
cg02155884	3.52E-13	1.65E-14	8.3	22.0579831	2.07E-01	112715454	112715577	NC_000013.10
cg20737554	3.53E-13	1.65E-14	8.3	22.0567423	1.64E-01	115612464	115612587	NC_000010.10
cg12778178	3.53E-13	1.65E-14	8.3	22.0563559	4.91E-02	62583555	62583678	NC_000020.10
cg08624445	3.53E-13	1.65E-14	8.3	22.0561575	8.22E-02	46306688	46306811	NC_000021.8
cg21250061	3.53E-13	1.65E-14	8.3	22.0555837	1.38E-01	37463344	37463467	NC_000009.11
cg14131755	3.54E-13	1.66E-14	8.3	22.0544236	2.39E-01	50606043	50606166	NC_000010.10
cg22457642	3.54E-13	1.66E-14	8.3	22.0525137	1.20E-01	109307774	109307897	NC_000006.11

cg16822666	3.55E-13	1.66E-14	8.3	22.050537	1.77E-01	35656660	35656783	NC_000017.10
cg16883063	3.55E-13	1.66E-14	8.3	22.0494034	1.70E-01	1801495	1801618	NC_000007.13
cg05417127	3.55E-13	1.66E-14	8.3	22.0484948	2.67E-01	1968709	1968832	NC_000016.9
cg03156260	3.56E-13	1.67E-14	8.3	22.0478875	9.91E-02	75721361	75721484	NC_000004.11
cg24436715	3.56E-13	1.67E-14	8.3	22.0471181	2.10E-01	31848130	31848253	NC_000019.9
cg14582400	3.56E-13	1.67E-14	8.3	22.046291	1.80E-01	112726118	112726241	NC_000013.10
cg07035046	3.57E-13	1.67E-14	8.3	22.0436161	1.15E-01	79791064	79791187	NC_000009.11
cg06237151	3.58E-13	1.68E-14	8.3	22.0420832	1.22E-01	46123153	46123276	NC_000010.10
cg14157417	3.60E-13	1.69E-14	8.3	22.0359326	6.86E-02	158265485	158265608	NC_000007.13
cg24851364	3.60E-13	1.69E-14	8.3	22.0347537	2.35E-01	57390306	57390429	NC_000010.10
cg16256902	3.60E-13	1.69E-14	8.3	22.0346698	1.32E-01	183826405	183826528	NC_000004.11
cg26917694	3.60E-13	1.69E-14	8.3	22.0338183	1.83E-01	221061058	221061181	NC_000001.10
cg16522412	3.61E-13	1.69E-14	8.3	22.033152	6.81E-02	33926811	33926934	NC_000013.10
cg22399458	3.61E-13	1.69E-14	8.3	22.0329245	5.31E-02	112038039	112038162	NC_000011.9
cg04077662	3.61E-13	1.70E-14	8.3	22.0305785	2.00E-01	77333229	77333352	NC_000001.10
cg02909136	3.62E-13	1.70E-14	8.3	22.0300345	2.09E-01	44941542	44941665	NC_000020.10
cg16426537	3.62E-13	1.70E-14	8.3	22.029019	2.49E-01	18813556	18813679	NC_000011.9
cg16984485	3.62E-13	1.70E-14	8.3	22.0287879	4.28E-02	3218116	3218239	NC_000003.11
cg14761227	3.63E-13	1.70E-14	8.3	22.025778	1.51E-01	130021088	130021211	NC_000007.13
cg01440497	3.64E-13	1.71E-14	8.3	22.0245717	7.60E-02	1273484	1273607	NC_000016.9
cg14180004	3.64E-13	1.71E-14	8.3	22.0224138	1.09E-01	46722777	46722900	NC_000021.8
cg12993163	3.65E-13	1.71E-14	8.3	22.0215349	2.06E-01	157821407	157821530	NC_000003.11
cg17982346	3.65E-13	1.71E-14	8.3	22.0215199	6.78E-02	58512825	58512948	NC_000017.10
cg12343182	3.65E-13	1.71E-14	8.3	22.0209208	3.21E-01	43439387	43439510	NC_000020.10
cg11232868	3.65E-13	1.71E-14	8.3	22.0198401	1.35E-01	12495801	12495924	NC_000003.11

cg03456651	3.65E-13	1.71E-14	8.3	22.0193745	6.07E-02	73479833	73479956	NC_000010.10
cg18146843	3.65E-13	1.72E-14	8.3	22.0190889	2.01E-01	53099248	53099371	NC_000001.10
cg12939425	3.66E-13	1.72E-14	8.3	22.0168386	1.11E-01	130344188	130344311	NC_000005.9
cg11628880	3.67E-13	1.72E-14	8.3	22.0152995	1.75E-01	37852492	37852615	NC_000021.8
cg10625575	3.67E-13	1.72E-14	8.3	22.0149212	9.77E-02	4645224	4645347	NC_000012.11
cg14651705	3.69E-13	1.73E-14	8.3	22.008768	5.93E-02	136226035	136226158	NC_000009.11
cg19633004	3.69E-13	1.73E-14	8.3	22.0079671	2.11E-01	10704044	10704167	NC_000005.9
cg09579310	3.71E-13	1.74E-14	8.3	22.0046797	2.49E-01	10395947	10396070	NC_000006.11
cg17480760	3.71E-13	1.74E-14	8.3	22.0042053	1.69E-01	86350889	86351012	NC_000008.10
cg11539780	3.71E-13	1.74E-14	8.3	22.0033996	2.66E-01	57019016	57019139	NC_000019.9
cg10008947	3.71E-13	1.74E-14	8.3	22.0031551	9.68E-02	73500739	73500862	NC_000017.10
cg25442600	3.71E-13	1.74E-14	8.3	22.0030107	1.54E-01	1888033	1888156	NC_000005.9
cg01818151	3.72E-13	1.75E-14	8.29	21.9999185	1.73E-01	46797042	46797165	NC_000017.10
cg26649504	3.74E-13	1.76E-14	8.29	21.9946072	1.14E-01	112196397	112196520	NC_000012.11
cg09968723	3.75E-13	1.76E-14	8.29	21.9923483	2.71E-01	143545789	143545912	NC_000008.10
cg08507422	3.75E-13	1.76E-14	8.29	21.991561	2.35E-01	1272512	1272635	NC_000007.13
cg26609631	3.76E-13	1.77E-14	8.29	21.9906165	2.45E-01	28366814	28366937	NC_000013.10
cg00333512	3.76E-13	1.77E-14	8.29	21.9882164	2.20E-01	25909599	25909722	NC_000008.10
cg17896776	3.77E-13	1.77E-14	8.29	21.9869098	2.07E-01	172733760	172733883	NC_000004.11
cg11326255	3.77E-13	1.77E-14	8.29	21.98572	1.79E-01	3786583	3786706	NC_000019.9
cg17889618	3.78E-13	1.78E-14	8.29	21.98456	8.02E-02	233085132	233085255	NC_000001.10
cg17463083	3.79E-13	1.78E-14	8.29	21.9800902	1.64E-01	62153284	62153407	NC_000017.10
cg11532431	3.81E-13	1.79E-14	8.29	21.9767163	2.12E-01	27169674	27169797	NC_000007.13
cg07135732	3.81E-13	1.79E-14	8.29	21.9764269	1.83E-01	91184290	91184413	NC_000001.10
cg16845394	3.81E-13	1.79E-14	8.29	21.9744919	2.42E-01	109096007	109096130	NC_000008.10

cg23807215	3.82E-13	1.80E-14	8.29	21.9716879	3.56E-02	101666942	101667065	NC_000002.11
cg00249511	3.83E-13	1.80E-14	8.29	21.9688617	2.45E-01	627152	627275	NC_000011.9
cg02441747	3.84E-13	1.81E-14	8.29	21.9669195	2.29E-01	110223646	110223769	NC_000004.11
cg03222176	3.85E-13	1.81E-14	8.29	21.9659113	1.18E-01	96948323	96948446	NC_000001.10
cg13434411	3.87E-13	1.82E-14	8.29	21.9590147	1.91E-01	970297	970420	NC_000009.11
cg18449734	3.87E-13	1.82E-14	8.29	21.9586834	1.88E-01	7853429	7853552	NC_000019.9
cg11880367	3.88E-13	1.83E-14	8.29	21.9572108	1.16E-01	80141181	80141304	NC_000010.10
cg00795616	3.90E-13	1.84E-14	8.29	21.9516282	1.04E-01	6867074	6867197	NC_000007.13
cg08332074	3.91E-13	1.84E-14	8.29	21.9482273	2.13E-01	51189941	51190064	NC_000016.9
cg15039102	3.91E-13	1.84E-14	8.29	21.9474515	1.89E-01	3121953	3122076	NC_000005.9
cg00395625	3.94E-13	1.86E-14	8.29	21.9417566	1.43E-01	138664249	138664372	NC_000007.13
cg22605415	3.94E-13	1.86E-14	8.29	21.9411575	1.65E-01	175135997	175136120	NC_000004.11
cg11781977	3.94E-13	1.86E-14	8.29	21.9406042	5.85E-02	40123107	40123230	NC_000021.8
cg01520571	3.95E-13	1.86E-14	8.28	21.9387267	2.19E-01	35296481	35296604	NC_000017.10
cg13149573	3.95E-13	1.86E-14	8.28	21.9385592	3.77E-02	3234519	3234642	NC_000004.11
cg12292648	3.95E-13	1.86E-14	8.28	21.9366859	8.19E-02	11207723	11207846	NC_000008.10
cg05596265	3.96E-13	1.87E-14	8.28	21.9351435	1.76E-01	2141382	2141505	NC_000003.11
cg10234511	3.97E-13	1.87E-14	8.28	21.9322163	8.12E-02	10788801	10788924	NC_000001.10
cg25781162	3.98E-13	1.88E-14	8.28	21.9290886	1.50E-01	44065996	44066119	NC_000002.11
cg22679728	3.99E-13	1.88E-14	8.28	21.9276778	2.08E-01	242688523	242688646	NC_000001.10
cg20744625	4.00E-13	1.89E-14	8.28	21.9250221	2.32E-01	64578469	64578592	NC_000010.10
cg06866140	4.00E-13	1.89E-14	8.28	21.9248839	1.05E-01	16993566	16993689	NC_000019.9
cg10287461	4.01E-13	1.89E-14	8.28	21.9230039	7.33E-02	119510240	119510363	NC_000011.9
cg17451493	4.01E-13	1.89E-14	8.28	21.9224631	8.21E-02	131132745	131132868	NC_000010.10
cg25092681	4.01E-13	1.89E-14	8.28	21.9209965	2.12E-01	16180033	16180156	NC_000005.9

cg02481498	4.02E-13	1.90E-14	8.28	21.9190779	6.45E-02	101956421	101956544	NC_000002.11
cg00839579	4.02E-13	1.90E-14	8.28	21.9178051	2.46E-01	15727441	15727564	NC_000007.13
cg06746118	4.03E-13	1.90E-14	8.28	21.9168303	2.53E-01	118922887	118923010	NC_000010.10
cg27267617	4.04E-13	1.91E-14	8.28	21.9138299	9.32E-02	42445462	42445585	NC_000019.9
cg13985135	4.08E-13	1.93E-14	8.28	21.9032162	1.60E-01	155641827	155641950	NC_000001.10
cg13285698	4.09E-13	1.93E-14	8.28	21.9014297	8.41E-02	141455681	141455804	NC_000008.10
cg19018435	4.10E-13	1.94E-14	8.28	21.8998516	1.50E-01	183797630	183797753	NC_000001.10
cg26411367	4.10E-13	1.94E-14	8.28	21.8984143	5.38E-02	155988060	155988183	NC_000001.10
cg04950301	4.10E-13	1.94E-14	8.28	21.8976014	1.55E-01	12750392	12750515	NC_000006.11
cg24982697	4.10E-13	1.94E-14	8.28	21.8975809	9.19E-02	113521532	113521655	NC_000013.10
cg00125455	4.11E-13	1.94E-14	8.28	21.8951349	1.51E-01	44574271	44574394	NC_000020.10
cg19764599	4.12E-13	1.95E-14	8.28	21.8933124	2.14E-01	75601276	75601399	NC_000001.10
cg25580656	4.12E-13	1.95E-14	8.28	21.8929926	2.37E-01	104197160	104197283	NC_000014.8
cg16280141	4.13E-13	1.95E-14	8.28	21.8917119	1.84E-01	63257753	63257876	NC_000005.9
cg21190038	4.13E-13	1.95E-14	8.28	21.8904789	1.27E-01	44086848	44086971	NC_000015.9
cg05288253	4.15E-13	1.96E-14	8.28	21.8869512	1.44E-01	89552259	89552382	NC_000016.9
cg26967100	4.15E-13	1.96E-14	8.28	21.8853577	1.28E-01	61104032	61104155	NC_000014.8
cg25345178	4.16E-13	1.97E-14	8.28	21.8843196	2.18E-01	72459908	72460031	NC_000008.10
cg18037808	4.16E-13	1.97E-14	8.28	21.8840157	5.16E-02	109059679	109059802	NC_000012.11
cg02672332	4.16E-13	1.97E-14	8.28	21.8823489	1.52E-01	134723970	134724093	NC_000010.10
cg09444036	4.17E-13	1.97E-14	8.28	21.8814211	1.44E-01	658638	658761	NC_000010.10
cg06015733	4.17E-13	1.97E-14	8.28	21.8802296	1.23E-01	38090440	38090563	NC_000008.10
cg00964997	4.18E-13	1.98E-14	8.27	21.8783105	1.52E-01	13531195	13531318	NC_000004.11
cg10639428	4.20E-13	1.99E-14	8.27	21.8731738	1.04E-01	25138879	25139002	NC_000002.11
cg00984146	4.21E-13	1.99E-14	8.27	21.8702458	2.59E-01	38258333	38258456	NC_000005.9

cg04479219	4.22E-13	2.00E-14	8.27	21.8685066	1.76E-01	88326918	88327041	NC_000013.10
cg14603046	4.23E-13	2.00E-14	8.27	21.8665026	2.06E-01	152157902	152158025	NC_000007.13
cg01719433	4.24E-13	2.01E-14	8.27	21.8624964	3.78E-02	62583564	62583687	NC_000020.10
cg20963741	4.24E-13	2.01E-14	8.27	21.8620172	1.87E-01	95359162	95359285	NC_000013.10
cg07687548	4.25E-13	2.01E-14	8.27	21.8604788	2.31E-01	172965280	172965403	NC_000002.11
cg22236485	4.25E-13	2.02E-14	8.27	21.8592121	6.67E-02	128742664	128742787	NC_000008.10
cg07330481	4.26E-13	2.02E-14	8.27	21.8589703	1.82E-01	37322330	37322453	NC_000017.10
cg06002516	4.26E-13	2.02E-14	8.27	21.858215	1.26E-01	57666521	57666644	NC_000012.11
cg09395562	4.26E-13	2.02E-14	8.27	21.8581995	1.16E-01	123501051	123501174	NC_000003.11
cg10813908	4.26E-13	2.02E-14	8.27	21.8576937	6.85E-02	138863041	138863164	NC_000005.9
cg24683222	4.27E-13	2.02E-14	8.27	21.8554645	1.38E-01	41880440	41880563	NC_000004.11
cg17159890	4.27E-13	2.03E-14	8.27	21.854613	1.07E-01	105233093	105233216	NC_000010.10
cg09792204	4.28E-13	2.03E-14	8.27	21.852812	2.14E-01	28342183	28342306	NC_000015.9
cg13831570	4.29E-13	2.03E-14	8.27	21.8503549	6.73E-02	39020053	39020176	NC_000017.10
cg02861380	4.30E-13	2.04E-14	8.27	21.8472031	1.88E-01	75596758	75596881	NC_000001.10
cg16014725	4.32E-13	2.05E-14	8.27	21.8434518	1.38E-01	70124422	70124545	NC_000017.10
cg10141715	4.32E-13	2.05E-14	8.27	21.8420937	1.87E-01	101603453	101603576	NC_000012.11
cg10624122	4.35E-13	2.07E-14	8.27	21.8357585	2.04E-01	19158747	19158870	NC_000007.13
cg01914621	4.36E-13	2.07E-14	8.27	21.8330963	2.10E-01	155260474	155260597	NC_000007.13
cg19707040	4.37E-13	2.07E-14	8.27	21.8318843	2.03E-01	79740185	79740308	NC_000002.11
cg18521914	4.37E-13	2.07E-14	8.27	21.8317245	1.57E-01	50887566	50887689	NC_000010.10
cg16516919	4.37E-13	2.08E-14	8.27	21.8298839	1.35E-01	203297711	203297834	NC_000001.10
cg01940297	4.38E-13	2.08E-14	8.27	21.8289584	1.38E-01	129061191	129061314	NC_000008.10
cg14492337	4.38E-13	2.08E-14	8.27	21.8275508	1.67E-01	79813507	79813630	NC_000017.10
cg08992499	4.40E-13	2.09E-14	8.27	21.8240354	9.58E-02	63956514	63956637	NC_000003.11

cg06759374	4.40E-13	2.09E-14	8.27	21.8238715	1.62E-01	22414364	22414487	NC_000003.11
cg06194960	4.42E-13	2.10E-14	8.27	21.8201607	9.37E-02	111362	111485	NC_000016.9
cg21001487	4.42E-13	2.10E-14	8.27	21.8180084	2.43E-01	53853112	53853235	NC_000008.10
cg17112076	4.43E-13	2.10E-14	8.26	21.8173722	1.20E-01	33170692	33170815	NC_000006.11
cg11281641	4.43E-13	2.10E-14	8.26	21.8170846	2.83E-01	171674855	171674978	NC_000002.11
cg09073398	4.45E-13	2.12E-14	8.26	21.8116453	2.91E-01	168727762	168727885	NC_000005.9
cg04945331	4.45E-13	2.12E-14	8.26	21.8106602	2.41E-01	137483992	137484115	NC_000003.11
cg00058329	4.46E-13	2.12E-14	8.26	21.8084152	2.52E-01	133195094	133195217	NC_000012.11
cg08149193	4.47E-13	2.12E-14	8.26	21.8077389	2.32E-01	44333067	44333190	NC_000011.9
cg04281204	4.47E-13	2.13E-14	8.26	21.8075228	1.52E-01	115612802	115612925	NC_000010.10
cg07127888	4.47E-13	2.13E-14	8.26	21.8072551	2.25E-01	41400789	41400912	NC_000008.10
cg17972100	4.47E-13	2.13E-14	8.26	21.8070715	1.57E-01	8046547	8046670	NC_000004.11
cg00088797	4.48E-13	2.13E-14	8.26	21.8052029	1.69E-01	12976288	12976411	NC_000003.11
cg03874978	4.48E-13	2.13E-14	8.26	21.8051712	1.20E-01	1800448	1800571	NC_000004.11
cg20120512	4.48E-13	2.13E-14	8.26	21.8044231	1.43E-01	47151769	47151892	NC_000019.9
cg07095995	4.48E-13	2.13E-14	8.26	21.8037336	2.43E-01	110223980	110224103	NC_000004.11
cg19706803	4.49E-13	2.14E-14	8.26	21.8013283	1.13E-01	3126033	3126156	NC_000001.10
cg12836958	4.51E-13	2.15E-14	8.26	21.7983016	1.96E-01	186132412	186132535	NC_000004.11
cg08947167	4.51E-13	2.15E-14	8.26	21.7973641	1.07E-01	63671692	63671815	NC_000015.9
cg17003293	4.54E-13	2.16E-14	8.26	21.7893719	2.34E-01	36003826	36003949	NC_000014.8
cg22021022	4.55E-13	2.17E-14	8.26	21.7883102	1.12E-01	183275320	183275443	NC_000001.10
cg25588274	4.55E-13	2.17E-14	8.26	21.7883018	1.85E-01	4230567	4230690	NC_000005.9
cg23437459	4.55E-13	2.17E-14	8.26	21.7882425	1.95E-01	124907457	124907580	NC_000010.10
cg26307359	4.55E-13	2.17E-14	8.26	21.7872099	2.33E-01	27332646	27332769	NC_000017.10
cg18077387	4.55E-13	2.17E-14	8.26	21.7870323	1.05E-01	70699636	70699759	NC_000015.9

cg26591066	4.56E-13	2.17E-14	8.26	21.7863853	1.06E-01	3922863	3922986	NC_000017.10
cg12908522	4.57E-13	2.18E-14	8.26	21.7825066	1.91E-01	5019091	5019214	NC_000012.11
cg22941086	4.58E-13	2.18E-14	8.26	21.7816243	9.31E-02	40019883	40020006	NC_000012.11
cg04775890	4.58E-13	2.18E-14	8.26	21.7810493	8.23E-02	19586046	19586169	NC_000019.9
cg12818557	4.59E-13	2.19E-14	8.26	21.7794795	2.52E-01	89340139	89340262	NC_000008.10
cg25119743	4.59E-13	2.19E-14	8.26	21.7777306	1.42E-01	11059290	11059413	NC_000010.10
cg10935498	4.61E-13	2.20E-14	8.26	21.7743398	1.24E-01	79898777	79898900	NC_000006.11
cg10507508	4.62E-13	2.20E-14	8.26	21.7731886	2.46E-01	30719828	30719951	NC_000019.9
cg23595601	4.63E-13	2.21E-14	8.26	21.7707607	4.90E-02	47690213	47690336	NC_000007.13
cg07799157	4.63E-13	2.21E-14	8.26	21.7705034	6.28E-02	2475103	2475226	NC_000004.11
cg25088758	4.63E-13	2.21E-14	8.26	21.7701856	2.17E-01	110223598	110223721	NC_000004.11
cg22748799	4.63E-13	2.21E-14	8.26	21.7697343	2.43E-01	58570790	58570913	NC_000019.9
cg10074775	4.64E-13	2.21E-14	8.26	21.7682425	2.18E-01	77595027	77595150	NC_000008.10
cg00204748	4.64E-13	2.21E-14	8.26	21.7674878	1.31E-01	29376779	29376902	NC_000012.11
cg05328670	4.64E-13	2.21E-14	8.26	21.7673034	1.23E-01	84096367	84096490	NC_000016.9
cg16962683	4.64E-13	2.21E-14	8.26	21.7671937	1.63E-01	97602671	97602794	NC_000008.10
cg27562682	4.64E-13	2.21E-14	8.26	21.7667275	2.10E-01	243053865	243053988	NC_000001.10
cg20553016	4.66E-13	2.22E-14	8.26	21.7633404	5.07E-02	235950085	235950208	NC_000002.11
cg02759846	4.66E-13	2.22E-14	8.26	21.7631987	2.14E-01	51227943	51228066	NC_000019.9
cg18143243	4.66E-13	2.22E-14	8.26	21.7623528	2.73E-01	54155663	54155786	NC_000003.11
cg26822782	4.67E-13	2.23E-14	8.26	21.7613645	1.10E-01	130621905	130622028	NC_000010.10
cg14645721	4.67E-13	2.23E-14	8.26	21.7597376	4.30E-02	78163589	78163712	NC_000017.10
cg06682185	4.69E-13	2.24E-14	8.25	21.7557046	1.10E-01	61157615	61157738	NC_000018.9
cg12352960	4.69E-13	2.24E-14	8.25	21.7550991	2.15E-01	43428578	43428701	NC_000010.10
cg17270257	4.70E-13	2.24E-14	8.25	21.7547195	2.23E-01	166074870	166074993	NC_000006.11

cg07815836	4.71E-13	2.25E-14	8.25	21.7518487	1.25E-01	58715351	58715474	NC_000019.9
cg12162138	4.72E-13	2.25E-14	8.25	21.7502882	2.35E-01	78673073	78673196	NC_000011.9
cg13405887	4.73E-13	2.26E-14	8.25	21.7481015	2.90E-01	132382812	132382935	NC_000009.11
cg23712594	4.74E-13	2.27E-14	8.25	21.7445663	1.29E-01	226591890	226592013	NC_000001.10
cg03716313	4.76E-13	2.28E-14	8.25	21.7401444	5.94E-02	19355725	19355848	NC_000017.10
cg02081006	4.77E-13	2.28E-14	8.25	21.7392391	2.14E-01	122430434	122430557	NC_000005.9
cg25763393	4.77E-13	2.28E-14	8.25	21.7390338	2.10E-01	52956832	52956955	NC_000019.9
cg25951210	4.78E-13	2.29E-14	8.25	21.7350175	1.07E-01	177016619	177016742	NC_000002.11
cg22851781	4.79E-13	2.29E-14	8.25	21.7331183	1.08E-01	133799053	133799176	NC_000009.11
cg05452406	4.81E-13	2.30E-14	8.25	21.7292356	2.24E-01	10881891	10882014	NC_000006.11
cg14344955	4.81E-13	2.30E-14	8.25	21.7291065	1.40E-01	71744725	71744848	NC_000017.10
cg08745329	4.82E-13	2.31E-14	8.25	21.7268677	5.72E-02	6189073	6189196	NC_000010.10
cg27430637	4.83E-13	2.31E-14	8.25	21.7259614	1.15E-01	26646801	26646924	NC_000001.10
cg17889439	4.83E-13	2.31E-14	8.25	21.7239832	6.44E-02	77501954	77502077	NC_000013.10
cg26667205	4.83E-13	2.31E-14	8.25	21.7239146	2.10E-01	56803778	56803901	NC_000020.10
cg04290586	4.84E-13	2.32E-14	8.25	21.7220865	1.76E-01	108168987	108169110	NC_000012.11
cg18700013	4.85E-13	2.32E-14	8.25	21.7209191	1.55E-01	29285119	29285242	NC_000019.9
cg08144931	4.85E-13	2.32E-14	8.25	21.7205233	5.91E-02	39115589	39115712	NC_000012.11
cg18325622	4.85E-13	2.32E-14	8.25	21.7201427	1.90E-01	16180259	16180382	NC_000005.9
cg00885682	4.85E-13	2.32E-14	8.25	21.7200763	1.81E-01	22825749	22825872	NC_000016.9
cg04849842	4.85E-13	2.32E-14	8.25	21.7193654	2.03E-01	140714610	140714733	NC_000008.10
cg08276289	4.87E-13	2.33E-14	8.25	21.7165036	2.17E-01	28617854	28617977	NC_000003.11
cg23288078	4.87E-13	2.33E-14	8.25	21.7163063	3.48E-02	124268173	124268296	NC_000008.10
cg15308062	4.87E-13	2.33E-14	8.25	21.7160495	2.37E-01	21680981	21681104	NC_000012.11
cg10187894	4.87E-13	2.33E-14	8.25	21.7153377	1.62E-01	38015525	38015648	NC_000010.10

cg01506806	4.88E-13	2.34E-14	8.25	21.7133155	7.61E-02	119956180	119956303	NC_000004.11
cg05420994	4.91E-13	2.35E-14	8.25	21.707915	1.05E-01	32807296	32807419	NC_000006.11
cg25769043	4.92E-13	2.36E-14	8.25	21.7057957	1.71E-01	57035948	57036071	NC_000004.11
cg14684434	4.92E-13	2.36E-14	8.25	21.7055964	1.60E-01	53829957	53830080	NC_000005.9
cg05547777	4.92E-13	2.36E-14	8.25	21.7054101	1.97E-01	50694888	50695011	NC_000005.9
cg10546977	4.93E-13	2.36E-14	8.25	21.7030904	1.30E-01	13915689	13915812	NC_000019.9
cg18772588	4.93E-13	2.36E-14	8.25	21.7030143	2.23E-01	61809932	61810055	NC_000020.10
cg06020661	4.93E-13	2.36E-14	8.25	21.7026181	5.42E-02	47472138	47472261	NC_000013.10
cg24496841	4.94E-13	2.37E-14	8.25	21.7015844	2.45E-01	19152017	19152140	NC_000007.13
cg12811072	4.94E-13	2.37E-14	8.25	21.7005942	9.47E-02	53849892	53850015	NC_000012.11
cg23990435	4.94E-13	2.37E-14	8.25	21.6999672	3.36E-02	139043443	139043566	NC_000005.9
cg09830575	4.96E-13	2.38E-14	8.25	21.6972781	7.93E-02	71444937	71445060	NC_000015.9
cg00654888	4.96E-13	2.38E-14	8.25	21.6966888	1.21E-01	119603133	119603256	NC_000002.11
cg09072052	4.96E-13	2.38E-14	8.25	21.695914	5.94E-02	144070960	144071083	NC_000008.10
cg03215422	4.97E-13	2.39E-14	8.24	21.6934878	1.70E-01	135266509	135266632	NC_000005.9
cg13558371	4.97E-13	2.39E-14	8.24	21.6934278	1.36E-01	126135610	126135733	NC_000009.11
cg10295609	4.97E-13	2.39E-14	8.24	21.6929507	1.37E-01	143132934	143133057	NC_000006.11
cg23306464	4.97E-13	2.39E-14	8.24	21.6929169	1.73E-01	4868356	4868479	NC_000004.11
cg02268225	4.98E-13	2.39E-14	8.24	21.6920695	2.20E-01	100437639	100437762	NC_000014.8
cg24676817	4.98E-13	2.39E-14	8.24	21.6912281	2.80E-01	42995369	42995492	NC_000005.9
cg04961101	4.98E-13	2.39E-14	8.24	21.6910459	1.59E-01	37303484	37303607	NC_000020.10
cg23371436	4.98E-13	2.39E-14	8.24	21.6908669	1.27E-01	20111534	20111657	NC_000011.9
cg04043795	4.99E-13	2.39E-14	8.24	21.6895972	1.77E-01	5836366	5836489	NC_000002.11
cg22289581	4.99E-13	2.40E-14	8.24	21.6889849	1.89E-01	122850227	122850350	NC_000011.9
cg05945059	5.00E-13	2.40E-14	8.24	21.6869217	1.70E-01	56940463	56940586	NC_000018.9

cg25225340	5.05E-13	2.42E-14	8.24	21.6777092	2.15E-02	26937534	26937657	NC_000022.10
cg08410604	5.06E-13	2.43E-14	8.24	21.6757764	8.08E-02	138556982	138557105	NC_000007.13
cg17298516	5.06E-13	2.43E-14	8.24	21.675508	9.58E-02	94131533	94131656	NC_000001.10
cg21002957	5.06E-13	2.43E-14	8.24	21.6752021	2.09E-01	15568894	15569017	NC_000019.9
cg23945382	5.06E-13	2.43E-14	8.24	21.6739075	8.26E-02	51748922	51749045	NC_000018.9
cg01157070	5.07E-13	2.44E-14	8.24	21.6718357	2.17E-01	56228511	56228634	NC_000016.9
cg10652393	5.09E-13	2.45E-14	8.24	21.6686904	2.27E-01	27112430	27112553	NC_000015.9
cg16096531	5.10E-13	2.45E-14	8.24	21.6672344	1.10E-01	53722216	53722339	NC_000012.11
cg01802380	5.10E-13	2.45E-14	8.24	21.666984	9.08E-02	107865407	107865530	NC_000013.10
cg11437784	5.10E-13	2.45E-14	8.24	21.6662226	2.14E-01	112710823	112710946	NC_000013.10
cg18621091	5.11E-13	2.46E-14	8.24	21.6642038	2.01E-01	75612056	75612179	NC_000018.9
cg20140735	5.11E-13	2.46E-14	8.24	21.6639835	1.40E-01	1494726	1494849	NC_000001.10
cg00596508	5.12E-13	2.46E-14	8.24	21.6620713	2.13E-01	41086800	41086923	NC_000012.11
cg25463688	5.13E-13	2.46E-14	8.24	21.6613214	8.52E-02	235254025	235254148	NC_000001.10
cg12270822	5.13E-13	2.47E-14	8.24	21.6607156	2.35E-01	87229722	87229845	NC_000007.13
cg06251181	5.15E-13	2.48E-14	8.24	21.656775	6.82E-02	109046825	109046948	NC_000012.11
cg24611446	5.16E-13	2.48E-14	8.24	21.6546655	2.20E-01	113917737	113917860	NC_000012.11
cg13762320	5.16E-13	2.48E-14	8.24	21.6545568	1.26E-01	2292905	2293028	NC_000011.9
cg02111951	5.16E-13	2.48E-14	8.24	21.6545557	1.27E-01	61544854	61544977	NC_000011.9
cg23571839	5.21E-13	2.51E-14	8.24	21.6431879	1.95E-01	1431343	1431466	NC_000011.9
cg07777790	5.22E-13	2.51E-14	8.24	21.6413896	1.89E-01	229570188	229570311	NC_000001.10
cg27473895	5.22E-13	2.52E-14	8.24	21.6410435	1.91E-01	102497354	102497477	NC_000010.10
cg15009331	5.23E-13	2.52E-14	8.24	21.6402101	6.23E-02	80502431	80502554	NC_000010.10
cg18225412	5.23E-13	2.52E-14	8.24	21.6397814	7.45E-02	240927227	240927350	NC_000001.10
cg03565241	5.24E-13	2.52E-14	8.24	21.6376494	1.08E-01	9286157	9286280	NC_000002.11

cg09413950	5.24E-13	2.53E-14	8.24	21.6371555	1.53E-01	49582872	49582995	NC_000011.9
cg08657492	5.26E-13	2.53E-14	8.23	21.6337597	1.51E-01	27170832	27170955	NC_000007.13
cg11438134	5.26E-13	2.53E-14	8.23	21.6336987	7.65E-02	46269016	46269139	NC_000021.8
cg06161915	5.26E-13	2.53E-14	8.23	21.6334701	4.00E-02	127451984	127452107	NC_000007.13
cg09208611	5.26E-13	2.54E-14	8.23	21.6328245	1.76E-01	63544319	63544442	NC_000012.11
cg17178268	5.27E-13	2.54E-14	8.23	21.6307502	1.05E-01	90048444	90048567	NC_000015.9
cg07164047	5.29E-13	2.55E-14	8.23	21.6266175	6.51E-02	236486625	236486748	NC_000002.11
cg27265170	5.30E-13	2.55E-14	8.23	21.6260435	1.61E-01	50889510	50889633	NC_000001.10
cg01566213	5.32E-13	2.56E-14	8.23	21.6224504	9.66E-02	1579287	1579410	NC_000004.11
cg02773188	5.32E-13	2.56E-14	8.23	21.622357	5.05E-02	152332936	152333059	NC_000004.11
cg14475381	5.33E-13	2.57E-14	8.23	21.6193936	1.31E-01	131401268	131401391	NC_000010.10
cg06479254	5.33E-13	2.57E-14	8.23	21.6192912	1.09E-01	176983285	176983408	NC_000002.11
cg21045915	5.33E-13	2.57E-14	8.23	21.6186306	8.44E-02	98629374	98629497	NC_000014.8
cg03863908	5.34E-13	2.58E-14	8.23	21.6173602	7.24E-02	1123738	1123861	NC_000005.9
cg04034583	5.37E-13	2.59E-14	8.23	21.6121361	9.59E-02	119603108	119603231	NC_000002.11
cg06195293	5.38E-13	2.60E-14	8.23	21.6100034	6.14E-02	30104885	30105008	NC_000006.11
cg24761507	5.39E-13	2.60E-14	8.23	21.6085341	2.65E-01	35293930	35294053	NC_000017.10
cg11074814	5.41E-13	2.61E-14	8.23	21.6033728	2.15E-01	43601969	43602092	NC_000011.9
cg26694724	5.42E-13	2.62E-14	8.23	21.602045	4.65E-02	1992028	1992151	NC_000007.13
cg18263365	5.43E-13	2.62E-14	8.23	21.5996767	1.71E-01	136834453	136834576	NC_000005.9
cg19988426	5.44E-13	2.62E-14	8.23	21.5990197	1.79E-01	130064021	130064144	NC_000010.10
cg07783282	5.44E-13	2.63E-14	8.23	21.597751	2.57E-01	95942964	95943087	NC_000012.11
cg01360628	5.44E-13	2.63E-14	8.23	21.5975997	1.57E-01	89156118	89156241	NC_000003.11
cg08912058	5.46E-13	2.64E-14	8.23	21.5939535	4.32E-02	6313858	6313981	NC_000001.10
cg24961583	5.48E-13	2.65E-14	8.23	21.5900666	2.41E-01	27765409	27765532	NC_000003.11

cg10541517	5.48E-13	2.65E-14	8.23	21.5900051	2.00E-01	128753103	128753226	NC_000012.11
cg26025557	5.48E-13	2.65E-14	8.23	21.5895004	1.21E-01	42842344	42842467	NC_000003.11
cg08650890	5.49E-13	2.65E-14	8.23	21.588411	1.67E-01	32064613	32064736	NC_000006.11
cg12352896	5.52E-13	2.67E-14	8.23	21.5821824	9.65E-02	77271034	77271157	NC_000018.9
cg24387472	5.52E-13	2.67E-14	8.23	21.582081	1.96E-01	142528745	142528868	NC_000008.10
cg21591624	5.53E-13	2.67E-14	8.23	21.5813575	1.21E-01	20623199	20623322	NC_000011.9
cg08288406	5.53E-13	2.67E-14	8.23	21.5811376	2.75E-01	1523917	1524040	NC_000019.9
cg21129253	5.54E-13	2.68E-14	8.23	21.5795819	1.05E-01	36638564	36638687	NC_000014.8
cg11569159	5.55E-13	2.69E-14	8.23	21.5764927	1.81E-01	115172959	115173082	NC_000012.11
cg26282384	5.58E-13	2.70E-14	8.22	21.5721868	2.04E-01	140767200	140767323	NC_000005.9
cg04316624	5.58E-13	2.70E-14	8.22	21.5716885	1.37E-01	177036809	177036932	NC_000002.11
cg23060747	5.60E-13	2.71E-14	8.22	21.5685105	1.08E-01	239962958	239963081	NC_000002.11
cg01049530	5.61E-13	2.72E-14	8.22	21.5649354	2.83E-01	81952008	81952131	NC_000004.11
cg07136998	5.62E-13	2.72E-14	8.22	21.5640395	3.06E-01	168728081	168728204	NC_000005.9
cg24051786	5.65E-13	2.74E-14	8.22	21.5578823	1.81E-01	47514673	47514796	NC_000003.11
cg13652513	5.67E-13	2.74E-14	8.22	21.5550881	1.70E-01	70654597	70654720	NC_000014.8
cg14029580	5.67E-13	2.75E-14	8.22	21.5547499	1.04E-01	1363708	1363831	NC_000004.11
cg15461413	5.71E-13	2.77E-14	8.22	21.547595	8.51E-02	62160543	62160666	NC_000020.10
cg24909548	5.72E-13	2.77E-14	8.22	21.5453404	2.03E-01	36736272	36736395	NC_000019.9
cg19001226	5.72E-13	2.77E-14	8.22	21.5453255	2.76E-01	177053345	177053468	NC_000002.11
cg14243778	5.73E-13	2.78E-14	8.22	21.5429078	1.92E-01	41086879	41087002	NC_000012.11
cg22285438	5.74E-13	2.78E-14	8.22	21.5414572	9.47E-02	915895	916018	NC_000017.10
cg04452311	5.75E-13	2.79E-14	8.22	21.5394788	1.59E-01	176936377	176936500	NC_000002.11
cg04543077	5.76E-13	2.79E-14	8.22	21.5377738	6.91E-02	2879544	2879667	NC_000016.9
cg02377021	5.76E-13	2.79E-14	8.22	21.5372409	8.72E-02	71537334	71537457	NC_000005.9

cg13954457	5.77E-13	2.80E-14	8.22	21.535153	2.08E-01	167956819	167956942	NC_000005.9
cg23913679	5.78E-13	2.80E-14	8.22	21.5345807	2.42E-01	7116700	7116823	NC_000018.9
cg10111292	5.80E-13	2.81E-14	8.22	21.5309035	1.92E-01	106975112	106975235	NC_000012.11
cg25362630	5.80E-13	2.82E-14	8.22	21.5297865	1.99E-01	42544650	42544773	NC_000020.10
cg02459389	5.81E-13	2.82E-14	8.22	21.5295951	7.31E-02	3923012	3923135	NC_000017.10
cg17966560	5.82E-13	2.82E-14	8.22	21.5270432	1.42E-01	156357871	156357994	NC_000001.10
cg19698137	5.82E-13	2.82E-14	8.22	21.5268481	1.27E-01	35990997	35991120	NC_000005.9
cg17123690	5.82E-13	2.82E-14	8.22	21.5267333	1.66E-01	55951801	55951924	NC_000019.9
cg12268575	5.83E-13	2.83E-14	8.22	21.524803	1.64E-01	177133536	177133659	NC_000001.10
cg23929194	5.86E-13	2.84E-14	8.22	21.519644	1.85E-01	44662296	44662419	NC_000007.13
cg24198558	5.87E-13	2.85E-14	8.22	21.5189664	2.31E-01	132757102	132757225	NC_000003.11
cg02387554	5.87E-13	2.85E-14	8.22	21.5183413	1.13E-01	1233285	1233408	NC_000007.13
cg08217589	5.87E-13	2.85E-14	8.22	21.5176378	1.11E-01	31025866	31025989	NC_000020.10
cg16697438	5.87E-13	2.85E-14	8.22	21.5172705	1.65E-01	72043538	72043661	NC_000010.10
cg09540629	5.88E-13	2.85E-14	8.22	21.5168619	1.62E-01	134981759	134981882	NC_000010.10
cg15106420	5.88E-13	2.86E-14	8.22	21.5160302	7.07E-02	109042237	109042360	NC_000005.9
cg25106645	5.88E-13	2.86E-14	8.22	21.5155588	1.62E-01	115135560	115135683	NC_000012.11
cg09183671	5.89E-13	2.86E-14	8.22	21.5148075	1.48E-01	112630780	112630903	NC_000010.10
cg00767948	5.90E-13	2.87E-14	8.22	21.5124932	1.25E-01	61940979	61941102	NC_000003.11
cg01817364	5.92E-13	2.87E-14	8.21	21.5098483	1.96E-01	43037411	43037534	NC_000005.9
cg23365071	5.92E-13	2.88E-14	8.21	21.5087698	5.04E-02	30793845	30793968	NC_000016.9
cg09852619	5.92E-13	2.88E-14	8.21	21.5082486	8.19E-02	25217537	25217660	NC_000008.10
cg02530022	5.93E-13	2.88E-14	8.21	21.5080552	2.25E-01	96642708	96642831	NC_000007.13
cg14331619	5.93E-13	2.88E-14	8.21	21.5076745	6.31E-02	35909996	35910119	NC_000009.11
cg25266888	5.94E-13	2.88E-14	8.21	21.5061716	1.71E-01	2482594	2482717	NC_000011.9

cg12598606	5.94E-13	2.88E-14	8.21	21.5059673	1.30E-01	155018256	155018379	NC_000007.13
cg01876531	5.94E-13	2.89E-14	8.21	21.5049388	1.63E-01	208405868	208405991	NC_000001.10
cg21707172	5.96E-13	2.89E-14	8.21	21.5023691	1.50E-01	155537944	155538067	NC_000006.11
cg02355885	5.96E-13	2.90E-14	8.21	21.5013801	2.03E-01	30142990	30143113	NC_000002.11
cg13716619	5.99E-13	2.91E-14	8.21	21.4972145	1.00E-01	31829895	31830018	NC_000006.11
cg01957901	6.00E-13	2.92E-14	8.21	21.4950843	1.01E-01	132116174	132116297	NC_000011.9
cg16689609	6.02E-13	2.92E-14	8.21	21.4923798	7.29E-02	144682208	144682331	NC_000006.11
cg18832348	6.02E-13	2.93E-14	8.21	21.4910086	9.61E-02	94844511	94844634	NC_000005.9
cg17877704	6.03E-13	2.93E-14	8.21	21.4900352	1.82E-01	127940483	127940606	NC_000012.11
cg20987369	6.03E-13	2.93E-14	8.21	21.4899404	1.14E-01	1579572	1579695	NC_000004.11
cg14657525	6.04E-13	2.94E-14	8.21	21.4884576	2.32E-01	32859099	32859222	NC_000003.11
cg13818654	6.04E-13	2.94E-14	8.21	21.4871636	2.16E-01	78493126	78493249	NC_000013.10
cg10789281	6.05E-13	2.94E-14	8.21	21.4864149	2.21E-01	81471882	81472005	NC_000012.11
cg17362861	6.07E-13	2.95E-14	8.21	21.4821987	3.03E-01	151107257	151107380	NC_000007.13
cg06841832	6.07E-13	2.96E-14	8.21	21.4821053	8.42E-02	140175142	140175265	NC_000006.11
cg10701252	6.08E-13	2.96E-14	8.21	21.4810741	1.32E-01	55508585	55508708	NC_000003.11
cg04485988	6.10E-13	2.97E-14	8.21	21.4776931	1.15E-01	118168275	118168398	NC_000001.10
cg00268840	6.10E-13	2.97E-14	8.21	21.4775482	2.86E-01	44332653	44332776	NC_000011.9
cg16661209	6.10E-13	2.97E-14	8.21	21.4775298	2.61E-01	29107069	29107192	NC_000013.10
cg13729816	6.11E-13	2.98E-14	8.21	21.4750123	1.44E-01	52954722	52954845	NC_000019.9
cg09338032	6.12E-13	2.98E-14	8.21	21.4748389	1.76E-01	113916609	113916732	NC_000012.11
cg12459514	6.13E-13	2.98E-14	8.21	21.4726712	4.55E-02	23914071	23914194	NC_000002.11
cg15784124	6.13E-13	2.99E-14	8.21	21.4716129	1.78E-01	111543114	111543237	NC_000004.11
cg02214096	6.13E-13	2.99E-14	8.21	21.4714966	1.80E-01	77822350	77822473	NC_000016.9
cg26071016	6.14E-13	2.99E-14	8.21	21.4706586	1.97E-01	111217785	111217908	NC_000001.10

cg13726463	6.14E-13	2.99E-14	8.21	21.4700392	6.84E-02	31439681	31439804	NC_000016.9
cg04891961	6.14E-13	2.99E-14	8.21	21.470028	1.45E-01	27939900	27940023	NC_000017.10
cg10089801	6.14E-13	2.99E-14	8.21	21.4694587	1.32E-01	203036235	203036358	NC_000002.11
cg20600210	6.15E-13	3.00E-14	8.21	21.4679283	1.85E-01	57090013	57090136	NC_000020.10
cg19277389	6.15E-13	3.00E-14	8.21	21.4678008	6.44E-02	7802545	7802668	NC_000004.11
cg09823576	6.16E-13	3.00E-14	8.21	21.4673063	1.30E-01	164640367	164640490	NC_000001.10
cg10863741	6.16E-13	3.00E-14	8.21	21.46668	2.33E-01	122686038	122686161	NC_000004.11
cg21194776	6.16E-13	3.00E-14	8.21	21.4664757	4.17E-02	75314128	75314251	NC_000010.10
cg21170796	6.19E-13	3.01E-14	8.21	21.4622902	1.67E-01	119528164	119528287	NC_000001.10
cg20099830	6.19E-13	3.02E-14	8.21	21.4609298	2.04E-01	114842031	114842154	NC_000012.11
cg09267051	6.19E-13	3.02E-14	8.21	21.4608926	2.49E-01	100943468	100943591	NC_000007.13
cg14631386	6.20E-13	3.02E-14	8.21	21.4598299	1.94E-01	93114305	93114428	NC_000008.10
cg07830534	6.22E-13	3.03E-14	8.21	21.4569102	1.10E-01	38953285	38953408	NC_000017.10
cg09354309	6.24E-13	3.04E-14	8.21	21.4532415	2.46E-01	22487437	22487560	NC_000012.11
cg16458868	6.24E-13	3.04E-14	8.21	21.4528718	5.16E-02	88260834	88260957	NC_000016.9
cg19718882	6.26E-13	3.05E-14	8.21	21.4505569	1.91E-01	32458714	32458837	NC_000011.9
cg11034763	6.26E-13	3.05E-14	8.21	21.4504355	8.16E-02	963020	963143	NC_000005.9
cg02069715	6.28E-13	3.07E-14	8.2	21.4459894	1.82E-01	27215962	27216085	NC_000015.9
cg25303831	6.30E-13	3.07E-14	8.2	21.4429294	1.44E-01	31544911	31545034	NC_000013.10
cg13069237	6.32E-13	3.08E-14	8.2	21.4403345	7.49E-02	6063456	6063579	NC_000001.10
cg02079908	6.34E-13	3.09E-14	8.2	21.4366031	5.19E-02	42411760	42411883	NC_000002.11
cg13823136	6.34E-13	3.10E-14	8.2	21.4361971	3.01E-01	77333138	77333261	NC_000001.10
cg04032066	6.39E-13	3.12E-14	8.2	21.4292863	1.91E-01	20622721	20622844	NC_000011.9
cg02259047	6.40E-13	3.12E-14	8.2	21.4274635	5.56E-02	104175555	104175678	NC_000014.8
cg15775921	6.40E-13	3.13E-14	8.2	21.4260056	1.58E-01	223156136	223156259	NC_000002.11

cg02339542	6.41E-13	3.13E-14	8.2	21.4254474	9.56E-02	92823161	92823284	NC_000012.11
cg02350767	6.42E-13	3.13E-14	8.2	21.4241543	7.73E-02	99250527	99250650	NC_000015.9
cg16307705	6.42E-13	3.13E-14	8.2	21.4240073	2.29E-01	3602025	3602148	NC_000011.9
cg01591037	6.44E-13	3.15E-14	8.2	21.4203641	1.17E-01	15134481	15134604	NC_000012.11
cg08115732	6.44E-13	3.15E-14	8.2	21.419939	1.39E-01	76171794	76171917	NC_000017.10
cg10068417	6.45E-13	3.15E-14	8.2	21.4178896	1.62E-01	50706583	50706706	NC_000013.10
cg10079740	6.46E-13	3.16E-14	8.2	21.416265	2.12E-01	55513480	55513603	NC_000016.9
cg18783033	6.49E-13	3.17E-14	8.2	21.4125421	9.09E-02	90488297	90488420	NC_000010.10
cg10521851	6.49E-13	3.17E-14	8.2	21.4121867	2.08E-01	130297771	130297894	NC_000011.9
cg20791178	6.53E-13	3.19E-14	8.2	21.4059806	6.48E-02	38246696	38246819	NC_000008.10
cg05676622	6.53E-13	3.19E-14	8.2	21.4054714	1.24E-01	66410998	66411121	NC_000011.9
cg15306794	6.54E-13	3.20E-14	8.2	21.404875	8.78E-02	16998450	16998573	NC_000011.9
cg05728754	6.54E-13	3.20E-14	8.2	21.4040816	2.35E-01	47225326	47225449	NC_000012.11
cg12080391	6.55E-13	3.20E-14	8.2	21.4022494	2.15E-01	5018984	5019107	NC_000012.11
cg20400592	6.57E-13	3.22E-14	8.2	21.3987617	1.25E-01	51418041	51418164	NC_000013.10
cg16732787	6.59E-13	3.22E-14	8.2	21.3958731	1.39E-01	228785992	228786115	NC_000001.10
cg17489750	6.64E-13	3.25E-14	8.19	21.3882667	1.54E-01	54504774	54504897	NC_000008.10
cg00039441	6.65E-13	3.25E-14	8.19	21.3869775	2.75E-01	176957473	176957596	NC_000002.11
cg02172492	6.65E-13	3.26E-14	8.19	21.3863616	1.55E-01	19553422	19553545	NC_000002.11
cg01159623	6.65E-13	3.26E-14	8.19	21.3862654	1.68E-01	70596377	70596500	NC_000007.13
cg14682419	6.66E-13	3.26E-14	8.19	21.3857155	9.89E-02	141994495	141994618	NC_000005.9
cg06093355	6.66E-13	3.26E-14	8.19	21.3855177	2.00E-01	46547693	46547816	NC_000002.11
cg23004527	6.66E-13	3.26E-14	8.19	21.3841477	1.36E-01	3390353	3390476	NC_000019.9
cg26057971	6.67E-13	3.27E-14	8.19	21.3826786	1.45E-01	62898351	62898474	NC_000001.10
cg12973591	6.69E-13	3.28E-14	8.19	21.3805882	2.66E-01	93519473	93519596	NC_000007.13

cg07014126	6.69E-13	3.28E-14	8.19	21.3804491	1.49E-01	169717713	169717836	NC_000006.11
cg20929476	6.70E-13	3.28E-14	8.19	21.3784552	1.32E-01	56284168	56284291	NC_000015.9
cg11876524	6.73E-13	3.30E-14	8.19	21.3742954	6.92E-02	82177159	82177282	NC_000010.10
cg15728861	6.74E-13	3.30E-14	8.19	21.3732197	2.11E-01	46961008	46961131	NC_000013.10
cg10959198	6.74E-13	3.30E-14	8.19	21.3718935	2.21E-01	93632896	93633019	NC_000015.9
cg16318271	6.75E-13	3.30E-14	8.19	21.371721	2.19E-01	41748193	41748316	NC_000004.11
cg07111988	6.75E-13	3.31E-14	8.19	21.3713231	1.75E-01	24971764	24971887	NC_000001.10
cg06460568	6.75E-13	3.31E-14	8.19	21.3713017	2.03E-01	2162211	2162334	NC_000011.9
cg10794439	6.77E-13	3.32E-14	8.19	21.36837	7.42E-02	41518051	41518174	NC_000008.10
cg17924021	6.78E-13	3.32E-14	8.19	21.3663036	7.46E-02	53638473	53638596	NC_000017.10
cg04184744	6.78E-13	3.32E-14	8.19	21.3660422	1.46E-01	31562255	31562378	NC_000014.8
cg12964647	6.80E-13	3.33E-14	8.19	21.3630478	7.09E-02	46234525	46234648	NC_000017.10
cg21851351	6.81E-13	3.34E-14	8.19	21.3616189	1.95E-01	28368087	28368210	NC_000013.10
cg21905819	6.82E-13	3.34E-14	8.19	21.3603235	8.89E-02	5972032	5972155	NC_000017.10
cg25267072	6.82E-13	3.34E-14	8.19	21.3597413	1.88E-01	23299889	23300012	NC_000019.9
cg01297020	6.83E-13	3.35E-14	8.19	21.3593062	1.44E-01	106117425	106117548	NC_000013.10
cg06263250	6.83E-13	3.35E-14	8.19	21.3591795	9.37E-02	10930482	10930605	NC_000011.9
cg09601175	6.83E-13	3.35E-14	8.19	21.3585129	1.33E-01	177025608	177025731	NC_000002.11
cg14311495	6.83E-13	3.35E-14	8.19	21.3585114	4.85E-02	95764742	95764865	NC_000009.11
cg07609862	6.85E-13	3.36E-14	8.19	21.3549995	1.36E-01	92702912	92703035	NC_000011.9
cg13273396	6.85E-13	3.36E-14	8.19	21.3549121	2.61E-01	81471867	81471990	NC_000012.11
cg02416510	6.86E-13	3.36E-14	8.19	21.3543701	1.46E-01	54791763	54791886	NC_000008.10
cg27084654	6.86E-13	3.37E-14	8.19	21.3532654	1.11E-01	141118615	141118738	NC_000003.11
cg01278387	6.88E-13	3.38E-14	8.19	21.3501005	1.80E-01	58152281	58152404	NC_000020.10
cg20014049	6.89E-13	3.38E-14	8.19	21.3492642	2.53E-01	23481385	23481508	NC_000010.10

cg19190699	6.91E-13	3.39E-14	8.19	21.3466222	1.18E-01	101514156	101514279	NC_000015.9
cg00376480	6.92E-13	3.39E-14	8.19	21.3455798	9.85E-02	46892832	46892955	NC_000021.8
cg26759954	6.92E-13	3.40E-14	8.19	21.3449801	2.06E-01	13925420	13925543	NC_000006.11
cg03223076	6.93E-13	3.40E-14	8.19	21.3427301	1.36E-01	48698558	48698681	NC_000003.11
cg08812889	6.94E-13	3.41E-14	8.19	21.3420367	1.29E-01	174439925	174440048	NC_000004.11
cg26721264	6.94E-13	3.41E-14	8.19	21.3414489	2.20E-01	74961727	74961850	NC_000018.9
cg13484329	6.96E-13	3.42E-14	8.19	21.3384593	9.04E-02	110342823	110342946	NC_000012.11
cg04184232	6.96E-13	3.42E-14	8.19	21.3381177	2.10E-01	29243234	29243357	NC_000014.8
cg15202738	6.97E-13	3.42E-14	8.19	21.3377237	2.71E-01	53636108	53636231	NC_000019.9
cg00145253	6.97E-13	3.42E-14	8.19	21.3374239	1.34E-01	75602857	75602980	NC_000001.10
cg17312004	6.98E-13	3.43E-14	8.19	21.3361139	1.94E-01	43569076	43569199	NC_000011.9
cg15699226	6.99E-13	3.43E-14	8.19	21.3343994	1.60E-01	78492678	78492801	NC_000013.10
cg17524637	7.01E-13	3.45E-14	8.19	21.3303867	2.14E-01	10458454	10458577	NC_000004.11
cg07286357	7.02E-13	3.45E-14	8.19	21.3296934	5.68E-02	96675199	96675322	NC_000002.11
cg18085117	7.02E-13	3.45E-14	8.19	21.3290853	7.18E-02	125806214	125806337	NC_000010.10
cg08787769	7.03E-13	3.46E-14	8.18	21.327601	7.01E-02	2869479	2869602	NC_000016.9
cg04078195	7.04E-13	3.46E-14	8.18	21.3270578	8.29E-02	20641735	20641858	NC_000022.10
cg24189904	7.06E-13	3.47E-14	8.18	21.3239903	1.98E-01	9473781	9473904	NC_000019.9
cg15773539	7.06E-13	3.47E-14	8.18	21.3234996	1.16E-01	61520112	61520235	NC_000017.10
cg24563570	7.08E-13	3.48E-14	8.18	21.3212804	1.82E-01	60936801	60936924	NC_000010.10
cg25563456	7.08E-13	3.48E-14	8.18	21.3211033	1.66E-01	32456069	32456192	NC_000011.9
cg01274324	7.08E-13	3.48E-14	8.18	21.3209188	6.46E-02	33948002	33948125	NC_000021.8
cg10203922	7.09E-13	3.49E-14	8.18	21.3191679	1.72E-01	145566200	145566323	NC_000004.11
cg07482257	7.09E-13	3.49E-14	8.18	21.3182308	1.62E-01	174135761	174135884	NC_000005.9
cg01183965	7.11E-13	3.50E-14	8.18	21.3163191	6.64E-02	134855243	134855366	NC_000010.10

cg19149681	7.12E-13	3.50E-14	8.18	21.3151335	1.95E-01	57359399	57359522	NC_000008.10
cg26947831	7.12E-13	3.50E-14	8.18	21.3148391	1.08E-01	86018811	86018934	NC_000016.9
cg06153765	7.13E-13	3.51E-14	8.18	21.3125172	9.44E-02	106638076	106638199	NC_000012.11
cg01860459	7.13E-13	3.51E-14	8.18	21.3124402	7.42E-02	123419554	123419677	NC_000003.11
cg12488111	7.16E-13	3.52E-14	8.18	21.3086626	1.12E-01	105945699	105945822	NC_000014.8
cg03713642	7.20E-13	3.54E-14	8.18	21.3032775	2.10E-01	22825100	22825223	NC_000016.9
cg19650157	7.21E-13	3.55E-14	8.18	21.3018987	2.43E-01	78493297	78493420	NC_000013.10
cg05345448	7.21E-13	3.55E-14	8.18	21.3011458	8.16E-02	105688412	105688535	NC_000014.8
cg25189808	7.22E-13	3.56E-14	8.18	21.2994697	9.82E-02	45802621	45802744	NC_000007.13
cg13663793	7.25E-13	3.57E-14	8.18	21.2951831	2.49E-01	32457124	32457247	NC_000011.9
cg02478907	7.26E-13	3.57E-14	8.18	21.294662	1.81E-01	143545940	143546063	NC_000008.10
cg13924715	7.26E-13	3.58E-14	8.18	21.2937627	1.55E-01	10750890	10751013	NC_000011.9
cg17240815	7.27E-13	3.58E-14	8.18	21.2921467	1.90E-01	55039382	55039505	NC_000006.11
cg07805777	7.28E-13	3.58E-14	8.18	21.2915467	1.57E-01	15962932	15963055	NC_000011.9
cg16288089	7.28E-13	3.58E-14	8.18	21.2914575	2.25E-01	97361408	97361531	NC_000007.13
cg02426611	7.29E-13	3.59E-14	8.18	21.2889195	1.53E-01	87681276	87681399	NC_000016.9
cg09791743	7.30E-13	3.60E-14	8.18	21.2876708	9.94E-02	42898508	42898631	NC_000006.11
cg09123431	7.31E-13	3.60E-14	8.18	21.2865621	1.66E-01	52209811	52209934	NC_000020.10
cg14192957	7.32E-13	3.61E-14	8.18	21.2848538	2.45E-01	133536793	133536916	NC_000009.11
cg17881203	7.34E-13	3.62E-14	8.18	21.2824654	8.53E-02	990398	990521	NC_000019.9
cg03903246	7.34E-13	3.62E-14	8.18	21.2819142	1.32E-01	240257032	240257155	NC_000001.10
cg13418631	7.34E-13	3.62E-14	8.18	21.2818053	4.39E-02	39636904	39637027	NC_000017.10
cg18370682	7.35E-13	3.63E-14	8.18	21.280376	9.17E-02	158239759	158239882	NC_000005.9
cg11942956	7.36E-13	3.63E-14	8.18	21.2788084	2.00E-01	133562470	133562593	NC_000006.11
cg05290820	7.37E-13	3.63E-14	8.18	21.2786115	1.88E-01	137392877	137393000	NC_000009.11

cg26980244	7.37E-13	3.63E-14	8.18	21.2776778	1.76E-01	24772513	24772636	NC_000008.10
cg26442210	7.39E-13	3.64E-14	8.18	21.2754381	5.03E-02	129932033	129932156	NC_000007.13
cg23835476	7.40E-13	3.65E-14	8.18	21.2734582	6.29E-02	155633298	155633421	NC_000007.13
cg12018140	7.42E-13	3.66E-14	8.18	21.2710987	1.17E-01	122434326	122434449	NC_000005.9
cg23518039	7.43E-13	3.66E-14	8.18	21.2696143	9.72E-02	148579190	148579313	NC_000005.9
cg16574154	7.46E-13	3.68E-14	8.17	21.2651577	1.50E-01	10652376	10652499	NC_000001.10
cg26247841	7.48E-13	3.69E-14	8.17	21.262866	1.62E-01	114252967	114253090	NC_000004.11
cg18087266	7.50E-13	3.70E-14	8.17	21.2603979	1.39E-01	25257629	25257752	NC_000001.10
cg15042811	7.51E-13	3.71E-14	8.17	21.2582346	2.32E-01	144621971	144622094	NC_000004.11
cg04503318	7.52E-13	3.71E-14	8.17	21.2570756	1.15E-01	15789862	15789985	NC_000001.10
cg10864603	7.52E-13	3.71E-14	8.17	21.256731	8.84E-02	122302722	122302845	NC_000008.10
cg24466241	7.53E-13	3.72E-14	8.17	21.256164	1.39E-01	53308908	53309031	NC_000001.10
cg13054534	7.54E-13	3.72E-14	8.17	21.2541852	1.12E-01	113980128	113980251	NC_000013.10
cg03705912	7.54E-13	3.72E-14	8.17	21.2535822	1.75E-01	16179210	16179333	NC_000005.9
cg13563903	7.55E-13	3.73E-14	8.17	21.2530401	2.11E-01	108924132	108924255	NC_000010.10
cg05384102	7.55E-13	3.73E-14	8.17	21.2522186	2.24E-01	84419329	84419452	NC_000006.11
cg04025150	7.56E-13	3.73E-14	8.17	21.2509179	1.96E-01	34642862	34642985	NC_000001.10
cg05348321	7.57E-13	3.74E-14	8.17	21.2493219	5.73E-02	121666879	121667002	NC_000012.11
cg03960424	7.58E-13	3.74E-14	8.17	21.2490836	1.24E-01	57042304	57042427	NC_000020.10
cg20348858	7.59E-13	3.75E-14	8.17	21.2473562	1.35E-01	119305335	119305458	NC_000010.10
cg21255438	7.62E-13	3.76E-14	8.17	21.2432975	1.43E-01	70983760	70983883	NC_000008.10
cg16319192	7.62E-13	3.77E-14	8.17	21.2429189	5.96E-02	142251291	142251414	NC_000008.10
cg16137147	7.62E-13	3.77E-14	8.17	21.2425044	1.74E-01	221067896	221068019	NC_000001.10
cg24655669	7.62E-13	3.77E-14	8.17	21.2423078	9.37E-02	2151788	2151911	NC_000007.13
cg04167903	7.65E-13	3.78E-14	8.17	21.2388526	2.43E-01	27135746	27135869	NC_000007.13

cg17978293	7.65E-13	3.78E-14	8.17	21.238359	1.04E-01	217614	217737	NC_000017.10
cg06726820	7.66E-13	3.79E-14	8.17	21.2376143	1.92E-01	139259742	139259865	NC_000003.11
cg12317601	7.67E-13	3.79E-14	8.17	21.2364833	1.97E-01	119301760	119301883	NC_000010.10
cg14542554	7.67E-13	3.79E-14	8.17	21.236452	1.78E-01	61122327	61122450	NC_000014.8
cg26632409	7.68E-13	3.80E-14	8.17	21.2341047	1.23E-01	134886312	134886435	NC_000010.10
cg15644756	7.69E-13	3.80E-14	8.17	21.2326358	2.02E-01	69198743	69198866	NC_000012.11
cg14377152	7.71E-13	3.82E-14	8.17	21.2298316	9.96E-02	45431690	45431813	NC_000015.9
cg21124497	7.72E-13	3.82E-14	8.17	21.2289645	1.87E-01	32859377	32859500	NC_000003.11
cg22950163	7.74E-13	3.83E-14	8.17	21.2258309	1.63E-01	55513525	55513648	NC_000016.9
cg26973607	7.74E-13	3.83E-14	8.17	21.2257484	1.27E-01	1549475	1549598	NC_000005.9
cg10264692	7.76E-13	3.84E-14	8.17	21.2234797	7.99E-02	61314827	61314950	NC_000020.10
cg13437581	7.79E-13	3.86E-14	8.17	21.2191794	1.86E-01	44777799	44777922	NC_000018.9
cg21768604	7.79E-13	3.86E-14	8.17	21.2191447	1.61E-01	2742325	2742448	NC_000007.13
cg09427417	7.80E-13	3.86E-14	8.17	21.218536	7.96E-02	112194570	112194693	NC_000012.11
cg00525681	7.82E-13	3.87E-14	8.17	21.2151272	1.29E-01	88329151	88329274	NC_000013.10
cg25118879	7.82E-13	3.87E-14	8.17	21.2147266	5.79E-02	72094780	72094903	NC_000015.9
cg05399244	7.83E-13	3.88E-14	8.17	21.2143265	1.19E-01	179611	179734	NC_000017.10
cg06501366	7.83E-13	3.88E-14	8.17	21.2141042	1.22E-01	78365687	78365810	NC_000005.9
cg11370259	7.83E-13	3.88E-14	8.17	21.2139848	1.30E-01	145580736	145580859	NC_000008.10
cg24626079	7.84E-13	3.88E-14	8.17	21.2126464	2.58E-01	4382171	4382294	NC_000012.11
cg15967847	7.85E-13	3.89E-14	8.17	21.211073	2.07E-01	15090746	15090869	NC_000019.9
cg22989103	7.85E-13	3.89E-14	8.17	21.2108644	1.93E-01	48972331	48972454	NC_000022.10
cg22052056	7.87E-13	3.90E-14	8.17	21.2088967	8.96E-02	31351813	31351936	NC_000020.10
cg19854521	7.90E-13	3.91E-14	8.16	21.2050224	2.08E-01	93520452	93520575	NC_000007.13
cg21424940	7.92E-13	3.92E-14	8.16	21.2020975	1.78E-01	19158666	19158789	NC_000007.13

cg07628855	7.92E-13	3.93E-14	8.16	21.2017368	1.38E-01	25100356	25100479	NC_000007.13
cg14159026	7.94E-13	3.94E-14	8.16	21.1989989	2.92E-01	105584551	105584674	NC_000006.11
cg09117841	7.94E-13	3.94E-14	8.16	21.1989815	3.32E-02	38654722	38654845	NC_000006.11
cg09488200	7.96E-13	3.95E-14	8.16	21.1966065	5.68E-02	213781204	213781327	NC_000002.11
cg26130023	7.96E-13	3.95E-14	8.16	21.1956918	7.57E-02	45453609	45453732	NC_000018.9
cg08828036	7.97E-13	3.95E-14	8.16	21.194933	1.37E-01	26109204	26109327	NC_000015.9
cg10797737	7.98E-13	3.96E-14	8.16	21.1938537	9.04E-02	105965379	105965502	NC_000014.8
cg10018233	8.02E-13	3.98E-14	8.16	21.1886404	1.42E-01	150070692	150070815	NC_000007.13
cg12373659	8.04E-13	3.99E-14	8.16	21.1860439	8.95E-02	178973820	178973943	NC_000005.9
cg26266934	8.05E-13	4.00E-14	8.16	21.184349	8.03E-02	93199519	93199642	NC_000015.9
cg04134755	8.06E-13	4.00E-14	8.16	21.1830649	1.08E-01	80828253	80828376	NC_000017.10
cg04228468	8.06E-13	4.00E-14	8.16	21.1827488	1.70E-01	164913627	164913750	NC_000003.11
cg14186066	8.07E-13	4.01E-14	8.16	21.1813995	2.10E-01	60976115	60976238	NC_000014.8
cg10448438	8.07E-13	4.01E-14	8.16	21.1813198	8.52E-02	121515019	121515142	NC_000011.9
cg09074938	8.09E-13	4.02E-14	8.16	21.1792755	8.12E-02	144757347	144757470	NC_000008.10
cg17156227	8.11E-13	4.02E-14	8.16	21.1771512	6.34E-02	3209945	3210068	NC_000020.10
cg12433277	8.12E-13	4.03E-14	8.16	21.1757056	2.00E-01	151106990	151107113	NC_000007.13
cg14969899	8.12E-13	4.03E-14	8.16	21.1749838	8.89E-02	104569581	104569704	NC_000014.8
cg08259168	8.13E-13	4.04E-14	8.16	21.1740193	1.57E-01	78509494	78509617	NC_000001.10
cg12417775	8.17E-13	4.06E-14	8.16	21.1692342	1.65E-01	212463238	212463361	NC_000001.10
cg06895103	8.19E-13	4.07E-14	8.16	21.1668404	4.10E-02	30160580	30160703	NC_000006.11
cg05334656	8.19E-13	4.07E-14	8.16	21.1663382	1.74E-01	4866047	4866170	NC_000004.11
cg02126753	8.19E-13	4.07E-14	8.16	21.166125	2.37E-01	44143998	44144121	NC_000007.13
cg24192660	8.21E-13	4.08E-14	8.16	21.1639285	1.60E-01	17439877	17440000	NC_000019.9
cg22534145	8.21E-13	4.08E-14	8.16	21.1639191	2.11E-01	23015936	23016059	NC_000020.10

cg27315341	8.23E-13	4.09E-14	8.16	21.1614147	1.65E-01	97887034	97887157	NC_000012.11
cg22287492	8.24E-13	4.09E-14	8.16	21.1603524	1.88E-01	2161411	2161534	NC_000011.9
cg00830252	8.26E-13	4.10E-14	8.16	21.1580848	1.78E-01	16039824	16039947	NC_000012.11
cg14519150	8.26E-13	4.10E-14	8.16	21.1577068	1.15E-01	20985249	20985372	NC_000001.10
cg18140268	8.26E-13	4.11E-14	8.16	21.1573342	1.61E-01	147781827	147781950	NC_000001.10
cg12951849	8.27E-13	4.11E-14	8.16	21.1566178	2.03E-01	11148932	11149055	NC_000018.9
cg26699569	8.28E-13	4.12E-14	8.16	21.1547035	2.08E-01	81952653	81952776	NC_000004.11
cg03556669	8.28E-13	4.12E-14	8.16	21.1545057	1.51E-01	32064497	32064620	NC_000006.11
cg16345915	8.30E-13	4.13E-14	8.16	21.1522909	1.04E-01	80371099	80371222	NC_000017.10
cg21144941	8.33E-13	4.14E-14	8.16	21.1489763	1.35E-01	17680575	17680698	NC_000022.10
cg19426594	8.34E-13	4.15E-14	8.16	21.1472726	1.19E-01	490116	490239	NC_000005.9
cg19267457	8.34E-13	4.15E-14	8.16	21.1471934	1.67E-01	64394702	64394825	NC_000001.10
cg22738219	8.36E-13	4.16E-14	8.15	21.144887	2.66E-01	13043313	13043436	NC_000010.10
cg09410453	8.36E-13	4.16E-14	8.15	21.1443441	1.78E-01	8815355	8815478	NC_000016.9
cg09692428	8.37E-13	4.16E-14	8.15	21.1439037	1.25E-01	84967481	84967604	NC_000016.9
cg05102668	8.37E-13	4.16E-14	8.15	21.1436627	1.11E-01	76541148	76541271	NC_000018.9
cg08380005	8.37E-13	4.17E-14	8.15	21.1432155	1.02E-01	816149	816272	NC_000016.9
cg17373442	8.38E-13	4.17E-14	8.15	21.1423469	2.94E-01	142839991	142840114	NC_000003.11
cg04106647	8.38E-13	4.17E-14	8.15	21.1419512	2.36E-01	12995603	12995726	NC_000016.9
cg05113927	8.38E-13	4.17E-14	8.15	21.1416562	1.19E-01	27531244	27531367	NC_000002.11
cg06873590	8.39E-13	4.18E-14	8.15	21.1403949	1.35E-01	167545249	167545372	NC_000005.9
cg22709008	8.44E-13	4.20E-14	8.15	21.135201	8.25E-02	45998083	45998206	NC_000019.9
cg09907439	8.44E-13	4.20E-14	8.15	21.1351812	7.73E-02	109031953	109032076	NC_000012.11
cg22134325	8.46E-13	4.21E-14	8.15	21.1328438	1.41E-01	66188745	66188868	NC_000011.9
cg01290710	8.47E-13	4.22E-14	8.15	21.1308945	9.79E-02	40889978	40890101	NC_000019.9

cg20171396	8.49E-13	4.22E-14	8.15	21.1292416	1.84E-01	13536910	13537033	NC_000004.11
cg15024592	8.50E-13	4.23E-14	8.15	21.1275513	5.83E-02	27771383	27771506	NC_000006.11
cg18921771	8.50E-13	4.23E-14	8.15	21.1272607	2.20E-01	60952196	60952319	NC_000014.8
cg11423556	8.51E-13	4.24E-14	8.15	21.1261948	1.10E-01	69728114	69728237	NC_000002.11
cg12380743	8.52E-13	4.24E-14	8.15	21.1254171	1.57E-01	149334007	149334130	NC_000001.10
cg09833545	8.52E-13	4.24E-14	8.15	21.1248335	1.51E-01	117160235	117160358	NC_000011.9
cg14361458	8.52E-13	4.24E-14	8.15	21.1246395	1.74E-01	189203962	189204085	NC_000004.11
cg13935577	8.54E-13	4.25E-14	8.15	21.1227264	1.08E-01	107974897	107975020	NC_000012.11
cg05860723	8.54E-13	4.26E-14	8.15	21.1221048	1.82E-01	81102486	81102609	NC_000012.11
cg01442601	8.54E-13	4.26E-14	8.15	21.1219015	2.01E-01	102030727	102030850	NC_000014.8
cg08034077	8.55E-13	4.26E-14	8.15	21.1211978	2.48E-01	50236087	50236210	NC_000017.10
cg13352836	8.56E-13	4.26E-14	8.15	21.120285	2.29E-01	31228263	31228386	NC_000016.9
cg05976168	8.58E-13	4.28E-14	8.15	21.1168184	5.43E-02	611508	611631	NC_000012.11
cg03308092	8.59E-13	4.28E-14	8.15	21.1157832	1.84E-01	17488275	17488398	NC_000022.10
cg08287175	8.59E-13	4.28E-14	8.15	21.1157332	1.13E-01	46895422	46895545	NC_000021.8
cg01076708	8.63E-13	4.30E-14	8.15	21.1116349	5.38E-02	129689897	129690020	NC_000011.9
cg10635895	8.63E-13	4.30E-14	8.15	21.1113434	1.97E-01	2339439	2339562	NC_000012.11
cg00714170	8.65E-13	4.31E-14	8.15	21.1091503	6.63E-02	195477854	195477977	NC_000003.11
cg10387551	8.65E-13	4.31E-14	8.15	21.1090063	2.11E-01	30038615	30038738	NC_000011.9
cg04910921	8.66E-13	4.32E-14	8.15	21.1078664	1.84E-01	26674563	26674686	NC_000014.8
cg23909834	8.66E-13	4.32E-14	8.15	21.1072573	6.00E-02	45838906	45839029	NC_000020.10
cg10803218	8.69E-13	4.33E-14	8.15	21.1045409	8.12E-02	177544994	177545117	NC_000005.9
cg06151165	8.69E-13	4.33E-14	8.15	21.1041511	2.44E-01	25062254	25062377	NC_000020.10
cg04384987	8.70E-13	4.34E-14	8.15	21.1029545	1.13E-01	81020862	81020985	NC_000016.9
cg05075562	8.70E-13	4.34E-14	8.15	21.1024301	1.14E-01	42123725	42123848	NC_000002.11

cg14290904	8.72E-13	4.35E-14	8.15	21.1006512	2.02E-01	36986585	36986708	NC_000014.8
cg23889730	8.72E-13	4.35E-14	8.15	21.1005244	1.35E-01	94254131	94254254	NC_000014.8
cg12991609	8.74E-13	4.36E-14	8.15	21.0978854	1.23E-01	1101551	1101674	NC_000008.10
cg04195548	8.76E-13	4.37E-14	8.15	21.0957556	1.03E-01	1206004	1206127	NC_000004.11
cg01866955	8.76E-13	4.37E-14	8.15	21.0955312	1.85E-01	19692217	19692340	NC_000006.11
cg24928391	8.80E-13	4.39E-14	8.15	21.090332	2.68E-01	55370336	55370459	NC_000008.10
cg13321077	8.81E-13	4.40E-14	8.15	21.0899481	1.33E-01	220196755	220196878	NC_000002.11
cg08215918	8.81E-13	4.40E-14	8.15	21.0891041	2.15E-01	37005557	37005680	NC_000013.10
cg13944468	8.82E-13	4.41E-14	8.15	21.0876868	2.28E-01	2753852	2753975	NC_000005.9
cg09126569	8.83E-13	4.41E-14	8.15	21.0871587	1.72E-01	93618390	93618513	NC_000008.10
cg17957183	8.84E-13	4.41E-14	8.15	21.0860019	1.03E-01	93803321	93803444	NC_000012.11
cg11108676	8.84E-13	4.41E-14	8.15	21.0858317	2.43E-01	88801004	88801127	NC_000015.9
cg24820783	8.86E-13	4.42E-14	8.14	21.0835704	2.08E-01	26504969	26505092	NC_000010.10
cg05524144	8.86E-13	4.43E-14	8.14	21.0835301	8.87E-02	134679383	134679506	NC_000010.10
cg02944007	8.87E-13	4.43E-14	8.14	21.082349	1.76E-01	79614795	79614918	NC_000017.10
cg04857033	8.89E-13	4.44E-14	8.14	21.0802465	1.66E-01	120258412	120258535	NC_000001.10
cg17746023	8.90E-13	4.45E-14	8.14	21.0785691	4.88E-02	46918393	46918516	NC_000021.8
cg02508651	8.92E-13	4.46E-14	8.14	21.076495	1.44E-01	46604554	46604677	NC_000017.10
cg26983874	8.93E-13	4.47E-14	8.14	21.0743558	1.45E-01	16736532	16736655	NC_000019.9
cg02961004	8.94E-13	4.47E-14	8.14	21.0740242	1.75E-01	57387702	57387825	NC_000010.10
cg00118229	8.97E-13	4.48E-14	8.14	21.0705637	8.80E-02	44088420	44088543	NC_000021.8
cg17953764	8.98E-13	4.49E-14	8.14	21.0694381	1.05E-01	48492845	48492968	NC_000004.11
cg21880079	8.98E-13	4.49E-14	8.14	21.0687488	3.18E-02	8419587	8419710	NC_000008.10
cg08252804	8.99E-13	4.50E-14	8.14	21.0678233	5.47E-02	202788592	202788715	NC_000002.11
cg00196414	9.00E-13	4.50E-14	8.14	21.0665969	1.88E-01	176947764	176947887	NC_000002.11

cg17735563	9.02E-13	4.51E-14	8.14	21.0642113	1.00E-01	33738079	33738202	NC_000002.11
cg02720691	9.02E-13	4.51E-14	8.14	21.0639015	1.57E-01	39366468	39366591	NC_000004.11
cg10435849	9.05E-13	4.53E-14	8.14	21.0613137	2.76E-01	47518747	47518870	NC_000021.8
cg24458300	9.05E-13	4.53E-14	8.14	21.0612859	3.94E-02	1302168	1302291	NC_000011.9
cg20455175	9.05E-13	4.53E-14	8.14	21.0605625	5.93E-02	304191	304314	NC_000011.9
cg00883123	9.07E-13	4.54E-14	8.14	21.0587137	1.05E-01	154373513	154373636	NC_000004.11
cg10838260	9.08E-13	4.55E-14	8.14	21.0567824	5.16E-02	88110197	88110320	NC_000016.9
cg00579520	9.09E-13	4.55E-14	8.14	21.0564957	1.63E-01	128752586	128752709	NC_000012.11
cg23239870	9.09E-13	4.55E-14	8.14	21.0559096	1.28E-01	3313940	3314063	NC_000002.11
cg17742359	9.10E-13	4.55E-14	8.14	21.0550219	7.83E-02	6312102	6312225	NC_000001.10
cg08283882	9.10E-13	4.56E-14	8.14	21.0542723	2.59E-01	25901017	25901140	NC_000008.10
cg24838839	9.10E-13	4.56E-14	8.14	21.0540253	6.02E-02	61031569	61031692	NC_000005.9
cg13845049	9.11E-13	4.56E-14	8.14	21.0530538	1.60E-01	77701327	77701450	NC_000009.11
cg06420903	9.11E-13	4.56E-14	8.14	21.0529307	1.64E-01	88186669	88186792	NC_000016.9
cg16905199	9.11E-13	4.56E-14	8.14	21.0528542	8.25E-02	149676378	149676501	NC_000001.10
cg26877715	9.12E-13	4.57E-14	8.14	21.0523598	2.18E-01	37353907	37354030	NC_000020.10
cg08962087	9.12E-13	4.57E-14	8.14	21.051742	1.80E-01	29107120	29107243	NC_000013.10
cg10402804	9.14E-13	4.58E-14	8.14	21.0493732	9.94E-02	105522348	105522471	NC_000014.8
cg03168582	9.16E-13	4.59E-14	8.14	21.0471921	2.60E-01	841850	841973	NC_000009.11
cg10526374	9.17E-13	4.60E-14	8.14	21.0459103	2.12E-01	2292525	2292648	NC_000011.9
cg25941299	9.20E-13	4.61E-14	8.14	21.0426377	1.63E-01	113727916	113728039	NC_000007.13
cg23076299	9.21E-13	4.61E-14	8.14	21.0423817	2.19E-01	85954312	85954435	NC_000010.10
cg19008133	9.22E-13	4.62E-14	8.14	21.0409445	8.26E-02	123124015	123124138	NC_000003.11
cg05306903	9.22E-13	4.62E-14	8.14	21.0408481	1.53E-01	109551804	109551927	NC_000012.11
cg17657179	9.22E-13	4.62E-14	8.14	21.0402804	1.84E-01	52227309	52227432	NC_000019.9

cg04700292	9.23E-13	4.63E-14	8.14	21.0398843	1.61E-01	73090723	73090846	NC_000016.9
cg19878194	9.24E-13	4.63E-14	8.14	21.0381509	1.49E-01	172744882	172745005	NC_000005.9
cg01873311	9.25E-13	4.64E-14	8.14	21.0368865	2.46E-01	89268689	89268812	NC_000016.9
cg04297067	9.25E-13	4.64E-14	8.14	21.0367028	1.24E-01	86550450	86550573	NC_000016.9
cg18581378	9.26E-13	4.64E-14	8.14	21.0363386	1.89E-01	11059443	11059566	NC_000010.10
cg04213910	9.26E-13	4.65E-14	8.14	21.0356514	4.82E-02	43238881	43239004	NC_000017.10
cg01025774	9.29E-13	4.66E-14	8.14	21.0327862	1.43E-01	42944457	42944580	NC_000005.9
cg04145890	9.29E-13	4.66E-14	8.14	21.0322087	5.48E-02	1007657	1007780	NC_000004.11
cg02176858	9.30E-13	4.66E-14	8.14	21.031916	9.21E-02	122286796	122286919	NC_000002.11
cg24659171	9.31E-13	4.67E-14	8.14	21.0305877	8.03E-02	67817691	67817814	NC_000011.9
cg21276413	9.31E-13	4.67E-14	8.14	21.0305598	2.02E-01	27112287	27112410	NC_000015.9
cg19624444	9.31E-13	4.67E-14	8.14	21.0296788	8.18E-02	2048497	2048620	NC_000007.13
cg25266629	9.32E-13	4.67E-14	8.14	21.0293761	2.34E-01	102894148	102894271	NC_000010.10
cg09317403	9.32E-13	4.68E-14	8.14	21.0284573	7.19E-02	92994936	92995059	NC_000014.8
cg11514604	9.33E-13	4.68E-14	8.14	21.0276611	1.53E-01	122901047	122901170	NC_000011.9
cg16277169	9.33E-13	4.68E-14	8.14	21.0274243	1.34E-01	115112189	115112312	NC_000012.11
cg07168204	9.34E-13	4.69E-14	8.14	21.0264022	8.58E-02	149864599	149864722	NC_000005.9
cg27308329	9.35E-13	4.69E-14	8.14	21.0256444	2.38E-01	48886043	48886166	NC_000022.10
cg13482308	9.35E-13	4.69E-14	8.14	21.0254934	2.00E-01	37002685	37002808	NC_000009.11
cg08924415	9.36E-13	4.70E-14	8.14	21.024685	1.16E-01	144902836	144902959	NC_000008.10
cg03318667	9.37E-13	4.71E-14	8.13	21.0227678	1.30E-01	104975516	104975639	NC_000012.11
cg15730491	9.39E-13	4.71E-14	8.13	21.0213367	1.71E-01	221068834	221068957	NC_000001.10
cg00836007	9.43E-13	4.74E-14	8.13	21.0164979	1.02E-01	52801982	52802105	NC_000008.10
cg02026062	9.47E-13	4.76E-14	8.13	21.0124616	1.99E-01	117086479	117086602	NC_000006.11
cg14984684	9.47E-13	4.76E-14	8.13	21.0123445	2.22E-01	79382708	79382831	NC_000015.9

cg18938204	9.47E-13	4.76E-14	8.13	21.0117081	2.05E-01	39994857	39994980	NC_000020.10
cg25723050	9.48E-13	4.76E-14	8.13	21.0105469	3.13E-01	224804222	224804345	NC_000001.10
cg08704536	9.52E-13	4.78E-14	8.13	21.0067829	1.33E-01	53638566	53638689	NC_000017.10
cg12412079	9.52E-13	4.78E-14	8.13	21.006746	2.34E-01	68981877	68982000	NC_000003.11
cg13610769	9.59E-13	4.82E-14	8.13	20.998856	4.66E-02	202484583	202484706	NC_000002.11
cg26116950	9.60E-13	4.82E-14	8.13	20.9984881	2.08E-01	41582017	41582140	NC_000012.11
cg00971369	9.64E-13	4.85E-14	8.13	20.9940344	8.41E-02	142263802	142263925	NC_000008.10
cg24842086	9.64E-13	4.85E-14	8.13	20.9937677	1.88E-01	119531857	119531980	NC_000001.10
cg07627556	9.64E-13	4.85E-14	8.13	20.9935782	1.88E-01	115530590	115530713	NC_000011.9
cg02488942	9.64E-13	4.85E-14	8.13	20.9933245	1.38E-01	105480687	105480810	NC_000002.11
cg11435225	9.67E-13	4.86E-14	8.13	20.9908411	9.37E-02	32014422	32014545	NC_000022.10
cg17137424	9.68E-13	4.87E-14	8.13	20.9891433	1.35E-01	231299352	231299475	NC_000001.10
cg26403494	9.68E-13	4.87E-14	8.13	20.9889503	5.79E-02	231360004	231360127	NC_000001.10
cg11830436	9.70E-13	4.88E-14	8.13	20.9869853	1.83E-01	34943260	34943383	NC_000010.10
cg19064523	9.70E-13	4.88E-14	8.13	20.9868603	1.62E-01	19158210	19158333	NC_000007.13
cg06694734	9.72E-13	4.89E-14	8.13	20.984578	1.61E-01	4871166	4871289	NC_000004.11
cg25092670	9.77E-13	4.92E-14	8.13	20.9798869	1.45E-01	108837620	108837743	NC_000003.11
cg14276286	9.78E-13	4.92E-14	8.13	20.9786681	1.86E-01	111782364	111782487	NC_000011.9
cg07709314	9.79E-13	4.93E-14	8.13	20.9769322	4.28E-02	114882877	114883000	NC_000013.10
cg18775702	9.82E-13	4.94E-14	8.13	20.9746008	9.21E-02	75501277	75501400	NC_000015.9
cg26639906	9.87E-13	4.97E-14	8.13	20.9691092	1.13E-01	48680554	48680677	NC_000017.10
cg19075717	9.90E-13	4.99E-14	8.13	20.9657848	1.64E-01	48670940	48671063	NC_000017.10
cg18334915	9.90E-13	4.99E-14	8.13	20.965695	1.44E-01	46758194	46758317	NC_000022.10
cg01856145	9.92E-13	5.00E-14	8.13	20.9638599	6.97E-02	63952230	63952353	NC_000003.11
cg21532913	9.94E-13	5.01E-14	8.12	20.9617499	5.10E-02	98950148	98950271	NC_000002.11

cg11600596	9.94E-13	5.01E-14	8.12	20.9611353	2.20E-01	2617199	2617322	NC_000002.11
cg19005102	9.95E-13	5.01E-14	8.12	20.960703	6.79E-02	168758843	168758966	NC_000006.11
cg02527199	9.96E-13	5.02E-14	8.12	20.9599038	1.63E-01	175085245	175085368	NC_000005.9
cg15565065	9.97E-13	5.02E-14	8.12	20.9587105	2.58E-01	46655476	46655599	NC_000017.10
cg24718971	9.98E-13	5.03E-14	8.12	20.9574773	2.37E-01	80256166	80256289	NC_000005.9
cg27042584	9.98E-13	5.03E-14	8.12	20.9572405	1.85E-01	51168485	51168608	NC_000016.9
cg03507218	1.00E-12	5.04E-14	8.12	20.9556594	1.37E-01	75736090	75736213	NC_000014.8
cg26786893	1.00E-12	5.05E-14	8.12	20.9530146	1.24E-01	43705792	43705915	NC_000011.9
cg06549216	1.00E-12	5.05E-14	8.12	20.9524677	2.00E-01	154710224	154710347	NC_000004.11
cg12188526	1.00E-12	5.06E-14	8.12	20.9515065	6.00E-02	71896386	71896509	NC_000014.8
cg20920097	1.01E-12	5.07E-14	8.12	20.9490686	1.13E-01	127057149	127057272	NC_000003.11
cg17526175	1.01E-12	5.08E-14	8.12	20.9482616	1.78E-01	33624542	33624665	NC_000019.9
cg12810152	1.01E-12	5.08E-14	8.12	20.9468149	5.52E-02	141994213	141994336	NC_000005.9
cg02527112	1.01E-12	5.08E-14	8.12	20.9464978	1.90E-01	176971353	176971476	NC_000002.11
cg02996570	1.01E-12	5.10E-14	8.12	20.9440611	5.63E-02	70001114	70001237	NC_000011.9
cg04700930	1.01E-12	5.10E-14	8.12	20.9438501	1.17E-01	98030706	98030829	NC_000012.11
cg21167761	1.01E-12	5.10E-14	8.12	20.9436736	1.57E-01	77903617	77903740	NC_000011.9
cg00566320	1.01E-12	5.10E-14	8.12	20.9429238	1.42E-01	32172204	32172327	NC_000001.10
cg04591469	1.01E-12	5.11E-14	8.12	20.9418824	1.71E-01	17810299	17810422	NC_000005.9
cg26551913	1.01E-12	5.11E-14	8.12	20.9414598	2.38E-01	102508286	102508409	NC_000010.10
cg04062040	1.01E-12	5.11E-14	8.12	20.9411797	1.93E-01	103343421	103343544	NC_000002.11
cg24191821	1.02E-12	5.12E-14	8.12	20.9388344	8.63E-02	73008025	73008148	NC_000011.9
cg06614044	1.02E-12	5.14E-14	8.12	20.9364128	1.64E-01	42324631	42324754	NC_000018.9
cg26012482	1.02E-12	5.14E-14	8.12	20.936195	6.89E-02	8423155	8423278	NC_000002.11
cg05538980	1.02E-12	5.14E-14	8.12	20.9348063	7.57E-02	41581845	41581968	NC_000008.10

cg02649597	1.02E-12	5.15E-14	8.12	20.9340946	1.64E-01	45171621	45171744	NC_000002.11
cg13426632	1.02E-12	5.16E-14	8.12	20.9319229	9.00E-02	94858277	94858400	NC_000008.10
cg10945079	1.02E-12	5.16E-14	8.12	20.9312584	1.64E-01	162283856	162283979	NC_000002.11
cg24710309	1.03E-12	5.18E-14	8.12	20.9279403	1.27E-01	235423736	235423859	NC_000001.10
cg14612428	1.03E-12	5.18E-14	8.12	20.9277078	1.71E-01	11751566	11751689	NC_000018.9
cg02700360	1.03E-12	5.19E-14	8.12	20.9265932	1.68E-01	11874932	11875055	NC_000003.11
cg05377041	1.03E-12	5.19E-14	8.12	20.9257741	1.28E-01	88473713	88473836	NC_000016.9
cg04660422	1.03E-12	5.19E-14	8.12	20.9254777	1.43E-01	4860551	4860674	NC_000004.11
cg15235798	1.03E-12	5.22E-14	8.12	20.9202054	1.86E-01	112720244	112720367	NC_000013.10
cg21158087	1.04E-12	5.24E-14	8.12	20.9167863	1.30E-01	58216415	58216538	NC_000017.10
cg20160996	1.04E-12	5.25E-14	8.12	20.9154679	1.39E-01	63990575	63990698	NC_000011.9
cg23097534	1.04E-12	5.25E-14	8.12	20.9154134	2.28E-01	37836748	37836871	NC_000005.9
cg23884241	1.04E-12	5.25E-14	8.12	20.9146092	9.21E-02	27169957	27170080	NC_000007.13
cg21167905	1.04E-12	5.27E-14	8.12	20.9118886	1.18E-01	172505895	172506018	NC_000001.10
cg15282819	1.04E-12	5.27E-14	8.12	20.9112021	2.43E-01	12997592	12997715	NC_000016.9
cg03630821	1.04E-12	5.27E-14	8.12	20.910307	1.34E-01	58861702	58861825	NC_000019.9
cg08063745	1.05E-12	5.30E-14	8.12	20.9048365	4.48E-02	44690652	44690775	NC_000020.10
cg24057218	1.05E-12	5.31E-14	8.12	20.9038899	9.84E-02	68660680	68660803	NC_000014.8
cg19475870	1.05E-12	5.31E-14	8.12	20.9036924	1.64E-01	27038605	27038728	NC_000005.9
cg05186311	1.05E-12	5.33E-14	8.11	20.9007341	1.60E-01	131758454	131758577	NC_000010.10
cg04009342	1.05E-12	5.33E-14	8.11	20.9006731	8.02E-02	25204113	25204236	NC_000012.11
cg24033330	1.05E-12	5.33E-14	8.11	20.8993315	1.98E-01	39317034	39317157	NC_000020.10
cg19264163	1.05E-12	5.34E-14	8.11	20.8987373	8.51E-02	19316990	19317113	NC_000003.11
cg02081733	1.06E-12	5.34E-14	8.11	20.8976821	1.47E-01	68120412	68120535	NC_000015.9
cg23520688	1.06E-12	5.35E-14	8.11	20.895777	2.16E-01	103294740	103294863	NC_000014.8

cg10319942	1.06E-12	5.37E-14	8.11	20.8927848	6.44E-02	127441767	127441890	NC_000003.11
cg12065366	1.06E-12	5.37E-14	8.11	20.8917487	2.30E-01	238618	238741	NC_000003.11
cg18639956	1.07E-12	5.41E-14	8.11	20.8859183	1.03E-01	77927982	77928105	NC_000017.10
cg17822911	1.07E-12	5.41E-14	8.11	20.8858141	9.09E-02	145625497	145625620	NC_000008.10
cg02318567	1.07E-12	5.42E-14	8.11	20.8834692	1.58E-01	31548908	31549031	NC_000016.9
cg26716839	1.07E-12	5.43E-14	8.11	20.8813669	5.94E-02	121146848	121146971	NC_000012.11
cg04156606	1.08E-12	5.46E-14	8.11	20.876174	1.77E-01	134859458	134859581	NC_000010.10
cg23814691	1.08E-12	5.46E-14	8.11	20.8752758	2.01E-02	50649161	50649284	NC_000003.11
cg25922637	1.08E-12	5.47E-14	8.11	20.8750643	1.38E-01	128076308	128076431	NC_000010.10
cg17357285	1.08E-12	5.47E-14	8.11	20.8745792	2.78E-01	28602938	28603061	NC_000006.11
cg06094607	1.08E-12	5.48E-14	8.11	20.8730405	1.32E-01	144561886	144562009	NC_000008.10
cg22348290	1.08E-12	5.48E-14	8.11	20.8727778	1.16E-01	72459499	72459622	NC_000008.10
cg10524033	1.08E-12	5.49E-14	8.11	20.87161	2.21E-01	134070433	134070556	NC_000004.11
cg24913868	1.08E-12	5.49E-14	8.11	20.8708607	1.72E-01	101296069	101296192	NC_000010.10
cg27505273	1.08E-12	5.49E-14	8.11	20.870759	1.78E-01	137483543	137483666	NC_000003.11
cg20925955	1.08E-12	5.49E-14	8.11	20.8705118	5.76E-02	155412182	155412305	NC_000006.11
cg26983177	1.08E-12	5.49E-14	8.11	20.8701102	1.83E-01	35395973	35396096	NC_000019.9
cg04641171	1.08E-12	5.50E-14	8.11	20.8691563	1.78E-01	79381805	79381928	NC_000015.9
cg24499516	1.09E-12	5.53E-14	8.11	20.8635456	7.85E-02	49638562	49638685	NC_000020.10
cg15012214	1.09E-12	5.54E-14	8.11	20.8625004	1.15E-01	56810320	56810443	NC_000003.11
cg00532455	1.09E-12	5.54E-14	8.11	20.8609992	1.11E-01	148617336	148617459	NC_000002.11
cg24978630	1.10E-12	5.57E-14	8.11	20.8568453	2.07E-01	57389676	57389799	NC_000010.10
cg21325485	1.10E-12	5.57E-14	8.11	20.8560507	1.21E-01	158364932	158365055	NC_000007.13
cg09726726	1.10E-12	5.57E-14	8.11	20.8556971	7.61E-02	23850540	23850663	NC_000002.11
cg06703635	1.10E-12	5.60E-14	8.11	20.8507386	7.97E-02	201488410	201488533	NC_000001.10

cg24035245	1.10E-12	5.60E-14	8.11	20.8505705	1.95E-01	119535928	119536051	NC_000001.10
cg13491584	1.11E-12	5.63E-14	8.11	20.8456632	2.43E-01	88752836	88752959	NC_000002.11
cg12694372	1.11E-12	5.64E-14	8.11	20.8445704	1.99E-01	32064582	32064705	NC_000006.11
cg09347306	1.11E-12	5.64E-14	8.11	20.8441379	6.06E-02	79078315	79078438	NC_000017.10
cg13075444	1.11E-12	5.64E-14	8.11	20.8436587	7.22E-02	33680427	33680550	NC_000011.9
cg20464804	1.11E-12	5.65E-14	8.11	20.8420938	2.24E-01	33454491	33454614	NC_000022.10
cg10366797	1.11E-12	5.66E-14	8.1	20.8407192	1.39E-01	54441509	54441632	NC_000012.11
cg15777781	1.11E-12	5.66E-14	8.1	20.8406615	1.75E-01	11047271	11047394	NC_000010.10
cg08317694	1.11E-12	5.66E-14	8.1	20.8403175	1.55E-01	78632144	78632267	NC_000015.9
cg12869688	1.11E-12	5.66E-14	8.1	20.8401871	4.71E-02	90169025	90169148	NC_000015.9
cg16361649	1.12E-12	5.67E-14	8.1	20.83883	7.45E-02	47424543	47424666	NC_000021.8
cg12339328	1.12E-12	5.68E-14	8.1	20.8374892	1.36E-01	18491484	18491607	NC_000020.10
cg26291543	1.12E-12	5.68E-14	8.1	20.8368022	5.92E-02	26895578	26895701	NC_000022.10
cg22024657	1.12E-12	5.68E-14	8.1	20.8367103	8.82E-02	122839955	122840078	NC_000007.13
cg25662463	1.12E-12	5.70E-14	8.1	20.8342668	2.18E-01	118897857	118897980	NC_000010.10
cg19461621	1.12E-12	5.71E-14	8.1	20.8327912	2.61E-01	500979	501102	NC_000018.9
cg05132222	1.12E-12	5.71E-14	8.1	20.8319109	1.44E-01	41304870	41304993	NC_000015.9
cg18181607	1.12E-12	5.72E-14	8.1	20.8310583	1.51E-01	155109364	155109487	NC_000005.9
cg01815529	1.13E-12	5.74E-14	8.1	20.8265094	2.32E-01	17206946	17207069	NC_000020.10
cg25131551	1.13E-12	5.76E-14	8.1	20.823795	7.35E-02	110607342	110607465	NC_000001.10
cg08214192	1.13E-12	5.76E-14	8.1	20.8237321	8.64E-02	189841723	189841846	NC_000003.11
cg21706183	1.14E-12	5.78E-14	8.1	20.8198719	1.40E-01	124785827	124785950	NC_000012.11
cg02256049	1.14E-12	5.79E-14	8.1	20.8179544	2.18E-01	115531001	115531124	NC_000011.9
cg22028785	1.14E-12	5.80E-14	8.1	20.8173001	1.08E-01	45678406	45678529	NC_000021.8
cg08523865	1.14E-12	5.80E-14	8.1	20.8164684	1.65E-01	124782253	124782376	NC_000002.11

cg23523535	1.15E-12	5.86E-14	8.1	20.806908	8.50E-02	161572308	161572431	NC_000002.11
cg04031411	1.15E-12	5.86E-14	8.1	20.806884	5.18E-02	11043956	11044079	NC_000016.9
cg01139861	1.15E-12	5.87E-14	8.1	20.8054076	1.33E-01	50343298	50343421	NC_000007.13
cg01156392	1.16E-12	5.89E-14	8.1	20.8017057	1.36E-01	5850005	5850128	NC_000006.11
cg05924540	1.16E-12	5.89E-14	8.1	20.801124	1.99E-01	140200723	140200846	NC_000004.11
cg23809442	1.16E-12	5.90E-14	8.1	20.799808	2.39E-01	102569876	102569999	NC_000013.10
cg12684209	1.16E-12	5.93E-14	8.1	20.7946752	2.05E-01	393110	393233	NC_000006.11
cg03585912	1.17E-12	5.94E-14	8.1	20.792961	1.94E-01	10385776	10385899	NC_000006.11
cg10520672	1.17E-12	5.95E-14	8.1	20.7908937	1.44E-01	86018680	86018803	NC_000016.9
cg13225427	1.17E-12	5.96E-14	8.1	20.7893714	4.19E-02	77797899	77798022	NC_000014.8
cg24390913	1.17E-12	5.97E-14	8.1	20.7882279	1.76E-01	118899788	118899911	NC_000010.10
cg25334575	1.17E-12	5.97E-14	8.1	20.7880788	1.77E-01	100641744	100641867	NC_000013.10
cg15836635	1.17E-12	5.97E-14	8.1	20.7877063	2.87E-01	57025662	57025785	NC_000008.10
cg08049853	1.17E-12	5.98E-14	8.1	20.7855672	1.85E-01	41875784	41875907	NC_000004.11
cg24150949	1.18E-12	5.99E-14	8.1	20.7841424	4.54E-02	36623419	36623542	NC_000017.10
cg26345916	1.18E-12	6.01E-14	8.1	20.7807218	1.57E-01	193815787	193815910	NC_000003.11
cg19444925	1.18E-12	6.02E-14	8.09	20.7801883	3.38E-02	62507590	62507713	NC_000020.10
cg04723401	1.18E-12	6.03E-14	8.09	20.7788876	1.82E-01	29229249	29229372	NC_000014.8
cg13242070	1.18E-12	6.03E-14	8.09	20.7786976	1.48E-01	228652067	228652190	NC_000001.10
cg21590264	1.18E-12	6.03E-14	8.09	20.7775653	1.57E-01	37834850	37834973	NC_000005.9
cg06943420	1.18E-12	6.04E-14	8.09	20.777216	2.29E-01	1566699	1566822	NC_000001.10
cg16084830	1.19E-12	6.05E-14	8.09	20.7747352	8.43E-02	134734693	134734816	NC_000010.10
cg02036261	1.19E-12	6.07E-14	8.09	20.7723396	1.94E-01	57276057	57276180	NC_000014.8
cg02881570	1.19E-12	6.07E-14	8.09	20.7714079	2.59E-01	77737495	77737618	NC_000014.8
cg26822097	1.19E-12	6.07E-14	8.09	20.7712442	1.65E-01	17082581	17082704	NC_000022.10

cg21915313	1.20E-12	6.10E-14	8.09	20.7667634	1.54E-01	140774426	140774549	NC_000005.9
cg03306615	1.20E-12	6.10E-14	8.09	20.766738	1.05E-01	2292890	2293013	NC_000011.9
cg09827433	1.20E-12	6.10E-14	8.09	20.7661415	1.53E-01	177215619	177215742	NC_000001.10
cg14580287	1.20E-12	6.10E-14	8.09	20.7661026	5.25E-02	25173338	25173461	NC_000010.10
cg09735723	1.20E-12	6.12E-14	8.09	20.7627505	2.27E-01	106402042	106402165	NC_000010.10
cg21537915	1.20E-12	6.14E-14	8.09	20.7598936	1.03E-01	7882943	7883066	NC_000006.11
cg04955877	1.21E-12	6.16E-14	8.09	20.7565977	6.30E-02	123856590	123856713	NC_000008.10
cg17333767	1.21E-12	6.16E-14	8.09	20.7565258	1.46E-01	17680561	17680684	NC_000022.10
cg14974749	1.21E-12	6.18E-14	8.09	20.7539989	1.71E-01	27181480	27181603	NC_000007.13
cg02009585	1.21E-12	6.20E-14	8.09	20.7504375	1.84E-01	179754529	179754652	NC_000003.11
cg00282249	1.21E-12	6.20E-14	8.09	20.7502151	2.62E-01	37005566	37005689	NC_000013.10
cg02071795	1.22E-12	6.22E-14	8.09	20.7479397	5.38E-02	23442517	23442640	NC_000014.8
cg25898550	1.22E-12	6.22E-14	8.09	20.7477544	1.63E-01	110225930	110226053	NC_000010.10
cg17838816	1.22E-12	6.23E-14	8.09	20.746285	8.43E-02	135015675	135015798	NC_000010.10
cg00954875	1.22E-12	6.25E-14	8.09	20.7431194	5.86E-02	245628858	245628981	NC_000001.10
cg00375146	1.22E-12	6.25E-14	8.09	20.7429686	1.01E-01	1221812	1221935	NC_000011.9
cg07782808	1.23E-12	6.26E-14	8.09	20.7406395	6.65E-02	39479702	39479825	NC_000017.10
cg15911806	1.23E-12	6.29E-14	8.09	20.7363098	2.50E-01	97685576	97685699	NC_000014.8
cg01819512	1.23E-12	6.29E-14	8.09	20.7360006	1.48E-01	27207996	27208119	NC_000007.13
cg14240300	1.24E-12	6.32E-14	8.09	20.7310832	1.57E-01	46705018	46705141	NC_000017.10
cg22883919	1.24E-12	6.34E-14	8.09	20.7294448	6.34E-02	48154459	48154582	NC_000012.11
cg19969104	1.24E-12	6.34E-14	8.09	20.7285377	9.70E-02	493746	493869	NC_000005.9
cg03694713	1.24E-12	6.34E-14	8.09	20.7281665	1.96E-01	23564156	23564279	NC_000008.10
cg00935653	1.24E-12	6.35E-14	8.09	20.7273793	1.26E-01	25897725	25897848	NC_000007.13
cg19509303	1.24E-12	6.35E-14	8.09	20.7266852	5.08E-02	101318703	101318826	NC_000014.8

cg09992746	1.25E-12	6.41E-14	8.08	20.7183382	1.48E-01	60285821	60285944	NC_000015.9
cg13899017	1.25E-12	6.42E-14	8.08	20.7166742	2.31E-01	159158444	159158567	NC_000001.10
cg04602007	1.25E-12	6.42E-14	8.08	20.7163583	1.83E-01	87080824	87080947	NC_000016.9
cg07125469	1.26E-12	6.43E-14	8.08	20.7141909	8.88E-02	125623573	125623696	NC_000006.11
cg05800416	1.26E-12	6.45E-14	8.08	20.7120078	1.36E-01	19460097	19460220	NC_000008.10
cg03970899	1.27E-12	6.48E-14	8.08	20.7066338	1.36E-01	19710091	19710214	NC_000022.10
cg19914620	1.27E-12	6.50E-14	8.08	20.7048215	9.15E-02	134811603	134811726	NC_000010.10
cg13655674	1.27E-12	6.50E-14	8.08	20.7039501	2.08E-01	119522386	119522509	NC_000001.10
cg01704198	1.27E-12	6.51E-14	8.08	20.7030448	1.51E-01	33757893	33758016	NC_000003.11
cg19511386	1.27E-12	6.51E-14	8.08	20.7028231	9.24E-02	192535635	192535758	NC_000003.11
cg27576241	1.27E-12	6.51E-14	8.08	20.7026556	1.37E-01	155034570	155034693	NC_000001.10
cg03245632	1.27E-12	6.53E-14	8.08	20.6998527	1.74E-01	12254148	12254271	NC_000018.9
cg00674679	1.27E-12	6.53E-14	8.08	20.699466	2.12E-01	176931680	176931803	NC_000002.11
cg19729338	1.28E-12	6.54E-14	8.08	20.6976502	5.29E-02	54909872	54909995	NC_000014.8
cg05406791	1.28E-12	6.55E-14	8.08	20.6966417	1.14E-01	10875874	10875997	NC_000012.11
cg07954075	1.28E-12	6.55E-14	8.08	20.6964105	7.49E-02	1633258	1633381	NC_000012.11
cg19811761	1.28E-12	6.56E-14	8.08	20.6951015	2.22E-01	57019022	57019145	NC_000019.9
cg03983058	1.29E-12	6.59E-14	8.08	20.6898163	1.39E-01	77369724	77369847	NC_000016.9
cg16334004	1.29E-12	6.60E-14	8.08	20.6893177	1.14E-01	134722168	134722291	NC_000010.10
cg10047595	1.29E-12	6.61E-14	8.08	20.6876679	2.33E-01	15500913	15501036	NC_000005.9
cg07622493	1.29E-12	6.62E-14	8.08	20.6862189	1.96E-01	93114294	93114417	NC_000008.10
cg13518270	1.29E-12	6.64E-14	8.08	20.6834826	1.39E-01	50415588	50415711	NC_000020.10
cg00314966	1.29E-12	6.64E-14	8.08	20.68314	2.41E-01	86544346	86544469	NC_000016.9
cg02785870	1.30E-12	6.68E-14	8.08	20.677731	3.07E-02	24744825	24744948	NC_000016.9
cg03779241	1.30E-12	6.68E-14	8.08	20.6770618	2.73E-01	45405061	45405184	NC_000022.10

cg05588496	1.30E-12	6.68E-14	8.08	20.6766672	1.73E-01	67067721	67067844	NC_000018.9
cg01784128	1.30E-12	6.69E-14	8.08	20.6753876	5.37E-02	66519888	66520011	NC_000016.9
cg13012868	1.30E-12	6.69E-14	8.08	20.675284	7.27E-02	91720578	91720701	NC_000014.8
cg26014391	1.31E-12	6.72E-14	8.08	20.6712043	1.87E-01	60936451	60936574	NC_000010.10
cg11842610	1.31E-12	6.73E-14	8.08	20.6701223	2.28E-01	118033762	118033885	NC_000010.10
cg05128922	1.31E-12	6.75E-14	8.08	20.6666343	1.71E-01	114882879	114883002	NC_000012.11
cg00766964	1.32E-12	6.76E-14	8.08	20.6646862	1.60E-01	24693844	24693967	NC_000013.10
cg20249071	1.32E-12	6.78E-14	8.08	20.6631542	1.49E-01	122684258	122684381	NC_000011.9
cg09568464	1.32E-12	6.78E-14	8.08	20.6627095	2.75E-01	56904901	56905024	NC_000019.9
cg10037494	1.32E-12	6.78E-14	8.08	20.6620423	2.48E-01	2161135	2161258	NC_000011.9
cg21515305	1.32E-12	6.79E-14	8.08	20.6612937	1.17E-01	37769171	37769294	NC_000022.10
cg08497530	1.32E-12	6.80E-14	8.08	20.6602011	1.77E-01	82660434	82660557	NC_000016.9
cg13802013	1.33E-12	6.82E-14	8.07	20.6560767	1.27E-01	37303351	37303474	NC_000020.10
cg12398314	1.33E-12	6.83E-14	8.07	20.654832	7.78E-02	71172133	71172256	NC_000001.10
cg24828322	1.34E-12	6.87E-14	8.07	20.6496397	1.30E-01	96830111	96830234	NC_000010.10
cg21928406	1.34E-12	6.88E-14	8.07	20.6486111	9.65E-02	140802669	140802792	NC_000005.9
cg12539415	1.34E-12	6.88E-14	8.07	20.6484737	1.75E-01	177016167	177016290	NC_000002.11
cg16457786	1.34E-12	6.88E-14	8.07	20.6483053	2.40E-01	904851	904974	NC_000018.9
cg25532099	1.34E-12	6.89E-14	8.07	20.6472753	2.69E-01	127874466	127874589	NC_000005.9
cg26456435	1.34E-12	6.89E-14	8.07	20.6471821	1.19E-01	31053788	31053911	NC_000016.9
cg23086519	1.34E-12	6.89E-14	8.07	20.646799	8.44E-02	75703717	75703840	NC_000014.8
cg09758273	1.34E-12	6.90E-14	8.07	20.6454513	1.49E-01	1237995	1238118	NC_000004.11
cg20801476	1.34E-12	6.91E-14	8.07	20.6441185	1.58E-01	27281465	27281588	NC_000007.13
cg24754689	1.34E-12	6.91E-14	8.07	20.6439382	1.20E-01	77702997	77703120	NC_000017.10
cg09201151	1.35E-12	6.95E-14	8.07	20.6385461	2.70E-01	84116151	84116274	NC_000015.9

cg06800040	1.35E-12	6.95E-14	8.07	20.6384712	7.81E-02	42427647	42427770	NC_000017.10
cg20214477	1.35E-12	6.95E-14	8.07	20.6381009	2.34E-01	166795401	166795524	NC_000004.11
cg19777067	1.35E-12	6.96E-14	8.07	20.6359303	1.72E-01	174440103	174440226	NC_000004.11
cg15092168	1.36E-12	6.98E-14	8.07	20.6338016	2.40E-01	63257873	63257996	NC_000005.9
cg08149747	1.36E-12	6.98E-14	8.07	20.6336739	1.71E-01	33761600	33761723	NC_000006.11
cg13940693	1.36E-12	6.99E-14	8.07	20.6324184	2.43E-01	907605	907728	NC_000018.9
cg25987923	1.36E-12	7.02E-14	8.07	20.6283084	1.61E-01	119302050	119302173	NC_000010.10
cg09283154	1.36E-12	7.03E-14	8.07	20.6269004	1.64E-01	118229641	118229764	NC_000006.11
cg24469980	1.37E-12	7.04E-14	8.07	20.6254648	1.44E-01	221064953	221065076	NC_000001.10
cg11357746	1.37E-12	7.05E-14	8.07	20.6240146	1.94E-01	114837887	114838010	NC_000012.11
cg08390877	1.37E-12	7.06E-14	8.07	20.6223626	1.81E-01	119615998	119616121	NC_000002.11
cg00020052	1.37E-12	7.07E-14	8.07	20.6218279	2.51E-01	68546899	68547022	NC_000002.11
cg17149103	1.38E-12	7.10E-14	8.07	20.6173845	1.27E-01	147789480	147789603	NC_000001.10
cg14270292	1.38E-12	7.10E-14	8.07	20.6163725	2.46E-01	133562494	133562617	NC_000006.11
cg13049432	1.38E-12	7.11E-14	8.07	20.6153782	6.07E-02	40141809	40141932	NC_000021.8
cg02279719	1.38E-12	7.11E-14	8.07	20.6152424	1.92E-01	204474	204597	NC_000016.9
cg00974598	1.38E-12	7.11E-14	8.07	20.6151095	4.99E-02	44223902	44224025	NC_000019.9
cg13961142	1.38E-12	7.12E-14	8.07	20.6143828	7.00E-02	11712098	11712221	NC_000019.9
cg22623133	1.38E-12	7.13E-14	8.07	20.6127913	2.03E-01	27195960	27196083	NC_000007.13
cg00290355	1.39E-12	7.15E-14	8.07	20.609683	1.22E-01	150996599	150996722	NC_000003.11
cg02378847	1.39E-12	7.16E-14	8.07	20.60821	2.07E-01	74422750	74422873	NC_000015.9
cg16294013	1.39E-12	7.17E-14	8.07	20.6076298	1.96E-01	66258022	66258145	NC_000001.10
cg16990168	1.39E-12	7.18E-14	8.07	20.6052672	1.62E-01	119526060	119526183	NC_000001.10
cg01648784	1.39E-12	7.19E-14	8.07	20.6042787	7.70E-02	30385444	30385567	NC_000016.9
cg07117311	1.40E-12	7.20E-14	8.07	20.6032295	1.23E-01	67966314	67966437	NC_000016.9

cg00505045	1.40E-12	7.20E-14	8.07	20.6028127	1.63E-01	147561669	147561792	NC_000004.11
cg12334488	1.40E-12	7.21E-14	8.07	20.6011226	1.08E-01	20871173	20871296	NC_000002.11
cg14725719	1.40E-12	7.24E-14	8.06	20.5975679	8.20E-02	43601916	43602039	NC_000010.10
cg11248896	1.41E-12	7.28E-14	8.06	20.5919915	1.98E-01	177003747	177003870	NC_000002.11
cg20189244	1.41E-12	7.28E-14	8.06	20.5916942	1.62E-01	96648205	96648328	NC_000007.13
cg07068674	1.41E-12	7.29E-14	8.06	20.5913299	2.02E-01	37123778	37123901	NC_000014.8
cg07438617	1.41E-12	7.29E-14	8.06	20.5912055	2.94E-01	46655289	46655412	NC_000017.10
cg26552477	1.41E-12	7.30E-14	8.06	20.5891053	1.65E-01	143029	143152	NC_000007.13
cg00765828	1.41E-12	7.31E-14	8.06	20.5888151	2.58E-01	130389013	130389136	NC_000012.11
cg14573448	1.41E-12	7.31E-14	8.06	20.58844	1.84E-01	27165187	27165310	NC_000007.13
cg12590346	1.42E-12	7.32E-14	8.06	20.587303	1.33E-01	1327962	1328085	NC_000011.9
cg12417685	1.42E-12	7.34E-14	8.06	20.5843963	2.37E-01	68981852	68981975	NC_000003.11
cg17290136	1.42E-12	7.36E-14	8.06	20.5819638	1.22E-01	79823345	79823468	NC_000017.10
cg02763671	1.43E-12	7.37E-14	8.06	20.5802085	1.14E-01	20103541	20103664	NC_000022.10
cg25740602	1.43E-12	7.39E-14	8.06	20.5776138	9.32E-02	60052464	60052587	NC_000018.9
cg12417071	1.43E-12	7.40E-14	8.06	20.5767974	2.50E-01	37005489	37005612	NC_000013.10
cg14307443	1.43E-12	7.42E-14	8.06	20.5740621	1.81E-01	172734507	172734630	NC_000004.11
cg14648534	1.44E-12	7.45E-14	8.06	20.5695422	1.31E-01	132315842	132315965	NC_000009.11
cg01826858	1.45E-12	7.50E-14	8.06	20.5633439	7.30E-02	30958494	30958617	NC_000016.9
cg11215296	1.46E-12	7.53E-14	8.06	20.5585609	4.26E-02	206224554	206224677	NC_000001.10
cg11229513	1.46E-12	7.56E-14	8.06	20.5547053	1.85E-01	70984559	70984682	NC_000008.10
cg10146008	1.46E-12	7.58E-14	8.06	20.5530073	6.39E-02	3042310	3042433	NC_000001.10
cg16491960	1.47E-12	7.61E-14	8.06	20.5481643	1.54E-01	56297490	56297613	NC_000007.13
cg16927416	1.47E-12	7.62E-14	8.06	20.5473052	7.84E-02	142295141	142295264	NC_000008.10
cg02471897	1.48E-12	7.64E-14	8.06	20.5443029	2.55E-01	1281200	1281323	NC_000007.13

cg27593384	1.48E-12	7.65E-14	8.06	20.542987	2.14E-01	25755608	25755731	NC_000018.9
cg26271895	1.48E-12	7.67E-14	8.06	20.5407498	3.43E-02	36629950	36630073	NC_000002.11
cg18096722	1.49E-12	7.71E-14	8.05	20.5357907	2.08E-01	70947218	70947341	NC_000008.10
cg09016610	1.49E-12	7.73E-14	8.05	20.5327682	1.36E-01	246203591	246203714	NC_000001.10
cg06264922	1.49E-12	7.74E-14	8.05	20.5319956	9.51E-02	1319648	1319771	NC_000011.9
cg22872478	1.49E-12	7.74E-14	8.05	20.5314521	1.35E-01	52030685	52030808	NC_000015.9
cg26899113	1.49E-12	7.74E-14	8.05	20.5312446	1.21E-01	22625733	22625856	NC_000010.10
cg01245966	1.50E-12	7.75E-14	8.05	20.5299874	2.10E-01	132053823	132053946	NC_000008.10
cg00715696	1.50E-12	7.76E-14	8.05	20.5296077	1.75E-01	85660534	85660657	NC_000016.9
cg14101302	1.50E-12	7.77E-14	8.05	20.5281828	2.37E-01	72596557	72596680	NC_000006.11
cg24490133	1.50E-12	7.78E-14	8.05	20.5271782	1.69E-01	245607970	245608093	NC_000001.10
cg06122871	1.50E-12	7.78E-14	8.05	20.526443	2.00E-01	85476210	85476333	NC_000006.11
cg26252167	1.50E-12	7.78E-14	8.05	20.5262806	1.60E-01	110300573	110300696	NC_000006.11
cg03369382	1.50E-12	7.79E-14	8.05	20.5250492	1.84E-01	139427635	139427758	NC_000007.13
cg07555125	1.51E-12	7.82E-14	8.05	20.5219857	7.68E-02	140175691	140175814	NC_000005.9
cg21402921	1.51E-12	7.84E-14	8.05	20.5196567	2.58E-01	27112305	27112428	NC_000015.9
cg15093216	1.51E-12	7.84E-14	8.05	20.5196354	6.30E-02	951747	951870	NC_000017.10
cg14830748	1.51E-12	7.85E-14	8.05	20.5184871	9.03E-02	97592199	97592322	NC_000008.10
cg18944010	1.51E-12	7.85E-14	8.05	20.5182119	1.84E-01	119522855	119522978	NC_000001.10
cg25762974	1.51E-12	7.85E-14	8.05	20.5173368	9.44E-02	50293815	50293938	NC_000012.11
cg07906331	1.52E-12	7.87E-14	8.05	20.5152279	1.34E-01	15783158	15783281	NC_000001.10
cg08840441	1.52E-12	7.87E-14	8.05	20.514904	8.69E-02	19748461	19748584	NC_000019.9
cg18936550	1.52E-12	7.88E-14	8.05	20.5145282	7.02E-02	3368447	3368570	NC_000001.10
cg07780095	1.52E-12	7.88E-14	8.05	20.5141255	2.37E-01	85674296	85674419	NC_000012.11
cg12673774	1.52E-12	7.88E-14	8.05	20.5135781	1.16E-01	114960970	114961093	NC_000013.10

cg03200537	1.52E-12	7.89E-14	8.05	20.5128812	1.20E-01	87022121	87022244	NC_000016.9
cg09360770	1.52E-12	7.89E-14	8.05	20.5123774	2.62E-01	86600326	86600449	NC_000016.9
cg07702100	1.52E-12	7.90E-14	8.05	20.5111008	5.82E-02	88178390	88178513	NC_000011.9
cg03394150	1.53E-12	7.92E-14	8.05	20.509298	3.25E-01	28074384	28074507	NC_000016.9
cg00158122	1.53E-12	7.92E-14	8.05	20.5092038	9.53E-02	101290029	101290152	NC_000010.10
cg02671880	1.53E-12	7.93E-14	8.05	20.5080033	2.09E-01	95355117	95355240	NC_000013.10
cg24908814	1.53E-12	7.95E-14	8.05	20.5057503	2.90E-01	132947725	132947848	NC_000005.9
cg02298220	1.53E-12	7.96E-14	8.05	20.5046676	1.07E-01	35914908	35915031	NC_000020.10
cg07444323	1.53E-12	7.97E-14	8.05	20.5033433	1.22E-01	3654504	3654627	NC_000020.10
cg26034516	1.53E-12	7.97E-14	8.05	20.5031964	2.31E-01	76228121	76228244	NC_000017.10
cg03329976	1.53E-12	7.97E-14	8.05	20.5027916	1.80E-01	55370283	55370406	NC_000008.10
cg17832364	1.54E-12	7.97E-14	8.05	20.5026978	7.09E-02	94847294	94847417	NC_000010.10
cg04213384	1.54E-12	7.98E-14	8.05	20.5012814	2.12E-01	98960589	98960712	NC_000013.10
cg11750592	1.54E-12	8.01E-14	8.05	20.4974952	1.67E-01	65289227	65289350	NC_000008.10
cg22459630	1.54E-12	8.02E-14	8.05	20.4962171	1.85E-01	68121028	68121151	NC_000015.9
cg20598039	1.54E-12	8.02E-14	8.05	20.4962155	5.90E-02	61914343	61914466	NC_000011.9
cg14428146	1.54E-12	8.03E-14	8.05	20.4959166	1.95E-01	23563925	23564048	NC_000008.10
cg15014975	1.55E-12	8.05E-14	8.05	20.4935248	1.68E-01	25257547	25257670	NC_000001.10
cg08520148	1.55E-12	8.06E-14	8.05	20.4917819	4.06E-02	42662138	42662261	NC_000022.10
cg11115431	1.55E-12	8.06E-14	8.05	20.4914631	1.03E-01	168661137	168661260	NC_000006.11
cg19470290	1.55E-12	8.07E-14	8.05	20.4911066	7.78E-02	98926570	98926693	NC_000013.10
cg11311843	1.55E-12	8.07E-14	8.05	20.4900647	1.32E-01	47302003	47302126	NC_000017.10
cg01292846	1.55E-12	8.08E-14	8.05	20.4895198	1.29E-01	33672543	33672666	NC_000017.10
cg06657852	1.55E-12	8.08E-14	8.05	20.4894697	4.92E-02	86786582	86786705	NC_000016.9
cg05130129	1.56E-12	8.09E-14	8.05	20.4881241	7.58E-02	185122266	185122389	NC_000004.11

cg19951663	1.56E-12	8.09E-14	8.05	20.4880739	1.84E-01	134871634	134871757	NC_000005.9
cg08404009	1.56E-12	8.09E-14	8.05	20.4875987	1.79E-01	109793152	109793275	NC_000013.10
cg21301240	1.56E-12	8.11E-14	8.05	20.4861932	8.83E-02	117727493	117727616	NC_000008.10
cg15679813	1.56E-12	8.11E-14	8.05	20.4858244	3.03E-01	45405627	45405750	NC_000022.10
cg25241559	1.56E-12	8.13E-14	8.05	20.4835573	1.30E-01	241989379	241989502	NC_000002.11
cg15060599	1.56E-12	8.13E-14	8.05	20.4831923	1.45E-01	13974467	13974590	NC_000003.11
cg00094585	1.56E-12	8.14E-14	8.05	20.4822379	1.11E-01	53631322	53631445	NC_000001.10
cg19026817	1.57E-12	8.15E-14	8.05	20.4804173	1.17E-01	56782259	56782382	NC_000020.10
cg02043159	1.57E-12	8.19E-14	8.04	20.4764	2.04E-01	134070235	134070358	NC_000004.11
cg27494087	1.57E-12	8.19E-14	8.04	20.4762708	2.00E-01	74070431	74070554	NC_000017.10
cg14479198	1.58E-12	8.24E-14	8.04	20.470441	7.38E-02	80255793	80255916	NC_000005.9
cg20636352	1.59E-12	8.26E-14	8.04	20.4682177	1.73E-01	200468832	200468955	NC_000002.11
cg08411443	1.59E-12	8.26E-14	8.04	20.4681381	8.80E-02	43958160	43958283	NC_000011.9
cg11891843	1.59E-12	8.27E-14	8.04	20.4659018	1.17E-01	61403381	61403504	NC_000020.10
cg12743359	1.59E-12	8.28E-14	8.04	20.4655794	8.47E-02	31980043	31980166	NC_000006.11
cg02451691	1.59E-12	8.29E-14	8.04	20.4643951	1.04E-01	105721586	105721709	NC_000006.11
cg08833577	1.60E-12	8.32E-14	8.04	20.4600449	1.80E-01	118899288	118899411	NC_000010.10
cg08876441	1.60E-12	8.33E-14	8.04	20.4591159	2.27E-01	190447421	190447544	NC_000001.10
cg07333191	1.61E-12	8.36E-14	8.04	20.4552729	1.70E-01	13526769	13526892	NC_000004.11
cg08280383	1.61E-12	8.37E-14	8.04	20.4545673	1.77E-01	20085358	20085481	NC_000016.9
cg10903903	1.61E-12	8.40E-14	8.04	20.4506852	2.79E-01	27647843	27647966	NC_000006.11
cg12486498	1.61E-12	8.41E-14	8.04	20.4501214	7.29E-02	230975106	230975229	NC_000001.10
cg23284720	1.62E-12	8.43E-14	8.04	20.4480204	1.49E-01	2862144	2862267	NC_000012.11
cg04936030	1.62E-12	8.43E-14	8.04	20.4476415	4.81E-02	63935046	63935169	NC_000011.9
cg20973720	1.62E-12	8.44E-14	8.04	20.4469095	2.96E-01	79383167	79383290	NC_000015.9

cg14494620	1.62E-12	8.44E-14	8.04	20.4463579	1.68E-01	96904912	96905035	NC_000015.9
cg11445123	1.62E-12	8.45E-14	8.04	20.4450306	2.18E-01	2781478	2781601	NC_000020.10
cg03472349	1.62E-12	8.46E-14	8.04	20.4440192	1.72E-01	31319218	31319341	NC_000015.9
cg07026560	1.62E-12	8.47E-14	8.04	20.4433932	1.38E-01	823839	823962	NC_000011.9
cg02315271	1.63E-12	8.51E-14	8.04	20.4382515	2.08E-01	72469987	72470110	NC_000008.10
cg23318812	1.63E-12	8.53E-14	8.04	20.4355434	2.66E-01	66123127	66123250	NC_000012.11
cg17677402	1.64E-12	8.55E-14	8.04	20.4339113	7.87E-02	61889979	61890102	NC_000011.9
cg13149442	1.64E-12	8.56E-14	8.04	20.4328899	1.26E-01	43303777	43303900	NC_000018.9
cg06306791	1.64E-12	8.56E-14	8.04	20.4322158	1.38E-01	32854970	32855093	NC_000006.11
cg24180975	1.64E-12	8.56E-14	8.04	20.4319145	9.96E-02	53883752	53883875	NC_000006.11
cg08247921	1.65E-12	8.59E-14	8.04	20.4285696	1.29E-01	103683115	103683238	NC_000004.11
cg20633956	1.65E-12	8.59E-14	8.04	20.4285649	3.30E-02	95353748	95353871	NC_000010.10
cg18338046	1.65E-12	8.61E-14	8.04	20.4264214	1.12E-01	133452188	133452311	NC_000005.9
cg25471802	1.65E-12	8.62E-14	8.04	20.4258888	1.07E-01	168193840	168193963	NC_000006.11
cg01386735	1.65E-12	8.63E-14	8.04	20.4240887	1.20E-01	70501081	70501204	NC_000006.11
cg22629762	1.65E-12	8.65E-14	8.04	20.4226087	4.01E-02	1864390	1864513	NC_000008.10
cg13764248	1.66E-12	8.65E-14	8.04	20.4216989	4.00E-02	3234933	3235056	NC_000004.11
cg14375924	1.66E-12	8.67E-14	8.04	20.4200931	9.31E-02	24168200	24168323	NC_000012.11
cg11124364	1.66E-12	8.68E-14	8.04	20.4192263	2.08E-01	19812850	19812973	NC_000007.13
cg18391747	1.67E-12	8.72E-14	8.03	20.4138389	7.05E-02	161375390	161375513	NC_000001.10
cg26425305	1.67E-12	8.73E-14	8.03	20.4133823	1.44E-01	3592526	3592649	NC_000005.9
cg06896987	1.67E-12	8.73E-14	8.03	20.4131498	2.03E-01	177003735	177003858	NC_000002.11
cg25060243	1.67E-12	8.73E-14	8.03	20.4129998	7.89E-02	234626464	234626587	NC_000002.11
cg07714757	1.67E-12	8.74E-14	8.03	20.4113887	1.92E-01	143532138	143532261	NC_000008.10
cg04528240	1.68E-12	8.77E-14	8.03	20.4088698	1.08E-01	56504657	56504780	NC_000004.11

cg09701582	1.68E-12	8.78E-14	8.03	20.4071499	1.29E-01	177017797	177017920	NC_000002.11
cg15864104	1.69E-12	8.82E-14	8.03	20.4028264	1.69E-01	55254508	55254631	NC_000018.9
cg00299105	1.69E-12	8.83E-14	8.03	20.4019684	6.59E-02	113243817	113243940	NC_000011.9
cg12777730	1.70E-12	8.91E-14	8.03	20.3924656	1.52E-01	63657985	63658108	NC_000010.10
cg16348742	1.71E-12	8.94E-14	8.03	20.3898923	6.10E-02	2584446	2584569	NC_000011.9
cg01689438	1.71E-12	8.97E-14	8.03	20.3863787	2.32E-01	147140930	147141053	NC_000003.11
cg00926267	1.71E-12	8.97E-14	8.03	20.3860094	1.21E-01	41030561	41030684	NC_000022.10
cg00690148	1.71E-12	8.98E-14	8.03	20.3857121	2.55E-01	119916017	119916140	NC_000002.11
cg12974545	1.72E-12	9.01E-14	8.03	20.3817609	1.36E-01	11198695	11198818	NC_000003.11
cg23460809	1.72E-12	9.01E-14	8.03	20.3817518	1.07E-01	134472105	134472228	NC_000010.10
cg11315991	1.72E-12	9.02E-14	8.03	20.3810893	1.88E-01	601283	601406	NC_000007.13
cg05234958	1.72E-12	9.03E-14	8.03	20.3791944	1.02E-01	41557710	41557833	NC_000008.10
cg12242474	1.73E-12	9.05E-14	8.03	20.3779226	7.21E-02	1293682	1293805	NC_000020.10
cg01794426	1.73E-12	9.05E-14	8.03	20.3778762	1.93E-01	37132490	37132613	NC_000014.8
cg11500797	1.73E-12	9.06E-14	8.03	20.376977	2.18E-01	96652123	96652246	NC_000007.13
cg23619577	1.73E-12	9.06E-14	8.03	20.3764577	8.11E-02	65297524	65297647	NC_000001.10
cg16703956	1.74E-12	9.09E-14	8.03	20.3728872	2.36E-01	1446217	1446340	NC_000005.9
cg07264726	1.74E-12	9.11E-14	8.03	20.370749	1.22E-01	142666293	142666416	NC_000003.11
cg24080008	1.74E-12	9.13E-14	8.03	20.3691612	1.46E-01	38600199	38600322	NC_000001.10
cg05485968	1.74E-12	9.13E-14	8.03	20.3686329	7.82E-02	79681568	79681691	NC_000017.10
cg14370314	1.75E-12	9.16E-14	8.03	20.3659013	1.82E-01	23834891	23835014	NC_000014.8
cg02631468	1.75E-12	9.16E-14	8.03	20.3652263	2.21E-01	25062777	25062900	NC_000020.10
cg26721877	1.75E-12	9.17E-14	8.03	20.3648811	1.69E-01	172757074	172757197	NC_000005.9
cg06803257	1.75E-12	9.19E-14	8.03	20.3624622	4.79E-02	67999044	67999167	NC_000016.9
cg15355341	1.76E-12	9.22E-14	8.03	20.3595535	8.28E-02	50430136	50430259	NC_000012.11

cg15095581	1.76E-12	9.22E-14	8.03	20.3590346	9.35E-02	46776775	46776898	NC_000003.11
cg04098866	1.76E-12	9.22E-14	8.03	20.3586657	1.78E-01	51190201	51190324	NC_000016.9
cg20399965	1.77E-12	9.27E-14	8.02	20.3542822	1.09E-01	6471712	6471835	NC_000004.11
cg23733386	1.77E-12	9.27E-14	8.02	20.3539926	4.21E-02	169630505	169630628	NC_000001.10
cg24148931	1.77E-12	9.28E-14	8.02	20.3531764	7.82E-02	169290459	169290582	NC_000006.11
cg11246938	1.77E-12	9.28E-14	8.02	20.3523452	1.77E-01	115112433	115112556	NC_000012.11
cg09982510	1.77E-12	9.30E-14	8.02	20.3507457	5.48E-02	233565164	233565287	NC_000002.11
cg13279673	1.77E-12	9.31E-14	8.02	20.3491692	1.95E-01	50674522	50674645	NC_000006.11
cg03345925	1.78E-12	9.34E-14	8.02	20.346028	9.55E-02	144599347	144599470	NC_000008.10
cg12647837	1.78E-12	9.35E-14	8.02	20.345409	6.13E-02	61820842	61820965	NC_000007.13
cg24041541	1.78E-12	9.36E-14	8.02	20.3443205	1.86E-01	77586395	77586518	NC_000008.10
cg10853543	1.78E-12	9.36E-14	8.02	20.3441971	1.47E-01	60763626	60763749	NC_000002.11
cg21639114	1.79E-12	9.39E-14	8.02	20.3413871	1.74E-01	130687884	130688007	NC_000006.11
cg14351526	1.79E-12	9.39E-14	8.02	20.3407362	1.20E-01	72086737	72086860	NC_000006.11
cg26673975	1.79E-12	9.41E-14	8.02	20.3393656	9.58E-02	66420974	66421097	NC_000016.9
cg08969532	1.80E-12	9.43E-14	8.02	20.336524	2.37E-01	99790438	99790561	NC_000010.10
cg16374093	1.80E-12	9.44E-14	8.02	20.3360259	5.64E-02	69931514	69931637	NC_000011.9
cg23130254	1.80E-12	9.46E-14	8.02	20.3334695	1.66E-01	176964588	176964711	NC_000002.11
cg23676682	1.81E-12	9.49E-14	8.02	20.3310615	1.53E-01	105480792	105480915	NC_000011.9
cg06578800	1.81E-12	9.50E-14	8.02	20.3301714	5.62E-02	21368039	21368162	NC_000017.10
cg12065138	1.81E-12	9.50E-14	8.02	20.3296024	1.96E-01	4771201	4771324	NC_000001.10
cg27090062	1.82E-12	9.56E-14	8.02	20.3239606	1.42E-01	92939842	92939965	NC_000005.9
cg26556624	1.82E-12	9.57E-14	8.02	20.322328	1.07E-01	9099862	9099985	NC_000018.9
cg23493016	1.82E-12	9.59E-14	8.02	20.3207789	1.90E-01	106440778	106440901	NC_000010.10
cg00982919	1.83E-12	9.60E-14	8.02	20.3190411	1.91E-01	126775263	126775386	NC_000009.11

cg15183083	1.83E-12	9.61E-14	8.02	20.3178245	2.03E-01	30038310	30038433	NC_000011.9
cg06134471	1.83E-12	9.64E-14	8.02	20.315186	9.49E-02	51560134	51560257	NC_000014.8
cg10134509	1.84E-12	9.67E-14	8.02	20.3122512	5.84E-02	33171319	33171442	NC_000006.11
cg07441518	1.84E-12	9.70E-14	8.02	20.3095701	1.50E-01	31846849	31846972	NC_000011.9
cg04093932	1.85E-12	9.71E-14	8.02	20.3078776	2.04E-01	77179778	77179901	NC_000017.10
cg10778337	1.85E-12	9.74E-14	8.02	20.3048013	7.98E-02	8786784	8786907	NC_000003.11
cg19091245	1.86E-12	9.77E-14	8.02	20.3025056	9.46E-02	133508648	133508771	NC_000010.10
cg11291712	1.86E-12	9.77E-14	8.02	20.302074	8.07E-02	95328682	95328805	NC_000004.11
cg13207326	1.86E-12	9.78E-14	8.02	20.300757	1.76E-01	4860190	4860313	NC_000004.11
cg15245095	1.86E-12	9.78E-14	8.02	20.3005375	2.14E-01	79258438	79258561	NC_000012.11
cg23893898	1.86E-12	9.79E-14	8.02	20.2999761	1.83E-01	6247571	6247694	NC_000004.11
cg21419585	1.86E-12	9.80E-14	8.02	20.2994246	1.57E-01	118166117	118166240	NC_000001.10
cg02894966	1.86E-12	9.80E-14	8.02	20.299283	4.45E-02	192700717	192700840	NC_000002.11
cg02764571	1.86E-12	9.80E-14	8.02	20.2990506	7.75E-02	42894332	42894455	NC_000005.9
cg09626193	1.86E-12	9.80E-14	8.02	20.2985819	1.96E-01	55370192	55370315	NC_000008.10
cg03366858	1.87E-12	9.83E-14	8.02	20.296153	1.02E-01	53742322	53742445	NC_000001.10
cg20416874	1.87E-12	9.85E-14	8.01	20.2943469	1.79E-01	8611966	8612089	NC_000001.10
cg04035209	1.87E-12	9.85E-14	8.01	20.2940575	2.10E-01	134069670	134069793	NC_000004.11
cg13102348	1.87E-12	9.85E-14	8.01	20.2936357	1.65E-01	97629221	97629344	NC_000015.9
cg21185686	1.87E-12	9.87E-14	8.01	20.2924257	1.93E-01	140810433	140810556	NC_000005.9
cg13875506	1.88E-12	9.91E-14	8.01	20.2878046	1.51E-01	16943573	16943696	NC_000011.9
cg19145710	1.89E-12	9.96E-14	8.01	20.283372	7.09E-02	36758130	36758253	NC_000002.11
cg04057238	1.90E-12	1.00E-13	8.01	20.2792361	6.71E-02	71713446	71713569	NC_000005.9
cg08370082	1.90E-12	1.00E-13	8.01	20.2780702	7.96E-02	196616876	196616999	NC_000003.11
cg23424003	1.90E-12	1.00E-13	8.01	20.2776903	1.98E-01	101006035	101006158	NC_000007.13

cg03625109	1.90E-12	1.00E-13	8.01	20.2776632	2.00E-01	122132103	122132226	NC_000009.11
cg00454862	1.90E-12	1.00E-13	8.01	20.2765489	1.45E-01	192983012	192983135	NC_000001.10
cg09341491	1.90E-12	1.00E-13	8.01	20.2760114	1.31E-01	50707795	50707918	NC_000016.9
cg15949044	1.91E-12	1.01E-13	8.01	20.2733618	2.06E-01	140864549	140864672	NC_000005.9
cg26708319	1.91E-12	1.01E-13	8.01	20.2719148	1.98E-01	111552040	111552163	NC_000004.11
cg20130213	1.91E-12	1.01E-13	8.01	20.2708561	2.27E-01	13934169	13934292	NC_000010.10
cg12511214	1.91E-12	1.01E-13	8.01	20.2703585	2.32E-01	40058293	40058416	NC_000022.10
cg09569850	1.91E-12	1.01E-13	8.01	20.2703092	1.70E-01	64955034	64955157	NC_000002.11
cg25391117	1.91E-12	1.01E-13	8.01	20.2693618	1.45E-01	34043284	34043407	NC_000005.9
cg24317827	1.92E-12	1.01E-13	8.01	20.2660617	1.10E-01	45106404	45106527	NC_000001.10
cg26336790	1.93E-12	1.02E-13	8.01	20.2620686	6.79E-02	1264568	1264691	NC_000005.9
cg10043865	1.94E-12	1.02E-13	8.01	20.2568806	3.14E-01	35294481	35294604	NC_000017.10
cg17422774	1.94E-12	1.02E-13	8.01	20.2562519	1.92E-01	124901928	124902051	NC_000010.10
cg13390101	1.94E-12	1.02E-13	8.01	20.2556891	7.23E-02	117557209	117557332	NC_000009.11
cg16042149	1.94E-12	1.02E-13	8.01	20.2554392	2.07E-01	29875947	29876070	NC_000022.10
cg12606911	1.94E-12	1.02E-13	8.01	20.2551701	1.82E-01	87036626	87036749	NC_000002.11
cg07837260	1.94E-12	1.03E-13	8.01	20.2536573	2.22E-01	19788564	19788687	NC_000009.11
cg11436222	1.95E-12	1.03E-13	8.01	20.2507792	9.09E-02	26225520	26225643	NC_000006.11
cg08835113	1.95E-12	1.03E-13	8.01	20.2505401	2.35E-01	96650192	96650315	NC_000007.13
cg08888487	1.95E-12	1.03E-13	8.01	20.2503113	1.14E-01	1124759	1124882	NC_000005.9
cg04005793	1.95E-12	1.03E-13	8.01	20.2487072	6.04E-02	56622893	56623016	NC_000012.11
cg27282264	1.96E-12	1.04E-13	8.01	20.2430637	2.04E-01	7353499	7353622	NC_000016.9
cg23469417	1.97E-12	1.04E-13	8.01	20.241114	1.02E-01	95041542	95041665	NC_000012.11
cg20805407	1.97E-12	1.04E-13	8.01	20.2381933	1.27E-01	156878319	156878442	NC_000001.10
cg09070142	1.97E-12	1.04E-13	8.01	20.2372548	1.49E-01	121532396	121532519	NC_000007.13

cg18541254	1.98E-12	1.05E-13	8	20.2330058	1.42E-01	112709550	112709673	NC_000013.10
cg15285250	1.98E-12	1.05E-13	8	20.2325472	2.25E-01	8482107	8482230	NC_000007.13
cg24876345	1.98E-12	1.05E-13	8	20.2324021	1.09E-01	56798053	56798176	NC_000004.11
cg13128937	1.98E-12	1.05E-13	8	20.2320128	1.76E-01	95357743	95357866	NC_000013.10
cg18023283	1.99E-12	1.05E-13	8	20.2289414	2.62E-01	85306828	85306951	NC_000012.11
cg15045292	1.99E-12	1.05E-13	8	20.2286125	1.27E-01	5403149	5403272	NC_000017.10
cg07213145	2.00E-12	1.06E-13	8	20.2238883	7.47E-02	72893945	72894068	NC_000016.9
cg15299997	2.00E-12	1.06E-13	8	20.2224257	8.58E-02	234620463	234620586	NC_000002.11
cg17453460	2.00E-12	1.06E-13	8	20.2222122	2.10E-01	118369423	118369546	NC_000010.10
cg07428136	2.00E-12	1.06E-13	8	20.221885	1.80E-01	46811492	46811615	NC_000017.10
cg10666926	2.01E-12	1.06E-13	8	20.2186797	6.66E-02	173945243	173945366	NC_000005.9
cg17786697	2.01E-12	1.07E-13	8	20.2169199	1.43E-01	122430509	122430632	NC_000005.9
cg04560225	2.01E-12	1.07E-13	8	20.2154004	1.94E-01	91195336	91195459	NC_000001.10
cg02855576	2.02E-12	1.07E-13	8	20.2143431	1.19E-01	153749964	153750087	NC_000007.13
cg07962620	2.02E-12	1.07E-13	8	20.2138562	8.00E-02	229415135	229415258	NC_000001.10
cg20077169	2.02E-12	1.07E-13	8	20.2130345	2.24E-02	71632188	71632311	NC_000003.11
cg11592503	2.02E-12	1.07E-13	8	20.2124541	2.30E-01	145813417	145813540	NC_000007.13
cg18682731	2.02E-12	1.07E-13	8	20.2114528	7.03E-02	134680867	134680990	NC_000010.10
cg24513224	2.02E-12	1.07E-13	8	20.2104712	1.92E-01	10102439	10102562	NC_000017.10
cg11192191	2.03E-12	1.08E-13	8	20.2066294	6.75E-02	69291998	69292121	NC_000011.9
cg01663016	2.03E-12	1.08E-13	8	20.2065742	2.42E-01	168727752	168727875	NC_000005.9
cg26624914	2.04E-12	1.08E-13	8	20.2018429	1.34E-01	33448382	33448505	NC_000009.11
cg27005794	2.04E-12	1.08E-13	8	20.2009938	2.38E-01	109746251	109746374	NC_000002.11
cg17371081	2.04E-12	1.08E-13	8	20.1996422	2.61E-01	20690957	20691080	NC_000011.9
cg18574396	2.04E-12	1.08E-13	8	20.1993942	6.69E-02	166245546	166245669	NC_000004.11

cg03392571	2.05E-12	1.09E-13	8	20.1956801	1.30E-01	46367939	46368062	NC_000021.8
cg20787173	2.05E-12	1.09E-13	8	20.1942775	1.90E-01	133562475	133562598	NC_000006.11
cg11325970	2.06E-12	1.09E-13	8	20.1913987	1.68E-01	87979642	87979765	NC_000005.9
cg18278744	2.07E-12	1.10E-13	8	20.1848766	3.38E-02	32085092	32085215	NC_000006.11
cg03501539	2.08E-12	1.10E-13	8	20.1833977	2.06E-01	114760598	114760721	NC_000011.9
cg25371634	2.08E-12	1.10E-13	8	20.1829743	2.09E-01	176981422	176981545	NC_000002.11
cg09246749	2.08E-12	1.10E-13	8	20.1822687	4.40E-02	79926242	79926365	NC_000006.11
cg00250743	2.08E-12	1.10E-13	8	20.1816688	7.38E-02	123264130	123264253	NC_000010.10
cg15265085	2.09E-12	1.11E-13	8	20.1768693	1.83E-01	32064588	32064711	NC_000006.11
cg26339943	2.09E-12	1.11E-13	7.99	20.1748401	1.34E-01	145571234	145571357	NC_000004.11
cg17926940	2.10E-12	1.11E-13	7.99	20.1733602	2.24E-01	97685060	97685183	NC_000014.8
cg08083009	2.10E-12	1.11E-13	7.99	20.1732486	4.87E-02	123826474	123826597	NC_000012.11
cg09548051	2.10E-12	1.12E-13	7.99	20.1716527	2.24E-01	1286324	1286447	NC_000007.13
cg08506931	2.11E-12	1.12E-13	7.99	20.1674838	1.58E-01	223168861	223168984	NC_000002.11
cg17974166	2.11E-12	1.12E-13	7.99	20.1661375	1.68E-01	23280069	23280192	NC_000001.10
cg09187936	2.11E-12	1.12E-13	7.99	20.1646664	7.43E-02	122268950	122269073	NC_000012.11
cg02173970	2.12E-12	1.12E-13	7.99	20.163963	1.38E-01	134723505	134723628	NC_000010.10
cg13208438	2.12E-12	1.13E-13	7.99	20.1604486	1.70E-01	78296041	78296164	NC_000015.9
cg24411961	2.13E-12	1.13E-13	7.99	20.1575326	1.59E-01	34642396	34642519	NC_000001.10
cg24804195	2.13E-12	1.13E-13	7.99	20.1559902	2.16E-01	87968844	87968967	NC_000005.9
cg17669334	2.13E-12	1.13E-13	7.99	20.1555101	8.96E-02	22474634	22474757	NC_000007.13
cg03789420	2.14E-12	1.14E-13	7.99	20.1534481	2.25E-01	159141717	159141840	NC_000001.10
cg21844856	2.14E-12	1.14E-13	7.99	20.153228	1.72E-01	75896645	75896768	NC_000008.10
cg12531838	2.14E-12	1.14E-13	7.99	20.1526533	1.35E-01	531914	532037	NC_000010.10
cg12541478	2.14E-12	1.14E-13	7.99	20.1526068	1.59E-01	52444020	52444143	NC_000012.11

cg16391955	2.14E-12	1.14E-13	7.99	20.1508161	1.69E-01	50881185	50881308	NC_000001.10
cg04980590	2.14E-12	1.14E-13	7.99	20.1492659	1.79E-01	3590820	3590943	NC_000005.9
cg22175873	2.14E-12	1.14E-13	7.99	20.1489564	1.72E-01	140801482	140801605	NC_000005.9
cg19716643	2.15E-12	1.14E-13	7.99	20.1465952	1.90E-01	52735026	52735149	NC_000014.8
cg02157467	2.15E-12	1.14E-13	7.99	20.1459002	1.87E-01	36004888	36005011	NC_000014.8
cg09229061	2.15E-12	1.15E-13	7.99	20.1452405	9.89E-02	29789103	29789226	NC_000016.9
cg08550785	2.15E-12	1.15E-13	7.99	20.1445189	2.55E-01	104513909	104514032	NC_000008.10
cg19463199	2.16E-12	1.15E-13	7.99	20.1437507	1.24E-01	197401858	197401981	NC_000003.11
cg15706424	2.16E-12	1.15E-13	7.99	20.1428488	1.31E-01	469781	469904	NC_000002.11
cg03399271	2.16E-12	1.15E-13	7.99	20.141764	2.24E-01	56228385	56228508	NC_000016.9
cg00983904	2.16E-12	1.15E-13	7.99	20.1416077	2.07E-01	6665288	6665411	NC_000012.11
cg26731327	2.16E-12	1.15E-13	7.99	20.1407739	2.25E-01	143591819	143591942	NC_000008.10
cg07014523	2.16E-12	1.15E-13	7.99	20.1401854	2.09E-01	50721313	50721436	NC_000020.10
cg11314569	2.17E-12	1.15E-13	7.99	20.1384233	1.47E-01	33419158	33419281	NC_000017.10
cg01385679	2.17E-12	1.15E-13	7.99	20.1377066	1.32E-01	45033155	45033278	NC_000020.10
cg17078253	2.17E-12	1.16E-13	7.99	20.1349295	1.95E-01	176944273	176944396	NC_000002.11
cg03797660	2.18E-12	1.16E-13	7.99	20.1336087	8.24E-02	89035228	89035351	NC_000016.9
cg04268624	2.18E-12	1.16E-13	7.99	20.1313032	1.87E-01	43037285	43037408	NC_000005.9
cg21988465	2.18E-12	1.16E-13	7.99	20.1303208	1.09E-01	57429277	57429400	NC_000020.10
cg01119875	2.18E-12	1.16E-13	7.99	20.1297191	1.54E-01	159737324	159737447	NC_000005.9
cg10604993	2.18E-12	1.16E-13	7.99	20.1294679	4.69E-02	33255569	33255692	NC_000006.11
cg07965326	2.18E-12	1.16E-13	7.99	20.1291781	1.02E-01	157522398	157522521	NC_000006.11
cg10804656	2.18E-12	1.16E-13	7.99	20.1291643	1.25E-01	22623460	22623583	NC_000010.10
cg13667638	2.19E-12	1.16E-13	7.99	20.128613	1.80E-01	5831147	5831270	NC_000002.11
cg19792599	2.19E-12	1.17E-13	7.99	20.1264864	1.85E-01	125774406	125774529	NC_000011.9

cg20669572	2.19E-12	1.17E-13	7.99	20.1255828	1.08E-01	140773483	140773606	NC_000009.11
cg06594404	2.19E-12	1.17E-13	7.99	20.1246301	1.89E-01	380396	380519	NC_000004.11
cg04012266	2.20E-12	1.17E-13	7.99	20.1218571	2.35E-01	20522384	20522507	NC_000012.11
cg08758887	2.20E-12	1.17E-13	7.99	20.1209952	1.31E-01	154373479	154373602	NC_000004.11
cg13707729	2.21E-12	1.18E-13	7.99	20.1183301	7.95E-02	123326490	123326613	NC_000010.10
cg24161311	2.21E-12	1.18E-13	7.99	20.116073	4.68E-02	69407863	69407986	NC_000011.9
cg13706544	2.21E-12	1.18E-13	7.99	20.1157381	1.71E-01	103969268	103969391	NC_000007.13
cg04673590	2.21E-12	1.18E-13	7.98	20.1150502	2.50E-01	27136191	27136314	NC_000007.13
cg09120987	2.21E-12	1.18E-13	7.98	20.1148074	1.32E-01	33813708	33813831	NC_000010.10
cg09447924	2.22E-12	1.18E-13	7.98	20.1137508	6.82E-02	48697823	48697946	NC_000003.11
cg25825488	2.22E-12	1.18E-13	7.98	20.1132581	2.28E-01	15761876	15761999	NC_000010.10
cg06159603	2.22E-12	1.18E-13	7.98	20.1119385	2.41E-02	122526836	122526959	NC_000007.13
cg13400168	2.22E-12	1.19E-13	7.98	20.1114995	1.92E-01	54481620	54481743	NC_000019.9
cg18035255	2.22E-12	1.19E-13	7.98	20.1106605	1.61E-01	76933375	76933498	NC_000005.9
cg14917589	2.23E-12	1.19E-13	7.98	20.105976	6.42E-02	31692760	31692883	NC_000006.11
cg19029181	2.24E-12	1.19E-13	7.98	20.1044136	1.65E-01	147130536	147130659	NC_000003.11
cg01412518	2.24E-12	1.19E-13	7.98	20.1042539	8.43E-02	3456118	3456241	NC_000001.10
cg10451262	2.24E-12	1.20E-13	7.98	20.1026509	1.47E-01	124284176	124284299	NC_000008.10
cg04744409	2.24E-12	1.20E-13	7.98	20.1021307	1.58E-01	105388191	105388314	NC_000006.11
cg10052561	2.25E-12	1.20E-13	7.98	20.0991953	1.87E-01	40137185	40137308	NC_000001.10
cg26973414	2.25E-12	1.20E-13	7.98	20.0984423	1.38E-01	169290629	169290752	NC_000006.11
cg12847240	2.26E-12	1.21E-13	7.98	20.0933224	1.20E-01	10382088	10382211	NC_000006.11
cg00803827	2.27E-12	1.21E-13	7.98	20.0887995	1.76E-01	43393846	43393969	NC_000010.10
cg06528559	2.28E-12	1.22E-13	7.98	20.0851302	1.76E-01	63275509	63275632	NC_000002.11
cg06428196	2.29E-12	1.22E-13	7.98	20.0805591	1.43E-01	19338174	19338297	NC_000002.11

cg07931391	2.29E-12	1.22E-13	7.98	20.0803495	2.50E-01	100608263	100608386	NC_000013.10
cg17710536	2.29E-12	1.22E-13	7.98	20.0800587	5.57E-02	43918101	43918224	NC_000007.13
cg21656751	2.29E-12	1.22E-13	7.98	20.0797392	1.75E-01	43153201	43153324	NC_000007.13
cg23557906	2.30E-12	1.23E-13	7.98	20.0737187	1.15E-01	467112	467235	NC_000002.11
cg23248357	2.31E-12	1.23E-13	7.98	20.0729525	1.75E-01	2755376	2755499	NC_000005.9
cg04306992	2.31E-12	1.23E-13	7.98	20.0723181	1.35E-01	4712355	4712478	NC_000001.10
cg04206351	2.31E-12	1.23E-13	7.98	20.0711085	1.47E-01	49582375	49582498	NC_000011.9
cg18673954	2.31E-12	1.23E-13	7.98	20.0710461	2.39E-01	105036611	105036734	NC_000010.10
cg01939274	2.32E-12	1.24E-13	7.98	20.0670167	2.81E-01	127874478	127874601	NC_000005.9
cg10877086	2.32E-12	1.24E-13	7.98	20.0655811	1.54E-01	114881844	114881967	NC_000012.11
cg07379550	2.32E-12	1.24E-13	7.98	20.0651039	1.86E-01	227204743	227204866	NC_000001.10
cg00511674	2.32E-12	1.24E-13	7.98	20.0650476	1.48E-01	78080068	78080191	NC_000016.9
cg08806153	2.33E-12	1.25E-13	7.98	20.0605852	1.79E-01	176971961	176972084	NC_000002.11
cg11245719	2.34E-12	1.25E-13	7.98	20.0578892	5.43E-02	8194731	8194854	NC_000017.10
cg18656708	2.34E-12	1.25E-13	7.98	20.0566637	1.11E-01	153859973	153860096	NC_000005.9
cg03583979	2.34E-12	1.25E-13	7.98	20.0557567	1.53E-01	3032345	3032468	NC_000001.10
cg11161150	2.34E-12	1.25E-13	7.98	20.0552524	1.08E-01	98119892	98120015	NC_000005.9
cg10096177	2.35E-12	1.26E-13	7.98	20.054922	2.75E-01	103889516	103889639	NC_000012.11
cg02085786	2.35E-12	1.26E-13	7.97	20.0532213	1.11E-01	16797770	16797893	NC_000007.13
cg02980079	2.35E-12	1.26E-13	7.97	20.0508024	2.51E-01	50323244	50323367	NC_000010.10
cg24617504	2.36E-12	1.26E-13	7.97	20.0495507	1.24E-01	155164762	155164885	NC_000007.13
cg09221951	2.36E-12	1.26E-13	7.97	20.0482548	1.07E-01	224543865	224543988	NC_000001.10
cg22894301	2.36E-12	1.26E-13	7.97	20.0473482	1.60E-01	147077665	147077788	NC_000003.11
cg21200539	2.36E-12	1.27E-13	7.97	20.0468434	2.47E-01	147562073	147562196	NC_000004.11
cg07814886	2.37E-12	1.27E-13	7.97	20.0447581	9.94E-02	36086032	36086155	NC_000007.13

cg12614105	2.37E-12	1.27E-13	7.97	20.0431605	2.02E-01	24324435	24324558	NC_000007.13
cg00590139	2.38E-12	1.27E-13	7.97	20.0411938	2.50E-01	7354254	7354377	NC_000016.9
cg11751707	2.38E-12	1.27E-13	7.97	20.0410219	1.84E-01	38302587	38302710	NC_000002.11
cg16580499	2.38E-12	1.27E-13	7.97	20.0408876	1.79E-01	76739559	76739682	NC_000018.9
cg19118812	2.38E-12	1.27E-13	7.97	20.0405686	2.68E-01	37488438	37488561	NC_000007.13
cg00532157	2.38E-12	1.27E-13	7.97	20.0400083	2.31E-01	178771329	178771452	NC_000005.9
cg24696183	2.38E-12	1.28E-13	7.97	20.0393106	1.28E-01	2891360	2891483	NC_000011.9
cg07092985	2.38E-12	1.28E-13	7.97	20.0378804	1.01E-01	55533887	55534010	NC_000008.10
cg03983336	2.38E-12	1.28E-13	7.97	20.0375917	1.39E-01	108602542	108602665	NC_000002.11
cg01686617	2.38E-12	1.28E-13	7.97	20.0374953	3.42E-02	4127256	4127379	NC_000020.10
cg05257275	2.39E-12	1.28E-13	7.97	20.0363885	1.39E-01	65547049	65547172	NC_000011.9
cg18143296	2.39E-12	1.28E-13	7.97	20.0361471	1.71E-01	157812763	157812886	NC_000003.11
cg11644479	2.39E-12	1.28E-13	7.97	20.0356952	1.07E-01	2292914	2293037	NC_000011.9
cg14644846	2.39E-12	1.28E-13	7.97	20.0354444	1.55E-01	128073866	128073989	NC_000003.11
cg11385338	2.39E-12	1.28E-13	7.97	20.0348674	1.54E-01	63250817	63250940	NC_000007.13
cg18146152	2.39E-12	1.28E-13	7.97	20.0336131	1.00E-01	1400772	1400895	NC_000016.9
cg19846622	2.39E-12	1.28E-13	7.97	20.0332978	1.25E-01	4859960	4860083	NC_000004.11
cg03355524	2.39E-12	1.28E-13	7.97	20.0331068	1.90E-01	54578898	54579021	NC_000020.10
cg12079322	2.40E-12	1.29E-13	7.97	20.0303976	2.74E-01	154728468	154728591	NC_000002.11
cg22336004	2.40E-12	1.29E-13	7.97	20.0288803	2.11E-01	66258035	66258158	NC_000001.10
cg07787450	2.40E-12	1.29E-13	7.97	20.0286933	1.20E-01	61405614	61405737	NC_000020.10
cg12948543	2.40E-12	1.29E-13	7.97	20.0285141	7.38E-02	105851564	105851687	NC_000014.8
cg11947423	2.41E-12	1.29E-13	7.97	20.0248615	8.12E-02	42329660	42329783	NC_000008.10
cg06010588	2.42E-12	1.30E-13	7.97	20.0233562	2.18E-01	35092876	35092999	NC_000008.10
cg16172031	2.42E-12	1.30E-13	7.97	20.021035	7.79E-02	39718940	39719063	NC_000022.10

cg27645345	2.43E-12	1.30E-13	7.97	20.0176451	1.02E-01	31829840	31829963	NC_000006.11
cg09221269	2.43E-12	1.30E-13	7.97	20.017571	1.88E-02	36402132	36402255	NC_000021.8
cg05379302	2.43E-12	1.30E-13	7.97	20.0173373	1.95E-01	11540200	11540323	NC_000001.10
cg20800117	2.43E-12	1.30E-13	7.97	20.0168146	9.68E-02	7308366	7308489	NC_000001.10
cg24882324	2.43E-12	1.31E-13	7.97	20.0164338	1.77E-01	32064508	32064631	NC_000006.11
cg01634458	2.43E-12	1.31E-13	7.97	20.0150003	2.33E-01	51168553	51168676	NC_000016.9
cg10656664	2.43E-12	1.31E-13	7.97	20.014781	6.09E-02	853447	853570	NC_000005.9
cg21547690	2.44E-12	1.31E-13	7.97	20.0130409	1.86E-01	42077327	42077450	NC_000014.8
cg18323912	2.45E-12	1.31E-13	7.97	20.0102169	2.65E-01	42994709	42994832	NC_000005.9
cg09636024	2.45E-12	1.31E-13	7.97	20.0096915	5.95E-02	1067228	1067351	NC_000017.10
cg26476173	2.45E-12	1.32E-13	7.97	20.0074283	1.25E-01	48334459	48334582	NC_000016.9
cg02505681	2.45E-12	1.32E-13	7.97	20.0071537	9.91E-02	39615285	39615408	NC_000019.9
cg16318253	2.46E-12	1.32E-13	7.97	20.0054112	1.45E-01	75377474	75377597	NC_000018.9
cg05771459	2.46E-12	1.32E-13	7.97	20.0045986	1.22E-01	144994318	144994441	NC_000001.10
cg20552236	2.46E-12	1.32E-13	7.97	20.0038954	1.23E-01	75090958	75091081	NC_000015.9
cg00494735	2.49E-12	1.34E-13	7.96	19.9920408	6.70E-02	2293593	2293716	NC_000011.9
cg08860622	2.49E-12	1.34E-13	7.96	19.991229	9.43E-02	43141679	43141802	NC_000017.10
cg06641366	2.49E-12	1.34E-13	7.96	19.9911633	2.12E-01	90097604	90097727	NC_000001.10
cg12498181	2.50E-12	1.34E-13	7.96	19.9890753	9.04E-02	113436309	113436432	NC_000013.10
cg13631916	2.50E-12	1.35E-13	7.96	19.9860338	2.54E-01	117086931	117087054	NC_000006.11
cg04839897	2.51E-12	1.35E-13	7.96	19.9844152	9.56E-02	28589957	28590080	NC_000001.10
cg05791173	2.51E-12	1.35E-13	7.96	19.9834434	1.13E-01	130508478	130508601	NC_000010.10
cg15845792	2.51E-12	1.35E-13	7.96	19.9824934	2.15E-01	28175446	28175569	NC_000006.11
cg06330593	2.51E-12	1.35E-13	7.96	19.9821058	1.09E-01	39537455	39537578	NC_000017.10
cg07037852	2.52E-12	1.35E-13	7.96	19.9794247	1.62E-01	41109565	41109688	NC_000013.10

cg22637867	2.52E-12	1.36E-13	7.96	19.9791699	1.71E-01	12624466	12624589	NC_000019.9
cg12219082	2.52E-12	1.36E-13	7.96	19.9789693	2.13E-01	68981854	68981977	NC_000003.11
cg01064265	2.52E-12	1.36E-13	7.96	19.9779891	2.09E-01	55363058	55363181	NC_000016.9
cg00615473	2.52E-12	1.36E-13	7.96	19.9778384	2.66E-01	32930423	32930546	NC_000021.8
cg22500428	2.52E-12	1.36E-13	7.96	19.9774773	2.92E-01	4381882	4382005	NC_000012.11
cg25942940	2.52E-12	1.36E-13	7.96	19.9763141	6.43E-02	8270645	8270768	NC_000001.10
cg21222350	2.53E-12	1.36E-13	7.96	19.9742516	1.36E-01	127602615	127602738	NC_000009.11
cg23332586	2.54E-12	1.37E-13	7.96	19.970841	1.21E-01	49042468	49042591	NC_000022.10
cg06040811	2.54E-12	1.37E-13	7.96	19.9702206	1.63E-01	88018605	88018728	NC_000005.9
cg21678461	2.54E-12	1.37E-13	7.96	19.9687798	2.02E-01	20180700	20180823	NC_000011.9
cg27539806	2.55E-12	1.37E-13	7.96	19.9666012	3.66E-02	88829700	88829823	NC_000016.9
cg18501647	2.55E-12	1.37E-13	7.96	19.9654002	9.82E-02	32117379	32117502	NC_000006.11
cg14602530	2.55E-12	1.38E-13	7.96	19.9645146	1.93E-01	4859772	4859895	NC_000004.11
cg19048251	2.55E-12	1.38E-13	7.96	19.9643551	1.78E-01	119311636	119311759	NC_000010.10
cg02624701	2.56E-12	1.38E-13	7.96	19.9625351	1.47E-01	49937176	49937299	NC_000019.9
cg03726556	2.56E-12	1.38E-13	7.96	19.9615034	1.57E-01	119607379	119607502	NC_000002.11
cg02022733	2.56E-12	1.38E-13	7.96	19.9614038	1.83E-01	140864578	140864701	NC_000005.9
cg09011256	2.56E-12	1.38E-13	7.96	19.9605991	7.68E-02	37074631	37074754	NC_000020.10
cg06475633	2.56E-12	1.38E-13	7.96	19.9599585	2.00E-01	3805065	3805188	NC_000017.10
cg16606320	2.56E-12	1.38E-13	7.96	19.9599573	1.97E-01	56685972	56686095	NC_000004.11
cg09695430	2.56E-12	1.38E-13	7.96	19.9596651	1.58E-01	32447944	32448067	NC_000011.9
cg05347158	2.57E-12	1.38E-13	7.96	19.9591039	5.29E-02	32148832	32148955	NC_000001.10
cg12133554	2.57E-12	1.39E-13	7.96	19.9559895	1.18E-01	299309	299432	NC_000004.11
cg18369516	2.59E-12	1.39E-13	7.96	19.9511868	1.76E-01	62406721	62406844	NC_000020.10
cg02969141	2.59E-12	1.39E-13	7.96	19.9509478	2.16E-01	15727598	15727721	NC_000007.13

cg09840386	2.60E-12	1.40E-13	7.96	19.9461207	1.50E-01	79058751	79058874	NC_000017.10
cg04606861	2.61E-12	1.41E-13	7.96	19.9419559	1.49E-01	132905735	132905858	NC_000012.11
cg09010107	2.61E-12	1.41E-13	7.96	19.9416132	1.90E-01	170638807	170638930	NC_000001.10
cg13641591	2.61E-12	1.41E-13	7.96	19.9411179	1.46E-01	75528534	75528657	NC_000011.9
cg06821075	2.61E-12	1.41E-13	7.96	19.9404684	7.81E-02	99933209	99933332	NC_000010.10
cg16718710	2.61E-12	1.41E-13	7.96	19.9400627	8.76E-02	2249145	2249268	NC_000004.11
cg12253830	2.62E-12	1.42E-13	7.96	19.9359546	2.05E-01	22487668	22487791	NC_000012.11
cg16705627	2.62E-12	1.42E-13	7.96	19.9356747	1.60E-01	112721950	112722073	NC_000013.10
cg22002216	2.62E-12	1.42E-13	7.96	19.9353969	6.66E-02	48502923	48503046	NC_000017.10
cg12368612	2.63E-12	1.42E-13	7.96	19.9348356	1.71E-01	115135999	115136122	NC_000012.11
cg15143788	2.63E-12	1.42E-13	7.95	19.9335672	1.53E-01	92909434	92909557	NC_000005.9
cg18609078	2.63E-12	1.42E-13	7.95	19.9333912	1.67E-01	13538807	13538930	NC_000004.11
cg24154271	2.63E-12	1.42E-13	7.95	19.9326675	1.17E-01	140957775	140957898	NC_000006.11
cg03302801	2.63E-12	1.42E-13	7.95	19.932559	8.15E-02	241567835	241567958	NC_000002.11
cg18007463	2.63E-12	1.42E-13	7.95	19.9322411	2.14E-01	166795913	166796036	NC_000004.11
cg11725101	2.63E-12	1.42E-13	7.95	19.9319122	8.46E-02	148739457	148739580	NC_000001.10
cg00602326	2.64E-12	1.42E-13	7.95	19.9308852	8.46E-02	31427700	31427823	NC_000005.9
cg09887220	2.64E-12	1.42E-13	7.95	19.9305195	1.47E-01	95356202	95356325	NC_000013.10
cg15602633	2.64E-12	1.43E-13	7.95	19.9277242	6.03E-02	134698661	134698784	NC_000010.10
cg15576918	2.64E-12	1.43E-13	7.95	19.9274499	1.22E-01	45834369	45834492	NC_000003.11
cg16894439	2.64E-12	1.43E-13	7.95	19.927351	7.90E-02	228634806	228634929	NC_000001.10
cg14623754	2.65E-12	1.43E-13	7.95	19.9266457	1.06E-01	117228122	117228245	NC_000009.11
cg25645268	2.65E-12	1.43E-13	7.95	19.9236901	2.18E-01	154710598	154710721	NC_000004.11
cg04658615	2.66E-12	1.44E-13	7.95	19.9224961	8.56E-02	53181709	53181832	NC_000006.11
cg20882455	2.66E-12	1.44E-13	7.95	19.9222868	8.76E-02	107777859	107777982	NC_000006.11

cg02719956	2.67E-12	1.44E-13	7.95	19.9172512	5.33E-02	306324	306447	NC_000011.9
cg21147648	2.67E-12	1.45E-13	7.95	19.9157836	1.27E-01	68118964	68119087	NC_000015.9
cg02357541	2.67E-12	1.45E-13	7.95	19.9151802	1.61E-01	127769619	127769742	NC_000003.11
cg09698471	2.68E-12	1.45E-13	7.95	19.914032	1.43E-01	63257847	63257970	NC_000005.9
cg09077319	2.68E-12	1.45E-13	7.95	19.9137361	1.04E-01	69285899	69286022	NC_000011.9
cg14213517	2.68E-12	1.45E-13	7.95	19.9134906	7.49E-02	102972205	102972328	NC_000002.11
cg16656232	2.71E-12	1.46E-13	7.95	19.9026408	6.36E-02	167543888	167544011	NC_000005.9
cg05346963	2.71E-12	1.46E-13	7.95	19.9024325	8.82E-02	77892772	77892895	NC_000004.11
cg26885268	2.71E-12	1.47E-13	7.95	19.9004809	1.58E-01	149389941	149390064	NC_000007.13
cg26383057	2.72E-12	1.47E-13	7.95	19.899612	6.57E-02	168591386	168591509	NC_000006.11
cg18550381	2.72E-12	1.47E-13	7.95	19.8977344	1.55E-01	97844391	97844514	NC_000007.13
cg21587238	2.72E-12	1.47E-13	7.95	19.8976328	2.14E-01	42953590	42953713	NC_000005.9
cg09656363	2.72E-12	1.47E-13	7.95	19.8972847	1.21E-01	53775035	53775158	NC_000013.10
cg26019584	2.73E-12	1.48E-13	7.95	19.8940378	1.18E-01	1302689	1302812	NC_000019.9
cg21591742	2.73E-12	1.48E-13	7.95	19.8935539	2.28E-01	176980998	176981121	NC_000002.11
cg21815337	2.74E-12	1.48E-13	7.95	19.8913817	1.21E-01	4348344	4348467	NC_000003.11
cg24724664	2.74E-12	1.48E-13	7.95	19.8911437	1.46E-01	172945383	172945506	NC_000002.11
cg14208839	2.74E-12	1.48E-13	7.95	19.8907694	2.60E-02	128080980	128081103	NC_000003.11
cg18361098	2.76E-12	1.49E-13	7.95	19.8836822	2.03E-01	37136391	37136514	NC_000014.8
cg15965134	2.76E-12	1.49E-13	7.95	19.8831984	1.53E-01	137480671	137480794	NC_000003.11
cg22850587	2.76E-12	1.49E-13	7.95	19.8831267	1.74E-01	4867638	4867761	NC_000004.11
cg05290780	2.77E-12	1.50E-13	7.95	19.8788108	1.56E-01	39609377	39609500	NC_000013.10
cg15760257	2.78E-12	1.51E-13	7.95	19.8753594	2.86E-01	26699169	26699292	NC_000017.10
cg25585712	2.79E-12	1.51E-13	7.94	19.8709141	1.77E-01	41869213	41869336	NC_000004.11
cg14419187	2.79E-12	1.51E-13	7.94	19.8699264	2.42E-01	210637094	210637217	NC_000002.11

cg04508575	2.80E-12	1.52E-13	7.94	19.8670357	1.27E-01	31323107	31323230	NC_000005.9
cg26140120	2.80E-12	1.52E-13	7.94	19.8662143	1.25E-01	124219575	124219698	NC_000008.10
cg17391620	2.81E-12	1.52E-13	7.94	19.8653682	1.16E-01	31734471	31734594	NC_000006.11
cg06567373	2.81E-12	1.52E-13	7.94	19.86439	1.45E-01	102483843	102483966	NC_000010.10
cg13921352	2.81E-12	1.52E-13	7.94	19.8633993	2.45E-01	68981890	68982013	NC_000003.11
cg05349062	2.81E-12	1.53E-13	7.94	19.8624231	2.25E-01	74741853	74741976	NC_000002.11
cg16665335	2.81E-12	1.53E-13	7.94	19.8618894	2.82E-02	205775	205898	NC_000005.9
cg03108697	2.82E-12	1.53E-13	7.94	19.8605502	5.46E-02	9732066	9732189	NC_000011.9
cg23504022	2.82E-12	1.53E-13	7.94	19.8595111	2.96E-02	35227391	35227514	NC_000006.11
cg05458079	2.83E-12	1.53E-13	7.94	19.8568113	2.62E-01	68119521	68119644	NC_000015.9
cg27323784	2.83E-12	1.54E-13	7.94	19.8551792	2.36E-01	116451730	116451853	NC_000011.9
cg15235999	2.83E-12	1.54E-13	7.94	19.8546037	1.92E-01	52996352	52996475	NC_000019.9
cg27443071	2.84E-12	1.54E-13	7.94	19.853587	1.92E-01	190939090	190939213	NC_000004.11
cg25092838	2.84E-12	1.54E-13	7.94	19.851486	1.99E-01	57279115	57279238	NC_000014.8
cg02151120	2.84E-12	1.54E-13	7.94	19.8504303	1.08E-01	71724287	71724410	NC_000011.9
cg15412759	2.85E-12	1.55E-13	7.94	19.8490221	2.27E-01	50683213	50683336	NC_000006.11
cg02165355	2.85E-12	1.55E-13	7.94	19.8478296	2.14E-01	178368071	178368194	NC_000005.9
cg27633530	2.86E-12	1.55E-13	7.94	19.8454101	2.35E-01	50344294	50344417	NC_000007.13
cg07033624	2.86E-12	1.55E-13	7.94	19.8448873	1.77E-01	112716623	112716746	NC_000013.10
cg07251046	2.86E-12	1.55E-13	7.94	19.8446997	1.26E-01	97791023	97791146	NC_000012.11
cg06838584	2.86E-12	1.55E-13	7.94	19.8442993	1.98E-01	2504581	2504704	NC_000011.9
cg24529500	2.86E-12	1.55E-13	7.94	19.8439746	1.39E-01	30540984	30541107	NC_000006.11
cg15829969	2.86E-12	1.55E-13	7.94	19.8439108	1.33E-01	147801025	147801148	NC_000001.10
cg06936155	2.87E-12	1.56E-13	7.94	19.8404818	1.43E-01	91192451	91192574	NC_000001.10
cg25937155	2.88E-12	1.56E-13	7.94	19.8376414	4.46E-02	20408772	20408895	NC_000017.10

cg01391022	2.88E-12	1.57E-13	7.94	19.8370319	8.26E-02	122360665	122360788	NC_000012.11
cg10057940	2.89E-12	1.57E-13	7.94	19.8352954	1.63E-01	6805506	6805629	NC_000010.10
cg03227184	2.89E-12	1.57E-13	7.94	19.8344286	2.55E-01	122686432	122686555	NC_000004.11
cg07935902	2.89E-12	1.57E-13	7.94	19.8330209	1.00E-01	10024364	10024487	NC_000004.11
cg21894728	2.89E-12	1.57E-13	7.94	19.8329578	8.68E-02	47453656	47453779	NC_000021.8
cg19394047	2.89E-12	1.57E-13	7.94	19.8329083	1.57E-01	131223487	131223610	NC_000010.10
cg06482019	2.89E-12	1.57E-13	7.94	19.8325464	2.10E-01	51147902	51148025	NC_000016.9
cg12218771	2.89E-12	1.57E-13	7.94	19.8321393	5.63E-02	6696166	6696289	NC_000001.10
cg06182584	2.90E-12	1.57E-13	7.94	19.8313358	1.37E-01	30614329	30614452	NC_000006.11
cg21390082	2.91E-12	1.58E-13	7.94	19.8264068	1.36E-01	33842255	33842378	NC_000017.10
cg09141965	2.94E-12	1.60E-13	7.94	19.8176627	1.49E-01	1269385	1269508	NC_000007.13
cg26623263	2.94E-12	1.60E-13	7.94	19.8149232	4.77E-02	202987140	202987263	NC_000002.11
cg03731131	2.95E-12	1.60E-13	7.93	19.813203	1.54E-01	44278274	44278397	NC_000019.9
cg22327646	2.95E-12	1.60E-13	7.93	19.8129136	1.85E-01	114878570	114878693	NC_000012.11
cg15800975	2.95E-12	1.61E-13	7.93	19.8111878	1.54E-01	21541652	21541775	NC_000013.10
cg09465698	2.96E-12	1.61E-13	7.93	19.8082337	1.88E-01	140787623	140787746	NC_000005.9
cg06772736	2.97E-12	1.62E-13	7.93	19.8062069	7.64E-02	93397394	93397517	NC_000012.11
cg01843946	2.97E-12	1.62E-13	7.93	19.8049762	1.64E-01	37124434	37124557	NC_000014.8
cg23462956	2.97E-12	1.62E-13	7.93	19.804883	1.72E-01	61119262	61119385	NC_000014.8
cg18895476	2.98E-12	1.62E-13	7.93	19.8009383	7.89E-02	26962071	26962194	NC_000022.10
cg18185554	2.99E-12	1.63E-13	7.93	19.7998993	1.68E-01	103294574	103294697	NC_000014.8
cg19475108	2.99E-12	1.63E-13	7.93	19.799588	4.05E-02	33758486	33758609	NC_000020.10
cg22473973	3.00E-12	1.63E-13	7.93	19.7964316	2.38E-01	133794911	133795034	NC_000010.10
cg11934170	3.00E-12	1.63E-13	7.93	19.7958433	1.65E-01	155254326	155254449	NC_000004.11
cg24054525	3.00E-12	1.63E-13	7.93	19.7950291	6.00E-02	49685846	49685969	NC_000019.9

cg15403900	3.00E-12	1.63E-13	7.93	19.7941727	1.09E-01	85667849	85667972	NC_000016.9
cg20146105	3.00E-12	1.64E-13	7.93	19.7940307	7.94E-02	205547082	205547205	NC_000001.10
cg20690922	3.00E-12	1.64E-13	7.93	19.7940072	1.24E-01	74498083	74498206	NC_000017.10
cg14214262	3.00E-12	1.64E-13	7.93	19.7930971	1.64E-01	114843188	114843311	NC_000012.11
cg13523818	3.01E-12	1.64E-13	7.93	19.7924107	9.25E-02	19371900	19372023	NC_000019.9
cg23030069	3.01E-12	1.64E-13	7.93	19.7911173	2.35E-01	2162183	2162306	NC_000011.9
cg05703996	3.01E-12	1.64E-13	7.93	19.790679	1.67E-01	335830	335953	NC_000016.9
cg00574913	3.01E-12	1.64E-13	7.93	19.7900148	1.09E-01	13761579	13761702	NC_000020.10
cg26324366	3.01E-12	1.64E-13	7.93	19.7898606	1.19E-01	99283652	99283775	NC_000006.11
cg25740301	3.01E-12	1.64E-13	7.93	19.7896526	4.38E-02	128403710	128403833	NC_000002.11
cg11605835	3.03E-12	1.65E-13	7.93	19.7856001	1.65E-01	3597695	3597818	NC_000005.9
cg15307022	3.03E-12	1.65E-13	7.93	19.7848083	1.21E-01	108675287	108675410	NC_000011.9
cg03430846	3.03E-12	1.65E-13	7.93	19.7830001	2.28E-01	31497082	31497205	NC_000008.10
cg05735547	3.06E-12	1.67E-13	7.93	19.7751555	7.42E-02	41830291	41830414	NC_000017.10
cg21718267	3.06E-12	1.67E-13	7.93	19.7743419	2.02E-01	113916473	113916596	NC_000012.11
cg26631178	3.07E-12	1.67E-13	7.93	19.7721514	4.46E-02	45773680	45773803	NC_000017.10
cg11837391	3.07E-12	1.68E-13	7.93	19.7690748	7.28E-02	128199781	128199904	NC_000003.11
cg09943716	3.08E-12	1.68E-13	7.93	19.7679825	1.98E-01	55202236	55202359	NC_000020.10
cg00366037	3.09E-12	1.68E-13	7.93	19.7656087	1.68E-01	76781121	76781244	NC_000010.10
cg14508508	3.09E-12	1.69E-13	7.93	19.7640857	1.70E-01	27113322	27113445	NC_000015.9
cg16492597	3.09E-12	1.69E-13	7.93	19.7626032	1.68E-01	133332095	133332218	NC_000005.9
cg04476839	3.10E-12	1.69E-13	7.93	19.7605796	5.99E-02	66014369	66014492	NC_000012.11
cg01718322	3.11E-12	1.70E-13	7.93	19.7565672	2.22E-01	96342365	96342488	NC_000014.8
cg10415122	3.11E-12	1.70E-13	7.93	19.7565632	4.87E-02	75981489	75981612	NC_000015.9
cg19773855	3.13E-12	1.71E-13	7.92	19.7502811	1.97E-01	172754417	172754540	NC_000005.9

cg14636534	3.13E-12	1.71E-13	7.92	19.7499845	1.80E-01	87968528	87968651	NC_000005.9
cg13103303	3.13E-12	1.71E-13	7.92	19.7493805	2.69E-01	71955113	71955236	NC_000011.9
cg06095631	3.13E-12	1.71E-13	7.92	19.749115	3.24E-02	4230707	4230830	NC_000010.10
cg16257051	3.14E-12	1.71E-13	7.92	19.7480895	2.03E-01	153862183	153862306	NC_000005.9
cg04656327	3.14E-12	1.72E-13	7.92	19.7467801	7.57E-02	58505303	58505426	NC_000020.10
cg04842472	3.14E-12	1.72E-13	7.92	19.7465349	8.68E-02	39872851	39872974	NC_000007.13
cg04003051	3.16E-12	1.72E-13	7.92	19.741811	7.11E-02	1219430	1219553	NC_000016.9
cg20954460	3.16E-12	1.73E-13	7.92	19.7410307	9.30E-02	239594118	239594241	NC_000002.11
cg19793376	3.16E-12	1.73E-13	7.92	19.7407789	1.97E-01	135462522	135462645	NC_000009.11
cg15964309	3.16E-12	1.73E-13	7.92	19.7407322	1.96E-01	114886561	114886684	NC_000012.11
cg18689958	3.16E-12	1.73E-13	7.92	19.7395497	2.57E-01	33936232	33936355	NC_000005.9
cg26387689	3.17E-12	1.73E-13	7.92	19.7380476	1.92E-01	20521814	20521937	NC_000012.11
cg19622581	3.18E-12	1.74E-13	7.92	19.7352383	4.40E-02	3305106	3305229	NC_000011.9
cg17928883	3.18E-12	1.74E-13	7.92	19.7344517	1.85E-01	2038528	2038651	NC_000005.9
cg05842480	3.18E-12	1.74E-13	7.92	19.7338708	1.84E-01	7795192	7795315	NC_000019.9
cg01994205	3.18E-12	1.74E-13	7.92	19.7333173	2.01E-01	79177467	79177590	NC_000013.10
cg20291855	3.19E-12	1.74E-13	7.92	19.7312739	2.15E-01	13524143	13524266	NC_000004.11
cg26174583	3.19E-12	1.74E-13	7.92	19.7303656	1.30E-01	1392624	1392747	NC_000006.11
cg04267526	3.19E-12	1.74E-13	7.92	19.7300127	1.70E-01	109674297	109674420	NC_000010.10
cg05787556	3.20E-12	1.75E-13	7.92	19.7283622	1.48E-01	170735186	170735309	NC_000005.9
cg00618450	3.21E-12	1.75E-13	7.92	19.7242707	1.81E-01	26565951	26566074	NC_000022.10
cg25889711	3.23E-12	1.76E-13	7.92	19.7188745	1.53E-01	102473609	102473732	NC_000010.10
cg20375342	3.23E-12	1.77E-13	7.92	19.7179876	2.28E-01	68981895	68982018	NC_000003.11
cg07618462	3.23E-12	1.77E-13	7.92	19.7170711	9.74E-02	142499847	142499970	NC_000005.9
cg16421616	3.23E-12	1.77E-13	7.92	19.716184	8.02E-02	151444499	151444622	NC_000002.11

cg02621130	3.24E-12	1.77E-13	7.92	19.7144238	1.75E-01	166267729	166267852	NC_000006.11
cg06497612	3.24E-12	1.77E-13	7.92	19.7135693	7.44E-02	239140610	239140733	NC_000002.11
cg16660899	3.24E-12	1.77E-13	7.92	19.7135239	6.81E-02	167095593	167095716	NC_000001.10
cg23008238	3.24E-12	1.78E-13	7.92	19.7129405	2.25E-01	2112459	2112582	NC_000005.9
cg01479738	3.25E-12	1.78E-13	7.92	19.7107898	5.87E-02	60741560	60741683	NC_000013.10
cg22300619	3.25E-12	1.78E-13	7.92	19.7097944	1.31E-01	40699809	40699932	NC_000017.10
cg25682299	3.26E-12	1.78E-13	7.92	19.7078957	1.56E-01	79177886	79178009	NC_000013.10
cg00620476	3.26E-12	1.79E-13	7.92	19.7071459	7.63E-02	31938176	31938299	NC_000006.11
cg11599981	3.26E-12	1.79E-13	7.92	19.707073	1.24E-01	6475902	6476025	NC_000019.9
cg02818409	3.27E-12	1.79E-13	7.92	19.7053452	5.27E-02	143116253	143116376	NC_000007.13
cg12031863	3.27E-12	1.79E-13	7.92	19.7046966	1.86E-01	4587854	4587977	NC_000016.9
cg22375610	3.27E-12	1.79E-13	7.92	19.704207	1.17E-01	41021161	41021284	NC_000006.11
cg03532026	3.27E-12	1.79E-13	7.92	19.7037258	1.29E-01	57829153	57829276	NC_000019.9
cg04395377	3.28E-12	1.80E-13	7.92	19.7019525	1.63E-01	17841862	17841985	NC_000016.9
cg02562761	3.28E-12	1.80E-13	7.92	19.6997442	1.11E-01	4307906	4308029	NC_000016.9
cg11908453	3.29E-12	1.80E-13	7.92	19.6985375	1.39E-01	242209506	242209629	NC_000002.11
cg20028827	3.29E-12	1.80E-13	7.92	19.6977517	1.49E-01	79169823	79169946	NC_000013.10
cg07832473	3.29E-12	1.80E-13	7.92	19.6976762	2.41E-01	33936253	33936376	NC_000005.9
cg04838147	3.29E-12	1.80E-13	7.92	19.696558	1.88E-01	26505069	26505192	NC_000010.10
cg11713800	3.30E-12	1.81E-13	7.92	19.6962634	1.28E-01	41620951	41621074	NC_000006.11
cg26684131	3.30E-12	1.81E-13	7.92	19.6949031	1.13E-01	32410365	32410488	NC_000006.11
cg05078091	3.30E-12	1.81E-13	7.92	19.6943046	1.16E-01	129984345	129984468	NC_000011.9
cg23004758	3.30E-12	1.81E-13	7.92	19.694249	2.47E-01	58204221	58204344	NC_000013.10
cg13733008	3.30E-12	1.81E-13	7.91	19.6932252	6.57E-02	78088735	78088858	NC_000004.11
cg27019929	3.31E-12	1.81E-13	7.91	19.6929212	1.49E-01	32421518	32421641	NC_000011.9

cg13040816	3.32E-12	1.82E-13	7.91	19.6894956	3.65E-02	30681089	30681212	NC_000006.11
cg13837295	3.32E-12	1.82E-13	7.91	19.6886038	1.02E-01	134826531	134826654	NC_000010.10
cg03712816	3.32E-12	1.82E-13	7.91	19.6884932	1.87E-01	76741157	76741280	NC_000018.9
cg07827420	3.33E-12	1.83E-13	7.91	19.684956	1.18E-01	75371764	75371887	NC_000017.10
cg04747226	3.33E-12	1.83E-13	7.91	19.6834698	2.36E-01	105481319	105481442	NC_000011.9
cg00418216	3.35E-12	1.84E-13	7.91	19.6787525	2.12E-01	27227847	27227970	NC_000007.13
cg25952581	3.36E-12	1.84E-13	7.91	19.6753647	1.55E-01	27170961	27171084	NC_000007.13
cg26935685	3.36E-12	1.85E-13	7.91	19.6746943	1.38E-01	15502881	15503004	NC_000001.10
cg14205791	3.36E-12	1.85E-13	7.91	19.6742485	6.80E-02	124800227	124800350	NC_000012.11
cg25462190	3.38E-12	1.85E-13	7.91	19.6700194	8.11E-02	177547067	177547190	NC_000005.9
cg15605746	3.38E-12	1.86E-13	7.91	19.6684865	1.37E-01	52584919	52585042	NC_000015.9
cg20859731	3.40E-12	1.86E-13	7.91	19.6646936	1.55E-01	36226820	36226943	NC_000020.10
cg07215504	3.40E-12	1.86E-13	7.91	19.6644942	1.89E-01	88497007	88497130	NC_000016.9
cg15089219	3.41E-12	1.87E-13	7.91	19.6597354	1.72E-01	119915368	119915491	NC_000002.11
cg08327884	3.41E-12	1.87E-13	7.91	19.6590894	1.51E-01	84798113	84798236	NC_000006.11
cg01717727	3.42E-12	1.88E-13	7.91	19.6585187	1.70E-01	5140406	5140529	NC_000005.9
cg06715976	3.43E-12	1.88E-13	7.91	19.6544825	2.54E-01	27135769	27135892	NC_000007.13
cg17767285	3.43E-12	1.88E-13	7.91	19.6539916	2.39E-01	84419202	84419325	NC_000006.11
cg24282829	3.43E-12	1.88E-13	7.91	19.6539371	5.58E-02	8596320	8596443	NC_000012.11
cg10725720	3.44E-12	1.89E-13	7.91	19.6518636	2.04E-01	55039232	55039355	NC_000006.11
cg20646280	3.44E-12	1.89E-13	7.91	19.651173	2.14E-01	44449738	44449861	NC_000004.11
cg15924285	3.44E-12	1.89E-13	7.91	19.650481	1.50E-01	93465688	93465811	NC_000011.9
cg13915566	3.44E-12	1.89E-13	7.91	19.6501811	5.87E-02	2286006	2286129	NC_000011.9
cg19577779	3.44E-12	1.89E-13	7.91	19.6499675	2.65E-01	88326635	88326758	NC_000013.10
cg19786627	3.45E-12	1.90E-13	7.91	19.6483988	1.51E-01	29235904	29236027	NC_000014.8

cg06292124	3.45E-12	1.90E-13	7.91	19.6483631	6.56E-02	52408520	52408643	NC_000012.11
cg05560333	3.45E-12	1.90E-13	7.91	19.6464523	1.17E-01	233432602	233432725	NC_000001.10
cg17632299	3.46E-12	1.90E-13	7.91	19.6451361	2.07E-01	53312966	53313089	NC_000013.10
cg13610307	3.47E-12	1.91E-13	7.91	19.6416575	1.66E-01	140917431	140917554	NC_000009.11
cg01382864	3.47E-12	1.91E-13	7.91	19.641514	1.37E-01	220174251	220174374	NC_000002.11
cg16433156	3.47E-12	1.91E-13	7.91	19.6409327	2.00E-01	76740091	76740214	NC_000018.9
cg16057262	3.47E-12	1.91E-13	7.91	19.6400899	1.95E-01	182322699	182322822	NC_000002.11
cg18484679	3.48E-12	1.91E-13	7.91	19.6396659	1.14E-01	142740314	142740437	NC_000005.9
cg02115041	3.48E-12	1.92E-13	7.91	19.6381795	1.58E-01	53852101	53852224	NC_000008.10
cg22619784	3.48E-12	1.92E-13	7.91	19.6376865	1.27E-01	32793355	32793478	NC_000006.11
cg10454246	3.49E-12	1.92E-13	7.91	19.6362937	2.45E-01	41883890	41884013	NC_000004.11
cg09133026	3.49E-12	1.92E-13	7.91	19.6359179	6.17E-02	75388105	75388228	NC_000014.8
cg20784098	3.49E-12	1.92E-13	7.91	19.6358522	6.12E-02	32157059	32157182	NC_000001.10
cg15066337	3.49E-12	1.92E-13	7.91	19.6354744	4.24E-02	190105668	190105791	NC_000003.11
cg10919664	3.49E-12	1.92E-13	7.91	19.6353067	1.89E-01	28945322	28945445	NC_000006.11
cg18013765	3.49E-12	1.92E-13	7.9	19.6338468	4.44E-02	4499993	4500116	NC_000010.10
cg18948125	3.51E-12	1.93E-13	7.9	19.6304997	1.40E-01	31805970	31806093	NC_000002.11
cg24713204	3.51E-12	1.93E-13	7.9	19.6290102	1.58E-01	57019373	57019496	NC_000019.9
cg18519264	3.51E-12	1.93E-13	7.9	19.6282801	3.36E-02	169666498	169666621	NC_000002.11
cg02534659	3.52E-12	1.94E-13	7.9	19.6264395	2.31E-01	2112561	2112684	NC_000005.9
cg17525102	3.52E-12	1.94E-13	7.9	19.6262351	1.93E-01	29236221	29236344	NC_000014.8
cg07676002	3.52E-12	1.94E-13	7.9	19.6257516	1.32E-01	44880000	44880123	NC_000020.10
cg17386911	3.52E-12	1.94E-13	7.9	19.6252975	8.21E-02	176523180	176523303	NC_000005.9
cg10239860	3.56E-12	1.96E-13	7.9	19.6153036	8.70E-02	71172023	71172146	NC_000001.10
cg09764984	3.56E-12	1.96E-13	7.9	19.6144358	7.29E-02	3702447	3702570	NC_000016.9

cg14041283	3.56E-12	1.96E-13	7.9	19.6136828	1.96E-01	17906338	17906461	NC_000009.11
cg23964057	3.56E-12	1.96E-13	7.9	19.6136606	1.94E-01	69590113	69590236	NC_000011.9
cg03324821	3.56E-12	1.96E-13	7.9	19.6132201	2.61E-01	51142933	51143056	NC_000019.9
cg18761549	3.57E-12	1.97E-13	7.9	19.6113893	1.91E-01	115134391	115134514	NC_000012.11
cg11601523	3.57E-12	1.97E-13	7.9	19.6100933	2.03E-01	2756361	2756484	NC_000005.9
cg14749465	3.58E-12	1.97E-13	7.9	19.6082168	2.21E-01	20690720	20690843	NC_000011.9
cg21551182	3.58E-12	1.97E-13	7.9	19.608204	4.02E-02	106399873	106399996	NC_000002.11
cg00581595	3.58E-12	1.97E-13	7.9	19.6080094	8.11E-02	58545346	58545469	NC_000016.9
cg18907363	3.59E-12	1.98E-13	7.9	19.6064273	8.89E-02	100067361	100067484	NC_000014.8
cg00908197	3.59E-12	1.98E-13	7.9	19.6038458	4.49E-02	41619666	41619789	NC_000006.11
cg23217097	3.60E-12	1.99E-13	7.9	19.6017702	1.84E-01	42074635	42074758	NC_000014.8
cg05330604	3.61E-12	1.99E-13	7.9	19.600059	1.14E-01	91837300	91837423	NC_000015.9
cg07009376	3.61E-12	1.99E-13	7.9	19.6000034	2.32E-01	28793219	28793342	NC_000006.11
cg21139046	3.61E-12	1.99E-13	7.9	19.5998359	1.34E-01	14339700	14339823	NC_000003.11
cg04552470	3.62E-12	2.00E-13	7.9	19.5978318	1.51E-01	20669792	20669915	NC_000001.10
cg10201141	3.62E-12	2.00E-13	7.9	19.595736	1.42E-01	7032793	7032916	NC_000012.11
cg05225634	3.63E-12	2.00E-13	7.9	19.593834	1.07E-01	156718288	156718411	NC_000006.11
cg11985360	3.63E-12	2.01E-13	7.9	19.5926997	2.11E-01	19138209	19138332	NC_000022.10
cg05944477	3.64E-12	2.01E-13	7.9	19.5918146	1.15E-01	205189476	205189599	NC_000002.11
cg12601945	3.64E-12	2.01E-13	7.9	19.5911586	2.05E-01	18958924	18959047	NC_000001.10
cg14445507	3.65E-12	2.01E-13	7.9	19.5888149	1.97E-01	152890360	152890483	NC_000007.13
cg23874437	3.65E-12	2.02E-13	7.9	19.5879321	1.94E-01	38886110	38886233	NC_000019.9
cg14484885	3.67E-12	2.03E-13	7.9	19.5823722	8.26E-02	111540404	111540527	NC_000013.10
cg06926314	3.67E-12	2.03E-13	7.9	19.5809892	1.38E-01	10650351	10650474	NC_000005.9
cg04804618	3.68E-12	2.04E-13	7.9	19.5782219	2.20E-01	131761386	131761509	NC_000010.10

cg07160277	3.70E-12	2.04E-13	7.9	19.5744903	1.59E-01	165304855	165304978	NC_000004.11
cg11739758	3.70E-12	2.04E-13	7.89	19.5735163	2.02E-01	46670663	46670786	NC_000017.10
cg03767531	3.71E-12	2.05E-13	7.89	19.5709077	1.56E-01	182545572	182545695	NC_000002.11
cg16875711	3.71E-12	2.05E-13	7.89	19.5708266	4.80E-02	51798822	51798945	NC_000018.9
cg10742917	3.71E-12	2.05E-13	7.89	19.5706677	1.28E-01	56242801	56242924	NC_000007.13
cg26067203	3.71E-12	2.05E-13	7.89	19.5699374	2.37E-01	1935289	1935412	NC_000001.10
cg08455099	3.72E-12	2.06E-13	7.89	19.5669494	1.84E-01	95663959	95664082	NC_000002.11
cg01833122	3.73E-12	2.06E-13	7.89	19.5659344	8.34E-02	167436541	167436664	NC_000001.10
cg21859594	3.73E-12	2.06E-13	7.89	19.5648005	1.42E-01	4872801	4872924	NC_000012.11
cg23503075	3.74E-12	2.07E-13	7.89	19.5625226	4.80E-02	3564803	3564926	NC_000010.10
cg18803251	3.75E-12	2.08E-13	7.89	19.5583747	5.49E-02	7374622	7374745	NC_000004.11
cg13587180	3.76E-12	2.08E-13	7.89	19.5555732	1.37E-01	42403248	42403371	NC_000004.11
cg22615203	3.77E-12	2.08E-13	7.89	19.5550732	1.20E-01	41111493	41111616	NC_000013.10
cg20495645	3.77E-12	2.08E-13	7.89	19.5550561	2.57E-01	45405624	45405747	NC_000022.10
cg24242823	3.77E-12	2.08E-13	7.89	19.5549531	2.33E-01	24323675	24323798	NC_000007.13
cg14734994	3.77E-12	2.09E-13	7.89	19.5538265	4.97E-02	88468372	88468495	NC_000002.11
cg10380328	3.77E-12	2.09E-13	7.89	19.5533223	2.11E-01	188953126	188953249	NC_000004.11
cg17019070	3.77E-12	2.09E-13	7.89	19.5529707	1.23E-01	73466639	73466762	NC_000010.10
cg09728607	3.78E-12	2.09E-13	7.89	19.5515257	1.45E-01	51190065	51190188	NC_000016.9
cg17904129	3.78E-12	2.09E-13	7.89	19.5499688	3.59E-02	54195800	54195923	NC_000001.10
cg10151685	3.78E-12	2.09E-13	7.89	19.5498077	1.71E-01	89312863	89312986	NC_000015.9
cg05991820	3.79E-12	2.10E-13	7.89	19.5491211	1.54E-01	11785161	11785284	NC_000010.10
cg16478737	3.79E-12	2.10E-13	7.89	19.5473584	1.13E-01	1442040	1442163	NC_000016.9
cg01646718	3.79E-12	2.10E-13	7.89	19.5471362	1.77E-01	145580727	145580850	NC_000008.10
cg22010052	3.80E-12	2.10E-13	7.89	19.5462167	1.09E-01	115298704	115298827	NC_000005.9

cg07260592	3.80E-12	2.10E-13	7.89	19.5460208	1.57E-01	161100122	161100245	NC_000006.11
cg01378812	3.81E-12	2.11E-13	7.89	19.5409096	1.57E-01	36004794	36004917	NC_000017.10
cg14633910	3.82E-12	2.11E-13	7.89	19.5403694	1.16E-01	10897204	10897327	NC_000006.11
cg21139325	3.82E-12	2.12E-13	7.89	19.5400874	8.83E-02	32729470	32729593	NC_000006.11
cg17648101	3.82E-12	2.12E-13	7.89	19.539726	8.55E-02	17438324	17438447	NC_000019.9
cg06575391	3.82E-12	2.12E-13	7.89	19.5394926	3.93E-02	100023123	100023246	NC_000008.10
cg21630370	3.82E-12	2.12E-13	7.89	19.5386062	4.09E-02	45141979	45142102	NC_000007.13
cg13933080	3.83E-12	2.12E-13	7.89	19.5377375	1.27E-01	22634145	22634268	NC_000010.10
cg02738641	3.84E-12	2.13E-13	7.89	19.5343207	1.31E-01	114303736	114303859	NC_000013.10
cg06506966	3.84E-12	2.13E-13	7.89	19.5336083	2.30E-01	53758289	53758412	NC_000019.9
cg06610259	3.85E-12	2.14E-13	7.89	19.5304845	1.35E-01	122515332	122515455	NC_000012.11
cg16043357	3.85E-12	2.14E-13	7.89	19.5301938	1.47E-01	118897904	118898027	NC_000010.10
cg13944871	3.86E-12	2.14E-13	7.89	19.5292544	8.15E-02	3461718	3461841	NC_000001.10
cg27025384	3.86E-12	2.14E-13	7.89	19.5279848	8.88E-02	90386331	90386454	NC_000005.9
cg07531228	3.88E-12	2.15E-13	7.89	19.5231805	1.31E-01	149745299	149745422	NC_000007.13
cg05484788	3.88E-12	2.15E-13	7.89	19.5229798	2.30E-01	58180357	58180480	NC_000020.10
cg18653282	3.88E-12	2.15E-13	7.89	19.5228993	1.81E-01	104604739	104604862	NC_000014.8
cg07813142	3.89E-12	2.16E-13	7.89	19.5211564	1.89E-01	171573223	171573346	NC_000002.11
cg16236779	3.89E-12	2.16E-13	7.89	19.521089	1.51E-01	104905387	104905510	NC_000010.10
cg16701059	3.89E-12	2.16E-13	7.89	19.5198456	1.31E-01	101513798	101513921	NC_000015.9
cg09953583	3.89E-12	2.16E-13	7.89	19.5188595	7.65E-02	22974144	22974267	NC_000014.8
cg08575537	3.90E-12	2.16E-13	7.89	19.5183943	2.06E-01	100318626	100318749	NC_000007.13
cg09223489	3.90E-12	2.16E-13	7.89	19.5181932	8.42E-02	86785970	86786093	NC_000016.9
cg00600617	3.90E-12	2.16E-13	7.89	19.5176606	1.46E-01	107221887	107222010	NC_000006.11
cg10332700	3.90E-12	2.17E-13	7.89	19.5161969	2.32E-01	53636077	53636200	NC_000019.9

cg24889512	3.91E-12	2.17E-13	7.89	19.5155274	2.07E-01	22816950	22817073	NC_000019.9
cg16277479	3.91E-12	2.17E-13	7.89	19.5144809	2.46E-01	13910209	13910332	NC_000001.10
cg02100008	3.92E-12	2.18E-13	7.88	19.5117703	6.73E-02	56658357	56658480	NC_000004.11
cg21297493	3.92E-12	2.18E-13	7.88	19.5114449	1.63E-01	156357905	156358028	NC_000001.10
cg01030406	3.92E-12	2.18E-13	7.88	19.5110617	1.10E-01	113374553	113374676	NC_000004.11
cg09367901	3.93E-12	2.18E-13	7.88	19.5092577	1.97E-01	54418851	54418974	NC_000014.8
cg13490729	3.93E-12	2.19E-13	7.88	19.5077135	6.32E-02	23779307	23779430	NC_000014.8
cg09071040	3.94E-12	2.19E-13	7.88	19.5074479	5.39E-02	49358690	49358813	NC_000012.11
cg03596113	3.94E-12	2.19E-13	7.88	19.506707	3.71E-02	3129969	3130092	NC_000001.10
cg19203333	3.94E-12	2.19E-13	7.88	19.5060466	1.80E-01	134175398	134175521	NC_000006.11
cg17680089	3.94E-12	2.19E-13	7.88	19.5058859	1.60E-01	228346338	228346461	NC_000001.10
cg00852414	3.94E-12	2.19E-13	7.88	19.5053837	1.26E-01	101543050	101543173	NC_000014.8
cg16952918	3.94E-12	2.19E-13	7.88	19.5050775	4.77E-02	69078072	69078195	NC_000012.11
cg00601368	3.95E-12	2.19E-13	7.88	19.5047311	1.14E-01	149318081	149318204	NC_000007.13
cg09048530	3.95E-12	2.19E-13	7.88	19.5038347	2.38E-01	130646022	130646145	NC_000012.11
cg05678033	3.96E-12	2.20E-13	7.88	19.5016638	1.67E-01	115104591	115104714	NC_000012.11
cg14400886	3.96E-12	2.20E-13	7.88	19.5013674	2.14E-01	134070441	134070564	NC_000004.11
cg19082560	3.96E-12	2.20E-13	7.88	19.4997957	1.44E-01	3603280	3603403	NC_000005.9
cg09229912	3.98E-12	2.21E-13	7.88	19.4963245	2.30E-01	111471963	111472086	NC_000012.11
cg10201791	3.98E-12	2.21E-13	7.88	19.4955705	4.45E-02	111855154	111855277	NC_000007.13
cg27273310	3.98E-12	2.21E-13	7.88	19.4951079	8.89E-02	131642913	131643036	NC_000010.10
cg14490428	3.99E-12	2.22E-13	7.88	19.4934777	1.74E-01	89841521	89841644	NC_000010.10
cg22190438	3.99E-12	2.22E-13	7.88	19.493322	1.38E-01	42733976	42734099	NC_000017.10
cg07921384	4.00E-12	2.22E-13	7.88	19.4910037	2.55E-01	220299740	220299863	NC_000002.11
cg08864310	4.01E-12	2.23E-13	7.88	19.4876225	9.72E-02	64402469	64402592	NC_000005.9

cg21057266	4.02E-12	2.24E-13	7.88	19.4848865	9.56E-02	189200528	189200651	NC_000004.11
cg00376979	4.04E-12	2.25E-13	7.88	19.4803504	1.32E-01	34350727	34350850	NC_000021.8
cg19041857	4.05E-12	2.25E-13	7.88	19.4784985	1.21E-01	27730383	27730506	NC_000006.11
cg23063647	4.05E-12	2.25E-13	7.88	19.4783569	1.84E-01	206339	206462	NC_000004.11
cg18237616	4.05E-12	2.25E-13	7.88	19.4776196	1.69E-01	151191667	151191790	NC_000007.13
cg11355658	4.05E-12	2.25E-13	7.88	19.4772271	1.20E-01	132453670	132453793	NC_000006.11
cg05368341	4.05E-12	2.26E-13	7.88	19.4760153	1.99E-01	114696465	114696588	NC_000001.10
cg20011402	4.05E-12	2.26E-13	7.88	19.4759946	3.16E-01	17496239	17496362	NC_000010.10
cg18036763	4.07E-12	2.26E-13	7.88	19.4731698	2.32E-01	45404910	45405033	NC_000022.10
cg18446069	4.07E-12	2.26E-13	7.88	19.4730944	1.11E-01	111644279	111644402	NC_000013.10
cg02101773	4.07E-12	2.26E-13	7.88	19.4730193	1.95E-01	20347593	20347716	NC_000020.10
cg05455720	4.08E-12	2.27E-13	7.88	19.4692292	1.89E-01	65291703	65291826	NC_000008.10
cg25657713	4.08E-12	2.27E-13	7.88	19.4691053	1.49E-01	100038708	100038831	NC_000006.11
cg05678100	4.10E-12	2.28E-13	7.88	19.4656048	1.44E-01	172734031	172734154	NC_000004.11
cg08535058	4.12E-12	2.30E-13	7.88	19.4589229	1.23E-01	12920991	12921114	NC_000019.9
cg10815835	4.13E-12	2.30E-13	7.88	19.4574232	1.08E-01	170396327	170396450	NC_000006.11
cg20892260	4.14E-12	2.31E-13	7.87	19.4543088	1.72E-01	23564025	23564148	NC_000008.10
cg24900963	4.14E-12	2.31E-13	7.87	19.4541235	1.03E-01	28948266	28948389	NC_000016.9
cg20738665	4.14E-12	2.31E-13	7.87	19.4531199	9.09E-02	61874249	61874372	NC_000020.10
cg23685953	4.14E-12	2.31E-13	7.87	19.4530459	6.66E-02	52216479	52216602	NC_000014.8
cg27436952	4.15E-12	2.31E-13	7.87	19.4524148	1.30E-01	8895870	8895993	NC_000016.9
cg22274813	4.15E-12	2.31E-13	7.87	19.4520204	1.92E-01	38067101	38067224	NC_000021.8
cg15420634	4.15E-12	2.31E-13	7.87	19.4515556	8.79E-02	485359	485482	NC_000005.9
cg01398502	4.16E-12	2.32E-13	7.87	19.4493413	1.05E-01	85990190	85990313	NC_000016.9
cg01951637	4.18E-12	2.33E-13	7.87	19.4446079	2.69E-01	89007466	89007589	NC_000016.9

cg10534507	4.18E-12	2.33E-13	7.87	19.4442711	1.66E-01	82336761	82336884	NC_000015.9
cg12182408	4.18E-12	2.33E-13	7.87	19.4439949	1.19E-01	104618441	104618564	NC_000014.8
cg19829856	4.19E-12	2.34E-13	7.87	19.4417983	1.14E-01	44237658	44237781	NC_000018.9
cg14419975	4.19E-12	2.34E-13	7.87	19.441196	2.52E-01	91196332	91196455	NC_000001.10
cg11373604	4.20E-12	2.34E-13	7.87	19.4389139	1.58E-01	2291260	2291383	NC_000011.9
cg17566175	4.21E-12	2.35E-13	7.87	19.4349664	1.35E-01	56389866	56389989	NC_000006.11
cg18705776	4.22E-12	2.36E-13	7.87	19.4337396	6.72E-02	5565829	5565952	NC_000010.10
cg17367901	4.22E-12	2.36E-13	7.87	19.4335037	5.85E-02	619032	619155	NC_000019.9
cg08850461	4.22E-12	2.36E-13	7.87	19.4328486	2.22E-01	87990082	87990205	NC_000005.9
cg07592254	4.23E-12	2.36E-13	7.87	19.4312803	1.55E-01	63444750	63444873	NC_000008.10
cg05624932	4.24E-12	2.37E-13	7.87	19.4288118	2.47E-01	75897310	75897433	NC_000008.10
cg14601981	4.24E-12	2.37E-13	7.87	19.4279913	1.39E-01	79145228	79145351	NC_000010.10
cg16018474	4.24E-12	2.37E-13	7.87	19.4279094	2.46E-01	158119501	158119624	NC_000001.10
cg05200250	4.24E-12	2.37E-13	7.87	19.4272767	1.08E-01	75264574	75264697	NC_000016.9
cg10013343	4.25E-12	2.37E-13	7.87	19.4268446	2.28E-01	29106503	29106626	NC_000013.10
cg20982735	4.25E-12	2.37E-13	7.87	19.4266861	1.45E-01	87082023	87082146	NC_000008.10
cg11321433	4.25E-12	2.37E-13	7.87	19.4262515	1.83E-01	32338542	32338665	NC_000007.13
cg03490331	4.25E-12	2.37E-13	7.87	19.4260629	1.32E-01	172672817	172672940	NC_000005.9
cg18358136	4.25E-12	2.38E-13	7.87	19.4252128	1.49E-01	46119636	46119759	NC_000019.9
cg20557801	4.25E-12	2.38E-13	7.87	19.4248673	1.69E-01	68121177	68121300	NC_000015.9
cg00851770	4.26E-12	2.38E-13	7.87	19.422538	2.36E-01	12254175	12254298	NC_000018.9
cg08190291	4.27E-12	2.39E-13	7.87	19.4212962	2.38E-01	28339442	28339565	NC_000021.8
cg12080492	4.27E-12	2.39E-13	7.87	19.420908	1.27E-01	203145656	203145779	NC_000001.10
cg08295543	4.27E-12	2.39E-13	7.87	19.4201162	1.43E-01	55357842	55357965	NC_000016.9
cg21230745	4.27E-12	2.39E-13	7.87	19.4201149	2.16E-01	87974414	87974537	NC_000005.9

cg12213062	4.27E-12	2.39E-13	7.87	19.4198781	1.85E-01	220299653	220299776	NC_000002.11
cg18744668	4.28E-12	2.39E-13	7.87	19.41938	7.69E-02	99962347	99962470	NC_000008.10
cg19959917	4.28E-12	2.39E-13	7.87	19.4181249	1.10E-01	44878373	44878496	NC_000010.10
cg24996278	4.28E-12	2.39E-13	7.87	19.4178939	3.65E-02	235327092	235327215	NC_000001.10
cg11854491	4.29E-12	2.40E-13	7.87	19.4160004	2.26E-01	46961115	46961238	NC_000013.10
cg03909500	4.29E-12	2.40E-13	7.87	19.4155245	1.56E-01	33936597	33936720	NC_000005.9
cg16295725	4.29E-12	2.40E-13	7.87	19.4147829	2.54E-01	10459219	10459342	NC_000004.11
cg02943604	4.31E-12	2.41E-13	7.87	19.4098845	1.47E-01	122217348	122217471	NC_000010.10
cg23720732	4.32E-12	2.42E-13	7.87	19.4083524	2.51E-01	44650380	44650503	NC_000020.10
cg18498241	4.32E-12	2.42E-13	7.87	19.4071372	1.21E-01	69286352	69286475	NC_000011.9
cg08214518	4.34E-12	2.43E-13	7.87	19.4025249	6.88E-02	1990114	1990237	NC_000007.13
cg17026582	4.35E-12	2.43E-13	7.87	19.4013808	6.04E-02	1298573	1298696	NC_000005.9
cg02726819	4.36E-12	2.44E-13	7.87	19.3985809	1.24E-01	158800869	158800992	NC_000007.13
cg02000318	4.36E-12	2.44E-13	7.87	19.3979801	2.01E-01	27197713	27197836	NC_000007.13
cg03830490	4.37E-12	2.45E-13	7.87	19.3970692	9.56E-02	45926669	45926792	NC_000003.11
cg26763877	4.37E-12	2.45E-13	7.87	19.3956791	1.83E-01	4851751	4851874	NC_000008.10
cg06925060	4.38E-12	2.45E-13	7.87	19.3948976	7.54E-02	10489288	10489411	NC_000001.10
cg25633678	4.38E-12	2.46E-13	7.86	19.3931487	2.11E-01	65284473	65284596	NC_000008.10
cg22052659	4.38E-12	2.46E-13	7.86	19.3929694	2.14E-01	114444989	114445112	NC_000008.10
cg00713567	4.39E-12	2.46E-13	7.86	19.3908518	1.51E-01	143545949	143546072	NC_000008.10
cg09501959	4.39E-12	2.46E-13	7.86	19.3902841	1.36E-01	193058203	193058326	NC_000002.11
cg01871526	4.40E-12	2.46E-13	7.86	19.3896839	4.93E-02	23867258	23867381	NC_000002.11
cg01663563	4.41E-12	2.47E-13	7.86	19.3872742	1.23E-01	90775513	90775636	NC_000015.9
cg08750504	4.41E-12	2.47E-13	7.86	19.3861416	1.42E-01	172946193	172946316	NC_000002.11
cg25316512	4.42E-12	2.48E-13	7.86	19.3842548	1.15E-01	7032991	7033114	NC_000012.11

cg03212183	4.43E-12	2.48E-13	7.86	19.3821442	8.77E-02	81565917	81566040	NC_000016.9
cg04466022	4.43E-12	2.48E-13	7.86	19.3817503	9.91E-02	37618123	37618246	NC_000006.11
cg01145544	4.44E-12	2.49E-13	7.86	19.3802078	7.90E-02	46889296	46889419	NC_000021.8
cg17131837	4.44E-12	2.49E-13	7.86	19.3785933	1.43E-01	66251661	66251784	NC_000012.11
cg21500133	4.45E-12	2.49E-13	7.86	19.3776641	2.82E-01	43439451	43439574	NC_000020.10
cg02086307	4.45E-12	2.50E-13	7.86	19.3761334	1.62E-01	53390878	53391001	NC_000001.10
cg03905134	4.46E-12	2.50E-13	7.86	19.3757823	1.15E-01	76146565	76146688	NC_000012.11
cg22234419	4.46E-12	2.50E-13	7.86	19.3748372	1.55E-01	145875907	145876030	NC_000003.11
cg05803265	4.47E-12	2.51E-13	7.86	19.3730928	6.36E-02	54352207	54352330	NC_000001.10
cg01876288	4.47E-12	2.51E-13	7.86	19.3725144	1.26E-01	158800934	158801057	NC_000007.13
cg18451114	4.47E-12	2.51E-13	7.86	19.3714505	1.40E-01	38511677	38511800	NC_000001.10
cg01656470	4.49E-12	2.52E-13	7.86	19.3688089	1.01E-01	57350096	57350219	NC_000019.9
cg01843018	4.50E-12	2.52E-13	7.86	19.366445	1.52E-01	123813144	123813267	NC_000009.11
cg02161292	4.51E-12	2.53E-13	7.86	19.364038	1.22E-01	140821687	140821810	NC_000005.9
cg15174552	4.51E-12	2.53E-13	7.86	19.3632407	1.60E-01	118982352	118982475	NC_000002.11
cg04130185	4.52E-12	2.54E-13	7.86	19.3608039	1.63E-01	102988251	102988374	NC_000010.10
cg13400209	4.52E-12	2.54E-13	7.86	19.3602913	4.90E-02	117813666	117813789	NC_000002.11
cg08710861	4.53E-12	2.54E-13	7.86	19.358789	7.26E-02	128493066	128493189	NC_000007.13
cg00627846	4.54E-12	2.55E-13	7.86	19.3560581	1.01E-01	49141122	49141245	NC_000022.10
cg18832738	4.55E-12	2.55E-13	7.86	19.3539362	1.40E-01	95210053	95210176	NC_000012.11
cg23808270	4.55E-12	2.56E-13	7.86	19.3536638	7.74E-02	223148963	223149086	NC_000001.10
cg10140638	4.57E-12	2.57E-13	7.86	19.3495755	1.47E-01	220174207	220174330	NC_000002.11
cg08691159	4.57E-12	2.57E-13	7.86	19.349054	1.32E-01	85635763	85635886	NC_000016.9
cg26205771	4.58E-12	2.57E-13	7.86	19.3472111	2.23E-01	53851156	53851279	NC_000008.10
cg07329360	4.58E-12	2.57E-13	7.86	19.3470616	1.68E-01	31841768	31841891	NC_000011.9

cg21434530	4.59E-12	2.58E-13	7.86	19.3456773	1.88E-01	4870486	4870609	NC_000004.11
cg07309238	4.59E-12	2.58E-13	7.86	19.344201	7.33E-02	110229803	110229926	NC_000005.9
cg21858485	4.60E-12	2.59E-13	7.86	19.3420664	2.27E-01	130236027	130236150	NC_000003.11
cg09890891	4.60E-12	2.59E-13	7.86	19.341954	1.15E-01	96904723	96904846	NC_000015.9
cg19316405	4.61E-12	2.59E-13	7.86	19.3398247	4.39E-02	770410	770533	NC_000011.9
cg00286188	4.61E-12	2.59E-13	7.86	19.3394406	9.95E-02	33493596	33493719	NC_000002.11
cg16209664	4.63E-12	2.60E-13	7.86	19.3364786	1.30E-01	147131146	147131269	NC_000003.11
cg06550340	4.63E-12	2.60E-13	7.86	19.3354552	2.02E-01	61638420	61638543	NC_000020.10
cg12845990	4.63E-12	2.60E-13	7.86	19.335023	3.20E-02	33391288	33391411	NC_000006.11
cg13728003	4.63E-12	2.60E-13	7.86	19.3348925	1.76E-01	1387836	1387959	NC_000006.11
cg01313313	4.64E-12	2.61E-13	7.85	19.333963	1.92E-01	43040590	43040713	NC_000005.9
cg16359550	4.65E-12	2.61E-13	7.85	19.3320379	2.02E-01	109292809	109292932	NC_000011.9
cg18689614	4.65E-12	2.61E-13	7.85	19.3311614	5.96E-02	77029386	77029509	NC_000017.10
cg25944479	4.65E-12	2.61E-13	7.85	19.3311368	1.17E-01	8195062	8195185	NC_000006.11
cg15836374	4.65E-12	2.62E-13	7.85	19.3301471	7.89E-02	132938027	132938150	NC_000012.11
cg21072457	4.66E-12	2.62E-13	7.85	19.3293001	6.38E-02	151192481	151192604	NC_000005.9
cg15936446	4.66E-12	2.62E-13	7.85	19.3287277	1.89E-01	42952369	42952492	NC_000005.9
cg23542449	4.66E-12	2.62E-13	7.85	19.3284775	1.33E-01	22602447	22602570	NC_000012.11
cg19695166	4.66E-12	2.62E-13	7.85	19.3277053	9.66E-02	16462492	16462615	NC_000005.9
cg07618581	4.66E-12	2.62E-13	7.85	19.3273808	1.27E-01	92512048	92512171	NC_000015.9
cg05840573	4.67E-12	2.63E-13	7.85	19.3262217	1.65E-01	24124045	24124168	NC_000018.9
cg00034468	4.67E-12	2.63E-13	7.85	19.3251471	1.41E-01	229568077	229568200	NC_000001.10
cg19595505	4.68E-12	2.63E-13	7.85	19.3237373	1.84E-01	155596322	155596445	NC_000007.13
cg21500166	4.69E-12	2.64E-13	7.85	19.3225776	1.23E-01	75603184	75603307	NC_000012.11
cg04732596	4.69E-12	2.64E-13	7.85	19.3205402	1.32E-01	66789740	66789863	NC_000002.11

cg17011276	4.72E-12	2.66E-13	7.85	19.3158357	1.84E-01	140797521	140797644	NC_000005.9
cg17943279	4.72E-12	2.66E-13	7.85	19.3150072	1.35E-01	72469560	72469683	NC_000008.10
cg05859308	4.73E-12	2.66E-13	7.85	19.3125835	1.20E-01	71712645	71712768	NC_000014.8
cg17207266	4.73E-12	2.67E-13	7.85	19.3120042	1.29E-01	25562957	25563080	NC_000002.11
cg23953290	4.74E-12	2.67E-13	7.85	19.3113372	4.09E-02	111048024	111048147	NC_000012.11
cg00303641	4.74E-12	2.67E-13	7.85	19.3112757	7.61E-02	23712764	23712887	NC_000016.9
cg00114132	4.74E-12	2.67E-13	7.85	19.3094294	1.02E-01	8657945	8658068	NC_000018.9
cg07602740	4.74E-12	2.67E-13	7.85	19.3093881	9.35E-02	32025960	32026083	NC_000006.11
cg00458395	4.74E-12	2.67E-13	7.85	19.309359	5.55E-02	13104674	13104797	NC_000019.9
cg06696905	4.75E-12	2.68E-13	7.85	19.3080606	1.01E-01	57539284	57539407	NC_000003.11
cg11338643	4.75E-12	2.68E-13	7.85	19.3077978	2.46E-01	166580983	166581106	NC_000006.11
cg14489474	4.76E-12	2.68E-13	7.85	19.3064888	1.48E-01	905611	905734	NC_000018.9
cg03801902	4.76E-12	2.68E-13	7.85	19.3055547	2.81E-01	4381800	4381923	NC_000012.11
cg22130262	4.76E-12	2.68E-13	7.85	19.3052677	1.80E-01	57026502	57026625	NC_000008.10
cg02696910	4.77E-12	2.69E-13	7.85	19.303413	9.79E-02	234683776	234683899	NC_000002.11
cg27377339	4.77E-12	2.69E-13	7.85	19.3032951	8.66E-02	155952301	155952424	NC_000007.13
cg02013148	4.78E-12	2.70E-13	7.85	19.301272	1.41E-01	149238478	149238601	NC_000003.11
cg18082638	4.78E-12	2.70E-13	7.85	19.3005446	1.95E-01	31827757	31827880	NC_000011.9
cg02483484	4.78E-12	2.70E-13	7.85	19.3002945	2.03E-01	4716537	4716660	NC_000001.10
cg15694715	4.79E-12	2.70E-13	7.85	19.2997593	2.18E-01	118402280	118402403	NC_000011.9
cg13334277	4.79E-12	2.70E-13	7.85	19.2993858	1.04E-01	88757878	88758001	NC_000006.11
cg06997381	4.79E-12	2.70E-13	7.85	19.2988152	2.65E-01	6449090	6449213	NC_000005.9
cg19079845	4.81E-12	2.71E-13	7.85	19.2948954	1.97E-01	21686308	21686431	NC_000020.10
cg23381863	4.81E-12	2.71E-13	7.85	19.2947864	5.83E-02	147165164	147165287	NC_000004.11
cg24862548	4.82E-12	2.72E-13	7.85	19.2919727	5.09E-02	97346037	97346160	NC_000008.10

cg10996058	4.82E-12	2.72E-13	7.85	19.2918858	1.69E-01	119915729	119915852	NC_000002.11
cg00708598	4.83E-12	2.73E-13	7.85	19.2901691	1.53E-01	53313895	53314018	NC_000013.10
cg26613361	4.83E-12	2.73E-13	7.85	19.2897905	1.23E-01	31830668	31830791	NC_000016.9
cg17291500	4.83E-12	2.73E-13	7.85	19.2893665	5.24E-02	550508	550631	NC_000007.13
cg05371498	4.84E-12	2.73E-13	7.85	19.2884157	9.93E-02	10989530	10989653	NC_000016.9
cg13914083	4.84E-12	2.73E-13	7.85	19.2878206	2.08E-01	158141526	158141649	NC_000004.11
cg25272065	4.84E-12	2.73E-13	7.85	19.2875474	1.83E-01	77334678	77334801	NC_000001.10
cg09194778	4.84E-12	2.73E-13	7.85	19.2874044	5.98E-02	792749	792872	NC_000006.11
cg16028175	4.84E-12	2.73E-13	7.85	19.2870083	1.76E-01	50059164	50059287	NC_000019.9
cg24580782	4.85E-12	2.74E-13	7.85	19.2845299	1.83E-01	27240365	27240488	NC_000001.10
cg21733531	4.86E-12	2.74E-13	7.85	19.2836815	1.12E-01	63560711	63560834	NC_000007.13
cg13211683	4.86E-12	2.75E-13	7.85	19.282708	2.15E-01	97171827	97171950	NC_000008.10
cg03646291	4.87E-12	2.75E-13	7.85	19.2819106	1.27E-01	41109598	41109721	NC_000013.10
cg22907258	4.87E-12	2.75E-13	7.85	19.2808761	3.82E-02	75141096	75141219	NC_000017.10
cg07964097	4.87E-12	2.75E-13	7.85	19.2798322	4.67E-02	131708081	131708204	NC_000012.11
cg19530178	4.88E-12	2.75E-13	7.85	19.2797435	6.09E-02	37578528	37578651	NC_000022.10
cg17902947	4.88E-12	2.75E-13	7.85	19.2796666	1.02E-01	57040807	57040930	NC_000001.10
cg13327545	4.88E-12	2.76E-13	7.85	19.279155	1.09E-01	22623548	22623671	NC_000010.10
cg15514848	4.88E-12	2.76E-13	7.85	19.2775527	1.80E-01	171217555	171217678	NC_000001.10
cg10514714	4.89E-12	2.77E-13	7.85	19.2754892	8.38E-02	111015299	111015422	NC_000001.10
cg18110883	4.90E-12	2.77E-13	7.84	19.2735231	5.10E-02	9009066	9009189	NC_000003.11
cg11113066	4.91E-12	2.78E-13	7.84	19.2716566	1.26E-01	101281981	101282104	NC_000010.10
cg05771369	4.92E-12	2.79E-13	7.84	19.2688312	1.52E-01	58021713	58021836	NC_000012.11
cg19227485	4.93E-12	2.79E-13	7.84	19.2672266	4.63E-02	33773898	33774021	NC_000017.10
cg22735419	4.93E-12	2.79E-13	7.84	19.2670904	3.82E-02	1360208	1360331	NC_000019.9

cg05375309	4.93E-12	2.79E-13	7.84	19.2669879	1.15E-01	79621517	79621640	NC_000006.11
cg10099105	4.94E-12	2.80E-13	7.84	19.265229	1.10E-01	155093457	155093580	NC_000007.13
cg21114773	4.94E-12	2.80E-13	7.84	19.2652157	2.15E-01	12254452	12254575	NC_000018.9
cg25631688	4.94E-12	2.80E-13	7.84	19.2645361	1.08E-01	93502004	93502127	NC_000012.11
cg13187820	4.95E-12	2.80E-13	7.84	19.2635187	1.19E-01	98960562	98960685	NC_000013.10
cg24154937	4.95E-12	2.80E-13	7.84	19.2630439	1.43E-01	5153711	5153834	NC_000012.11
cg04154502	4.95E-12	2.80E-13	7.84	19.262398	7.20E-02	3831293	3831416	NC_000019.9
cg20524972	4.96E-12	2.80E-13	7.84	19.2620569	4.96E-02	107219895	107220018	NC_000013.10
cg07717083	4.96E-12	2.81E-13	7.84	19.2615354	1.25E-01	32051857	32051980	NC_000006.11
cg22273168	4.96E-12	2.81E-13	7.84	19.2606572	5.98E-02	60827525	60827648	NC_000017.10
cg03821418	4.96E-12	2.81E-13	7.84	19.2602346	9.93E-02	124859683	124859806	NC_000012.11
cg12631713	4.96E-12	2.81E-13	7.84	19.2600732	7.98E-02	4771985	4772108	NC_000001.10
cg11670651	4.97E-12	2.81E-13	7.84	19.2596731	2.43E-01	119419106	119419229	NC_000012.11
cg22645209	4.97E-12	2.81E-13	7.84	19.259454	3.72E-02	97300809	97300932	NC_000002.11
cg20345915	4.97E-12	2.81E-13	7.84	19.2591967	1.11E-01	121296042	121296165	NC_000010.10
cg00027083	4.97E-12	2.81E-13	7.84	19.258828	2.24E-01	5543801	5543924	NC_000018.9
cg12505146	4.97E-12	2.82E-13	7.84	19.2577908	2.49E-01	72322388	72322511	NC_000017.10
cg22029275	4.98E-12	2.82E-13	7.84	19.2575741	2.83E-01	25745784	25745907	NC_000013.10
cg16197202	4.99E-12	2.83E-13	7.84	19.2546144	7.49E-02	133643702	133643825	NC_000010.10
cg26649904	4.99E-12	2.83E-13	7.84	19.2538669	1.92E-01	236407361	236407484	NC_000002.11
cg08928882	4.99E-12	2.83E-13	7.84	19.2537103	2.22E-01	77593362	77593485	NC_000008.10
cg12315466	5.02E-12	2.84E-13	7.84	19.2490995	7.44E-02	233923814	233923937	NC_000002.11
cg13069385	5.02E-12	2.84E-13	7.84	19.2490208	1.17E-01	38822397	38822520	NC_000017.10
cg12899423	5.03E-12	2.85E-13	7.84	19.2464941	1.17E-01	44328154	44328277	NC_000011.9
cg04307561	5.04E-12	2.86E-13	7.84	19.2434585	2.66E-01	72460119	72460242	NC_000008.10

cg16766249	5.06E-12	2.87E-13	7.84	19.2393224	1.95E-01	79745154	79745277	NC_000014.8
cg09657963	5.07E-12	2.87E-13	7.84	19.2387433	2.14E-01	124790875	124790998	NC_000011.9
cg09305096	5.07E-12	2.87E-13	7.84	19.2379041	1.51E-01	189191655	189191778	NC_000002.11
cg16424082	5.08E-12	2.88E-13	7.84	19.235963	8.75E-02	51323740	51323863	NC_000012.11
cg08082810	5.08E-12	2.88E-13	7.84	19.2350504	1.51E-01	61108391	61108514	NC_000014.8
cg10274108	5.09E-12	2.88E-13	7.84	19.2342667	1.25E-01	38082931	38083054	NC_000004.11
cg08086724	5.10E-12	2.89E-13	7.84	19.2316473	1.83E-01	36973204	36973327	NC_000014.8
cg08512345	5.10E-12	2.89E-13	7.84	19.2314871	2.02E-02	112043275	112043398	NC_000005.9
cg23633700	5.11E-12	2.90E-13	7.84	19.2304614	1.47E-01	48016735	48016858	NC_000007.13
cg14129589	5.14E-12	2.91E-13	7.84	19.2240795	1.15E-01	3240109	3240232	NC_000012.11
cg26620450	5.15E-12	2.92E-13	7.84	19.2216619	1.93E-01	133195061	133195184	NC_000012.11
cg20820063	5.16E-12	2.93E-13	7.84	19.2191893	1.74E-01	31020627	31020750	NC_000018.9
cg15067340	5.17E-12	2.93E-13	7.84	19.2187195	2.39E-01	37488671	37488794	NC_000007.13
cg22711111	5.17E-12	2.93E-13	7.84	19.2184232	1.86E-01	30139979	30140102	NC_000006.11
cg12279518	5.18E-12	2.94E-13	7.84	19.215574	1.28E-01	85190400	85190523	NC_000001.10
cg04420991	5.19E-12	2.94E-13	7.83	19.2141059	6.59E-02	77067214	77067337	NC_000004.11
cg08309183	5.19E-12	2.95E-13	7.83	19.2128677	1.25E-01	150674375	150674498	NC_000007.13
cg06682197	5.19E-12	2.95E-13	7.83	19.2128609	2.04E-01	88324879	88325002	NC_000013.10
cg15020425	5.20E-12	2.95E-13	7.83	19.2119021	1.68E-01	69633716	69633839	NC_000011.9
cg02646491	5.22E-12	2.96E-13	7.83	19.2074302	1.41E-01	2890710	2890833	NC_000011.9
cg13644317	5.24E-12	2.97E-13	7.83	19.2048504	1.83E-01	70536461	70536584	NC_000018.9
cg03120091	5.24E-12	2.97E-13	7.83	19.204227	1.96E-01	132052779	132052902	NC_000008.10
cg22123464	5.24E-12	2.97E-13	7.83	19.2041285	2.11E-01	47974901	47975024	NC_000019.9
cg08512702	5.25E-12	2.98E-13	7.83	19.2018121	2.35E-01	13548915	13549038	NC_000004.11
cg23708361	5.25E-12	2.98E-13	7.83	19.20123	2.17E-01	145813432	145813555	NC_000007.13

cg26210445	5.26E-12	2.99E-13	7.83	19.1999881	2.39E-01	162273326	162273449	NC_000002.11
cg13590117	5.27E-12	2.99E-13	7.83	19.1986803	1.13E-01	138057965	138058088	NC_000009.11
cg05658491	5.28E-12	3.00E-13	7.83	19.1964345	1.78E-01	240256014	240256137	NC_000001.10
cg04653893	5.28E-12	3.00E-13	7.83	19.1952741	1.03E-01	20996560	20996683	NC_000016.9
cg02379973	5.29E-12	3.00E-13	7.83	19.1941634	9.52E-02	57478107	57478230	NC_000012.11
cg19003797	5.31E-12	3.01E-13	7.83	19.1907178	1.99E-01	137819267	137819390	NC_000006.11
cg24645214	5.31E-12	3.02E-13	7.83	19.1895043	2.24E-01	54789978	54790101	NC_000008.10
cg11138769	5.32E-12	3.02E-13	7.83	19.1879608	3.90E-02	134117039	134117162	NC_000011.9
cg22976218	5.32E-12	3.02E-13	7.83	19.1875696	1.91E-01	182544321	182544444	NC_000002.11
cg26464221	5.32E-12	3.02E-13	7.83	19.1875259	1.75E-01	119801431	119801554	NC_000005.9
cg03269976	5.33E-12	3.03E-13	7.83	19.1866222	8.15E-02	240658243	240658366	NC_000002.11
cg21296676	5.33E-12	3.03E-13	7.83	19.1865295	1.41E-01	133561660	133561783	NC_000006.11
cg08996678	5.33E-12	3.03E-13	7.83	19.1853592	7.94E-02	30113783	30113906	NC_000006.11
cg05855917	5.33E-12	3.03E-13	7.83	19.1851774	2.52E-01	147113700	147113823	NC_000003.11
cg01678091	5.34E-12	3.03E-13	7.83	19.1843777	1.11E-01	23890686	23890809	NC_000015.9
cg22400703	5.34E-12	3.04E-13	7.83	19.1839213	1.48E-01	68545995	68546118	NC_000002.11
cg07790279	5.34E-12	3.04E-13	7.83	19.183539	1.28E-01	125851132	125851255	NC_000010.10
cg06897145	5.34E-12	3.04E-13	7.83	19.1831139	1.22E-01	3238937	3239060	NC_000016.9
cg23973626	5.35E-12	3.04E-13	7.83	19.1820191	2.15E-02	18451326	18451449	NC_000019.9
cg07035659	5.37E-12	3.05E-13	7.83	19.1781322	1.45E-01	4859975	4860098	NC_000004.11
cg22631350	5.37E-12	3.06E-13	7.83	19.1773635	7.63E-02	22555243	22555366	NC_000008.10
cg26717983	5.38E-12	3.06E-13	7.83	19.1762875	1.89E-01	72596472	72596595	NC_000006.11
cg01045835	5.38E-12	3.06E-13	7.83	19.1760806	1.01E-01	82969530	82969653	NC_000005.9
cg10297191	5.39E-12	3.06E-13	7.83	19.1749187	9.41E-02	46620661	46620784	NC_000006.11
cg25460731	5.40E-12	3.07E-13	7.83	19.172847	9.58E-02	67549363	67549486	NC_000002.11

cg25913520	5.41E-12	3.08E-13	7.83	19.1697092	1.43E-01	13524041	13524164	NC_000004.11
cg15400629	5.42E-12	3.08E-13	7.83	19.1683958	1.44E-01	13548243	13548366	NC_000004.11
cg24891539	5.42E-12	3.08E-13	7.83	19.1683898	1.95E-01	55370407	55370530	NC_000008.10
cg07464977	5.44E-12	3.10E-13	7.83	19.1647784	1.90E-01	2097136	2097259	NC_000005.9
cg14660788	5.45E-12	3.10E-13	7.83	19.1630688	9.45E-02	3625312	3625435	NC_000001.10
cg11919837	5.45E-12	3.10E-13	7.83	19.1618245	1.38E-01	57350735	57350858	NC_000008.10
cg14553338	5.47E-12	3.11E-13	7.83	19.1590662	8.70E-02	66689807	66689930	NC_000002.11
cg21920775	5.47E-12	3.11E-13	7.83	19.1586714	2.25E-01	114696641	114696764	NC_000001.10
cg11850773	5.47E-12	3.12E-13	7.83	19.1581687	2.05E-01	904963	905086	NC_000018.9
cg11718030	5.47E-12	3.12E-13	7.83	19.1580083	1.97E-01	27744363	27744486	NC_000011.9
cg12625300	5.48E-12	3.12E-13	7.83	19.156765	3.94E-02	2959621	2959744	NC_000006.11
cg11929898	5.50E-12	3.13E-13	7.82	19.1531034	1.14E-01	111760010	111760133	NC_000003.11
cg17004711	5.51E-12	3.14E-13	7.82	19.1509694	7.79E-02	4875911	4876034	NC_000007.13
cg11346128	5.51E-12	3.14E-13	7.82	19.1505667	9.28E-02	142465233	142465356	NC_000006.11
cg19754554	5.52E-12	3.14E-13	7.82	19.149381	1.73E-01	66258687	66258810	NC_000001.10
cg02228111	5.53E-12	3.15E-13	7.82	19.1472684	2.14E-01	132905819	132905942	NC_000012.11
cg17838420	5.53E-12	3.15E-13	7.82	19.1472327	1.44E-01	1877906	1878029	NC_000005.9
cg13294846	5.54E-12	3.16E-13	7.82	19.1458526	2.39E-01	2112351	2112474	NC_000005.9
cg08301572	5.54E-12	3.16E-13	7.82	19.1450546	2.26E-01	23300243	23300366	NC_000019.9
cg09879526	5.55E-12	3.16E-13	7.82	19.1429292	3.56E-02	243652762	243652885	NC_000001.10
cg25367890	5.57E-12	3.18E-13	7.82	19.1395356	2.99E-02	67815048	67815171	NC_000011.9
cg01519784	5.57E-12	3.18E-13	7.82	19.1391891	1.83E-01	36450896	36451019	NC_000019.9
cg12930573	5.57E-12	3.18E-13	7.82	19.1389442	2.08E-02	182971009	182971132	NC_000003.11
cg27648738	5.57E-12	3.18E-13	7.82	19.1388594	2.99E-01	84115811	84115934	NC_000015.9
cg20611276	5.58E-12	3.18E-13	7.82	19.1368464	1.77E-01	133561866	133561989	NC_000006.11

cg01791874	5.59E-12	3.19E-13	7.82	19.1360276	2.23E-01	16180055	16180178	NC_000005.9
cg22052948	5.59E-12	3.19E-13	7.82	19.135513	1.79E-01	140778342	140778465	NC_000005.9
cg22717825	5.59E-12	3.19E-13	7.82	19.1353622	7.87E-02	20250298	20250421	NC_000001.10
cg16208657	5.62E-12	3.21E-13	7.82	19.130315	1.42E-01	68119293	68119416	NC_000015.9
cg13210260	5.62E-12	3.21E-13	7.82	19.1296157	1.76E-01	50889294	50889417	NC_000001.10
cg03935956	5.63E-12	3.21E-13	7.82	19.1278849	1.00E-01	23511855	23511978	NC_000016.9
cg21688264	5.64E-12	3.22E-13	7.82	19.1264834	2.19E-01	84418724	84418847	NC_000006.11
cg08084616	5.64E-12	3.22E-13	7.82	19.1256316	1.83E-01	132110831	132110954	NC_000002.11
cg23407561	5.65E-12	3.22E-13	7.82	19.1248243	6.54E-02	113544232	113544355	NC_000002.11
cg26123605	5.65E-12	3.22E-13	7.82	19.1244631	2.09E-01	147141614	147141737	NC_000003.11
cg17479280	5.66E-12	3.23E-13	7.82	19.1228764	1.24E-01	156466088	156466211	NC_000001.10
cg19580752	5.66E-12	3.23E-13	7.82	19.1226972	2.50E-01	135050355	135050478	NC_000010.10
cg05735384	5.69E-12	3.25E-13	7.82	19.1176065	1.67E-01	32639426	32639549	NC_000015.9
cg18848394	5.69E-12	3.25E-13	7.82	19.1173462	1.13E-01	39597909	39598032	NC_000017.10
cg02039465	5.69E-12	3.25E-13	7.82	19.1172989	7.18E-02	252450	252573	NC_000012.11
cg17457637	5.69E-12	3.25E-13	7.82	19.1159525	1.58E-01	27170717	27170840	NC_000007.13
cg02644867	5.69E-12	3.25E-13	7.82	19.1158427	1.63E-01	1271057	1271180	NC_000019.9
cg02964287	5.70E-12	3.26E-13	7.82	19.1141273	9.51E-02	28887830	28887953	NC_000016.9
cg25175900	5.71E-12	3.26E-13	7.82	19.1129599	8.50E-02	22854001	22854124	NC_000012.11
cg15213604	5.71E-12	3.26E-13	7.82	19.1128824	1.17E-01	79890769	79890892	NC_000017.10
cg23089838	5.71E-12	3.26E-13	7.82	19.1128682	1.91E-01	13424419	13424542	NC_000008.10
cg12676149	5.71E-12	3.26E-13	7.82	19.1123301	1.93E-01	30139634	30139757	NC_000006.11
cg07639287	5.73E-12	3.27E-13	7.82	19.1098568	1.50E-01	17850911	17851034	NC_000022.10
cg01891966	5.73E-12	3.28E-13	7.82	19.109121	1.37E-01	27202344	27202467	NC_000007.13
cg26498616	5.74E-12	3.28E-13	7.82	19.1079382	1.24E-01	1223240	1223363	NC_000005.9

cg01972843	5.74E-12	3.28E-13	7.82	19.107504	1.26E-01	17680706	17680829	NC_000022.10
cg00923458	5.75E-12	3.29E-13	7.82	19.1057104	4.09E-02	168514532	168514655	NC_000001.10
cg12246165	5.77E-12	3.30E-13	7.82	19.1014556	5.45E-02	46972489	46972612	NC_000021.8
cg20996351	5.78E-12	3.31E-13	7.82	19.100049	1.45E-01	27687232	27687355	NC_000001.10
cg19084726	5.79E-12	3.31E-13	7.82	19.0991397	1.98E-01	145075538	145075661	NC_000001.10
cg09809416	5.80E-12	3.32E-13	7.82	19.0960364	1.34E-01	151970131	151970254	NC_000001.10
cg04854226	5.81E-12	3.32E-13	7.81	19.0947672	1.56E-01	96910513	96910636	NC_000015.9
cg03835158	5.82E-12	3.33E-13	7.81	19.0935508	3.68E-02	52773607	52773730	NC_000008.10
cg19445269	5.84E-12	3.34E-13	7.81	19.089954	7.97E-02	39171830	39171953	NC_000002.11
cg08202494	5.84E-12	3.34E-13	7.81	19.0896745	1.41E-01	99961545	99961668	NC_000008.10
cg24843380	5.84E-12	3.34E-13	7.81	19.0891045	2.48E-01	178367827	178367950	NC_000005.9
cg04217778	5.86E-12	3.35E-13	7.81	19.0865213	1.96E-01	5111916	5112039	NC_000007.13
cg23055499	5.87E-12	3.36E-13	7.81	19.0840959	9.21E-02	51847363	51847486	NC_000012.11
cg21645973	5.87E-12	3.36E-13	7.81	19.0840405	9.52E-02	17914070	17914193	NC_000001.10
cg05470523	5.87E-12	3.36E-13	7.81	19.0836238	1.55E-01	49814781	49814904	NC_000007.13
cg15617139	5.89E-12	3.37E-13	7.81	19.0805565	4.19E-02	130731583	130731706	NC_000011.9
cg10989326	5.90E-12	3.38E-13	7.81	19.0793302	6.29E-02	93258908	93259031	NC_000001.10
cg10082589	5.90E-12	3.38E-13	7.81	19.0789556	1.18E-01	192712920	192713043	NC_000002.11
cg24985672	5.90E-12	3.38E-13	7.81	19.0777798	1.41E-01	67600231	67600354	NC_000015.9
cg06800421	5.91E-12	3.38E-13	7.81	19.0769081	5.78E-02	93854668	93854791	NC_000010.10
cg18404513	5.91E-12	3.39E-13	7.81	19.0760524	1.00E-01	48587440	48587563	NC_000008.10
cg27134816	5.92E-12	3.39E-13	7.81	19.0758857	1.96E-01	102569815	102569938	NC_000013.10
cg07326665	5.92E-12	3.39E-13	7.81	19.075253	1.14E-01	334445	334568	NC_000016.9
cg01352175	5.92E-12	3.39E-13	7.81	19.0751607	1.50E-01	44773985	44774108	NC_000018.9
cg22043168	5.92E-12	3.39E-13	7.81	19.0742633	2.19E-01	27741077	27741200	NC_000011.9

cg24359109	5.94E-12	3.40E-13	7.81	19.0715125	1.60E-01	245772794	245772917	NC_000001.10
cg19397321	5.94E-12	3.40E-13	7.81	19.0710739	3.02E-02	43404741	43404864	NC_000018.9
cg06607919	5.94E-12	3.40E-13	7.81	19.0707723	1.75E-01	80549797	80549920	NC_000002.11
cg08884928	5.94E-12	3.41E-13	7.81	19.070597	1.53E-01	57431996	57432119	NC_000017.10
cg26220673	5.96E-12	3.42E-13	7.81	19.0674799	1.49E-01	221064700	221064823	NC_000001.10
cg04178266	5.97E-12	3.42E-13	7.81	19.0665898	1.77E-01	41258790	41258913	NC_000004.11
cg05393563	5.97E-12	3.42E-13	7.81	19.0659208	1.65E-02	45672071	45672194	NC_000001.10
cg13404421	5.97E-12	3.42E-13	7.81	19.0654522	2.62E-01	4382181	4382304	NC_000012.11
cg15190742	5.98E-12	3.43E-13	7.81	19.0647581	2.07E-01	42154140	42154263	NC_000004.11
cg06129556	5.98E-12	3.43E-13	7.81	19.0643187	3.94E-02	64634880	64635003	NC_000015.9
cg18646207	5.99E-12	3.43E-13	7.81	19.0631019	1.92E-01	118897836	118897959	NC_000010.10
cg03982801	5.99E-12	3.43E-13	7.81	19.0622436	1.33E-01	27168688	27168811	NC_000007.13
cg00133595	5.99E-12	3.44E-13	7.81	19.0617271	2.65E-01	31228059	31228182	NC_000016.9
cg04875212	6.00E-12	3.44E-13	7.81	19.0612234	1.02E-01	67830554	67830677	NC_000014.8
cg00436254	6.01E-12	3.44E-13	7.81	19.0595703	1.12E-01	127862614	127862737	NC_000002.11
cg16103732	6.01E-12	3.45E-13	7.81	19.0585577	4.81E-02	86370663	86370786	NC_000013.10
cg15805540	6.03E-12	3.46E-13	7.81	19.055385	1.87E-01	64286415	64286538	NC_000006.11
cg21293902	6.03E-12	3.46E-13	7.81	19.0550701	1.11E-01	49994097	49994220	NC_000019.9
cg19529273	6.08E-12	3.49E-13	7.81	19.0477542	4.41E-02	63274743	63274866	NC_000011.9
cg24842760	6.08E-12	3.49E-13	7.81	19.0476616	1.63E-01	133561719	133561842	NC_000006.11
cg07197785	6.09E-12	3.49E-13	7.81	19.0459375	1.94E-01	111813690	111813813	NC_000001.10
cg04886672	6.09E-12	3.49E-13	7.81	19.0457896	3.75E-02	79215047	79215170	NC_000017.10
cg01755562	6.09E-12	3.49E-13	7.81	19.0454105	1.97E-01	139653545	139653668	NC_000003.11
cg26522297	6.09E-12	3.50E-13	7.81	19.0443456	1.46E-01	115829838	115829961	NC_000011.9
cg10680051	6.10E-12	3.50E-13	7.81	19.0436215	2.50E-01	124735089	124735212	NC_000011.9

cg11142705	6.12E-12	3.51E-13	7.81	19.0401623	2.12E-01	42306974	42307097	NC_000003.11
cg08916646	6.12E-12	3.51E-13	7.81	19.0400243	6.52E-02	122526031	122526154	NC_000008.10
cg15790526	6.15E-12	3.53E-13	7.8	19.03428	1.36E-01	123711842	123711965	NC_000003.11
cg01650245	6.15E-12	3.53E-13	7.8	19.0340437	7.43E-02	105626788	105626911	NC_000012.11
cg01883425	6.16E-12	3.54E-13	7.8	19.0337344	1.47E-01	41606770	41606893	NC_000006.11
cg11231143	6.16E-12	3.54E-13	7.8	19.0336306	1.11E-01	26554245	26554368	NC_000017.10
cg00339556	6.16E-12	3.54E-13	7.8	19.0331813	2.21E-01	16180048	16180171	NC_000005.9
cg08506654	6.20E-12	3.56E-13	7.8	19.0267196	1.50E-01	172673098	172673221	NC_000005.9
cg17633592	6.21E-12	3.57E-13	7.8	19.0241992	1.84E-01	112758599	112758722	NC_000013.10
cg00041641	6.21E-12	3.57E-13	7.8	19.0237972	7.09E-02	151024614	151024737	NC_000001.10
cg27107094	6.23E-12	3.58E-13	7.8	19.0220676	1.04E-01	37768915	37769038	NC_000022.10
cg17453780	6.24E-12	3.59E-13	7.8	19.0187684	2.08E-01	113013048	113013171	NC_000012.11
cg13389502	6.26E-12	3.60E-13	7.8	19.0165059	1.11E-01	1961440	1961563	NC_000017.10
cg18063493	6.26E-12	3.60E-13	7.8	19.0153649	2.23E-02	43196221	43196344	NC_000017.10
cg26866452	6.28E-12	3.61E-13	7.8	19.0126561	8.52E-02	77857909	77858032	NC_000015.9
cg06457317	6.29E-12	3.62E-13	7.8	19.0106071	2.71E-01	20185267	20185390	NC_000011.9
cg25881804	6.31E-12	3.63E-13	7.8	19.0081662	2.43E-01	21081901	21082024	NC_000020.10
cg08702941	6.33E-12	3.64E-13	7.8	19.004687	8.43E-02	42155308	42155431	NC_000004.11
cg22601348	6.34E-12	3.64E-13	7.8	19.0036358	2.39E-01	69243904	69244027	NC_000008.10
cg16499166	6.35E-12	3.65E-13	7.8	19.00099	4.20E-02	88808981	88809104	NC_000016.9
cg11754900	6.35E-12	3.66E-13	7.8	19.0007388	6.86E-02	101758860	101758983	NC_000004.11
cg26236177	6.37E-12	3.66E-13	7.8	18.9982185	1.95E-01	61110649	61110772	NC_000014.8
cg27072996	6.37E-12	3.67E-13	7.8	18.9981483	1.93E-01	822635	822758	NC_000019.9
cg22340747	6.39E-12	3.68E-13	7.8	18.9952833	1.44E-01	45683210	45683333	NC_000015.9
cg06103218	6.40E-12	3.69E-13	7.8	18.9925657	1.58E-01	2073751	2073874	NC_000004.11

cg25114781	6.41E-12	3.69E-13	7.8	18.990574	1.45E-01	92996919	92997042	NC_000014.8
cg13543106	6.42E-12	3.70E-13	7.8	18.9899862	1.27E-01	90086806	90086929	NC_000003.11
cg17037101	6.42E-12	3.70E-13	7.8	18.989655	2.09E-01	2161314	2161437	NC_000011.9
cg01287975	6.45E-12	3.71E-13	7.8	18.9851856	2.14E-01	97361241	97361364	NC_000007.13
cg25144111	6.46E-12	3.72E-13	7.8	18.9834062	6.28E-02	127796312	127796435	NC_000006.11
cg27345346	6.47E-12	3.73E-13	7.8	18.9813509	7.82E-02	36259144	36259267	NC_000019.9
cg12612393	6.48E-12	3.73E-13	7.8	18.9803599	1.35E-01	3474376	3474499	NC_000001.10
cg07872979	6.50E-12	3.75E-13	7.79	18.9761723	1.09E-01	94771165	94771288	NC_000011.9
cg13951074	6.52E-12	3.76E-13	7.79	18.9730561	1.47E-01	1045628	1045751	NC_000009.11
cg11655243	6.52E-12	3.76E-13	7.79	18.9726921	1.82E-01	140778396	140778519	NC_000005.9
cg09832102	6.53E-12	3.77E-13	7.79	18.9713194	1.26E-01	75503269	75503392	NC_000017.10
cg20411075	6.53E-12	3.77E-13	7.79	18.9711476	4.18E-02	21677723	21677846	NC_000013.10
cg26202847	6.54E-12	3.77E-13	7.79	18.9696893	1.68E-01	153855353	153855476	NC_000005.9
cg11732619	6.55E-12	3.77E-13	7.79	18.9692316	2.10E-01	168728076	168728199	NC_000005.9
cg17192381	6.55E-12	3.78E-13	7.79	18.9688136	1.19E-01	60792598	60792721	NC_000018.9
cg23434186	6.55E-12	3.78E-13	7.79	18.9684143	1.38E-01	67068730	67068853	NC_000018.9
cg04419610	6.56E-12	3.78E-13	7.79	18.9676697	1.88E-01	48946564	48946687	NC_000019.9
cg20887073	6.56E-12	3.78E-13	7.79	18.9671799	1.63E-01	10381764	10381887	NC_000006.11
cg17856495	6.57E-12	3.79E-13	7.79	18.9654613	5.33E-02	1691867	1691990	NC_000012.11
cg24977338	6.57E-12	3.79E-13	7.79	18.9652271	1.15E-01	113188963	113189086	NC_000002.11
cg02509825	6.59E-12	3.80E-13	7.79	18.9618897	1.55E-01	56015399	56015522	NC_000008.10
cg20139338	6.59E-12	3.80E-13	7.79	18.9617154	1.74E-01	64120656	64120779	NC_000011.9
cg10217445	6.59E-12	3.80E-13	7.79	18.9613866	1.65E-01	34397784	34397907	NC_000021.8
cg17018021	6.60E-12	3.81E-13	7.79	18.9608631	7.43E-02	1029394	1029517	NC_000017.10
cg21748223	6.62E-12	3.82E-13	7.79	18.9578896	1.96E-01	164264853	164264976	NC_000004.11

cg02005600	6.62E-12	3.82E-13	7.79	18.957879	1.51E-01	27183686	27183809	NC_000007.13
cg11573170	6.62E-12	3.82E-13	7.79	18.9568379	1.40E-01	556762	556885	NC_000010.10
cg02216246	6.64E-12	3.83E-13	7.79	18.9540047	2.20E-01	128564756	128564879	NC_000011.9
cg17277892	6.67E-12	3.85E-13	7.79	18.9499048	2.14E-01	69591803	69591926	NC_000003.11
cg00997079	6.67E-12	3.85E-13	7.79	18.949453	1.55E-01	80529990	80530113	NC_000002.11
cg06959053	6.67E-12	3.85E-13	7.79	18.949224	1.98E-01	79382548	79382671	NC_000015.9
cg07895132	6.67E-12	3.85E-13	7.79	18.9490992	1.28E-01	33825172	33825295	NC_000017.10
cg25967612	6.67E-12	3.85E-13	7.79	18.9486821	9.08E-02	59482691	59482814	NC_000011.9
cg27599792	6.68E-12	3.85E-13	7.79	18.9483882	1.22E-01	140792992	140793115	NC_000005.9
cg07751669	6.68E-12	3.86E-13	7.79	18.9482244	1.28E-01	114546247	114546370	NC_000009.11
cg26638505	6.68E-12	3.86E-13	7.79	18.9477145	1.27E-01	31806183	31806306	NC_000002.11
cg27129048	6.69E-12	3.86E-13	7.79	18.9461412	1.46E-01	92938576	92938699	NC_000015.9
cg14549051	6.69E-12	3.87E-13	7.79	18.9457272	7.60E-02	201131810	201131933	NC_000002.11
cg19819285	6.69E-12	3.87E-13	7.79	18.9455909	1.59E-01	58545290	58545413	NC_000019.9
cg26282762	6.71E-12	3.88E-13	7.79	18.9430877	1.50E-01	1356444	1356567	NC_000019.9
cg10967023	6.72E-12	3.88E-13	7.79	18.9408988	2.12E-01	115134886	115135009	NC_000012.11
cg14492293	6.74E-12	3.90E-13	7.79	18.9374329	1.83E-01	139093852	139093975	NC_000009.11
cg22063654	6.75E-12	3.90E-13	7.79	18.9372354	1.60E-01	72758701	72758824	NC_000008.10
cg22544416	6.75E-12	3.90E-13	7.79	18.9370042	6.41E-02	46859899	46860022	NC_000022.10
cg26988213	6.77E-12	3.91E-13	7.79	18.9340018	8.30E-02	128496531	128496654	NC_000007.13
cg13891768	6.78E-12	3.92E-13	7.79	18.932195	1.97E-01	111536024	111536147	NC_000004.11
cg19418922	6.78E-12	3.92E-13	7.79	18.932072	6.56E-02	44265840	44265963	NC_000011.9
cg06302566	6.79E-12	3.92E-13	7.79	18.9307531	1.29E-01	121150686	121150809	NC_000012.11
cg22867315	6.79E-12	3.92E-13	7.79	18.9306744	9.06E-02	153476415	153476538	NC_000006.11
cg01667646	6.80E-12	3.93E-13	7.79	18.9288573	2.06E-01	84116018	84116141	NC_000015.9

cg13790906	6.80E-12	3.93E-13	7.79	18.928251	4.60E-02	20564145	20564268	NC_000015.9
cg09491536	6.80E-12	3.93E-13	7.79	18.9281937	1.36E-01	3787915	3788038	NC_000005.9
cg18257839	6.81E-12	3.94E-13	7.79	18.9276345	4.33E-02	63369284	63369407	NC_000015.9
cg20249252	6.81E-12	3.94E-13	7.79	18.9271176	2.10E-01	114852308	114852431	NC_000012.11
cg23272088	6.82E-12	3.95E-13	7.79	18.9255898	2.23E-01	2504813	2504936	NC_000011.9
cg01255486	6.83E-12	3.95E-13	7.79	18.9247691	1.42E-01	112199323	112199446	NC_000005.9
cg25461249	6.83E-12	3.95E-13	7.79	18.9241373	1.54E-01	1019317	1019440	NC_000008.10
cg17081489	6.86E-12	3.97E-13	7.79	18.919865	7.78E-02	177953700	177953823	NC_000001.10
cg19846491	6.86E-12	3.97E-13	7.79	18.9191881	1.62E-01	79322924	79323047	NC_000017.10
cg20727555	6.88E-12	3.98E-13	7.78	18.9165163	4.57E-02	57910480	57910603	NC_000012.11
cg16512661	6.88E-12	3.98E-13	7.78	18.9163744	1.24E-01	2743620	2743743	NC_000005.9
cg18693395	6.89E-12	3.99E-13	7.78	18.9148474	2.37E-01	52735421	52735544	NC_000014.8
cg03417156	6.89E-12	3.99E-13	7.78	18.9148031	8.70E-02	14043646	14043769	NC_000019.9
cg00768993	6.89E-12	3.99E-13	7.78	18.9144629	1.90E-01	200335874	200335997	NC_000002.11
cg20449726	6.90E-12	3.99E-13	7.78	18.9137508	1.33E-01	15276987	15277110	NC_000001.10
cg06204735	6.90E-12	3.99E-13	7.78	18.9133223	2.50E-01	74892986	74893109	NC_000014.8
cg14925024	6.90E-12	4.00E-13	7.78	18.9126764	1.56E-01	228291705	228291828	NC_000001.10
cg09246253	6.91E-12	4.00E-13	7.78	18.9117798	8.40E-02	5643021	5643144	NC_000007.13
cg14392725	6.92E-12	4.01E-13	7.78	18.9106278	7.58E-02	109893226	109893349	NC_000009.11
cg00326251	6.92E-12	4.01E-13	7.78	18.9096919	1.33E-01	23262648	23262771	NC_000008.10
cg19613624	6.93E-12	4.01E-13	7.78	18.9085124	8.68E-02	56223757	56223880	NC_000020.10
cg02936752	6.93E-12	4.01E-13	7.78	18.9083082	1.37E-01	54963326	54963449	NC_000019.9
cg00178562	6.96E-12	4.03E-13	7.78	18.9041343	7.77E-02	16966354	16966477	NC_000001.10
cg13939055	6.96E-12	4.03E-13	7.78	18.9039127	1.25E-01	244094869	244094992	NC_000001.10
cg24345260	6.97E-12	4.04E-13	7.78	18.9018951	5.62E-02	2952979	2953102	NC_000007.13

cg22398359	6.98E-12	4.05E-13	7.78	18.9008576	5.73E-02	53558066	53558189	NC_000001.10
cg19707326	6.99E-12	4.05E-13	7.78	18.8991686	7.77E-02	24787611	24787734	NC_000014.8
cg23730788	6.99E-12	4.05E-13	7.78	18.8988054	8.97E-02	241581277	241581400	NC_000002.11
cg26276327	7.00E-12	4.06E-13	7.78	18.8977153	1.49E-01	247511469	247511592	NC_000001.10
cg19807257	7.01E-12	4.06E-13	7.78	18.8970624	2.32E-01	46951354	46951477	NC_000001.10
cg15126179	7.01E-12	4.06E-13	7.78	18.8968222	1.27E-01	79181338	79181461	NC_000013.10
cg12277789	7.02E-12	4.07E-13	7.78	18.8954544	1.13E-01	125033770	125033893	NC_000010.10
cg07828169	7.02E-12	4.07E-13	7.78	18.895129	1.14E-01	132114623	132114746	NC_000005.9
cg26368049	7.03E-12	4.08E-13	7.78	18.8932742	9.60E-02	49572730	49572853	NC_000003.11
cg11973479	7.06E-12	4.09E-13	7.78	18.8892958	1.66E-01	19152073	19152196	NC_000007.13
cg24651824	7.07E-12	4.10E-13	7.78	18.8874501	4.91E-02	104117048	104117171	NC_000004.11
cg19103770	7.09E-12	4.11E-13	7.78	18.8850808	1.71E-01	93520566	93520689	NC_000007.13
cg04396462	7.09E-12	4.11E-13	7.78	18.8850609	3.56E-02	63082613	63082736	NC_000003.11
cg20275739	7.10E-12	4.12E-13	7.78	18.8829097	5.09E-02	26692284	26692407	NC_000017.10
cg22265452	7.11E-12	4.12E-13	7.78	18.8821273	7.42E-02	213697031	213697154	NC_000002.11
cg05707116	7.11E-12	4.12E-13	7.78	18.8821115	1.11E-01	88924001	88924124	NC_000002.11
cg06539804	7.11E-12	4.13E-13	7.78	18.8812372	2.31E-01	2781489	2781612	NC_000020.10
cg12616941	7.11E-12	4.13E-13	7.78	18.8809018	1.63E-01	22612797	22612920	NC_000010.10
cg06889951	7.15E-12	4.15E-13	7.78	18.8759927	4.42E-02	69625218	69625341	NC_000011.9
cg00011459	7.15E-12	4.15E-13	7.78	18.8759272	4.67E-02	8890425	8890548	NC_000016.9
cg03202077	7.15E-12	4.15E-13	7.78	18.8752675	1.99E-01	28338836	28338959	NC_000021.8
cg01986717	7.17E-12	4.17E-13	7.78	18.871889	1.34E-01	35535021	35535144	NC_000019.9
cg06132028	7.19E-12	4.17E-13	7.78	18.8699023	1.96E-01	133562466	133562589	NC_000006.11
cg15917438	7.19E-12	4.18E-13	7.78	18.8696601	1.16E-01	170169088	170169211	NC_000005.9
cg26649384	7.19E-12	4.18E-13	7.78	18.8694124	2.11E-01	154334651	154334774	NC_000002.11

cg07595776	7.20E-12	4.18E-13	7.78	18.8675554	1.55E-01	77253990	77254113	NC_000005.9
cg06377964	7.21E-12	4.19E-13	7.78	18.866929	7.05E-02	129429172	129429295	NC_000012.11
cg17426084	7.21E-12	4.19E-13	7.78	18.8666529	1.07E-01	2293552	2293675	NC_000011.9
cg00557959	7.21E-12	4.19E-13	7.78	18.8663769	1.65E-01	113296414	113296537	NC_000013.10
cg21127999	7.22E-12	4.19E-13	7.78	18.8653044	7.69E-02	168476501	168476624	NC_000006.11
cg20987323	7.22E-12	4.20E-13	7.78	18.864855	7.89E-02	1474011	1474134	NC_000007.13
cg05467160	7.23E-12	4.20E-13	7.78	18.8643831	1.65E-01	176945526	176945649	NC_000002.11
cg21112485	7.23E-12	4.20E-13	7.78	18.8642337	2.52E-02	11808413	11808536	NC_000001.10
cg03428109	7.23E-12	4.20E-13	7.78	18.8641859	8.84E-02	207118288	207118411	NC_000002.11
cg06942701	7.23E-12	4.20E-13	7.78	18.8628827	1.04E-01	162280009	162280132	NC_000002.11
cg13301733	7.25E-12	4.21E-13	7.78	18.8613245	6.90E-02	114050884	114051007	NC_000002.11
cg27569203	7.26E-12	4.22E-13	7.78	18.8597949	4.64E-02	46835135	46835258	NC_000022.10
cg08697689	7.27E-12	4.23E-13	7.77	18.8575855	1.68E-01	84457340	84457463	NC_000013.10
cg19675103	7.30E-12	4.24E-13	7.77	18.8537515	1.08E-01	47513375	47513498	NC_000004.11
cg06053376	7.30E-12	4.25E-13	7.77	18.8528623	6.23E-02	139055122	139055245	NC_000005.9
cg16406892	7.31E-12	4.25E-13	7.77	18.852282	1.77E-01	115118777	115118900	NC_000012.11
cg22686132	7.35E-12	4.28E-13	7.77	18.8460083	2.00E-01	28725957	28726080	NC_000006.11
cg00905524	7.38E-12	4.29E-13	7.77	18.8422431	1.08E-01	27206907	27207030	NC_000007.13
cg02562431	7.38E-12	4.30E-13	7.77	18.8417321	2.17E-01	134070369	134070492	NC_000004.11
cg17307479	7.40E-12	4.31E-13	7.77	18.8386583	2.11E-01	65494101	65494224	NC_000008.10
cg18394881	7.40E-12	4.31E-13	7.77	18.8384643	6.25E-02	3512777	3512900	NC_000001.10
cg10636403	7.41E-12	4.31E-13	7.77	18.8377726	2.16E-01	27136486	27136609	NC_000007.13
cg15180789	7.42E-12	4.32E-13	7.77	18.8359058	1.43E-01	159557797	159557920	NC_000003.11
cg02339752	7.43E-12	4.32E-13	7.77	18.8353709	9.82E-02	86549705	86549828	NC_000016.9
cg09418063	7.43E-12	4.32E-13	7.77	18.8352786	1.91E-01	22193540	22193663	NC_000019.9

cg07546139	7.43E-12	4.33E-13	7.77	18.8345743	1.66E-01	92907553	92907676	NC_000005.9
cg09788416	7.44E-12	4.33E-13	7.77	18.8332833	2.08E-01	39539408	39539531	NC_000012.11
cg17039428	7.45E-12	4.34E-13	7.77	18.8314671	1.60E-01	115190758	115190881	NC_000012.11
cg13910989	7.45E-12	4.34E-13	7.77	18.8311583	8.53E-02	176020401	176020524	NC_000005.9
cg03846892	7.46E-12	4.34E-13	7.77	18.8310003	3.90E-02	178675021	178675144	NC_000001.10
cg01106926	7.47E-12	4.35E-13	7.77	18.8290325	1.50E-01	13539255	13539378	NC_000004.11
cg08010619	7.47E-12	4.35E-13	7.77	18.8285739	6.14E-02	9769192	9769315	NC_000011.9
cg25971347	7.49E-12	4.36E-13	7.77	18.8264996	2.47E-01	86544339	86544462	NC_000016.9
cg01567634	7.51E-12	4.38E-13	7.77	18.8231751	1.53E-01	114696671	114696794	NC_000001.10
cg03213967	7.54E-12	4.39E-13	7.77	18.8195164	5.28E-02	227729463	227729586	NC_000001.10
cg14765959	7.55E-12	4.40E-13	7.77	18.8173281	2.36E-01	106429316	106429439	NC_000006.11
cg24805559	7.56E-12	4.41E-13	7.77	18.8168225	5.09E-02	115786178	115786301	NC_000012.11
cg20699463	7.58E-12	4.42E-13	7.77	18.8131584	6.21E-02	23785143	23785266	NC_000002.11
cg24740218	7.60E-12	4.43E-13	7.77	18.8113703	1.42E-01	114075947	114076070	NC_000012.11
cg08528826	7.60E-12	4.43E-13	7.77	18.8109958	2.09E-01	139168305	139168428	NC_000007.13
cg27232145	7.60E-12	4.43E-13	7.77	18.8108758	1.59E-01	130647289	130647412	NC_000012.11
cg26483809	7.60E-12	4.44E-13	7.77	18.8102309	6.11E-02	90377257	90377380	NC_000015.9
cg27570892	7.62E-12	4.44E-13	7.77	18.8086366	1.33E-01	14160542	14160665	NC_000003.11
cg15657785	7.63E-12	4.45E-13	7.77	18.8059976	7.85E-02	155613360	155613483	NC_000007.13
cg20347882	7.66E-12	4.47E-13	7.77	18.8030989	1.85E-01	155166554	155166677	NC_000007.13
cg01458605	7.69E-12	4.48E-13	7.77	18.7992729	2.01E-01	147128679	147128802	NC_000003.11
cg09012125	7.69E-12	4.48E-13	7.77	18.7992193	1.55E-01	128779608	128779731	NC_000011.9
cg16544704	7.69E-12	4.49E-13	7.77	18.7990218	1.10E-01	44037277	44037400	NC_000021.8
cg03133987	7.69E-12	4.49E-13	7.77	18.7988727	1.19E-01	2053563	2053686	NC_000016.9
cg16770832	7.69E-12	4.49E-13	7.76	18.7980504	1.39E-01	56604516	56604639	NC_000014.8

cg05801246	7.71E-12	4.50E-13	7.76	18.7960476	1.67E-01	125651549	125651672	NC_000010.10
cg26790247	7.72E-12	4.50E-13	7.76	18.7949953	1.81E-01	147108512	147108635	NC_000003.11
cg21609339	7.72E-12	4.51E-13	7.76	18.7944976	1.45E-01	44650270	44650393	NC_000020.10
cg16001495	7.72E-12	4.51E-13	7.76	18.7940409	1.85E-01	38080526	38080649	NC_000021.8
cg13502741	7.73E-12	4.51E-13	7.76	18.793296	2.04E-01	31848488	31848611	NC_000011.9
cg06649639	7.75E-12	4.52E-13	7.76	18.7908592	1.03E-01	154983995	154984118	NC_000001.10
cg19694010	7.76E-12	4.53E-13	7.76	18.7886684	2.43E-01	130645520	130645643	NC_000012.11
cg08944029	7.79E-12	4.55E-13	7.76	18.7852961	1.37E-01	112553445	112553568	NC_000005.9
cg17211890	7.80E-12	4.55E-13	7.76	18.7841519	1.37E-01	94906892	94907015	NC_000010.10
cg00674681	7.80E-12	4.55E-13	7.76	18.7840134	8.59E-02	50517357	50517480	NC_000014.8
cg13013975	7.82E-12	4.57E-13	7.76	18.7803606	3.73E-02	98581010	98581133	NC_000007.13
cg19537969	7.83E-12	4.57E-13	7.76	18.7801015	1.06E-01	91357909	91358032	NC_000012.11
cg13411015	7.83E-12	4.57E-13	7.76	18.7798958	1.65E-01	57274900	57275023	NC_000014.8
cg12746059	7.83E-12	4.58E-13	7.76	18.7792888	1.94E-01	134070389	134070512	NC_000004.11
cg10586883	7.83E-12	4.58E-13	7.76	18.7791055	1.04E-01	79312681	79312804	NC_000017.10
cg05476568	7.83E-12	4.58E-13	7.76	18.7787545	2.14E-01	153840109	153840232	NC_000003.11
cg11943209	7.85E-12	4.59E-13	7.76	18.7769966	1.40E-01	19553321	19553444	NC_000002.11
cg00676030	7.85E-12	4.59E-13	7.76	18.7768239	1.03E-01	38307962	38308085	NC_000008.10
cg23502639	7.85E-12	4.59E-13	7.76	18.7761411	1.56E-01	5402883	5403006	NC_000017.10
cg17746625	7.88E-12	4.61E-13	7.76	18.7725686	1.17E-01	150247007	150247130	NC_000006.11
cg13786089	7.89E-12	4.61E-13	7.76	18.771266	1.56E-01	3869086	3869209	NC_000019.9
cg04032181	7.91E-12	4.63E-13	7.76	18.7687901	7.37E-02	49382422	49382545	NC_000008.10
cg12907702	7.92E-12	4.63E-13	7.76	18.767736	1.50E-01	177133734	177133857	NC_000001.10
cg23076550	7.93E-12	4.64E-13	7.76	18.7663141	9.87E-02	5843661	5843784	NC_000019.9
cg26348254	7.93E-12	4.64E-13	7.76	18.7662391	6.56E-02	49265006	49265129	NC_000015.9

cg18268547	7.94E-12	4.64E-13	7.76	18.7651232	1.65E-01	79615552	79615675	NC_000017.10
cg25121513	7.94E-12	4.64E-13	7.76	18.7647961	1.50E-01	25898206	25898329	NC_000007.13
cg04180823	7.96E-12	4.66E-13	7.76	18.761614	7.88E-02	167652028	167652151	NC_000006.11
cg04618219	7.97E-12	4.66E-13	7.76	18.7609846	5.66E-02	144902581	144902704	NC_000008.10
cg13434989	7.99E-12	4.68E-13	7.76	18.7581314	2.54E-01	78493305	78493428	NC_000013.10
cg10126234	8.02E-12	4.69E-13	7.76	18.7546898	1.41E-01	104195533	104195656	NC_000014.8
cg05230054	8.03E-12	4.70E-13	7.76	18.7527105	1.91E-01	34175419	34175542	NC_000019.9
cg23244790	8.03E-12	4.70E-13	7.76	18.7526678	1.99E-01	140811102	140811225	NC_000005.9
cg24722073	8.09E-12	4.73E-13	7.76	18.74575	1.81E-01	41882163	41882286	NC_000004.11
cg05396987	8.09E-12	4.74E-13	7.76	18.7454767	1.92E-01	122686269	122686392	NC_000004.11
cg20936172	8.09E-12	4.74E-13	7.76	18.7454625	8.11E-02	142533635	142533758	NC_000008.10
cg13042636	8.10E-12	4.74E-13	7.76	18.7442287	4.66E-02	122218807	122218930	NC_000012.11
cg19721867	8.10E-12	4.74E-13	7.76	18.7441221	2.15E-01	119001066	119001189	NC_000010.10
cg16392691	8.11E-12	4.75E-13	7.76	18.7429424	1.46E-01	28587771	28587894	NC_000001.10
cg16500497	8.11E-12	4.75E-13	7.76	18.7419801	8.70E-02	42427639	42427762	NC_000017.10
cg08582701	8.12E-12	4.75E-13	7.76	18.7417664	1.50E-01	6070005	6070128	NC_000016.9
cg03785456	8.13E-12	4.76E-13	7.76	18.7405064	9.42E-02	134255543	134255666	NC_000002.11
cg02380878	8.13E-12	4.76E-13	7.76	18.7404138	9.77E-02	1136618	1136741	NC_000005.9
cg21819468	8.13E-12	4.76E-13	7.76	18.740398	2.16E-01	13910569	13910692	NC_000001.10
cg25514273	8.15E-12	4.78E-13	7.75	18.7372464	1.84E-01	70681804	70681927	NC_000013.10
cg09551147	8.15E-12	4.78E-13	7.75	18.7368224	2.07E-01	106399957	106400080	NC_000010.10
cg25959472	8.16E-12	4.78E-13	7.75	18.7357283	1.46E-01	171384415	171384538	NC_000005.9
cg26584743	8.17E-12	4.79E-13	7.75	18.7348431	6.27E-02	31711736	31711859	NC_000006.11
cg04211018	8.17E-12	4.79E-13	7.75	18.7341027	1.30E-01	62783533	62783656	NC_000001.10
cg12964065	8.19E-12	4.80E-13	7.75	18.7315612	5.01E-02	77638022	77638145	NC_000018.9

cg00650809	8.21E-12	4.81E-13	7.75	18.7294255	1.72E-01	17439339	17439462	NC_000019.9
cg22022798	8.23E-12	4.83E-13	7.75	18.7268561	1.56E-01	221065961	221066084	NC_000001.10
cg14914552	8.26E-12	4.84E-13	7.75	18.7232784	1.88E-01	97340188	97340311	NC_000008.10
cg13890706	8.26E-12	4.85E-13	7.75	18.7227086	2.24E-01	118033115	118033238	NC_000010.10
cg05074517	8.28E-12	4.85E-13	7.75	18.721122	9.16E-02	133901760	133901883	NC_000009.11
cg04210082	8.29E-12	4.86E-13	7.75	18.7199772	4.21E-02	109459746	109459869	NC_000008.10
cg06911487	8.29E-12	4.87E-13	7.75	18.7189435	1.41E-01	10024282	10024405	NC_000019.9
cg10059942	8.30E-12	4.87E-13	7.75	18.7184853	1.92E-01	124908488	124908611	NC_000010.10
cg15215348	8.31E-12	4.87E-13	7.75	18.7170021	1.64E-01	18344520	18344643	NC_000019.9
cg00609966	8.31E-12	4.88E-13	7.75	18.716975	2.43E-01	1202819	1202942	NC_000016.9
cg04198308	8.31E-12	4.88E-13	7.75	18.716233	2.24E-01	48971959	48972082	NC_000022.10
cg06221712	8.32E-12	4.88E-13	7.75	18.7157025	7.38E-02	29169423	29169546	NC_000016.9
cg02460314	8.32E-12	4.88E-13	7.75	18.7153789	1.83E-01	101841285	101841408	NC_000010.10
cg24494114	8.33E-12	4.89E-13	7.75	18.713901	1.29E-01	131769343	131769466	NC_000012.11
cg04380873	8.34E-12	4.90E-13	7.75	18.712697	1.05E-01	184089743	184089866	NC_000003.11
cg04914126	8.37E-12	4.91E-13	7.75	18.7094716	1.66E-01	143086405	143086528	NC_000008.10
cg08979895	8.42E-12	4.94E-13	7.75	18.7034614	1.43E-01	111540126	111540249	NC_000004.11
cg16055159	8.42E-12	4.94E-13	7.75	18.7033488	3.79E-02	101446936	101447059	NC_000003.11
cg21481021	8.47E-12	4.97E-13	7.75	18.6972124	7.13E-02	21037291	21037414	NC_000018.9
cg06288251	8.47E-12	4.97E-13	7.75	18.6970108	2.21E-01	132757087	132757210	NC_000003.11
cg22837767	8.49E-12	4.99E-13	7.75	18.6946258	2.35E-01	21686251	21686374	NC_000020.10
cg16910830	8.53E-12	5.01E-13	7.75	18.6899338	2.37E-01	145813437	145813560	NC_000007.13
cg11532863	8.53E-12	5.01E-13	7.75	18.689336	1.07E-01	19613430	19613553	NC_000001.10
cg00837677	8.55E-12	5.02E-13	7.75	18.6872104	2.81E-02	48372681	48372804	NC_000010.10
cg00984540	8.56E-12	5.03E-13	7.75	18.6859534	7.92E-02	155589293	155589416	NC_000007.13

cg27319123	8.57E-12	5.04E-13	7.75	18.6846273	2.10E-01	30038286	30038409	NC_000011.9
cg08091601	8.60E-12	5.05E-13	7.75	18.6812863	1.65E-01	38248464	38248587	NC_000022.10
cg03603951	8.61E-12	5.06E-13	7.75	18.6797165	2.44E-01	47797488	47797611	NC_000002.11
cg25019648	8.62E-12	5.07E-13	7.74	18.6785477	2.23E-01	72353261	72353384	NC_000017.10
cg13066658	8.62E-12	5.07E-13	7.74	18.6777721	1.83E-01	32449163	32449286	NC_000011.9
cg16420199	8.65E-12	5.09E-13	7.74	18.6742735	2.09E-01	19797965	19798088	NC_000008.10
cg07076845	8.66E-12	5.09E-13	7.74	18.6738634	1.01E-01	103553804	103553927	NC_000014.8
cg08396985	8.67E-12	5.10E-13	7.74	18.6721374	1.69E-01	5896489	5896612	NC_000002.11
cg24364282	8.69E-12	5.11E-13	7.74	18.6703649	7.24E-02	114126759	114126882	NC_000006.11
cg24277791	8.69E-12	5.11E-13	7.74	18.6698041	1.53E-01	103968615	103968738	NC_000007.13
cg13544027	8.69E-12	5.11E-13	7.74	18.6697339	8.21E-02	20465288	20465411	NC_000011.9
cg21713614	8.70E-12	5.12E-13	7.74	18.6681622	1.17E-01	79126719	79126842	NC_000004.11
cg01769521	8.71E-12	5.12E-13	7.74	18.6679764	1.32E-01	142179603	142179726	NC_000008.10
cg02072532	8.71E-12	5.13E-13	7.74	18.6674327	3.56E-02	53926416	53926539	NC_000014.8
cg26552621	8.73E-12	5.14E-13	7.74	18.6656163	1.70E-01	1271019	1271142	NC_000019.9
cg13435820	8.73E-12	5.14E-13	7.74	18.6653367	1.08E-01	115907128	115907251	NC_000012.11
cg04030615	8.73E-12	5.14E-13	7.74	18.6647108	2.64E-01	229569902	229570025	NC_000001.10
cg22337128	8.75E-12	5.15E-13	7.74	18.6631278	2.17E-01	29243404	29243527	NC_000014.8
cg13193455	8.75E-12	5.15E-13	7.74	18.6630875	1.76E-01	38511455	38511578	NC_000001.10
cg06081917	8.83E-12	5.20E-13	7.74	18.6529111	7.32E-02	14762377	14762500	NC_000016.9
cg05466469	8.84E-12	5.21E-13	7.74	18.651672	6.28E-02	157278622	157278745	NC_000006.11
cg23679332	8.86E-12	5.22E-13	7.74	18.6495011	1.30E-01	38129652	38129775	NC_000008.10
cg26843110	8.86E-12	5.22E-13	7.74	18.6494725	1.82E-01	74935742	74935865	NC_000015.9
cg05045332	8.91E-12	5.25E-13	7.74	18.6435826	8.13E-02	32012897	32013020	NC_000006.11
cg25291004	8.92E-12	5.26E-13	7.74	18.6427568	1.64E-01	165305314	165305437	NC_000004.11

cg02569509	8.94E-12	5.27E-13	7.74	18.6405135	1.34E-01	142423070	142423193	NC_000007.13
cg13575339	8.94E-12	5.27E-13	7.74	18.6398505	1.82E-01	74708313	74708436	NC_000014.8
cg24041917	8.96E-12	5.28E-13	7.74	18.637581	1.84E-01	119535986	119536109	NC_000001.10
cg24895052	8.98E-12	5.29E-13	7.74	18.6356734	1.10E-01	41909601	41909724	NC_000017.10
cg24856658	8.98E-12	5.30E-13	7.74	18.6352319	1.82E-01	78533917	78534040	NC_000005.9
cg10230442	8.99E-12	5.30E-13	7.74	18.6346223	1.39E-01	186383752	186383875	NC_000003.11
cg07142201	8.99E-12	5.30E-13	7.74	18.6341003	2.01E-01	109293216	109293339	NC_000011.9
cg03495340	9.00E-12	5.31E-13	7.74	18.6327029	1.71E-01	97685551	97685674	NC_000014.8
cg05487954	9.04E-12	5.33E-13	7.74	18.6285445	1.30E-01	38144887	38145010	NC_000010.10
cg18151410	9.06E-12	5.34E-13	7.74	18.6264027	1.50E-01	92367692	92367815	NC_000015.9
cg05184395	9.09E-12	5.37E-13	7.74	18.622559	1.10E-01	169192573	169192696	NC_000006.11
cg18530551	9.11E-12	5.38E-13	7.74	18.6204784	2.13E-01	172670879	172671002	NC_000005.9
cg14012310	9.11E-12	5.38E-13	7.73	18.6202599	1.41E-01	79181947	79182070	NC_000013.10
cg26595643	9.11E-12	5.38E-13	7.73	18.6201708	1.69E-01	118899291	118899414	NC_000010.10
cg01181227	9.13E-12	5.39E-13	7.73	18.6185133	2.02E-01	1399135	1399258	NC_000004.11
cg00150837	9.14E-12	5.40E-13	7.73	18.616874	1.09E-01	52721831	52721954	NC_000003.11
cg26796679	9.14E-12	5.40E-13	7.73	18.6168616	2.53E-01	17849639	17849762	NC_000022.10
cg13267931	9.14E-12	5.40E-13	7.73	18.6164266	2.53E-01	101006308	101006431	NC_000007.13
cg07539798	9.15E-12	5.40E-13	7.73	18.6157087	1.41E-01	140811312	140811435	NC_000005.9
cg09893305	9.17E-12	5.41E-13	7.73	18.6138689	1.15E-01	83017184	83017307	NC_000005.9
cg12565585	9.18E-12	5.42E-13	7.73	18.6129012	1.81E-01	105235943	105236066	NC_000008.10
cg12825070	9.19E-12	5.43E-13	7.73	18.6115983	2.57E-01	148033708	148033831	NC_000005.9
cg22750155	9.20E-12	5.43E-13	7.73	18.610185	2.13E-01	80524253	80524376	NC_000008.10
cg22958047	9.21E-12	5.44E-13	7.73	18.6090548	1.24E-01	96909322	96909445	NC_000015.9
cg00368687	9.23E-12	5.45E-13	7.73	18.6068086	2.01E-01	112758379	112758502	NC_000013.10

cg18106312	9.23E-12	5.45E-13	7.73	18.6067201	2.09E-01	51560488	51560611	NC_000014.8
cg23662448	9.23E-12	5.45E-13	7.73	18.6067019	5.54E-02	109797184	109797307	NC_000012.11
cg20839206	9.23E-12	5.45E-13	7.73	18.6062784	1.17E-01	28584121	28584244	NC_000006.11
cg16537483	9.23E-12	5.46E-13	7.73	18.60599	1.42E-01	152019539	152019662	NC_000003.11
cg06172939	9.24E-12	5.46E-13	7.73	18.6045706	8.38E-02	88127334	88127457	NC_000016.9
cg07961015	9.25E-12	5.47E-13	7.73	18.6033761	1.90E-01	1876215	1876338	NC_000005.9
cg01552711	9.29E-12	5.49E-13	7.73	18.5992014	1.42E-01	21291353	21291476	NC_000013.10
cg20070618	9.30E-12	5.50E-13	7.73	18.5986596	1.68E-01	17934070	17934193	NC_000006.11
cg10056096	9.30E-12	5.50E-13	7.73	18.5976672	7.58E-02	9450411	9450534	NC_000011.9
cg22471726	9.32E-12	5.51E-13	7.73	18.5961765	1.91E-01	46719276	46719399	NC_000017.10
cg01088410	9.33E-12	5.52E-13	7.73	18.5943958	2.78E-01	170739179	170739302	NC_000005.9
cg21805880	9.34E-12	5.53E-13	7.73	18.593571	1.30E-01	4464294	4464417	NC_000017.10
cg26612735	9.35E-12	5.53E-13	7.73	18.592073	1.76E-01	56988806	56988929	NC_000019.9
cg21217024	9.39E-12	5.55E-13	7.73	18.5887794	2.29E-01	105481406	105481529	NC_000011.9
cg05481243	9.39E-12	5.56E-13	7.73	18.5878102	6.95E-02	18258640	18258763	NC_000022.10
cg08587845	9.40E-12	5.56E-13	7.73	18.5875516	1.37E-01	134870506	134870629	NC_000005.9
cg16568131	9.41E-12	5.57E-13	7.73	18.5864159	1.28E-01	28983079	28983202	NC_000006.11
cg17663463	9.41E-12	5.57E-13	7.73	18.5858782	9.95E-02	57349709	57349832	NC_000019.9
cg15311814	9.44E-12	5.59E-13	7.73	18.582896	1.78E-01	35296213	35296336	NC_000007.13
cg24553170	9.44E-12	5.59E-13	7.73	18.5826441	1.36E-01	49243472	49243595	NC_000001.10
cg23453372	9.44E-12	5.59E-13	7.73	18.5821391	1.45E-01	4866619	4866742	NC_000004.11
cg02038213	9.46E-12	5.60E-13	7.73	18.5800003	7.10E-02	95662442	95662565	NC_000002.11
cg07405021	9.46E-12	5.60E-13	7.73	18.5798763	1.63E-01	115152019	115152142	NC_000005.9
cg10241484	9.46E-12	5.60E-13	7.73	18.5797643	1.61E-01	62070219	62070342	NC_000016.9
cg02245378	9.47E-12	5.61E-13	7.73	18.5786266	1.54E-01	223161771	223161894	NC_000002.11

cg03229561	9.49E-12	5.62E-13	7.73	18.5769214	1.31E-01	63498186	63498309	NC_000007.13
cg15648859	9.52E-12	5.64E-13	7.73	18.5739406	9.08E-02	124986509	124986632	NC_000012.11
cg13321605	9.53E-12	5.64E-13	7.73	18.5725953	1.13E-01	91298666	91298789	NC_000005.9
cg07907386	9.53E-12	5.64E-13	7.73	18.5725415	2.10E-01	41749443	41749566	NC_000004.11
cg11056406	9.53E-12	5.65E-13	7.73	18.5719298	3.25E-02	144903651	144903774	NC_000008.10
cg00955307	9.53E-12	5.65E-13	7.73	18.5717222	1.16E-01	166889789	166889912	NC_000001.10
cg27552679	9.54E-12	5.65E-13	7.73	18.5716066	1.57E-01	74425757	74425880	NC_000015.9
cg27047243	9.54E-12	5.65E-13	7.73	18.5712434	1.99E-01	214360753	214360876	NC_000001.10
cg27638196	9.54E-12	5.65E-13	7.73	18.5709798	1.99E-01	219826870	219826993	NC_000002.11
cg07992457	9.55E-12	5.66E-13	7.73	18.5696067	1.42E-01	71744924	71745047	NC_000017.10
cg14389139	9.56E-12	5.67E-13	7.73	18.5686059	9.11E-02	1007218	1007341	NC_000011.9
cg10630680	9.57E-12	5.67E-13	7.73	18.5678734	2.50E-02	30521320	30521443	NC_000006.11
cg04574034	9.59E-12	5.68E-13	7.73	18.566002	1.76E-01	46799745	46799868	NC_000017.10
cg10808414	9.60E-12	5.69E-13	7.73	18.5647936	6.10E-02	77942302	77942425	NC_000014.8
cg22874255	9.60E-12	5.69E-13	7.73	18.5640512	1.71E-01	1768630	1768753	NC_000019.9
cg14321743	9.60E-12	5.69E-13	7.73	18.5639455	9.60E-02	20445662	20445785	NC_000001.10
cg04254681	9.61E-12	5.70E-13	7.73	18.5636193	1.09E-01	9679744	9679867	NC_000002.11
cg18844382	9.61E-12	5.70E-13	7.73	18.5634082	2.12E-01	23834977	23835100	NC_000014.8
cg15723536	9.62E-12	5.71E-13	7.73	18.5616405	2.18E-01	54926617	54926740	NC_000019.9
cg23540651	9.63E-12	5.71E-13	7.72	18.5609447	8.29E-02	89490034	89490157	NC_000001.10
cg26855689	9.63E-12	5.71E-13	7.72	18.560694	9.76E-02	79668668	79668791	NC_000017.10
cg02708185	9.63E-12	5.71E-13	7.72	18.5606518	1.32E-01	146620097	146620220	NC_000002.11
cg08836199	9.64E-12	5.72E-13	7.72	18.5594458	1.62E-01	194469052	194469175	NC_000003.11
cg16651126	9.69E-12	5.75E-13	7.72	18.5539655	1.23E-01	27170552	27170675	NC_000007.13
cg12641165	9.71E-12	5.76E-13	7.72	18.5526096	1.38E-01	3051529	3051652	NC_000001.10

cg04131583	9.71E-12	5.76E-13	7.72	18.552471	1.59E-01	86529961	86530084	NC_000016.9
cg11430783	9.71E-12	5.76E-13	7.72	18.552266	7.71E-02	3703792	3703915	NC_000001.10
cg04065304	9.71E-12	5.76E-13	7.72	18.5517438	6.11E-02	53322501	53322624	NC_000008.10
cg09728487	9.72E-12	5.77E-13	7.72	18.5514317	1.49E-01	141079462	141079585	NC_000004.11
cg14413996	9.73E-12	5.78E-13	7.72	18.5494796	8.02E-02	138592162	138592285	NC_000009.11
cg09947844	9.75E-12	5.79E-13	7.72	18.5480507	1.34E-01	4163819	4163942	NC_000016.9
cg13390484	9.75E-12	5.79E-13	7.72	18.5472611	4.98E-02	80765924	80766047	NC_000009.11
cg15986555	9.76E-12	5.80E-13	7.72	18.5462192	1.30E-01	123243400	123243523	NC_000010.10
cg26237003	9.78E-12	5.81E-13	7.72	18.5443319	1.00E-01	43413209	43413332	NC_000005.9
cg22544863	9.79E-12	5.81E-13	7.72	18.5432481	1.83E-01	15580400	15580523	NC_000019.9
cg09636525	9.82E-12	5.83E-13	7.72	18.5400481	1.71E-01	7052612	7052735	NC_000004.11
cg10956056	9.82E-12	5.83E-13	7.72	18.5399208	1.10E-01	35890214	35890337	NC_000017.10
cg20249882	9.83E-12	5.84E-13	7.72	18.539623	9.29E-02	1364046	1364169	NC_000004.11
cg18239753	9.86E-12	5.86E-13	7.72	18.5364496	2.01E-01	62995963	62996086	NC_000006.11
cg09821218	9.89E-12	5.87E-13	7.72	18.5334063	1.09E-01	68691561	68691684	NC_000016.9
cg14830601	9.90E-12	5.88E-13	7.72	18.5323666	1.59E-01	118893602	118893725	NC_000010.10
cg25249539	9.90E-12	5.88E-13	7.72	18.5314978	1.40E-01	56803358	56803481	NC_000020.10
cg13753550	9.90E-12	5.89E-13	7.72	18.5313232	3.56E-02	79892876	79892999	NC_000017.10
cg14843030	9.91E-12	5.89E-13	7.72	18.5306141	1.26E-01	32716051	32716174	NC_000001.10
cg21099419	9.91E-12	5.89E-13	7.72	18.530451	2.19E-01	97844473	97844596	NC_000007.13
cg11881754	9.91E-12	5.89E-13	7.72	18.5303162	2.18E-01	75889293	75889416	NC_000007.13
cg14714046	9.91E-12	5.89E-13	7.72	18.5301765	1.75E-01	41341654	41341777	NC_000006.11
cg16440299	9.98E-12	5.93E-13	7.72	18.5236936	1.50E-01	56779141	56779264	NC_000016.9
cg24724633	9.98E-12	5.94E-13	7.72	18.5230145	2.79E-01	36909413	36909536	NC_000019.9
cg03854198	9.99E-12	5.94E-13	7.72	18.5224477	1.32E-01	5541311	5541434	NC_000012.11

cg09664054	1.00E-11	5.95E-13	7.72	18.5203569	1.11E-01	42179097	42179220	NC_000001.10
cg11374732	1.00E-11	5.96E-13	7.72	18.5195921	9.97E-02	234113197	234113320	NC_000002.11
cg00468846	1.00E-11	5.96E-13	7.72	18.5185621	7.07E-02	31831421	31831544	NC_000016.9
cg16620608	1.00E-11	5.97E-13	7.72	18.5175617	8.01E-02	100841473	100841596	NC_000007.13
cg26083752	1.00E-11	5.98E-13	7.72	18.516274	4.25E-02	10590587	10590710	NC_000011.9
cg15506157	1.00E-11	5.98E-13	7.72	18.515779	3.09E-01	139168484	139168607	NC_000007.13
cg13174898	1.01E-11	5.99E-13	7.72	18.514312	1.31E-01	202104055	202104178	NC_000001.10
cg09870004	1.01E-11	5.99E-13	7.72	18.5135453	5.90E-02	88050251	88050374	NC_000016.9
cg03293976	1.01E-11	6.00E-13	7.72	18.5130024	1.07E-01	152554195	152554318	NC_000003.11
cg15990972	1.01E-11	6.00E-13	7.72	18.5129095	1.60E-01	177012372	177012495	NC_000002.11
cg27533288	1.01E-11	6.00E-13	7.72	18.5123197	2.45E-01	118896776	118896899	NC_000010.10
cg20325850	1.01E-11	6.01E-13	7.72	18.5113845	2.65E-02	56977724	56977847	NC_000001.10
cg02070077	1.01E-11	6.02E-13	7.72	18.5096418	7.57E-02	168199753	168199876	NC_000006.11
cg04521583	1.01E-11	6.02E-13	7.72	18.5090967	1.48E-01	44087089	44087212	NC_000015.9
cg10058779	1.01E-11	6.03E-13	7.72	18.5066407	1.90E-01	23821435	23821558	NC_000014.8
cg26619043	1.01E-11	6.04E-13	7.72	18.5059066	1.01E-01	66287492	66287615	NC_000017.10
cg18161327	1.02E-11	6.05E-13	7.72	18.5046577	1.82E-01	100627703	100627826	NC_000013.10
cg05660670	1.02E-11	6.05E-13	7.72	18.5043332	2.17E-01	20521635	20521758	NC_000012.11
cg00333919	1.02E-11	6.06E-13	7.72	18.502931	1.38E-01	155087199	155087322	NC_000007.13
cg01939562	1.02E-11	6.06E-13	7.72	18.5025108	2.46E-01	3105012	3105135	NC_000005.9
cg00626856	1.02E-11	6.07E-13	7.71	18.5006168	1.78E-01	1885950	1886073	NC_000005.9
cg18502142	1.02E-11	6.07E-13	7.71	18.5005033	2.15E-01	96622709	96622832	NC_000007.13
cg19057986	1.02E-11	6.09E-13	7.71	18.4980124	2.20E-01	46802871	46802994	NC_000017.10
cg16970851	1.02E-11	6.09E-13	7.71	18.4977118	2.17E-01	22487658	22487781	NC_000012.11
cg20169913	1.03E-11	6.11E-13	7.71	18.4949514	1.40E-01	184014083	184014206	NC_000003.11

cg23325963	1.03E-11	6.12E-13	7.71	18.4928467	1.96E-01	45160093	45160216	NC_000002.11
cg06032735	1.03E-11	6.13E-13	7.71	18.4908577	6.77E-02	84227227	84227350	NC_000006.11
cg12668953	1.03E-11	6.14E-13	7.71	18.4891718	1.09E-01	138679653	138679776	NC_000005.9
cg18810983	1.03E-11	6.15E-13	7.71	18.48828	1.13E-01	192632111	192632234	NC_000003.11
cg10176110	1.03E-11	6.15E-13	7.71	18.4881537	1.68E-01	168841653	168841776	NC_000006.11
cg23954787	1.03E-11	6.15E-13	7.71	18.4878893	1.80E-01	72470700	72470823	NC_000008.10
cg01336878	1.03E-11	6.17E-13	7.71	18.4851795	1.53E-01	54963396	54963519	NC_000019.9
cg26857670	1.03E-11	6.17E-13	7.71	18.4850954	1.87E-01	27225448	27225571	NC_000007.13
cg00970558	1.04E-11	6.18E-13	7.71	18.4827577	2.03E-01	7854469	7854592	NC_000019.9
cg13814990	1.04E-11	6.19E-13	7.71	18.4822718	5.55E-02	46016683	46016806	NC_000007.13
cg19871940	1.04E-11	6.19E-13	7.71	18.4822531	2.00E-01	57264287	57264410	NC_000014.8
cg00621899	1.04E-11	6.19E-13	7.71	18.4817278	1.54E-01	100486423	100486546	NC_000007.13
cg08431529	1.04E-11	6.20E-13	7.71	18.4807901	6.80E-02	6619138	6619261	NC_000012.11
cg07205670	1.04E-11	6.20E-13	7.71	18.4795975	7.81E-02	87442901	87443024	NC_000016.9
cg22604316	1.04E-11	6.20E-13	7.71	18.4794237	2.21E-01	46958801	46958924	NC_000001.10
cg23810282	1.04E-11	6.23E-13	7.71	18.4759297	1.62E-01	43037519	43037642	NC_000005.9
cg18632637	1.04E-11	6.23E-13	7.71	18.47484	1.51E-01	134893591	134893714	NC_000010.10
cg21213430	1.05E-11	6.24E-13	7.71	18.4741729	7.79E-02	35859254	35859377	NC_000009.11
cg14615946	1.05E-11	6.24E-13	7.71	18.4732933	7.21E-02	74466087	74466210	NC_000017.10
cg13829680	1.05E-11	6.25E-13	7.71	18.4725289	1.89E-01	136746975	136747098	NC_000009.11
cg18249580	1.05E-11	6.26E-13	7.71	18.4708827	2.37E-01	65281496	65281619	NC_000008.10
cg16251457	1.05E-11	6.27E-13	7.71	18.4697012	5.82E-02	79210394	79210517	NC_000017.10
cg16620382	1.05E-11	6.28E-13	7.71	18.4671055	2.93E-01	43211213	43211336	NC_000006.11
cg25089357	1.05E-11	6.29E-13	7.71	18.4653635	1.63E-01	131649255	131649378	NC_000010.10
cg01794802	1.06E-11	6.31E-13	7.71	18.4629401	1.80E-01	31096931	31097054	NC_000006.11

cg17264670	1.06E-11	6.31E-13	7.71	18.462055	2.81E-01	153452732	153452855	NC_000006.11
cg25635251	1.06E-11	6.32E-13	7.71	18.4611041	1.29E-01	2234043	2234166	NC_000011.9
cg18826572	1.06E-11	6.34E-13	7.71	18.4586218	1.69E-01	144510343	144510466	NC_000008.10
cg15762399	1.06E-11	6.34E-13	7.71	18.4577714	1.08E-01	80109047	80109170	NC_000013.10
cg25950112	1.07E-11	6.37E-13	7.71	18.4534415	1.99E-01	170740870	170740993	NC_000005.9
cg23986620	1.07E-11	6.38E-13	7.71	18.451375	1.68E-01	177012706	177012829	NC_000002.11
cg04863892	1.07E-11	6.39E-13	7.71	18.4505422	1.81E-01	27183375	27183498	NC_000007.13
cg20426532	1.07E-11	6.41E-13	7.71	18.4479133	8.81E-02	64185918	64186041	NC_000006.11
cg13416889	1.07E-11	6.41E-13	7.71	18.44787	1.66E-01	64814395	64814518	NC_000015.9
cg16067696	1.07E-11	6.41E-13	7.71	18.4467745	4.96E-02	119522486	119522609	NC_000011.9
cg07392385	1.08E-11	6.43E-13	7.71	18.4445111	7.60E-02	108925222	108925345	NC_000010.10
cg00909646	1.08E-11	6.43E-13	7.71	18.4444861	2.17E-01	67874250	67874373	NC_000008.10
cg03769939	1.08E-11	6.44E-13	7.7	18.4425817	1.41E-01	25254129	25254252	NC_000001.10
cg13611347	1.08E-11	6.44E-13	7.7	18.4421266	1.95E-01	31739207	31739330	NC_000018.9
cg12201988	1.08E-11	6.44E-13	7.7	18.4419346	1.07E-01	19982870	19982993	NC_000020.10
cg05272349	1.08E-11	6.45E-13	7.7	18.4409102	1.41E-01	122434379	122434502	NC_000005.9
cg10751726	1.08E-11	6.45E-13	7.7	18.4407993	1.67E-01	132121314	132121437	NC_000002.11
cg11857997	1.08E-11	6.47E-13	7.7	18.4384512	2.53E-01	3591992	3592115	NC_000005.9
cg04092437	1.08E-11	6.47E-13	7.7	18.4384488	7.84E-02	92971269	92971392	NC_000010.10
cg10606834	1.08E-11	6.47E-13	7.7	18.4377676	1.69E-01	59532197	59532320	NC_000017.10
cg16142855	1.09E-11	6.50E-13	7.7	18.433985	1.59E-01	379900	380023	NC_000004.11
cg06459847	1.09E-11	6.50E-13	7.7	18.4327565	1.97E-01	204601	204724	NC_000016.9
cg17757602	1.09E-11	6.51E-13	7.7	18.4325026	2.01E-01	42952113	42952236	NC_000005.9
cg23239840	1.09E-11	6.51E-13	7.7	18.4322624	4.23E-02	114052325	114052448	NC_000002.11
cg14902389	1.09E-11	6.53E-13	7.7	18.4289718	1.24E-01	99342685	99342808	NC_000002.11

cg27242132	1.09E-11	6.55E-13	7.7	18.426272	1.67E-01	81119178	81119301	NC_000004.11
cg02182210	1.10E-11	6.56E-13	7.7	18.4238999	1.16E-01	30619096	30619219	NC_000020.10
cg18199753	1.10E-11	6.57E-13	7.7	18.4234224	9.46E-02	245755356	245755479	NC_000001.10
cg07491828	1.10E-11	6.57E-13	7.7	18.4232122	1.14E-01	14227116	14227239	NC_000016.9
cg07388347	1.10E-11	6.57E-13	7.7	18.4229607	1.74E-01	133481466	133481589	NC_000012.11
cg23367608	1.10E-11	6.57E-13	7.7	18.4226954	2.18E-01	117086601	117086724	NC_000006.11
cg05274650	1.10E-11	6.58E-13	7.7	18.4210065	3.82E-02	17928213	17928336	NC_000011.9
cg25143359	1.10E-11	6.60E-13	7.7	18.4190837	1.32E-01	245533949	245534072	NC_000001.10
cg13565138	1.10E-11	6.60E-13	7.7	18.4189302	1.18E-01	91614156	91614279	NC_000009.11
cg06261500	1.10E-11	6.61E-13	7.7	18.4176318	9.98E-02	127484663	127484786	NC_000007.13
cg24066316	1.10E-11	6.61E-13	7.7	18.4165505	1.70E-01	37132764	37132887	NC_000014.8
cg23219859	1.11E-11	6.63E-13	7.7	18.4138259	2.66E-01	50799316	50799439	NC_000001.10
cg10201402	1.11E-11	6.63E-13	7.7	18.4133932	1.07E-01	31938527	31938650	NC_000006.11
cg16925678	1.11E-11	6.65E-13	7.7	18.410839	7.37E-02	61871067	61871190	NC_000020.10
cg07145531	1.11E-11	6.66E-13	7.7	18.4090154	7.05E-02	141993716	141993839	NC_000005.9
cg19544662	1.11E-11	6.67E-13	7.7	18.4081358	3.00E-01	88752056	88752179	NC_000002.11
cg16646743	1.11E-11	6.68E-13	7.7	18.4065494	1.65E-01	9608947	9609070	NC_000019.9
cg21099470	1.11E-11	6.68E-13	7.7	18.4063223	1.92E-01	10459141	10459264	NC_000004.11
cg09373511	1.12E-11	6.68E-13	7.7	18.405872	8.96E-02	127560294	127560417	NC_000010.10
cg08446824	1.12E-11	6.69E-13	7.7	18.4055293	1.94E-01	1720184	1720307	NC_000004.11
cg03794213	1.12E-11	6.69E-13	7.7	18.4048974	1.22E-01	22220727	22220850	NC_000016.9
cg14348706	1.12E-11	6.69E-13	7.7	18.4044459	1.06E-01	33170405	33170528	NC_000006.11
cg19347790	1.12E-11	6.71E-13	7.7	18.4023487	1.06E-01	81332050	81332173	NC_000012.11
cg11064034	1.12E-11	6.71E-13	7.7	18.4019201	1.90E-01	114877421	114877544	NC_000012.11
cg00467420	1.12E-11	6.74E-13	7.7	18.3984739	2.09E-01	1779432	1779555	NC_000010.10

cg20504856	1.13E-11	6.75E-13	7.7	18.3957225	1.60E-01	19692011	19692134	NC_000006.11
cg03202564	1.13E-11	6.75E-13	7.7	18.3956991	1.20E-01	50977130	50977253	NC_000010.10
cg07137336	1.13E-11	6.77E-13	7.7	18.3936028	1.66E-01	501137	501260	NC_000018.9
cg04545398	1.13E-11	6.77E-13	7.7	18.3932704	5.20E-02	105819003	105819126	NC_000010.10
cg08234507	1.13E-11	6.78E-13	7.7	18.3922461	5.07E-02	64990959	64991082	NC_000011.9
cg15649193	1.13E-11	6.78E-13	7.7	18.3919781	2.61E-02	14640403	14640526	NC_000019.9
cg25750389	1.13E-11	6.78E-13	7.7	18.3915755	1.90E-01	111544016	111544139	NC_000004.11
cg22700148	1.13E-11	6.78E-13	7.7	18.3913992	1.01E-01	65041201	65041324	NC_000017.10
cg16609297	1.13E-11	6.79E-13	7.7	18.3910038	9.11E-02	66437039	66437162	NC_000016.9
cg15527308	1.13E-11	6.79E-13	7.7	18.3897577	1.01E-01	54813067	54813190	NC_000018.9
cg26203336	1.13E-11	6.80E-13	7.7	18.3883231	5.61E-02	303166	303289	NC_000011.9
cg07919775	1.14E-11	6.82E-13	7.7	18.385729	1.42E-01	991161	991284	NC_000019.9
cg09516476	1.14E-11	6.83E-13	7.7	18.3840497	1.87E-01	10881925	10882048	NC_000006.11
cg25213546	1.14E-11	6.84E-13	7.69	18.3830505	2.62E-01	84116920	84117043	NC_000015.9
cg19999212	1.15E-11	6.88E-13	7.69	18.3778627	8.94E-02	131096964	131097087	NC_000003.11
cg24975564	1.15E-11	6.89E-13	7.69	18.3757938	2.48E-01	20522236	20522359	NC_000012.11
cg17386977	1.15E-11	6.90E-13	7.69	18.3745042	9.17E-02	44109743	44109866	NC_000021.8
cg04622775	1.15E-11	6.91E-13	7.69	18.3735288	6.24E-02	89442186	89442309	NC_000015.9
cg01764252	1.15E-11	6.92E-13	7.69	18.3720399	2.07E-01	87991071	87991194	NC_000005.9
cg18067859	1.15E-11	6.93E-13	7.69	18.3709402	1.50E-01	33776345	33776468	NC_000017.10
cg11785240	1.15E-11	6.94E-13	7.69	18.3692278	7.24E-02	240049338	240049461	NC_000002.11
cg08346159	1.16E-11	6.95E-13	7.69	18.3672346	2.55E-01	164265012	164265135	NC_000004.11
cg20174393	1.16E-11	6.96E-13	7.69	18.3659547	1.23E-01	106258511	106258634	NC_000007.13
cg16235582	1.16E-11	6.96E-13	7.69	18.3654983	1.65E-01	78673212	78673335	NC_000011.9
cg03127416	1.16E-11	6.97E-13	7.69	18.3653202	1.52E-01	22548006	22548129	NC_000008.10

cg18132228	1.16E-11	6.97E-13	7.69	18.3652492	1.18E-01	56035476	56035599	NC_000015.9
cg09643544	1.16E-11	6.98E-13	7.69	18.3634676	1.49E-01	9473696	9473819	NC_000019.9
cg21485303	1.16E-11	6.99E-13	7.69	18.3614849	1.95E-01	22094699	22094822	NC_000012.11
cg27517681	1.17E-11	7.01E-13	7.69	18.3597118	1.58E-01	119528848	119528971	NC_000001.10
cg18138206	1.17E-11	7.01E-13	7.69	18.3590044	1.87E-01	133110731	133110854	NC_000010.10
cg03315869	1.17E-11	7.02E-13	7.69	18.3581817	2.99E-01	134146676	134146799	NC_000011.9
cg05403655	1.17E-11	7.03E-13	7.69	18.3565911	1.81E-01	125215457	125215580	NC_000011.9
cg17761419	1.17E-11	7.03E-13	7.69	18.3558939	9.56E-02	57350749	57350872	NC_000008.10
cg13788381	1.17E-11	7.05E-13	7.69	18.3540133	1.93E-01	17907265	17907388	NC_000001.10
cg02663382	1.17E-11	7.05E-13	7.69	18.3537121	2.17E-01	38308458	38308581	NC_000019.9
cg24502027	1.17E-11	7.05E-13	7.69	18.3534771	9.42E-02	556470	556593	NC_000010.10
cg14095438	1.17E-11	7.05E-13	7.69	18.353236	2.03E-01	49468684	49468807	NC_000008.10
cg08208899	1.17E-11	7.07E-13	7.69	18.3509967	1.52E-01	181931215	181931338	NC_000002.11
cg05478407	1.18E-11	7.09E-13	7.69	18.3474088	1.14E-01	60554897	60555020	NC_000017.10
cg22867608	1.18E-11	7.10E-13	7.69	18.3471362	6.33E-02	64573356	64573479	NC_000010.10
cg18396042	1.18E-11	7.10E-13	7.69	18.3461654	9.81E-02	1567137	1567260	NC_000002.11
cg09935282	1.18E-11	7.11E-13	7.69	18.3446862	2.45E-01	118897280	118897403	NC_000010.10
cg04155086	1.18E-11	7.12E-13	7.69	18.343191	1.20E-01	69207281	69207404	NC_000004.11
cg02633036	1.18E-11	7.13E-13	7.69	18.342956	1.47E-01	92661549	92661672	NC_000015.9
cg09913956	1.19E-11	7.14E-13	7.69	18.3409346	1.80E-01	1776600	1776723	NC_000019.9
cg24005685	1.19E-11	7.15E-13	7.69	18.3401795	1.93E-01	111549880	111550003	NC_000004.11
cg16254309	1.19E-11	7.15E-13	7.69	18.3400122	2.11E-01	145814152	145814275	NC_000007.13
cg02457312	1.19E-11	7.18E-13	7.69	18.3351348	4.97E-02	60347626	60347749	NC_000017.10
cg22823122	1.19E-11	7.19E-13	7.69	18.3339228	2.91E-02	170034076	170034199	NC_000004.11
cg02450613	1.20E-11	7.21E-13	7.69	18.3319754	2.19E-01	37840089	37840212	NC_000005.9

cg01066451	1.20E-11	7.23E-13	7.69	18.3287776	4.06E-02	79083504	79083627	NC_000017.10
cg05415020	1.20E-11	7.24E-13	7.69	18.3276951	2.38E-01	110797797	110797920	NC_000006.11
cg25679864	1.20E-11	7.24E-13	7.69	18.3266943	1.64E-01	134844753	134844876	NC_000010.10
cg13066682	1.21E-11	7.26E-13	7.68	18.3241368	7.43E-02	69786026	69786149	NC_000009.11
cg16533767	1.21E-11	7.27E-13	7.68	18.3226813	1.44E-01	23736552	23736675	NC_000002.11
cg26536401	1.21E-11	7.28E-13	7.68	18.322395	4.02E-02	6956432	6956555	NC_000012.11
cg23369685	1.21E-11	7.29E-13	7.68	18.3203563	1.22E-01	235442529	235442652	NC_000001.10
cg12686847	1.21E-11	7.31E-13	7.68	18.318241	6.19E-02	31779716	31779839	NC_000006.11
cg22685409	1.22E-11	7.33E-13	7.68	18.3155624	1.76E-01	30038692	30038815	NC_000011.9
cg20337384	1.22E-11	7.34E-13	7.68	18.3142745	2.44E-01	39015705	39015828	NC_000007.13
cg16600634	1.22E-11	7.34E-13	7.68	18.3138202	1.51E-01	92907881	92908004	NC_000005.9
cg04684246	1.22E-11	7.34E-13	7.68	18.3136006	8.59E-02	110385989	110386112	NC_000012.11
cg12402555	1.22E-11	7.35E-13	7.68	18.3121291	5.25E-02	17281819	17281942	NC_000001.10
cg02842227	1.22E-11	7.36E-13	7.68	18.3106681	1.64E-01	22370444	22370567	NC_000021.8
cg03257575	1.22E-11	7.37E-13	7.68	18.3096861	2.35E-01	46951318	46951441	NC_000001.10
cg21553182	1.22E-11	7.37E-13	7.68	18.3092788	1.64E-01	52956821	52956944	NC_000019.9
cg03202804	1.22E-11	7.39E-13	7.68	18.3073405	2.27E-01	154710353	154710476	NC_000004.11
cg03237606	1.23E-11	7.40E-13	7.68	18.305899	1.61E-01	63212265	63212388	NC_000010.10
cg01407254	1.23E-11	7.40E-13	7.68	18.3053756	1.47E-01	71787808	71787931	NC_000002.11
cg14993464	1.23E-11	7.42E-13	7.68	18.3029327	4.68E-02	236867278	236867401	NC_000002.11
cg18028483	1.23E-11	7.43E-13	7.68	18.3021827	1.10E-01	109307801	109307924	NC_000006.11
cg13351143	1.23E-11	7.44E-13	7.68	18.3000991	5.76E-02	69292064	69292187	NC_000011.9
cg12077664	1.23E-11	7.45E-13	7.68	18.2993478	1.02E-01	125145446	125145569	NC_000012.11
cg03804272	1.24E-11	7.46E-13	7.68	18.2979251	1.61E-01	59189375	59189498	NC_000005.9
cg08698796	1.24E-11	7.48E-13	7.68	18.2956361	1.17E-01	47561199	47561322	NC_000011.9

cg22910449	1.24E-11	7.48E-13	7.68	18.2955097	1.51E-01	7274234	7274357	NC_000011.9
cg03146993	1.24E-11	7.48E-13	7.68	18.2953218	2.15E-01	35297146	35297269	NC_000017.10
cg13106484	1.24E-11	7.48E-13	7.68	18.2946197	1.59E-01	157692127	157692250	NC_000007.13
cg01982597	1.24E-11	7.49E-13	7.68	18.2936134	5.21E-02	50733420	50733543	NC_000010.10
cg09380135	1.24E-11	7.49E-13	7.68	18.2932837	1.63E-01	40789866	40789989	NC_000019.9
cg04627496	1.24E-11	7.50E-13	7.68	18.2919994	1.72E-01	88799973	88800096	NC_000015.9
cg04249559	1.24E-11	7.51E-13	7.68	18.2908641	1.31E-01	655964	656087	NC_000012.11
cg19284039	1.24E-11	7.52E-13	7.68	18.2899424	1.23E-01	2292895	2293018	NC_000011.9
cg18342866	1.24E-11	7.52E-13	7.68	18.2898702	1.06E-01	114468988	114469111	NC_000001.10
cg25079102	1.25E-11	7.54E-13	7.68	18.2869522	1.95E-01	114843435	114843558	NC_000012.11
cg00346951	1.25E-11	7.56E-13	7.68	18.2850861	3.77E-02	108979731	108979854	NC_000002.11
cg06086198	1.25E-11	7.57E-13	7.68	18.2838713	1.46E-01	29021552	29021675	NC_000012.11
cg08330224	1.25E-11	7.57E-13	7.68	18.2829601	2.32E-01	12996889	12997012	NC_000016.9
cg05535398	1.26E-11	7.59E-13	7.68	18.2809831	1.08E-01	43034257	43034380	NC_000005.9
cg21283167	1.26E-11	7.60E-13	7.68	18.2799996	8.55E-02	47986245	47986368	NC_000001.10
cg00993881	1.26E-11	7.62E-13	7.68	18.2771946	8.43E-02	59681199	59681322	NC_000014.8
cg02886408	1.26E-11	7.62E-13	7.68	18.2769316	1.90E-01	133796164	133796287	NC_000010.10
cg08901089	1.26E-11	7.62E-13	7.68	18.2764302	4.20E-02	148895445	148895568	NC_000007.13
cg19007200	1.26E-11	7.63E-13	7.68	18.275456	1.16E-01	24553899	24554022	NC_000013.10
cg17287235	1.26E-11	7.63E-13	7.68	18.2754325	1.70E-01	131768149	131768272	NC_000010.10
cg14667505	1.26E-11	7.65E-13	7.68	18.273487	1.21E-01	7734159	7734282	NC_000017.10
cg00848728	1.27E-11	7.65E-13	7.68	18.2724253	1.47E-01	58716018	58716141	NC_000001.10
cg23367107	1.27E-11	7.67E-13	7.68	18.2701837	1.81E-01	115136287	115136410	NC_000012.11
cg17859502	1.27E-11	7.67E-13	7.68	18.2700353	6.73E-02	71586893	71587016	NC_000015.9
cg07861373	1.27E-11	7.69E-13	7.68	18.2676998	7.46E-02	73501230	73501353	NC_000017.10

cg11552585	1.28E-11	7.73E-13	7.67	18.262119	6.75E-02	3196206	3196329	NC_000019.9
cg23932873	1.28E-11	7.74E-13	7.67	18.2609475	1.63E-01	113615136	113615259	NC_000001.10
cg17891759	1.28E-11	7.74E-13	7.67	18.2609042	1.64E-01	299121	299244	NC_000004.11
cg22861116	1.28E-11	7.75E-13	7.67	18.2606074	1.89E-01	140762582	140762705	NC_000005.9
cg03171361	1.28E-11	7.75E-13	7.67	18.2600944	1.86E-01	47075758	47075881	NC_000017.10
cg17846334	1.28E-11	7.76E-13	7.67	18.2590239	1.97E-01	49639134	49639257	NC_000020.10
cg03627350	1.28E-11	7.78E-13	7.67	18.2569605	1.26E-01	134694425	134694548	NC_000010.10
cg20612128	1.28E-11	7.78E-13	7.67	18.2567384	1.05E-01	56290261	56290384	NC_000015.9
cg07605285	1.29E-11	7.78E-13	7.67	18.256108	1.46E-01	26680729	26680852	NC_000010.10
cg19628148	1.29E-11	7.79E-13	7.67	18.2550904	1.68E-01	126113918	126114041	NC_000003.11
cg08076158	1.29E-11	7.81E-13	7.67	18.2520003	1.65E-01	86539022	86539145	NC_000016.9
cg10123293	1.29E-11	7.83E-13	7.67	18.2498826	1.31E-01	99228465	99228588	NC_000002.11
cg27434368	1.29E-11	7.84E-13	7.67	18.2490286	1.30E-01	19650978	19651101	NC_000019.9
cg14500776	1.29E-11	7.84E-13	7.67	18.2488518	8.21E-02	27256176	27256299	NC_000007.13
cg18076755	1.29E-11	7.84E-13	7.67	18.248299	5.16E-02	133743453	133743576	NC_000010.10
cg13807970	1.30E-11	7.86E-13	7.67	18.2466185	2.58E-01	145274441	145274564	NC_000002.11
cg19137748	1.30E-11	7.86E-13	7.67	18.2461591	1.30E-01	69466054	69466177	NC_000011.9
cg25915982	1.30E-11	7.86E-13	7.67	18.2459854	1.30E-01	50849415	50849538	NC_000007.13
cg07943548	1.30E-11	7.86E-13	7.67	18.2457057	1.07E-01	86304357	86304480	NC_000015.9
cg23082339	1.30E-11	7.87E-13	7.67	18.2454549	2.39E-01	140810051	140810174	NC_000005.9
cg04620091	1.30E-11	7.87E-13	7.67	18.2452547	2.30E-01	134871805	134871928	NC_000005.9
cg08638184	1.30E-11	7.87E-13	7.67	18.2450745	1.56E-01	19183563	19183686	NC_000007.13
cg03893692	1.30E-11	7.91E-13	7.67	18.2404025	1.13E-01	149669484	149669607	NC_000005.9
cg27112565	1.31E-11	7.92E-13	7.67	18.238232	2.14E-01	51259552	51259675	NC_000002.11
cg01663407	1.31E-11	7.94E-13	7.67	18.2361083	1.35E-01	64754308	64754431	NC_000002.11

cg02264082	1.31E-11	7.97E-13	7.67	18.2321161	1.95E-01	130235850	130235973	NC_000003.11
cg24110839	1.32E-11	7.99E-13	7.67	18.2305796	5.16E-02	36896577	36896700	NC_000017.10
cg27125849	1.32E-11	8.00E-13	7.67	18.2290935	1.63E-01	59473674	59473797	NC_000017.10
cg24152605	1.32E-11	8.00E-13	7.67	18.2283361	2.66E-01	57050359	57050482	NC_000019.9
cg03729337	1.32E-11	8.01E-13	7.67	18.2281932	1.42E-01	122430019	122430142	NC_000005.9
cg17494034	1.32E-11	8.01E-13	7.67	18.2280079	1.50E-01	12513497	12513620	NC_000001.10
cg03631065	1.32E-11	8.01E-13	7.67	18.227045	1.38E-01	96672440	96672563	NC_000013.10
cg08648286	1.32E-11	8.02E-13	7.67	18.225918	9.94E-02	23549467	23549590	NC_000020.10
cg12394777	1.32E-11	8.03E-13	7.67	18.2253896	1.17E-01	171224431	171224554	NC_000002.11
cg05707833	1.33E-11	8.06E-13	7.67	18.221538	9.40E-02	77203371	77203494	NC_000018.9
cg13442811	1.33E-11	8.08E-13	7.67	18.2194375	8.99E-02	86131100	86131223	NC_000010.10
cg10824107	1.33E-11	8.09E-13	7.67	18.2184364	1.10E-01	10382069	10382192	NC_000006.11
cg25839023	1.33E-11	8.10E-13	7.67	18.2165869	1.46E-01	22414349	22414472	NC_000003.11
cg09307530	1.33E-11	8.10E-13	7.67	18.2161925	1.49E-01	2739257	2739380	NC_000005.9
cg15126544	1.33E-11	8.10E-13	7.67	18.2160966	2.14E-01	171678954	171679077	NC_000002.11
cg16737409	1.34E-11	8.11E-13	7.67	18.2154524	8.80E-02	57428366	57428489	NC_000020.10
cg08145262	1.34E-11	8.14E-13	7.67	18.2121589	8.43E-02	69869974	69870097	NC_000012.11
cg03828874	1.34E-11	8.14E-13	7.67	18.2115222	1.51E-01	3605954	3606077	NC_000005.9
cg12272198	1.34E-11	8.15E-13	7.67	18.2109269	1.09E-01	47808805	47808928	NC_000018.9
cg03921753	1.34E-11	8.15E-13	7.67	18.2103361	2.33E-01	28074670	28074793	NC_000016.9
cg08421768	1.34E-11	8.16E-13	7.67	18.209559	1.25E-01	123797144	123797267	NC_000004.11
cg04617640	1.35E-11	8.18E-13	7.67	18.207032	1.50E-01	46874243	46874366	NC_000021.8
cg26345105	1.35E-11	8.19E-13	7.67	18.206299	1.31E-01	228634265	228634388	NC_000001.10
cg11686427	1.35E-11	8.20E-13	7.66	18.2044368	1.85E-01	140777473	140777596	NC_000005.9
cg18294610	1.35E-11	8.20E-13	7.66	18.204369	9.01E-02	18138837	18138960	NC_000011.9

cg25636665	1.35E-11	8.21E-13	7.66	18.2036013	2.09E-01	80549579	80549702	NC_000002.11
cg23337631	1.35E-11	8.21E-13	7.66	18.2034691	1.11E-01	52994933	52995056	NC_000012.11
cg18806980	1.35E-11	8.23E-13	7.66	18.2007618	8.61E-02	84438123	84438246	NC_000016.9
cg06083330	1.36E-11	8.24E-13	7.66	18.1999322	2.04E-01	147561203	147561326	NC_000004.11
cg21776577	1.36E-11	8.24E-13	7.66	18.1998827	1.61E-01	180811774	180811897	NC_000001.10
cg11212312	1.36E-11	8.26E-13	7.66	18.1977198	9.55E-02	131395683	131395806	NC_000005.9
cg25230117	1.36E-11	8.26E-13	7.66	18.197598	1.08E-01	146104495	146104618	NC_000004.11
cg14411266	1.36E-11	8.27E-13	7.66	18.1960525	1.82E-01	40679582	40679705	NC_000002.11
cg12551635	1.36E-11	8.28E-13	7.66	18.1947556	5.75E-02	27994779	27994902	NC_000001.10
cg03181618	1.36E-11	8.29E-13	7.66	18.1935577	1.28E-01	160113343	160113466	NC_000002.11
cg26161820	1.36E-11	8.29E-13	7.66	18.1934407	5.83E-02	37792777	37792900	NC_000017.10
cg08575722	1.36E-11	8.29E-13	7.66	18.1933339	1.60E-01	32421845	32421968	NC_000011.9
cg19270505	1.37E-11	8.32E-13	7.66	18.1901395	2.29E-01	25256705	25256828	NC_000001.10
cg26787096	1.37E-11	8.32E-13	7.66	18.1901328	7.26E-02	72094989	72095112	NC_000015.9
cg09406856	1.37E-11	8.33E-13	7.66	18.1895338	1.25E-01	66491863	66491986	NC_000003.11
cg04455430	1.37E-11	8.33E-13	7.66	18.1890311	1.30E-01	50886920	50887043	NC_000001.10
cg04883903	1.37E-11	8.34E-13	7.66	18.1883346	2.03E-01	117798954	117799077	NC_000012.11
cg26333224	1.37E-11	8.35E-13	7.66	18.1863684	1.21E-01	57388283	57388406	NC_000010.10
cg10287814	1.37E-11	8.36E-13	7.66	18.1853034	7.59E-02	74019633	74019756	NC_000017.10
cg07466705	1.37E-11	8.36E-13	7.66	18.1852345	1.68E-01	11034749	11034872	NC_000003.11
cg18252903	1.37E-11	8.37E-13	7.66	18.1847003	1.95E-01	147072643	147072766	NC_000003.11
cg21225430	1.38E-11	8.37E-13	7.66	18.1838647	9.36E-02	39792959	39793082	NC_000022.10
cg26292442	1.38E-11	8.40E-13	7.66	18.1813226	1.14E-01	10693912	10694035	NC_000019.9
cg06780703	1.38E-11	8.40E-13	7.66	18.1808399	3.91E-02	161358133	161358256	NC_000001.10
cg15870334	1.38E-11	8.40E-13	7.66	18.1807244	1.65E-01	5891982	5892105	NC_000004.11

cg03227021	1.38E-11	8.41E-13	7.66	18.1800867	1.44E-01	112711380	112711503	NC_000013.10
cg27522078	1.38E-11	8.42E-13	7.66	18.1782454	1.07E-01	22290005	22290128	NC_000001.10
cg04130427	1.38E-11	8.43E-13	7.66	18.1772487	1.66E-01	77113915	77114038	NC_000009.11
cg01363324	1.39E-11	8.44E-13	7.66	18.175866	1.29E-01	124979353	124979476	NC_000009.11
cg23170850	1.39E-11	8.44E-13	7.66	18.1756964	1.80E-01	134145889	134146012	NC_000011.9
cg00840310	1.39E-11	8.45E-13	7.66	18.1754284	2.16E-01	74061514	74061637	NC_000009.11
cg19759481	1.39E-11	8.46E-13	7.66	18.1736793	1.81E-01	27183401	27183524	NC_000007.13
cg20980055	1.39E-11	8.47E-13	7.66	18.1726952	1.93E-01	133562258	133562381	NC_000006.11
cg25888561	1.39E-11	8.48E-13	7.66	18.1710711	1.49E-01	44163067	44163190	NC_000010.10
cg02178032	1.40E-11	8.50E-13	7.66	18.1687063	8.14E-02	11213424	11213547	NC_000008.10
cg24878115	1.40E-11	8.51E-13	7.66	18.1680341	1.24E-01	18545062	18545185	NC_000019.9
cg26822480	1.40E-11	8.53E-13	7.66	18.1653525	8.52E-02	84341323	84341446	NC_000016.9
cg01238073	1.40E-11	8.55E-13	7.66	18.1631443	8.40E-02	50766441	50766564	NC_000007.13
cg18582342	1.40E-11	8.55E-13	7.66	18.1631439	1.34E-01	11591989	11592112	NC_000019.9
cg19239040	1.40E-11	8.56E-13	7.66	18.1625757	1.46E-01	35298664	35298787	NC_000007.13
cg27089736	1.41E-11	8.58E-13	7.66	18.1603299	5.25E-02	3714278	3714401	NC_000002.11
cg01572884	1.41E-11	8.59E-13	7.66	18.1590065	1.14E-01	110610266	110610389	NC_000001.10
cg02093902	1.41E-11	8.60E-13	7.66	18.1572835	2.05E-01	45501532	45501655	NC_000002.11
cg14470398	1.41E-11	8.62E-13	7.66	18.1551978	2.07E-01	657536	657659	NC_000004.11
cg16435923	1.41E-11	8.62E-13	7.66	18.1549352	9.40E-02	140744155	140744278	NC_000005.9
cg03355526	1.41E-11	8.63E-13	7.66	18.1546913	1.44E-01	178368415	178368538	NC_000005.9
cg09970120	1.42E-11	8.63E-13	7.66	18.154028	1.83E-01	155167844	155167967	NC_000007.13
cg00026546	1.42E-11	8.64E-13	7.66	18.1533645	5.79E-02	33354785	33354908	NC_000001.10
cg14273502	1.42E-11	8.65E-13	7.66	18.1519346	1.21E-01	113013494	113013617	NC_000012.11
cg12864235	1.42E-11	8.67E-13	7.66	18.1501565	2.07E-01	27038782	27038905	NC_000005.9

cg01090089	1.43E-11	8.71E-13	7.65	18.1447975	2.00E-01	6570974	6571097	NC_000007.13
cg16435571	1.43E-11	8.71E-13	7.65	18.1446953	1.93E-01	10858003	10858126	NC_000003.11
cg26661718	1.44E-11	8.76E-13	7.65	18.1389079	9.51E-02	36718014	36718137	NC_000001.10
cg24650501	1.44E-11	8.77E-13	7.65	18.1381897	1.77E-01	165324990	165325113	NC_000001.10
cg02348151	1.44E-11	8.77E-13	7.65	18.1380812	2.03E-01	34442257	34442380	NC_000021.8
cg24975581	1.44E-11	8.78E-13	7.65	18.1376802	1.33E-01	121128268	121128391	NC_000012.11
cg01076459	1.44E-11	8.78E-13	7.65	18.1372151	1.28E-01	2908230	2908353	NC_000017.10
cg07887163	1.44E-11	8.78E-13	7.65	18.1367223	5.17E-02	65958288	65958411	NC_000012.11
cg22635491	1.44E-11	8.80E-13	7.65	18.1348931	1.85E-01	115109934	115110057	NC_000012.11
cg19364068	1.44E-11	8.80E-13	7.65	18.1346917	1.13E-01	46734104	46734227	NC_000019.9
cg00646019	1.45E-11	8.83E-13	7.65	18.1319676	1.69E-01	29034163	29034286	NC_000015.9
cg12252868	1.45E-11	8.83E-13	7.65	18.1311126	9.35E-02	112383396	112383519	NC_000002.11
cg26541867	1.45E-11	8.84E-13	7.65	18.1305428	2.56E-01	37246688	37246811	NC_000004.11
cg24287189	1.45E-11	8.84E-13	7.65	18.1303906	5.31E-02	98676253	98676376	NC_000007.13
cg01297721	1.45E-11	8.86E-13	7.65	18.1282909	1.68E-01	19373566	19373689	NC_000019.9
cg20924470	1.45E-11	8.86E-13	7.65	18.1279489	2.08E-01	135265849	135265972	NC_000005.9
cg22624827	1.45E-11	8.88E-13	7.65	18.1261149	1.39E-01	64058180	64058303	NC_000001.10
cg11525643	1.45E-11	8.88E-13	7.65	18.1260182	2.09E-01	35046765	35046888	NC_000015.9
cg13978542	1.45E-11	8.88E-13	7.65	18.1259776	6.51E-02	138973152	138973275	NC_000009.11
cg16997312	1.46E-11	8.90E-13	7.65	18.1237705	6.25E-02	160314505	160314628	NC_000001.10
cg09022943	1.46E-11	8.93E-13	7.65	18.1205747	1.79E-01	98511210	98511333	NC_000001.10
cg19058585	1.46E-11	8.94E-13	7.65	18.1188867	4.60E-02	112944983	112945106	NC_000002.11
cg03130248	1.46E-11	8.95E-13	7.65	18.1183986	1.48E-01	245524538	245524661	NC_000001.10
cg09744068	1.47E-11	9.01E-13	7.65	18.1117137	1.38E-01	125732787	125732910	NC_000010.10
cg17080163	1.48E-11	9.03E-13	7.65	18.1097443	1.89E-01	123418757	123418880	NC_000002.11

cg09674093	1.48E-11	9.06E-13	7.65	18.1063592	6.26E-02	23646462	23646585	NC_000002.11
cg21947116	1.48E-11	9.06E-13	7.65	18.1059682	4.87E-02	46918189	46918312	NC_000021.8
cg14855877	1.48E-11	9.07E-13	7.65	18.1054115	2.94E-02	12905834	12905957	NC_000017.10
cg24119717	1.48E-11	9.08E-13	7.65	18.1042977	1.59E-01	139488731	139488854	NC_000005.9
cg14977066	1.49E-11	9.09E-13	7.65	18.1034156	2.18E-01	50891195	50891318	NC_000001.10
cg21893358	1.49E-11	9.09E-13	7.65	18.1028702	1.15E-01	120434880	120435003	NC_000011.9
cg22745397	1.49E-11	9.10E-13	7.65	18.1022068	1.46E-01	42122161	42122284	NC_000004.11
cg12864185	1.49E-11	9.11E-13	7.65	18.1012911	1.60E-01	240160972	240161095	NC_000001.10
cg26872137	1.49E-11	9.12E-13	7.65	18.0993786	1.84E-01	35092870	35092993	NC_000008.10
cg24298311	1.49E-11	9.13E-13	7.65	18.098162	2.12E-01	158119610	158119733	NC_000001.10
cg17442776	1.49E-11	9.14E-13	7.65	18.0971523	8.85E-02	147718540	147718663	NC_000001.10
cg07919443	1.50E-11	9.15E-13	7.65	18.0964937	2.00E-01	102419409	102419532	NC_000010.10
cg18789663	1.50E-11	9.17E-13	7.65	18.094235	2.24E-01	242688591	242688714	NC_000001.10
cg02446647	1.50E-11	9.18E-13	7.65	18.0933718	1.98E-01	133562101	133562224	NC_000006.11
cg07147166	1.50E-11	9.18E-13	7.65	18.0930443	1.68E-01	147569185	147569308	NC_000004.11
cg15428578	1.50E-11	9.18E-13	7.65	18.0929358	2.21E-01	33603312	33603435	NC_000015.9
cg00802168	1.50E-11	9.19E-13	7.65	18.091812	5.14E-02	32047005	32047128	NC_000006.11
cg13675957	1.50E-11	9.20E-13	7.65	18.0914475	3.18E-02	39672463	39672586	NC_000017.10
cg17277529	1.50E-11	9.21E-13	7.65	18.0898238	1.78E-01	69633959	69634082	NC_000011.9
cg25366582	1.51E-11	9.23E-13	7.64	18.0882932	2.58E-01	25621027	25621150	NC_000013.10
cg22274539	1.51E-11	9.23E-13	7.64	18.0880522	1.84E-01	103696209	103696332	NC_000012.11
cg23323341	1.51E-11	9.24E-13	7.64	18.0869801	7.96E-02	46722583	46722706	NC_000021.8
cg07291445	1.51E-11	9.24E-13	7.64	18.0863774	1.89E-01	39015928	39016051	NC_000007.13
cg14526047	1.51E-11	9.25E-13	7.64	18.0858689	1.85E-01	50887616	50887739	NC_000010.10
cg17827208	1.52E-11	9.31E-13	7.64	18.0799847	1.12E-01	99470414	99470537	NC_000015.9

cg26016241	1.52E-11	9.33E-13	7.64	18.0772217	7.29E-02	17571656	17571779	NC_000019.9
cg10797129	1.53E-11	9.37E-13	7.64	18.0735394	4.92E-02	100745314	100745437	NC_000011.9
cg13702053	1.53E-11	9.37E-13	7.64	18.0730291	1.61E-01	2752601	2752724	NC_000005.9
cg24366314	1.53E-11	9.38E-13	7.64	18.0715794	1.46E-01	88329680	88329803	NC_000013.10
cg17918002	1.54E-11	9.41E-13	7.64	18.0685295	1.81E-01	28035719	28035842	NC_000010.10
cg17478016	1.54E-11	9.42E-13	7.64	18.0674132	9.61E-02	169846686	169846809	NC_000006.11
cg03724423	1.55E-11	9.47E-13	7.64	18.0624118	1.51E-01	27170755	27170878	NC_000007.13
cg07092157	1.55E-11	9.48E-13	7.64	18.0611944	4.11E-02	89353329	89353452	NC_000012.11
cg25494992	1.55E-11	9.49E-13	7.64	18.0609766	6.12E-02	130757650	130757773	NC_000010.10
cg16191088	1.55E-11	9.49E-13	7.64	18.0603745	9.57E-02	8654389	8654512	NC_000019.9
cg17152436	1.55E-11	9.50E-13	7.64	18.0595605	1.29E-01	1318418	1318541	NC_000011.9
cg07798593	1.55E-11	9.50E-13	7.64	18.0594852	7.40E-02	80890238	80890361	NC_000010.10
cg03151319	1.55E-11	9.50E-13	7.64	18.0593996	5.58E-02	31637636	31637759	NC_000006.11
cg14871414	1.55E-11	9.52E-13	7.64	18.0579439	2.51E-01	130687581	130687704	NC_000006.11
cg27325190	1.55E-11	9.52E-13	7.64	18.0569895	4.70E-02	74668981	74669104	NC_000011.9
cg26791464	1.55E-11	9.53E-13	7.64	18.0569565	9.56E-02	40769744	40769867	NC_000001.10
cg15212418	1.55E-11	9.53E-13	7.64	18.0565768	1.15E-01	155045	155168	NC_000017.10
cg12786548	1.55E-11	9.53E-13	7.64	18.0564103	2.04E-01	102507733	102507856	NC_000010.10
cg12307237	1.56E-11	9.55E-13	7.64	18.0542965	1.38E-01	178735241	178735364	NC_000005.9
cg02981663	1.56E-11	9.55E-13	7.64	18.054152	1.52E-01	28232083	28232206	NC_000013.10
cg16994506	1.56E-11	9.56E-13	7.64	18.0538095	2.35E-01	4383724	4383847	NC_000012.11
cg08985029	1.56E-11	9.56E-13	7.64	18.0529796	2.33E-01	114843296	114843419	NC_000012.11
cg06911354	1.57E-11	9.62E-13	7.64	18.0469714	2.05E-01	27197455	27197578	NC_000007.13
cg08607209	1.57E-11	9.62E-13	7.64	18.0469473	1.71E-01	52610808	52610931	NC_000006.11
cg02065182	1.57E-11	9.65E-13	7.64	18.044554	4.63E-02	2637029	2637152	NC_000006.11

cg17676835	1.57E-11	9.65E-13	7.64	18.0438576	4.36E-02	1007395	1007518	NC_000011.9
cg04178787	1.58E-11	9.68E-13	7.64	18.0412932	1.98E-01	123418787	123418910	NC_000002.11
cg06498720	1.58E-11	9.68E-13	7.64	18.0406323	1.46E-01	73147481	73147604	NC_000002.11
cg00656603	1.59E-11	9.74E-13	7.64	18.0351349	1.35E-01	1070957	1071080	NC_000016.9
cg02536995	1.59E-11	9.75E-13	7.64	18.0338296	5.74E-02	107222842	107222965	NC_000006.11
cg12167688	1.59E-11	9.75E-13	7.64	18.0336073	1.02E-01	2771225	2771348	NC_000017.10
cg02420615	1.59E-11	9.76E-13	7.64	18.033174	3.72E-02	149150718	149150841	NC_000007.13
cg07531356	1.59E-11	9.76E-13	7.64	18.0325976	1.10E-01	5185951	5186074	NC_000009.11
cg01942816	1.59E-11	9.77E-13	7.64	18.0322938	1.61E-01	5535463	5535586	NC_000007.13
cg14558529	1.59E-11	9.77E-13	7.64	18.0315398	1.84E-01	27648341	27648464	NC_000006.11
cg14442746	1.59E-11	9.79E-13	7.64	18.0294966	5.70E-02	56264572	56264695	NC_000014.8
cg14768785	1.59E-11	9.80E-13	7.63	18.0292076	1.99E-01	172166517	172166640	NC_000003.11
cg07994987	1.59E-11	9.80E-13	7.63	18.0286428	2.06E-01	6007769	6007892	NC_000006.11
cg10381520	1.60E-11	9.81E-13	7.63	18.0283264	4.98E-02	33396942	33397065	NC_000010.10
cg14101623	1.60E-11	9.81E-13	7.63	18.0283011	5.92E-02	138350387	138350510	NC_000007.13
cg26917367	1.60E-11	9.81E-13	7.63	18.0276988	9.01E-02	57352807	57352930	NC_000019.9
cg05375680	1.60E-11	9.83E-13	7.63	18.0260268	4.61E-02	53521504	53521627	NC_000017.10
cg13926358	1.60E-11	9.86E-13	7.63	18.0228142	1.24E-01	13915659	13915782	NC_000019.9
cg07498275	1.60E-11	9.87E-13	7.63	18.0222735	1.69E-01	51184886	51185009	NC_000016.9
cg15549216	1.60E-11	9.87E-13	7.63	18.0220889	1.45E-01	118368264	118368387	NC_000010.10
cg12301921	1.61E-11	9.89E-13	7.63	18.01993	9.54E-02	1122879	1123002	NC_000005.9
cg21448471	1.62E-11	9.95E-13	7.63	18.0142529	9.79E-02	10407538	10407661	NC_000019.9
cg07676709	1.62E-11	9.95E-13	7.63	18.0141227	1.57E-01	46673442	46673565	NC_000017.10
cg11689813	1.62E-11	9.95E-13	7.63	18.0139306	2.39E-01	229570052	229570175	NC_000001.10
cg10324224	1.62E-11	9.96E-13	7.63	18.0132805	9.49E-02	231115997	231116120	NC_000001.10

cg18328894	1.62E-11	9.96E-13	7.63	18.013176	2.21E-01	175224563	175224686	NC_000005.9
cg09559950	1.62E-11	9.98E-13	7.63	18.0113572	1.36E-01	4873514	4873637	NC_000004.11
cg24873464	1.62E-11	9.98E-13	7.63	18.0107746	2.20E-01	2139977	2140100	NC_000003.11
cg23676551	1.62E-11	9.99E-13	7.63	18.0095845	1.82E-01	2161374	2161497	NC_000011.9
cg11398452	1.62E-11	1.00E-12	7.63	18.0091705	2.36E-01	118896755	118896878	NC_000010.10
cg13283952	1.62E-11	1.00E-12	7.63	18.0090411	1.30E-01	121412005	121412128	NC_000002.11
cg09412850	1.63E-11	1.00E-12	7.63	18.006505	5.82E-02	132835834	132835957	NC_000012.11
cg18591945	1.63E-11	1.00E-12	7.63	18.0064802	1.24E-01	105962171	105962294	NC_000014.8
cg13842293	1.63E-11	1.00E-12	7.63	18.0052632	1.07E-01	35859090	35859213	NC_000009.11
cg11532513	1.63E-11	1.01E-12	7.63	18.0039691	1.24E-01	54962242	54962365	NC_000003.11
cg20016673	1.63E-11	1.01E-12	7.63	18.0029524	1.40E-01	108023366	108023489	NC_000001.10
cg08196642	1.64E-11	1.01E-12	7.63	18.0022995	1.09E-01	138515409	138515532	NC_000005.9
cg05859777	1.64E-11	1.01E-12	7.63	18.001899	2.03E-01	2161102	2161225	NC_000011.9
cg04069351	1.64E-11	1.01E-12	7.63	18.001018	1.11E-01	46732972	46733095	NC_000022.10
cg11260904	1.64E-11	1.01E-12	7.63	17.9996232	1.96E-01	134872029	134872152	NC_000005.9
cg04711622	1.65E-11	1.01E-12	7.63	17.9946189	1.20E-01	64578638	64578761	NC_000005.9
cg04908789	1.65E-11	1.02E-12	7.63	17.9930935	1.84E-01	59001676	59001799	NC_000018.9
cg22762492	1.65E-11	1.02E-12	7.63	17.9925804	1.83E-01	3591171	3591294	NC_000005.9
cg27279508	1.66E-11	1.02E-12	7.63	17.9894842	9.66E-02	31476672	31476795	NC_000013.10
cg07775420	1.66E-11	1.02E-12	7.63	17.9850759	7.27E-02	110159458	110159581	NC_000012.11
cg19287858	1.66E-11	1.03E-12	7.63	17.9838383	1.25E-01	130181841	130181964	NC_000011.9
cg26473844	1.67E-11	1.03E-12	7.63	17.9834516	1.24E-01	37834909	37835032	NC_000005.9
cg10883064	1.67E-11	1.03E-12	7.63	17.9821926	1.71E-01	60757346	60757469	NC_000017.10
cg07071978	1.67E-11	1.03E-12	7.63	17.9810167	2.12E-01	13933652	13933775	NC_000010.10
cg23829949	1.67E-11	1.03E-12	7.63	17.9805726	1.76E-01	244214679	244214802	NC_000001.10

cg09000583	1.67E-11	1.03E-12	7.63	17.9785434	2.37E-01	46802888	46803011	NC_000017.10
cg16137498	1.67E-11	1.03E-12	7.63	17.9781396	1.29E-01	101286541	101286664	NC_000010.10
cg18409732	1.68E-11	1.03E-12	7.63	17.9758474	1.60E-01	4866454	4866577	NC_000004.11
cg07373578	1.68E-11	1.04E-12	7.63	17.9738713	8.01E-02	55993519	55993642	NC_000017.10
cg18476557	1.68E-11	1.04E-12	7.63	17.9728468	9.62E-02	77386989	77387112	NC_000018.9
cg12047941	1.69E-11	1.04E-12	7.62	17.9703378	1.17E-01	315908	316031	NC_000011.9
cg23541422	1.69E-11	1.04E-12	7.62	17.9696771	1.42E-01	8714363	8714486	NC_000002.11
cg17939805	1.70E-11	1.05E-12	7.62	17.9646749	1.51E-01	44773988	44774111	NC_000018.9
cg14645017	1.70E-11	1.05E-12	7.62	17.9614947	1.55E-01	132977408	132977531	NC_000010.10
cg19240319	1.70E-11	1.05E-12	7.62	17.9601014	8.92E-02	161427101	161427224	NC_000001.10
cg25903072	1.70E-11	1.05E-12	7.62	17.9588535	9.82E-02	63334703	63334826	NC_000015.9
cg21532325	1.71E-11	1.05E-12	7.62	17.9582678	2.27E-01	151107400	151107523	NC_000007.13
cg10919204	1.71E-11	1.05E-12	7.62	17.9577817	1.36E-01	31193340	31193463	NC_000005.9
cg16321280	1.71E-11	1.05E-12	7.62	17.957717	1.64E-01	44332972	44333095	NC_000011.9
cg08569517	1.71E-11	1.05E-12	7.62	17.956661	1.09E-01	2424793	2424916	NC_000019.9
cg13604337	1.71E-11	1.06E-12	7.62	17.9557836	1.93E-01	54321653	54321776	NC_000012.11
cg02539855	1.71E-11	1.06E-12	7.62	17.9530442	2.33E-01	144241434	144241557	NC_000008.10
cg25142390	1.72E-11	1.06E-12	7.62	17.9523774	9.21E-02	6534951	6535074	NC_000001.10
cg23524195	1.72E-11	1.06E-12	7.62	17.9516315	1.94E-01	118034031	118034154	NC_000010.10
cg19685491	1.72E-11	1.06E-12	7.62	17.9515477	1.47E-01	2800278	2800401	NC_000012.11
cg12113984	1.72E-11	1.06E-12	7.62	17.9513678	2.10E-01	108238461	108238584	NC_000012.11
cg10815420	1.72E-11	1.06E-12	7.62	17.9497507	1.16E-01	105599835	105599958	NC_000008.10
cg27423216	1.72E-11	1.06E-12	7.62	17.9488514	1.53E-01	5543269	5543392	NC_000012.11
cg13392861	1.73E-11	1.07E-12	7.62	17.9455838	8.77E-02	37914861	37914984	NC_000009.11
cg15286180	1.73E-11	1.07E-12	7.62	17.9444987	1.06E-01	134679435	134679558	NC_000010.10

cg19746567	1.73E-11	1.07E-12	7.62	17.9443126	1.01E-01	228495111	228495234	NC_000001.10
cg07345734	1.73E-11	1.07E-12	7.62	17.9442948	2.67E-01	36986363	36986486	NC_000014.8
cg15089111	1.73E-11	1.07E-12	7.62	17.943825	1.40E-01	34270113	34270236	NC_000014.8
cg06133145	1.74E-11	1.07E-12	7.62	17.9401665	2.15E-01	94254351	94254474	NC_000014.8
cg24239329	1.74E-11	1.07E-12	7.62	17.9399674	2.59E-01	36705622	36705745	NC_000013.10
cg11362010	1.74E-11	1.08E-12	7.62	17.9354708	2.09E-01	181442713	181442836	NC_000003.11
cg11988604	1.75E-11	1.08E-12	7.62	17.9328031	1.25E-01	48551938	48552061	NC_000017.10
cg02635449	1.75E-11	1.08E-12	7.62	17.9306483	1.16E-01	109926	110049	NC_000017.10
cg25802465	1.75E-11	1.08E-12	7.62	17.9301201	7.54E-02	107865768	107865891	NC_000013.10
cg23588462	1.76E-11	1.09E-12	7.62	17.928198	1.79E-01	95357223	95357346	NC_000013.10
cg14496450	1.76E-11	1.09E-12	7.62	17.9277353	2.19E-01	77178924	77179047	NC_000017.10
cg14405924	1.76E-11	1.09E-12	7.62	17.9271617	1.27E-01	131758112	131758235	NC_000010.10
cg01870995	1.76E-11	1.09E-12	7.62	17.9263946	2.10E-01	3602546	3602669	NC_000012.11
cg13671919	1.76E-11	1.09E-12	7.62	17.924741	1.35E-01	5196874	5196997	NC_000018.9
cg07547000	1.76E-11	1.09E-12	7.62	17.9247082	2.05E-01	154728210	154728333	NC_000002.11
cg23373966	1.76E-11	1.09E-12	7.62	17.9246544	7.51E-02	20668719	20668842	NC_000006.11
cg13912115	1.76E-11	1.09E-12	7.62	17.9230534	2.19E-01	33936362	33936485	NC_000005.9
cg01810713	1.77E-11	1.09E-12	7.62	17.9205545	8.54E-02	85966031	85966154	NC_000016.9
cg08393041	1.77E-11	1.10E-12	7.62	17.9190453	1.83E-01	147109426	147109549	NC_000003.11
cg05694095	1.77E-11	1.10E-12	7.62	17.9188606	1.13E-01	169659745	169659868	NC_000005.9
cg16202340	1.77E-11	1.10E-12	7.62	17.9185519	1.58E-01	4867839	4867962	NC_000004.11
cg16163765	1.77E-11	1.10E-12	7.62	17.9183466	1.22E-01	40365499	40365622	NC_000001.10
cg08422054	1.77E-11	1.10E-12	7.62	17.9181688	1.39E-01	100875565	100875688	NC_000003.11
cg00324748	1.77E-11	1.10E-12	7.62	17.9179846	1.83E-01	80505324	80505447	NC_000010.10
cg08875705	1.78E-11	1.10E-12	7.62	17.9135925	2.18E-01	6665330	6665453	NC_000012.11

cg11523290	1.78E-11	1.10E-12	7.62	17.9130216	2.06E-01	10463256	10463379	NC_000004.11
cg18274896	1.78E-11	1.10E-12	7.62	17.9129198	7.59E-02	140395754	140395877	NC_000009.11
cg12389397	1.78E-11	1.10E-12	7.62	17.9127377	5.23E-02	110313543	110313666	NC_000001.10
cg12847373	1.78E-11	1.10E-12	7.62	17.9127102	1.95E-01	78493349	78493472	NC_000013.10
cg14100973	1.78E-11	1.10E-12	7.61	17.9107936	1.64E-01	87280816	87280939	NC_000004.11
cg17634650	1.78E-11	1.10E-12	7.61	17.910691	8.20E-02	10966220	10966343	NC_000004.11
cg19430967	1.79E-11	1.11E-12	7.61	17.9097364	1.91E-01	132757741	132757864	NC_000003.11
cg24056153	1.79E-11	1.11E-12	7.61	17.909355	2.48E-02	90988694	90988817	NC_000014.8
cg00589550	1.79E-11	1.11E-12	7.61	17.9086233	2.30E-02	57099057	57099180	NC_000016.9
cg05031741	1.79E-11	1.11E-12	7.61	17.9080803	2.24E-01	89314419	89314542	NC_000016.9
cg05423392	1.79E-11	1.11E-12	7.61	17.9075578	1.15E-01	34586837	34586960	NC_000016.9
cg17194154	1.79E-11	1.11E-12	7.61	17.907356	1.66E-01	95359974	95360097	NC_000013.10
cg18436984	1.79E-11	1.11E-12	7.61	17.906281	1.53E-01	27198374	27198497	NC_000007.13
cg14688579	1.80E-11	1.11E-12	7.61	17.9042946	1.79E-01	223168453	223168576	NC_000002.11
cg04622777	1.80E-11	1.12E-12	7.61	17.9013059	1.90E-01	55357776	55357899	NC_000016.9
cg05832509	1.80E-11	1.12E-12	7.61	17.9002281	7.94E-02	99515080	99515203	NC_000007.13
cg18651870	1.80E-11	1.12E-12	7.61	17.8999122	1.49E-01	70535005	70535128	NC_000018.9
cg00185566	1.80E-11	1.12E-12	7.61	17.8995271	2.35E-01	14925856	14925979	NC_000001.10
cg01396176	1.80E-11	1.12E-12	7.61	17.8992951	1.56E-01	114881783	114881906	NC_000012.11
cg01831805	1.80E-11	1.12E-12	7.61	17.8986747	4.64E-02	24254624	24254747	NC_000022.10
cg04794372	1.81E-11	1.12E-12	7.61	17.8984311	8.37E-02	113773201	113773324	NC_000003.11
cg00931558	1.81E-11	1.12E-12	7.61	17.8982281	2.06E-01	77594664	77594787	NC_000008.10
cg14244013	1.81E-11	1.12E-12	7.61	17.8966388	1.16E-01	59473124	59473247	NC_000017.10
cg03763508	1.81E-11	1.12E-12	7.61	17.8956139	1.86E-01	27140942	27141065	NC_000007.13
cg02409090	1.81E-11	1.12E-12	7.61	17.8946696	9.52E-02	709725	709848	NC_000006.11

cg16332936	1.81E-11	1.12E-12	7.61	17.8945014	1.59E-01	19748910	19749033	NC_000022.10
cg22749589	1.81E-11	1.12E-12	7.61	17.8934523	1.92E-01	21501710	21501833	NC_000020.10
cg12851526	1.82E-11	1.13E-12	7.61	17.8908183	1.28E-01	100609034	100609157	NC_000007.13
cg24196693	1.82E-11	1.13E-12	7.61	17.8881527	1.96E-01	85954958	85955081	NC_000010.10
cg18349835	1.82E-11	1.13E-12	7.61	17.888029	1.74E-01	158937107	158937230	NC_000007.13
cg26263234	1.82E-11	1.13E-12	7.61	17.8872183	2.00E-01	26666060	26666183	NC_000003.11
cg16783478	1.83E-11	1.13E-12	7.61	17.8862572	9.46E-02	9943657	9943780	NC_000016.9
cg00030420	1.83E-11	1.13E-12	7.61	17.8852101	7.55E-02	140683772	140683895	NC_000005.9
cg21360079	1.83E-11	1.13E-12	7.61	17.884879	1.17E-01	107162445	107162568	NC_000012.11
cg23686983	1.83E-11	1.14E-12	7.61	17.8835199	6.92E-02	101880640	101880763	NC_000007.13
cg02108033	1.83E-11	1.14E-12	7.61	17.8834018	2.20E-01	17783294	17783417	NC_000004.11
cg00569447	1.83E-11	1.14E-12	7.61	17.8831107	1.42E-01	50695224	50695347	NC_000005.9
cg24864413	1.83E-11	1.14E-12	7.61	17.8825016	1.05E-01	160542732	160542855	NC_000006.11
cg21747876	1.83E-11	1.14E-12	7.61	17.8813662	1.51E-01	178687984	178688107	NC_000005.9
cg01307684	1.84E-11	1.14E-12	7.61	17.8805674	4.46E-02	80886143	80886266	NC_000004.11
cg05255522	1.84E-11	1.14E-12	7.61	17.8789098	1.87E-01	50682710	50682833	NC_000006.11
cg01072388	1.84E-11	1.14E-12	7.61	17.8782457	1.11E-01	85620244	85620367	NC_000016.9
cg04307702	1.84E-11	1.14E-12	7.61	17.8778646	6.03E-02	3714315	3714438	NC_000002.11
cg12939049	1.84E-11	1.14E-12	7.61	17.8776557	9.89E-02	10602753	10602876	NC_000004.11
cg14845689	1.84E-11	1.14E-12	7.61	17.8761891	1.58E-01	119306193	119306316	NC_000010.10
cg10091994	1.84E-11	1.14E-12	7.61	17.8761699	2.29E-01	4381803	4381926	NC_000012.11
cg15133041	1.84E-11	1.15E-12	7.61	17.8754948	5.05E-02	761765	761888	NC_000019.9
cg06322218	1.85E-11	1.15E-12	7.61	17.8742548	1.14E-01	9044663	9044786	NC_000016.9
cg13209298	1.85E-11	1.15E-12	7.61	17.8738486	1.11E-01	50303807	50303930	NC_000003.11
cg22471517	1.85E-11	1.15E-12	7.61	17.8719404	1.66E-01	136553682	136553805	NC_000007.13

cg00449067	1.85E-11	1.15E-12	7.61	17.8711806	1.15E-01	188269443	188269566	NC_000002.11
cg04840051	1.86E-11	1.15E-12	7.61	17.8684121	1.41E-01	112582591	112582714	NC_000010.10
cg21186708	1.86E-11	1.16E-12	7.61	17.8660734	2.06E-01	112716728	112716851	NC_000013.10
cg16123707	1.86E-11	1.16E-12	7.61	17.8658779	3.88E-02	134049802	134049925	NC_000010.10
cg14856220	1.86E-11	1.16E-12	7.61	17.8655001	9.34E-02	123353500	123353623	NC_000010.10
cg20499290	1.86E-11	1.16E-12	7.61	17.8654745	1.29E-01	38015579	38015702	NC_000010.10
cg09273030	1.87E-11	1.16E-12	7.61	17.860547	4.20E-02	242566905	242567028	NC_000002.11
cg17255450	1.87E-11	1.16E-12	7.61	17.8601304	2.06E-01	5710372	5710495	NC_000004.11
cg21656685	1.87E-11	1.16E-12	7.61	17.8600506	9.75E-02	2947843	2947966	NC_000018.9
cg04453140	1.87E-11	1.16E-12	7.61	17.8595233	1.65E-01	46692021	46692144	NC_000017.10
cg19599451	1.87E-11	1.17E-12	7.61	17.8582721	4.31E-02	11374377	11374500	NC_000004.11
cg25347264	1.88E-11	1.17E-12	7.61	17.8560785	1.88E-01	5540220	5540343	NC_000012.11
cg15888663	1.88E-11	1.17E-12	7.61	17.8543827	5.35E-02	207992084	207992207	NC_000001.10
cg16697024	1.88E-11	1.17E-12	7.61	17.8535215	4.92E-02	126715891	126716014	NC_000010.10
cg24720571	1.89E-11	1.17E-12	7.6	17.8517768	1.85E-01	6004328	6004451	NC_000006.11
cg22974236	1.89E-11	1.18E-12	7.6	17.8492456	1.56E-01	4867979	4868102	NC_000004.11
cg02252795	1.89E-11	1.18E-12	7.6	17.848979	7.38E-02	85877243	85877366	NC_000016.9
cg11213520	1.89E-11	1.18E-12	7.6	17.8484293	2.35E-01	113901529	113901652	NC_000012.11
cg25649039	1.90E-11	1.18E-12	7.6	17.8457774	1.71E-01	64335475	64335598	NC_000017.10
cg12821804	1.90E-11	1.18E-12	7.6	17.8456962	1.61E-01	2890705	2890828	NC_000011.9
cg15786900	1.90E-11	1.18E-12	7.6	17.8434726	1.29E-01	44333131	44333254	NC_000011.9
cg04779860	1.90E-11	1.18E-12	7.6	17.842328	1.26E-01	131763929	131764052	NC_000010.10
cg11731238	1.91E-11	1.19E-12	7.6	17.8378098	9.96E-02	69603848	69603971	NC_000011.9
cg15808426	1.91E-11	1.19E-12	7.6	17.8372134	1.56E-01	57154302	57154425	NC_000019.9
cg23007574	1.91E-11	1.19E-12	7.6	17.837041	8.36E-02	41942070	41942193	NC_000019.9

cg17470095	1.92E-11	1.19E-12	7.6	17.8341386	7.08E-02	21318106	21318229	NC_000022.10
cg05844247	1.92E-11	1.20E-12	7.6	17.8326336	1.73E-01	21694428	21694551	NC_000020.10
cg00048759	1.92E-11	1.20E-12	7.6	17.8321841	9.55E-02	99775422	99775545	NC_000007.13
cg08374596	1.92E-11	1.20E-12	7.6	17.8319247	3.18E-02	50744349	50744472	NC_000016.9
cg18191688	1.92E-11	1.20E-12	7.6	17.8309696	6.78E-02	144994933	144995056	NC_000001.10
cg03718620	1.93E-11	1.20E-12	7.6	17.8300243	6.15E-02	8771680	8771803	NC_000016.9
cg15630071	1.93E-11	1.20E-12	7.6	17.8281571	1.11E-01	32861318	32861441	NC_000003.11
cg24934400	1.93E-11	1.20E-12	7.6	17.8276624	2.13E-01	4873966	4874089	NC_000004.11
cg09896622	1.93E-11	1.21E-12	7.6	17.8251072	1.88E-01	102568555	102568678	NC_000013.10
cg20552688	1.94E-11	1.21E-12	7.6	17.8246166	2.38E-02	105956371	105956494	NC_000014.8
cg15889913	1.94E-11	1.21E-12	7.6	17.8226323	1.56E-01	131770762	131770885	NC_000010.10
cg14349667	1.95E-11	1.21E-12	7.6	17.818858	1.84E-01	81189706	81189829	NC_000004.11
cg18920423	1.95E-11	1.21E-12	7.6	17.8187057	2.07E-01	23481389	23481512	NC_000010.10
cg21595409	1.95E-11	1.21E-12	7.6	17.8179453	1.40E-01	81014522	81014645	NC_000008.10
cg20388729	1.95E-11	1.22E-12	7.6	17.8169261	1.28E-01	99200492	99200615	NC_000015.9
cg17786496	1.95E-11	1.22E-12	7.6	17.8167383	1.01E-01	22051566	22051689	NC_000017.10
cg04880138	1.95E-11	1.22E-12	7.6	17.8148215	1.59E-01	1007119	1007242	NC_000011.9
cg16747109	1.95E-11	1.22E-12	7.6	17.814706	1.99E-01	111544463	111544586	NC_000004.11
cg01882535	1.96E-11	1.22E-12	7.6	17.8140377	1.08E-01	56643145	56643268	NC_000011.9
cg21187554	1.96E-11	1.22E-12	7.6	17.8121098	1.24E-01	38620311	38620434	NC_000009.11
cg00474713	1.96E-11	1.22E-12	7.6	17.8119778	7.33E-02	28888747	28888870	NC_000016.9
cg25203704	1.96E-11	1.22E-12	7.6	17.8113902	2.01E-01	17216922	17217045	NC_000005.9
cg24064506	1.96E-11	1.22E-12	7.6	17.8111372	1.03E-01	117052397	117052520	NC_000011.9
cg06739029	1.96E-11	1.22E-12	7.6	17.8099221	1.69E-01	19837421	19837544	NC_000006.11
cg13295262	1.98E-11	1.23E-12	7.6	17.8029054	1.75E-01	177355072	177355195	NC_000002.11

cg10023530	1.98E-11	1.23E-12	7.6	17.8020601	1.88E-01	11148783	11148906	NC_000018.9
cg08153621	1.98E-11	1.23E-12	7.6	17.801531	1.22E-01	53561441	53561564	NC_000019.9
cg08400962	1.98E-11	1.24E-12	7.6	17.8008371	1.70E-01	34112316	34112439	NC_000019.9
cg16815850	1.98E-11	1.24E-12	7.6	17.7995597	1.62E-01	4577320	4577443	NC_000001.10
cg27641532	1.99E-11	1.24E-12	7.6	17.7974647	1.59E-01	27648317	27648440	NC_000006.11
cg11442877	1.99E-11	1.24E-12	7.6	17.796293	1.30E-01	25961909	25962032	NC_000015.9
cg05579549	1.99E-11	1.24E-12	7.6	17.7943003	5.80E-02	75889403	75889526	NC_000017.10
cg01997590	1.99E-11	1.24E-12	7.59	17.7942522	1.40E-01	223177473	223177596	NC_000002.11
cg13304274	1.99E-11	1.25E-12	7.59	17.7927926	2.00E-01	36909606	36909729	NC_000019.9
cg23331484	1.99E-11	1.25E-12	7.59	17.792789	1.34E-01	33718148	33718271	NC_000019.9
cg25386426	2.00E-11	1.25E-12	7.59	17.7916793	9.06E-02	2459224	2459347	NC_000001.10
cg10019916	2.00E-11	1.25E-12	7.59	17.7895359	2.09E-01	228196376	228196499	NC_000001.10
cg02625536	2.00E-11	1.25E-12	7.59	17.7885217	1.19E-01	47749901	47750024	NC_000020.10
cg23210521	2.00E-11	1.25E-12	7.59	17.7874711	1.50E-01	95680414	95680537	NC_000004.11
cg15331500	2.01E-11	1.25E-12	7.59	17.7870854	1.63E-01	41779351	41779474	NC_000022.10
cg08482531	2.01E-11	1.25E-12	7.59	17.7870132	1.44E-01	8078127	8078250	NC_000010.10
cg09596722	2.01E-11	1.26E-12	7.59	17.7852542	1.75E-01	232525772	232525895	NC_000002.11
cg03547797	2.01E-11	1.26E-12	7.59	17.7839081	1.69E-01	22689644	22689767	NC_000011.9
cg24385568	2.01E-11	1.26E-12	7.59	17.7838969	5.72E-02	53547962	53548085	NC_000012.11
cg11751213	2.02E-11	1.26E-12	7.59	17.7820131	1.29E-01	65547023	65547146	NC_000011.9
cg21235151	2.02E-11	1.26E-12	7.59	17.7810537	2.43E-01	177001256	177001379	NC_000002.11
cg10662314	2.02E-11	1.26E-12	7.59	17.7785564	2.41E-01	54384666	54384789	NC_000012.11
cg23549358	2.02E-11	1.26E-12	7.59	17.7778564	1.70E-01	20692791	20692914	NC_000011.9
cg24483247	2.03E-11	1.27E-12	7.59	17.7769001	2.17E-01	5890411	5890534	NC_000004.11
cg08000731	2.03E-11	1.27E-12	7.59	17.776247	1.28E-01	316247	316370	NC_000011.9

cg11700800	2.03E-11	1.27E-12	7.59	17.7744603	1.50E-01	92906633	92906756	NC_000005.9
cg27619646	2.03E-11	1.27E-12	7.59	17.7742381	1.05E-01	41583018	41583141	NC_000008.10
cg10486998	2.04E-11	1.27E-12	7.59	17.771175	2.14E-01	74961787	74961910	NC_000018.9
cg06055873	2.04E-11	1.27E-12	7.59	17.7703182	1.62E-01	27142204	27142327	NC_000007.13
cg02946934	2.04E-11	1.28E-12	7.59	17.768171	2.11E-01	17496681	17496804	NC_000010.10
cg02637318	2.04E-11	1.28E-12	7.59	17.7667427	2.36E-01	38965252	38965375	NC_000008.10
cg27567761	2.05E-11	1.28E-12	7.59	17.7656883	4.98E-02	64573184	64573307	NC_000010.10
cg21306240	2.05E-11	1.29E-12	7.59	17.7619543	2.31E-01	38230845	38230968	NC_000001.10
cg11718886	2.06E-11	1.29E-12	7.59	17.7585213	7.83E-02	219345052	219345175	NC_000001.10
cg22948684	2.06E-11	1.29E-12	7.59	17.7583493	7.40E-02	15222078	15222201	NC_000001.10
cg24428099	2.06E-11	1.29E-12	7.59	17.7580683	5.50E-02	25065702	25065825	NC_000002.11
cg26949694	2.07E-11	1.29E-12	7.59	17.7557481	1.56E-01	27742060	27742183	NC_000011.9
cg09259595	2.07E-11	1.29E-12	7.59	17.75558	7.55E-02	1779056	1779179	NC_000008.10
cg03577505	2.07E-11	1.29E-12	7.59	17.75474	4.40E-02	159486976	159487099	NC_000003.11
cg27159583	2.07E-11	1.29E-12	7.59	17.754553	1.91E-01	38289905	38290028	NC_000021.8
cg14209823	2.07E-11	1.30E-12	7.59	17.754052	6.72E-02	136029078	136029201	NC_000009.11
cg19971716	2.07E-11	1.30E-12	7.59	17.7534411	2.18E-01	122301740	122301863	NC_000004.11
cg10923662	2.07E-11	1.30E-12	7.59	17.7529811	2.01E-01	32064258	32064381	NC_000006.11
cg12433423	2.08E-11	1.30E-12	7.59	17.7499238	4.91E-02	225659296	225659419	NC_000001.10
cg24042242	2.08E-11	1.30E-12	7.59	17.7498031	1.92E-01	43044660	43044783	NC_000017.10
cg11439671	2.08E-11	1.30E-12	7.59	17.7476653	4.29E-02	73515429	73515552	NC_000017.10
cg12618475	2.08E-11	1.30E-12	7.59	17.7473997	8.08E-02	110732361	110732484	NC_000006.11
cg07142797	2.08E-11	1.31E-12	7.59	17.74678	1.93E-01	50605613	50605736	NC_000010.10
cg07012926	2.08E-11	1.31E-12	7.59	17.7462269	1.87E-01	151304488	151304611	NC_000005.9
cg24550525	2.09E-11	1.31E-12	7.59	17.7459186	1.29E-01	69181420	69181543	NC_000002.11

cg23648991	2.09E-11	1.31E-12	7.59	17.744917	5.44E-02	158586534	158586657	NC_000006.11
cg04478223	2.09E-11	1.31E-12	7.59	17.7441912	7.79E-02	49699726	49699849	NC_000005.9
cg18127481	2.09E-11	1.31E-12	7.59	17.7440984	1.11E-01	49880645	49880768	NC_000005.9
cg12252412	2.09E-11	1.31E-12	7.59	17.7439287	3.10E-02	26513782	26513905	NC_000008.10
cg03760589	2.09E-11	1.31E-12	7.59	17.7434758	1.48E-01	170630397	170630520	NC_000001.10
cg25793387	2.10E-11	1.31E-12	7.59	17.7404187	9.65E-02	56554318	56554441	NC_000017.10
cg13147664	2.10E-11	1.32E-12	7.59	17.73869	9.02E-02	1029744	1029867	NC_000017.10
cg18271789	2.10E-11	1.32E-12	7.59	17.7367404	1.16E-01	4832112	4832235	NC_000007.13
cg01291920	2.10E-11	1.32E-12	7.59	17.7367318	8.41E-02	31915077	31915200	NC_000006.11
cg11917579	2.11E-11	1.32E-12	7.58	17.7347466	1.46E-01	2815429	2815552	NC_000010.10
cg03219768	2.11E-11	1.32E-12	7.58	17.7328547	4.98E-02	76641090	76641213	NC_000017.10
cg20718170	2.11E-11	1.32E-12	7.58	17.7325772	6.72E-02	123322268	123322391	NC_000007.13
cg07554664	2.11E-11	1.32E-12	7.58	17.7322894	1.11E-01	68431982	68432105	NC_000016.9
cg07688213	2.12E-11	1.33E-12	7.58	17.7305093	5.20E-02	76013203	76013326	NC_000014.8
cg00197006	2.12E-11	1.33E-12	7.58	17.7283006	1.21E-01	84616559	84616682	NC_000016.9
cg27441182	2.12E-11	1.33E-12	7.58	17.7272989	1.39E-01	44484200	44484323	NC_000015.9
cg27509347	2.12E-11	1.33E-12	7.58	17.726908	1.40E-01	28385657	28385780	NC_000001.10
cg03497399	2.12E-11	1.33E-12	7.58	17.7264642	1.48E-01	33260934	33261057	NC_000003.11
cg10314411	2.13E-11	1.33E-12	7.58	17.7258842	2.57E-02	206279954	206280077	NC_000001.10
cg19098268	2.13E-11	1.34E-12	7.58	17.7241368	1.07E-01	57352269	57352392	NC_000019.9
cg19814400	2.13E-11	1.34E-12	7.58	17.722891	1.80E-01	158936632	158936755	NC_000007.13
cg01189604	2.13E-11	1.34E-12	7.58	17.7222513	5.17E-02	134391939	134392062	NC_000010.10
cg10228777	2.14E-11	1.34E-12	7.58	17.7205008	1.98E-01	14925424	14925547	NC_000001.10
cg15825417	2.14E-11	1.34E-12	7.58	17.7186443	7.94E-02	15689957	15690080	NC_000017.10
cg10247840	2.14E-11	1.34E-12	7.58	17.7176488	1.49E-01	40034535	40034658	NC_000001.10

cg26181334	2.14E-11	1.35E-12	7.58	17.7170521	1.07E-01	4832535	4832658	NC_000007.13
cg15882353	2.14E-11	1.35E-12	7.58	17.7164856	5.80E-02	67023811	67023934	NC_000008.10
cg17362661	2.15E-11	1.35E-12	7.58	17.7144018	1.13E-01	100210490	100210613	NC_000002.11
cg06714480	2.15E-11	1.35E-12	7.58	17.711132	2.03E-01	182544307	182544430	NC_000002.11
cg26570233	2.16E-11	1.35E-12	7.58	17.7103552	2.02E-01	93475962	93476085	NC_000011.9
cg20474425	2.16E-11	1.35E-12	7.58	17.710227	1.16E-01	54746067	54746190	NC_000012.11
cg26988423	2.16E-11	1.36E-12	7.58	17.7083159	1.68E-01	3238423	3238546	NC_000016.9
cg24884703	2.16E-11	1.36E-12	7.58	17.7071293	2.15E-01	91185422	91185545	NC_000001.10
cg05866021	2.17E-11	1.36E-12	7.58	17.7046845	1.11E-01	57285278	57285401	NC_000001.10
cg24464397	2.17E-11	1.37E-12	7.58	17.7018216	1.67E-01	56988778	56988901	NC_000019.9
cg24394856	2.17E-11	1.37E-12	7.58	17.7018074	1.73E-01	134175900	134176023	NC_000006.11
cg12743978	2.18E-11	1.37E-12	7.58	17.7005548	1.44E-01	19617145	19617268	NC_000021.8
cg05345286	2.18E-11	1.37E-12	7.58	17.6993714	1.55E-01	41606641	41606764	NC_000006.11
cg15637527	2.18E-11	1.37E-12	7.58	17.6984862	8.76E-02	46700234	46700357	NC_000011.9
cg00036788	2.18E-11	1.37E-12	7.58	17.6967563	1.37E-01	54974682	54974805	NC_000004.11
cg11758945	2.19E-11	1.37E-12	7.58	17.6959442	2.79E-01	143533532	143533655	NC_000008.10
cg18023096	2.19E-11	1.37E-12	7.58	17.6954675	1.37E-01	17090586	17090709	NC_000022.10
cg10343742	2.19E-11	1.38E-12	7.58	17.6927259	1.12E-01	137483506	137483629	NC_000003.11
cg20846447	2.19E-11	1.38E-12	7.58	17.6924582	1.55E-01	177140305	177140428	NC_000001.10
cg09562045	2.19E-11	1.38E-12	7.58	17.6920774	2.57E-01	5890424	5890547	NC_000004.11
cg05733181	2.20E-11	1.38E-12	7.58	17.6913481	1.85E-01	117847088	117847211	NC_000004.11
cg20552282	2.20E-11	1.38E-12	7.58	17.6911301	1.69E-01	21686728	21686851	NC_000020.10
cg26149244	2.20E-11	1.38E-12	7.58	17.6911152	2.28E-01	80329964	80330087	NC_000017.10
cg10036918	2.20E-11	1.38E-12	7.58	17.6884959	1.72E-01	103969166	103969289	NC_000007.13
cg08145698	2.21E-11	1.39E-12	7.58	17.6845465	1.59E-01	87636539	87636662	NC_000016.9

cg26666835	2.22E-11	1.40E-12	7.58	17.6805922	1.77E-01	170742118	170742241	NC_000005.9
cg13938187	2.22E-11	1.40E-12	7.58	17.6803403	9.69E-02	64980038	64980161	NC_000015.9
cg00890363	2.22E-11	1.40E-12	7.58	17.6796119	1.70E-01	13540673	13540796	NC_000004.11
cg20668644	2.23E-11	1.40E-12	7.57	17.6767782	3.08E-01	38347603	38347726	NC_000017.10
cg04726360	2.23E-11	1.40E-12	7.57	17.6766439	1.09E-01	71708613	71708736	NC_000004.11
cg15202353	2.23E-11	1.40E-12	7.57	17.676317	8.87E-02	162831613	162831736	NC_000001.10
cg27650540	2.24E-11	1.41E-12	7.57	17.6723857	1.08E-01	111664582	111664705	NC_000012.11
cg21493633	2.24E-11	1.41E-12	7.57	17.671558	1.85E-01	166795596	166795719	NC_000004.11
cg03114255	2.24E-11	1.41E-12	7.57	17.6714181	2.25E-01	150358985	150359108	NC_000006.11
cg24361256	2.24E-11	1.41E-12	7.57	17.6696397	6.52E-02	50637945	50638068	NC_000022.10
cg19492047	2.24E-11	1.41E-12	7.57	17.6689138	1.94E-01	217236040	217236163	NC_000002.11
cg14714797	2.25E-11	1.42E-12	7.57	17.6655933	1.77E-01	343927	344050	NC_000005.9
cg17303756	2.25E-11	1.42E-12	7.57	17.6642572	1.94E-01	99279108	99279231	NC_000006.11
cg08093277	2.25E-11	1.42E-12	7.57	17.664085	1.02E-01	29595299	29595422	NC_000006.11
cg07995570	2.26E-11	1.42E-12	7.57	17.6619239	1.49E-01	91192025	91192148	NC_000001.10
cg07913430	2.26E-11	1.42E-12	7.57	17.6612962	1.21E-01	115609343	115609466	NC_000004.11
cg11641702	2.27E-11	1.43E-12	7.57	17.6582949	3.48E-02	107601656	107601779	NC_000007.13
cg04864807	2.27E-11	1.43E-12	7.57	17.6555938	1.27E-01	121412139	121412262	NC_000002.11
cg05597504	2.27E-11	1.43E-12	7.57	17.6553432	6.70E-02	150210742	150210865	NC_000007.13
cg12299478	2.27E-11	1.43E-12	7.57	17.6545362	1.36E-01	111935236	111935359	NC_000010.10
cg04227278	2.27E-11	1.43E-12	7.57	17.6545346	1.23E-01	134301020	134301143	NC_000008.10
cg18404112	2.28E-11	1.43E-12	7.57	17.6534366	4.32E-02	144893173	144893296	NC_000008.10
cg16038120	2.28E-11	1.44E-12	7.57	17.6525907	1.88E-01	29235196	29235319	NC_000014.8
cg27580953	2.28E-11	1.44E-12	7.57	17.6514126	5.01E-02	134119745	134119868	NC_000010.10
cg09473282	2.29E-11	1.44E-12	7.57	17.648968	1.07E-01	35115955	35116078	NC_000006.11

cg09913449	2.29E-11	1.44E-12	7.57	17.6487623	3.72E-02	42400586	42400709	NC_000008.10
cg08254399	2.29E-11	1.44E-12	7.57	17.6473954	5.66E-02	45671659	45671782	NC_000006.11
cg25435255	2.29E-11	1.44E-12	7.57	17.6472261	4.93E-02	104086857	104086980	NC_000010.10
cg17311074	2.29E-11	1.45E-12	7.57	17.6456173	1.11E-01	71507695	71507818	NC_000015.9
cg03032214	2.30E-11	1.45E-12	7.57	17.6445729	2.06E-01	74961809	74961932	NC_000018.9
cg20914464	2.30E-11	1.45E-12	7.57	17.6440432	1.47E-01	31803674	31803797	NC_000018.9
cg18091615	2.30E-11	1.45E-12	7.57	17.6437681	1.13E-01	137675881	137676004	NC_000007.13
cg13550802	2.30E-11	1.45E-12	7.57	17.6437569	6.53E-02	134935854	134935977	NC_000010.10
cg00144335	2.30E-11	1.45E-12	7.57	17.6427795	1.23E-01	107176	107299	NC_000004.11
cg12450016	2.30E-11	1.45E-12	7.57	17.6423789	1.69E-01	98112437	98112560	NC_000005.9
cg11567608	2.30E-11	1.45E-12	7.57	17.6417065	1.33E-01	135271518	135271641	NC_000006.11
cg23733384	2.30E-11	1.45E-12	7.57	17.64086	1.51E-01	228346347	228346470	NC_000001.10
cg02681442	2.30E-11	1.45E-12	7.57	17.640568	1.81E-01	29236008	29236131	NC_000014.8
cg14947466	2.30E-11	1.45E-12	7.57	17.6400879	5.34E-02	2583705	2583828	NC_000011.9
cg24453600	2.31E-11	1.46E-12	7.57	17.6388122	7.24E-02	71921678	71921801	NC_000015.9
cg01546793	2.31E-11	1.46E-12	7.57	17.6380397	1.34E-01	116913720	116913843	NC_000011.9
cg04670802	2.31E-11	1.46E-12	7.57	17.6364262	1.66E-01	100050812	100050935	NC_000006.11
cg00541104	2.31E-11	1.46E-12	7.57	17.6362429	1.64E-01	158359935	158360058	NC_000007.13
cg22809920	2.31E-11	1.46E-12	7.57	17.6355652	1.53E-01	43935551	43935674	NC_000020.10
cg12744722	2.32E-11	1.46E-12	7.57	17.6348631	1.66E-01	111473809	111473932	NC_000012.11
cg03414318	2.32E-11	1.46E-12	7.57	17.6330873	2.75E-01	34894648	34894771	NC_000020.10
cg23982890	2.32E-11	1.47E-12	7.57	17.6325409	4.75E-02	68869531	68869654	NC_000011.9
cg09648467	2.33E-11	1.47E-12	7.57	17.6284171	1.61E-01	156955280	156955403	NC_000005.9
cg20002207	2.33E-11	1.47E-12	7.57	17.626903	1.00E-01	57957811	57957934	NC_000004.11
cg24465685	2.34E-11	1.48E-12	7.57	17.6224622	1.73E-01	47009809	47009932	NC_000001.10

cg01142710	2.34E-11	1.48E-12	7.57	17.6224608	1.72E-01	145713754	145713877	NC_000005.9
cg01540949	2.34E-11	1.48E-12	7.57	17.6219841	1.06E-01	126736629	126736752	NC_000011.9
cg08495617	2.35E-11	1.48E-12	7.57	17.6197497	1.79E-01	171791143	171791266	NC_000003.11
cg13943217	2.35E-11	1.49E-12	7.57	17.618541	2.19E-01	133542125	133542248	NC_000009.11
cg01650904	2.35E-11	1.49E-12	7.56	17.6176136	1.64E-01	37354889	37355012	NC_000020.10
cg23217126	2.36E-11	1.49E-12	7.56	17.6158886	1.73E-01	67068268	67068391	NC_000018.9
cg01409163	2.36E-11	1.49E-12	7.56	17.6157036	4.61E-02	670353	670476	NC_000008.10
cg17448335	2.36E-11	1.49E-12	7.56	17.6131771	2.22E-01	76249776	76249899	NC_000005.9
cg14566959	2.37E-11	1.50E-12	7.56	17.6126447	1.10E-01	140772681	140772804	NC_000005.9
cg19628343	2.37E-11	1.50E-12	7.56	17.6125052	7.20E-02	17873806	17873929	NC_000019.9
cg26569771	2.37E-11	1.50E-12	7.56	17.6113823	7.85E-02	4448522	4448645	NC_000019.9
cg09827048	2.37E-11	1.50E-12	7.56	17.6111478	9.60E-02	46113067	46113190	NC_000001.10
cg16392754	2.37E-11	1.50E-12	7.56	17.6110003	8.25E-02	172388746	172388869	NC_000005.9
cg03429689	2.38E-11	1.50E-12	7.56	17.6069649	9.10E-02	93199536	93199659	NC_000015.9
cg26069745	2.38E-11	1.50E-12	7.56	17.6065615	1.53E-01	27142200	27142323	NC_000007.13
cg25743486	2.39E-11	1.51E-12	7.56	17.603924	5.10E-02	1051843	1051966	NC_000017.10
cg02858606	2.39E-11	1.51E-12	7.56	17.6026683	1.81E-01	62068447	62068570	NC_000016.9
cg05935800	2.39E-11	1.51E-12	7.56	17.601986	1.61E-01	226023590	226023713	NC_000001.10
cg01454487	2.39E-11	1.51E-12	7.56	17.6011781	1.64E-01	45427577	45427700	NC_000015.9
cg18669209	2.39E-11	1.51E-12	7.56	17.6004726	2.26E-01	44777736	44777859	NC_000018.9
cg25887739	2.39E-11	1.51E-12	7.56	17.6002694	2.73E-02	101675766	101675889	NC_000002.11
cg03425110	2.40E-11	1.52E-12	7.56	17.5994623	2.10E-01	71333037	71333160	NC_000010.10
cg20892990	2.40E-11	1.52E-12	7.56	17.5989463	1.67E-01	69589460	69589583	NC_000011.9
cg00486604	2.40E-11	1.52E-12	7.56	17.5971954	1.24E-01	43739515	43739638	NC_000003.11
cg15060366	2.40E-11	1.52E-12	7.56	17.5968953	2.34E-01	17206529	17206652	NC_000020.10

cg17217421	2.41E-11	1.52E-12	7.56	17.5937719	4.43E-02	244504355	244504478	NC_000001.10
cg21270847	2.41E-11	1.52E-12	7.56	17.5936979	2.39E-01	164604806	164604929	NC_000001.10
cg02066682	2.41E-11	1.52E-12	7.56	17.5933714	1.39E-01	79634037	79634160	NC_000009.11
cg21156684	2.41E-11	1.53E-12	7.56	17.5930043	5.35E-02	6411612	6411735	NC_000001.10
cg11494841	2.41E-11	1.53E-12	7.56	17.5908986	8.78E-02	80402750	80402873	NC_000017.10
cg11832543	2.42E-11	1.53E-12	7.56	17.590647	4.43E-02	178481818	178481941	NC_000001.10
cg00092644	2.42E-11	1.53E-12	7.56	17.5903083	5.23E-02	95360379	95360502	NC_000001.10
cg22161784	2.42E-11	1.53E-12	7.56	17.5901512	1.41E-01	78337534	78337657	NC_000017.10
cg15648792	2.42E-11	1.53E-12	7.56	17.58984	1.13E-01	11146255	11146378	NC_000018.9
cg01106338	2.42E-11	1.53E-12	7.56	17.5876684	1.29E-01	44790514	44790637	NC_000018.9
cg04828133	2.42E-11	1.53E-12	7.56	17.5873587	2.47E-01	155259845	155259968	NC_000007.13
cg20482521	2.42E-11	1.54E-12	7.56	17.5867055	2.10E-01	106400667	106400790	NC_000010.10
cg17251713	2.43E-11	1.54E-12	7.56	17.5821248	1.80E-01	61443038	61443161	NC_000018.9
cg03799283	2.44E-11	1.54E-12	7.56	17.5809722	1.22E-01	96910011	96910134	NC_000015.9
cg24814707	2.44E-11	1.55E-12	7.56	17.5804096	1.66E-01	57035957	57036080	NC_000004.11
cg02484391	2.44E-11	1.55E-12	7.56	17.5795616	1.70E-01	22889301	22889424	NC_000001.10
cg05212802	2.44E-11	1.55E-12	7.56	17.5786378	1.78E-01	142053623	142053746	NC_000004.11
cg10042106	2.44E-11	1.55E-12	7.56	17.5785624	1.71E-01	57274739	57274862	NC_000014.8
cg19600750	2.45E-11	1.55E-12	7.56	17.5775445	1.43E-01	57274677	57274800	NC_000014.8
cg19258062	2.46E-11	1.56E-12	7.56	17.5729248	1.22E-01	152133423	152133546	NC_000007.13
cg11101926	2.46E-11	1.56E-12	7.56	17.5725473	1.62E-01	32483711	32483834	NC_000017.10
cg09167553	2.46E-11	1.56E-12	7.56	17.5721485	2.41E-01	70034475	70034598	NC_000001.10
cg08204023	2.46E-11	1.56E-12	7.56	17.5717785	1.91E-01	37835109	37835232	NC_000005.9
cg26747317	2.46E-11	1.56E-12	7.56	17.5709465	1.23E-01	31829793	31829916	NC_000006.11
cg21495612	2.47E-11	1.56E-12	7.56	17.5685502	2.24E-01	96342250	96342373	NC_000014.8

cg04128880	2.47E-11	1.57E-12	7.56	17.5668839	4.28E-02	22198056	22198179	NC_000016.9
cg21096050	2.48E-11	1.57E-12	7.56	17.5649563	5.68E-02	85502665	85502788	NC_000004.11
cg20912770	2.48E-11	1.57E-12	7.56	17.5642412	1.77E-01	2292428	2292551	NC_000011.9
cg26621770	2.49E-11	1.58E-12	7.56	17.5600712	1.66E-01	115131447	115131570	NC_000012.11
cg17295225	2.49E-11	1.58E-12	7.55	17.5568538	1.34E-01	137814971	137815094	NC_000006.11
cg22183731	2.50E-11	1.58E-12	7.55	17.5558519	8.81E-02	102221100	102221223	NC_000011.9
cg09614724	2.50E-11	1.59E-12	7.55	17.5536298	2.31E-01	30719547	30719670	NC_000019.9
cg21931419	2.51E-11	1.59E-12	7.55	17.5511769	1.64E-01	74893169	74893292	NC_000014.8
cg13031171	2.51E-11	1.59E-12	7.55	17.5511725	1.90E-01	29106870	29106993	NC_000013.10
cg03650282	2.51E-11	1.59E-12	7.55	17.5503748	6.17E-02	64451448	64451571	NC_000017.10
cg01427575	2.51E-11	1.59E-12	7.55	17.5497065	1.74E-01	51171712	51171835	NC_000019.9
cg18578080	2.52E-11	1.60E-12	7.55	17.5480744	7.24E-02	82405786	82405909	NC_000006.11
cg24074033	2.52E-11	1.60E-12	7.55	17.5477615	1.92E-01	20180437	20180560	NC_000011.9
cg16026922	2.52E-11	1.60E-12	7.55	17.5475225	1.19E-01	41259044	41259167	NC_000004.11
cg23929344	2.52E-11	1.60E-12	7.55	17.5469403	1.69E-01	36975402	36975525	NC_000014.8
cg16257029	2.52E-11	1.60E-12	7.55	17.546904	1.23E-01	128077369	128077492	NC_000010.10
cg19019371	2.52E-11	1.60E-12	7.55	17.545529	1.58E-01	140770984	140771107	NC_000003.11
cg24785473	2.53E-11	1.61E-12	7.55	17.5428666	1.16E-01	35681883	35682006	NC_000003.11
cg12710552	2.53E-11	1.61E-12	7.55	17.5423173	7.57E-02	18270955	18271078	NC_000011.9
cg04946910	2.53E-11	1.61E-12	7.55	17.5410743	8.86E-02	3243392	3243515	NC_000016.9
cg14116122	2.54E-11	1.61E-12	7.55	17.5386097	1.94E-01	85673884	85674007	NC_000012.11
cg00501711	2.54E-11	1.62E-12	7.55	17.5367165	7.45E-02	43538180	43538303	NC_000020.10
cg22012299	2.54E-11	1.62E-12	7.55	17.5360854	6.86E-02	102360643	102360766	NC_000013.10
cg16629702	2.54E-11	1.62E-12	7.55	17.5360731	1.96E-01	55690248	55690371	NC_000016.9
cg18813599	2.55E-11	1.62E-12	7.55	17.5351328	1.39E-01	29937472	29937595	NC_000008.10

cg15852891	2.56E-11	1.63E-12	7.55	17.530507	1.79E-01	76934946	76935069	NC_000005.9
cg16852955	2.56E-11	1.63E-12	7.55	17.5303751	1.11E-01	227773531	227773654	NC_000002.11
cg08089301	2.56E-11	1.63E-12	7.55	17.5280106	2.48E-01	46655561	46655684	NC_000017.10
cg18112708	2.57E-11	1.63E-12	7.55	17.527519	7.13E-02	160169450	160169573	NC_000006.11
cg13915754	2.57E-11	1.63E-12	7.55	17.526039	2.05E-01	37355965	37356088	NC_000020.10
cg16831361	2.57E-11	1.63E-12	7.55	17.5250543	1.07E-01	39668954	39669077	NC_000021.8
cg12353207	2.57E-11	1.64E-12	7.55	17.5247494	1.87E-01	113444295	113444418	NC_000004.11
cg00432789	2.57E-11	1.64E-12	7.55	17.5238115	8.19E-02	211650394	211650517	NC_000001.10
cg26104293	2.58E-11	1.64E-12	7.55	17.5208315	1.12E-01	91499812	91499935	NC_000006.11
cg15428435	2.58E-11	1.64E-12	7.55	17.5195408	2.19E-01	102247681	102247804	NC_000014.8
cg01224715	2.59E-11	1.64E-12	7.55	17.5191487	1.32E-01	140811520	140811643	NC_000005.9
cg19092317	2.59E-11	1.64E-12	7.55	17.51891	1.71E-01	72739768	72739891	NC_000005.9
cg09622420	2.59E-11	1.65E-12	7.55	17.5177337	6.60E-02	11033670	11033793	NC_000016.9
cg00284386	2.59E-11	1.65E-12	7.55	17.5164024	1.01E-01	1786207	1786330	NC_000008.10
cg02939292	2.59E-11	1.65E-12	7.55	17.5151006	5.54E-02	60716250	60716373	NC_000011.9
cg09059267	2.60E-11	1.65E-12	7.55	17.5139213	1.48E-01	220223082	220223205	NC_000002.11
cg22660120	2.60E-11	1.66E-12	7.55	17.5115326	4.19E-02	33424102	33424225	NC_000006.11
cg21667878	2.61E-11	1.66E-12	7.55	17.5093242	2.37E-01	2160980	2161103	NC_000011.9
cg04030444	2.62E-11	1.67E-12	7.55	17.506171	1.20E-01	170581971	170582094	NC_000006.11
cg22902505	2.62E-11	1.67E-12	7.55	17.5055384	1.56E-01	81119473	81119596	NC_000004.11
cg05153474	2.63E-11	1.67E-12	7.55	17.5023059	7.17E-02	39884449	39884572	NC_000022.10
cg26098399	2.63E-11	1.67E-12	7.55	17.5013569	6.05E-02	56716944	56717067	NC_000004.11
cg13071208	2.63E-11	1.68E-12	7.54	17.4998458	1.23E-01	145686887	145687010	NC_000008.10
cg10016380	2.64E-11	1.68E-12	7.54	17.498607	2.23E-01	78493313	78493436	NC_000013.10
cg04575524	2.64E-11	1.68E-12	7.54	17.4966943	8.48E-02	84820574	84820697	NC_000016.9

cg13325066	2.64E-11	1.68E-12	7.54	17.4966321	1.07E-01	2499932	2500055	NC_000007.13
cg08111446	2.64E-11	1.68E-12	7.54	17.4960169	2.40E-01	61886079	61886202	NC_000020.10
cg07028533	2.64E-11	1.68E-12	7.54	17.4958942	2.34E-01	145813439	145813562	NC_000007.13
cg07589773	2.64E-11	1.68E-12	7.54	17.4951049	2.57E-01	50343883	50344006	NC_000007.13
cg06740372	2.65E-11	1.69E-12	7.54	17.4941479	1.44E-01	7957779	7957902	NC_000003.11
cg19623717	2.65E-11	1.69E-12	7.54	17.493962	2.17E-01	5296442	5296565	NC_000020.10
cg15452135	2.65E-11	1.69E-12	7.54	17.4929439	5.54E-02	105233106	105233229	NC_000010.10
cg10517535	2.65E-11	1.69E-12	7.54	17.4914108	1.70E-01	9929698	9929821	NC_000017.10
cg21765359	2.66E-11	1.69E-12	7.54	17.4907056	8.92E-02	75246756	75246879	NC_000017.10
cg10934407	2.66E-11	1.69E-12	7.54	17.4900264	7.43E-02	168053081	168053204	NC_000006.11
cg02745636	2.66E-11	1.70E-12	7.54	17.488813	1.72E-01	61104571	61104694	NC_000014.8
cg13234863	2.67E-11	1.70E-12	7.54	17.4856458	1.74E-01	130389138	130389261	NC_000012.11
cg21848407	2.67E-11	1.70E-12	7.54	17.4851305	1.05E-01	61499094	61499217	NC_000017.10
cg01208537	2.67E-11	1.70E-12	7.54	17.4849454	8.23E-02	64597387	64597510	NC_000011.9
cg09430445	2.67E-11	1.70E-12	7.54	17.4847291	1.59E-01	42918546	42918669	NC_000003.11
cg23036804	2.67E-11	1.70E-12	7.54	17.4837459	1.70E-01	23734763	23734886	NC_000013.10
cg21156851	2.69E-11	1.71E-12	7.54	17.4788015	3.19E-02	45607281	45607404	NC_000006.11
cg05599109	2.69E-11	1.71E-12	7.54	17.4786949	7.29E-02	6348943	6349066	NC_000010.10
cg25023596	2.69E-11	1.71E-12	7.54	17.4782348	1.84E-01	118627943	118628066	NC_000012.11
cg09471204	2.69E-11	1.71E-12	7.54	17.4779214	1.19E-01	42347991	42348114	NC_000022.10
cg26130488	2.70E-11	1.72E-12	7.54	17.4751653	1.79E-01	59530104	59530227	NC_000017.10
cg01328444	2.70E-11	1.72E-12	7.54	17.4747209	1.38E-01	155967739	155967862	NC_000006.11
cg04557184	2.70E-11	1.72E-12	7.54	17.4744857	1.17E-01	42971330	42971453	NC_000005.9
cg11766283	2.70E-11	1.72E-12	7.54	17.4722256	7.09E-02	24525070	24525193	NC_000013.10
cg12028548	2.71E-11	1.73E-12	7.54	17.4707517	1.71E-01	590799	590922	NC_000020.10

cg24477315	2.71E-11	1.73E-12	7.54	17.4689873	1.24E-01	164570052	164570175	NC_000001.10
cg00324366	2.71E-11	1.73E-12	7.54	17.468853	1.32E-01	32199183	32199306	NC_000008.10
cg04201750	2.72E-11	1.74E-12	7.54	17.4662145	1.47E-01	3591012	3591135	NC_000005.9
cg02469871	2.72E-11	1.74E-12	7.54	17.464883	2.00E-01	103218581	103218704	NC_000012.11
cg03014829	2.73E-11	1.74E-12	7.54	17.4637523	1.31E-01	55516872	55516995	NC_000007.13
cg09003833	2.73E-11	1.74E-12	7.54	17.4629947	1.56E-01	147079053	147079176	NC_000003.11
cg26266308	2.73E-11	1.74E-12	7.54	17.4627314	6.47E-02	66701194	66701317	NC_000008.10
cg09942248	2.73E-11	1.74E-12	7.54	17.4617955	1.90E-01	182545625	182545748	NC_000002.11
cg20404387	2.73E-11	1.74E-12	7.54	17.4617366	1.82E-01	20879296	20879419	NC_000001.10
cg14312661	2.73E-11	1.75E-12	7.54	17.4599907	5.92E-02	3064385	3064508	NC_000011.9
cg16929041	2.73E-11	1.75E-12	7.54	17.4598329	1.51E-01	26648951	26649074	NC_000017.10
cg18877285	2.74E-11	1.75E-12	7.54	17.4591398	1.76E-01	124790815	124790938	NC_000011.9
cg03006175	2.74E-11	1.75E-12	7.54	17.4570858	4.38E-02	105412417	105412540	NC_000014.8
cg24058446	2.74E-11	1.75E-12	7.54	17.4568875	8.11E-02	147834002	147834125	NC_000006.11
cg16172814	2.74E-11	1.75E-12	7.54	17.456732	2.06E-01	50969997	50970120	NC_000010.10
cg19360104	2.74E-11	1.75E-12	7.54	17.4560602	1.23E-01	154475269	154475392	NC_000001.10
cg24046864	2.75E-11	1.75E-12	7.54	17.455235	7.71E-02	54427219	54427342	NC_000014.8
cg22767348	2.75E-11	1.76E-12	7.54	17.4545249	6.78E-02	4669484	4669607	NC_000004.11
cg12714746	2.76E-11	1.76E-12	7.54	17.4496343	6.89E-02	4509054	4509177	NC_000017.10
cg18348494	2.76E-11	1.77E-12	7.54	17.4493084	1.27E-01	62781737	62781860	NC_000001.10
cg02408850	2.77E-11	1.77E-12	7.54	17.4481912	9.52E-02	79793206	79793329	NC_000017.10
cg11287292	2.77E-11	1.77E-12	7.54	17.447262	8.77E-02	178277261	178277384	NC_000004.11
cg22674717	2.77E-11	1.77E-12	7.54	17.4467419	1.73E-01	51185394	51185517	NC_000016.9
cg15072319	2.77E-11	1.77E-12	7.54	17.446262	2.81E-01	156405924	156406047	NC_000001.10
cg05629821	2.77E-11	1.77E-12	7.54	17.4462595	8.30E-02	113436400	113436523	NC_000013.10

cg17088290	2.77E-11	1.77E-12	7.54	17.4456454	2.54E-02	53818182	53818305	NC_000012.11
cg09976876	2.77E-11	1.77E-12	7.54	17.4452983	5.13E-02	57184713	57184836	NC_000016.9
cg06573787	2.77E-11	1.77E-12	7.54	17.4449362	2.31E-01	143070187	143070310	NC_000008.10
cg18947175	2.78E-11	1.78E-12	7.54	17.4433817	1.53E-01	77093804	77093927	NC_000017.10
cg07873155	2.79E-11	1.78E-12	7.53	17.439344	1.13E-01	28197499	28197622	NC_000013.10
cg04936639	2.79E-11	1.78E-12	7.53	17.4385084	7.17E-02	48922035	48922158	NC_000011.9
cg05056133	2.80E-11	1.79E-12	7.53	17.4338463	9.30E-02	220491676	220491799	NC_000002.11
cg09558850	2.80E-11	1.79E-12	7.53	17.4336801	1.94E-01	93520445	93520568	NC_000007.13
cg11831711	2.80E-11	1.79E-12	7.53	17.4336714	1.11E-02	91966971	91967094	NC_000001.10
cg22095604	2.81E-11	1.80E-12	7.53	17.432498	1.67E-01	31803241	31803364	NC_000018.9
cg02095401	2.81E-11	1.80E-12	7.53	17.4318608	1.15E-01	167545264	167545387	NC_000005.9
cg20175390	2.81E-11	1.80E-12	7.53	17.4317715	1.32E-01	119599459	119599582	NC_000002.11
cg02423014	2.81E-11	1.80E-12	7.53	17.4316971	8.53E-02	64404647	64404770	NC_000015.9
cg05047908	2.81E-11	1.80E-12	7.53	17.4296297	9.08E-02	157084809	157084932	NC_000007.13
cg27385729	2.82E-11	1.80E-12	7.53	17.4284193	5.83E-02	118619582	118619705	NC_000011.9
cg11763866	2.82E-11	1.81E-12	7.53	17.4262027	1.22E-01	70310112	70310235	NC_000011.9
cg26102801	2.83E-11	1.81E-12	7.53	17.4254249	7.38E-02	155793769	155793892	NC_000007.13
cg01369033	2.83E-11	1.81E-12	7.53	17.422796	1.13E-01	3292386	3292509	NC_000004.11
cg09405238	2.84E-11	1.82E-12	7.53	17.419362	6.98E-02	183994500	183994623	NC_000003.11
cg20529344	2.85E-11	1.82E-12	7.53	17.4172595	1.20E-01	50707427	50707550	NC_000013.10
cg17967577	2.85E-11	1.82E-12	7.53	17.4172312	2.32E-01	88324711	88324834	NC_000013.10
cg01950845	2.85E-11	1.83E-12	7.53	17.4162471	2.63E-01	93632730	93632853	NC_000015.9
cg03843978	2.86E-11	1.83E-12	7.53	17.4127099	1.10E-01	4864625	4864748	NC_000004.11
cg02649987	2.87E-11	1.84E-12	7.53	17.4082619	2.00E-01	106888392	106888515	NC_000011.9
cg07744841	2.88E-11	1.84E-12	7.53	17.4065003	1.79E-01	10857703	10857826	NC_000003.11

cg18607529	2.89E-11	1.85E-12	7.53	17.4036881	2.41E-01	50343869	50343992	NC_000007.13
cg07183447	2.89E-11	1.85E-12	7.53	17.4036775	1.53E-01	102484107	102484230	NC_000010.10
cg15626350	2.89E-11	1.85E-12	7.53	17.4032062	1.67E-01	152130207	152130330	NC_000006.11
cg17715521	2.89E-11	1.85E-12	7.53	17.4028069	9.50E-02	45417414	45417537	NC_000015.9
cg25061701	2.89E-11	1.85E-12	7.53	17.4021073	7.71E-02	62608856	62608979	NC_000017.10
cg15126975	2.89E-11	1.85E-12	7.53	17.4004494	2.63E-01	175208605	175208728	NC_000002.11
cg26276395	2.90E-11	1.86E-12	7.53	17.3976884	1.20E-01	107496342	107496465	NC_000001.10
cg03924483	2.91E-11	1.87E-12	7.53	17.394965	1.11E-01	26553567	26553690	NC_000017.10
cg14262681	2.91E-11	1.87E-12	7.53	17.3934781	2.52E-01	21084420	21084543	NC_000020.10
cg08668316	2.92E-11	1.87E-12	7.53	17.3902246	1.33E-01	380157	380280	NC_000004.11
cg10764918	2.92E-11	1.88E-12	7.53	17.389473	1.59E-01	113541683	113541806	NC_000012.11
cg00092551	2.93E-11	1.88E-12	7.53	17.3880981	3.33E-02	127371565	127371688	NC_000007.13
cg08496953	2.94E-11	1.88E-12	7.53	17.385396	1.49E-01	42733527	42733650	NC_000017.10
cg26175428	2.94E-11	1.89E-12	7.53	17.3844457	7.62E-02	77631756	77631879	NC_000018.9
cg24524057	2.94E-11	1.89E-12	7.52	17.3841559	1.46E-01	125732842	125732965	NC_000010.10
cg07928255	2.94E-11	1.89E-12	7.52	17.3822588	8.30E-02	11343164	11343287	NC_000016.9
cg20976694	2.94E-11	1.89E-12	7.52	17.382043	1.69E-01	55714052	55714175	NC_000018.9
cg27320658	2.95E-11	1.89E-12	7.52	17.3817907	6.47E-02	176519389	176519512	NC_000005.9
cg13799919	2.95E-11	1.89E-12	7.52	17.3800874	1.18E-01	28212848	28212971	NC_000013.10
cg26449787	2.96E-11	1.90E-12	7.52	17.3771048	2.13E-01	133562087	133562210	NC_000006.11
cg20449590	2.96E-11	1.90E-12	7.52	17.376182	2.09E-01	146551201	146551324	NC_000001.10
cg07769732	2.96E-11	1.90E-12	7.52	17.3754133	8.66E-02	8815465	8815588	NC_000002.11
cg09705759	2.97E-11	1.91E-12	7.52	17.3727765	3.95E-02	140555532	140555655	NC_000004.11
cg05079767	2.98E-11	1.92E-12	7.52	17.3680194	7.04E-02	37770944	37771067	NC_000022.10
cg04147272	2.99E-11	1.92E-12	7.52	17.3670633	1.30E-01	79261496	79261619	NC_000015.9

cg00209038	2.99E-11	1.92E-12	7.52	17.366461	1.94E-01	48982683	48982806	NC_000002.11
cg01211137	2.99E-11	1.92E-12	7.52	17.3663828	1.67E-01	13993807	13993930	NC_000011.9
cg19269666	2.99E-11	1.92E-12	7.52	17.3643977	1.77E-01	229046590	229046713	NC_000002.11
cg06579738	3.00E-11	1.93E-12	7.52	17.3638006	9.38E-02	111165311	111165434	NC_000011.9
cg01881308	3.00E-11	1.93E-12	7.52	17.3632137	9.93E-02	1389301	1389424	NC_000016.9
cg15697036	3.00E-11	1.93E-12	7.52	17.3623732	7.62E-02	1808923	1809046	NC_000004.11
cg06479755	3.00E-11	1.93E-12	7.52	17.3622695	2.03E-01	27012313	27012436	NC_000021.8
cg09444818	3.01E-11	1.93E-12	7.52	17.3606215	1.22E-01	102097226	102097349	NC_000004.11
cg10029842	3.02E-11	1.94E-12	7.52	17.3562492	1.75E-01	75595919	75596042	NC_000001.10
cg22977397	3.02E-11	1.94E-12	7.52	17.3557101	3.99E-02	141542551	141542674	NC_000008.10
cg25925252	3.02E-11	1.94E-12	7.52	17.3551154	1.18E-01	178913683	178913806	NC_000005.9
cg06513139	3.03E-11	1.95E-12	7.52	17.3536353	1.54E-01	4455923	4456046	NC_000019.9
cg06611922	3.03E-11	1.95E-12	7.52	17.3535663	1.81E-01	118893174	118893297	NC_000010.10
cg14861089	3.03E-11	1.95E-12	7.52	17.3535502	2.64E-01	102895043	102895166	NC_000010.10
cg01630925	3.03E-11	1.95E-12	7.52	17.3519163	8.60E-02	7615827	7615950	NC_000019.9
cg24309040	3.04E-11	1.95E-12	7.52	17.3492762	1.56E-01	111664600	111664723	NC_000012.11
cg09354454	3.04E-11	1.96E-12	7.52	17.348182	1.19E-01	86795853	86795976	NC_000016.9
cg17982165	3.05E-11	1.96E-12	7.52	17.3465716	1.56E-01	30484753	30484876	NC_000015.9
cg07661899	3.05E-11	1.96E-12	7.52	17.3460278	1.52E-01	53443057	53443180	NC_000012.11
cg26843074	3.05E-11	1.96E-12	7.52	17.344351	2.12E-01	36422061	36422184	NC_000003.11
cg21269062	3.06E-11	1.97E-12	7.52	17.342238	6.26E-02	159898557	159898680	NC_000001.10
cg09549073	3.06E-11	1.97E-12	7.52	17.3416166	1.55E-01	27183274	27183397	NC_000007.13
cg24556660	3.07E-11	1.98E-12	7.52	17.3369041	1.68E-01	106842632	106842755	NC_000007.13
cg02361878	3.08E-11	1.98E-12	7.52	17.3360338	1.40E-01	165326327	165326450	NC_000001.10
cg07539601	3.08E-11	1.98E-12	7.52	17.3339936	9.34E-02	8277249	8277372	NC_000010.10

cg15814157	3.08E-11	1.98E-12	7.52	17.3337498	5.22E-02	56041511	56041634	NC_000019.9
cg19742623	3.08E-11	1.98E-12	7.52	17.3337374	6.34E-02	109810228	109810351	NC_000013.10
cg04760604	3.08E-11	1.99E-12	7.52	17.3336993	9.24E-02	22436179	22436302	NC_000016.9
cg17469276	3.09E-11	1.99E-12	7.52	17.3320968	1.13E-01	116925411	116925534	NC_000001.10
cg12969193	3.09E-11	1.99E-12	7.52	17.3319642	1.71E-01	176988505	176988628	NC_000002.11
cg09016806	3.10E-11	2.00E-12	7.52	17.3280681	4.25E-02	30120365	30120488	NC_000006.11
cg00418068	3.10E-11	2.00E-12	7.52	17.3267447	1.33E-01	33895505	33895628	NC_000001.10
cg23141355	3.11E-11	2.00E-12	7.51	17.3253663	2.34E-01	44063593	44063716	NC_000003.11
cg08571918	3.11E-11	2.00E-12	7.51	17.3245039	1.01E-01	105275920	105276043	NC_000002.11
cg23671279	3.12E-11	2.01E-12	7.51	17.3225752	1.70E-01	140969608	140969731	NC_000008.10
cg03495660	3.12E-11	2.01E-12	7.51	17.3220651	1.17E-01	1074500	1074623	NC_000016.9
cg09659661	3.12E-11	2.01E-12	7.51	17.3208786	1.80E-01	55358072	55358195	NC_000016.9
cg08811309	3.12E-11	2.01E-12	7.51	17.3208246	1.81E-01	87968749	87968872	NC_000005.9
cg02002583	3.13E-11	2.01E-12	7.51	17.3192165	2.03E-01	53811940	53812063	NC_000019.9
cg11227822	3.13E-11	2.02E-12	7.51	17.3174994	6.69E-02	53774359	53774482	NC_000013.10
cg01775921	3.13E-11	2.02E-12	7.51	17.316608	8.67E-02	30138213	30138336	NC_000006.11
cg23080354	3.14E-11	2.02E-12	7.51	17.315913	2.70E-01	45405880	45406003	NC_000022.10
cg19290410	3.14E-11	2.02E-12	7.51	17.3149571	2.07E-01	114847438	114847561	NC_000012.11
cg10484756	3.14E-11	2.03E-12	7.51	17.3140845	9.20E-02	139614104	139614227	NC_000006.11
cg10462643	3.14E-11	2.03E-12	7.51	17.3133585	1.78E-01	127296973	127297096	NC_000006.11
cg06272611	3.15E-11	2.03E-12	7.51	17.3115814	2.22E-01	29068567	29068690	NC_000013.10
cg07019315	3.15E-11	2.03E-12	7.51	17.3111292	1.31E-01	93344381	93344504	NC_000014.8
cg07360021	3.17E-11	2.04E-12	7.51	17.3053921	3.30E-02	151186904	151187027	NC_000006.11
cg08132837	3.18E-11	2.05E-12	7.51	17.3007443	1.60E-01	60975811	60975934	NC_000014.8
cg11882304	3.19E-11	2.05E-12	7.51	17.2996149	1.29E-01	159456907	159457030	NC_000005.9

cg15906675	3.19E-11	2.06E-12	7.51	17.2991107	1.28E-01	90171420	90171543	NC_000004.11
cg09545894	3.19E-11	2.06E-12	7.51	17.2986113	7.50E-02	24008532	24008655	NC_000013.10
cg14210862	3.19E-11	2.06E-12	7.51	17.2980762	1.36E-01	91193195	91193318	NC_000009.11
cg04401986	3.19E-11	2.06E-12	7.51	17.2979026	1.95E-01	81189927	81190050	NC_000004.11
cg14520424	3.19E-11	2.06E-12	7.51	17.2977805	2.30E-01	70034491	70034614	NC_000001.10
cg02726739	3.19E-11	2.06E-12	7.51	17.2973385	7.91E-02	40745035	40745158	NC_000015.9
cg20146536	3.19E-11	2.06E-12	7.51	17.2972695	1.14E-01	102096205	102096328	NC_000002.11
cg01779043	3.20E-11	2.06E-12	7.51	17.2961222	7.83E-02	21456586	21456709	NC_000014.8
cg18121355	3.20E-11	2.06E-12	7.51	17.2960813	2.09E-01	37840438	37840561	NC_000005.9
cg00089091	3.20E-11	2.06E-12	7.51	17.2952333	2.48E-01	115919950	115920073	NC_000002.11
cg00823843	3.20E-11	2.07E-12	7.51	17.2939517	1.16E-01	244094885	244095008	NC_000001.10
cg11366849	3.21E-11	2.07E-12	7.51	17.2921228	1.99E-01	42267547	42267670	NC_000007.13
cg19536929	3.21E-11	2.07E-12	7.51	17.2919324	1.66E-01	115134594	115134717	NC_000012.11
cg08721908	3.21E-11	2.07E-12	7.51	17.2914243	1.42E-01	95522902	95523025	NC_000002.11
cg21765730	3.21E-11	2.08E-12	7.51	17.289978	1.38E-01	155777175	155777298	NC_000006.11
cg09854828	3.22E-11	2.08E-12	7.51	17.2892813	4.42E-02	88841557	88841680	NC_000016.9
cg24218295	3.22E-11	2.08E-12	7.51	17.2883649	1.73E-01	30015820	30015943	NC_000019.9
cg00573338	3.23E-11	2.09E-12	7.51	17.2848294	1.08E-01	38479099	38479222	NC_000022.10
cg05139043	3.23E-11	2.09E-12	7.51	17.2842245	4.31E-02	72979531	72979654	NC_000011.9
cg24112562	3.23E-11	2.09E-12	7.51	17.2841208	1.25E-01	38070041	38070164	NC_000021.8
cg25555087	3.23E-11	2.09E-12	7.51	17.2832579	9.12E-02	20148758	20148881	NC_000015.9
cg13296809	3.24E-11	2.09E-12	7.51	17.2830389	7.16E-02	69709095	69709218	NC_000011.9
cg04550737	3.24E-11	2.09E-12	7.51	17.2826169	2.79E-01	119530600	119530723	NC_000001.10
cg14400672	3.24E-11	2.09E-12	7.51	17.2820821	1.12E-01	21393116	21393239	NC_000009.11
cg05201145	3.24E-11	2.09E-12	7.51	17.2817683	1.44E-01	259373	259496	NC_000012.11

cg12120326	3.24E-11	2.09E-12	7.51	17.2815745	4.27E-02	30237309	30237432	NC_000022.10
cg04044720	3.24E-11	2.09E-12	7.51	17.2810263	1.56E-01	22413680	22413803	NC_000003.11
cg17619823	3.24E-11	2.10E-12	7.51	17.279698	1.68E-01	37823475	37823598	NC_000008.10
cg09354241	3.25E-11	2.10E-12	7.51	17.2789161	2.06E-01	13540850	13540973	NC_000004.11
cg03303640	3.26E-11	2.11E-12	7.51	17.275685	9.44E-02	94840650	94840773	NC_000010.10
cg11856542	3.26E-11	2.11E-12	7.51	17.2746625	2.22E-01	37245707	37245830	NC_000004.11
cg04225551	3.26E-11	2.11E-12	7.51	17.2736553	1.52E-01	102526827	102526950	NC_000008.10
cg07020690	3.27E-11	2.11E-12	7.51	17.2723445	7.64E-02	92057672	92057795	NC_000015.9
cg13139972	3.27E-11	2.12E-12	7.51	17.2702669	2.12E-01	107957430	107957553	NC_000004.11
cg02065637	3.28E-11	2.12E-12	7.51	17.2684389	1.64E-01	61809035	61809158	NC_000020.10
cg06926146	3.28E-11	2.12E-12	7.51	17.2681169	1.62E-01	170629502	170629625	NC_000001.10
cg00731886	3.28E-11	2.12E-12	7.51	17.2678146	7.63E-02	1363008	1363131	NC_000004.11
cg18424634	3.29E-11	2.13E-12	7.5	17.2634699	1.62E-01	147127097	147127220	NC_000003.11
cg00683332	3.30E-11	2.14E-12	7.5	17.2607407	1.58E-01	51188697	51188820	NC_000016.9
cg08285446	3.31E-11	2.14E-12	7.5	17.2581833	1.21E-01	1273856	1273979	NC_000008.10
cg06797925	3.33E-11	2.16E-12	7.5	17.252161	2.44E-01	8548978	8549101	NC_000012.11
cg01643624	3.33E-11	2.16E-12	7.5	17.2515938	6.87E-02	8954893	8955016	NC_000011.9
cg20072171	3.33E-11	2.16E-12	7.5	17.2515388	2.44E-01	62356962	62357085	NC_000003.11
cg16430099	3.34E-11	2.16E-12	7.5	17.2503331	9.38E-02	118049898	118050021	NC_000001.10
cg13725172	3.34E-11	2.16E-12	7.5	17.2501489	1.31E-01	109267293	109267416	NC_000006.11
cg24129193	3.34E-11	2.16E-12	7.5	17.2501127	1.18E-01	89428192	89428315	NC_000015.9
cg13424940	3.35E-11	2.17E-12	7.5	17.2468374	1.17E-01	136844397	136844520	NC_000009.11
cg11481223	3.35E-11	2.17E-12	7.5	17.2464255	8.28E-02	147625024	147625147	NC_000002.11
cg26277657	3.36E-11	2.17E-12	7.5	17.2438119	6.12E-02	110430371	110430494	NC_000012.11
cg09032973	3.36E-11	2.18E-12	7.5	17.2429068	1.14E-01	140165998	140166121	NC_000005.9

cg17191518	3.36E-11	2.18E-12	7.5	17.242337	2.93E-02	52468850	52468973	NC_000015.9
cg00957516	3.36E-11	2.18E-12	7.5	17.2417182	1.68E-01	159141747	159141870	NC_000001.10
cg01249384	3.37E-11	2.18E-12	7.5	17.2392257	5.36E-02	11374254	11374377	NC_000004.11
cg17363629	3.38E-11	2.19E-12	7.5	17.2371724	1.40E-01	47704221	47704344	NC_000015.9
cg16915576	3.38E-11	2.19E-12	7.5	17.2368266	5.79E-02	89978468	89978591	NC_000007.13
cg10411146	3.38E-11	2.19E-12	7.5	17.2360087	1.43E-01	289966	290089	NC_000011.9
cg08981421	3.39E-11	2.20E-12	7.5	17.2345832	1.46E-01	27199956	27200079	NC_000007.13
cg08183074	3.39E-11	2.20E-12	7.5	17.2331708	1.10E-01	113296369	113296492	NC_000013.10
cg12149986	3.39E-11	2.20E-12	7.5	17.2321337	1.84E-01	50059027	50059150	NC_000019.9
cg03263618	3.40E-11	2.21E-12	7.5	17.2294566	1.63E-01	71802574	71802697	NC_000007.13
cg01824349	3.41E-11	2.21E-12	7.5	17.228041	7.26E-02	98260405	98260528	NC_000005.9
cg03289548	3.41E-11	2.21E-12	7.5	17.2262592	1.24E-01	114580989	114581112	NC_000010.10
cg15313459	3.41E-11	2.21E-12	7.5	17.2258528	2.35E-01	68547052	68547175	NC_000002.11
cg11452391	3.42E-11	2.22E-12	7.5	17.2249405	1.72E-01	16180315	16180438	NC_000005.9
cg09223396	3.42E-11	2.22E-12	7.5	17.2248282	5.22E-02	72893924	72894047	NC_000016.9
cg13138552	3.43E-11	2.22E-12	7.5	17.2224277	1.13E-01	66305468	66305591	NC_000012.11
cg20339692	3.43E-11	2.23E-12	7.5	17.2204917	6.83E-02	28886062	28886185	NC_000017.10
cg27403635	3.44E-11	2.23E-12	7.5	17.2182156	2.06E-01	113698291	113698414	NC_000005.9
cg01514490	3.44E-11	2.23E-12	7.5	17.2173958	1.55E-01	7455338	7455461	NC_000010.10
cg16496217	3.45E-11	2.24E-12	7.5	17.21625	1.09E-01	134651975	134652098	NC_000010.10
cg02725370	3.45E-11	2.24E-12	7.5	17.2155564	1.85E-01	111551829	111551952	NC_000004.11
cg12657297	3.45E-11	2.24E-12	7.5	17.2151032	1.29E-01	234667363	234667486	NC_000001.10
cg12112529	3.45E-11	2.24E-12	7.5	17.2149107	2.43E-01	118922736	118922859	NC_000010.10
cg22276258	3.45E-11	2.24E-12	7.5	17.214619	5.30E-02	65179503	65179626	NC_000012.11
cg10389936	3.46E-11	2.25E-12	7.5	17.2115348	1.44E-01	115741652	115741775	NC_000001.10

cg25723866	3.46E-11	2.25E-12	7.5	17.2110914	7.56E-02	41099080	41099203	NC_000019.9
cg15579567	3.47E-11	2.25E-12	7.5	17.2099315	3.93E-02	138952482	138952605	NC_000003.11
cg00717995	3.47E-11	2.25E-12	7.5	17.2094209	1.03E-01	44819838	44819961	NC_000001.10
cg01882870	3.47E-11	2.25E-12	7.49	17.2085923	1.80E-01	89910748	89910871	NC_000015.9
cg10904109	3.48E-11	2.26E-12	7.49	17.2057858	1.51E-01	146755494	146755617	NC_000006.11
cg05006349	3.48E-11	2.26E-12	7.49	17.2053692	2.26E-01	14925517	14925640	NC_000001.10
cg18535611	3.49E-11	2.27E-12	7.49	17.2030179	8.88E-02	28505434	28505557	NC_000013.10
cg10143811	3.49E-11	2.27E-12	7.49	17.202269	2.14E-01	16757985	16758108	NC_000012.11
cg05437823	3.50E-11	2.27E-12	7.49	17.2006114	1.64E-01	50813676	50813799	NC_000006.11
cg22060073	3.50E-11	2.28E-12	7.49	17.1990105	1.87E-01	132052942	132053065	NC_000008.10
cg24122068	3.51E-11	2.28E-12	7.49	17.197326	1.34E-01	23845240	23845363	NC_000003.11
cg10166277	3.51E-11	2.28E-12	7.49	17.1963367	1.42E-01	104752828	104752951	NC_000007.13
cg09170553	3.51E-11	2.28E-12	7.49	17.1960952	1.11E-01	298743	298866	NC_000004.11
cg15295166	3.51E-11	2.28E-12	7.49	17.1952462	4.89E-02	36040771	36040894	NC_000021.8
cg04673825	3.52E-11	2.29E-12	7.49	17.1930878	2.33E-01	10459183	10459306	NC_000004.11
cg19269029	3.52E-11	2.29E-12	7.49	17.1929651	7.69E-02	143614797	143614920	NC_000008.10
cg22138735	3.52E-11	2.29E-12	7.49	17.19288	1.77E-01	62679713	62679836	NC_000020.10
cg23201008	3.52E-11	2.29E-12	7.49	17.1924527	1.23E-01	99939568	99939691	NC_000014.8
cg21282549	3.52E-11	2.29E-12	7.49	17.1921386	2.73E-01	51925128	51925251	NC_000019.9
cg15158086	3.54E-11	2.30E-12	7.49	17.187934	1.05E-01	1221572	1221695	NC_000011.9
cg22506548	3.54E-11	2.31E-12	7.49	17.1864114	1.45E-01	2996949	2997072	NC_000001.10
cg09089421	3.54E-11	2.31E-12	7.49	17.1862878	1.45E-01	127803668	127803791	NC_000003.11
cg13397057	3.56E-11	2.31E-12	7.49	17.1823771	1.48E-01	62070246	62070369	NC_000016.9
cg08836825	3.56E-11	2.32E-12	7.49	17.1822643	9.52E-02	82726757	82726880	NC_000006.11
cg25045746	3.56E-11	2.32E-12	7.49	17.180391	1.61E-01	98963768	98963891	NC_000002.11

cg19184449	3.56E-11	2.32E-12	7.49	17.1801962	2.76E-02	52700707	52700830	NC_000012.11
cg14292870	3.56E-11	2.32E-12	7.49	17.1801865	1.48E-01	6887543	6887666	NC_000012.11
cg27183007	3.56E-11	2.32E-12	7.49	17.180087	1.01E-01	50132975	50133098	NC_000007.13
cg01615333	3.57E-11	2.32E-12	7.49	17.1784894	1.04E-01	127765195	127765318	NC_000012.11
cg07403228	3.57E-11	2.33E-12	7.49	17.177334	1.04E-01	61792641	61792764	NC_000017.10
cg08965744	3.57E-11	2.33E-12	7.49	17.1766815	6.72E-02	6993580	6993703	NC_000012.11
cg20365867	3.58E-11	2.33E-12	7.49	17.1761267	8.56E-02	42917340	42917463	NC_000003.11
cg22986947	3.59E-11	2.34E-12	7.49	17.1728848	9.53E-02	7907918	7908041	NC_000006.11
cg04386206	3.59E-11	2.34E-12	7.49	17.1727559	1.80E-01	170303640	170303763	NC_000003.11
cg26963356	3.59E-11	2.34E-12	7.49	17.1721988	2.17E-01	55443833	55443956	NC_000006.11
cg14999001	3.59E-11	2.34E-12	7.49	17.1717836	2.62E-01	45197441	45197564	NC_000007.13
cg20645601	3.60E-11	2.35E-12	7.49	17.1682002	1.45E-01	2742371	2742494	NC_000007.13
cg23253448	3.60E-11	2.35E-12	7.49	17.1677307	1.08E-01	22548828	22548951	NC_000008.10
cg10152449	3.61E-11	2.35E-12	7.49	17.1665473	1.26E-01	2444534	2444657	NC_000007.13
cg14644752	3.61E-11	2.36E-12	7.49	17.1654148	2.15E-01	89340331	89340454	NC_000008.10
cg03760839	3.61E-11	2.36E-12	7.49	17.1650523	1.11E-01	119531718	119531841	NC_000001.10
cg03331175	3.62E-11	2.36E-12	7.49	17.1644117	8.48E-02	99069044	99069167	NC_000007.13
cg12958813	3.62E-11	2.36E-12	7.49	17.1637959	1.25E-01	198509669	198509792	NC_000001.10
cg21113740	3.62E-11	2.36E-12	7.49	17.1627399	1.41E-01	79472343	79472466	NC_000001.10
cg01557549	3.62E-11	2.36E-12	7.49	17.1616606	1.50E-01	53168665	53168788	NC_000003.11
cg23284338	3.63E-11	2.36E-12	7.49	17.1612651	9.28E-02	73569605	73569728	NC_000010.10
cg04478905	3.63E-11	2.37E-12	7.49	17.1598851	1.44E-01	37915091	37915214	NC_000021.8
cg09311683	3.65E-11	2.38E-12	7.49	17.1556803	1.86E-01	2801995	2802118	NC_000020.10
cg15433534	3.65E-11	2.38E-12	7.49	17.1553649	2.66E-01	75612223	75612346	NC_000018.9
cg11755405	3.65E-11	2.38E-12	7.49	17.1548903	2.07E-01	63511872	63511995	NC_000014.8

cg20176142	3.65E-11	2.38E-12	7.49	17.1546867	1.65E-01	81408357	81408480	NC_000008.10
cg02268156	3.65E-11	2.38E-12	7.49	17.1539626	1.28E-01	66162107	66162230	NC_000016.9
cg26164907	3.65E-11	2.39E-12	7.49	17.1528177	5.56E-02	155616192	155616315	NC_000007.13
cg25449440	3.66E-11	2.39E-12	7.49	17.1517788	1.52E-01	147116420	147116543	NC_000003.11
cg18720151	3.66E-11	2.39E-12	7.49	17.1516209	1.48E-01	16763480	16763603	NC_000012.11
cg13537237	3.66E-11	2.39E-12	7.49	17.1515081	1.59E-01	241908681	241908804	NC_000002.11
cg27361577	3.66E-11	2.39E-12	7.48	17.1500933	8.49E-02	234662759	234662882	NC_000002.11
cg05194552	3.67E-11	2.40E-12	7.48	17.148776	5.18E-02	3873444	3873567	NC_000016.9
cg12041387	3.70E-11	2.42E-12	7.48	17.1403619	2.10E-01	96650171	96650294	NC_000007.13
cg27103296	3.71E-11	2.42E-12	7.48	17.1384316	2.49E-01	121940951	121941074	NC_000007.13
cg05133706	3.71E-11	2.43E-12	7.48	17.1360228	1.06E-01	234667230	234667353	NC_000001.10
cg08234308	3.73E-11	2.44E-12	7.48	17.1309497	2.63E-01	65991176	65991299	NC_000001.10
cg26195495	3.74E-11	2.44E-12	7.48	17.1298832	1.51E-01	1906338	1906461	NC_000008.10
cg10725227	3.74E-11	2.44E-12	7.48	17.129254	5.81E-02	107012207	107012330	NC_000006.11
cg13102079	3.74E-11	2.44E-12	7.48	17.1285973	1.42E-01	136554731	136554854	NC_000007.13
cg08115682	3.74E-11	2.45E-12	7.48	17.1284775	1.32E-01	5153729	5153852	NC_000012.11
cg22305346	3.74E-11	2.45E-12	7.48	17.127896	3.69E-02	7830039	7830162	NC_000017.10
cg25042860	3.75E-11	2.45E-12	7.48	17.1259303	9.88E-02	62467034	62467157	NC_000017.10
cg03671597	3.75E-11	2.45E-12	7.48	17.1257035	1.64E-01	27333463	27333586	NC_000017.10
cg24838174	3.75E-11	2.45E-12	7.48	17.125293	6.47E-02	15990592	15990715	NC_000019.9
cg09176256	3.76E-11	2.46E-12	7.48	17.1241687	1.33E-01	119293967	119294090	NC_000010.10
cg26417713	3.76E-11	2.46E-12	7.48	17.1233307	9.14E-02	36497719	36497842	NC_000017.10
cg27067575	3.76E-11	2.46E-12	7.48	17.1224198	9.42E-02	41866725	41866848	NC_000005.9
cg19161859	3.76E-11	2.46E-12	7.48	17.1219334	1.25E-01	84327426	84327549	NC_000001.10
cg11562502	3.77E-11	2.46E-12	7.48	17.1207915	2.11E-01	113441626	113441749	NC_000004.11

cg19480263	3.77E-11	2.46E-12	7.48	17.1207246	9.30E-02	5743854	5743977	NC_000007.13
cg21535000	3.78E-11	2.47E-12	7.48	17.1185849	2.41E-01	9760877	9761000	NC_000008.10
cg19095600	3.78E-11	2.47E-12	7.48	17.1178614	1.73E-01	155108933	155109056	NC_000005.9
cg05320944	3.79E-11	2.48E-12	7.48	17.1145525	1.09E-01	76018865	76018988	NC_000014.8
cg17534564	3.79E-11	2.48E-12	7.48	17.1142941	7.38E-02	124600518	124600641	NC_000012.11
cg22569496	3.79E-11	2.48E-12	7.48	17.1132291	1.13E-01	13409671	13409794	NC_000019.9
cg27043010	3.80E-11	2.49E-12	7.48	17.1121274	1.61E-01	99712380	99712503	NC_000014.8
cg20172627	3.80E-11	2.49E-12	7.48	17.1119646	2.79E-01	25439110	25439233	NC_000002.11
cg19400601	3.80E-11	2.49E-12	7.48	17.1112897	8.31E-02	117691210	117691333	NC_000011.9
cg05112120	3.80E-11	2.49E-12	7.48	17.1104578	1.37E-01	10119723	10119846	NC_000017.10
cg13242000	3.81E-11	2.49E-12	7.48	17.1096738	1.42E-01	74426086	74426209	NC_000015.9
cg04888037	3.81E-11	2.49E-12	7.48	17.1086387	1.24E-01	126677203	126677326	NC_000012.11
cg12475507	3.82E-11	2.50E-12	7.48	17.1062804	1.32E-01	3567852	3567975	NC_000001.10
cg13792596	3.82E-11	2.50E-12	7.48	17.1062259	8.28E-02	64660940	64661063	NC_000014.8
cg05911990	3.82E-11	2.50E-12	7.48	17.1059506	1.50E-01	112063449	112063572	NC_000007.13
cg16664496	3.82E-11	2.50E-12	7.48	17.1055883	6.59E-02	87740413	87740536	NC_000016.9
cg15836660	3.82E-11	2.50E-12	7.48	17.105559	1.83E-01	78493128	78493251	NC_000013.10
cg23420260	3.82E-11	2.50E-12	7.48	17.1053644	2.45E-01	177053285	177053408	NC_000002.11
cg14823763	3.82E-11	2.50E-12	7.48	17.1048473	1.93E-01	1884365	1884488	NC_000005.9
cg27199820	3.83E-11	2.51E-12	7.48	17.1044976	1.91E-01	6903019	6903142	NC_000003.11
cg18774195	3.83E-11	2.51E-12	7.48	17.104303	1.70E-01	170303830	170303953	NC_000003.11
cg01906054	3.83E-11	2.51E-12	7.48	17.1042554	4.45E-02	14683649	14683772	NC_000017.10
cg14056644	3.83E-11	2.51E-12	7.48	17.1023099	1.44E-01	111559105	111559228	NC_000004.11
cg17398252	3.84E-11	2.51E-12	7.48	17.101826	2.75E-01	105585179	105585302	NC_000006.11
cg23241914	3.84E-11	2.52E-12	7.48	17.1002618	9.38E-02	161193393	161193516	NC_000001.10

cg09662034	3.85E-11	2.52E-12	7.48	17.0988023	1.35E-01	55206467	55206590	NC_000020.10
cg21060817	3.85E-11	2.52E-12	7.48	17.0983479	3.54E-02	28792031	28792154	NC_000002.11
cg04243827	3.85E-11	2.52E-12	7.48	17.0973914	1.81E-01	3103718	3103841	NC_000005.9
cg04062391	3.85E-11	2.52E-12	7.48	17.0968624	1.87E-01	9609204	9609327	NC_000019.9
cg02694427	3.86E-11	2.53E-12	7.48	17.0953268	1.64E-01	176964512	176964635	NC_000002.11
cg02320740	3.87E-11	2.54E-12	7.47	17.0920435	1.91E-01	106400259	106400382	NC_000010.10
cg09538809	3.88E-11	2.55E-12	7.47	17.0889034	1.04E-01	140782948	140783071	NC_000005.9
cg01726792	3.88E-11	2.55E-12	7.47	17.088606	9.68E-02	21509062	21509185	NC_000014.8
cg05682333	3.89E-11	2.55E-12	7.47	17.0861253	8.77E-02	233274291	233274414	NC_000002.11
cg09753749	3.89E-11	2.55E-12	7.47	17.0859438	8.02E-02	184359437	184359560	NC_000001.10
cg16560824	3.90E-11	2.55E-12	7.47	17.0852676	2.19E-01	142189535	142189658	NC_000008.10
cg15708175	3.90E-11	2.56E-12	7.47	17.0841485	5.57E-02	145814244	145814367	NC_000008.10
cg18984809	3.90E-11	2.56E-12	7.47	17.0838733	5.70E-02	973184	973307	NC_000017.10
cg15037137	3.91E-11	2.56E-12	7.47	17.0827287	1.20E-01	27144302	27144425	NC_000007.13
cg04328762	3.91E-11	2.56E-12	7.47	17.0822257	1.99E-01	19706186	19706309	NC_000022.10
cg27532847	3.91E-11	2.56E-12	7.47	17.0821673	2.24E-01	35047401	35047524	NC_000015.9
cg14014964	3.91E-11	2.56E-12	7.47	17.0817892	1.16E-01	94443692	94443815	NC_000009.11
cg19030607	3.91E-11	2.56E-12	7.47	17.0815828	1.36E-01	48885506	48885629	NC_000022.10
cg04178316	3.91E-11	2.57E-12	7.47	17.0804713	1.61E-01	170738059	170738182	NC_000005.9
cg00960395	3.93E-11	2.58E-12	7.47	17.0769272	1.79E-01	46799948	46800071	NC_000017.10
cg06263341	3.93E-11	2.58E-12	7.47	17.0768341	1.75E-01	75525151	75525274	NC_000017.10
cg19675288	3.93E-11	2.58E-12	7.47	17.0764152	2.51E-01	166582206	166582329	NC_000006.11
cg06032349	3.93E-11	2.58E-12	7.47	17.0750485	1.21E-01	40032331	40032454	NC_000021.8
cg10589825	3.94E-11	2.58E-12	7.47	17.0740504	1.14E-01	105362890	105363013	NC_000002.11
cg08441822	3.94E-11	2.59E-12	7.47	17.0730171	1.39E-01	58723879	58724002	NC_000015.9

cg03169018	3.94E-11	2.59E-12	7.47	17.0721589	1.65E-01	24772435	24772558	NC_000008.10
cg05027554	3.95E-11	2.59E-12	7.47	17.0705264	1.12E-01	646579	646702	NC_000016.9
cg26560222	3.95E-11	2.59E-12	7.47	17.0704366	1.89E-01	50888826	50888949	NC_000001.10
cg04028634	3.95E-11	2.59E-12	7.47	17.0700479	1.39E-01	45613825	45613948	NC_000007.13
cg19855489	3.95E-11	2.60E-12	7.47	17.0695692	4.95E-02	132257551	132257674	NC_000003.11
cg06832406	3.95E-11	2.60E-12	7.47	17.069525	1.05E-01	4230482	4230605	NC_000010.10
cg00201192	3.96E-11	2.60E-12	7.47	17.06875	8.50E-02	48829788	48829911	NC_000004.11
cg03085321	3.96E-11	2.60E-12	7.47	17.0685404	9.51E-02	140133752	140133875	NC_000009.11
cg11418681	3.96E-11	2.60E-12	7.47	17.0675588	1.27E-01	14855132	14855255	NC_000003.11
cg02345060	3.96E-11	2.60E-12	7.47	17.0668063	5.77E-02	3165341	3165464	NC_000010.10
cg01591369	3.96E-11	2.60E-12	7.47	17.0667002	6.13E-02	10687325	10687448	NC_000006.11
cg00444059	3.97E-11	2.61E-12	7.47	17.0656233	7.50E-02	31439672	31439795	NC_000016.9
cg11983612	3.97E-11	2.61E-12	7.47	17.0651591	7.85E-02	123442240	123442363	NC_000010.10
cg09062663	3.99E-11	2.62E-12	7.47	17.0608594	8.75E-02	10262804	10262927	NC_000005.9
cg14398860	3.99E-11	2.62E-12	7.47	17.0604401	1.17E-01	134491205	134491328	NC_000010.10
cg17310600	3.99E-11	2.63E-12	7.47	17.058513	1.53E-01	165305334	165305457	NC_000004.11
cg26717554	4.00E-11	2.63E-12	7.47	17.0577518	1.42E-01	853334	853457	NC_000019.9
cg20033070	4.00E-11	2.63E-12	7.47	17.0573875	1.03E-01	24685656	24685779	NC_000014.8
cg01838443	4.00E-11	2.63E-12	7.47	17.0572715	1.09E-01	15752932	15753055	NC_000021.8
cg18448949	4.01E-11	2.64E-12	7.47	17.0543819	2.22E-01	176993089	176993212	NC_000002.11
cg01397057	4.01E-11	2.64E-12	7.47	17.0540579	4.59E-02	130344094	130344217	NC_000005.9
cg13371788	4.01E-11	2.64E-12	7.47	17.0535495	2.10E-01	117586817	117586940	NC_000006.11
cg06961473	4.01E-11	2.64E-12	7.47	17.0532522	1.18E-01	65369801	65369924	NC_000015.9
cg08370178	4.01E-11	2.64E-12	7.47	17.0531362	1.35E-01	43034043	43034166	NC_000005.9
cg15046304	4.01E-11	2.64E-12	7.47	17.0530277	9.11E-02	75205489	75205612	NC_000017.10

cg17434309	4.02E-11	2.64E-12	7.47	17.052404	2.42E-01	2161079	2161202	NC_000011.9
cg05114861	4.02E-11	2.65E-12	7.47	17.0502952	1.48E-01	15366034	15366157	NC_000003.11
cg06722407	4.03E-11	2.65E-12	7.47	17.049011	1.72E-01	19918586	19918709	NC_000013.10
cg08301307	4.03E-11	2.65E-12	7.47	17.0484502	7.58E-02	103599204	103599327	NC_000014.8
cg01449266	4.03E-11	2.65E-12	7.47	17.0482435	6.55E-02	2109999	2110122	NC_000001.10
cg05065572	4.04E-11	2.66E-12	7.47	17.047327	1.86E-01	114833912	114834035	NC_000012.11
cg06143897	4.04E-11	2.66E-12	7.47	17.0472176	1.73E-01	112404754	112404877	NC_000010.10
cg00089798	4.04E-11	2.66E-12	7.47	17.0460293	2.02E-01	62585204	62585327	NC_000012.11
cg22961278	4.04E-11	2.66E-12	7.47	17.0459531	1.67E-01	81107087	81107210	NC_000004.11
cg08254359	4.04E-11	2.66E-12	7.47	17.0455134	1.78E-01	54398518	54398641	NC_000012.11
cg11060276	4.05E-11	2.66E-12	7.47	17.0440091	1.69E-01	57359414	57359537	NC_000008.10
cg02034102	4.06E-11	2.67E-12	7.47	17.0409466	1.68E-01	37026739	37026862	NC_000009.11
cg11591516	4.06E-11	2.67E-12	7.47	17.0403658	2.49E-01	85954316	85954439	NC_000010.10
cg24032056	4.07E-11	2.68E-12	7.47	17.0376751	6.32E-02	75961704	75961827	NC_000012.11
cg26531286	4.07E-11	2.68E-12	7.47	17.0376719	1.29E-01	98799358	98799481	NC_000013.10
cg13839391	4.07E-11	2.68E-12	7.47	17.0371513	6.79E-02	121719066	121719189	NC_000012.11
cg15622556	4.09E-11	2.69E-12	7.46	17.0340641	1.29E-01	12251820	12251943	NC_000001.10
cg14415034	4.09E-11	2.69E-12	7.46	17.0338144	2.42E-02	45730717	45730840	NC_000003.11
cg26770200	4.09E-11	2.69E-12	7.46	17.0337114	5.32E-02	119752914	119753037	NC_000008.10
cg27117509	4.09E-11	2.69E-12	7.46	17.0330788	1.68E-01	19551398	19551521	NC_000002.11
cg00037457	4.09E-11	2.69E-12	7.46	17.0327182	1.51E-01	59531744	59531867	NC_000017.10
cg15922174	4.09E-11	2.70E-12	7.46	17.0318927	2.54E-01	126135998	126136121	NC_000009.11
cg25549459	4.10E-11	2.70E-12	7.46	17.0298813	1.76E-01	105471252	105471375	NC_000002.11
cg18161956	4.10E-11	2.70E-12	7.46	17.0296412	1.34E-01	115112626	115112749	NC_000012.11
cg06101841	4.11E-11	2.71E-12	7.46	17.0282481	9.20E-02	48758048	48758171	NC_000003.11

cg22374233	4.11E-11	2.71E-12	7.46	17.0277304	1.64E-01	906726	906849	NC_000018.9
cg21850852	4.12E-11	2.72E-12	7.46	17.0249051	1.01E-01	73746186	73746309	NC_000017.10
cg19944763	4.13E-11	2.72E-12	7.46	17.0240827	2.21E-01	15761854	15761977	NC_000010.10
cg03249890	4.13E-11	2.72E-12	7.46	17.0226878	1.33E-01	175199803	175199926	NC_000002.11
cg16937268	4.14E-11	2.73E-12	7.46	17.0210878	2.06E-01	21684417	21684540	NC_000020.10
cg22047387	4.14E-11	2.73E-12	7.46	17.0202164	1.97E-01	55413872	55413995	NC_000012.11
cg19099050	4.15E-11	2.74E-12	7.46	17.018017	1.97E-01	9594382	9594505	NC_000003.11
cg24709745	4.17E-11	2.75E-12	7.46	17.0135851	3.02E-02	1049864	1049987	NC_000001.10
cg05171933	4.17E-11	2.75E-12	7.46	17.0135849	1.40E-01	147205668	147205791	NC_000005.9
cg13280612	4.17E-11	2.75E-12	7.46	17.0135787	3.16E-02	126133599	126133722	NC_000010.10
cg04067139	4.17E-11	2.75E-12	7.46	17.0134084	2.58E-01	58673167	58673290	NC_000011.9
cg12597281	4.18E-11	2.76E-12	7.46	17.0106388	4.18E-02	208266216	208266339	NC_000001.10
cg05630016	4.18E-11	2.76E-12	7.46	17.0104436	2.29E-01	51815281	51815404	NC_000019.9
cg25901381	4.18E-11	2.76E-12	7.46	17.009499	1.65E-01	27225485	27225608	NC_000007.13
cg00376022	4.20E-11	2.77E-12	7.46	17.0064119	1.56E-01	19183880	19184003	NC_000007.13
cg11689732	4.20E-11	2.77E-12	7.46	17.0046491	1.13E-01	79139096	79139219	NC_000017.10
cg11525479	4.21E-11	2.78E-12	7.46	17.0025147	1.44E-01	88799523	88799646	NC_000015.9
cg00709112	4.22E-11	2.79E-12	7.46	16.999503	1.45E-01	118369983	118370106	NC_000010.10
cg25648436	4.23E-11	2.79E-12	7.46	16.9984191	1.43E-01	58554527	58554650	NC_000019.9
cg09141300	4.23E-11	2.79E-12	7.46	16.9983653	7.33E-02	30346020	30346143	NC_000005.9
cg17455088	4.23E-11	2.79E-12	7.46	16.9982795	1.38E-01	206551466	206551589	NC_000002.11
cg08603125	4.23E-11	2.79E-12	7.46	16.9981089	1.44E-01	35890197	35890320	NC_000006.11
cg01328892	4.24E-11	2.80E-12	7.46	16.9962529	2.33E-01	133562332	133562455	NC_000006.11
cg25201047	4.24E-11	2.80E-12	7.46	16.9958011	1.40E-01	1282630	1282753	NC_000007.13
cg27573549	4.24E-11	2.80E-12	7.46	16.9952379	7.62E-02	56189952	56190075	NC_000019.9

cg09317928	4.24E-11	2.80E-12	7.46	16.9949725	1.17E-01	61716830	61716953	NC_000011.9
cg09834343	4.24E-11	2.80E-12	7.46	16.9949215	2.15E-01	32942910	32943033	NC_000015.9
cg05686379	4.24E-11	2.80E-12	7.46	16.9947079	6.82E-02	36101779	36101902	NC_000007.13
cg03514545	4.24E-11	2.80E-12	7.46	16.9946029	1.93E-01	114696698	114696821	NC_000001.10
cg23141855	4.25E-11	2.80E-12	7.46	16.9937174	2.14E-01	93519892	93520015	NC_000007.13
cg13801381	4.25E-11	2.81E-12	7.46	16.9919032	2.24E-01	4384022	4384145	NC_000012.11
cg21908972	4.26E-11	2.81E-12	7.46	16.9905526	9.00E-02	281291	281414	NC_000019.9
cg05527856	4.26E-11	2.81E-12	7.46	16.9903742	8.54E-02	11603196	11603319	NC_000011.9
cg16397021	4.26E-11	2.82E-12	7.46	16.9894085	1.30E-01	25953388	25953511	NC_000015.9
cg06928695	4.27E-11	2.82E-12	7.46	16.9884993	7.47E-02	6384119	6384242	NC_000017.10
cg10672108	4.27E-11	2.82E-12	7.46	16.9870052	1.22E-01	58389987	58390110	NC_000011.9
cg06701796	4.28E-11	2.83E-12	7.46	16.9842004	5.43E-02	47745582	47745705	NC_000019.9
cg09346993	4.29E-11	2.83E-12	7.46	16.9838967	7.42E-02	31938358	31938481	NC_000006.11
cg26126740	4.30E-11	2.84E-12	7.46	16.9809946	9.35E-02	42574332	42574455	NC_000020.10
cg26156687	4.30E-11	2.84E-12	7.46	16.9798061	1.79E-01	102586168	102586291	NC_000010.10
cg27484483	4.30E-11	2.84E-12	7.46	16.9794784	1.46E-01	103249853	103249976	NC_000008.10
cg19198993	4.30E-11	2.85E-12	7.46	16.9792313	5.83E-02	131708300	131708423	NC_000012.11
cg00970396	4.31E-11	2.85E-12	7.46	16.978704	1.91E-01	9107199	9107322	NC_000016.9
cg26795012	4.31E-11	2.85E-12	7.46	16.9785636	6.58E-02	245874750	245874873	NC_000001.10
cg02883229	4.31E-11	2.85E-12	7.46	16.9782298	7.43E-02	155616337	155616460	NC_000007.13
cg05529500	4.31E-11	2.85E-12	7.46	16.9776266	3.28E-02	79298678	79298801	NC_000015.9
cg24716664	4.31E-11	2.85E-12	7.46	16.9775586	1.51E-01	1006121	1006244	NC_000011.9
cg10038618	4.31E-11	2.85E-12	7.46	16.9768696	1.50E-01	3567719	3567842	NC_000001.10
cg08376020	4.32E-11	2.85E-12	7.45	16.9762211	8.79E-02	8154317	8154440	NC_000004.11
cg04049225	4.33E-11	2.86E-12	7.45	16.9731315	1.00E-01	85672175	85672298	NC_000016.9

cg27331863	4.34E-11	2.87E-12	7.45	16.9703049	1.17E-01	66062176	66062299	NC_000015.9
cg24569447	4.35E-11	2.88E-12	7.45	16.9671889	1.98E-01	89312795	89312918	NC_000015.9
cg07230107	4.36E-11	2.88E-12	7.45	16.9659835	1.76E-01	224804396	224804519	NC_000001.10
cg08686425	4.36E-11	2.89E-12	7.45	16.9645195	7.28E-02	67722909	67723032	NC_000011.9
cg22398616	4.38E-11	2.90E-12	7.45	16.9620048	1.06E-01	53314203	53314326	NC_000013.10
cg04584301	4.39E-11	2.90E-12	7.45	16.9595641	2.12E-01	17208740	17208863	NC_000020.10
cg07748767	4.39E-11	2.90E-12	7.45	16.9594697	1.31E-01	112659978	112660101	NC_000002.11
cg26053346	4.40E-11	2.91E-12	7.45	16.9567868	1.19E-01	66454856	66454979	NC_000003.11
cg01864361	4.40E-11	2.92E-12	7.45	16.9548161	1.02E-01	54188647	54188770	NC_000019.9
cg17543488	4.41E-11	2.92E-12	7.45	16.9544421	1.75E-01	172672959	172673082	NC_000005.9
cg00854242	4.42E-11	2.92E-12	7.45	16.9521072	1.71E-01	74961724	74961847	NC_000018.9
cg08572734	4.42E-11	2.93E-12	7.45	16.9515477	1.93E-01	133485298	133485421	NC_000012.11
cg19046783	4.43E-11	2.94E-12	7.45	16.9484113	1.03E-01	99382086	99382209	NC_000007.13
cg14173476	4.43E-11	2.94E-12	7.45	16.9479668	5.91E-02	19190476	19190599	NC_000017.10
cg08472142	4.44E-11	2.94E-12	7.45	16.9471941	7.47E-02	155007166	155007289	NC_000001.10
cg04688351	4.44E-11	2.94E-12	7.45	16.947044	1.87E-01	223154140	223154263	NC_000002.11
cg20709984	4.44E-11	2.94E-12	7.45	16.9465688	4.48E-02	37922198	37922321	NC_000017.10
cg24912438	4.45E-11	2.95E-12	7.45	16.9451895	2.22E-01	122686564	122686687	NC_000004.11
cg12798775	4.45E-11	2.95E-12	7.45	16.9445996	1.15E-01	26350473	26350596	NC_000011.9
cg11092616	4.45E-11	2.95E-12	7.45	16.9437528	2.80E-01	72596493	72596616	NC_000006.11
cg00769012	4.45E-11	2.95E-12	7.45	16.9430251	4.63E-02	39776397	39776520	NC_000022.10
cg18984002	4.46E-11	2.95E-12	7.45	16.942506	1.38E-01	36876514	36876637	NC_000017.10
cg26019112	4.46E-11	2.96E-12	7.45	16.9418476	1.61E-01	31821313	31821436	NC_000011.9
cg19881992	4.46E-11	2.96E-12	7.45	16.9417251	9.99E-02	216491679	216491802	NC_000002.11
cg17254701	4.47E-11	2.96E-12	7.45	16.9392872	6.17E-02	78158076	78158199	NC_000017.10

cg25073967	4.47E-11	2.97E-12	7.45	16.9385271	1.09E-01	105360327	105360450	NC_000014.8
cg12346874	4.48E-11	2.97E-12	7.45	16.9375949	1.81E-01	137523644	137523767	NC_000002.11
cg26832142	4.48E-11	2.97E-12	7.45	16.9363737	1.38E-01	66258441	66258564	NC_000001.10
cg00335735	4.48E-11	2.97E-12	7.45	16.9361218	1.42E-01	222355484	222355607	NC_000002.11
cg00397635	4.49E-11	2.98E-12	7.45	16.9337071	1.95E-01	53309162	53309285	NC_000001.10
cg22227621	4.50E-11	2.98E-12	7.45	16.9326806	9.04E-02	95464471	95464594	NC_000001.10
cg08274552	4.50E-11	2.98E-12	7.45	16.9326314	1.50E-01	220491834	220491957	NC_000002.11
cg12791492	4.51E-11	2.99E-12	7.45	16.9295812	2.05E-01	57522094	57522217	NC_000004.11
cg12630714	4.51E-11	2.99E-12	7.45	16.9294338	2.01E-01	54975894	54976017	NC_000004.11
cg15594205	4.52E-11	3.00E-12	7.45	16.9274023	2.34E-01	105470794	105470917	NC_000002.11
cg17616537	4.52E-11	3.00E-12	7.45	16.9269764	1.65E-01	46628663	46628786	NC_000017.10
cg25734198	4.52E-11	3.00E-12	7.45	16.9266948	1.11E-01	49670744	49670867	NC_000006.11
cg00908291	4.53E-11	3.01E-12	7.45	16.9253311	2.04E-01	140771786	140771909	NC_000003.11
cg04181150	4.53E-11	3.01E-12	7.45	16.924801	1.83E-01	33603883	33604006	NC_000015.9
cg12734852	4.54E-11	3.01E-12	7.45	16.9238319	8.15E-02	79410138	79410261	NC_000010.10
cg12627071	4.54E-11	3.01E-12	7.45	16.9236574	1.63E-01	237072673	237072796	NC_000002.11
cg10261191	4.54E-11	3.01E-12	7.45	16.9228347	1.51E-01	125364744	125364867	NC_000011.9
cg22693493	4.54E-11	3.01E-12	7.45	16.9222832	7.73E-02	172044942	172045065	NC_000005.9
cg19714132	4.54E-11	3.02E-12	7.45	16.9217593	2.01E-01	29235984	29236107	NC_000014.8
cg13379763	4.55E-11	3.02E-12	7.45	16.9214289	7.81E-02	219892516	219892639	NC_000002.11
cg22597340	4.55E-11	3.02E-12	7.45	16.919916	3.01E-02	3208678	3208801	NC_000004.11
cg23358479	4.55E-11	3.02E-12	7.45	16.9197234	6.89E-02	1110784	1110907	NC_000007.13
cg02710485	4.56E-11	3.03E-12	7.44	16.9179139	1.47E-01	21911128	21911251	NC_000008.10
cg14537179	4.56E-11	3.03E-12	7.44	16.9178218	1.25E-01	115915892	115916015	NC_000009.11
cg04996873	4.56E-11	3.03E-12	7.44	16.917497	2.23E-01	14925992	14926115	NC_000001.10

cg10043313	4.57E-11	3.03E-12	7.44	16.9165843	7.49E-02	32212399	32212522	NC_000016.9
cg02960347	4.57E-11	3.04E-12	7.44	16.9149194	1.38E-01	61715662	61715785	NC_000003.11
cg05621259	4.58E-11	3.04E-12	7.44	16.913917	1.07E-01	39836007	39836130	NC_000008.10
cg17469434	4.58E-11	3.04E-12	7.44	16.9130176	6.69E-02	64748689	64748812	NC_000002.11
cg25490241	4.58E-11	3.04E-12	7.44	16.9126705	1.33E-01	65073816	65073939	NC_000015.9
cg02075820	4.59E-11	3.05E-12	7.44	16.9117245	1.88E-01	17300909	17301032	NC_000011.9
cg09342766	4.59E-11	3.05E-12	7.44	16.9108539	1.69E-01	72596291	72596414	NC_000006.11
cg11416338	4.60E-11	3.05E-12	7.44	16.9093543	1.96E-01	20523143	20523266	NC_000012.11
cg14929805	4.60E-11	3.06E-12	7.44	16.9089126	1.37E-01	134232964	134233087	NC_000008.10
cg07035503	4.61E-11	3.06E-12	7.44	16.906235	2.65E-01	134871966	134872089	NC_000005.9
cg24530250	4.63E-11	3.08E-12	7.44	16.9024512	2.38E-01	71955599	71955722	NC_000011.9
cg04302570	4.63E-11	3.08E-12	7.44	16.9014708	1.14E-01	21640162	21640285	NC_000008.10
cg17567560	4.64E-11	3.09E-12	7.44	16.8992518	2.35E-01	105036863	105036986	NC_000010.10
cg07142833	4.66E-11	3.10E-12	7.44	16.8962232	9.20E-02	48944598	48944721	NC_000022.10
cg13494489	4.66E-11	3.10E-12	7.44	16.8961451	1.04E-01	124535465	124535588	NC_000009.11
cg26106921	4.66E-11	3.10E-12	7.44	16.8957371	6.11E-02	44875053	44875176	NC_000021.8
cg20366397	4.66E-11	3.10E-12	7.44	16.8955958	1.27E-01	59759843	59759966	NC_000003.11
cg22310279	4.66E-11	3.10E-12	7.44	16.8945454	1.65E-01	78493232	78493355	NC_000013.10
cg26104752	4.66E-11	3.10E-12	7.44	16.8942738	1.72E-01	119527377	119527500	NC_000001.10
cg00481629	4.67E-11	3.10E-12	7.44	16.893838	1.94E-01	48972850	48972973	NC_000022.10
cg22512651	4.68E-11	3.11E-12	7.44	16.8911107	1.39E-01	42603117	42603240	NC_000019.9
cg15279413	4.68E-11	3.11E-12	7.44	16.8903572	8.04E-02	18961206	18961329	NC_000022.10
cg07651242	4.68E-11	3.11E-12	7.44	16.890198	1.74E-01	45614720	45614843	NC_000007.13
cg20933239	4.68E-11	3.11E-12	7.44	16.8901271	1.86E-01	2112381	2112504	NC_000005.9
cg01408654	4.70E-11	3.13E-12	7.44	16.8867281	2.16E-01	134070437	134070560	NC_000004.11

cg02639634	4.70E-11	3.13E-12	7.44	16.8865388	1.89E-01	54792161	54792284	NC_000008.10
cg00601311	4.70E-11	3.13E-12	7.44	16.8864722	8.03E-02	2186129	2186252	NC_000007.13
cg16304215	4.70E-11	3.13E-12	7.44	16.8852269	1.94E-01	76928810	76928933	NC_000005.9
cg01274028	4.70E-11	3.13E-12	7.44	16.8847225	6.85E-02	66633768	66633891	NC_000011.9
cg25013011	4.71E-11	3.13E-12	7.44	16.8846174	2.26E-01	130389010	130389133	NC_000012.11
cg12087643	4.72E-11	3.14E-12	7.44	16.8824161	1.83E-01	118033370	118033493	NC_000010.10
cg12152919	4.72E-11	3.14E-12	7.44	16.8819262	1.63E-01	133561886	133562009	NC_000006.11
cg06410057	4.73E-11	3.15E-12	7.44	16.8802085	2.00E-01	25907218	25907341	NC_000008.10
cg05474726	4.73E-11	3.15E-12	7.44	16.8780959	1.93E-01	65291523	65291646	NC_000008.10
cg12037895	4.74E-11	3.15E-12	7.44	16.8776683	5.21E-02	3058175	3058298	NC_000010.10
cg24418063	4.74E-11	3.16E-12	7.44	16.8775035	9.77E-02	7050950	7051073	NC_000012.11
cg02568902	4.74E-11	3.16E-12	7.44	16.8766695	6.17E-02	6302992	6303115	NC_000004.11
cg01663052	4.74E-11	3.16E-12	7.44	16.8764079	9.15E-02	31923667	31923790	NC_000014.8
cg23812665	4.75E-11	3.16E-12	7.44	16.8754923	3.84E-02	117061545	117061668	NC_000001.10
cg26501369	4.75E-11	3.16E-12	7.44	16.8752684	1.73E-01	133561814	133561937	NC_000006.11
cg19715410	4.75E-11	3.17E-12	7.44	16.8740423	2.56E-01	29139021	29139144	NC_000001.10
cg05062192	4.76E-11	3.17E-12	7.44	16.8718333	1.02E-01	45678303	45678426	NC_000021.8
cg06650419	4.76E-11	3.17E-12	7.44	16.8713463	1.29E-01	177133946	177134069	NC_000001.10
cg24236409	4.77E-11	3.18E-12	7.44	16.8708401	1.88E-01	78493282	78493405	NC_000013.10
cg11723713	4.77E-11	3.18E-12	7.44	16.8705966	1.49E-01	131721477	131721600	NC_000002.11
cg13832669	4.77E-11	3.18E-12	7.44	16.8701051	2.08E-01	119609429	119609552	NC_000002.11
cg15039604	4.77E-11	3.18E-12	7.44	16.8698516	6.89E-02	3063192	3063315	NC_000010.10
cg26080154	4.77E-11	3.18E-12	7.44	16.8696019	5.18E-02	1314228	1314351	NC_000011.9
cg11084334	4.77E-11	3.18E-12	7.44	16.8694302	1.07E-01	9594264	9594387	NC_000003.11
cg06631944	4.78E-11	3.19E-12	7.44	16.8681636	1.51E-01	158365160	158365283	NC_000007.13

cg19313408	4.78E-11	3.19E-12	7.44	16.8675753	1.02E-01	46827585	46827708	NC_000021.8
cg16597689	4.78E-11	3.19E-12	7.44	16.867199	5.22E-02	75848879	75849002	NC_000017.10
cg18265435	4.78E-11	3.19E-12	7.44	16.866812	5.67E-02	129828798	129828921	NC_000010.10
cg03901532	4.78E-11	3.19E-12	7.44	16.8666196	9.00E-02	36718460	36718583	NC_000002.11
cg06245037	4.80E-11	3.20E-12	7.44	16.8641864	1.93E-01	44332938	44333061	NC_000011.9
cg07291387	4.80E-11	3.20E-12	7.44	16.8622465	1.14E-01	3445288	3445411	NC_000006.11
cg23423423	4.82E-11	3.21E-12	7.43	16.8599014	6.76E-02	37603155	37603278	NC_000022.10
cg03226844	4.82E-11	3.21E-12	7.43	16.8591196	1.88E-01	185102	185225	NC_000017.10
cg07752026	4.82E-11	3.22E-12	7.43	16.858091	1.94E-01	3786483	3786606	NC_000019.9
cg05670717	4.83E-11	3.22E-12	7.43	16.8572937	1.30E-01	46368052	46368175	NC_000021.8
cg01436128	4.83E-11	3.22E-12	7.43	16.8571455	1.43E-01	22605186	22605309	NC_000019.9
cg14635586	4.83E-11	3.22E-12	7.43	16.85695	1.20E-01	91321898	91322021	NC_000006.11
cg13912573	4.83E-11	3.23E-12	7.43	16.8557675	1.26E-01	92293297	92293420	NC_000009.11
cg25154609	4.84E-11	3.23E-12	7.43	16.8555978	8.21E-02	57404184	57404307	NC_000012.11
cg11108474	4.84E-11	3.23E-12	7.43	16.8537932	9.48E-02	19919156	19919279	NC_000013.10
cg14301191	4.85E-11	3.24E-12	7.43	16.8516848	1.87E-01	158616065	158616188	NC_000006.11
cg23647616	4.85E-11	3.24E-12	7.43	16.851526	1.08E-01	169238245	169238368	NC_000006.11
cg10577511	4.86E-11	3.24E-12	7.43	16.8511954	1.21E-01	122277240	122277363	NC_000002.11
cg11908057	4.86E-11	3.24E-12	7.43	16.8504711	1.55E-01	27171154	27171277	NC_000007.13
cg09793619	4.87E-11	3.25E-12	7.43	16.8481759	1.21E-01	131650331	131650454	NC_000010.10
cg07428004	4.88E-11	3.26E-12	7.43	16.8456934	1.34E-01	127912372	127912495	NC_000007.13
cg17168456	4.88E-11	3.26E-12	7.43	16.8455139	2.03E-01	50717667	50717790	NC_000001.10
cg20011248	4.88E-11	3.26E-12	7.43	16.8454415	9.82E-02	227747268	227747391	NC_000001.10
cg09074113	4.88E-11	3.26E-12	7.43	16.8452346	1.12E-01	20870087	20870210	NC_000002.11
cg22224462	4.89E-11	3.26E-12	7.43	16.8438667	7.46E-02	6214772	6214895	NC_000001.10

cg26963797	4.89E-11	3.27E-12	7.43	16.843212	1.79E-01	51189291	51189414	NC_000016.9
cg14056110	4.89E-11	3.27E-12	7.43	16.8430825	1.90E-01	50236465	50236588	NC_000017.10
cg02421962	4.90E-11	3.28E-12	7.43	16.8404849	8.64E-02	815975	816098	NC_000016.9
cg10308578	4.91E-11	3.28E-12	7.43	16.838425	9.75E-02	8053246	8053369	NC_000011.9
cg26139953	4.93E-11	3.29E-12	7.43	16.8355674	5.20E-02	237973085	237973208	NC_000002.11
cg14510333	4.94E-11	3.30E-12	7.43	16.8322354	1.80E-01	51168503	51168626	NC_000016.9
cg11142389	4.95E-11	3.31E-12	7.43	16.8300374	1.85E-01	147125765	147125888	NC_000003.11
cg17677678	4.96E-11	3.32E-12	7.43	16.8281828	5.00E-02	31735443	31735566	NC_000006.11
cg06908778	4.96E-11	3.32E-12	7.43	16.8280485	1.56E-01	22634602	22634725	NC_000010.10
cg21911021	4.96E-11	3.32E-12	7.43	16.8279649	1.65E-01	58095011	58095134	NC_000019.9
cg27112264	4.97E-11	3.32E-12	7.43	16.8277351	2.12E-01	58400504	58400627	NC_000019.9
cg25433222	4.97E-11	3.32E-12	7.43	16.8276357	6.44E-02	3155143	3155266	NC_000006.11
cg09180848	4.97E-11	3.32E-12	7.43	16.8265487	1.58E-01	83953780	83953903	NC_000015.9
cg15759937	4.98E-11	3.33E-12	7.43	16.8236847	2.49E-01	58400325	58400448	NC_000019.9
cg19291576	4.99E-11	3.33E-12	7.43	16.8231778	2.05E-01	969530	969653	NC_000009.11
cg13307880	4.99E-11	3.33E-12	7.43	16.8229356	1.67E-01	140744367	140744490	NC_000005.9
cg19518845	4.99E-11	3.34E-12	7.43	16.8227	5.46E-02	183338346	183338469	NC_000001.10
cg21590967	4.99E-11	3.34E-12	7.43	16.821519	1.57E-02	120517099	120517222	NC_000002.11
cg15765724	5.00E-11	3.34E-12	7.43	16.8208479	2.05E-01	237077151	237077274	NC_000002.11
cg06966113	5.01E-11	3.35E-12	7.43	16.8173588	1.38E-01	4873263	4873386	NC_000004.11
cg14582763	5.01E-11	3.35E-12	7.43	16.8170324	1.89E-01	52956683	52956806	NC_000019.9
cg26897313	5.02E-11	3.36E-12	7.43	16.8153093	1.93E-01	57903521	57903644	NC_000015.9
cg24304919	5.02E-11	3.36E-12	7.43	16.8151591	1.83E-01	113699194	113699317	NC_000005.9
cg07727170	5.02E-11	3.36E-12	7.43	16.8149697	1.63E-01	70458214	70458337	NC_000015.9
cg02154269	5.05E-11	3.38E-12	7.43	16.8098953	7.37E-02	4228637	4228760	NC_000004.11

cg27069753	5.05E-11	3.38E-12	7.43	16.8092187	8.78E-02	22303571	22303694	NC_000001.10
cg09907521	5.05E-11	3.38E-12	7.43	16.8090516	7.32E-02	240566442	240566565	NC_000002.11
cg21135560	5.05E-11	3.38E-12	7.43	16.8089183	5.11E-02	144946659	144946782	NC_000008.10
cg27550984	5.06E-11	3.38E-12	7.43	16.8086755	8.11E-02	156027790	156027913	NC_000001.10
cg26354493	5.06E-11	3.38E-12	7.43	16.808447	1.72E-01	4305072	4305195	NC_000019.9
cg13912311	5.06E-11	3.38E-12	7.43	16.8083399	1.16E-01	127265348	127265471	NC_000009.11
cg24653263	5.07E-11	3.39E-12	7.43	16.8065581	1.94E-01	38258335	38258458	NC_000005.9
cg25017620	5.07E-11	3.39E-12	7.43	16.8055157	1.75E-01	247802802	247802925	NC_000001.10
cg24900666	5.07E-11	3.40E-12	7.43	16.8051951	8.82E-02	46608329	46608452	NC_000017.10
cg10160975	5.08E-11	3.40E-12	7.43	16.8045809	1.86E-01	42075810	42075933	NC_000014.8
cg23009921	5.08E-11	3.40E-12	7.43	16.8041792	5.62E-02	134219740	134219863	NC_000010.10
cg14409023	5.09E-11	3.41E-12	7.42	16.8019542	2.20E-01	57283436	57283559	NC_000014.8
cg01901262	5.09E-11	3.41E-12	7.42	16.8016327	2.32E-01	54966837	54966960	NC_000016.9
cg02109605	5.09E-11	3.41E-12	7.42	16.8014715	1.37E-01	13028573	13028696	NC_000003.11
cg11403046	5.10E-11	3.41E-12	7.42	16.799902	5.84E-02	40628070	40628193	NC_000020.10
cg07532353	5.10E-11	3.42E-12	7.42	16.7989186	1.10E-01	85608659	85608782	NC_000016.9
cg04797496	5.10E-11	3.42E-12	7.42	16.7984272	1.07E-01	141339086	141339209	NC_000005.9
cg08659667	5.11E-11	3.42E-12	7.42	16.7980386	2.10E-01	242688226	242688349	NC_000001.10
cg21548955	5.12E-11	3.43E-12	7.42	16.7953514	1.17E-01	140794993	140795116	NC_000005.9
cg16792842	5.12E-11	3.43E-12	7.42	16.7952648	1.69E-01	25899459	25899582	NC_000008.10
cg17495719	5.14E-11	3.44E-12	7.42	16.7917688	2.12E-01	144621945	144622068	NC_000004.11
cg14659346	5.14E-11	3.45E-12	7.42	16.7909496	2.05E-01	233246431	233246554	NC_000002.11
cg15104081	5.14E-11	3.45E-12	7.42	16.7909225	7.05E-02	77560291	77560414	NC_000018.9
cg06569421	5.14E-11	3.45E-12	7.42	16.7908173	4.12E-02	101509114	101509237	NC_000007.13
cg11199172	5.14E-11	3.45E-12	7.42	16.7907999	1.62E-01	46707338	46707461	NC_000017.10

cg03382304	5.14E-11	3.45E-12	7.42	16.7899586	2.28E-01	27012176	27012299	NC_000021.8
cg19602485	5.17E-11	3.46E-12	7.42	16.7853368	1.24E-01	84327319	84327442	NC_000001.10
cg03020554	5.17E-11	3.47E-12	7.42	16.7845441	1.79E-01	99951730	99951853	NC_000008.10
cg11251215	5.17E-11	3.47E-12	7.42	16.784101	9.04E-02	45103933	45104056	NC_000001.10
cg26225694	5.17E-11	3.47E-12	7.42	16.7840627	1.76E-01	180205253	180205376	NC_000001.10
cg05433039	5.17E-11	3.47E-12	7.42	16.7840517	1.55E-01	79397075	79397198	NC_000010.10
cg07224726	5.18E-11	3.47E-12	7.42	16.7835687	1.78E-01	115298511	115298634	NC_000005.9
cg26517262	5.18E-11	3.47E-12	7.42	16.7834261	3.96E-02	109774738	109774861	NC_000006.11
cg09410830	5.18E-11	3.47E-12	7.42	16.7831223	8.18E-02	112041335	112041458	NC_000006.11
cg22536150	5.19E-11	3.48E-12	7.42	16.7800611	1.40E-01	142840129	142840252	NC_000003.11
cg06330323	5.19E-11	3.48E-12	7.42	16.7800515	1.87E-01	2132315	2132438	NC_000016.9
cg27021357	5.20E-11	3.49E-12	7.42	16.779389	2.34E-01	84115969	84116092	NC_000015.9
cg22838882	5.20E-11	3.49E-12	7.42	16.7783118	1.10E-01	138952118	138952241	NC_000003.11
cg02621694	5.21E-11	3.50E-12	7.42	16.7767513	2.40E-01	81951472	81951595	NC_000004.11
cg10698355	5.22E-11	3.50E-12	7.42	16.7752695	1.53E-01	119801193	119801316	NC_000005.9
cg20304988	5.23E-11	3.51E-12	7.42	16.7726255	1.39E-01	29237766	29237889	NC_000007.13
cg00318347	5.23E-11	3.51E-12	7.42	16.7720174	1.01E-01	25812500	25812623	NC_000008.10
cg22920418	5.24E-11	3.51E-12	7.42	16.7714064	1.22E-01	58902394	58902517	NC_000019.9
cg17296482	5.24E-11	3.52E-12	7.42	16.7708319	2.39E-01	4382188	4382311	NC_000012.11
cg11389011	5.24E-11	3.52E-12	7.42	16.7703815	2.87E-02	30576923	30577046	NC_000006.11
cg09621472	5.25E-11	3.52E-12	7.42	16.7691019	7.19E-02	113439314	113439437	NC_000003.11
cg22005656	5.26E-11	3.53E-12	7.42	16.7663702	6.35E-02	159691948	159692071	NC_000005.9
cg26855234	5.26E-11	3.53E-12	7.42	16.7658381	5.83E-02	33798089	33798212	NC_000019.9
cg16161135	5.27E-11	3.53E-12	7.42	16.765638	2.38E-02	179330491	179330614	NC_000005.9
cg11645631	5.27E-11	3.54E-12	7.42	16.7641644	2.20E-01	63986656	63986779	NC_000005.9

cg05311410	5.28E-11	3.54E-12	7.42	16.7635073	2.38E-01	27225523	27225646	NC_000007.13
cg07877257	5.28E-11	3.54E-12	7.42	16.7634651	8.96E-02	125411922	125412045	NC_000012.11
cg18869700	5.28E-11	3.55E-12	7.42	16.7626941	6.36E-02	20907205	20907328	NC_000014.8
cg10813070	5.28E-11	3.55E-12	7.42	16.7616871	7.78E-02	2228344	2228467	NC_000016.9
cg24006721	5.29E-11	3.55E-12	7.42	16.7605088	1.27E-01	25258225	25258348	NC_000001.10
cg13554714	5.32E-11	3.58E-12	7.42	16.7543264	6.48E-02	15698215	15698338	NC_000012.11
cg23756251	5.32E-11	3.58E-12	7.42	16.7538993	1.79E-01	179754521	179754644	NC_000003.11
cg16973098	5.33E-11	3.58E-12	7.42	16.7534533	1.63E-01	95359203	95359326	NC_000013.10
cg16186001	5.33E-11	3.58E-12	7.42	16.7534252	7.08E-02	159448398	159448521	NC_000005.9
cg14292834	5.34E-11	3.59E-12	7.42	16.7502971	1.16E-01	36691116	36691239	NC_000014.8
cg05630812	5.35E-11	3.59E-12	7.42	16.7492541	9.16E-02	202612712	202612835	NC_000001.10
cg10648179	5.36E-11	3.60E-12	7.42	16.7470332	1.49E-01	73986783	73986906	NC_000002.11
cg11245681	5.36E-11	3.60E-12	7.42	16.7468521	1.46E-01	176166754	176166877	NC_000005.9
cg05123019	5.37E-11	3.61E-12	7.42	16.7447774	9.16E-02	97417411	97417534	NC_000010.10
cg08114265	5.37E-11	3.61E-12	7.42	16.7443257	4.70E-02	112052356	112052479	NC_000006.11
cg04157587	5.40E-11	3.63E-12	7.41	16.7397102	1.24E-01	25895751	25895874	NC_000007.13
cg22494346	5.40E-11	3.63E-12	7.41	16.7393385	1.64E-01	68852996	68853119	NC_000005.9
cg05232496	5.40E-11	3.64E-12	7.41	16.7378395	1.03E-01	1438225	1438348	NC_000005.9
cg14932124	5.43E-11	3.65E-12	7.41	16.7337624	3.45E-02	32885430	32885553	NC_000013.10
cg00622837	5.43E-11	3.65E-12	7.41	16.7334504	1.67E-01	166421992	166422115	NC_000006.11
cg26339504	5.44E-11	3.66E-12	7.41	16.73046	1.63E-01	118897859	118897982	NC_000010.10
cg11606570	5.44E-11	3.66E-12	7.41	16.730439	1.25E-01	117126698	117126821	NC_000007.13
cg00816936	5.44E-11	3.66E-12	7.41	16.7303297	3.12E-02	68631105	68631228	NC_000001.10
cg12457683	5.44E-11	3.67E-12	7.41	16.7300181	8.66E-02	221393426	221393549	NC_000002.11
cg09317239	5.45E-11	3.67E-12	7.41	16.7291436	9.44E-02	21818060	21818183	NC_000010.10

cg26618618	5.45E-11	3.67E-12	7.41	16.7281106	7.51E-02	95661178	95661301	NC_000011.9
cg24743310	5.46E-11	3.68E-12	7.41	16.7263659	4.01E-02	19619905	19620028	NC_000017.10
cg08497917	5.47E-11	3.69E-12	7.41	16.7245274	1.15E-01	101880285	101880408	NC_000004.11
cg09838442	5.48E-11	3.69E-12	7.41	16.7236028	9.74E-02	74875644	74875767	NC_000017.10
cg06358218	5.48E-11	3.69E-12	7.41	16.7228851	1.54E-01	15766930	15767053	NC_000001.10
cg03336086	5.48E-11	3.69E-12	7.41	16.722557	1.85E-01	79331047	79331170	NC_000005.9
cg20294962	5.48E-11	3.69E-12	7.41	16.7223594	8.88E-02	135126559	135126682	NC_000010.10
cg14843337	5.50E-11	3.70E-12	7.41	16.719467	5.19E-02	12194691	12194814	NC_000003.11
cg12099546	5.50E-11	3.71E-12	7.41	16.7187564	7.26E-02	72763849	72763972	NC_000015.9
cg12873928	5.51E-11	3.71E-12	7.41	16.7174048	7.60E-02	112120720	112120843	NC_000006.11
cg02835823	5.52E-11	3.72E-12	7.41	16.7164081	9.04E-02	85979060	85979183	NC_000016.9
cg05874806	5.52E-11	3.72E-12	7.41	16.7152188	1.19E-01	102350276	102350399	NC_000002.11
cg00002145	5.52E-11	3.72E-12	7.41	16.7150224	8.18E-02	238249536	238249659	NC_000002.11
cg20089491	5.52E-11	3.72E-12	7.41	16.7149027	1.25E-01	92257477	92257600	NC_000012.11
cg22679316	5.54E-11	3.73E-12	7.41	16.7122264	1.39E-01	54396990	54397113	NC_000012.11
cg25205699	5.54E-11	3.74E-12	7.41	16.7113423	1.83E-01	37246280	37246403	NC_000004.11
cg20568045	5.55E-11	3.74E-12	7.41	16.7095818	3.04E-02	169642014	169642137	NC_000006.11
cg14081737	5.55E-11	3.74E-12	7.41	16.7092177	1.30E-01	116111377	116111500	NC_000009.11
cg26048630	5.56E-11	3.75E-12	7.41	16.7078399	1.90E-01	170640675	170640798	NC_000001.10
cg07086492	5.56E-11	3.75E-12	7.41	16.7071825	1.36E-01	17439938	17440061	NC_000019.9
cg15059743	5.57E-11	3.75E-12	7.41	16.7063229	8.66E-02	39580880	39581003	NC_000017.10
cg15325373	5.58E-11	3.76E-12	7.41	16.7047719	1.34E-01	119302275	119302398	NC_000010.10
cg27135510	5.58E-11	3.76E-12	7.41	16.7044357	7.82E-02	2423571	2423694	NC_000011.9
cg09868336	5.58E-11	3.76E-12	7.41	16.7042429	2.31E-01	68981866	68981989	NC_000003.11
cg02522906	5.61E-11	3.78E-12	7.41	16.6991478	9.16E-02	123450196	123450319	NC_000010.10

cg19950556	5.61E-11	3.78E-12	7.41	16.6990831	7.66E-02	46439470	46439593	NC_000021.8
cg25136408	5.62E-11	3.79E-12	7.41	16.697193	1.22E-01	5265254	5265377	NC_000017.10
cg08024992	5.62E-11	3.79E-12	7.41	16.6964389	3.56E-02	2133441	2133564	NC_000007.13
cg06371452	5.62E-11	3.79E-12	7.41	16.6960749	5.21E-02	38109879	38110002	NC_000010.10
cg14627348	5.62E-11	3.80E-12	7.41	16.6957087	2.09E-01	79630987	79631110	NC_000009.11
cg07060006	5.64E-11	3.80E-12	7.41	16.6937241	1.95E-01	60296469	60296592	NC_000015.9
cg23730181	5.64E-11	3.80E-12	7.41	16.693585	2.27E-01	70033739	70033862	NC_000001.10
cg00502665	5.66E-11	3.82E-12	7.41	16.6901113	1.81E-01	56988813	56988936	NC_000019.9
cg11789740	5.66E-11	3.82E-12	7.41	16.6895838	1.86E-01	33602824	33602947	NC_000015.9
cg14507337	5.66E-11	3.82E-12	7.41	16.6893397	2.18E-01	60978161	60978284	NC_000014.8
cg02248486	5.66E-11	3.82E-12	7.41	16.6885987	1.62E-01	27183196	27183319	NC_000007.13
cg04697262	5.66E-11	3.82E-12	7.41	16.6885597	1.62E-01	130338886	130339009	NC_000010.10
cg06860555	5.70E-11	3.85E-12	7.4	16.6823781	8.03E-02	112672711	112672834	NC_000010.10
cg27259320	5.70E-11	3.85E-12	7.4	16.6822406	1.34E-01	32460587	32460710	NC_000011.9
cg19911801	5.71E-11	3.85E-12	7.4	16.680635	9.06E-02	2435632	2435755	NC_000001.10
cg03723247	5.71E-11	3.85E-12	7.4	16.6804412	1.56E-01	115135238	115135361	NC_000012.11
cg11128216	5.71E-11	3.85E-12	7.4	16.6804073	1.93E-01	31825833	31825956	NC_000011.9
cg18032190	5.71E-11	3.86E-12	7.4	16.6801608	2.03E-01	78493229	78493352	NC_000013.10
cg18209212	5.71E-11	3.86E-12	7.4	16.6794588	2.47E-01	130646256	130646379	NC_000012.11
cg03524083	5.71E-11	3.86E-12	7.4	16.6793091	1.63E-01	70534316	70534439	NC_000018.9
cg15885628	5.71E-11	3.86E-12	7.4	16.6792358	1.18E-01	28344718	28344841	NC_000015.9
cg19677607	5.73E-11	3.87E-12	7.4	16.6762566	1.54E-01	24772270	24772393	NC_000008.10
cg00120505	5.74E-11	3.88E-12	7.4	16.6748857	9.95E-02	5655609	5655732	NC_000009.11
cg24177433	5.74E-11	3.88E-12	7.4	16.6742564	1.13E-01	123535751	123535874	NC_000006.11
cg26333618	5.74E-11	3.88E-12	7.4	16.6735777	1.19E-01	48336655	48336778	NC_000012.11

cg04291079	5.75E-11	3.89E-12	7.4	16.6720044	8.61E-02	101294430	101294553	NC_000014.8
cg13398291	5.75E-11	3.89E-12	7.4	16.6715983	1.62E-01	41166169	41166292	NC_000008.10
cg10290764	5.76E-11	3.89E-12	7.4	16.671007	1.77E-01	4849399	4849522	NC_000008.10
cg25094126	5.77E-11	3.90E-12	7.4	16.6690041	1.31E-01	5541253	5541376	NC_000012.11
cg06308258	5.78E-11	3.91E-12	7.4	16.6673465	1.13E-01	117069282	117069405	NC_000012.11
cg04020079	5.78E-11	3.91E-12	7.4	16.666678	1.37E-01	68546688	68546811	NC_000002.11
cg17138769	5.78E-11	3.91E-12	7.4	16.666415	2.00E-01	118892211	118892334	NC_000010.10
cg14925328	5.79E-11	3.91E-12	7.4	16.665749	1.25E-01	207913718	207913841	NC_000002.11
cg23371746	5.79E-11	3.91E-12	7.4	16.6652246	1.51E-01	119532925	119533048	NC_000001.10
cg00395501	5.79E-11	3.92E-12	7.4	16.664532	1.19E-01	93296860	93296983	NC_000001.10
cg09737421	5.80E-11	3.92E-12	7.4	16.6641227	1.71E-01	7855622	7855745	NC_000016.9
cg18506390	5.81E-11	3.93E-12	7.4	16.6613642	1.86E-01	25903554	25903677	NC_000008.10
cg22248052	5.82E-11	3.94E-12	7.4	16.6598391	1.82E-01	50791419	50791542	NC_000006.11
cg04875614	5.82E-11	3.94E-12	7.4	16.6593986	7.77E-02	2008706	2008829	NC_000004.11
cg16896031	5.84E-11	3.95E-12	7.4	16.6570076	5.77E-02	26148105	26148228	NC_000008.10
cg08464190	5.84E-11	3.95E-12	7.4	16.6563089	2.38E-01	49315302	49315425	NC_000016.9
cg13677800	5.84E-11	3.95E-12	7.4	16.65627	8.33E-02	125426598	125426721	NC_000010.10
cg23503101	5.84E-11	3.95E-12	7.4	16.6557032	5.33E-02	1056579	1056702	NC_000005.9
cg10279872	5.84E-11	3.95E-12	7.4	16.65563	1.03E-01	120118684	120118807	NC_000008.10
cg07822928	5.84E-11	3.95E-12	7.4	16.6556198	1.03E-01	616454	616577	NC_000019.9
cg27528660	5.86E-11	3.97E-12	7.4	16.6523248	1.76E-01	72987924	72988047	NC_000008.10
cg19370054	5.86E-11	3.97E-12	7.4	16.6516884	2.17E-01	98964186	98964309	NC_000002.11
cg09109520	5.87E-11	3.97E-12	7.4	16.6510733	1.33E-01	57673258	57673381	NC_000016.9
cg24787130	5.88E-11	3.98E-12	7.4	16.6488142	1.22E-01	146948683	146948806	NC_000005.9
cg17486860	5.89E-11	3.98E-12	7.4	16.648019	1.73E-01	32454840	32454963	NC_000011.9

cg06294856	5.89E-11	3.99E-12	7.4	16.6475904	1.15E-01	18776340	18776463	NC_000009.11
cg27361134	5.89E-11	3.99E-12	7.4	16.6466463	2.36E-01	43439545	43439668	NC_000020.10
cg12442902	5.90E-11	4.00E-12	7.4	16.6448324	1.03E-01	7427446	7427569	NC_000004.11
cg03816687	5.91E-11	4.00E-12	7.4	16.643897	1.64E-01	21683745	21683868	NC_000020.10
cg07342845	5.91E-11	4.00E-12	7.4	16.6438638	6.34E-02	60555591	60555714	NC_000020.10
cg13758543	5.92E-11	4.01E-12	7.4	16.6417261	1.69E-01	79169714	79169837	NC_000013.10
cg11751806	5.93E-11	4.01E-12	7.4	16.6408224	1.95E-01	43945680	43945803	NC_000012.11
cg12065562	5.98E-11	4.05E-12	7.4	16.6321356	7.54E-02	140615656	140615779	NC_000005.9
cg03591285	5.98E-11	4.05E-12	7.4	16.6315703	1.60E-01	1667404	1667527	NC_000002.11
cg13419986	5.98E-11	4.05E-12	7.4	16.6312882	1.09E-01	218769406	218769529	NC_000002.11
cg18507138	5.98E-11	4.05E-12	7.4	16.6307222	1.54E-01	26665242	26665365	NC_000003.11
cg02004723	6.00E-11	4.06E-12	7.39	16.6284182	7.80E-02	42574341	42574464	NC_000020.10
cg23695133	6.00E-11	4.06E-12	7.39	16.6282533	2.47E-01	21087075	21087198	NC_000020.10
cg18625538	6.00E-11	4.07E-12	7.39	16.6271075	1.29E-01	87832609	87832732	NC_000006.11
cg09563777	6.01E-11	4.07E-12	7.39	16.6261209	1.31E-01	55885870	55885993	NC_000004.11
cg04546041	6.02E-11	4.08E-12	7.39	16.6252833	2.07E-01	57863210	57863333	NC_000019.9
cg15364355	6.03E-11	4.08E-12	7.39	16.6235008	1.32E-01	127567722	127567845	NC_000007.13
cg01588748	6.03E-11	4.09E-12	7.39	16.6218915	1.71E-01	79258386	79258509	NC_000012.11
cg04891086	6.03E-11	4.09E-12	7.39	16.6218693	2.10E-01	5018513	5018636	NC_000012.11
cg16374906	6.04E-11	4.09E-12	7.39	16.6214531	6.92E-02	221838383	221838506	NC_000001.10
cg17002645	6.05E-11	4.10E-12	7.39	16.6189269	1.35E-01	6717799	6717922	NC_000005.9
cg18860812	6.05E-11	4.10E-12	7.39	16.6188852	4.64E-02	6423371	6423494	NC_000004.11
cg00447018	6.06E-11	4.11E-12	7.39	16.6179385	1.18E-01	106938320	106938443	NC_000014.8
cg13231921	6.06E-11	4.11E-12	7.39	16.6176884	1.60E-01	95360542	95360665	NC_000013.10
cg03464473	6.06E-11	4.11E-12	7.39	16.6175487	4.61E-02	72343742	72343865	NC_000007.13

cg11927738	6.06E-11	4.11E-12	7.39	16.6174631	3.88E-02	68850580	68850703	NC_000011.9
cg26425256	6.06E-11	4.11E-12	7.39	16.6171547	1.32E-01	119611362	119611485	NC_000002.11
cg24696861	6.06E-11	4.11E-12	7.39	16.6168253	5.00E-02	46871582	46871705	NC_000017.10
cg22714668	6.06E-11	4.11E-12	7.39	16.6165863	1.06E-01	145453107	145453230	NC_000005.9
cg25405145	6.08E-11	4.12E-12	7.39	16.6145629	1.60E-01	51142580	51142703	NC_000019.9
cg14295306	6.08E-11	4.12E-12	7.39	16.6142843	7.45E-02	71355182	71355305	NC_000017.10
cg19406736	6.08E-11	4.12E-12	7.39	16.6140603	2.14E-01	37096010	37096133	NC_000019.9
cg16669607	6.08E-11	4.13E-12	7.39	16.6135318	1.26E-01	202965759	202965882	NC_000002.11
cg19188855	6.08E-11	4.13E-12	7.39	16.6132891	1.56E-01	54355476	54355599	NC_000012.11
cg24934408	6.10E-11	4.14E-12	7.39	16.6108504	7.86E-02	17530321	17530444	NC_000001.10
cg24842733	6.10E-11	4.14E-12	7.39	16.6103417	1.65E-01	84419237	84419360	NC_000006.11
cg17942639	6.10E-11	4.14E-12	7.39	16.610281	2.72E-01	154335184	154335307	NC_000002.11
cg08310608	6.15E-11	4.17E-12	7.39	16.6020144	2.94E-02	133278223	133278346	NC_000012.11
cg10452060	6.15E-11	4.17E-12	7.39	16.6019001	7.57E-02	154617645	154617768	NC_000006.11
cg00452257	6.15E-11	4.17E-12	7.39	16.6018801	2.40E-01	40006451	40006574	NC_000019.9
cg10963949	6.16E-11	4.18E-12	7.39	16.6005359	1.34E-01	173445463	173445586	NC_000001.10
cg26128079	6.16E-11	4.18E-12	7.39	16.599634	1.54E-01	127212416	127212539	NC_000012.11
cg09384610	6.16E-11	4.19E-12	7.39	16.5993051	1.84E-01	113229534	113229657	NC_000012.11
cg10981909	6.17E-11	4.19E-12	7.39	16.5985438	2.57E-01	63986392	63986515	NC_000005.9
cg08622369	6.17E-11	4.19E-12	7.39	16.5978645	1.03E-01	23724130	23724253	NC_000002.11
cg04897041	6.17E-11	4.19E-12	7.39	16.5976195	5.79E-02	1536462	1536585	NC_000016.9
cg03917492	6.20E-11	4.21E-12	7.39	16.5938527	8.50E-02	49663159	49663282	NC_000010.10
cg02895639	6.20E-11	4.22E-12	7.39	16.5923934	2.27E-01	48970993	48971116	NC_000022.10
cg21807313	6.21E-11	4.22E-12	7.39	16.591766	6.33E-02	176931111	176931234	NC_000005.9
cg21026830	6.21E-11	4.22E-12	7.39	16.5917615	2.33E-01	60973517	60973640	NC_000014.8

cg10153214	6.21E-11	4.22E-12	7.39	16.5917211	8.13E-02	71571146	71571269	NC_000016.9
cg03117544	6.21E-11	4.22E-12	7.39	16.5915202	5.83E-02	27526147	27526270	NC_000001.10
cg04534926	6.21E-11	4.22E-12	7.39	16.5914152	1.88E-01	49314257	49314380	NC_000016.9
cg25567594	6.23E-11	4.23E-12	7.39	16.5883205	2.18E-01	21683478	21683601	NC_000020.10
cg09746736	6.23E-11	4.24E-12	7.39	16.5876113	2.32E-01	55690130	55690253	NC_000016.9
cg20347647	6.23E-11	4.24E-12	7.39	16.5871673	2.88E-02	69364031	69364154	NC_000015.9
cg03465861	6.25E-11	4.25E-12	7.39	16.5850774	1.46E-01	76630660	76630783	NC_000015.9
cg22278296	6.25E-11	4.25E-12	7.39	16.5848005	2.25E-01	154335199	154335322	NC_000002.11
cg02140171	6.27E-11	4.27E-12	7.39	16.5804025	9.17E-02	9509896	9510019	NC_000004.11
cg17641861	6.28E-11	4.27E-12	7.39	16.578803	2.36E-01	570361	570484	NC_000004.11
cg21407117	6.28E-11	4.27E-12	7.39	16.5787928	1.24E-01	228548708	228548831	NC_000001.10
cg00745389	6.29E-11	4.28E-12	7.39	16.5784963	1.65E-01	32467435	32467558	NC_000007.13
cg20965743	6.30E-11	4.28E-12	7.39	16.5765893	1.27E-01	104552209	104552332	NC_000014.8
cg21158633	6.31E-11	4.29E-12	7.39	16.57451	1.65E-01	1272526	1272649	NC_000007.13
cg02852670	6.33E-11	4.30E-12	7.39	16.5718661	1.38E-01	20903410	20903533	NC_000014.8
cg11272874	6.33E-11	4.31E-12	7.38	16.5704021	5.05E-02	44264948	44265071	NC_000011.9
cg23125492	6.34E-11	4.32E-12	7.38	16.5687571	1.34E-01	31821298	31821421	NC_000011.9
cg20618622	6.35E-11	4.32E-12	7.38	16.568017	2.35E-01	164265004	164265127	NC_000004.11
cg19939997	6.36E-11	4.33E-12	7.38	16.5668411	2.08E-01	97173335	97173458	NC_000008.10
cg08920252	6.36E-11	4.33E-12	7.38	16.5658622	1.09E-01	85262820	85262943	NC_000016.9
cg20064452	6.37E-11	4.34E-12	7.38	16.5646984	1.43E-01	59807178	59807301	NC_000014.8
cg09676860	6.37E-11	4.34E-12	7.38	16.5637116	1.64E-01	130737664	130737787	NC_000002.11
cg05222924	6.38E-11	4.34E-12	7.38	16.5631205	1.56E-01	32450486	32450609	NC_000011.9
cg13844591	6.38E-11	4.35E-12	7.38	16.5620948	1.72E-01	158150675	158150798	NC_000001.10
cg20887241	6.41E-11	4.36E-12	7.38	16.5586434	9.59E-02	23522636	23522759	NC_000001.10

cg02925556	6.41E-11	4.37E-12	7.38	16.5574457	3.90E-02	132414758	132414881	NC_000002.11
cg04543938	6.42E-11	4.37E-12	7.38	16.5568876	2.52E-01	28956426	28956549	NC_000006.11
cg15661415	6.42E-11	4.37E-12	7.38	16.5560713	1.35E-01	54825247	54825370	NC_000007.13
cg05328547	6.42E-11	4.38E-12	7.38	16.555477	2.02E-01	19185139	19185262	NC_000007.13
cg15794859	6.43E-11	4.38E-12	7.38	16.5548115	1.64E-01	134214134	134214257	NC_000006.11
cg06638913	6.43E-11	4.38E-12	7.38	16.5547532	1.20E-01	235883680	235883803	NC_000002.11
cg18906596	6.43E-11	4.38E-12	7.38	16.5544645	1.21E-01	4151473	4151596	NC_000017.10
cg10775733	6.43E-11	4.38E-12	7.38	16.5538822	7.77E-02	110498910	110499033	NC_000006.11
cg19632594	6.44E-11	4.39E-12	7.38	16.5531445	1.51E-01	15500077	15500200	NC_000005.9
cg06756436	6.44E-11	4.39E-12	7.38	16.5530962	1.62E-01	615936	616059	NC_000018.9
cg21024112	6.45E-11	4.39E-12	7.38	16.5518016	6.95E-02	41376993	41377116	NC_000006.11
cg18085407	6.47E-11	4.41E-12	7.38	16.5472572	1.07E-01	244080114	244080237	NC_000001.10
cg10783149	6.48E-11	4.41E-12	7.38	16.5471406	1.01E-01	55707866	55707989	NC_000014.8
cg17714025	6.48E-11	4.41E-12	7.38	16.5470865	1.73E-01	30038619	30038742	NC_000011.9
cg06578117	6.48E-11	4.42E-12	7.38	16.5467294	1.18E-01	61444070	61444193	NC_000010.10
cg15738933	6.49E-11	4.43E-12	7.38	16.5444503	1.17E-01	75181373	75181496	NC_000017.10
cg24057615	6.50E-11	4.43E-12	7.38	16.5429488	1.52E-01	43732208	43732331	NC_000021.8
cg17809748	6.50E-11	4.43E-12	7.38	16.5426432	1.50E-01	13548336	13548459	NC_000004.11
cg24186674	6.50E-11	4.43E-12	7.38	16.5425565	2.35E-01	115631116	115631239	NC_000011.9
cg14486346	6.50E-11	4.43E-12	7.38	16.5424443	9.46E-02	102000131	102000254	NC_000002.11
cg02162286	6.51E-11	4.44E-12	7.38	16.5414083	1.71E-01	5887361	5887484	NC_000005.9
cg17932810	6.51E-11	4.44E-12	7.38	16.541295	1.05E-01	46602117	46602240	NC_000001.10
cg07073391	6.52E-11	4.45E-12	7.38	16.5389935	1.99E-01	15761869	15761992	NC_000010.10
cg07826574	6.53E-11	4.45E-12	7.38	16.5381556	7.28E-02	241581281	241581404	NC_000002.11
cg12138450	6.54E-11	4.46E-12	7.38	16.5359348	1.36E-01	34320864	34320987	NC_000014.8

cg24463006	6.55E-11	4.47E-12	7.38	16.534676	1.60E-01	37303096	37303219	NC_000020.10
cg19726546	6.55E-11	4.47E-12	7.38	16.5343601	1.41E-01	979220	979343	NC_000008.10
cg01226811	6.56E-11	4.47E-12	7.38	16.5338785	1.61E-01	21927612	21927735	NC_000012.11
cg20586163	6.57E-11	4.49E-12	7.38	16.531018	1.08E-01	1136538	1136661	NC_000008.10
cg02860732	6.58E-11	4.49E-12	7.38	16.5303454	2.29E-01	12995292	12995415	NC_000016.9
cg09125430	6.58E-11	4.49E-12	7.38	16.5295604	2.81E-01	21927433	21927556	NC_000012.11
cg05721839	6.58E-11	4.49E-12	7.38	16.5292727	6.19E-02	134751598	134751721	NC_000010.10
cg20219100	6.58E-11	4.49E-12	7.38	16.5292375	9.18E-02	63776397	63776520	NC_000011.9
cg02370605	6.59E-11	4.50E-12	7.38	16.5285374	1.71E-01	63417776	63417899	NC_000018.9
cg15930596	6.59E-11	4.50E-12	7.38	16.5282244	1.81E-01	5018229	5018352	NC_000012.11
cg25481073	6.60E-11	4.50E-12	7.38	16.5274287	1.74E-01	39995542	39995665	NC_000020.10
cg02203711	6.60E-11	4.50E-12	7.38	16.5272617	6.80E-02	39855863	39855986	NC_000022.10
cg13281868	6.60E-11	4.51E-12	7.38	16.526167	7.91E-02	53883888	53884011	NC_000006.11
cg06239616	6.60E-11	4.51E-12	7.38	16.5259702	2.16E-01	154705941	154706064	NC_000007.13
cg12724384	6.61E-11	4.51E-12	7.38	16.5254828	9.05E-02	71267300	71267423	NC_000008.10
cg19999645	6.61E-11	4.51E-12	7.38	16.5247607	2.04E-01	35047394	35047517	NC_000015.9
cg25652029	6.63E-11	4.53E-12	7.38	16.5215264	1.94E-01	182321786	182321909	NC_000002.11
cg23457166	6.65E-11	4.54E-12	7.38	16.5185652	5.68E-02	127797286	127797409	NC_000006.11
cg10375890	6.66E-11	4.55E-12	7.38	16.5175711	2.29E-01	50892511	50892634	NC_000001.10
cg09940861	6.66E-11	4.55E-12	7.38	16.517377	9.55E-02	101201649	101201772	NC_000014.8
cg08207378	6.67E-11	4.56E-12	7.38	16.5159458	4.56E-02	6313762	6313885	NC_000001.10
cg02106385	6.68E-11	4.57E-12	7.38	16.5137281	9.43E-02	36202339	36202462	NC_000001.10
cg14021523	6.69E-11	4.57E-12	7.37	16.5126337	6.58E-02	92959873	92959996	NC_000014.8
cg07934812	6.69E-11	4.57E-12	7.37	16.5126085	1.84E-01	133109013	133109136	NC_000010.10
cg01596674	6.70E-11	4.58E-12	7.37	16.5117472	1.40E-01	130342290	130342413	NC_000009.11

cg27349460	6.70E-11	4.58E-12	7.37	16.5115541	8.02E-02	76000984	76001107	NC_000018.9
cg03841638	6.71E-11	4.58E-12	7.37	16.5100267	1.23E-01	77911850	77911973	NC_000017.10
cg04897683	6.71E-11	4.58E-12	7.37	16.5099453	1.87E-01	134871462	134871585	NC_000005.9
cg18371424	6.71E-11	4.59E-12	7.37	16.5094024	1.29E-01	64654259	64654382	NC_000014.8
cg19479744	6.73E-11	4.60E-12	7.37	16.5065727	6.27E-02	156022315	156022438	NC_000006.11
cg15908093	6.73E-11	4.60E-12	7.37	16.5055616	7.61E-02	175463643	175463766	NC_000005.9
cg16000017	6.76E-11	4.62E-12	7.37	16.5023599	6.85E-02	95639346	95639469	NC_000001.10
cg02363202	6.76E-11	4.62E-12	7.37	16.5013171	1.43E-01	57653226	57653349	NC_000012.11
cg11841394	6.77E-11	4.63E-12	7.37	16.500804	2.28E-01	114841536	114841659	NC_000012.11
cg16443866	6.78E-11	4.63E-12	7.37	16.4993933	2.74E-01	172756558	172756681	NC_000005.9
cg03425785	6.78E-11	4.64E-12	7.37	16.4984442	1.40E-01	62221387	62221510	NC_000020.10
cg11841771	6.79E-11	4.64E-12	7.37	16.4973313	1.60E-01	115134956	115135079	NC_000012.11
cg07658280	6.81E-11	4.66E-12	7.37	16.4937558	1.74E-01	145728490	145728613	NC_000008.10
cg16316394	6.83E-11	4.67E-12	7.37	16.4911067	1.37E-01	140805841	140805964	NC_000005.9
cg11388325	6.84E-11	4.68E-12	7.37	16.4900842	1.70E-01	5018815	5018938	NC_000012.11
cg02745719	6.84E-11	4.68E-12	7.37	16.4892116	6.93E-02	112251076	112251199	NC_000005.9
cg16658020	6.85E-11	4.69E-12	7.37	16.4878623	1.21E-01	39203641	39203764	NC_000017.10
cg02179455	6.85E-11	4.69E-12	7.37	16.4876238	2.08E-01	19183732	19183855	NC_000007.13
cg01565291	6.87E-11	4.70E-12	7.37	16.4855553	1.73E-01	245812674	245812797	NC_000001.10
cg11352339	6.87E-11	4.70E-12	7.37	16.4850209	7.19E-02	43482436	43482559	NC_000006.11
cg25969984	6.87E-11	4.70E-12	7.37	16.484827	1.20E-01	683832	683955	NC_000008.10
cg14256587	6.87E-11	4.70E-12	7.37	16.4842699	1.98E-01	946010	946133	NC_000002.11
cg25021182	6.88E-11	4.71E-12	7.37	16.4832052	1.20E-01	15802594	15802717	NC_000001.10
cg03607359	6.88E-11	4.71E-12	7.37	16.4830296	2.43E-01	176057465	176057588	NC_000005.9
cg02786019	6.89E-11	4.71E-12	7.37	16.4824237	1.51E-01	142582859	142582982	NC_000007.13

cg05625471	6.90E-11	4.72E-12	7.37	16.4806568	1.48E-01	31342748	31342871	NC_000016.9
cg18944176	6.90E-11	4.72E-12	7.37	16.4802859	9.53E-02	27271688	27271811	NC_000007.13
cg18793661	6.90E-11	4.73E-12	7.37	16.479848	1.20E-01	192897	193020	NC_000011.9
cg21215550	6.90E-11	4.73E-12	7.37	16.4794561	2.35E-01	969544	969667	NC_000009.11
cg12232463	6.91E-11	4.73E-12	7.37	16.4788812	1.30E-01	100926650	100926773	NC_000002.11
cg23900070	6.91E-11	4.73E-12	7.37	16.4786886	7.67E-02	68853595	68853718	NC_000014.8
cg17489939	6.91E-11	4.73E-12	7.37	16.478066	1.22E-01	176981919	176982042	NC_000002.11
cg13412545	6.92E-11	4.74E-12	7.37	16.4766775	1.26E-01	172175765	172175888	NC_000005.9
cg08132931	6.93E-11	4.75E-12	7.37	16.4748887	1.85E-01	70994962	70995085	NC_000002.11
cg09998861	6.94E-11	4.76E-12	7.37	16.473635	1.37E-01	86538106	86538229	NC_000016.9
cg18646864	6.95E-11	4.76E-12	7.37	16.4721653	4.60E-02	52866643	52866766	NC_000001.10
cg04154276	6.95E-11	4.76E-12	7.37	16.4717244	1.97E-01	23983888	23984011	NC_000010.10
cg14156650	6.97E-11	4.78E-12	7.37	16.4693226	1.28E-01	2054287	2054410	NC_000016.9
cg25638626	7.02E-11	4.81E-12	7.37	16.4624572	1.84E-01	68117903	68118026	NC_000015.9
cg22013966	7.02E-11	4.81E-12	7.37	16.462265	1.37E-01	95106170	95106293	NC_000014.8
cg12202913	7.02E-11	4.82E-12	7.37	16.4612413	8.03E-02	12846618	12846741	NC_000019.9
cg19678828	7.02E-11	4.82E-12	7.37	16.4611887	1.46E-01	27945413	27945536	NC_000021.8
cg05617551	7.03E-11	4.82E-12	7.37	16.4601803	1.59E-01	54209183	54209306	NC_000016.9
cg01002529	7.04E-11	4.83E-12	7.37	16.4585522	1.90E-01	69591807	69591930	NC_000003.11
cg09671060	7.05E-11	4.84E-12	7.37	16.4570303	1.84E-01	47062953	47063076	NC_000021.8
cg01210642	7.06E-11	4.84E-12	7.37	16.4560034	1.06E-01	36921499	36921622	NC_000006.11
cg20225749	7.06E-11	4.84E-12	7.37	16.4554949	2.28E-01	946127	946250	NC_000002.11
cg08620606	7.07E-11	4.85E-12	7.36	16.4548882	1.47E-01	40005611	40005734	NC_000019.9
cg06759189	7.08E-11	4.85E-12	7.36	16.4533532	2.01E-01	77777947	77778070	NC_000017.10
cg07169289	7.08E-11	4.86E-12	7.36	16.453044	3.80E-02	136534480	136534603	NC_000006.11

cg23768829	7.09E-11	4.86E-12	7.36	16.4513609	1.33E-01	8089653	8089776	NC_000010.10
cg00196787	7.09E-11	4.87E-12	7.36	16.4509776	1.82E-01	32907132	32907255	NC_000017.10
cg00361017	7.10E-11	4.87E-12	7.36	16.4503164	1.20E-01	166073666	166073789	NC_000006.11
cg25480336	7.10E-11	4.87E-12	7.36	16.4500618	2.26E-01	50720908	50721031	NC_000020.10
cg27362525	7.11E-11	4.88E-12	7.36	16.4488092	1.81E-01	5019638	5019761	NC_000017.10
cg12053971	7.11E-11	4.88E-12	7.36	16.4477376	8.34E-02	134917266	134917389	NC_000010.10
cg03700135	7.11E-11	4.88E-12	7.36	16.4477125	1.39E-01	707059	707182	NC_000017.10
cg09347413	7.12E-11	4.89E-12	7.36	16.4466939	2.57E-01	23734299	23734422	NC_000013.10
cg01150936	7.14E-11	4.90E-12	7.36	16.4442773	5.37E-02	67235960	67236083	NC_000016.9
cg24288310	7.14E-11	4.90E-12	7.36	16.4440865	1.94E-01	92936678	92936801	NC_000015.9
cg17306116	7.14E-11	4.90E-12	7.36	16.4437341	2.94E-02	77528194	77528317	NC_000014.8
cg07675169	7.14E-11	4.90E-12	7.36	16.4436811	1.77E-01	61886262	61886385	NC_000020.10
cg04439218	7.14E-11	4.91E-12	7.36	16.4429409	1.33E-01	37839407	37839530	NC_000005.9
cg25474372	7.15E-11	4.91E-12	7.36	16.4417421	1.89E-01	28762125	28762248	NC_000006.11
cg25103417	7.16E-11	4.92E-12	7.36	16.440659	1.42E-01	157812018	157812141	NC_000003.11
cg05950570	7.17E-11	4.93E-12	7.36	16.4391764	2.53E-01	132261418	132261541	NC_000007.13
cg02805814	7.17E-11	4.93E-12	7.36	16.4385731	8.11E-02	53088482	53088605	NC_000019.9
cg26815414	7.17E-11	4.93E-12	7.36	16.4384105	8.42E-02	113300120	113300243	NC_000013.10
cg20072442	7.19E-11	4.94E-12	7.36	16.4362406	2.23E-01	80530255	80530378	NC_000002.11
cg25429672	7.19E-11	4.94E-12	7.36	16.4358693	1.50E-01	73692155	73692278	NC_000011.9
cg14689623	7.20E-11	4.95E-12	7.36	16.4342544	2.67E-01	20691161	20691284	NC_000011.9
cg20114732	7.21E-11	4.95E-12	7.36	16.4338298	2.06E-01	166579135	166579258	NC_000006.11
cg16757332	7.21E-11	4.95E-12	7.36	16.4338223	9.05E-02	92956197	92956320	NC_000014.8
cg09520393	7.21E-11	4.96E-12	7.36	16.4331158	1.31E-01	9783059	9783182	NC_000004.11
cg00228708	7.21E-11	4.96E-12	7.36	16.4328121	7.11E-02	135048062	135048185	NC_000010.10

cg09528449	7.22E-11	4.96E-12	7.36	16.4324533	1.63E-01	144241120	144241243	NC_000008.10
cg22250390	7.23E-11	4.97E-12	7.36	16.4309301	7.53E-02	5246986	5247109	NC_000003.11
cg04043571	7.24E-11	4.98E-12	7.36	16.4288398	1.38E-01	127265989	127266112	NC_000009.11
cg23706449	7.25E-11	4.99E-12	7.36	16.4271165	7.19E-02	47046101	47046224	NC_000008.10
cg19596297	7.26E-11	4.99E-12	7.36	16.4257796	8.02E-02	45021974	45022097	NC_000003.11
cg22138627	7.27E-11	5.00E-12	7.36	16.4251028	4.38E-02	42163505	42163628	NC_000006.11
cg16436782	7.28E-11	5.01E-12	7.36	16.4228029	9.92E-02	110917824	110917947	NC_000013.10
cg02985445	7.29E-11	5.01E-12	7.36	16.4214294	1.31E-01	97908505	97908628	NC_000007.13
cg09110241	7.30E-11	5.02E-12	7.36	16.4207222	6.23E-02	227079364	227079487	NC_000002.11
cg24987745	7.31E-11	5.02E-12	7.36	16.4196399	9.72E-02	17819167	17819290	NC_000002.11
cg21126844	7.31E-11	5.03E-12	7.36	16.4187682	3.71E-02	69254520	69254643	NC_000011.9
cg02446487	7.34E-11	5.05E-12	7.36	16.4145619	3.55E-02	49177504	49177627	NC_000019.9
cg21004604	7.34E-11	5.05E-12	7.36	16.4143287	7.06E-02	4378085	4378208	NC_000017.10
cg25865467	7.36E-11	5.07E-12	7.36	16.4114103	1.58E-01	73673231	73673354	NC_000003.11
cg02641288	7.36E-11	5.07E-12	7.36	16.4112018	9.94E-02	1883011	1883134	NC_000005.9
cg27462210	7.37E-11	5.07E-12	7.36	16.4104263	6.49E-02	44905012	44905135	NC_000021.8
cg03642328	7.37E-11	5.07E-12	7.36	16.4104225	6.88E-02	69624973	69625096	NC_000011.9
cg15556781	7.39E-11	5.09E-12	7.36	16.4074663	7.50E-02	25756688	25756811	NC_000022.10
cg14209344	7.39E-11	5.09E-12	7.36	16.4073604	7.67E-02	87886892	87887015	NC_000004.11
cg00107772	7.42E-11	5.11E-12	7.36	16.4033361	1.69E-01	57261886	57262009	NC_000014.8
cg09798090	7.43E-11	5.11E-12	7.36	16.4025056	5.60E-02	47472140	47472263	NC_000013.10
cg08495115	7.44E-11	5.12E-12	7.36	16.4007899	2.19E-01	33010399	33010522	NC_000015.9
cg18824585	7.44E-11	5.12E-12	7.36	16.400113	1.71E-01	114445084	114445207	NC_000008.10
cg21760040	7.45E-11	5.13E-12	7.36	16.3990689	7.75E-02	19813493	19813616	NC_000007.13
cg10176687	7.45E-11	5.13E-12	7.36	16.3989953	1.94E-01	123229073	123229196	NC_000011.9

cg03623178	7.45E-11	5.13E-12	7.36	16.3989584	2.01E-01	28175578	28175701	NC_000006.11
cg19125584	7.45E-11	5.13E-12	7.36	16.3986452	9.81E-02	601828	601951	NC_000007.13
cg24410255	7.46E-11	5.14E-12	7.36	16.3973804	1.17E-01	218098593	218098716	NC_000001.10
cg09765463	7.47E-11	5.14E-12	7.35	16.3967769	1.28E-01	65393430	65393553	NC_000001.10
cg16085649	7.47E-11	5.14E-12	7.35	16.3964971	1.14E-01	86186011	86186134	NC_000015.9
cg01055099	7.48E-11	5.15E-12	7.35	16.3947714	8.00E-02	3516099	3516222	NC_000016.9
cg00280345	7.50E-11	5.17E-12	7.35	16.3921673	1.27E-01	145728388	145728511	NC_000008.10
cg02005873	7.55E-11	5.20E-12	7.35	16.3848774	1.92E-01	10385839	10385962	NC_000006.11
cg06733347	7.55E-11	5.21E-12	7.35	16.3845178	5.36E-02	72453427	72453550	NC_000015.9
cg17743939	7.56E-11	5.21E-12	7.35	16.3835117	3.11E-02	46444050	46444173	NC_000019.9
cg01937650	7.59E-11	5.23E-12	7.35	16.3801098	1.97E-01	75896836	75896959	NC_000008.10
cg19615102	7.59E-11	5.23E-12	7.35	16.3792393	8.61E-02	104335436	104335559	NC_000012.11
cg04645914	7.60E-11	5.24E-12	7.35	16.3777475	1.73E-01	52452317	52452440	NC_000019.9
cg25470148	7.63E-11	5.26E-12	7.35	16.3744471	1.51E-01	25257931	25258054	NC_000001.10
cg04367345	7.68E-11	5.30E-12	7.35	16.3675915	1.32E-01	44332636	44332759	NC_000011.9
cg15745284	7.69E-11	5.30E-12	7.35	16.3665313	1.62E-01	32064923	32065046	NC_000006.11
cg15174623	7.69E-11	5.30E-12	7.35	16.3662113	1.37E-01	35494345	35494468	NC_000007.13
cg23902471	7.69E-11	5.31E-12	7.35	16.3658121	1.12E-01	49039001	49039124	NC_000022.10
cg22925751	7.70E-11	5.31E-12	7.35	16.3646271	8.74E-02	93509137	93509260	NC_000012.11
cg21980107	7.70E-11	5.32E-12	7.35	16.3640134	5.63E-02	42520181	42520304	NC_000021.8
cg23291886	7.70E-11	5.32E-12	7.35	16.3639292	1.37E-01	174440681	174440804	NC_000004.11
cg01942962	7.71E-11	5.32E-12	7.35	16.3627612	1.95E-01	158143439	158143562	NC_000004.11
cg12780678	7.71E-11	5.32E-12	7.35	16.3626049	1.70E-01	1746846	1746969	NC_000002.11
cg22878622	7.72E-11	5.33E-12	7.35	16.3619078	1.98E-01	67873476	67873599	NC_000008.10
cg09355771	7.73E-11	5.33E-12	7.35	16.3607176	1.89E-01	177043147	177043270	NC_000002.11

cg24595510	7.73E-11	5.34E-12	7.35	16.3603974	2.29E-01	644302	644425	NC_000020.10
cg16914674	7.74E-11	5.35E-12	7.35	16.3583518	2.35E-01	90349662	90349785	NC_000015.9
cg19054219	7.74E-11	5.35E-12	7.35	16.3582525	3.54E-02	46805820	46805943	NC_000022.10
cg18482892	7.76E-11	5.36E-12	7.35	16.3560726	1.56E-01	56833426	56833549	NC_000003.11
cg05605603	7.77E-11	5.37E-12	7.35	16.3546378	1.60E-01	35602594	35602717	NC_000011.9
cg20599066	7.77E-11	5.37E-12	7.35	16.3544481	1.35E-01	23287420	23287543	NC_000007.13
cg18845960	7.78E-11	5.37E-12	7.35	16.3536478	1.23E-01	37387350	37387473	NC_000002.11
cg00914726	7.78E-11	5.37E-12	7.35	16.353253	2.01E-01	60539400	60539523	NC_000001.10
cg06120379	7.78E-11	5.38E-12	7.35	16.3525897	3.90E-02	197357859	197357982	NC_000003.11
cg02092141	7.79E-11	5.38E-12	7.35	16.3516207	1.15E-01	66411184	66411307	NC_000011.9
cg15743922	7.80E-11	5.39E-12	7.35	16.3505945	1.88E-01	22930545	22930668	NC_000018.9
cg13891539	7.81E-11	5.39E-12	7.35	16.3497225	1.43E-01	30322730	30322853	NC_000012.11
cg15559979	7.81E-11	5.40E-12	7.35	16.3494686	5.36E-02	49049574	49049697	NC_000003.11
cg18511930	7.81E-11	5.40E-12	7.35	16.3493605	4.59E-02	42371788	42371911	NC_000004.11
cg25461801	7.82E-11	5.41E-12	7.35	16.3476623	1.38E-01	100630316	100630439	NC_000013.10
cg06029846	7.82E-11	5.41E-12	7.35	16.3472765	1.88E-01	3600317	3600440	NC_000012.11
cg10334354	7.83E-11	5.41E-12	7.35	16.3463659	1.63E-01	66535203	66535326	NC_000004.11
cg07805604	7.83E-11	5.41E-12	7.35	16.34636	1.67E-01	56158885	56159008	NC_000019.9
cg20886017	7.84E-11	5.42E-12	7.35	16.3455288	1.48E-01	131002896	131003019	NC_000003.11
cg02613418	7.85E-11	5.43E-12	7.35	16.3439329	1.78E-01	68723914	68724037	NC_000015.9
cg03530744	7.87E-11	5.44E-12	7.35	16.3412265	1.31E-01	111270991	111271114	NC_000013.10
cg20664313	7.87E-11	5.44E-12	7.35	16.3409841	1.46E-01	133071297	133071420	NC_000012.11
cg15212349	7.87E-11	5.44E-12	7.35	16.3408288	1.45E-01	1444852	1444975	NC_000005.9
cg05038288	7.87E-11	5.44E-12	7.35	16.3408038	9.26E-02	41800491	41800614	NC_000017.10
cg07929029	7.88E-11	5.45E-12	7.35	16.3403288	1.40E-01	150623203	150623326	NC_000001.10

cg27486624	7.89E-11	5.45E-12	7.34	16.3390961	7.93E-02	43542382	43542505	NC_000006.11
cg27320005	7.89E-11	5.46E-12	7.34	16.3380815	1.15E-01	140683767	140683890	NC_000005.9
cg06490627	7.90E-11	5.46E-12	7.34	16.3373585	9.17E-02	50344198	50344321	NC_000016.9
cg08050115	7.90E-11	5.46E-12	7.34	16.3372957	1.34E-01	31341479	31341602	NC_000016.9
cg23001370	7.93E-11	5.48E-12	7.34	16.3337241	1.05E-01	53817073	53817196	NC_000005.9
cg23161999	7.93E-11	5.49E-12	7.34	16.3331328	2.03E-01	162283189	162283312	NC_000002.11
cg08716217	7.94E-11	5.49E-12	7.34	16.3323513	3.27E-02	2483719	2483842	NC_000001.10
cg18679410	7.94E-11	5.50E-12	7.34	16.331414	1.86E-01	26664308	26664431	NC_000003.11
cg20655220	7.95E-11	5.50E-12	7.34	16.3308619	1.24E-01	102246737	102246860	NC_000005.9
cg13338137	7.95E-11	5.50E-12	7.34	16.3307749	1.69E-01	79169966	79170089	NC_000013.10
cg01838004	7.95E-11	5.50E-12	7.34	16.3305983	1.35E-01	100070007	100070130	NC_000009.11
cg10724940	7.95E-11	5.50E-12	7.34	16.3305576	5.33E-02	50656595	50656718	NC_000022.10
cg00210249	7.96E-11	5.51E-12	7.34	16.3289839	1.83E-01	71135679	71135802	NC_000010.10
cg02257517	7.97E-11	5.52E-12	7.34	16.3273048	5.58E-02	136340262	136340385	NC_000009.11
cg19593680	7.99E-11	5.53E-12	7.34	16.325795	9.56E-02	7374669	7374792	NC_000004.11
cg19023320	7.99E-11	5.53E-12	7.34	16.3257754	1.53E-01	13033587	13033710	NC_000003.11
cg02943074	7.99E-11	5.53E-12	7.34	16.3248238	4.95E-02	79906185	79906308	NC_000017.10
cg13875518	7.99E-11	5.53E-12	7.34	16.3246085	1.93E-01	21502218	21502341	NC_000020.10
cg17218713	7.99E-11	5.53E-12	7.34	16.3244495	2.28E-01	71513114	71513237	NC_000001.10
cg16048781	8.00E-11	5.54E-12	7.34	16.3239977	7.01E-02	15296155	15296278	NC_000019.9
cg06082745	8.00E-11	5.54E-12	7.34	16.3239426	2.17E-01	133795139	133795262	NC_000010.10
cg05722981	8.00E-11	5.54E-12	7.34	16.3238541	1.44E-01	111533509	111533632	NC_000004.11
cg06201642	8.00E-11	5.54E-12	7.34	16.3237185	1.91E-01	77333198	77333321	NC_000001.10
cg09155219	8.01E-11	5.54E-12	7.34	16.3225596	1.22E-01	48144280	48144403	NC_000014.8
cg21047695	8.02E-11	5.55E-12	7.34	16.3207682	1.62E-01	63614552	63614675	NC_000008.10

cg10769146	8.04E-11	5.57E-12	7.34	16.3186803	1.65E-01	214156644	214156767	NC_000001.10
cg02653559	8.04E-11	5.57E-12	7.34	16.3179968	2.26E-01	147112081	147112204	NC_000003.11
cg18405727	8.06E-11	5.58E-12	7.34	16.3157152	1.75E-01	32860004	32860127	NC_000003.11
cg12420104	8.06E-11	5.58E-12	7.34	16.3156558	2.03E-01	977109	977232	NC_000009.11
cg11155579	8.07E-11	5.59E-12	7.34	16.3141486	7.21E-02	87582125	87582248	NC_000001.10
cg26239984	8.08E-11	5.60E-12	7.34	16.3127179	1.58E-01	1278428	1278551	NC_000007.13
cg09994035	8.11E-11	5.62E-12	7.34	16.308887	1.50E-01	241826199	241826322	NC_000002.11
cg22523852	8.13E-11	5.63E-12	7.34	16.3073758	1.90E-01	27112049	27112172	NC_000015.9
cg19193956	8.13E-11	5.63E-12	7.34	16.3070732	1.76E-01	19148187	19148310	NC_000007.13
cg17189167	8.14E-11	5.64E-12	7.34	16.3053844	7.99E-02	161087506	161087629	NC_000006.11
cg25306579	8.16E-11	5.65E-12	7.34	16.3034479	1.02E-01	134468976	134469099	NC_000010.10
cg04018622	8.16E-11	5.65E-12	7.34	16.3033561	1.51E-01	3602291	3602414	NC_000012.11
cg16703762	8.16E-11	5.66E-12	7.34	16.3025215	1.48E-01	76932047	76932170	NC_000005.9
cg14701597	8.17E-11	5.66E-12	7.34	16.3022547	7.30E-02	130757617	130757740	NC_000010.10
cg20740249	8.17E-11	5.66E-12	7.34	16.3018101	6.05E-02	130018834	130018957	NC_000007.13
cg16647921	8.18E-11	5.67E-12	7.34	16.300438	2.11E-01	41867533	41867656	NC_000004.11
cg00162231	8.20E-11	5.69E-12	7.34	16.2973523	1.79E-01	11035357	11035480	NC_000003.11
cg02019774	8.20E-11	5.69E-12	7.34	16.2972075	1.84E-01	138658470	138658593	NC_000003.11
cg05022105	8.21E-11	5.70E-12	7.34	16.2961905	1.44E-01	32460980	32461103	NC_000011.9
cg12938003	8.22E-11	5.70E-12	7.34	16.2951202	2.34E-01	109092724	109092847	NC_000004.11
cg13888886	8.23E-11	5.71E-12	7.34	16.2940254	6.50E-02	55904640	55904763	NC_000020.10
cg24749866	8.24E-11	5.72E-12	7.34	16.2921138	1.33E-01	235883655	235883778	NC_000002.11
cg27420236	8.25E-11	5.72E-12	7.34	16.2911794	1.85E-01	154334716	154334839	NC_000002.11
cg01214573	8.25E-11	5.73E-12	7.34	16.2909198	7.24E-02	49257931	49258054	NC_000015.9
cg10155304	8.26E-11	5.73E-12	7.34	16.2898885	1.12E-01	109107559	109107682	NC_000012.11

cg08692733	8.26E-11	5.73E-12	7.34	16.2897248	1.53E-01	112404738	112404861	NC_000010.10
cg16880856	8.30E-11	5.76E-12	7.34	16.2845967	1.57E-01	152133898	152134021	NC_000007.13
cg14597534	8.33E-11	5.79E-12	7.33	16.2808051	2.56E-01	43601442	43601565	NC_000011.9
cg00182534	8.33E-11	5.79E-12	7.33	16.2805382	1.30E-01	5541234	5541357	NC_000012.11
cg03157531	8.33E-11	5.79E-12	7.33	16.280531	2.19E-01	133795006	133795129	NC_000010.10
cg07171111	8.33E-11	5.79E-12	7.33	16.2803239	1.57E-01	10462903	10463026	NC_000004.11
cg25654705	8.34E-11	5.79E-12	7.33	16.2800014	1.03E-01	174424942	174425065	NC_000004.11
cg03384579	8.34E-11	5.79E-12	7.33	16.2797873	2.08E-01	70995440	70995563	NC_000002.11
cg18482164	8.36E-11	5.80E-12	7.33	16.2776284	1.90E-01	37764118	37764241	NC_000017.10
cg26721627	8.36E-11	5.81E-12	7.33	16.2767165	1.11E-01	1319713	1319836	NC_000011.9
cg02487331	8.37E-11	5.82E-12	7.33	16.2753694	1.58E-01	146550467	146550590	NC_000001.10
cg04536844	8.38E-11	5.82E-12	7.33	16.2750251	1.12E-01	155724260	155724383	NC_000007.13
cg25399352	8.39E-11	5.83E-12	7.33	16.2728622	1.73E-01	114837572	114837695	NC_000012.11
cg27053177	8.41E-11	5.84E-12	7.33	16.2713958	6.43E-02	169508778	169508901	NC_000002.11
cg00527805	8.41E-11	5.85E-12	7.33	16.2705229	6.97E-02	76541071	76541194	NC_000018.9
cg00902104	8.42E-11	5.85E-12	7.33	16.2694725	2.09E-01	124901908	124902031	NC_000010.10
cg27366617	8.42E-11	5.85E-12	7.33	16.2691537	1.59E-01	76459785	76459908	NC_000018.9
cg15810304	8.43E-11	5.86E-12	7.33	16.2681235	9.13E-02	57183618	57183741	NC_000004.11
cg22827833	8.44E-11	5.86E-12	7.33	16.2677072	6.32E-02	13295841	13295964	NC_000011.9
cg06837568	8.44E-11	5.87E-12	7.33	16.2666604	1.35E-01	108634212	108634335	NC_000012.11
cg17554604	8.45E-11	5.87E-12	7.33	16.2664683	2.20E-01	140774522	140774645	NC_000007.13
cg18764804	8.45E-11	5.87E-12	7.33	16.2663218	1.14E-01	226596668	226596791	NC_000001.10
cg22619018	8.45E-11	5.87E-12	7.33	16.2656469	2.35E-01	4852624	4852747	NC_000008.10
cg18801045	8.46E-11	5.88E-12	7.33	16.2645974	1.61E-01	10751081	10751204	NC_000005.9
cg19408467	8.46E-11	5.88E-12	7.33	16.264071	6.24E-02	43232908	43233031	NC_000006.11

cg01541178	8.47E-11	5.89E-12	7.33	16.2628748	1.32E-01	95358498	95358621	NC_000013.10
cg18811597	8.48E-11	5.90E-12	7.33	16.2618847	7.62E-02	39873001	39873124	NC_000007.13
cg26123256	8.50E-11	5.91E-12	7.33	16.260024	1.33E-01	27530670	27530793	NC_000002.11
cg09341793	8.50E-11	5.91E-12	7.33	16.2592122	2.00E-01	94405681	94405804	NC_000014.8
cg14356190	8.53E-11	5.93E-12	7.33	16.2561372	1.42E-01	14908132	14908255	NC_000009.11
cg19974854	8.55E-11	5.95E-12	7.33	16.253699	1.67E-01	145718491	145718614	NC_000005.9
cg22630755	8.57E-11	5.96E-12	7.33	16.2516139	2.82E-01	134871807	134871930	NC_000005.9
cg11986861	8.58E-11	5.97E-12	7.33	16.2492834	1.24E-01	29284199	29284322	NC_000022.10
cg08655760	8.59E-11	5.98E-12	7.33	16.2481271	5.38E-02	116941251	116941374	NC_000001.10
cg15779837	8.60E-11	5.98E-12	7.33	16.2474384	2.33E-01	48918116	48918239	NC_000019.9
cg22883332	8.60E-11	5.99E-12	7.33	16.2472061	1.02E-01	5566260	5566383	NC_000010.10
cg19601293	8.64E-11	6.02E-12	7.33	16.2424131	2.14E-02	2029752	2029875	NC_000007.13
cg15863598	8.64E-11	6.02E-12	7.33	16.242217	1.15E-01	76708663	76708786	NC_000010.10
cg23275776	8.64E-11	6.02E-12	7.33	16.242213	5.09E-02	102496501	102496624	NC_000011.9
cg12187687	8.65E-11	6.02E-12	7.33	16.241285	9.95E-02	27253834	27253957	NC_000007.13
cg05298458	8.65E-11	6.03E-12	7.33	16.2406627	1.35E-01	19749188	19749311	NC_000022.10
cg21384797	8.67E-11	6.04E-12	7.33	16.2388726	8.64E-02	163054771	163054894	NC_000002.11
cg09918296	8.68E-11	6.05E-12	7.33	16.2374915	1.88E-01	54024649	54024772	NC_000019.9
cg14218374	8.68E-11	6.05E-12	7.33	16.2374708	7.24E-02	33747192	33747315	NC_000006.11
cg05139788	8.70E-11	6.06E-12	7.33	16.2355908	1.92E-01	50690647	50690770	NC_000006.11
cg22399133	8.70E-11	6.06E-12	7.33	16.2354217	2.23E-01	208989248	208989371	NC_000002.11
cg23890204	8.72E-11	6.07E-12	7.33	16.2331092	4.82E-02	26247238	26247361	NC_000006.11
cg00790098	8.74E-11	6.09E-12	7.33	16.2309417	1.99E-01	223167400	223167523	NC_000002.11
cg03896611	8.74E-11	6.09E-12	7.33	16.2302091	1.34E-01	99986344	99986467	NC_000008.10
cg08512142	8.76E-11	6.10E-12	7.33	16.2284779	1.77E-01	88796227	88796350	NC_000011.9

cg20699586	8.78E-11	6.12E-12	7.33	16.2260807	1.36E-01	227748719	227748842	NC_000001.10
cg14703605	8.78E-11	6.12E-12	7.33	16.2255888	1.93E-01	72987916	72988039	NC_000008.10
cg23445461	8.79E-11	6.12E-12	7.33	16.2247155	1.84E-01	140864733	140864856	NC_000005.9
cg06745753	8.79E-11	6.13E-12	7.32	16.2244491	7.36E-02	45190297	45190420	NC_000001.10
cg08384637	8.79E-11	6.13E-12	7.32	16.22442	2.79E-01	86601133	86601256	NC_000016.9
cg23221737	8.79E-11	6.13E-12	7.32	16.2243379	1.06E-01	34543055	34543178	NC_000006.11
cg08332990	8.80E-11	6.13E-12	7.32	16.2234815	1.15E-01	997351	997474	NC_000004.11
cg14285365	8.80E-11	6.13E-12	7.32	16.223439	1.16E-01	36643743	36643866	NC_000001.10
cg02416781	8.80E-11	6.13E-12	7.32	16.2233152	1.20E-01	82331856	82331979	NC_000005.9
cg24626310	8.80E-11	6.14E-12	7.32	16.2229641	1.28E-01	25276739	25276862	NC_000020.10
cg09047290	8.80E-11	6.14E-12	7.32	16.2229555	3.71E-02	81978370	81978493	NC_000016.9
cg01487195	8.82E-11	6.15E-12	7.32	16.2204242	3.33E-02	88529573	88529696	NC_000010.10
cg08477106	8.83E-11	6.16E-12	7.32	16.2194244	1.45E-01	75895596	75895719	NC_000004.11
cg26637881	8.84E-11	6.16E-12	7.32	16.2184077	7.76E-02	87879698	87879821	NC_000016.9
cg16790849	8.87E-11	6.19E-12	7.32	16.2144923	6.34E-02	31829511	31829634	NC_000006.11
cg18185958	8.87E-11	6.19E-12	7.32	16.2143083	1.43E-01	223176899	223177022	NC_000002.11
cg14251870	8.88E-11	6.19E-12	7.32	16.2137571	1.88E-01	137809529	137809652	NC_000006.11
cg16102793	8.88E-11	6.20E-12	7.32	16.2131233	2.88E-02	117484975	117485098	NC_000012.11
cg20330472	8.89E-11	6.20E-12	7.32	16.2125657	2.04E-01	133561932	133562055	NC_000006.11
cg08977371	8.90E-11	6.21E-12	7.32	16.2109253	1.39E-01	82660490	82660613	NC_000016.9
cg15840638	8.91E-11	6.22E-12	7.32	16.2099756	2.67E-02	79298691	79298814	NC_000015.9
cg13264394	8.91E-11	6.22E-12	7.32	16.2093336	7.64E-02	152066605	152066728	NC_000003.11
cg05681560	8.92E-11	6.23E-12	7.32	16.2083677	1.02E-01	164288814	164288937	NC_000006.11
cg13463054	8.93E-11	6.23E-12	7.32	16.2076384	2.22E-01	77333159	77333282	NC_000001.10
cg21123160	8.93E-11	6.23E-12	7.32	16.2074612	1.39E-01	10857719	10857842	NC_000003.11

cg07547549	8.94E-11	6.24E-12	7.32	16.2068271	1.44E-01	44658225	44658348	NC_000020.10
cg10109590	8.94E-11	6.24E-12	7.32	16.2065233	5.97E-02	93090866	93090989	NC_000014.8
cg18115215	8.95E-11	6.24E-12	7.32	16.2056554	2.10E-01	132757090	132757213	NC_000003.11
cg01599925	8.95E-11	6.25E-12	7.32	16.205519	9.91E-02	35488024	35488147	NC_000020.10
cg07352438	8.95E-11	6.25E-12	7.32	16.2049818	2.91E-01	5543548	5543671	NC_000018.9
cg02114924	8.97E-11	6.26E-12	7.32	16.2027609	1.55E-01	134069304	134069427	NC_000004.11
cg14866595	8.98E-11	6.27E-12	7.32	16.2020555	1.89E-01	4873442	4873565	NC_000004.11
cg25590826	8.98E-11	6.27E-12	7.32	16.2011014	1.43E-01	74557537	74557660	NC_000015.9
cg06871074	8.99E-11	6.28E-12	7.32	16.200508	1.39E-01	146551565	146551688	NC_000001.10
cg17023149	8.99E-11	6.28E-12	7.32	16.2001583	1.52E-01	13536504	13536627	NC_000004.11
cg01717201	9.00E-11	6.28E-12	7.32	16.199352	8.13E-02	48174262	48174385	NC_000017.10
cg15638055	9.01E-11	6.30E-12	7.32	16.1976609	1.62E-01	110986974	110987097	NC_000008.10
cg15522957	9.02E-11	6.30E-12	7.32	16.1964861	1.33E-01	44324925	44325048	NC_000011.9
cg14709826	9.03E-11	6.31E-12	7.32	16.1957404	1.18E-01	151267707	151267830	NC_000002.11
cg13745279	9.04E-11	6.31E-12	7.32	16.1949185	8.95E-02	111788810	111788933	NC_000011.9
cg06651450	9.04E-11	6.32E-12	7.32	16.1945028	6.86E-02	32860909	32861032	NC_000003.11
cg25535257	9.05E-11	6.32E-12	7.32	16.1938433	6.03E-02	4936114	4936237	NC_000017.10
cg20084504	9.05E-11	6.32E-12	7.32	16.1936176	1.60E-01	28369514	28369637	NC_000013.10
cg24509898	9.05E-11	6.32E-12	7.32	16.1931992	1.04E-01	185070553	185070676	NC_000001.10
cg17891968	9.05E-11	6.33E-12	7.32	16.1927237	1.00E-01	17673737	17673860	NC_000020.10
cg21287054	9.10E-11	6.36E-12	7.32	16.1874885	1.76E-01	54928012	54928135	NC_000019.9
cg11598178	9.12E-11	6.38E-12	7.32	16.1851421	8.99E-02	8602619	8602742	NC_000004.11
cg05194864	9.13E-11	6.38E-12	7.32	16.1845488	1.58E-01	15100977	15101100	NC_000012.11
cg16818395	9.14E-11	6.39E-12	7.32	16.1827863	1.69E-01	128274084	128274207	NC_000003.11
cg16924337	9.19E-11	6.43E-12	7.32	16.1771973	2.36E-01	153452724	153452847	NC_000006.11

cg01150982	9.20E-11	6.43E-12	7.32	16.1763798	7.51E-02	33664555	33664678	NC_000021.8
cg15622891	9.20E-11	6.44E-12	7.32	16.1757854	1.57E-01	163862445	163862568	NC_000006.11
cg00755751	9.21E-11	6.44E-12	7.32	16.1752527	7.58E-02	64677007	64677130	NC_000014.8
cg23543123	9.23E-11	6.46E-12	7.32	16.1727072	1.50E-01	48485569	48485692	NC_000004.11
cg04812475	9.24E-11	6.46E-12	7.32	16.1717145	2.43E-01	17906561	17906684	NC_000009.11
cg00525277	9.24E-11	6.46E-12	7.32	16.1715763	1.63E-01	32064239	32064362	NC_000006.11
cg24072474	9.27E-11	6.48E-12	7.32	16.1686812	1.25E-01	140770013	140770136	NC_000003.11
cg22694191	9.27E-11	6.49E-12	7.32	16.1680481	1.88E-01	19373575	19373698	NC_000019.9
cg19156335	9.29E-11	6.50E-12	7.31	16.1663029	3.57E-02	158497539	158497662	NC_000007.13
cg15105326	9.29E-11	6.50E-12	7.31	16.1660727	1.15E-01	147108916	147109039	NC_000003.11
cg25819026	9.33E-11	6.53E-12	7.31	16.1620332	1.24E-01	41060392	41060515	NC_000019.9
cg20421482	9.36E-11	6.56E-12	7.31	16.1577072	1.22E-01	132169009	132169132	NC_000012.11
cg08595804	9.37E-11	6.56E-12	7.31	16.1566263	1.57E-01	112712424	112712547	NC_000013.10
cg22295864	9.38E-11	6.57E-12	7.31	16.1559844	1.25E-01	74233472	74233595	NC_000011.9
cg05616122	9.39E-11	6.57E-12	7.31	16.1550939	7.02E-02	77982987	77983110	NC_000017.10
cg12977135	9.39E-11	6.58E-12	7.31	16.1544366	7.20E-02	119036478	119036601	NC_000008.10
cg11612852	9.41E-11	6.59E-12	7.31	16.1528977	1.35E-01	21699745	21699868	NC_000004.11
cg21390624	9.41E-11	6.59E-12	7.31	16.1528033	1.47E-01	133561677	133561800	NC_000006.11
cg01078989	9.42E-11	6.59E-12	7.31	16.1519663	1.53E-01	15761657	15761780	NC_000010.10
cg13565656	9.42E-11	6.60E-12	7.31	16.151882	2.61E-01	94543449	94543572	NC_000012.11
cg11700356	9.46E-11	6.63E-12	7.31	16.1468498	1.42E-01	23287560	23287683	NC_000007.13
cg08826251	9.47E-11	6.64E-12	7.31	16.1458244	2.16E-01	58332909	58333032	NC_000014.8
cg23213872	9.48E-11	6.65E-12	7.31	16.1443222	1.27E-01	242613203	242613326	NC_000001.10
cg19662846	9.49E-11	6.65E-12	7.31	16.1434393	1.30E-01	8650410	8650533	NC_000019.9
cg09919917	9.50E-11	6.66E-12	7.31	16.1423461	1.47E-01	242834074	242834197	NC_000002.11

cg21290042	9.50E-11	6.66E-12	7.31	16.1421575	2.14E-01	224804053	224804176	NC_000001.10
cg10059660	9.51E-11	6.67E-12	7.31	16.1411586	1.86E-01	44450234	44450357	NC_000004.11
cg06086731	9.52E-11	6.67E-12	7.31	16.1400886	1.81E-01	38942075	38942198	NC_000001.10
cg06389888	9.52E-11	6.68E-12	7.31	16.1400197	1.42E-01	135011991	135012114	NC_000002.11
cg10600647	9.55E-11	6.69E-12	7.31	16.1374814	1.27E-01	60763808	60763931	NC_000002.11
cg12382902	9.55E-11	6.70E-12	7.31	16.1366047	2.25E-01	4383699	4383822	NC_000012.11
cg14031402	9.56E-11	6.70E-12	7.31	16.1362751	1.57E-01	30353957	30354080	NC_000012.11
cg20303399	9.57E-11	6.71E-12	7.31	16.1350115	1.59E-01	26722965	26723088	NC_000008.10
cg04796828	9.58E-11	6.72E-12	7.31	16.1334595	9.86E-02	10452854	10452977	NC_000008.10
cg03099291	9.59E-11	6.72E-12	7.31	16.133121	5.41E-02	6809939	6810062	NC_000012.11
cg01310875	9.60E-11	6.73E-12	7.31	16.131753	9.14E-02	27938995	27939118	NC_000008.10
cg12460187	9.62E-11	6.75E-12	7.31	16.1289176	7.35E-02	36318437	36318560	NC_000011.9
cg03716908	9.63E-11	6.75E-12	7.31	16.1285463	6.17E-02	81149002	81149125	NC_000005.9
cg25769127	9.66E-11	6.78E-12	7.31	16.1253199	1.04E-01	61543023	61543146	NC_000003.11
cg24463664	9.66E-11	6.78E-12	7.31	16.1252636	1.09E-01	38160704	38160827	NC_000008.10
cg27392802	9.66E-11	6.78E-12	7.31	16.1252468	1.64E-01	332705	332828	NC_000016.9
cg11315168	9.70E-11	6.81E-12	7.31	16.1204314	1.22E-01	129905368	129905491	NC_000007.13
cg27100227	9.74E-11	6.84E-12	7.31	16.1166016	1.88E-01	27281687	27281810	NC_000007.13
cg01307526	9.76E-11	6.85E-12	7.31	16.1142556	7.25E-02	122252471	122252594	NC_000012.11
cg14769792	9.77E-11	6.86E-12	7.31	16.1135692	3.67E-02	89629747	89629870	NC_000004.11
cg25976257	9.77E-11	6.86E-12	7.31	16.1127398	1.99E-01	45405621	45405744	NC_000022.10
cg02138264	9.78E-11	6.86E-12	7.31	16.1126192	1.82E-01	172754465	172754588	NC_000005.9
cg23976499	9.78E-11	6.87E-12	7.31	16.1120951	1.99E-01	11207527	11207650	NC_000010.10
cg09704152	9.79E-11	6.88E-12	7.31	16.1105311	4.40E-02	100028166	100028289	NC_000008.10
cg05511722	9.80E-11	6.88E-12	7.31	16.1102117	1.88E-01	55379566	55379689	NC_000008.10

cg16448037	9.82E-11	6.90E-12	7.3	16.1081038	2.85E-02	72172333	72172456	NC_000010.10
cg00492118	9.82E-11	6.90E-12	7.3	16.1080638	1.50E-01	140771184	140771307	NC_000003.11
cg11016563	9.82E-11	6.90E-12	7.3	16.1076503	1.78E-01	101454626	101454749	NC_000011.9
cg07957294	9.83E-11	6.90E-12	7.3	16.1068448	1.77E-01	170638917	170639040	NC_000001.10
cg26014036	9.84E-11	6.92E-12	7.3	16.1051502	1.92E-01	147112096	147112219	NC_000003.11
cg26784596	9.85E-11	6.92E-12	7.3	16.1040315	1.17E-01	140769814	140769937	NC_000003.11
cg10439742	9.85E-11	6.92E-12	7.3	16.1038797	1.39E-01	83845465	83845588	NC_000016.9
cg08182191	9.86E-11	6.93E-12	7.3	16.1035807	3.50E-02	106714396	106714519	NC_000002.11
cg10634182	9.88E-11	6.94E-12	7.3	16.1015119	7.83E-02	572373	572496	NC_000005.9
cg08116462	9.88E-11	6.94E-12	7.3	16.1012842	1.88E-01	31823141	31823264	NC_000011.9
cg14228146	9.88E-11	6.95E-12	7.3	16.1010059	1.42E-01	119607395	119607518	NC_000002.11
cg26938597	9.90E-11	6.96E-12	7.3	16.0992796	1.80E-01	44778021	44778144	NC_000018.9
cg05236348	9.91E-11	6.96E-12	7.3	16.0983409	4.45E-02	73767717	73767840	NC_000010.10
cg05422049	9.91E-11	6.96E-12	7.3	16.0982712	1.57E-01	1595734	1595857	NC_000002.11
cg04982193	9.91E-11	6.96E-12	7.3	16.0982352	7.76E-02	146278795	146278918	NC_000008.10
cg00021476	9.93E-11	6.98E-12	7.3	16.0956092	1.56E-01	85660215	85660338	NC_000016.9
cg17171215	9.93E-11	6.98E-12	7.3	16.0955794	1.84E-01	237078223	237078346	NC_000002.11
cg25912911	9.94E-11	6.99E-12	7.3	16.095039	1.91E-01	20523121	20523244	NC_000012.11
cg06678594	9.94E-11	6.99E-12	7.3	16.094367	6.59E-02	43573198	43573321	NC_000002.11
cg26694386	9.95E-11	7.00E-12	7.3	16.0936101	1.35E-01	244868765	244868888	NC_000001.10
cg26802291	9.95E-11	7.00E-12	7.3	16.0935565	1.99E-01	19372387	19372510	NC_000019.9
cg10520887	9.96E-11	7.01E-12	7.3	16.0924351	2.20E-01	96470094	96470217	NC_000004.11
cg19688761	9.97E-11	7.01E-12	7.3	16.0916611	8.15E-02	129785464	129785587	NC_000010.10
cg01984304	9.98E-11	7.02E-12	7.3	16.0906805	1.68E-01	91191813	91191936	NC_000001.10
cg24367850	9.99E-11	7.03E-12	7.3	16.089443	5.28E-02	27217401	27217524	NC_000001.10

cg23018092	9.99E-11	7.03E-12	7.3	16.0890094	1.66E-01	126626364	126626487	NC_000005.9
cg04830191	9.99E-11	7.03E-12	7.3	16.0889052	8.90E-02	212868520	212868643	NC_000001.10
cg20632871	1.00E-10	7.04E-12	7.3	16.0873414	5.18E-02	2318918	2319041	NC_000001.10
cg23688510	1.00E-10	7.04E-12	7.3	16.0870855	2.55E-01	166581929	166582052	NC_000006.11
cg02564291	1.00E-10	7.05E-12	7.3	16.0858965	1.54E-01	122430235	122430358	NC_000005.9
cg22240704	1.00E-10	7.06E-12	7.3	16.0844217	4.15E-02	20202403	20202526	NC_000003.11
cg10094078	1.01E-10	7.08E-12	7.3	16.0815616	1.44E-01	1467925	1468048	NC_000019.9
cg00723275	1.01E-10	7.10E-12	7.3	16.0794636	9.31E-02	216973091	216973214	NC_000002.11
cg07886195	1.01E-10	7.11E-12	7.3	16.0781988	1.76E-01	11263615	11263738	NC_000019.9
cg09382096	1.01E-10	7.11E-12	7.3	16.077788	1.61E-01	31827593	31827716	NC_000011.9
cg05595832	1.01E-10	7.11E-12	7.3	16.0775407	1.90E-01	104567372	104567495	NC_000007.13
cg00601711	1.01E-10	7.11E-12	7.3	16.0774148	1.56E-01	1089507	1089630	NC_000005.9
cg06821079	1.01E-10	7.11E-12	7.3	16.07724	1.46E-01	39143567	39143690	NC_000017.10
cg14289246	1.01E-10	7.12E-12	7.3	16.0762552	1.59E-01	154710475	154710598	NC_000004.11
cg10715265	1.01E-10	7.12E-12	7.3	16.0760204	2.13E-01	58715499	58715622	NC_000001.10
cg06288154	1.01E-10	7.13E-12	7.3	16.0756879	1.52E-01	130737494	130737617	NC_000002.11
cg01523474	1.01E-10	7.13E-12	7.3	16.0748407	1.59E-01	45365651	45365774	NC_000014.8
cg15880016	1.01E-10	7.15E-12	7.3	16.0725485	8.36E-02	100661648	100661771	NC_000011.9
cg08640361	1.02E-10	7.16E-12	7.3	16.0712392	1.61E-01	134494620	134494743	NC_000006.11
cg01835308	1.02E-10	7.17E-12	7.3	16.0701734	2.26E-01	12997160	12997283	NC_000016.9
cg18706841	1.02E-10	7.20E-12	7.3	16.0657902	6.65E-02	77186157	77186280	NC_000012.11
cg23881458	1.02E-10	7.20E-12	7.3	16.0650528	1.25E-01	42754477	42754600	NC_000006.11
cg13888600	1.02E-10	7.21E-12	7.3	16.0645302	1.57E-01	89911317	89911440	NC_000015.9
cg07468782	1.02E-10	7.21E-12	7.3	16.0639563	8.02E-02	5023846	5023969	NC_000017.10
cg02117086	1.02E-10	7.22E-12	7.3	16.0628398	1.29E-01	232265670	232265793	NC_000002.11

cg09567439	1.02E-10	7.22E-12	7.3	16.0626884	2.08E-01	60297694	60297817	NC_000015.9
cg24530147	1.03E-10	7.23E-12	7.3	16.0612298	1.34E-01	138763894	138764017	NC_000003.11
cg05229403	1.03E-10	7.26E-12	7.3	16.0577211	2.05E-01	133252433	133252556	NC_000010.10
cg12315391	1.03E-10	7.26E-12	7.3	16.05749	1.47E-01	157815145	157815268	NC_000003.11
cg00362285	1.03E-10	7.28E-12	7.3	16.0546321	1.55E-01	236444212	236444335	NC_000001.10
cg10192893	1.03E-10	7.29E-12	7.3	16.0527157	1.90E-01	41747895	41748018	NC_000004.11
cg25895961	1.03E-10	7.30E-12	7.3	16.0523335	1.74E-01	29106422	29106545	NC_000013.10
cg15753178	1.03E-10	7.30E-12	7.3	16.05231	4.72E-02	124535611	124535734	NC_000009.11
cg18470456	1.04E-10	7.31E-12	7.29	16.0512812	1.93E-01	96204493	96204616	NC_000013.10
cg26089160	1.04E-10	7.32E-12	7.29	16.0498953	1.59E-01	104170217	104170340	NC_000010.10
cg12470092	1.04E-10	7.33E-12	7.29	16.0483323	1.02E-01	37948808	37948931	NC_000021.8
cg11979589	1.04E-10	7.33E-12	7.29	16.0480169	2.40E-01	110223967	110224090	NC_000004.11
cg27516404	1.04E-10	7.33E-12	7.29	16.0479909	1.10E-01	155640019	155640142	NC_000007.13
cg11023989	1.04E-10	7.33E-12	7.29	16.0476953	6.46E-02	168758239	168758362	NC_000006.11
cg25552435	1.04E-10	7.33E-12	7.29	16.0473674	2.33E-01	178017300	178017423	NC_000005.9
cg15805568	1.04E-10	7.34E-12	7.29	16.0460123	1.36E-01	51199000	51199123	NC_000019.9
cg16834823	1.04E-10	7.34E-12	7.29	16.045974	1.79E-01	32064218	32064341	NC_000006.11
cg05797097	1.04E-10	7.35E-12	7.29	16.0457372	1.14E-01	14720183	14720306	NC_000006.11
cg12035092	1.04E-10	7.35E-12	7.29	16.0455475	2.43E-01	149633226	149633349	NC_000002.11
cg01386831	1.04E-10	7.35E-12	7.29	16.0453372	4.83E-02	79906364	79906487	NC_000017.10
cg16055052	1.04E-10	7.36E-12	7.29	16.0444171	7.02E-02	30657275	30657398	NC_000006.11
cg24361162	1.04E-10	7.36E-12	7.29	16.0432735	1.63E-01	156358116	156358239	NC_000001.10
cg13955436	1.04E-10	7.37E-12	7.29	16.0430297	1.98E-01	6449153	6449276	NC_000005.9
cg05998109	1.04E-10	7.37E-12	7.29	16.0428421	7.28E-02	5644940	5645063	NC_000007.13
cg22827250	1.05E-10	7.38E-12	7.29	16.0416092	1.06E-01	134363823	134363946	NC_000005.9

cg03310503	1.05E-10	7.39E-12	7.29	16.0396201	6.46E-02	45609836	45609959	NC_000006.11
cg05663341	1.05E-10	7.40E-12	7.29	16.0392503	2.60E-01	96634660	96634783	NC_000007.13
cg04553838	1.05E-10	7.40E-12	7.29	16.0388477	1.59E-01	101454733	101454856	NC_000011.9
cg01477985	1.05E-10	7.40E-12	7.29	16.0387292	2.02E-01	112727160	112727283	NC_000013.10
cg06805348	1.05E-10	7.40E-12	7.29	16.0384261	1.18E-01	37245195	37245318	NC_000004.11
cg14752361	1.05E-10	7.40E-12	7.29	16.0379464	9.33E-02	20205176	20205299	NC_000007.13
cg14991764	1.05E-10	7.41E-12	7.29	16.0372035	1.03E-01	133486947	133487070	NC_000006.11
cg07124117	1.05E-10	7.41E-12	7.29	16.0371894	1.92E-01	31827775	31827898	NC_000011.9
cg26654798	1.05E-10	7.41E-12	7.29	16.0371633	1.27E-01	83953864	83953987	NC_000015.9
cg27634876	1.05E-10	7.42E-12	7.29	16.036532	1.83E-01	52735485	52735608	NC_000014.8
cg21279601	1.05E-10	7.42E-12	7.29	16.0365085	1.27E-01	9782931	9783054	NC_000004.11
cg16023545	1.05E-10	7.42E-12	7.29	16.0360502	1.74E-01	237076726	237076849	NC_000002.11
cg06419761	1.05E-10	7.42E-12	7.29	16.0359147	2.11E-01	72043661	72043784	NC_000010.10
cg02501779	1.05E-10	7.43E-12	7.29	16.0351693	1.46E-01	54579355	54579478	NC_000020.10
cg27205687	1.05E-10	7.43E-12	7.29	16.0342024	2.38E-01	106888787	106888910	NC_000011.9
cg02765859	1.05E-10	7.44E-12	7.29	16.0339315	1.90E-01	58152311	58152434	NC_000020.10
cg07586272	1.05E-10	7.44E-12	7.29	16.0333201	1.98E-01	94134580	94134703	NC_000011.9
cg05943996	1.05E-10	7.45E-12	7.29	16.0323523	1.73E-01	42075975	42076098	NC_000014.8
cg23994112	1.05E-10	7.45E-12	7.29	16.0319951	1.90E-01	75373457	75373580	NC_000017.10
cg26465405	1.05E-10	7.45E-12	7.29	16.0314381	1.44E-01	3039745	3039868	NC_000019.9
cg22948791	1.06E-10	7.46E-12	7.29	16.0302157	7.26E-02	71404101	71404224	NC_000015.9
cg08002883	1.06E-10	7.47E-12	7.29	16.029237	2.85E-01	192127457	192127580	NC_000003.11
cg15947096	1.06E-10	7.48E-12	7.29	16.0280109	2.70E-01	37488835	37488958	NC_000007.13
cg26337020	1.06E-10	7.48E-12	7.29	16.0277331	1.84E-01	100239022	100239145	NC_000005.9
cg01417432	1.06E-10	7.49E-12	7.29	16.0261017	6.57E-02	63913731	63913854	NC_000010.10

cg15787744	1.06E-10	7.50E-12	7.29	16.02519	1.08E-01	44234175	44234298	NC_000006.11
cg04509682	1.06E-10	7.50E-12	7.29	16.0250894	1.33E-01	41056288	41056411	NC_000013.10
cg13420320	1.06E-10	7.51E-12	7.29	16.0236689	1.18E-01	131193104	131193227	NC_000010.10
cg21150026	1.06E-10	7.53E-12	7.29	16.021798	1.36E-01	207904284	207904407	NC_000002.11
cg05489292	1.07E-10	7.56E-12	7.29	16.0170394	1.62E-01	112331187	112331310	NC_000012.11
cg01265068	1.07E-10	7.58E-12	7.29	16.014983	1.84E-01	52052945	52053068	NC_000013.10
cg04666975	1.07E-10	7.59E-12	7.29	16.0134143	1.58E-01	60977353	60977476	NC_000014.8
cg00129651	1.07E-10	7.60E-12	7.29	16.0123677	2.58E-01	38347710	38347833	NC_000017.10
cg10716862	1.08E-10	7.61E-12	7.29	16.0108699	1.07E-01	65674876	65674999	NC_000001.10
cg27213024	1.08E-10	7.62E-12	7.29	16.0102644	6.92E-02	143913327	143913450	NC_000006.11
cg05967403	1.08E-10	7.62E-12	7.29	16.0097385	1.39E-01	28367245	28367368	NC_000013.10
cg26135388	1.08E-10	7.64E-12	7.29	16.0066589	1.55E-01	130831162	130831285	NC_000010.10
cg16582419	1.08E-10	7.66E-12	7.29	16.0046391	1.08E-01	30038998	30039121	NC_000006.11
cg19818271	1.08E-10	7.66E-12	7.29	16.0041932	1.24E-01	77468418	77468541	NC_000007.13
cg01775414	1.09E-10	7.69E-12	7.29	16.001204	2.21E-01	45405404	45405527	NC_000022.10
cg08033114	1.09E-10	7.69E-12	7.29	16.0007353	1.76E-01	32907919	32908042	NC_000017.10
cg10602757	1.09E-10	7.70E-12	7.29	15.998886	1.40E-01	114256781	114256904	NC_000002.11
cg24229589	1.09E-10	7.72E-12	7.29	15.9970547	1.40E-01	19651962	19652085	NC_000019.9
cg00003305	1.09E-10	7.72E-12	7.29	15.9967164	2.06E-01	30715645	30715768	NC_000019.9
cg10982364	1.09E-10	7.72E-12	7.29	15.9963449	1.62E-01	200329670	200329793	NC_000002.11
cg13503268	1.09E-10	7.73E-12	7.29	15.9954435	4.02E-02	74387214	74387337	NC_000009.11
cg05674475	1.09E-10	7.73E-12	7.29	15.9954375	1.72E-01	42154385	42154508	NC_000004.11
cg04760348	1.09E-10	7.73E-12	7.29	15.9950502	1.04E-01	86608910	86609033	NC_000016.9
cg11186405	1.09E-10	7.74E-12	7.29	15.9947912	1.96E-01	112728206	112728329	NC_000013.10
cg16566400	1.09E-10	7.74E-12	7.28	15.9942579	2.42E-01	119293869	119293992	NC_000011.9

cg02078690	1.10E-10	7.76E-12	7.28	15.9915815	1.95E-01	122852454	122852577	NC_000011.9
cg15915658	1.10E-10	7.78E-12	7.28	15.9898011	1.29E-01	65262358	65262481	NC_000011.9
cg26332740	1.10E-10	7.78E-12	7.28	15.9892139	1.72E-01	228196227	228196350	NC_000001.10
cg16830930	1.10E-10	7.78E-12	7.28	15.9889168	1.11E-01	55371392	55371515	NC_000008.10
cg02846316	1.10E-10	7.80E-12	7.28	15.9873255	1.08E-01	31340340	31340463	NC_000016.9
cg17035996	1.10E-10	7.80E-12	7.28	15.9870181	1.02E-01	63985489	63985612	NC_000005.9
cg17432748	1.10E-10	7.80E-12	7.28	15.9863091	1.47E-01	235959556	235959679	NC_000002.11
cg22517020	1.10E-10	7.80E-12	7.28	15.9862818	6.38E-02	193082085	193082208	NC_000001.10
cg17695504	1.10E-10	7.81E-12	7.28	15.9856529	9.85E-02	29923120	29923243	NC_000007.13
cg21517792	1.10E-10	7.81E-12	7.28	15.9852388	1.04E-01	105885531	105885654	NC_000014.8
cg10109500	1.10E-10	7.82E-12	7.28	15.9838656	1.95E-01	172165884	172166007	NC_000003.11
cg19839825	1.10E-10	7.83E-12	7.28	15.9833059	1.50E-01	7616011	7616134	NC_000019.9
cg00507008	1.10E-10	7.83E-12	7.28	15.982756	1.84E-01	115131421	115131544	NC_000012.11
cg10091144	1.10E-10	7.84E-12	7.28	15.9821476	1.00E-01	105966148	105966271	NC_000014.8
cg03923071	1.11E-10	7.84E-12	7.28	15.9815118	1.39E-01	153852929	153853052	NC_000005.9
cg13542368	1.11E-10	7.85E-12	7.28	15.9804575	1.07E-01	95049539	95049662	NC_000010.10
cg07057177	1.11E-10	7.86E-12	7.28	15.9791692	2.17E-01	132261393	132261516	NC_000007.13
cg03839714	1.11E-10	7.86E-12	7.28	15.9791393	1.07E-01	31361534	31361657	NC_000010.10
cg23684973	1.11E-10	7.87E-12	7.28	15.9782963	2.19E-01	1780003	1780126	NC_000010.10
cg09807215	1.11E-10	7.87E-12	7.28	15.9782318	1.70E-01	144510884	144511007	NC_000008.10
cg06654537	1.11E-10	7.87E-12	7.28	15.9780711	1.42E-01	100641317	100641440	NC_000013.10
cg06730678	1.11E-10	7.87E-12	7.28	15.9779171	1.34E-01	6166943	6167066	NC_000012.11
cg26934034	1.11E-10	7.88E-12	7.28	15.976572	8.07E-02	113861110	113861233	NC_000012.11
cg01073765	1.11E-10	7.90E-12	7.28	15.974011	8.67E-02	20568217	20568340	NC_000001.10
cg00814733	1.11E-10	7.90E-12	7.28	15.9737631	1.79E-01	4382051	4382174	NC_000012.11

cg13677415	1.11E-10	7.90E-12	7.28	15.9737205	1.96E-01	35291900	35292023	NC_000017.10
cg04531425	1.11E-10	7.91E-12	7.28	15.9725181	1.59E-01	182322841	182322964	NC_000002.11
cg03075050	1.12E-10	7.92E-12	7.28	15.9720986	8.58E-02	46462790	46462913	NC_000016.9
cg24772753	1.12E-10	7.92E-12	7.28	15.9715403	1.43E-01	171573419	171573542	NC_000002.11
cg11368281	1.12E-10	7.94E-12	7.28	15.969098	5.91E-02	35170588	35170711	NC_000019.9
cg02319149	1.12E-10	7.96E-12	7.28	15.9668609	1.47E-01	99336246	99336369	NC_000013.10
cg08711858	1.12E-10	7.97E-12	7.28	15.9661883	2.58E-01	172756550	172756673	NC_000005.9
cg07442409	1.12E-10	7.98E-12	7.28	15.9641192	1.41E-01	60973773	60973896	NC_000014.8
cg10724965	1.13E-10	8.00E-12	7.28	15.962075	9.42E-02	25543202	25543325	NC_000008.10
cg23434461	1.13E-10	8.00E-12	7.28	15.9620546	9.14E-02	119803683	119803806	NC_000008.10
cg13526221	1.13E-10	8.00E-12	7.28	15.9614278	1.27E-01	987389	987512	NC_000008.10
cg24092282	1.13E-10	8.00E-12	7.28	15.9614005	1.57E-01	54413371	54413494	NC_000014.8
cg20386316	1.13E-10	8.01E-12	7.28	15.9612205	1.93E-01	72470982	72471105	NC_000008.10
cg20748533	1.13E-10	8.01E-12	7.28	15.9603077	1.41E-01	51189975	51190098	NC_000019.9
cg19594840	1.13E-10	8.02E-12	7.28	15.9600198	1.21E-01	157934391	157934514	NC_000006.11
cg11439390	1.13E-10	8.04E-12	7.28	15.9566789	7.99E-02	7506552	7506675	NC_000006.11
cg19870698	1.13E-10	8.06E-12	7.28	15.9549782	1.87E-01	95239570	95239693	NC_000014.8
cg02362467	1.13E-10	8.06E-12	7.28	15.9545309	1.72E-01	104513683	104513806	NC_000008.10
cg18293662	1.13E-10	8.06E-12	7.28	15.9543207	1.74E-01	27766054	27766177	NC_000003.11
cg17750109	1.14E-10	8.09E-12	7.28	15.9508939	1.01E-01	115844232	115844355	NC_000001.10
cg03769383	1.14E-10	8.10E-12	7.28	15.9501268	1.76E-01	41583367	41583490	NC_000012.11
cg01271485	1.14E-10	8.10E-12	7.28	15.9498596	1.26E-01	55650017	55650140	NC_000002.11
cg11959316	1.14E-10	8.10E-12	7.28	15.9496998	1.74E-01	57001742	57001865	NC_000001.10
cg04578774	1.14E-10	8.12E-12	7.28	15.9471895	2.31E-01	44332664	44332787	NC_000011.9
cg17381030	1.14E-10	8.12E-12	7.28	15.9470428	1.10E-01	118369843	118369966	NC_000010.10

cg19631064	1.14E-10	8.14E-12	7.28	15.9448799	1.13E-01	197807355	197807478	NC_000003.11
cg25728362	1.15E-10	8.16E-12	7.28	15.9428145	4.75E-02	105222945	105223068	NC_000007.13
cg06868481	1.15E-10	8.17E-12	7.28	15.9408938	9.72E-02	29192086	29192209	NC_000001.10
cg04285275	1.15E-10	8.17E-12	7.28	15.9406719	1.78E-01	31549141	31549264	NC_000016.9
cg25886284	1.15E-10	8.18E-12	7.28	15.9400331	2.36E-01	36909418	36909541	NC_000019.9
cg23665824	1.15E-10	8.19E-12	7.28	15.9390132	1.72E-01	80530701	80530824	NC_000002.11
cg11076668	1.15E-10	8.19E-12	7.28	15.9389106	3.29E-02	82336109	82336232	NC_000015.9
cg26739849	1.15E-10	8.19E-12	7.28	15.9388479	5.94E-02	111165419	111165542	NC_000013.10
cg05358729	1.16E-10	8.24E-12	7.27	15.9322305	1.79E-01	29719159	29719282	NC_000017.10
cg17912835	1.16E-10	8.25E-12	7.27	15.9311654	1.53E-01	105478804	105478927	NC_000002.11
cg07381141	1.16E-10	8.26E-12	7.27	15.9301549	1.20E-01	15783239	15783362	NC_000001.10
cg00916536	1.16E-10	8.26E-12	7.27	15.9298827	2.05E-01	87017419	87017542	NC_000002.11
cg23335916	1.16E-10	8.27E-12	7.27	15.929216	1.03E-01	42578842	42578965	NC_000008.10
cg12211161	1.16E-10	8.27E-12	7.27	15.9288183	1.24E-01	157448659	157448782	NC_000007.13
cg00287096	1.16E-10	8.28E-12	7.27	15.9283277	1.15E-01	63642709	63642832	NC_000007.13
cg06793562	1.16E-10	8.29E-12	7.27	15.9271953	1.96E-01	85482570	85482693	NC_000006.11
cg22927788	1.17E-10	8.31E-12	7.27	15.9239734	1.60E-01	20521984	20522107	NC_000012.11
cg13818075	1.17E-10	8.32E-12	7.27	15.9236628	1.16E-01	48391043	48391166	NC_000013.10
cg08008088	1.17E-10	8.32E-12	7.27	15.9235538	1.25E-01	143609509	143609632	NC_000008.10
cg14460403	1.17E-10	8.32E-12	7.27	15.9235491	1.42E-01	119301766	119301889	NC_000010.10
cg08750534	1.17E-10	8.32E-12	7.27	15.9233253	1.32E-01	65630259	65630382	NC_000002.11
cg01210554	1.17E-10	8.34E-12	7.27	15.921372	1.78E-01	27191564	27191687	NC_000007.13
cg03322752	1.17E-10	8.34E-12	7.27	15.9204491	8.67E-02	54824520	54824643	NC_000007.13
cg15448736	1.17E-10	8.36E-12	7.27	15.9188715	8.17E-02	132852389	132852512	NC_000012.11
cg12921750	1.17E-10	8.36E-12	7.27	15.918633	1.60E-01	70535336	70535459	NC_000018.9

cg01156374	1.17E-10	8.37E-12	7.27	15.9179919	1.36E-01	4130800	4130923	NC_000006.11
cg12320750	1.17E-10	8.37E-12	7.27	15.9176908	7.22E-02	17944975	17945098	NC_000017.10
cg06262098	1.17E-10	8.37E-12	7.27	15.9176585	7.57E-02	5413872	5413995	NC_000007.13
cg19333100	1.17E-10	8.37E-12	7.27	15.9176374	1.69E-01	123229032	123229155	NC_000011.9
cg15697019	1.18E-10	8.37E-12	7.27	15.9169444	1.03E-01	77357984	77358107	NC_000007.13
cg04822518	1.18E-10	8.38E-12	7.27	15.9157398	1.44E-01	29936075	29936198	NC_000012.11
cg11319389	1.18E-10	8.41E-12	7.27	15.9124212	1.82E-01	42544648	42544771	NC_000020.10
cg19924619	1.18E-10	8.42E-12	7.27	15.9112924	1.63E-01	165323692	165323815	NC_000001.10
cg20717123	1.18E-10	8.43E-12	7.27	15.910906	1.99E-01	133195520	133195643	NC_000012.11
cg03290827	1.18E-10	8.44E-12	7.27	15.9089746	1.08E-01	75180760	75180883	NC_000017.10
cg18392016	1.19E-10	8.45E-12	7.27	15.9077009	2.19E-01	117591684	117591807	NC_000006.11
cg13946520	1.19E-10	8.45E-12	7.27	15.9074986	2.03E-01	87017689	87017812	NC_000002.11
cg25228422	1.19E-10	8.48E-12	7.27	15.9051133	1.90E-01	19152335	19152458	NC_000007.13
cg07704934	1.19E-10	8.48E-12	7.27	15.9042572	1.63E-01	44332940	44333063	NC_000011.9
cg11805552	1.19E-10	8.49E-12	7.27	15.903876	1.08E-01	16175128	16175251	NC_000006.11
cg05093169	1.19E-10	8.49E-12	7.27	15.9036764	1.76E-01	71955401	71955524	NC_000011.9
cg13425135	1.19E-10	8.49E-12	7.27	15.9036117	7.00E-02	137330570	137330693	NC_000009.11
cg16325502	1.19E-10	8.49E-12	7.27	15.903432	1.45E-01	223166435	223166558	NC_000002.11
cg22095128	1.19E-10	8.50E-12	7.27	15.9022713	1.44E-01	33754450	33754573	NC_000013.10
cg10237419	1.19E-10	8.50E-12	7.27	15.9020234	1.66E-01	110224235	110224358	NC_000004.11
cg04079301	1.19E-10	8.51E-12	7.27	15.9006437	1.59E-01	147106635	147106758	NC_000003.11
cg09914029	1.20E-10	8.53E-12	7.27	15.8991225	1.02E-01	117859175	117859298	NC_000008.10
cg24725933	1.20E-10	8.54E-12	7.27	15.8979653	5.41E-02	98999128	98999251	NC_000002.11
cg26396815	1.20E-10	8.55E-12	7.27	15.896891	4.11E-02	102878132	102878255	NC_000004.11
cg18448695	1.20E-10	8.55E-12	7.27	15.8960988	7.90E-02	56781117	56781240	NC_000020.10

cg25580634	1.20E-10	8.56E-12	7.27	15.8956443	3.71E-02	205947	206070	NC_000005.9
cg17960934	1.20E-10	8.56E-12	7.27	15.89553	1.16E-01	70096791	70096914	NC_000007.13
cg19433697	1.20E-10	8.58E-12	7.27	15.8930631	2.16E-01	60294787	60294910	NC_000015.9
cg00563824	1.20E-10	8.58E-12	7.27	15.8925201	1.74E-01	209405064	209405187	NC_000001.10
cg19635505	1.20E-10	8.59E-12	7.27	15.8914129	1.22E-01	20903555	20903678	NC_000014.8
cg21016855	1.21E-10	8.62E-12	7.27	15.8881605	1.64E-01	31819444	31819567	NC_000011.9
cg01658421	1.21E-10	8.63E-12	7.27	15.8877605	1.58E-01	176945899	176946022	NC_000002.11
cg13486820	1.21E-10	8.63E-12	7.27	15.8876289	1.40E-01	91182288	91182411	NC_000001.10
cg08765199	1.21E-10	8.68E-12	7.27	15.8821953	1.52E-01	130021519	130021642	NC_000007.13
cg12653796	1.22E-10	8.70E-12	7.26	15.8791085	1.54E-01	87016720	87016843	NC_000002.11
cg26616526	1.22E-10	8.71E-12	7.26	15.8779586	9.79E-02	3165100	3165223	NC_000010.10
cg09681167	1.22E-10	8.72E-12	7.26	15.8775945	2.22E-01	237078026	237078149	NC_000002.11
cg12064276	1.22E-10	8.73E-12	7.26	15.8763216	1.38E-01	79182205	79182328	NC_000013.10
cg15562022	1.22E-10	8.74E-12	7.26	15.8754027	8.15E-02	31167427	31167550	NC_000006.11
cg14004602	1.22E-10	8.74E-12	7.26	15.874731	9.67E-02	34330410	34330533	NC_000017.10
cg07241917	1.22E-10	8.74E-12	7.26	15.8747087	1.16E-01	221069699	221069822	NC_000001.10
cg21336288	1.22E-10	8.74E-12	7.26	15.8745332	1.43E-01	117742444	117742567	NC_000008.10
cg11367354	1.23E-10	8.76E-12	7.26	15.8721119	1.35E-01	96651983	96652106	NC_000007.13
cg03445310	1.23E-10	8.78E-12	7.26	15.8698984	8.21E-02	39728478	39728601	NC_000017.10
cg04855961	1.23E-10	8.79E-12	7.26	15.869633	1.03E-01	159719849	159719972	NC_000003.11
cg25122369	1.23E-10	8.79E-12	7.26	15.869447	1.01E-01	168801790	168801913	NC_000003.11
cg20192747	1.23E-10	8.80E-12	7.26	15.8679449	1.15E-01	44774846	44774969	NC_000018.9
cg22682811	1.24E-10	8.83E-12	7.26	15.864404	1.53E-01	21372462	21372585	NC_000020.10
cg07589393	1.24E-10	8.84E-12	7.26	15.8640374	1.40E-01	26984305	26984428	NC_000007.13
cg10039163	1.24E-10	8.85E-12	7.26	15.8628751	5.31E-02	144593087	144593210	NC_000008.10

cg22753340	1.24E-10	8.86E-12	7.26	15.8615433	2.37E-01	31831489	31831612	NC_000006.11
cg22392666	1.24E-10	8.87E-12	7.26	15.8607786	1.59E-01	35634167	35634290	NC_000019.9
cg03826688	1.24E-10	8.87E-12	7.26	15.8606215	6.68E-02	16756662	16756785	NC_000012.11
cg00852921	1.24E-10	8.88E-12	7.26	15.8591506	1.17E-01	62120544	62120667	NC_000020.10
cg13928709	1.24E-10	8.88E-12	7.26	15.8590889	2.89E-01	176237221	176237344	NC_000005.9
cg08271804	1.24E-10	8.89E-12	7.26	15.8579896	2.00E-01	22816896	22817019	NC_000019.9
cg04988216	1.24E-10	8.90E-12	7.26	15.8573977	1.07E-01	64471626	64471749	NC_000001.10
cg25026755	1.24E-10	8.91E-12	7.26	15.8563509	1.08E-01	57702195	57702318	NC_000005.9
cg05229355	1.25E-10	8.93E-12	7.26	15.8542206	1.61E-01	132052702	132052825	NC_000008.10
cg20510600	1.25E-10	8.93E-12	7.26	15.8536546	6.94E-02	54820824	54820947	NC_000001.10
cg15828353	1.25E-10	8.95E-12	7.26	15.8515618	7.77E-02	114004106	114004229	NC_000013.10
cg10266060	1.25E-10	8.95E-12	7.26	15.8513243	1.50E-01	28043215	28043338	NC_000015.9
cg16961816	1.25E-10	8.95E-12	7.26	15.8512251	1.29E-01	96633530	96633653	NC_000007.13
cg27040709	1.25E-10	8.96E-12	7.26	15.8508428	1.23E-01	19191723	19191846	NC_000016.9
cg11822175	1.25E-10	8.97E-12	7.26	15.8493299	1.54E-01	102637177	102637300	NC_000010.10
cg01104724	1.25E-10	8.98E-12	7.26	15.8477859	1.97E-01	56297564	56297687	NC_000007.13
cg04195774	1.26E-10	9.00E-12	7.26	15.8465129	1.57E-01	118677172	118677295	NC_000005.9
cg10983544	1.26E-10	9.00E-12	7.26	15.8464555	1.86E-01	248020377	248020500	NC_000001.10
cg01037890	1.26E-10	9.00E-12	7.26	15.8463998	1.70E-01	60937048	60937171	NC_000010.10
cg21041447	1.26E-10	9.00E-12	7.26	15.8461161	9.60E-02	42924694	42924817	NC_000005.9
cg07719077	1.26E-10	9.02E-12	7.26	15.8435925	4.93E-02	57178318	57178441	NC_000004.11
cg16812621	1.26E-10	9.04E-12	7.26	15.8414784	5.43E-02	19888434	19888557	NC_000014.8
cg05525305	1.26E-10	9.05E-12	7.26	15.8400575	5.11E-02	14459174	14459297	NC_000018.9
cg05034973	1.26E-10	9.06E-12	7.26	15.8399816	1.79E-01	119294332	119294455	NC_000010.10
cg03480337	1.26E-10	9.06E-12	7.26	15.8392307	1.93E-01	65284519	65284642	NC_000008.10

cg05952786	1.26E-10	9.07E-12	7.26	15.8387703	3.70E-02	48559485	48559608	NC_000017.10
cg07220032	1.27E-10	9.07E-12	7.26	15.8386338	1.30E-01	24742701	24742824	NC_000013.10
cg26885858	1.27E-10	9.08E-12	7.26	15.8377211	9.95E-02	21766368	21766491	NC_000001.10
cg25084878	1.27E-10	9.09E-12	7.26	15.8365164	2.16E-01	127873711	127873834	NC_000005.9
cg20274006	1.27E-10	9.11E-12	7.26	15.8340359	6.73E-02	29778956	29779079	NC_000008.10
cg14306734	1.27E-10	9.11E-12	7.26	15.8338377	2.50E-01	34833938	34834061	NC_000018.9
cg03978514	1.27E-10	9.13E-12	7.26	15.8314176	6.62E-02	241494647	241494770	NC_000002.11
cg18880500	1.27E-10	9.14E-12	7.26	15.8311699	1.21E-01	53392492	53392615	NC_000001.10
cg03067613	1.27E-10	9.14E-12	7.26	15.8308405	9.10E-02	1785527	1785650	NC_000019.9
cg17295053	1.27E-10	9.14E-12	7.26	15.8307093	1.33E-01	101194096	101194219	NC_000010.10
cg26641935	1.27E-10	9.14E-12	7.26	15.8304218	9.23E-02	74423256	74423379	NC_000006.11
cg02377941	1.28E-10	9.15E-12	7.26	15.8295761	1.06E-01	54973385	54973508	NC_000016.9
cg04881642	1.28E-10	9.15E-12	7.26	15.8294587	5.15E-02	69048156	69048279	NC_000004.11
cg25479732	1.28E-10	9.16E-12	7.26	15.8292437	1.32E-01	80528698	80528821	NC_000005.9
cg24299740	1.28E-10	9.16E-12	7.26	15.8285032	1.05E-01	134252804	134252927	NC_000011.9
cg16277306	1.28E-10	9.18E-12	7.26	15.8266503	8.57E-02	105351751	105351874	NC_000012.11
cg11530960	1.28E-10	9.19E-12	7.26	15.8254363	1.67E-01	1052239	1052362	NC_000009.11
cg11556846	1.28E-10	9.20E-12	7.26	15.8248191	1.44E-01	200468728	200468851	NC_000002.11
cg08533773	1.28E-10	9.20E-12	7.26	15.8241495	1.57E-01	113392004	113392127	NC_000005.9
cg15895543	1.28E-10	9.21E-12	7.26	15.8231595	2.91E-02	105516255	105516378	NC_000014.8
cg03639897	1.28E-10	9.22E-12	7.25	15.8224878	1.23E-01	897545	897668	NC_000020.10
cg14042851	1.29E-10	9.23E-12	7.25	15.8207568	2.12E-01	57019005	57019128	NC_000019.9
cg09821685	1.29E-10	9.24E-12	7.25	15.8201101	4.81E-02	109034944	109035067	NC_000003.11
cg02331262	1.29E-10	9.26E-12	7.25	15.8184578	1.63E-01	87988631	87988754	NC_000016.9
cg19429599	1.29E-10	9.26E-12	7.25	15.8181588	1.46E-01	50266099	50266222	NC_000005.9

cg02340083	1.29E-10	9.26E-12	7.25	15.8179464	2.04E-01	50882006	50882129	NC_000001.10
cg10365769	1.29E-10	9.27E-12	7.25	15.8172481	7.89E-02	7589937	7590060	NC_000004.11
cg16804165	1.29E-10	9.27E-12	7.25	15.8170076	9.91E-02	43989074	43989197	NC_000015.9
cg15347156	1.29E-10	9.28E-12	7.25	15.8159161	7.84E-02	88702795	88702918	NC_000010.10
cg07861790	1.29E-10	9.29E-12	7.25	15.8148973	1.09E-01	112761844	112761967	NC_000013.10
cg00910616	1.30E-10	9.30E-12	7.25	15.8134279	7.41E-02	41245733	41245856	NC_000001.10
cg20667964	1.30E-10	9.31E-12	7.25	15.8127935	7.44E-02	139553002	139553125	NC_000007.13
cg20208394	1.30E-10	9.31E-12	7.25	15.8126009	1.25E-01	7052425	7052548	NC_000004.11
cg23984434	1.30E-10	9.32E-12	7.25	15.812162	2.52E-01	106888656	106888779	NC_000011.9
cg26660654	1.30E-10	9.32E-12	7.25	15.8119223	5.05E-02	90580274	90580397	NC_000010.10
cg27579233	1.30E-10	9.34E-12	7.25	15.8097439	1.37E-01	22827348	22827471	NC_000001.10
cg25206536	1.30E-10	9.37E-12	7.25	15.8059361	1.47E-01	11369007	11369130	NC_000004.11
cg08296191	1.31E-10	9.38E-12	7.25	15.8054636	1.41E-01	51159555	51159678	NC_000022.10
cg26917673	1.31E-10	9.41E-12	7.25	15.8019924	2.72E-01	81952024	81952147	NC_000004.11
cg05166820	1.31E-10	9.41E-12	7.25	15.8017462	1.40E-01	55444153	55444276	NC_000006.11
cg02620013	1.31E-10	9.42E-12	7.25	15.80124	1.38E-01	49794681	49794804	NC_000013.10
cg22524998	1.31E-10	9.42E-12	7.25	15.8010474	1.25E-01	17877846	17877969	NC_000019.9
cg06852058	1.31E-10	9.43E-12	7.25	15.7998569	1.11E-01	34498249	34498372	NC_000015.9
cg20181739	1.31E-10	9.44E-12	7.25	15.7993529	9.63E-02	109907962	109908085	NC_000002.11
cg22524061	1.31E-10	9.44E-12	7.25	15.7986245	1.61E-01	99959430	99959553	NC_000008.10
cg13898875	1.31E-10	9.45E-12	7.25	15.797688	1.05E-01	36980923	36981046	NC_000002.11
cg01410615	1.32E-10	9.46E-12	7.25	15.7970402	1.10E-01	242164067	242164190	NC_000002.11
cg16145209	1.32E-10	9.46E-12	7.25	15.7970074	1.35E-01	2481909	2482032	NC_000011.9
cg16985652	1.32E-10	9.50E-12	7.25	15.7931396	1.26E-01	27241897	27242020	NC_000001.10
cg16046833	1.32E-10	9.50E-12	7.25	15.7925169	9.32E-02	99136054	99136177	NC_000001.10

cg17380661	1.32E-10	9.52E-12	7.25	15.7911402	2.39E-01	100912946	100913069	NC_000006.11
cg01072076	1.32E-10	9.52E-12	7.25	15.7906538	1.93E-01	1930801	1930924	NC_000005.9
cg25950325	1.33E-10	9.53E-12	7.25	15.789279	1.90E-01	41753712	41753835	NC_000004.11
cg27553162	1.33E-10	9.54E-12	7.25	15.7886553	1.58E-01	30582980	30583103	NC_000020.10
cg08231710	1.33E-10	9.54E-12	7.25	15.7884899	1.25E-01	1566687	1566810	NC_000001.10
cg24845595	1.33E-10	9.54E-12	7.25	15.7884614	1.16E-01	156814488	156814611	NC_000001.10
cg00134776	1.33E-10	9.55E-12	7.25	15.7875018	1.64E-01	139653573	139653696	NC_000003.11
cg03442378	1.33E-10	9.56E-12	7.25	15.7871185	1.64E-01	44332385	44332508	NC_000011.9
cg02381539	1.33E-10	9.56E-12	7.25	15.7865689	2.00E-01	65290109	65290232	NC_000008.10
cg17696160	1.33E-10	9.58E-12	7.25	15.7842295	1.56E-01	37136192	37136315	NC_000014.8
cg10050816	1.33E-10	9.59E-12	7.25	15.7839571	1.39E-01	86549237	86549360	NC_000016.9
cg18560204	1.33E-10	9.60E-12	7.25	15.7827633	1.30E-01	83953709	83953832	NC_000015.9
cg03540912	1.33E-10	9.60E-12	7.25	15.7826187	1.65E-01	71792711	71792834	NC_000016.9
cg01420765	1.33E-10	9.60E-12	7.25	15.7825213	5.46E-02	54971610	54971733	NC_000001.10
cg16558432	1.34E-10	9.64E-12	7.25	15.7789363	2.09E-01	5403805	5403928	NC_000017.10
cg10119075	1.34E-10	9.65E-12	7.25	15.7777187	1.97E-01	7709738	7709861	NC_000010.10
cg05001145	1.34E-10	9.66E-12	7.25	15.7769428	5.02E-02	95297731	95297854	NC_000005.9
cg08642771	1.34E-10	9.67E-12	7.25	15.775872	1.15E-01	198392758	198392881	NC_000001.10
cg07463541	1.35E-10	9.69E-12	7.25	15.773	1.30E-01	140181074	140181197	NC_000005.9
cg00073244	1.35E-10	9.70E-12	7.25	15.7719804	8.96E-02	3529108	3529231	NC_000001.10
cg22957360	1.35E-10	9.71E-12	7.25	15.7715521	1.37E-01	101282572	101282695	NC_000010.10
cg09803262	1.35E-10	9.71E-12	7.25	15.7714227	1.72E-01	96622608	96622731	NC_000007.13
cg12186618	1.36E-10	9.77E-12	7.24	15.7648934	1.32E-01	54515274	54515397	NC_000019.9
cg18197392	1.36E-10	9.78E-12	7.24	15.7645053	2.30E-01	23015908	23016031	NC_000020.10
cg05325182	1.36E-10	9.78E-12	7.24	15.764353	1.22E-01	13915751	13915874	NC_000019.9

cg03134573	1.36E-10	9.79E-12	7.24	15.7632253	6.54E-02	144457905	144458028	NC_000008.10
cg19554461	1.36E-10	9.80E-12	7.24	15.7626094	2.15E-01	4998470	4998593	NC_000007.13
cg07032488	1.36E-10	9.80E-12	7.24	15.7618786	7.88E-02	48201121	48201244	NC_000016.9
cg13011388	1.36E-10	9.80E-12	7.24	15.7617831	1.56E-01	23834909	23835032	NC_000014.8
cg02552945	1.36E-10	9.82E-12	7.24	15.7604357	1.35E-01	123811614	123811737	NC_000009.11
cg08120263	1.37E-10	9.85E-12	7.24	15.7574366	1.65E-01	29236013	29236136	NC_000014.8
cg00705613	1.37E-10	9.85E-12	7.24	15.757331	8.01E-02	132661528	132661651	NC_000012.11
cg01720455	1.37E-10	9.85E-12	7.24	15.7572187	1.61E-01	176948343	176948466	NC_000002.11
cg12647497	1.37E-10	9.86E-12	7.24	15.7558331	1.28E-01	110210265	110210388	NC_000001.10
cg27389185	1.37E-10	9.89E-12	7.24	15.7537119	1.27E-01	38042123	38042246	NC_000019.9
cg16918905	1.38E-10	9.95E-12	7.24	15.7476571	1.48E-01	220361609	220361732	NC_000002.11
cg00548268	1.38E-10	9.97E-12	7.24	15.745418	1.14E-01	98245830	98245953	NC_000007.13
cg15082983	1.38E-10	9.99E-12	7.24	15.7438107	1.25E-01	39894780	39894903	NC_000017.10
cg22366088	1.39E-10	9.99E-12	7.24	15.7430789	2.18E-01	154713789	154713912	NC_000004.11
cg01880541	1.39E-10	1.00E-11	7.24	15.7424282	1.32E-01	2146870	2146993	NC_000008.10
cg18313661	1.39E-10	1.00E-11	7.24	15.7413013	1.16E-01	63560590	63560713	NC_000007.13
cg04324666	1.39E-10	1.00E-11	7.24	15.7410374	1.26E-01	23386784	23386907	NC_000019.9
cg20525183	1.39E-10	1.00E-11	7.24	15.7410272	2.38E-01	45405768	45405891	NC_000022.10
cg16799926	1.39E-10	1.00E-11	7.24	15.7394559	1.55E-01	70546695	70546818	NC_000014.8
cg21634100	1.39E-10	1.00E-11	7.24	15.7378512	9.99E-02	55254527	55254650	NC_000018.9
cg05865769	1.39E-10	1.01E-11	7.24	15.7372165	9.66E-02	11943841	11943964	NC_000017.10
cg16122796	1.39E-10	1.01E-11	7.24	15.7370638	6.68E-02	32145414	32145537	NC_000001.10
cg16540704	1.39E-10	1.01E-11	7.24	15.7365422	6.86E-02	117067416	117067539	NC_000007.13
cg05679108	1.39E-10	1.01E-11	7.24	15.7357404	1.60E-01	1395061	1395184	NC_000006.11
cg26823535	1.39E-10	1.01E-11	7.24	15.7355333	4.61E-02	11881420	11881543	NC_000002.11

cg12444450	1.40E-10	1.01E-11	7.24	15.7330746	1.03E-01	45763846	45763969	NC_000001.10
cg26199241	1.40E-10	1.01E-11	7.24	15.7306035	2.08E-01	59529486	59529609	NC_000017.10
cg16444701	1.40E-10	1.01E-11	7.24	15.7303404	7.69E-02	134790855	134790978	NC_000010.10
cg10756127	1.40E-10	1.01E-11	7.24	15.7288973	2.53E-01	166795524	166795647	NC_000004.11
cg15408073	1.40E-10	1.01E-11	7.24	15.7288751	1.87E-01	33936322	33936445	NC_000005.9
cg08244181	1.40E-10	1.01E-11	7.24	15.7281667	1.52E-01	130021807	130021930	NC_000007.13
cg13168877	1.41E-10	1.02E-11	7.24	15.7254113	8.29E-02	1896470	1896593	NC_000001.10
cg16536450	1.41E-10	1.02E-11	7.24	15.7252785	8.71E-02	139658896	139659019	NC_000009.11
cg03556497	1.41E-10	1.02E-11	7.24	15.7239346	1.69E-01	37352792	37352915	NC_000020.10
cg02697402	1.41E-10	1.02E-11	7.24	15.7218231	1.09E-01	87616984	87617107	NC_000016.9
cg27206367	1.41E-10	1.02E-11	7.24	15.7216314	1.08E-01	76398311	76398434	NC_000018.9
cg08095770	1.41E-10	1.02E-11	7.24	15.7214693	3.22E-02	7619558	7619681	NC_000019.9
cg09562322	1.41E-10	1.02E-11	7.24	15.7211833	1.70E-01	119419143	119419266	NC_000012.11
cg09815769	1.41E-10	1.02E-11	7.24	15.7209157	1.20E-01	119539738	119539861	NC_000001.10
cg01345315	1.42E-10	1.02E-11	7.24	15.7202433	1.49E-01	43945998	43946121	NC_000012.11
cg04916485	1.42E-10	1.02E-11	7.24	15.7193564	4.46E-02	126128143	126128266	NC_000009.11
cg26654934	1.42E-10	1.03E-11	7.24	15.7167785	2.49E-01	28603230	28603353	NC_000006.11
cg19909239	1.42E-10	1.03E-11	7.24	15.7152358	1.40E-01	111555503	111555626	NC_000004.11
cg18848917	1.42E-10	1.03E-11	7.24	15.7141547	3.17E-02	110359164	110359287	NC_000004.11
cg14200170	1.42E-10	1.03E-11	7.24	15.7139112	2.01E-01	905252	905375	NC_000018.9
cg08270023	1.42E-10	1.03E-11	7.24	15.7136546	1.53E-01	132619174	132619297	NC_000012.11
cg03234186	1.43E-10	1.03E-11	7.24	15.7128489	2.12E-01	58220657	58220780	NC_000019.9
cg08697503	1.43E-10	1.03E-11	7.24	15.7111398	1.43E-01	223166946	223167069	NC_000002.11
cg24285903	1.43E-10	1.04E-11	7.23	15.7083698	1.25E-01	25187166	25187289	NC_000006.11
cg22633280	1.43E-10	1.04E-11	7.23	15.7081444	1.68E-01	110671907	110672030	NC_000010.10

cg17448336	1.43E-10	1.04E-11	7.23	15.7080501	1.34E-01	147141588	147141711	NC_000003.11
cg23557788	1.43E-10	1.04E-11	7.23	15.7074633	7.07E-02	74119120	74119243	NC_000002.11
cg21852117	1.43E-10	1.04E-11	7.23	15.7069953	1.29E-01	25898296	25898419	NC_000007.13
cg22586996	1.44E-10	1.04E-11	7.23	15.7054717	1.39E-01	158523907	158524030	NC_000005.9
cg04312119	1.44E-10	1.04E-11	7.23	15.7045886	1.17E-01	116839146	116839269	NC_000006.11
cg09778596	1.44E-10	1.04E-11	7.23	15.7038656	1.15E-01	162282922	162283045	NC_000002.11
cg10877183	1.44E-10	1.04E-11	7.23	15.7018997	7.14E-02	133528844	133528967	NC_000010.10
cg21751085	1.44E-10	1.04E-11	7.23	15.7011786	6.32E-02	43935439	43935562	NC_000002.11
cg08644365	1.44E-10	1.04E-11	7.23	15.6997895	8.99E-02	203120194	203120317	NC_000001.10
cg20084577	1.44E-10	1.04E-11	7.23	15.6997598	1.71E-01	33780913	33781036	NC_000013.10
cg24245418	1.44E-10	1.04E-11	7.23	15.6995526	2.33E-01	24324976	24325099	NC_000007.13
cg13696436	1.44E-10	1.04E-11	7.23	15.6995268	5.01E-02	131695094	131695217	NC_000010.10
cg03899315	1.45E-10	1.05E-11	7.23	15.6966569	7.75E-02	117149598	117149721	NC_000001.10
cg06206902	1.45E-10	1.05E-11	7.23	15.6963457	1.75E-01	27191226	27191349	NC_000007.13
cg23319012	1.45E-10	1.05E-11	7.23	15.6948128	5.45E-02	134007935	134008058	NC_000011.9
cg00919016	1.45E-10	1.05E-11	7.23	15.6944664	2.02E-01	139168487	139168610	NC_000007.13
cg11475550	1.45E-10	1.05E-11	7.23	15.6939232	1.94E-01	24324570	24324693	NC_000007.13
cg12562479	1.45E-10	1.05E-11	7.23	15.6937884	6.22E-02	63479057	63479180	NC_000015.9
cg17458693	1.45E-10	1.05E-11	7.23	15.6937645	9.40E-02	6543527	6543650	NC_000012.11
cg24844423	1.45E-10	1.05E-11	7.23	15.691107	1.09E-01	57352465	57352588	NC_000019.9
cg12128839	1.46E-10	1.06E-11	7.23	15.6895892	1.84E-01	27183436	27183559	NC_000007.13
cg02675083	1.46E-10	1.06E-11	7.23	15.689531	5.75E-02	16972917	16973040	NC_000001.10
cg19598567	1.46E-10	1.06E-11	7.23	15.6893025	1.77E-01	141931207	141931330	NC_000005.9
cg25485734	1.46E-10	1.06E-11	7.23	15.6874284	9.09E-02	30981289	30981412	NC_000010.10
cg07910986	1.46E-10	1.06E-11	7.23	15.6873904	1.56E-01	45961311	45961434	NC_000007.13

cg19008693	1.46E-10	1.06E-11	7.23	15.6849244	2.02E-02	48270864	48270987	NC_000012.11
cg11876022	1.46E-10	1.06E-11	7.23	15.6842645	2.08E-01	19812479	19812602	NC_000007.13
cg08613597	1.46E-10	1.06E-11	7.23	15.6841619	2.21E-01	56224627	56224750	NC_000016.9
cg21202529	1.46E-10	1.06E-11	7.23	15.6837895	1.15E-01	20656503	20656626	NC_000007.13
cg16154416	1.47E-10	1.06E-11	7.23	15.6820414	1.14E-01	79382767	79382890	NC_000015.9
cg01941585	1.47E-10	1.06E-11	7.23	15.6815428	3.94E-02	66240388	66240511	NC_000012.11
cg03234732	1.47E-10	1.07E-11	7.23	15.6794269	2.02E-01	178368123	178368246	NC_000005.9
cg01024168	1.47E-10	1.07E-11	7.23	15.6789607	2.07E-01	27209281	27209404	NC_000007.13
cg26032198	1.47E-10	1.07E-11	7.23	15.6780486	2.01E-01	27192339	27192462	NC_000007.13
cg02952809	1.48E-10	1.07E-11	7.23	15.6739146	1.12E-01	128094710	128094833	NC_000007.13
cg23833461	1.48E-10	1.07E-11	7.23	15.6728775	1.43E-01	9929528	9929651	NC_000017.10
cg19122901	1.49E-10	1.08E-11	7.23	15.6688495	1.57E-01	101293790	101293913	NC_000010.10
cg17832029	1.49E-10	1.08E-11	7.23	15.6671432	2.06E-02	98574111	98574234	NC_000007.13
cg14476745	1.49E-10	1.08E-11	7.23	15.666497	1.35E-01	124979356	124979479	NC_000009.11
cg23045602	1.49E-10	1.08E-11	7.23	15.6652762	1.85E-01	140770213	140770336	NC_000003.11
cg22846067	1.49E-10	1.08E-11	7.23	15.6651772	1.19E-01	75252565	75252688	NC_000015.9
cg27540038	1.49E-10	1.08E-11	7.23	15.6630906	5.42E-02	71385496	71385619	NC_000015.9
cg19925639	1.50E-10	1.09E-11	7.23	15.6616822	7.74E-02	75470039	75470162	NC_000005.9
cg21928701	1.50E-10	1.09E-11	7.23	15.6597273	2.41E-01	822086	822209	NC_000019.9
cg07664027	1.50E-10	1.09E-11	7.23	15.6591257	7.17E-02	49990137	49990260	NC_000019.9
cg21608519	1.50E-10	1.09E-11	7.23	15.6588427	1.67E-01	27199752	27199875	NC_000007.13
cg16618417	1.50E-10	1.09E-11	7.23	15.657245	5.35E-02	7535526	7535649	NC_000019.9
cg08060353	1.50E-10	1.09E-11	7.23	15.6571031	5.73E-02	77889974	77890097	NC_000015.9
cg16385939	1.50E-10	1.09E-11	7.23	15.6562873	9.56E-02	144513999	144514122	NC_000001.10
cg05451094	1.50E-10	1.09E-11	7.23	15.6552468	1.66E-01	26557385	26557508	NC_000017.10

cg00351047	1.51E-10	1.09E-11	7.23	15.6541396	1.22E-01	15234963	15235086	NC_000019.9
cg27480727	1.51E-10	1.09E-11	7.23	15.6541363	1.66E-01	223164831	223164954	NC_000002.11
cg20647690	1.51E-10	1.09E-11	7.23	15.6541053	6.45E-02	107191808	107191931	NC_000013.10
cg00317879	1.51E-10	1.09E-11	7.23	15.653621	1.19E-01	68920611	68920734	NC_000011.9
cg20253172	1.51E-10	1.09E-11	7.23	15.6534806	1.01E-01	3107290	3107413	NC_000001.10
cg20968821	1.51E-10	1.10E-11	7.23	15.6515708	9.03E-02	105522684	105522807	NC_000014.8
cg04510512	1.51E-10	1.10E-11	7.22	15.6511207	2.39E-01	86601520	86601643	NC_000016.9
cg21453840	1.51E-10	1.10E-11	7.22	15.648379	1.63E-01	1101471	1101594	NC_000008.10
cg15988950	1.51E-10	1.10E-11	7.22	15.6479866	6.77E-02	166128633	166128756	NC_000006.11
cg10999780	1.52E-10	1.10E-11	7.22	15.6470088	7.48E-02	18464717	18464840	NC_000022.10
cg02311725	1.52E-10	1.10E-11	7.22	15.6458065	1.18E-01	30639816	30639939	NC_000020.10
cg18397405	1.52E-10	1.10E-11	7.22	15.6452674	9.91E-02	94965245	94965368	NC_000013.10
cg07499259	1.52E-10	1.11E-11	7.22	15.6438842	4.74E-02	12188502	12188625	NC_000001.10
cg12699728	1.52E-10	1.11E-11	7.22	15.6434976	9.82E-02	45461636	45461759	NC_000015.9
cg17869514	1.53E-10	1.11E-11	7.22	15.6396476	1.75E-01	1042770	1042893	NC_000009.11
cg09019936	1.53E-10	1.11E-11	7.22	15.6389748	2.05E-01	162283434	162283557	NC_000002.11
cg25653995	1.53E-10	1.11E-11	7.22	15.6377675	4.72E-02	32015707	32015830	NC_000006.11
cg18175690	1.53E-10	1.11E-11	7.22	15.6373007	1.00E-01	40580770	40580893	NC_000015.9
cg23293999	1.53E-10	1.11E-11	7.22	15.6360699	7.02E-02	106826042	106826165	NC_000007.13
cg06426416	1.53E-10	1.11E-11	7.22	15.6353733	1.71E-01	5710403	5710526	NC_000004.11
cg20148210	1.53E-10	1.12E-11	7.22	15.6348387	1.56E-01	38326358	38326481	NC_000008.10
cg20771595	1.54E-10	1.12E-11	7.22	15.6338077	1.00E-01	80523387	80523510	NC_000007.13
cg24463320	1.54E-10	1.12E-11	7.22	15.6335269	6.90E-02	33121236	33121359	NC_000001.10
cg12552771	1.54E-10	1.12E-11	7.22	15.6332678	1.58E-01	29709320	29709443	NC_000022.10
cg16643851	1.54E-10	1.12E-11	7.22	15.6302123	2.10E-01	8542839	8542962	NC_000012.11

cg18105179	1.54E-10	1.12E-11	7.22	15.6280051	6.66E-02	242741407	242741530	NC_000002.11
cg17863312	1.54E-10	1.12E-11	7.22	15.6279052	1.17E-01	14207968	14208091	NC_000017.10
cg03315649	1.55E-10	1.12E-11	7.22	15.6271765	1.10E-01	5093541	5093664	NC_000019.9
cg13032463	1.55E-10	1.13E-11	7.22	15.6262072	2.20E-01	166582585	166582708	NC_000006.11
cg08079580	1.55E-10	1.13E-11	7.22	15.6238597	1.80E-01	133795885	133796008	NC_000010.10
cg12521267	1.55E-10	1.13E-11	7.22	15.6233432	1.29E-01	52412667	52412790	NC_000012.11
cg08203284	1.55E-10	1.13E-11	7.22	15.6229091	1.68E-01	27171528	27171651	NC_000007.13
cg23510026	1.55E-10	1.13E-11	7.22	15.621453	6.89E-02	134915537	134915660	NC_000005.9
cg26207423	1.56E-10	1.13E-11	7.22	15.6191477	1.31E-01	28787952	28788075	NC_000008.10
cg08448589	1.56E-10	1.13E-11	7.22	15.6185234	1.95E-01	69242921	69243044	NC_000008.10
cg21408915	1.56E-10	1.13E-11	7.22	15.6178852	1.18E-01	69286231	69286354	NC_000011.9
cg16626580	1.56E-10	1.14E-11	7.22	15.6173879	7.52E-02	89713163	89713286	NC_000011.9
cg10538797	1.56E-10	1.14E-11	7.22	15.6153144	1.26E-01	105480546	105480669	NC_000002.11
cg07448060	1.56E-10	1.14E-11	7.22	15.6152295	1.53E-01	79083753	79083876	NC_000007.13
cg23275958	1.56E-10	1.14E-11	7.22	15.6150863	1.25E-01	75503249	75503372	NC_000017.10
cg13759674	1.57E-10	1.14E-11	7.22	15.6134006	2.60E-01	140051205	140051328	NC_000009.11
cg14283758	1.57E-10	1.14E-11	7.22	15.6132964	1.87E-01	299370	299493	NC_000004.11
cg17924366	1.57E-10	1.14E-11	7.22	15.6115359	1.23E-01	25042656	25042779	NC_000020.10
cg14306956	1.57E-10	1.15E-11	7.22	15.6090152	2.20E-01	37027384	37027507	NC_000009.11
cg21522988	1.57E-10	1.15E-11	7.22	15.6088817	1.25E-01	29376872	29376995	NC_000012.11
cg26381878	1.57E-10	1.15E-11	7.22	15.6081068	1.07E-01	45071340	45071463	NC_000022.10
cg15910662	1.57E-10	1.15E-11	7.22	15.6080932	1.72E-01	105460108	105460231	NC_000002.11
cg01729717	1.57E-10	1.15E-11	7.22	15.607284	1.84E-01	84456486	84456609	NC_000013.10
cg20008332	1.58E-10	1.15E-11	7.22	15.603749	1.66E-01	5833169	5833292	NC_000002.11
cg19478371	1.58E-10	1.15E-11	7.22	15.6029109	4.56E-02	102310830	102310953	NC_000014.8

cg09552213	1.58E-10	1.15E-11	7.22	15.6015999	1.76E-01	87636782	87636905	NC_000016.9
cg24638819	1.58E-10	1.15E-11	7.22	15.6015503	2.87E-02	33515115	33515238	NC_000010.10
cg11809014	1.58E-10	1.15E-11	7.22	15.6013263	5.12E-02	39067081	39067204	NC_000017.10
cg01892547	1.58E-10	1.15E-11	7.22	15.6012276	8.16E-02	47040292	47040415	NC_000003.11
cg11021744	1.59E-10	1.16E-11	7.22	15.5999938	1.63E-01	11034279	11034402	NC_000003.11
cg27252696	1.59E-10	1.16E-11	7.22	15.5990332	2.56E-01	100912940	100913063	NC_000006.11
cg08589354	1.59E-10	1.16E-11	7.22	15.5960844	1.65E-01	86548895	86549018	NC_000016.9
cg24865270	1.59E-10	1.16E-11	7.22	15.5958141	1.45E-01	10422447	10422570	NC_000006.11
cg05763664	1.59E-10	1.16E-11	7.22	15.5944837	6.25E-02	67816794	67816917	NC_000011.9
cg21902160	1.59E-10	1.16E-11	7.21	15.5941497	8.54E-02	29689075	29689198	NC_000007.13
cg22732749	1.60E-10	1.16E-11	7.21	15.5938142	2.05E-01	140624888	140625011	NC_000005.9
cg05151803	1.60E-10	1.16E-11	7.21	15.5930803	1.81E-01	155167283	155167406	NC_000007.13
cg27529004	1.60E-10	1.16E-11	7.21	15.5927161	1.34E-01	237490808	237490931	NC_000002.11
cg03788227	1.60E-10	1.16E-11	7.21	15.5923133	1.26E-01	88155031	88155154	NC_000016.9
cg20497704	1.60E-10	1.17E-11	7.21	15.5916294	1.47E-01	43913147	43913270	NC_000018.9
cg17279125	1.60E-10	1.17E-11	7.21	15.5886487	1.19E-01	8736209	8736332	NC_000017.10
cg23767994	1.61E-10	1.17E-11	7.21	15.587311	1.50E-01	96621323	96621446	NC_000007.13
cg08787251	1.61E-10	1.17E-11	7.21	15.5845733	1.61E-01	27281964	27282087	NC_000007.13
cg07058694	1.61E-10	1.18E-11	7.21	15.5829221	7.30E-02	111909145	111909268	NC_000012.11
cg26897717	1.61E-10	1.18E-11	7.21	15.582861	1.47E-01	39691363	39691486	NC_000019.9
cg14750066	1.62E-10	1.18E-11	7.21	15.5794799	1.56E-01	41582063	41582186	NC_000012.11
cg03722184	1.62E-10	1.18E-11	7.21	15.5765395	4.50E-02	51353941	51354064	NC_000013.10
cg18505401	1.63E-10	1.19E-11	7.21	15.5742021	2.33E-01	19812570	19812693	NC_000007.13
cg05044185	1.63E-10	1.19E-11	7.21	15.5708059	1.62E-01	27112197	27112320	NC_000015.9
cg10378032	1.63E-10	1.19E-11	7.21	15.570301	1.36E-01	65214497	65214620	NC_000001.10

cg14665523	1.63E-10	1.19E-11	7.21	15.5702474	1.29E-01	89421684	89421807	NC_000010.10
cg08472243	1.63E-10	1.19E-11	7.21	15.570029	4.53E-02	427912	428035	NC_000016.9
cg23498106	1.63E-10	1.19E-11	7.21	15.5694213	1.01E-01	208280643	208280766	NC_000002.11
cg10680235	1.64E-10	1.19E-11	7.21	15.5678871	8.69E-02	85430038	85430161	NC_000015.9
cg27341472	1.64E-10	1.20E-11	7.21	15.5667335	1.93E-01	19812592	19812715	NC_000007.13
cg18252360	1.64E-10	1.20E-11	7.21	15.5665544	1.69E-01	105461192	105461315	NC_000002.11
cg13826890	1.64E-10	1.20E-11	7.21	15.5649884	1.87E-01	57275282	57275405	NC_000014.8
cg25985263	1.64E-10	1.20E-11	7.21	15.5635972	9.87E-02	134996895	134997018	NC_000010.10
cg14790860	1.64E-10	1.20E-11	7.21	15.5624633	2.51E-02	66433618	66433741	NC_000003.11
cg21316236	1.64E-10	1.20E-11	7.21	15.562094	9.18E-02	40125781	40125904	NC_000017.10
cg02582754	1.64E-10	1.20E-11	7.21	15.5617681	8.10E-02	160327345	160327468	NC_000006.11
cg07955752	1.65E-10	1.21E-11	7.21	15.5587171	1.88E-01	30018436	30018559	NC_000019.9
cg12122041	1.65E-10	1.21E-11	7.21	15.5563691	5.20E-02	3234814	3234937	NC_000004.11
cg19620724	1.66E-10	1.21E-11	7.21	15.5552128	1.58E-01	50810852	50810975	NC_000006.11
cg14103680	1.66E-10	1.22E-11	7.21	15.5501422	2.50E-01	28603059	28603182	NC_000006.11
cg22871870	1.66E-10	1.22E-11	7.21	15.5493277	1.14E-01	77348431	77348554	NC_000015.9
cg06690644	1.67E-10	1.22E-11	7.21	15.548529	2.74E-02	93454869	93454992	NC_000011.9
cg03232620	1.67E-10	1.22E-11	7.21	15.547608	2.14E-01	27197879	27198002	NC_000007.13
cg27012396	1.67E-10	1.22E-11	7.21	15.5468122	7.33E-02	240233043	240233166	NC_000002.11
cg02134720	1.67E-10	1.22E-11	7.21	15.5462563	8.56E-02	119948501	119948624	NC_000008.10
cg12639663	1.67E-10	1.22E-11	7.21	15.5459991	5.85E-02	207063173	207063296	NC_000001.10
cg23933241	1.67E-10	1.22E-11	7.21	15.5456692	1.55E-01	33823690	33823813	NC_000017.10
cg06487194	1.67E-10	1.22E-11	7.21	15.5447825	1.53E-01	15345097	15345220	NC_000006.11
cg00688810	1.67E-10	1.22E-11	7.21	15.5432151	1.08E-01	7517138	7517261	NC_000017.10
cg04549679	1.68E-10	1.23E-11	7.21	15.541918	6.22E-02	3524425	3524548	NC_000001.10

cg14907310	1.68E-10	1.23E-11	7.21	15.5412367	1.09E-01	2287734	2287857	NC_000011.9
cg05477823	1.68E-10	1.23E-11	7.21	15.5410685	1.62E-01	59481085	59481208	NC_000017.10
cg09292354	1.68E-10	1.23E-11	7.21	15.539955	9.00E-02	134956328	134956451	NC_000010.10
cg22007163	1.68E-10	1.23E-11	7.21	15.5384288	1.83E-01	28602927	28603050	NC_000006.11
cg04255044	1.69E-10	1.24E-11	7.2	15.534131	1.58E-01	87415168	87415291	NC_000016.9
cg18295183	1.69E-10	1.24E-11	7.2	15.5321563	1.66E-01	161494711	161494834	NC_000005.9
cg24903887	1.69E-10	1.24E-11	7.2	15.5316681	1.95E-01	4580777	4580900	NC_000019.9
cg24594293	1.69E-10	1.24E-11	7.2	15.5308244	6.28E-02	156564330	156564453	NC_000001.10
cg05062333	1.70E-10	1.24E-11	7.2	15.5302785	1.90E-01	133562269	133562392	NC_000006.11
cg16462462	1.70E-10	1.24E-11	7.2	15.5300284	2.46E-01	117950825	117950948	NC_000008.10
cg06197024	1.70E-10	1.24E-11	7.2	15.5281346	5.84E-02	134940464	134940587	NC_000010.10
cg01250490	1.70E-10	1.24E-11	7.2	15.5280625	1.04E-01	12251718	12251841	NC_000001.10
cg09524508	1.70E-10	1.24E-11	7.2	15.5276985	1.33E-01	49202530	49202653	NC_000003.11
cg18122493	1.70E-10	1.24E-11	7.2	15.5274587	1.27E-01	149380909	149381032	NC_000001.10
cg25346915	1.70E-10	1.24E-11	7.2	15.5273684	2.12E-01	49230036	49230159	NC_000011.9
cg04163637	1.70E-10	1.25E-11	7.2	15.5267591	4.47E-02	203869223	203869346	NC_000001.10
cg03746851	1.70E-10	1.25E-11	7.2	15.5264195	1.27E-01	21282502	21282625	NC_000017.10
cg02023345	1.70E-10	1.25E-11	7.2	15.5259125	2.10E-01	63212226	63212349	NC_000010.10
cg21836370	1.70E-10	1.25E-11	7.2	15.5240684	1.48E-01	85667451	85667574	NC_000012.11
cg22797270	1.70E-10	1.25E-11	7.2	15.5240187	1.13E-01	64398015	64398138	NC_000011.9
cg18319612	1.71E-10	1.25E-11	7.2	15.5229696	1.50E-01	203257946	203258069	NC_000001.10
cg01245224	1.71E-10	1.25E-11	7.2	15.5217284	1.52E-01	101313190	101313313	NC_000011.9
cg00802855	1.71E-10	1.25E-11	7.2	15.5205253	1.04E-01	134911097	134911220	NC_000010.10
cg09603381	1.71E-10	1.25E-11	7.2	15.5203857	9.24E-02	49954066	49954189	NC_000010.10
cg23517116	1.71E-10	1.25E-11	7.2	15.5200961	1.11E-01	65499841	65499964	NC_000008.10

cg19742055	1.72E-10	1.26E-11	7.2	15.5169632	1.80E-01	78493365	78493488	NC_000013.10
cg23237276	1.72E-10	1.26E-11	7.2	15.5157431	1.08E-01	101827449	101827572	NC_000014.8
cg02384857	1.72E-10	1.26E-11	7.2	15.514424	2.20E-01	27197614	27197737	NC_000007.13
cg22676401	1.72E-10	1.26E-11	7.2	15.5141214	1.79E-01	21496507	21496630	NC_000020.10
cg17190608	1.72E-10	1.26E-11	7.2	15.5128096	1.77E-01	111471489	111471612	NC_000012.11
cg25182501	1.73E-10	1.27E-11	7.2	15.5103267	6.69E-02	74747143	74747266	NC_000002.11
cg21063758	1.73E-10	1.27E-11	7.2	15.5101655	1.55E-01	74422761	74422884	NC_000015.9
cg11099684	1.73E-10	1.27E-11	7.2	15.5075979	1.53E-01	26402889	26403012	NC_000004.11
cg13811168	1.73E-10	1.27E-11	7.2	15.5063264	6.38E-02	1581473	1581596	NC_000011.9
cg26465391	1.74E-10	1.27E-11	7.2	15.5051068	1.82E-01	58220773	58220896	NC_000019.9
cg04936274	1.74E-10	1.28E-11	7.2	15.5022422	8.79E-02	134672412	134672535	NC_000010.10
cg27244482	1.74E-10	1.28E-11	7.2	15.5019586	1.36E-01	149669488	149669611	NC_000005.9
cg04897900	1.75E-10	1.28E-11	7.2	15.4985168	1.26E-01	157245108	157245231	NC_000003.11
cg14522034	1.75E-10	1.29E-11	7.2	15.4948904	1.06E-01	46706759	46706882	NC_000021.8
cg03892356	1.76E-10	1.29E-11	7.2	15.4933216	2.30E-01	54420837	54420960	NC_000012.11
cg14861857	1.76E-10	1.29E-11	7.2	15.4932038	1.81E-02	24584179	24584302	NC_000022.10
cg12901917	1.76E-10	1.29E-11	7.2	15.4924461	8.99E-02	116196207	116196330	NC_000012.11
cg14955103	1.76E-10	1.29E-11	7.2	15.4916056	8.03E-02	31864106	31864229	NC_000006.11
cg24989181	1.76E-10	1.29E-11	7.2	15.489694	8.72E-02	30781821	30781944	NC_000020.10
cg13239420	1.76E-10	1.29E-11	7.2	15.4882823	2.19E-01	240161249	240161372	NC_000001.10
cg01790083	1.77E-10	1.30E-11	7.2	15.4875265	1.27E-01	33671181	33671304	NC_000021.8
cg16269488	1.77E-10	1.30E-11	7.2	15.4862456	5.67E-02	1858639	1858762	NC_000007.13
cg15425541	1.77E-10	1.30E-11	7.2	15.4857555	2.30E-01	61118751	61118874	NC_000014.8
cg04954559	1.77E-10	1.30E-11	7.2	15.4846329	2.03E-01	22365428	22365551	NC_000011.9
cg00692780	1.77E-10	1.30E-11	7.2	15.4842346	1.04E-01	222434778	222434901	NC_000002.11

cg07159023	1.78E-10	1.30E-11	7.2	15.4810259	1.39E-01	39615988	39616111	NC_000017.10
cg13862210	1.78E-10	1.31E-11	7.19	15.4800666	1.08E-02	11796076	11796199	NC_000001.10
cg25560333	1.78E-10	1.31E-11	7.19	15.4787307	1.65E-01	11060104	11060227	NC_000010.10
cg03940525	1.78E-10	1.31E-11	7.19	15.477004	4.48E-02	61698089	61698212	NC_000005.9
cg16589299	1.79E-10	1.31E-11	7.19	15.4754055	1.80E-01	131758786	131758909	NC_000010.10
cg08675717	1.79E-10	1.31E-11	7.19	15.4753647	2.35E-01	58180241	58180364	NC_000020.10
cg24818566	1.79E-10	1.31E-11	7.19	15.4749097	9.51E-02	122132054	122132177	NC_000009.11
cg11148130	1.79E-10	1.31E-11	7.19	15.4734091	1.81E-01	73674068	73674191	NC_000003.11
cg25705519	1.79E-10	1.32E-11	7.19	15.4728479	1.31E-01	159557552	159557675	NC_000003.11
cg01749213	1.79E-10	1.32E-11	7.19	15.4724492	8.99E-02	219906749	219906872	NC_000002.11
cg16535144	1.79E-10	1.32E-11	7.19	15.4721506	2.07E-01	155259524	155259647	NC_000007.13
cg18312113	1.79E-10	1.32E-11	7.19	15.4713987	7.10E-02	1487538	1487661	NC_000001.10
cg19610905	1.80E-10	1.32E-11	7.19	15.4689498	1.95E-01	61596333	61596456	NC_000011.9
cg20562147	1.80E-10	1.32E-11	7.19	15.4679831	1.87E-01	143086459	143086582	NC_000008.10
cg22979783	1.80E-10	1.32E-11	7.19	15.4668852	1.34E-01	72371261	72371384	NC_000002.11
cg01525017	1.80E-10	1.32E-11	7.19	15.4667742	1.12E-01	181182210	181182333	NC_000003.11
cg27549878	1.80E-10	1.32E-11	7.19	15.4660806	1.69E-01	38886554	38886677	NC_000019.9
cg08996579	1.80E-10	1.33E-11	7.19	15.4652988	2.33E-01	50817804	50817927	NC_000010.10
cg07483462	1.80E-10	1.33E-11	7.19	15.4649561	1.12E-01	104928289	104928412	NC_000014.8
cg16772035	1.80E-10	1.33E-11	7.19	15.4647058	1.38E-01	87229880	87230003	NC_000007.13
cg00219282	1.80E-10	1.33E-11	7.19	15.4643444	1.26E-01	159654898	159655021	NC_000006.11
cg16968596	1.81E-10	1.33E-11	7.19	15.4636002	2.67E-01	89346629	89346752	NC_000015.9
cg00078259	1.81E-10	1.33E-11	7.19	15.4635954	4.75E-02	2656232	2656355	NC_000006.11
cg11657203	1.81E-10	1.33E-11	7.19	15.4613772	1.70E-01	41087101	41087224	NC_000012.11
cg10085824	1.81E-10	1.33E-11	7.19	15.4606418	5.66E-02	71800315	71800438	NC_000016.9

cg13019143	1.81E-10	1.33E-11	7.19	15.4590381	1.44E-01	96633060	96633183	NC_000007.13
cg15148691	1.82E-10	1.34E-11	7.19	15.4572995	8.05E-02	29404733	29404856	NC_000006.11
cg04657000	1.82E-10	1.34E-11	7.19	15.4567905	6.73E-02	112115430	112115553	NC_000006.11
cg08260406	1.82E-10	1.34E-11	7.19	15.4554037	1.54E-01	248100407	248100530	NC_000001.10
cg04798919	1.82E-10	1.34E-11	7.19	15.4546654	9.90E-02	44039475	44039598	NC_000019.9
cg08644463	1.83E-10	1.34E-11	7.19	15.4521468	1.09E-01	110106962	110107085	NC_000001.10
cg23066159	1.83E-10	1.34E-11	7.19	15.4519564	1.18E-01	51481472	51481595	NC_000019.9
cg05896193	1.83E-10	1.35E-11	7.19	15.449319	1.08E-01	25563141	25563264	NC_000002.11
cg05492075	1.83E-10	1.35E-11	7.19	15.4478107	6.50E-02	12881563	12881686	NC_000010.10
cg17514558	1.83E-10	1.35E-11	7.19	15.4474339	1.59E-01	140621375	140621498	NC_000005.9
cg23300372	1.84E-10	1.35E-11	7.19	15.4465229	1.89E-01	71015432	71015555	NC_000005.9
cg12149299	1.84E-10	1.36E-11	7.19	15.4433203	1.30E-01	96762309	96762432	NC_000012.11
cg11980129	1.85E-10	1.36E-11	7.19	15.4399641	1.75E-01	79815639	79815762	NC_000003.11
cg25090499	1.85E-10	1.36E-11	7.19	15.4396367	1.85E-01	26043165	26043288	NC_000013.10
cg13559439	1.85E-10	1.36E-11	7.19	15.4393276	1.55E-01	12989237	12989360	NC_000008.10
cg20732160	1.85E-10	1.36E-11	7.19	15.4386376	1.17E-01	48590040	48590163	NC_000003.11
cg08597067	1.85E-10	1.36E-11	7.19	15.4383624	1.01E-01	53147641	53147764	NC_000006.11
cg01038597	1.86E-10	1.37E-11	7.19	15.4355811	1.10E-01	31120649	31120772	NC_000016.9
cg01519508	1.86E-10	1.37E-11	7.19	15.4344318	1.15E-01	86411222	86411345	NC_000016.9
cg22982368	1.86E-10	1.37E-11	7.19	15.4339333	1.71E-01	31825969	31826092	NC_000011.9
cg08610862	1.86E-10	1.37E-11	7.19	15.4335416	1.83E-01	197888469	197888592	NC_000001.10
cg07396817	1.86E-10	1.37E-11	7.19	15.4315151	1.28E-01	185453355	185453478	NC_000003.11
cg11555666	1.87E-10	1.38E-11	7.19	15.4292164	8.64E-02	78172397	78172520	NC_000017.10
cg04645150	1.87E-10	1.38E-11	7.19	15.4283326	1.71E-01	28584172	28584295	NC_000006.11
cg10606059	1.87E-10	1.38E-11	7.19	15.4283311	1.55E-01	72987838	72987961	NC_000008.10

cg10370599	1.87E-10	1.38E-11	7.19	15.427353	1.37E-01	22825158	22825281	NC_000016.9
cg18274510	1.87E-10	1.38E-11	7.19	15.427001	1.49E-01	69736743	69736866	NC_000010.10
cg06829830	1.87E-10	1.38E-11	7.19	15.4260693	1.97E-01	80256087	80256210	NC_000005.9
cg24184636	1.87E-10	1.38E-11	7.19	15.4254757	1.64E-01	25906789	25906912	NC_000008.10
cg13318256	1.87E-10	1.38E-11	7.19	15.4254264	1.61E-01	67885605	67885728	NC_000011.9
cg09561125	1.88E-10	1.38E-11	7.19	15.423824	1.42E-01	108097477	108097600	NC_000007.13
cg08361684	1.88E-10	1.38E-11	7.19	15.4236737	2.21E-01	35641255	35641378	NC_000011.9
cg09400566	1.88E-10	1.38E-11	7.18	15.4232343	1.44E-01	1271371	1271494	NC_000019.9
cg13000912	1.88E-10	1.38E-11	7.18	15.4225735	1.83E-01	100056060	100056183	NC_000006.11
cg04001802	1.88E-10	1.39E-11	7.18	15.4221072	1.28E-01	15085279	15085402	NC_000010.10
cg07213060	1.88E-10	1.39E-11	7.18	15.4215238	1.85E-01	21694827	21694950	NC_000020.10
cg13586599	1.88E-10	1.39E-11	7.18	15.4207669	1.65E-01	25652383	25652506	NC_000006.11
cg22053159	1.88E-10	1.39E-11	7.18	15.420052	1.38E-01	2478543	2478666	NC_000006.11
cg24592972	1.89E-10	1.39E-11	7.18	15.4154077	4.96E-02	134828571	134828694	NC_000010.10
cg02589791	1.89E-10	1.39E-11	7.18	15.415131	1.42E-01	63955666	63955789	NC_000007.13
cg04191300	1.89E-10	1.40E-11	7.18	15.4147777	1.34E-01	128729652	128729775	NC_000011.9
cg13789236	1.89E-10	1.40E-11	7.18	15.4135745	3.64E-02	79009433	79009556	NC_000009.11
cg14275836	1.90E-10	1.40E-11	7.18	15.4131268	4.19E-02	2941313	2941436	NC_000011.9
cg18355533	1.90E-10	1.40E-11	7.18	15.4123695	6.35E-02	3961679	3961802	NC_000019.9
cg06556929	1.90E-10	1.40E-11	7.18	15.4118151	7.45E-02	142368169	142368292	NC_000008.10
cg07095836	1.90E-10	1.40E-11	7.18	15.4090932	6.24E-02	62574925	62575048	NC_000007.13
cg09802627	1.91E-10	1.41E-11	7.18	15.4075519	5.69E-02	228613207	228613330	NC_000001.10
cg27119877	1.91E-10	1.41E-11	7.18	15.406919	5.24E-02	30122036	30122159	NC_000006.11
cg00709966	1.91E-10	1.41E-11	7.18	15.4053593	1.11E-01	27253143	27253266	NC_000007.13
cg27020216	1.91E-10	1.41E-11	7.18	15.4048	1.49E-01	153840347	153840470	NC_000003.11

cg26332310	1.91E-10	1.41E-11	7.18	15.4036452	1.83E-01	91182305	91182428	NC_000001.10
cg24718866	1.91E-10	1.41E-11	7.18	15.402712	1.85E-01	25391274	25391397	NC_000002.11
cg05021643	1.91E-10	1.41E-11	7.18	15.4024018	1.11E-01	177029608	177029731	NC_000002.11
cg18644235	1.92E-10	1.41E-11	7.18	15.4015619	1.02E-01	57352746	57352869	NC_000019.9
cg02335141	1.92E-10	1.41E-11	7.18	15.4010783	5.59E-02	167551083	167551206	NC_000006.11
cg22557662	1.92E-10	1.42E-11	7.18	15.4004896	2.14E-01	38747374	38747497	NC_000019.9
cg00060715	1.92E-10	1.42E-11	7.18	15.3997187	5.33E-02	214779657	214779780	NC_000001.10
cg06424097	1.92E-10	1.42E-11	7.18	15.3993772	1.52E-01	122848244	122848367	NC_000011.9
cg07104209	1.92E-10	1.42E-11	7.18	15.3979789	1.72E-01	27281872	27281995	NC_000007.13
cg22167065	1.92E-10	1.42E-11	7.18	15.3979142	9.66E-02	61814422	61814545	NC_000020.10
cg12276203	1.92E-10	1.42E-11	7.18	15.3968539	1.03E-01	46917565	46917688	NC_000021.8
cg12635937	1.93E-10	1.42E-11	7.18	15.3958605	1.08E-01	81184925	81185048	NC_000004.11
cg06480138	1.93E-10	1.43E-11	7.18	15.3938696	8.18E-02	91477148	91477271	NC_000015.9
cg23316599	1.93E-10	1.43E-11	7.18	15.3935288	1.04E-01	57271379	57271502	NC_000007.13
cg01904169	1.93E-10	1.43E-11	7.18	15.3925357	6.43E-02	27884654	27884777	NC_000002.11
cg26296364	1.93E-10	1.43E-11	7.18	15.3922893	1.62E-01	63212206	63212329	NC_000010.10
cg15116918	1.94E-10	1.43E-11	7.18	15.3909141	1.47E-01	78669552	78669675	NC_000017.10
cg21982455	1.94E-10	1.43E-11	7.18	15.3894247	1.65E-01	2757976	2758099	NC_000005.9
cg21813063	1.94E-10	1.44E-11	7.18	15.3866633	5.59E-02	10264192	10264315	NC_000012.11
cg13962212	1.95E-10	1.44E-11	7.18	15.3848815	1.55E-01	30325907	30326030	NC_000006.11
cg24329658	1.95E-10	1.44E-11	7.18	15.3845048	4.45E-02	30122023	30122146	NC_000006.11
cg16370491	1.95E-10	1.44E-11	7.18	15.3844525	2.02E-01	75601928	75602051	NC_000012.11
cg24341611	1.95E-10	1.44E-11	7.18	15.3840522	2.14E-01	50235279	50235402	NC_000017.10
cg08552214	1.95E-10	1.44E-11	7.18	15.3829863	1.81E-01	176931864	176931987	NC_000002.11
cg20831708	1.95E-10	1.44E-11	7.18	15.3828877	1.61E-01	102279373	102279496	NC_000010.10

cg02548290	1.95E-10	1.44E-11	7.18	15.3825548	4.64E-02	25126153	25126276	NC_000016.9
cg06518233	1.95E-10	1.44E-11	7.18	15.381297	2.91E-02	102765350	102765473	NC_000010.10
cg12613383	1.96E-10	1.45E-11	7.18	15.3800104	1.63E-01	242688681	242688804	NC_000001.10
cg20116099	1.96E-10	1.45E-11	7.18	15.3775747	1.02E-01	25416204	25416327	NC_000017.10
cg10130718	1.96E-10	1.45E-11	7.18	15.3774328	7.33E-02	53925368	53925491	NC_000001.10
cg04616218	1.96E-10	1.45E-11	7.18	15.3764672	4.85E-02	29789904	29790027	NC_000016.9
cg22489153	1.96E-10	1.45E-11	7.18	15.37636	1.55E-01	115136175	115136298	NC_000012.11
cg10866298	1.96E-10	1.45E-11	7.18	15.3756188	2.11E-01	9760869	9760992	NC_000008.10
cg25008858	1.96E-10	1.45E-11	7.18	15.3754116	1.09E-01	220313657	220313780	NC_000002.11
cg24304249	1.96E-10	1.45E-11	7.18	15.3748899	1.56E-01	181455181	181455304	NC_000001.10
cg09052626	1.97E-10	1.45E-11	7.18	15.3745567	1.56E-01	86603583	86603706	NC_000016.9
cg26239391	1.97E-10	1.46E-11	7.18	15.373616	1.97E-01	8869285	8869408	NC_000017.10
cg16198450	1.97E-10	1.46E-11	7.18	15.3727419	6.30E-02	159655853	159655976	NC_000005.9
cg10156366	1.97E-10	1.46E-11	7.18	15.3727379	2.26E-01	79220381	79220504	NC_000002.11
cg24562814	1.97E-10	1.46E-11	7.18	15.3723845	9.00E-02	26375698	26375821	NC_000008.10
cg18963171	1.97E-10	1.46E-11	7.18	15.3718549	1.01E-01	43442940	43443063	NC_000021.8
cg01311170	1.97E-10	1.46E-11	7.18	15.3712562	7.90E-02	39169407	39169530	NC_000017.10
cg10453420	1.97E-10	1.46E-11	7.18	15.3700405	1.79E-01	3874006	3874129	NC_000004.11
cg01187920	1.98E-10	1.46E-11	7.18	15.3683564	1.58E-01	71015162	71015285	NC_000005.9
cg01718116	1.98E-10	1.46E-11	7.18	15.3683058	2.18E-01	115919829	115919952	NC_000002.11
cg06235438	1.98E-10	1.46E-11	7.18	15.3675664	8.38E-02	30485966	30486089	NC_000016.9
cg23189410	1.98E-10	1.46E-11	7.18	15.3669668	1.95E-01	147125712	147125835	NC_000003.11
cg25670060	1.98E-10	1.47E-11	7.17	15.3659342	2.51E-01	98891544	98891667	NC_000011.9
cg24314877	1.98E-10	1.47E-11	7.17	15.3655549	8.82E-02	128656055	128656178	NC_000008.10
cg21251140	1.98E-10	1.47E-11	7.17	15.3644267	8.69E-02	181544642	181544765	NC_000002.11

cg26108017	1.98E-10	1.47E-11	7.17	15.3641097	7.01E-02	97548061	97548184	NC_000002.11
cg09466667	1.98E-10	1.47E-11	7.17	15.364054	5.87E-02	42140775	42140898	NC_000003.11
cg01886514	1.98E-10	1.47E-11	7.17	15.3638862	1.29E-01	105478395	105478518	NC_000002.11
cg21215767	1.98E-10	1.47E-11	7.17	15.3638006	1.55E-01	119599819	119599942	NC_000002.11
cg09675604	1.99E-10	1.48E-11	7.17	15.3597926	1.22E-01	107591096	107591219	NC_000007.13
cg04441477	1.99E-10	1.48E-11	7.17	15.3592608	2.11E-01	24267399	24267522	NC_000016.9
cg09473180	2.00E-10	1.48E-11	7.17	15.3583914	9.64E-02	217403190	217403313	NC_000002.11
cg15665203	2.00E-10	1.48E-11	7.17	15.3579789	1.14E-01	112282481	112282604	NC_000013.10
cg20268522	2.00E-10	1.48E-11	7.17	15.3571583	1.94E-01	112832152	112832275	NC_000011.9
cg01596963	2.00E-10	1.48E-11	7.17	15.3569366	1.06E-01	62455830	62455953	NC_000011.9
cg16081457	2.00E-10	1.48E-11	7.17	15.3551205	1.13E-01	81103680	81103803	NC_000012.11
cg18341512	2.00E-10	1.48E-11	7.17	15.3547618	1.43E-01	2757023	2757146	NC_000005.9
cg05050657	2.02E-10	1.49E-11	7.17	15.3479079	1.71E-01	46957105	46957228	NC_000001.10
cg24462247	2.02E-10	1.50E-11	7.17	15.3460758	9.31E-02	1770837	1770960	NC_000004.11
cg13580382	2.02E-10	1.50E-11	7.17	15.3448146	1.22E-01	140081032	140081155	NC_000009.11
cg19875375	2.02E-10	1.50E-11	7.17	15.3442596	1.34E-01	46740666	46740789	NC_000011.9
cg20442232	2.02E-10	1.50E-11	7.17	15.3438092	1.10E-01	134465552	134465675	NC_000005.9
cg21133251	2.03E-10	1.50E-11	7.17	15.3426374	2.94E-02	242566890	242567013	NC_000002.11
cg01105375	2.03E-10	1.50E-11	7.17	15.3424362	7.88E-02	20748025	20748148	NC_000013.10
cg02337614	2.03E-10	1.50E-11	7.17	15.3407348	1.29E-01	112609986	112610109	NC_000013.10
cg13224075	2.03E-10	1.51E-11	7.17	15.3392357	3.02E-02	30107081	30107204	NC_000013.10
cg13097816	2.03E-10	1.51E-11	7.17	15.3389057	8.83E-02	241568439	241568562	NC_000002.11
cg16154629	2.03E-10	1.51E-11	7.17	15.3385165	4.71E-02	15125907	15126030	NC_000012.11
cg17949813	2.04E-10	1.51E-11	7.17	15.3371887	2.03E-01	1801521	1801644	NC_000007.13
cg20366906	2.04E-10	1.51E-11	7.17	15.3357727	1.15E-01	53422381	53422504	NC_000013.10

cg25875953	2.04E-10	1.51E-11	7.17	15.3350878	4.09E-02	3001128	3001251	NC_000001.10
cg00303989	2.04E-10	1.51E-11	7.17	15.3348265	4.70E-02	8302769	8302892	NC_000010.10
cg10486439	2.04E-10	1.52E-11	7.17	15.3337326	5.29E-02	68919970	68920093	NC_000011.9
cg13256308	2.04E-10	1.52E-11	7.17	15.333513	7.60E-02	93106286	93106409	NC_000014.8
cg00574530	2.04E-10	1.52E-11	7.17	15.3325551	1.74E-01	134872064	134872187	NC_000005.9
cg02720618	2.05E-10	1.52E-11	7.17	15.3317342	1.70E-01	152129791	152129914	NC_000006.11
cg07709475	2.05E-10	1.52E-11	7.17	15.331293	7.67E-02	105423558	105423681	NC_000010.10
cg17130982	2.05E-10	1.52E-11	7.17	15.3311585	1.08E-01	1946008	1946131	NC_000001.10
cg12014886	2.05E-10	1.53E-11	7.17	15.3269528	4.98E-02	132890865	132890988	NC_000010.10
cg12069446	2.06E-10	1.53E-11	7.17	15.3263926	4.45E-02	118420562	118420685	NC_000001.10
cg06806958	2.06E-10	1.53E-11	7.17	15.3257425	2.04E-01	23983686	23983809	NC_000010.10
cg24679043	2.06E-10	1.53E-11	7.17	15.3257304	8.13E-02	30128404	30128527	NC_000006.11
cg25290307	2.06E-10	1.53E-11	7.17	15.3255828	1.27E-01	101005910	101006033	NC_000007.13
cg22300615	2.06E-10	1.53E-11	7.17	15.3245749	7.35E-02	57620819	57620942	NC_000020.10
cg10159630	2.06E-10	1.53E-11	7.17	15.3245724	1.82E-01	177037364	177037487	NC_000002.11
cg11572080	2.06E-10	1.53E-11	7.17	15.3224168	1.34E-01	111925158	111925281	NC_000006.11
cg03431738	2.07E-10	1.53E-11	7.17	15.3213871	1.05E-01	40031295	40031418	NC_000021.8
cg03077492	2.07E-10	1.54E-11	7.17	15.3203446	7.23E-02	43413095	43413218	NC_000005.9
cg25820062	2.07E-10	1.54E-11	7.17	15.3186126	9.89E-02	3730325	3730448	NC_000020.10
cg04495670	2.07E-10	1.54E-11	7.17	15.3171197	2.01E-01	69634296	69634419	NC_000011.9
cg15249639	2.07E-10	1.54E-11	7.17	15.3169764	2.19E-01	4382174	4382297	NC_000012.11
cg18154954	2.08E-10	1.54E-11	7.17	15.3156831	1.03E-01	39055500	39055623	NC_000019.9
cg11982577	2.08E-10	1.55E-11	7.17	15.3132546	6.74E-02	1299755	1299878	NC_000019.9
cg22385702	2.08E-10	1.55E-11	7.17	15.3132176	1.25E-01	45175881	45176004	NC_000002.11
cg12662072	2.08E-10	1.55E-11	7.17	15.3126041	2.56E-01	63986793	63986916	NC_000005.9

cg02869459	2.09E-10	1.55E-11	7.16	15.309007	2.28E-01	220349208	220349331	NC_000002.11
cg20374595	2.09E-10	1.55E-11	7.16	15.3087467	1.33E-01	100627956	100628079	NC_000013.10
cg00891278	2.09E-10	1.56E-11	7.16	15.3069115	1.60E-01	126113784	126113907	NC_000003.11
cg20568196	2.09E-10	1.56E-11	7.16	15.3067907	2.58E-01	45811015	45811138	NC_000017.10
cg18602919	2.10E-10	1.56E-11	7.16	15.3062622	7.30E-02	23931674	23931797	NC_000015.9
cg06381463	2.10E-10	1.56E-11	7.16	15.3056801	3.80E-02	129100506	129100629	NC_000009.11
cg11473876	2.10E-10	1.56E-11	7.16	15.3046912	2.02E-01	109292803	109292926	NC_000011.9
cg13198321	2.10E-10	1.56E-11	7.16	15.3032635	2.18E-01	58357891	58358014	NC_000015.9
cg08286196	2.11E-10	1.57E-11	7.16	15.3005647	1.45E-01	114847882	114848005	NC_000013.10
cg16521605	2.11E-10	1.57E-11	7.16	15.2998889	1.39E-01	46363340	46363463	NC_000019.9
cg19590445	2.11E-10	1.57E-11	7.16	15.2998258	7.14E-02	174155345	174155468	NC_000005.9
cg04200411	2.11E-10	1.57E-11	7.16	15.2971402	4.52E-02	80449551	80449674	NC_000017.10
cg24805875	2.11E-10	1.57E-11	7.16	15.2968013	2.10E-01	117086941	117087064	NC_000006.11
cg06449094	2.11E-10	1.57E-11	7.16	15.2964388	1.08E-01	822397	822520	NC_000011.9
cg23520119	2.12E-10	1.57E-11	7.16	15.2958236	1.04E-01	121249868	121249991	NC_000010.10
cg14803869	2.12E-10	1.58E-11	7.16	15.2945153	1.26E-01	131237682	131237805	NC_000012.11
cg06970510	2.12E-10	1.58E-11	7.16	15.293173	1.70E-01	52401448	52401571	NC_000012.11
cg15908627	2.12E-10	1.58E-11	7.16	15.2931175	1.13E-01	1007420	1007543	NC_000011.9
cg08528231	2.12E-10	1.58E-11	7.16	15.2929354	1.88E-01	12997261	12997384	NC_000016.9
cg16297171	2.12E-10	1.58E-11	7.16	15.2918624	1.26E-01	99017690	99017813	NC_000008.10
cg15063116	2.13E-10	1.58E-11	7.16	15.2911272	1.71E-01	12254365	12254488	NC_000018.9
cg13954022	2.13E-10	1.59E-11	7.16	15.2881206	4.61E-02	106825650	106825773	NC_000013.10
cg23814714	2.13E-10	1.59E-11	7.16	15.2871498	7.28E-02	32056735	32056858	NC_000006.11
cg07405182	2.14E-10	1.59E-11	7.16	15.2861997	1.61E-01	40598455	40598578	NC_000001.10
cg26802289	2.14E-10	1.59E-11	7.16	15.28568	2.58E-01	20817859	20817982	NC_000007.13

cg14520944	2.14E-10	1.59E-11	7.16	15.2846251	5.85E-02	169256780	169256903	NC_000006.11
cg17754769	2.14E-10	1.59E-11	7.16	15.284295	9.43E-02	50735417	50735540	NC_000022.10
cg00757082	2.14E-10	1.60E-11	7.16	15.2828586	2.26E-01	30719149	30719272	NC_000019.9
cg05621401	2.15E-10	1.60E-11	7.16	15.2812886	2.73E-01	43572065	43572188	NC_000010.10
cg13822256	2.15E-10	1.60E-11	7.16	15.2804036	1.08E-01	24811266	24811389	NC_000008.10
cg03634479	2.15E-10	1.60E-11	7.16	15.2801922	1.44E-01	18430412	18430535	NC_000010.10
cg17200320	2.15E-10	1.60E-11	7.16	15.2800377	4.72E-02	176931422	176931545	NC_000005.9
cg26157451	2.15E-10	1.60E-11	7.16	15.2782223	6.88E-02	89547102	89547225	NC_000015.9
cg01678383	2.15E-10	1.60E-11	7.16	15.2774866	5.71E-02	187903164	187903287	NC_000003.11
cg27434245	2.15E-10	1.61E-11	7.16	15.2767025	2.64E-01	89007493	89007616	NC_000016.9
cg20337815	2.16E-10	1.61E-11	7.16	15.2762004	7.41E-02	8736232	8736355	NC_000002.11
cg02792168	2.16E-10	1.61E-11	7.16	15.2744744	1.61E-01	101281924	101282047	NC_000010.10
cg23551455	2.16E-10	1.61E-11	7.16	15.273838	4.21E-02	80977010	80977133	NC_000017.10
cg13512069	2.16E-10	1.61E-11	7.16	15.2735797	1.36E-01	82555113	82555236	NC_000015.9
cg08041350	2.16E-10	1.61E-11	7.16	15.2731254	1.17E-01	25819112	25819235	NC_000017.10
cg21384645	2.16E-10	1.61E-11	7.16	15.2726002	1.57E-01	176957304	176957427	NC_000002.11
cg02979203	2.17E-10	1.61E-11	7.16	15.2715688	1.13E-01	118365961	118366084	NC_000010.10
cg09271882	2.17E-10	1.62E-11	7.16	15.2706463	4.85E-02	6214849	6214972	NC_000001.10
cg10474350	2.17E-10	1.62E-11	7.16	15.2704791	2.02E-01	27196555	27196678	NC_000007.13
cg09835239	2.17E-10	1.62E-11	7.16	15.267888	2.18E-01	66188203	66188326	NC_000011.9
cg04103250	2.18E-10	1.62E-11	7.16	15.2667782	1.48E-01	29840320	29840443	NC_000010.10
cg22851295	2.18E-10	1.62E-11	7.16	15.2667077	1.58E-01	10881949	10882072	NC_000006.11
cg21022447	2.18E-10	1.62E-11	7.16	15.2666641	3.88E-02	30521181	30521304	NC_000006.11
cg23148701	2.18E-10	1.62E-11	7.16	15.2659403	1.89E-01	64062498	64062621	NC_000012.11
cg01781662	2.18E-10	1.62E-11	7.16	15.2657164	1.42E-01	132387805	132387928	NC_000009.11

cg26744375	2.18E-10	1.62E-11	7.16	15.2652255	2.37E-01	27135723	27135846	NC_000007.13
cg07145988	2.18E-10	1.63E-11	7.16	15.2643827	7.94E-02	8692312	8692435	NC_000001.10
cg13496492	2.18E-10	1.63E-11	7.16	15.2635396	7.65E-02	132463186	132463309	NC_000009.11
cg15309953	2.19E-10	1.63E-11	7.16	15.2593356	1.47E-01	5813258	5813381	NC_000002.11
cg02166450	2.19E-10	1.64E-11	7.16	15.2581007	1.86E-01	112712795	112712918	NC_000013.10
cg00632188	2.19E-10	1.64E-11	7.16	15.2577578	4.82E-02	31730389	31730512	NC_000006.11
cg06043908	2.19E-10	1.64E-11	7.16	15.2572355	1.04E-01	49467831	49467954	NC_000008.10
cg26398921	2.20E-10	1.64E-11	7.16	15.2567281	1.63E-01	221065480	221065603	NC_000001.10
cg04844692	2.20E-10	1.64E-11	7.16	15.2566464	6.41E-02	117157450	117157573	NC_000012.11
cg21806832	2.20E-10	1.64E-11	7.16	15.2562571	5.26E-02	21262831	21262954	NC_000017.10
cg08814405	2.20E-10	1.64E-11	7.16	15.2553296	2.22E-01	19183753	19183876	NC_000007.13
cg25391162	2.20E-10	1.64E-11	7.16	15.254238	1.01E-01	118091	118214	NC_000009.11
cg20156659	2.20E-10	1.64E-11	7.16	15.254124	1.64E-01	48982832	48982955	NC_000002.11
cg07395354	2.20E-10	1.65E-11	7.15	15.2524252	1.98E-01	15727127	15727250	NC_000007.13
cg26866482	2.21E-10	1.65E-11	7.15	15.2519862	2.54E-01	103351443	103351566	NC_000012.11
cg05106058	2.21E-10	1.65E-11	7.15	15.2507845	1.03E-01	172735924	172736047	NC_000004.11
cg15685941	2.21E-10	1.65E-11	7.15	15.2492485	8.02E-02	52723104	52723227	NC_000003.11
cg15923139	2.21E-10	1.65E-11	7.15	15.2490366	1.57E-01	186801896	186802019	NC_000004.11
cg25738714	2.21E-10	1.65E-11	7.15	15.2484294	2.06E-01	50893658	50893781	NC_000001.10
cg20090194	2.22E-10	1.65E-11	7.15	15.2471157	8.44E-02	75401258	75401381	NC_000010.10
cg12274508	2.22E-10	1.66E-11	7.15	15.244599	6.48E-02	33170770	33170893	NC_000006.11
cg02706018	2.22E-10	1.66E-11	7.15	15.2444216	1.24E-01	27168780	27168903	NC_000007.13
cg21541833	2.22E-10	1.66E-11	7.15	15.2438214	2.17E-01	207507096	207507219	NC_000002.11
cg06553975	2.22E-10	1.66E-11	7.15	15.2432872	6.37E-02	133085262	133085385	NC_000012.11
cg06721255	2.23E-10	1.66E-11	7.15	15.2423902	1.99E-01	114878877	114879000	NC_000012.11

cg12523924	2.23E-10	1.66E-11	7.15	15.2412209	1.81E-01	63255359	63255482	NC_000005.9
cg05959980	2.23E-10	1.67E-11	7.15	15.2409521	8.29E-02	182083019	182083142	NC_000001.10
cg25796050	2.23E-10	1.67E-11	7.15	15.2407543	1.24E-01	234621572	234621695	NC_000002.11
cg02240936	2.23E-10	1.67E-11	7.15	15.2401202	7.05E-02	134584660	134584783	NC_000010.10
cg21182616	2.23E-10	1.67E-11	7.15	15.2401096	5.21E-02	1363182	1363305	NC_000004.11
cg04047221	2.23E-10	1.67E-11	7.15	15.2385536	1.61E-01	112723477	112723600	NC_000013.10
cg09579081	2.23E-10	1.67E-11	7.15	15.2380304	7.03E-02	81109237	81109360	NC_000015.9
cg13763061	2.24E-10	1.67E-11	7.15	15.2373404	1.78E-01	84814656	84814779	NC_000007.13
cg06709756	2.24E-10	1.67E-11	7.15	15.2362335	1.65E-01	1339049	1339172	NC_000008.10
cg12601909	2.24E-10	1.67E-11	7.15	15.235823	1.46E-01	1324478	1324601	NC_000011.9
cg10827650	2.25E-10	1.68E-11	7.15	15.2327907	5.24E-02	139511	139634	NC_000017.10
cg27665860	2.25E-10	1.68E-11	7.15	15.232535	5.92E-02	68970673	68970796	NC_000011.9
cg19002907	2.25E-10	1.68E-11	7.15	15.2314633	1.22E-01	115939579	115939702	NC_000010.10
cg20703261	2.25E-10	1.68E-11	7.15	15.2310128	4.85E-02	41375153	41375276	NC_000006.11
cg22967542	2.25E-10	1.68E-11	7.15	15.2294531	1.32E-01	96647483	96647606	NC_000007.13
cg05941819	2.25E-10	1.69E-11	7.15	15.2291006	2.03E-02	32568405	32568528	NC_000001.10
cg01145054	2.25E-10	1.69E-11	7.15	15.2289536	2.20E-01	47063285	47063408	NC_000021.8
cg14037937	2.26E-10	1.69E-11	7.15	15.2275126	1.06E-01	7987530	7987653	NC_000004.11
cg24286765	2.26E-10	1.69E-11	7.15	15.2267798	1.65E-01	137361532	137361655	NC_000003.11
cg10908752	2.26E-10	1.69E-11	7.15	15.2252665	5.68E-02	98044144	98044267	NC_000013.10
cg08399444	2.26E-10	1.69E-11	7.15	15.2247532	1.25E-01	13248548	13248671	NC_000012.11
cg05036656	2.26E-10	1.69E-11	7.15	15.2240535	1.63E-01	41875470	41875593	NC_000004.11
cg24133115	2.27E-10	1.70E-11	7.15	15.222806	2.14E-01	166075520	166075643	NC_000006.11
cg16091162	2.27E-10	1.70E-11	7.15	15.2193337	3.53E-02	71850401	71850524	NC_000004.11
cg27397850	2.28E-10	1.70E-11	7.15	15.2188922	1.84E-01	27281941	27282064	NC_000007.13

cg24659538	2.28E-10	1.71E-11	7.15	15.21593	3.00E-02	160222618	160222741	NC_000001.10
cg05164933	2.29E-10	1.71E-11	7.15	15.2140179	1.95E-01	154710418	154710541	NC_000004.11
cg03301200	2.29E-10	1.72E-11	7.15	15.210773	2.05E-01	99954557	99954680	NC_000008.10
cg26186239	2.30E-10	1.72E-11	7.15	15.2095711	1.34E-01	62119492	62119615	NC_000020.10
cg23933044	2.30E-10	1.72E-11	7.15	15.208992	1.36E-01	37249909	37250032	NC_000005.9
cg25153629	2.30E-10	1.72E-11	7.15	15.2072707	8.14E-02	3135375	3135498	NC_000001.10
cg05099387	2.30E-10	1.73E-11	7.15	15.2058465	2.19E-01	177053292	177053415	NC_000002.11
cg06539116	2.31E-10	1.73E-11	7.15	15.2054687	1.32E-01	148597365	148597488	NC_000005.9
cg12388007	2.31E-10	1.73E-11	7.15	15.204934	1.56E-01	147110499	147110622	NC_000003.11
cg26467918	2.31E-10	1.73E-11	7.15	15.2048391	7.99E-02	63932088	63932211	NC_000011.9
cg13291704	2.31E-10	1.73E-11	7.15	15.2047801	1.84E-01	15761498	15761621	NC_000010.10
cg04921989	2.31E-10	1.73E-11	7.15	15.2039915	2.26E-01	132183100	132183223	NC_000002.11
cg12818107	2.31E-10	1.73E-11	7.15	15.2035334	1.25E-01	142852265	142852388	NC_000008.10
cg11388320	2.31E-10	1.73E-11	7.15	15.2028962	8.97E-02	81119299	81119422	NC_000004.11
cg24033224	2.31E-10	1.73E-11	7.15	15.2020818	1.30E-01	131438016	131438139	NC_000012.11
cg06738242	2.31E-10	1.73E-11	7.15	15.2017949	1.78E-01	239755703	239755826	NC_000002.11
cg14329039	2.31E-10	1.73E-11	7.15	15.2011535	3.12E-02	3058109	3058232	NC_000010.10
cg24116870	2.32E-10	1.73E-11	7.15	15.2008518	1.82E-01	43946284	43946407	NC_000012.11
cg12454814	2.32E-10	1.73E-11	7.15	15.200846	1.60E-01	58332480	58332603	NC_000014.8
cg02115911	2.32E-10	1.73E-11	7.15	15.200815	2.11E-01	34395548	34395671	NC_000021.8
cg20295353	2.32E-10	1.74E-11	7.15	15.2003994	7.59E-02	64989846	64989969	NC_000014.8
cg14051662	2.33E-10	1.74E-11	7.14	15.1950231	1.64E-01	137279180	137279303	NC_000009.11
cg13544883	2.33E-10	1.75E-11	7.14	15.1947273	9.72E-02	7852051	7852174	NC_000019.9
cg15888290	2.33E-10	1.75E-11	7.14	15.1929652	1.78E-01	119600348	119600471	NC_000002.11
cg10851698	2.34E-10	1.75E-11	7.14	15.1910491	1.67E-01	54024744	54024867	NC_000019.9

cg13939185	2.34E-10	1.75E-11	7.14	15.1908675	1.34E-01	77116084	77116207	NC_000009.11
cg04765483	2.34E-10	1.75E-11	7.14	15.1897717	1.30E-01	134288552	134288675	NC_000011.9
cg20643952	2.34E-10	1.75E-11	7.14	15.1893289	2.27E-01	54398809	54398932	NC_000012.11
cg01037683	2.34E-10	1.76E-11	7.14	15.1882736	7.94E-02	20056493	20056616	NC_000017.10
cg03196627	2.35E-10	1.76E-11	7.14	15.1873342	3.40E-02	79298606	79298729	NC_000015.9
cg23151024	2.35E-10	1.76E-11	7.14	15.1867656	2.01E-01	84815940	84816063	NC_000007.13
cg23259813	2.35E-10	1.76E-11	7.14	15.186374	5.51E-02	18572503	18572626	NC_000019.9
cg05336698	2.35E-10	1.76E-11	7.14	15.1860526	1.96E-01	102497298	102497421	NC_000010.10
cg11662340	2.35E-10	1.76E-11	7.14	15.1855228	8.53E-02	32166839	32166962	NC_000006.11
cg21195256	2.35E-10	1.76E-11	7.14	15.1838911	1.85E-01	29244278	29244401	NC_000014.8
cg24500832	2.35E-10	1.76E-11	7.14	15.1838102	2.01E-01	54394212	54394335	NC_000012.11
cg21653184	2.36E-10	1.77E-11	7.14	15.1828741	2.56E-01	58609361	58609484	NC_000019.9
cg11708721	2.36E-10	1.77E-11	7.14	15.1816563	1.28E-01	117530009	117530132	NC_000001.10
cg17583413	2.36E-10	1.77E-11	7.14	15.1806783	1.67E-01	71332804	71332927	NC_000010.10
cg01565438	2.36E-10	1.77E-11	7.14	15.1799495	1.32E-01	33776554	33776677	NC_000017.10
cg00412805	2.36E-10	1.77E-11	7.14	15.1795328	4.28E-02	42726970	42727093	NC_000003.11
cg01325552	2.36E-10	1.77E-11	7.14	15.1786772	1.57E-01	187025420	187025543	NC_000004.11
cg03625287	2.37E-10	1.78E-11	7.14	15.1775424	1.72E-01	24121359	24121482	NC_000013.10
cg26687406	2.37E-10	1.78E-11	7.14	15.1773335	1.36E-01	169717976	169718099	NC_000006.11
cg14358699	2.37E-10	1.78E-11	7.14	15.1770101	8.67E-02	133521882	133522005	NC_000010.10
cg23526504	2.37E-10	1.78E-11	7.14	15.1769758	9.07E-02	29186780	29186903	NC_000010.10
cg02845071	2.37E-10	1.78E-11	7.14	15.1767763	5.10E-02	18571145	18571268	NC_000017.10
cg06263193	2.37E-10	1.78E-11	7.14	15.1753298	7.90E-02	28622912	28623035	NC_000018.9
cg23067299	2.37E-10	1.78E-11	7.14	15.1742457	1.13E-01	323907	324030	NC_000005.9
cg15799353	2.38E-10	1.79E-11	7.14	15.1719592	1.19E-01	363112	363235	NC_000020.10

cg09020927	2.38E-10	1.79E-11	7.14	15.1704008	4.59E-02	150058090	150058213	NC_000005.9
cg12383964	2.39E-10	1.79E-11	7.14	15.169217	4.16E-02	134681298	134681421	NC_000010.10
cg18984724	2.39E-10	1.79E-11	7.14	15.1679433	1.15E-01	220299604	220299727	NC_000002.11
cg18335068	2.39E-10	1.80E-11	7.14	15.166418	2.04E-01	53757910	53758033	NC_000019.9
cg15055817	2.40E-10	1.80E-11	7.14	15.1635492	2.08E-01	186079142	186079265	NC_000003.11
cg18431910	2.40E-10	1.80E-11	7.14	15.1624346	9.34E-02	78614239	78614362	NC_000011.9
cg25137711	2.41E-10	1.81E-11	7.14	15.1595784	1.38E-01	48059173	48059296	NC_000001.10
cg06993118	2.41E-10	1.81E-11	7.14	15.1595375	2.73E-01	127873980	127874103	NC_000005.9
cg18568534	2.41E-10	1.81E-11	7.14	15.1584747	1.40E-01	23240117	23240240	NC_000014.8
cg13056524	2.41E-10	1.81E-11	7.14	15.1575035	8.19E-02	15448614	15448737	NC_000006.11
cg13307782	2.41E-10	1.81E-11	7.14	15.156876	6.57E-02	77291146	77291269	NC_000005.9
cg02388319	2.41E-10	1.81E-11	7.14	15.1566454	1.15E-01	155019321	155019444	NC_000007.13
cg24033278	2.41E-10	1.81E-11	7.14	15.1564984	4.51E-02	41510272	41510395	NC_000019.9
cg11932225	2.42E-10	1.82E-11	7.14	15.1523988	2.98E-02	7802347	7802470	NC_000004.11
cg19788371	2.42E-10	1.82E-11	7.14	15.1519592	1.63E-01	143086298	143086421	NC_000008.10
cg06444755	2.43E-10	1.82E-11	7.14	15.1511819	1.57E-01	8473990	8474113	NC_000007.13
cg18302637	2.43E-10	1.83E-11	7.14	15.1503501	6.34E-02	1254777	1254900	NC_000005.9
cg08501735	2.43E-10	1.83E-11	7.14	15.1495214	4.02E-02	172992719	172992842	NC_000003.11
cg18402615	2.43E-10	1.83E-11	7.14	15.1495159	1.99E-01	124896659	124896782	NC_000010.10
cg11325267	2.43E-10	1.83E-11	7.14	15.148284	1.26E-01	137480853	137480976	NC_000003.11
cg27470208	2.43E-10	1.83E-11	7.14	15.1475032	6.49E-02	8373416	8373539	NC_000010.10
cg13870866	2.44E-10	1.83E-11	7.14	15.1470236	1.53E-01	35293130	35293253	NC_000007.13
cg00873601	2.44E-10	1.83E-11	7.14	15.1455811	1.37E-01	116044025	116044148	NC_000012.11
cg26958838	2.44E-10	1.84E-11	7.14	15.1453515	1.27E-01	2416025	2416148	NC_000005.9
cg08969274	2.44E-10	1.84E-11	7.14	15.1450407	1.09E-01	75848810	75848933	NC_000017.10

cg11850508	2.45E-10	1.84E-11	7.14	15.1418862	7.51E-02	78172311	78172434	NC_000017.10
cg19035496	2.46E-10	1.85E-11	7.13	15.1369178	9.12E-02	67090792	67090915	NC_000008.10
cg01256089	2.46E-10	1.85E-11	7.13	15.1364292	1.80E-01	77822500	77822623	NC_000016.9
cg00017489	2.46E-10	1.85E-11	7.13	15.1356831	1.76E-01	153583318	153583441	NC_000007.13
cg14747563	2.47E-10	1.86E-11	7.13	15.1340645	2.33E-01	134871962	134872085	NC_000005.9
cg03265991	2.47E-10	1.86E-11	7.13	15.1328802	3.25E-02	66646895	66647018	NC_000016.9
cg26597242	2.47E-10	1.86E-11	7.13	15.1323847	1.98E-01	161274540	161274663	NC_000005.9
cg03847768	2.47E-10	1.86E-11	7.13	15.1313347	8.74E-02	32111398	32111521	NC_000012.11
cg07434271	2.48E-10	1.87E-11	7.13	15.1267991	1.54E-01	31840698	31840821	NC_000011.9
cg10715460	2.48E-10	1.87E-11	7.13	15.1266788	1.06E-01	21278879	21279002	NC_000017.10
cg15151929	2.49E-10	1.87E-11	7.13	15.1250625	7.86E-02	1896429	1896552	NC_000001.10
cg02222728	2.49E-10	1.87E-11	7.13	15.1248186	1.36E-01	55371829	55371952	NC_000008.10
cg23827284	2.49E-10	1.88E-11	7.13	15.1239256	1.41E-01	30407145	30407268	NC_000020.10
cg25732462	2.49E-10	1.88E-11	7.13	15.1237263	2.27E-01	44324898	44325021	NC_000019.9
cg10583000	2.49E-10	1.88E-11	7.13	15.1234537	1.70E-01	98963134	98963257	NC_000002.11
cg06991392	2.49E-10	1.88E-11	7.13	15.1231791	1.22E-01	147789842	147789965	NC_000001.10
cg21433231	2.49E-10	1.88E-11	7.13	15.1231517	1.30E-01	34820712	34820835	NC_000017.10
cg17306747	2.49E-10	1.88E-11	7.13	15.1229766	1.68E-01	1446165	1446288	NC_000005.9
cg19226007	2.50E-10	1.88E-11	7.13	15.1218208	1.13E-01	43045068	43045191	NC_000017.10
cg19905251	2.50E-10	1.88E-11	7.13	15.1196689	1.08E-01	168728495	168728618	NC_000005.9
cg10909790	2.51E-10	1.89E-11	7.13	15.1174155	1.24E-01	45925455	45925578	NC_000010.10
cg19504702	2.51E-10	1.89E-11	7.13	15.1164683	1.62E-01	153862394	153862517	NC_000005.9
cg13688769	2.51E-10	1.89E-11	7.13	15.1160095	1.48E-01	73674363	73674486	NC_000003.11
cg11085282	2.51E-10	1.90E-11	7.13	15.1138584	1.70E-01	46262203	46262326	NC_000022.10
cg22507154	2.52E-10	1.90E-11	7.13	15.1122676	1.32E-01	91185233	91185356	NC_000001.10

cg00522588	2.52E-10	1.90E-11	7.13	15.1116063	1.53E-01	49449063	49449186	NC_000012.11
cg12089570	2.52E-10	1.90E-11	7.13	15.1099775	1.36E-01	9473715	9473838	NC_000019.9
cg14130459	2.52E-10	1.90E-11	7.13	15.1099632	9.75E-02	60718568	60718691	NC_000011.9
cg13050556	2.53E-10	1.90E-11	7.13	15.1087885	4.79E-02	80180068	80180191	NC_000017.10
cg14069928	2.53E-10	1.91E-11	7.13	15.108347	1.22E-01	136845816	136845939	NC_000009.11
cg10717463	2.53E-10	1.91E-11	7.13	15.1082398	1.51E-01	76740962	76741085	NC_000018.9
cg02188358	2.53E-10	1.91E-11	7.13	15.1081465	1.65E-01	29068150	29068273	NC_000013.10
cg13681800	2.53E-10	1.91E-11	7.13	15.1069951	1.41E-01	168498470	168498593	NC_000006.11
cg08422172	2.54E-10	1.91E-11	7.13	15.1046267	2.04E-01	11886668	11886791	NC_000016.9
cg05432034	2.54E-10	1.91E-11	7.13	15.1045604	9.85E-02	86743939	86744062	NC_000016.9
cg14453201	2.54E-10	1.91E-11	7.13	15.1038827	2.07E-01	320888	321011	NC_000005.9
cg04290835	2.54E-10	1.92E-11	7.13	15.1032903	5.71E-02	5350284	5350407	NC_000017.10
cg01797106	2.54E-10	1.92E-11	7.13	15.1023542	9.81E-02	129431954	129432077	NC_000012.11
cg14448169	2.55E-10	1.92E-11	7.13	15.0998117	1.73E-01	120968904	120969027	NC_000007.13
cg25759507	2.55E-10	1.93E-11	7.13	15.0981277	3.83E-02	12008553	12008676	NC_000011.9
cg19133958	2.55E-10	1.93E-11	7.13	15.0976084	4.83E-02	59560599	59560722	NC_000017.10
cg26794997	2.56E-10	1.93E-11	7.13	15.095072	8.90E-02	76189257	76189380	NC_000018.9
cg03774463	2.56E-10	1.93E-11	7.13	15.0947733	1.53E-01	96636616	96636739	NC_000007.13
cg21983192	2.56E-10	1.93E-11	7.13	15.0940898	5.83E-02	28935651	28935774	NC_000018.9
cg13096405	2.56E-10	1.93E-11	7.13	15.0934207	6.52E-02	1120954	1121077	NC_000011.9
cg09705456	2.57E-10	1.94E-11	7.13	15.0919275	1.25E-01	63839479	63839602	NC_000011.9
cg02229261	2.58E-10	1.94E-11	7.13	15.0883606	1.34E-01	52222431	52222554	NC_000019.9
cg04866029	2.58E-10	1.95E-11	7.13	15.0875995	6.98E-02	2707161	2707284	NC_000001.10
cg13187764	2.58E-10	1.95E-11	7.13	15.0873632	2.21E-01	28677521	28677644	NC_000006.11
cg13546514	2.58E-10	1.95E-11	7.13	15.0867189	8.97E-02	9712113	9712236	NC_000003.11

cg10108296	2.58E-10	1.95E-11	7.13	15.0866466	1.42E-01	30139592	30139715	NC_000006.11
cg07962143	2.58E-10	1.95E-11	7.13	15.0853053	1.99E-01	57149436	57149559	NC_000019.9
cg03421195	2.59E-10	1.96E-11	7.12	15.0815536	1.80E-01	7517215	7517338	NC_000010.10
cg08284926	2.60E-10	1.97E-11	7.12	15.0773749	1.03E-01	134826760	134826883	NC_000010.10
cg05852143	2.60E-10	1.97E-11	7.12	15.0765803	1.85E-01	5540116	5540239	NC_000012.11
cg11824639	2.61E-10	1.97E-11	7.12	15.0759615	1.69E-01	67218080	67218203	NC_000001.10
cg23662142	2.61E-10	1.97E-11	7.12	15.0754643	1.60E-01	55199914	55200037	NC_000020.10
cg12226948	2.61E-10	1.97E-11	7.12	15.0752349	1.38E-01	153504453	153504576	NC_000002.11
cg15897102	2.61E-10	1.97E-11	7.12	15.0750139	1.26E-01	115677211	115677334	NC_000001.10
cg02318784	2.61E-10	1.97E-11	7.12	15.0739003	1.52E-01	27213174	27213297	NC_000015.9
cg21127068	2.61E-10	1.97E-11	7.12	15.0735029	1.58E-01	147124417	147124540	NC_000003.11
cg26647200	2.61E-10	1.97E-11	7.12	15.0734901	8.48E-02	2482775	2482898	NC_000016.9
cg01489177	2.61E-10	1.97E-11	7.12	15.073336	8.07E-02	55828178	55828301	NC_000019.9
cg13723431	2.62E-10	1.98E-11	7.12	15.0684798	1.13E-01	1444395	1444518	NC_000005.9
cg13361749	2.62E-10	1.98E-11	7.12	15.0683682	4.78E-02	5824196	5824319	NC_000019.9
cg04771413	2.63E-10	1.99E-11	7.12	15.0677924	1.52E-01	5896549	5896672	NC_000002.11
cg09616857	2.63E-10	1.99E-11	7.12	15.0675192	4.73E-02	169122917	169123040	NC_000003.11
cg04605987	2.63E-10	1.99E-11	7.12	15.0666175	1.38E-01	200007403	200007526	NC_000001.10
cg07084163	2.63E-10	1.99E-11	7.12	15.0661874	1.28E-01	36066783	36066906	NC_000005.9
cg15998779	2.63E-10	1.99E-11	7.12	15.0659628	1.51E-01	159756690	159756813	NC_000003.11
cg07537370	2.63E-10	1.99E-11	7.12	15.0658498	1.06E-01	27531124	27531247	NC_000002.11
cg14515032	2.63E-10	1.99E-11	7.12	15.0652029	7.26E-02	2900269	2900392	NC_000017.10
cg20710709	2.64E-10	2.00E-11	7.12	15.0623071	1.40E-01	37135671	37135794	NC_000014.8
cg14144305	2.64E-10	2.00E-11	7.12	15.0615724	1.27E-01	44325960	44326083	NC_000011.9
cg07612928	2.64E-10	2.00E-11	7.12	15.0613405	1.11E-01	51956044	51956167	NC_000014.8

cg15140798	2.64E-10	2.00E-11	7.12	15.0606877	1.16E-01	46782485	46782608	NC_000021.8
cg23553912	2.64E-10	2.00E-11	7.12	15.0601057	1.41E-01	159558031	159558154	NC_000003.11
cg21678377	2.65E-10	2.00E-11	7.12	15.0594646	2.50E-01	115919009	115919132	NC_000002.11
cg13828758	2.65E-10	2.00E-11	7.12	15.0592804	8.96E-02	23932373	23932496	NC_000015.9
cg00527489	2.65E-10	2.00E-11	7.12	15.0588972	2.02E-01	214360954	214361077	NC_000001.10
cg25724837	2.65E-10	2.01E-11	7.12	15.0574962	3.63E-02	123493091	123493214	NC_000010.10
cg16828491	2.65E-10	2.01E-11	7.12	15.0568813	1.05E-01	8230829	8230952	NC_000004.11
cg09451457	2.66E-10	2.01E-11	7.12	15.0552776	1.60E-01	87440246	87440369	NC_000005.9
cg20892492	2.66E-10	2.01E-11	7.12	15.0551827	9.67E-02	129300912	129301035	NC_000004.11
cg05131483	2.66E-10	2.01E-11	7.12	15.0549321	1.67E-01	23706242	23706365	NC_000016.9
cg27664390	2.66E-10	2.02E-11	7.12	15.0530921	2.01E-02	59171928	59172051	NC_000015.9
cg06756919	2.66E-10	2.02E-11	7.12	15.0528303	1.60E-01	37135898	37136021	NC_000014.8
cg15057046	2.66E-10	2.02E-11	7.12	15.0522104	5.81E-02	4483078	4483201	NC_000006.11
cg02633600	2.67E-10	2.02E-11	7.12	15.0506655	8.11E-02	1074159	1074282	NC_000016.9
cg26124318	2.67E-10	2.02E-11	7.12	15.0490222	1.84E-01	38965122	38965245	NC_000008.10
cg21069500	2.67E-10	2.02E-11	7.12	15.0487891	1.12E-01	139050159	139050282	NC_000005.9
cg17864046	2.67E-10	2.03E-11	7.12	15.0485966	1.52E-01	166422341	166422464	NC_000006.11
cg25655234	2.67E-10	2.03E-11	7.12	15.0483028	1.96E-01	7194841	7194964	NC_000004.11
cg02662277	2.68E-10	2.03E-11	7.12	15.0476638	1.61E-01	60936383	60936506	NC_000010.10
cg03634394	2.68E-10	2.03E-11	7.12	15.0453539	6.50E-02	16144375	16144498	NC_000019.9
cg07710481	2.68E-10	2.03E-11	7.12	15.0451425	2.37E-01	88325384	88325507	NC_000013.10
cg02350677	2.68E-10	2.03E-11	7.12	15.0451251	1.58E-01	23254381	23254504	NC_000019.9
cg06997259	2.68E-10	2.03E-11	7.12	15.0447458	7.51E-02	235956326	235956449	NC_000002.11
cg19916212	2.68E-10	2.03E-11	7.12	15.0440533	2.24E-01	78493203	78493326	NC_000013.10
cg09092054	2.69E-10	2.04E-11	7.12	15.0423958	2.15E-01	19511987	19512110	NC_000022.10

cg06554200	2.69E-10	2.04E-11	7.12	15.0423833	1.45E-01	1270134	1270257	NC_000007.13
cg25829961	2.69E-10	2.04E-11	7.12	15.0420746	6.43E-02	46900754	46900877	NC_000021.8
cg07636952	2.69E-10	2.04E-11	7.12	15.0420616	5.68E-02	124596397	124596520	NC_000010.10
cg26014905	2.69E-10	2.04E-11	7.12	15.0419832	3.07E-02	50601263	50601386	NC_000003.11
cg08797892	2.70E-10	2.04E-11	7.12	15.039553	2.98E-02	119885187	119885310	NC_000003.11
cg27474175	2.70E-10	2.05E-11	7.12	15.0376825	1.50E-01	80363992	80364115	NC_000015.9
cg17527819	2.71E-10	2.05E-11	7.12	15.0358385	1.10E-01	30140409	30140532	NC_000006.11
cg15074709	2.71E-10	2.06E-11	7.12	15.0325006	1.15E-01	112547653	112547776	NC_000013.10
cg22687748	2.72E-10	2.06E-11	7.12	15.0317868	1.72E-01	71017211	71017334	NC_000002.11
cg13169072	2.72E-10	2.06E-11	7.12	15.0310202	2.69E-02	36350271	36350394	NC_000014.8
cg12426234	2.72E-10	2.07E-11	7.12	15.0292452	1.72E-01	22370347	22370470	NC_000021.8
cg19011844	2.73E-10	2.07E-11	7.12	15.0277257	2.19E-01	10102232	10102355	NC_000017.10
cg23760687	2.73E-10	2.07E-11	7.12	15.027494	1.44E-01	25621735	25621858	NC_000013.10
cg12728097	2.73E-10	2.07E-11	7.12	15.0269148	8.37E-02	28154501	28154624	NC_000001.10
cg23367392	2.74E-10	2.08E-11	7.11	15.0238748	1.45E-01	42267337	42267460	NC_000007.13
cg26055770	2.75E-10	2.08E-11	7.11	15.0204619	1.77E-01	73674070	73674193	NC_000003.11
cg18893528	2.76E-10	2.09E-11	7.11	15.0168446	7.15E-02	4296406	4296529	NC_000010.10
cg23859051	2.76E-10	2.10E-11	7.11	15.015173	1.15E-01	1478585	1478708	NC_000017.10
cg11346837	2.76E-10	2.10E-11	7.11	15.0135552	1.67E-01	147077671	147077794	NC_000003.11
cg15444648	2.76E-10	2.10E-11	7.11	15.0134867	1.51E-01	92907851	92907974	NC_000005.9
cg07057434	2.76E-10	2.10E-11	7.11	15.0133873	5.65E-02	70372625	70372748	NC_000008.10
cg27318318	2.77E-10	2.10E-11	7.11	15.0129865	1.37E-01	44332849	44332972	NC_000011.9
cg14150751	2.77E-10	2.10E-11	7.11	15.0129614	5.72E-02	76341733	76341856	NC_000006.11
cg25349034	2.77E-10	2.10E-11	7.11	15.0127444	9.24E-02	149265300	149265423	NC_000003.11
cg27315742	2.77E-10	2.10E-11	7.11	15.012664	1.67E-01	117586681	117586804	NC_000006.11

cg24216555	2.77E-10	2.10E-11	7.11	15.0112264	1.26E-01	55814143	55814266	NC_000019.9
cg18683586	2.78E-10	2.11E-11	7.11	15.0080266	5.81E-02	155620659	155620782	NC_000007.13
cg23440816	2.78E-10	2.11E-11	7.11	15.0071845	8.04E-02	223948910	223949033	NC_000001.10
cg01999539	2.78E-10	2.11E-11	7.11	15.0071096	1.42E-01	150259860	150259983	NC_000006.11
cg09825093	2.78E-10	2.11E-11	7.11	15.0068543	1.53E-01	82660505	82660628	NC_000016.9
cg15699085	2.78E-10	2.11E-11	7.11	15.0065516	1.45E-01	140457609	140457732	NC_000005.9
cg01785568	2.79E-10	2.12E-11	7.11	15.0027742	1.29E-01	4864833	4864956	NC_000004.11
cg11800117	2.80E-10	2.13E-11	7.11	15.0006057	1.43E-01	93270375	93270498	NC_000011.9
cg22177309	2.80E-10	2.13E-11	7.11	15.000099	8.04E-02	48260285	48260408	NC_000019.9
cg08014499	2.80E-10	2.13E-11	7.11	14.9987292	1.33E-01	2162545	2162668	NC_000011.9
cg02118194	2.80E-10	2.13E-11	7.11	14.9982159	8.73E-02	46404538	46404661	NC_000019.9
cg18073471	2.81E-10	2.13E-11	7.11	14.9967392	1.41E-01	81119198	81119321	NC_000004.11
cg14744743	2.81E-10	2.14E-11	7.11	14.9960092	1.28E-01	13871595	13871718	NC_000018.9
cg26922697	2.81E-10	2.14E-11	7.11	14.9959781	8.82E-02	33284542	33284665	NC_000006.11
cg26829322	2.81E-10	2.14E-11	7.11	14.9948017	1.49E-01	11713867	11713990	NC_000001.10
cg09353985	2.82E-10	2.14E-11	7.11	14.9922958	8.34E-02	207062674	207062797	NC_000001.10
cg10471437	2.82E-10	2.15E-11	7.11	14.9912811	2.78E-01	28074462	28074585	NC_000016.9
cg22909138	2.82E-10	2.15E-11	7.11	14.990817	2.51E-02	79803493	79803616	NC_000017.10
cg16931822	2.82E-10	2.15E-11	7.11	14.9907463	8.35E-02	66475150	66475273	NC_000003.11
cg27053763	2.83E-10	2.15E-11	7.11	14.9881473	3.00E-02	180653815	180653938	NC_000005.9
cg14483771	2.83E-10	2.15E-11	7.11	14.9877368	5.61E-02	134998463	134998586	NC_000010.10
cg14884793	2.83E-10	2.16E-11	7.11	14.987348	1.58E-01	109148553	109148676	NC_000013.10
cg12995800	2.84E-10	2.16E-11	7.11	14.9863174	1.96E-01	151304312	151304435	NC_000005.9
cg26530758	2.84E-10	2.16E-11	7.11	14.9857344	2.97E-01	41754060	41754183	NC_000008.10
cg05375728	2.84E-10	2.16E-11	7.11	14.9852549	2.10E-01	58715539	58715662	NC_000001.10

cg04008601	2.84E-10	2.16E-11	7.11	14.9845479	1.68E-01	103696126	103696249	NC_000012.11
cg17062917	2.84E-10	2.16E-11	7.11	14.983113	4.65E-02	70054539	70054662	NC_000015.9
cg00716257	2.85E-10	2.16E-11	7.11	14.9829854	1.73E-01	75897417	75897540	NC_000014.8
cg18262937	2.85E-10	2.17E-11	7.11	14.9811761	8.72E-02	17002291	17002414	NC_000022.10
cg01586959	2.85E-10	2.17E-11	7.11	14.97969	1.80E-01	86541607	86541730	NC_000016.9
cg12796916	2.85E-10	2.17E-11	7.11	14.9796887	5.73E-02	67471698	67471821	NC_000011.9
cg25623524	2.85E-10	2.17E-11	7.11	14.9793477	1.25E-01	58862063	58862186	NC_000019.9
cg12926938	2.86E-10	2.18E-11	7.11	14.9777191	2.01E-01	93114150	93114273	NC_000008.10
cg09854011	2.86E-10	2.18E-11	7.11	14.9767101	1.89E-01	17907024	17907147	NC_000001.10
cg05327835	2.86E-10	2.18E-11	7.11	14.9762885	2.36E-01	996175	996298	NC_000004.11
cg07416237	2.86E-10	2.18E-11	7.11	14.9761007	1.13E-01	100548078	100548201	NC_000013.10
cg03804255	2.87E-10	2.18E-11	7.11	14.9748183	9.97E-02	90278335	90278458	NC_000012.11
cg22534621	2.87E-10	2.19E-11	7.11	14.9735812	1.09E-01	82276730	82276853	NC_000001.10
cg14230397	2.87E-10	2.19E-11	7.11	14.9735229	1.72E-01	46799986	46800109	NC_000017.10
cg24937675	2.87E-10	2.19E-11	7.11	14.9729927	1.46E-01	134639482	134639605	NC_000006.11
cg27583671	2.88E-10	2.19E-11	7.11	14.9701776	7.63E-02	151670624	151670747	NC_000006.11
cg07883722	2.88E-10	2.19E-11	7.11	14.9700159	3.58E-02	95246404	95246527	NC_000005.9
cg24920126	2.88E-10	2.20E-11	7.1	14.9690904	8.58E-02	5087266	5087389	NC_000006.11
cg05707655	2.88E-10	2.20E-11	7.1	14.9685995	1.55E-01	908690	908813	NC_000017.10
cg19752627	2.89E-10	2.20E-11	7.1	14.9675557	2.52E-01	98467380	98467503	NC_000007.13
cg12459280	2.89E-10	2.20E-11	7.1	14.9667102	4.59E-02	36165083	36165206	NC_000021.8
cg07839533	2.90E-10	2.21E-11	7.1	14.964459	2.16E-01	63257885	63258008	NC_000005.9
cg08119884	2.90E-10	2.21E-11	7.1	14.9619637	1.98E-01	79739863	79739986	NC_000002.11
cg18372548	2.90E-10	2.21E-11	7.1	14.9613248	2.84E-02	26913852	26913975	NC_000004.11
cg01937068	2.91E-10	2.22E-11	7.1	14.9599572	1.72E-01	156406349	156406472	NC_000001.10

cg06567920	2.91E-10	2.22E-11	7.1	14.9599494	1.05E-01	129582864	129582987	NC_000004.11
cg19519747	2.91E-10	2.22E-11	7.1	14.9597915	1.28E-01	177014555	177014678	NC_000002.11
cg01181415	2.91E-10	2.22E-11	7.1	14.9592275	1.44E-01	16757954	16758077	NC_000012.11
cg24024424	2.91E-10	2.22E-11	7.1	14.9581228	1.39E-01	140858046	140858169	NC_000005.9
cg18468394	2.92E-10	2.22E-11	7.1	14.9567936	1.38E-01	170736021	170736144	NC_000005.9
cg06293049	2.92E-10	2.22E-11	7.1	14.9564564	3.70E-02	53622101	53622224	NC_000012.11
cg13119928	2.92E-10	2.22E-11	7.1	14.9562217	6.60E-02	53228661	53228784	NC_000012.11
cg02723372	2.92E-10	2.23E-11	7.1	14.955146	4.61E-02	25228582	25228705	NC_000001.10
cg03677056	2.93E-10	2.23E-11	7.1	14.9539916	1.51E-01	33817217	33817340	NC_000017.10
cg26800786	2.93E-10	2.23E-11	7.1	14.9522101	1.54E-01	105922059	105922182	NC_000011.9
cg10157251	2.93E-10	2.24E-11	7.1	14.9505559	1.71E-01	89914608	89914731	NC_000015.9
cg20648149	2.94E-10	2.24E-11	7.1	14.949094	1.42E-01	64319089	64319212	NC_000014.8
cg12341314	2.94E-10	2.24E-11	7.1	14.9486051	1.18E-01	229568467	229568590	NC_000001.10
cg01910869	2.94E-10	2.24E-11	7.1	14.9484988	1.52E-01	78493182	78493305	NC_000013.10
cg08464003	2.94E-10	2.24E-11	7.1	14.9474925	1.52E-01	139623012	139623135	NC_000005.9
cg18221336	2.95E-10	2.25E-11	7.1	14.9464241	5.52E-02	31215287	31215410	NC_000001.10
cg24394631	2.95E-10	2.25E-11	7.1	14.9459983	1.59E-01	43863000	43863123	NC_000017.10
cg13450944	2.95E-10	2.25E-11	7.1	14.9448379	1.24E-01	66533056	66533179	NC_000003.11
cg17528559	2.96E-10	2.25E-11	7.1	14.9432192	6.92E-02	113571025	113571148	NC_000002.11
cg07232003	2.96E-10	2.25E-11	7.1	14.9431549	1.54E-01	28971331	28971454	NC_000010.10
cg22522939	2.96E-10	2.26E-11	7.1	14.94263	7.96E-02	144210636	144210759	NC_000008.10
cg18597940	2.96E-10	2.26E-11	7.1	14.9400839	7.00E-02	76327105	76327228	NC_000014.8
cg11465466	2.97E-10	2.26E-11	7.1	14.9386978	1.23E-01	39549192	39549315	NC_000017.10
cg22378717	2.97E-10	2.27E-11	7.1	14.9369528	6.14E-02	143200284	143200407	NC_000008.10
cg24171907	2.98E-10	2.27E-11	7.1	14.9356586	2.55E-01	68546579	68546702	NC_000002.11

cg14102880	2.98E-10	2.27E-11	7.1	14.9349365	1.09E-01	915634	915757	NC_000017.10
cg24161501	2.98E-10	2.28E-11	7.1	14.9340105	1.58E-01	67218275	67218398	NC_000001.10
cg07760204	2.98E-10	2.28E-11	7.1	14.933389	1.56E-01	147576547	147576670	NC_000004.11
cg14641018	2.99E-10	2.28E-11	7.1	14.9318378	5.35E-02	42676114	42676237	NC_000021.8
cg04607032	2.99E-10	2.28E-11	7.1	14.9301706	6.07E-02	75503069	75503192	NC_000017.10
cg06571387	3.01E-10	2.30E-11	7.1	14.9243321	1.25E-01	176964101	176964224	NC_000002.11
cg11788234	3.02E-10	2.31E-11	7.1	14.9209908	1.69E-01	29393811	29393934	NC_000013.10
cg13833437	3.02E-10	2.31E-11	7.1	14.9209074	1.12E-01	27531236	27531359	NC_000002.11
cg13694749	3.03E-10	2.31E-11	7.1	14.9177149	1.06E-01	62050038	62050161	NC_000017.10
cg04350645	3.03E-10	2.31E-11	7.1	14.9171542	1.01E-01	26620797	26620920	NC_000013.10
cg01733599	3.03E-10	2.31E-11	7.1	14.9171183	1.50E-01	58723851	58723974	NC_000015.9
cg13389958	3.03E-10	2.32E-11	7.1	14.9167743	1.34E-01	27280924	27281047	NC_000007.13
cg21757872	3.04E-10	2.32E-11	7.1	14.9154717	1.18E-01	107974453	107974576	NC_000012.11
cg20964248	3.04E-10	2.32E-11	7.1	14.914632	1.80E-01	111564787	111564910	NC_000011.9
cg19671533	3.04E-10	2.32E-11	7.1	14.9138539	1.56E-01	84419234	84419357	NC_000006.11
cg14218542	3.04E-10	2.32E-11	7.1	14.9133927	9.91E-02	134853281	134853404	NC_000010.10
cg14559422	3.04E-10	2.32E-11	7.1	14.9129682	9.26E-02	26148138	26148261	NC_000001.10
cg13784080	3.05E-10	2.33E-11	7.09	14.9108465	2.03E-01	57275413	57275536	NC_000014.8
cg21339581	3.05E-10	2.33E-11	7.09	14.9100304	6.10E-02	1800987	1801110	NC_000004.11
cg26714129	3.05E-10	2.33E-11	7.09	14.909836	9.00E-02	142310710	142310833	NC_000008.10
cg15637465	3.05E-10	2.33E-11	7.09	14.9097782	2.07E-01	168728270	168728393	NC_000005.9
cg07927072	3.05E-10	2.33E-11	7.09	14.9092366	7.71E-02	28117087	28117210	NC_000002.11
cg22345692	3.06E-10	2.34E-11	7.09	14.9086303	1.45E-01	32712879	32713002	NC_000005.9
cg22143289	3.06E-10	2.34E-11	7.09	14.9075046	1.84E-01	30019898	30020021	NC_000019.9
cg25482132	3.06E-10	2.34E-11	7.09	14.9072546	5.99E-02	41873172	41873295	NC_000006.11

cg02936553	3.06E-10	2.34E-11	7.09	14.9058944	9.05E-02	3134745	3134868	NC_000017.10
cg14499413	3.07E-10	2.34E-11	7.09	14.9051966	1.82E-01	10749947	10750070	NC_000003.11
cg27328839	3.07E-10	2.35E-11	7.09	14.9034628	9.31E-02	79679462	79679585	NC_000010.10
cg03927893	3.07E-10	2.35E-11	7.09	14.9025612	2.01E-01	17850130	17850253	NC_000022.10
cg02524954	3.08E-10	2.35E-11	7.09	14.901013	1.21E-01	32454964	32455087	NC_000011.9
cg17400476	3.09E-10	2.36E-11	7.09	14.8978914	1.83E-01	27231491	27231614	NC_000007.13
cg22874988	3.10E-10	2.37E-11	7.09	14.8947495	1.78E-01	111451090	111451213	NC_000003.11
cg21346679	3.10E-10	2.37E-11	7.09	14.8934304	1.14E-01	169888373	169888496	NC_000002.11
cg25970832	3.11E-10	2.38E-11	7.09	14.8906071	1.69E-01	28621651	28621774	NC_000018.9
cg06181518	3.11E-10	2.38E-11	7.09	14.8898654	2.39E-01	133562196	133562319	NC_000006.11
cg11107116	3.11E-10	2.38E-11	7.09	14.8896384	1.00E-01	100804081	100804204	NC_000015.9
cg21117426	3.11E-10	2.38E-11	7.09	14.8895976	7.38E-02	126162682	126162805	NC_000011.9
cg23831876	3.12E-10	2.39E-11	7.09	14.8871085	1.29E-01	65225988	65226111	NC_000002.11
cg16486132	3.12E-10	2.39E-11	7.09	14.8867194	8.58E-02	74400946	74401069	NC_000016.9
cg18100702	3.12E-10	2.39E-11	7.09	14.8855653	1.45E-01	3600572	3600695	NC_000005.9
cg01464484	3.13E-10	2.39E-11	7.09	14.884992	7.52E-02	67089352	67089475	NC_000011.9
cg07347645	3.13E-10	2.39E-11	7.09	14.8846074	8.97E-02	58507171	58507294	NC_000020.10
cg08222293	3.13E-10	2.39E-11	7.09	14.8840753	7.02E-02	180628689	180628812	NC_000005.9
cg10601940	3.13E-10	2.40E-11	7.09	14.8817581	1.29E-01	223159305	223159428	NC_000002.11
cg05724240	3.13E-10	2.40E-11	7.09	14.8817426	4.60E-02	134822001	134822124	NC_000010.10
cg02487987	3.14E-10	2.40E-11	7.09	14.8803802	1.16E-01	52153575	52153698	NC_000005.9
cg17214502	3.14E-10	2.41E-11	7.09	14.8791224	2.19E-01	4869172	4869295	NC_000004.11
cg05537653	3.14E-10	2.41E-11	7.09	14.8791206	1.34E-01	47612070	47612193	NC_000011.9
cg02149965	3.14E-10	2.41E-11	7.09	14.8787226	1.10E-01	30883203	30883326	NC_000006.11
cg19771465	3.15E-10	2.41E-11	7.09	14.8759439	1.68E-01	237145587	237145710	NC_000002.11

cg24252708	3.15E-10	2.41E-11	7.09	14.8758103	2.78E-02	32046663	32046786	NC_000006.11
cg01461410	3.16E-10	2.42E-11	7.09	14.874669	1.96E-01	85473773	85473896	NC_000006.11
cg06878741	3.16E-10	2.42E-11	7.09	14.8744287	1.58E-01	601349	601472	NC_000007.13
cg10422233	3.16E-10	2.42E-11	7.09	14.872375	1.17E-01	103565915	103566038	NC_000014.8
cg05926722	3.16E-10	2.42E-11	7.09	14.8715474	1.41E-01	26674202	26674325	NC_000014.8
cg01410314	3.17E-10	2.43E-11	7.09	14.870844	1.45E-01	77641437	77641560	NC_000009.11
cg16206813	3.17E-10	2.43E-11	7.09	14.8686713	1.90E-01	3122162	3122285	NC_000005.9
cg07369094	3.18E-10	2.43E-11	7.09	14.8676294	1.10E-01	107471174	107471297	NC_000005.9
cg01569346	3.18E-10	2.44E-11	7.09	14.8673676	1.08E-01	32064148	32064271	NC_000006.11
cg07642499	3.18E-10	2.44E-11	7.09	14.8650832	1.45E-01	69957602	69957725	NC_000011.9
cg06115835	3.19E-10	2.44E-11	7.09	14.864721	5.47E-02	110259173	110259296	NC_000002.11
cg05455234	3.19E-10	2.44E-11	7.09	14.8637346	3.31E-02	47847919	47848042	NC_000021.8
cg00457403	3.19E-10	2.45E-11	7.09	14.8628385	1.75E-01	187387734	187387857	NC_000003.11
cg14062977	3.19E-10	2.45E-11	7.09	14.8620348	1.11E-01	135380213	135380336	NC_000009.11
cg26582803	3.20E-10	2.45E-11	7.09	14.8609471	4.48E-02	32259626	32259749	NC_000020.10
cg00904966	3.20E-10	2.45E-11	7.09	14.8599356	2.31E-01	1122937	1123060	NC_000016.9
cg12528713	3.20E-10	2.46E-11	7.09	14.8593552	1.98E-01	81187605	81187728	NC_000004.11
cg15245038	3.20E-10	2.46E-11	7.09	14.8590627	2.55E-02	138917011	138917134	NC_000006.11
cg26454433	3.21E-10	2.46E-11	7.09	14.8576714	1.28E-01	237084695	237084818	NC_000002.11
cg18516833	3.21E-10	2.46E-11	7.09	14.8574866	6.44E-02	57350313	57350436	NC_000019.9
cg23556443	3.21E-10	2.46E-11	7.08	14.8564003	1.80E-01	11148776	11148899	NC_000018.9
cg22398259	3.21E-10	2.47E-11	7.08	14.8550427	6.36E-02	144255688	144255811	NC_000008.10
cg13816321	3.22E-10	2.47E-11	7.08	14.853752	1.61E-01	973198	973321	NC_000009.11
cg00781110	3.22E-10	2.47E-11	7.08	14.8535364	1.76E-01	74421535	74421658	NC_000015.9
cg11236727	3.22E-10	2.47E-11	7.08	14.8526554	2.26E-01	119609592	119609715	NC_000002.11

cg18759960	3.22E-10	2.47E-11	7.08	14.8520345	1.71E-01	23481776	23481899	NC_000010.10
cg02345991	3.23E-10	2.48E-11	7.08	14.8509539	2.06E-01	119418676	119418799	NC_000012.11
cg23637385	3.23E-10	2.48E-11	7.08	14.8503028	1.92E-01	45161510	45161633	NC_000002.11
cg18565783	3.23E-10	2.48E-11	7.08	14.8500129	1.82E-01	57390704	57390827	NC_000010.10
cg01152168	3.23E-10	2.48E-11	7.08	14.8487725	7.43E-02	18280011	18280134	NC_000017.10
cg26938561	3.24E-10	2.49E-11	7.08	14.8472714	1.41E-01	22193724	22193847	NC_000019.9
cg21389119	3.24E-10	2.49E-11	7.08	14.8468401	1.02E-01	202848791	202848914	NC_000002.11
cg04741853	3.24E-10	2.49E-11	7.08	14.8463524	2.50E-01	44037189	44037312	NC_000003.11
cg25148589	3.24E-10	2.49E-11	7.08	14.8460757	1.41E-01	158141936	158142059	NC_000004.11
cg26043460	3.24E-10	2.49E-11	7.08	14.8459938	1.15E-01	39593631	39593754	NC_000017.10
cg05701791	3.25E-10	2.50E-11	7.08	14.8434236	1.46E-01	97172825	97172948	NC_000008.10
cg06633061	3.25E-10	2.50E-11	7.08	14.8431269	1.52E-01	57261601	57261724	NC_000014.8
cg12400664	3.25E-10	2.50E-11	7.08	14.8423169	5.34E-02	31779521	31779644	NC_000006.11
cg25678815	3.25E-10	2.50E-11	7.08	14.8422758	2.09E-01	85673915	85674038	NC_000012.11
cg08313393	3.25E-10	2.50E-11	7.08	14.8422477	1.28E-01	216637593	216637716	NC_000002.11
cg19883384	3.25E-10	2.50E-11	7.08	14.841726	1.81E-01	25905478	25905601	NC_000008.10
cg06593507	3.26E-10	2.51E-11	7.08	14.8394546	1.35E-01	5290324	5290447	NC_000016.9
cg25617725	3.26E-10	2.51E-11	7.08	14.8392058	1.54E-01	118033290	118033413	NC_000010.10
cg24354101	3.26E-10	2.51E-11	7.08	14.8390893	1.56E-01	124383535	124383658	NC_000003.11
cg05990479	3.27E-10	2.51E-11	7.08	14.8375472	7.61E-02	50575784	50575907	NC_000005.9
cg18395499	3.27E-10	2.51E-11	7.08	14.8366359	1.04E-01	149194510	149194633	NC_000001.10
cg15134033	3.28E-10	2.52E-11	7.08	14.8345389	1.23E-01	10274415	10274538	NC_000016.9
cg13952556	3.28E-10	2.52E-11	7.08	14.8343709	1.66E-01	109294044	109294167	NC_000011.9
cg14548272	3.28E-10	2.52E-11	7.08	14.8326521	1.59E-01	99961122	99961245	NC_000008.10
cg17543296	3.29E-10	2.53E-11	7.08	14.8314379	1.86E-01	20880378	20880501	NC_000001.10

cg00803088	3.29E-10	2.53E-11	7.08	14.8307175	2.72E-01	43600706	43600829	NC_000010.10
cg10429922	3.29E-10	2.53E-11	7.08	14.8293918	1.37E-01	33010143	33010266	NC_000015.9
cg07493435	3.29E-10	2.53E-11	7.08	14.8292268	9.41E-02	128556140	128556263	NC_000007.13
cg07102705	3.30E-10	2.54E-11	7.08	14.8262819	1.98E-01	148033896	148034019	NC_000005.9
cg02840199	3.30E-10	2.54E-11	7.08	14.8259415	1.11E-01	4165257	4165380	NC_000016.9
cg25182716	3.31E-10	2.54E-11	7.08	14.8240784	7.91E-02	13622875	13622998	NC_000020.10
cg15810859	3.31E-10	2.55E-11	7.08	14.8233482	3.65E-02	127628206	127628329	NC_000007.13
cg09231862	3.31E-10	2.55E-11	7.08	14.8232325	2.05E-01	29235376	29235499	NC_000014.8
cg17725019	3.31E-10	2.55E-11	7.08	14.8226189	3.08E-02	31677931	31678054	NC_000022.10
cg05584235	3.31E-10	2.55E-11	7.08	14.8225737	5.97E-02	111183275	111183398	NC_000002.11
cg06760710	3.32E-10	2.55E-11	7.08	14.8215888	1.67E-01	46692008	46692131	NC_000017.10
cg17852221	3.32E-10	2.55E-11	7.08	14.821242	8.50E-02	140263655	140263778	NC_000005.9
cg10601616	3.32E-10	2.56E-11	7.08	14.8194711	2.20E-01	106400702	106400825	NC_000010.10
cg22899100	3.33E-10	2.56E-11	7.08	14.8187918	2.52E-01	23462237	23462360	NC_000010.10
cg01811325	3.33E-10	2.57E-11	7.08	14.8159161	1.22E-01	131757672	131757795	NC_000010.10
cg13260278	3.34E-10	2.57E-11	7.08	14.8149637	6.64E-02	121265587	121265710	NC_000010.10
cg26235490	3.34E-10	2.57E-11	7.08	14.8140688	1.08E-01	1047567	1047690	NC_000017.10
cg25707805	3.34E-10	2.57E-11	7.08	14.8138874	1.11E-01	113285387	113285510	NC_000001.10
cg20974609	3.34E-10	2.57E-11	7.08	14.8129147	1.50E-01	27181671	27181794	NC_000007.13
cg02502025	3.35E-10	2.58E-11	7.08	14.8115121	1.13E-01	42151282	42151405	NC_000004.11
cg13536757	3.35E-10	2.58E-11	7.08	14.8112186	1.95E-01	185463583	185463706	NC_000002.11
cg14094983	3.35E-10	2.58E-11	7.08	14.8106454	1.65E-01	8482099	8482222	NC_000007.13
cg24263062	3.35E-10	2.58E-11	7.08	14.8104279	1.39E-01	2730191	2730314	NC_000020.10
cg03662064	3.35E-10	2.58E-11	7.08	14.8098992	1.67E-01	29069888	29070011	NC_000013.10
cg03819089	3.36E-10	2.59E-11	7.08	14.8071987	1.09E-01	66298217	66298340	NC_000002.11

cg05484508	3.36E-10	2.59E-11	7.08	14.8071353	4.26E-02	89589025	89589148	NC_000016.9
cg24605023	3.36E-10	2.59E-11	7.08	14.8069483	1.05E-01	62654733	62654856	NC_000003.11
cg00027114	3.37E-10	2.60E-11	7.08	14.8046586	1.61E-01	42740486	42740609	NC_000007.13
cg17775765	3.37E-10	2.60E-11	7.08	14.8044707	8.87E-02	95354021	95354144	NC_000013.10
cg21998983	3.37E-10	2.60E-11	7.08	14.8042209	1.89E-01	154727925	154728048	NC_000002.11
cg12980692	3.38E-10	2.60E-11	7.08	14.8024061	1.62E-01	51142613	51142736	NC_000019.9
cg17783244	3.38E-10	2.60E-11	7.08	14.8018856	1.24E-01	31738549	31738672	NC_000022.10
cg06491139	3.38E-10	2.60E-11	7.08	14.8012317	2.87E-02	74467828	74467951	NC_000017.10
cg12494277	3.38E-10	2.61E-11	7.08	14.8008231	4.35E-02	9514840	9514963	NC_000004.11
cg08274518	3.38E-10	2.61E-11	7.08	14.8003426	8.34E-02	33758023	33758146	NC_000003.11
cg04506928	3.39E-10	2.61E-11	7.07	14.7992273	8.76E-02	31647876	31647999	NC_000013.10
cg20415809	3.39E-10	2.61E-11	7.07	14.799192	2.35E-01	182321855	182321978	NC_000002.11
cg21636168	3.39E-10	2.61E-11	7.07	14.7976998	3.98E-02	61914232	61914355	NC_000011.9
cg21833107	3.40E-10	2.62E-11	7.07	14.7962776	4.83E-02	194057457	194057580	NC_000003.11
cg19266635	3.40E-10	2.62E-11	7.07	14.7948293	1.87E-01	46776389	46776512	NC_000012.11
cg07680965	3.40E-10	2.62E-11	7.07	14.7939929	8.51E-02	41563429	41563552	NC_000008.10
cg27563985	3.40E-10	2.62E-11	7.07	14.7939108	1.95E-01	54580677	54580800	NC_000020.10
cg03671700	3.41E-10	2.63E-11	7.07	14.7932766	1.51E-01	62068673	62068796	NC_000016.9
cg22983290	3.41E-10	2.63E-11	7.07	14.7925829	4.92E-02	134593727	134593850	NC_000010.10
cg14331316	3.41E-10	2.63E-11	7.07	14.7910259	1.14E-01	99139396	99139519	NC_000009.11
cg20712263	3.42E-10	2.64E-11	7.07	14.7894855	1.85E-01	131438527	131438650	NC_000012.11
cg24530041	3.42E-10	2.64E-11	7.07	14.7891693	1.49E-01	90349551	90349674	NC_000015.9
cg07335294	3.42E-10	2.64E-11	7.07	14.788752	2.09E-01	84417933	84418056	NC_000006.11
cg05007697	3.42E-10	2.64E-11	7.07	14.7885907	1.03E-01	169718132	169718255	NC_000006.11
cg09195198	3.43E-10	2.64E-11	7.07	14.7873864	2.05E-01	86602083	86602206	NC_000016.9

cg05499272	3.43E-10	2.64E-11	7.07	14.7872624	6.64E-02	32020587	32020710	NC_000006.11
cg17611193	3.43E-10	2.64E-11	7.07	14.7866375	1.57E-01	35395942	35396065	NC_000019.9
cg13877260	3.43E-10	2.64E-11	7.07	14.7862782	3.14E-02	105885896	105886019	NC_000002.11
cg25853960	3.43E-10	2.65E-11	7.07	14.785494	9.74E-02	34650841	34650964	NC_000013.10
cg17222500	3.44E-10	2.65E-11	7.07	14.7834216	1.56E-01	85306741	85306864	NC_000012.11
cg05100846	3.44E-10	2.65E-11	7.07	14.7826664	6.66E-02	102647731	102647854	NC_000014.8
cg12180613	3.45E-10	2.66E-11	7.07	14.7807083	1.21E-01	132158560	132158683	NC_000005.9
cg17772028	3.46E-10	2.67E-11	7.07	14.7780745	2.20E-01	61806651	61806774	NC_000020.10
cg18538958	3.46E-10	2.67E-11	7.07	14.7777548	1.80E-01	104640662	104640785	NC_000004.11
cg22185643	3.46E-10	2.67E-11	7.07	14.7767952	9.46E-02	72961	73084	NC_000009.11
cg12152384	3.47E-10	2.68E-11	7.07	14.7743838	1.59E-01	47164566	47164689	NC_000012.11
cg09107912	3.47E-10	2.68E-11	7.07	14.7740169	2.08E-01	159590059	159590182	NC_000006.11
cg07893575	3.47E-10	2.68E-11	7.07	14.7738798	1.35E-01	65289751	65289874	NC_000008.10
cg03532037	3.47E-10	2.68E-11	7.07	14.7726913	9.30E-02	106959205	106959328	NC_000002.11
cg03682421	3.49E-10	2.69E-11	7.07	14.7681386	1.55E-01	14320549	14320672	NC_000019.9
cg02743342	3.49E-10	2.70E-11	7.07	14.7676516	1.36E-01	126984564	126984687	NC_000005.9
cg25044651	3.49E-10	2.70E-11	7.07	14.7664936	1.18E-01	115298720	115298843	NC_000005.9
cg05544500	3.50E-10	2.70E-11	7.07	14.7662622	1.02E-01	54324346	54324469	NC_000016.9
cg11286436	3.50E-10	2.70E-11	7.07	14.7644738	1.68E-01	88798582	88798705	NC_000015.9
cg00126889	3.51E-10	2.71E-11	7.07	14.7620096	1.13E-01	42103037	42103160	NC_000008.10
cg12841525	3.51E-10	2.71E-11	7.07	14.7619545	5.60E-02	38953403	38953526	NC_000017.10
cg08667569	3.52E-10	2.72E-11	7.07	14.7601128	1.59E-01	147240402	147240525	NC_000005.9
cg05578056	3.52E-10	2.72E-11	7.07	14.7577685	1.41E-01	962909	963032	NC_000016.9
cg14051973	3.53E-10	2.73E-11	7.07	14.7563926	7.08E-02	1973545	1973668	NC_000011.9
cg24368848	3.53E-10	2.73E-11	7.07	14.756239	2.13E-01	58545160	58545283	NC_000019.9

cg10991599	3.54E-10	2.73E-11	7.07	14.7541051	1.38E-01	26506539	26506662	NC_000010.10
cg15180867	3.54E-10	2.73E-11	7.07	14.7539698	1.24E-01	591076	591199	NC_000020.10
cg02228403	3.54E-10	2.74E-11	7.07	14.7514368	9.72E-02	11522965	11523088	NC_000003.11
cg03182917	3.56E-10	2.75E-11	7.07	14.7482499	1.71E-01	176944601	176944724	NC_000002.11
cg16211684	3.56E-10	2.75E-11	7.07	14.7480884	1.82E-01	85954622	85954745	NC_000010.10
cg21007852	3.56E-10	2.75E-11	7.07	14.7473226	1.38E-01	27203546	27203669	NC_000007.13
cg19547319	3.56E-10	2.76E-11	7.07	14.7457612	1.45E-01	102987993	102988116	NC_000010.10
cg13356709	3.57E-10	2.76E-11	7.07	14.7451363	1.20E-01	35338762	35338885	NC_000005.9
cg11202634	3.57E-10	2.76E-11	7.07	14.7438758	7.86E-02	37184569	37184692	NC_000008.10
cg13290745	3.57E-10	2.76E-11	7.06	14.743188	5.82E-02	159655866	159655989	NC_000005.9
cg03109101	3.58E-10	2.77E-11	7.06	14.7416644	1.37E-01	119305095	119305218	NC_000010.10
cg26567423	3.58E-10	2.77E-11	7.06	14.7408553	1.82E-01	54500619	54500742	NC_000012.11
cg06869796	3.58E-10	2.77E-11	7.06	14.7402623	7.89E-02	135412196	135412319	NC_000007.13
cg15292275	3.58E-10	2.77E-11	7.06	14.7397335	2.22E-01	109149011	109149134	NC_000013.10
cg07492910	3.59E-10	2.77E-11	7.06	14.7393636	9.44E-02	27235514	27235637	NC_000010.10
cg05529899	3.59E-10	2.77E-11	7.06	14.7393031	5.95E-02	45206768	45206891	NC_000021.8
cg17418463	3.59E-10	2.78E-11	7.06	14.7380116	2.26E-01	35395541	35395664	NC_000001.10
cg00102726	3.59E-10	2.78E-11	7.06	14.7372651	9.18E-02	46897181	46897304	NC_000021.8
cg19628603	3.59E-10	2.78E-11	7.06	14.7368868	1.08E-01	32847596	32847719	NC_000006.11
cg25472589	3.60E-10	2.79E-11	7.06	14.7335898	1.33E-01	107995927	107996050	NC_000011.9
cg21973405	3.61E-10	2.79E-11	7.06	14.7325346	6.26E-02	54826547	54826670	NC_000005.9
cg21319323	3.62E-10	2.80E-11	7.06	14.7292537	1.81E-01	177001263	177001386	NC_000002.11
cg26866174	3.62E-10	2.81E-11	7.06	14.7280663	7.34E-02	71177163	71177286	NC_000010.10
cg18027632	3.63E-10	2.81E-11	7.06	14.7272226	3.69E-02	142278163	142278286	NC_000008.10
cg05667158	3.63E-10	2.81E-11	7.06	14.7271431	2.08E-01	4381890	4382013	NC_000012.11

cg08717880	3.63E-10	2.81E-11	7.06	14.7264015	1.39E-01	177014849	177014972	NC_000002.11
cg24740531	3.63E-10	2.81E-11	7.06	14.7254762	2.22E-01	60985732	60985855	NC_000018.9
cg26975086	3.64E-10	2.82E-11	7.06	14.7222631	1.73E-01	42533282	42533405	NC_000007.13
cg09231171	3.65E-10	2.82E-11	7.06	14.7220241	2.00E-01	49518414	49518537	NC_000016.9
cg03929570	3.66E-10	2.83E-11	7.06	14.7181226	1.16E-01	70484978	70485101	NC_000016.9
cg01622930	3.66E-10	2.84E-11	7.06	14.7178072	4.95E-02	3960266	3960389	NC_000004.11
cg09542210	3.66E-10	2.84E-11	7.06	14.7172672	2.01E-01	157821536	157821659	NC_000003.11
cg09775582	3.66E-10	2.84E-11	7.06	14.7164624	2.95E-01	45810865	45810988	NC_000017.10
cg16324314	3.67E-10	2.84E-11	7.06	14.7158249	1.35E-01	77480847	77480970	NC_000014.8
cg22369879	3.67E-10	2.84E-11	7.06	14.7155697	1.73E-01	137228636	137228759	NC_000003.11
cg11946666	3.67E-10	2.85E-11	7.06	14.7142835	1.06E-01	3247595	3247718	NC_000001.10
cg15678861	3.67E-10	2.85E-11	7.06	14.7140365	5.60E-02	144462502	144462625	NC_000008.10
cg16543094	3.67E-10	2.85E-11	7.06	14.7133562	1.90E-01	20229517	20229640	NC_000011.9
cg22355517	3.67E-10	2.85E-11	7.06	14.713113	1.57E-01	20522674	20522797	NC_000012.11
cg20905516	3.68E-10	2.85E-11	7.06	14.7127949	2.49E-01	106889364	106889487	NC_000011.9
cg27422759	3.68E-10	2.85E-11	7.06	14.7119201	5.19E-02	69970464	69970587	NC_000011.9
cg01101663	3.68E-10	2.85E-11	7.06	14.7118252	1.13E-01	140564161	140564284	NC_000005.9
cg14568217	3.70E-10	2.87E-11	7.06	14.7059801	1.98E-01	176057061	176057184	NC_000005.9
cg26132511	3.70E-10	2.87E-11	7.06	14.7059112	7.94E-02	38045143	38045266	NC_000006.11
cg22891500	3.70E-10	2.87E-11	7.06	14.705466	1.93E-01	23653996	23654119	NC_000019.9
cg00648020	3.71E-10	2.88E-11	7.06	14.7040475	9.79E-02	134992340	134992463	NC_000010.10
cg18030960	3.71E-10	2.88E-11	7.06	14.7025829	1.60E-01	13530948	13531071	NC_000004.11
cg19547547	3.71E-10	2.88E-11	7.06	14.7019233	1.04E-01	140166059	140166182	NC_000005.9
cg27440715	3.71E-10	2.88E-11	7.06	14.7018789	1.72E-01	165325169	165325292	NC_000001.10
cg04558424	3.73E-10	2.89E-11	7.06	14.6986215	9.56E-02	85743497	85743620	NC_000016.9

cg10397765	3.73E-10	2.89E-11	7.06	14.6980415	2.05E-01	61113109	61113232	NC_000014.8
cg16240162	3.74E-10	2.91E-11	7.06	14.6936348	1.70E-01	147106890	147107013	NC_000003.11
cg09986292	3.74E-10	2.91E-11	7.06	14.6935486	1.29E-01	13525605	13525728	NC_000004.11
cg00816406	3.75E-10	2.91E-11	7.06	14.6920864	1.94E-01	240286626	240286749	NC_000001.10
cg20440891	3.75E-10	2.91E-11	7.06	14.6916221	1.13E-01	28448103	28448226	NC_000007.13
cg11417701	3.75E-10	2.91E-11	7.06	14.6913485	1.48E-01	401311	401434	NC_000006.11
cg10029213	3.76E-10	2.92E-11	7.06	14.6905513	1.98E-01	117716312	117716435	NC_000003.11
cg04651781	3.76E-10	2.92E-11	7.06	14.6896597	1.77E-01	1398798	1398921	NC_000004.11
cg23337845	3.76E-10	2.92E-11	7.06	14.6896579	4.04E-02	170641374	170641497	NC_000004.11
cg13882665	3.78E-10	2.93E-11	7.05	14.6846958	6.21E-02	169560079	169560202	NC_000006.11
cg23627057	3.78E-10	2.94E-11	7.05	14.6833206	5.83E-02	140154394	140154517	NC_000007.13
cg18087672	3.78E-10	2.94E-11	7.05	14.6825066	2.03E-01	46824915	46825038	NC_000017.10
cg26692818	3.79E-10	2.94E-11	7.05	14.681644	6.01E-02	87108066	87108189	NC_000001.10
cg22704696	3.79E-10	2.94E-11	7.05	14.6815516	1.02E-01	47302476	47302599	NC_000017.10
cg07139946	3.80E-10	2.95E-11	7.05	14.6786805	8.87E-02	32037339	32037462	NC_000006.11
cg08312626	3.80E-10	2.95E-11	7.05	14.6784306	1.78E-01	27534137	27534260	NC_000006.11
cg17795158	3.80E-10	2.95E-11	7.05	14.6781169	1.69E-01	124908427	124908550	NC_000010.10
cg13067553	3.80E-10	2.95E-11	7.05	14.6774022	1.93E-01	147782452	147782575	NC_000001.10
cg06757662	3.80E-10	2.95E-11	7.05	14.6773679	1.83E-01	101924596	101924719	NC_000014.8
cg00992850	3.81E-10	2.96E-11	7.05	14.6758257	1.62E-01	25900703	25900826	NC_000007.13
cg01598007	3.81E-10	2.96E-11	7.05	14.6757299	1.02E-01	167063581	167063704	NC_000001.10
cg10890302	3.81E-10	2.96E-11	7.05	14.6746877	1.86E-01	32064246	32064369	NC_000006.11
cg00861610	3.82E-10	2.97E-11	7.05	14.6724159	1.36E-01	62065095	62065218	NC_000004.11
cg05843457	3.82E-10	2.97E-11	7.05	14.6718588	1.94E-01	158147849	158147972	NC_000001.10
cg23256459	3.83E-10	2.97E-11	7.05	14.6709341	6.05E-02	66713103	66713226	NC_000005.9

cg00493304	3.83E-10	2.98E-11	7.05	14.6705755	1.01E-01	48844908	48845031	NC_000002.11
cg25405962	3.84E-10	2.98E-11	7.05	14.66779	1.37E-01	9852785	9852908	NC_000001.10
cg26517663	3.84E-10	2.98E-11	7.05	14.6674372	1.61E-01	60295294	60295417	NC_000015.9
cg02615582	3.84E-10	2.99E-11	7.05	14.6670866	1.16E-01	49939549	49939672	NC_000019.9
cg20406528	3.84E-10	2.99E-11	7.05	14.6667948	6.70E-02	94980962	94981085	NC_000010.10
cg13634602	3.84E-10	2.99E-11	7.05	14.6656744	2.16E-01	76739226	76739349	NC_000018.9
cg16170346	3.85E-10	2.99E-11	7.05	14.6655773	1.09E-01	2424006	2424129	NC_000019.9
cg14197923	3.86E-10	3.00E-11	7.05	14.6622264	1.93E-01	81187906	81188029	NC_000004.11
cg13755204	3.86E-10	3.00E-11	7.05	14.6622026	1.02E-01	136844768	136844891	NC_000009.11
cg14978307	3.86E-10	3.00E-11	7.05	14.6621156	1.33E-01	155242684	155242807	NC_000007.13
cg06672163	3.86E-10	3.00E-11	7.05	14.6617395	9.93E-02	88455674	88455797	NC_000016.9
cg26657675	3.86E-10	3.00E-11	7.05	14.6615373	1.04E-01	85575407	85575530	NC_000016.9
cg00733780	3.86E-10	3.01E-11	7.05	14.6604924	2.11E-01	26505127	26505250	NC_000010.10
cg21749556	3.87E-10	3.01E-11	7.05	14.6600054	1.21E-01	51680555	51680678	NC_000007.13
cg03719026	3.87E-10	3.01E-11	7.05	14.6594305	3.48E-02	6619479	6619602	NC_000012.11
cg15122993	3.87E-10	3.01E-11	7.05	14.6590802	1.07E-01	140256817	140256940	NC_000005.9
cg13264183	3.87E-10	3.01E-11	7.05	14.6581605	7.60E-02	126194992	126195115	NC_000003.11
cg23306526	3.87E-10	3.01E-11	7.05	14.6581517	1.44E-01	45171583	45171706	NC_000002.11
cg03753331	3.87E-10	3.01E-11	7.05	14.6575673	2.30E-01	115919006	115919129	NC_000002.11
cg16698674	3.88E-10	3.02E-11	7.05	14.6572272	7.81E-02	230272358	230272481	NC_000001.10
cg07636117	3.88E-10	3.02E-11	7.05	14.6559427	1.37E-01	237072957	237073080	NC_000002.11
cg05315047	3.89E-10	3.03E-11	7.05	14.6540337	8.08E-02	137766727	137766850	NC_000003.11
cg20528583	3.89E-10	3.03E-11	7.05	14.6530732	2.03E-01	81187610	81187733	NC_000004.11
cg15726245	3.89E-10	3.03E-11	7.05	14.652598	6.93E-02	27333506	27333629	NC_000013.10
cg01406506	3.89E-10	3.03E-11	7.05	14.652292	1.23E-01	147938667	147938790	NC_000005.9

cg05659187	3.90E-10	3.03E-11	7.05	14.6513693	1.99E-01	70995522	70995645	NC_000002.11
cg23387863	3.90E-10	3.04E-11	7.05	14.650005	1.34E-01	77472416	77472539	NC_000015.9
cg02304353	3.90E-10	3.04E-11	7.05	14.6494604	9.01E-02	79215938	79216061	NC_000017.10
cg24680632	3.90E-10	3.04E-11	7.05	14.64922	1.60E-01	116044032	116044155	NC_000012.11
cg13699245	3.91E-10	3.04E-11	7.05	14.6479258	1.19E-01	137485382	137485505	NC_000009.11
cg21693015	3.91E-10	3.05E-11	7.05	14.647584	3.23E-02	30303537	30303660	NC_000006.11
cg14565439	3.91E-10	3.05E-11	7.05	14.6471582	4.73E-02	107702185	107702308	NC_000005.9
cg23197945	3.91E-10	3.05E-11	7.05	14.6471303	1.80E-01	101841303	101841426	NC_000010.10
cg22175764	3.91E-10	3.05E-11	7.05	14.6468745	2.11E-01	56803964	56804087	NC_000020.10
cg03446973	3.91E-10	3.05E-11	7.05	14.6463467	2.50E-02	186524572	186524695	NC_000003.11
cg09658678	3.92E-10	3.05E-11	7.05	14.6450911	8.30E-02	148448256	148448379	NC_000003.11
cg22195627	3.92E-10	3.06E-11	7.05	14.6438865	2.16E-01	12305869	12305992	NC_000019.9
cg14319235	3.92E-10	3.06E-11	7.05	14.6436717	1.55E-01	79472282	79472405	NC_000001.10
cg19713959	3.93E-10	3.06E-11	7.05	14.6431257	5.93E-02	112092994	112093117	NC_000006.11
cg07252933	3.94E-10	3.07E-11	7.05	14.6408147	9.56E-02	44184114	44184237	NC_000006.11
cg24094706	3.94E-10	3.07E-11	7.05	14.6406771	1.85E-01	48972536	48972659	NC_000022.10
cg12376271	3.95E-10	3.08E-11	7.05	14.6375722	4.83E-02	95799144	95799267	NC_000011.9
cg23848624	3.95E-10	3.08E-11	7.05	14.6370872	1.89E-01	100622582	100622705	NC_000013.10
cg00842299	3.95E-10	3.08E-11	7.05	14.6360263	1.40E-01	147129278	147129401	NC_000003.11
cg04012282	3.95E-10	3.08E-11	7.05	14.635743	8.65E-02	138739807	138739930	NC_000003.11
cg09166898	3.95E-10	3.08E-11	7.05	14.6356903	5.19E-02	41167165	41167288	NC_000015.9
cg26385256	3.95E-10	3.08E-11	7.05	14.6356103	1.88E-01	38326334	38326457	NC_000008.10
cg22225179	3.96E-10	3.08E-11	7.05	14.6351437	8.44E-02	56072363	56072486	NC_000017.10
cg17896808	3.96E-10	3.09E-11	7.05	14.6338622	7.86E-02	22992842	22992965	NC_000015.9
cg22060087	3.96E-10	3.09E-11	7.05	14.6336197	3.65E-02	32892208	32892331	NC_000020.10

cg21319053	3.96E-10	3.09E-11	7.05	14.6336041	1.84E-01	37840725	37840848	NC_000005.9
cg04375784	3.96E-10	3.09E-11	7.05	14.6331977	1.07E-01	133137810	133137933	NC_000006.11
cg11518846	3.98E-10	3.10E-11	7.04	14.6298562	1.88E-01	133562246	133562369	NC_000006.11
cg22692507	3.98E-10	3.10E-11	7.04	14.6291986	7.14E-02	81446503	81446626	NC_000005.9
cg03182688	3.99E-10	3.11E-11	7.04	14.6275387	1.29E-01	33825300	33825423	NC_000017.10
cg25831075	3.99E-10	3.11E-11	7.04	14.6266326	1.65E-01	164915196	164915319	NC_000003.11
cg05417162	3.99E-10	3.11E-11	7.04	14.6264052	1.56E-01	89959854	89959977	NC_000015.9
cg01445580	3.99E-10	3.12E-11	7.04	14.62512	1.94E-01	34631891	34632014	NC_000001.10
cg03294619	3.99E-10	3.12E-11	7.04	14.6250236	1.72E-01	172661803	172661926	NC_000005.9
cg23174932	3.99E-10	3.12E-11	7.04	14.625009	2.34E-01	31831502	31831625	NC_000006.11
cg22160472	4.00E-10	3.12E-11	7.04	14.6238678	1.33E-01	42944494	42944617	NC_000005.9
cg06940705	4.00E-10	3.12E-11	7.04	14.6232639	1.68E-01	14924712	14924835	NC_000001.10
cg02921923	4.01E-10	3.13E-11	7.04	14.6211941	1.45E-01	92651425	92651548	NC_000011.9
cg24399395	4.01E-10	3.13E-11	7.04	14.6199574	1.79E-01	34392203	34392326	NC_000021.8
cg25558103	4.02E-10	3.14E-11	7.04	14.6190829	1.53E-01	42835859	42835982	NC_000017.10
cg19719547	4.02E-10	3.14E-11	7.04	14.6170237	1.95E-01	242688144	242688267	NC_000001.10
cg19392831	4.03E-10	3.14E-11	7.04	14.6159861	9.28E-02	120355756	120355879	NC_000010.10
cg11460364	4.03E-10	3.15E-11	7.04	14.6156967	9.84E-02	162931397	162931520	NC_000002.11
cg24884519	4.03E-10	3.15E-11	7.04	14.6153169	2.35E-01	27209790	27209913	NC_000007.13
cg22339356	4.04E-10	3.15E-11	7.04	14.6137833	1.98E-01	8473385	8473508	NC_000007.13
cg16939112	4.04E-10	3.15E-11	7.04	14.6136812	1.16E-01	1807305	1807428	NC_000012.11
cg11870560	4.04E-10	3.16E-11	7.04	14.6125261	1.81E-01	112709480	112709603	NC_000013.10
cg08381013	4.05E-10	3.16E-11	7.04	14.6108797	3.69E-02	1862572	1862695	NC_000008.10
cg03791159	4.05E-10	3.16E-11	7.04	14.6106466	1.29E-01	155018144	155018267	NC_000007.13
cg02180189	4.05E-10	3.16E-11	7.04	14.6103039	1.10E-01	2710486	2710609	NC_000011.9

cg18434718	4.05E-10	3.17E-11	7.04	14.6095284	2.13E-01	15090872	15090995	NC_000019.9
cg27256528	4.06E-10	3.17E-11	7.04	14.6085805	1.72E-01	6533667	6533790	NC_000016.9
cg14015706	4.06E-10	3.17E-11	7.04	14.6068917	2.53E-01	132382433	132382556	NC_000009.11
cg18499321	4.07E-10	3.18E-11	7.04	14.6055335	1.41E-01	130941214	130941337	NC_000012.11
cg17231999	4.07E-10	3.18E-11	7.04	14.6037076	2.21E-01	41882564	41882687	NC_000004.11
cg00945234	4.08E-10	3.19E-11	7.04	14.6025769	1.62E-01	50886411	50886534	NC_000001.10
cg20627174	4.09E-10	3.20E-11	7.04	14.5994669	1.80E-01	119599744	119599867	NC_000002.11
cg03622666	4.09E-10	3.20E-11	7.04	14.5993078	9.28E-02	49499091	49499214	NC_000016.9
cg08134803	4.10E-10	3.20E-11	7.04	14.5975676	6.07E-02	1218913	1219036	NC_000011.9
cg22272713	4.11E-10	3.21E-11	7.04	14.5947358	1.37E-01	21780820	21780943	NC_000008.10
cg06899530	4.11E-10	3.22E-11	7.04	14.5942005	1.59E-01	166916866	166916989	NC_000001.10
cg25194720	4.11E-10	3.22E-11	7.04	14.5939303	1.24E-01	128712417	128712540	NC_000011.9
cg26181818	4.12E-10	3.22E-11	7.04	14.5932983	1.33E-01	233323935	233324058	NC_000002.11
cg21901718	4.12E-10	3.22E-11	7.04	14.5932332	2.09E-01	16180076	16180199	NC_000005.9
cg00040427	4.12E-10	3.22E-11	7.04	14.5926013	9.35E-02	14044197	14044320	NC_000019.9
cg04425632	4.12E-10	3.22E-11	7.04	14.5924185	1.83E-01	137531612	137531735	NC_000007.13
cg08828403	4.12E-10	3.22E-11	7.04	14.592142	1.69E-01	21681006	21681129	NC_000012.11
cg00395579	4.13E-10	3.23E-11	7.04	14.5897505	1.58E-01	103243598	103243721	NC_000008.10
cg18359596	4.13E-10	3.23E-11	7.04	14.5896152	7.96E-02	89875471	89875594	NC_000014.8
cg16019558	4.14E-10	3.24E-11	7.04	14.5880052	8.46E-02	110104633	110104756	NC_000006.11
cg21347353	4.14E-10	3.24E-11	7.04	14.5869507	1.94E-01	25678439	25678562	NC_000022.10
cg08370882	4.14E-10	3.24E-11	7.04	14.5869133	1.36E-01	219522648	219522771	NC_000002.11
cg08595806	4.15E-10	3.25E-11	7.04	14.583388	7.44E-02	160512464	160512587	NC_000006.11
cg02332525	4.16E-10	3.25E-11	7.04	14.5830297	1.28E-01	6903153	6903276	NC_000003.11
cg00402352	4.17E-10	3.26E-11	7.04	14.579569	9.54E-02	134373160	134373283	NC_000011.9

cg19947439	4.17E-10	3.27E-11	7.04	14.578655	1.09E-01	186795552	186795675	NC_000003.11
cg03152428	4.19E-10	3.28E-11	7.03	14.5738672	1.08E-01	46850691	46850814	NC_000021.8
cg13833923	4.19E-10	3.28E-11	7.03	14.5735854	1.92E-01	79746627	79746750	NC_000014.8
cg02741229	4.20E-10	3.29E-11	7.03	14.5728784	1.86E-01	140767197	140767320	NC_000005.9
cg15412401	4.20E-10	3.29E-11	7.03	14.5727907	6.37E-02	33170699	33170822	NC_000006.11
cg21342910	4.20E-10	3.29E-11	7.03	14.5727581	1.76E-01	48042779	48042902	NC_000017.10
cg16475255	4.20E-10	3.29E-11	7.03	14.5723851	6.52E-02	72671985	72672108	NC_000015.9
cg10306035	4.21E-10	3.30E-11	7.03	14.5691095	1.06E-01	23464518	23464641	NC_000010.10
cg00333703	4.22E-10	3.30E-11	7.03	14.5681384	1.56E-01	123384406	123384529	NC_000007.13
cg03969515	4.23E-10	3.31E-11	7.03	14.5649452	1.20E-01	16553267	16553390	NC_000001.10
cg00907505	4.23E-10	3.31E-11	7.03	14.5645757	6.90E-02	77715338	77715461	NC_000017.10
cg14765646	4.24E-10	3.32E-11	7.03	14.5615348	2.15E-01	154334450	154334573	NC_000002.11
cg23916167	4.25E-10	3.33E-11	7.03	14.560221	2.14E-01	37027267	37027390	NC_000009.11
cg09303778	4.25E-10	3.33E-11	7.03	14.5592361	5.65E-02	33269832	33269955	NC_000006.11
cg26363038	4.26E-10	3.33E-11	7.03	14.5585307	7.30E-02	155970301	155970424	NC_000007.13
cg05291588	4.26E-10	3.33E-11	7.03	14.5583496	9.40E-02	1607090	1607213	NC_000002.11
cg01541600	4.26E-10	3.34E-11	7.03	14.5578566	9.59E-02	87988715	87988838	NC_000016.9
cg11021321	4.26E-10	3.34E-11	7.03	14.5574082	1.19E-01	57471660	57471783	NC_000020.10
cg19280364	4.26E-10	3.34E-11	7.03	14.5572926	1.09E-01	73077442	73077565	NC_000015.9
cg12530503	4.27E-10	3.34E-11	7.03	14.5558495	1.17E-01	89911148	89911271	NC_000015.9
cg15657100	4.27E-10	3.34E-11	7.03	14.5558434	1.23E-01	161420987	161421110	NC_000002.11
cg22848598	4.29E-10	3.36E-11	7.03	14.5508847	2.25E-01	38965026	38965149	NC_000008.10
cg13320291	4.29E-10	3.36E-11	7.03	14.5499995	2.25E-01	118030970	118031093	NC_000010.10
cg19252741	4.29E-10	3.36E-11	7.03	14.5499121	1.89E-01	68456607	68456730	NC_000011.9
cg01458510	4.29E-10	3.36E-11	7.03	14.5499119	1.23E-01	109752622	109752745	NC_000002.11

cg15373880	4.29E-10	3.37E-11	7.03	14.5492839	1.03E-01	3783345	3783468	NC_000006.11
cg02749843	4.30E-10	3.37E-11	7.03	14.5479917	8.21E-02	19712623	19712746	NC_000022.10
cg08864344	4.30E-10	3.37E-11	7.03	14.5473429	1.22E-01	33772997	33773120	NC_000001.10
cg12829687	4.31E-10	3.38E-11	7.03	14.5450209	1.14E-01	114581004	114581127	NC_000010.10
cg10385303	4.32E-10	3.39E-11	7.03	14.5426637	1.64E-01	36983146	36983269	NC_000014.8
cg01161204	4.32E-10	3.39E-11	7.03	14.5412928	1.68E-01	115530932	115531055	NC_000011.9
cg00865541	4.33E-10	3.40E-11	7.03	14.5404381	8.44E-02	56279934	56280057	NC_000018.9
cg02122467	4.33E-10	3.40E-11	7.03	14.540394	3.71E-02	43027820	43027943	NC_000015.9
cg25495735	4.33E-10	3.40E-11	7.03	14.5399208	5.26E-02	169284053	169284176	NC_000006.11
cg06824157	4.33E-10	3.40E-11	7.03	14.5396039	1.31E-01	14457501	14457624	NC_000005.9
cg05475524	4.33E-10	3.40E-11	7.03	14.5392852	1.84E-01	66326798	66326921	NC_000011.9
cg19946376	4.34E-10	3.40E-11	7.03	14.5380528	1.05E-01	184035137	184035260	NC_000003.11
cg16526961	4.34E-10	3.41E-11	7.03	14.5374823	8.40E-02	43965849	43965972	NC_000012.11
cg12699286	4.34E-10	3.41E-11	7.03	14.5362976	2.18E-01	54394194	54394317	NC_000012.11
cg20086208	4.35E-10	3.42E-11	7.03	14.5345162	1.99E-01	20085214	20085337	NC_000016.9
cg02773588	4.35E-10	3.42E-11	7.03	14.5341786	9.65E-02	48698519	48698642	NC_000003.11
cg26194089	4.36E-10	3.42E-11	7.03	14.532854	1.25E-01	2862360	2862483	NC_000012.11
cg00728907	4.37E-10	3.43E-11	7.03	14.5313812	5.75E-02	1285487	1285610	NC_000005.9
cg23563866	4.38E-10	3.44E-11	7.03	14.5288445	1.19E-01	22246575	22246698	NC_000008.10
cg13551706	4.38E-10	3.44E-11	7.03	14.5277768	7.56E-02	46323481	46323604	NC_000020.10
cg15976883	4.39E-10	3.45E-11	7.03	14.525613	1.02E-01	54960972	54961095	NC_000004.11
cg21932452	4.39E-10	3.45E-11	7.03	14.5244859	6.38E-02	150702471	150702594	NC_000007.13
cg15929698	4.40E-10	3.46E-11	7.03	14.5235438	1.52E-01	24323792	24323915	NC_000007.13
cg08551532	4.40E-10	3.46E-11	7.03	14.5234529	1.70E-01	39998270	39998393	NC_000019.9
cg16531275	4.40E-10	3.46E-11	7.03	14.5233332	1.07E-01	89927907	89928030	NC_000016.9

cg23030159	4.40E-10	3.46E-11	7.03	14.5227906	8.63E-02	1358467	1358590	NC_000007.13
cg10793234	4.42E-10	3.47E-11	7.03	14.5192649	5.13E-02	194062061	194062184	NC_000003.11
cg06161964	4.42E-10	3.47E-11	7.03	14.5190707	1.54E-01	1884513	1884636	NC_000005.9
cg18335607	4.43E-10	3.48E-11	7.02	14.5169356	1.50E-01	172755413	172755536	NC_000005.9
cg05928053	4.43E-10	3.48E-11	7.02	14.5165449	1.46E-01	182549565	182549688	NC_000002.11
cg17412258	4.44E-10	3.49E-11	7.02	14.5133664	1.39E-01	101193017	101193140	NC_000014.8
cg09780768	4.44E-10	3.49E-11	7.02	14.5133354	1.18E-01	54498824	54498947	NC_000019.9
cg12378888	4.44E-10	3.49E-11	7.02	14.5128583	1.83E-01	4867102	4867225	NC_000004.11
cg03612765	4.45E-10	3.50E-11	7.02	14.5114634	5.24E-02	89552576	89552699	NC_000001.10
cg01957732	4.46E-10	3.50E-11	7.02	14.5100043	1.70E-01	133561716	133561839	NC_000006.11
cg04048339	4.46E-10	3.51E-11	7.02	14.509263	8.33E-02	31496644	31496767	NC_000006.11
cg11123303	4.46E-10	3.51E-11	7.02	14.5082643	9.05E-02	132946216	132946339	NC_000006.11
cg07033549	4.46E-10	3.51E-11	7.02	14.508009	3.96E-02	101944576	101944699	NC_000014.8
cg25472539	4.47E-10	3.52E-11	7.02	14.50573	9.62E-02	32037978	32038101	NC_000006.11
cg06008912	4.48E-10	3.52E-11	7.02	14.5045962	2.37E-01	193059645	193059768	NC_000002.11
cg13485685	4.48E-10	3.52E-11	7.02	14.5043415	1.38E-01	20255805	20255928	NC_000004.11
cg15169469	4.49E-10	3.53E-11	7.02	14.502737	8.01E-02	21398575	21398698	NC_000022.10
cg24549161	4.49E-10	3.53E-11	7.02	14.5016267	9.56E-02	120278049	120278172	NC_000002.11
cg13844402	4.50E-10	3.54E-11	7.02	14.4993683	1.41E-01	112716025	112716148	NC_000013.10
cg00535403	4.51E-10	3.55E-11	7.02	14.497515	9.96E-02	120570282	120570405	NC_000002.11
cg21038156	4.51E-10	3.55E-11	7.02	14.497394	1.53E-01	158936739	158936862	NC_000007.13
cg19680222	4.51E-10	3.55E-11	7.02	14.4964555	4.85E-02	160253463	160253586	NC_000001.10
cg23144681	4.51E-10	3.55E-11	7.02	14.4964296	1.92E-01	23488710	23488833	NC_000010.10
cg08682532	4.51E-10	3.55E-11	7.02	14.4961719	1.38E-01	74427499	74427622	NC_000015.9
cg17223809	4.52E-10	3.55E-11	7.02	14.4959677	1.20E-01	86620676	86620799	NC_000001.10

cg03290530	4.52E-10	3.56E-11	7.02	14.4950988	1.70E-01	137361526	137361649	NC_000003.11
cg13243168	4.52E-10	3.56E-11	7.02	14.4947589	1.13E-01	61915833	61915956	NC_000017.10
cg18452695	4.52E-10	3.56E-11	7.02	14.4946277	1.50E-01	9608637	9608760	NC_000019.9
cg24956250	4.53E-10	3.57E-11	7.02	14.4928061	2.05E-01	139168481	139168604	NC_000007.13
cg19656950	4.53E-10	3.57E-11	7.02	14.4922096	8.60E-02	100607772	100607895	NC_000013.10
cg16649888	4.54E-10	3.57E-11	7.02	14.4911547	1.34E-01	96302476	96302599	NC_000010.10
cg12411917	4.54E-10	3.57E-11	7.02	14.4903132	7.80E-02	8014323	8014446	NC_000006.11
cg15451075	4.54E-10	3.58E-11	7.02	14.4900079	6.12E-02	148966942	148967065	NC_000003.11
cg06740600	4.54E-10	3.58E-11	7.02	14.4892949	2.23E-01	38924436	38924559	NC_000019.9
cg00710196	4.55E-10	3.58E-11	7.02	14.4885762	3.96E-02	36870572	36870695	NC_000017.10
cg15975122	4.55E-10	3.58E-11	7.02	14.4878559	5.45E-02	195379862	195379985	NC_000003.11
cg05929882	4.55E-10	3.58E-11	7.02	14.4877168	1.92E-01	114847508	114847631	NC_000012.11
cg05269473	4.55E-10	3.59E-11	7.02	14.4871116	3.96E-02	224801070	224801193	NC_000002.11
cg05207973	4.55E-10	3.59E-11	7.02	14.4867022	8.89E-02	176823642	176823765	NC_000005.9
cg19700380	4.55E-10	3.59E-11	7.02	14.4866473	8.10E-02	36897562	36897685	NC_000017.10
cg07018645	4.56E-10	3.59E-11	7.02	14.4864103	8.60E-02	75108826	75108949	NC_000015.9
cg17603689	4.57E-10	3.60E-11	7.02	14.4841707	1.25E-01	50883013	50883136	NC_000001.10
cg01625087	4.57E-10	3.60E-11	7.02	14.4822703	1.69E-01	101841008	101841131	NC_000006.11
cg08663552	4.59E-10	3.62E-11	7.02	14.4788389	1.16E-01	20755508	20755631	NC_000017.10
cg17028658	4.59E-10	3.62E-11	7.02	14.4786353	1.27E-01	186321177	186321300	NC_000004.11
cg09198601	4.59E-10	3.62E-11	7.02	14.4782556	1.06E-01	10319180	10319303	NC_000006.11
cg25300694	4.59E-10	3.62E-11	7.02	14.4782047	1.14E-01	151184358	151184481	NC_000002.11
cg01082843	4.60E-10	3.62E-11	7.02	14.4770041	2.00E-01	210636844	210636967	NC_000002.11
cg00718418	4.60E-10	3.63E-11	7.02	14.4752069	1.03E-01	50678441	50678564	NC_000012.11
cg06176201	4.61E-10	3.63E-11	7.02	14.4738816	2.65E-02	40963033	40963156	NC_000017.10

cg20392615	4.61E-10	3.63E-11	7.02	14.4738345	1.36E-01	125732565	125732688	NC_000008.10
cg22282405	4.61E-10	3.64E-11	7.02	14.473651	2.02E-01	50810682	50810805	NC_000006.11
cg06462347	4.61E-10	3.64E-11	7.02	14.4736028	2.18E-01	5404508	5404631	NC_000017.10
cg22517388	4.62E-10	3.64E-11	7.02	14.4727419	1.02E-01	71014918	71015041	NC_000005.9
cg08194313	4.62E-10	3.65E-11	7.02	14.4706232	2.36E-01	118032892	118033015	NC_000010.10
cg10455938	4.63E-10	3.65E-11	7.02	14.4705708	2.14E-01	40058150	40058273	NC_000022.10
cg00065215	4.63E-10	3.65E-11	7.02	14.4701	2.20E-01	18959268	18959391	NC_000001.10
cg06017559	4.64E-10	3.66E-11	7.02	14.4662036	9.25E-02	1026760	1026883	NC_000016.9
cg06912966	4.65E-10	3.67E-11	7.02	14.464888	2.11E-01	114852359	114852482	NC_000012.11
cg00573235	4.65E-10	3.67E-11	7.02	14.4639589	1.26E-01	56883532	56883655	NC_000016.9
cg12427264	4.66E-10	3.68E-11	7.02	14.4624986	5.95E-02	144508349	144508472	NC_000006.11
cg06971129	4.66E-10	3.68E-11	7.01	14.461753	2.74E-01	78493066	78493189	NC_000013.10
cg24725789	4.67E-10	3.68E-11	7.01	14.4613299	1.91E-01	119310288	119310411	NC_000010.10
cg20015986	4.67E-10	3.68E-11	7.01	14.4610654	1.17E-01	62072253	62072376	NC_000004.11
cg17886536	4.67E-10	3.68E-11	7.01	14.4610004	4.20E-02	16320218	16320341	NC_000003.11
cg03775416	4.67E-10	3.69E-11	7.01	14.4597303	9.77E-02	19368775	19368898	NC_000019.9
cg17015844	4.68E-10	3.69E-11	7.01	14.458752	1.29E-01	103352694	103352817	NC_000012.11
cg17101165	4.68E-10	3.70E-11	7.01	14.4574819	1.52E-01	115136226	115136349	NC_000012.11
cg04181032	4.69E-10	3.70E-11	7.01	14.4554287	1.17E-01	147165097	147165220	NC_000004.11
cg21549415	4.69E-10	3.70E-11	7.01	14.455326	2.07E-02	79803535	79803658	NC_000017.10
cg07129803	4.69E-10	3.70E-11	7.01	14.455312	9.14E-02	88370583	88370706	NC_000016.9
cg09961254	4.69E-10	3.71E-11	7.01	14.4546532	8.02E-02	627679	627802	NC_000010.10
cg24314474	4.72E-10	3.72E-11	7.01	14.4501522	4.63E-02	40627134	40627257	NC_000003.11
cg18479593	4.73E-10	3.74E-11	7.01	14.4468412	2.25E-01	57265560	57265683	NC_000014.8
cg06558502	4.73E-10	3.74E-11	7.01	14.4462335	1.39E-01	57610699	57610822	NC_000019.9

cg05859076	4.75E-10	3.75E-11	7.01	14.4433786	1.33E-01	128838350	128838473	NC_000003.11
cg26492514	4.75E-10	3.75E-11	7.01	14.4430704	1.76E-01	168728236	168728359	NC_000005.9
cg02211449	4.76E-10	3.76E-11	7.01	14.4415045	2.04E-01	110671609	110671732	NC_000010.10
cg08110874	4.76E-10	3.76E-11	7.01	14.4414628	1.88E-01	36704955	36705078	NC_000013.10
cg11564981	4.76E-10	3.76E-11	7.01	14.439352	1.87E-01	6007670	6007793	NC_000006.11
cg08061040	4.76E-10	3.76E-11	7.01	14.4393464	9.60E-02	149746077	149746200	NC_000007.13
cg04171052	4.77E-10	3.77E-11	7.01	14.4385921	5.76E-02	87879581	87879704	NC_000016.9
cg04600170	4.77E-10	3.77E-11	7.01	14.4385176	1.80E-01	102484499	102484622	NC_000010.10
cg27128734	4.77E-10	3.77E-11	7.01	14.4380147	8.42E-02	51411940	51412063	NC_000019.9
cg03290281	4.77E-10	3.77E-11	7.01	14.4379012	1.06E-01	2632607	2632730	NC_000006.11
cg24010859	4.77E-10	3.77E-11	7.01	14.4376307	1.28E-01	110868153	110868276	NC_000012.11
cg07090057	4.78E-10	3.77E-11	7.01	14.4366109	8.51E-02	108837484	108837607	NC_000003.11
cg19507206	4.78E-10	3.78E-11	7.01	14.4361084	2.27E-01	103044008	103044131	NC_000010.10
cg22235018	4.78E-10	3.78E-11	7.01	14.4351134	2.19E-01	50692150	50692273	NC_000006.11
cg25725843	4.78E-10	3.78E-11	7.01	14.4348727	1.19E-01	107504169	107504292	NC_000002.11
cg11802553	4.79E-10	3.79E-11	7.01	14.4333375	1.61E-01	65203330	65203453	NC_000014.8
cg05640361	4.80E-10	3.79E-11	7.01	14.4324321	5.40E-02	79073545	79073668	NC_000017.10
cg05647503	4.80E-10	3.79E-11	7.01	14.4322044	4.09E-02	10475589	10475712	NC_000011.9
cg08263708	4.80E-10	3.80E-11	7.01	14.430912	1.55E-01	85476373	85476496	NC_000006.11
cg25512912	4.81E-10	3.80E-11	7.01	14.4299415	4.51E-02	64791606	64791729	NC_000015.9
cg16879608	4.81E-10	3.80E-11	7.01	14.429627	8.97E-02	13356921	13357044	NC_000012.11
cg23009508	4.81E-10	3.80E-11	7.01	14.4294576	6.99E-02	23913568	23913691	NC_000002.11
cg02522485	4.82E-10	3.81E-11	7.01	14.4273328	3.32E-02	49531973	49532096	NC_000003.11
cg11536474	4.82E-10	3.81E-11	7.01	14.4264068	2.38E-01	63286049	63286172	NC_000002.11
cg00558702	4.83E-10	3.82E-11	7.01	14.4255242	1.15E-01	16726818	16726941	NC_000012.11

cg03450948	4.83E-10	3.82E-11	7.01	14.4243039	2.05E-01	177053274	177053397	NC_000002.11
cg07807395	4.84E-10	3.83E-11	7.01	14.4226293	1.35E-01	4698416	4698539	NC_000001.10
cg12592691	4.84E-10	3.83E-11	7.01	14.4224695	1.36E-01	51102023	51102146	NC_000007.13
cg18524739	4.84E-10	3.83E-11	7.01	14.421845	1.42E-01	105471348	105471471	NC_000002.11
cg10132917	4.85E-10	3.84E-11	7.01	14.4202056	1.50E-01	30951801	30951924	NC_000007.13
cg10384245	4.86E-10	3.84E-11	7.01	14.4191692	1.83E-01	909086	909209	NC_000018.9
cg26298855	4.86E-10	3.85E-11	7.01	14.4181925	7.41E-02	99635663	99635786	NC_000010.10
cg22350070	4.86E-10	3.85E-11	7.01	14.4181615	1.95E-01	79738639	79738762	NC_000002.11
cg10614856	4.86E-10	3.85E-11	7.01	14.4175495	9.38E-02	50025115	50025238	NC_000019.9
cg01014029	4.87E-10	3.85E-11	7.01	14.4166477	5.71E-02	5646477	5646600	NC_000007.13
cg05086314	4.87E-10	3.85E-11	7.01	14.4163362	8.17E-02	122648997	122649120	NC_000004.11
cg02402565	4.87E-10	3.86E-11	7.01	14.4155514	1.08E-01	57590030	57590153	NC_000015.9
cg22983903	4.88E-10	3.86E-11	7.01	14.4134999	1.54E-01	5140935	5141058	NC_000005.9
cg07209931	4.88E-10	3.87E-11	7.01	14.4129003	6.96E-02	176304988	176305111	NC_000005.9
cg03325407	4.88E-10	3.87E-11	7.01	14.412877	1.52E-01	114423726	114423849	NC_000001.10
cg18884037	4.89E-10	3.87E-11	7.01	14.4125988	1.97E-01	62996214	62996337	NC_000006.11
cg15372603	4.89E-10	3.87E-11	7.01	14.4121877	1.73E-01	27197239	27197362	NC_000007.13
cg13433891	4.90E-10	3.88E-11	7.01	14.410532	1.15E-01	26264492	26264615	NC_000012.11
cg22277567	4.90E-10	3.88E-11	7.01	14.4104641	1.60E-01	240226201	240226324	NC_000002.11
cg17492965	4.90E-10	3.88E-11	7.01	14.4087073	9.35E-02	8602594	8602717	NC_000004.11
cg10103266	4.90E-10	3.88E-11	7.01	14.4084605	1.49E-01	176977458	176977581	NC_000002.11
cg26478297	4.91E-10	3.89E-11	7.01	14.4076187	1.50E-01	54387435	54387558	NC_000019.9
cg18139178	4.92E-10	3.90E-11	7	14.405185	8.08E-02	134997342	134997465	NC_000010.10
cg18290624	4.92E-10	3.90E-11	7	14.4051331	1.51E-01	10857699	10857822	NC_000003.11
cg12614213	4.93E-10	3.91E-11	7	14.4030838	1.93E-01	38324613	38324736	NC_000008.10

cg26263664	4.93E-10	3.91E-11	7	14.4022453	7.56E-02	73501058	73501181	NC_000017.10
cg26481402	4.94E-10	3.91E-11	7	14.4017997	7.50E-02	28853050	28853173	NC_000006.11
cg23509641	4.94E-10	3.91E-11	7	14.4009636	1.33E-01	30583001	30583124	NC_000020.10
cg21814870	4.94E-10	3.92E-11	7	14.4000587	1.50E-01	62383240	62383363	NC_000004.11
cg18020547	4.94E-10	3.92E-11	7	14.4000551	1.10E-01	223538957	223539080	NC_000001.10
cg10486098	4.95E-10	3.93E-11	7	14.3980259	9.96E-02	147826960	147827083	NC_000001.10
cg06878709	4.96E-10	3.93E-11	7	14.3975816	1.05E-01	95862342	95862465	NC_000010.10
cg00629116	4.96E-10	3.93E-11	7	14.3958883	6.23E-02	4705360	4705483	NC_000020.10
cg00816770	4.96E-10	3.93E-11	7	14.395882	1.50E-01	144621089	144621212	NC_000004.11
cg10716518	4.96E-10	3.93E-11	7	14.3958271	9.54E-02	32166722	32166845	NC_000006.11
cg20026576	4.97E-10	3.94E-11	7	14.3952111	1.01E-01	4481538	4481661	NC_000006.11
cg19352193	4.97E-10	3.94E-11	7	14.39402	2.24E-01	46425508	46425631	NC_000013.10
cg16954727	4.99E-10	3.96E-11	7	14.3906062	1.16E-01	3440330	3440453	NC_000010.10
cg08618204	4.99E-10	3.96E-11	7	14.3906036	1.41E-01	115135076	115135199	NC_000012.11
cg19122389	4.99E-10	3.96E-11	7	14.3899375	1.41E-01	21687378	21687501	NC_000020.10
cg06531379	5.01E-10	3.97E-11	7	14.3871103	1.91E-01	47911449	47911572	NC_000001.10
cg21781988	5.01E-10	3.97E-11	7	14.3870434	1.71E-01	101287742	101287865	NC_000010.10
cg02076785	5.01E-10	3.97E-11	7	14.3864247	1.78E-01	98963582	98963705	NC_000002.11
cg06632797	5.02E-10	3.98E-11	7	14.3834518	9.98E-02	68968215	68968338	NC_000011.9
cg24729221	5.02E-10	3.99E-11	7	14.383193	1.05E-01	63497644	63497767	NC_000007.13
cg05900538	5.03E-10	3.99E-11	7	14.3828376	9.57E-02	95756907	95757030	NC_000011.9
cg06648455	5.03E-10	3.99E-11	7	14.382828	9.16E-02	69962465	69962588	NC_000011.9
cg02295992	5.03E-10	3.99E-11	7	14.3828203	1.03E-01	205255619	205255742	NC_000001.10
cg20618826	5.03E-10	3.99E-11	7	14.3823645	8.21E-02	81148896	81149019	NC_000010.10
cg05616379	5.04E-10	4.00E-11	7	14.3809115	1.18E-01	26943660	26943783	NC_000002.11

cg26405128	5.04E-10	4.00E-11	7	14.3808056	1.15E-01	31655265	31655388	NC_000003.11
cg16562275	5.04E-10	4.00E-11	7	14.3790817	1.85E-01	133481390	133481513	NC_000012.11
cg00595030	5.06E-10	4.01E-11	7	14.3765668	9.47E-02	10398582	10398705	NC_000019.9
cg01953289	5.06E-10	4.01E-11	7	14.3760483	6.30E-02	60874239	60874362	NC_000020.10
cg04740046	5.06E-10	4.02E-11	7	14.3756772	6.47E-02	55330842	55330965	NC_000017.10
cg04616566	5.06E-10	4.02E-11	7	14.374804	3.99E-02	72020560	72020683	NC_000015.9
cg13408597	5.07E-10	4.02E-11	7	14.3746522	1.29E-01	97865092	97865215	NC_000009.11
cg17210933	5.07E-10	4.02E-11	7	14.3739695	1.86E-01	3428696	3428819	NC_000005.9
cg05595088	5.11E-10	4.06E-11	7	14.3654547	8.47E-02	3243426	3243549	NC_000016.9
cg20668096	5.13E-10	4.07E-11	7	14.3615424	1.27E-01	151500192	151500315	NC_000004.11
cg22932482	5.13E-10	4.08E-11	7	14.361173	1.13E-01	144790992	144791115	NC_000008.10
cg11554266	5.13E-10	4.08E-11	7	14.3608852	1.51E-01	99288473	99288596	NC_000012.11
cg23065934	5.15E-10	4.09E-11	7	14.3570619	1.96E-01	16180266	16180389	NC_000005.9
cg15380511	5.18E-10	4.12E-11	7	14.3518523	6.92E-02	124835828	124835951	NC_000005.9
cg02363960	5.18E-10	4.12E-11	7	14.3513828	1.37E-01	79080535	79080658	NC_000007.13
cg13451356	5.20E-10	4.13E-11	6.99	14.3474462	1.01E-01	40598648	40598771	NC_000001.10
cg19455306	5.21E-10	4.14E-11	6.99	14.345708	8.04E-02	45154734	45154857	NC_000003.11
cg14041462	5.21E-10	4.14E-11	6.99	14.3448929	6.49E-02	130551579	130551702	NC_000009.11
cg04591032	5.22E-10	4.15E-11	6.99	14.3446447	1.78E-01	46827458	46827581	NC_000017.10
cg24047810	5.22E-10	4.15E-11	6.99	14.3430937	9.83E-02	2162341	2162464	NC_000011.9
cg00733328	5.23E-10	4.15E-11	6.99	14.3424642	1.05E-01	101001145	101001268	NC_000014.8
cg24017995	5.23E-10	4.16E-11	6.99	14.3422422	1.45E-01	65652792	65652915	NC_000014.8
cg19209066	5.23E-10	4.16E-11	6.99	14.3408727	2.22E-01	8085349	8085472	NC_000010.10
cg09072859	5.25E-10	4.17E-11	6.99	14.338803	1.87E-01	132934128	132934251	NC_000011.9
cg21231964	5.25E-10	4.17E-11	6.99	14.3378393	1.11E-01	77505378	77505501	NC_000017.10

cg04393060	5.25E-10	4.18E-11	6.99	14.3368924	4.88E-02	35919432	35919555	NC_000001.10
cg17025841	5.26E-10	4.18E-11	6.99	14.335918	1.11E-01	11575501	11575624	NC_000003.11
cg06003187	5.26E-10	4.19E-11	6.99	14.335254	9.78E-02	42629798	42629921	NC_000001.10
cg17032989	5.28E-10	4.20E-11	6.99	14.3323579	9.61E-02	153259061	153259184	NC_000002.11
cg26132493	5.29E-10	4.20E-11	6.99	14.330652	1.11E-01	108634275	108634398	NC_000012.11
cg16104384	5.29E-10	4.21E-11	6.99	14.33032	1.13E-01	49935689	49935812	NC_000022.10
cg01831896	5.29E-10	4.21E-11	6.99	14.3298774	1.39E-01	32638319	32638442	NC_000015.9
cg02348751	5.30E-10	4.22E-11	6.99	14.3279055	2.20E-01	183064035	183064158	NC_000004.11
cg02163943	5.31E-10	4.23E-11	6.99	14.3254438	1.43E-01	42217859	42217982	NC_000020.10
cg13081009	5.31E-10	4.23E-11	6.99	14.3253439	1.75E-01	8430745	8430868	NC_000001.10
cg07566931	5.32E-10	4.23E-11	6.99	14.3250734	7.94E-02	39374990	39375113	NC_000005.9
cg01064903	5.32E-10	4.23E-11	6.99	14.3238163	5.61E-02	8001477	8001600	NC_000019.9
cg04496204	5.32E-10	4.23E-11	6.99	14.3236843	5.77E-02	72249085	72249208	NC_000013.10
cg12003026	5.33E-10	4.24E-11	6.99	14.3229019	9.31E-02	26273370	26273493	NC_000006.11
cg21680474	5.33E-10	4.24E-11	6.99	14.3221965	9.45E-02	28463306	28463429	NC_000001.10
cg18335796	5.33E-10	4.24E-11	6.99	14.3217203	1.31E-01	91094472	91094595	NC_000008.10
cg01126576	5.35E-10	4.26E-11	6.99	14.318626	9.58E-02	120949145	120949268	NC_000010.10
cg02517686	5.35E-10	4.26E-11	6.99	14.3177867	9.44E-02	132900761	132900884	NC_000012.11
cg05298922	5.36E-10	4.27E-11	6.99	14.3166818	2.86E-01	175191045	175191168	NC_000002.11
cg18841298	5.37E-10	4.28E-11	6.99	14.3141588	1.22E-01	101604258	101604381	NC_000012.11
cg05835982	5.38E-10	4.28E-11	6.99	14.3127777	1.88E-01	129693791	129693914	NC_000003.11
cg07740640	5.38E-10	4.28E-11	6.99	14.3126706	1.06E-01	97841941	97842064	NC_000007.13
cg03127713	5.38E-10	4.28E-11	6.99	14.3122035	2.38E-01	224804071	224804194	NC_000001.10
cg06141004	5.38E-10	4.29E-11	6.99	14.3118165	1.06E-01	6867070	6867193	NC_000007.13
cg07738031	5.39E-10	4.29E-11	6.99	14.3105645	6.52E-02	61871027	61871150	NC_000020.10

cg19414591	5.40E-10	4.30E-11	6.99	14.3084198	1.52E-01	91190582	91190705	NC_000001.10
cg17306661	5.40E-10	4.30E-11	6.99	14.3078787	3.88E-02	6888415	6888538	NC_000007.13
cg06972188	5.40E-10	4.30E-11	6.99	14.3077952	1.21E-01	81565706	81565829	NC_000016.9
cg13927566	5.41E-10	4.31E-11	6.99	14.3069329	1.62E-01	91172288	91172411	NC_000001.10
cg23603003	5.41E-10	4.31E-11	6.99	14.3061031	4.57E-02	147760353	147760476	NC_000001.10
cg10238972	5.41E-10	4.31E-11	6.99	14.3060724	1.63E-01	10398754	10398877	NC_000006.11
cg11062417	5.41E-10	4.31E-11	6.99	14.3055222	1.45E-01	76578232	76578355	NC_000005.9
cg25308742	5.42E-10	4.31E-11	6.99	14.3053136	1.56E-01	3590983	3591106	NC_000005.9
cg10171357	5.42E-10	4.32E-11	6.99	14.3051105	1.51E-01	96910540	96910663	NC_000015.9
cg15893895	5.42E-10	4.32E-11	6.99	14.3047371	9.99E-02	51104413	51104536	NC_000007.13
cg02063560	5.42E-10	4.32E-11	6.99	14.3044545	5.66E-02	62120405	62120528	NC_000001.10
cg02935338	5.42E-10	4.32E-11	6.99	14.3040788	1.56E-01	25755847	25755970	NC_000018.9
cg05656566	5.45E-10	4.35E-11	6.99	14.2977034	8.08E-02	178593924	178594047	NC_000005.9
cg00359110	5.47E-10	4.36E-11	6.99	14.2951577	7.35E-02	75699252	75699375	NC_000012.11
cg26690075	5.47E-10	4.36E-11	6.99	14.2949355	1.34E-01	120968919	120969042	NC_000007.13
cg13187827	5.47E-10	4.36E-11	6.99	14.2947447	1.11E-01	31734192	31734315	NC_000006.11
cg05029822	5.47E-10	4.36E-11	6.99	14.2943922	8.71E-02	2891118	2891241	NC_000011.9
cg00909993	5.48E-10	4.37E-11	6.98	14.2934925	1.41E-01	120516537	120516660	NC_000002.11
cg21011621	5.48E-10	4.37E-11	6.98	14.2932477	1.03E-01	48471643	48471766	NC_000017.10
cg26620530	5.48E-10	4.37E-11	6.98	14.2928946	1.85E-01	115102726	115102849	NC_000012.11
cg01416563	5.48E-10	4.37E-11	6.98	14.2928717	1.09E-01	140237151	140237274	NC_000005.9
cg05387399	5.52E-10	4.40E-11	6.98	14.2860074	1.45E-01	124893766	124893889	NC_000010.10
cg23792093	5.52E-10	4.40E-11	6.98	14.2853766	6.24E-02	47012437	47012560	NC_000013.10
cg22771904	5.52E-10	4.40E-11	6.98	14.28527	1.49E-01	169051407	169051530	NC_000006.11
cg07519816	5.52E-10	4.40E-11	6.98	14.2850049	1.86E-01	53447596	53447719	NC_000018.9

cg26958236	5.52E-10	4.41E-11	6.98	14.2847757	9.95E-02	46785338	46785461	NC_000021.8
cg23805360	5.53E-10	4.41E-11	6.98	14.2835645	2.16E-01	976947	977070	NC_000009.11
cg15248835	5.53E-10	4.41E-11	6.98	14.2829075	2.09E-01	9761171	9761294	NC_000008.10
cg21199093	5.54E-10	4.42E-11	6.98	14.2820674	1.46E-01	35836009	35836132	NC_000001.10
cg18115248	5.54E-10	4.42E-11	6.98	14.2808139	6.57E-02	41495942	41496065	NC_000019.9
cg26099164	5.55E-10	4.43E-11	6.98	14.2794468	1.35E-01	7574782	7574905	NC_000010.10
cg24677321	5.55E-10	4.43E-11	6.98	14.2794214	6.05E-02	25116377	25116500	NC_000003.11
cg01218896	5.57E-10	4.45E-11	6.98	14.2751162	1.22E-01	96911988	96912111	NC_000015.9
cg15218775	5.58E-10	4.46E-11	6.98	14.2734484	1.72E-01	27135679	27135802	NC_000007.13
cg08442334	5.59E-10	4.46E-11	6.98	14.2728421	9.20E-02	66411384	66411507	NC_000011.9
cg01204927	5.59E-10	4.46E-11	6.98	14.2727719	1.34E-01	28505394	28505517	NC_000006.11
cg23647410	5.59E-10	4.46E-11	6.98	14.2720452	8.12E-02	221051066	221051189	NC_000001.10
cg14768459	5.60E-10	4.47E-11	6.98	14.2708481	9.49E-02	19672432	19672555	NC_000002.11
cg06967496	5.60E-10	4.47E-11	6.98	14.2704921	4.36E-02	71677658	71677781	NC_000005.9
cg01685883	5.61E-10	4.48E-11	6.98	14.2688172	1.68E-01	155254497	155254620	NC_000004.11
cg10022526	5.62E-10	4.49E-11	6.98	14.2664736	1.89E-01	27744557	27744680	NC_000011.9
cg00304388	5.63E-10	4.49E-11	6.98	14.2653626	1.74E-01	181454744	181454867	NC_000001.10
cg06049868	5.63E-10	4.49E-11	6.98	14.2653379	1.50E-01	112717707	112717830	NC_000013.10
cg08244443	5.63E-10	4.49E-11	6.98	14.2653164	1.24E-01	58013900	58014023	NC_000012.11
cg02503981	5.63E-10	4.50E-11	6.98	14.2638125	1.98E-01	112720182	112720305	NC_000013.10
cg04794430	5.64E-10	4.50E-11	6.98	14.2634967	1.52E-01	3290071	3290194	NC_000017.10
cg20871586	5.64E-10	4.51E-11	6.98	14.2618325	1.70E-01	134175678	134175801	NC_000006.11
cg12883980	5.64E-10	4.51E-11	6.98	14.261778	1.18E-01	50707586	50707709	NC_000013.10
cg13910746	5.65E-10	4.51E-11	6.98	14.2616716	6.06E-02	144365795	144365918	NC_000008.10
cg24169822	5.65E-10	4.52E-11	6.98	14.2604101	1.49E-01	27170994	27171117	NC_000007.13

cg04961235	5.66E-10	4.52E-11	6.98	14.2592266	1.27E-01	4901628	4901751	NC_000007.13
cg25743819	5.66E-10	4.52E-11	6.98	14.259015	1.50E-01	173245704	173245827	NC_000002.11
cg01936839	5.66E-10	4.52E-11	6.98	14.2588236	1.05E-01	27198896	27199019	NC_000007.13
cg23986671	5.68E-10	4.54E-11	6.98	14.2550395	2.01E-01	28339262	28339385	NC_000021.8
cg15619820	5.69E-10	4.55E-11	6.98	14.2531164	1.12E-01	3133763	3133886	NC_000001.10
cg17851748	5.70E-10	4.56E-11	6.98	14.2516971	6.33E-02	83264140	83264263	NC_000012.11
cg00797142	5.70E-10	4.56E-11	6.98	14.2512018	5.40E-02	90998279	90998402	NC_000008.10
cg02803775	5.70E-10	4.56E-11	6.98	14.2509328	7.94E-02	41937252	41937375	NC_000022.10
cg01769968	5.71E-10	4.57E-11	6.98	14.2494736	1.43E-01	155891244	155891367	NC_000001.10
cg06294766	5.72E-10	4.57E-11	6.98	14.2481298	1.00E-01	154680314	154680437	NC_000004.11
cg00383384	5.72E-10	4.58E-11	6.98	14.247573	1.46E-01	57391271	57391394	NC_000010.10
cg08155325	5.72E-10	4.58E-11	6.98	14.2471982	1.23E-01	140857813	140857936	NC_000005.9
cg04850976	5.74E-10	4.59E-11	6.98	14.2446906	9.62E-02	3782990	3783113	NC_000016.9
cg20260127	5.74E-10	4.59E-11	6.98	14.2444151	1.08E-01	56355681	56355804	NC_000007.13
cg24830738	5.74E-10	4.59E-11	6.98	14.2438798	1.55E-01	113698445	113698568	NC_000005.9
cg20353780	5.75E-10	4.60E-11	6.98	14.2433447	6.83E-02	118588556	118588679	NC_000012.11
cg08058544	5.75E-10	4.60E-11	6.98	14.2416322	1.73E-01	237080267	237080390	NC_000002.11
cg00031105	5.75E-10	4.60E-11	6.98	14.2415828	1.06E-01	33166117	33166240	NC_000006.11
cg02283366	5.76E-10	4.60E-11	6.98	14.2414485	1.81E-01	63785544	63785667	NC_000001.10
cg15835091	5.77E-10	4.62E-11	6.98	14.2391743	5.14E-02	16468340	16468463	NC_000022.10
cg07906632	5.77E-10	4.62E-11	6.98	14.2390369	2.01E-01	147074073	147074196	NC_000003.11
cg22344727	5.77E-10	4.62E-11	6.98	14.2389872	1.68E-01	81102035	81102158	NC_000012.11
cg03764965	5.77E-10	4.62E-11	6.98	14.2389861	1.20E-01	117971251	117971374	NC_000002.11
cg10491556	5.78E-10	4.62E-11	6.97	14.2377101	2.31E-01	124895886	124896009	NC_000010.10
cg16993043	5.78E-10	4.62E-11	6.97	14.2373888	2.29E-01	200008026	200008149	NC_000001.10

cg20297360	5.78E-10	4.62E-11	6.97	14.2372311	5.43E-02	2976098	2976221	NC_000017.10
cg06248560	5.78E-10	4.62E-11	6.97	14.2372249	1.82E-01	35104681	35104804	NC_000018.9
cg25837277	5.78E-10	4.63E-11	6.97	14.2367526	6.04E-02	46999366	46999489	NC_000019.9
cg02151752	5.78E-10	4.63E-11	6.97	14.2366582	3.68E-02	32798417	32798540	NC_000006.11
cg16202331	5.79E-10	4.63E-11	6.97	14.2349748	1.86E-01	13532655	13532778	NC_000004.11
cg09479783	5.79E-10	4.64E-11	6.97	14.2343938	3.73E-02	124796409	124796532	NC_000012.11
cg25984524	5.79E-10	4.64E-11	6.97	14.2341192	1.18E-01	104871849	104871972	NC_000010.10
cg18579879	5.80E-10	4.64E-11	6.97	14.2336972	1.27E-01	120968913	120969036	NC_000007.13
cg16268315	5.80E-10	4.64E-11	6.97	14.2331124	1.41E-01	103491087	103491210	NC_000008.10
cg23208791	5.80E-10	4.64E-11	6.97	14.2331034	8.50E-02	99123733	99123856	NC_000001.10
cg21135533	5.81E-10	4.65E-11	6.97	14.2319533	1.65E-01	70982935	70983058	NC_000008.10
cg10672343	5.82E-10	4.66E-11	6.97	14.2288177	6.13E-02	169718117	169718240	NC_000006.11
cg26390660	5.83E-10	4.67E-11	6.97	14.227047	1.35E-01	110344165	110344288	NC_000003.11
cg27426442	5.84E-10	4.67E-11	6.97	14.2267725	6.79E-02	35181113	35181236	NC_000006.11
cg00585714	5.84E-10	4.68E-11	6.97	14.2255641	1.18E-01	97156859	97156982	NC_000008.10
cg21429087	5.85E-10	4.69E-11	6.97	14.223474	1.96E-01	166074814	166074937	NC_000006.11
cg14216940	5.85E-10	4.69E-11	6.97	14.2233005	1.16E-01	50343131	50343254	NC_000007.13
cg21184115	5.87E-10	4.70E-11	6.97	14.2213866	3.58E-02	108931012	108931135	NC_000006.11
cg22218447	5.87E-10	4.70E-11	6.97	14.2212772	4.67E-02	21221791	21221914	NC_000017.10
cg01800455	5.87E-10	4.70E-11	6.97	14.2208457	3.48E-02	20996671	20996794	NC_000016.9
cg10852177	5.87E-10	4.70E-11	6.97	14.2207493	2.02E-01	84816454	84816577	NC_000007.13
cg23483069	5.88E-10	4.71E-11	6.97	14.2194533	5.38E-02	17825778	17825901	NC_000002.11
cg11268834	5.89E-10	4.72E-11	6.97	14.2173678	1.58E-01	224824500	224824623	NC_000002.11
cg25098068	5.90E-10	4.73E-11	6.97	14.2156222	5.06E-02	62462051	62462174	NC_000017.10
cg23302570	5.90E-10	4.73E-11	6.97	14.2148532	1.80E-01	139538601	139538724	NC_000002.11

cg27050763	5.91E-10	4.73E-11	6.97	14.2141595	8.00E-02	247081594	247081717	NC_000001.10
cg00397479	5.91E-10	4.74E-11	6.97	14.2131673	2.19E-01	31831510	31831633	NC_000006.11
cg23226129	5.92E-10	4.74E-11	6.97	14.2124609	1.72E-01	39995539	39995662	NC_000020.10
cg04565337	5.92E-10	4.74E-11	6.97	14.2124391	1.38E-01	38071768	38071891	NC_000014.8
cg20756891	5.92E-10	4.75E-11	6.97	14.2115594	1.44E-01	170743951	170744074	NC_000005.9
cg26157386	5.92E-10	4.75E-11	6.97	14.21133	1.85E-01	36994270	36994393	NC_000014.8
cg23181844	5.93E-10	4.76E-11	6.97	14.2096643	1.77E-01	19812548	19812671	NC_000007.13
cg18612627	5.93E-10	4.76E-11	6.97	14.2095713	1.22E-01	14135203	14135326	NC_000012.11
cg10055471	5.94E-10	4.76E-11	6.97	14.2080088	1.48E-01	27240319	27240442	NC_000001.10
cg19994157	5.95E-10	4.77E-11	6.97	14.2066238	3.08E-02	32825852	32825975	NC_000006.11
cg21375294	5.95E-10	4.77E-11	6.97	14.2061747	1.53E-01	35065040	35065163	NC_000018.9
cg15182939	5.95E-10	4.78E-11	6.97	14.2056931	4.02E-02	67701995	67702118	NC_000003.11
cg02227188	5.95E-10	4.78E-11	6.97	14.2055174	2.04E-01	54396354	54396477	NC_000012.11
cg15829642	5.97E-10	4.79E-11	6.97	14.2034155	1.37E-01	140744983	140745106	NC_000005.9
cg09504874	5.97E-10	4.79E-11	6.97	14.2024653	3.06E-02	224713	224836	NC_000006.11
cg23338503	5.97E-10	4.79E-11	6.97	14.2023649	1.97E-01	169064530	169064653	NC_000005.9
cg06183256	5.98E-10	4.80E-11	6.97	14.2013978	9.91E-02	53124971	53125094	NC_000003.11
cg19909679	5.98E-10	4.80E-11	6.97	14.200519	1.36E-01	9812135	9812258	NC_000017.10
cg15215830	5.98E-10	4.80E-11	6.97	14.200396	1.09E-01	125999805	125999928	NC_000011.9
cg18867480	5.98E-10	4.80E-11	6.97	14.2003276	1.62E-01	27744816	27744939	NC_000011.9
cg18128969	5.98E-10	4.80E-11	6.97	14.2001805	1.36E-01	22369908	22370031	NC_000021.8
cg10675138	5.98E-10	4.80E-11	6.97	14.1998652	1.91E-01	166075504	166075627	NC_000006.11
cg09431525	5.98E-10	4.80E-11	6.97	14.1998202	8.18E-02	39993313	39993436	NC_000019.9
cg13835436	6.00E-10	4.82E-11	6.97	14.1969799	6.58E-02	69809807	69809930	NC_000011.9
cg02919936	6.01E-10	4.82E-11	6.97	14.1962734	1.94E-01	70982285	70982408	NC_000008.10

cg12610239	6.01E-10	4.82E-11	6.97	14.1958206	6.26E-02	2382748	2382871	NC_000001.10
cg17183752	6.01E-10	4.83E-11	6.97	14.1953524	8.87E-02	3508884	3509007	NC_000001.10
cg08581953	6.01E-10	4.83E-11	6.97	14.1952173	1.09E-01	25593057	25593180	NC_000013.10
cg12859211	6.02E-10	4.83E-11	6.97	14.1938777	2.39E-01	124735105	124735228	NC_000011.9
cg16499415	6.03E-10	4.84E-11	6.97	14.1925808	4.51E-02	61669675	61669798	NC_000014.8
cg00635522	6.03E-10	4.84E-11	6.97	14.1922677	5.17E-02	45396744	45396867	NC_000013.10
cg11635029	6.05E-10	4.86E-11	6.97	14.1878996	1.20E-01	130830829	130830952	NC_000010.10
cg05313279	6.06E-10	4.87E-11	6.97	14.1872809	5.44E-02	234627401	234627524	NC_000002.11
cg20417527	6.06E-10	4.87E-11	6.97	14.187066	1.41E-01	128300708	128300831	NC_000005.9
cg02058924	6.07E-10	4.88E-11	6.97	14.1850868	6.24E-02	133465592	133465715	NC_000012.11
cg18808506	6.07E-10	4.88E-11	6.97	14.184914	1.00E-01	142078020	142078143	NC_000005.9
cg07379703	6.07E-10	4.88E-11	6.97	14.1843592	1.20E-01	78279424	78279547	NC_000005.9
cg07930539	6.08E-10	4.88E-11	6.97	14.1834847	1.59E-01	177025975	177026098	NC_000002.11
cg15323936	6.08E-10	4.89E-11	6.97	14.1827485	9.37E-02	194996295	194996418	NC_000003.11
cg07439157	6.09E-10	4.90E-11	6.96	14.1809792	2.92E-02	74527263	74527386	NC_000017.10
cg20577477	6.10E-10	4.90E-11	6.96	14.1807349	1.36E-01	11668491	11668614	NC_000008.10
cg23232188	6.10E-10	4.90E-11	6.96	14.1804781	9.86E-02	121556543	121556666	NC_000003.11
cg22433574	6.10E-10	4.90E-11	6.96	14.1794728	1.80E-02	35849808	35849931	NC_000017.10
cg04131153	6.10E-10	4.91E-11	6.96	14.1793015	7.45E-02	19614541	19614664	NC_000014.8
cg04297507	6.11E-10	4.91E-11	6.96	14.1778204	1.07E-01	108749432	108749555	NC_000004.11
cg06962768	6.12E-10	4.92E-11	6.96	14.1770374	8.26E-02	37959782	37959905	NC_000007.13
cg22308726	6.12E-10	4.92E-11	6.96	14.176331	1.46E-01	4566575	4566698	NC_000019.9
cg11389960	6.12E-10	4.92E-11	6.96	14.1759391	1.09E-01	31277661	31277784	NC_000015.9
cg12577003	6.14E-10	4.93E-11	6.96	14.1736971	6.46E-02	2261337	2261460	NC_000007.13
cg03276401	6.14E-10	4.93E-11	6.96	14.1735458	1.27E-01	62225407	62225530	NC_000017.10

cg08356522	6.14E-10	4.94E-11	6.96	14.1727546	7.72E-02	4445948	4446071	NC_000010.10
cg26532358	6.14E-10	4.94E-11	6.96	14.1721917	2.30E-01	118228871	118228994	NC_000006.11
cg10497345	6.15E-10	4.95E-11	6.96	14.170969	1.16E-01	90172038	90172161	NC_000004.11
cg17146473	6.15E-10	4.95E-11	6.96	14.1709564	4.95E-02	32052428	32052551	NC_000006.11
cg07523788	6.15E-10	4.95E-11	6.96	14.1705815	2.66E-02	242572342	242572465	NC_000002.11
cg26576890	6.17E-10	4.96E-11	6.96	14.1685897	1.17E-01	41374939	41375062	NC_000006.11
cg05328667	6.17E-10	4.96E-11	6.96	14.1685196	8.73E-02	28015357	28015480	NC_000015.9
cg00743269	6.17E-10	4.96E-11	6.96	14.1678674	9.79E-02	60747654	60747777	NC_000017.10
cg03164800	6.17E-10	4.96E-11	6.96	14.1677823	5.31E-02	80836552	80836675	NC_000004.11
cg15531403	6.17E-10	4.96E-11	6.96	14.167677	1.95E-01	53852184	53852307	NC_000008.10
cg22672282	6.17E-10	4.97E-11	6.96	14.1670089	1.20E-01	44222974	44223097	NC_000019.9
cg04145134	6.18E-10	4.97E-11	6.96	14.1659231	1.46E-01	99697672	99697795	NC_000014.8
cg22605303	6.18E-10	4.98E-11	6.96	14.1652809	1.64E-01	91172472	91172595	NC_000001.10
cg07695835	6.20E-10	4.99E-11	6.96	14.1627701	1.76E-01	20985984	20986107	NC_000004.11
cg26014723	6.20E-10	4.99E-11	6.96	14.1626181	1.29E-01	71055476	71055599	NC_000002.11
cg15343119	6.21E-10	5.00E-11	6.96	14.1611747	1.83E-01	74961785	74961908	NC_000018.9
cg03834286	6.21E-10	5.00E-11	6.96	14.1611393	1.42E-01	3590482	3590605	NC_000005.9
cg11337915	6.21E-10	5.00E-11	6.96	14.1607684	1.27E-01	40719088	40719211	NC_000021.8
cg00449767	6.22E-10	5.00E-11	6.96	14.1597387	1.05E-01	45737603	45737726	NC_000019.9
cg09298920	6.22E-10	5.00E-11	6.96	14.1596975	7.70E-02	35859139	35859262	NC_000009.11
cg01445399	6.22E-10	5.00E-11	6.96	14.1596691	1.14E-01	87596934	87597057	NC_000001.10
cg24172509	6.22E-10	5.01E-11	6.96	14.159217	1.62E-01	140778424	140778547	NC_000005.9
cg00630237	6.23E-10	5.01E-11	6.96	14.1580711	2.80E-02	91433507	91433630	NC_000015.9
cg25324047	6.25E-10	5.03E-11	6.96	14.154719	2.03E-01	22765840	22765963	NC_000010.10
cg16332927	6.25E-10	5.03E-11	6.96	14.1541569	1.31E-01	37461350	37461473	NC_000006.11

cg00162806	6.25E-10	5.04E-11	6.96	14.1536028	1.25E-01	135051929	135052052	NC_000010.10
cg05680531	6.26E-10	5.04E-11	6.96	14.1533315	1.91E-01	136553327	136553450	NC_000007.13
cg15131934	6.26E-10	5.04E-11	6.96	14.1526045	1.02E-01	53659328	53659451	NC_000001.10
cg09571082	6.27E-10	5.05E-11	6.96	14.1508747	1.49E-01	14549941	14550064	NC_000006.11
cg10454766	6.27E-10	5.05E-11	6.96	14.1507016	1.87E-01	109293945	109294068	NC_000011.9
cg05296773	6.27E-10	5.05E-11	6.96	14.1502292	6.03E-02	93449339	93449462	NC_000012.11
cg02155048	6.28E-10	5.06E-11	6.96	14.1497004	7.78E-02	10721597	10721720	NC_000016.9
cg25787956	6.28E-10	5.06E-11	6.96	14.1492577	9.13E-02	77548882	77549005	NC_000018.9
cg02186782	6.28E-10	5.06E-11	6.96	14.1483696	1.69E-01	33602817	33602940	NC_000015.9
cg07927308	6.29E-10	5.07E-11	6.96	14.1477175	6.72E-02	84886961	84887084	NC_000016.9
cg27587310	6.30E-10	5.07E-11	6.96	14.1463173	1.10E-01	29204994	29205117	NC_000016.9
cg08141424	6.30E-10	5.08E-11	6.96	14.1458171	1.63E-01	73674087	73674210	NC_000003.11
cg16930746	6.30E-10	5.08E-11	6.96	14.1451394	1.38E-01	33780951	33781074	NC_000013.10
cg02732804	6.32E-10	5.09E-11	6.96	14.1422979	2.31E-01	28075269	28075392	NC_000016.9
cg04237666	6.32E-10	5.09E-11	6.96	14.1421864	1.28E-01	83674615	83674738	NC_000004.11
cg15921949	6.33E-10	5.10E-11	6.96	14.141342	1.71E-01	137486213	137486336	NC_000003.11
cg07422329	6.33E-10	5.10E-11	6.96	14.1411204	1.21E-01	186642182	186642305	NC_000001.10
cg16737018	6.34E-10	5.11E-11	6.96	14.13986	8.07E-02	103914581	103914704	NC_000014.8
cg06529761	6.34E-10	5.11E-11	6.96	14.1389147	9.22E-02	3702486	3702609	NC_000016.9
cg26775087	6.35E-10	5.11E-11	6.96	14.1382657	6.51E-02	15382808	15382931	NC_000003.11
cg14868994	6.35E-10	5.12E-11	6.96	14.1378515	1.60E-01	45155672	45155795	NC_000002.11
cg21657955	6.35E-10	5.12E-11	6.96	14.1374707	1.85E-01	19812578	19812701	NC_000007.13
cg07542000	6.36E-10	5.13E-11	6.96	14.1351974	5.90E-02	31258602	31258725	NC_000017.10
cg03701266	6.38E-10	5.14E-11	6.96	14.133409	6.99E-02	10649678	10649801	NC_000005.9
cg05418042	6.38E-10	5.14E-11	6.96	14.1329514	1.15E-01	16063771	16063894	NC_000012.11

cg18231690	6.38E-10	5.15E-11	6.96	14.1320875	6.29E-02	85294586	85294709	NC_000015.9
cg11229185	6.40E-10	5.16E-11	6.96	14.1297185	1.40E-01	22625274	22625397	NC_000010.10
cg22675745	6.41E-10	5.17E-11	6.96	14.1276296	9.42E-02	218577902	218578025	NC_000002.11
cg06110297	6.41E-10	5.17E-11	6.96	14.1270102	1.49E-01	22589830	22589953	NC_000007.13
cg04901179	6.41E-10	5.17E-11	6.96	14.1270008	5.89E-02	111430583	111430706	NC_000007.13
cg06330691	6.42E-10	5.18E-11	6.96	14.126595	1.81E-01	78673290	78673413	NC_000011.9
cg19876232	6.42E-10	5.18E-11	6.95	14.1260768	2.26E-02	52174504	52174627	NC_000010.10
cg13548946	6.43E-10	5.18E-11	6.95	14.1251957	9.06E-02	123350077	123350200	NC_000012.11
cg02655195	6.43E-10	5.18E-11	6.95	14.1249812	1.01E-01	166511914	166512037	NC_000006.11
cg21051964	6.44E-10	5.19E-11	6.95	14.1231127	5.70E-02	58022620	58022743	NC_000012.11
cg18182111	6.44E-10	5.20E-11	6.95	14.1224494	1.76E-01	37835111	37835234	NC_000005.9
cg26618965	6.44E-10	5.20E-11	6.95	14.1220955	1.91E-01	67874206	67874329	NC_000008.10
cg14573411	6.45E-10	5.20E-11	6.95	14.1217247	2.11E-01	126135992	126136115	NC_000009.11
cg08776908	6.45E-10	5.20E-11	6.95	14.1216092	1.63E-01	157898347	157898470	NC_000005.9
cg14339043	6.46E-10	5.21E-11	6.95	14.1199858	1.83E-01	4198058	4198181	NC_000019.9
cg24971112	6.47E-10	5.22E-11	6.95	14.1174076	1.25E-01	153385052	153385175	NC_000003.11
cg08247297	6.49E-10	5.24E-11	6.95	14.1150658	8.06E-02	46917541	46917664	NC_000021.8
cg05297328	6.49E-10	5.24E-11	6.95	14.1145493	5.02E-02	101532225	101532348	NC_000014.8
cg05928290	6.52E-10	5.26E-11	6.95	14.1104668	1.27E-01	54418728	54418851	NC_000014.8
cg10318313	6.52E-10	5.26E-11	6.95	14.1101346	6.78E-02	3014937	3015060	NC_000011.9
cg25148347	6.52E-10	5.26E-11	6.95	14.1101055	6.25E-02	42074193	42074316	NC_000006.11
cg00847277	6.54E-10	5.28E-11	6.95	14.1071642	1.47E-01	142528956	142529079	NC_000008.10
cg04836851	6.54E-10	5.28E-11	6.95	14.1071202	1.14E-01	140562819	140562942	NC_000005.9
cg15272362	6.54E-10	5.29E-11	6.95	14.1059316	1.84E-01	25062823	25062946	NC_000020.10
cg25225155	6.55E-10	5.29E-11	6.95	14.1050461	9.70E-02	140222253	140222376	NC_000005.9

cg00779065	6.56E-10	5.30E-11	6.95	14.1035899	1.83E-01	61119218	61119341	NC_000014.8
cg05403433	6.57E-10	5.31E-11	6.95	14.1018282	1.17E-01	87088843	87088966	NC_000002.11
cg21053529	6.57E-10	5.31E-11	6.95	14.1012465	1.74E-01	35046760	35046883	NC_000015.9
cg00044796	6.57E-10	5.31E-11	6.95	14.1011017	1.67E-01	146552390	146552513	NC_000001.10
cg00264591	6.57E-10	5.31E-11	6.95	14.1009968	1.54E-01	1883514	1883637	NC_000005.9
cg24521010	6.58E-10	5.31E-11	6.95	14.1007445	3.21E-02	16155290	16155413	NC_000022.10
cg02641337	6.58E-10	5.31E-11	6.95	14.1006455	2.17E-01	4867387	4867510	NC_000004.11
cg17958315	6.58E-10	5.32E-11	6.95	14.0995921	1.92E-01	127873552	127873675	NC_000005.9
cg00693401	6.58E-10	5.32E-11	6.95	14.0993562	3.24E-02	80655583	80655706	NC_000017.10
cg24981593	6.59E-10	5.32E-11	6.95	14.0991447	2.11E-01	45160253	45160376	NC_000002.11
cg15020132	6.59E-10	5.32E-11	6.95	14.0986767	1.06E-01	34122682	34122805	NC_000007.13
cg12581627	6.59E-10	5.33E-11	6.95	14.0980692	1.88E-01	61987935	61988058	NC_000013.10
cg03087912	6.59E-10	5.33E-11	6.95	14.0980135	1.63E-01	55379034	55379157	NC_000008.10
cg26051413	6.59E-10	5.33E-11	6.95	14.097849	1.26E-01	2292902	2293025	NC_000011.9
cg11586570	6.62E-10	5.35E-11	6.95	14.0932594	1.47E-01	63498133	63498256	NC_000007.13
cg22719241	6.63E-10	5.36E-11	6.95	14.0925127	1.41E-01	36524058	36524181	NC_000019.9
cg17431391	6.64E-10	5.37E-11	6.95	14.0900234	1.14E-01	38042109	38042232	NC_000019.9
cg02593924	6.64E-10	5.37E-11	6.95	14.0899371	1.11E-01	1844998	1845121	NC_000016.9
cg16584393	6.65E-10	5.38E-11	6.95	14.0887946	1.32E-01	14747888	14748011	NC_000018.9
cg06218338	6.66E-10	5.38E-11	6.95	14.0881291	1.45E-01	27231894	27232017	NC_000007.13
cg19594666	6.66E-10	5.39E-11	6.95	14.0868279	1.06E-01	127881280	127881403	NC_000007.13
cg05063374	6.67E-10	5.40E-11	6.95	14.0858268	5.86E-02	38953512	38953635	NC_000017.10
cg22710398	6.67E-10	5.40E-11	6.95	14.0856225	5.34E-02	86349279	86349402	NC_000006.11
cg03826642	6.68E-10	5.40E-11	6.95	14.0846438	1.66E-01	10268858	10268981	NC_000006.11
cg16051195	6.69E-10	5.41E-11	6.95	14.0826236	1.55E-01	166268183	166268306	NC_000006.11

cg01231921	6.70E-10	5.42E-11	6.95	14.0807677	1.11E-01	241611991	241612114	NC_000002.11
cg15553500	6.71E-10	5.42E-11	6.95	14.0804263	1.07E-01	41880987	41881110	NC_000004.11
cg16384492	6.71E-10	5.43E-11	6.95	14.0797641	1.40E-01	40034481	40034604	NC_000001.10
cg23602453	6.71E-10	5.43E-11	6.95	14.079627	1.04E-01	246958862	246958985	NC_000001.10
cg18035571	6.71E-10	5.43E-11	6.95	14.079058	7.75E-02	176580675	176580798	NC_000005.9
cg11248812	6.73E-10	5.44E-11	6.95	14.0768115	6.54E-02	75502917	75503040	NC_000017.10
cg22856324	6.73E-10	5.45E-11	6.95	14.0763542	1.21E-01	72371255	72371378	NC_000002.11
cg12456799	6.73E-10	5.45E-11	6.95	14.0762351	1.81E-01	106441036	106441159	NC_000010.10
cg00183067	6.73E-10	5.45E-11	6.95	14.0757164	1.13E-01	151075493	151075616	NC_000006.11
cg07498975	6.74E-10	5.45E-11	6.95	14.0751101	4.00E-02	101338750	101338873	NC_000001.10
cg14529086	6.74E-10	5.46E-11	6.95	14.0741368	7.36E-02	35226828	35226951	NC_000019.9
cg01998089	6.75E-10	5.47E-11	6.95	14.0728895	5.40E-02	91205834	91205957	NC_000014.8
cg22707381	6.76E-10	5.47E-11	6.95	14.0724387	8.43E-02	66458942	66459065	NC_000011.9
cg07637213	6.76E-10	5.47E-11	6.95	14.071483	1.07E-01	50820604	50820727	NC_000010.10
cg00359590	6.77E-10	5.49E-11	6.94	14.0694824	1.12E-01	27469673	27469796	NC_000008.10
cg19049860	6.79E-10	5.49E-11	6.94	14.067864	1.01E-01	53040267	53040390	NC_000006.11
cg08729318	6.79E-10	5.50E-11	6.94	14.0676145	2.25E-01	29936839	29936962	NC_000012.11
cg07592136	6.79E-10	5.50E-11	6.94	14.066821	8.38E-02	51473480	51473603	NC_000019.9
cg11219883	6.79E-10	5.50E-11	6.94	14.0667413	7.67E-02	130501758	130501881	NC_000012.11
cg08580014	6.79E-10	5.50E-11	6.94	14.0665423	1.32E-01	118368634	118368757	NC_000010.10
cg03192598	6.80E-10	5.51E-11	6.94	14.0659113	2.43E-01	12305854	12305977	NC_000019.9
cg11403027	6.80E-10	5.51E-11	6.94	14.0657183	8.64E-02	42971411	42971534	NC_000005.9
cg01433520	6.80E-10	5.51E-11	6.94	14.0656157	2.30E-02	10363474	10363597	NC_000003.11
cg22799141	6.80E-10	5.51E-11	6.94	14.0650841	1.90E-01	114885621	114885744	NC_000012.11
cg05281894	6.81E-10	5.52E-11	6.94	14.0636107	2.07E-01	13910698	13910821	NC_000001.10

cg15624376	6.81E-10	5.52E-11	6.94	14.0635752	1.51E-01	27171391	27171514	NC_000007.13
cg20820996	6.81E-10	5.52E-11	6.94	14.0630402	6.55E-02	33749849	33749972	NC_000001.10
cg07986943	6.83E-10	5.53E-11	6.94	14.0611225	1.20E-01	176932579	176932702	NC_000002.11
cg17777676	6.83E-10	5.53E-11	6.94	14.0608105	1.25E-01	140186895	140187018	NC_000005.9
cg05457075	6.83E-10	5.54E-11	6.94	14.06046	3.17E-02	1676296	1676419	NC_000016.9
cg15028514	6.84E-10	5.54E-11	6.94	14.0592598	1.67E-01	61809841	61809964	NC_000020.10
cg14094639	6.84E-10	5.54E-11	6.94	14.0592136	1.09E-01	1309477	1309600	NC_000001.10
cg16035667	6.84E-10	5.55E-11	6.94	14.0586668	5.31E-02	197359987	197360110	NC_000003.11
cg18044383	6.86E-10	5.57E-11	6.94	14.055332	1.30E-01	59001158	59001281	NC_000018.9
cg07616499	6.87E-10	5.57E-11	6.94	14.0540025	1.04E-01	30324789	30324912	NC_000010.10
cg24494227	6.89E-10	5.58E-11	6.94	14.0519472	1.17E-01	168070460	168070583	NC_000006.11
cg10832304	6.89E-10	5.59E-11	6.94	14.0512192	2.09E-01	39998018	39998141	NC_000019.9
cg21390574	6.90E-10	5.60E-11	6.94	14.0499794	9.57E-02	122675597	122675720	NC_000003.11
cg03461296	6.90E-10	5.60E-11	6.94	14.0497819	6.38E-02	60637948	60638071	NC_000020.10
cg05121480	6.90E-10	5.60E-11	6.94	14.0489892	1.68E-01	187387601	187387724	NC_000003.11
cg11522612	6.91E-10	5.61E-11	6.94	14.0476707	9.11E-02	31687637	31687760	NC_000006.11
cg14504259	6.92E-10	5.61E-11	6.94	14.046918	1.31E-01	970882	971005	NC_000009.11
cg23064554	6.92E-10	5.62E-11	6.94	14.0462709	1.05E-01	15764095	15764218	NC_000001.10
cg19589939	6.93E-10	5.62E-11	6.94	14.0458462	1.86E-01	103355326	103355449	NC_000012.11
cg05402793	6.93E-10	5.62E-11	6.94	14.0455421	1.39E-01	149633660	149633783	NC_000002.11
cg11345811	6.93E-10	5.62E-11	6.94	14.0454388	1.07E-01	3283643	3283766	NC_000010.10
cg14032025	6.93E-10	5.62E-11	6.94	14.0448371	2.17E-01	50721129	50721252	NC_000020.10
cg23895340	6.93E-10	5.63E-11	6.94	14.0447965	1.74E-01	176973275	176973398	NC_000002.11
cg04853284	6.93E-10	5.63E-11	6.94	14.0447075	2.87E-02	91435964	91436087	NC_000015.9
cg07755203	6.94E-10	5.63E-11	6.94	14.044098	6.54E-02	150177004	150177127	NC_000002.11

cg02259324	6.94E-10	5.63E-11	6.94	14.0433646	1.64E-01	78111196	78111319	NC_000015.9
cg11557636	6.96E-10	5.65E-11	6.94	14.0408782	1.99E-01	37246751	37246874	NC_000004.11
cg21435375	6.96E-10	5.65E-11	6.94	14.0408208	9.30E-02	172878103	172878226	NC_000002.11
cg13021619	6.98E-10	5.66E-11	6.94	14.0380729	1.82E-01	23563859	23563982	NC_000008.10
cg08545287	6.99E-10	5.67E-11	6.94	14.0364973	1.55E-01	6069047	6069170	NC_000016.9
cg01181485	6.99E-10	5.67E-11	6.94	14.0362367	8.47E-02	153454027	153454150	NC_000004.11
cg07118985	6.99E-10	5.68E-11	6.94	14.036116	9.75E-02	51424481	51424604	NC_000003.11
cg18952647	6.99E-10	5.68E-11	6.94	14.0360997	1.65E-01	83953744	83953867	NC_000015.9
ch.2.216208170F	6.99E-10	5.68E-11	6.94	14.035607	6.07E-02	216499925	216500048	NC_000002.11
cg26232187	7.00E-10	5.68E-11	6.94	14.0347441	1.71E-01	88799300	88799423	NC_000015.9
cg16386984	7.01E-10	5.69E-11	6.94	14.0333667	3.17E-02	187216224	187216347	NC_000004.11
cg25019989	7.01E-10	5.69E-11	6.94	14.0329243	1.62E-01	54961835	54961958	NC_000004.11
cg01508023	7.02E-10	5.70E-11	6.94	14.0312769	1.89E-01	82136116	82136239	NC_000004.11
cg26217572	7.02E-10	5.71E-11	6.94	14.0308113	1.09E-01	27540240	27540363	NC_000013.10
cg27398401	7.03E-10	5.71E-11	6.94	14.0303108	1.13E-01	56976548	56976671	NC_000001.10
cg04370634	7.03E-10	5.71E-11	6.94	14.0298061	1.21E-01	31721446	31721569	NC_000003.11
cg24608684	7.04E-10	5.72E-11	6.94	14.028679	2.36E-01	130686865	130686988	NC_000006.11
cg27264049	7.05E-10	5.73E-11	6.94	14.0274563	1.79E-01	13537872	13537995	NC_000004.11
cg24705960	7.05E-10	5.73E-11	6.94	14.0267308	1.76E-01	62359456	62359579	NC_000003.11
cg10056728	7.06E-10	5.73E-11	6.94	14.0260731	8.76E-02	56497073	56497196	NC_000012.11
cg09960109	7.06E-10	5.74E-11	6.94	14.0251319	1.33E-01	134072237	134072360	NC_000004.11
cg12424785	7.06E-10	5.74E-11	6.94	14.0247364	1.47E-01	154728133	154728256	NC_000002.11
cg18762727	7.07E-10	5.74E-11	6.94	14.0244979	1.81E-01	101006089	101006212	NC_000007.13
cg07369950	7.07E-10	5.74E-11	6.94	14.0241339	1.81E-01	58609987	58610110	NC_000019.9
cg12158272	7.08E-10	5.75E-11	6.94	14.0228949	1.45E-01	119800972	119801095	NC_000005.9

cg20962532	7.09E-10	5.76E-11	6.94	14.0216621	1.30E-01	128712462	128712585	NC_000011.9
cg00314343	7.09E-10	5.76E-11	6.94	14.0213302	6.61E-02	92428182	92428305	NC_000015.9
cg04832466	7.10E-10	5.77E-11	6.94	14.0195285	1.50E-01	112760088	112760211	NC_000013.10
cg09396704	7.11E-10	5.78E-11	6.94	14.0184541	1.30E-01	203045229	203045352	NC_000001.10
cg04365721	7.11E-10	5.78E-11	6.94	14.0178472	1.34E-01	3600391	3600514	NC_000005.9
cg22076941	7.11E-10	5.78E-11	6.94	14.0173373	1.07E-01	70036106	70036229	NC_000001.10
cg06632762	7.12E-10	5.79E-11	6.94	14.0171055	2.18E-01	110230545	110230668	NC_000001.10
cg09906145	7.12E-10	5.79E-11	6.94	14.0169675	1.02E-01	67562424	67562547	NC_000016.9
cg03632245	7.13E-10	5.80E-11	6.93	14.0148587	8.26E-02	72590921	72591044	NC_000005.9
cg00070680	7.13E-10	5.80E-11	6.93	14.0146835	1.83E-01	137531956	137532079	NC_000007.13
cg03672342	7.14E-10	5.80E-11	6.93	14.0142373	2.13E-01	8094093	8094216	NC_000010.10
cg24669786	7.15E-10	5.81E-11	6.93	14.0127218	7.80E-02	92731118	92731241	NC_000007.13
cg07109453	7.15E-10	5.82E-11	6.93	14.0120652	1.74E-01	234669825	234669948	NC_000001.10
cg22876812	7.15E-10	5.82E-11	6.93	14.0120243	1.88E-01	71116188	71116311	NC_000002.11
cg04920689	7.15E-10	5.82E-11	6.93	14.0117886	1.60E-01	88800510	88800633	NC_000015.9
cg06697536	7.16E-10	5.82E-11	6.93	14.0105564	1.63E-01	177053496	177053619	NC_000002.11
cg20357628	7.16E-10	5.83E-11	6.93	14.010461	2.30E-01	58179847	58179970	NC_000020.10
cg08570686	7.16E-10	5.83E-11	6.93	14.0101653	6.80E-02	2287843	2287966	NC_000011.9
cg18783886	7.17E-10	5.83E-11	6.93	14.0096751	4.06E-02	148964538	148964661	NC_000007.13
cg10211298	7.17E-10	5.83E-11	6.93	14.0094411	1.46E-01	132757894	132758017	NC_000003.11
cg05266564	7.17E-10	5.83E-11	6.93	14.0088572	1.39E-01	177024502	177024625	NC_000002.11
cg15399466	7.19E-10	5.85E-11	6.93	14.0059446	1.40E-01	50696059	50696182	NC_000005.9
cg14823851	7.21E-10	5.87E-11	6.93	14.0033668	1.04E-01	59532306	59532429	NC_000017.10
cg10765171	7.21E-10	5.87E-11	6.93	14.0029735	8.95E-02	23270757	23270880	NC_000013.10
cg07598580	7.21E-10	5.87E-11	6.93	14.0027744	1.06E-01	86620525	86620648	NC_000001.10

cg22549437	7.22E-10	5.88E-11	6.93	14.001828	1.14E-01	18466223	18466346	NC_000006.11
cg04974290	7.22E-10	5.88E-11	6.93	14.0013544	1.75E-01	5001047	5001170	NC_000017.10
cg08599259	7.24E-10	5.89E-11	6.93	13.9990442	1.81E-01	38510933	38511056	NC_000001.10
cg24844202	7.24E-10	5.89E-11	6.93	13.9988894	3.10E-02	95072875	95072998	NC_000008.10
cg07923686	7.24E-10	5.90E-11	6.93	13.9986032	1.73E-01	65491318	65491441	NC_000008.10
cg00842325	7.24E-10	5.90E-11	6.93	13.9982086	1.89E-01	21503536	21503659	NC_000020.10
cg09784428	7.27E-10	5.92E-11	6.93	13.9949685	1.08E-01	134791450	134791573	NC_000010.10
cg22538780	7.27E-10	5.92E-11	6.93	13.9943846	1.75E-01	77585448	77585571	NC_000008.10
cg06847624	7.27E-10	5.92E-11	6.93	13.9943673	1.76E-01	176827671	176827794	NC_000005.9
cg20284937	7.27E-10	5.92E-11	6.93	13.9942129	3.62E-02	45002564	45002687	NC_000001.10
cg12973930	7.28E-10	5.93E-11	6.93	13.9927118	2.29E-01	57049695	57049818	NC_000019.9
cg11152033	7.29E-10	5.94E-11	6.93	13.9911913	3.67E-02	61542496	61542619	NC_000020.10
cg04865110	7.30E-10	5.94E-11	6.93	13.9905602	2.46E-01	6202558	6202681	NC_000004.11
cg12894776	7.31E-10	5.95E-11	6.93	13.9889505	4.64E-02	79792334	79792457	NC_000017.10
cg00949635	7.31E-10	5.95E-11	6.93	13.9888665	1.49E-01	103634624	103634747	NC_000004.11
cg04756597	7.31E-10	5.96E-11	6.93	13.9884381	7.65E-02	44730607	44730730	NC_000022.10
cg19109538	7.31E-10	5.96E-11	6.93	13.9882457	2.08E-01	88325263	88325386	NC_000013.10
cg04918696	7.31E-10	5.96E-11	6.93	13.9881863	1.34E-01	1004106	1004229	NC_000008.10
cg10739095	7.32E-10	5.97E-11	6.93	13.986854	1.96E-01	139653928	139654051	NC_000003.11
cg05390356	7.36E-10	5.99E-11	6.93	13.9823109	1.35E-01	10277983	10278106	NC_000001.10
cg14283944	7.36E-10	6.00E-11	6.93	13.9820962	1.93E-01	114883316	114883439	NC_000012.11
cg22987590	7.36E-10	6.00E-11	6.93	13.9815883	1.74E-01	21645524	21645647	NC_000008.10
cg14476670	7.37E-10	6.01E-11	6.93	13.9796331	9.67E-02	21352224	21352347	NC_000013.10
cg19579167	7.38E-10	6.01E-11	6.93	13.9792261	2.24E-01	5543878	5544001	NC_000018.9
cg14329508	7.38E-10	6.02E-11	6.93	13.978995	4.89E-02	18168289	18168412	NC_000020.10

cg11626195	7.39E-10	6.02E-11	6.93	13.9774345	3.65E-02	4823045	4823168	NC_000007.13
cg11368643	7.39E-10	6.03E-11	6.93	13.9769762	1.42E-01	140625059	140625182	NC_000005.9
cg11999288	7.41E-10	6.04E-11	6.93	13.9750332	4.72E-02	31637244	31637367	NC_000006.11
cg05860190	7.41E-10	6.04E-11	6.93	13.9746488	1.13E-01	30794065	30794188	NC_000020.10
cg27136844	7.41E-10	6.04E-11	6.93	13.9745334	1.80E-01	147142355	147142478	NC_000003.11
cg02442900	7.43E-10	6.06E-11	6.93	13.9712427	1.31E-01	234296642	234296765	NC_000002.11
cg23444265	7.44E-10	6.07E-11	6.93	13.9705083	1.98E-01	48918205	48918328	NC_000019.9
cg09042791	7.45E-10	6.08E-11	6.93	13.9692056	1.07E-01	235904300	235904423	NC_000002.11
cg01889284	7.46E-10	6.09E-11	6.93	13.9673596	9.23E-02	81890512	81890635	NC_000005.9
cg27116061	7.47E-10	6.10E-11	6.93	13.9659463	1.99E-01	3239638	3239761	NC_000016.9
cg00436633	7.47E-10	6.10E-11	6.93	13.9657823	3.90E-02	73061407	73061530	NC_000007.13
cg02718516	7.48E-10	6.10E-11	6.93	13.9651139	1.72E-01	13424094	13424217	NC_000008.10
cg03036592	7.48E-10	6.10E-11	6.93	13.9650836	1.65E-01	101287839	101287962	NC_000010.10
cg19358493	7.49E-10	6.11E-11	6.93	13.9637608	1.50E-01	119300942	119301065	NC_000010.10
cg27207796	7.49E-10	6.11E-11	6.93	13.9630742	1.96E-01	17783322	17783445	NC_000004.11
cg21929183	7.50E-10	6.12E-11	6.93	13.962251	1.60E-01	140807225	140807348	NC_000005.9
cg18121601	7.50E-10	6.12E-11	6.93	13.9618807	5.29E-02	1462838	1462961	NC_000017.10
cg14904115	7.50E-10	6.12E-11	6.93	13.9617417	4.41E-02	113488382	113488505	NC_000013.10
cg18987491	7.54E-10	6.15E-11	6.92	13.956879	1.07E-01	135033926	135034049	NC_000010.10
cg02110411	7.54E-10	6.15E-11	6.92	13.9566324	3.62E-02	38659139	38659262	NC_000017.10
cg23673980	7.55E-10	6.16E-11	6.92	13.9555897	5.58E-02	5641966	5642089	NC_000007.13
cg16306898	7.55E-10	6.16E-11	6.92	13.9554263	1.98E-01	1475675	1475798	NC_000001.10
cg12109455	7.55E-10	6.16E-11	6.92	13.9553034	1.49E-01	134000737	134000860	NC_000010.10
cg08768569	7.55E-10	6.17E-11	6.92	13.9543775	2.03E-01	106400565	106400688	NC_000010.10
cg12521353	7.57E-10	6.18E-11	6.92	13.9527117	2.49E-01	55204593	55204716	NC_000020.10

cg02758612	7.57E-10	6.18E-11	6.92	13.9526745	2.09E-01	53541186	53541309	NC_000019.9
cg08157228	7.57E-10	6.18E-11	6.92	13.9519314	1.71E-01	86544308	86544431	NC_000016.9
cg22368664	7.57E-10	6.18E-11	6.92	13.9518295	2.06E-01	133195196	133195319	NC_000012.11
cg01571977	7.59E-10	6.20E-11	6.92	13.9498674	8.54E-02	2337318	2337441	NC_000019.9
cg18420965	7.59E-10	6.20E-11	6.92	13.9494682	1.91E-01	66535145	66535268	NC_000004.11
cg05324776	7.59E-10	6.20E-11	6.92	13.9489995	9.60E-02	70002020	70002143	NC_000011.9
cg07256732	7.59E-10	6.20E-11	6.92	13.9488567	1.17E-01	621771	621894	NC_000016.9
cg05574272	7.63E-10	6.23E-11	6.92	13.943948	1.05E-01	33938213	33938336	NC_000005.9
cg21960859	7.64E-10	6.24E-11	6.92	13.9425219	2.00E-01	166795649	166795772	NC_000004.11
cg14148055	7.65E-10	6.25E-11	6.92	13.941892	2.38E-01	54411773	54411896	NC_000019.9
cg15372218	7.65E-10	6.25E-11	6.92	13.9406514	1.24E-01	156837707	156837830	NC_000007.13
cg17557284	7.66E-10	6.26E-11	6.92	13.939852	1.14E-01	93865479	93865602	NC_000003.11
cg11396585	7.67E-10	6.26E-11	6.92	13.9390431	6.14E-02	33273477	33273600	NC_000006.11
cg13654588	7.67E-10	6.27E-11	6.92	13.9389631	1.31E-01	120354081	120354204	NC_000010.10
cg00019678	7.68E-10	6.28E-11	6.92	13.9373814	9.46E-02	110154229	110154352	NC_000012.11
cg14030940	7.68E-10	6.28E-11	6.92	13.9373492	7.91E-02	101906274	101906397	NC_000014.8
cg24723331	7.68E-10	6.28E-11	6.92	13.9371167	1.81E-01	22487219	22487342	NC_000012.11
cg17362052	7.71E-10	6.30E-11	6.92	13.9334613	1.97E-01	107955762	107955885	NC_000004.11
cg05520940	7.71E-10	6.30E-11	6.92	13.9333054	1.55E-01	119534765	119534888	NC_000011.9
cg20824189	7.72E-10	6.31E-11	6.92	13.9312203	2.08E-01	1704082	1704205	NC_000007.13
cg07591403	7.73E-10	6.32E-11	6.92	13.9308669	2.10E-01	89267679	89267802	NC_000016.9
cg12568898	7.73E-10	6.32E-11	6.92	13.9308521	1.58E-01	53424049	53424172	NC_000013.10
cg04843067	7.73E-10	6.32E-11	6.92	13.9305986	1.24E-01	177016037	177016160	NC_000002.11
cg00920970	7.73E-10	6.32E-11	6.92	13.9299352	1.90E-01	152129388	152129511	NC_000006.11
cg24857620	7.74E-10	6.32E-11	6.92	13.9296525	2.14E-01	53851707	53851830	NC_000008.10

cg14286208	7.74E-10	6.33E-11	6.92	13.9294885	1.07E-01	20903445	20903568	NC_000014.8
cg25353171	7.74E-10	6.33E-11	6.92	13.9284859	1.35E-01	9755804	9755927	NC_000008.10
cg08160350	7.75E-10	6.34E-11	6.92	13.9278053	1.94E-01	996052	996175	NC_000004.11
cg02800810	7.75E-10	6.34E-11	6.92	13.9275982	1.24E-01	28340139	28340262	NC_000021.8
cg07903370	7.76E-10	6.34E-11	6.92	13.9266903	1.39E-01	3595106	3595229	NC_000005.9
cg17288397	7.76E-10	6.34E-11	6.92	13.9265904	1.77E-01	72715731	72715854	NC_000005.9
cg18438777	7.76E-10	6.35E-11	6.92	13.9260314	2.33E-01	164265745	164265868	NC_000004.11
cg04503968	7.76E-10	6.35E-11	6.92	13.9256254	1.43E-01	122423530	122423653	NC_000005.9
cg03792491	7.78E-10	6.36E-11	6.92	13.9236616	3.14E-02	95324966	95325089	NC_000010.10
cg16108230	7.78E-10	6.37E-11	6.92	13.9232506	1.45E-01	117584815	117584938	NC_000006.11
cg22001110	7.79E-10	6.37E-11	6.92	13.9221679	6.69E-02	31626313	31626436	NC_000006.11
cg26352161	7.81E-10	6.39E-11	6.92	13.9200891	9.13E-02	43071742	43071865	NC_000006.11
cg04272011	7.83E-10	6.40E-11	6.92	13.9175072	2.01E-01	79746359	79746482	NC_000014.8
cg11343449	7.84E-10	6.42E-11	6.92	13.9157234	2.54E-02	203001461	203001584	NC_000002.11
cg23820885	7.85E-10	6.42E-11	6.92	13.9146427	1.53E-01	114840942	114841065	NC_000012.11
cg27082921	7.86E-10	6.43E-11	6.92	13.9129308	1.51E-01	1800150	1800273	NC_000019.9
cg06652199	7.86E-10	6.44E-11	6.92	13.9126157	2.15E-01	5018715	5018838	NC_000012.11
cg02104456	7.86E-10	6.44E-11	6.92	13.9125935	7.76E-02	83726483	83726606	NC_000015.9
cg01518607	7.87E-10	6.44E-11	6.92	13.9116378	1.81E-01	27235891	27236014	NC_000006.11
cg07077013	7.87E-10	6.44E-11	6.92	13.9114928	1.34E-01	177025198	177025321	NC_000002.11
cg03552025	7.87E-10	6.44E-11	6.92	13.9112528	7.51E-02	52437451	52437574	NC_000012.11
cg06753057	7.87E-10	6.45E-11	6.92	13.9109975	6.80E-02	2382774	2382897	NC_000001.10
cg13797670	7.88E-10	6.45E-11	6.92	13.9104944	7.91E-02	116135281	116135404	NC_000009.11
cg13882606	7.88E-10	6.45E-11	6.92	13.9104839	9.12E-02	17101011	17101134	NC_000021.8
cg03775422	7.88E-10	6.45E-11	6.92	13.9102796	1.97E-01	54610192	54610315	NC_000007.13

cg04532057	7.88E-10	6.45E-11	6.92	13.9102634	1.08E-01	48704329	48704452	NC_000017.10
cg05348870	7.88E-10	6.45E-11	6.92	13.9097961	6.53E-02	6671045	6671168	NC_000019.9
cg06784991	7.91E-10	6.48E-11	6.92	13.9056722	1.13E-01	53308768	53308891	NC_000001.10
cg00187933	7.92E-10	6.48E-11	6.92	13.9054256	1.81E-01	996785	996908	NC_000004.11
cg11122195	7.92E-10	6.48E-11	6.92	13.9051252	6.12E-02	40732511	40732634	NC_000017.10
cg20855565	7.93E-10	6.50E-11	6.91	13.9033034	1.79E-01	248020350	248020473	NC_000001.10
cg27202646	7.93E-10	6.50E-11	6.91	13.9029694	6.04E-02	112071383	112071506	NC_000006.11
cg23760574	7.94E-10	6.50E-11	6.91	13.9027304	7.92E-02	32038097	32038220	NC_000006.11
cg02767613	7.95E-10	6.51E-11	6.91	13.9010698	2.08E-01	229569894	229570017	NC_000001.10
cg10161743	7.95E-10	6.52E-11	6.91	13.9004512	1.23E-01	48917816	48917939	NC_000019.9
cg16438688	7.96E-10	6.52E-11	6.91	13.8996433	1.16E-01	156815529	156815652	NC_000001.10
cg03209130	7.96E-10	6.52E-11	6.91	13.8993362	9.39E-02	107621307	107621430	NC_000011.9
cg16710348	7.96E-10	6.52E-11	6.91	13.8993242	1.30E-01	121660655	121660778	NC_000003.11
cg03295417	7.97E-10	6.53E-11	6.91	13.8983605	1.40E-01	14397892	14398015	NC_000016.9
cg18839609	7.97E-10	6.53E-11	6.91	13.8976197	1.22E-01	60936070	60936193	NC_000010.10
cg06974388	7.99E-10	6.55E-11	6.91	13.8951844	3.01E-02	161065862	161065985	NC_000001.10
cg09087087	8.01E-10	6.56E-11	6.91	13.8934062	8.09E-02	1656273	1656396	NC_000012.11
cg15911612	8.01E-10	6.57E-11	6.91	13.8926747	3.00E-02	25516343	25516466	NC_000014.8
cg01644518	8.01E-10	6.57E-11	6.91	13.8923802	1.04E-01	40889848	40889971	NC_000019.9
cg18998365	8.02E-10	6.57E-11	6.91	13.8916035	1.46E-01	142781532	142781655	NC_000005.9
cg20930366	8.03E-10	6.58E-11	6.91	13.8908414	1.48E-01	113698847	113698970	NC_000005.9
cg05923197	8.03E-10	6.58E-11	6.91	13.8907914	1.47E-01	54418804	54418927	NC_000014.8
cg07775293	8.04E-10	6.59E-11	6.91	13.8894638	5.79E-02	25572639	25572762	NC_000018.9
cg08980837	8.05E-10	6.60E-11	6.91	13.887352	1.58E-01	91172337	91172460	NC_000001.10
cg09639964	8.05E-10	6.60E-11	6.91	13.8872009	1.63E-01	909300	909423	NC_000017.10

cg19302831	8.05E-10	6.60E-11	6.91	13.8871594	1.56E-01	107502251	107502374	NC_000002.11
cg23248205	8.05E-10	6.61E-11	6.91	13.8869531	4.96E-02	24475283	24475406	NC_000013.10
cg19524824	8.07E-10	6.62E-11	6.91	13.8846759	1.20E-01	50023313	50023436	NC_000012.11
cg06550629	8.07E-10	6.62E-11	6.91	13.8844249	1.30E-01	131439172	131439295	NC_000012.11
cg23867997	8.08E-10	6.63E-11	6.91	13.8834454	2.32E-01	61810624	61810747	NC_000020.10
cg15557878	8.12E-10	6.66E-11	6.91	13.8790103	1.36E-01	110406049	110406172	NC_000005.9
cg13472735	8.13E-10	6.67E-11	6.91	13.8774921	5.20E-02	1215781	1215904	NC_000011.9
cg09759699	8.14E-10	6.68E-11	6.91	13.8757346	1.15E-01	23334250	23334373	NC_000011.9
cg12023513	8.15E-10	6.69E-11	6.91	13.8749927	5.46E-02	72483686	72483809	NC_000007.13
cg20027296	8.15E-10	6.69E-11	6.91	13.8746271	1.37E-01	170737396	170737519	NC_000005.9
cg22696426	8.15E-10	6.69E-11	6.91	13.8744513	1.24E-01	121250007	121250130	NC_000010.10
cg19094243	8.16E-10	6.70E-11	6.91	13.8735586	1.66E-01	193058628	193058751	NC_000002.11
cg15312264	8.17E-10	6.71E-11	6.91	13.8716319	1.66E-01	55907299	55907422	NC_000014.8
cg02168436	8.17E-10	6.71E-11	6.91	13.8715563	9.50E-02	37644675	37644798	NC_000014.8
cg27313642	8.18E-10	6.71E-11	6.91	13.8711298	1.55E-01	65291542	65291665	NC_000008.10
cg20518362	8.20E-10	6.73E-11	6.91	13.8687644	9.33E-02	80039295	80039418	NC_000011.9
cg12045715	8.20E-10	6.73E-11	6.91	13.8681468	4.44E-02	901805	901928	NC_000019.9
cg18491080	8.20E-10	6.73E-11	6.91	13.8680361	1.80E-01	60286463	60286586	NC_000015.9
cg02899206	8.21E-10	6.74E-11	6.91	13.8671788	2.33E-01	1779835	1779958	NC_000010.10
cg14298200	8.21E-10	6.74E-11	6.91	13.8667975	1.49E-01	65498812	65498935	NC_000008.10
cg08448886	8.21E-10	6.74E-11	6.91	13.8665733	4.16E-02	68560728	68560851	NC_000016.9
cg18139575	8.21E-10	6.75E-11	6.91	13.8662407	4.84E-02	195354978	195355101	NC_000003.11
cg04881036	8.23E-10	6.76E-11	6.91	13.864655	5.18E-02	65179732	65179855	NC_000012.11
cg27125439	8.24E-10	6.76E-11	6.91	13.8636723	1.10E-01	29311888	29312011	NC_000010.10
cg00745606	8.24E-10	6.77E-11	6.91	13.8627132	1.88E-01	89268847	89268970	NC_000016.9

cg03001942	8.25E-10	6.78E-11	6.91	13.8621064	1.60E-01	71115787	71115910	NC_000002.11
cg03502630	8.26E-10	6.79E-11	6.91	13.8605436	1.32E-01	126306654	126306777	NC_000006.11
cg27175491	8.27E-10	6.79E-11	6.91	13.8596632	2.77E-02	70425656	70425779	NC_000012.11
cg01097071	8.27E-10	6.79E-11	6.91	13.8596599	7.36E-02	77643816	77643939	NC_000018.9
cg19507160	8.27E-10	6.79E-11	6.91	13.8596507	1.27E-01	28227321	28227444	NC_000008.10
cg14522800	8.30E-10	6.82E-11	6.91	13.8558451	9.84E-02	4648566	4648689	NC_000017.10
cg22908000	8.31E-10	6.83E-11	6.91	13.8546256	1.52E-01	237489107	237489230	NC_000002.11
cg00177875	8.31E-10	6.83E-11	6.91	13.8537769	7.15E-02	75478404	75478527	NC_000011.9
cg14224762	8.33E-10	6.84E-11	6.91	13.8523599	1.52E-01	72596566	72596689	NC_000006.11
cg07138793	8.34E-10	6.85E-11	6.91	13.8510447	8.44E-02	28148330	28148453	NC_000015.9
cg15621651	8.35E-10	6.86E-11	6.91	13.8495945	4.74E-02	129796199	129796322	NC_000010.10
cg18208742	8.35E-10	6.86E-11	6.91	13.8495665	5.25E-02	42992872	42992995	NC_000017.10
cg10982031	8.36E-10	6.87E-11	6.91	13.8482615	6.84E-02	129330389	129330512	NC_000012.11
cg18021286	8.37E-10	6.88E-11	6.9	13.8467637	1.43E-01	115530371	115530494	NC_000011.9
cg23300659	8.37E-10	6.88E-11	6.9	13.8465635	1.63E-01	136553822	136553945	NC_000007.13
cg20770516	8.38E-10	6.89E-11	6.9	13.8456576	1.05E-01	104681396	104681519	NC_000007.13
cg26653392	8.40E-10	6.91E-11	6.9	13.8430825	1.12E-01	53001117	53001240	NC_000015.9
cg13101087	8.40E-10	6.91E-11	6.9	13.8427759	1.86E-01	154729059	154729182	NC_000002.11
cg09528660	8.41E-10	6.92E-11	6.9	13.8418095	8.19E-02	89568662	89568785	NC_000004.11
cg05227131	8.42E-10	6.92E-11	6.9	13.8409426	1.81E-01	21378220	21378343	NC_000020.10
cg09146197	8.42E-10	6.92E-11	6.9	13.8406916	1.40E-01	118033581	118033704	NC_000010.10
cg10421002	8.43E-10	6.93E-11	6.9	13.8392944	1.47E-01	33603610	33603733	NC_000015.9
cg08481543	8.43E-10	6.94E-11	6.9	13.8387574	7.92E-02	44223976	44224099	NC_000019.9
cg23288973	8.44E-10	6.95E-11	6.9	13.8374298	1.81E-01	135701050	135701173	NC_000005.9
cg15242330	8.45E-10	6.95E-11	6.9	13.837234	4.85E-02	27422532	27422655	NC_000002.11

cg02064106	8.45E-10	6.95E-11	6.9	13.8366096	2.24E-01	165722858	165722981	NC_000006.11
cg12670347	8.47E-10	6.97E-11	6.9	13.8346569	1.22E-01	114847578	114847701	NC_000012.11
cg06384463	8.48E-10	6.98E-11	6.9	13.8330902	1.49E-01	91182708	91182831	NC_000001.10
cg04154986	8.49E-10	6.99E-11	6.9	13.8319773	8.83E-02	16734819	16734942	NC_000017.10
cg17099397	8.49E-10	6.99E-11	6.9	13.8318054	2.00E-01	108508185	108508308	NC_000001.10
cg02663265	8.49E-10	6.99E-11	6.9	13.831764	1.96E-01	104513186	104513309	NC_000008.10
cg23922454	8.49E-10	6.99E-11	6.9	13.8316666	1.83E-01	113910545	113910668	NC_000012.11
cg03587978	8.50E-10	6.99E-11	6.9	13.8309272	1.31E-01	172165696	172165819	NC_000003.11
cg24549277	8.50E-10	7.00E-11	6.9	13.8307197	1.79E-01	28778121	28778244	NC_000006.11
cg02606327	8.50E-10	7.00E-11	6.9	13.830491	1.63E-01	31079406	31079529	NC_000012.11
cg18712755	8.50E-10	7.00E-11	6.9	13.8299278	2.34E-02	31600642	31600765	NC_000006.11
cg01695225	8.52E-10	7.01E-11	6.9	13.8283658	1.72E-01	73152672	73152795	NC_000002.11
cg03052301	8.54E-10	7.03E-11	6.9	13.8257504	1.64E-01	32459954	32460077	NC_000011.9
cg18424776	8.57E-10	7.06E-11	6.9	13.8215022	9.51E-02	8456316	8456439	NC_000019.9
cg16564033	8.59E-10	7.08E-11	6.9	13.8193309	1.90E-01	38067741	38067864	NC_000021.8
cg07362003	8.60E-10	7.08E-11	6.9	13.8186797	1.16E-01	157682747	157682870	NC_000002.11
cg19741945	8.60E-10	7.08E-11	6.9	13.8183268	1.70E-01	32859587	32859710	NC_000003.11
cg13977320	8.63E-10	7.11E-11	6.9	13.8148657	8.24E-02	116859631	116859754	NC_000009.11
cg01904776	8.63E-10	7.11E-11	6.9	13.8147948	4.15E-02	2153834	2153957	NC_000016.9
cg05571905	8.63E-10	7.11E-11	6.9	13.8144413	7.16E-02	125767795	125767918	NC_000010.10
cg13020868	8.64E-10	7.12E-11	6.9	13.8132959	1.60E-01	25522307	25522430	NC_000014.8
cg21032564	8.67E-10	7.15E-11	6.9	13.8093749	1.11E-01	65381956	65382079	NC_000015.9
cg19883813	8.68E-10	7.15E-11	6.9	13.8090963	1.52E-01	8098005	8098128	NC_000010.10
cg26553789	8.68E-10	7.16E-11	6.9	13.808234	5.08E-02	1616767	1616890	NC_000008.10
cg14028400	8.68E-10	7.16E-11	6.9	13.8081533	1.57E-01	28793656	28793779	NC_000006.11

cg19117365	8.70E-10	7.17E-11	6.9	13.8065291	1.64E-01	139653915	139654038	NC_000003.11
cg20521389	8.71E-10	7.18E-11	6.9	13.8052706	1.86E-01	21645509	21645632	NC_000008.10
cg23883058	8.74E-10	7.21E-11	6.9	13.801712	1.54E-01	5813866	5813989	NC_000002.11
cg09257824	8.74E-10	7.21E-11	6.9	13.8016578	1.65E-01	32455642	32455765	NC_000011.9
cg08737296	8.74E-10	7.21E-11	6.9	13.8014679	1.95E-01	61808691	61808814	NC_000020.10
cg25191332	8.74E-10	7.21E-11	6.9	13.8012734	1.41E-01	112062419	112062542	NC_000007.13
cg16786808	8.75E-10	7.21E-11	6.9	13.8004733	1.53E-01	222434497	222434620	NC_000002.11
cg15085447	8.76E-10	7.22E-11	6.9	13.79926	1.61E-01	137490846	137490969	NC_000003.11
cg01331196	8.77E-10	7.23E-11	6.9	13.7982931	1.28E-01	11287078	11287201	NC_000003.11
cg18066515	8.78E-10	7.24E-11	6.9	13.7971184	3.79E-02	124791616	124791739	NC_000012.11
cg11127874	8.78E-10	7.25E-11	6.9	13.7961995	6.71E-02	62214996	62215119	NC_000011.9
cg22216302	8.79E-10	7.25E-11	6.9	13.7960446	1.23E-01	33468207	33468330	NC_000020.10
cg03048654	8.79E-10	7.25E-11	6.9	13.7958326	2.09E-01	118893138	118893261	NC_000010.10
cg01456626	8.80E-10	7.26E-11	6.9	13.7947441	8.38E-02	38611497	38611620	NC_000017.10
cg24082871	8.80E-10	7.26E-11	6.9	13.7944888	1.51E-01	27333407	27333530	NC_000017.10
cg10864319	8.80E-10	7.26E-11	6.9	13.7944787	2.09E-01	164593498	164593621	NC_000002.11
cg13297560	8.80E-10	7.26E-11	6.9	13.7939846	1.73E-01	99320054	99320177	NC_000015.9
cg23725321	8.82E-10	7.27E-11	6.89	13.7922707	1.14E-01	102279690	102279813	NC_000010.10
cg09854003	8.82E-10	7.28E-11	6.89	13.7921436	1.76E-01	170743459	170743582	NC_000005.9
cg26369484	8.84E-10	7.29E-11	6.89	13.7898081	7.82E-02	46522464	46522587	NC_000017.10
cg14570291	8.85E-10	7.30E-11	6.89	13.7888607	1.40E-01	110672231	110672354	NC_000010.10
cg09416908	8.85E-10	7.30E-11	6.89	13.7884644	1.47E-01	86384670	86384793	NC_000011.9
cg20019163	8.86E-10	7.31E-11	6.89	13.7873906	5.67E-02	133809962	133810085	NC_000010.10
cg10157715	8.86E-10	7.31E-11	6.89	13.7871425	1.28E-01	35060679	35060802	NC_000017.10
cg07781035	8.86E-10	7.31E-11	6.89	13.7870202	2.27E-01	134871778	134871901	NC_000005.9

cg19284211	8.87E-10	7.32E-11	6.89	13.786028	1.41E-01	20347495	20347618	NC_000020.10
cg03940688	8.87E-10	7.32E-11	6.89	13.7858166	7.06E-02	46988236	46988359	NC_000020.10
cg01466017	8.88E-10	7.33E-11	6.89	13.7847775	2.26E-01	17496720	17496843	NC_000010.10
cg13906669	8.90E-10	7.35E-11	6.89	13.7819926	1.60E-01	100999399	100999522	NC_000011.9
cg08217234	8.91E-10	7.35E-11	6.89	13.7815232	1.24E-01	4042688	4042811	NC_000019.9
cg24357161	8.91E-10	7.36E-11	6.89	13.7809419	9.50E-02	9810114	9810237	NC_000017.10
cg03208044	8.94E-10	7.38E-11	6.89	13.7777488	5.78E-02	113337251	113337374	NC_000001.10
cg12069856	8.95E-10	7.39E-11	6.89	13.776852	3.60E-02	89438504	89438627	NC_000016.9
cg03874542	8.95E-10	7.40E-11	6.89	13.7760432	1.49E-01	8338503	8338626	NC_000010.10
cg14171414	8.97E-10	7.41E-11	6.89	13.774368	1.29E-01	53422474	53422597	NC_000013.10
cg23683620	8.97E-10	7.41E-11	6.89	13.7741038	7.48E-02	2753457	2753580	NC_000019.9
cg01285926	8.97E-10	7.41E-11	6.89	13.7737361	1.05E-01	56242554	56242677	NC_000007.13
cg13004587	8.98E-10	7.42E-11	6.89	13.7731514	2.46E-01	180017349	180017472	NC_000005.9
cg13288164	8.99E-10	7.43E-11	6.89	13.7721886	1.56E-01	140501380	140501503	NC_000005.9
cg19215671	8.99E-10	7.43E-11	6.89	13.7721022	1.52E-01	237081839	237081962	NC_000002.11
cg04146259	9.00E-10	7.43E-11	6.89	13.7710787	1.29E-01	85058259	85058382	NC_000014.8
cg16363146	9.02E-10	7.45E-11	6.89	13.7687512	1.01E-01	22689281	22689404	NC_000011.9
cg02841875	9.04E-10	7.47E-11	6.89	13.7660015	4.11E-02	6056063	6056186	NC_000001.10
cg22589822	9.05E-10	7.48E-11	6.89	13.7654423	6.01E-02	91912699	91912822	NC_000015.9
cg14023737	9.05E-10	7.48E-11	6.89	13.7645219	1.16E-01	132386878	132387001	NC_000009.11
cg07304923	9.06E-10	7.49E-11	6.89	13.7639876	9.60E-02	10323048	10323171	NC_000006.11
cg24754598	9.06E-10	7.49E-11	6.89	13.7637617	7.60E-02	95966488	95966611	NC_000014.8
cg18320917	9.08E-10	7.50E-11	6.89	13.7617309	5.29E-02	20644440	20644563	NC_000015.9
cg01148781	9.08E-10	7.51E-11	6.89	13.7613376	1.61E-01	99963021	99963144	NC_000008.10
cg14180839	9.09E-10	7.51E-11	6.89	13.7604801	1.26E-01	139142049	139142172	NC_000009.11

cg21000072	9.11E-10	7.53E-11	6.89	13.758846	1.27E-01	3567408	3567531	NC_000001.10
cg17368254	9.11E-10	7.53E-11	6.89	13.7582468	2.29E-01	95942847	95942970	NC_000012.11
cg02630045	9.11E-10	7.53E-11	6.89	13.7577882	4.54E-02	30303579	30303702	NC_000006.11
cg12950645	9.12E-10	7.54E-11	6.89	13.7573028	1.28E-01	71410605	71410728	NC_000015.9
cg10472919	9.12E-10	7.54E-11	6.89	13.75658	1.56E-01	135266805	135266928	NC_000005.9
cg11460282	9.14E-10	7.56E-11	6.89	13.7549735	1.37E-01	56297661	56297784	NC_000007.13
cg15091407	9.16E-10	7.57E-11	6.89	13.7527529	1.93E-01	57149499	57149622	NC_000019.9
cg22640193	9.16E-10	7.57E-11	6.89	13.7525773	8.51E-02	74884487	74884610	NC_000017.10
cg00569982	9.17E-10	7.59E-11	6.89	13.7510999	9.85E-02	75947572	75947695	NC_000006.11
cg12781568	9.17E-10	7.59E-11	6.89	13.750824	9.16E-02	32452038	32452161	NC_000011.9
cg18971671	9.18E-10	7.60E-11	6.89	13.7498844	5.46E-02	49401721	49401844	NC_000019.9
cg21446000	9.19E-10	7.61E-11	6.89	13.7484176	1.23E-01	115641974	115642097	NC_000004.11
cg20495183	9.20E-10	7.61E-11	6.89	13.7480868	1.57E-01	132814206	132814329	NC_000011.9
cg09813301	9.20E-10	7.62E-11	6.89	13.7471827	6.36E-02	411182	411305	NC_000016.9
cg11857704	9.22E-10	7.63E-11	6.89	13.7451554	1.73E-01	3750952	3751075	NC_000002.11
cg24886076	9.23E-10	7.64E-11	6.89	13.7442822	6.13E-02	192542576	192542699	NC_000001.10
cg19081437	9.23E-10	7.64E-11	6.89	13.7441832	1.78E-01	46655796	46655919	NC_000017.10
cg06908550	9.24E-10	7.65E-11	6.89	13.7433558	9.98E-02	222279701	222279824	NC_000001.10
cg14614811	9.24E-10	7.65E-11	6.89	13.7427765	1.74E-01	159399681	159399804	NC_000005.9
cg10612285	9.25E-10	7.65E-11	6.89	13.7423661	2.70E-02	59394689	59394812	NC_000015.9
cg01792403	9.25E-10	7.66E-11	6.89	13.7417864	1.58E-01	100626995	100627118	NC_000013.10
cg12486635	9.26E-10	7.67E-11	6.89	13.740899	9.02E-02	57828461	57828584	NC_000012.11
cg11194118	9.26E-10	7.67E-11	6.89	13.7407775	1.01E-01	134790922	134791045	NC_000010.10
cg12308631	9.26E-10	7.67E-11	6.89	13.7406851	7.03E-02	158454502	158454625	NC_000006.11
cg21872037	9.26E-10	7.67E-11	6.89	13.7402953	1.72E-01	6617470	6617593	NC_000017.10

cg22799321	9.27E-10	7.68E-11	6.89	13.739536	2.43E-01	93519621	93519744	NC_000007.13
cg24269991	9.27E-10	7.68E-11	6.89	13.7393998	7.09E-02	141069186	141069309	NC_000004.11
cg10687936	9.28E-10	7.69E-11	6.89	13.7378194	1.66E-01	125085322	125085445	NC_000003.11
cg17538280	9.28E-10	7.69E-11	6.89	13.7377542	2.00E-01	49812836	49812959	NC_000007.13
cg11499970	9.29E-10	7.70E-11	6.88	13.7366221	2.06E-01	167956581	167956704	NC_000005.9
cg13369817	9.31E-10	7.71E-11	6.88	13.7354628	9.96E-02	55116866	55116989	NC_000001.10
cg17188046	9.32E-10	7.72E-11	6.88	13.7335102	2.11E-01	166582197	166582320	NC_000006.11
cg00054971	9.33E-10	7.73E-11	6.88	13.7332422	5.21E-02	129796937	129797060	NC_000010.10
cg05374756	9.33E-10	7.73E-11	6.88	13.7327603	5.33E-02	37586943	37587066	NC_000020.10
cg08635822	9.33E-10	7.73E-11	6.88	13.7326609	9.27E-02	82958259	82958382	NC_000004.11
cg08110693	9.34E-10	7.74E-11	6.88	13.7319109	1.69E-01	36407533	36407656	NC_000006.11
cg17647597	9.34E-10	7.74E-11	6.88	13.7315494	3.52E-02	20935799	20935922	NC_000012.11
cg14210765	9.35E-10	7.75E-11	6.88	13.730343	7.61E-02	73628830	73628953	NC_000018.9
cg02884176	9.36E-10	7.76E-11	6.88	13.7294314	2.24E-01	88752219	88752342	NC_000002.11
cg02062404	9.37E-10	7.77E-11	6.88	13.7280556	3.21E-02	228295382	228295505	NC_000001.10
cg22885558	9.37E-10	7.77E-11	6.88	13.7276801	1.73E-01	137484220	137484343	NC_000003.11
cg12388397	9.38E-10	7.78E-11	6.88	13.7268022	4.72E-02	79517983	79518106	NC_000017.10
cg15157241	9.39E-10	7.78E-11	6.88	13.725842	1.18E-01	44396204	44396327	NC_000021.8
cg27172488	9.40E-10	7.79E-11	6.88	13.7251905	5.82E-02	157219499	157219622	NC_000007.13
cg01198033	9.42E-10	7.81E-11	6.88	13.7223632	1.54E-01	155302342	155302465	NC_000007.13
cg10577241	9.43E-10	7.82E-11	6.88	13.7212976	1.45E-01	77456283	77456406	NC_000015.9
cg27341926	9.44E-10	7.83E-11	6.88	13.7198249	2.31E-01	118032905	118033028	NC_000010.10
cg13033990	9.45E-10	7.83E-11	6.88	13.7194898	1.90E-01	28617304	28617427	NC_000003.11
cg18062333	9.45E-10	7.84E-11	6.88	13.7186942	1.23E-01	110446221	110446344	NC_000003.11
cg01899911	9.48E-10	7.86E-11	6.88	13.7157212	7.95E-02	1039070	1039193	NC_000007.13

cg24198889	9.48E-10	7.87E-11	6.88	13.715586	7.82E-02	2423215	2423338	NC_000011.9
cg11705975	9.49E-10	7.87E-11	6.88	13.7147942	7.92E-02	120354248	120354371	NC_000010.10
cg10490742	9.51E-10	7.89E-11	6.88	13.7120827	1.08E-01	127765504	127765627	NC_000012.11
cg13298963	9.52E-10	7.90E-11	6.88	13.7116025	1.77E-01	32483938	32484061	NC_000017.10
cg07401378	9.52E-10	7.90E-11	6.88	13.7113097	1.64E-01	37248675	37248798	NC_000013.10
cg02445664	9.52E-10	7.90E-11	6.88	13.7110266	1.78E-01	47210599	47210722	NC_000017.10
cg22734397	9.52E-10	7.90E-11	6.88	13.7108276	2.03E-01	134871942	134872065	NC_000005.9
cg16852704	9.53E-10	7.91E-11	6.88	13.7106377	1.29E-01	74743243	74743366	NC_000002.11
cg05843621	9.53E-10	7.91E-11	6.88	13.7102888	1.14E-01	80231999	80232122	NC_000017.10
cg25238480	9.56E-10	7.94E-11	6.88	13.7066836	1.19E-01	11335633	11335756	NC_000011.9
cg07875818	9.57E-10	7.94E-11	6.88	13.70622	2.73E-01	132261765	132261888	NC_000007.13
cg17677030	9.57E-10	7.94E-11	6.88	13.7061538	1.92E-01	50575069	50575192	NC_000002.11
cg21784383	9.57E-10	7.94E-11	6.88	13.7058041	1.69E-01	217311172	217311295	NC_000001.10
cg14950072	9.59E-10	7.96E-11	6.88	13.7039343	1.78E-01	7118122	7118245	NC_000018.9
cg06187770	9.60E-10	7.98E-11	6.88	13.7019933	1.64E-01	85484863	85484986	NC_000006.11
cg20595271	9.60E-10	7.98E-11	6.88	13.7019452	1.02E-01	111889200	111889323	NC_000012.11
cg20570313	9.63E-10	8.00E-11	6.88	13.6988441	8.05E-02	68772808	68772931	NC_000011.9
cg19175386	9.66E-10	8.02E-11	6.88	13.6961211	1.79E-01	115134496	115134619	NC_000012.11
cg01944044	9.66E-10	8.03E-11	6.88	13.6958339	1.72E-01	20084851	20084974	NC_000016.9
cg07618048	9.67E-10	8.03E-11	6.88	13.6949138	7.03E-02	75024638	75024761	NC_000007.13
cg09802835	9.67E-10	8.03E-11	6.88	13.694805	1.36E-01	71952131	71952254	NC_000011.9
cg07047280	9.68E-10	8.04E-11	6.88	13.6935651	1.28E-01	41516394	41516517	NC_000021.8
cg24018520	9.69E-10	8.05E-11	6.88	13.6931529	1.56E-01	175133103	175133226	NC_000004.11
cg27158341	9.69E-10	8.05E-11	6.88	13.6929861	4.42E-02	52959510	52959633	NC_000006.11
cg05981335	9.70E-10	8.06E-11	6.88	13.6920877	1.43E-01	81102562	81102685	NC_000012.11

cg09659227	9.70E-10	8.06E-11	6.88	13.6918463	1.57E-01	46797291	46797414	NC_000017.10
cg24511738	9.70E-10	8.06E-11	6.88	13.6911211	1.62E-01	9762716	9762839	NC_000008.10
cg09764761	9.72E-10	8.07E-11	6.88	13.6898492	1.22E-01	44105544	44105667	NC_000017.10
cg12409982	9.72E-10	8.08E-11	6.88	13.6895582	1.51E-01	137489312	137489435	NC_000003.11
cg19542816	9.73E-10	8.09E-11	6.88	13.688165	2.02E-01	177053295	177053418	NC_000002.11
cg07733257	9.75E-10	8.10E-11	6.88	13.6862225	1.36E-01	1882520	1882643	NC_000005.9
cg02763409	9.78E-10	8.13E-11	6.88	13.6834884	7.63E-02	239695552	239695675	NC_000002.11
cg25611723	9.78E-10	8.13E-11	6.88	13.6830474	8.12E-02	10453624	10453747	NC_000018.9
cg27439396	9.80E-10	8.15E-11	6.87	13.6805906	2.04E-02	21304091	21304214	NC_000013.10
cg05949020	9.81E-10	8.16E-11	6.87	13.6800854	1.70E-01	110693757	110693880	NC_000001.10
cg09435415	9.82E-10	8.17E-11	6.87	13.6785194	1.35E-01	105479383	105479506	NC_000008.10
cg05875410	9.83E-10	8.18E-11	6.87	13.6774169	1.32E-01	51187572	51187695	NC_000016.9
cg22819171	9.84E-10	8.18E-11	6.87	13.6771436	1.08E-01	53742252	53742375	NC_000001.10
cg07941953	9.84E-10	8.18E-11	6.87	13.6769327	9.76E-02	10508668	10508791	NC_000020.10
cg25160286	9.88E-10	8.22E-11	6.87	13.6728972	1.61E-01	27282308	27282431	NC_000007.13
cg24998791	9.89E-10	8.23E-11	6.87	13.6713007	9.69E-02	10454100	10454223	NC_000008.10
cg16108388	9.91E-10	8.24E-11	6.87	13.6697458	8.17E-02	41704631	41704754	NC_000006.11
cg03004999	9.91E-10	8.24E-11	6.87	13.6697075	1.19E-01	43152125	43152248	NC_000007.13
cg05005577	9.91E-10	8.24E-11	6.87	13.6695291	4.63E-02	18669132	18669255	NC_000022.10
cg01580681	9.91E-10	8.25E-11	6.87	13.6692799	1.67E-01	174450016	174450139	NC_000004.11
cg00259618	9.92E-10	8.25E-11	6.87	13.6686347	1.99E-01	103889655	103889778	NC_000012.11
cg05091519	9.92E-10	8.26E-11	6.87	13.6680045	1.67E-01	31839552	31839675	NC_000011.9
cg13585586	9.93E-10	8.26E-11	6.87	13.6674375	1.44E-01	104199266	104199389	NC_000009.11
cg07598199	9.95E-10	8.28E-11	6.87	13.6649236	1.06E-01	120056829	120056952	NC_000004.11
cg12869912	9.96E-10	8.29E-11	6.87	13.6643257	2.10E-01	58152803	58152926	NC_000020.10

cg14327228	9.96E-10	8.29E-11	6.87	13.6636806	9.90E-02	36343530	36343653	NC_000019.9
cg23421023	9.97E-10	8.30E-11	6.87	13.6626077	1.71E-01	40755165	40755288	NC_000008.10
cg13466809	9.98E-10	8.31E-11	6.87	13.6617488	7.26E-02	160542944	160543067	NC_000006.11
cg07419968	9.99E-10	8.31E-11	6.87	13.6613456	6.05E-02	106339081	106339204	NC_000001.10
cg06329574	1.00E-09	8.32E-11	6.87	13.6600822	1.70E-01	69634334	69634457	NC_000011.9
cg11196175	1.00E-09	8.34E-11	6.87	13.6586389	7.10E-02	21560541	21560664	NC_000014.8
cg22581933	1.00E-09	8.34E-11	6.87	13.6584977	5.38E-02	109797277	109797400	NC_000012.11
cg10277811	1.00E-09	8.35E-11	6.87	13.6569151	1.34E-01	68049474	68049597	NC_000017.10
cg10325478	1.00E-09	8.36E-11	6.87	13.6558325	1.77E-01	88793021	88793144	NC_000014.8
cg08749917	1.00E-09	8.36E-11	6.87	13.6557948	8.88E-02	186915320	186915443	NC_000003.11
cg11488401	1.01E-09	8.37E-11	6.87	13.654244	9.73E-02	77468373	77468496	NC_000016.9
cg25562106	1.01E-09	8.38E-11	6.87	13.6534227	7.61E-02	149891579	149891702	NC_000006.11
cg27483455	1.01E-09	8.38E-11	6.87	13.653392	8.31E-02	50475360	50475483	NC_000012.11
cg27279583	1.01E-09	8.39E-11	6.87	13.6526071	1.26E-01	61514753	61514876	NC_000017.10
cg18485627	1.01E-09	8.40E-11	6.87	13.6515522	3.61E-02	101340188	101340311	NC_000014.8
cg24151423	1.01E-09	8.40E-11	6.87	13.6514834	3.15E-02	61270712	61270835	NC_000020.10
cg17108388	1.01E-09	8.40E-11	6.87	13.651429	7.31E-02	204226566	204226689	NC_000001.10
cg04460847	1.01E-09	8.42E-11	6.87	13.6489807	1.36E-01	127841335	127841458	NC_000007.13
cg01653005	1.01E-09	8.43E-11	6.87	13.6473774	2.23E-01	8869105	8869228	NC_000017.10
cg00953201	1.01E-09	8.44E-11	6.87	13.6461046	1.68E-01	30156486	30156609	NC_000013.10
cg01015662	1.01E-09	8.44E-11	6.87	13.6460722	8.43E-02	40598394	40598517	NC_000001.10
cg22390041	1.01E-09	8.45E-11	6.87	13.6451552	1.32E-01	19226319	19226442	NC_000001.10
cg18543610	1.01E-09	8.46E-11	6.87	13.6442246	9.90E-02	100627929	100628052	NC_000013.10
cg14645027	1.02E-09	8.46E-11	6.87	13.6436063	1.00E-01	52642706	52642829	NC_000012.11
cg15335860	1.02E-09	8.47E-11	6.87	13.6429766	4.91E-02	56879323	56879446	NC_000007.13

cg07223180	1.02E-09	8.47E-11	6.87	13.6429724	9.25E-02	20989142	20989265	NC_000013.10
cg10553204	1.02E-09	8.47E-11	6.87	13.6429516	1.25E-01	20871195	20871318	NC_000002.11
cg10000952	1.02E-09	8.47E-11	6.87	13.6426212	1.72E-01	116451628	116451751	NC_000011.9
cg01033191	1.02E-09	8.49E-11	6.87	13.6408615	1.57E-01	62488850	62488973	NC_000010.10
cg23405575	1.02E-09	8.50E-11	6.87	13.6398469	1.78E-01	11034281	11034404	NC_000003.11
cg07827796	1.02E-09	8.51E-11	6.87	13.6385415	5.29E-02	33622959	33623082	NC_000019.9
cg21217540	1.02E-09	8.51E-11	6.87	13.6384305	1.09E-01	110752307	110752430	NC_000001.10
cg20122586	1.02E-09	8.51E-11	6.87	13.6381176	1.29E-01	53522821	53522944	NC_000004.11
cg12432236	1.02E-09	8.55E-11	6.87	13.6342392	1.55E-01	58209133	58209256	NC_000013.10
cg06091094	1.03E-09	8.55E-11	6.87	13.6335936	7.33E-02	44927766	44927889	NC_000011.9
cg00919971	1.03E-09	8.56E-11	6.87	13.6328894	1.43E-01	1286149	1286272	NC_000007.13
cg23765754	1.03E-09	8.56E-11	6.87	13.6324936	1.40E-01	30717766	30717889	NC_000019.9
cg20792294	1.03E-09	8.57E-11	6.87	13.6310926	1.77E-01	2165425	2165548	NC_000011.9
cg26575637	1.03E-09	8.58E-11	6.87	13.6306019	8.42E-02	16760056	16760179	NC_000012.11
cg03817667	1.03E-09	8.59E-11	6.87	13.6293942	1.87E-01	54609953	54610076	NC_000007.13
cg12106728	1.03E-09	8.59E-11	6.87	13.6291293	2.02E-01	115136258	115136381	NC_000012.11
cg00272971	1.03E-09	8.59E-11	6.87	13.6290407	1.74E-01	102419564	102419687	NC_000010.10
cg24827660	1.03E-09	8.60E-11	6.87	13.6279874	2.09E-01	58179538	58179661	NC_000020.10
cg17545182	1.03E-09	8.60E-11	6.87	13.6276479	1.21E-01	247694041	247694164	NC_000001.10
cg25121292	1.03E-09	8.61E-11	6.87	13.627237	1.07E-01	203036093	203036216	NC_000002.11
cg07817024	1.03E-09	8.62E-11	6.86	13.6258482	1.82E-01	119535500	119535623	NC_000001.10
cg04546557	1.04E-09	8.65E-11	6.86	13.6228198	1.14E-01	27262483	27262606	NC_000007.13
cg06088623	1.04E-09	8.65E-11	6.86	13.6225008	1.18E-01	8274890	8275013	NC_000019.9
cg08316825	1.04E-09	8.65E-11	6.86	13.622294	1.09E-01	151304547	151304670	NC_000005.9
cg20501434	1.04E-09	8.65E-11	6.86	13.6222653	1.23E-01	30868167	30868290	NC_000022.10

cg18972811	1.04E-09	8.67E-11	6.86	13.6205214	1.53E-01	20254736	20254859	NC_000004.11
cg25782229	1.04E-09	8.68E-11	6.86	13.6190065	1.55E-01	32450692	32450815	NC_000011.9
cg07213870	1.04E-09	8.68E-11	6.86	13.6184745	9.76E-02	123613331	123613454	NC_000011.9
cg16946271	1.04E-09	8.69E-11	6.86	13.6177634	5.68E-02	119666271	119666394	NC_000006.11
cg08121453	1.04E-09	8.69E-11	6.86	13.6177565	8.51E-02	248855364	248855487	NC_000001.10
cg20337028	1.04E-09	8.69E-11	6.86	13.6174858	5.26E-02	75181836	75181959	NC_000017.10
cg04600411	1.04E-09	8.70E-11	6.86	13.6167031	3.13E-02	40269556	40269679	NC_000017.10
cg20634967	1.04E-09	8.71E-11	6.86	13.6151079	2.44E-01	58204224	58204347	NC_000013.10
cg01524278	1.04E-09	8.71E-11	6.86	13.6149711	1.42E-01	54355460	54355583	NC_000012.11
cg02985568	1.04E-09	8.72E-11	6.86	13.6139382	1.66E-01	15753565	15753688	NC_000021.8
cg26952362	1.05E-09	8.75E-11	6.86	13.6110869	6.29E-02	72820053	72820176	NC_000010.10
cg06173889	1.05E-09	8.75E-11	6.86	13.6109157	1.30E-01	5835853	5835976	NC_000002.11
cg08777316	1.05E-09	8.77E-11	6.86	13.6083697	1.23E-01	158531511	158531634	NC_000005.9
cg24855045	1.05E-09	8.78E-11	6.86	13.6081338	1.01E-01	18938449	18938572	NC_000010.10
cg24914278	1.05E-09	8.79E-11	6.86	13.606974	2.03E-01	109799739	109799862	NC_000008.10
cg03167413	1.05E-09	8.79E-11	6.86	13.6062467	1.82E-01	113723093	113723216	NC_000007.13
cg11469051	1.05E-09	8.81E-11	6.86	13.6042122	1.10E-01	24804131	24804254	NC_000004.11
cg10776230	1.06E-09	8.82E-11	6.86	13.6037057	1.29E-01	245851226	245851349	NC_000001.10
cg09536516	1.06E-09	8.82E-11	6.86	13.6033843	3.83E-02	30934475	30934598	NC_000007.13
cg10977667	1.06E-09	8.85E-11	6.86	13.5996638	1.07E-01	31053799	31053922	NC_000016.9
cg04123357	1.06E-09	8.85E-11	6.86	13.5992908	7.93E-02	46929500	46929623	NC_000021.8
cg22048474	1.06E-09	8.86E-11	6.86	13.5990842	1.70E-01	55366834	55366957	NC_000008.10
cg05033560	1.06E-09	8.86E-11	6.86	13.5987632	5.89E-02	172173145	172173268	NC_000005.9
cg14185341	1.06E-09	8.87E-11	6.86	13.5980596	6.27E-02	51829712	51829835	NC_000010.10
cg24928333	1.06E-09	8.88E-11	6.86	13.596991	1.80E-01	44790141	44790264	NC_000018.9

cg01590753	1.06E-09	8.88E-11	6.86	13.5969057	1.90E-01	177714373	177714496	NC_000004.11
cg21948716	1.06E-09	8.88E-11	6.86	13.5967453	1.10E-01	145562756	145562879	NC_000008.10
cg03042281	1.06E-09	8.89E-11	6.86	13.5958915	8.00E-02	17608394	17608517	NC_000019.9
cg27109006	1.06E-09	8.89E-11	6.86	13.5956303	1.40E-01	61378423	61378546	NC_000015.9
cg09251429	1.06E-09	8.90E-11	6.86	13.5945551	1.62E-01	124735128	124735251	NC_000011.9
cg19850348	1.06E-09	8.90E-11	6.86	13.594229	2.05E-01	55880997	55881120	NC_000015.9
cg17152757	1.06E-09	8.90E-11	6.86	13.5940685	1.58E-01	172166258	172166381	NC_000003.11
cg21773872	1.07E-09	8.91E-11	6.86	13.592864	2.02E-01	30722320	30722443	NC_000007.13
cg11675630	1.07E-09	8.91E-11	6.86	13.5927069	7.85E-02	29255881	29256004	NC_000002.11
cg02153747	1.07E-09	8.92E-11	6.86	13.5922966	1.03E-01	29917507	29917630	NC_000010.10
cg06733215	1.07E-09	8.92E-11	6.86	13.5920084	3.11E-02	70317506	70317629	NC_000016.9
cg26990102	1.07E-09	8.94E-11	6.86	13.5900362	1.74E-01	119606746	119606869	NC_000002.11
cg03935991	1.07E-09	8.94E-11	6.86	13.5898611	5.39E-02	33592746	33592869	NC_000010.10
cg00147850	1.07E-09	8.96E-11	6.86	13.5878397	5.46E-02	144994715	144994838	NC_000001.10
cg15748507	1.07E-09	8.96E-11	6.86	13.587715	1.21E-01	120354627	120354750	NC_000010.10
cg13004897	1.07E-09	8.97E-11	6.86	13.5869988	8.64E-02	177225404	177225527	NC_000005.9
cg03734437	1.07E-09	8.97E-11	6.86	13.5868959	2.20E-01	97173526	97173649	NC_000008.10
cg14289511	1.07E-09	8.98E-11	6.86	13.585795	1.67E-01	24682446	24682569	NC_000016.9
cg06209197	1.07E-09	8.98E-11	6.86	13.5855922	5.72E-02	32106926	32107049	NC_000001.10
cg00863378	1.07E-09	8.98E-11	6.86	13.585579	1.02E-01	56549757	56549880	NC_000016.9
cg25173574	1.07E-09	8.98E-11	6.86	13.5853813	3.81E-02	154112549	154112672	NC_000004.11
cg04909529	1.07E-09	8.98E-11	6.86	13.5852517	5.17E-02	104218302	104218425	NC_000012.11
cg01824466	1.08E-09	9.00E-11	6.86	13.583716	1.70E-01	95959531	95959654	NC_000008.10
cg06014958	1.08E-09	9.00E-11	6.86	13.5836337	1.64E-01	10425553	10425676	NC_000006.11
cg23564700	1.08E-09	9.00E-11	6.86	13.5833287	2.17E-01	5544169	5544292	NC_000018.9

cg08801446	1.08E-09	9.01E-11	6.86	13.5824933	1.17E-01	36626126	36626249	NC_000002.11
cg03151469	1.08E-09	9.01E-11	6.86	13.5824554	1.39E-01	176936091	176936214	NC_000002.11
cg24332743	1.08E-09	9.01E-11	6.86	13.5820348	1.39E-01	114610614	114610737	NC_000010.10
cg04175027	1.08E-09	9.01E-11	6.86	13.5819783	1.59E-01	123418722	123418845	NC_000002.11
cg17523945	1.08E-09	9.03E-11	6.86	13.5801894	1.28E-01	46732351	46732474	NC_000019.9
cg19523085	1.08E-09	9.04E-11	6.86	13.5792631	1.86E-01	28339477	28339600	NC_000021.8
cg13617198	1.08E-09	9.05E-11	6.86	13.5783057	1.83E-01	77594256	77594379	NC_000008.10
cg19803052	1.08E-09	9.06E-11	6.86	13.5771444	1.40E-01	19813244	19813367	NC_000007.13
cg01005365	1.08E-09	9.08E-11	6.86	13.5748533	1.47E-01	76029522	76029645	NC_000005.9
cg09916357	1.08E-09	9.08E-11	6.86	13.5743719	1.02E-01	157397131	157397254	NC_000004.11
cg14017655	1.09E-09	9.09E-11	6.86	13.5734152	2.32E-01	133562193	133562316	NC_000006.11
cg02923490	1.09E-09	9.10E-11	6.86	13.5726732	8.75E-02	37872019	37872142	NC_000006.11
cg22333214	1.09E-09	9.11E-11	6.86	13.5709301	2.36E-01	98511789	98511912	NC_000001.10
cg25950278	1.09E-09	9.12E-11	6.86	13.5708018	1.29E-01	49388412	49388535	NC_000012.11
cg27454064	1.09E-09	9.12E-11	6.85	13.5705199	1.28E-01	64215611	64215734	NC_000012.11
cg20573931	1.09E-09	9.12E-11	6.85	13.5704973	9.93E-02	79710803	79710926	NC_000008.10
cg00216693	1.09E-09	9.12E-11	6.85	13.5704381	5.91E-02	131913247	131913370	NC_000002.11
cg04405466	1.09E-09	9.12E-11	6.85	13.5698675	6.63E-02	105851087	105851210	NC_000010.10
cg14515453	1.09E-09	9.14E-11	6.85	13.568523	1.58E-01	19158041	19158164	NC_000007.13
cg25456468	1.09E-09	9.14E-11	6.85	13.5683019	2.12E-01	54394116	54394239	NC_000012.11
cg15659537	1.09E-09	9.14E-11	6.85	13.5678029	1.05E-01	75712028	75712151	NC_000012.11
cg04856042	1.09E-09	9.15E-11	6.85	13.5675564	6.68E-02	115752704	115752827	NC_000003.11
cg18594777	1.09E-09	9.15E-11	6.85	13.5673273	3.03E-02	53342353	53342476	NC_000001.10
cg13407144	1.09E-09	9.17E-11	6.85	13.5645264	5.28E-02	33468337	33468460	NC_000010.10
cg26157385	1.10E-09	9.18E-11	6.85	13.5634002	1.31E-01	248020641	248020764	NC_000001.10

cg15993083	1.10E-09	9.19E-11	6.85	13.5630587	2.07E-01	4381788	4381911	NC_000012.11
cg08326300	1.10E-09	9.19E-11	6.85	13.5623399	9.07E-02	80319770	80319893	NC_000010.10
cg00056541	1.10E-09	9.20E-11	6.85	13.5621009	1.10E-01	164288623	164288746	NC_000006.11
cg23413924	1.10E-09	9.23E-11	6.85	13.5581259	9.61E-02	45427734	45427857	NC_000015.9
cg00648184	1.10E-09	9.25E-11	6.85	13.5566372	1.41E-01	60828157	60828280	NC_000017.10
cg09008705	1.10E-09	9.25E-11	6.85	13.5563246	2.32E-01	115880865	115880988	NC_000001.10
cg06392574	1.10E-09	9.26E-11	6.85	13.5552109	1.35E-01	37212272	37212395	NC_000006.11
cg17354303	1.11E-09	9.28E-11	6.85	13.5532388	1.04E-01	96716665	96716788	NC_000009.11
cg03175305	1.11E-09	9.29E-11	6.85	13.552721	1.56E-01	179754483	179754606	NC_000003.11
cg06110728	1.11E-09	9.29E-11	6.85	13.5521602	2.41E-01	45270886	45271009	NC_000012.11
cg08733933	1.11E-09	9.30E-11	6.85	13.5513239	1.08E-01	2954429	2954552	NC_000001.10
cg19003412	1.11E-09	9.30E-11	6.85	13.5512965	1.11E-01	74277799	74277922	NC_000004.11
cg19764326	1.11E-09	9.30E-11	6.85	13.5509825	4.74E-02	67509003	67509126	NC_000001.10
cg16698845	1.11E-09	9.31E-11	6.85	13.5499792	4.39E-02	588478	588601	NC_000007.13
cg18745131	1.11E-09	9.32E-11	6.85	13.5491178	1.28E-01	1269963	1270086	NC_000007.13
cg05921138	1.11E-09	9.32E-11	6.85	13.5486729	7.69E-02	121440849	121440972	NC_000011.9
cg09255966	1.11E-09	9.33E-11	6.85	13.548383	3.65E-02	2334136	2334259	NC_000001.10
cg17959850	1.11E-09	9.34E-11	6.85	13.5464456	3.67E-02	49708563	49708686	NC_000005.9
cg04606210	1.12E-09	9.38E-11	6.85	13.542937	2.04E-01	55205571	55205694	NC_000020.10
cg14646081	1.12E-09	9.38E-11	6.85	13.542435	1.03E-01	71691305	71691428	NC_000009.11
cg02965712	1.12E-09	9.39E-11	6.85	13.5416107	6.84E-02	77746028	77746151	NC_000001.10
cg11909748	1.12E-09	9.40E-11	6.85	13.5409855	1.49E-01	52956674	52956797	NC_000019.9
cg20635409	1.12E-09	9.42E-11	6.85	13.5382601	1.38E-01	103359273	103359396	NC_000012.11
cg15376615	1.12E-09	9.42E-11	6.85	13.5382102	2.11E-01	153858822	153858945	NC_000005.9
cg23473285	1.12E-09	9.43E-11	6.85	13.5380344	1.79E-01	72596698	72596821	NC_000006.11

cg02746161	1.12E-09	9.44E-11	6.85	13.536724	2.45E-02	114117367	114117490	NC_000010.10
cg26923754	1.12E-09	9.44E-11	6.85	13.5363815	9.69E-02	16063122	16063245	NC_000001.10
cg09298852	1.12E-09	9.44E-11	6.85	13.5362188	5.08E-02	1574816	1574939	NC_000019.9
cg19352038	1.13E-09	9.45E-11	6.85	13.5357412	1.45E-01	223164869	223164992	NC_000002.11
cg25490475	1.13E-09	9.46E-11	6.85	13.5339358	7.24E-02	28702222	28702345	NC_000011.9
cg12465710	1.13E-09	9.47E-11	6.85	13.5337923	1.27E-01	135042885	135043008	NC_000010.10
cg05978864	1.13E-09	9.47E-11	6.85	13.5335196	3.28E-02	30105174	30105297	NC_000006.11
cg09694722	1.13E-09	9.47E-11	6.85	13.5331846	1.22E-01	2161318	2161441	NC_000011.9
cg20813996	1.13E-09	9.47E-11	6.85	13.5331106	1.97E-01	166422741	166422864	NC_000006.11
cg09825327	1.13E-09	9.47E-11	6.85	13.53308	1.30E-01	211503219	211503342	NC_000001.10
cg00647217	1.13E-09	9.48E-11	6.85	13.5328334	1.52E-01	165086866	165086989	NC_000001.10
cg23864514	1.13E-09	9.49E-11	6.85	13.5316516	8.64E-02	22882512	22882635	NC_000007.13
cg21678813	1.13E-09	9.50E-11	6.85	13.5305784	1.81E-01	20229879	20230002	NC_000011.9
cg13575161	1.13E-09	9.51E-11	6.85	13.5289441	2.26E-01	4381792	4381915	NC_000012.11
cg11635125	1.13E-09	9.51E-11	6.85	13.5288502	2.75E-02	108728931	108729054	NC_000011.9
cg08500128	1.14E-09	9.56E-11	6.85	13.5245136	1.29E-01	10763670	10763793	NC_000012.11
cg23262036	1.14E-09	9.56E-11	6.85	13.5244653	1.92E-01	96647021	96647144	NC_000007.13
cg25827102	1.14E-09	9.57E-11	6.85	13.5232389	5.06E-02	89880727	89880850	NC_000015.9
cg11200635	1.14E-09	9.57E-11	6.85	13.5226705	1.83E-01	43573195	43573318	NC_000010.10
cg16203271	1.14E-09	9.59E-11	6.85	13.5210523	6.01E-02	27306681	27306804	NC_000002.11
cg14611112	1.14E-09	9.59E-11	6.85	13.5210134	1.51E-01	139643351	139643474	NC_000009.11
cg10897631	1.14E-09	9.60E-11	6.85	13.5204941	1.15E-01	195773232	195773355	NC_000003.11
cg01403123	1.14E-09	9.60E-11	6.85	13.5201964	3.75E-02	240287795	240287918	NC_000002.11
cg27122213	1.14E-09	9.61E-11	6.85	13.5193943	1.87E-01	57274763	57274886	NC_000014.8
cg03284392	1.14E-09	9.61E-11	6.85	13.5191271	1.34E-01	141071438	141071561	NC_000005.9

cg03545536	1.15E-09	9.64E-11	6.85	13.5162211	2.67E-02	99905334	99905457	NC_000007.13
cg09515953	1.15E-09	9.65E-11	6.84	13.515054	1.65E-01	38747355	38747478	NC_000019.9
cg27513979	1.15E-09	9.65E-11	6.84	13.5145189	1.73E-01	96743895	96744018	NC_000013.10
cg13478228	1.15E-09	9.67E-11	6.84	13.5131764	2.10E-01	70209432	70209555	NC_000018.9
cg21728840	1.15E-09	9.68E-11	6.84	13.5118393	1.18E-01	57829009	57829132	NC_000019.9
cg00320608	1.15E-09	9.68E-11	6.84	13.5116528	1.15E-01	29116256	29116379	NC_000002.11
cg04884908	1.15E-09	9.69E-11	6.84	13.5112914	1.77E-01	72374840	72374963	NC_000002.11
cg14306161	1.15E-09	9.69E-11	6.84	13.5105637	8.84E-02	71102111	71102234	NC_000015.9
cg08074528	1.15E-09	9.70E-11	6.84	13.5099066	6.48E-02	50528586	50528709	NC_000022.10
cg17016922	1.15E-09	9.71E-11	6.84	13.5090114	9.50E-02	141195365	141195488	NC_000005.9
cg04191403	1.15E-09	9.71E-11	6.84	13.5088056	1.45E-01	87637999	87638122	NC_000016.9
cg27628784	1.16E-09	9.74E-11	6.84	13.5059257	1.70E-01	131767387	131767510	NC_000010.10
cg23850212	1.16E-09	9.74E-11	6.84	13.5054287	2.14E-01	57049857	57049980	NC_000019.9
cg24625984	1.16E-09	9.75E-11	6.84	13.5049124	1.20E-01	130279707	130279830	NC_000010.10
cg25486757	1.16E-09	9.77E-11	6.84	13.5027557	1.60E-01	73674074	73674197	NC_000003.11
cg20492034	1.16E-09	9.78E-11	6.84	13.5021359	1.78E-01	6313058	6313181	NC_000007.13
cg10441640	1.16E-09	9.78E-11	6.84	13.5020194	5.69E-02	33031065	33031188	NC_000012.11
cg23732781	1.16E-09	9.79E-11	6.84	13.5006849	1.13E-01	29595016	29595139	NC_000006.11
cg00025647	1.16E-09	9.79E-11	6.84	13.5005736	1.43E-01	147077127	147077250	NC_000003.11
cg23098693	1.16E-09	9.79E-11	6.84	13.5004753	1.35E-01	118150813	118150936	NC_000001.10
cg19673705	1.16E-09	9.81E-11	6.84	13.4991938	3.62E-02	7532359	7532482	NC_000011.9
cg21529323	1.17E-09	9.81E-11	6.84	13.4986399	1.89E-01	89910860	89910983	NC_000015.9
cg18548400	1.17E-09	9.83E-11	6.84	13.4967947	6.78E-02	182506874	182506997	NC_000003.11
cg20550790	1.17E-09	9.84E-11	6.84	13.4953429	1.33E-01	92906240	92906363	NC_000005.9
cg14248715	1.17E-09	9.85E-11	6.84	13.494724	1.84E-01	57284096	57284219	NC_000014.8

cg05966228	1.17E-09	9.85E-11	6.84	13.4945518	8.95E-02	67666442	67666565	NC_000016.9
cg24891434	1.17E-09	9.86E-11	6.84	13.4939676	1.13E-01	69934031	69934154	NC_000011.9
cg11743470	1.17E-09	9.86E-11	6.84	13.4936385	6.99E-02	74327277	74327400	NC_000005.9
cg24461964	1.17E-09	9.86E-11	6.84	13.4935272	2.08E-01	149633112	149633235	NC_000002.11
cg25456849	1.17E-09	9.86E-11	6.84	13.4935141	1.26E-01	79575334	79575457	NC_000015.9
cg08092513	1.17E-09	9.87E-11	6.84	13.4932009	3.75E-02	32886140	32886263	NC_000013.10
cg23542968	1.17E-09	9.89E-11	6.84	13.4903349	1.49E-01	36983129	36983252	NC_000014.8
cg11503274	1.17E-09	9.90E-11	6.84	13.4897762	1.18E-01	149546934	149547057	NC_000005.9
cg05970777	1.18E-09	9.91E-11	6.84	13.4889506	1.21E-01	34490033	34490156	NC_000016.9
cg12707907	1.18E-09	9.91E-11	6.84	13.4885046	7.41E-02	86370772	86370895	NC_000013.10
cg10859329	1.18E-09	9.92E-11	6.84	13.4877264	7.95E-02	54413218	54413341	NC_000014.8
cg24884142	1.18E-09	9.92E-11	6.84	13.4877039	1.45E-01	119532056	119532179	NC_000001.10
cg26308133	1.18E-09	9.93E-11	6.84	13.4865425	4.61E-02	8809360	8809483	NC_000019.9
cg19356082	1.18E-09	9.94E-11	6.84	13.4860166	6.03E-02	68862916	68863039	NC_000012.11
cg14486857	1.18E-09	9.97E-11	6.84	13.4831881	5.58E-02	977048	977171	NC_000011.9
cg18387156	1.18E-09	9.97E-11	6.84	13.4831382	1.56E-01	31496644	31496767	NC_000008.10
cg11260097	1.18E-09	9.98E-11	6.84	13.4822147	1.36E-01	121940647	121940770	NC_000007.13
cg11812928	1.19E-09	1.00E-10	6.84	13.4780517	7.49E-02	227164459	227164582	NC_000001.10
cg16175560	1.19E-09	1.00E-10	6.84	13.4763926	6.52E-02	119685050	119685173	NC_000004.11
cg00736523	1.19E-09	1.00E-10	6.84	13.4763413	1.44E-01	32783202	32783325	NC_000009.11
cg04675040	1.19E-09	1.00E-10	6.84	13.4756588	7.69E-02	93316104	93316227	NC_000015.9
cg15475851	1.19E-09	1.01E-10	6.84	13.4748991	1.39E-01	105037349	105037472	NC_000010.10
cg22058708	1.19E-09	1.01E-10	6.84	13.4742281	1.70E-01	28602970	28603093	NC_000006.11
cg11556489	1.19E-09	1.01E-10	6.84	13.4740336	1.33E-01	30178724	30178847	NC_000006.11
cg11235741	1.19E-09	1.01E-10	6.84	13.4732451	1.99E-01	82336869	82336992	NC_000015.9

cg06664258	1.19E-09	1.01E-10	6.84	13.4726787	2.12E-01	103889903	103890026	NC_000012.11
cg26064247	1.19E-09	1.01E-10	6.84	13.4726723	7.45E-02	33792972	33793095	NC_000011.9
cg17879912	1.20E-09	1.01E-10	6.84	13.4713145	3.65E-02	118626530	118626653	NC_000005.9
cg08726446	1.20E-09	1.01E-10	6.84	13.4704137	1.52E-01	108237937	108238060	NC_000012.11
cg19683251	1.20E-09	1.01E-10	6.84	13.4698771	3.27E-02	60762219	60762342	NC_000017.10
cg20306574	1.20E-09	1.01E-10	6.84	13.4698033	1.18E-01	24601499	24601622	NC_000014.8
cg02604524	1.20E-09	1.01E-10	6.84	13.4685348	2.82E-01	128720884	128721007	NC_000003.11
cg08031211	1.20E-09	1.01E-10	6.84	13.4673541	7.00E-02	68751981	68752104	NC_000012.11
cg06808751	1.20E-09	1.01E-10	6.84	13.4664261	1.97E-01	54163693	54163816	NC_000008.10
cg10939650	1.20E-09	1.01E-10	6.84	13.4661561	3.26E-02	76013186	76013309	NC_000014.8
cg18493027	1.20E-09	1.01E-10	6.84	13.4659802	1.83E-01	70346151	70346274	NC_000014.8
cg16135698	1.20E-09	1.01E-10	6.84	13.4656411	1.59E-01	46825179	46825302	NC_000017.10
cg18032969	1.21E-09	1.02E-10	6.84	13.4624343	1.35E-01	145725385	145725508	NC_000005.9
cg20307896	1.21E-09	1.02E-10	6.84	13.4623062	1.63E-01	114883063	114883186	NC_000012.11
cg02329038	1.21E-09	1.02E-10	6.84	13.4620472	1.46E-01	46710001	46710124	NC_000017.10
cg23437733	1.21E-09	1.02E-10	6.84	13.4604534	2.43E-01	178017571	178017694	NC_000005.9
cg15604467	1.21E-09	1.02E-10	6.84	13.4601397	1.62E-01	79177925	79178048	NC_000013.10
cg16712438	1.21E-09	1.02E-10	6.83	13.4599583	9.41E-02	3187397	3187520	NC_000011.9
cg01287820	1.21E-09	1.02E-10	6.83	13.4588393	3.35E-02	1065285	1065408	NC_000007.13
cg02916312	1.21E-09	1.02E-10	6.83	13.4567777	2.17E-01	72596135	72596258	NC_000006.11
cg14563954	1.22E-09	1.03E-10	6.83	13.453593	1.39E-01	147141286	147141409	NC_000003.11
cg04461228	1.22E-09	1.03E-10	6.83	13.4524689	1.44E-01	92906678	92906801	NC_000005.9
cg00233943	1.22E-09	1.03E-10	6.83	13.4490218	7.32E-02	59553911	59554034	NC_000017.10
cg00403874	1.22E-09	1.03E-10	6.83	13.4484691	5.04E-02	57088851	57088974	NC_000007.13
cg19426522	1.22E-09	1.03E-10	6.83	13.4469109	1.26E-01	27766009	27766132	NC_000003.11

cg09318375	1.22E-09	1.03E-10	6.83	13.4464866	1.55E-01	67218090	67218213	NC_000001.10
cg24761366	1.23E-09	1.04E-10	6.83	13.4444403	1.47E-01	60936866	60936989	NC_000010.10
cg23938300	1.23E-09	1.04E-10	6.83	13.4427382	1.46E-01	60292055	60292178	NC_000015.9
cg06912355	1.23E-09	1.04E-10	6.83	13.4423445	4.77E-02	90002255	90002378	NC_000016.9
cg11430157	1.23E-09	1.04E-10	6.83	13.4409562	2.59E-01	118228869	118228992	NC_000006.11
cg12601118	1.23E-09	1.04E-10	6.83	13.4401838	5.69E-02	9859123	9859246	NC_000017.10
cg13252583	1.23E-09	1.04E-10	6.83	13.4394441	2.35E-01	36909831	36909954	NC_000019.9
cg12742826	1.24E-09	1.04E-10	6.83	13.4369312	4.44E-02	26365331	26365454	NC_000001.10
cg11723565	1.24E-09	1.05E-10	6.83	13.4362423	3.05E-02	55990708	55990831	NC_000019.9
cg02387803	1.24E-09	1.05E-10	6.83	13.4356396	1.89E-01	147123457	147123580	NC_000003.11
cg09938462	1.24E-09	1.05E-10	6.83	13.4337518	2.00E-01	19706365	19706488	NC_000022.10
cg08460041	1.24E-09	1.05E-10	6.83	13.4329602	1.80E-01	165325136	165325259	NC_000001.10
cg20711695	1.24E-09	1.05E-10	6.83	13.4327982	3.19E-02	124698165	124698288	NC_000004.11
cg15740790	1.24E-09	1.05E-10	6.83	13.4322135	5.55E-02	22225034	22225157	NC_000008.10
cg07262139	1.24E-09	1.05E-10	6.83	13.4321215	1.04E-01	134253609	134253732	NC_000011.9
cg10603004	1.24E-09	1.05E-10	6.83	13.4319651	1.46E-01	23564294	23564417	NC_000008.10
cg16025492	1.24E-09	1.05E-10	6.83	13.4313005	1.01E-01	114764748	114764871	NC_000005.9
cg08436224	1.24E-09	1.05E-10	6.83	13.4306053	3.60E-02	1122753	1122876	NC_000005.9
cg10764068	1.24E-09	1.05E-10	6.83	13.429897	1.17E-01	27289829	27289952	NC_000007.13
cg04240553	1.24E-09	1.05E-10	6.83	13.4296133	8.71E-02	67188614	67188737	NC_000016.9
cg04850423	1.25E-09	1.05E-10	6.83	13.4286069	1.78E-01	20880461	20880584	NC_000001.10
cg14014955	1.25E-09	1.05E-10	6.83	13.4282365	1.31E-01	27183701	27183824	NC_000007.13
cg09263059	1.25E-09	1.06E-10	6.83	13.4273444	1.22E-01	43065876	43065999	NC_000017.10
cg10718130	1.25E-09	1.06E-10	6.83	13.4267267	9.74E-02	145539893	145540016	NC_000008.10
cg01716474	1.25E-09	1.06E-10	6.83	13.4260383	3.35E-02	56615274	56615397	NC_000006.11

cg12640000	1.25E-09	1.06E-10	6.83	13.425233	1.30E-01	62660741	62660864	NC_000001.10
cg24584367	1.25E-09	1.06E-10	6.83	13.4246251	7.73E-02	156565633	156565756	NC_000001.10
cg15769764	1.25E-09	1.06E-10	6.83	13.42443	1.73E-01	996790	996913	NC_000004.11
cg13964105	1.25E-09	1.06E-10	6.83	13.4232593	1.70E-01	26663885	26664008	NC_000003.11
cg25026529	1.25E-09	1.06E-10	6.83	13.4218554	1.95E-01	91183051	91183174	NC_000001.10
cg17851134	1.25E-09	1.06E-10	6.83	13.4211514	7.78E-02	170622699	170622822	NC_000006.11
cg10806639	1.26E-09	1.07E-10	6.83	13.4168876	1.73E-01	33780078	33780201	NC_000013.10
cg26504422	1.26E-09	1.07E-10	6.83	13.4160388	8.07E-02	44277951	44278074	NC_000019.9
cg27356296	1.26E-09	1.07E-10	6.83	13.4157874	1.02E-01	134565928	134566051	NC_000007.13
cg25772658	1.26E-09	1.07E-10	6.83	13.4151297	8.18E-02	32038027	32038150	NC_000006.11
cg19461644	1.26E-09	1.07E-10	6.83	13.4140514	2.00E-01	101706617	101706740	NC_000009.11
cg07122245	1.26E-09	1.07E-10	6.83	13.412264	2.34E-01	37488428	37488551	NC_000007.13
cg03111114	1.27E-09	1.07E-10	6.83	13.4116693	9.41E-02	149886185	149886308	NC_000005.9
cg11022519	1.27E-09	1.07E-10	6.83	13.4115767	1.00E-01	135967896	135968019	NC_000003.11
cg22942846	1.27E-09	1.07E-10	6.83	13.4114854	1.06E-01	161646918	161647041	NC_000001.10
cg18935108	1.27E-09	1.07E-10	6.83	13.4114255	9.63E-02	113660932	113661055	NC_000011.9
cg21239317	1.27E-09	1.07E-10	6.83	13.4108927	8.69E-02	23640510	23640633	NC_000007.13
cg13574488	1.27E-09	1.07E-10	6.83	13.4102564	1.24E-01	119306246	119306369	NC_000010.10
cg16703135	1.27E-09	1.08E-10	6.83	13.4087879	1.05E-01	55517132	55517255	NC_000007.13
cg07622833	1.27E-09	1.08E-10	6.83	13.4075401	7.63E-02	33640167	33640290	NC_000011.9
cg23827572	1.27E-09	1.08E-10	6.83	13.4053642	2.19E-01	114846313	114846436	NC_000012.11
cg08291069	1.27E-09	1.08E-10	6.83	13.4053092	1.44E-01	10531543	10531666	NC_000019.9
cg00495775	1.27E-09	1.08E-10	6.83	13.4051942	1.24E-01	176972041	176972164	NC_000002.11
cg17610790	1.28E-09	1.08E-10	6.82	13.4034475	7.12E-02	32276752	32276875	NC_000020.10
cg04511041	1.28E-09	1.08E-10	6.82	13.4029622	1.88E-01	140613869	140613992	NC_000005.9

cg13731636	1.28E-09	1.08E-10	6.82	13.4025002	6.68E-02	77287213	77287336	NC_000018.9
cg09557462	1.28E-09	1.08E-10	6.82	13.4010626	1.74E-01	26721736	26721859	NC_000008.10
cg03464224	1.28E-09	1.08E-10	6.82	13.4009871	1.65E-01	154439980	154440103	NC_000004.11
cg03486157	1.28E-09	1.08E-10	6.82	13.4006789	3.45E-02	29208275	29208398	NC_000008.10
cg08535260	1.28E-09	1.08E-10	6.82	13.4005816	1.89E-01	127643971	127644094	NC_000002.11
cg07592963	1.28E-09	1.09E-10	6.82	13.3998067	1.95E-01	2890649	2890772	NC_000011.9
cg20066391	1.28E-09	1.09E-10	6.82	13.3961258	3.82E-02	22853979	22854102	NC_000012.11
cg26415343	1.29E-09	1.09E-10	6.82	13.3955852	1.09E-01	30683404	30683527	NC_000007.13
cg04923928	1.29E-09	1.09E-10	6.82	13.3952022	1.46E-01	30721797	30721920	NC_000007.13
cg19153763	1.29E-09	1.09E-10	6.82	13.3942045	1.39E-01	27196371	27196494	NC_000007.13
cg03030984	1.29E-09	1.09E-10	6.82	13.3940212	8.58E-02	134833874	134833997	NC_000010.10
cg04954615	1.29E-09	1.09E-10	6.82	13.3940059	5.20E-02	56913133	56913256	NC_000004.11
cg02482497	1.29E-09	1.09E-10	6.82	13.3934572	1.90E-01	153610672	153610795	NC_000001.10
cg03911502	1.29E-09	1.09E-10	6.82	13.393407	2.09E-01	5890402	5890525	NC_000004.11
cg00015319	1.29E-09	1.09E-10	6.82	13.3925659	1.02E-01	54500832	54500955	NC_000012.11
cg23683254	1.29E-09	1.09E-10	6.82	13.3913954	1.74E-01	99584667	99584790	NC_000014.8
cg01823440	1.29E-09	1.09E-10	6.82	13.3912311	4.64E-02	120870729	120870852	NC_000008.10
cg25781202	1.29E-09	1.09E-10	6.82	13.39113	2.18E-02	33424350	33424473	NC_000008.10
cg07441319	1.29E-09	1.10E-10	6.82	13.3897588	1.18E-01	8117467	8117590	NC_000010.10
cg10454514	1.29E-09	1.10E-10	6.82	13.3892687	1.63E-01	136553778	136553901	NC_000007.13
cg09801824	1.30E-09	1.10E-10	6.82	13.3872344	1.19E-01	74076930	74077053	NC_000010.10
cg01684901	1.30E-09	1.10E-10	6.82	13.3866092	2.39E-02	184058405	184058528	NC_000003.11
cg14510359	1.30E-09	1.10E-10	6.82	13.3860325	1.81E-01	2559930	2560053	NC_000001.10
cg25856228	1.30E-09	1.10E-10	6.82	13.3851839	7.63E-02	73901184	73901307	NC_000015.9
cg03104309	1.30E-09	1.10E-10	6.82	13.3846248	6.22E-02	51052080	51052203	NC_000019.9

cg26159541	1.30E-09	1.10E-10	6.82	13.3831881	1.18E-01	58326121	58326244	NC_000020.10
cg07420190	1.30E-09	1.11E-10	6.82	13.3811296	2.95E-01	220702225	220702348	NC_000001.10
cg05578358	1.30E-09	1.11E-10	6.82	13.3807651	1.99E-01	115631100	115631223	NC_000011.9
cg08561286	1.30E-09	1.11E-10	6.82	13.3807635	9.61E-02	121567875	121567998	NC_000002.11
cg07974511	1.30E-09	1.11E-10	6.82	13.3801254	1.63E-01	63283013	63283136	NC_000002.11
cg13713148	1.30E-09	1.11E-10	6.82	13.3798298	4.15E-02	22545322	22545445	NC_000008.10
cg07405771	1.30E-09	1.11E-10	6.82	13.3797523	1.43E-01	124930625	124930748	NC_000003.11
cg05435993	1.30E-09	1.11E-10	6.82	13.3793505	1.09E-01	50239406	50239529	NC_000003.11
cg23532828	1.30E-09	1.11E-10	6.82	13.3793338	7.77E-02	54544119	54544242	NC_000019.9
cg14964285	1.31E-09	1.11E-10	6.82	13.3791601	6.17E-02	4435533	4435656	NC_000012.11
cg23093016	1.31E-09	1.11E-10	6.82	13.3782427	4.11E-02	113486498	113486621	NC_000002.11
cg10765336	1.31E-09	1.11E-10	6.82	13.3781084	1.12E-01	359491	359614	NC_000019.9
cg00417823	1.31E-09	1.11E-10	6.82	13.3774273	9.39E-02	56330268	56330391	NC_000016.9
cg20689135	1.31E-09	1.11E-10	6.82	13.3771822	1.39E-01	113764225	113764348	NC_000013.10
cg08538258	1.31E-09	1.11E-10	6.82	13.3769049	1.62E-01	46706820	46706943	NC_000021.8
cg26272623	1.31E-09	1.11E-10	6.82	13.3753779	1.24E-01	119532320	119532443	NC_000001.10
cg23234261	1.31E-09	1.11E-10	6.82	13.3753443	1.02E-01	55582407	55582530	NC_000014.8
cg05618934	1.31E-09	1.11E-10	6.82	13.3740075	1.28E-01	1407592	1407715	NC_000004.11
cg05791136	1.31E-09	1.11E-10	6.82	13.3734707	1.94E-01	110223969	110224092	NC_000004.11
cg20472643	1.31E-09	1.12E-10	6.82	13.3727734	1.52E-01	33591899	33592022	NC_000012.11
cg14956197	1.32E-09	1.12E-10	6.82	13.3704254	1.91E-01	36993831	36993954	NC_000014.8
cg03631455	1.32E-09	1.12E-10	6.82	13.3689434	1.17E-01	140595072	140595195	NC_000005.9
cg02401978	1.32E-09	1.12E-10	6.82	13.3687885	1.05E-01	149195465	149195588	NC_000007.13
cg12700755	1.32E-09	1.12E-10	6.82	13.3685281	6.74E-02	2258373	2258496	NC_000001.10
cg00522018	1.32E-09	1.12E-10	6.82	13.3662937	1.88E-01	139091378	139091501	NC_000009.11

cg06604249	1.32E-09	1.12E-10	6.82	13.3654202	1.42E-01	8814755	8814878	NC_000016.9
cg01113073	1.32E-09	1.12E-10	6.82	13.3645973	9.33E-02	155686868	155686991	NC_000006.11
cg10049708	1.32E-09	1.12E-10	6.82	13.3645378	1.60E-01	6201080	6201203	NC_000004.11
cg04762890	1.33E-09	1.13E-10	6.82	13.3624795	7.14E-02	58077795	58077918	NC_000016.9
cg10054984	1.33E-09	1.13E-10	6.82	13.3623208	5.47E-02	129329790	129329913	NC_000012.11
cg08316417	1.33E-09	1.13E-10	6.82	13.361805	2.12E-01	5890430	5890553	NC_000004.11
cg22841810	1.33E-09	1.13E-10	6.82	13.359447	1.79E-01	25902284	25902407	NC_000008.10
cg08854366	1.33E-09	1.13E-10	6.82	13.3594304	2.29E-02	142229820	142229943	NC_000008.10
cg16414660	1.33E-09	1.13E-10	6.82	13.3587973	1.07E-01	234338088	234338211	NC_000002.11
cg06651752	1.33E-09	1.13E-10	6.82	13.3583382	1.12E-01	3592976	3593099	NC_000017.10
cg00942495	1.33E-09	1.13E-10	6.82	13.3581546	1.33E-01	76214616	76214739	NC_000015.9
cg23189276	1.33E-09	1.13E-10	6.82	13.357231	1.65E-01	37245916	37246039	NC_000004.11
cg23685759	1.33E-09	1.13E-10	6.82	13.3558881	2.17E-01	98945131	98945254	NC_000010.10
cg20457544	1.34E-09	1.14E-10	6.82	13.3547635	9.48E-02	2920603	2920726	NC_000001.10
cg06377278	1.34E-09	1.14E-10	6.82	13.3546269	2.27E-01	25256369	25256492	NC_000001.10
cg18198999	1.34E-09	1.14E-10	6.82	13.3544485	1.85E-01	50890153	50890276	NC_000001.10
cg06535156	1.34E-09	1.14E-10	6.82	13.3516707	9.29E-02	155538055	155538178	NC_000006.11
cg15701208	1.34E-09	1.14E-10	6.82	13.3515615	5.95E-02	139465825	139465948	NC_000002.11
cg06003986	1.34E-09	1.14E-10	6.81	13.3487763	1.87E-01	35104868	35104991	NC_000018.9
cg01416521	1.34E-09	1.14E-10	6.81	13.3483366	7.08E-02	56047632	56047755	NC_000019.9
cg15136870	1.34E-09	1.14E-10	6.81	13.3478208	1.30E-01	18717036	18717159	NC_000019.9
cg04642306	1.34E-09	1.14E-10	6.81	13.3477949	1.12E-01	112834466	112834589	NC_000011.9
cg26095658	1.35E-09	1.15E-10	6.81	13.3461423	9.42E-02	71017887	71018010	NC_000002.11
cg23638252	1.35E-09	1.15E-10	6.81	13.3458839	1.36E-01	50891750	50891873	NC_000001.10
cg06233930	1.35E-09	1.15E-10	6.81	13.3440064	9.03E-02	76899379	76899502	NC_000017.10

cg13086467	1.35E-09	1.15E-10	6.81	13.3431049	8.75E-02	12779373	12779496	NC_000019.9
cg20359994	1.35E-09	1.15E-10	6.81	13.3417693	1.44E-01	27599141	27599264	NC_000006.11
cg06724462	1.35E-09	1.15E-10	6.81	13.3412923	7.03E-02	41266297	41266420	NC_000001.10
cg23178550	1.35E-09	1.15E-10	6.81	13.3404662	1.12E-01	27127576	27127699	NC_000007.13
cg02715006	1.35E-09	1.15E-10	6.81	13.3403047	9.94E-02	140803130	140803253	NC_000005.9
cg18206017	1.36E-09	1.15E-10	6.81	13.3392775	1.90E-01	84563387	84563510	NC_000006.11
cg27344587	1.36E-09	1.15E-10	6.81	13.338609	1.18E-01	63275602	63275725	NC_000002.11
cg13678470	1.36E-09	1.16E-10	6.81	13.3369082	1.07E-01	140137615	140137738	NC_000009.11
cg20708909	1.36E-09	1.16E-10	6.81	13.3368567	9.24E-02	132812607	132812730	NC_000011.9
cg04407470	1.36E-09	1.16E-10	6.81	13.3367673	2.68E-01	108489977	108490100	NC_000006.11
cg08516217	1.36E-09	1.16E-10	6.81	13.3356434	9.69E-02	38887799	38887922	NC_000019.9
cg01836687	1.36E-09	1.16E-10	6.81	13.335435	1.71E-01	17081987	17082110	NC_000022.10
cg10121058	1.36E-09	1.16E-10	6.81	13.3348721	1.14E-01	84553063	84553186	NC_000016.9
cg27365825	1.36E-09	1.16E-10	6.81	13.3340209	1.90E-01	18059130	18059253	NC_000002.11
cg23132327	1.36E-09	1.16E-10	6.81	13.3330052	9.43E-02	80167599	80167722	NC_000011.9
cg05780883	1.37E-09	1.16E-10	6.81	13.3306216	6.07E-02	49883097	49883220	NC_000022.10
cg05961935	1.37E-09	1.17E-10	6.81	13.3298921	1.69E-01	145274125	145274248	NC_000002.11
cg10279055	1.37E-09	1.17E-10	6.81	13.3274702	4.20E-02	134117051	134117174	NC_000011.9
cg02026246	1.37E-09	1.17E-10	6.81	13.3266126	1.12E-01	22687589	22687712	NC_000011.9
cg14063945	1.37E-09	1.17E-10	6.81	13.3256351	5.39E-02	24470966	24471089	NC_000013.10
cg14377593	1.38E-09	1.17E-10	6.81	13.3234873	1.95E-01	93520183	93520306	NC_000007.13
cg08377398	1.38E-09	1.17E-10	6.81	13.3228155	2.60E-01	6202553	6202676	NC_000004.11
cg03746765	1.38E-09	1.18E-10	6.81	13.3207095	1.08E-01	15288099	15288222	NC_000017.10
cg16557178	1.38E-09	1.18E-10	6.81	13.3206285	1.57E-01	46804309	46804432	NC_000017.10
cg06621480	1.38E-09	1.18E-10	6.81	13.3206224	8.35E-02	49812410	49812533	NC_000022.10

cg09515947	1.38E-09	1.18E-10	6.81	13.3203384	1.61E-01	62070005	62070128	NC_000016.9
cg00131983	1.38E-09	1.18E-10	6.81	13.3195672	1.75E-01	4867424	4867547	NC_000004.11
cg15729404	1.38E-09	1.18E-10	6.81	13.3193974	1.66E-01	133561614	133561737	NC_000006.11
cg21533331	1.38E-09	1.18E-10	6.81	13.3190476	1.54E-01	31848049	31848172	NC_000019.9
cg25513073	1.38E-09	1.18E-10	6.81	13.3189883	1.29E-01	2481641	2481764	NC_000011.9
cg19049837	1.38E-09	1.18E-10	6.81	13.3175871	1.02E-01	85293337	85293460	NC_000002.11
cg20586076	1.38E-09	1.18E-10	6.81	13.3175025	1.19E-01	133252332	133252455	NC_000010.10
cg18710462	1.38E-09	1.18E-10	6.81	13.3173118	1.42E-01	108486123	108486246	NC_000006.11
cg26823505	1.39E-09	1.18E-10	6.81	13.3149543	1.09E-01	35765589	35765712	NC_000006.11
cg04504062	1.39E-09	1.18E-10	6.81	13.3144833	5.21E-02	8840829	8840952	NC_000005.9
cg26266274	1.39E-09	1.18E-10	6.81	13.3144527	9.70E-02	132482399	132482522	NC_000009.11
cg04560545	1.39E-09	1.18E-10	6.81	13.3134528	2.48E-02	66448589	66448712	NC_000003.11
cg02696327	1.39E-09	1.19E-10	6.81	13.312879	1.53E-01	49312543	49312666	NC_000016.9
cg21349674	1.39E-09	1.19E-10	6.81	13.3128575	1.99E-01	50323369	50323492	NC_000010.10
cg07926895	1.39E-09	1.19E-10	6.81	13.3120049	1.68E-01	200005833	200005956	NC_000001.10
cg20833090	1.39E-09	1.19E-10	6.81	13.3115571	1.05E-01	143053037	143053160	NC_000007.13
cg04076968	1.40E-09	1.20E-10	6.81	13.3048202	4.81E-02	61289326	61289449	NC_000002.11
cg20576153	1.40E-09	1.20E-10	6.81	13.3045127	1.67E-01	60975841	60975964	NC_000014.8
cg02192204	1.40E-09	1.20E-10	6.81	13.303758	1.05E-01	88947943	88948066	NC_000016.9
cg15255946	1.40E-09	1.20E-10	6.81	13.3036522	1.22E-01	32729563	32729686	NC_000006.11
cg14241879	1.40E-09	1.20E-10	6.81	13.3032879	5.08E-02	1048922	1049045	NC_000007.13
cg26517004	1.40E-09	1.20E-10	6.81	13.3026542	8.00E-02	110903494	110903617	NC_000012.11
cg12128119	1.40E-09	1.20E-10	6.81	13.302646	3.54E-02	134212891	134213014	NC_000011.9
cg26494542	1.41E-09	1.20E-10	6.81	13.3009338	8.28E-02	963219	963342	NC_000016.9
cg09424526	1.41E-09	1.20E-10	6.81	13.3006215	1.97E-01	223163809	223163932	NC_000002.11

cg09179347	1.41E-09	1.20E-10	6.81	13.3005229	3.97E-02	67517453	67517576	NC_000016.9
cg14569778	1.41E-09	1.20E-10	6.81	13.3003552	3.70E-02	28952111	28952234	NC_000017.10
cg11563844	1.41E-09	1.20E-10	6.81	13.2992239	1.75E-01	33780307	33780430	NC_000013.10
cg05442408	1.41E-09	1.20E-10	6.81	13.2986022	1.60E-01	2706173	2706296	NC_000001.10
cg16913789	1.41E-09	1.20E-10	6.81	13.2980839	1.61E-01	27204005	27204128	NC_000007.13
cg04272613	1.41E-09	1.20E-10	6.81	13.2975124	1.49E-01	59721173	59721296	NC_000014.8
cg01236132	1.41E-09	1.21E-10	6.81	13.2968857	1.48E-01	112723581	112723704	NC_000013.10
cg26724232	1.41E-09	1.21E-10	6.81	13.2963237	9.94E-02	60885912	60886035	NC_000015.9
cg12782180	1.41E-09	1.21E-10	6.81	13.2962504	1.13E-01	127880932	127881055	NC_000007.13
cg26282299	1.41E-09	1.21E-10	6.81	13.2961176	8.92E-02	142189729	142189852	NC_000008.10
cg13959523	1.41E-09	1.21E-10	6.8	13.2945754	1.69E-01	34175481	34175604	NC_000019.9
cg22245862	1.41E-09	1.21E-10	6.8	13.2938234	9.59E-02	942544	942667	NC_000004.11
cg23371877	1.41E-09	1.21E-10	6.8	13.293646	1.31E-01	152067904	152068027	NC_000007.13
cg17955761	1.41E-09	1.21E-10	6.8	13.2935367	9.38E-02	11628430	11628553	NC_000008.10
cg18118685	1.42E-09	1.22E-10	6.8	13.2869445	2.11E-01	154729622	154729745	NC_000002.11
cg07249459	1.43E-09	1.22E-10	6.8	13.2850203	1.80E-01	86600309	86600432	NC_000016.9
cg01355739	1.43E-09	1.22E-10	6.8	13.282925	8.79E-02	57416888	57417011	NC_000020.10
cg04935434	1.43E-09	1.22E-10	6.8	13.2815173	1.12E-01	177024947	177025070	NC_000002.11
cg22604188	1.43E-09	1.22E-10	6.8	13.2813922	8.31E-02	140774054	140774177	NC_000005.9
cg04427498	1.43E-09	1.23E-10	6.8	13.2804422	1.27E-01	20830657	20830780	NC_000007.13
cg01466678	1.43E-09	1.23E-10	6.8	13.2802683	2.05E-01	58497395	58497518	NC_000016.9
cg08874512	1.43E-09	1.23E-10	6.8	13.2800591	1.62E-01	51831274	51831397	NC_000019.9
cg11827910	1.43E-09	1.23E-10	6.8	13.2796642	1.81E-01	31825756	31825879	NC_000011.9
cg13453374	1.43E-09	1.23E-10	6.8	13.2791132	1.61E-01	147072597	147072720	NC_000003.11
cg22510337	1.43E-09	1.23E-10	6.8	13.2791058	2.21E-01	58399967	58400090	NC_000019.9

cg05488770	1.44E-09	1.23E-10	6.8	13.2785361	1.73E-01	103969940	103970063	NC_000007.13
cg08363193	1.44E-09	1.23E-10	6.8	13.2778015	1.33E-01	89690234	89690357	NC_000010.10
cg15055553	1.44E-09	1.23E-10	6.8	13.2774721	1.18E-01	1697406	1697529	NC_000012.11
cg14655590	1.44E-09	1.23E-10	6.8	13.2771886	1.51E-01	95993065	95993188	NC_000009.11
cg04551776	1.44E-09	1.23E-10	6.8	13.2769423	1.02E-01	393366	393489	NC_000005.9
cg23618344	1.44E-09	1.23E-10	6.8	13.2766782	1.64E-01	54423428	54423551	NC_000012.11
cg11657744	1.44E-09	1.23E-10	6.8	13.2742191	2.51E-02	33236869	33236992	NC_000006.11
cg01569601	1.44E-09	1.23E-10	6.8	13.2741928	9.56E-02	46811242	46811365	NC_000021.8
cg10785263	1.44E-09	1.23E-10	6.8	13.2735984	1.85E-01	63785867	63785990	NC_000001.10
cg18143566	1.44E-09	1.24E-10	6.8	13.2720514	1.19E-01	77398897	77399020	NC_000018.9
cg00933210	1.44E-09	1.24E-10	6.8	13.2718409	1.36E-01	3233117	3233240	NC_000016.9
cg26413683	1.44E-09	1.24E-10	6.8	13.271423	9.89E-02	89520886	89521009	NC_000011.9
cg05544940	1.45E-09	1.24E-10	6.8	13.2707297	1.10E-01	33170339	33170462	NC_000006.11
cg02809707	1.45E-09	1.24E-10	6.8	13.2692284	2.56E-01	175191069	175191192	NC_000002.11
cg14401466	1.45E-09	1.24E-10	6.8	13.2682206	1.43E-01	166405523	166405646	NC_000005.9
cg18044663	1.45E-09	1.24E-10	6.8	13.2670752	2.34E-01	118032081	118032204	NC_000010.10
cg06689045	1.45E-09	1.24E-10	6.8	13.2669719	5.92E-02	120025420	120025543	NC_000003.11
cg03368634	1.45E-09	1.24E-10	6.8	13.2667053	1.03E-01	3824553	3824676	NC_000016.9
cg12861034	1.45E-09	1.24E-10	6.8	13.2657673	2.53E-01	38347816	38347939	NC_000017.10
cg10116864	1.45E-09	1.25E-10	6.8	13.2642694	1.79E-01	219826743	219826866	NC_000002.11
cg22504898	1.45E-09	1.25E-10	6.8	13.2641943	4.93E-02	52294622	52294745	NC_000012.11
cg23508701	1.46E-09	1.25E-10	6.8	13.2633157	5.54E-02	76331877	76332000	NC_000012.11
cg11228052	1.46E-09	1.25E-10	6.8	13.2626677	1.48E-01	103350170	103350293	NC_000012.11
cg11971789	1.46E-09	1.25E-10	6.8	13.2621227	5.78E-02	42545623	42545746	NC_000019.9
cg00559963	1.46E-09	1.25E-10	6.8	13.2611094	3.18E-02	40424484	40424607	NC_000001.10

cg07850592	1.46E-09	1.25E-10	6.8	13.260806	1.65E-01	231299396	231299519	NC_000001.10
cg10016770	1.46E-09	1.25E-10	6.8	13.2597521	4.51E-02	244805996	244806119	NC_000001.10
cg13652336	1.46E-09	1.25E-10	6.8	13.2593051	1.88E-01	68864012	68864135	NC_000008.10
cg10501976	1.46E-09	1.25E-10	6.8	13.2592554	1.33E-01	10883834	10883957	NC_000006.11
cg11480800	1.46E-09	1.25E-10	6.8	13.2583931	2.00E-01	55881075	55881198	NC_000015.9
cg19627145	1.46E-09	1.25E-10	6.8	13.2579243	3.21E-02	145662110	145662233	NC_000008.10
cg08785317	1.46E-09	1.25E-10	6.8	13.2574521	7.77E-02	86604854	86604977	NC_000016.9
cg01630940	1.47E-09	1.26E-10	6.8	13.2561409	4.23E-02	32020498	32020621	NC_000006.11
cg15737177	1.47E-09	1.26E-10	6.8	13.2557925	1.33E-01	8717467	8717590	NC_000012.11
cg10081304	1.47E-09	1.26E-10	6.8	13.2554816	4.97E-02	177952737	177952860	NC_000005.9
cg14123543	1.47E-09	1.26E-10	6.8	13.2543665	1.73E-01	114877063	114877186	NC_000012.11
cg24878374	1.47E-09	1.26E-10	6.8	13.2532343	9.61E-02	71946066	71946189	NC_000017.10
cg26937943	1.47E-09	1.26E-10	6.8	13.252313	1.80E-01	8800248	8800371	NC_000018.9
cg05617469	1.47E-09	1.26E-10	6.8	13.2516246	1.21E-01	100624281	100624404	NC_000013.10
cg06894334	1.47E-09	1.26E-10	6.8	13.251227	1.30E-01	1398599	1398722	NC_000004.11
cg05040472	1.47E-09	1.26E-10	6.8	13.2506915	1.51E-01	63255687	63255810	NC_000005.9
cg25218417	1.47E-09	1.26E-10	6.8	13.2499551	1.03E-01	12374100	12374223	NC_000001.10
cg23647248	1.48E-09	1.27E-10	6.8	13.2485136	7.90E-02	42265874	42265997	NC_000008.10
cg07592012	1.48E-09	1.27E-10	6.8	13.2471618	6.91E-02	79977307	79977430	NC_000017.10
cg02878284	1.48E-09	1.27E-10	6.8	13.2470723	8.85E-02	32825701	32825824	NC_000006.11
cg24068372	1.48E-09	1.27E-10	6.8	13.2467961	2.35E-01	151107261	151107384	NC_000007.13
cg19506535	1.48E-09	1.27E-10	6.8	13.2444407	5.77E-02	4853363	4853486	NC_000017.10
cg18652346	1.48E-09	1.27E-10	6.8	13.2444225	1.90E-01	28733673	28733796	NC_000006.11
cg04837104	1.48E-09	1.27E-10	6.8	13.2432073	1.55E-01	46129620	46129743	NC_000021.8
cg03912954	1.49E-09	1.28E-10	6.8	13.2409282	1.14E-01	140773129	140773252	NC_000009.11

cg16554447	1.49E-09	1.28E-10	6.8	13.2395651	8.98E-02	54058452	54058575	NC_000006.11
cg25406138	1.49E-09	1.28E-10	6.79	13.2385859	1.80E-01	147560770	147560893	NC_000004.11
cg16400647	1.49E-09	1.28E-10	6.79	13.2382739	9.35E-02	150069707	150069830	NC_000007.13
cg25223771	1.49E-09	1.28E-10	6.79	13.2362633	1.32E-01	145105503	145105626	NC_000008.10
cg01317569	1.50E-09	1.28E-10	6.79	13.2342046	1.37E-01	60140824	60140947	NC_000005.9
cg12652634	1.50E-09	1.28E-10	6.79	13.2340853	6.76E-02	71310320	71310443	NC_000005.9
cg09568001	1.50E-09	1.29E-10	6.79	13.2334268	6.80E-02	114383949	114384072	NC_000006.11
cg22873539	1.50E-09	1.29E-10	6.79	13.233191	1.40E-01	147554210	147554333	NC_000004.11
cg02520281	1.50E-09	1.29E-10	6.79	13.2329989	1.23E-01	140535668	140535791	NC_000005.9
cg01364674	1.50E-09	1.29E-10	6.79	13.2325275	1.30E-01	24348512	24348635	NC_000001.10
cg05655837	1.50E-09	1.29E-10	6.79	13.2318782	2.13E-01	166582188	166582311	NC_000006.11
cg25012919	1.50E-09	1.29E-10	6.79	13.2311124	1.90E-01	36705606	36705729	NC_000013.10
cg14574066	1.50E-09	1.29E-10	6.79	13.22973	7.85E-02	68984142	68984265	NC_000011.9
cg25667490	1.50E-09	1.29E-10	6.79	13.2295297	2.03E-02	72052924	72053047	NC_000004.11
cg11811610	1.51E-09	1.29E-10	6.79	13.2283563	1.27E-01	10390811	10390934	NC_000006.11
cg00445591	1.51E-09	1.29E-10	6.79	13.2273589	6.47E-02	127999762	127999885	NC_000003.11
cg15345206	1.51E-09	1.29E-10	6.79	13.2267753	1.57E-01	23463706	23463829	NC_000010.10
cg15242630	1.51E-09	1.29E-10	6.79	13.2265742	5.13E-02	18488478	18488601	NC_000022.10
cg02147617	1.51E-09	1.30E-10	6.79	13.2251385	6.73E-02	45073463	45073586	NC_000003.11
cg01889237	1.51E-09	1.30E-10	6.79	13.2247179	1.12E-01	137816623	137816746	NC_000006.11
cg13988209	1.51E-09	1.30E-10	6.79	13.2246802	8.48E-02	69683043	69683166	NC_000011.9
cg26983835	1.51E-09	1.30E-10	6.79	13.2244379	1.48E-01	59481280	59481403	NC_000017.10
cg00713400	1.51E-09	1.30E-10	6.79	13.2241748	1.21E-01	22233206	22233329	NC_000007.13
cg05048475	1.51E-09	1.30E-10	6.79	13.2231975	1.23E-01	109926115	109926238	NC_000001.10
cg16658337	1.51E-09	1.30E-10	6.79	13.222707	5.99E-02	1468749	1468872	NC_000005.9

cg16759952	1.51E-09	1.30E-10	6.79	13.222542	7.52E-02	7330658	7330781	NC_000017.10
cg25832305	1.51E-09	1.30E-10	6.79	13.2221688	1.33E-01	46860683	46860806	NC_000021.8
cg10451253	1.51E-09	1.30E-10	6.79	13.2219628	1.66E-01	32907705	32907828	NC_000017.10
cg13496979	1.52E-09	1.30E-10	6.79	13.2207957	2.06E-01	133308888	133309011	NC_000009.11
cg21745537	1.52E-09	1.30E-10	6.79	13.2203444	3.78E-02	121233743	121233866	NC_000011.9
cg21079946	1.52E-09	1.30E-10	6.79	13.2203133	1.00E-01	58622915	58623038	NC_000003.11
cg22605919	1.52E-09	1.30E-10	6.79	13.2196584	1.26E-01	42733542	42733665	NC_000017.10
cg02932314	1.52E-09	1.30E-10	6.79	13.2195812	1.13E-01	42733698	42733821	NC_000017.10
cg05314394	1.52E-09	1.30E-10	6.79	13.2190697	1.66E-01	96957070	96957193	NC_000015.9
cg14828404	1.52E-09	1.30E-10	6.79	13.2189852	1.24E-01	56267246	56267369	NC_000002.11
cg11334165	1.52E-09	1.30E-10	6.79	13.2189052	1.37E-01	145758782	145758905	NC_000005.9
cg14051264	1.52E-09	1.31E-10	6.79	13.2182622	1.34E-01	1886309	1886432	NC_000005.9
cg02058267	1.52E-09	1.31E-10	6.79	13.2163342	1.66E-01	76605088	76605211	NC_000014.8
cg14871932	1.53E-09	1.31E-10	6.79	13.2116524	1.17E-01	44185323	44185446	NC_000007.13
cg24642825	1.53E-09	1.31E-10	6.79	13.2112269	2.32E-01	89248793	89248916	NC_000015.9
cg08661751	1.53E-09	1.32E-10	6.79	13.2096827	8.96E-02	142001770	142001893	NC_000005.9
cg19736503	1.53E-09	1.32E-10	6.79	13.2085468	1.83E-01	84563604	84563727	NC_000006.11
cg24093176	1.54E-09	1.32E-10	6.79	13.2075602	7.53E-02	73716853	73716976	NC_000014.8
cg14270434	1.54E-09	1.32E-10	6.79	13.2073313	1.47E-01	100613647	100613770	NC_000009.11
cg26451188	1.54E-09	1.32E-10	6.79	13.2070942	9.08E-02	8295971	8296094	NC_000017.10
cg24173182	1.54E-09	1.32E-10	6.79	13.2066932	1.05E-01	1961286	1961409	NC_000017.10
cg24831051	1.54E-09	1.32E-10	6.79	13.2063516	1.59E-01	88018481	88018604	NC_000005.9
cg24058566	1.55E-09	1.33E-10	6.79	13.2004069	1.53E-01	87974100	87974223	NC_000005.9
cg23058901	1.55E-09	1.33E-10	6.79	13.2000282	1.08E-01	44324759	44324882	NC_000011.9
cg05264816	1.55E-09	1.33E-10	6.79	13.1991691	8.46E-02	44237023	44237146	NC_000018.9

cg12691679	1.55E-09	1.33E-10	6.79	13.1987143	1.29E-01	41594904	41595027	NC_000013.10
cg18647989	1.55E-09	1.33E-10	6.79	13.1981231	1.25E-01	39260335	39260458	NC_000022.10
cg13891576	1.55E-09	1.33E-10	6.79	13.1970485	5.93E-02	136107649	136107772	NC_000009.11
cg13360215	1.55E-09	1.34E-10	6.79	13.1960792	8.83E-02	67997425	67997548	NC_000018.9
cg01109065	1.55E-09	1.34E-10	6.79	13.194981	9.79E-02	55508379	55508502	NC_000003.11
cg25200586	1.55E-09	1.34E-10	6.79	13.1948237	1.41E-01	148000763	148000886	NC_000001.10
cg14627172	1.56E-09	1.34E-10	6.79	13.1937458	2.37E-01	28510339	28510462	NC_000006.11
cg08971724	1.56E-09	1.34E-10	6.79	13.1937057	2.30E-02	2983426	2983549	NC_000012.11
cg22499215	1.56E-09	1.34E-10	6.79	13.1929269	3.49E-02	19772241	19772364	NC_000022.10
cg11885589	1.56E-09	1.34E-10	6.79	13.1926411	3.23E-02	155463114	155463237	NC_000006.11
cg08473752	1.56E-09	1.34E-10	6.79	13.1918703	1.31E-01	26517057	26517180	NC_000017.10
cg13558899	1.56E-09	1.34E-10	6.79	13.1909692	6.33E-02	55665232	55665355	NC_000017.10
cg06339542	1.56E-09	1.34E-10	6.79	13.1903187	1.89E-01	10102237	10102360	NC_000017.10
cg25053336	1.56E-09	1.34E-10	6.79	13.1898355	2.12E-01	45161333	45161456	NC_000002.11
cg09651522	1.56E-09	1.34E-10	6.79	13.1890317	1.50E-01	1384780	1384903	NC_000006.11
cg16405012	1.56E-09	1.35E-10	6.79	13.1880553	1.80E-01	5542395	5542518	NC_000012.11
cg13184019	1.56E-09	1.35E-10	6.79	13.1877248	8.69E-02	32169790	32169913	NC_000006.11
cg03190505	1.57E-09	1.35E-10	6.79	13.1859467	1.09E-01	137522287	137522410	NC_000002.11
cg00341950	1.57E-09	1.35E-10	6.79	13.1857945	1.53E-01	1431735	1431858	NC_000011.9
cg10944379	1.57E-09	1.35E-10	6.79	13.184913	2.76E-02	35744528	35744651	NC_000010.10
cg14933807	1.57E-09	1.35E-10	6.79	13.184775	5.58E-02	129883528	129883651	NC_000010.10
cg00032805	1.57E-09	1.35E-10	6.78	13.1843767	1.42E-01	165304473	165304596	NC_000004.11
cg07371530	1.57E-09	1.35E-10	6.78	13.1839937	9.51E-02	839581	839704	NC_000016.9
cg05024939	1.57E-09	1.35E-10	6.78	13.1839682	1.27E-01	113442251	113442374	NC_000004.11
cg24612124	1.57E-09	1.35E-10	6.78	13.1817729	5.87E-02	93106264	93106387	NC_000014.8

cg07353303	1.58E-09	1.36E-10	6.78	13.1804105	1.63E-01	145713747	145713870	NC_000005.9
cg01461840	1.58E-09	1.36E-10	6.78	13.1797222	1.61E-01	130085088	130085211	NC_000010.10
cg18699337	1.58E-09	1.36E-10	6.78	13.1777155	1.16E-01	44159144	44159267	NC_000017.10
cg16580073	1.58E-09	1.36E-10	6.78	13.1769122	7.63E-02	64446603	64446726	NC_000005.9
cg13131329	1.58E-09	1.36E-10	6.78	13.1758526	7.95E-02	1801148	1801271	NC_000004.11
cg16234557	1.58E-09	1.36E-10	6.78	13.1756011	2.05E-01	119292062	119292185	NC_000010.10
cg02052895	1.58E-09	1.37E-10	6.78	13.1745335	1.58E-01	5540702	5540825	NC_000012.11
cg07003542	1.59E-09	1.37E-10	6.78	13.1733812	1.85E-01	176056682	176056805	NC_000005.9
cg02283487	1.59E-09	1.37E-10	6.78	13.1732416	6.27E-02	20904020	20904143	NC_000014.8
cg08342194	1.59E-09	1.37E-10	6.78	13.1732313	6.33E-02	31683075	31683198	NC_000006.11
cg26647524	1.59E-09	1.37E-10	6.78	13.1731594	6.03E-02	42726812	42726935	NC_000003.11
cg14452947	1.59E-09	1.37E-10	6.78	13.1730289	1.66E-01	87970640	87970763	NC_000005.9
cg10111115	1.59E-09	1.37E-10	6.78	13.1723957	1.22E-01	73674146	73674269	NC_000003.11
cg05969567	1.59E-09	1.37E-10	6.78	13.1723853	7.67E-02	169655497	169655620	NC_000006.11
cg03949391	1.59E-09	1.37E-10	6.78	13.1720869	1.68E-01	78956845	78956968	NC_000001.10
cg21817012	1.59E-09	1.37E-10	6.78	13.171618	6.42E-02	159924541	159924664	NC_000001.10
cg24186398	1.59E-09	1.37E-10	6.78	13.1706767	8.88E-02	1321530	1321653	NC_000011.9
cg03754403	1.59E-09	1.37E-10	6.78	13.1696147	1.56E-01	40567579	40567702	NC_000006.11
cg26363695	1.59E-09	1.37E-10	6.78	13.1692561	1.37E-01	80203299	80203422	NC_000004.11
cg10922530	1.59E-09	1.37E-10	6.78	13.1686205	2.96E-02	124457083	124457206	NC_000012.11
cg08017323	1.59E-09	1.37E-10	6.78	13.168145	1.20E-01	3240167	3240290	NC_000012.11
cg06042608	1.60E-09	1.38E-10	6.78	13.1670087	8.51E-02	46918356	46918479	NC_000003.11
cg17546247	1.60E-09	1.38E-10	6.78	13.1664418	1.43E-01	147125714	147125837	NC_000003.11
cg18809597	1.60E-09	1.38E-10	6.78	13.1653618	6.90E-02	151748660	151748783	NC_000001.10
cg25892587	1.60E-09	1.38E-10	6.78	13.1648152	1.10E-01	3666169	3666292	NC_000010.10

cg09420439	1.60E-09	1.38E-10	6.78	13.1627509	2.60E-01	27136424	27136547	NC_000007.13
cg07678904	1.60E-09	1.38E-10	6.78	13.1621706	2.02E-01	4304577	4304700	NC_000019.9
cg05617027	1.60E-09	1.38E-10	6.78	13.1610392	3.73E-02	40437086	40437209	NC_000004.11
cg08691273	1.60E-09	1.38E-10	6.78	13.1608365	5.34E-02	144333467	144333590	NC_000008.10
cg09556043	1.60E-09	1.38E-10	6.78	13.1608239	1.87E-01	144621952	144622075	NC_000004.11
cg22967396	1.61E-09	1.38E-10	6.78	13.1604294	1.31E-01	57283942	57284065	NC_000014.8
cg09111482	1.61E-09	1.38E-10	6.78	13.1602706	1.02E-01	134890692	134890815	NC_000010.10
cg24482024	1.61E-09	1.39E-10	6.78	13.1597773	6.59E-02	74660566	74660689	NC_000015.9
cg21946667	1.61E-09	1.39E-10	6.78	13.1588167	6.17E-02	40839643	40839766	NC_000017.10
cg04599297	1.61E-09	1.39E-10	6.78	13.1587246	1.52E-01	26505442	26505565	NC_000010.10
cg05961272	1.61E-09	1.39E-10	6.78	13.1583787	5.51E-02	77399130	77399253	NC_000013.10
cg02543268	1.61E-09	1.39E-10	6.78	13.1579293	7.69E-02	160201261	160201384	NC_000005.9
cg20695433	1.62E-09	1.40E-10	6.78	13.1531871	1.63E-01	30019102	30019225	NC_000019.9
cg07105285	1.62E-09	1.40E-10	6.78	13.1529844	1.01E-01	92702663	92702786	NC_000011.9
cg06701529	1.62E-09	1.40E-10	6.78	13.1522254	1.29E-01	32008841	32008964	NC_000011.9
cg08495770	1.62E-09	1.40E-10	6.78	13.1518748	1.74E-01	106400824	106400947	NC_000010.10
cg12159837	1.62E-09	1.40E-10	6.78	13.1517193	3.55E-02	1069335	1069458	NC_000017.10
cg14994639	1.62E-09	1.40E-10	6.78	13.1506781	4.16E-02	121422921	121423044	NC_000011.9
cg00301033	1.62E-09	1.40E-10	6.78	13.1492636	6.33E-02	43390036	43390159	NC_000011.9
cg05696406	1.62E-09	1.40E-10	6.78	13.1490682	1.04E-01	27599888	27600011	NC_000002.11
cg13854495	1.62E-09	1.40E-10	6.78	13.1489395	1.21E-01	131846350	131846473	NC_000009.11
cg02318629	1.62E-09	1.40E-10	6.78	13.1482083	1.62E-01	47797415	47797538	NC_000002.11
cg25377802	1.62E-09	1.40E-10	6.78	13.148177	1.61E-01	28616847	28616970	NC_000003.11
cg26030804	1.63E-09	1.40E-10	6.78	13.1470289	1.79E-01	794764	794887	NC_000011.9
cg00594011	1.63E-09	1.41E-10	6.78	13.1446034	2.00E-01	107956556	107956679	NC_000004.11

cg17875028	1.63E-09	1.41E-10	6.78	13.1421268	6.98E-02	233321828	233321951	NC_000002.11
cg10648197	1.63E-09	1.41E-10	6.78	13.1416659	1.76E-01	22634578	22634701	NC_000010.10
cg08053686	1.64E-09	1.41E-10	6.78	13.1408247	1.41E-01	6223581	6223704	NC_000004.11
cg14654613	1.64E-09	1.41E-10	6.78	13.1402908	1.29E-01	23185804	23185927	NC_000019.9
cg00177290	1.64E-09	1.42E-10	6.78	13.1391704	1.60E-01	110161285	110161408	NC_000001.10
cg11728738	1.64E-09	1.42E-10	6.78	13.1386579	1.53E-01	21711700	21711823	NC_000006.11
cg18128212	1.64E-09	1.42E-10	6.78	13.1382745	3.53E-02	26199190	26199313	NC_000001.10
cg17078427	1.64E-09	1.42E-10	6.78	13.1375572	1.59E-01	170137552	170137675	NC_000003.11
cg02894328	1.64E-09	1.42E-10	6.78	13.1373112	3.66E-02	30919516	30919639	NC_000006.11
cg00255759	1.64E-09	1.42E-10	6.78	13.1370296	4.25E-02	73699748	73699871	NC_000010.10
cg03401096	1.64E-09	1.42E-10	6.78	13.1367361	2.87E-01	123301171	123301294	NC_000011.9
cg06282596	1.64E-09	1.42E-10	6.78	13.1363826	1.00E-01	97175479	97175602	NC_000010.10
cg24970879	1.64E-09	1.42E-10	6.78	13.1345822	1.40E-01	38030068	38030191	NC_000007.13
cg00654296	1.65E-09	1.42E-10	6.78	13.1330459	7.84E-02	7230819	7230942	NC_000017.10
cg01656394	1.65E-09	1.42E-10	6.78	13.1326787	1.59E-01	244080781	244080904	NC_000001.10
cg07057579	1.65E-09	1.43E-10	6.78	13.132068	9.46E-02	95870112	95870235	NC_000015.9
cg00387941	1.65E-09	1.43E-10	6.78	13.1316321	4.60E-02	26661686	26661809	NC_000001.10
cg15641463	1.65E-09	1.43E-10	6.78	13.1312762	3.37E-02	30129346	30129469	NC_000006.11
cg10093805	1.65E-09	1.43E-10	6.78	13.1307256	9.55E-02	154296135	154296258	NC_000001.10
cg14362758	1.65E-09	1.43E-10	6.78	13.130253	1.76E-01	139090991	139091114	NC_000009.11
cg17662941	1.65E-09	1.43E-10	6.78	13.1299198	1.19E-01	63560973	63561096	NC_000007.13
cg05424678	1.65E-09	1.43E-10	6.78	13.1298411	8.41E-02	105910233	105910356	NC_000007.13
cg05176667	1.66E-09	1.43E-10	6.77	13.1277172	1.67E-01	102029207	102029330	NC_000014.8
cg20107759	1.66E-09	1.43E-10	6.77	13.1270259	2.30E-01	12936491	12936614	NC_000019.9
cg10828577	1.66E-09	1.43E-10	6.77	13.1268206	6.48E-02	61507612	61507735	NC_000017.10

cg10543574	1.66E-09	1.43E-10	6.77	13.1266156	1.67E-01	9763159	9763282	NC_000008.10
cg01582980	1.66E-09	1.44E-10	6.77	13.1254325	1.48E-01	20825545	20825668	NC_000007.13
cg24599434	1.66E-09	1.44E-10	6.77	13.1253711	1.41E-01	172166242	172166365	NC_000003.11
cg23591139	1.66E-09	1.44E-10	6.77	13.1247831	5.57E-02	1120557	1120680	NC_000017.10
cg07842403	1.66E-09	1.44E-10	6.77	13.1241582	1.79E-01	134515143	134515266	NC_000003.11
cg10927449	1.66E-09	1.44E-10	6.77	13.1233167	1.40E-01	63286621	63286744	NC_000002.11
cg13631444	1.66E-09	1.44E-10	6.77	13.1218031	1.18E-01	110098866	110098989	NC_000008.10
cg22271921	1.66E-09	1.44E-10	6.77	13.1217627	1.41E-01	40755383	40755506	NC_000008.10
cg22242585	1.67E-09	1.44E-10	6.77	13.1211196	4.88E-02	216659791	216659914	NC_000002.11
cg05416180	1.67E-09	1.44E-10	6.77	13.1210705	6.08E-02	188920619	188920742	NC_000003.11
cg11312408	1.67E-09	1.44E-10	6.77	13.1198933	1.59E-01	58207013	58207136	NC_000013.10
cg03804500	1.67E-09	1.44E-10	6.77	13.1186698	8.02E-02	140081972	140082095	NC_000009.11
cg25420952	1.67E-09	1.45E-10	6.77	13.1173726	9.20E-02	116841084	116841207	NC_000009.11
cg13906377	1.67E-09	1.45E-10	6.77	13.1173458	2.01E-01	63792695	63792818	NC_000001.10
cg13893543	1.67E-09	1.45E-10	6.77	13.1172824	1.50E-01	224805631	224805754	NC_000001.10
cg05422683	1.67E-09	1.45E-10	6.77	13.1158422	1.40E-01	145814003	145814126	NC_000007.13
cg10711871	1.67E-09	1.45E-10	6.77	13.1156632	9.03E-02	13860812	13860935	NC_000003.11
cg04904385	1.67E-09	1.45E-10	6.77	13.1151636	1.54E-01	19158692	19158815	NC_000007.13
cg12493107	1.67E-09	1.45E-10	6.77	13.1150691	7.86E-02	23344079	23344202	NC_000020.10
cg25436152	1.67E-09	1.45E-10	6.77	13.1149486	7.08E-02	954490	954613	NC_000017.10
cg06793679	1.68E-09	1.45E-10	6.77	13.1142691	6.23E-02	103054085	103054208	NC_000014.8
cg14633742	1.68E-09	1.45E-10	6.77	13.1141561	1.51E-01	98032587	98032710	NC_000009.11
cg05491166	1.68E-09	1.45E-10	6.77	13.1120682	1.61E-01	19885901	19886024	NC_000011.9
cg27264051	1.68E-09	1.46E-10	6.77	13.1111281	6.84E-02	133383895	133384018	NC_000003.11
cg03460245	1.69E-09	1.46E-10	6.77	13.1086625	4.47E-02	31475984	31476107	NC_000006.11

cg06598091	1.69E-09	1.46E-10	6.77	13.1081044	2.52E-01	11653600	11653723	NC_000012.11
cg21373739	1.69E-09	1.46E-10	6.77	13.1078002	1.59E-02	45730686	45730809	NC_000003.11
cg17315500	1.69E-09	1.46E-10	6.77	13.1077792	1.68E-01	103359572	103359695	NC_000012.11
cg00623774	1.69E-09	1.46E-10	6.77	13.1068793	5.82E-02	155674426	155674549	NC_000007.13
cg07749724	1.69E-09	1.46E-10	6.77	13.1061346	1.98E-01	112832480	112832603	NC_000011.9
cg05307923	1.69E-09	1.46E-10	6.77	13.1061288	1.82E-01	1779667	1779790	NC_000010.10
cg14216285	1.69E-09	1.47E-10	6.77	13.103626	1.73E-01	28778124	28778247	NC_000006.11
cg26660316	1.70E-09	1.47E-10	6.77	13.1012324	5.68E-02	110077272	110077395	NC_000005.9
cg09531729	1.70E-09	1.47E-10	6.77	13.1003308	6.29E-02	88124215	88124338	NC_000016.9
cg06737666	1.70E-09	1.47E-10	6.77	13.0986327	1.31E-01	78080026	78080149	NC_000016.9
cg01185626	1.70E-09	1.48E-10	6.77	13.0974814	1.76E-01	75594016	75594139	NC_000001.10
cg10994149	1.70E-09	1.48E-10	6.77	13.0971191	1.38E-01	43037123	43037246	NC_000005.9
cg01511557	1.70E-09	1.48E-10	6.77	13.0964329	1.14E-01	50514319	50514442	NC_000001.10
cg15907965	1.70E-09	1.48E-10	6.77	13.0963994	5.01E-02	89430272	89430395	NC_000010.10
cg11597131	1.71E-09	1.48E-10	6.77	13.0953145	1.72E-01	176988939	176989062	NC_000002.11
cg01549404	1.71E-09	1.48E-10	6.77	13.0942475	1.42E-01	55358636	55358759	NC_000016.9
cg22629111	1.71E-09	1.48E-10	6.77	13.0931233	1.54E-01	166135208	166135331	NC_000001.10
cg23733502	1.71E-09	1.48E-10	6.77	13.0929628	1.21E-01	577629	577752	NC_000003.11
cg23402821	1.71E-09	1.49E-10	6.77	13.0908457	1.65E-01	35292159	35292282	NC_000017.10
cg11735997	1.71E-09	1.49E-10	6.77	13.0907015	1.38E-01	30039113	30039236	NC_000011.9
cg17603132	1.72E-09	1.49E-10	6.77	13.0893019	1.65E-01	8190152	8190275	NC_000011.9
cg01644731	1.72E-09	1.49E-10	6.77	13.0884534	9.04E-02	58236712	58236835	NC_000012.11
cg06340987	1.72E-09	1.49E-10	6.77	13.0882487	1.59E-01	20182695	20182818	NC_000011.9
cg27383873	1.72E-09	1.49E-10	6.77	13.0881245	2.58E-02	50724286	50724409	NC_000010.10
cg00585733	1.72E-09	1.49E-10	6.77	13.0878715	1.52E-01	69064884	69065007	NC_000007.13

cg02943578	1.72E-09	1.49E-10	6.77	13.0861174	1.98E-01	86600499	86600622	NC_000016.9
cg02048657	1.72E-09	1.49E-10	6.77	13.0858496	9.52E-02	1293231	1293354	NC_000005.9
cg21548464	1.73E-09	1.50E-10	6.77	13.0836445	2.82E-02	50707937	50708060	NC_000013.10
cg21871746	1.73E-09	1.50E-10	6.77	13.0827432	1.37E-01	99616820	99616943	NC_000007.13
cg06829299	1.73E-09	1.50E-10	6.77	13.0826873	1.58E-01	65286632	65286755	NC_000008.10
cg04081402	1.73E-09	1.50E-10	6.77	13.0821259	7.38E-02	31003587	31003710	NC_000022.10
cg19526568	1.73E-09	1.50E-10	6.77	13.0820278	1.11E-01	100643087	100643210	NC_000013.10
cg24948792	1.73E-09	1.50E-10	6.77	13.0815344	5.03E-02	102789961	102790084	NC_000010.10
cg08732659	1.74E-09	1.51E-10	6.77	13.0777158	9.74E-02	54973966	54974089	NC_000016.9
cg27263903	1.74E-09	1.51E-10	6.77	13.07675	1.23E-01	160314452	160314575	NC_000001.10
cg27467929	1.74E-09	1.51E-10	6.76	13.0730516	2.34E-01	68864529	68864652	NC_000008.10
cg22675937	1.74E-09	1.51E-10	6.76	13.0729787	1.45E-01	59481571	59481694	NC_000017.10
cg14905657	1.74E-09	1.51E-10	6.76	13.0726786	9.69E-02	16760048	16760171	NC_000012.11
cg23411869	1.74E-09	1.51E-10	6.76	13.0722982	1.41E-01	74846412	74846535	NC_000017.10
cg22060367	1.75E-09	1.52E-10	6.76	13.0702207	2.39E-02	161957720	161957843	NC_000002.11
cg15974867	1.75E-09	1.52E-10	6.76	13.0690047	7.74E-02	69464012	69464135	NC_000011.9
cg17967673	1.75E-09	1.52E-10	6.76	13.068957	1.19E-01	38040009	38040132	NC_000019.9
cg04618333	1.75E-09	1.52E-10	6.76	13.0687515	2.27E-01	46800674	46800797	NC_000017.10
cg22654517	1.75E-09	1.52E-10	6.76	13.0662081	7.54E-02	96458247	96458370	NC_000002.11
cg24496745	1.76E-09	1.53E-10	6.76	13.0655702	9.51E-02	57285156	57285279	NC_000001.10
cg03444245	1.76E-09	1.53E-10	6.76	13.0652143	1.84E-01	45377079	45377202	NC_000011.9
cg15707093	1.76E-09	1.53E-10	6.76	13.0651102	2.06E-01	174450353	174450476	NC_000004.11
cg10091996	1.76E-09	1.53E-10	6.76	13.0646544	1.16E-01	31548640	31548763	NC_000016.9
cg19357275	1.76E-09	1.53E-10	6.76	13.0646072	4.47E-02	248855040	248855163	NC_000001.10
cg14463912	1.76E-09	1.53E-10	6.76	13.0643702	7.94E-02	102789979	102790102	NC_000010.10

cg03887163	1.76E-09	1.53E-10	6.76	13.0624862	1.46E-01	165324100	165324223	NC_000001.10
cg03510605	1.76E-09	1.53E-10	6.76	13.062047	1.28E-01	115104734	115104857	NC_000012.11
cg05158615	1.76E-09	1.53E-10	6.76	13.0616188	1.76E-01	24323559	24323682	NC_000007.13
cg01619107	1.76E-09	1.53E-10	6.76	13.0610432	7.49E-02	52515354	52515477	NC_000012.11
cg26348521	1.76E-09	1.53E-10	6.76	13.0609129	1.09E-01	1375657	1375780	NC_000011.9
cg08230105	1.76E-09	1.53E-10	6.76	13.0602907	1.13E-01	41692501	41692624	NC_000017.10
cg17507751	1.76E-09	1.53E-10	6.76	13.0601236	7.65E-02	41275792	41275915	NC_000005.9
cg19186145	1.77E-09	1.54E-10	6.76	13.0566485	2.64E-01	45169562	45169685	NC_000002.11
cg13415865	1.77E-09	1.54E-10	6.76	13.056072	9.27E-02	2831573	2831696	NC_000017.10
cg25745379	1.78E-09	1.54E-10	6.76	13.0539358	1.09E-01	27333481	27333604	NC_000013.10
cg13180920	1.78E-09	1.55E-10	6.76	13.0496194	8.88E-02	46896174	46896297	NC_000021.8
cg05385163	1.79E-09	1.55E-10	6.76	13.0477899	1.50E-01	96717349	96717472	NC_000009.11
cg14805347	1.79E-09	1.55E-10	6.76	13.0468547	1.89E-01	58357879	58358002	NC_000015.9
cg10394047	1.79E-09	1.56E-10	6.76	13.0462819	5.67E-02	47575416	47575539	NC_000021.8
cg02370232	1.79E-09	1.56E-10	6.76	13.0453483	9.59E-02	97175365	97175488	NC_000010.10
cg27423323	1.79E-09	1.56E-10	6.76	13.0443301	1.02E-01	2963112	2963235	NC_000020.10
cg02647941	1.79E-09	1.56E-10	6.76	13.0432223	1.49E-01	147127143	147127266	NC_000003.11
cg07712493	1.80E-09	1.56E-10	6.76	13.0419134	1.46E-01	115151427	115151550	NC_000005.9
cg20864608	1.80E-09	1.56E-10	6.76	13.041536	2.31E-01	143533499	143533622	NC_000008.10
cg20220255	1.80E-09	1.56E-10	6.76	13.0411406	5.61E-02	95228096	95228219	NC_000012.11
cg14561530	1.80E-09	1.56E-10	6.76	13.0404897	6.05E-02	3129958	3130081	NC_000001.10
cg19410796	1.80E-09	1.56E-10	6.76	13.0404055	2.78E-02	45966279	45966402	NC_000013.10
cg19308769	1.80E-09	1.57E-10	6.76	13.0397341	9.94E-02	31289850	31289973	NC_000001.10
cg04446501	1.80E-09	1.57E-10	6.76	13.0389381	6.17E-02	3742328	3742451	NC_000004.11
cg18404374	1.80E-09	1.57E-10	6.76	13.0383631	1.62E-01	26663551	26663674	NC_000003.11

cg26619987	1.80E-09	1.57E-10	6.76	13.0368653	2.81E-02	78153772	78153895	NC_000017.10
cg23182200	1.81E-09	1.57E-10	6.76	13.0362361	9.09E-02	97345907	97346030	NC_000008.10
cg20688791	1.81E-09	1.57E-10	6.76	13.0359264	6.28E-02	154415062	154415185	NC_000001.10
cg09056181	1.81E-09	1.57E-10	6.76	13.0354217	1.76E-01	26505503	26505626	NC_000010.10
cg01291404	1.81E-09	1.57E-10	6.76	13.0353399	1.75E-01	48397872	48397995	NC_000012.11
cg02602455	1.81E-09	1.58E-10	6.76	13.0339518	1.36E-01	36739781	36739904	NC_000009.11
cg24448870	1.81E-09	1.58E-10	6.76	13.0336119	4.12E-02	126668133	126668256	NC_000003.11
cg27314569	1.81E-09	1.58E-10	6.76	13.0330008	1.66E-01	122852461	122852584	NC_000011.9
cg13661740	1.81E-09	1.58E-10	6.76	13.0321978	1.79E-01	79635912	79636035	NC_000009.11
cg13919631	1.82E-09	1.58E-10	6.76	13.0296906	7.47E-02	38071571	38071694	NC_000001.10
cg14614211	1.82E-09	1.58E-10	6.76	13.0287944	1.33E-01	28034247	28034370	NC_000010.10
cg25316663	1.82E-09	1.58E-10	6.76	13.0279817	2.01E-01	147569938	147570061	NC_000004.11
cg03796781	1.82E-09	1.59E-10	6.76	13.0271046	1.38E-01	137818836	137818959	NC_000006.11
cg05583608	1.82E-09	1.59E-10	6.76	13.0267703	1.51E-01	51147536	51147659	NC_000016.9
cg00916854	1.82E-09	1.59E-10	6.76	13.026417	1.02E-01	123350361	123350484	NC_000012.11
cg26953640	1.82E-09	1.59E-10	6.76	13.0260236	1.70E-01	1174473	1174596	NC_000017.10
cg09473826	1.82E-09	1.59E-10	6.76	13.0249703	9.70E-02	175568216	175568339	NC_000001.10
cg13996750	1.83E-09	1.59E-10	6.76	13.0246285	9.45E-02	139419958	139420081	NC_000009.11
cg14653284	1.83E-09	1.59E-10	6.76	13.0232555	1.47E-01	11679879	11680002	NC_000002.11
cg03320255	1.83E-09	1.59E-10	6.76	13.0231829	1.31E-01	38042154	38042277	NC_000019.9
cg15712323	1.83E-09	1.60E-10	6.76	13.0203354	1.13E-01	11565399	11565522	NC_000019.9
cg12492157	1.83E-09	1.60E-10	6.76	13.0202242	7.18E-02	27498310	27498433	NC_000007.13
cg14770827	1.83E-09	1.60E-10	6.76	13.0200364	1.17E-01	1775275	1775398	NC_000019.9
cg06489433	1.83E-09	1.60E-10	6.75	13.019478	3.99E-02	89491229	89491352	NC_000016.9
cg07147573	1.84E-09	1.60E-10	6.75	13.0186795	1.39E-01	42918806	42918929	NC_000003.11

cg13918613	1.84E-09	1.60E-10	6.75	13.0167193	1.75E-01	18429126	18429249	NC_000010.10
cg02086467	1.84E-09	1.60E-10	6.75	13.0160918	1.53E-01	57276527	57276650	NC_000014.8
cg11694490	1.84E-09	1.61E-10	6.75	13.0140788	1.78E-01	21927823	21927946	NC_000012.11
cg12816979	1.84E-09	1.61E-10	6.75	13.0138541	1.26E-01	59001398	59001521	NC_000018.9
cg03160135	1.84E-09	1.61E-10	6.75	13.0137591	1.32E-01	117586918	117587041	NC_000006.11
cg27350402	1.85E-09	1.61E-10	6.75	13.0124727	1.44E-01	27550691	27550814	NC_000006.11
cg25985013	1.85E-09	1.61E-10	6.75	13.012099	1.05E-01	21925112	21925235	NC_000008.10
cg04002235	1.85E-09	1.61E-10	6.75	13.0117739	1.33E-01	128560443	128560566	NC_000011.9
cg17708167	1.85E-09	1.61E-10	6.75	13.0108479	1.69E-01	68117917	68118040	NC_000015.9
cg10581650	1.85E-09	1.61E-10	6.75	13.0105618	5.58E-02	100155032	100155155	NC_000010.10
cg23500122	1.85E-09	1.61E-10	6.75	13.0095764	1.82E-01	172167610	172167733	NC_000003.11
cg23602974	1.85E-09	1.62E-10	6.75	13.0090966	1.26E-01	35293884	35294007	NC_000007.13
cg07531516	1.86E-09	1.62E-10	6.75	13.0077044	9.93E-02	4566644	4566767	NC_000019.9
cg02630553	1.86E-09	1.62E-10	6.75	13.0068857	1.27E-01	60296809	60296932	NC_000015.9
cg16909495	1.86E-09	1.62E-10	6.75	13.0065185	1.50E-01	49940062	49940185	NC_000019.9
cg03015433	1.86E-09	1.62E-10	6.75	13.0054899	1.28E-01	56623111	56623234	NC_000016.9
cg15467646	1.86E-09	1.62E-10	6.75	13.0048285	1.99E-01	22141014	22141137	NC_000001.10
cg03057072	1.86E-09	1.62E-10	6.75	13.0046868	8.27E-02	32186554	32186677	NC_000010.10
cg03986574	1.86E-09	1.62E-10	6.75	13.004261	7.26E-02	64544080	64544203	NC_000011.9
cg26578682	1.86E-09	1.62E-10	6.75	13.0041635	1.30E-01	2750163	2750286	NC_000005.9
cg13140167	1.86E-09	1.63E-10	6.75	13.0026157	1.46E-01	33454504	33454627	NC_000022.10
cg16869622	1.86E-09	1.63E-10	6.75	13.0026026	3.81E-02	133102529	133102652	NC_000012.11
cg10972873	1.86E-09	1.63E-10	6.75	13.002309	1.90E-01	200007592	200007715	NC_000001.10
cg02571816	1.87E-09	1.63E-10	6.75	13.001689	1.81E-01	38747378	38747501	NC_000019.9
cg22783747	1.87E-09	1.63E-10	6.75	13.0005732	2.14E-02	69614817	69614940	NC_000014.8

cg08468913	1.87E-09	1.63E-10	6.75	13.0003219	1.05E-01	122632352	122632475	NC_000003.11
cg13798842	1.87E-09	1.63E-10	6.75	13.0001929	6.83E-02	91450714	91450837	NC_000015.9
cg10243196	1.87E-09	1.63E-10	6.75	12.9999004	1.65E-01	23945996	23946119	NC_000019.9
cg08073312	1.87E-09	1.63E-10	6.75	12.9997432	1.59E-01	137483555	137483678	NC_000003.11
cg15911153	1.87E-09	1.63E-10	6.75	12.9989355	8.93E-02	138730783	138730906	NC_000005.9
cg02305723	1.87E-09	1.63E-10	6.75	12.9987675	1.25E-01	25093456	25093579	NC_000015.9
cg02920396	1.87E-09	1.63E-10	6.75	12.9978568	7.54E-02	68782211	68782334	NC_000011.9
cg16772031	1.88E-09	1.64E-10	6.75	12.9960403	9.27E-02	132600233	132600356	NC_000010.10
cg14700304	1.88E-09	1.64E-10	6.75	12.9959913	2.29E-01	105760890	105761013	NC_000002.11
cg15117739	1.88E-09	1.64E-10	6.75	12.995898	1.17E-01	46698881	46699004	NC_000017.10
cg20399871	1.88E-09	1.64E-10	6.75	12.9950997	1.38E-01	27204663	27204786	NC_000007.13
cg02380531	1.88E-09	1.64E-10	6.75	12.9949171	1.56E-01	99279097	99279220	NC_000006.11
cg05268122	1.88E-09	1.64E-10	6.75	12.9941701	9.03E-02	2288808	2288931	NC_000016.9
cg09660365	1.89E-09	1.65E-10	6.75	12.9907555	1.21E-01	10858395	10858518	NC_000003.11
cg25734951	1.89E-09	1.65E-10	6.75	12.9904319	4.67E-02	49526053	49526176	NC_000004.11
cg03724896	1.89E-09	1.65E-10	6.75	12.9888751	1.20E-01	3854465	3854588	NC_000005.9
cg22212691	1.89E-09	1.65E-10	6.75	12.9887627	2.19E-01	220417868	220417991	NC_000002.11
cg04961920	1.89E-09	1.65E-10	6.75	12.9869559	7.49E-02	71482267	71482390	NC_000010.10
cg03399210	1.89E-09	1.65E-10	6.75	12.9867807	9.99E-02	37616064	37616187	NC_000008.10
cg26419265	1.89E-09	1.65E-10	6.75	12.9866984	1.85E-01	151303982	151304105	NC_000005.9
cg11141380	1.89E-09	1.66E-10	6.75	12.9853304	1.88E-01	122852417	122852540	NC_000011.9
cg11385933	1.90E-09	1.66E-10	6.75	12.9848817	9.70E-02	45431839	45431962	NC_000007.13
cg20814312	1.90E-09	1.66E-10	6.75	12.9846874	1.30E-01	133339746	133339869	NC_000002.11
cg12328333	1.90E-09	1.66E-10	6.75	12.9846343	1.96E-01	36705041	36705164	NC_000013.10
cg27021368	1.90E-09	1.66E-10	6.75	12.9842219	9.48E-02	155639080	155639203	NC_000006.11

cg20194811	1.90E-09	1.66E-10	6.75	12.9827586	1.39E-01	112720491	112720614	NC_000013.10
cg20581674	1.90E-09	1.66E-10	6.75	12.9824489	9.22E-02	24887420	24887543	NC_000003.11
cg14346872	1.90E-09	1.66E-10	6.75	12.9813477	3.26E-02	31500033	31500156	NC_000014.8
cg15594967	1.90E-09	1.66E-10	6.75	12.9810369	1.28E-01	55444079	55444202	NC_000006.11
cg01579950	1.90E-09	1.66E-10	6.75	12.9809425	1.94E-01	64481018	64481141	NC_000011.9
cg24073994	1.90E-09	1.66E-10	6.75	12.9808572	6.28E-02	38511770	38511893	NC_000017.10
cg00232735	1.90E-09	1.66E-10	6.75	12.9799139	2.04E-01	132934139	132934262	NC_000011.9
cg19179801	1.90E-09	1.66E-10	6.75	12.9796586	1.55E-01	37381836	37381959	NC_000017.10
cg22963915	1.90E-09	1.67E-10	6.75	12.9794078	1.38E-01	3785855	3785978	NC_000019.9
cg27010214	1.91E-09	1.67E-10	6.75	12.9792155	9.04E-02	74826663	74826786	NC_000014.8
cg05513806	1.91E-09	1.67E-10	6.75	12.9774423	1.71E-01	181438216	181438339	NC_000003.11
cg12697796	1.91E-09	1.67E-10	6.75	12.977217	1.18E-01	49954016	49954139	NC_000010.10
cg11076814	1.91E-09	1.67E-10	6.75	12.9756473	1.27E-01	11225303	11225426	NC_000004.11
cg00571870	1.91E-09	1.67E-10	6.75	12.9747872	8.40E-02	109349597	109349720	NC_000006.11
cg05985317	1.91E-09	1.67E-10	6.75	12.9745791	1.02E-01	32118208	32118331	NC_000006.11
cg09526758	1.92E-09	1.68E-10	6.75	12.9734768	1.54E-01	16758047	16758170	NC_000012.11
cg05856556	1.92E-09	1.68E-10	6.75	12.9723925	8.87E-02	150104705	150104828	NC_000007.13
cg09413529	1.92E-09	1.68E-10	6.75	12.9719396	1.39E-01	115112225	115112348	NC_000012.11
cg03268697	1.92E-09	1.68E-10	6.75	12.9690652	5.86E-02	75160330	75160453	NC_000017.10
cg01546568	1.93E-09	1.68E-10	6.75	12.968492	2.08E-01	79740322	79740445	NC_000002.11
cg02339891	1.93E-09	1.68E-10	6.75	12.9682434	1.46E-01	1704031	1704154	NC_000007.13
cg08797606	1.93E-09	1.69E-10	6.75	12.9673444	1.55E-01	87956769	87956892	NC_000005.9
cg22813366	1.93E-09	1.69E-10	6.75	12.9665288	1.23E-01	17440072	17440195	NC_000019.9
cg14649424	1.93E-09	1.69E-10	6.75	12.9661458	9.10E-02	98115754	98115877	NC_000009.11
cg15509361	1.93E-09	1.69E-10	6.75	12.9656664	1.28E-01	19183325	19183448	NC_000007.13

cg14769991	1.93E-09	1.69E-10	6.75	12.9653056	8.36E-02	112688311	112688434	NC_000006.11
cg16259571	1.93E-09	1.69E-10	6.74	12.9643995	7.86E-02	220384841	220384964	NC_000002.11
cg09561029	1.94E-09	1.69E-10	6.74	12.9622485	4.56E-02	3273364	3273487	NC_000006.11
cg02252248	1.94E-09	1.70E-10	6.74	12.9617051	1.77E-01	47073164	47073287	NC_000017.10
cg06038944	1.94E-09	1.70E-10	6.74	12.9602433	8.84E-02	181010312	181010435	NC_000002.11
cg04723721	1.94E-09	1.70E-10	6.74	12.9594289	4.64E-02	139915388	139915511	NC_000009.11
cg13305657	1.94E-09	1.70E-10	6.74	12.959287	7.55E-02	25258181	25258304	NC_000001.10
cg07085827	1.94E-09	1.70E-10	6.74	12.9589907	1.99E-01	132121676	132121799	NC_000002.11
cg09497409	1.95E-09	1.70E-10	6.74	12.9566288	1.54E-01	8275083	8275206	NC_000019.9
cg23998650	1.95E-09	1.71E-10	6.74	12.9551661	1.38E-01	3787948	3788071	NC_000005.9
cg18557131	1.95E-09	1.71E-10	6.74	12.9544407	1.45E-01	100624207	100624330	NC_000013.10
cg01753198	1.95E-09	1.71E-10	6.74	12.9539343	1.20E-01	70560099	70560222	NC_000016.9
cg17374433	1.95E-09	1.71E-10	6.74	12.9538793	9.58E-02	119876327	119876450	NC_000010.10
cg09614300	1.96E-09	1.71E-10	6.74	12.9519851	1.54E-01	124894076	124894199	NC_000010.10
cg12338097	1.96E-09	1.71E-10	6.74	12.9518699	1.72E-01	22929407	22929530	NC_000018.9
cg18063312	1.96E-09	1.71E-10	6.74	12.9515708	1.61E-01	72740737	72740860	NC_000005.9
cg05470554	1.96E-09	1.72E-10	6.74	12.9499833	1.53E-01	120969079	120969202	NC_000007.13
cg01957629	1.96E-09	1.72E-10	6.74	12.9498617	1.10E-01	63613490	63613613	NC_000011.9
cg24987667	1.96E-09	1.72E-10	6.74	12.9488107	1.01E-01	8518709	8518832	NC_000004.11
cg04104489	1.96E-09	1.72E-10	6.74	12.9476884	8.00E-02	64063351	64063474	NC_000001.10
cg00018128	1.96E-09	1.72E-10	6.74	12.9474994	1.74E-01	162275746	162275869	NC_000002.11
cg26456021	1.97E-09	1.72E-10	6.74	12.9461838	2.46E-02	2580073	2580196	NC_000016.9
cg18180569	1.97E-09	1.72E-10	6.74	12.9452145	2.12E-01	44883362	44883485	NC_000001.10
cg12085812	1.97E-09	1.72E-10	6.74	12.9449157	2.48E-02	45768215	45768338	NC_000001.10
cg02367144	1.97E-09	1.73E-10	6.74	12.9440077	1.83E-01	237146137	237146260	NC_000002.11

cg19078576	1.97E-09	1.73E-10	6.74	12.9439801	2.02E-01	17217877	17218000	NC_000005.9
cg23091758	1.97E-09	1.73E-10	6.74	12.9422689	1.68E-01	9025767	9025890	NC_000011.9
cg13778709	1.97E-09	1.73E-10	6.74	12.9416944	1.50E-01	57089961	57090084	NC_000020.10
cg05166490	1.98E-09	1.73E-10	6.74	12.9415311	1.88E-01	41754172	41754295	NC_000008.10
cg22374485	1.98E-09	1.73E-10	6.74	12.9411625	2.36E-01	21927425	21927548	NC_000012.11
cg21184415	1.98E-09	1.73E-10	6.74	12.9409531	9.49E-02	39996039	39996162	NC_000020.10
cg08522176	1.98E-09	1.74E-10	6.74	12.9390472	1.98E-01	124735398	124735521	NC_000011.9
cg17586860	1.98E-09	1.74E-10	6.74	12.938914	1.78E-01	23015906	23016029	NC_000020.10
cg18008880	1.98E-09	1.74E-10	6.74	12.9381783	4.80E-02	10132097	10132220	NC_000018.9
cg09414264	1.99E-09	1.74E-10	6.74	12.9353963	5.50E-02	3113709	3113832	NC_000011.9
cg05639246	1.99E-09	1.74E-10	6.74	12.9352063	2.28E-01	10393778	10393901	NC_000006.11
cg03692563	1.99E-09	1.74E-10	6.74	12.9342787	1.36E-01	31846414	31846537	NC_000011.9
cg08894131	1.99E-09	1.74E-10	6.74	12.9340137	6.08E-02	147231061	147231184	NC_000001.10
cg02401399	1.99E-09	1.75E-10	6.74	12.9329545	2.14E-01	36523584	36523707	NC_000019.9
cg17058616	1.99E-09	1.75E-10	6.74	12.9316227	1.40E-01	73102145	73102268	NC_000017.10
cg14212412	2.00E-09	1.75E-10	6.74	12.9301319	5.38E-02	105918992	105919115	NC_000006.11
cg06033268	2.00E-09	1.75E-10	6.74	12.929359	7.57E-02	174125205	174125328	NC_000001.10
cg12883523	2.00E-09	1.76E-10	6.74	12.9278485	1.37E-01	55372392	55372515	NC_000008.10
cg25328184	2.00E-09	1.76E-10	6.74	12.9275904	1.60E-01	110406414	110406537	NC_000005.9
cg24938830	2.01E-09	1.76E-10	6.74	12.9254242	2.09E-01	210636539	210636662	NC_000002.11
cg04108438	2.01E-09	1.76E-10	6.74	12.9246215	8.83E-02	64427959	64428082	NC_000003.11
cg04513214	2.01E-09	1.77E-10	6.74	12.922367	1.33E-01	17057446	17057569	NC_000005.9
cg07160420	2.01E-09	1.77E-10	6.74	12.9217243	6.15E-02	48863591	48863714	NC_000017.10
cg04001014	2.01E-09	1.77E-10	6.74	12.9216556	2.31E-01	238318	238441	NC_000003.11
cg05997350	2.01E-09	1.77E-10	6.74	12.9213667	1.59E-01	50817104	50817227	NC_000010.10

cg22794704	2.02E-09	1.77E-10	6.74	12.9202861	1.75E-01	162273437	162273560	NC_000002.11
cg24648061	2.02E-09	1.77E-10	6.74	12.9195943	4.10E-02	107114615	107114738	NC_000007.13
cg11787346	2.02E-09	1.77E-10	6.74	12.9193153	2.08E-01	55367611	55367734	NC_000008.10
cg19860245	2.02E-09	1.77E-10	6.74	12.9191523	8.49E-02	74300557	74300680	NC_000014.8
cg00592781	2.02E-09	1.77E-10	6.74	12.9189049	1.61E-01	66122874	66122997	NC_000012.11
cg06733311	2.02E-09	1.77E-10	6.74	12.9176711	4.97E-02	33381680	33381803	NC_000006.11
cg13229857	2.03E-09	1.78E-10	6.74	12.9149524	1.25E-01	200006247	200006370	NC_000001.10
cg15727287	2.03E-09	1.78E-10	6.74	12.9143819	1.63E-01	176046980	176047103	NC_000005.9
cg14625175	2.03E-09	1.78E-10	6.74	12.9123453	2.41E-01	27214056	27214179	NC_000007.13
cg03733219	2.03E-09	1.78E-10	6.74	12.9120803	1.62E-01	38886319	38886442	NC_000019.9
cg15795787	2.04E-09	1.79E-10	6.73	12.9098771	1.45E-01	72470415	72470538	NC_000008.10
cg08050967	2.04E-09	1.79E-10	6.73	12.9098283	4.83E-02	3464931	3465054	NC_000002.11
cg09745791	2.04E-09	1.79E-10	6.73	12.9093432	9.84E-02	75252619	75252742	NC_000016.9
cg02727566	2.04E-09	1.79E-10	6.73	12.9091474	4.06E-02	40718426	40718549	NC_000019.9
cg10902717	2.04E-09	1.79E-10	6.73	12.9079097	1.63E-01	178368620	178368743	NC_000005.9
cg00779167	2.04E-09	1.79E-10	6.73	12.9076685	7.90E-02	109753001	109753124	NC_000001.10
cg10929873	2.04E-09	1.79E-10	6.73	12.9070224	5.46E-02	124972783	124972906	NC_000012.11
cg14810013	2.04E-09	1.80E-10	6.73	12.9054323	1.09E-01	66535412	66535535	NC_000004.11
cg14472366	2.04E-09	1.80E-10	6.73	12.9052983	1.24E-01	20875915	20876038	NC_000013.10
cg04730685	2.05E-09	1.80E-10	6.73	12.9047892	1.34E-01	95360278	95360401	NC_000013.10
cg04779631	2.05E-09	1.80E-10	6.73	12.9038678	2.36E-01	84115895	84116018	NC_000015.9
cg25673112	2.05E-09	1.80E-10	6.73	12.9029411	5.34E-02	126824398	126824521	NC_000010.10
cg02996801	2.05E-09	1.80E-10	6.73	12.9027401	1.65E-01	113441944	113442067	NC_000004.11
cg21296602	2.05E-09	1.80E-10	6.73	12.9021254	1.70E-01	93476060	93476183	NC_000011.9
cg07039180	2.05E-09	1.80E-10	6.73	12.9008806	1.98E-01	49814435	49814558	NC_000007.13

cg16802159	2.06E-09	1.81E-10	6.73	12.8993633	1.48E-01	86457719	86457842	NC_000016.9
cg02014853	2.06E-09	1.81E-10	6.73	12.8992755	1.05E-01	29595335	29595458	NC_000006.11
cg11963233	2.06E-09	1.81E-10	6.73	12.8989421	1.38E-01	25754301	25754424	NC_000018.9
cg00656478	2.06E-09	1.81E-10	6.73	12.8982165	4.47E-02	132480963	132481086	NC_000009.11
cg05133323	2.06E-09	1.81E-10	6.73	12.8964017	9.37E-02	116682493	116682616	NC_000012.11
cg07560587	2.06E-09	1.81E-10	6.73	12.8952461	1.09E-01	88016574	88016697	NC_000016.9
cg09182138	2.06E-09	1.81E-10	6.73	12.8951925	1.16E-01	156612436	156612559	NC_000001.10
cg04264908	2.07E-09	1.82E-10	6.73	12.8933916	2.16E-01	35303330	35303453	NC_000017.10
cg23766724	2.07E-09	1.82E-10	6.73	12.8933842	1.09E-01	27172631	27172754	NC_000007.13
cg05670472	2.07E-09	1.82E-10	6.73	12.8933639	1.36E-01	140805732	140805855	NC_000005.9
cg13564533	2.07E-09	1.82E-10	6.73	12.8923252	1.68E-01	55379081	55379204	NC_000008.10
cg22536554	2.07E-09	1.82E-10	6.73	12.8923178	2.26E-01	94834201	94834324	NC_000010.10
cg16701893	2.07E-09	1.82E-10	6.73	12.8907346	5.62E-02	164670259	164670382	NC_000001.10
cg22340508	2.08E-09	1.83E-10	6.73	12.8866972	1.41E-01	22891978	22892101	NC_000019.9
cg06327407	2.08E-09	1.83E-10	6.73	12.88613	1.81E-01	2781399	2781522	NC_000020.10
cg14784009	2.09E-09	1.84E-10	6.73	12.8840845	6.65E-02	94964696	94964819	NC_000012.11
cg13986215	2.09E-09	1.84E-10	6.73	12.8835145	4.94E-02	129828770	129828893	NC_000010.10
cg15161225	2.09E-09	1.84E-10	6.73	12.8821736	6.68E-02	36013678	36013801	NC_000022.10
cg06773295	2.09E-09	1.84E-10	6.73	12.8814433	3.93E-02	3028577	3028700	NC_000001.10
cg12110677	2.09E-09	1.84E-10	6.73	12.8809525	1.34E-01	2781483	2781606	NC_000020.10
cg03349184	2.09E-09	1.84E-10	6.73	12.8800538	2.33E-01	28740982	28741105	NC_000006.11
cg08924917	2.09E-09	1.84E-10	6.73	12.8795906	5.86E-02	17054295	17054418	NC_000003.11
cg27311970	2.10E-09	1.84E-10	6.73	12.8789121	6.79E-02	54195830	54195953	NC_000001.10
cg07664005	2.10E-09	1.85E-10	6.73	12.8769511	1.20E-01	172671068	172671191	NC_000005.9
cg16834320	2.10E-09	1.85E-10	6.73	12.8755393	8.25E-02	195167302	195167425	NC_000003.11

cg09034646	2.11E-09	1.85E-10	6.73	12.8746183	1.15E-01	1309654	1309777	NC_000008.10
cg18133300	2.11E-09	1.85E-10	6.73	12.8745977	2.77E-02	62376474	62376597	NC_000011.9
cg08130144	2.11E-09	1.85E-10	6.73	12.8741537	1.81E-01	41883493	41883616	NC_000004.11
cg14558568	2.11E-09	1.85E-10	6.73	12.8740445	1.17E-01	41621052	41621175	NC_000006.11
cg07008600	2.11E-09	1.85E-10	6.73	12.873743	1.30E-01	33261204	33261327	NC_000003.11
cg19556854	2.11E-09	1.86E-10	6.73	12.872962	1.04E-01	32945549	32945672	NC_000006.11
cg15854847	2.11E-09	1.86E-10	6.73	12.8728412	1.42E-01	23563970	23564093	NC_000008.10
cg24886267	2.11E-09	1.86E-10	6.73	12.8725805	1.66E-01	167956306	167956429	NC_000005.9
cg07518714	2.11E-09	1.86E-10	6.73	12.8725077	1.09E-01	32064945	32065068	NC_000006.11
cg21838979	2.11E-09	1.86E-10	6.73	12.8712726	1.20E-01	106681945	106682068	NC_000002.11
cg22408430	2.11E-09	1.86E-10	6.73	12.8709885	1.11E-01	63883266	63883389	NC_000011.9
cg11944480	2.11E-09	1.86E-10	6.73	12.8709677	1.11E-01	23452035	23452158	NC_000007.13
cg15996043	2.12E-09	1.86E-10	6.73	12.8691306	2.01E-01	70346221	70346344	NC_000014.8
cg20101398	2.12E-09	1.86E-10	6.73	12.868562	4.96E-02	114384045	114384168	NC_000006.11
cg15812020	2.12E-09	1.86E-10	6.73	12.8685174	1.13E-01	25506235	25506358	NC_000013.10
cg26499363	2.12E-09	1.87E-10	6.73	12.8682251	1.56E-01	46796119	46796242	NC_000017.10
cg08047376	2.12E-09	1.87E-10	6.73	12.8681088	1.83E-01	79739940	79740063	NC_000002.11
cg04716530	2.12E-09	1.87E-10	6.73	12.8680231	1.01E-01	30485684	30485807	NC_000016.9
cg18892424	2.12E-09	1.87E-10	6.73	12.8675218	1.13E-01	131034796	131034919	NC_000010.10
cg04751631	2.12E-09	1.87E-10	6.73	12.8675097	1.52E-01	149917170	149917293	NC_000007.13
cg17172761	2.12E-09	1.87E-10	6.73	12.8660128	9.63E-02	62120439	62120562	NC_000017.10
cg12270738	2.12E-09	1.87E-10	6.73	12.8650857	1.54E-02	19282546	19282669	NC_000001.10
cg16521213	2.12E-09	1.87E-10	6.73	12.8647873	5.36E-02	277384	277507	NC_000002.11
cg04674956	2.12E-09	1.87E-10	6.73	12.8646612	1.44E-01	65499048	65499171	NC_000008.10
cg26181583	2.13E-09	1.88E-10	6.73	12.8618847	7.87E-02	38907470	38907593	NC_000017.10

cg18774692	2.13E-09	1.88E-10	6.73	12.8616734	9.39E-02	89033280	89033403	NC_000012.11
cg15075709	2.14E-09	1.89E-10	6.73	12.8577715	1.15E-01	1217335	1217458	NC_000011.9
cg18649503	2.14E-09	1.89E-10	6.73	12.8573995	8.61E-02	28187510	28187633	NC_000006.11
cg12243597	2.14E-09	1.89E-10	6.73	12.8558383	2.34E-01	62585877	62586000	NC_000012.11
cg04329125	2.15E-09	1.89E-10	6.72	12.854382	1.21E-01	105945022	105945145	NC_000014.8
cg16559361	2.15E-09	1.89E-10	6.72	12.8541355	1.12E-01	88543440	88543563	NC_000016.9
cg01450705	2.15E-09	1.89E-10	6.72	12.8540461	1.40E-01	175300027	175300150	NC_000005.9
cg18854392	2.15E-09	1.89E-10	6.72	12.8535837	1.49E-01	167191873	167191996	NC_000003.11
cg15946312	2.15E-09	1.89E-10	6.72	12.8535714	5.90E-02	180646880	180647003	NC_000005.9
cg17418784	2.15E-09	1.89E-10	6.72	12.8535136	8.30E-02	4004409	4004532	NC_000001.10
cg12848639	2.15E-09	1.90E-10	6.72	12.8523305	6.22E-02	1438375	1438498	NC_000005.9
cg14395791	2.15E-09	1.90E-10	6.72	12.8522469	3.81E-02	140166606	140166729	NC_000009.11
cg10319857	2.15E-09	1.90E-10	6.72	12.8515607	1.30E-01	69653345	69653468	NC_000016.9
cg05171693	2.15E-09	1.90E-10	6.72	12.8501687	6.92E-02	1117775	1117898	NC_000007.13
cg05208249	2.15E-09	1.90E-10	6.72	12.8497166	9.71E-02	81135497	81135620	NC_000015.9
cg25271672	2.16E-09	1.90E-10	6.72	12.8495518	1.03E-01	156462624	156462747	NC_000001.10
cg11698180	2.16E-09	1.90E-10	6.72	12.8483322	5.79E-02	46019376	46019499	NC_000021.8
cg03369269	2.16E-09	1.91E-10	6.72	12.8473151	1.65E-01	25745946	25746069	NC_000013.10
cg22244305	2.16E-09	1.91E-10	6.72	12.8470867	3.58E-02	33892662	33892785	NC_000019.9
cg23726408	2.16E-09	1.91E-10	6.72	12.8467113	1.19E-01	123498880	123499003	NC_000003.11
cg01727686	2.16E-09	1.91E-10	6.72	12.8462338	9.54E-02	63997306	63997429	NC_000011.9
cg05688651	2.16E-09	1.91E-10	6.72	12.8447867	1.85E-01	75612141	75612264	NC_000018.9
cg22584618	2.16E-09	1.91E-10	6.72	12.8447852	1.27E-01	35298212	35298335	NC_000007.13
cg07078225	2.17E-09	1.91E-10	6.72	12.844327	1.69E-01	52652377	52652500	NC_000012.11
cg27352992	2.17E-09	1.92E-10	6.72	12.841708	1.70E-01	41087092	41087215	NC_000012.11

cg03506028	2.18E-09	1.92E-10	6.72	12.8397763	1.32E-01	35494689	35494812	NC_000007.13
cg10206882	2.18E-09	1.92E-10	6.72	12.839515	9.85E-02	25411743	25411866	NC_000014.8
cg03479211	2.18E-09	1.92E-10	6.72	12.8394745	1.20E-01	8117508	8117631	NC_000010.10
cg14385245	2.18E-09	1.92E-10	6.72	12.8388867	1.28E-01	83953765	83953888	NC_000015.9
cg07900958	2.18E-09	1.92E-10	6.72	12.8384847	1.15E-01	49139512	49139635	NC_000022.10
cg04685747	2.18E-09	1.92E-10	6.72	12.8375348	9.19E-02	8458793	8458916	NC_000019.9
cg17862113	2.18E-09	1.93E-10	6.72	12.8371364	1.36E-01	17743282	17743405	NC_000011.9
cg21540621	2.18E-09	1.93E-10	6.72	12.8366024	1.69E-01	115531033	115531156	NC_000011.9
cg00183415	2.18E-09	1.93E-10	6.72	12.8363997	4.64E-02	71008388	71008511	NC_000010.10
cg13689003	2.18E-09	1.93E-10	6.72	12.8356285	2.11E-01	156406102	156406225	NC_000001.10
cg15123692	2.18E-09	1.93E-10	6.72	12.8354088	1.77E-01	23886357	23886480	NC_000002.11
cg12170868	2.19E-09	1.93E-10	6.72	12.8336116	4.69E-02	33275470	33275593	NC_000006.11
cg12857541	2.20E-09	1.94E-10	6.72	12.8292293	1.19E-01	24583375	24583498	NC_000004.11
cg19706446	2.20E-09	1.94E-10	6.72	12.8289351	1.00E-01	3440313	3440436	NC_000010.10
cg04492459	2.20E-09	1.94E-10	6.72	12.8286899	6.76E-02	4520501	4520624	NC_000017.10
cg20467114	2.20E-09	1.94E-10	6.72	12.8279106	4.22E-02	3365744	3365867	NC_000012.11
cg23082773	2.20E-09	1.95E-10	6.72	12.8260692	4.53E-02	77445972	77446095	NC_000011.9
cg26405376	2.20E-09	1.95E-10	6.72	12.8257226	1.43E-01	175961105	175961228	NC_000005.9
cg06982190	2.21E-09	1.95E-10	6.72	12.8253314	1.43E-01	27199815	27199938	NC_000007.13
cg25234732	2.21E-09	1.95E-10	6.72	12.824327	1.48E-01	56183885	56184008	NC_000007.13
cg24924958	2.21E-09	1.95E-10	6.72	12.8238395	1.63E-01	142409993	142410116	NC_000006.11
cg22115168	2.21E-09	1.95E-10	6.72	12.8226611	6.95E-02	114567943	114568066	NC_000006.11
cg11108269	2.22E-09	1.96E-10	6.72	12.8204959	1.90E-01	36989404	36989527	NC_000014.8
cg08201421	2.22E-09	1.96E-10	6.72	12.8203336	5.02E-02	44456151	44456274	NC_000020.10
cg11442052	2.22E-09	1.96E-10	6.72	12.819712	1.30E-01	35723321	35723444	NC_000003.11

cg23475625	2.22E-09	1.96E-10	6.72	12.8196472	1.73E-01	155259680	155259803	NC_000007.13
cg10111335	2.22E-09	1.96E-10	6.72	12.8177766	1.38E-01	105935915	105936038	NC_000006.11
cg19555331	2.22E-09	1.96E-10	6.72	12.8170958	1.81E-01	147561900	147562023	NC_000004.11
cg17361203	2.22E-09	1.97E-10	6.72	12.8168441	1.37E-01	117666889	117667012	NC_000011.9
cg00366531	2.23E-09	1.97E-10	6.72	12.8151039	9.75E-02	130970940	130971063	NC_000002.11
cg25957599	2.23E-09	1.97E-10	6.72	12.8150096	1.15E-01	166635593	166635716	NC_000006.11
cg07450037	2.23E-09	1.97E-10	6.72	12.8131086	1.75E-01	27135147	27135270	NC_000007.13
cg00073543	2.23E-09	1.97E-10	6.72	12.8126765	1.26E-01	38941882	38942005	NC_000001.10
cg00053086	2.23E-09	1.98E-10	6.72	12.8118446	3.36E-02	233119296	233119419	NC_000001.10
cg08569180	2.23E-09	1.98E-10	6.72	12.8117307	1.46E-01	2773427	2773550	NC_000002.11
cg04217082	2.23E-09	1.98E-10	6.72	12.8117188	1.37E-01	37889752	37889875	NC_000008.10
cg22204479	2.24E-09	1.98E-10	6.72	12.8109098	1.58E-01	21378227	21378350	NC_000020.10
cg18134312	2.24E-09	1.98E-10	6.72	12.8107676	1.68E-01	62996183	62996306	NC_000006.11
cg14909325	2.24E-09	1.98E-10	6.72	12.8084249	9.07E-02	1674770	1674893	NC_000011.9
cg19035186	2.25E-09	1.99E-10	6.72	12.8061916	8.29E-02	239948889	239949012	NC_000002.11
cg19178853	2.25E-09	1.99E-10	6.72	12.8060877	2.13E-01	174450408	174450531	NC_000004.11
cg03035213	2.25E-09	1.99E-10	6.72	12.8057135	2.51E-01	88497077	88497200	NC_000016.9
cg20695936	2.25E-09	1.99E-10	6.72	12.8047783	1.24E-01	25257624	25257747	NC_000001.10
cg17208467	2.25E-09	1.99E-10	6.72	12.8042956	7.28E-02	23025348	23025471	NC_000008.10
cg13644811	2.25E-09	1.99E-10	6.72	12.8037521	9.37E-02	90771714	90771837	NC_000015.9
cg01686933	2.25E-09	1.99E-10	6.72	12.8035874	8.81E-02	119596104	119596227	NC_000011.9
cg13101887	2.26E-09	2.00E-10	6.71	12.8007531	9.21E-02	29890938	29891061	NC_000010.10
cg23308184	2.26E-09	2.00E-10	6.71	12.80074	5.97E-02	132977221	132977344	NC_000010.10
cg18470427	2.26E-09	2.00E-10	6.71	12.8004787	1.41E-01	33842301	33842424	NC_000017.10
cg19003884	2.26E-09	2.00E-10	6.71	12.7998387	1.38E-01	100060945	100061068	NC_000006.11

cg05552184	2.26E-09	2.01E-10	6.71	12.7969934	8.87E-02	71548685	71548808	NC_000017.10
cg14670629	2.26E-09	2.01E-10	6.71	12.7969104	7.28E-02	29769556	29769679	NC_000010.10
cg01464093	2.27E-09	2.01E-10	6.71	12.7949973	9.79E-02	125242501	125242624	NC_000003.11
cg00054771	2.27E-09	2.01E-10	6.71	12.7942334	1.60E-01	175202397	175202520	NC_000002.11
cg23295454	2.27E-09	2.01E-10	6.71	12.7930366	1.54E-01	100437951	100438074	NC_000014.8
cg10435816	2.27E-09	2.02E-10	6.71	12.7924455	1.75E-01	140797280	140797403	NC_000005.9
cg26864230	2.28E-09	2.02E-10	6.71	12.7921054	1.61E-01	47911508	47911631	NC_000001.10
cg13421062	2.28E-09	2.02E-10	6.71	12.7918343	1.72E-01	35301069	35301192	NC_000007.13
cg22315453	2.28E-09	2.02E-10	6.71	12.7916853	1.02E-01	10402330	10402453	NC_000006.11
cg17969084	2.28E-09	2.02E-10	6.71	12.7912015	1.94E-01	27191097	27191220	NC_000007.13
cg04523661	2.28E-09	2.02E-10	6.71	12.7906698	9.21E-02	1302808	1302931	NC_000019.9
cg21865844	2.28E-09	2.02E-10	6.71	12.7892663	6.89E-02	69661197	69661320	NC_000011.9
cg02881274	2.28E-09	2.02E-10	6.71	12.7888764	1.36E-01	49935948	49936071	NC_000019.9
cg05335975	2.28E-09	2.02E-10	6.71	12.7882195	9.72E-02	24045702	24045825	NC_000014.8
cg05396425	2.29E-09	2.03E-10	6.71	12.7857219	6.47E-02	4587038	4587161	NC_000016.9
cg05774290	2.29E-09	2.03E-10	6.71	12.7848909	8.67E-02	31811203	31811326	NC_000012.11
cg07488338	2.29E-09	2.03E-10	6.71	12.7831715	1.05E-01	89663024	89663147	NC_000005.9
cg04203587	2.30E-09	2.04E-10	6.71	12.7825036	1.23E-01	75895612	75895735	NC_000004.11
cg16122424	2.30E-09	2.04E-10	6.71	12.7824789	1.05E-01	41258337	41258460	NC_000015.9
cg06070229	2.30E-09	2.04E-10	6.71	12.7823856	3.76E-02	133348826	133348949	NC_000007.13
cg23405172	2.30E-09	2.04E-10	6.71	12.7814373	4.88E-02	49165394	49165517	NC_000019.9
cg12172441	2.30E-09	2.04E-10	6.71	12.7808278	1.80E-01	28176163	28176286	NC_000006.11
cg06943005	2.30E-09	2.04E-10	6.71	12.7797865	1.92E-02	144897576	144897699	NC_000008.10
cg01746118	2.30E-09	2.04E-10	6.71	12.7794128	4.50E-02	169569064	169569187	NC_000006.11
cg18215217	2.31E-09	2.04E-10	6.71	12.7784668	1.51E-01	84456410	84456533	NC_000013.10

cg10625533	2.31E-09	2.04E-10	6.71	12.7782601	1.53E-01	233498682	233498805	NC_000002.11
cg08199013	2.31E-09	2.05E-10	6.71	12.7778128	1.05E-01	330010	330133	NC_000016.9
cg05452899	2.31E-09	2.05E-10	6.71	12.7767585	1.96E-01	2161133	2161256	NC_000011.9
cg24926465	2.31E-09	2.05E-10	6.71	12.7762372	1.40E-01	156007787	156007910	NC_000003.11
cg19404354	2.31E-09	2.05E-10	6.71	12.7753278	9.77E-02	202093479	202093602	NC_000001.10
cg22740492	2.31E-09	2.05E-10	6.71	12.7751934	1.38E-01	31820658	31820781	NC_000011.9
cg01149940	2.32E-09	2.06E-10	6.71	12.7699636	7.09E-02	50632967	50633090	NC_000022.10
cg13392078	2.32E-09	2.06E-10	6.71	12.7696927	1.74E-01	36036514	36036637	NC_000009.11
cg04932544	2.33E-09	2.06E-10	6.71	12.769051	1.53E-01	210111403	210111526	NC_000001.10
cg23432345	2.33E-09	2.07E-10	6.71	12.7667942	1.76E-01	27196153	27196276	NC_000007.13
cg00850481	2.33E-09	2.07E-10	6.71	12.7657602	7.61E-02	228891306	228891429	NC_000001.10
cg06856528	2.33E-09	2.07E-10	6.71	12.7657412	1.69E-01	193059405	193059528	NC_000002.11
cg08959182	2.33E-09	2.07E-10	6.71	12.7652869	1.36E-01	87661076	87661199	NC_000005.9
cg21566642	2.34E-09	2.08E-10	6.71	12.7636148	1.27E-01	233284661	233284784	NC_000002.11
cg22280475	2.34E-09	2.08E-10	6.71	12.7620112	1.43E-01	25897534	25897657	NC_000008.10
cg23330204	2.34E-09	2.08E-10	6.71	12.7608918	9.57E-02	91321677	91321800	NC_000006.11
cg09876519	2.34E-09	2.08E-10	6.71	12.7605142	1.49E-01	50675610	50675733	NC_000006.11
cg21242918	2.35E-09	2.08E-10	6.71	12.7596572	2.12E-01	118897194	118897317	NC_000010.10
cg16648632	2.35E-09	2.08E-10	6.71	12.7590602	1.42E-01	6003584	6003707	NC_000006.11
cg21582253	2.36E-09	2.09E-10	6.71	12.7546672	1.42E-01	16181039	16181162	NC_000005.9
cg21705897	2.36E-09	2.10E-10	6.71	12.7536926	2.13E-01	55444061	55444184	NC_000006.11
cg13534450	2.36E-09	2.10E-10	6.71	12.7522297	2.07E-01	75897297	75897420	NC_000008.10
cg02316596	2.37E-09	2.10E-10	6.71	12.750735	1.56E-01	120434979	120435102	NC_000011.9
cg03576469	2.37E-09	2.10E-10	6.71	12.7505564	2.02E-01	46917061	46917184	NC_000019.9
cg06307913	2.37E-09	2.10E-10	6.71	12.7503531	1.62E-01	81118794	81118917	NC_000004.11

cg27318000	2.37E-09	2.10E-10	6.71	12.7501379	8.38E-02	3845667	3845790	NC_000018.9
cg26555777	2.37E-09	2.11E-10	6.71	12.7491225	1.01E-01	38885050	38885173	NC_000019.9
cg13860360	2.37E-09	2.11E-10	6.71	12.7487432	1.29E-01	33846010	33846133	NC_000013.10
cg07658503	2.37E-09	2.11E-10	6.71	12.748024	1.97E-01	30722177	30722300	NC_000007.13
cg09965733	2.38E-09	2.11E-10	6.71	12.7462576	1.92E-01	42079131	42079254	NC_000014.8
cg19721730	2.38E-09	2.11E-10	6.7	12.7454442	6.01E-02	50173685	50173808	NC_000019.9
cg22458832	2.38E-09	2.11E-10	6.7	12.7452815	1.16E-01	43506497	43506620	NC_000017.10
cg08465346	2.38E-09	2.11E-10	6.7	12.7450803	1.39E-01	54441458	54441581	NC_000012.11
cg21266547	2.38E-09	2.12E-10	6.7	12.7448127	5.04E-02	61654618	61654741	NC_000018.9
cg16376000	2.38E-09	2.12E-10	6.7	12.7442766	2.50E-01	192127330	192127453	NC_000003.11
cg01478234	2.38E-09	2.12E-10	6.7	12.7438909	1.03E-01	107975299	107975422	NC_000012.11
cg18720533	2.38E-09	2.12E-10	6.7	12.7431675	1.05E-01	134833372	134833495	NC_000010.10
cg22620111	2.38E-09	2.12E-10	6.7	12.7430651	1.04E-01	14849245	14849368	NC_000003.11
cg17623854	2.38E-09	2.12E-10	6.7	12.7428416	6.28E-02	134791660	134791783	NC_000010.10
cg02899219	2.38E-09	2.12E-10	6.7	12.7428233	1.06E-01	51448944	51449067	NC_000014.8
cg06457577	2.39E-09	2.12E-10	6.7	12.7412305	4.27E-02	74631030	74631153	NC_000015.9
cg06473363	2.39E-09	2.12E-10	6.7	12.7410674	1.26E-01	31631797	31631920	NC_000006.11
cg24625634	2.39E-09	2.13E-10	6.7	12.7400459	1.52E-02	117484948	117485071	NC_000012.11
cg03679715	2.39E-09	2.13E-10	6.7	12.7391651	4.75E-02	176295869	176295992	NC_000005.9
cg00500705	2.39E-09	2.13E-10	6.7	12.7390781	1.38E-01	114029659	114029782	NC_000012.11
cg13318787	2.39E-09	2.13E-10	6.7	12.738776	1.35E-01	50262177	50262300	NC_000005.9
cg24216701	2.40E-09	2.13E-10	6.7	12.7378376	7.41E-02	149545928	149546051	NC_000005.9
cg12983839	2.40E-09	2.13E-10	6.7	12.7363629	6.61E-02	2334615	2334738	NC_000019.9
cg03293883	2.40E-09	2.13E-10	6.7	12.7359782	5.63E-02	86124742	86124865	NC_000006.11
cg21213617	2.40E-09	2.14E-10	6.7	12.7353357	9.02E-02	124989241	124989364	NC_000009.11

cg21255942	2.40E-09	2.14E-10	6.7	12.733978	2.20E-01	39954186	39954309	NC_000022.10
cg02300114	2.41E-09	2.14E-10	6.7	12.733124	1.00E-01	130604047	130604170	NC_000012.11
cg05233155	2.41E-09	2.14E-10	6.7	12.733081	5.33E-02	10408528	10408651	NC_000011.9
cg07979271	2.41E-09	2.14E-10	6.7	12.7319217	1.07E-01	50921353	50921476	NC_000019.9
cg06984025	2.41E-09	2.14E-10	6.7	12.7312308	1.45E-01	971757	971880	NC_000009.11
cg16080876	2.41E-09	2.15E-10	6.7	12.7303794	1.48E-01	24813866	24813989	NC_000008.10
cg27105390	2.41E-09	2.15E-10	6.7	12.7302756	1.41E-01	23253316	23253439	NC_000019.9
cg01259029	2.41E-09	2.15E-10	6.7	12.7301493	1.50E-01	10564690	10564813	NC_000005.9
cg24776343	2.41E-09	2.15E-10	6.7	12.7301087	1.08E-01	24189786	24189909	NC_000013.10
cg03943081	2.42E-09	2.15E-10	6.7	12.7279898	1.37E-01	133110646	133110769	NC_000010.10
cg00942219	2.42E-09	2.15E-10	6.7	12.7276676	1.23E-01	85203635	85203758	NC_000016.9
cg19876649	2.42E-09	2.16E-10	6.7	12.7248147	1.18E-01	3220273	3220396	NC_000018.9
cg25593631	2.43E-09	2.16E-10	6.7	12.7239429	1.07E-01	9687880	9688003	NC_000001.10
cg05653707	2.43E-09	2.16E-10	6.7	12.7238712	1.90E-01	119304938	119305061	NC_000010.10
cg15835058	2.43E-09	2.16E-10	6.7	12.7227359	1.28E-01	39022380	39022503	NC_000006.11
cg14861047	2.43E-09	2.17E-10	6.7	12.7217255	6.30E-02	48657326	48657449	NC_000003.11
cg16977035	2.43E-09	2.17E-10	6.7	12.7213777	8.25E-02	44330474	44330597	NC_000011.9
cg14443467	2.44E-09	2.17E-10	6.7	12.7198366	1.32E-01	140604406	140604529	NC_000005.9
cg02285263	2.44E-09	2.17E-10	6.7	12.7194809	1.35E-01	152129749	152129872	NC_000006.11
cg09540565	2.44E-09	2.17E-10	6.7	12.7184434	4.01E-02	88212683	88212806	NC_000016.9
cg25966908	2.44E-09	2.17E-10	6.7	12.7182472	1.22E-01	55516751	55516874	NC_000007.13
cg11659501	2.44E-09	2.17E-10	6.7	12.7175189	1.89E-01	81471678	81471801	NC_000012.11
cg00306395	2.44E-09	2.18E-10	6.7	12.7169394	4.71E-02	45832603	45832726	NC_000011.9
cg26814075	2.44E-09	2.18E-10	6.7	12.7167291	9.75E-02	127881298	127881421	NC_000007.13
cg25911551	2.45E-09	2.18E-10	6.7	12.7147036	1.34E-01	144266241	144266364	NC_000004.11

cg19435264	2.45E-09	2.18E-10	6.7	12.7139676	1.97E-01	149569537	149569660	NC_000005.9
cg00554173	2.45E-09	2.18E-10	6.7	12.7136074	8.68E-02	3149777	3149900	NC_000020.10
cg01454215	2.45E-09	2.19E-10	6.7	12.7129204	1.39E-01	27530363	27530486	NC_000002.11
cg05372242	2.46E-09	2.19E-10	6.7	12.7112573	1.50E-01	36983542	36983665	NC_000014.8
cg26115216	2.46E-09	2.19E-10	6.7	12.7098894	1.13E-01	138739916	138740039	NC_000003.11
cg07763376	2.46E-09	2.19E-10	6.7	12.7086297	1.25E-01	178016195	178016318	NC_000005.9
cg18304339	2.46E-09	2.20E-10	6.7	12.7080662	4.66E-02	52213734	52213857	NC_000014.8
cg14222245	2.46E-09	2.20E-10	6.7	12.7080489	1.11E-01	58861502	58861625	NC_000019.9
cg05715492	2.47E-09	2.20E-10	6.7	12.7069326	1.48E-01	98991138	98991261	NC_000007.13
cg07498624	2.47E-09	2.20E-10	6.7	12.7057642	1.58E-01	204831	204954	NC_000016.9
cg24975244	2.47E-09	2.20E-10	6.7	12.7055953	1.02E-01	106091410	106091533	NC_000014.8
cg08559648	2.47E-09	2.20E-10	6.7	12.7043406	5.31E-02	45889692	45889815	NC_000013.10
cg12757011	2.47E-09	2.21E-10	6.7	12.7030286	1.08E-01	162281111	162281234	NC_000002.11
cg23059310	2.48E-09	2.21E-10	6.7	12.7017705	4.94E-02	32807327	32807450	NC_000006.11
cg11310950	2.48E-09	2.21E-10	6.7	12.7001747	1.86E-01	71217407	71217530	NC_000007.13
cg08567000	2.48E-09	2.22E-10	6.7	12.6995924	3.91E-02	31084107	31084230	NC_000006.11
cg19442470	2.48E-09	2.22E-10	6.7	12.6994712	1.17E-01	27470225	27470348	NC_000008.10
cg27021722	2.48E-09	2.22E-10	6.7	12.6987917	7.53E-02	22999077	22999200	NC_000004.11
cg06876311	2.49E-09	2.22E-10	6.7	12.6959942	9.71E-02	38479181	38479304	NC_000022.10
cg20419735	2.50E-09	2.23E-10	6.7	12.6935298	1.44E-01	46824292	46824415	NC_000017.10
cg01911708	2.50E-09	2.23E-10	6.7	12.6919345	1.73E-01	61886082	61886205	NC_000020.10
cg04689048	2.50E-09	2.23E-10	6.69	12.6913298	1.18E-01	46999289	46999412	NC_000019.9
cg06541501	2.50E-09	2.23E-10	6.69	12.6911591	9.41E-02	25903852	25903975	NC_000008.10
cg06564388	2.50E-09	2.23E-10	6.69	12.6910911	1.02E-01	153855257	153855380	NC_000005.9
cg16495265	2.51E-09	2.24E-10	6.69	12.6894164	2.04E-01	46671010	46671133	NC_000017.10

cg22399111	2.51E-09	2.24E-10	6.69	12.6893142	9.86E-02	142295325	142295448	NC_000008.10
cg09953520	2.51E-09	2.24E-10	6.69	12.6868573	9.57E-02	25812278	25812401	NC_000008.10
cg26202195	2.51E-09	2.24E-10	6.69	12.686521	1.01E-01	105379368	105379491	NC_000008.10
cg05970033	2.52E-09	2.25E-10	6.69	12.6855232	6.73E-02	78441950	78442073	NC_000005.9
cg13974394	2.52E-09	2.25E-10	6.69	12.6842548	1.16E-01	1882188	1882311	NC_000005.9
cg03849279	2.52E-09	2.25E-10	6.69	12.6840218	7.82E-02	67751821	67751944	NC_000016.9
cg04469059	2.52E-09	2.25E-10	6.69	12.6836641	1.83E-01	134872096	134872219	NC_000005.9
cg25756586	2.52E-09	2.25E-10	6.69	12.6826518	5.37E-02	78172613	78172736	NC_000017.10
cg17069590	2.53E-09	2.26E-10	6.69	12.6795706	1.10E-01	112647810	112647933	NC_000003.11
cg22942897	2.53E-09	2.26E-10	6.69	12.6795613	1.27E-01	3680345	3680468	NC_000001.10
cg21922574	2.53E-09	2.26E-10	6.69	12.678651	2.00E-01	70535925	70536048	NC_000018.9
cg08480826	2.54E-09	2.27E-10	6.69	12.6770133	1.83E-01	87981253	87981376	NC_000005.9
cg26981593	2.54E-09	2.27E-10	6.69	12.6752239	4.13E-02	33232239	33232362	NC_000006.11
cg09937190	2.54E-09	2.27E-10	6.69	12.6744848	1.14E-01	15177509	15177632	NC_000020.10
cg08241373	2.54E-09	2.27E-10	6.69	12.6738232	3.57E-02	33139529	33139652	NC_000006.11
cg25537993	2.55E-09	2.27E-10	6.69	12.6736863	1.82E-01	58545182	58545305	NC_000019.9
cg16657397	2.55E-09	2.28E-10	6.69	12.6723071	1.93E-01	186079367	186079490	NC_000003.11
cg13482432	2.55E-09	2.28E-10	6.69	12.6722906	1.99E-01	79633350	79633473	NC_000009.11
cg08367801	2.55E-09	2.28E-10	6.69	12.6714439	1.89E-01	98111529	98111652	NC_000005.9
cg10281977	2.55E-09	2.28E-10	6.69	12.6711063	1.98E-01	105070864	105070987	NC_000014.8
cg06818710	2.55E-09	2.28E-10	6.69	12.6710054	2.23E-01	28411271	28411394	NC_000006.11
cg06707286	2.55E-09	2.28E-10	6.69	12.6709526	6.52E-02	102010195	102010318	NC_000015.9
cg23718462	2.55E-09	2.28E-10	6.69	12.6707639	1.24E-01	25621118	25621241	NC_000013.10
cg07594636	2.55E-09	2.28E-10	6.69	12.6699503	2.19E-01	68547088	68547211	NC_000002.11
cg19700658	2.56E-09	2.29E-10	6.69	12.66776	4.64E-02	73719872	73719995	NC_000011.9

cg04444661	2.56E-09	2.29E-10	6.69	12.6667678	9.75E-02	38552952	38553075	NC_000005.9
cg16255914	2.57E-09	2.30E-10	6.69	12.6644599	1.33E-01	53832438	53832561	NC_000019.9
cg19313043	2.57E-09	2.30E-10	6.69	12.6644287	1.95E-01	115880207	115880330	NC_000001.10
cg06324785	2.57E-09	2.30E-10	6.69	12.6642366	1.14E-01	25960219	25960342	NC_000022.10
cg20894270	2.57E-09	2.30E-10	6.69	12.6631999	6.07E-02	21200993	21201116	NC_000018.9
cg07791834	2.57E-09	2.30E-10	6.69	12.6622359	6.95E-02	48800382	48800505	NC_000019.9
cg01098939	2.58E-09	2.30E-10	6.69	12.6610481	4.80E-02	2742274	2742397	NC_000007.13
cg17610169	2.58E-09	2.30E-10	6.69	12.6610361	2.64E-02	10750973	10751096	NC_000011.9
cg14524956	2.58E-09	2.31E-10	6.69	12.6589877	1.01E-01	215804508	215804631	NC_000001.10
cg27530370	2.58E-09	2.31E-10	6.69	12.6587386	1.19E-01	154589288	154589411	NC_000001.10
cg08956010	2.58E-09	2.31E-10	6.69	12.6580794	8.05E-02	4479889	4480012	NC_000012.11
cg25770267	2.59E-09	2.31E-10	6.69	12.6567874	8.46E-02	7596685	7596808	NC_000001.10
cg05860313	2.59E-09	2.32E-10	6.69	12.655325	2.29E-01	35395663	35395786	NC_000001.10
cg07858908	2.59E-09	2.32E-10	6.69	12.6549719	1.64E-01	76733537	76733660	NC_000018.9
cg21651328	2.60E-09	2.32E-10	6.69	12.6531912	1.82E-01	21657756	21657879	NC_000019.9
cg11328303	2.60E-09	2.32E-10	6.69	12.6526179	1.66E-01	26505440	26505563	NC_000010.10
cg06954658	2.60E-09	2.32E-10	6.69	12.6525553	1.73E-01	76740093	76740216	NC_000018.9
cg25095187	2.60E-09	2.33E-10	6.69	12.6517282	1.06E-01	29335792	29335915	NC_000002.11
cg09714379	2.60E-09	2.33E-10	6.69	12.6504427	1.51E-01	135044040	135044163	NC_000010.10
cg19763461	2.60E-09	2.33E-10	6.69	12.6500503	1.87E-01	63284557	63284680	NC_000002.11
cg00303982	2.60E-09	2.33E-10	6.69	12.6499977	2.21E-01	145556952	145557075	NC_000008.10
cg11005250	2.61E-09	2.33E-10	6.69	12.6486079	1.70E-01	108297742	108297865	NC_000012.11
cg19402371	2.61E-09	2.34E-10	6.69	12.6470795	9.89E-02	55889013	55889136	NC_000019.9
cg10879397	2.61E-09	2.34E-10	6.69	12.6462402	7.09E-02	148555266	148555389	NC_000005.9
cg08612384	2.61E-09	2.34E-10	6.69	12.646111	5.83E-02	27228511	27228634	NC_000017.10

cg02736395	2.62E-09	2.34E-10	6.69	12.6451192	1.01E-01	227748424	227748547	NC_000001.10
cg02565715	2.62E-09	2.35E-10	6.69	12.6423742	2.07E-01	75525001	75525124	NC_000017.10
cg20647257	2.62E-09	2.35E-10	6.69	12.6417739	1.22E-01	601713	601836	NC_000007.13
cg21970789	2.62E-09	2.35E-10	6.69	12.6417737	9.60E-02	134826388	134826511	NC_000005.9
cg09804380	2.62E-09	2.35E-10	6.69	12.6414424	2.07E-01	13910700	13910823	NC_000001.10
cg24452128	2.63E-09	2.35E-10	6.69	12.6410041	1.92E-01	10390919	10391042	NC_000006.11
cg18119735	2.63E-09	2.35E-10	6.69	12.6396995	9.89E-02	236850308	236850431	NC_000001.10
cg20568390	2.63E-09	2.36E-10	6.69	12.6391979	6.69E-02	54723340	54723463	NC_000001.10
cg02952984	2.63E-09	2.36E-10	6.69	12.6375347	1.80E-01	58724041	58724164	NC_000015.9
cg02593932	2.64E-09	2.36E-10	6.68	12.6363051	1.56E-01	154728308	154728431	NC_000002.11
cg10323051	2.64E-09	2.36E-10	6.68	12.6361713	5.18E-02	92570771	92570894	NC_000015.9
cg09697644	2.64E-09	2.36E-10	6.68	12.6357659	7.11E-02	34375039	34375162	NC_000016.9
cg21971807	2.64E-09	2.37E-10	6.68	12.6353116	7.80E-02	57471654	57471777	NC_000020.10
cg05332960	2.64E-09	2.37E-10	6.68	12.6350698	1.58E-01	31020418	31020541	NC_000018.9
cg05791946	2.64E-09	2.37E-10	6.68	12.6350127	1.08E-01	3160851	3160974	NC_000001.10
cg17368114	2.65E-09	2.37E-10	6.68	12.6327247	7.28E-02	138268312	138268435	NC_000005.9
cg14002226	2.65E-09	2.37E-10	6.68	12.6324556	1.23E-01	138676624	138676747	NC_000009.11
cg03706436	2.65E-09	2.37E-10	6.68	12.6321619	1.86E-01	30717225	30717348	NC_000019.9
cg25229306	2.65E-09	2.37E-10	6.68	12.6316386	1.00E-01	57426374	57426497	NC_000020.10
cg03007524	2.65E-09	2.38E-10	6.68	12.6307289	8.97E-02	50620975	50621098	NC_000022.10
cg19851909	2.66E-09	2.38E-10	6.68	12.627384	1.81E-01	94134827	94134950	NC_000011.9
cg21494680	2.67E-09	2.39E-10	6.68	12.625527	1.09E-01	375849	375972	NC_000019.9
cg02940165	2.67E-09	2.39E-10	6.68	12.6242594	1.58E-01	57149563	57149686	NC_000019.9
cg14586939	2.67E-09	2.40E-10	6.68	12.6223268	1.71E-01	156130206	156130329	NC_000004.11
cg04166913	2.68E-09	2.40E-10	6.68	12.6208929	1.18E-01	32945336	32945459	NC_000006.11

cg17975177	2.68E-09	2.40E-10	6.68	12.6199295	5.46E-02	53816548	53816671	NC_000001.10
cg00059737	2.68E-09	2.40E-10	6.68	12.6193105	1.23E-01	12400434	12400557	NC_000001.10
cg01973394	2.68E-09	2.41E-10	6.68	12.6180726	9.62E-02	82335947	82336070	NC_000015.9
cg04115740	2.69E-09	2.41E-10	6.68	12.6160214	1.45E-01	6690948	6691071	NC_000017.10
cg10934129	2.69E-09	2.42E-10	6.68	12.6148314	1.67E-01	172655841	172655964	NC_000005.9
cg26660800	2.70E-09	2.42E-10	6.68	12.6133251	1.24E-01	59480926	59481049	NC_000017.10
cg15100100	2.70E-09	2.42E-10	6.68	12.6129444	2.17E-01	77332993	77333116	NC_000001.10
cg01938476	2.70E-09	2.42E-10	6.68	12.6116965	1.19E-01	7076853	7076976	NC_000012.11
cg22090592	2.71E-09	2.43E-10	6.68	12.6098404	8.16E-02	105174642	105174765	NC_000014.8
cg11804932	2.71E-09	2.43E-10	6.68	12.60939	1.19E-01	106402507	106402630	NC_000010.10
cg21582956	2.71E-09	2.43E-10	6.68	12.6081712	6.69E-02	57422435	57422558	NC_000012.11
cg24669528	2.71E-09	2.43E-10	6.68	12.6081386	1.83E-01	37248838	37248961	NC_000013.10
cg13582542	2.71E-09	2.44E-10	6.68	12.6066885	7.49E-02	132795405	132795528	NC_000002.11
cg01410472	2.72E-09	2.44E-10	6.68	12.6060856	1.53E-01	75896975	75897098	NC_000008.10
cg24006667	2.72E-09	2.44E-10	6.68	12.6055007	1.18E-01	86758571	86758694	NC_000007.13
cg27627493	2.72E-09	2.44E-10	6.68	12.6037844	1.46E-01	63767360	63767483	NC_000015.9
cg19095981	2.72E-09	2.44E-10	6.68	12.6036385	1.60E-01	53516701	53516824	NC_000005.9
cg21507528	2.72E-09	2.44E-10	6.68	12.6028089	4.07E-02	18747315	18747438	NC_000011.9
cg13436968	2.73E-09	2.45E-10	6.68	12.5993819	1.31E-01	54622973	54623096	NC_000007.13
cg04091712	2.74E-09	2.46E-10	6.68	12.5975701	6.44E-02	4563767	4563890	NC_000016.9
cg11185802	2.74E-09	2.46E-10	6.68	12.596955	5.84E-02	9494396	9494519	NC_000004.11
cg08718490	2.74E-09	2.46E-10	6.68	12.5968617	1.64E-01	52943099	52943222	NC_000004.11
cg02520639	2.74E-09	2.46E-10	6.68	12.5954149	1.50E-01	82021210	82021333	NC_000008.10
cg23601515	2.74E-09	2.46E-10	6.68	12.5951825	7.42E-02	74017805	74017928	NC_000017.10
cg01514341	2.74E-09	2.46E-10	6.68	12.5951638	8.45E-02	29142291	29142414	NC_000016.9

cg24495052	2.74E-09	2.46E-10	6.68	12.5950256	6.26E-02	8189468	8189591	NC_000017.10
cg04788769	2.75E-09	2.47E-10	6.68	12.5934127	7.61E-02	102800604	102800727	NC_000002.11
cg14611174	2.75E-09	2.47E-10	6.68	12.5923639	1.68E-01	60975837	60975960	NC_000014.8
cg02919615	2.75E-09	2.47E-10	6.68	12.5920102	1.21E-01	1295656	1295779	NC_000019.9
cg00870778	2.76E-09	2.48E-10	6.68	12.5900731	1.36E-01	97598492	97598615	NC_000007.13
cg09226986	2.76E-09	2.48E-10	6.68	12.5891682	1.05E-01	160852328	160852451	NC_000006.11
cg19760917	2.76E-09	2.48E-10	6.68	12.5888022	6.47E-02	2411643	2411766	NC_000001.10
cg23264443	2.76E-09	2.48E-10	6.68	12.5883109	2.96E-02	4854690	4854813	NC_000019.9
cg16139785	2.76E-09	2.48E-10	6.68	12.588195	1.03E-01	103088924	103089047	NC_000014.8
cg10311806	2.76E-09	2.48E-10	6.68	12.5881705	2.49E-01	7708670	7708793	NC_000010.10
cg15391359	2.76E-09	2.48E-10	6.68	12.5874533	1.78E-01	14995536	14995659	NC_000011.9
cg14196143	2.77E-09	2.49E-10	6.68	12.5865266	2.78E-02	13711924	13712047	NC_000006.11
cg23243982	2.77E-09	2.49E-10	6.68	12.5856311	1.02E-01	132974568	132974691	NC_000012.11
cg08811259	2.77E-09	2.49E-10	6.68	12.5832874	1.20E-01	56878665	56878788	NC_000006.11
cg10800833	2.78E-09	2.50E-10	6.67	12.5815595	2.05E-01	57521657	57521780	NC_000004.11
cg13386839	2.78E-09	2.50E-10	6.67	12.5814383	3.34E-02	12250343	12250466	NC_000001.10
cg03082580	2.78E-09	2.50E-10	6.67	12.5810445	1.01E-01	89911298	89911421	NC_000015.9
cg21084260	2.78E-09	2.50E-10	6.67	12.5796355	6.44E-02	195311868	195311991	NC_000003.11
cg15504461	2.79E-09	2.51E-10	6.67	12.5786758	1.31E-01	147140880	147141003	NC_000003.11
cg23153481	2.79E-09	2.51E-10	6.67	12.5772777	1.55E-01	112547871	112547994	NC_000013.10
cg16594581	2.79E-09	2.51E-10	6.67	12.5761345	6.96E-02	140531865	140531988	NC_000005.9
cg19950455	2.79E-09	2.51E-10	6.67	12.5759005	2.03E-01	44495595	44495718	NC_000021.8
cg27383651	2.79E-09	2.51E-10	6.67	12.5758391	1.10E-01	28888809	28888932	NC_000006.11
cg21402417	2.80E-09	2.51E-10	6.67	12.5754914	4.90E-02	234926093	234926216	NC_000002.11
cg07803284	2.80E-09	2.51E-10	6.67	12.575396	7.86E-02	185812471	185812594	NC_000003.11

cg19828791	2.80E-09	2.52E-10	6.67	12.5738267	1.07E-01	100823400	100823523	NC_000007.13
cg00469814	2.80E-09	2.52E-10	6.67	12.5738243	1.61E-01	188953677	188953800	NC_000004.11
cg00924685	2.80E-09	2.52E-10	6.67	12.5732511	7.98E-02	17475656	17475779	NC_000001.10
cg20027133	2.80E-09	2.52E-10	6.67	12.5731372	1.65E-01	35547514	35547637	NC_000011.9
cg26781392	2.80E-09	2.52E-10	6.67	12.5723724	8.68E-02	126984891	126985014	NC_000006.11
cg22300320	2.80E-09	2.52E-10	6.67	12.571998	2.80E-02	79027117	79027240	NC_000017.10
cg25915307	2.81E-09	2.52E-10	6.67	12.5713929	2.06E-01	50064736	50064859	NC_000022.10
cg09415639	2.81E-09	2.53E-10	6.67	12.5707738	1.30E-01	72987836	72987959	NC_000008.10
cg10511467	2.81E-09	2.53E-10	6.67	12.5707209	4.47E-02	81529963	81530086	NC_000016.9
cg15414924	2.81E-09	2.53E-10	6.67	12.5684129	1.08E-01	36291501	36291624	NC_000014.8
cg12104689	2.82E-09	2.53E-10	6.67	12.5681076	3.86E-02	58600131	58600254	NC_000017.10
cg13526007	2.82E-09	2.53E-10	6.67	12.5679368	1.79E-01	42076710	42076833	NC_000014.8
cg15746583	2.82E-09	2.53E-10	6.67	12.5679226	1.18E-01	87088834	87088957	NC_000002.11
cg15199678	2.82E-09	2.54E-10	6.67	12.5671641	1.55E-01	96642510	96642633	NC_000007.13
cg00414354	2.82E-09	2.54E-10	6.67	12.5662371	4.92E-02	174788002	174788125	NC_000002.11
cg25544164	2.82E-09	2.54E-10	6.67	12.5655019	1.31E-01	5824159	5824282	NC_000019.9
cg11192416	2.82E-09	2.54E-10	6.67	12.5648271	1.48E-01	231296968	231297091	NC_000001.10
cg09736968	2.83E-09	2.54E-10	6.67	12.5641808	1.68E-01	233351609	233351732	NC_000002.11
cg11672037	2.83E-09	2.54E-10	6.67	12.5641128	2.30E-01	48010808	48010931	NC_000015.9
cg04662609	2.83E-09	2.54E-10	6.67	12.5637822	8.27E-02	180646976	180647099	NC_000005.9
cg06163425	2.84E-09	2.56E-10	6.67	12.5584304	1.67E-01	131761150	131761273	NC_000010.10
cg11739633	2.84E-09	2.56E-10	6.67	12.5575894	1.24E-01	156459986	156460109	NC_000001.10
cg08678851	2.84E-09	2.56E-10	6.67	12.5574986	7.21E-02	91710951	91711074	NC_000005.9
cg00254133	2.85E-09	2.56E-10	6.67	12.5563053	1.87E-01	61340542	61340665	NC_000020.10
cg17139666	2.85E-09	2.56E-10	6.67	12.5562569	1.12E-01	174421440	174421563	NC_000004.11

cg13846998	2.85E-09	2.57E-10	6.67	12.5547238	1.44E-01	45406295	45406418	NC_000010.10
cg23673761	2.85E-09	2.57E-10	6.67	12.5546452	1.39E-01	196865686	196865809	NC_000001.10
cg17299899	2.85E-09	2.57E-10	6.67	12.5535718	1.65E-01	76738478	76738601	NC_000018.9
cg10762626	2.85E-09	2.57E-10	6.67	12.553422	1.63E-01	28344715	28344838	NC_000015.9
cg06507579	2.86E-09	2.57E-10	6.67	12.5526561	1.63E-01	1282819	1282942	NC_000007.13
cg20312179	2.86E-09	2.57E-10	6.67	12.5520437	1.03E-01	51981505	51981628	NC_000001.10
cg02639927	2.86E-09	2.58E-10	6.67	12.5518744	1.42E-01	77334748	77334871	NC_000001.10
cg18476690	2.86E-09	2.58E-10	6.67	12.550723	4.26E-02	61125255	61125378	NC_000011.9
cg09314708	2.86E-09	2.58E-10	6.67	12.5498678	1.36E-01	132812991	132813114	NC_000011.9
cg05030481	2.87E-09	2.58E-10	6.67	12.5489462	1.83E-01	1445593	1445716	NC_000005.9
cg04355077	2.87E-09	2.59E-10	6.67	12.5469539	1.84E-01	50059946	50060069	NC_000019.9
cg05620923	2.87E-09	2.59E-10	6.67	12.5462589	7.71E-02	1466647	1466770	NC_000019.9
cg18646979	2.88E-09	2.59E-10	6.67	12.5459666	2.60E-02	72012470	72012593	NC_000010.10
cg20586900	2.88E-09	2.59E-10	6.67	12.5450665	6.56E-02	156256229	156256352	NC_000003.11
cg15513163	2.88E-09	2.59E-10	6.67	12.5448907	1.06E-01	70034139	70034262	NC_000001.10
cg21253087	2.88E-09	2.59E-10	6.67	12.5447322	7.99E-02	139290292	139290415	NC_000009.11
cg09619624	2.88E-09	2.60E-10	6.67	12.543792	1.44E-01	18985632	18985755	NC_000022.10
cg12769519	2.88E-09	2.60E-10	6.67	12.5425132	5.15E-02	76249217	76249340	NC_000017.10
cg05890785	2.89E-09	2.60E-10	6.67	12.541116	1.65E-01	53811704	53811827	NC_000019.9
cg09151754	2.90E-09	2.62E-10	6.67	12.5358144	1.27E-01	5019310	5019433	NC_000017.10
cg13503413	2.90E-09	2.62E-10	6.67	12.5354989	1.46E-01	60937257	60937380	NC_000010.10
cg09271277	2.91E-09	2.62E-10	6.67	12.5351306	4.24E-02	6590947	6591070	NC_000007.13
cg09872088	2.91E-09	2.62E-10	6.67	12.5349143	3.61E-02	121265893	121266016	NC_000010.10
cg27155163	2.91E-09	2.62E-10	6.67	12.5342093	5.25E-02	151026011	151026134	NC_000007.13
cg02207386	2.91E-09	2.62E-10	6.67	12.5341606	1.43E-01	25895834	25895957	NC_000007.13

cg25140773	2.91E-09	2.62E-10	6.67	12.5338023	9.73E-02	128448807	128448930	NC_000005.9
cg11676902	2.91E-09	2.62E-10	6.67	12.5334756	1.75E-01	100061972	100062095	NC_000006.11
cg16795066	2.91E-09	2.62E-10	6.67	12.5330749	8.14E-02	66437078	66437201	NC_000016.9
cg24351410	2.91E-09	2.63E-10	6.67	12.5323346	1.68E-01	58716033	58716156	NC_000001.10
cg16403344	2.92E-09	2.63E-10	6.67	12.5306899	2.10E-01	113229392	113229515	NC_000012.11
cg03906434	2.92E-09	2.63E-10	6.67	12.5293876	1.53E-01	27231819	27231942	NC_000007.13
cg03454705	2.92E-09	2.64E-10	6.67	12.528465	7.75E-02	80535428	80535551	NC_000017.10
cg08780398	2.92E-09	2.64E-10	6.67	12.5284522	1.40E-01	77585381	77585504	NC_000008.10
cg11496364	2.92E-09	2.64E-10	6.66	12.5279749	8.49E-02	114258120	114258243	NC_000002.11
cg00143362	2.93E-09	2.64E-10	6.66	12.5277286	5.36E-02	15000025	15000148	NC_000010.10
cg13660942	2.93E-09	2.64E-10	6.66	12.5275731	1.04E-01	2031922	2032045	NC_000012.11
cg16117653	2.93E-09	2.64E-10	6.66	12.5269174	1.33E-01	4891192	4891315	NC_000019.9
cg12188410	2.93E-09	2.64E-10	6.66	12.5268979	1.24E-01	112460467	112460590	NC_000006.11
cg13769223	2.93E-09	2.64E-10	6.66	12.5268186	1.95E-01	27011963	27012086	NC_000021.8
cg16344173	2.93E-09	2.64E-10	6.66	12.5259546	6.18E-02	2158623	2158746	NC_000007.13
cg03347444	2.93E-09	2.65E-10	6.66	12.5246846	1.42E-01	32926880	32927003	NC_000011.9
cg12514789	2.93E-09	2.65E-10	6.66	12.524384	1.58E-01	19158123	19158246	NC_000007.13
cg20387341	2.93E-09	2.65E-10	6.66	12.524315	1.77E-01	44389282	44389405	NC_000005.9
cg14870792	2.94E-09	2.65E-10	6.66	12.523286	1.59E-01	2163532	2163655	NC_000012.11
cg06872514	2.95E-09	2.66E-10	6.66	12.5203031	8.30E-02	139746715	139746838	NC_000009.11
cg01104047	2.95E-09	2.66E-10	6.66	12.5190506	1.33E-01	136834155	136834278	NC_000005.9
cg09829551	2.95E-09	2.66E-10	6.66	12.5188183	1.35E-01	86599111	86599234	NC_000016.9
cg12163132	2.95E-09	2.67E-10	6.66	12.5178925	9.46E-02	59475917	59476040	NC_000017.10
cg13153708	2.95E-09	2.67E-10	6.66	12.5172916	1.39E-01	65078521	65078644	NC_000014.8
cg13489127	2.95E-09	2.67E-10	6.66	12.5172403	4.08E-02	33781501	33781624	NC_000021.8

cg22533573	2.96E-09	2.67E-10	6.66	12.516833	1.06E-01	32452771	32452894	NC_000011.9
cg01791778	2.97E-09	2.68E-10	6.66	12.5132996	7.27E-02	583901	584024	NC_000002.11
cg06043419	2.97E-09	2.68E-10	6.66	12.5130955	1.50E-01	80256137	80256260	NC_000005.9
cg01557332	2.97E-09	2.68E-10	6.66	12.5128032	8.45E-02	169641045	169641168	NC_000006.11
cg03348397	2.97E-09	2.68E-10	6.66	12.51276	1.60E-01	54789648	54789771	NC_000008.10
cg10129041	2.97E-09	2.69E-10	6.66	12.5101501	1.33E-01	129494526	129494649	NC_000002.11
cg01405404	2.98E-09	2.69E-10	6.66	12.5093937	1.69E-01	37282556	37282679	NC_000019.9
cg23905216	2.98E-09	2.70E-10	6.66	12.5068551	1.55E-01	2161390	2161513	NC_000011.9
cg21769640	2.99E-09	2.70E-10	6.66	12.5060666	1.03E-01	23300066	23300189	NC_000019.9
cg11910759	2.99E-09	2.70E-10	6.66	12.504681	8.64E-02	7011519	7011642	NC_000017.10
cg05492170	2.99E-09	2.70E-10	6.66	12.5042589	1.35E-01	111536341	111536464	NC_000004.11
cg04539957	2.99E-09	2.70E-10	6.66	12.5042506	7.80E-02	44650068	44650191	NC_000020.10
cg19346371	3.00E-09	2.71E-10	6.66	12.5021249	1.75E-01	115120789	115120912	NC_000012.11
cg15744026	3.01E-09	2.72E-10	6.66	12.4979022	9.58E-02	32258302	32258425	NC_000017.10
cg03048432	3.01E-09	2.72E-10	6.66	12.4972627	1.47E-01	51290751	51290874	NC_000014.8
cg04651603	3.01E-09	2.72E-10	6.66	12.496418	1.50E-01	54321302	54321425	NC_000012.11
cg16672956	3.02E-09	2.73E-10	6.66	12.4958025	9.47E-02	88091767	88091890	NC_000016.9
cg12385482	3.03E-09	2.74E-10	6.66	12.4927171	6.00E-02	125802442	125802565	NC_000011.9
cg03958798	3.03E-09	2.74E-10	6.66	12.4910316	1.95E-01	106400686	106400809	NC_000010.10
cg21300719	3.04E-09	2.75E-10	6.66	12.4889817	1.38E-01	201097122	201097245	NC_000001.10
cg18031880	3.04E-09	2.75E-10	6.66	12.4878317	1.46E-01	115829932	115830055	NC_000011.9
cg00089100	3.04E-09	2.75E-10	6.66	12.4877821	3.82E-02	26922951	26923074	NC_000022.10
cg27554156	3.04E-09	2.75E-10	6.66	12.4872535	1.57E-01	13248725	13248848	NC_000012.11
cg22346073	3.04E-09	2.75E-10	6.66	12.4871251	1.12E-01	122402890	122403013	NC_000002.11
cg18045133	3.04E-09	2.75E-10	6.66	12.4859081	7.16E-02	133183688	133183811	NC_000012.11

cg07547695	3.05E-09	2.76E-10	6.66	12.4832124	1.24E-01	111881075	111881198	NC_000002.11
cg18416585	3.05E-09	2.76E-10	6.66	12.4827675	1.05E-01	62756100	62756223	NC_000004.11
cg22934200	3.06E-09	2.77E-10	6.66	12.4814414	1.13E-01	61407270	61407393	NC_000002.11
cg04140663	3.06E-09	2.77E-10	6.66	12.4814308	1.56E-01	83315922	83316045	NC_000015.9
cg20124587	3.06E-09	2.77E-10	6.66	12.4810635	1.66E-01	176971355	176971478	NC_000002.11
cg18043195	3.07E-09	2.78E-10	6.66	12.4781139	9.97E-02	19033231	19033354	NC_000009.11
cg24234899	3.07E-09	2.78E-10	6.66	12.4762891	2.00E-01	118922493	118922616	NC_000010.10
cg09830083	3.08E-09	2.79E-10	6.66	12.4750457	9.60E-02	131561058	131561181	NC_000005.9
cg22920300	3.08E-09	2.79E-10	6.66	12.4749911	1.82E-01	181441364	181441487	NC_000003.11
cg17173166	3.08E-09	2.79E-10	6.66	12.4745089	4.91E-02	129692887	129693010	NC_000003.11
cg23786545	3.08E-09	2.79E-10	6.66	12.4744596	7.47E-02	130580518	130580641	NC_000011.9
cg22147660	3.08E-09	2.79E-10	6.66	12.4740099	1.74E-01	5895006	5895129	NC_000018.9
cg27308754	3.09E-09	2.79E-10	6.65	12.4720172	1.09E-01	123318999	123319122	NC_000006.11
cg14618923	3.09E-09	2.80E-10	6.65	12.4711343	1.52E-01	108978075	108978198	NC_000006.11
cg06721601	3.09E-09	2.80E-10	6.65	12.4707373	1.20E-01	101762633	101762756	NC_000007.13
cg12562967	3.09E-09	2.80E-10	6.65	12.4698427	2.02E-01	40006192	40006315	NC_000019.9
cg14780990	3.09E-09	2.80E-10	6.65	12.4692555	1.18E-01	93181182	93181305	NC_000015.9
cg02390569	3.10E-09	2.81E-10	6.65	12.4674458	4.93E-02	145624983	145625106	NC_000008.10
cg14493920	3.10E-09	2.81E-10	6.65	12.4668733	1.63E-01	37038053	37038176	NC_000009.11
cg14724471	3.11E-09	2.81E-10	6.65	12.4653791	1.38E-01	57089461	57089584	NC_000020.10
cg10634317	3.11E-09	2.81E-10	6.65	12.4649957	2.86E-02	56657854	56657977	NC_000019.9
cg02626307	3.11E-09	2.81E-10	6.65	12.4645684	7.19E-02	156305725	156305848	NC_000001.10
cg12248614	3.12E-09	2.82E-10	6.65	12.4617867	2.30E-01	41018880	41019003	NC_000019.9
cg09403249	3.12E-09	2.82E-10	6.65	12.4615902	1.61E-01	45444203	45444326	NC_000012.11
cg17331296	3.12E-09	2.83E-10	6.65	12.4604946	2.27E-01	41754181	41754304	NC_000008.10

cg08262496	3.12E-09	2.83E-10	6.65	12.4592645	8.77E-02	22023163	22023286	NC_000016.9
cg06212471	3.13E-09	2.84E-10	6.65	12.4558877	5.60E-02	72458478	72458601	NC_000004.11
cg05423257	3.14E-09	2.85E-10	6.65	12.4539603	2.66E-02	233321600	233321723	NC_000002.11
cg07153966	3.15E-09	2.85E-10	6.65	12.4516419	1.36E-01	27147590	27147713	NC_000007.13
cg11877726	3.15E-09	2.85E-10	6.65	12.4515817	2.50E-02	99637027	99637150	NC_000014.8
cg24430580	3.15E-09	2.85E-10	6.65	12.451067	1.55E-01	111544235	111544358	NC_000004.11
cg19035107	3.16E-09	2.86E-10	6.65	12.4484728	1.50E-01	26745662	26745785	NC_000006.11
cg11659025	3.16E-09	2.86E-10	6.65	12.4480154	4.02E-02	147767989	147768112	NC_000001.10
cg23401210	3.16E-09	2.86E-10	6.65	12.4479896	5.55E-02	57630628	57630751	NC_000019.9
cg19764540	3.16E-09	2.87E-10	6.65	12.447174	9.74E-02	26782423	26782546	NC_000002.11
cg16790847	3.16E-09	2.87E-10	6.65	12.4458404	1.11E-01	147123429	147123552	NC_000003.11
cg07143418	3.17E-09	2.87E-10	6.65	12.4444738	1.97E-01	23567548	23567671	NC_000008.10
cg06274671	3.17E-09	2.88E-10	6.65	12.4437927	1.27E-01	51185236	51185359	NC_000016.9
cg27067781	3.18E-09	2.88E-10	6.65	12.4418518	1.56E-01	32116853	32116976	NC_000006.11
cg04142555	3.18E-09	2.89E-10	6.65	12.4399359	1.00E-01	23892656	23892779	NC_000015.9
cg01604138	3.18E-09	2.89E-10	6.65	12.4393449	4.65E-02	71202794	71202917	NC_000011.9
cg20318156	3.18E-09	2.89E-10	6.65	12.4390779	8.41E-02	99469323	99469446	NC_000001.10
cg21859603	3.19E-09	2.90E-10	6.65	12.4357988	7.40E-02	33247092	33247215	NC_000006.11
cg23886314	3.20E-09	2.90E-10	6.65	12.4354557	8.14E-02	116272127	116272250	NC_000007.13
cg23509896	3.20E-09	2.90E-10	6.65	12.4339219	1.79E-01	62680345	62680468	NC_000020.10
cg17520027	3.20E-09	2.91E-10	6.65	12.4327024	1.40E-01	200010708	200010831	NC_000001.10
cg15996950	3.21E-09	2.91E-10	6.65	12.4318879	9.58E-02	112282703	112282826	NC_000013.10
cg21764921	3.21E-09	2.91E-10	6.65	12.43057	3.92E-02	32825040	32825163	NC_000006.11
cg18888520	3.21E-09	2.92E-10	6.65	12.4299838	2.13E-01	58609806	58609929	NC_000019.9
cg19078067	3.21E-09	2.92E-10	6.65	12.4296937	1.19E-01	71586227	71586350	NC_000017.10

cg23818921	3.22E-09	2.92E-10	6.65	12.4288161	6.33E-02	76871621	76871744	NC_000004.11
cg03587843	3.22E-09	2.92E-10	6.65	12.4286171	1.36E-01	1885516	1885639	NC_000005.9
cg27413632	3.22E-09	2.93E-10	6.65	12.4268976	3.75E-02	63963913	63964036	NC_000003.11
cg01696226	3.22E-09	2.93E-10	6.65	12.4266053	1.60E-01	79448527	79448650	NC_000017.10
cg21532103	3.22E-09	2.93E-10	6.65	12.4264703	7.33E-02	238535538	238535661	NC_000002.11
cg18099189	3.23E-09	2.93E-10	6.65	12.4255267	1.06E-01	54960259	54960382	NC_000004.11
cg18030133	3.23E-09	2.93E-10	6.65	12.4251058	8.72E-02	143929406	143929529	NC_000006.11
cg00205340	3.23E-09	2.94E-10	6.65	12.4230509	5.19E-02	79044277	79044400	NC_000015.9
cg16808373	3.24E-09	2.94E-10	6.65	12.4222212	1.39E-01	122850140	122850263	NC_000011.9
cg03053597	3.24E-09	2.94E-10	6.65	12.4211524	1.09E-01	17231486	17231609	NC_000001.10
cg20313969	3.24E-09	2.94E-10	6.65	12.420985	1.20E-01	112939562	112939685	NC_000002.11
cg07301944	3.24E-09	2.94E-10	6.65	12.4206863	1.16E-01	82660464	82660587	NC_000016.9
cg11112161	3.24E-09	2.95E-10	6.65	12.4202305	1.55E-01	33823004	33823127	NC_000017.10
cg24534926	3.24E-09	2.95E-10	6.65	12.4202192	1.31E-01	36667484	36667607	NC_000017.10
cg07782451	3.24E-09	2.95E-10	6.65	12.4201992	1.55E-01	494156	494279	NC_000008.10
cg23781495	3.25E-09	2.95E-10	6.64	12.4189239	1.38E-01	118917109	118917232	NC_000009.11
cg22586192	3.25E-09	2.95E-10	6.64	12.4185766	1.36E-02	57146928	57147051	NC_000012.11
cg14108380	3.25E-09	2.95E-10	6.64	12.4174274	8.56E-02	139299357	139299480	NC_000009.11
cg17062109	3.25E-09	2.95E-10	6.64	12.417115	1.77E-01	46670029	46670152	NC_000017.10
cg07073096	3.26E-09	2.96E-10	6.64	12.4160535	1.05E-01	2870441	2870564	NC_000007.13
cg20817902	3.26E-09	2.96E-10	6.64	12.4156457	2.37E-01	81124121	81124244	NC_000004.11
cg08601899	3.26E-09	2.96E-10	6.64	12.4155066	4.64E-02	114379217	114379340	NC_000006.11
cg14807945	3.26E-09	2.96E-10	6.64	12.4152448	9.60E-02	145189102	145189225	NC_000002.11
cg23861668	3.26E-09	2.96E-10	6.64	12.4150526	2.50E-01	20691126	20691249	NC_000011.9
cg06602847	3.26E-09	2.96E-10	6.64	12.4144235	1.34E-01	122131497	122131620	NC_000009.11

cg04609859	3.27E-09	2.97E-10	6.64	12.4115599	1.74E-01	46655736	46655859	NC_000017.10
cg00703513	3.27E-09	2.97E-10	6.64	12.4111849	9.98E-02	145925258	145925381	NC_000008.10
cg01555907	3.28E-09	2.98E-10	6.64	12.4097498	9.78E-02	32571504	32571627	NC_000009.11
cg27287745	3.28E-09	2.98E-10	6.64	12.4085288	1.36E-01	61104967	61105090	NC_000014.8
cg12545687	3.28E-09	2.98E-10	6.64	12.4082663	1.58E-01	53423262	53423385	NC_000013.10
cg00577070	3.28E-09	2.98E-10	6.64	12.4078639	2.55E-02	47049880	47050003	NC_000003.11
cg08200869	3.29E-09	2.99E-10	6.64	12.4058878	8.23E-02	140807666	140807789	NC_000005.9
cg22858210	3.29E-09	2.99E-10	6.64	12.4039453	7.04E-02	65685787	65685910	NC_000011.9
cg23978968	3.29E-09	2.99E-10	6.64	12.4038091	1.05E-01	101704656	101704779	NC_000001.10
cg12426652	3.29E-09	3.00E-10	6.64	12.4034275	1.53E-01	37915056	37915179	NC_000021.8
cg18965213	3.30E-09	3.00E-10	6.64	12.4020735	4.51E-02	62989995	62990118	NC_000015.9
cg16681268	3.30E-09	3.00E-10	6.64	12.4015789	6.98E-02	117067616	117067739	NC_000007.13
cg11686208	3.30E-09	3.00E-10	6.64	12.4009294	8.76E-02	60763357	60763480	NC_000002.11
cg09895009	3.30E-09	3.01E-10	6.64	12.400487	8.33E-02	152595992	152596115	NC_000001.10
cg23952663	3.31E-09	3.01E-10	6.64	12.3998842	1.54E-01	75601120	75601243	NC_000001.10
cg12571423	3.31E-09	3.01E-10	6.64	12.3990697	1.32E-01	37006748	37006871	NC_000013.10
cg06535308	3.31E-09	3.01E-10	6.64	12.398889	1.56E-01	17497693	17497816	NC_000011.9
cg20253551	3.31E-09	3.01E-10	6.64	12.3986541	1.69E-01	152129400	152129523	NC_000006.11
cg18277764	3.31E-09	3.01E-10	6.64	12.3986142	1.75E-01	239188	239311	NC_000003.11
cg11630154	3.31E-09	3.01E-10	6.64	12.3973952	1.76E-01	137484364	137484487	NC_000003.11
cg03731303	3.31E-09	3.02E-10	6.64	12.3968996	1.60E-01	181443009	181443132	NC_000003.11
cg20405893	3.32E-09	3.02E-10	6.64	12.3956336	1.91E-01	50822158	50822281	NC_000010.10
cg26676559	3.32E-09	3.02E-10	6.64	12.395476	2.10E-01	14995167	14995290	NC_000011.9
cg11706835	3.32E-09	3.02E-10	6.64	12.3947521	1.33E-01	31580560	31580683	NC_000016.9
cg01749436	3.34E-09	3.04E-10	6.64	12.3904448	1.69E-01	9756178	9756301	NC_000008.10

cg10089028	3.35E-09	3.04E-10	6.64	12.3876001	7.32E-02	95286216	95286339	NC_000013.10
cg00981837	3.35E-09	3.05E-10	6.64	12.3855374	1.80E-01	33936262	33936385	NC_000005.9
cg15742840	3.35E-09	3.05E-10	6.64	12.3848834	7.90E-02	21935133	21935256	NC_000017.10
cg01403246	3.35E-09	3.05E-10	6.64	12.3845091	1.30E-01	144512192	144512315	NC_000008.10
cg19694200	3.37E-09	3.07E-10	6.64	12.3797472	2.21E-01	55204351	55204474	NC_000020.10
cg22403273	3.37E-09	3.07E-10	6.64	12.378627	2.16E-01	81951956	81952079	NC_000004.11
cg04005969	3.38E-09	3.07E-10	6.64	12.3782149	1.77E-01	51171247	51171370	NC_000019.9
cg01566235	3.38E-09	3.08E-10	6.64	12.3755691	1.54E-01	36991101	36991224	NC_000014.8
cg01139553	3.39E-09	3.08E-10	6.64	12.3752763	6.00E-02	193493033	193493156	NC_000003.11
cg14074305	3.39E-09	3.08E-10	6.64	12.3748944	1.64E-01	106260513	106260636	NC_000002.11
cg16617817	3.39E-09	3.09E-10	6.64	12.3744607	8.69E-02	1582686	1582809	NC_000007.13
cg21932416	3.39E-09	3.09E-10	6.64	12.3741871	1.79E-01	176997707	176997830	NC_000002.11
cg04837832	3.39E-09	3.09E-10	6.64	12.3728679	1.41E-01	101290958	101291081	NC_000010.10
cg10741308	3.40E-09	3.10E-10	6.64	12.3707292	7.75E-02	97175272	97175395	NC_000010.10
cg14757228	3.40E-09	3.10E-10	6.64	12.3705628	1.78E-01	32116858	32116981	NC_000006.11
cg17979595	3.41E-09	3.10E-10	6.64	12.3688003	8.52E-02	175995850	175995973	NC_000005.9
cg13223938	3.41E-09	3.11E-10	6.64	12.3678982	4.52E-02	132171201	132171324	NC_000012.11
cg04988476	3.41E-09	3.11E-10	6.64	12.3669433	1.64E-01	192444985	192445108	NC_000003.11
cg25657218	3.41E-09	3.11E-10	6.64	12.3662866	1.13E-01	22017062	22017185	NC_000017.10
cg19134945	3.42E-09	3.11E-10	6.64	12.3655534	2.10E-01	111558566	111558689	NC_000004.11
cg13290643	3.42E-09	3.12E-10	6.63	12.3645313	4.96E-02	2093631	2093754	NC_000011.9
cg17234150	3.42E-09	3.12E-10	6.63	12.3645202	1.75E-01	24266905	24267028	NC_000016.9
cg06397845	3.42E-09	3.12E-10	6.63	12.3635844	7.41E-02	35732737	35732860	NC_000017.10
cg24951286	3.42E-09	3.12E-10	6.63	12.3633561	1.50E-01	140625068	140625191	NC_000005.9
cg17202109	3.43E-09	3.12E-10	6.63	12.3629322	2.93E-02	77508977	77509100	NC_000017.10

cg25950625	3.43E-09	3.13E-10	6.63	12.3612708	2.12E-01	41510116	41510239	NC_000005.9
cg07015195	3.43E-09	3.13E-10	6.63	12.3611059	1.55E-01	109093450	109093573	NC_000008.10
cg01466330	3.43E-09	3.13E-10	6.63	12.3603463	8.24E-02	39835697	39835820	NC_000008.10
cg13653963	3.43E-09	3.13E-10	6.63	12.3599472	8.83E-02	135632595	135632718	NC_000009.11
cg05462359	3.43E-09	3.13E-10	6.63	12.3599164	1.83E-01	37837788	37837911	NC_000005.9
cg23573900	3.44E-09	3.14E-10	6.63	12.3583376	1.30E-01	139056794	139056917	NC_000005.9
cg22614787	3.44E-09	3.14E-10	6.63	12.3580073	4.66E-02	13919708	13919831	NC_000019.9
cg09784259	3.44E-09	3.14E-10	6.63	12.3577261	6.52E-02	169703422	169703545	NC_000001.10
cg18803257	3.44E-09	3.14E-10	6.63	12.3573267	8.25E-02	77158025	77158148	NC_000010.10
cg06842954	3.45E-09	3.15E-10	6.63	12.3546942	1.58E-01	111559066	111559189	NC_000004.11
cg06391932	3.45E-09	3.15E-10	6.63	12.354643	1.28E-01	45159894	45160017	NC_000002.11
cg00146249	3.45E-09	3.15E-10	6.63	12.3539302	2.04E-02	79042364	79042487	NC_000017.10
cg22797653	3.46E-09	3.15E-10	6.63	12.3536801	5.11E-02	33466255	33466378	NC_000003.11
cg17929687	3.46E-09	3.15E-10	6.63	12.3536635	1.87E-01	52734325	52734448	NC_000014.8
cg25600049	3.46E-09	3.15E-10	6.63	12.3533113	1.68E-01	53419729	53419852	NC_000013.10
cg10238917	3.46E-09	3.15E-10	6.63	12.3530894	5.60E-02	30942621	30942744	NC_000016.9
cg19643097	3.46E-09	3.16E-10	6.63	12.3525358	5.40E-02	105794085	105794208	NC_000014.8
cg23818621	3.46E-09	3.16E-10	6.63	12.3513472	1.94E-01	4850551	4850674	NC_000008.10
cg13117164	3.46E-09	3.16E-10	6.63	12.3509259	1.60E-01	54960232	54960355	NC_000004.11
cg18366919	3.47E-09	3.16E-10	6.63	12.3502078	1.48E-01	15344364	15344487	NC_000019.9
cg10143543	3.47E-09	3.17E-10	6.63	12.3496054	6.31E-02	76437005	76437128	NC_000004.11
cg22656521	3.47E-09	3.17E-10	6.63	12.3494278	6.30E-02	19118165	19118288	NC_000022.10
cg14975429	3.47E-09	3.17E-10	6.63	12.3480406	1.20E-01	182549604	182549727	NC_000002.11
cg18534345	3.48E-09	3.17E-10	6.63	12.3472927	1.39E-01	120355342	120355465	NC_000010.10
cg08990926	3.48E-09	3.18E-10	6.63	12.3463137	1.70E-01	41086365	41086488	NC_000012.11

cg23554956	3.48E-09	3.18E-10	6.63	12.3453223	2.03E-01	65489040	65489163	NC_000008.10
cg07347205	3.49E-09	3.19E-10	6.63	12.3432115	1.34E-01	87159274	87159397	NC_000016.9
cg21201099	3.49E-09	3.19E-10	6.63	12.3431711	1.34E-01	59482446	59482569	NC_000017.10
cg19961480	3.49E-09	3.19E-10	6.63	12.3427379	1.55E-01	88877216	88877339	NC_000006.11
cg01653446	3.49E-09	3.19E-10	6.63	12.3426119	4.24E-02	20904169	20904292	NC_000014.8
cg04063906	3.49E-09	3.19E-10	6.63	12.3424744	5.59E-02	588539	588662	NC_000007.13
cg23527920	3.49E-09	3.19E-10	6.63	12.3422352	6.66E-02	32906857	32906980	NC_000006.11
cg25387861	3.49E-09	3.19E-10	6.63	12.3422186	5.87E-02	234731684	234731807	NC_000001.10
cg14778576	3.49E-09	3.19E-10	6.63	12.3421834	3.86E-02	160477458	160477581	NC_000003.11
cg23432707	3.49E-09	3.19E-10	6.63	12.3421557	9.28E-02	97278556	97278679	NC_000008.10
cg00319545	3.49E-09	3.19E-10	6.63	12.3420982	1.35E-01	177133737	177133860	NC_000001.10
cg17098255	3.49E-09	3.19E-10	6.63	12.341833	4.69E-02	92636767	92636890	NC_000006.11
cg15698066	3.50E-09	3.19E-10	6.63	12.3415047	2.08E-01	100438647	100438770	NC_000014.8
cg19694460	3.50E-09	3.20E-10	6.63	12.3391944	8.91E-02	6797497	6797620	NC_000007.13
cg05444816	3.51E-09	3.20E-10	6.63	12.3376069	1.37E-01	2162406	2162529	NC_000011.9
cg18811621	3.51E-09	3.21E-10	6.63	12.3373141	7.21E-02	208207188	208207311	NC_000002.11
cg23816049	3.51E-09	3.21E-10	6.63	12.3372952	2.27E-01	224804081	224804204	NC_000001.10
cg05634149	3.51E-09	3.21E-10	6.63	12.3369906	1.98E-01	34395317	34395440	NC_000021.8
cg04422562	3.51E-09	3.21E-10	6.63	12.3365347	1.33E-01	155247170	155247293	NC_000007.13
cg08555552	3.51E-09	3.21E-10	6.63	12.3364228	1.49E-01	13536409	13536532	NC_000004.11
cg27160130	3.51E-09	3.21E-10	6.63	12.3357209	1.65E-01	237073518	237073641	NC_000002.11
cg03397031	3.52E-09	3.21E-10	6.63	12.3351625	4.89E-02	29189308	29189431	NC_000001.10
cg21050013	3.52E-09	3.21E-10	6.63	12.3349902	7.00E-02	48306664	48306787	NC_000019.9
cg04974130	3.52E-09	3.21E-10	6.63	12.3346027	1.17E-01	21951046	21951169	NC_000004.11
cg02867216	3.52E-09	3.21E-10	6.63	12.334367	1.60E-01	72925513	72925636	NC_000018.9

cg03648116	3.52E-09	3.21E-10	6.63	12.3343271	5.44E-02	105945234	105945357	NC_000011.9
cg08697092	3.52E-09	3.22E-10	6.63	12.334092	1.94E-01	56879933	56880056	NC_000019.9
cg00192136	3.52E-09	3.22E-10	6.63	12.3334437	1.90E-01	20178496	20178619	NC_000011.9
cg09071155	3.53E-09	3.22E-10	6.63	12.3314977	1.48E-01	27280914	27281037	NC_000007.13
cg06284231	3.53E-09	3.23E-10	6.63	12.331158	1.59E-01	38724255	38724378	NC_000014.8
cg09781028	3.53E-09	3.23E-10	6.63	12.330339	1.35E-01	90355905	90356028	NC_000007.13
cg23448348	3.53E-09	3.23E-10	6.63	12.3302231	1.66E-01	139257907	139258030	NC_000003.11
cg26195583	3.53E-09	3.23E-10	6.63	12.3299239	1.60E-01	41869348	41869471	NC_000004.11
cg23658462	3.54E-09	3.23E-10	6.63	12.3285665	1.15E-01	1290552	1290675	NC_000019.9
cg10811426	3.54E-09	3.24E-10	6.63	12.3273116	1.32E-01	234667461	234667584	NC_000001.10
cg17319774	3.54E-09	3.24E-10	6.63	12.3271931	1.49E-01	79171053	79171176	NC_000013.10
cg09355652	3.54E-09	3.24E-10	6.63	12.32652	4.69E-02	64668475	64668598	NC_000011.9
cg07105117	3.55E-09	3.24E-10	6.63	12.3261763	3.85E-02	234652601	234652724	NC_000002.11
cg09234672	3.55E-09	3.24E-10	6.63	12.3258261	1.24E-01	51187750	51187873	NC_000016.9
cg01341655	3.55E-09	3.25E-10	6.63	12.3239952	1.19E-01	62202294	62202417	NC_000004.11
cg04629595	3.56E-09	3.26E-10	6.63	12.3219363	1.08E-01	74592566	74592689	NC_000015.9
cg15832905	3.57E-09	3.26E-10	6.63	12.3203232	3.54E-02	1707625	1707748	NC_000011.9
cg06907391	3.57E-09	3.26E-10	6.63	12.320081	1.84E-01	161275138	161275261	NC_000005.9
cg10321146	3.57E-09	3.26E-10	6.63	12.3195235	1.34E-01	6822469	6822592	NC_000019.9
cg22340475	3.57E-09	3.26E-10	6.63	12.3195176	1.27E-01	55529845	55529968	NC_000008.10
cg11240043	3.58E-09	3.27E-10	6.63	12.3173911	8.09E-02	20250501	20250624	NC_000001.10
cg04066265	3.58E-09	3.27E-10	6.63	12.3171704	1.35E-01	19460243	19460366	NC_000008.10
cg07265743	3.58E-09	3.27E-10	6.63	12.3166387	2.32E-01	59535253	59535376	NC_000017.10
cg09103619	3.58E-09	3.27E-10	6.63	12.3161894	2.15E-01	79575048	79575171	NC_000015.9
cg02984614	3.58E-09	3.28E-10	6.63	12.3153565	2.31E-01	31618409	31618532	NC_000017.10

cg10567498	3.59E-09	3.28E-10	6.63	12.3144176	8.22E-02	41020537	41020660	NC_000006.11
cg24181062	3.59E-09	3.28E-10	6.63	12.3138789	1.02E-01	115705401	115705524	NC_000003.11
cg13981287	3.59E-09	3.29E-10	6.63	12.3129831	3.93E-02	207081344	207081467	NC_000001.10
cg08250918	3.59E-09	3.29E-10	6.63	12.3126031	1.07E-01	50467921	50468044	NC_000007.13
cg27179353	3.60E-09	3.29E-10	6.63	12.3113222	1.11E-01	156364125	156364248	NC_000004.11
cg24582338	3.60E-09	3.29E-10	6.62	12.3107641	6.62E-02	106594431	106594554	NC_000006.11
cg11665613	3.60E-09	3.29E-10	6.62	12.31057	7.24E-02	12268883	12269006	NC_000001.10
cg18315960	3.60E-09	3.30E-10	6.62	12.3100968	1.57E-01	58204028	58204151	NC_000013.10
cg18217632	3.61E-09	3.30E-10	6.62	12.3086597	1.36E-01	3602937	3603060	NC_000012.11
cg12908399	3.61E-09	3.30E-10	6.62	12.3082349	1.25E-01	229570288	229570411	NC_000001.10
cg09851650	3.61E-09	3.30E-10	6.62	12.3079323	5.71E-02	20465286	20465409	NC_000001.10
cg18399218	3.61E-09	3.30E-10	6.62	12.3075237	1.06E-01	170561359	170561482	NC_000004.11
cg25184481	3.61E-09	3.30E-10	6.62	12.3074329	1.53E-01	19813001	19813124	NC_000007.13
cg19478558	3.61E-09	3.31E-10	6.62	12.307141	7.09E-02	44260048	44260171	NC_000018.9
cg19223129	3.62E-09	3.31E-10	6.62	12.3055561	1.29E-01	7052403	7052526	NC_000004.11
cg12493218	3.62E-09	3.31E-10	6.62	12.3050842	1.37E-01	101453358	101453481	NC_000011.9
cg21476463	3.62E-09	3.31E-10	6.62	12.3046497	5.04E-02	139259926	139260049	NC_000003.11
cg04898026	3.62E-09	3.32E-10	6.62	12.3039958	8.56E-02	149487677	149487800	NC_000007.13
cg08408994	3.62E-09	3.32E-10	6.62	12.3035596	2.20E-01	44330958	44331081	NC_000011.9
cg01322214	3.62E-09	3.32E-10	6.62	12.3030212	1.27E-01	25219198	25219321	NC_000007.13
cg05157791	3.63E-09	3.33E-10	6.62	12.3008659	8.63E-02	113551036	113551159	NC_000012.11
cg08172044	3.63E-09	3.33E-10	6.62	12.300847	5.05E-02	126070462	126070585	NC_000006.11
cg10826043	3.63E-09	3.33E-10	6.62	12.3002325	1.53E-01	17100121	17100244	NC_000006.11
cg26020513	3.64E-09	3.33E-10	6.62	12.2996513	1.25E-01	11568356	11568479	NC_000008.10
cg26332285	3.64E-09	3.33E-10	6.62	12.2991579	7.80E-02	134915746	134915869	NC_000010.10

cg12940886	3.64E-09	3.33E-10	6.62	12.299102	1.60E-01	59989928	59990051	NC_000013.10
cg09996789	3.64E-09	3.33E-10	6.62	12.2984651	1.06E-01	1310584	1310707	NC_000016.9
cg11969556	3.65E-09	3.34E-10	6.62	12.2967567	1.03E-01	176994498	176994621	NC_000002.11
cg27228433	3.65E-09	3.34E-10	6.62	12.2963342	9.66E-02	71475716	71475839	NC_000016.9
cg21756485	3.65E-09	3.34E-10	6.62	12.2956912	1.53E-01	36805370	36805493	NC_000003.11
cg16366685	3.65E-09	3.35E-10	6.62	12.2951353	7.29E-02	80535398	80535521	NC_000017.10
cg05616010	3.66E-09	3.35E-10	6.62	12.2935641	1.05E-01	58630089	58630212	NC_000019.9
cg02095498	3.66E-09	3.35E-10	6.62	12.2933584	4.90E-02	142368264	142368387	NC_000008.10
cg15381162	3.66E-09	3.36E-10	6.62	12.2922444	2.47E-02	23517736	23517859	NC_000014.8
cg07549735	3.67E-09	3.36E-10	6.62	12.2895913	1.68E-02	6495256	6495379	NC_000011.9
cg07090701	3.67E-09	3.37E-10	6.62	12.28902	1.53E-01	119914553	119914676	NC_000002.11
cg20125971	3.68E-09	3.37E-10	6.62	12.2886318	8.84E-02	56458333	56458456	NC_000004.11
cg08031492	3.68E-09	3.37E-10	6.62	12.2873059	1.33E-01	469214	469337	NC_000002.11
cg06741594	3.68E-09	3.38E-10	6.62	12.2863672	2.01E-01	132260475	132260598	NC_000007.13
cg19740353	3.68E-09	3.38E-10	6.62	12.2862546	5.15E-02	53394492	53394615	NC_000006.11
cg02988984	3.68E-09	3.38E-10	6.62	12.2858944	1.12E-01	180579844	180579967	NC_000001.10
cg22989843	3.69E-09	3.38E-10	6.62	12.2854598	2.10E-01	223154201	223154324	NC_000002.11
cg22793136	3.69E-09	3.38E-10	6.62	12.2842743	1.67E-01	94543560	94543683	NC_000012.11
cg05619587	3.69E-09	3.38E-10	6.62	12.2839323	1.80E-01	85306021	85306144	NC_000012.11
cg04344749	3.70E-09	3.39E-10	6.62	12.282492	8.72E-02	25871814	25871937	NC_000001.10
cg21684012	3.70E-09	3.39E-10	6.62	12.2816062	1.84E-01	100912906	100913029	NC_000006.11
cg06976250	3.70E-09	3.39E-10	6.62	12.2811955	2.26E-01	113258750	113258873	NC_000011.9
cg04091180	3.70E-09	3.39E-10	6.62	12.2809542	5.60E-02	38822199	38822322	NC_000017.10
cg03401738	3.70E-09	3.40E-10	6.62	12.2808372	1.67E-01	65991664	65991787	NC_000001.10
cg18255550	3.70E-09	3.40E-10	6.62	12.2804223	8.48E-02	35246954	35247077	NC_000017.10

cg03811905	3.70E-09	3.40E-10	6.62	12.2802701	5.07E-02	239070208	239070331	NC_000002.11
cg12717612	3.71E-09	3.40E-10	6.62	12.2795681	8.14E-02	56321500	56321623	NC_000020.10
cg00513100	3.71E-09	3.40E-10	6.62	12.2781033	4.51E-02	66487501	66487624	NC_000003.11
cg08233331	3.73E-09	3.42E-10	6.62	12.2744103	7.57E-02	1359656	1359779	NC_000018.9
cg04037585	3.73E-09	3.42E-10	6.62	12.2729296	1.26E-01	56231292	56231415	NC_000016.9
cg17136073	3.73E-09	3.42E-10	6.62	12.2725344	9.20E-02	23310675	23310798	NC_000013.10
cg06728579	3.74E-09	3.43E-10	6.62	12.2715714	2.83E-01	56224901	56225024	NC_000016.9
cg12820681	3.74E-09	3.43E-10	6.62	12.2712538	1.86E-01	155166290	155166413	NC_000007.13
cg05269013	3.74E-09	3.43E-10	6.62	12.2712316	1.08E-01	56728520	56728643	NC_000020.10
cg17971015	3.74E-09	3.43E-10	6.62	12.2711142	1.82E-01	12306198	12306321	NC_000019.9
cg25652953	3.74E-09	3.43E-10	6.62	12.2708466	4.64E-02	106713261	106713384	NC_000005.9
cg17630392	3.74E-09	3.43E-10	6.62	12.2708418	1.29E-01	32712623	32712746	NC_000005.9
cg17526483	3.74E-09	3.43E-10	6.62	12.2704773	1.15E-01	27281404	27281527	NC_000007.13
cg08842907	3.74E-09	3.43E-10	6.62	12.2697537	8.79E-02	62470818	62470941	NC_000011.9
cg24829430	3.74E-09	3.44E-10	6.62	12.2690754	5.08E-02	44471057	44471180	NC_000007.13
cg15546071	3.74E-09	3.44E-10	6.62	12.2689453	7.03E-02	135691756	135691879	NC_000002.11
cg07150036	3.75E-09	3.44E-10	6.62	12.2684744	5.73E-02	90017616	90017739	NC_000016.9
cg17561452	3.75E-09	3.44E-10	6.62	12.2684603	6.80E-02	84224707	84224830	NC_000016.9
cg13537240	3.76E-09	3.45E-10	6.62	12.2650381	1.06E-01	2761549	2761672	NC_000012.11
cg15108727	3.76E-09	3.45E-10	6.62	12.2640843	1.47E-01	3104873	3104996	NC_000005.9
cg22286936	3.76E-09	3.45E-10	6.62	12.263756	1.53E-01	50683593	50683716	NC_000006.11
cg02212009	3.77E-09	3.46E-10	6.62	12.2620063	1.44E-01	12820860	12820983	NC_000011.9
cg02902882	3.78E-09	3.47E-10	6.62	12.2598602	9.19E-02	798345	798468	NC_000016.9
cg27491557	3.78E-09	3.47E-10	6.62	12.2598577	1.08E-01	3165885	3166008	NC_000006.11
cg01690182	3.78E-09	3.47E-10	6.62	12.2590922	1.58E-01	27018874	27018997	NC_000015.9

cg06836725	3.78E-09	3.47E-10	6.62	12.2588273	9.22E-02	217515425	217515548	NC_000002.11
cg11826327	3.78E-09	3.47E-10	6.62	12.2587299	6.01E-02	1669926	1670049	NC_000011.9
cg13692655	3.78E-09	3.47E-10	6.62	12.25869	1.14E-01	130713088	130713211	NC_000009.11
cg06849452	3.79E-09	3.48E-10	6.62	12.257001	2.67E-02	68732297	68732420	NC_000016.9
cg15815435	3.79E-09	3.48E-10	6.61	12.2556282	1.10E-01	11605526	11605649	NC_000004.11
cg00592871	3.79E-09	3.49E-10	6.61	12.2550845	7.62E-02	118738517	118738640	NC_000008.10
cg09693004	3.80E-09	3.49E-10	6.61	12.2536466	1.19E-01	134072931	134073054	NC_000004.11
cg23998071	3.80E-09	3.49E-10	6.61	12.2530327	1.90E-01	27289669	27289792	NC_000007.13
cg07253769	3.81E-09	3.50E-10	6.61	12.250657	1.11E-01	113447342	113447465	NC_000012.11
cg18531176	3.81E-09	3.51E-10	6.61	12.2495749	8.03E-02	131706744	131706867	NC_000010.10
cg16960402	3.81E-09	3.51E-10	6.61	12.249312	8.68E-02	2032075	2032198	NC_000007.13
cg10098582	3.82E-09	3.51E-10	6.61	12.2471778	6.52E-02	28877881	28878004	NC_000016.9
cg19696317	3.82E-09	3.52E-10	6.61	12.2465861	2.16E-01	84456171	84456294	NC_000013.10
cg25496947	3.83E-09	3.52E-10	6.61	12.2462439	3.09E-02	46192911	46193034	NC_000007.13
cg18739950	3.84E-09	3.53E-10	6.61	12.2426578	1.45E-01	95870440	95870563	NC_000015.9
cg21962423	3.84E-09	3.53E-10	6.61	12.2417494	1.42E-01	113913887	113914010	NC_000012.11
cg03886898	3.84E-09	3.53E-10	6.61	12.241406	4.92E-02	54359132	54359255	NC_000001.10
cg05930000	3.85E-09	3.54E-10	6.61	12.2395668	8.86E-02	23691424	23691547	NC_000002.11
cg00966398	3.85E-09	3.54E-10	6.61	12.2384891	1.40E-01	3602933	3603056	NC_000012.11
cg17929065	3.86E-09	3.55E-10	6.61	12.2372551	1.26E-01	192747379	192747502	NC_000002.11
cg11974937	3.86E-09	3.55E-10	6.61	12.2367531	6.69E-02	22052128	22052251	NC_000017.10
cg12364786	3.86E-09	3.55E-10	6.61	12.2365541	1.40E-01	64056664	64056787	NC_000015.9
cg11267829	3.86E-09	3.55E-10	6.61	12.2361893	1.98E-01	60289417	60289540	NC_000015.9
cg22810684	3.86E-09	3.55E-10	6.61	12.2361813	9.69E-02	80401665	80401788	NC_000017.10
cg19402039	3.86E-09	3.55E-10	6.61	12.2358247	1.25E-01	7546211	7546334	NC_000006.11

cg12451099	3.86E-09	3.56E-10	6.61	12.23569	1.36E-01	37356466	37356589	NC_000020.10
cg00426486	3.87E-09	3.56E-10	6.61	12.2341057	1.27E-01	86605100	86605223	NC_000016.9
cg26189021	3.87E-09	3.56E-10	6.61	12.2338584	1.14E-01	110757770	110757893	NC_000001.10
cg06509598	3.87E-09	3.56E-10	6.61	12.2334027	5.62E-02	129436051	129436174	NC_000003.11
cg04511534	3.87E-09	3.56E-10	6.61	12.2333575	1.46E-01	4463371	4463494	NC_000017.10
cg11764900	3.87E-09	3.56E-10	6.61	12.2331221	1.59E-01	38683035	38683158	NC_000006.11
cg10997906	3.88E-09	3.57E-10	6.61	12.2322912	1.24E-01	178753803	178753926	NC_000005.9
cg01618914	3.88E-09	3.57E-10	6.61	12.23128	3.45E-02	33267907	33268030	NC_000020.10
cg27013696	3.88E-09	3.57E-10	6.61	12.2306533	1.21E-01	19158030	19158153	NC_000007.13
cg20634288	3.88E-09	3.57E-10	6.61	12.2303466	5.23E-02	239834567	239834690	NC_000002.11
cg05365320	3.89E-09	3.58E-10	6.61	12.2301228	1.45E-01	200971339	200971462	NC_000001.10
cg24945701	3.89E-09	3.58E-10	6.61	12.2300547	1.36E-01	62362783	62362906	NC_000003.11
cg15837212	3.89E-09	3.58E-10	6.61	12.2295677	1.41E-01	63795970	63796093	NC_000001.10
cg27039662	3.90E-09	3.59E-10	6.61	12.2253917	1.52E-01	73163696	73163819	NC_000008.10
cg08627720	3.91E-09	3.60E-10	6.61	12.224289	1.56E-01	96959977	96960100	NC_000015.9
cg21383810	3.91E-09	3.60E-10	6.61	12.224029	2.32E-01	65515470	65515593	NC_000012.11
cg12632832	3.91E-09	3.60E-10	6.61	12.2239755	9.43E-02	157013346	157013469	NC_000001.10
cg20778071	3.91E-09	3.60E-10	6.61	12.2235328	7.27E-02	43729725	43729848	NC_000006.11
cg00682069	3.91E-09	3.60E-10	6.61	12.2222571	1.29E-01	1667458	1667581	NC_000002.11
cg14487379	3.92E-09	3.61E-10	6.61	12.2219647	9.97E-02	135481870	135481993	NC_000009.11
cg06962275	3.92E-09	3.61E-10	6.61	12.2207819	8.80E-02	30010203	30010326	NC_000006.11
cg01718222	3.92E-09	3.61E-10	6.61	12.2199925	9.19E-02	298277	298400	NC_000004.11
cg09992387	3.92E-09	3.61E-10	6.61	12.2195077	1.01E-01	55331285	55331408	NC_000001.10
cg12214908	3.93E-09	3.62E-10	6.61	12.2184412	1.33E-01	47910549	47910672	NC_000019.9
cg19211851	3.93E-09	3.62E-10	6.61	12.217914	1.35E-01	43555466	43555589	NC_000002.11

cg09176274	3.93E-09	3.62E-10	6.61	12.2176742	5.04E-02	32398912	32399035	NC_000010.10
cg06186727	3.93E-09	3.62E-10	6.61	12.2171727	7.11E-02	152591343	152591466	NC_000007.13
cg16702191	3.93E-09	3.62E-10	6.61	12.2167771	3.77E-02	195355586	195355709	NC_000003.11
cg24672116	3.94E-09	3.63E-10	6.61	12.2158722	1.13E-01	130124853	130124976	NC_000010.10
cg15561613	3.94E-09	3.63E-10	6.61	12.2158447	1.25E-01	245851610	245851733	NC_000001.10
cg19823847	3.94E-09	3.63E-10	6.61	12.2152504	8.61E-02	138539706	138539829	NC_000006.11
cg16709433	3.94E-09	3.63E-10	6.61	12.2151388	8.45E-02	53832593	53832716	NC_000006.11
cg20885782	3.94E-09	3.63E-10	6.61	12.2145863	1.52E-01	41875606	41875729	NC_000004.11
cg04276007	3.95E-09	3.64E-10	6.61	12.2137933	9.20E-02	107386512	107386635	NC_000007.13
cg08748948	3.95E-09	3.64E-10	6.61	12.2122895	1.88E-01	20792476	20792599	NC_000022.10
cg18482268	3.95E-09	3.64E-10	6.61	12.2116815	1.64E-01	145718965	145719088	NC_000005.9
cg00095859	3.96E-09	3.65E-10	6.61	12.210833	1.08E-01	1336729	1336852	NC_000007.13
cg11469061	3.96E-09	3.65E-10	6.61	12.2098173	1.68E-01	31826421	31826544	NC_000011.9
cg14215689	3.96E-09	3.65E-10	6.61	12.2091871	1.19E-01	139334807	139334930	NC_000009.11
cg05825073	3.96E-09	3.65E-10	6.61	12.2090443	1.92E-01	27282237	27282360	NC_000007.13
cg24083469	3.97E-09	3.65E-10	6.61	12.2086752	1.59E-01	85673879	85674002	NC_000012.11
cg16347279	3.97E-09	3.66E-10	6.61	12.2072341	1.49E-01	72667709	72667832	NC_000017.10
cg09414627	3.97E-09	3.66E-10	6.61	12.206818	8.69E-02	90225171	90225294	NC_000001.10
cg20198684	3.97E-09	3.66E-10	6.61	12.2065305	7.22E-02	45742032	45742155	NC_000019.9
cg15987088	3.97E-09	3.66E-10	6.61	12.206205	1.31E-01	172166234	172166357	NC_000003.11
cg08372051	3.97E-09	3.66E-10	6.61	12.2061771	2.91E-02	88294446	88294569	NC_000016.9
cg26035366	3.98E-09	3.67E-10	6.61	12.2049915	1.96E-01	26664621	26664744	NC_000003.11
cg19197905	3.98E-09	3.67E-10	6.61	12.2047464	1.01E-01	98716488	98716611	NC_000010.10
cg11723066	3.98E-09	3.67E-10	6.61	12.2045179	1.20E-01	133937475	133937598	NC_000011.9
cg02012576	3.98E-09	3.67E-10	6.61	12.2044183	2.10E-01	133485691	133485814	NC_000012.11

cg06925502	3.98E-09	3.67E-10	6.61	12.2039253	3.63E-02	106848012	106848135	NC_000005.9
cg04277893	3.98E-09	3.67E-10	6.61	12.2037314	5.69E-02	208128984	208129107	NC_000001.10
cg25617340	3.98E-09	3.67E-10	6.61	12.2035603	1.38E-01	37128011	37128134	NC_000014.8
cg11959676	3.99E-09	3.68E-10	6.6	12.2023581	3.16E-02	49719536	49719659	NC_000012.11
cg10900455	3.99E-09	3.68E-10	6.6	12.2023371	1.48E-01	42545099	42545222	NC_000020.10
cg10074544	3.99E-09	3.68E-10	6.6	12.2020756	1.66E-01	110223700	110223823	NC_000004.11
cg15461335	4.00E-09	3.69E-10	6.6	12.200149	1.45E-01	88324432	88324555	NC_000013.10
cg25325949	4.00E-09	3.69E-10	6.6	12.2000029	7.46E-02	82969524	82969647	NC_000005.9
cg00919055	4.00E-09	3.69E-10	6.6	12.1995797	7.90E-02	149546946	149547069	NC_000005.9
cg24630195	4.00E-09	3.69E-10	6.6	12.1991971	1.37E-01	2748369	2748492	NC_000005.9
cg04446870	4.00E-09	3.69E-10	6.6	12.1989121	1.35E-01	64440273	64440396	NC_000017.10
cg10208609	4.01E-09	3.70E-10	6.6	12.1978483	6.32E-02	90331229	90331352	NC_000001.10
cg13690873	4.01E-09	3.70E-10	6.6	12.1960311	5.48E-02	36587439	36587562	NC_000001.10
cg02756106	4.01E-09	3.70E-10	6.6	12.1959736	2.50E-01	45270707	45270830	NC_000012.11
cg16134717	4.01E-09	3.70E-10	6.6	12.1958846	1.74E-01	11752494	11752617	NC_000001.10
cg08328513	4.01E-09	3.70E-10	6.6	12.1956022	1.28E-01	78365691	78365814	NC_000005.9
cg24612667	4.02E-09	3.70E-10	6.6	12.1952929	1.40E-01	124735554	124735677	NC_000011.9
cg15233681	4.02E-09	3.71E-10	6.6	12.1939116	3.75E-02	102770602	102770725	NC_000002.11
cg25437410	4.03E-09	3.71E-10	6.6	12.1927726	2.28E-01	178017578	178017701	NC_000005.9
cg14971597	4.03E-09	3.72E-10	6.6	12.1910254	2.04E-01	159590578	159590701	NC_000006.11
cg00423167	4.03E-09	3.72E-10	6.6	12.1904727	2.18E-02	31937355	31937478	NC_000006.11
cg11610754	4.04E-09	3.72E-10	6.6	12.1899923	1.99E-01	18722595	18722718	NC_000019.9
cg05584070	4.04E-09	3.73E-10	6.6	12.1895642	7.80E-02	111329221	111329344	NC_000013.10
cg06240465	4.04E-09	3.73E-10	6.6	12.1875853	1.12E-01	46853413	46853536	NC_000021.8
cg23291534	4.05E-09	3.74E-10	6.6	12.186588	2.24E-01	110988043	110988166	NC_000008.10

cg01910478	4.05E-09	3.74E-10	6.6	12.1858029	1.03E-01	76373678	76373801	NC_000014.8
cg04919234	4.06E-09	3.75E-10	6.6	12.1842051	1.49E-01	99392478	99392601	NC_000006.11
cg11089489	4.06E-09	3.75E-10	6.6	12.183937	1.46E-01	37124313	37124436	NC_000014.8
cg24269074	4.07E-09	3.76E-10	6.6	12.1819022	1.63E-01	119614489	119614612	NC_000002.11
cg17577122	4.07E-09	3.76E-10	6.6	12.1818965	1.80E-01	19511967	19512090	NC_000022.10
cg04439622	4.07E-09	3.76E-10	6.6	12.1818469	1.31E-01	105944942	105945065	NC_000014.8
cg00514284	4.07E-09	3.76E-10	6.6	12.180678	6.38E-02	106855077	106855200	NC_000014.8
cg21136104	4.08E-09	3.76E-10	6.6	12.1795236	1.67E-01	142666320	142666443	NC_000003.11
cg05901522	4.08E-09	3.77E-10	6.6	12.1789993	8.96E-02	82169480	82169603	NC_000010.10
cg07057320	4.08E-09	3.77E-10	6.6	12.1785938	1.41E-01	37852479	37852602	NC_000021.8
cg21672843	4.09E-09	3.78E-10	6.6	12.1762393	1.91E-01	41818905	41819028	NC_000020.10
cg22738281	4.10E-09	3.79E-10	6.6	12.1738162	2.74E-02	8248444	8248567	NC_000004.11
cg21047938	4.10E-09	3.79E-10	6.6	12.1736498	1.99E-02	10192422	10192545	NC_000002.11
cg04374393	4.11E-09	3.79E-10	6.6	12.172053	9.92E-02	137483503	137483626	NC_000003.11
cg09177131	4.11E-09	3.80E-10	6.6	12.1711562	1.46E-01	114696462	114696585	NC_000001.10
cg23239971	4.11E-09	3.80E-10	6.6	12.1704115	3.96E-02	169630201	169630324	NC_000004.11
cg22142205	4.12E-09	3.80E-10	6.6	12.1692477	5.73E-02	80132787	80132910	NC_000017.10
cg06000330	4.12E-09	3.81E-10	6.6	12.1677226	1.37E-01	19183231	19183354	NC_000007.13
cg19857737	4.13E-09	3.82E-10	6.6	12.1656843	9.27E-02	39044238	39044361	NC_000001.10
cg23997911	4.14E-09	3.82E-10	6.6	12.1641689	6.17E-02	161575382	161575505	NC_000006.11
cg20383794	4.14E-09	3.83E-10	6.6	12.1639589	7.93E-02	2445271	2445394	NC_000011.9
cg10197263	4.14E-09	3.83E-10	6.6	12.1626447	1.01E-01	61718852	61718975	NC_000020.10
cg12060499	4.15E-09	3.83E-10	6.6	12.1622838	1.32E-01	102172296	102172419	NC_000014.8
cg26954843	4.15E-09	3.84E-10	6.6	12.1609071	6.42E-02	4310489	4310612	NC_000004.11
cg16399589	4.16E-09	3.84E-10	6.6	12.1597271	1.16E-01	26824423	26824546	NC_000017.10

cg01815567	4.16E-09	3.85E-10	6.6	12.1579508	1.50E-01	4866091	4866214	NC_000004.11
cg01273256	4.17E-09	3.85E-10	6.6	12.1565841	3.59E-02	251829	251952	NC_000020.10
cg00655498	4.18E-09	3.87E-10	6.6	12.1534747	9.32E-02	52022386	52022509	NC_000013.10
cg08659494	4.18E-09	3.87E-10	6.6	12.1529998	4.28E-02	89167990	89168113	NC_000016.9
cg01257889	4.18E-09	3.87E-10	6.6	12.1526794	1.51E-01	113230065	113230188	NC_000012.11
cg15939357	4.19E-09	3.87E-10	6.6	12.1522065	6.59E-02	44223465	44223588	NC_000019.9
cg23821145	4.19E-09	3.88E-10	6.6	12.1507239	8.23E-02	170696031	170696154	NC_000004.11
cg11367401	4.20E-09	3.88E-10	6.6	12.1495853	1.81E-02	134496777	134496900	NC_000010.10
cg17074353	4.21E-09	3.89E-10	6.59	12.1470829	9.57E-02	69590852	69590975	NC_000015.9
cg24603969	4.22E-09	3.90E-10	6.59	12.1447935	6.72E-02	143223013	143223136	NC_000006.11
cg21229079	4.22E-09	3.90E-10	6.59	12.1446006	1.43E-01	111158814	111158937	NC_000013.10
cg09995736	4.22E-09	3.90E-10	6.59	12.1436843	6.02E-02	95702136	95702259	NC_000012.11
cg26811852	4.23E-09	3.91E-10	6.59	12.1423007	1.27E-01	179051885	179052008	NC_000005.9
cg14556896	4.23E-09	3.91E-10	6.59	12.1420185	1.81E-01	22094963	22095086	NC_000012.11
cg19324698	4.23E-09	3.91E-10	6.59	12.1412322	3.07E-02	134652709	134652832	NC_000010.10
cg22289831	4.24E-09	3.92E-10	6.59	12.1392112	1.69E-01	38076709	38076832	NC_000021.8
cg13179074	4.24E-09	3.93E-10	6.59	12.138399	8.32E-02	167064091	167064214	NC_000001.10
cg08461451	4.24E-09	3.93E-10	6.59	12.1383633	1.30E-01	2295092	2295215	NC_000019.9
cg05872306	4.26E-09	3.94E-10	6.59	12.1346017	1.51E-01	29236535	29236658	NC_000014.8
cg00508158	4.26E-09	3.95E-10	6.59	12.1336286	8.83E-02	48189514	48189637	NC_000001.10
cg11723848	4.26E-09	3.95E-10	6.59	12.1334056	1.95E-01	96470286	96470409	NC_000004.11
cg19181479	4.26E-09	3.95E-10	6.59	12.132831	7.81E-02	48373736	48373859	NC_000010.10
cg23999695	4.26E-09	3.95E-10	6.59	12.1327052	2.25E-01	89943625	89943748	NC_000015.9
cg13308063	4.27E-09	3.95E-10	6.59	12.1321218	4.34E-02	32042344	32042467	NC_000006.11
cg07769957	4.27E-09	3.95E-10	6.59	12.1318553	1.06E-01	46443657	46443780	NC_000019.9

cg04447756	4.27E-09	3.95E-10	6.59	12.1316348	9.95E-02	138644986	138645109	NC_000005.9
cg09514401	4.28E-09	3.97E-10	6.59	12.1282131	1.31E-01	72988022	72988145	NC_000008.10
cg27421117	4.29E-09	3.97E-10	6.59	12.1266457	1.47E-01	74215659	74215782	NC_000002.11
cg22698998	4.29E-09	3.97E-10	6.59	12.126494	5.69E-02	211344378	211344501	NC_000002.11
cg17330515	4.29E-09	3.98E-10	6.59	12.1254168	9.96E-02	13031369	13031492	NC_000019.9
cg12456269	4.29E-09	3.98E-10	6.59	12.1252281	2.01E-01	142887725	142887848	NC_000002.11
cg17875935	4.29E-09	3.98E-10	6.59	12.125068	1.35E-01	45666384	45666507	NC_000015.9
cg10750934	4.30E-09	3.98E-10	6.59	12.1244652	4.92E-02	45652975	45653098	NC_000019.9
cg14128735	4.31E-09	4.00E-10	6.59	12.1213306	1.06E-01	44324976	44325099	NC_000011.9
cg14757661	4.31E-09	4.00E-10	6.59	12.1205137	1.31E-01	92684308	92684431	NC_000005.9
cg06212670	4.33E-09	4.01E-10	6.59	12.1171128	1.27E-01	88543627	88543750	NC_000016.9
cg02704103	4.33E-09	4.02E-10	6.59	12.1164262	9.45E-02	72846991	72847114	NC_000003.11
cg17167920	4.34E-09	4.03E-10	6.59	12.1135896	9.02E-02	154127537	154127660	NC_000001.10
cg06830692	4.34E-09	4.03E-10	6.59	12.1135803	1.45E-01	44954192	44954315	NC_000017.10
cg25561904	4.34E-09	4.03E-10	6.59	12.1135546	1.24E-01	104165401	104165524	NC_000014.8
cg24836607	4.34E-09	4.03E-10	6.59	12.113246	6.97E-02	117067752	117067875	NC_000007.13
cg19078037	4.35E-09	4.03E-10	6.59	12.1119682	1.59E-01	77329049	77329172	NC_000018.9
cg10207181	4.35E-09	4.03E-10	6.59	12.1117753	7.75E-02	147801090	147801213	NC_000001.10
cg21827986	4.36E-09	4.04E-10	6.59	12.1105786	6.70E-02	105176581	105176704	NC_000014.8
cg09227150	4.36E-09	4.05E-10	6.59	12.1088324	1.61E-01	102475578	102475701	NC_000010.10
cg25425140	4.37E-09	4.05E-10	6.59	12.1079188	1.87E-01	147098450	147098573	NC_000003.11
cg19389884	4.37E-09	4.05E-10	6.59	12.1078514	1.54E-01	1358515	1358638	NC_000011.9
cg20546002	4.37E-09	4.05E-10	6.59	12.1078333	1.86E-01	178017376	178017499	NC_000005.9
cg19801921	4.38E-09	4.06E-10	6.59	12.1045954	1.83E-01	63784864	63784987	NC_000001.10
cg01934709	4.39E-09	4.07E-10	6.59	12.1032142	5.20E-02	39357454	39357577	NC_000001.10

cg00629427	4.39E-09	4.07E-10	6.59	12.1023428	1.18E-01	95357638	95357761	NC_000013.10
cg25363679	4.39E-09	4.08E-10	6.59	12.1018705	7.55E-02	71031202	71031325	NC_000012.11
cg10964994	4.39E-09	4.08E-10	6.59	12.1015035	2.97E-02	79892921	79893044	NC_000017.10
cg27051315	4.40E-09	4.08E-10	6.59	12.099578	1.08E-01	238864959	238865082	NC_000002.11
cg04166812	4.41E-09	4.09E-10	6.59	12.0987291	1.53E-01	61122051	61122174	NC_000014.8
cg12506930	4.42E-09	4.10E-10	6.59	12.096091	2.04E-01	58220718	58220841	NC_000019.9
cg19141258	4.42E-09	4.10E-10	6.59	12.0954465	9.85E-02	183993368	183993491	NC_000003.11
cg16031338	4.42E-09	4.10E-10	6.59	12.0951994	6.05E-02	2430136	2430259	NC_000001.10
cg08933939	4.43E-09	4.11E-10	6.58	12.0930577	2.07E-01	100061504	100061627	NC_000006.11
cg21352158	4.43E-09	4.12E-10	6.58	12.092073	8.49E-02	832917	833040	NC_000008.10
cg20648847	4.44E-09	4.12E-10	6.58	12.0916741	1.57E-01	66326767	66326890	NC_000011.9
cg00472801	4.44E-09	4.12E-10	6.58	12.0916164	1.41E-01	62995876	62995999	NC_000006.11
cg08822616	4.44E-09	4.12E-10	6.58	12.0905501	7.85E-02	134257514	134257637	NC_000011.9
cg13661519	4.44E-09	4.12E-10	6.58	12.0904648	1.30E-01	27141088	27141211	NC_000007.13
cg05792861	4.45E-09	4.13E-10	6.58	12.0886288	5.10E-02	18145434	18145557	NC_000003.11
cg09149894	4.45E-09	4.14E-10	6.58	12.0873347	1.10E-01	32097233	32097356	NC_000006.11
cg10418524	4.46E-09	4.14E-10	6.58	12.0864084	1.77E-01	176956678	176956801	NC_000002.11
cg12582959	4.46E-09	4.14E-10	6.58	12.0852495	1.46E-01	56159199	56159322	NC_000019.9
cg06882544	4.46E-09	4.15E-10	6.58	12.0850104	7.10E-02	298926	299049	NC_000004.11
cg23237183	4.47E-09	4.15E-10	6.58	12.0830459	1.82E-01	73429791	73429914	NC_000002.11
cg03273420	4.48E-09	4.16E-10	6.58	12.0817379	8.63E-02	18166322	18166445	NC_000017.10
cg08332148	4.48E-09	4.16E-10	6.58	12.0816949	3.81E-02	75551697	75551820	NC_000010.10
cg20704330	4.49E-09	4.17E-10	6.58	12.079295	4.62E-02	131411549	131411672	NC_000005.9
cg06908244	4.50E-09	4.18E-10	6.58	12.0770628	1.64E-01	100126358	100126481	NC_000014.8
cg16590190	4.50E-09	4.18E-10	6.58	12.0764726	1.06E-01	13909522	13909645	NC_000001.10

cg17694719	4.50E-09	4.19E-10	6.58	12.075693	6.23E-02	150554915	150555038	NC_000007.13
cg11100804	4.51E-09	4.19E-10	6.58	12.0745502	1.27E-01	140783173	140783296	NC_000005.9
cg00008564	4.52E-09	4.20E-10	6.58	12.0714199	9.52E-02	27830391	27830514	NC_000006.11
cg12007399	4.53E-09	4.21E-10	6.58	12.0707683	1.55E-01	3868876	3868999	NC_000019.9
cg06365303	4.53E-09	4.21E-10	6.58	12.068912	1.56E-01	2801844	2801967	NC_000020.10
cg14339760	4.54E-09	4.22E-10	6.58	12.0685895	3.91E-02	130550808	130550931	NC_000009.11
cg16049690	4.55E-09	4.23E-10	6.58	12.0664366	1.25E-01	180486679	180486802	NC_000005.9
cg24609884	4.55E-09	4.23E-10	6.58	12.064929	1.09E-01	208148998	208149121	NC_000002.11
cg18220921	4.55E-09	4.23E-10	6.58	12.0648175	1.25E-01	242687527	242687650	NC_000001.10
cg21679169	4.56E-09	4.24E-10	6.58	12.0639011	3.67E-02	150518766	150518889	NC_000001.10
cg25460563	4.56E-09	4.24E-10	6.58	12.063816	5.48E-02	53839886	53840009	NC_000005.9
cg14084056	4.56E-09	4.24E-10	6.58	12.0621309	2.25E-02	48744539	48744662	NC_000012.11
cg09275869	4.58E-09	4.26E-10	6.58	12.0582794	1.94E-01	44337099	44337222	NC_000018.9
cg21534423	4.58E-09	4.26E-10	6.58	12.0576226	1.92E-01	24154607	24154730	NC_000019.9
cg00860372	4.59E-09	4.27E-10	6.58	12.0568677	7.85E-02	13912327	13912450	NC_000019.9
cg04120171	4.59E-09	4.27E-10	6.58	12.0567963	2.00E-01	83775850	83775973	NC_000015.9
cg25902187	4.60E-09	4.28E-10	6.58	12.0547447	2.36E-01	55881044	55881167	NC_000015.9
cg14758740	4.60E-09	4.28E-10	6.58	12.0545044	5.84E-02	941872	941995	NC_000017.10
cg04551925	4.61E-09	4.29E-10	6.58	12.0515001	1.14E-01	30951740	30951863	NC_000007.13
cg03630832	4.62E-09	4.29E-10	6.58	12.0506274	6.56E-02	62443059	62443182	NC_000016.9
cg27277532	4.62E-09	4.29E-10	6.58	12.0504933	1.49E-01	86321612	86321735	NC_000016.9
cg05884032	4.62E-09	4.30E-10	6.58	12.0503086	1.57E-01	76740088	76740211	NC_000018.9
cg16302049	4.62E-09	4.30E-10	6.58	12.0501217	1.13E-01	175349051	175349174	NC_000002.11
cg21305475	4.62E-09	4.30E-10	6.58	12.0499122	5.48E-02	34595432	34595555	NC_000017.10
cg23117727	4.62E-09	4.30E-10	6.58	12.0497983	1.20E-01	23019450	23019573	NC_000020.10

cg02091100	4.63E-09	4.31E-10	6.58	12.0479188	9.39E-02	42123029	42123152	NC_000006.11
cg06643056	4.63E-09	4.31E-10	6.58	12.0474284	6.04E-02	170875683	170875806	NC_000005.9
cg05085336	4.63E-09	4.31E-10	6.58	12.0473384	1.28E-01	73523729	73523852	NC_000003.11
cg22945387	4.63E-09	4.31E-10	6.58	12.0468516	1.31E-01	36983404	36983527	NC_000014.8
cg11334818	4.64E-09	4.32E-10	6.58	12.0454449	2.28E-01	18813191	18813314	NC_000011.9
cg25955816	4.65E-09	4.33E-10	6.58	12.0431637	5.79E-02	228612876	228612999	NC_000001.10
cg23357150	4.65E-09	4.33E-10	6.58	12.0428694	4.62E-02	134470225	134470348	NC_000010.10
cg15983520	4.65E-09	4.33E-10	6.58	12.0421849	1.31E-01	145729106	145729229	NC_000008.10
cg03186333	4.66E-09	4.34E-10	6.58	12.041258	1.54E-01	72470352	72470475	NC_000008.10
cg02578368	4.66E-09	4.34E-10	6.57	12.0406443	2.51E-01	26250744	26250867	NC_000006.11
cg19721065	4.68E-09	4.35E-10	6.57	12.0368408	8.58E-02	113549986	113550109	NC_000012.11
cg18083764	4.69E-09	4.37E-10	6.57	12.0344397	1.29E-01	4230848	4230971	NC_000005.9
cg13041244	4.69E-09	4.37E-10	6.57	12.0337459	1.24E-01	19284694	19284817	NC_000011.9
cg20653075	4.69E-09	4.37E-10	6.57	12.0335911	1.46E-01	65291682	65291805	NC_000008.10
cg01650618	4.69E-09	4.37E-10	6.57	12.0334135	5.43E-02	20210743	20210866	NC_000013.10
cg17625032	4.69E-09	4.37E-10	6.57	12.0330289	9.68E-02	57054740	57054863	NC_000016.9
cg12626956	4.69E-09	4.37E-10	6.57	12.0325748	1.38E-01	79634509	79634632	NC_000009.11
cg25558218	4.70E-09	4.38E-10	6.57	12.0318073	8.09E-02	51445694	51445817	NC_000005.9
cg12011136	4.70E-09	4.38E-10	6.57	12.0306703	1.12E-01	4901750	4901873	NC_000007.13
cg20703242	4.71E-09	4.39E-10	6.57	12.0300411	8.45E-02	230279135	230279258	NC_000001.10
cg05671386	4.71E-09	4.39E-10	6.57	12.0295157	1.20E-01	3550239	3550362	NC_000016.9
cg00660105	4.71E-09	4.39E-10	6.57	12.0289224	1.72E-01	82135784	82135907	NC_000004.11
cg22070855	4.72E-09	4.40E-10	6.57	12.027455	1.73E-01	172167527	172167650	NC_000003.11
cg06022179	4.72E-09	4.40E-10	6.57	12.0270636	1.29E-01	89903506	89903629	NC_000015.9
cg15161691	4.73E-09	4.41E-10	6.57	12.0250796	4.19E-02	134257429	134257552	NC_000011.9

cg16729832	4.74E-09	4.42E-10	6.57	12.0221135	1.53E-01	21645078	21645201	NC_000008.10
cg03091752	4.74E-09	4.42E-10	6.57	12.0220266	1.79E-01	51221605	51221728	NC_000019.9
cg13740815	4.75E-09	4.43E-10	6.57	12.0205409	1.45E-01	51170356	51170479	NC_000019.9
cg05221167	4.75E-09	4.43E-10	6.57	12.019391	1.57E-01	9609397	9609520	NC_000019.9
cg05730365	4.76E-09	4.43E-10	6.57	12.0192752	2.04E-01	37838563	37838686	NC_000005.9
cg19149132	4.76E-09	4.43E-10	6.57	12.0190951	1.24E-01	130854436	130854559	NC_000010.10
cg02221765	4.76E-09	4.44E-10	6.57	12.0174	4.46E-02	89760916	89761039	NC_000014.8
cg16459735	4.77E-09	4.45E-10	6.57	12.0162639	1.30E-01	134999791	134999914	NC_000010.10
cg16596755	4.77E-09	4.45E-10	6.57	12.015971	1.08E-01	5021778	5021901	NC_000004.11
cg04554613	4.77E-09	4.45E-10	6.57	12.0153835	8.53E-02	169945470	169945593	NC_000002.11
cg05097899	4.77E-09	4.45E-10	6.57	12.0149289	3.37E-02	5080743	5080866	NC_000020.10
cg02472348	4.78E-09	4.46E-10	6.57	12.0140345	1.70E-01	22487781	22487904	NC_000012.11
cg22778957	4.78E-09	4.46E-10	6.57	12.0138193	1.10E-01	60639054	60639177	NC_000020.10
cg21575295	4.78E-09	4.46E-10	6.57	12.0130578	1.35E-01	101193432	101193555	NC_000014.8
cg01539133	4.78E-09	4.46E-10	6.57	12.0128541	1.49E-01	4607324	4607447	NC_000019.9
cg20981451	4.78E-09	4.46E-10	6.57	12.0127519	1.13E-01	52954615	52954738	NC_000019.9
cg13275321	4.79E-09	4.47E-10	6.57	12.011155	8.70E-02	307848	307971	NC_000005.9
cg03721362	4.80E-09	4.48E-10	6.57	12.0098208	2.22E-01	126626298	126626421	NC_000005.9
cg15055165	4.80E-09	4.48E-10	6.57	12.0091234	9.33E-02	2964849	2964972	NC_000002.11
cg25404650	4.80E-09	4.48E-10	6.57	12.0087102	2.11E-02	155616381	155616504	NC_000007.13
cg16573328	4.80E-09	4.48E-10	6.57	12.008291	1.91E-01	185463588	185463711	NC_000002.11
cg23056539	4.81E-09	4.49E-10	6.57	12.0076358	1.12E-01	242977619	242977742	NC_000002.11
cg15484988	4.81E-09	4.49E-10	6.57	12.0063536	1.79E-01	74961494	74961617	NC_000018.9
cg21539988	4.82E-09	4.50E-10	6.57	12.0054645	3.63E-02	118312424	118312547	NC_000012.11
cg08196032	4.82E-09	4.50E-10	6.57	12.0052114	1.71E-01	95239751	95239874	NC_000014.8

cg11809342	4.82E-09	4.50E-10	6.57	12.0048414	1.65E-01	160007430	160007553	NC_000002.11
cg11433319	4.82E-09	4.50E-10	6.57	12.0047291	1.69E-01	19617873	19617996	NC_000021.8
cg06506868	4.82E-09	4.50E-10	6.57	12.0045833	6.56E-02	197648437	197648560	NC_000003.11
cg22510079	4.82E-09	4.50E-10	6.57	12.0038204	2.26E-02	32809403	32809526	NC_000006.11
cg05477514	4.82E-09	4.50E-10	6.57	12.0037718	1.23E-01	23564008	23564131	NC_000008.10
cg20669414	4.83E-09	4.51E-10	6.57	12.0034281	1.58E-01	37808629	37808752	NC_000017.10
cg13585776	4.83E-09	4.51E-10	6.57	12.0029614	1.09E-01	126783547	126783670	NC_000009.11
cg05512684	4.83E-09	4.51E-10	6.57	12.0019581	8.05E-02	37879858	37879981	NC_000017.10
cg27387239	4.84E-09	4.52E-10	6.57	12.0004973	1.33E-01	218667548	218667671	NC_000001.10
cg08889797	4.84E-09	4.52E-10	6.57	12.0002602	1.74E-01	147106489	147106612	NC_000003.11
cg08605458	4.84E-09	4.52E-10	6.57	12.0000287	1.14E-01	26949714	26949837	NC_000002.11
cg26718878	4.84E-09	4.52E-10	6.57	11.9994942	1.74E-01	96647882	96648005	NC_000007.13
cg18794475	4.85E-09	4.53E-10	6.57	11.9987913	6.49E-02	55533829	55533952	NC_000008.10
cg24037938	4.85E-09	4.53E-10	6.57	11.9985134	9.81E-02	4697967	4698090	NC_000001.10
cg16705138	4.85E-09	4.53E-10	6.57	11.9976572	7.24E-02	94508740	94508863	NC_000014.8
cg13718539	4.86E-09	4.54E-10	6.57	11.9966447	1.35E-01	140801354	140801477	NC_000005.9
cg09113440	4.86E-09	4.54E-10	6.57	11.9953343	5.85E-02	18999313	18999436	NC_000010.10
cg09571858	4.86E-09	4.54E-10	6.57	11.9952965	2.29E-01	17496221	17496344	NC_000010.10
cg10078898	4.87E-09	4.55E-10	6.57	11.9948685	5.97E-02	21491045	21491168	NC_000014.8
cg26105156	4.87E-09	4.55E-10	6.57	11.9947843	2.00E-01	81951953	81952076	NC_000004.11
cg02892367	4.87E-09	4.55E-10	6.57	11.9935342	1.17E-01	148724141	148724264	NC_000007.13
cg11241627	4.87E-09	4.55E-10	6.57	11.9933452	1.48E-01	19185165	19185288	NC_000007.13
cg04931256	4.87E-09	4.55E-10	6.57	11.9931662	1.36E-01	147077636	147077759	NC_000003.11
cg01268824	4.87E-09	4.55E-10	6.57	11.9928874	1.97E-01	58220818	58220941	NC_000019.9
cg25171856	4.88E-09	4.56E-10	6.57	11.9919611	3.39E-02	75402751	75402874	NC_000010.10

cg23814172	4.88E-09	4.56E-10	6.57	11.9917309	1.02E-01	687493	687616	NC_000010.10
cg04774110	4.88E-09	4.57E-10	6.57	11.9906184	3.24E-02	80857351	80857474	NC_000017.10
cg17718960	4.90E-09	4.58E-10	6.57	11.9874327	1.12E-01	6063138	6063261	NC_000001.10
cg11793646	4.91E-09	4.59E-10	6.56	11.9860951	3.83E-02	44087129	44087252	NC_000021.8
cg09156097	4.91E-09	4.59E-10	6.56	11.9859841	1.42E-01	138729283	138729406	NC_000005.9
cg06573378	4.91E-09	4.59E-10	6.56	11.9850042	1.27E-01	66907862	66907985	NC_000002.11
cg20271517	4.92E-09	4.60E-10	6.56	11.9823033	1.98E-01	156129862	156129985	NC_000004.11
cg07249209	4.94E-09	4.62E-10	6.56	11.979595	6.65E-02	19183930	19184053	NC_000013.10
cg01476003	4.94E-09	4.62E-10	6.56	11.9785766	1.62E-01	71460486	71460609	NC_000016.9
cg01761256	4.94E-09	4.62E-10	6.56	11.9780375	6.53E-02	72243817	72243940	NC_000014.8
cg16081281	4.95E-09	4.63E-10	6.56	11.977178	1.55E-01	23559839	23559962	NC_000008.10
cg06371489	4.95E-09	4.63E-10	6.56	11.9770607	7.33E-02	86309582	86309705	NC_000015.9
cg00047501	4.95E-09	4.63E-10	6.56	11.9763022	6.17E-02	201680860	201680983	NC_000002.11
cg08259925	4.95E-09	4.63E-10	6.56	11.9760599	1.36E-01	63257813	63257936	NC_000005.9
cg14420668	4.95E-09	4.64E-10	6.56	11.9755532	4.70E-02	1054275	1054398	NC_000007.13
cg02231404	4.96E-09	4.64E-10	6.56	11.9753147	1.20E-01	62679635	62679758	NC_000020.10
cg01718065	4.96E-09	4.64E-10	6.56	11.9746921	1.05E-01	51774429	51774552	NC_000019.9
cg16868298	4.96E-09	4.64E-10	6.56	11.9746289	1.35E-01	120969033	120969156	NC_000007.13
cg26344392	4.96E-09	4.64E-10	6.56	11.9745484	8.98E-02	167825464	167825587	NC_000001.10
cg14078678	4.96E-09	4.64E-10	6.56	11.9744469	7.44E-02	46584148	46584271	NC_000020.10
cg17623793	4.96E-09	4.64E-10	6.56	11.9740904	1.21E-01	4230872	4230995	NC_000005.9
cg06046819	4.96E-09	4.64E-10	6.56	11.9737712	5.64E-02	4500281	4500404	NC_000017.10
cg24164238	4.98E-09	4.66E-10	6.56	11.9706218	7.30E-02	114199055	114199178	NC_000013.10
cg23381749	4.98E-09	4.66E-10	6.56	11.9702259	1.51E-01	130645423	130645546	NC_000012.11
cg11388277	4.99E-09	4.67E-10	6.56	11.9689553	1.15E-01	328132	328255	NC_000004.11

cg10500406	4.99E-09	4.67E-10	6.56	11.9685534	1.74E-02	99036512	99036635	NC_000007.13
cg23237765	4.99E-09	4.67E-10	6.56	11.9677936	6.52E-02	921845	921968	NC_000007.13
cg26994225	5.00E-09	4.68E-10	6.56	11.9670285	1.46E-01	119616769	119616892	NC_000002.11
cg16810605	5.00E-09	4.68E-10	6.56	11.9664911	1.64E-01	65283263	65283386	NC_000008.10
cg09711746	5.00E-09	4.68E-10	6.56	11.9659343	1.25E-01	32460375	32460498	NC_000011.9
cg25020286	5.00E-09	4.68E-10	6.56	11.9659016	1.64E-01	18956947	18957070	NC_000001.10
cg04352272	5.00E-09	4.68E-10	6.56	11.9658115	1.45E-01	3289798	3289921	NC_000017.10
cg10835823	5.00E-09	4.69E-10	6.56	11.9650533	8.97E-02	169659763	169659886	NC_000005.9
cg21778348	5.01E-09	4.69E-10	6.56	11.9642465	1.31E-01	27194614	27194737	NC_000007.13
cg23913421	5.01E-09	4.69E-10	6.56	11.9640332	2.11E-01	50674663	50674786	NC_000006.11
cg12105941	5.01E-09	4.69E-10	6.56	11.9633677	1.63E-01	133657466	133657589	NC_000011.9
cg02104434	5.01E-09	4.69E-10	6.56	11.9632437	8.85E-02	247694406	247694529	NC_000001.10
cg06795523	5.01E-09	4.69E-10	6.56	11.9631144	1.11E-01	58358974	58359097	NC_000002.11
cg24393316	5.01E-09	4.70E-10	6.56	11.9630196	2.03E-01	100616469	100616592	NC_000009.11
cg11752440	5.02E-09	4.70E-10	6.56	11.9616902	2.02E-01	102980466	102980589	NC_000011.9
cg06202276	5.02E-09	4.71E-10	6.56	11.9610156	1.57E-01	154713333	154713456	NC_000004.11
cg04878000	5.03E-09	4.71E-10	6.56	11.9596589	2.24E-01	32237923	32238046	NC_000001.10
cg10202544	5.03E-09	4.71E-10	6.56	11.9595625	1.13E-01	92303685	92303808	NC_000014.8
cg23452498	5.03E-09	4.72E-10	6.56	11.9586567	1.59E-01	144889607	144889730	NC_000005.9
cg06809364	5.03E-09	4.72E-10	6.56	11.9584961	8.56E-02	152555206	152555329	NC_000003.11
cg25098479	5.04E-09	4.72E-10	6.56	11.9584146	7.19E-02	124763800	124763923	NC_000012.11
cg24167841	5.04E-09	4.72E-10	6.56	11.9581525	3.59E-02	57389041	57389164	NC_000012.11
cg08230910	5.04E-09	4.72E-10	6.56	11.9577999	5.02E-02	80507149	80507272	NC_000007.13
cg12945194	5.04E-09	4.72E-10	6.56	11.9573797	9.16E-02	64703312	64703435	NC_000011.9
cg24084681	5.04E-09	4.72E-10	6.56	11.9573113	2.15E-01	93520288	93520411	NC_000007.13

cg27331241	5.04E-09	4.72E-10	6.56	11.9570763	2.09E-01	751830	751953	NC_000007.13
cg16056164	5.04E-09	4.73E-10	6.56	11.9566836	7.10E-02	233211572	233211695	NC_000001.10
cg05666607	5.04E-09	4.73E-10	6.56	11.9564575	1.40E-01	145718405	145718528	NC_000005.9
cg09583939	5.06E-09	4.75E-10	6.56	11.9525659	9.25E-02	140166213	140166336	NC_000005.9
cg17838029	5.06E-09	4.75E-10	6.56	11.9524513	1.25E-01	133562334	133562457	NC_000006.11
cg02046160	5.07E-09	4.75E-10	6.56	11.9521726	4.50E-02	88874667	88874790	NC_000009.11
cg02853358	5.07E-09	4.75E-10	6.56	11.9516332	5.87E-02	74010290	74010413	NC_000002.11
cg04210284	5.07E-09	4.75E-10	6.56	11.9515507	2.27E-01	1445561	1445684	NC_000005.9
cg23030903	5.07E-09	4.76E-10	6.56	11.9504096	5.06E-02	9908764	9908887	NC_000003.11
cg18549575	5.08E-09	4.76E-10	6.56	11.949709	4.03E-02	128494568	128494691	NC_000007.13
cg00158333	5.09E-09	4.77E-10	6.56	11.9469194	1.45E-01	47075855	47075978	NC_000017.10
cg04874129	5.09E-09	4.77E-10	6.56	11.9468914	1.35E-01	55690873	55690996	NC_000016.9
cg14741939	5.10E-09	4.78E-10	6.56	11.9452891	1.63E-01	147127137	147127260	NC_000003.11
cg00706802	5.10E-09	4.78E-10	6.56	11.9451571	6.29E-02	47322368	47322491	NC_000003.11
cg08932381	5.11E-09	4.79E-10	6.56	11.9433754	1.12E-01	138078989	138079112	NC_000003.11
cg01471384	5.11E-09	4.79E-10	6.56	11.9429977	1.62E-01	107957265	107957388	NC_000004.11
cg15831296	5.12E-09	4.80E-10	6.56	11.940606	6.00E-02	101192591	101192714	NC_000014.8
cg00460286	5.13E-09	4.81E-10	6.56	11.9391658	7.87E-02	77320261	77320384	NC_000015.9
cg09014354	5.13E-09	4.81E-10	6.56	11.9386052	1.86E-01	55413878	55414001	NC_000012.11
cg26250093	5.13E-09	4.81E-10	6.56	11.9385514	5.86E-02	24397649	24397772	NC_000002.11
cg09580592	5.14E-09	4.82E-10	6.56	11.936451	1.12E-01	907643	907766	NC_000017.10
cg07039560	5.15E-09	4.83E-10	6.56	11.935733	1.03E-01	140683681	140683804	NC_000005.9
cg17772342	5.15E-09	4.83E-10	6.56	11.9348487	1.57E-01	134871686	134871809	NC_000005.9
cg14146100	5.15E-09	4.84E-10	6.56	11.9340648	1.40E-01	134069236	134069359	NC_000004.11
cg06370771	5.16E-09	4.84E-10	6.56	11.9338466	1.91E-01	18960940	18961063	NC_000001.10

cg10229905	5.16E-09	4.84E-10	6.56	11.9336337	7.62E-02	50715703	50715826	NC_000019.9
cg22131691	5.16E-09	4.84E-10	6.55	11.9328469	2.10E-01	32110988	32111111	NC_000007.13
cg13704497	5.17E-09	4.85E-10	6.55	11.9316224	1.25E-01	31590622	31590745	NC_000006.11
cg07538634	5.17E-09	4.85E-10	6.55	11.930294	1.05E-01	3825141	3825264	NC_000019.9
cg17807172	5.17E-09	4.86E-10	6.55	11.9300547	1.82E-01	122620929	122621052	NC_000005.9
cg07541744	5.18E-09	4.86E-10	6.55	11.9297206	1.68E-01	55363277	55363400	NC_000016.9
cg21600537	5.18E-09	4.86E-10	6.55	11.9295945	1.47E-01	8115260	8115383	NC_000019.9
cg03259887	5.18E-09	4.86E-10	6.55	11.9288905	1.01E-01	85608894	85609017	NC_000016.9
cg15179472	5.18E-09	4.86E-10	6.55	11.928335	3.28E-02	70736858	70736981	NC_000015.9
cg13859860	5.18E-09	4.87E-10	6.55	11.9281692	1.10E-01	51569878	51570001	NC_000010.10
cg15994026	5.18E-09	4.87E-10	6.55	11.9279825	2.50E-01	15780306	15780429	NC_000004.11
cg26856475	5.19E-09	4.87E-10	6.55	11.9269585	1.09E-01	140740503	140740626	NC_000005.9
cg06122148	5.20E-09	4.88E-10	6.55	11.9258425	2.45E-01	98891553	98891676	NC_000011.9
cg08397978	5.20E-09	4.88E-10	6.55	11.9251504	1.91E-01	89340205	89340328	NC_000008.10
cg04821895	5.20E-09	4.88E-10	6.55	11.9251102	1.26E-01	102462585	102462708	NC_000015.9
cg01414185	5.20E-09	4.88E-10	6.55	11.9243956	1.99E-01	177017449	177017572	NC_000002.11
cg19069039	5.20E-09	4.89E-10	6.55	11.9238853	5.38E-02	52238665	52238788	NC_000012.11
cg03383508	5.21E-09	4.89E-10	6.55	11.9233817	1.36E-01	119591097	119591220	NC_000002.11
cg05452066	5.21E-09	4.89E-10	6.55	11.9228559	3.97E-02	138298086	138298209	NC_000005.9
cg24778248	5.21E-09	4.89E-10	6.55	11.9222296	1.48E-01	98963062	98963185	NC_000002.11
cg21795221	5.22E-09	4.90E-10	6.55	11.9214204	1.25E-01	156718262	156718385	NC_000006.11
cg27171201	5.23E-09	4.91E-10	6.55	11.9195864	6.15E-02	3365137	3365260	NC_000019.9
cg01966891	5.23E-09	4.91E-10	6.55	11.9188278	1.31E-01	95870990	95871113	NC_000015.9
cg03299740	5.24E-09	4.92E-10	6.55	11.9175554	4.19E-02	17997779	17997902	NC_000002.11
cg21382800	5.24E-09	4.92E-10	6.55	11.9171276	1.84E-02	29661771	29661894	NC_000022.10

cg22344703	5.24E-09	4.93E-10	6.55	11.9158761	1.42E-01	219736312	219736435	NC_000002.11
cg04737042	5.25E-09	4.93E-10	6.55	11.9150718	5.82E-02	29153994	29154117	NC_000016.9
cg14020278	5.25E-09	4.93E-10	6.55	11.9149763	2.59E-02	139727398	139727521	NC_000009.11
cg21472506	5.25E-09	4.93E-10	6.55	11.9145896	1.96E-01	63283967	63284090	NC_000002.11
cg15140703	5.25E-09	4.94E-10	6.55	11.9141694	1.71E-01	99775532	99775655	NC_000007.13
cg03270995	5.25E-09	4.94E-10	6.55	11.9141292	3.04E-02	4790537	4790660	NC_000016.9
cg19888509	5.26E-09	4.95E-10	6.55	11.9119127	1.03E-01	125145641	125145764	NC_000012.11
cg12345501	5.27E-09	4.95E-10	6.55	11.9115412	8.24E-02	180936706	180936829	NC_000002.11
cg19600494	5.27E-09	4.95E-10	6.55	11.9112931	9.50E-02	106959525	106959648	NC_000002.11
cg17174980	5.27E-09	4.95E-10	6.55	11.9106547	6.80E-02	14109514	14109637	NC_000012.11
cg23100720	5.28E-09	4.96E-10	6.55	11.9095519	1.31E-01	155770386	155770509	NC_000006.11
cg00971110	5.28E-09	4.96E-10	6.55	11.9087437	6.31E-02	84814140	84814263	NC_000007.13
cg17110559	5.29E-09	4.97E-10	6.55	11.9066537	9.85E-02	32111392	32111515	NC_000012.11
cg23288962	5.30E-09	4.98E-10	6.55	11.9048897	1.25E-01	35517516	35517639	NC_000013.10
cg19571106	5.31E-09	4.99E-10	6.55	11.903049	1.58E-01	9762850	9762973	NC_000008.10
cg18788932	5.31E-09	4.99E-10	6.55	11.9025357	1.57E-01	115135700	115135823	NC_000012.11
cg22622123	5.32E-09	5.00E-10	6.55	11.9015659	1.58E-01	118902351	118902474	NC_000011.9
cg23639692	5.32E-09	5.01E-10	6.55	11.8999787	1.26E-01	27175152	27175275	NC_000007.13
cg11134052	5.33E-09	5.01E-10	6.55	11.8997036	1.42E-01	61119625	61119748	NC_000014.8
cg14578500	5.34E-09	5.02E-10	6.55	11.8974858	5.30E-02	43191793	43191916	NC_000017.10
cg00582316	5.34E-09	5.02E-10	6.55	11.8966719	1.89E-01	175201430	175201553	NC_000002.11
cg02501166	5.34E-09	5.03E-10	6.55	11.8963642	1.42E-01	128866698	128866821	NC_000012.11
cg08655206	5.35E-09	5.03E-10	6.55	11.8952878	1.13E-01	221058198	221058321	NC_000001.10
cg09521719	5.35E-09	5.04E-10	6.55	11.8945155	1.47E-01	237416370	237416493	NC_000002.11
cg11825642	5.36E-09	5.05E-10	6.55	11.892475	1.64E-01	75798196	75798319	NC_000017.10

cg05390968	5.37E-09	5.05E-10	6.55	11.89183	1.28E-01	86598792	86598915	NC_000016.9
cg22041590	5.38E-09	5.06E-10	6.55	11.8897166	6.16E-02	158747855	158747978	NC_000002.11
cg23605284	5.38E-09	5.06E-10	6.55	11.8896451	1.59E-01	131764280	131764403	NC_000010.10
cg06321962	5.38E-09	5.07E-10	6.55	11.8884451	9.61E-02	38528511	38528634	NC_000022.10
cg26669806	5.40E-09	5.08E-10	6.55	11.8854313	1.53E-01	18899483	18899606	NC_000019.9
cg10863075	5.40E-09	5.08E-10	6.55	11.8854292	1.32E-01	5296267	5296390	NC_000020.10
cg16065899	5.40E-09	5.08E-10	6.55	11.8850608	1.25E-01	44332958	44333081	NC_000011.9
cg13087761	5.40E-09	5.09E-10	6.55	11.8847118	1.36E-01	5645778	5645901	NC_000007.13
cg23085167	5.41E-09	5.10E-10	6.55	11.8828507	1.05E-01	3160739	3160862	NC_000001.10
cg24905248	5.42E-09	5.10E-10	6.55	11.8822385	8.05E-02	44219907	44220030	NC_000002.11
cg16457413	5.42E-09	5.10E-10	6.55	11.881477	5.79E-02	52269736	52269859	NC_000003.11
cg24730564	5.42E-09	5.11E-10	6.55	11.8810378	9.44E-02	106938322	106938445	NC_000014.8
cg06372015	5.42E-09	5.11E-10	6.55	11.8807238	2.05E-01	43602000	43602123	NC_000011.9
cg07212852	5.43E-09	5.11E-10	6.55	11.8799872	1.46E-01	21684348	21684471	NC_000020.10
cg20773033	5.43E-09	5.12E-10	6.54	11.8791045	1.16E-01	39635187	39635310	NC_000022.10
cg11431402	5.43E-09	5.12E-10	6.54	11.8789753	9.56E-02	126135408	126135531	NC_000009.11
cg00624617	5.43E-09	5.12E-10	6.54	11.8787849	9.46E-02	153852554	153852677	NC_000005.9
cg13192938	5.43E-09	5.12E-10	6.54	11.8785769	1.25E-01	105739124	105739247	NC_000014.8
cg27540865	5.43E-09	5.12E-10	6.54	11.8784116	1.33E-01	243053934	243054057	NC_000001.10
cg04936260	5.44E-09	5.13E-10	6.54	11.8765771	9.83E-02	74888889	74889012	NC_000014.8
cg24757553	5.45E-09	5.13E-10	6.54	11.8764269	1.08E-01	27127501	27127624	NC_000007.13
cg10080155	5.46E-09	5.14E-10	6.54	11.8744277	2.40E-01	45405904	45406027	NC_000022.10
cg21277243	5.47E-09	5.15E-10	6.54	11.8716252	1.38E-01	5996331	5996454	NC_000006.11
cg07922924	5.47E-09	5.16E-10	6.54	11.8709146	1.14E-01	11211274	11211397	NC_000001.10
cg15409712	5.48E-09	5.16E-10	6.54	11.8700598	1.17E-01	103426288	103426411	NC_000004.11

cg17240133	5.48E-09	5.17E-10	6.54	11.8689334	1.20E-01	57977245	57977368	NC_000015.9
cg10123247	5.49E-09	5.18E-10	6.54	11.8670492	2.02E-01	117950929	117951052	NC_000008.10
cg10249507	5.50E-09	5.18E-10	6.54	11.86623	4.29E-02	17758229	17758352	NC_000019.9
cg06562644	5.50E-09	5.19E-10	6.54	11.8656219	1.00E-01	134992590	134992713	NC_000010.10
cg06005396	5.50E-09	5.19E-10	6.54	11.8655073	1.17E-01	590541	590664	NC_000019.9
cg22882539	5.51E-09	5.19E-10	6.54	11.8646255	1.52E-01	131438523	131438646	NC_000012.11
cg10170678	5.52E-09	5.20E-10	6.54	11.862829	3.89E-02	92994057	92994180	NC_000014.8
cg07742404	5.53E-09	5.21E-10	6.54	11.8612503	1.09E-01	130648987	130649110	NC_000012.11
cg20006729	5.53E-09	5.21E-10	6.54	11.8609368	1.15E-01	7033146	7033269	NC_000012.11
cg16424920	5.53E-09	5.21E-10	6.54	11.8603934	1.57E-01	9760855	9760978	NC_000008.10
cg12556586	5.53E-09	5.21E-10	6.54	11.8603728	4.22E-02	59733552	59733675	NC_000015.9
cg00630958	5.54E-09	5.22E-10	6.54	11.8591697	1.64E-01	65991461	65991584	NC_000001.10
cg22755445	5.54E-09	5.22E-10	6.54	11.8587029	1.19E-01	102772036	102772159	NC_000002.11
cg15426006	5.54E-09	5.22E-10	6.54	11.8584173	8.22E-02	99869897	99870020	NC_000013.10
cg06695286	5.54E-09	5.23E-10	6.54	11.8579261	1.17E-01	42880445	42880568	NC_000012.11
cg08069899	5.54E-09	5.23E-10	6.54	11.8578588	1.57E-01	4383770	4383893	NC_000012.11
cg20984972	5.56E-09	5.24E-10	6.54	11.8551535	1.50E-01	3390125	3390248	NC_000019.9
cg08563299	5.56E-09	5.25E-10	6.54	11.8542571	8.99E-02	16922592	16922715	NC_000019.9
cg24146349	5.57E-09	5.25E-10	6.54	11.853309	9.65E-02	1542769	1542892	NC_000007.13
cg20228495	5.57E-09	5.26E-10	6.54	11.8524818	4.95E-02	74945369	74945492	NC_000002.11
cg26429925	5.57E-09	5.26E-10	6.54	11.8520945	1.41E-01	83953775	83953898	NC_000015.9
cg23832237	5.57E-09	5.26E-10	6.54	11.8520394	1.17E-01	72667208	72667331	NC_000017.10
cg20639942	5.57E-09	5.26E-10	6.54	11.8519469	4.40E-02	54271958	54272081	NC_000019.9
cg24286165	5.58E-09	5.27E-10	6.54	11.8507515	1.72E-01	32458966	32459089	NC_000011.9
cg11666559	5.58E-09	5.27E-10	6.54	11.8503526	1.02E-01	221067662	221067785	NC_000001.10

cg14857764	5.59E-09	5.27E-10	6.54	11.849701	1.55E-01	50706663	50706786	NC_000013.10
cg23448486	5.59E-09	5.27E-10	6.54	11.8492004	1.52E-01	147129213	147129336	NC_000003.11
cg04608722	5.59E-09	5.28E-10	6.54	11.8484324	6.89E-02	46919549	46919672	NC_000022.10
cg15456742	5.59E-09	5.28E-10	6.54	11.8480901	1.60E-01	207991729	207991852	NC_000001.10
cg22359606	5.60E-09	5.28E-10	6.54	11.847668	1.67E-01	182545492	182545615	NC_000002.11
cg09981884	5.60E-09	5.29E-10	6.54	11.8469822	1.22E-01	1720110	1720233	NC_000004.11
cg09737407	5.61E-09	5.29E-10	6.54	11.8457343	3.54E-02	31079698	31079821	NC_000006.11
cg10659382	5.61E-09	5.30E-10	6.54	11.8450736	2.31E-01	155166856	155166979	NC_000007.13
cg17174275	5.61E-09	5.30E-10	6.54	11.844758	9.85E-02	26109429	26109552	NC_000015.9
cg27260684	5.62E-09	5.30E-10	6.54	11.844001	1.39E-01	85063742	85063865	NC_000016.9
cg11257429	5.62E-09	5.30E-10	6.54	11.8439997	1.52E-01	178016796	178016919	NC_000005.9
cg25733898	5.62E-09	5.30E-10	6.54	11.8437788	5.64E-02	71330058	71330181	NC_000015.9
cg05457480	5.62E-09	5.31E-10	6.54	11.8433931	1.53E-01	139139201	139139324	NC_000005.9
cg16774479	5.63E-09	5.31E-10	6.54	11.8416751	1.14E-01	33529461	33529584	NC_000018.9
cg09462445	5.63E-09	5.32E-10	6.54	11.8407839	2.06E-01	217559131	217559254	NC_000002.11
cg00319168	5.63E-09	5.32E-10	6.54	11.8404501	1.46E-01	176958174	176958297	NC_000002.11
cg00958225	5.64E-09	5.32E-10	6.54	11.840365	8.61E-02	44972477	44972600	NC_000001.10
cg27327911	5.64E-09	5.33E-10	6.54	11.8395299	1.67E-01	54969146	54969269	NC_000016.9
cg13858975	5.65E-09	5.33E-10	6.54	11.8382548	5.80E-02	52016644	52016767	NC_000015.9
cg06509140	5.65E-09	5.33E-10	6.54	11.8379324	6.65E-02	1729975	1730098	NC_000017.10
cg10623853	5.65E-09	5.34E-10	6.54	11.8373249	8.88E-02	42573639	42573762	NC_000020.10
cg21054473	5.66E-09	5.35E-10	6.54	11.8359744	2.06E-01	100438957	100439080	NC_000014.8
cg10947457	5.67E-09	5.35E-10	6.54	11.8346948	6.87E-02	32096945	32097068	NC_000006.11
cg06934774	5.67E-09	5.36E-10	6.54	11.8338975	1.56E-01	27198025	27198148	NC_000007.13
cg21823149	5.68E-09	5.36E-10	6.54	11.8327675	1.68E-01	207309003	207309126	NC_000002.11

cg05319305	5.70E-09	5.38E-10	6.54	11.8290228	1.98E-01	101111927	101112050	NC_000004.11
cg25781755	5.70E-09	5.39E-10	6.54	11.8285265	1.55E-01	13537089	13537212	NC_000004.11
cg03700171	5.71E-09	5.40E-10	6.54	11.8262915	4.72E-02	78748272	78748395	NC_000015.9
cg12878812	5.72E-09	5.41E-10	6.53	11.8249867	1.25E-01	119419696	119419819	NC_000012.11
cg22411207	5.72E-09	5.41E-10	6.53	11.8246386	1.05E-01	57026301	57026424	NC_000008.10
cg10988573	5.72E-09	5.41E-10	6.53	11.8246189	1.02E-01	61104928	61105051	NC_000014.8
cg10001374	5.72E-09	5.41E-10	6.53	11.8245001	7.59E-02	134254128	134254251	NC_000011.9
cg13160967	5.74E-09	5.43E-10	6.53	11.821402	1.11E-01	100730303	100730426	NC_000006.11
cg24218935	5.74E-09	5.43E-10	6.53	11.8204716	4.47E-02	1686150	1686273	NC_000011.9
cg23976464	5.75E-09	5.44E-10	6.53	11.8193842	1.79E-01	32483950	32484073	NC_000017.10
cg18404308	5.75E-09	5.44E-10	6.53	11.8191072	1.75E-01	98962900	98963023	NC_000002.11
cg22449370	5.76E-09	5.45E-10	6.53	11.8170862	9.61E-02	153586022	153586145	NC_000007.13
cg19563062	5.76E-09	5.45E-10	6.53	11.8170328	7.38E-02	34097075	34097198	NC_000017.10
cg05771261	5.77E-09	5.45E-10	6.53	11.8164922	1.30E-01	20879286	20879409	NC_000001.10
cg00470955	5.77E-09	5.46E-10	6.53	11.8157142	1.26E-01	7354432	7354555	NC_000016.9
cg14371590	5.77E-09	5.46E-10	6.53	11.8153998	1.07E-01	58013915	58014038	NC_000012.11
cg03683003	5.78E-09	5.46E-10	6.53	11.8147512	9.74E-02	138874004	138874127	NC_000009.11
cg09503234	5.78E-09	5.46E-10	6.53	11.8145558	1.26E-01	2151721	2151844	NC_000011.9
cg12758973	5.78E-09	5.47E-10	6.53	11.8139087	1.08E-01	247694275	247694398	NC_000001.10
cg05028467	5.78E-09	5.47E-10	6.53	11.8138999	1.80E-01	176057097	176057220	NC_000005.9
cg03860252	5.79E-09	5.48E-10	6.53	11.8118597	9.33E-02	1799705	1799828	NC_000004.11
cg15736169	5.79E-09	5.48E-10	6.53	11.8112021	1.83E-01	112761025	112761148	NC_000013.10
cg14225450	5.80E-09	5.49E-10	6.53	11.810299	8.70E-02	169637832	169637955	NC_000006.11
cg14319657	5.80E-09	5.49E-10	6.53	11.8095088	9.73E-02	48027921	48028044	NC_000022.10
cg26992603	5.81E-09	5.50E-10	6.53	11.8088233	1.58E-01	115130994	115131117	NC_000012.11

cg04882273	5.81E-09	5.50E-10	6.53	11.8079716	1.37E-01	62070365	62070488	NC_000016.9
cg13242092	5.81E-09	5.50E-10	6.53	11.8079507	6.79E-02	46271421	46271544	NC_000021.8
cg17108748	5.81E-09	5.50E-10	6.53	11.8079481	1.18E-01	89065330	89065453	NC_000002.11
cg01070987	5.81E-09	5.50E-10	6.53	11.8075202	1.69E-01	149687002	149687125	NC_000003.11
cg21210758	5.81E-09	5.50E-10	6.53	11.8075009	1.89E-01	5713005	5713128	NC_000004.11
cg03350814	5.82E-09	5.51E-10	6.53	11.8057692	2.00E-01	104512423	104512546	NC_000008.10
cg25965185	5.82E-09	5.51E-10	6.53	11.8057123	5.79E-02	66480641	66480764	NC_000014.8
cg26685352	5.82E-09	5.51E-10	6.53	11.8056326	1.39E-01	48485328	48485451	NC_000004.11
cg02617791	5.83E-09	5.52E-10	6.53	11.8053362	6.47E-02	27235627	27235750	NC_000010.10
cg19457237	5.85E-09	5.54E-10	6.53	11.8016632	1.21E-01	34500585	34500708	NC_000012.11
cg01189783	5.85E-09	5.54E-10	6.53	11.8011881	1.34E-01	100441581	100441704	NC_000006.11
cg16245844	5.85E-09	5.54E-10	6.53	11.8005338	1.53E-02	1800488	1800611	NC_000012.11
cg24059119	5.86E-09	5.55E-10	6.53	11.7998885	1.65E-01	30715575	30715698	NC_000019.9
cg09018383	5.86E-09	5.55E-10	6.53	11.7997734	4.77E-02	31504385	31504508	NC_000006.11
cg02063817	5.86E-09	5.55E-10	6.53	11.7996739	1.00E-01	27253521	27253644	NC_000007.13
cg20747380	5.86E-09	5.55E-10	6.53	11.7987981	1.31E-01	27142275	27142398	NC_000007.13
cg06832820	5.86E-09	5.55E-10	6.53	11.7986647	1.04E-01	64543849	64543972	NC_000011.9
cg20938085	5.87E-09	5.55E-10	6.53	11.7982918	9.66E-02	140768230	140768353	NC_000005.9
cg15409616	5.87E-09	5.56E-10	6.53	11.7976949	5.48E-02	106936446	106936569	NC_000014.8
cg26209005	5.87E-09	5.56E-10	6.53	11.7975566	1.54E-01	132262526	132262649	NC_000007.13
cg26040761	5.87E-09	5.56E-10	6.53	11.796895	4.10E-02	110307829	110307952	NC_000001.10
cg03350439	5.87E-09	5.56E-10	6.53	11.7965306	1.11E-01	231299033	231299156	NC_000001.10
cg01837630	5.88E-09	5.57E-10	6.53	11.7950658	3.19E-02	128385752	128385875	NC_000002.11
cg08111284	5.88E-09	5.57E-10	6.53	11.7950577	1.21E-01	32057125	32057248	NC_000006.11
cg00318000	5.88E-09	5.57E-10	6.53	11.7949176	3.55E-02	151128630	151128753	NC_000001.10

cg07665387	5.89E-09	5.58E-10	6.53	11.7943904	1.39E-01	134069388	134069511	NC_000004.11
cg04205655	5.89E-09	5.58E-10	6.53	11.7937422	1.10E-01	67965756	67965879	NC_000016.9
cg23378722	5.89E-09	5.58E-10	6.53	11.7935789	1.29E-01	49882500	49882623	NC_000013.10
cg22296612	5.89E-09	5.58E-10	6.53	11.7931899	3.26E-02	15457699	15457822	NC_000006.11
cg09295799	5.90E-09	5.59E-10	6.53	11.7924143	1.39E-01	5994235	5994358	NC_000006.11
cg08278741	5.90E-09	5.59E-10	6.53	11.7923412	1.90E-01	36736477	36736600	NC_000019.9
cg25758978	5.90E-09	5.59E-10	6.53	11.7922431	1.32E-01	8454161	8454284	NC_000019.9
cg20711812	5.90E-09	5.59E-10	6.53	11.7914827	1.32E-01	1775422	1775545	NC_000019.9
cg24411302	5.91E-09	5.60E-10	6.53	11.7904486	9.36E-02	74893183	74893306	NC_000014.8
cg27532621	5.91E-09	5.60E-10	6.53	11.7903192	6.03E-02	164593763	164593886	NC_000001.10
cg01085125	5.92E-09	5.61E-10	6.53	11.7885053	1.43E-01	122423433	122423556	NC_000005.9
cg15244223	5.92E-09	5.61E-10	6.53	11.7881717	1.82E-01	118228493	118228616	NC_000006.11
cg00068750	5.92E-09	5.62E-10	6.53	11.7874336	4.53E-02	2535955	2536078	NC_000016.9
cg01438868	5.92E-09	5.62E-10	6.53	11.7873506	1.40E-01	177802381	177802504	NC_000003.11
cg07682547	5.92E-09	5.62E-10	6.53	11.7872553	1.49E-01	153863417	153863540	NC_000005.9
cg08346922	5.93E-09	5.62E-10	6.53	11.7867338	1.05E-01	31228299	31228422	NC_000016.9
cg07063890	5.95E-09	5.64E-10	6.53	11.7826692	5.92E-02	88496241	88496364	NC_000016.9
cg23498221	5.96E-09	5.65E-10	6.53	11.7819001	3.01E-02	26681583	26681706	NC_000017.10
cg06118384	5.96E-09	5.65E-10	6.53	11.7816407	1.60E-01	149645798	149645921	NC_000002.11
cg06460717	5.96E-09	5.65E-10	6.53	11.7808475	1.69E-01	53420135	53420258	NC_000013.10
cg03486475	5.97E-09	5.66E-10	6.53	11.7800794	9.28E-02	140796240	140796363	NC_000005.9
cg00090936	5.97E-09	5.66E-10	6.53	11.7794635	1.72E-01	69592090	69592213	NC_000003.11
cg11539296	5.97E-09	5.66E-10	6.53	11.7794457	6.69E-02	140216145	140216268	NC_000005.9
cg11516654	5.98E-09	5.67E-10	6.53	11.7782314	4.38E-02	19783852	19783975	NC_000011.9
cg01856384	5.98E-09	5.67E-10	6.53	11.7779498	1.03E-01	41376904	41377027	NC_000006.11

cg21331821	5.98E-09	5.67E-10	6.53	11.7778513	1.66E-01	58545149	58545272	NC_000019.9
cg13030332	5.99E-09	5.69E-10	6.53	11.7755022	1.07E-01	115117351	115117474	NC_000012.11
cg12013685	5.99E-09	5.69E-10	6.53	11.7751664	1.05E-01	63243220	63243343	NC_000015.9
cg02119755	6.00E-09	5.69E-10	6.53	11.7748343	1.01E-01	2589556	2589679	NC_000016.9
cg24407243	6.00E-09	5.69E-10	6.53	11.7742018	1.30E-01	21541756	21541879	NC_000019.9
cg25561792	6.00E-09	5.69E-10	6.53	11.7741343	7.95E-02	62050205	62050328	NC_000017.10
cg24939733	6.01E-09	5.70E-10	6.53	11.7726323	9.18E-02	85397684	85397807	NC_000011.9
cg08453036	6.01E-09	5.71E-10	6.52	11.7717729	1.52E-01	215255572	215255695	NC_000001.10
cg00302587	6.01E-09	5.71E-10	6.52	11.7714351	1.35E-01	51576241	51576364	NC_000010.10
cg24250393	6.02E-09	5.71E-10	6.52	11.7707716	1.18E-01	23846838	23846961	NC_000016.9
cg27278953	6.02E-09	5.71E-10	6.52	11.7706846	1.70E-01	77737661	77737784	NC_000014.8
cg11954332	6.02E-09	5.71E-10	6.52	11.7706046	1.67E-01	170639289	170639412	NC_000001.10
cg02186542	6.02E-09	5.71E-10	6.52	11.7705627	1.31E-01	77469167	77469290	NC_000016.9
cg08902698	6.02E-09	5.72E-10	6.52	11.770293	9.57E-02	1271174	1271297	NC_000019.9
cg27462519	6.03E-09	5.73E-10	6.52	11.7683084	1.15E-01	3242639	3242762	NC_000016.9
cg03146683	6.04E-09	5.73E-10	6.52	11.7674104	7.04E-02	22011878	22012001	NC_000006.11
cg21837669	6.04E-09	5.74E-10	6.52	11.7665905	9.78E-02	103889307	103889430	NC_000012.11
cg25285090	6.05E-09	5.74E-10	6.52	11.7658496	2.20E-01	28510313	28510436	NC_000006.11
cg20491726	6.05E-09	5.75E-10	6.52	11.7652971	7.65E-02	242820622	242820745	NC_000002.11
cg09988738	6.06E-09	5.75E-10	6.52	11.7637437	1.38E-01	4857255	4857378	NC_000004.11
cg02109484	6.06E-09	5.76E-10	6.52	11.763006	1.27E-01	74875253	74875376	NC_000002.11
cg10998570	6.07E-09	5.77E-10	6.52	11.761346	7.57E-02	965862	965985	NC_000005.9
cg09312576	6.07E-09	5.77E-10	6.52	11.7612357	1.09E-01	1385351	1385474	NC_000006.11
cg04716021	6.08E-09	5.78E-10	6.52	11.7592181	1.01E-01	80981119	80981242	NC_000010.10
cg18120979	6.09E-09	5.78E-10	6.52	11.7590815	1.02E-01	313464	313587	NC_000006.11

cg11842953	6.09E-09	5.79E-10	6.52	11.7582545	5.51E-02	1218561	1218684	NC_000005.9
cg09866983	6.10E-09	5.80E-10	6.52	11.7563635	1.64E-01	46799755	46799878	NC_000017.10
cg00976097	6.11E-09	5.80E-10	6.52	11.7556958	8.89E-02	421733	421856	NC_000005.9
cg08819023	6.11E-09	5.81E-10	6.52	11.7548394	8.57E-02	6329091	6329214	NC_000017.10
cg10476112	6.12E-09	5.81E-10	6.52	11.7538534	1.82E-01	45241222	45241345	NC_000002.11
cg15908006	6.12E-09	5.82E-10	6.52	11.7526824	1.69E-01	4850180	4850303	NC_000008.10
cg25797887	6.13E-09	5.82E-10	6.52	11.7522854	1.47E-01	96627821	96627944	NC_000007.13
cg07229938	6.13E-09	5.83E-10	6.52	11.7515122	1.23E-01	124907600	124907723	NC_000010.10
cg04008703	6.16E-09	5.86E-10	6.52	11.7463787	1.52E-01	27280585	27280708	NC_000007.13
cg17825173	6.17E-09	5.87E-10	6.52	11.7450725	3.82E-02	71164116	71164239	NC_000010.10
cg18739865	6.17E-09	5.87E-10	6.52	11.7443629	1.42E-01	50684720	50684843	NC_000005.9
cg27084746	6.17E-09	5.87E-10	6.52	11.7442017	1.70E-01	31831532	31831655	NC_000006.11
cg08207660	6.18E-09	5.87E-10	6.52	11.743994	4.54E-02	71694874	71694997	NC_000014.8
cg26919347	6.19E-09	5.89E-10	6.52	11.7415194	6.24E-02	77231677	77231800	NC_000004.11
cg11711816	6.19E-09	5.89E-10	6.52	11.741129	1.16E-01	131764932	131765055	NC_000010.10
cg09061632	6.20E-09	5.89E-10	6.52	11.740542	1.14E-01	101923621	101923744	NC_000014.8
cg01620486	6.20E-09	5.89E-10	6.52	11.7405391	7.96E-02	47015640	47015763	NC_000008.10
cg09649610	6.20E-09	5.89E-10	6.52	11.7403572	2.04E-01	235814039	235814162	NC_000001.10
cg17291521	6.20E-09	5.89E-10	6.52	11.7403117	1.00E-01	50260045	50260168	NC_000005.9
cg23165899	6.20E-09	5.90E-10	6.52	11.7388627	1.94E-01	19837095	19837218	NC_000006.11
cg27231068	6.21E-09	5.91E-10	6.52	11.7381993	8.61E-02	19620291	19620414	NC_000017.10
cg06922606	6.23E-09	5.92E-10	6.52	11.7351888	1.65E-01	10274632	10274755	NC_000016.9
cg03547017	6.24E-09	5.94E-10	6.52	11.7329944	1.11E-01	37031281	37031404	NC_000017.10
cg03638172	6.25E-09	5.95E-10	6.52	11.7315138	2.00E-01	4922944	4923067	NC_000007.13
cg19850728	6.25E-09	5.95E-10	6.52	11.7308068	1.04E-01	97688465	97688588	NC_000003.11

cg23904955	6.25E-09	5.95E-10	6.52	11.7305011	9.95E-02	101282759	101282882	NC_000010.10
cg22149137	6.26E-09	5.96E-10	6.52	11.7300563	1.51E-01	46804414	46804537	NC_000017.10
cg24353217	6.27E-09	5.97E-10	6.52	11.7279729	8.74E-02	111358188	111358311	NC_000012.11
cg22345214	6.27E-09	5.97E-10	6.52	11.7278903	1.36E-01	46995509	46995632	NC_000004.11
cg04609952	6.28E-09	5.97E-10	6.52	11.726915	4.51E-02	16085893	16086016	NC_000002.11
cg16775752	6.28E-09	5.98E-10	6.52	11.7267439	1.12E-01	49674530	49674653	NC_000010.10
cg10838385	6.28E-09	5.98E-10	6.52	11.7254949	1.46E-01	26674370	26674493	NC_000014.8
cg06493930	6.30E-09	5.99E-10	6.52	11.7236289	1.57E-01	22689814	22689937	NC_000011.9
cg15189764	6.30E-09	6.00E-10	6.52	11.723116	7.40E-02	27282064	27282187	NC_000017.10
cg07955004	6.30E-09	6.00E-10	6.52	11.72255	2.53E-02	79803515	79803638	NC_000017.10
cg01637289	6.30E-09	6.00E-10	6.52	11.7222441	6.86E-02	118769157	118769280	NC_000011.9
cg05509659	6.31E-09	6.01E-10	6.52	11.7218872	1.41E-01	124738871	124738994	NC_000011.9
cg14134497	6.31E-09	6.01E-10	6.52	11.7213745	1.63E-01	32074251	32074374	NC_000018.9
cg13633659	6.31E-09	6.01E-10	6.52	11.7211995	1.49E-01	98012426	98012549	NC_000009.11
cg14743594	6.32E-09	6.02E-10	6.52	11.720033	2.03E-01	167956394	167956517	NC_000005.9
cg17787084	6.33E-09	6.03E-10	6.52	11.7183515	1.37E-01	165323896	165324019	NC_000001.10
cg17466131	6.34E-09	6.04E-10	6.51	11.7162208	7.30E-02	186578190	186578313	NC_000004.11
cg19459876	6.34E-09	6.04E-10	6.51	11.7156328	1.48E-01	2292477	2292600	NC_000011.9
cg13930544	6.35E-09	6.05E-10	6.51	11.7144573	8.82E-02	247694061	247694184	NC_000001.10
cg00741624	6.36E-09	6.05E-10	6.51	11.7139307	1.59E-01	93897389	93897512	NC_000014.8
cg20616836	6.37E-09	6.07E-10	6.51	11.7121822	2.77E-02	3064281	3064404	NC_000019.9
cg14888369	6.37E-09	6.07E-10	6.51	11.7119655	3.67E-02	25039081	25039204	NC_000008.10
cg00532449	6.37E-09	6.07E-10	6.51	11.7115794	1.98E-01	2375420	2375543	NC_000001.10
cg13070193	6.38E-09	6.08E-10	6.51	11.7090394	1.53E-01	45613752	45613875	NC_000007.13
cg23346516	6.39E-09	6.09E-10	6.51	11.7089043	7.08E-02	76421646	76421769	NC_000017.10

cg07901351	6.39E-09	6.09E-10	6.51	11.7078533	5.93E-02	224625023	224625146	NC_000001.10
cg01647314	6.40E-09	6.10E-10	6.51	11.7072954	1.13E-01	23280066	23280189	NC_000001.10
cg16198468	6.40E-09	6.10E-10	6.51	11.706797	1.58E-01	76708051	76708174	NC_000010.10
cg01626112	6.40E-09	6.10E-10	6.51	11.7065316	1.21E-01	76653220	76653343	NC_000012.11
cg01667319	6.42E-09	6.12E-10	6.51	11.7038398	1.80E-01	2161128	2161251	NC_000011.9
cg18097850	6.42E-09	6.12E-10	6.51	11.7036214	1.29E-01	40005602	40005725	NC_000019.9
cg03205252	6.43E-09	6.13E-10	6.51	11.7025701	1.51E-01	51187536	51187659	NC_000016.9
cg26674479	6.43E-09	6.13E-10	6.51	11.7021889	1.09E-01	27281415	27281538	NC_000007.13
cg14076024	6.45E-09	6.15E-10	6.51	11.6982606	3.75E-02	104897608	104897731	NC_000014.8
cg21811143	6.45E-09	6.15E-10	6.51	11.6980908	1.56E-01	119599748	119599871	NC_000002.11
cg12107189	6.46E-09	6.16E-10	6.51	11.6971948	6.40E-02	125765152	125765275	NC_000008.10
cg16523839	6.46E-09	6.16E-10	6.51	11.6968953	1.36E-01	10077134	10077257	NC_000019.9
cg20687825	6.46E-09	6.16E-10	6.51	11.6967589	5.05E-02	79005609	79005732	NC_000004.11
cg19247475	6.47E-09	6.17E-10	6.51	11.6959738	1.03E-01	140789745	140789868	NC_000005.9
cg11717054	6.47E-09	6.17E-10	6.51	11.6957074	1.81E-01	279423	279546	NC_000011.9
cg05127995	6.47E-09	6.17E-10	6.51	11.695472	4.98E-02	2735245	2735368	NC_000004.11
cg19955284	6.47E-09	6.17E-10	6.51	11.6950136	7.92E-02	31833747	31833870	NC_000006.11
cg17833265	6.47E-09	6.17E-10	6.51	11.694968	1.37E-01	48042135	48042258	NC_000017.10
cg09274961	6.48E-09	6.18E-10	6.51	11.6939434	1.19E-01	85243231	85243354	NC_000016.9
cg16273546	6.50E-09	6.20E-10	6.51	11.6900482	7.21E-02	123070392	123070515	NC_000010.10
cg19047265	6.50E-09	6.21E-10	6.51	11.6897393	1.72E-01	177025998	177026121	NC_000002.11
cg05490712	6.51E-09	6.21E-10	6.51	11.6892759	1.44E-01	31838674	31838797	NC_000011.9
cg20245520	6.51E-09	6.21E-10	6.51	11.6892184	7.24E-02	157185279	157185402	NC_000007.13
cg06213468	6.51E-09	6.21E-10	6.51	11.6889014	1.48E-01	129534411	129534534	NC_000010.10
cg19140834	6.53E-09	6.23E-10	6.51	11.6860223	6.84E-02	131217668	131217791	NC_000007.13

cg24089118	6.53E-09	6.23E-10	6.51	11.6855208	1.53E-01	52734251	52734374	NC_000014.8
cg21097283	6.53E-09	6.24E-10	6.51	11.6847859	1.68E-01	76934461	76934584	NC_000005.9
cg10625851	6.54E-09	6.24E-10	6.51	11.6840627	4.05E-02	100084567	100084690	NC_000007.13
cg17080902	6.54E-09	6.24E-10	6.51	11.6838442	7.27E-02	51762495	51762618	NC_000015.9
cg27535380	6.57E-09	6.27E-10	6.51	11.6796313	6.12E-02	30795743	30795866	NC_000016.9
cg14188639	6.57E-09	6.27E-10	6.51	11.6794171	1.84E-01	2161544	2161667	NC_000011.9
cg15489250	6.57E-09	6.27E-10	6.51	11.6791156	2.29E-01	36994906	36995029	NC_000014.8
cg05545672	6.57E-09	6.27E-10	6.51	11.6789638	1.52E-01	170739541	170739664	NC_000005.9
cg19211915	6.57E-09	6.28E-10	6.51	11.6787722	1.13E-01	32452513	32452636	NC_000011.9
cg12002303	6.58E-09	6.28E-10	6.51	11.6774394	2.09E-01	68113478	68113601	NC_000015.9
cg16427239	6.58E-09	6.29E-10	6.51	11.6772321	8.81E-02	6623995	6624118	NC_000016.9
cg08015447	6.59E-09	6.29E-10	6.51	11.6764696	1.52E-01	6070188	6070311	NC_000016.9
cg20396436	6.59E-09	6.29E-10	6.51	11.6759885	1.54E-01	159173195	159173318	NC_000006.11
cg00316312	6.61E-09	6.31E-10	6.51	11.6732274	9.69E-02	134916087	134916210	NC_000010.10
cg12850078	6.61E-09	6.31E-10	6.51	11.6731302	1.83E-01	89922071	89922194	NC_000015.9
cg11959156	6.63E-09	6.33E-10	6.51	11.6699113	1.58E-01	62865391	62865514	NC_000020.10
cg27665767	6.63E-09	6.34E-10	6.51	11.6694267	1.00E-01	140764485	140764608	NC_000005.9
cg24639902	6.64E-09	6.34E-10	6.51	11.6682053	8.27E-02	22774971	22775094	NC_000008.10
cg26593267	6.64E-09	6.34E-10	6.51	11.6681781	1.90E-01	113764871	113764994	NC_000013.10
cg22441141	6.64E-09	6.34E-10	6.51	11.6681714	6.48E-02	63632453	63632576	NC_000015.9
cg17029156	6.65E-09	6.36E-10	6.51	11.6658998	1.96E-01	27070458	27070581	NC_000002.11
cg03144619	6.65E-09	6.36E-10	6.51	11.665724	4.05E-02	230415668	230415791	NC_000001.10
cg10284425	6.66E-09	6.36E-10	6.51	11.6652446	8.75E-02	86457686	86457809	NC_000016.9
cg26343001	6.66E-09	6.36E-10	6.51	11.6651662	1.51E-01	115104274	115104397	NC_000012.11
cg24938743	6.66E-09	6.37E-10	6.5	11.6645739	9.18E-02	98960620	98960743	NC_000013.10

cg13577628	6.66E-09	6.37E-10	6.5	11.664337	1.40E-01	119534899	119535022	NC_000001.10
cg01952742	6.67E-09	6.37E-10	6.5	11.6637608	7.80E-02	169558925	169559048	NC_000001.10
cg17913306	6.67E-09	6.38E-10	6.5	11.6627765	1.63E-01	92220385	92220508	NC_000014.8
cg11002686	6.68E-09	6.38E-10	6.5	11.6622777	1.13E-01	55412682	55412805	NC_000007.13
cg27207308	6.68E-09	6.38E-10	6.5	11.6619949	2.09E-01	47935683	47935806	NC_000020.10
cg26987597	6.69E-09	6.40E-10	6.5	11.6603127	2.07E-01	1391207	1391330	NC_000006.11
cg08533221	6.69E-09	6.40E-10	6.5	11.6600278	4.77E-02	112076633	112076756	NC_000013.10
cg24456365	6.69E-09	6.40E-10	6.5	11.6593711	1.79E-01	89340480	89340603	NC_000008.10
cg10426017	6.70E-09	6.41E-10	6.5	11.6580186	5.73E-02	167591040	167591163	NC_000006.11
cg04189838	6.71E-09	6.42E-10	6.5	11.6572036	6.08E-02	96523347	96523470	NC_000010.10
cg01974875	6.72E-09	6.43E-10	6.5	11.6555743	6.99E-02	6617580	6617703	NC_000017.10
cg21699252	6.72E-09	6.43E-10	6.5	11.6547254	3.67E-02	16079013	16079136	NC_000002.11
cg27000368	6.72E-09	6.43E-10	6.5	11.654625	4.34E-02	30122213	30122336	NC_000006.11
cg24224304	6.74E-09	6.45E-10	6.5	11.6525189	8.95E-02	89313120	89313243	NC_000015.9
cg16455972	6.75E-09	6.46E-10	6.5	11.6510276	8.05E-02	18286412	18286535	NC_000017.10
cg00117335	6.75E-09	6.46E-10	6.5	11.6505961	2.02E-02	117206442	117206565	NC_000001.10
cg00468146	6.76E-09	6.46E-10	6.5	11.6496936	1.49E-01	19838211	19838334	NC_000006.11
cg03308399	6.76E-09	6.47E-10	6.5	11.6485104	1.05E-01	27146237	27146360	NC_000007.13
cg26319169	6.77E-09	6.47E-10	6.5	11.6482958	4.89E-02	65870540	65870663	NC_000015.9
cg02717061	6.77E-09	6.47E-10	6.5	11.6482467	2.44E-01	110959356	110959479	NC_000013.10
cg01777812	6.78E-09	6.49E-10	6.5	11.6464191	1.28E-01	90117697	90117820	NC_000004.11
cg24061208	6.78E-09	6.49E-10	6.5	11.646128	1.67E-01	14748250	14748373	NC_000018.9
cg27169263	6.78E-09	6.49E-10	6.5	11.6454438	2.50E-02	1633373	1633496	NC_000001.10
cg23467079	6.79E-09	6.49E-10	6.5	11.6452308	1.09E-01	124219425	124219548	NC_000008.10
cg25303427	6.79E-09	6.49E-10	6.5	11.6451663	9.89E-02	128482679	128482802	NC_000007.13

cg19370653	6.79E-09	6.49E-10	6.5	11.6451425	1.81E-01	111550040	111550163	NC_000004.11
cg01617933	6.80E-09	6.51E-10	6.5	11.6435613	1.04E-01	29316555	29316678	NC_000007.13
cg15946904	6.80E-09	6.51E-10	6.5	11.6429881	2.73E-02	957248	957371	NC_000017.10
cg02480685	6.80E-09	6.51E-10	6.5	11.6422652	1.21E-01	96163530	96163653	NC_000008.10
cg09267787	6.81E-09	6.51E-10	6.5	11.6421255	4.03E-02	11200441	11200564	NC_000006.11
cg02388865	6.81E-09	6.52E-10	6.5	11.6418665	1.62E-01	26737318	26737441	NC_000001.10
cg24341452	6.83E-09	6.54E-10	6.5	11.6386912	1.45E-01	182911633	182911756	NC_000001.10
cg25559313	6.83E-09	6.54E-10	6.5	11.6383493	1.72E-01	19138267	19138390	NC_000022.10
cg23690528	6.83E-09	6.54E-10	6.5	11.6382618	2.02E-01	94285270	94285393	NC_000007.13
cg02120774	6.83E-09	6.54E-10	6.5	11.6382593	2.07E-01	45961473	45961596	NC_000007.13
cg21942490	6.83E-09	6.54E-10	6.5	11.6378302	1.36E-01	27206237	27206360	NC_000007.13
cg24049616	6.84E-09	6.55E-10	6.5	11.6370142	1.35E-01	55359029	55359152	NC_000016.9
cg14509403	6.85E-09	6.56E-10	6.5	11.6359529	1.06E-01	30476281	30476404	NC_000022.10
cg04787317	6.85E-09	6.56E-10	6.5	11.6347552	2.53E-02	38009934	38010057	NC_000003.11
cg23093609	6.85E-09	6.57E-10	6.5	11.6345197	1.28E-01	32572422	32572545	NC_000022.10
cg21075378	6.86E-09	6.57E-10	6.5	11.6339446	1.22E-01	44495876	44495999	NC_000018.9
cg03962821	6.86E-09	6.57E-10	6.5	11.6338256	7.99E-02	1315051	1315174	NC_000007.13
cg20239017	6.86E-09	6.57E-10	6.5	11.633306	9.53E-02	41961110	41961233	NC_000001.10
cg18326562	6.87E-09	6.59E-10	6.5	11.631447	1.01E-01	67413885	67414008	NC_000017.10
cg13736376	6.88E-09	6.59E-10	6.5	11.631247	1.35E-01	111539291	111539414	NC_000004.11
cg16428357	6.88E-09	6.59E-10	6.5	11.6306686	9.75E-02	34190258	34190381	NC_000020.10
cg00064336	6.88E-09	6.60E-10	6.5	11.6298805	1.10E-01	88143970	88144093	NC_000016.9
cg21550580	6.90E-09	6.62E-10	6.5	11.6271559	1.11E-01	65755382	65755505	NC_000003.11
cg06275859	6.91E-09	6.62E-10	6.5	11.6262722	1.39E-01	1407694	1407817	NC_000004.11
cg13884568	6.91E-09	6.62E-10	6.5	11.6257985	1.40E-01	79630305	79630428	NC_000009.11

cg20833182	6.91E-09	6.63E-10	6.5	11.6254753	4.74E-02	7741012	7741135	NC_000017.10
cg02393907	6.93E-09	6.64E-10	6.5	11.6229981	1.08E-01	150011063	150011186	NC_000007.13
cg25035939	6.94E-09	6.65E-10	6.5	11.6221756	6.25E-02	245674287	245674410	NC_000001.10
cg15368455	6.94E-09	6.66E-10	6.5	11.6212001	1.11E-01	37835958	37836081	NC_000005.9
cg01121554	6.95E-09	6.66E-10	6.5	11.6203754	7.68E-02	23253797	23253920	NC_000019.9
cg17102807	6.96E-09	6.67E-10	6.5	11.6185629	5.15E-02	43483382	43483505	NC_000006.11
cg21609106	6.96E-09	6.67E-10	6.5	11.6185118	1.63E-01	38259214	38259337	NC_000005.9
cg20667845	6.96E-09	6.67E-10	6.5	11.6184142	1.09E-01	152132086	152132209	NC_000007.13
cg05743982	6.96E-09	6.68E-10	6.5	11.6182933	4.00E-02	30018158	30018281	NC_000016.9
cg15724417	6.96E-09	6.68E-10	6.5	11.6177758	3.50E-02	66433521	66433644	NC_000003.11
cg07997434	6.97E-09	6.68E-10	6.5	11.6174427	1.79E-01	238864969	238865092	NC_000002.11
cg03683946	6.97E-09	6.68E-10	6.5	11.6174117	7.25E-02	38051826	38051949	NC_000022.10
cg10095242	6.97E-09	6.68E-10	6.5	11.6173135	2.20E-01	54163622	54163745	NC_000008.10
cg23109174	6.98E-09	6.69E-10	6.5	11.6159559	4.69E-02	24998564	24998687	NC_000022.10
cg08654915	6.98E-09	6.70E-10	6.5	11.6147387	1.23E-01	157813417	157813540	NC_000003.11
cg22015128	6.98E-09	6.70E-10	6.5	11.6147307	9.22E-02	202612948	202613071	NC_000001.10
cg00035316	7.00E-09	6.71E-10	6.5	11.6125919	1.61E-01	176993017	176993140	NC_000002.11
cg23799901	7.00E-09	6.71E-10	6.5	11.6125055	1.56E-01	124404431	124404554	NC_000007.13
cg04112211	7.01E-09	6.73E-10	6.49	11.61083	1.60E-01	87436327	87436450	NC_000005.9
cg08789022	7.02E-09	6.73E-10	6.49	11.6097706	1.69E-01	27484942	27485065	NC_000002.11
cg02374745	7.03E-09	6.74E-10	6.49	11.608546	2.11E-01	6679564	6679687	NC_000017.10
cg11188483	7.03E-09	6.75E-10	6.49	11.608089	7.27E-02	39542821	39542944	NC_000003.11
cg05289253	7.03E-09	6.75E-10	6.49	11.607895	2.01E-01	123301674	123301797	NC_000011.9
cg22572159	7.03E-09	6.75E-10	6.49	11.6072519	2.02E-01	115880646	115880769	NC_000001.10
cg25538809	7.03E-09	6.75E-10	6.49	11.6071968	8.10E-02	21186789	21186912	NC_000017.10

cg14204462	7.04E-09	6.75E-10	6.49	11.6071424	1.49E-01	119540044	119540167	NC_000001.10
cg07247419	7.04E-09	6.76E-10	6.49	11.6060407	1.38E-01	21376484	21376607	NC_000020.10
cg04089240	7.05E-09	6.76E-10	6.49	11.6052954	4.27E-02	2437789	2437912	NC_000011.9
cg24147428	7.06E-09	6.78E-10	6.49	11.6037112	1.11E-01	65409760	65409883	NC_000011.9
cg01060089	7.06E-09	6.78E-10	6.49	11.6036506	1.17E-01	131783940	131784063	NC_000012.11
cg01822600	7.06E-09	6.78E-10	6.49	11.6031725	5.49E-02	245751692	245751815	NC_000001.10
cg21910390	7.06E-09	6.78E-10	6.49	11.6028514	9.40E-02	52718946	52719069	NC_000004.11
cg04452785	7.07E-09	6.79E-10	6.49	11.6012976	3.63E-02	128809028	128809151	NC_000003.11
cg03532904	7.08E-09	6.80E-10	6.49	11.6005988	1.75E-01	114877894	114878017	NC_000012.11
cg09538651	7.10E-09	6.82E-10	6.49	11.5978156	6.30E-02	70001896	70002019	NC_000011.9
cg27345924	7.10E-09	6.82E-10	6.49	11.5972204	1.38E-01	2727758	2727881	NC_000005.9
cg16765487	7.11E-09	6.83E-10	6.49	11.5958771	9.32E-02	89548175	89548298	NC_000016.9
cg05363903	7.11E-09	6.83E-10	6.49	11.5954927	1.14E-01	237415899	237416022	NC_000002.11
cg02650317	7.12E-09	6.83E-10	6.49	11.5952793	2.45E-01	61808888	61809011	NC_000020.10
cg04569615	7.12E-09	6.84E-10	6.49	11.5943734	1.22E-01	20622198	20622321	NC_000011.9
cg06119016	7.13E-09	6.85E-10	6.49	11.5932554	8.56E-02	80373277	80373400	NC_000017.10
cg05864140	7.13E-09	6.85E-10	6.49	11.5930987	1.05E-01	220196530	220196653	NC_000002.11
cg05737535	7.13E-09	6.85E-10	6.49	11.5928168	1.20E-01	130316398	130316521	NC_000010.10
cg08032924	7.14E-09	6.86E-10	6.49	11.5916734	1.50E-01	66613096	66613219	NC_000016.9
cg03149560	7.15E-09	6.87E-10	6.49	11.5905261	1.71E-01	29796379	29796502	NC_000006.11
cg22677715	7.15E-09	6.87E-10	6.49	11.5901782	1.37E-01	162284644	162284767	NC_000002.11
cg08372619	7.16E-09	6.88E-10	6.49	11.588202	1.99E-01	109799518	109799641	NC_000008.10
cg15164708	7.16E-09	6.88E-10	6.49	11.5881667	1.14E-01	49936274	49936397	NC_000019.9
cg05099491	7.16E-09	6.88E-10	6.49	11.5880419	1.84E-01	165205352	165205475	NC_000001.10
cg17569124	7.17E-09	6.89E-10	6.49	11.5876247	1.42E-01	27183643	27183766	NC_000007.13

cg03210257	7.17E-09	6.89E-10	6.49	11.5869206	1.28E-01	68118127	68118250	NC_000015.9
cg04549460	7.17E-09	6.89E-10	6.49	11.5867522	1.59E-01	109095975	109096098	NC_000008.10
cg00499237	7.17E-09	6.89E-10	6.49	11.586621	1.22E-01	97169033	97169156	NC_000002.11
cg23309947	7.17E-09	6.89E-10	6.49	11.5865915	5.28E-02	44129997	44130120	NC_000021.8
cg10117884	7.20E-09	6.92E-10	6.49	11.5834626	1.43E-01	166955898	166956021	NC_000001.10
cg10021878	7.21E-09	6.93E-10	6.49	11.5818139	1.46E-01	207669922	207670045	NC_000001.10
cg18463891	7.21E-09	6.93E-10	6.49	11.5816057	7.93E-02	593650	593773	NC_000007.13
cg15613761	7.21E-09	6.93E-10	6.49	11.5813084	8.56E-02	46404710	46404833	NC_000019.9
cg24714905	7.21E-09	6.93E-10	6.49	11.5812279	1.51E-01	100637241	100637364	NC_000013.10
cg27648006	7.22E-09	6.94E-10	6.49	11.580425	6.02E-02	131526535	131526658	NC_000012.11
cg21269934	7.23E-09	6.95E-10	6.49	11.5788419	1.46E-01	2060219	2060342	NC_000004.11
cg09207077	7.23E-09	6.95E-10	6.49	11.5785934	8.96E-02	62708202	62708325	NC_000010.10
cg26841114	7.23E-09	6.95E-10	6.49	11.5785425	6.08E-02	88871987	88872110	NC_000016.9
cg08815081	7.23E-09	6.96E-10	6.49	11.577812	1.51E-01	28035731	28035854	NC_000010.10
cg10319893	7.23E-09	6.96E-10	6.49	11.5776925	1.61E-01	37248953	37249076	NC_000013.10
cg10377921	7.25E-09	6.97E-10	6.49	11.5755404	1.37E-01	15391946	15392069	NC_000019.9
cg11435943	7.25E-09	6.98E-10	6.49	11.575219	1.05E-01	61222687	61222810	NC_000018.9
cg01922936	7.25E-09	6.98E-10	6.49	11.5750357	1.35E-01	100239017	100239140	NC_000005.9
cg11741356	7.26E-09	6.98E-10	6.49	11.5746271	9.13E-02	97490784	97490907	NC_000015.9
cg12354318	7.26E-09	6.98E-10	6.49	11.5738726	5.61E-02	14582415	14582538	NC_000010.10
cg14908170	7.27E-09	6.99E-10	6.49	11.5729961	1.19E-01	240405317	240405440	NC_000002.11
cg18840956	7.27E-09	6.99E-10	6.49	11.5728018	1.88E-01	95769005	95769128	NC_000005.9
cg10828337	7.28E-09	7.00E-10	6.49	11.5713468	1.49E-01	29236476	29236599	NC_000014.8
cg14716990	7.28E-09	7.01E-10	6.49	11.5704867	1.86E-01	129533731	129533854	NC_000010.10
cg07152755	7.29E-09	7.01E-10	6.49	11.5701476	1.47E-01	105474472	105474595	NC_000002.11

cg20303561	7.29E-09	7.01E-10	6.49	11.5700498	1.26E-01	91881497	91881620	NC_000014.8
cg06650206	7.30E-09	7.03E-10	6.49	11.5681579	1.02E-01	166068485	166068608	NC_000006.11
cg24199834	7.31E-09	7.04E-10	6.49	11.5664456	1.38E-01	147560126	147560249	NC_000004.11
cg18466173	7.33E-09	7.05E-10	6.49	11.5646027	1.50E-01	46726267	46726390	NC_000021.8
cg19742687	7.33E-09	7.05E-10	6.49	11.5644889	1.01E-01	88025975	88026098	NC_000011.9
cg08941355	7.33E-09	7.06E-10	6.49	11.5636865	9.20E-02	27133106	27133229	NC_000007.13
cg01354961	7.34E-09	7.07E-10	6.49	11.5624948	1.64E-01	8473462	8473585	NC_000007.13
cg01656244	7.36E-09	7.09E-10	6.49	11.559388	1.74E-01	65284080	65284203	NC_000008.10
cg21467371	7.38E-09	7.10E-10	6.48	11.557539	9.38E-02	79648722	79648845	NC_000017.10
cg18780412	7.38E-09	7.10E-10	6.48	11.5574824	1.80E-01	179755086	179755209	NC_000003.11
cg07991479	7.38E-09	7.11E-10	6.48	11.5571043	3.77E-02	40265765	40265888	NC_000017.10
cg13269527	7.38E-09	7.11E-10	6.48	11.55692	5.88E-02	25572674	25572797	NC_000018.9
cg22054362	7.39E-09	7.12E-10	6.48	11.5556644	1.82E-01	57050367	57050490	NC_000019.9
cg10031614	7.39E-09	7.12E-10	6.48	11.5556238	1.56E-01	81187601	81187724	NC_000004.11
cg08314208	7.39E-09	7.12E-10	6.48	11.5554325	9.04E-02	67682810	67682933	NC_000016.9
cg08823240	7.39E-09	7.12E-10	6.48	11.555405	1.47E-01	43388897	43389020	NC_000017.10
cg05616550	7.41E-09	7.13E-10	6.48	11.5531724	1.75E-01	54675154	54675277	NC_000017.10
cg18325439	7.41E-09	7.14E-10	6.48	11.5529414	1.56E-01	71217320	71217443	NC_000007.13
cg09511846	7.41E-09	7.14E-10	6.48	11.5522476	2.22E-01	77332991	77333114	NC_000001.10
cg03535239	7.43E-09	7.16E-10	6.48	11.5499935	8.25E-02	77241424	77241547	NC_000014.8
cg24114014	7.44E-09	7.17E-10	6.48	11.5485584	1.58E-01	28584167	28584290	NC_000006.11
cg04970570	7.45E-09	7.18E-10	6.48	11.5473882	1.62E-01	87990145	87990268	NC_000005.9
cg23808946	7.46E-09	7.19E-10	6.48	11.5454577	2.33E-01	45169548	45169671	NC_000002.11
cg07295678	7.47E-09	7.20E-10	6.48	11.5444163	1.53E-01	134000009	134000132	NC_000010.10
cg09258813	7.47E-09	7.20E-10	6.48	11.5443903	2.33E-01	37823409	37823532	NC_000008.10

cg14392929	7.47E-09	7.20E-10	6.48	11.5443315	7.14E-02	136758143	136758266	NC_000009.11
cg16195569	7.47E-09	7.20E-10	6.48	11.5439522	1.38E-01	57390536	57390659	NC_000010.10
cg01322405	7.48E-09	7.21E-10	6.48	11.5428863	1.20E-01	40757317	40757440	NC_000021.8
cg04166294	7.48E-09	7.21E-10	6.48	11.5426446	1.16E-01	54322984	54323107	NC_000016.9
cg18536148	7.50E-09	7.23E-10	6.48	11.5397877	1.13E-01	59533844	59533967	NC_000017.10
cg24310904	7.51E-09	7.24E-10	6.48	11.5389651	8.04E-02	47421270	47421393	NC_000021.8
cg22954645	7.51E-09	7.24E-10	6.48	11.538465	7.91E-02	168972438	168972561	NC_000006.11
cg02093692	7.51E-09	7.24E-10	6.48	11.5383979	1.23E-01	193798490	193798613	NC_000003.11
cg15805104	7.51E-09	7.24E-10	6.48	11.5383975	1.63E-01	56988935	56989058	NC_000019.9
cg07434402	7.51E-09	7.24E-10	6.48	11.5381916	2.13E-01	84431535	84431658	NC_000011.9
cg27329091	7.51E-09	7.25E-10	6.48	11.5380224	9.49E-02	213698049	213698172	NC_000002.11
cg12300382	7.51E-09	7.25E-10	6.48	11.5379043	8.80E-02	50467567	50467690	NC_000007.13
cg15759056	7.52E-09	7.26E-10	6.48	11.5363126	1.41E-01	87229552	87229675	NC_000007.13
cg10573018	7.53E-09	7.26E-10	6.48	11.5357328	1.79E-01	38100837	38100960	NC_000001.10
cg23164183	7.55E-09	7.28E-10	6.48	11.532697	1.23E-01	2754894	2755017	NC_000005.9
cg14048487	7.55E-09	7.28E-10	6.48	11.5326912	4.68E-02	41273439	41273562	NC_000017.10
cg19437183	7.56E-09	7.29E-10	6.48	11.5319768	1.82E-01	77585222	77585345	NC_000008.10
cg07558006	7.56E-09	7.30E-10	6.48	11.5311988	3.14E-02	132874263	132874386	NC_000006.11
cg00342530	7.56E-09	7.30E-10	6.48	11.5309983	1.25E-01	130027020	130027143	NC_000009.11
cg17991695	7.56E-09	7.30E-10	6.48	11.5308284	1.36E-01	10882974	10883097	NC_000006.11
cg23024136	7.57E-09	7.31E-10	6.48	11.5295838	1.32E-01	153853154	153853277	NC_000005.9
cg10678266	7.58E-09	7.31E-10	6.48	11.5288786	1.48E-01	24614348	24614471	NC_000007.13
cg12351433	7.58E-09	7.32E-10	6.48	11.5284595	1.82E-01	48982957	48983080	NC_000002.11
cg02185464	7.58E-09	7.32E-10	6.48	11.5279577	1.29E-01	84867338	84867461	NC_000016.9
cg06169131	7.59E-09	7.32E-10	6.48	11.5274858	1.24E-01	103536348	103536471	NC_000010.10

cg10906284	7.59E-09	7.33E-10	6.48	11.5268904	1.08E-01	63544430	63544553	NC_000012.11
cg01028849	7.59E-09	7.33E-10	6.48	11.5268599	1.43E-01	65490422	65490545	NC_000008.10
cg11409285	7.60E-09	7.33E-10	6.48	11.5260838	9.14E-02	76641147	76641270	NC_000017.10
cg23847381	7.60E-09	7.34E-10	6.48	11.5251781	1.95E-01	5544031	5544154	NC_000018.9
cg20374054	7.60E-09	7.34E-10	6.48	11.5250863	1.46E-01	124735126	124735249	NC_000011.9
cg23820302	7.62E-09	7.36E-10	6.48	11.5225096	5.33E-02	19172577	19172700	NC_000013.10
cg26658465	7.63E-09	7.36E-10	6.48	11.5222412	1.82E-01	178594195	178594318	NC_000005.9
cg07908679	7.66E-09	7.40E-10	6.48	11.5173788	6.57E-02	156371426	156371549	NC_000007.13
cg12111758	7.66E-09	7.40E-10	6.48	11.517246	1.59E-01	111472375	111472498	NC_000012.11
cg00266286	7.67E-09	7.41E-10	6.48	11.5158359	1.49E-01	32713723	32713846	NC_000005.9
cg04157161	7.67E-09	7.41E-10	6.48	11.5157601	9.34E-02	7906847	7906970	NC_000017.10
cg17443668	7.68E-09	7.42E-10	6.48	11.5148179	9.41E-02	152130772	152130895	NC_000001.10
cg16690286	7.68E-09	7.42E-10	6.48	11.5146813	6.74E-02	31870244	31870367	NC_000020.10
cg13826749	7.69E-09	7.42E-10	6.48	11.5140528	5.03E-02	43867568	43867691	NC_000021.8
cg09919743	7.69E-09	7.43E-10	6.48	11.5135862	3.00E-02	31663851	31663974	NC_000015.9
cg26343532	7.69E-09	7.43E-10	6.48	11.513508	1.03E-01	144207101	144207224	NC_000008.10
cg19105442	7.70E-09	7.44E-10	6.48	11.5126348	7.70E-02	110965775	110965898	NC_000013.10
cg15778437	7.71E-09	7.45E-10	6.48	11.511044	2.04E-01	31839521	31839644	NC_000011.9
cg13062455	7.71E-09	7.45E-10	6.48	11.5105427	1.35E-01	81978021	81978144	NC_000014.8
cg21530453	7.72E-09	7.46E-10	6.48	11.509996	2.01E-01	56226346	56226469	NC_000016.9
cg06066137	7.73E-09	7.47E-10	6.48	11.508746	1.31E-01	57359200	57359323	NC_000008.10
cg23155932	7.73E-09	7.47E-10	6.48	11.5085418	4.21E-02	134877706	134877829	NC_000010.10
cg07802350	7.73E-09	7.47E-10	6.48	11.5078493	1.75E-01	176956573	176956696	NC_000002.11
cg13041355	7.73E-09	7.47E-10	6.48	11.507549	7.65E-02	178622080	178622203	NC_000005.9
cg01011748	7.73E-09	7.48E-10	6.48	11.5074536	2.16E-01	154729200	154729323	NC_000002.11

cg21762788	7.74E-09	7.48E-10	6.48	11.5070293	1.60E-01	9756501	9756624	NC_000008.10
cg20296493	7.76E-09	7.50E-10	6.48	11.5044147	1.07E-01	124219725	124219848	NC_000008.10
cg17220749	7.76E-09	7.50E-10	6.48	11.5043217	1.17E-01	230249292	230249415	NC_000001.10
cg23409774	7.76E-09	7.50E-10	6.48	11.5042744	1.47E-01	175300141	175300264	NC_000005.9
cg16210375	7.76E-09	7.50E-10	6.47	11.5035461	3.42E-02	26682730	26682853	NC_000002.11
cg21591693	7.77E-09	7.51E-10	6.47	11.503181	6.62E-02	159647782	159647905	NC_000004.11
cg22399023	7.77E-09	7.51E-10	6.47	11.5029903	3.63E-02	5950951	5951074	NC_000001.10
cg09598590	7.77E-09	7.51E-10	6.47	11.502954	5.56E-02	46563704	46563827	NC_000013.10
cg07133479	7.77E-09	7.52E-10	6.47	11.5019999	4.61E-02	86420600	86420723	NC_000002.11
cg26843278	7.78E-09	7.52E-10	6.47	11.5015142	1.35E-01	131194523	131194646	NC_000010.10
cg10894566	7.78E-09	7.52E-10	6.47	11.5012267	1.54E-01	89905901	89906024	NC_000015.9
cg21251125	7.79E-09	7.53E-10	6.47	11.5002462	1.46E-01	126771781	126771904	NC_000009.11
cg08787039	7.79E-09	7.54E-10	6.47	11.4994673	1.03E-01	182921857	182921980	NC_000001.10
cg20194689	7.80E-09	7.54E-10	6.47	11.4988056	9.42E-02	178003633	178003756	NC_000005.9
cg02954212	7.80E-09	7.54E-10	6.47	11.4986459	2.02E-01	56226350	56226473	NC_000016.9
cg27662246	7.80E-09	7.55E-10	6.47	11.4983033	8.10E-02	107974539	107974662	NC_000012.11
cg22537280	7.80E-09	7.55E-10	6.47	11.497916	8.95E-02	54723213	54723336	NC_000001.10
cg03957235	7.80E-09	7.55E-10	6.47	11.4978244	6.83E-02	3512243	3512366	NC_000001.10
cg17466857	7.81E-09	7.55E-10	6.47	11.4975357	1.72E-01	27225528	27225651	NC_000007.13
cg00211337	7.81E-09	7.56E-10	6.47	11.496781	1.49E-01	230670550	230670673	NC_000002.11
cg14164099	7.81E-09	7.56E-10	6.47	11.4967397	1.27E-01	95870475	95870598	NC_000015.9
cg21869027	7.82E-09	7.56E-10	6.47	11.4963101	6.53E-02	29902402	29902525	NC_000017.10
cg22159514	7.82E-09	7.57E-10	6.47	11.4954935	1.95E-01	11591185	11591308	NC_000019.9
cg15972331	7.83E-09	7.58E-10	6.47	11.4941445	1.46E-01	102569894	102570017	NC_000013.10
cg25482900	7.83E-09	7.58E-10	6.47	11.4937103	1.31E-01	167956911	167957034	NC_000005.9

cg06399148	7.83E-09	7.58E-10	6.47	11.4935848	1.89E-01	104153148	104153271	NC_000008.10
cg19905757	7.84E-09	7.58E-10	6.47	11.4934525	1.10E-01	68924127	68924250	NC_000015.9
cg15792957	7.84E-09	7.58E-10	6.47	11.4931972	9.33E-02	47604206	47604329	NC_000002.11
cg00406023	7.85E-09	7.59E-10	6.47	11.4920438	8.77E-02	120090023	120090146	NC_000011.9
cg11012061	7.87E-09	7.61E-10	6.47	11.489547	1.49E-01	145722468	145722591	NC_000005.9
cg08862830	7.87E-09	7.62E-10	6.47	11.4892762	9.38E-02	5507336	5507459	NC_000019.9
cg15239123	7.87E-09	7.62E-10	6.47	11.4891667	1.89E-01	70346413	70346536	NC_000014.8
cg26976732	7.87E-09	7.62E-10	6.47	11.4890026	2.12E-01	216100	216223	NC_000016.9
cg13651561	7.87E-09	7.62E-10	6.47	11.488966	5.99E-02	134663491	134663614	NC_000009.11
cg19838506	7.89E-09	7.64E-10	6.47	11.4861705	6.14E-02	13025242	13025365	NC_000007.13
cg15196197	7.90E-09	7.64E-10	6.47	11.4856218	1.67E-01	32064573	32064696	NC_000006.11
cg01532436	7.90E-09	7.65E-10	6.47	11.4853157	1.96E-01	48983942	48984065	NC_000019.9
cg02886806	7.90E-09	7.65E-10	6.47	11.4850442	7.56E-02	1906992	1907115	NC_000002.11
cg09447105	7.91E-09	7.66E-10	6.47	11.4839151	7.60E-02	15126020	15126143	NC_000012.11
cg26327666	7.92E-09	7.67E-10	6.47	11.4818729	1.55E-01	76937963	76938086	NC_000005.9
cg01255417	7.93E-09	7.68E-10	6.47	11.480914	1.32E-01	10422636	10422759	NC_000006.11
cg03754311	7.95E-09	7.70E-10	6.47	11.4788879	1.55E-01	20879029	20879152	NC_000001.10
cg06539449	7.95E-09	7.70E-10	6.47	11.4781242	8.67E-02	69461008	69461131	NC_000011.9
cg07647726	7.97E-09	7.72E-10	6.47	11.4760355	1.58E-01	109149171	109149294	NC_000013.10
cg16134678	7.97E-09	7.72E-10	6.47	11.4756792	1.13E-01	25670187	25670310	NC_000013.10
cg23071120	7.98E-09	7.73E-10	6.47	11.4749465	2.15E-01	13840480	13840603	NC_000001.10
cg17940495	7.98E-09	7.73E-10	6.47	11.4749178	8.94E-02	14282882	14283005	NC_000006.11
cg04299439	7.98E-09	7.73E-10	6.47	11.4745773	1.40E-01	182547599	182547722	NC_000002.11
cg22294267	7.98E-09	7.73E-10	6.47	11.4744338	7.91E-02	57352252	57352375	NC_000019.9
cg05812266	7.98E-09	7.73E-10	6.47	11.4740262	9.78E-02	29595194	29595317	NC_000006.11

cg12514620	7.98E-09	7.74E-10	6.47	11.473838	7.89E-02	54871858	54871981	NC_000001.10
cg02214745	7.99E-09	7.74E-10	6.47	11.4732098	1.82E-01	130645645	130645768	NC_000012.11
cg01164789	7.99E-09	7.74E-10	6.47	11.4729327	2.32E-02	77424274	77424397	NC_000012.11
cg14270789	8.00E-09	7.75E-10	6.47	11.4723837	1.75E-01	25620954	25621077	NC_000013.10
cg14301844	8.00E-09	7.75E-10	6.47	11.4721917	6.38E-02	100904721	100904844	NC_000014.8
cg25400229	8.01E-09	7.76E-10	6.47	11.4711143	1.88E-01	11752592	11752715	NC_000018.9
cg26833667	8.02E-09	7.77E-10	6.47	11.4696211	1.63E-02	15005466	15005589	NC_000004.11
cg10481019	8.03E-09	7.78E-10	6.47	11.468069	5.00E-02	81095002	81095125	NC_000016.9
cg09232937	8.04E-09	7.79E-10	6.47	11.4670314	1.37E-01	3595970	3596093	NC_000005.9
cg24504927	8.04E-09	7.79E-10	6.47	11.4667611	1.98E-01	85667353	85667476	NC_000012.11
cg12298268	8.04E-09	7.79E-10	6.47	11.4664288	1.77E-01	139257866	139257989	NC_000003.11
cg24811864	8.05E-09	7.80E-10	6.47	11.4651889	1.50E-01	55019711	55019834	NC_000018.9
cg16786581	8.05E-09	7.81E-10	6.47	11.4650152	9.68E-02	10320712	10320835	NC_000006.11
cg14186846	8.06E-09	7.82E-10	6.47	11.4635198	1.14E-01	15256907	15257030	NC_000006.11
cg23563443	8.07E-09	7.83E-10	6.47	11.4623013	2.86E-02	22408989	22409112	NC_000008.10
cg06679347	8.08E-09	7.83E-10	6.47	11.4615032	2.00E-01	177714350	177714473	NC_000004.11
cg25035988	8.09E-09	7.85E-10	6.47	11.4599226	1.44E-01	57387978	57388101	NC_000010.10
cg17811167	8.09E-09	7.85E-10	6.47	11.4598449	1.14E-01	36861499	36861622	NC_000017.10
cg09359907	8.10E-09	7.85E-10	6.47	11.4593617	2.37E-01	8869099	8869222	NC_000017.10
cg19358442	8.11E-09	7.86E-10	6.47	11.4578744	1.13E-01	44325687	44325810	NC_000011.9
cg07520649	8.11E-09	7.86E-10	6.47	11.4578108	1.50E-01	140743575	140743698	NC_000005.9
cg25586361	8.11E-09	7.86E-10	6.47	11.4577729	1.11E-01	32455444	32455567	NC_000011.9
cg17880014	8.11E-09	7.87E-10	6.47	11.4573047	1.43E-01	71693165	71693288	NC_000002.11
cg06553513	8.12E-09	7.87E-10	6.47	11.456553	1.31E-01	131193272	131193395	NC_000010.10
cg12690369	8.12E-09	7.88E-10	6.47	11.4559892	1.72E-01	71314144	71314267	NC_000012.11

cg16933248	8.12E-09	7.88E-10	6.47	11.4559016	1.03E-01	132932245	132932368	NC_000012.11
cg01384163	8.13E-09	7.88E-10	6.47	11.4553362	1.62E-01	47073969	47074092	NC_000017.10
cg07922007	8.13E-09	7.89E-10	6.47	11.4551063	2.10E-01	67874858	67874981	NC_000008.10
cg12110911	8.14E-09	7.89E-10	6.47	11.4542402	1.32E-01	207506991	207507114	NC_000002.11
cg05432213	8.14E-09	7.89E-10	6.47	11.4541381	7.73E-02	35086985	35087108	NC_000015.9
cg13824385	8.15E-09	7.91E-10	6.47	11.4519293	7.55E-02	132584750	132584873	NC_000009.11
cg23497569	8.16E-09	7.92E-10	6.47	11.4509819	1.70E-01	19417585	19417708	NC_000007.13
cg12405231	8.17E-09	7.93E-10	6.46	11.4493311	9.23E-02	211749023	211749146	NC_000001.10
cg16691196	8.18E-09	7.93E-10	6.46	11.4490793	6.38E-02	20508651	20508774	NC_000001.10
cg22147084	8.18E-09	7.94E-10	6.46	11.4487243	1.62E-01	179779664	179779787	NC_000005.9
cg02919960	8.19E-09	7.94E-10	6.46	11.4478552	1.22E-01	27192549	27192672	NC_000007.13
cg05306173	8.19E-09	7.94E-10	6.46	11.4477467	8.66E-02	118885636	118885759	NC_000010.10
cg22961241	8.19E-09	7.95E-10	6.46	11.4471586	1.08E-01	32917502	32917625	NC_000006.11
cg27534599	8.20E-09	7.96E-10	6.46	11.4461205	1.36E-01	17680477	17680600	NC_000022.10
cg18760835	8.20E-09	7.96E-10	6.46	11.4457528	1.09E-01	5996245	5996368	NC_000006.11
cg24252925	8.21E-09	7.97E-10	6.46	11.4449842	1.99E-01	116854028	116854151	NC_000010.10
cg23634417	8.21E-09	7.97E-10	6.46	11.4444946	2.41E-02	156255479	156255602	NC_000001.10
cg11078221	8.22E-09	7.98E-10	6.46	11.444043	1.52E-01	115135934	115136057	NC_000012.11
cg08550101	8.22E-09	7.98E-10	6.46	11.4432048	1.31E-01	20823981	20824104	NC_000007.13
cg13240116	8.22E-09	7.98E-10	6.46	11.4431331	9.74E-02	27147513	27147636	NC_000007.13
cg12600346	8.23E-09	7.99E-10	6.46	11.4426896	4.55E-02	82753800	82753923	NC_000002.11
cg23038594	8.23E-09	7.99E-10	6.46	11.4426869	1.41E-01	170254369	170254492	NC_000001.10
cg19888869	8.24E-09	8.00E-10	6.46	11.4404293	1.09E-01	54967674	54967797	NC_000004.11
cg12620706	8.24E-09	8.00E-10	6.46	11.4403982	6.29E-02	155917159	155917282	NC_000007.13
cg12042864	8.25E-09	8.01E-10	6.46	11.4393782	4.57E-02	99066260	99066383	NC_000007.13

cg26410450	8.25E-09	8.01E-10	6.46	11.4392872	1.76E-01	17740967	17741090	NC_000011.9
cg17250160	8.25E-09	8.01E-10	6.46	11.4392499	1.13E-01	156919811	156919934	NC_000006.11
cg16479539	8.27E-09	8.03E-10	6.46	11.4377777	1.54E-01	54345212	54345335	NC_000012.11
cg02711212	8.27E-09	8.03E-10	6.46	11.4375046	1.68E-01	69854024	69854147	NC_000015.9
cg15684724	8.27E-09	8.03E-10	6.46	11.4369074	2.38E-01	67875033	67875156	NC_000008.10
cg11429658	8.28E-09	8.04E-10	6.46	11.4362854	1.34E-01	15646635	15646758	NC_000021.8
cg09909151	8.28E-09	8.04E-10	6.46	11.4358468	1.13E-01	47494258	47494381	NC_000016.9
cg25260543	8.28E-09	8.04E-10	6.46	11.4357711	1.43E-01	100946792	100946915	NC_000007.13
cg05523911	8.29E-09	8.06E-10	6.46	11.4342697	1.48E-01	152081002	152081125	NC_000001.10
cg17861230	8.30E-09	8.06E-10	6.46	11.4333407	1.33E-01	18343901	18344024	NC_000019.9
cg01016119	8.31E-09	8.08E-10	6.46	11.4317515	1.66E-01	56835282	56835405	NC_000003.11
cg27317433	8.33E-09	8.10E-10	6.46	11.4291072	1.99E-01	1401781	1401904	NC_000019.9
cg13686115	8.34E-09	8.10E-10	6.46	11.4284539	1.40E-01	60936626	60936749	NC_000010.10
cg12135286	8.34E-09	8.11E-10	6.46	11.4279359	7.27E-02	4035711	4035834	NC_000010.10
cg16099210	8.35E-09	8.11E-10	6.46	11.427308	1.75E-01	119001250	119001373	NC_000010.10
cg12031031	8.35E-09	8.11E-10	6.46	11.4270144	5.75E-02	103980882	103981005	NC_000012.11
cg12391690	8.36E-09	8.13E-10	6.46	11.4252305	1.71E-01	38100811	38100934	NC_000001.10
cg26341453	8.37E-09	8.13E-10	6.46	11.4248978	1.05E-01	28593732	28593855	NC_000001.10
cg09865386	8.38E-09	8.15E-10	6.46	11.4229252	4.98E-02	101192599	101192722	NC_000014.8
cg14102055	8.39E-09	8.16E-10	6.46	11.4220365	6.34E-02	133715238	133715361	NC_000009.11
cg00146655	8.39E-09	8.16E-10	6.46	11.4219388	7.41E-02	7517194	7517317	NC_000003.11
cg08882547	8.39E-09	8.16E-10	6.46	11.4215177	1.25E-01	45737792	45737915	NC_000019.9
cg12405265	8.39E-09	8.16E-10	6.46	11.4214987	4.57E-02	24655961	24656084	NC_000001.10
cg13494428	8.40E-09	8.16E-10	6.46	11.4213172	9.38E-02	19639494	19639617	NC_000001.10
cg26536189	8.40E-09	8.17E-10	6.46	11.4206349	1.95E-01	13840494	13840617	NC_000001.10

cg25365014	8.41E-09	8.17E-10	6.46	11.4199583	1.30E-01	2727713	2727836	NC_000005.9
cg20289911	8.41E-09	8.18E-10	6.46	11.4195466	9.92E-02	816362	816485	NC_000020.10
cg21622568	8.43E-09	8.19E-10	6.46	11.4175523	3.53E-02	111091243	111091366	NC_000013.10
cg12129983	8.43E-09	8.20E-10	6.46	11.4165824	3.90E-02	69462660	69462783	NC_000011.9
cg08270734	8.44E-09	8.21E-10	6.46	11.4158208	2.23E-02	108951574	108951697	NC_000012.11
cg21483883	8.45E-09	8.21E-10	6.46	11.4151441	9.50E-02	37487352	37487475	NC_000007.13
cg06849515	8.46E-09	8.23E-10	6.46	11.4137538	1.76E-01	13549607	13549730	NC_000004.11
cg19511748	8.46E-09	8.23E-10	6.46	11.4136305	6.11E-02	94220272	94220395	NC_000005.9
cg14118515	8.46E-09	8.23E-10	6.46	11.4128643	1.96E-01	176948728	176948851	NC_000002.11
cg05193407	8.47E-09	8.24E-10	6.46	11.4123469	6.16E-02	57036298	57036421	NC_000004.11
cg17537466	8.47E-09	8.24E-10	6.46	11.4116472	1.08E-01	14673322	14673445	NC_000005.9
cg24601327	8.48E-09	8.25E-10	6.46	11.4106937	1.25E-01	96526353	96526476	NC_000001.10
cg23134869	8.48E-09	8.25E-10	6.46	11.4106462	1.60E-01	77593233	77593356	NC_000008.10
cg20390515	8.49E-09	8.26E-10	6.46	11.41008	2.00E-01	945697	945820	NC_000002.11
cg01204606	8.49E-09	8.26E-10	6.46	11.4092886	1.93E-01	116451788	116451911	NC_000011.9
cg19832521	8.49E-09	8.27E-10	6.46	11.4090468	2.01E-01	27065974	27066097	NC_000014.8
cg06497668	8.50E-09	8.27E-10	6.46	11.4083957	1.55E-01	45155703	45155826	NC_000002.11
cg02370395	8.50E-09	8.28E-10	6.46	11.4077259	1.84E-01	35641505	35641628	NC_000011.9
cg05778440	8.51E-09	8.28E-10	6.46	11.4070516	1.10E-01	66196160	66196283	NC_000017.10
cg10356657	8.51E-09	8.28E-10	6.46	11.4068525	1.02E-01	99978574	99978697	NC_000014.8
cg24801123	8.51E-09	8.28E-10	6.46	11.4068296	1.40E-01	45615503	45615626	NC_000007.13
cg04123685	8.51E-09	8.29E-10	6.46	11.4064173	2.87E-02	36897340	36897463	NC_000022.10
cg19389953	8.53E-09	8.31E-10	6.46	11.4041299	1.29E-01	121939827	121939950	NC_000007.13
cg05933997	8.55E-09	8.32E-10	6.46	11.402568	7.31E-02	86735122	86735245	NC_000016.9
cg00797104	8.55E-09	8.33E-10	6.46	11.4015863	1.03E-01	32847605	32847728	NC_000006.11

cg14985989	8.55E-09	8.33E-10	6.46	11.4014194	2.01E-01	496428	496551	NC_000019.9
cg26986911	8.56E-09	8.34E-10	6.46	11.4007626	1.93E-01	33936292	33936415	NC_000005.9
cg24645221	8.56E-09	8.34E-10	6.46	11.4004796	1.03E-01	57358928	57359051	NC_000008.10
cg27326661	8.57E-09	8.35E-10	6.46	11.3996176	9.27E-02	80922579	80922702	NC_000008.10
cg05870140	8.57E-09	8.35E-10	6.46	11.399523	1.46E-01	101923427	101923550	NC_000014.8
cg14562851	8.57E-09	8.35E-10	6.46	11.3992444	4.98E-02	12660012	12660135	NC_000001.10
cg23752086	8.58E-09	8.36E-10	6.46	11.3981199	1.58E-01	20522177	20522300	NC_000012.11
cg23489627	8.58E-09	8.36E-10	6.46	11.3978137	1.22E-01	73674339	73674462	NC_000003.11
cg26780138	8.59E-09	8.37E-10	6.45	11.3967176	1.77E-01	477087	477210	NC_000016.9
cg17453671	8.61E-09	8.38E-10	6.45	11.3951465	4.13E-02	207109202	207109325	NC_000001.10
cg18932809	8.61E-09	8.39E-10	6.45	11.3946444	1.76E-01	130686826	130686949	NC_000006.11
cg07703364	8.61E-09	8.39E-10	6.45	11.3945915	2.20E-02	6992514	6992637	NC_000012.11
cg07413251	8.62E-09	8.40E-10	6.45	11.3933832	1.91E-01	27771667	27771790	NC_000003.11
cg02770983	8.63E-09	8.40E-10	6.45	11.3926944	1.37E-01	31822341	31822464	NC_000011.9
cg23526676	8.63E-09	8.41E-10	6.45	11.3925905	8.48E-02	31734458	31734581	NC_000006.11
cg15135914	8.63E-09	8.41E-10	6.45	11.392532	1.97E-02	65430544	65430667	NC_000011.9
cg24875029	8.63E-09	8.41E-10	6.45	11.3924714	1.43E-01	105471438	105471561	NC_000002.11
cg16664523	8.63E-09	8.41E-10	6.45	11.3924687	1.70E-01	67586170	67586293	NC_000005.9
cg14657728	8.63E-09	8.41E-10	6.45	11.3920034	2.20E-01	130498701	130498824	NC_000012.11
cg01308258	8.63E-09	8.41E-10	6.45	11.391749	1.22E-01	124783243	124783366	NC_000002.11
cg07351192	8.64E-09	8.42E-10	6.45	11.391292	1.42E-01	5137773	5137896	NC_000003.11
cg03345448	8.64E-09	8.42E-10	6.45	11.3909301	1.16E-01	40722516	40722639	NC_000006.11
cg23300486	8.65E-09	8.43E-10	6.45	11.3893431	1.98E-01	103294375	103294498	NC_000014.8
cg14716686	8.66E-09	8.44E-10	6.45	11.3885533	1.27E-01	30452862	30452985	NC_000021.8
cg14074052	8.67E-09	8.45E-10	6.45	11.3869809	1.25E-01	22094226	22094349	NC_000012.11

cg22587479	8.67E-09	8.46E-10	6.45	11.3866998	1.22E-01	219738226	219738349	NC_000002.11
cg05340085	8.67E-09	8.46E-10	6.45	11.3866767	6.33E-02	47134186	47134309	NC_000007.13
cg04037640	8.68E-09	8.47E-10	6.45	11.3854518	9.93E-02	32065034	32065157	NC_000006.11
cg14921743	8.68E-09	8.47E-10	6.45	11.3854489	2.29E-01	44650449	44650572	NC_000020.10
cg05628390	8.69E-09	8.47E-10	6.45	11.3845697	4.49E-02	2259411	2259534	NC_000001.10
cg04755999	8.69E-09	8.48E-10	6.45	11.3844862	5.46E-02	39274491	39274614	NC_000017.10
cg14297966	8.70E-09	8.48E-10	6.45	11.3840445	6.94E-02	35101708	35101831	NC_000009.11
cg09885735	8.70E-09	8.49E-10	6.45	11.3831399	1.69E-01	60297747	60297870	NC_000015.9
cg07967630	8.71E-09	8.49E-10	6.45	11.3826894	1.16E-01	58149317	58149440	NC_000006.11
cg08344360	8.72E-09	8.51E-10	6.45	11.3808981	9.51E-02	34701049	34701172	NC_000009.11
cg02868244	8.72E-09	8.51E-10	6.45	11.3807803	6.07E-02	134855441	134855564	NC_000010.10
cg12748607	8.74E-09	8.53E-10	6.45	11.3786713	1.94E-01	40678691	40678814	NC_000002.11
cg05371438	8.76E-09	8.54E-10	6.45	11.3769677	9.14E-02	184234	184357	NC_000012.11
cg24336989	8.76E-09	8.55E-10	6.45	11.3758768	1.78E-01	10385903	10386026	NC_000006.11
cg19276343	8.76E-09	8.55E-10	6.45	11.3758143	9.90E-02	41303922	41304045	NC_000001.10
cg04880737	8.77E-09	8.56E-10	6.45	11.3752679	9.50E-02	238617378	238617501	NC_000002.11
cg07481380	8.77E-09	8.56E-10	6.45	11.3749731	5.22E-02	3834519	3834642	NC_000019.9
cg11365913	8.77E-09	8.56E-10	6.45	11.3745551	1.40E-01	19318160	19318283	NC_000008.10
cg19181160	8.78E-09	8.57E-10	6.45	11.3740792	3.27E-02	3187593	3187716	NC_000011.9
cg08784934	8.78E-09	8.57E-10	6.45	11.3738059	6.65E-02	664994	665117	NC_000010.10
cg24468955	8.79E-09	8.58E-10	6.45	11.3728649	5.73E-02	90577545	90577668	NC_000008.10
cg21602520	8.79E-09	8.58E-10	6.45	11.3723362	1.40E-01	60985380	60985503	NC_000018.9
cg23350350	8.80E-09	8.59E-10	6.45	11.3718367	1.48E-01	16652008	16652131	NC_000008.10
cg25185429	8.80E-09	8.59E-10	6.45	11.3718084	5.49E-02	4739461	4739584	NC_000003.11
cg22231400	8.80E-09	8.59E-10	6.45	11.3716431	1.01E-01	49935823	49935946	NC_000019.9

cg09736669	8.81E-09	8.60E-10	6.45	11.3704654	1.55E-01	1851828	1851951	NC_000005.9
cg22530255	8.81E-09	8.60E-10	6.45	11.3698924	5.95E-02	3051692	3051815	NC_000001.10
cg02543772	8.82E-09	8.61E-10	6.45	11.3691201	1.72E-01	128850630	128850753	NC_000012.11
cg19115272	8.82E-09	8.61E-10	6.45	11.3690731	1.46E-01	30139538	30139661	NC_000006.11
cg09493063	8.82E-09	8.61E-10	6.45	11.3687298	2.71E-01	142494985	142495108	NC_000007.13
cg13300509	8.82E-09	8.61E-10	6.45	11.3685996	9.54E-02	57146833	57146956	NC_000012.11
cg10743378	8.84E-09	8.63E-10	6.45	11.3668262	5.54E-02	102028800	102028923	NC_000010.10
cg15829006	8.85E-09	8.64E-10	6.45	11.3655696	1.37E-01	137967652	137967775	NC_000009.11
cg27343616	8.86E-09	8.65E-10	6.45	11.3644995	9.78E-02	17207697	17207820	NC_000020.10
cg21596825	8.88E-09	8.67E-10	6.45	11.3625391	1.13E-01	3818723	3818846	NC_000002.11
cg17522727	8.89E-09	8.68E-10	6.45	11.3610663	1.59E-01	794867	794990	NC_000011.9
cg23675401	8.89E-09	8.68E-10	6.45	11.3608271	9.77E-02	4867481	4867604	NC_000004.11
cg00002809	8.91E-09	8.70E-10	6.45	11.3589705	3.31E-02	76482353	76482476	NC_000017.10
cg17633170	8.92E-09	8.71E-10	6.45	11.3580191	1.32E-01	131217151	131217274	NC_000012.11
cg23801969	8.92E-09	8.72E-10	6.45	11.3570698	7.44E-02	39051805	39051928	NC_000019.9
cg04920270	8.96E-09	8.76E-10	6.45	11.3523532	1.39E-01	112832249	112832372	NC_000011.9
cg16710124	8.97E-09	8.76E-10	6.45	11.3518484	7.62E-02	44716058	44716181	NC_000001.10
cg09420412	8.97E-09	8.76E-10	6.45	11.351598	8.60E-02	14683948	14684071	NC_000017.10
cg11052202	8.97E-09	8.77E-10	6.45	11.3515344	1.63E-01	99290546	99290669	NC_000006.11
cg07005778	8.97E-09	8.77E-10	6.45	11.3514095	1.42E-01	188953586	188953709	NC_000004.11
cg02601140	8.98E-09	8.78E-10	6.45	11.3500345	1.16E-01	63250517	63250640	NC_000007.13
cg13787438	8.98E-09	8.78E-10	6.45	11.3500247	1.55E-01	26664115	26664238	NC_000003.11
cg12661427	9.00E-09	8.80E-10	6.45	11.3479316	2.56E-02	44437166	44437289	NC_000001.10
cg11147215	9.00E-09	8.80E-10	6.45	11.3478807	8.64E-02	105379726	105379849	NC_000008.10
cg01460694	9.01E-09	8.80E-10	6.45	11.3471933	2.83E-02	97477428	97477551	NC_000002.11

cg03979754	9.01E-09	8.81E-10	6.45	11.346934	1.44E-01	110703460	110703583	NC_000008.10
cg04588165	9.03E-09	8.83E-10	6.45	11.3442851	1.89E-01	35300874	35300997	NC_000017.10
cg11858450	9.04E-09	8.84E-10	6.44	11.343703	1.15E-01	15132184	15132307	NC_000019.9
cg07962128	9.04E-09	8.84E-10	6.44	11.3431808	1.77E-01	37004812	37004935	NC_000013.10
cg06804433	9.04E-09	8.84E-10	6.44	11.3430916	1.69E-01	50682485	50682608	NC_000006.11
cg15819780	9.05E-09	8.84E-10	6.44	11.3427721	1.13E-01	53038915	53039038	NC_000017.10
cg21488538	9.06E-09	8.86E-10	6.44	11.3413137	1.71E-01	98964436	98964559	NC_000002.11
cg00547441	9.07E-09	8.87E-10	6.44	11.3400915	1.63E-01	50344724	50344847	NC_000007.13
cg09560096	9.07E-09	8.87E-10	6.44	11.339988	8.06E-02	219527018	219527141	NC_000002.11
cg19043574	9.08E-09	8.88E-10	6.44	11.3390075	1.18E-01	99439533	99439656	NC_000002.11
cg06877669	9.09E-09	8.89E-10	6.44	11.3381681	1.57E-01	25904216	25904339	NC_000008.10
cg08160862	9.12E-09	8.92E-10	6.44	11.3348135	2.03E-02	1270137	1270260	NC_000001.10
cg22488849	9.12E-09	8.92E-10	6.44	11.3342404	1.14E-01	3607134	3607257	NC_000005.9
cg16168668	9.13E-09	8.93E-10	6.44	11.3331584	1.24E-01	19158566	19158689	NC_000007.13
cg24156796	9.14E-09	8.94E-10	6.44	11.3319858	1.35E-01	114029646	114029769	NC_000012.11
cg11597832	9.14E-09	8.95E-10	6.44	11.3315501	1.13E-01	105993747	105993870	NC_000014.8
cg23546750	9.15E-09	8.95E-10	6.44	11.3313501	1.47E-01	3716787	3716910	NC_000020.10
cg27157619	9.15E-09	8.95E-10	6.44	11.3307732	6.94E-02	74714463	74714586	NC_000015.9
cg21580016	9.15E-09	8.96E-10	6.44	11.3304293	6.04E-02	121887392	121887515	NC_000012.11
cg16946687	9.16E-09	8.96E-10	6.44	11.3298322	9.84E-02	133172465	133172588	NC_000012.11
cg08056990	9.17E-09	8.97E-10	6.44	11.3292048	2.61E-02	800186	800309	NC_000011.9
cg15825311	9.18E-09	8.98E-10	6.44	11.3281015	1.97E-02	49948429	49948552	NC_000003.11
cg14920503	9.19E-09	8.99E-10	6.44	11.3268575	4.83E-02	45429155	45429278	NC_000021.8
cg10615150	9.19E-09	8.99E-10	6.44	11.3265414	1.69E-01	30582838	30582961	NC_000020.10
cg04462627	9.19E-09	9.00E-10	6.44	11.325917	2.13E-01	58019123	58019246	NC_000016.9

cg14614314	9.21E-09	9.01E-10	6.44	11.3245699	2.18E-01	135150428	135150551	NC_000010.10
cg26477573	9.22E-09	9.02E-10	6.44	11.3230323	2.17E-01	15342915	15343038	NC_000019.9
cg17167253	9.23E-09	9.03E-10	6.44	11.3219317	1.35E-01	119603383	119603506	NC_000002.11
cg07602571	9.23E-09	9.03E-10	6.44	11.3218707	4.36E-02	77644868	77644991	NC_000007.13
cg13340245	9.23E-09	9.04E-10	6.44	11.3214909	2.29E-02	239333713	239333836	NC_000002.11
cg10778441	9.24E-09	9.05E-10	6.44	11.3206359	1.53E-01	35293279	35293402	NC_000007.13
cg19447025	9.25E-09	9.05E-10	6.44	11.3197801	7.45E-02	33335436	33335559	NC_000006.11
cg15843929	9.25E-09	9.06E-10	6.44	11.3190931	1.05E-01	59182305	59182428	NC_000002.11
cg11197015	9.26E-09	9.06E-10	6.44	11.3187965	1.61E-01	133795796	133795919	NC_000010.10
cg21105319	9.27E-09	9.08E-10	6.44	11.316994	2.00E-02	118307072	118307195	NC_000011.9
cg00216061	9.28E-09	9.09E-10	6.44	11.3163305	5.96E-02	88547579	88547702	NC_000016.9
cg07892785	9.28E-09	9.09E-10	6.44	11.3160534	2.34E-01	39851788	39851911	NC_000003.11
cg24431667	9.28E-09	9.09E-10	6.44	11.3157635	1.33E-01	2162387	2162510	NC_000011.9
cg13973086	9.31E-09	9.12E-10	6.44	11.3127937	1.29E-01	469105	469228	NC_000002.11
cg12978433	9.31E-09	9.12E-10	6.44	11.3121426	1.85E-01	52789956	52790079	NC_000020.10
cg13448092	9.32E-09	9.14E-10	6.44	11.3110297	1.39E-01	181135878	181136001	NC_000003.11
cg07462053	9.32E-09	9.14E-10	6.44	11.310906	8.72E-02	32712829	32712952	NC_000021.8
cg02928664	9.33E-09	9.14E-10	6.44	11.3108083	1.49E-01	54976506	54976629	NC_000019.9
cg25717239	9.34E-09	9.16E-10	6.44	11.3087327	1.64E-01	43153595	43153718	NC_000007.13
cg14043803	9.35E-09	9.16E-10	6.44	11.3081942	7.55E-02	3627796	3627919	NC_000010.10
cg04083466	9.36E-09	9.17E-10	6.44	11.3074079	9.97E-02	25753732	25753855	NC_000006.11
cg12699044	9.36E-09	9.17E-10	6.44	11.3073922	3.54E-02	113703999	113704122	NC_000012.11
cg26293182	9.40E-09	9.22E-10	6.44	11.3022371	1.74E-01	57389779	57389902	NC_000010.10
cg11930908	9.42E-09	9.23E-10	6.44	11.3007061	1.60E-01	23500752	23500875	NC_000013.10
cg10390435	9.42E-09	9.23E-10	6.44	11.300482	6.07E-02	142368026	142368149	NC_000008.10

cg04470984	9.42E-09	9.24E-10	6.44	11.3000194	7.14E-02	100692757	100692880	NC_000011.9
cg16423505	9.43E-09	9.24E-10	6.44	11.2996539	2.31E-01	92051400	92051523	NC_000013.10
cg21537383	9.43E-09	9.25E-10	6.44	11.2992468	2.03E-01	22034799	22034922	NC_000019.9
cg03586793	9.45E-09	9.26E-10	6.44	11.2976352	1.20E-01	104312558	104312681	NC_000014.8
cg22070406	9.45E-09	9.26E-10	6.44	11.2975305	1.47E-01	55019708	55019831	NC_000018.9
cg03563308	9.45E-09	9.27E-10	6.44	11.2965935	1.69E-01	56887279	56887402	NC_000018.9
cg05619622	9.51E-09	9.33E-10	6.43	11.2907787	5.83E-02	62365104	62365227	NC_000011.9
cg16307674	9.51E-09	9.33E-10	6.43	11.290729	1.86E-01	155170868	155170991	NC_000007.13
cg26811065	9.51E-09	9.33E-10	6.43	11.2906811	4.34E-02	32825360	32825483	NC_000006.11
cg11354906	9.51E-09	9.33E-10	6.43	11.2900625	1.92E-01	154710371	154710494	NC_000004.11
cg23737208	9.52E-09	9.34E-10	6.43	11.2896033	6.45E-02	25499464	25499587	NC_000006.11
cg15962336	9.52E-09	9.34E-10	6.43	11.2891603	9.19E-02	53932577	53932700	NC_000001.10
cg02155658	9.53E-09	9.35E-10	6.43	11.2884725	1.64E-01	21690036	21690159	NC_000020.10
cg21815258	9.53E-09	9.35E-10	6.43	11.2884537	3.58E-02	1336833	1336956	NC_000005.9
cg07871971	9.56E-09	9.38E-10	6.43	11.2852216	5.21E-02	40839766	40839889	NC_000017.10
cg01962676	9.56E-09	9.38E-10	6.43	11.2851346	1.79E-01	18436969	18437092	NC_000001.10
cg27030744	9.56E-09	9.38E-10	6.43	11.2846864	3.88E-02	44496130	44496253	NC_000006.11
cg23286081	9.57E-09	9.39E-10	6.43	11.2841367	4.82E-02	125775778	125775901	NC_000010.10
cg07519873	9.57E-09	9.39E-10	6.43	11.2838451	1.85E-01	54483188	54483311	NC_000019.9
cg24040751	9.58E-09	9.40E-10	6.43	11.2831156	3.10E-02	56661627	56661750	NC_000014.8
cg20014304	9.58E-09	9.41E-10	6.43	11.2823924	3.35E-02	79804327	79804450	NC_000017.10
cg16666329	9.60E-09	9.42E-10	6.43	11.2806334	1.28E-01	76932526	76932649	NC_000005.9
cg12145018	9.60E-09	9.42E-10	6.43	11.2806023	6.84E-02	103912507	103912630	NC_000014.8
cg25603409	9.61E-09	9.43E-10	6.43	11.2796275	3.72E-02	50532834	50532957	NC_000019.9
cg04599946	9.62E-09	9.44E-10	6.43	11.2783756	1.51E-01	85936480	85936603	NC_000016.9

cg02721902	9.62E-09	9.45E-10	6.43	11.2781904	1.17E-01	46825371	46825494	NC_000017.10
cg06344952	9.64E-09	9.47E-10	6.43	11.2760547	4.38E-02	50707292	50707415	NC_000016.9
cg24085946	9.66E-09	9.49E-10	6.43	11.2741787	1.65E-01	204797839	204797962	NC_000001.10
cg01611862	9.67E-09	9.49E-10	6.43	11.2736722	1.29E-01	36982649	36982772	NC_000014.8
cg10341154	9.67E-09	9.50E-10	6.43	11.2728682	1.98E-01	164265442	164265565	NC_000004.11
cg21184206	9.68E-09	9.50E-10	6.43	11.2726783	8.22E-02	181769759	181769882	NC_000002.11
cg09271157	9.68E-09	9.50E-10	6.43	11.2726251	1.18E-01	122217376	122217499	NC_000010.10
cg09012411	9.68E-09	9.50E-10	6.43	11.2726195	1.00E-01	95167783	95167906	NC_000001.10
cg13951490	9.68E-09	9.51E-10	6.43	11.2721024	8.84E-02	140799290	140799413	NC_000005.9
cg05893614	9.68E-09	9.51E-10	6.43	11.2718311	1.86E-01	68981956	68982079	NC_000003.11
cg05099596	9.69E-09	9.52E-10	6.43	11.2710143	1.09E-01	108604750	108604873	NC_000002.11
cg14089267	9.70E-09	9.53E-10	6.43	11.2697589	1.37E-01	23412409	23412532	NC_000013.10
cg08005774	9.76E-09	9.59E-10	6.43	11.2636652	1.27E-01	18434106	18434229	NC_000001.10
cg07622877	9.76E-09	9.59E-10	6.43	11.2633298	1.00E-01	26434164	26434287	NC_000004.11
cg08996459	9.77E-09	9.59E-10	6.43	11.2631628	1.12E-01	125617230	125617353	NC_000010.10
cg14517390	9.77E-09	9.60E-10	6.43	11.2622395	2.94E-02	78463330	78463453	NC_000015.9
cg08276295	9.78E-09	9.61E-10	6.43	11.2617032	1.84E-01	27017174	27017297	NC_000015.9
cg09309112	9.78E-09	9.61E-10	6.43	11.2615685	6.14E-02	53571325	53571448	NC_000017.10
cg02853497	9.78E-09	9.61E-10	6.43	11.2613591	1.27E-01	46113000	46113123	NC_000001.10
cg25905674	9.78E-09	9.61E-10	6.43	11.2610789	2.21E-01	43047856	43047979	NC_000017.10
cg00512279	9.79E-09	9.62E-10	6.43	11.2604003	1.60E-01	119000991	119001114	NC_000010.10
cg01735384	9.79E-09	9.62E-10	6.43	11.2602076	1.97E-01	28603292	28603415	NC_000006.11
cg27310319	9.80E-09	9.63E-10	6.43	11.2593935	1.89E-01	160975978	160976101	NC_000005.9
cg21350073	9.80E-09	9.63E-10	6.43	11.2590323	6.60E-02	138724834	138724957	NC_000003.11
cg04061117	9.81E-09	9.64E-10	6.43	11.2582204	1.40E-01	9594385	9594508	NC_000003.11

cg20140110	9.82E-09	9.65E-10	6.43	11.2577306	1.81E-01	29936845	29936968	NC_000012.11
cg00132008	9.82E-09	9.65E-10	6.43	11.2573783	3.43E-02	8752348	8752471	NC_000008.10
cg20095233	9.83E-09	9.66E-10	6.43	11.2563064	1.88E-01	118033902	118034025	NC_000010.10
cg04157263	9.83E-09	9.67E-10	6.43	11.2556743	1.46E-01	37752991	37753114	NC_000017.10
cg13264018	9.84E-09	9.67E-10	6.43	11.2553259	3.58E-02	127788509	127788632	NC_000003.11
cg13402635	9.85E-09	9.68E-10	6.43	11.2543913	1.37E-01	97669802	97669925	NC_000009.11
cg03808577	9.85E-09	9.68E-10	6.43	11.2541433	1.06E-01	100219429	100219552	NC_000007.13
cg02829680	9.85E-09	9.68E-10	6.43	11.2541227	1.57E-01	21086336	21086459	NC_000020.10
cg15251598	9.85E-09	9.69E-10	6.43	11.2535783	1.31E-01	131731416	131731539	NC_000010.10
cg23262218	9.86E-09	9.69E-10	6.43	11.2530701	1.41E-01	101290270	101290393	NC_000010.10
cg05724271	9.88E-09	9.71E-10	6.43	11.2510741	8.21E-02	8151066	8151189	NC_000007.13
cg19549469	9.91E-09	9.75E-10	6.43	11.2476915	1.89E-02	45668950	45669073	NC_000012.11
cg03610852	9.91E-09	9.75E-10	6.43	11.2474065	1.83E-01	65287050	65287173	NC_000008.10
cg26115667	9.95E-09	9.79E-10	6.43	11.2437243	1.67E-01	103294656	103294779	NC_000014.8
cg05143887	9.95E-09	9.79E-10	6.43	11.2431982	1.71E-01	81471938	81472061	NC_000012.11
cg22821908	9.96E-09	9.80E-10	6.43	11.2426396	1.55E-01	38510522	38510645	NC_000001.10
cg20050826	9.97E-09	9.81E-10	6.43	11.2415474	1.08E-01	52995295	52995418	NC_000012.11
cg01448155	9.97E-09	9.81E-10	6.43	11.241113	3.32E-02	3564782	3564905	NC_000010.10
cg24989962	9.98E-09	9.81E-10	6.43	11.2409058	1.58E-01	52734286	52734409	NC_000014.8
cg06958563	9.98E-09	9.82E-10	6.43	11.2407083	1.55E-01	87980911	87981034	NC_000005.9
cg22727965	1.00E-08	9.84E-10	6.43	11.2385119	1.05E-01	25898770	25898893	NC_000007.13
cg06951677	1.00E-08	9.84E-10	6.43	11.2384775	1.20E-01	221061837	221061960	NC_000001.10
cg14628260	1.00E-08	9.84E-10	6.43	11.2381934	9.70E-02	10449096	10449219	NC_000008.10
cg26301143	1.00E-08	9.85E-10	6.42	11.2375233	2.76E-02	52430120	52430243	NC_000006.11
cg00708580	1.00E-08	9.85E-10	6.42	11.2369472	1.98E-02	239333832	239333955	NC_000002.11

cg01050266	1.00E-08	9.86E-10	6.42	11.2361375	9.39E-02	239948729	239948852	NC_000002.11
cg00489485	1.00E-08	9.86E-10	6.42	11.2359633	7.29E-02	84341364	84341487	NC_000016.9
cg15556599	1.00E-08	9.86E-10	6.42	11.2358911	8.75E-02	44733804	44733927	NC_000011.9
cg04057674	1.00E-08	9.87E-10	6.42	11.235749	5.77E-02	50708021	50708144	NC_000016.9
cg24100636	1.00E-08	9.89E-10	6.42	11.2334176	1.51E-01	3600087	3600210	NC_000012.11
cg23657185	1.01E-08	9.89E-10	6.42	11.2330018	2.05E-01	99272559	99272682	NC_000006.11
cg07370959	1.01E-08	9.90E-10	6.42	11.2324239	3.80E-02	51434293	51434416	NC_000001.10
cg02305377	1.01E-08	9.90E-10	6.42	11.2320503	1.84E-01	103355958	103356081	NC_000012.11
cg18129621	1.01E-08	9.91E-10	6.42	11.2310237	1.72E-01	29875991	29876114	NC_000022.10
cg15120882	1.01E-08	9.91E-10	6.42	11.2308613	6.46E-02	122257644	122257767	NC_000012.11
cg15473155	1.01E-08	9.91E-10	6.42	11.230834	1.01E-01	57349204	57349327	NC_000019.9
cg21387147	1.01E-08	9.93E-10	6.42	11.2291932	6.05E-02	43888271	43888394	NC_000001.10
cg12582965	1.01E-08	9.94E-10	6.42	11.2287955	9.29E-02	25972328	25972451	NC_000015.9
cg21997555	1.01E-08	9.94E-10	6.42	11.2283572	2.02E-02	78157897	78158020	NC_000017.10
cg07150445	1.01E-08	9.95E-10	6.42	11.2271757	1.19E-01	29235448	29235571	NC_000014.8
cg01657380	1.01E-08	9.97E-10	6.42	11.2258024	2.21E-02	53901523	53901646	NC_000012.11
cg10396546	1.01E-08	9.97E-10	6.42	11.2252779	1.95E-01	187713902	187714025	NC_000002.11
cg26724798	1.01E-08	9.97E-10	6.42	11.2249348	5.64E-02	113844828	113844951	NC_000011.9
cg21064916	1.02E-08	1.00E-09	6.42	11.2213196	1.60E-01	44340839	44340962	NC_000011.9
cg27505472	1.02E-08	1.00E-09	6.42	11.2212491	1.92E-02	121036705	121036828	NC_000002.11
cg11629830	1.02E-08	1.00E-09	6.42	11.2194586	1.46E-01	75701460	75701583	NC_000005.9
cg11312087	1.02E-08	1.00E-09	6.42	11.2194554	1.30E-01	100944053	100944176	NC_000013.10
cg00849713	1.02E-08	1.00E-09	6.42	11.2183322	1.54E-01	166267744	166267867	NC_000006.11
cg00531453	1.02E-08	1.00E-09	6.42	11.2181602	9.08E-02	123673926	123674049	NC_000007.13
cg01320748	1.02E-08	1.01E-09	6.42	11.2163965	8.55E-02	105681962	105682085	NC_000004.11

cg03131298	1.02E-08	1.01E-09	6.42	11.2163016	1.15E-01	31580889	31581012	NC_000016.9
cg15909981	1.03E-08	1.01E-09	6.42	11.2121759	1.19E-01	37465178	37465301	NC_000022.10
cg04735481	1.03E-08	1.01E-09	6.42	11.2121248	7.97E-02	90172816	90172939	NC_000016.9
cg19070159	1.03E-08	1.01E-09	6.42	11.2109742	9.79E-02	31829644	31829767	NC_000006.11
cg25361850	1.03E-08	1.01E-09	6.42	11.2098694	1.22E-01	52391789	52391912	NC_000019.9
cg09515921	1.03E-08	1.01E-09	6.42	11.2093672	1.12E-01	221057558	221057681	NC_000001.10
cg17031926	1.03E-08	1.02E-09	6.42	11.2072183	1.22E-01	99830489	99830612	NC_000004.11
cg01550017	1.03E-08	1.02E-09	6.42	11.206852	7.96E-02	42924215	42924338	NC_000005.9
cg06333307	1.03E-08	1.02E-09	6.42	11.2064448	1.21E-01	43934146	43934269	NC_000013.10
cg12386721	1.03E-08	1.02E-09	6.42	11.2061796	1.21E-01	68265892	68266015	NC_000011.9
cg23589617	1.03E-08	1.02E-09	6.42	11.2058278	1.71E-01	43600728	43600851	NC_000011.9
cg23368787	1.03E-08	1.02E-09	6.42	11.2051041	9.46E-02	36049342	36049465	NC_000019.9
cg02675646	1.03E-08	1.02E-09	6.42	11.2043974	6.72E-02	40996118	40996241	NC_000019.9
cg19988449	1.03E-08	1.02E-09	6.42	11.2042415	1.73E-01	83952808	83952931	NC_000015.9
cg11811513	1.04E-08	1.02E-09	6.42	11.202304	1.81E-01	185462408	185462531	NC_000002.11
cg10997627	1.04E-08	1.02E-09	6.42	11.2000777	1.53E-01	97363080	97363203	NC_000007.13
cg00461685	1.04E-08	1.02E-09	6.42	11.1996816	9.10E-02	74198210	74198333	NC_000008.10
cg06687772	1.04E-08	1.02E-09	6.42	11.1996795	9.68E-02	46383540	46383663	NC_000019.9
cg16227072	1.04E-08	1.02E-09	6.42	11.1990624	4.88E-02	77320503	77320626	NC_000011.9
cg11684930	1.04E-08	1.03E-09	6.42	11.1981041	1.16E-01	135000940	135001063	NC_000010.10
cg26390156	1.04E-08	1.03E-09	6.42	11.1980372	1.14E-01	24101543	24101666	NC_000014.8
cg25381737	1.04E-08	1.03E-09	6.42	11.1974693	6.28E-02	111022774	111022897	NC_000001.10
cg03610849	1.04E-08	1.03E-09	6.42	11.1970861	1.35E-01	129080113	129080236	NC_000011.9
cg10398682	1.04E-08	1.03E-09	6.42	11.1970803	1.15E-01	83952019	83952142	NC_000015.9
cg15022308	1.04E-08	1.03E-09	6.42	11.1959188	7.43E-02	43447071	43447194	NC_000010.10

cg21554569	1.04E-08	1.03E-09	6.42	11.1945907	7.48E-02	52586721	52586844	NC_000005.9
cg17393267	1.04E-08	1.03E-09	6.42	11.1945765	1.98E-01	192127356	192127479	NC_000003.11
cg23015155	1.04E-08	1.03E-09	6.42	11.1945534	2.91E-02	102698092	102698215	NC_000014.8
cg16896008	1.04E-08	1.03E-09	6.42	11.1937807	9.10E-02	24206177	24206300	NC_000006.11
cg10077401	1.04E-08	1.03E-09	6.42	11.1937208	1.88E-01	70346798	70346921	NC_000014.8
cg12817107	1.04E-08	1.03E-09	6.42	11.1930502	1.57E-01	157013368	157013491	NC_000002.11
cg11334822	1.04E-08	1.03E-09	6.42	11.1925967	7.31E-02	30128686	30128809	NC_000006.11
cg08258978	1.04E-08	1.03E-09	6.42	11.1924704	4.07E-02	6667276	6667399	NC_000005.9
cg27408184	1.04E-08	1.03E-09	6.42	11.1919417	8.25E-02	8015494	8015617	NC_000017.10
cg07580827	1.05E-08	1.03E-09	6.42	11.1915853	4.20E-02	111943186	111943309	NC_000011.9
cg11205072	1.05E-08	1.03E-09	6.42	11.1907617	2.21E-01	28510306	28510429	NC_000006.11
cg15404858	1.05E-08	1.03E-09	6.42	11.1905875	1.54E-01	21495476	21495599	NC_000020.10
cg06142324	1.05E-08	1.03E-09	6.42	11.1900802	7.65E-02	124805538	124805661	NC_000011.9
cg24460126	1.05E-08	1.03E-09	6.42	11.1896785	9.82E-02	179713828	179713951	NC_000001.10
cg11560672	1.05E-08	1.03E-09	6.42	11.1887895	7.43E-02	92526239	92526362	NC_000006.11
cg15961693	1.05E-08	1.04E-09	6.42	11.1879058	8.39E-02	139689053	139689176	NC_000006.11
cg24567600	1.05E-08	1.04E-09	6.42	11.1871301	8.47E-02	63154696	63154819	NC_000007.13
cg02957173	1.05E-08	1.04E-09	6.42	11.1859808	6.02E-02	208196958	208197081	NC_000002.11
cg07897169	1.05E-08	1.04E-09	6.42	11.1855114	1.53E-01	85549951	85550074	NC_000016.9
cg02613624	1.05E-08	1.04E-09	6.42	11.1851934	9.05E-02	2154386	2154509	NC_000011.9
cg02087289	1.05E-08	1.04E-09	6.41	11.1846174	6.02E-02	45622096	45622219	NC_000021.8
cg25585172	1.05E-08	1.04E-09	6.41	11.1835855	1.46E-01	181454975	181455098	NC_000001.10
cg01594233	1.05E-08	1.04E-09	6.41	11.1827837	1.55E-01	175135937	175136060	NC_000004.11
cg01881966	1.05E-08	1.04E-09	6.41	11.1827096	7.80E-02	177504062	177504185	NC_000002.11
cg27293357	1.05E-08	1.04E-09	6.41	11.1820441	1.15E-01	241174071	241174194	NC_000001.10

cg07814541	1.06E-08	1.04E-09	6.41	11.1814045	8.59E-02	71329680	71329803	NC_000005.9
cg08428452	1.06E-08	1.04E-09	6.41	11.1813	1.41E-01	20255343	20255466	NC_000004.11
cg12601933	1.06E-08	1.04E-09	6.41	11.1798782	1.08E-01	12248895	12249018	NC_000001.10
cg07287444	1.06E-08	1.04E-09	6.41	11.1795665	6.07E-02	35717168	35717291	NC_000007.13
cg27580689	1.06E-08	1.05E-09	6.41	11.1791332	1.05E-01	115939651	115939774	NC_000010.10
cg18105529	1.06E-08	1.05E-09	6.41	11.1791156	1.21E-01	7643190	7643313	NC_000017.10
cg03450842	1.06E-08	1.05E-09	6.41	11.1785539	1.14E-01	80834947	80835070	NC_000010.10
cg20041710	1.06E-08	1.05E-09	6.41	11.1782535	1.47E-01	57729383	57729506	NC_000015.9
cg07841173	1.06E-08	1.05E-09	6.41	11.1774268	1.26E-01	128210150	128210273	NC_000003.11
cg00850505	1.06E-08	1.05E-09	6.41	11.1773435	1.65E-01	166580460	166580583	NC_000006.11
cg01247522	1.06E-08	1.05E-09	6.41	11.1758289	1.87E-01	140716991	140717114	NC_000008.10
cg13614286	1.06E-08	1.05E-09	6.41	11.1754939	1.00E-01	131038327	131038450	NC_000009.11
cg16555417	1.06E-08	1.05E-09	6.41	11.1744189	1.38E-01	40598772	40598895	NC_000001.10
cg17035770	1.06E-08	1.05E-09	6.41	11.1742262	1.53E-01	657538	657661	NC_000004.11
cg14430151	1.06E-08	1.05E-09	6.41	11.173176	1.26E-01	8440600	8440723	NC_000004.11
cg13237147	1.06E-08	1.05E-09	6.41	11.1728399	4.72E-02	188745304	188745427	NC_000003.11
cg27034576	1.06E-08	1.05E-09	6.41	11.1725483	1.67E-01	118031654	118031777	NC_000010.10
cg24039816	1.06E-08	1.05E-09	6.41	11.1722015	8.85E-02	51220098	51220221	NC_000019.9
cg17222000	1.07E-08	1.06E-09	6.41	11.1699717	9.41E-02	168937817	168937940	NC_000004.11
cg21503275	1.07E-08	1.06E-09	6.41	11.1694764	8.62E-02	68944441	68944564	NC_000014.8
cg00840960	1.07E-08	1.06E-09	6.41	11.1688181	1.85E-01	148034030	148034153	NC_000005.9
cg24405381	1.07E-08	1.06E-09	6.41	11.1678873	1.06E-01	52464193	52464316	NC_000001.10
cg26015416	1.07E-08	1.06E-09	6.41	11.1677339	1.43E-01	108023482	108023605	NC_000001.10
cg03774803	1.07E-08	1.06E-09	6.41	11.1667733	2.06E-01	198650880	198651003	NC_000002.11
cg13572093	1.07E-08	1.06E-09	6.41	11.1658944	6.23E-02	30333911	30334034	NC_000015.9

cg04974913	1.07E-08	1.06E-09	6.41	11.1656659	1.22E-01	13839662	13839785	NC_000001.10
cg13439463	1.07E-08	1.06E-09	6.41	11.1655446	2.84E-02	100566588	100566711	NC_000009.11
cg16719466	1.07E-08	1.06E-09	6.41	11.1653194	1.73E-01	32338569	32338692	NC_000007.13
cg07555084	1.07E-08	1.06E-09	6.41	11.1642411	9.54E-02	27186199	27186322	NC_000006.11
cg18161950	1.07E-08	1.06E-09	6.41	11.1642164	1.94E-01	42995203	42995326	NC_000005.9
cg12666279	1.07E-08	1.06E-09	6.41	11.1639631	2.22E-01	115918745	115918868	NC_000002.11
cg06306198	1.07E-08	1.06E-09	6.41	11.1638659	1.31E-01	147128998	147129121	NC_000003.11
cg12303582	1.07E-08	1.06E-09	6.41	11.1638583	1.48E-01	3169516	3169639	NC_000010.10
cg09251680	1.07E-08	1.06E-09	6.41	11.1627706	6.94E-02	45801144	45801267	NC_000003.11
cg21869586	1.07E-08	1.06E-09	6.41	11.1618386	1.57E-01	3197992	3198115	NC_000005.9
cg15301316	1.08E-08	1.06E-09	6.41	11.1610342	1.49E-01	23461580	23461703	NC_000010.10
cg21607141	1.08E-08	1.06E-09	6.41	11.1608861	8.98E-02	119722917	119723040	NC_000004.11
cg16857858	1.08E-08	1.07E-09	6.41	11.1605638	2.40E-01	27213984	27214107	NC_000007.13
cg13365591	1.08E-08	1.07E-09	6.41	11.160163	1.12E-01	133930663	133930786	NC_000010.10
cg01490578	1.08E-08	1.07E-09	6.41	11.1583421	1.09E-01	148248077	148248200	NC_000001.10
cg08267489	1.08E-08	1.07E-09	6.41	11.1583305	1.97E-01	166400656	166400779	NC_000006.11
cg25335557	1.08E-08	1.07E-09	6.41	11.1582231	1.45E-01	237082393	237082516	NC_000002.11
cg02979923	1.08E-08	1.07E-09	6.41	11.1576645	1.73E-01	17207337	17207460	NC_000020.10
cg22251569	1.08E-08	1.07E-09	6.41	11.1575524	1.66E-01	22562236	22562359	NC_000008.10
cg17392043	1.08E-08	1.07E-09	6.41	11.1563149	6.13E-02	188495102	188495225	NC_000003.11
cg14263924	1.08E-08	1.07E-09	6.41	11.1562645	3.12E-02	33163202	33163325	NC_000006.11
cg26270899	1.08E-08	1.07E-09	6.41	11.1561079	1.14E-01	139590760	139590883	NC_000009.11
cg18538961	1.08E-08	1.07E-09	6.41	11.1560202	9.06E-02	101363192	101363315	NC_000014.8
cg04902669	1.08E-08	1.07E-09	6.41	11.1557912	9.76E-02	6329050	6329173	NC_000017.10
cg10794058	1.08E-08	1.07E-09	6.41	11.1557132	1.43E-01	85673918	85674041	NC_000012.11

cg03659340	1.08E-08	1.07E-09	6.41	11.1550114	1.49E-01	73555254	73555377	NC_000017.10
cg07872448	1.08E-08	1.07E-09	6.41	11.1546508	2.40E-02	88806661	88806784	NC_000016.9
cg26525127	1.08E-08	1.07E-09	6.41	11.1544503	2.15E-01	233350365	233350488	NC_000002.11
cg00573330	1.08E-08	1.07E-09	6.41	11.1543972	2.37E-01	330651	330774	NC_000004.11
cg26660002	1.08E-08	1.07E-09	6.41	11.1532769	1.74E-01	154729763	154729886	NC_000002.11
cg26199445	1.08E-08	1.07E-09	6.41	11.153008	9.47E-02	134844771	134844894	NC_000010.10
cg23353952	1.08E-08	1.07E-09	6.41	11.1518994	1.53E-01	29876945	29877068	NC_000022.10
cg00305878	1.09E-08	1.08E-09	6.41	11.1509875	6.97E-02	45694301	45694424	NC_000010.10
cg26185859	1.09E-08	1.08E-09	6.41	11.150025	1.29E-01	60691266	60691389	NC_000002.11
cg04521510	1.09E-08	1.08E-09	6.41	11.1493173	1.92E-01	27242044	27242167	NC_000007.13
cg18661205	1.09E-08	1.08E-09	6.41	11.1481308	2.00E-01	140613884	140614007	NC_000005.9
cg12942690	1.09E-08	1.08E-09	6.41	11.1478965	4.31E-02	123957029	123957152	NC_000012.11
cg19696103	1.09E-08	1.08E-09	6.41	11.1476185	6.84E-02	132354130	132354253	NC_000005.9
cg06127801	1.09E-08	1.08E-09	6.41	11.1472354	1.31E-01	239625367	239625490	NC_000002.11
cg26585560	1.09E-08	1.08E-09	6.41	11.1472075	1.42E-01	139509708	139509831	NC_000008.10
cg03542938	1.09E-08	1.08E-09	6.41	11.146547	1.14E-01	62677009	62677132	NC_000020.10
cg03638905	1.09E-08	1.08E-09	6.41	11.1462191	1.05E-01	72595625	72595748	NC_000005.9
cg07185854	1.09E-08	1.08E-09	6.41	11.1459294	1.04E-01	65071589	65071712	NC_000016.9
cg20346284	1.09E-08	1.08E-09	6.41	11.1440853	1.08E-01	3280503	3280626	NC_000006.11
cg18723341	1.09E-08	1.09E-09	6.41	11.1425755	8.29E-02	2141614	2141737	NC_000003.11
cg22038700	1.09E-08	1.09E-09	6.41	11.1425078	5.02E-02	62654536	62654659	NC_000015.9
cg06398881	1.10E-08	1.09E-09	6.41	11.1416862	6.23E-02	28035524	28035647	NC_000022.10
cg02380585	1.10E-08	1.09E-09	6.41	11.1413144	1.48E-01	33776683	33776806	NC_000017.10
cg01263407	1.10E-08	1.09E-09	6.41	11.1412477	9.70E-02	8138058	8138181	NC_000010.10
cg05310990	1.10E-08	1.09E-09	6.41	11.1411611	1.27E-01	21106043	21106166	NC_000019.9

cg07181528	1.10E-08	1.09E-09	6.41	11.1404819	8.96E-02	14552306	14552429	NC_000016.9
cg08768435	1.10E-08	1.09E-09	6.41	11.140384	5.70E-02	84215035	84215158	NC_000016.9
cg02758463	1.10E-08	1.09E-09	6.41	11.1403338	9.75E-02	89982122	89982245	NC_000016.9
cg05921820	1.10E-08	1.09E-09	6.41	11.1399712	3.68E-02	32141778	32141901	NC_000006.11
cg15143024	1.10E-08	1.09E-09	6.41	11.1399284	4.94E-02	91199642	91199765	NC_000007.13
cg02267483	1.10E-08	1.09E-09	6.41	11.1392499	1.41E-01	140810404	140810527	NC_000005.9
cg08016336	1.10E-08	1.09E-09	6.41	11.1389992	1.50E-01	41181318	41181441	NC_000004.11
cg02500806	1.10E-08	1.09E-09	6.41	11.1378965	2.54E-02	89349976	89350099	NC_000016.9
cg18983604	1.10E-08	1.09E-09	6.41	11.1377823	2.36E-01	1710516	1710639	NC_000007.13
cg13076829	1.10E-08	1.09E-09	6.41	11.1374148	1.09E-01	45530698	45530821	NC_000018.9
cg07829289	1.10E-08	1.09E-09	6.41	11.1365635	5.89E-02	158894390	158894513	NC_000005.9
cg10252600	1.10E-08	1.09E-09	6.41	11.1359337	6.31E-02	53897344	53897467	NC_000012.11
cg27642554	1.10E-08	1.09E-09	6.41	11.1358517	1.63E-01	146755622	146755745	NC_000006.11
cg21074959	1.10E-08	1.09E-09	6.41	11.134541	1.25E-01	203044287	203044410	NC_000002.11
cg24115032	1.10E-08	1.09E-09	6.41	11.1337698	1.27E-01	57089953	57090076	NC_000020.10
cg16052198	1.10E-08	1.10E-09	6.41	11.1335037	1.14E-01	52264411	52264534	NC_000019.9
cg00739139	1.11E-08	1.10E-09	6.41	11.1325529	6.91E-02	65233991	65234114	NC_000007.13
cg03495868	1.11E-08	1.10E-09	6.41	11.1325288	1.69E-01	78956905	78957028	NC_000001.10
cg25306277	1.11E-08	1.10E-09	6.4	11.1314902	1.26E-01	200003267	200003390	NC_000001.10
cg07382132	1.11E-08	1.10E-09	6.4	11.1314316	1.23E-01	101280887	101281010	NC_000010.10
cg09939375	1.11E-08	1.10E-09	6.4	11.1305775	4.02E-02	40755027	40755150	NC_000004.11
cg01750714	1.11E-08	1.10E-09	6.4	11.1300299	2.00E-01	119418367	119418490	NC_000012.11
cg07912003	1.11E-08	1.10E-09	6.4	11.1297462	1.07E-01	75797838	75797961	NC_000002.11
cg24830367	1.11E-08	1.10E-09	6.4	11.1284929	6.56E-02	108083606	108083729	NC_000012.11
cg06672696	1.11E-08	1.10E-09	6.4	11.1284303	1.40E-01	57774266	57774389	NC_000003.11

cg17214351	1.11E-08	1.10E-09	6.4	11.1278955	4.09E-02	3567533	3567656	NC_000004.11
cg01281904	1.11E-08	1.10E-09	6.4	11.1273789	1.12E-01	54164757	54164880	NC_000008.10
cg01501819	1.11E-08	1.11E-09	6.4	11.1242475	2.03E-01	49576200	49576323	NC_000019.9
cg08848774	1.11E-08	1.11E-09	6.4	11.1239325	2.38E-01	43047733	43047856	NC_000017.10
cg27047406	1.11E-08	1.11E-09	6.4	11.1234161	1.35E-01	55689534	55689657	NC_000016.9
cg15004938	1.12E-08	1.11E-09	6.4	11.1214718	7.57E-02	33195943	33196066	NC_000022.10
cg19091779	1.12E-08	1.11E-09	6.4	11.1212278	1.56E-01	13200089	13200212	NC_000012.11
cg17152135	1.12E-08	1.11E-09	6.4	11.1207923	4.36E-02	33381867	33381990	NC_000006.11
cg03171651	1.12E-08	1.11E-09	6.4	11.120709	7.35E-02	20409125	20409248	NC_000017.10
cg09544728	1.12E-08	1.11E-09	6.4	11.1192134	2.08E-01	601503	601626	NC_000007.13
cg23975975	1.12E-08	1.11E-09	6.4	11.1185278	1.40E-01	76192780	76192903	NC_000018.9
cg08463932	1.12E-08	1.11E-09	6.4	11.1182842	6.03E-02	31231488	31231611	NC_000001.10
cg21523908	1.12E-08	1.11E-09	6.4	11.1178305	1.64E-01	177140609	177140732	NC_000001.10
cg21292909	1.12E-08	1.11E-09	6.4	11.1176336	2.01E-01	50059937	50060060	NC_000019.9
cg21557030	1.12E-08	1.11E-09	6.4	11.1176325	1.61E-01	88793311	88793434	NC_000014.8
cg12559228	1.12E-08	1.11E-09	6.4	11.1173582	4.50E-02	50191882	50192005	NC_000019.9
cg17977173	1.12E-08	1.12E-09	6.4	11.115153	1.31E-01	129076335	129076458	NC_000002.11
cg18119885	1.12E-08	1.12E-09	6.4	11.1145906	1.94E-01	2617271	2617394	NC_000002.11
cg26292232	1.13E-08	1.12E-09	6.4	11.1122746	8.49E-02	74047853	74047976	NC_000010.10
cg25818292	1.13E-08	1.12E-09	6.4	11.1121	1.75E-01	22370758	22370881	NC_000021.8
cg13599062	1.13E-08	1.12E-09	6.4	11.1111883	6.36E-02	37985583	37985706	NC_000009.11
cg16209873	1.13E-08	1.12E-09	6.4	11.1105995	4.40E-02	6219739	6219862	NC_000011.9
cg12131906	1.13E-08	1.12E-09	6.4	11.1096684	1.28E-01	61360359	61360482	NC_000001.10
cg07217924	1.13E-08	1.12E-09	6.4	11.1096582	1.49E-01	18814078	18814201	NC_000011.9
cg09295573	1.13E-08	1.12E-09	6.4	11.1091578	1.71E-01	37245514	37245637	NC_000004.11

cg19741688	1.13E-08	1.12E-09	6.4	11.1087272	1.48E-01	70346849	70346972	NC_000014.8
cg27202641	1.13E-08	1.12E-09	6.4	11.1082736	1.09E-01	4383739	4383862	NC_000007.13
cg20021506	1.13E-08	1.13E-09	6.4	11.1060861	9.16E-02	156718546	156718669	NC_000006.11
cg18672939	1.13E-08	1.13E-09	6.4	11.1060179	2.35E-01	118032879	118033002	NC_000010.10
cg24362716	1.13E-08	1.13E-09	6.4	11.1045792	9.43E-02	1219189	1219312	NC_000011.9
cg12884605	1.14E-08	1.13E-09	6.4	11.1022114	1.26E-01	66123314	66123437	NC_000012.11
cg07468062	1.14E-08	1.13E-09	6.4	11.1020634	6.12E-02	128703912	128704035	NC_000003.11
cg08597345	1.14E-08	1.13E-09	6.4	11.101703	1.13E-01	18124823	18124946	NC_000019.9
cg17518825	1.14E-08	1.13E-09	6.4	11.1015286	1.12E-01	46523461	46523584	NC_000002.11
cg07256668	1.14E-08	1.13E-09	6.4	11.1015087	3.50E-02	64398137	64398260	NC_000011.9
cg01755551	1.14E-08	1.13E-09	6.4	11.0996999	1.16E-01	99615764	99615887	NC_000009.11
cg06826760	1.14E-08	1.13E-09	6.4	11.09942	6.93E-02	193076307	193076430	NC_000001.10
cg18101022	1.14E-08	1.14E-09	6.4	11.097699	1.81E-01	26916149	26916272	NC_000002.11
cg23224633	1.14E-08	1.14E-09	6.4	11.0974782	5.63E-02	19355762	19355885	NC_000017.10
cg16326674	1.14E-08	1.14E-09	6.4	11.097113	2.06E-01	122301816	122301939	NC_000004.11
cg18858331	1.15E-08	1.14E-09	6.4	11.0948689	1.13E-01	140857889	140858012	NC_000005.9
cg12570712	1.15E-08	1.14E-09	6.4	11.0943737	1.91E-01	54412885	54413008	NC_000019.9
cg15690696	1.15E-08	1.14E-09	6.4	11.0937209	1.28E-01	120217718	120217841	NC_000011.9
cg21236379	1.15E-08	1.14E-09	6.4	11.0932582	5.56E-02	132328596	132328719	NC_000012.11
cg11454719	1.15E-08	1.14E-09	6.4	11.0926872	1.74E-01	223155841	223155964	NC_000002.11
cg10026317	1.15E-08	1.14E-09	6.4	11.0926735	1.15E-01	47694919	47695042	NC_000001.10
cg17936495	1.15E-08	1.14E-09	6.4	11.09253	1.90E-02	155233051	155233174	NC_000001.10
cg12892747	1.15E-08	1.14E-09	6.4	11.0914445	6.40E-02	187813459	187813582	NC_000004.11
cg01182159	1.15E-08	1.15E-09	6.4	11.0890947	3.88E-02	89762169	89762292	NC_000016.9
cg04305319	1.15E-08	1.15E-09	6.4	11.0887275	7.62E-02	149698023	149698146	NC_000005.9

cg02378335	1.15E-08	1.15E-09	6.4	11.0878446	3.31E-02	134449269	134449392	NC_000005.9
cg03199745	1.15E-08	1.15E-09	6.4	11.0870899	9.49E-02	54454445	54454568	NC_000012.11
cg02918557	1.15E-08	1.15E-09	6.4	11.0870394	1.02E-01	4901337	4901460	NC_000007.13
cg21165221	1.15E-08	1.15E-09	6.4	11.0868514	1.37E-01	237078166	237078289	NC_000002.11
cg06745656	1.15E-08	1.15E-09	6.4	11.0864386	7.95E-02	37305728	37305851	NC_000017.10
cg12534424	1.16E-08	1.15E-09	6.4	11.085459	1.26E-01	127992316	127992439	NC_000007.13
cg09816180	1.16E-08	1.15E-09	6.4	11.0852317	1.46E-01	72650088	72650211	NC_000004.11
cg04014889	1.16E-08	1.15E-09	6.4	11.0852051	8.54E-02	23890593	23890716	NC_000015.9
cg07559319	1.16E-08	1.15E-09	6.4	11.0848771	6.91E-02	2282854	2282977	NC_000001.10
cg03894242	1.16E-08	1.15E-09	6.4	11.0839664	3.44E-02	57513121	57513244	NC_000012.11
cg04220455	1.16E-08	1.15E-09	6.4	11.0837095	5.79E-02	151344746	151344869	NC_000002.11
cg26124242	1.16E-08	1.15E-09	6.4	11.0834525	1.92E-01	106888757	106888880	NC_000011.9
cg09168808	1.16E-08	1.15E-09	6.4	11.0831828	1.30E-01	115124584	115124707	NC_000012.11
cg27169020	1.16E-08	1.15E-09	6.4	11.0830499	9.01E-02	83954229	83954352	NC_000015.9
cg09576074	1.16E-08	1.15E-09	6.4	11.0828239	7.40E-02	84341327	84341450	NC_000016.9
cg13610138	1.16E-08	1.15E-09	6.4	11.0825699	1.06E-01	59281920	59282043	NC_000012.11
cg14347436	1.16E-08	1.15E-09	6.4	11.0824604	6.16E-02	92748248	92748371	NC_000007.13
cg11403823	1.16E-08	1.15E-09	6.4	11.0821621	1.00E-01	66856314	66856437	NC_000007.13
cg06483795	1.16E-08	1.15E-09	6.4	11.0821141	6.12E-02	511719	511842	NC_000005.9
cg01157046	1.16E-08	1.16E-09	6.4	11.0812249	1.20E-01	223419421	223419544	NC_000002.11
cg17475733	1.16E-08	1.16E-09	6.4	11.080588	1.13E-01	96830030	96830153	NC_000001.10
cg08052120	1.16E-08	1.16E-09	6.4	11.0793781	9.72E-02	52409580	52409703	NC_000019.9
cg24850445	1.16E-08	1.16E-09	6.39	11.0778912	4.31E-02	46306794	46306917	NC_000021.8
cg12111104	1.16E-08	1.16E-09	6.39	11.0767389	6.80E-02	33860756	33860879	NC_000013.10
cg02248514	1.17E-08	1.16E-09	6.39	11.0764983	4.50E-02	32842070	32842193	NC_000014.8

cg27000659	1.17E-08	1.16E-09	6.39	11.0759652	4.53E-02	31617530	31617653	NC_000006.11
cg26186331	1.17E-08	1.16E-09	6.39	11.0749468	1.06E-01	39415351	39415474	NC_000005.9
cg13443797	1.17E-08	1.16E-09	6.39	11.0746542	6.72E-02	50133024	50133147	NC_000007.13
cg25268537	1.17E-08	1.16E-09	6.39	11.0745542	4.10E-02	72860402	72860525	NC_000017.10
cg12669271	1.17E-08	1.16E-09	6.39	11.0739623	6.93E-02	118987299	118987422	NC_000008.10
cg20624923	1.17E-08	1.16E-09	6.39	11.0739576	1.30E-01	120355427	120355550	NC_000010.10
cg03827835	1.17E-08	1.16E-09	6.39	11.0738166	1.33E-01	134388224	134388347	NC_000005.9
cg13975503	1.17E-08	1.17E-09	6.39	11.0728141	1.33E-01	91182215	91182338	NC_000001.10
cg22660147	1.17E-08	1.17E-09	6.39	11.0726225	1.72E-01	46656093	46656216	NC_000017.10
cg20630410	1.17E-08	1.17E-09	6.39	11.0713551	5.96E-02	78952751	78952874	NC_000017.10
cg26746936	1.17E-08	1.17E-09	6.39	11.0703286	1.20E-01	42503392	42503515	NC_000019.9
cg24591082	1.17E-08	1.17E-09	6.39	11.0691616	8.70E-02	56143542	56143665	NC_000006.11
cg01860378	1.18E-08	1.17E-09	6.39	11.0668909	9.16E-02	133792985	133793108	NC_000009.11
cg05241015	1.18E-08	1.17E-09	6.39	11.0667945	1.28E-01	1748876	1748999	NC_000002.11
cg15058253	1.18E-08	1.17E-09	6.39	11.0666869	1.12E-01	149744935	149745058	NC_000007.13
cg02499281	1.18E-08	1.17E-09	6.39	11.0666566	3.88E-02	155573752	155573875	NC_000007.13
cg27639782	1.18E-08	1.17E-09	6.39	11.0666464	6.81E-02	169241606	169241729	NC_000006.11
cg10918202	1.18E-08	1.17E-09	6.39	11.0663027	1.40E-01	104153173	104153296	NC_000008.10
cg26912105	1.18E-08	1.17E-09	6.39	11.0659814	6.69E-02	84553585	84553708	NC_000016.9
cg18382893	1.18E-08	1.17E-09	6.39	11.0647657	1.46E-01	30139641	30139764	NC_000006.11
cg00902417	1.18E-08	1.17E-09	6.39	11.0646921	9.81E-02	15492168	15492291	NC_000017.10
cg06977823	1.18E-08	1.18E-09	6.39	11.0635046	6.49E-02	69778399	69778522	NC_000001.10
cg10815625	1.18E-08	1.18E-09	6.39	11.0633097	1.36E-01	165323768	165323891	NC_000001.10
cg11100386	1.18E-08	1.18E-09	6.39	11.063246	1.69E-01	8093416	8093539	NC_000010.10
cg14700504	1.18E-08	1.18E-09	6.39	11.0630181	8.68E-02	64136222	64136345	NC_000010.10

cg24495585	1.18E-08	1.18E-09	6.39	11.0625778	1.58E-01	56659985	56660108	NC_000004.11
cg25618731	1.18E-08	1.18E-09	6.39	11.0622487	1.16E-01	2848409	2848532	NC_000016.9
cg01210589	1.18E-08	1.18E-09	6.39	11.0621278	9.31E-02	89895742	89895865	NC_000014.8
cg01523600	1.18E-08	1.18E-09	6.39	11.0621147	6.69E-02	22959713	22959836	NC_000008.10
cg10103187	1.18E-08	1.18E-09	6.39	11.062001	4.09E-02	165183299	165183422	NC_000001.10
cg11599539	1.18E-08	1.18E-09	6.39	11.0605776	1.86E-01	72459614	72459737	NC_000008.10
cg16689238	1.18E-08	1.18E-09	6.39	11.0597463	5.36E-02	34649152	34649275	NC_000016.9
cg22593177	1.18E-08	1.18E-09	6.39	11.0594342	1.34E-01	82174376	82174499	NC_000010.10
cg05501958	1.19E-08	1.18E-09	6.39	11.0583082	2.16E-02	45411873	45411996	NC_000019.9
cg15556943	1.19E-08	1.18E-09	6.39	11.0581974	2.06E-01	21694617	21694740	NC_000020.10
cg17293161	1.19E-08	1.18E-09	6.39	11.0567565	1.12E-01	80141089	80141212	NC_000010.10
cg09068031	1.19E-08	1.19E-09	6.39	11.0556993	9.60E-02	11500492	11500615	NC_000011.9
cg10136216	1.19E-08	1.19E-09	6.39	11.0555176	1.02E-01	39623664	39623787	NC_000022.10
cg03156396	1.19E-08	1.19E-09	6.39	11.0552515	6.38E-02	816177	816300	NC_000016.9
cg05803596	1.19E-08	1.19E-09	6.39	11.0550196	8.42E-02	203098085	203098208	NC_000001.10
cg13478928	1.19E-08	1.19E-09	6.39	11.0545691	1.53E-01	75596724	75596847	NC_000001.10
cg14662009	1.19E-08	1.19E-09	6.39	11.054355	8.74E-02	50300243	50300366	NC_000012.11
cg07082452	1.19E-08	1.19E-09	6.39	11.053171	8.70E-02	22547688	22547811	NC_000008.10
cg18930976	1.19E-08	1.19E-09	6.39	11.0531653	1.19E-01	131697980	131698103	NC_000010.10
cg04100843	1.19E-08	1.19E-09	6.39	11.0525762	1.10E-01	4860655	4860778	NC_000004.11
cg04336159	1.19E-08	1.19E-09	6.39	11.0525471	4.79E-02	113609512	113609635	NC_000002.11
cg17628540	1.19E-08	1.19E-09	6.39	11.0508006	9.88E-02	27829199	27829322	NC_000013.10
cg21033116	1.20E-08	1.19E-09	6.39	11.0496676	4.58E-02	49265283	49265406	NC_000015.9
cg06324373	1.20E-08	1.19E-09	6.39	11.0495125	1.38E-01	99734805	99734928	NC_000010.10
cg22553634	1.20E-08	1.19E-09	6.39	11.0494791	5.95E-02	193489233	193489356	NC_000003.11

cg07066369	1.20E-08	1.19E-09	6.39	11.049034	1.56E-01	4384888	4385011	NC_000012.11
cg06301178	1.20E-08	1.20E-09	6.39	11.0478938	1.23E-01	31580150	31580273	NC_000016.9
cg02020829	1.20E-08	1.20E-09	6.39	11.0473446	1.11E-01	92908771	92908894	NC_000005.9
cg07179872	1.20E-08	1.20E-09	6.39	11.0467012	1.29E-01	140802135	140802258	NC_000005.9
cg12310850	1.20E-08	1.20E-09	6.39	11.0465758	1.34E-01	6533700	6533823	NC_000016.9
cg06780839	1.20E-08	1.20E-09	6.39	11.0462517	1.32E-01	56228577	56228700	NC_000016.9
cg06452875	1.20E-08	1.20E-09	6.39	11.0451444	6.96E-02	72133551	72133674	NC_000016.9
cg25147622	1.20E-08	1.20E-09	6.39	11.0447109	1.08E-01	1403502	1403625	NC_000008.10
cg07362168	1.20E-08	1.20E-09	6.39	11.0439622	1.81E-01	50684985	50685108	NC_000005.9
cg13451048	1.20E-08	1.20E-09	6.39	11.0439188	7.57E-02	44820073	44820196	NC_000001.10
cg00684248	1.20E-08	1.20E-09	6.39	11.0437069	1.12E-01	124907409	124907532	NC_000010.10
cg22870092	1.20E-08	1.20E-09	6.39	11.0434024	4.38E-02	7918691	7918814	NC_000004.11
cg24481801	1.20E-08	1.20E-09	6.39	11.0425045	6.05E-02	110197235	110197358	NC_000001.10
cg24928687	1.20E-08	1.20E-09	6.39	11.0424042	1.70E-01	226012913	226013036	NC_000001.10
cg04101351	1.20E-08	1.20E-09	6.39	11.0420696	1.39E-01	20522672	20522795	NC_000012.11
cg05255275	1.20E-08	1.20E-09	6.39	11.0417803	1.88E-01	60952097	60952220	NC_000014.8
cg08454334	1.21E-08	1.20E-09	6.39	11.0400085	8.38E-02	175472678	175472801	NC_000005.9
cg15746696	1.21E-08	1.21E-09	6.39	11.0397059	2.21E-01	58400494	58400617	NC_000019.9
cg24946911	1.21E-08	1.21E-09	6.39	11.0386051	5.04E-02	2788654	2788777	NC_000012.11
cg02318282	1.21E-08	1.21E-09	6.39	11.0355062	6.73E-02	82789386	82789509	NC_000005.9
cg14267095	1.21E-08	1.21E-09	6.39	11.0339798	2.11E-01	37026445	37026568	NC_000009.11
cg04864990	1.21E-08	1.21E-09	6.39	11.0334682	5.66E-02	32168694	32168817	NC_000006.11
cg00820171	1.21E-08	1.21E-09	6.39	11.0328975	8.87E-02	31340805	31340928	NC_000016.9
cg21900624	1.21E-08	1.21E-09	6.39	11.0324528	1.48E-01	113013363	113013486	NC_000012.11
cg07428323	1.22E-08	1.22E-09	6.39	11.0311974	1.55E-01	109294233	109294356	NC_000011.9

cg18445760	1.22E-08	1.22E-09	6.39	11.0307093	4.99E-02	31837445	31837568	NC_000006.11
cg24221541	1.22E-08	1.22E-09	6.39	11.0305217	1.89E-01	46974662	46974785	NC_000019.9
cg15889424	1.22E-08	1.22E-09	6.39	11.030521	7.77E-02	33749529	33749652	NC_000021.8
cg22852830	1.22E-08	1.22E-09	6.39	11.029644	1.21E-01	105989959	105990082	NC_000002.11
cg14755066	1.22E-08	1.22E-09	6.39	11.0291321	8.30E-02	74239328	74239451	NC_000017.10
cg27087622	1.22E-08	1.22E-09	6.39	11.0278006	2.39E-01	87970114	87970237	NC_000005.9
cg26132144	1.22E-08	1.22E-09	6.38	11.0254424	8.23E-02	120777192	120777315	NC_000002.11
cg04718304	1.22E-08	1.22E-09	6.38	11.0242678	9.93E-02	134703407	134703530	NC_000010.10
cg11293800	1.22E-08	1.23E-09	6.38	11.0237632	8.80E-02	48412924	48413047	NC_000003.11
cg06070625	1.22E-08	1.23E-09	6.38	11.0235763	9.19E-02	69812798	69812921	NC_000003.11
cg08055786	1.23E-08	1.23E-09	6.38	11.0232594	8.08E-02	206857579	206857702	NC_000001.10
cg02677946	1.23E-08	1.23E-09	6.38	11.019572	1.41E-01	132864357	132864480	NC_000011.9
cg25067242	1.23E-08	1.23E-09	6.38	11.0192977	1.13E-01	115881259	115881382	NC_000001.10
cg01003245	1.23E-08	1.23E-09	6.38	11.0189078	9.05E-02	151428287	151428410	NC_000001.10
cg18542304	1.23E-08	1.23E-09	6.38	11.0188612	8.71E-02	99288216	99288339	NC_000015.9
cg00484358	1.23E-08	1.23E-09	6.38	11.0173026	1.03E-01	110610995	110611118	NC_000001.10
cg06538774	1.23E-08	1.23E-09	6.38	11.0170705	1.27E-02	24584247	24584370	NC_000022.10
cg26828131	1.23E-08	1.23E-09	6.38	11.0163118	2.90E-02	249106207	249106330	NC_000001.10
cg08799997	1.23E-08	1.23E-09	6.38	11.015828	6.67E-02	167737937	167738060	NC_000006.11
cg07905119	1.24E-08	1.24E-09	6.38	11.014918	7.93E-02	1779084	1779207	NC_000007.13
cg23267987	1.24E-08	1.24E-09	6.38	11.0149166	6.18E-02	120414862	120414985	NC_000001.10
cg09074625	1.24E-08	1.24E-09	6.38	11.0149116	8.71E-02	143664102	143664225	NC_000001.10
cg01544213	1.24E-08	1.24E-09	6.38	11.0145853	8.98E-02	140774440	140774563	NC_000005.9
cg03760759	1.24E-08	1.24E-09	6.38	11.0135767	4.66E-02	156549533	156549656	NC_000001.10
cg17617843	1.24E-08	1.24E-09	6.38	11.0122775	1.57E-01	34043454	34043577	NC_000005.9

cg08872064	1.24E-08	1.24E-09	6.38	11.0114144	8.92E-02	8787638	8787761	NC_000003.11
cg24635112	1.24E-08	1.24E-09	6.38	11.0094144	1.51E-01	114908901	114909024	NC_000013.10
cg02315483	1.24E-08	1.24E-09	6.38	11.0088481	5.53E-02	44487129	44487252	NC_000019.9
cg13866910	1.24E-08	1.24E-09	6.38	11.008708	8.23E-02	1222028	1222151	NC_000016.9
cg16781264	1.24E-08	1.25E-09	6.38	11.0077897	1.71E-01	101087575	101087698	NC_000002.11
cg16312972	1.24E-08	1.25E-09	6.38	11.0077685	9.87E-02	24302860	24302983	NC_000001.10
cg13975106	1.25E-08	1.25E-09	6.38	11.0065603	1.42E-01	106939472	106939595	NC_000013.10
cg10674476	1.25E-08	1.25E-09	6.38	11.0061099	5.38E-02	97498336	97498459	NC_000002.11
cg04212229	1.25E-08	1.26E-09	6.38	10.9996005	8.64E-02	148460131	148460254	NC_000003.11
cg16549957	1.25E-08	1.26E-09	6.38	10.999113	9.86E-02	12218933	12219056	NC_000001.10
cg03897139	1.26E-08	1.26E-09	6.38	10.9982912	6.24E-02	134018809	134018932	NC_000010.10
cg09424138	1.26E-08	1.26E-09	6.38	10.9981554	8.23E-02	143700425	143700548	NC_000007.13
cg26060583	1.26E-08	1.26E-09	6.38	10.9970699	5.06E-02	71311300	71311423	NC_000008.10
cg09199698	1.26E-08	1.26E-09	6.38	10.9961355	1.27E-01	31311797	31311920	NC_000021.8
cg02765328	1.26E-08	1.26E-09	6.38	10.9941596	1.76E-01	4383281	4383404	NC_000012.11
cg25236277	1.26E-08	1.26E-09	6.38	10.9940891	8.07E-02	119070599	119070722	NC_000003.11
cg02546690	1.26E-08	1.26E-09	6.38	10.9938417	7.95E-02	17834480	17834603	NC_000011.9
cg10988187	1.26E-08	1.26E-09	6.38	10.992962	6.98E-02	71031813	71031936	NC_000012.11
cg23655970	1.26E-08	1.27E-09	6.38	10.9909233	2.10E-01	37126582	37126705	NC_000014.8
cg07888205	1.26E-08	1.27E-09	6.38	10.9906277	7.77E-02	34257432	34257555	NC_000016.9
cg16367103	1.26E-08	1.27E-09	6.38	10.9906206	5.57E-02	105681416	105681539	NC_000014.8
cg23347399	1.27E-08	1.27E-09	6.38	10.9886341	1.34E-01	140766911	140767034	NC_000005.9
cg21607030	1.27E-08	1.27E-09	6.38	10.986706	2.12E-01	133563532	133563655	NC_000006.11
cg09062550	1.27E-08	1.27E-09	6.38	10.9865959	2.16E-01	20350481	20350604	NC_000020.10
cg06500337	1.27E-08	1.27E-09	6.38	10.9859428	2.05E-01	161100242	161100365	NC_000006.11

cg08552198	1.27E-08	1.27E-09	6.38	10.9849333	1.23E-01	49309124	49309247	NC_000016.9
cg14836313	1.27E-08	1.27E-09	6.38	10.984761	8.08E-02	1975560	1975683	NC_000011.9
cg26072058	1.27E-08	1.28E-09	6.38	10.9845477	1.62E-01	91191055	91191178	NC_000001.10
cg04150055	1.27E-08	1.28E-09	6.38	10.9833008	1.65E-01	133536855	133536978	NC_000009.11
cg18439535	1.27E-08	1.28E-09	6.38	10.9830394	9.14E-02	131304342	131304465	NC_000012.11
cg23129930	1.27E-08	1.28E-09	6.38	10.9827726	1.15E-01	27186993	27187116	NC_000007.13
cg22541520	1.27E-08	1.28E-09	6.38	10.9822905	1.45E-01	6703533	6703656	NC_000007.13
cg06815630	1.27E-08	1.28E-09	6.38	10.9821747	5.73E-02	88156829	88156952	NC_000016.9
cg20516620	1.27E-08	1.28E-09	6.38	10.9819785	2.06E-02	99197040	99197163	NC_000008.10
cg03921860	1.27E-08	1.28E-09	6.38	10.9819168	8.76E-02	57458733	57458856	NC_000004.11
cg22843684	1.28E-08	1.28E-09	6.38	10.9785508	9.73E-02	10806114	10806237	NC_000003.11
cg17801930	1.28E-08	1.28E-09	6.38	10.9784581	1.58E-01	56009405	56009528	NC_000005.9
cg13531886	1.28E-08	1.28E-09	6.38	10.9776459	5.29E-02	3281434	3281557	NC_000010.10
cg04034767	1.28E-08	1.29E-09	6.38	10.9769153	2.53E-01	52400907	52401030	NC_000012.11
cg06679209	1.28E-08	1.29E-09	6.38	10.9768405	6.36E-02	1073679	1073802	NC_000019.9
cg20030310	1.28E-08	1.29E-09	6.38	10.976188	1.99E-01	158119489	158119612	NC_000001.10
cg08604097	1.28E-08	1.29E-09	6.38	10.975346	1.73E-01	8480865	8480988	NC_000007.13
cg26568372	1.28E-08	1.29E-09	6.38	10.9752737	1.58E-01	91190173	91190296	NC_000001.10
cg09917979	1.28E-08	1.29E-09	6.38	10.9751049	1.20E-01	1275630	1275753	NC_000007.13
cg27620681	1.29E-08	1.29E-09	6.38	10.9729263	7.82E-02	69349490	69349613	NC_000016.9
cg05936219	1.29E-08	1.29E-09	6.37	10.9726817	7.75E-02	4228644	4228767	NC_000004.11
cg12718579	1.29E-08	1.29E-09	6.37	10.9708278	9.19E-02	53322451	53322574	NC_000008.10
cg21106407	1.29E-08	1.30E-09	6.37	10.9678834	1.65E-01	57262177	57262300	NC_000014.8
cg03803071	1.29E-08	1.30E-09	6.37	10.967338	2.16E-01	22034523	22034646	NC_000019.9
cg23954465	1.29E-08	1.30E-09	6.37	10.9667625	1.78E-01	8078038	8078161	NC_000010.10

cg09235562	1.29E-08	1.30E-09	6.37	10.9664351	1.32E-01	384251	384374	NC_000019.9
cg21715431	1.29E-08	1.30E-09	6.37	10.9658863	7.35E-02	8601423	8601546	NC_000019.9
cg21587472	1.29E-08	1.30E-09	6.37	10.9656331	7.29E-02	11705530	11705653	NC_000019.9
cg12742799	1.30E-08	1.30E-09	6.37	10.9652811	9.76E-02	62959193	62959316	NC_000020.10
cg13597574	1.30E-08	1.30E-09	6.37	10.9642062	3.97E-02	107696634	107696757	NC_000009.11
cg26697474	1.30E-08	1.30E-09	6.37	10.9640415	2.06E-02	62185223	62185346	NC_000011.9
cg14677698	1.30E-08	1.30E-09	6.37	10.9629933	1.87E-01	3197730	3197853	NC_000005.9
cg08883066	1.30E-08	1.31E-09	6.37	10.9608218	1.11E-01	114696822	114696945	NC_000001.10
cg23990012	1.30E-08	1.31E-09	6.37	10.9606091	1.46E-01	95870409	95870532	NC_000015.9
cg21875946	1.30E-08	1.31E-09	6.37	10.9589179	8.64E-02	206663985	206664108	NC_000001.10
cg05453358	1.30E-08	1.31E-09	6.37	10.95828	1.32E-01	47529280	47529403	NC_000008.10
cg12563644	1.30E-08	1.31E-09	6.37	10.958035	1.89E-01	87956996	87957119	NC_000005.9
cg03540794	1.31E-08	1.31E-09	6.37	10.956726	1.90E-01	2112109	2112232	NC_000005.9
cg14432910	1.31E-08	1.31E-09	6.37	10.9553731	1.34E-01	68121931	68122054	NC_000015.9
cg26419728	1.31E-08	1.31E-09	6.37	10.9551339	1.35E-01	43604860	43604983	NC_000011.9
cg24146331	1.31E-08	1.31E-09	6.37	10.9550246	1.11E-01	102590225	102590348	NC_000010.10
cg09387867	1.31E-08	1.31E-09	6.37	10.9547322	3.86E-02	144879954	144880077	NC_000008.10
cg05007549	1.31E-08	1.32E-09	6.37	10.9525666	1.14E-01	81103968	81104091	NC_000004.11
cg01150433	1.31E-08	1.32E-09	6.37	10.95193	1.23E-01	147142182	147142305	NC_000003.11
cg14382976	1.31E-08	1.32E-09	6.37	10.950151	7.77E-02	117067624	117067747	NC_000007.13
cg09561419	1.31E-08	1.32E-09	6.37	10.9496129	2.15E-02	57055798	57055921	NC_000016.9
cg24693341	1.32E-08	1.32E-09	6.37	10.9488883	1.69E-01	41746947	41747070	NC_000004.11
cg23737737	1.32E-08	1.32E-09	6.37	10.9474627	1.88E-01	6665370	6665493	NC_000012.11
cg09752583	1.32E-08	1.32E-09	6.37	10.9471508	7.68E-02	53058587	53058710	NC_000006.11
cg26886277	1.32E-08	1.33E-09	6.37	10.945093	6.90E-02	86566099	86566222	NC_000002.11

cg07697895	1.32E-08	1.33E-09	6.37	10.9444402	2.32E-01	116963259	116963382	NC_000007.13
cg03434886	1.32E-08	1.33E-09	6.37	10.9443723	1.09E-01	48555361	48555484	NC_000020.10
cg08215400	1.32E-08	1.33E-09	6.37	10.944356	7.91E-02	7592407	7592530	NC_000012.11
cg05013064	1.32E-08	1.33E-09	6.37	10.9441472	2.10E-01	55204633	55204756	NC_000020.10
cg01656588	1.32E-08	1.33E-09	6.37	10.943114	1.14E-01	3187100	3187223	NC_000005.9
cg05722918	1.32E-08	1.33E-09	6.37	10.9425976	1.31E-01	101603929	101604052	NC_000012.11
cg18077160	1.32E-08	1.33E-09	6.37	10.9423554	3.37E-02	48987205	48987328	NC_000019.9
cg24954590	1.32E-08	1.33E-09	6.37	10.9415211	1.18E-01	140777611	140777734	NC_000005.9
[truncated: 372,021 more chars]
