# Supplementary material for: circRNA circ_102049 Implicates in Pancreatic Ductal Adenocarcinoma Progression through Activating CD80 by Targeting miR-455-3p
Source: Mediators Inflamm. 2021 Jan 7;2021:8819990. doi: 10.1155/2021/8819990 (PMC7811564; doi:10.1155/2021/8819990)
Supplement: Supplementary 10 — Table S8 The differentially expressed genes of GSE14245, GSE27890, GSE32676, GSE41372, GSE62165, GSE62452, and GSE71989. [file 8819990.f10.pdf]

Supplementary Table S8 The differentially expressed genes of GSE14245, GSE27890, GSE32676, GSE41372, GSE62165, GSE62452, and GSE71989

GSE14245

ID	adj.P.Val	P.Value	t	B	logFC	Gene.symbol	Gene.title
219441_s_at	0.14	0.00000356	5.8628901	-3.12	6.2572679	LRRK1	leucine rich repeat kinase 1
220227_at	0.14	0.00000879	5.5150701	-3.21	2.8356585	CDH4	cadherin 4
211628_x_at	0.167	0.00001545	5.2993286	-3.27	4.2741316	FTH1P5	ferritin heavy chain 1 pseudogene 5
204351_at	0.167	0.00001837	5.2787255	-3.35	3.2890457	S100P	S100 calcium binding protein P
201809_s_at	0.259	0.00003792	4.9578361	-3.37	2.6487957	ENG	endoglin
235721_at	0.274	0.00005509	4.8161369	-3.41	2.3564623	DTX3	deltex E3 ubiquitin ligase 3
207706_at	0.601	0.00015389	4.4262983	-3.53	2.5315866	USH2A	usherin
208022_s_at	0.605	0.00016589	4.3977555	-3.54	2.8320087	CDC14B	cell division cycle 14B
214091_s_at	0.641	0.00020031	4.3260309	-3.56	2.0954102	GPX3	glutathione peroxidase 3
209517_s_at	0.574	0.00013649	4.5022248	-3.56	7.3796552	ASH2L	ASH2 like histone lysine methyltransferase complex subunit
216956_s_at	0.641	0.00021096	4.3063005	-3.57	2.1488537	ITGA2B	integrin subunit alpha 2b
208275_x_at	0.669	0.00024192	4.2541157	-3.58	3.5111417	UTF1	undifferentiated embryonic cell transcription factor 1
221525_at	0.726	0.00031849	4.149146	-3.62	4.1659539	ZMIZ2	zinc finger MIZ-type containing 2
240797_at	0.726	0.00030815	4.1869756	-3.65	3.5617015	PERP	PERP, TP53 apoptosis effector

212790_x_at	0.759	0.00038059	4.1049615	-3.68	4.4630929	RPL13AP5///SNORD32A///SNORD33///SNORD34///SNORD35A///RPL13A	ribosomal protein L13a pseudogene 5///small nucleolar RNA, C/D box 32A///small nucleolar RNA, C/D box 33///small nucleolar RNA, C/D box 34///small nucleolar RNA, C/D box 35A///ribosomal protein L13a
225792_at	0.262	0.00004795	5.1004271	-3.68	4.6107373	HOOK1	hook microtubule tethering protein 1
207004_at	0.715	0.00028791	4.2414073	-3.69	4.1355238	BCL2	BCL2, apoptosis regulator
1555960_at	0.192	0.00002457	5.4589671	-3.71	5.8192231	HINT1	histidine triad nucleotide binding protein 1
1569867_at	0.945	0.00077264	3.8084481	-3.73	2.7758833	EME2	essential meiotic structure-specific endonuclease subunit 2
210646_x_at	0.641	0.00019782	4.4602862	-3.74	5.6372141	RPL13AP5///SNORD32A///SNORD33///SNORD34///SNORD35A///RPL13A	ribosomal protein L13a pseudogene 5///small nucleolar RNA, C/D box 32A///small nucleolar RNA, C/D box 33///small nucleolar RNA, C/D box 34///small nucleolar RNA, C/D box 35A///ribosomal protein L13a
207857_at	0.759	0.00041573	4.1245217	-3.77	4.0999276	LILRA2	leukocyte immunoglobulin like receptor A2
1553811_at	0.945	0.00104382	3.6916558	-3.77	2.2149003	FAM222A-AS1	FAM222A antisense RNA 1
224941_at	0.945	0.0011382	3.6579108	-3.78	1.9922369	PAPPA	pappalysin 1
1558621_at	0.945	0.00073723	3.8690723	-3.8	5.5755548	CABLES1	Cdk5 and Abl enzyme substrate 1
225246_at	0.945	0.00060976	3.9701402	-3.81	3.5954745	STIM2	stromal interaction molecule 2
235004_at	0.945	0.00119703	3.6560192	-3.82	4.4004428	RBM24	RNA binding motif protein 24

1556114_a_at	0.945	0.00121402	3.6504442	-3.82	2.0874283	LOC101928841	collagen alpha-1(II) chain-like
216158_at	0.945	0.00164009	3.5147508	-3.83	3.0272459		
220006_at	0.945	0.00198696	3.4390107	-3.86	3.0364632	EFCC1	EF-hand and coiled-coil domain containing 1
1562062_at	0.945	0.00202838	3.4308413	-3.86	2.0745352		
207842_s_at	0.945	0.00137737	3.6190953	-3.87	5.4167854	MIR6866///CASC3	microRNA 6866///cancer susceptibility candidate 3
235416_at	0.945	0.00114561	3.7146619	-3.88	4.4591786	LOC643201	centrosomal protein 192kDa pseudogene
220620_at	0.945	0.00267633	3.3205726	-3.9	4.7500217	CRCT1	cysteine rich C-terminal 1
232937_at	0.945	0.00276631	3.3073521	-3.9	2.0061428		
1557021_s_at	0.945	0.00227324	3.4003574	-3.91	2.1687517	LOC100507250	uncharacterized LOC100507250
207990_x_at	0.945	0.0023738	3.3829462	-3.91	5.4681509	ACRV1	acrosomal vesicle protein 1
233220_at	0.945	0.00189265	3.4908377	-3.91	2.0518303	GRIN3A	glutamate ionotropic receptor NMDA type subunit 3A
206367_at	0.945	0.00323942	3.2440256	-3.93	2.1974967	REN	renin
202173_s_at	0.945	0.00324289	3.2435958	-3.93	2.4741884	VEZF1	vascular endothelial zinc finger 1
207768_at	0.759	0.00037106	4.3223599	-3.93	3.5437297	EGR4	early growth response 4
243540_at	0.945	0.00240747	3.3930861	-3.94	2.0466775		
218617_at	0.945	0.00356915	3.2049669	-3.94	2.0778704	TRIT1	tRNA isopentenyltransferase 1
201878_at	0.945	0.00298653	3.2901974	-3.94	1.6990611	ARIH1	ariadne RBR E3 ubiquitin protein ligase 1
1563002_at	0.945	0.00366512	3.1942503	-3.94	1.509713		
235215_at	0.669	0.00025699	4.4843196	-3.95	3.9639499	ERCC4	ERCC excision repair 4, endonuclease catalytic subunit

206037_at	0.669	0.00025667	4.5403052	-3.95	4.0499587	KYAT1	kynurenine aminotransferase 1
230931_at	0.759	0.00041046	4.2779519	-3.95	4.1885334	PLG	plasminogen
212345_s_at	0.945	0.0039337	3.1656344	-3.95	2.0422238	CREB3L2	cAMP responsive element binding protein 3 like 2
207660_at	0.945	0.00333064	3.245908	-3.96	2.2492209	DMD	dystrophin
230096_at	0.945	0.00402314	3.1565207	-3.96	1.9064681		
202236_s_at	0.945	0.00126742	3.722307	-3.96	3.8348051	SLC16A1	solute carrier family 16 member 1
212999_x_at	0.945	0.00366014	3.2074581	-3.97	4.4548634	LOC101060835///HLA-DQB1	HLA class II histocompatibility antigen, DQ beta 1 chain-like///major histocompatibility complex, class II, DQ beta 1
230578_at	0.945	0.00392487	3.1789132	-3.98	4.6816465	ZNF471	zinc finger protein 471
1557256_a_at	0.945	0.00478466	3.0859679	-3.98	1.5099947	GABRB1	gamma-aminobutyric acid type A receptor beta1 subunit
211927_x_at	0.945	0.00267659	3.3665972	-3.98	1.9473813	MIR3654///EEF1G	microRNA 3654///eukaryotic translation elongation factor 1 gamma
1563143_at	0.945	0.00202503	3.5018628	-3.98	3.3608001	LOC100507065	uncharacterized LOC100507065
1562349_at	0.945	0.00343076	3.2479656	-3.98	5.2297629	TRAPPC2	trafficking protein particle complex 2
236474_at	0.945	0.00498652	3.0690734	-3.99	1.936127		
204437_s_at	0.945	0.00371776	3.2148255	-4	4.0392946	FOLR1	folate receptor 1
1558120_at	0.945	0.00553205	3.0264927	-4	3.548794	DDX3X	DEAD-box helicase 3, X-linked
208795_s_at	0.945	0.00421492	3.162867	-4.01	2.1425809	MCM7	minichromosome maintenance complex component 7
239666_at	0.945	0.00413411	3.1708969	-4.01	2.9719892	PYGO2	pygopus family PHD finger 2
230285_at	0.945	0.0060044	2.9927478	-4.01	1.765866	SVIP	small VCP interacting protein

224375_at	0.945	0.00510277	3.0709424	-4.01	2.8616601	LOC102724870	uncharacterized LOC102724870
204158_s_at	0.945	0.00215376	3.4979991	-4.02	3.5567106	TCIRG1	T-cell immune regulator 1, ATPase H+ transporting V0 subunit a3
237635_at	0.945	0.00524923	3.0592325	-4.02	2.358315	LOC100128164	four and a half LIM domains 1 pseudogene
241489_at	0.945	0.00619553	2.9798074	-4.02	2.4849523		
202295_s_at	0.945	0.00620916	2.9788994	-4.02	2.1243945	CTSH	cathepsin H
232210_at	0.945	0.00433669	3.1510449	-4.02	3.0957672		
1559140_at	0.945	0.00227507	3.4747112	-4.02	3.4569083	FAM87B///FAM87A	family with sequence similarity 87 member B///family with sequence similarity 87 member A
209058_at	0.945	0.00461013	3.1256212	-4.02	3.414579	EDF1	endothelial differentiation related factor 1
1554976_a_at	0.945	0.00548709	3.0408651	-4.02	3.7706456	LINC00051	long intergenic non-protein coding RNA 51
225780_at	0.945	0.00387076	3.2130146	-4.03	2.1591636	DDI2///RSC1A1	DNA damage inducible 1 homolog 2///regulatory solute carrier protein, family 1, member 1
237199_at	0.945	0.00175683	3.6103096	-4.03	4.1609479		
1557709_at	0.945	0.00673651	2.9451426	-4.03	4.9805348		
203611_at	0.945	0.00078377	4.0352996	-4.03	4.7797117	TERF2	telomeric repeat binding factor 2
220288_at	0.945	0.00190494	3.5753891	-4.03	4.4088833	MYO15A	myosin XVA
1552456_a_at	0.945	0.00585476	3.0139209	-4.03	2.7474998	MBD3L2	methyl-CpG binding domain protein 3 like 2
1557775_a_at	0.945	0.00302434	3.333677	-4.04	3.5123764		

225606_at	0.945	0.00198322	3.5580004	-4.04	4.7182517	BCL2L11	BCL2 like 11
207993_s_at	0.945	0.00278045	3.3892446	-4.04	3.6351799	CHP1	calcineurin like EF-hand protein 1
219733_s_at	0.945	0.007321	2.9105464	-4.04	1.8051581	SLC27A5	solute carrier family 27 member 5
224667_x_at	0.945	0.00633966	2.9807587	-4.04	3.5240787	ANAPC16	anaphase promoting complex subunit 16
207368_at	0.945	0.00537045	3.0618862	-4.05	1.6842462	HTR1D	5-hydroxytryptamine receptor 1D
232726_at	0.945	0.00757869	2.8961199	-4.05	2.1345401		
1562694_at	0.945	0.00764284	2.8926005	-4.05	4.3410509	IGF2BP2-AS1	IGF2BP2 antisense RNA 1
221589_s_at	0.945	0.00567758	3.0385706	-4.05	3.7287706	ALDH6A1	aldehyde dehydrogenase 6 family member A1
1562481_at	0.945	0.00780303	2.8839343	-4.05	1.9150281	PCBP1-AS1	PCBP1 antisense RNA 1
220443_s_at	0.945	0.0057672	3.0319949	-4.05	3.1800273	VAX2	ventral anterior homeobox 2
222968_at	0.945	0.00679731	2.9516094	-4.05	3.7266926		
220547_s_at	0.945	0.00795232	2.8760078	-4.05	1.8983849	FAM35A	family with sequence similarity 35 member A
244600_at	0.945	0.00070169	4.1338078	-4.06	3.3016308	LOC100996583	uncharacterized LOC100996583
219822_at	0.945	0.00591707	3.0212142	-4.06	3.3430804	MTRF1	mitochondrial translational release factor 1
227249_at	0.945	0.0082666	2.8597721	-4.06	1.5567359	NDE1	nudE neurodevelopment protein 1
210441_at	0.945	0.00114566	3.864465	-4.06	5.2222572	CDC14A	cell division cycle 14A
223099_s_at	0.945	0.00250256	3.4573743	-4.07	4.6715694	LOC100507577 ///LONP2	uncharacterized LOC100507577///lon peptidase 2, peroxisomal
221274_s_at	0.945	0.00743639	2.9138877	-4.07	1.9489288	LMAN2L	lectin, mannose binding 2 like
223132_s_at	0.945	0.00254119	3.4507349	-4.07	3.2764218	TRIM8	tripartite motif containing 8
1552660_a_at	0.945	0.00865534	2.8404796	-4.07	4.0834448	C5orf22	chromosome 5 open reading frame 22

207927_at	0.945	0.00353589	3.2863491	-4.07	3.8709022	HTR7	5-hydroxytryptamine receptor 7
235469_at	0.945	0.00281909	3.4057014	-4.07	3.4312552	FAM133DP///FAM133B	family with sequence similarity 133, member A pseudogene///family with sequence similarity 133 member B
210169_at	0.945	0.00889775	2.8288599	-4.07	1.196474	SEC14L5	SEC14 like lipid binding 5
206396_at	0.945	0.0089126	2.828158	-4.07	2.6649666	SLC1A1	solute carrier family 1 member 1
216678_at	0.945	0.00784383	2.8914171	-4.07	2.0896876	IFT122	intraflagellar transport 122
244456_at	0.945	0.00794369	2.8860797	-4.08	2.3187646		
1557701_s_at	0.945	0.00919605	2.8149652	-4.08	1.7653064	POLH	DNA polymerase eta
1554039_at	0.945	0.00930102	2.8101767	-4.08	1.8787058	C14orf159	chromosome 14 open reading frame 159
224256_at	0.945	0.00933457	2.8086568	-4.08	1.8951218	LOC100129449	PRO2055
239115_at	0.945	0.00935549	2.8077115	-4.08	1.8674944	TMED3	transmembrane p24 trafficking protein 3
239468_at	0.945	0.00687008	2.9582235	-4.08	4.3978812	MKX	mohawk homeobox
237572_at	0.945	0.00075208	4.1016393	-4.08	4.5212226	UGT3A1	UDP glycosyltransferase family 3 member A1
228904_at	0.945	0.00829293	2.8678989	-4.08	2.1018097	HOXB3	homeobox B3
241978_at	0.945	0.00364034	3.273846	-4.08	3.3290257	AKR1A1	aldo-keto reductase family 1 member A1
227254_at	0.945	0.00302326	3.3753124	-4.08	2.6538361	POU2F1	POU class 2 homeobox 1
209048_s_at	0.945	0.00980123	2.7880366	-4.09	1.7977196	ZMYND8	zinc finger MYND-type containing 8
217393_x_at	0.945	0.00625801	3.0101852	-4.09	4.4254492	UBE2NL	ubiquitin conjugating enzyme E2 N like (gene/pseudogene)
201633_s_at	0.945	0.00986208	2.7854164	-4.09	1.6936404	CYB5B	cytochrome b5 type B

204613_at	0.945	0.00995107	2.7816117	-4.09	1.278237	PLCG2	phospholipase C gamma 2
220902_at	0.945	0.00597312	3.0300179	-4.09	3.7202788		
201622_at	0.945	0.00514026	3.1085125	-4.09	4.2740387	SND1	staphylococcal nuclease and tudor domain containing 1
1566695_at	0.945	0.01013695	2.7737669	-4.09	2.4947945		
237183_at	0.945	0.00548855	3.0804162	-4.09	3.0492631	GALNT5	polypeptide N-acetylgalactosaminyltransferase 5
1556158_at	0.945	0.00532819	3.0931323	-4.09	3.3046421	SAXO2	stabilizer of axonemal microtubules 2
1553641_a_at	0.945	0.0040921	3.2235078	-4.09	3.4817933	TSGA13	testis specific 13
218010_x_at	0.945	0.00571791	3.0628381	-4.1	3.2364713	PPDPF	pancreatic progenitor cell differentiation and proliferation factor
241245_at	0.945	0.00685997	2.9709807	-4.1	1.8440856	SRSF4	serine and arginine rich splicing factor 4
229837_s_at	0.945	0.00686904	2.9704159	-4.1	3.2913242	KCTD5	potassium channel tetramerization domain containing 5
239541_at	0.945	0.00581328	3.0557289	-4.1	4.0954486		
231486_x_at	0.945	0.00082941	4.0098134	-4.1	5.0131573		
223745_at	0.945	0.01086334	2.7443568	-4.1	3.070645	C16orf95	chromosome 16 open reading frame 95
208736_at	0.945	0.01089641	2.7430622	-4.1	2.7153287	ARPC3	actin related protein 2/3 complex subunit 3
214937_x_at	0.945	0.00820592	2.8827168	-4.1	2.7204545	PCM1	pericentriolar material 1
1555632_at	0.945	0.00607662	3.0366645	-4.1	3.8825	PIK3IP1	phosphoinositide-3-kinase interacting protein 1

1554745_at	0.945	0.01116632	2.732633	-4.1	1.3087685	RALGPS1	Ral GEF with PH domain and SH3 binding motif 1
236821_at	0.945	0.01117768	2.7321994	-4.11	1.5276741		
239811_at	0.945	0.01124919	2.7294787	-4.11	1.9476992		
1553743_at	0.945	0.00069494	4.1382952	-4.11	4.2852389	METTL21A	methyltransferase like 21A
203073_at	0.945	0.01163514	2.7150687	-4.11	1.6028398	COG2	component of oligomeric golgi complex 2
230196_x_at	0.945	0.00645464	3.0106446	-4.11	2.2035261	ARHGAP23	Rho GTPase activating protein 23
1566284_at	0.945	0.01029503	2.7758932	-4.11	1.9325943		
1556078_at	0.945	0.01182072	2.7082989	-4.11	1.6152816	LOC143286	uncharacterized LOC143286
233798_at	0.945	0.00916698	2.8353219	-4.11	3.8396496		
1559332_at	0.945	0.01191227	2.7049959	-4.11	1.9924258		
224865_at	0.945	0.00537701	3.1053448	-4.11	4.4263844	FAR1	fatty acyl-CoA reductase 1
232510_s_at	0.945	0.00538892	3.1043831	-4.11	3.9552364	DPP3	dipeptidyl peptidase 3
224458_at	0.945	0.00439795	3.2116492	-4.12	4.0425579	TMEM246	transmembrane protein 246
222785_x_at	0.945	0.01083402	2.7540223	-4.12	4.8876387	C11orf1	chromosome 11 open reading frame 1
227615_at	0.945	0.0125325	2.6832264	-4.12	3.5510088	SRRD	SRR1 domain containing
209948_at	0.945	0.01255936	2.6823068	-4.12	1.4310932	KCNMB1	potassium calcium-activated channel subfamily M regulatory beta subunit 1
1556306_at	0.945	0.00953563	2.818385	-4.12	5.006019		
208984_x_at	0.945	0.01260179	2.6808573	-4.12	4.1162751	RBM10	RNA binding motif protein 10
240310_at	0.945	0.01265632	2.6790015	-4.12	1.979111	TOR1AIP1	torsin 1A interacting protein 1
1553461_at	0.945	0.01270875	2.6772244	-4.12	5.0346891	FAM9B	family with sequence similarity 9 member B
208439_s_at	0.945	0.00976747	2.8080484	-4.13	2.5700099	FCN2	ficolin 2

234349_at	0.945	0.00178989	3.7007162	-4.13	3.5813033	SSPO	SCO-spondin
230390_at	0.945	0.01298175	2.6680798	-4.13	1.6714704	LOC101928222	uncharacterized LOC101928222
239344_at	0.945	0.0087073	2.8684505	-4.13	2.8812435		
225385_s_at	0.945	0.00742633	2.9499433	-4.13	3.1509119	HNRNPLL	heterogeneous nuclear ribonucleoprotein L like
1565820_x_at	0.945	0.01311702	2.6636151	-4.13	1.7289342		
212277_at	0.945	0.00377117	3.3016445	-4.13	2.9594633	MTMR4	myotubularin related protein 4
241657_at	0.945	0.00284435	3.4548546	-4.13	3.9913495		
241742_at	0.945	0.0133293	2.656695	-4.13	1.5420824	PRAM1	PML-RARA regulated adaptor molecule 1
211282_x_at	0.945	0.01338731	2.6548218	-4.13	1.6633295	TNFRSF25	TNF receptor superfamily member 25
1553148_a_at	0.945	0.00906528	2.8510148	-4.13	2.5600428	SNX13	sorting nexin 13
242932_at	0.945	0.01355045	2.6495944	-4.13	4.7801261		
207726_at	0.945	0.01369113	2.6451336	-4.14	1.5586909	ESRRB	estrogen related receptor beta
1555037_a_at	0.945	0.01373096	2.6438782	-4.14	1.6051533	IDH1	isocitrate dehydrogenase (NADP(+)) 1, cytosolic
243045_at	0.945	0.01382867	2.6408136	-4.14	1.3275857	SMYD1	SET and MYND domain containing 1
235547_at	0.945	0.01382928	2.6407943	-4.14	2.5045842	N4BP2L2	NEDD4 binding protein 2 like 2
242630_at	0.945	0.00935644	2.8373097	-4.14	1.6971852		
202913_at	0.945	0.01405547	2.6337769	-4.14	3.1933711	ARHGEF11	Rho guanine nucleotide exchange factor 11
221385_s_at	0.945	0.0140827	2.6329393	-4.14	1.8239352	GPR42///FFAR3	G protein-coupled receptor 42 (gene/pseudogene)///free fatty acid receptor 3
210872_x_at	0.945	0.01408948	2.632731	-4.14	1.4009781	GAS7	growth arrest specific 7

233769_at	0.945	0.0141728	2.630178	-4.14	3.2709782	LOC284561	uncharacterized LOC284561
1560524_at	0.945	0.01422394	2.6286182	-4.14	3.8728058	GRAPL	GRB2 related adaptor protein like
224724_at	0.945	0.00687844	2.9978865	-4.14	3.1347923	SULF2	sulfatase 2
222066_at	0.945	0.01436295	2.6244044	-4.14	1.9072126	EPB41L1	erythrocyte membrane protein band 4.1 like 1
244381_at	0.945	0.01438593	2.6237115	-4.14	1.5314011		
1554198_at	0.945	0.00819007	2.9073613	-4.14	1.5543561	SH3YL1	SH3 and SYLF domain containing 1
228248_at	0.945	0.0069845	2.9911782	-4.14	1.8953242	RICTOR	RPTOR independent companion of MTOR complex 2
221494_x_at	0.945	0.01452766	2.6194607	-4.14	1.4601586	EIF3K	eukaryotic translation initiation factor 3 subunit K
228120_at	0.945	0.00226076	3.5928631	-4.14	4.0821132	AGO1	argonaute 1, RISC catalytic component
1558168_at	0.945	0.01147913	2.7382199	-4.14	3.7246616	H1FX-AS1	H1FX antisense RNA 1
217252_at	0.945	0.01467029	2.6152216	-4.15	1.170494		
243113_at	0.945	0.00555465	3.1089166	-4.15	4.1737219		
214892_x_at	0.945	0.01006062	2.8057705	-4.15	3.1741052	CACTIN	cactin, spliceosome C complex subunit
225861_at	0.945	0.01314359	2.6706049	-4.15	4.8415034	MCRIP2	MAPK regulated corepressor interacting protein 2
244111_at	0.945	0.00715706	2.980471	-4.15	3.9487203	KRT222	keratin 222
222197_s_at	0.945	0.01319205	2.6690068	-4.15	2.2689888		
225967_s_at	0.945	0.01489555	2.6086046	-4.15	1.7736048	C17orf89	chromosome 17 open reading frame 89

240603_s_at	0.945	0.01495869	2.6067665	-4.15	1.1214736	ERI2	ERI1 exoribonuclease family member 2
204535_s_at	0.945	0.00462222	3.2109567	-4.15	4.6924031	REST	RE1 silencing transcription factor
224329_s_at	0.945	0.01353818	2.6577495	-4.15	3.6357022	CNFN	cornifelin
208632_at	0.945	0.01523939	2.5986818	-4.15	2.7983916	RNF10	ring finger protein 10
234829_at	0.945	0.01369782	2.6526478	-4.15	1.452572	DUSP15	dual specificity phosphatase 15
218167_at	0.945	0.00619764	3.0604832	-4.15	4.3952313	AMZ2	archaelysin family metallopeptidase 2
225242_s_at	0.945	0.0122658	2.7093725	-4.15	1.6089473	CCDC80	coiled-coil domain containing 80
221627_at	0.945	0.01235002	2.7063882	-4.15	2.0395539	TRIM10	tripartite motif containing 10
1566979_at	0.945	0.00812998	2.9105707	-4.15	3.1849425		
1552553_a_at	0.945	0.01395052	2.6446851	-4.16	3.5500865	NLRC4	NLR family CARD domain containing 4
211129_x_at	0.945	0.01079242	2.7751409	-4.16	2.3968025	EDA	ectodysplasin A
208628_s_at	0.945	0.00904606	2.8639321	-4.16	3.1315364	YBX1	Y-box binding protein 1
211061_s_at	0.945	0.01410224	2.6399691	-4.16	3.3147718	MGAT2	mannosyl (alpha-1,6-)-glycoprotein beta-1,2-N-acetylglucosaminyltransferase
1559139_at	0.945	0.01036874	2.792625	-4.16	1.6602189	NOC2L	NOC2 like nucleolar associated transcriptional repressor
219892_at	0.945	0.01095098	2.7687643	-4.16	2.8624704	TM6SF1	transmembrane 6 superfamily member 1
213796_at	0.945	0.01418196	2.6375101	-4.16	3.730082	SPRR1A	small proline rich protein 1A
212905_at	0.945	0.01420365	2.6368433	-4.16	2.2155973	CSTF2T	cleavage stimulation factor subunit 2, tau variant

226090_x_at	0.945	0.0045536	3.2176362	-4.16	3.5307023	RABL3	RAB, member of RAS oncogene family like 3
220016_at	0.945	0.00457215	3.2158208	-4.16	3.7932369	AHNAK	AHNAK nucleoprotein
217510_at	0.945	0.01434858	2.632412	-4.16	1.2439582	CRX	cone-rod homeobox
206137_at	0.945	0.01607509	2.5754076	-4.16	1.1632683	RIMS2	regulating synaptic membrane exocytosis 2
234622_at	0.945	0.01438147	2.6314122	-4.16	3.5404593		
210983_s_at	0.945	0.01611983	2.5741936	-4.16	1.8854286	MCM7	minichromosome maintenance complex component 7
222711_s_at	0.945	0.0046289	3.210312	-4.16	1.8450539	RHBDF1	rhomboid 5 homolog 1
208486_at	0.945	0.0017062	3.7658734	-4.16	4.5667271	DRD5	dopamine receptor D5
220268_at	0.945	0.00684927	3.0161303	-4.16	3.4885833	AAMDC	adipogenesis associated Mth938 domain containing
216835_s_at	0.945	0.01292859	2.6863899	-4.16	3.0525887	DOK1	docking protein 1
209388_at	0.945	0.00386519	3.3160153	-4.16	3.3731545	PAPOLA	poly(A) polymerase alpha
208800_at	0.945	0.00541696	3.139957	-4.16	4.0248618	SRP72	signal recognition particle 72
232507_at	0.945	0.01683956	2.5550827	-4.17	1.6024121	C15orf41	chromosome 15 open reading frame 41
1558839_at	0.945	0.00322671	3.3978218	-4.17	3.5562085	MAPKBP1	mitogen-activated protein kinase binding protein 1
1554616_at	0.945	0.01523526	2.6061795	-4.17	4.7923998	SERPINB8	serpin family B member 8
1561459_at	0.945	0.01194539	2.7306612	-4.17	2.5277884	LOC101929526	uncharacterized LOC101929526
231883_at	0.945	0.0041223	3.2867796	-4.17	2.1466425	FBXW8	F-box and WD repeat domain containing 8
202562_s_at	0.945	0.01549292	2.5988237	-4.17	1.3378176	C14orf1	chromosome 14 open reading frame 1

220552_at	0.945	0.00314267	3.4405944	-4.17	3.7949273	TRPC5	transient receptor potential cation channel subfamily C member 5
226871_s_at	0.945	0.0135969	2.6643111	-4.17	3.4174576	ATG4D	autophagy related 4D cysteine peptidase
204943_at	0.945	0.01554633	2.5973132	-4.17	2.23529	ADAM12	ADAM metallopeptidase domain 12
238314_x_at	0.945	0.00520614	3.1577473	-4.17	3.5967931		
218328_at	0.945	0.01026987	2.8082075	-4.17	1.7408922	COQ4	coenzyme Q4
201139_s_at	0.945	0.01563079	2.5949346	-4.17	2.2562821	SSB	Sjogren syndrome antigen B
1564449_at	0.945	0.01062323	2.7932932	-4.17	2.7494921	USP2-AS1	USP2 antisense RNA 1 (head to head)
219407_s_at	0.945	0.01375136	2.6593536	-4.17	2.1694621	LAMC3	laminin subunit gamma 3
1562777_at	0.945	0.01750737	2.5380172	-4.17	4.5661323	ERV3-1	endogenous retrovirus group 3 member 1
232428_at	0.945	0.01234378	2.7162318	-4.17	2.8814993	MOGAT2	monoacylglycerol O-acyltransferase 2
237283_at	0.945	0.0159326	2.5865316	-4.17	1.6159425		
219511_s_at	0.945	0.00329919	3.4180967	-4.17	4.922431	SNCAIP	synuclein alpha interacting protein
205436_s_at	0.945	0.01780613	2.5305772	-4.17	1.5091366	H2AFX	H2A histone family member X
1563179_at	0.945	0.0160013	2.5846395	-4.17	1.9041253		
242670_at	0.945	0.01607395	2.5826468	-4.18	1.8514969	LGI4	leucine rich repeat LGI family member 4
234590_x_at	0.945	0.01446828	2.6370061	-4.18	1.9710836		
213542_at	0.945	0.01446833	2.6370048	-4.18	3.1714665	ZNF710	zinc finger protein 710
235603_at	0.945	0.01420529	2.6450807	-4.18	2.1256619	HNRNPU	heterogeneous nuclear ribonucleoprotein U
231053_at	0.945	0.0107967	2.7861429	-4.18	2.3968159	KCNB1	potassium voltage-gated channel subfamily B member 1

230158_at	0.945	0.01080201	2.7859262	-4.18	3.2668851	DPY19L2	dpy-19 like 2
223104_at	0.945	0.01817644	2.5215144	-4.18	1.7207247	JAGN1	jagunal homolog 1
226615_at	0.945	0.00751953	2.9745745	-4.18	3.0486429	XPR1	xenotropic and polytropic retrovirus receptor 1
230587_at	0.945	0.01285028	2.6985085	-4.18	1.5783148	STGC3	uncharacterized STGC3
1565911_at	0.945	0.00944376	2.8580986	-4.18	3.087752	ACTG1P17	actin gamma 1 pseudogene 17
211167_s_at	0.945	0.00486989	3.2109711	-4.18	3.4923016	GCK	glucokinase
231373_at	0.945	0.01830653	2.5183713	-4.18	1.6590976	RNF133	ring finger protein 133
218736_s_at	0.945	0.01097378	2.7789561	-4.18	4.7379651	PALMD	palmdelphin
206898_at	0.945	0.01453524	2.6349724	-4.18	1.363374	CDH19	cadherin 19
240283_at	0.945	0.01854314	2.5127071	-4.18	1.699352		
228751_at	0.945	0.01672668	2.5651115	-4.18	2.0894413	CLK4	CDC like kinase 4
243567_at	0.945	0.01862499	2.5107633	-4.18	1.8310224		
219745_at	0.945	0.01870627	2.508841	-4.18	3.0603317	MFSD13A	major facilitator superfamily domain containing 13A
230986_at	0.945	0.00784899	2.9554411	-4.18	3.1866588	KLF8	Kruppel like factor 8
215012_at	0.945	0.00791164	2.9518905	-4.18	3.4716332	ZNF451	zinc finger protein 451
33323_r_at	0.945	0.01701618	2.5575373	-4.18	1.861531	SFN	stratifin
232037_at	0.945	0.01352739	2.675817	-4.18	2.6512939	IGDCC3	immunoglobulin superfamily DCC subclass member 3
243926_at	0.945	0.01363444	2.6723278	-4.19	2.8631061		
232242_at	0.945	0.00115759	4.0065976	-4.19	4.1975817	PTPRG-AS1	PTPRG antisense RNA 1
235950_at	0.945	0.01305793	2.6914322	-4.19	3.2165595	ZNF688	zinc finger protein 688
231371_at	0.945	0.0157267	2.6001771	-4.19	2.6087681	TDRD10	tudor domain containing 10
1570021_at	0.945	0.00415559	3.2831262	-4.19	3.9559778		

1552689_at	0.945	0.01952621	2.4898682	-4.19	1.4653575	CASKIN1	CASK interacting protein 1
212959_s_at	0.945	0.01588192	2.5958271	-4.19	2.4517992	GNPTAB	N-acetylglucosamine-1-phosphate transferase alpha and beta subunits
239132_at	0.945	0.01975712	2.4846579	-4.19	1.2197128	NOS1	nitric oxide synthase 1
235398_at	0.945	0.01789465	2.5352664	-4.19	2.844048	ZNF805	zinc finger protein 805
216756_at	0.945	0.01985525	2.4824609	-4.19	1.1042156		
231604_at	0.945	0.01113524	2.7724985	-4.19	2.4671499		
208697_s_at	0.945	0.01801234	2.5323602	-4.19	2.4007749	EIF3E	eukaryotic translation initiation factor 3 subunit E
222117_s_at	0.945	0.0071649	3.0140801	-4.19	2.7762139	ADCK2	aarF domain containing kinase 2
233615_at	0.945	0.02008104	2.4774432	-4.19	1.463051	CGA	glycoprotein hormones, alpha polypeptide
216667_at	0.945	0.02017723	2.4753214	-4.19	1.7387006	ECRP	ribonuclease A family member 2 pseudogene
218525_s_at	0.945	0.02021094	2.4745802	-4.19	1.2356879	HIF1AN	hypoxia inducible factor 1 alpha subunit inhibitor
1557569_at	0.945	0.01144305	2.7604295	-4.19	2.8651657	MPDU1	mannose-P-dolichol utilization defect 1
235898_at	0.945	0.02042056	2.4699954	-4.2	1.8629345	LOC102723845	uncharacterized LOC102723845
210379_s_at	0.945	0.00670951	3.0437295	-4.2	3.4763236	TLK1	tousled like kinase 1
1555420_a_at	0.945	0.02052021	2.4678312	-4.2	1.1114276	KLF7	Kruppel like factor 7
234382_x_at	0.945	0.02053228	2.4675698	-4.2	1.7900557	HHCM	Mahlavu hepatocellular carcinoma
239644_at	0.945	0.02055576	2.4670616	-4.2	1.2100882	ZC3H8	zinc finger CCCH-type containing 8
202630_at	0.945	0.02067628	2.464461	-4.2	1.1687982	APPBP2	amyloid beta precursor protein binding protein 2

218427_at	0.945	0.00600003	3.1156819	-4.2	2.0003022	SDCCAG3	serologically defined colon cancer antigen 3
210615_at	0.945	0.0207601	2.4626608	-4.2	1.4314232	NRP1	neuropilin 1
202104_s_at	0.945	0.0104591	2.8126596	-4.2	2.9754035	LOC101930112///SPG7	uncharacterized LOC101930112///SPG7, paraplegin matrix AAA peptidase subunit
236192_at	0.945	0.00915158	2.8866804	-4.2	3.4046098	HOOK3	hook microtubule tethering protein 3
220341_s_at	0.945	0.0091815	2.885214	-4.2	1.520651	C5orf45	chromosome 5 open reading frame 45
1563097_at	0.945	0.0211174	2.4550609	-4.2	3.4474813	DNAH12	dynein axonemal heavy chain 12
1569502_s_at	0.945	0.0212091	2.4531295	-4.2	2.3213491	TP73-AS1	TP73 antisense RNA 1
1561940_at	0.945	0.02125606	2.4521435	-4.2	2.095517	LOC100128843///CHFR	uncharacterized LOC100128843///checkpoint with forkhead and ring finger domains, E3 ubiquitin protein ligase
202595_s_at	0.945	0.00460707	3.2632222	-4.2	4.1581527	LEPROTL1	leptin receptor overlapping transcript-like 1
218846_at	0.945	0.01930461	2.5015599	-4.2	2.0058696	MED23	mediator complex subunit 23
203893_at	0.945	0.01935576	2.5003805	-4.2	2.5038344	AK6	adenylate kinase 6
217471_at	0.945	0.01752408	2.5520905	-4.2	3.1532334	LINC01482	long intergenic non-protein coding RNA 1482
211051_s_at	0.945	0.00955563	2.8672573	-4.2	2.8039086	EXTL3	exostosin like glycosyltransferase 3
210848_at	0.945	0.00728721	3.006428	-4.2	2.5919511		
220095_at	0.945	0.02158854	2.4452188	-4.2	1.2218537	CNTLN	centlein
241020_at	0.945	0.01567056	2.6104459	-4.2	2.1681616		
233532_x_at	0.945	0.02168825	2.4431613	-4.2	1.527141	IFT52	intraflagellar transport 52

229618_at	0.945	0.01744345	2.5541468	-4.2	2.6552975	SNX16	sorting nexin 16
219708_at	0.945	0.01748913	2.5529808	-4.2	2.996482	NT5M	5',3'-nucleotidase, mitochondrial
222165_x_at	0.945	0.01976249	2.4911039	-4.21	1.5543159	C9orf16	chromosome 9 open reading frame 16
1569722_s_at	0.945	0.01786987	2.5433701	-4.21	2.6404527	PROSER2-AS1	PROSER2 antisense RNA 1
227004_at	0.945	0.00480723	3.2434484	-4.21	3.622585	CDKL5	cyclin dependent kinase like 5
232770_at	0.945	0.01394253	2.6724665	-4.21	3.4153958	TUSC3	tumor suppressor candidate 3
203896_s_at	0.945	0.02183141	2.4402223	-4.21	1.5383028	PLCB4	phospholipase C beta 4
1562645_at	0.945	0.00618058	3.1021085	-4.21	3.5428928	LOC401176	uncharacterized LOC401176
209524_at	0.945	0.01200827	2.7508539	-4.21	3.0614794	HDGFRP3	hepatoma-derived growth factor, related protein 3
230172_at	0.945	0.02202835	2.4362083	-4.21	1.9269955	IFI27L1	interferon alpha inducible protein 27 like 1
227352_at	0.945	0.01807161	2.5383549	-4.21	2.4997459	SWSAP1	SWIM-type zinc finger 7 associated protein 1
223622_s_at	0.945	0.00829218	2.9478843	-4.21	3.0553187	HYI	hydroxypyruvate isomerase (putative)
236698_at	0.945	0.02207965	2.4351681	-4.21	1.9339883	DYNC1I2	dynein cytoplasmic 1 intermediate chain 2
1565934_at	0.945	0.01781116	2.5448398	-4.21	2.0150055	ZGRF1	zinc finger GRF-type containing 1
1559229_at	0.945	0.01611574	2.5979272	-4.21	2.1796351		
1554914_at	0.945	0.00883501	2.9024821	-4.21	4.2645367	PLA2G4D	phospholipase A2 group IVD
232465_at	0.945	0.01617248	2.5963552	-4.21	2.9159813		
41386_i_at	0.945	0.01433891	2.6599071	-4.21	3.3665817	KDM6B	lysine demethylase 6B
237493_at	0.945	0.00679341	3.0587587	-4.21	1.8968439	IL22RA2	interleukin 22 receptor subunit alpha 2
227359_at	0.945	0.02239097	2.4289027	-4.21	1.644668	OSCP1	organic solute carrier partner 1
239092_at	0.945	0.00724902	2.9908978	-4.21	3.2684164	ITGA8	integrin subunit alpha 8

206151_x_at	0.945	0.0226296	2.4241543	-4.21	2.0287169	CELA3B	chymotrypsin like elastase family member 3B
228805_at	0.945	0.01653656	2.586388	-4.21	2.4582405	SIMC1	SUMO interacting motifs containing 1
218054_s_at	0.945	0.016621	2.5841058	-4.21	1.9479609	KXD1	KxDL motif containing 1
211073_x_at	0.945	0.01033282	2.8320151	-4.21	3.214621	SNORD83B///SNORD139///RPL3	small nucleolar RNA, C/D box 83B///small nucleolar RNA, C/D box 139///ribosomal protein L3
244743_x_at	0.945	0.02072417	2.4698546	-4.21	2.6072876	ZNF138	zinc finger protein 138
217606_at	0.945	0.0103915	2.8294585	-4.21	2.5940235		
1555196_at	0.945	0.02304684	2.4159614	-4.21	1.6808184	LINC00421	long intergenic non-protein coding RNA 421
223083_s_at	0.945	0.02313514	2.4142449	-4.21	1.0297324	RAB4B-EGLN2///EGLN2///RAB4B	RAB4B-EGLN2 readthrough (NMD candidate)///egl-9 family hypoxia inducible factor 2///RAB4B, member RAS oncogene family
1552584_at	0.945	0.00564361	3.1436897	-4.21	3.6575497	IL12RB1	interleukin 12 receptor subunit beta 1
203302_at	0.945	0.0128715	2.7196317	-4.21	3.35718	DCK	deoxycytidine kinase
229330_at	0.945	0.01288102	2.7192985	-4.21	2.3265667		
222132_s_at	0.945	0.01210744	2.7471606	-4.21	1.7341629	AGK	acylglycerol kinase
233941_at	0.945	0.0232184	2.4126322	-4.21	1.6796882	LRRC74A	leucine rich repeat containing 74A
229016_s_at	0.945	0.01504774	2.638242	-4.21	1.5689451	TRERF1	transcriptional regulating factor 1
209240_at	0.945	0.01929101	2.5091063	-4.22	1.4765232	OGT	O-linked N-acetylglucosamine (GlcNAc) transferase
218222_x_at	0.945	0.01714657	2.5701396	-4.22	2.7541222	ARNT	aryl hydrocarbon receptor nuclear translocator

234396_at	0.945	0.02345913	2.4079984	-4.22	1.4217		
233560_x_at	0.945	0.01524792	2.6322976	-4.22	3.1976251	MCM8	minichromosome maintenance 8 homologous recombination repair factor
241009_at	0.945	0.01948546	2.5046018	-4.22	3.5912061	FOXN4	forkhead box N4
232339_at	0.945	0.02362572	2.4048173	-4.22	1.8653809	DCAF8	DDB1 and CUL4 associated factor 8
212428_at	0.945	0.01504607	2.6382921	-4.22	2.2880745	KIAA0368	KIAA0368
1552767_a_at	0.945	0.00925651	2.8978263	-4.22	2.6515871	HS6ST2	heparan sulfate 6-O-sulfotransferase 2
226521_s_at	0.945	0.02374475	2.402557	-4.22	1.8520601	FAM175A	family with sequence similarity 175 member A
228826_at	0.945	0.02163691	2.4505136	-4.22	2.3517793	TSPOAP1-AS1	TSPOAP1 antisense RNA 1
213924_at	0.945	0.02381833	2.4011651	-4.22	1.4765409	GNAL	G protein subunit alpha L
227090_at	0.945	0.01988128	2.4955602	-4.22	2.1990991	PHF21A	PHD finger protein 21A
203703_s_at	0.945	0.02196591	2.4437265	-4.22	1.7889432	TTLL4	tubulin tyrosine ligase like 4
240446_at	0.945	0.00867248	2.9275039	-4.22	3.7389708		
221697_at	0.945	0.02419574	2.3940863	-4.22	1.6512808	MAP1LC3C	microtubule associated protein 1 light chain 3 gamma
219614_s_at	0.945	0.01287642	2.7194594	-4.22	2.5214762	SLC6A20	solute carrier family 6 member 20
227165_at	0.945	0.02446574	2.3890841	-4.22	1.8446802	SKA3	spindle and kinetochore associated complex subunit 3
205216_s_at	0.945	0.01561782	2.621504	-4.22	2.1029278	APOH	apolipoprotein H
241032_at	0.945	0.02233715	2.4361798	-4.22	2.1687907	ANKRD40	ankyrin repeat domain 40
202128_at	0.945	0.00746735	3.0152789	-4.22	2.8455606	AREL1	apoptosis resistant E3 ubiquitin protein ligase 1
215604_x_at	0.945	0.02238031	2.4353099	-4.22	4.1306733		

237623_at	0.945	0.01815274	2.5444841	-4.22	1.6650271	CST3	cystatin C
221648_s_at	0.945	0.01389381	2.6851273	-4.22	2.9991206	AGMAT	agmatinase
209345_s_at	0.945	0.02466511	2.3854227	-4.22	1.6426178	PI4K2A	phosphatidylinositol 4-kinase type 2 alpha
229134_at	0.945	0.02482725	2.382465	-4.22	1.6685137	VANGL1	VANGL planar cell polarity protein 1
1558876_at	0.945	0.01839265	2.5385633	-4.23	3.366392		
213335_s_at	0.945	0.02509437	2.3776308	-4.23	2.5683936	ST3GAL6	ST3 beta-galactoside alpha-2,3-sialyltransferase 6
231797_at	0.945	0.00313291	3.4773505	-4.23	2.4775914	SIX4	SIX homeobox 4
204375_at	0.945	0.00424205	3.3333422	-4.23	4.3500325	CLSTN3	calsyntenin 3
208610_s_at	0.945	0.02537517	2.3725995	-4.23	1.6941875	SRRM2	serine/arginine repetitive matrix 2
213745_at	0.945	0.01208	2.7612437	-4.23	3.2591571	ATRNL1	attractin like 1
239524_at	0.945	0.0125531	2.7437631	-4.23	2.3680786	TSSK6	testis specific serine kinase 6
219771_at	0.945	0.02334101	2.4163372	-4.23	2.1173292	TBC1D8B	TBC1 domain family member 8B
200090_at	0.945	0.01539895	2.6278613	-4.23	3.2082793	FNTA	farnesyltransferase, CAAX box, alpha
231744_at	0.945	0.02585361	2.3641433	-4.23	1.7344921	CELF5	CUGBP, Elav-like family member 5
205086_s_at	0.945	0.02121498	2.4662774	-4.23	2.5810799	NCAPH2	non-SMC condensin II complex subunit H2
242007_at	0.945	0.00863588	2.9482203	-4.23	3.839944	FBXO30	F-box protein 30
213185_at	0.945	0.02609307	2.3599645	-4.23	1.1956214	KIAA0556	KIAA0556
224320_s_at	0.945	0.02614525	2.3590586	-4.23	1.4434729	MCM8	minichromosome maintenance 8 homologous recombination repair factor

215078_at	0.945	0.01251624	2.7451023	-4.23	3.8037355	LOC100129518///SOD2	uncharacterized LOC100129518///superoxide dismutase 2, mitochondrial
1563318_s_at	0.945	0.01951157	2.5118661	-4.23	2.3334906	MAGIX	MAGI family member, X-linked
200839_s_at	0.945	0.02623334	2.357533	-4.23	1.6526091	CTSB	cathepsin B
206036_s_at	0.945	0.01703828	2.582173	-4.23	2.7000178	REL	REL proto-oncogene, NF-kB subunit
215552_s_at	0.945	0.00672116	3.0871296	-4.23	4.1438932	ESR1	estrogen receptor 1
211841_s_at	0.945	0.02634959	2.3555269	-4.23	1.0762903	TNFRSF25	TNF receptor superfamily member 25
1561171_a_at	0.945	0.02408584	2.402116	-4.23	1.6126719	GOLGA6L6///GOLGA6L22///GOLGA6L1	golgin A6 family-like 6///golgin A6 family-like 22///golgin A6 family-like 1
231104_at	0.945	0.02639159	2.3548041	-4.23	1.5557988	TDRD5	tudor domain containing 5
206823_at	0.945	0.02422293	2.3995427	-4.23	2.1785755	L3MBTL1	l(3)mbt-like 1 (Drosophila)
1564164_at	0.945	0.02651729	2.352647	-4.23	2.27891	DENND1B	DENN domain containing 1B
227482_at	0.945	0.00349275	3.4257168	-4.23	4.3681364	ADCK1	aarF domain containing kinase 1
202296_s_at	0.945	0.01727351	2.5759588	-4.24	4.9434878	RER1	retention in endoplasmic reticulum sorting receptor 1
201162_at	0.945	0.02660497	2.351148	-4.24	1.6814502	IGFBP7	insulin like growth factor binding protein 7
214081_at	0.945	0.01738148	2.5731328	-4.24	3.8611761	PLXDC1	plexin domain containing 1
233625_x_at	0.945	0.02243399	2.4409676	-4.24	2.6053836	MIR6727///CPSF3L	microRNA 6727///cleavage and polyadenylation specific factor 3-like
1558573_at	0.945	0.02686033	2.3468078	-4.24	1.1681304	MCTS1	MCTS1, re-initiation and release factor

228609_at	0.945	0.00345753	3.4699629	-4.24	4.6083931	LOC102723600///MIR1302-11///MIR1302-10///MIR1302-9///MIR1302-2	uncharacterized LOC102723600///microRNA 1302-11///microRNA 1302-10///microRNA 1302-9///microRNA 1302-2
228292_at	0.945	0.02693877	2.345482	-4.24	1.6833599		
203606_at	0.945	0.0179905	2.5574947	-4.24	3.2433154	NDUFS6	NADH:ubiquinone oxidoreductase subunit S6
229499_at	0.945	0.02257114	2.4382	-4.24	2.7016728	CAPN13	calpain 13
1563165_at	0.945	0.02724889	2.3402751	-4.24	1.1611175	LOC101928978	uncharacterized LOC101928978
228214_at	0.945	0.02046231	2.4902791	-4.24	1.215908	SOX6	SRY-box 6
233863_at	0.945	0.01505193	2.6488301	-4.24	2.6990828	CASZ1	castor zinc finger 1
219446_at	0.945	0.02252728	2.4390835	-4.24	1.8757131	RIC8B	RIC8 guanine nucleotide exchange factor B
206304_at	0.945	0.00722476	3.0532922	-4.24	4.4811235	MYBPH	myosin binding protein H
230999_at	0.945	0.00361572	3.448098	-4.24	4.8675941	ST3GAL4-AS1	ST3GAL4 antisense RNA 1 (head to head)
234571_at	0.945	0.02749653	2.336156	-4.24	1.0799208		
213080_x_at	0.945	0.0275003	2.3360937	-4.24	1.5915912	RPL5///SNORD21	ribosomal protein L5///small nucleolar RNA, C/D box 21
1562165_at	0.945	0.02761973	2.3341195	-4.24	2.1484115		
1553775_at	0.945	0.00640379	3.1097532	-4.24	2.8465299	LOC152048	uncharacterized FLJ31715
225080_at	0.945	0.01159335	2.7947509	-4.24	4.0951463	MYO1C	myosin IC
216699_s_at	0.945	0.02316931	2.4263086	-4.24	2.6381387	KLK1	kallikrein 1
234146_at	0.945	0.01617749	2.6159974	-4.24	2.8400641	B3GAT2	beta-1,3-glucuronyltransferase 2

207376_at	0.945	0.01867701	2.5404504	-4.24	4.9601663	VENTX	VENT homeobox
232821_at	0.945	0.02336763	2.4224285	-4.24	2.8364892	GTSF1L	gametocyte specific factor 1 like
203956_at	0.945	0.01637047	2.6105854	-4.24	3.6077605	MORC2	MORC family CW-type zinc finger 2
220723_s_at	0.945	0.02794918	2.3287138	-4.24	1.7405396	CWH43	cell wall biogenesis 43 C-terminal homolog
242965_at	0.945	0.0155225	2.6348309	-4.24	4.8865655		
213085_s_at	0.945	0.02803662	2.3272887	-4.24	2.061494	WWC1	WW and C2 domain containing 1
223568_s_at	0.945	0.02355288	2.4188314	-4.24	2.593975	PLPP5	phospholipid phosphatase 5
243052_at	0.945	0.02359768	2.4179655	-4.24	2.1321629	MOB3C	MOB kinase activator 3C
238330_s_at	0.945	0.01386662	2.6860136	-4.24	2.9013561		
235583_at	0.945	0.02824232	2.3239523	-4.24	1.3345325	ILDR1	immunoglobulin like domain containing receptor 1
212382_at	0.945	0.01105227	2.8167141	-4.24	2.1798583	TCF4	transcription factor 4
233197_at	0.945	0.025992	2.3674839	-4.24	2.5177569	KLHL9	kelch like family member 9
212789_at	0.945	0.02341731	2.4214611	-4.25	1.8453018	NCAPD3	non-SMC condensin II complex subunit D3
209117_at	0.945	0.02606243	2.3662495	-4.25	1.7614786	WBP2	WW domain binding protein 2
234168_at	0.945	0.02851473	2.319568	-4.25	1.2297805	TAF15	TATA-box binding protein associated factor 15
244356_at	0.945	0.01683231	2.5978737	-4.25	3.0301644		
244373_at	0.945	0.02858581	2.3184303	-4.25	1.450961		
239619_at	0.945	0.01219204	2.7715651	-4.25	2.6363888		
230318_at	0.945	0.01282273	2.7340794	-4.25	2.2987742	SERPINA1	serpin family A member 1
230125_at	0.945	0.01132395	2.8055606	-4.25	3.2103481	GUSB	glucuronidase beta

221074_at	0.945	0.01230822	2.7671923	-4.25	2.9745335	NCR2	natural cytotoxicity triggering receptor 2
237964_at	0.945	0.02882174	2.3146724	-4.25	1.737253		
238396_at	0.945	0.00975264	2.8918925	-4.25	3.0635002		
1564240_at	0.945	0.01438443	2.6815343	-4.25	4.6105644	LOC100130856 ///PSPN	uncharacterized LOC100130856///persephin
220663_at	0.945	0.02898466	2.3120935	-4.25	2.024243	IL1RAPL1	interleukin 1 receptor accessory protein like 1
221245_s_at	0.945	0.02662949	2.3564206	-4.25	2.3839218	FZD5	frizzled class receptor 5
225912_at	0.945	0.01718006	2.5885168	-4.25	2.9657258	TP53INP1	tumor protein p53 inducible nuclear protein 1
1555299_s_at	0.945	0.00982646	2.8883922	-4.25	3.4421674	ERVW-1	endogenous retrovirus group W member 1
205755_at	0.945	0.01249606	2.760205	-4.25	2.2552541	ITIH3	inter-alpha-trypsin inhibitor heavy chain 3
1561529_at	0.945	0.0098626	2.8866882	-4.25	3.649888		
204387_x_at	0.945	0.0197971	2.5138596	-4.25	3.3506953	MRPL57	mitochondrial ribosomal protein L57
1559629_at	0.945	0.02922102	2.3083757	-4.25	2.5668484	LOC101927710	uncharacterized LOC101927710
222148_s_at	0.945	0.02924267	2.3080366	-4.25	3.4833808	RHOT1	ras homolog family member T1
217558_at	0.945	0.0126051	2.7561949	-4.25	3.2754113	CYP2C9	cytochrome P450 family 2 subfamily C member 9
236427_at	0.945	0.01262847	2.7553397	-4.25	3.4670766		
239735_at	0.945	0.00299037	3.4635794	-4.25	4.2464262		
227844_at	0.945	0.02427085	2.4051327	-4.25	1.4822712	FMNL3	formin like 3
204266_s_at	0.945	0.01049662	2.85773	-4.25	4.1090072	CHKA	choline kinase alpha

238819_at	0.945	0.01275169	2.7508557	-4.25	3.1250603	ZNF347	zinc finger protein 347
208149_x_at	0.945	0.02951844	2.3037363	-4.25	1.0147036	DDX11	DEAD/H-box helicase 11
242325_at	0.945	0.00826377	2.9902118	-4.25	3.4435576	YWHAH	tyrosine 3-monooxygenase/tryptophan 5-monooxygenase activation protein eta
211848_s_at	0.945	0.00140142	4.0481891	-4.25	5.3825543	CEACAM7	carcinoembryonic antigen related cell adhesion molecule 7
230979_at	0.945	0.02968369	2.3011768	-4.25	1.6589206	BMPR1A	bone morphogenetic protein receptor type 1A
1562310_at	0.945	0.02972655	2.3005152	-4.25	2.1304955	LOC101927165	uncharacterized LOC101927165
210052_s_at	0.945	0.01844675	2.5461009	-4.25	1.5617557	TPX2	TPX2, microtubule nucleation factor
1563867_at	0.945	0.01986987	2.512181	-4.25	3.3046109	LOC283194	uncharacterized LOC283194
1559668_s_at	0.945	0.00591277	3.1751475	-4.25	6.660839		
212247_at	0.945	0.01687523	2.596709	-4.25	3.043596	NUP205	nucleoporin 205
200954_at	0.945	0.02988511	2.2980748	-4.25	1.6695182	ATP6V0C	ATPase H+ transporting V0 subunit c
1562557_at	0.945	0.02993354	2.2973317	-4.25	1.6943504	LOC101928144	uncharacterized LOC101928144
233927_at	0.945	0.0275369	2.3410844	-4.25	1.6727241		
240549_at	0.945	0.02997261	2.296733	-4.25	1.5196652	NRSN2-AS1	NRSN2 antisense RNA 1
235380_at	0.945	0.02758427	2.3402965	-4.25	2.0756257		
242596_at	0.945	0.02759829	2.3400635	-4.25	2.1678603		
1554119_at	0.945	0.01317454	2.7357767	-4.25	2.2089755	USB1	U6 snRNA biogenesis phosphodiesterase 1
203519_s_at	0.945	0.03022274	2.2929171	-4.25	1.6144633	UPF2	UPF2 regulator of nonsense transcripts homolog (yeast)

211774_s_at	0.945	0.02781601	2.33646	-4.25	3.2207862	MMACHC	methylmalonic aciduria (cobalamin deficiency) cblC type, with homocystinuria
213840_s_at	0.945	0.03026885	2.292217	-4.25	1.2321406	MRPS12	mitochondrial ribosomal protein S12
223044_at	0.945	0.01103693	2.8343536	-4.26	3.7794588	SLC40A1	solute carrier family 40 member 1
227065_at	0.945	0.03031885	2.2914587	-4.26	2.994797	RNF216	ring finger protein 216
219423_x_at	0.945	0.03033705	2.2911829	-4.26	1.4716762	TNFRSF25	TNF receptor superfamily member 25
242686_at	0.945	0.03035691	2.2908822	-4.26	1.2523821	STARD13	StAR related lipid transfer domain containing 13
1556937_at	0.945	0.02319507	2.4330502	-4.26	2.4106045		
224625_x_at	0.945	0.02323675	2.4322269	-4.26	3.0533647	MIR1282///HYPK///SERF2	microRNA 1282///huntingtin interacting protein K///small EDRK-rich factor 2
204243_at	0.945	0.02577975	2.3775196	-4.26	2.2771269	RLF	rearranged L-myc fusion
222920_s_at	0.945	0.03053814	2.2881464	-4.26	1.4962699	TESPA1	thymocyte expressed, positive selection associated 1
212013_at	0.945	0.03056171	2.2877917	-4.26	1.0856733	PXDN	peroxidasin
217874_at	0.945	0.01604311	2.6313503	-4.26	3.7604641	SUCLG1	succinate-CoA ligase alpha subunit
219245_s_at	0.945	0.01352055	2.7237778	-4.26	3.2414681	OGFOD2	2-oxoglutarate and iron dependent oxygenase domain containing 2
208244_at	0.945	0.02059209	2.4958257	-4.26	1.6461318	BMP3	bone morphogenetic protein 3
208952_s_at	0.945	0.03081945	2.2839288	-4.26	1.3901704	LARP4B	La ribonucleoprotein domain family member 4B
235925_at	0.945	0.02359782	2.42515	-4.26	1.5735988		

205948_at	0.945	0.01864903	2.55086	-4.26	2.5104653	PTPRT	protein tyrosine phosphatase, receptor type T
225277_at	0.945	0.02369598	2.4232434	-4.26	1.3694715	SLC39A13	solute carrier family 39 member 13
203052_at	0.945	0.01436039	2.6823011	-4.26	3.5924542	C2	complement component 2
215937_at	0.945	0.00643956	3.1343827	-4.26	4.2766765	PTGDR	prostaglandin D2 receptor
214672_at	0.945	0.03117891	2.2785902	-4.26	1.4122175	TTLL5	tubulin tyrosine ligase like 5
234127_at	0.945	0.0214598	2.4768685	-4.26	2.5725921	LOC105378749	uncharacterized LOC105378749
230974_at	0.945	0.03125474	2.2774711	-4.26	1.6854711	DDX19B	DEAD-box helicase 19B
223661_at	0.945	0.03132306	2.276465	-4.26	1.7885021	NUCKS1	nuclear casein kinase and cyclin dependent kinase substrate 1
234216_at	0.945	0.0215863	2.4741645	-4.26	2.6904498	FLJ21408	uncharacterized LOC400512
225703_at	0.945	0.00914998	2.9422425	-4.26	4.0714152	FBRSL1	fibrosin like 1
237115_at	0.945	0.01898153	2.542724	-4.26	3.7069959		
205710_at	0.945	0.01402177	2.7069057	-4.26	3.3486199	LRP2	LDL receptor related protein 2
244888_at	0.945	0.03147497	2.274235	-4.26	2.0308761		
203597_s_at	0.945	0.02907345	2.3161386	-4.26	3.7828318	WBP4	WW domain binding protein 4
236411_at	0.945	0.02425088	2.4126009	-4.26	1.1207885		
244595_at	0.945	0.03166695	2.2714307	-4.26	1.0769184		
209337_at	0.945	0.02934141	2.3119121	-4.26	2.6357994	PSIP1	PC4 and SFRS1 interacting protein 1
202934_at	0.945	0.01643899	2.6201007	-4.26	2.2291618	HK2	hexokinase 2
212245_at	0.945	0.00520598	3.2358651	-4.26	4.0809461	MCFD2	multiple coagulation factor deficiency 2
1556347_at	0.945	0.01716879	2.6000173	-4.26	1.8822843		
201769_at	0.945	0.0268889	2.3581543	-4.26	4.0081386	CLINT1	clathrin interactor 1
207984_s_at	0.945	0.02971494	2.3060788	-4.26	1.8776414	MPP2	membrane palmitoylated protein 2

233180_at	0.945	0.0274201	2.3491382	-4.26	2.0013894		
208763_s_at	0.945	0.03232663	2.2619108	-4.26	1.4538786	TSC22D3	TSC22 domain family member 3
240701_at	0.945	0.0323312	2.2618455	-4.26	1.1838749	GSDMB	gasdermin B
215052_at	0.945	0.02704262	2.3555285	-4.26	1.3327633	FRMPD4	FERM and PDZ domain containing 4
241495_at	0.945	0.01227166	2.7848203	-4.27	2.6148402	CCNL1	cyclin L1
223175_s_at	0.945	0.03254776	2.258759	-4.27	2.1573828	FEM1A	fem-1 homolog A
231539_at	0.945	0.03258838	2.2581822	-4.27	1.1853492		
233580_at	0.945	0.03269448	2.2566785	-4.27	3.9551235		
201187_s_at	0.945	0.02078272	2.4915981	-4.27	2.6502334	ITPR3	inositol 1,4,5-trisphosphate receptor type 3
228777_at	0.945	0.02440698	2.4096478	-4.27	2.8710809	KBTBD3	kelch repeat and BTB domain containing 3
209905_at	0.945	0.00718458	3.0820102	-4.27	4.7398213	HOXA10-HOXA9///MIR196B///HOXA9	HOXA10-HOXA9 readthrough///microRNA 196b///homeobox A9
213962_s_at	0.945	0.00439953	3.352198	-4.27	4.2157209	ANKLE2	ankyrin repeat and LEM domain containing 2
233054_at	0.945	0.01209352	2.7916628	-4.27	2.7031928	CNOT2	CCR4-NOT transcription complex subunit 2
1558301_a_at	0.945	0.01511337	2.6720645	-4.27	4.3315881	EFCAB5	EF-hand calcium binding domain 5
233081_at	0.945	0.01512834	2.6716036	-4.27	3.041576		
244802_at	0.945	0.01044021	2.8602369	-4.27	3.1365146		
1560227_at	0.945	0.02834537	2.3338093	-4.27	1.1793785	GDPD1	glycerophosphodiester phosphodiesterase domain containing 1

1553747_at	0.945	0.01014831	2.8933377	-4.27	3.4582179	MGC16025	uncharacterized LOC85009
204961_s_at	0.945	0.01804363	2.5769779	-4.27	2.9561224	NCF1C///NCF1B///NCF1	neutrophil cytosolic factor 1C pseudogene///neutrophil cytosolic factor 1B pseudogene///neutrophil cytosolic factor 1
1560457_x_at	0.945	0.02334477	2.4380291	-4.27	3.0302854	PLIN5	perilipin 5
225205_at	0.945	0.02061433	2.5046123	-4.27	3.8447581	KIF3B	kinesin family member 3B
227034_at	0.945	0.01225712	2.7691107	-4.27	2.9805646	SOWAHC	sosondowah ankyrin repeat domain family member C
237510_at	0.945	0.03395447	2.239152	-4.27	1.0882838	MYNN	myoneurin
1566288_at	0.945	0.03138999	2.2807155	-4.27	2.2005689	OR2M4	olfactory receptor family 2 subfamily M member 4
211600_at	0.945	0.0262891	2.3753486	-4.27	2.2821079	PTPRO	protein tyrosine phosphatase, receptor type O
1559706_at	0.945	0.02101498	2.4956929	-4.27	3.1450056	ARHGEF28	Rho guanine nucleotide exchange factor 28
220781_at	0.945	0.02905896	2.3222971	-4.27	3.2461487	1-Dec	deleted in esophageal cancer 1
203298_s_at	0.945	0.00599395	3.1686375	-4.27	3.0376131	JARID2	jumonji and AT-rich interaction domain containing 2
229920_at	0.945	0.03411588	2.2369494	-4.27	2.5663512	RBCK1	RANBP2-type and C3HC4-type zinc finger containing 1
203022_at	0.945	0.03158054	2.2779087	-4.27	2.1081202	RNASEH2A	ribonuclease H2 subunit A
214820_at	0.945	0.03421999	2.2355338	-4.27	1.1676454	BRWD1	bromodomain and WD repeat domain containing 1
241568_at	0.945	0.01064109	2.8708941	-4.27	3.6381469	LOC100506319	uncharacterized LOC100506319

231825_x_at	0.945	0.03426927	2.234865	-4.27	3.1332424	ATF7IP	activating transcription factor 7 interacting protein
1562220_at	0.945	0.03176402	2.2752206	-4.27	3.0709792		
225145_at	0.945	0.02363337	2.4323415	-4.27	3.1129018	NCOA5	nuclear receptor coactivator 5
216524_x_at	0.945	0.02212096	2.4628966	-4.27	4.276496		
234077_at	0.945	0.0344059	2.2330154	-4.27	3.8949796		
208386_x_at	0.945	0.00953259	2.9229141	-4.27	3.6409863	DMC1	DNA meiotic recombinase 1
241882_at	0.945	0.03441829	2.2328481	-4.27	2.1289279	CAMTA1	calmodulin binding transcription activator 1
223243_s_at	0.945	0.01288034	2.7621401	-4.27	3.5804977	EDEM3	ER degradation enhancing alpha-mannosidase like protein 3
229240_at	0.945	0.03186225	2.2737874	-4.27	1.3247466	ZDHHC21	zinc finger DHHC-type containing 21
240885_at	0.945	0.02912607	2.3212279	-4.28	1.9513162		
230516_at	0.945	0.01616892	2.6405793	-4.28	2.7040914	MALSU1	mitochondrial assembly of ribosomal large subunit 1
237981_at	0.945	0.03214837	2.2696352	-4.28	2.5452636	CMYA5	cardiomyopathy associated 5
226016_at	0.945	0.03476667	2.2281635	-4.28	1.4141087	CD47	CD47 molecule
43544_at	0.945	0.03218993	2.2690349	-4.28	2.2895534	MED16	mediator complex subunit 16
228966_at	0.945	0.01624195	2.6384738	-4.28	2.5696187	PANK2	pantothenate kinase 2
224497_x_at	0.945	0.0322466	2.2682174	-4.28	3.9193214	HSD17B14	hydroxysteroid 17-beta dehydrogenase 14
229876_at	0.945	0.01513291	2.6714629	-4.28	2.7033643	PHKA1	phosphorylase kinase regulatory subunit alpha 1
207609_s_at	0.945	0.03232105	2.2671455	-4.28	1.7904629	CYP1A2	cytochrome P450 family 1 subfamily A member 2

226853_at	0.945	0.02069156	2.5028805	-4.28	2.6714106	BMP2K	BMP2 inducible kinase
1555225_at	0.945	0.01140485	2.8190583	-4.28	3.5420054	C1orf43	chromosome 1 open reading frame 43
201967_at	0.945	0.03494599	2.2257688	-4.28	1.4352299	RBM6	RNA binding motif protein 6
203365_s_at	0.945	0.01694496	2.6060899	-4.28	2.4483034	MMP15	matrix metallopeptidase 15
243815_at	0.945	0.0351313	2.2233057	-4.28	1.3136839	PGBD4	piggyBac transposable element derived 4
203889_at	0.945	0.03015504	2.3051085	-4.28	2.8880353	SCG5	secretogranin V
200885_at	0.945	0.0352818	2.2213138	-4.28	1.5350551	RHOC	ras homolog family member C
205977_s_at	0.945	0.03020462	2.3043447	-4.28	2.3312348	EPHA1	EPH receptor A1
230985_at	0.945	0.03529936	2.221082	-4.28	1.6665756	C9orf131	chromosome 9 open reading frame 131
209103_s_at	0.945	0.03531221	2.2209123	-4.28	2.2629722	UFD1L	ubiquitin fusion degradation 1 like (yeast)
244822_at	0.945	0.03531222	2.2209123	-4.28	1.3861372	GART	phosphoribosylglycinamide formyltransferase, phosphoribosylglycinamide synthetase, phosphoribosylaminoimidazole synthetase
221711_s_at	0.945	0.01342009	2.742872	-4.28	3.3771193	BABAM1	BRISC and BRCA1 A complex member 1
220759_at	0.945	0.01891864	2.5549634	-4.28	3.1235824	EDDM3B	epididymal protein 3B
231951_at	0.945	0.03025655	2.3035457	-4.28	2.1044003	GNAO1	G protein subunit alpha o1
219153_s_at	0.945	0.03543765	2.2192594	-4.28	3.0005517	THSD4	thrombospondin type 1 domain containing 4
241223_x_at	0.945	0.03548002	2.2187023	-4.28	1.2986649		

215076_s_at	0.945	0.00520647	3.269844	-4.28	2.8166707	COL3A1	collagen type III alpha 1 chain
1560712_at	0.945	0.01670647	2.6252878	-4.28	2.6081374	TMPRSS11B	transmembrane protease, serine 11B
242783_at	0.945	0.01355152	2.7382928	-4.28	2.8508224		
210110_x_at	0.945	0.02466541	2.412516	-4.28	2.9852665	HNRNPH3	heterogeneous nuclear ribonucleoprotein H3
213949_s_at	0.945	0.03009403	2.3060502	-4.28	2.2889701	DOHH	deoxyhypusine hydroxylase/monooxygenase
239856_at	0.945	0.03569511	2.2158832	-4.28	1.5319125		
235176_at	0.945	0.03574281	2.21526	-4.28	1.7176105	ZFP82	ZFP82 zinc finger protein
235889_at	0.945	0.03589132	2.2133248	-4.28	2.8570162	RSPH3	radial spoke 3 homolog
221653_x_at	0.945	0.01702031	2.6165744	-4.28	2.7515205	APOL2	apolipoprotein L2
201971_s_at	0.945	0.03598848	2.2120627	-4.28	1.5672704	ATP6V1A	ATPase H+ transporting V1 subunit A
214134_at	0.945	0.02497896	2.4066448	-4.28	2.4756064	KIAA1211L	KIAA1211 like
212568_s_at	0.945	0.00538748	3.2531227	-4.28	3.9836443	DLAT	dihydrolipoamide S-acetyltransferase
214095_at	0.945	0.02558024	2.3955742	-4.28	1.8382864	SHMT2	serine hydroxymethyltransferase 2
225552_x_at	0.945	0.03344877	2.2511771	-4.28	2.6146959	AURKAIP1	aurora kinase A interacting protein 1
222950_at	0.945	0.03613831	2.2101224	-4.28	1.2154653	NIPAL2	NIPA like domain containing 2
224068_x_at	0.945	0.03617755	2.2096155	-4.28	1.606548	RBM22	RNA binding motif protein 22
239589_at	0.945	0.02827236	2.3415721	-4.28	1.6807649		
224874_at	0.945	0.01170913	2.8255174	-4.28	1.931243	POLR1D	RNA polymerase I subunit D
212210_at	0.945	0.02197775	2.4748924	-4.28	2.0636241	INTS1	integrator complex subunit 1
212081_x_at	0.945	0.01194963	2.8158528	-4.28	3.2912858	PRRC2A	proline rich coiled-coil 2A
204509_at	0.945	0.03400175	2.2435247	-4.28	2.1394087	CA12	carbonic anhydrase 12
213930_at	0.945	0.01071106	2.8677892	-4.28	2.7405003	ATG12	autophagy related 12
222192_s_at	0.945	0.03409296	2.2422733	-4.28	1.6446041	LDAH	lipid droplet associated hydrolase

202619_s_at	0.945	0.03679626	2.2016866	-4.28	1.768398	PLOD2	procollagen-lysine,2-oxoglutarate 5-dioxygenase 2
225997_at	0.945	0.03166797	2.2822975	-4.28	1.6829698	MOB1B	MOB kinase activator 1B
238440_at	0.945	0.03690847	2.2002617	-4.29	1.5641828	CLYBL	citrate lyase beta like
231680_at	0.945	0.0200956	2.5268164	-4.29	1.4679454		
201306_s_at	0.945	0.01772052	2.5976695	-4.29	3.3374522	ANP32B	acidic nuclear phosphoprotein 32 family member B
225151_at	0.945	0.01409906	2.7043552	-4.29	3.1044021	RTKN	rhotekin
206441_s_at	0.945	0.00700487	3.124516	-4.29	4.0045453	COMMD4	COMM domain containing 4
203475_at	0.945	0.03710635	2.1977581	-4.29	1.5618955	CYP19A1	cytochrome P450 family 19 subfamily A member 1
225931_s_at	0.945	0.03712182	2.1975628	-4.29	1.9687422	RNF213	ring finger protein 213
1554736_at	0.945	0.02595729	2.3887541	-4.29	1.5994688	ARHGAP29	Rho GTPase activating protein 29
203543_s_at	0.945	0.01786864	2.5937613	-4.29	2.2769166	KLF9	Kruppel like factor 9
201757_at	0.945	0.03450194	2.2366994	-4.29	2.1696626	RPL10///NDUFS5	ribosomal protein L10///NADH:ubiquinone oxidoreductase subunit S5
206940_s_at	0.945	0.02915052	2.3273072	-4.29	2.0614884	POU4F1	POU class 4 homeobox 1
224137_at	0.945	0.02090671	2.5083062	-4.29	2.510928	CACNG7	calcium voltage-gated channel auxiliary subunit gamma 7
228336_at	0.945	0.0372985	2.1953384	-4.29	1.1994304	PWWP2A	PWWP domain containing 2A
239545_at	0.945	0.01466746	2.70104	-4.29	1.6541564		
1561424_at	0.945	0.01432487	2.6969795	-4.29	2.6471189	LOC101928907	uncharacterized LOC101928907
47550_at	0.945	0.03740811	2.1939633	-4.29	2.4280294	LZTS1	leucine zipper tumor suppressor 1

233111_at	0.945	0.03747806	2.1930875	-4.29	1.4460501	PTCSC1	papillary thyroid carcinoma susceptibility candidate 1 (non-protein coding)
224808_s_at	0.945	0.02680931	2.373674	-4.29	3.338387	GET4	golgi to ER traffic protein 4
212257_s_at	0.945	0.02847518	2.3382417	-4.29	2.7321767	SMARCA2	SWI/SNF related, matrix associated, actin dependent regulator of chromatin, subfamily a, member 2
225949_at	0.945	0.02634836	2.3817766	-4.29	3.6642275	MIR6845///NRBP2	microRNA 6845///nuclear receptor binding protein 2
207356_at	0.945	0.02398625	2.4341024	-4.29	2.5155018	DEFB4B///DEFB4A	defensin beta 4B///defensin beta 4A
1561516_at	0.945	0.00989329	2.8852455	-4.29	3.5883464	LOC101928995	uncharacterized LOC101928995
1569763_at	0.945	0.03774962	2.1897017	-4.29	1.433348	KCNMA1-AS1	KCNMA1 antisense RNA 1
220270_at	0.945	0.02071028	2.5127262	-4.29	2.7761494	RNF17	ring finger protein 17
229307_at	0.945	0.03511172	2.2284989	-4.29	2.5976812	ANKRD28	ankyrin repeat domain 28
224151_s_at	0.945	0.03787902	2.1880959	-4.29	1.7504604	AK3	adenylate kinase 3
201016_at	0.945	0.02659925	2.3773503	-4.29	2.4743096	EIF1AX	eukaryotic translation initiation factor 1A, X-linked
1554069_at	0.945	0.02143612	2.4965844	-4.29	2.6014048	EPHA8	EPH receptor A8
201506_at	0.945	0.03795509	2.1871543	-4.29	1.162434	TGFBI	transforming growth factor beta induced
209701_at	0.945	0.03800204	2.186574	-4.29	1.058275	ERAP1	endoplasmic reticulum aminopeptidase 1
219998_at	0.945	0.03807328	2.1856946	-4.29	2.0478566	LGALSL	galectin like
219678_x_at	0.945	0.03812888	2.1850094	-4.29	1.9176962	DCLRE1C	DNA cross-link repair 1C

212384_at	0.945	0.01145501	2.8359397	-4.29	3.9124651	ATP6V1G2-DDX39B///SNORD84///DDX39B	ATP6V1G2-DDX39B readthrough (NMD candidate)///small nucleolar RNA, C/D box 84///DEAD-box helicase 39B
225367_at	0.945	0.02684001	2.3731389	-4.29	1.9721032	PGM2	phosphoglucomutase 2
242414_at	0.945	0.03005831	2.3129695	-4.29	2.2148046	QPRT	quinolinate phosphoribosyltransferase
201689_s_at	0.945	0.0186002	2.5748966	-4.29	2.8139502	TPD52	tumor protein D52
237780_at	0.945	0.03298056	2.2632983	-4.29	3.739573		
217918_at	0.945	0.02690226	2.3720556	-4.29	2.7217627	DYNLRB1	dynein light chain roadblock-type 1
1556186_s_at	0.945	0.00764754	3.0520915	-4.29	4.0571492	EMC1	ER membrane protein complex subunit 1
220968_s_at	0.945	0.01866855	2.5731701	-4.29	3.8486031	TSPAN9	tetraspanin 9
205968_at	0.945	0.01867019	2.5731288	-4.29	3.1344907	KCNS3	potassium voltage-gated channel modifier subfamily S member 3
212996_s_at	0.945	0.03835479	2.1822344	-4.29	1.2861503	URB1	URB1 ribosome biogenesis 1 homolog (S. cerevisiae)
227830_at	0.945	0.02453559	2.4235	-4.29	2.7966666	GABRB3	gamma-aminobutyric acid type A receptor beta3 subunit
210658_s_at	0.945	0.01293759	2.7780268	-4.29	3.3158127	GGA2	golgi associated, gamma adaptin ear containing, ARF binding protein 2
211901_s_at	0.945	0.02763757	2.3594324	-4.29	2.5040289	PDE4A	phosphodiesterase 4A
218953_s_at	0.945	0.03862852	2.1788916	-4.29	1.5846839	PCYOX1L	prenylcysteine oxidase 1 like
209144_s_at	0.945	0.02720243	2.3668646	-4.29	2.8326487	CBFA2T2	CBFA2/RUNX1 translocation partner 2
1563367_at	0.945	0.01612329	2.6563168	-4.29	2.5777217	MAPT-AS1	MAPT antisense RNA 1

210684_s_at	0.945	0.03287913	2.2647417	-4.29	1.6382538	DLG4	discs large MAGUK scaffold protein 4
1561557_at	0.945	0.03867129	2.1783713	-4.29	1.7831297	LOC105378419	uncharacterized LOC105378419
217579_x_at	0.945	0.03871575	2.1778308	-4.29	3.1445273		
224077_at	0.945	0.02480938	2.4182978	-4.29	3.1571298	WHSC1L1	Wolf-Hirschhorn syndrome candidate 1-like 1
240880_at	0.945	0.0360131	2.2166101	-4.29	3.1141208	LOC101927089	uncharacterized LOC101927089
218435_at	0.945	0.03874712	2.1774499	-4.29	2.0798786	DNAJC15	DnaJ heat shock protein family (Hsp40) member C15
238506_at	0.945	0.01316174	2.769833	-4.29	3.0567294	LRRC58	leucine rich repeat containing 58
1560696_x_at	0.945	0.01902987	2.564141	-4.29	2.5371333	QTRT2	queuine tRNA-ribosyltransferase accessory subunit 2
225163_at	0.945	0.03352881	2.2555659	-4.29	2.4074592	FRMD4A	FERM domain containing 4A
227530_at	0.945	0.02491711	2.4162656	-4.29	2.7304437	AKAP12	A-kinase anchoring protein 12
228184_at	0.945	0.03359413	2.2546522	-4.29	1.515277	DISP1	dispatched RND transporter family member 1
202599_s_at	0.945	0.03069873	2.3030913	-4.29	2.2735249	NRIP1	nuclear receptor interacting protein 1
239987_at	0.945	0.03075198	2.3022784	-4.29	2.6806965		
1555194_at	0.945	0.03905279	2.1737522	-4.29	1.1350729		
205122_at	0.945	0.03631916	2.2126345	-4.29	2.1384227	MSANTD3-TMEFF1///TMEFF1	MSANTD3-TMEFF1 readthrough///transmembrane protein with EGF like and two follistatin like domains 1
204908_s_at	0.945	0.036397	2.2116282	-4.29	1.8560248	MIR8085///BCL3	microRNA 8085///B-cell CLL/lymphoma 3

1559870_at	0.945	0.02174149	2.4899459	-4.29	2.6859103	LOC100129129	uncharacterized LOC100129129
221192_x_at	0.945	0.03641022	2.2114575	-4.29	3.7208794	MFSD11	major facilitator superfamily domain containing 11
226719_at	0.945	0.03920365	2.171937	-4.29	1.3584773	DERL2	derlin 2
236101_at	0.945	0.01929971	2.557503	-4.29	2.5670834		
213726_x_at	0.945	0.03924614	2.1714268	-4.29	1.2362894	TUBB4B	tubulin beta 4B class IVb
211241_at	0.945	0.0219076	2.4863712	-4.29	3.1769329	ANXA2P3	annexin A2 pseudogene 3
1562601_at	0.945	0.03401695	2.2487773	-4.29	2.4798485	LINC01121	long intergenic non-protein coding RNA 1121
243654_at	0.945	0.03940068	2.1695754	-4.29	2.3503358		
211213_at	0.945	0.03943235	2.1691969	-4.3	1.2637599	ORC5	origin recognition complex subunit 5
239756_at	0.945	0.03359492	2.2546413	-4.3	1.5822524		
236635_at	0.945	0.03670118	2.2077144	-4.3	2.0615975	ZNF667	zinc finger protein 667
213957_s_at	0.945	0.02848134	2.3453232	-4.3	2.5813836	CEP350	centrosomal protein 350
216483_s_at	0.945	0.0159928	2.6601647	-4.3	1.7612158	MYDGF	myeloid derived growth factor
212172_at	0.945	0.02543887	2.4065364	-4.3	2.9517084	AK2	adenylate kinase 2
239536_at	0.945	0.03686122	2.205667	-4.3	2.2070925		
210970_s_at	0.945	0.02211098	2.4820291	-4.3	1.5355525	IBTK	inhibitor of Bruton tyrosine kinase
232067_at	0.945	0.02868055	2.3420483	-4.3	2.5914262	FAXC	failed axon connections homolog
1558712_at	0.945	0.01969256	2.5479938	-4.3	2.2806719	TMEM134	transmembrane protein 134
234861_at	0.945	0.03393569	2.2499011	-4.3	1.3408373	LOC93463	uncharacterized LOC93463
210746_s_at	0.945	0.03712073	2.202364	-4.3	1.8021612	EPB42	erythrocyte membrane protein band 4.2
205871_at	0.945	0.02829523	2.3484018	-4.3	2.8182537	PLGLB1///PLGLB2	plasminogen-like B1///plasminogen-like B2

1564369_at	0.945	0.02836093	2.3473129	-4.3	2.8417408		
1558019_at	0.945	0.04000312	2.1624203	-4.3	1.2138241		
224664_at	0.945	0.02468045	2.420741	-4.3	2.3766638	ANAPC16	anaphase promoting complex subunit 16
229257_at	0.945	0.03171462	2.287802	-4.3	2.9020535	TNRC18	trinucleotide repeat containing 18
234015_at	0.945	0.01712291	2.6277859	-4.3	4.5210039		
230565_at	0.945	0.03740499	2.1987699	-4.3	1.1686926	ATP6V1G3	ATPase H+ transporting V1 subunit G3
225038_s_at	0.945	0.02852409	2.3446186	-4.3	2.1625569	SURF6	surfeit 6
227280_s_at	0.945	0.03430697	2.2447858	-4.3	1.9591623	CCNYL1	cyclin Y like 1
224408_at	0.945	0.011289	2.8645852	-4.3	2.9309934	MCHR2	melanin concentrating hormone receptor 2
234853_s_at	0.945	0.01873972	2.5713786	-4.3	3.354039	EFCAB6	EF-hand calcium binding domain 6
244481_at	0.945	0.02920118	2.3335873	-4.3	1.9410388		
233015_at	0.945	0.02328781	2.4576138	-4.3	2.2092602	MBNL1	muscleblind like splicing regulator 1
220359_s_at	0.945	0.04036492	2.1581695	-4.3	1.6052987	ARPP21	cAMP regulated phosphoprotein 21
1555497_a_at	0.945	0.04038336	2.1579538	-4.3	1.9558808	CYP4B1	cytochrome P450 family 4 subfamily B member 1
239635_at	0.945	0.03760538	2.1962509	-4.3	1.5288549	RBM14	RNA binding motif protein 14
202714_s_at	0.945	0.04038923	2.1578851	-4.3	2.0927062	KIAA0391	KIAA0391
231738_at	0.945	0.03501787	2.23513	-4.3	2.2165331	PCDHB7	protocadherin beta 7
228692_at	0.945	0.01411506	2.7364214	-4.3	2.1464612	PREX2	phosphatidylinositol-3,4,5-trisphosphate dependent Rac exchange factor 2

229129_at	0.945	0.01453646	2.7052698	-4.3	3.3085845	HNRNPD	heterogeneous nuclear ribonucleoprotein D
1568686_at	0.945	0.03212561	2.2817435	-4.3	1.9358204	ATP8B5P	ATPase phospholipid transporting 8B5, pseudogene
212820_at	0.945	0.03777791	2.1940919	-4.3	4.2364165	DMXL2	Dmx like 2
210908_s_at	0.945	0.03119565	2.2955557	-4.3	3.3030088	PFDN5	prefoldin subunit 5
209563_x_at	0.945	0.01006291	2.8973342	-4.3	3.6348067	CALM3///CALM2///CALM1	calmodulin 3///calmodulin 2///calmodulin 1
233039_at	0.945	0.02905774	2.3359045	-4.3	2.5599276		
243702_at	0.945	0.0108628	2.8831957	-4.3	3.5710064		
218174_s_at	0.945	0.0324915	2.2764091	-4.3	3.0931452	TMEM254	transmembrane protein 254
209352_s_at	0.945	0.02746736	2.3623266	-4.3	3.6068851	SIN3B	SIN3 transcription regulator family member B
230284_at	0.945	0.03503085	2.2349553	-4.3	4.2352776	MYOM3	myomesin 3
202725_at	0.945	0.03256687	2.275317	-4.3	1.6925594	POLR2A	RNA polymerase II subunit A
228569_at	0.945	0.0267202	2.3834077	-4.3	1.6078542	PAPOLA	poly(A) polymerase alpha
237844_at	0.945	0.04108718	2.149785	-4.3	1.030945	LOC100506639///ZNF131	uncharacterized LOC100506639///zinc finger protein 131
231032_at	0.945	0.03834617	2.1870431	-4.3	2.1613553	LOC286071	uncharacterized LOC286071
220828_s_at	0.945	0.0193232	2.5569293	-4.3	1.5622023	LOC55338	uncharacterized LOC55338
239876_at	0.945	0.04115944	2.1489535	-4.3	1.3351794		
227130_s_at	0.945	0.02685486	2.381037	-4.3	3.9118777	TLE1	transducin like enhancer of split 1
232537_x_at	0.945	0.0207471	2.5233253	-4.3	1.9383699	MARK3	microtubule affinity regulating kinase 3

214935_at	0.945	0.00889795	2.979381	-4.3	2.749165	IL4I1///NUP62	interleukin 4 induced 1///nucleoporin 62
230658_at	0.945	0.03852922	2.1847926	-4.3	1.451371	SLC7A2	solute carrier family 7 member 2
211359_s_at	0.945	0.02410452	2.4413385	-4.3	2.5268296	OPRM1	opioid receptor mu 1
240952_at	0.945	0.04147129	2.1453796	-4.3	1.0468885		
230776_at	0.945	0.0386616	2.1831711	-4.3	1.7209565	RNF157-AS1	RNF157 antisense RNA 1
226707_at	0.945	0.02969593	2.3256745	-4.3	2.3627787	NAPRT	nicotinate phosphoribosyltransferase
207733_x_at	0.945	0.03874277	2.1821793	-4.3	1.6240047	PSG9	pregnancy specific beta-1-glycoprotein 9
220647_s_at	0.945	0.02971465	2.3253775	-4.3	2.4961333	COA4	cytochrome c oxidase assembly factor 4 homolog
238181_at	0.945	0.00377479	3.4716897	-4.3	3.9300692		
241948_at	0.945	0.01971628	2.5474252	-4.3	3.4356032		
1554522_at	0.945	0.02359662	2.4417601	-4.3	2.6957488	CNNM2	cyclin and CBS domain divalent metal cation transport mediator 2
204541_at	0.945	0.03223512	2.2801412	-4.3	2.2444457	SEC14L2	SEC14 like lipid binding 2
240430_at	0.945	0.01495789	2.7086418	-4.3	2.8155164	KCMF1	potassium channel modulatory factor 1
208169_s_at	0.945	0.02118884	2.5133411	-4.3	2.9778828	PTGER3	prostaglandin E receptor 3
204676_at	0.945	0.02119881	2.513118	-4.3	2.9404072	TMEM186	transmembrane protein 186
211195_s_at	0.945	0.02383009	2.4467491	-4.3	2.8093181	TP63	tumor protein p63
221408_x_at	0.945	0.02743488	2.3709498	-4.3	2.5875072	PCDHB12	protocadherin beta 12
240314_at	0.945	0.02750088	2.3698145	-4.3	2.8262921		
214214_s_at	0.945	0.0360517	2.2214006	-4.3	2.4891523	C1QBP	complement C1q binding protein
205186_at	0.945	0.03248332	2.2765278	-4.31	2.3239214	DNALI1	dynein axonemal light intermediate chain 1

206955_at	0.945	0.01154152	2.8538785	-4.31	3.9360021	LOC101930168///LOC100509620///AQP7	putative aquaporin-7-like protein 3///putative aquaporin-7-like protein 3///aquaporin 7
223671_x_at	0.945	0.04237276	2.1351827	-4.31	3.1463144	DPH5	diphthamide biosynthesis 5
204663_at	0.945	0.04239263	2.1349601	-4.31	1.6210076	ME3	malic enzyme 3
241718_x_at	0.945	0.04247075	2.134086	-4.31	2.3013574		
225328_at	0.945	0.01533471	2.6967048	-4.31	3.6755036	FBXO32	F-box protein 32
1553566_at	0.945	0.03645904	2.2160891	-4.31	3.048731	PIANP	PILR alpha associated neural protein
210044_s_at	0.945	0.04257164	2.1329592	-4.31	2.6228328	LYL1	LYL1, basic helix-loop-helix family member
207572_at	0.945	0.03399756	2.2550135	-4.31	1.3101176	EFCAB2	EF-hand calcium binding domain 2
236816_at	0.945	0.02176112	2.5006936	-4.31	3.5598451	NAA25	N(alpha)-acetyltransferase 25, NatB auxiliary subunit
208200_at	0.945	0.02805329	2.3604101	-4.31	2.0062097	IL1A	interleukin 1 alpha
208977_x_at	0.945	0.03986831	2.168616	-4.31	1.4301783	TUBB4B	tubulin beta 4B class IVb
218226_s_at	0.945	0.03718577	2.2067446	-4.31	3.5047982	NDUFB4	NADH:ubiquinone oxidoreductase subunit B4
218670_at	0.945	0.04278422	2.1305929	-4.31	1.0050674	PUS1	pseudouridylate synthase 1
1554208_at	0.945	0.02813706	2.3589989	-4.31	2.3235155	MEI1	meiotic double-stranded break formation protein 1
243454_at	0.945	0.04289122	2.1294057	-4.31	1.2642756		
234944_s_at	0.945	0.04289731	2.1293382	-4.31	1.4544066	MTFR2	mitochondrial fission regulator 2
237151_s_at	0.945	0.03689794	2.2104257	-4.31	2.6433591	CFAP221	cilia and flagella associated protein 221
1562396_at	0.945	0.01903332	2.5774101	-4.31	3.0939194		

237395_at	0.945	0.02551773	2.4143538	-4.31	2.8359403	CYP4Z1	cytochrome P450 family 4 subfamily Z member 1
210461_s_at	0.945	0.01205291	2.832875	-4.31	3.8510284	ABLIM1	actin binding LIM protein 1
227019_at	0.945	0.03461688	2.2464652	-4.31	2.2533947	C1orf226	chromosome 1 open reading frame 226
240263_at	0.945	0.04042505	2.162034	-4.31	3.2833473		
235463_s_at	0.945	0.02858228	2.3515633	-4.31	2.5400339	CERS6	ceramide synthase 6
226343_at	0.945	0.04342808	2.1234892	-4.31	2.2159607	DPP8	dipeptidyl peptidase 8
200716_x_at	0.945	0.04065671	2.1593192	-4.31	1.8930335	RPL13AP5///SNORD32A///SNORD33///SNORD34///SNORD35A///RPL13A	ribosomal protein L13a pseudogene 5///small nucleolar RNA, C/D box 32A///small nucleolar RNA, C/D box 33///small nucleolar RNA, C/D box 34///small nucleolar RNA, C/D box 35A///ribosomal protein L13a
1554455_at	0.945	0.00981005	2.9589092	-4.31	3.0564771	LINS1	lines homolog 1
201877_s_at	0.945	0.01601962	2.6757103	-4.31	2.3472662	PPP2R5C	protein phosphatase 2 regulatory subunit B'gamma
203844_at	0.945	0.04364236	2.1211459	-4.31	1.3849703	VHL	von Hippel-Lindau tumor suppressor
214275_at	0.945	0.01832219	2.5819815	-4.31	2.9475583	MED12	mediator complex subunit 12
242671_at	0.945	0.03160572	2.2962248	-4.31	2.8094583		
203686_at	0.945	0.03403807	2.2544501	-4.31	2.1992645	MPG	N-methylpurine DNA glycosylase
233239_at	0.945	0.03182772	2.292908	-4.31	3.8306868		
201979_s_at	0.945	0.03546486	2.2349838	-4.31	1.7933333	PPP5C	protein phosphatase 5 catalytic subunit
213136_at	0.945	0.03574325	2.2312689	-4.31	2.227072	PTPN2	protein tyrosine phosphatase, non-receptor type 2

211417_x_at	0.945	0.03284993	2.2779044	-4.31	2.4561354	GGT2///GGTLC1///GGTLC2///GGT1	gamma-glutamyltransferase 2///gamma-glutamyltransferase light chain 1///gamma-glutamyltransferase light chain 2///gamma-glutamyltransferase 1
224391_s_at	0.945	0.03578074	2.2307707	-4.31	2.5076171	SIAE	sialic acid acetylesterase
229059_at	0.945	0.02657345	2.3950884	-4.31	2.4831778	FAM225B///FAM225A	family with sequence similarity 225 member B (non-protein coding)///family with sequence similarity 225 member A (non-protein coding)
218667_at	0.945	0.02841618	2.3543247	-4.31	2.1245439	PJA1	praja ring finger ubiquitin ligase 1
212441_at	0.945	0.03848094	2.1904937	-4.31	1.6215216	KIAA0232	KIAA0232
228279_s_at	0.945	0.02609211	2.4037822	-4.31	2.2532056		
1570263_at	0.945	0.04484449	2.1081877	-4.31	1.0580362		
1557828_a_at	0.945	0.02200287	2.4954448	-4.32	3.3343116	TMEM267	transmembrane protein 267
1557636_a_at	0.945	0.01530062	2.697773	-4.32	2.7608318	C7orf57	chromosome 7 open reading frame 57
1563001_at	0.945	0.03272306	2.2797431	-4.32	1.9418412		
1569630_a_at	0.945	0.01533511	2.6966921	-4.32	3.5217145	RUFY2	RUN and FYVE domain containing 2
1569739_at	0.945	0.03892628	2.1850199	-4.32	2.2271954	LOC221946	uncharacterized LOC221946
1555855_at	0.945	0.04513854	2.1050653	-4.32	1.3335747	AKR1C1	aldo-keto reductase family 1 member C1
215439_x_at	0.945	0.03341716	2.2697621	-4.32	3.2112266		
241013_at	0.945	0.04516786	2.1047549	-4.32	2.9791111	FAM124A	family with sequence similarity 124 member A

223092_at	0.945	0.0452419	2.103972	-4.32	1.4506559	ANKH	ANKH inorganic pyrophosphate transport regulator
239267_at	0.945	0.02372478	2.4595513	-4.32	1.4899651	NEK6	NIMA related kinase 6
212226_s_at	0.945	0.02720677	2.38387	-4.32	3.0456063	PLPP3	phospholipid phosphatase 3
1566183_at	0.945	0.04535373	2.1027917	-4.32	1.3399139		
214323_s_at	0.945	0.04541806	2.1021139	-4.32	1.3296009	UPF3A	UPF3 regulator of nonsense transcripts homolog A (yeast)
240659_x_at	0.945	0.04541942	2.1020996	-4.32	1.0618681		
210942_s_at	0.945	0.04544898	2.1017885	-4.32	3.0573948	ST3GAL6	ST3 beta-galactoside alpha-2,3-sialyltransferase 6
219285_s_at	0.945	0.04544957	2.1017823	-4.32	1.3224678	NIN	ninein
225849_s_at	0.945	0.02383944	2.4572488	-4.32	3.1529308	SFT2D1	SFT2 domain containing 1
227668_at	0.945	0.04548652	2.1013936	-4.32	1.1558752	TEPSIN	TEPSIN, adaptor related protein complex 4 accessory protein
204310_s_at	0.945	0.01718388	2.6419189	-4.32	3.0297433	NPR2	natriuretic peptide receptor 2
228375_at	0.945	0.04258715	2.1372219	-4.32	1.9426202	IGSF11	immunoglobulin superfamily member 11
203880_at	0.945	0.02466702	2.4304255	-4.32	2.3955059	POPDC2///COX17	popeye domain containing 2///COX17, cytochrome c oxidase copper chaperone
204182_s_at	0.945	0.03326108	2.2719899	-4.32	2.5917129	ZBTB43	zinc finger and BTB domain containing 43
213929_at	0.945	0.04565909	2.0995819	-4.32	4.0915886	EXPH5	exophilin 5
1566947_at	0.945	0.03391256	2.2627539	-4.32	2.4108297		
212399_s_at	0.945	0.02093456	2.5318277	-4.32	3.2987332	VGLL4	vestigial like family member 4

1562910_at	0.945	0.03696634	2.215253	-4.32	1.8330145	SH3PXD2B	SH3 and PX domains 2B
215602_at	0.945	0.02016668	2.5497465	-4.32	2.5918232	FGD2	FYVE, RhoGEF and PH domain containing 2
211345_x_at	0.945	0.03959843	2.1768645	-4.32	1.3488006	MIR3654///EEF1G	microRNA 3654///eukaryotic translation elongation factor 1 gamma
207891_s_at	0.945	0.01743108	2.6350268	-4.32	2.5107948	HAUS7///TREX2	HAUS augmin like complex subunit 7///three prime repair exonuclease 2
239788_at	0.945	0.01584376	2.6810175	-4.32	3.8428971		
233521_at	0.945	0.01986527	2.5438695	-4.32	3.3964305		
211372_s_at	0.945	0.04611939	2.0947796	-4.32	1.0043787	IL1R2	interleukin 1 receptor type 2
228862_at	0.945	0.02784894	2.3727396	-4.32	2.1420976	TSNARE1	t-SNARE domain containing 1
226709_at	0.945	0.04617284	2.0942247	-4.32	1.0734348	ROBO2	roundabout guidance receptor 2
204922_at	0.945	0.03994018	2.1727657	-4.32	3.0115754	C11orf80	chromosome 11 open reading frame 80
242147_at	0.945	0.02291913	2.47603	-4.32	3.0478573		
206104_at	0.945	0.02050622	2.5417449	-4.32	2.8389944	ISL1	ISL LIM homeobox 1
231699_at	0.945	0.03743697	2.2092182	-4.32	3.14023	NFKBIA	NFKB inhibitor alpha
209280_at	0.945	0.01774799	2.6263263	-4.32	3.2689306	MRC2	mannose receptor C type 2
237373_at	0.945	0.02137732	2.5217762	-4.32	2.7066295	RAB20	RAB20, member RAS oncogene family
224891_at	0.945	0.0465248	2.0905851	-4.32	1.5899563	FOXO3	forkhead box O3
1556828_at	0.945	0.04659415	2.0898708	-4.32	1.4085482		
212498_at	0.945	0.01387694	2.7644117	-4.32	3.6906412	6-Mar	membrane associated ring-CH-type finger 6

230320_at	0.945	0.04366491	2.1252732	-4.32	2.43734	TBRG1	transforming growth factor beta regulator 1
212613_at	0.945	0.03136618	2.3073574	-4.32	2.5904881	BTN3A2	butyrophilin subfamily 3 member A2
1556821_x_at	0.945	0.02072973	2.5365459	-4.32	3.5988118	DLEU2	deleted in lymphocytic leukemia 2 (non-protein coding)
240646_at	0.945	0.0179258	2.6215094	-4.32	2.0026677	GIMAP8	GTPase, IMAP family member 8
237998_at	0.945	0.02325892	2.4690147	-4.32	3.5925471		
1565633_at	0.945	0.01399705	2.7602147	-4.32	3.1477061		
230655_at	0.945	0.03433372	2.256869	-4.32	2.0385708		
209508_x_at	0.945	0.04689695	2.0867631	-4.32	2.8955263	CFLAR	CASP8 and FADD like apoptosis regulator
203648_at	0.945	0.04690028	2.0867291	-4.32	2.0255751	TATDN2	TatD DNase domain containing 2
233866_at	0.945	0.03037483	2.322669	-4.32	2.7374918	KLHL5	kelch like family member 5
208216_at	0.945	0.02786616	2.3724444	-4.32	2.3810391	DLX4	distal-less homeobox 4
243733_at	0.945	0.03263488	2.2810249	-4.32	2.5515577		
224755_at	0.945	0.03453243	2.2541152	-4.32	2.2680393	TM9SF3	transmembrane 9 superfamily member 3
216724_at	0.945	0.04716572	2.0840198	-4.32	1.3723627	DCLK2	doublecortin like kinase 2
1569132_s_at	0.945	0.0408651	2.1618294	-4.32	1.6738466	ARSK	arylsulfatase family member K
231553_s_at	0.945	0.03461814	2.2529319	-4.32	2.6390377	MICAL3	microtubule associated monooxygenase, calponin and LIM domain containing 3
1553569_at	0.945	0.0219215	2.5096878	-4.32	2.5176768	COX2	cytochrome c oxidase subunit II
237188_x_at	0.945	0.04427325	2.1186449	-4.32	1.2550605	SUN5	Sad1 and UNC84 domain containing 5
215229_at	0.945	0.01510514	2.7230563	-4.32	3.3423561	LOC100129973	uncharacterized LOC100129973

219731_at	0.945	0.04738013	2.0818412	-4.32	1.6843068	ENTPD1-AS1	ENTPD1 antisense RNA 1
236260_at	0.945	0.04740801	2.0815586	-4.32	1.3387525		
211040_x_at	0.945	0.03849664	2.1958783	-4.32	2.7760099	GTSE1	G2 and S-phase expressed 1
217101_at	0.945	0.02131725	2.5231282	-4.32	2.4732877	TTC39A	tetratricopeptide repeat domain 39A
240033_at	0.945	0.02839347	2.3634865	-4.32	2.4993959	PLG	plasminogen
1557030_at	0.945	0.03099257	2.3130748	-4.32	2.9178093	GAB1	GRB2 associated binding protein 1
232590_at	0.945	0.0186421	2.6025552	-4.32	3.3136088		
1555431_a_at	0.945	0.04783247	2.0772737	-4.32	1.2305081	IL31RA	interleukin 31 receptor A
1561689_at	0.945	0.04784159	2.0771821	-4.32	1.4805485		
233455_at	0.945	0.01456641	2.7407813	-4.32	3.5343189		
1557066_at	0.945	0.04789729	2.0766224	-4.32	1.1267185	LUC7L	LUC7 like
236879_at	0.945	0.02927078	2.348919	-4.32	2.7144713		
206744_s_at	0.945	0.04793835	2.0762102	-4.32	1.4111413	ZMYM5	zinc finger MYM-type containing 5
1558779_at	0.945	0.03891696	2.1906792	-4.32	1.5175738	H2AFY	H2A histone family member Y
217838_s_at	0.945	0.02870907	2.3581985	-4.33	2.5527767	EVL	Enah/Vasp-like
218643_s_at	0.945	0.02123866	2.5122275	-4.33	2.7369707	CRIPT	CXXC repeat containing interactor of PDZ3 domain
237746_at	0.945	0.04517345	2.1089846	-4.33	1.6225625		
239342_at	0.945	0.03916353	2.1876527	-4.33	2.3755097	DGKZ	diacylglycerol kinase zeta
219314_s_at	0.945	0.0327412	2.2868407	-4.33	2.1270423	ZNF219	zinc finger protein 219
210212_x_at	0.945	0.0226876	2.493141	-4.33	2.8402365	CMC4	C-X9-C motif containing 4
1570607_at	0.945	0.03924446	2.1866631	-4.33	2.0171351		
223260_s_at	0.945	0.04831606	2.0724331	-4.33	1.4894806	POLK	DNA polymerase kappa
205516_x_at	0.945	0.01547324	2.6757892	-4.33	3.2573788	CIZ1	CDKN1A interacting zinc finger protein 1

236483_at	0.945	0.04834869	2.0721079	-4.33	1.1102974		
227391_x_at	0.945	0.04536188	2.1069843	-4.33	2.1801303	LRRFIP1	LRR binding FLII interacting protein 1
1563528_at	0.945	0.03935046	2.1853698	-4.33	2.6155984	RAPGEF4-AS1	RAPGEF4 antisense RNA 1
215998_at	0.945	0.04842746	2.071324	-4.33	2.7571697		
211849_s_at	0.945	0.04543537	2.1062063	-4.33	1.3895409	RNGTT	RNA guanylyltransferase and 5'-phosphatase
242343_x_at	0.945	0.02683625	2.3904034	-4.33	3.0264658		
214522_x_at	0.945	0.01493369	2.7286303	-4.33	3.6175257	HIST1H3D///HIST1H2AD	histone cluster 1, H3d///histone cluster 1, H2ad
202906_s_at	0.945	0.01218431	2.8517924	-4.33	3.6891292	NBN	nibrin
237293_at	0.945	0.0329837	2.2833049	-4.33	1.8890737		
211817_s_at	0.945	0.04271109	2.1406529	-4.33	2.0618876	KCNJ5	potassium voltage-gated channel subfamily J member 5
238201_at	0.945	0.04858164	2.0697929	-4.33	3.4872195	LOC105376975	uncharacterized LOC105376975
205066_s_at	0.945	0.04220756	2.1463462	-4.33	1.9757266	ENPP1	ectonucleotide pyrophosphatase/phosphodiesterase 1
56829_at	0.945	0.02297294	2.4871126	-4.33	2.6794198	TRAPPC9	trafficking protein particle complex 9
1553338_at	0.945	0.04235857	2.1446324	-4.33	2.4531746	SDE2	SDE2 telomere maintenance homolog
223306_at	0.945	0.04877963	2.067833	-4.33	1.5229489	EBPL	emopamil binding protein like
1558920_at	0.945	0.04878361	2.0677936	-4.33	1.2616179	SLC8A1-AS1	SLC8A1 antisense RNA 1
217863_at	0.945	0.03006781	2.3360306	-4.33	2.1848861	PIAS1	protein inhibitor of activated STAT 1
244666_at	0.945	0.0264388	2.4076517	-4.33	2.7402882		
1558354_s_at	0.945	0.04252573	2.1427417	-4.33	3.421413	PLCG1-AS1	PLCG1 antisense RNA 1
243840_at	0.945	0.02921958	2.3411037	-4.33	2.677362	CLSPN	claspin
1559156_at	0.945	0.0489467	2.0661846	-4.33	1.520058		

204239_s_at	0.945	0.04895627	2.0660903	-4.33	1.1665925	NNAT	neuronatin
210935_s_at	0.945	0.04600077	2.1002571	-4.33	1.1675607	WDR1	WD repeat domain 1
236207_at	0.945	0.01620984	2.6885409	-4.33	3.7106613	SSFA2	sperm specific antigen 2
241239_at	0.945	0.04915357	2.0641505	-4.33	1.6041053	EPN2-IT1///EPN2	EPN2 intronic transcript 1///epsin 2
234249_at	0.945	0.04614176	2.0987838	-4.33	2.8852116		
213142_x_at	0.945	0.02512372	2.4321444	-4.33	3.0028075	GSAP	gamma-secretase activating protein
218302_at	0.945	0.03036252	2.331345	-4.33	2.8993765	PSENEN	presenilin enhancer gamma-secretase subunit
234250_at	0.945	0.03360774	2.274314	-4.33	2.3829455	LOC100506405	uncharacterized LOC100506405
1557652_a_at	0.945	0.04933389	2.0623836	-4.33	1.4221713	CCDC13-AS1	CCDC13 antisense RNA 1
216340_s_at	0.945	0.02522015	2.4303083	-4.33	3.710516	CYP2A7P1	cytochrome P450 family 2 subfamily A member 7 pseudogene 1
1559800_a_at	0.945	0.04022834	2.174778	-4.33	1.7827923		
240077_at	0.945	0.01971301	2.5754736	-4.33	3.3249033		
206077_at	0.945	0.03239984	2.2918593	-4.33	4.8643027	KEL	Kell blood group, metallo-endopeptidase
229586_at	0.945	0.04949215	2.0608376	-4.33	2.3146835	CHD9	chromodomain helicase DNA binding protein 9
216950_s_at	0.945	0.01284443	2.8020128	-4.33	3.1476293	FCGR1CP///FCGR1B///FCGR1A	Fc fragment of IgG receptor Ic, pseudogene///Fc fragment of IgG receptor Ib///Fc fragment of IgG receptor Ia
200060_s_at	0.945	0.02992233	2.3383594	-4.33	2.1856376	RNPS1	RNA binding protein with serine rich domain 1

223086_x_at	0.945	0.04046763	2.1719272	-4.33	2.3357746	MRPL51	mitochondrial ribosomal protein L51
228031_at	0.945	0.00308362	3.6986861	-4.33	5.1490482	TTPAL	alpha tocopherol transfer protein like
208123_at	0.945	0.03257219	2.2893194	-4.33	2.5177589	KCNB2	potassium voltage-gated channel subfamily B member 2
1557720_s_at	0.945	0.0438034	2.1285068	-4.33	2.0770323	MYO16	myosin XVI
208181_at	0.945	0.02036183	2.5451323	-4.33	3.2429252	HIST2H4B///HIST4H4///HIST2H4A///HIST1H4L///HIST1H4E///HIST1H4B///HIST1H4H///HIST1H4C///HIST1H4J///HIST1H4K///HIST1H4F///HIST1H4D///HIST1H4A///HIST1H4I	histone cluster 2, H4b///histone cluster 4, H4///histone cluster 2, H4a///histone cluster 1, H4l///histone cluster 1, H4e///histone cluster 1, H4b///histone cluster 1, H4h///histone cluster 1, H4c///histone cluster 1, H4j///histone cluster 1, H4k///histone cluster 1, H4f///histone cluster 1, H4d///histone cluster 1, H4a///histone cluster 1, H4i
241915_at	0.945	0.04054703	2.1709847	-4.33	1.965803	ACSM2B	acyl-CoA synthetase medium-chain family member 2B
213430_at	0.945	0.04974126	2.0584131	-4.33	2.0865779	RUFY3	RUN and FYVE domain containing 3
211673_s_at	0.945	0.04675174	2.0924556	-4.33	2.4727814	MOCS1	molybdenum cofactor synthesis 1
244078_at	0.945	0.04980541	2.0577904	-4.33	1.4275301		
1563828_at	0.945	0.03406322	2.2678479	-4.33	2.6252162		
222517_at	0.945	0.03755816	2.213878	-4.33	1.8489222	AP3M1	adaptor related protein complex 3 mu 1 subunit
231209_at	0.945	0.02789081	2.3720222	-4.33	2.4234446	EXOC3L4	exocyst complex component 3 like 4

243500_at	0.945	0.04403713	2.1259432	-4.33	2.0649075		
210320_s_at	0.945	0.03705713	2.2203319	-4.33	2.3687258	DDX52	DEAD-box helicase 52
201464_x_at	0.945	0.03284941	2.28526	-4.33	2.5075115	JUN	Jun proto-oncogene, AP-1 transcription factor subunit
207048_at	0.945	0.02734042	2.391508	-4.33	1.5619507	SLC6A11	solute carrier family 6 member 11
213299_at	0.945	0.04423777	2.1237523	-4.33	1.6291186	ZBTB7A	zinc finger and BTB domain containing 7A
206877_at	0.945	0.04713891	2.088477	-4.33	1.3765242	MXD1	MAX dimerization protein 1
235193_at	0.945	0.04719135	2.0879404	-4.33	2.2517994	FAM214A	family with sequence similarity 214 member A
243641_at	0.945	0.02587654	2.417981	-4.33	1.4244259		
217394_at	0.945	0.03120212	2.3182228	-4.33	2.7046805		
239608_at	0.945	0.04723354	2.0875091	-4.33	1.5686814	LPCAT4	lysophosphatidylcholine acyltransferase 4
234725_s_at	0.945	0.02330469	2.4801912	-4.33	3.3809213	SEMA4B	semaphorin 4B
1555794_at	0.945	0.03053176	2.3286732	-4.33	2.5985833	ARL17B///ARL17A	ADP ribosylation factor like GTPase 17B///ADP ribosylation factor like GTPase 17A
214314_s_at	0.945	0.01320241	2.8119932	-4.33	3.0643305	EIF5B	eukaryotic translation initiation factor 5B
203220_s_at	0.945	0.0474792	2.0850042	-4.33	1.1944273	TLE1	transducin like enhancer of split 1
201734_at	0.945	0.0446418	2.1193672	-4.33	3.8334628	CLCN3	chloride voltage-gated channel 3
1558821_s_at	0.945	0.04768627	2.0829018	-4.33	2.9575661	LOC102723694	uncharacterized LOC102723694

229865_at	0.945	0.02444812	2.4570266	-4.33	3.3443935	LOC101928615///FNDC3B	uncharacterized LOC101928615///fibronectin type III domain containing 3B
241680_at	0.945	0.03841022	2.2030799	-4.33	2.4282089		
219626_at	0.945	0.03086904	2.3233891	-4.33	3.0921147	MAP7D3	MAP7 domain containing 3
1567320_at	0.945	0.03490614	2.2560878	-4.33	2.8833443		
222397_at	0.945	0.02795503	2.3807852	-4.33	1.5686346	EFTUD2	elongation factor Tu GTP binding domain containing 2
238754_at	0.945	0.03788882	2.2096614	-4.33	1.9534474	PTCH1	patched 1
212003_at	0.945	0.02082118	2.5488849	-4.33	3.6445185	SZRD1	SUZ RNA binding domain containing 1
231311_at	0.945	0.03117301	2.3186723	-4.33	2.1094936		
233920_at	0.945	0.044701	2.1187277	-4.33	1.7619715		
216360_x_at	0.945	0.03136963	2.3156438	-4.34	2.653345	RRP12	ribosomal RNA processing 12 homolog
241625_at	0.945	0.02409672	2.4640352	-4.34	2.4212162	LOC389834	ankyrin repeat domain 57 pseudogene
236015_at	0.945	0.04839205	2.0757966	-4.34	1.8154521	PCNX4	pecanex homolog 4 (Drosophila)
231599_x_at	0.945	0.04842135	2.0755036	-4.34	1.7369627	DPF1	double PHD fingers 1
240245_at	0.945	0.04494659	2.1160828	-4.34	1.8756621		
208335_s_at	0.945	0.02682644	2.4006491	-4.34	2.7568657	ACKR1	atypical chemokine receptor 1 (Duffy blood group)
229734_at	0.945	0.02845161	2.3722808	-4.34	4.0786302	MIR4697///MIR4697HG	microRNA 4697///MIR4697 host gene
204857_at	0.945	0.04502792	2.1152098	-4.34	1.8012021	MAD1L1	MAD1 mitotic arrest deficient like 1
1558680_s_at	0.945	0.03921589	2.1930682	-4.34	2.2217556	PDE1A	phosphodiesterase 1A

1566709_at	0.945	0.02427875	2.4603927	-4.34	3.4564454		
236091_at	0.945	0.03164364	2.3114525	-4.34	3.0623658	HMGB2	high mobility group box 2
220350_at	0.945	0.04243879	2.1490061	-4.34	2.2896345	ZNF235	zinc finger protein 235
205885_s_at	0.945	0.02432733	2.459425	-4.34	3.6606467	ITGA4	integrin subunit alpha 4
243637_at	0.945	0.04263552	2.1467716	-4.34	2.0547066		
224565_at	0.945	0.04268833	2.1461733	-4.34	2.3539163	MIR612///NEAT1	microRNA 612///nuclear paraspeckle assembly transcript 1 (non-protein coding)
1556988_s_at	0.945	0.04602713	2.1045974	-4.34	4.0146723	CHD1L	chromodomain helicase DNA binding protein 1 like
1555179_at	0.945	0.01415773	2.7546543	-4.34	2.3943297	IGHV7-81	immunoglobulin heavy variable 7-81 (non-functional)
238008_at	0.945	0.03609336	2.2399576	-4.34	2.5146031	PRR18	proline rich 18
240455_at	0.945	0.02900319	2.3629949	-4.34	1.7515582		
1563681_at	0.945	0.04916964	2.0680746	-4.34	1.5684753		
234185_at	0.945	0.02161308	2.5306955	-4.34	2.9284406		
241270_at	0.945	0.04299586	2.1427026	-4.34	4.1173323		
243860_at	0.945	0.03627668	2.2375103	-4.34	2.5026397		
220686_s_at	0.945	0.0372336	2.2180498	-4.34	2.3294938	PIWIL2	piwi like RNA-mediated gene silencing 2
230726_at	0.945	0.03207823	2.296632	-4.34	2.2532832	MRPL38	mitochondrial ribosomal protein L38
209212_s_at	0.945	0.04319684	2.1404464	-4.34	1.2732476	KLF5	Kruppel like factor 5
232791_at	0.945	0.04954949	2.0643419	-4.34	2.2356196		
1561045_a_at	0.945	0.03651563	2.2343372	-4.34	2.9683797	LOC101928973	uncharacterized LOC101928973
236606_at	0.945	0.03222982	2.294377	-4.34	1.9890417		

1554314_at	0.945	0.02776539	2.3840703	-4.34	2.7070128	C6orf141	chromosome 6 open reading frame 141
242392_at	0.945	0.0395445	2.1890382	-4.34	1.9598692	UBE2U	ubiquitin conjugating enzyme E2 U (putative)
226086_at	0.945	0.0366376	2.2327249	-4.34	2.6143222	SYT13	synaptotagmin 13
227769_at	0.945	0.03342177	2.2850398	-4.34	2.3805513	GPR27	G protein-coupled receptor 27
233774_at	0.945	0.03979055	2.1860406	-4.34	1.2020796		
214493_s_at	0.945	0.04370337	2.1348019	-4.34	2.1653329	PATJ	PATJ, crumbs cell polarity complex component
209638_x_at	0.945	0.04707295	2.0937088	-4.34	2.5368324	RGS12	regulator of G-protein signaling 12
1561414_at	0.945	0.02622269	2.4230173	-4.34	2.2619158	LINC01242	long intergenic non-protein coding RNA 1242
216016_at	0.945	0.04654888	2.0991378	-4.34	1.8626402	NLRP3	NLR family pyrin domain containing 3
203985_at	0.945	0.04062739	2.1759696	-4.34	2.3592559	ZNF212	zinc finger protein 212
225404_at	0.945	0.04063467	2.1758828	-4.34	2.2348976	SMIM12	small integral membrane protein 12
228149_at	0.945	0.02630284	2.4215327	-4.34	3.3488033	BMT2	base methyltransferase of 25S rRNA 2 homolog
219749_at	0.945	0.04261371	2.1470189	-4.34	2.5835285	SH2D4A	SH2 domain containing 4A
240718_at	0.945	0.04722474	2.0921465	-4.34	1.8644165	LRMP	lymphoid restricted membrane protein
232762_at	0.945	0.03298624	2.2913873	-4.34	2.1022894	KIAA1217	KIAA1217
216804_s_at	0.945	0.01146685	2.910691	-4.34	3.6052674	PDLIM5	PDZ and LIM domain 5
226143_at	0.945	0.03011633	2.3447442	-4.34	2.9421348	RAI1	retinoic acid induced 1
211909_x_at	0.945	0.02255706	2.5098211	-4.34	2.9856027	PTGER3	prostaglandin E receptor 3
232453_at	0.945	0.04761918	2.0881074	-4.34	3.9877446		
217142_at	0.945	0.03413028	2.2748747	-4.34	2.2753271		

1569538_at	0.945	0.0302569	2.3424842	-4.34	2.4010888		
36566_at	0.945	0.04718822	2.0925219	-4.34	2.654784	CTNS	cystinosin, lysosomal cystine transporter
229700_at	0.945	0.01915086	2.6066313	-4.34	4.1873924	ZNF738	zinc finger protein 738
224087_at	0.945	0.04794757	2.0847675	-4.34	2.1934284		
210679_x_at	0.945	0.04138151	2.1670539	-4.34	2.7271914		
241303_x_at	0.945	0.04079931	2.173924	-4.34	2.0422628		
233151_s_at	0.945	0.02886281	2.3653425	-4.34	3.0236013	TTTY7B///TTTY7	testis-specific transcript, Y-linked 7B (non-protein coding)///testis-specific transcript, Y-linked 7 (non-protein coding)
213715_s_at	0.945	0.02351812	2.4757875	-4.34	2.2541501	KANK3	KN motif and ankyrin repeat domains 3
1566893_at	0.945	0.04100715	2.1714612	-4.34	1.3188349		
212763_at	0.945	0.0410108	2.1714181	-4.34	2.2706518	CAMSAP2	calmodulin regulated spectrin associated protein family member 2
1555526_a_at	0.945	0.0307853	2.3340751	-4.34	1.8315659	SEPT6	septin 6
214251_s_at	0.945	0.04841291	2.0800696	-4.34	1.9365809	NUMA1	nuclear mitotic apparatus protein 1
1563016_at	0.945	0.03084529	2.3331287	-4.34	2.6174236		
234841_x_at	0.945	0.02578256	2.4197281	-4.34	2.9543725	OBP2A	odorant binding protein 2A
233333_x_at	0.945	0.0391673	2.1936667	-4.34	2.7728103	AVIL	advillin
244101_at	0.945	0.03170462	2.3105242	-4.34	2.1463559		
234518_at	0.945	0.03831711	2.2110076	-4.35	2.2616458		

215223_s_at	0.945	0.03687716	2.2295723	-4.35	3.2709315	LOC100129518///SOD2	uncharacterized LOC100129518///superoxide dismutase 2, mitochondrial
1568689_at	0.945	0.04522205	2.1182245	-4.35	2.4806867	LOC100631378	uncharacterized 100631378
1564463_at	0.945	0.01865175	2.619642	-4.35	3.1172561	KLF13	Kruppel like factor 13
1563397_at	0.945	0.0265928	2.416197	-4.35	3.0016277	LOC105374768	uncharacterized LOC105374768
202333_s_at	0.945	0.04409623	2.1304645	-4.35	2.0344536	UBE2B	ubiquitin conjugating enzyme E2 B
218707_at	0.945	0.03702804	2.2275963	-4.35	2.9872769	ZNF444	zinc finger protein 444
242760_x_at	0.945	0.0187389	2.6173464	-4.35	4.3544717	PIGB	phosphatidylinositol glycan anchor biosynthesis class B
227156_at	0.945	0.04219368	2.157615	-4.35	2.0675137	CASK	calcium/calmodulin dependent serine protein kinase
211851_x_at	0.945	0.03853348	2.2082732	-4.35	2.1396171	BRCA1	BRCA1, DNA repair associated
1554677_s_at	0.945	0.04549717	2.1152751	-4.35	2.2358093	CMTM4	CKLF like MARVEL transmembrane domain containing 4
1553437_at	0.945	0.04891591	2.0750366	-4.35	1.6950117	KLHDC7A	kelch domain containing 7A
244500_s_at	0.945	0.04557584	2.1144346	-4.35	1.9246375	EVI5L	ecotropic viral integration site 5 like
239890_s_at	0.945	0.02358785	2.4879513	-4.35	2.1833969	SERP2	stress-associated endoplasmic reticulum protein family member 2
203449_s_at	0.945	0.02619865	2.4120382	-4.35	1.7867887	TERF1	telomeric repeat binding factor 1
242092_at	0.945	0.0277437	2.3955426	-4.35	2.2728616	EPB41L2	erythrocyte membrane protein band 4.1 like 2
1563259_at	0.945	0.03873938	2.205684	-4.35	2.2893798		
225265_at	0.945	0.04179007	2.1622849	-4.35	2.5168903	RBMS1	RNA binding motif single stranded interacting protein 1

242759_at	0.945	0.01588973	2.6983036	-4.35	3.8480967		
242735_x_at	0.945	0.03538716	2.2573102	-4.35	2.2694345	ELF2	E74 like ETS transcription factor 2
234045_x_at	0.945	0.02377962	2.4839824	-4.35	2.6293364	BHLHE23	basic helix-loop-helix family member e23
1555078_at	0.945	0.04201099	2.1597237	-4.35	3.0332951	ZNF843	zinc finger protein 843
229228_at	0.945	0.02803046	2.3905211	-4.35	2.6419016	LOC401317///CREB5	uncharacterized LOC401317///cAMP responsive element binding protein 5
215601_at	0.945	0.01916046	2.6063843	-4.35	5.5256131		
1559151_at	0.945	0.02721098	2.4050013	-4.35	3.4313673		
228087_at	0.945	0.02399187	2.4796249	-4.35	3.6234569	CCDC126	coiled-coil domain containing 126
214247_s_at	0.945	0.0349856	2.2628587	-4.35	3.1064649	DKK3	dickkopf WNT signaling pathway inhibitor 3
233984_at	0.945	0.0301215	2.3446609	-4.35	2.893417		
209672_s_at	0.945	0.04302135	2.1481633	-4.35	1.9522018	MIOS	meiosis regulator for oocyte development
1564749_at	0.945	0.02732705	2.4029256	-4.35	1.5369815	LOC100289045	uncharacterized LOC100289045
217228_s_at	0.945	0.04979831	2.0663178	-4.35	2.180471	ASB4	ankyrin repeat and SOCS box containing 4
235559_at	0.945	0.03199027	2.3153883	-4.35	2.7642765	INMT-FAM188B///FAM188B	INMT-FAM188B readthrough (NMD candidate)///family with sequence similarity 188 member B
227704_at	0.945	0.03518142	2.2601458	-4.35	2.232176	C19orf12	chromosome 19 open reading frame 12
1553216_at	0.945	0.02053116	2.5722703	-4.35	3.3785907	ZNF41	zinc finger protein 41
1561217_at	0.945	0.0360831	2.2478296	-4.35	2.519835		

237626_at	0.945	0.01949567	2.597831	-4.35	3.9288281		
1558868_a_at	0.945	0.03964772	2.1944094	-4.35	2.3154282	DSE	dermatan sulfate epimerase
235877_at	0.945	0.03620654	2.2461656	-4.35	3.0111131		
224507_s_at	0.945	0.04069171	2.1752033	-4.35	3.7099067	MGC12916	uncharacterized protein MGC12916
222034_at	0.945	0.04959024	2.0683612	-4.35	1.1953863	SNORD96A///SNORD95///RACK1	small nucleolar RNA, C/D box 96A///small nucleolar RNA, C/D box 95///receptor for activated C kinase 1
1554722_at	0.945	0.02248038	2.5114855	-4.35	2.1259853	TRPM3	transient receptor potential cation channel subfamily M member 3
217056_at	0.945	0.04679169	2.1016085	-4.35	1.157439	YME1L1	YME1 like 1 ATPase
1568805_at	0.945	0.02869948	2.37899	-4.35	2.9091493	NCOA7	nuclear receptor coactivator 7
1553918_at	0.945	0.02873228	2.3784312	-4.35	1.8932154	LINC00479	long intergenic non-protein coding RNA 479
214336_s_at	0.945	0.03250841	2.3075527	-4.35	2.647477	COPA	coatomer protein complex subunit alpha
237678_at	0.945	0.04002374	2.1898108	-4.35	1.9705041		
220151_at	0.945	0.04308379	2.147457	-4.35	2.2588764	C19orf73	chromosome 19 open reading frame 73
1569274_at	0.945	0.04314147	2.1468053	-4.35	1.99232		
235205_at	0.945	0.04995964	2.0647386	-4.35	1.6205826	OXR1	oxidation resistance 1
230394_at	0.945	0.03090226	2.3322318	-4.35	2.790917	TCP10L	t-complex 10-like
203243_s_at	0.945	0.0366712	2.2399479	-4.35	3.2447048	PDLIM5	PDZ and LIM domain 5
210002_at	0.945	0.02907024	2.3727072	-4.35	2.310585	GATA6	GATA binding protein 6
224815_at	0.945	0.02910415	2.3721363	-4.35	1.288628	COMMD7	COMM domain containing 7
1568666_at	0.945	0.04142235	2.1665753	-4.35	2.4538788	PLIN5	perilipin 5

222039_at	0.945	0.03292428	2.3013468	-4.35	2.3679367	KIF18B	kinesin family member 18B
211662_s_at	0.945	0.02829566	2.3859197	-4.35	2.4794105	VDAC2	voltage dependent anion channel 2
243336_at	0.945	0.00881548	3.0438126	-4.35	5.9187705	LOC101928042///LOC101927579	uncharacterized LOC101928042///dynamin-1-like
209312_x_at	0.945	0.03297654	2.300572	-4.35	2.1495226	LOC101060835///LOC100996809///HLA-DRB5///HLA-DRB4///HLA-DRB1///HLA-DQB1	HLA class II histocompatibility antigen, DQ beta 1 chain-like///HLA class II histocompatibility antigen, DRB1-10 beta chain-like///major histocompatibility complex, class II, DR beta 5///major histocompatibility complex, class II, DR beta 4///major histocompatibility complex, class II, DR beta 1///major histocompatibility complex, class II, DQ beta 1
220680_at	0.945	0.040833	2.1800458	-4.35	1.3762137	RAVER2	ribonucleoprotein, PTB binding 2
1554761_a_at	0.945	0.02960062	2.3638486	-4.35	2.3491868	DNAAF5	dynein axonemal assembly factor 5
226174_at	0.945	0.02536085	2.4523644	-4.35	2.7488968	USP42	ubiquitin specific peptidase 42
211451_s_at	0.945	0.03416723	2.2743499	-4.35	2.5481482	KCNJ4	potassium voltage-gated channel subfamily J member 4
231381_at	0.945	0.02049546	2.5731307	-4.35	3.266501	MIR4454///ESRG	microRNA 4454///embryonic stem cell related (non-protein coding)
225905_s_at	0.945	0.04806034	2.0885402	-4.35	1.8610885	ST3GAL3	ST3 beta-galactoside alpha-2,3-sialyltransferase 3
219662_at	0.945	0.03751893	2.2287882	-4.35	2.5113554	C2orf49	chromosome 2 open reading frame 49
210721_s_at	0.945	0.03434658	2.2718101	-4.35	2.6999088	PAK5	p21 (RAC1) activated kinase 5

241327_at	0.945	0.03965026	2.1943782	-4.35	2.7240306	LINC01339	long intergenic non-protein coding RNA 1339
1552275_s_at	0.945	0.04217403	2.1578414	-4.35	3.4748819	PXK	PX domain containing serine/threonine kinase like
1569139_s_at	0.945	0.02355266	2.4886827	-4.35	1.9640484	FAM53A	family with sequence similarity 53 member A
231660_at	0.945	0.04222169	2.1572923	-4.35	1.3003581		
226103_at	0.945	0.01742659	2.6736168	-4.35	3.7333004	NEXN	nexilin F-actin binding protein
240067_at	0.945	0.0337515	2.2892149	-4.35	3.0378816		
204050_s_at	0.945	0.0174792	2.6721074	-4.35	2.7677864	CLTA	clathrin light chain A
223386_at	0.945	0.04138443	2.173492	-4.35	2.4023272	FAM118B	family with sequence similarity 118 member B
218998_at	0.945	0.03378645	2.2887084	-4.35	2.6263695	FAM206A	family with sequence similarity 206 member A
205506_at	0.945	0.02191479	2.5399725	-4.35	3.4830125	VIL1	villin 1
231224_x_at	0.945	0.04859329	2.0831423	-4.35	1.894464	PRKAG2	protein kinase AMP-activated non-catalytic subunit gamma 2
221785_at	0.945	0.04002482	2.1897977	-4.35	2.5460384	WIZ	widely interspaced zinc finger motifs
242595_at	0.945	0.04155362	2.1714971	-4.35	2.5073351	TSSK4	testis specific serine kinase 4
1557293_at	0.945	0.0372039	2.2329082	-4.35	1.6303457	LINC00969	long intergenic non-protein coding RNA 969
216662_at	0.945	0.04899007	2.0791579	-4.36	1.746888	MYO7B	myosin VIIB
226383_at	0.945	0.04193205	2.1670617	-4.36	2.2266414	ARL14EP	ADP ribosylation factor like GTPase 14 effector protein
218022_at	0.945	0.04222359	2.1636693	-4.36	2.0486605	VRK3	vaccinia related kinase 3

228517_at	0.945	0.04224684	2.1633998	-4.36	2.2997409	MEAF6	MYST/Esa1 associated factor 6
204409_s_at	0.945	0.01817497	2.6323859	-4.36	4.1148754	EIF1AY	eukaryotic translation initiation factor 1A, Y-linked
217448_s_at	0.945	0.02457922	2.4677537	-4.36	3.5909061	TOX4	TOX high mobility group box family member 4
206611_at	0.945	0.02279218	2.5204839	-4.36	3.3386556	C2orf27A	chromosome 2 open reading frame 27A
222127_s_at	0.945	0.04269661	2.1582103	-4.36	2.1418231	EXOC1	exocyst complex component 1
231321_s_at	0.945	0.02475717	2.4642099	-4.36	2.2222751	ACER3	alkaline ceramidase 3
210281_s_at	0.945	0.03845354	2.2167492	-4.36	2.0033983	ZMYM2	zinc finger MYM-type containing 2
219846_at	0.945	0.0384658	2.2165931	-4.36	2.6667744	GON4L	gon-4 like
239748_x_at	0.945	0.02191179	2.5400403	-4.36	4.4391662	OCIAD1	OCIA domain containing 1
225612_s_at	0.945	0.03518706	2.2687982	-4.36	2.7588431	B3GNT5	UDP-GlcNAc:betaGal beta-1,3-N-acetylglucosaminyltransferase 5
243259_at	0.945	0.02747861	2.4002278	-4.36	2.7566243	ATXN7	ataxin 7
224108_at	0.945	0.03599596	2.2490075	-4.36	3.3559356		
242054_s_at	0.945	0.03143435	2.3343203	-4.36	3.9678828	SIX3	SIX homeobox 3
225809_at	0.945	0.0186673	2.6391396	-4.36	3.4128069	PARM1	prostate androgen-regulated mucin-like protein 1
203856_at	0.945	0.04699744	2.1049279	-4.36	1.9331256	VRK1	vaccinia related kinase 1
1554241_at	0.945	0.0463075	2.1121878	-4.36	2.5261263	COCH	cochlin
227449_at	0.945	0.0441755	2.1352541	-4.36	1.4806703	EPHA4	EPH receptor A4
1553140_at	0.945	0.04706309	2.1042421	-4.36	1.9575102	PELO	pelota homolog (Drosophila)
214231_s_at	0.945	0.04640865	2.1111174	-4.36	2.7702762	VWA8	von Willebrand factor A domain containing 8

216565_x_at	0.945	0.04329045	2.1514339	-4.36	2.1593526		
206677_at	0.945	0.04646729	2.1104979	-4.36	1.2103678	KRT31	keratin 31
236130_at	0.945	0.03558821	2.2632304	-4.36	2.5879551	SNORA37	small nucleolar RNA, H/ACA box 37
237667_at	0.945	0.02525883	2.4543474	-4.36	3.4264625		
229166_s_at	0.945	0.04666391	2.1084254	-4.36	2.1600001	GATAD2A	GATA zinc finger domain containing 2A
1554711_at	0.945	0.02238212	2.5295017	-4.36	2.7608163	CALHM3	calcium homeostasis modulator 3
220880_at	0.945	0.04211458	2.1649352	-4.36	2.6987252		
52731_at	0.945	0.03921876	2.2070898	-4.36	2.9890385	AMBRA1	autophagy and beclin 1 regulator 1
207307_at	0.945	0.04762476	2.0984097	-4.36	2.9044663	HTR2C	5-hydroxytryptamine receptor 2C
216630_at	0.945	0.04764323	2.0982189	-4.36	2.1318826		
229009_at	0.945	0.04694573	2.1054687	-4.36	1.9320041	SIX5	SIX homeobox 5
203979_at	0.945	0.03118673	2.3382126	-4.36	2.260145	CYP27A1	cytochrome P450 family 27 subfamily A member 1
208247_at	0.945	0.04390501	2.1445091	-4.36	1.9641367	ERC2-IT1	ERC2 intronic transcript 1
214531_s_at	0.945	0.04031725	2.1935203	-4.36	2.2631345	SNX1	sorting nexin 1
241178_at	0.945	0.04783965	2.0961946	-4.36	4.5450333		
206128_at	0.945	0.0042753	3.4593007	-4.36	4.4345546	ADRA2C	adrenoceptor alpha 2C
244548_at	0.945	0.0395672	2.2027484	-4.36	2.0879966		
231948_s_at	0.945	0.03963953	2.2018515	-4.36	2.1061004	UBE2F	ubiquitin conjugating enzyme E2 F (putative)
225856_at	0.945	0.03638935	2.2522818	-4.36	3.1218982	CLOCK	clock circadian regulator
244394_at	0.945	0.02814762	2.4009103	-4.36	1.5822753		
217688_at	0.945	0.03995059	2.1980116	-4.36	2.3657316		
241874_at	0.945	0.02873056	2.3784604	-4.36	2.7381389	IGIP	IgA inducing protein

213410_at	0.945	0.03677034	2.2471527	-4.36	2.2296143	EDRF1	erythroid differentiation regulatory factor 1
1557052_at	0.945	0.04465859	2.1361367	-4.36	2.6191401		
214991_s_at	0.945	0.03191861	2.3267902	-4.36	2.5752299	PIGO	phosphatidylinositol glycan anchor biosynthesis class O
1559891_at	0.945	0.03194393	2.3263994	-4.36	2.7218281	HMGA2	high mobility group AT-hook 2
214094_at	0.945	0.0486694	2.0877245	-4.36	2.9575488	FUBP1	far upstream element binding protein 1
218642_s_at	0.945	0.04028054	2.1939683	-4.36	2.2958318	CHCHD7	coiled-coil-helix-coiled-coil-helix domain containing 7
234821_at	0.945	0.04480458	2.1345296	-4.36	2.8350985	LOC101929645	uncharacterized LOC101929645
1565733_at	0.945	0.03782343	2.2248361	-4.36	1.9539649		
203216_s_at	0.945	0.02861614	2.3927333	-4.36	2.746608	MYO6	myosin VI
205008_s_at	0.945	0.04044723	2.1919371	-4.36	1.9461705	CIB2	calcium and integrin binding family member 2
241042_at	0.945	0.03531984	2.2669489	-4.36	2.0124793		
202325_s_at	0.945	0.03801088	2.2224174	-4.36	1.6458703	ATP5J	ATP synthase, H+ transporting, mitochondrial Fo complex subunit F6
232138_at	0.945	0.04516549	2.1305767	-4.37	2.115603	MBNL2	muscleblind like splicing regulator 2
229667_s_at	0.945	0.03735467	2.2393802	-4.37	1.9745486	HOXB8	homeobox B8
240815_at	0.945	0.02672957	2.4264621	-4.37	3.0731043		
1553929_at	0.945	0.02893622	2.3872189	-4.37	2.7851517	ACER1	alkaline ceramidase 1
242011_at	0.945	0.02901522	2.3858667	-4.37	2.9475804		
233355_at	0.945	0.04181496	2.1755528	-4.37	2.6955946	LINC00176	long intergenic non-protein coding RNA 176
216204_at	0.945	0.02959203	2.3639909	-4.37	2.6153918	COMT	catechol-O-methyltransferase

230440_at	0.945	0.04395779	2.1439185	-4.37	2.2711252	ZNF469	zinc finger protein 469
214188_at	0.945	0.01790122	2.6601562	-4.37	3.6589367		
244823_at	0.945	0.0131314	2.8146706	-4.37	3.7232624	LOC100129034	uncharacterized LOC100129034
226442_at	0.945	0.0035289	3.6246792	-4.37	4.942281	ABTB1	ankyrin repeat and BTB domain containing 1
211993_at	0.945	0.04414904	2.1417837	-4.37	2.6707542	WNK1	WNK lysine deficient protein kinase 1
222510_s_at	0.945	0.04581644	2.1235186	-4.37	1.8500891	MKRN2	makorin ring finger protein 2
232672_x_at	0.945	0.04130615	2.1815901	-4.37	2.2174133	CTXN2///SLC24A5	cortexin 2///solute carrier family 24 member 5
1559883_s_at	0.945	0.0379304	2.2318309	-4.37	2.9580531	SAMHD1	SAM and HD domain containing deoxynucleoside triphosphate triphosphohydrolase 1
1566585_at	0.945	0.02054458	2.590957	-4.37	2.8958761		
218551_at	0.945	0.01807511	2.6553106	-4.37	3.04569	MIIP	migration and invasion inhibitory protein
220850_at	0.945	0.04931314	2.0812421	-4.37	2.8273302	MORC1	MORC family CW-type zinc finger 1
1560396_at	0.945	0.02740783	2.4140856	-4.37	3.7874066	KLHL6	kelch like family member 6
226953_at	0.945	0.0382297	2.2279476	-4.37	1.5118944	CADM3///ACKR1	cell adhesion molecule 3///atypical chemokine receptor 1 (Duffy blood group)
221845_s_at	0.945	0.03829689	2.2270798	-4.37	2.8098063	CLPB	ClpB homolog, mitochondrial AAA ATPase chaperonin
239154_at	0.945	0.04268494	2.1653834	-4.37	1.709534		
218140_x_at	0.945	0.02457135	2.4830637	-4.37	3.5660059	SRPRB	SRP receptor beta subunit
244288_s_at	0.945	0.04974271	2.0769583	-4.37	2.1044992	SIX3	SIX homeobox 3

217934_x_at	0.945	0.02466689	2.4811279	-4.37	3.4166146	STUB1	STIP1 homology and U-box containing protein 1
238698_at	0.945	0.0420864	2.1723594	-4.37	2.3404548	CASK	calcium/calmodulin dependent serine protein kinase
216964_at	0.945	0.02471548	2.480146	-4.37	1.7957715	USP22	ubiquitin specific peptidase 22
206932_at	0.945	0.03697416	2.2444288	-4.37	3.1256282	CH25H	cholesterol 25-hydroxylase
214900_at	0.945	0.03889592	2.2194033	-4.37	1.8969781	ZKSCAN1	zinc finger with KRAB and SCAN domains 1
1552662_a_at	0.945	0.03500216	2.2811919	-4.37	2.1039363	PCDHGB7	protocadherin gamma subfamily B, 7
206832_s_at	0.945	0.01581629	2.7470648	-4.37	3.0707603	SEMA3F	semaphorin 3F
226229_s_at	0.945	0.04571267	2.1246376	-4.37	3.0213668	SSU72	SSU72 homolog, RNA polymerase II CTD phosphatase
1562957_at	0.945	0.0342614	2.2917957	-4.37	3.0524101		
238395_at	0.945	0.0474667	2.1060232	-4.37	2.3932437		
235419_at	0.945	0.04747696	2.1059161	-4.37	2.3232768		
244673_at	0.945	0.0343685	2.2902495	-4.37	2.44026	KIAA1841	KIAA1841
240062_at	0.945	0.04289637	2.1629404	-4.37	2.2443211	FAM3C	family with sequence similarity 3 member C
243580_at	0.945	0.03079004	2.3563631	-4.37	2.827943	GNA14	G protein subunit alpha 14
1559633_a_at	0.945	0.03445184	2.2890494	-4.37	3.131888	CHRM3	cholinergic receptor muscarinic 3
243031_at	0.945	0.03552633	2.2738135	-4.37	1.9263783		
211521_s_at	0.945	0.02544418	2.465637	-4.37	3.4363041	CYTH4	cytohesin 4
211474_s_at	0.945	0.0477233	2.1033523	-4.37	1.4244836	SERPINB6	serpin family B member 6
225734_at	0.945	0.0477526	2.1030481	-4.37	1.9859369	FBXO22	F-box protein 22
225335_at	0.945	0.03099091	2.3531249	-4.37	1.8619834	ZNF496	zinc finger protein 496

221664_s_at	0.945	0.03469121	2.2856176	-4.37	2.969847	F11R	F11 receptor
220223_at	0.945	0.04423005	2.1477781	-4.37	2.5160364	ATAD5	ATPase family, AAA domain containing 5
1558722_at	0.945	0.03990407	2.2067254	-4.37	2.6850541	ZNF252P	zinc finger protein 252, pseudogene
224165_s_at	0.945	0.04816788	2.0987551	-4.37	2.3391264	IQCH	IQ motif containing H
244074_at	0.945	0.0435761	2.15516	-4.37	1.8866005		
233456_at	0.945	0.02712589	2.4336008	-4.37	2.8108378		
211029_x_at	0.945	0.02911901	2.3840956	-4.37	3.1465028	FGF18	fibroblast growth factor 18
241777_x_at	0.945	0.01794187	2.6825658	-4.37	3.2240998		
235493_at	0.945	0.03145448	2.3457259	-4.37	2.2468493		
228542_at	0.945	0.02726907	2.4309606	-4.37	3.2246238	MRS2	MRS2, magnesium transporter
228448_at	0.945	0.04848511	2.0954982	-4.37	2.9212864	MAP6	microtubule associated protein 6
232873_at	0.945	0.04687693	2.1122117	-4.37	1.9775376	ZNF33A	zinc finger protein 33A
217582_at	0.945	0.04474175	2.1420708	-4.37	2.6089525		
1554582_a_at	0.945	0.04403501	2.1499694	-4.37	2.5152881	ETFBKMT	electron transfer flavoprotein beta subunit lysine methyltransferase
220919_s_at	0.945	0.02503024	2.4588183	-4.37	3.3467228	CFAP43	cilia and flagella associated protein 43
237269_at	0.945	0.04750824	2.1055899	-4.38	2.0192167		
1569886_a_at	0.945	0.04448246	2.1449554	-4.38	2.7480086	GLB1L3	galactosidase beta 1 like 3
203805_s_at	0.945	0.04932213	2.0869958	-4.38	1.5018977	FANCA	Fanconi anemia complementation group A
1561589_a_at	0.945	0.0279807	2.4180301	-4.38	1.9916975	NBEAL1	neurobeachin like 1
242439_s_at	0.945	0.03617421	2.2648317	-4.38	3.4784724	ASXL1	additional sex combs like 1, transcriptional regulator

214199_at	0.945	0.04962485	2.0839528	-4.38	2.4517815	SFTPD	surfactant protein D
204597_x_at	0.945	0.03627389	2.2634629	-4.38	3.5530544	STC1	stanniocalcin 1
1560025_at	0.945	0.03630966	2.2629726	-4.38	1.7850716		
1561014_at	0.945	0.03951354	2.2116012	-4.38	1.8988054		
1557374_at	0.945	0.0498342	2.0818581	-4.38	2.2815897	ABCC9	ATP binding cassette subfamily C member 9
208523_x_at	0.945	0.04180804	2.1835626	-4.38	2.30974	HIST1H2BC///HIST1H2BI///HIST1H2BE///HIST1H2BF///HIST1H2BG	histone cluster 1, H2bc///histone cluster 1, H2bi///histone cluster 1, H2be///histone cluster 1, H2bf///histone cluster 1, H2bg
1569840_at	0.945	0.0329509	2.3225181	-4.38	3.1889127	LOC101927798	uncharacterized LOC101927798
231431_s_at	0.945	0.02740437	2.4284778	-4.38	3.4025433	INAFM2	InaF motif containing 2
223465_at	0.945	0.0400453	2.2049729	-4.38	1.4703469	COL4A3BP	collagen type IV alpha 3 binding protein
231875_at	0.945	0.03093114	2.3540865	-4.38	2.0305308	KIF21A	kinesin family member 21A
226388_at	0.945	0.04669565	2.1208088	-4.38	2.6344285	TCEA3	transcription elongation factor A3
209615_s_at	0.945	0.03706386	2.2527373	-4.38	2.1446778	PAK1	p21 (RAC1) activated kinase 1
214657_s_at	0.945	0.02769672	2.4231524	-4.38	1.9860238	MIR612///NEAT1	microRNA 612///nuclear paraspeckle assembly transcript 1 (non-protein coding)
228879_at	0.945	0.03824803	2.2370481	-4.38	2.4347251	SNORD104///SNORA50C	small nucleolar RNA, C/D box 104///small nucleolar RNA, H/ACA box 50C
220384_at	0.945	0.03723984	2.2503767	-4.38	2.5715209	NME8	NME/NM23 family member 8
222293_at	0.945	0.02912826	2.3978181	-4.38	2.6127843	CADM4	cell adhesion molecule 4

232180_at	0.945	0.02787271	2.4199722	-4.38	2.2001314	UGP2	UDP-glucose pyrophosphorylase 2
222079_at	0.945	0.03126003	2.348817	-4.38	3.7228644	ERG	ERG, ETS transcription factor
237422_at	0.945	0.03132083	2.3478487	-4.38	2.2476575		
211560_s_at	0.945	0.04277795	2.1721316	-4.38	2.9303393	ALAS2	5'-aminolevulinate synthase 2
206148_at	0.945	0.04080391	2.1956551	-4.38	1.6928055	IL3RA	interleukin 3 receptor subunit alpha
1561038_at	0.945	0.04740999	2.1132363	-4.38	2.3287237	ZNF81	zinc finger protein 81
201624_at	0.945	0.03155374	2.3441549	-4.38	2.597147	DARS	aspartyl-tRNA synthetase
46167_at	0.945	0.03168798	2.3420376	-4.38	1.6456777	MROH7-TTC4///TTC4	MROH7-TTC4 readthrough (NMD candidate)///tetratricopeptide repeat domain 4
202157_s_at	0.945	0.02463493	2.4991429	-4.38	2.8210032	CELF2	CUGBP, Elav-like family member 2
234128_at	0.945	0.03486283	2.2831704	-4.38	3.0229134		
208392_x_at	0.945	0.04148092	2.1874726	-4.38	2.4638288	SP110	SP110 nuclear body protein
224419_x_at	0.945	0.04349522	2.1638295	-4.38	2.3575444	PMCHL1	pro-melanin concentrating hormone like 1 (pseudogene)
208659_at	0.945	0.03449474	2.2995829	-4.38	3.5076019	CLIC1	chloride intracellular channel 1
243998_at	0.945	0.04443929	2.1454371	-4.38	3.1579542	KRT222	keratin 222
237010_at	0.945	0.03953218	2.2205328	-4.38	1.7737047		
235096_at	0.945	0.02043103	2.6158633	-4.38	3.817633	LEO1	LEO1 homolog, Paf1/RNA polymerase II complex component
244687_at	0.945	0.04459022	2.1437548	-4.38	2.3038765	DBT	dihydrolipoamide branched chain transacylase E2
207841_at	0.945	0.02963705	2.3632455	-4.38	3.3019288	SPIN2A	spindlin family member 2A
213783_at	0.945	0.02530161	2.4855777	-4.38	3.4573741	MFNG	MFNG O-fucosylpeptide 3-beta-N-acetylglucosaminyltransferase

235794_at	0.945	0.0400677	2.2137911	-4.38	2.8163936	MOBP	myelin-associated oligodendrocyte basic protein
218785_s_at	0.945	0.03901567	2.2271154	-4.38	2.2358391	IFT22	intraflagellar transport 22
1562863_at	0.945	0.03286753	2.3237849	-4.38	2.3953663		
220817_at	0.945	0.04447803	2.1526543	-4.38	2.4645862	TRPC4	transient receptor potential cation channel subfamily C member 4
1568848_at	0.945	0.04457996	2.1515082	-4.38	2.6644738	LOC101927820	uncharacterized LOC101927820
206965_at	0.945	0.04488892	2.1480488	-4.39	2.3734667	KLF12	Kruppel like factor 12
205633_s_at	0.945	0.0395982	2.2196971	-4.39	2.6595992	ALAS1	5'-aminolevulinate synthase 1
241880_x_at	0.945	0.04512761	2.145391	-4.39	2.3881131		
232567_at	0.945	0.0359846	2.2783382	-4.39	2.8653316	PRR5-ARHGAP8///ARHGAP8	PRR5-ARHGAP8 readthrough///Rho GTPase activating protein 8
244588_at	0.945	0.04529381	2.143548	-4.39	2.3715881		
206467_x_at	0.945	0.03157055	2.3571874	-4.39	3.1024897	RTEL1-TNFRSF6B///TNFRSF6B	RTEL1-TNFRSF6B readthrough (NMD candidate)///TNF receptor superfamily member 6b
211285_s_at	0.945	0.04915065	2.0952074	-4.39	2.7068406	UBE3A	ubiquitin protein ligase E3A
201585_s_at	0.945	0.01573878	2.7495734	-4.39	3.8791603	SFPQ	splicing factor proline and glutamine rich
212300_at	0.945	0.04941915	2.0924781	-4.39	2.2083355	TXLNA	taxilin alpha
230274_s_at	0.945	0.04585184	2.1374045	-4.39	2.5916597		
223938_at	0.945	0.04049868	2.2084254	-4.39	2.7229241	TEX35	testis expressed 35
233490_at	0.945	0.03665233	2.2690808	-4.39	4.6027467	DCTN4	dynactin subunit 4
1570359_at	0.945	0.03208603	2.3489908	-4.39	3.1669907	DDX3Y	DEAD-box helicase 3, Y-linked

211631_x_at	0.945	0.04930459	2.0871727	-4.39	3.0560491	B4GALT1	beta-1,4-galactosyltransferase 1
222757_s_at	0.945	0.0460223	2.1355414	-4.39	2.4914832	ZAK	sterile alpha motif and leucine zipper containing kinase AZK
211660_at	0.945	0.00423693	3.5988428	-4.39	4.9202073	POU2F2	POU class 2 homeobox 2
207528_s_at	0.945	0.02933432	2.3804397	-4.39	2.9801833	SLC7A11	solute carrier family 7 member 11
214259_s_at	0.945	0.0498045	2.088584	-4.39	2.0524527	AKR7A2	aldo-keto reductase family 7 member A2
221353_at	0.945	0.02689286	2.4379259	-4.39	2.7498502	OR3A1	olfactory receptor family 3 subfamily A member 1
243424_at	0.945	0.04621492	2.1334436	-4.39	2.2987114		
235512_at	0.945	0.02223988	2.5721552	-4.39	3.4195817	CDKL1	cyclin dependent kinase like 1
218602_s_at	0.945	0.04101473	2.2020692	-4.39	2.9729879	HAUS6	HAUS augmin like complex subunit 6
221735_at	0.945	0.03729171	2.2603621	-4.39	3.3305916	WDR48	WD repeat domain 48
1570225_at	0.945	0.04670477	2.1281445	-4.39	2.5603668	TMEM150B	transmembrane protein 150B
213648_at	0.945	0.04467059	2.1504912	-4.39	2.2310794	EXOSC7	exosome component 7
227415_at	0.945	0.04244036	2.184884	-4.39	2.2339908	DGKH	diacylglycerol kinase eta
213639_s_at	0.945	0.03742437	2.2585705	-4.39	3.0291161	ZNF500	zinc finger protein 500
231804_at	0.945	0.03805116	2.2396252	-4.39	1.7435685	RXFP1	relaxin/insulin like family peptide receptor 1
1554855_at	0.945	0.02273521	2.5607845	-4.39	3.1112796	PARK2	parkin RBR E3 ubiquitin protein ligase
1562695_at	0.945	0.02079767	2.565891	-4.39	3.2873931	FOXN4	forkhead box N4
231090_s_at	0.945	0.03771866	2.2546169	-4.39	3.5185494	ARID2	AT-rich interaction domain 2
1558295_a_at	0.945	0.04715848	2.1232812	-4.39	2.5055779	PPFIA2	PTPRF interacting protein alpha 2
233722_at	0.945	0.03777628	2.2538462	-4.39	1.5711356		

212726_at	0.945	0.01401869	2.8396256	-4.39	2.7501596	PHF2	PHD finger protein 2
1556586_x_at	0.945	0.04179219	2.1926306	-4.39	2.6407964		
213413_at	0.945	0.03189711	2.3519803	-4.39	2.4899785	STON1	stonin 1
213354_s_at	0.945	0.04730442	2.121726	-4.39	2.7035	NR2F6	nuclear receptor subfamily 2 group F member 6
230477_at	0.945	0.03194348	2.351245	-4.39	2.9307854	PARD6G-AS1	PARD6G antisense RNA 1
1553272_at	0.945	0.03196697	2.3508729	-4.39	4.134192	SLC36A1	solute carrier family 36 member 1
226109_at	0.945	0.03797585	2.2511852	-4.39	2.2788175	C21orf91	chromosome 21 open reading frame 91
213519_s_at	0.945	0.02491396	2.4934233	-4.39	3.56132	LAMA2	laminin subunit alpha 2
242598_at	0.945	0.04199921	2.1901444	-4.39	2.808375		
222513_s_at	0.945	0.03559099	2.2838703	-4.39	2.1506987	SORBS1	sorbin and SH3 domain containing 1
233522_at	0.945	0.03349072	2.3272663	-4.39	1.7629631	MEF2C-AS1	MEF2C antisense RNA 1
210369_at	0.945	0.0111279	3.0013849	-4.39	5.0495648	SWAP70	SWAP switching B-cell complex 70kDa subunit
207567_at	0.945	0.04759205	2.1186733	-4.39	2.4873289	SLC13A2	solute carrier family 13 member 2
229225_at	0.945	0.04778571	2.1166275	-4.39	2.4060892	NRP2	neuropilin 2
210479_s_at	0.945	0.03838997	2.2457044	-4.39	2.9580352	RORA	RAR related orphan receptor A
232579_at	0.945	0.02838998	2.4107333	-4.39	2.9526133	JHDM1D-AS1	JHDM1D antisense RNA 1 (head to head)
200966_x_at	0.945	0.04383438	2.1685845	-4.39	3.0373821	ALDOA	aldolase, fructose-bisphosphate A
236447_at	0.945	0.04388548	2.1679962	-4.39	2.9157193	GIGYF1	GRB10 interacting GYF protein 1
222499_at	0.945	0.03627084	2.2743505	-4.39	2.7566533	MRPS16	mitochondrial ribosomal protein S16
204903_x_at	0.945	0.03634022	2.2733883	-4.39	2.7943215	ATG4B	autophagy related 4B cysteine peptidase

244884_at	0.945	0.03420592	2.3165312	-4.39	2.1890749		
207544_s_at	0.945	0.02225833	2.5717273	-4.39	4.4424914	ADH6	alcohol dehydrogenase 6 (class V)
227514_at	0.945	0.03911674	2.2362169	-4.39	2.5646366	ITPRIPL2	inositol 1,4,5-trisphosphate receptor interacting protein like 2
1560917_at	0.945	0.048757	2.1064787	-4.39	1.8469106		
230542_at	0.945	0.04334857	2.1742102	-4.39	2.389391	ZNF597	zinc finger protein 597
219059_s_at	0.945	0.04892175	2.1047756	-4.39	2.6622099	LYVE1	lymphatic vessel endothelial hyaluronan receptor 1
221987_s_at	0.945	0.0347712	2.3081941	-4.4	2.8164165	TSR1	TSR1, ribosome maturation factor
208730_x_at	0.945	0.0470391	2.1245568	-4.4	2.4305938	RAB2A	RAB2A, member RAS oncogene family
1558044_s_at	0.945	0.03498751	2.3050373	-4.4	2.8010306	EXOSC6	exosome component 6
210667_s_at	0.945	0.04042766	2.209306	-4.4	2.7728908	C21orf33	chromosome 21 open reading frame 33
235208_at	0.945	0.02295186	2.5558858	-4.4	3.3457561	CCDC112	coiled-coil domain containing 112
216602_s_at	0.945	0.04953266	2.0985049	-4.4	2.2890189	FARSA	phenylalanyl-tRNA synthetase alpha subunit
234984_at	0.945	0.02653474	2.4613612	-4.4	2.8568504	NEDD1	neural precursor cell expressed, developmentally down-regulated 1
221561_at	0.945	0.040059	2.2241561	-4.4	2.7645278	SOAT1	sterol O-acyltransferase 1
1556017_at	0.945	0.04049503	2.2186635	-4.4	1.8279982	NBEAL2	neurobeachin like 2
1566768_at	0.945	0.02506152	2.5103835	-4.4	3.7706657		
224990_at	0.945	0.03435723	2.314287	-4.4	2.1232649	SMIM14	small integral membrane protein 14
1554534_at	0.945	0.04059768	2.2173783	-4.4	3.403483	DPYD	dihydropyrimidine dehydrogenase
225601_at	0.945	0.03026741	2.3784834	-4.4	3.5408248	HMGB3	high mobility group box 3

1567656_at	0.945	0.04618299	2.1421674	-4.4	3.0270978	OR2H1	olfactory receptor family 2 subfamily H member 1
231770_x_at	0.945	0.02764263	2.4405006	-4.4	2.2943408	WDPCP	WD repeat containing planar cell polarity effector
238293_at	0.945	0.03869817	2.241661	-4.4	2.7907081		
1557071_s_at	0.945	0.02403529	2.5320378	-4.4	2.167737	NUB1	negative regulator of ubiquitin like proteins 1
219751_at	0.945	0.0454629	2.1501338	-4.4	2.447368	SETD6	SET domain containing 6
217462_at	0.945	0.04560727	2.1485274	-4.4	1.6540257	MYRF	myelin regulatory factor
208403_x_at	0.945	0.03537116	2.2994829	-4.4	3.0740505	MAX	MYC associated factor X
222082_at	0.945	0.03119422	2.3786009	-4.4	3.0692548	ZBTB7A	zinc finger and BTB domain containing 7A
1554579_a_at	0.945	0.00799489	3.180028	-4.4	4.8463832	MYO18B	myosin XVIIIB
220621_at	0.945	0.03391045	2.3081484	-4.4	2.4285108	FOXE3	forkhead box E3
220396_at	0.945	0.04189823	2.2013508	-4.4	3.1700168	SLC35E3	solute carrier family 35 member E3
218629_at	0.945	0.04721058	2.1309933	-4.4	1.9434442	SMO	smoothened, frizzled class receptor
235857_at	0.945	0.04207772	2.199175	-4.4	3.4340403	KCTD11	potassium channel tetramerization domain containing 11
208603_s_at	0.945	0.03955008	2.2306371	-4.4	2.9680969	MAPK8IP2	mitogen-activated protein kinase 8 interacting protein 2
205811_at	0.945	0.04758778	2.1269473	-4.4	3.0846101	POLG2	DNA polymerase gamma 2, accessory subunit
241708_at	0.945	0.03599485	2.2905725	-4.4	2.7523938	DOCK1	dedicator of cytokinesis 1
224241_s_at	0.945	0.03967662	2.2290185	-4.4	2.6217077		

201198_s_at	0.945	0.03607088	2.2894961	-4.4	2.8948878	PSMD1	proteasome 26S subunit, non-ATPase 1
201767_s_at	0.945	0.04669861	2.1365324	-4.4	2.331615	ELAC2	elaC ribonuclease Z 2
1559467_at	0.945	0.0319315	2.3665919	-4.4	2.5734084		
1570391_at	0.945	0.02085096	2.6310949	-4.4	2.3669572	CRAT37	cervical cancer-associated transcript 37
226968_at	0.945	0.03219219	2.3624083	-4.4	2.3704273	KIF1B	kinesin family member 1B
219687_at	0.945	0.04704355	2.1327944	-4.4	1.5125333	HHAT	hedgehog acyltransferase
230347_at	0.945	0.04660745	2.1291933	-4.4	2.1443398	ORAI2	ORAI calcium release-activated calcium modulator 2
242251_at	0.945	0.03849577	2.2562321	-4.4	2.788517	SPATA5	spermatogenesis associated 5
206273_at	0.945	0.0205345	2.6132642	-4.4	4.1185667	PRELID3A	PRELI domain containing 3A
223235_s_at	0.945	0.03726581	2.2728522	-4.4	2.5916504	SMOC2	SPARC related modular calcium binding 2
237675_at	0.945	0.03294786	2.3504594	-4.4	3.4985735	LINC01351	long intergenic non-protein coding RNA 1351
1553772_at	0.945	0.04391537	2.1773777	-4.41	1.3988217	GK5	glycerol kinase 5 (putative)
1567079_at	0.945	0.03751057	2.2695044	-4.41	3.0679042	CLN6	ceroid-lipofuscinosis, neuronal 6, late infantile, variant
218149_s_at	0.945	0.03309978	2.3332253	-4.41	2.5438431	ZNF395	zinc finger protein 395
1556432_at	0.945	0.0333046	2.3300951	-4.41	2.3101355		
225939_at	0.945	0.04440249	2.1717405	-4.41	2.224469	EIF4E3	eukaryotic translation initiation factor 4E family member 3
204508_s_at	0.945	0.02656497	2.4801484	-4.41	2.9930835	CA12	carbonic anhydrase 12

235511_at	0.945	0.03815017	2.2608516	-4.41	3.2532529	RBM14-RBM4///RBM4	RBM14-RBM4 readthrough///RNA binding motif protein 4
1555753_x_at	0.945	0.0396402	2.2412058	-4.41	3.0694724	ERVH-6	endogenous retrovirus group H member 6
215572_at	0.945	0.0367209	2.2681391	-4.41	2.3390758		
201096_s_at	0.945	0.02160465	2.5871004	-4.41	3.27806	ARF4	ADP ribosylation factor 4
204874_x_at	0.945	0.04930099	2.1089304	-4.41	1.9138411	BAIAP3	BAI1 associated protein 3
218797_s_at	0.945	0.04930322	2.1089073	-4.41	2.2160517	SIRT7	sirtuin 7
203729_at	0.945	0.03422912	2.3161864	-4.41	2.830458	EMP3	epithelial membrane protein 3
203230_at	0.945	0.03894763	2.2502501	-4.41	3.0781639	DVL1	dishevelled segment polarity protein 1
241545_x_at	0.945	0.03441086	2.3134938	-4.41	2.0147319		
220864_s_at	0.945	0.02766032	2.4591357	-4.41	2.6163888	NDUFA13	NADH:ubiquinone oxidoreductase subunit A13
219898_at	0.945	0.04595162	2.1541836	-4.41	2.3902563	GPR85	G protein-coupled receptor 85
1552389_at	0.945	0.0394439	2.2437543	-4.41	4.2513852	ERICH5	glutamate rich 5
238623_at	0.945	0.04605975	2.1529786	-4.41	3.0606541		
203967_at	0.945	0.0411016	2.2225933	-4.41	2.3627756	CDC6	cell division cycle 6
236950_s_at	0.945	0.03526722	2.3153337	-4.41	2.479372	LINC00964	long intergenic non-protein coding RNA 964
226830_x_at	0.945	0.04364251	2.1805605	-4.41	3.0732378	LINC01578	long intergenic non-protein coding RNA 1578
202910_s_at	0.945	0.03998395	2.2367712	-4.41	2.8295326	ADGRE5	adhesion G protein-coupled receptor E5
218078_s_at	0.945	0.03213544	2.3633163	-4.41	2.9630649	ZDHHC3	zinc finger DHHC-type containing 3
236428_at	0.945	0.04692022	2.1434801	-4.41	2.4806278		

225689_at	0.945	0.02859163	2.4418864	-4.41	1.9687516	POMGNT2	protein O-linked mannose N-acetylglucosaminyltransferase 2 (beta 1,4-)
221193_s_at	0.945	0.04762257	2.1265756	-4.41	3.0620812	ZCCHC10	zinc finger CCHC-type containing 10
1554883_a_at	0.945	0.047233	2.1400671	-4.41	2.2763432	ERCC8	ERCC excision repair 8, CSA ubiquitin ligase complex subunit
222828_at	0.945	0.04788749	2.1237532	-4.41	2.1692492	IL20RA	interleukin 20 receptor subunit alpha
202275_at	0.945	0.03638367	2.2991938	-4.41	1.9768533	G6PD	glucose-6-phosphate dehydrogenase
1562762_at	0.945	0.03653356	2.2970624	-4.41	3.1527355	C3orf56	chromosome 3 open reading frame 56
209866_s_at	0.945	0.03311726	2.3478162	-4.41	1.7711055	ADGRL3	adhesion G protein-coupled receptor L3
228815_s_at	0.945	0.02921744	2.4305944	-4.41	2.7496738	REPIN1	replication initiator 1
208727_s_at	0.945	0.04785807	2.1333078	-4.41	1.6470421	CDC42	cell division cycle 42
218373_at	0.945	0.0263584	2.4647578	-4.41	2.8942071	AKTIP	AKT interacting protein
211981_at	0.945	0.0413351	2.2196766	-4.41	1.5448609	COL4A1	collagen type IV alpha 1 chain
217112_at	0.945	0.01087833	3.0136344	-4.41	5.0318615	PDGFB	platelet derived growth factor subunit B
216275_at	0.945	0.04836081	2.1279298	-4.41	2.6958807	BUB1	BUB1 mitotic checkpoint serine/threonine kinase
201565_s_at	0.945	0.04838657	2.1276556	-4.41	1.6781528	ID2	inhibitor of DNA binding 2, HLH protein
206636_at	0.945	0.04584444	2.1553806	-4.42	2.7308889	RASA2	RAS p21 protein activator 2
212707_s_at	0.945	0.04585235	2.1552922	-4.42	3.0335564	RASA4B///RASA4CP///RASA4	RAS p21 protein activator 4B///RAS p21 protein activator 4C, pseudogene///RAS p21 protein activator 4

228153_at	0.945	0.04873752	2.1239335	-4.42	1.9909815	RNF144B	ring finger protein 144B
221840_at	0.945	0.03397662	2.334601	-4.42	3.9194626	PTPRE	protein tyrosine phosphatase, receptor type E
206819_at	0.945	0.03760714	2.2681892	-4.42	2.7334591	POM121L9P	
214551_s_at	0.14	0.00000577	-5.9357216	-3.48	-3.7612548	CD7	CD7 molecule
223982_s_at	0.388	0.00008523	-4.6841501	-3.51	-4.0378781	PNPLA8	patatin like phospholipase domain containing 8
212289_at	0.14	0.00001027	-5.946845	-3.68	-9.2218269	ANKRD12	ankyrin repeat domain 12
208903_at	0.945	0.00056035	-3.9324669	-3.69	-6.1426433	RPS28	ribosomal protein S28
217807_s_at	0.945	0.00064532	-3.9220138	-3.78	-3.8508459	SNORD23///GLTSCR2	small nucleolar RNA, C/D box 23///glioma tumor suppressor candidate region gene 2
1557727_at	0.759	0.00040445	-4.1669806	-3.81	-5.1416916	PCBP1-AS1	PCBP1 antisense RNA 1
1556086_at	0.262	0.00004757	-5.3196395	-3.87	-5.4123506	HPF1	histone PARylation factor 1
1555847_a_at	0.945	0.00225578	-3.4034563	-3.9	-5.5476469	MIR24-2///MIR23A///LOC284454	microRNA 24-2///microRNA 23a///uncharacterized LOC284454
220863_at	0.945	0.00171133	-3.5508167	-3.93	-4.1623142	MIP	major intrinsic protein of lens fiber
1560352_at	0.945	0.00340678	-3.2237461	-3.93	-4.9771081	LOC101927104///ENTPD1-AS1	uncharacterized LOC101927104///ENTPD1 antisense RNA 1
226824_at	0.945	0.00287535	-3.3055684	-3.94	-5.3617075	CPXM2	carboxypeptidase X, M14 family member 2
218638_s_at	0.945	0.00250332	-3.377164	-3.94	-2.0959783	LOC100130872///SPON2	uncharacterized LOC100130872///spondin 2
203407_at	0.945	0.00308315	-3.2772832	-3.95	-2.2835954	PPL	periplakin

201810_s_at	0.945	0.00390837	-3.168252	-3.95	-4.678479	SH3BP5	SH3 domain binding protein 5
234074_at	0.945	0.00462435	-3.0998779	-3.98	-5.5369681		
244291_x_at	0.945	0.00215053	-3.476745	-3.98	-1.9203633		
206433_s_at	0.945	0.00433684	-3.1379817	-3.99	-1.5505655	SPOCK3	sparc/osteonectin, cwcv and kazal-like domains proteoglycan (testican) 3
1558780_a_at	0.945	0.00226857	-3.4543968	-3.99	-2.8803517	H2AFY	H2A histone family member Y
216563_at	0.945	0.00372678	-3.2138238	-4	-3.0164265	ANKRD12	ankyrin repeat domain 12
243999_at	0.945	0.00313133	-3.3014728	-4	-1.9305816	SLFN5	schlafen family member 5
233846_at	0.945	0.00551695	-3.0276168	-4	-4.0214016	CCDC85A	coiled-coil domain containing 85A
236017_at	0.945	0.0007049	-4.0026175	-4	-3.2869003	CDKL3	cyclin dependent kinase like 3
1563362_at	0.945	0.00401964	-3.1825324	-4.01	-2.6305849	D21S2090E	uncharacterized D21S2090E
224306_at	0.945	0.00434103	-3.1506291	-4.02	-2.3568062	NPC1L1	NPC1 like intracellular cholesterol transporter 1
223625_at	0.945	0.00201184	-3.5269378	-4.02	-5.6981479	FAM126A	family with sequence similarity 126 member A
241831_at	0.945	0.0018596	-3.5857852	-4.03	-4.2486458		
232345_at	0.945	0.00189962	-3.5765953	-4.03	-4.0636287	C18orf8	chromosome 18 open reading frame 8
218925_s_at	0.945	0.00709819	-2.9234142	-4.04	-4.6934479	C11orf1	chromosome 11 open reading frame 1
231479_at	0.945	0.00618839	-2.9908363	-4.04	-2.4825276	TTC33	tetratricopeptide repeat domain 33
222322_at	0.945	0.00368769	-3.2499067	-4.05	-3.5937722		
242873_at	0.945	0.00657428	-2.9655728	-4.05	-2.4552752	LOC101928100	uncharacterized LOC101928100
227232_at	0.945	0.00802976	-2.8719514	-4.06	-3.7594618	EVL	Enah/Vasp-like
228241_at	0.945	0.00392945	-3.2229845	-4.06	-3.0692526	AGR3	anterior gradient 3, protein disulphide isomerase family member
230894_s_at	0.945	0.00699453	-2.9396205	-4.06	-2.7940088		

212340_at	0.945	0.00812165	-2.8671864	-4.06	-1.9667685	YIPF6	Yip1 domain family member 6
201826_s_at	0.945	0.00818047	-2.8641627	-4.06	-1.7637497	SCCPDH	saccharopine dehydrogenase (putative)
207036_x_at	0.945	0.00361643	-3.2581707	-4.06	-3.4453032	GRIN2D	glutamate ionotropic receptor NMDA type subunit 2D
227413_at	0.945	0.00536829	-3.0753228	-4.07	-3.5759759	UBLCP1	ubiquitin like domain containing CTD phosphatase 1
225494_at	0.945	0.00634867	-2.9915689	-4.07	-3.7041385	DYNLL2	dynein light chain LC8-type 2
218792_s_at	0.945	0.00450308	-3.1650372	-4.07	-1.5745779	BSPRY	B-box and SPRY domain containing
229588_at	0.945	0.00272725	-3.4200822	-4.07	-4.3252113	DNAJC10	DnaJ heat shock protein family (Hsp40) member C10
202172_at	0.945	0.00797257	-2.8845476	-4.08	-5.2618737	VEZF1	vascular endothelial zinc finger 1
227035_x_at	0.945	0.00927931	-2.8111628	-4.08	-4.1879283	RP9P	retinitis pigmentosa 9 pseudogene
235147_at	0.945	0.00611228	-3.0202197	-4.08	-3.4435369		
211550_at	0.945	0.00578374	-3.0437104	-4.08	-3.363365	EGFR	epidermal growth factor receptor
232198_at	0.945	0.00238589	-3.5044559	-4.09	-3.5586686		
236198_at	0.945	0.00241446	-3.4991998	-4.09	-4.7680824	LINC01215	long intergenic non-protein coding RNA 1215
216797_at	0.945	0.00388734	-3.2456159	-4.09	-2.9763304		
215333_x_at	0.945	0.01022337	-2.7701656	-4.09	-3.6337054	GSTM1	glutathione S-transferase mu 1
41387_r_at	0.945	0.01033137	-2.7657055	-4.09	-2.8650417	KDM6B	lysine demethylase 6B
1552365_at	0.945	0.00238464	-3.5046858	-4.1	-3.290059	SCIN	scinderin
201583_s_at	0.945	0.01073845	-2.7492791	-4.1	-3.9237016	SEC23B	Sec23 homolog B, coat complex II component

1555447_at	0.945	0.00279272	-3.4348702	-4.1	-4.2518005	ADGRG5	adhesion G protein-coupled receptor G5
233558_s_at	0.945	0.00959105	-2.8061465	-4.1	-3.3437602	TRAPPC11	trafficking protein particle complex 11
218614_at	0.945	0.01094385	-2.7412117	-4.1	-2.9176911	KIAA1551	KIAA1551
210226_at	0.945	0.00846293	-2.8695458	-4.1	-4.9598848	NR4A1	nuclear receptor subfamily 4 group A member 1
239984_at	0.945	0.0010653	-3.9404301	-4.1	-5.0831345		
232093_at	0.945	0.00678207	-2.9758643	-4.1	-2.885526	SPACA6	sperm acrosome associated 6
235882_at	0.945	0.0061062	-3.0345733	-4.1	-1.7609932	VPS53	VPS53, GARP complex subunit
202109_at	0.945	0.00843345	-2.8710374	-4.11	-4.6872358	ARFIP2	ADP ribosylation factor interacting protein 2
242275_at	0.945	0.00459304	-3.1736535	-4.11	-3.6955139		
210245_at	0.945	0.00400691	-3.252443	-4.11	-6.3493887	ABCC8	ATP binding cassette subfamily C member 8
206685_at	0.945	0.01149258	-2.7203382	-4.11	-2.6201346	HCG4	HLA complex group 4 (non-protein coding)
239120_at	0.945	0.00639269	-3.0148067	-4.11	-2.5160997	TMEM51-AS1	TMEM51 antisense RNA 1
229160_at	0.945	0.0031223	-3.3854707	-4.11	-4.6418853	MUM1L1	MUM1 like 1
210263_at	0.945	0.00481512	-3.153221	-4.11	-3.6227665	KCNF1	potassium voltage-gated channel modifier subfamily F member 1
243119_at	0.945	0.00121056	-3.8813376	-4.11	-4.7534993		
1555135_at	0.945	0.00918375	-2.8345377	-4.11	-3.9222899		
209893_s_at	0.945	0.0105402	-2.7658142	-4.12	-1.815104	FUT4	fucosyltransferase 4
203876_s_at	0.945	0.00325105	-3.3675545	-4.12	-3.7689774	MMP11	matrix metallopeptidase 11
224533_s_at	0.945	0.0064574	-3.0104602	-4.12	-2.9739963		

235959_at	0.945	0.00096834	-4.0356152	-4.12	-2.8416927		
222157_s_at	0.945	0.01066431	-2.7607959	-4.12	-1.5054785	WDR48	WD repeat domain 48
1560043_at	0.945	0.00945228	-2.8221592	-4.12	-3.5962315	CYB5R1	cytochrome b5 reductase 1
236677_at	0.945	0.0108002	-2.7553643	-4.12	-2.5050392	NGB	neuroglobin
207775_at	0.945	0.00565026	-3.0837871	-4.12	-2.2217721	MGC4859	uncharacterized LOC79150
1556951_at	0.945	0.00845377	-2.8812169	-4.12	-3.3947959		
228175_at	0.945	0.00804628	-2.9025207	-4.13	-2.6776812	SLC4A8	solute carrier family 4 member 8
210583_at	0.945	0.00703785	-2.9732411	-4.13	-2.9190998	POLDIP3	DNA polymerase delta interacting protein 3
213007_at	0.945	0.01001467	-2.79728	-4.13	-3.1239522	FANCI	Fanconi anemia complementation group I
201426_s_at	0.945	0.00598107	-3.0589993	-4.13	-3.1527669	VIM	vimentin
209660_at	0.945	0.01292915	-2.6698275	-4.13	-4.6387756	TTR	transthyretin
225415_at	0.945	0.00828129	-2.8901121	-4.13	-4.4824547	DTX3L	deltex E3 ubiquitin ligase 3L
232343_at	0.945	0.00878072	-2.8648194	-4.13	-2.0601045	DCTN5	dynactin subunit 5
213196_at	0.945	0.01314916	-2.6625606	-4.13	-4.0377517	ZNF629	zinc finger protein 629
230134_s_at	0.945	0.0133068	-2.6574236	-4.13	-3.5644873	RC3H2	ring finger and CCCH-type domains 2
236241_at	0.945	0.00634143	-3.0334599	-4.13	-3.5133155	MED31	mediator complex subunit 31
223195_s_at	0.945	0.0052249	-3.1359074	-4.13	-3.5809623	SESN2	sestrin 2
237993_at	0.945	0.00920102	-2.8445741	-4.14	-2.5427167	CHCHD5	coiled-coil-helix-coiled-coil-helix domain containing 5
200765_x_at	0.945	0.00934092	-2.8380301	-4.14	-2.0416078	CTNNA1	catenin alpha 1
231887_s_at	0.945	0.00174836	-3.7115563	-4.14	-4.6038777	PALD1	phosphatase domain containing, paladin 1
210242_x_at	0.945	0.01110389	-2.7526435	-4.14	-3.2897928	ST20	suppressor of tumorigenicity 20

237393_at	0.945	0.01438545	-2.6237259	-4.14	-1.8906325	TPBGL	trophoblast glycoprotein-like
215042_at	0.945	0.00690969	-2.9958996	-4.14	-3.7655916	BMP6	bone morphogenetic protein 6
205082_s_at	0.945	0.0092663	-2.8415089	-4.14	-5.4735352	AOX1	aldehyde oxidase 1
212147_at	0.945	0.00539447	-3.1218274	-4.14	-3.2698472	SMG5	SMG5, nonsense mediated mRNA decay factor
244545_at	0.945	0.01447167	-2.6211354	-4.14	-2.6884369		
207414_s_at	0.945	0.00438214	-3.2347653	-4.14	-3.5803919	LOC100507472 ///PCSK6	uncharacterized LOC100507472///proprotein convertase subtilisin/kexin type 6
235340_at	0.945	0.01459445	-2.617471	-4.14	-4.2276432	GANC	glucosidase alpha, neutral C
232048_at	0.945	0.01474994	-2.612871	-4.15	-4.2375081	FAM76B	family with sequence similarity 76 member B
227114_at	0.945	0.01018944	-2.8002286	-4.15	-2.6827461	RNF214	ring finger protein 214
234383_x_at	0.945	0.00861759	-2.8851555	-4.15	-2.2338294	ATP7B	ATPase copper transporting beta
1555342_a_at	0.945	0.0047835	-3.1956315	-4.15	-4.1208612	UNC5C	unc-5 netrin receptor C
239040_at	0.945	0.00907579	-2.8624952	-4.15	-2.8931797	HNRNPD	heterogeneous nuclear ribonucleoprotein D
242281_at	0.759	0.00041641	-4.4411354	-4.15	-6.0673958	GLUL	glutamate-ammonia ligase
240687_at	0.945	0.00597883	-3.0763953	-4.15	-5.0550325	PASD1	PAS domain containing 1
219799_s_at	0.945	0.01579875	-2.5829765	-4.16	-1.2693705	DHRS9	dehydrogenase/reductase 9
235847_at	0.945	0.0072658	-2.9738503	-4.16	-2.6401848		
227322_s_at	0.945	0.00785377	-2.9396234	-4.16	-3.1544864	BCCIP	BRCA2 and CDKN1A interacting protein
222226_at	0.945	0.01096739	-2.7681095	-4.16	-2.9036474	SAA3P	serum amyloid A3 pseudogene
205696_s_at	0.945	0.01240309	-2.7045177	-4.16	-1.8629211	GFRA1	GDNF family receptor alpha 1

241326_at	0.945	0.01269986	-2.6941933	-4.16	-2.3434276	AK7	adenylate kinase 7
1561596_at	0.945	0.00555474	-3.1089089	-4.16	-3.929858		
238628_s_at	0.945	0.01636181	-2.5676814	-4.16	-4.0874737	TRAPPC2L	trafficking protein particle complex 2 like
242997_at	0.945	0.01134221	-2.7533974	-4.16	-3.8976901		
1554686_at	0.945	0.00774084	-2.9460021	-4.16	-3.2236388	STAU2	staufen double-stranded RNA binding protein 2
213610_s_at	0.945	0.00838695	-2.9106502	-4.16	-1.9261983	PHOSPHO2-KLHL23///KLHL23	PHOSPHO2-KLHL23 readthrough///kelch like family member 23
206798_x_at	0.945	0.0168086	-2.5558888	-4.17	-3.2968877	DLEC1	deleted in lung and esophageal cancer 1
1555040_at	0.945	0.01184407	-2.7344036	-4.17	-2.8470804	LINC00612	long intergenic non-protein coding RNA 612
204992_s_at	0.945	0.01185447	-2.734018	-4.17	-4.7996465	PFN2	profilin 2
1553835_a_at	0.945	0.00803929	-2.9293338	-4.17	-3.7966758	COL6A5	collagen type VI alpha 5 chain
217757_at	0.945	0.01732373	-2.5426487	-4.17	-1.575474	A2M	alpha-2-macroglobulin
227877_at	0.945	0.00418028	-3.2804359	-4.17	-4.6251849	ANXA2R	annexin A2 receptor
226859_at	0.945	0.01745223	-2.539403	-4.17	-4.0608592	DNAJC25-GNG10///DNAJC25	DNAJC25-GNG10 readthrough///DnaJ heat shock protein family (Hsp40) member C25
1562467_at	0.945	0.00730494	-2.9874756	-4.17	-2.0715688		
220439_at	0.945	0.01455232	-2.6344548	-4.18	-2.6849974	RIN3	Ras and Rab interactor 3
1562189_at	0.945	0.00616056	-3.0821827	-4.18	-3.2043433		

205850_s_at	0.945	0.01438956	-2.6394086	-4.18	-2.8022954	GABRB3	gamma-aminobutyric acid type A receptor beta3 subunit
218118_s_at	0.945	0.00624224	-3.0762552	-4.18	-3.2660403	TIMM23B///TIMM23	translocase of inner mitochondrial membrane 23 homolog B///translocase of inner mitochondrial membrane 23
1561846_s_at	0.945	0.01288937	-2.697168	-4.18	-2.7631822	CCDC168	coiled-coil domain containing 168
227194_at	0.945	0.01836015	-2.5170818	-4.18	-2.739815	FAM3B	family with sequence similarity 3 member B
200748_s_at	0.945	0.01836082	-2.5170658	-4.18	-3.1820722	FTH1	ferritin heavy chain 1
224027_at	0.945	0.00217746	-3.6500448	-4.18	-4.2540396	CCL28	C-C motif chemokine ligand 28
1566518_at	0.945	0.01659111	-2.5687006	-4.18	-1.4661863		
214324_at	0.945	0.01311014	-2.6896698	-4.18	-2.7748421	GP2	glycoprotein 2
202673_at	0.945	0.00686109	-3.0153639	-4.18	-2.5024647	DPM1	dolichyl-phosphate mannosyltransferase subunit 1, catalytic
215073_s_at	0.945	0.00985801	-2.8390218	-4.18	-2.8675905	NR2F2	nuclear receptor subfamily 2 group F member 2
224288_x_at	0.945	0.01364107	-2.6721127	-4.19	-3.9330699	FKSG49	FKSG49
226471_at	0.945	0.01367209	-2.6711068	-4.19	-3.788192	GGT7	gamma-glutamyltransferase 7
235474_at	0.945	0.01012412	-2.8271671	-4.19	-4.0425555	NEU3	neuraminidase 3
241216_at	0.945	0.00054973	-4.3765477	-4.19	-6.0251674		
239676_x_at	0.945	0.01534301	-2.6111049	-4.19	-1.4120915	FBRSL1	fibrosin like 1
1569476_at	0.945	0.01022063	-2.822941	-4.19	-3.0776437	DKFZP434L187	uncharacterized LOC26082

204723_at	0.945	0.01766722	-2.540933	-4.19	-5.0757753	SCN3B	sodium voltage-gated channel beta subunit 3
203947_at	0.945	0.00838568	-2.925871	-4.19	-3.6084535	CSTF3	cleavage stimulation factor subunit 3
210836_x_at	0.945	0.00554518	-3.1517291	-4.19	-3.1024571	PDE4D	phosphodiesterase 4D
204656_at	0.945	0.01411999	-2.656819	-4.19	-2.0242433	SHB	SH2 domain containing adaptor protein B
233663_s_at	0.945	0.01237012	-2.7258562	-4.19	-3.1522189	CDH26	cadherin 26
229585_at	0.945	0.00848041	-2.9208414	-4.19	-3.1638765	ADAMTSL1	ADAMTS like 1
226756_at	0.945	0.00404341	-3.3238245	-4.19	-4.6111175	CCDC71L	coiled-coil domain containing 71-like
243141_at	0.945	0.01580863	-2.5978762	-4.19	-2.3848026	SGMS2	sphingomyelin synthase 2
201859_at	0.945	0.01994777	-2.4803986	-4.19	-4.6219718	SRGN	serglycin
241695_s_at	0.945	0.01814533	-2.5290974	-4.19	-2.0979157		
222400_s_at	0.945	0.01264681	-2.7160125	-4.19	-3.2237123	ADI1	acireductone dioxygenase 1
210359_at	0.945	0.02049848	-2.4683025	-4.2	-2.0273929	MTSS1	metastasis suppressor 1
210184_at	0.945	0.00432426	-3.2926537	-4.2	-3.3152176	ITGAX	integrin subunit alpha X
232557_at	0.945	0.01881072	-2.513099	-4.2	-2.6674254	UBAP1L	ubiquitin associated protein 1 like
243834_at	0.945	0.00806271	-2.9434394	-4.2	-3.5133698	TNRC6A	trinucleotide repeat containing 6A
210459_at	0.945	0.01891201	-2.5107098	-4.2	-2.3270469	PSMD4	proteasome 26S subunit, non-ATPase 4
208175_s_at	0.945	0.01328351	-2.6941131	-4.2	-4.9587717	DMP1	dentin matrix acidic phosphoprotein 1
203261_at	0.945	0.00576208	-3.134193	-4.2	-3.673786	DCTN6	dynactin subunit 6
227712_at	0.945	0.01919913	-2.5040012	-4.2	-1.9190376	LYRM2	LYR motif containing 2
218019_s_at	0.945	0.0093523	-2.8769308	-4.2	-3.4340661	PDXK	pyridoxal (pyridoxine, vitamin B6) kinase
1560185_at	0.945	0.00949545	-2.8556752	-4.2	-2.0404878	LOC105376360	uncharacterized LOC105376360

224432_at	0.945	0.01096112	-2.7917274	-4.2	-2.8386839	SH3GLB2	SH3 domain containing GRB2 like endophilin B2
236335_at	0.945	0.01766066	-2.5486272	-4.2	-2.2461184	GUCY1A2	guanylate cyclase 1 soluble subunit alpha 2
212473_s_at	0.945	0.00964124	-2.8632431	-4.2	-4.1354723	MICAL2	microtubule associated monooxygenase, calponin and LIM domain containing 2
228728_at	0.945	0.00968053	-2.8614126	-4.2	-3.3603207	CPED1	cadherin like and PC-esterase domain containing 1
204880_at	0.945	0.01750234	-2.5526439	-4.2	-1.8482699	MGMT	O-6-methylguanine-DNA methyltransferase
219829_at	0.945	0.02177884	-2.4412995	-4.2	-2.5423063	ITGB1BP2	integrin subunit beta 1 binding protein 2
227982_at	0.945	0.00614006	-3.1051205	-4.21	-2.7951986	SEPSECS	Sep (O-phosphoserine) tRNA:Sec (selenocysteine) tRNA synthase
218750_at	0.945	0.01599348	-2.6013326	-4.21	-2.8487109	TAF1D	TATA-box binding protein associated factor, RNA polymerase I subunit D
220420_at	0.945	0.01528468	-2.6215697	-4.21	-4.1345145	LMAN1L	lectin, mannose binding 1 like
203690_at	0.945	0.01541064	-2.6179099	-4.21	-2.8142944	TUBGCP3	tubulin gamma complex associated protein 3
1570422_at	0.945	0.01541682	-2.6177311	-4.21	-3.7450773	SNTG2	syntrophin gamma 2
235650_at	0.945	0.01388484	-2.6743222	-4.21	-2.4708683	CDHR3	cadherin related family member 3
244889_at	0.945	0.02026401	-2.4799058	-4.21	-2.7410477	LOC400499///LOC388210	vitellogenin///uncharacterized LOC388210
215715_at	0.945	0.0223643	-2.4294362	-4.21	-3.6158015	SLC6A2	solute carrier family 6 member 2

223256_at	0.945	0.02243066	-2.4281096	-4.21	-1.417033	G2E3	G2/M-phase specific E3 ubiquitin protein ligase
215221_at	0.945	0.02245573	-2.4276095	-4.21	-4.0592299		
208616_s_at	0.945	0.01643685	-2.589097	-4.21	-4.2500632	PTP4A2	protein tyrosine phosphatase type IVA, member 2
243625_at	0.945	0.01019545	-2.8380556	-4.21	-2.9062802		
231688_at	0.945	0.00246191	-3.5917646	-4.21	-4.1239513	MMP8	matrix metallopeptidase 8
218227_at	0.945	0.01463997	-2.6505848	-4.21	-2.1490365	NUBP2	nucleotide binding protein 2
242555_at	0.945	0.01181529	-2.758125	-4.21	-3.2446932	C16orf87	chromosome 16 open reading frame 87
221399_at	0.945	0.01481369	-2.6452874	-4.21	-3.4870817	EDA2R	ectodysplasin A2 receptor
222934_s_at	0.945	0.01192982	-2.7537962	-4.21	-2.1223253	CLEC4E	C-type lectin domain family 4 member E
1564881_at	0.945	0.01896814	-2.5166797	-4.21	-4.0028566	LINC01618	long intergenic non-protein coding RNA 1618
207894_s_at	0.945	0.0210448	-2.4629725	-4.21	-3.5135528	TCL6	T-cell leukemia/lymphoma 6 (non-protein coding)
233198_at	0.945	0.0231854	-2.4132707	-4.21	-2.0736346	GOLGA2P5	golgin A2 pseudogene 5
211799_x_at	0.945	0.00682438	-3.0566701	-4.21	-3.2339879	HLA-C	major histocompatibility complex, class I, C
1557698_at	0.945	0.01300376	-2.7150242	-4.22	-2.9107779		
235911_at	0.945	0.00907004	-2.907101	-4.22	-3.3981188	MELTF	melanotransferrin
1562103_at	0.945	0.02347606	-2.4076742	-4.22	-1.6291031	LINC01359	long intergenic non-protein coding RNA 1359
223394_at	0.945	0.02370837	-2.4032468	-4.22	-2.54459	SERTAD1	SERTA domain containing 1

218829_s_at	0.945	0.01260712	-2.7289774	-4.22	-2.9553262	CHD7	chromodomain helicase DNA binding protein 7
1560810_at	0.945	0.02406406	-2.3965445	-4.22	-4.2592342	LOC101927735	uncharacterized LOC101927735
221398_at	0.945	0.00573992	-3.1608596	-4.22	-3.7827812	TAS2R8	taste 2 receptor member 8
218408_at	0.945	0.01773799	-2.5548947	-4.22	-2.2487634	TIMM10	translocase of inner mitochondrial membrane 10 homolog (yeast)
223684_s_at	0.945	0.01722145	-2.5681825	-4.22	-2.7039525	SMUG1	single-strand-selective monofunctional uracil-DNA glycosylase 1
238519_at	0.945	0.02020705	-2.4882431	-4.22	-3.1132126	DDI2///RSC1A1	DNA damage inducible 1 homolog 2///regulatory solute carrier protein, family 1, member 1
238901_at	0.945	0.01801473	-2.5479234	-4.22	-1.4907127	LINC01133	long intergenic non-protein coding RNA 1133
244496_at	0.945	0.01180166	-2.7718356	-4.22	-2.9247818		
243974_at	0.945	0.02227543	-2.4374264	-4.22	-1.5505569		
241840_at	0.945	0.01809705	-2.545869	-4.22	-1.6562344		
224662_at	0.945	0.02032772	-2.4855606	-4.22	-3.2112476	KIF5B	kinesin family member 5B
244041_at	0.945	0.01020847	-2.8374795	-4.22	-3.0975411	STX6	syntaxin 6
236856_x_at	0.945	0.00975916	-2.8736943	-4.22	-3.301036		
211947_s_at	0.945	0.02001977	-2.4924361	-4.22	-2.2497411	PRRC2C	proline rich coiled-coil 2C
214779_s_at	0.945	0.01309635	-2.7118254	-4.22	-2.2138887	SGSM3	small G protein signaling modulator 3
214523_at	0.945	0.01197639	-2.7651593	-4.22	-3.5972092	CEBPE	CCAAT/enhancer binding protein epsilon
1553428_at	0.945	0.02253448	-2.4322152	-4.22	-1.8219361	LY86-AS1	LY86 antisense RNA 1

236237_at	0.945	0.01206303	-2.7618829	-4.22	-3.4059112		
236498_s_at	0.945	0.02483956	-2.3822413	-4.22	-1.9506514	FAAP20	Fanconi anemia core complex associated protein 20
230574_at	0.945	0.01837475	-2.5390025	-4.22	-2.7887897	LOC100130938	uncharacterized LOC100130938
215759_at	0.945	0.0080583	-2.9801855	-4.23	-3.2857998	ANKRD53	ankyrin repeat domain 53
213537_at	0.945	0.01764492	-2.5572618	-4.23	-2.6547492	HLA-DPA1	major histocompatibility complex, class II, DP alpha 1
240207_at	0.945	0.01648383	-2.5971452	-4.23	-2.7481305		
243510_at	0.945	0.02507638	-2.3779549	-4.23	-1.836234		
1555100_at	0.945	0.02287975	-2.4253548	-4.23	-1.5923241	APLF	aprataxin and PNKP like factor
1557895_at	0.945	0.0100854	-2.8586634	-4.23	-2.991644	FLJ35934	FLJ35934
229270_x_at	0.945	0.02306341	-2.4217443	-4.23	-1.4843268	SSBP4	single stranded DNA binding protein 4
1554459_s_at	0.945	0.01447523	-2.6665617	-4.23	-3.0808833	CFHR3	complement factor H related 3
223486_at	0.945	0.02541751	-2.3718454	-4.23	-2.1226261	GTPBP8	GTP binding protein 8 (putative)
239448_at	0.945	0.02123181	-2.4659189	-4.23	-1.6427273		
234255_at	0.945	0.01919505	-2.5192703	-4.23	-2.9686099		
1556696_s_at	0.945	0.01923634	-2.518298	-4.23	-2.6163498	NR2F1-AS1	NR2F1 antisense RNA 1
241063_at	0.945	0.01551952	-2.6243488	-4.23	-2.1166266		
211419_s_at	0.945	0.01105611	-2.8014262	-4.23	-3.0607438	CHN2	chimerin 2
227641_at	0.945	0.01735188	-2.5739061	-4.23	-2.0276122	FBXL16	F-box and leucine rich repeat protein 16
204930_s_at	0.945	0.02399748	-2.4037818	-4.23	-1.7051899	BNIP1	BCL2 interacting protein 1
242695_at	0.945	0.00990848	-2.8667554	-4.23	-2.9918652		
208043_at	0.945	0.02161837	-2.4577563	-4.23	-2.4261934		

202907_s_at	0.945	0.02644745	-2.3538444	-4.23	-2.0768666	NBN	nibrin
208412_s_at	0.945	0.01776253	-2.5632896	-4.24	-3.0454614	RARB	retinoic acid receptor beta
239720_at	0.945	0.01108654	-2.815293	-4.24	-2.9559879	ZGLP1	zinc finger, GATA-like protein 1
241547_at	0.945	0.02452786	-2.3938666	-4.24	-2.4888278	A1CF	APOBEC1 complementation factor
1563721_at	0.945	0.02200708	-2.4496835	-4.24	-2.0897329		
210681_s_at	0.945	0.0268199	-2.3474923	-4.24	-1.9064407	MIR6125///USP15	microRNA 6125///ubiquitin specific peptidase 15
1565666_s_at	0.945	0.01796866	-2.5580469	-4.24	-2.7040447	MUC6	mucin 6, oligomeric mucus/gel-forming
244082_at	0.945	0.02705077	-2.3435953	-4.24	-1.0162711		
1555988_a_at	0.945	0.01484773	-2.6550341	-4.24	-2.5254045	LINC00661	long intergenic non-protein coding RNA 661
212833_at	0.945	0.01814004	-2.5537307	-4.24	-2.6477171	SLC25A46	solute carrier family 25 member 46
231764_at	0.945	0.02484297	-2.3880687	-4.24	-1.2082859	CHRAC1	chromatin accessibility complex 1
209439_s_at	0.945	0.02231199	-2.4434425	-4.24	-3.0779668	PHKA2	phosphorylase kinase regulatory subunit alpha 2
1552372_at	0.945	0.00535466	-3.2224427	-4.24	-2.4103318	C4orf33	chromosome 4 open reading frame 33
204020_at	0.945	0.02733433	-2.3388501	-4.24	-2.4874595	PURA	purine rich element binding protein A
241477_at	0.945	0.01591559	-2.6234403	-4.24	-3.2124616	MIR631///NEIL1	microRNA 631///nei like DNA glycosylase 1
231003_at	0.945	0.02743184	-2.3372289	-4.24	-1.8397009	SLC35B3	solute carrier family 35 member B3
204202_at	0.945	0.01527319	-2.6421971	-4.24	-2.7213802	IQCE	IQ motif containing E
230345_at	0.945	0.02536442	-2.378621	-4.24	-3.3780287	SEMA7A	semaphorin 7A (John Milton Hagen blood group)
1568406_at	0.945	0.0051084	-3.2448814	-4.24	-4.4983798		

235846_at	0.945	0.02094317	-2.4797138	-4.24	-2.2836192	FSBP///RAD54B	fibrinogen silencer binding protein///RAD54 homolog B (S. cerevisiae)
219753_at	0.945	0.01632194	-2.6119406	-4.24	-2.6490541	STAG3	stromal antigen 3
230499_at	0.945	0.01004516	-2.8447535	-4.24	-3.635711	BIRC3	baculoviral IAP repeat containing 3
230063_at	0.945	0.00238807	-3.7032063	-4.24	-2.699812	ZNF264	zinc finger protein 264
1556201_at	0.945	0.0202328	-2.4954034	-4.24	-1.4198943	RNASET2	ribonuclease T2
211162_x_at	0.945	0.02040223	-2.4916155	-4.24	-2.1140046	SCD	stearoyl-CoA desaturase
1557215_at	0.945	0.0020893	-3.771017	-4.24	-4.826786	LINC00648	long intergenic non-protein coding RNA 648
218714_at	0.945	0.00939129	-2.9094039	-4.24	-3.7610122	PRR14	proline rich 14
1555790_a_at	0.945	0.01906763	-2.531012	-4.24	-2.3670518	TMEM192///ZNF320	transmembrane protein 192///zinc finger protein 320
213230_at	0.945	0.00962594	-2.8979602	-4.25	-3.0900238	CDR2L	cerebellar degeneration related protein 2 like
216974_at	0.945	0.02412078	-2.4079648	-4.25	-2.4596806	KITLG	KIT ligand
218369_s_at	0.945	0.01602708	-2.6202577	-4.25	-2.6219189	EXOSC1	exosome component 1
209396_s_at	0.945	0.0238133	-2.4138188	-4.25	-2.2678126	CHI3L1	chitinase 3 like 1
203136_at	0.945	0.02177115	-2.4620386	-4.25	-2.6267415	RABAC1	Rab acceptor 1
240506_at	0.945	0.01236815	-2.7649518	-4.25	-3.1883309		
224687_at	0.945	0.01713716	-2.5896613	-4.25	-2.9536433	ANKIB1	ankyrin repeat and IBR domain containing 1
202281_at	0.945	0.02660918	-2.3567693	-4.25	-1.9730272	GAK	cyclin G associated kinase
202312_s_at	0.945	0.02933037	-2.306665	-4.25	-3.1011569	COL1A1	collagen type I alpha 1 chain
238370_x_at	0.945	0.01950032	-2.5207654	-4.25	-2.4876062		

237959_at	0.945	0.01650305	-2.6069021	-4.25	-2.6501324		
241069_at	0.945	0.01268101	-2.7534229	-4.25	-5.1522907		
218778_x_at	0.945	0.01973354	-2.5153304	-4.25	-2.4325489	EPS8L1	EPS8 like 1
1561890_at	0.945	0.01768961	-2.5751234	-4.25	-2.5115008	C10orf128	chromosome 10 open reading frame 128
211640_x_at	0.945	0.02733552	-2.3444474	-4.25	-4.1960792	IGHM///IGHG1	immunoglobulin heavy constant mu///immunoglobulin heavy constant gamma 1 (G1m marker)
210964_s_at	0.945	0.01495192	-2.6637746	-4.25	-2.8400234	GYG2	glycogenin 2
1557139_at	0.945	0.01776367	-2.573207	-4.25	-2.6528347	DDX11-AS1	DDX11 antisense RNA 1
235039_x_at	0.945	0.01990893	-2.5112823	-4.25	-2.4291121	LIN9	lin-9 DREAM MuvB core complex component
209324_s_at	0.945	0.01794115	-2.5686448	-4.25	-2.2852652	RGS16	regulator of G-protein signaling 16
211462_s_at	0.945	0.02499897	-2.3916165	-4.25	-2.2663836	TBL1Y	transducin (beta)-like 1, Y-linked
236634_at	0.945	0.02292898	-2.4383398	-4.25	-2.0865906	C8orf48	chromosome 8 open reading frame 48
225216_at	0.945	0.03020335	-2.293212	-4.25	-1.7450436	FAM199X	family with sequence similarity 199, X-linked
218521_s_at	0.945	0.02298823	-2.437157	-4.25	-2.2688558	UBE2W	ubiquitin conjugating enzyme E2 W (putative)
212764_at	0.945	0.02778935	-2.3368998	-4.25	-2.6348676	ZEB1	zinc finger E-box binding homeobox 1
1564798_at	0.945	0.0279336	-2.3345243	-4.26	-2.4416266		
201669_s_at	0.945	0.03039321	-2.290333	-4.26	-1.7735377	MARCKS	myristoylated alanine rich protein kinase C substrate
243913_at	0.945	0.02810888	-2.331653	-4.26	-1.6306041		
204717_s_at	0.945	0.01345766	-2.7259368	-4.26	-3.3112487	SLC29A2	solute carrier family 29 member 2

213539_at	0.945	0.01845256	-2.5557317	-4.26	-3.0695649	CD3D	CD3d molecule
207301_at	0.945	0.02823991	-2.3295172	-4.26	-1.7917881	EFNA5	ephrin A5
1553024_at	0.945	0.02561095	-2.3805336	-4.26	-1.7435062	DAOA-AS1	DAOA antisense RNA 1
233370_at	0.945	0.0227997	-2.4409302	-4.26	-1.3248549		
1563250_at	0.945	0.02632012	-2.3679912	-4.26	-1.6233143	LOC101927362	uncharacterized LOC101927362
226132_s_at	0.945	0.01639989	-2.6212004	-4.26	-1.6541454	MANEAL	mannosidase endo-alpha like
220553_s_at	0.945	0.00796103	-3.0077548	-4.26	-4.1926326	PRPF39	pre-mRNA processing factor 39
226105_at	0.945	0.01389145	-2.7112366	-4.26	-3.0957547	C1GALT1	core 1 synthase, glycoprotein-N-acetylgalactosamine 3-beta-galactosyltransferase 1
204421_s_at	0.945	0.02396877	-2.4179832	-4.26	-2.4841285	FGF2	fibroblast growth factor 2
226180_at	0.945	0.02307442	-2.4354417	-4.26	-2.3975952	WDR36	WD repeat domain 36
35666_at	0.945	0.0267107	-2.3612156	-4.26	-3.9869003	SEMA3F	semaphorin 3F
230853_at	0.945	0.02415786	-2.4143692	-4.26	-2.7914672	FEM1A	fem-1 homolog A
1570007_at	0.945	0.02916199	-2.3147381	-4.26	-2.2795894	LOC101927933///LRRC8C	uncharacterized LOC101927933///leucine rich repeat containing 8 family member C
1563073_at	0.945	0.03171124	-2.2707859	-4.26	-3.5704505	LINC01282	long intergenic non-protein coding RNA 1282
211856_x_at	0.945	0.02429943	-2.4116806	-4.26	-2.8988276	CD28	CD28 molecule
240972_at	0.945	0.02696874	-2.3567887	-4.26	-1.6882752		
237690_at	0.945	0.0143284	-2.696865	-4.26	-3.3293191	ADGRF4	adhesion G protein-coupled receptor F4
207031_at	0.945	0.01649881	-2.6184231	-4.26	-2.3726761	NKX3-2	NK3 homeobox 2
241450_at	0.945	0.01848831	-2.5548416	-4.26	-2.5472099	RSPO1	R-spondin 1

222745_s_at	0.945	0.02220368	-2.4611759	-4.26	-3.1084102	KATNBL1	katanin regulatory subunit B1 like 1
1557812_a_at	0.945	0.01954286	-2.5292869	-4.26	-2.884553		
218351_at	0.945	0.00520602	-3.2698865	-4.26	-4.260662	COMMD8	COMM domain containing 8
37145_at	0.945	0.03241473	-2.2606528	-4.27	-1.8445154	GNLY	granulysin
231418_at	0.945	0.01466453	-2.6860907	-4.27	-3.3550175		
238037_at	0.945	0.02495653	-2.3993876	-4.27	-4.0854693	LMLN	leishmanolysin like peptidase
212213_x_at	0.945	0.03249531	-2.2595048	-4.27	-2.3012435	OPA1	OPA1, mitochondrial dynamin like GTPase
222843_at	0.945	0.02722144	-2.3524911	-4.27	-1.8477589	FIGNL1	fidgetin like 1
1556405_s_at	0.945	0.03258539	-2.2582246	-4.27	-2.4571497	LOC374890	uncharacterized LOC374890
233827_s_at	0.945	0.01698886	-2.6048929	-4.27	-3.0893924	SUPT16H	SPT16 homolog, facilitates chromatin remodeling subunit
237316_at	0.945	0.01703405	-2.6036639	-4.27	-3.7983912	ANKDD1A	ankyrin repeat and death domain containing 1A
225862_at	0.945	0.01486006	-2.679931	-4.27	-3.0218965	SLC25A26	solute carrier family 25 member 26
209507_at	0.945	0.01194098	-2.7975991	-4.27	-3.768616	RPA3	replication protein A3
234434_at	0.945	0.01382441	-2.7134795	-4.27	-3.5902951		
234866_s_at	0.945	0.00877588	-2.9619155	-4.27	-3.8655348	ZNRD1ASP	zinc ribbon domain containing 1 antisense, pseudogene
208619_at	0.945	0.03033792	-2.2964971	-4.27	-1.9299884	DDB1	damage specific DNA binding protein 1
1553281_at	0.945	0.03296524	-2.2528612	-4.27	-1.5497281	PLCXD2	phosphatidylinositol specific phospholipase C X domain containing 2
242088_at	0.945	0.0202672	-2.5124721	-4.27	-3.2580544	KLHL24	kelch like family member 24

221087_s_at	0.945	0.0246271	-2.4055126	-4.27	-2.3716138	APOL3	apolipoprotein L3
204885_s_at	0.945	0.0078503	-3.0395433	-4.27	-2.1891582	MSLN	mesothelin
237691_x_at	0.945	0.01738464	-2.5942317	-4.27	-3.1491009	ENO3	enolase 3
1552745_at	0.945	0.01522364	-2.6686792	-4.27	-3.6429988	SLCO6A1	solute carrier organic anion transporter family member 6A1
1561064_a_at	0.945	0.01226826	-2.78495	-4.27	-3.5505403		
1559190_s_at	0.945	0.01593221	-2.6345488	-4.27	-3.3749654	RDH13	retinol dehydrogenase 13
205564_at	0.945	0.03098896	-2.2866735	-4.27	-1.0495321	PAGE4	PAGE family member 4
218007_s_at	0.945	0.02070641	-2.5025482	-4.27	-2.0350804	RPS27L	ribosomal protein S27 like
1556015_a_at	0.945	0.01240399	-2.7797992	-4.27	-4.0145418	MESP2	mesoderm posterior bHLH transcription factor 2
222129_at	0.945	0.02084224	-2.4995188	-4.27	-3.3814978	FAM134A	family with sequence similarity 134 member A
220704_at	0.945	0.02092126	-2.497765	-4.27	-2.8723811	IKZF1	IKAROS family zinc finger 1
238829_at	0.945	0.02092702	-2.4976373	-4.27	-2.5659276		
1553329_at	0.945	0.02898143	-2.3235353	-4.27	-2.1662265	SSMEM1	serine rich single-pass membrane protein 1
217884_at	0.945	0.01322158	-2.7498705	-4.27	-3.5197693	NAT10	N-acetyltransferase 10
1556486_at	0.945	0.03145	-2.2798299	-4.27	-1.960021	LOC105378577	basic proline-rich protein-like
209651_at	0.945	0.02634692	-2.3743312	-4.27	-2.6044215	TGFB1I1	transforming growth factor beta 1 induced transcript 1
206350_at	0.945	0.02386134	-2.4278941	-4.27	-2.0950915	APCS	amyloid P component, serum
215503_at	0.945	0.00359252	-3.4123372	-4.27	-4.3154568	SPINT3	serine peptidase inhibitor, Kunitz type 3
219040_at	0.945	0.02119538	-2.4917286	-4.27	-3.0126792	CORO7	coronin 7

213615_at	0.945	0.03168132	-2.2764305	-4.27	-1.8741541	LPCAT3	lysophosphatidylcholine acyltransferase 3
1570337_at	0.945	0.03429735	-2.2344843	-4.27	-2.4013764	FIGLA	folliculogenesis specific bHLH transcription factor
206934_at	0.945	0.01654044	-2.6172592	-4.27	-4.1306009	SIRPB1	signal regulatory protein beta 1
224712_x_at	0.945	0.02590933	-2.3820829	-4.28	-1.8187803	SMIM7	small integral membrane protein 7
219148_at	0.945	0.0296166	-2.3134796	-4.28	-2.474251	PBK	PDZ binding kinase
220189_s_at	0.945	0.03477632	-2.2280344	-4.28	-1.6413002	MGAT4B	mannosyl (alpha-1,3-)-glycoprotein beta-1,4-N-acetylglucosaminyltransferase, isozyme B
1556872_s_at	0.945	0.0216824	-2.4811814	-4.28	-2.6012664	IQSEC3	IQ motif and Sec7 domain 3
226190_at	0.945	0.01627241	-2.6375984	-4.28	-2.9782253	MAP3K13	mitogen-activated protein kinase kinase kinase 13
238039_at	0.945	0.03491366	-2.2261997	-4.28	-4.3400776	SCAMP1-AS1	SCAMP1 antisense RNA 1
218577_at	0.945	0.02186222	-2.4773429	-4.28	-2.916613	LRRC40	leucine rich repeat containing 40
207109_at	0.945	0.02198635	-2.4747104	-4.28	-2.7613433	POU2F3	POU class 2 homeobox 3
228514_at	0.945	0.01336047	-2.7449636	-4.28	-2.8589989		
1564482_at	0.945	0.01947484	-2.5414628	-4.28	-2.12274	ATP5O	ATP synthase, H+ transporting, mitochondrial F1 complex, O subunit
235253_at	0.945	0.03026508	-2.3034146	-4.28	-1.5086653	RAD1	RAD1 checkpoint DNA exonuclease
225208_s_at	0.945	0.02505987	-2.4051408	-4.28	-2.5243887	FAM103A1	family with sequence similarity 103 member A1
242508_at	0.945	0.03286506	-2.2593802	-4.28	-2.5753944	USF3	upstream transcription factor family member 3

223910_at	0.945	0.02763978	-2.3521023	-4.28	-2.2047671	ERN2	endoplasmic reticulum to nucleus signaling 2
228429_x_at	0.945	0.01681322	-2.6223067	-4.28	-3.3613108	KIF9	kinesin family member 9
236185_at	0.945	0.01143769	-2.8366582	-4.28	-3.1421238	NHLRC2	NHL repeat containing 2
205417_s_at	0.945	0.02246711	-2.4646436	-4.28	-2.6055676	DAG1	dystroglycan 1
1562718_at	0.945	0.02493872	-2.4073944	-4.28	-2.2960348	LOC102724449	uncharacterized LOC102724449
237142_at	0.945	0.0138301	-2.7287256	-4.28	-2.6940575	PPARA	peroxisome proliferator activated receptor alpha
1552612_at	0.945	0.03043565	-2.3007999	-4.28	-1.5971049	CDC42SE2	CDC42 small effector 2
238735_at	0.945	0.00796981	-3.0072365	-4.28	-3.8569891		
227055_at	0.945	0.03611985	-2.210361	-4.28	-3.1809083	METTL7B	methyltransferase like 7B
204929_s_at	0.945	0.02272937	-2.4592364	-4.28	-2.7907804	VAMP5	vesicle associated membrane protein 5
242028_at	0.945	0.02825749	-2.3418172	-4.28	-2.2286047	ZNF709	zinc finger protein 709
211595_s_at	0.945	0.01959966	-2.5384829	-4.28	-2.983146	MRPS11	mitochondrial ribosomal protein S11
238097_at	0.945	0.02523375	-2.4019238	-4.28	-1.9635581	GAS6-AS1	GAS6 antisense RNA 1
1567055_at	0.945	0.03071702	-2.2965159	-4.28	-1.439421	OR1C1	olfactory receptor family 1 subfamily C member 1
231394_at	0.945	0.01187183	-2.8189586	-4.28	-3.5860524	PCDHB3	protocadherin beta 3
1559789_a_at	0.945	0.01746946	-2.6043651	-4.28	-2.7925702	ZNF781	zinc finger protein 781
203403_s_at	0.945	0.02868675	-2.3347908	-4.28	-2.6610476	RNF6	ring finger protein 6
217184_s_at	0.945	0.01068694	-2.8688575	-4.28	-4.1796276	LTK	leukocyte receptor tyrosine kinase
1565923_at	0.945	0.01424844	-2.7146993	-4.28	-3.4103103		
227311_at	0.945	0.03407776	-2.2424816	-4.28	-2.6659271	SNX25	sorting nexin 25

218588_s_at	0.945	0.01990038	-2.520916	-4.28	-2.908155	FAM114A2	family with sequence similarity 114 member A2
211579_at	0.945	0.03681902	-2.2013974	-4.28	-1.4166816	ITGB3	integrin subunit beta 3
206964_at	0.945	0.00831435	-2.9873422	-4.28	-3.2510101	NAT8B	N-acetyltransferase 8B (putative, gene/pseudogene)
239825_at	0.945	0.02568409	-2.3936865	-4.28	-2.7173685		
242418_at	0.945	0.03684599	-2.2010547	-4.28	-1.7737382	C2orf27A	chromosome 2 open reading frame 27A
1556567_at	0.945	0.01642659	-2.6331902	-4.28	-2.1104964	NAP1L4	nucleosome assembly protein 1 like 4
234247_at	0.945	0.01249184	-2.7764941	-4.29	-2.9163926		
207530_s_at	0.945	0.01651364	-2.6307189	-4.29	-3.2884824	CDKN2B	cyclin dependent kinase inhibitor 2B
217736_s_at	0.945	0.00697995	-3.1262642	-4.29	-3.6700239	EIF2AK1	eukaryotic translation initiation factor 2 alpha kinase 1
215240_at	0.945	0.00185947	-3.897558	-4.29	-4.449741	ITGB3	integrin subunit beta 3
1552892_at	0.945	0.03200516	-2.2773494	-4.29	-2.104182	TNFRSF13C	TNF receptor superfamily member 13C
230874_at	0.945	0.01468296	-2.7005419	-4.29	-3.0377649	SLC36A4	solute carrier family 36 member 4
211126_s_at	0.945	0.02051333	-2.5171977	-4.29	-3.1396701	CSRP2	cysteine and glycine rich protein 2
230609_at	0.945	0.0205345	-2.5167152	-4.29	-2.4160133	CLINT1	clathrin interactor 1
1561467_at	0.945	0.037624	-2.1912652	-4.29	-1.1830555		
222239_s_at	0.945	0.03244518	-2.2709631	-4.29	-2.6991431	INTS6	integrator complex subunit 6
214249_at	0.945	0.02488119	-2.4084681	-4.29	-2.6453156	TRIM2	tripartite motif containing 2
215722_s_at	0.945	0.02704303	-2.3696146	-4.29	-2.4195972	SNRPA1	small nuclear ribonucleoprotein polypeptide A'
210657_s_at	0.945	0.01836641	-2.580848	-4.29	-2.9970685	SEPT4	septin 4

1553369_at	0.945	0.02984413	-2.316316	-4.29	-2.3256879	FAM129C	family with sequence similarity 129 member C
244399_at	0.945	0.03810834	-2.1852625	-4.29	-1.9948048		
1569975_at	0.945	0.01578851	-2.6662498	-4.29	-3.1573982		
203709_at	0.945	0.02099847	-2.5062546	-4.29	-2.8245978	PHKG2	phosphorylase kinase catalytic subunit gamma 2
238909_at	0.945	0.01854506	-2.5762939	-4.29	-3.1852169	S100A10	S100 calcium binding protein A10
231324_at	0.945	0.03016194	-2.311358	-4.29	-1.8626014		
243067_at	0.945	0.02451154	-2.4239594	-4.29	-2.6409482		
1564911_at	0.945	0.02451261	-2.423939	-4.29	-2.574269	SNHG4	small nucleolar RNA host gene 4
228982_s_at	0.945	0.02451786	-2.4238386	-4.29	-2.6239116	USP42	ubiquitin specific peptidase 42
211488_s_at	0.945	0.03259808	-2.2687624	-4.29	-2.3179311	ITGB8	integrin subunit beta 8
212680_x_at	0.945	0.02116351	-2.502586	-4.29	-2.0993228	PPP1R14B	protein phosphatase 1 regulatory inhibitor subunit 14B
242655_at	0.945	0.01934801	-2.5445093	-4.29	-2.911855	BARD1	BRCA1 associated RING domain 1
242758_x_at	0.945	0.02458853	-2.42249	-4.29	-2.9119888	KDM3A	lysine demethylase 3A
214416_at	0.945	0.02760653	-2.3599589	-4.29	-2.7025464		
204911_s_at	0.945	0.0357518	-2.2200286	-4.29	-2.0967121	TRIM3	tripartite motif containing 3
211531_x_at	0.945	0.03853731	-2.1800032	-4.29	-2.3251742	PRB1	proline rich protein BstNI subfamily 1
210083_at	0.945	0.03853913	-2.1799809	-4.29	-2.6678852	SEMA7A	semaphorin 7A (John Milton Hagen blood group)
237638_at	0.945	0.01888976	-2.5676228	-4.29	-2.5913462		
228401_at	0.945	0.0278091	-2.3565322	-4.29	-2.3560882	ATAD2	ATPase family, AAA domain containing 2
1555595_at	0.945	0.02479187	-2.418629	-4.29	-3.768228	SCRN3	secernin 3

1555745_a_at	0.945	0.02783522	-2.3560921	-4.29	-2.4634857	LYZ	lysozyme
224727_at	0.945	0.03056534	-2.3051331	-4.29	-1.4670885	EMC10	ER membrane protein complex subunit 10
226774_at	0.945	0.02215863	-2.4810171	-4.29	-2.4053029	FAM120B	family with sequence similarity 120B
201899_s_at	0.945	0.03645321	-2.2109027	-4.29	-2.2916784	UBE2A	ubiquitin conjugating enzyme E2 A
1560028_at	0.945	0.0311441	-2.2963323	-4.3	-2.2124644	C11orf57	chromosome 11 open reading frame 57
207600_at	0.945	0.01945426	-2.5537403	-4.3	-2.5614819	KCNC3	potassium voltage-gated channel subfamily C member 3
223032_x_at	0.945	0.02540305	-2.4071985	-4.3	-1.8675321	PRELID1	PRELI domain containing 1
1553849_at	0.945	0.02546189	-2.4061115	-4.3	-2.5166372	CCDC26	CCDC26 long non-coding RNA
215670_s_at	0.945	0.00815257	-3.0214154	-4.3	-2.1804389	SCAND2P	SCAN domain containing 2 pseudogene
1564807_at	0.945	0.02204755	-2.4833793	-4.3	-3.1070965		
234366_x_at	0.945	0.03680347	-2.2064049	-4.3	-1.491655	IGLJ3///CKAP2///IGLV@///IGLC1	immunoglobulin lambda joining 3///cytoskeleton associated protein 2///immunoglobulin lambda variable cluster///immunoglobulin lambda constant 1
218854_at	0.945	0.02212117	-2.4818125	-4.3	-2.7871	DSE	dermatan sulfate epimerase
1553639_a_at	0.945	0.01960781	-2.5500298	-4.3	-2.6298011	PPARGC1B	PPARG coactivator 1 beta
239587_at	0.945	0.03138763	-2.2926738	-4.3	-1.8469398	TLR3	toll like receptor 3
227420_at	0.945	0.02291329	-2.4652562	-4.3	-3.2091366	TNFAIP8L1	TNF alpha induced protein 8 like 1
1559494_at	0.945	0.00816462	-3.0492906	-4.3	-3.90075	SLC35G2	solute carrier family 35 member G2
222356_at	0.945	0.0340349	-2.2485293	-4.3	-1.7555101		

243541_at	0.945	0.02460408	-2.4221936	-4.3	-2.2481353	IL31RA	interleukin 31 receptor A
217677_at	0.945	0.03167915	-2.2883282	-4.3	-2.5446298	PLEKHA2	pleckstrin homology domain containing A2
1566294_at	0.945	0.0111649	-2.8699327	-4.3	-2.7680973		
1556334_s_at	0.945	0.02473303	-2.4197432	-4.3	-2.4594618		
1566973_at	0.945	0.04016184	-2.1605513	-4.3	-2.0740455		
238745_at	0.945	0.01864994	-2.5736395	-4.3	-2.551347		
223736_at	0.945	0.01714779	-2.6270962	-4.3	-2.9388991	IFT81	intraflagellar transport 81
1557218_s_at	0.945	0.04021879	-2.1598822	-4.3	-3.5208731	FANCB	Fanconi anemia complementation group B
217364_x_at	0.945	0.01400274	-2.7402434	-4.3	-2.6980727		
239841_at	0.945	0.02003036	-2.5399589	-4.3	-3.3068094		
230467_at	0.945	0.01717924	-2.6262256	-4.3	-2.6562521	TMEM52	transmembrane protein 52
223210_at	0.945	0.03489444	-2.2367936	-4.3	-1.9849573	CHURC1	churchill domain containing 1
238912_x_at	0.945	0.0172831	-2.6233615	-4.3	-2.9011249	C9orf85	chromosome 9 open reading frame 85
221263_s_at	0.945	0.02335353	-2.4562846	-4.3	-2.0416243	SF3B5	splicing factor 3b subunit 5
216103_at	0.945	0.02874391	-2.3410111	-4.3	-2.4241864	ACOT11	acyl-CoA thioesterase 11
242231_at	0.945	0.03454091	-2.2415884	-4.3	-1.5080804		
204552_at	0.945	0.0320905	-2.2822582	-4.3	-2.5853074	INPP4A	inositol polyphosphate-4-phosphatase type I A
214395_x_at	0.945	0.04049766	-2.1566186	-4.3	-1.3691457	EEF1D	eukaryotic translation elongation factor 1 delta
236070_at	0.945	0.03458301	-2.2410151	-4.3	-2.6251292	C2orf81	chromosome 2 open reading frame 81
1553378_a_at	0.945	0.0351186	-2.2337762	-4.3	-1.6761653	HEATR9	HEAT repeat containing 9

224752_at	0.945	0.02348206	-2.4536951	-4.3	-3.2248607	LOC101930655///C7orf73	uncharacterized LOC101930655///chromosome 7 open reading frame 73
227215_at	0.945	0.01897459	-2.5655119	-4.3	-3.1492189	GOPC	golgi associated PDZ and coiled-coil motif containing
235057_at	0.945	0.04065949	-2.1547338	-4.3	-2.8765789	ITCH	itchy E3 ubiquitin protein ligase
1562648_at	0.945	0.03526419	-2.2318257	-4.3	-2.5460442	CCDC88A	coiled-coil domain containing 88A
1565756_a_at	0.945	0.03798317	-2.1915349	-4.3	-1.9283316	METAP1D	methionyl aminopeptidase type 1D (mitochondrial)
1556613_s_at	0.945	0.02299842	-2.463509	-4.3	-1.2168084	DPY19L4	dpy-19 like 4 (C. elegans)
212914_at	0.945	0.0291104	-2.3350526	-4.3	-2.8172675	CBX7	chromobox 7
215766_at	0.945	0.04094702	-2.1514017	-4.3	-1.6488632	GSTA1	glutathione S-transferase alpha 1
223313_s_at	0.945	0.02994635	-2.3217155	-4.3	-3.4182433	SNORA11E///SNORA11D///MAGED4///MAGED4B	small nucleolar RNA, H/ACA box 11E///small nucleolar RNA, H/ACA box 11D///MAGE family member D4///MAGE family member D4B
209867_s_at	0.945	0.03285519	-2.2711608	-4.3	-2.3757929	ADGRL3	adhesion G protein-coupled receptor L3
214569_at	0.945	0.03287353	-2.2708975	-4.3	-2.6817207	IFNA5	interferon alpha 5
226947_at	0.945	0.03188368	-2.2853011	-4.3	-2.7326053	GUSBP1///GUSBP4	glucuronidase, beta pseudogene 1///glucuronidase, beta pseudogene 4
1556469_s_at	0.945	0.03296717	-2.2695555	-4.3	-2.3169787	LOC105376166	uncharacterized LOC105376166
213023_at	0.945	0.041466	-2.1454401	-4.3	-2.6405176	UTRN	utrophin
212296_at	0.945	0.02586591	-2.3987105	-4.3	-1.4225047	PSMD14	proteasome 26S subunit, non-ATPase 14
1554219_at	0.945	0.03307433	-2.268024	-4.3	-3.9721192		

215271_at	0.945	0.04156559	-2.1443037	-4.3	-1.7817763	TNN	tenascin N
212100_s_at	0.945	0.03317875	-2.266536	-4.3	-2.0661905	POLDIP3	DNA polymerase delta interacting protein 3
215490_at	0.945	0.03890572	-2.1801939	-4.3	-2.2265601	IBA57	IBA57 homolog, iron-sulfur cluster assembly
1561550_at	0.945	0.02438177	-2.4359303	-4.3	-3.1774022	LOC101928012	uncharacterized LOC101928012
218193_s_at	0.945	0.02121686	-2.5127146	-4.3	-2.9261774	GOLT1B	golgi transport 1B
226355_at	0.945	0.01070205	-2.8681881	-4.3	-2.950388	POC1A	POC1 centriolar protein A
223444_at	0.945	0.01762995	-2.6139143	-4.3	-2.6507506	SENP7	SUMO1/sentrin specific peptidase 7
1555874_x_at	0.945	0.03011617	-2.319048	-4.3	-1.4666909	GLIDR	glioblastoma down-regulated RNA
228492_at	0.945	0.03654088	-2.2150283	-4.3	-2.4799165	USP9Y	ubiquitin specific peptidase 9, Y-linked
1570630_at	0.945	0.04207832	-2.1384917	-4.3	-4.0238073		
1568821_at	0.945	0.03023495	-2.3171903	-4.31	-2.5343863	TTC23	tetratricopeptide repeat domain 23
1561104_at	0.945	0.02394367	-2.4349337	-4.31	-3.1783602	LOC101926962	uncharacterized LOC101926962
234881_at	0.945	0.03083968	-2.3078359	-4.31	-2.1867108		
211930_at	0.945	0.0214617	-2.5072714	-4.31	-3.4484707	HNRNPA3	heterogeneous nuclear ribonucleoprotein A3
221010_s_at	0.945	0.03032406	-2.3158013	-4.31	-3.016591	SIRT5	sirtuin 5
224710_at	0.945	0.02012035	-2.53784	-4.31	-2.5351158	NARR///RAB34	nine-amino acid residue-repeats///RAB34, member RAS oncogene family
232808_at	0.945	0.03273586	-2.272877	-4.31	-1.9301949		
1562011_at	0.945	0.01528196	-2.6983588	-4.31	-3.7775681	DOCK4-AS1	DOCK4 antisense RNA 1
239845_at	0.945	0.02807463	-2.3600502	-4.31	-1.5175388		

206099_at	0.945	0.04294162	-2.1288475	-4.31	-2.9999667	PRKCH	protein kinase C eta
232169_x_at	0.945	0.02831126	-2.3560767	-4.31	-2.9303554	MIR7113///MIR4691///NDUFS8	microRNA 7113///microRNA 4691///NADH:ubiquinone oxidoreductase core subunit S8
205049_s_at	0.945	0.04313818	-2.1266759	-4.31	-3.3443849	CD79A	CD79a molecule
1553185_at	0.945	0.01838241	-2.5940121	-4.31	-2.8448829	RASEF	RAS and EF-hand domain containing
223013_at	0.945	0.04044439	-2.1618069	-4.31	-1.7258207	TBL1XR1	transducin (beta)-like 1 X-linked receptor 1
1555772_a_at	0.945	0.02859082	-2.3514217	-4.31	-2.5066432	CDC25A	cell division cycle 25A
1566169_at	0.945	0.02498431	-2.4243718	-4.31	-2.1170819		
228259_s_at	0.945	0.04054237	-2.1606574	-4.31	-3.6714484	EPB41L4A-AS1	EPB41L4A antisense RNA 1
211836_s_at	0.945	0.04074827	-2.1582501	-4.31	-2.147612	MOG	myelin oligodendrocyte glycoprotein
209118_s_at	0.945	0.03806196	-2.195696	-4.31	-3.0151141	TUBA1A	tubulin alpha 1a
241219_at	0.945	0.02897198	-2.3451423	-4.31	-3.0506794		
242458_at	0.945	0.04093199	-2.1561113	-4.31	-4.3507352	RALGPS2	Ral GEF with PH domain and SH3 binding motif 2
221874_at	0.945	0.01887367	-2.5814319	-4.31	-3.4631679	KIAA1324	KIAA1324
1570180_at	0.945	0.01001019	-2.9489489	-4.31	-2.5057094		
203088_at	0.945	0.02921157	-2.3412339	-4.31	-3.0122369	FBLN5	fibulin 5
218224_at	0.945	0.01899923	-2.5782661	-4.31	-3.5220396	PNMA1	paraneoplastic Ma antigen 1
206451_at	0.945	0.02797239	-2.3617766	-4.31	-2.0460571	TBCCD1	TBCC domain containing 1
224740_at	0.945	0.03798186	-2.1966964	-4.31	-2.4988186	SMIM15	small integral membrane protein 15
1568897_at	0.945	0.02291182	-2.4761819	-4.31	-3.9305366	LOC100422212	eukaryotic translation initiation factor 3 subunit J pseudogene

213619_at	0.945	0.02937191	-2.338635	-4.31	-2.8700504	HNRNPH1	heterogeneous nuclear ribonucleoprotein H1 (H)
208012_x_at	0.945	0.03559654	-2.2332234	-4.31	-2.7072437	SP110	SP110 nuclear body protein
238025_at	0.945	0.04152824	-2.1492289	-4.31	-4.3567804	MLKL	mixed lineage kinase domain like
228092_at	0.945	0.02973198	-2.3328458	-4.31	-2.1459489	CREM	cAMP responsive element modulator
1563475_s_at	0.945	0.0260438	-2.404663	-4.31	-1.5385892	ETFBKMT	electron transfer flavoprotein beta subunit lysine methyltransferase
207042_at	0.945	0.0284992	-2.3529426	-4.31	-3.3749288	E2F2	E2F transcription factor 2
243276_at	0.945	0.01946069	-2.5667998	-4.31	-2.527208	ALS2CL	ALS2 C-terminal like
234958_at	0.945	0.02990897	-2.3300238	-4.31	-2.9967655		
239074_at	0.945	0.02345377	-2.4650352	-4.32	-2.2067619	GRAPL	GRB2 related adaptor protein like
223478_at	0.945	0.01963837	-2.5624534	-4.32	-3.2985719	TIMM8B	translocase of inner mitochondrial membrane 8 homolog B
1556798_a_at	0.945	0.04505451	-2.1059557	-4.32	-1.5552632	RNF144A-AS1	RNF144A antisense RNA 1
205758_at	0.945	0.02639553	-2.3982848	-4.32	-2.189581	CD8A	CD8a molecule
237117_at	0.945	0.03527859	-2.2374842	-4.32	-1.9751059		
243553_x_at	0.945	0.03650229	-2.2212721	-4.32	-2.3202356	TRAF3IP2-AS1	TRAF3IP2 antisense RNA 1
225278_at	0.945	0.03043453	-2.3217338	-4.32	-2.3193256	PRKAB2	protein kinase AMP-activated non-catalytic subunit beta 2
203554_x_at	0.945	0.03931211	-2.1803231	-4.32	-1.527422	PTTG1	pituitary tumor-transforming 1
228323_at	0.945	0.03683876	-2.216901	-4.32	-2.4935735	KNL1	kinetochore scaffold 1
210985_s_at	0.945	0.01777184	-2.6101005	-4.32	-3.209949	SP100	SP100 nuclear antigen
238535_at	0.945	0.02420598	-2.4499572	-4.32	-2.4045197	CEP152	centrosomal protein 152
220512_at	0.945	0.03595811	-2.2284197	-4.32	-2.1009997	DLC1	DLC1 Rho GTPase activating protein

1569030_s_at	0.945	0.02111118	-2.5277943	-4.32	-2.5357252	NUB1	negative regulator of ubiquitin like proteins 1
224204_x_at	0.945	0.03980368	-2.1743991	-4.32	-1.256194	ARNTL2	aryl hydrocarbon receptor nuclear translocator like 2
211044_at	0.945	0.02504393	-2.4232422	-4.32	-2.5111677	TRIM14	tripartite motif containing 14
1561953_at	0.945	0.0343669	-2.256408	-4.32	-2.4755536		
1553984_s_at	0.945	0.03995701	-2.1725648	-4.32	-2.184519	DTYMK	deoxythymidylate kinase
1558470_at	0.945	0.03733839	-2.2104766	-4.32	-4.2069228	VWA3B	von Willebrand factor A domain containing 3B
215652_at	0.945	0.00240615	-3.8354771	-4.32	-4.6580649	SDHD	succinate dehydrogenase complex subunit D
217963_s_at	0.945	0.03738176	-2.2099226	-4.32	-1.992524	BEX3	brain expressed X-linked 3
208184_s_at	0.945	0.03740385	-2.2096407	-4.32	-2.1652345	LOC102724200///TRAPPC10	trafficking protein particle complex subunit 10-like///trafficking protein particle complex 10
220601_at	0.945	0.04337122	-2.1285028	-4.32	-4.0766562	C16orf70	chromosome 16 open reading frame 70
203151_at	0.945	0.03392983	-2.2625112	-4.32	-1.4861765	MAP1A	microtubule associated protein 1A
1558077_s_at	0.945	0.0434942	-2.127148	-4.32	-2.3470303	MDH1B	malate dehydrogenase 1B
219507_at	0.945	0.0375478	-2.2078071	-4.32	-2.9034854	RSRC1	arginine and serine rich coiled-coil 1
203346_s_at	0.945	0.03121695	-2.3096336	-4.32	-2.7254703	MTF2	metal response element binding transcription factor 2
1553086_at	0.945	0.025323	-2.4179881	-4.32	-3.3375257	C11orf40	chromosome 11 open reading frame 40
1565703_at	0.945	0.02014185	-2.5373351	-4.32	-2.4861732	SMAD4	SMAD family member 4

1554420_at	0.945	0.03465485	-2.2524259	-4.32	-2.2241689	ATF3	activating transcription factor 3
227534_at	0.945	0.03467504	-2.2521478	-4.32	-2.7830417	AAED1	AhpC/TSA antioxidant enzyme domain containing 1
214368_at	0.945	0.0403753	-2.1675927	-4.32	-2.2865163	RASGRP2	RAS guanyl releasing protein 2
205226_at	0.945	0.03139057	-2.3069862	-4.32	-2.3518104	PDGFRL	platelet derived growth factor receptor like
223528_s_at	0.945	0.02325928	-2.4690073	-4.32	-2.9017994	METTL17	methyltransferase like 17
217020_at	0.945	0.04380638	-2.1237245	-4.32	-1.4795068	RARB	retinoic acid receptor beta
207846_at	0.945	0.02835171	-2.3641902	-4.32	-2.5838	POU1F1	POU class 1 homeobox 1
1562010_x_at	0.945	0.03432068	-2.2570502	-4.32	-1.7425103		
1559946_s_at	0.945	0.03494291	-2.2484722	-4.32	-2.465924	RUVBL2	RuvB like AAA ATPase 2
226770_at	0.945	0.03028482	-2.3240818	-4.32	-2.6919994	MAGI3	membrane associated guanylate kinase, WW and PDZ domain containing 3
219993_at	0.945	0.03683024	-2.2170112	-4.32	-2.2376774	SOX17	SRY-box 17
218763_at	0.945	0.03165137	-2.3030349	-4.32	-2.4688814	STX18	syntaxin 18
209740_s_at	0.945	0.0278137	-2.3733441	-4.32	-2.8195884	PNPLA4	patatin like phospholipase domain containing 4
227075_at	0.945	0.01412003	-2.7559534	-4.32	-3.6846087	ELP3	elongator acetyltransferase complex subunit 3
1564421_at	0.945	0.04415681	-2.1199073	-4.32	-1.9763794	DLGAP2-AS1	DLGAP2 antisense RNA 1
207272_at	0.945	0.03180789	-2.3006779	-4.32	-2.9761787	ZNF80	zinc finger protein 80
215001_s_at	0.945	0.04097224	-2.1605771	-4.32	-3.0504858	GLUL	glutamate-ammonia ligase
233384_at	0.945	0.04432961	-2.118035	-4.32	-1.7968435		
1552368_at	0.945	0.02883957	-2.3560278	-4.32	-2.2434303	CTCFL	CCCTC-binding factor like

218675_at	0.945	0.03200041	-2.2977934	-4.32	-2.2407217	SLC22A17	solute carrier family 22 member 17
221674_s_at	0.945	0.04443556	-2.1168902	-4.32	-1.705278	CHRD	chordin
204227_s_at	0.945	0.03726349	-2.2114347	-4.32	-3.5774261	TK2	thymidine kinase 2, mitochondrial
224193_s_at	0.945	0.01348331	-2.7583099	-4.32	-2.8084394	FCRL2	Fc receptor like 2
243177_at	0.945	0.03853567	-2.1953934	-4.32	-3.789374		
205812_s_at	0.945	0.03496752	-2.2481359	-4.32	-2.987758	TMED9	transmembrane p24 trafficking protein 9
1566843_at	0.945	0.02394561	-2.4551261	-4.32	-3.2247468	PER4	period circadian clock 3 pseudogene
205286_at	0.945	0.01863562	-2.6027235	-4.32	-3.4956853	TFAP2C	transcription factor AP-2 gamma
201507_at	0.945	0.04812761	-2.0743142	-4.33	-2.5695729	PFDN1	prefoldin subunit 1
239829_at	0.945	0.03265003	-2.2881764	-4.33	-4.1218062		
220202_s_at	0.945	0.02277016	-2.4913896	-4.33	-3.4343927	RC3H2	ring finger and CCCH-type domains 2
214570_x_at	0.945	0.04282761	-2.139344	-4.33	-2.4768229	LOC102725315///LOC102725072///LOC727983///POM121L10P///POM121L4P///POM121L8P///POM121L9P///POM121L1P	putative POM121-like protein 1-like///uncharacterized LOC102725072///putative POM121-like protein 1-like///POM121 transmembrane nucleoporin like 10, pseudogene///POM121 transmembrane nucleoporin like 4, pseudogene///POM121 transmembrane nucleoporin like 8, pseudogene///POM121 transmembrane nucleoporin like 9, pseudogene///POM121 transmembrane nucleoporin like 1, pseudogene

235537_at	0.945	0.04576283	-2.1027527	-4.33	-1.4298386	OCIAD1	OCIA domain containing 1
243696_at	0.945	0.04886186	-2.067021	-4.33	-4.0475604	CEP104	centrosomal protein 104
1561128_at	0.945	0.02937848	-2.3471586	-4.33	-2.6181074		
242384_at	0.945	0.02493378	-2.4357806	-4.33	-2.4972042		
1556999_at	0.945	0.01242806	-2.8419767	-4.33	-3.9119669	LOC100271832	uncharacterized LOC100271832
243359_at	0.945	0.02183298	-2.4991277	-4.33	-3.7034826		
208187_s_at	0.945	0.02240498	-2.4991831	-4.33	-3.4769052		
225375_at	0.945	0.04012818	-2.1759758	-4.33	-3.0762337	MIR4723///TMEM199	microRNA 4723///transmembrane protein 199
1569540_at	0.945	0.02338942	-2.4784384	-4.33	-2.8981484		
226691_at	0.945	0.03361893	-2.2741542	-4.33	-1.9157202	TNRC18	trinucleotide repeat containing 18
207576_x_at	0.945	0.04633883	-2.0967311	-4.33	-4.1222469	OXT	oxytocin/neurophysin I prepropeptide
234368_at	0.945	0.01983594	-2.5724549	-4.33	-2.0821096		
205566_at	0.945	0.04373022	-2.129312	-4.33	-1.9530903	ABHD2	abhydrolase domain containing 2
238533_at	0.945	0.02284307	-2.4898476	-4.33	-2.9227339	EPHA7	EPH receptor A7
243715_at	0.945	0.0496988	-2.0588255	-4.33	-1.2778955		
1560372_at	0.945	0.03078236	-2.3247419	-4.33	-2.5842894	LOC101929713	uncharacterized LOC101929713
234047_at	0.945	0.0438719	-2.1277542	-4.33	-1.1973617		
206720_at	0.945	0.01824454	-2.6129876	-4.33	-1.9434007	MGAT5	mannosyl (alpha-1,6-)-glycoprotein beta-1,6-N-acetyl-glucosaminyltransferase
215979_s_at	0.945	0.0110328	-2.9009194	-4.33	-3.2013625	SLC7A1	solute carrier family 7 member 1
226160_at	0.945	0.02006754	-2.5668158	-4.33	-3.5015173	H6PD	hexose-6-phosphate dehydrogenase/glucose 1-dehydrogenase

208302_at	0.945	0.03771302	-2.2118991	-4.33	-2.1786108	HMHB1	histocompatibility minor HB-1
1561527_at	0.945	0.02574566	-2.4204155	-4.33	-2.2521429	LINC01514	long intergenic non-protein coding RNA 1514
237435_at	0.945	0.03432307	-2.2641944	-4.33	-1.8820342		
234720_s_at	0.945	0.03786262	-2.2099943	-4.33	-2.1865113	TOB2	transducer of ERBB2, 2
1562353_x_at	0.945	0.03118755	-2.3184477	-4.33	-2.1694658	LOC101928583	uncharacterized LOC101928583
216510_x_at	0.945	0.03307107	-2.2820367	-4.33	-2.304141	IGHV3-23///IGHV4-31///IGHM///IGHG1///IGHA1	immunoglobulin heavy variable 3-23///immunoglobulin heavy variable 4-31///immunoglobulin heavy constant mu///immunoglobulin heavy constant gamma 1 (G1m marker)///immunoglobulin heavy constant alpha 1
219663_s_at	0.945	0.03135763	-2.3158282	-4.33	-3.3156965	TMEM121	transmembrane protein 121
203323_at	0.945	0.04743315	-2.0854729	-4.33	-1.7257837	CAV2	caveolin 2
224572_s_at	0.945	0.04759963	-2.0837805	-4.33	-1.9615417	IRF2BP2	interferon regulatory factor 2 binding protein 2
231563_at	0.945	0.03077058	-2.3249261	-4.33	-2.3102828	LOC100505824	uncharacterized LOC100505824
220616_at	0.945	0.0476584	-2.0831843	-4.33	-1.4388905	ZNF384	zinc finger protein 384
210912_x_at	0.945	0.02357575	-2.4746049	-4.33	-2.673687	GSTM4	glutathione S-transferase mu 4
45633_at	0.945	0.04027878	-2.1741758	-4.33	-2.4718056	GINS3	GINS complex subunit 3
227624_at	0.945	0.0414803	-2.1600289	-4.33	-2.1745298	TET2	tet methylcytosine dioxygenase 2
204655_at	0.945	0.04165156	-2.1580426	-4.33	-2.1577037	CCL5	C-C motif chemokine ligand 5
215769_at	0.945	0.02117463	-2.5263529	-4.33	-3.8141829	YME1L1	YME1 like 1 ATPase
238171_at	0.945	0.04054477	-2.1710115	-4.33	-2.0784512	SLC25A30	solute carrier family 25 member 30

1556747_a_at	0.945	0.04796627	-2.0800718	-4.33	-1.0924435	LOC101928343	uncharacterized LOC101928343
1563405_at	0.945	0.04506454	-2.1148171	-4.33	-1.6769528	ATP4B	ATPase H+/K+ transporting beta subunit
1555741_at	0.945	0.02078414	-2.5497517	-4.33	-2.9587114	MRAP	melanocortin 2 receptor accessory protein
1554951_at	0.945	0.02814423	-2.3775285	-4.33	-3.2127556		
211050_x_at	0.945	0.04807295	-2.0789975	-4.33	-2.0028771	LOC101929540///LOC101928670///LOC101928344///LOC100996442///LOC100288069///LOC100134822	uncharacterized LOC101929540///uncharacterized LOC101928670///uncharacterized LOC101928344///uncharacterized LOC100996442///uncharacterized LOC100288069///uncharacterized LOC100134822
205203_at	0.945	0.02388157	-2.4683739	-4.33	-3.3603853	PLD1	phospholipase D1
212954_at	0.945	0.03878259	-2.1984292	-4.33	-1.9806475	DYRK4	dual specificity tyrosine phosphorylation regulated kinase 4
218991_at	0.945	0.0419598	-2.1544862	-4.34	-1.7453684	HEATR6	HEAT repeat containing 6
238187_at	0.945	0.03533531	-2.2501997	-4.34	-2.9893218	C2orf57	chromosome 2 open reading frame 57
216921_s_at	0.945	0.02099132	-2.5449223	-4.34	-2.6776639	KRT35	keratin 35
239946_at	0.945	0.01658884	-2.6772192	-4.34	-3.4501275		
205447_s_at	0.945	0.03541487	-2.2491154	-4.34	-2.4893419	MAP3K12	mitogen-activated protein kinase kinase kinase 12
239098_at	0.945	0.03841642	-2.2030023	-4.34	-2.1835014	KCNRG	potassium channel regulator

201014_s_at	0.945	0.03920437	-2.1932101	-4.34	-2.5655525	PAICS	phosphoribosylaminoimidazole carboxylase; phosphoribosylaminoimidazolesuccinocarboxamide synthase
210112_at	0.945	0.04579795	-2.1070131	-4.34	-2.0593771	HPS1	HPS1, biogenesis of lysosomal organelles complex 3 subunit 1
219271_at	0.945	0.04875089	-2.0722194	-4.34	-1.3769215	GALNT14	polypeptide N-acetylgalactosaminyltransferase 14
236285_at	0.945	0.03944233	-2.190288	-4.34	-2.6646107	KLHDC7B	kelch domain containing 7B
208124_s_at	0.945	0.04886244	-2.0711122	-4.34	-2.1788578	SEMA4F	ssemaphorin 4F
1557867_s_at	0.945	0.03948656	-2.1897466	-4.34	-2.2160264	CFAP157	cilia and flagella associated protein 157
243432_at	0.945	0.04888486	-2.0708899	-4.34	-1.6293541		
243907_at	0.945	0.04546048	-2.1105901	-4.34	-2.1506761	GREB1L	growth regulation by estrogen in breast cancer 1 like
215452_x_at	0.945	0.04280086	-2.1449007	-4.34	-2.2562211	SUMO4	small ubiquitin-like modifier 4
38964_r_at	0.945	0.04619177	-2.1028687	-4.34	-2.1965178	WAS	Wiskott-Aldrich syndrome
212676_at	0.945	0.04915966	-2.068173	-4.34	-1.4952792	NF1P9///NF1	neurofibromin 1 pseudogene 9///neurofibromin 1
217647_at	0.945	0.02561552	-2.4344038	-4.34	-2.642494	DHODH	dihydroorotate dehydrogenase (quinone)
234073_at	0.945	0.03986259	-2.1851662	-4.34	-2.4875469	SDK2	sidekick cell adhesion molecule 2
223966_at	0.945	0.04311974	-2.1413109	-4.34	-2.4810294	UBE2D3	ubiquitin conjugating enzyme E2 D3
218873_at	0.945	0.03507677	-2.2537389	-4.34	-2.5502487	GON4L	gon-4 like
224150_s_at	0.945	0.04955026	-2.0643343	-4.34	-1.786726	CEP70	centrosomal protein 70

234295_at	0.945	0.0143634	-2.7701016	-4.34	-4.2717494	DBR1	debranching RNA lariats 1
214604_at	0.945	0.02935631	-2.3571358	-4.34	-2.5748949	HOXD11	homeobox D11
201206_s_at	0.945	0.03243208	-2.2995764	-4.34	-3.0501675	RRBP1	ribosome binding protein 1
228255_at	0.945	0.03654859	-2.2339011	-4.34	-2.6062696	TMEM237	transmembrane protein 237
226609_at	0.945	0.04610137	-2.1038172	-4.34	-1.8752178	DCBLD1	discoidin, CUB and LCCL domain containing 1
221632_s_at	0.945	0.02589974	-2.4290425	-4.34	-2.3166665	WDR4	WD repeat domain 4
244015_at	0.945	0.04025372	-2.1804431	-4.34	-3.7863009		
235122_at	0.945	0.02509198	-2.4444271	-4.34	-3.0142755	HIVEP3	human immunodeficiency virus type I enhancer binding protein 3
241974_at	0.945	0.04682113	-2.0963107	-4.34	-1.6776527		
233843_at	0.945	0.02602374	-2.4267207	-4.34	-3.289622	ZBTB12	zinc finger and BTB domain containing 12
210218_s_at	0.945	0.04349715	-2.1370928	-4.34	-2.1978371	SP100	SP100 nuclear antigen
215244_at	0.945	0.04983075	-2.0615943	-4.34	-2.1604744	DGCR5	DiGeorge syndrome critical region gene 5 (non-protein coding)
221967_at	0.945	0.04632455	-2.1014784	-4.34	-2.2518439	NXPH4	neurexophilin 4
230708_at	0.945	0.04238992	-2.1495627	-4.34	-1.9244714	PRICKLE1	prickle planar cell polarity protein 1
226713_at	0.945	0.02025673	-2.5622553	-4.34	-3.0168116	CCDC50	coiled-coil domain containing 50
232935_at	0.945	0.02215446	-2.5186208	-4.34	-2.8424157		
241209_at	0.945	0.02028609	-2.5615511	-4.34	-2.726211	DCAF6	DDB1 and CUL4 associated factor 6
203943_at	0.945	0.0424834	-2.1484986	-4.34	-1.4664444	KIF3B	kinesin family member 3B
201679_at	0.945	0.02620947	-2.4232626	-4.34	-3.439566	SRRT	serrate, RNA effector molecule
239537_at	0.945	0.03991242	-2.1845621	-4.34	-2.1998405	ST8SIA2	ST8 alpha-N-acetyl-neuraminide alpha-2,8-sialyltransferase 2

220055_at	0.945	0.02810408	-2.378218	-4.34	-2.0289283	ZNF287	zinc finger protein 287
224964_s_at	0.945	0.04719138	-2.0924894	-4.34	-1.9481421	GNG2	G protein subunit gamma 2
220784_s_at	0.945	0.04719219	-2.0924811	-4.34	-1.7818486	UTS2	urotensin 2
216918_s_at	0.945	0.04391117	-2.1325033	-4.34	-2.0577545	DST	dystonin
1562016_at	0.945	0.02248203	-2.5114497	-4.34	-3.1090706		
204520_x_at	0.945	0.04029065	-2.1799994	-4.34	-2.847125	BRD1	bromodomain containing 1
213320_at	0.945	0.0332846	-2.2870308	-4.34	-4.5124187	PRMT3	protein arginine methyltransferase 3
235294_at	0.945	0.03746938	-2.2218584	-4.34	-1.9392491	SIKE1	suppressor of IKBKE 1
244339_at	0.945	0.02071161	-2.5514532	-4.34	-1.9377672		
224652_at	0.945	0.04316831	-2.1407661	-4.34	-2.2065372	CCNY	cyclin Y
201552_at	0.945	0.02080342	-2.5493003	-4.34	-1.7752216	LAMP1	lysosomal associated membrane protein 1
213374_x_at	0.945	0.02272722	-2.5061466	-4.34	-3.2888572	HIBCH	3-hydroxyisobutyryl-CoA hydrolase
207854_at	0.945	0.04073708	-2.1746635	-4.34	-3.2879412	GYPE	glycophorin E (MNS blood group)
1557506_a_at	0.945	0.02605102	-2.4262113	-4.34	-1.7222199		
229104_s_at	0.945	0.04466682	-2.1242263	-4.34	-2.5397713	GPR39	G protein-coupled receptor 39
233402_at	0.945	0.04106196	-2.1708137	-4.34	-3.6856808		
243051_at	0.945	0.04497035	-2.120937	-4.34	-2.1615046	CNIH4	cornichon family AMPA receptor auxiliary protein 4
229047_at	0.945	0.04499033	-2.1207212	-4.34	-1.488164	PLEKHB1	pleckstrin homology domain containing B1
1560762_at	0.945	0.03164316	-2.3114598	-4.34	-2.4570942	LINC00996	long intergenic non-protein coding RNA 996
200906_s_at	0.945	0.01958048	-2.5956893	-4.34	-1.9905397	PALLD	palladin, cytoskeletal associated protein

243672_at	0.945	0.04862977	-2.077894	-4.35	-1.9366906	SALL3	spalt like transcription factor 3
206983_at	0.945	0.04203068	-2.159496	-4.35	-2.313435	CCR6	C-C motif chemokine receptor 6
212116_at	0.945	0.03853282	-2.2082815	-4.35	-2.5660992	TRIM27	tripartite motif containing 27
244109_at	0.945	0.012266	-2.8034216	-4.35	-3.8686124	WDR38	WD repeat domain 38
233176_at	0.945	0.0387166	-2.2059698	-4.35	-1.6517102	LOC100507642	uncharacterized LOC100507642
224290_at	0.945	0.04924381	-2.0717803	-4.35	-1.6891365	VN1R3	vomeronasal 1 receptor 3 (gene/pseudogene)
204964_s_at	0.945	0.04929577	-2.0712662	-4.35	-1.8494737	SSPN	sarcospan
232744_x_at	0.945	0.03156539	-2.3219018	-4.35	-3.1199555		
205450_at	0.945	0.04268652	-2.1519669	-4.35	-2.0520309	PHKA1	phosphorylase kinase regulatory subunit alpha 1
244421_at	0.945	0.02016233	-2.5812306	-4.35	-2.9968776		
1557571_at	0.945	0.04945959	-2.0696481	-4.35	-2.5356288	VPS13D	vacuolar protein sorting 13 homolog D
228129_at	0.945	0.03176586	-2.3188185	-4.35	-3.1414191	SERBP1	SERPINE1 mRNA binding protein 1
1556891_at	0.945	0.03499522	-2.2627252	-4.35	-2.4321384	SORCS1	sortilin related VPS10 domain containing receptor 1
236953_s_at	0.945	0.03192579	-2.3163717	-4.35	-2.6720914	NHLRC3	NHL repeat containing 3
219719_at	0.945	0.03789128	-2.2164304	-4.35	-1.9787658	HIGD1B	HIG1 hypoxia inducible domain family member 1B
215285_s_at	0.945	0.02743047	-2.4010832	-4.35	-3.2879822	PHTF1	putative homeodomain transcription factor 1
229550_at	0.945	0.03946647	-2.1966402	-4.35	-2.6555401	UNC79	unc-79 homolog (C. elegans)
1563809_a_at	0.945	0.0274814	-2.4001784	-4.35	-2.7236	MCF2L	MCF.2 cell line derived transforming sequence like

244739_at	0.945	0.03542704	-2.2567623	-4.35	-2.8389599	RDX	radixin
240868_at	0.945	0.02713661	-2.3951135	-4.35	-2.8075604	LOC100129406	uncharacterized LOC100129406
1555856_s_at	0.945	0.0435698	-2.1419904	-4.35	-3.8826272	AKR1C1	aldo-keto reductase family 1 member C1
224029_x_at	0.945	0.02871915	-2.3786547	-4.35	-2.4350453	SCN11A	sodium voltage-gated channel alpha subunit 11
220045_at	0.945	0.04566263	-2.1135089	-4.35	-1.7944995	NEUROD6	neuronal differentiation 6
240744_at	0.945	0.04987029	-2.0656126	-4.35	-2.7600045	CPA5	carboxypeptidase A5
237081_at	0.945	0.03655886	-2.2414446	-4.35	-1.3827688		
216547_at	0.945	0.03856404	-2.207888	-4.35	-2.4621442		
205563_at	0.945	0.04998347	-2.0645057	-4.35	-4.1019801	KISS1	KiSS-1 metastasis-suppressor
204966_at	0.945	0.01668182	-2.6954694	-4.35	-3.6516931	ADGRB2	adhesion G protein-coupled receptor B2
1556718_s_at	0.945	0.04322203	-2.1458965	-4.35	-1.7814608	MIR194-2	microRNA 194-2
219535_at	0.945	0.03302762	-2.2998159	-4.35	-2.1413859	HUNK	hormonally up-regulated Neu-associated kinase
219035_s_at	0.945	0.03637551	-2.2438962	-4.35	-2.9256358	RNF34	ring finger protein 34
244719_at	0.945	0.03318345	-2.2975159	-4.35	-2.6228421		
1563903_x_at	0.945	0.033214	-2.297066	-4.35	-2.5431531		
228298_at	0.945	0.02325978	-2.4948121	-4.35	-2.5993422	PCED1B	PC-esterase domain containing 1B
205772_s_at	0.945	0.04797425	-2.0894171	-4.35	-1.9750109	AKAP7	A-kinase anchoring protein 7
233755_at	0.945	0.03662197	-2.2406032	-4.35	-2.5112896		
235330_at	0.945	0.02123489	-2.5249879	-4.35	-3.2241061	CCDC117	coiled-coil domain containing 117
235172_at	0.945	0.04217452	-2.1578356	-4.35	-2.3354038	GABPB1-AS1	GABPB1 antisense RNA 1
207421_at	0.945	0.03362142	-2.2911043	-4.35	-2.3889429	CA5A	carbonic anhydrase 5A

1552287_s_at	0.945	0.02367711	-2.4861001	-4.35	-2.5996787	AFG3L1P	AFG3 like matrix AAA peptidase subunit 1, pseudogene
216043_x_at	0.945	0.04853362	-2.0837441	-4.35	-1.9572851		
237369_at	0.945	0.0485426	-2.0836534	-4.35	-2.3745596		
202574_s_at	0.945	0.0291729	-2.3709809	-4.35	-2.7579928	CSNK1G2	casein kinase 1 gamma 2
1560332_at	0.945	0.01774639	-2.6441181	-4.36	-2.9170859		
210996_s_at	0.945	0.03035472	-2.3515047	-4.36	-3.0509303	YWHAE	tyrosine 3-monooxygenase/tryptophan 5-monooxygenase activation protein epsilon
237080_at	0.945	0.03744308	-2.2297773	-4.36	-2.0003561		
242751_at	0.945	0.04567954	-2.1188806	-4.36	-2.2019231		
1561864_at	0.945	0.0457188	-2.1184598	-4.36	-2.3206153		
1569861_at	0.945	0.04903404	-2.0787181	-4.36	-2.0205188	TRAF5	TNF receptor associated factor 5
227320_at	0.945	0.02619166	-2.4364894	-4.36	-2.4465208	ZNF664-FAM101A///FAM101A	filamin-interacting protein FAM101A///family with sequence similarity 101 member A
239027_at	0.945	0.03757654	-2.2280382	-4.36	-1.9122738	DOCK8	dedicator of cytokinesis 8
241341_at	0.945	0.04579125	-2.117684	-4.36	-2.2065201		
230595_at	0.945	0.02623832	-2.4356119	-4.36	-2.5542643	PGM5-AS1	PGM5 antisense RNA 1
232752_at	0.945	0.01794967	-2.6588015	-4.36	-4.5466041	LOXL1-AS1	LOXL1 antisense RNA 1
234793_at	0.945	0.01139817	-2.8848095	-4.36	-3.1707102		
237651_x_at	0.945	0.04633129	-2.1119359	-4.36	-1.7475321	LINC00518	long intergenic non-protein coding RNA 518
200607_s_at	0.945	0.04099868	-2.1780683	-4.36	-3.1364673	RAD21	RAD21 cohesin complex component
219324_at	0.945	0.04970383	-2.0720626	-4.36	-2.0277695	NOL12	nucleolar protein 12

232818_at	0.945	0.03112338	-2.3392129	-4.36	-2.5192908		
216305_s_at	0.945	0.04980181	-2.0710957	-4.36	-2.4378787	GCFC2	GC-rich sequence DNA-binding factor 2
219110_at	0.945	0.04268006	-2.1584003	-4.36	-2.0751775	GAR1	GAR1 ribonucleoprotein
232381_s_at	0.945	0.04594069	-2.1160873	-4.36	-4.0710861	DNAH5	dynein axonemal heavy chain 5
240670_at	0.945	0.03040623	-2.3506719	-4.36	-3.8324122		
222511_x_at	0.945	0.02702225	-2.4210856	-4.36	-3.0380515	FAF1	Fas associated factor 1
221333_at	0.945	0.03603744	-2.2484465	-4.36	-2.64022	FOXP3	forkhead box P3
215642_at	0.945	0.01865683	-2.6195081	-4.36	-3.9098333		
223575_at	0.945	0.04630488	-2.1122156	-4.36	-2.3770162	KIAA1549	KIAA1549
201258_at	0.945	0.03062573	-2.3471381	-4.36	-2.7060168	RPS16	ribosomal protein S16
203264_s_at	0.945	0.04322798	-2.1521426	-4.36	-1.8670864	ARHGEF9	Cdc42 guanine nucleotide exchange factor 9
1569472_s_at	0.945	0.01877362	-2.6362889	-4.36	-3.6093031	TTC3P1///TTC3	tetratricopeptide repeat domain 3 pseudogene 1///tetratricopeptide repeat domain 3
207033_at	0.945	0.0317509	-2.329386	-4.36	-2.709441	GIF	gastric intrinsic factor
231397_at	0.945	0.03192906	-2.326629	-4.36	-2.0321436	PLPPR5	phospholipid phosphatase related 5
218708_at	0.945	0.0391191	-2.2083381	-4.36	-2.387054	NXT1	nuclear transport factor 2 like export factor 1
221345_at	0.945	0.04690921	-2.105851	-4.36	-2.9450177	FFAR2	free fatty acid receptor 2
227883_at	0.945	0.04024755	-2.1943713	-4.36	-2.2346339	CCDC71L	coiled-coil domain containing 71-like
233635_at	0.945	0.02138689	-2.5358256	-4.36	-3.8738265		
227960_s_at	0.945	0.01529398	-2.6979814	-4.36	-3.2436729	FAHD1	fumarylacetoacetate hydrolase domain containing 1

216973_s_at	0.945	0.03449558	-2.2785346	-4.36	-3.0393917	HOXB7	homeobox B7
228709_at	0.945	0.04070035	-2.1888672	-4.36	-2.0728915	TPR	translocated promoter region, nuclear basket protein
206536_s_at	0.945	0.04779652	-2.0966385	-4.36	-2.3565013	XIAP	X-linked inhibitor of apoptosis
207076_s_at	0.945	0.03503008	-2.270993	-4.36	-2.3266048	ASS1	argininosuccinate synthase 1
203604_at	0.945	0.04322882	-2.1521331	-4.36	-2.9560665	ZNF516	zinc finger protein 516
242731_x_at	0.945	0.01085091	-2.9386829	-4.36	-4.4135575	BMPR1A	bone morphogenetic protein receptor type 1A
235337_at	0.945	0.03546549	-2.2649276	-4.37	-2.7397415	SERTAD4	SERTA domain containing 4
221025_x_at	0.945	0.01474955	-2.7826918	-4.37	-4.391187	PUS7L	pseudouridylate synthase 7 like
1570628_at	0.945	0.03244659	-2.3187003	-4.37	-1.9004321		
203883_s_at	0.945	0.04171698	-2.1767102	-4.37	-2.2570152	RAB11FIP2	RAB11 family interacting protein 2
221536_s_at	0.945	0.04185539	-2.175076	-4.37	-1.9718592	LSG1	large 60S subunit nuclear export GTPase 1
238917_s_at	0.945	0.0407975	-2.1804704	-4.37	-4.2325106	DENND5B	DENN domain containing 5B
238374_at	0.945	0.02918408	-2.382988	-4.37	-2.7617941	H1FNT	H1 histone family member N, testis specific
1555491_a_at	0.945	0.04885467	-2.085851	-4.37	-2.3650405	C19orf66	chromosome 19 open reading frame 66
213040_s_at	0.945	0.0297886	-2.3607443	-4.37	-2.3746582	NPTXR	neuronal pentraxin receptor
219873_at	0.945	0.00678442	-3.2688704	-4.37	-4.5313046	COLEC11	collectin subfamily member 11
223687_s_at	0.945	0.02941139	-2.3791373	-4.37	-2.4255748	LY6K	lymphocyte antigen 6 complex, locus K
231164_at	0.945	0.020654	-2.5693201	-4.37	-3.4141657	ABCA17P	ATP binding cassette subfamily A member 17, pseudogene

1567222_x_at	0.945	0.03826051	-2.2275494	-4.37	-2.5426915	ELOVL5	ELOVL fatty acid elongase 5
200688_at	0.945	0.04168679	-2.1770672	-4.37	-2.8155846	SF3B3	splicing factor 3b subunit 3
208089_s_at	0.945	0.04956001	-2.0787762	-4.37	-1.3938614	TDRD3	tudor domain containing 3
215795_at	0.945	0.02448969	-2.4847241	-4.37	-2.5236611	MYH7B	myosin heavy chain 7B
239915_at	0.945	0.03359425	-2.301529	-4.37	-2.9059609	HOXA-AS3	HOXA cluster antisense RNA 3
228057_at	0.945	0.042034	-2.1729744	-4.37	-2.1269297	DDIT4L	DNA damage inducible transcript 4 like
1565758_at	0.945	0.02782566	-2.4066037	-4.37	-3.7073512		
206935_at	0.945	0.03869786	-2.2219293	-4.37	-2.0869596	PCDH8	protocadherin 8
214562_at	0.945	0.04520205	-2.1301778	-4.37	-2.3251267	HIST1H4L	histone cluster 1, H4l
235184_at	0.945	0.04697179	-2.1112117	-4.37	-1.7614439	AEBP2	AE binding protein 2
202522_at	0.945	0.04724804	-2.1083095	-4.37	-2.3426209	PITPNB	phosphatidylinositol transfer protein beta
244058_at	0.945	0.0392295	-2.2151754	-4.37	-2.5141885	VSTM4	V-set and transmembrane domain containing 4
227020_at	0.945	0.04277616	-2.164328	-4.37	-2.3313432	YPEL2	yippee like 2
231498_at	0.945	0.03940811	-2.2129252	-4.37	-2.7034657		
236401_at	0.945	0.04748151	-2.1058687	-4.37	-2.4945838	GIMAP1	GTPase, IMAP family member 1
1553605_a_at	0.945	0.01909364	-2.6278006	-4.37	-2.948526	ABCA13	ATP binding cassette subfamily A member 13
219369_s_at	0.945	0.04026875	-2.1941123	-4.37	-3.1570079	OTUB2	OTU deubiquitinase, ubiquitin aldehyde binding 2
237324_s_at	0.945	0.03945834	-2.2122939	-4.37	-2.3129546	HKDC1	hexokinase domain containing 1
1569669_at	0.945	0.04032008	-2.1934858	-4.37	-2.7193111	FOXR2	forkhead box R2

220436_at	0.945	0.03557178	-2.2731785	-4.37	-3.0998914	CNTNAP3P2	contactin associated protein-like 3 pseudogene 2
226118_at	0.945	0.0218773	-2.5408216	-4.37	-1.7679099	CENPO	centromere protein O
205744_at	0.945	0.02555656	-2.4634348	-4.37	-2.9520963	DOC2A	double C2 domain alpha
212548_s_at	0.945	0.02889321	-2.3879566	-4.37	-2.4608582	FRYL	FRY like transcription coactivator
218454_at	0.945	0.04072224	-2.1886026	-4.37	-2.2315606	PLBD1	phospholipase B domain containing 1
1557944_s_at	0.945	0.03993916	-2.2062894	-4.37	-3.0654749	TMX2-CTNND1///CTNND1	TMX2-CTNND1 readthrough (NMD candidate)///catenin delta 1
210770_s_at	0.945	0.01646427	-2.7265506	-4.37	-4.0801256	LOC100507353///CACNA1A	uncharacterized LOC100507353///calcium voltage-gated channel subunit alpha1 A
1553082_at	0.945	0.03606655	-2.2663139	-4.37	-2.6116393	CRYGN	crystallin gamma N
236488_s_at	0.945	0.03821748	-2.2281057	-4.37	-2.1156294		
227744_s_at	0.945	0.04832147	-2.0971758	-4.37	-2.0033096	HNRNPD	heterogeneous nuclear ribonucleoprotein D
1568711_a_at	0.945	0.02916859	-2.3832515	-4.37	-2.6598062	LOC105369860	uncharacterized LOC105369860
239691_at	0.945	0.03621945	-2.26421	-4.37	-2.642759	C12orf77	chromosome 12 open reading frame 77
1552671_a_at	0.945	0.0437802	-2.1528454	-4.37	-2.3316392	SLC9A7	solute carrier family 9 member A7
221352_at	0.945	0.01976714	-2.6103754	-4.37	-3.170051		
216814_at	0.945	0.02934257	-2.3803003	-4.37	-2.4783312		
242121_at	0.945	0.02477936	-2.4637697	-4.37	-3.5044457	FTX	FTX transcript, XIST regulator (non-protein coding)
1569234_at	0.945	0.03181373	-2.3400617	-4.37	-2.6070158		

1553728_at	0.945	0.0356285	-2.2723871	-4.37	-3.03688	LRRC43	leucine rich repeat containing 43
220074_at	0.945	0.01059883	-2.9857295	-4.37	-2.6431477	CDHR5	cadherin related family member 5
1569661_at	0.945	0.03206164	-2.3361877	-4.38	-2.7874877	NPAS3	neuronal PAS domain protein 3
217742_s_at	0.945	0.04173521	-2.1764946	-4.38	-3.0106894	WAC	WW domain containing adaptor with coiled-coil
222395_s_at	0.945	0.02658994	-2.4436016	-4.38	-2.9650916	UBE2Z	ubiquitin conjugating enzyme E2 Z
221381_s_at	0.945	0.04754246	-2.1052332	-4.38	-1.1728617	MORF4L1	mortality factor 4 like 1
213433_at	0.945	0.04451145	-2.1446322	-4.38	-2.2063079	ARL3	ADP ribosylation factor like GTPase 3
205611_at	0.945	0.04543246	-2.1344601	-4.38	-1.910898	TNFSF12-TNFSF13///TNFSF12	TNFSF12-TNFSF13 readthrough///tumor necrosis factor superfamily member 12
1557783_at	0.945	0.04484795	-2.1408938	-4.38	-2.7915551	MAP3K14-AS1	MAP3K14 antisense RNA 1
209379_s_at	0.945	0.03249944	-2.3294142	-4.38	-3.91362	CCSER2	coiled-coil serine rich protein 2
244222_at	0.945	0.04139407	-2.1885154	-4.38	-3.0330493		
1553276_at	0.945	0.04155175	-2.1866235	-4.38	-2.2895475	ZNF560	zinc finger protein 560
236939_at	0.945	0.04156166	-2.1865049	-4.38	-2.1066387	HACD4	3-hydroxyacyl-CoA dehydratase 4
229227_at	0.945	0.01336013	-2.8330723	-4.38	-4.1480322	DICER1-AS1	DICER1 antisense RNA 1
219742_at	0.945	0.03981539	-2.2078287	-4.38	-3.1458757	PRR7	proline rich 7 (synaptic)
243115_at	0.945	0.0400708	-2.204657	-4.38	-2.5556343	THCAT158	thyroid cancer-associated transcript 158
217096_at	0.945	0.0336968	-2.3000212	-4.38	-3.640837	PCLO	piccolo presynaptic cytomatrix protein
222647_at	0.945	0.04880124	-2.0922716	-4.38	-3.180882	SLC35C1	solute carrier family 35 member C1
228056_s_at	0.945	0.0457919	-2.130541	-4.38	-1.9263778	NAPSB	napsin B aspartic peptidase, pseudogene

231163_at	0.945	0.04589019	-2.1294742	-4.38	-1.1473411	C1orf111	chromosome 1 open reading frame 111
1557590_at	0.945	0.04043935	-2.2001129	-4.38	-2.4239656	PARD6G-AS1	PARD6G antisense RNA 1
242234_at	0.945	0.03400852	-2.2954642	-4.38	-2.567069	XAF1	XIAP associated factor 1
234998_at	0.945	0.03727083	-2.249962	-4.38	-1.9622269	RAB11A	RAB11A, member RAS oncogene family
224487_at	0.945	0.02918318	-2.3968699	-4.38	-3.1795707		
231720_s_at	0.945	0.03665772	-2.2486637	-4.38	-3.0407247	JAM3	junctional adhesion molecule 3
206414_s_at	0.945	0.03373504	-2.3107467	-4.38	-2.3903885	ASAP2	ArfGAP with SH3 domain, ankyrin repeat and PH domain 2
242683_at	0.945	0.04276071	-2.1723328	-4.38	-1.7952784	LINC00668	long intergenic non-protein coding RNA 668
212278_x_at	0.945	0.04282186	-2.1716197	-4.38	-2.4376225	UBE3A	ubiquitin protein ligase E3A
214266_s_at	0.945	0.04974151	-2.0827846	-4.38	-2.8421607	PDLIM7	PDZ and LIM domain 7
221676_s_at	0.945	0.04316437	-2.1676432	-4.38	-2.0677024	CORO1C	coronin 1C
202625_at	0.945	0.04675821	-2.1201414	-4.38	-2.9949811	LYN	LYN proto-oncogene, Src family tyrosine kinase
204807_at	0.945	0.04400675	-2.1502876	-4.38	-2.2703702	TMEM5	transmembrane protein 5
1557192_at	0.945	0.04348043	-2.1639994	-4.38	-2.5169977	COX10-AS1	COX10 antisense RNA 1
204036_at	0.945	0.04708363	-2.1166831	-4.38	-1.5789763	LPAR1	lysophosphatidic acid receptor 1
241437_s_at	0.945	0.04350931	-2.1636676	-4.38	-2.7727137	EP400NL	EP400 N-terminal like
1556402_at	0.945	0.03941683	-2.221996	-4.38	-2.9801973		
228400_at	0.945	0.04724145	-2.1150136	-4.38	-2.6319572	SHROOM3	shroom family member 3
244187_at	0.945	0.04376361	-2.160755	-4.38	-2.6367165		

1568658_at	0.945	0.03519095	-2.2785227	-4.38	-2.3538884	C2orf74///KIAA1841	chromosome 2 open reading frame 74///KIAA1841
213291_s_at	0.945	0.03781697	-2.2333098	-4.38	-3.5160408	UBE3A	ubiquitin protein ligase E3A
203828_s_at	0.945	0.00628373	-3.3739611	-4.38	-4.861958	IL32	interleukin 32
202220_at	0.945	0.04855493	-2.1013118	-4.38	-1.7070336	KIAA0907	KIAA0907
233751_at	0.945	0.03545115	-2.2748656	-4.38	-2.4389242	ZFHX3	zinc finger homeobox 3
1559513_a_at	0.945	0.01230297	-2.9078884	-4.38	-3.2616232	FANCC	Fanconi anemia complementation group C
242600_at	0.945	0.02247302	-2.545678	-4.38	-3.2042964	FRMD3	FERM domain containing 3
230479_at	0.945	0.04879882	-2.0988046	-4.38	-2.5563569	EIF3F	eukaryotic translation initiation factor 3 subunit F
205511_at	0.945	0.04429176	-2.1547548	-4.38	-2.4328769	FLJ10038	uncharacterized protein FLJ10038
232281_at	0.945	0.04229234	-2.1778248	-4.38	-1.6216259	LINC00662	long intergenic non-protein coding RNA 662
220892_s_at	0.945	0.0328204	-2.3245024	-4.38	-2.9839874	PSAT1	phosphoserine aminotransferase 1
226818_at	0.945	0.0480087	-2.1069687	-4.38	-2.3659349	MPEG1	macrophage expressed 1
218458_at	0.945	0.02283082	-2.5376884	-4.38	-3.6544384	GMCL1	germ cell-less, spermatogenesis associated 1
209516_at	0.945	0.04477301	-2.1493441	-4.39	-2.185432	SMYD5	SMYD family member 5
233035_at	0.945	0.03623964	-2.2639328	-4.39	-1.9773237		
207531_at	0.945	0.0396449	-2.2191069	-4.39	-2.7444806	CRYGC	crystallin gamma C
201562_s_at	0.945	0.0430924	-2.1684764	-4.39	-1.7931324	SORD	sorbitol dehydrogenase
232599_at	0.945	0.0099731	-3.0605558	-4.39	-3.8485356	EXOC6	exocyst complex component 6
236775_s_at	0.945	0.04889856	-2.0977825	-4.39	-2.4130224		

223176_at	0.945	0.03021329	-2.3793863	-4.39	-2.562337	KCTD20	potassium channel tetramerization domain containing 20
205541_s_at	0.945	0.04894826	-2.0972738	-4.39	-2.1638128	GSPT2	G1 to S phase transition 2
229437_at	0.945	0.04536168	-2.1427971	-4.39	-2.0518452	MIR155///MIR155HG	microRNA 155///MIR155 host gene
240164_at	0.945	0.03026525	-2.3785194	-4.39	-3.4029633	MUC4	mucin 4, cell surface associated
216671_x_at	0.945	0.04018611	-2.2123117	-4.39	-1.6404533	MUC8	mucin 8
1558815_at	0.945	0.04643827	-2.123563	-4.39	-2.5358324	SORBS2	sorbin and SH3 domain containing 2
1561461_at	0.945	0.02646928	-2.4458793	-4.39	-2.4185401		
207165_at	0.945	0.02657872	-2.4605174	-4.39	-2.8251108	HMMR	hyaluronan mediated motility receptor
205173_x_at	0.945	0.03207002	-2.3492435	-4.39	-1.4943883	CD58	CD58 molecule
207394_at	0.945	0.04199716	-2.1901691	-4.39	-1.5460859	ZNF137P	zinc finger protein 137, pseudogene
221325_at	0.945	0.03719001	-2.2617397	-4.39	-3.5735477	KCNK13	potassium two pore domain channel subfamily K member 13
221357_at	0.945	0.0411251	-2.2007193	-4.39	-2.6637503	CHRM4	cholinergic receptor muscarinic 4
1559167_x_at	0.945	0.04691571	-2.1258782	-4.39	-1.8546247	MPV17L	MPV17 mitochondrial inner membrane protein like
1561306_s_at	0.945	0.0414964	-2.1962024	-4.39	-1.5017369	LOC101930630///STRC	putative stereocilin-like protein-like///stereocilin
1558649_at	0.945	0.02773215	-2.4225106	-4.39	-3.2228628	ALDH1A3	aldehyde dehydrogenase 1 family member A3
239201_at	0.945	0.04816609	-2.1053327	-4.39	-1.976641	CDK15	cyclin dependent kinase 15
205697_at	0.945	0.03871224	-2.2310199	-4.39	-3.2264983	SCGN	secretagogin, EF-hand calcium binding protein
243559_at	0.945	0.03361411	-2.325399	-4.39	-1.8887298		

1554108_at	0.945	0.04364869	-2.1707281	-4.39	-2.5525019		
239329_at	0.945	0.02354543	-2.5426897	-4.39	-2.5624358	RAB21	RAB21, member RAS oncogene family
214880_x_at	0.945	0.03394916	-2.3203608	-4.39	-3.6255338	CALD1	caldesmon 1
1556138_a_at	0.945	0.02844532	-2.4258771	-4.39	-2.9469498		
214209_s_at	0.945	0.04269161	-2.1819104	-4.39	-2.4799525	ABCB9	ATP binding cassette subfamily B member 9
230686_s_at	0.945	0.04275079	-2.1812124	-4.39	-2.7647045	SLC13A3	solute carrier family 13 member 3
244562_s_at	0.945	0.03277712	-2.3381934	-4.39	-3.4270128		
1556595_at	0.945	0.04282927	-2.180288	-4.39	-2.215733		
244261_at	0.945	0.03283407	-2.3373133	-4.39	-1.7828724	IFNLR1	interferon lambda receptor 1
1569310_at	0.945	0.04851115	-2.1090302	-4.39	-3.0748676	IRGQ	immunity related GTPase Q
230788_at	0.945	0.02224705	-2.571989	-4.39	-4.4235847	GCNT2	glucosaminyl (N-acetyl) transferase 2, I-branching enzyme (I blood group)
1569949_at	0.945	0.03683389	-2.2665908	-4.39	-3.806148	GRK5	G protein-coupled receptor kinase 5
225261_x_at	0.945	0.03339631	-2.3286993	-4.4	-3.3327231	NELFCD	negative elongation factor complex member C/D
242777_at	0.945	0.04476862	-2.1579247	-4.4	-2.8724857		
202616_s_at	0.945	0.03192834	-2.3382673	-4.4	-2.4114789	MECP2	methyl-CpG binding protein 2
233668_at	0.945	0.02440545	-2.5241264	-4.4	-1.997826		
1561564_at	0.945	0.01868579	-2.6888582	-4.4	-2.2038974		
242074_at	0.945	0.04503859	-2.1548822	-4.4	-3.0754231		
233872_x_at	0.945	0.0399872	-2.2250658	-4.4	-2.4117939	ARHGAP5	Rho GTPase activating protein 5
226504_at	0.945	0.02966592	-2.3886054	-4.4	-2.9721365	FAM109B	family with sequence similarity 109 member B

1556322_a_at	0.945	0.04410252	-2.1655039	-4.4	-2.4557365		
243247_at	0.945	0.0443378	-2.1628148	-4.4	-2.2878168		
242566_at	0.945	0.03556361	-2.2967178	-4.4	-2.8360525	VASH1	vasohibin 1
1570267_at	0.945	0.03003758	-2.3979923	-4.4	-2.8443919		
204133_at	0.945	0.04375729	-2.1608272	-4.4	-2.7499536	RRP9	ribosomal RNA processing 9, small subunit (SSU) processome component, homolog (yeast)
244372_at	0.945	0.03814613	-2.2489248	-4.4	-2.5571502	LOC101060019	uncharacterized LOC101060019
202903_at	0.945	0.03456698	-2.3111913	-4.4	-2.2406774	LSM5	LSM5 homolog, U6 small nuclear RNA and mRNA degradation associated
1563693_at	0.945	0.04602212	-2.1439372	-4.4	-2.5634762		
242749_at	0.945	0.03461948	-2.3104192	-4.4	-2.9215165		
223662_x_at	0.945	0.01947779	-2.6670058	-4.4	-2.6486399	DDX59	DEAD-box helicase 59
212767_at	0.945	0.03873709	-2.2411523	-4.4	-2.7249275	MTG1	mitochondrial ribosome associated GTPase 1
1553703_at	0.945	0.04577626	-2.1466531	-4.4	-2.5280688	ZNF791	zinc finger protein 791
221973_at	0.945	0.04702135	-2.1330343	-4.4	-2.6936453	LOC100506123///LOC100506076	uncharacterized LOC100506123///uncharacterized LOC100506076
221336_at	0.945	0.03552981	-2.2972025	-4.4	-2.0739176	ATOH1	atonal bHLH transcription factor 1
213899_at	0.945	0.04738712	-2.129096	-4.4	-2.2468514	METAP2	methionyl aminopeptidase 2
223717_s_at	0.945	0.04218353	-2.1978964	-4.4	-2.8486159	ACRBP	acrosin binding protein
206192_at	0.945	0.04629212	-2.14097	-4.4	-2.9381032	CDSN	corneodesmosin

210926_at	0.945	0.04750198	-2.127865	-4.4	-3.2220605	POTEKP	POTE ankyrin domain family member K, pseudogene
214713_at	0.945	0.0423455	-2.1959448	-4.4	-2.9720634	YLPM1	YLP motif containing 1
222572_at	0.945	0.0425115	-2.1939518	-4.4	-2.8405491	PDP1	pyruvate dehyrogenase phosphatase catalytic subunit 1
201911_s_at	0.945	0.03456262	-2.2985966	-4.4	-3.0503346	FARP1	FERM, ARH/RhoGEF and pleckstrin domain protein 1
243679_at	0.945	0.04790711	-2.1235447	-4.4	-3.1875543	JPH3	junctophilin 3
224022_x_at	0.945	0.04680398	-2.135388	-4.4	-2.1202379	WNT16	Wnt family member 16
202605_at	0.945	0.04334506	-2.1742511	-4.4	-2.6002701	GUSB	glucuronidase beta
1554276_at	0.945	0.02703996	-2.4709364	-4.4	-3.233297	SART3	squamous cell carcinoma antigen recognized by T-cells 3
235151_at	0.945	0.03686455	-2.278385	-4.4	-2.6485437	LOC283357	uncharacterized LOC283357
1552990_at	0.945	0.04355938	-2.1815337	-4.4	-2.784845	FAM71E2	family with sequence similarity 71 member E2
232886_at	0.945	0.04685711	-2.1265069	-4.4	-2.9990091		
1556607_at	0.945	0.04781547	-2.124519	-4.4	-3.1850548	EHD4	EH domain containing 4
229762_at	0.945	0.04785475	-2.1241012	-4.4	-2.0338793		
238684_at	0.945	0.0491408	-2.1105907	-4.41	-1.3335723	SETDB2	SET domain bifurcated 2
235890_at	0.945	0.03807029	-2.2393742	-4.41	-2.2163695	TBL1XR1	transducin (beta)-like 1 X-linked receptor 1
238951_at	0.945	0.03333317	-2.3444657	-4.41	-2.9831432		
210250_x_at	0.945	0.04850848	-2.1171927	-4.41	-3.1692516	ADSL	adenylosuccinate lyase
227902_at	0.945	0.0285714	-2.4422554	-4.41	-3.5695933	ZFP41	ZFP41 zinc finger protein
214720_x_at	0.945	0.03390741	-2.3356536	-4.41	-2.527258	SEPT10	septin 10

1556528_at	0.945	0.03074522	-2.3860465	-4.41	-3.4212965	LINC00692	long intergenic non-protein coding RNA 692
239501_at	0.945	0.04262818	-2.1925552	-4.41	-2.9982996		
220848_x_at	0.945	0.02271353	-2.5859006	-4.41	-3.4682047	OBP2A	odorant binding protein 2A
201919_at	0.945	0.04970225	-2.1047931	-4.41	-2.6706551	SLC25A36	solute carrier family 25 member 36
1554542_at	0.945	0.0390525	-2.2488711	-4.41	-2.583707	SLC25A48	solute carrier family 25 member 48
1561384_a_at	0.945	0.04352501	-2.1819367	-4.41	-2.2483783	LOC284661	uncharacterized LOC284661
212786_at	0.945	0.04631186	-2.1501789	-4.41	-2.1325381	CLEC16A	C-type lectin domain family 16 member A
224720_at	0.945	0.03526197	-2.3010579	-4.41	-2.6204698	MIB1	mindbomb E3 ubiquitin protein ligase 1
244317_at	0.945	0.04401738	-2.1761924	-4.41	-2.8346423	KIAA1324L	KIAA1324 like
211390_at	0.945	0.0467757	-2.1450642	-4.41	-2.9993511	N4BP2L1	NEDD4 binding protein 2 like 1
218608_at	0.945	0.04694964	-2.1431582	-4.41	-3.5615472	ATP13A2	ATPase 13A2
237947_at	0.945	0.04203686	-2.2110016	-4.41	-2.3882784		
222385_x_at	0.945	0.03606183	-2.3037983	-4.41	-2.5580015	SEC61A1	Sec61 translocon alpha 1 subunit
220700_at	0.945	0.04733412	-2.1389681	-4.41	-3.1863998		
238410_x_at	0.945	0.04091812	-2.2248961	-4.41	-2.5822259		
200621_at	0.945	0.02346959	-2.5443578	-4.41	-2.6825626	CSRP1	cysteine and glycine rich protein 1
210405_x_at	0.945	0.0369159	-2.2916627	-4.41	-3.7149785	TNFRSF10B	TNF receptor superfamily member 10b
221606_s_at	0.945	0.04558896	-2.1582445	-4.41	-2.3060273	HMGN5	high mobility group nucleosome binding domain 5
218948_at	0.945	0.03158942	-2.389791	-4.41	-3.352703	QRSL1	glutaminyl-tRNA synthase (glutamine-hydrolyzing)-like 1

225738_at	0.945	0.02500194	-2.5350598	-4.41	-2.6250048	RAPGEF1	Rap guanine nucleotide exchange factor 1
1561480_a_at	0.945	0.04934932	-2.1084305	-4.42	-2.6056534		

GSE27890

ID	adj.P.Val	P.Value	t	B	logFC	Gene.symbol
229552_at	0.759	0.0000958	6.0186579	-4.54	4.3593317	LOC283454///HRK
229638_at	0.759	0.0001081	5.9334785	-4.54	4.1043461	IRX3
241141_at	0.759	0.000141	5.7480908	-4.54	5.4058304	BMP6
242164_s_at	0.759	0.0001893	5.5463347	-4.54	5.1784128	LRIG2
210729_at	0.759	0.0002153	5.4590468	-4.54	5.0229993	NPY2R
229889_at	0.759	0.0004462	4.9796686	-4.55	3.2610511	LRRC75A
1559134_a_at	0.759	0.0004562	4.9654116	-4.55	3.6428686	ZNF546
1552484_at	0.759	0.000464	4.9545872	-4.55	3.2132517	SMCR8
1557297_at	0.759	0.0004999	4.9068388	-4.55	3.7057512	
234358_at	0.759	0.0005307	4.8686045	-4.55	5.0061014	
211055_s_at	0.759	0.0005987	4.7921026	-4.55	4.1945591	INVS
1552763_at	0.759	0.0006415	4.7485124	-4.55	4.2883563	TMEM67
237982_at	0.759	0.0006636	4.7271671	-4.55	4.1373508	LOC101928327
243877_at	0.759	0.0007088	4.6858018	-4.55	5.0003844	
240093_x_at	0.759	0.0007437	4.65574	-4.55	3.8524909	

216907_x_at	0.759	0.0008111	4.6016266	-4.55	4.023287	KIR3DL2///KIR3DL1
229271_x_at	0.759	0.0008657	4.5611252	-4.55	4.4542988	COL11A1
232655_at	0.759	0.0008879	4.5454648	-4.55	4.1552847	UGT1A1
217595_at	0.759	0.0009756	4.4872923	-4.55	4.6263943	GSPT1
1562850_at	0.759	0.0010383	4.448927	-4.55	4.1682162	LOC101928283
234145_at	0.759	0.001131	4.3965243	-4.55	3.3697183	
244613_at	0.759	0.0012432	4.3388464	-4.55	4.3902145	
1569439_at	0.759	0.0013879	4.2719919	-4.55	4.3933241	
1568923_at	0.759	0.0014264	4.2554141	-4.55	3.0555606	
1567334_at	0.759	0.0014546	4.2435833	-4.55	4.4830209	
1556394_a_at	0.759	0.0014601	4.2412968	-4.55	2.9059188	
217528_at	0.759	0.0014978	4.2259296	-4.55	4.1050767	CLCA2
216891_at	0.759	0.0015064	4.2224658	-4.55	2.7863461	
206149_at	0.759	0.0015273	4.2141534	-4.55	3.8669066	CHP2
237939_at	0.759	0.0015823	4.1928346	-4.55	4.28844	EPHA5
1557427_at	0.759	0.001632	4.1742555	-4.55	3.1803648	
211269_s_at	0.759	0.0017109	4.145898	-4.55	4.0878713	IL2RA
1569504_at	0.759	0.0017622	4.1281956	-4.55	4.3806527	LILRB4
239900_x_at	0.759	0.0018579	4.096569	-4.55	4.0384386	
240606_at	0.759	0.0018713	4.0922704	-4.55	3.3695003	

214580_x_at	0.759	0.0018928	4.0854601	-4.55	3.3895201	KRT6C///KRT6B///KRT6A
238490_at	0.759	0.0018928	4.0854551	-4.55	2.8847903	KIAA2026
230508_at	0.759	0.0018955	4.0846075	-4.55	3.3924225	DKK3
1561983_at	0.759	0.0019408	4.0705103	-4.55	3.2325989	LINC01206
1557838_at	0.759	0.0019443	4.0694412	-4.55	3.9812802	FAM135B
1561919_at	0.759	0.0019939	4.0544333	-4.55	2.9589809	
207935_s_at	0.759	0.0020427	4.0400202	-4.55	3.5912971	KRT13
219827_at	0.759	0.00205	4.0379212	-4.55	3.0562615	UCP3
1554059_at	0.759	0.0020823	4.0286212	-4.55	3.335112	SETMAR
1559298_a_at	0.759	0.0020843	4.0280426	-4.55	2.793844	SPATA41
208333_at	0.759	0.002107	4.0216079	-4.55	3.9648009	LHX5
1552661_at	0.759	0.0021737	4.0030997	-4.55	3.4362059	PCDHGB7
234473_at	0.759	0.0021846	4.0001217	-4.55	3.3123828	LOC101928697
238611_at	0.759	0.0021951	3.9972858	-4.55	3.1334572	
1555394_at	0.759	0.0022113	3.9929202	-4.55	3.5637169	PIGK
1554371_at	0.759	0.002253	3.9818579	-4.55	3.7071936	PKD1L2
238250_at	0.759	0.0022653	3.9786314	-4.55	4.02193	
1561062_a_at	0.759	0.0022951	3.9708722	-4.55	3.2364854	LOC101928850
238284_at	0.759	0.0023009	3.9694029	-4.55	3.6688939	

212084_at	0.759	0.00231	3.9670454	-4.55	2.9605327	TEX261
234540_at	0.759	0.002335	3.9606945	-4.55	3.4965731	GCNT7
1563271_at	0.759	0.0024452	3.9334236	-4.55	3.6620089	
206840_at	0.759	0.002447	3.9329813	-4.55	4.149817	AFM
244051_at	0.759	0.0024787	3.9253946	-4.55	3.2496967	
215469_at	0.759	0.0025031	3.9196089	-4.55	3.5219181	SLITRK5
1553526_at	0.759	0.0025258	3.914278	-4.55	2.8833931	NLRP8
1553111_a_at	0.759	0.0025332	3.9125545	-4.55	4.6570242	KBTBD6
224154_at	0.759	0.002535	3.912144	-4.55	3.0243459	B3GNT2
234129_at	0.759	0.0025397	3.9110619	-4.55	2.5997741	ARHGEF12
237512_at	0.759	0.0025634	3.9055658	-4.55	3.4380213	NAV2-AS2
1561371_at	0.759	0.0026116	3.8946075	-4.55	2.9754282	MIR2052HG
1552890_a_at	0.759	0.0026408	3.8880516	-4.55	2.6040219	CABP4
208317_at	0.759	0.0026682	3.8819783	-4.55	3.4344407	XYLB
1565936_a_at	0.759	0.0026693	3.8817294	-4.55	3.0885164	LMO3
216868_s_at	0.759	0.0026746	3.8805614	-4.55	3.6603297	
1563074_at	0.759	0.0026873	3.8777706	-4.55	3.7910833	LOC255654
217953_at	0.759	0.002696	3.8758811	-4.55	3.2736856	PHF3
1563894_at	0.759	0.0027099	3.8728485	-4.55	3.0063074	LOC441178
237645_at	0.759	0.0027363	3.8671619	-4.56	3.0966303	
1570235_at	0.759	0.002741	3.8661529	-4.56	3.6657733	MGC27382
206159_at	0.759	0.002745	3.8652857	-4.56	3.8026528	GDF10
1556062_at	0.759	0.00278	3.8578472	-4.56	4.717014	RPP30

1563290_at	0.759	0.0027865	3.856467	-4.56	2.9728262	DNAH3
241492_at	0.759	0.0028821	3.836657	-4.56	3.0779363	
1568639_a_at	0.759	0.0028859	3.835897	-4.56	2.2882212	GATA2-AS1
234776_at	0.759	0.0028889	3.8352898	-4.56	3.9397779	DMBX1
220811_at	0.759	0.0028992	3.8331866	-4.56	3.5039691	PRG3
241915_at	0.759	0.0029352	3.8259558	-4.56	4.1317176	ACSM2B
231304_at	0.759	0.0029434	3.8243255	-4.56	3.3948012	PPP3R2
1566832_at	0.759	0.0029463	3.8237343	-4.56	3.6993637	TOP1P2
219085_s_at	0.759	0.0029481	3.8233844	-4.56	3.6683079	GEMIN7
1554918_a_at	0.759	0.0030407	3.8052666	-4.56	3.0382499	ABCC4
211491_at	0.759	0.0030568	3.8021773	-4.56	3.6311794	ADRA1A
217578_at	0.759	0.0030591	3.8017327	-4.56	3.0147292	
209125_at	0.759	0.0030883	3.7961656	-4.56	4.1738869	KRT6A
209986_at	0.759	0.0030948	3.7949502	-4.56	3.0482343	LOC101929036///PAH
1562644_at	0.759	0.0031184	3.7904979	-4.56	2.6548298	MTHFD2L
240946_at	0.759	0.0031463	3.7852807	-4.56	3.1085693	
1555048_a_at	0.759	0.0031718	3.7805669	-4.56	2.9902037	TSPEAR
233722_at	0.759	0.0032434	3.767521	-4.56	3.7863775	
238561_s_at	0.759	0.003259	3.7647206	-4.56	3.0146599	UTP23
243107_at	0.759	0.0032631	3.7639782	-4.56	3.3907873	

1561517_at	0.759	0.0033005	3.7573396	-4.56	3.7523541	SSPN
217068_at	0.759	0.0033119	3.7553263	-4.56	3.3221992	
231712_at	0.759	0.0033128	3.7551563	-4.56	4.0778395	ASCL5
205710_at	0.759	0.0033315	3.7518742	-4.56	3.2893057	LRP2
220664_at	0.759	0.0033618	3.7466001	-4.56	2.7639682	SPRR2C
214189_s_at	0.759	0.0033627	3.7464374	-4.56	3.4836462	GGA2
208122_x_at	0.759	0.0033832	3.7428991	-4.56	2.3545366	KIR2DS3
242138_at	0.759	0.0033887	3.7419384	-4.56	3.4490332	DLX1
238947_at	0.759	0.0034133	3.7377243	-4.56	2.5778957	
1555662_s_at	0.759	0.0034172	3.7370685	-4.56	3.217673	DAOA
236857_at	0.759	0.0035456	3.7155659	-4.56	2.783815	SLC38A1
1562301_at	0.759	0.0036693	3.6956176	-4.56	3.3019419	C8orf34
1559603_at	0.759	0.0037306	3.6859765	-4.56	3.8506821	GPR12
1567377_at	0.759	0.0037331	3.6855937	-4.56	3.6194284	DNAH1
1570627_at	0.759	0.003766	3.6804942	-4.56	3.3110263	TCEB3
220425_x_at	0.759	0.0038301	3.6706779	-4.56	3.6218321	ROPN1B
226260_x_at	0.759	0.0038311	3.670528	-4.56	2.9519487	ZNF358
224120_at	0.759	0.0038693	3.6647743	-4.56	2.7209983	
208339_at	0.759	0.0038711	3.6644985	-4.56	3.7723479	XKRY2///XKRY
1561402_at	0.759	0.0038733	3.6641789	-4.56	3.1654898	LINC00880

219346_at	0.759	0.0038894	3.6617708	-4.56	2.8954356	LRFN3
1569879_a_at	0.759	0.0039191	3.6573479	-4.56	2.32899	MEGF11
238355_at	0.759	0.0039215	3.6569932	-4.56	3.5321059	
1565338_x_at	0.759	0.0039502	3.6527681	-4.56	2.815719	DNAH6
1562937_at	0.759	0.0039827	3.6480049	-4.56	2.5387483	
204738_s_at	0.759	0.0039833	3.6479194	-4.56	3.5189801	KRIT1
1570391_at	0.759	0.004007	3.644485	-4.56	2.6533418	CRAT37
236693_at	0.759	0.0040257	3.6417827	-4.56	3.7613698	MIR124-2HG
232722_at	0.759	0.0040444	3.639094	-4.56	2.7299972	RNASET2
241142_at	0.759	0.0040664	3.6359586	-4.56	3.6890597	
1570013_at	0.759	0.0041237	3.6278499	-4.56	2.783448	LOC101927411
215916_at	0.759	0.0041299	3.6269714	-4.56	3.5470838	CHRNE
233774_at	0.759	0.0041659	3.6219505	-4.56	3.5320852	
208305_at	0.759	0.0042089	3.6160014	-4.56	4.2823887	PGR
1562738_a_at	0.759	0.0042536	3.6098908	-4.56	2.8293685	USP3-AS1
1561944_at	0.759	0.004269	3.6077922	-4.56	3.0322475	
211525_s_at	0.759	0.0042769	3.6067179	-4.56	2.7363449	GP5
215540_at	0.759	0.004278	3.6065681	-4.56	2.4702023	YME1L1
215878_at	0.759	0.0042814	3.6061102	-4.56	4.0274608	ITGB1
242792_at	0.759	0.0043052	3.6029026	-4.56	2.9350141	
237879_at	0.759	0.0043495	3.5969854	-4.56	2.9046297	
237828_at	0.759	0.0044018	3.5900791	-4.56	4.0005311	SRRM4
234119_at	0.759	0.0044054	3.5895963	-4.56	2.9247195	

1554424_at	0.759	0.0044319	3.586134	-4.56	2.592822	FIP1L1
204270_at	0.759	0.0045091	3.5761624	-4.56	3.3910395	SKI
233284_at	0.759	0.004592	3.5656371	-4.56	3.0850712	
240959_at	0.759	0.0046364	3.5600814	-4.56	3.4776147	
1562528_at	0.759	0.0046459	3.5589	-4.56	2.9065904	
234087_at	0.759	0.0046683	3.5561242	-4.56	2.7741828	
212275_s_at	0.759	0.0046739	3.5554338	-4.56	3.5484397	SRCAP
238388_x_at	0.759	0.0046801	3.5546606	-4.56	2.6494142	
217697_at	0.759	0.0047328	3.5482069	-4.56	3.0277705	
231523_at	0.759	0.0047915	3.5410929	-4.56	3.4231098	FGF14
1563671_at	0.759	0.0047986	3.5402456	-4.56	3.2510952	LACE1
236567_at	0.759	0.0048196	3.53773	-4.56	3.1574472	
212258_s_at	0.759	0.0048278	3.5367489	-4.56	3.527381	SMARCA2
1556456_at	0.759	0.0048323	3.5362052	-4.56	2.6429915	LINC01138
216553_x_at	0.759	0.0048655	3.532266	-4.56	2.9529788	
241029_at	0.759	0.0049221	3.5255992	-4.56	2.7016726	
217429_at	0.759	0.0049599	3.5211868	-4.56	3.0794417	
1558601_at	0.759	0.0049964	3.5169652	-4.56	3.2756567	TUSC7
1556447_at	0.759	0.0050116	3.515217	-4.56	4.5683941	LINC00606
239478_x_at	0.759	0.005044	3.5115023	-4.56	3.2428161	GPATCH2L
237660_at	0.759	0.0051342	3.5012988	-4.56	3.6922435	UNC80
207962_at	0.759	0.0051981	3.4941819	-4.56	2.8097081	CAPN11

241481_at	0.759	0.005217	3.4921023	-4.56	2.6100941	FAM81A
1565484_x_at	0.759	0.0052444	3.4890903	-4.56	3.3504333	EGFR
1554394_at	0.759	0.0052485	3.4886345	-4.56	4.1036375	NELL1
202198_s_at	0.759	0.0053287	3.4799195	-4.56	2.7672087	MTMR3
218899_s_at	0.759	0.0053343	3.4793186	-4.56	3.5244172	BAALC
AFFX-r2-Bs-dap-5_at	0.759	0.0053535	3.4772513	-4.56	2.4000887	
231935_at	0.759	0.0053631	3.4762171	-4.56	2.3563935	ARPP21
206256_at	0.759	0.0054223	3.4699098	-4.56	2.5706572	CPN1
233504_at	0.759	0.0054367	3.468387	-4.56	2.4231696	C9orf84
1553805_at	0.759	0.005498	3.4619487	-4.56	2.7422417	C3orf49
233335_at	0.759	0.0055321	3.4583992	-4.56	3.4899037	ITGA11
243620_at	0.759	0.0055577	3.4557455	-4.56	2.3444361	
1560425_s_at	0.759	0.0055667	3.4548194	-4.56	3.5922052	PTPRD-AS1
1553822_at	0.759	0.0056076	3.4506164	-4.56	3.3293188	RTP1
1560758_at	0.759	0.0056433	3.4469647	-4.56	3.9204252	
239537_at	0.759	0.0057078	3.4404519	-4.56	5.1091809	ST8SIA2
243932_at	0.759	0.0057417	3.437048	-4.56	3.1816412	
205243_at	0.759	0.005801	3.4311534	-4.56	2.2078263	SLC13A3
240817_at	0.759	0.0058328	3.4280203	-4.56	3.0095317	LOC101929034
224185_at	0.759	0.0058573	3.4256188	-4.56	2.3334796	
237123_x_at	0.759	0.0059396	3.4176193	-4.56	3.6149455	KLHL9
1557480_a_at	0.759	0.0059528	3.4163464	-4.56	3.289825	PPP1R27

1558654_at	0.759	0.0059572	3.4159269	-4.56	2.3215711	PPM1H
208026_at	0.759	0.0060029	3.4115424	-4.56	3.6948119	HIST1H4G///HIST1H4F
208299_at	0.759	0.0060394	3.4080675	-4.56	2.6529409	CACNA1I
1562974_at	0.759	0.0060432	3.4077065	-4.56	3.1814828	
240411_at	0.759	0.0060473	3.4073249	-4.56	3.8239171	MROH2B
244551_at	0.759	0.0060493	3.4071321	-4.56	2.8426317	ZNF736
229244_at	0.759	0.0060566	3.406445	-4.56	2.8217935	LSAMP
1557354_at	0.759	0.0060598	3.4061391	-4.56	2.4581375	SOS1
1568898_at	0.759	0.0060799	3.4042417	-4.56	3.0850654	LOC101928140
233597_at	0.759	0.0061034	3.4020354	-4.56	3.3149174	PNPLA5
224110_at	0.759	0.0061203	3.4004452	-4.56	3.0123638	PRO1804
1569459_a_at	0.759	0.0061644	3.3963322	-4.56	3.7344566	
238224_at	0.759	0.0061852	3.3944064	-4.56	2.9789002	
237116_at	0.759	0.0062002	3.3930194	-4.56	3.0088735	LOC646903
216877_at	0.759	0.0062132	3.3918192	-4.56	2.640657	LOC100505498
220783_at	0.759	0.0062726	3.3863705	-4.56	3.0778447	MMP27
202828_s_at	0.759	0.0062918	3.3846244	-4.56	2.7495774	MMP14
1564022_at	0.759	0.0063196	3.3821005	-4.56	2.9329645	ZNF804B
1560107_at	0.759	0.006336	3.3806192	-4.56	3.5543233	LOC105372793
207841_at	0.759	0.0063409	3.3801774	-4.56	2.5669618	SPIN2A

1556916_a_at	0.759	0.0063813	3.3765448	-4.56	3.3699477	LOC101928651
1559204_x_at	0.759	0.0063871	3.3760238	-4.56	2.4589622	KRAS
217477_at	0.759	0.0064116	3.3738319	-4.56	2.4869325	PIP5K1B
209680_s_at	0.759	0.0064302	3.3721709	-4.56	2.6383798	KIFC1
211772_x_at	0.759	0.006438	3.3714776	-4.56	2.877623	CHRNA3
210760_x_at	0.759	0.0064415	3.3711685	-4.56	3.0356196	TRIP11
1562601_at	0.759	0.0064511	3.3703219	-4.56	2.697659	LINC01121
213823_at	0.759	0.006463	3.369266	-4.56	3.466264	HOXA11
210206_s_at	0.759	0.0064663	3.3689707	-4.56	3.2619941	DDX11
221993_s_at	0.759	0.0064954	3.3664037	-4.56	2.3266752	TSR3
1555262_a_at	0.759	0.0065424	3.3622785	-4.56	3.1815005	MAGI1
1554743_x_at	0.759	0.0065585	3.3608746	-4.56	2.7627525	PMS1
1554963_at	0.759	0.006616	3.3558892	-4.56	3.1375427	
207262_at	0.759	0.006625	3.3551114	-4.56	2.5139778	APOF
229072_at	0.759	0.006653	3.3526977	-4.56	2.4386985	RAB30
1564333_a_at	0.759	0.0067296	3.3461516	-4.56	2.8570945	PSAPL1
237611_at	0.759	0.0067336	3.345815	-4.56	3.7793775	
224103_at	0.759	0.0067555	3.3439585	-4.56	3.4703032	LOC100132661
237217_at	0.759	0.0067805	3.3418516	-4.56	3.3012789	ADAMTSL1

223674_s_at	0.759	0.0067927	3.340821	-4.56	2.5523453	CDC42SE1
228417_at	0.759	0.0067947	3.340655	-4.56	2.3709795	ISYNA1
208528_x_at	0.759	0.0068033	3.3399309	-4.56	2.6485445	SSX7///SSX3///SSX5
244405_s_at	0.759	0.0068151	3.3389412	-4.56	2.7335035	
1568897_at	0.759	0.0068227	3.3383045	-4.56	3.171237	LOC100422212
240442_at	0.759	0.0068268	3.3379661	-4.56	3.0592086	
215958_at	0.759	0.0068431	3.336598	-4.56	3.977628	
1563061_at	0.759	0.0068789	3.3336235	-4.56	3.4234813	
1560467_at	0.759	0.0069354	3.3289497	-4.56	4.2432893	PAQR9
1562655_at	0.759	0.0069539	3.3274271	-4.56	4.0208617	LOC101927447
1562057_at	0.759	0.0069615	3.3268052	-4.56	3.8740521	
208243_s_at	0.759	0.0069989	3.3237473	-4.56	3.0283055	CNR1
227496_at	0.759	0.007007	3.3230864	-4.56	2.1953101	NR6A1
1555338_s_at	0.759	0.0070249	3.3216321	-4.56	3.0056603	AQP10
231555_at	0.759	0.0070336	3.3209184	-4.56	2.7976389	
232329_at	0.759	0.0070562	3.3190944	-4.56	3.0869734	RANBP10
237885_at	0.759	0.0070722	3.317797	-4.56	2.2472153	SOX21-AS1
236273_at	0.759	0.0070879	3.3165348	-4.56	2.1468326	NBPF1
212257_s_at	0.759	0.0071301	3.3131459	-4.56	2.6731431	SMARCA2
1553282_at	0.759	0.0071439	3.3120437	-4.56	2.778154	UMODL1-AS1

231385_at	0.759	0.007168	3.3101161	-4.56	3.6048588	LOC101060236///DPPA3P2///DPPA3
206098_at	0.759	0.0072177	3.3061759	-4.56	3.8755409	ZBTB6
1556465_at	0.759	0.0072598	3.3028587	-4.56	2.5385139	
222567_s_at	0.759	0.0073116	3.2988023	-4.56	2.4086437	MEX3C
1553491_at	0.759	0.0073128	3.2987086	-4.56	3.3215311	KSR2
1567286_at	0.759	0.0073177	3.2983259	-4.56	3.1172108	OR5L2
243925_at	0.759	0.0073457	3.2961496	-4.56	3.2227633	
222948_s_at	0.759	0.0073467	3.2960693	-4.56	2.872141	MRPS18C
237876_at	0.759	0.0073625	3.2948444	-4.56	2.3842862	
1566821_at	0.759	0.0073737	3.2939773	-4.56	2.3886772	
238483_at	0.759	0.0073744	3.2939305	-4.56	3.3477449	SSBP2
214452_at	0.759	0.0073973	3.2921551	-4.56	3.5803296	BCAT1
206670_s_at	0.759	0.0074051	3.2915589	-4.56	2.9833141	GAD1
1565694_at	0.759	0.0074673	3.2867878	-4.56	3.5345709	DTYMK
241493_at	0.759	0.0074981	3.2844428	-4.56	3.1956555	
210493_s_at	0.759	0.0075974	3.2769452	-4.56	3.2736141	MFAP3L
213955_at	0.759	0.007624	3.2749511	-4.56	3.4352422	MYOZ3
244047_at	0.759	0.0077058	3.2688707	-4.56	3.4255727	
200865_at	0.759	0.007711	3.2684839	-4.56	3.4341907	EIF3F
237631_at	0.759	0.0077306	3.2670409	-4.56	3.6310557	

237063_at	0.759	0.0077329	3.266868	-4.56	2.7363799	
242401_x_at	0.759	0.0077332	3.2668479	-4.56	3.6014437	
221373_x_at	0.759	0.007735	3.2667166	-4.56	2.5424143	PSPN
216455_at	0.759	0.0077523	3.2654462	-4.56	2.9938274	LOC101927181
1570533_at	0.759	0.0077597	3.2649002	-4.56	2.7145645	CMPK1
1563001_at	0.759	0.0078187	3.2605881	-4.56	2.9164532	
1566969_at	0.759	0.007837	3.2592564	-4.56	4.3326793	
1569205_at	0.759	0.0078416	3.2589222	-4.56	3.2469343	PRCP
232414_at	0.759	0.0078745	3.2565332	-4.56	3.2090115	HEATR1
243549_at	0.759	0.0078771	3.256348	-4.56	3.8240275	
1554717_a_at	0.759	0.0078914	3.2553129	-4.56	2.8579592	PDE4D
238680_at	0.759	0.0079585	3.2504967	-4.56	3.7201702	METTL12///SNORA57
1559388_a_at	0.759	0.0079686	3.2497749	-4.56	3.0072956	LYNX1
239457_at	0.759	0.007977	3.2491742	-4.56	2.3563321	ATP8B3
242072_at	0.759	0.0079884	3.2483607	-4.56	4.1806911	
221785_at	0.759	0.0079976	3.2477021	-4.56	2.5561115	WIZ
203441_s_at	0.759	0.0080208	3.2460524	-4.56	2.4649304	CDH2
221092_at	0.759	0.0080994	3.2405065	-4.56	2.95808	IKZF3
1559837_at	0.759	0.0081148	3.2394223	-4.56	2.8411549	LINC01490
1561033_at	0.759	0.0081581	3.2363966	-4.56	3.261917	LOC101927211
206801_at	0.759	0.0081709	3.2355008	-4.56	2.9566674	NPPB
243155_at	0.759	0.0082432	3.2304874	-4.56	3.005101	

1561055_at	0.759	0.0082861	3.2275346	-4.56	3.5882624	LOC100507534
224020_at	0.759	0.0082887	3.2273583	-4.56	3.1046091	LINC00626
243884_at	0.759	0.0083156	3.2255167	-4.56	2.7740957	TEX26
238240_at	0.759	0.0084167	3.2186389	-4.56	2.8198847	
205062_x_at	0.759	0.0084853	3.2140277	-4.56	2.9820494	ARID4A
208871_at	0.759	0.0085012	3.2129595	-4.56	3.5801173	ATN1
1559949_at	0.759	0.0085407	3.210328	-4.56	2.3885016	
234026_at	0.759	0.0085519	3.2095811	-4.56	2.7138889	EML2-AS1
1567359_at	0.759	0.0085869	3.2072561	-4.56	3.4901105	BDNF-AS
208577_at	0.759	0.008627	3.2046097	-4.56	2.5322951	HIST1H3C
1552372_at	0.759	0.0086439	3.2034959	-4.56	3.4753502	C4orf33
231334_at	0.759	0.0086486	3.2031895	-4.56	2.1665117	LOC101929480
232672_x_at	0.759	0.00865	3.2030988	-4.56	3.0919491	CTXN2///SLC24A5
220304_s_at	0.759	0.0086839	3.2008758	-4.56	3.0609662	CNGB3
237685_at	0.759	0.0086984	3.199927	-4.56	3.0282279	LOC101929926
238932_at	0.759	0.0087253	3.1981661	-4.56	2.41075	TSC22D2
238499_at	0.759	0.0087795	3.1946521	-4.56	2.5122534	SLC45A3
239620_at	0.759	0.0087938	3.193727	-4.56	2.6198075	TTC23L
218513_at	0.759	0.0087987	3.1934108	-4.56	2.6938934	TMA16
231398_at	0.759	0.0088444	3.1904666	-4.56	2.3132085	SLC22A7
238118_s_at	0.759	0.0088468	3.1903083	-4.56	2.7709816	PPOX

232200_at	0.759	0.0088844	3.1878995	-4.56	3.0029372	HSPA9
217306_at	0.759	0.0089349	3.1846801	-4.56	3.1210105	
229179_at	0.759	0.0089413	3.1842725	-4.56	1.9588213	RUFY1
233620_at	0.759	0.0089481	3.1838434	-4.56	3.7450992	ARHGEF12
1553740_a_at	0.759	0.0089759	3.1820796	-4.56	2.5163758	IRAK2
240739_at	0.759	0.0089911	3.1811182	-4.56	3.4875938	
1555947_at	0.759	0.009014	3.179673	-4.56	2.5578993	FAM120A
215655_at	0.759	0.0090187	3.1793783	-4.56	2.5936612	GRIK2
216365_x_at	0.759	0.0090707	3.1761148	-4.56	2.8068931	IGLJ3///CKAP2
1560177_at	0.759	0.0090778	3.1756687	-4.56	3.2150249	CCDC66
241042_at	0.759	0.009079	3.1755943	-4.56	3.1523563	
1563620_at	0.759	0.0090824	3.1753787	-4.56	3.5436691	BTRC
237831_x_at	0.759	0.0091024	3.1741293	-4.56	3.3589427	MMAA
1562786_at	0.759	0.0091055	3.173938	-4.56	2.8270128	LOC101928886
208521_at	0.759	0.0091254	3.1726946	-4.56	3.5445554	OR5I1
241638_at	0.759	0.0091963	3.1682993	-4.56	2.9152956	
221618_s_at	0.759	0.009205	3.1677668	-4.56	3.0406993	TAF9B
204931_at	0.759	0.009262	3.1642614	-4.56	2.842089	TCF21
239992_at	0.759	0.0092737	3.1635463	-4.56	3.6765925	

1554400_at	0.759	0.0092812	3.1630847	-4.56	3.5162394	TCTE3
224966_s_at	0.759	0.0092899	3.1625519	-4.56	3.1910204	DUS3L
214503_x_at	0.759	0.009293	3.1623651	-4.56	3.4582575	GPR135
1557948_at	0.759	0.0092949	3.1622486	-4.56	3.2651476	PHLDB3
234659_at	0.759	0.009359	3.1583431	-4.56	3.0046327	
1558852_at	0.759	0.0094372	3.1536249	-4.56	2.7430469	
1556914_at	0.759	0.0094765	3.1512646	-4.56	2.3500317	LOC100652911
244143_at	0.759	0.0095131	3.1490767	-4.56	3.2552562	
229217_at	0.759	0.0095145	3.1489905	-4.56	2.4436915	SP3
215796_at	0.759	0.0095257	3.1483234	-4.56	2.4625949	
244262_x_at	0.759	0.0095918	3.144398	-4.56	3.1937646	
210704_at	0.759	0.0096311	3.1420829	-4.56	2.7913987	FEZ2
233704_at	0.759	0.009637	3.1417304	-4.56	3.8162549	
219990_at	0.759	0.0096454	3.1412366	-4.56	2.3248355	E2F8
234481_at	0.759	0.0096843	3.1389548	-4.56	2.2160096	
234647_at	0.759	0.0097221	3.1367461	-4.56	2.6729503	PROM2
234845_at	0.759	0.0097421	3.1355804	-4.56	2.6070851	LOC100130370
204653_at	0.759	0.0098069	3.1318195	-4.56	1.9680905	TFAP2A
240893_at	0.759	0.009836	3.1301361	-4.56	2.8698049	
234300_s_at	0.759	0.0098691	3.1282342	-4.56	2.7187117	ZFP28

208235_x_at	0.759	0.0099092	3.1259324	-4.56	2.6338922	GAGE12F///GAGE12B///GAGE12G///GAGE12I///GAGE7///GAGE6///GAG[illegible]
1570274_at	0.759	0.0099148	3.1256103	-4.56	2.4827258	PRSS55
1562585_at	0.759	0.0099218	3.1252114	-4.56	3.0295333	LOC284263
231330_at	0.759	0.0099317	3.1246444	-4.56	2.5372349	LINC00445
1553915_at	0.759	0.0099757	3.1221398	-4.56	2.9866091	C10orf126
242250_at	0.759	0.0100161	3.1198451	-4.56	2.5545758	
241658_at	0.759	0.0100419	3.1183872	-4.56	2.7603129	ARIH1
1563872_at	0.759	0.0100962	3.1153315	-4.56	2.2130973	LOC284395
236746_at	0.759	0.0101013	3.1150423	-4.56	3.0847283	GALNT1
1558636_s_at	0.759	0.0101199	3.1140016	-4.56	3.6524709	ADAMTS5
233552_at	0.759	0.0101661	3.111417	-4.56	1.9999284	
1553893_at	0.759	0.0101717	3.1111055	-4.56	3.4224259	CCDC105
216777_at	0.759	0.0101824	3.110509	-4.56	3.401056	
1566598_at	0.759	0.0102067	3.1091573	-4.56	2.7036056	
1562074_a_at	0.759	0.010216	3.1086407	-4.56	3.2354178	UNC13C
237106_at	0.759	0.0102193	3.1084572	-4.56	2.9426974	SLC11A2
240488_at	0.759	0.0102498	3.1067684	-4.56	3.3489889	

212427_at	0.759	0.0102551	3.1064738	-4.56	2.1910234	KIAA0368
206759_at	0.759	0.0102608	3.1061606	-4.56	2.9157113	FCER2
206418_at	0.759	0.0103652	3.1004252	-4.56	2.6199073	NOX1
1554203_at	0.759	0.0103776	3.0997452	-4.56	2.5445676	GRIK1-AS1
210945_at	0.759	0.0103851	3.0993341	-4.56	3.9658289	COL4A6
236493_at	0.759	0.010395	3.098796	-4.56	3.3027165	NKAPP1
1566403_at	0.759	0.0104192	3.0974793	-4.56	2.5118463	SNORA68///RPL18A
243214_at	0.759	0.0104381	3.0964487	-4.56	2.8084972	ZBTB46
207427_at	0.759	0.0104806	3.0941501	-4.56	2.6724974	ACR
224026_at	0.759	0.0105084	3.0926477	-4.56	2.3382332	
243542_at	0.759	0.0105085	3.0926406	-4.56	4.27008	PREPL
235356_at	0.759	0.0105181	3.092126	-4.56	3.0372159	NHLRC2
216961_s_at	0.759	0.010539	3.0909976	-4.56	2.6863738	RPAIN
229411_at	0.759	0.0105419	3.0908415	-4.56	2.2332999	PNCK
241103_at	0.759	0.0105971	3.0878859	-4.56	3.7190769	PPP4R3B
1568732_at	0.759	0.0106314	3.0860533	-4.56	3.085656	
1558440_at	0.759	0.0107442	3.0800705	-4.56	2.6723066	LOC100506351
220895_at	0.759	0.0108201	3.0760867	-4.56	3.8436366	USP29
1561473_at	0.759	0.0108275	3.0756962	-4.56	3.0483271	LOC102546226
1558400_x_at	0.759	0.0108476	3.0746495	-4.56	2.1239863	ANKRD24
1552672_a_at	0.759	0.0108598	3.0740091	-4.56	3.6477162	IGSF3

1562562_at	0.759	0.0108667	3.073652	-4.56	2.8564825	ANKUB1
1557908_at	0.759	0.0108778	3.0730748	-4.56	2.1422565	HP11014
243457_s_at	0.759	0.0109311	3.0703043	-4.56	2.9553119	ZNF214
1560510_at	0.759	0.0109348	3.0701131	-4.56	2.3328153	ARHGEF7-IT1
220656_at	0.759	0.0109359	3.070053	-4.56	2.9764578	NAA16
233911_s_at	0.759	0.0109519	3.0692288	-4.56	2.6166743	PPM1H
207599_at	0.759	0.0109907	3.0672244	-4.56	3.1810743	MMP20
1555460_a_at	0.759	0.0110667	3.0633212	-4.56	2.709302	SLC39A6
215289_at	0.759	0.0110758	3.0628557	-4.56	3.1422051	ZNF749
224401_s_at	0.759	0.0110899	3.0621357	-4.56	2.30318	FCRL4
1560325_at	0.759	0.0111099	3.0611142	-4.56	3.0401826	LOC105370612
237596_at	0.759	0.0111353	3.0598238	-4.56	2.7346992	
1560241_at	0.759	0.0111619	3.0584682	-4.56	2.4789353	LOC101928682
234507_at	0.759	0.0111746	3.0578253	-4.56	3.2266231	
1559029_at	0.759	0.011183	3.0573988	-4.56	2.4876058	LOC286154
211032_at	0.759	0.0111947	3.0568077	-4.56	3.7624034	COBLL1
1555273_at	0.759	0.0111982	3.0566315	-4.56	2.8257502	GALNTL6
233181_at	0.759	0.011203	3.0563896	-4.56	2.1209483	
216193_at	0.759	0.0112678	3.0531248	-4.56	2.8996835	
1559722_at	0.759	0.0113233	3.0503419	-4.56	2.8128468	C9orf3

215637_at	0.759	0.0113514	3.0489362	-4.56	3.4501377	CEP41
1566042_at	0.759	0.0113518	3.0489208	-4.56	3.399581	
1564413_at	0.759	0.0114281	3.0451279	-4.56	2.1484459	LINC01356
232533_at	0.759	0.0114879	3.0421696	-4.56	2.5073704	METTL8
244632_at	0.759	0.0114881	3.0421619	-4.56	3.2558856	CNTN5
238166_s_at	0.759	0.0114952	3.0418113	-4.56	3.0162662	C17orf51
211079_s_at	0.759	0.0115282	3.0401902	-4.56	2.6912904	DYRK1A
232673_at	0.759	0.0115315	3.0400269	-4.56	2.4498667	LRRFIP2
211815_s_at	0.759	0.0115668	3.0382983	-4.56	2.6440853	GGA3
219764_at	0.759	0.0115815	3.037578	-4.56	2.9241302	FZD10
1567558_at	0.759	0.0115904	3.0371444	-4.56	2.2657687	TREML4
220478_at	0.759	0.0116151	3.0359396	-4.56	2.8120504	APOL5
1554348_s_at	0.759	0.0116418	3.0346413	-4.56	2.5105815	CDKN2AIPNL
1558839_at	0.759	0.0116845	3.0325655	-4.56	2.3717864	MAPKBP1
206484_s_at	0.759	0.0116862	3.0324836	-4.56	2.5200748	XPNPEP2
1560021_at	0.759	0.0116911	3.0322476	-4.56	3.4836622	
217314_at	0.759	0.0117188	3.0309077	-4.56	2.5164562	
218413_s_at	0.759	0.0117264	3.0305392	-4.56	2.1461694	ZNF639
237209_s_at	0.759	0.0118168	3.026193	-4.56	2.5834684	NFRKB
236695_at	0.759	0.011833	3.0254187	-4.56	2.5427079	STK4-AS1
1553776_at	0.759	0.0118384	3.025161	-4.56	2.9939107	UBE2U

1556986_at	0.759	0.0118393	3.0251174	-4.56	2.6734486	OR2H1
241077_at	0.759	0.011877	3.0233214	-4.56	2.8405284	
240358_at	0.759	0.0118824	3.0230601	-4.56	2.7860507	
1553605_a_at	0.759	0.011936	3.0205134	-4.56	2.204606	ABCA13
230579_at	0.759	0.0119671	3.0190413	-4.57	2.8940736	LOC728705///TMEM263
216605_s_at	0.759	0.0119732	3.0187551	-4.57	2.6974851	CEACAM21
1556421_at	0.759	0.0119809	3.0183935	-4.57	2.8658939	C8orf34-AS1
232197_x_at	0.759	0.0119929	3.0178237	-4.57	2.2909938	ARSB
236613_at	0.759	0.0120349	3.0158455	-4.57	2.9761155	RBM25
214557_at	0.759	0.0120755	3.01394	-4.57	2.7712089	PTTG2
237129_at	0.759	0.0120771	3.0138651	-4.57	2.579606	CEP152
234407_s_at	0.759	0.0121366	3.0110856	-4.57	2.4668994	TRPC7
1562812_at	0.759	0.0121558	3.0101928	-4.57	2.5155832	ZNF32-AS3
1559595_at	0.759	0.0122071	3.0078109	-4.57	3.032972	LOC728084
230272_at	0.759	0.0122183	3.0072924	-4.57	3.4129156	LINC00461///MIR9-2
244539_at	0.759	0.0122424	3.0061746	-4.57	3.8708007	
1559105_at	0.759	0.0122427	3.0061635	-4.57	2.3524963	LEKR1
1561453_at	0.759	0.0122564	3.0055305	-4.57	2.5695336	
1560962_at	0.759	0.0122605	3.0053402	-4.57	2.9970626	
1566952_at	0.759	0.0122754	3.004655	-4.57	2.3305837	

234406_at	0.759	0.0122831	3.0043003	-4.57	2.2362942	RGMA
202103_at	0.759	0.012293	3.0038421	-4.57	2.221667	BRD4
1562715_at	0.759	0.0123324	3.0020319	-4.57	3.0981564	
207105_s_at	0.759	0.0123923	2.9992914	-4.57	3.1704701	PIK3R2
243234_at	0.759	0.0124004	2.9989215	-4.57	2.9327269	TBX3
1552849_at	0.759	0.0124278	2.9976754	-4.57	3.2478425	M1AP
230445_at	0.759	0.0125069	2.9940856	-4.57	3.1723892	BTBD17
1561765_at	0.759	0.0125263	2.9932066	-4.57	3.9895001	LOC105377925
234381_at	0.759	0.0125302	2.9930319	-4.57	2.2895746	
222335_at	0.759	0.012545	2.9923667	-4.57	1.8071095	
243232_at	0.759	0.012594	2.9901588	-4.57	2.7440146	
240375_at	0.759	0.012599	2.9899357	-4.57	2.04186	PIGV
243788_at	0.759	0.0126024	2.9897848	-4.57	3.5440441	
203461_at	0.759	0.0126514	2.9875861	-4.57	2.536837	CHD2
224074_at	0.759	0.0126953	2.9856307	-4.57	3.1571743	VSX1
204936_at	0.759	0.0126999	2.9854232	-4.57	2.9721826	MAP4K2
207371_at	0.759	0.0127042	2.9852323	-4.57	2.7339753	
1562988_at	0.759	0.0127091	2.9850132	-4.57	2.8025911	ZSCAN30
211672_s_at	0.759	0.0127095	2.9849962	-4.57	2.6527659	ARPC4-TTLL3///ARPC4
206701_x_at	0.759	0.0127158	2.9847156	-4.57	4.2356942	EDNRB

243298_at	0.759	0.0127203	2.9845191	-4.57	2.6693277	
216799_at	0.759	0.0127341	2.9839054	-4.57	2.4646747	
1557886_at	0.759	0.0127665	2.9824681	-4.57	2.7090873	CEP112
231534_at	0.759	0.012767	2.9824425	-4.57	3.0308543	CDK1
231670_at	0.759	0.0127732	2.9821722	-4.57	2.393441	
1560317_s_at	0.759	0.0128057	2.980735	-4.57	2.5168311	HIP1
216948_at	0.759	0.0128413	2.9791617	-4.57	3.4578033	
213147_at	0.759	0.0128815	2.9773954	-4.57	2.7732288	HOXA10
216407_at	0.759	0.0129134	2.9759973	-4.57	2.7443994	VAC14
1559166_at	0.759	0.0129261	2.9754428	-4.57	2.7894332	
1565817_at	0.759	0.0129312	2.97522	-4.57	2.5457954	IKZF1
205732_s_at	0.759	0.0129334	2.9751224	-4.57	2.3719019	NCOA2
235663_at	0.759	0.0129358	2.9750178	-4.57	2.2847571	
1554386_at	0.759	0.0129586	2.9740224	-4.57	3.5832101	CST9
232661_s_at	0.759	0.012965	2.9737425	-4.57	2.5505202	RBM48
244733_at	0.759	0.0129679	2.9736169	-4.57	3.4371808	
201130_s_at	0.759	0.0129738	2.9733584	-4.57	2.4368384	CDH1
1557627_at	0.759	0.0129775	2.9731991	-4.57	3.9231065	LOC283745
228808_s_at	0.759	0.0129853	2.9728577	-4.57	2.6240143	LOXL2
217163_at	0.759	0.0129907	2.9726233	-4.57	2.7211821	ESR1
1552486_s_at	0.759	0.0130028	2.9720969	-4.57	2.9834878	LACTB
235590_at	0.759	0.0130053	2.9719874	-4.57	2.1714835	SLF2
232660_at	0.759	0.0130247	2.9711453	-4.57	2.8856055	BAD
1570479_at	0.759	0.0130496	2.9700658	-4.57	2.6830457	ART1
1552878_at	0.759	0.0130694	2.9692078	-4.57	3.1384881	ATOH7

221351_at	0.759	0.013092	2.9682322	-4.57	2.666527	HTR1A
1557314_at	0.759	0.0131303	2.9665788	-4.57	2.2056943	DPY19L2P3
233858_at	0.759	0.0131369	2.9662953	-4.57	2.5783858	
1561670_at	0.759	0.0131457	2.965917	-4.57	2.2125943	
238411_x_at	0.759	0.0131604	2.965286	-4.57	3.2221747	NREP
237080_at	0.759	0.0132403	2.9618617	-4.57	2.2901686	
235524_at	0.759	0.0132467	2.9615886	-4.57	2.250066	RALGAPA1
233533_at	0.759	0.0132492	2.961484	-4.57	2.9476784	KRTAP1-5
230137_at	0.759	0.0132614	2.9609611	-4.57	2.3651509	TMEM155
1560371_at	0.759	0.0132649	2.9608159	-4.57	1.9685852	LINC00997
233735_at	0.759	0.0132694	2.9606233	-4.57	3.3602452	
243845_at	0.759	0.0132708	2.9605646	-4.57	2.5266482	HSPD1
230910_s_at	0.759	0.0132847	2.9599723	-4.57	3.3682322	LOC100288181
241326_at	0.759	0.0133548	2.9569943	-4.57	2.4686914	AK7
214571_at	0.759	0.0133588	2.9568249	-4.57	1.97895	FGF3
1558532_at	0.759	0.0134058	2.9548429	-4.57	2.6082108	TPM1
224244_s_at	0.759	0.0134413	2.9533456	-4.57	2.3434715	APOA5
234152_at	0.759	0.0134529	2.9528595	-4.57	2.8226337	
232252_at	0.759	0.0134709	2.9521049	-4.57	2.9211013	DUSP27
1563484_at	0.759	0.0134761	2.9518859	-4.57	2.1978693	LOC101927766
207946_at	0.759	0.0135051	2.9506686	-4.57	2.4436029	MAML3
217338_at	0.759	0.0135079	2.9505529	-4.57	2.3022973	KRT19P2

233795_at	0.759	0.0135283	2.9497013	-4.57	2.5550323	ODF3
1563805_a_at	0.759	0.0135347	2.9494325	-4.57	2.8888508	FAM83C
232566_at	0.759	0.0135717	2.9478891	-4.57	2.1352201	NOL6
1563539_at	0.759	0.0135993	2.946739	-4.57	2.5276247	LOC101927610
1563143_at	0.759	0.01361	2.9462956	-4.57	2.8487316	LOC100507065
233020_at	0.759	0.0136201	2.9458781	-4.57	2.108644	SEC22B
1557569_at	0.759	0.0136205	2.9458615	-4.57	2.1816663	MPDU1
210520_at	0.759	0.0136283	2.9455377	-4.57	2.4563631	FETUB
228178_s_at	0.759	0.0136457	2.9448157	-4.57	2.7626316	
1558481_s_at	0.759	0.0136475	2.9447423	-4.57	3.1137535	TMCC1-AS1
219308_s_at	0.759	0.0136654	2.9439997	-4.57	2.2077556	AK5
204637_at	0.759	0.0136786	2.9434527	-4.57	3.1190706	CGA
207710_at	0.759	0.0136838	2.9432384	-4.57	2.6648768	LCE2B
1554187_at	0.759	0.0136939	2.9428233	-4.57	2.0414796	LOC554206
217343_at	0.759	0.0137022	2.9424821	-4.57	3.9080769	
1564351_at	0.759	0.0137095	2.942181	-4.57	2.8058892	
91580_at	0.759	0.0137098	2.9421653	-4.57	2.5686802	LRTM1
240736_at	0.759	0.0137546	2.9403219	-4.57	3.1632315	
229225_at	0.759	0.0137984	2.938526	-4.57	2.2066509	NRP2
1566834_at	0.759	0.0138142	2.9378787	-4.57	3.4753942	

1562270_at	0.759	0.0138144	2.937871	-4.57	2.9874828	ARHGEF7
236397_at	0.759	0.0138147	2.9378588	-4.57	2.4018007	
233886_at	0.759	0.0138406	2.9368025	-4.57	3.082341	
1570331_at	0.759	0.0138435	2.9366834	-4.57	3.1061892	
222124_at	0.759	0.0138436	2.9366771	-4.57	3.7007421	HIF3A
238023_at	0.759	0.0138854	2.934973	-4.57	2.0888274	
207151_at	0.759	0.0139071	2.9340929	-4.57	2.6235047	ADCYAP1R1
241235_at	0.759	0.0139159	2.9337331	-4.57	2.9184977	
240043_at	0.759	0.0139222	2.9334777	-4.57	2.2510517	PRR32
1556516_at	0.759	0.0139227	2.9334591	-4.57	2.6779267	LOC100506331
1563065_at	0.759	0.013934	2.932998	-4.57	3.3527752	
1561880_a_at	0.759	0.0139375	2.932858	-4.57	3.4677295	SIGLEC16
1554041_at	0.759	0.013967	2.9316632	-4.57	3.0193787	TMEM239
1554964_x_at	0.759	0.0139953	2.9305175	-4.57	1.9833015	
1569689_s_at	0.759	0.0140049	2.9301318	-4.57	2.9117303	GABRB3
237602_at	0.759	0.014005	2.9301296	-4.57	4.0470649	
208368_s_at	0.759	0.0140364	2.9288627	-4.57	2.2023532	BRCA2
217139_at	0.759	0.0140908	2.9266768	-4.57	2.7529648	VDAC1
237271_at	0.759	0.0141023	2.9262142	-4.57	3.4131296	C7orf77
222194_at	0.759	0.0141275	2.9252075	-4.57	3.0958279	FAM66D
1555717_at	0.759	0.0141728	2.9233973	-4.57	3.5062094	
1570301_at	0.759	0.0141864	2.9228571	-4.57	2.8065195	

219488_at	0.759	0.0142333	2.9209912	-4.57	2.7007229	A4GALT
230883_at	0.759	0.0142516	2.9202628	-4.57	2.5400355	NXPH2
231623_at	0.759	0.0142578	2.9200195	-4.57	2.8520165	TMEM174
206887_at	0.759	0.0142737	2.9193902	-4.57	3.0255716	ACKR2
1564718_at	0.759	0.0142741	2.9193722	-4.57	3.3679009	
207609_s_at	0.759	0.0142897	2.9187553	-4.57	1.9826743	CYP1A2
233683_at	0.759	0.0142908	2.9187142	-4.57	2.8410427	
241801_at	0.759	0.0143056	2.9181276	-4.57	2.4487259	PGAP1
241966_at	0.759	0.014358	2.9160608	-4.57	2.1438368	MYO5A
241868_at	0.759	0.0144066	2.9141521	-4.57	2.7421451	
1554774_at	0.759	0.0144365	2.9129824	-4.57	2.2884083	MINA
1559732_at	0.759	0.0144483	2.91252	-4.57	2.7937893	NUB1
227588_s_at	0.759	0.0144697	2.9116814	-4.57	2.4775082	GET4
1559843_s_at	0.759	0.0145152	2.9099089	-4.57	3.0271442	
1566540_at	0.759	0.0145355	2.9091213	-4.57	2.6806066	
1554610_at	0.759	0.014554	2.9084019	-4.57	2.6349643	ANKMY1
214804_at	0.759	0.0145799	2.9073954	-4.57	3.0551611	CENPI
241016_at	0.759	0.0145803	2.9073825	-4.57	2.8951398	
1564319_at	0.759	0.0146191	2.9058812	-4.57	2.8156159	FAM71E2
243269_s_at	0.759	0.0146357	2.9052377	-4.57	2.9571041	FAM205BP///FAM205A
234579_at	0.759	0.0146585	2.9043577	-4.57	3.7441348	
1556417_a_at	0.759	0.0146897	2.9031583	-4.57	2.9355222	

240803_at	0.759	0.0146971	2.9028742	-4.57	2.6109951	
232359_at	0.759	0.0147026	2.9026627	-4.57	2.5852319	RDH11
221982_x_at	0.759	0.0147171	2.9021068	-4.57	2.4991876	LAGE3
220269_at	0.759	0.0147517	2.9007774	-4.57	3.2000487	ZBBX
220317_at	0.759	0.0147694	2.9000996	-4.57	2.9618847	LRAT
243832_at	0.759	0.0148058	2.8987118	-4.57	2.4872087	
1553346_a_at	0.759	0.0148516	2.8969671	-4.57	2.5918639	TNRC6A
240517_at	0.759	0.0148596	2.8966604	-4.57	2.8985033	CBS
230583_s_at	0.759	0.01486	2.8966449	-4.57	3.3033801	LOC100287728///ZNF75D
211403_x_at	0.759	0.0148756	2.8960527	-4.57	2.3881467	VCX2
1560735_s_at	0.759	0.0148901	2.8955021	-4.57	1.9331225	LOC102723532///LOC101927079///LOC727924///OR4N4
1554405_a_at	0.759	0.0149059	2.8949034	-4.57	2.5825354	LINC00161
229502_at	0.759	0.0149103	2.8947383	-4.57	2.9319281	CHDH
240918_at	0.759	0.0149357	2.8937752	-4.57	2.8551772	LOC101928631///ZNF77
240957_at	0.759	0.0149643	2.8926938	-4.57	3.3421289	
229051_at	0.759	0.0149685	2.8925362	-4.57	2.1128986	

206447_at	0.759	0.0149713	2.8924295	-4.57	3.5334837	CELA2A///CELA2B
1553362_at	0.759	0.0149796	2.8921181	-4.57	3.0985805	DNAH6
1562006_at	0.759	0.0150221	2.8905174	-4.57	2.9593244	
1561523_at	0.759	0.0150523	2.8893816	-4.57	3.2524255	CCDC65
1560480_at	0.759	0.0150862	2.8881135	-4.57	2.7070742	
1570146_at	0.759	0.0151083	2.887284	-4.57	2.9188051	
215897_at	0.759	0.0151102	2.8872143	-4.57	2.5464627	MED25
1560979_a_at	0.759	0.0151301	2.8864684	-4.57	3.0346402	
1553701_a_at	0.759	0.015174	2.884834	-4.57	2.3743326	DUSP18
204539_s_at	0.759	0.0151911	2.8841972	-4.57	3.4791111	CELSR1
237514_at	0.759	0.0152265	2.8828817	-4.57	3.3751143	LINC01210
1567378_x_at	0.759	0.015249	2.8820475	-4.57	3.0742608	DNAH1
210077_s_at	0.759	0.0152565	2.8817709	-4.57	3.1189711	SRSF5
240715_at	0.759	0.0152829	2.880795	-4.57	2.7893217	TBX5
218711_s_at	0.759	0.0153403	2.8786749	-4.57	1.8938907	SDPR
214923_at	0.759	0.0153607	2.8779267	-4.57	2.5250579	ATP6V1D
221109_at	0.759	0.0153633	2.8778291	-4.57	2.7220922	LOC100506571
241564_at	0.759	0.0153933	2.87673	-4.57	2.5270603	GLOD5
207136_at	0.759	0.0154346	2.8752136	-4.57	2.9269696	ARR3
37953_s_at	0.759	0.0154461	2.8747948	-4.57	2.5736515	ASIC1

235794_at	0.759	0.01546	2.8742852	-4.57	2.6820283	MOBP
233517_s_at	0.759	0.0154675	2.8740106	-4.57	2.5826693	HIF3A
244223_at	0.759	0.0154789	2.8735958	-4.57	2.3464272	
206681_x_at	0.759	0.0155097	2.8724731	-4.57	2.5413127	GP2
220359_s_at	0.759	0.0155246	2.8719311	-4.57	3.6203576	ARPP21
208334_at	0.759	0.0155349	2.8715549	-4.57	2.4234007	NDST4
1553552_at	0.759	0.0155388	2.8714155	-4.57	3.3892989	TAAR8
1566527_at	0.759	0.0155458	2.8711617	-4.57	2.8167856	
216721_at	0.759	0.015548	2.8710819	-4.57	2.5296367	SLC25A30
217476_at	0.759	0.0155688	2.8703242	-4.57	3.1526691	NR1D1///THRA
240671_at	0.759	0.0155848	2.8697465	-4.57	2.8609857	
215282_at	0.759	0.0156155	2.8686317	-4.57	2.5978116	ANAPC13
1556319_at	0.759	0.0156189	2.8685097	-4.57	2.2076117	TRIM44
220567_at	0.759	0.0156223	2.8683883	-4.57	3.1767243	IKZF2
221302_at	0.759	0.0156239	2.8683302	-4.57	2.2286921	KLF15
1561140_at	0.759	0.0156745	2.8665015	-4.57	3.1438429	
1563224_at	0.759	0.0157028	2.8654845	-4.57	2.9138074	
203180_at	0.759	0.0157185	2.864921	-4.57	2.3109027	ALDH1A3
242395_at	0.759	0.0157235	2.8647394	-4.57	3.1756997	
223587_s_at	0.759	0.0157455	2.8639515	-4.57	2.1199332	AMN
1556144_at	0.759	0.0157638	2.863293	-4.57	2.4487962	DHX30
1554728_at	0.759	0.0157648	2.8632605	-4.57	2.5526803	SLC9A1
242782_x_at	0.759	0.0157741	2.8629272	-4.57	3.1263576	TMEM198

237818_at	0.759	0.0157821	2.8626405	-4.57	3.3590537	LARP6
1553080_at	0.759	0.015783	2.8626067	-4.57	3.2159939	CSN1S2AP
210743_s_at	0.759	0.015796	2.8621434	-4.57	3.2835973	CDC14A
209888_s_at	0.759	0.0158013	2.8619517	-4.57	3.509737	MYL1
243187_at	0.759	0.015838	2.8606428	-4.57	3.2030381	
233366_at	0.759	0.0158387	2.8606165	-4.57	3.16726	FBXO4
1555510_at	0.759	0.0158466	2.8603357	-4.57	3.1484	ZNF215
228401_at	0.759	0.0158783	2.859208	-4.57	2.4377905	ATAD2
224055_x_at	0.759	0.0159366	2.8571369	-4.57	3.3173227	KCNK7
239332_at	0.759	0.0160643	2.8526287	-4.57	4.4792498	LOC105371809
211380_s_at	0.759	0.016079	2.8521124	-4.57	2.8343571	PRKG1
1569237_at	0.759	0.0160918	2.8516624	-4.57	3.1544905	
221372_s_at	0.759	0.0161689	2.8489624	-4.57	3.1736978	P2RX2
233368_s_at	0.759	0.0161728	2.8488288	-4.57	2.9090773	DNAJC27
1552631_a_at	0.759	0.0162014	2.8478306	-4.57	2.3207243	MAP3K6
1564250_at	0.759	0.0162166	2.847301	-4.57	3.135131	LINC00693
239622_at	0.759	0.016267	2.8455481	-4.57	3.0731912	TRAP1
1557128_at	0.759	0.0162972	2.8444987	-4.57	2.6312838	FAM111B
228576_s_at	0.759	0.0162996	2.8444153	-4.57	3.1789239	MXRA8
215150_at	0.759	0.0163072	2.8441544	-4.57	2.2892661	YOD1
1559658_at	0.759	0.01632	2.8437108	-4.57	2.1298166	KATNBL1

210333_at	0.759	0.0163663	2.8421097	-4.57	2.5203977	NR5A1
208180_s_at	0.759	0.0163747	2.8418194	-4.57	2.8039878	HIST1H4H
1566709_at	0.759	0.0163943	2.841145	-4.57	2.4590725	
231651_at	0.759	0.0163977	2.8410294	-4.57	2.7352972	EFCAB13
216712_at	0.759	0.0164012	2.8409064	-4.57	3.6179202	SLC25A30
61734_at	0.759	0.0164137	2.840478	-4.57	2.4096278	RCN3
233680_at	0.759	0.0164282	2.83998	-4.57	2.905113	LOC101929454
242219_at	0.759	0.0164331	2.839809	-4.57	2.4616079	LINC01588
224034_at	0.759	0.0164436	2.8394492	-4.57	2.4583745	
235465_at	0.759	0.016453	2.8391273	-4.57	2.7417329	AMER2
1564567_at	0.759	0.0164567	2.8389981	-4.57	3.2337644	
210969_at	0.759	0.0164568	2.8389975	-4.57	2.6959881	PKN2
1560133_at	0.759	0.016458	2.8389564	-4.57	2.7484879	
231336_at	0.759	0.0164865	2.8379777	-4.57	3.2798184	CPNE4
239430_at	0.759	0.0164898	2.8378639	-4.57	3.758933	IGFL1
230869_at	0.759	0.0165736	2.8350013	-4.57	2.3705877	FAM155A
1556459_at	0.759	0.0166014	2.8340543	-4.57	2.8372901	ARHGAP22-IT1
231064_s_at	0.759	0.016602	2.8340354	-4.57	2.8573642	NUP50
209465_x_at	0.759	0.0166335	2.8329652	-4.57	2.3607141	PTN
216169_at	0.759	0.0166405	2.832727	-4.57	2.5594835	
207272_at	0.759	0.0166416	2.8326899	-4.57	3.4589446	ZNF80
1556742_at	0.759	0.0166569	2.8321717	-4.57	2.8831819	GUSBP1///GUSBP4

220876_at	0.759	0.0166846	2.831231	-4.57	2.9350474	
220586_at	0.759	0.0167123	2.8302954	-4.57	2.9968757	CHD9
232399_at	0.759	0.0167305	2.8296797	-4.57	3.1138167	DCLK3
238268_at	0.759	0.016797	2.8274405	-4.57	3.3010711	
1556453_at	0.759	0.0168175	2.8267521	-4.57	3.1480594	LOC100506274
1554591_at	0.759	0.0168231	2.826565	-4.57	2.0793426	PCAT4
242860_at	0.759	0.0168464	2.8257813	-4.57	2.5064149	
220915_s_at	0.759	0.0168652	2.8251534	-4.57	2.4155292	
228626_at	0.759	0.0169153	2.8234782	-4.57	2.0433067	URM1
201564_s_at	0.759	0.0169344	2.8228407	-4.57	2.8284091	FSCN1
219650_at	0.759	0.016937	2.8227538	-4.57	2.5610545	ERCC6L
1555282_a_at	0.759	0.01697	2.8216524	-4.57	2.1272416	PPARGC1B
202707_at	0.759	0.0169767	2.8214294	-4.57	2.1811154	UMPS
228368_at	0.759	0.0169837	2.8211982	-4.57	2.3807135	ARHGAP20
242906_at	0.759	0.0169925	2.8209065	-4.57	3.118949	SESN3
235230_at	0.759	0.0169963	2.82078	-4.57	2.1310896	PLCXD2
1563062_at	0.759	0.0170153	2.8201489	-4.57	2.6939054	LINC00460
244066_at	0.759	0.0170221	2.8199222	-4.57	2.0805559	
237170_at	0.759	0.017031	2.8196256	-4.57	3.2426493	LOC100507384
210228_at	0.759	0.0170442	2.8191893	-4.57	1.9295231	CSF2

225700_at	0.759	0.0170516	2.8189434	-4.57	2.648324	GLCCI1
236323_at	0.759	0.0170654	2.8184868	-4.57	2.21096	PVRL3-AS1
220103_s_at	0.759	0.0170684	2.8183882	-4.57	3.1759425	MRPS18C
236827_at	0.759	0.0170736	2.8182156	-4.57	2.7475073	
1556760_a_at	0.759	0.0170994	2.8173637	-4.57	2.9814891	
1559965_at	0.759	0.0171099	2.8170173	-4.57	3.873535	ZFHX4-AS1
1562383_at	0.759	0.0171106	2.8169943	-4.57	2.4681028	MIR4313
1564772_at	0.759	0.0171267	2.8164632	-4.57	2.1607052	
240020_at	0.759	0.0171305	2.8163389	-4.57	1.8336279	CYB5R4
231748_at	0.759	0.0171421	2.8159549	-4.57	2.4507627	ULBP3
229827_at	0.759	0.0171486	2.8157417	-4.57	3.0188266	BUB3
241005_at	0.759	0.0171563	2.8154862	-4.57	2.9954461	
207145_at	0.759	0.0171658	2.8151758	-4.57	3.1709471	MSTN
235090_at	0.759	0.0171698	2.8150437	-4.57	2.6133032	ZNF710
236455_at	0.759	0.0172148	2.8135665	-4.57	2.4922115	
219748_at	0.759	0.0172155	2.8135412	-4.57	3.0413521	TREML2
223897_at	0.759	0.0172166	2.8135075	-4.57	2.9003259	ZNF765
236068_s_at	0.759	0.0172633	2.8119746	-4.57	2.1852381	C3orf14
218839_at	0.759	0.0172635	2.8119687	-4.57	1.8105216	HEY1
1562457_at	0.759	0.0172815	2.8113799	-4.57	2.9331955	NCKAP5L
237744_at	0.759	0.0172956	2.8109209	-4.57	2.7465006	
1556498_at	0.759	0.017302	2.8107107	-4.57	2.7581641	FAM69A
1569006_at	0.759	0.0173345	2.8096516	-4.57	2.2701997	LOC284379

220588_at	0.759	0.017347	2.8092438	-4.57	2.8984915	BCAS4
234889_at	0.759	0.0173619	2.8087601	-4.57	2.5797014	
240255_at	0.759	0.0173656	2.8086394	-4.57	2.063779	LOC100507395
1561776_at	0.759	0.017391	2.8078127	-4.57	2.9808791	
228126_x_at	0.759	0.0174128	2.8071069	-4.57	2.8239175	CTXN1
220585_at	0.759	0.0174369	2.8063239	-4.57	2.2210977	HKDC1
234818_at	0.759	0.0174521	2.8058328	-4.57	2.6179875	TMEM108
1561386_at	0.759	0.017453	2.8058055	-4.57	2.9153875	LOC102723701
1556941_a_at	0.759	0.0174542	2.8057656	-4.57	3.0936301	LOC283484
202210_x_at	0.759	0.0174741	2.8051217	-4.57	2.2757052	GSK3A
1560944_at	0.759	0.017485	2.8047708	-4.57	2.4772553	SLC25A3P1
239449_at	0.759	0.017494	2.8044803	-4.57	3.2392836	
235331_x_at	0.759	0.0174958	2.8044222	-4.57	2.2949275	PCGF5
1554920_at	0.759	0.0174969	2.804387	-4.57	3.4276942	SCEL
242570_at	0.759	0.0175025	2.8042056	-4.57	2.6534098	
238125_at	0.759	0.0175072	2.804054	-4.57	2.9620406	ADAMTS16
1561939_at	0.759	0.0175223	2.8035663	-4.57	2.8481144	DYNC2H1
207458_at	0.759	0.0175327	2.8032318	-4.57	1.9432924	RHPN1-AS1
242063_s_at	0.759	0.0175755	2.8018539	-4.57	3.0212048	
244853_at	0.759	0.0175952	2.8012215	-4.57	2.9784494	
239369_at	0.759	0.0175955	2.8012128	-4.57	2.3317312	LCN8
221394_at	0.759	0.0176349	2.7999478	-4.57	2.6191877	TAAR2

1561591_at	0.759	0.0176669	2.7989253	-4.57	3.0691394	
244541_x_at	0.759	0.0176695	2.7988417	-4.57	3.3025797	
1552982_a_at	0.759	0.0176882	2.798244	-4.57	3.0867822	FGF4
237851_at	0.759	0.0176886	2.7982303	-4.57	2.3464174	
1560411_at	0.759	0.0177127	2.7974623	-4.57	2.4395218	
1559715_at	0.759	0.0177346	2.7967645	-4.57	2.5012039	LOC100507391
1563523_at	0.759	0.0177832	2.7952196	-4.57	3.1656259	
238872_at	0.759	0.0178171	2.7941436	-4.57	3.148833	LOC100128239
1562820_at	0.759	0.0178815	2.792105	-4.57	3.2633627	
218355_at	0.759	0.0179099	2.79121	-4.57	3.69583	KIF4A
1562122_at	0.759	0.0179534	2.7898392	-4.57	2.5728843	
238276_at	0.759	0.0180004	2.788362	-4.57	2.3765378	
206029_at	0.759	0.0180119	2.7880022	-4.57	3.3691562	ANKRD1
234247_at	0.759	0.0180219	2.7876903	-4.57	3.9466459	
237810_at	0.759	0.0180272	2.7875236	-4.57	2.0144169	CLDN6
234784_at	0.759	0.018057	2.7865904	-4.57	2.0734324	
1568949_at	0.759	0.0180682	2.7862413	-4.57	2.3227107	PITPNC1
234368_at	0.759	0.0180952	2.7853983	-4.57	1.9273119	
210311_at	0.759	0.0181017	2.7851954	-4.57	2.7157662	FGF5
1566784_at	0.759	0.0181421	2.7839352	-4.57	2.0294308	NSF
244452_at	0.759	0.0181587	2.783418	-4.57	2.8038751	ERLEC1

1559551_at	0.759	0.0182434	2.7807893	-4.57	2.3012044	
237677_at	0.759	0.0182441	2.7807685	-4.57	3.3477506	
219780_at	0.759	0.0182585	2.7803221	-4.57	2.9338629	ZNF771
232880_at	0.759	0.0182609	2.7802484	-4.57	2.64259	
234196_at	0.759	0.0182618	2.7802195	-4.57	2.4165193	
206356_s_at	0.759	0.0182957	2.7791736	-4.57	3.4153145	GNAL
230963_at	0.759	0.0183922	2.7762025	-4.57	2.0893229	EMX2OS
1559510_at	0.759	0.0184015	2.7759165	-4.57	2.3521447	LINC00630
214560_at	0.759	0.0184259	2.7751683	-4.57	2.8540222	FPR3
231663_s_at	0.759	0.0184284	2.7750923	-4.57	3.0689894	ARG1
241229_at	0.759	0.0184746	2.7736783	-4.57	2.0621197	
1553032_at	0.759	0.0185091	2.7726225	-4.57	1.8716258	IL31RA
209812_x_at	0.759	0.01851	2.7725964	-4.57	1.9265174	CASP2
211917_s_at	0.759	0.0185481	2.7714337	-4.57	2.3542973	PRLR
221320_at	0.759	0.0185558	2.7711995	-4.57	2.4759049	BCL2L10
237313_at	0.759	0.0185691	2.7707974	-4.57	2.3827839	C1QTNF1-AS1
241530_at	0.759	0.0185734	2.7706657	-4.57	2.3401641	
217333_at	0.759	0.0186052	2.7696997	-4.57	1.9816969	
241876_at	0.759	0.0186526	2.768262	-4.57	2.0406714	MDM4
231772_x_at	0.759	0.0186729	2.7676475	-4.57	2.833752	CENPH
224043_s_at	0.759	0.0186751	2.7675823	-4.57	2.7044393	UPB1
1552424_at	0.759	0.018698	2.7668894	-4.57	3.3470191	KLHL10
1570190_at	0.759	0.0188169	2.7633074	-4.57	2.1214792	LSAMP
1554636_at	0.759	0.0188343	2.7627861	-4.57	2.0176209	

215132_at	0.759	0.0188511	2.7622829	-4.57	2.0075005	
219864_s_at	0.759	0.0188626	2.7619398	-4.57	2.163073	RCAN3
239467_at	0.759	0.0188657	2.7618453	-4.57	2.3306291	
226876_at	0.759	0.0188871	2.7612041	-4.57	2.2109333	FAM101B
1561924_at	0.759	0.0189119	2.7604652	-4.57	3.5828827	
234236_at	0.759	0.0189524	2.7592556	-4.57	2.7814177	AMBRA1
207779_at	0.759	0.0189608	2.7590055	-4.57	2.634218	
1555794_at	0.759	0.0189786	2.7584762	-4.57	3.8560894	ARL17B///ARL17A
1557309_at	0.759	0.0190315	2.7569049	-4.57	2.3374074	DENND1B
220118_at	0.759	0.0190333	2.7568515	-4.57	2.6426841	ZBTB32
1567333_at	0.759	0.0190333	2.7568487	-4.57	2.3007735	
243152_at	0.759	0.0190447	2.756511	-4.57	2.3705244	COPS8
219537_x_at	0.759	0.0190552	2.7562001	-4.57	2.8121006	DLL3
224411_at	0.759	0.0190703	2.7557543	-4.57	3.1218112	PLA2G12B
211797_s_at	0.759	0.019125	2.7541356	-4.57	2.802915	NFYC
1552521_a_at	0.759	0.0191252	2.7541283	-4.57	2.9898182	TMEM74
233697_at	0.759	0.0191389	2.7537247	-4.57	3.005709	
222966_at	0.759	0.0191591	2.7531278	-4.57	2.421148	TLK1
233048_at	0.759	0.0191795	2.752529	-4.57	3.1110809	
243692_at	0.759	0.0191969	2.7520157	-4.57	2.569228	GATA4
1565896_at	0.759	0.0192437	2.7506394	-4.57	3.5018209	
1566735_at	0.759	0.0192493	2.7504756	-4.57	2.9386248	LINC01346
220787_at	0.759	0.0192551	2.7503074	-4.57	3.0914136	

208054_at	0.759	0.0192727	2.74979	-4.57	3.9917299	HERC4
1553505_at	0.759	0.0192964	2.7490952	-4.57	2.3874579	A2ML1
1564760_at	0.759	0.0193195	2.7484196	-4.57	2.583069	
1559469_s_at	0.759	0.0193389	2.7478539	-4.57	1.9204156	SIPA1L2
1553458_at	0.759	0.0193628	2.7471554	-4.57	2.2391003	VSTM4
236502_at	0.759	0.0194226	2.7454146	-4.57	3.8695165	
223963_s_at	0.759	0.0194343	2.7450724	-4.57	2.5189514	IGF2BP2
238364_x_at	0.759	0.0194375	2.7449798	-4.57	3.2229226	GLI4
237213_at	0.759	0.019484	2.7436314	-4.57	2.275618	
234520_at	0.759	0.0194889	2.7434871	-4.57	2.6123721	FLI1
1553592_x_at	0.759	0.0195036	2.7430622	-4.57	2.1185885	BCRP3
204473_s_at	0.759	0.0195055	2.743008	-4.57	2.1255969	ZNF592
1570536_at	0.759	0.019516	2.7427024	-4.57	2.2899015	
1553602_at	0.759	0.0195219	2.7425336	-4.57	2.0551159	MUCL1
229680_at	0.759	0.0195387	2.7420457	-4.57	2.1472881	TACO1
228194_s_at	0.759	0.0195399	2.7420115	-4.57	3.3054159	SORCS1
216703_at	0.759	0.0195425	2.7419378	-4.57	2.4280308	
1553765_a_at	0.759	0.0195959	2.7403952	-4.57	3.3101101	KLHL32
1568190_at	0.759	0.0195971	2.7403596	-4.57	3.0393802	
1557431_at	0.759	0.0196343	2.7392888	-4.57	2.914103	
236973_at	0.759	0.0196462	2.7389475	-4.57	1.9744818	LOC100131662

237887_at	0.759	0.0196624	2.7384828	-4.57	2.1973737	
240416_at	0.759	0.0196758	2.7380959	-4.57	2.8874212	
1562761_at	0.759	0.0196827	2.7378996	-4.57	2.2010663	NMRK1
1560407_at	0.759	0.0197174	2.7369035	-4.57	3.0582996	MARK1
211045_s_at	0.759	0.0197294	2.7365592	-4.57	2.7282215	KCNH6
207635_s_at	0.759	0.0197381	2.73631	-4.57	2.187183	KCNH1
1554884_at	0.759	0.0197622	2.7356205	-4.57	3.128082	PRIM2B///PRIM2
207395_at	0.759	0.0198118	2.734205	-4.57	2.5944895	BTN1A1
237612_at	0.759	0.0198349	2.7335469	-4.57	2.6348154	SLC14A2-AS1
206912_at	0.759	0.0198556	2.7329592	-4.57	3.0758943	FOXE1
234098_at	0.759	0.0198593	2.7328529	-4.57	2.5235495	SOBP
237682_at	0.759	0.0198601	2.7328314	-4.57	2.8142889	
220090_at	0.759	0.01988	2.7322634	-4.57	1.9319061	CRNN
207854_at	0.759	0.0199107	2.7313932	-4.57	2.5171393	GYPE
1555068_at	0.759	0.0199262	2.7309533	-4.57	1.9463507	WNK1
240978_at	0.759	0.0199402	2.7305561	-4.57	3.9350221	
1561230_at	0.759	0.0199444	2.7304373	-4.57	2.5728216	
206615_s_at	0.759	0.0199562	2.7301044	-4.57	2.8539916	ADAM22
207451_at	0.759	0.0199786	2.72947	-4.57	2.2841122	NKX2-8
1568845_at	0.759	0.0200688	2.7269236	-4.57	2.4312335	
1557864_x_at	0.759	0.0200699	2.7268932	-4.57	2.8211876	PLA2G4E-AS1

244517_x_at	0.759	0.0200801	2.726606	-4.57	1.9256677	
229188_s_at	0.759	0.0201119	2.7257124	-4.57	2.1270157	ZNRF2
1563536_at	0.759	0.0201464	2.7247441	-4.57	2.0361526	
1553366_s_at	0.759	0.020154	2.7245316	-4.57	2.6143326	ANKRD23///ANKRD39
208346_at	0.759	0.020169	2.7241103	-4.57	2.28452	PPBPP2
243128_at	0.759	0.0201838	2.7236957	-4.57	2.0659809	ZNF175
1557595_at	0.759	0.0202251	2.7225424	-4.57	2.0420324	GINS2
236837_x_at	0.759	0.0202458	2.7219642	-4.57	2.0429824	MIPEPP3
209942_x_at	0.759	0.0202586	2.7216062	-4.57	2.8689517	MAGEA6///MAGEA3
215715_at	0.759	0.020292	2.7206766	-4.57	2.4118625	SLC6A2
1553533_at	0.759	0.0203185	2.7199377	-4.57	2.5012857	JPH1
241120_s_at	0.759	0.0203378	2.7194018	-4.57	2.6714897	CDC20B
1564933_at	0.759	0.0203429	2.7192614	-4.57	2.8069438	
1560033_at	0.759	0.0203509	2.7190384	-4.57	2.4162984	MCCC2
242552_x_at	0.759	0.0203654	2.7186365	-4.57	2.2856505	ZBED5
233789_at	0.759	0.0203706	2.7184929	-4.57	2.4296422	
1568836_at	0.759	0.0204102	2.7173939	-4.57	3.6863222	CLK4
232364_at	0.759	0.0204663	2.7158432	-4.57	2.7075464	LOC100506235
207155_at	0.759	0.0204939	2.7150807	-4.57	3.0464282	TBX5
1564763_at	0.759	0.0205132	2.7145496	-4.57	2.5303355	

214541_s_at	0.759	0.0205624	2.7131956	-4.57	1.8944371	QKI
217636_at	0.759	0.0205624	2.7131953	-4.57	2.7021822	POLG
237526_at	0.759	0.0205698	2.7129947	-4.57	2.8844521	
1554942_a_at	0.759	0.0205705	2.7129733	-4.57	2.2571793	KLHL14
1560851_at	0.759	0.0206022	2.7121037	-4.57	2.9482897	LINC00619
221048_x_at	0.759	0.0206116	2.7118451	-4.57	2.9639982	C17orf80
1566505_at	0.759	0.020612	2.7118356	-4.57	3.1465783	ERVK13-1
238182_at	0.759	0.0206382	2.711117	-4.57	1.816046	
1568672_at	0.759	0.0206424	2.7110024	-4.57	2.2416434	EAF2
235400_at	0.759	0.0206585	2.7105626	-4.57	3.6743797	FCRLA
1556081_at	0.759	0.0207255	2.7087336	-4.57	2.9737752	
233989_at	0.759	0.0207398	2.7083428	-4.57	2.411662	
231895_at	0.759	0.0207445	2.708215	-4.57	1.8645103	SASS6
1567704_at	0.759	0.0207585	2.7078326	-4.57	2.1488944	TFE3
207061_at	0.759	0.020796	2.7068124	-4.57	2.0501259	ERN1
1561673_at	0.759	0.0208222	2.7061028	-4.57	3.1093604	LOC101929151
1553523_at	0.759	0.0208278	2.7059499	-4.57	2.1905753	NLRP14
1556357_s_at	0.759	0.0208405	2.7056062	-4.57	2.5296926	ERICH1
238799_at	0.759	0.0208786	2.7045736	-4.57	3.2237543	ATP5S
1560563_at	0.759	0.0208947	2.7041392	-4.57	2.0421968	LOC101929552
236169_at	0.759	0.0209353	2.7030415	-4.57	2.3033589	
1553789_a_at	0.759	0.0209483	2.7026915	-4.57	3.3507358	C21orf58

205768_s_at	0.759	0.0209586	2.7024118	-4.57	2.2218067	SLC27A2
205468_s_at	0.759	0.0209673	2.7021785	-4.57	2.1156098	IRF5
231054_at	0.759	0.0209695	2.7021179	-4.57	2.4201032	SPACA4
1558565_at	0.759	0.0209779	2.7018931	-4.57	1.8978109	AKNA
244337_at	0.759	0.0210202	2.7007545	-4.57	2.2152303	
1562323_at	0.759	0.021024	2.7006531	-4.57	2.519016	
243344_at	0.759	0.0210351	2.7003548	-4.57	2.6227034	
1561092_at	0.759	0.0210377	2.7002831	-4.57	2.725013	
211873_s_at	0.759	0.0210429	2.7001448	-4.57	2.5665037	PCDHGA9
227060_at	0.759	0.0210675	2.699485	-4.57	2.3738383	RELT
235312_s_at	0.759	0.0210803	2.6991413	-4.57	2.3170033	NRAP
207748_at	0.759	0.0210912	2.6988488	-4.57	1.8258533	
235215_at	0.759	0.0210964	2.6987089	-4.57	1.7073548	ERCC4
240378_at	0.759	0.0211058	2.6984568	-4.57	2.2627055	
242042_s_at	0.759	0.0211134	2.6982546	-4.57	2.6470908	HAGLR
244488_at	0.759	0.0211773	2.6965452	-4.57	2.7866263	LSM14B
229039_at	0.759	0.0211927	2.6961368	-4.57	1.9596827	SYN2
211024_s_at	0.759	0.0212019	2.6958915	-4.57	1.9763546	NKX2-1
1562351_at	0.759	0.0212385	2.6949165	-4.57	3.157856	
1563532_at	0.759	0.0212413	2.6948419	-4.57	1.9305778	HMCN2
1553730_x_at	0.759	0.0212523	2.694548	-4.57	2.4037409	LRRC43
1566748_at	0.759	0.0212616	2.6943022	-4.57	2.6365848	
234745_at	0.759	0.021331	2.6924587	-4.57	2.9861573	

234600_at	0.759	0.0213334	2.6923972	-4.57	3.2826729	
228202_at	0.759	0.0213377	2.6922811	-4.57	2.9001185	
1564391_at	0.759	0.0213719	2.691376	-4.57	2.6274371	
240939_x_at	0.759	0.0213912	2.6908667	-4.57	3.553253	
1553371_at	0.759	0.0213917	2.6908536	-4.57	2.1700256	EPHA10
1553517_at	0.759	0.0213929	2.6908207	-4.57	2.1619868	FERD3L
1556766_at	0.759	0.0214104	2.6903611	-4.57	1.8598254	ERICH1
1565558_at	0.759	0.0214259	2.6899495	-4.57	3.2184271	
233509_at	0.759	0.0214531	2.689234	-4.57	2.3792911	HERC4
1569013_s_at	0.759	0.021483	2.6884479	-4.57	1.825919	LOC101929959///BMS1P20
1561692_at	0.759	0.0215013	2.6879654	-4.57	2.9809493	
243965_at	0.759	0.0215156	2.6875883	-4.57	2.9656717	
1554954_at	0.759	0.0215186	2.6875109	-4.57	2.9362121	TSPEAR-AS2
1556929_at	0.759	0.0215337	2.6871148	-4.57	2.4066693	PAPOLG
216421_at	0.759	0.0215608	2.686404	-4.57	2.406415	
216636_at	0.759	0.021576	2.6860043	-4.57	3.3270078	
1555074_a_at	0.759	0.0216089	2.6851446	-4.57	2.9406281	KCNH5
1569235_a_at	0.759	0.021609	2.685141	-4.57	2.5301902	
234601_x_at	0.759	0.0216209	2.6848305	-4.57	3.8053603	
211601_at	0.759	0.0216263	2.6846884	-4.57	2.8304553	CATR1
230420_at	0.759	0.0216623	2.68375	-4.57	2.5092848	CASZ1

1552946_at	0.759	0.0216745	2.6834304	-4.57	1.8385765	ZNF114
1562829_at	0.759	0.021681	2.6832621	-4.57	3.2735686	LOC339568
235292_at	0.759	0.0217114	2.6824693	-4.57	1.8214811	FLJ32255
1569665_at	0.759	0.0217256	2.6820986	-4.57	2.4768516	
1562835_at	0.759	0.0217804	2.6806758	-4.57	2.0058687	
1570206_at	0.759	0.0217902	2.6804215	-4.57	2.2864841	LOC101928797
240186_at	0.759	0.0217907	2.6804086	-4.57	2.9884662	
239994_at	0.759	0.021847	2.6789492	-4.57	2.1232245	B3GALT5
1569124_at	0.759	0.0218543	2.6787615	-4.57	2.6087345	LRRC63
1557386_at	0.759	0.0218621	2.6785585	-4.57	2.7616954	LOC100507600
238971_at	0.759	0.0218718	2.6783083	-4.57	2.8462297	
236005_at	0.759	0.0219159	2.6771695	-4.57	2.8154437	
244349_at	0.759	0.0219586	2.676069	-4.57	2.891201	
226704_at	0.759	0.021961	2.6760082	-4.57	2.2984559	UBE2J2
235722_at	0.759	0.0219682	2.6758222	-4.57	2.04701	SYNJ2BP-COX16///SYNJ2BP
1561238_at	0.759	0.0219894	2.6752768	-4.57	2.321466	PEX2
1561244_at	0.759	0.0219921	2.6752084	-4.57	2.8913429	
227355_at	0.759	0.0219954	2.6751235	-4.57	2.561115	RBM26
204858_s_at	0.759	0.0219993	2.675023	-4.57	2.8360594	TYMP
235877_at	0.759	0.0220108	2.6747267	-4.57	3.5101758	
1554681_a_at	0.759	0.0220174	2.6745594	-4.57	2.1746455	CCDC187

205782_at	0.759	0.0220232	2.6744101	-4.57	3.3555524	FGF7
1554264_at	0.759	0.0220288	2.6742663	-4.57	2.0607573	CKAP2
1561204_at	0.759	0.0220353	2.6740994	-4.57	2.7688678	
242944_at	0.759	0.0220578	2.673521	-4.57	2.3228207	FAM83A
219491_at	0.759	0.0220592	2.673485	-4.57	2.5286241	LRFN4
1561398_at	0.759	0.0220733	2.6731249	-4.57	2.6097744	LINC01505
1562532_at	0.759	0.0220942	2.6725901	-4.57	1.7399781	
207093_s_at	0.759	0.0221501	2.6711612	-4.57	2.0356582	LOC101927057///OMG
1553086_at	0.759	0.0221534	2.6710775	-4.57	2.7563513	C11orf40
239447_at	0.759	0.022164	2.6708076	-4.57	2.1102946	TRA2B
231924_at	0.759	0.0221652	2.6707769	-4.57	3.295157	LINC00958
244727_at	0.759	0.0221743	2.6705434	-4.57	1.9290745	LOC101927338///KCNQ1OT1
217044_s_at	0.759	0.0221818	2.6703541	-4.57	2.3325832	PLEKHG3
237597_at	0.759	0.0221916	2.6701026	-4.57	3.13213	
1561073_at	0.759	0.0222019	2.6698411	-4.57	2.4276061	
207864_at	0.759	0.0222151	2.6695053	-4.57	3.1050837	SCN7A
1556395_at	0.759	0.0222361	2.6689709	-4.57	3.5480577	
238123_at	0.759	0.022238	2.6689219	-4.57	1.7039337	GABRQ

244009_at	0.759	0.0223396	2.6663457	-4.57	2.7843114	
211109_at	0.759	0.0223444	2.6662249	-4.57	2.1102162	JAK3
1555513_at	0.759	0.0223815	2.6652856	-4.57	2.9482243	PIAS2
211736_at	0.759	0.0223928	2.6649996	-4.57	2.7736845	SP2
220578_at	0.759	0.022433	2.6639855	-4.57	2.4690479	ADAMTSL4
1561450_at	0.759	0.0224627	2.6632389	-4.57	2.6103933	LOC101928618
239117_at	0.759	0.0225204	2.6617888	-4.57	2.4210307	
242044_at	0.759	0.0225632	2.6607158	-4.57	2.5150323	
229378_at	0.759	0.0225647	2.6606779	-4.57	2.7829729	STOX1
237062_at	0.759	0.0225647	2.6606778	-4.57	2.1826834	
237263_at	0.759	0.0225855	2.6601568	-4.57	2.1523146	
237746_at	0.759	0.0227492	2.6560721	-4.57	2.9775365	
242597_at	0.759	0.0227509	2.6560302	-4.57	2.8360936	ZMYM2
224249_at	0.759	0.022774	2.6554564	-4.57	2.6634711	
244650_at	0.759	0.0228096	2.6545728	-4.57	2.9894776	FAM105A
1557215_at	0.759	0.0228562	2.6534199	-4.57	2.2160893	LINC00648
216451_at	0.759	0.0228908	2.6525639	-4.57	3.423862	STK38
1565947_a_at	0.759	0.0228956	2.6524458	-4.57	2.5542737	CHML
242184_s_at	0.759	0.0228979	2.6523896	-4.57	1.8750233	SREK1
206040_s_at	0.759	0.0228995	2.652349	-4.57	2.8298297	MAPK11
235650_at	0.759	0.022908	2.652138	-4.57	2.2118088	CDHR3
207390_s_at	0.759	0.0229193	2.6518594	-4.57	3.0894315	SMTN
232327_at	0.759	0.0229308	2.6515756	-4.57	2.4855066	THSD7B

AFFX-ThrX-M_at	0.759	0.022935	2.651472	-4.57	2.6018318	
244325_at	0.759	0.0229491	2.6511255	-4.57	3.3943658	LINC00690
220752_at	0.759	0.0229745	2.6504989	-4.57	3.4350524	LOC51145
240539_at	0.759	0.0230296	2.6491464	-4.57	2.7111719	
1569741_at	0.759	0.0230448	2.6487715	-4.57	2.1719963	LINC01477
1561861_a_at	0.759	0.0230885	2.6477018	-4.57	2.5735205	
1553328_a_at	0.759	0.0231037	2.6473277	-4.57	2.4328004	SLC18A2
37831_at	0.759	0.0231278	2.646739	-4.57	1.9638469	SIPA1L3
239643_at	0.759	0.0231587	2.6459838	-4.57	2.4347686	LOC100129516
243914_at	0.759	0.0231651	2.645827	-4.57	2.1787364	
233747_at	0.759	0.0231951	2.6450964	-4.57	2.5750593	
213700_s_at	0.759	0.0232181	2.6445354	-4.57	2.0307215	PKM
236763_at	0.759	0.0232226	2.6444248	-4.57	2.8973525	
206751_s_at	0.759	0.0232301	2.6442429	-4.57	2.0846273	PCYT1B
1553579_a_at	0.759	0.0232305	2.6442323	-4.57	3.2313778	SPAG11A///SPAG11B
216896_at	0.759	0.0232453	2.6438733	-4.57	1.8232953	COL4A3
1569297_at	0.759	0.0232497	2.6437659	-4.57	1.8764074	LINC01300
243043_at	0.759	0.0232731	2.6431971	-4.57	2.664969	EFCAB10
242813_at	0.759	0.0232872	2.6428551	-4.57	2.83788	
235873_at	0.759	0.0233065	2.6423872	-4.57	3.3379587	LOC100506235
234193_at	0.759	0.0233198	2.6420627	-4.57	2.5594398	

240958_at	0.759	0.0233211	2.6420311	-4.57	2.3869833	UNC5C
215661_at	0.759	0.0233372	2.6416421	-4.57	2.9006161	MAST2
216168_at	0.759	0.023352	2.6412823	-4.57	2.4381215	
223465_at	0.759	0.0233763	2.6406939	-4.57	2.4338845	COL4A3BP
236852_at	0.759	0.0233889	2.6403903	-4.57	2.5575221	FBXO43
237908_at	0.759	0.0233992	2.6401404	-4.57	2.5861263	
237929_at	0.759	0.0234024	2.6400634	-4.57	2.0560792	C17orf50
205838_at	0.759	0.0234373	2.6392206	-4.57	2.1046359	GYPA
1552965_a_at	0.759	0.0234736	2.6383463	-4.57	2.6295596	B3GNT7
206935_at	0.759	0.0235277	2.6370438	-4.57	2.3872492	PCDH8
233966_at	0.759	0.0235488	2.6365364	-4.57	2.1353856	NTNG1
1562944_at	0.759	0.0235608	2.6362483	-4.57	2.6508222	LOC101928160///ZNF707
220830_at	0.759	0.023566	2.6361244	-4.57	2.4235136	IMPG2
239829_at	0.759	0.0236328	2.6345216	-4.57	2.074011	
207142_at	0.759	0.0236496	2.6341196	-4.57	2.6953396	KCNJ3
239536_at	0.759	0.0236533	2.6340316	-4.57	1.8338742	
1556494_at	0.759	0.0236561	2.6339646	-4.57	2.6161966	LINC01365
201820_at	0.759	0.0236577	2.6339265	-4.57	2.7770329	KRT5
216107_at	0.759	0.0236591	2.6338931	-4.57	1.9236804	LOC100129503
232205_at	0.759	0.0236627	2.6338066	-4.57	1.597391	

1568879_a_at	0.759	0.0237141	2.6325785	-4.57	2.4357656	LAMA3
1569823_at	0.759	0.0237245	2.6323305	-4.57	3.1228282	LOC440346
1556808_at	0.759	0.0237314	2.6321667	-4.57	2.1013669	RAP2A
232255_at	0.759	0.0237358	2.6320621	-4.57	2.1854875	LINC00997
233577_at	0.759	0.0237375	2.632022	-4.57	3.5496222	
235889_at	0.759	0.0237397	2.6319687	-4.57	1.9276587	RSPH3
1565544_at	0.759	0.0237625	2.631427	-4.57	2.7414436	RNF141
210713_at	0.759	0.0237819	2.630965	-4.57	1.9928067	ITSN1
1563474_at	0.759	0.0237843	2.6309074	-4.57	2.6411396	ETFBKMT
1558383_at	0.759	0.0237914	2.6307379	-4.57	2.464905	KPNA4
1558708_at	0.759	0.0238046	2.6304258	-4.57	4.0589216	NRXN1
217509_x_at	0.759	0.0238328	2.6297548	-4.57	2.5809146	GRIK5
237854_at	0.759	0.0238685	2.6289091	-4.57	1.6107308	
1561015_at	0.759	0.0238845	2.6285288	-4.57	2.2457112	
1569838_at	0.759	0.0239396	2.6272247	-4.57	2.1276401	LOC101928597
205021_s_at	0.759	0.023964	2.6266494	-4.57	1.7753811	FOXN3
220515_at	0.759	0.0239819	2.6262259	-4.57	2.8903736	DUSP21
241691_at	0.759	0.024007	2.6256363	-4.57	2.2453993	SCARF2
243277_x_at	0.759	0.0240177	2.6253839	-4.57	2.6591954	MECOM
225655_at	0.759	0.0240239	2.6252381	-4.57	1.9388826	UHRF1
244218_at	0.759	0.0240378	2.6249089	-4.57	1.7221396	

201787_at	0.759	0.0240583	2.6244281	-4.57	2.6037308	FBLN1
221416_at	0.759	0.0240692	2.624171	-4.57	2.8959738	PLA2G2F
241166_at	0.759	0.0240718	2.6241109	-4.57	2.7667714	CEP41
1569058_at	0.759	0.0240739	2.6240612	-4.57	2.5838566	GTF3C3
242181_at	0.759	0.0241124	2.6231578	-4.57	1.7081048	
221204_s_at	0.759	0.0241156	2.6230819	-4.57	2.0439293	CRTAC1
210742_at	0.759	0.0241311	2.6227186	-4.57	2.8081668	CDC14A
244617_at	0.759	0.0241781	2.6216178	-4.57	2.4975144	GPR26
1556885_at	0.759	0.0241997	2.6211112	-4.57	3.0201316	LAYN
243211_at	0.759	0.0242031	2.6210334	-4.57	2.7494981	
231430_at	0.759	0.02421	2.6208712	-4.57	2.2806922	FAM181B
1567380_at	0.759	0.0242128	2.620806	-4.57	3.5976456	
210810_s_at	0.759	0.0242462	2.6200269	-4.57	2.0347441	SLC6A5
222009_at	0.759	0.0242726	2.6194102	-4.57	2.2485356	CEMP1
221623_at	0.759	0.0242796	2.619248	-4.57	2.2361536	BCAN
1570490_at	0.759	0.0242985	2.6188056	-4.57	2.8698815	
221762_s_at	0.759	0.0243494	2.6176235	-4.57	2.1417231	PCIF1
1553138_a_at	0.759	0.0244091	2.6162378	-4.57	2.2612937	ANKLE1
240662_at	0.759	0.0244204	2.6159759	-4.57	3.0674593	
216570_x_at	0.759	0.0244418	2.6154803	-4.57	1.8352239	
227675_at	0.759	0.0244791	2.6146176	-4.57	2.2463752	LRSAM1
238823_at	0.759	0.0245001	2.6141311	-4.57	2.4259511	FMNL3
215476_at	0.759	0.0245279	2.6134886	-4.57	1.7773704	
244466_at	0.759	0.0245384	2.6132477	-4.57	2.7882563	ZNF544

211591_s_at	0.759	0.0245393	2.6132268	-4.57	2.2140607	PDE4A
216801_at	0.759	0.0245801	2.6122862	-4.57	2.284114	CROCC
244437_at	0.759	0.0245944	2.611957	-4.57	2.2920701	LOC101927340
1569909_at	0.759	0.0246312	2.6111102	-4.57	2.0480089	KRT79
231879_at	0.759	0.0246492	2.6106972	-4.57	3.1797016	COL12A1
220645_at	0.759	0.0246587	2.6104801	-4.57	2.7341951	NXPE4
241247_at	0.759	0.0246808	2.6099719	-4.57	2.5858675	LINC01213
243831_at	0.759	0.0246849	2.6098799	-4.57	2.0663406	MAPK6
237986_at	0.759	0.0246937	2.6096778	-4.57	2.6511788	
1563088_a_at	0.759	0.0247204	2.6090657	-4.57	2.4154809	AATBC
201822_at	0.759	0.0247294	2.6088598	-4.57	2.7197566	TIMM17A
1560959_a_at	0.759	0.024763	2.6080913	-4.57	3.3429119	
206505_at	0.759	0.0247947	2.6073679	-4.57	3.7264834	UGT2B4
1566927_at	0.759	0.0248204	2.6067814	-4.57	2.3401226	LINC00527
207012_at	0.759	0.0248388	2.6063622	-4.57	3.2127816	MMP16
225385_s_at	0.759	0.0248438	2.6062485	-4.57	1.6786208	HNRNPLL
228966_at	0.759	0.0248756	2.6055234	-4.57	2.2250801	PANK2
1561125_at	0.759	0.0248921	2.605148	-4.57	2.7728927	MTHFD1L
231581_at	0.759	0.0249003	2.6049623	-4.57	2.8092527	LINC00664

1556199_a_at	0.759	0.0249027	2.6049072	-4.57	2.3799053	RGS9BP
224383_at	0.759	0.0249451	2.6039434	-4.57	2.7214647	RNF17
220747_at	0.759	0.0249769	2.603224	-4.57	2.9476826	LINC00652
232055_at	0.759	0.0249983	2.602738	-4.57	1.6711415	SFXN1
236439_at	0.759	0.0250181	2.6022907	-4.57	2.8101432	LOC106146153
232982_at	0.759	0.0250204	2.6022384	-4.57	1.800062	
216254_at	0.759	0.0250372	2.6018586	-4.57	1.7672712	PARVB
211191_at	0.759	0.025039	2.6018184	-4.57	2.1385497	CD84
239844_x_at	0.759	0.0250556	2.6014417	-4.57	2.5036967	C1orf228
237555_at	0.759	0.0251157	2.6000866	-4.57	2.9179452	CARS2
242574_at	0.759	0.0251533	2.5992393	-4.57	1.9946241	FKBP15
1555517_at	0.759	0.0251749	2.598754	-4.57	2.763751	GABRG3
224005_at	0.759	0.0251941	2.5983223	-4.57	2.5332778	
1552609_s_at	0.759	0.0251979	2.5982373	-4.57	2.6494317	IFNL3///IFNL2
241350_at	0.759	0.025209	2.5979877	-4.57	3.7392352	FBXL22
201926_s_at	0.759	0.0252245	2.5976388	-4.57	1.9141418	CD55
237608_at	0.759	0.0252417	2.5972547	-4.57	2.78906	LOC101929570
211021_s_at	0.759	0.0252842	2.5963014	-4.57	3.0554008	RGS14
237755_s_at	0.759	0.0252961	2.5960356	-4.57	2.8808523	CFAP52
244013_at	0.759	0.0253519	2.5947871	-4.57	2.1832274	

220065_at	0.759	0.0253608	2.5945899	-4.57	2.6146956	TNMD
216616_at	0.759	0.0253814	2.5941297	-4.57	1.957628	
1561424_at	0.759	0.0253885	2.5939706	-4.57	2.7277188	LOC101928907
1554831_x_at	0.759	0.0253959	2.5938065	-4.57	2.1685609	ALS2CR11
234990_at	0.759	0.0254004	2.5937059	-4.57	2.7483824	CBX5
234365_at	0.759	0.0254023	2.5936641	-4.57	2.5051351	
215575_at	0.759	0.0254411	2.5928003	-4.57	2.5138964	PDE4DIP
1559589_a_at	0.759	0.0254412	2.5927967	-4.57	2.3907992	
234739_at	0.759	0.0254549	2.5924925	-4.57	2.979815	
243258_at	0.759	0.0254822	2.5918847	-4.57	2.1668208	CLUH
235304_at	0.759	0.0254903	2.5917057	-4.57	2.4244823	
1558814_s_at	0.759	0.0254927	2.5916529	-4.57	2.2675285	TMED5
1570202_a_at	0.759	0.0255188	2.5910722	-4.57	2.6567772	MKL2
1555488_at	0.759	0.0255253	2.5909277	-4.57	2.2013032	
230580_at	0.759	0.0255254	2.5909257	-4.57	2.1773217	
217295_at	0.759	0.0255376	2.5906558	-4.57	2.3455453	MUC8
205235_s_at	0.759	0.0255399	2.5906051	-4.57	1.84831	KIF20B
230594_at	0.759	0.0256191	2.5888523	-4.57	2.7663907	
217683_at	0.759	0.0256229	2.5887668	-4.57	2.5387047	HBE1
1569212_at	0.759	0.0256286	2.5886421	-4.57	2.1203366	SCART1
1566203_at	0.759	0.0256339	2.5885258	-4.57	3.6463127	
233381_at	0.759	0.0256429	2.5883272	-4.57	2.1926482	RUFY1
1555926_a_at	0.759	0.0256464	2.5882479	-4.57	3.0662657	

1564683_at	0.759	0.0256504	2.5881613	-4.57	2.3199173	TBL3
215644_at	0.759	0.0256517	2.5881328	-4.57	3.1218474	ZNF518A
207680_x_at	0.759	0.0256577	2.5879989	-4.57	2.0344822	PAX3
237950_s_at	0.759	0.0256644	2.5878519	-4.57	2.4011989	
1561065_at	0.759	0.0256672	2.5877908	-4.57	2.764223	
207454_at	0.759	0.0257111	2.5868219	-4.57	2.5423618	GRIK3
224191_x_at	0.759	0.0257646	2.5856447	-4.57	1.8449188	ROPN1
207369_at	0.759	0.0257662	2.5856091	-4.57	2.3017549	BRS3
232995_at	0.759	0.0257675	2.5855808	-4.57	2.7964025	
240860_at	0.759	0.0257679	2.5855717	-4.57	1.965101	
1562509_at	0.759	0.0257846	2.5852069	-4.57	2.097767	TMEM108-AS1
215681_at	0.759	0.0258045	2.5847699	-4.57	2.9730619	KIAA1654
1569722_s_at	0.759	0.025821	2.5844073	-4.57	2.1143021	PROSER2-AS1
1569660_at	0.759	0.0258555	2.5836512	-4.57	1.9127087	PP13439
1553192_at	0.759	0.025901	2.582655	-4.57	3.1796835	ZNF441
1554886_a_at	0.759	0.0259346	2.581922	-4.57	2.7963204	MLXIP
241232_x_at	0.759	0.0259425	2.5817497	-4.57	2.7927006	
207817_at	0.759	0.0259522	2.5815368	-4.57	2.3692112	IFNW1
1560946_at	0.759	0.0259657	2.581243	-4.57	2.4520364	
202101_s_at	0.759	0.025972	2.5811042	-4.57	2.2388472	RALB
220836_at	0.759	0.0259733	2.5810765	-4.57	3.7086782	ZNF407
230155_x_at	0.759	0.0259851	2.5808187	-4.57	2.1975197	MSL1

228233_at	0.759	0.026004	2.5804082	-4.57	2.6729842	FREM1
234412_at	0.759	0.0260121	2.5802304	-4.57	1.7538927	
207872_s_at	0.759	0.0260268	2.5799123	-4.57	1.9884557	LILRA1
228741_s_at	0.759	0.0260556	2.5792851	-4.57	1.7538677	HCN3
1559221_at	0.759	0.0260931	2.5784705	-4.57	1.687399	LINC01013
219699_at	0.759	0.0261228	2.5778273	-4.57	2.5423139	LGI2
223243_s_at	0.759	0.026137	2.5775181	-4.57	2.4358617	EDEM3
1570574_at	0.759	0.0262192	2.5757412	-4.57	2.6525682	
236373_at	0.759	0.026232	2.575463	-4.57	2.2348038	
221032_s_at	0.759	0.0262516	2.5750399	-4.57	2.4190244	TMPRSS5
1563101_at	0.759	0.0262588	2.5748863	-4.57	2.9731696	ARNTL2-AS1
242041_at	0.759	0.0262655	2.5747403	-4.57	2.9328057	CSPP1
243272_at	0.759	0.0262723	2.5745947	-4.57	2.6470267	LOC101593348
1555950_a_at	0.759	0.0263056	2.5738778	-4.57	1.8411421	CD55
231942_at	0.759	0.0263134	2.5737094	-4.57	1.824011	LINC01128
208359_s_at	0.759	0.0263139	2.5736977	-4.57	2.8853388	KCNJ4
1564149_at	0.759	0.0263204	2.5735573	-4.57	3.1133515	LOC102723927
1569322_at	0.759	0.0263313	2.5733237	-4.57	2.4722425	LINC00857
241138_at	0.759	0.0263366	2.5732093	-4.57	2.4376001	

1569112_at	0.759	0.0263594	2.5727205	-4.57	2.5762045	SLC44A5
210929_s_at	0.759	0.0264015	2.5718156	-4.57	2.861219	AHSG
236464_at	0.759	0.0264446	2.5708922	-4.57	2.1959048	
1554228_a_at	0.759	0.0264519	2.5707354	-4.57	1.7432832	BAALC-AS2
208408_at	0.759	0.0264585	2.5705933	-4.57	2.3242251	PTN
234728_s_at	0.759	0.0264695	2.5703585	-4.57	1.6674146	DHX35
211973_at	0.759	0.0264697	2.5703545	-4.57	2.7700117	NUDT3
240413_at	0.759	0.0264749	2.5702427	-4.57	2.9491917	PYHIN1
237670_at	0.759	0.026505	2.5695989	-4.57	2.8520995	MIR663AHG
1559863_a_at	0.759	0.0265127	2.5694357	-4.57	2.1342805	AFG3L1P
1566861_at	0.759	0.0265234	2.5692069	-4.57	3.226452	GATM
207743_at	0.759	0.0265474	2.5686952	-4.57	2.2854779	
49051_g_at	0.759	0.0265478	2.5686859	-4.57	2.8152299	DTX3
1553451_at	0.759	0.0265665	2.5682876	-4.57	2.0328631	TERB1
223955_at	0.759	0.0265698	2.5682164	-4.57	2.1594947	CRACR2A
1553016_at	0.759	0.0266135	2.5672861	-4.57	2.1957879	ADGRF3
240597_at	0.759	0.0266328	2.5668751	-4.57	3.9152036	
203294_s_at	0.759	0.0266328	2.5668748	-4.57	2.9241801	LMAN1
1560679_at	0.759	0.0266331	2.5668676	-4.57	2.5887688	LINC01127
1558021_at	0.759	0.0266732	2.5660169	-4.57	2.7868792	RABEPK
237346_at	0.759	0.0266814	2.5658427	-4.57	2.0910109	TGDS
200861_at	0.759	0.0266893	2.5656748	-4.57	2.2006932	CNOT1

239118_at	0.759	0.0266957	2.5655376	-4.57	2.2876248	KCNA2
242869_at	0.759	0.0267103	2.5652278	-4.57	2.5235827	
1554317_s_at	0.759	0.026711	2.5652134	-4.57	1.9960726	TRMT61A
206206_at	0.759	0.0267257	2.5649018	-4.57	2.3852327	CD180
220729_at	0.759	0.0267454	2.5644836	-4.57	1.9248576	
220433_at	0.759	0.0267638	2.5640943	-4.57	2.6754133	PRRG3
210292_s_at	0.759	0.0267662	2.5640446	-4.57	3.4117467	PCDH11Y///PCDH11X
211466_at	0.759	0.026767	2.5640273	-4.57	2.2210638	NFIB
239840_at	0.759	0.0267749	2.5638607	-4.57	2.2901232	TMEM161B-AS1
1555722_at	0.759	0.0267916	2.5635065	-4.57	2.3766089	SCAMPER
1566041_at	0.759	0.0268307	2.5626806	-4.57	2.1726376	
240224_at	0.759	0.0268349	2.562592	-4.57	2.1871151	
233925_at	0.759	0.0268358	2.5625733	-4.57	2.8302364	
1570244_at	0.759	0.0268479	2.5623179	-4.57	3.2032879	
1554909_at	0.759	0.0268593	2.5620766	-4.57	2.5821947	CCDC36
1562022_s_at	0.759	0.0268724	2.5617997	-4.57	2.4425747	LOC100130987///RAD9A
235379_at	0.759	0.0269006	2.5612075	-4.57	1.7836071	LOC100506114
216271_x_at	0.759	0.0269346	2.5604904	-4.57	2.2247466	SYDE1
241251_at	0.759	0.0270021	2.5590734	-4.57	2.5492491	

228897_at	0.759	0.0270043	2.5590262	-4.57	2.3198698	DERL3
1568795_at	0.759	0.0270348	2.5583876	-4.57	1.8797193	LOC105373495
203864_s_at	0.759	0.0270375	2.5583293	-4.57	2.3930984	ACTN2
221375_at	0.759	0.0270444	2.5581854	-4.57	2.1601747	OR1G1
228322_at	0.759	0.0270661	2.5577303	-4.57	3.0806344	NAA30
1560281_a_at	0.759	0.0270838	2.5573616	-4.57	2.3582204	TMEM95
242408_at	0.759	0.0270911	2.5572085	-4.57	2.2926335	STYX
1553376_a_at	0.759	0.0271079	2.5568569	-4.57	2.2881443	AXDND1
1566824_at	0.759	0.0271155	2.5566978	-4.57	2.3941109	
1566831_at	0.759	0.0271237	2.556527	-4.57	3.1737298	SBF2-AS1
1556037_s_at	0.759	0.0271237	2.5565263	-4.57	2.7722013	HHIP
222963_s_at	0.759	0.0271442	2.5560983	-4.57	2.794089	IL1RAPL1
231971_at	0.759	0.0271456	2.5560694	-4.57	2.9083103	FANCM
213362_at	0.759	0.0271478	2.556023	-4.57	3.5154073	PTPRD
232426_at	0.759	0.0271532	2.5559102	-4.57	3.1882822	SV2B
229270_x_at	0.759	0.0271897	2.5551486	-4.57	2.3647976	SSBP4
233896_s_at	0.759	0.0271921	2.5550993	-4.57	1.9155107	PAPLN
228383_at	0.759	0.0271929	2.5550824	-4.57	2.2169996	PNPLA7
217700_at	0.759	0.0272057	2.5548162	-4.57	2.3560836	CNPY4
1566438_at	0.759	0.0272217	2.5544823	-4.57	2.5606109	LOC283475
1569551_at	0.759	0.0272536	2.5538188	-4.57	2.5015629	

1568801_at	0.759	0.0272547	2.5537953	-4.57	2.1950511	VWA9
218029_at	0.759	0.0272654	2.5535732	-4.57	2.1674109	FAM65A
1559049_a_at	0.759	0.0272753	2.5533677	-4.57	2.6329078	
219042_at	0.759	0.027295	2.5529595	-4.57	2.6029118	LZTS1
237239_at	0.759	0.0273058	2.5527354	-4.57	2.330208	
203059_s_at	0.759	0.0273296	2.5522407	-4.57	2.2036781	PAPSS2
238552_at	0.759	0.0273303	2.5522268	-4.57	1.9820172	
231238_at	0.759	0.0273692	2.5514194	-4.57	2.638894	
221869_at	0.759	0.0273873	2.5510454	-4.57	2.5377653	ZNF512B
216084_at	0.759	0.0274072	2.5506342	-4.57	2.9194672	FAM205BP
215522_at	0.759	0.0274371	2.5500151	-4.57	2.6295082	SORCS3
1560893_at	0.759	0.027442	2.5499143	-4.57	3.0707305	
237801_at	0.759	0.0275491	2.547707	-4.57	2.8748744	
1561638_at	0.759	0.0275518	2.5476521	-4.57	3.4720311	
206626_x_at	0.759	0.0275717	2.5472422	-4.57	2.8055453	SSX1
220805_at	0.759	0.0275832	2.5470065	-4.57	2.1729928	HRH2
242952_at	0.759	0.0276009	2.5466415	-4.57	2.2541566	
236161_at	0.759	0.0276349	2.5459446	-4.57	2.6705084	KCCAT333
239334_at	0.759	0.0276452	2.5457325	-4.57	2.4603726	
208202_s_at	0.759	0.0276939	2.5447366	-4.57	2.7034305	JADE2
237294_at	0.759	0.0277065	2.5444776	-4.57	2.4090389	RASSF4
222608_s_at	0.759	0.0277789	2.542998	-4.57	1.6656505	ANLN

231885_at	0.759	0.0278065	2.5424349	-4.57	2.5718206	ZNF451
241063_at	0.759	0.0278272	2.5420147	-4.57	2.3975772	
240996_at	0.759	0.0278629	2.5412877	-4.57	2.4049281	
235336_at	0.759	0.0278644	2.541256	-4.57	2.478238	
209908_s_at	0.759	0.027873	2.5410825	-4.57	2.908624	TGFB2
1562623_at	0.759	0.0278833	2.5408722	-4.57	2.3511442	LOC146513
1553208_s_at	0.759	0.0278859	2.5408198	-4.57	1.8896128	ARL10
231156_at	0.759	0.0278935	2.5406655	-4.57	2.7177921	HAO2
230135_at	0.759	0.0279121	2.5402865	-4.57	3.3789441	HHIP
232958_at	0.759	0.0279288	2.5399477	-4.57	2.9006353	
221386_at	0.759	0.0279302	2.5399189	-4.57	2.3939767	OR3A2
217081_at	0.759	0.0279346	2.5398297	-4.57	2.0251401	OR2H2
234021_at	0.759	0.0279398	2.5397249	-4.57	2.2580072	EML2
213496_at	0.759	0.0279512	2.5394932	-4.57	3.5444594	PLPPR4
216937_s_at	0.759	0.0279616	2.5392826	-4.57	2.279882	RS1
1570198_x_at	0.759	0.0279858	2.5387915	-4.57	2.2188574	
1555595_at	0.759	0.0280081	2.5383402	-4.57	2.7735291	SCRN3
219170_at	0.759	0.0280258	2.5379818	-4.57	2.2441008	FSD1
231267_at	0.759	0.0280441	2.5376113	-4.57	1.9249443	FAM163A
210552_s_at	0.759	0.0280781	2.5369241	-4.57	1.9246642	RALGPS1

244718_at	0.759	0.0280896	2.5366925	-4.57	2.5315388	MTMR9LP
235426_at	0.759	0.0281018	2.5364467	-4.57	2.4071288	GATM
221100_at	0.759	0.0281329	2.5358195	-4.57	2.5445849	C6orf15
243013_at	0.759	0.0281357	2.5357632	-4.57	1.9369553	
239712_at	0.759	0.0281478	2.5355198	-4.57	2.8399924	CCDC171
1570186_at	0.759	0.0281647	2.5351796	-4.57	2.3819051	GRASPOS
239359_at	0.759	0.0281941	2.5345865	-4.57	2.5968756	11-Mar
207260_at	0.759	0.0282022	2.5344251	-4.57	2.6148224	FEV
231320_at	0.759	0.0282113	2.5342425	-4.57	1.9850313	EBP
240979_at	0.759	0.0282254	2.5339577	-4.57	2.314116	YWHAEP7
242432_at	0.759	0.0282356	2.5337531	-4.57	2.5452546	LRRC37A2
209352_s_at	0.759	0.0282436	2.5335923	-4.57	2.7185291	SIN3B
1553298_at	0.759	0.0282472	2.5335201	-4.57	2.8526449	C17orf77
214865_at	0.759	0.0282612	2.5332395	-4.57	2.2255617	DOT1L
223998_at	0.759	0.0282851	2.5327594	-4.57	3.2883899	TTLL2
1561240_at	0.759	0.0282989	2.5324836	-4.57	3.1484169	
233343_at	0.759	0.0283166	2.5321284	-4.57	2.4242509	DEFB127
240654_at	0.759	0.028332	2.5318213	-4.57	1.9662562	
217434_at	0.759	0.0283638	2.5311846	-4.57	2.5325655	MC2R
234355_s_at	0.759	0.0283854	2.5307525	-4.57	2.4872668	DISP3
1564051_at	0.759	0.0284337	2.5297895	-4.57	2.5323108	
234427_at	0.759	0.0284781	2.5289041	-4.57	2.5222693	YME1L1

221421_s_at	0.759	0.0284893	2.5286806	-4.57	2.5563694	ADAMTS12
207420_at	0.759	0.0285037	2.5283941	-4.57	3.0039006	COLEC10
224051_at	0.759	0.0285041	2.5283867	-4.57	2.1308214	
234851_at	0.759	0.0285124	2.5282216	-4.57	2.5991157	IGLC1
1568903_at	0.759	0.0285173	2.5281246	-4.57	2.0176659	
1556976_s_at	0.759	0.0285181	2.528109	-4.57	2.2907724	LOC105375273
240864_at	0.759	0.0285193	2.5280843	-4.57	1.8232489	
216408_at	0.759	0.0285475	2.5275228	-4.57	1.8987404	OR2B2
1565242_at	0.759	0.0285608	2.5272589	-4.57	3.0061299	TNXB
230367_at	0.759	0.0285698	2.5270802	-4.57	2.1680649	SMTNL1
1554863_s_at	0.759	0.0286194	2.5260968	-4.57	1.9832024	DOK5
243641_at	0.759	0.0286384	2.5257199	-4.57	2.6714837	
233566_at	0.759	0.0286717	2.5250612	-4.57	3.1751776	LINC00839
227912_s_at	0.759	0.0286787	2.5249222	-4.57	1.8185243	EXOSC3
237370_at	0.759	0.0286812	2.5248745	-4.57	2.4656302	
234102_at	0.759	0.0286838	2.5248215	-4.57	1.9385359	RASL11B
210536_s_at	0.759	0.0286972	2.5245576	-4.57	2.9640991	SPAM1
210012_s_at	0.759	0.0287389	2.5237339	-4.57	2.4017884	EWSR1
1569290_s_at	0.759	0.028775	2.5230211	-4.57	2.6015065	GRIA3
206837_at	0.759	0.0288058	2.5224149	-4.57	2.1082097	ALX1
1560098_at	0.759	0.0288144	2.5222465	-4.57	2.3474775	

207796_x_at	0.759	0.0288278	2.5219823	-4.57	2.0862174	KLRD1
1568690_a_at	0.759	0.0288558	2.5214312	-4.57	2.8538855	LOC100631378
233617_at	0.759	0.02892	2.5201706	-4.57	2.4982013	TSPY26P
218756_s_at	0.759	0.0289382	2.5198141	-4.57	1.9702408	DHRS11
243499_at	0.759	0.0289423	2.519734	-4.57	1.6868142	
243862_at	0.759	0.0289572	2.5194404	-4.57	2.4840267	RASEF
232826_at	0.759	0.0289911	2.5187772	-4.57	1.6477856	
208538_at	0.759	0.0290353	2.5179133	-4.57	2.6189474	ANP32C
228671_at	0.759	0.0290563	2.5175022	-4.57	2.0540371	TMEM201
223873_s_at	0.759	0.0290669	2.5172969	-4.57	2.8828397	
216220_s_at	0.759	0.0290892	2.5168611	-4.57	2.80703	ADORA1
1565580_s_at	0.759	0.0291045	2.5165625	-4.57	2.6643284	TATDN2
241612_at	0.759	0.029116	2.5163391	-4.57	1.9467752	FOXD3
1561728_a_at	0.759	0.0291677	2.5153313	-4.57	2.7889659	LINC00642
221608_at	0.759	0.0291699	2.5152891	-4.57	1.9700724	WNT6
242592_at	0.759	0.02917	2.5152869	-4.57	3.1036394	GPR137C
233158_at	0.759	0.0292048	2.5146104	-4.57	1.5579299	KRT82
1562923_at	0.759	0.0292104	2.5145022	-4.57	2.2125598	LOC101928449
241130_at	0.759	0.029213	2.5144524	-4.57	2.5393477	
239905_at	0.759	0.0292713	2.51332	-4.57	3.409978	YTHDC1
239950_at	0.759	0.0292772	2.5132071	-4.57	3.1767286	HOXA11-AS
234093_at	0.759	0.0292873	2.5130112	-4.57	1.901812	ZNF326

216119_s_at	0.759	0.0292941	2.5128797	-4.57	2.4214861	SPEF1
1552749_a_at	0.759	0.0293262	2.5122572	-4.57	2.2964623	KLC3
221411_at	0.759	0.0293499	2.5117984	-4.57	1.982381	HOXD12
230183_at	0.759	0.0293559	2.5116831	-4.57	1.715241	EXT1
237675_at	0.759	0.0293678	2.5114538	-4.57	2.8985218	LINC01351
1556445_at	0.759	0.0293721	2.5113692	-4.57	2.7850129	
225438_at	0.759	0.0293799	2.5112197	-4.57	2.0595296	NUDCD1
1560996_at	0.759	0.0294302	2.5102482	-4.57	2.7678769	
1562215_at	0.759	0.0294379	2.5100997	-4.57	2.1912081	
216869_at	0.759	0.0294597	2.509681	-4.57	2.7097328	PDE1C
242267_x_at	0.759	0.0294765	2.5093566	-4.57	1.8626466	LOC105369595
1569430_at	0.759	0.0294784	2.5093203	-4.57	2.0952173	PTRH1
1553202_at	0.759	0.0295348	2.5082352	-4.57	2.4619074	STOX1
1560129_at	0.759	0.0295636	2.5076831	-4.57	2.3353636	
233298_at	0.759	0.0295752	2.5074594	-4.57	2.7126766	CCDC169
1552412_a_at	0.759	0.0295832	2.5073054	-4.57	4.1058243	DEFB106B///DEFB106A
1559254_at	0.759	0.0296007	2.5069706	-4.57	2.4499062	PICSAR
233050_at	0.759	0.0296066	2.5068573	-4.57	3.1445071	SOGA3///KIAA0408
231448_at	0.759	0.0296156	2.5066859	-4.57	1.6995471	ADAD1
238634_x_at	0.759	0.0296157	2.5066828	-4.57	2.6223097	LIPJ
211188_at	0.759	0.0296158	2.5066818	-4.57	2.8082985	CD84

1561521_at	0.759	0.0296202	2.5065967	-4.57	2.3228319	S100B
1561225_at	0.759	0.0296691	2.5056616	-4.57	2.2014048	ANKRD30BP3
1560581_at	0.759	0.0297052	2.5049706	-4.57	3.1974026	
224234_at	0.759	0.0297293	2.5045112	-4.57	2.2929728	
206000_at	0.759	0.0297327	2.5044463	-4.57	2.9010973	MEP1A
1558391_s_at	0.759	0.0297772	2.5035973	-4.57	2.5622741	ZNF599
237532_at	0.759	0.029806	2.5030482	-4.57	1.7906114	
1553301_a_at	0.759	0.0298494	2.5022225	-4.57	3.0474886	TMEM182
1561345_at	0.759	0.0298498	2.502214	-4.57	2.4444005	LOC101928009
229987_at	0.759	0.0298636	2.5019518	-4.57	2.51186	
1557832_at	0.759	0.0298874	2.5015	-4.57	3.2351525	LOC101926960
1559926_at	0.759	0.0298972	2.5013144	-4.57	2.1439937	LOC728353
239368_at	0.759	0.0299125	2.5010235	-4.57	2.2824674	
226898_s_at	0.759	0.0299252	2.5007824	-4.57	1.9195715	SFPQ
233904_at	0.759	0.0299339	2.5006171	-4.57	2.2446389	
243345_at	0.759	0.0299527	2.500262	-4.57	1.785877	RNF14
239962_at	0.759	0.0299535	2.5002452	-4.57	2.732238	
233215_s_at	0.759	0.0299702	2.4999304	-4.57	2.4421966	ZDHHC21
1558589_at	0.759	0.0299794	2.4997563	-4.57	1.8703489	LINC00417
241593_x_at	0.759	0.0300444	2.4985258	-4.57	2.8068709	
1569105_at	0.759	0.0300532	2.49836	-4.57	2.0781744	SETD5
1566209_at	0.759	0.0300609	2.4982136	-4.57	2.951942	
239548_at	0.759	0.0300642	2.498152	-4.57	2.1947864	NEGR1

1554384_at	0.759	0.0301191	2.4971169	-4.57	2.2950522	PADI2
230013_s_at	0.759	0.03012	2.4970996	-4.57	2.0062657	ARHGAP23
1560977_a_at	0.759	0.0301338	2.4968387	-4.57	1.935683	BCL2L13
1565192_at	0.759	0.0301547	2.4964458	-4.57	2.8723653	
207882_at	0.759	0.0301579	2.4963852	-4.57	2.3645286	
1564883_a_at	0.759	0.0301634	2.4962818	-4.57	2.093337	TAS1R1
207089_at	0.759	0.0301792	2.4959848	-4.57	2.4674938	NRAP
244006_at	0.759	0.0302191	2.495235	-4.57	2.907523	POU2F1
1553464_at	0.759	0.030225	2.4951228	-4.57	3.0361091	FLJ40288
1558573_at	0.759	0.0302259	2.4951069	-4.57	2.1108797	MCTS1
237852_at	0.759	0.0302329	2.4949762	-4.57	2.8590958	
217540_at	0.759	0.0302557	2.4945467	-4.57	2.8431375	NXPE3
1563376_at	0.759	0.0302645	2.4943818	-4.57	2.4212931	LOC101929174
1566949_at	0.759	0.0302983	2.493749	-4.57	1.7915071	
218952_at	0.759	0.0302991	2.4937334	-4.57	2.6612644	PCSK1N
214522_x_at	0.759	0.0303096	2.493536	-4.57	1.9659726	HIST1H3D///HIST1H2AD
237509_at	0.759	0.0303149	2.4934381	-4.57	2.5168454	LINC01616
1556233_s_at	0.759	0.0303652	2.4924969	-4.57	2.9845819	KIF6
1567274_at	0.759	0.0303745	2.4923216	-4.57	2.8070225	
227819_at	0.759	0.0303768	2.4922801	-4.57	2.4194031	LGR6

237332_at	0.759	0.0303885	2.4920613	-4.57	2.3298626	MON2
237277_at	0.759	0.0304073	2.4917093	-4.57	3.3270391	
222175_s_at	0.759	0.0304199	2.4914747	-4.57	2.084436	MED15
239479_x_at	0.759	0.0304217	2.4914399	-4.57	2.8650866	
210823_s_at	0.759	0.0304397	2.4911055	-4.57	2.4569942	PTPRS
1562739_at	0.759	0.0304479	2.4909519	-4.57	2.6103259	LOC285593
237592_at	0.759	0.030451	2.4908939	-4.57	2.5348567	ZC2HC1B
206439_at	0.759	0.030454	2.4908383	-4.57	4.3502169	EPYC
241213_x_at	0.759	0.0304648	2.490637	-4.57	1.9845477	
1556737_at	0.759	0.030465	2.4906339	-4.57	2.6062948	LINC00671
232428_at	0.759	0.0304753	2.4904411	-4.57	2.6701572	MOGAT2
1559433_at	0.759	0.0305058	2.4898734	-4.57	2.6994255	APCDD1L-AS1
209877_at	0.759	0.0305182	2.4896419	-4.57	2.3873242	SNCG
205048_s_at	0.759	0.0305309	2.4894059	-4.57	2.1352952	PSPH
220676_at	0.759	0.0305377	2.4892796	-4.57	2.2851791	ADAMTS8
238763_at	0.759	0.0305388	2.4892589	-4.57	2.7618885	RBM20
233979_s_at	0.759	0.0306273	2.4876168	-4.57	2.6900696	ESPN
239989_at	0.759	0.0306669	2.4868828	-4.57	2.1840878	CNTLN
1570006_at	0.759	0.0306914	2.4864283	-4.57	2.1977696	LOC400958
215396_at	0.759	0.0307106	2.4860743	-4.57	3.3163991	ADGRV1
207450_s_at	0.759	0.0307144	2.4860025	-4.57	2.1930983	POU6F2
228699_at	0.759	0.0307426	2.4854817	-4.57	1.9664719	NRP2

1556619_at	0.759	0.0307877	2.4846501	-4.57	2.953367	SHISA9
1569002_x_at	0.759	0.0307961	2.4844951	-4.57	2.6490255	BMP1
239662_x_at	0.759	0.0308156	2.484134	-4.57	2.246231	TMCC1
219789_at	0.759	0.0308507	2.483489	-4.57	3.2034523	NPR3
210654_at	0.759	0.0308861	2.4828362	-4.57	2.1596733	TNFRSF10D
1565998_at	0.759	0.0309294	2.4820406	-4.57	2.671134	
243378_at	0.759	0.0309469	2.4817192	-4.57	1.6455865	
241364_at	0.759	0.030963	2.4814253	-4.57	1.8916542	TMEM57
1557739_at	0.759	0.0309793	2.4811257	-4.57	3.8054089	LOC105372547
222972_at	0.759	0.0309807	2.4810993	-4.57	2.7261109	VSX1
1556542_a_at	0.759	0.0310487	2.4798549	-4.57	2.3253183	
234018_s_at	0.759	0.0311348	2.4782814	-4.57	2.4290414	SEL1L2
239034_at	0.759	0.0311844	2.4773774	-4.57	1.5171893	LINC01560
222820_at	0.759	0.0312175	2.4767754	-4.57	2.2334747	TNRC6C
239330_at	0.759	0.031232	2.4765116	-4.57	3.3161182	DCAF12L2
1553608_a_at	0.759	0.031253	2.4761293	-4.57	2.5827994	LINC00189
1559707_at	0.759	0.03129	2.4754576	-4.57	2.231055	
202654_x_at	0.759	0.0312949	2.4753685	-4.57	1.8407226	7-Mar
1554074_s_at	0.759	0.0313013	2.4752518	-4.57	2.372675	SLFNL1
221307_at	0.759	0.0313016	2.475246	-4.57	2.7852964	KCNIP1
219589_s_at	0.759	0.031305	2.4751845	-4.57	2.4748148	TMEM143
207481_at	0.759	0.0313217	2.4748805	-4.57	2.0304731	

213108_at	0.759	0.0313303	2.4747246	-4.57	2.6461845	CAMK2A
210226_at	0.759	0.0313327	2.4746821	-4.57	1.8326749	NR4A1
221038_at	0.759	0.0313366	2.4746108	-4.57	2.1147694	
240051_at	0.759	0.0313495	2.474377	-4.57	1.7778819	TPD52L3
232924_at	0.759	0.0313502	2.4743654	-4.57	2.4596083	LRRC17
1559992_a_at	0.759	0.0313653	2.4740911	-4.57	2.00371	LINC00645
216473_x_at	0.759	0.0313691	2.474022	-4.57	1.8013769	DUX4L24///DBET///LOC100291626///DUX4///LOC100288289///DUX4L2// /DUX4L1
1552964_at	0.759	0.0313859	2.4737188	-4.57	2.6717166	CFAP46
223083_s_at	0.759	0.0313973	2.4735121	-4.57	2.3050935	RAB4B-EGLN2///EGLN2///RAB4B
244048_x_at	0.759	0.0314056	2.4733624	-4.57	3.3294117	
243545_at	0.759	0.0314403	2.4727339	-4.57	2.8973109	

242910_x_at	0.759	0.0314421	2.472701	-4.57	3.1622846	
1562645_at	0.759	0.0314885	2.4718634	-4.57	2.2493202	LOC401176
220499_at	0.759	0.0315085	2.4715029	-4.57	2.1109277	FNDC8
230710_at	0.759	0.031527	2.4711702	-4.57	2.2363086	MIR210HG
1555774_at	0.759	0.0315948	2.4699479	-4.57	2.6054287	ZAR1
222558_at	0.759	0.0316032	2.469797	-4.57	2.539108	RPRD1A
222260_at	0.759	0.0316397	2.4691423	-4.57	2.1874516	
1566475_at	0.759	0.0316457	2.4690336	-4.57	2.8101031	
237856_at	0.759	0.0316899	2.4682407	-4.57	2.3552163	RAP1GDS1
1558251_a_at	0.759	0.0316928	2.4681886	-4.57	2.1865523	ZNF417///ZNF587
234499_at	0.759	0.0317062	2.4679484	-4.57	2.3838915	
231745_at	0.759	0.0317416	2.4673138	-4.57	2.7262956	GPR55
239796_x_at	0.759	0.0317492	2.4671783	-4.57	2.6400632	TIRAP
1561459_at	0.759	0.0317503	2.4671578	-4.57	2.0423095	LOC101929526
231471_at	0.759	0.031753	2.4671094	-4.57	2.2849946	
240735_at	0.759	0.0317571	2.4670373	-4.57	3.2894692	CDC42BPA
206254_at	0.759	0.0317852	2.4665338	-4.57	2.9518408	EGF
241576_at	0.759	0.0317887	2.466472	-4.57	2.2179567	
202455_at	0.759	0.0318011	2.4662507	-4.57	1.5583626	HDAC5
242594_at	0.759	0.0318024	2.4662265	-4.57	1.7958807	BOD1L1
222484_s_at	0.759	0.0318273	2.465782	-4.57	2.6427921	CXCL14

237538_at	0.759	0.0318806	2.4648305	-4.57	1.9768498	
228860_at	0.759	0.0318917	2.4646334	-4.57	2.0973236	UBE3B
223729_at	0.759	0.0318978	2.4645238	-4.57	2.2603232	CECR2
1556355_x_at	0.759	0.0319166	2.46419	-4.57	1.8092889	RGL3
1552425_a_at	0.759	0.0319481	2.4636287	-4.57	2.5284426	KLHL10
1561136_at	0.759	0.0319555	2.4634978	-4.57	3.7495422	GYPE
244331_at	0.759	0.0319611	2.4633984	-4.57	3.218799	
1563821_at	0.759	0.0319615	2.463391	-4.57	2.2931469	LINC00858
1561368_at	0.759	0.0319666	2.4632994	-4.57	2.5450706	LOC105376081
216673_at	0.759	0.0319749	2.4631518	-4.57	2.4330339	TTTY1B///TTTY1
1553508_at	0.759	0.0320037	2.4626398	-4.57	1.9000151	MDS2
1564962_at	0.759	0.0320169	2.4624053	-4.57	3.16624	ZNF92
236143_at	0.759	0.032041	2.4619793	-4.57	1.9559141	
220978_at	0.759	0.0320458	2.4618925	-4.57	2.0773476	KRTAP1-3
1570001_at	0.759	0.0320952	2.4610183	-4.57	2.5343506	CASP8AP2
210408_s_at	0.759	0.0321016	2.4609043	-4.57	2.5169882	CPNE6
219427_at	0.759	0.0321045	2.460853	-4.57	1.8604529	FAT4
216728_at	0.759	0.0321094	2.4607653	-4.57	1.8181375	
211091_s_at	0.759	0.0321276	2.4604445	-4.57	2.6613746	NF2
242631_x_at	0.759	0.0321641	2.4597989	-4.57	3.2800726	DLC1

1554885_a_at	0.759	0.0321662	2.4597622	-4.57	3.0659877	PRIM2B///PRIM2
1570024_at	0.759	0.0321716	2.4596665	-4.57	2.3546078	
216087_at	0.759	0.032226	2.458706	-4.57	2.8064276	
1554324_s_at	0.759	0.0322308	2.4586204	-4.57	2.0598539	DYNC2LI1
224216_at	0.759	0.0322439	2.4583908	-4.57	3.3896161	
207555_s_at	0.759	0.0322599	2.4581072	-4.57	2.3765816	TBXA2R
220631_at	0.759	0.0323241	2.4569779	-4.57	2.4126902	OSGEPL1
213946_s_at	0.759	0.0323528	2.4564732	-4.57	3.0028719	OBSL1
235184_at	0.759	0.032387	2.4558719	-4.57	2.3123881	AEBP2
214599_at	0.759	0.0323952	2.4557292	-4.57	2.1495156	IVL
229298_at	0.759	0.0324142	2.4553944	-4.57	2.2715038	KBTBD7
1554578_at	0.759	0.0324282	2.4551497	-4.57	2.1937374	ZNF70
1553094_at	0.759	0.0324287	2.455141	-4.57	2.4262718	FLJ45513///TAC4
244195_at	0.759	0.0324489	2.4547863	-4.57	1.989302	
237791_at	0.759	0.0324909	2.454052	-4.57	1.9345855	LOC105370178
221293_s_at	0.759	0.0325025	2.453848	-4.57	2.2350392	DEF6
220594_at	0.759	0.0325455	2.453097	-4.57	2.7798577	OGT
220129_at	0.759	0.0325568	2.4528985	-4.57	3.2299076	CCDC169-SOHLH2///SOHLH2
237826_at	0.759	0.0326141	2.4518993	-4.57	2.242249	

218002_s_at	0.759	0.0326407	2.4514359	-4.57	2.7995349	CXCL14
1565722_at	0.759	0.0326503	2.4512687	-4.57	2.6952709	LOC100128281
1567223_at	0.759	0.032661	2.4510826	-4.57	2.6131378	HMGA2
1553156_at	0.759	0.0327045	2.4503256	-4.57	2.1409662	LHX4
1565814_at	0.759	0.0327076	2.4502718	-4.57	2.7726456	TRIM36
214382_at	0.759	0.03274	2.4497083	-4.57	2.5786465	UNC93A
231303_at	0.759	0.0327456	2.449611	-4.57	2.6731267	LINC00158
1557754_at	0.759	0.0327681	2.4492209	-4.57	1.6667845	LOC401068
1554762_a_at	0.759	0.0327792	2.4490272	-4.57	1.8652687	WWC2///CLDN22
234074_at	0.759	0.032788	2.448875	-4.57	2.254793	
239403_at	0.759	0.0328205	2.4483114	-4.57	2.5645486	CCDC120
234022_at	0.759	0.0328374	2.4480186	-4.57	2.8298998	
238607_at	0.759	0.0328485	2.447826	-4.57	1.9495813	ZNF296
206358_at	0.759	0.0328583	2.4476576	-4.57	1.6956648	PRM1
1563015_at	0.759	0.0328653	2.4475364	-4.57	2.6425655	LOC101928135
244820_at	0.759	0.0328776	2.4473234	-4.57	1.9037281	
217351_at	0.759	0.0328921	2.4470722	-4.57	1.884899	
242382_at	0.759	0.0329082	2.4467938	-4.57	2.3042334	LOC100506207
211870_s_at	0.759	0.0329422	2.4462073	-4.57	2.324457	PCDHA2///PCDHA3
219705_at	0.759	0.0329609	2.4458838	-4.57	1.6433822	QSER1

1555445_at	0.759	0.0329668	2.4457824	-4.57	2.1132273	CA8
240724_at	0.759	0.0330291	2.4447085	-4.57	2.6714187	
244882_at	0.759	0.0330451	2.444432	-4.57	1.7338783	TNRC18
1552791_a_at	0.759	0.0330473	2.4443949	-4.57	2.6837945	TRDN
1568826_at	0.759	0.0330534	2.4442902	-4.57	2.4389967	LINC01012
239697_x_at	0.759	0.0330765	2.443892	-4.57	2.3836393	C3orf67
1569750_at	0.759	0.0330814	2.4438087	-4.57	2.8949456	
216173_at	0.759	0.0331113	2.4432943	-4.57	2.6651978	
231689_at	0.759	0.033127	2.4430248	-4.57	1.5584674	TRPM7
243144_at	0.759	0.0331357	2.4428759	-4.57	2.095009	
233203_at	0.759	0.0331427	2.4427547	-4.57	2.4281414	ROPN1
1561083_at	0.759	0.0331567	2.4425143	-4.57	2.0138112	LINC01254
240208_at	0.759	0.0331574	2.4425029	-4.57	2.6473372	
242475_at	0.759	0.0331617	2.4424288	-4.57	2.5337743	
1566653_at	0.759	0.0331753	2.4421948	-4.57	2.5013997	
217229_at	0.759	0.0331794	2.4421244	-4.57	4.2001877	ASB4
237245_at	0.759	0.0331996	2.4417791	-4.57	2.2322514	BUB3
234027_at	0.759	0.0332002	2.4417686	-4.57	1.9883238	CCDC129
237162_at	0.759	0.0332043	2.4416987	-4.57	2.1474658	KANK1
243382_at	0.759	0.0332062	2.4416652	-4.57	2.5030128	LOC101928647
228668_x_at	0.759	0.0332631	2.4406927	-4.57	1.9933624	CCDC71L
221128_at	0.759	0.0332844	2.4403281	-4.57	2.0926056	ADAM19
1566599_at	0.759	0.0332945	2.440155	-4.57	2.1311666	

210128_s_at	0.759	0.0333457	2.4392812	-4.57	2.3504504	LTB4R
234585_at	0.759	0.0333583	2.4390668	-4.57	1.90506	
240569_at	0.759	0.0333621	2.4390018	-4.57	2.9227606	
233165_at	0.759	0.0333735	2.4388072	-4.57	2.6387682	NCKIPSD
230232_at	0.759	0.0334004	2.4383485	-4.57	1.9349704	SEL1L3
1566897_at	0.759	0.0334508	2.4374901	-4.57	2.0616937	SPPL3
1563091_at	0.759	0.0334522	2.4374668	-4.57	2.0365163	
1556181_at	0.759	0.033469	2.4371806	-4.57	1.8727316	ANKRD65
209856_x_at	0.759	0.0334983	2.4366831	-4.57	2.1312678	ABI2
219701_at	0.759	0.0335406	2.4359654	-4.57	3.1413405	TMOD2
240360_at	0.759	0.0335498	2.4358095	-4.57	2.1568481	BRD4
233415_at	0.759	0.0335542	2.4357336	-4.57	2.3426995	
1566215_at	0.759	0.0336056	2.4348636	-4.57	3.2444406	
222774_s_at	0.759	0.0336178	2.4346564	-4.57	2.1343621	NETO2
244326_at	0.759	0.0336253	2.43453	-4.57	2.4299454	
239000_at	0.759	0.0336434	2.4342242	-4.57	1.885552	BRD4
235491_at	0.759	0.0336441	2.4342124	-4.57	1.9901958	ZBTB10
244369_at	0.759	0.0336677	2.4338122	-4.57	2.2293519	C21orf59
206212_at	0.759	0.0336713	2.4337527	-4.57	2.0656198	CPA2
243058_at	0.759	0.033696	2.4333349	-4.57	1.8670532	
242265_at	0.759	0.0337324	2.4327209	-4.57	1.7796314	BRD8
218657_at	0.759	0.0337392	2.4326058	-4.57	2.3348399	RAPGEFL1
206668_s_at	0.759	0.0337726	2.4320422	-4.57	2.4191617	SCAMP1
216829_at	0.759	0.0337761	2.431983	-4.57	3.2940962	IGK///IGKC

240350_at	0.759	0.0337774	2.4319622	-4.57	2.7427727	
233659_at	0.759	0.0337788	2.4319385	-4.57	2.0217204	
1561262_at	0.759	0.033794	2.4316814	-4.57	2.0490614	LINC01516
233200_at	0.759	0.0337968	2.4316348	-4.57	2.0455093	LOC731157
1552360_a_at	0.759	0.0338016	2.4315533	-4.57	1.552567	TIRAP
1560097_at	0.759	0.0338106	2.431403	-4.57	2.8806085	FHAD1
243363_at	0.759	0.0338161	2.4313101	-4.57	2.735049	LEF1-AS1
1562112_at	0.759	0.0338168	2.4312982	-4.57	2.744375	
240504_at	0.759	0.0338606	2.430561	-4.57	2.260793	
1557417_s_at	0.759	0.0338611	2.4305528	-4.57	2.7179069	RSPH10B2///RSPH10B
233166_at	0.759	0.0338694	2.4304137	-4.57	3.3627279	DEFB126
241296_at	0.759	0.0338746	2.4303257	-4.57	3.6121535	
207804_s_at	0.759	0.0338766	2.4302919	-4.57	2.4222518	FCN2
1559535_s_at	0.759	0.0338826	2.4301909	-4.57	2.0095994	LOC100996419
231505_s_at	0.759	0.0339007	2.4298884	-4.57	1.8717117	SFXN4
205485_at	0.759	0.033915	2.4296478	-4.57	1.7888372	RYR1
224082_at	0.759	0.0339306	2.429386	-4.57	1.9240532	
1568749_at	0.759	0.0339347	2.4293176	-4.57	2.2234377	LOC105374928
213150_at	0.759	0.0339565	2.4289521	-4.57	2.5280067	HOXA10
222611_s_at	0.759	0.0339727	2.4286797	-4.57	2.6164041	PSPC1
232231_at	0.759	0.0339761	2.4286233	-4.57	1.9699638	RUNX2

240269_at	0.759	0.0339791	2.4285737	-4.57	1.7386935	
216240_at	0.759	0.0340085	2.4280802	-4.57	2.0552767	MIR1204///PVT1
217061_s_at	0.759	0.0340278	2.4277574	-4.57	1.6363174	ETV1
238543_x_at	0.759	0.0340285	2.4277458	-4.57	2.6841595	MDGA1
216959_x_at	0.759	0.0340359	2.4276228	-4.57	1.9735038	NRCAM
213023_at	0.759	0.0340808	2.426871	-4.57	1.7153002	UTRN
1554652_s_at	0.759	0.034132	2.4260168	-4.57	2.0178055	MAST4
210670_at	0.759	0.0341464	2.4257767	-4.57	3.1653943	PPY
234619_at	0.759	0.0341888	2.4250713	-4.57	2.5629081	
1560142_at	0.759	0.0341943	2.4249789	-4.57	2.3925509	GRIK2
237781_at	0.759	0.03425	2.4240521	-4.57	2.8049166	HORMAD2
236179_at	0.759	0.0342567	2.4239408	-4.57	2.4075061	CDH11
233074_at	0.759	0.0342751	2.4236347	-4.57	1.9667725	LOC100421494
242411_at	0.759	0.0342792	2.4235676	-4.57	1.6341866	ARL10
215349_at	0.759	0.0342966	2.4232791	-4.57	2.6004	BTBD18
214729_at	0.759	0.0343006	2.4232123	-4.57	1.8905554	TWISTNB
244714_at	0.759	0.0343062	2.4231187	-4.57	2.3338548	
1565836_at	0.759	0.0343463	2.4224532	-4.57	2.4241895	LOC100506411
244891_x_at	0.759	0.0343563	2.4222885	-4.57	1.9863949	

216580_at	0.759	0.0343894	2.4217396	-4.57	1.5535259	
230699_at	0.759	0.0344235	2.4211748	-4.57	2.4343689	PGLS
203382_s_at	0.759	0.0344484	2.4207638	-4.57	2.6077684	APOE
1569311_at	0.759	0.0344651	2.4204877	-4.57	2.4852462	
244844_at	0.759	0.0344759	2.4203099	-4.57	2.1365006	
1567241_at	0.759	0.0344874	2.4201195	-4.57	2.5044471	OR2L1P
207036_x_at	0.759	0.0345047	2.4198333	-4.57	2.8199913	GRIN2D
238254_at	0.759	0.0345384	2.4192787	-4.57	2.6222562	ZNF677
241948_at	0.759	0.0345949	2.418348	-4.57	1.9792984	
227023_at	0.759	0.0346099	2.4180998	-4.57	2.485304	ZFP41///GLI4
241487_at	0.759	0.0346366	2.4176609	-4.57	2.3444138	
223074_s_at	0.759	0.0346684	2.4171389	-4.57	2.4129834	AIF1L
208044_s_at	0.759	0.0346701	2.4171105	-4.57	2.4572935	PPARD
1558322_a_at	0.759	0.0346776	2.4169874	-4.57	2.669533	PAQR9
220601_at	0.759	0.0347039	2.4165556	-4.57	1.9913432	C16orf70
239752_at	0.759	0.0347276	2.4161673	-4.57	3.2239163	CECR2
204960_at	0.759	0.0347362	2.4160253	-4.57	2.5164333	PTPRCAP
203798_s_at	0.759	0.0347374	2.4160072	-4.57	2.5810708	VSNL1
1553027_a_at	0.759	0.0347446	2.4158881	-4.57	1.893762	KLHL4
208111_at	0.759	0.0347608	2.415623	-4.57	2.5355926	AVPR2
1554086_at	0.759	0.0347612	2.4156155	-4.57	2.3777143	TUBGCP3

216946_at	0.759	0.03477	2.4154722	-4.57	2.4699485	HLA-DOA
216478_at	0.759	0.0347743	2.4154011	-4.57	2.6914964	
241096_at	0.759	0.0347744	2.4154006	-4.57	2.4488597	
205986_at	0.759	0.0347772	2.4153536	-4.57	1.6620237	AATK
236702_at	0.759	0.0347938	2.4150832	-4.57	1.7882994	RTFDC1
202987_at	0.759	0.0348157	2.4147238	-4.57	1.5352658	TRAF3IP2
242115_at	0.759	0.0348201	2.414652	-4.57	2.716642	
238713_at	0.759	0.0348248	2.4145759	-4.57	1.6420594	
216674_at	0.759	0.0348546	2.4140874	-4.57	2.5163492	HES2
1556781_at	0.759	0.0348609	2.4139851	-4.57	3.2178146	
207007_at	0.759	0.0349069	2.4132344	-4.57	2.4238753	NR1I3
1558247_s_at	0.759	0.0349135	2.4131269	-4.57	2.6601091	NTN5
233603_at	0.759	0.0349227	2.412976	-4.57	2.4046827	
224342_x_at	0.759	0.0349285	2.4128816	-4.57	1.9998187	BMS1P20
244244_at	0.759	0.0349417	2.4126659	-4.57	2.3926746	CD300LG
1559785_at	0.759	0.0349574	2.4124106	-4.57	2.4177292	TPPP2
217179_x_at	0.759	0.0349644	2.4122968	-4.57	1.8279768	BMS1P20
242322_at	0.759	0.0349991	2.4117322	-4.57	2.3285554	
238755_at	0.759	0.035004	2.4116518	-4.57	2.4958016	RASSF10
204888_s_at	0.759	0.0350309	2.4112142	-4.57	1.9691932	NEURL1

209776_s_at	0.759	0.0350599	2.4107431	-4.57	1.9650254	SLC19A1
210913_at	0.759	0.0350622	2.4107055	-4.57	2.097542	CDH20
224290_at	0.759	0.0350818	2.4103874	-4.57	2.5329039	VN1R3
215801_at	0.759	0.0350861	2.4103178	-4.57	2.2800499	
206091_at	0.759	0.0351301	2.4096029	-4.57	1.9842983	MATN3
211467_s_at	0.759	0.0351417	2.4094148	-4.57	2.3117636	NFIB
211262_at	0.759	0.0351558	2.4091874	-4.57	2.3890695	PCSK6
217440_at	0.759	0.035157	2.4091675	-4.57	3.428358	
1562945_at	0.759	0.0351915	2.4086093	-4.57	2.5674601	LOC105378130
234518_at	0.759	0.0352026	2.4084286	-4.57	2.9097498	
210173_at	0.759	0.0352047	2.4083947	-4.57	2.5470597	PTPRJ
234716_at	0.759	0.03522	2.4081479	-4.57	2.0940516	ZIC1
207800_at	0.759	0.0352419	2.4077928	-4.57	2.7520922	AKAP5
208439_s_at	0.759	0.0352826	2.4071359	-4.57	1.7530956	FCN2
1565576_at	0.759	0.035298	2.4068864	-4.57	1.9945488	
220437_at	0.759	0.0353329	2.4063244	-4.57	2.4907641	ANGPTL8
1562732_at	0.759	0.0353441	2.4061435	-4.57	2.0212676	TMEM232
1553909_x_at	0.759	0.0353506	2.4060392	-4.57	1.9867928	SLF2
1552903_at	0.759	0.0353692	2.4057386	-4.57	2.2973588	B4GALNT2
231325_at	0.759	0.0353794	2.4055751	-4.57	2.0109344	UNC5D
216611_s_at	0.759	0.03539	2.4054036	-4.57	2.8211218	SLC6A2
1561857_at	0.759	0.035402	2.4052109	-4.57	2.7538509	
205924_at	0.759	0.0354089	2.4050999	-4.57	1.9422463	RAB3B
229154_at	0.759	0.0354213	2.4049005	-4.57	2.6176408	WNT10A

228321_s_at	0.759	0.035431	2.404744	-4.57	2.2163649	NAA30
227103_s_at	0.759	0.035463	2.4042303	-4.57	2.1554589	ECE2
1570110_at	0.759	0.0354772	2.4040016	-4.57	2.4921641	LOC101928730
204727_at	0.759	0.0354779	2.40399	-4.57	2.0999527	WDHD1
230926_s_at	0.759	0.0354829	2.4039093	-4.57	1.7784997	ODF2L
238445_x_at	0.759	0.0354877	2.403833	-4.57	2.2266006	MGAT5B
229722_at	0.759	0.0354986	2.4036586	-4.57	2.1817193	LOC100270804
217355_at	0.759	0.0355147	2.4034001	-4.57	2.0675115	
234830_at	0.759	0.0355264	2.4032111	-4.57	2.1869883	FRG2EP
238379_x_at	0.759	0.0355332	2.4031036	-4.57	2.4285342	
236110_at	0.759	0.0355346	2.4030806	-4.57	1.9793675	ST8SIA5
207678_s_at	0.759	0.0355637	2.4026135	-4.57	2.455276	SOX30
1570065_at	0.759	0.035587	2.4022407	-4.57	2.0354087	C22orf34
224008_s_at	0.759	0.0356035	2.4019758	-4.57	2.430076	KCNK7
207663_x_at	0.759	0.0356063	2.4019323	-4.57	2.8132113	GAGE3
214012_at	0.759	0.0356067	2.4019256	-4.57	1.5919961	ERAP1

237481_at	0.759	0.0356152	2.4017896	-4.57	2.0801626	ACAD10
223453_s_at	0.759	0.0356385	2.401416	-4.57	1.900202	ATL3
217134_at	0.759	0.0356415	2.401368	-4.57	2.1492759	MTAP
1553472_at	0.759	0.0356437	2.4013339	-4.57	2.584361	LOC150596
224069_x_at	0.759	0.0356638	2.4010128	-4.57	2.0836922	P2RX2
230483_at	0.759	0.0357224	2.4000764	-4.57	2.2265004	
243221_at	0.759	0.0357235	2.4000581	-4.57	2.3434132	FAM20A
216177_at	0.759	0.035731	2.3999398	-4.57	2.1372888	
234614_at	0.759	0.0357332	2.3999046	-4.57	2.4517039	FLI1
211822_s_at	0.759	0.0357405	2.3997871	-4.57	1.9576355	NLRP1
244589_at	0.759	0.0357716	2.3992921	-4.57	1.9627895	LOC105378470
1554492_at	0.759	0.0357914	2.3989762	-4.57	3.4171204	THADA
1560756_at	0.759	0.035801	2.3988239	-4.57	2.3281751	
1564691_at	0.759	0.0358273	2.398406	-4.57	2.3635682	LL0XNC01-116E7.2
240540_at	0.759	0.0358469	2.3980935	-4.57	1.7791729	
212283_at	0.759	0.0358572	2.3979298	-4.57	2.2466956	AGRN
236440_at	0.759	0.0358732	2.3976753	-4.57	2.717071	NETO1
238227_at	0.759	0.0358747	2.3976518	-4.57	1.8949257	
240510_at	0.759	0.0358752	2.3976442	-4.57	1.7904986	NBN
226638_at	0.759	0.0358878	2.3974436	-4.57	1.5766524	ARHGAP23
237132_at	0.759	0.0358936	2.3973522	-4.57	2.4404805	TJP2
231479_at	0.759	0.0359166	2.3969863	-4.57	1.5542285	TTC33
222528_s_at	0.759	0.0359189	2.3969492	-4.57	2.6826332	SLC25A37

241013_at	0.759	0.035923	2.3968846	-4.57	2.4302142	FAM124A
229148_at	0.759	0.0359267	2.3968256	-4.57	2.2319605	HOTAIRM1///HOXA2
241147_at	0.759	0.0359513	2.3964356	-4.57	2.4676111	
1553973_a_at	0.759	0.0359737	2.3960806	-4.57	2.8162354	SPINK6
237905_at	0.759	0.0359978	2.3956994	-4.57	2.1336571	KRT25
216004_s_at	0.759	0.0360065	2.395561	-4.57	2.0061139	PKNOX1
AFFX-r2-Bs-lys-5_at	0.759	0.0360521	2.3948395	-4.57	2.5282647	
231997_at	0.759	0.036062	2.3946831	-4.57	1.9761252	TBCEL
243785_at	0.759	0.0360786	2.3944203	-4.57	2.2485932	LOC100272217
231812_x_at	0.759	0.0360979	2.3941156	-4.57	1.6259112	PHAX
244005_at	0.759	0.0361081	2.3939551	-4.57	3.043245	
241858_at	0.759	0.0361092	2.3939383	-4.57	1.6290944	TNNI3K
241113_at	0.759	0.0361455	2.3933642	-4.57	2.7514536	
207217_s_at	0.759	0.0361461	2.393356	-4.57	2.3989191	NOX1
1570044_at	0.759	0.0361487	2.3933142	-4.57	2.81975	GTF2IP7
206802_at	0.759	0.036159	2.3931517	-4.57	2.5849346	PAX5
221348_at	0.759	0.0361763	2.3928787	-4.57	2.6800428	NPPC
223621_at	0.759	0.0361834	2.3927667	-4.57	1.8083727	PNMA3
1562730_a_at	0.759	0.0361899	2.3926646	-4.57	2.669431	
243340_at	0.759	0.0362019	2.3924762	-4.57	2.4676606	
213335_s_at	0.759	0.036227	2.392081	-4.57	2.9085156	ST3GAL6

242723_at	0.759	0.0362308	2.3920212	-4.57	2.2906052	
206795_at	0.759	0.0362441	2.3918113	-4.57	2.150372	F2RL2
1569577_x_at	0.759	0.0362976	2.3909713	-4.57	2.3251105	LOC102724927
228901_at	0.759	0.0363108	2.3907637	-4.57	3.4903652	CDK9
220420_at	0.759	0.0363278	2.3904972	-4.57	1.7800003	LMAN1L
242971_at	0.759	0.0363324	2.3904247	-4.57	2.127088	
1556768_at	0.759	0.0363645	2.389921	-4.57	1.7372315	LINC00930
229485_x_at	0.759	0.0363964	2.3894207	-4.57	2.6230027	SHISA3
215880_at	0.759	0.0364149	2.3891305	-4.57	2.4661332	NAGLU
1555741_at	0.759	0.0364646	2.3883525	-4.57	2.1895535	MRAP
216314_at	0.759	0.0364824	2.3880755	-4.57	2.8916733	CRISP1
1557984_s_at	0.759	0.0365253	2.3874054	-4.57	2.5252712	RPAP3
1553685_s_at	0.759	0.0365342	2.387266	-4.57	1.9716767	SP1
233303_at	0.759	0.0365452	2.3870946	-4.57	2.6401187	
1561215_at	0.759	0.0365783	2.3865771	-4.57	2.743241	
1555025_at	0.759	0.0365885	2.386419	-4.57	2.5138755	TMEM26
1555197_a_at	0.759	0.0365964	2.3862955	-4.57	3.4492877	C21orf58
239446_x_at	0.759	0.0366775	2.385033	-4.57	2.0244694	DCBLD2
215014_at	0.759	0.0366813	2.3849735	-4.57	3.233512	KCND3
224253_at	0.759	0.0366874	2.3848787	-4.57	2.7405597	EXOC5

1560734_at	0.759	0.0366913	2.3848182	-4.57	2.8567381	LOC101927079///LOC727924///OR4N4
204795_at	0.759	0.0366953	2.3847571	-4.57	2.2635257	PRR3
1568286_at	0.759	0.0367167	2.3844245	-4.57	1.8610931	HMGA2
240667_at	0.759	0.036717	2.3844188	-4.57	2.287642	SERP2
235251_at	0.759	0.0367298	2.3842202	-4.57	1.9737386	
243227_at	0.759	0.03676	2.3837513	-4.57	2.8437788	
1567457_at	0.759	0.0367696	2.3836022	-4.57	2.2893367	RAC1
236187_s_at	0.759	0.0368074	2.3830173	-4.57	2.2944326	
1557012_a_at	0.759	0.0368271	2.3827108	-4.57	2.3588708	
1558177_at	0.759	0.0368302	2.3826641	-4.57	1.9308946	TMEM229B
206146_s_at	0.759	0.036843	2.3824651	-4.57	2.278757	RHAG
204455_at	0.759	0.0368831	2.3818444	-4.57	1.9324437	DST
207144_s_at	0.759	0.0368991	2.3815971	-4.57	2.9527157	CITED1
1558290_a_at	0.759	0.036906	2.3814904	-4.57	1.8455937	MIR1204///PVT1
1559678_s_at	0.759	0.0369174	2.3813147	-4.57	2.3699751	GS1-24F4.2
214625_s_at	0.759	0.0369304	2.381113	-4.57	2.3969633	MINK1
244547_at	0.759	0.0369662	2.3805616	-4.57	1.9312647	SGK494

244630_at	0.759	0.0369813	2.3803282	-4.57	2.1870758	LOC101060747///PDPK1
219849_at	0.759	0.0369949	2.3801187	-4.57	1.5859706	ZNF671
241464_s_at	0.759	0.0370365	2.3794765	-4.57	1.7959755	
231141_at	0.759	0.0370531	2.3792212	-4.57	2.0471691	LOC105370962
206280_at	0.759	0.0370552	2.3791888	-4.57	1.8255094	CDH18
221182_at	0.759	0.0370592	2.3791271	-4.57	2.3363734	MROH9
208500_x_at	0.759	0.0370724	2.3789247	-4.57	2.9064113	FOXD3
243323_s_at	0.759	0.0371035	2.3784455	-4.57	1.5600525	ZFHX3
216997_x_at	0.759	0.037105	2.3784236	-4.57	2.330168	TLE4
235869_at	0.759	0.0371372	2.3779285	-4.57	2.2107062	SCOC-AS1
243341_at	0.759	0.0371873	2.3771593	-4.57	2.5119537	
244520_at	0.759	0.0372044	2.3768959	-4.57	1.5833866	
234915_s_at	0.759	0.0372064	2.3768654	-4.57	1.8816808	DENR
229924_s_at	0.759	0.0372136	2.3767561	-4.57	2.6096681	JAG1
231624_s_at	0.759	0.0372156	2.3767245	-4.57	2.0906977	
237167_at	0.759	0.0372246	2.3765872	-4.57	1.7412313	KIAA1217
211832_s_at	0.759	0.0372352	2.3764245	-4.57	1.6086941	MDM2
229960_at	0.759	0.0372469	2.3762448	-4.57	2.1976582	MAP3K6
230689_at	0.759	0.0372948	2.3755116	-4.57	1.7337695	
1561850_at	0.759	0.0373291	2.3749873	-4.57	2.1742708	LOC100133669

1559579_at	0.759	0.0373298	2.3749762	-4.57	2.1101289	LINC00927
217053_x_at	0.759	0.03733	2.3749736	-4.57	1.8351021	ETV1
1560678_at	0.759	0.0373317	2.3749476	-4.57	2.2805169	
1569122_at	0.759	0.0373517	2.3746421	-4.57	3.2839328	LOC105370697
240980_at	0.759	0.0373657	2.3744277	-4.57	3.3647194	
231737_at	0.759	0.037367	2.3744087	-4.57	2.1432113	CACNG4
221546_at	0.759	0.0373765	2.3742625	-4.57	2.8616126	PRPF18
216038_x_at	0.759	0.0374132	2.3737023	-4.57	2.0828342	DAXX
205333_s_at	0.759	0.037452	2.3731109	-4.57	2.3535334	RCE1
1567856_x_at	0.759	0.0374607	2.3729792	-4.57	2.4821861	ZNF29P
1552960_at	0.759	0.0374849	2.3726109	-4.57	2.7369664	LRRC15
236306_at	0.759	0.0374879	2.3725649	-4.57	2.323096	KIAA2026
236203_at	0.759	0.0375099	2.37223	-4.57	3.1196876	LOC100509457///HLA-DQA1
219779_at	0.759	0.0375457	2.3716848	-4.57	2.0834729	ZFHX4
221340_at	0.759	0.03755	2.3716199	-4.57	2.8064274	CDX4
223925_s_at	0.759	0.0375514	2.3715982	-4.57	2.1268031	MTPN
239423_at	0.759	0.0375934	2.3709601	-4.57	1.7073527	
239055_at	0.759	0.0376245	2.3704894	-4.57	1.9881728	LINC-PINT
241496_at	0.759	0.0376463	2.3701576	-4.57	2.161957	BTNL9
1553697_at	0.759	0.0376488	2.3701201	-4.57	2.8225192	CCSAP

217268_at	0.759	0.037651	2.3700875	-4.57	3.3658617	RAB7A
1554440_at	0.759	0.0376966	2.3693961	-4.57	2.0873049	KIAA0513
202744_at	0.759	0.0377391	2.3687528	-4.57	2.1400536	SLC20A2
1566032_at	0.759	0.0377467	2.3686375	-4.57	2.1655043	
202234_s_at	0.759	0.0377596	2.3684423	-4.57	1.5384164	SLC16A1
213395_at	0.759	0.0377769	2.3681821	-4.57	2.0201413	MLC1
212682_s_at	0.759	0.0377866	2.3680355	-4.57	2.7338214	LMF2
209639_s_at	0.759	0.0377886	2.368005	-4.57	1.6933965	RGS12
235682_s_at	0.759	0.0377958	2.3678967	-4.57	1.874915	HSPB7
204188_s_at	0.759	0.0378063	2.3677377	-4.57	2.4872372	RARG
1564300_at	0.759	0.0378128	2.3676391	-4.57	1.8767999	IQCF5-AS1
241039_at	0.759	0.0378154	2.3676003	-4.57	2.2538886	
231619_at	0.759	0.0378383	2.3672551	-4.57	1.8723194	
234148_at	0.759	0.0378445	2.3671604	-4.57	1.8653388	
240391_at	0.759	0.0378526	2.367039	-4.57	2.1910006	NDUFB2
242244_at	0.759	0.0378582	2.3669541	-4.57	2.8951376	
214543_x_at	0.759	0.0378788	2.3666432	-4.57	2.2438338	QKI
218908_at	0.759	0.0378941	2.3664126	-4.57	2.4158246	ASPSCR1
1567859_at	0.759	0.0379	2.3663251	-4.57	2.8790752	
244164_at	0.759	0.0379231	2.3659762	-4.57	1.875398	FAM223B
1552321_a_at	0.759	0.0379357	2.3657874	-4.57	2.3696268	CCDC65
230094_at	0.759	0.0379757	2.3651845	-4.57	2.2967427	KLF13

1560935_s_at	0.759	0.0380157	2.3645846	-4.57	1.8919022	LOC284669
236374_at	0.759	0.0380263	2.3644253	-4.57	2.8519037	CTXN3
1564122_at	0.759	0.0380337	2.3643145	-4.57	2.042871	LINC00514
1552393_at	0.759	0.0380537	2.3640145	-4.57	2.4771281	ENTHD1
207359_at	0.759	0.0380637	2.3638643	-4.57	2.8910718	CAMKK2
1555772_a_at	0.759	0.0380647	2.3638482	-4.57	2.4918979	CDC25A
241011_at	0.759	0.0380771	2.3636632	-4.57	1.8112548	
220965_s_at	0.759	0.0380981	2.3633479	-4.57	2.1525574	RSPH6A
1563704_at	0.759	0.0381035	2.3632664	-4.57	2.3980934	
234725_s_at	0.759	0.0381042	2.3632569	-4.57	1.868628	SEMA4B
216788_at	0.759	0.0381154	2.3630892	-4.57	1.8090712	ZNF443
240625_at	0.759	0.038132	2.3628404	-4.57	2.2035321	
221091_at	0.759	0.0381699	2.3622733	-4.57	2.3259242	INSL5
208173_at	0.759	0.0381797	2.3621259	-4.57	2.3541832	IFNB1
240338_at	0.759	0.0381838	2.3620645	-4.57	2.0189598	
220862_s_at	0.759	0.0382145	2.3616057	-4.57	1.6993201	
216270_at	0.759	0.0382485	2.3610986	-4.57	2.8071858	ILVBL
1569289_at	0.759	0.0382535	2.3610242	-4.57	3.0469671	BIVM
224153_s_at	0.759	0.0382547	2.3610051	-4.57	1.7761353	DHRS4-AS1
203862_s_at	0.759	0.0382796	2.3606338	-4.57	2.7133454	ACTN2
1570383_at	0.759	0.0383103	2.3601759	-4.57	2.5329008	BMP1
242038_at	0.759	0.038314	2.3601216	-4.57	2.2760889	LRRC8B

231309_at	0.759	0.0383186	2.3600521	-4.57	1.734362	
1552933_at	0.759	0.0383247	2.3599623	-4.57	3.2439597	AKNAD1
220142_at	0.759	0.0383451	2.3596582	-4.57	1.6041138	HAPLN2
210918_at	0.759	0.038354	2.3595254	-4.57	3.0078922	
206320_s_at	0.759	0.0383599	2.3594378	-4.57	2.2180986	SMAD9
237224_at	0.759	0.0383663	2.3593422	-4.57	2.3643726	LINC00853
1569188_s_at	0.759	0.0383908	2.3589779	-4.57	1.9149608	
1553023_a_at	0.759	0.0384166	2.3585939	-4.57	2.1410073	NOX5
232993_at	0.759	0.0384173	2.3585831	-4.57	3.3926058	SYNJ1
231184_at	0.759	0.0384309	2.3583808	-4.57	1.8581022	DHX8
241231_at	0.759	0.0384529	2.3580551	-4.57	1.8436498	
208332_at	0.759	0.0384934	2.3574534	-4.57	1.8117097	LOC100509646///PRY2///PRY
243565_at	0.759	0.0385052	2.3572783	-4.57	2.6428067	CCDC150
241855_s_at	0.759	0.0385128	2.3571654	-4.57	1.6687389	
236625_at	0.759	0.0385187	2.3570784	-4.57	2.3033676	
243035_at	0.759	0.0385399	2.3567635	-4.57	2.5708076	LOC101927038
236085_at	0.759	0.0385623	2.3564325	-4.57	2.556852	CAPSL
1555277_a_at	0.759	0.0385684	2.356342	-4.57	1.9380717	SLC4A5
214717_at	0.759	0.038583	2.3561259	-4.57	2.0763979	PKI55
244329_at	0.759	0.038586	2.3560809	-4.57	2.3560236	
238275_at	0.759	0.0386169	2.3556237	-4.57	2.7820135	HAP1

221595_at	0.759	0.0386489	2.3551501	-4.57	2.0951271	RBM48
240917_at	0.759	0.0386634	2.3549365	-4.57	2.2851641	LOC101927640
243165_at	0.759	0.0386661	2.3548971	-4.57	3.0301157	BTF3
243730_at	0.759	0.0386662	2.3548951	-4.57	3.0336231	
234375_x_at	0.759	0.0386816	2.354668	-4.57	2.5124908	
240691_at	0.759	0.03869	2.3545439	-4.57	2.9691895	
233005_at	0.759	0.0386962	2.3544524	-4.57	1.9175821	ZNHIT6
1553727_at	0.759	0.038722	2.354072	-4.57	2.1134937	B4GALNT3
209832_s_at	0.759	0.0387274	2.3539914	-4.57	2.4581574	CDT1
1553006_at	0.759	0.0387445	2.35374	-4.57	2.5973246	ADGRG4
234220_at	0.759	0.0387531	2.3536129	-4.57	1.8508818	CADPS
217294_s_at	0.759	0.0387539	2.3536005	-4.57	1.6219719	ENO1
232393_at	0.759	0.0387919	2.3530407	-4.57	3.0011261	ZNF462
206370_at	0.759	0.0388235	2.3525762	-4.57	2.3823973	PIK3CG
1561360_at	0.759	0.0388271	2.3525233	-4.57	1.8268605	LOC101928661
216564_at	0.759	0.0388382	2.3523596	-4.57	1.7574728	
206679_at	0.759	0.0388414	2.3523128	-4.57	1.7331666	APBA1
223537_s_at	0.759	0.0388699	2.351894	-4.57	2.0801162	WNT5B
207415_at	0.759	0.0388781	2.3517724	-4.57	1.8134576	PLA2R1
236103_at	0.759	0.0388951	2.3515236	-4.57	1.4287838	

1565657_at	0.759	0.0389196	2.351164	-4.57	2.1851288	CMTM6
216197_at	0.759	0.0389267	2.3510586	-4.57	2.6060051	
211314_at	0.759	0.0389315	2.3509895	-4.57	1.9613723	CACNA1G
242182_x_at	0.759	0.0389462	2.3507736	-4.57	2.4361517	
214270_s_at	0.759	0.0389605	2.3505636	-4.57	2.0678828	MAPRE3
240185_at	0.759	0.0389728	2.3503825	-4.57	1.9055326	LOC100147773
1554534_at	0.759	0.0390715	2.348938	-4.57	2.0782241	DPYD
203919_at	0.759	0.0390974	2.3485592	-4.57	1.5881931	TCEA2
244258_at	0.759	0.0391143	2.3483127	-4.57	3.4874367	
205889_s_at	0.759	0.0391441	2.3478765	-4.57	2.3549387	JAKMIP2
229299_at	0.759	0.0391624	2.3476106	-4.57	1.8264786	NADK2
1553711_a_at	0.759	0.0391659	2.347559	-4.57	2.3348138	FAM218A
231731_at	0.759	0.039173	2.3474551	-4.57	1.923298	OTX2
223643_at	0.759	0.0391953	2.3471296	-4.57	2.2454891	CRYGS
208242_at	0.759	0.0392026	2.3470243	-4.57	1.7243015	RAX
227310_at	0.759	0.0392079	2.3469461	-4.57	2.6785709	ADSS
241565_at	0.759	0.0392427	2.3464396	-4.57	2.3417185	COL4A4
1565612_at	0.759	0.0392633	2.346139	-4.57	1.8794057	DYNLRB1
1568488_at	0.759	0.0392989	2.345622	-4.57	2.4668548	
215455_at	0.759	0.0393079	2.3454901	-4.57	1.7088033	TIMELESS

241304_at	0.759	0.0393091	2.3454738	-4.57	2.5344114	PIK3C3
1555560_at	0.759	0.0393174	2.3453531	-4.57	3.9196977	UGGT2
1561889_at	0.759	0.03933	2.3451698	-4.57	3.1679766	LOC105375527
242867_x_at	0.759	0.0393358	2.3450851	-4.57	2.2214397	LOC105372179
207322_at	0.759	0.0393382	2.3450501	-4.57	2.2595191	ITSN1
1568638_a_at	0.759	0.0393519	2.3448516	-4.57	2.5438047	IDO2
202523_s_at	0.759	0.0394383	2.3435981	-4.57	2.1301011	SPOCK2
1553810_a_at	0.759	0.039497	2.3427473	-4.57	2.0162091	KIAA1524
214348_at	0.759	0.0395651	2.3417635	-4.57	2.7581143	TACR2
241121_at	0.759	0.0395723	2.3416596	-4.57	1.7680536	
233119_at	0.759	0.0395841	2.341488	-4.57	1.8378421	PRR5L
214645_at	0.759	0.0396763	2.3401588	-4.57	1.9155329	
236433_at	0.759	0.0396812	2.3400883	-4.57	2.6126522	ALOX12-AS1
203699_s_at	0.759	0.0396838	2.3400501	-4.57	3.0023635	DIO2
214708_at	0.759	0.0396854	2.3400281	-4.57	1.7357129	SNTB1
235662_at	0.759	0.0397097	2.3396772	-4.57	1.7016138	
243212_at	0.759	0.0397106	2.339664	-4.57	1.7462602	
217782_s_at	0.759	0.0397163	2.3395832	-4.57	1.6923679	GPS1
1566339_at	0.759	0.0397164	2.3395811	-4.57	3.1476569	SNORD8
228460_at	0.759	0.0397292	2.3393977	-4.57	1.5461038	ZNF319

1559069_at	0.759	0.0397349	2.3393149	-4.57	1.7250674	ST20-MTHFS///MTHFS
243146_at	0.759	0.0397421	2.339211	-4.57	3.3099763	
215521_at	0.759	0.0397838	2.3386125	-4.57	1.9902958	PHC3
230153_at	0.759	0.0398001	2.3383776	-4.57	2.037924	NEK9
1566145_s_at	0.759	0.0398257	2.3380102	-4.57	2.0683599	LOC101930100///LOC101928669///LOC644450
230568_x_at	0.759	0.0398438	2.3377502	-4.57	3.3454559	DLL3
211305_x_at	0.759	0.0398442	2.3377448	-4.57	2.3775199	FCAR
1565525_a_at	0.759	0.0398454	2.3377266	-4.57	2.7206237	TCP11L2
210327_s_at	0.759	0.0398538	2.3376063	-4.57	2.0967564	AGXT
211399_at	0.759	0.0398695	2.3373819	-4.57	1.4778179	FGFR2
211178_s_at	0.759	0.0399099	2.3368026	-4.57	1.5919564	PSTPIP1
212091_s_at	0.759	0.0399301	2.3365133	-4.57	2.1910509	COL6A1
206778_at	0.759	0.0399325	2.3364794	-4.57	2.2468594	CRYBB2
207531_at	0.759	0.03996	2.336085	-4.57	1.8324793	CRYGC
207096_at	0.759	0.0399612	2.3360676	-4.57	1.744546	SAA2-SAA4///SAA4
244460_at	0.759	0.0399627	2.3360465	-4.57	2.1931094	TMEM225
1553399_a_at	0.759	0.0399758	2.3358595	-4.57	1.5616763	MGC57346///CRHR1-IT1

1558052_at	0.759	0.0400241	2.3351692	-4.57	1.9885461	TMED4
211459_at	0.759	0.0400346	2.3350193	-4.57	2.484463	
208241_at	0.759	0.0400741	2.3344545	-4.57	2.7321639	NRG1
230700_at	0.759	0.0400751	2.3344405	-4.57	1.80232	RTN4RL1
240096_at	0.759	0.0400783	2.3343945	-4.57	2.3933489	LOC100506236
201971_s_at	0.759	0.0401379	2.3335449	-4.57	2.0064433	ATP6V1A
1561144_at	0.759	0.0401429	2.3334732	-4.57	1.6795605	
207022_s_at	0.759	0.0401498	2.3333757	-4.57	1.8967067	LDHC
1567853_at	0.759	0.0401509	2.3333593	-4.57	2.7996688	ZNF28
204166_at	0.759	0.0401545	2.333308	-4.57	2.6420788	SBNO2
224425_x_at	0.759	0.0401709	2.3330748	-4.57	2.0314404	ACTR3BP2
234852_at	0.759	0.0402187	2.3323953	-4.57	2.8590963	
1561171_a_at	0.759	0.040237	2.3321349	-4.57	1.5896514	GOLGA6L6///GOLGA6L22///GOLGA6L1
208375_at	0.759	0.040247	2.3319929	-4.57	2.9684476	IFNA1
216842_x_at	0.759	0.0402625	2.3317726	-4.57	2.2499506	
1570405_at	0.759	0.040273	2.3316237	-4.57	2.0080037	LOC101929076
233549_at	0.759	0.0402797	2.3315284	-4.57	2.6426414	PDE1A
230555_s_at	0.759	0.0403212	2.3309387	-4.57	1.8022533	MED30
217248_s_at	0.759	0.0403575	2.3304238	-4.57	1.686545	SLC7A8
242833_at	0.759	0.0403905	2.3299565	-4.57	2.3926271	
236661_at	0.759	0.0404416	2.3292338	-4.57	2.081776	IQCF6

219651_at	0.759	0.0404605	2.3289668	-4.57	3.0948946	DPPA4
222695_s_at	0.759	0.0404751	2.3287604	-4.57	2.0821307	AXIN2
220167_s_at	0.759	0.0404923	2.3285174	-4.57	2.5970713	TP53TG3B///TP53TG3D///TP53TG3C///TP53TG3
224048_at	0.759	0.0405188	2.3281422	-4.57	2.4115353	USP44
232590_at	0.759	0.0405286	2.3280037	-4.57	1.7459772	
207634_at	0.759	0.0405383	2.3278682	-4.58	2.2047807	PDCD1
1554288_at	0.759	0.0405397	2.3278484	-4.58	2.0455664	FAM160B1
1560405_at	0.759	0.0405505	2.3276949	-4.58	2.5894942	LOC101928632
242490_at	0.759	0.0405522	2.3276713	-4.58	2.1853465	
1569787_at	0.759	0.0405898	2.3271411	-4.58	2.4476492	RFTN1
210736_x_at	0.759	0.0406032	2.3269521	-4.58	2.7750019	DTNA
1563252_at	0.759	0.0406083	2.3268809	-4.58	1.8632665	ERBB3
205602_x_at	0.759	0.0406255	2.3266379	-4.58	1.917106	PSG7
205092_x_at	0.759	0.0406347	2.3265087	-4.58	1.8380933	ZBTB1
233292_s_at	0.759	0.0406466	2.3263414	-4.58	2.5534719	ANKHD1-EIF4EBP3///ANKHD1
235928_at	0.759	0.0406511	2.3262785	-4.58	2.2442613	ZNF503-AS2
1557727_at	0.759	0.0406573	2.3261902	-4.58	1.8474383	PCBP1-AS1
1560148_at	0.759	0.0406687	2.3260297	-4.58	2.4344918	MIA3

220750_s_at	0.759	0.0406859	2.3257878	-4.58	1.5499216	P3H1
216683_at	0.759	0.0406999	2.3255911	-4.58	1.9682742	
1570185_at	0.759	0.0407111	2.3254341	-4.58	1.6377673	LOC105378568
237882_at	0.759	0.0407154	2.3253734	-4.58	2.0759876	
217401_at	0.759	0.040764	2.324691	-4.58	1.9935126	
1561261_at	0.759	0.0407726	2.3245707	-4.58	2.4778202	
1559141_s_at	0.759	0.0408	2.3241867	-4.58	3.0491364	FAM87B///FAM87A
1557079_at	0.759	0.0408117	2.3240213	-4.58	2.2185666	ITGBL1
1554857_at	0.759	0.0408244	2.3238439	-4.58	1.7714077	ELMO2
231384_at	0.759	0.04084	2.3236246	-4.58	2.3613929	GRIN2A
236207_at	0.759	0.040851	2.3234717	-4.58	2.4787682	SSFA2
227664_at	0.759	0.0408524	2.3234509	-4.58	1.6707826	FLJ37453
210578_at	0.759	0.0408564	2.3233952	-4.58	2.2313878	TRIM10
241653_x_at	0.759	0.0408957	2.3228452	-4.58	1.8662827	
213481_at	0.759	0.0409372	2.3222644	-4.58	2.3918168	S100A13
1569736_at	0.759	0.0409429	2.3221859	-4.58	2.1977128	EYA1
226113_at	0.759	0.0409492	2.322097	-4.58	1.4084178	ZNF436
234109_x_at	0.759	0.04096	2.3219464	-4.58	1.9000936	ONECUT3
207147_at	0.759	0.0409679	2.3218356	-4.58	2.6435451	DLX2
205038_at	0.759	0.0409881	2.3215535	-4.58	1.7099194	IKZF1

244634_at	0.759	0.0410031	2.3213439	-4.58	1.8728244	
236120_at	0.759	0.0410458	2.3207493	-4.58	1.93388	
1570126_at	0.759	0.0410587	2.3205684	-4.58	2.0030489	
1556504_at	0.759	0.0411361	2.319491	-4.58	2.0716992	
217368_at	0.759	0.0411571	2.3191997	-4.58	1.6709077	ATP5G2P1
231803_at	0.759	0.0411582	2.3191832	-4.58	1.8397192	FGF11
205365_at	0.759	0.0411736	2.3189703	-4.58	2.3168822	HOXB6
1564053_a_at	0.759	0.0411997	2.3186067	-4.58	2.1427487	YTHDF3
240215_at	0.759	0.0412012	2.3185867	-4.58	3.4855516	
1569254_s_at	0.759	0.0412238	2.318272	-4.58	1.9625811	INTS4
234957_at	0.759	0.0412388	2.3180644	-4.58	2.6320291	
240129_at	0.759	0.0412412	2.3180313	-4.58	2.3478604	
218812_s_at	0.759	0.0412444	2.3179867	-4.58	1.9054603	ORAI2
1563253_s_at	0.759	0.0412511	2.3178927	-4.58	1.9339824	ERBB3
1561192_at	0.759	0.0412512	2.3178923	-4.58	2.4588044	
1562573_at	0.759	0.0412662	2.3176837	-4.58	2.8321657	CYP17A1
244107_at	0.759	0.0413149	2.3170093	-4.58	3.0845808	DSC3
233639_at	0.759	0.0413213	2.31692	-4.58	2.6247744	POMGNT1
1558390_at	0.759	0.0413574	2.3164195	-4.58	2.4989658	ZNF599

1556381_at	0.759	0.0413621	2.3163549	-4.58	1.5202227	NAA15
210274_at	0.759	0.0413809	2.3160949	-4.58	1.775553	MAGEA8
1566844_at	0.759	0.0413865	2.3160176	-4.58	1.8451863	PER4
234500_at	0.759	0.0413997	2.3158343	-4.58	1.9282103	
1557775_a_at	0.759	0.0414126	2.3156568	-4.58	2.1358359	
222509_s_at	0.759	0.0414216	2.3155318	-4.58	3.7247136	ZNF672
207591_s_at	0.759	0.041452	2.3151126	-4.58	2.4383659	ARID1A
237395_at	0.759	0.0414638	2.3149498	-4.58	1.9487189	CYP4Z1
233945_at	0.759	0.041471	2.3148499	-4.58	3.1626272	UGGT2
244163_at	0.759	0.0415375	2.3139327	-4.58	1.7186309	SEMA3A
238922_at	0.759	0.0415426	2.3138619	-4.58	2.5719552	
230732_s_at	0.759	0.0415461	2.313814	-4.58	2.5557558	MAPK4
243105_at	0.759	0.0415482	2.3137847	-4.58	2.5392059	
1553460_at	0.759	0.0415671	2.3135241	-4.58	1.9404789	C2orf61
228008_at	0.759	0.0416371	2.3125615	-4.58	1.6840086	ARID3A
1558603_at	0.759	0.04165	2.3123835	-4.58	2.3441349	PLGLB2
235387_at	0.759	0.041686	2.3118898	-4.58	1.8216718	GSTCD
1560347_at	0.759	0.0417266	2.3113314	-4.58	2.1898597	
228691_at	0.759	0.0417406	2.31114	-4.58	2.2049028	
243605_at	0.759	0.0417413	2.3111295	-4.58	3.3844563	
1560912_at	0.759	0.0417569	2.3109158	-4.58	2.8615272	LINC01120
1570068_at	0.759	0.0417585	2.3108938	-4.58	1.7690408	

1567527_at	0.759	0.0417639	2.3108206	-4.58	2.7477093	
1568754_at	0.759	0.041768	2.3107636	-4.58	1.9918102	LOC101927814
204825_at	0.759	0.0417904	2.3104564	-4.58	1.9582747	MELK
238921_at	0.759	0.0418032	2.3102815	-4.58	1.7998295	LOC644794
219082_at	0.759	0.041843	2.3097367	-4.58	2.33841	CEMP1///AMDHD2
209516_at	0.759	0.0418558	2.3095615	-4.58	1.7750995	SMYD5
237316_at	0.759	0.0418591	2.3095157	-4.58	2.0071557	ANKDD1A
1560994_x_at	0.759	0.0418698	2.3093697	-4.58	2.4715316	LOC400590
207424_at	0.759	0.0418816	2.309209	-4.58	2.0329265	MYF5
223324_s_at	0.759	0.0418965	2.3090053	-4.58	1.7509535	TRPM7
240930_at	0.759	0.0419169	2.3087257	-4.58	2.2243791	CRAMP1
244465_at	0.759	0.0419313	2.3085301	-4.58	2.4410376	
220833_at	0.759	0.0419315	2.3085269	-4.58	2.378343	
205637_s_at	0.759	0.0419647	2.3080731	-4.58	1.9650661	SH3GL3
209930_s_at	0.759	0.0419904	2.3077222	-4.58	2.5367802	NFE2
229719_s_at	0.759	0.0419914	2.307709	-4.58	2.927985	DERL3
233092_s_at	0.759	0.0420031	2.3075496	-4.58	2.5314785	
207403_at	0.759	0.0420496	2.3069156	-4.58	1.8511319	IRS4
1562910_at	0.759	0.0420762	2.3065539	-4.58	2.0614928	SH3PXD2B
1560300_a_at	0.759	0.0421258	2.3058794	-4.58	2.1812687	DMRTC1B///DMRTC1

216412_x_at	0.759	0.0421556	2.3054742	-4.58	2.1513259	CKAP2///IGLC1
234577_at	0.759	0.0421626	2.3053783	-4.58	1.4496287	
242781_at	0.759	0.0421678	2.3053086	-4.58	2.763518	FAM199X
237902_at	0.759	0.0421692	2.3052889	-4.58	3.650215	
AFFX-r2-Bs-phe-3_at	0.759	0.0421893	2.3050163	-4.58	2.6695039	
205666_at	0.759	0.0422023	2.3048392	-4.58	2.3584891	FMO1
238531_x_at	0.759	0.0422056	2.3047951	-4.58	1.920916	
241853_at	0.759	0.0422148	2.3046697	-4.58	2.9201508	
1553666_at	0.759	0.0422151	2.3046658	-4.58	2.1753574	CCDC34
1560348_at	0.759	0.0422158	2.3046568	-4.58	1.72044	ARHGEF28
226608_at	0.759	0.0422257	2.3045219	-4.58	2.0024825	C16orf87
1561556_at	0.759	0.0422524	2.3041605	-4.58	1.7213725	LOC101929648
234924_s_at	0.759	0.0422649	2.3039906	-4.58	1.9465398	ZNF687
1556643_at	0.759	0.0423023	2.3034845	-4.58	1.7522317	
1565661_x_at	0.759	0.0423037	2.3034646	-4.58	2.4291492	FUT6
214115_at	0.759	0.0423579	2.302732	-4.58	2.9102074	VAMP5
240925_at	0.759	0.0423692	2.3025783	-4.58	1.7685364	
227389_x_at	0.759	0.0423729	2.3025282	-4.58	2.3756218	IRF2BP2
234076_at	0.759	0.0423738	2.3025159	-4.58	2.5868581	
216252_x_at	0.759	0.0424338	2.3017056	-4.58	2.1520377	FAS
1553211_at	0.759	0.0424493	2.3014968	-4.58	2.6037451	ANKFN1

1552948_at	0.759	0.0424749	2.3011509	-4.58	1.852288	CCDC27
215665_at	0.759	0.0424876	2.3009791	-4.58	1.7149125	
207928_s_at	0.759	0.0424909	2.3009357	-4.58	2.5087521	GLRA3
1562281_at	0.759	0.0424934	2.3009014	-4.58	1.8566536	
236844_at	0.759	0.042494	2.3008933	-4.58	2.9298628	DHX30
1561770_at	0.759	0.0425115	2.3006577	-4.58	2.4230481	
242149_at	0.759	0.0425479	2.3001665	-4.58	1.6054248	FAM210A
224320_s_at	0.759	0.0426046	2.2994044	-4.58	1.9311333	MCM8
232045_at	0.759	0.0426179	2.2992257	-4.58	2.0048671	PHACTR1
218850_s_at	0.759	0.0426307	2.2990534	-4.58	2.2662686	LIMD1
241744_x_at	0.759	0.0426914	2.2982376	-4.58	2.7814228	
240876_x_at	0.759	0.0427019	2.2980971	-4.58	2.5556093	TERB2
241171_at	0.759	0.0427443	2.2975285	-4.58	2.3672279	
1566814_at	0.759	0.0427737	2.2971344	-4.58	2.7035604	FGF22
234754_at	0.759	0.0427824	2.2970168	-4.58	1.9606636	SLC37A1
1555441_at	0.759	0.0427951	2.2968472	-4.58	3.2344044	UBA6
1569604_at	0.759	0.0428175	2.2965469	-4.58	1.9360955	LINC01134
1564323_at	0.759	0.0428267	2.2964247	-4.58	2.2381854	LOC101928405
217109_at	0.759	0.0428577	2.2960092	-4.58	1.8089859	MUC4
215414_at	0.759	0.0428957	2.2955017	-4.58	2.4685002	FARS2
1563149_at	0.759	0.042898	2.2954711	-4.58	2.4825634	
221679_s_at	0.759	0.0429456	2.2948352	-4.58	2.2110973	ABHD6

1560262_at	0.759	0.0429581	2.2946689	-4.58	2.2401325	
221107_at	0.759	0.0429658	2.2945655	-4.58	2.4810269	CHRNA9
236536_at	0.759	0.0429815	2.2943557	-4.58	1.4737139	GALNT13
237967_at	0.759	0.0429882	2.2942666	-4.58	2.1660343	HAL
213072_at	0.759	0.0430355	2.2936362	-4.58	2.1834962	CYHR1
211876_x_at	0.759	0.0430383	2.2935997	-4.58	2.4047563	PCDHGA3///PCDHGA5///PCDHGA6///PCDHGA10///PCDHGA11///PCDH[illegible]
211582_x_at	0.759	0.0430596	2.2933155	-4.58	2.3231706	LST1
209250_at	0.759	0.0430714	2.2931587	-4.58	1.5223757	DEGS1
215814_at	0.759	0.0430721	2.2931495	-4.58	2.1654995	DST
1555133_at	0.759	0.0430904	2.2929061	-4.58	2.7120519	FAM9A
1561289_at	0.759	0.0430913	2.2928941	-4.58	3.7085396	MIR4290HG
219148_at	0.759	0.0431055	2.2927045	-4.58	1.9365845	PBK
207478_at	0.759	0.0431312	2.292363	-4.58	3.0109532	PRO2958
232295_at	0.759	0.0431708	2.2918377	-4.58	2.7100557	GFM1
1560646_at	0.759	0.0431796	2.2917207	-4.58	1.9822281	METTL21EP
207899_at	0.759	0.0432117	2.2912944	-4.58	2.4005781	GIP
1555842_at	0.759	0.0432166	2.2912291	-4.58	1.7876451	CYTH2
1553620_at	0.759	0.0432366	2.2909642	-4.58	2.0548792	TRIM42

215717_s_at	0.759	0.0432473	2.2908216	-4.58	2.639896	FBN2
1557755_at	0.759	0.0432722	2.2904924	-4.58	2.0735439	CEP128
235047_x_at	0.759	0.0432793	2.2903978	-4.58	1.824152	NACC1
241687_at	0.759	0.0432902	2.2902534	-4.58	1.5576541	WDR12
219836_at	0.759	0.0432913	2.2902397	-4.58	2.2870973	ZBED2
208568_at	0.759	0.0433661	2.28925	-4.58	2.0926995	MC2R
240244_at	0.759	0.0433805	2.2890589	-4.58	2.2183185	
237582_at	0.759	0.0434083	2.2886914	-4.58	1.5599989	
242084_at	0.759	0.0434293	2.2884144	-4.58	1.9391886	THEG5
1566873_at	0.759	0.043465	2.2879428	-4.58	1.6375768	
1557341_x_at	0.759	0.0434666	2.2879218	-4.58	1.8364409	PIK3CD-AS2
1570361_a_at	0.759	0.0434863	2.2876626	-4.58	2.4085717	
220102_at	0.759	0.0434952	2.2875445	-4.58	1.9680312	FOXL2
213837_at	0.759	0.0434954	2.2875423	-4.58	1.9971701	L3MBTL1
244394_at	0.759	0.0435035	2.2874354	-4.58	1.9619589	
1560836_at	0.759	0.0435486	2.2868411	-4.58	2.5072939	LINC01241
237853_x_at	0.759	0.0435609	2.2866794	-4.58	2.1301396	KRTAP10-12///KRTAP10-7
241361_at	0.759	0.0435632	2.2866489	-4.58	1.7259062	
241556_at	0.759	0.0435815	2.2864088	-4.58	2.1095927	
243018_at	0.759	0.0436019	2.2861395	-4.58	2.3896738	BBOX1-AS1
1570189_at	0.759	0.0436067	2.2860761	-4.58	1.9090975	LINC00671

213831_at	0.759	0.0436097	2.2860368	-4.58	1.7449753	LOC100509457///HLA-DQA1
209829_at	0.759	0.0436217	2.2858796	-4.58	2.0997859	FAM65B
216327_s_at	0.759	0.0436353	2.2857008	-4.58	2.4491524	SIGLEC8
1561085_at	0.759	0.0437109	2.2847077	-4.58	2.6104141	LOC153910
238855_at	0.759	0.0437164	2.2846361	-4.58	2.3694667	AHNAK
217481_x_at	0.759	0.04374	2.2843264	-4.58	1.9529735	SHISA6
1554451_s_at	0.759	0.0437519	2.2841705	-4.58	1.8175143	DNAJC14
239910_at	0.759	0.0437707	2.2839239	-4.58	2.0087542	PSG6
1561305_at	0.759	0.0437757	2.2838578	-4.58	1.6516364	LOC101927849
237841_at	0.759	0.0437903	2.2836677	-4.58	3.7657475	
241836_x_at	0.759	0.0437945	2.2836126	-4.58	2.012944	CEP97
220721_at	0.759	0.043798	2.2835664	-4.58	1.9783837	ZNF614
1567253_at	0.759	0.0438363	2.2830653	-4.58	1.98699	OR10D3
243430_at	0.759	0.0438431	2.2829759	-4.58	2.0365625	SEZ6
217207_s_at	0.759	0.0438781	2.2825179	-4.58	1.9709962	BTNL3
217332_at	0.759	0.0438786	2.2825114	-4.58	1.705663	CTAGE11P
219075_at	0.759	0.0439018	2.2822085	-4.58	2.1032622	YIPF2
1557021_s_at	0.759	0.043922	2.2819439	-4.58	2.3074588	LOC100507250
1555189_a_at	0.759	0.0439698	2.2813208	-4.58	2.6848515	TAT

233845_at	0.759	0.0439758	2.2812428	-4.58	2.941396	
202311_s_at	0.759	0.044045	2.2803408	-4.58	2.7769131	COL1A1
1561307_at	0.759	0.0440583	2.2801665	-4.58	2.3110343	LOC105372550
1555571_at	0.759	0.0440771	2.2799224	-4.58	2.1718905	IMMP2L
234892_at	0.759	0.0440805	2.2798776	-4.58	1.9314606	
1567247_at	0.759	0.0441098	2.2794973	-4.58	2.3806085	OR5H1
1564072_at	0.759	0.0441131	2.2794539	-4.58	2.9921684	MYH16
241012_at	0.759	0.044125	2.2792993	-4.58	2.0073371	
222485_at	0.759	0.0441564	2.2788906	-4.58	1.6431135	FKBP3
1554614_a_at	0.759	0.0441766	2.2786288	-4.58	2.2551041	PTBP2
236386_at	0.759	0.0442425	2.2777737	-4.58	2.0199014	SUZ12P1
212193_s_at	0.759	0.0442623	2.2775164	-4.58	2.1890165	LARP1
242278_at	0.759	0.0442643	2.277491	-4.58	1.8670685	LOC101930097
1556903_at	0.759	0.044282	2.2772614	-4.58	2.6304841	LOC101928335
244271_at	0.759	0.0442908	2.2771472	-4.58	1.8971083	
1566664_at	0.759	0.0443276	2.2766709	-4.58	2.111791	CCDC168
1554071_at	0.759	0.0443411	2.2764964	-4.58	3.3644858	DEUP1
1566721_at	0.759	0.0443888	2.2758794	-4.58	1.6911122	SVEP1

232289_at	0.759	0.0444045	2.275676	-4.58	1.7189127	LOC100996843///KCNJ18///KCNJ12
243642_x_at	0.759	0.044438	2.2752434	-4.58	1.9632632	
1552408_at	0.759	0.0444863	2.27462	-4.58	2.2131717	ODF4
1569518_at	0.759	0.0444882	2.2745958	-4.58	2.1624742	
209905_at	0.759	0.0445039	2.2743923	-4.58	3.7660301	HOXA10-HOXA9///MIR196B///HOXA9
1555990_at	0.759	0.0445536	2.2737516	-4.58	2.6390178	C22orf42
47560_at	0.759	0.0445756	2.2734685	-4.58	1.6876728	ADGRL1
227774_s_at	0.759	0.0445975	2.2731871	-4.58	3.452708	IMPAD1
1553226_at	0.759	0.044608	2.2730516	-4.58	2.0815259	LINC00052
243948_at	0.759	0.044638	2.272666	-4.58	1.8588704	ZC3H14
200894_s_at	0.759	0.0446393	2.2726491	-4.58	1.6926204	FKBP4
240509_s_at	0.759	0.0446517	2.2724892	-4.58	3.3033081	GREM2
AFFX-r2-Bs-phe-M_at	0.759	0.0446667	2.2722964	-4.58	3.0723841	
1559993_at	0.759	0.0446846	2.2720675	-4.58	2.313851	SFXN3
237451_x_at	0.759	0.0446985	2.2718886	-4.58	1.8852076	
239009_at	0.759	0.0446997	2.271873	-4.58	2.089391	KIAA0754
203686_at	0.759	0.0446998	2.2718722	-4.58	1.7287148	MPG
1564701_at	0.759	0.0447023	2.2718394	-4.58	2.0331036	

216238_s_at	0.759	0.0447263	2.2715315	-4.58	2.6642771	FGB
1554686_at	0.759	0.0447503	2.2712235	-4.58	2.1270062	STAU2
1555868_at	0.759	0.0447511	2.2712133	-4.58	1.8551207	LOC100507477
238667_at	0.759	0.0447591	2.2711113	-4.58	2.3824278	
241647_x_at	0.759	0.0447646	2.2710405	-4.58	2.4346965	
217396_at	0.759	0.0447719	2.2709466	-4.58	2.2518734	
1560485_at	0.759	0.0447731	2.2709319	-4.58	1.9171115	HIVEP1
211248_s_at	0.759	0.0447918	2.2706911	-4.58	2.2955902	CHRD
231305_at	0.759	0.0448417	2.2700524	-4.58	1.825658	LOC100507520
224193_s_at	0.759	0.0448562	2.2698669	-4.58	1.6624901	FCRL2
227165_at	0.759	0.044893	2.2693955	-4.58	1.9758762	SKA3
1565601_at	0.759	0.0449093	2.2691874	-4.58	2.4497457	
231554_at	0.759	0.0449124	2.2691478	-4.58	2.7247252	TPD52L3
206940_s_at	0.759	0.0449184	2.2690717	-4.58	2.5680978	POU4F1
1570587_at	0.759	0.0449358	2.268849	-4.58	2.1843102	
203784_s_at	0.759	0.0449562	2.2685881	-4.58	2.2426474	DDX28
239424_at	0.759	0.0449592	2.2685495	-4.58	2.1364747	
1564003_at	0.759	0.0450203	2.2677697	-4.58	1.9379442	
241217_x_at	0.759	0.0450277	2.2676763	-4.58	2.0659631	
1564281_at	0.759	0.0450468	2.2674325	-4.58	2.000459	LINC00491
201276_at	0.759	0.0450492	2.2674014	-4.58	1.6770809	RAB5B

222319_at	0.759	0.0450674	2.2671698	-4.58	2.0691943	
1566499_at	0.759	0.0450729	2.2670995	-4.58	2.4003267	
223906_s_at	0.759	0.0450949	2.2668198	-4.58	1.5772928	TEX101
236588_at	0.759	0.0450981	2.2667786	-4.58	1.309723	CFDP1
206786_at	0.759	0.0451017	2.2667327	-4.58	2.4434636	HTN3
1554232_a_at	0.759	0.0451062	2.2666754	-4.58	2.4228525	
240970_x_at	0.759	0.0451217	2.2664781	-4.58	2.7190534	LOC102723684
241291_at	0.759	0.0451364	2.2662911	-4.58	2.1853814	LINC01541
219188_s_at	0.759	0.045143	2.2662072	-4.58	2.0084345	MACROD1
208543_at	0.759	0.0451439	2.2661963	-4.58	2.4072574	OR10H2
240497_at	0.759	0.0451538	2.2660699	-4.58	1.9381362	
216049_at	0.759	0.0451954	2.2655416	-4.58	2.2164392	RHOBTB3
1558381_a_at	0.759	0.0451964	2.2655284	-4.58	2.0449744	TMEM147-AS1
207129_at	0.759	0.0452072	2.2653918	-4.58	1.9742937	CA5B
1564545_a_at	0.759	0.04523	2.2651017	-4.58	2.6419016	
1552520_at	0.759	0.0452492	2.2648587	-4.58	2.2933389	TMEM74
244725_at	0.759	0.0452611	2.2647067	-4.58	1.7157368	DMRTA1
206387_at	0.759	0.0452855	2.2643975	-4.58	1.8598131	CDX2
229538_s_at	0.759	0.0453009	2.2642018	-4.58	2.0962894	IQGAP3
242030_at	0.759	0.0453349	2.2637718	-4.58	1.9109434	
208331_at	0.759	0.0453522	2.2635519	-4.58	2.3068799	BPY2
1562928_at	0.759	0.0453977	2.2629764	-4.58	2.3655534	

220568_at	0.759	0.0454135	2.2627772	-4.58	2.5203657	PRO1483
201958_s_at	0.759	0.0454151	2.2627566	-4.58	2.4332343	PPP1R12B
235428_at	0.759	0.0454339	2.2625185	-4.58	1.4673266	MINCR
242161_at	0.759	0.0454934	2.2617665	-4.58	2.5595338	
1569241_a_at	0.759	0.0455171	2.2614678	-4.58	2.8771124	ZNF93
237796_at	0.759	0.045518	2.2614562	-4.58	2.9656803	
215939_at	0.759	0.0455242	2.2613778	-4.58	1.8155689	
1570261_at	0.759	0.045554	2.261002	-4.58	2.2004476	
217359_s_at	0.759	0.0455613	2.2609098	-4.58	1.7006056	NCAM1
1562537_at	0.759	0.0455881	2.2605727	-4.58	2.3110402	FCER1A
244099_at	0.759	0.0456022	2.2603951	-4.58	2.1883996	CACNG2
1556999_at	0.759	0.0456103	2.2602931	-4.58	1.9515369	LOC100271832
233507_at	0.759	0.0456132	2.2602561	-4.58	1.6326095	
242690_at	0.759	0.0456433	2.2598778	-4.58	3.2227731	ATP11C
1553465_a_at	0.759	0.045654	2.2597432	-4.58	1.8322903	CES5A
231175_at	0.759	0.0456622	2.2596402	-4.58	2.550813	BEND6
217062_at	0.759	0.0456884	2.2593094	-4.58	1.9018812	DMPK
217507_at	0.759	0.0456951	2.2592261	-4.58	2.3978468	SLC11A1
216577_at	0.759	0.0457121	2.2590123	-4.58	1.6211283	
205699_at	0.759	0.0457183	2.2589344	-4.58	2.5030959	MAP2K6
1556584_at	0.759	0.0457425	2.2586301	-4.58	1.5289158	
1566948_at	0.759	0.0457691	2.2582961	-4.58	2.2598628	
231679_at	0.759	0.0457716	2.2582643	-4.58	2.1325793	

208363_s_at	0.759	0.0457932	2.2579936	-4.58	2.068579	INPP4A
215491_at	0.759	0.0458341	2.2574814	-4.58	2.0408418	MYCL
220218_at	0.759	0.0458467	2.2573233	-4.58	1.633014	SPATA6L
216601_at	0.759	0.0458475	2.2573123	-4.58	2.2858986	AOC4P
220810_at	0.759	0.0458578	2.2571839	-4.58	2.654352	CLCA3P
230853_at	0.759	0.0458588	2.2571709	-4.58	1.8780731	FEM1A
210464_at	0.759	0.0458782	2.2569279	-4.58	3.3274771	LINC00588
1564008_at	0.759	0.0458887	2.2567973	-4.58	1.6398188	COL27A1
234705_at	0.759	0.0459031	2.2566162	-4.58	1.8555106	CATX-1
237449_at	0.759	0.0459114	2.2565126	-4.58	2.1216546	SP8
242348_at	0.759	0.0459239	2.2563561	-4.58	3.1320735	FAM19A4
242035_at	0.759	0.0459561	2.2559532	-4.58	1.6639041	LMBR1
1561280_at	0.759	0.0459722	2.2557517	-4.58	3.1243624	LEMD1-AS1
214236_at	0.759	0.0459801	2.2556532	-4.58	1.6667972	
201265_at	0.759	0.0460066	2.2553227	-4.58	2.3096888	
211496_s_at	0.759	0.0460145	2.2552235	-4.58	2.9078541	PDC
1558163_at	0.759	0.046026	2.2550804	-4.58	2.2870618	PEX13
240083_at	0.759	0.0460395	2.2549113	-4.58	2.7432932	SNORD112///MEG8
232894_at	0.759	0.0460497	2.2547849	-4.58	2.6297402	SEC14L2
1556786_at	0.759	0.0460531	2.254742	-4.58	3.0407753	PDE5A
243947_s_at	0.759	0.0460622	2.2546283	-4.58	2.4118028	
1564373_a_at	0.759	0.046064	2.2546061	-4.58	2.210897	LOC283887

1553060_at	0.759	0.0460916	2.2542615	-4.58	1.7577433	PSKH2
236479_at	0.759	0.0461095	2.2540387	-4.58	1.7293872	
208080_at	0.759	0.0461199	2.2539085	-4.58	2.3848106	AURKA
219989_s_at	0.759	0.0461473	2.2535673	-4.58	2.104795	ANKS1B
1563612_at	0.759	0.0461577	2.2534378	-4.58	2.4324002	
224860_at	0.759	0.0461705	2.2532787	-4.58	1.5885297	TMEM261
237308_at	0.759	0.0461767	2.2532013	-4.58	2.0372018	
1557641_at	0.759	0.0461835	2.2531173	-4.58	2.3806565	
206990_at	0.759	0.0461925	2.2530046	-4.58	2.483291	TNR
206248_at	0.759	0.046215	2.2527247	-4.58	1.6015616	PRKCE
1556205_at	0.759	0.0462182	2.2526857	-4.58	1.586366	
1560842_a_at	0.759	0.0462191	2.2526738	-4.58	1.914456	TEX26-AS1
237185_at	0.759	0.0462218	2.2526403	-4.58	2.0938657	
1553710_at	0.759	0.0463146	2.2514877	-4.58	2.4279242	FAM218A
221445_at	0.759	0.0463322	2.2512693	-4.58	3.3105081	OR1A2
243007_at	0.759	0.0463454	2.2511058	-4.58	1.8474219	TTC5
207501_s_at	0.759	0.0463507	2.2510396	-4.58	2.1857564	FGF12
221013_s_at	0.759	0.0463578	2.2509512	-4.58	1.4871479	APOL2
205143_at	0.759	0.0463655	2.250856	-4.58	1.7533644	NCAN
1556086_at	0.759	0.0463678	2.2508282	-4.58	2.3869155	HPF1
228272_at	0.759	0.0463792	2.2506863	-4.58	2.9098919	DNLZ
235517_at	0.759	0.0464034	2.2503871	-4.58	2.1545694	PACRGL
1556679_at	0.759	0.046415	2.2502431	-4.58	2.1266786	

1561392_at	0.759	0.0464165	2.2502248	-4.58	1.9561332	
244000_at	0.759	0.0464288	2.2500717	-4.58	3.0610035	
238514_at	0.759	0.0464363	2.2499792	-4.58	2.5640781	TMEM25
240421_x_at	0.759	0.046444	2.2498838	-4.58	1.684479	SAV1
1553755_at	0.759	0.0464672	2.249597	-4.58	2.4300546	NXNL1
1565650_at	0.759	0.0464672	2.2495963	-4.58	2.8768588	
230955_s_at	0.759	0.0465011	2.2491776	-4.58	2.1126071	NOL4L
213908_at	0.759	0.0465333	2.2487797	-4.58	1.5389971	WHAMMP2///WHAMMP3
224300_x_at	0.759	0.0465402	2.2486946	-4.58	2.0682162	FTCD
236903_at	0.759	0.0465498	2.2485757	-4.58	1.6862696	
233189_at	0.759	0.0465636	2.248405	-4.58	2.1829038	
222177_s_at	0.759	0.0466105	2.2478271	-4.58	2.7689408	SCAND2P
1556935_at	0.759	0.0466173	2.2477432	-4.58	2.7039033	
244305_at	0.759	0.0466595	2.2472223	-4.58	2.2907124	GGN
240519_at	0.759	0.0466639	2.2471687	-4.58	2.0642963	
1568736_s_at	0.759	0.0466949	2.2467863	-4.58	2.3123206	DLGAP1
206964_at	0.759	0.0467031	2.2466856	-4.58	2.1110076	NAT8B
237521_x_at	0.759	0.0467296	2.2463595	-4.58	2.7267085	
1569987_at	0.759	0.0468248	2.2451894	-4.58	2.3447762	DLEU7-AS1

221359_at	0.759	0.0468345	2.2450702	-4.58	1.7150648	GDNF
216851_at	0.759	0.0468352	2.2450612	-4.58	1.6291586	IGLL5///IGLJ3///IGLV3-19
229224_x_at	0.759	0.0468477	2.2449086	-4.58	1.8071342	LOC643085
230159_at	0.759	0.0468517	2.2448595	-4.58	1.9338961	C1orf115
207632_at	0.759	0.0468612	2.2447419	-4.58	2.5873996	MUSK
207658_s_at	0.759	0.0468888	2.2444041	-4.58	2.1290814	FOXG1
230574_at	0.759	0.0468981	2.2442898	-4.58	1.6421503	LOC100130938
1555697_at	0.759	0.0469102	2.2441412	-4.58	1.8870841	KLK4
1555205_at	0.759	0.0469319	2.2438755	-4.58	2.1214457	
238881_at	0.759	0.0469533	2.2436127	-4.58	1.4754463	
226286_at	0.759	0.0469535	2.2436109	-4.58	2.1282733	ELMOD3
229110_at	0.759	0.0470081	2.2429424	-4.58	2.1899935	SLC24A2
1557779_at	0.759	0.0470261	2.2427221	-4.58	2.6426894	LINC01468
243789_at	0.759	0.0470577	2.2423357	-4.58	3.7920141	
233089_at	0.759	0.0470632	2.2422693	-4.58	1.8190111	QRSL1
237989_at	0.759	0.0470657	2.2422384	-4.58	2.1595289	IGFBP1
1561002_at	0.759	0.0471081	2.2417199	-4.58	2.5456499	
216826_at	0.759	0.0471161	2.2416221	-4.58	2.1350287	
228675_at	0.759	0.0471225	2.241544	-4.58	1.748671	USP30-AS1
206304_at	0.759	0.0471679	2.2409908	-4.58	2.0360377	MYBPH
239216_at	0.759	0.0471728	2.2409306	-4.58	2.0949167	TEKT1

236774_at	0.759	0.0471846	2.2407866	-4.58	2.2011059	
230567_at	0.759	0.0471859	2.2407709	-4.58	2.5143935	CFAP97
223745_at	0.759	0.0471969	2.240637	-4.58	2.4486381	C16orf95
234677_at	0.759	0.0472281	2.2402573	-4.58	1.6514759	LOC105376689
244372_at	0.759	0.0472717	2.2397257	-4.58	1.9850437	LOC101060019
221975_s_at	0.759	0.0472902	2.2395008	-4.58	2.1461075	C21orf2
1554468_s_at	0.759	0.0472906	2.2394963	-4.58	2.0144336	FBF1
235596_at	0.759	0.0473157	2.2391915	-4.58	2.2808906	
208597_at	0.759	0.0473186	2.2391553	-4.58	2.252616	CNTF
1562294_x_at	0.759	0.0473555	2.2387068	-4.58	2.5067517	ANKRD30B
211426_x_at	0.759	0.0473667	2.2385707	-4.58	1.7156875	GNAQ
220779_at	0.759	0.0473851	2.2383473	-4.58	2.066644	PADI3
1552480_s_at	0.759	0.0474259	2.2378521	-4.58	2.8008119	PTPRC
230544_at	0.759	0.0474729	2.2372827	-4.58	2.2104744	RPS6KA4
1568865_at	0.759	0.0474763	2.2372416	-4.58	2.2730599	FNTB
239729_at	0.759	0.0474875	2.2371055	-4.58	2.2462259	
220404_at	0.759	0.0474949	2.2370166	-4.58	2.1114837	ADGRG3
241748_x_at	0.759	0.0475002	2.2369524	-4.58	2.0344733	DGCR14
214640_at	0.759	0.0475061	2.2368805	-4.58	2.3976438	UNC93A
230315_at	0.759	0.0475479	2.2363742	-4.58	2.0578978	
234525_at	0.759	0.0475556	2.236281	-4.58	3.1488635	DKFZP761C1711
215762_at	0.759	0.047556	2.2362765	-4.58	2.0948427	

215207_x_at	0.759	0.047565	2.2361672	-4.58	1.4924607	NUS1///NUS1P3
1554834_a_at	0.759	0.0475669	2.2361451	-4.58	1.9035527	RASSF5
233574_at	0.759	0.0475702	2.2361048	-4.58	2.3904209	BCORL1
229919_at	0.759	0.0476451	2.2351998	-4.58	1.7022343	NTPCR
1553169_at	0.759	0.0476554	2.2350744	-4.58	2.5302969	LRRN4
237168_at	0.759	0.0476677	2.2349265	-4.58	1.7611486	NEK5
241662_x_at	0.759	0.0476796	2.2347832	-4.58	1.704072	
236654_s_at	0.759	0.0476937	2.234613	-4.58	1.6189298	
235150_at	0.759	0.0476969	2.2345738	-4.58	1.9212323	SESN3
1555626_a_at	0.759	0.0476984	2.2345562	-4.58	1.9424546	SLAMF1
244660_at	0.759	0.0477254	2.2342307	-4.58	1.3729215	ELAVL1
233695_s_at	0.759	0.047731	2.2341633	-4.58	1.8162079	CECR2
218887_at	0.759	0.0477477	2.2339613	-4.58	2.0279838	MRPL2
1558896_at	0.759	0.0477537	2.2338892	-4.58	2.0973117	IBA57
241054_at	0.759	0.0477679	2.2337179	-4.58	1.8972798	
1563638_at	0.759	0.0478017	2.2333115	-4.58	2.6721928	TVP23A
227419_x_at	0.759	0.0478073	2.233243	-4.58	2.5745391	PLAC9
208085_s_at	0.759	0.0478079	2.2332362	-4.58	3.1640883	ARHGAP6
207867_at	0.759	0.0478246	2.2330348	-4.58	1.8586378	PAX4
240415_at	0.759	0.0478317	2.2329505	-4.58	1.9790002	

1553004_at	0.759	0.047855	2.23267	-4.58	1.9316945	PKD1L1
1560265_at	0.759	0.0478825	2.2323394	-4.58	2.819697	GRIK2
1564292_at	0.759	0.0479002	2.2321266	-4.58	2.4536667	
1562710_at	0.759	0.0479052	2.2320666	-4.58	2.3859532	LOC101928553
223687_s_at	0.759	0.0479081	2.2320312	-4.58	2.0540259	LY6K
230803_s_at	0.759	0.0479168	2.2319269	-4.58	1.4667421	ARHGAP24
1556361_s_at	0.759	0.0479553	2.231465	-4.58	3.0463594	ANKRD13C
228637_at	0.759	0.0479589	2.2314212	-4.58	1.6407788	ZDHHC1
231461_at	0.759	0.0479723	2.2312607	-4.58	2.0879725	KRT71
1557506_a_at	0.759	0.0479832	2.2311301	-4.58	2.111356	
1554808_at	0.759	0.0479854	2.2311031	-4.58	2.2323596	ACP1
207286_at	0.759	0.0479889	2.231061	-4.58	2.8540246	CEP135
211923_s_at	0.759	0.0480206	2.2306818	-4.58	2.4325429	ZNF471
1570448_at	0.759	0.0480257	2.2306196	-4.58	2.3655469	
232154_at	0.759	0.0480428	2.2304148	-4.58	2.1451616	ADM5
211363_s_at	0.759	0.0480589	2.2302221	-4.58	1.4800718	MTAP
239180_at	0.759	0.0480695	2.2300951	-4.58	1.6311908	MMP25-AS1
221199_at	0.759	0.048097	2.2297669	-4.58	2.0845807	GFRA4
241196_at	0.759	0.0481091	2.2296216	-4.58	1.391603	
206228_at	0.759	0.0481203	2.2294871	-4.58	1.9080132	PAX2
237835_at	0.759	0.0481248	2.2294332	-4.58	2.274373	
216573_at	0.759	0.0481342	2.2293215	-4.58	2.2984335	IGLV1-44

1566502_at	0.759	0.0481357	2.2293029	-4.58	2.255253	
234682_at	0.759	0.0481496	2.2291365	-4.58	2.812771	BTBD9
229290_at	0.759	0.048153	2.2290964	-4.58	1.9763932	DAPL1
234038_at	0.759	0.048155	2.2290721	-4.58	2.2235629	
242444_at	0.759	0.0481558	2.2290624	-4.58	1.8777657	C1QTNF6
244736_at	0.759	0.0482386	2.2280745	-4.58	2.8341164	
233856_at	0.759	0.0482614	2.2278017	-4.58	2.383577	
215050_x_at	0.759	0.0482621	2.2277931	-4.58	2.2439441	MAPKAPK2
232100_at	0.759	0.0482712	2.2276848	-4.58	2.3578331	RNF217
216969_s_at	0.759	0.0482722	2.2276726	-4.58	1.4830276	KIF22
242437_at	0.759	0.0482955	2.2273948	-4.58	1.6012058	
1552769_at	0.759	0.0483147	2.2271661	-4.58	2.1906912	ZNF625
242523_at	0.759	0.0483203	2.2270999	-4.58	1.5647507	LINC00938
206937_at	0.759	0.0483437	2.2268207	-4.58	1.8862539	SPTA1
230500_at	0.759	0.0483513	2.22673	-4.58	2.9203912	PDE7A
228740_at	0.759	0.0483551	2.2266848	-4.58	2.5455316	
228821_at	0.759	0.0483712	2.2264937	-4.58	2.4466056	ST6GAL2
216185_at	0.759	0.0484016	2.2261311	-4.58	2.4487043	FUT9
238267_s_at	0.759	0.0484268	2.2258314	-4.58	1.9975907	
1564985_a_at	0.759	0.0484605	2.225431	-4.58	2.3041601	SLC8A1
241696_at	0.759	0.0484706	2.2253116	-4.58	1.9875727	CNTLN
229092_at	0.759	0.0484929	2.2250469	-4.58	1.6005139	NR2F2
238160_at	0.759	0.0485006	2.2249546	-4.58	1.8328968	ACOT12

239105_at	0.759	0.0485118	2.2248218	-4.58	1.7930872	
1569910_at	0.759	0.0485745	2.2240788	-4.58	2.7798987	
1564816_at	0.759	0.0485905	2.2238882	-4.58	1.850592	C14orf178
1562055_at	0.759	0.0486102	2.2236556	-4.58	2.2296397	ZNF91
226643_s_at	0.759	0.0486124	2.2236289	-4.58	1.3325207	NUDCD2
1563250_at	0.759	0.0486215	2.2235212	-4.58	1.8602597	LOC101927362
231443_at	0.759	0.0486229	2.2235047	-4.58	2.728421	ELFN1-AS1
1556901_s_at	0.759	0.0486488	2.2231981	-4.58	2.4173329	APCDD1L-AS1
217613_at	0.759	0.0486787	2.2228439	-4.58	2.19845	TMEM144
230849_at	0.759	0.0486803	2.2228252	-4.58	2.4441559	KCNA1
233182_x_at	0.759	0.0486804	2.2228247	-4.58	1.5223416	ATXN3
221297_at	0.759	0.0486857	2.2227612	-4.58	1.6660225	GPRC5D
1562905_at	0.759	0.0487295	2.2222441	-4.58	2.4285138	
1553630_at	0.759	0.0487312	2.2222232	-4.58	2.2257144	C10orf107
243274_x_at	0.759	0.0487344	2.2221857	-4.58	2.9135677	
238386_x_at	0.759	0.0487467	2.2220406	-4.58	2.4306384	
240167_at	0.759	0.0487655	2.2218184	-4.58	2.4651466	LINC01085
1567679_at	0.759	0.0487871	2.2215635	-4.58	2.9794606	SNORA74A
240354_at	0.759	0.0487981	2.2214334	-4.58	2.3557167	C12orf54
1552919_at	0.759	0.0488013	2.2213958	-4.58	3.0260762	C4orf36
1569675_at	0.759	0.0488042	2.2213607	-4.58	2.3642664	POU2AF1

236538_at	0.759	0.0488199	2.2211763	-4.58	2.5071234	GRIA2
207428_x_at	0.759	0.0488248	2.2211183	-4.58	1.8648146	CDK11A///CDK11B
1554880_at	0.759	0.0488434	2.2208987	-4.58	2.7486287	DKFZP434K028
232980_at	0.759	0.0488472	2.2208539	-4.58	1.8096406	LMBRD1
230090_at	0.759	0.048865	2.2206446	-4.58	2.3024162	GDNF
1562719_at	0.759	0.0489	2.2202312	-4.58	1.8087709	LOC101928476
237604_at	0.759	0.0489202	2.2199937	-4.58	1.8166276	
1553844_a_at	0.759	0.0489292	2.2198884	-4.58	2.1344246	C10orf67
1561324_at	0.759	0.0489702	2.2194051	-4.58	2.6870507	
244118_at	0.759	0.0490104	2.2189323	-4.58	1.8025613	GABRA1
233503_at	0.759	0.0490468	2.2185047	-4.58	2.406714	CCDC169
235770_at	0.759	0.0490503	2.2184643	-4.58	1.9662279	MASP1
1559226_x_at	0.759	0.0490542	2.2184178	-4.58	1.6078496	LCE1E
228717_at	0.759	0.0490574	2.2183806	-4.58	1.6813466	
232040_at	0.759	0.049084	2.2180681	-4.58	1.5818756	LOC157860
242699_at	0.759	0.0491308	2.2175192	-4.58	2.1375103	
216909_at	0.759	0.0491473	2.2173251	-4.58	1.8562696	RRP12
1557311_at	0.759	0.0491577	2.2172038	-4.58	1.7198214	
211562_s_at	0.759	0.0491685	2.2170774	-4.58	2.6175333	LMOD1
1569004_at	0.759	0.0491783	2.2169616	-4.58	2.2594851	CARD8-AS1

204898_at	0.759	0.0491916	2.216807	-4.58	1.8670682	SAP30
229079_at	0.759	0.0492251	2.2164144	-4.58	1.7080374	EHMT2
220112_at	0.759	0.0492602	2.2160037	-4.58	1.9110941	ANKRD55
223932_at	0.759	0.0492684	2.2159074	-4.58	2.0724912	
228683_s_at	0.759	0.0492767	2.2158108	-4.58	2.2062388	KCTD15
232852_at	0.759	0.0492945	2.2156025	-4.58	1.9203163	
1557050_at	0.759	0.0493092	2.2154302	-4.58	2.369722	HOTAIRM1
217464_at	0.759	0.0493235	2.2152633	-4.58	1.4351928	
237686_at	0.759	0.0493244	2.2152526	-4.58	2.571281	RNF219
208280_at	0.759	0.0493384	2.215089	-4.58	1.9254933	CDRT1
241525_at	0.759	0.0493542	2.2149048	-4.58	2.4534473	LOC200772
1561545_at	0.759	0.0493904	2.2144822	-4.58	1.7701065	EPN2-AS1
235975_at	0.759	0.0493943	2.2144369	-4.58	1.4021271	MTO1

231568_at	0.759	0.0494021	2.2143457	-4.58	2.4828613	CT47A12///CT47A1///CT47A2///CT47A3///CT47A4///CT47A5///CT47A6///[illegible]
240559_at	0.759	0.0494345	2.2139678	-4.58	2.5633411	IZUMO2
1554316_at	0.759	0.049456	2.213718	-4.58	1.6307957	PGLS
1556850_at	0.759	0.0494792	2.213447	-4.58	1.7018897	CEP290
215061_at	0.759	0.0494848	2.2133814	-4.58	2.65402	METTL10
243607_at	0.759	0.0494876	2.2133499	-4.58	2.5872977	
211645_x_at	0.759	0.0494952	2.2132607	-4.58	1.7717793	
1559538_at	0.759	0.0495252	2.212912	-4.58	2.8044187	LOC101929549
216136_at	0.759	0.049537	2.2127747	-4.58	2.9222607	
231410_at	0.759	0.0495397	2.2127424	-4.58	2.4975502	
1553433_at	0.759	0.0495566	2.2125461	-4.58	2.2537349	CCDC171
244229_at	0.759	0.0495678	2.2124159	-4.58	2.0047581	PARVG
1561152_a_at	0.759	0.0495767	2.2123125	-4.58	2.175767	

242009_at	0.759	0.0495805	2.2122685	-4.58	2.6462754	SLC6A4
221303_at	0.759	0.0496286	2.2117093	-4.58	1.7937818	PCDHB1
212770_at	0.759	0.0496997	2.2108842	-4.58	2.3460193	TLE3
1570219_at	0.759	0.0497025	2.2108523	-4.58	2.641143	LOXL4
243393_at	0.759	0.0497161	2.2106937	-4.58	2.4221999	
1570320_at	0.759	0.0497227	2.2106179	-4.58	1.9835348	
236714_at	0.759	0.0497385	2.2104349	-4.58	2.3411031	
241971_at	0.759	0.049745	2.2103594	-4.58	1.9859263	
243141_at	0.759	0.0497484	2.2103192	-4.58	1.5846815	SGMS2
1570534_a_at	0.759	0.0497829	2.2099198	-4.58	1.9218138	ZNF483
208570_at	0.759	0.0497834	2.2099143	-4.58	2.7384361	WNT1
235943_at	0.759	0.0498275	2.2094036	-4.58	2.6620667	
213635_s_at	0.759	0.0498341	2.2093272	-4.58	1.7663928	SAFB
223825_at	0.759	0.0498484	2.2091622	-4.58	1.3257749	RIC1
1570625_at	0.759	0.0498553	2.2090823	-4.58	2.200998	TCEB3
1555085_at	0.759	0.0498889	2.2086935	-4.58	2.9307383	LOC101928751
237842_at	0.759	0.0499054	2.2085029	-4.58	2.0470847	MAP7D3
215079_at	0.759	0.049908	2.2084732	-4.58	2.510603	
220171_x_at	0.759	0.049913	2.2084152	-4.58	1.493027	GPALPP1
1558195_at	0.759	0.0499597	2.2078767	-4.58	2.0229033	LINC00592
235032_at	0.759	0.0499615	2.2078556	-4.58	1.5976868	DNAJC21
230984_s_at	0.759	0.049983	2.2076074	-4.58	2.0538001	
233145_at	0.759	0.0499866	2.2075659	-4.58	1.9137818	CSMD2

37020_at	0.759	0.0003611	-5.1167939	-4.54	-6.0844282	CRP
223509_at	0.759	0.0003628	-5.1136843	-4.54	-3.7944973	CLDN2
204470_at	0.759	0.0004381	-4.9915188	-4.55	-3.1940919	CXCL1
202376_at	0.759	0.0005087	-4.8956934	-4.55	-3.5278451	SERPINA3
232850_at	0.759	0.0006023	-4.7883576	-4.55	-2.9273507	
202859_x_at	0.759	0.000784	-4.6227586	-4.55	-3.4634357	CXCL8
213680_at	0.759	0.0008018	-4.6087933	-4.55	-4.1763175	KRT6B
212768_s_at	0.759	0.0008402	-4.5796777	-4.55	-4.6057903	OLFM4
231154_x_at	0.759	0.0008851	-4.5474015	-4.55	-4.0402097	TINAG
238103_at	0.759	0.0011589	-4.381618	-4.55	-4.1455515	LINC01207
208383_s_at	0.759	0.0015515	-4.2046642	-4.55	-3.4410908	PCK1
207430_s_at	0.759	0.0017284	-4.1398113	-4.55	-3.865359	MSMB
235229_at	0.759	0.0017519	-4.1317087	-4.55	-4.4004387	
231180_at	0.759	0.0019785	-4.0590567	-4.55	-3.0922052	
229761_at	0.759	0.0019821	-4.0579562	-4.55	-2.8982171	LOC440173
1564451_at	0.759	0.0019935	-4.0545378	-4.55	-3.5636411	
38037_at	0.759	0.0022353	-3.9865121	-4.55	-4.0768594	HBEGF
205815_at	0.759	0.0023532	-3.9560856	-4.55	-3.8318924	REG3A
202833_s_at	0.759	0.0024545	-3.9311877	-4.55	-2.9819832	SERPINA1
209774_x_at	0.759	0.0025949	-3.8983709	-4.55	-4.0622593	CXCL2
209752_at	0.759	0.0026158	-3.8936506	-4.55	-3.3740768	REG1A
222303_at	0.759	0.0029392	-3.8251573	-4.56	-3.3014003	
243483_at	0.759	0.0030619	-3.8012035	-4.56	-3.4750853	TRPM8
222934_s_at	0.759	0.0032403	-3.7680911	-4.56	-2.9078842	CLEC4E

205890_s_at	0.759	0.0032467	-3.7669322	-4.56	-3.1927073	UBD///GABBR1
1553062_at	0.759	0.003258	-3.7649021	-4.56	-4.0567449	MOGAT1
204621_s_at	0.759	0.0033807	-3.7433283	-4.56	-3.6763384	NR4A2
203821_at	0.759	0.0035936	-3.7077462	-4.56	-2.4612493	HBEGF
202672_s_at	0.759	0.0037608	-3.6812936	-4.56	-4.5751806	ATF3
222256_s_at	0.759	0.0039512	-3.6526235	-4.56	-2.5202953	JMJD7///JMJD7-PLA2G4B
239093_at	0.759	0.0041145	-3.6291429	-4.56	-2.9377574	HOGA1
205513_at	0.759	0.0041443	-3.6249606	-4.56	-2.8254585	TCN1
210121_at	0.759	0.0043179	-3.601206	-4.56	-3.0089341	B3GALT2
240259_at	0.759	0.0045427	-3.5718688	-4.56	-2.7081583	LOC100506718///FLRT2
221545_x_at	0.759	0.0045987	-3.564794	-4.56	-2.4518582	MED16
209641_s_at	0.759	0.0049167	-3.5262329	-4.56	-2.330948	ABCC3
215807_s_at	0.759	0.0050091	-3.5154991	-4.56	-2.2795857	PLXNB1
229764_at	0.759	0.0050245	-3.5137341	-4.56	-3.4612991	TPRG1
222271_at	0.759	0.0050379	-3.5122039	-4.56	-3.4157441	
232593_at	0.759	0.0051065	-3.5044198	-4.56	-2.9503577	NEURL3
223722_at	0.759	0.0054485	-3.467138	-4.56	-3.551959	DNAJC12
208450_at	0.759	0.0055491	-3.4566304	-4.56	-4.037357	LGALS2
241694_at	0.759	0.0055679	-3.4546879	-4.56	-2.9429478	PKHD1
238625_at	0.759	0.0056452	-3.4467785	-4.56	-2.3933973	C1orf168

243237_at	0.759	0.0058552	-3.4258257	-4.56	-2.4001448	CFAP221
207392_x_at	0.759	0.0060214	-3.4097802	-4.56	-3.504236	UGT2B15
206535_at	0.759	0.0060387	-3.4081374	-4.56	-3.5894674	SLC2A2
205969_at	0.759	0.0063811	-3.3765642	-4.56	-2.7482063	AADAC
206239_s_at	0.759	0.0063846	-3.3762489	-4.56	-3.6728666	SPINK1
228360_at	0.759	0.0068302	-3.3376823	-4.56	-2.5548479	LYPD6B
208961_s_at	0.759	0.007196	-3.3078949	-4.56	-2.9837931	KLF6
210180_s_at	0.759	0.0072382	-3.3045574	-4.56	-2.834716	TRA2B
205844_at	0.759	0.0073324	-3.2971824	-4.56	-3.7683408	VNN1
210168_at	0.759	0.0073809	-3.293423	-4.56	-4.0975526	C6
206197_at	0.759	0.0074033	-3.2916991	-4.56	-2.572105	NME5
221265_s_at	0.759	0.0075719	-3.2788634	-4.56	-3.0449919	VWA9
223721_s_at	0.759	0.0076308	-3.2744406	-4.56	-2.7429889	DNAJC12
231412_at	0.759	0.0078937	-3.2551492	-4.56	-2.6261008	LOC101929709
205043_at	0.759	0.0079274	-3.2527251	-4.56	-3.0417812	CFTR
240861_at	0.759	0.0079352	-3.2521652	-4.56	-2.0660138	
230480_at	0.759	0.0080315	-3.2452957	-4.56	-2.0966316	PIWIL4
224606_at	0.759	0.0081121	-3.2396115	-4.56	-2.1953866	KLF6
239014_at	0.759	0.0086728	-3.2015994	-4.56	-2.0473163	CCAR1
204041_at	0.759	0.0087362	-3.1974567	-4.56	-3.0316327	MAOB
202831_at	0.759	0.0087824	-3.1944615	-4.56	-2.4779228	GPX2

201041_s_at	0.759	0.0089384	-3.1844605	-4.56	-2.7236015	DUSP1
212935_at	0.759	0.0090386	-3.1781226	-4.56	-2.6762506	MCF2L
209189_at	0.759	0.0090452	-3.1777111	-4.56	-3.7175145	FOS
242558_at	0.759	0.0093368	-3.1596915	-4.56	-2.9675113	
1559170_at	0.759	0.0093611	-3.158221	-4.56	-2.2373155	ANKRD20A5P
239747_s_at	0.759	0.0094833	-3.1508572	-4.56	-1.973941	
210999_s_at	0.759	0.0095098	-3.1492739	-4.56	-2.192876	GRB10
202151_s_at	0.759	0.0095166	-3.1488671	-4.56	-2.4332352	UBAC1
202768_at	0.759	0.0097715	-3.1338709	-4.56	-4.0907942	FOSB
1560527_at	0.759	0.0098341	-3.1302449	-4.56	-2.8498886	NFE4
233030_at	0.759	0.0098793	-3.1276469	-4.56	-1.9419038	PNPLA3
238909_at	0.759	0.0100732	-3.1166207	-4.56	-3.4282032	S100A10
227742_at	0.759	0.0103331	-3.1021825	-4.56	-3.0902246	CLIC6
233223_at	0.759	0.0105537	-3.0902066	-4.56	-2.3176634	
1568924_a_at	0.759	0.0105873	-3.088408	-4.56	-2.2118676	IQUB
206350_at	0.759	0.0111138	-3.0609142	-4.56	-2.4592995	APCS
244353_s_at	0.759	0.0113091	-3.0510532	-4.56	-2.5258787	SLC2A12
225516_at	0.759	0.0113996	-3.0465378	-4.56	-3.8722788	SLC7A2
231836_at	0.759	0.0114782	-3.0426511	-4.56	-2.2376444	HKR1
221577_x_at	0.759	0.0115137	-3.0409012	-4.56	-2.5283876	GDF15
220518_at	0.759	0.0115964	-3.0368529	-4.56	-3.5898445	ABI3BP

237554_at	0.759	0.012022	-3.0164513	-4.57	-2.8277218	
222314_x_at	0.759	0.0121482	-3.0105436	-4.57	-2.205956	EGOT
210382_at	0.759	0.0122159	-3.0073993	-4.57	-2.9212583	SCTR
220376_at	0.759	0.0122987	-3.0035782	-4.57	-2.3688521	LRRC19
207655_s_at	0.759	0.012318	-3.0026912	-4.57	-3.1569226	BLNK
201236_s_at	0.759	0.0123217	-3.0025223	-4.57	-2.1680175	BTG2
226248_s_at	0.759	0.0123298	-3.0021514	-4.57	-3.0750971	KIAA1324
226796_at	0.759	0.012751	-2.9831528	-4.57	-2.3121339	ABHD15
230776_at	0.759	0.0128454	-2.9789834	-4.57	-2.9463943	RNF157-AS1
205799_s_at	0.759	0.012918	-2.9757974	-4.57	-3.003607	SLC3A1
202150_s_at	0.759	0.0129953	-2.9724247	-4.57	-2.3189966	NEDD9
225911_at	0.759	0.0129975	-2.972325	-4.57	-3.0576398	NPNT
223686_at	0.759	0.0130051	-2.9719973	-4.57	-2.042914	TPK1
243231_at	0.759	0.0131245	-2.9668305	-4.57	-2.8990854	SLC38A11
201170_s_at	0.759	0.0131678	-2.9649657	-4.57	-2.1521266	BHLHE40
236561_at	0.759	0.0131935	-2.9638646	-4.57	-2.3910418	TGFBR1
227763_at	0.759	0.0133191	-2.9585078	-4.57	-2.3566109	LYPD6
212533_at	0.759	0.0133595	-2.9567964	-4.57	-2.4906527	WEE1
203491_s_at	0.759	0.0133887	-2.9555637	-4.57	-2.8059228	CEP57
217626_at	0.759	0.0135304	-2.9496135	-4.57	-2.9098215	AKR1C1
233317_at	0.759	0.0135777	-2.9476383	-4.57	-2.4267842	CD9
229033_s_at	0.759	0.0136527	-2.9445258	-4.57	-2.2659807	MUM1

210119_at	0.759	0.0137486	-2.9405686	-4.57	-2.6271925	KCNJ15
203836_s_at	0.759	0.013757	-2.9402239	-4.57	-2.2715125	MAP3K5
1552755_at	0.759	0.0137629	-2.9399818	-4.57	-2.1674	C9orf66
1565705_x_at	0.759	0.0139037	-2.9342311	-4.57	-2.2708672	
218510_x_at	0.759	0.0142569	-2.9200534	-4.57	-2.8757806	FAM134B
212771_at	0.759	0.014325	-2.9173603	-4.57	-2.3672909	FAM171A1
205221_at	0.759	0.0143308	-2.9171326	-4.57	-3.2291899	HGD
222348_at	0.759	0.0143865	-2.914942	-4.57	-2.8799777	MAST4
237151_s_at	0.759	0.0144024	-2.9143187	-4.57	-2.3616573	CFAP221
228773_at	0.759	0.0144486	-2.9125064	-4.57	-2.9350636	LOC100506100
1557905_s_at	0.759	0.0144503	-2.9124429	-4.57	-2.6544296	CD44
215702_s_at	0.759	0.0145206	-2.9096995	-4.57	-2.0230366	CFTR
243296_at	0.759	0.014551	-2.9085183	-4.57	-2.3542057	NAMPT
204714_s_at	0.759	0.0145981	-2.9066932	-4.57	-2.2219441	F5
210390_s_at	0.759	0.0146926	-2.9030481	-4.57	-2.2509977	CCL15-CCL14///CCL15
214890_s_at	0.759	0.0148177	-2.8982544	-4.57	-2.7264588	FAM149A
205044_at	0.759	0.0148918	-2.8954368	-4.57	-3.0953665	GABRP

208670_s_at	0.759	0.0148947	-2.8953295	-4.57	-2.6770607	EID1
228674_s_at	0.759	0.0150396	-2.8898596	-4.57	-1.7993742	EML4
210619_s_at	0.759	0.0150727	-2.8886165	-4.57	-2.9110491	HYAL1
210830_s_at	0.759	0.0152941	-2.8803813	-4.57	-2.8171262	PON2
243168_at	0.759	0.0152995	-2.8801806	-4.57	-3.2193756	
205476_at	0.759	0.0154375	-2.8751087	-4.57	-2.0934875	CCL20
225660_at	0.759	0.0154692	-2.8739511	-4.57	-1.9085597	SEMA6A
220017_x_at	0.759	0.0155103	-2.8724515	-4.57	-2.3715885	CYP2C9
219956_at	0.759	0.0162383	-2.8465452	-4.57	-2.8093008	GALNT6
1569607_s_at	0.759	0.0162771	-2.8451954	-4.57	-3.4817392	LOC102725051///LOC102723891///ANKRD20A4///ANKRD20A2///ANKRI
239656_at	0.759	0.0163461	-2.842807	-4.57	-1.9919868	LHFPL3-AS2
201169_s_at	0.759	0.0164538	-2.8390998	-4.57	-2.1782958	BHLHE40
212225_at	0.759	0.0167594	-2.8287057	-4.57	-2.0843377	EIF1

240156_at	0.759	0.0168284	-2.8263849	-4.57	-2.099548	
208699_x_at	0.759	0.0168575	-2.8254092	-4.57	-1.8498117	TKT
208791_at	0.759	0.0169521	-2.8222507	-4.57	-2.1123408	CLU
204993_at	0.759	0.0170622	-2.818595	-4.57	-2.0021086	GNAZ
216219_at	0.759	0.0171002	-2.8173364	-4.57	-2.1984442	AQP6
205328_at	0.759	0.0172018	-2.8139912	-4.57	-2.674278	CLDN10
219759_at	0.759	0.0176687	-2.7988659	-4.57	-1.887685	ERAP2
223055_s_at	0.759	0.0176768	-2.7986098	-4.57	-2.0133177	XPO5
201328_at	0.759	0.0178161	-2.7941762	-4.57	-2.1298177	ETS2
218541_s_at	0.759	0.0182126	-2.7817459	-4.57	-2.254706	C8orf4
203946_s_at	0.759	0.0183513	-2.7774599	-4.57	-2.3659921	ARG2
213664_at	0.759	0.0184141	-2.7755293	-4.57	-3.1466529	SLC1A1
237328_at	0.759	0.0184258	-2.7751718	-4.57	-4.0567011	C14orf105
211806_s_at	0.759	0.018586	-2.7702832	-4.57	-2.2122767	KCNJ15
217997_at	0.759	0.0187877	-2.7641865	-4.57	-2.0043858	PHLDA1
235942_at	0.759	0.0190908	-2.755145	-4.57	-2.1880062	FAM224A///FAM224B
1558549_s_at	0.759	0.0191297	-2.7539956	-4.57	-2.736953	VNN1
215388_s_at	0.759	0.0191856	-2.7523483	-4.57	-3.0284496	CFHR1///CFH
209835_x_at	0.759	0.0192541	-2.7503357	-4.57	-1.9522804	CD44
230027_s_at	0.759	0.0192634	-2.7500618	-4.57	-1.5686466	MRPL43

236251_at	0.759	0.0194914	-2.7434174	-4.57	-1.7546029	
228382_at	0.759	0.0199596	-2.7300083	-4.57	-1.891683	OTULIN
225738_at	0.759	0.0200309	-2.7279913	-4.57	-2.0650566	RAPGEF1
206204_at	0.759	0.0202225	-2.7226147	-4.57	-2.901046	GRB14
206710_s_at	0.759	0.0203357	-2.7194617	-4.57	-2.1871471	EPB41L3
229866_at	0.759	0.020408	-2.7174546	-4.57	-2.0134258	STK32A
216248_s_at	0.759	0.0204126	-2.7173266	-4.57	-2.0862421	NR4A2
1563498_s_at	0.759	0.0206049	-2.7120296	-4.57	-1.9802237	SLC25A45
222925_at	0.759	0.0207316	-2.7085666	-4.57	-2.5932221	DCDC2
215672_s_at	0.759	0.020734	-2.708502	-4.57	-2.2261229	AHCYL2
203203_s_at	0.759	0.0207372	-2.7084124	-4.57	-2.0395092	KRR1
243184_at	0.759	0.0209216	-2.7034113	-4.57	-1.9620218	
218182_s_at	0.759	0.0209927	-2.701494	-4.57	-1.7781306	CLDN1
1556364_at	0.759	0.0210481	-2.7000045	-4.57	-2.063102	ADAMTS9-AS2
206947_at	0.759	0.0211062	-2.6984468	-4.57	-1.8891419	B3GALT5
227081_at	0.759	0.0211937	-2.6961094	-4.57	-2.5427228	DNALI1
242011_at	0.759	0.0215285	-2.6872509	-4.57	-1.7700923	
219500_at	0.759	0.0218793	-2.6781161	-4.57	-2.517657	CLCF1
205080_at	0.759	0.0220183	-2.6745356	-4.57	-1.7549744	RARB
213186_at	0.759	0.0220428	-2.6739053	-4.57	-2.51721	DZIP3
226900_at	0.759	0.0220433	-2.673894	-4.57	-2.2493987	GABPB1-AS1
206010_at	0.759	0.0220677	-2.6732671	-4.57	-1.9616537	HABP2

227764_at	0.759	0.0221595	-2.6709219	-4.57	-2.7996789	LYPD6
221058_s_at	0.759	0.022184	-2.6702966	-4.57	-2.1406137	CKLF
1569898_a_at	0.759	0.0222103	-2.6696258	-4.57	-1.7042217	PAXIP1-AS2
230892_at	0.759	0.0222337	-2.6690308	-4.57	-2.5706846	
224889_at	0.759	0.0222341	-2.6690211	-4.57	-2.5778417	FOXO3
204622_x_at	0.759	0.0223296	-2.6665976	-4.57	-2.183536	NR4A2
209410_s_at	0.759	0.0223371	-2.6664082	-4.57	-1.8191031	GRB10
244054_at	0.759	0.0223736	-2.6654858	-4.57	-2.7104292	
229221_at	0.759	0.0226708	-2.6580242	-4.57	-2.1374695	CD44
232959_at	0.759	0.0227007	-2.6572794	-4.57	-1.8001481	
236952_at	0.759	0.0229607	-2.6508398	-4.57	-2.3598951	
214889_at	0.759	0.0233732	-2.6407692	-4.57	-3.0171732	FAM149A
204285_s_at	0.759	0.0235672	-2.6360939	-4.57	-2.2615166	PMAIP1
1559039_at	0.759	0.0235961	-2.6354017	-4.57	-2.0287329	DHX36
208627_s_at	0.759	0.0237802	-2.6310044	-4.57	-2.3832458	YBX1
216841_s_at	0.759	0.0238116	-2.6302582	-4.57	-2.2834497	LOC100129518///SOD2
240233_at	0.759	0.0239304	-2.627444	-4.57	-1.8440819	NUP50-AS1

211942_x_at	0.759	0.0240497	-2.6246289	-4.57	-1.8467786	RPL13AP5///RPL13AP6///SNORD32A///SNORD33///SNORD34///SNORD3
230964_at	0.759	0.0241663	-2.6218945	-4.57	-2.134109	FREM2
204490_s_at	0.759	0.0243069	-2.6186108	-4.57	-1.7587247	CD44
242904_x_at	0.759	0.0243659	-2.6172395	-4.57	-2.624664	
211833_s_at	0.759	0.0244121	-2.6161682	-4.57	-2.0201063	BAX
201693_s_at	0.759	0.0244426	-2.615461	-4.57	-1.6564883	EGR1
1562898_at	0.759	0.0246753	-2.6100992	-4.57	-1.8787089	
203346_s_at	0.759	0.0247023	-2.6094808	-4.57	-2.2680776	MTF2
204015_s_at	0.759	0.0249786	-2.6031856	-4.57	-1.9576271	DUSP4
215089_s_at	0.759	0.0250631	-2.6012741	-4.57	-2.3752986	RBM10
208900_s_at	0.759	0.0251817	-2.5986008	-4.57	-1.9942747	TOP1
211682_x_at	0.759	0.0254632	-2.5923067	-4.57	-2.1513731	UGT2B28
243837_x_at	0.759	0.0254954	-2.5915926	-4.57	-1.881624	
211429_s_at	0.759	0.0255653	-2.5900419	-4.57	-1.9512512	SERPINA1
208456_s_at	0.759	0.0256654	-2.5878298	-4.57	-2.1711228	RRAS2
230432_at	0.759	0.0258605	-2.5835418	-4.57	-3.1017405	LOC100422737

218801_at	0.759	0.0258735	-2.5832563	-4.57	-1.9134713	UGGT2
244216_at	0.759	0.0259059	-2.582548	-4.57	-2.6283953	
35147_at	0.759	0.0259102	-2.5824535	-4.57	-1.9860891	MCF2L
225826_at	0.759	0.0260989	-2.5783458	-4.57	-1.7372986	MMAB
219564_at	0.759	0.0262759	-2.5745169	-4.57	-2.635269	KCNJ16
1555495_a_at	0.759	0.0263507	-2.5729059	-4.57	-2.3896775	CWC27
229596_at	0.759	0.0264531	-2.5707092	-4.57	-2.4386981	AMDHD1
206396_at	0.759	0.0265562	-2.5685065	-4.57	-2.0246599	SLC1A1
201694_s_at	0.759	0.0266209	-2.5671288	-4.57	-1.6807136	EGR1
239806_at	0.759	0.0266988	-2.5654736	-4.57	-2.3204814	
209959_at	0.759	0.0267794	-2.563764	-4.57	-2.7567962	NR4A3
217974_at	0.759	0.0267802	-2.5637485	-4.57	-1.8263759	TM7SF3
240200_at	0.759	0.0268638	-2.561981	-4.57	-2.2425868	SULT1C2
233607_at	0.759	0.0268906	-2.561417	-4.57	-3.585302	
210355_at	0.759	0.0269019	-2.5611783	-4.57	-2.363955	PTHLH
227690_at	0.759	0.0269336	-2.5605113	-4.57	-2.3414221	GABRB3
203304_at	0.759	0.0269462	-2.5602469	-4.57	-1.5670406	BAMBI
201751_at	0.759	0.0271443	-2.5560974	-4.57	-2.2326413	JOSD1
233982_x_at	0.759	0.0273954	-2.5508782	-4.57	-1.6981916	STYXL1
1557222_at	0.759	0.027486	-2.5490069	-4.57	-1.8120171	

213988_s_at	0.759	0.027516	-2.5483887	-4.57	-2.7526425	SAT1
215193_x_at	0.759	0.0275301	-2.5480983	-4.57	-3.0176122	LOC101060835///LOC100996809///HLA-DRB4///HLA-DRB3///HLA-DRB1
1562020_s_at	0.759	0.0276977	-2.5446574	-4.57	-1.8699556	NT5DC4
1569020_at	0.759	0.0277935	-2.5427008	-4.57	-2.2695103	NEDD9
212871_at	0.759	0.0278121	-2.5423218	-4.57	-2.3189236	MAPKAPK5
224832_at	0.759	0.0280315	-2.5378667	-4.57	-2.6991029	DUSP16
227608_at	0.759	0.0282923	-2.5326165	-4.57	-1.8570549	DAP3
230381_at	0.759	0.028296	-2.5325412	-4.57	-1.9482398	LOC101929219///C1orf186
235823_at	0.759	0.0284906	-2.5286551	-4.57	-2.0562527	ACSF3
204148_s_at	0.759	0.028537	-2.5277325	-4.57	-2.0480467	POMZP3///ZP3
1556608_a_at	0.759	0.0285456	-2.527561	-4.57	-2.3750579	EHD4
1557136_at	0.759	0.0286482	-2.5255257	-4.57	-2.4236732	ATP13A4
230318_at	0.759	0.0286804	-2.5248889	-4.57	-2.2701062	SERPINA1
244447_at	0.759	0.0287018	-2.5244657	-4.57	-2.5869442	

1554140_at	0.759	0.0287171	-2.524165	-4.57	-2.0760168	WDR78
210174_at	0.759	0.0288276	-2.5219863	-4.57	-2.7348214	NR5A2
226086_at	0.759	0.0289813	-2.5189696	-4.57	-2.0671472	SYT13
221437_s_at	0.759	0.0291839	-2.5150174	-4.57	-2.2598494	MRPS15
231677_at	0.759	0.0292341	-2.5140416	-4.57	-2.4663213	LOC105377924
227251_at	0.759	0.0292707	-2.5133328	-4.57	-1.8085582	DCAF5
229972_at	0.759	0.0293091	-2.5125886	-4.57	-1.9935101	LOC101926963
219024_at	0.759	0.029407	-2.5106958	-4.57	-2.257116	PLEKHA1
213659_at	0.759	0.0295199	-2.5085221	-4.57	-2.8591222	ZNF75D
200984_s_at	0.759	0.0296998	-2.5050732	-4.57	-1.8852787	CD59
225062_at	0.759	0.0298752	-2.5017311	-4.57	-2.8580551	LOC102724951///LOC389831
204734_at	0.759	0.0299211	-2.5008601	-4.57	-2.3887894	KRT15
200831_s_at	0.759	0.0299634	-2.5000575	-4.57	-1.866177	SCD
218976_at	0.759	0.029972	-2.4998947	-4.57	-2.339339	DNAJC12
207850_at	0.759	0.0300565	-2.4982966	-4.57	-2.0345754	CXCL3
220106_at	0.759	0.0302257	-2.4951113	-4.57	-2.3569325	NPC1L1
222773_s_at	0.759	0.0302352	-2.4949317	-4.57	-2.2331478	GALNT12
201042_at	0.759	0.0302942	-2.4938256	-4.57	-1.8570315	TGM2

212014_x_at	0.759	0.030337	-2.4930242	-4.57	-1.6283484	CD44
219822_at	0.759	0.0304238	-2.491402	-4.57	-2.6752763	MTRF1
203692_s_at	0.759	0.0308221	-2.4840154	-4.57	-2.0630044	E2F3
203912_s_at	0.759	0.030964	-2.481406	-4.57	-2.2991632	DNASE1L1
209042_s_at	0.759	0.0311044	-2.4788373	-4.57	-1.8082294	UBE2G2
1555136_at	0.759	0.031116	-2.4786249	-4.57	-2.8388594	FGD6
1570291_at	0.759	0.0311348	-2.4782818	-4.57	-1.8077087	CLDN10-AS1
209091_s_at	0.759	0.0313281	-2.4747656	-4.57	-2.7171136	SH3GLB1
232013_at	0.759	0.0314429	-2.4726863	-4.57	-1.8729857	ERCC6L2
238113_at	0.759	0.0314732	-2.4721407	-4.57	-2.1205446	SMIM2-AS1
203113_s_at	0.759	0.0315425	-2.4708898	-4.57	-2.2548085	EEF1D
239157_at	0.759	0.0315935	-2.4699715	-4.57	-1.8551868	ZSCAN12P1
209875_s_at	0.759	0.0316373	-2.4691854	-4.57	-2.6665146	SPP1
240382_at	0.759	0.0316969	-2.468115	-4.57	-2.6216894	LOC101928076
214875_x_at	0.759	0.0317286	-2.4675478	-4.57	-1.593228	APLP2
223658_at	0.759	0.0317675	-2.4668509	-4.57	-1.9633197	KCNK6
206683_at	0.759	0.031807	-2.466144	-4.57	-2.8945931	ZNF165
1552370_at	0.759	0.0318529	-2.4653253	-4.57	-2.2434418	C4orf33
237242_at	0.759	0.0319048	-2.4643995	-4.57	-1.737599	LINC00661
243637_at	0.759	0.0319648	-2.4633322	-4.57	-2.0740219	
205348_s_at	0.759	0.0321708	-2.4596809	-4.57	-1.5819201	DYNC1I1

200920_s_at	0.759	0.0321834	-2.4594577	-4.57	-3.2282111	BTG1
201892_s_at	0.759	0.0322812	-2.4577327	-4.57	-1.9925267	IMPDH2
224918_x_at	0.759	0.032369	-2.4561887	-4.57	-2.3304544	MGST1
221215_s_at	0.759	0.0324709	-2.4544012	-4.57	-2.1004107	RIPK4
201411_s_at	0.759	0.0325598	-2.4528466	-4.57	-1.6489971	PLEKHB2
220107_s_at	0.759	0.0326345	-2.4515437	-4.57	-1.9251751	ZC2HC1C
224566_at	0.759	0.0327726	-2.449142	-4.57	-2.3510653	MIR612///NEAT1
218843_at	0.759	0.0329114	-2.4467383	-4.57	-2.2309996	FNDC4
229682_at	0.759	0.0330329	-2.4446431	-4.57	-1.5290933	MAPRE3
227647_at	0.759	0.0331003	-2.4434838	-4.57	-2.3208154	KCNE3
202696_at	0.759	0.0332954	-2.4401396	-4.57	-2.7824418	OXSR1
209699_x_at	0.759	0.0334354	-2.4377525	-4.57	-2.2763867	LOC101930400///AKR1C2
240045_at	0.759	0.0334908	-2.4368098	-4.57	-1.9892442	
226034_at	0.759	0.0336139	-2.4347221	-4.57	-1.9664144	DUSP4
221781_s_at	0.759	0.0337272	-2.4328086	-4.57	-1.8891626	DNAJC10
237056_at	0.759	0.0338813	-2.4302127	-4.57	-1.7493497	INSC
219474_at	0.759	0.0339499	-2.4290614	-4.57	-2.9268533	C3orf52
210609_s_at	0.759	0.0340734	-2.4269949	-4.57	-1.7434743	TP53I3
204340_at	0.759	0.0340883	-2.4267462	-4.57	-2.0375992	TMEM187
240242_at	0.759	0.0343464	-2.4224524	-4.57	-2.059335	

204363_at	0.759	0.0343933	-2.4216751	-4.57	-1.8812052	F3
234592_at	0.759	0.0345263	-2.4194775	-4.57	-1.8372909	
220987_s_at	0.759	0.0345399	-2.4192533	-4.57	-2.2324657	NUAK2///AKIP1
211924_s_at	0.759	0.0345401	-2.4192505	-4.57	-1.9488328	PLAUR
235628_x_at	0.759	0.0345944	-2.4183562	-4.57	-1.5099574	LOC101929964///LINC01184
226783_at	0.759	0.03472	-2.4162914	-4.57	-2.2036175	PHYKPL
223395_at	0.759	0.0347286	-2.41615	-4.57	-2.0167021	ABI3BP
235652_at	0.759	0.0348809	-2.413658	-4.57	-2.07561	
32811_at	0.759	0.0352716	-2.4073135	-4.57	-1.5117971	MYO1C
213174_at	0.759	0.0352761	-2.407241	-4.57	-2.3251101	TTC9
203963_at	0.759	0.0352945	-2.4069441	-4.57	-1.7732014	CA12
223737_x_at	0.759	0.0354579	-2.4043111	-4.57	-2.5573772	CHST9
204952_at	0.759	0.0355627	-2.4026305	-4.57	-2.1901789	LYPD3
239350_at	0.759	0.0356889	-2.4006105	-4.57	-1.7978568	MARVELD3
239345_at	0.759	0.0357471	-2.3996818	-4.57	-2.4588716	SLC19A3
236340_at	0.759	0.0357973	-2.3988826	-4.57	-1.9325368	LINC01006///C7orf13
204151_x_at	0.759	0.0358004	-2.3988328	-4.57	-2.1753219	AKR1C1
205727_at	0.759	0.0358064	-2.3987385	-4.57	-1.7037369	TEP1
230757_at	0.759	0.0358275	-2.3984023	-4.57	-1.5470379	
232769_at	0.759	0.0358364	-2.3982608	-4.57	-1.8582954	
219583_s_at	0.759	0.0358531	-2.3979952	-4.57	-2.0210123	SPATA7
226992_at	0.759	0.0358672	-2.397771	-4.57	-1.6504311	NOSTRIN

237204_at	0.759	0.0358702	-2.3977224	-4.57	-2.1433487	
220954_s_at	0.759	0.0358804	-2.3975608	-4.57	-1.9370934	MIR6840///STAG3L5P-PVRIG2P-PILRB///PILRB
226320_at	0.759	0.0359512	-2.3964373	-4.57	-1.7125136	ALYREF
223136_at	0.759	0.036072	-2.3945259	-4.57	-1.8260577	AIG1
209600_s_at	0.759	0.0361116	-2.3939002	-4.57	-1.8330638	ACOX1
209021_x_at	0.759	0.03616	-2.3931363	-4.57	-2.0677149	ATG13
223206_s_at	0.759	0.0363556	-2.3900604	-4.57	-1.9674043	NMRAL1
213967_at	0.759	0.036383	-2.38963	-4.57	-3.0614153	RALYL
211504_x_at	0.759	0.0365155	-2.3875574	-4.57	-2.0935368	ROCK2
202149_at	0.759	0.0366793	-2.3850059	-4.57	-1.7268495	NEDD9
221815_at	0.759	0.0367034	-2.384631	-4.57	-2.7559937	ABHD2
244535_at	0.759	0.0367725	-2.3835585	-4.57	-1.9086086	
223449_at	0.759	0.0369176	-2.3813116	-4.57	-2.0234535	SEMA6A
224896_s_at	0.759	0.0372511	-2.3761811	-4.57	-2.8448723	TTL
235911_at	0.759	0.0372884	-2.3756097	-4.57	-2.1599279	MELTF
215358_x_at	0.759	0.0374727	-2.372796	-4.57	-1.6728068	ZNF37BP
228359_at	0.759	0.0375533	-2.3715692	-4.57	-1.8936455	UBASH3B

226485_at	0.759	0.0375695	-2.3713235	-4.57	-2.3743443	LOC101928274///VSIG10
231808_at	0.759	0.0376206	-2.3705487	-4.57	-1.8124306	OIP5-AS1
208310_s_at	0.759	0.0377526	-2.3685492	-4.57	-1.8565295	CCZ1B///CCZ1
223358_s_at	0.759	0.0378567	-2.3669771	-4.57	-1.824646	PDE7A
224209_s_at	0.759	0.0378871	-2.3665185	-4.57	-3.2144226	GDA
205421_at	0.759	0.0380711	-2.3637531	-4.57	-2.3057938	SLC22A3
203423_at	0.759	0.0381125	-2.3631329	-4.57	-2.0403895	RBP1
232472_at	0.759	0.0381217	-2.3629943	-4.57	-1.9788871	
225611_at	0.759	0.03814	-2.3627208	-4.57	-1.9532423	MAST4
204818_at	0.759	0.0381987	-2.3618418	-4.57	-2.6183053	HSD17B2
230084_at	0.759	0.0383749	-2.3592144	-4.57	-2.3179802	SLC30A2
236427_at	0.759	0.0384359	-2.358307	-4.57	-2.502694	
206027_at	0.759	0.0384804	-2.3576463	-4.57	-1.782188	S100A3
229790_at	0.759	0.0385471	-2.3566571	-4.57	-2.2176207	TERF2
209160_at	0.759	0.0385956	-2.3559384	-4.57	-1.968567	AKR1C3
202628_s_at	0.759	0.0386397	-2.3552862	-4.57	-2.2164131	SERPINE1
213649_at	0.759	0.0387434	-2.3537562	-4.57	-1.9493397	SRSF7
212912_at	0.759	0.0387688	-2.3533809	-4.57	-1.7376893	RPS6KA2
201481_s_at	0.759	0.0388205	-2.3526207	-4.57	-1.788211	PYGB
239721_at	0.759	0.0390382	-2.3494244	-4.57	-1.9720689	

201878_at	0.759	0.0390625	-2.3490692	-4.57	-1.9442529	ARIH1
205590_at	0.759	0.039078	-2.3488422	-4.57	-1.9365687	RASGRP1
217517_x_at	0.759	0.0392007	-2.3470508	-4.57	-1.6063606	SRPK2
211890_x_at	0.759	0.0392469	-2.3463786	-4.57	-1.7288457	CAPN3
228214_at	0.759	0.0393008	-2.3455944	-4.57	-2.1864967	SOX6
227944_at	0.759	0.0393295	-2.345177	-4.57	-2.1290151	PTPN3
225331_at	0.759	0.039374	-2.3445301	-4.57	-1.7297205	CCDC50
226575_at	0.759	0.0394264	-2.3437699	-4.57	-2.3270289	ZNF462
217968_at	0.759	0.0394652	-2.3432088	-4.57	-1.9690555	TSSC1
1565715_at	0.759	0.0395786	-2.3415683	-4.57	-1.5154786	
229845_at	0.759	0.0398011	-2.3383629	-4.57	-1.6082792	MAPKAP1
227113_at	0.759	0.0398105	-2.3382282	-4.57	-1.7840482	ADHFE1
244261_at	0.759	0.0398764	-2.3372823	-4.57	-1.8126989	IFNLR1
1569477_at	0.759	0.0399417	-2.336347	-4.57	-2.1989706	
1562698_x_at	0.759	0.0400984	-2.3341082	-4.57	-2.1582807	LOC339988
244383_at	0.759	0.0401081	-2.3339697	-4.57	-2.8765397	
208306_x_at	0.759	0.0401948	-2.332735	-4.57	-1.841931	HLA-DRB1
240038_at	0.759	0.0403429	-2.3306314	-4.57	-2.8714187	
230585_at	0.759	0.0403464	-2.3305819	-4.57	-2.2400567	KCNJ15
230791_at	0.759	0.0403654	-2.3303124	-4.57	-1.627046	
218898_at	0.759	0.0404147	-2.329614	-4.57	-1.501267	FAM57A

242134_at	0.759	0.0404395	-2.329263	-4.57	-2.4045636	
219469_at	0.759	0.040617	-2.3267583	-4.58	-1.5726398	DYNC2H1
223568_s_at	0.759	0.0406734	-2.3259645	-4.58	-1.9264692	PLPP5
209040_s_at	0.759	0.0407528	-2.324848	-4.58	-2.704386	PSMB8
232762_at	0.759	0.0408137	-2.3239945	-4.58	-2.6760833	KIAA1217
1565149_at	0.759	0.0408496	-2.3234904	-4.58	-1.5979166	DYNC2H1
207249_s_at	0.759	0.0409128	-2.3226058	-4.58	-2.3056421	SLC28A2
219049_at	0.759	0.0409877	-2.3215591	-4.58	-2.1015941	CSGALNACT1
219718_at	0.759	0.0412211	-2.3183094	-4.58	-1.5986475	FGGY
207784_at	0.759	0.0412903	-2.3173497	-4.58	-1.7079029	ARSD
218995_s_at	0.759	0.041296	-2.3172705	-4.58	-1.4339932	EDN1
242467_at	0.759	0.0413251	-2.3168675	-4.58	-2.0476201	
204965_at	0.759	0.0413865	-2.3160173	-4.58	-3.0286175	GC
205778_at	0.759	0.041488	-2.3146157	-4.58	-1.6730499	KLK7
244636_at	0.759	0.0415704	-2.3134795	-4.58	-2.1230095	
217811_at	0.759	0.0417263	-2.3113366	-4.58	-1.9997067	SELT
225484_at	0.759	0.0420271	-2.307222	-4.58	-1.8522068	CEP41
226851_at	0.759	0.0420437	-2.3069957	-4.58	-2.2571766	LYPLAL1
201510_at	0.759	0.0420494	-2.3069191	-4.58	-1.749283	ELF3
210461_s_at	0.759	0.0420925	-2.3063322	-4.58	-1.6390577	ABLIM1
243718_at	0.759	0.0421197	-2.305962	-4.58	-1.4113315	
218631_at	0.759	0.0421545	-2.3054892	-4.58	-1.6075894	AVPI1
223033_s_at	0.759	0.042434	-2.3017029	-4.58	-2.0952849	SCYL1
209605_at	0.759	0.0424598	-2.3013547	-4.58	-1.8222214	TST

39402_at	0.759	0.042489	-2.3009615	-4.58	-2.2277329	IL1B
209164_s_at	0.759	0.0424899	-2.3009481	-4.58	-2.1017823	CYB561
202893_at	0.759	0.0425961	-2.2995178	-4.58	-1.7942862	UNC13B
209116_x_at	0.759	0.0426954	-2.2981837	-4.58	-3.6783971	HBB
209233_at	0.759	0.0427595	-2.297324	-4.58	-2.50629	EMG1
216526_x_at	0.759	0.0427679	-2.2972116	-4.58	-1.4506713	HLA-C
1561817_at	0.759	0.0427971	-2.2968204	-4.58	-2.2788601	
1554708_s_at	0.759	0.0428382	-2.2962703	-4.58	-1.4826206	SPATA6L
242403_at	0.759	0.0428462	-2.2961633	-4.58	-2.2574531	
239638_at	0.759	0.0428497	-2.2961168	-4.58	-1.8148322	
217414_x_at	0.759	0.0428898	-2.2955806	-4.58	-1.7843482	HBA2///HBA1
240383_at	0.759	0.0429401	-2.2949084	-4.58	-2.158497	UBE2D3
213674_x_at	0.759	0.0429855	-2.2943035	-4.58	-2.8776855	IGHD
220040_x_at	0.759	0.0429958	-2.2941651	-4.58	-1.6684596	ZC4H2
236390_at	0.759	0.0430435	-2.2935304	-4.58	-2.8307382	SLX4IP
228325_at	0.759	0.0432067	-2.2913601	-4.58	-2.7483973	SPIDR
228391_at	0.759	0.0432606	-2.2906457	-4.58	-1.7232675	CYP4V2
240024_at	0.759	0.0435036	-2.2874337	-4.58	-2.0580252	SEC14L2
243395_at	0.759	0.0436035	-2.2861187	-4.58	-1.8298309	
209409_at	0.759	0.0437669	-2.2839738	-4.58	-1.8586007	GRB10
1555579_s_at	0.759	0.0437988	-2.2835561	-4.58	-1.7061477	PTPRM
202067_s_at	0.759	0.0438084	-2.2834303	-4.58	-1.8856508	LDLR

217185_s_at	0.759	0.0438274	-2.2831819	-4.58	-1.4234647	ZNF259P1///ZPR1
228846_at	0.759	0.0438368	-2.2830583	-4.58	-1.590111	MXD1
203632_s_at	0.759	0.044012	-2.2807704	-4.58	-3.2774442	GPRC5B
217767_at	0.759	0.0440383	-2.2804277	-4.58	-1.7134824	C3
202464_s_at	0.759	0.0440448	-2.2803427	-4.58	-1.4671461	PFKFB3
221627_at	0.759	0.0440654	-2.2800747	-4.58	-2.108247	TRIM10
208623_s_at	0.759	0.0441318	-2.2792103	-4.58	-1.4941821	EZR
213423_x_at	0.759	0.0443379	-2.2765379	-4.58	-2.3899645	TUSC3
210987_x_at	0.759	0.0444017	-2.2757116	-4.58	-1.6695419	TPM1
215963_x_at	0.759	0.0444163	-2.2755237	-4.58	-1.9407379	RPL3
201876_at	0.759	0.0444618	-2.2749362	-4.58	-1.6801597	PON2
200838_at	0.759	0.0445676	-2.2735718	-4.58	-1.5067624	CTSB
223569_at	0.759	0.044611	-2.2730128	-4.58	-2.2551637	PLPP5
218307_at	0.759	0.0446339	-2.2727193	-4.58	-2.1559597	RSAD1
200983_x_at	0.759	0.0446979	-2.2718965	-4.58	-1.7289109	CD59
213508_at	0.759	0.0447631	-2.2710597	-4.58	-2.240826	SPTSSA
1556950_s_at	0.759	0.0448303	-2.2701981	-4.58	-1.9017044	SERPINB6
215931_s_at	0.759	0.0448686	-2.2697077	-4.58	-1.9096868	ARFGEF2
242055_at	0.759	0.0449001	-2.2693049	-4.58	-1.6542785	PSMG4
213695_at	0.759	0.0449439	-2.2687451	-4.58	-1.9714329	PON3

228058_at	0.759	0.045038	-2.2675445	-4.58	-1.8524339	ZG16B
225503_at	0.759	0.045055	-2.2673274	-4.58	-1.826418	DHRSX
201502_s_at	0.759	0.0450972	-2.26679	-4.58	-1.6592502	NFKBIA
218723_s_at	0.759	0.0451152	-2.2665613	-4.58	-1.790702	RGCC
205650_s_at	0.759	0.0451586	-2.2660086	-4.58	-1.8535028	FGA
229899_s_at	0.759	0.0452182	-2.2652522	-4.58	-2.0961193	ZFAS1
239561_at	0.759	0.0453315	-2.2638147	-4.58	-1.7327897	
205992_s_at	0.759	0.0453521	-2.2635538	-4.58	-1.9334478	IL15
1555884_at	0.759	0.0453739	-2.2632781	-4.58	-1.9622822	PSMD6
219499_at	0.759	0.0454426	-2.2624087	-4.58	-2.1374077	SEC61A2
202284_s_at	0.759	0.0454489	-2.2623288	-4.58	-1.4400156	CDKN1A
221449_s_at	0.759	0.0455146	-2.2614991	-4.58	-2.1333196	ITFG1
228030_at	0.759	0.0455164	-2.2614766	-4.58	-2.3746251	RBM6
226499_at	0.759	0.0455331	-2.2612655	-4.58	-2.4112998	NRARP
202761_s_at	0.759	0.0456721	-2.259515	-4.58	-2.6983052	SYNE2
218864_at	0.759	0.0456743	-2.2594871	-4.58	-1.902209	TNS1
222764_at	0.759	0.0457772	-2.2581949	-4.58	-1.5834999	ASRGL1
226557_at	0.759	0.0457894	-2.2580412	-4.58	-1.6933865	TTC5
202951_at	0.759	0.0459045	-2.2565993	-4.58	-1.6770872	STK38
232357_at	0.759	0.0459196	-2.2564101	-4.58	-1.5179623	TTLL9
204674_at	0.759	0.045991	-2.255517	-4.58	-2.0425423	LRMP
209336_at	0.759	0.0460463	-2.2548273	-4.58	-2.0520374	PWP2
206764_x_at	0.759	0.0460804	-2.2544019	-4.58	-1.83355	MPPE1

1560486_at	0.759	0.046116	-2.2539573	-4.58	-1.6568112	STXBP3
223723_at	0.759	0.0461659	-2.2533361	-4.58	-2.0681969	MELTF
219247_s_at	0.759	0.0461748	-2.2532251	-4.58	-2.0984854	ZDHHC14
222450_at	0.759	0.0463052	-2.2516048	-4.58	-1.7271873	PMEPA1
244080_at	0.759	0.0463949	-2.250492	-4.58	-2.1723057	
209538_at	0.759	0.0464103	-2.2503016	-4.58	-1.8532463	ZNF32
204627_s_at	0.759	0.0464162	-2.2502279	-4.58	-1.9698923	ITGB3
201576_s_at	0.759	0.046641	-2.24745	-4.58	-2.4077513	TMPPE///GLB1
201739_at	0.759	0.0467858	-2.2456685	-4.58	-1.3478515	SGK1
200732_s_at	0.759	0.0468378	-2.245029	-4.58	-1.6137835	PTP4A1
205380_at	0.759	0.0472882	-2.2395252	-4.58	-2.3695558	PDZK1
203696_s_at	0.759	0.0473048	-2.2393236	-4.58	-1.8422285	RFC2
238053_at	0.759	0.0474307	-2.237794	-4.58	-1.9751768	DHRSX
210949_s_at	0.759	0.0475778	-2.2360122	-4.58	-1.4400771	EIF3CL///EIF3C
201650_at	0.759	0.0476743	-2.2348466	-4.58	-2.3570703	KRT19
237223_at	0.759	0.0477468	-2.2339718	-4.58	-1.6410237	
203854_at	0.759	0.0481186	-2.2295076	-4.58	-1.475211	CFI
202083_s_at	0.759	0.048135	-2.2293112	-4.58	-2.0817293	SEC14L1
227438_at	0.759	0.0482378	-2.228084	-4.58	-1.5817052	ALPK1

209850_s_at	0.759	0.0482768	-2.2276181	-4.58	-1.6637406	CDC42EP2
219181_at	0.759	0.0483654	-2.2265622	-4.58	-1.7298922	LIPG
211653_x_at	0.759	0.0483909	-2.2262589	-4.58	-2.214124	LOC101930400///AKR1C2
1569041_at	0.759	0.0484765	-2.2252414	-4.58	-2.078822	
218584_at	0.759	0.0485418	-2.2244657	-4.58	-1.6566287	TCTN1
208679_s_at	0.759	0.0486769	-2.2228656	-4.58	-1.8698733	ARPC2
218784_s_at	0.759	0.0487135	-2.2224322	-4.58	-1.9955822	SAYSD1
229202_at	0.759	0.0493137	-2.2153781	-4.58	-1.5104987	PCNX2
202487_s_at	0.759	0.0495808	-2.212265	-4.58	-1.2989011	H2AFV
201061_s_at	0.759	0.0496137	-2.211882	-4.58	-1.5094857	STOM
220444_at	0.759	0.0498368	-2.209296	-4.58	-1.7472994	ZNF557
202508_s_at	0.759	0.0498481	-2.2091655	-4.58	-2.7700075	SNAP25
1557384_at	0.759	0.0498519	-2.209122	-4.58	-1.9278884	LOC100506639///ZNF131
206696_at	0.759	0.049876	-2.208843	-4.58	-2.0486888	GPR143
226082_s_at	0.759	0.0499336	-2.2081772	-4.58	-1.8693486	SCAF4
232406_at	0.759	0.0499417	-2.2080838	-4.58	-1.7905386	LOC105372526
203233_at	0.759	0.0499682	-2.2077783	-4.58	-1.8208763	IL4R

GSE32676

ID	adj.P.Val	P.Value	t	B	logFC	Gene.symbol
201416_at	0.0000529	1.71E-09	8.46	11.732533	2.34	SOX4

223631_s_at	0.0000529	2.15E-09	8.37	11.514199	5.67	C19orf33
204885_s_at	0.0000529	2.90E-09	8.25	11.227863	6.44	MSLN
204855_at	0.0000736	5.39E-09	8.01	10.638039	5.62	SERPINB5
223484_at	0.0000903	8.26E-09	7.85	10.229196	3.77	C15orf48
218960_at	0.000136	1.49E-08	7.63	9.662555	5.76	TMPRSS4
201650_at	0.0001693	2.17E-08	7.49	9.305284	6.22	KRT19
202489_s_at	0.0001866	2.73E-08	7.4	9.083566	5.39	FXYD3
231211_s_at	0.0002011	3.31E-08	7.33	8.899194	4.54	YIF1B
209173_at	0.0002504	6.00E-08	7.11	8.327266	5.69	AGR2

213571_s_at	0.0002504	6.11E-08	7.1	8.311296	1.01	EIF4E2
206482_at	0.0002504	7.06E-08	7.05	8.17135	4.64	PTK6
219010_at	0.0002504	7.32E-08	7.04	8.137425	4.18	C1orf106
209270_at	0.0002728	9.10E-08	6.96	7.928002	4.87	LAMB3
202267_at	0.0002728	9.44E-08	6.95	7.892391	4.93	LAMC2
226988_s_at	0.0002728	9.79E-08	6.93	7.857852	2.67	MYH14
209212_s_at	0.0002728	1.05E-07	6.91	7.792327	4.14	KLF5
218677_at	0.0002995	1.30E-07	6.83	7.587519	3.12	S100A14
203878_s_at	0.0002995	1.37E-07	6.81	7.534758	5.44	MMP11
204351_at	0.0003364	1.60E-07	6.75	7.385237	6.75	S100P
218804_at	0.0004249	2.18E-07	6.64	7.089446	2.83	ANO1
209016_s_at	0.0004267	2.34E-07	6.62	7.019069	4.65	KRT7
211574_s_at	0.0004468	2.59E-07	6.58	6.921763	1.58	CD46
232361_s_at	0.0004468	2.67E-07	6.57	6.891907	2.99	EHF
207549_x_at	0.0004468	2.70E-07	6.56	6.882927	1.32	CD46

224650_at	0.0004557	2.83E-07	6.55	6.835116	3.34	MAL2
232530_at	0.0005182	3.39E-07	6.48	6.661376	2.57	PLD1
205032_at	0.0005182	3.58E-07	6.46	6.608884	3.25	ITGA2
218186_at	0.0005568	3.97E-07	6.43	6.510245	3.96	RAB25
205640_at	0.0006144	4.64E-07	6.37	6.360182	2.16	ALDH3B1
219684_at	0.0006144	4.72E-07	6.36	6.344084	2.77	RTP4
201548_s_at	0.0006221	4.89E-07	6.35	6.309498	1.49	KDM5B
205076_s_at	0.0006905	5.56E-07	6.31	6.186881	3.58	MTMR11

239370_at	0.0006964	5.73E-07	6.29	6.157027	4.23	LINC01133
223000_s_at	0.0007451	6.29E-07	6.26	6.067611	1.41	F11R
223540_at	0.0007451	6.61E-07	6.24	6.019985	3.26	NECTIN4
225436_at	0.0007451	6.71E-07	6.24	6.004807	3.21	ABHD17C
209114_at	0.0007451	6.91E-07	6.23	5.977471	4.41	TSPAN1
33323_r_at	0.0007451	6.95E-07	6.23	5.971381	5	SFN
235871_at	0.000778	7.54E-07	6.2	5.892747	3.09	LIPH
223232_s_at	0.0008784	8.68E-07	6.15	5.757788	3.32	CGN
232164_s_at	0.0009141	9.19E-07	6.13	5.701797	4.08	EPPK1
227372_s_at	0.000915	9.45E-07	6.12	5.675138	3.45	BAIAP2L1
242722_at	0.000915	9.74E-07	6.11	5.646243	2.93	LMO7
1552797_s_at	0.000915	9.87E-07	6.1	5.633221	4.07	PROM2
213668_s_at	0.0010596	1.16E-06	6.04	5.475659	2.69	SOX4
201286_at	0.0010609	1.18E-06	6.04	5.458571	3.43	SDC1
60474_at	0.0010705	1.21E-06	6.03	5.434267	3.71	FERMT1
218796_at	0.0011006	1.28E-06	6.01	5.379772	3.92	FERMT1

223949_at	0.00111	1.35E-06	5.99	5.33385	3.55	TMPRSS3
229997_at	0.00111	1.35E-06	5.99	5.332549	1.63	VANGL1
218211_s_at	0.00111	1.36E-06	5.99	5.322552	4.15	MLPH
227371_at	0.00111	1.40E-06	5.98	5.296281	3.07	BAIAP2L1
201884_at	0.0011281	1.45E-06	5.96	5.260882	6.81	CEACAM5
205807_s_at	0.0011281	1.46E-06	5.96	5.253195	2.13	TUFT1
223278_at	0.0011318	1.49E-06	5.95	5.236554	4.54	GJB2
204990_s_at	0.0011795	1.57E-06	5.93	5.183468	2.87	ITGB4
232360_at	0.0011854	1.61E-06	5.93	5.163302	3	EHF
219215_s_at	0.0011854	1.63E-06	5.92	5.152703	2.55	SLC39A4
203021_at	0.0012104	1.68E-06	5.91	5.119778	3.79	SLPI

222608_s_at	0.0012258	1.73E-06	5.9	5.095018	3.97	ANLN
219901_at	0.001255	1.84E-06	5.88	5.035436	2.18	FGD6
226961_at	0.001255	1.85E-06	5.88	5.029048	3.67	PRR15
214088_s_at	0.001255	1.86E-06	5.88	5.023566	2.42	FUT3
226535_at	0.0012901	1.93E-06	5.86	4.985174	4.96	LOC100505984///ITGB6
202504_at	0.0012938	1.99E-06	5.85	4.95924	5.31	TRIM29
220177_s_at	0.0013039	2.03E-06	5.85	4.939633	3.46	TMPRSS3
209792_s_at	0.0013039	2.05E-06	5.84	4.92904	5.15	KLK10
209211_at	0.001448	2.36E-06	5.79	4.795072	3.09	KLF5
218451_at	0.0014512	2.39E-06	5.79	4.782159	2.65	CDCP1
222904_s_at	0.0014658	2.45E-06	5.78	4.75689	3.83	TMC5

204678_s_at	0.0014658	2.47E-06	5.78	4.751341	3.6	KCNK1
1558378_a_at	0.0014868	2.53E-06	5.77	4.727279	3.65	AHNAK2
33322_i_at	0.0014886	2.59E-06	5.76	4.705289	3.76	SFN
230206_at	0.0014886	2.61E-06	5.76	4.697072	2.19	DOCK5
227475_at	0.0014886	2.61E-06	5.76	4.695537	4.22	FOXQ1
226803_at	0.001559	2.79E-06	5.73	4.633131	2.87	CHMP4C
201015_s_at	0.0015708	2.84E-06	5.73	4.614093	2.86	JUP
223233_s_at	0.0016163	3.02E-06	5.71	4.557183	2.77	CGN
226622_at	0.0016163	3.03E-06	5.7	4.552386	2.93	MUC20
206224_at	0.0016163	3.04E-06	5.7	4.548465	4.9	CST1
210715_s_at	0.0016624	3.16E-06	5.69	4.512081	1.96	SPINT2

201830_s_at	0.0016641	3.22E-06	5.68	4.494397	1.71	NET1
91826_at	0.0016641	3.23E-06	5.68	4.492769	3.8	EPS8L1
229150_at	0.0016713	3.32E-06	5.67	4.465857	3.89	
203510_at	0.0016713	3.34E-06	5.67	4.460129	2.71	MET
202826_at	0.0016713	3.35E-06	5.67	4.455237	2.72	SPINT1
227196_at	0.0016713	3.38E-06	5.67	4.448521	2.45	RHPN2
209678_s_at	0.0016713	3.39E-06	5.66	4.444222	1.48	PRKCI
226799_at	0.0017064	3.50E-06	5.65	4.415547	2.03	FGD6
201829_at	0.0017231	3.56E-06	5.65	4.397603	1.44	NET1
205016_at	0.0017469	3.67E-06	5.64	4.367483	1.63	TGFA

201474_s_at	0.0017638	3.75E-06	5.63	4.349122	2.59	ITGA3
202286_s_at	0.0017638	3.77E-06	5.63	4.34165	3.98	TACSTD2
227314_at	0.0018777	4.05E-06	5.6	4.273161	3.33	ITGA2
225314_at	0.0019231	4.29E-06	5.58	4.218769	2.27	OCIAD2
203287_at	0.0019231	4.32E-06	5.58	4.211162	2.75	LAD1
203892_at	0.0019231	4.41E-06	5.57	4.191612	3.61	WFDC2
1566766_a_at	0.0019231	4.45E-06	5.57	4.18248	2.91	MACC1
65517_at	0.0019231	4.47E-06	5.57	4.179381	2.27	AP1M2
203726_s_at	0.0019654	4.67E-06	5.55	4.135979	3.75	LAMA3
210791_s_at	0.0019987	4.79E-06	5.54	4.112428	2.19	ARHGAP32

226129_at	0.0020512	4.95E-06	5.53	4.080151	2.87	FAM83H
238439_at	0.0020542	5.12E-06	5.52	4.04867	3.13	ANKRD22
201287_s_at	0.0020542	5.14E-06	5.52	4.043887	2.68	SDC1
213590_at	0.0020542	5.18E-06	5.52	4.037711	1.88	SLC16A5
202488_s_at	0.0020542	5.21E-06	5.51	4.032148	3.75	FXYD3
228865_at	0.0020542	5.26E-06	5.51	4.022061	2.94	C1orf116
205597_at	0.00206	5.33E-06	5.5	4.009254	4.06	SLC44A4
219795_at	0.0020798	5.52E-06	5.49	3.976363	5.88	SLC6A14

1562440_at	0.0021533	5.79E-06	5.48	3.92973	2.43	MAP3K13
230263_s_at	0.0021938	5.96E-06	5.47	3.901584	1.68	DOCK5
212992_at	0.0021938	6.02E-06	5.46	3.892489	3.76	AHNAK2
208886_at	0.0022115	6.13E-06	5.46	3.874437	1.97	H1F0
232151_at	0.0022115	6.17E-06	5.45	3.868399	3.07	MACC1
214435_x_at	0.0022115	6.19E-06	5.45	3.865587	1.22	RALA
209369_at	0.0022201	6.29E-06	5.45	3.850572	2.58	ANXA3
202708_s_at	0.0022201	6.30E-06	5.45	3.849081	3.08	HIST2H2BE
219580_s_at	0.0022201	6.33E-06	5.44	3.843131	3.95	TMC5
241898_at	0.0022628	6.50E-06	5.44	3.818635	2.7	LIPH
206600_s_at	0.002271	6.59E-06	5.43	3.805449	2.06	SLC16A5
224097_s_at	0.002271	6.68E-06	5.43	3.792412	1.34	F11R

201340_s_at	0.002271	6.73E-06	5.42	3.784987	2.18	ENC1
241994_at	0.0022892	6.82E-06	5.42	3.771397	4.02	XDH
213235_at	0.0023145	6.94E-06	5.41	3.754922	1.49	KNOP1
230336_at	0.0023728	7.16E-06	5.4	3.72513	1.28	
219429_at	0.0024556	7.46E-06	5.39	3.686313	3.21	FA2H
202005_at	0.0024661	7.53E-06	5.38	3.676418	1.69	ST14
219360_s_at	0.0024732	7.60E-06	5.38	3.6679	2.44	TRPM4
203431_s_at	0.0024847	7.68E-06	5.38	3.657719	2.12	ARHGAP32
205308_at	0.0025624	8.01E-06	5.36	3.616795	1.34	ZC2HC1A
222344_at	0.0025792	8.16E-06	5.35	3.599318	2.39	

218261_at	0.002603	8.29E-06	5.35	3.584301	2.26	AP1M2
205190_at	0.002603	8.38E-06	5.35	3.574059	3.48	PLS1
201590_x_at	0.002603	8.59E-06	5.34	3.550539	1.08	ANXA2
208711_s_at	0.002603	8.59E-06	5.34	3.549736	2.13	CCND1
219476_at	0.002603	8.60E-06	5.34	3.548818	3.35	C1orf116
227725_at	0.002603	8.62E-06	5.34	3.546962	3.75	ST6GALNAC1
228969_at	0.0026291	8.82E-06	5.33	3.52505	3.96	AGR2
231941_s_at	0.0026291	8.88E-06	5.33	3.518447	2.88	MUC20
35148_at	0.0026291	8.90E-06	5.32	3.516345	2.77	TJP3
213503_x_at	0.0026354	8.99E-06	5.32	3.505707	1.1	ANXA2
201059_at	0.0026354	9.01E-06	5.32	3.503704	1.23	CTTN

213412_at	0.0026645	9.39E-06	5.31	3.46465	2.44	TJP3
236979_at	0.0026645	9.41E-06	5.3	3.462749	3.2	BCL2L15
232165_at	0.0026665	9.46E-06	5.3	3.457055	3.54	EPPK1
209008_x_at	0.002672	9.53E-06	5.3	3.45011	2.95	KRT8
201417_at	0.0026927	9.65E-06	5.3	3.437758	1.49	SOX4
238017_at	0.0026992	9.73E-06	5.29	3.430537	4.78	SDR16C5
203256_at	0.0028073	1.02E-05	5.28	3.387891	3.65	CDH3
34726_at	0.0028379	1.04E-05	5.27	3.370355	1.49	CACNB3
218644_at	0.0028379	1.04E-05	5.27	3.366514	2.71	PLEK2
210427_x_at	0.0028379	1.05E-05	5.27	3.357554	1.09	ANXA2
229377_at	0.0028379	1.06E-05	5.26	3.350476	2.97	GRTP1
219121_s_at	0.0028379	1.06E-05	5.26	3.344167	2.71	ESRP1
201562_s_at	0.0028379	1.06E-05	5.26	3.344057	1.63	SORD

224762_at	0.0028637	1.08E-05	5.26	3.330657	2.68	SERINC2
228708_at	0.0029003	1.10E-05	5.25	3.313083	3.23	RAB27B
211465_x_at	0.0029003	1.10E-05	5.25	3.309162	2.13	FUT6
211657_at	0.0029107	1.11E-05	5.25	3.299509	5.72	CEACAM6
218412_s_at	0.0029107	1.12E-05	5.24	3.29195	1.65	GTF2IRD1
239196_at	0.0029448	1.14E-05	5.24	3.276209	2.54	ANKRD22
202597_at	0.0029862	1.16E-05	5.23	3.258256	2.88	IRF6
229030_at	0.0030735	1.20E-05	5.22	3.226015	5.28	CAPN8
241397_at	0.0031122	1.23E-05	5.21	3.205049	2.29	
223748_at	0.0033301	1.35E-05	5.18	3.115046	3.12	SLC4A11
240304_s_at	0.0033501	1.37E-05	5.17	3.103539	4.32	TMC5

222392_x_at	0.0035875	1.48E-05	5.15	3.029117	2.19	PERP
219024_at	0.0036455	1.51E-05	5.14	3.009437	1.38	PLEKHA1
216836_s_at	0.0036739	1.53E-05	5.13	2.993906	2.25	ERBB2
238513_at	0.0036739	1.54E-05	5.13	2.988929	2.25	PRRG4
203757_s_at	0.0036739	1.54E-05	5.13	2.986818	5.85	CEACAM6
218686_s_at	0.0036739	1.55E-05	5.13	2.98095	1.79	RHBDF1
201418_s_at	0.0037041	1.57E-05	5.12	2.968926	1.63	SOX4
221664_s_at	0.0037353	1.59E-05	5.12	2.956739	1.33	F11R
225645_at	0.0038955	1.67E-05	5.1	2.912241	2.87	EHF
207517_at	0.0038994	1.70E-05	5.1	2.89499	2.25	LAMC2

204679_at	0.0040172	1.76E-05	5.09	2.862353	2.79	KCNK1
220030_at	0.0040779	1.80E-05	5.08	2.839926	3.34	STYK1
230207_s_at	0.0040864	1.81E-05	5.08	2.833952	1.56	DOCK5
215243_s_at	0.0040877	1.82E-05	5.07	2.826752	2.41	GJB3
201579_at	0.0041557	1.88E-05	5.06	2.798531	1.71	FAT1
208949_s_at	0.0041557	1.88E-05	5.06	2.797508	1.24	LGALS3
225412_at	0.004202	1.92E-05	5.05	2.775889	1.11	TMEM87B
205455_at	0.0043654	2.02E-05	5.04	2.728408	3.39	MST1R
217728_at	0.0043654	2.02E-05	5.04	2.727349	1.46	S100A6

1552477_a_at	0.0043654	2.03E-05	5.03	2.723974	2.78	IRF6
209035_at	0.0044634	2.10E-05	5.02	2.690223	2.35	MDK
207291_at	0.0044634	2.11E-05	5.02	2.684855	1.39	PRRG4
229927_at	0.0044634	2.11E-05	5.02	2.683924	3.74	LEMD1
208407_s_at	0.0045033	2.16E-05	5.01	2.665341	1.12	TMX2-CTNND1///CTNND1
201876_at	0.0045368	2.20E-05	5.01	2.647089	1.23	PON2
210827_s_at	0.0045368	2.20E-05	5.01	2.64454	3.19	ELF3
226185_at	0.0045531	2.23E-05	5	2.63219	3.03	CDS1
210830_s_at	0.0046014	2.28E-05	4.99	2.611157	1.44	PON2

226726_at	0.0046766	2.33E-05	4.99	2.592047	2.1	MBOAT2
223948_s_at	0.0047088	2.35E-05	4.98	2.581944	2.29	TMPRSS3
227272_at	0.0047374	2.38E-05	4.98	2.56911	2.46	C15orf52
238593_at	0.0047465	2.42E-05	4.97	2.553282	1.72	C11orf80
210608_s_at	0.0047465	2.43E-05	4.97	2.550127	2.48	FUT2
213288_at	0.0048512	2.50E-05	4.96	2.523626	2.82	MBOAT2
208190_s_at	0.0048512	2.50E-05	4.96	2.522169	2.79	LSR
228707_at	0.0049007	2.54E-05	4.96	2.507176	3.21	CLDN23
213272_s_at	0.0049771	2.60E-05	4.95	2.484044	1.42	TMEM159

227046_at	0.0051636	2.71E-05	4.93	2.445363	1.43	SLC39A11
220468_at	0.0051811	2.74E-05	4.93	2.43624	4.08	ARL14
225864_at	0.0051811	2.74E-05	4.93	2.435443	1.44	FAM84B
206414_s_at	0.0052525	2.79E-05	4.92	2.418999	1.47	ASAP2
214168_s_at	0.0054262	2.90E-05	4.91	2.381151	1.17	TJP1
221696_s_at	0.0055452	2.97E-05	4.9	2.357046	2.75	STYK1
202440_s_at	0.0055717	3.00E-05	4.9	2.349198	1.1	ST5
200635_s_at	0.0057965	3.14E-05	4.88	2.304717	1.7	PTPRF
217744_s_at	0.0058129	3.16E-05	4.88	2.298779	2.74	PERP

243444_at	0.0059112	3.22E-05	4.87	2.279453	1.63	SRD5A3
201131_s_at	0.0059962	3.30E-05	4.86	2.256134	1.99	CDH1
224598_at	0.0060524	3.35E-05	4.86	2.242384	1.91	MGAT4B
238021_s_at	0.0060524	3.35E-05	4.86	2.240824	3.12	CRNDE
226213_at	0.0061396	3.43E-05	4.85	2.219689	3.34	ERBB3
222154_s_at	0.0061396	3.44E-05	4.85	2.217639	1.19	SPATS2L
1556034_s_at	0.0061949	3.48E-05	4.85	2.205913	1.85	MTMR11

203228_at	0.0062181	3.50E-05	4.84	2.199207	1.12	PAFAH1B3
230518_at	0.0063541	3.60E-05	4.83	2.172236	2.53	MPZL2
205709_s_at	0.0063763	3.63E-05	4.83	2.16375	2.84	CDS1
201467_s_at	0.0063763	3.64E-05	4.83	2.161278	3.69	NQO1
202454_s_at	0.0063763	3.67E-05	4.83	2.15478	3.15	ERBB3
204856_at	0.0063763	3.67E-05	4.83	2.154666	2.39	B3GNT3
202525_at	0.0063763	3.67E-05	4.83	2.153526	2.73	PRSS8
1554438_at	0.006583	3.82E-05	4.81	2.116859	1.55	KIAA1217

37117_at	0.0066153	3.85E-05	4.81	2.109137	2.73	PRR5-ARHGAP8///ARHGAP8
238673_at	0.0066352	3.87E-05	4.81	2.102787	2.14	SAMD12
201596_x_at	0.0066795	3.94E-05	4.8	2.086802	2.67	KRT18
236215_at	0.0066795	3.95E-05	4.8	2.084908	1.68	
219630_at	0.0067273	4.00E-05	4.8	2.072149	3.39	PDZK1IP1
208300_at	0.0067373	4.02E-05	4.79	2.067768	2.64	PTPRH
203215_s_at	0.0068118	4.11E-05	4.79	2.044915	1.66	MYO6
238049_at	0.0068118	4.12E-05	4.79	2.042606	1.51	GRAMD3
240303_at	0.0068118	4.13E-05	4.78	2.041637	3.33	TMC5
226767_s_at	0.0068118	4.14E-05	4.78	2.038654	1.04	FAHD1

232476_at	0.0068118	4.16E-05	4.78	2.034139	1.85	
232977_x_at	0.0068118	4.16E-05	4.78	2.033977	1.9	MYH14
209803_s_at	0.0068819	4.24E-05	4.78	2.015103	3.04	PHLDA2
1553589_a_at	0.0068819	4.25E-05	4.77	2.012737	3.22	PDZK1IP1
214464_at	0.0068897	4.28E-05	4.77	2.008088	1.54	CDC42BPA
219946_x_at	0.0068897	4.28E-05	4.77	2.005984	1.67	MYH14
222675_s_at	0.0069313	4.34E-05	4.77	1.994284	1.86	BAIAP2L1
1555137_a_at	0.0069313	4.35E-05	4.77	1.991787	1.81	FGD6
214074_s_at	0.0069802	4.39E-05	4.76	1.982255	1.77	CTTN

225846_at	0.0070216	4.45E-05	4.76	1.969816	3.05	ESRP1
229490_s_at	0.0070216	4.47E-05	4.76	1.964969	2.48	
203397_s_at	0.0070216	4.48E-05	4.76	1.962753	2.25	GALNT3
235651_at	0.0070395	4.51E-05	4.75	1.957557	2.28	TTC22
211004_s_at	0.0070679	4.54E-05	4.75	1.950967	1.64	ALDH3B1
229105_at	0.0070906	4.59E-05	4.75	1.940276	2.14	GPR39
205490_x_at	0.0070906	4.59E-05	4.75	1.939726	2.26	GJB3
222549_at	0.0071236	4.63E-05	4.74	1.932581	2.8	CLDN1
203453_at	0.0071731	4.68E-05	4.74	1.920513	3.39	SCNN1A
228450_at	0.0071851	4.73E-05	4.74	1.910896	1.31	PLEKHA7

233565_s_at	0.0071851	4.76E-05	4.73	1.904507	2.64	FKBP1A-SDCBP2///SDCBP2
204641_at	0.0072015	4.79E-05	4.73	1.898149	2.87	NEK2
216905_s_at	0.0072551	4.84E-05	4.73	1.888409	1.84	ST14
219250_s_at	0.0073219	4.90E-05	4.72	1.876371	2.14	FLRT3
205601_s_at	0.0073219	4.94E-05	4.72	1.869192	1.36	HOXB5
215066_at	0.0073219	4.95E-05	4.72	1.866943	2.23	PTPRF
213425_at	0.0073219	4.98E-05	4.72	1.86187	2.29	WNT5A
227753_at	0.0073219	5.01E-05	4.72	1.856226	2.48	TMEM139

222773_s_at	0.0073219	5.04E-05	4.71	1.850157	2.11	GALNT12
218779_x_at	0.0073219	5.04E-05	4.71	1.849551	2.9	EPS8L1
239272_at	0.0073219	5.06E-05	4.71	1.846069	3.22	MMP28
226324_s_at	0.0073475	5.12E-05	4.71	1.834549	1.46	IFT172
202528_at	0.0073475	5.13E-05	4.71	1.833297	2.41	GALE
244056_at	0.0073639	5.16E-05	4.71	1.827992	4.14	SFTA2
207525_s_at	0.0074365	5.22E-05	4.7	1.816087	1.23	GIPC1
218963_s_at	0.0074453	5.24E-05	4.7	1.812469	4.41	KRT23
212531_at	0.0075448	5.34E-05	4.69	1.794325	4.59	LCN2
229553_at	0.0075448	5.38E-05	4.69	1.78845	1.4	PGM2L1
209911_x_at	0.0075448	5.38E-05	4.69	1.788187	3.02	HIST1H2BD
214782_at	0.0075952	5.45E-05	4.69	1.77609	1.53	CTTN

200660_at	0.0076293	5.49E-05	4.68	1.768992	1.35	S100A11
210399_x_at	0.0076293	5.50E-05	4.68	1.766109	1.85	FUT6
201904_s_at	0.0076377	5.54E-05	4.68	1.759591	1.79	CTDSPL
219508_at	0.0076377	5.55E-05	4.68	1.758592	3.93	GCNT3
221794_at	0.0076455	5.57E-05	4.68	1.755211	1.37	DOCK6
210519_s_at	0.0077605	5.69E-05	4.67	1.733704	3.56	NQO1
218849_s_at	0.0079049	5.84E-05	4.66	1.708896	2.33	PPP1R13L

220189_s_at	0.0080061	5.96E-05	4.66	1.689626	1.95	MGAT4B
243409_at	0.0080642	6.02E-05	4.65	1.680342	3.32	FOXL1
204702_s_at	0.0080839	6.05E-05	4.65	1.675667	2.23	NFE2L3
203407_at	0.0082137	6.16E-05	4.64	1.658058	2.7	PPL
232105_at	0.0083007	6.24E-05	4.64	1.645633	2.95	BLACAT1
216222_s_at	0.0084146	6.34E-05	4.63	1.630253	1.76	MYO10
203691_at	0.0084767	6.42E-05	4.63	1.618568	3.13	PI3
202129_s_at	0.0085038	6.50E-05	4.62	1.606439	1.65	RIOK3
201563_at	0.0085038	6.50E-05	4.62	1.606302	1.59	SORD
228437_at	0.008521	6.53E-05	4.62	1.601773	1.08	CNIH4
242013_at	0.008521	6.55E-05	4.62	1.599798	2.38	BCL2L15

202239_at	0.0086284	6.64E-05	4.62	1.585527	1.17	PARP4
1559125_at	0.0086471	6.67E-05	4.62	1.581186	2.4	
227998_at	0.0086713	6.73E-05	4.61	1.572716	1.67	S100A16
55583_at	0.0086713	6.74E-05	4.61	1.571723	1.05	DOCK6
212925_at	0.0086954	6.77E-05	4.61	1.566816	2.39	MISP
218280_x_at	0.0087552	6.86E-05	4.61	1.554705	2.43	HIST2H2AA4///HIST2H2AA3
221927_s_at	0.0087552	6.86E-05	4.61	1.554704	1.66	ABHD11
232762_at	0.0089575	7.07E-05	4.6	1.526566	1.98	KIAA1217
224595_at	0.0089575	7.08E-05	4.59	1.52427	1.35	SLC44A1
218856_at	0.0089774	7.13E-05	4.59	1.517557	1.74	TNFRSF21
209691_s_at	0.0090348	7.20E-05	4.59	1.507929	2.2	DOK4

218454_at	0.00907	7.25E-05	4.59	1.502012	1.4	PLBD1
221610_s_at	0.0090718	7.27E-05	4.59	1.499634	1.54	STAP2
222456_s_at	0.009099	7.31E-05	4.58	1.494583	2.06	LIMA1
228058_at	0.0091133	7.33E-05	4.58	1.490903	3.94	ZG16B
204825_at	0.0091571	7.39E-05	4.58	1.484138	2.56	MELK
219330_at	0.0091729	7.43E-05	4.58	1.478159	1.28	VANGL1
232046_at	0.0091826	7.46E-05	4.58	1.474986	1.9	KIAA1217
226697_at	0.0091995	7.50E-05	4.57	1.468922	1.29	FAM114A1
205927_s_at	0.009362	7.67E-05	4.57	1.447878	5.12	CTSE

205980_s_at	0.0093944	7.72E-05	4.56	1.441875	2.63	PRR5-ARHGAP8///ARHGAP8
241903_at	0.0093944	7.73E-05	4.56	1.440309	1.19	
202381_at	0.0094716	7.81E-05	4.56	1.430351	1.32	ADAM9
241763_s_at	0.0095169	7.87E-05	4.56	1.423665	2.44	FBXO32
202023_at	0.0095937	7.99E-05	4.55	1.408368	2.01	EFNA1
206994_at	0.0095937	8.02E-05	4.55	1.405811	2.42	CST4
209950_s_at	0.0097686	8.20E-05	4.54	1.384006	2.98	VILL
219787_s_at	0.0097686	8.20E-05	4.54	1.383978	2.4	ECT2
225119_at	0.009825	8.30E-05	4.54	1.372237	1	CHMP4B
225536_at	0.0098814	8.37E-05	4.54	1.364388	2.15	TMEM54
201428_at	0.0098814	8.40E-05	4.53	1.361132	2	CLDN4
213693_s_at	0.0098814	8.40E-05	4.53	1.360559	3.14	MUC1

209448_at	0.0099214	8.47E-05	4.53	1.352595	1.27	HTATIP2
239723_at	0.0099843	8.55E-05	4.53	1.344493	2.6	
215071_s_at	0.0102152	8.82E-05	4.52	1.314478	2.3	HIST1H2AC
209679_s_at	0.0104007	9.00E-05	4.51	1.295235	2.46	SMAGP
229163_at	0.0105739	9.22E-05	4.5	1.27212	2.66	CAMK2N1
204653_at	0.0105739	9.24E-05	4.5	1.269509	3.07	TFAP2A
202786_at	0.0105739	9.25E-05	4.5	1.268726	1.31	STK39
201468_s_at	0.0107335	9.44E-05	4.49	1.249066	3.09	NQO1
208613_s_at	0.0108032	9.53E-05	4.49	1.240638	1.76	FLNB
229492_at	0.0108032	9.54E-05	4.49	1.238911	1.16	VANGL1

1553099_at	0.0108309	9.59E-05	4.49	1.233981	1.7	TIGD1
236094_at	0.0108309	9.61E-05	4.49	1.232504	1.73	TCF7L2
207847_s_at	0.0108386	9.64E-05	4.49	1.22934	3.83	MUC1
214374_s_at	0.0108386	9.65E-05	4.48	1.227894	1.15	PPFIBP1
40472_at	0.0109017	9.75E-05	4.48	1.218422	2.52	LPCAT4
1555136_at	0.0109209	9.79E-05	4.48	1.214785	1.46	FGD6
209498_at	0.0109235	9.82E-05	4.48	1.211435	3.47	CEACAM1
1553768_a_at	0.0109235	9.83E-05	4.48	1.210658	2.26	DCBLD1

204026_s_at	0.0110719	1.00E-04	4.47	1.192526	2.3	ZWINT
240502_at	0.0110719	1.01E-04	4.47	1.187533	2.22	
202604_x_at	0.0110762	1.01E-04	4.47	1.182046	1.19	ADAM10
229538_s_at	0.011282	1.04E-04	4.46	1.158642	2.95	IQGAP3
215444_s_at	0.0113192	1.04E-04	4.46	1.153595	3.1	TRIM31
228904_at	0.0113736	1.05E-04	4.45	1.145224	2.08	HOXB3
202669_s_at	0.0114824	1.06E-04	4.45	1.134234	1.92	EFNB2
226637_at	0.0115914	1.08E-04	4.44	1.119567	1.13	UBE2H
214833_at	0.0116003	1.09E-04	4.44	1.11509	1.72	TMEM63A
218542_at	0.0116108	1.09E-04	4.44	1.112363	2.46	CEP55
208712_at	0.0118134	1.11E-04	4.43	1.091702	1.61	CCND1

235165_at	0.01185	1.12E-04	4.43	1.083595	1.9	PARD6B
220638_s_at	0.0118899	1.13E-04	4.43	1.077024	2.42	CBLC
202687_s_at	0.0118899	1.13E-04	4.43	1.076699	1.4	TNFSF10
222721_at	0.0119011	1.14E-04	4.43	1.072765	1.52	CNIH4
41469_at	0.0119684	1.14E-04	4.42	1.064914	3.47	PI3
213506_at	0.0120203	1.15E-04	4.42	1.057132	2.22	F2RL1
223779_at	0.0121113	1.17E-04	4.42	1.044477	2.88	AFAP1-AS1
203713_s_at	0.012144	1.18E-04	4.41	1.037399	1.88	LLGL2
202421_at	0.012144	1.18E-04	4.41	1.036865	1.5	IGSF3

205780_at	0.0123809	1.21E-04	4.4	1.010855	2.63	BIK
221884_at	0.0123918	1.22E-04	4.4	1.000893	2.46	MECOM
215177_s_at	0.0123918	1.22E-04	4.4	1.0008	1.96	ITGA6
232471_at	0.0123918	1.23E-04	4.4	0.999887	1.07	
200923_at	0.0123918	1.23E-04	4.4	0.994078	1.36	LGALS3BP
222587_s_at	0.0125929	1.26E-04	4.39	0.9717	1.56	GALNT7
206023_at	0.012781	1.29E-04	4.38	0.952659	3.97	NMU
209260_at	0.012781	1.29E-04	4.38	0.952323	2.64	SFN
235286_at	0.0128581	1.30E-04	4.38	0.941392	1.76	
218816_at	0.0129937	1.32E-04	4.37	0.926213	1.46	LRRC1
203585_at	0.0130123	1.33E-04	4.37	0.923131	1.75	ZNF185
225548_at	0.0130493	1.33E-04	4.37	0.91871	2.66	SHROOM3
201291_s_at	0.0133645	1.37E-04	4.36	0.894213	3.95	TOP2A

228923_at	0.013467	1.38E-04	4.36	0.883509	3.11	S100A6
202954_at	0.0135113	1.40E-04	4.35	0.875788	2.44	UBE2C
227473_at	0.0135113	1.40E-04	4.35	0.873186	1.06	
241812_at	0.0135113	1.40E-04	4.35	0.871885	1.51	SPATS2L
202790_at	0.0135113	1.41E-04	4.35	0.869289	2.31	CLDN7
204779_s_at	0.0135113	1.41E-04	4.35	0.867919	2.53	HOXB7
228241_at	0.0136175	1.43E-04	4.35	0.854394	3.76	AGR3
222853_at	0.0137031	1.44E-04	4.34	0.846747	2.42	FLRT3
205009_at	0.0137165	1.44E-04	4.34	0.844152	5.06	TFF1
213929_at	0.0137504	1.46E-04	4.34	0.83353	2.32	EXPH5

227960_s_at	0.0138289	1.47E-04	4.33	0.823168	1.14	FAHD1
242196_at	0.0139381	1.49E-04	4.33	0.812389	1.68	ARHGAP32
202949_s_at	0.0140029	1.50E-04	4.33	0.804665	1.58	FHL2
206515_at	0.0140087	1.51E-04	4.32	0.798036	2.85	CYP4F3
223249_at	0.0140087	1.51E-04	4.32	0.797828	1.88	CLDN12
219411_at	0.014039	1.52E-04	4.32	0.794153	1.93	ELMO3
217208_s_at	0.0140889	1.54E-04	4.32	0.784341	1.41	DLG1
224596_at	0.0141464	1.55E-04	4.32	0.774201	1.31	SLC44A1
201633_s_at	0.0141573	1.57E-04	4.31	0.762265	1.11	CYB5B

214352_s_at	0.0143225	1.59E-04	4.31	0.749618	1.07	KRAS
1555326_a_at	0.0144209	1.62E-04	4.3	0.735746	1.98	ADAM9
226403_at	0.0144209	1.62E-04	4.3	0.733924	2.53	TMC4
205990_s_at	0.0144209	1.62E-04	4.3	0.730614	2.19	WNT5A
202411_at	0.0144464	1.63E-04	4.3	0.72678	2.1	IFI27
218885_s_at	0.0144994	1.65E-04	4.29	0.717467	1.77	GALNT12
202831_at	0.01459	1.66E-04	4.29	0.70871	4.2	GPX2
227492_at	0.0146398	1.67E-04	4.29	0.702201	1.78	OCLN
218693_at	0.0146398	1.68E-04	4.29	0.699665	1.61	TSPAN15
230398_at	0.0146398	1.68E-04	4.29	0.699351	2.98	TNS4
218815_s_at	0.0147117	1.69E-04	4.28	0.691644	1.09	TMEM51
208540_x_at	0.0148732	1.72E-04	4.28	0.677038	1.04	

228232_s_at	0.0148732	1.72E-04	4.28	0.676455	2.97	VSIG2
213893_x_at	0.0152078	1.77E-04	4.27	0.649447	1.03	PMS2P9///PMS2P5
214290_s_at	0.0153048	1.78E-04	4.27	0.641793	2.21	HIST2H2AA4///HIST2H2AA3
217901_at	0.0153377	1.79E-04	4.26	0.63685	2.33	DSG2
225177_at	0.0156774	1.84E-04	4.25	0.611357	1.3	RAB11FIP1
202145_at	0.0156774	1.85E-04	4.25	0.608524	2.16	LY6E

203108_at	0.015766	1.86E-04	4.25	0.600197	3.14	GPRC5A
51192_at	0.0160211	1.90E-04	4.24	0.580473	1.47	SSH3
225750_at	0.0160945	1.91E-04	4.24	0.574648	1.4	ERO1A
218585_s_at	0.0161756	1.93E-04	4.24	0.566929	2.35	DTL
225320_at	0.0163506	1.96E-04	4.23	0.553873	1.92	MCU
205483_s_at	0.0163506	1.96E-04	4.23	0.553746	1.79	ISG15
204136_at	0.0164007	1.97E-04	4.23	0.547631	1.92	COL7A1
226552_at	0.0164007	1.98E-04	4.23	0.542093	1.51	IER5L
202668_at	0.0164007	1.99E-04	4.23	0.539088	1.43	EFNB2
228400_at	0.0164007	1.99E-04	4.23	0.538819	2.88	SHROOM3

232202_at	0.0164007	1.99E-04	4.23	0.535514	2.39	FAM83B
217109_at	0.0164007	1.99E-04	4.23	0.534933	4.3	MUC4
208170_s_at	0.0165164	2.01E-04	4.22	0.526802	2.86	TRIM31
1553105_s_at	0.0167085	2.05E-04	4.22	0.508398	2.71	DSG2
212876_at	0.01677	2.06E-04	4.21	0.502311	1.16	B4GALT4
219404_at	0.0170772	2.12E-04	4.2	0.47883	4.19	EPS8L3
210143_at	0.0170772	2.12E-04	4.2	0.476831	4.32	ANXA10
213078_x_at	0.0170772	2.12E-04	4.2	0.476555	2.62	LPCAT4
236513_at	0.0173383	2.17E-04	4.2	0.452797	1.67	PRELID2
223229_at	0.0173495	2.18E-04	4.19	0.448908	2.17	UBE2T
216641_s_at	0.0173495	2.19E-04	4.19	0.447551	1.66	LAD1
201549_x_at	0.0174172	2.21E-04	4.19	0.437846	1.09	KDM5B

235152_at	0.0174172	2.21E-04	4.19	0.435976	1.16	LOC101927204
217140_s_at	0.0174657	2.22E-04	4.19	0.431562	1.06	VDAC1
225803_at	0.0176426	2.26E-04	4.18	0.416508	1.88	FBXO32
203236_s_at	0.0177116	2.28E-04	4.18	0.408714	1.02	LGALS9
201341_at	0.0177138	2.29E-04	4.18	0.405456	1.61	ENC1
223078_s_at	0.0177939	2.30E-04	4.17	0.398355	1.19	TMOD3
215903_s_at	0.0179704	2.33E-04	4.17	0.386071	1.07	MAST2
211002_s_at	0.0179704	2.33E-04	4.17	0.385142	2.81	TRIM29
227334_at	0.0179704	2.34E-04	4.17	0.382095	1.7	USP54
242649_x_at	0.0180357	2.36E-04	4.17	0.374744	1.41	HMGN2P46
1559360_at	0.0180357	2.39E-04	4.16	0.364378	2.79	
230323_s_at	0.0180357	2.39E-04	4.16	0.364309	3.63	TMEM45B

203213_at	0.0180357	2.39E-04	4.16	0.362632	2.6	CDK1
212236_x_at	0.0180357	2.40E-04	4.16	0.358276	3.52	KRT17///JUP
205339_at	0.0180357	2.40E-04	4.16	0.357184	1.51	STIL
240735_at	0.0180357	2.40E-04	4.16	0.35686	1.78	CDC42BPA
205406_s_at	0.0180375	2.41E-04	4.16	0.355455	1.32	SPA17
227431_at	0.0181164	2.43E-04	4.16	0.348696	1.42	APTR
234725_s_at	0.0183601	2.46E-04	4.15	0.334675	1.6	SEMA4B
210159_s_at	0.0188915	2.55E-04	4.14	0.299751	2.56	TRIM31
210253_at	0.0189552	2.57E-04	4.14	0.295259	1.47	HTATIP2
222750_s_at	0.0190941	2.59E-04	4.13	0.287026	1.93	SRD5A3

202935_s_at	0.0192543	2.62E-04	4.13	0.276504	2.42	SOX9
201656_at	0.0194031	2.65E-04	4.12	0.264069	1.37	ITGA6
219749_at	0.0194034	2.66E-04	4.12	0.262122	1.76	SH2D4A
219513_s_at	0.019433	2.68E-04	4.12	0.252828	1.89	SH2D3A
218180_s_at	0.0196711	2.73E-04	4.11	0.237105	1.94	EPS8L2
238666_at	0.0198336	2.76E-04	4.11	0.225517	1.82	
222810_s_at	0.0201577	2.81E-04	4.1	0.20885	1.3	RASAL2
236616_at	0.0203725	2.85E-04	4.1	0.196271	1.72	PSD4
229545_at	0.0204231	2.86E-04	4.1	0.192669	2.13	FERMT1
230229_at	0.0207506	2.91E-04	4.09	0.17381	1.11	DLG1
211184_s_at	0.0209309	2.95E-04	4.09	0.16311	2.74	USH1C
202869_at	0.0209813	2.96E-04	4.08	0.159586	2.51	OAS1

213008_at	0.0209991	2.97E-04	4.08	0.157546	2.24	FANCI
239381_at	0.0210657	2.98E-04	4.08	0.153307	3.31	KLK7
201310_s_at	0.0212139	3.01E-04	4.08	0.144212	1.53	NREP
212070_at	0.0212139	3.01E-04	4.08	0.144182	1.65	ADGRG1
217419_x_at	0.0213166	3.03E-04	4.08	0.138364	1.86	AGRN
216568_x_at	0.0213707	3.04E-04	4.07	0.132283	1.6	
238028_at	0.0213897	3.06E-04	4.07	0.128998	1.96	C6orf132
204765_at	0.0214401	3.07E-04	4.07	0.123117	1.59	ARHGEF5
241762_at	0.0215962	3.12E-04	4.06	0.107916	2.32	FBXO32
214476_at	0.0215962	3.13E-04	4.06	0.106567	5.19	TFF2
218182_s_at	0.0216886	3.16E-04	4.06	0.097718	1.97	CLDN1
229254_at	0.0218487	3.19E-04	4.06	0.088341	2.37	MFSD4A

239273_s_at	0.0219116	3.21E-04	4.06	0.083227	3.06	MMP28
234156_at	0.02201	3.24E-04	4.05	0.07376	1.5	
202185_at	0.02201	3.24E-04	4.05	0.073047	1.05	PLOD3
202096_s_at	0.02201	3.24E-04	4.05	0.072515	1.26	TSPO
214329_x_at	0.02201	3.24E-04	4.05	0.071865	1.34	TNFSF10
208890_s_at	0.0220388	3.26E-04	4.05	0.068266	1.08	PLXNB2
221655_x_at	0.0221411	3.29E-04	4.05	0.059173	2.26	EPS8L1
205660_at	0.0221724	3.30E-04	4.05	0.05666	2.55	OASL
212285_s_at	0.022521	3.37E-04	4.04	0.036006	1.82	AGRN
239609_s_at	0.022546	3.38E-04	4.04	0.03379	2.15	LPCAT4
242881_x_at	0.0226129	3.39E-04	4.04	0.029817	1.91	DUXAP10

235148_at	0.0226375	3.40E-04	4.03	0.026465	1.82	KRTCAP3
235927_at	0.0226869	3.42E-04	4.03	0.022085	1.27	XPO1
201243_s_at	0.0229415	3.47E-04	4.03	0.007755	1.84	ATP1B1
230660_at	0.02304	3.50E-04	4.02	0.000189	2.17	SERTAD4
239022_at	0.0230679	3.51E-04	4.02	-0.00406	1.02	
214898_x_at	0.0232811	3.57E-04	4.02	-0.019244	1.99	MUC3B
209222_s_at	0.0234156	3.62E-04	4.01	-0.032305	1.08	OSBPL2
214073_at	0.0234156	3.62E-04	4.01	-0.033229	2.31	CTTN
204401_at	0.0234156	3.63E-04	4.01	-0.034103	2.8	KCNN4
222396_at	0.0236263	3.66E-04	4.01	-0.043734	1.31	HN1

206949_s_at	0.0237987	3.71E-04	4	-0.054438	1.17	RUSC1
218309_at	0.0238918	3.76E-04	4	-0.068598	2.08	CAMK2N1
231311_at	0.0238918	3.76E-04	4	-0.069386	2.07	
222067_x_at	0.0239589	3.79E-04	3.99	-0.075858	2.61	HIST1H2BD
204073_s_at	0.0241775	3.83E-04	3.99	-0.0856	2.75	MYRF
221665_s_at	0.0241775	3.84E-04	3.99	-0.088864	2.1	EPS8L1
214235_at	0.0242543	3.87E-04	3.99	-0.096125	3.55	CYP3A5
201193_at	0.0242543	3.88E-04	3.99	-0.097107	1.09	IDH1
220800_s_at	0.0242543	3.88E-04	3.99	-0.098576	1.01	TMOD3
1552502_s_at	0.0244111	3.94E-04	3.98	-0.11221	2.6	RHBDL2

207169_x_at	0.0244512	3.96E-04	3.98	-0.117955	2.11	MIR4640///DDR1
219439_at	0.0244512	3.96E-04	3.98	-0.11834	1.46	C1GALT1
225224_at	0.0244512	3.97E-04	3.98	-0.119019	1.1	NOL4L
226609_at	0.0247553	4.03E-04	3.97	-0.134302	1.45	DCBLD1
230424_at	0.0247553	4.03E-04	3.97	-0.135023	1.65	NREP
214234_s_at	0.0247748	4.05E-04	3.97	-0.138711	3.57	CYP3A5
227556_at	0.0249437	4.09E-04	3.97	-0.147529	2.14	NME7
236083_at	0.0249995	4.11E-04	3.97	-0.152823	2.66	BCL2L15

210706_s_at	0.0251074	4.16E-04	3.96	-0.163222	1.28	RNF24
209605_at	0.0251682	4.18E-04	3.96	-0.167766	1.73	TST
226560_at	0.0251682	4.18E-04	3.96	-0.169702	2.83	SGPP2
231835_at	0.0252551	4.23E-04	3.96	-0.179304	1.93	FAM213B
202071_at	0.0252551	4.23E-04	3.96	-0.179762	1.39	SDC4
227452_at	0.0252551	4.23E-04	3.96	-0.180253	2.57	LINC00673///LINC00511
218657_at	0.0252692	4.24E-04	3.95	-0.183206	1.98	RAPGEFL1
231227_at	0.0252692	4.25E-04	3.95	-0.184326	1.88	

201189_s_at	0.0252692	4.25E-04	3.95	-0.184917	1.86	ITPR3
226485_at	0.0253334	4.27E-04	3.95	-0.188355	1.36	LOC101928274///VSIG10
1554897_s_at	0.0253856	4.29E-04	3.95	-0.192367	2.4	RHBDL2
221879_at	0.0256441	4.33E-04	3.95	-0.203008	2.21	CALML4
203824_at	0.0257764	4.36E-04	3.94	-0.208913	3.02	TSPAN8
235583_at	0.0258692	4.38E-04	3.94	-0.213348	1.74	ILDR1
234290_x_at	0.0259171	4.41E-04	3.94	-0.219255	1.53	MYH14
1558216_at	0.0259171	4.43E-04	3.94	-0.224387	3.02	AFAP1-AS1
1568857_a_at	0.0259171	4.44E-04	3.94	-0.226538	1	NBR1
238967_at	0.0259171	4.45E-04	3.94	-0.228674	1.68	

221843_s_at	0.0259171	4.46E-04	3.94	-0.229337	1.46	TLDC1
204378_at	0.0259812	4.47E-04	3.94	-0.23269	2.74	BCAS1
224746_at	0.0261329	4.51E-04	3.93	-0.241294	1.65	KIAA1522

208596_s_at	0.0261329	4.51E-04	3.93	-0.241534	2.5	UGT1A3///UGT1A1///UGT1A4///UGT1A9///UGT1A5///UGT1A6///UGT1A

202503_s_at	0.0261329	4.53E-04	3.93	-0.244096	2.68	KIAA0101
214734_at	0.0261329	4.53E-04	3.93	-0.245243	1.68	EXPH5
206102_at	0.0261625	4.54E-04	3.93	-0.247316	1.97	GINS1
200824_at	0.0262039	4.55E-04	3.93	-0.249815	1.02	GSTP1
209529_at	0.0263133	4.59E-04	3.93	-0.257039	2.27	PLPP2
206043_s_at	0.0263133	4.59E-04	3.93	-0.25775	2.73	ATP2C2
225278_at	0.0263457	4.61E-04	3.92	-0.260902	1.1	PRKAB2
209635_at	0.0266312	4.69E-04	3.92	-0.278043	1.28	AP1S1
226292_at	0.0266437	4.70E-04	3.92	-0.27947	2.8	CAPN5
229887_at	0.0266704	4.71E-04	3.92	-0.282385	1.42	ALS2CL
201681_s_at	0.0267263	4.74E-04	3.91	-0.28731	1.07	DLG5
209873_s_at	0.0268222	4.76E-04	3.91	-0.291684	1.75	PKP3

210480_s_at	0.0268806	4.79E-04	3.91	-0.297298	1.57	MYO6
205157_s_at	0.0268806	4.79E-04	3.91	-0.297489	4.01	KRT17///JUP
212473_s_at	0.0268806	4.79E-04	3.91	-0.297649	1.24	MICAL2
226456_at	0.0269408	4.81E-04	3.91	-0.301713	1.84	RMI2
207828_s_at	0.027127	4.85E-04	3.91	-0.309215	2.44	CENPF
226455_at	0.0272611	4.89E-04	3.9	-0.31761	1.52	CREB3L4
200885_at	0.0272611	4.90E-04	3.9	-0.318864	1.18	RHOC
39549_at	0.0272611	4.90E-04	3.9	-0.319694	1.51	NPAS2
226226_at	0.0273176	4.93E-04	3.9	-0.324741	3.14	TMEM45B
206659_at	0.0273176	4.93E-04	3.9	-0.325505	1.6	

65438_at	0.0273235	4.95E-04	3.9	-0.328803	1.42	TLDC1
219919_s_at	0.0273472	4.97E-04	3.9	-0.33273	1.32	SSH3
200636_s_at	0.027399	5.00E-04	3.89	-0.33788	1.4	PTPRF
201976_s_at	0.0275307	5.06E-04	3.89	-0.349998	1.38	MYO10
238022_at	0.0277006	5.11E-04	3.89	-0.358653	2.35	CRNDE
219722_s_at	0.0277302	5.12E-04	3.89	-0.360934	1.66	GDPD3
210540_s_at	0.0277302	5.12E-04	3.89	-0.361793	1.12	B4GALT4
204608_at	0.0277302	5.13E-04	3.89	-0.36248	1.33	ASL

214355_x_at	0.0277506	5.14E-04	3.88	-0.36547	1.58	CTAGE8///CTAGE4///CTAGE9///CTAGE15
212611_at	0.0277506	5.15E-04	3.88	-0.36609	1.43	DTX4
226507_at	0.0277506	5.15E-04	3.88	-0.366921	1.33	PAK1
207076_s_at	0.0277828	5.16E-04	3.88	-0.368954	1.6	ASS1
213462_at	0.028118	5.27E-04	3.88	-0.388671	1.39	NPAS2

204532_x_at	0.0282436	5.30E-04	3.87	-0.393817	2.62	UGT1A1///UGT1A4///UGT1A9///UGT1A6///UGT1A8///UGT1A10

213365_at	0.0282821	5.32E-04	3.87	-0.396952	1.14	ERI2
225467_s_at	0.0283938	5.34E-04	3.87	-0.401709	1.1	RDH13
204272_at	0.0283938	5.35E-04	3.87	-0.402529	3.84	LGALS4
243695_at	0.0284651	5.37E-04	3.87	-0.406248	1.2	
1007_s_at	0.0284651	5.38E-04	3.87	-0.407392	1.85	MIR4640///DDR1
242979_at	0.0284651	5.38E-04	3.87	-0.407659	1.68	IRS1
1558356_at	0.0285074	5.40E-04	3.87	-0.411813	1.34	UACA
219395_at	0.0285095	5.41E-04	3.87	-0.41371	2.34	MIR6773///ESRP2

225325_at	0.028714	5.46E-04	3.86	-0.421393	1.09	MFSD6
238715_at	0.0288165	5.49E-04	3.86	-0.426316	1.24	LOC646014
218942_at	0.0288221	5.49E-04	3.86	-0.427697	1.03	PIP4K2C
210609_s_at	0.0288688	5.52E-04	3.86	-0.431583	1.31	TP53I3
223382_s_at	0.0288688	5.52E-04	3.86	-0.431937	1.11	ZNRF1
221256_s_at	0.0289554	5.54E-04	3.86	-0.436355	1.92	HDHD3
207705_s_at	0.0289554	5.56E-04	3.86	-0.438411	1.19	NINL
242310_at	0.0289554	5.56E-04	3.86	-0.438495	1.36	

222847_s_at	0.0289554	5.57E-04	3.86	-0.440101	2.7	EGLN3
205137_x_at	0.0294827	5.74E-04	3.84	-0.468536	2.49	USH1C
225009_at	0.0297619	5.81E-04	3.84	-0.481502	1.35	CMTM4
243462_s_at	0.0297989	5.84E-04	3.84	-0.485234	1.59	
224204_x_at	0.0300739	5.92E-04	3.83	-0.498339	1.94	ARNTL2
225150_s_at	0.0301	5.93E-04	3.83	-0.50004	1.76	RTKN
227943_at	0.0301851	5.96E-04	3.83	-0.504468	1.56	
207606_s_at	0.0303105	5.99E-04	3.83	-0.510146	1.48	ARHGAP12
204669_s_at	0.030426	6.02E-04	3.83	-0.51462	1.34	RNF24
235452_at	0.0304709	6.04E-04	3.83	-0.517929	1.48	

226706_at	0.0307313	6.15E-04	3.82	-0.535023	1.19	FLJ23867///QSOX1
211043_s_at	0.030735	6.17E-04	3.82	-0.537214	1.06	CLTB
226420_at	0.0307637	6.18E-04	3.82	-0.539485	2.13	MECOM
211202_s_at	0.0309034	6.24E-04	3.81	-0.549012	1.03	KDM5B
229648_at	0.0310125	6.28E-04	3.81	-0.554305	1.74	ARHGAP32
227177_at	0.0310674	6.31E-04	3.81	-0.559618	2.66	CORO2A
214483_s_at	0.0310674	6.32E-04	3.81	-0.5602	1.26	ARFIP1
201242_s_at	0.0310674	6.33E-04	3.81	-0.561497	2.21	ATP1B1
210749_x_at	0.0310674	6.33E-04	3.81	-0.562188	2.01	MIR4640///DDR1

223586_at	0.0310674	6.33E-04	3.81	-0.562374	1.82	ARNTL2
212336_at	0.0313069	6.40E-04	3.8	-0.572598	1.71	EPB41L1

207126_x_at	0.0313274	6.42E-04	3.8	-0.574907	3.19	UGT1A1///UGT1A4///UGT1A9///UGT1A6///UGT1A8///UGT1A10

235141_at	0.0313424	6.43E-04	3.8	-0.576203	2.14	MARVELD2
222819_at	0.0313812	6.45E-04	3.8	-0.579061	1.15	CTPS2
201927_s_at	0.0314038	6.46E-04	3.8	-0.580583	1.24	PKP4
221911_at	0.0314797	6.48E-04	3.8	-0.584632	2.18	ETV1
204778_x_at	0.0314797	6.49E-04	3.8	-0.585658	1.93	HOXB7
228236_at	0.0314797	6.52E-04	3.8	-0.589387	1.66	SLC52A3
201481_s_at	0.0314797	6.52E-04	3.8	-0.590107	1.47	PYGB
202674_s_at	0.0314797	6.53E-04	3.8	-0.591991	1.88	LMO7
218313_s_at	0.0314797	6.55E-04	3.8	-0.59383	1.18	GALNT7
231882_at	0.0314797	6.55E-04	3.8	-0.594136	1.85	
219127_at	0.0314797	6.59E-04	3.79	-0.599395	2.44	PRR15L
235110_at	0.0314797	6.59E-04	3.79	-0.600375	1.75	PLA2G16
221173_at	0.0315538	6.62E-04	3.79	-0.604	2.61	USH1C
243184_at	0.0316022	6.65E-04	3.79	-0.60817	1.48	

220658_s_at	0.031948	6.77E-04	3.78	-0.625013	2.08	ARNTL2
243582_at	0.0320699	6.83E-04	3.78	-0.633898	2.67	SH3RF2
217755_at	0.0322796	6.95E-04	3.77	-0.650405	1.7	HN1
1558738_at	0.0322796	6.96E-04	3.77	-0.651393	1.4	NOL3
242546_at	0.0322796	6.96E-04	3.77	-0.652063	1.73	LINC01296///DUXAP10
205778_at	0.0322796	6.97E-04	3.77	-0.652574	3.43	KLK7
223363_at	0.0324516	7.02E-04	3.77	-0.659998	1.11	PSMG3

227676_at	0.0324539	7.03E-04	3.77	-0.660863	3.27	FAM3D
224623_at	0.0324763	7.04E-04	3.77	-0.662314	1.21	LOC728554///THOC3
205366_s_at	0.03265	7.11E-04	3.77	-0.671337	1.48	HOXB6
223849_s_at	0.0329487	7.19E-04	3.76	-0.682134	1.09	MOV10
204927_at	0.0329487	7.19E-04	3.76	-0.682324	1.72	RASSF7
201292_at	0.0330342	7.24E-04	3.76	-0.688727	2.76	TOP2A
218009_s_at	0.0331429	7.29E-04	3.76	-0.695547	2.04	PRC1
204259_at	0.0331429	7.29E-04	3.76	-0.695766	3.3	MMP7
207180_s_at	0.0332566	7.32E-04	3.75	-0.699789	1.41	HTATIP2
236471_at	0.0333912	7.37E-04	3.75	-0.705172	1.68	NFE2L3

232017_at	0.033553	7.42E-04	3.75	-0.711578	1.35	TJP2
90265_at	0.0335702	7.45E-04	3.75	-0.716064	1.98	ADAP1
205968_at	0.0337925	7.52E-04	3.75	-0.724341	1.83	KCNS3
223452_s_at	0.0337925	7.53E-04	3.74	-0.726495	1.21	ATL3
218788_s_at	0.0341021	7.62E-04	3.74	-0.737528	1.18	SMYD3
202516_s_at	0.0341842	7.66E-04	3.74	-0.741674	1.15	DLG1
205107_s_at	0.0342116	7.69E-04	3.74	-0.745952	1.23	EFNA4

215125_s_at	0.0343666	7.75E-04	3.73	-0.753153	3.58	UGT1A3///UGT1A1///UGT1A4///UGT1A9///UGT1A5///UGT1A6///UGT1A

211026_s_at	0.0344067	7.77E-04	3.73	-0.75516	1.94	MGLL
229097_at	0.0347292	7.86E-04	3.73	-0.767027	1.17	DIAPH3
201839_s_at	0.0347785	7.88E-04	3.73	-0.769129	2.6	EPCAM
218077_s_at	0.0350232	7.97E-04	3.72	-0.779178	1.04	ZDHHC3
221024_s_at	0.0350446	7.98E-04	3.72	-0.781191	1.64	SLC2A10
213518_at	0.0350446	7.99E-04	3.72	-0.781853	1.08	PRKCI
218826_at	0.0350454	8.01E-04	3.72	-0.784696	1.26	SLC35F2
242234_at	0.0352393	8.09E-04	3.72	-0.793675	1.37	XAF1
1552619_a_at	0.0353205	8.13E-04	3.72	-0.798124	2.39	ANLN
203876_s_at	0.0353683	8.16E-04	3.71	-0.802375	2.88	MMP11

1556009_at	0.0354197	8.19E-04	3.71	-0.805411	1.09	PEX13
201906_s_at	0.0354339	8.22E-04	3.71	-0.809283	1.22	CTDSPL
203559_s_at	0.0354477	8.23E-04	3.71	-0.810459	3.76	AOC1
1555842_at	0.0355727	8.29E-04	3.71	-0.81717	1.32	CYTH2
244780_at	0.0355727	8.30E-04	3.71	-0.818027	2.69	SGPP2
228892_at	0.0355727	8.30E-04	3.71	-0.818232	1.7	SH3RF2
201510_at	0.0355997	8.33E-04	3.71	-0.821346	2.31	ELF3
201188_s_at	0.0355997	8.34E-04	3.71	-0.822017	1.87	ITPR3
238567_at	0.0356759	8.38E-04	3.71	-0.826864	2.01	SGPP2

1567458_s_at	0.0357095	8.39E-04	3.7	-0.828488	1.04	RAC1
201890_at	0.0358383	8.45E-04	3.7	-0.834546	2.4	RRM2
200872_at	0.0358383	8.48E-04	3.7	-0.837983	1.07	S100A10
211518_s_at	0.0358383	8.48E-04	3.7	-0.838769	2.83	BMP4
218800_at	0.0358383	8.49E-04	3.7	-0.839006	1.35	SRD5A3
235955_at	0.0358468	8.50E-04	3.7	-0.840156	1.81	MARVELD2
1558695_at	0.0359796	8.54E-04	3.7	-0.8451	1.79	
209921_at	0.036051	8.57E-04	3.7	-0.848058	2.15	SLC7A11

229842_at	0.036051	8.59E-04	3.7	-0.850313	2.49	ELF3
208161_s_at	0.036051	8.60E-04	3.7	-0.851324	2.07	ABCC3
1553764_a_at	0.0360716	8.61E-04	3.7	-0.852588	1.37	AJUBA
214070_s_at	0.0360731	8.62E-04	3.69	-0.853349	2.67	ATP10B
1553530_a_at	0.0361671	8.65E-04	3.69	-0.857249	1.16	ITGB1
201201_at	0.0362227	8.68E-04	3.69	-0.860267	1.12	CSTB
216973_s_at	0.0364123	8.75E-04	3.69	-0.867942	1.95	HOXB7
201825_s_at	0.0364316	8.76E-04	3.69	-0.869159	1.4	SCCPDH
229964_at	0.0366016	8.81E-04	3.69	-0.874269	3.49	C9orf152
219959_at	0.0367625	8.86E-04	3.68	-0.880088	2.04	MOCOS

239010_at	0.0367625	8.87E-04	3.68	-0.880556	1.56	DUXAP10
219918_s_at	0.0367761	8.88E-04	3.68	-0.881621	2.8	ASPM
202894_at	0.0371169	9.03E-04	3.68	-0.897775	1.22	EPHB4
225301_s_at	0.0371169	9.04E-04	3.68	-0.898371	1.9	MYO5B
212022_s_at	0.0371169	9.05E-04	3.68	-0.899904	2.2	MKI67
203216_s_at	0.0371621	9.08E-04	3.68	-0.902835	1.19	MYO6
219263_at	0.0372438	9.13E-04	3.67	-0.908217	2.68	RNF128
210220_at	0.0372438	9.14E-04	3.67	-0.909491	1.38	FZD2
230892_at	0.0372438	9.17E-04	3.67	-0.911559	1.04	
209160_at	0.0375896	9.28E-04	3.67	-0.923454	2.2	AKR1C3
220021_at	0.0377058	9.34E-04	3.67	-0.929156	1.34	TMC7
226187_at	0.0377394	9.35E-04	3.66	-0.93069	1.52	CDS1

209596_at	0.0379712	9.42E-04	3.66	-0.937684	1.88	MXRA5
204818_at	0.0382953	9.58E-04	3.66	-0.953242	2.72	HSD17B2
219332_at	0.0385411	9.69E-04	3.65	-0.963989	1.53	MICALL2
215017_s_at	0.0385673	9.72E-04	3.65	-0.96702	1.1	FNBP1L
201850_at	0.0386414	9.75E-04	3.65	-0.970196	1.62	CAPG
220520_s_at	0.0386649	9.77E-04	3.65	-0.971452	1.77	NUP62CL
214375_at	0.0387248	9.80E-04	3.65	-0.974276	1.36	PPFIBP1
237159_x_at	0.0390521	9.92E-04	3.64	-0.98662	1.73	AP1S3
218900_at	0.0391135	9.97E-04	3.64	-0.990478	1.25	CNNM4

218400_at	0.0391695	1.00E-03	3.64	-0.993764	1.44	OAS3
218687_s_at	0.0391695	1.00E-03	3.64	-0.994158	2.6	MUC13
226287_at	0.0392613	1.01E-03	3.64	-0.999453	1.1	CCDC34
236129_at	0.0392613	1.01E-03	3.64	-1.000775	2.78	GALNT5
240770_at	0.0394427	1.01E-03	3.63	-1.00713	1.94	TMEM171
235144_at	0.0397664	1.02E-03	3.63	-1.016735	2.36	RASEF
222647_at	0.0397664	1.02E-03	3.63	-1.01684	1.01	SLC35C1
208779_x_at	0.0397698	1.03E-03	3.63	-1.01759	1.66	MIR4640///DDR1
64408_s_at	0.0398793	1.03E-03	3.63	-1.020848	1.95	CALML4
227955_s_at	0.0399515	1.03E-03	3.63	-1.024553	2.12	EFNA5

237554_at	0.0400022	1.04E-03	3.63	-1.028408	1.08	
203917_at	0.0400482	1.04E-03	3.63	-1.030154	1.52	CXADR
228323_at	0.0401248	1.04E-03	3.62	-1.03336	2.22	KNL1
226899_at	0.040209	1.05E-03	3.62	-1.036583	1.39	UNC5B
222387_s_at	0.0403636	1.05E-03	3.62	-1.042564	1.02	VPS35
201251_at	0.0403636	1.05E-03	3.62	-1.04284	1.32	PKM
244241_x_at	0.0406375	1.07E-03	3.62	-1.056544	1.5	
239559_at	0.0406682	1.07E-03	3.61	-1.058463	1.73	
230682_x_at	0.040673	1.07E-03	3.61	-1.059229	2.02	ABCC3
232961_at	0.0407665	1.08E-03	3.61	-1.064001	1.06	SPATS2L
210150_s_at	0.0410062	1.09E-03	3.61	-1.070824	1.19	LAMA5
211778_s_at	0.0411187	1.09E-03	3.61	-1.0769	1.72	OVOL2

209403_at	0.0411187	1.09E-03	3.61	-1.077609	1.04	TBC1D3I///TBC1D3E///LOC101060389///TBC1D3L///TBC1D3K///TBC1D[illegible]

209373_at	0.0411187	1.09E-03	3.61	-1.077675	2.13	MALL
213007_at	0.0411187	1.09E-03	3.61	-1.078595	1.86	FANCI
216243_s_at	0.0412402	1.10E-03	3.6	-1.083312	1.23	IL1RN
239433_at	0.0413877	1.11E-03	3.6	-1.089255	1.49	LRRC8E
207265_s_at	0.0417435	1.12E-03	3.6	-1.0986	1.49	KDELR3
212339_at	0.041771	1.12E-03	3.6	-1.099889	1.76	EPB41L1
221815_at	0.0419161	1.13E-03	3.59	-1.108893	1.53	ABHD2

237411_at	0.0419523	1.13E-03	3.59	-1.110344	1.29	ADAMTS6
231807_at	0.0420814	1.14E-03	3.59	-1.115548	1.22	KIAA1217
229369_at	0.0421383	1.14E-03	3.59	-1.118328	2.36	VSIG2
244476_at	0.042621	1.16E-03	3.58	-1.133487	1.2	
231646_at	0.0431839	1.18E-03	3.58	-1.150318	4.23	DPCR1
1553114_a_at	0.0436602	1.20E-03	3.57	-1.166216	1.65	PTK6
1562416_at	0.0439162	1.21E-03	3.57	-1.172965	1.24	
209262_s_at	0.0441697	1.22E-03	3.57	-1.180874	1.52	NR2F6
204034_at	0.0444119	1.23E-03	3.56	-1.189748	1.03	ETHE1

221215_s_at	0.0445459	1.24E-03	3.56	-1.195058	1.86	RIPK4
1555037_a_at	0.0446852	1.25E-03	3.56	-1.200467	1	IDH1
214697_s_at	0.0450241	1.26E-03	3.55	-1.210653	1.17	PTBP3
219916_s_at	0.0450942	1.27E-03	3.55	-1.215185	1.5	RNF39
1554609_at	0.0451772	1.27E-03	3.55	-1.217527	1.33	LOC100287896
210087_s_at	0.0457864	1.29E-03	3.54	-1.235582	1.08	MPZL1
205552_s_at	0.0457864	1.29E-03	3.54	-1.235615	1.97	OAS1
209048_s_at	0.0458678	1.30E-03	3.54	-1.238502	1.02	ZMYND8

202740_at	0.0458699	1.30E-03	3.54	-1.239153	1.32	ABHD14A-ACY1///ACY1
36936_at	0.0458719	1.30E-03	3.54	-1.240722	1.39	TSTA3
218755_at	0.0459804	1.31E-03	3.54	-1.245657	1.87	KIF20A
228696_at	0.0463527	1.32E-03	3.54	-1.255653	2.09	SLC45A3
242417_at	0.0467428	1.34E-03	3.53	-1.269542	1.67	PLEKHA7
229396_at	0.0469413	1.35E-03	3.53	-1.274976	1.59	OVOL1
227614_at	0.0473872	1.36E-03	3.52	-1.285399	2.33	HKDC1
224209_s_at	0.0474517	1.37E-03	3.52	-1.288351	2.99	GDA
238884_at	0.0474517	1.37E-03	3.52	-1.288487	1.16	
1556211_a_at	0.0478778	1.39E-03	3.52	-1.302215	1.56	

212762_s_at	0.0481092	1.40E-03	3.51	-1.310292	1.25	TCF7L2
205513_at	0.0481311	1.40E-03	3.51	-1.3119	4.18	TCN1
209446_s_at	0.0487657	1.43E-03	3.51	-1.328781	1.49	
243618_s_at	0.0487657	1.43E-03	3.51	-1.329987	1.17	ZNF827
214581_x_at	0.0487657	1.43E-03	3.51	-1.33184	1.73	TNFRSF21
203370_s_at	0.0489162	1.44E-03	3.5	-1.337756	1.47	PDLIM7
222600_s_at	0.0489757	1.44E-03	3.5	-1.339953	1.07	UBA6
239155_at	0.0490766	1.45E-03	3.5	-1.342467	1.62	CXADR
229795_at	0.0490984	1.45E-03	3.5	-1.345165	1.29	
206560_s_at	0.0490984	1.45E-03	3.5	-1.346368	3.2	MIA
229689_s_at	0.0491789	1.46E-03	3.5	-1.348486	1.08	

210026_s_at	0.0494165	1.47E-03	3.5	-1.354167	1.84	CARD10
210813_s_at	0.0495089	1.47E-03	3.5	-1.356804	1.26	XRCC4
217875_s_at	0.0496078	1.48E-03	3.49	-1.361259	1.54	PMEPA1
226997_at	0.0497022	1.48E-03	3.49	-1.364196	2.69	ADAMTS12
226707_at	0.0499882	1.49E-03	3.49	-1.371881	1.47	NAPRT
231832_at	0.0499926	1.50E-03	3.49	-1.373876	1.75	POC1B-GALNT4///GALNT4

213230_at	0.0499926	1.50E-03	3.49	-1.37483	1.53	CDR2L
225496_s_at	0.050164	1.51E-03	3.49	-1.379828	1.11	SYTL2
224799_at	0.0503506	1.52E-03	3.48	-1.387671	1.09	NDFIP2
220289_s_at	0.0503506	1.52E-03	3.48	-1.388816	1.92	AIM1L
205765_at	0.0505159	1.53E-03	3.48	-1.393612	3.26	CYP3A5
226245_at	0.0505159	1.53E-03	3.48	-1.393714	1.34	KCTD1
234995_at	0.0506022	1.54E-03	3.48	-1.398144	1.26	SPICE1

242662_at	0.0507052	1.54E-03	3.48	-1.401181	1.32	PCSK6
218883_s_at	0.050955	1.55E-03	3.48	-1.408856	2.28	CENPU
229276_at	0.050955	1.56E-03	3.47	-1.409732	1.78	IGSF9
1569150_x_at	0.0514856	1.58E-03	3.47	-1.424709	1.83	PDLIM7
217730_at	0.0515236	1.58E-03	3.47	-1.426829	1.05	MIR6513///TMBIM1
230269_at	0.0515236	1.59E-03	3.47	-1.42686	1.87	B3GALT5
235245_at	0.0518216	1.60E-03	3.46	-1.438581	1.53	TMEM92
225806_at	0.0518216	1.61E-03	3.46	-1.438728	1.61	AJUBA
237183_at	0.0519023	1.61E-03	3.46	-1.442598	2.41	GALNT5

204268_at	0.0519023	1.61E-03	3.46	-1.443025	4.07	S100A2
1569542_at	0.0519437	1.62E-03	3.46	-1.444461	1.11	ADGRA3
209925_at	0.0524147	1.63E-03	3.46	-1.455129	1.79	OCLN
218039_at	0.0527747	1.65E-03	3.45	-1.465392	2.06	NUSAP1
235476_at	0.0528438	1.66E-03	3.45	-1.468808	1.64	TRIM59///IFT80
202721_s_at	0.0528699	1.66E-03	3.45	-1.47159	1.04	GFPT1
218498_s_at	0.0528699	1.66E-03	3.45	-1.472043	1.64	ERO1A
217518_at	0.0528699	1.67E-03	3.45	-1.47309	1.28	MYOF

228150_at	0.0529053	1.67E-03	3.45	-1.475893	1.42	SEC16B
212444_at	0.0529573	1.68E-03	3.45	-1.479462	2.23	GPRC5A
204254_s_at	0.0529573	1.68E-03	3.45	-1.481695	1.04	VDR
244356_at	0.0529573	1.68E-03	3.45	-1.482159	1.18	
235924_at	0.0529573	1.68E-03	3.45	-1.48278	1.78	FRK
210007_s_at	0.0529573	1.68E-03	3.45	-1.483299	1.14	GPD2
219233_s_at	0.0529573	1.69E-03	3.44	-1.484215	1.59	GSDMB
208555_x_at	0.0532485	1.70E-03	3.44	-1.492215	1.53	CST2
221582_at	0.0533107	1.71E-03	3.44	-1.497226	1.62	HIST3H2A
203502_at	0.0534342	1.72E-03	3.44	-1.501368	1.16	BPGM
203779_s_at	0.0537202	1.73E-03	3.44	-1.506933	1.41	MPZL2

218928_s_at	0.0537204	1.73E-03	3.44	-1.508644	1.11	SLC37A1
217620_s_at	0.0537204	1.73E-03	3.43	-1.50957	1.14	PIK3CB
213116_at	0.0538213	1.74E-03	3.43	-1.512392	1.05	NEK3
222714_s_at	0.0538386	1.74E-03	3.43	-1.513224	1.33	LACTB2
242759_at	0.0538433	1.74E-03	3.43	-1.513837	1.05	
228481_at	0.0539281	1.74E-03	3.43	-1.516819	2.53	
215549_x_at	0.0540569	1.75E-03	3.43	-1.519667	1.42	CTAGE8///CTAGE4///CTAGE9
217234_s_at	0.0542897	1.76E-03	3.43	-1.52475	1.1	EZR
220952_s_at	0.0543717	1.77E-03	3.43	-1.528841	1.25	PLEKHA5
1555858_at	0.0543717	1.77E-03	3.43	-1.52886	1.27	THUMPD3-AS1

209205_s_at	0.0546227	1.78E-03	3.42	-1.5347	1.17	LMO4
217771_at	0.0546372	1.78E-03	3.42	-1.535475	1.96	GOLM1
223165_s_at	0.0548331	1.79E-03	3.42	-1.542874	1.19	IP6K2
241726_at	0.0548331	1.79E-03	3.42	-1.543026	1.61	
242635_s_at	0.0548389	1.80E-03	3.42	-1.543649	1.26	NAPEPLD
209627_s_at	0.0548614	1.80E-03	3.42	-1.544556	1.63	OSBPL3
203955_at	0.0549981	1.80E-03	3.42	-1.54803	1.08	PPP1R26
228273_at	0.0550183	1.81E-03	3.42	-1.550266	1.83	PRR11
212472_at	0.0550183	1.81E-03	3.42	-1.550901	1.29	MICAL2

227420_at	0.0551653	1.82E-03	3.42	-1.555467	1.28	TNFAIP8L1
230100_x_at	0.0552497	1.82E-03	3.42	-1.557936	1.62	PAK1
208614_s_at	0.0554296	1.84E-03	3.41	-1.565641	1.11	FLNB
225655_at	0.0557009	1.85E-03	3.41	-1.571335	2.02	UHRF1
209365_s_at	0.0557009	1.85E-03	3.41	-1.571761	2.27	ECM1
235952_at	0.0557457	1.85E-03	3.41	-1.573159	1.23	DGKH
226189_at	0.0557457	1.85E-03	3.41	-1.574218	1.7	ITGB8
213667_at	0.0557457	1.86E-03	3.41	-1.575586	1.22	TMEM265///SRCAP

202712_s_at	0.0559023	1.87E-03	3.41	-1.579874	2.7	CKMT1A///CKMT1B
243594_x_at	0.0559023	1.87E-03	3.41	-1.580766	1.94	SPIRE2
225599_s_at	0.0559153	1.87E-03	3.41	-1.581863	1.07	TRIQK
201826_s_at	0.0559153	1.87E-03	3.41	-1.582037	1.5	SCCPDH
209326_at	0.0559389	1.87E-03	3.41	-1.582944	1.14	SLC35A2
234000_s_at	0.0560549	1.88E-03	3.4	-1.586651	1.1	HACD3
209790_s_at	0.0560549	1.88E-03	3.4	-1.586927	1.08	CASP6
212650_at	0.0560595	1.88E-03	3.4	-1.588024	1.01	EHBP1

206094_x_at	0.0563373	1.90E-03	3.4	-1.59469	2.6	UGT1A3///UGT1A1///UGT1A4///UGT1A9///UGT1A5///UGT1A6///UGT1A

1568877_a_at	0.0570688	1.93E-03	3.39	-1.609934	1.43	ACBD5
224624_at	0.0572197	1.93E-03	3.39	-1.613297	1.05	LRRC8A
230778_at	0.057546	1.95E-03	3.39	-1.62178	1.03	
224825_at	0.057546	1.95E-03	3.39	-1.622054	1.18	DNTTIP1
225590_at	0.0576707	1.97E-03	3.39	-1.628255	1.46	SH3RF1
225871_at	0.0576707	1.97E-03	3.39	-1.628848	1.31	STEAP2
214974_x_at	0.0576707	1.97E-03	3.39	-1.629715	3.83	CXCL5
212446_s_at	0.0580947	1.99E-03	3.38	-1.63892	1.19	CERS6
232406_at	0.0582095	2.00E-03	3.38	-1.643416	1.1	LOC105372526

203794_at	0.0585357	2.01E-03	3.38	-1.650214	1.02	CDC42BPA
238075_at	0.0585357	2.01E-03	3.38	-1.651352	1.14	CHEK1
219978_s_at	0.0588257	2.03E-03	3.37	-1.658239	2.29	NUSAP1
206249_at	0.0593304	2.05E-03	3.37	-1.670032	1.37	MAP3K13
223307_at	0.0593678	2.06E-03	3.37	-1.671346	2.04	CDCA3
202962_at	0.0598137	2.08E-03	3.37	-1.682009	1.15	KIF13B
222449_at	0.0598554	2.09E-03	3.36	-1.686294	1.56	PMEPA1
205319_at	0.0600105	2.10E-03	3.36	-1.691416	3.03	PSCA
204519_s_at	0.0600105	2.11E-03	3.36	-1.69262	1.98	PLLP

227850_x_at	0.0600582	2.11E-03	3.36	-1.694825	2.13	CDC42EP5
222848_at	0.0602998	2.13E-03	3.36	-1.701494	2.26	CENPK
213865_at	0.0604754	2.14E-03	3.35	-1.708084	1.85	DCBLD2
227672_at	0.0606907	2.15E-03	3.35	-1.7143	1.34	MROH6
205534_at	0.060778	2.16E-03	3.35	-1.716711	1.75	PCDH7
63825_at	0.060778	2.16E-03	3.35	-1.717077	1.31	ABHD2
230831_at	0.060778	2.16E-03	3.35	-1.717087	1.33	FRMD5
206084_at	0.0608482	2.16E-03	3.35	-1.718647	2.16	PTPRR
236279_at	0.0610527	2.18E-03	3.35	-1.723703	1.56	
210797_s_at	0.0611878	2.18E-03	3.35	-1.726726	1.51	OASL

209969_s_at	0.0611914	2.18E-03	3.35	-1.727261	1.18	STAT1
222036_s_at	0.0611982	2.19E-03	3.35	-1.728321	1.14	MCM4
224458_at	0.061775	2.22E-03	3.34	-1.741381	1.64	TMEM246
229450_at	0.0618726	2.22E-03	3.34	-1.744283	1.23	IFIT3
1555870_at	0.0621993	2.25E-03	3.33	-1.755934	1.94	RNF207
204686_at	0.0621993	2.25E-03	3.33	-1.756304	1.24	IRS1
205466_s_at	0.0624792	2.27E-03	3.33	-1.762975	2.16	HS3ST1
214135_at	0.0625972	2.28E-03	3.33	-1.768838	5.29	CLDN18
215108_x_at	0.0628527	2.30E-03	3.33	-1.774046	3.07	TOX3

204675_at	0.0630989	2.31E-03	3.33	-1.779098	1.82	SRD5A1
202338_at	0.0634532	2.34E-03	3.32	-1.789909	1.39	TK1
210530_s_at	0.0636727	2.35E-03	3.32	-1.794987	1.15	NR2C1
220945_x_at	0.0638286	2.36E-03	3.32	-1.799272	1.26	MANSC1
212442_s_at	0.0639075	2.37E-03	3.32	-1.803169	1.27	CERS6
201905_s_at	0.0639075	2.37E-03	3.32	-1.803501	1.03	CTDSPL
232103_at	0.0639495	2.37E-03	3.32	-1.804576	1.32	BPNT1
201250_s_at	0.0641161	2.39E-03	3.31	-1.810471	2.31	SLC2A1
201005_at	0.0643293	2.40E-03	3.31	-1.814692	1.08	CD9
229256_at	0.0643737	2.41E-03	3.31	-1.818884	1.17	PGM2L1

206576_s_at	0.0643737	2.41E-03	3.31	-1.819068	2.2	CEACAM1
205044_at	0.064687	2.43E-03	3.31	-1.826255	4.44	GABRP
202856_s_at	0.0651298	2.45E-03	3.3	-1.836069	2.06	MIR6787///SLC16A3
221530_s_at	0.0651298	2.46E-03	3.3	-1.838511	1.18	BHLHE41
239435_x_at	0.0651574	2.46E-03	3.3	-1.839809	1.53	SHROOM1
210675_s_at	0.0655465	2.48E-03	3.3	-1.846717	1.83	PTPRR
209677_at	0.0656353	2.49E-03	3.3	-1.848431	1.17	PRKCI

227062_at	0.066032	2.51E-03	3.29	-1.856748	1.07	MIR612///NEAT1
203780_at	0.0663967	2.53E-03	3.29	-1.864537	1.4	MPZL2
203035_s_at	0.0665104	2.54E-03	3.29	-1.868407	1.03	PIAS3
46323_at	0.0671913	2.58E-03	3.28	-1.883038	1.01	CANT1
1568865_at	0.0673587	2.59E-03	3.28	-1.88741	1.08	FNTB
208002_s_at	0.0673587	2.60E-03	3.28	-1.888671	1.13	ACOT7
227566_at	0.0673806	2.60E-03	3.28	-1.889416	2.27	LOC102725271///NTM
204602_at	0.0675761	2.62E-03	3.28	-1.89523	2.73	DKK1
230256_at	0.0676586	2.63E-03	3.28	-1.899424	1.07	RUSC1-AS1

230319_at	0.0677465	2.63E-03	3.28	-1.901892	2.24	
206857_s_at	0.0677465	2.64E-03	3.27	-1.902508	1.44	FKBP1B
204304_s_at	0.0677465	2.64E-03	3.27	-1.903109	2.71	PROM1
225687_at	0.0678255	2.65E-03	3.27	-1.905928	2.35	FAM83D
200612_s_at	0.0678759	2.65E-03	3.27	-1.908985	1.27	AP2B1
206284_x_at	0.0678759	2.65E-03	3.27	-1.909081	1.03	CLTB
203918_at	0.0679993	2.66E-03	3.27	-1.912412	1.07	PCDH1
214059_at	0.068024	2.67E-03	3.27	-1.913186	2.07	IFI44
208209_s_at	0.068055	2.67E-03	3.27	-1.91437	1.87	C4BPB
210117_at	0.068055	2.67E-03	3.27	-1.915349	1.45	SPAG1
229555_at	0.068271	2.69E-03	3.27	-1.920471	1.49	GALNT5

216074_x_at	0.0683643	2.70E-03	3.27	-1.924337	1.34	WWC1
205941_s_at	0.0685163	2.71E-03	3.26	-1.927271	3.83	COL10A1
202589_at	0.0687606	2.72E-03	3.26	-1.932742	1.93	TYMS
219388_at	0.068904	2.73E-03	3.26	-1.936628	1.56	GRHL2
212021_s_at	0.068904	2.73E-03	3.26	-1.936834	1.54	MKI67
203354_s_at	0.069494	2.77E-03	3.26	-1.949916	1.66	PSD3
218567_x_at	0.0695016	2.78E-03	3.26	-1.950811	1.05	DPP3
204981_at	0.0695016	2.78E-03	3.25	-1.951297	1.8	SLC22A18
225601_at	0.0695842	2.78E-03	3.25	-1.953257	1.04	HMGB3
210177_at	0.0696447	2.79E-03	3.25	-1.954846	1.3	TRIM15

1566465_at	0.0697156	2.79E-03	3.25	-1.956715	1.76	KCNK1
241455_at	0.0698572	2.81E-03	3.25	-1.961362	1.62	C6orf132
225345_s_at	0.0702839	2.84E-03	3.25	-1.971898	1.56	FBXO32
204017_at	0.0703294	2.85E-03	3.25	-1.973766	1.33	KDELR3
224715_at	0.0703629	2.85E-03	3.24	-1.975891	1.16	WDR34
202936_s_at	0.0706887	2.87E-03	3.24	-1.980613	1.72	SOX9
219637_at	0.0707734	2.87E-03	3.24	-1.982148	1.16	ARMC9
228490_at	0.0708886	2.88E-03	3.24	-1.984897	1.43	ABHD2
200644_at	0.0710472	2.89E-03	3.24	-1.988259	1.36	MARCKSL1
220770_s_at	0.0712753	2.90E-03	3.24	-1.992196	1.25	ZBED8

218847_at	0.0712753	2.91E-03	3.24	-1.993335	1.55	IGF2BP2
226246_at	0.0712799	2.91E-03	3.24	-1.995495	1.35	KCTD1
242354_at	0.0712799	2.92E-03	3.24	-1.997474	1.53	
223186_at	0.0713036	2.92E-03	3.24	-1.999155	1.02	TMEM189-UBE2V1///TMEM189///UBE2V1
1558154_at	0.0714232	2.93E-03	3.23	-2.002338	1.2	
214924_s_at	0.0715079	2.94E-03	3.23	-2.004683	1.49	TRAK1
222258_s_at	0.0719399	2.97E-03	3.23	-2.014007	1.14	SH3BP4

227647_at	0.0719585	2.97E-03	3.23	-2.015071	2.39	KCNE3
223185_s_at	0.07208	2.99E-03	3.23	-2.018982	1.39	BHLHE41
227415_at	0.07208	2.99E-03	3.23	-2.01911	1.13	DGKH
228742_at	0.072088	2.99E-03	3.23	-2.019624	1.46	
212727_at	0.0720917	2.99E-03	3.23	-2.020684	1.17	DLG3
238722_x_at	0.0724565	3.02E-03	3.22	-2.030662	1.05	NAPEPLD
228729_at	0.0724651	3.03E-03	3.22	-2.032245	2.16	CCNB1
202998_s_at	0.0725749	3.04E-03	3.22	-2.035554	1.87	LOXL2
205342_s_at	0.072732	3.05E-03	3.22	-2.038511	3.31	SULT1C2

230135_at	0.0729221	3.07E-03	3.22	-2.044603	1.83	HHIP
240861_at	0.0730355	3.09E-03	3.21	-2.049081	1.74	
219148_at	0.0730809	3.09E-03	3.21	-2.051119	1.92	PBK
228116_at	0.0731277	3.10E-03	3.21	-2.054709	1.56	LINC01296///DUXAP10///DUXAP8
210473_s_at	0.0731277	3.10E-03	3.21	-2.054929	1.21	ADGRA3
231822_at	0.0731277	3.11E-03	3.21	-2.057157	1.04	CTTNBP2NL
1557651_x_at	0.0734018	3.13E-03	3.21	-2.06219	1.41	GALE
225362_at	0.073449	3.14E-03	3.21	-2.066802	1.34	FAM122B

204162_at	0.0736713	3.16E-03	3.21	-2.071397	1.77	NDC80
225062_at	0.0736713	3.17E-03	3.2	-2.073285	1.47	LOC102724951///LOC389831
229479_at	0.0740684	3.19E-03	3.2	-2.08105	2.93	
218252_at	0.0741493	3.20E-03	3.2	-2.082459	1.22	CKAP2
239834_at	0.0742914	3.21E-03	3.2	-2.086152	1.3	
204415_at	0.0745158	3.22E-03	3.2	-2.089799	1.91	IFI6
214774_x_at	0.0745402	3.23E-03	3.2	-2.090911	3.01	TOX3
218638_s_at	0.0746371	3.24E-03	3.2	-2.095411	1.36	LOC100130872///SPON2
210719_s_at	0.0746371	3.24E-03	3.2	-2.095555	1.05	HMG20B

242800_at	0.0746371	3.24E-03	3.2	-2.095624	1.78	NHS
216623_x_at	0.0749279	3.26E-03	3.19	-2.10093	2.97	TOX3
226702_at	0.0749574	3.27E-03	3.19	-2.10195	1.39	CMPK2
218689_at	0.0749833	3.27E-03	3.19	-2.104208	1.02	FANCF
220613_s_at	0.0752275	3.29E-03	3.19	-2.110037	1.2	SYTL2
203362_s_at	0.0752275	3.30E-03	3.19	-2.110352	1.87	MAD2L1
209468_at	0.0754025	3.31E-03	3.19	-2.115223	1.54	LRP5
236390_at	0.0754734	3.32E-03	3.19	-2.11692	1.07	SLX4IP
235606_at	0.0756445	3.34E-03	3.18	-2.122569	1.08	DUBR

226041_at	0.0756445	3.35E-03	3.18	-2.125625	1.41	NAPEPLD
31637_s_at	0.0756445	3.35E-03	3.18	-2.125783	1.24	NR1D1///THRA
1555976_s_at	0.0756592	3.35E-03	3.18	-2.126469	1.02	MYL12A
219150_s_at	0.0757768	3.36E-03	3.18	-2.129445	1.9	ADAP1
209204_at	0.0758833	3.37E-03	3.18	-2.131133	1.44	LMO4
205538_at	0.0760599	3.38E-03	3.18	-2.134058	1.62	CORO2A
233487_s_at	0.0764017	3.43E-03	3.17	-2.146716	1.44	LRRC8A
1559190_s_at	0.0767613	3.45E-03	3.17	-2.153005	1.42	RDH13

201609_x_at	0.0768298	3.46E-03	3.17	-2.155565	1.09	ICMT
227994_x_at	0.0769476	3.47E-03	3.17	-2.157365	1.16	PPDPF
203074_at	0.0769896	3.47E-03	3.17	-2.158369	3.08	ANXA8L1///ANXA8
206539_s_at	0.0769896	3.47E-03	3.17	-2.159412	1.58	CYP4F12
211089_s_at	0.0769896	3.48E-03	3.17	-2.160883	1.17	NEK3
201644_at	0.0770238	3.48E-03	3.17	-2.162063	1.13	TSTA3
1555968_a_at	0.0770238	3.49E-03	3.17	-2.162422	1.46	
241137_at	0.0770587	3.49E-03	3.17	-2.163218	3.64	DPCR1
206884_s_at	0.0772573	3.50E-03	3.17	-2.166903	2.97	SCEL

210652_s_at	0.0772573	3.50E-03	3.17	-2.166904	1.87	TTC39A
233908_x_at	0.0772573	3.51E-03	3.16	-2.168087	1.27	
1553992_s_at	0.0772573	3.51E-03	3.16	-2.168231	1.21	NBR2
227949_at	0.077818	3.54E-03	3.16	-2.177932	2.33	PHACTR3
204404_at	0.0781704	3.57E-03	3.16	-2.184787	2.1	SLC12A2
201798_s_at	0.0781704	3.58E-03	3.16	-2.186218	1.14	MYOF
202705_at	0.0782441	3.59E-03	3.16	-2.188637	2.18	CCNB2
225337_at	0.0783801	3.60E-03	3.15	-2.19375	1.14	ABHD2
1555420_a_at	0.0783801	3.61E-03	3.15	-2.193907	1.13	KLF7

220602_s_at	0.0785649	3.62E-03	3.15	-2.198283	1.19	LOC101929479///LOC727751///LOC642423///GOLGA2P7///GOLGA2P10
209255_at	0.0787686	3.64E-03	3.15	-2.202525	1.39	KLHDC10
222646_s_at	0.078802	3.64E-03	3.15	-2.20341	1.55	ERO1A
209398_at	0.0790608	3.66E-03	3.15	-2.208529	2.09	HIST1H1C
239579_at	0.0792536	3.67E-03	3.15	-2.211623	1.35	EPHX4
219691_at	0.0793972	3.69E-03	3.15	-2.215394	1.48	SAMD9

226107_at	0.0794892	3.70E-03	3.14	-2.218027	1.55	C1GALT1
229661_at	0.0794892	3.70E-03	3.14	-2.218961	1.96	SALL4
204740_at	0.0795464	3.72E-03	3.14	-2.222869	1.27	CNKSR1
222020_s_at	0.0795796	3.73E-03	3.14	-2.226245	2.17	LOC102725271///NTM
211695_x_at	0.0797635	3.75E-03	3.14	-2.229832	1.88	MUC1
235638_at	0.0799497	3.76E-03	3.14	-2.232716	1.58	RASSF6
235463_s_at	0.0800139	3.77E-03	3.14	-2.234182	1.44	CERS6

222528_s_at	0.0802284	3.78E-03	3.14	-2.238825	1.48	SLC25A37
206685_at	0.080245	3.79E-03	3.14	-2.239377	1.31	HCG4
222830_at	0.0803238	3.79E-03	3.13	-2.240646	1.75	GRHL1
225735_at	0.0805493	3.81E-03	3.13	-2.243964	1.12	ANKRD50
226132_s_at	0.0807325	3.82E-03	3.13	-2.247148	1.69	MANEAL
225568_at	0.0807774	3.84E-03	3.13	-2.2514	1.04	TMEM141
231849_at	0.0810864	3.86E-03	3.13	-2.257992	1.57	KRT80
1553976_a_at	0.0812462	3.88E-03	3.13	-2.260883	1.39	DPCD
1552575_a_at	0.0817486	3.92E-03	3.12	-2.272379	1.47	C6orf141
228531_at	0.0817486	3.92E-03	3.12	-2.272622	1.4	SAMD9

238881_at	0.0822656	3.97E-03	3.12	-2.283044	1.11	
203458_at	0.0825406	3.99E-03	3.11	-2.28719	1.25	SPR
226829_at	0.0826447	4.00E-03	3.11	-2.289696	1.26	AFAP1L2
217867_x_at	0.0826503	4.00E-03	3.11	-2.290173	1.31	BACE2
230493_at	0.0828337	4.01E-03	3.11	-2.292927	2.65	SHISA2
212768_s_at	0.0828581	4.01E-03	3.11	-2.29355	3.81	OLFM4
209626_s_at	0.0829489	4.02E-03	3.11	-2.295613	1.54	OSBPL3
236462_at	0.0830645	4.04E-03	3.11	-2.298649	1.19	
204822_at	0.083473	4.08E-03	3.11	-2.30857	1.75	TTK
209885_at	0.0836771	4.10E-03	3.1	-2.312057	1.59	RHOD
229720_at	0.083715	4.10E-03	3.1	-2.313169	1.65	BAG1

203755_at	0.0840185	4.13E-03	3.1	-2.3193	2.12	BUB1B
236010_at	0.0840268	4.13E-03	3.1	-2.320409	1.23	
202357_s_at	0.0841122	4.14E-03	3.1	-2.322932	1.37	CFB
221526_x_at	0.0841122	4.15E-03	3.1	-2.323421	1.1	PARD3
222077_s_at	0.0841122	4.15E-03	3.1	-2.323543	1.31	RACGAP1
227966_s_at	0.0841122	4.15E-03	3.1	-2.323758	1.08	CCDC74B///CCDC74A
218966_at	0.0845514	4.18E-03	3.1	-2.330658	1.27	MYO5C
219494_at	0.0846984	4.19E-03	3.1	-2.333441	1.2	RAD54B
218663_at	0.0847355	4.20E-03	3.09	-2.336415	1.94	NCAPG

204235_s_at	0.0853817	4.25E-03	3.09	-2.346175	1.26	GULP1
231022_at	0.0857384	4.27E-03	3.09	-2.351086	1.18	OCLN
1552257_a_at	0.0866925	4.34E-03	3.08	-2.365704	1.16	TTLL12
203797_at	0.0873103	4.38E-03	3.08	-2.374758	1.4	VSNL1
205668_at	0.0874991	4.40E-03	3.08	-2.378322	1.39	LY75
218701_at	0.0879611	4.46E-03	3.07	-2.390902	1.31	LACTB2
209546_s_at	0.0880117	4.47E-03	3.07	-2.392655	1.27	APOL1
1558748_at	0.0880117	4.47E-03	3.07	-2.392683	1.27	
226374_at	0.0884324	4.51E-03	3.07	-2.400943	1.76	CXADR
207202_s_at	0.0884324	4.51E-03	3.07	-2.401131	2.44	NR1I2
233177_s_at	0.0884324	4.51E-03	3.07	-2.401854	1.46	PNKD

218662_s_at	0.0890944	4.56E-03	3.06	-2.41148	1.62	NCAPG
220192_x_at	0.0891326	4.57E-03	3.06	-2.413256	1.9	SPDEF
202688_at	0.0891691	4.58E-03	3.06	-2.415911	1.01	TNFSF10
212759_s_at	0.0898683	4.64E-03	3.06	-2.42725	1.2	TCF7L2
220230_s_at	0.0898683	4.64E-03	3.06	-2.42738	1.21	CYB5R2
219909_at	0.0898683	4.64E-03	3.06	-2.428019	2.16	MMP28
222446_s_at	0.0898683	4.64E-03	3.06	-2.428067	1.14	BACE2
225418_at	0.0899008	4.65E-03	3.05	-2.429756	1.02	NECTIN2

240081_at	0.0899008	4.65E-03	3.05	-2.42983	1.22	
204341_at	0.0900132	4.67E-03	3.05	-2.433007	1.35	TRIM16
210625_s_at	0.0902715	4.70E-03	3.05	-2.439885	1.22	AKAP1
207165_at	0.0903128	4.71E-03	3.05	-2.441587	2.31	HMMR
204623_at	0.0904559	4.74E-03	3.05	-2.446852	3.06	TFF3
213352_at	0.090915	4.77E-03	3.04	-2.453847	1.08	TMCC1
218795_at	0.0910672	4.79E-03	3.04	-2.457061	1.36	ACP6
204078_at	0.0913614	4.83E-03	3.04	-2.465285	1.07	P3H4
1556105_at	0.0916474	4.86E-03	3.04	-2.471216	1.57	
225598_at	0.0917517	4.87E-03	3.04	-2.473221	1.14	SLC45A4
1553995_a_at	0.0925361	4.94E-03	3.03	-2.484876	1.72	NT5E

234207_at	0.0930895	4.99E-03	3.03	-2.495131	1.18	
229669_at	0.0931377	5.00E-03	3.03	-2.497496	1.28	CCDC144NL-AS1
220180_at	0.0932107	5.01E-03	3.03	-2.498955	1.63	CCDC68
204202_at	0.0934891	5.04E-03	3.02	-2.504359	1.16	IQCE
229674_at	0.0934891	5.04E-03	3.02	-2.504573	1.39	SERTAD4
228933_at	0.0934891	5.04E-03	3.02	-2.504801	1.63	NHS
222760_at	0.0935919	5.07E-03	3.02	-2.510174	1.38	ZNF703
229223_at	0.0935919	5.08E-03	3.02	-2.510432	1.64	
244612_at	0.0937782	5.10E-03	3.02	-2.515066	1.96	
204751_x_at	0.0940263	5.14E-03	3.02	-2.521858	1.42	DSC2
1553986_at	0.0946526	5.20E-03	3.01	-2.532096	2.01	RASEF
1562527_at	0.0946526	5.20E-03	3.01	-2.533184	1.75	LOC441666

1566764_at	0.0948937	5.22E-03	3.01	-2.53708	1.44	MACC1
59625_at	0.0948968	5.23E-03	3.01	-2.537598	1.12	NOL3
238718_at	0.0951146	5.25E-03	3.01	-2.541153	1.94	
205566_at	0.0951146	5.25E-03	3.01	-2.541429	1.41	ABHD2
240690_at	0.0951602	5.26E-03	3.01	-2.542976	1.43	
223251_s_at	0.0952347	5.27E-03	3.01	-2.545279	1.08	ANKRD10
208079_s_at	0.0954742	5.31E-03	3	-2.551451	1.96	AURKA
220920_at	0.0954742	5.31E-03	3	-2.552716	1.32	ATP10B
203358_s_at	0.0955351	5.32E-03	3	-2.553654	1.47	EZH2
217110_s_at	0.0956338	5.33E-03	3	-2.5555	2.57	MUC4
238806_at	0.0959312	5.36E-03	3	-2.561272	1.03	

213324_at	0.0961725	5.40E-03	3	-2.567625	1.12	SRC
219727_at	0.0965452	5.43E-03	2.99	-2.572995	3.22	DUOX2
229090_at	0.0966151	5.45E-03	2.99	-2.575269	1.12	ZEB1-AS1
218990_s_at	0.0966657	5.46E-03	2.99	-2.578331	3.33	SPRR3
235521_at	0.0968776	5.49E-03	2.99	-2.582721	1.69	HOXA3
219856_at	0.097263	5.53E-03	2.99	-2.589868	1.11	C1orf116
234942_s_at	0.0978276	5.57E-03	2.98	-2.596474	1.23	DNTTIP1
232860_x_at	0.0978861	5.58E-03	2.98	-2.597321	1	RBM41
228302_x_at	0.0980192	5.59E-03	2.98	-2.599179	2.57	CAMK2N1

236009_at	0.0980416	5.59E-03	2.98	-2.599967	1.45	PERP
225102_at	0.0984983	5.64E-03	2.98	-2.607401	1.56	MGLL
221666_s_at	0.0984983	5.64E-03	2.98	-2.607492	1.11	PYCARD
219850_s_at	0.0988205	5.67E-03	2.98	-2.611883	1.43	EHF
232322_x_at	0.0990823	5.69E-03	2.97	-2.616274	1.58	STARD10
211864_s_at	0.0991466	5.70E-03	2.97	-2.617647	1.29	MYOF
201674_s_at	0.0992055	5.71E-03	2.97	-2.618487	1.04	AKAP1
228617_at	0.0998293	5.78E-03	2.97	-2.63097	1.08	XAF1
214373_at	0.0999562	5.80E-03	2.97	-2.632759	1.28	
219771_at	0.0999562	5.80E-03	2.97	-2.632792	1.02	TBC1D8B
243205_at	0.1002699	5.83E-03	2.97	-2.638049	1.44	

235085_at	0.1002888	5.84E-03	2.96	-2.640568	1.13	SGK223
241036_at	0.100291	5.86E-03	2.96	-2.643285	1.21	
208546_x_at	0.1005199	5.89E-03	2.96	-2.648296	1.8	HIST1H2BH
215101_s_at	0.100714	5.91E-03	2.96	-2.650646	3.41	CXCL5
230972_at	0.1009703	5.94E-03	2.96	-2.654992	1.06	ANKRD9
225299_at	0.101239	5.96E-03	2.96	-2.658375	1.78	MYO5B
227140_at	0.1013579	5.97E-03	2.96	-2.660806	2.27	INHBA
41660_at	0.101366	5.98E-03	2.96	-2.661164	1.61	CELSR1
227342_s_at	0.1014437	5.99E-03	2.95	-2.662726	2.13	MYEOV
202734_at	0.102776	6.10E-03	2.95	-2.679566	1.04	TRIP10
211470_s_at	0.1028597	6.11E-03	2.95	-2.681159	3.01	SULT1C2

219529_at	0.1029404	6.12E-03	2.95	-2.682736	2.19	CLIC3
214981_at	0.1033929	6.17E-03	2.94	-2.689879	2.27	POSTN
232459_at	0.1035877	6.18E-03	2.94	-2.692456	1.26	
210978_s_at	0.1036856	6.20E-03	2.94	-2.695014	1.01	TAGLN2
228088_at	0.1037502	6.21E-03	2.94	-2.696149	1.04	SESTD1
242271_at	0.1037947	6.21E-03	2.94	-2.696825	3.34	SLC26A9
215807_s_at	0.1039026	6.22E-03	2.94	-2.698342	1.24	PLXNB1
202219_at	0.1039666	6.23E-03	2.94	-2.699189	2.2	SLC6A8
208156_x_at	0.1042967	6.28E-03	2.94	-2.706449	1.11	EPPK1
238827_at	0.1042967	6.28E-03	2.94	-2.706736	1.85	SH3RF2
232231_at	0.1046045	6.31E-03	2.93	-2.710688	1.69	RUNX2
1569149_at	0.1046456	6.32E-03	2.93	-2.712412	1.5	PDLIM7
236422_at	0.1046456	6.32E-03	2.93	-2.712627	1.46	

239246_at	0.1049359	6.36E-03	2.93	-2.71777	1.13	FARP1
219789_at	0.1050768	6.37E-03	2.93	-2.719718	2.21	NPR3
218986_s_at	0.1051995	6.39E-03	2.93	-2.722503	1	DDX60
233571_x_at	0.1052407	6.40E-03	2.93	-2.72376	1.12	PPDPF
204709_s_at	0.1057463	6.44E-03	2.93	-2.729822	1.49	KIF23
211883_x_at	0.1058114	6.45E-03	2.92	-2.731719	1.64	CEACAM1
224707_at	0.1058114	6.47E-03	2.92	-2.733393	1.25	CYSTM1
227946_at	0.1058114	6.47E-03	2.92	-2.733832	1.65	OSBPL7

206377_at	0.1058114	6.47E-03	2.92	-2.734581	1.47	FOXF2
239091_at	0.1058114	6.49E-03	2.92	-2.736439	1.04	
217428_s_at	0.1058623	6.49E-03	2.92	-2.737156	3.44	COL10A1
224998_at	0.1062534	6.52E-03	2.92	-2.741092	1.27	CMTM4
210610_at	0.1070324	6.58E-03	2.92	-2.749172	1.44	CEACAM1
37005_at	0.1072077	6.60E-03	2.92	-2.75227	1.1	MINOS1-NBL1///NBL1
207222_at	0.1074285	6.62E-03	2.91	-2.754749	1.99	LOC100652777///PLA2G10
215501_s_at	0.1084458	6.75E-03	2.91	-2.773112	1.1	DUSP10

227801_at	0.1084458	6.75E-03	2.91	-2.773169	1.34	TRIM59
233689_at	0.1099108	6.90E-03	2.9	-2.793301	1.27	
200752_s_at	0.1104011	6.96E-03	2.89	-2.801627	1	CAPN1
227163_at	0.1104641	6.98E-03	2.89	-2.803209	1.62	GSTO2
210511_s_at	0.111084	7.05E-03	2.89	-2.812325	2.21	INHBA
209213_at	0.111178	7.06E-03	2.89	-2.813895	1.04	CBR1
219352_at	0.1128227	7.22E-03	2.88	-2.834254	1.06	HERC6
236489_at	0.1129567	7.25E-03	2.88	-2.83891	2.25	ADGRF1
232056_at	0.1134994	7.33E-03	2.87	-2.848442	2.47	SCEL
204345_at	0.1136469	7.36E-03	2.87	-2.852605	1.04	COL16A1

238689_at	0.1136469	7.40E-03	2.87	-2.856669	1.58	ADGRF1
204503_at	0.1136469	7.40E-03	2.87	-2.856688	1.24	EVPL
213797_at	0.1136469	7.40E-03	2.87	-2.856734	1.32	RSAD2
225835_at	0.1139743	7.44E-03	2.87	-2.862713	1.39	SLC12A2
204324_s_at	0.1144325	7.49E-03	2.86	-2.86844	1.02	GOLIM4
201896_s_at	0.1144674	7.50E-03	2.86	-2.868976	1.55	PSRC1
209773_s_at	0.1146419	7.54E-03	2.86	-2.873926	2.36	RRM2
207109_at	0.1146419	7.54E-03	2.86	-2.874248	1.81	POU2F3
230343_at	0.1146419	7.55E-03	2.86	-2.875399	1.33	CST3
231033_at	0.1148752	7.59E-03	2.86	-2.88064	1.76	DSC2

207069_s_at	0.1149686	7.60E-03	2.86	-2.881638	1.5	SMAD6
230795_at	0.115034	7.61E-03	2.86	-2.882642	1.43	
238996_x_at	0.115034	7.61E-03	2.86	-2.882919	1.15	ALDOA
214036_at	0.1154478	7.67E-03	2.86	-2.889746	1.43	EFNA5
213700_s_at	0.1158084	7.71E-03	2.85	-2.895372	1.41	PKM
232481_s_at	0.1158908	7.73E-03	2.85	-2.897832	1.67	SLITRK6
228596_at	0.1158908	7.74E-03	2.85	-2.897922	1.47	ARHGEF5
235425_at	0.1158908	7.74E-03	2.85	-2.898253	1.2	SGO2
204237_at	0.115976	7.76E-03	2.85	-2.900792	1.15	GULP1
228221_at	0.1161988	7.79E-03	2.85	-2.904468	1.66	SLC44A3
208025_s_at	0.1170322	7.87E-03	2.84	-2.914002	1.91	HMGA2

201926_s_at	0.1170417	7.88E-03	2.84	-2.914325	1.37	CD55
224428_s_at	0.1171121	7.88E-03	2.84	-2.915124	2.35	CDCA7
209215_at	0.1173259	7.92E-03	2.84	-2.919438	1.19	MFSD10
212489_at	0.1173259	7.93E-03	2.84	-2.920132	1.88	COL5A1
209641_s_at	0.1173259	7.93E-03	2.84	-2.920144	1.36	ABCC3
239694_at	0.1173259	7.93E-03	2.84	-2.920368	2.08	TRIM7
203325_s_at	0.1173259	7.93E-03	2.84	-2.920516	1.77	COL5A1
1565149_at	0.1174263	7.95E-03	2.84	-2.923031	1.37	DYNC2H1
239350_at	0.1177354	7.99E-03	2.84	-2.927778	1.24	MARVELD3

232994_s_at	0.1177354	8.00E-03	2.84	-2.928773	1.2	ARHGEF28
230710_at	0.1182379	8.06E-03	2.84	-2.935001	1.54	MIR210HG
214455_at	0.1183176	8.08E-03	2.83	-2.938071	2.13	HIST1H2BC///HIST1H2BI///HIST1H2BE///HIST1H2BF///HIST1H2BG
236725_at	0.1183776	8.10E-03	2.83	-2.939569	1.24	WWC1
212686_at	0.1184312	8.11E-03	2.83	-2.941061	1.44	PPM1H
205073_at	0.1184545	8.12E-03	2.83	-2.942063	1.77	CYP2J2

226764_at	0.1185199	8.13E-03	2.83	-2.943787	1	ZNF827
241607_at	0.1192314	8.21E-03	2.83	-2.952854	1.42	LOC730102
1568633_a_at	0.1192314	8.22E-03	2.83	-2.953287	1.14	LOC101927809
234608_at	0.1195722	8.28E-03	2.82	-2.959877	1.27	LAMA3
204146_at	0.1196029	8.28E-03	2.82	-2.960367	1.64	RAD51AP1
232567_at	0.1200409	8.33E-03	2.82	-2.965387	1.3	PRR5-ARHGAP8///ARHGAP8
1555950_a_at	0.1210482	8.44E-03	2.82	-2.977739	1.41	CD55
214895_s_at	0.1210482	8.45E-03	2.82	-2.978313	1.04	ADAM10
215059_at	0.1213213	8.50E-03	2.81	-2.984511	1.03	

209615_s_at	0.1213213	8.52E-03	2.81	-2.986086	1.15	PAK1
205542_at	0.1213213	8.53E-03	2.81	-2.986997	1.23	STEAP1
1552930_at	0.1213213	8.53E-03	2.81	-2.987312	1.12	MMEL1
40020_at	0.1219984	8.63E-03	2.81	-2.997829	1.28	CELSR3
206133_at	0.1221228	8.65E-03	2.81	-2.99994	1.16	XAF1
225830_at	0.1223793	8.68E-03	2.8	-3.003507	1.13	PDZD8
238617_at	0.122593	8.70E-03	2.8	-3.005867	1.83	KIF26B
232465_at	0.1226281	8.72E-03	2.8	-3.007009	1.1	
1554003_at	0.1227174	8.73E-03	2.8	-3.008143	1.17	ARHGEF28

206326_at	0.1228553	8.76E-03	2.8	-3.011283	1.82	GRP
213094_at	0.1230735	8.79E-03	2.8	-3.01454	1.27	ADGRG6
205618_at	0.1235607	8.85E-03	2.8	-3.020717	1.11	PRRG1
221241_s_at	0.123835	8.88E-03	2.8	-3.023674	1.63	BCL2L14
231148_at	0.1242306	8.94E-03	2.79	-3.030538	2.26	IGFL2
228485_s_at	0.1247772	9.02E-03	2.79	-3.038504	1.17	SLC44A1
237864_at	0.1247772	9.02E-03	2.79	-3.038559	1.01	
232238_at	0.1247772	9.03E-03	2.79	-3.039529	1.63	ASPM
240390_at	0.1248216	9.05E-03	2.79	-3.040947	1.8	GALNT5
200637_s_at	0.1250483	9.08E-03	2.79	-3.044605	1.03	PTPRF
232478_at	0.1253217	9.12E-03	2.79	-3.04795	1.02	MIR181A2HG
238501_at	0.1255346	9.15E-03	2.78	-3.050968	1.13	

208394_x_at	0.1262503	9.24E-03	2.78	-3.059902	1.26	ESM1
216251_s_at	0.1265255	9.28E-03	2.78	-3.064008	1.08	TTLL12
226553_at	0.1276661	9.42E-03	2.77	-3.078087	1.77	TMPRSS2
219747_at	0.1277248	9.44E-03	2.77	-3.079851	1.67	NDNF
210868_s_at	0.1277248	9.44E-03	2.77	-3.079916	1.7	ELOVL6
1553994_at	0.128121	9.51E-03	2.77	-3.086164	1.45	NT5E
213577_at	0.128121	9.51E-03	2.77	-3.086832	1.5	SQLE
209238_at	0.1281229	9.53E-03	2.77	-3.088637	1.03	STX3
231881_at	0.1284356	9.59E-03	2.76	-3.094247	1.21	CALD1
237483_at	0.1287615	9.64E-03	2.76	-3.098543	1.46	
223854_at	0.1288105	9.65E-03	2.76	-3.099916	1.39	PCDHB9///PCDHB10
228033_at	0.1288368	9.66E-03	2.76	-3.101197	1.25	E2F7

219474_at	0.1292715	9.71E-03	2.76	-3.105443	1.25	C3orf52
225618_at	0.1292715	9.71E-03	2.76	-3.105946	1.14	ARHGAP27
210233_at	0.129768	9.77E-03	2.76	-3.110864	1.36	IL1RAP
218704_at	0.1298161	9.77E-03	2.76	-3.111423	1.68	RNF43
215409_at	0.1301721	9.82E-03	2.75	-3.115846	1.32	LPCAT4
223284_at	0.1301721	9.82E-03	2.75	-3.115918	1.07	NAT14
221566_s_at	0.1305814	9.89E-03	2.75	-3.122513	1.01	NOL3
1554408_a_at	0.1313122	9.99E-03	2.75	-3.131325	1.21	TK1
228873_at	0.1314342	1.00E-02	2.75	-3.134697	1.29	COL22A1
1562608_at	0.1316107	1.00E-02	2.75	-3.136234	1.21	

212488_at	0.1328923	1.02E-02	2.74	-3.152994	1.76	COL5A1
227041_at	0.1328923	1.02E-02	2.74	-3.153092	1.07	SESTD1
234605_at	0.1330954	1.03E-02	2.74	-3.155346	1.12	CDC14B
213285_at	0.1333564	1.03E-02	2.73	-3.159502	1.29	TMEM30B
213010_at	0.1334849	1.03E-02	2.73	-3.16079	1.19	PRKCDBP
206364_at	0.1338642	1.04E-02	2.73	-3.164036	1.51	KIF14
213308_at	0.1341178	1.04E-02	2.73	-3.167836	1.35	SHANK2
201621_at	0.1342578	1.04E-02	2.73	-3.170146	1.09	NBL1
225046_at	0.13479	1.05E-02	2.73	-3.18089	1.33	LOC102724951///LOC389831

204285_s_at	0.13479	1.06E-02	2.72	-3.181421	1.53	PMAIP1
228654_at	0.1353387	1.06E-02	2.72	-3.186813	1.22	SPIN4
208165_s_at	0.1355207	1.06E-02	2.72	-3.188664	1.42	PRSS16
206558_at	0.1355409	1.07E-02	2.72	-3.190288	1.2	SIM2
223672_at	0.1355524	1.07E-02	2.72	-3.190951	1.37	SGIP1
213107_at	0.1358125	1.07E-02	2.72	-3.19368	1.44	TNIK
240200_at	0.1358125	1.07E-02	2.72	-3.19416	2.61	SULT1C2
202404_s_at	0.1362805	1.08E-02	2.72	-3.200295	1.53	COL1A2
228919_at	0.1364992	1.08E-02	2.71	-3.203433	1.27	
242181_at	0.1367712	1.09E-02	2.71	-3.207898	1.75	

207414_s_at	0.1367712	1.09E-02	2.71	-3.207966	1.22	LOC100507472///PCSK6
205071_x_at	0.1370895	1.09E-02	2.71	-3.211522	1.1	XRCC4
219858_s_at	0.1370895	1.09E-02	2.71	-3.211773	1.2	MFSD6
208579_x_at	0.1395683	1.13E-02	2.7	-3.240649	1.57	H2BFS
205376_at	0.1395683	1.13E-02	2.7	-3.241089	1.12	INPP4B
214710_s_at	0.1395683	1.13E-02	2.7	-3.242109	1.76	CCNB1
243252_at	0.1397713	1.13E-02	2.7	-3.243839	1.4	
203554_x_at	0.1398594	1.13E-02	2.7	-3.245303	1.77	PTTG1
227862_at	0.1398594	1.13E-02	2.7	-3.245973	2.19	TRNP1
203499_at	0.140406	1.14E-02	2.69	-3.253886	1.76	EPHA2

223385_at	0.1407258	1.15E-02	2.69	-3.256953	2.32	CYP2S1
243386_at	0.1408077	1.15E-02	2.69	-3.259125	1.47	CASZ1
231070_at	0.141352	1.16E-02	2.69	-3.265923	1.7	IYD
213085_s_at	0.141352	1.16E-02	2.69	-3.267011	1.2	WWC1
204475_at	0.141352	1.16E-02	2.69	-3.267289	3.13	MMP1
206548_at	0.1414218	1.16E-02	2.69	-3.268338	1.17	
236193_at	0.1417005	1.17E-02	2.68	-3.271744	2.12	HIST1H2BC
222039_at	0.1419976	1.17E-02	2.68	-3.275257	1.73	KIF18B
229053_at	0.1421674	1.17E-02	2.68	-3.276743	1.19	SYT17
227019_at	0.1422039	1.17E-02	2.68	-3.277378	1.23	C1orf226

223103_at	0.142667	1.18E-02	2.68	-3.282174	1.62	STARD10
202934_at	0.1428471	1.18E-02	2.68	-3.284148	1.54	HK2
229245_at	0.1428471	1.18E-02	2.68	-3.284282	1.89	PLEKHA6
221922_at	0.143288	1.19E-02	2.68	-3.290079	1.27	GPSM2
201984_s_at	0.1434178	1.19E-02	2.67	-3.29114	1.04	EGFR
232578_at	0.1436241	1.19E-02	2.67	-3.293452	3.99	CLDN18
204092_s_at	0.1436241	1.19E-02	2.67	-3.2936	1.3	AURKA
219232_s_at	0.1436243	1.20E-02	2.67	-3.294797	1.99	EGLN3
226434_at	0.1437451	1.20E-02	2.67	-3.296635	1.02	PPP1R35
224189_x_at	0.1440327	1.20E-02	2.67	-3.299161	1.09	EHF
221245_s_at	0.1442332	1.21E-02	2.67	-3.302836	1.22	FZD5

210904_s_at	0.1442898	1.21E-02	2.67	-3.303865	1.19	IL13RA1
234331_s_at	0.1446456	1.21E-02	2.67	-3.307767	1.41	FAM84A
235911_at	0.1453436	1.22E-02	2.66	-3.316163	1.56	MELTF
210390_s_at	0.1454556	1.23E-02	2.66	-3.318228	1.97	CCL15-CCL14///CCL15
226086_at	0.1459714	1.24E-02	2.66	-3.324171	2.76	SYT13
202095_s_at	0.1462432	1.24E-02	2.66	-3.327155	1.42	BIRC5
229500_at	0.1465453	1.24E-02	2.66	-3.330209	1.01	SLC30A9
235153_at	0.1467051	1.25E-02	2.66	-3.331964	1.95	RNF183
236044_at	0.1475036	1.26E-02	2.65	-3.340772	2.43	PLPP4

204320_at	0.1478649	1.26E-02	2.65	-3.344661	3.55	COL11A1
236055_at	0.1484456	1.28E-02	2.65	-3.354399	1.66	DQX1
227260_at	0.1487307	1.29E-02	2.64	-3.360015	1.57	
213150_at	0.1489846	1.29E-02	2.64	-3.362536	2.39	HOXA10
219194_at	0.1492726	1.29E-02	2.64	-3.366958	1.63	SEMA4G
1558508_a_at	0.1493837	1.30E-02	2.64	-3.368529	1	C1orf53
226013_at	0.1494198	1.30E-02	2.64	-3.369226	1.35	TRAK1
204444_at	0.1496142	1.30E-02	2.64	-3.371502	1.67	KIF11
1554110_at	0.149705	1.30E-02	2.64	-3.37252	1.31	CDCP1
208527_x_at	0.1501038	1.31E-02	2.64	-3.375788	1.62	HIST1H2BE
238134_at	0.1506826	1.32E-02	2.63	-3.381923	1.69	
225681_at	0.1512751	1.32E-02	2.63	-3.386734	1.97	CTHRC1
238942_at	0.1512751	1.32E-02	2.63	-3.386956	1.17	

227240_at	0.1526386	1.34E-02	2.63	-3.398303	1.27	NGEF
214068_at	0.1527147	1.34E-02	2.62	-3.399507	1.17	BEAN1
229796_at	0.1531036	1.35E-02	2.62	-3.403497	1.21	SIX4
1556221_a_at	0.1534364	1.36E-02	2.62	-3.413147	1.32	
204033_at	0.1534364	1.36E-02	2.62	-3.413493	1.85	TRIP13
204989_s_at	0.1536229	1.37E-02	2.62	-3.415886	1.13	ITGB4
219045_at	0.1546404	1.38E-02	2.61	-3.427407	1.06	RHOF
239979_at	0.1553791	1.40E-02	2.61	-3.43576	1.06	
214319_at	0.1558012	1.41E-02	2.61	-3.440941	1.33	FRY
226869_at	0.1566023	1.42E-02	2.6	-3.448512	1.15	MEGF6
201301_s_at	0.157123	1.42E-02	2.6	-3.453148	1.16	ANXA4
AFFX-M27830_5_at	0.157246	1.43E-02	2.6	-3.45555	1.4	
229659_s_at	0.1574774	1.43E-02	2.6	-3.458089	2.14	

222262_s_at	0.1581383	1.44E-02	2.59	-3.464765	1.01	ETNK1
208490_x_at	0.1583263	1.45E-02	2.59	-3.467102	1.38	HIST1H2BC///HIST1H2BI///HIST1H2BE///HIST1H2BF///HIST1H2BG
211488_s_at	0.1585926	1.45E-02	2.59	-3.470392	1.02	ITGB8
204256_at	0.1593555	1.47E-02	2.59	-3.479224	1.27	ELOVL6
203997_at	0.1594163	1.47E-02	2.59	-3.480801	1.1	PTPN3
202575_at	0.160552	1.49E-02	2.58	-3.490772	1.99	CRABP2
219054_at	0.1609526	1.49E-02	2.58	-3.495015	1.88	NPR3

87100_at	0.1610015	1.49E-02	2.58	-3.495644	1.14	ABHD2
202311_s_at	0.1611124	1.50E-02	2.58	-3.497849	2.05	COL1A1
209806_at	0.1612314	1.50E-02	2.58	-3.499446	1.25	HIST1H2BK
1553993_s_at	0.1615285	1.51E-02	2.58	-3.504435	1.34	MIR6800///MED25
220022_at	0.1616216	1.51E-02	2.57	-3.506551	1.12	ZNF334
228624_at	0.1620759	1.52E-02	2.57	-3.511181	1.06	TMEM144
202341_s_at	0.1622482	1.52E-02	2.57	-3.512629	1.46	TRIM2
1559035_a_at	0.1623357	1.52E-02	2.57	-3.513503	1.29	
213787_s_at	0.1628359	1.53E-02	2.57	-3.519326	1.29	EBP
201663_s_at	0.1628718	1.53E-02	2.57	-3.519976	1.07	SMC4

203524_s_at	0.1628934	1.53E-02	2.57	-3.520271	1.07	MPST
226405_s_at	0.1635152	1.54E-02	2.57	-3.52632	1.07	ARRDC1
213059_at	0.16434	1.56E-02	2.56	-3.534675	1.68	CREB3L1
208083_s_at	0.1651493	1.57E-02	2.56	-3.543339	1.66	LOC100505984///ITGB6
212814_at	0.1659761	1.59E-02	2.55	-3.551497	1.04	AHCYL2
229634_at	0.1663566	1.59E-02	2.55	-3.554968	1.36	TMEM139
219703_at	0.1664625	1.60E-02	2.55	-3.556102	1.24	MNS1
233446_at	0.1670136	1.60E-02	2.55	-3.560056	2.2	ONECUT2
214993_at	0.1673027	1.61E-02	2.55	-3.563152	1.75	ASPHD1

230076_at	0.168568	1.63E-02	2.54	-3.572453	1.15	PITPNM3
219956_at	0.168653	1.63E-02	2.54	-3.573158	1.43	GALNT6
222740_at	0.168653	1.63E-02	2.54	-3.573282	1.01	ATAD2
202086_at	0.1689508	1.63E-02	2.54	-3.575038	1.13	MX1
227660_at	0.1691144	1.63E-02	2.54	-3.576419	1.37	ANTXR1
219874_at	0.1693523	1.64E-02	2.54	-3.579541	1.07	SLC12A8
230430_at	0.1693523	1.64E-02	2.54	-3.579553	1.55	ENTPD2
241140_at	0.169984	1.65E-02	2.54	-3.585128	1.55	LMO7

208523_x_at	0.170915	1.67E-02	2.53	-3.594566	1.12	HIST1H2BC///HIST1H2BI///HIST1H2BE///HIST1H2BF///HIST1H2BG
218355_at	0.170915	1.67E-02	2.53	-3.594742	1.16	KIF4A
205774_at	0.1717675	1.68E-02	2.53	-3.601897	1.41	F12
201309_x_at	0.1718219	1.69E-02	2.53	-3.604482	1.14	NREP
243276_at	0.1718288	1.69E-02	2.53	-3.605129	1.05	ALS2CL
1554195_a_at	0.1723422	1.70E-02	2.53	-3.609886	1.94	C5orf46
205068_s_at	0.17299	1.71E-02	2.52	-3.616114	1.1	ARHGAP26
227155_at	0.1730535	1.71E-02	2.52	-3.616955	1.23	LMO4

212942_s_at	0.173799	1.72E-02	2.52	-3.623635	2.58	CEMIP
228824_s_at	0.1748335	1.74E-02	2.52	-3.63165	1.13	PTGR1
213802_at	0.1749344	1.74E-02	2.51	-3.633281	1.56	PRSS12
243084_at	0.1750604	1.74E-02	2.51	-3.634587	1.12	CALD1
213680_at	0.1753878	1.75E-02	2.51	-3.637087	2.82	KRT6B
243435_at	0.1764936	1.77E-02	2.51	-3.646205	1.13	LOC101927338///KCNQ1OT1
223839_s_at	0.1772315	1.78E-02	2.51	-3.6527	1.29	
210365_at	0.1773417	1.78E-02	2.51	-3.653584	1	LOC101928269///LOC100506403///RUNX1

237654_at	0.1777393	1.79E-02	2.5	-3.656898	1.1	PPP1R36
222712_s_at	0.1780522	1.79E-02	2.5	-3.65929	1.58	MUC13
205623_at	0.1784932	1.80E-02	2.5	-3.66281	1.23	ALDH3A1
1552680_a_at	0.1796051	1.82E-02	2.5	-3.671212	1.09	KNL1
244665_at	0.1796051	1.82E-02	2.5	-3.671288	1.11	
229147_at	0.1806937	1.84E-02	2.49	-3.680917	1.41	RASSF6
236429_at	0.1811584	1.84E-02	2.49	-3.68499	1.05	ZNF83
228956_at	0.1818422	1.86E-02	2.49	-3.690776	1.57	UGT8
231766_s_at	0.1825747	1.87E-02	2.48	-3.697099	2.04	COL12A1
226424_at	0.1832233	1.88E-02	2.48	-3.702176	1.31	CAPS

219990_at	0.1836525	1.89E-02	2.48	-3.704923	1.53	E2F8
227609_at	0.1857762	1.93E-02	2.47	-3.72441	1.16	EPSTI1
236263_at	0.1859667	1.93E-02	2.47	-3.726034	1.3	SHH
204636_at	0.1870486	1.95E-02	2.47	-3.736486	1.22	COL17A1
219531_at	0.187436	1.96E-02	2.46	-3.739119	1.26	CEP72
206595_at	0.1879238	1.97E-02	2.46	-3.7439	1.88	CST6
203764_at	0.1879935	1.97E-02	2.46	-3.744429	1.76	DLGAP5
230772_at	0.1881792	1.97E-02	2.46	-3.745623	1.75	HNF4A
214385_s_at	0.1883182	1.98E-02	2.46	-3.747218	2.76	MUC5AC
204926_at	0.1884492	1.98E-02	2.46	-3.748774	1.6	INHBA
228876_at	0.1885832	1.98E-02	2.46	-3.749875	1.73	BAIAP2L2

226757_at	0.1886645	1.98E-02	2.46	-3.751098	1.14	IFIT2
232290_at	0.1886936	1.99E-02	2.46	-3.753308	1.5	
226582_at	0.1890544	2.00E-02	2.46	-3.755829	1.17	LOC400043
209581_at	0.1898222	2.01E-02	2.45	-3.762848	1.16	PLA2G16
229740_at	0.1899103	2.02E-02	2.45	-3.764956	1.09	SMIM5
221133_s_at	0.1901069	2.02E-02	2.45	-3.767111	3.29	CLDN18
204914_s_at	0.1905601	2.03E-02	2.45	-3.770772	1.53	SOX11
202870_s_at	0.1913512	2.04E-02	2.45	-3.776839	1.97	CDC20
223383_at	0.1913512	2.04E-02	2.45	-3.777074	1.12	ZNRF1
202079_s_at	0.1915243	2.05E-02	2.45	-3.778411	1.25	TRAK1
204750_s_at	0.1919729	2.06E-02	2.44	-3.782647	1.58	DSC2
238632_at	0.1920951	2.06E-02	2.44	-3.784959	1.79	

227055_at	0.1921343	2.06E-02	2.44	-3.785294	1.57	METTL7B
230285_at	0.192676	2.07E-02	2.44	-3.789912	1.2	SVIP
220082_at	0.1929176	2.08E-02	2.44	-3.794153	1.41	PPP1R14D
233030_at	0.1933346	2.09E-02	2.44	-3.797212	1.27	PNPLA3
206276_at	0.1938676	2.10E-02	2.43	-3.801361	2.94	LY6D
223381_at	0.1939407	2.10E-02	2.43	-3.801964	1.71	NUF2
201283_s_at	0.1946811	2.12E-02	2.43	-3.809117	1.09	TRAK1
231726_at	0.1955473	2.14E-02	2.43	-3.816365	1.13	PCDHB14

205180_s_at	0.1959676	2.14E-02	2.43	-3.819472	1.14	ADAM8
235456_at	0.1971429	2.16E-02	2.42	-3.828519	1.79	
229777_at	0.1972324	2.17E-02	2.42	-3.829369	2.57	CLRN3
227921_at	0.1972334	2.17E-02	2.42	-3.82967	1.13	LOC105379499///FLJ45482
205535_s_at	0.1973503	2.17E-02	2.42	-3.830703	1.13	PCDH7
37892_at	0.1973503	2.17E-02	2.42	-3.830834	3.56	COL11A1
204286_s_at	0.1974424	2.17E-02	2.42	-3.831799	1.27	PMAIP1
204972_at	0.1975165	2.18E-02	2.42	-3.832931	1.37	OAS2
239332_at	0.1975165	2.18E-02	2.42	-3.833287	2.36	LOC105371809
227626_at	0.1977151	2.18E-02	2.42	-3.835125	1.44	PAQR8
243141_at	0.1977651	2.18E-02	2.42	-3.835619	1.06	SGMS2

204455_at	0.1978101	2.18E-02	2.42	-3.836707	1.99	DST
238178_at	0.0002098	3.84E-08	-7.28	8.75717	-2.49	
237204_at	0.0002504	5.54E-08	-7.14	8.404259	-2.36	
1568768_s_at	0.0002728	1.02E-07	-6.92	7.818845	-3.23	BRE-AS1
215838_at	0.0002995	1.24E-07	-6.84	7.626961	-1.45	LILRA5
41386_i_at	0.0002995	1.32E-07	-6.82	7.572848	-1.74	KDM6B
41387_r_at	0.000383	1.89E-07	-6.69	7.224267	-1.32	KDM6B
229404_at	0.0005182	3.48E-07	-6.47	6.637707	-2.16	TWIST2
210162_s_at	0.0005182	3.60E-07	-6.46	6.604376	-2.13	NFATC1
206211_at	0.0007451	6.62E-07	-6.24	6.0182	-4.07	SELE
208707_at	0.0007455	7.09E-07	-6.22	5.952157	-1.47	EIF5
39402_at	0.000915	9.85E-07	-6.1	5.635074	-2.93	IL1B
205067_at	0.00111	1.40E-06	-5.98	5.296319	-3.4	IL1B
205207_at	0.0012466	1.78E-06	-5.89	5.066364	-5.19	IL6

220027_s_at	0.0014303	2.30E-06	-5.8	4.817833	-1.56	RASIP1
226833_at	0.001559	2.79E-06	-5.73	4.631137	-1.77	CYB5D1
1569136_at	0.0015857	2.90E-06	-5.72	4.595355	-1.55	MGAT4A
206157_at	0.0017333	3.61E-06	-5.64	4.383422	-5.02	PTX3
205863_at	0.0019075	4.15E-06	-5.59	4.249908	-2.98	S100A12
206359_at	0.0019219	4.22E-06	-5.59	4.234597	-2.01	SOCS3
229407_at	0.0019231	4.35E-06	-5.58	4.205396	-1.12	SDK1
1552711_a_at	0.0019231	4.43E-06	-5.57	4.187559	-1.26	CYB5D1

204575_s_at	0.0019468	4.56E-06	-5.56	4.160054	-3	MMP19
207316_at	0.0019574	4.62E-06	-5.56	4.147335	-1.51	HAS1
213068_at	0.0020542	5.02E-06	-5.53	4.0668	-3.89	DPT
240806_at	0.0020542	5.13E-06	-5.52	4.046409	-1.19	RPL15
227859_at	0.00206	5.39E-06	-5.5	3.998943	-1.39	DNAJC27
228399_at	0.0021938	6.02E-06	-5.46	3.892351	-2.21	OSR1
210873_x_at	0.0025792	8.14E-06	-5.36	3.60149	-2.05	APOBEC3A_B///APOBEC3A

235597_s_at	0.002603	8.59E-06	-5.34	3.550427	-1.85	RGPD2///RGPD1
210146_x_at	0.0026291	8.81E-06	-5.33	3.525732	-2.45	LILRB2
213146_at	0.0026645	9.23E-06	-5.31	3.481086	-1.82	KDM6B
202431_s_at	0.0026645	9.24E-06	-5.31	3.47966	-1.55	MYC
226370_at	0.0026645	9.34E-06	-5.31	3.469521	-1.26	KLHL15
1562275_at	0.0028379	1.05E-05	-5.27	3.355965	-4.19	
207977_s_at	0.0031893	1.27E-05	-5.2	3.172656	-2.97	DPT
205876_at	0.0033301	1.35E-05	-5.18	3.113606	-1.6	LIFR
212196_at	0.0035026	1.44E-05	-5.16	3.05643	-1.32	IL6ST

238624_at	0.0036739	1.55E-05	-5.13	2.984105	-1.17	LOC102724517///NLK
203812_at	0.0038994	1.69E-05	-5.1	2.901515	-2.07	SLIT3
218012_at	0.0040779	1.80E-05	-5.08	2.840842	-1.2	TSPYL2
213071_at	0.0040877	1.82E-05	-5.07	2.82573	-3.12	DPT
213791_at	0.0040896	1.83E-05	-5.07	2.821359	-2.86	PENK
209959_at	0.004202	1.92E-05	-5.05	2.776058	-4.06	NR4A3
219059_s_at	0.0044205	2.06E-05	-5.03	2.70816	-2.72	LYVE1
205237_at	0.0045033	2.16E-05	-5.01	2.664302	-1.38	FCN1
204513_s_at	0.0045368	2.19E-05	-5.01	2.649112	-1.78	ELMO1
241272_at	0.0045531	2.24E-05	-5	2.628648	-2.13	
239823_at	0.0047374	2.38E-05	-4.98	2.570977	-1.52	LOC101927841
237252_at	0.0047465	2.43E-05	-4.97	2.549958	-2.34	THBD
222317_at	0.0049007	2.55E-05	-4.96	2.505634	-2.32	PDE3B

209074_s_at	0.0049451	2.58E-05	-4.95	2.493601	-3.01	FAM107A
241369_at	0.0059962	3.29E-05	-4.86	2.259166	-1.89	ADAMTS9-AS1
210432_s_at	0.0061344	3.41E-05	-4.85	2.224747	-1.77	SCN3A
216233_at	0.0066352	3.88E-05	-4.81	2.100236	-1.8	CD163
238071_at	0.0066795	3.94E-05	-4.8	2.087273	-1.55	LCN10
226573_at	0.0068316	4.20E-05	-4.78	2.025454	-1.03	DIRAS1
201531_at	0.0069313	4.34E-05	-4.77	1.994012	-1.52	ZFP36
226397_s_at	0.0070003	4.42E-05	-4.76	1.97672	-1.69	PHACTR1
243813_at	0.0070216	4.47E-05	-4.76	1.964924	-2.34	LINC00968
207978_s_at	0.0070906	4.57E-05	-4.75	1.94499	-1.77	NR4A3

221031_s_at	0.0071731	4.68E-05	-4.74	1.920477	-2.16	APOLD1
239301_at	0.0071822	4.72E-05	-4.74	1.913982	-1.56	
239042_at	0.0073219	4.96E-05	-4.72	1.865798	-1.02	TSR1
241722_x_at	0.0073219	4.97E-05	-4.72	1.863579	-1.82	
229121_at	0.0073219	5.03E-05	-4.71	1.851264	-1.1	CMKLR1
229452_at	0.0073219	5.08E-05	-4.71	1.843541	-1.06	TMEM88
219448_at	0.0073475	5.13E-05	-4.71	1.832634	-1.15	TMEM70
205384_at	0.0075481	5.40E-05	-4.69	1.784504	-2.03	FXYD1
240165_at	0.0076772	5.60E-05	-4.68	1.748844	-1.72	
1559362_at	0.0079049	5.84E-05	-4.66	1.709007	-1.41	
235086_at	0.0079051	5.86E-05	-4.66	1.706503	-3.11	THBS1
210375_at	0.0084584	6.39E-05	-4.63	1.622965	-1.69	PTGER3
200790_at	0.0084841	6.44E-05	-4.63	1.615432	-1.3	ODC1

229839_at	0.0086713	6.74E-05	-4.61	1.572292	-3.7	SCARA5
238649_at	0.0087552	6.87E-05	-4.61	1.553532	-1.42	PITPNC1
235798_at	0.0089575	7.09E-05	-4.59	1.522772	-2.21	TMEM170B
236090_at	0.0091995	7.50E-05	-4.57	1.469501	-1.27	
1569484_s_at	0.00928	7.59E-05	-4.57	1.458446	-1.06	MDN1
215311_at	0.0095534	7.92E-05	-4.55	1.417889	-1.52	NTRK3
219993_at	0.0100591	8.64E-05	-4.52	1.333677	-1.73	SOX17
228193_s_at	0.0102152	8.80E-05	-4.52	1.316265	-1.67	RGCC
238695_s_at	0.0105739	9.19E-05	-4.5	1.275008	-1.65	RAB39B
214913_at	0.011052	9.97E-05	-4.47	1.197533	-1.95	ADAMTS3

235849_at	0.0110719	1.00E-04	-4.47	1.189935	-2.63	SCARA5
217833_at	0.0110762	1.01E-04	-4.47	1.181962	-1.08	SYNCRIP
236383_at	0.0115866	1.08E-04	-4.45	1.122804	-1.91	
1556989_at	0.0118134	1.12E-04	-4.43	1.088853	-2.59	
229414_at	0.0119011	1.14E-04	-4.43	1.072131	-1.7	PITPNC1
206432_at	0.0119764	1.15E-04	-4.42	1.062449	-2.41	HAS2
231907_at	0.0120209	1.16E-04	-4.42	1.053452	-1.2	ABL2
1569540_at	0.012144	1.18E-04	-4.41	1.032893	-1.06	
214582_at	0.0123856	1.21E-04	-4.4	1.00871	-1.88	PDE3B
227613_at	0.0123858	1.22E-04	-4.4	1.006911	-2.35	ZNF331

228831_s_at	0.0123918	1.23E-04	-4.4	0.994466	-1.64	GNG7
218305_at	0.0124044	1.24E-04	-4.4	0.991351	-1.08	IPO4
1556321_a_at	0.0124554	1.25E-04	-4.39	0.984138	-1.38	MESDC1
208609_s_at	0.0124554	1.25E-04	-4.39	0.983932	-1.57	TNXB///TNXA
207075_at	0.013467	1.38E-04	-4.36	0.88509	-1.35	NLRP3
209841_s_at	0.0135113	1.41E-04	-4.35	0.866877	-1.99	LRRN3
225570_at	0.0137504	1.45E-04	-4.34	0.836647	-1.2	SLC41A1
203888_at	0.0137504	1.46E-04	-4.34	0.835136	-2.5	THBD
227697_at	0.0137768	1.47E-04	-4.34	0.828408	-2.43	SOCS3
218723_s_at	0.0140029	1.51E-04	-4.33	0.80146	-1.68	RGCC
229934_at	0.0140548	1.53E-04	-4.32	0.789861	-1.5	
235831_at	0.0141166	1.54E-04	-4.32	0.780867	-1.54	
1565577_s_at	0.0141464	1.55E-04	-4.32	0.774841	-1.23	

209789_at	0.0141464	1.56E-04	-4.31	0.770773	-1.04	CORO2B
211663_x_at	0.0141464	1.56E-04	-4.31	0.769752	-2.5	PTGDS
213895_at	0.0141464	1.56E-04	-4.31	0.76931	-2.72	EMP1
236936_at	0.014148	1.56E-04	-4.31	0.767242	-2.32	
203887_s_at	0.0141573	1.57E-04	-4.31	0.763502	-2.38	THBD
237496_at	0.0144209	1.62E-04	-4.3	0.732791	-1.91	
214091_s_at	0.0144209	1.62E-04	-4.3	0.732626	-2.85	GPX3
206701_x_at	0.0144464	1.63E-04	-4.3	0.725454	-2.22	EDNRB
230711_at	0.0148732	1.72E-04	-4.28	0.67518	-1.55	
205839_s_at	0.0151299	1.76E-04	-4.27	0.655848	-1.15	TSPOAP1
213247_at	0.0154487	1.81E-04	-4.26	0.628479	-2.73	SVEP1
223467_at	0.0155534	1.82E-04	-4.26	0.620548	-2.62	RASD1

227058_at	0.0156774	1.84E-04	-4.25	0.609842	-3.52	MEDAG
229926_at	0.0158074	1.87E-04	-4.25	0.596221	-1.46	MIR3682
214696_at	0.0164007	1.99E-04	-4.23	0.538279	-1.77	MIR22///MIR22HG
220370_s_at	0.0166459	2.04E-04	-4.22	0.514816	-1.19	USP36
205792_at	0.0172326	2.15E-04	-4.2	0.463724	-1.82	WISP2
239544_at	0.0173358	2.17E-04	-4.2	0.456305	-1.17	
244251_at	0.0173358	2.17E-04	-4.2	0.455244	-1.01	LCP2
215330_at	0.0173646	2.19E-04	-4.19	0.445343	-1.32	
202768_at	0.0174172	2.21E-04	-4.19	0.435581	-3.97	FOSB
225557_at	0.0176164	2.25E-04	-4.18	0.419998	-1.84	CSRNP1

207697_x_at	0.0176164	2.25E-04	-4.18	0.419281	-1.62	LILRB2
205945_at	0.0176683	2.27E-04	-4.18	0.412737	-1.49	IL6R
211143_x_at	0.0180357	2.37E-04	-4.16	0.372263	-1.51	NR4A1
212230_at	0.0180357	2.38E-04	-4.16	0.368544	-1.61	PLPP3
242384_at	0.0180357	2.40E-04	-4.16	0.359625	-1.47	
230372_at	0.0180357	2.40E-04	-4.16	0.358316	-2.16	HAS2
201739_at	0.0187698	2.53E-04	-4.14	0.308485	-1.81	SGK1
201348_at	0.019216	2.61E-04	-4.13	0.279681	-2.83	GPX3
236621_at	0.0193784	2.64E-04	-4.13	0.269112	-1.32	RPS27
223504_at	0.0193824	2.64E-04	-4.13	0.267636	-1.16	DNAJC27

230511_at	0.0194031	2.65E-04	-4.12	0.264941	-1.86	CREM
207072_at	0.0194236	2.67E-04	-4.12	0.257746	-1.42	IL18RAP
225539_at	0.0194236	2.67E-04	-4.12	0.256718	-1.17	ZBTB21
207547_s_at	0.0194922	2.70E-04	-4.12	0.248314	-1.73	FAM107A
230075_at	0.0198336	2.76E-04	-4.11	0.226721	-1.44	RAB39B
1564776_at	0.0203349	2.84E-04	-4.1	0.199274	-1.18	
229544_at	0.02043	2.86E-04	-4.1	0.1911	-1.77	
220287_at	0.0207506	2.91E-04	-4.09	0.174893	-2.82	ADAMTS9
205382_s_at	0.0213707	3.04E-04	-4.07	0.13231	-2.83	CFD

206380_s_at	0.0213897	3.05E-04	-4.07	0.129121	-1.31	CFP
227993_at	0.021514	3.09E-04	-4.07	0.118631	-1.14	METAP2
203665_at	0.0216886	3.16E-04	-4.06	0.097828	-2.22	HMOX1
243473_at	0.0218352	3.18E-04	-4.06	0.090118	-1.08	
244546_at	0.0221111	3.28E-04	-4.05	0.062624	-1.46	CYCS
211105_s_at	0.0221111	3.28E-04	-4.05	0.061631	-1.99	NFATC1
230380_at	0.0224437	3.35E-04	-4.04	0.041598	-1.99	THAP2
228442_at	0.0230679	3.51E-04	-4.02	-0.004	-1.93	NFATC2
206170_at	0.023186	3.54E-04	-4.02	-0.01005	-1.78	ADRB2
206067_s_at	0.0234156	3.61E-04	-4.01	-0.029853	-2.42	WT1
238106_at	0.0234156	3.63E-04	-4.01	-0.033575	-1.16	

214805_at	0.0236571	3.67E-04	-4.01	-0.04609	-1.13	SNORD10///SNORA48///SNORA67///EIF4A1
208335_s_at	0.0237987	3.71E-04	-4	-0.055108	-3.52	ACKR1
216333_x_at	0.0238061	3.72E-04	-4	-0.057511	-2.19	TNXB///TNXA
226991_at	0.0238061	3.72E-04	-4	-0.058568	-1.86	NFATC2
210152_at	0.0238061	3.72E-04	-4	-0.058739	-1.22	LILRB4
240103_at	0.0238783	3.74E-04	-4	-0.062729	-1.17	

222486_s_at	0.0238918	3.76E-04	-4	-0.068831	-2.83	ADAMTS1
214038_at	0.0238918	3.76E-04	-4	-0.069409	-3.23	CCL8
204642_at	0.0241775	3.84E-04	-3.99	-0.088483	-1.9	S1PR1
244025_at	0.0242543	3.88E-04	-3.99	-0.096963	-1.14	ITPRIP
232307_at	0.0242543	3.89E-04	-3.99	-0.099482	-1.06	
214961_at	0.0242543	3.89E-04	-3.99	-0.101547	-2.32	MTUS2
1569597_at	0.0242725	3.90E-04	-3.98	-0.103453	-1.13	
243509_at	0.0249544	4.09E-04	-3.97	-0.148996	-2.33	
220117_at	0.0251016	4.14E-04	-3.96	-0.158888	-1.09	ZNF385D

209967_s_at	0.0251016	4.15E-04	-3.96	-0.161501	-1.62	CREM
222164_at	0.0251682	4.18E-04	-3.96	-0.169346	-1.93	FGFR1
226694_at	0.0251692	4.19E-04	-3.96	-0.170786	-1.27	PALM2-AKAP2///AKAP2
239648_at	0.0252195	4.21E-04	-3.96	-0.175801	-1.32	DCUN1D3
235030_at	0.0253342	4.27E-04	-3.95	-0.189416	-1.37	NXPE3
227791_at	0.025909	4.39E-04	-3.94	-0.215833	-1.63	SLC9A9

226856_at	0.0259171	4.44E-04	-3.94	-0.226445	-2.47	TMEM110-MUSTN1///MUSTN1///TMEM110
213515_x_at	0.0259171	4.45E-04	-3.94	-0.227445	-1.14	HBG2///HBG1
204184_s_at	0.0261065	4.50E-04	-3.93	-0.23826	-1.05	GRK3
213451_x_at	0.0261329	4.53E-04	-3.93	-0.245159	-2.36	TNXB///TNXA
206093_x_at	0.0262067	4.56E-04	-3.93	-0.250912	-2.03	TNXB///TNXA
204591_at	0.0262796	4.58E-04	-3.93	-0.254543	-2.81	CHL1
233127_at	0.026497	4.65E-04	-3.92	-0.269305	-2.22	

216248_s_at	0.0267029	4.73E-04	-3.91	-0.285501	-2.31	NR4A2
206876_at	0.0268806	4.78E-04	-3.91	-0.295051	-1.57	SIM1
1553133_at	0.0269408	4.81E-04	-3.91	-0.301178	-1.39	C9orf72
225571_at	0.0271893	4.87E-04	-3.9	-0.312666	-2.07	LIFR
201236_s_at	0.0273235	4.94E-04	-3.9	-0.326858	-2.17	BTG2
238835_at	0.0273472	4.98E-04	-3.9	-0.334184	-2.6	AVPR1A
220244_at	0.0275175	5.04E-04	-3.89	-0.346974	-1.85	LINC00312
227419_x_at	0.0275267	5.05E-04	-3.89	-0.348916	-1.9	PLAC9

217540_at	0.028001	5.21E-04	-3.88	-0.378003	-1.44	NXPE3
202340_x_at	0.028001	5.22E-04	-3.88	-0.3797	-2.17	NR4A1
235122_at	0.028001	5.23E-04	-3.88	-0.381869	-1.42	HIVEP3
1553645_at	0.028001	5.24E-04	-3.88	-0.382126	-1.39	CCDC141
233952_s_at	0.0285074	5.40E-04	-3.87	-0.411331	-1.16	ZBTB21
209304_x_at	0.0294827	5.70E-04	-3.85	-0.462465	-1.98	GADD45B

219563_at	0.0294827	5.70E-04	-3.85	-0.46318	-2.25	SYNE3///LINC00341
236495_at	0.0294827	5.73E-04	-3.84	-0.468183	-2.1	
202861_at	0.0297394	5.80E-04	-3.84	-0.478911	-1.27	MIR6883///PER1
205225_at	0.0297619	5.82E-04	-3.84	-0.48229	-1.47	ESR1
205612_at	0.029775	5.83E-04	-3.84	-0.483589	-2.35	MMRN1
213715_s_at	0.0304709	6.04E-04	-3.83	-0.518117	-1.04	KANK3
209840_s_at	0.030594	6.11E-04	-3.82	-0.528537	-1.86	LRRN3
202242_at	0.0307313	6.15E-04	-3.82	-0.535372	-2.66	TSPAN7
243847_at	0.0307637	6.18E-04	-3.82	-0.53982	-1.23	
219334_s_at	0.0307825	6.19E-04	-3.82	-0.541259	-1.49	NABP1

216598_s_at	0.0307905	6.20E-04	-3.82	-0.542363	-3.71	CCL2
229487_at	0.0310674	6.32E-04	-3.81	-0.560844	-1.87	EBF1
225575_at	0.0310674	6.34E-04	-3.81	-0.562791	-2.01	LIFR
209763_at	0.0314618	6.47E-04	-3.8	-0.58317	-3.85	CHRDL1
206932_at	0.0314797	6.50E-04	-3.8	-0.587283	-2.42	CH25H
1569477_at	0.0314797	6.52E-04	-3.8	-0.590604	-1.25	
227439_at	0.0314797	6.54E-04	-3.8	-0.593026	-1.01	ANKS1B
219343_at	0.0314797	6.58E-04	-3.79	-0.598147	-1	CDC37L1
204273_at	0.0315846	6.63E-04	-3.79	-0.606104	-2.13	EDNRB
204753_s_at	0.0316971	6.68E-04	-3.79	-0.613471	-1.23	HLF

238600_at	0.0319009	6.74E-04	-3.79	-0.62117	-1.19	JAKMIP1
32625_at	0.031948	6.77E-04	-3.78	-0.625017	-1.1	NPR1
205756_s_at	0.0320699	6.81E-04	-3.78	-0.630814	-1.56	F8
240467_at	0.0320699	6.82E-04	-3.78	-0.632138	-1.2	
214370_at	0.0321266	6.86E-04	-3.78	-0.63842	-2.76	S100A8
208056_s_at	0.0321729	6.88E-04	-3.78	-0.640689	-1.26	CBFA2T3
208438_s_at	0.0322419	6.91E-04	-3.78	-0.645037	-2.01	FGR
207442_at	0.0325979	7.08E-04	-3.77	-0.668238	-1.62	CSF3
232355_at	0.0330252	7.23E-04	-3.76	-0.68768	-1.53	SNORD114-3

221795_at	0.0330647	7.25E-04	-3.76	-0.690386	-2.55	NTRK2
209614_at	0.0331429	7.27E-04	-3.76	-0.693418	-2.23	ADH1B
224925_at	0.0333908	7.36E-04	-3.75	-0.704379	-1.43	PREX1
226022_at	0.033553	7.42E-04	-3.75	-0.71209	-1.38	SASH1
204271_s_at	0.0338378	7.55E-04	-3.74	-0.728632	-1.74	EDNRB

210676_x_at	0.0341051	7.63E-04	-3.74	-0.738384	-1.08	RGPD6///RGPD8///RGPD3///RGPD4///RGPD5
228771_at	0.0342362	7.71E-04	-3.74	-0.747796	-1.59	GRK3
239250_at	0.0343666	7.75E-04	-3.73	-0.753293	-1.48	ZNF542P
222162_s_at	0.0347906	7.89E-04	-3.73	-0.770221	-3.02	ADAMTS1

1557459_at	0.0350232	7.97E-04	-3.72	-0.779553	-1.62	
1556185_a_at	0.0350446	8.00E-04	-3.72	-0.783708	-1.85	
200798_x_at	0.0350973	8.03E-04	-3.72	-0.786848	-1.39	MCL1
212187_x_at	0.0351709	8.07E-04	-3.72	-0.791087	-2.97	PTGDS
232606_at	0.0353683	8.16E-04	-3.71	-0.802096	-1.38	ANK2
206896_s_at	0.0354339	8.22E-04	-3.71	-0.808458	-1.22	GNG7
1553243_at	0.0354339	8.22E-04	-3.71	-0.809341	-1.64	ITIH5
232257_s_at	0.0355851	8.31E-04	-3.71	-0.819299	-1.28	
223533_at	0.0356159	8.35E-04	-3.71	-0.823804	-1.47	LOC101927933///LRRC8C
229465_s_at	0.036051	8.58E-04	-3.7	-0.849552	-1.39	PTPRS

209613_s_at	0.036051	8.59E-04	-3.7	-0.850713	-4.3	ADH1B
209612_s_at	0.0362568	8.70E-04	-3.69	-0.862466	-3.97	ADH1B
218032_at	0.0364123	8.75E-04	-3.69	-0.867628	-1.11	SNN
209193_at	0.0367625	8.87E-04	-3.68	-0.880513	-1.25	PIM1
64064_at	0.0367947	8.89E-04	-3.68	-0.882812	-1.68	GIMAP1-GIMAP5///GIMAP5
214153_at	0.0369242	8.95E-04	-3.68	-0.888978	-1.1	ELOVL5
214508_x_at	0.0371169	9.05E-04	-3.68	-0.899174	-1.95	CREM

1564796_at	0.0372438	9.12E-04	-3.67	-0.907068	-2.63	EMP1
206030_at	0.0372438	9.16E-04	-3.67	-0.911106	-1.91	ASPA
229004_at	0.0372438	9.18E-04	-3.67	-0.91302	-1.96	ADAMTS15
215303_at	0.0373807	9.22E-04	-3.67	-0.917501	-1.53	DCLK1
204622_x_at	0.0376484	9.31E-04	-3.67	-0.926385	-2.28	NR4A2
214156_at	0.0379712	9.45E-04	-3.66	-0.939937	-2.26	MYRIP
209307_at	0.0383115	9.60E-04	-3.66	-0.955257	-1.11	SWAP70
220037_s_at	0.038377	9.63E-04	-3.65	-0.958243	-2.1	LYVE1

228325_at	0.0387406	9.81E-04	-3.65	-0.975342	-1.96	SPIDR
203574_at	0.0388107	9.83E-04	-3.65	-0.977725	-1.75	NFIL3
205813_s_at	0.038833	9.84E-04	-3.65	-0.978948	-1.13	MAT1A
1560049_at	0.0389922	9.89E-04	-3.64	-0.983483	-1.48	
229580_at	0.0390521	9.92E-04	-3.64	-0.986207	-2.36	CLSTN2
221796_at	0.0390521	9.93E-04	-3.64	-0.987492	-3.44	NTRK2
205027_s_at	0.0390785	9.95E-04	-3.64	-0.988958	-1.29	MAP3K8
205498_at	0.0391695	1.00E-03	-3.64	-0.99453	-2.38	GHR
213236_at	0.0391933	1.00E-03	-3.64	-0.995776	-1.09	SASH1
218730_s_at	0.0392402	1.00E-03	-3.64	-0.997576	-3.2	OGN
224450_s_at	0.039675	1.02E-03	-3.63	-1.013333	-1.13	RIOK1
232629_at	0.0403853	1.05E-03	-3.62	-1.044007	-1.06	PROK2

209681_at	0.0405468	1.06E-03	-3.62	-1.048425	-1.3	SLC19A2
36829_at	0.0405752	1.06E-03	-3.62	-1.051387	-1.12	MIR6883///PER1
234987_at	0.0406375	1.07E-03	-3.62	-1.05682	-1.55	SAMHD1
226333_at	0.0407184	1.07E-03	-3.61	-1.061252	-1.46	IL6R
231031_at	0.0408763	1.08E-03	-3.61	-1.067185	-1.05	FGF7P3
213960_at	0.0412034	1.10E-03	-3.61	-1.081181	-1.74	NTRK3
244860_at	0.0418454	1.13E-03	-3.6	-1.106024	-1.37	
213006_at	0.0418928	1.13E-03	-3.59	-1.10773	-2.03	CEBPD

242680_at	0.0420814	1.14E-03	-3.59	-1.115784	-2.36	AVPR1A
226911_at	0.0421464	1.15E-03	-3.59	-1.121365	-1.42	EGFLAM
230867_at	0.0421464	1.15E-03	-3.59	-1.121611	-1.62	COL6A6
38487_at	0.0427566	1.17E-03	-3.58	-1.137739	-1.51	STAB1
228749_at	0.0428373	1.17E-03	-3.58	-1.140776	-1.5	ZDBF2
211776_s_at	0.0432353	1.18E-03	-3.58	-1.153254	-1.77	EPB41L3
235359_at	0.0445059	1.24E-03	-3.56	-1.193596	-1.14	NRROS
216979_at	0.0446203	1.24E-03	-3.56	-1.197865	-1.81	NR4A3
211748_x_at	0.0450942	1.26E-03	-3.55	-1.214904	-2.79	PTGDS

227550_at	0.0450942	1.27E-03	-3.55	-1.215153	-1.84	GFRA1
220088_at	0.0454334	1.28E-03	-3.55	-1.224679	-1.87	C5AR1
218625_at	0.0459804	1.31E-03	-3.54	-1.244846	-1.56	NRN1
240410_at	0.0462908	1.32E-03	-3.54	-1.25319	-1.93	
1554182_at	0.0463857	1.32E-03	-3.54	-1.257889	-1.35	TRIM74///TRIM73
235735_at	0.0463857	1.32E-03	-3.54	-1.258027	-2.3	TNFSF8
223623_at	0.0465883	1.34E-03	-3.53	-1.265831	-2.97	C2orf40
239262_at	0.0467455	1.34E-03	-3.53	-1.270195	-2.2	
201963_at	0.0477459	1.38E-03	-3.52	-1.29606	-1.16	ACSL1

209305_s_at	0.0477792	1.38E-03	-3.52	-1.298175	-1.93	GADD45B
205858_at	0.0477792	1.38E-03	-3.52	-1.299061	-1.2	NGFR
233057_at	0.0477792	1.38E-03	-3.52	-1.299092	-1.21	HSPB8
204154_at	0.0479137	1.39E-03	-3.52	-1.303511	-2.39	CDO1
226811_at	0.0479984	1.39E-03	-3.52	-1.305762	-1.99	FAM46C
229687_s_at	0.0482026	1.41E-03	-3.51	-1.313882	-1.06	PRDM11
1569482_at	0.0487657	1.43E-03	-3.51	-1.330899	-1.03	TNRC6C
205478_at	0.0487657	1.43E-03	-3.51	-1.331932	-2.42	PPP1R1A

239427_at	0.0487841	1.44E-03	-3.51	-1.333941	-1.08	SLAMF1
227178_at	0.0488625	1.44E-03	-3.5	-1.336032	-1.5	CELF2
223960_s_at	0.0490984	1.45E-03	-3.5	-1.345159	-1.26	CDIP1
235024_at	0.0490984	1.45E-03	-3.5	-1.346179	-1.75	JADE1
220358_at	0.0493953	1.47E-03	-3.5	-1.353186	-1.28	BATF3
233674_at	0.0496078	1.48E-03	-3.49	-1.360977	-1.11	
244654_at	0.0497022	1.48E-03	-3.49	-1.364024	-1.63	MYO1G
233648_at	0.0499926	1.50E-03	-3.49	-1.373939	-1.25	
219298_at	0.050164	1.51E-03	-3.49	-1.379205	-1.25	ECHDC3

218805_at	0.0503506	1.52E-03	-3.48	-1.388599	-2.05	GIMAP1-GIMAP5///GIMAP5
228376_at	0.050551	1.53E-03	-3.48	-1.396628	-2.21	GGTA1P
235885_at	0.0509017	1.55E-03	-3.48	-1.406268	-1.84	P2RY12
212486_s_at	0.0509818	1.56E-03	-3.47	-1.410788	-1.58	FYN
229367_s_at	0.0514856	1.58E-03	-3.47	-1.425041	-2.15	GIMAP6
220416_at	0.0516957	1.59E-03	-3.47	-1.431407	-1.23	ATP8B4
203845_at	0.0516957	1.60E-03	-3.46	-1.434438	-1.12	KAT2B

227708_at	0.0518535	1.61E-03	-3.46	-1.440067	-1.3	EEF1A1
203320_at	0.0520514	1.62E-03	-3.46	-1.446954	-1.2	SH2B3
210858_x_at	0.0528438	1.66E-03	-3.45	-1.467736	-1.01	ATM
207574_s_at	0.0528699	1.66E-03	-3.45	-1.470513	-1.89	GADD45B
212226_s_at	0.0528699	1.66E-03	-3.45	-1.471923	-1.31	PLPP3
1562194_at	0.0528699	1.66E-03	-3.45	-1.472316	-1.57	
207610_s_at	0.0528699	1.66E-03	-3.45	-1.472953	-1.25	ADGRE2
235529_x_at	0.0528746	1.67E-03	-3.45	-1.474805	-1.07	SAMHD1

220038_at	0.0529532	1.67E-03	-3.45	-1.477284	-1.19	C8orf44-SGK3///SGK3
236651_at	0.0533107	1.71E-03	-3.44	-1.495592	-1.12	KALRN
219183_s_at	0.0533107	1.71E-03	-3.44	-1.496693	-1.06	CYTH4
229910_at	0.0537202	1.73E-03	-3.44	-1.507437	-1.75	SHE
213221_s_at	0.0539281	1.74E-03	-3.43	-1.516252	-1.2	SIK2
234072_at	0.054131	1.75E-03	-3.43	-1.52148	-1.22	SEMA4A
219228_at	0.0547656	1.79E-03	-3.42	-1.538776	-1.96	ZNF331
1566518_at	0.0547656	1.79E-03	-3.42	-1.53925	-1.85	
204829_s_at	0.0548066	1.79E-03	-3.42	-1.541525	-1.79	FOLR2
214617_at	0.0550183	1.81E-03	-3.42	-1.551405	-1.55	PRF1
241679_at	0.0551653	1.82E-03	-3.42	-1.555605	-1.76	
223316_at	0.0551653	1.82E-03	-3.42	-1.555847	-2.13	CCDC3

213245_at	0.0553033	1.83E-03	-3.41	-1.559881	-1.72	ADCY1
204621_s_at	0.0553119	1.83E-03	-3.41	-1.560545	-1.99	NR4A2
212681_at	0.0554032	1.83E-03	-3.41	-1.562608	-1.75	EPB41L3
205200_at	0.0554296	1.84E-03	-3.41	-1.565465	-1.83	EXOSC7///CLEC3B
213974_at	0.0557457	1.85E-03	-3.41	-1.573695	-1.81	ADAMTSL3
201325_s_at	0.0557457	1.86E-03	-3.41	-1.575541	-1.75	EMP1
217504_at	0.0557457	1.86E-03	-3.41	-1.575603	-1.93	ABCA6
227243_s_at	0.0559023	1.87E-03	-3.41	-1.580794	-1.25	EBF3

202759_s_at	0.0560549	1.88E-03	-3.4	-1.586903	-1.41	PALM2-AKAP2///AKAP2
201427_s_at	0.0560595	1.88E-03	-3.4	-1.587889	-1.55	SEPP1
223640_at	0.0561916	1.89E-03	-3.4	-1.590737	-1.43	HCST
233289_at	0.0562057	1.89E-03	-3.4	-1.591482	-1.36	
202920_at	0.0576707	1.96E-03	-3.39	-1.62704	-3.05	ANK2
227771_at	0.0576707	1.97E-03	-3.39	-1.62968	-1.51	LIFR
229497_at	0.0580527	1.98E-03	-3.38	-1.637394	-1.35	ANKDD1A
243296_at	0.0581091	1.99E-03	-3.38	-1.641302	-1.9	NAMPT
226931_at	0.0585357	2.01E-03	-3.38	-1.651317	-1.28	TMTC1

209542_x_at	0.0586382	2.02E-03	-3.38	-1.65426	-1.97	IGF1
226571_s_at	0.0589972	2.04E-03	-3.37	-1.661459	-1.56	PTPRS
219368_at	0.0593304	2.05E-03	-3.37	-1.668573	-1.61	NAP1L2
244464_at	0.0593304	2.05E-03	-3.37	-1.669546	-1.26	
238780_s_at	0.0593678	2.06E-03	-3.37	-1.671765	-2.3	KCNJ5
235964_x_at	0.0598137	2.08E-03	-3.36	-1.682211	-1.07	SAMHD1
215151_at	0.0602127	2.12E-03	-3.36	-1.69925	-1.07	DOCK10

238018_at	0.060379	2.13E-03	-3.36	-1.70369	-3.47	FAM150B
229912_at	0.060444	2.13E-03	-3.36	-1.705181	-1.16	SDK1
228063_s_at	0.0604754	2.14E-03	-3.35	-1.707714	-1.33	NAP1L5
224341_x_at	0.060687	2.15E-03	-3.35	-1.713762	-1.4	TLR4
239808_at	0.0611878	2.18E-03	-3.35	-1.726269	-1.29	
211000_s_at	0.0612445	2.19E-03	-3.35	-1.729506	-1.05	IL6ST
232313_at	0.0612558	2.19E-03	-3.35	-1.730634	-1.11	TMEM132C
240652_at	0.0612879	2.20E-03	-3.34	-1.732078	-1.23	
224822_at	0.0617768	2.22E-03	-3.34	-1.742012	-1.62	DLC1
225747_at	0.0620138	2.24E-03	-3.34	-1.749804	-1.02	COQ10A
237176_at	0.062085	2.25E-03	-3.34	-1.753609	-1.05	
229312_s_at	0.0623643	2.26E-03	-3.33	-1.760639	-1.16	GKAP1

205098_at	0.0624792	2.27E-03	-3.33	-1.763571	-2.06	CCR1
1553494_at	0.0624841	2.27E-03	-3.33	-1.764336	-1.82	TDH
213733_at	0.0625972	2.28E-03	-3.33	-1.767906	-1.16	MYO1F
210762_s_at	0.0625972	2.28E-03	-3.33	-1.768465	-1.66	DLC1
235593_at	0.0626811	2.29E-03	-3.33	-1.770719	-1.49	ZEB2
230052_s_at	0.0630989	2.31E-03	-3.33	-1.778863	-1.22	NFKBID
205932_s_at	0.0632358	2.32E-03	-3.32	-1.782054	-1.24	MSX1
1554462_a_at	0.0632384	2.32E-03	-3.32	-1.783026	-1.24	DNAJB9
204521_at	0.063277	2.32E-03	-3.32	-1.784526	-1.02	FAM216A
211456_x_at	0.0633565	2.33E-03	-3.32	-1.788022	-1.61	MT1HL1

1566825_at	0.0638286	2.36E-03	-3.32	-1.798959	-1.87	
1556300_s_at	0.0638286	2.36E-03	-3.32	-1.799456	-2.06	SIM1
227923_at	0.0638286	2.36E-03	-3.32	-1.800458	-1.22	SHANK3
244592_at	0.0638286	2.36E-03	-3.32	-1.800595	-1.37	
214735_at	0.0641161	2.39E-03	-3.31	-1.810678	-1.43	IPCEF1
228766_at	0.0643737	2.41E-03	-3.31	-1.819775	-2.95	CD36
220327_at	0.0643737	2.41E-03	-3.31	-1.820358	-1.66	VGLL3
219820_at	0.064606	2.42E-03	-3.31	-1.824175	-1.11	SLC6A16
230943_at	0.0646702	2.43E-03	-3.31	-1.825557	-1.61	SOX17
1556474_a_at	0.0651298	2.46E-03	-3.3	-1.836741	-2.29	LOC285097
242809_at	0.0651724	2.47E-03	-3.3	-1.840474	-1.28	IL1RL1

201694_s_at	0.0654256	2.48E-03	-3.3	-1.844544	-1.69	EGR1
215049_x_at	0.0656424	2.49E-03	-3.3	-1.848981	-2.41	CD163
210155_at	0.0656981	2.49E-03	-3.3	-1.850222	-1.41	MYOC
1552509_a_at	0.0660873	2.51E-03	-3.29	-1.857979	-1.54	CD300LG
207277_at	0.0663967	2.53E-03	-3.29	-1.863525	-1.57	CD209
241925_x_at	0.0664736	2.54E-03	-3.29	-1.867445	-1.57	SLC16A7
230962_at	0.0665771	2.54E-03	-3.29	-1.869789	-1.11	DCLK1
227811_at	0.0666797	2.55E-03	-3.29	-1.872117	-1.59	FGD3
218711_s_at	0.0669306	2.56E-03	-3.29	-1.876858	-1.01	SDPR
202760_s_at	0.0671913	2.58E-03	-3.28	-1.883248	-1.2	PALM2-AKAP2///AKAP2
201041_s_at	0.0672101	2.58E-03	-3.28	-1.883953	-1.73	DUSP1

227646_at	0.0672959	2.59E-03	-3.28	-1.88597	-1.59	EBF1
209291_at	0.0676235	2.62E-03	-3.28	-1.897566	-2.01	ID4
235077_at	0.0677465	2.63E-03	-3.28	-1.902189	-2.33	MEG3
216813_at	0.0678759	2.65E-03	-3.27	-1.908496	-1.25	
230158_at	0.0678759	2.66E-03	-3.27	-1.909849	-1.4	DPY19L2
216920_s_at	0.0685163	2.71E-03	-3.26	-1.92716	-1.7	TARP///TRGV9///TRGC2
242868_at	0.0685685	2.71E-03	-3.26	-1.928519	-1.49	
216005_at	0.0690429	2.74E-03	-3.26	-1.939138	-2.33	TNC
212558_at	0.0690531	2.74E-03	-3.26	-1.939851	-1.24	SPRY1

207630_s_at	0.0693349	2.76E-03	-3.26	-1.946498	-1.74	CREM
226322_at	0.0693845	2.77E-03	-3.26	-1.947591	-1.74	TMTC1
204897_at	0.0696602	2.79E-03	-3.25	-1.95555	-1.7	PTGER4
214056_at	0.0697161	2.80E-03	-3.25	-1.957147	-1.21	MCL1
203710_at	0.0697174	2.80E-03	-3.25	-1.958086	-1.54	ITPR1
1554676_at	0.0701946	2.83E-03	-3.25	-1.969448	-1.73	SRGN
222722_at	0.0710813	2.89E-03	-3.24	-1.989124	-3.49	OGN
206710_s_at	0.0712753	2.90E-03	-3.24	-1.992982	-2.02	EPB41L3
214470_at	0.0712799	2.91E-03	-3.24	-1.994295	-2.24	KLRB1

202747_s_at	0.0712799	2.91E-03	-3.24	-1.995705	-2.34	ITM2A
215058_at	0.0712799	2.92E-03	-3.24	-1.996852	-1.39	DENND5B

228370_at	0.0712799	2.92E-03	-3.24	-1.997723	-1.75	LOC101930404///SNORD116-28///SNORD115-26///SNORD115-13///SNOR[illegible] SNORD107///IPW

204036_at	0.0712818	2.92E-03	-3.24	-1.998418	-1.4	LPAR1
202842_s_at	0.0713036	2.93E-03	-3.23	-1.999533	-1.23	DNAJB9
205119_s_at	0.0714177	2.93E-03	-3.23	-2.001436	-1.83	FPR1
201289_at	0.071523	2.94E-03	-3.23	-2.005707	-1.83	CYR61
205099_s_at	0.0715335	2.95E-03	-3.23	-2.006258	-1.42	CCR1
201324_at	0.07208	2.99E-03	-3.23	-2.018393	-1.38	EMP1
205883_at	0.0722711	3.00E-03	-3.22	-2.024444	-2.55	ZBTB16
229177_at	0.0722981	3.01E-03	-3.22	-2.025201	-1.98	C16orf89
226778_at	0.0724565	3.03E-03	-3.22	-2.031133	-1.12	TDRP

229800_at	0.0724578	3.03E-03	-3.22	-2.031745	-2.47	DCLK1
209292_at	0.0725749	3.04E-03	-3.22	-2.035676	-1.9	ID4
244313_at	0.0730355	3.08E-03	-3.22	-2.047086	-2.11	CR1
213560_at	0.0730355	3.08E-03	-3.21	-2.047737	-1.35	GADD45B
219471_at	0.0730355	3.08E-03	-3.21	-2.048341	-1.94	KIAA0226L
211685_s_at	0.0730355	3.09E-03	-3.21	-2.049257	-1.31	NCALD
209760_at	0.0730809	3.10E-03	-3.21	-2.052477	-1.85	KIAA0922
203685_at	0.0731277	3.11E-03	-3.21	-2.056346	-1.77	BCL2

232291_at	0.0731277	3.11E-03	-3.21	-2.056842	-1.42	MIR17HG///MIR92A1///MIR20A///MIR19B1///MIR19A///MIR18A///MIR1[illegible]
209355_s_at	0.0734018	3.13E-03	-3.21	-2.061922	-1.58	PLPP3
1553102_a_at	0.0734018	3.14E-03	-3.21	-2.064719	-2.31	CCDC69
221928_at	0.0734393	3.14E-03	-3.21	-2.065592	-1.09	ACACB
226773_at	0.0737551	3.17E-03	-3.2	-2.074843	-1.36	PPM1K
228532_at	0.0738308	3.18E-03	-3.2	-2.076628	-1.81	C1orf162

1556606_at	0.0740342	3.19E-03	-3.2	-2.079831	-1.06	NAV2
211577_s_at	0.0740684	3.19E-03	-3.2	-2.080944	-1.67	IGF1
228007_at	0.0742164	3.20E-03	-3.2	-2.083694	-1.22	CEP85L
206488_s_at	0.0742895	3.21E-03	-3.2	-2.085004	-3	CD36
235979_at	0.0745402	3.23E-03	-3.2	-2.091272	-2.32	C7
212886_at	0.0749279	3.26E-03	-3.19	-2.101194	-2.14	CCDC69
210222_s_at	0.0753136	3.30E-03	-3.19	-2.112391	-1.1	RTN1
229116_at	0.0754734	3.32E-03	-3.19	-2.117643	-1.14	CNKSR2
232951_at	0.0755386	3.33E-03	-3.18	-2.11997	-1.01	
220170_at	0.0755386	3.33E-03	-3.18	-2.12017	-1.66	FHL5
209447_at	0.0756445	3.35E-03	-3.18	-2.124644	-1.57	SYNE1
241791_at	0.0758977	3.37E-03	-3.18	-2.131692	-1.62	TTN

207822_at	0.0761712	3.39E-03	-3.18	-2.137325	-1.37	FGFR1
231067_s_at	0.0762066	3.41E-03	-3.18	-2.140804	-1.67	AKAP12
204042_at	0.0762826	3.41E-03	-3.18	-2.14287	-1.44	WASF3
203543_s_at	0.076359	3.42E-03	-3.17	-2.145317	-1.4	KLF9
226099_at	0.0764017	3.43E-03	-3.17	-2.14631	-1.38	ELL2
201811_x_at	0.0764017	3.43E-03	-3.17	-2.146973	-1.23	SH3BP5
205392_s_at	0.0768298	3.46E-03	-3.17	-2.155232	-2.6	CCL15-CCL14///CCL14
222820_at	0.0769896	3.47E-03	-3.17	-2.15899	-1	TNRC6C
227099_s_at	0.0769896	3.47E-03	-3.17	-2.159434	-1.61	C11orf96

224646_x_at	0.0777487	3.54E-03	-3.16	-2.176732	-2.42	MIR675///H19
205639_at	0.0781704	3.57E-03	-3.16	-2.185447	-1.27	AOAH
236075_s_at	0.0781704	3.57E-03	-3.16	-2.185922	-1.18	LOC101928000
205083_at	0.0782441	3.58E-03	-3.16	-2.187834	-2.96	AOX1
202746_at	0.0782441	3.59E-03	-3.16	-2.188947	-2.41	ITM2A
206210_s_at	0.0786821	3.63E-03	-3.15	-2.200771	-1.63	CETP
227478_at	0.0787014	3.63E-03	-3.15	-2.201365	-1.43	SETBP1
226982_at	0.078802	3.64E-03	-3.15	-2.20402	-1.38	ELL2
228854_at	0.0792876	3.68E-03	-3.15	-2.213173	-2.74	

238488_at	0.0794892	3.70E-03	-3.14	-2.217699	-1.25	LRRC70///IPO11
1557419_a_at	0.0795464	3.71E-03	-3.14	-2.221119	-1.28	ACSL4
203799_at	0.0795475	3.72E-03	-3.14	-2.223754	-1.44	LY75-CD302///CD302///LY75
214032_at	0.0795475	3.73E-03	-3.14	-2.225404	-1.49	ZAP70
227265_at	0.0795475	3.73E-03	-3.14	-2.225508	-1.97	FGL2
41644_at	0.0801824	3.78E-03	-3.14	-2.237495	-1.11	SASH1
203761_at	0.0807132	3.81E-03	-3.13	-2.246208	-1.8	SLA
210764_s_at	0.0807439	3.82E-03	-3.13	-2.247995	-1.99	CYR61
218517_at	0.0807774	3.83E-03	-3.13	-2.250979	-1.43	JADE1

213933_at	0.0808544	3.84E-03	-3.13	-2.253196	-1.67	PTGER3
202877_s_at	0.0810254	3.85E-03	-3.13	-2.255512	-1.22	CD93
237442_at	0.0810864	3.86E-03	-3.13	-2.257893	-1.5	
222717_at	0.0812801	3.88E-03	-3.13	-2.262691	-2.14	SDPR
228410_at	0.0813302	3.89E-03	-3.12	-2.263617	-1.16	GAB3
230276_at	0.0820147	3.95E-03	-3.12	-2.278579	-1.07	FAM49A
207526_s_at	0.0825465	3.99E-03	-3.11	-2.287606	-1.66	IL1RL1
243601_at	0.0826544	4.00E-03	-3.11	-2.290569	-1.02	LOC285957
215785_s_at	0.0829489	4.02E-03	-3.11	-2.295372	-1.49	CYFIP2
203645_s_at	0.0833855	4.06E-03	-3.11	-2.303268	-2.35	CD163
237230_at	0.0834629	4.07E-03	-3.11	-2.305172	-1.78	GPHA2
235670_at	0.083473	4.07E-03	-3.11	-2.306206	-1.71	STX11
225820_at	0.083473	4.07E-03	-3.11	-2.306627	-1.49	JADE1

43427_at	0.083473	4.07E-03	-3.11	-2.307097	-1.37	ACACB
217738_at	0.083473	4.08E-03	-3.11	-2.308103	-1.25	NAMPT
227762_at	0.083473	4.08E-03	-3.11	-2.30878	-1.69	
207761_s_at	0.0838445	4.11E-03	-3.1	-2.315983	-1.4	METTL7A
212185_x_at	0.0839507	4.12E-03	-3.1	-2.318191	-1.53	MT2A
207275_s_at	0.0843005	4.16E-03	-3.1	-2.326516	-1.26	ACSL1
203881_s_at	0.0847039	4.20E-03	-3.09	-2.335386	-2.02	DMD
227645_at	0.0847087	4.20E-03	-3.09	-2.335781	-1.74	PIK3R5
242388_x_at	0.084952	4.22E-03	-3.09	-2.339656	-1.93	TAGAP

215806_x_at	0.084952	4.22E-03	-3.09	-2.339801	-1.55	TARP///TRGV9///TRGC2
1557418_at	0.0860042	4.29E-03	-3.09	-2.355617	-1.5	ACSL4
208581_x_at	0.0862774	4.31E-03	-3.08	-2.359931	-1.69	MT1X
219312_s_at	0.0867561	4.35E-03	-3.08	-2.367058	-1.48	ZBTB10
209894_at	0.0873103	4.38E-03	-3.08	-2.374976	-1.15	LEPROT///LEPR
1552318_at	0.0875592	4.41E-03	-3.08	-2.380639	-1.16	GIMAP1
203438_at	0.0878011	4.43E-03	-3.07	-2.383872	-1.24	STC2

202843_at	0.0878011	4.43E-03	-3.07	-2.384199	-1.5	DNAJB9
203542_s_at	0.0879611	4.45E-03	-3.07	-2.389343	-1.02	KLF9
244358_at	0.0879611	4.45E-03	-3.07	-2.3896	-1.15	
209683_at	0.0880117	4.47E-03	-3.07	-2.392768	-1.63	FAM49A
207092_at	0.0880647	4.47E-03	-3.07	-2.393657	-2.34	LEP
204502_at	0.0882548	4.48E-03	-3.07	-2.395986	-1.11	SAMHD1
206115_at	0.0883142	4.49E-03	-3.07	-2.39694	-2.07	EGR3
239835_at	0.0883895	4.50E-03	-3.07	-2.399259	-1.78	KBTBD8

235740_at	0.0884324	4.51E-03	-3.07	-2.401517	-1.56	MCTP1
217739_s_at	0.0884324	4.51E-03	-3.07	-2.401642	-1.23	NAMPT
217546_at	0.0888861	4.55E-03	-3.06	-2.40855	-3.15	MT1M
239946_at	0.0889179	4.55E-03	-3.06	-2.409212	-2.27	
217552_x_at	0.0891326	4.57E-03	-3.06	-2.4142	-1.71	CR1
206181_at	0.0892105	4.59E-03	-3.06	-2.416867	-1.35	SLAMF1
205374_at	0.0895139	4.61E-03	-3.06	-2.421701	-1.44	SLN
204236_at	0.0895634	4.62E-03	-3.06	-2.422817	-1.66	FLI1

206208_at	0.0898683	4.64E-03	-3.06	-2.426884	-1.58	CA4
206856_at	0.0899329	4.66E-03	-3.05	-2.430857	-1.19	LILRB5
230003_at	0.0899329	4.66E-03	-3.05	-2.431729	-1.73	SLC16A7
226933_s_at	0.0899329	4.66E-03	-3.05	-2.431856	-1.57	ID4
225516_at	0.0902715	4.70E-03	-3.05	-2.439871	-2.31	SLC7A2
238559_at	0.0904288	4.72E-03	-3.05	-2.443438	-1.05	LOC101927402
208078_s_at	0.0904559	4.73E-03	-3.05	-2.445967	-1.5	SIK1
214467_at	0.0904559	4.74E-03	-3.05	-2.446609	-1.59	GPR65
235497_at	0.0905263	4.74E-03	-3.05	-2.448088	-1.21	LINC01128

206118_at	0.090587	4.75E-03	-3.05	-2.449897	-1.87	STAT4
204037_at	0.0910672	4.79E-03	-3.04	-2.457677	-1.12	LPAR1
1555938_x_at	0.0912719	4.81E-03	-3.04	-2.460763	-1.74	VIM
204150_at	0.0913614	4.83E-03	-3.04	-2.465036	-1.43	STAB1
205100_at	0.0913614	4.84E-03	-3.04	-2.466147	-2.33	GFPT2
204719_at	0.0915501	4.86E-03	-3.04	-2.469598	-3.02	ABCA8
203813_s_at	0.0922412	4.91E-03	-3.03	-2.479287	-1.55	SLIT3
204326_x_at	0.0926473	4.95E-03	-3.03	-2.486632	-1.67	MT1X
219777_at	0.0927307	4.96E-03	-3.03	-2.488307	-1.68	GIMAP6
232068_s_at	0.0927307	4.96E-03	-3.03	-2.4889	-1.42	TLR4
202917_s_at	0.0927307	4.96E-03	-3.03	-2.488999	-2.97	S100A8

1569004_at	0.0927509	4.96E-03	-3.03	-2.489875	-1.26	CARD8-AS1
232383_at	0.092879	4.97E-03	-3.03	-2.491466	-1.22	TFEC
220306_at	0.0929358	4.98E-03	-3.03	-2.492883	-1.42	FAM46C
216080_s_at	0.0930701	4.99E-03	-3.03	-2.494624	-1.22	FADS3
219064_at	0.0931023	5.00E-03	-3.03	-2.495708	-1.85	ITIH5
1557797_a_at	0.0933807	5.03E-03	-3.02	-2.5021	-1.28	
228062_at	0.0935877	5.05E-03	-3.02	-2.50661	-1.54	NAP1L5
209687_at	0.0936307	5.08E-03	-3.02	-2.511126	-2.77	CXCL12
209555_s_at	0.0939473	5.12E-03	-3.02	-2.517938	-2.76	CD36
218764_at	0.0939473	5.12E-03	-3.02	-2.517969	-1.23	PRKCH

208116_s_at	0.0939616	5.13E-03	-3.02	-2.519732	-1.23	MAN1A1
203373_at	0.0939616	5.13E-03	-3.02	-2.519995	-1.54	SOCS2
202974_at	0.0939616	5.13E-03	-3.02	-2.520145	-1.59	MPP1
229584_at	0.09423	5.16E-03	-3.01	-2.525378	-1.54	LRRK2
210184_at	0.0947002	5.21E-03	-3.01	-2.534276	-1.11	ITGAX
209829_at	0.0949917	5.24E-03	-3.01	-2.539257	-2.38	FAM65B
229521_at	0.0950893	5.24E-03	-3.01	-2.54051	-1.24	CCDC71L
228551_at	0.0951799	5.27E-03	-3.01	-2.544443	-1.13	DENND5B
205752_s_at	0.0954742	5.29E-03	-3	-2.548706	-1	GSTM5
201859_at	0.0954742	5.30E-03	-3	-2.550419	-1.26	SRGN

210299_s_at	0.0954742	5.31E-03	-3	-2.552433	-2.34	FHL1
44790_s_at	0.0956338	5.33E-03	-3	-2.555515	-2.02	KIAA0226L
210090_at	0.0956691	5.33E-03	-3	-2.556158	-1.03	ARC
202878_s_at	0.0959312	5.36E-03	-3	-2.561176	-1.3	CD93
225092_at	0.0959312	5.36E-03	-3	-2.5614	-1.06	RABEP1
229383_at	0.0959736	5.37E-03	-3	-2.563245	-1.56	1-Mar
201060_x_at	0.0959736	5.38E-03	-3	-2.564217	-1	STOM
235121_at	0.0965017	5.43E-03	-2.99	-2.57198	-1.09	ZNF542P
230866_at	0.0966151	5.44E-03	-2.99	-2.574943	-1.08	CYSLTR1
224964_s_at	0.0966167	5.45E-03	-2.99	-2.5759	-1.94	GNG2
232330_at	0.0966657	5.46E-03	-2.99	-2.578014	-1.47	COA1

203088_at	0.0967286	5.47E-03	-2.99	-2.580024	-2.16	FBLN5
228233_at	0.096925	5.49E-03	-2.99	-2.583482	-1.23	FREM1
226771_at	0.0971892	5.52E-03	-2.99	-2.5875	-1.11	ATP8B2
224840_at	0.097263	5.53E-03	-2.99	-2.588846	-1.5	FKBP5
201810_s_at	0.0975221	5.55E-03	-2.98	-2.592704	-1.35	SH3BP5
205307_s_at	0.0980971	5.60E-03	-2.98	-2.601078	-1.04	KMO
1569940_at	0.0981003	5.60E-03	-2.98	-2.601819	-1.11	SLC6A16
1564077_at	0.0989191	5.68E-03	-2.98	-2.613771	-1.37	
213438_at	0.0989254	5.68E-03	-2.98	-2.614123	-2	NFASC
243606_at	0.099253	5.71E-03	-2.97	-2.61922	-1.46	NXPE3

1569599_at	0.0993331	5.74E-03	-2.97	-2.624047	-1.46	SAMSN1
231484_at	0.0995369	5.76E-03	-2.97	-2.627449	-1.75	
226673_at	0.1002048	5.82E-03	-2.97	-2.63617	-1.03	SH2D3C
235674_at	0.1002699	5.83E-03	-2.97	-2.637981	-1.03	
235020_at	0.1002699	5.83E-03	-2.97	-2.638067	-1.11	TAF4B
213262_at	0.1002888	5.84E-03	-2.96	-2.639244	-1.1	SACS
205758_at	0.1002888	5.84E-03	-2.96	-2.640456	-2.28	CD8A
203666_at	0.100291	5.86E-03	-2.96	-2.643414	-1.98	CXCL12
206978_at	0.1002926	5.87E-03	-2.96	-2.644775	-1.68	CCR2
203372_s_at	0.1004341	5.89E-03	-2.96	-2.647044	-1.54	SOCS2
231947_at	0.1005507	5.90E-03	-2.96	-2.648865	-1.33	MYCT1

207735_at	0.1009395	5.93E-03	-2.96	-2.653565	-1.28	RNF125
235306_at	0.1009529	5.93E-03	-2.96	-2.654261	-1.61	GIMAP8
209220_at	0.1012945	5.97E-03	-2.96	-2.659944	-1.84	GPC3
213436_at	0.1015642	6.00E-03	-2.95	-2.664388	-1.35	CNR1
203400_s_at	0.1024818	6.07E-03	-2.95	-2.675225	-1.56	TF
239803_at	0.1029431	6.13E-03	-2.95	-2.68365	-1.03	
206974_at	0.1037975	6.22E-03	-2.94	-2.697131	-1.01	CXCR6
226806_s_at	0.1041718	6.25E-03	-2.94	-2.702685	-1.01	NFIA
228097_at	0.1042561	6.26E-03	-2.94	-2.703708	-1.02	MYLIP
1561181_at	0.1046456	6.32E-03	-2.93	-2.712722	-1.16	
215630_at	0.1048194	6.33E-03	-2.93	-2.714526	-1.11	
208914_at	0.1051279	6.38E-03	-2.93	-2.720559	-1.04	GGA2
49452_at	0.1051967	6.39E-03	-2.93	-2.721929	-1.44	ACACB

206461_x_at	0.1051995	6.39E-03	-2.93	-2.722667	-1.62	MT1H
204959_at	0.1051995	6.39E-03	-2.93	-2.722847	-1.64	MNDA
220012_at	0.1057196	6.44E-03	-2.93	-2.729314	-2.51	ERO1B
210031_at	0.1058114	6.48E-03	-2.92	-2.734794	-1.28	CD247
223395_at	0.1058114	6.48E-03	-2.92	-2.736042	-2.57	ABI3BP
228339_at	0.1068265	6.56E-03	-2.92	-2.746581	-1.33	ECSCR
227404_s_at	0.1076068	6.63E-03	-2.91	-2.757039	-1.57	EGR1
227239_at	0.1083805	6.74E-03	-2.91	-2.771005	-1.35	FAM126A

227799_at	0.1084458	6.75E-03	-2.91	-2.772704	-1.11	MYO1G
239205_s_at	0.1087731	6.78E-03	-2.9	-2.776848	-1.35	CR1L///CR1
228357_at	0.1088072	6.79E-03	-2.9	-2.77784	-1.06	UNK
224856_at	0.1088574	6.79E-03	-2.9	-2.778802	-1.69	FKBP5
230081_at	0.1090987	6.82E-03	-2.9	-2.782179	-2.21	PLCXD3
228665_at	0.1096473	6.87E-03	-2.9	-2.789378	-1.36	CYYR1
201743_at	0.1096759	6.88E-03	-2.9	-2.790188	-1.72	CD14
209539_at	0.1099108	6.90E-03	-2.9	-2.793383	-1.47	ARHGEF6
232712_at	0.1102781	6.95E-03	-2.89	-2.79977	-1.21	

201693_s_at	0.1102781	6.95E-03	-2.89	-2.799805	-1.81	EGR1
224909_s_at	0.1104005	6.96E-03	-2.89	-2.801316	-1.12	PREX1
201110_s_at	0.1104641	6.97E-03	-2.89	-2.802587	-1.58	THBS1
210401_at	0.1104641	6.98E-03	-2.89	-2.803213	-2.15	P2RX1
207008_at	0.111084	7.03E-03	-2.89	-2.810473	-1.16	CXCR2
216766_at	0.111084	7.05E-03	-2.89	-2.812207	-1.22	
238987_at	0.1123655	7.16E-03	-2.88	-2.827069	-1.16	B4GALT1
225816_at	0.1126493	7.20E-03	-2.88	-2.832011	-1.43	JADE1
229937_x_at	0.1128227	7.22E-03	-2.88	-2.83447	-1.75	
232204_at	0.1129567	7.25E-03	-2.88	-2.838208	-1.5	EBF1
205741_s_at	0.1134793	7.32E-03	-2.87	-2.84736	-1.01	DTNA

205933_at	0.1134994	7.33E-03	-2.87	-2.848821	-1.11	SETBP1
212956_at	0.1135015	7.34E-03	-2.87	-2.849464	-1	TBC1D9
206209_s_at	0.1135015	7.34E-03	-2.87	-2.849616	-1.83	CA4
230309_at	0.1136469	7.39E-03	-2.87	-2.855977	-1.47	BHMT2
235061_at	0.1139392	7.43E-03	-2.87	-2.861225	-1.28	PPM1K
226959_at	0.1140799	7.46E-03	-2.87	-2.864312	-1.24	
1554636_at	0.1146419	7.53E-03	-2.86	-2.87327	-1.47	
235708_at	0.1146419	7.53E-03	-2.86	-2.873388	-1.21	KLB
1556451_at	0.1146419	7.54E-03	-2.86	-2.874025	-1.32	
202957_at	0.1146419	7.54E-03	-2.86	-2.874728	-1.83	HCLS1
41577_at	0.1147782	7.57E-03	-2.86	-2.877581	-1.75	PPP1R16B

221760_at	0.1148258	7.57E-03	-2.86	-2.87847	-1.19	MAN1A1
209774_x_at	0.1148506	7.58E-03	-2.86	-2.879307	-2.2	CXCL2
204955_at	0.1148752	7.59E-03	-2.86	-2.880393	-2.31	SRPX
244885_at	0.1154478	7.65E-03	-2.86	-2.887704	-1.82	EBF2
225389_at	0.1154478	7.65E-03	-2.86	-2.888026	-1.1	BTBD6
242563_at	0.1155688	7.69E-03	-2.85	-2.892217	-1.12	
226810_at	0.1158792	7.73E-03	-2.85	-2.896937	-1.08	OGFRL1
201540_at	0.115976	7.76E-03	-2.85	-2.900756	-1.93	FHL1
208788_at	0.1160356	7.77E-03	-2.85	-2.901931	-1.06	ELOVL5
204103_at	0.1162643	7.80E-03	-2.85	-2.905732	-1.94	CCL4

228636_at	0.1164206	7.82E-03	-2.85	-2.907461	-1.39	BHLHE22
207175_at	0.1166249	7.84E-03	-2.85	-2.910561	-3.3	ADIPOQ
203865_s_at	0.1171303	7.89E-03	-2.84	-2.915515	-1.23	ADARB1
208195_at	0.1172525	7.90E-03	-2.84	-2.917464	-1.18	TTN
1558686_at	0.1173259	7.93E-03	-2.84	-2.920496	-1.1	MPV17L
229656_s_at	0.117446	7.96E-03	-2.84	-2.923927	-1.27	EML6
228314_at	0.1178527	8.02E-03	-2.84	-2.93054	-1.05	LOC101927933///LRRC8C

226304_at	0.1183776	8.10E-03	-2.83	-2.939758	-1.12	HSPB6
228750_at	0.1185199	8.13E-03	-2.83	-2.94365	-2.55	COL14A1
209243_s_at	0.1190678	8.19E-03	-2.83	-2.949931	-1.2	PEG3
226627_at	0.1193498	8.24E-03	-2.83	-2.956001	-1.15	SEPT8
222326_at	0.1203275	8.35E-03	-2.82	-2.968291	-1.46	
225809_at	0.1204869	8.37E-03	-2.82	-2.970464	-1.51	PARM1
229560_at	0.1211548	8.46E-03	-2.82	-2.979889	-1.57	TLR8
244276_at	0.1212736	8.48E-03	-2.81	-2.982266	-1.33	KLB
205440_s_at	0.1212736	8.48E-03	-2.81	-2.982404	-2.04	NPY1R
213547_at	0.1212736	8.49E-03	-2.81	-2.982769	-1.11	CAND2
242836_at	0.1213213	8.51E-03	-2.81	-2.984722	-1.88	
206049_at	0.1213213	8.52E-03	-2.81	-2.985774	-1.29	SELP

211144_x_at	0.1213213	8.53E-03	-2.81	-2.987343	-1.23	TARP///TRGV9///TRGC2
226382_at	0.1213213	8.53E-03	-2.81	-2.987531	-1.71	
209357_at	0.1214682	8.56E-03	-2.81	-2.990549	-1.26	CITED2
1563498_s_at	0.1215379	8.58E-03	-2.81	-2.992245	-1.21	SLC25A45
209795_at	0.121722	8.59E-03	-2.81	-2.994101	-2.26	CD69
213541_s_at	0.1218038	8.61E-03	-2.81	-2.995425	-1.64	ERG
200811_at	0.1218705	8.62E-03	-2.81	-2.996398	-1.08	CIRBP

215671_at	0.122159	8.66E-03	-2.81	-3.000854	-1.15	PDE4B
232614_at	0.1225719	8.70E-03	-2.8	-3.005414	-1.33	
203657_s_at	0.1228085	8.74E-03	-2.8	-3.009582	-1	CTSF
241844_x_at	0.1229953	8.77E-03	-2.8	-3.013024	-1.27	TMEM156
242751_at	0.1231199	8.80E-03	-2.8	-3.015352	-1.14	
219191_s_at	0.1232798	8.81E-03	-2.8	-3.017238	-1.25	BIN2
204796_at	0.1233475	8.82E-03	-2.8	-3.017974	-1.29	EML1
225987_at	0.1235607	8.85E-03	-2.8	-3.020582	-1.71	STEAP4
235213_at	0.1240385	8.91E-03	-2.79	-3.026951	-1.13	ITPKB
32502_at	0.1243657	8.96E-03	-2.79	-3.031762	-1.03	GDPD5
229686_at	0.1246263	8.99E-03	-2.79	-3.035294	-1.86	P2RY8
241302_at	0.1247772	9.01E-03	-2.79	-3.037084	-1.39	

226625_at	0.1247772	9.01E-03	-2.79	-3.037154	-1.14	TGFBR3
206002_at	0.1247772	9.03E-03	-2.79	-3.039187	-1.61	ADGRG2
207980_s_at	0.125095	9.09E-03	-2.79	-3.045176	-1.12	CITED2
205399_at	0.1253472	9.12E-03	-2.78	-3.048391	-1.08	DCLK1
209821_at	0.1254258	9.13E-03	-2.78	-3.049651	-2.2	IL33
244370_at	0.1254977	9.14E-03	-2.78	-3.050402	-1.33	KIAA2022
235209_at	0.1260111	9.20E-03	-2.78	-3.056416	-1.01	SBSPON
1565228_s_at	0.1262503	9.24E-03	-2.78	-3.0602	-2.15	ALB
1559663_at	0.1269301	9.32E-03	-2.78	-3.068395	-1.26	

215332_s_at	0.1274147	9.39E-03	-2.77	-3.075162	-1.03	LOC100996919///CD8B
209568_s_at	0.1274147	9.39E-03	-2.77	-3.075331	-1.32	RGL1
203485_at	0.1276661	9.42E-03	-2.77	-3.07788	-1.82	RTN1
209199_s_at	0.1277248	9.44E-03	-2.77	-3.080046	-1.49	MEF2C
204745_x_at	0.1280004	9.48E-03	-2.77	-3.083404	-1.73	MT1G
232473_at	0.128121	9.50E-03	-2.77	-3.08601	-1.25	LOC101928524
231181_at	0.128121	9.51E-03	-2.77	-3.086139	-1.25	
242521_at	0.1281397	9.54E-03	-2.77	-3.089113	-1.11	CARD8-AS1
241505_at	0.1282043	9.55E-03	-2.77	-3.090468	-2.41	
232543_x_at	0.1284356	9.58E-03	-2.76	-3.093174	-1.71	ARHGAP9

220330_s_at	0.1286183	9.62E-03	-2.76	-3.096756	-1.96	SAMSN1
204430_s_at	0.1288105	9.64E-03	-2.76	-3.099434	-1.25	SLC2A5
203680_at	0.1288261	9.66E-03	-2.76	-3.100891	-2.15	PRKAR2B
203471_s_at	0.1288748	9.67E-03	-2.76	-3.101688	-1.77	PLEK
202075_s_at	0.1293097	9.72E-03	-2.76	-3.106533	-1.46	PLTP
239006_at	0.1301722	9.83E-03	-2.75	-3.116641	-1.32	SLC26A7
205518_s_at	0.1303516	9.87E-03	-2.75	-3.120029	-1.62	CMAHP
230958_s_at	0.1308698	9.93E-03	-2.75	-3.12628	-1.01	

219505_at	0.1310845	9.95E-03	-2.75	-3.128168	-1.99	CECR1
221541_at	0.1311943	9.97E-03	-2.75	-3.129193	-1.27	CRISPLD2
204501_at	0.1312256	9.98E-03	-2.75	-3.130286	-1.43	NOV
204912_at	0.1313122	9.99E-03	-2.75	-3.131138	-1.76	IL10RA
202644_s_at	0.1314342	1.00E-02	-2.75	-3.134522	-1.45	TNFAIP3
227530_at	0.1320298	1.01E-02	-2.74	-3.141961	-1.71	AKAP12
210839_s_at	0.132074	1.01E-02	-2.74	-3.1427	-1.84	ENPP2
210039_s_at	0.1324566	1.02E-02	-2.74	-3.146429	-1.02	PRKCQ
219279_at	0.1324566	1.02E-02	-2.74	-3.146854	-1.92	DOCK10

244598_at	0.1326866	1.02E-02	-2.74	-3.149614	-1.02	LCP2
209541_at	0.133289	1.03E-02	-2.74	-3.158459	-2.6	IGF1
201858_s_at	0.1338402	1.03E-02	-2.73	-3.163442	-1.74	SRGN
237624_at	0.1340184	1.04E-02	-2.73	-3.165944	-1.29	
202992_at	0.1340358	1.04E-02	-2.73	-3.166277	-2.75	C7
239533_at	0.1340395	1.04E-02	-2.73	-3.166749	-1.39	GPR155
206618_at	0.1342553	1.04E-02	-2.73	-3.169669	-1.46	IL18R1
234192_s_at	0.1342578	1.04E-02	-2.73	-3.170387	-1.16	GKAP1
1558397_at	0.1343458	1.04E-02	-2.73	-3.171381	-1.08	PECAM1
219155_at	0.1344314	1.05E-02	-2.73	-3.174329	-1.1	PITPNC1

221667_s_at	0.13479	1.05E-02	-2.73	-3.180214	-1.85	HSPB8
229477_at	0.1347949	1.06E-02	-2.72	-3.181666	-1.36	THRSP
213135_at	0.1353249	1.06E-02	-2.72	-3.186508	-1.51	TIAM1
205961_s_at	0.1355207	1.06E-02	-2.72	-3.188706	-1	PSIP1
1559776_at	0.1358125	1.07E-02	-2.72	-3.19398	-1	
232687_at	0.1358125	1.07E-02	-2.72	-3.194089	-1.26	GPRIN3
227232_at	0.1358283	1.07E-02	-2.72	-3.194803	-1.01	EVL
229011_at	0.1358283	1.07E-02	-2.72	-3.195136	-1.32	EMP1
226906_s_at	0.1364191	1.08E-02	-2.72	-3.20248	-1.5	ARHGAP9
210613_s_at	0.1364992	1.08E-02	-2.71	-3.203421	-1.18	SYNGR1
205419_at	0.1366426	1.08E-02	-2.71	-3.205436	-2.08	GPR183

209277_at	0.1371662	1.09E-02	-2.71	-3.214079	-2.69	TFPI2
203104_at	0.1378069	1.10E-02	-2.71	-3.22045	-1.52	CSF1R
235978_at	0.1383089	1.11E-02	-2.7	-3.225414	-2.06	FABP4
203185_at	0.1383379	1.11E-02	-2.7	-3.226798	-1.41	RASSF2
209813_x_at	0.1385039	1.11E-02	-2.7	-3.228352	-1.32	TARP///TRGV9///TRGC2
224997_x_at	0.1385599	1.11E-02	-2.7	-3.228938	-2.01	MIR675///H19

226705_at	0.1385599	1.11E-02	-2.7	-3.229276	-1.5	FGFR1
1555638_a_at	0.1386423	1.11E-02	-2.7	-3.2305	-1.64	SAMSN1
204834_at	0.1391789	1.12E-02	-2.7	-3.236034	-1.97	FGL2
205357_s_at	0.1391789	1.12E-02	-2.7	-3.236073	-1.63	AGTR1
228434_at	0.1395683	1.13E-02	-2.7	-3.241523	-1.18	BTNL9
206366_x_at	0.1395683	1.13E-02	-2.7	-3.241794	-1.39	XCL1
212750_at	0.1395683	1.13E-02	-2.7	-3.241817	-1.68	PPP1R16B
239183_at	0.1395683	1.13E-02	-2.7	-3.241985	-1.71	ANGPTL1
209828_s_at	0.1398624	1.13E-02	-2.7	-3.247096	-1.03	IL16
224451_x_at	0.1399168	1.14E-02	-2.69	-3.247858	-1.76	ARHGAP9

217966_s_at	0.140406	1.14E-02	-2.69	-3.25252	-1.31	FAM129A
206707_x_at	0.140406	1.14E-02	-2.69	-3.253038	-1.44	FAM65B
220187_at	0.1407152	1.15E-02	-2.69	-3.256493	-1.35	STEAP4
238458_at	0.1407258	1.15E-02	-2.69	-3.25697	-1.12	MICU3
240173_at	0.1408995	1.15E-02	-2.69	-3.26078	-1.2	GPRIN3
227929_at	0.1408995	1.15E-02	-2.69	-3.261341	-1.03	LIN7A
236907_at	0.1414218	1.16E-02	-2.69	-3.267957	-1.12	
206761_at	0.1414218	1.16E-02	-2.69	-3.268543	-1.26	CD96
1566079_at	0.1416897	1.17E-02	-2.68	-3.271474	-1.44	RPS16P5

212636_at	0.1422039	1.17E-02	-2.68	-3.277298	-1.46	QKI
241278_at	0.1425052	1.18E-02	-2.68	-3.280683	-1.2	
218870_at	0.1436243	1.20E-02	-2.67	-3.29461	-1.84	LOC101928361///ARHGAP15
222043_at	0.1437451	1.20E-02	-2.67	-3.296753	-1.96	CLU
215775_at	0.1442023	1.20E-02	-2.67	-3.300825	-1.06	THBS1
202156_s_at	0.1442332	1.21E-02	-2.67	-3.302538	-1.62	CELF2
243915_at	0.1442367	1.21E-02	-2.67	-3.303223	-1.29	
239336_at	0.1442898	1.21E-02	-2.67	-3.303952	-1.5	THBS1
209540_at	0.1445516	1.21E-02	-2.67	-3.306387	-2.25	IGF1
216944_s_at	0.1447355	1.21E-02	-2.67	-3.309121	-1.48	ITPR1
203980_at	0.1449386	1.22E-02	-2.67	-3.311181	-3.35	FABP4

1557166_at	0.1454674	1.23E-02	-2.66	-3.318614	-1.33	PDCD4
225282_at	0.1454857	1.23E-02	-2.66	-3.318925	-1.29	SMAP2
201109_s_at	0.1459714	1.24E-02	-2.66	-3.324514	-1.78	THBS1
228528_at	0.1465168	1.24E-02	-2.66	-3.32963	-1.09	MIR29C///MIR29B2
229127_at	0.1467686	1.25E-02	-2.66	-3.332551	-1.52	JAM2
231773_at	0.1471895	1.25E-02	-2.65	-3.336256	-2.17	ANGPTL1
211535_s_at	0.1475036	1.26E-02	-2.65	-3.340495	-1.17	FGFR1
205901_at	0.1476793	1.26E-02	-2.65	-3.342238	-1.51	PNOC
235666_at	0.1484456	1.28E-02	-2.65	-3.354501	-1.58	ITGA8
214434_at	0.1484456	1.28E-02	-2.65	-3.355203	-1.23	HSPA12A

202628_s_at	0.1484456	1.28E-02	-2.65	-3.355389	-1.85	SERPINE1
225602_at	0.1486538	1.28E-02	-2.64	-3.357664	-1.15	GLIPR2
235626_at	0.1487307	1.29E-02	-2.64	-3.360036	-1.02	CAMK1D
241926_s_at	0.1491152	1.29E-02	-2.64	-3.364285	-1.16	ERG
223809_at	0.1492726	1.29E-02	-2.64	-3.366024	-1.05	RGS18
213309_at	0.1493546	1.30E-02	-2.64	-3.368027	-1.22	PLCL2
244026_at	0.1493837	1.30E-02	-2.64	-3.368629	-1.29	
237459_at	0.1494198	1.30E-02	-2.64	-3.369374	-1.03	
205821_at	0.1496142	1.30E-02	-2.64	-3.371362	-1.69	KLRC4-KLRK1///KLRK1
202037_s_at	0.149705	1.30E-02	-2.64	-3.372621	-2.47	SFRP1
220065_at	0.1502304	1.31E-02	-2.64	-3.377072	-1.51	TNMD
214954_at	0.1512751	1.32E-02	-2.63	-3.387209	-1.53	SUSD5

231944_at	0.1513406	1.33E-02	-2.63	-3.387943	-2.31	ERO1B
205653_at	0.1518182	1.33E-02	-2.63	-3.392301	-1.18	CTSG
224348_s_at	0.1527147	1.34E-02	-2.63	-3.399099	-1.22	HOTS
205269_at	0.1528911	1.34E-02	-2.62	-3.401249	-1.41	LCP2
244289_at	0.1529514	1.35E-02	-2.62	-3.402121	-1.39	ZNF300P1
214063_s_at	0.153195	1.35E-02	-2.62	-3.404974	-1.04	TF
219689_at	0.1533298	1.36E-02	-2.62	-3.408163	-1.6	SEMA3G
203708_at	0.1533298	1.36E-02	-2.62	-3.409521	-1.68	PDE4B
202743_at	0.1533298	1.36E-02	-2.62	-3.40978	-1.05	PIK3R3
231192_at	0.1533298	1.36E-02	-2.62	-3.409793	-1.22	LPAR3
230048_at	0.1533298	1.36E-02	-2.62	-3.410694	-1.38	IFRD1

220423_at	0.1534364	1.36E-02	-2.62	-3.413336	-1.12	PLA2G2D
226271_at	0.1535306	1.36E-02	-2.62	-3.414605	-1.09	GDAP1
211138_s_at	0.1536229	1.37E-02	-2.62	-3.415891	-1.34	KMO
227113_at	0.1537813	1.37E-02	-2.62	-3.417717	-1.13	ADHFE1
214567_s_at	0.1544115	1.38E-02	-2.61	-3.425203	-1.27	XCL2///XCL1
226591_at	0.1544115	1.38E-02	-2.61	-3.42551	-1.48	PWAR6
229623_at	0.1554222	1.40E-02	-2.61	-3.436614	-1.18	TMEM150C

214078_at	0.1554222	1.40E-02	-2.61	-3.436907	-2	PAK3
219574_at	0.1554222	1.40E-02	-2.61	-3.436928	-1.48	1-Mar
225212_at	0.1557688	1.40E-02	-2.61	-3.440405	-1.1	SLC25A25
203296_s_at	0.1561804	1.41E-02	-2.6	-3.443989	-2.13	ATP1A2
200670_at	0.1561804	1.41E-02	-2.6	-3.444068	-1.05	XBP1
203130_s_at	0.1565971	1.42E-02	-2.6	-3.448134	-1.17	KIF5C
230237_at	0.1565971	1.42E-02	-2.6	-3.4483	-1.68	ADCYAP1
232843_s_at	0.1567473	1.42E-02	-2.6	-3.449894	-1.44	DOCK8
202498_s_at	0.1568253	1.42E-02	-2.6	-3.450708	-1.56	SLC2A3

202158_s_at	0.1568461	1.42E-02	-2.6	-3.451374	-1.58	CELF2
235129_at	0.1573546	1.43E-02	-2.6	-3.456477	-1.12	PPP1R1A
229199_at	0.1574774	1.43E-02	-2.6	-3.457785	-1.03	SCN9A
230550_at	0.1577829	1.44E-02	-2.6	-3.460583	-1.6	MS4A6A
1563469_at	0.1577985	1.44E-02	-2.6	-3.46192	-1.23	
205354_at	0.1581774	1.44E-02	-2.59	-3.465711	-1.33	GAMT
228409_at	0.1585318	1.45E-02	-2.59	-3.469715	-1.56	PLIN4
228737_at	0.1586475	1.45E-02	-2.59	-3.470914	-1.39	TOX2
227084_at	0.1586575	1.45E-02	-2.59	-3.471151	-1.66	DTNA

203603_s_at	0.1590683	1.46E-02	-2.59	-3.474964	-1.35	ZEB2
210517_s_at	0.1590683	1.46E-02	-2.59	-3.475196	-1.69	AKAP12
206296_x_at	0.1590683	1.46E-02	-2.59	-3.475785	-1.51	MAP4K1
206702_at	0.1590683	1.46E-02	-2.59	-3.476312	-1.15	TEK
205407_at	0.1598289	1.48E-02	-2.59	-3.484796	-1.5	RECK
209392_at	0.1603768	1.48E-02	-2.58	-3.489292	-1.76	ENPP2
203470_s_at	0.1607346	1.49E-02	-2.58	-3.492548	-1.25	PLEK
226641_at	0.1609416	1.49E-02	-2.58	-3.494471	-1.39	ANKRD44
222872_x_at	0.1609416	1.49E-02	-2.58	-3.494598	-1.05	NABP1

228333_at	0.1610344	1.50E-02	-2.58	-3.496865	-1.39	ZEB2
206682_at	0.1610344	1.50E-02	-2.58	-3.496894	-1.29	CLEC10A
218559_s_at	0.1612314	1.50E-02	-2.58	-3.499192	-1.33	MAFB
230391_at	0.1632	1.54E-02	-2.57	-3.522838	-1.8	CD84
229024_at	0.1635055	1.54E-02	-2.57	-3.525958	-1.59	RNF150
238668_at	0.164063	1.55E-02	-2.56	-3.530202	-1.71	NCKAP1L
203413_at	0.1641796	1.55E-02	-2.56	-3.531213	-1.76	NELL2
228176_at	0.1641796	1.56E-02	-2.56	-3.532188	-1.38	S1PR3
222670_s_at	0.1641796	1.56E-02	-2.56	-3.532417	-1.15	MAFB
220276_at	0.1641796	1.56E-02	-2.56	-3.532442	-1.47	RERGL
202157_s_at	0.1643212	1.56E-02	-2.56	-3.534399	-1.55	CELF2

240665_at	0.1645056	1.56E-02	-2.56	-3.536622	-1.47	
213924_at	0.1645676	1.56E-02	-2.56	-3.537135	-1.02	GNAL
230030_at	0.1648027	1.57E-02	-2.56	-3.538939	-1.22	HS6ST2
1552474_a_at	0.1651493	1.57E-02	-2.56	-3.543029	-1.56	GAMT
227826_s_at	0.1651493	1.57E-02	-2.56	-3.543423	-2.32	SORBS2
232262_at	0.1652283	1.58E-02	-2.56	-3.544337	-1.11	PIGL
205913_at	0.1652491	1.58E-02	-2.56	-3.545002	-2	PLIN1
241765_at	0.1659337	1.59E-02	-2.55	-3.550616	-1.06	CPM
235042_at	0.166093	1.59E-02	-2.55	-3.552763	-1.22	XIRP1
210889_s_at	0.1670522	1.61E-02	-2.55	-3.560968	-1.64	FCGR2B
236506_at	0.1676925	1.62E-02	-2.55	-3.56627	-1.02	

212845_at	0.168568	1.63E-02	-2.54	-3.572487	-1.2	SAMD4A
217967_s_at	0.1692692	1.64E-02	-2.54	-3.578262	-1.29	FAM129A
235385_at	0.1693845	1.64E-02	-2.54	-3.579893	-1.75	1-Mar
204224_s_at	0.1694896	1.64E-02	-2.54	-3.58062	-1.01	GCH1
237201_at	0.1695378	1.64E-02	-2.54	-3.581555	-1.05	
236359_at	0.1696174	1.64E-02	-2.54	-3.582316	-1.58	SCN4B
221602_s_at	0.169984	1.65E-02	-2.54	-3.585273	-1.51	FCMR
205270_s_at	0.1700314	1.65E-02	-2.54	-3.585693	-1.25	LCP2
222168_at	0.1700644	1.65E-02	-2.54	-3.586206	-1.08	
218236_s_at	0.1703625	1.66E-02	-2.54	-3.589367	-1.02	PRKD3
1552497_a_at	0.1703625	1.66E-02	-2.54	-3.589471	-1.17	SLAMF6

227061_at	0.171139	1.67E-02	-2.53	-3.596423	-2.37	LINC01279
217165_x_at	0.1718064	1.68E-02	-2.53	-3.603936	-1.49	MT1F
231109_at	0.1723506	1.70E-02	-2.53	-3.610469	-1.22	
205771_s_at	0.1724499	1.70E-02	-2.53	-3.611153	-2.06	AKAP7
226909_at	0.172652	1.70E-02	-2.52	-3.613538	-1.47	ZNF518B
222088_s_at	0.172947	1.71E-02	-2.52	-3.615486	-1.83	SLC2A14///SLC2A3
210439_at	0.172947	1.71E-02	-2.52	-3.61557	-1.49	ICOS
220518_at	0.1730535	1.71E-02	-2.52	-3.616791	-1.51	ABI3BP
229476_s_at	0.1733044	1.71E-02	-2.52	-3.619086	-1.46	THRSP
228071_at	0.1736824	1.72E-02	-2.52	-3.622535	-1.77	GIMAP7

227002_at	0.17549	1.75E-02	-2.51	-3.638377	-1.03	LOC101927137///FAM78A
238429_at	0.1758548	1.75E-02	-2.51	-3.640789	-1.38	TMEM71
222073_at	0.176366	1.76E-02	-2.51	-3.644214	-1.65	COL4A3
223553_s_at	0.176732	1.77E-02	-2.51	-3.649025	-1.39	DOK3
244375_at	0.1780522	1.79E-02	-2.5	-3.659202	-1.02	
229670_at	0.1781117	1.79E-02	-2.5	-3.659915	-1.55	
225502_at	0.178775	1.80E-02	-2.5	-3.664712	-1.39	DOCK8
221933_at	0.1794648	1.81E-02	-2.5	-3.66995	-1.37	NLGN4X
227529_s_at	0.1802927	1.83E-02	-2.49	-3.677312	-1.99	AKAP12
205820_s_at	0.180497	1.83E-02	-2.49	-3.678976	-1.78	APOC3
209906_at	0.1813041	1.85E-02	-2.49	-3.685872	-1.34	C3AR1

229461_x_at	0.1818405	1.85E-02	-2.49	-3.689573	-1.83	NEGR1
1552280_at	0.1818422	1.85E-02	-2.49	-3.690305	-1.09	TIMD4
227554_at	0.1821398	1.86E-02	-2.49	-3.694324	-1.37	MAGI2-AS3
202643_s_at	0.1822501	1.86E-02	-2.49	-3.695026	-1.46	TNFAIP3
212713_at	0.1836752	1.89E-02	-2.48	-3.705194	-2.22	MFAP4
204204_at	0.1843899	1.90E-02	-2.48	-3.713281	-1.08	SLC31A2
220418_at	0.1846258	1.91E-02	-2.48	-3.715376	-1	UBASH3A
235438_at	0.184799	1.91E-02	-2.48	-3.716551	-1.22	CYP7B1

214265_at	0.1848415	1.91E-02	-2.47	-3.717053	-1.18	ITGA8
215039_at	0.1857762	1.93E-02	-2.47	-3.724354	-1.44	LINC01140
205789_at	0.185821	1.93E-02	-2.47	-3.724783	-1.64	CD1D
217227_x_at	0.1858377	1.93E-02	-2.47	-3.725021	-2.41	IGLV1-44
221259_s_at	0.1859667	1.93E-02	-2.47	-3.72643	-1.69	TEX11
210658_s_at	0.1859677	1.93E-02	-2.47	-3.726868	-1.01	GGA2
227030_at	0.1859677	1.93E-02	-2.47	-3.727537	-1.4	IKZF3
228828_at	0.1860845	1.93E-02	-2.47	-3.728255	-1	TSPOAP1-AS1
236796_at	0.1867129	1.94E-02	-2.47	-3.732821	-1.47	
213160_at	0.1867323	1.95E-02	-2.47	-3.73359	-1.68	DOCK2

206028_s_at	0.1869847	1.95E-02	-2.47	-3.735711	-1.19	MERTK
227817_at	0.1869975	1.95E-02	-2.47	-3.735929	-1.47	PRKCB
212977_at	0.187513	1.96E-02	-2.46	-3.740111	-1.09	ACKR3
224061_at	0.1879238	1.97E-02	-2.46	-3.743942	-1.38	INMT
239122_at	0.1881983	1.97E-02	-2.46	-3.74587	-1.16	
237945_at	0.1883129	1.98E-02	-2.46	-3.747037	-1.2	
226818_at	0.1886645	1.99E-02	-2.46	-3.752678	-1.95	MPEG1
236307_at	0.1893365	2.00E-02	-2.45	-3.758089	-1.45	
205159_at	0.1898259	2.01E-02	-2.45	-3.763482	-1.79	CSF2RB
209191_at	0.1898645	2.01E-02	-2.45	-3.763971	-1.15	TUBB6
239237_at	0.1911259	2.04E-02	-2.45	-3.774443	-1.03	TRG-AS1

243357_at	0.1913512	2.04E-02	-2.45	-3.776923	-1.54	NEGR1
205551_at	0.1918459	2.05E-02	-2.44	-3.781516	-1.6	SV2B
1559425_at	0.1919852	2.06E-02	-2.44	-3.783079	-1.16	
229309_at	0.192676	2.07E-02	-2.44	-3.789932	-1.32	ADRB1
200962_at	0.1926825	2.07E-02	-2.44	-3.790361	-1.11	RPL31
243771_at	0.1926825	2.07E-02	-2.44	-3.790509	-1.13	
203760_s_at	0.1927368	2.08E-02	-2.44	-3.792041	-1.52	SLA
209734_at	0.1927368	2.08E-02	-2.44	-3.792485	-1.2	NCKAP1L
225424_at	0.1928928	2.08E-02	-2.44	-3.793812	-1.33	GPAM
226344_at	0.1933809	2.09E-02	-2.44	-3.797728	-1.01	ZMAT1
213407_at	0.193488	2.09E-02	-2.44	-3.798975	-1.05	PHLPP2
215275_at	0.1949521	2.12E-02	-2.43	-3.811555	-1.07	TRAF3IP3

202497_x_at	0.195068	2.13E-02	-2.43	-3.812708	-1.59	SLC2A3
212732_at	0.1958648	2.14E-02	-2.43	-3.818408	-1.9	MEG3
208981_at	0.1959878	2.14E-02	-2.43	-3.819751	-1.02	PECAM1
227344_at	0.1959878	2.15E-02	-2.42	-3.820574	-1.23	IKZF1
240450_at	0.1959878	2.15E-02	-2.42	-3.820607	-1.26	
216331_at	0.196505	2.15E-02	-2.42	-3.823849	-1.27	ITGA7
203387_s_at	0.1970468	2.16E-02	-2.42	-3.827694	-1.11	TBC1D4
228826_at	0.1975165	2.17E-02	-2.42	-3.832438	-1.27	TSPOAP1-AS1
207002_s_at	0.1975827	2.18E-02	-2.42	-3.834354	-1.08	PLAGL1
227677_at	0.19777	2.18E-02	-2.42	-3.835789	-1.39	JAK3

GSE41372

ID	adj.P.Val	P.Value	t	B	logFC	Gene.symbol	Gene.title

7971077	4.00E-08	1.38E-12	2.57E+01	18.390565	6.14	POSTN	periostin
7953603	5.17E-08	4.16E-12	2.35E+01	17.528426	2.6	C1S	complement component 1, s subcomponent
7920128	7.34E-08	1.02E-11	2.20E+01	16.801415	2.43	S100A11	S100 calcium binding protein A11
7908072	1.50E-07	2.60E-11	2.04E+01	16.01109	4.43	LAMC2	laminin subunit gamma 2
8146863	2.14E-07	4.43E-11	1.96E+01	15.553041	4.96	SULF1	sulfatase 1
8056860	2.19E-07	5.30E-11	1.93E+01	15.397494	2.26	WIPF1	WAS/WASL interacting protein family member 1
7995681	4.01E-07	1.25E-10	1.80E+01	14.643232	3.41	MMP2	matrix metallopeptidase 2

8058765	6.45E-07	2.23E-10	1.72E+01	14.121596	4.6	FN1	fibronectin 1
7919815	7.83E-07	2.98E-10	1.68E+01	13.858927	3.32	CTSK	cathepsin K
7971296	8.26E-07	3.62E-10	1.66E+01	13.681818	2.77	EPSTI1	epithelial stromal interaction 1 (breast)
7965403	8.26E-07	3.71E-10	1.65E+01	13.658242	2.08	LUM	lumican
8170648	8.74E-07	4.42E-10	1.63E+01	13.498322	3.35	BGN	biglycan
8059905	8.74E-07	4.53E-10	1.63E+01	13.47535	3.52	COL6A3	collagen type VI alpha 3 chain
8066214	1.27E-06	7.50E-10	1.56E+01	13.009744	1.88	TGM2	transglutaminase 2
8106743	1.27E-06	8.02E-10	1.55E+01	12.948021	4.18	VCAN	versican

8179041	1.27E-06	8.31E-10	1.55E+01	12.914649	1.66	HLA-H///HLA-C///HLA-B///HLA-A///HLA-B///HLA-A	major histocompatibility complex, class I, H (pseudogene)///major histocompatibility complex, class I, C///major histocompatibility complex, class I, B///major histocompatibility complex, class I, A///major histocompatibility complex, class I, B///major histocompatibility complex, class I, A

8115327	1.27E-06	8.31E-10	1.55E+01	12.913892	2.61	SPARC	secreted protein acidic and cysteine rich
8020110	1.29E-06	8.92E-10	1.54E+01	12.848629	3.49	RAB31	RAB31, member RAS oncogene family
8045688	1.40E-06	1.07E-09	1.52E+01	12.676725	4.35	TNFAIP6	TNF alpha induced protein 6
8150509	1.40E-06	1.13E-09	1.51E+01	12.630084	1.88	PLAT	plasminogen activator, tissue type
8106393	1.40E-06	1.24E-09	1.50E+01	12.537268	2.78	F2R	coagulation factor II thrombin receptor

7960744	1.40E-06	1.28E-09	1.50E+01	12.508682	2.38	C1R	complement C1r subcomponent
7950933	1.40E-06	1.33E-09	1.49E+01	12.473807	3.56	NOX4	NADPH oxidase 4
8139087	1.40E-06	1.39E-09	1.49E+01	12.434215	3.89	SFRP4	secreted frizzled related protein 4
7903893	1.40E-06	1.40E-09	1.49E+01	12.424007	3.27	CD53	CD53 molecule
7902553	1.41E-06	1.50E-09	1.48E+01	12.364264	2.34	IFI44	interferon induced protein 44
8001800	1.41E-06	1.52E-09	1.48E+01	12.352359	3.52	CDH11	cadherin 11
8167185	1.51E-06	1.76E-09	1.46E+01	12.210357	3.52	TIMP1	TIMP metallopeptidase inhibitor 1
8102232	1.51E-06	1.77E-09	1.46E+01	12.206293	3.69	LEF1	lymphoid enhancer binding factor 1

7926545	1.55E-06	1.88E-09	1.45E+01	12.148668	2.57	PLXDC2	plexin domain containing 2
8162394	1.57E-06	1.97E-09	1.45E+01	12.105202	2.41	ASPN	asporin
8042439	1.60E-06	2.10E-09	1.44E+01	12.04605	3.55	ANTXR1	anthrax toxin receptor 1
8012896	1.78E-06	2.46E-09	1.42E+01	11.894451	2.37	PMP22	peripheral myelin protein 22
8046861	1.82E-06	2.58E-09	1.41E+01	11.84773	1.33	ITGAV	integrin subunit alpha V

8117777	1.82E-06	2.64E-09	1.41E+01	11.828505	1.41	HLA-H///HLA-G///HLA-A///HLA-A	major histocompatibility complex, class I, H (pseudogene)///major histocompatibility complex, class I, G///major histocompatibility complex, class I, A///major histocompatibility complex, class I, A
8103563	1.84E-06	2.86E-09	1.40E+01	11.752255	2.06	DDX60	DEXD/H-box helicase 60

7918902	1.84E-06	2.86E-09	1.40E+01	11.750886	1.79	CD58	CD58 molecule
8131844	1.84E-06	2.89E-09	1.40E+01	11.740938	2.85	GPNMB	glycoprotein nmb
8155849	1.84E-06	2.98E-09	1.40E+01	11.713739	2.46	ANXA1	annexin A1
8167965	1.84E-06	3.05E-09	1.40E+01	11.68888	2.21	MSN	moesin
8089835	1.84E-06	3.06E-09	1.40E+01	11.68595	2.26	FSTL1	follistatin like 1
8115147	1.93E-06	3.34E-09	1.39E+01	11.602787	2.68	CD74///ROS1	CD74 molecule///ROS proto-oncogene 1, receptor tyrosine kinase
8091715	1.99E-06	3.51E-09	1.38E+01	11.555699	1.9	LXN	latexin

8044333	2.01E-06	3.68E-09	1.37E+01	11.511915	1.36	LIMS4///LIMS3-LOC440895///LIMS3///LIMS1	LIM zinc finger domain containing 4///LIMS3-LOC440895 readthrough///LIM zinc finger domain containing 3///LIM zinc finger domain containing 1

8054519	2.01E-06	3.68E-09	1.37E+01	11.511915	1.36	LIMS4///LIMS3-LOC440895///LIMS3///LIMS1	LIM zinc finger domain containing 4///LIMS3-LOC440895 readthrough///LIM zinc finger domain containing 3///LIM zinc finger domain containing 1
7943413	2.28E-06	4.26E-09	1.36E+01	11.370403	2.42	BIRC3	baculoviral IAP repeat containing 3

7910680	2.36E-06	4.56E-09	1.35E+01	11.306624	1.83	GPR137B	G protein-coupled receptor 137B
7956878	2.36E-06	4.56E-09	1.35E+01	11.305173	3.02	IRAK3	interleukin 1 receptor associated kinase 3
7937330	2.39E-06	4.72E-09	1.35E+01	11.273564	1.43	IFITM2	interferon induced transmembrane protein 2
7904018	2.62E-06	5.31E-09	1.33E+01	11.15935	1.69	CTTNBP2NL	CTTNBP2 N-terminal like
8097692	2.62E-06	5.35E-09	1.33E+01	11.152544	3.14	EDNRA	endothelin receptor type A
8129573	2.75E-06	5.69E-09	1.33E+01	11.092881	3.64	MOXD1	monooxygenase DBH like 1

8043945	2.80E-06	6.04E-09	1.32E+01	11.036779	1.71	MAP4K4	mitogen-activated protein kinase kinase kinase kinase 4
8124911	2.98E-06	6.59E-09	1.31E+01	10.952728	1.79	HLA-C///HLA-B///HLA-B	major histocompatibility complex, class I, C///major histocompatibility complex, class I, B///major histocompatibility complex, class I, B
7935058	2.98E-06	6.71E-09	1.31E+01	10.935458	2.76	MYOF	myoferlin
8146957	3.08E-06	7.13E-09	1.30E+01	10.876068	3.06	PI15	peptidase inhibitor 15

8101260	3.08E-06	7.18E-09	1.30E+01	10.8696	1.8	ANTXR2	anthrax toxin receptor 2
8066822	3.08E-06	7.24E-09	1.30E+01	10.86138	2.86	SULF2	sulfatase 2
7908614	3.23E-06	7.86E-09	1.29E+01	10.782699	1.4	CAMSAP2	calmodulin regulated spectrin associated protein family member 2

8179731	3.23E-06	7.91E-09	1.29E+01	10.775797	1.78	HLA-C///HLA-B///HLA-C///HLA-B	major histocompatibility complex, class I, C///major histocompatibility complex, class I, B///major histocompatibility complex, class I, C///major histocompatibility complex, class I, B

8115099	3.23E-06	8.01E-09	1.29E+01	10.764405	1.21	PDGFRB///PDGFRB	platelet derived growth factor receptor beta///platelet derived growth factor receptor beta
7968800	3.23E-06	8.05E-09	1.29E+01	10.759606	2.31	DGKH	diacylglycerol kinase eta
8092251	3.38E-06	8.53E-09	1.28E+01	10.703419	2.11	GNB4	G protein subunit beta 4
7983360	3.46E-06	8.96E-09	1.28E+01	10.656213	1.14	B2M	beta-2-microglobulin
7988467	3.46E-06	8.98E-09	1.28E+01	10.653534	3.28	FBN1	fibrillin 1
7918426	3.65E-06	9.71E-09	1.27E+01	10.577934	2.71	SLC16A4	solute carrier family 16 member 4

8122756	3.65E-06	9.87E-09	1.27E+01	10.561857	1.86	PLEKHG1///PLEKHG1	pleckstrin homology and RhoGEF domain containing G1///pleckstrin homology and RhoGEF domain containing G1

8178498	3.65E-06	9.96E-09	1.27E+01	10.553398	1.8	HLA-C///HLA-B///HLA-B	major histocompatibility complex, class I, C///major histocompatibility complex, class I, B///major histocompatibility complex, class I, B
7896722	3.78E-06	1.05E-08	1.26E+01	10.505468	1.71		
8117760	3.82E-06	1.07E-08	1.26E+01	10.484182	1.6	HLA-F///HLA-B	major histocompatibility complex, class I, F///major histocompatibility complex, class I, B

8010454	3.83E-06	1.10E-08	1.26E+01	10.45308	1.17	RNF213	ring finger protein 213
7900382	3.83E-06	1.11E-08	1.26E+01	10.44461	1.43	CAP1	adenylate cyclase associated protein 1
8111739	3.83E-06	1.13E-08	1.25E+01	10.433726	2.81	FYB	FYN binding protein
7908459	4.01E-06	1.19E-08	1.25E+01	10.37977	2.38	CFH	complement factor H
8063444	4.21E-06	1.26E-08	1.24E+01	10.32144	1.88	TSHZ2	teashirt zinc finger homeobox 2
8101126	4.21E-06	1.28E-08	1.24E+01	10.309021	4.59	CXCL10	C-X-C motif chemokine ligand 10

8057599	4.40E-06	1.35E-08	1.24E+01	10.256361	1.61	TFPI	tissue factor pathway inhibitor
7917532	4.66E-06	1.47E-08	1.23E+01	10.177767	2.77	GBP2	guanylate binding protein 2
8021653	4.69E-06	1.49E-08	1.23E+01	10.161682	1.47	SERPINB8	serpin family B member 8
8173444	4.70E-06	1.51E-08	1.22E+01	10.148243	3.7	IL2RG	interleukin 2 receptor subunit gamma
7921882	4.71E-06	1.53E-08	1.22E+01	10.135061	2.47	OLFML2B	olfactomedin like 2B
7901175	4.97E-06	1.69E-08	1.21E+01	10.039095	3.64	TSPAN1	tetraspanin 1
8113709	4.97E-06	1.72E-08	1.21E+01	10.019377	3.03	LOX	lysyl oxidase

8113130	4.97E-06	1.74E-08	1.21E+01	10.010933	2.39	MCTP1	multiple C2 and transmembrane domain containing 1
7945371	4.97E-06	1.75E-08	1.21E+01	10.007545	1.42	IFITM3	interferon induced transmembrane protein 3
8105267	4.97E-06	1.77E-08	1.21E+01	9.995237	2.94	ITGA2	integrin subunit alpha 2
8082100	4.97E-06	1.77E-08	1.21E+01	9.994116	1.4	PARP14	poly(ADP-ribose) polymerase family member 14
8009417	4.97E-06	1.77E-08	1.21E+01	9.993792	1.87	KPNA2	karyopherin subunit alpha 2

7920258	5.08E-06	1.83E-08	1.21E+01	9.962607	2.72	S100A6	S100 calcium binding protein A6
8134263	5.19E-06	1.90E-08	1.20E+01	9.924118	3.04	COL1A2///COL1A2	collagen type I alpha 2 chain///collagen type I alpha 2 chain
7938035	5.32E-06	1.98E-08	1.20E+01	9.884455	2.02	TRIM22	tripartite motif containing 22
8019737	5.32E-06	1.99E-08	1.20E+01	9.880666	1.81	KPNA2	karyopherin subunit alpha 2
8088919	5.37E-06	2.03E-08	1.20E+01	9.862417	1.77	ROBO1	roundabout guidance receptor 1

8177788	5.53E-06	2.16E-08	1.19E+01	9.798811	1.44	HLA-H///HLA-F///HLA-E///HLA-B///HLA-A///HLA-A	major histocompatibility complex, class I, H (pseudogene)///major histocompatibility complex, class I, F///major histocompatibility complex, class I, E///major histocompatibility complex, class I, B///major histocompatibility complex, class I, A///major histocompatibility complex, class I, A

8179103	5.53E-06	2.16E-08	1.19E+01	9.798811	1.44	HLA-H///HLA-F///HLA-E///HLA-B///HLA-A///HLA-A	major histocompatibility complex, class I, H (pseudogene)///major histocompatibility complex, class I, F///major histocompatibility complex, class I, E///major histocompatibility complex, class I, B///major histocompatibility complex, class I, A///major histocompatibility complex, class I, A

8066905	5.53E-06	2.16E-08	1.19E+01	9.798746	1.28	ZNFX1	zinc finger NFX1-type containing 1
8085914	5.77E-06	2.28E-08	1.18E+01	9.748419	1.92	SLC4A7	solute carrier family 4 member 7

8177732	5.78E-06	2.31E-08	1.18E+01	9.735364	1.89	HLA-H///HLA-B///HLA-A///HLA-B///HLA-A	major histocompatibility complex, class I, H (pseudogene)///major histocompatibility complex, class I, B///major histocompatibility complex, class I, A///major histocompatibility complex, class I, B///major histocompatibility complex, class I, A

8021442	5.78E-06	2.32E-08	1.18E+01	9.730397	1.66	ZNF532	zinc finger protein 532

8117800	5.84E-06	2.36E-08	1.18E+01	9.712067	1.87	HLA-H///HLA-C///HLA-B///HLA-A///HLA-A	major histocompatibility complex, class I, H (pseudogene)///major histocompatibility complex, class I, C///major histocompatibility complex, class I, B///major histocompatibility complex, class I, A///major histocompatibility complex, class I, A

8103601	5.84E-06	2.40E-08	1.18E+01	9.696883	1.67	DDX60L	DEAD-box helicase 60-like
8122336	5.84E-06	2.42E-08	1.18E+01	9.688575	1.85	ABRACL	ABRA C-terminal like
8022674	5.84E-06	2.46E-08	1.18E+01	9.673705	2.09	CDH2	cadherin 2
8105061	6.45E-06	2.74E-08	1.17E+01	9.565441	3.14	FYB	FYN binding protein
8174610	6.56E-06	2.83E-08	1.16E+01	9.534816	2.1	LRCH2	leucine rich repeats and calponin homology domain containing 2
8130867	6.56E-06	2.84E-08	1.16E+01	9.531489	3.8	THBS2	thrombospondin 2

8089544	6.62E-06	2.93E-08	1.16E+01	9.50103	2.17	CCDC80	coiled-coil domain containing 80
8092392	6.65E-06	2.97E-08	1.16E+01	9.48859	2.14	KLHL6	kelch like family member 6
8104901	6.74E-06	3.04E-08	1.16E+01	9.464205	3.29	IL7R	interleukin 7 receptor
7963054	6.74E-06	3.06E-08	1.15E+01	9.458422	1.49	TUBA1A	tubulin alpha 1a
8058477	6.74E-06	3.08E-08	1.15E+01	9.452599	1.39	KLF7	Kruppel like factor 7
8146000	6.80E-06	3.15E-08	1.15E+01	9.429335	1.73	ADAM9	ADAM metallopeptidase domain 9

8052355	6.80E-06	3.15E-08	1.15E+01	9.428941	1.82	EFEMP1	EGF containing fibulin like extracellular matrix protein 1
7964757	6.80E-06	3.17E-08	1.15E+01	9.422728	2.35		
7954711	6.84E-06	3.21E-08	1.15E+01	9.40945	1.22	KIAA1551	KIAA1551
8179019	6.99E-06	3.33E-08	1.15E+01	9.3748	1.7	HLA-F///HLA-B	major histocompatibility complex, class I, F///major histocompatibility complex, class I, B
8155214	6.99E-06	3.36E-08	1.15E+01	9.365246	1.7	MELK	maternal embryonic leucine zipper kinase

8048926	6.99E-06	3.39E-08	1.14E+01	9.357343	1.45	SP140L	SP140 nuclear body protein like

8117890	6.99E-06	3.41E-08	1.14E+01	9.352802	1.41	HLA-H///HLA-F///HLA-E///HLA-B///HLA-A///HLA-A	major histocompatibility complex, class I, H (pseudogene)///major histocompatibility complex, class I, F///major histocompatibility complex, class I, E///major histocompatibility complex, class I, B///major histocompatibility complex, class I, A///major histocompatibility complex, class I, A

8046333	7.00E-06	3.48E-08	1.14E+01	9.33134	2.04	CYBRD1	cytochrome b reductase 1
8056257	7.00E-06	3.50E-08	1.14E+01	9.32608	3.92	FAP	fibroblast activation protein alpha
8115734	7.00E-06	3.50E-08	1.14E+01	9.324694	2.59	LCP2	lymphocyte cytosolic protein 2
7920123	7.00E-06	3.51E-08	1.14E+01	9.323732	1.71	S100A10	S100 calcium binding protein A10

8178193	7.20E-06	3.68E-08	1.14E+01	9.277265	2.33	HLA-DRA///HLA-DQA1	major histocompatibility complex, class II, DR alpha///major histocompatibility complex, class II, DQ alpha 1
8015349	7.20E-06	3.68E-08	1.14E+01	9.275824	2.2	KRT19	keratin 19
8053064	7.45E-06	3.86E-08	1.13E+01	9.228753	1.18	MOB1A	MOB kinase activator 1A
8050007	7.82E-06	4.08E-08	1.13E+01	9.174533	2.06	PXDN	peroxidasin
8108873	7.82E-06	4.16E-08	1.13E+01	9.154737	1.77	ARHGAP26	Rho GTPase activating protein 26

8046922	7.82E-06	4.19E-08	1.12E+01	9.147778	2.73	COL3A1	collagen type III alpha 1 chain
8118158	7.82E-06	4.23E-08	1.12E+01	9.139796	2.28	AIF1	allograft inflammatory factor 1
8177996	7.82E-06	4.23E-08	1.12E+01	9.139796	2.28	AIF1	allograft inflammatory factor 1
8179276	7.82E-06	4.23E-08	1.12E+01	9.139796	2.28	AIF1	allograft inflammatory factor 1
7965941	7.82E-06	4.25E-08	1.12E+01	9.135616	2.75	GLT8D2	glycosyltransferase 8 domain containing 2

8177717	7.96E-06	4.35E-08	1.12E+01	9.112331	1.7	HLA-F///HLA-B	major histocompatibility complex, class I, F///major histocompatibility complex, class I, B
8137979	7.96E-06	4.38E-08	1.12E+01	9.104969	1.83	ACTB	actin beta
8179481	7.96E-06	4.41E-08	1.12E+01	9.097925	2.44	HLA-DRA///HLA-DQA1	major histocompatibility complex, class II, DR alpha///major histocompatibility complex, class II, DQ alpha 1

8055702	7.96E-06	4.48E-08	1.12E+01	9.083508	1.65	NMI	N-myc and STAT interactor
8103415	7.96E-06	4.49E-08	1.12E+01	9.081049	1.86	FAM198B	family with sequence similarity 198 member B
8047763	8.19E-06	4.67E-08	1.11E+01	9.041364	3.47	NRP2	neuropilin 2
8056184	8.35E-06	4.82E-08	1.11E+01	9.011329	3.29	ITGB6///ITGB6	integrin subunit beta 6///integrin subunit beta 6
8128956	8.35E-06	4.83E-08	1.11E+01	9.008906	1.74	FYN	FYN proto-oncogene, Src family tyrosine kinase

7914270	8.35E-06	4.85E-08	1.11E+01	9.00476	3.13	LAPTM5	lysosomal protein transmembrane 5
7986214	8.35E-06	4.88E-08	1.11E+01	8.998595	1.47	SLCO3A1///SLCO3A1	solute carrier organic anion transporter family member 3A1///solute carrier organic anion transporter family member 3A1
7931951	8.36E-06	4.91E-08	1.11E+01	8.991104	1.38	SFMBT2	Scm-like with four mbt domains 2

8118548	8.71E-06	5.18E-08	1.11E+01	8.939691	2.46	HLA-DRA///HLA-DQA1	major histocompatibility complex, class II, DR alpha///major histocompatibility complex, class II, DQ alpha 1
7926916	9.01E-06	5.39E-08	1.10E+01	8.900078	1.88	ZEB1	zinc finger E-box binding homeobox 1
8056113	9.09E-06	5.48E-08	1.10E+01	8.882916	3.02	LY75-CD302///LY75	LY75-CD302 readthrough///lymphocyte antigen 75

7952268	9.09E-06	5.50E-08	1.10E+01	8.880553	2.52	THY1	Thy-1 cell surface antigen
7957570	9.10E-06	5.56E-08	1.10E+01	8.868894	2.43	PLXNC1	plexin C1
7929511	9.10E-06	5.57E-08	1.10E+01	8.867706	2.42	ENTPD1	ectonucleoside triphosphate diphosphohydrolase 1
7985493	9.21E-06	5.67E-08	1.10E+01	8.850048	2.66	TM6SF1	transmembrane 6 superfamily member 1
8063437	9.34E-06	5.78E-08	1.09E+01	8.830885	1.73	TSHZ2	teashirt zinc finger homeobox 2

7954527	9.36E-06	5.86E-08	1.09E+01	8.817737	2.32	ARNTL2	aryl hydrocarbon receptor nuclear translocator like 2
8089011	9.81E-06	6.22E-08	1.09E+01	8.75814	2.05	PROS1	protein S (alpha)
8104788	9.81E-06	6.22E-08	1.09E+01	8.757947	1.69	RAI14	retinoic acid induced 14
8100541	9.81E-06	6.24E-08	1.09E+01	8.75476	1.21	IGFBP7	insulin like growth factor binding protein 7
7961532	9.83E-06	6.29E-08	1.09E+01	8.747573	2.39	ARHGDIB	Rho GDP dissociation inhibitor beta

8169541	9.98E-06	6.41E-08	1.09E+01	8.727771	3.46	DOCK11	dedicator of cytokinesis 11
8149448	1.07E-05	6.93E-08	1.08E+01	8.651269	2.52	MSR1	macrophage scavenger receptor 1
8108217	1.07E-05	6.95E-08	1.08E+01	8.647885	2.09	TGFBI	transforming growth factor beta induced
7972750	1.07E-05	7.00E-08	1.08E+01	8.64057	1.1	COL4A1	collagen type IV alpha 1 chain
7945262	1.07E-05	7.01E-08	1.08E+01	8.639334	1.56	JAM3	junctional adhesion molecule 3

8179049	1.08E-05	7.10E-08	1.08E+01	8.62737	1.47	HLA-J///HLA-H///HLA-A///HLA-A	major histocompatibility complex, class I, J (pseudogene)///major histocompatibility complex, class I, H (pseudogene)///major histocompatibility complex, class I, A///major histocompatibility complex, class I, A
7980958	1.10E-05	7.32E-08	1.07E+01	8.597132	1.22	LGMN	legumain

8004184	1.12E-05	7.50E-08	1.07E+01	8.572437	2.49	XAF1	XIAP associated factor 1
8158167	1.12E-05	7.53E-08	1.07E+01	8.568579	3.5	LCN2	lipocalin 2
7908940	1.14E-05	7.70E-08	1.07E+01	8.546928	1.34	ATP2B4	ATPase plasma membrane Ca2+ transporting 4
8113358	1.15E-05	7.80E-08	1.07E+01	8.533732	2.62	ST8SIA4	ST8 alpha-N-acetyl-neuraminide alpha-2,8-sialyltransferase 4
7934906	1.15E-05	7.82E-08	1.07E+01	8.531605	3.01	ACTA2	actin, alpha 2, smooth muscle, aorta

8068238	1.15E-05	7.93E-08	1.07E+01	8.516967	1.47	IFNAR2	interferon alpha and beta receptor subunit 2
7906564	1.15E-05	7.99E-08	1.07E+01	8.510219	1.47	PEA15	phosphoprotein enriched in astrocytes 15
8045539	1.15E-05	8.03E-08	1.06E+01	8.504948	3.64	KYNU	kynureninase
8141076	1.15E-05	8.07E-08	1.06E+01	8.499708	1.12	PON2	paraoxonase 2
7956301	1.15E-05	8.10E-08	1.06E+01	8.496594	1.66	LRP1	LDL receptor related protein 1
7969861	1.15E-05	8.13E-08	1.06E+01	8.49305	2.07	ITGBL1	integrin subunit beta like 1

8125530	1.15E-05	8.22E-08	1.06E+01	8.481645	2.64	HLA-DMB	major histocompatibility complex, class II, DM beta
7907893	1.16E-05	8.44E-08	1.06E+01	8.455745	1.56	MR1	major histocompatibility complex, class I-related
8105229	1.16E-05	8.47E-08	1.06E+01	8.452415	1.65	PELO///ITGA1	pelota homolog (Drosophila)///integrin subunit alpha 1
7969613	1.16E-05	8.47E-08	1.06E+01	8.451633	2.2	GPC6	glypican 6

8096808	1.16E-05	8.48E-08	1.06E+01	8.451013	2.12	MCUB	mitochondrial calcium uniporter dominant negative beta subunit
8136347	1.19E-05	8.85E-08	1.06E+01	8.408629	2.31	CALD1	caldesmon 1
8027778	1.19E-05	8.88E-08	1.06E+01	8.405339	2.01	FXYD5	FXYD domain containing ion transport regulator 5
7953218	1.19E-05	8.91E-08	1.06E+01	8.402199	1.88	RAD51AP1	RAD51 associated protein 1
8131614	1.20E-05	8.99E-08	1.05E+01	8.39295	2.16	AHR	aryl hydrocarbon receptor

7907092	1.23E-05	9.25E-08	1.05E+01	8.364317	1.07	MPZL1	myelin protein zero like 1
8160260	1.25E-05	9.51E-08	1.05E+01	8.337367	2.41	BNC2	basonuclin 2
8056005	1.25E-05	9.56E-08	1.05E+01	8.331725	1.11	ACVR1	activin A receptor type 1
8059413	1.25E-05	9.58E-08	1.05E+01	8.329675	2.61	DOCK10	dedicator of cytokinesis 10
8103254	1.26E-05	9.69E-08	1.05E+01	8.31841	3.13	SFRP2	secreted frizzled related protein 2
8096602	1.27E-05	9.79E-08	1.05E+01	8.308178	2.24	DAPP1	dual adaptor of phosphotyrosine and 3-phosphoinositides 1

7905147	1.34E-05	1.05E-07	1.04E+01	8.243051	2.74	C1orf54	chromosome 1 open reading frame 54
8121588	1.37E-05	1.08E-07	1.04E+01	8.212101	2.36	DSE	dermatan sulfate epimerase
7908553	1.38E-05	1.09E-07	1.04E+01	8.202867	2.97	PTPRC	protein tyrosine phosphatase, receptor type C
7908488	1.39E-05	1.10E-07	1.04E+01	8.191416	1.92	CFHR1	complement factor H related 1
8014974	1.39E-05	1.10E-07	1.04E+01	8.188682	2.76	TOP2A	topoisomerase (DNA) II alpha
8155192	1.48E-05	1.19E-07	1.03E+01	8.116176	1.6	GLIPR2	GLI pathogenesis related 2

8164607	1.51E-05	1.22E-07	1.03E+01	8.092347	1.37	FNBP1	formin binding protein 1
7907222	1.51E-05	1.22E-07	1.03E+01	8.087959	2.59	PRRX1	paired related homeobox 1
8180078	1.52E-05	1.23E-07	1.03E+01	8.078142	2.59	HLA-DMB	major histocompatibility complex, class II, DM beta
8059650	1.59E-05	1.30E-07	1.02E+01	8.02618	1.7	SP110	SP110 nuclear body protein
8140967	1.61E-05	1.32E-07	1.02E+01	8.014391	2.6	SAMD9	sterile alpha motif domain containing 9

8056206	1.62E-05	1.33E-07	1.02E+01	8.002482	1.23	RBMS1	RNA binding motif single stranded interacting protein 1
8138613	1.68E-05	1.40E-07	1.02E+01	7.952929	2.2	OSBPL3	oxysterol binding protein like 3

8178811	1.70E-05	1.42E-07	1.01E+01	7.937115	2.28	LOC105369230///HLA-DRB6///HLA-DRB5///HLA-DRB4///HLA-DRB3///HLA-DRB1///LOC105369230///HLA-DRB5///HLA-DRB4///HLA-DRB3///HLA-DRB1///HLA-DQB1	HLA class II histocompatibility antigen, DRB1-7 beta chain///major histocompatibility complex, class II, DR beta 6 (pseudogene)///major histocompatibility complex, class II, DR beta 5///major histocompatibility complex, class II, DR beta 4///major histocompatibility complex, class II, DR beta 3///major histocompatibility complex, class II, DR beta 1///HLA class II histocompatibility antigen, DRB1-7 beta chain///major histocompatibility complex, class II, DR beta 5///major histocompatibility complex, class II, DR beta 4///major histocompatibility complex, class II, DR beta 3///major histocompatibility complex, class II, DR beta 1///major histocompatibility complex, class II, DQ beta 1

8041383	1.70E-05	1.43E-07	1.01E+01	7.931535	2.25	LTBP1	latent transforming growth factor beta binding protein 1
8043100	1.70E-05	1.43E-07	1.01E+01	7.929231	1.41	TMSB10	thymosin beta 10
8128991	1.72E-05	1.46E-07	1.01E+01	7.91143	2.55	LAMA4	laminin subunit alpha 4
8050160	1.73E-05	1.47E-07	1.01E+01	7.903994	2.26	MBOAT2	membrane bound O-acyltransferase domain containing 2
7951217	1.73E-05	1.48E-07	1.01E+01	7.899347	2.84	MMP7	matrix metallopeptidase 7

7952601	1.77E-05	1.52E-07	1.01E+01	7.872687	1.44	ETS1	ETS proto-oncogene 1, transcription factor
8140971	1.77E-05	1.52E-07	1.01E+01	7.870096	2.1	SAMD9L	sterile alpha motif domain containing 9 like
8105191	1.78E-05	1.54E-07	1.01E+01	7.855749	1.12	PARP8	poly(ADP-ribose) polymerase family member 8
8018975	1.78E-05	1.56E-07	1.01E+01	7.845281	1.45	LGALS3BP	galectin 3 binding protein
8138689	1.78E-05	1.56E-07	1.01E+01	7.841846	1.57	SKAP2	src kinase associated phosphoprotein 2

7957260	1.78E-05	1.58E-07	1.01E+01	7.830999	2.38	GLIPR1	GLI pathogenesis related 1
7948399	1.81E-05	1.61E-07	1.00E+01	7.811491	1	PATL1	PAT1 homolog 1, processing body mRNA decay factor
7906400	1.81E-05	1.62E-07	1.00E+01	7.807935	2.68	IFI16	interferon gamma inducible protein 16

8178199	1.82E-05	1.65E-07	1.00E+01	7.7857	2.71	HLA-DQA2///HLA-DQA1	major histocompatibility complex, class II, DQ alpha 2///major histocompatibility complex, class II, DQ alpha 1
8179489	1.82E-05	1.65E-07	1.00E+01	7.7857	2.71	HLA-DQA2///HLA-DQA1	major histocompatibility complex, class II, DQ alpha 2///major histocompatibility complex, class II, DQ alpha 1

8011713	1.82E-05	1.66E-07	1.00E+01	7.782856	2.17	CXCL16	C-X-C motif chemokine ligand 16
7943998	1.82E-05	1.66E-07	1.00E+01	7.779845	2.14	NNMT	nicotinamide N-methyltransferase
7902541	1.82E-05	1.67E-07	1.00E+01	7.774633	2.8	IFI44L	interferon induced protein 44 like
7979658	1.82E-05	1.68E-07	1.00E+01	7.771398	3.31	GPX2	glutathione peroxidase 2
8152703	1.84E-05	1.70E-07	9.99	7.758177	2.39	FBXO32	F-box protein 32

8117813	1.84E-05	1.71E-07	9.99	7.752723	1.38	HLA-J///HLA-H///HLA-B///HLA-A///HLA-A	major histocompatibility complex, class I, J (pseudogene)///major histocompatibility complex, class I, H (pseudogene)///major histocompatibility complex, class I, B///major histocompatibility complex, class I, A///major histocompatibility complex, class I, A

8082058	1.87E-05	1.74E-07	9.97	7.733885	2.74	CSTA	cystatin A
7917530	1.87E-05	1.75E-07	9.97	7.7301	2.8	GBP2	guanylate binding protein 2
7930498	1.89E-05	1.77E-07	9.96	7.718431	2.46	ACSL5	acyl-CoA synthetase long-chain family member 5
7929032	1.89E-05	1.78E-07	9.95	7.711246	1.92	FAS	Fas cell surface death receptor
8010426	1.94E-05	1.84E-07	9.93	7.681676	1.15	RNF213	ring finger protein 213
8063458	1.96E-05	1.87E-07	9.91	7.664975	2.25	DOK5	docking protein 5
7955179	1.96E-05	1.87E-07	9.91	7.664737	1.54	TUBA1C	tubulin alpha 1c

7996290	2.21E-05	2.14E-07	9.8	7.527498	1.16	CKLF-CMTM1///CMTM1///CKLF///RHOQ///CMTM1///CKLF	CKLF-CMTM1 readthrough///CKLF like MARVEL transmembrane domain containing 1///chemokine like factor///ras homolog family member Q///CKLF like MARVEL transmembrane domain containing 1///chemokine like factor

8126784	2.21E-05	2.15E-07	9.79	7.523951	3.43	PLA2G7	phospholipase A2 group VII
8046695	2.24E-05	2.18E-07	9.78	7.507192	2.45	ITGA4	integrin subunit alpha 4

8178802	2.28E-05	2.23E-07	9.76	7.486633	2.25	LOC105369230///HLA-DRB6///HLA-DRB5///HLA-DRB4///HLA-DRB3///HLA-DRB1///LOC105369230///HLA-DRB5///HLA-DRB4///HLA-DRB3///HLA-DRB1///HLA-DQB1	HLA class II histocompatibility antigen, DRB1-7 beta chain///major histocompatibility complex, class II, DR beta 6 (pseudogene)///major histocompatibility complex, class II, DR beta 5///major histocompatibility complex, class II, DR beta 4///major histocompatibility complex, class II, DR beta 3///major histocompatibility complex, class II, DR beta 1///HLA class II histocompatibility antigen, DRB1-7 beta chain///major histocompatibility complex, class II, DR beta 5///major histocompatibility complex, class II, DR beta 4///major histocompatibility complex, class II, DR beta 3///major histocompatibility complex, class II, DR beta 1///major histocompatibility complex, class II, DQ beta 1

8030866	2.28E-05	2.24E-07	9.76	7.481946	2.67	FPR3	formyl peptide receptor 3
8155169	2.29E-05	2.25E-07	9.75	7.475735	1.22	RECK	reversion inducing cysteine rich protein with kazal motifs
7997642	2.31E-05	2.29E-07	9.74	7.460684	2	CRISPLD2	cysteine rich secretory protein LCCL domain containing 2
8168749	2.32E-05	2.32E-07	9.73	7.446617	1.86	SRPX2	sushi repeat containing protein, X-linked 2

7971461	2.32E-05	2.32E-07	9.73	7.445065	2.71	LCP1	lymphocyte cytosolic protein 1
8023415	2.37E-05	2.39E-07	9.7	7.4182	1.9	TCF4	transcription factor 4
7973101	2.45E-05	2.51E-07	9.66	7.369503	2.62	RNASE6	ribonuclease A family member k6
7966135	2.45E-05	2.51E-07	9.66	7.369258	1.97	CORO1C	coronin 1C
8086125	2.45E-05	2.51E-07	9.66	7.366974	1.41	TRANK1	tetratricopeptide repeat and ankyrin repeat containing 1
7974816	2.45E-05	2.52E-07	9.66	7.36368	1.25	SLC38A6	solute carrier family 38 member 6

8149330	2.47E-05	2.56E-07	9.65	7.349764	1.89	CTSB	cathepsin B
8171684	2.48E-05	2.57E-07	9.64	7.344595	1.88	SH3KBP1	SH3 domain containing kinase binding protein 1
8127563	2.48E-05	2.60E-07	9.63	7.333794	3.64	COL12A1	collagen type XII alpha 1 chain
8098263	2.48E-05	2.61E-07	9.63	7.330068	1.75	PALLD	palladin, cytoskeletal associated protein
8135363	2.48E-05	2.61E-07	9.63	7.328787	2.4	PIK3CG	phosphatidylinositol-4,5-bisphosphate 3-kinase catalytic subunit gamma

8140668	2.51E-05	2.64E-07	9.62	7.316756	3.02	SEMA3A	semaphorin 3A
8047738	2.52E-05	2.68E-07	9.61	7.300775	1.93	NRP2	neuropilin 2
7937335	2.52E-05	2.69E-07	9.61	7.299621	1.99	IFITM1	interferon induced transmembrane protein 1
7937020	2.54E-05	2.72E-07	9.6	7.288401	1.87	MKI67	marker of proliferation Ki-67
8151447	2.54E-05	2.73E-07	9.59	7.285364	1.56	IL7	interleukin 7
8089299	2.54E-05	2.73E-07	9.59	7.282782	1.17	CD47	CD47 molecule
8134030	2.57E-05	2.78E-07	9.58	7.265692	2.15	STEAP1	six transmembrane epithelial antigen of the prostate 1
8140556	2.58E-05	2.80E-07	9.57	7.260342	1.73	HGF	hepatocyte growth factor

8162388	2.65E-05	2.90E-07	9.54	7.224905	2.48	OMD	osteomodulin
8091723	2.80E-05	3.12E-07	9.48	7.150398	2.52	RARRES1	retinoic acid receptor responder 1
8147019	2.80E-05	3.13E-07	9.48	7.145984	1.06	ZC2HC1A	zinc finger C2HC-type containing 1A
7960874	2.80E-05	3.14E-07	9.48	7.143163	2.83	C3AR1	complement component 3a receptor 1
7923086	2.82E-05	3.19E-07	9.46	7.128141	2.04	ASPM	abnormal spindle microtubule assembly

8109843	2.83E-05	3.21E-07	9.46	7.120415	2.01	DOCK2	dedicator of cytokinesis 2
8133788	2.84E-05	3.24E-07	9.45	7.111818	1.35	PTPN12	protein tyrosine phosphatase, non-receptor type 12
7980438	2.88E-05	3.31E-07	9.43	7.091055	1.04	SPTLC2	serine palmitoyltransferase long chain base subunit 2
8178295	2.89E-05	3.34E-07	9.43	7.082565	2.49	UBD	ubiquitin D

8180003	2.89E-05	3.35E-07	9.42	7.079164	2.26	LOC105369230///HLA-DRB6///HLA-DRB5///HLA-DRB4///HLA-DRB3///HLA-DRB1///LOC105369230///HLA-DRB5///HLA-DRB4///HLA-DRB3///HLA-DRB1///HLA-DQB1	HLA class II histocompatibility antigen, DRB1-7 beta chain///major histocompatibility complex, class II, DR beta 6 (pseudogene)///major histocompatibility complex, class II, DR beta 5///major histocompatibility complex, class II, DR beta 4///major histocompatibility complex, class II, DR beta 3///major histocompatibility complex, class II, DR beta 1///HLA class II histocompatibility antigen, DRB1-7 beta chain///major histocompatibility complex, class II, DR beta 5///major histocompatibility complex, class II, DR beta 4///major histocompatibility complex, class II, DR beta 3///major histocompatibility complex, class II, DR beta 1///major histocompatibility complex, class II, DQ beta 1

7908204	2.96E-05	3.47E-07	9.4	7.044158	2.77	HMCN1	hemicentin 1
8152453	2.96E-05	3.48E-07	9.39	7.039378	1.86	TRPS1	transcriptional repressor GATA binding 1
7925320	2.96E-05	3.49E-07	9.39	7.038986	1.52	NID1	nidogen 1
8163637	2.96E-05	3.50E-07	9.39	7.033683	2.04	TNC	tenascin C
8165735	2.96E-05	3.53E-07	9.38	7.02484	1.35	CSF2RA	colony stimulating factor 2 receptor alpha subunit
8176306	2.96E-05	3.53E-07	9.38	7.02484	1.35	CSF2RA	colony stimulating factor 2 receptor alpha subunit
8157605	2.96E-05	3.56E-07	9.37	7.018008	1.4		

8033257	2.96E-05	3.61E-07	9.36	7.004098	2.85	C3	complement component 3
8103226	2.96E-05	3.62E-07	9.36	7.002026	2.84	TMEM154	transmembrane protein 154
8118571	2.96E-05	3.62E-07	9.36	7.000942	2.06	PSMB9	proteasome subunit beta 9
8178211	2.96E-05	3.62E-07	9.36	7.000942	2.06	PSMB9	proteasome subunit beta 9
8179495	2.96E-05	3.62E-07	9.36	7.000942	2.06	PSMB9	proteasome subunit beta 9

8114287	3.05E-05	3.74E-07	9.33	6.968106	1.24	SPOCK1	sparc/osteonectin, cwcv and kazal-like domains proteoglycan (testican) 1
8124650	3.05E-05	3.76E-07	9.33	6.9639	2.5	UBD	ubiquitin D
8037205	3.11E-05	3.84E-07	9.31	6.94042	1.76	CEACAM1	carcinoembryonic antigen related cell adhesion molecule 1
8132318	3.18E-05	3.94E-07	9.29	6.914923	2.84	ANLN	anillin actin binding protein

7955908	3.19E-05	4.00E-07	9.28	6.900092	2.47	NCKAP1L	NCK associated protein 1 like
8109830	3.24E-05	4.08E-07	9.26	6.879996	1.28	SPDL1	spindle apparatus coiled-coil protein 1
8094556	3.25E-05	4.11E-07	9.26	6.873436	1.25	PGM2	phosphoglucomutase 2
7938485	3.25E-05	4.11E-07	9.26	6.872649	1.6	MICAL2	microtubule associated monooxygenase, calponin and LIM domain containing 2

8127145	3.26E-05	4.14E-07	9.25	6.866401	1.9	ELOVL5	ELOVL fatty acid elongase 5
7972682	3.29E-05	4.20E-07	9.24	6.851532	2.58	KDELC1	KDEL motif containing 1
7951397	3.30E-05	4.22E-07	9.24	6.847225	2.27	CASP1	caspase 1
7961083	3.35E-05	4.29E-07	9.22	6.82965	2.38	CLEC2B	C-type lectin domain family 2 member B
8140534	3.38E-05	4.35E-07	9.21	6.816407	3.52	SEMA3C	semaphorin 3C

7989094	3.38E-05	4.36E-07	9.21	6.814385	1.37	NEDD4	neural precursor cell expressed, developmentally down-regulated 4, E3 ubiquitin protein ligase
7909175	3.42E-05	4.42E-07	9.2	6.79929	1.42	SRGAP2	SLIT-ROBO Rho GTPase activating protein 2
7961546	3.44E-05	4.47E-07	9.19	6.788559	1.5	EPS8	epidermal growth factor receptor pathway substrate 8

7906307	3.49E-05	4.57E-07	9.17	6.766705	1.22	KIRREL	kin of IRRE like (Drosophila)
7993638	3.49E-05	4.57E-07	9.17	6.766195	2.69	TMC5	transmembrane channel like 5
7970844	3.49E-05	4.57E-07	9.17	6.766181	1.62	KATNAL1	katanin catalytic subunit A1 like 1

7904452	3.50E-05	4.60E-07	9.17	6.76064	1.39	FAM72A///FAM72D///FAM72B	family with sequence similarity 72 member A///family with sequence similarity 72 member D///family with sequence similarity 72 member B
8004510	3.53E-05	4.65E-07	9.16	6.749435	2.38	CD68	CD68 molecule
7971565	3.58E-05	4.79E-07	9.14	6.720194	1.7	LPAR6	lysophosphatidic acid receptor 6

8177725	3.58E-05	4.79E-07	9.14	6.71923	1.28	HLA-H///HLA-G///HLA-C///HLA-A///HLA-A	major histocompatibility complex, class I, H (pseudogene)///major histocompatibility complex, class I, G///major histocompatibility complex, class I, C///major histocompatibility complex, class I, A///major histocompatibility complex, class I, A

8179034	3.58E-05	4.79E-07	9.14	6.71923	1.28	HLA-H///HLA-G///HLA-C///HLA-A///HLA-A	major histocompatibility complex, class I, H (pseudogene)///major histocompatibility complex, class I, G///major histocompatibility complex, class I, C///major histocompatibility complex, class I, A///major histocompatibility complex, class I, A

7957850	3.64E-05	4.88E-07	9.12	6.700664	1.68	GAS2L3	growth arrest specific 2 like 3
7964701	3.64E-05	4.89E-07	9.12	6.697838	1.13	GNS	glucosamine (N-acetyl)-6-sulfatase
8102328	3.65E-05	4.92E-07	9.11	6.691343	1.19	CFI	complement factor I
8050190	3.68E-05	4.97E-07	9.11	6.682001	1.22	ADAM17	ADAM metallopeptidase domain 17
7939120	3.69E-05	5.00E-07	9.1	6.676098	1.08	RCN1	reticulocalbin 1
7902874	3.69E-05	5.01E-07	9.1	6.674158	1.35	LRRC8C	leucine rich repeat containing 8 family member C

7974851	3.71E-05	5.06E-07	9.09	6.664399	1.23	HIF1A	hypoxia inducible factor 1 alpha subunit
8147837	3.80E-05	5.21E-07	9.07	6.634688	2.28	ZFPM2	zinc finger protein, FOG family member 2
7979133	3.81E-05	5.23E-07	9.07	6.631233	1.52	NID2	nidogen 2
8056151	3.99E-05	5.54E-07	9.02	6.572829	1.74	PLA2R1	phospholipase A2 receptor 1
8160274	3.99E-05	5.55E-07	9.02	6.570928	2.51	MGC24103	uncharacterized MGC24103
7906720	4.02E-05	5.62E-07	9.01	6.557648	2.97	FCER1G	Fc fragment of IgE receptor Ig
8011759	4.02E-05	5.71E-07	9	6.541986	1.02	PFN1	profilin 1

7979824	4.02E-05	5.71E-07	9	6.541314	1.68	ACTN1	actinin alpha 1
8125500	4.02E-05	5.72E-07	8.99	6.541133	1.59	PSMB8	proteasome subunit beta 8
8178855	4.02E-05	5.72E-07	8.99	6.541133	1.59	PSMB8	proteasome subunit beta 8
8180049	4.02E-05	5.72E-07	8.99	6.541133	1.59	PSMB8	proteasome subunit beta 8
8138977	4.05E-05	5.76E-07	8.99	6.532632	1.41	DPY19L1	dpy-19 like 1
8072710	4.06E-05	5.80E-07	8.98	6.527193	1.18	APOL6	apolipoprotein L6
8049246	4.10E-05	5.87E-07	8.97	6.513519	1.25	INPP5D	inositol polyphosphate-5-phosphatase D

8014233	4.16E-05	5.97E-07	8.96	6.496623	1.94	SLFN11	schlafen family member 11
8135601	4.23E-05	6.14E-07	8.94	6.469706	1.51	MET	MET proto-oncogene, receptor tyrosine kinase
8134552	4.26E-05	6.20E-07	8.93	6.45894	1.39	ARPC1B	actin related protein 2/3 complex subunit 1B
7944722	4.32E-05	6.35E-07	8.91	6.434975	1.32	UBASH3B	ubiquitin associated and SH3 domain containing B

7974366	4.32E-05	6.37E-07	8.91	6.432197	1.43	PTGER2	prostaglandin E receptor 2
7936673	4.32E-05	6.38E-07	8.91	6.430885	1.68	RGS10	regulator of G-protein signaling 10
8127051	4.33E-05	6.41E-07	8.9	6.42576	1.44	TRAM2	translocation associated membrane protein 2
8166730	4.39E-05	6.52E-07	8.89	6.40912	2.72	CYBB	cytochrome b-245 beta chain
7903507	4.42E-05	6.58E-07	8.88	6.399127	1.27	FAM102B	family with sequence similarity 102 member B
8006531	4.42E-05	6.59E-07	8.88	6.397172	1.59	SLFN5	schlafen family member 5

8055465	4.65E-05	6.98E-07	8.84	6.340235	3.06	CXCR4	C-X-C motif chemokine receptor 4

8124901	4.65E-05	7.00E-07	8.84	6.337406	1.32	HLA-C///HLA-B///HLA-A///HLA-B///HLA-A	major histocompatibility complex, class I, C///major histocompatibility complex, class I, B///major histocompatibility complex, class I, A///major histocompatibility complex, class I, B///major histocompatibility complex, class I, A

7960919	4.68E-05	7.07E-07	8.83	6.326709	3.76	MFAP5	microfibrillar associated protein 5
7896726	4.74E-05	7.18E-07	8.82	6.311538	2.35		
8121749	4.74E-05	7.19E-07	8.82	6.309981	2.19	GJA1	gap junction protein alpha 1
7972713	4.74E-05	7.21E-07	8.81	6.306907	1.94	EFNB2	ephrin B2
8068697	4.77E-05	7.29E-07	8.8	6.29571	1.95	MX2	MX dynamin like GTPase 2
8166805	4.79E-05	7.34E-07	8.8	6.289238	1.01	ATP6AP2	ATPase H+ transporting accessory protein 2

7909146	4.81E-05	7.38E-07	8.79	6.283587	1.35	FAM72A///FAM72D///FAM72B	family with sequence similarity 72 member A///family with sequence similarity 72 member D///family with sequence similarity 72 member B
8043995	4.85E-05	7.50E-07	8.78	6.26752	1.41	IL1R1	interleukin 1 receptor type 1
8178115	4.89E-05	7.60E-07	8.77	6.253355	2.29	CFB	complement factor B

8089015	4.89E-05	7.63E-07	8.77	6.249675	1.68	PROS1	protein S (alpha)
7906900	4.89E-05	7.63E-07	8.77	6.249204	2.08	DDR2	discoidin domain receptor tyrosine kinase 2
7927658	4.89E-05	7.65E-07	8.77	6.247057	1.33	UBE2D1	ubiquitin conjugating enzyme E2 D1
8043981	4.94E-05	7.78E-07	8.75	6.229493	1.59	IL1R2	interleukin 1 receptor type 2

8178489	5.01E-05	7.91E-07	8.74	6.213407	1.34	HLA-C///HLA-B///HLA-A///HLA-B///HLA-A	major histocompatibility complex, class I, C///major histocompatibility complex, class I, B///major histocompatibility complex, class I, A///major histocompatibility complex, class I, B///major histocompatibility complex, class I, A

8152867	5.02E-05	7.95E-07	8.74	6.208395	1.48	ASAP1	ArfGAP with SH3 domain, ankyrin repeat and PH domain 1
8178220	5.03E-05	7.97E-07	8.73	6.20513	2.53	HLA-DPB1	major histocompatibility complex, class II, DP beta 1
8127346	5.07E-05	8.06E-07	8.73	6.193847	2.19	RAB23	RAB23, member RAS oncogene family
8104022	5.12E-05	8.17E-07	8.72	6.1801	2.05	PDLIM3	PDZ and LIM domain 3

8118594	5.15E-05	8.29E-07	8.71	6.166465	2.51	HLA-DPB1	major histocompatibility complex, class II, DP beta 1
8099982	5.15E-05	8.32E-07	8.7	6.162484	1.23	APBB2	amyloid beta precursor protein binding family B member 2
8117321	5.15E-05	8.32E-07	8.7	6.162425	1.39	TRIM38	tripartite motif containing 38
8118345	5.15E-05	8.33E-07	8.7	6.161318	2.27	CFB	complement factor B
8126018	5.15E-05	8.34E-07	8.7	6.160116	1.2	STK38	serine/threonine kinase 38

8045835	5.23E-05	8.49E-07	8.69	6.142298	3.99	GALNT5///GALNT5	polypeptide N-acetylgalactosaminyltransferase 5///polypeptide N-acetylgalactosaminyltransferase 5
7958913	5.27E-05	8.57E-07	8.68	6.131981	2.11	OAS2	2'-5'-oligoadenylate synthetase 2
7974316	5.28E-05	8.63E-07	8.67	6.125752	1.74	FRMD6	FERM domain containing 6

8085999	5.28E-05	8.63E-07	8.67	6.125409	1.08	CMTM6	CKLF like MARVEL transmembrane domain containing 6
8089372	5.34E-05	8.75E-07	8.66	6.111006	1.47	KIAA1524	KIAA1524
8150698	5.46E-05	9.07E-07	8.64	6.075331	1.99	SNAI2	snail family transcriptional repressor 2
8151816	5.46E-05	9.10E-07	8.63	6.072252	2.05	GEM	GTP binding protein overexpressed in skeletal muscle

7965723	5.50E-05	9.18E-07	8.63	6.062457	1.13	UHRF1BP1L	UHRF1 binding protein 1 like
7958202	5.52E-05	9.24E-07	8.62	6.056409	1.84	CHST11	carbohydrate (chondroitin 4) sulfotransferase 11
8050427	5.54E-05	9.31E-07	8.62	6.048832	1.74	FAM49A	family with sequence similarity 49 member A
8120043	5.59E-05	9.44E-07	8.61	6.034829	1.32	RUNX2	runt related transcription factor 2
7950307	5.62E-05	9.52E-07	8.6	6.026612	1.89	UCP2	uncoupling protein 2

7965166	5.66E-05	9.64E-07	8.59	6.013739	2.12	PPFIA2	PTPRF interacting protein alpha 2
8016832	5.66E-05	9.65E-07	8.59	6.012911	1.71	MMD	monocyte to macrophage differentiation associated
8129497	5.70E-05	9.74E-07	8.58	6.003256	1.26	EPB41L2	erythrocyte membrane protein band 4.1 like 2
8163402	5.77E-05	9.88E-07	8.57	5.989075	1.12	PTBP3	polypyrimidine tract binding protein 3
7995258	5.82E-05	9.98E-07	8.56	5.978816	1.65	ZNF267	zinc finger protein 267

8095680	5.82E-05	1.01E-06	8.56	5.970857	3.04	CXCL8	C-X-C motif chemokine ligand 8
7927964	5.82E-05	1.01E-06	8.56	5.969595	1.92	SRGN	serglycin
7909708	5.83E-05	1.01E-06	8.55	5.966267	2.09	CENPF	centromere protein F
8007100	5.83E-05	1.01E-06	8.55	5.964698	1.28	IGFBP4	insulin like growth factor binding protein 4
8035896	5.85E-05	1.02E-06	8.55	5.958342	1.61	TSHZ3	teashirt zinc finger homeobox 3
7906878	5.90E-05	1.03E-06	8.54	5.947854	2.14	DDR2	discoidin domain receptor tyrosine kinase 2

7908041	5.93E-05	1.03E-06	8.54	5.941882	1.14	LAMC1	laminin subunit gamma 1
8179519	5.99E-05	1.05E-06	8.52	5.928139	2.57	HLA-DPB1	major histocompatibility complex, class II, DP beta 1
8089911	6.02E-05	1.06E-06	8.51	5.914191	2.03	HCLS1	hematopoietic cell-specific Lyn substrate 1
7982889	6.07E-05	1.07E-06	8.51	5.903688	1.2	NUSAP1	nucleolar and spindle associated protein 1
7989335	6.07E-05	1.08E-06	8.51	5.90243	1.12	ANXA2	annexin A2
8146550	6.18E-05	1.10E-06	8.49	5.8799	1.13	SDCBP	syndecan binding protein

8018966	6.18E-05	1.10E-06	8.49	5.878994	1.56	TIMP2	TIMP metallopeptidase inhibitor 2
8113214	6.19E-05	1.11E-06	8.48	5.87475	2.38	GLRX	glutaredoxin
8068305	6.24E-05	1.12E-06	8.48	5.864701	1.18	ITSN1	intersectin 1
8129618	6.29E-05	1.13E-06	8.47	5.852503	2.9	VNN1	vanin 1
7919800	6.30E-05	1.14E-06	8.47	5.848335	2.34	CTSS	cathepsin S
7910001	6.40E-05	1.16E-06	8.45	5.828538	1.11	DEGS1	delta 4-desaturase, sphingolipid 1
8103769	6.44E-05	1.17E-06	8.44	5.819979	2.26	HPGD	hydroxyprostaglandin dehydrogenase 15-(NAD)

8090018	6.44E-05	1.17E-06	8.44	5.817865	1.32	PARP9	poly(ADP-ribose) polymerase family member 9
7958884	6.45E-05	1.17E-06	8.44	5.814382	2.79	OAS1	2'-5'-oligoadenylate synthetase 1
8047419	6.47E-05	1.18E-06	8.43	5.807394	1.01	CASP8	caspase 8
8123315	6.47E-05	1.18E-06	8.43	5.806796	1.07	QKI	QKI, KH domain containing RNA binding
8154836	6.48E-05	1.19E-06	8.43	5.802598	1.01	ANXA2P2	annexin A2 pseudogene 2
7995697	6.52E-05	1.20E-06	8.43	5.794635	1.21	LPCAT2	lysophosphatidylcholine acyltransferase 2

8146934	6.52E-05	1.20E-06	8.42	5.793267	2.24	LY96	lymphocyte antigen 96
8139207	6.59E-05	1.22E-06	8.41	5.778277	3.37	INHBA///INHBA	inhibin beta A subunit///inhibin beta A subunit
7952341	6.71E-05	1.24E-06	8.4	5.757847	1.52	CLMP	CXADR like membrane protein
8055624	6.74E-05	1.25E-06	8.39	5.749121	2.17	ZEB2	zinc finger E-box binding homeobox 2
8047174	6.76E-05	1.26E-06	8.39	5.744602	1.05	SLC39A10	solute carrier family 39 member 10

7982564	6.76E-05	1.26E-06	8.39	5.74095	1.24	SPRED1	sprouty related EVH1 domain containing 1
8132557	6.76E-05	1.27E-06	8.38	5.737317	1.9	AEBP1	AE binding protein 1
8014063	6.88E-05	1.29E-06	8.37	5.715371	1.43	EVI2B	ecotropic viral integration site 2B
8171248	6.88E-05	1.30E-06	8.37	5.71433	2.52	ANOS1	anosmin 1
7989277	6.88E-05	1.30E-06	8.37	5.713837	1.73	MYO1E	myosin IE
7940259	6.93E-05	1.31E-06	8.36	5.704921	2.09	MS4A14///MS4A7	membrane spanning 4-domains A14///membrane spanning 4-domains A7

8180100	7.02E-05	1.33E-06	8.34	5.684708	2.06	HLA-DPA1	major histocompatibility complex, class II, DP alpha 1
8094259	7.02E-05	1.34E-06	8.34	5.681726	1.08	LAP3	leucine aminopeptidase 3
8064790	7.03E-05	1.34E-06	8.34	5.678726	1.71	RASSF2	Ras association domain family member 2
7951686	7.21E-05	1.38E-06	8.32	5.649574	1.59	IL18	interleukin 18
7932966	7.33E-05	1.41E-06	8.3	5.630473	1.13	ITGB1	integrin subunit beta 1
7931914	7.33E-05	1.41E-06	8.3	5.630082	2.33	IL2RA	interleukin 2 receptor subunit alpha

7970602	7.41E-05	1.43E-06	8.29	5.614281	1.07	PARP4	poly(ADP-ribose) polymerase family member 4
8145317	7.49E-05	1.46E-06	8.28	5.5963	2.68	ADAMDEC1	ADAM like decysin 1
7906486	7.49E-05	1.46E-06	8.28	5.591441	1.82	SLAMF8	SLAM family member 8
7938608	7.49E-05	1.47E-06	8.27	5.58887	2.01	SPON1	spondin 1
8122261	7.51E-05	1.47E-06	8.27	5.58512	1.11		
7948455	7.52E-05	1.48E-06	8.27	5.580128	2.31	MS4A6A	membrane spanning 4-domains A6A
7909164	7.56E-05	1.49E-06	8.26	5.571024	4.13	CTSE	cathepsin E
8058857	7.62E-05	1.51E-06	8.25	5.560104	3.1	IGFBP5	insulin like growth factor binding protein 5

8009075	7.70E-05	1.53E-06	8.24	5.546093	1.1	TANC2	tetratricopeptide repeat, ankyrin repeat and coiled-coil containing 2
7909371	7.70E-05	1.53E-06	8.24	5.546063	1.87	CR1	complement component 3b/4b receptor 1 (Knops blood group)

8178826	7.77E-05	1.55E-06	8.23	5.530941	3	HLA-DQB2///<br>HLA-DQB1	major histocompatibility complex, class II, DQ beta 2///major histocompatibility complex, class II, DQ beta 1
8180022	7.77E-05	1.55E-06	8.23	5.530941	3	HLA-DQB2///<br>HLA-DQB1	major histocompatibility complex, class II, DQ beta 2///major histocompatibility complex, class II, DQ beta 1

8048898	7.92E-05	1.60E-06	8.21	5.503046	2.16	SP140	SP140 nuclear body protein
7974689	7.94E-05	1.61E-06	8.21	5.497244	1.14	DACT1	dishevelled binding antagonist of beta catenin 1
7917516	7.97E-05	1.61E-06	8.2	5.491433	2.17	GBP1P1///GBP3///GBP1	guanylate binding protein 1 pseudogene 1///guanylate binding protein 3///guanylate binding protein 1

8083941	8.03E-05	1.63E-06	8.2	5.48264	2.31	ECT2	epithelial cell transforming 2
8039928	8.06E-05	1.64E-06	8.19	5.476127	1.32	FAM72A///FAM72D///FAM72B	family with sequence similarity 72 member A///family with sequence similarity 72 member D///family with sequence similarity 72 member B
8040365	8.14E-05	1.66E-06	8.18	5.463338	1.14	TRIB2	tribbles pseudokinase 2

7940237	8.26E-05	1.69E-06	8.17	5.442538	2.83	MS4A4A	membrane spanning 4-domains A4A
8107307	8.29E-05	1.70E-06	8.16	5.4373	2.04	CAMK4	calcium/calmodulin dependent protein kinase IV
7913169	8.37E-05	1.73E-06	8.15	5.424157	1.46	CAPZB	capping actin protein of muscle Z-line beta subunit
7950906	8.43E-05	1.74E-06	8.15	5.415879	1.54	CTSC	cathepsin C
7923426	8.52E-05	1.76E-06	8.14	5.403029	2.13	UBE2T	ubiquitin conjugating enzyme E2 T

7921868	8.53E-05	1.77E-06	8.14	5.400414	3.12	FCGR3A	Fc fragment of IgG receptor IIIa
7916584	8.56E-05	1.78E-06	8.13	5.392776	1.1	TACSTD2	tumor-associated calcium signal transducer 2
8156826	8.57E-05	1.78E-06	8.13	5.390374	1.63	TGFBR1	transforming growth factor beta receptor 1
8008646	8.69E-05	1.82E-06	8.12	5.372549	1.17	SCPEP1	serine carboxypeptidase 1
8123744	8.74E-05	1.83E-06	8.11	5.365871	2.87	F13A1	coagulation factor XIII A chain

8014066	8.82E-05	1.85E-06	8.1	5.352795	2.48	EVI2A	ecotropic viral integration site 2A
8083876	8.82E-05	1.85E-06	8.1	5.352674	1.52	SKIL	SKI-like proto-oncogene
7945245	9.03E-05	1.91E-06	8.08	5.320823	1.91	NTM	neurotrimin
8057620	9.19E-05	1.95E-06	8.06	5.298381	2.1	COL5A2	collagen type V alpha 2 chain
7906061	9.23E-05	1.97E-06	8.06	5.29057	1.21	SYT11	synaptotagmin 11
8059680	9.33E-05	2.00E-06	8.05	5.275894	1.93	HTR2B	5-hydroxytryptamine receptor 2B
7961654	9.38E-05	2.01E-06	8.04	5.269332	1.07	RECQL	RecQ like helicase

8100870	9.38E-05	2.02E-06	8.04	5.266275	1.2	ADAMTS3	ADAM metallopeptidase with thrombospondin type 1 motif 3
8160346	9.38E-05	2.02E-06	8.04	5.266077	1.86	HACD4	3-hydroxyacyl-CoA dehydratase 4
8138289	9.43E-05	2.04E-06	8.03	5.25575	2.19	ETV1	ETS variant 1
8102076	9.53E-05	2.06E-06	8.02	5.242645	1.27	CENPE	centromere protein E
8046086	9.61E-05	2.08E-06	8.01	5.232642	1.1	CERS6	ceramide synthase 6
8117476	9.78E-05	2.14E-06	8	5.20762	1.42	BTN3A3	butyrophilin subfamily 3 member A3

8152988	9.81E-05	2.15E-06	7.99	5.201304	1.83	SLA	Src-like-adaptor
8014241	9.83E-05	2.16E-06	7.99	5.197396	1.78	SLFN12	schlafen family member 12
8065359	9.83E-05	2.16E-06	7.99	5.196003	1.36	CD93	CD93 molecule
8025601	9.87E-05	2.18E-06	7.98	5.188345	1.33	ICAM1	intercellular adhesion molecule 1
7963046	9.89E-05	2.19E-06	7.98	5.183963	1.47	TUBA1B	tubulin alpha 1b
8085984	9.89E-05	2.19E-06	7.98	5.181998	1.55	OSBPL10	oxysterol binding protein like 10
8121257	9.89E-05	2.20E-06	7.98	5.179359	2.74	PRDM1	PR/SET domain 1
8097288	9.93E-05	2.21E-06	7.97	5.173955	1.48	FAT4	FAT atypical cadherin 4

7938225	9.97E-05	2.22E-06	7.97	5.168054	2.11	OLFML1	olfactomedin like 1
8017599	9.97E-05	2.22E-06	7.97	5.166189	1.53	PECAM1	platelet and endothelial cell adhesion molecule 1
8127234	9.97E-05	2.23E-06	7.96	5.161932	1.04	DST	dystonin
7932530	1.02E-04	2.30E-06	7.94	5.131966	1.57	PIP4K2A	phosphatidylinositol-5-phosphate 4-kinase type 2 alpha
7954065	1.03E-04	2.34E-06	7.93	5.116936	3.35	GPRC5A	G protein-coupled receptor class C group 5 member A

7931353	1.04E-04	2.35E-06	7.93	5.110711	1.47	PTPRE	protein tyrosine phosphatase, receptor type E
8095854	1.05E-04	2.39E-06	7.92	5.09554	1.02	SEPT11	septin 11
8176026	1.05E-04	2.40E-06	7.91	5.089989	1.73	FLNA	filamin A
8095080	1.06E-04	2.44E-06	7.9	5.074312	1.95	PDGFRA	platelet derived growth factor receptor alpha
7943349	1.08E-04	2.49E-06	7.89	5.051821	2.11	ARHGAP42	Rho GTPase activating protein 42
7925525	1.09E-04	2.50E-06	7.88	5.046237	1.83	CEP170	centrosomal protein 170
8036710	1.09E-04	2.53E-06	7.88	5.037207	2.41	GMFG	glia maturation factor gamma

7996393	1.09E-04	2.53E-06	7.88	5.037003	1.03	CBFB	core-binding factor beta subunit
8125512	1.09E-04	2.54E-06	7.87	5.032175	1.14	TAP1	transporter 1, ATP binding cassette subfamily B member
8178867	1.09E-04	2.54E-06	7.87	5.032175	1.14	TAP1	transporter 1, ATP binding cassette subfamily B member
8180061	1.09E-04	2.54E-06	7.87	5.032175	1.14	TAP1	transporter 1, ATP binding cassette subfamily B member

7968344	1.12E-04	2.61E-06	7.85	5.004131	3.07	ALOX5AP	arachidonate 5-lipoxygenase activating protein
7981514	1.13E-04	2.65E-06	7.84	4.988335	1.92	AHNAK2	AHNAK nucleoprotein 2
7957023	1.13E-04	2.65E-06	7.84	4.987198	2.66	LYZ	lysozyme
8057486	1.14E-04	2.70E-06	7.83	4.969434	2.06	PDE1A	phosphodiesterase 1A
7922773	1.15E-04	2.73E-06	7.82	4.958627	2.33	NCF2	neutrophil cytosolic factor 2
8117435	1.16E-04	2.75E-06	7.82	4.952224	1.3	BTN3A2	butyrophilin subfamily 3 member A2
8137250	1.16E-04	2.75E-06	7.82	4.951802	2.38	GIMAP2	GTPase, IMAP family member 2

8149071	1.16E-04	2.76E-06	7.81	4.945735	1.99	ANGPT2	angiopoietin 2
7927786	1.16E-04	2.78E-06	7.81	4.938594	1.23	REEP3	receptor accessory protein 3
7927146	1.17E-04	2.80E-06	7.8	4.932262	1.52	CSGALNACT2	chondroitin sulfate N-acetylgalactosaminyltransferase 2
8043363	1.17E-04	2.80E-06	7.8	4.931104	1.72	MIR4435-2HG///LINC00152	MIR4435-2 host gene///long intergenic non-protein coding RNA 152

8122365	1.17E-04	2.81E-06	7.8	4.929111	1.33	ADGRG6	adhesion G protein-coupled receptor G6
7979400	1.17E-04	2.82E-06	7.8	4.925636	1.54	C14orf37	chromosome 14 open reading frame 37
8111892	1.18E-04	2.83E-06	7.79	4.920205	1.21	OXCT1	3-oxoacid CoA-transferase 1
8104663	1.18E-04	2.84E-06	7.79	4.916768	1.84	CDH6	cadherin 6
8070194	1.18E-04	2.85E-06	7.79	4.915571	1.44	RUNX1	runt related transcription factor 1
7898057	1.18E-04	2.85E-06	7.79	4.913877	1.76	PDPN	podoplanin

8126839	1.18E-04	2.86E-06	7.79	4.911527	1.46	TNFRSF21	TNF receptor superfamily member 21
8120967	1.19E-04	2.89E-06	7.78	4.901057	2.41	NT5E	5'-nucleotidase ecto
8082035	1.19E-04	2.90E-06	7.78	4.896119	2.67	CD86	CD86 molecule
8066493	1.21E-04	2.93E-06	7.77	4.885114	2.58	SLPI	secretory leukocyte peptidase inhibitor
7978776	1.21E-04	2.94E-06	7.77	4.883113	1.01	MIS18BP1	MIS18 binding protein 1
8169504	1.21E-04	2.94E-06	7.77	4.88239	4.02	SLC6A14	solute carrier family 6 member 14

8049544	1.21E-04	2.96E-06	7.76	4.875092	1.35	LRRFIP1	LRR binding FLII interacting protein 1
7898988	1.24E-04	3.06E-06	7.74	4.840741	1.05	CLIC4	chloride intracellular channel 4
7945132	1.25E-04	3.09E-06	7.73	4.831395	1.35	FLI1	Fli-1 proto-oncogene, ETS transcription factor
8156538	1.26E-04	3.15E-06	7.72	4.813014	1.15	MFSD14B	major facilitator superfamily domain containing 14B
8062908	1.27E-04	3.16E-06	7.72	4.808597	1.42	STK4	serine/threonine kinase 4

8112376	1.28E-04	3.20E-06	7.71	4.797453	1.61	CENPK	centromere protein K
8105077	1.29E-04	3.23E-06	7.7	4.787033	1.25	CARD6	caspase recruitment domain family member 6
8138231	1.30E-04	3.28E-06	7.69	4.773132	1.49	THSD7A	thrombospondin type 1 domain containing 7A
7905329	1.30E-04	3.28E-06	7.69	4.771111	1.57	MLLT11	myeloid/lymphoid or mixed-lineage leukemia; translocated to, 11
8151101	1.31E-04	3.30E-06	7.69	4.765042	1.88	MYBL1	MYB proto-oncogene like 1

7923043	1.33E-04	3.37E-06	7.67	4.742918	2.2	KCNT2	potassium sodium-activated channel subfamily T member 2
8055688	1.35E-04	3.42E-06	7.66	4.727943	2.25	RND3	Rho family GTPase 3
8147000	1.36E-04	3.45E-06	7.65	4.720177	1.88	ZFHX4	zinc finger homeobox 4
8105348	1.37E-04	3.48E-06	7.65	4.711838	1.98	GPX8	glutathione peroxidase 8 (putative)
8174692	1.37E-04	3.48E-06	7.65	4.711442	1.68	SEPT6	septin 6

8054611	1.38E-04	3.53E-06	7.64	4.695735	1.74	MIR4435-2HG ///LINC00152	MIR4435-2 host gene///long intergenic non-protein coding RNA 152
8139033	1.38E-04	3.55E-06	7.63	4.692079	2.69	AOAH	acyloxyacyl hydrolase
8163896	1.39E-04	3.57E-06	7.63	4.685898	1.14	STOM	stomatin
8142452	1.40E-04	3.61E-06	7.62	4.674039	2.66	TFEC	transcription factor EC
8069541	1.40E-04	3.62E-06	7.62	4.670942	2.63	SAMSN1	SAM domain, SH3 domain and nuclear localization signals 1
7896700	1.41E-04	3.65E-06	7.61	4.663625	2.8		

8047538	1.47E-04	3.82E-06	7.58	4.617217	1.15	BMPR2	bone morphogenetic protein receptor type 2
7949588	1.49E-04	3.90E-06	7.57	4.595187	1.65	CD248	CD248 molecule
7987145	1.49E-04	3.92E-06	7.56	4.591403	1.63	FMN1	formin 1
8113504	1.50E-04	3.93E-06	7.56	4.589042	1.77	NREP	neuronal regeneration related protein
7982757	1.50E-04	3.93E-06	7.56	4.587333	1.51	KNL1	kinetochore scaffold 1
8016094	1.51E-04	3.98E-06	7.55	4.574893	1.59	GJC1	gap junction protein gamma 1
8129590	1.52E-04	4.00E-06	7.55	4.570126	1.02	STX7	syntaxin 7
7994131	1.52E-04	4.01E-06	7.55	4.567917	1.72	PRKCB	protein kinase C beta

8151756	1.52E-04	4.04E-06	7.54	4.560952	1.4	TMEM55A	transmembrane protein 55A
8116734	1.52E-04	4.04E-06	7.54	4.558722	2.01	LY86	lymphocyte antigen 86
8048139	1.52E-04	4.06E-06	7.54	4.555309	1.88	DNAPTP3	histone demethylase UTY-like
8088952	1.52E-04	4.06E-06	7.54	4.55458	1.52		
7979307	1.54E-04	4.11E-06	7.53	4.541688	1.85	DLGAP5	DLG associated protein 5
8171297	1.54E-04	4.12E-06	7.53	4.539432	1.06	MID1	midline 1
8040080	1.55E-04	4.16E-06	7.52	4.530775	2.16	RSAD2	radical S-adenosyl methionine domain containing 2
8122807	1.59E-04	4.27E-06	7.51	4.5041	1.62	AKAP12	A-kinase anchoring protein 12

7961142	1.60E-04	4.32E-06	7.5	4.490646	3.78	OLR1	oxidized low density lipoprotein receptor 1
7984112	1.60E-04	4.33E-06	7.49	4.489108	1.74	RAB8B	RAB8B, member RAS oncogene family
8049512	1.61E-04	4.37E-06	7.49	4.479534	1.1	LRRFIP1	LRR binding FLII interacting protein 1
8099340	1.61E-04	4.37E-06	7.49	4.479088	1.22	WDR1	WD repeat domain 1

8125556	1.61E-04	4.38E-06	7.49	4.477242	2.08	HLA-DPA1	major histocompatibility complex, class II, DP alpha 1
8178891	1.61E-04	4.38E-06	7.49	4.477242	2.08	HLA-DPA1	major histocompatibility complex, class II, DP alpha 1
7973067	1.62E-04	4.40E-06	7.48	4.473261	1.84	PNP	purine nucleoside phosphorylase
7961120	1.62E-04	4.43E-06	7.48	4.466217	1.85	CLEC7A	C-type lectin domain family 7 member A

7930162	1.63E-04	4.47E-06	7.47	4.45777	1.12	WBP1L	WW domain binding protein 1-like
8145470	1.63E-04	4.47E-06	7.47	4.457118	1.16	DPYSL2	dihydropyrimidinase like 2
7900426	1.65E-04	4.52E-06	7.47	4.446003	1.18	SMAP2	small ArfGAP2
8142345	1.65E-04	4.52E-06	7.46	4.444889	1.23	DOCK4	dedicator of cytokinesis 4
8041179	1.66E-04	4.58E-06	7.46	4.431464	1.17	CLIP4	CAP-Gly domain containing linker protein family member 4

8080810	1.67E-04	4.60E-06	7.45	4.427839	1.01	PTPRG	protein tyrosine phosphatase, receptor type G
7953878	1.67E-04	4.62E-06	7.45	4.422841	1.62	CLEC2D	C-type lectin domain family 2 member D
8063078	1.69E-04	4.68E-06	7.44	4.409211	1.18	CTSA	cathepsin A
8103389	1.70E-04	4.74E-06	7.43	4.397279	1.03	CTSO	cathepsin O
7982377	1.70E-04	4.74E-06	7.43	4.396477	3.16	GREM1	gremlin 1, DAN family BMP antagonist
7946228	1.72E-04	4.80E-06	7.42	4.383981	1.13	TPP1	tripeptidyl peptidase 1

8121277	1.72E-04	4.81E-06	7.42	4.383183	1.03	AIM1	absent in melanoma 1
8094625	1.72E-04	4.84E-06	7.42	4.376818	1.09	KLHL5	kelch like family member 5
8040113	1.72E-04	4.85E-06	7.42	4.374416	1.19	ASAP2	ArfGAP with SH3 domain, ankyrin repeat and PH domain 2
7965410	1.72E-04	4.86E-06	7.41	4.370868	1.6	DCN	decorin
8152845	1.73E-04	4.87E-06	7.41	4.368571	1.13	FAM49B	family with sequence similarity 49 member B
7927732	1.74E-04	4.92E-06	7.41	4.358344	1.39	ARID5B	AT-rich interaction domain 5B

8057578	1.74E-04	4.94E-06	7.4	4.355245	2.13	CALCRL	calcitonin receptor like receptor
7963786	1.75E-04	4.97E-06	7.4	4.348944	1.33	ITGA5	integrin subunit alpha 5
7969438	1.77E-04	5.03E-06	7.39	4.335896	1.09	LMO7	LIM domain 7
8178598	1.78E-04	5.08E-06	7.38	4.327216	1.44	CLIC1	chloride intracellular channel 1
8179351	1.79E-04	5.12E-06	7.38	4.319151	1.92	CFB	complement factor B
8089261	1.79E-04	5.12E-06	7.38	4.319014	1.31	CBLB	Cbl proto-oncogene B

7957478	1.79E-04	5.12E-06	7.38	4.318475	1.1	TMTC3	transmembrane and tetratricopeptide repeat containing 3
8066925	1.79E-04	5.14E-06	7.38	4.314875	2.71	PTGIS	prostaglandin I2 (prostacyclin) synthase
7938777	1.81E-04	5.21E-06	7.37	4.300434	1.21	LDHA	lactate dehydrogenase A
8142194	1.82E-04	5.26E-06	7.36	4.290591	1.25	LAMB1	laminin subunit beta 1
7975799	1.82E-04	5.28E-06	7.36	4.288005	1.2	FLVCR2	feline leukemia virus subgroup C cellular receptor family member 2

8105040	1.85E-04	5.35E-06	7.35	4.273419	2.13	OSMR	oncostatin M receptor
8010983	1.85E-04	5.37E-06	7.35	4.270256	1.27	ABR	active BCR-related
7956795	1.85E-04	5.40E-06	7.34	4.264519	1.01	TBK1	TANK binding kinase 1
8131475	1.86E-04	5.43E-06	7.34	4.259232	1.64	C1GALT1	core 1 synthase, glycoprotein-N-acetylgalactosamine 3-beta-galactosyltransferase 1
7896718	1.89E-04	5.55E-06	7.32	4.237117	2.78		

7966202	1.90E-04	5.58E-06	7.32	4.231242	1.13	KCTD10	potassium channel tetramerization domain containing 10
7958644	1.90E-04	5.59E-06	7.32	4.228685	1	ATP2A2	ATPase sarcoplasmic/endoplasmic reticulum Ca2+ transporting 2
8137264	1.93E-04	5.69E-06	7.31	4.210674	2.03	TMEM176A	transmembrane protein 176A
8059279	1.94E-04	5.72E-06	7.3	4.206266	1.66	EPHA4	EPH receptor A4
8003068	1.95E-04	5.78E-06	7.3	4.195972	1.24	MPHOSPH6	M-phase phosphoprotein 6

8022612	1.98E-04	5.89E-06	7.28	4.175574	2.14	ZNF521	zinc finger protein 521
8125059	1.99E-04	5.91E-06	7.28	4.171897	1.38	CLIC1	chloride intracellular channel 1
8051583	2.02E-04	6.04E-06	7.27	4.150569	3.06	CYP1B1	cytochrome P450 family 1 subfamily B member 1
8084742	2.03E-04	6.09E-06	7.26	4.142351	1.16	LPP	LIM domain containing preferred translocation partner in lipoma
8179827	2.05E-04	6.15E-06	7.25	4.131619	1.43	CLIC1	chloride intracellular channel 1

8012906	2.06E-04	6.19E-06	7.25	4.125235	1.91		
8063000	2.10E-04	6.36E-06	7.23	4.097841	1.04	WFDC2	WAP four-disulfide core domain 2
7970954	2.10E-04	6.39E-06	7.23	4.093581	1.66	DCLK1	doublecortin like kinase 1
7915184	2.12E-04	6.46E-06	7.22	4.081642	1.64	RHBDL2	rhomboid like 2
8067985	2.14E-04	6.55E-06	7.21	4.067387	1.39	NCAM2	neural cell adhesion molecule 2
8079950	2.16E-04	6.64E-06	7.2	4.054083	1.26	GNAI2	G protein subunit alpha i2
7969243	2.16E-04	6.66E-06	7.2	4.051136	1.6	CKAP2	cytoskeleton associated protein 2

8056201	2.16E-04	6.68E-06	7.2	4.046986	1.5	RBMS1	RNA binding motif single stranded interacting protein 1

8055151	2.19E-04	6.80E-06	7.19	4.029302	1.38	POTEF///POTEJ///POTEM///POTEE///POTEKP	POTE ankyrin domain family member F///POTE ankyrin domain family member J///POTE ankyrin domain family member M///POTE ankyrin domain family member E///POTE ankyrin domain family member K, pseudogene

8101762	2.19E-04	6.84E-06	7.18	4.02424	1.03	SNCA	synuclein alpha
8166906	2.21E-04	6.91E-06	7.17	4.013466	2.33	GPR34	G protein-coupled receptor 34
7953211	2.21E-04	6.91E-06	7.17	4.012485	1.09	TIGAR	TP53 induced glycolysis regulatory phosphatase
7942957	2.25E-04	7.03E-06	7.16	3.995401	1.58	PRSS23	protease, serine 23
8112980	2.27E-04	7.11E-06	7.16	3.983864	3.17	EDIL3	EGF like repeats and discoidin domains 3

7906767	2.30E-04	7.24E-06	7.14	3.965297	2.3	FCGR2C///FCGR2B	Fc fragment of IgG receptor IIc (gene/pseudogene)///Fc fragment of IgG receptor IIb
7926410	2.31E-04	7.29E-06	7.14	3.958702	2.91	MRC1	mannose receptor, C type 1
7926451	2.31E-04	7.29E-06	7.14	3.958702	2.91	MRC1	mannose receptor, C type 1
7979085	2.31E-04	7.29E-06	7.14	3.958021	1.6	PYGL	phosphorylase, glycogen, liver
8068713	2.34E-04	7.43E-06	7.13	3.939487	1.91	MX1	MX dynamin like GTPase 1

8152522	2.35E-04	7.47E-06	7.12	3.933807	1.6	ENPP2	ectonucleotide pyrophosphatase/phosphodiesterase 2
7940775	2.36E-04	7.50E-06	7.12	3.929006	2.54	RARRES3///RARRES3	retinoic acid receptor responder 3///retinoic acid receptor responder 3
7908694	2.39E-04	7.60E-06	7.11	3.915641	1.03	NAV1	neuron navigator 1
7962250	2.40E-04	7.68E-06	7.1	3.904883	1.15	CPNE8	copine 8

7919591	2.40E-04	7.69E-06	7.1	3.90428	2.29	FAM72A///FAM72D///FAM72B	family with sequence similarity 72 member A///family with sequence similarity 72 member D///family with sequence similarity 72 member B
8002303	2.45E-04	7.91E-06	7.08	3.875976	2.75	NQO1	NAD(P)H quinone dehydrogenase 1
8090433	2.46E-04	7.93E-06	7.08	3.872641	1.32	MGLL	monoglyceride lipase

8118564	2.46E-04	7.95E-06	7.08	3.87045	2.25	HLA-DQA2///<br>HLA-DQA1	major histocompatibility complex, class II, DQ alpha 2///major histocompatibility complex, class II, DQ alpha 1
8057394	2.46E-04	7.95E-06	7.08	3.869998	1.08	SESTD1	SEC14 and spectrin domain containing 1
8085665	2.46E-04	7.96E-06	7.08	3.868983	1.1	RFTN1	raftlin, lipid raft linker 1
8073775	2.47E-04	8.03E-06	7.07	3.859858	1.46	FBLN1	fibulin 1

8124307	2.48E-04	8.09E-06	7.07	3.851907	1.79	CMAHP	cytidine monophospho-N-acetylneuraminic acid hydroxylase, pseudogene
8161044	2.50E-04	8.18E-06	7.06	3.841092	1.99	TPM2	tropomyosin 2 (beta)
7942135	2.51E-04	8.23E-06	7.06	3.834701	1.28	ANO1	anoctamin 1
7898805	2.51E-04	8.24E-06	7.06	3.833351	2.1	C1QB	complement C1q B chain
8099841	2.51E-04	8.27E-06	7.05	3.830394	1.74	TLR6	toll like receptor 6

8178205	2.57E-04	8.53E-06	7.03	3.798872	2.25	HLA-DQA2/// HLA-DQA1	major histocompatibility complex, class II, DQ alpha 2///major histocompatibility complex, class II, DQ alpha 1
8095187	2.58E-04	8.55E-06	7.03	3.796558	1.28	CEP135	centrosomal protein 135
8140463	2.59E-04	8.63E-06	7.03	3.786677	1.89	FGL2	fibrinogen like 2
7971150	2.60E-04	8.67E-06	7.02	3.78244	1.14	LHFP	lipoma HMGIC fusion partner
7922669	2.60E-04	8.67E-06	7.02	3.781558	1.13	STX6	syntaxin 6
7921625	2.60E-04	8.68E-06	7.02	3.780222	1.7	SLAMF6	SLAM family member 6

7918593	2.61E-04	8.71E-06	7.02	3.77754	1.07	RHOC	ras homolog family member C
8043468	2.66E-04	9.00E-06	7	3.744201	3.57	IGKC	immunoglobulin kappa constant
8114920	2.66E-04	9.01E-06	7	3.742117	1.83	DPYSL3	dihydropyrimidinase like 3
8152976	2.66E-04	9.02E-06	7	3.741103	1.6	TMEM71	transmembrane protein 71
8135594	2.70E-04	9.20E-06	6.98	3.721855	1.39	CAV1	caveolin 1
7898799	2.73E-04	9.30E-06	6.98	3.709833	1.98	C1QC	complement C1q C chain
8059854	2.75E-04	9.44E-06	6.97	3.69527	1.7	ARL4C	ADP ribosylation factor like GTPase 4C

8122554	2.77E-04	9.52E-06	6.96	3.686116	1.32	RAB32	RAB32, member RAS oncogene family
8112428	2.79E-04	9.59E-06	6.96	3.679499	2.31	CD180	CD180 molecule
8094278	2.79E-04	9.61E-06	6.95	3.676839	1.28	NCAPG	non-SMC condensin I complex subunit G
8103508	2.79E-04	9.65E-06	6.95	3.672469	1.19	1-Mar	membrane associated ring-CH-type finger 1
8084794	2.81E-04	9.74E-06	6.94	3.663055	2.4	IL1RAP	interleukin 1 receptor accessory protein
7917954	2.81E-04	9.75E-06	6.94	3.661787	1.42	FRRS1	ferric chelate reductase 1

7903980	2.81E-04	9.77E-06	6.94	3.660083	1.85	C1orf162	chromosome 1 open reading frame 162
8154178	2.81E-04	9.77E-06	6.94	3.65982	1.14	JAK2	Janus kinase 2
7899160	2.81E-04	9.79E-06	6.94	3.658138	2.47	CD52	CD52 molecule
8137244	2.81E-04	9.80E-06	6.94	3.657392	2.23	GIMAP4	GTPase, IMAP family member 4
8163383	2.83E-04	9.89E-06	6.93	3.64787	1.22	SUSD1	sushi domain containing 1
7910950	2.84E-04	9.95E-06	6.93	3.641773	1.69	KMO	kynurenine 3-monooxygenase (kynurenine 3-hydroxylase)

7977786	2.84E-04	9.97E-06	6.93	3.639437	1.66	SLC7A7	solute carrier family 7 member 7
8053417	2.90E-04	1.02E-05	6.91	3.612348	1.78	CAPG	capping actin protein, gelsolin like
8178095	2.90E-04	1.03E-05	6.91	3.610418	2.47	C2	complement component 2
8179331	2.90E-04	1.03E-05	6.91	3.610418	2.47	C2	complement component 2
7940028	2.90E-04	1.03E-05	6.91	3.610261	1.33	SERPING1	serpin family G member 1
8096635	2.91E-04	1.03E-05	6.91	3.604411	1.02	NFKB1	nuclear factor kappa B subunit 1
7955613	2.92E-04	1.04E-05	6.9	3.599212	2.1	KRT7	keratin 7
7896724	2.92E-04	1.04E-05	6.9	3.59852	3.15		

8096004	2.92E-04	1.04E-05	6.9	3.598209	1.14	BMP2K	BMP2 inducible kinase
8046895	2.92E-04	1.04E-05	6.9	3.597052	1.26	FAM171B	family with sequence similarity 171 member B
8175393	2.93E-04	1.04E-05	6.9	3.59331	1.82	ARHGEF6	Rac/Cdc42 guanine nucleotide exchange factor 6
8121685	2.95E-04	1.05E-05	6.89	3.582795	1.15	DCBLD1	discoidin, CUB and LCCL domain containing 1
7952022	2.98E-04	1.07E-05	6.88	3.569395	2.64	JAML	junction adhesion molecule like

7983940	2.99E-04	1.08E-05	6.88	3.561634	1.2	FAM63B	family with sequence similarity 63 member B
7990839	3.02E-04	1.09E-05	6.87	3.548226	1.09	STARD5	StAR related lipid transfer domain containing 5
8072735	3.04E-04	1.10E-05	6.87	3.539843	2.56	APOL1	apolipoprotein L1
8102950	3.04E-04	1.10E-05	6.86	3.53851	1.43	INPP4B	inositol polyphosphate-4-phosphatase type II B
8045563	3.10E-04	1.13E-05	6.85	3.512775	2.27	ARHGAP15	Rho GTPase activating protein 15

7990632	3.10E-04	1.13E-05	6.85	3.510813	1.46	PEAK1	pseudopodium enriched atypical kinase 1
8140579	3.15E-04	1.15E-05	6.83	3.489374	2.12	CACNA2D1	calcium voltage-gated channel auxiliary subunit alpha2delta 1
8145293	3.15E-04	1.16E-05	6.83	3.486367	2.12	ADAM28	ADAM metallopeptidase domain 28
8167973	3.16E-04	1.16E-05	6.83	3.483248	1.47	HEPH	hephaestin
8113433	3.16E-04	1.17E-05	6.83	3.478836	1.53	EFNA5	ephrin A5
7914603	3.19E-04	1.18E-05	6.82	3.468569	1.32	RNF19B	ring finger protein 19B

7906757	3.25E-04	1.21E-05	6.8	3.441045	2.89	FCGR2A	Fc fragment of IgG receptor IIa
8101118	3.25E-04	1.21E-05	6.8	3.43962	2.38	CXCL9	C-X-C motif chemokine ligand 9
8084732	3.26E-04	1.22E-05	6.8	3.431885	1.33	RTP4	receptor transporter protein 4
8118324	3.29E-04	1.23E-05	6.79	3.42215	2.36	C2	complement component 2
8174474	3.32E-04	1.25E-05	6.78	3.411787	1.52	ACSL4	acyl-CoA synthetase long-chain family member 4

7961059	3.32E-04	1.25E-05	6.78	3.411236	2.04	KLRB1	killer cell lectin like receptor B1
8023043	3.33E-04	1.25E-05	6.78	3.408316	1.38	PSTPIP2	proline-serine-threonine phosphatase interacting protein 2
8101780	3.33E-04	1.26E-05	6.78	3.404366	2.4	HPGDS	hematopoietic prostaglandin D synthase
7957665	3.34E-04	1.27E-05	6.77	3.395621	1.67	ELK3	ELK3, ETS transcription factor

7948229	3.35E-04	1.27E-05	6.77	3.393613	1.41	SLC43A3///PRG2	solute carrier family 43 member 3///proteoglycan 2, pro eosinophil major basic protein
8016646	3.35E-04	1.27E-05	6.77	3.391333	1.69	COL1A1	collagen type I alpha 1 chain
7954717	3.36E-04	1.27E-05	6.77	3.388925	1.25	BICD1	BICD cargo adaptor 1
8068280	3.37E-04	1.28E-05	6.76	3.38269	1.06	IFNGR2	interferon gamma receptor 2 (interferon gamma transducer 1)

8043431	3.39E-04	1.30E-05	6.76	3.372245	3.93	IGKC	immunoglobulin kappa constant
8106730	3.44E-04	1.32E-05	6.75	3.3538	1.21	XRCC4	X-ray repair cross complementing 4
7978595	3.44E-04	1.32E-05	6.75	3.35275	1.07	BAZ1A	bromodomain adjacent to zinc finger domain 1A
8057045	3.45E-04	1.33E-05	6.74	3.347229	1.55	FKBP7	FK506 binding protein 7
7934920	3.46E-04	1.33E-05	6.74	3.344581	1.39	LIPA	lipase A, lysosomal acid type
8115814	3.47E-04	1.34E-05	6.74	3.340437	1.26	SH3PXD2B	SH3 and PX domains 2B

8024572	3.47E-04	1.34E-05	6.74	3.338828	1.02	GNA15	G protein subunit alpha 15
8029098	3.53E-04	1.37E-05	6.72	3.316915	3.93	CEACAM6	carcinoembryonic antigen related cell adhesion molecule 6
7979281	3.53E-04	1.37E-05	6.72	3.315697	1.37	WDHD1	WD repeat and HMG-box DNA binding protein 1
7927215	3.65E-04	1.43E-05	6.69	3.274059	1.13	ALOX5	arachidonate 5-lipoxygenase

7917182	3.66E-04	1.44E-05	6.69	3.26723	2.21	ADGRL4	adhesion G protein-coupled receptor L4
7951408	3.68E-04	1.44E-05	6.69	3.260902	1.9	CARD16	caspase recruitment domain family member 16
8077899	3.69E-04	1.45E-05	6.68	3.255107	2.2	PPARG	peroxisome proliferator activated receptor gamma
8084717	3.70E-04	1.46E-05	6.68	3.25039	2.3	ST6GAL1	ST6 beta-galactoside alpha-2,6-sialyltransferase 1

7982597	3.74E-04	1.48E-05	6.67	3.239397	2.14	THBS1	thrombospondin 1
8070297	3.77E-04	1.49E-05	6.67	3.228969	1.66	ERG	ERG, ETS transcription factor
7932867	3.80E-04	1.51E-05	6.66	3.218776	1.07	ZNF438///ZNF 438	zinc finger protein 438///zinc finger protein 438
8166469	3.80E-04	1.51E-05	6.66	3.215206	1.24	SAT1	spermidine/spermine N1-acetyltransferase 1
7907135	3.85E-04	1.53E-05	6.65	3.199324	1	SFT2D2	SFT2 domain containing 2
8157524	3.86E-04	1.54E-05	6.65	3.195782	2.23	TLR4	toll like receptor 4

8060940	3.92E-04	1.57E-05	6.63	3.174969	2.41	LAMP5	lysosomal associated membrane protein family member 5
8111387	3.94E-04	1.59E-05	6.63	3.164711	1.81	ADAMTS12	ADAM metallopeptidase with thrombospondin type 1 motif 12
7963289	3.96E-04	1.59E-05	6.62	3.160274	1.46	BIN2	bridging integrator 2

8017210	3.96E-04	1.60E-05	6.62	3.158455	1.04	LOC653653///AP1S2	adaptor related protein complex 1 sigma 2 subunit pseudogene///adaptor related protein complex 1 sigma 2 subunit
8072876	3.97E-04	1.60E-05	6.62	3.154824	2.28	LGALS1	galectin 1
7928444	3.99E-04	1.62E-05	6.61	3.145391	1.06	VCL	vinculin
8094228	4.02E-04	1.63E-05	6.61	3.134781	1.04	BST1	bone marrow stromal cell antigen 1
8129861	4.03E-04	1.64E-05	6.6	3.131018	1.08	IFNGR1	interferon gamma receptor 1
7990151	4.03E-04	1.64E-05	6.6	3.129998	1.6	PKM	pyruvate kinase, muscle

8100318	4.13E-04	1.70E-05	6.58	3.096968	1.22	SGCB	sarcoglycan beta
7943369	4.14E-04	1.71E-05	6.58	3.090556	1.93	TMEM133	transmembrane protein 133
8081235	4.16E-04	1.72E-05	6.57	3.082359	1.5	COL8A1	collagen type VIII alpha 1 chain
8047161	4.17E-04	1.73E-05	6.57	3.079396	1.57	NABP1	nucleic acid binding protein 1
7914342	4.19E-04	1.73E-05	6.57	3.074309	2.19	FABP3	fatty acid binding protein 3
8000184	4.19E-04	1.74E-05	6.57	3.073013	2.31	IGSF6	immunoglobulin superfamily member 6
7915733	4.24E-04	1.76E-05	6.56	3.058462	1.04	PRDX1	peroxiredoxin 1

7908924	4.25E-04	1.77E-05	6.56	3.053163	1.33	PRELP	proline and arginine rich end leucine rich repeat protein
8078330	4.25E-04	1.77E-05	6.55	3.051366	1.19	RBMS3	RNA binding motif single stranded interacting protein 3
7972557	4.25E-04	1.77E-05	6.55	3.05116	2	GPR183	G protein-coupled receptor 183
8123104	4.27E-04	1.78E-05	6.55	3.045236	2.26	FNDC1	fibronectin type III domain containing 1

8173287	4.29E-04	1.80E-05	6.55	3.036994	2.51	VSIG4	V-set and immunoglobulin domain containing 4
8011826	4.33E-04	1.83E-05	6.53	3.01985	1.91	SCIMP	SLP adaptor and CSK interacting membrane protein
8139488	4.35E-04	1.84E-05	6.53	3.014111	2.05	IGFBP3	insulin like growth factor binding protein 3
8112007	4.35E-04	1.84E-05	6.53	3.012251	2.05	EMB	embigin
8178884	4.35E-04	1.85E-05	6.53	3.009515	1.89	HLA-DMA	major histocompatibility complex, class II, DM alpha

8180086	4.35E-04	1.85E-05	6.53	3.009515	1.89	HLA-DMA	major histocompatibility complex, class II, DM alpha
7896714	4.36E-04	1.85E-05	6.53	3.007124	2		
8161563	4.36E-04	1.85E-05	6.53	3.006702	1.15	IGKC	immunoglobulin kappa constant
7972003	4.36E-04	1.86E-05	6.52	3.001826	1.46	KLF12	Kruppel like factor 12
7925876	4.41E-04	1.89E-05	6.52	2.988595	1.41	PFKP	phosphofructokinase, platelet
8171172	4.42E-04	1.89E-05	6.51	2.984662	2.09	MXRA5	matrix remodeling associated 5
7943749	4.43E-04	1.90E-05	6.51	2.980951	1.63	LAYN	layilin

7968976	4.43E-04	1.91E-05	6.51	2.978261	1.18	LRCH1	leucine rich repeats and calponin homology domain containing 1
8074237	4.45E-04	1.92E-05	6.5	2.971416	1.33	CECR1	cat eye syndrome chromosome region, candidate 1
7948364	4.45E-04	1.92E-05	6.5	2.971219	1.91	MPEG1	macrophage expressed 1
7958019	4.49E-04	1.95E-05	6.5	2.956591	1.3	DRAM1	DNA damage regulated autophagy modulator 1

8101322	4.49E-04	1.95E-05	6.5	2.956532	1.89	MOP-1	MOP-1
8125537	4.49E-04	1.95E-05	6.49	2.95504	1.83	HLA-DMA	major histocompatibility complex, class II, DM alpha
8055426	4.51E-04	1.96E-05	6.49	2.949663	1.13	MCM6	minichromosome maintenance complex component 6
8091385	4.54E-04	1.98E-05	6.48	2.938527	3.07	CP	ceruloplasmin (ferroxidase)
8018305	4.54E-04	1.98E-05	6.48	2.937707	1.28	HN1	hematological and neurological expressed 1

8057898	4.55E-04	1.98E-05	6.48	2.936651	1.33	HECW2	HECT, C2 and WW domain containing E3 ubiquitin protein ligase 2
8078014	4.55E-04	1.99E-05	6.48	2.933467	1.87	SLC6A6	solute carrier family 6 member 6
8062312	4.57E-04	2.01E-05	6.48	2.926004	1.57	MYL9	myosin light chain 9
7917433	4.58E-04	2.02E-05	6.47	2.920192	1.22	ODF2L	outer dense fiber of sperm tails 2 like
7949496	4.58E-04	2.02E-05	6.47	2.918373	1.07	CFL1	cofilin 1

8081537	4.64E-04	2.06E-05	6.46	2.900209	2.91	TRAT1	T cell receptor associated transmembrane adaptor 1
7965541	4.66E-04	2.07E-05	6.46	2.894703	1.16	FGD6	FYVE, RhoGEF and PH domain containing 6
8133818	4.70E-04	2.09E-05	6.45	2.885944	1.03	PHTF2	putative homeodomain transcription factor 2
7906930	4.73E-04	2.10E-05	6.45	2.877396	1.26	NUF2	NUF2, NDC80 kinetochore complex component

8058708	4.77E-04	2.13E-05	6.44	2.865346	1.37	ABCA12	ATP binding cassette subfamily A member 12
7956046	4.82E-04	2.16E-05	6.43	2.84897	1.29	DGKA	diacylglycerol kinase alpha
8021584	4.84E-04	2.17E-05	6.43	2.844484	2.08	SERPINB5	serpin family B member 5
7943293	4.85E-04	2.18E-05	6.42	2.841018	1.11	ENDOD1	endonuclease domain containing 1
8057744	4.85E-04	2.18E-05	6.42	2.838575	1.14	STAT1	signal transducer and activator of transcription 1

7953291	4.86E-04	2.19E-05	6.42	2.836638	1.38	CD9	CD9 molecule
7974461	4.88E-04	2.21E-05	6.42	2.828563	1.11	LGALS3	lectin, galactoside binding soluble 3
8162404	4.89E-04	2.22E-05	6.41	2.822999	1.09	ECM2	extracellular matrix protein 2
8097903	4.91E-04	2.23E-05	6.41	2.817395	1.9	TLR2	toll like receptor 2
7918622	4.93E-04	2.24E-05	6.41	2.81197	1.69	SLC16A1	solute carrier family 16 member 1
8146159	5.05E-04	2.31E-05	6.39	2.78085	1.2	AP3M2	adaptor related protein complex 3 mu 2 subunit
8131631	5.10E-04	2.33E-05	6.38	2.770711	1.01	HDAC9	histone deacetylase 9

7988644	5.17E-04	2.38E-05	6.37	2.753134	1.93	ATP8B4	ATPase phospholipid transporting 8B4 (putative)
7980044	5.22E-04	2.41E-05	6.36	2.738604	1.1	PNMA1	paraneoplastic Ma antigen 1
7929334	5.25E-04	2.43E-05	6.35	2.730204	1.74	CEP55	centrosomal protein 55
8101659	5.29E-04	2.47E-05	6.35	2.715173	1.39	SPARCL1	SPARC like 1
7917576	5.31E-04	2.48E-05	6.34	2.710587	2.79	GBP5	guanylate binding protein 5
7933312	5.34E-04	2.50E-05	6.34	2.701662	1.56	ANXA8L1///ANXA8	annexin A8-like 1///annexin A8
8026971	5.36E-04	2.51E-05	6.33	2.697873	1.82	IFI30	IFI30, lysosomal thiol reductase

8169115	5.38E-04	2.52E-05	6.33	2.691847	2.21	NRK	Nik related kinase
8106098	5.39E-04	2.53E-05	6.33	2.687623	1.62	MAP1B	microtubule associated protein 1B
8151219	5.39E-04	2.53E-05	6.33	2.686733	1.34		
8151684	5.39E-04	2.54E-05	6.33	2.685375	1.07	MMP16	matrix metallopeptidase 16
7990818	5.40E-04	2.54E-05	6.33	2.683686	2.93	BCL2A1	BCL2 related protein A1
8134814	5.45E-04	2.57E-05	6.32	2.672598	1.29	PILRA	paired immunoglobin like type 2 receptor alpha
7960794	5.48E-04	2.59E-05	6.31	2.663796	2.28	CD163	CD163 molecule

7969986	5.53E-04	2.62E-05	6.31	2.652419	1.17	TNFSF13B	tumor necrosis factor superfamily member 13b
8167006	5.53E-04	2.63E-05	6.31	2.649567	1.32	RP2	retinitis pigmentosa 2 (X-linked recessive)
7927367	5.57E-04	2.65E-05	6.3	2.640164	1.01	ANXA8L1///ANXA8	annexin A8-like 1///annexin A8

7981728	5.65E-04	2.70E-05	6.29	2.624125	3.97	IGHM///IGHV3-48///IGHV3-69-1	immunoglobulin heavy constant mu///immunoglobulin heavy variable 3-48///immunoglobulin heavy variable 3-69-1 (pseudogene)
7929258	5.67E-04	2.71E-05	6.29	2.61732	1.32	KIF11	kinesin family member 11

7929052	5.75E-04	2.76E-05	6.27	2.59845	1.61	IFIT3	interferon induced protein with tetratricopeptide repeats 3
7927307	5.75E-04	2.76E-05	6.27	2.598202	1.07	ANXA8L1///ANXA8	annexin A8-like 1///annexin A8
7931097	5.79E-04	2.80E-05	6.27	2.585352	1.05	HTRA1	HtrA serine peptidase 1
7951077	5.82E-04	2.82E-05	6.26	2.577771	1.21	SESN3	sestrin 3
7902127	5.84E-04	2.83E-05	6.26	2.573874	1.69	SGIP1	SH3 domain GRB2 like endophilin interacting protein 1

8081564	5.84E-04	2.84E-05	6.26	2.571861	2.17	CD96	CD96 molecule
8014891	5.93E-04	2.88E-05	6.25	2.555453	1.61	IKZF3	IKAROS family zinc finger 3
8133983	5.93E-04	2.89E-05	6.25	2.554385	1.68	ADAM22	ADAM metallopeptidase domain 22

7904469	5.96E-04	2.90E-05	6.24	2.548581	1.3	SRGAP2D///SRGAP2C///SRGAP2B///SRGAP2	SLIT-ROBO Rho GTPase activating protein 2D (pseudogene)///SLIT-ROBO Rho GTPase activating protein 2C///SLIT-ROBO Rho GTPase activating protein 2B///SLIT-ROBO Rho GTPase activating protein 2

8174051	5.96E-04	2.91E-05	6.24	2.546833	1.5	BTK	Bruton tyrosine kinase
8147777	6.06E-04	2.97E-05	6.23	2.525346	1.04	CTHRC1	collagen triple helix repeat containing 1
8075635	6.08E-04	2.98E-05	6.23	2.519896	1.62	TIMP3	TIMP metallopeptidase inhibitor 3
7984540	6.08E-04	2.99E-05	6.22	2.517655	1.45	KIF23	kinesin family member 23
8104930	6.10E-04	3.00E-05	6.22	2.513258	2.17	SLC1A3	solute carrier family 1 member 3
8023757	6.10E-04	3.01E-05	6.22	2.512238	1.96	CD226	CD226 molecule

8083094	6.10E-04	3.01E-05	6.22	2.510911	1.09	RASA2	RAS p21 protein activator 2
8082086	6.12E-04	3.02E-05	6.22	2.506662	1.43	PARP15	poly(ADP-ribose) polymerase family member 15
8102050	6.19E-04	3.08E-05	6.21	2.488555	2.02	SLC9B2	solute carrier family 9 member B2
8156290	6.26E-04	3.14E-05	6.19	2.468533	2.04	CKS2	CDC28 protein kinase regulatory subunit 2
7929026	6.28E-04	3.15E-05	6.19	2.464743	1.41	ACTA2-AS1	ACTA2 antisense RNA 1
8160297	6.34E-04	3.20E-05	6.18	2.449546	1.75	PLIN2	perilipin 2

8072626	6.35E-04	3.20E-05	6.18	2.44834	1.4	TIMP3	TIMP metallopeptidase inhibitor 3
8022283	6.35E-04	3.21E-05	6.18	2.446527	2.32	PIEZO2	piezo type mechanosensitive ion channel component 2
8083166	6.36E-04	3.22E-05	6.18	2.443735	1.58	TRPC1	transient receptor potential cation channel subfamily C member 1
8031047	6.37E-04	3.23E-05	6.18	2.439958	1.4	MYADM	myeloid associated differentiation marker

8146500	6.37E-04	3.23E-05	6.18	2.438803	1.41	LYN	LYN proto-oncogene, Src family tyrosine kinase
7917561	6.38E-04	3.24E-05	6.18	2.436433	2.02	GBP4	guanylate binding protein 4
8085774	6.39E-04	3.25E-05	6.17	2.433033	1.42	ZNF385D	zinc finger protein 385D
8101304	6.46E-04	3.30E-05	6.16	2.418612	1.54	RASGEF1B	RasGEF domain family member 1B
8120719	6.47E-04	3.30E-05	6.16	2.41681	2.08	CD109	CD109 molecule

8112342	6.56E-04	3.36E-05	6.15	2.399338	1.32	ADAMTS6	ADAM metallopeptidase with thrombospondin type 1 motif 6
8144931	6.60E-04	3.39E-05	6.15	2.390734	1.02	ATP6V1B2	ATPase H+ transporting V1 subunit B2
8057990	6.60E-04	3.39E-05	6.15	2.38885	1.14	ANKRD44	ankyrin repeat domain 44
8131867	6.70E-04	3.45E-05	6.14	2.370685	1.24		
8169473	6.72E-04	3.48E-05	6.13	2.363321	1.04	PLS3	plastin 3
8034873	6.83E-04	3.55E-05	6.12	2.342678	1.12	ADGRE2	adhesion G protein-coupled receptor E2

8065412	6.88E-04	3.58E-05	6.11	2.332815	3.34	CST1	cystatin SN
7904482	6.88E-04	3.59E-05	6.11	2.33126	1.01	SRGAP2C///SRGAP2B///SRGAP2	SLIT-ROBO Rho GTPase activating protein 2C///SLIT-ROBO Rho GTPase activating protein 2B///SLIT-ROBO Rho GTPase activating protein 2
8056890	6.89E-04	3.60E-05	6.11	2.328286	1.85	CHN1	chimerin 1
8161580	6.99E-04	3.66E-05	6.1	2.312607	1.03		

8086600	7.00E-04	3.67E-05	6.1	2.30911	1.25	CCR1	C-C motif chemokine receptor 1
8174076	7.01E-04	3.68E-05	6.1	2.305393	1.13	GLA	galactosidase alpha
8180029	7.02E-04	3.69E-05	6.1	2.304007	1.51	HLA-DQB2///<br>HLA-DQB1	major histocompatibility complex, class II, DQ beta 2///major histocompatibility complex, class II, DQ beta 1
8053690	7.02E-04	3.69E-05	6.09	2.303027	3.29		
8051762	7.06E-04	3.72E-05	6.09	2.294153	1.63	SLC8A1	solute carrier family 8 member A1

8135705	7.08E-04	3.74E-05	6.09	2.288301	1.59	KCND2	potassium voltage-gated channel subfamily D member 2
8153959	7.08E-04	3.75E-05	6.09	2.2874	1.23	DOCK8	dedicator of cytokinesis 8
7926786	7.09E-04	3.76E-05	6.08	2.284748	1.22	APBB1IP	amyloid beta precursor protein binding family B member 1 interacting protein
7953901	7.15E-04	3.81E-05	6.07	2.269603	1.79	CLEC12A	C-type lectin domain family 12 member A

8027748	7.17E-04	3.83E-05	6.07	2.265961	1.63	FXYD3	FXYD domain containing ion transport regulator 3
8102200	7.22E-04	3.86E-05	6.07	2.258122	1.11	DKK2	dickkopf WNT signaling pathway inhibitor 2
7978718	7.24E-04	3.87E-05	6.07	2.254706	1.38	SEC23A	Sec23 homolog A, coat complex II component
8122265	7.33E-04	3.93E-05	6.06	2.238762	1.85	TNFAIP3	TNF alpha induced protein 3

8054580	7.43E-04	4.00E-05	6.04	2.219763	1.35	BUB1	BUB1 mitotic checkpoint serine/threonine kinase
8043480	7.45E-04	4.02E-05	6.04	2.215185	2.38	IGKC	immunoglobulin kappa constant
8152512	7.45E-04	4.03E-05	6.04	2.213418	1.71	TNFRSF11B	TNF receptor superfamily member 11b
8105331	7.45E-04	4.03E-05	6.04	2.21315	3.5	GZMK	granzyme K
7976443	7.46E-04	4.05E-05	6.04	2.208373	2.05	IFI27	interferon alpha inducible protein 27

8092702	7.47E-04	4.07E-05	6.04	2.204066	1.27	LOC100132319	uncharacterized LOC100132319
8036591	7.47E-04	4.07E-05	6.04	2.203954	1.67	LGALS4	galectin 4
8106475	7.48E-04	4.08E-05	6.03	2.201485	1.23		
8113039	7.49E-04	4.09E-05	6.03	2.19888	1.41	MEF2C	myocyte enhancer factor 2C
7999468	7.52E-04	4.11E-05	6.03	2.193141	1.04	LITAF	lipopolysaccharide induced TNF factor
8061579	7.64E-04	4.18E-05	6.02	2.17531	1.5	TPX2	TPX2, microtubule nucleation factor
7909568	7.67E-04	4.20E-05	6.01	2.170223	1.8	DTL	denticleless E3 ubiquitin protein ligase homolog

8090098	7.70E-04	4.22E-05	6.01	2.164841	1.54	MYLK	myosin light chain kinase
7958207	7.77E-04	4.27E-05	6	2.153659	1.33		
8043436	7.85E-04	4.34E-05	6	2.138203	3.93	IGKC	immunoglobulin kappa constant
7999553	7.96E-04	4.40E-05	5.99	2.122533	1.18	CPPED1	calcineurin like phosphoesterase domain containing 1
7927799	7.99E-04	4.43E-05	5.98	2.117399	1.35	REEP3	receptor accessory protein 3
7991234	8.02E-04	4.45E-05	5.98	2.111084	1.55	MFGE8	milk fat globule-EGF factor 8 protein

8089714	8.02E-04	4.46E-05	5.98	2.109968	1.49	LSAMP	limbic system-associated membrane protein
8145365	8.05E-04	4.47E-05	5.98	2.106624	1.05	DOCK5	dedicator of cytokinesis 5
8043476	8.08E-04	4.50E-05	5.97	2.100418	2.94		
8089082	8.14E-04	4.54E-05	5.97	2.091709	1.36	DCBLD2	discoidin, CUB and LCCL domain containing 2
8164200	8.18E-04	4.57E-05	5.96	2.084784	1.41	ANGPTL2	angiopoietin like 2
8052654	8.21E-04	4.59E-05	5.96	2.079545	1.57	PELI1	pellino E3 ubiquitin protein ligase 1

8094301	8.26E-04	4.63E-05	5.96	2.071316	1.6	SLIT2	slit guidance ligand 2
8012475	8.28E-04	4.65E-05	5.95	2.06644	1.28	MYH10	myosin, heavy chain 10, non-muscle
8125463	8.30E-04	4.67E-05	5.95	2.062387	1.79	HLA-DQB2/// HLA-DQB1	major histocompatibility complex, class II, DQ beta 2///major histocompatibility complex, class II, DQ beta 1
7921434	8.32E-04	4.69E-05	5.95	2.058174	1.74	AIM2	absent in melanoma 2

8017843	8.32E-04	4.69E-05	5.95	2.057488	1.93	SLC16A6	solute carrier family 16 member 6
8109712	8.33E-04	4.71E-05	5.95	2.054829	1.56	HMMR	hyaluronan mediated motility receptor
8114612	8.45E-04	4.79E-05	5.94	2.037231	1.39	CD14	CD14 molecule
8143471	8.51E-04	4.83E-05	5.93	2.027318	2.45	CLEC5A	C-type lectin domain family 5 member A

7981720	8.51E-04	4.84E-05	5.93	2.026745	1.63	IGHA1///SCFV///C14orf99///IGHV3-48///IGHV3-69-1	immunoglobulin heavy constant alpha 1///single-chain Fv fragment///uncharacterized LOC317730///immunoglobulin heavy variable 3-48///immunoglobulin heavy variable 3-69-1 (pseudogene)

8075728	8.54E-04	4.86E-05	5.93	2.021321	1	MYH9	myosin, heavy chain 9, non-muscle
8103166	8.54E-04	4.87E-05	5.93	2.020518	1.03	SH3D19	SH3 domain containing 19
8057506	8.57E-04	4.89E-05	5.92	2.015268	1.85	FRZB	frizzled-related protein
7944185	8.59E-04	4.90E-05	5.92	2.012965	1.83	CD3G	CD3g molecule
8101429	8.77E-04	5.03E-05	5.91	1.986884	2.61	PLAC8	placenta specific 8
7905220	8.79E-04	5.05E-05	5.9	1.982359	1.36	ECM1	extracellular matrix protein 1
7912198	8.83E-04	5.09E-05	5.9	1.97361	1.4	ENO1	enolase 1
7927606	8.84E-04	5.11E-05	5.9	1.97013	1.1	PRKG1	protein kinase, cGMP-dependent, type I

8053735	8.85E-04	5.13E-05	5.89	1.966392	2.24	IGKC	immunoglobulin kappa constant
7933855	8.85E-04	5.13E-05	5.89	1.965985	1.48	RTKN2	rhotekin 2
8017867	8.87E-04	5.15E-05	5.89	1.961704	1.09	FAM20A	family with sequence similarity 20 member A
8019046	8.95E-04	5.22E-05	5.88	1.949068	1.08	EIF4A3	eukaryotic translation initiation factor 4A3
8111772	9.01E-04	5.25E-05	5.88	1.942282	1.04	DAB2	DAB2, clathrin adaptor protein
7921667	9.03E-04	5.27E-05	5.88	1.938288	1.93	CD48	CD48 molecule

8171896	9.10E-04	5.36E-05	5.87	1.922304	1.54	CXorf21	chromosome X open reading frame 21

7981730	9.14E-04	5.40E-05	5.86	1.914189	3.1	SCFV///IGLJ3///IGHV3-23///IGHV4-31///IGHM///IGHG2///IGHG1///IGHA1///SCFV///C14orf99///IGK///IGKV3-20///IGHV3-23///IGHV3-30///IGHV3-48///IGHV3-75///IGHV3-69-1///IGHV3OR16-7	single-chain Fv fragment///immunoglobulin lambda joining 3///immunoglobulin heavy variable 3-23///immunoglobulin heavy variable 4-31///immunoglobulin heavy constant mu///immunoglobulin heavy constant gamma 2 (G2m marker)///immunoglobulin heavy constant gamma 1 (G1m marker)///immunoglobulin heavy constant alpha 1///single-chain Fv fragment///uncharacterized LOC317730///immunoglobulin kappa locus///immunoglobulin kappa variable 3-20///immunoglobulin heavy variable 3-23///immunoglobulin heavy variable 3-30///immunoglobulin heavy variable 3-48///immunoglobulin heavy variable 3-75 (pseudogene)///immunoglobulin heavy variable 3-69-1 (pseudogene)///immunoglobulin heavy variable 3/OR16-7 (pseudogene)

7929078	9.19E-04	5.44E-05	5.86	1.906782	1.3	KIF20B	kinesin family member 20B
8096617	9.26E-04	5.50E-05	5.85	1.895895	2.2	BANK1	B-cell scaffold protein with ankyrin repeats 1
8081081	9.26E-04	5.50E-05	5.85	1.895622	1.95	EPHA3	EPH receptor A3
8037374	9.27E-04	5.51E-05	5.85	1.893821	2.11	PLAUR	plasminogen activator, urokinase receptor
8043470	9.37E-04	5.58E-05	5.84	1.880807	3.19		
7906613	9.38E-04	5.60E-05	5.84	1.877049	2.3	SLAMF7	SLAM family member 7

8175492	9.40E-04	5.62E-05	5.84	1.873354	1.12	ATP11C	ATPase phospholipid transporting 11C
8036224	9.45E-04	5.69E-05	5.83	1.86139	1.47	TYROBP	TYRO protein tyrosine kinase binding protein
8146967	9.45E-04	5.69E-05	5.83	1.861203	2.04	CRISPLD1	cysteine rich secretory protein LCCL domain containing 1
8043484	9.45E-04	5.70E-05	5.83	1.859737	1.21	IGKC	immunoglobulin kappa constant
8090162	9.46E-04	5.70E-05	5.83	1.85813	1.1	ITGB5	integrin subunit beta 5

8094240	9.48E-04	5.72E-05	5.83	1.854476	1.52	CD38	CD38 molecule
8078252	9.58E-04	5.79E-05	5.82	1.842157	1.09	UBE2E2	ubiquitin conjugating enzyme E2 E2
8006594	9.63E-04	5.84E-05	5.82	1.834905	2.68	CCL18	C-C motif chemokine ligand 18
8048864	9.73E-04	5.91E-05	5.81	1.822238	3.02	CCL20	C-C motif chemokine ligand 20
8014248	9.73E-04	5.91E-05	5.81	1.822141	1.33	SLFN13	schlafen family member 13
8157105	9.76E-04	5.94E-05	5.8	1.816156	1.16	ZNF462	zinc finger protein 462
7919669	9.80E-04	5.98E-05	5.8	1.809528	1.24	MTMR11	myotubularin related protein 11

8107044	9.82E-04	6.00E-05	5.8	1.805776	2.34	ERAP2	endoplasmic reticulum aminopeptidase 2
8097991	1.00E-03	6.15E-05	5.78	1.781921	2.67	TDO2	tryptophan 2,3-dioxygenase
8117458	1.01E-03	6.23E-05	5.78	1.767336	1	BTN3A1	butyrophilin subfamily 3 member A1
7925492	1.01E-03	6.24E-05	5.78	1.766965	1.21	OPN3	opsin 3
8043459	1.02E-03	6.29E-05	5.77	1.759084	2.92	IGKC	immunoglobulin kappa constant
8091515	1.02E-03	6.37E-05	5.76	1.745376	2.04	GPR87	G protein-coupled receptor 87

8162940	1.03E-03	6.45E-05	5.76	1.731989	1.53	ABCA1	ATP binding cassette subfamily A member 1
7909332	1.04E-03	6.48E-05	5.75	1.728037	1.35	CD55	CD55 molecule (Cromer blood group)
7923978	1.04E-03	6.50E-05	5.75	1.725388	1.14	CD34	CD34 molecule
8020090	1.04E-03	6.51E-05	5.75	1.723168	1.39	TWSG1	twisted gastrulation BMP signaling modulator 1
8078286	1.05E-03	6.60E-05	5.74	1.709558	1.31	RARB	retinoic acid receptor beta

8109639	1.05E-03	6.61E-05	5.74	1.707951	1.2	PTTG1	pituitary tumor-transforming 1
7913237	1.06E-03	6.66E-05	5.74	1.700502	1.36	CAMK2N1	calcium/calmodulin dependent protein kinase II inhibitor 1
8116418	1.06E-03	6.69E-05	5.73	1.695354	2.17	GFPT2	glutamine-fructose-6-phosphate transaminase 2
8146660	1.07E-03	6.79E-05	5.73	1.680504	1.42	DNAJC5B	DnaJ heat shock protein family (Hsp40) member C5 beta
8109490	1.08E-03	6.83E-05	5.72	1.67472	1.57	SGCD	sarcoglycan delta

8012931	1.11E-03	7.12E-05	5.7	1.631541	1.07	CDRT1///FBXW10	CMT1A duplicated region transcript 1///F-box and WD repeat domain containing 10
8154233	1.12E-03	7.16E-05	5.69	1.625541	1.2	CD274	CD274 molecule
8166079	1.14E-03	7.38E-05	5.68	1.595731	2.26	EGFL6	EGF like domain multiple 6
7929065	1.15E-03	7.42E-05	5.67	1.589827	1.61	IFIT1	interferon induced protein with tetratricopeptide repeats 1
7973336	1.15E-03	7.43E-05	5.67	1.588504	1.23	MMP14	matrix metallopeptidase 14

8008237	1.15E-03	7.48E-05	5.67	1.580856	1.27	ITGA3	integrin subunit alpha 3
7908388	1.16E-03	7.51E-05	5.66	1.576864	2.82	RGS1	regulator of G-protein signaling 1
7953723	1.16E-03	7.52E-05	5.66	1.575283	1.66	CLEC4A	C-type lectin domain family 4 member A
8043449	1.16E-03	7.53E-05	5.66	1.574876	3.33	IGK///IGKC	immunoglobulin kappa locus///immunoglobulin kappa constant
7950810	1.16E-03	7.53E-05	5.66	1.574037	2	SYTL2	synaptotagmin like 2

8151768	1.16E-03	7.59E-05	5.66	1.566288	1.7	RUNX1T1	RUNX1 translocation partner 1
8075910	1.18E-03	7.78E-05	5.64	1.541488	1.17	RAC2	ras-related C3 botulinum toxin substrate 2 (rho family, small GTP binding protein Rac2)
7914127	1.19E-03	7.87E-05	5.64	1.529918	1.59	IFI6	interferon alpha inducible protein 6
8147112	1.20E-03	7.95E-05	5.63	1.519717	1.04	CA13	carbonic anhydrase 13

8020551	1.20E-03	7.96E-05	5.63	1.518342	1.87	LAMA3	laminin subunit alpha 3
8157153	1.20E-03	7.97E-05	5.63	1.516438	1.02	PALM2-AKAP2///PALM2///AKAP2	PALM2-AKAP2 readthrough///paralemmin 2///A-kinase anchoring protein 2
8139212	1.21E-03	8.08E-05	5.62	1.502693	1.5	GLI3	GLI family zinc finger 3
8058390	1.22E-03	8.10E-05	5.62	1.500325	1.25	RAPH1	Ras association (RalGDS/AF-6) and pleckstrin homology domains 1

7918533	1.22E-03	8.11E-05	5.62	1.499242	1.79	TMIGD3///ADORA3///TMIGD3///ADORA3	transmembrane and immunoglobulin domain containing 3///adenosine A3 receptor///transmembrane and immunoglobulin domain containing 3///adenosine A3 receptor
8093950	1.24E-03	8.27E-05	5.61	1.478966	2.37	S100P	S100 calcium binding protein P
8043465	1.24E-03	8.33E-05	5.6	1.471719	2.63	IGKC	immunoglobulin kappa constant

8103399	1.26E-03	8.46E-05	5.59	1.455075	1.03	PDGFC	platelet derived growth factor C

7981724	1.27E-03	8.56E-05	5.59	1.443417	3.32	SCFV///IGLJ3///IGHM///IGHG1///IGHD///C14orf99///IGHV3-48///IGHV3-69-1	single-chain Fv fragment///immunoglobulin lambda joining 3///immunoglobulin heavy constant mu///immunoglobulin heavy constant gamma 1 (G1m marker)///immunoglobulin heavy constant delta///uncharacterized LOC317730///immunoglobulin heavy variable 3-48///immunoglobulin heavy variable 3-69-1 (pseudogene)

7981718	1.27E-03	8.58E-05	5.59	1.440932	3.04	SCFV///IGHM///SCFV///IGHV3-30	single-chain Fv fragment///immunoglobulin heavy constant mu///single-chain Fv fragment///immunoglobulin heavy variable 3-30
8094520	1.27E-03	8.59E-05	5.59	1.439487	1.32	PCDH7	protocadherin 7
7909306	1.35E-03	9.24E-05	5.54	1.366099	1.13	C4BPB	complement component 4 binding protein beta

7951297	1.38E-03	9.53E-05	5.52	1.334234	2.75	MMP12	matrix metallopeptidase 12
7896708	1.41E-03	9.84E-05	5.51	1.301181	2.7		
7994826	1.42E-03	9.91E-05	5.5	1.293985	1.01	ITGAL	integrin subunit alpha L
7908003	1.42E-03	9.92E-05	5.5	1.292792	1.83	NPL	N-acetylneuraminate pyruvate lyase
7968883	1.43E-03	9.97E-05	5.5	1.287743	1.24	LACC1	laccase domain containing 1
8163629	1.43E-03	9.98E-05	5.5	1.287032	1.23	TNFSF8	tumor necrosis factor superfamily member 8

8099807	1.43E-03	1.00E-04	5.49	1.281775	1.02	GAFA3	FGF-2 activity-associated protein 3
8134869	1.43E-03	1.01E-04	5.49	1.277797	1.48	PCOLCE	procollagen C-endopeptidase enhancer
8043360	1.44E-03	1.01E-04	5.49	1.275551	2.79	IGK///IGKC	immunoglobulin kappa locus///immunoglobulin kappa constant
7896717	1.44E-03	1.01E-04	5.49	1.269966	1.6		
8131957	1.45E-03	1.02E-04	5.48	1.261704	1.42	SNX10	sorting nexin 10

7925062	1.47E-03	1.04E-04	5.48	1.249647	1.02	SIPA1L2	signal induced proliferation associated 1 like 2
8122426	1.47E-03	1.04E-04	5.47	1.244401	1.08	PHACTR2	phosphatase and actin regulator 2
8070826	1.49E-03	1.06E-04	5.46	1.228087	1.23	ITGB2	integrin subunit beta 2
8109507	1.51E-03	1.08E-04	5.45	1.209999	1.67	ITK	IL2 inducible T-cell kinase
7896701	1.52E-03	1.09E-04	5.45	1.200944	1.55		
8042391	1.52E-03	1.09E-04	5.45	1.196031	2.56	PLEK	pleckstrin
8092177	1.53E-03	1.10E-04	5.44	1.189003	1.19	NCEH1	neutral cholesterol ester hydrolase 1

8060736	1.53E-03	1.10E-04	5.44	1.187159	1.01	PANK2	pantothenate kinase 2
8019857	1.53E-03	1.10E-04	5.44	1.185816	1.74	NDC80	NDC80, kinetochore complex component
8098259	1.53E-03	1.10E-04	5.44	1.184046	1.81		
7920291	1.53E-03	1.11E-04	5.44	1.181401	1.48	S100A16	S100 calcium binding protein A16
8084757	1.54E-03	1.11E-04	5.44	1.178326	1.44	TPRG1	tumor protein p63 regulated 1

7981722	1.54E-03	1.11E-04	5.43	1.176618	2.68	IGLJ3///IGHV3-23///IGHV4-31///IGHM///IGHG1///IGHA1///SCFV///C14orf99///IGK///IGKV3-20///IGHV3-23///IGHV3-48///IGHV3-69-1///IGHV3OR16-7	immunoglobulin lambda joining 3///immunoglobulin heavy variable 3-23///immunoglobulin heavy variable 4-31///immunoglobulin heavy constant mu///immunoglobulin heavy constant gamma 1 (G1m marker)///immunoglobulin heavy constant alpha 1///single-chain Fv fragment///uncharacterized LOC317730///immunoglobulin kappa locus///immunoglobulin kappa variable 3-20///immunoglobulin heavy variable 3-23///immunoglobulin heavy variable 3-48///immunoglobulin heavy variable 3-69-1 (pseudogene)///immunoglobulin heavy variable 3/OR16-7 (pseudogene)

8129037	1.55E-03	1.13E-04	5.43	1.163832	1.32		
7950391	1.56E-03	1.13E-04	5.42	1.159773	1.25	PGM2L1	phosphoglucomutase 2 like 1
7939839	1.56E-03	1.14E-04	5.42	1.153949	1.07	PTPRJ	protein tyrosine phosphatase, receptor type J
7904287	1.56E-03	1.14E-04	5.42	1.153472	1.66	CD2	CD2 molecule
7946661	1.57E-03	1.14E-04	5.42	1.148769	1.01	DKK3	dickkopf WNT signaling pathway inhibitor 3
8176234	1.58E-03	1.15E-04	5.42	1.143472	1.86	CLIC2	chloride intracellular channel 2
8173745	1.58E-03	1.15E-04	5.41	1.140725	1.66	CYSLTR1	cysteinyl leukotriene receptor 1

7902913	1.58E-03	1.15E-04	5.41	1.140617	1.45	CDC7	cell division cycle 7
8160637	1.58E-03	1.15E-04	5.41	1.13962	1.05	B4GALT1	beta-1,4-galactosyltransferase 1
8136557	1.58E-03	1.16E-04	5.41	1.135279	1.24	TBXAS1	thromboxane A synthase 1
8139057	1.58E-03	1.16E-04	5.41	1.135186	1.44	ELMO1	engulfment and cell motility 1
8091283	1.58E-03	1.16E-04	5.41	1.134191	1.12	PLOD2	procollagen-lysine,2-oxoglutarate 5-dioxygenase 2
8107769	1.58E-03	1.16E-04	5.41	1.133725	1.41	SLC12A2	solute carrier family 12 member 2

7946275	1.60E-03	1.18E-04	5.4	1.119888	1.51	GVINP1	GTPase, very large interferon inducible pseudogene 1
8030007	1.61E-03	1.19E-04	5.4	1.110629	1.67	EMP3	epithelial membrane protein 3
8166632	1.62E-03	1.19E-04	5.39	1.107278	1.39	GK3P///GK	glycerol kinase 3 pseudogene///glycerol kinase
8127534	1.62E-03	1.19E-04	5.39	1.104579	1.21	MB21D1	Mab-21 domain containing 1
7956856	1.63E-03	1.20E-04	5.39	1.095317	1.2	MSRB3	methionine sulfoxide reductase B3

7944769	1.64E-03	1.22E-04	5.38	1.086068	1.45	GRAMD1B	GRAM domain containing 1B
8115464	1.68E-03	1.26E-04	5.36	1.052502	1.22	HAVCR2	hepatitis A virus cellular receptor 2
8094743	1.69E-03	1.27E-04	5.36	1.042182	1.41	RHOH	ras homolog family member H
8091260	1.72E-03	1.29E-04	5.35	1.024852	1.71	SLC9A9	solute carrier family 9 member A9
8169792	1.72E-03	1.29E-04	5.35	1.024416	1.48	SH2D1A	SH2 domain containing 1A
7922174	1.72E-03	1.30E-04	5.34	1.018616	1.94	F5	coagulation factor V

8080964	1.74E-03	1.31E-04	5.34	1.005795	1.48	GXYLT2	glucoside xylosyltransferase 2
7928318	1.75E-03	1.32E-04	5.33	0.998941	1.42	MCU	mitochondrial calcium uniporter
7896699	1.76E-03	1.34E-04	5.33	0.988168	1.47		
7960947	1.76E-03	1.34E-04	5.33	0.984187	1.23	A2M	alpha-2-macroglobulin
8081818	1.77E-03	1.35E-04	5.32	0.980238	2.2		
8095744	1.77E-03	1.35E-04	5.32	0.977544	2.84	AREG	amphiregulin
8081838	1.77E-03	1.35E-04	5.32	0.975889	1.2	ARHGAP31	Rho GTPase activating protein 31
8159961	1.78E-03	1.36E-04	5.32	0.971175	1.3		
7918657	1.79E-03	1.37E-04	5.31	0.963822	1.51	PTPN22	protein tyrosine phosphatase, non-receptor type 22

7936968	1.79E-03	1.38E-04	5.31	0.959062	2.4	ADAM12	ADAM metallopeptidase domain 12
8022118	1.79E-03	1.38E-04	5.31	0.95787	1.38	EPB41L3	erythrocyte membrane protein band 4.1 like 3
8106660	1.82E-03	1.40E-04	5.3	0.939477	1.52	RASGRF2	Ras protein specific guanine nucleotide releasing factor 2
8092654	1.82E-03	1.41E-04	5.3	0.936015	1.06	RPL39L	ribosomal protein L39 like

7909446	1.87E-03	1.46E-04	5.28	0.901303	1.44	HSD11B1	hydroxysteroid 11-beta dehydrogenase 1
8044035	1.92E-03	1.51E-04	5.26	0.865898	1.15	IL18R1///IL18R1	interleukin 18 receptor 1///interleukin 18 receptor 1
8173917	1.94E-03	1.52E-04	5.25	0.856705	1.12	NAP1L3	nucleosome assembly protein 1 like 3
7896727	1.94E-03	1.53E-04	5.25	0.852108	1.49		
7896687	1.96E-03	1.55E-04	5.24	0.837785	2.06		
8091327	1.97E-03	1.56E-04	5.24	0.834052	1.01	PLSCR1	phospholipid scramblase 1
7970441	1.98E-03	1.57E-04	5.24	0.82727	1.98	GJB2	gap junction protein beta 2

8118556	1.98E-03	1.57E-04	5.24	0.824683	2.46	HLA-DQA1	major histocompatibility complex, class II, DQ alpha 1
7947248	2.01E-03	1.60E-04	5.22	0.806303	1.23	KIF18A	kinesin family member 18A
8013341	2.02E-03	1.61E-04	5.22	0.798784	1.54	MFAP4	microfibrillar associated protein 4
8125149	2.04E-03	1.63E-04	5.21	0.78484	2.09	SLC44A4	solute carrier family 44 member 4
8178653	2.04E-03	1.63E-04	5.21	0.78484	2.09	SLC44A4	solute carrier family 44 member 4

8179861	2.04E-03	1.63E-04	5.21	0.78484	2.09	SLC44A4	solute carrier family 44 member 4
7921487	2.06E-03	1.66E-04	5.2	0.766626	1.15	TAGLN2	transgelin 2
7944164	2.07E-03	1.68E-04	5.2	0.756265	1.7	TMPRSS4	transmembrane protease, serine 4
8174103	2.10E-03	1.71E-04	5.19	0.738804	1.31	GK3P///GK	glycerol kinase 3 pseudogene///glycerol kinase
7906386	2.14E-03	1.74E-04	5.18	0.71789	1.63	PYHIN1	pyrin and HIN domain family member 1
8059350	2.21E-03	1.82E-04	5.15	0.672807	1.31	AP1S3	adaptor related protein complex 1 sigma 3 subunit

7930980	2.22E-03	1.84E-04	5.15	0.665231	1.85	PLPP4	phospholipid phosphatase 4
7909789	2.23E-03	1.84E-04	5.14	0.660044	1.39	TGFB2	transforming growth factor beta 2
8121601	2.25E-03	1.88E-04	5.13	0.643331	1.65	FAM26E	family with sequence similarity 26 member E
7953428	2.27E-03	1.90E-04	5.13	0.632682	1.13	CD4	CD4 molecule
8102720	2.27E-03	1.90E-04	5.13	0.630023	1.03	ANKRD50	ankyrin repeat domain 50
8043474	2.29E-03	1.92E-04	5.12	0.616894	2.55	IGKC	immunoglobulin kappa constant

8016414	2.30E-03	1.93E-04	5.12	0.611957	1.07	SKAP1	src kinase associated phosphoprotein 1
8116983	2.31E-03	1.94E-04	5.11	0.608641	1.45	CD83	CD83 molecule
8041940	2.36E-03	2.00E-04	5.1	0.578227	1.48	STON1-GTF2A1L///STON1///GTF2A1L	STON1-GTF2A1L readthrough///stonin 1///general transcription factor IIA subunit 1 like
7929012	2.36E-03	2.00E-04	5.1	0.575538	1.3	STAMBPL1	STAM binding protein like 1

8152617	2.42E-03	2.06E-04	5.08	0.545224	2.9	HAS2	hyaluronan synthase 2
7940147	2.44E-03	2.09E-04	5.07	0.531207	1.55	FAM111B	family with sequence similarity 111 member B
7896703	2.47E-03	2.12E-04	5.06	0.518169	2.24		
7929047	2.47E-03	2.12E-04	5.06	0.516249	1.68	IFIT2	interferon induced protein with tetratricopeptide repeats 2
7974404	2.50E-03	2.15E-04	5.06	0.502268	1.34	CDKN3	cyclin dependent kinase inhibitor 3

8155930	2.50E-03	2.16E-04	5.05	0.501215	1.53	GCNT1	glucosaminyl (N-acetyl) transferase 1, core 2
8157216	2.50E-03	2.16E-04	5.05	0.500693	1.14	UGCG	UDP-glucose ceramide glucosyltransferase
8028924	2.51E-03	2.17E-04	5.05	0.496032	1.81	MIA-RAB4B///MIA	MIA-RAB4B readthrough (NMD candidate)///melanoma inhibitory activity
8144802	2.52E-03	2.19E-04	5.05	0.486321	1.53	PDGFRL	platelet derived growth factor receptor like

7922219	2.53E-03	2.20E-04	5.04	0.482632	1.97	SELL	selectin L
7976080	2.54E-03	2.21E-04	5.04	0.47728	1.61	GPR65	G protein-coupled receptor 65
7979131	2.54E-03	2.21E-04	5.04	0.475355	1.42	GNG2	G protein subunit gamma 2
7902104	2.54E-03	2.22E-04	5.04	0.473257	1.33	PDE4B	phosphodiesterase 4B
7950671	2.54E-03	2.22E-04	5.04	0.471855	1.07	GAB2	GRB2 associated binding protein 2
7980316	2.58E-03	2.26E-04	5.03	0.454479	1.44	TGFB3	transforming growth factor beta 3
8043438	2.63E-03	2.31E-04	5.01	0.428879	3.16	IGKC	immunoglobulin kappa constant

8178833	2.68E-03	2.38E-04	5	0.402536	1.12	HLA-DOB	major histocompatibility complex, class II, DO beta
7919888	2.69E-03	2.38E-04	5	0.399064	1.15	CDC42SE1	CDC42 small effector 1
7920297	2.70E-03	2.40E-04	4.99	0.392563	1.26	S100A14	S100 calcium binding protein A14
8042416	2.71E-03	2.41E-04	4.99	0.386621	1.26	ARHGAP25	Rho GTPase activating protein 25
8168531	2.76E-03	2.48E-04	4.98	0.359661	2.13	GPR174	G protein-coupled receptor 174
8105340	2.76E-03	2.48E-04	4.98	0.358303	2.18	GZMA	granzyme A
8043441	2.76E-03	2.48E-04	4.97	0.356773	4.16		

7944082	2.77E-03	2.49E-04	4.97	0.353041	1.91	TAGLN	transgelin
7903358	2.78E-03	2.51E-04	4.97	0.347623	1.97	VCAM1	vascular cell adhesion molecule 1
7906777	2.79E-03	2.51E-04	4.97	0.344786	1.72	FCGR2B	Fc fragment of IgG receptor IIb
8148280	2.80E-03	2.53E-04	4.96	0.336685	1.28	SQLE	squalene epoxidase
7896719	2.80E-03	2.54E-04	4.96	0.336163	1.52		
7952046	2.80E-03	2.54E-04	4.96	0.332754	1.17	MPZL2	myelin protein zero like 2
8001102	2.81E-03	2.55E-04	4.96	0.3303	1.21		
8043433	2.85E-03	2.59E-04	4.95	0.313117	2.96	IGKC	immunoglobulin kappa constant

8156905	2.87E-03	2.62E-04	4.94	0.301584	1.25	MSANTD3-TMEFF1///TMEFF1	MSANTD3-TMEFF1 readthrough///transmembrane protein with EGF like and two follistatin like domains 1
7896721	2.89E-03	2.65E-04	4.94	0.292003	2.42		
8022295	2.90E-03	2.66E-04	4.94	0.288467	1.45	PIEZO2	piezo type mechanosensitive ion channel component 2

8105481	2.90E-03	2.66E-04	4.94	0.286816	1.98	GAPT	GRB2-binding adaptor protein, transmembrane
7937772	2.92E-03	2.69E-04	4.93	0.275914	2.15		
8151686	2.95E-03	2.73E-04	4.92	0.261806	1.14	MMP16	matrix metallopeptidase 16
8053713	2.98E-03	2.77E-04	4.91	0.245668	3.02	IGKC	immunoglobulin kappa constant
8130674	3.08E-03	2.89E-04	4.89	0.201937	1.1	PDE10A	phosphodiesterase 10A

8001104	3.10E-03	2.92E-04	4.88	0.19309	2.13	IGHV3-48///IGHV3-69-1///IGHV3OR16-7	immunoglobulin heavy variable 3-48///immunoglobulin heavy variable 3-69-1 (pseudogene)///immunoglobulin heavy variable 3/OR16-7 (pseudogene)

7905047	3.11E-03	2.93E-04	4.88	0.190115	2.13	FCGR1CP///FCGR1B///FCGR1A	Fc fragment of IgG receptor Ic, pseudogene///Fc fragment of IgG receptor Ib///Fc fragment of IgG receptor Ia
8129082	3.16E-03	2.99E-04	4.87	0.16794	1.21	COL10A1	collagen type X alpha 1 chain
8115076	3.19E-03	3.03E-04	4.86	0.155451	1.15	CSF1R	colony stimulating factor 1 receptor

7922343	3.20E-03	3.05E-04	4.86	0.149459	1.01	TNFSF4	tumor necrosis factor superfamily member 4
7962375	3.21E-03	3.05E-04	4.86	0.147599	1.36	PRICKLE1	prickle planar cell polarity protein 1
8022531	3.24E-03	3.09E-04	4.85	0.133914	1.59	NPC1	NPC intracellular cholesterol transporter 1

7981601	3.24E-03	3.10E-04	4.85	0.131341	1.52	IGHM///IGHG1///IGHD///IGHA2///IGHA1///IGHA2///IGHA1///IGH	immunoglobulin heavy constant mu///immunoglobulin heavy constant gamma 1 (G1m marker)///immunoglobulin heavy constant delta///immunoglobulin heavy constant alpha 2 (A2m marker)///immunoglobulin heavy constant alpha 1///immunoglobulin heavy constant alpha 2 (A2m marker)///immunoglobulin heavy constant alpha 1///immunoglobulin heavy locus

8014316	3.25E-03	3.11E-04	4.85	0.128116	1.78	CCL5	C-C motif chemokine ligand 5
8047692	3.32E-03	3.20E-04	4.83	0.099745	1.45	CTLA4	cytotoxic T-lymphocyte associated protein 4
8021470	3.33E-03	3.21E-04	4.83	0.094832	1.71	PMAIP1	phorbol-12-myristate-13-acetate-induced protein 1
7977270	3.36E-03	3.25E-04	4.82	0.083771	1.49	LOC388022	uncharacterized LOC388022
8090180	3.38E-03	3.28E-04	4.82	0.074587	2.74	MUC13	mucin 13, cell surface associated

8015607	3.40E-03	3.31E-04	4.81	0.064316	1.03	STAT3	signal transducer and activator of transcription 3
7974902	3.40E-03	3.31E-04	4.81	0.063997	1.07	RHOJ	ras homolog family member J
7942998	3.40E-03	3.32E-04	4.81	0.062198	1.4	FOLH1B///FOLH1	folate hydrolase 1B///folate hydrolase (prostate-specific membrane antigen) 1
7942372	3.42E-03	3.34E-04	4.81	0.055299	1.64	FOLR2	folate receptor beta
7924029	3.42E-03	3.34E-04	4.81	0.055013	1.11	LAMB3	laminin subunit beta 3

7989985	3.42E-03	3.35E-04	4.81	0.052375	1.56	ITGA11	integrin subunit alpha 11
7905060	3.48E-03	3.42E-04	4.8	0.030696	1.88	FCGR1CP///FCGR1B///FCGR1A	Fc fragment of IgG receptor Ic, pseudogene///Fc fragment of IgG receptor Ib///Fc fragment of IgG receptor Ia
8095343	3.48E-03	3.43E-04	4.79	0.028554	2.05	STAP1	signal transducing adaptor family member 1
7952056	3.52E-03	3.48E-04	4.79	0.013005	1.66	CD3D	CD3d molecule

8095697	3.52E-03	3.49E-04	4.78	0.010847	1.01	CXCL1	C-X-C motif chemokine ligand 1
8163908	3.53E-03	3.50E-04	4.78	0.007531	1.5	GGTA1P	glycoprotein, alpha-galactosyltransferase 1 pseudogene
8041422	3.54E-03	3.51E-04	4.78	0.00455	1.2	RASGRP3	RAS guanyl releasing protein 3
8124430	3.54E-03	3.52E-04	4.78	0.002298	2.02	HIST1H1D	histone cluster 1, H1d
8081657	3.55E-03	3.53E-04	4.78	-0.000664	1.22	CD200	CD200 molecule

8125470	3.55E-03	3.53E-04	4.78	-0.000834	1.04	HLA-DOB	major histocompatibility complex, class II, DO beta
8047677	3.56E-03	3.55E-04	4.78	-0.006552	1.64	CD28	CD28 molecule
8168524	3.68E-03	3.70E-04	4.75	-0.04974	2.21	P2RY10	purinergic receptor P2Y10
7920285	3.71E-03	3.76E-04	4.74	-0.064969	1.04	S100A2	S100 calcium binding protein A2
8021946	3.73E-03	3.79E-04	4.74	-0.072247	1.17	COLEC12	collectin subfamily member 12

7948058	3.76E-03	3.83E-04	4.73	-0.084321	1.33	FOLH1B///FOLH1	folate hydrolase 1B///folate hydrolase (prostate-specific membrane antigen) 1
8122038	3.77E-03	3.84E-04	4.73	-0.085626	1.22	TMEM200A	transmembrane protein 200A
7935776	3.78E-03	3.87E-04	4.73	-0.092998	2.04		
8102792	3.83E-03	3.93E-04	4.72	-0.108943	1.85	PCDH18	protocadherin 18
7962183	3.84E-03	3.95E-04	4.72	-0.114064	1.22	AK4	adenylate kinase 4
8050537	3.85E-03	3.96E-04	4.71	-0.118204	1.63	MATN3	matrilin 3
8112841	3.88E-03	4.00E-04	4.71	-0.128188	1.13	HOMER1	homer scaffolding protein 1

8123936	3.89E-03	4.02E-04	4.71	-0.133931	1.03	NEDD9	neural precursor cell expressed, developmentally down-regulated 9

7981740	3.92E-03	4.06E-04	4.7	-0.141813	2.26	SCFV///IGLJ3///IGHM///IGHA1///SCFV///C14orf99///IGK///IGKV3-20///IGHV3-23///IGHV3-30///IGHV3-48///IGHV3-69-1	single-chain Fv fragment///immunoglobulin lambda joining 3///immunoglobulin heavy constant mu///immunoglobulin heavy constant alpha 1///single-chain Fv fragment///uncharacterized LOC317730///immunoglobulin kappa locus///immunoglobulin kappa variable 3-20///immunoglobulin heavy variable 3-23///immunoglobulin heavy variable 3-30///immunoglobulin heavy variable 3-48///immunoglobulin heavy variable 3-69-1 (pseudogene)

8163672	3.92E-03	4.07E-04	4.7	-0.144774	1.5	PAPPA-AS1	PAPPA antisense RNA 1
7923917	3.93E-03	4.08E-04	4.7	-0.148731	1.46	FCMR	Fc fragment of IgM receptor
8124280	3.94E-03	4.09E-04	4.7	-0.150735	1.27	FAM65B	family with sequence similarity 65 member B
7965156	3.95E-03	4.11E-04	4.69	-0.154809	1.3	LIN7A	lin-7 homolog A, crumbs cell polarity complex component
8092095	3.99E-03	4.17E-04	4.69	-0.169038	1.07	TNIK	TRAF2 and NCK interacting kinase
8163000	4.01E-03	4.18E-04	4.68	-0.173608	1.23		

8029086	4.01E-03	4.20E-04	4.68	-0.176435	3.37	CEACAM5	carcinoembryonic antigen related cell adhesion molecule 5
7964834	4.05E-03	4.24E-04	4.68	-0.187971	1.32	CPM	carboxypeptidase M
7917503	4.05E-03	4.25E-04	4.68	-0.189644	2.15	GBP3	guanylate binding protein 3
7972601	4.05E-03	4.26E-04	4.68	-0.190608	1.09	NALCN	sodium leak channel, non-selective
7984001	4.06E-03	4.27E-04	4.67	-0.192942	2.34	GCNT3	glucosaminyl (N-acetyl) transferase 3, mucin type

8130556	4.14E-03	4.39E-04	4.66	-0.222395	1.49	SOD2	superoxide dismutase 2, mitochondrial
8068593	4.19E-03	4.46E-04	4.65	-0.238028	1.54	ETS2	ETS proto-oncogene 2, transcription factor
7932254	4.22E-03	4.51E-04	4.64	-0.249401	1.45	ITGA8	integrin subunit alpha 8
7942596	4.23E-03	4.52E-04	4.64	-0.250921	1.52	SERPINH1	serpin family H member 1
7983630	4.24E-03	4.53E-04	4.64	-0.254136	2.11	FGF7	fibroblast growth factor 7

8092552	4.25E-03	4.56E-04	4.64	-0.260124	1.03	IGF2BP2	insulin like growth factor 2 mRNA binding protein 2
7962579	4.30E-03	4.62E-04	4.63	-0.27484	1.25	AMIGO2	adhesion molecule with Ig like domain 2
8067932	4.30E-03	4.63E-04	4.63	-0.275317	1.56	MIR99AHG	mir-99a-let-7c cluster host gene
7957298	4.31E-03	4.64E-04	4.63	-0.278299	1.31	NAV3	neuron navigator 3
7981068	4.32E-03	4.65E-04	4.63	-0.281175	1.33	SERPINA1	serpin family A member 1
7896702	4.35E-03	4.69E-04	4.62	-0.288789	1.03		

7981732	4.37E-03	4.72E-04	4.62	-0.294984	2.58	LOC102723407///IGHV4-31///IGHM///IGHD///IGHA2///LOC102723407///SKAP2///IGHA2///IGHA1///IGH	putative V-set and immunoglobulin domain-containing-like protein IGHV4OR15-8///immunoglobulin heavy variable 4-31///immunoglobulin heavy constant mu///immunoglobulin heavy constant delta///immunoglobulin heavy constant alpha 2 (A2m marker)///putative V-set and immunoglobulin domain-containing-like protein IGHV4OR15-8///src kinase associated phosphoprotein 2///immunoglobulin heavy constant alpha 2 (A2m marker)///immunoglobulin heavy constant alpha 1///immunoglobulin heavy locus

7993588	4.39E-03	4.75E-04	4.62	-0.301691	1.21	TMC7	transmembrane channel like 7
8097080	4.39E-03	4.75E-04	4.62	-0.30174	1.63	SYNPO2	synaptopodin 2
7919133	4.41E-03	4.78E-04	4.61	-0.307961	1.83	FCGR1CP///FCGR1B///FCGR1A	Fc fragment of IgG receptor Ic, pseudogene///Fc fragment of IgG receptor Ib///Fc fragment of IgG receptor Ia

7917728	4.42E-03	4.80E-04	4.61	-0.312456	1.15	FAM69A	family with sequence similarity 69 member A
7981737	4.43E-03	4.81E-04	4.61	-0.315962	1.96	IGHV3-69-1///IGHV3OR16-7	immunoglobulin heavy variable 3-69-1 (pseudogene)///immunoglobulin heavy variable 3/OR16-7 (pseudogene)
7963817	4.45E-03	4.84E-04	4.6	-0.321984	1.33	GTSF1	gametocyte specific factor 1

7995263	4.47E-03	4.88E-04	4.6	-0.329582	2.22	SCFV///IGLJ3///IGHV3-23///IGHV3-41///IGHV4-31///IGHM///IGHG2///IGHG1///IGHA1///IGH///C14orf99///IGK///IGKV3-20///IGHV3-23///IGHV3-48///IGHV3-75///IGHV3-69-1///IGHV3OR16-7	single-chain Fv fragment///immunoglobulin lambda joining 3///immunoglobulin heavy variable 3-23///immunoglobulin heavy variable 3-41 (pseudogene)///immunoglobulin heavy variable 4-31///immunoglobulin heavy constant mu///immunoglobulin heavy constant gamma 2 (G2m marker)///immunoglobulin heavy constant gamma 1 (G1m marker)///immunoglobulin heavy constant alpha 1///immunoglobulin heavy locus///uncharacterized LOC317730///immunoglobulin kappa locus///immunoglobulin kappa variable 3-20///immunoglobulin heavy variable 3-23///immunoglobulin heavy variable 3-48///immunoglobulin heavy variable 3-75 (pseudogene)///immunoglobulin heavy variable 3-69-1 (pseudogene)///immunoglobulin heavy variable 3/OR16-7 (pseudogene)

7904131	4.53E-03	4.96E-04	4.59	-0.346826	1.08	DCLRE1B	DNA cross-link repair 1B
7923386	4.54E-03	4.98E-04	4.59	-0.349887	1.12	LMOD1	leiomodin 1
7983478	4.54E-03	4.99E-04	4.59	-0.352061	2.02	C15orf48	chromosome 15 open reading frame 48
8031238	4.55E-03	4.99E-04	4.59	-0.352459	1.17	LILRB4	leukocyte immunoglobulin like receptor B4
7918457	4.55E-03	5.00E-04	4.59	-0.353683	1.22	KCNA3	potassium voltage-gated channel subfamily A member 3
7960514	4.56E-03	5.02E-04	4.58	-0.358639	2.32		

8129482	4.57E-03	5.04E-04	4.58	-0.363287	1.45	SAMD3	sterile alpha motif domain containing 3
8057887	4.57E-03	5.05E-04	4.58	-0.364096	1.05	STK17B	serine/threonine kinase 17b
8166065	4.58E-03	5.05E-04	4.58	-0.365249	1.48	TLR8	toll like receptor 8
8049528	4.61E-03	5.10E-04	4.58	-0.37407	1.17	LRRFIP1	LRR binding FLII interacting protein 1
8150962	4.62E-03	5.12E-04	4.57	-0.378339	1.08	TOX	thymocyte selection associated high mobility group box

7995096	4.62E-03	5.12E-04	4.57	-0.379252	1.34	ITGAM	integrin subunit alpha M
7921652	4.68E-03	5.21E-04	4.56	-0.396105	1	SLAMF1	signaling lymphocytic activation molecule family member 1
8112033	4.73E-03	5.28E-04	4.56	-0.409711	1.03	ARL15	ADP ribosylation factor like GTPase 15
8038899	4.76E-03	5.34E-04	4.55	-0.420829	2.13	FPR1	formyl peptide receptor 1
8155898	4.78E-03	5.37E-04	4.55	-0.425952	1.03	PCSK5	proprotein convertase subtilisin/kexin type 5

8095736	4.85E-03	5.48E-04	4.54	-0.447427	2.19	AREG	amphiregulin
7960771	4.85E-03	5.49E-04	4.54	-0.449466	1.46	CD163L1	CD163 molecule like 1
8041206	4.85E-03	5.49E-04	4.54	-0.449706	1.45	LBH	limb bud and heart development
8112018	4.90E-03	5.58E-04	4.53	-0.465488	1.07	PELO	pelota homolog (Drosophila)
8091503	4.96E-03	5.67E-04	4.52	-0.482712	1.46	GPR171	G protein-coupled receptor 171

8122202	4.98E-03	5.70E-04	4.52	-0.486684	1.09	MYB///MYB	MYB proto-oncogene, transcription factor///MYB proto-oncogene, transcription factor
7915472	5.15E-03	5.96E-04	4.49	-0.533196	1.5	SLC2A1	solute carrier family 2 member 1
8054712	5.15E-03	5.97E-04	4.49	-0.535003	2	IL1A	interleukin 1 alpha
7969493	5.16E-03	6.00E-04	4.49	-0.539264	1.91	SCEL	sciellin
8129410	5.21E-03	6.10E-04	4.48	-0.555338	1.66	THEMIS	thymocyte selection associated
7928429	5.23E-03	6.12E-04	4.48	-0.560185	1.03	PLAU	plasminogen activator, urokinase
7896729	5.28E-03	6.21E-04	4.47	-0.573529	2.17		

8138805	5.34E-03	6.31E-04	4.46	-0.590847	1.11	CPVL	carboxypeptidase, vitellogenic like
8048717	5.36E-03	6.35E-04	4.46	-0.596463	1.24	SGPP2	sphingosine-1-phosphate phosphatase 2
8089771	5.43E-03	6.44E-04	4.45	-0.610831	1.04	CD80	CD80 molecule
8021685	5.49E-03	6.56E-04	4.44	-0.629417	1.35	CCDC102B	coiled-coil domain containing 102B
8048761	5.50E-03	6.57E-04	4.44	-0.631421	1.16		
8136938	5.69E-03	6.89E-04	4.41	-0.678929	1.09		
8143599	5.69E-03	6.89E-04	4.41	-0.678929	1.09		
7979710	5.69E-03	6.90E-04	4.41	-0.680951	1.16	PLEK2	pleckstrin 2
8126428	5.72E-03	6.95E-04	4.41	-0.68872	1.15	TRERF1	transcriptional regulating factor 1

7933872	5.82E-03	7.12E-04	4.39	-0.713484	1.46	EGR2	early growth response 2
7986068	5.83E-03	7.13E-04	4.39	-0.714743	1.27	BLM	Bloom syndrome RecQ like helicase
7948904	5.83E-03	7.13E-04	4.39	-0.714864	1.16	SNORD28	small nucleolar RNA, C/D box 28
8166705	5.91E-03	7.28E-04	4.38	-0.735	1.13	PRRG1	proline rich and Gla domain 1
8172270	5.95E-03	7.35E-04	4.38	-0.744485	1.59		
8120335	5.95E-03	7.35E-04	4.38	-0.745696	1.1	FAM83B	family with sequence similarity 83 member B
8091735	6.07E-03	7.55E-04	4.36	-0.772303	1.48		

8078442	6.13E-03	7.65E-04	4.36	-0.785884	1.37	CCR4	C-C motif chemokine receptor 4
7896710	6.14E-03	7.66E-04	4.36	-0.78739	2.51		
8006602	6.14E-03	7.67E-04	4.35	-0.788578	1.98	CCL4	C-C motif chemokine ligand 4
8096959	6.17E-03	7.73E-04	4.35	-0.79624	1.27	ANK2	ankyrin 2, neuronal
8126820	6.31E-03	7.98E-04	4.33	-0.828602	1.42	ADGRF1	adhesion G protein-coupled receptor F1
7989073	6.35E-03	8.06E-04	4.33	-0.838095	1.03	PRTG	protogenin
8029536	6.43E-03	8.18E-04	4.32	-0.853669	1.57	APOC1	apolipoprotein C1

8006621	6.50E-03	8.32E-04	4.31	-0.870049	2.15	CCL4L1///CCL4L2///CCL4///CCL4L1	C-C motif chemokine ligand 4 like 1///C-C motif chemokine ligand 4 like 2///C-C motif chemokine ligand 4///C-C motif chemokine ligand 4 like 1

8019651	6.50E-03	8.32E-04	4.31	-0.870049	2.15	CCL4L1///CCL4L2///CCL4///CCL4L1	C-C motif chemokine ligand 4 like 1///C-C motif chemokine ligand 4 like 2///C-C motif chemokine ligand 4///C-C motif chemokine ligand 4 like 1
8035304	6.60E-03	8.51E-04	4.3	-0.893636	1.14	BST2	bone marrow stromal cell antigen 2

8100798	6.61E-03	8.53E-04	4.3	-0.895862	2.51	SULT1B1	sulfotransferase family 1B member 1
7971311	6.71E-03	8.67E-04	4.29	-0.912799	1.06	ENOX1	ecto-NOX disulfide-thiol exchanger 1
8105300	6.71E-03	8.68E-04	4.29	-0.913075	1.94		
8166059	6.84E-03	8.90E-04	4.27	-0.939277	1.39	TLR7	toll like receptor 7
8148070	6.92E-03	9.02E-04	4.27	-0.95196	1.85	COL14A1	collagen type XIV alpha 1 chain
8092541	6.95E-03	9.08E-04	4.26	-0.958676	1.62	LIPH	lipase H

8135015	7.03E-03	9.21E-04	4.26	-0.973614	1.06	MUC3B///MUC3A	mucin 3B, cell surface associated///mucin 3A, cell surface associated
7954090	7.07E-03	9.31E-04	4.25	-0.984823	1.27	EMP1	epithelial membrane protein 1
8057771	7.16E-03	9.48E-04	4.24	-1.002205	1.05	STAT4	signal transducer and activator of transcription 4
7906377	7.16E-03	9.49E-04	4.24	-1.003464	1.91	MNDA	myeloid cell nuclear differentiation antigen

8114249	7.16E-03	9.50E-04	4.24	-1.00461	1.91	CXCL14	C-X-C motif chemokine ligand 14
8146092	7.16E-03	9.50E-04	4.24	-1.005152	1.45	IDO1	indoleamine 2,3-dioxygenase 1
7927631	7.17E-03	9.52E-04	4.24	-1.006345	1.77	DKK1	dickkopf WNT signaling pathway inhibitor 1
7997504	7.20E-03	9.57E-04	4.24	-1.012136	1.06	CDH13	cadherin 13
8049542	7.24E-03	9.64E-04	4.23	-1.019632	1.15	LRRFIP1	LRR binding FLII interacting protein 1
8101828	7.28E-03	9.72E-04	4.23	-1.027554	1.39	TSPAN5	tetraspanin 5

8135734	7.31E-03	9.78E-04	4.22	-1.034464	1.46	CPED1	cadherin like and PC-esterase domain containing 1
8123246	7.33E-03	9.82E-04	4.22	-1.038602	1.39	SLC22A3	solute carrier family 22 member 3
7921873	7.35E-03	9.87E-04	4.22	-1.043191	2.61	FCGR3B///FCGR3A	Fc fragment of IgG receptor IIIb///Fc fragment of IgG receptor IIIa
7965873	7.36E-03	9.89E-04	4.22	-1.045769	2.53	IGF1	insulin like growth factor 1
8119076	7.61E-03	1.04E-03	4.19	-1.094094	1.18		
7986637	7.69E-03	1.05E-03	4.18	-1.110197	2.03		

7999674	7.69E-03	1.05E-03	4.18	-1.110623	2.09	MYH11	myosin heavy chain 11
8015133	7.74E-03	1.06E-03	4.18	-1.119629	2.16	KRT23	keratin 23
7915504	8.18E-03	1.15E-03	4.14	-1.19768	1.02	ELOVL1	ELOVL fatty acid elongase 1
8091523	8.22E-03	1.16E-03	4.13	-1.20595	1.83	P2RY13	purinergic receptor P2Y13
8125447	8.27E-03	1.17E-03	4.13	-1.214034	2.89	HLA-DRB1///HLA-DQB1	major histocompatibility complex, class II, DR beta 1///major histocompatibility complex, class II, DQ beta 1

8135048	8.27E-03	1.17E-03	4.13	-1.214287	2.02	MUC17	mucin 17, cell surface associated
8044080	8.33E-03	1.18E-03	4.12	-1.225045	1.51	SLC9A2	solute carrier family 9 member A2
7896697	8.34E-03	1.18E-03	4.12	-1.225869	1.9		
8134036	8.36E-03	1.19E-03	4.12	-1.23413	1.08	STEAP2	STEAP2 metalloreductase
7944179	8.48E-03	1.21E-03	4.11	-1.253237	1.39	CD3E	CD3e molecule
8083136	8.53E-03	1.23E-03	4.1	-1.261727	1.47	ATP1B3	ATPase Na+/K+ transporting subunit beta 3
8047788	8.57E-03	1.23E-03	4.1	-1.267398	1.04	ADAM23	ADAM metallopeptidase domain 23

8062927	8.59E-03	1.23E-03	4.1	-1.269728	1.84	PI3	peptidase inhibitor 3
7961151	8.68E-03	1.25E-03	4.09	-1.285374	1.76	KLRC4-KLRK1///KLRK1	KLRC4-KLRK1 readthrough///killer cell lectin like receptor K1
7918064	8.74E-03	1.27E-03	4.09	-1.295283	1.64	COL11A1	collagen type XI alpha 1 chain
8131550	8.97E-03	1.31E-03	4.07	-1.332524	1.29	SCIN	scinderin
8039257	9.14E-03	1.35E-03	4.05	-1.359556	1.08	LAIR1	leukocyte associated immunoglobulin like receptor 1

8089519	9.44E-03	1.41E-03	4.03	-1.405175	1.18	BTLA	B and T lymphocyte associated
8043443	9.56E-03	1.44E-03	4.02	-1.42178	2.33		
8115074	9.57E-03	1.44E-03	4.02	-1.423013	1.34		
8084810	9.76E-03	1.48E-03	4	-1.449746	1.39		
7922200	9.86E-03	1.50E-03	4	-1.466852	1.13	SELP	selectin P
7922229	1.01E-02	1.55E-03	3.98	-1.501498	1.51	SELE	selectin E

8006608	1.01E-02	1.56E-03	3.98	-1.504041	1.75	CCL4L1///CCL4L2///CCL4///CCL4L1	C-C motif chemokine ligand 4 like 1///C-C motif chemokine ligand 4 like 2///C-C motif chemokine ligand 4///C-C motif chemokine ligand 4 like 1
8059376	1.02E-02	1.56E-03	3.97	-1.508224	1.85	SERPINE2	serpin family E member 2
8056222	1.02E-02	1.58E-03	3.97	-1.519554	1.55	DPP4	dipeptidyl peptidase 4

8022488	1.04E-02	1.61E-03	3.96	-1.535512	1.4	ABHD3	abhydrolase domain containing 3
8154245	1.04E-02	1.62E-03	3.96	-1.540675	1.22	PDCD1LG2	programmed cell death 1 ligand 2
7933084	1.04E-02	1.62E-03	3.96	-1.541289	1.09	NAMPT	nicotinamide phosphoribosyltransferase
8055980	1.05E-02	1.63E-03	3.95	-1.546737	1.81	CYTIP	cytohesin 1 interacting protein
8165866	1.05E-02	1.63E-03	3.95	-1.54966	1	STS	steroid sulfatase (microsomal), isozyme S
7934916	1.05E-02	1.64E-03	3.95	-1.552517	1.32	CH25H	cholesterol 25-hydroxylase

7896720	1.05E-02	1.64E-03	3.95	-1.556744	1.15		
8020164	1.05E-02	1.65E-03	3.95	-1.558822	1.06	GNAL	G protein subunit alpha L
7896693	1.06E-02	1.67E-03	3.94	-1.570979	1.91		
7961075	1.06E-02	1.67E-03	3.94	-1.570979	1.91	CD69	CD69 molecule
8072587	1.06E-02	1.67E-03	3.94	-1.576279	1.11	SLC5A1	solute carrier family 5 member 1
8126303	1.08E-02	1.69E-03	3.93	-1.588604	2.55	TREM1	triggering receptor expressed on myeloid cells 1
7896728	1.09E-02	1.73E-03	3.92	-1.612143	1.84		
8042942	1.10E-02	1.74E-03	3.92	-1.614236	1.05	HK2	hexokinase 2
8148325	1.10E-02	1.75E-03	3.91	-1.622229	1.24		
8007990	1.11E-02	1.78E-03	3.91	-1.637151	1.33		

8123951	1.14E-02	1.84E-03	3.89	-1.671035	2.03	ADTRP	androgen dependent TFPI regulating protein
8100474	1.15E-02	1.85E-03	3.88	-1.678679	1.35		
8014391	1.15E-02	1.86E-03	3.88	-1.679482	1.39	CCL3L3///CCL3L1///CCL3	C-C motif chemokine ligand 3 like 3///C-C motif chemokine ligand 3 like 1///C-C motif chemokine ligand 3

8014414	1.15E-02	1.86E-03	3.88	-1.679482	1.39	CCL3L3///CCL3L1///CCL3	C-C motif chemokine ligand 3 like 3///C-C motif chemokine ligand 3 like 1///C-C motif chemokine ligand 3
8019731	1.15E-02	1.86E-03	3.88	-1.679482	1.39	CCL3L3///CCL3L1///CCL3	C-C motif chemokine ligand 3 like 3///C-C motif chemokine ligand 3 like 1///C-C motif chemokine ligand 3

8111443	1.15E-02	1.86E-03	3.88	-1.681649	1.29	C1QTNF3-AMACR///C1QTNF3	C1QTNF3-AMACR readthrough (NMD candidate)///C1q and tumor necrosis factor related protein 3
8043036	1.15E-02	1.88E-03	3.88	-1.690552	1.01	LOC1720///DHFR	dihydrofolate reductase pseudogene///dihydrofolate reductase
8100827	1.16E-02	1.89E-03	3.87	-1.697994	2.66	JCHAIN	joining chain of multimeric IgA and IgM
7908312	1.17E-02	1.93E-03	3.86	-1.717478	1.53	PRG4	proteoglycan 4

7910611	1.19E-02	1.96E-03	3.86	-1.735369	1.41	KCNK1	potassium two pore domain channel subfamily K member 1
8137240	1.20E-02	1.98E-03	3.85	-1.744864	1.51	GIMAP7	GTPase, IMAP family member 7
7973303	1.21E-02	2.00E-03	3.84	-1.755408	1.13	OR6C4	olfactory receptor family 6 subfamily C member 4
8115455	1.22E-02	2.04E-03	3.84	-1.773182	1.51	HAVCR1	hepatitis A virus cellular receptor 1
8038683	1.22E-02	2.04E-03	3.83	-1.776623	2.01	KLK6	kallikrein related peptidase 6

7913216	1.24E-02	2.10E-03	3.82	-1.803683	2.27	PLA2G2A	phospholipase A2 group IIA
8083494	1.24E-02	2.10E-03	3.82	-1.805317	1.37	MME	membrane metallo-endopeptidase
7953835	1.30E-02	2.23E-03	3.79	-1.866026	1.26	KLRG1	killer cell lectin like receptor G1
7940226	1.30E-02	2.24E-03	3.79	-1.869234	1.51	MS4A2	membrane spanning 4-domains A2
8099834	1.31E-02	2.26E-03	3.78	-1.877064	1.24	TLR1	toll like receptor 1
7923547	1.36E-02	2.39E-03	3.75	-1.931971	1.34	CHI3L1	chitinase 3 like 1
8071642	1.36E-02	2.40E-03	3.75	-1.937206	1.43		
7923929	1.37E-02	2.41E-03	3.75	-1.941019	2	PIGR	polymeric immunoglobulin receptor

7929816	1.37E-02	2.42E-03	3.75	-1.944262	1.47	SCD	stearoyl-CoA desaturase
8168622	1.38E-02	2.44E-03	3.74	-1.955688	1.34	KLHL4	kelch like family member 4
7908376	1.41E-02	2.52E-03	3.72	-1.98544	1.19	RGS18	regulator of G-protein signaling 18
7964927	1.45E-02	2.61E-03	3.7	-2.02379	1.25	TSPAN8	tetraspanin 8
7996022	1.47E-02	2.66E-03	3.7	-2.040451	1.55	CCL22	C-C motif chemokine ligand 22
8098287	1.48E-02	2.69E-03	3.69	-2.052932	1.1		
8143790	1.50E-02	2.73E-03	3.68	-2.068572	1.08	TMEM176B	transmembrane protein 176B
8094028	1.50E-02	2.74E-03	3.68	-2.071977	1.52	AFAP1-AS1	AFAP1 antisense RNA 1

8117018	1.52E-02	2.80E-03	3.67	-2.09218	1.96		
7960865	1.53E-02	2.81E-03	3.67	-2.096274	1.76	SLC2A3	solute carrier family 2 member 3
8038695	1.53E-02	2.81E-03	3.67	-2.096776	1.1	KLK7	kallikrein related peptidase 7
8125436	1.53E-02	2.83E-03	3.66	-2.103163	2.38	HLA-DRB5	major histocompatibility complex, class II, DR beta 5
8097857	1.54E-02	2.86E-03	3.66	-2.112089	1.57	MND1	meiotic nuclear divisions 1
7907249	1.55E-02	2.88E-03	3.65	-2.121965	1.06	FMO3	flavin containing monooxygenase 3

7934278	1.56E-02	2.89E-03	3.65	-2.125125	1.04	P4HA1///P4HA1	prolyl 4-hydroxylase subunit alpha 1///prolyl 4-hydroxylase subunit alpha 1
8112731	1.59E-02	2.98E-03	3.64	-2.155805	1.44	F2RL2	coagulation factor II thrombin receptor like 2
8109926	1.59E-02	3.01E-03	3.63	-2.162987	2.9	GABRP	gamma-aminobutyric acid type A receptor pi subunit
8042788	1.60E-02	3.01E-03	3.63	-2.16374	2.93	ACTG2	actin, gamma 2, smooth muscle, enteric

8137257	1.61E-02	3.04E-03	3.63	-2.174287	1.15	GIMAP1-GIMAP5///GIMAP5	GIMAP1-GIMAP5 readthrough///GTPase, IMAP family member 5
8077499	1.64E-02	3.10E-03	3.62	-2.193731	1.35	LINC00312	long intergenic non-protein coding RNA 312
8044499	1.66E-02	3.16E-03	3.61	-2.212774	1.14	SLC20A1	solute carrier family 20 member 1

8067839	1.66E-02	3.17E-03	3.6	-2.217778	1.26	FGF7P3///FGF7P6///FGF7	fibroblast growth factor 7 pseudogene 3///fibroblast growth factor 7 pseudogene 6///fibroblast growth factor 7
8047702	1.69E-02	3.23E-03	3.59	-2.236252	1.14	ICOS	inducible T-cell costimulator

8049349	1.70E-02	3.27E-03	3.59	-2.246428	1.1	UGT1A3///UGT1A1///UGT1A4///UGT1A9///UGT1A5///UGT1A6///UGT1A7///UGT1A8///UGT1A10	UDP glucuronosyltransferase family 1 member A3///UDP glucuronosyltransferase family 1 member A1///UDP glucuronosyltransferase family 1 member A4///UDP glucuronosyltransferase family 1 member A9///UDP glucuronosyltransferase family 1 member A5///UDP glucuronosyltransferase family 1 member A6///UDP glucuronosyltransferase family 1 member A7///UDP glucuronosyltransferase family 1 member A8///UDP glucuronosyltransferase family 1 member A10

8094533	1.75E-02	3.40E-03	3.57	-2.286113	1.19	DTHD1	death domain containing 1
7909946	1.78E-02	3.49E-03	3.55	-2.312229	2.39	FAM177B	family with sequence similarity 177 member B
8152863	1.80E-02	3.53E-03	3.55	-2.324727	1.14		
8089145	1.80E-02	3.54E-03	3.55	-2.325652	1.45	ABI3BP	ABI family member 3 binding protein
7957431	1.83E-02	3.62E-03	3.54	-2.348745	1		

8118409	1.86E-02	3.70E-03	3.52	-2.371025	1.33	C4B_2///C4B///C4A	complement component 4B (Chido blood group), copy 2///complement component 4B (Chido blood group)///complement component 4A (Rodgers blood group)

8118455	1.86E-02	3.70E-03	3.52	-2.371025	1.33	C4B_2///C4B///C4A	complement component 4B (Chido blood group), copy 2///complement component 4B (Chido blood group)///complement component 4A (Rodgers blood group)

8179399	1.86E-02	3.70E-03	3.52	-2.371025	1.33	C4B_2///C4B///C4A	complement component 4B (Chido blood group), copy 2///complement component 4B (Chido blood group)///complement component 4A (Rodgers blood group)

7968351	1.87E-02	3.73E-03	3.52	-2.379821	1.54	MEDAG	mesenteric estrogen dependent adipogenesis
7896715	1.88E-02	3.75E-03	3.52	-2.383942	1.65		
8121911	1.90E-02	3.82E-03	3.51	-2.402795	1.07	CENPW	centromere protein W
8044605	1.91E-02	3.85E-03	3.5	-2.410548	1.46	PAX8-AS1	PAX8 antisense RNA 1
7922976	1.92E-02	3.86E-03	3.5	-2.413646	2.04	PTGS2	prostaglandin-endoperoxide synthase 2
8060897	1.93E-02	3.89E-03	3.5	-2.419915	1.23	PLCB4	phospholipase C beta 4
8082928	1.93E-02	3.89E-03	3.5	-2.420963	2.18	CLDN18	claudin 18

7985317	1.95E-02	3.95E-03	3.49	-2.435267	1.04	CEMIP	cell migration inducing hyaluronan binding protein
7929466	1.97E-02	4.01E-03	3.48	-2.450992	1.93	CYP2C18	cytochrome P450 family 2 subfamily C member 18
8100154	1.97E-02	4.01E-03	3.48	-2.451812	1.26	CORIN	corin, serine peptidase
8160879	1.98E-02	4.05E-03	3.48	-2.460263	2.55	CCL19	C-C motif chemokine ligand 19
8117286	1.98E-02	4.06E-03	3.48	-2.462236	1.21		

8084206	2.01E-02	4.13E-03	3.47	-2.479456	1	B3GNT5	UDP-GlcNAc:betaGal beta-1,3-N-acetylglucosaminyltransferase 5
8092970	2.01E-02	4.13E-03	3.47	-2.479925	1.1	APOD	apolipoprotein D
7939052	2.02E-02	4.15E-03	3.46	-2.485975	1.2	FIBIN	fin bud initiation factor homolog (zebrafish)
8095886	2.03E-02	4.18E-03	3.46	-2.493106	1.1	CXCL13	C-X-C motif chemokine ligand 13
8128553	2.03E-02	4.20E-03	3.46	-2.496441	1.37	BVES	blood vessel epicardial substance

7980485	2.06E-02	4.29E-03	3.45	-2.518024	1.56	DIO2	deiodinase, iodothyronine, type II
7983239	2.07E-02	4.31E-03	3.44	-2.522642	1.18	CKMT1A///CKMT1B///CKMT1A///CKMT1B	creatine kinase, mitochondrial 1A///creatine kinase, mitochondrial 1B///creatine kinase, mitochondrial 1A///creatine kinase, mitochondrial 1B

7983256	2.07E-02	4.31E-03	3.44	-2.522642	1.18	CKMT1A///CKMT1B///CKMT1A///CKMT1B	creatine kinase, mitochondrial 1A///creatine kinase, mitochondrial 1B///creatine kinase, mitochondrial 1A///creatine kinase, mitochondrial 1B
8125461	2.08E-02	4.35E-03	3.44	-2.530873	1.77	HLA-DQB1	major histocompatibility complex, class II, DQ beta 1

7923772	2.14E-02	4.52E-03	3.42	-2.569354	1.71	LEMD1	LEM domain containing 1
8138392	2.14E-02	4.52E-03	3.42	-2.569619	1.74	AGR3	anterior gradient 3, protein disulphide isomerase family member
8099467	2.15E-02	4.55E-03	3.42	-2.576804	1.47	FGFBP1	fibroblast growth factor binding protein 1
8100977	2.18E-02	4.65E-03	3.41	-2.597356	2.67	CXCL5	C-X-C motif chemokine ligand 5
8179617	2.21E-02	4.73E-03	3.4	-2.614918	1.01	TRIM31	tripartite motif containing 31

7920271	2.21E-02	4.74E-03	3.4	-2.616593	2.11	S100A4	S100 calcium binding protein A4
8095467	2.22E-02	4.79E-03	3.39	-2.627855	1.01	FDCSP	follicular dendritic cell secreted protein
7983910	2.23E-02	4.80E-03	3.39	-2.630629	1.91	AQP9	aquaporin 9
8067125	2.23E-02	4.81E-03	3.39	-2.632002	1.62	BCAS1	breast carcinoma amplified sequence 1
8053715	2.23E-02	4.81E-03	3.39	-2.632171	1.55		
8160889	2.24E-02	4.84E-03	3.38	-2.638545	1.37	CCL21	C-C motif chemokine ligand 21
8021635	2.25E-02	4.89E-03	3.38	-2.648255	1.9	SERPINB2	serpin family B member 2

8077366	2.28E-02	4.96E-03	3.37	-2.662083	1.16	LRRN1	leucine rich repeat neuronal 1
7978544	2.30E-02	5.05E-03	3.36	-2.680101	1.11	EGLN3	egl-9 family hypoxia inducible factor 3
8129637	2.31E-02	5.08E-03	3.36	-2.685401	1.68	VNN2	vanin 2
7909730	2.32E-02	5.11E-03	3.36	-2.692102	1.13	KCNK2	potassium two pore domain channel subfamily K member 2
8028652	2.32E-02	5.12E-03	3.36	-2.692762	1.03	ZFP36	ZFP36 ring finger protein
8077323	2.34E-02	5.17E-03	3.35	-2.703274	1.09	CNTN4	contactin 4

7997712	2.36E-02	5.23E-03	3.34	-2.715323	1.15	IRF8	interferon regulatory factor 8
8098246	2.37E-02	5.25E-03	3.34	-2.719392	2.72	ANXA10	annexin A10
8104746	2.37E-02	5.28E-03	3.34	-2.723549	1.9	NPR3	natriuretic peptide receptor 3
7951309	2.38E-02	5.29E-03	3.34	-2.726651	1.59	MMP13	matrix metallopeptidase 13
7963946	2.39E-02	5.34E-03	3.33	-2.734485	1.16	MMP19	matrix metallopeptidase 19
8070574	2.39E-02	5.34E-03	3.33	-2.735742	1.14	TFF2	trefoil factor 2
8157650	2.42E-02	5.40E-03	3.33	-2.746452	1.01	PTGS1	prostaglandin-endoperoxide synthase 1
8091511	2.46E-02	5.53E-03	3.32	-2.769224	1.53	P2RY14	purinergic receptor P2Y14

8118061	2.50E-02	5.66E-03	3.3	-2.793765	3.3	DPCR1	diffuse panbronchiolitis critical region 1
8091411	2.50E-02	5.67E-03	3.3	-2.795623	1.11	TM4SF1	transmembrane 4 L six family member 1
7988350	2.50E-02	5.68E-03	3.3	-2.795686	1.68	DUOX2	dual oxidase 2
7993267	2.53E-02	5.77E-03	3.29	-2.811688	1.51	TNFRSF17	TNF receptor superfamily member 17
8138337	2.54E-02	5.77E-03	3.29	-2.812744	1.2	AGMO	alkylglycerol monooxygenase
8099850	2.59E-02	5.93E-03	3.28	-2.839092	1.39	TMEM156	transmembrane protein 156

8095110	2.62E-02	6.02E-03	3.27	-2.853408	1.13	KIT	KIT proto-oncogene receptor tyrosine kinase
8121916	2.74E-02	6.41E-03	3.24	-2.915973	1.3	RSPO3	R-spondin 3
8043743	2.75E-02	6.46E-03	3.23	-2.924038	1.03		
7956271	2.81E-02	6.65E-03	3.22	-2.952071	1.64	HSD17B6	hydroxysteroid 17-beta dehydrogenase 6
8073088	2.84E-02	6.76E-03	3.21	-2.969036	1.11	APOBEC3G	apolipoprotein B mRNA editing enzyme catalytic subunit 3G
8020762	2.85E-02	6.80E-03	3.21	-2.974257	1.33	DSG3	desmoglein 3

8049540	2.86E-02	6.82E-03	3.21	-2.977426	1.27	LRRFIP1	LRR binding FLII interacting protein 1
7962312	2.86E-02	6.83E-03	3.21	-2.979009	1.16	ABCD2	ATP binding cassette subfamily D member 2
7940287	2.93E-02	7.10E-03	3.19	-3.017249	1.78	MS4A1	membrane spanning 4-domains A1
8071036	3.00E-02	7.33E-03	3.17	-3.048171	1.19	S100B	S100 calcium binding protein B
8042503	3.04E-02	7.48E-03	3.16	-3.068134	1.24	MXD1	MAX dimerization protein 1

7906348	3.04E-02	7.48E-03	3.16	-3.068161	1.46	CD1C	CD1c molecule
8162117	3.05E-02	7.52E-03	3.16	-3.073254	1.06	GOLM1	golgi membrane protein 1
8169263	3.08E-02	7.62E-03	3.15	-3.086864	3.25	VSIG1	V-set and immunoglobulin domain containing 1
8105596	3.09E-02	7.64E-03	3.15	-3.089809	1.17	RGS7BP	regulator of G-protein signaling 7 binding protein
7906919	3.13E-02	7.79E-03	3.14	-3.107993	1.14	RGS4	regulator of G-protein signaling 4
8079237	3.17E-02	7.96E-03	3.13	-3.129857	1.26	KIF15	kinesin family member 15

8179221	3.17E-02	7.97E-03	3.13	-3.130433	3.09	DPCR1	diffuse panbronchiolitis critical region 1
8155487	3.25E-02	8.21E-03	3.11	-3.160679	1.16	FGF7P3///FGF7P6///FGF7	fibroblast growth factor 7 pseudogene 3///fibroblast growth factor 7 pseudogene 6///fibroblast growth factor 7

8161423	3.25E-02	8.21E-03	3.11	-3.160679	1.16	FGF7P3///FGF7P6///FGF7	fibroblast growth factor 7 pseudogene 3///fibroblast growth factor 7 pseudogene 6///fibroblast growth factor 7

8161455	3.25E-02	8.21E-03	3.11	-3.160679	1.16	FGF7P3///FGF7P6///FGF7	fibroblast growth factor 7 pseudogene 3///fibroblast growth factor 7 pseudogene 6///fibroblast growth factor 7
8083229	3.26E-02	8.26E-03	3.11	-3.165894	1.03		
8026490	3.27E-02	8.30E-03	3.1	-3.17089	1.84	UCA1	urothelial cancer associated 1 (non-protein coding)
8099826	3.29E-02	8.35E-03	3.1	-3.176957	1.04	TLR10	toll like receptor 10

8163002	3.31E-02	8.44E-03	3.1	-3.187646	1.25	KLF4	Kruppel like factor 4
7948444	3.31E-02	8.46E-03	3.1	-3.18913	2.72	TCN1	transcobalamin 1
8150373	3.32E-02	8.48E-03	3.09	-3.191431	1.27		
7909350	3.34E-02	8.54E-03	3.09	-3.198729	1.3	CR2	complement component 3d receptor 2
8077441	3.41E-02	8.81E-03	3.07	-3.229719	1.12	BHLHE40	basic helix-loop-helix family member e40
7896706	3.43E-02	8.88E-03	3.07	-3.237397	1.42		
8151795	3.45E-02	8.95E-03	3.07	-3.245248	1.7	CDH17	cadherin 17
8102587	3.49E-02	9.10E-03	3.06	-3.261807	1.16	NDNF	neuron derived neurotrophic factor

8006459	3.62E-02	9.59E-03	3.03	-3.312996	1.86	CCL13	C-C motif chemokine ligand 13
7929487	3.64E-02	9.68E-03	3.03	-3.321464	1.36	CYP2C9	cytochrome P450 family 2 subfamily C member 9
7896712	3.65E-02	9.70E-03	3.02	-3.324355	1.37		
7957140	3.72E-02	9.94E-03	3.01	-3.347617	1.95	LGR5	leucine rich repeat containing G protein-coupled receptor 5
8053718	3.78E-02	1.02E-02	3	-3.37421	1.36		
8043456	3.80E-02	1.03E-02	2.99	-3.381492	1.18		
8089568	4.13E-02	1.15E-02	2.93	-3.494459	1.13	CD200R1	CD200 receptor 1

7964907	4.15E-02	1.16E-02	2.93	-3.500579	1.76	PTPRR	protein tyrosine phosphatase, receptor type R
7934898	4.22E-02	1.19E-02	2.92	-3.521014	1.86	ANKRD22	ankyrin repeat domain 22
8104758	4.24E-02	1.20E-02	2.91	-3.531635	1.57	NPR3	natriuretic peptide receptor 3
8030860	4.31E-02	1.23E-02	2.9	-3.554246	1.11	FPR2	formyl peptide receptor 2
8135069	4.31E-02	1.23E-02	2.9	-3.554382	1.22	SERPINE1	serpin family E member 1
8046124	4.47E-02	1.29E-02	2.88	-3.603821	1.54	DHRS9	dehydrogenase/reductase 9

7908397	4.48E-02	1.29E-02	2.87	-3.606141	1.13	RGS13	regulator of G-protein signaling 13
8043446	4.58E-02	1.34E-02	2.86	-3.637127	2.17		
8088560	4.61E-02	1.35E-02	2.85	-3.64422	1.02	ADAMTS9	ADAM metallopeptidase with thrombospondin type 1 motif 9
8070579	4.63E-02	1.36E-02	2.85	-3.651736	2.36	TFF1	trefoil factor 1
7961069	4.71E-02	1.39E-02	2.84	-3.673267	1.13	CLECL1	C-type lectin like 1
7951271	4.78E-02	1.42E-02	2.83	-3.692985	2.33	MMP1	matrix metallopeptidase 1
7931108	4.81E-02	1.43E-02	2.82	-3.701886	1.56	DMBT1	deleted in malignant brain tumors 1

8114572	4.86E-02	1.45E-02	2.82	-3.714967	1.26	HBEGF	heparin binding EGF like growth factor
7902808	5.04E-02	1.53E-02	2.79	-3.766121	1.43	LINC01140	long intergenic non-protein coding RNA 1140
8136336	5.17E-02	1.59E-02	2.77	-3.803345	1.29	AKR1B10	aldo-keto reductase family 1 member B10
7954899	5.20E-02	1.60E-02	2.77	-3.809985	1.25	CNTN1	contactin 1
7929478	5.33E-02	1.66E-02	2.75	-3.845281	1.59	CYP2C19	cytochrome P450 family 2 subfamily C member 19

8070584	5.41E-02	1.69E-02	2.73	-3.865767	1.11	TMPRSS3	transmembrane protease, serine 3
7965398	5.52E-02	1.73E-02	2.72	-3.889631	1.15	KERA	keratocan
8054722	5.73E-02	1.84E-02	2.69	-3.945971	1.2	IL1B	interleukin 1 beta
8041508	5.79E-02	1.86E-02	2.68	-3.959308	1.25	QPCT	glutaminyl-peptide cyclotransferase
8040430	5.82E-02	1.88E-02	2.68	-3.96606	1.4	VSNL1	visinin like 1
8006433	5.94E-02	1.94E-02	2.66	-3.996682	1.28	CCL2///CCL2	C-C motif chemokine ligand 2///C-C motif chemokine ligand 2
8101957	6.06E-02	1.99E-02	2.65	-4.023767	1.07	EMCN	endomucin
8154295	6.08E-02	2.00E-02	2.65	-4.027845	1.23	IL33	interleukin 33
7969288	6.10E-02	2.01E-02	2.64	-4.032131	1.78	OLFM4	olfactomedin 4

8044021	6.11E-02	2.02E-02	2.64	-4.036748	1.44	IL1RL1	interleukin 1 receptor like 1
7918794	6.16E-02	2.04E-02	2.64	-4.047933	1.29	AMPD1	adenosine monophosphate deaminase 1
8140686	6.42E-02	2.17E-02	2.61	-4.103799	1.21	SEMA3D	semaphorin 3D
7963406	6.46E-02	2.18E-02	2.6	-4.111158	1.04	KRT6B	keratin 6B
7907286	6.78E-02	2.34E-02	2.56	-4.178007	1.04	FMO1	flavin containing monooxygenase 1

7985555	6.91E-02	2.41E-02	2.55	-4.206489	1.09	EFTUD1P1///EFL1	elongation factor Tu GTP binding domain containing 1 pseudogene 1///elongation factor like GTPase 1
7967322	6.95E-02	2.43E-02	2.54	-4.214471	1.11	HCAR3	hydroxycarboxylic acid receptor 3
8163181	7.00E-02	2.46E-02	2.54	-4.224024	1.1	C9orf152	chromosome 9 open reading frame 152

7961413	7.15E-02	2.54E-02	2.52	-4.254062	1.01	LINC01559	long intergenic non-protein coding RNA 1559
7921099	7.17E-02	2.55E-02	2.52	-4.258317	1.02	CRABP2	cellular retinoic acid binding protein 2
8044574	7.17E-02	2.55E-02	2.52	-4.258458	1	IL1RN	interleukin 1 receptor antagonist
8096415	7.46E-02	2.69E-02	2.49	-4.311839	1.02	MMRN1	multimerin 1
8157446	7.53E-02	2.74E-02	2.48	-4.326455	1.35	ORM1	orosomucoid 1
8043993	7.53E-02	2.74E-02	2.48	-4.326594	1	LOC100131131	AHPA9419
7965390	7.98E-02	2.98E-02	2.44	-4.407264	2.12	EPYC	epiphycan
8124160	8.79E-02	3.43E-02	2.36	-4.539618	1.32		
8144917	8.98E-02	3.53E-02	2.35	-4.566607	1.19	LPL	lipoprotein lipase

7923440	9.05E-02	3.57E-02	2.34	-4.577344	1.24		
8138381	9.08E-02	3.59E-02	2.34	-4.582914	1.71	AGR2	anterior gradient 2, protein disulphide isomerase family member
7960900	9.46E-02	3.81E-02	2.31	-4.638274	1.16	CLEC4E	C-type lectin domain family 4 member E
8074980	9.76E-02	3.97E-02	2.28	-4.678681	1.25	GSTT1	glutathione S-transferase theta 1
8037387	9.90E-02	4.06E-02	2.27	-4.699993	1.05		
7946579	1.01E-01	4.16E-02	2.26	-4.722221	1.02	LYVE1	lymphatic vessel endothelial hyaluronan receptor 1

8079392	1.05E-01	4.44E-02	2.22	-4.782644	1.04	CCR2	C-C motif chemokine receptor 2
7938687	5.17E-08	5.36E-12	-2.31E+01	17.323072	-1.93	NUCB2	nucleobindin 2
8020197	3.61E-07	9.98E-11	-1.83E+01	14.840861	-1.77	ANKRD62	ankyrin repeat domain 62
8166455	1.29E-06	9.38E-10	-1.53E+01	12.801212	-2.09	PRDX4	peroxiredoxin 4
8116402	1.40E-06	1.21E-09	-1.50E+01	12.560241	-1.35	MAPK9	mitogen-activated protein kinase 9
7951910	1.49E-06	1.65E-09	-1.47E+01	12.271307	-2.73	BACE1	beta-secretase 1
7949320	1.57E-06	2.00E-09	-1.44E+01	12.088469	-2.79	GPHA2	glycoprotein hormone alpha 2

8079615	1.71E-06	2.31E-09	-1.43E+01	11.954263	-2.13	FBXW12	F-box and WD repeat domain containing 12
8150276	1.84E-06	3.11E-09	-1.39E+01	11.671244	-1.62	PLPP5	phospholipid phosphatase 5
7965979	2.80E-06	5.99E-09	-1.32E+01	11.04436	-1.9	ALDH1L2	aldehyde dehydrogenase 1 family member L2
7917276	2.80E-06	6.09E-09	-1.32E+01	11.028053	-2.47	LPAR3	lysophosphatidic acid receptor 3
8092277	3.65E-06	9.74E-09	-1.27E+01	10.574622	-2.49	PEX5L	peroxisomal biogenesis factor 5 like

7922846	3.83E-06	1.11E-08	-1.26E+01	10.450329	-1.98	FAM129A	family with sequence similarity 129 member A
8021453	4.42E-06	1.38E-08	-1.23E+01	10.238955	-1.7	SEC11C	SEC11 homolog C, signal peptidase complex subunit
8173924	4.88E-06	1.60E-08	-1.22E+01	10.090968	-3.08		
8144656	5.19E-06	1.90E-08	-1.20E+01	9.926234	-2.66	C8orf49	chromosome 8 open reading frame 49
7962895	5.53E-06	2.11E-08	-1.19E+01	9.820782	-1.08	FKBP11	FK506 binding protein 11
8091637	5.84E-06	2.46E-08	-1.18E+01	9.671162	-1.44	SLC33A1	solute carrier family 33 member 1

8060126	6.56E-06	2.86E-08	-1.16E+01	9.524873	-1.22	AQP12B///AQP12A	aquaporin 12B///aquaporin 12A
7948973	6.62E-06	2.91E-08	-1.16E+01	9.505823	-2.06	HRASLS5	HRAS like suppressor family member 5
8122242	6.99E-06	3.35E-08	-1.15E+01	9.367579	-1.49	PEX7	peroxisomal biogenesis factor 7
8088535	7.10E-06	3.59E-08	-1.14E+01	9.302115	-1.37	PSMD6	proteasome 26S subunit, non-ATPase 6
8156043	7.26E-06	3.74E-08	-1.14E+01	9.260232	-3.69	PSAT1	phosphoserine aminotransferase 1
8144239	7.96E-06	4.44E-08	-1.12E+01	9.091596	-1.09	FBXO25	F-box protein 25
7954398	8.18E-06	4.64E-08	-1.12E+01	9.048742	-3.5	SPX	spexin hormone

8123695	8.44E-06	4.99E-08	-1.11E+01	8.975686	-2.19	ECI2	enoyl-CoA delta isomerase 2
8013581	9.36E-06	5.84E-08	-1.09E+01	8.820977	-1.98	IFT20	intraflagellar transport 20
7904433	1.15E-05	7.95E-08	-1.07E+01	8.515555	-1.45	PHGDH	phosphoglycerate dehydrogenase
7992934	1.15E-05	8.12E-08	-1.06E+01	8.493718	-1.83	DNASE1	deoxyribonuclease 1
7980547	1.16E-05	8.48E-08	-1.06E+01	8.450497	-2.18	SEL1L	SEL1L ERAD E3 ligase adaptor subunit
8049729	1.19E-05	8.70E-08	-1.06E+01	8.425886	-1.25	AQP12B///AQP12A	aquaporin 12B///aquaporin 12A

8164535	1.19E-05	8.76E-08	-1.06E+01	8.418597	-1.2	CRAT	carnitine O-acetyltransferase
8163839	1.34E-05	1.04E-07	-1.04E+01	8.246839	-3.3	C5	complement component 5
7977615	1.41E-05	1.12E-07	-1.04E+01	8.170789	-1.87	RNASE1	ribonuclease A family member 1, pancreatic
7904340	1.63E-05	1.35E-07	-1.02E+01	7.988699	-1.02	MAN1A2	mannosidase alpha class 1A member 2

8111998	1.68E-05	1.40E-07	-1.02E+01	7.950819	-1.4	HCN1	hyperpolarization activated cyclic nucleotide gated potassium channel 1
8044700	1.78E-05	1.56E-07	-1.01E+01	7.841059	-3.53	DPP10	dipeptidyl peptidase like 10
7948249	1.78E-05	1.57E-07	-1.01E+01	7.837828	-2.49	SLC43A1	solute carrier family 43 member 1
8108905	1.78E-05	1.57E-07	-1.01E+01	7.835693	-1.61	KCTD16	potassium channel tetramerization domain containing 16
7956229	1.78E-05	1.58E-07	-1.01E+01	7.832527	-1.65	SLC39A5	solute carrier family 39 member 5

8065136	1.81E-05	1.63E-07	-1.00E+01	7.802263	-1.38	RRBP1	ribosome binding protein 1
8020209	1.89E-05	1.77E-07	-9.95	7.715385	-2.52		
8036033	2.02E-05	1.93E-07	-9.88	7.628783	-1.35	SCGB2B2	secretoglobin family 2B member 2
8154100	2.13E-05	2.04E-07	-9.83	7.573761	-1.74	VLDLR	very low density lipoprotein receptor
7926672	2.21E-05	2.13E-07	-9.8	7.531169	-2.58		
8062948	2.32E-05	2.30E-07	-9.73	7.454645	-2.07	RBPJL	recombination signal binding protein for immunoglobulin kappa J region like

7926638	2.32E-05	2.33E-07	-9.73	7.443797	-2.65	ARMC3	armadillo repeat containing 3
8023855	2.43E-05	2.46E-07	-9.68	7.387769	-1.85	CYB5A	cytochrome b5 type A
7991815	2.48E-05	2.58E-07	-9.64	7.339747	-2.86	PDIA2	protein disulfide isomerase family A member 2
7961829	2.51E-05	2.66E-07	-9.62	7.311251	-2.03	BCAT1	branched chain amino acid transaminase 1
8088908	2.57E-05	2.78E-07	-9.58	7.266779	-1.44		
7910030	2.59E-05	2.82E-07	-9.56	7.250353	-1.48	DNAH14	dynein axonemal heavy chain 14
7994252	2.67E-05	2.93E-07	-9.53	7.21326	-2.75	AQP8	aquaporin 8

7944799	2.79E-05	3.09E-07	-9.49	7.160709	-1.78	OR10G7///OR10G4///OR10G9///OR10G8	olfactory receptor family 10 subfamily G member 7///olfactory receptor family 10 subfamily G member 4///olfactory receptor family 10 subfamily G member 9///olfactory receptor family 10 subfamily G member 8

8102567	2.79E-05	3.09E-07	-9.49	7.159488	-1.14	PRDM5	PR/SET domain 5
7898736	2.82E-05	3.18E-07	-9.47	7.132108	-2.71	LINC00339	long intergenic non-protein coding RNA 339
8078933	2.85E-05	3.26E-07	-9.45	7.107355	-1.11	MYRIP	myosin VIIA and Rab interacting protein
8075106	2.90E-05	3.37E-07	-9.42	7.072809	-2.24	TPST2	tyrosylprotein sulfotransferase 2
8141371	2.96E-05	3.57E-07	-9.37	7.015406	-1.36	GJC3	gap junction protein gamma 3
8072931	2.96E-05	3.60E-07	-9.36	7.005117	-1.19	GCAT	glycine C-acetyltransferase

8118622	3.19E-05	4.00E-07	-9.28	6.899897	-1.35	HSD17B8	hydroxysteroid 17-beta dehydrogenase 8
8178234	3.19E-05	4.00E-07	-9.28	6.899897	-1.35	HSD17B8	hydroxysteroid 17-beta dehydrogenase 8
8179534	3.19E-05	4.00E-07	-9.28	6.899897	-1.35	HSD17B8	hydroxysteroid 17-beta dehydrogenase 8
8097388	3.58E-05	4.73E-07	-9.15	6.73267	-1.51	LARP1B	La ribonucleoprotein domain family member 1B
8159850	3.58E-05	4.76E-07	-9.14	6.725945	-1.7	VLDLR-AS1	VLDLR antisense RNA 1

8170775	3.80E-05	5.19E-07	-9.07	6.637561	-1.42	SSR4	signal sequence receptor subunit 4
8078397	3.88E-05	5.34E-07	-9.05	6.609519	-1.54	CMTM8	CKLF like MARVEL transmembrane domain containing 8
8053278	3.88E-05	5.36E-07	-9.05	6.606105	-1.32	EVA1A	eva-1 homolog A, regulator of programmed cell death
8171161	3.88E-05	5.37E-07	-9.04	6.604225	-2.89	ARSE	arylsulfatase E (chondrodysplasia punctata 1)

8093451	3.99E-05	5.55E-07	-9.02	6.569936	-1.93		
8145532	3.99E-05	5.57E-07	-9.02	6.567719	-2.12	EPHX2	epoxide hydrolase 2
7930181	4.16E-05	5.99E-07	-8.96	6.493326	-1.45	BORCS7-ASMT///AS3MT	BORCS7-ASMT readthrough (NMD candidate)///arsenite methyltransferase
8074201	4.23E-05	6.12E-07	-8.94	6.472079	-2.89	ANKRD62	ankyrin repeat domain 62
8111490	4.32E-05	6.33E-07	-8.91	6.438746	-2.6	PRLR	prolactin receptor
7928695	4.32E-05	6.33E-07	-8.91	6.437936	-1.07	FAM213A	family with sequence similarity 213 member A

8046726	4.43E-05	6.62E-07	-8.88	6.393297	-1.39	SSFA2	sperm specific antigen 2
8049957	4.55E-05	6.81E-07	-8.86	6.3652	-1.93	LOC285097	uncharacterized FLJ38379
7952390	4.84E-05	7.45E-07	-8.79	6.273565	-2.16		
7923824	4.89E-05	7.59E-07	-8.77	6.25532	-1.74	SLC41A1	solute carrier family 41 member 1
8094759	4.93E-05	7.73E-07	-8.76	6.236288	-1.26	NSUN7	NOP2/Sun RNA methyltransferase family member 7
7940914	4.94E-05	7.79E-07	-8.75	6.229329	-1.17	FKBP2	FK506 binding protein 2
8049961	5.07E-05	8.09E-07	-8.72	6.191239	-1.36	LOC728323///FBXO25	uncharacterized LOC728323///F-box protein 25

8104014	5.23E-05	8.50E-07	-8.69	6.140939	-2.77	CCDC110	coiled-coil domain containing 110
8116696	5.34E-05	8.77E-07	-8.66	6.109653	-1.15	C6orf201	chromosome 6 open reading frame 201
8000574	5.39E-05	8.87E-07	-8.65	6.097799	-1.84	NUPR1	nuclear protein 1, transcriptional regulator
7997525	5.40E-05	8.91E-07	-8.65	6.092782	-1.02	MLYCD	malonyl-CoA decarboxylase
8075956	5.40E-05	8.93E-07	-8.65	6.091074	-3.31	LGALS2	galectin 2
7968734	5.41E-05	8.96E-07	-8.65	6.08735	-2.11	SLC25A15	solute carrier family 25 member 15

7915846	5.46E-05	9.09E-07	-8.63	6.072357	-1.57	MKNK1	MAP kinase interacting serine/threonine kinase 1
8162823	5.54E-05	9.33E-07	-8.61	6.046504	-1.22		
7938090	5.99E-05	1.05E-06	-8.52	5.926649	-1.02	CCKBR	cholecystokinin B receptor
8144213	6.28E-05	1.13E-06	-8.47	5.856158	-1.04	VIPR2	vasoactive intestinal peptide receptor 2
8166202	6.46E-05	1.18E-06	-8.44	5.811533	-3.17	GRPR	gastrin releasing peptide receptor
7993126	6.96E-05	1.32E-06	-8.35	5.698644	-2.48	ABAT	4-aminobutyrate aminotransferase

7952382	7.35E-05	1.42E-06	-8.3	5.625008	-3.29	OR6T1	olfactory receptor family 6 subfamily T member 1
8096845	7.49E-05	1.46E-06	-8.28	5.594318	-4.56	EGF	epidermal growth factor
8061122	7.52E-05	1.48E-06	-8.27	5.577982	-2.5	BANF2	barrier to autointegration factor 2
8082066	7.61E-05	1.50E-06	-8.25	5.562784	-1.5	FAM162A	family with sequence similarity 162 member A
7998637	7.73E-05	1.54E-06	-8.24	5.539177	-1.1	MSRB1	methionine sulfoxide reductase B1

8052940	7.78E-05	1.56E-06	-8.23	5.525823	-3.36	PAIP2B///VPS36	poly(A) binding protein interacting protein 2B///vacuolar protein sorting 36 homolog
8092328	7.88E-05	1.58E-06	-8.22	5.511074	-1.42	MCCC1	methylcrotonoyl-CoA carboxylase 1
8172425	7.94E-05	1.61E-06	-8.21	5.497289	-1.87	SLC38A5	solute carrier family 38 member 5
8111552	8.12E-05	1.65E-06	-8.18	5.467882	-1.02	NADK2	NAD kinase 2, mitochondrial

8089402	8.20E-05	1.68E-06	-8.17	5.453939	-5.25	GUCA1C	guanylate cyclase activator 1C
8122099	8.33E-05	1.71E-06	-8.16	5.431396	-1.66	ENPP1	ectonucleotide pyrophosphatase/phosphodiesterase 1
7910792	8.58E-05	1.79E-06	-8.13	5.387298	-2.41	RYR2	ryanodine receptor 2
8019250	8.82E-05	1.86E-06	-8.1	5.349487	-1.72	P4HB	prolyl 4-hydroxylase subunit beta
8004416	8.95E-05	1.89E-06	-8.09	5.333095	-1.05	CHRNB1	cholinergic receptor nicotinic beta 1 subunit
8036699	9.09E-05	1.93E-06	-8.07	5.31038	-4.18	SYCN	syncollin

7934334	9.28E-05	1.98E-06	-8.05	5.28263	-1.35	CFAP70	cilia and flagella associated protein 70
7986394	9.65E-05	2.10E-06	-8.01	5.223412	-1.06	LRRC28	leucine rich repeat containing 28
7993453	9.97E-05	2.23E-06	-7.96	5.162226	-2.06	MPV17L	MPV17 mitochondrial inner membrane protein like
7975081	1.02E-04	2.30E-06	-7.94	5.130577	-1.44	PPP1R36	protein phosphatase 1 regulatory subunit 36

7904843	1.06E-04	2.42E-06	-7.91	5.08264	-3.08	PDZK1P1///PDZK1	PDZ domain containing 1 pseudogene 1///PDZ domain containing 1
7947199	1.07E-04	2.45E-06	-7.9	5.067627	-1.48	LGR4	leucine rich repeat containing G protein-coupled receptor 4
7925229	1.07E-04	2.47E-06	-7.89	5.060591	-1.16	B3GALNT2	beta-1,3-N-acetylgalactosaminyltransferase 2

7944793	1.09E-04	2.55E-06	-7.87	5.026929	-2.12	OR8D4	olfactory receptor family 8 subfamily D member 4
8085358	1.13E-04	2.64E-06	-7.84	4.991608	-1.43		
7934755	1.13E-04	2.67E-06	-7.84	4.981197	-1.16	MAT1A	methionine adenosyltransferase 1A
7936949	1.14E-04	2.69E-06	-7.83	4.973457	-1.03	DHX32	DEAH-box helicase 32 (putative)
8096917	1.16E-04	2.74E-06	-7.82	4.955169	-2.04		
8002283	1.16E-04	2.79E-06	-7.81	4.937676	-4.55	TMED6	transmembrane p24 trafficking protein 6
8091241	1.22E-04	2.99E-06	-7.76	4.867535	-1.21		

7933982	1.23E-04	3.03E-06	-7.75	4.851765	-2.08	PBLD	phenazine biosynthesis like protein domain containing
7932094	1.23E-04	3.04E-06	-7.74	4.848208	-1.09	PHYH	phytanoyl-CoA 2-hydroxylase
8057377	1.24E-04	3.06E-06	-7.74	4.843223	-2.19	CCDC141	coiled-coil domain containing 141
8006319	1.25E-04	3.11E-06	-7.73	4.827503	-1.18		
8157300	1.36E-04	3.45E-06	-7.65	4.720877	-1.23	BSPRY	B-box and SPRY domain containing
8146225	1.37E-04	3.49E-06	-7.65	4.709059	-1.14	SMIM19	small integral membrane protein 19

7944795	1.38E-04	3.52E-06	-7.64	4.699973	-1.98	OR4D5	olfactory receptor family 4 subfamily D member 5
8095628	1.41E-04	3.64E-06	-7.62	4.666759	-4.92	ALB	albumin
7961983	1.46E-04	3.78E-06	-7.59	4.628625	-1.12	TM7SF3	transmembrane 7 superfamily member 3
8083779	1.47E-04	3.83E-06	-7.58	4.614731	-2.08	SERPINI1	serpin family I member 1
8082816	1.52E-04	4.01E-06	-7.55	4.566778	-1.34	SRPRB	SRP receptor beta subunit
8065351	1.52E-04	4.04E-06	-7.54	4.559429	-1.01		
7952557	1.54E-04	4.11E-06	-7.53	4.541474	-1.1	SRPRA	SRP receptor alpha subunit

8057719	1.55E-04	4.16E-06	-7.52	4.530887	-1.11	HIBCH	3-hydroxyisobutyryl-CoA hydrolase
8019762	1.60E-04	4.32E-06	-7.5	4.491919	-1.61	P4HB	prolyl 4-hydroxylase subunit beta
8146427	1.63E-04	4.47E-06	-7.47	4.456807	-1.04		
7943605	1.78E-04	5.08E-06	-7.38	4.327287	-1.67	ACAT1	acetyl-CoA acetyltransferase 1
8096765	1.79E-04	5.11E-06	-7.38	4.321455	-1.22	RPL34	ribosomal protein L34
8119620	1.80E-04	5.18E-06	-7.37	4.307083	-3.26	GNMT	glycine N-methyltransferase
7944049	1.81E-04	5.22E-06	-7.37	4.299376	-1.7	SIDT2	SID1 transmembrane family member 2

7990564	1.84E-04	5.32E-06	-7.35	4.280126	-2.28		
8096251	1.85E-04	5.40E-06	-7.34	4.265322	-1.12	NUDT9	nudix hydrolase 9
7905283	1.86E-04	5.43E-06	-7.34	4.258777	-1.32	ANXA9	annexin A9
8015712	1.95E-04	5.80E-06	-7.29	4.192183	-1.23	COA3	cytochrome c oxidase assembly factor 3
7913593	2.00E-04	5.96E-06	-7.27	4.163732	-2.37	TCEA3	transcription elongation factor A3
8150580	2.08E-04	6.28E-06	-7.24	4.110622	-1.62		
8067305	2.10E-04	6.37E-06	-7.23	4.096038	-1.88	SYCP2	synaptonemal complex protein 2
7910111	2.11E-04	6.42E-06	-7.22	4.088558	-1.46	EPHX1	epoxide hydrolase 1

8098671	2.12E-04	6.49E-06	-7.22	4.077656	-4.17	F11	coagulation factor XI
8139640	2.16E-04	6.63E-06	-7.2	4.055974	-2.3	DDC	dopa decarboxylase
8070632	2.16E-04	6.64E-06	-7.2	4.053939	-1.14	CBSL///CBS	cystathionine-beta-synthase like///cystathionine-beta-synthase
7997962	2.17E-04	6.72E-06	-7.19	4.04199	-1.35	DPEP1	dipeptidase 1 (renal)
8062728	2.18E-04	6.75E-06	-7.19	4.037477	-1.13	SGK2	SGK2, serine/threonine kinase 2
8023228	2.26E-04	7.08E-06	-7.16	3.988734	-1.23	DYM	dymeclin
8149725	2.28E-04	7.15E-06	-7.15	3.979089	-1.89	PEBP4	phosphatidylethanolamine binding protein 4

8099860	2.39E-04	7.63E-06	-7.11	3.912588	-1.06	RFC1	replication factor C subunit 1
8127087	2.40E-04	7.68E-06	-7.1	3.906072	-1.38	GSTA3	glutathione S-transferase alpha 3
8168179	2.40E-04	7.70E-06	-7.1	3.903512	-1.14	DLG3	discs large MAGUK scaffold protein 3
7942647	2.41E-04	7.75E-06	-7.1	3.895608	-1.36	LOC100506127	putative uncharacterized protein FLJ37770-like
7902290	2.46E-04	7.95E-06	-7.08	3.870814	-1.71	CTH	cystathionine gamma-lyase
7995362	2.46E-04	7.99E-06	-7.08	3.865524	-2.04	GPT2	glutamic--pyruvic transaminase 2

8092750	2.47E-04	8.05E-06	-7.07	3.858101	-1.23	FGF12	fibroblast growth factor 12
7952377	2.47E-04	8.06E-06	-7.07	3.856785	-2.99	TMEM225	transmembrane protein 225
7924309	2.48E-04	8.08E-06	-7.07	3.853642	-2.14	ESRRG	estrogen related receptor gamma
8097801	2.50E-04	8.20E-06	-7.06	3.838566	-2.18	FAM160A1	family with sequence similarity 160 member A1
8051998	2.53E-04	8.35E-06	-7.05	3.820006	-1.13	MCFD2	multiple coagulation factor deficiency 2
7938989	2.54E-04	8.37E-06	-7.05	3.817473	-2.79	GAS2	growth arrest specific 2

8056837	2.54E-04	8.40E-06	-7.04	3.814297	-1.26	GPR155	G protein-coupled receptor 155
8038633	2.59E-04	8.63E-06	-7.03	3.786716	-3.87	KLK1	kallikrein 1
8106827	2.62E-04	8.80E-06	-7.01	3.76646	-1.41	ADGRV1	adhesion G protein-coupled receptor V1
8154841	2.64E-04	8.88E-06	-7.01	3.757737	-1.17		
7956826	2.66E-04	9.00E-06	-7	3.743643	-1.48	TBC1D30	TBC1 domain family member 30
8048205	2.68E-04	9.11E-06	-6.99	3.731445	-1.49	IGFBP2	insulin like growth factor binding protein 2

8146687	2.79E-04	9.62E-06	-6.95	3.676328	-1.88	ADHFE1	alcohol dehydrogenase, iron containing 1
7994615	2.81E-04	9.71E-06	-6.95	3.665902	-1	ZG16	zymogen granule protein 16
7947496	2.81E-04	9.80E-06	-6.94	3.657239	-2.41	SLC1A2	solute carrier family 1 member 2
7980906	2.83E-04	9.87E-06	-6.94	3.649168	-3.16		
7956242	2.85E-04	1.00E-05	-6.93	3.632677	-1.06	COQ10A	coenzyme Q10A

8138151	2.86E-04	1.01E-05	-6.92	3.626907	-1.09	OR7E37P///MGC72080///DAPL1///OR7E12P	olfactory receptor family 7 subfamily E member 37 pseudogene///MGC72080 pseudogene///death associated protein like 1///olfactory receptor family 7 subfamily E member 12 pseudogene

7937079	2.94E-04	1.05E-05	-6.9	3.58804	-1.97	BNIP3	BCL2 interacting protein 3
7936507	2.97E-04	1.06E-05	-6.89	3.575195	-1.27	C10orf82	chromosome 10 open reading frame 82
8047784	2.98E-04	1.07E-05	-6.88	3.568442	-1.11	ZDBF2	zinc finger DBF-type containing 2
7961024	2.98E-04	1.07E-05	-6.88	3.565924	-1.07		
8134018	2.99E-04	1.07E-05	-6.88	3.56388	-1.07	ZNF804B	zinc finger protein 804B
8117225	3.00E-04	1.08E-05	-6.88	3.555821	-2.58	GMNN	geminin, DNA replication inhibitor

7946365	3.01E-04	1.09E-05	-6.87	3.551821	-1.4	STK33	serine/threonine kinase 33
8143461	3.06E-04	1.11E-05	-6.86	3.528378	-1.02		
8151423	3.11E-04	1.14E-05	-6.84	3.504793	-1.25	JPH1	junctophilin 1
7961702	3.15E-04	1.15E-05	-6.83	3.489552	-1.43	KCNJ8	potassium voltage-gated channel subfamily J member 8
8135876	3.16E-04	1.17E-05	-6.83	3.478573	-1.03	SND1	staphylococcal nuclease and tudor domain containing 1
8089489	3.19E-04	1.18E-05	-6.82	3.469356	-1.05	SLC9C1	solute carrier family 9 member C1

7971027	3.20E-04	1.19E-05	-6.81	3.461482	-1.26	ALG5///ALG5	ALG5, dolichyl-phosphate beta-glucosyltransferase///ALG5, dolichyl-phosphate beta-glucosyltransferase
8058627	3.24E-04	1.20E-05	-6.81	3.447842	-1.61	ERBB4	erb-b2 receptor tyrosine kinase 4
8102321	3.25E-04	1.21E-05	-6.8	3.443745	-1.15	PLA2G12AP1///PLA2G12A	phospholipase A2 group XIIA pseudogene 1///phospholipase A2 group XIIA

8152355	3.33E-04	1.25E-05	-6.78	3.406581	-2.41	SYBU	syntabulin
8102362	3.34E-04	1.26E-05	-6.78	3.400577	-1.49	TIFA	TRAF interacting protein with forkhead associated domain
7972231	3.34E-04	1.26E-05	-6.77	3.397742	-1.08	SLITRK1	SLIT and NTRK like family member 1
8024238	3.38E-04	1.29E-05	-6.76	3.377789	-1.39	CIRBP	cold inducible RNA binding protein
8003578	3.38E-04	1.29E-05	-6.76	3.375004	-1.27	ZG16	zymogen granule protein 16
8127065	3.42E-04	1.31E-05	-6.75	3.360626	-4.23	GSTA2	glutathione S-transferase alpha 2
8176566	3.45E-04	1.32E-05	-6.74	3.349557	-1.31		

8011499	3.50E-04	1.35E-05	-6.73	3.32983	-1.04	P2RX1	purinergic receptor P2X 1
7912638	3.51E-04	1.36E-05	-6.73	3.325323	-1.08	TMEM51-AS1	TMEM51 antisense RNA 1
8102948	3.53E-04	1.37E-05	-6.72	3.312734	-1.94		
7991034	3.57E-04	1.39E-05	-6.71	3.297459	-2.94	HOMER2	homer scaffolding protein 2
8127094	3.59E-04	1.40E-05	-6.71	3.293257	-1.12	GSTA4	glutathione S-transferase alpha 4
8060949	3.66E-04	1.43E-05	-6.69	3.268296	-2.29	ANKEF1	ankyrin repeat and EF-hand domain containing 1

7934936	3.70E-04	1.46E-05	-6.68	3.25153	-2.71	SLC16A12	solute carrier family 16 member 12
8101728	3.75E-04	1.48E-05	-6.67	3.235101	-1.03	FAM13A	family with sequence similarity 13 member A
8141150	3.86E-04	1.54E-05	-6.65	3.195878	-1.6	ASNS	asparagine synthetase (glutamine-hydrolyzing)
7990555	3.90E-04	1.56E-05	-6.64	3.180383	-3.34	NRG4	neuregulin 4
7954436	3.91E-04	1.57E-05	-6.63	3.17782	-1.12	LRMP	lymphoid restricted membrane protein

8009241	3.96E-04	1.60E-05	-6.62	3.158831	-2.01	SNORD104	small nucleolar RNA, C/D box 104
8044882	3.96E-04	1.60E-05	-6.62	3.157165	-1.2	EPB41L5	erythrocyte membrane protein band 4.1 like 5
7968761	3.99E-04	1.62E-05	-6.61	3.144213	-1.44	NAA16	N(alpha)-acetyltransferase 16, NatA auxiliary subunit
8005839	4.20E-04	1.74E-05	-6.57	3.068436	-2.85	TMEM97	transmembrane protein 97
8027744	4.25E-04	1.77E-05	-6.55	3.050864	-1.11	HPN-AS1	HPN antisense RNA 1

7957221	4.28E-04	1.79E-05	-6.55	3.041343	-3.74	TRHDE	thyrotropin releasing hormone degrading enzyme
8002121	4.30E-04	1.81E-05	-6.54	3.033146	-4.27	CTRL	chymotrypsin like
7917304	4.31E-04	1.81E-05	-6.54	3.031511	-1.89	MCOLN3	mucolipin 3
7952386	4.31E-04	1.82E-05	-6.54	3.02622	-2.87		
8144121	4.34E-04	1.83E-05	-6.53	3.017356	-1.29	PTPRN2	protein tyrosine phosphatase, receptor type N2
8052372	4.36E-04	1.86E-05	-6.52	3.001963	-2.32	MIR217	microRNA 217
8020183	4.42E-04	1.89E-05	-6.51	2.986614	-2.08	IMPA2	inositol monophosphatase 2
7912473	4.42E-04	1.89E-05	-6.51	2.986537	-1.34	FBXO2	F-box protein 2

7929383	4.44E-04	1.91E-05	-6.51	2.97595	-1.7	SLC35G1	solute carrier family 35 member G1
8103494	4.64E-04	2.05E-05	-6.46	2.901424	-1.81	NPY1R///NPY1R	neuropeptide Y receptor Y1///neuropeptide Y receptor Y1
8006181	4.70E-04	2.09E-05	-6.45	2.884729	-1.11		
7895139	4.77E-04	2.13E-05	-6.44	2.864077	-1.1		
8160769	4.82E-04	2.16E-05	-6.43	2.849736	-1.2		
7904361	4.85E-04	2.18E-05	-6.42	2.841464	-1.33	FAM46C	family with sequence similarity 46 member C
7938951	4.87E-04	2.20E-05	-6.42	2.832265	-1.72	ANO5	anoctamin 5
8057056	4.87E-04	2.20E-05	-6.42	2.831468	-1.92	TTN	titin

8053668	4.99E-04	2.28E-05	-6.39	2.795262	-1.24	EIF2AK3	eukaryotic translation initiation factor 2 alpha kinase 3

7945873	5.15E-04	2.37E-05	-6.37	2.757399	-1.17	OR7E37P///OR7E35P///MGC72080///DAPL1///OR7E47P///OR7E12P///OR7E14P	olfactory receptor family 7 subfamily E member 37 pseudogene///olfactory receptor family 7 subfamily E member 35 pseudogene///MGC72080 pseudogene///death associated protein like 1///olfactory receptor family 7 subfamily E member 47 pseudogene///olfactory receptor family 7 subfamily E member 12 pseudogene///olfactory receptor family 7 subfamily E member 14 pseudogene

7991335	5.20E-04	2.39E-05	-6.36	2.745185	-2.91	ANPEP	alanyl aminopeptidase, membrane
8020847	5.34E-04	2.50E-05	-6.34	2.701815	-1.54	DTNA	dystrobrevin alpha
8091678	5.42E-04	2.56E-05	-6.32	2.678501	-2.47	VEPH1	ventricular zone expressed PH domain containing 1
8123685	5.60E-04	2.67E-05	-6.3	2.634546	-1.31	FAM217A	family with sequence similarity 217 member A
8101992	5.69E-04	2.72E-05	-6.28	2.612998	-1.66	SLC39A8	solute carrier family 39 member 8

7929388	5.75E-04	2.76E-05	-6.27	2.599945	-1.28	PLCE1	phospholipase C epsilon 1
8091910	5.75E-04	2.77E-05	-6.27	2.596149	-4.68	SERPINI2	serpin family I member 2

8082248	5.79E-04	2.79E-05	-6.27	2.587365	-1.09	OR7E37P///OR7E35P///MGC72080///OR7E5P///DAPL1///OR7E24///OR7E47P///OR7E12P///OR7E14P	olfactory receptor family 7 subfamily E member 37 pseudogene///olfactory receptor family 7 subfamily E member 35 pseudogene///MGC72080 pseudogene///olfactory receptor family 7 subfamily E member 5 pseudogene///death associated protein like 1///olfactory receptor family 7 subfamily E member 24///olfactory receptor family 7 subfamily E member 47 pseudogene///olfactory receptor family 7 subfamily E member 12 pseudogene///olfactory receptor family 7 subfamily E member 14 pseudogene

7974533	5.96E-04	2.91E-05	-6.24	2.545675	-1.28	PELI2	pellino E3 ubiquitin protein ligase family member 2
8053366	6.02E-04	2.94E-05	-6.23	2.533679	-1.01	SUCLG1	succinate-CoA ligase alpha subunit
8077526	6.13E-04	3.03E-05	-6.22	2.505667	-1.26		
7898167	6.15E-04	3.05E-05	-6.21	2.497608	-3.85	CTRC	chymotrypsin C
8121515	6.17E-04	3.07E-05	-6.21	2.492015	-2.9	SLC16A10	solute carrier family 16 member 10
8091554	6.19E-04	3.08E-05	-6.21	2.487549	-1.06		

8082244	6.22E-04	3.11E-05	-6.2	2.477353	-1	OR7E37P///OR7E35P///MGC72080///OR7E5P///DAPL1///OR7E24///OR7E47P///OR7E12P///OR7E14P	olfactory receptor family 7 subfamily E member 37 pseudogene///olfactory receptor family 7 subfamily E member 35 pseudogene///MGC72080 pseudogene///olfactory receptor family 7 subfamily E member 5 pseudogene///death associated protein like 1///olfactory receptor family 7 subfamily E member 24///olfactory receptor family 7 subfamily E member 47 pseudogene///olfactory receptor family 7 subfamily E member 12 pseudogene///olfactory receptor family 7 subfamily E member 14 pseudogene

8105067	6.31E-04	3.17E-05	-6.19	2.457398	-1.08	PTGER4	prostaglandin E receptor 4
8052374	6.32E-04	3.18E-05	-6.19	2.454587	-2.16	MIR216A	microRNA 216a
8140852	6.32E-04	3.18E-05	-6.19	2.453532	-2.4	C7orf62	chromosome 7 open reading frame 62
7929497	6.38E-04	3.24E-05	-6.17	2.435297	-1.66	ACSM6	acyl-CoA synthetase medium-chain family member 6
7902518	6.39E-04	3.25E-05	-6.17	2.433412	-2.09	GIPC2	GIPC PDZ domain containing family member 2
7903959	6.42E-04	3.27E-05	-6.17	2.426412	-1.49	PIFO	primary cilia formation

8137526	6.72E-04	3.47E-05	-6.13	2.365987	-1.38	INSIG1	insulin induced gene 1

7968787	6.81E-04	3.54E-05	-6.12	2.345282	-1.1	OR7E37P///OR7E35P///MGC72080///DAPL1///OR7E24///OR7E47P///OR7E12P///OR7E14P	olfactory receptor family 7 subfamily E member 37 pseudogene///olfactory receptor family 7 subfamily E member 35 pseudogene///MGC72080 pseudogene///death associated protein like 1///olfactory receptor family 7 subfamily E member 24///olfactory receptor family 7 subfamily E member 47 pseudogene///olfactory receptor family 7 subfamily E member 12 pseudogene///olfactory receptor family 7 subfamily E member 14 pseudogene

7917322	6.84E-04	3.56E-05	-6.12	2.339139	-1.3	SYDE2	synapse defective Rho GTPase homolog 2
8006423	6.84E-04	3.56E-05	-6.12	2.33908	-1.2	SPACA3	sperm acrosome associated 3
7910047	6.99E-04	3.66E-05	-6.1	2.311408	-1.34	DNAH14	dynein axonemal heavy chain 14
8163535	7.08E-04	3.75E-05	-6.09	2.287542	-1.93	AMBP	alpha-1-microglobulin/bikunin precursor
8075182	7.08E-04	3.75E-05	-6.08	2.28712	-1.34	XBP1	X-box binding protein 1

8076515	7.12E-04	3.78E-05	-6.08	2.278741	-1.13	ARFGAP3	ADP ribosylation factor GTPase activating protein 3
8137448	7.12E-04	3.78E-05	-6.08	2.277761	-1.06	GALNT11	polypeptide N-acetylgalactosaminyltransferase 11

8156319	7.12E-04	3.78E-05	-6.08	2.277372	-1.04	OR7E37P///MGC72080///OR7E5P///DAPL1///OR7E24///OR7E47P///OR7E12P///OR7E14P	olfactory receptor family 7 subfamily E member 37 pseudogene///MGC72080 pseudogene///olfactory receptor family 7 subfamily E member 5 pseudogene///death associated protein like 1///olfactory receptor family 7 subfamily E member 24///olfactory receptor family 7 subfamily E member 47 pseudogene///olfactory receptor family 7 subfamily E member 12 pseudogene///olfactory receptor family 7 subfamily E member 14 pseudogene

7988876	7.12E-04	3.79E-05	-6.08	2.276481	-1.58	MYO5C	myosin VC

7971241	7.29E-04	3.91E-05	-6.06	2.245002	-1.09	OR7E37P///OR7E35P///MGC72080///OR7E5P///DAPL1///OR7E24///OR7E47P///OR7E12P///OR7E14P	olfactory receptor family 7 subfamily E member 37 pseudogene///olfactory receptor family 7 subfamily E member 35 pseudogene///MGC72080 pseudogene///olfactory receptor family 7 subfamily E member 5 pseudogene///death associated protein like 1///olfactory receptor family 7 subfamily E member 24///olfactory receptor family 7 subfamily E member 47 pseudogene///olfactory receptor family 7 subfamily E member 12 pseudogene///olfactory receptor family 7 subfamily E member 14 pseudogene

7933045	7.34E-04	3.94E-05	-6.05	2.236898	-1.11

8045887	7.43E-04	4.00E-05	-6.04	2.220219	-1.1	OR7E37P///OR7E35P///MGC72080///OR7E5P///DAPL1///OR7E24///OR7E47P///OR7E12P///OR7E14P	olfactory receptor family 7 subfamily E member 37 pseudogene///olfactory receptor family 7 subfamily E member 35 pseudogene///MGC72080 pseudogene///olfactory receptor family 7 subfamily E member 5 pseudogene///death associated protein like 1///olfactory receptor family 7 subfamily E member 24///olfactory receptor family 7 subfamily E member 47 pseudogene///olfactory receptor family 7 subfamily E member 12 pseudogene///olfactory receptor family 7 subfamily E member 14 pseudogene

8015735	7.43E-04	4.01E-05	-6.04	2.218836	-2.26		
8097809	7.45E-04	4.04E-05	-6.04	2.211077	-1.4		
8135514	7.48E-04	4.08E-05	-6.03	2.200866	-1.59	IFRD1	interferon related developmental regulator 1
8103094	7.72E-04	4.24E-05	-6.01	2.161453	-1.23	NR3C2	nuclear receptor subfamily 3 group C member 2
7933640	7.77E-04	4.28E-05	-6	2.152422	-2.2	A1CF	APOBEC1 complementation factor
7973054	7.82E-04	4.31E-05	-6	2.143378	-1.48		
8121838	7.82E-04	4.32E-05	-6	2.142534	-1.42	TPD52L1	tumor protein D52-like 1

8141374	7.99E-04	4.42E-05	-5.98	2.117662	-3.5	AZGP1	alpha-2-glycoprotein 1, zinc-binding
8163116	8.15E-04	4.54E-05	-5.97	2.090611	-1.75	EPB41L4B	erythrocyte membrane protein band 4.1 like 4B
7952375	8.24E-04	4.61E-05	-5.96	2.075643	-1.89	OR6M1	olfactory receptor family 6 subfamily M member 1
8142497	8.33E-04	4.70E-05	-5.95	2.055692	-1.22	CTTNBP2	cortactin binding protein 2
7910915	8.33E-04	4.70E-05	-5.95	2.055497	-2.15	CHRM3	cholinergic receptor muscarinic 3

8040211	8.42E-04	4.77E-05	-5.94	2.040744	-1.09	KLF11	Kruppel like factor 11
8100362	8.48E-04	4.81E-05	-5.93	2.032879	-1.15	LNX1	ligand of numb-protein X 1

8138145	8.54E-04	4.86E-05	-5.93	2.020732	-1.06	OR7E37P///MGC72080///DAPL1///OR7E47P	olfactory receptor family 7 subfamily E member 37 pseudogene///MGC72080 pseudogene///death associated protein like 1///olfactory receptor family 7 subfamily E member 47 pseudogene

8098856	8.65E-04	4.94E-05	-5.92	2.004453	-1.5	RNF212	ring finger protein 212
8041727	8.80E-04	5.06E-05	-5.9	1.980131	-2.46	SLC3A1	solute carrier family 3 member 1
8134024	9.10E-04	5.36E-05	-5.87	1.922258	-1.12		
8167027	9.17E-04	5.42E-05	-5.86	1.911181	-2.33	RGN	regucalcin
7898723	9.33E-04	5.55E-05	-5.85	1.885477	-2.47		
7925342	9.33E-04	5.55E-05	-5.85	1.885435	-2.77	ERO1B	endoplasmic reticulum oxidoreductase 1 beta
8169389	9.37E-04	5.58E-05	-5.84	1.880584	-2.26	PAK3	p21 (RAC1) activated kinase 3
8094719	9.50E-04	5.74E-05	-5.83	1.852066	-1.03	N4BP2	NEDD4 binding protein 2

8074969	9.74E-04	5.92E-05	-5.81	1.820985	-1.2	DDT	D-dopachrome tautomerase
7944867	1.01E-03	6.22E-05	-5.78	1.769665	-1.48		
7944147	1.03E-03	6.45E-05	-5.76	1.733029	-2.23		
8145334	1.04E-03	6.54E-05	-5.75	1.718873	-1.58	ADAM7	ADAM metallopeptidase domain 7
7961524	1.05E-03	6.60E-05	-5.74	1.708928	-3.78	ERP27	endoplasmic reticulum protein 27
8113483	1.07E-03	6.80E-05	-5.72	1.678678	-1.19	TMEM232	transmembrane protein 232
8127072	1.08E-03	6.88E-05	-5.72	1.66711	-3.34	GSTA1	glutathione S-transferase alpha 1
7910636	1.10E-03	6.99E-05	-5.71	1.650236	-1.23		

8027685	1.12E-03	7.15E-05	-5.69	1.626948	-1.1	ZNF181	zinc finger protein 181
7951966	1.14E-03	7.34E-05	-5.68	1.600782	-1.44	FXYD6-FXYD2///FXYD2	FXYD6-FXYD2 readthrough///FXYD domain containing ion transport regulator 2
7898184	1.14E-03	7.39E-05	-5.67	1.594161	-3.86	CELA2B	chymotrypsin like elastase family member 2B
7973446	1.15E-03	7.46E-05	-5.67	1.583755	-1.13	DHRS4-AS1	DHRS4 antisense RNA 1
8158976	1.16E-03	7.54E-05	-5.66	1.572813	-3.67	CEL	carboxyl ester lipase

8122125	1.16E-03	7.55E-05	-5.66	1.572228	-1.49		
7969640	1.17E-03	7.68E-05	-5.65	1.55488	-2.94	CLDN10	claudin 10
8056343	1.19E-03	7.87E-05	-5.64	1.529981	-1.14	COBLL1	cordon-bleu WH2 repeat protein like 1
8081903	1.20E-03	7.89E-05	-5.64	1.526605	-2.16	MAATS1	MYCBP associated and testis expressed 1
8047379	1.21E-03	8.08E-05	-5.62	1.502708	-1.08		
8111677	1.24E-03	8.34E-05	-5.6	1.470059	-1.52	LIFR	leukemia inhibitory factor receptor alpha
7981383	1.26E-03	8.48E-05	-5.59	1.453524	-1		
7933139	1.31E-03	8.98E-05	-5.56	1.395162	-1.33	ZNF33B	zinc finger protein 33B

7968254	1.32E-03	9.02E-05	-5.56	1.389923	-1.06	POLR1D	RNA polymerase I subunit D
7903162	1.33E-03	9.07E-05	-5.55	1.384809	-1.93	TMEM56-RWDD3///TMEM56	TMEM56-RWDD3 readthrough///transmembrane protein 56
8056327	1.33E-03	9.11E-05	-5.55	1.380163	-2.94	GRB14	growth factor receptor bound protein 14
7970842	1.34E-03	9.19E-05	-5.55	1.371269	-1.06		
8111136	1.35E-03	9.30E-05	-5.54	1.359108	-1.58	FAM134B	family with sequence similarity 134 member B
7980891	1.36E-03	9.39E-05	-5.53	1.349275	-1.53	TC2N	tandem C2 domains, nuclear

8158987	1.37E-03	9.42E-05	-5.53	1.346093	-1.47	CELP	carboxyl ester lipase pseudogene
7898176	1.37E-03	9.47E-05	-5.53	1.340685	-3.32	CELA2A	chymotrypsin like elastase family member 2A
8174141	1.43E-03	9.98E-05	-5.5	1.286895	-2.35	BEX5	brain expressed X-linked 5
7995895	1.45E-03	1.02E-04	-5.49	1.267345	-1.1	HERPUD1	homocysteine inducible ER protein with ubiquitin like domain 1
7964183	1.47E-03	1.04E-04	-5.47	1.244552	-1.56	GLS2	glutaminase 2

7898734	1.47E-03	1.04E-04	-5.47	1.240766	-2.66		
8134351	1.49E-03	1.06E-04	-5.46	1.223396	-1.43	PPP1R9A	protein phosphatase 1 regulatory subunit 9A
8095562	1.50E-03	1.06E-04	-5.46	1.22268	-1.07		
8081758	1.53E-03	1.11E-04	-5.44	1.182907	-1.44	GRAMD1C	GRAM domain containing 1C
8092523	1.53E-03	1.11E-04	-5.44	1.182129	-1.02	EHHADH	enoyl-CoA, hydratase/3-hydroxyacyl CoA dehydrogenase
8124498	1.58E-03	1.15E-04	-5.41	1.141166	-1.49	ZNF204P	zinc finger protein 204, pseudogene

7970716	1.58E-03	1.15E-04	-5.41	1.140106	-1.21	LNX2	ligand of numb-protein X 2
7979378	1.61E-03	1.18E-04	-5.4	1.113231	-2.03	C14orf105	chromosome 14 open reading frame 105
7906954	1.62E-03	1.19E-04	-5.4	1.108104	-1.12	PBX1	PBX homeobox 1
8162006	1.67E-03	1.25E-04	-5.37	1.058888	-1.08	GKAP1	G kinase anchoring protein 1
7950641	1.70E-03	1.27E-04	-5.36	1.037305	-1.35	NDUFC2-KCTD14///KCTD14	NDUFC2-KCTD14 readthrough///potassium channel tetramerization domain containing 14

7903592	1.70E-03	1.28E-04	-5.35	1.034834	-2.88	KIAA1324	KIAA1324
7976481	1.76E-03	1.34E-04	-5.33	0.989499	-1.57	SERPINA4	serpin family A member 4
8090988	1.78E-03	1.36E-04	-5.32	0.974775	-1.39	CEP70	centrosomal protein 70
7923974	1.80E-03	1.39E-04	-5.31	0.951087	-1.93	C1orf132///MIR29C	chromosome 1 open reading frame 132///microRNA 29c
8144604	1.81E-03	1.39E-04	-5.3	0.947331	-2.28	TDH	L-threonine dehydrogenase (pseudogene)
7962557	1.82E-03	1.41E-04	-5.3	0.937706	-1.32		
8063174	1.83E-03	1.42E-04	-5.29	0.92745	-1.76		

7910022	1.86E-03	1.44E-04	-5.28	0.910876	-1.46	CNIH3	cornichon family AMPA receptor auxiliary protein 3
8079931	1.86E-03	1.45E-04	-5.28	0.90655	-1.48	SLC38A3	solute carrier family 38 member 3
8058591	1.90E-03	1.49E-04	-5.27	0.880305	-3.28	ACADL	acyl-CoA dehydrogenase, long chain
7913768	1.92E-03	1.51E-04	-5.26	0.866336	-2.52	IL22RA1	interleukin 22 receptor subunit alpha 1
8050111	1.96E-03	1.55E-04	-5.24	0.837953	-1.51		

7987279	1.97E-03	1.56E-04	-5.24	0.828668	-1.53	GOLGA8DP///GOLGA8CP///GOLGA8B///GOLGA8A	golgin A8 family member D, pseudogene///golgin A8 family member C, pseudogene///golgin A8 family member B///golgin A8 family member A
7910600	1.97E-03	1.56E-04	-5.24	0.828495	-1.27	KIAA1804	mixed lineage kinase 4

7943162	2.01E-03	1.60E-04	-5.22	0.806286	-1.14	C11orf54	chromosome 11 open reading frame 54
7952384	2.04E-03	1.64E-04	-5.21	0.780459	-1.5	OR10S1	olfactory receptor family 10 subfamily S member 1
8145122	2.05E-03	1.65E-04	-5.21	0.77532	-1.35	SLC39A14	solute carrier family 39 member 14

7909866	2.08E-03	1.68E-04	-5.19	0.752542	-1.15	MARC2///MARC2	mitochondrial amidoxime reducing component 2///mitochondrial amidoxime reducing component 2
8098326	2.08E-03	1.69E-04	-5.19	0.751652	-1.16		
8135480	2.10E-03	1.71E-04	-5.19	0.740343	-1.11	DNAJB9	DnaJ heat shock protein family (Hsp40) member B9

8088192	2.13E-03	1.74E-04	-5.18	0.722329	-1.03	ERC2	ELKS/RAB6-interacting/CAST family member 2
8110990	2.20E-03	1.81E-04	-5.15	0.679794	-1.62	CTNND2	catenin delta 2
7915207	2.23E-03	1.85E-04	-5.14	0.65744	-1.56	PABPC4	poly(A) binding protein cytoplasmic 4
8161839	2.25E-03	1.87E-04	-5.14	0.648172	-1.28	NMRK1	nicotinamide riboside kinase 1
8089851	2.26E-03	1.89E-04	-5.13	0.638046	-2.09	HGD	homogentisate 1,2-dioxygenase
8093278	2.26E-03	1.89E-04	-5.13	0.638046	-2.09	HGD	homogentisate 1,2-dioxygenase

7961580	2.26E-03	1.89E-04	-5.13	0.636758	-2.59	LMO3	LIM domain only 3
8031933	2.30E-03	1.93E-04	-5.12	0.615142	-1.06	RPS5	ribosomal protein S5
8142270	2.42E-03	2.06E-04	-5.08	0.547037	-2.76	NRCAM	neuronal cell adhesion molecule
8135544	2.43E-03	2.08E-04	-5.07	0.537148	-1.57	FOXP2	forkhead box P2
7908917	2.51E-03	2.16E-04	-5.05	0.49792	-1.84	BTG2	BTG anti-proliferation factor 2
7998643	2.51E-03	2.17E-04	-5.05	0.494854	-1.04	RPL3L	ribosomal protein L3 like
7893306	2.52E-03	2.17E-04	-5.05	0.492343	-1.09		
7956668	2.54E-03	2.21E-04	-5.04	0.477514	-1.02		
7901765	2.54E-03	2.21E-04	-5.04	0.473966	-1.77	HOOK1	hook microtubule tethering protein 1

7947156	2.54E-03	2.22E-04	-5.04	0.472146	-2.85	MUC15	mucin 15, cell surface associated
7908610	2.55E-03	2.23E-04	-5.04	0.467877	-3.45		
8132013	2.62E-03	2.29E-04	-5.02	0.438372	-1.58	CHN2	chimerin 2
8072946	2.68E-03	2.37E-04	-5	0.406703	-1.04	EIF3L	eukaryotic translation initiation factor 3 subunit L
7910146	2.68E-03	2.37E-04	-5	0.404898	-1.09	PSEN2	presenilin 2
8031867	2.78E-03	2.50E-04	-4.97	0.349326	-1.26		
7973869	2.80E-03	2.53E-04	-4.96	0.337656	-1.12		
7908597	2.80E-03	2.54E-04	-4.96	0.334347	-2.79	NR5A2	nuclear receptor subfamily 5 group A member 2

8156935	2.83E-03	2.57E-04	-4.96	0.321589	-1.06	ZNF189	zinc finger protein 189
7960863	2.87E-03	2.62E-04	-4.94	0.302442	-1.08		
7983650	2.89E-03	2.65E-04	-4.94	0.291933	-1.5	SLC27A2	solute carrier family 27 member 2
7993756	2.93E-03	2.70E-04	-4.93	0.271535	-2.04	ACSM3	acyl-CoA synthetase medium-chain family member 3
8144621	2.95E-03	2.74E-04	-4.92	0.258164	-1.2	FAM167A-AS1	FAM167A antisense RNA 1
8027437	2.96E-03	2.75E-04	-4.92	0.254463	-1.12		
8047300	2.99E-03	2.79E-04	-4.91	0.240322	-2.73	AOX1	aldehyde oxidase 1
7932727	2.99E-03	2.79E-04	-4.91	0.238965	-1.54	PTCHD3	patched domain containing 3

8087224	3.00E-03	2.81E-04	-4.91	0.232256	-1.14	SLC25A20	solute carrier family 25 member 20
7930790	3.04E-03	2.85E-04	-4.9	0.217022	-3.49	PNLIPRP1	pancreatic lipase related protein 1
8107996	3.09E-03	2.90E-04	-4.89	0.199187	-1.23		
7930454	3.12E-03	2.94E-04	-4.88	0.18587	-1.37	PDCD4	programmed cell death 4 (neoplastic transformation inhibitor)
8129231	3.12E-03	2.94E-04	-4.88	0.183749	-1.05	FAM184A	family with sequence similarity 184 member A

8106986	3.15E-03	2.98E-04	-4.87	0.172802	-1.11	RHOBTB3	Rho related BTB domain containing 3
7898732	3.19E-03	3.02E-04	-4.86	0.157632	-4.15		
8126954	3.19E-03	3.02E-04	-4.86	0.156942	-2.8	PKHD1	polycystic kidney and hepatic disease 1 (autosomal recessive)
8154848	3.22E-03	3.06E-04	-4.86	0.143238	-2.34	PRSS3	protease, serine 3
7898721	3.24E-03	3.09E-04	-4.85	0.133383	-3.67		
8009493	3.45E-03	3.39E-04	-4.8	0.041194	-3.5	KCNJ16	potassium voltage-gated channel subfamily J member 16

7936734	3.50E-03	3.46E-04	-4.79	0.019795	-1.07	FGFR2	fibroblast growth factor receptor 2
8097417	3.52E-03	3.48E-04	-4.79	0.013015	-1.28	JADE1	jade family PHD finger 1
8139816	3.54E-03	3.52E-04	-4.78	0.00268	-1.43		
8095907	3.54E-03	3.52E-04	-4.78	0.001405	-1.29	FRAS1	Fraser extracellular matrix complex subunit 1
8117304	3.56E-03	3.55E-04	-4.78	-0.00595	-2.6	SLC17A4	solute carrier family 17 member 4
7954377	3.62E-03	3.63E-04	-4.76	-0.02909	-4.27	IAPP	islet amyloid polypeptide
8073680	3.62E-03	3.64E-04	-4.76	-0.031103	-1.04		

7931643	3.63E-03	3.65E-04	-4.76	-0.034463	-1.1	CYP2E1	cytochrome P450 family 2 subfamily E member 1
7965884	3.66E-03	3.69E-04	-4.75	-0.044978	-2.14	PAH	phenylalanine hydroxylase
8135943	3.69E-03	3.72E-04	-4.75	-0.053934	-1.63		
8091078	3.75E-03	3.82E-04	-4.74	-0.080047	-1.94	RBP1	retinol binding protein 1

8053744	3.78E-03	3.86E-04	-4.73	-0.092698	-1.04	ANKRD20A21P///ANKRD20A8P///ANKRD20A4///ANKRD20A2///ANKRD20A3///ANKRD20A5P///ANKRD20A1	ankyrin repeat domain 20 family member A21, pseudogene///ankyrin repeat domain 20 family member A8, pseudogene///ankyrin repeat domain 20 family member A4///ankyrin repeat domain 20 family member A2///ankyrin repeat domain 20 family member A3///ankyrin repeat domain 20 family member A5, pseudogene///ankyrin repeat domain 20 family member A1

8130422	3.79E-03	3.87E-04	-4.73	-0.094965	-1.38	CNKSR3///CNKSR3	CNKSR family member 3///CNKSR family member 3
8157246	3.83E-03	3.93E-04	-4.72	-0.108554	-1.02	KIAA1958	KIAA1958
8145776	3.89E-03	4.02E-04	-4.71	-0.132241	-1.09		
7923991	3.95E-03	4.11E-04	-4.69	-0.155173	-1.18	PLXNA2	plexin A2
8163149	3.96E-03	4.12E-04	-4.69	-0.157021	-1.14	PTPN3	protein tyrosine phosphatase, non-receptor type 3
8113369	4.01E-03	4.19E-04	-4.68	-0.175499	-1.57	SLCO4C1	solute carrier organic anion transporter family member 4C1

7919067	4.06E-03	4.27E-04	-4.67	-0.193288	-1.16	REG4	regenerating family member 4
7987248	4.08E-03	4.30E-04	-4.67	-0.200735	-1.4	GOLGA8DP///GOLGA8CP///GOLGA8B///GOLGA8A	golgin A8 family member D, pseudogene///golgin A8 family member C, pseudogene///golgin A8 family member B///golgin A8 family member A
8027312	4.08E-03	4.30E-04	-4.67	-0.201452	-1.2	ZNF429	zinc finger protein 429

8002845	4.10E-03	4.33E-04	-4.67	-0.207543	-2.89	CTRB2///CTRB1	chymotrypsinogen B2///chymotrypsinogen B1
8016739	4.14E-03	4.39E-04	-4.66	-0.221574	-1.03	TOB1	transducer of ERBB2, 1
8141066	4.16E-03	4.42E-04	-4.65	-0.229228	-1.17	PON3	paraoxonase 3
8143772	4.17E-03	4.43E-04	-4.65	-0.231698	-1.46	RARRES2	retinoic acid receptor responder 2
7973797	4.20E-03	4.47E-04	-4.65	-0.241055	-1.45	COCH	cochlin
7981059	4.22E-03	4.49E-04	-4.65	-0.246153	-1.2	SERPINA6	serpin family A member 6
8149521	4.24E-03	4.54E-04	-4.64	-0.255313	-4.01	FGL1	fibrinogen like 1

8095751	4.24E-03	4.54E-04	-4.64	-0.256379	-1.05	PARM1	prostate androgen-regulated mucin-like protein 1
8125936	4.36E-03	4.70E-04	-4.62	-0.292515	-2.55	CLPS	colipase
8009515	4.37E-03	4.72E-04	-4.62	-0.295327	-1.38	LINC01152	long intergenic non-protein coding RNA 1152
8081645	4.44E-03	4.83E-04	-4.61	-0.318989	-1.4	C3orf52	chromosome 3 open reading frame 52
7997264	4.47E-03	4.87E-04	-4.6	-0.326642	-2.63	CTRB2///CTRB1	chymotrypsinogen B2///chymotrypsinogen B1

8049969	4.65E-03	5.17E-04	-4.57	-0.387621	-1.3	SH3YL1	SH3 and SYLF domain containing 1
8057821	4.71E-03	5.26E-04	-4.56	-0.404947	-1.14	DNAH7	dynein axonemal heavy chain 7
8104068	4.75E-03	5.32E-04	-4.55	-0.417868	-1.75		
7984985	4.82E-03	5.42E-04	-4.54	-0.435434	-1.01		
7983867	4.83E-03	5.45E-04	-4.54	-0.442119	-1.05	CGNL1	cingulin like 1
8132998	4.98E-03	5.70E-04	-4.52	-0.486955	-1.51		
8095585	5.00E-03	5.73E-04	-4.51	-0.492439	-2.8	SLC4A4	solute carrier family 4 member 4
8078956	5.04E-03	5.80E-04	-4.51	-0.504291	-2.32		
8102468	5.05E-03	5.81E-04	-4.51	-0.50618	-1.14	PRSS12	protease, serine 12
7995825	5.15E-03	5.96E-04	-4.49	-0.533127	-1.77	MT1F	metallothionein 1F

7983890	5.16E-03	5.98E-04	-4.49	-0.536847	-1.35	MYZAP///GCOM1	myocardial zonula adherens protein///GRINL1A complex locus 1
7965565	5.19E-03	6.06E-04	-4.48	-0.549029	-1.22	USP44	ubiquitin specific peptidase 44
8097867	5.26E-03	6.18E-04	-4.47	-0.56925	-1.04	KIAA0922	KIAA0922
8149097	5.29E-03	6.22E-04	-4.47	-0.575715	-2.77	DEFB1	defensin beta 1
8079163	5.34E-03	6.30E-04	-4.46	-0.588969	-1.01		

8147351	5.49E-03	6.54E-04	-4.44	-0.627428	-1.14	ESRP1///RAF1	epithelial splicing regulatory protein 1///Raf-1 proto-oncogene, serine/threonine kinase
8104066	5.49E-03	6.55E-04	-4.44	-0.628196	-1.46	SORBS2	sorbin and SH3 domain containing 2
7988414	5.51E-03	6.59E-04	-4.44	-0.634808	-2.83	GATM	glycine amidinotransferase
7898713	5.64E-03	6.79E-04	-4.42	-0.664935	-2.5	CELA3B	chymotrypsin like elastase family member 3B

7897953	5.65E-03	6.81E-04	-4.42	-0.667993	-1.01	SNORA59A///SNORA59B	small nucleolar RNA, H/ACA box 59A///small nucleolar RNA, H/ACA box 59B
8005626	5.65E-03	6.81E-04	-4.42	-0.667993	-1.01	SNORA59A///SNORA59B	small nucleolar RNA, H/ACA box 59A///small nucleolar RNA, H/ACA box 59B

7946340	5.68E-03	6.88E-04	-4.41	-0.677853	-1.29	RIC3	RIC3 acetylcholine receptor chaperone
7922130	5.69E-03	6.88E-04	-4.41	-0.678609	-1.09	DPT	dermatopontin
7999920	5.72E-03	6.97E-04	-4.41	-0.690971	-3.36	GP2	glycoprotein 2
8139656	5.84E-03	7.15E-04	-4.39	-0.717442	-1.33	GRB10	growth factor receptor bound protein 10
7965573	5.95E-03	7.36E-04	-4.38	-0.746348	-1.3	NTN4	netrin 4
8108697	5.97E-03	7.38E-04	-4.38	-0.749652	-1.02	PCDHB5	protocadherin beta 5
8068684	5.99E-03	7.41E-04	-4.37	-0.753673	-2.27	FAM3B	family with sequence similarity 3 member B

8110971	6.19E-03	7.76E-04	-4.35	-0.799851	-1.14	CMBL	carboxymethylenebutenolidase homolog
8102869	6.30E-03	7.97E-04	-4.33	-0.826832	-1		
8021376	6.30E-03	7.97E-04	-4.33	-0.827644	-1.19	NEDD4L	neural precursor cell expressed, developmentally down-regulated 4-like, E3 ubiquitin protein ligase
8148317	6.47E-03	8.25E-04	-4.32	-0.861859	-1.14	MYC	v-myc avian myelocytomatosis viral oncogene homolog

8150689	6.64E-03	8.57E-04	-4.29	-0.900604	-1.06		
8114209	6.72E-03	8.70E-04	-4.29	-0.916104	-1.01		
8123303	6.76E-03	8.77E-04	-4.28	-0.923325	-1.19	PACRG	PARK2 coregulated
8052370	6.84E-03	8.90E-04	-4.27	-0.938739	-1.51		
7952249	6.90E-03	8.99E-04	-4.27	-0.948556	-1.19	USP2	ubiquitin specific peptidase 2
7981943	6.99E-03	9.13E-04	-4.26	-0.964706	-1.15	SNORD64///PWAR5	small nucleolar RNA, C/D box 64///Prader Willi/Angelman region RNA 5
8055486	6.99E-03	9.15E-04	-4.26	-0.966895	-1.17		
7939024	7.13E-03	9.41E-04	-4.24	-0.995412	-1.12	ANO3	anoctamin 3

8117288	7.16E-03	9.49E-04	-4.24	-1.003517	-2.49	SCGN	secretagogin, EF-hand calcium binding protein
8174201	7.27E-03	9.71E-04	-4.23	-1.026397	-2.34	BEX1	brain expressed X-linked 1
7917199	7.32E-03	9.80E-04	-4.22	-1.03606	-1.3	TTLL7	tubulin tyrosine ligase like 7
7932765	7.36E-03	9.89E-04	-4.22	-1.045012	-1.59	MPP7	membrane palmitoylated protein 7
7923837	7.39E-03	9.94E-04	-4.22	-1.050112	-3.46	PM20D1	peptidase M20 domain containing 1
7944970	7.49E-03	1.01E-03	-4.21	-1.069577	-1.08		
8169291	7.52E-03	1.02E-03	-4.2	-1.075076	-1.85		

8146024	7.53E-03	1.02E-03	-4.2	-1.078222	-1.19	ADAM32	ADAM metallopeptidase domain 32
8084589	7.55E-03	1.02E-03	-4.2	-1.081302	-1.1	MAP3K13	mitogen-activated protein kinase kinase kinase 13
8078971	7.61E-03	1.04E-03	-4.19	-1.09415	-1.3	ENTPD3	ectonucleoside triphosphate diphosphohydrolase 3
8174239	7.66E-03	1.05E-03	-4.19	-1.103947	-1	BEX2	brain expressed X-linked 2

8104035	7.68E-03	1.05E-03	-4.19	-1.106339	-1.53	SORBS2	sorbin and SH3 domain containing 2
8095819	7.70E-03	1.06E-03	-4.18	-1.112327	-1.83	FAM47E-STBD1///FAM47E	FAM47E-STBD1 readthrough///family with sequence similarity 47 member E
7983051	7.88E-03	1.09E-03	-4.17	-1.145345	-1.07	LOC105370792	uncharacterized LOC105370792
8088642	7.96E-03	1.11E-03	-4.16	-1.162501	-1.24	LRIG1	leucine rich repeats and immunoglobulin like domains 1

8005753	8.02E-03	1.12E-03	-4.15	-1.172779	-1.16		
8135661	8.09E-03	1.13E-03	-4.15	-1.182995	-2.68	CFTR	cystic fibrosis transmembrane conductance regulator
8046018	8.20E-03	1.16E-03	-4.14	-1.202481	-1.82		
7936798	8.25E-03	1.16E-03	-4.13	-1.210787	-3.68	FAM24B-CUZD1///CUZD1	FAM24B-CUZD1 readthrough///CUB and zona pellucida like domains 1
7933263	8.26E-03	1.17E-03	-4.13	-1.21142	-1.29	PTPN20	protein tyrosine phosphatase, non-receptor type 20
7960355	8.27E-03	1.17E-03	-4.13	-1.214154	-1.1		

7993371	8.35E-03	1.19E-03	-4.12	-1.230165	-1.06	NOMO3///NOMO2///NOMO1	NODAL modulator 3///NODAL modulator 2///NODAL modulator 1
7993511	8.35E-03	1.19E-03	-4.12	-1.230165	-1.06	NOMO3///NOMO2///NOMO1	NODAL modulator 3///NODAL modulator 2///NODAL modulator 1

7999791	8.35E-03	1.19E-03	-4.12	-1.230165	-1.06	NOMO3///NOMO2///NOMO1	NODAL modulator 3///NODAL modulator 2///NODAL modulator 1
8059708	8.35E-03	1.19E-03	-4.12	-1.231153	-1.58	SNORA75	small nucleolar RNA, H/ACA box 75
8124385	8.46E-03	1.21E-03	-4.11	-1.249562	-1.05	HIST1H4B	histone cluster 1, H4b
8027354	8.48E-03	1.22E-03	-4.11	-1.253588	-1	RPSAP58	ribosomal protein SA pseudogene 58
7981333	8.49E-03	1.22E-03	-4.11	-1.254835	-3.14		
7978376	8.52E-03	1.22E-03	-4.1	-1.260728	-1.15	STXBP6	syntaxin binding protein 6

8098620	8.54E-03	1.23E-03	-4.1	-1.263148	-1.12	FAM149A	family with sequence similarity 149 member A
8156134	8.73E-03	1.26E-03	-4.09	-1.293528	-1.54	NTRK2	neurotrophic receptor tyrosine kinase 2
7976350	8.87E-03	1.29E-03	-4.08	-1.315283	-1.36	UNC79	unc-79 homolog (C. elegans)
7996260	8.91E-03	1.30E-03	-4.07	-1.322525	-2.13		
8166747	8.93E-03	1.31E-03	-4.07	-1.327295	-1.52	SYTL5	synaptotagmin like 5
8106448	9.03E-03	1.32E-03	-4.06	-1.340338	-1.17	PDE8B	phosphodiesterase 8B
7996943	9.25E-03	1.37E-03	-4.04	-1.375446	-1.04		

8162492	9.36E-03	1.39E-03	-4.04	-1.392382	-1.21	FBP2	fructose-bisphosphatase 2
8042466	9.37E-03	1.40E-03	-4.03	-1.395033	-1.58		
7908409	9.43E-03	1.41E-03	-4.03	-1.403822	-1.46	RGS2	regulator of G-protein signaling 2
8113346	9.61E-03	1.45E-03	-4.02	-1.428158	-1.25		
7931405	9.81E-03	1.49E-03	-4	-1.458301	-1.27	PPP2R2D	protein phosphatase 2 regulatory subunit Bdelta
8168079	9.81E-03	1.49E-03	-4	-1.458394	-1.06		
8092800	1.00E-02	1.53E-03	-3.99	-1.484707	-1.14	ATP13A4	ATPase 13A4
7933379	1.01E-02	1.56E-03	-3.98	-1.503491	-1.27	PTPN20	protein tyrosine phosphatase, non-receptor type 20

8083000	1.03E-02	1.60E-03	-3.96	-1.529343	-1.12	FAIM	Fas apoptotic inhibitory molecule
8068620	1.05E-02	1.64E-03	-3.95	-1.55719	-1.02	SH3BGR	SH3 domain binding glutamate rich protein
8143499	1.09E-02	1.72E-03	-3.92	-1.604597	-1.14	TRPV6	transient receptor potential cation channel subfamily V member 6
8038326	1.10E-02	1.74E-03	-3.92	-1.613257	-1	SLC6A16	solute carrier family 6 member 16

8155397	1.10E-02	1.74E-03	-3.92	-1.614724	-1.13	ANKRD20A21P///ANKRD20A8P///ANKRD20A4///ANKRD20A2///ANKRD20A3///ANKRD20A5P///ANKRD20A1	ankyrin repeat domain 20 family member A21, pseudogene///ankyrin repeat domain 20 family member A8, pseudogene///ankyrin repeat domain 20 family member A4///ankyrin repeat domain 20 family member A2///ankyrin repeat domain 20 family member A3///ankyrin repeat domain 20 family member A5, pseudogene///ankyrin repeat domain 20 family member A1

8161388	1.10E-02	1.74E-03	-3.92	-1.614724	-1.13	ANKRD20A21P///ANKRD20A8P///ANKRD20A4///ANKRD20A2///ANKRD20A3///ANKRD20A5P///ANKRD20A1	ankyrin repeat domain 20 family member A21, pseudogene///ankyrin repeat domain 20 family member A8, pseudogene///ankyrin repeat domain 20 family member A4///ankyrin repeat domain 20 family member A2///ankyrin repeat domain 20 family member A3///ankyrin repeat domain 20 family member A5, pseudogene///ankyrin repeat domain 20 family member A1

8155574	1.10E-02	1.74E-03	-3.92	-1.615896	-1.1	ANKRD20A21P///ANKRD20A8P///ANKRD20A4///ANKRD20A2///ANKRD20A3///ANKRD20A5P///ANKRD20A1	ankyrin repeat domain 20 family member A21, pseudogene///ankyrin repeat domain 20 family member A8, pseudogene///ankyrin repeat domain 20 family member A4///ankyrin repeat domain 20 family member A2///ankyrin repeat domain 20 family member A3///ankyrin repeat domain 20 family member A5, pseudogene///ankyrin repeat domain 20 family member A1

7903407	1.11E-02	1.77E-03	-3.91	-1.630545	-1.03	RNPC3///AMY2B	RNA binding region (RNP1, RRM) containing 3///amylase, alpha 2B (pancreatic)
8018038	1.11E-02	1.77E-03	-3.91	-1.630771	-1.2	ABCA5	ATP binding cassette subfamily A member 5
8160168	1.12E-02	1.79E-03	-3.9	-1.641935	-1.21	FREM1	FRAS1 related extracellular matrix 1
8117187	1.12E-02	1.79E-03	-3.9	-1.643584	-1		

8095395	1.15E-02	1.87E-03	-3.88	-1.685082	-1.61	UGT2B7	UDP glucuronosyltransferase family 2 member B7
8082797	1.15E-02	1.88E-03	-3.88	-1.691185	-1.94	TF	transferrin
8161913	1.16E-02	1.88E-03	-3.88	-1.692408	-1.03		
7927694	1.16E-02	1.89E-03	-3.87	-1.700299	-1.97	PHYHIPL	phytanoyl-CoA 2-hydroxylase interacting protein like
8177222	1.18E-02	1.94E-03	-3.86	-1.726806	-1.12	CD24	CD24 molecule
7919055	1.19E-02	1.97E-03	-3.85	-1.738697	-1.8	HMGCS2	3-hydroxy-3-methylglutaryl-CoA synthase 2

7918936	1.22E-02	2.05E-03	-3.83	-1.780785	-1.46	VTCN1	V-set domain containing T cell activation inhibitor 1
7961844	1.22E-02	2.06E-03	-3.83	-1.782703	-1.16	CASC1	cancer susceptibility candidate 1
8168274	1.23E-02	2.07E-03	-3.83	-1.790029	-1.15	GJB1	gap junction protein beta 1
8035793	1.23E-02	2.08E-03	-3.82	-1.796467	-1.34	ZNF737///ZNF737	zinc finger protein 737///zinc finger protein 737
7992893	1.24E-02	2.10E-03	-3.82	-1.801818	-1.14		

8098146	1.24E-02	2.10E-03	-3.82	-1.806123	-1.15	NPY5R	neuropeptide Y receptor Y5
7976488	1.24E-02	2.11E-03	-3.82	-1.809468	-1.77	SERPINA5	serpin family A member 5

8053741	1.26E-02	2.15E-03	-3.81	-1.828681	-1.44	ANKRD20A8P///ANKRD20A4///ANKRD20A2///ANKRD20A3///ANKRD20A5P///ANKRD20A1	ankyrin repeat domain 20 family member A8, pseudogene///ankyrin repeat domain 20 family member A4///ankyrin repeat domain 20 family member A2///ankyrin repeat domain 20 family member A3///ankyrin repeat domain 20 family member A5, pseudogene///ankyrin repeat domain 20 family member A1

7909681	1.26E-02	2.15E-03	-3.81	-1.829179	-1.9	PROX1	prospero homeobox 1
8121559	1.27E-02	2.16E-03	-3.8	-1.834458	-1.41		
8103360	1.29E-02	2.22E-03	-3.79	-1.860662	-1.16		
8169145	1.31E-02	2.27E-03	-3.78	-1.881204	-1.1	MUM1L1	MUM1 like 1
7898725	1.31E-02	2.28E-03	-3.78	-1.88475	-2.37	CELA3A	chymotrypsin like elastase family member 3A
8114964	1.33E-02	2.32E-03	-3.77	-1.905361	-2.35	SPINK1	serine peptidase inhibitor, Kazal type 1

8155602	1.38E-02	2.43E-03	-3.74	-1.952251	-1.22	LOC102725104///ANKRD20A21P///LOC101059935///ANKRD20A12P///ANKRD20A8P///ANKRD20A4///ANKRD20A2///ANKRD20A3///ANKRD20A5P///ANKRD20A1	putative ankyrin repeat domain-containing protein 20A12 pseudogene///ankyrin repeat domain 20 family member A21, pseudogene///ankyrin repeat domain-containing protein 18A-like///ankyrin repeat domain 20 family member A12, pseudogene///ankyrin repeat domain 20 family member A8, pseudogene///ankyrin repeat domain 20 family member A4///ankyrin repeat domain 20 family member A2///ankyrin repeat domain 20 family member A3///ankyrin repeat domain 20 family member A5, pseudogene///ankyrin repeat domain 20 family member A1

8173156	1.44E-02	2.60E-03	-3.71	-2.016873	-1.28		
7930777	1.51E-02	2.75E-03	-3.68	-2.0742	-1.19	PNLIP///COL1A2	pancreatic lipase///collagen type I alpha 2 chain
7969091	1.51E-02	2.76E-03	-3.68	-2.076785	-1.31		
8022045	1.57E-02	2.93E-03	-3.65	-2.137306	-1.17	MYOM1	myomesin 1
7999362	1.61E-02	3.04E-03	-3.63	-2.174584	-1.34		
8147697	1.67E-02	3.19E-03	-3.6	-2.221223	-1.11	GRHL2	grainyhead like transcription factor 2

7981953	1.71E-02	3.30E-03	-3.58	-2.257319	-1.01	SNORD116-9///SNORD116-8///SNORD116-7///SNORD116-5///SNORD116-3	small nucleolar RNA, C/D box 116-9///small nucleolar RNA, C/D box 116-8///small nucleolar RNA, C/D box 116-7///small nucleolar RNA, C/D box 116-5///small nucleolar RNA, C/D box 116-3

7981966	1.71E-02	3.30E-03	-3.58	-2.257319	-1.01	SNORD116-9///SNORD116-8///SNORD116-7///SNORD116-5///SNORD116-3	small nucleolar RNA, C/D box 116-9///small nucleolar RNA, C/D box 116-8///small nucleolar RNA, C/D box 116-7///small nucleolar RNA, C/D box 116-5///small nucleolar RNA, C/D box 116-3

8136235	1.71E-02	3.31E-03	-3.58	-2.259828	-1.85	CPA1	carboxypeptidase A1
7930804	1.72E-02	3.33E-03	-3.58	-2.265783	-2.78	PNLIPRP2	pancreatic lipase related protein 2 (gene/pseudogene)
7981976	1.73E-02	3.34E-03	-3.58	-2.268956	-1.27	SNORD116-14	small nucleolar RNA, C/D box 116-14
8017766	1.74E-02	3.37E-03	-3.57	-2.277227	-2.07	APOH	apolipoprotein H
8124196	1.76E-02	3.43E-03	-3.56	-2.296368	-2.26	DCDC2	doublecortin domain containing 2
8151559	1.92E-02	3.87E-03	-3.5	-2.414334	-1.26	SLC10A5	solute carrier family 10 member 5

7986195	1.93E-02	3.89E-03	-3.5	-2.420835	-1.49	SV2B	synaptic vesicle glycoprotein 2B
8064098	1.94E-02	3.93E-03	-3.49	-2.430533	-1.02		
8043276	1.95E-02	3.94E-03	-3.49	-2.434467	-1.11	SNORD94	small nucleolar RNA, C/D box 94

7981958	1.96E-02	3.99E-03	-3.49	-2.444748	-1.08	SNORD116-9///SNORD116-7///SNORD116-5///SNORD116-3	small nucleolar RNA, C/D box 116-9///small nucleolar RNA, C/D box 116-7///small nucleolar RNA, C/D box 116-5///small nucleolar RNA, C/D box 116-3

7981962	1.96E-02	3.99E-03	-3.49	-2.444748	-1.08	SNORD116-9///SNORD116-7///SNORD116-5///SNORD116-3	small nucleolar RNA, C/D box 116-9///small nucleolar RNA, C/D box 116-7///small nucleolar RNA, C/D box 116-5///small nucleolar RNA, C/D box 116-3
8096682	1.98E-02	4.05E-03	-3.48	-2.461822	-1.19	ARHGEF38	Rho guanine nucleotide exchange factor 38

8165656	2.10E-02	4.39E-03	-3.44	-2.540441	-1.01		
7971239	2.10E-02	4.40E-03	-3.43	-2.543626	-1.05		
7933437	2.11E-02	4.42E-03	-3.43	-2.547303	-1.37	PTPN20	protein tyrosine phosphatase, non-receptor type 20
8157231	2.11E-02	4.43E-03	-3.43	-2.550579	-1.84		
8086752	2.13E-02	4.49E-03	-3.42	-2.564335	-1.35	SNORD13P3	small nucleolar RNA, C/D box 13 pseudogene 3
8054846	2.15E-02	4.55E-03	-3.42	-2.577415	-1.4	SCTR	secretin receptor
8136187	2.15E-02	4.57E-03	-3.41	-2.580951	-1.98	CPA2	carboxypeptidase A2
8156358	2.16E-02	4.58E-03	-3.41	-2.58264	-1.02		

7981982	2.19E-02	4.67E-03	-3.4	-2.602846	-1.15	SNORD116-22///SNORD116-21///SNORD116-20///SNORD116-18///SNORD116-17///SNORD116-16///SNORD116-15///SNORD116-14///SNORD116-19///SNRPN	small nucleolar RNA, C/D box 116-22///small nucleolar RNA, C/D box 116-21///small nucleolar RNA, C/D box 116-20///small nucleolar RNA, C/D box 116-18///small nucleolar RNA, C/D box 116-17///small nucleolar RNA, C/D box 116-16///small nucleolar RNA, C/D box 116-15///small nucleolar RNA, C/D box 116-14///small nucleolar RNA, C/D box 116-19///small nuclear ribonucleoprotein polypeptide N

7981986	2.19E-02	4.67E-03	-3.4	-2.602846	-1.15	SNORD116-22///SNORD116-21///SNORD116-20///SNORD116-18///SNORD116-17///SNORD116-16///SNORD116-15///SNORD116-14///SNORD116-19///SNRPN	small nucleolar RNA, C/D box 116-22///small nucleolar RNA, C/D box 116-21///small nucleolar RNA, C/D box 116-20///small nucleolar RNA, C/D box 116-18///small nucleolar RNA, C/D box 116-17///small nucleolar RNA, C/D box 116-16///small nucleolar RNA, C/D box 116-15///small nucleolar RNA, C/D box 116-14///small nucleolar RNA, C/D box 116-19///small nuclear ribonucleoprotein polypeptide N

8107129	2.19E-02	4.68E-03	-3.4	-2.604162	-1.23	SLCO4C1	solute carrier organic anion transporter family member 4C1
7933080	2.20E-02	4.70E-03	-3.4	-2.608795	-1.16		

8053737	2.20E-02	4.71E-03	-3.4	-2.609888	-1.17	LOC102725104///LOC101059935///ANKRD20A8P	putative ankyrin repeat domain-containing protein 20A12 pseudogene///ankyrin repeat domain-containing protein 18A-like///ankyrin repeat domain 20 family member A8, pseudogene

8136709	2.20E-02	4.71E-03	-3.4	-2.611728	-1.52	MGAM2	maltase-glucoamylase 2 (putative)
8095535	2.25E-02	4.87E-03	-3.38	-2.644743	-1.35	ENAM	enamelin
7976073	2.25E-02	4.87E-03	-3.38	-2.644949	-1.1	FLRT2	fibronectin leucine rich transmembrane protein 2
8126770	2.28E-02	5.00E-03	-3.37	-2.669282	-1	CYP39A1	cytochrome P450 family 39 subfamily A member 1

8155414	2.36E-02	5.25E-03	-3.34	-2.718234	-1.31	LOC102725104///LOC101059935///ANKRD20A8P	putative ankyrin repeat domain-containing protein 20A12 pseudogene///ankyrin repeat domain-containing protein 18A-like///ankyrin repeat domain 20 family member A8, pseudogene

8161384	2.36E-02	5.25E-03	-3.34	-2.718234	-1.31	LOC102725104///LOC101059935///ANKRD20A8P	putative ankyrin repeat domain-containing protein 20A12 pseudogene///ankyrin repeat domain-containing protein 18A-like///ankyrin repeat domain 20 family member A8, pseudogene
8098654	2.41E-02	5.38E-03	-3.33	-2.743573	-1.76	KLKB1	kallikrein B1

8136801	2.43E-02	5.45E-03	-3.32	-2.755012	-1.36	PRSS3P2	protease, serine 3 pseudogene 2
8097955	2.45E-02	5.49E-03	-3.32	-2.763651	-1.13		
8106512	2.45E-02	5.51E-03	-3.32	-2.767317	-1.05		
8147988	2.49E-02	5.64E-03	-3.31	-2.789562	-1.56		
8022666	2.55E-02	5.80E-03	-3.29	-2.817898	-2.17	CHST9	carbohydrate sulfotransferase 9
8001531	2.57E-02	5.87E-03	-3.28	-2.829114	-1.96	MT1G	metallothionein 1G
8045533	2.61E-02	6.00E-03	-3.27	-2.850723	-1.37		
8013354	2.61E-02	6.01E-03	-3.27	-2.851782	-1.06		
7930714	2.61E-02	6.01E-03	-3.27	-2.852008	-1.39	ATRNL1	attractin like 1
8055202	2.65E-02	6.11E-03	-3.26	-2.868961	-1.42		
7896752	2.71E-02	6.32E-03	-3.25	-2.9021	-1.15		
8165667	2.71E-02	6.32E-03	-3.25	-2.9021	-1.15		
8165663	2.77E-02	6.52E-03	-3.23	-2.933648	-2.18		

8046116	2.83E-02	6.73E-03	-3.21	-2.963991	-1.95	G6PC2	glucose-6-phosphatase catalytic subunit 2
8136795	2.83E-02	6.74E-03	-3.21	-2.965865	-1.04	PRSS1	protease, serine 1
7918923	2.84E-02	6.75E-03	-3.21	-2.966999	-1.08		
8176193	2.84E-02	6.78E-03	-3.21	-2.971833	-1.7	F8	coagulation factor VIII
8112611	2.85E-02	6.81E-03	-3.21	-2.975578	-1.42		
8100760	2.86E-02	6.82E-03	-3.21	-2.977175	-2.42	UGT2A3	UDP glucuronosyltransferase family 2 member A3
8133012	2.92E-02	7.04E-03	-3.19	-3.008234	-1.01		

7923976	2.96E-02	7.19E-03	-3.18	-3.028845	-1	C1orf132///MIR29B2	chromosome 1 open reading frame 132///microRNA 29b-2
7981990	2.97E-02	7.22E-03	-3.18	-3.034026	-1.17	SNORD116-21	small nucleolar RNA, C/D box 116-21
8133038	2.98E-02	7.26E-03	-3.17	-3.039264	-1.17		
7896750	3.00E-02	7.33E-03	-3.17	-3.047977	-2.04		
7967034	3.05E-02	7.50E-03	-3.16	-3.071569	-1.51	PLA2G1B	phospholipase A2 group IB
8128892	3.05E-02	7.50E-03	-3.16	-3.071613	-1.19		
8141094	3.11E-02	7.71E-03	-3.14	-3.098672	-1.65	PDK4	pyruvate dehydrogenase kinase 4
7900652	3.18E-02	7.98E-03	-3.13	-3.131796	-1.25		

8161415	3.19E-02	8.00E-03	-3.12	-3.135263	-1.13	LOC101059935///ANKRD20A8P	ankyrin repeat domain-containing protein 18A-like///ankyrin repeat domain 20 family member A8, pseudogene
7995829	3.23E-02	8.13E-03	-3.12	-3.150983	-1.72	MT1H	metallothionein 1H
8043375	3.23E-02	8.16E-03	-3.11	-3.154116	-1.04		
7968678	3.24E-02	8.19E-03	-3.11	-3.157257	-1.28	FREM2	FRAS1 related extracellular matrix protein 2

8155591	3.32E-02	8.47E-03	-3.09	-3.190756	-1.22	LOC102725104///LOC101059935///ANKRD20A8P	putative ankyrin repeat domain-containing protein 20A12 pseudogene///ankyrin repeat domain-containing protein 18A-like///ankyrin repeat domain 20 family member A8, pseudogene
8114213	3.43E-02	8.87E-03	-3.07	-3.235913	-1.08		
8130660	3.43E-02	8.88E-03	-3.07	-3.236985	-1.03		

8042986	3.44E-02	8.92E-03	-3.07	-3.241835	-2	REG1A	regenerating family member 1 alpha
7989501	3.56E-02	9.35E-03	-3.04	-3.288353	-1.68	CA12	carbonic anhydrase 12
8053057	3.60E-02	9.50E-03	-3.03	-3.303722	-1.18		

8100758	3.61E-02	9.54E-03	-3.03	-3.307498	-2.04	UGT2B11///UGT2B17///UGT2B15///UGT2B10///UGT2B7	UDP glucuronosyltransferase family 2 member B11///UDP glucuronosyltransferase family 2 member B17///UDP glucuronosyltransferase family 2 member B15///UDP glucuronosyltransferase family 2 member B10///UDP glucuronosyltransferase family 2 member B7

8099633	3.65E-02	9.68E-03	-3.03	-3.321927	-1.08	PPARGC1A	PPARG coactivator 1 alpha
8083246	3.75E-02	1.01E-02	-3.01	-3.359274	-1.1	CPB1	carboxypeptidase B1
8045882	3.76E-02	1.01E-02	-3	-3.366041	-1.43	DAPL1	death associated protein like 1
7965036	3.76E-02	1.01E-02	-3	-3.366876	-1.05		
8045279	3.88E-02	1.06E-02	-2.98	-3.407163	-1.13		
7991171	3.95E-02	1.08E-02	-2.97	-3.429993	-1.2		
7970737	4.01E-02	1.10E-02	-2.96	-3.450343	-1.13	FLT3	fms related tyrosine kinase 3
8033362	4.07E-02	1.13E-02	-2.95	-3.47138	-1.1	INSR	insulin receptor
8114006	4.07E-02	1.13E-02	-2.95	-3.471752	-1		
8058104	4.07E-02	1.13E-02	-2.94	-3.474499	-1.26		
7981949	4.12E-02	1.15E-02	-2.94	-3.489112	-1.04	SNORD116-1	small nucleolar RNA, C/D box 116-1

8113234	4.18E-02	1.17E-02	-2.93	-3.509678	-1.06	PCSK1	proprotein convertase subtilisin/kexin type 1
8092682	4.36E-02	1.25E-02	-2.89	-3.570574	-2.15	SST	somatostatin
7924307	4.63E-02	1.36E-02	-2.85	-3.651461	-1.16		
8105899	4.72E-02	1.39E-02	-2.84	-3.67833	-1.04	MARVELD2	MARVEL domain containing 2
8177498	4.72E-02	1.39E-02	-2.84	-3.67833	-1.04	MARVELD2	MARVEL domain containing 2
8003605	4.74E-02	1.40E-02	-2.83	-3.681916	-1.29		
7912157	4.86E-02	1.45E-02	-2.82	-3.716033	-1.22	ERRFI1	ERBB receptor feedback inhibitor 1
8128618	4.88E-02	1.46E-02	-2.81	-3.722233	-1.18		

8115851	4.95E-02	1.49E-02	-2.8	-3.742402	-1.11	STC2	stanniocalcin 2
7986327	5.06E-02	1.54E-02	-2.79	-3.772182	-1.04		
8121152	5.10E-02	1.55E-02	-2.78	-3.783121	-1.27	FUT9	fucosyltransferase 9
7982593	5.10E-02	1.56E-02	-2.78	-3.785511	-1.48	C15orf54	chromosome 15 open reading frame 54
8165672	5.17E-02	1.58E-02	-2.77	-3.802012	-1.4		
8111864	5.70E-02	1.82E-02	-2.7	-3.938373	-2.08	C6	complement component 6
8101131	5.75E-02	1.85E-02	-2.69	-3.949685	-1.2	CXCL11	C-X-C motif chemokine ligand 11
7952339	5.76E-02	1.85E-02	-2.69	-3.951957	-1.51	SNORD14C	small nucleolar RNA, C/D box 14C
8104348	5.88E-02	1.91E-02	-2.67	-3.983089	-1.06		

8053330	6.01E-02	1.97E-02	-2.66	-4.011136	-3.15	REG1B	regenerating family member 1 beta
8113356	6.41E-02	2.16E-02	-2.61	-4.099829	-1.65		
7928308	6.59E-02	2.25E-02	-2.59	-4.139566	-1.06	DDIT4	DNA damage inducible transcript 4
8145793	6.64E-02	2.27E-02	-2.58	-4.149066	-1.01	SNORD13	small nucleolar RNA, C/D box 13
7983718	6.66E-02	2.28E-02	-2.58	-4.153482	-1.86	SCG3	secretogranin III
8020795	6.79E-02	2.35E-02	-2.56	-4.181168	-2.51	TTR	transthyretin
8056250	7.07E-02	2.49E-02	-2.53	-4.236225	-3.42	GCG	glucagon

8068583	7.47E-02	2.70E-02	-2.49	-4.315699	-1.37	KCNJ15	potassium voltage-gated channel subfamily J member 15
8166769	7.78E-02	2.88E-02	-2.46	-4.37385	-1.09	OTC	ornithine carbamoyltransferase
7981992	7.97E-02	2.97E-02	-2.44	-4.40485	-1.24	SNORD116-22	small nucleolar RNA, C/D box 116-22
8103483	8.10E-02	3.04E-02	-2.43	-4.427807	-1.03		
8113344	8.10E-02	3.04E-02	-2.43	-4.427807	-1.03		
8114207	8.10E-02	3.04E-02	-2.43	-4.427807	-1.03		

7980861	8.36E-02	3.18E-02	-2.4	-4.470499	-1.4	CATSPERB	cation channel sperm associated auxiliary subunit beta
8129876	8.43E-02	3.23E-02	-2.39	-4.482724	-1.21	PBOV1	prostate and breast cancer overexpressed 1
7896748	8.70E-02	3.38E-02	-2.37	-4.527132	-1.19		
8139482	9.05E-02	3.57E-02	-2.34	-4.578608	-1.21	SNORA5A	small nucleolar RNA, H/ACA box 5A
8144786	9.06E-02	3.57E-02	-2.34	-4.579306	-1.39	SLC7A2	solute carrier family 7 member 2
7973867	9.59E-02	3.87E-02	-2.3	-4.653861	-1.32		
7995797	9.88E-02	4.05E-02	-2.27	-4.696503	-1.07	MT1E	metallothionein 1E

8165684	1.05E-01	4.42E-02	-2.23	-4.779286	-1.27		
7999999	1.08E-01	4.63E-02	-2.2	-4.820953	-1.67		
**GSE62165**							
ID	adj.P.Val	P.Value	t	B	logFC	Gene.Symbol	GB_LIST
11743282_a_at	1.87E-44	3.79E-49	2.36E+01	101.235356	4.74	SULF1	NM_001128204,NM_001128205,NM_001128206,NM_015170
11758071_s_at	9.90E-43	4.01E-47	2.26E+01	96.647773	4.49	SULF1	NM_001128204,NM_001128205,NM_001128206,NM_015170
11737874_a_at	6.53E-41	3.97E-45	2.16E+01	92.120645	4.76	SULF1	NM_001128204,NM_001128205,NM_001128206,NM_015170
11748905_a_at	9.52E-41	7.71E-45	2.15E+01	91.46556	4.97	CTHRC1	NM_138455
11716018_s_at	1.26E-37	1.79E-41	1.99E+01	83.817419	3.4	RAB31	NM_006868
11756071_x_at	5.13E-36	1.14E-39	1.91E+01	79.709076	1.77	ACTB	NM_001101
11739534_a_at	5.14E-36	1.25E-39	1.90E+01	79.620827	4.51	FN1	NM_002026,NM_054034,NM_212474,NM_212475,NM_212476,NM_212478,NM_212482
11748311_s_at	9.38E-36	2.47E-39	1.89E+01	78.947316	4.04	VCAN	NM_001126336,NM_001164097,NM_001164098,NM_004385
11749485_x_at	2.37E-35	7.66E-39	1.87E+01	77.827301	2.4	SEC23A	NM_006364

11746931_a_at	2.67E-35	9.21E-39	1.87E+01	77.645827	4.54	INHBA	NM_002192
AFFX-HSAC07/X00351_3_at	3.48E-35	1.41E-38	1.86E+01	77.225823	1.32	ACTB	NM_001101
11738808_a_at	3.58E-35	1.59E-38	1.86E+01	77.10315	3.3	OSBPL10	NM_001174060,NM_017784
11739535_s_at	6.34E-35	2.95E-38	1.84E+01	76.492946	3.98	FN1	NM_002026,NM_054034,NM_212474,NM_212475,NM_212476,NM_212478,NM_212482
AFFX-HSAC07/X00351_M_at	7.04E-35	3.42E-38	1.84E+01	76.347817	1.53	ACTB	NM_001101
11739536_x_at	2.66E-34	1.67E-37	1.81E+01	74.778686	4.59	FN1	NM_002026,NM_054034,NM_212474,NM_212475,NM_212476,NM_212478,NM_212482
11757833_a_at	2.97E-34	1.98E-37	1.81E+01	74.609026	2.94	RAB31	NM_006868
11749460_x_at	3.73E-34	2.66E-37	1.80E+01	74.317123	4.6	FN1	NM_002026,NM_054034,NM_212474,NM_212475,NM_212476,NM_212478,NM_212482
11745837_x_at	3.73E-34	2.72E-37	1.80E+01	74.297876	4.65	FN1	NM_002026,NM_054034,NM_212474,NM_212475,NM_212476,NM_212478,NM_212482
11748164_a_at	4.35E-34	3.35E-37	1.80E+01	74.090474	4.03	VCAN	NM_001126336,NM_001164097,NM_001164098,NM_004385
11730864_s_at	5.65E-34	4.46E-37	1.79E+01	73.805779	1.59	CTTNBP2NL	NM_018704

11716019_at	5.86E-34	4.75E-37	1.79E+01	73.745264	3.18	RAB31	NM_006868
11721135_s_at	5.94E-34	4.93E-37	1.79E+01	73.707694	4.3	IGFBP3	NM_000598,NM_001013398
11726070_s_at	7.53E-34	6.56E-37	1.78E+01	73.425665	2.29	MAP4K4	NM_004834,NM_145686,NM_145687
11715604_x_at	1.33E-33	1.23E-36	1.77E+01	72.806267	2.61	SPARC	NM_003118
11757409_x_at	1.75E-33	1.70E-36	1.77E+01	72.481183	1.87	TMSB10	NM_021103
11728776_s_at	2.52E-33	2.65E-36	1.76E+01	72.043236	2.78	VDR	NM_000376,NM_001017535
11749980_x_at	2.66E-33	2.90E-36	1.76E+01	71.952577	4.13	HOPX	NM_001145459,NM_001145460,NM_032495, NM_139211,NM_139212
11743191_a_at	2.66E-33	2.91E-36	1.75E+01	71.949836	4.31	NTM	NM_001048209,NM_001144058,NM_0011440 59,NM_016522
11726333_s_at	3.70E-33	4.20E-36	1.75E+01	71.586957	4.16	LEF1	NM_001130713,NM_001130714,NM_0011661 19,NM_016269
11719377_x_at	5.16E-33	6.17E-36	1.74E+01	71.206731	1.6	CAP1	NM_001105530,NM_006367
11745004_x_at	6.65E-33	8.48E-36	1.73E+01	70.891305	3.92	HOPX	NM_001145459,NM_001145460,NM_032495, NM_139211,NM_139212
11747925_a_at	1.06E-32	1.39E-35	1.73E+01	70.399664	2.23	SEC23A	NM_006364
11757731_s_at	1.06E-32	1.42E-35	1.72E+01	70.379596	2.85	RAB31	NM_006868

11753549_a_at	1.14E-32	1.55E-35	1.72E+01	70.294018	3.02	CMTM3	NM_001048251,NM_144601,NM_181553,NM_181554
11727782_a_at	1.20E-32	1.65E-35	1.72E+01	70.231245	2.37	TPM4	NM_001145160,NM_003290
11743856_a_at	1.24E-32	1.73E-35	1.72E+01	70.183846	4.47	CTHRC1	NM_138455
11758546_x_at	1.37E-32	1.97E-35	1.72E+01	70.056228	2.67	VDR	NM_000376,NM_001017535
11727401_x_at	1.95E-32	2.92E-35	1.71E+01	69.666158	4.11	HOPX	NM_001145459,NM_001145460,NM_032495,NM_139211,NM_139212
11729190_a_at	2.38E-32	3.80E-35	1.71E+01	69.405126	1.72	MAX	NM_002382,NM_145112,NM_145113,NM_145114,NM_145116,NM_197957
11728374_s_at	3.04E-32	5.05E-35	1.70E+01	69.12388	1.72	ACTN1	NM_001102,NM_001130004,NM_001130005
11744718_a_at	4.03E-32	6.93E-35	1.70E+01	68.8109	4	CTHRC1	NM_138455
11728761_a_at	4.03E-32	7.07E-35	1.69E+01	68.791272	3.02	RUNX1	NM_001001890,NM_001122607,NM_001754
11718168_a_at	4.03E-32	7.11E-35	1.69E+01	68.786069	2.84	MYOF	NM_013451,NM_133337
11754928_x_at	4.51E-32	8.22E-35	1.69E+01	68.641779	2.85	MYOF	NM_013451,NM_133337
11740181_a_at	4.51E-32	8.29E-35	1.69E+01	68.633408	2.22	PTPN12	NM_001131008,NM_001131009,NM_002835
11742191_a_at	4.51E-32	8.30E-35	1.69E+01	68.63222	3.16	RUNX1	NM_001001890,NM_001122607,NM_001754

11715603_s_at	4.70E-32	8.75E-35	1.69E+01	68.580074	2.7	SPARC	NM_003118
11722354_at	7.74E-32	1.55E-34	1.68E+01	68.013364	1.66	SEC23A	NM_006364
11749275_a_at	8.42E-32	1.71E-34	1.68E+01	67.919329	2.15	SKAP2	NM_003930
11727783_s_at	8.55E-32	1.75E-34	1.68E+01	67.894816	2.1	LOC643634 /// TPM4	NM_001145160,NM_003290,XR_016808,XR_019021,XR_039579
11747841_a_at	9.61E-32	2.04E-34	1.68E+01	67.74012	1.97	ACVR1	NM_001105,NM_001111067
11740879_s_at	1.03E-31	2.21E-34	1.67E+01	67.663934	2.11	ANXA2	NM_001002857,NM_001002858,NM_001136015,NM_004039
11742712_a_at	1.17E-31	2.53E-34	1.67E+01	67.528851	4.23	THBS2	NM_003247
11718648_s_at	1.27E-31	2.80E-34	1.67E+01	67.428249	1.76	ACVR1	NM_001105,NM_001111067
11746471_s_at	1.29E-31	2.87E-34	1.67E+01	67.402701	2.37	MAP4K4	NM_004834,NM_145686,NM_145687
11755955_a_at	1.52E-31	3.42E-34	1.67E+01	67.230108	3.72	FAP	NM_004460
11718068_at	2.35E-31	5.51E-34	1.66E+01	66.757265	2.17	MYO1E	NM_004998
11735218_at	2.37E-31	5.61E-34	1.66E+01	66.740073	5.12	COL10A1	NM_000493
11739134_a_at	3.31E-31	7.97E-34	1.65E+01	66.39178	3.57	COL5A2	NM_000393

11737050_x_at	5.92E-31	1.51E-33	1.64E+01	65.757892	3.27	HRH1	NM_000861,NM_001098211,NM_001098212, NM_001098213
11739135_s_at	5.92E-31	1.52E-33	1.64E+01	65.751014	3.38	COL5A2	NM_000393
11740475_x_at	5.99E-31	1.55E-33	1.64E+01	65.731777	4.2	NOX4	NM_001143836,NM_001143837,NM_016931, NR_026571
11739435_a_at	6.18E-31	1.62E-33	1.64E+01	65.688547	4.28	FN1	NM_002026,NM_054034,NM_212474,NM_212 475,NM_212476,NM_212478,NM_212482
11723903_at	6.28E-31	1.68E-33	1.64E+01	65.654169	2.41	PLEKHG1	NM_001029884
11720754_x_at	6.50E-31	1.76E-33	1.64E+01	65.604603	1.24	ACTB	NM_001101
11747282_x_at	7.16E-31	1.96E-33	1.63E+01	65.502191	1.31	ANXA2P2	NR_003573
11744033_s_at	7.70E-31	2.15E-33	1.63E+01	65.407518	2.53	S100A11	NM_005620
11743031_a_at	7.79E-31	2.19E-33	1.63E+01	65.388574	2.1	S100A6	NM_014624
11747923_s_at	7.93E-31	2.25E-33	1.63E+01	65.364109	4.37	LAMC2	NM_005562,NM_018891
11743062_a_at	9.45E-31	2.72E-33	1.63E+01	65.176565	4.06	PLAUR	NM_001005376,NM_001005377,NM_002659
11722352_s_at	1.07E-30	3.09E-33	1.62E+01	65.047755	2.74	ACTA2	NM_001141945,NM_001613
11737219_a_at	1.24E-30	3.67E-33	1.62E+01	64.877768	1.98	ITGB1	NM_002211,NM_033666,NM_033667,NM_033 668,NM_033669,NM_133376

11743721_at	1.60E-30	4.86E-33	1.62E+01	64.601131	2.4	LGALS1	NM_002305
11759402_s_at	1.60E-30	4.89E-33	1.62E+01	64.593519	3.69	VCAN	NM_001126336,NM_001164097,NM_001164098,NM_004385
11733383_a_at	1.60E-30	4.92E-33	1.62E+01	64.587381	2.17	AFAP1	NM_001134647,NM_198595
11756577_a_at	1.77E-30	5.51E-33	1.61E+01	64.475504	1.82	PEA15	NM_003768
11749912_x_at	1.87E-30	5.86E-33	1.61E+01	64.414499	4.36	IGFBP3	NM_000598,NM_001013398
11735744_a_at	1.91E-30	6.06E-33	1.61E+01	64.382304	2.96	RAB34	NM_001142624,NM_001142625,NM_001144942,NM_001144943,NM_031934,NR_024575,NR_024579
11740133_a_at	1.91E-30	6.11E-33	1.61E+01	64.373401	2.94	CALD1	NM_004342,NM_033138,NM_033139,NM_033140,NM_033157
11759403_x_at	1.92E-30	6.18E-33	1.61E+01	64.361818	3.46	VCAN	NM_001126336,NM_001164097,NM_001164098,NM_004385
11716015_a_at	2.46E-30	8.03E-33	1.61E+01	64.102505	2.94	CMTM3	NM_001048251,NM_144601,NM_181553,NM_181554
11746476_x_at	2.57E-30	8.43E-33	1.61E+01	64.054815	2.62	CALD1	NM_004342,NM_033138,NM_033139,NM_033140,NM_033157
11756134_a_at	2.63E-30	8.69E-33	1.61E+01	64.024917	2.88	FHL2	NM_001039492,NM_001450,NM_201555,NM_201557
11724821_a_at	2.77E-30	9.24E-33	1.60E+01	63.96389	2	NEK6	NM_001145001,NM_001166167,NM_001166168,NM_001166169,NM_001166170,NM_001166171,NM_014397

11745932_x_at	2.79E-30	9.37E-33	1.60E+01	63.949817	1.47	TMSB10	NM_021103
11755009_s_at	2.92E-30	9.87E-33	1.60E+01	63.898045	3.44	VCAN	NM_001126336,NM_001164097,NM_001164098,NM_004385
11743969_s_at	2.97E-30	1.01E-32	1.60E+01	63.876348	2.47	ACTN1	NM_001102,NM_001130004,NM_001130005
11715746_a_at	3.08E-30	1.06E-32	1.60E+01	63.832204	2.89	NBL1	NM_005380,NM_182744
11735219_at	3.18E-30	1.09E-32	1.60E+01	63.796114	5.27	COL10A1	NM_000493
11741105_a_at	3.36E-30	1.17E-32	1.60E+01	63.729072	3.23	FHL2	NM_001039492,NM_001450,NM_201555,NM_201557
11742873_a_at	3.39E-30	1.19E-32	1.60E+01	63.715053	3.64	CDH11	NM_001797
11759497_at	3.92E-30	1.40E-32	1.60E+01	63.554842	3.01	CHST11	NM_001173982,NM_018413
11763872_x_at	4.01E-30	1.44E-32	1.60E+01	63.525199	2.89	SPARC	NM_003118
11757290_s_at	4.29E-30	1.55E-32	1.59E+01	63.44834	2.92	COL5A2	NM_000393
11741106_x_at	4.33E-30	1.58E-32	1.59E+01	63.432196	3.26	FHL2	NM_001039492,NM_001450,NM_201555,NM_201557
11742208_a_at	4.37E-30	1.60E-32	1.59E+01	63.419719	3.57	VCAN	NM_001126336,NM_001164097,NM_001164098,NM_004385
11715777_s_at	6.37E-30	2.39E-32	1.59E+01	63.022735	1.35	LUZP6 /// MTPN	NM_001128619,NM_145808

11733946_at	8.22E-30	3.20E-32	1.58E+01	62.733994	1.76	RAP2B	NM_002886
11742207_a_at	1.01E-29	4.08E-32	1.58E+01	62.492781	3.59	VCAN	NM_001126336,NM_001164097,NM_001164098,NM_004385
11718073_at	1.06E-29	4.32E-32	1.58E+01	62.434926	1.92	IL4R	NM_000418,NM_001008699
11743970_x_at	1.24E-29	5.22E-32	1.57E+01	62.247351	2.06	ACTN1	NM_001102,NM_001130004,NM_001130005
11740880_x_at	1.26E-29	5.33E-32	1.57E+01	62.226985	1.47	ANXA2	NM_001002857,NM_001002858,NM_001136015,NM_004039
11757941_s_at	1.26E-29	5.35E-32	1.57E+01	62.22392	3.67	THBS2	NM_003247
11755553_a_at	1.30E-29	5.60E-32	1.57E+01	62.178011	1.71	WDR1	NM_005112,NM_017491
11757818_x_at	1.79E-29	7.89E-32	1.57E+01	61.837762	1.88	CFL1	NM_005507
11749461_a_at	1.91E-29	8.56E-32	1.56E+01	61.757361	3.61	CDH11	NM_001797
11739641_a_at	2.13E-29	9.71E-32	1.56E+01	61.632489	2.13	AFAP1	NM_001134647,NM_198595
11755122_a_at	2.35E-29	1.08E-31	1.56E+01	61.530507	2.92	PALLD	NM_001166108,NM_001166109,NM_001166110,NM_016081
11718523_s_at	3.03E-29	1.40E-31	1.55E+01	61.271846	3.22	ANTXR1	NM_018153,NM_032208,NM_053034
11725180_a_at	3.10E-29	1.47E-31	1.55E+01	61.220897	3.7	RUNX2	NM_001015051,NM_001024630,NM_004348

11742711_at	3.32E-29	1.59E-31	1.55E+01	61.145698	3.68	THBS2	NM_003247
11727085_s_at	3.40E-29	1.64E-31	1.55E+01	61.109987	5.13	GJB2	NM_004004
11745801_s_at	3.86E-29	1.88E-31	1.55E+01	60.975657	1.54	CAP1	NM_001105530,NM_006367
11757783_s_at	4.68E-29	2.30E-31	1.55E+01	60.779519	3.5	VCAN	NM_001126336,NM_001164097,NM_001164098,NM_004385
11743618_at	5.03E-29	2.50E-31	1.54E+01	60.696066	3.53	ITGA2	NM_002203
11722759_a_at	5.23E-29	2.63E-31	1.54E+01	60.646309	2.28	CAMSAP1L1	NM_203459
11720044_at	5.32E-29	2.68E-31	1.54E+01	60.625769	1.96	SKAP2	NM_003930
11730862_at	5.48E-29	2.79E-31	1.54E+01	60.587507	1.73	CTTNBP2NL	NM_018704
11743617_at	5.53E-29	2.83E-31	1.54E+01	60.57115	3.69	ITGA2	NM_002203
11754266_s_at	5.57E-29	2.87E-31	1.54E+01	60.556802	2.07	E2F3	NM_001949
11736423_a_at	5.57E-29	2.89E-31	1.54E+01	60.552283	4.51	POSTN	NM_001135934,NM_001135935,NM_001135936,NM_006475
11746872_a_at	5.85E-29	3.04E-31	1.54E+01	60.499615	4.25	COL1A1	NM_000088
11718292_s_at	5.91E-29	3.09E-31	1.54E+01	60.484924	2.21	HK1	NM_000188,NM_033496,NM_033497,NM_033498,NM_033500

11743976_at	6.52E-29	3.45E-31	1.54E+01	60.376773	1.69	MYL12A	NM_006471
11747928_x_at	7.30E-29	3.95E-31	1.54E+01	60.242648	4.26	POSTN	NM_001135934,NM_001135935,NM_001135936,NM_006475
11743636_at	7.94E-29	4.33E-31	1.53E+01	60.151416	3.38	TNFAIP6	NM_007115
11747298_a_at	8.31E-29	4.56E-31	1.53E+01	60.099858	3.13	S100A16	NM_080388
11749094_a_at	9.27E-29	5.16E-31	1.53E+01	59.976256	2.7	DSE	NM_001080976,NM_013352
11721993_at	9.29E-29	5.19E-31	1.53E+01	59.970442	3.31	SLC6A6	NM_001134367,NM_001134368,NM_003043
11758648_s_at	1.04E-28	5.86E-31	1.53E+01	59.850484	1.92	KCTD10	NM_031954
11743515_s_at	1.06E-28	6.00E-31	1.53E+01	59.827169	4.35	LAMC2	NM_005562,NM_018891
11715716_at	1.08E-28	6.17E-31	1.53E+01	59.799752	2	MSN	NM_002444
11758189_s_at	1.08E-28	6.21E-31	1.53E+01	59.792781	1.56	ITGB1	NM_002211,NM_033666,NM_033667,NM_033668,NM_033669,NM_133376
11719488_at	1.17E-28	6.70E-31	1.53E+01	59.717119	3.24	EDNRA	NM_001166055,NM_001957,NR_028596
11715350_a_at	1.21E-28	7.01E-31	1.53E+01	59.673117	4.06	COL1A1	NM_000088
11715355_at	1.22E-28	7.08E-31	1.53E+01	59.662451	3.24	COL1A2	NM_000089

11717272_at	1.22E-28	7.11E-31	1.53E+01	59.658173	3.8	COL5A1	NM_000093
11721493_a_at	1.33E-28	7.84E-31	1.52E+01	59.561811	2.42	SULF2	NM_001161841,NM_018837,NM_198596
11744077_at	1.44E-28	8.58E-31	1.52E+01	59.472025	2.13	ARPC1B /// LOC653888	NM_005720,XM_936251
11753623_s_at	1.57E-28	9.44E-31	1.52E+01	59.378014	1.8	RAP1B	NM_001010942,NM_015646
11716883_s_at	1.58E-28	9.55E-31	1.52E+01	59.36677	1.51	RAP1B	NM_001010942,NM_015646
11740238_a_at	1.64E-28	9.94E-31	1.52E+01	59.326212	2.72	SULF2	NM_001161841,NM_018837,NM_198596
11715717_s_at	1.83E-28	1.12E-30	1.52E+01	59.208118	1.98	MSN	NM_002444
11731680_a_at	2.05E-28	1.27E-30	1.51E+01	59.083418	3.7	NOX4	NM_001143836,NM_001143837,NM_016931, NR_026571
11758114_x_at	2.77E-28	1.76E-30	1.51E+01	58.761465	1.92	SKAP2	NM_003930
11725988_at	2.90E-28	1.85E-30	1.51E+01	58.709166	2.25	MMP14	NM_004995
11740743_a_at	3.83E-28	2.50E-30	1.50E+01	58.41377	3.09	TPM2	NM_003289,NM_213674
11720849_a_at	3.99E-28	2.62E-30	1.50E+01	58.367002	2.87	RAB23	NM_016277,NM_183227

11757650_s_at	4.09E-28	2.70E-30	1.50E+01	58.337648	3.02	FN1	NM_002026,NM_054034,NM_212474,NM_212475,NM_212476,NM_212478,NM_212482
11715876_at	4.09E-28	2.70E-30	1.50E+01	58.335442	1.18	TAX1BP3	NM_014604
11754058_a_at	4.39E-28	2.94E-30	1.50E+01	58.252606	2.96	SPARC	NM_003118
11749527_a_at	4.87E-28	3.28E-30	1.50E+01	58.143807	4.48	POSTN	NM_001135934,NM_001135935,NM_001135936,NM_006475
11722532_s_at	5.38E-28	3.64E-30	1.50E+01	58.039488	2.18	CALD1	NM_004342,NM_033138,NM_033139,NM_033140,NM_033157
11754064_x_at	5.73E-28	3.90E-30	1.49E+01	57.971852	1.4	ANXA2	NM_001002857,NM_001002858,NM_001136015,NM_004039
11737127_a_at	6.43E-28	4.45E-30	1.49E+01	57.840095	2.53	ITGA1	NM_181501
11718154_a_at	6.72E-28	4.67E-30	1.49E+01	57.793319	3.29	HEPH	NM_001130860,NM_014799,NM_138737
11754593_x_at	7.09E-28	4.96E-30	1.49E+01	57.732067	3.15	TUBB3	NM_006086
11744277_x_at	7.91E-28	5.62E-30	1.49E+01	57.60913	2.7	CAPG	NM_001747
11755137_a_at	8.00E-28	5.72E-30	1.49E+01	57.591851	2.41	CCDC109B	NM_017918
11724983_at	8.26E-28	5.92E-30	1.49E+01	57.557664	3.96	PCDH7	NM_001173523,NM_002589,NM_032456,NM_032457
11744735_a_at	8.26E-28	5.94E-30	1.49E+01	57.554785	3.31	TMEM200A	NM_052913

11732701_a_at	8.28E-28	5.97E-30	1.49E+01	57.549466	1.89	CSGALNACT2	NM_018590
11727084_at	9.27E-28	6.72E-30	1.48E+01	57.432093	5	GJB2	NM_004004
11717275_s_at	1.01E-27	7.41E-30	1.48E+01	57.334881	3.46	COL5A1	NM_000093
11722531_a_at	1.01E-27	7.44E-30	1.48E+01	57.331362	2.74	CALD1	NM_004342,NM_033138,NM_033139,NM_033140,NM_033157
11744276_a_at	1.17E-27	8.63E-30	1.48E+01	57.183291	2.92	CAPG	NM_001747
11747927_a_at	1.22E-27	9.05E-30	1.48E+01	57.136837	4.43	POSTN	NM_001135934,NM_001135935,NM_001135936,NM_006475
11728375_x_at	1.26E-27	9.39E-30	1.48E+01	57.099485	1.64	ACTN1	NM_001102,NM_001130004,NM_001130005
11731500_a_at	1.34E-27	1.01E-29	1.48E+01	57.032214	3.29	KRT19	NM_002276
11740134_a_at	1.58E-27	1.20E-29	1.47E+01	56.85914	1.92	CALD1	NM_004342,NM_033138,NM_033139,NM_033140,NM_033157
11757405_a_at	1.70E-27	1.30E-29	1.47E+01	56.776325	1.53	MSRB2	NM_012228
11741143_x_at	1.85E-27	1.43E-29	1.47E+01	56.685912	3.6	NOX4	NM_001143836,NM_001143837,NM_016931,NR_026571
11725766_x_at	1.90E-27	1.47E-29	1.47E+01	56.658721	5.96	COL11A1	NM_001168249,NM_001854,NM_080629,NM_080630
11715354_a_at	2.27E-27	1.77E-29	1.47E+01	56.470361	3.42	COL1A2	NM_000089

11719447_s_at	2.46E-27	1.93E-29	1.47E+01	56.384876	2.42	GBP2	NM_004120
11725042_a_at	2.50E-27	1.97E-29	1.47E+01	56.363114	1.04	YWHAB	NM_003404,NM_139323
11736047_a_at	2.61E-27	2.07E-29	1.46E+01	56.315721	2.62	ANTXR2	NM_001145794,NM_058172
11736422_s_at	2.80E-27	2.23E-29	1.46E+01	56.243199	4.19	POSTN	NM_001135934,NM_001135935,NM_001135936,NM_006475
11715751_a_at	3.34E-27	2.68E-29	1.46E+01	56.058158	3.11	DPYSL3	NM_001387
11743063_x_at	3.37E-27	2.71E-29	1.46E+01	56.047926	3.31	PLAUR	NM_001005376,NM_001005377,NM_002659
11728809_a_at	3.38E-27	2.73E-29	1.46E+01	56.041454	3.75	COL8A1	NM_001850,NM_020351
11746555_x_at	3.90E-27	3.16E-29	1.46E+01	55.896302	2.69	SPARC	NM_003118
11717281_a_at	3.98E-27	3.26E-29	1.46E+01	55.867195	2.34	ASAP1	NM_018482
11739574_x_at	5.76E-27	4.84E-29	1.45E+01	55.473522	2.37	ZNF532	NM_018181
11724006_a_at	6.11E-27	5.16E-29	1.45E+01	55.411039	2.83	ENTPD1	NM_001098175,NM_001164178,NM_001164179,NM_001164181,NM_001164182,NM_001164183,NM_001776
11719487_a_at	6.13E-27	5.19E-29	1.45E+01	55.404534	4.14	LAMB3	NM_000228,NM_001017402,NM_001127641

11717131_a_at	6.33E-27	5.39E-29	1.45E+01	55.36779	4.68	AHNAK2	NM_138420
11742819_at	6.48E-27	5.53E-29	1.45E+01	55.341118	3.33	PLAUR	NM_001005376,NM_001005377,NM_002659
11746554_a_at	6.70E-27	5.76E-29	1.45E+01	55.300668	2.91	SPARC	NM_003118
11716870_a_at	7.15E-27	6.18E-29	1.45E+01	55.231726	1.47	PHC2	NM_004427,NM_198040
11726218_a_at	7.37E-27	6.40E-29	1.44E+01	55.196169	2.83	PRDM1	NM_001198,NM_182907
11725765_a_at	7.67E-27	6.70E-29	1.44E+01	55.151844	5.92	COL11A1	NM_001168249,NM_001854,NM_080629,NM_080630
11730900_s_at	8.01E-27	7.02E-29	1.44E+01	55.104601	3.6	MBOAT2	NM_138799
11754202_a_at	8.93E-27	7.94E-29	1.44E+01	54.982697	2.66	CAPG	NM_001747
11715356_x_at	8.95E-27	7.97E-29	1.44E+01	54.979163	3.08	COL1A2	NM_000089
11722530_a_at	9.10E-27	8.16E-29	1.44E+01	54.955331	2.02	CALD1	NM_004342,NM_033138,NM_033139,NM_033140,NM_033157
11722533_x_at	9.27E-27	8.35E-29	1.44E+01	54.933182	2.39	CALD1	NM_004342,NM_033138,NM_033139,NM_033140,NM_033157
11753213_x_at	1.05E-26	9.56E-29	1.44E+01	54.79833	1.76	GNAI2	NM_001166425,NM_002070
11757887_x_at	1.08E-26	9.95E-29	1.44E+01	54.759043	1.12	YWHAB	NM_003404,NM_139323

11720384_at	1.24E-26	1.15E-28	1.43E+01	54.616573	1.35	STX6	NM_005819
11763528_s_at	1.25E-26	1.16E-28	1.43E+01	54.610813	1.79	SLC12A4	NM_001145961,NM_001145962,NM_001145963,NM_001145964,NM_005072
11739573_s_at	1.32E-26	1.22E-28	1.43E+01	54.554577	3.05	ZNF532	NM_018181
11723439_at	1.35E-26	1.27E-28	1.43E+01	54.519893	2.97	SRPX2	NM_014467
11758282_s_at	1.41E-26	1.33E-28	1.43E+01	54.472288	1.5	TUBA1C	NM_032704
11758556_s_at	1.47E-26	1.38E-28	1.43E+01	54.432447	2.06	PGM2	NM_018290
11743516_s_at	1.49E-26	1.41E-28	1.43E+01	54.414913	4.29	LAMC2	NM_005562,NM_018891
11758342_s_at	1.64E-26	1.58E-28	1.43E+01	54.302219	2.66	COL3A1	NM_000090
11742870_at	1.67E-26	1.61E-28	1.43E+01	54.282074	1.02	ARPC2	NM_005731,NM_152862
11716687_x_at	1.85E-26	1.79E-28	1.43E+01	54.175078	1.96	ABR	NM_001092,NM_001159746,NM_021962
11758279_s_at	1.87E-26	1.81E-28	1.43E+01	54.164884	1.64	CORO1C	NM_014325
11729035_at	1.87E-26	1.82E-28	1.43E+01	54.160094	1.22	ITPRIPL2	NM_001034841,NR_028028
11726839_s_at	2.07E-26	2.03E-28	1.42E+01	54.051431	1.64	RELL1	NM_001085399,NM_001085400

11744209_s_at	2.09E-26	2.05E-28	1.42E+01	54.040742	2.48	SERPINH1	NM_001235
11723829_s_at	2.12E-26	2.10E-28	1.42E+01	54.020112	3.39	ANO1	NM_018043,NR_030691
11716104_a_at	2.28E-26	2.27E-28	1.42E+01	53.94304	1.55	MPZL1	NM_001146191,NM_003953,NM_024569
11725306_s_at	2.43E-26	2.42E-28	1.42E+01	53.878025	1.8	CALU	NM_001130674,NM_001219
11733707_x_at	2.50E-26	2.49E-28	1.42E+01	53.847434	5.66	COL11A1	NM_001168249,NM_001854,NM_080629,NM_080630
11753646_x_at	2.70E-26	2.72E-28	1.42E+01	53.761078	1.49	CFL1	NM_005507
11720382_at	2.83E-26	2.85E-28	1.42E+01	53.713979	1.93	STX6	NM_005819
11732331_s_at	3.17E-26	3.22E-28	1.42E+01	53.593539	2.4	ARL4C	NM_005737
11717726_s_at	3.19E-26	3.25E-28	1.42E+01	53.58585	2.81	HN1	NM_001002032,NM_001002033,NM_016185
11731496_at	3.38E-26	3.47E-28	1.41E+01	53.521089	3.67	INHBA	NM_002192
11739989_a_at	4.02E-26	4.17E-28	1.41E+01	53.337744	2	CPPED1	NM_001099455,NM_018340
11717274_s_at	4.07E-26	4.24E-28	1.41E+01	53.322615	3.22	COL5A1	NM_000093
11757843_s_at	4.14E-26	4.35E-28	1.41E+01	53.295964	1.42	PEA15	NM_003768

11756670_x_at	4.24E-26	4.47E-28	1.41E+01	53.268031	1.89	TUBA1C	NM_032704
11726004_a_at	4.41E-26	4.67E-28	1.41E+01	53.226188	2.4	LAYN	NM_178834
11724397_s_at	4.68E-26	4.98E-28	1.41E+01	53.162235	2.46	PRRG1	NM_000950,NM_001142395,NM_001173486,NM_001173489,NM_001173490
11743253_x_at	4.76E-26	5.08E-28	1.41E+01	53.141318	2.14	CALD1	NM_004342,NM_033138,NM_033139,NM_033140,NM_033157
11733084_a_at	5.09E-26	5.48E-28	1.41E+01	53.066598	2.27	PALLD	NM_001166108,NM_001166109,NM_001166110,NM_016081
11747922_a_at	5.44E-26	5.89E-28	1.41E+01	52.996217	1.61	RBM9	NM_001031695,NM_001082576,NM_001082577,NM_001082578,NM_001082579,NM_014309
11731491_a_at	5.50E-26	5.96E-28	1.40E+01	52.984101	1.58	HK1	NM_000188,NM_033496,NM_033497,NM_033498,NM_033500
11743960_a_at	5.69E-26	6.19E-28	1.40E+01	52.946929	3.89	SLC16A3	NM_001042422,NM_001042423,NM_004207
11732037_a_at	5.69E-26	6.20E-28	1.40E+01	52.944365	1.56	PTPN12	NM_001131008,NM_001131009,NM_002835
11718647_at	5.99E-26	6.55E-28	1.40E+01	52.890612	1.83	ACVR1	NM_001105,NM_001111067
11764247_s_at	6.11E-26	6.71E-28	1.40E+01	52.865519	3.55	---	AI917494
11724229_s_at	6.13E-26	6.76E-28	1.40E+01	52.859365	1.36	RBMS1	NM_002897,NM_016836

11716715_a_at	6.27E-26	6.93E-28	1.40E+01	52.834492	1.72	CTSB	NM_001908,NM_147780,NM_147781,NM_147782,NM_147783
11715353_s_at	6.29E-26	6.96E-28	1.40E+01	52.829564	2.55	COL1A2	NM_000089
11720404_at	6.35E-26	7.05E-28	1.40E+01	52.816509	2.63	EFNB2	NM_004093
11732330_s_at	6.53E-26	7.27E-28	1.40E+01	52.786734	1.39	MAP7D1	NM_018067
11715352_x_at	6.53E-26	7.28E-28	1.40E+01	52.784862	3.95	COL1A1	NM_000088
11723669_s_at	7.30E-26	8.21E-28	1.40E+01	52.666207	3.24	SEMA3C	NM_006379
11740496_x_at	7.52E-26	8.51E-28	1.40E+01	52.629981	1.86	ST3GAL2	NM_006927
11743514_a_at	7.56E-26	8.59E-28	1.40E+01	52.621691	4.09	LAMC2	NM_005562,NM_018891
11746874_a_at	7.67E-26	8.75E-28	1.40E+01	52.603311	1.88	GNAI2	NM_001166425,NM_002070
11737791_s_at	7.69E-26	8.78E-28	1.40E+01	52.599281	3.33	APOL1 /// APOL2	NM_001136540,NM_001136541,NM_003661,NM_030882,NM_145343,NM_145637
11715415_a_at	7.89E-26	9.04E-28	1.40E+01	52.570628	2.43	COL3A1	NM_000090
11744379_a_at	8.37E-26	9.64E-28	1.40E+01	52.506889	2.4	COL3A1	NM_000090
11744345_x_at	8.37E-26	9.66E-28	1.40E+01	52.504286	1.26	TUBA1B	NM_006082

11752865_x_at	9.22E-26	1.08E-27	1.39E+01	52.395297	1.38	ACTB	NM_001101
11719489_a_at	9.63E-26	1.13E-27	1.39E+01	52.346963	2.99	EDNRA	NM_001166055,NM_001957,NR_028596
11738684_x_at	9.67E-26	1.14E-27	1.39E+01	52.340549	2.38	RAB34	NM_001142624,NM_001142625,NM_001144942,NM_001144943,NM_031934,NR_024575,NR_024579
11724816_a_at	1.08E-25	1.30E-27	1.39E+01	52.210719	2.15	SH3KBP1	NM_001024666,NM_031892
11724387_at	1.10E-25	1.33E-27	1.39E+01	52.189457	3.03	HOXB2	NM_002145
11754313_s_at	1.11E-25	1.34E-27	1.39E+01	52.182939	2.76	ARL4C	NM_005737
11715359_a_at	1.15E-25	1.39E-27	1.39E+01	52.145386	3.06	TIMP1	NM_003254
11746028_x_at	1.16E-25	1.40E-27	1.39E+01	52.135582	1.82	NMI	NM_004688
11724611_a_at	1.18E-25	1.43E-27	1.39E+01	52.113533	1.7	DYRK2	NM_003583,NM_006482
11748492_a_at	1.19E-25	1.45E-27	1.39E+01	52.102921	2.29	LAYN	NM_178834
11745433_a_at	1.22E-25	1.49E-27	1.39E+01	52.071698	1.52	PHC2	NM_004427,NM_198040
11743681_at	1.22E-25	1.50E-27	1.39E+01	52.068008	2.68	S100A16	NM_080388

11757519_x_at	1.24E-25	1.53E-27	1.39E+01	52.046776	2.36	CAPG	NM_001747
11724901_s_at	1.28E-25	1.58E-27	1.39E+01	52.013694	2.5	SCHIP1	NM_014575
11723104_at	1.40E-25	1.75E-27	1.39E+01	51.917495	2.28	GNB4	NM_021629
11717886_a_at	1.51E-25	1.91E-27	1.38E+01	51.830781	3.31	PLAU	NM_001145031,NM_002658
11751921_s_at	1.53E-25	1.93E-27	1.38E+01	51.818868	2.01	AHR	NM_001621
11723668_at	1.59E-25	2.01E-27	1.38E+01	51.777004	3.32	SEMA3C	NM_006379
11715687_x_at	1.59E-25	2.01E-27	1.38E+01	51.776193	1.03	ARPC5	NM_005717
11746320_x_at	1.59E-25	2.02E-27	1.38E+01	51.773247	2.59	SERPINH1	NM_001235
11755062_a_at	1.59E-25	2.02E-27	1.38E+01	51.771096	2.09	KDELC1	NM_024089
11731650_a_at	1.66E-25	2.13E-27	1.38E+01	51.72176	3.12	NTM	NM_001048209,NM_001144058,NM_001144059,NM_016522
11731197_a_at	1.74E-25	2.23E-27	1.38E+01	51.672937	2.76	PRR5L	NM_001160167,NM_001160168,NM_001160169,NM_024841
11721124_s_at	1.78E-25	2.29E-27	1.38E+01	51.648026	4.86	MMP11	NM_005940
11753399_x_at	1.83E-25	2.37E-27	1.38E+01	51.615364	1.58	ANXA2	NM_001002857,NM_001002858,NM_001136015,NM_004039

11723830_x_at	1.88E-25	2.44E-27	1.38E+01	51.585186	3.32	ANO1	NM_018043,NR_030691
11756403_s_at	2.06E-25	2.70E-27	1.38E+01	51.484254	1.74	KCTD10	NM_031954
11746321_s_at	2.08E-25	2.74E-27	1.38E+01	51.469603	2.49	SERPINH1	NM_001235
11749744_a_at	2.24E-25	2.96E-27	1.38E+01	51.395174	1.48	TBL1XR1	NM_024665
11721574_a_at	2.30E-25	3.06E-27	1.38E+01	51.360931	2.04	ELF4	NM_001127197,NM_001421
11716262_a_at	2.31E-25	3.08E-27	1.38E+01	51.355385	1.77	AP2B1	NM_001030006,NM_001282
11751904_x_at	2.32E-25	3.09E-27	1.38E+01	51.352791	5.66	COL11A1	NM_001168249,NM_001854,NM_080629,NM_080630
AFFX-HSAC07/X00351_5_at	2.59E-25	3.46E-27	1.37E+01	51.238258	1.64	ACTB	NM_001101
11754059_x_at	2.67E-25	3.59E-27	1.37E+01	51.203572	2.66	SPARC	NM_003118
11753645_a_at	2.75E-25	3.71E-27	1.37E+01	51.170962	1.46	CFL1	NM_005507
11744380_s_at	2.76E-25	3.73E-27	1.37E+01	51.166004	1.66	COL3A1	NM_000090
11746875_x_at	2.91E-25	3.94E-27	1.37E+01	51.111012	1.78	GNAI2	NM_001166425,NM_002070

11744339_a_at	3.07E-25	4.17E-27	1.37E+01	51.053504	1.98	PRMT2	NM_001535,NM_206962
11717132_s_at	3.14E-25	4.27E-27	1.37E+01	51.02991	4.09	AHNAK2	NM_138420
11719018_at	3.16E-25	4.31E-27	1.37E+01	51.020776	1.27	CBFB	NM_001755,NM_022845
11742818_a_at	3.29E-25	4.51E-27	1.37E+01	50.976975	3.23	PLAUR	NM_001005376,NM_001005377,NM_002659
11743112_at	3.30E-25	4.52E-27	1.37E+01	50.974027	1.87	S100A10	NM_002966
11753294_a_at	3.52E-25	4.84E-27	1.37E+01	50.905432	2.93	COL1A2	NM_000089
11729870_x_at	3.65E-25	5.03E-27	1.37E+01	50.868506	2.28	EMILIN1	NM_007046
11715971_a_at	3.69E-25	5.11E-27	1.37E+01	50.853405	2.98	PFKP	NM_002627
11717154_a_at	4.00E-25	5.54E-27	1.37E+01	50.771869	3.79	PLAU	NM_001145031,NM_002658
11720441_x_at	4.18E-25	5.80E-27	1.36E+01	50.726139	3.26	OLFML2B	NM_015441
11740744_x_at	4.46E-25	6.22E-27	1.36E+01	50.657561	2.91	TPM2	NM_003289,NM_213674
11717057_x_at	4.70E-25	6.60E-27	1.36E+01	50.598962	1.97	CORO1C	NM_014325

11724008_a_at	4.72E-25	6.65E-27	1.36E+01	50.591563	2.26	ENTPD1	NM_001098175,NM_001164178,NM_001164179,NM_001164181,NM_001164182,NM_001164183,NM_001776
11715452_s_at	4.76E-25	6.73E-27	1.36E+01	50.580022	2.13	COL4A2	NM_001846
11737325_a_at	4.80E-25	6.81E-27	1.36E+01	50.56796	3.49	LAMC2	NM_005562,NM_018891
11728763_x_at	5.05E-25	7.17E-27	1.36E+01	50.516973	2.85	GAL3ST4	NM_024637
11719689_a_at	5.10E-25	7.25E-27	1.36E+01	50.505205	2.23	TWSG1	NM_020648
11720600_a_at	5.27E-25	7.52E-27	1.36E+01	50.46914	1.49	CAPZB	NM_004930
11749023_a_at	5.31E-25	7.59E-27	1.36E+01	50.460424	4.21	SLC16A4	NM_004696
11756947_a_at	5.43E-25	7.79E-27	1.36E+01	50.434187	1.54	CTSB	NM_001908,NM_147780,NM_147781,NM_147782,NM_147783
11758942_s_at	5.63E-25	8.08E-27	1.36E+01	50.397607	1.26	MOBKL1B	NM_018221
11757474_x_at	5.99E-25	8.63E-27	1.36E+01	50.332649	1.57	CLIC1	NM_001288
11738053_x_at	6.13E-25	8.86E-27	1.36E+01	50.307075	2.83	PLAUR	NM_001005376,NM_001005377,NM_002659
11756979_x_at	6.59E-25	9.60E-27	1.36E+01	50.226591	1.1	TUBA1B	NM_006082

11731397_a_at	7.07E-25	1.04E-26	1.35E+01	50.150689	1.08	YWHAB	NM_003404,NM_139323
11726433_a_at	7.20E-25	1.06E-26	1.35E+01	50.129383	2.81	OSBPL3	NM_015550,NM_145320,NM_145321,NM_145322
11744776_a_at	7.70E-25	1.14E-26	1.35E+01	50.057415	2.54	NNMT	NM_006169
11731181_a_at	7.79E-25	1.16E-26	1.35E+01	50.042093	3.26	EPSTI1	NM_001002264,NM_033255
11722849_a_at	7.82E-25	1.16E-26	1.35E+01	50.036535	1.26	PPP3CA	NM_000944,NM_001130691,NM_001130692
11727219_a_at	7.92E-25	1.18E-26	1.35E+01	50.022609	2.24	DSE	NM_001080976,NM_013352
11742924_a_at	8.17E-25	1.22E-26	1.35E+01	49.985611	1.13	PRDX1	NM_002574,NM_181696,NM_181697
11717067_a_at	8.21E-25	1.23E-26	1.35E+01	49.980255	4.35	GREM1	NM_013372
11724819_a_at	8.47E-25	1.27E-26	1.35E+01	49.947343	2.23	SH3KBP1	NM_001024666,NM_031892
11749188_a_at	8.90E-25	1.34E-26	1.35E+01	49.895896	2.58	ENTPD1	NM_001098175,NM_001164178,NM_001164179,NM_001164181,NM_001164182,NM_001164183,NM_001776
11715542_s_at	8.92E-25	1.35E-26	1.35E+01	49.88915	2.8	THY1	NM_006288
11716139_s_at	9.44E-25	1.44E-26	1.35E+01	49.826882	1.7	SMS	NM_004595

11718067_at	9.49E-25	1.45E-26	1.35E+01	49.820084	1.91	MYO1E	NM_004998
11754993_s_at	9.73E-25	1.49E-26	1.35E+01	49.792685	2.91	PXDN	NM_012293
11722178_at	1.05E-24	1.61E-26	1.35E+01	49.714679	1.95	DR1	NM_001938
11733078_a_at	1.14E-24	1.75E-26	1.35E+01	49.629693	1.53	PPP1R12A	NM_001143885,NM_001143886,NM_002480
11717401_at	1.34E-24	2.08E-26	1.34E+01	49.457914	1.42	ARF6	NM_001663
11715586_at	1.37E-24	2.13E-26	1.34E+01	49.436917	1.39	MAPRE1	NM_012325
11728289_a_at	1.38E-24	2.16E-26	1.34E+01	49.423223	2.69	TBC1D2	NM_018421
11756279_a_at	1.51E-24	2.37E-26	1.34E+01	49.330359	1.81	FAM118B	NM_024556
11717283_a_at	1.52E-24	2.39E-26	1.34E+01	49.32177	1.93	ASAP1	NM_018482
11756723_x_at	1.56E-24	2.47E-26	1.34E+01	49.289545	1.67	LDHA	NM_001135239,NM_001165414,NM_001165415,NM_001165416,NM_005566,NR_028500
11717727_s_at	1.68E-24	2.69E-26	1.34E+01	49.206233	2.44	HN1	NM_001002032,NM_001002033,NM_016185
11758995_at	1.72E-24	2.75E-26	1.34E+01	49.1823	3.07	LOX	NM_001178102,NM_002317

11716724_x_at	1.83E-24	2.92E-26	1.34E+01	49.123113	2.02	DPY19L1	NM_015283
11726432_a_at	1.87E-24	2.99E-26	1.34E+01	49.098935	2.66	OSBPL3	NM_015550,NM_145320,NM_145321,NM_145322
11754664_x_at	1.94E-24	3.13E-26	1.34E+01	49.053415	1.11	RAP2C	NM_021183
11725249_at	1.94E-24	3.13E-26	1.34E+01	49.053283	2.7	LY75	NM_002349
11754601_s_at	2.08E-24	3.37E-26	1.33E+01	48.982248	2.8	SPON2	NM_001128325,NM_012445
11733020_s_at	2.11E-24	3.42E-26	1.33E+01	48.966313	1.45	LDHA	NM_001135239,NM_001165414,NM_001165415,NM_001165416,NM_005566,NR_028500
11726331_a_at	2.12E-24	3.44E-26	1.33E+01	48.960182	1.69	CEP170	NM_001042404,NM_001042405,NM_014812
11715414_x_at	2.17E-24	3.53E-26	1.33E+01	48.935751	2.81	COL3A1	NM_000090
11719791_s_at	2.18E-24	3.55E-26	1.33E+01	48.929146	4.32	GPRC5A	NM_003979
11740170_s_at	2.19E-24	3.58E-26	1.33E+01	48.921564	2.52	ARHGAP42	NM_152432
11716941_at	2.22E-24	3.64E-26	1.33E+01	48.906109	2.2	TRIB2	NM_021643,NR_027303
11757652_x_at	2.39E-24	3.94E-26	1.33E+01	48.826202	2.06	LEPRE1	NM_001146289,NM_022356

11723947_a_at	2.72E-24	4.52E-26	1.33E+01	48.691134	3.75	ACSL5	NM_016234,NM_203379,NM_203380
11759022_s_at	2.84E-24	4.72E-26	1.33E+01	48.647157	1.47	ELK3	NM_005230
11717887_s_at	3.22E-24	5.41E-26	1.33E+01	48.512712	1.24	PTPN12	NM_001131008,NM_001131009,NM_002835
11743150_s_at	3.27E-24	5.49E-26	1.33E+01	48.496567	1.64	ZNF143	NM_003442
11732735_s_at	3.28E-24	5.52E-26	1.33E+01	48.491701	2.38	CHSY3	NM_175856
11717273_at	3.36E-24	5.66E-26	1.32E+01	48.467896	3.15	COL5A1	NM_000093
11745421_a_at	3.36E-24	5.67E-26	1.32E+01	48.465921	2.02	BID	NM_001196,NM_197966,NM_197967
11718050_a_at	3.44E-24	5.82E-26	1.32E+01	48.43999	2.67	BASP1	NM_006317
11735189_s_at	3.59E-24	6.09E-26	1.32E+01	48.394011	1.13	H2AFY	NM_001040158,NM_004893,NM_138609,NM_138610
11717889_s_at	3.64E-24	6.19E-26	1.32E+01	48.378715	1.65	GNAI2	NM_001166425,NM_002070
11759496_at	3.71E-24	6.33E-26	1.32E+01	48.355695	2.51	CHST11	NM_001173982,NM_018413
11751527_s_at	3.82E-24	6.54E-26	1.32E+01	48.323999	1.78	WDR1	NM_005112,NM_017491
11744054_a_at	3.82E-24	6.55E-26	1.32E+01	48.322852	1.74	ZNFX1	NM_021035

11749326_s_at	4.08E-24	7.04E-26	1.32E+01	48.25117	3.05	OLFML2B	NM_015441
11716638_s_at	4.09E-24	7.06E-26	1.32E+01	48.247979	1.92	COL4A1	NM_001845
11746059_a_at	4.22E-24	7.31E-26	1.32E+01	48.213318	1.73	PSMD7	NM_002811
11721596_s_at	4.22E-24	7.31E-26	1.32E+01	48.213209	1.31	CDYL	NM_001143970,NM_001143971,NM_004824, NR_026590
11739681_x_at	4.24E-24	7.35E-26	1.32E+01	48.207739	1.73	AHR	NM_001621
11752165_s_at	4.61E-24	8.05E-26	1.32E+01	48.118194	2.63	LOC652797 /// PKM2	NM_002654,NM_182470,NM_182471,XM_001719890
11723902_at	4.63E-24	8.10E-26	1.32E+01	48.111469	1.52	PLEKHG1	NM_001029884
11721245_s_at	5.31E-24	9.36E-26	1.32E+01	47.96868	2.66	BGN	NM_001711
11717480_a_at	5.37E-24	9.48E-26	1.32E+01	47.955977	2.55	FKBP10	NM_021939
11732299_at	5.64E-24	9.98E-26	1.31E+01	47.904556	1.91	KIAA1715	NM_030650
11716775_a_at	5.71E-24	1.01E-25	1.31E+01	47.891951	1.82	RAI14	NM_001145520,NM_001145521,NM_001145522,NM_001145523,NM_001145525,NM_015577
11720440_at	5.82E-24	1.03E-25	1.31E+01	47.869099	2.92	OLFML2B	NM_015441

11746251_x_at	6.06E-24	1.08E-25	1.31E+01	47.827216	1.56	CLIC1	NM_001288
11718875_a_at	6.19E-24	1.10E-25	1.31E+01	47.80368	1.89	PNMA1	NM_006029
11718656_at	6.52E-24	1.17E-25	1.31E+01	47.749308	2.81	SLC16A1	NM_001166496,NM_003051
11716587_at	6.64E-24	1.19E-25	1.31E+01	47.729509	2.3	AXL	NM_001699,NM_021913
11757950_s_at	6.68E-24	1.20E-25	1.31E+01	47.72017	1.81	CHSY1	NM_014918
11719227_x_at	6.69E-24	1.20E-25	1.31E+01	47.718156	3.59	KCNN4	NM_002250
11742116_x_at	6.79E-24	1.22E-25	1.31E+01	47.70247	1.39	LDHA	NM_001135239,NM_001165414,NM_001165415,NM_001165416,NM_005566,NR_028500
11742800_s_at	6.96E-24	1.26E-25	1.31E+01	47.67483	1.83	E2F3	NM_001949
11764077_s_at	6.98E-24	1.26E-25	1.31E+01	47.669925	3.32	EDIL3	NM_005711
11758751_at	7.53E-24	1.37E-25	1.31E+01	47.590742	1.32	YWHAZ	NM_001135699,NM_001135700,NM_001135701,NM_001135702,NM_003406,NM_145690
11734827_at	7.63E-24	1.39E-25	1.31E+01	47.574941	1.82	SPRED1	NM_152594
11726704_x_at	7.70E-24	1.41E-25	1.31E+01	47.563473	1.83	BCL10	NM_003921

11718843_s_at	8.00E-24	1.47E-25	1.31E+01	47.521456	1	DNAJC25-GNG10 /// GNG10	NM_001017998,NM_004125
11730863_at	8.10E-24	1.49E-25	1.31E+01	47.508754	1.57	CTTNBP2NL	NM_018704
11716639_a_at	8.11E-24	1.49E-25	1.31E+01	47.506831	2.33	COL4A1	NM_001845
11730922_at	1.45E-40	1.47E-44	-2.13E+01	90.827475	-1.8	NHLRC4	NM_176677
11759074_at	1.56E-40	1.90E-44	-2.13E+01	90.577781	-2.6	BRSK2	NM_003957
11715173_s_at	4.41E-37	7.53E-41	-1.96E+01	82.39686	-1.75	KRTAP10-12	NM_198699
11738461_x_at	4.41E-37	8.04E-41	-1.96E+01	82.332481	-1.46	FAM22F	NM_017561
11749010_a_at	5.13E-36	1.08E-39	-1.91E+01	79.768477	-2.77	BRSK2	NM_003957
11751277_a_at	1.08E-35	3.07E-39	-1.89E+01	78.733596	-2.46	AMHR2	NM_001164690,NM_001164691,NM_020547
11722293_a_at	2.14E-35	6.51E-39	-1.87E+01	77.988514	-1.67	NAPSA	NM_004851
11728434_a_at	2.94E-35	1.07E-38	-1.86E+01	77.495231	-1.64	GH2	NM_002059,NM_022556,NM_022557,NM_022558
11763240_s_at	2.96E-35	1.14E-38	-1.86E+01	77.435851	-3.43	TRAJ17	L34698.1

11732026_a_at	3.58E-35	1.56E-38	-1.86E+01	77.123936	-3.02	CRIP3	NM_206922
11759390_at	1.35E-34	6.85E-38	-1.83E+01	75.660975	-1.62	OR2A14	NM_001001659
11734227_at	1.40E-34	7.36E-38	-1.83E+01	75.589146	-1.98	PRDM9	NM_020227
11735888_at	1.56E-34	8.58E-38	-1.82E+01	75.438068	-1.5	NTN3	NM_006181
11752059_a_at	1.56E-34	8.83E-38	-1.82E+01	75.410003	-1.75	SARDH	NM_001134707,NM_007101
11759204_a_at	1.69E-34	9.92E-38	-1.82E+01	75.294341	-2.25	SLC2A14	NM_153449
11749011_x_at	2.03E-34	1.23E-37	-1.82E+01	75.080797	-2.92	BRSK2	NM_003957
11748134_x_at	2.74E-34	1.77E-37	-1.81E+01	74.719895	-1.48	SLC2A11	NM_001024938,NM_001024939,NM_030807
11731307_a_at	2.99E-34	2.06E-37	-1.81E+01	74.572345	-1.57	AMHR2	NM_001164690,NM_001164691,NM_020547
11735342_s_at	3.94E-34	2.95E-37	-1.80E+01	74.214929	-1.68	TBXA2R	NM_001060,NM_201636
11726825_at	7.12E-34	6.06E-37	-1.78E+01	73.50409	-1.79	VPS37D	NM_001077621
11740737_a_at	1.15E-33	1.02E-36	-1.77E+01	72.984783	-1.54	ECE2	NM_001037324,NM_001100120,NM_001100121,NM_014693,NM_032331
11761569_x_at	1.33E-33	1.24E-36	-1.77E+01	72.792096	-2.08	TMC6	NM_001127198,NM_007267

11725593_s_at	1.74E-33	1.65E-36	-1.77E+01	72.509494	-1.8	SPATA2	NM_001135773,NM_006038
11761568_at	1.92E-33	1.93E-36	-1.76E+01	72.356691	-2.33	TMC6	NM_001127198,NM_007267
11749743_a_at	1.92E-33	1.94E-36	-1.76E+01	72.350785	-1.37	MST1R	NM_002447
11762299_x_at	1.98E-33	2.04E-36	-1.76E+01	72.299829	-1.46	COMMD4	NM_017828
11749966_a_at	3.25E-33	3.62E-36	-1.75E+01	71.733347	-1.6	CCDC78	NM_001031737
11753948_a_at	4.72E-33	5.45E-36	-1.74E+01	71.329479	-1.23	FBXW8	NM_012174,NM_153348
11754372_x_at	4.95E-33	5.82E-36	-1.74E+01	71.264657	-2.12	LASS4	NM_024552
11730002_x_at	6.48E-33	7.88E-36	-1.74E+01	70.964262	-2.1	NRTN	NM_004558
11748743_a_at	6.65E-33	8.25E-36	-1.74E+01	70.918742	-1.86	ADD2	NM_001617,NM_017482,NM_017483,NM_017484,NM_017488
11756749_a_at	6.65E-33	8.42E-36	-1.73E+01	70.898255	-2.58	EGF	NM_001178130,NM_001178131,NM_001963
11761205_a_at	8.22E-33	1.07E-35	-1.73E+01	70.665535	-1.21	NUDT16	NM_001171905,NM_001171906,NM_152395,NR_033268
11715325_at	1.37E-32	1.95E-35	-1.72E+01	70.067863	-2.43	C11orf90	NM_001144871
11736078_a_at	1.89E-32	2.76E-35	-1.71E+01	69.723027	-1.7	NEK9	NM_033116

11733609_x_at	1.95E-32	2.90E-35	-1.71E+01	69.672461	-2.12	TRIM73 /// TRIM74	NM_198853,NM_198924
11746741_a_at	2.16E-32	3.28E-35	-1.71E+01	69.552736	-1.39	HRH3	NM_007232
11753644_a_at	2.21E-32	3.42E-35	-1.71E+01	69.510739	-1.4	ADM2	NM_024866
11763924_at	2.21E-32	3.48E-35	-1.71E+01	69.493733	-1.2	SNORD9	NR_003029
11737165_at	2.21E-32	3.48E-35	-1.71E+01	69.492457	-5.05	RBPJL	NM_014276
11741080_a_at	2.67E-32	4.32E-35	-1.70E+01	69.278058	-1.86	SARDH	NM_001134707,NM_007101
11760149_a_at	3.03E-32	4.96E-35	-1.70E+01	69.141539	-3.93	PRSS1	NM_002769
11738368_a_at	3.40E-32	5.72E-35	-1.70E+01	69.001961	-2.32	C6orf126	NM_207409
11759055_at	3.87E-32	6.59E-35	-1.70E+01	68.861306	-2.1	TMEM72	NM_001123376
11731279_a_at	4.51E-32	8.04E-35	-1.69E+01	68.663749	-1.68	IGFN1	NM_001164586,NM_178275
11738978_x_at	4.77E-32	8.99E-35	-1.69E+01	68.553181	-1.32	KRTAP23-1	NM_181624
11715243_x_at	4.91E-32	9.34E-35	-1.69E+01	68.515623	-1.67	LCE1F	NM_178354
11737576_at	5.45E-32	1.05E-34	-1.69E+01	68.401847	-2.54	NPHS1	NM_004646

11757270_x_at	5.93E-32	1.15E-34	-1.69E+01	68.307906	-1.25	SFTPC	NM_001172357,NM_001172410,NM_003018
11730460_a_at	7.64E-32	1.50E-34	-1.68E+01	68.045924	-1.37	C17orf74	NM_175734
11738679_a_at	7.74E-32	1.55E-34	-1.68E+01	68.014638	-1.24	NCR1	NM_001145457,NM_001145458,NM_004829, NR_027041,NR_027043
11763689_x_at	8.77E-32	1.81E-34	-1.68E+01	67.859271	-1.82	FMNL1	NM_005892
11734490_a_at	8.91E-32	1.87E-34	-1.68E+01	67.828643	-1.16	VSX1	NM_014588,NM_199425
11762917_at	8.91E-32	1.88E-34	-1.68E+01	67.824741	-1.28	CCDC159	NM_001080503
11763688_at	1.24E-31	2.72E-34	-1.67E+01	67.457946	-2.22	FMNL1	NM_005892
11716154_x_at	1.68E-31	3.81E-34	-1.66E+01	67.122776	-1.3	B4GALT1	NM_001497
11745019_a_at	2.04E-31	4.66E-34	-1.66E+01	66.922857	-3.07	CBS	NM_000071,NM_001178008,NM_001178009
11738579_s_at	2.33E-31	5.38E-34	-1.66E+01	66.780711	-1.39	OR10A2 /// OR10A5	NM_001004460,NM_178168
11748322_a_at	2.34E-31	5.44E-34	-1.66E+01	66.769391	-1.34	HEYL	NM_014571
11738564_at	3.07E-31	7.34E-34	-1.65E+01	66.472796	-1.59	OR8D4	NM_001005197
11738129_x_at	3.71E-31	9.10E-34	-1.65E+01	66.260693	-1.74	SARDH	NM_001134707,NM_007101

11725113_at	4.10E-31	1.01E-33	-1.65E+01	66.153609	-1.55	SLC38A3	NM_006841
11746926_a_at	4.38E-31	1.09E-33	-1.64E+01	66.081141	-3.76	CCDC110	NM_001145411,NM_152775
11736537_a_at	4.61E-31	1.16E-33	-1.64E+01	66.022264	-1.59	RHCE /// RHD	NM_001127691,NM_016124,NM_020485,NM_138616,NM_138617,NM_138618
11729342_a_at	5.24E-31	1.33E-33	-1.64E+01	65.886453	-1.61	POU2F2	NM_002698
11735728_x_at	6.18E-31	1.63E-33	-1.64E+01	65.681624	-1.32	ZNF385A	NM_001130967,NM_001130968,NM_015481
11731041_a_at	6.18E-31	1.64E-33	-1.64E+01	65.676568	-3.29	FBXO24	NM_001163499,NM_012172,NM_033506
11720341_at	6.43E-31	1.73E-33	-1.64E+01	65.622662	-2.66	NDUFA13 /// YJEFN3	NM_015965,NM_198537
11752785_a_at	7.31E-31	2.01E-33	-1.63E+01	65.474046	-1.52	ZBTB7C	NM_001039360
11737555_s_at	7.68E-31	2.13E-33	-1.63E+01	65.417524	-1.08	NUDT17	NM_001012758
11715996_a_at	8.09E-31	2.31E-33	-1.63E+01	65.337687	-1.98	NOSIP	NM_015953
11763092_at	1.07E-30	3.13E-33	-1.62E+01	65.037528	-1.46	GYPB	NM_002100
11736490_at	1.24E-30	3.68E-33	-1.62E+01	64.87702	-2.29	KCNJ5	NM_000890
11729311_a_at	1.28E-30	3.80E-33	-1.62E+01	64.843912	-3.96	C12orf39	NM_030572

11715178_s_at	1.57E-30	4.71E-33	-1.62E+01	64.631946	-1.79	OR2A14	NM_001001659
11715174_s_at	1.60E-30	4.86E-33	-1.62E+01	64.601202	-1.39	KRTAP10-1	NM_198691
11752505_x_at	1.67E-30	5.16E-33	-1.62E+01	64.541101	-1.43	MAPK8IP1	NM_005456
11738460_at	1.87E-30	5.91E-33	-1.61E+01	64.406642	-1.62	FAM22F /// FAM22G	NM_001045477,NM_001170741,NM_017561
11749069_a_at	2.35E-30	7.62E-33	-1.61E+01	64.155283	-1.55	PAQR4	NM_152341
11752600_a_at	2.75E-30	9.14E-33	-1.60E+01	63.97426	-1.71	IL22RA1	NM_021258
11742534_at	3.20E-30	1.11E-32	-1.60E+01	63.784281	-1.44	FAM58B	NM_001105517
11754701_a_at	3.46E-30	1.22E-32	-1.60E+01	63.688611	-1.15	SLC5A2	NM_003041
11725907_at	3.62E-30	1.28E-32	-1.60E+01	63.63851	-1.47	SIX2	NM_016932
11732516_a_at	4.09E-30	1.48E-32	-1.60E+01	63.499707	-2.39	LASS4	NM_024552
11739355_at	4.92E-30	1.81E-32	-1.59E+01	63.296623	-1.46	CHDH	NM_018397
11744287_x_at	5.91E-30	2.19E-32	-1.59E+01	63.109239	-3.81	CBS	NM_000071,NM_001178008,NM_001178009

11736205_a_at	6.71E-30	2.53E-32	-1.59E+01	62.966414	-1.98	OSBP2	NM_030758
11750267_x_at	6.87E-30	2.60E-32	-1.59E+01	62.937596	-1.45	INPP5J	NM_001002837
11727260_x_at	7.55E-30	2.87E-32	-1.58E+01	62.839591	-1.99	C19orf36	NM_001031735,NM_001039846
11727974_at	7.80E-30	2.99E-32	-1.58E+01	62.801205	-1.36	FAM171A2	NM_198475
11754882_x_at	7.85E-30	3.02E-32	-1.58E+01	62.789427	-2.64	BRSK2	NM_003957
11715175_x_at	7.94E-30	3.07E-32	-1.58E+01	62.773092	-1.29	KRTAP10-3	NM_198696
11755731_a_at	8.78E-30	3.45E-32	-1.58E+01	62.657943	-3.58	KLK3	NM_001030047,NM_001030048,NM_001030049,NM_001030050,NM_001648
11751278_x_at	9.43E-30	3.72E-32	-1.58E+01	62.582726	-2.22	AMHR2	NM_001164690,NM_001164691,NM_020547
11729205_a_at	9.97E-30	3.96E-32	-1.58E+01	62.521835	-2.11	DTNA	NM_001128175,NM_001390,NM_001391,NM_001392,NM_032975,NM_032978,NM_032979,NM_032980,NM_032981
11721848_at	1.01E-29	4.03E-32	-1.58E+01	62.50345	-1.33	REEP2	NM_016606
11751079_a_at	1.01E-29	4.04E-32	-1.58E+01	62.502438	-1.62	GPR114	NM_153837
11738408_a_at	1.06E-29	4.32E-32	-1.58E+01	62.434293	-3.84	SPACA3	NM_173847

11736330_a_at	1.06E-29	4.35E-32	-1.58E+01	62.428996	-1.31	SLC2A11	NM_001024938,NM_001024939,NM_030807
11741862_x_at	1.12E-29	4.58E-32	-1.58E+01	62.376265	-1.4	AGER	NM_001136,NM_172197
11761334_at	1.16E-29	4.78E-32	-1.57E+01	62.335898	-1.17	TROAP	NM_001100620,NM_005480
11749594_a_at	1.20E-29	4.99E-32	-1.57E+01	62.292268	-1.12	ZNF213	NM_001134655,NM_004220
11737163_a_at	1.20E-29	5.00E-32	-1.57E+01	62.290182	-1.32	ATP13A2	NM_001141973,NM_001141974,NM_022089
11730093_a_at	1.22E-29	5.13E-32	-1.57E+01	62.265125	-1.19	FTCD	NM_006657,NM_206965
11741512_a_at	1.28E-29	5.46E-32	-1.57E+01	62.20285	-1.99	SEC14L3	NM_174975
11740202_at	1.45E-29	6.30E-32	-1.57E+01	62.060592	-1.27	KISS1R	NM_032551
11750262_a_at	1.50E-29	6.52E-32	-1.57E+01	62.026979	-3.07	BRSK2	NM_003957
11727961_a_at	1.79E-29	7.88E-32	-1.57E+01	61.839171	-1.14	ING1	NM_005537,NM_198217,NM_198218,NM_198219
11735367_a_at	1.79E-29	7.95E-32	-1.57E+01	61.831232	-1.83	BANF2	NM_001014977,NM_001159495,NM_178477
11756033_x_at	1.85E-29	8.22E-32	-1.56E+01	61.796976	-1.21	AGER	NM_001136,NM_172197
11724400_a_at	2.08E-29	9.40E-32	-1.56E+01	61.664788	-2.3	C16orf48	NM_032140

11730119_at	2.08E-29	9.41E-32	-1.56E+01	61.663753	-1.36	CLDND2	NM_152353
11752816_a_at	2.08E-29	9.43E-32	-1.56E+01	61.660948	-1.18	LIG1	NM_000234
11753584_x_at	2.75E-29	1.26E-31	-1.56E+01	61.372549	-1.39	MADCAM1	NM_130760,NM_130762
11729012_at	3.08E-29	1.43E-31	-1.55E+01	61.249025	-1.24	CCDC108	NM_152389,NM_194302
11751325_a_at	3.10E-29	1.46E-31	-1.55E+01	61.230035	-1.41	FYCO1	NM_024513
11718812_a_at	3.10E-29	1.46E-31	-1.55E+01	61.229924	-2.02	MKNK1	NM_001135553,NM_003684,NM_198973,NR_024174,NR_024176
11754275_s_at	3.10E-29	1.46E-31	-1.55E+01	61.227199	-1.61	ST6GALNAC4	NM_175039,NM_175040
11747712_s_at	3.10E-29	1.47E-31	-1.55E+01	61.223209	-1.37	LOC100288299 /// LOC399491 /// NPIP /// PKD1 /// PKD1P1	NM_000296,NM_001009944,NM_006985,XM_002343423,XR_041580,XR_078513
11739181_a_at	3.32E-29	1.58E-31	-1.55E+01	61.151094	-1.17	ST6GALNAC4	NM_175039,NM_175040
11735345_at	3.32E-29	1.59E-31	-1.55E+01	61.140758	-1.05	C1QTNF5 /// MFRP	NM_015645,NM_031433
11762427_at	3.40E-29	1.64E-31	-1.55E+01	61.113861	-1.25	FLJ43826	XR_017719,XR_040619,XR_040620

11744035_x_at	3.53E-29	1.71E-31	-1.55E+01	61.068678	-1.36	ZNF358	NM_018083
11738128_a_at	4.80E-29	2.36E-31	-1.55E+01	60.750834	-1.82	SARDH	NM_001134707,NM_007101
11724896_a_at	5.03E-29	2.49E-31	-1.54E+01	60.699752	-1.28	FZR1	NM_001136197,NM_001136198,NM_016263
11749070_x_at	5.11E-29	2.54E-31	-1.54E+01	60.677744	-1.39	PAQR4	NM_152341
11761687_a_at	5.13E-29	2.57E-31	-1.54E+01	60.669442	-1.61	ENTPD6	NM_001114089,NM_001247
11737515_at	5.50E-29	2.81E-31	-1.54E+01	60.580343	-1.03	MEFV	NM_000243
11733272_x_at	5.57E-29	2.89E-31	-1.54E+01	60.551338	-1.07	NAT8L	NM_178557
11715201_s_at	6.72E-29	3.57E-31	-1.54E+01	60.342627	-1.22	DUX4 /// LOC441056 /// LOC653543 /// LOC653544 /// LOC653545 /// LOC653548 /// LOC72841	NM_001127386,NM_001127387,NM_001127388,NM_001127389,NM_001164467,NM_001177376,NM_033178,XM_496731,XM_928023

0

11748830_a_at	6.76E-29	3.60E-31	-1.54E+01	60.333629	-1.63	MKNK1	NM_001135553,NM_003684,NM_198973,NR_024174,NR_024176
11731280_x_at	6.93E-29	3.70E-31	-1.54E+01	60.305426	-1.91	IGFN1	NM_001164586,NM_178275
11748446_a_at	7.01E-29	3.76E-31	-1.54E+01	60.289962	-1.3	IL17RC	NM_032732,NM_153460,NM_153461
11744981_a_at	7.30E-29	3.94E-31	-1.54E+01	60.243172	-1.41	ZNF541	NM_001101419
11742325_s_at	8.31E-29	4.56E-31	-1.53E+01	60.098743	-1.23	OR10H1 /// OR10H5	NM_001004466,NM_013940
11755672_a_at	8.49E-29	4.67E-31	-1.53E+01	60.074805	-1.15	GRIK5	NM_002088
11740849_at	8.51E-29	4.71E-31	-1.53E+01	60.067791	-1.17	C19orf35	NM_198532

11763478_x_at	9.05E-29	5.02E-31	-1.53E+01	60.004187	-3.79	PIF1	NM_025049
11742876_a_at	9.79E-29	5.49E-31	-1.53E+01	59.91507	-1.56	SGSM3	NM_015705
11753120_x_at	9.89E-29	5.57E-31	-1.53E+01	59.900784	-1.36	MADCAM1	NM_130760,NM_130762
11761383_a_at	1.22E-28	7.12E-31	-1.53E+01	59.657432	-1.21	SPATA9	NM_031952
11720412_a_at	1.28E-28	7.47E-31	-1.52E+01	59.609966	-2.24	SLC41A1	NM_173854
11741325_x_at	1.32E-28	7.72E-31	-1.52E+01	59.576838	-1.03	SPRYD5	NM_032681
11723510_a_at	1.37E-28	8.12E-31	-1.52E+01	59.526581	-1.35	PLEKHH3	NM_024927
11750631_a_at	1.44E-28	8.59E-31	-1.52E+01	59.471768	-1.39	MAP3K9	NM_033141
11730598_at	1.52E-28	9.10E-31	-1.52E+01	59.414128	-1.01	CCDC13	NM_144719
11763241_x_at	1.54E-28	9.21E-31	-1.52E+01	59.402047	-1.5	TRAJ17	L34698.1
11715284_x_at	1.58E-28	9.59E-31	-1.52E+01	59.362041	-1.26	C15orf40	NM_001160113,NM_001160114,NM_001160115,NM_001160116,NM_144597,NR_027649,NR_027650
11744941_a_at	1.64E-28	1.00E-30	-1.52E+01	59.320043	-1.31	AQP7	NM_001170

11735023_s_at	1.99E-28	1.22E-30	-1.52E+01	59.123813	-1.21	ZBTB7B	NM_015872
11744213_a_at	2.05E-28	1.27E-30	-1.51E+01	59.082666	-2.48	TRAFD1	NM_001143906,NM_006700
11715200_s_at	2.14E-28	1.33E-30	-1.51E+01	59.036403	-1.16	DUX4 /// LOC441056 /// LOC653543 /// LOC653544 /// LOC653545 /// LOC653548 /// LOC728410	NM_001127386,NM_001127387,NM_001127388,NM_001127389,NM_001164467,NM_001177376,NM_033178,XM_496731,XM_928023
11726866_at	2.16E-28	1.35E-30	-1.51E+01	59.02446	-1.35	FOXO4	NM_001170931,NM_005938
11731484_s_at	2.56E-28	1.61E-30	-1.51E+01	58.84903	-1.21	NFIC	NM_005597,NM_205843
11741150_a_at	2.62E-28	1.65E-30	-1.51E+01	58.824404	-1.89	NRCAM	NM_001037132,NM_001037133,NM_005010
11732619_at	2.65E-28	1.68E-30	-1.51E+01	58.808623	-1.18	CD164L2	NM_207397

11760114_s_at	2.90E-28	1.84E-30	-1.51E+01	58.71499	-1.72	HERC2P2 /// HERC2P3	NR_002824
11756231_a_at	2.97E-28	1.90E-30	-1.51E+01	58.685152	-1.08	TAF6L	NM_006473
11750601_a_at	3.05E-28	1.96E-30	-1.51E+01	58.653283	-1.18	ZC3H12D	NM_207360
11715473_at	3.14E-28	2.02E-30	-1.51E+01	58.622449	-1.42	SRPR	NM_001177842,NM_003139
11755118_a_at	3.70E-28	2.40E-30	-1.50E+01	58.453632	-1.41	MYOZ3	NM_001122853,NM_133371
11754641_x_at	3.76E-28	2.44E-30	-1.50E+01	58.435937	-1.23	SFTPC	NM_001172357,NM_001172410,NM_003018
11758349_s_at	3.98E-28	2.60E-30	-1.50E+01	58.371869	-1.78	RORC	NM_001001523,NM_005060
11731162_a_at	4.21E-28	2.79E-30	-1.50E+01	58.304504	-1.42	VIPR2	NM_003382
11752656_a_at	4.32E-28	2.87E-30	-1.50E+01	58.276125	-1.76	CBS	NM_000071,NM_001178008,NM_001178009
11727771_a_at	4.37E-28	2.91E-30	-1.50E+01	58.260847	-4.14	KIRREL2	NM_032123,NM_199179,NM_199180
11755749_x_at	4.79E-28	3.21E-30	-1.50E+01	58.16478	-1.06	C5orf38	NM_178569
11733614_a_at	5.13E-28	3.46E-30	-1.50E+01	58.089712	-1.29	SEMA6D	NM_020858,NM_024966,NM_153616,NM_153617,NM_153618,NM_153619

11746684_a_at	5.50E-28	3.73E-30	-1.50E+01	58.014495	-1.14	PXK	NM_017771
11730001_at	5.99E-28	4.08E-30	-1.49E+01	57.925438	-1.84	NRTN	NM_004558
11731884_x_at	6.22E-28	4.29E-30	-1.49E+01	57.875888	-1.29	PTCRA	NM_138296
11730955_a_at	6.76E-28	4.71E-30	-1.49E+01	57.784294	-4.14	P2RX1	NM_002558
11746077_a_at	6.85E-28	4.78E-30	-1.49E+01	57.768907	-1.34	NARFL	NM_022493
11761772_at	7.09E-28	4.98E-30	-1.49E+01	57.729175	-1.41	PCOLCE	NM_002593
11728526_at	7.52E-28	5.31E-30	-1.49E+01	57.665047	-1.76	PEX5L	NM_016559
11741909_a_at	7.56E-28	5.36E-30	-1.49E+01	57.656396	-1.29	PKNOX2	NM_022062
11762208_a_at	7.91E-28	5.64E-30	-1.49E+01	57.605929	-1.31	ATG16L2	NM_033388
11751045_a_at	9.06E-28	6.55E-30	-1.49E+01	57.457751	-1.55	GPR114	NM_153837
11718099_a_at	9.55E-28	6.94E-30	-1.48E+01	57.399273	-1.96	IFT20	NM_174887
11738607_a_at	1.15E-27	8.46E-30	-1.48E+01	57.203095	-1.47	KCTD16	NM_020768
11764049_at	1.26E-27	9.42E-30	-1.48E+01	57.097306	-1.47	DPYSL4	NM_006426

11731250_a_at	1.27E-27	9.47E-30	-1.48E+01	57.091821	-1.03	EPHA8	NM_001006943,NM_020526
11737253_x_at	1.43E-27	1.08E-29	-1.48E+01	56.964171	-1.51	SLC16A11	NM_153357
11715337_s_at	1.53E-27	1.16E-29	-1.48E+01	56.894338	-1.14	GH1 /// GH2	NM_000515,NM_002059,NM_022556,NM_022557,NM_022558,NM_022559,NM_022560,NM_022561,NM_022562
11749296_a_at	1.61E-27	1.23E-29	-1.47E+01	56.835453	-1.14	GDAP1L1	NM_024034
11715199_s_at	1.62E-27	1.23E-29	-1.47E+01	56.829619	-1.15	DUX4 /// LOC441056 /// LOC653543 /// LOC653544 /// LOC653545 /// LOC653548 /// LOC728410	NM_001127386,NM_001127387,NM_001127388,NM_001127389,NM_001164467,NM_001177376,NM_033178,XM_496731,XM_928023
11741459_s_at	1.76E-27	1.35E-29	-1.47E+01	56.741917	-1.71	CYP4A11 /// CYP4A22	NM_000778,NM_001010969

11730209_x_at	1.85E-27	1.43E-29	-1.47E+01	56.685269	-1.32	TRIM46	NM_025058
11761556_at	2.01E-27	1.55E-29	-1.47E+01	56.600856	-2.24	ACADL	NM_001608
11751771_a_at	2.02E-27	1.56E-29	-1.47E+01	56.594321	-1.52	KLK2	NM_001002231,NM_005551
11735457_at	2.06E-27	1.60E-29	-1.47E+01	56.569202	-1.25	LENG9	NM_198988
11725204_a_at	2.28E-27	1.78E-29	-1.47E+01	56.463232	-1.94	RPH3AL	NM_006987
11763961_x_at	2.33E-27	1.83E-29	-1.47E+01	56.439658	-1.29	SFTPC	NM_001172357,NM_001172410,NM_003018
11722361_a_at	2.46E-27	1.94E-29	-1.47E+01	56.380202	-1.22	LMBR1L	NM_018113
11747955_a_at	2.50E-27	1.98E-29	-1.47E+01	56.361155	-1.85	PDZD3	NM_001168468,NM_024791,NR_033122
11748781_a_at	2.85E-27	2.28E-29	-1.46E+01	56.222147	-1.58	FBXO24	NM_001163499,NM_012172,NM_033506
11736322_a_at	3.25E-27	2.61E-29	-1.46E+01	56.087068	-1.14	BAIAP3	NM_003933
11748569_a_at	3.91E-27	3.18E-29	-1.46E+01	55.891367	-2.31	C19orf36	NM_001031735,NM_001039846
11732786_at	3.92E-27	3.19E-29	-1.46E+01	55.886779	-1.13	FFAR1	NM_005303
11749940_a_at	3.98E-27	3.25E-29	-1.46E+01	55.868082	-3.41	BACE1	NM_012104,NM_138971,NM_138972,NM_138973

11715303_s_at	4.05E-27	3.32E-29	-1.46E+01	55.84831	-1.02	CEMP1	NM_001048212
11752322_a_at	4.17E-27	3.43E-29	-1.46E+01	55.815254	-1.14	PANX2	NM_001160300,NM_052839,NR_027691
11715253_s_at	4.66E-27	3.85E-29	-1.45E+01	55.701007	-1.26	KRTAP10-10 /// KRTAP10-6	NM_181688,NM_198688
11715244_x_at	4.76E-27	3.94E-29	-1.45E+01	55.677791	-1.16	LCE1A	NM_178348
11748795_x_at	4.94E-27	4.10E-29	-1.45E+01	55.637737	-2.15	HEXDC	NM_173620
11756629_a_at	5.08E-27	4.22E-29	-1.45E+01	55.608781	-1.04	MTHFSD	NM_001159377,NM_001159378,NM_001159379,NM_001159380,NM_022764,NR_027489,NR_027490
11724069_at	5.47E-27	4.56E-29	-1.45E+01	55.532805	-1.68	PELI2	NM_021255
11726345_at	5.51E-27	4.61E-29	-1.45E+01	55.52312	-1.03	GPA33	NM_005814
11750448_a_at	5.56E-27	4.66E-29	-1.45E+01	55.511726	-1.32	SLC8A2	NM_015063
11763957_at	6.09E-27	5.13E-29	-1.45E+01	55.415644	-1.96	SCARNA20	NR_002999
11738787_a_at	6.32E-27	5.36E-29	-1.45E+01	55.372407	-1.02	DEFB119	NM_153289,NM_153323,NM_173460

11744514_a_at	6.48E-27	5.55E-29	-1.45E+01	55.33851	-1.46	YIPF2	NM_024029
11744560_s_at	6.53E-27	5.61E-29	-1.45E+01	55.32795	-1.7	IFT20	NM_174887
11756535_a_at	6.79E-27	5.85E-29	-1.45E+01	55.285317	-1.99	SLC25A45	NM_001077241,NM_182556
11736824_at	7.45E-27	6.48E-29	-1.44E+01	55.183677	-1.1	C10orf95	NM_024886
11718031_a_at	7.88E-27	6.90E-29	-1.44E+01	55.122762	-1.17	ATP6V0A1	NM_001130020,NM_001130021,NM_005177
11716570_a_at	8.23E-27	7.24E-29	-1.44E+01	55.074895	-1	NOTCH3	NM_000435
11762035_a_at	8.31E-27	7.32E-29	-1.44E+01	55.063775	-1.11	FLJ40448	XR_039971,XR_039972,XR_039973
11750508_a_at	8.51E-27	7.51E-29	-1.44E+01	55.038042	-1.11	PTPRN	NM_002846
11757877_a_at	8.76E-27	7.75E-29	-1.44E+01	55.006722	-1.5	KIF1A	NM_004321
11730448_a_at	8.76E-27	7.77E-29	-1.44E+01	55.003931	-1.96	DHRS12	NM_001031719,NM_024705
11741615_at	9.03E-27	8.06E-29	-1.44E+01	54.967792	-1.62	KPNA7	NM_001145715
11723306_s_at	9.10E-27	8.15E-29	-1.44E+01	54.956675	-1.76	PYGO2	NM_138300
11740738_x_at	9.20E-27	8.27E-29	-1.44E+01	54.942224	-1.04	ECE2	NM_001037324,NM_001100120,NM_001100121,NM_014693,NM_032331

11740313_s_at	9.27E-27	8.37E-29	-1.44E+01	54.93026	-1.31	MYH6 /// MYH7	NM_000257,NM_002471
11747950_a_at	9.51E-27	8.61E-29	-1.44E+01	54.902777	-1.11	DMRTC2	NM_001040283
11722294_x_at	1.03E-26	9.37E-29	-1.44E+01	54.818335	-1.03	NAPSA	NM_004851
11715296_s_at	1.05E-26	9.50E-29	-1.44E+01	54.804718	-1.61	KRT33A	NM_004138
11762050_x_at	1.07E-26	9.73E-29	-1.44E+01	54.781444	-1.14	ZFYVE28	NM_001172656,NM_001172657,NM_001172658,NM_001172659,NM_001172660,NM_020972
11722036_a_at	1.08E-26	9.84E-29	-1.44E+01	54.769681	-1.37	SND1	NM_014390
11749433_a_at	1.18E-26	1.08E-28	-1.44E+01	54.675067	-2.4	KIAA1324	NM_020775
11731410_a_at	1.23E-26	1.14E-28	-1.43E+01	54.628089	-1.24	SLC8A2	NM_015063
11735225_x_at	1.33E-26	1.24E-28	-1.43E+01	54.540202	-2.89	GAMT	NM_000156,NM_138924
11737351_a_at	1.34E-26	1.25E-28	-1.43E+01	54.535312	-1.08	ANO7	NM_001001666,NM_001001891
11759005_a_at	1.38E-26	1.30E-28	-1.43E+01	54.497249	-5.68	C12orf39	NM_030572
11760492_x_at	1.52E-26	1.44E-28	-1.43E+01	54.389752	-1.26	PRMT1	NM_001536,NM_198318,NM_198319,NR_033397

11761759_at	1.57E-26	1.49E-28	-1.43E+01	54.355818	-1.28	CAMK2G	NM_001222,NM_172169,NM_172170,NM_172171,NM_172173
11718343_a_at	1.57E-26	1.50E-28	-1.43E+01	54.351856	-1.25	TUBGCP2	NM_006659
11724009_a_at	1.57E-26	1.50E-28	-1.43E+01	54.350892	-1.04	GPR162	NM_014449,NM_019858
11755913_a_at	1.66E-26	1.59E-28	-1.43E+01	54.293035	-1.14	LPO	NM_001160102,NM_006151,NR_027647
11715313_s_at	1.84E-26	1.78E-28	-1.43E+01	54.184724	-1.31	LHB	NM_000894
11730693_a_at	1.92E-26	1.87E-28	-1.43E+01	54.131186	-1.2	C6orf146	NM_173563
11744285_a_at	2.04E-26	2.00E-28	-1.42E+01	54.068339	-4.15	CBS	NM_000071,NM_001178008,NM_001178009
11761773_x_at	2.05E-26	2.01E-28	-1.42E+01	54.061728	-1.5	PCOLCE	NM_002593
11730449_a_at	2.21E-26	2.19E-28	-1.42E+01	53.975884	-2.56	DHRS12	NM_001031719,NM_024705
11729744_a_at	2.41E-26	2.40E-28	-1.42E+01	53.886256	-3.74	SLC39A5	NM_001135195,NM_173596
11744040_a_at	2.60E-26	2.60E-28	-1.42E+01	53.805324	-1.49	CCDC56	NM_001040431
11743329_a_at	2.68E-26	2.68E-28	-1.42E+01	53.775889	-1.08	PGP	NM_001042371
11735852_s_at	2.70E-26	2.71E-28	-1.42E+01	53.764512	-1.1	OR10H1 /// OR10H5	NM_001004466,NM_013940

11738963_at	3.02E-26	3.05E-28	-1.42E+01	53.647695	-1.14	KRTAP12-3	NM_198697
11761220_x_at	3.04E-26	3.08E-28	-1.42E+01	53.637794	-1.03	EFCAB4A	NM_173584
11762014_at	3.07E-26	3.11E-28	-1.42E+01	53.628447	-1.04	PTCHD1	NM_173495
11742297_a_at	3.31E-26	3.38E-28	-1.42E+01	53.547116	-1.18	FBXO24	NM_001163499,NM_012172,NM_033506
11740275_a_at	3.34E-26	3.41E-28	-1.41E+01	53.537332	-1.16	LRRC25	NM_145256
11731844_a_at	3.35E-26	3.43E-28	-1.41E+01	53.531558	-4.36	DNASE1	NM_005223
11756728_a_at	3.61E-26	3.71E-28	-1.41E+01	53.454099	-2.4	GAS2	NM_001143830,NM_005256,NM_177553
11756057_a_at	3.64E-26	3.75E-28	-1.41E+01	53.443851	-1.34	MED16	NM_005481
11740825_a_at	3.67E-26	3.79E-28	-1.41E+01	53.433808	-1.14	C10orf27	NM_152710
11731163_at	3.72E-26	3.84E-28	-1.41E+01	53.41874	-1.52	VIPR2	NM_003382
11752811_a_at	4.10E-26	4.27E-28	-1.41E+01	53.314271	-1.4	TTYH1	NM_001005367,NM_020659
11750743_x_at	4.14E-26	4.33E-28	-1.41E+01	53.300457	-1.11	EPN1	NM_001130071,NM_001130072,NM_013333
11763650_a_at	4.14E-26	4.34E-28	-1.41E+01	53.297946	-1	GET4	NM_015949

11761409_at	4.22E-26	4.45E-28	-1.41E+01	53.273991	-1.52	---	AK300656.1
11741166_a_at	4.39E-26	4.64E-28	-1.41E+01	53.232958	-2.48	DPP10	NM_001004360,NM_001178034,NM_001178036,NM_001178037,NM_020868
11737375_x_at	4.68E-26	4.98E-28	-1.41E+01	53.16264	-1.6	RUNDC3A	NM_001144825,NM_001144826,NM_006695
11761584_x_at	4.85E-26	5.19E-28	-1.41E+01	53.120837	-1.14	DNM1	NM_001005336,NM_004408
11756339_x_at	4.85E-26	5.19E-28	-1.41E+01	53.120656	-2.4	SARDH	NM_001134707,NM_007101
11747028_at	4.94E-26	5.30E-28	-1.41E+01	53.100479	-1.06	HMCN2	XM_001715154,XM_001726942,XM_002347078
11732029_a_at	4.94E-26	5.31E-28	-1.41E+01	53.097599	-1.2	SPEM1	NM_199339
11753030_a_at	5.27E-26	5.69E-28	-1.41E+01	53.029664	-1.38	NLGN3	NM_001166660,NM_018977,NM_181303
11761321_at	5.56E-26	6.03E-28	-1.40E+01	52.971416	-1.37	ANXA8L1	NM_001098845
11723132_a_at	5.81E-26	6.34E-28	-1.40E+01	52.922953	-1.38	RGS9	NM_001081955,NM_001165933,NM_003835
11722228_at	6.05E-26	6.62E-28	-1.40E+01	52.879272	-1.88	DNAJC30	NM_032317
11726818_at	6.09E-26	6.68E-28	-1.40E+01	52.870779	-1.04	TMEM201	NM_001010866,NM_001130924
11750369_x_at	6.25E-26	6.89E-28	-1.40E+01	52.839487	-1.3	KRTAP5-2	NM_001004325

11751163_a_at	6.35E-26	7.05E-28	-1.40E+01	52.817329	-1.46	PSEN2	NM_000447,NM_012486
11718493_a_at	7.26E-26	8.13E-28	-1.40E+01	52.676141	-2.59	SLC1A2	NM_004171
11739520_a_at	7.26E-26	8.15E-28	-1.40E+01	52.673921	-1.46	CHID1	NM_001142674,NM_001142675,NM_001142676,NM_001142677,NM_023947
11748906_a_at	7.36E-26	8.29E-28	-1.40E+01	52.656672	-1.22	NPM2	NM_182795
11747273_a_at	7.52E-26	8.51E-28	-1.40E+01	52.630359	-1.22	BAIAP3	NM_003933
11751082_a_at	7.55E-26	8.56E-28	-1.40E+01	52.625111	-1.13	MLC1	NM_015166,NM_139202
11761191_a_at	7.66E-26	8.71E-28	-1.40E+01	52.607135	-1.06	C16orf57	NM_024598
11751161_a_at	7.88E-26	9.02E-28	-1.40E+01	52.573125	-1.07	TTYH1	NM_001005367,NM_020659
11722328_a_at	8.13E-26	9.33E-28	-1.40E+01	52.539405	-1.08	PGBD5	NM_024554
11753426_x_at	8.18E-26	9.41E-28	-1.40E+01	52.531032	-1.06	SLC25A45	NM_001077241,NM_182556
11759447_at	8.46E-26	9.78E-28	-1.40E+01	52.492781	-2.76	GJC3	NM_181538
11739130_a_at	8.53E-26	9.90E-28	-1.40E+01	52.480565	-1.64	ABAT	NM_000663,NM_001127448,NM_020686
11750340_s_at	8.58E-26	9.97E-28	-1.40E+01	52.473716	-1.14	GPR144	NM_001161808,XM_002342934,XM_002346195,XM_002347085

11763877_at	8.65E-26	1.01E-27	-1.40E+01	52.463964	-1.31	---	XM_001718745.1
11734566_a_at	8.88E-26	1.04E-27	-1.40E+01	52.435796	-1.3	FAM188B	NM_032222
11740538_at	9.16E-26	1.07E-27	-1.39E+01	52.402644	-1.83	LOC653486 /// SCGB1C1	NM_001097610,NM_145651
11745479_a_at	9.26E-26	1.09E-27	-1.39E+01	52.388621	-1.47	GLYCTK	NM_001144951,NM_145262,NR_026699,NR_026700,NR_026701,NR_026702
11730009_a_at	9.59E-26	1.13E-27	-1.39E+01	52.352181	-1.2	MTUS2	NM_001033602,NM_015233
11734561_a_at	9.70E-26	1.15E-27	-1.39E+01	52.335111	-1.75	WBSCR17	NM_022479
11719128_a_at	9.70E-26	1.15E-27	-1.39E+01	52.334326	-1.82	LMF2	NM_033200
11727259_a_at	9.89E-26	1.17E-27	-1.39E+01	52.313373	-1.59	C19orf36	NM_001031735,NM_001039846
11747174_x_at	1.01E-25	1.19E-27	-1.39E+01	52.294143	-2.93	ERO1LB	NM_019891
11747611_a_at	1.01E-25	1.20E-27	-1.39E+01	52.29048	-1.06	TMCO6	NM_018502
11760534_a_at	1.05E-25	1.25E-27	-1.39E+01	52.246012	-3.73	NRG4	NM_138573
11741281_a_at	1.05E-25	1.26E-27	-1.39E+01	52.241877	-1.6	AKAP7	NM_004842,NM_016377,NM_138633

11733168_at	1.18E-25	1.43E-27	-1.39E+01	52.11403	-1.12	TSSK3	NM_052841
11757090_s_at	1.19E-25	1.44E-27	-1.39E+01	52.105766	-1.13	AZGP1P1 /// GJC3 /// LOC100128334	NM_181538,XM_001718225,XM_001724501, XM_001725084,XM_002344373
11726098_a_at	1.20E-25	1.46E-27	-1.39E+01	52.095685	-1.41	NKAIN1	NM_024522
11756008_a_at	1.22E-25	1.49E-27	-1.39E+01	52.074508	-1.29	AGBL4	NM_032785
11715120_s_at	1.22E-25	1.49E-27	-1.39E+01	52.074309	-1.24	HIST1H2BE	NM_003523
11739449_s_at	1.22E-25	1.49E-27	-1.39E+01	52.07318	-1.18	ROCK1	NM_005406
11755566_a_at	1.27E-25	1.57E-27	-1.39E+01	52.02262	-2.47	HEATR7A	NM_001099280,NM_001099281,NM_032450
11739760_a_at	1.31E-25	1.62E-27	-1.39E+01	51.991823	-1.05	ASB10	NM_001142459,NM_001142460,NM_080871
11747934_x_at	1.31E-25	1.63E-27	-1.39E+01	51.985935	-1.04	PMS2L11	NR_023383
11752890_a_at	1.33E-25	1.66E-27	-1.39E+01	51.966452	-1.17	SNTA1	NM_003098
11762477_at	1.34E-25	1.67E-27	-1.39E+01	51.959558	-1.36	CPXM2	NM_198148
11756514_a_at	1.42E-25	1.79E-27	-1.39E+01	51.894785	-1.05	TEKT5	NM_144674

11760783_at	1.45E-25	1.82E-27	-1.39E+01	51.874142	-1.13	FN3K	NM_022158
11736534_s_at	1.64E-25	2.09E-27	-1.38E+01	51.738409	-1.99	NELL1	NM_006157,NM_201551
11748577_a_at	1.66E-25	2.12E-27	-1.38E+01	51.725966	-1.71	IFT20	NM_174887
11736040_a_at	1.69E-25	2.16E-27	-1.38E+01	51.707467	-2.82	KIF1A	NM_004321
11761547_at	1.70E-25	2.18E-27	-1.38E+01	51.695445	-1.15	NOP16	NM_016391
11737803_a_at	1.73E-25	2.22E-27	-1.38E+01	51.678592	-1.28	YIPF2	NM_024029
11724899_a_at	1.83E-25	2.36E-27	-1.38E+01	51.619288	-1.16	TMEM198	NM_001005209
11757171_x_at	1.83E-25	2.36E-27	-1.38E+01	51.617445	-1.51	SNORA71D	NR_003018
11751746_a_at	1.85E-25	2.40E-27	-1.38E+01	51.602685	-3.34	GAMT	NM_000156,NM_138924
11722724_a_at	1.85E-25	2.41E-27	-1.38E+01	51.599699	-1.24	CSF2RB	NM_000395
11728188_at	1.94E-25	2.53E-27	-1.38E+01	51.550521	-1.15	DHRS12	NM_001031719,NM_024705
11715230_s_at	2.05E-25	2.69E-27	-1.38E+01	51.490724	-2.2	PARD6A	NM_001037281,NM_016948
11743198_a_at	2.05E-25	2.69E-27	-1.38E+01	51.489075	-1.02	SLC6A16	NM_014037

11753063_a_at	2.05E-25	2.69E-27	-1.38E+01	51.488236	-1.09	SIDT2	NM_001040455
11763522_a_at	2.08E-25	2.73E-27	-1.38E+01	51.475242	-2.63	RYR2	NM_001035
11731813_a_at	2.10E-25	2.76E-27	-1.38E+01	51.462295	-1.48	GATA1	NM_002049
11747189_a_at	2.24E-25	2.95E-27	-1.38E+01	51.396564	-1.11	BCL11A	NM_018014,NM_022893,NM_138559
11745867_x_at	2.30E-25	3.05E-27	-1.38E+01	51.363565	-1.08	PSEN2	NM_000447,NM_012486
11754022_s_at	2.56E-25	3.43E-27	-1.37E+01	51.248781	-1.49	GAP43	NM_001130064,NM_002045
11742213_x_at	2.65E-25	3.55E-27	-1.37E+01	51.214458	-1.25	CDH24	NM_022478,NM_144985
11715321_x_at	2.95E-25	4.00E-27	-1.37E+01	51.095468	-1.22	KRTAP10-8	NM_198695
11742502_at	3.12E-25	4.25E-27	-1.37E+01	51.036436	-1.07	PRR25	NM_001013638,XM_002344945
11737969_a_at	3.20E-25	4.37E-27	-1.37E+01	51.007306	-1.08	PCDHGA7	NM_018920,NM_032087
11742367_at	3.30E-25	4.53E-27	-1.37E+01	50.971536	-1.34	OR4N2	NM_001004723
11757108_a_at	3.75E-25	5.20E-27	-1.37E+01	50.835706	-1.31	GSTTP1	NR_003081
11739858_a_at	4.58E-25	6.39E-27	-1.36E+01	50.630133	-1.14	PACSIN1	NM_020804

11744967_x_at	4.64E-25	6.50E-27	-1.36E+01	50.613579	-1.06	HSD11B1L	NM_198533,NM_198704,NM_198705,NM_198706,NM_198707,NM_198708
11749431_a_at	4.78E-25	6.77E-27	-1.36E+01	50.573919	-1.42	PIGQ	NM_004204,NM_148920
11749994_a_at	5.90E-25	8.48E-27	-1.36E+01	50.349687	-1.21	CD300LG	NM_001168322,NM_001168323,NM_001168324,NM_145273
11731411_a_at	6.03E-25	8.70E-27	-1.36E+01	50.324712	-1.21	SLC8A2	NM_015063
11762953_a_at	6.59E-25	9.59E-27	-1.36E+01	50.227889	-1.58	NARFL	NM_022493
11744171_a_at	6.71E-25	9.80E-27	-1.36E+01	50.206468	-1.4	DNAJC4	NM_005528
11729403_a_at	6.84E-25	1.00E-26	-1.36E+01	50.186547	-1.22	SLC30A3	NM_003459
11746089_a_at	7.15E-25	1.05E-26	-1.35E+01	50.139186	-1.15	LTBP4	NM_001042544,NM_001042545,NM_003573
11761993_a_at	7.70E-25	1.14E-26	-1.35E+01	50.055168	-1.2	THRA	NM_003250,NM_199334
11762195_a_at	7.82E-25	1.16E-26	-1.35E+01	50.037051	-1.35	TCEA3	NM_003196
11751809_a_at	7.99E-25	1.19E-26	-1.35E+01	50.01255	-1.54	LOC388387	NR_027254
11733775_a_at	8.15E-25	1.22E-26	-1.35E+01	49.989851	-1.25	UMOD	NM_001008389,NM_003361
11745106_x_at	8.77E-25	1.32E-26	-1.35E+01	49.911154	-1.31	MEIS3	NM_001009813,NM_020160

11745625_a_at	8.90E-25	1.34E-26	-1.35E+01	49.894257	-1.14	PCDHGA2	NM_018915,NM_032009
11756333_a_at	8.92E-25	1.35E-26	-1.35E+01	49.890243	-1.22	CROCC	NM_014675
11722060_at	9.29E-25	1.41E-26	-1.35E+01	49.846201	-1.55	PPP1R1A	NM_006741
11762663_at	9.43E-25	1.43E-26	-1.35E+01	49.829345	-1.29	SEL1L	NM_005065
11715150_s_at	9.51E-25	1.45E-26	-1.35E+01	49.81681	-1.15	PCDHA4	NM_018907,NM_031500
11722992_a_at	1.05E-24	1.61E-26	-1.35E+01	49.716448	-2.43	DMD	NM_000109,NM_004006,NM_004007,NM_004009,NM_004010,NM_004011,NM_004012,NM_004013,NM_004014,NM_004015,NM_004016,NM_004017,NM_004018,NM_004019,NM_004020,NM_004021,NM_004022,NM_004023
11742501_a_at	1.06E-24	1.63E-26	-1.35E+01	49.701107	-1.27	CLTA	NM_001076677,NM_001184760,NM_001184761,NM_001184762,NM_001833,NM_007096

11715198_s_at	1.10E-24	1.69E-26	-1.35E+01	49.665682	-1.18	DUX4 /// LOC441056 /// LOC653543 /// LOC653544 /// LOC653545 /// LOC653548 /// LOC728410	NM_001127386,NM_001127387,NM_001127388,NM_001127389,NM_001164467,NM_001177376,NM_033178,XM_496731,XM_928023
11742381_at	1.15E-24	1.77E-26	-1.35E+01	49.617927	-1.28	OR5H1	NM_001005338
11729577_a_at	1.16E-24	1.80E-26	-1.34E+01	49.603222	-1.02	ATP1A3	NM_152296
11717570_s_at	1.21E-24	1.87E-26	-1.34E+01	49.565539	-1.08	MYH7	NM_000257
11725434_a_at	1.21E-24	1.88E-26	-1.34E+01	49.562238	-1.27	RUNDC3A	NM_001144825,NM_001144826,NM_006695
11720059_a_at	1.21E-24	1.88E-26	-1.34E+01	49.559106	-1.91	TTYH1	NM_001005367,NM_020659
11759603_at	1.44E-24	2.25E-26	-1.34E+01	49.380058	-2.5	BCL2L13	NM_015367

11736188_a_at	1.46E-24	2.29E-26	-1.34E+01	49.363991	-1.34	ORMDL3	NM_139280
11728299_x_at	1.47E-24	2.30E-26	-1.34E+01	49.360194	-1	AP2A1	NM_014203,NM_130787
11745240_a_at	1.52E-24	2.40E-26	-1.34E+01	49.319539	-1.05	TIE1	NM_005424
11722364_at	1.56E-24	2.47E-26	-1.34E+01	49.287821	-1.04	COX6A2	NM_005205
11761606_at	1.62E-24	2.56E-26	-1.34E+01	49.254482	-1.13	CDH22	NM_021248
11760454_x_at	1.62E-24	2.56E-26	-1.34E+01	49.25246	-1.15	CASZ1	NM_001079843,NM_017766
11760626_at	1.68E-24	2.69E-26	-1.34E+01	49.205762	-1.02	DYRK3	NM_001004023,NM_003582
11762332_at	1.88E-24	3.02E-26	-1.34E+01	49.090418	-1.1	NDUFA13	NM_015965
11726221_a_at	1.89E-24	3.03E-26	-1.34E+01	49.087049	-1.11	SYPL2	NM_001040709
11752736_x_at	1.92E-24	3.09E-26	-1.34E+01	49.067753	-1.41	CHID1	NM_001142674,NM_001142675,NM_001142676,NM_001142677,NM_023947
11752706_x_at	2.06E-24	3.32E-26	-1.33E+01	48.994905	-1.12	SEC14L4	NM_001161368,NM_174977
11723794_a_at	2.11E-24	3.44E-26	-1.33E+01	48.962188	-1.09	SLC29A2	NM_001532
11730019_a_at	2.20E-24	3.60E-26	-1.33E+01	48.917058	-1.08	C6orf142	NM_138569

11760019_x_at	2.23E-24	3.65E-26	-1.33E+01	48.901039	-1.23	GALNT9	NM_001122636,NM_021808
11732650_a_at	2.27E-24	3.73E-26	-1.33E+01	48.880112	-2.18	HEXDC	NM_173620
11741926_x_at	2.29E-24	3.77E-26	-1.33E+01	48.871029	-1.14	FUT5	NM_002034
11741578_a_at	2.56E-24	4.22E-26	-1.33E+01	48.759042	-1.36	SLC30A2	NM_001004434,NM_032513
11734070_x_at	2.62E-24	4.32E-26	-1.33E+01	48.734038	-1.4	SSR3	NM_007107
11755685_s_at	2.63E-24	4.35E-26	-1.33E+01	48.729305	-1.46	LOC100130872	BC030552.1
11715320_s_at	2.71E-24	4.49E-26	-1.33E+01	48.697488	-2.63	TTN	NM_003319,NM_133378,NM_133379,NM_133432,NM_133437
11740551_a_at	3.53E-24	5.98E-26	-1.32E+01	48.412133	-1.1	ASPG	NM_001080464
11735652_at	3.69E-24	6.29E-26	-1.32E+01	48.363158	-1	TMEM105	NM_178520
AFFX-r2-Ec-bioB-3_at	3.74E-24	6.38E-26	-1.32E+01	48.348046	-1.33	---	
11731339_a_at	3.79E-24	6.47E-26	-1.32E+01	48.333767	-2.04	ECE2	NM_001037324,NM_001100120,NM_001100121,NM_014693,NM_032331
11750548_s_at	3.86E-24	6.62E-26	-1.32E+01	48.311242	-1.03	CYP4A11	NM_000778

11738097_x_at	3.87E-24	6.66E-26	-1.32E+01	48.305263	-1.3	C2orf62	NM_198559
11751800_a_at	3.87E-24	6.67E-26	-1.32E+01	48.304617	-1.47	MDC1	NM_014641
11715156_s_at	4.25E-24	7.38E-26	-1.32E+01	48.203683	-1.25	KRTAP6-1	NM_181602
11763786_x_at	4.30E-24	7.47E-26	-1.32E+01	48.191832	-1.26	NARFL	NM_022493
11735334_a_at	4.67E-24	8.17E-26	-1.32E+01	48.102954	-1.02	HRH3	NM_007232
11756776_a_at	4.88E-24	8.55E-26	-1.32E+01	48.057677	-1.09	GCGR	NM_000160
11734508_at	5.08E-24	8.93E-26	-1.32E+01	48.015418	-1.04	FOXI2	NM_207426
11745115_a_at	5.28E-24	9.29E-26	-1.32E+01	47.976015	-2.36	SLC1A2	NM_004171
11716738_at	5.45E-24	9.61E-26	-1.32E+01	47.941621	-2.39	SEPX1	NM_016332
11736677_a_at	5.84E-24	1.04E-25	-1.31E+01	47.865237	-3.46	DPP10	NM_001004360,NM_001178034,NM_001178036,NM_001178037,NM_020868
11754653_a_at	5.95E-24	1.06E-25	-1.31E+01	47.846133	-3.13	BACE1	NM_012104,NM_138971,NM_138972,NM_138973
11721325_a_at	6.21E-24	1.11E-25	-1.31E+01	47.799366	-3.11	GCAT	NM_001171690,NM_014291
11729851_a_at	6.64E-24	1.19E-25	-1.31E+01	47.729537	-1.71	RADIL	NM_018059

11728779_a_at	6.66E-24	1.20E-25	-1.31E+01	47.724872	-1.27	ZAP70	NM_001079,NM_207519
11747674_a_at	6.88E-24	1.24E-25	-1.31E+01	47.687795	-1.01	ATP1A2	NM_000702
11724628_a_at	6.95E-24	1.25E-25	-1.31E+01	47.677488	-4.08	MAT1A	NM_000429
11720545_at	7.06E-24	1.28E-25	-1.31E+01	47.658019	-1.77	ANGEL1	NM_015305
11760179_x_at	7.06E-24	1.28E-25	-1.31E+01	47.656626	-1.06	WASH3P	NR_003659
11747348_a_at	7.48E-24	1.36E-25	-1.31E+01	47.599245	-3.35	PSAT1	NM_021154,NM_058179
11726475_a_at	7.59E-24	1.38E-25	-1.31E+01	47.581942	-3.76	TMEM52	NM_178545
11737183_at	7.61E-24	1.39E-25	-1.31E+01	47.578413	-1.13	EXOC3L2	NM_138568
11732410_a_at	7.77E-24	1.42E-25	-1.31E+01	47.553075	-1.12	GPER	NM_001039966,NM_001098201,NM_001505
11729485_a_at	7.82E-24	1.43E-25	-1.31E+01	47.545371	-1.1	RGS6	NM_004296
11753147_a_at	8.28E-24	1.53E-25	-1.31E+01	47.48372	-1.08	GATA1	NM_002049

GSE62452

ID	adj.P.Val	P.Value	t	B	logFC	Gene.symbol	Gene.title
7908072	1.36E-16	4.10E-21	1.13E+01	37.271717	2.65	LAMC2	laminin subunit gamma 2

7924029	6.69E-16	4.02E-20	1.09E+01	35.059259	2.08	LAMB3	laminin subunit beta 3
7901175	1.44E-15	1.30E-19	1.07E+01	33.925447	2.38	TSPAN1	tetraspanin 1
8045835	2.24E-15	2.69E-19	1.06E+01	33.217026	2.46	GALNT5	polypeptide N-acetylgalactosaminyltransferase 5///polypeptide N-acetylgalactosaminyltransferase 5
8050160	3.69E-15	5.54E-19	1.05E+01	32.516171	1.55	MBOAT2	membrane bound O-acyltransferase domain containing 2
7981514	4.38E-15	7.88E-19	1.04E+01	32.174554	1.51	AHNAK2	AHNAK nucleoprotein 2
7915472	4.57E-15	9.61E-19	1.04E+01	31.982275	1.67	SLC2A1	solute carrier family 2 member 1
7944164	5.75E-15	1.38E-18	1.03E+01	31.630673	2.06	TMPRSS4	transmembrane protease, serine 4
8040113	9.43E-15	2.89E-18	1.02E+01	30.914733	1.03	ASAP2	ArfGAP with SH3 domain, ankyrin repeat and PH domain 2
8020551	9.43E-15	3.04E-18	1.02E+01	30.865807	1.24	LAMA3	laminin subunit alpha 3
8049487	1.03E-14	3.71E-18	1.01E+01	30.672266	1.24	MLPH	melanophilin
7909164	1.62E-14	6.32E-18	1.00E+01	30.155874	2.55	CTSE	cathepsin E
7936144	1.84E-14	7.85E-18	9.99	29.945471	1.65	COL17A1	collagen type XVII alpha 1 chain
8009951	1.97E-14	9.48E-18	9.96	29.762674	1.23	ITGB4	integrin subunit beta 4
7993588	2.58E-14	1.31E-17	9.9	29.445673	1.14	TMC7	transmembrane channel like 7

7996819	3.93E-14	2.25E-17	9.81	28.926889	1.43	CDH3	cadherin 3
7961215	7.07E-14	4.25E-17	9.7	28.308879	1.03	STYK1	serine/threonine/tyrosine kinase 1
8169504	1.63E-13	1.08E-16	9.54	27.406181	2.66	SLC6A14	solute carrier family 6 member 14
8152703	1.83E-13	1.26E-16	9.51	27.252543	1.45	FBXO32	F-box protein 32
8105267	1.85E-13	1.33E-16	9.5	27.198774	2.14	ITGA2	integrin subunit alpha 2
8129082	1.89E-13	1.42E-16	9.49	27.138301	1.42	COL10A1	collagen type X alpha 1 chain
8021584	1.98E-13	1.54E-16	9.47	27.057531	1.97	SERPINB5	serpin family B member 5
8015349	2.31E-13	1.87E-16	9.44	26.868885	1.83	KRT19	keratin 19
7938485	6.07E-13	5.47E-16	9.25	25.830046	1.13	MICAL2	microtubule associated monooxygenase, calponin and LIM domain containing 2
7950933	6.32E-13	6.10E-16	9.23	25.723257	1.41	NOX4	NADPH oxidase 4
7952290	6.32E-13	6.26E-16	9.23	25.698928	1.28	TRIM29	tripartite motif containing 29
8081818	8.72E-13	8.91E-16	9.16	25.356645	1.67		
7965541	1.21E-12	1.38E-15	9.08	24.930078	1.18	FGD6	FYVE, RhoGEF and PH domain containing 6
7918064	1.25E-12	1.46E-15	9.07	24.876163	1.85	COL11A1	collagen type XI alpha 1 chain
8029086	1.35E-12	1.62E-15	9.06	24.778976	2.79	CEACAM 5	carcinoembryonic antigen related cell adhesion molecule 5

8064904	1.80E-12	2.27E-15	9	24.451083	1.34	FERMT1	fermitin family member 1
8029098	1.99E-12	2.64E-15	8.97	24.302113	2.42	CEACAM 6	carcinoembryonic antigen related cell adhesion molecule 6
8042439	2.30E-12	3.30E-15	8.93	24.085885	1.55	ANTXR1	anthrax toxin receptor 1
8058765	2.30E-12	3.32E-15	8.93	24.081064	2.19	FN1	fibronectin 1
8146863	2.40E-12	3.54E-15	8.92	24.018439	1.98	SULF1	sulfatase 1
7971077	2.43E-12	3.76E-15	8.91	23.960814	2.5	POSTN	periostin
8102232	2.43E-12	3.80E-15	8.91	23.950557	1.23	LEF1	lymphoid enhancer binding factor 1
8120043	2.63E-12	4.31E-15	8.88	23.828197	1.03	RUNX2	runt related transcription factor 2
8150889	3.06E-12	5.14E-15	8.85	23.656643	1.2	SDR16C5	short chain dehydrogenase/reductase family 16C, member 5
8072735	3.34E-12	5.72E-15	8.83	23.553507	1.43	APOL1	apolipoprotein L1
7942135	4.40E-12	7.97E-15	8.77	23.231626	1.2	ANO1	anoctamin 1
8113433	4.40E-12	8.07E-15	8.77	23.219146	1	EFNA5	ephrin A5
7969493	5.30E-12	9.91E-15	8.73	23.019956	1.55	SCEL	sciellin
7989985	5.30E-12	1.01E-14	8.73	22.997307	1.09	ITGA11	integrin subunit alpha 11
8083941	5.30E-12	1.02E-14	8.73	22.993426	1.19	ECT2	epithelial cell transforming 2
7935058	6.27E-12	1.29E-14	8.69	22.76449	1.39	MYOF	myoferlin
7979710	6.70E-12	1.41E-14	8.67	22.678923	1.09	PLEK2	pleckstrin 2

7928429	7.60E-12	1.67E-14	8.64	22.515644	1.29	PLAU	plasminogen activator, urokinase
8053417	8.48E-12	1.90E-14	8.62	22.390788	1.22	CAPG	capping actin protein, gelsolin like
8056113	8.58E-12	1.96E-14	8.61	22.35927	1.39	LY75-CD302///LY75	LY75-CD302 readthrough///lymphocyte antigen 75
8138613	8.62E-12	1.99E-14	8.61	22.342452	1.16	OSBPL3	oxysterol binding protein like 3
8002303	9.16E-12	2.18E-14	8.59	22.256677	1.31	NQO1	NAD(P)H quinone dehydrogenase 1
8102950	9.16E-12	2.20E-14	8.59	22.246505	1.18	INPP4B	inositol polyphosphate-4-phosphatase type II B
8027748	9.18E-12	2.23E-14	8.59	22.232118	1.32	FXYD3	FXYD domain containing ion transport regulator 3
7978544	9.24E-12	2.28E-14	8.59	22.213336	1.39	EGLN3	egl-9 family hypoxia inducible factor 3
7920291	9.39E-12	2.34E-14	8.58	22.186523	1	S100A16	S100 calcium binding protein A16
8105300	1.24E-11	3.31E-14	8.52	21.851754	1.53		
8015016	1.54E-11	4.18E-14	8.48	21.625118	1.31	TNS4	tensin 4
7976443	1.55E-11	4.29E-14	8.47	21.599544	1.23	IFI27	interferon alpha inducible protein 27
8138381	1.87E-11	5.39E-14	8.43	21.377094	1.86	AGR2	anterior gradient 2, protein disulphide isomerase family member

7985317	2.06E-11	6.13E-14	8.41	21.252953	1.39	CEMIP	cell migration inducing hyaluronan binding protein
8008237	2.14E-11	6.48E-14	8.4	21.198815	1.43	ITGA3	integrin subunit alpha 3
8066493	2.37E-11	7.27E-14	8.38	21.088129	1.73	SLPI	secretory leukocyte peptidase inhibitor
7969438	2.43E-11	7.51E-14	8.37	21.055997	1.17	LMO7	LIM domain 7
8078014	2.47E-11	7.80E-14	8.36	21.019625	1.2	SLC6A6	solute carrier family 6 member 6
7954065	2.61E-11	8.40E-14	8.35	20.947177	1.01	GPRC5A	G protein-coupled receptor class C group 5 member A
8084794	3.55E-11	1.20E-13	8.28	20.598007	1.08	IL1RAP	interleukin 1 receptor accessory protein
8001800	4.77E-11	1.72E-13	8.22	20.254021	1.51	CDH11	cadherin 11
7950810	4.96E-11	1.82E-13	8.21	20.199139	1.06	SYTL2	synaptotagmin like 2
8135601	5.81E-11	2.14E-13	8.18	20.038763	1.35	MET	MET proto-oncogene, receptor tyrosine kinase
7973336	5.86E-11	2.18E-13	8.18	20.022426	1.19	MMP14	matrix metallopeptidase 14
8097692	6.46E-11	2.43E-13	8.16	19.919686	1.32	EDNRA	endothelin receptor type A
7964907	6.61E-11	2.54E-13	8.15	19.874288	1.01	PTPRR	protein tyrosine phosphatase, receptor type R
8056151	6.73E-11	2.61E-13	8.14	19.849209	1.11	PLA2R1	phospholipase A2 receptor 1

8056184	6.81E-11	2.66E-13	8.14	19.830819	2.02	ITGB6///ITGB6	integrin subunit beta 6///integrin subunit beta 6
8042942	6.93E-11	2.73E-13	8.14	19.806326	1.16	HK2	hexokinase 2
8067125	8.86E-11	3.73E-13	8.08	19.503367	1.21	BCAS1	breast carcinoma amplified sequence 1
8101429	1.04E-10	4.58E-13	8.04	19.30413	1.76	PLAC8	placenta specific 8
7958884	1.18E-10	5.28E-13	8.02	19.166342	1	OAS1	2'-5'-oligoadenylate synthetase 1
8132318	1.19E-10	5.39E-13	8.01	19.14491	1.47	ANLN	anillin actin binding protein

8049349	1.26E-10	5.88E-13	8	19.06186	1.28	UGT1A3///UGT1A1///UGT1A4///UGT1A9///UGT1A5///UGT1A6///UGT1A7///UGT1A8///UGT1A10	UDP glucuronosyltransferase family 1 member A3///UDP glucuronosyltransferase family 1 member A1///UDP glucuronosyltransferase family 1 member A4///UDP glucuronosyltransferase family 1 member A9///UDP glucuronosyltransferase family 1 member A5///UDP glucuronosyltransferase family 1 member A6///UDP glucuronosyltransferase family 1 member A7///UDP glucuronosyltransferase family 1 member A8///UDP glucuronosyltransferase family 1 member A10

8112980	1.42E-10	6.86E-13	7.97	18.91228	1.61	EDIL3	EGF like repeats and discoidin domains 3
8071758	1.47E-10	7.16E-13	7.96	18.871142	1.23	MMP11	matrix metallopeptidase 11
7934278	1.47E-10	7.22E-13	7.96	18.86196	1.05	P4HA1///P4HA1	prolyl 4-hydroxylase subunit alpha 1///prolyl 4-hydroxylase subunit alpha 1
8103563	1.73E-10	8.80E-13	7.92	18.67066	1.13	DDX60	DEXD/H-box helicase 60
8086517	1.86E-10	9.70E-13	7.9	18.57639	1.01	CDCP1	CUB domain containing protein 1
7958913	1.89E-10	1.00E-12	7.9	18.547007	1.04	OAS2	2'-5'-oligoadenylate synthetase 2
8015387	1.95E-10	1.04E-12	7.89	18.511522	1.45	KRT17	keratin 17
8028924	2.72E-10	1.54E-12	7.82	18.127458	1.04	MIA-RAB4B///MIA	MIA-RAB4B readthrough (NMD candidate)///melanoma inhibitory activity
7979179	2.83E-10	1.62E-12	7.81	18.077497	1.09	ERO1A	endoplasmic reticulum oxidoreductase 1 alpha
8089082	2.94E-10	1.72E-12	7.8	18.021056	1.1	DCBLD2	discoidin, CUB and LCCL domain containing 2
8167973	3.68E-10	2.21E-12	7.75	17.778873	1.07	HEPH	hephaestin
8111387	3.74E-10	2.27E-12	7.75	17.752562	1.14	ADAMTS12	ADAM metallopeptidase with thrombospondin type 1 motif 12

8065416	4.78E-10	3.03E-12	7.69	17.473631	1.04	CST2	cystatin SA
8130867	4.89E-10	3.13E-12	7.69	17.44194	1.53	THBS2	thrombospondin 2
8041383	5.22E-10	3.42E-12	7.67	17.355585	1.1	LTBP1	latent transforming growth factor beta binding protein 1
8126820	5.44E-10	3.62E-12	7.66	17.299374	1.38	ADGRF1	adhesion G protein-coupled receptor F1
8081235	5.50E-10	3.69E-12	7.66	17.283005	1.37	COL8A1	collagen type VIII alpha 1 chain
7954527	5.72E-10	3.90E-12	7.65	17.228588	1.08	ARNTL2	aryl hydrocarbon receptor nuclear translocator like 2
8120719	5.80E-10	4.00E-12	7.64	17.203495	1.25	CD109	CD109 molecule
8029773	5.89E-10	4.13E-12	7.64	17.172123	1.08	IGFL2	IGF like family member 2
8037205	6.20E-10	4.41E-12	7.62	17.109357	1.1	CEACAM1	carcinoembryonic antigen related cell adhesion molecule 1
8116579	6.20E-10	4.43E-12	7.62	17.105224	1	FOXQ1	forkhead box Q1
8101260	6.31E-10	4.53E-12	7.62	17.084413	1.03	ANTXR2	anthrax toxin receptor 2
7974335	6.41E-10	4.66E-12	7.61	17.057038	1.28		
8146000	7.47E-10	5.52E-12	7.58	16.892241	1.2	ADAM9	ADAM metallopeptidase domain 9
7930498	8.95E-10	6.85E-12	7.54	16.682951	1.42	ACSL5	acyl-CoA synthetase long-chain family member 5
8059279	1.08E-09	8.50E-12	7.5	16.47409	1.02	EPHA4	EPH receptor A4
8013042	1.10E-09	8.71E-12	7.5	16.450043	1.05	KRT17	keratin 17

8106743	1.18E-09	9.57E-12	7.48	16.359713	1.76	VCAN	versican
7909708	1.19E-09	9.65E-12	7.48	16.351204	1.12	CENPF	centromere protein F
7898413	1.25E-09	1.04E-11	7.46	16.282331	1.14	PADI1	peptidyl arginine deiminase 1
8066214	1.55E-09	1.38E-11	7.41	16.005562	1.26	TGM2	transglutaminase 2
8092541	1.77E-09	1.61E-11	7.38	15.853666	1.47	LIPH	lipase H
8138289	1.86E-09	1.70E-11	7.37	15.802511	1.02	ETV1	ETS variant 1
8005449	2.01E-09	1.86E-11	7.35	15.714914	1.03	KRT17	keratin 17
8145293	2.32E-09	2.25E-11	7.32	15.533236	1.23	ADAM28	ADAM metallopeptidase domain 28
8127563	2.57E-09	2.53E-11	7.3	15.417338	1.65	COL12A1	collagen type XII alpha 1 chain
8065412	2.63E-09	2.60E-11	7.29	15.393482	1.65	CST1	cystatin SN
8098246	2.82E-09	2.82E-11	7.28	15.315163	1.94	ANXA10	annexin A10
8150509	2.92E-09	2.92E-11	7.27	15.280626	1.41	PLAT	plasminogen activator, tissue type
8100298	3.24E-09	3.34E-11	7.24	15.148647	1	OCIAD2	OCIA domain containing 2
7968800	3.32E-09	3.46E-11	7.24	15.116654	1.02	DGKH	diacylglycerol kinase eta
8123246	3.68E-09	3.88E-11	7.21	15.005546	1.13	SLC22A3	solute carrier family 22 member 3
8132557	4.04E-09	4.33E-11	7.19	14.898971	1.07	AEBP1	AE binding protein 1
8014974	4.08E-09	4.39E-11	7.19	14.885159	1.16	TOP2A	topoisomerase (DNA) II alpha

7927307	4.37E-09	4.74E-11	7.18	14.812146	1.32	ANXA8L1///ANXA8	annexin A8-like 1///annexin A8
7927367	5.13E-09	5.68E-11	7.14	14.636391	1.32	ANXA8L1///ANXA8	annexin A8-like 1///annexin A8
7933312	5.28E-09	5.87E-11	7.14	14.605254	1.35	ANXA8L1///ANXA8	annexin A8-like 1///annexin A8
8139207	5.57E-09	6.24E-11	7.12	14.546103	1.38	INHBA///INHBA	inhibin beta A subunit///inhibin beta A subunit
7982377	6.05E-09	7.03E-11	7.1	14.430227	1.03	GREM1	gremlin 1, DAN family BMP antagonist
8170648	6.45E-09	7.58E-11	7.09	14.356857	1.23	BGN	biglycan
8179617	6.68E-09	7.88E-11	7.08	14.319741	1.35	TRIM31	tripartite motif containing 31
8033674	6.81E-09	8.10E-11	7.07	14.292992	1.19	MUC16	mucin 16, cell surface associated
8105348	7.26E-09	8.85E-11	7.06	14.208065	1.08	GPX8	glutathione peroxidase 8 (putative)
8109926	7.58E-09	9.35E-11	7.05	14.154231	1.86	GABRP	gamma-aminobutyric acid type A receptor pi subunit
7937020	8.82E-09	1.12E-10	7.01	13.979733	1	MKI67	marker of proliferation Ki-67
8058857	9.60E-09	1.23E-10	6.99	13.885735	1.49	IGFBP5	insulin like growth factor binding protein 5
8104746	9.68E-09	1.25E-10	6.99	13.871688	1.15	NPR3	natriuretic peptide receptor 3

8056257	1.11E-08	1.48E-10	6.96	13.708641	1.61	FAP	fibroblast activation protein alpha
8082928	1.16E-08	1.57E-10	6.95	13.655686	1.72	CLDN18	claudin 18
8120967	1.28E-08	1.76E-10	6.92	13.543377	1.24	NT5E	5'-nucleotidase ecto
8045539	1.35E-08	1.89E-10	6.91	13.472173	1.17	KYNU	kynureninase
7951297	1.38E-08	1.94E-10	6.91	13.447103	1.41	MMP12	matrix metallopeptidase 12
7920297	1.39E-08	1.97E-10	6.9	13.434686	1.08	S100A14	S100 calcium binding protein A14
8104758	1.42E-08	2.01E-10	6.9	13.415032	1.27	NPR3	natriuretic peptide receptor 3
8047763	1.48E-08	2.10E-10	6.89	13.371274	1.16	NRP2	neuropilin 2
8178330	1.62E-08	2.34E-10	6.87	13.265921	1.33	TRIM31	tripartite motif containing 31
8050537	1.90E-08	2.86E-10	6.83	13.07515	1.23	MATN3	matrilin 3
8138566	1.92E-08	2.92E-10	6.83	13.055466	1	IGF2BP3	insulin like growth factor 2 mRNA binding protein 3
8057620	2.15E-08	3.35E-10	6.8	12.922082	1.36	COL5A2	collagen type V alpha 2 chain
7955613	2.21E-08	3.49E-10	6.79	12.881495	1.49	KRT7	keratin 7
7993638	2.22E-08	3.51E-10	6.79	12.877252	1.55	TMC5	transmembrane channel like 5
8100977	2.68E-08	4.36E-10	6.75	12.667303	1.23	CXCL5	C-X-C motif chemokine ligand 5
7923086	3.07E-08	5.15E-10	6.72	12.506678	1.03	ASPM	abnormal spindle microtubule assembly

7902541	4.04E-08	7.29E-10	6.65	12.170561	1.42	IFI44L	interferon induced protein 44 like
8048864	5.25E-08	1.01E-09	6.58	11.855848	1.26	CCL20	C-C motif chemokine ligand 20
8123104	5.58E-08	1.08E-09	6.57	11.790541	1.31	FNDC1	fibronectin type III domain containing 1
8169263	5.94E-08	1.16E-09	6.56	11.723566	1.6	VSIG1	V-set and immunoglobulin domain containing 1
8134263	6.08E-08	1.19E-09	6.55	11.697614	1.53	COL1A2/// COL1A2	collagen type I alpha 2 chain///collagen type I alpha 2 chain
8124707	6.10E-08	1.20E-09	6.55	11.692335	1.13	TRIM31	tripartite motif containing 31
7902553	6.39E-08	1.27E-09	6.54	11.633895	1.09	IFI44	interferon induced protein 44
8046124	7.08E-08	1.43E-09	6.51	11.522859	1.25	DHRS9	dehydrogenase/reductase 9
7934906	8.15E-08	1.69E-09	6.48	11.358177	1.23	ACTA2	actin, alpha 2, smooth muscle, aorta
8066822	9.93E-08	2.14E-09	6.43	11.129558	1.09	SULF2	sulfatase 2
8114920	1.02E-07	2.24E-09	6.42	11.088701	1.1	DPYSL3	dihydropyrimidinase like 3
8060940	1.09E-07	2.46E-09	6.41	10.997131	1.16	LAMP5	lysosomal associated membrane protein family member 5

7945169	1.16E-07	2.66E-09	6.39	10.922201	1.12	TMEM45B	transmembrane protein 45B
8077366	1.19E-07	2.74E-09	6.38	10.892254	1	LRRN1	leucine rich repeat neuronal 1
8016646	1.19E-07	2.75E-09	6.38	10.89135	1.53	COL1A1	collagen type I alpha 1 chain
7984001	1.19E-07	2.77E-09	6.38	10.884419	1.34	GCNT3	glucosaminyl (N-acetyl) transferase 3, mucin type
8125149	1.24E-07	2.91E-09	6.37	10.836133	1.08	SLC44A4	solute carrier family 44 member 4
8178653	1.24E-07	2.91E-09	6.37	10.836133	1.08	SLC44A4	solute carrier family 44 member 4
8179861	1.24E-07	2.91E-09	6.37	10.836133	1.08	SLC44A4	solute carrier family 44 member 4
8118061	1.50E-07	3.67E-09	6.33	10.612372	1.81	DPCR1	diffuse panbronchiolitis critical region 1
8035517	1.70E-07	4.25E-09	6.3	10.470639	1.2	COMP	cartilage oligomeric matrix protein
7929466	1.70E-07	4.28E-09	6.29	10.463604	1.26	CYP2C18	cytochrome P450 family 2 subfamily C member 18
8070579	2.27E-07	6.08E-09	6.22	10.125091	1.51	TFF1	trefoil factor 1
8108217	2.31E-07	6.23E-09	6.22	10.101358	1.02	TGFBI	transforming growth factor beta induced
7979658	3.41E-07	1.00E-08	6.12	9.644416	1.07	GPX2	glutathione peroxidase 2
8179221	3.45E-07	1.02E-08	6.12	9.629002	1.75	DPCR1	diffuse panbronchiolitis critical region 1

7963410	3.87E-07	1.18E-08	6.09	9.48463	1.34	KRT6C///KRT6B///KRT6A///KRT5	keratin 6C///keratin 6B///keratin 6A///keratin 5
7957023	3.87E-07	1.19E-08	6.09	9.481407	1.05	LYZ	lysozyme
8046922	4.20E-07	1.33E-08	6.06	9.372749	1.42	COL3A1	collagen type III alpha 1 chain
8059905	4.22E-07	1.33E-08	6.06	9.368106	1.27	COL6A3	collagen type VI alpha 3 chain
8090180	4.82E-07	1.56E-08	6.03	9.217066	1.51	MUC13	mucin 13, cell surface associated
8162394	4.90E-07	1.59E-08	6.03	9.199761	1.16	ASPN	asporin
8168749	5.54E-07	1.87E-08	5.99	9.045678	1.07	SRPX2	sushi repeat containing protein, X-linked 2
7896722	6.87E-07	2.39E-08	5.94	8.808424	1.24		
8140534	7.29E-07	2.57E-08	5.93	8.738226	1.26	SEMA3C	semaphorin 3C
7934898	7.78E-07	2.78E-08	5.91	8.662651	1.14	ANKRD22	ankyrin repeat domain 22
7919815	8.70E-07	3.20E-08	5.88	8.526324	1.19	CTSK	cathepsin K
8115327	1.41E-06	5.79E-08	5.76	7.95792	1.03	SPARC	secreted protein acidic and cysteine rich
8091385	2.14E-06	9.59E-08	5.65	7.473538	1.67	CP	ceruloplasmin (ferroxidase)
8148220	2.15E-06	9.66E-08	5.65	7.466368	1.07	FER1L6	fer-1 like family member 6
8135048	2.57E-06	1.21E-07	5.6	7.252905	1.3	MUC17	mucin 17, cell surface associated

7964927	2.69E-06	1.28E-07	5.59	7.192772	1.39		TSPAN8	tetraspanin 8
7988467	3.92E-06	2.02E-07	5.49	6.761423	1.19		FBN1	fibrillin 1
8158167	4.54E-06	2.41E-07	5.45	6.592062	1.12		LCN2	lipocalin 2
7948444	4.71E-06	2.51E-07	5.44	6.549529	1.59		TCN1	transcobalamin 1
7961142	6.66E-06	3.79E-07	5.35	6.156738	1.24		OLR1	oxidized low density lipoprotein receptor 1
8103769	1.08E-05	6.90E-07	5.22	5.584379	1.06		HPGD	hydroxyprostaglandin dehydrogenase 15-(NAD)
8045688	1.73E-05	1.22E-06	5.09	5.037804	1.15		TNFAIP6	TNF alpha induced protein 6
8020762	2.53E-05	1.97E-06	4.98	4.58267	1.04		DSG3	desmoglein 3
7963421	3.39E-05	2.82E-06	4.9	4.241976	1.02		KRT6A	keratin 6A
7951271	9.21E-05	9.75E-06	4.6	3.062391	1.19		MMP1	matrix metallopeptidase 1
8023696	1.03E-04	1.11E-05	4.57	2.936007	1.1		SERPINB3	serpin family B member 3
7960919	1.13E-04	1.26E-05	4.54	2.816153	1.06		MFAP5	microfibrillar associated protein 5
8095744	1.57E-04	1.88E-05	4.44	2.440636	1.08		AREG	amphiregulin
7951217	2.63E-04	3.58E-05	4.28	1.832066	1.2		MMP7	matrix metallopeptidase 7
7988350	1.15E-03	2.29E-04	3.79	0.091088	1.03		DUOX2	dual oxidase 2
8144786	3.87E-11	1.34E-13	-8.27	20.497087		-1.39	SLC7A2	solute carrier family 7 member 2
8047300	1.04E-10	4.56E-13	-8.04	19.307694		-1.77	AOX1	aldehyde oxidase 1
8057056	3.18E-10	1.89E-12	-7.78	17.9292		-1.2	TTN	titin
7908917	3.23E-10	1.93E-12	-7.78	17.909334		-1.12	BTG2	BTG anti-proliferation factor 2

8145532	5.36E-10	3.54E-12	-7.66	17.321477	-1.05	EPHX2	epoxide hydrolase 2
7925342	1.37E-09	1.18E-11	-7.44	16.158242	-1.62	ERO1B	endoplasmic reticulum oxidoreductase 1 beta
7961580	1.48E-09	1.30E-11	-7.42	16.062702	-1.12	LMO3	LIM domain only 3
8141094	4.65E-09	5.08E-11	-7.16	14.744465	-1.52	PDK4	pyruvate dehydrogenase kinase 4
8058591	1.42E-08	2.01E-10	-6.9	13.413989	-1.45	ACADL	acyl-CoA dehydrogenase, long chain
7954377	1.92E-08	2.90E-10	-6.83	13.061002	-2.31	IAPP	islet amyloid polypeptide
7934936	2.39E-08	3.80E-10	-6.77	12.798368	-1.52	SLC16A12	solute carrier family 16 member 12
8002283	2.59E-08	4.20E-10	-6.76	12.702837	-1.73	TMED6	transmembrane p24 trafficking protein 6
8095628	3.52E-08	6.14E-10	-6.68	12.336439	-2.51	ALB	albumin
8163839	3.62E-08	6.42E-10	-6.67	12.293608	-1.13	C5	complement component 5
8174201	4.11E-08	7.50E-10	-6.64	12.143161	-1.07	BEX1	brain expressed X-linked 1
8110990	4.34E-08	8.07E-10	-6.63	12.072116	-1.19	CTNND2	catenin delta 2
8142270	5.75E-08	1.12E-09	-6.56	11.758801	-1.33	NRCAM	neuronal cell adhesion molecule
7991335	6.95E-08	1.40E-09	-6.52	11.543858	-1.78	ANPEP	alanyl aminopeptidase, membrane
8060126	8.15E-08	1.69E-09	-6.48	11.359367	-1.13	AQP12B///AQP12A	aquaporin 12B///aquaporin 12A
8049729	1.01E-07	2.21E-09	-6.43	11.101649	-1.11	AQP12B///AQP12A	aquaporin 12B///aquaporin 12A

7948249	1.12E-07	2.57E-09	-6.4	10.956249	-1.12	SLC43A1	solute carrier family 43 member 1
7908597	1.37E-07	3.29E-09	-6.35	10.715848	-1.85	NR5A2	nuclear receptor subfamily 5 group A member 2
8167027	1.49E-07	3.63E-09	-6.33	10.621448	-1.03	RGN	regucalcin
8052940	1.61E-07	3.99E-09	-6.31	10.532172	-1.54	PAIP2B///VPS36	poly(A) binding protein interacting protein 2B///vacuolar protein sorting 36 homolog
8127065	2.14E-07	5.66E-09	-6.24	10.19488	-1.38	GSTA2	glutathione S-transferase alpha 2
7951910	2.55E-07	7.03E-09	-6.19	9.985271	-1.14	BACE1	beta-secretase 1
7908610	2.66E-07	7.39E-09	-6.18	9.937381	-1.96		
7903592	2.69E-07	7.50E-09	-6.18	9.923568	-1.78	KIAA1324	KIAA1324
7989473	2.79E-07	7.82E-09	-6.17	9.882686	-1.12	C2CD4B	C2 calcium dependent domain containing 4B
8098671	2.81E-07	7.89E-09	-6.17	9.874503	-1.67	F11	coagulation factor XI
8044700	3.10E-07	8.95E-09	-6.14	9.752926	-1.1	DPP10	dipeptidyl peptidase like 10
8056327	3.80E-07	1.15E-08	-6.09	9.508555	-1.04	GRB14	growth factor receptor bound protein 14
7944793	4.82E-07	1.56E-08	-6.03	9.217983	-1.31	OR8D4	olfactory receptor family 8 subfamily D member 4
8089402	5.17E-07	1.70E-08	-6.01	9.136638	-1.17	GUCA1C	guanylate cyclase activator 1C

7957221	5.32E-07	1.77E-08	-6	9.097515	-1.61	TRHDE	thyrotropin releasing hormone degrading enzyme
7938687	6.18E-07	2.11E-08	-5.97	8.926266	-1.02	NUCB2	nucleobindin 2
7991815	6.56E-07	2.27E-08	-5.95	8.858128	-1.94	PDIA2	protein disulfide isomerase family A member 2
8015735	7.22E-07	2.53E-08	-5.93	8.754014	-1.34		
7956229	7.54E-07	2.67E-08	-5.92	8.699638	-1.29	SLC39A5	solute carrier family 39 member 5
8062948	7.75E-07	2.77E-08	-5.91	8.666585	-1.52	RBPJL	recombination signal binding protein for immunoglobulin kappa J region like
8169389	8.25E-07	2.99E-08	-5.9	8.592624	-1.19	PAK3	p21 (RAC1) activated kinase 3
8176193	8.43E-07	3.08E-08	-5.89	8.56514	-1.07	F8	coagulation factor VIII
8091910	8.58E-07	3.15E-08	-5.88	8.542364	-2.3	SERPINI2	serpin family I member 2
7991034	9.47E-07	3.55E-08	-5.86	8.428693	-1.15	HOMER2	homer scaffolding protein 2
8001531	1.03E-06	3.95E-08	-5.84	8.324385	-1.22	MT1G	metallothionein 1G
8074201	1.04E-06	3.98E-08	-5.84	8.318858	-1.2	ANKRD62	ankyrin repeat domain 62
7994252	1.15E-06	4.52E-08	-5.81	8.194617	-1.46	AQP8	aquaporin 8
7923837	1.25E-06	5.02E-08	-5.79	8.095404	-1.35	PM20D1	peptidase M20 domain containing 1
8052372	1.30E-06	5.28E-08	-5.78	8.045449	-1.3	MIR217	microRNA 217

8158987	1.52E-06	6.37E-08	-5.74	7.866466	-1.45	CELP	carboxyl ester lipase pseudogene
8002121	1.66E-06	7.07E-08	-5.71	7.766205	-2.06	CTRL	chymotrypsin like
8149521	1.78E-06	7.69E-08	-5.7	7.685459	-1.67	FGL1	fibrinogen like 1
7961524	1.97E-06	8.66E-08	-5.67	7.570978	-2.42	ERP27	endoplasmic reticulum protein 27
7937079	2.19E-06	9.88E-08	-5.64	7.444541	-1.13	BNIP3	BCL2 interacting protein 3
8038633	2.42E-06	1.13E-07	-5.61	7.319167	-1.79	KLK1	kallikrein 1
7990555	2.74E-06	1.31E-07	-5.58	7.171457	-1.53	NRG4	neuregulin 4
7930790	3.06E-06	1.51E-07	-5.55	7.037571	-2.84	PNLIPRP1	pancreatic lipase related protein 1
8096845	3.49E-06	1.76E-07	-5.52	6.890988	-1.82	EGF	epidermal growth factor
7949320	3.82E-06	1.96E-07	-5.5	6.790114	-1.05	GPHA2	glycoprotein hormone alpha 2
7910915	4.13E-06	2.14E-07	-5.48	6.702274	-1.02	CHRM3	cholinergic receptor muscarinic 3
8141016	5.08E-06	2.75E-07	-5.42	6.464718	-1.11	TFPI2	tissue factor pathway inhibitor 2
8166202	5.11E-06	2.77E-07	-5.42	6.456185	-1.2	GRPR	gastrin releasing peptide receptor
8111864	5.26E-06	2.87E-07	-5.41	6.423687	-1.18	C6	complement component 6
7898723	5.44E-06	2.99E-07	-5.4	6.383139	-1.23		
7898732	6.17E-06	3.47E-07	-5.37	6.242125	-2.34		
7898167	8.46E-06	5.05E-07	-5.29	5.882723	-2.57	CTRC	chymotrypsin C

7895058	8.98E-06	5.46E-07	-5.27	5.807845	-1.18		
8046116	1.01E-05	6.36E-07	-5.24	5.66178	-1.09	G6PC2	glucose-6-phosphatase catalytic subunit 2
8119620	1.17E-05	7.59E-07	-5.2	5.492879	-1.28	GNMT	glycine N-methyltransferase
8124196	1.32E-05	8.77E-07	-5.16	5.354794	-1.18	DCDC2	doublecortin domain containing 2
8036699	1.43E-05	9.75E-07	-5.14	5.253522	-1.25	SYCN	syncollin
7999920	1.53E-05	1.06E-06	-5.12	5.176254	-2.6	GP2	glycoprotein 2
7930804	1.59E-05	1.10E-06	-5.11	5.136382	-2.6	PNLIPRP2	pancreatic lipase related protein 2 (gene/pseudogene)
7898184	1.77E-05	1.26E-06	-5.08	5.006161	-2.09	CELA2B	chymotrypsin like elastase family member 2B
7898734	1.81E-05	1.30E-06	-5.07	4.980186	-1.58		
7936798	2.11E-05	1.58E-06	-5.03	4.793894	-2.52	FAM24B-CUZD1///CUZD1	FAM24B-CUZD1 readthrough///CUB and zona pellucida like domains 1
7980906	2.16E-05	1.62E-06	-5.02	4.771456	-1.31		
8169291	2.32E-05	1.78E-06	-5	4.681931	-1.5		
8092682	2.49E-05	1.94E-06	-4.98	4.59932	-1.03	SST	somatostatin
7898176	2.55E-05	2.00E-06	-4.98	4.568545	-2.14	CELA2A	chymotrypsin like elastase family member 2A
8117304	2.58E-05	2.03E-06	-4.97	4.554524	-1.15	SLC17A4	solute carrier family 17 member 4

7898713	2.60E-05	2.05E-06	-4.97	4.546923	-1.96	CELA3B	chymotrypsin like elastase family member 3B
8125936	2.66E-05	2.10E-06	-4.96	4.52213	-2.35	CLPS	colipase
7967034	2.75E-05	2.19E-06	-4.96	4.482931	-2.22	PLA2G1B	phospholipase A2 group IB
7898721	2.76E-05	2.20E-06	-4.95	4.478404	-1.97		
8117288	2.82E-05	2.25E-06	-4.95	4.455616	-1.28	SCGN	secretagogin, EF-hand calcium binding protein
8009493	3.06E-05	2.49E-06	-4.93	4.361013	-1.35	KCNJ16	potassium voltage-gated channel subfamily J member 16
8158976	3.11E-05	2.54E-06	-4.92	4.338912	-2.46	CEL	carboxyl ester lipase
8136187	4.51E-05	4.02E-06	-4.81	3.90361	-2.5	CPA2	carboxypeptidase A2
8154848	4.81E-05	4.34E-06	-4.8	3.831727	-1.25	PRSS3	protease, serine 3
7913768	4.91E-05	4.46E-06	-4.79	3.805736	-1.04	IL22RA1	interleukin 22 receptor subunit alpha 1
7898725	5.30E-05	4.89E-06	-4.77	3.717826	-2.12	CELA3A	chymotrypsin like elastase family member 3A
8101881	8.35E-05	8.64E-06	-4.63	3.176751	-1.11	ADH1B	alcohol dehydrogenase 1B (class I), beta polypeptide
7988987	9.40E-05	1.00E-05	-4.6	3.037957	-1.02	ONECUT1	one cut homeobox 1
8002845	1.12E-04	1.24E-05	-4.54	2.831995	-2.01	CTRB2///CTRB1	chymotrypsinogen B2///chymotrypsinogen B1
7988414	1.50E-04	1.78E-05	-4.46	2.495168	-1.54	GATM	glycine amidinotransferase

8068684	1.62E-04	1.96E-05	-4.43	2.400699	-1.03	FAM3B	family with sequence similarity 3 member B
7997264	1.80E-04	2.24E-05	-4.4	2.276569	-1.84	CTRB2///CTRB1	chymotrypsinogen B2///chymotrypsinogen B1
7930777	2.01E-04	2.57E-05	-4.36	2.146352	-2.03	PNLIP///COL1A2	pancreatic lipase///collagen type I alpha 2 chain
7918134	2.30E-04	3.03E-05	-4.32	1.990058	-1.19	AMY2B///AMY2A///AMY1C///AMY1B///AMY1A	amylase, alpha 2B (pancreatic)///amylase, alpha 2A (pancreatic)///amylase, alpha 1C (salivary)///amylase, alpha 1B (salivary)///amylase, alpha 1A (salivary)
7903425	2.30E-04	3.03E-05	-4.32	1.99005	-1.19	AMY2B///AMY2A///AMY1C///AMY1B///AMY1A	amylase, alpha 2B (pancreatic)///amylase, alpha 2A (pancreatic)///amylase, alpha 1C (salivary)///amylase, alpha 1B (salivary)///amylase, alpha 1A (salivary)

7903440	2.30E-04	3.03E-05	-4.32	1.99005	-1.19	AMY2B///<br>AMY2A///<br>AMY1C///<br>AMY1B///<br>AMY1A	amylase, alpha 2B (pancreatic)///amylase, alpha 2A (pancreatic)///amylase, alpha 1C (salivary)///amylase, alpha 1B (salivary)///amylase, alpha 1A (salivary)
8136235	2.36E-04	3.14E-05	-4.31	1.957935	-1.9	CPA1	carboxypeptidase A1
8136801	2.42E-04	3.24E-05	-4.3	1.925764	-1.5	PRSS3P2	protease, serine 3 pseudogene 2
8095585	3.37E-04	4.87E-05	-4.2	1.542518	-1.39	SLC4A4	solute carrier family 4 member 4
7903414	3.54E-04	5.17E-05	-4.19	1.48591	-1.49	AMY2B///<br>AMY2A///<br>AMY1C///<br>AMY1B///<br>AMY1A	amylase, alpha 2B (pancreatic)///amylase, alpha 2A (pancreatic)///amylase, alpha 1C (salivary)///amylase, alpha 1B (salivary)///amylase, alpha 1A (salivary)
8053337	3.98E-04	6.00E-05	-4.15	1.34618	-1.98	REG1CP	regenerating family member 1 gamma, pseudogene

8136790	5.27E-04	8.50E-05	-4.06	1.019337	-1.28	PRSS2///PRSS3P2///PRSS2	protease, serine 2///protease, serine 3 pseudogene 2///protease, serine 2
8136795	5.38E-04	8.74E-05	-4.05	0.993629	-1.64	PRSS1	protease, serine 1
8083246	5.61E-04	9.24E-05	-4.03	0.940974	-1.71	CPB1	carboxypeptidase B1
8053330	7.83E-04	1.41E-04	-3.92	0.546139	-1.96	REG1B	regenerating family member 1 beta
8042978	8.21E-04	1.49E-04	-3.91	0.492128	-1.27	REG3G	regenerating family member 3 gamma
8151532	8.56E-04	1.57E-04	-3.89	0.443075	-1.25	FABP4	fatty acid binding protein 4
8042986	9.91E-04	1.89E-04	-3.84	0.269753	-1.58	REG1A	regenerating family member 1 alpha
8126954	1.84E-03	4.13E-04	-3.62	-0.456741	-1.03	PKHD1	polycystic kidney and hepatic disease 1 (autosomal recessive)
8053341	3.03E-03	7.73E-04	-3.44	-1.036173	-1.49	REG3A	regenerating family member 3 alpha
8136807	3.04E-03	7.76E-04	-3.44	-1.039822	-1.06	PRSS3P2///PRSS2///PRSS3P2///PRSS2	protease, serine 3 pseudogene 2///protease, serine 2///protease, serine 3 pseudogene 2///protease, serine 2
8114964	4.71E-03	1.33E-03	-3.28	-1.534464	-1.3	SPINK1	serine peptidase inhibitor, Kazal type 1

8135661	5.31E-03	1.55E-03	-3.23	-1.672537	-1.28	CFTR	cystic fibrosis transmembrane conductance regulator
8020795	2.71E-02	1.18E-02	-2.55	-3.505797	-1.04	TTR	transthyretin

GSE71989

ID	adj.P.Val	P.Value	t	B	logFC	Gene.symbol	Gene.title
218248_at	5.05E-09	9.24E-14	15.9263679	21.101948	1.9487238	FAM111A	family with sequence similarity 111 member A
1558048_x_at	1.98E-08	7.24E-13	14.4130138	19.243497	3.727645		
208718_at	1.77E-07	1.05E-11	12.6255273	16.774251	1.3639446	DDX17	DEAD-box helicase 17
220917_s_at	1.77E-07	1.92E-11	12.2456161	16.206772	1.6357048	WDR19	WD repeat domain 19
239577_at	1.77E-07	2.16E-11	12.1716833	16.094482	2.2056275		

225505_s_at	1.77E-07	2.27E-11	12.1417796	16.04889	1.203835	PCED1A	PC-esterase domain containing 1A
208956_x_at	2.55E-07	4.09E-11	11.7821953	15.49269	1.4939541	DUT	deoxyuridine triphosphatase
227070_at	2.55E-07	4.20E-11	11.7650734	15.465835	3.4444686	GLT8D2	glycosyltransferase 8 domain containing 2
201744_s_at	3.24E-07	6.52E-11	11.5024448	15.049611	3.3916309	LUM	lumican
214241_at	3.29E-07	7.22E-11	11.4424381	14.953366	1.4113561	NDUFB8	NADH:ubiquinone oxidoreductase subunit B8

212359_s_at	3.41E-07	8.11E-11	11.3736743	14.842546	1.047027	ZSWIM8	zinc finger SWIM-type containing 8
205249_at	3.62E-07	9.94E-11	11.254864	14.649729	3.2356827	EGR2	early growth response 2
225017_at	3.79E-07	1.11E-10	11.1908305	14.5451	2.2986725	CCDC14	coiled-coil domain containing 14
211068_x_at	4.01E-07	1.25E-10	11.1234084	14.434394	1.1143021	LOC101930591///FAM21C	uncharacterized LOC101930591///family with sequence similarity 21 member C

219833_s_at	4.33E-07	1.42E-10	11.0464063	14.307275	2.2409971	EFHC1	EF-hand domain containing 1
221264_s_at	4.83E-07	1.79E-10	10.9155559	14.08958	1.405658	TARDBP	TAR DNA binding protein
232458_at	4.83E-07	1.85E-10	10.8957729	14.056481	4.4936971	COL3A1	collagen type III alpha 1 chain
225022_at	4.83E-07	1.94E-10	10.8683269	14.010482	1.4092152	GOPC	golgi associated PDZ and coiled-coil motif containing
1555938_x_at	5.41E-07	2.35E-10	10.7611418	13.829934	2.9063111	VIM	vimentin

209110_s_at	5.41E-07	2.67E-10	10.688843	13.707332	1.2844568	RGL2	ral guanine nucleotide dissociation stimulator like 2
1555847_a_at	5.41E-07	2.76E-10	10.6692769	13.674039	2.627478	MIR24-2///MIR23A///LOC284454	microRNA 24-2///microRNA 23a///uncharacterized LOC284454
205081_at	5.41E-07	2.78E-10	10.6656123	13.667798	4.323558	CRIP1	cysteine rich protein 1

221973_at	5.41E-07	2.85E-10	10.652253	13.645032	1.9550246	LOC100506123///LOC100506076	uncharacterized LOC100506123///uncharacterized LOC100506076
235094_at	5.41E-07	3.32E-10	10.5670913	13.49937	2.9863918		
225496_s_at	5.41E-07	3.33E-10	10.5640158	13.494092	3.1780438	SYTL2	synaptotagmin like 2
201289_at	5.41E-07	3.43E-10	10.5488114	13.467983	3.5708888	CYR61	cysteine rich angiogenic inducer 61
227025_at	5.41E-07	3.50E-10	10.5364024	13.446652	1.0978463	PPHLN1	periphilin 1
208922_s_at	5.41E-07	3.62E-10	10.5177396	13.414533	1.178697	NXF1	nuclear RNA export factor 1

220079_s_at	5.41E-07	3.75E-10	10.4980607	13.380618	1.4066898	USP48	ubiquitin specific peptidase 48
242558_at	5.41E-07	3.75E-10	10.4979299	13.380392	2.1821639		
219087_at	5.41E-07	3.87E-10	10.4817047	13.352391	3.8863545	ASPN	asporin
38241_at	5.41E-07	4.05E-10	10.4560219	13.307999	2.7723296	BTN3A3	butyrophilin subfamily 3 member A3
209101_at	5.41E-07	4.25E-10	10.4290603	13.261304	3.7809045	CTGF	connective tissue growth factor
209268_at	5.41E-07	4.45E-10	10.4037272	13.217345	1.5186529	VPS45	vacuolar protein sorting 45 homolog
209189_at	5.41E-07	4.53E-10	10.3943539	13.201059	3.5152686	FOS	Fos proto-oncogene, AP-1 transcription factor subunit

226334_s_at	5.41E-07	4.53E-10	10.3933142	13.199251	2.0158427	AHSA2	AHA1, activator of heat shock 90kDa protein ATPase homolog 2 (yeast)
242471_at	5.41E-07	4.75E-10	10.3677039	13.154692	2.3306711		
212621_at	5.41E-07	4.91E-10	10.3498485	13.123574	1.7204579	NEMP1	nuclear envelope integral membrane protein 1

205788_s_at	5.41E-07	4.96E-10	10.3442604	13.113827	1.0824573	LOC441155///ZC3H11A	zinc finger CCCH-type domain-containing-like///zinc finger CCCH-type containing 11A
236114_at	5.41E-07	5.05E-10	10.3342616	13.096377	3.2221148		
209272_at	5.62E-07	5.49E-10	10.2879905	13.015451	1.9960673	NAB1	NGFI-A binding protein 1
212725_s_at	5.62E-07	5.62E-10	10.2755112	12.993578	1.3595675	TUG1	taurine up-regulated 1 (non-protein coding)

201448_at	5.62E-07	5.68E-10	10.269421	12.982896	2.038793	TIA1	TIA1 cytotoxic granule-associated RNA binding protein
214494_s_at	5.62E-07	5.84E-10	10.2540718	12.955952	1.1017989	LOC101930112///SPG7	uncharacterized LOC101930112///SPG7, paraplegin matrix AAA peptidase subunit
235008_at	5.62E-07	5.86E-10	10.2519822	12.952282	3.231938		
201449_at	5.72E-07	6.07E-10	10.2330983	12.919085	1.3179755	TIA1	TIA1 cytotoxic granule-associated RNA binding protein

226363_at	5.86E-07	6.32E-10	10.2110704	12.880303	1.8272111	ABCC5	ATP binding cassette subfamily C member 5
201967_at	6.14E-07	6.82E-10	10.1693576	12.806689	1.5494605	RBM6	RNA binding motif protein 6
209884_s_at	6.15E-07	7.05E-10	10.1510572	12.774321	1.7941632	SLC4A7	solute carrier family 4 member 7
205583_s_at	6.24E-07	7.45E-10	10.1215292	12.722001	2.7998684	ALG13	ALG13, UDP-N-acetylglucosaminyltransferase subunit
201324_at	6.24E-07	7.75E-10	10.0998476	12.68351	3.3773405	EMP1	epithelial membrane protein 1

219151_s_at	6.24E-07	8.03E-10	10.0805252	12.649156	1.9743534	RABL2A///RABL2B	RAB, member of RAS oncogene family-like 2A///RAB, member of RAS oncogene family-like 2B
213238_at	6.24E-07	8.07E-10	10.0782239	12.645061	1.8217486	ATP10D	ATPase phospholipid transporting 10D (putative)
233223_at	6.27E-07	8.41E-10	10.0556028	12.604771	3.0574711		
201310_s_at	6.27E-07	8.48E-10	10.0512572	12.597023	3.4560536	NREP	neuronal regeneration related protein

225656_at	6.41E-07	8.91E-10	10.0244319	12.549142	1.8299502	EFHC1	EF-hand domain containing 1
235613_at	6.56E-07	9.24E-10	10.0047672	12.513982	1.5502939		
209360_s_at	7.08E-07	1.06E-09	9.9333594	12.38587	2.6380432	LOC101928269///LOC100506403///RUNX1	uncharacterized LOC101928269///uncharacterized LOC100506403///runt related transcription factor 1
227426_at	7.08E-07	1.06E-09	9.9317467	12.382969	1.3982507	SOS1	SOS Ras/Rac guanine nucleotide exchange factor 1
241786_at	7.08E-07	1.07E-09	9.9251559	12.371109	1.9265704		

228046_at	7.08E-07	1.07E-09	9.9239537	12.368945	1.9527138	ZNF827	zinc finger protein 827
227199_at	7.29E-07	1.12E-09	9.9017117	12.328873	1.4952888	DIP2A	disco interacting protein 2 homolog A
212153_at	7.45E-07	1.20E-09	9.8632735	12.259467	1.2419123	POGZ	pogo transposable element with ZNF domain
201936_s_at	7.45E-07	1.21E-09	9.8591209	12.251957	1.4047282	EIF4G3	eukaryotic translation initiation factor 4 gamma 3
227017_at	7.68E-07	1.28E-09	9.8311167	12.20125	1.5705968	ERICH1	glutamate rich 1

202949_s_at	7.68E-07	1.28E-09	9.8309393	12.200928	3.100067	FHL2	four and a half LIM domains 2
226098_at	7.85E-07	1.32E-09	9.8135258	12.169344	2.2306207	IFT80	intraflagellar transport 80
209335_at	8.07E-07	1.45E-09	9.7647456	12.08065	2.1670404	DCN	decorin
228617_at	8.07E-07	1.46E-09	9.7600643	12.072121	3.8262459	XAF1	XIAP associated factor 1
225180_at	8.13E-07	1.51E-09	9.7437383	12.042354	2.5269754	TTC14	tetratricopeptide repeat domain 14
212387_at	8.13E-07	1.52E-09	9.7398922	12.035337	2.2939507	TCF4	transcription factor 4
228623_at	8.13E-07	1.53E-09	9.7371456	12.030324	1.9666766		
213557_at	8.13E-07	1.53E-09	9.7353956	12.02713	1.237518	CDK12	cyclin dependent kinase 12

235432_at	8.26E-07	1.59E-09	9.7164115	11.99245	1.9965863	NPHP3	nephronophthisis 3 (adolescent)
226837_at	8.52E-07	1.67E-09	9.6904623	11.944967	2.2850386	SPRED1	sprouty related EVH1 domain containing 1
213891_s_at	8.57E-07	1.71E-09	9.6774202	11.921067	2.5126495	TCF4	transcription factor 4
226932_at	8.57E-07	1.71E-09	9.6768004	11.919931	2.0279771	SSPN	sarcospan
201719_s_at	8.57E-07	1.74E-09	9.6689964	11.905619	1.8476025	EPB41L2	erythrocyte membrane protein band 4.1 like 2
204791_at	8.57E-07	1.77E-09	9.6585926	11.886525	1.6564568	NR2C1	nuclear receptor subfamily 2 group C member 1

225636_at	8.89E-07	1.88E-09	9.6280984	11.830477	1.8367311	STAT2	signal transducer and activator of transcription 2
225814_at	8.89E-07	1.89E-09	9.624265	11.823422	1.75584	XRN1	5'-3' exoribonuclease 1
222150_s_at	8.89E-07	1.92E-09	9.6170831	11.8102	2.4459163	GSAP	gamma-secretase activating protein
1557081_at	8.89E-07	1.92E-09	9.6164764	11.809082	1.9809262	RBM25	RNA binding motif protein 25
203146_s_at	9.03E-07	1.96E-09	9.6041261	11.786327	1.2923857	GABBR1	gamma-aminobutyric acid type B receptor subunit 1
227576_at	9.11E-07	2.04E-09	9.5837102	11.748665	1.7835982		

227087_at	9.11E-07	2.09E-09	9.570879	11.724965	1.8969148	INPP4A	inositol polyphosphate-4-phosphatase type I A
225821_s_at	9.11E-07	2.10E-09	9.5695278	11.722468	1.7918123	BOD1L1	biorientation of chromosomes in cell division 1 like 1
1556658_a_at	9.42E-07	2.21E-09	9.5434982	11.674317	2.2792259		
232914_s_at	9.52E-07	2.32E-09	9.5173601	11.625872	3.5061923	SYTL2	synaptotagmin like 2
224635_s_at	9.52E-07	2.33E-09	9.5158837	11.623133	1.1756098	BIRC6	baculoviral IAP repeat containing 6

228670_at	1.01E-06	2.52E-09	9.4734349	11.544248	2.0525098	TEP1	telomerase associated protein 1
202771_at	1.01E-06	2.57E-09	9.4642724	11.527189	2.4127188	PIEZO1	piezo type mechanosensitive ion channel component 1
221221_s_at	1.01E-06	2.59E-09	9.4601227	11.519459	1.2313957	KLHL3	kelch like family member 3
238558_at	1.01E-06	2.60E-09	9.4570886	11.513805	2.1840432		
213142_x_at	1.01E-06	2.68E-09	9.4414686	11.484681	2.001918	GSAP	gamma-secretase activating protein
213241_at	1.01E-06	2.71E-09	9.4362278	11.474901	2.9741143	PLXNC1	plexin C1
202310_s_at	1.01E-06	2.77E-09	9.4242874	11.452606	5.199103	COL1A1	collagen type I alpha 1 chain

209607_x_at	1.01E-06	2.79E-09	9.4209408	11.446354	1.2544532	SLX1B-SULT1A4///SLX1A-SULT1A3///SULT1A4///SULT1A3	SLX1B-SULT1A4 readthrough (NMD candidate)///SLX1A-SULT1A3 readthrough (NMD candidate)///sulfotransferase family 1A member 4///sulfotransferase family 1A member 3
222803_at	1.01E-06	2.80E-09	9.4200025	11.444601	2.3111595	PRTFDC1	phosphoribosyl transferase domain containing 1

203856_at	1.01E-06	2.83E-09	9.4130269	11.431563	1.7653284	VRK1	vaccinia related kinase 1
227623_at	1.01E-06	2.85E-09	9.4092833	11.424563	2.4485796	CACNA2D1	calcium voltage-gated channel auxiliary subunit alpha2delta 1
203651_at	1.01E-06	2.86E-09	9.4085184	11.423132	2.0672254	ZFYVE16	zinc finger FYVE-type containing 16
222629_at	1.02E-06	2.96E-09	9.3913471	11.390998	1.4304245	REV1	REV1, DNA directed polymerase
201680_x_at	1.02E-06	2.99E-09	9.3855693	11.380177	1.1146139	SRRT	serrate, RNA effector molecule

224917_at	1.03E-06	3.03E-09	9.3791204	11.368093	3.6272073	MIR21///VMP1	microRNA 21///vacuole membrane protein 1
213761_at	1.03E-06	3.05E-09	9.374323	11.3591	1.5314059	MDM1	Mdm1 nuclear protein
221729_at	1.04E-06	3.12E-09	9.3633474	11.338513	4.6793121	COL5A2	collagen type V alpha 2 chain
227140_at	1.04E-06	3.18E-09	9.3538078	11.320606	6.3315538	INHBA	inhibin beta A subunit
214092_x_at	1.04E-06	3.19E-09	9.3514522	11.316183	1.5543266	SUGP2	SURP and G-patch domain containing 2

1294_at	1.04E-06	3.21E-09	9.3488238	11.311246	1.121712	MIR5193///UBA7	microRNA 5193///ubiquitin like modifier activating enzyme 7
203580_s_at	1.04E-06	3.22E-09	9.3469324	11.307693	1.4804646	SLC7A6	solute carrier family 7 member 6
207719_x_at	1.04E-06	3.24E-09	9.3435961	11.301424	2.4335139	CEP170P1///CEP170	centrosomal protein 170 pseudogene 1///centrosomal protein 170

219778_at	1.06E-06	3.35E-09	9.3261201	11.268563	2.7013005	ZFPM2	zinc finger protein, FOG family member 2
221427_s_at	1.06E-06	3.37E-09	9.3240034	11.26458	1.4765248	CCNL2	cyclin L2
221447_s_at	1.07E-06	3.39E-09	9.3203724	11.257746	2.1245782	GLT8D2	glycosyltransferase 8 domain containing 2
223122_s_at	1.07E-06	3.43E-09	9.3141271	11.245987	3.2896891	SFRP2	secreted frizzled related protein 2
213905_x_at	1.07E-06	3.47E-09	9.309093	11.236505	3.3294761	BGN	biglycan
203763_at	1.07E-06	3.48E-09	9.3074985	11.233501	1.4470161	DYNC2LI1	dynein cytoplasmic 2 light intermediate chain 1
225227_at	1.07E-06	3.49E-09	9.304924	11.228649	2.7812963	SKIL	SKI-like proto-oncogene

225883_at	1.08E-06	3.61E-09	9.2886091	11.197885	1.3811596	ATG16L2	autophagy related 16 like 2
207173_x_at	1.08E-06	3.63E-09	9.2847442	11.190592	4.4921082	CDH11	cadherin 11
222047_s_at	1.08E-06	3.64E-09	9.2838205	11.188849	1.4144475	SRRT	serrate, RNA effector molecule
228216_at	1.08E-06	3.65E-09	9.2825281	11.186409	1.35658	ZBTB37	zinc finger and BTB domain containing 37
1558956_s_at	1.09E-06	3.69E-09	9.276189	11.17444	2.7254779	IFT80	intraflagellar transport 80
202403_s_at	1.09E-06	3.71E-09	9.2736099	11.169569	3.8649573	COL1A2	collagen type I alpha 2 chain

228999_at	1.09E-06	3.72E-09	9.2726868	11.167826	2.3948693	CHD2	chromodomain helicase DNA binding protein 2
203229_s_at	1.09E-06	3.77E-09	9.2658392	11.154887	1.0130314	CLK2	CDC like kinase 2
228465_at	1.09E-06	3.77E-09	9.2654798	11.154208	1.5661063		
211986_at	1.10E-06	3.94E-09	9.2429489	11.11159	2.026938	AHNAK	AHNAK nucleoprotein
203753_at	1.10E-06	4.02E-09	9.2328172	11.092402	2.5353688	TCF4	transcription factor 4
209561_at	1.10E-06	4.10E-09	9.2228324	11.073478	1.3854939	THBS3	thrombospondin 3
202311_s_at	1.10E-06	4.11E-09	9.2213393	11.070647	5.8551877	COL1A1	collagen type I alpha 1 chain
202766_s_at	1.10E-06	4.13E-09	9.219635	11.067416	3.379587	FBN1	fibrillin 1

226939_at	1.10E-06	4.15E-09	9.2166338	11.061724	1.5555155	CPEB2	cytoplasmic polyadenylation element binding protein 2
211896_s_at	1.10E-06	4.16E-09	9.2156687	11.059893	2.3037148	DCN	decorin
225786_at	1.10E-06	4.18E-09	9.2123488	11.053595	2.8319859		
225269_s_at	1.11E-06	4.27E-09	9.2017588	11.033493	2.3198914	RBMS1	RNA binding motif single stranded interacting protein 1
203253_s_at	1.11E-06	4.31E-09	9.1967456	11.023972	1.632823	PPIP5K2	diphosphoinositol pentakisphosphate kinase 2
202450_s_at	1.12E-06	4.37E-09	9.1899246	11.011012	3.6342257	CTSK	cathepsin K

227003_at	1.13E-06	4.50E-09	9.1756478	10.983865	1.7320118	RAB28	RAB28, member RAS oncogene family
212386_at	1.13E-06	4.52E-09	9.1733489	10.979491	2.4632866	TCF4	transcription factor 4
235879_at	1.13E-06	4.58E-09	9.166579	10.966606	2.4334554		
226865_at	1.14E-06	4.66E-09	9.157712	10.949719	2.9159102	PLXDC2	plexin domain containing 2
230304_at	1.17E-06	4.86E-09	9.1363749	10.90904	1.3225082		
227074_at	1.17E-06	4.88E-09	9.1337509	10.904033	1.2452804	CCDC18-AS1	CCDC18 antisense RNA 1
230728_at	1.17E-06	4.91E-09	9.130594	10.898008	1.4504938	FKBP14	FK506 binding protein 14
227261_at	1.17E-06	4.95E-09	9.1265897	10.890364	1.9626525	KLF12	Kruppel like factor 12

212488_at	1.17E-06	4.97E-09	9.1247798	10.886908	4.5385884	COL5A1	collagen type V alpha 1 chain
225176_at	1.19E-06	5.09E-09	9.1124888	10.863426	1.7455521	LNPEP	leucyl and cystinyl aminopeptidase
212176_at	1.19E-06	5.15E-09	9.1066476	10.852259	2.0172991	PNISR	PNN interacting serine and arginine rich protein
211161_s_at	1.20E-06	5.28E-09	9.0941654	10.828381	4.8369	COL3A1	collagen type III alpha 1 chain
202963_at	1.20E-06	5.31E-09	9.0911306	10.822572	1.6714461	RFX5	regulatory factor X5
236561_at	1.21E-06	5.40E-09	9.0820606	10.805203	2.9257848	TGFBR1	transforming growth factor beta receptor 1

232180_at	1.21E-06	5.46E-09	9.0766633	10.794862	1.4347077	UGP2	UDP-glucose pyrophosphorylase 2
223227_at	1.21E-06	5.47E-09	9.0756086	10.792841	1.5043618	BBS2	Bardet-Biedl syndrome 2
211813_x_at	1.21E-06	5.53E-09	9.0703192	10.782702	2.1197107	DCN	decorin
215076_s_at	1.25E-06	5.93E-09	9.0349172	10.71474	3.840505	COL3A1	collagen type III alpha 1 chain
226330_s_at	1.25E-06	5.96E-09	9.0325263	10.710144	1.7244414	SUPT20H	SPT20 homolog, SAGA complex component
213703_at	1.25E-06	6.10E-09	9.0206842	10.687368	2.3041845	LINC00342	long intergenic non-protein coding RNA 342

201852_x_at	1.25E-06	6.12E-09	9.0187458	10.683638	4.5475064	COL3A1	collagen type III alpha 1 chain
228751_at	1.25E-06	6.14E-09	9.017403	10.681053	1.6480171	CLK4	CDC like kinase 4
220990_s_at	1.28E-06	6.36E-09	9.0000783	10.647688	2.3196479	MIR21///VMP1	microRNA 21///vacuole membrane protein 1
201221_s_at	1.30E-06	6.48E-09	8.9905799	10.629378	1.3862709	SNRNP70	small nuclear ribonucleoprotein U1 subunit 70
224694_at	1.30E-06	6.52E-09	8.9868479	10.62218	3.8526373	ANTXR1	anthrax toxin receptor 1
222999_s_at	1.30E-06	6.56E-09	8.9840453	10.616773	2.0840445	CCNL2	cyclin L2

225477_s_at	1.31E-06	6.71E-09	8.9723907	10.594278	1.1413289	NR2C2	nuclear receptor subfamily 2 group C member 2
201893_x_at	1.33E-06	6.89E-09	8.9596985	10.569758	1.9579304	DCN	decorin
212929_s_at	1.33E-06	7.02E-09	8.9500109	10.551028	1.056183	LOC101930591///FAM21A///FAM21C	uncharacterized LOC101930591///family with sequence similarity 21 member A///family with sequence similarity 21 member C
242008_at	1.33E-06	7.05E-09	8.9476822	10.546523	1.8056745		

226993_at	1.33E-06	7.06E-09	8.9474844	10.546141	1.5608734	TRIP12	thyroid hormone receptor interactor 12
226291_at	1.33E-06	7.06E-09	8.9471934	10.545578	1.2470413	ALS2	ALS2, alsin Rho guanine nucleotide exchange factor
218067_s_at	1.33E-06	7.13E-09	8.9418758	10.535289	2.2846955	ARGLU1	arginine and glutamate rich 1
36711_at	1.33E-06	7.15E-09	8.9407596	10.533129	2.8920845	MAFF	MAF bZIP transcription factor F
208030_s_at	1.33E-06	7.16E-09	8.9403407	10.532318	1.045367	ADD1	adducin 1

212307_s_at	1.33E-06	7.18E-09	8.9386642	10.529073	1.886815	OGT	O-linked N-acetylglucosamine (GlcNAc) transferase
244579_at	1.34E-06	7.27E-09	8.9323797	10.516905	2.0611618		
206011_at	1.34E-06	7.35E-09	8.9268711	10.506235	2.0933911	CASP1	caspase 1
225242_s_at	1.34E-06	7.40E-09	8.923582	10.499862	3.6640298	CCDC80	coiled-coil domain containing 80
202404_s_at	1.34E-06	7.43E-09	8.9216098	10.49604	5.3686193	COL1A2	collagen type I alpha 2 chain
204821_at	1.35E-06	7.55E-09	8.9136217	10.480553	1.8528621	BTN3A3	butyrophilin subfamily 3 member A3

210764_s_at	1.35E-06	7.61E-09	8.9097175	10.472981	3.3819279	CYR61	cysteine rich angiogenic inducer 61
217828_at	1.35E-06	7.63E-09	8.9082494	10.470133	1.6071277	SLTM	SAFB like transcription modulator
213684_s_at	1.38E-06	7.90E-09	8.8905572	10.435788	2.2056109	PDLIM5	PDZ and LIM domain 5
244015_at	1.39E-06	8.00E-09	8.8841488	10.423336	1.491762		
227900_at	1.41E-06	8.17E-09	8.8741998	10.403995	1.4028548	CBLB	Cbl proto-oncogene B
202081_at	1.46E-06	8.54E-09	8.8516519	10.360107	1.7339136	IER2	immediate early response 2
225931_s_at	1.46E-06	8.54E-09	8.8515064	10.359824	1.9774886	RNF213	ring finger protein 213

217591_at	1.46E-06	8.60E-09	8.8484425	10.353854	2.0154934	SKIL	SKI-like proto-oncogene
221730_at	1.46E-06	8.65E-09	8.8455849	10.348286	4.8402629	COL5A2	collagen type V alpha 2 chain
218428_s_at	1.46E-06	8.65E-09	8.8451851	10.347507	1.6806196	REV1	REV1, DNA directed polymerase
214247_s_at	1.46E-06	8.72E-09	8.841585	10.34049	2.8440939	DKK3	dickkopf WNT signaling pathway inhibitor 3
212513_s_at	1.46E-06	8.76E-09	8.8389589	10.33537	1.341498	USP33	ubiquitin specific peptidase 33

218659_at	1.46E-06	8.77E-09	8.8383594	10.334201	1.0505227	ASXL2	additional sex combs like 2, transcriptional regulator
202664_at	1.46E-06	8.88E-09	8.8324455	10.322667	2.8768577	WIPF1	WAS/WASL interacting protein family member 1
218871_x_at	1.46E-06	8.94E-09	8.8290566	10.316055	2.3116582	CSGALNACT2	chondroitin sulfate N-acetylgalactosaminyltransferase 2
213656_s_at	1.46E-06	8.95E-09	8.8283472	10.314671	1.4570286	KLC1	kinesin light chain 1

228793_at	1.46E-06	8.97E-09	8.8274095	10.312841	2.586398	JMJD1C	jumonji domain containing 1C
208988_at	1.46E-06	8.98E-09	8.8267897	10.311632	2.0006611	KDM2A	lysine demethylase 2A
228946_at	1.46E-06	9.02E-09	8.8242949	10.306763	1.7178164	INTU	inturned planar cell polarity protein
213387_at	1.46E-06	9.07E-09	8.8218051	10.301903	1.7782998	ATAD2B	ATPase family, AAA domain containing 2B
232113_at	1.46E-06	9.09E-09	8.8206721	10.299691	3.6342746		
223716_s_at	1.47E-06	9.27E-09	8.8106535	10.280123	1.6962305	ZRANB2	zinc finger RANBP2-type containing 2

202892_at	1.48E-06	9.39E-09	8.8042227	10.267556	1.4352841	CDC23	cell division cycle 23
204206_at	1.49E-06	9.56E-09	8.7954667	10.250435	1.0502005	MNT	MAX network transcriptional repressor
235444_at	1.49E-06	9.57E-09	8.7952441	10.25	1.5938464	FOXP1	forkhead box P1
225381_at	1.49E-06	9.57E-09	8.7949511	10.249427	2.4629682	MIR100HG	mir-100-let-7a-2 cluster host gene
232333_at	1.50E-06	9.87E-09	8.7796684	10.219517	1.8160352		
203668_at	1.50E-06	9.88E-09	8.7791692	10.218539	1.4492625	MAN2C1	mannosidase alpha class 2C member 1
223679_at	1.50E-06	9.91E-09	8.7778387	10.215934	2.3145611	CTNNB1	catenin beta 1

211990_at	1.50E-06	9.91E-09	8.7777288	10.215718	2.7469229	HLA-DPA1	major histocompatibility complex, class II, DP alpha 1
242405_at	1.53E-06	1.02E-08	8.7628447	10.186553	2.1312264		
226441_at	1.53E-06	1.02E-08	8.7626586	10.186188	1.49345	MAP3K2	mitogen-activated protein kinase kinase kinase 2
226795_at	1.54E-06	1.03E-08	8.758154	10.177355	2.2729275	LRCH1	leucine rich repeats and calponin homology domain containing 1
225724_at	1.54E-06	1.03E-08	8.7564056	10.173926	1.9277625	PSMA3-AS1	PSMA3 antisense RNA 1

200665_s_at	1.54E-06	1.04E-08	8.7556735	10.17249	3.6204684	SPARC	secreted protein acidic and cysteine rich
208851_s_at	1.54E-06	1.05E-08	8.7483518	10.158124	2.7073759	THY1	Thy-1 cell surface antigen
1556499_s_at	1.54E-06	1.05E-08	8.746995	10.155461	3.4945652	COL1A1	collagen type I alpha 1 chain
212724_at	1.54E-06	1.06E-08	8.7462417	10.153982	2.9004038	RND3	Rho family GTPase 3
205584_at	1.54E-06	1.06E-08	8.7449597	10.151465	1.4298589	ALG13	ALG13, UDP-N-acetylglucosaminyltransferase subunit

223121_s_at	1.55E-06	1.07E-08	8.7410593	10.143807	4.0162855	SFRP2	secreted frizzled related protein 2
200816_s_at	1.58E-06	1.09E-08	8.7300531	10.122187	1.406137	PAFAH1B1	platelet activating factor acetylhydrolase 1b regulatory subunit 1

211996_s_at	1.58E-06	1.10E-08	8.7274589	10.117088	2.278832	LOC101060275///NPIPA5///NPIPB5///NPIPB11///LOC613037///NPIPB4///NPIPB3	putative NPIP-like protein LOC613037///nuclear pore complex interacting protein family member A5///nuclear pore complex interacting protein family member B5///nuclear pore complex interacting protein family member B11///nuclear pore complex interacting protein member///nuclear pore complex interacting protein family member B4///nuclear pore complex interacting protein family member B3

218370_s_at	1.58E-06	1.11E-08	8.7203369	10.103085	1.6366743	S100PBP	S100P binding protein
211709_s_at	1.58E-06	1.11E-08	8.719557	10.101552	2.4330507	CLEC11A	C-type lectin domain family 11 member A
232231_at	1.61E-06	1.15E-08	8.7037286	10.070404	3.6707487	RUNX2	runt related transcription factor 2
203132_at	1.61E-06	1.15E-08	8.7031759	10.069316	1.9464629	RB1	RB transcriptional corepressor 1
225085_at	1.62E-06	1.16E-08	8.6980038	10.05913	1.4926779	USP40	ubiquitin specific peptidase 40

204049_s_at	1.62E-06	1.18E-08	8.692933	10.04914	1.8361188	PHACTR2	phosphatase and actin regulator 2
202369_s_at	1.62E-06	1.18E-08	8.6921818	10.047659	1.6073896	TRAM2	translocation associated membrane protein 2
219922_s_at	1.67E-06	1.23E-08	8.6721538	10.008164	2.0875871	LTBP3	latent transforming growth factor beta binding protein 3
208850_s_at	1.67E-06	1.25E-08	8.6640408	9.992149	3.5319196	THY1	Thy-1 cell surface antigen
212382_at	1.67E-06	1.25E-08	8.6637576	9.99159	2.1617443	TCF4	transcription factor 4

227395_at	1.67E-06	1.25E-08	8.6626496	9.989402	1.4341316	TBCEL	tubulin folding cofactor E like
218838_s_at	1.68E-06	1.26E-08	8.6579791	9.980177	1.0417475	TTC31	tetratricopeptide repeat domain 31
210561_s_at	1.68E-06	1.28E-08	8.6511097	9.966604	1.8781934	WSB1	WD repeat and SOCS box containing 1
217525_at	1.68E-06	1.28E-08	8.6501965	9.964799	2.2021625	OLFML1	olfactomedin like 1
201843_s_at	1.68E-06	1.29E-08	8.6457256	9.95596	2.4471921	EFEMP1	EGF containing fibulin like extracellular matrix protein 1

208306_x_at	1.68E-06	1.30E-08	8.6424883	9.949559	2.5498518	HLA-DRB1	major histocompatibility complex, class II, DR beta 1
204759_at	1.68E-06	1.31E-08	8.6410585	9.946731	1.8347891	RCBTB2	RCC1 and BTB domain containing protein 2
201935_s_at	1.68E-06	1.31E-08	8.6409776	9.946571	1.5093	EIF4G3	eukaryotic translation initiation factor 4 gamma 3
226164_x_at	1.69E-06	1.31E-08	8.6377061	9.9401	1.80464	RIMKLB	ribosomal modification protein rimK like family member B

225035_x_at	1.70E-06	1.34E-08	8.6279011	9.920695	1.3743146	LOC102723897///MIR6859-1///MIR6859-2///LOC101930154///LOC100288778///WASH1///WASH7P///WASH2P///WASH3P	WAS protein family homolog 2-like///microRNA 6859-1///microRNA 6859-2///WAS protein family homolog 6-like///WAS protein family homolog 1 pseudogene///WAS protein family homolog 1///WAS protein family homolog 7 pseudogene///WAS protein family homolog 2 pseudogene///WAS protein family homolog 3 pseudogene

213483_at	1.70E-06	1.35E-08	8.6252106	9.915368	1.6124821	PPWD1	peptidylprolyl isomerase domain and WD repeat containing 1
204464_s_at	1.70E-06	1.35E-08	8.6249301	9.914813	3.3575448	EDNRA	endothelin receptor type A
1552703_s_at	1.70E-06	1.35E-08	8.6244981	9.913958	2.8289086	CARD16///CASP1	caspase recruitment domain family member 16///caspase 1
233632_s_at	1.70E-06	1.35E-08	8.6229357	9.910863	1.3469334	XRN1	5'-3' exoribonuclease 1
215000_s_at	1.70E-06	1.36E-08	8.6209149	9.906861	1.7615959	FEZ2	fasciculation and elongation protein zeta 2

217317_s_at	1.70E-06	1.37E-08	8.6173825	9.899864	1.9716334	HERC2P9///HERC2P2	hect domain and RLD 2 pseudogene 9///hect domain and RLD 2 pseudogene 2
206115_at	1.70E-06	1.38E-08	8.6135593	9.892288	3.6955127	EGR3	early growth response 3
213293_s_at	1.71E-06	1.40E-08	8.6060685	9.877439	2.7332618	TRIM22	tripartite motif containing 22
212222_at	1.72E-06	1.41E-08	8.6034061	9.872159	1.2032652	PSME4	proteasome activator subunit 4

1555960_at	1.72E-06	1.41E-08	8.6020037	9.869378	1.7163246	HINT1	histidine triad nucleotide binding protein 1
200680_x_at	1.72E-06	1.42E-08	8.5988999	9.863221	1.1845536	HMGB1	high mobility group box 1
215287_at	1.74E-06	1.45E-08	8.5887165	9.843011	1.0880771	STRN	striatin
228397_at	1.75E-06	1.47E-08	8.5836572	9.832965	1.9580886	TUG1	taurine up-regulated 1 (non-protein coding)
240499_at	1.77E-06	1.51E-08	8.5704501	9.806723	1.9000782		

223197_s_at	1.77E-06	1.52E-08	8.5672108	9.800283	1.2448868	SMARCAD1	SWI/SNF-related, matrix-associated actin-dependent regulator of chromatin, subfamily a, containing DEAD/H box 1
209553_at	1.78E-06	1.53E-08	8.5617033	9.78933	1.2292327	VPS8	VPS8, CORVET complex subunit
203232_s_at	1.80E-06	1.56E-08	8.5544582	9.774915	1.722955	ATXN1	ataxin 1
203743_s_at	1.80E-06	1.56E-08	8.5542993	9.774599	2.0565495	TDG	thymine DNA glycosylase

213125_at	1.83E-06	1.61E-08	8.5387733	9.743682	3.5831832	OLFML2B	olfactomedin like 2B
40569_at	1.85E-06	1.63E-08	8.5333471	9.732868	1.0709334	MZF1	myeloid zinc finger 1
212899_at	1.85E-06	1.64E-08	8.5303707	9.726935	1.2536696	CDK19	cyclin dependent kinase 19
215388_s_at	1.85E-06	1.64E-08	8.5297721	9.725742	3.1373973	CFHR1///CFH	complement factor H related 1///complement factor H
213313_at	1.86E-06	1.65E-08	8.5251742	9.716574	1.050852	RABGAP1	RAB GTPase activating protein 1
220940_at	1.86E-06	1.66E-08	8.5239917	9.714215	2.2137121	ANKRD36B	ankyrin repeat domain 36B

225537_at	1.87E-06	1.67E-08	8.5205762	9.707402	1.2366743	TRAPPC6B	trafficking protein particle complex 6B
201041_s_at	1.87E-06	1.68E-08	8.5169746	9.700216	2.1513304	DUSP1	dual specificity phosphatase 1
214085_x_at	1.90E-06	1.72E-08	8.5064037	9.679114	2.3104325	GLIPR1	GLI pathogenesis related 1
225673_at	1.91E-06	1.73E-08	8.5023786	9.671074	2.0142202	MYADM	myeloid associated differentiation marker
1554168_a_at	1.92E-06	1.74E-08	8.4998769	9.666076	2.4114136	SH3KBP1	SH3 domain containing kinase binding protein 1

202270_at	1.93E-06	1.75E-08	8.4968928	9.660114	3.2740684	GBP1	guanylate binding protein 1
201694_s_at	1.94E-06	1.77E-08	8.4918595	9.650054	2.5372938	EGR1	early growth response 1
222127_s_at	1.94E-06	1.77E-08	8.4910647	9.648465	1.8684288	EXOC1	exocyst complex component 1
209780_at	1.96E-06	1.80E-08	8.4828622	9.632061	2.2612282	PHTF2	putative homeodomain transcription factor 2
231577_s_at	1.96E-06	1.81E-08	8.4823053	9.630947	3.5896504	GBP1	guanylate binding protein 1
201137_s_at	1.97E-06	1.82E-08	8.4785035	9.623341	2.3318348	HLA-DPB1	major histocompatibility complex, class II, DP beta 1

202465_at	1.98E-06	1.83E-08	8.4749574	9.616244	2.5793045	PCOLCE	procollagen C-endopeptidase enhancer
203567_s_at	2.00E-06	1.85E-08	8.4703083	9.606937	1.4997716	TRIM38	tripartite motif containing 38
202196_s_at	2.00E-06	1.85E-08	8.4691098	9.604538	2.8207855	DKK3	dickkopf WNT signaling pathway inhibitor 3
213243_at	2.01E-06	1.87E-08	8.46397	9.594244	1.449313	VPS13B	vacuolar protein sorting 13 homolog B
226237_at	2.01E-06	1.88E-08	8.4618531	9.590004	4.7713079	COL8A1	collagen type VIII alpha 1 chain
226203_at	2.01E-06	1.89E-08	8.4604906	9.587274	1.5908132	MYO9A	myosin IXA

211991_s_at	2.02E-06	1.91E-08	8.4547977	9.575865	3.1534925	HLA-DPA1	major histocompatibility complex, class II, DP alpha 1
225681_at	2.03E-06	1.93E-08	8.4494185	9.565081	5.1875159	CTHRC1	collagen triple helix repeat containing 1
212667_at	2.04E-06	1.95E-08	8.4446054	9.555429	2.8940602	SPARC	secreted protein acidic and cysteine rich
204319_s_at	2.04E-06	1.96E-08	8.4429544	9.552117	2.3879732	RGS10	regulator of G-protein signaling 10

227628_at	2.04E-06	1.96E-08	8.4420759	9.550355	1.880283	GPX8	glutathione peroxidase 8 (putative)
225361_x_at	2.04E-06	1.96E-08	8.4415106	9.54922	1.4171643	FAM122B	family with sequence similarity 122B
221833_at	2.05E-06	1.98E-08	8.4383179	9.542814	1.591212	LOC100507577///LONP2///SIAH1	uncharacterized LOC100507577///lon peptidase 2, peroxisomal///siah E3 ubiquitin protein ligase 1

213194_at	2.05E-06	1.98E-08	8.4374647	9.541102	2.8769312	ROBO1	roundabout guidance receptor 1
216526_x_at	2.07E-06	2.00E-08	8.431294	9.528716	1.4626429	HLA-C	major histocompatibility complex, class I, C
219078_at	2.07E-06	2.01E-08	8.4299322	9.525981	1.3567105	GPATCH2	G-patch domain containing 2
226676_at	2.08E-06	2.03E-08	8.42599	9.518065	2.3509848	ZNF521	zinc finger protein 521
200897_s_at	2.08E-06	2.03E-08	8.4253114	9.516702	2.790137	PALLD	palladin, cytoskeletal associated protein
224673_at	2.11E-06	2.06E-08	8.4175871	9.501182	1.218952	LENG8	leukocyte receptor cluster member 8

205803_s_at	2.11E-06	2.07E-08	8.4161421	9.498278	2.1501532	TRPC1	transient receptor potential cation channel subfamily C member 1
201296_s_at	2.11E-06	2.08E-08	8.4135237	9.493015	2.0728652	WSB1	WD repeat and SOCS box containing 1
203789_s_at	2.11E-06	2.08E-08	8.41258	9.491118	4.2743452	SEMA3C	semaphorin 3C
204872_at	2.11E-06	2.10E-08	8.4090681	9.484057	1.37501	TLE4	transducin like enhancer of split 4
1555832_s_at	2.11E-06	2.10E-08	8.4086206	9.483157	1.9478332	KLF6	Kruppel like factor 6
209795_at	2.11E-06	2.11E-08	8.4071045	9.480108	3.1997984	CD69	CD69 molecule

202642_s_at	2.11E-06	2.11E-08	8.4064828	9.478857	1.1170277	TRRAP	transformation/transcription domain associated protein
218683_at	2.11E-06	2.11E-08	8.4061661	9.47822	1.4772921	PTBP2	polypyrimidine tract binding protein 2
224606_at	2.13E-06	2.14E-08	8.3989984	9.4638	1.855737	KLF6	Kruppel like factor 6

210580_x_at	2.16E-06	2.19E-08	8.3879171	9.441492	1.4539246	SLX1B-SULT1A4///SLX1A-SULT1A3///SULT1A4///SULT1A3	SLX1B-SULT1A4 readthrough (NMD candidate)///SLX1A-SULT1A3 readthrough (NMD candidate)///sulfotransferase family 1A member 4///sulfotransferase family 1A member 3
213065_at	2.16E-06	2.20E-08	8.3859247	9.437479	1.7967886	ZFC3H1	zinc finger C3H1-type containing
226848_at	2.16E-06	2.21E-08	8.383468	9.43253	1.6038577		

1557049_at	2.17E-06	2.23E-08	8.3797026	9.424944	2.1775945	BTBD19	BTB domain containing 19
201012_at	2.18E-06	2.26E-08	8.373156	9.411749	3.7860636	ANXA1	annexin A1
219506_at	2.18E-06	2.27E-08	8.371684	9.408781	2.2981686	C1orf54	chromosome 1 open reading frame 54
241681_at	2.18E-06	2.29E-08	8.3669229	9.39918	2.3273393		
1556049_at	2.18E-06	2.29E-08	8.3668691	9.399071	1.4260286	RTN4	reticulon 4
216264_s_at	2.19E-06	2.31E-08	8.3631821	9.391634	1.7785479	LAMB2	laminin subunit beta 2
203903_s_at	2.19E-06	2.31E-08	8.362854	9.390972	3.3735573	HEPH	hephaestin
212918_at	2.20E-06	2.32E-08	8.3594154	9.384033	2.1792416	RECQL	RecQ like helicase
1568765_at	2.20E-06	2.34E-08	8.357099	9.379359	2.9773754	SERPINE1	serpin family E member 1
223513_at	2.20E-06	2.34E-08	8.3564192	9.377987	1.8493396	CENPJ	centromere protein J

203900_at	2.22E-06	2.39E-08	8.3453018	9.355538	1.0228948	SZT2	seizure threshold 2 homolog (mouse)
224591_at	2.23E-06	2.41E-08	8.3426815	9.350244	1.1408113	HP1BP3	heterochromatin protein 1 binding protein 3
227595_at	2.23E-06	2.41E-08	8.3417283	9.348319	1.2466545	ZMYM6	zinc finger MYM-type containing 6
235318_at	2.24E-06	2.44E-08	8.3359798	9.336701	2.7750227	FBN1	fibrillin 1
1564796_at	2.24E-06	2.46E-08	8.3321201	9.328898	2.9155905	EMP1	epithelial membrane protein 1
214736_s_at	2.24E-06	2.47E-08	8.3307869	9.326203	1.2824023	ADD1	adducin 1
226695_at	2.25E-06	2.48E-08	8.3288806	9.322348	2.8335555	PRRX1	paired related homeobox 1

226392_at	2.27E-06	2.52E-08	8.3201106	9.304606	2.2110677	RASA2	RAS p21 protein activator 2
227624_at	2.29E-06	2.57E-08	8.3115524	9.287282	1.6665559	TET2	tet methylcytosine dioxygenase 2
202499_s_at	2.30E-06	2.59E-08	8.3075769	9.279231	3.5660668	SLC2A3	solute carrier family 2 member 3
204051_s_at	2.31E-06	2.60E-08	8.3048491	9.273706	4.2392357	SFRP4	secreted frizzled related protein 4
226329_s_at	2.31E-06	2.61E-08	8.3037007	9.271379	2.173937	MITD1	microtubule interacting and trafficking domain containing 1

230000_at	2.31E-06	2.63E-08	8.2989104	9.261673	2.1417668	RNF213	ring finger protein 213
241435_at	2.31E-06	2.64E-08	8.2979338	9.259694	2.5397329		
213269_at	2.32E-06	2.65E-08	8.2964405	9.256667	1.0149982	ZNF248	zinc finger protein 248
226474_at	2.34E-06	2.69E-08	8.2886987	9.24097	2.0162039	NLRC5	NLR family CARD domain containing 5
204094_s_at	2.36E-06	2.75E-08	8.2777432	9.218742	1.6728002	TSC22D2	TSC22 domain family member 2
213994_s_at	2.37E-06	2.77E-08	8.2747222	9.21261	3.115313	SPON1	spondin 1
203603_s_at	2.37E-06	2.78E-08	8.2723626	9.20782	2.6593877	ZEB2	zinc finger E-box binding homeobox 2

229554_at	2.38E-06	2.82E-08	8.2660908	9.195082	3.5482943	LUM	lumican
228282_at	2.41E-06	2.87E-08	8.2576513	9.177934	1.4602943	MFSD8	major facilitator superfamily domain containing 8
224900_at	2.41E-06	2.88E-08	8.2556365	9.173838	1.1563314	ANKFY1	ankyrin repeat and FYVE domain containing 1
203325_s_at	2.41E-06	2.89E-08	8.2551472	9.172843	4.0726157	COL5A1	collagen type V alpha 1 chain
212177_at	2.44E-06	2.93E-08	8.2484457	9.159217	1.9583639	PNISR	PNN interacting serine and arginine rich protein

213931_at	2.45E-06	2.95E-08	8.2448113	9.151824	2.5612227	ID2B///ID2	inhibitor of DNA binding 2B, HLH protein (pseudogene)///inhibitor of DNA binding 2, HLH protein
201836_s_at	2.46E-06	2.98E-08	8.2395906	9.141201	1.1959814	SUPT7L	SPT7-like STAGA complex gamma subunit
212489_at	2.47E-06	3.01E-08	8.2354226	9.132718	4.4764364	COL5A1	collagen type V alpha 1 chain

228628_at	2.49E-06	3.04E-08	8.2307277	9.123158	2.0063518	SRGAP2C	SLIT-ROBO Rho GTPase activating protein 2C
215016_x_at	2.49E-06	3.07E-08	8.2256712	9.112859	1.5067596	DST	dystonin
228964_at	2.52E-06	3.15E-08	8.2138226	9.088712	3.0381125	PRDM1	PR/SET domain 1
211368_s_at	2.52E-06	3.15E-08	8.2130706	9.087179	2.6154854	CASP1	caspase 1
228837_at	2.52E-06	3.15E-08	8.2128583	9.086746	1.6961032	TCF4	transcription factor 4
204345_at	2.52E-06	3.17E-08	8.2100434	9.081006	1.9769213	COL16A1	collagen type XVI alpha 1 chain
212847_at	2.52E-06	3.17E-08	8.209978	9.080873	1.6137868	FUBP1	far upstream element binding protein 1
244414_at	2.54E-06	3.20E-08	8.204803	9.070317	2.2570546		

1552701_a_at	2.54E-06	3.21E-08	8.2044824	9.069663	2.069917	CARD16	caspase recruitment domain family member 16
202820_at	2.54E-06	3.22E-08	8.2026739	9.065974	1.9976373	AHR	aryl hydrocarbon receptor
219700_at	2.54E-06	3.28E-08	8.1942634	9.048808	2.7531388	PLXDC1	plexin domain containing 1
202628_s_at	2.54E-06	3.28E-08	8.1932036	9.046644	3.7111646	SERPINE1	serpin family E member 1
233480_at	2.54E-06	3.28E-08	8.1931772	9.04659	1.9842607	TMEM43	transmembrane protein 43
203024_s_at	2.54E-06	3.28E-08	8.1930176	9.046264	1.5007334	C5orf15	chromosome 5 open reading frame 15

238025_at	2.54E-06	3.29E-08	8.1917927	9.043763	1.30942	MLKL	mixed lineage kinase domain like
226251_at	2.54E-06	3.30E-08	8.1909403	9.042022	1.2667764	ASXL2	additional sex combs like 2, transcriptional regulator
220617_s_at	2.56E-06	3.32E-08	8.1873214	9.034631	1.9124702	ZNF532	zinc finger protein 532
222266_at	2.56E-06	3.34E-08	8.1849072	9.0297	1.3484711		
37005_at	2.56E-06	3.35E-08	8.1842219	9.0283	2.3856282	MINOS1-NBL1///NBL1	MINOS1-NBL1 readthrough///neuroblastoma 1, DAN family BMP antagonist

225639_at	2.56E-06	3.36E-08	8.1822137	9.024196	2.64336	SKAP2	src kinase associated phosphoprotein 2
206854_s_at	2.57E-06	3.39E-08	8.1782188	9.016032	1.3482882	MAP3K7	mitogen-activated protein kinase kinase kinase 7
212354_at	2.57E-06	3.39E-08	8.1773611	9.014279	5.0389423	SULF1	sulfatase 1
229310_at	2.57E-06	3.40E-08	8.1766784	9.012884	1.8200105	KLHL29	kelch like family member 29
209932_s_at	2.59E-06	3.46E-08	8.1678989	8.994932	1.1205802	DUT	deoxyuridine triphosphatase
222146_s_at	2.60E-06	3.49E-08	8.1635771	8.986091	2.2277305	TCF4	transcription factor 4

202656_s_at	2.61E-06	3.51E-08	8.1616501	8.982148	1.2470341	SERTAD2	SERTA domain containing 2
238534_at	2.63E-06	3.56E-08	8.1542875	8.967078	2.0363911	LRRFIP1	LRR binding FLII interacting protein 1
225913_at	2.65E-06	3.62E-08	8.1466606	8.951459	1.4194345	PEAK1	pseudopodium enriched atypical kinase 1
213800_at	2.65E-06	3.63E-08	8.1458804	8.949861	2.3955505	CFH	complement factor H
232597_x_at	2.65E-06	3.65E-08	8.1425786	8.943096	1.5957384	SCAF11	SR-related CTD associated factor 11

1555889_a_at	2.65E-06	3.65E-08	8.141981	8.941872	1.0842679	CRTAP	cartilage associated protein

233929_x_at	2.67E-06	3.70E-08	8.1364448	8.930526	1.2592945	MIR6859-1///MIR6859-2///LOC101930154///LOC100288778///WASH1///WASH7P///WASH2P///WASH3P	microRNA 6859-1///microRNA 6859-2///WAS protein family homolog 6-like///WAS protein family homolog 1 pseudogene///WAS protein family homolog 1///WAS protein family homolog 7 pseudogene///WAS protein family homolog 2 pseudogene///WAS protein family homolog 3 pseudogene

208634_s_at	2.68E-06	3.72E-08	8.1333938	8.924271	1.6511307	MACF1	microtubule-actin crosslinking factor 1
219073_s_at	2.68E-06	3.72E-08	8.1331085	8.923686	2.7948045	OSBPL10	oxysterol binding protein like 10
218396_at	2.68E-06	3.73E-08	8.1325952	8.922633	1.9094663	VPS13C	vacuolar protein sorting 13 homolog C
228722_at	2.68E-06	3.74E-08	8.1303877	8.918107	1.0911525	PRMT2	protein arginine methyltransferase 2
227947_at	2.68E-06	3.75E-08	8.1290889	8.915443	2.106268	PHACTR2	phosphatase and actin regulator 2

229694_at	2.68E-06	3.79E-08	8.1243131	8.905647	1.4962395	WDR11	WD repeat domain 11
218854_at	2.68E-06	3.81E-08	8.1215885	8.900056	2.9147591	DSE	dermatan sulfate epimerase
218456_at	2.71E-06	3.88E-08	8.1140562	8.884596	2.2522691	CAPRIN2	caprin family member 2
209115_at	2.71E-06	3.90E-08	8.1109824	8.878285	1.6584766	UBA3	ubiquitin like modifier activating enzyme 3

209412_at	2.73E-06	3.95E-08	8.1055045	8.867033	1.38170	LOC102724200///TRAPPC10	trafficking protein particle complex subunit 10-like///trafficking protein particle complex 10
218249_at	2.74E-06	3.97E-08	8.102533	8.860929	1.3533832	ZDHHC6	zinc finger DHHC-type containing 6
235072_s_at	2.74E-06	3.97E-08	8.1022238	8.860293	1.1660009	KIF13A	kinesin family member 13A
212820_at	2.75E-06	4.00E-08	8.0988745	8.85341	2.5732073	DMXL2	Dmx like 2
212254_s_at	2.76E-06	4.02E-08	8.0970895	8.849742	1.4121789	DST	dystonin

201938_at	2.77E-06	4.04E-08	8.0936929	8.842759	1.2528232	CDK2AP1	cyclin dependent kinase 2 associated protein 1
225275_at	2.78E-06	4.07E-08	8.09106	8.837345	3.0836384	EDIL3	EGF like repeats and discoidin domains 3
224817_at	2.78E-06	4.07E-08	8.0904601	8.836112	1.9753388	SH3PXD2A	SH3 and PX domains 2A

207856_s_at	2.78E-06	4.10E-08	8.0874453	8.829911	1.1249195	LOC150776///SMPD4	sphingomyelin phosphodiesterase 4, neutral membrane (neutral sphingomyelinase-3) pseudogene///sphingomyelin phosphodiesterase 4
227861_at	2.78E-06	4.10E-08	8.0873778	8.829772	1.3245987	TMEM161B	transmembrane protein 161B
221774_x_at	2.78E-06	4.11E-08	8.0864029	8.827767	1.0716836	SUPT20H	SPT20 homolog, SAGA complex component

217196_s_at	2.78E-06	4.12E-08	8.0848634	8.8246	1.8921664	CAMSAP2	calmodulin regulated spectrin associated protein family member 2
236251_at	2.80E-06	4.16E-08	8.0804963	8.815615	1.8162129		
225123_at	2.80E-06	4.16E-08	8.0802391	8.815085	1.8415721	SESN3	sestrin 3
209006_s_at	2.86E-06	4.33E-08	8.0616032	8.776709	2.3629536	RSRP1	arginine and serine rich protein 1
235421_at	2.86E-06	4.34E-08	8.0604528	8.774339	2.4365307	MAP3K8	mitogen-activated protein kinase kinase kinase 8
201655_s_at	2.87E-06	4.37E-08	8.0567269	8.76666	2.0490307	HSPG2	heparan sulfate proteoglycan 2

204472_at	2.87E-06	4.38E-08	8.055734	8.764613	3.3700168	GEM	GTP binding protein overexpressed in skeletal muscle
203518_at	2.88E-06	4.42E-08	8.0517483	8.756396	1.7954271	LYST	lysosomal trafficking regulator
230387_at	2.88E-06	4.44E-08	8.0488979	8.750518	2.2419018		
209341_s_at	2.88E-06	4.45E-08	8.0484373	8.749568	1.6873936	IKBKB	inhibitor of kappa light polypeptide gene enhancer in B-cells, kinase beta
212353_at	2.89E-06	4.49E-08	8.0443941	8.741228	5.3117462	SULF1	sulfatase 1
202269_x_at	2.90E-06	4.53E-08	8.039684	8.731509	3.2955534	GBP1	guanylate binding protein 1

1555630_a_at	2.91E-06	4.54E-08	8.0384619	8.728987	2.5383539	NARR///RAB34	nine-amino acid residue-repeats///RAB34, member RAS oncogene family
225021_at	2.93E-06	4.61E-08	8.0320537	8.715758	2.423737	ZNF532	zinc finger protein 532
234995_at	2.94E-06	4.62E-08	8.0305805	8.712716	1.5791588	SPICE1	spindle and centriole associated protein 1
222872_x_at	2.94E-06	4.65E-08	8.0277554	8.706882	2.6541836	NABP1	nucleic acid binding protein 1
221911_at	2.94E-06	4.67E-08	8.0256539	8.702541	2.3802818	ETV1	ETS variant 1

208882_s_at	2.94E-06	4.70E-08	8.0228836	8.696818	1.5908132	UBR5	ubiquitin protein ligase E3 component n-recognin 5
228640_at	2.94E-06	4.70E-08	8.0223598	8.695735	3.8660296	PCDH7	protocadherin 7
225646_at	2.94E-06	4.71E-08	8.0218094	8.694598	2.2717471	CTSC	cathepsin C
226875_at	2.96E-06	4.75E-08	8.0178689	8.686455	2.5500905	DOCK11	dedicator of cytokinesis 11

209312_x_at	2.96E-06	4.75E-08	8.0173312	8.685344	2.6476305	LOC101060835///LOC100996809///HLA-DRB5///HLA-DRB4///HLA-DRB1///HLA-DQB1	HLA class II histocompatibility antigen, DQ beta 1 chain-like///HLA class II histocompatibility antigen, DRB1-10 beta chain-like///major histocompatibility complex, class II, DR beta 5///major histocompatibility complex, class II, DR beta 4///major histocompatibility complex, class II, DR beta 1///major histocompatibility complex, class II, DQ beta 1

211675_s_at	2.96E-06	4.76E-08	8.0163111	8.683235	1.7420716	MDFIC	MyoD family inhibitor domain containing
240307_at	2.96E-06	4.78E-08	8.0142573	8.678989	1.6117057		
225524_at	2.96E-06	4.79E-08	8.0139022	8.678255	2.4591929	ANTXR2	anthrax toxin receptor 2
219165_at	2.96E-06	4.81E-08	8.0111104	8.672483	1.9113713	PDLIM2	PDZ and LIM domain 2
204176_at	2.97E-06	4.83E-08	8.0090095	8.668139	1.5960377	KLHL20	kelch like family member 20
221989_at	2.97E-06	4.84E-08	8.0085309	8.667149	1.9221452	SNORA70///RPL10	small nucleolar RNA, H/ACA box 70///ribosomal protein L10

209682_at	2.97E-06	4.85E-08	8.0073636	8.664734	1.9366429	CBLB	Cbl proto-oncogene B
215127_s_at	2.97E-06	4.86E-08	8.0068369	8.663645	1.9579821	RBMS1	RNA binding motif single stranded interacting protein 1
212895_s_at	2.98E-06	4.91E-08	8.0020417	8.653725	1.3234841	ABR	active BCR-related
224778_s_at	2.98E-06	4.92E-08	8.0006448	8.650835	1.4318318	TAOK1	TAO kinase 1
225077_at	2.98E-06	4.93E-08	7.9995242	8.648516	1.2368455	CHD2	chromodomain helicase DNA binding protein 2

209084_s_at	2.98E-06	4.93E-08	7.9994536	8.64837	1.3459888	RAB28	RAB28, member RAS oncogene family
212599_at	3.03E-06	5.04E-08	7.9892118	8.627167	2.0530213	AUTS2	autism susceptibility candidate 2
201987_at	3.04E-06	5.07E-08	7.9863158	8.621169	1.029962	MED13	mediator complex subunit 13
225045_at	3.04E-06	5.09E-08	7.984804	8.618038	2.2277888	CCDC88A	coiled-coil domain containing 88A

211656_x_at	3.04E-06	5.12E-08	7.9814747	8.61114	2.6261366	LOC101060835///HLA-DQB1	HLA class II histocompatibility antigen, DQ beta 1 chain-like///major histocompatibility complex, class II, DQ beta 1

215629_s_at	3.04E-06	5.13E-08	7.9808922	8.609933	1.4844768	MIR15A///DLEU2L///DLEU2	microRNA 15a///deleted in lymphocytic leukemia 2-like///deleted in lymphocytic leukemia 2 (non-protein coding)
229218_at	3.04E-06	5.15E-08	7.9794883	8.607024	3.6445152	COL1A2	collagen type I alpha 2 chain
201325_s_at	3.04E-06	5.16E-08	7.9786051	8.605194	2.4274943	EMP1	epithelial membrane protein 1

225461_at	3.06E-06	5.21E-08	7.9735784	8.594774	1.2363521	EHMT1	euchromatic histone lysine methyltransferase 1
218656_s_at	3.07E-06	5.27E-08	7.9683275	8.583886	1.795123	LHFP	lipoma HMGIC fusion partner
202202_s_at	3.07E-06	5.27E-08	7.9678683	8.582934	3.4489655	LAMA4	laminin subunit alpha 4
37408_at	3.07E-06	5.29E-08	7.9662268	8.57953	2.1001363	MRC2	mannose receptor C type 2
225364_at	3.09E-06	5.32E-08	7.9637058	8.5743	1.3289484	STK4	serine/threonine kinase 4
225921_at	3.11E-06	5.37E-08	7.9591412	8.564829	1.8047925	NIN	ninein
221011_s_at	3.11E-06	5.41E-08	7.9561512	8.558623	2.5653295	LBH	limb bud and heart development

226743_at	3.14E-06	5.50E-08	7.9479938	8.541687	1.6236661	SLFN11	schlafen family member 11
227061_at	3.14E-06	5.50E-08	7.9477659	8.541214	2.994443	LINC01279	long intergenic non-protein coding RNA 1279
232098_at	3.15E-06	5.54E-08	7.9447429	8.534935	2.162598	DST	dystonin
239014_at	3.15E-06	5.54E-08	7.9446942	8.534833	1.4654191	CCAR1	cell division cycle and apoptosis regulator 1
226831_at	3.15E-06	5.56E-08	7.9427551	8.530805	1.2697695	SLC25A46	solute carrier family 25 member 46
233314_at	3.15E-06	5.57E-08	7.942336	8.529935	1.6585493		

203688_at	3.16E-06	5.58E-08	7.9415377	8.528276	1.450078	PKD2	polycystin 2, transient receptor potential cation channel
209285_s_at	3.16E-06	5.61E-08	7.9390206	8.523046	1.5308964	FAM208A	family with sequence similarity 208 member A
205422_s_at	3.16E-06	5.61E-08	7.9386942	8.522367	3.5586421	ITGBL1	integrin subunit beta like 1
201664_at	3.18E-06	5.65E-08	7.935074	8.514843	2.691747	SMC4	structural maintenance of chromosomes 4
201141_at	3.18E-06	5.66E-08	7.9345417	8.513737	3.2287305	GPNMB	glycoprotein nmb

212511_at	3.20E-06	5.70E-08	7.9309048	8.506176	1.7831513	PICALM	phosphatidylinositol binding clathrin assembly protein
226208_at	3.20E-06	5.75E-08	7.9271671	8.498403	1.3981359	ZSWIM6	zinc finger SWIM-type containing 6
232207_at	3.20E-06	5.75E-08	7.927008	8.498072	1.0318489	LOC100653061///GUSBP4	putative inactive beta-glucuronidase-like protein SMA3///glucuronidase, beta pseudogene 4

221698_s_at	3.21E-06	5.77E-08	7.9257963	8.495552	2.515273	CLEC7A	C-type lectin domain family 7 member A
225886_at	3.21E-06	5.78E-08	7.9246042	8.493072	1.3904032	DDX5	DEAD-box helicase 5
221778_at	3.21E-06	5.79E-08	7.9235438	8.490867	1.2539682	KDM7A	lysine demethylase 7A
242467_at	3.21E-06	5.80E-08	7.9230618	8.489864	1.8435627		
219371_s_at	3.29E-06	6.00E-08	7.9067805	8.455974	2.3356196	KLF2	Kruppel like factor 2
200033_at	3.29E-06	6.02E-08	7.9052078	8.452698	1.8469327	MIR5047///MIR3064///DDX5	microRNA 5047///microRNA 3064///DEAD-box helicase 5

225758_s_at	3.29E-06	6.04E-08	7.9044219	8.451061	1.0418571	TUBGCP6	tubulin gamma complex associated protein 6
203706_s_at	3.29E-06	6.04E-08	7.9041095	8.450411	2.5915784	FZD7	frizzled class receptor 7
204963_at	3.30E-06	6.07E-08	7.9019107	8.44583	1.6610204	SSPN	sarcospan
227276_at	3.30E-06	6.10E-08	7.899591	8.440997	2.7398436	PLXDC2	plexin domain containing 2
227040_at	3.32E-06	6.13E-08	7.8967239	8.435022	2.1595416	NHLRC3	NHL repeat containing 3
215111_s_at	3.32E-06	6.14E-08	7.8965215	8.4346	1.7521625	TSC22D1	TSC22 domain family member 1
213883_s_at	3.33E-06	6.18E-08	7.8932308	8.427741	1.2290371	TM2D1	TM2 domain containing 1

224560_at	3.33E-06	6.22E-08	7.8902108	8.421445	2.2077486	TIMP2	TIMP metallopeptidase inhibitor 2
226103_at	3.33E-06	6.24E-08	7.8886512	8.418193	2.0347782	NEXN	nexilin F-actin binding protein
204881_s_at	3.33E-06	6.25E-08	7.8877033	8.416216	1.6668725	UGCG	UDP-glucose ceramide glucosyltransferase
215446_s_at	3.33E-06	6.26E-08	7.8873264	8.41543	3.4415623	LOX	lysyl oxidase
205976_at	3.33E-06	6.27E-08	7.8866439	8.414007	1.0719545	FASTKD2	FAST kinase domains 2
242907_at	3.33E-06	6.28E-08	7.8856708	8.411977	2.4575766	GBP2	guanylate binding protein 2
203156_at	3.35E-06	6.31E-08	7.8834244	8.407292	1.3765032	AKAP11	A-kinase anchoring protein 11

201431_s_at	3.35E-06	6.32E-08	7.8828713	8.406138	2.5968473	DPYSL3	dihydropyrimidinase like 3
204663_at	3.36E-06	6.39E-08	7.8778052	8.395568	1.1909305	ME3	malic enzyme 3
208611_s_at	3.40E-06	6.49E-08	7.8706316	8.380594	1.1842914	SPTAN1	spectrin alpha, non-erythrocytic 1
230389_at	3.41E-06	6.53E-08	7.867112	8.373245	1.5658146	FNBP1	formin binding protein 1
226294_x_at	3.41E-06	6.54E-08	7.8668115	8.372617	1.3917273	FAM91A1	family with sequence similarity 91 member A1
217728_at	3.41E-06	6.58E-08	7.863928	8.366595	2.9200175	S100A6	S100 calcium binding protein A6
201261_x_at	3.41E-06	6.59E-08	7.8632121	8.3651	2.8287811	BGN	biglycan

214927_at	3.41E-06	6.63E-08	7.8605303	8.359497	3.2088277	ITGBL1	integrin subunit beta like 1
212447_at	3.43E-06	6.66E-08	7.8579847	8.354178	1.3901339	KBTBD2	kelch repeat and BTB domain containing 2
213376_at	3.44E-06	6.73E-08	7.8535534	8.344917	1.6863463	ZBTB1	zinc finger and BTB domain containing 1
243874_at	3.45E-06	6.79E-08	7.8489715	8.335338	2.0132114		
207081_s_at	3.45E-06	6.79E-08	7.84897	8.335335	1.7581988	PI4KA	phosphatidylinositol 4-kinase alpha
236079_at	3.46E-06	6.83E-08	7.8462746	8.329699	1.3402802	LOC202025	uncharacterized LOC202025

238327_at	3.46E-06	6.84E-08	7.8456872	8.32847	2.539382	ODF3B	outer dense fiber of sperm tails 3B
201798_s_at	3.46E-06	6.85E-08	7.8448518	8.326723	3.0976388	MYOF	myoferlin
219372_at	3.46E-06	6.89E-08	7.8422553	8.321292	1.4021113	IFT81	intraflagellar transport 81
226040_at	3.46E-06	6.89E-08	7.8421125	8.320993	1.2898646	TRIM56	tripartite motif containing 56
224791_at	3.46E-06	6.90E-08	7.8416226	8.319968	2.158317	ASAP1	ArfGAP with SH3 domain, ankyrin repeat and PH domain 1
218397_at	3.54E-06	7.12E-08	7.8271885	8.289758	1.6630123	FANCL	Fanconi anemia complementation group L

222673_x_at	3.54E-06	7.14E-08	7.8257711	8.28679	1.2327341	FAM122B	family with sequence similarity 122B
227111_at	3.59E-06	7.24E-08	7.8189253	8.27245	1.0212188	ZBTB34	zinc finger and BTB domain containing 34
201899_s_at	3.60E-06	7.28E-08	7.8163863	8.267129	1.0348382	UBE2A	ubiquitin conjugating enzyme E2 A
225842_at	3.60E-06	7.29E-08	7.8158667	8.266041	2.1989338	PHLDA1	pleckstrin homology like domain family A member 1
209156_s_at	3.60E-06	7.30E-08	7.8150646	8.26436	3.0110911	COL6A2	collagen type VI alpha 2 chain

225147_at	3.61E-06	7.36E-08	7.8115278	8.256947	1.2513663	CYTH3	cytohesin 3
227566_at	3.61E-06	7.36E-08	7.81133	8.256532	4.0181595	LOC102725271///NTM	neurotrimin-like///neurotrimin
212785_s_at	3.61E-06	7.37E-08	7.8107775	8.255374	1.3271086	LARP7	La ribonucleoprotein domain family member 7
218962_s_at	3.61E-06	7.37E-08	7.8107574	8.255331	1.2273129	TMEM168	transmembrane protein 168
209348_s_at	3.61E-06	7.37E-08	7.8107399	8.255295	2.101618	MAF	MAF bZIP transcription factor
208782_at	3.62E-06	7.39E-08	7.8094345	8.252558	2.4026602	FSTL1	follistatin like 1
236297_at	3.63E-06	7.43E-08	7.80706	8.247579	2.9903973	PLXDC2	plexin domain containing 2

201450_s_at	3.63E-06	7.43E-08	7.807018	8.247491	1.8033007	TIA1	TIA1 cytotoxic granule-associated RNA binding protein
212622_at	3.63E-06	7.46E-08	7.8048657	8.242978	1.1297289	TMEM41B	transmembrane protein 41B
203241_at	3.63E-06	7.50E-08	7.8028862	8.238826	1.0887334	UVRAG	UV radiation resistance associated
218247_s_at	3.63E-06	7.50E-08	7.8025892	8.238203	1.1846382	MEX3C	mex-3 RNA binding family member C

215193_x_at	3.63E-06	7.51E-08	7.8020484	8.237069	2.8043129	LOC101060835///LOC100996809///HLA-DRB4///HLA-DRB3///HLA-DRB1///HLA-DQB1	HLA class II histocompatibility antigen, DQ beta 1 chain-like///HLA class II histocompatibility antigen, DRB1-10 beta chain-like///major histocompatibility complex, class II, DR beta 4///major histocompatibility complex, class II, DR beta 3///major histocompatibility complex, class II, DR beta 1///major histocompatibility complex, class II, DQ beta 1

208679_s_at	3.63E-06	7.51E-08	7.8018172	8.236584	1.6974714	ARPC2	actin related protein 2/3 complex subunit 2
231579_s_at	3.64E-06	7.57E-08	7.7979659	8.228504	2.1167527	TIMP2	TIMP metallopeptidase inhibitor 2
227930_at	3.64E-06	7.57E-08	7.79793	8.228429	1.3022016	AGO4	argonaute 4, RISC catalytic component
236179_at	3.66E-06	7.63E-08	7.7946443	8.221534	3.0590313	CDH11	cadherin 11
205269_at	3.67E-06	7.68E-08	7.7913672	8.214656	2.697222	LCP2	lymphocyte cytosolic protein 2
227485_at	3.67E-06	7.69E-08	7.790716	8.213289	1.3938386	INTS6L	integrator complex subunit 6 like

213261_at	3.68E-06	7.70E-08	7.790014	8.211815	1.2722409	TRANK1	tetratricopeptide repeat and ankyrin repeat containing 1
1556126_s_at	3.68E-06	7.72E-08	7.788888	8.209451	1.2226414		
217478_s_at	3.68E-06	7.72E-08	7.7888669	8.209407	2.5058036	HLA-DMA	major histocompatibility complex, class II, DM alpha
230071_at	3.72E-06	7.86E-08	7.7806019	8.19205	1.8067	SEPT11	septin 11
232568_at	3.73E-06	7.90E-08	7.7782723	8.187156	2.7249373	MGC24103	uncharacterized MGC24103
202066_at	3.73E-06	7.91E-08	7.7778061	8.186176	1.1998512	PPFIA1	PTPRF interacting protein alpha 1

236600_at	3.75E-06	7.98E-08	7.7735412	8.177214	1.1505073	SPG20	spastic paraplegia 20 (Troyer syndrome)
206133_at	3.76E-06	8.02E-08	7.7713158	8.172537	2.9713764	XAF1	XIAP associated factor 1
226917_s_at	3.78E-06	8.08E-08	7.7679101	8.165378	1.3854241	ANAPC4	anaphase promoting complex subunit 4
235925_at	3.78E-06	8.09E-08	7.7673876	8.164279	1.6116877		
204820_s_at	3.78E-06	8.10E-08	7.7666713	8.162773	2.0961588	BTN3A2///BTN3A3	butyrophilin subfamily 3 member A2///butyrophilin subfamily 3 member A3

223204_at	3.78E-06	8.10E-08	7.7666364	8.1627	2.2795018	FAM198B	family with sequence similarity 198 member B
218579_s_at	3.78E-06	8.12E-08	7.7653211	8.159934	1.3106552	DHX35	DEAH-box helicase 35
232304_at	3.78E-06	8.14E-08	7.7644838	8.158173	2.7208646	PELI1	pellino E3 ubiquitin protein ligase 1
243303_at	3.78E-06	8.15E-08	7.7639121	8.156971	1.178292		
235811_at	3.78E-06	8.19E-08	7.7616259	8.152163	1.2909811		
221517_s_at	3.78E-06	8.20E-08	7.7611852	8.151236	1.7342398	MED17	mediator complex subunit 17
216199_s_at	3.79E-06	8.25E-08	7.7583339	8.145238	1.0785455	MAP3K4	mitogen-activated protein kinase kinase kinase 4

210982_s_at	3.81E-06	8.37E-08	7.7516068	8.131082	3.0545405	HLA-DRA	major histocompatibility complex, class II, DR alpha
225302_at	3.82E-06	8.39E-08	7.7504788	8.128708	1.6356271	TMX3	thioredoxin related transmembrane protein 3
237006_at	3.82E-06	8.43E-08	7.7480708	8.123638	1.5754389		
201828_x_at	3.82E-06	8.44E-08	7.7474364	8.122303	1.2152659	FAM127A	family with sequence similarity 127 member A
226877_at	3.83E-06	8.49E-08	7.7449041	8.116971	1.1847202	RPL32P3	ribosomal protein L32 pseudogene 3

203960_s_at	3.85E-06	8.57E-08	7.7403938	8.107472	1.6363132	HSPB11	heat shock protein family B (small) member 11
212414_s_at	3.86E-06	8.61E-08	7.7383343	8.103134	2.2387073	GLYR1///SEPT6	glyoxylate reductase 1 homolog///septin 6
209140_x_at	3.90E-06	8.71E-08	7.7326939	8.091249	1.621025	HLA-B	major histocompatibility complex, class I, B
209662_at	3.91E-06	8.78E-08	7.7292245	8.083937	1.3560543	CETN3	centrin 3
202951_at	3.91E-06	8.80E-08	7.7283326	8.082056	1.9951793	STK38	serine/threonine kinase 38
214807_at	3.91E-06	8.82E-08	7.7271541	8.079572	2.2266968	PLXDC2	plexin domain containing 2
222303_at	3.91E-06	8.82E-08	7.7270463	8.079345	2.7869788		

223082_at	3.91E-06	8.82E-08	7.7268679	8.078969	1.6923071	SH3KBP1	SH3 domain containing kinase binding protein 1
214791_at	3.92E-06	9.00E-08	7.7178188	8.059885	1.677942	SP140L	SP140 nuclear body protein like
213552_at	3.92E-06	9.01E-08	7.7169464	8.058044	1.4242961	GLCE	glucuronic acid epimerase
212163_at	3.92E-06	9.02E-08	7.7164625	8.057024	1.4820202	KIDINS220	kinase D-interacting substrate 220kDa
229011_at	3.96E-06	9.21E-08	7.7071259	8.037319	2.1747925	EMP1	epithelial membrane protein 1
232797_at	3.97E-06	9.23E-08	7.7057216	8.034355	2.117298		

218589_at	3.97E-06	9.25E-08	7.7048933	8.032606	2.1652782	LPAR6	lysophosphatidic acid receptor 6
218729_at	3.97E-06	9.31E-08	7.7019338	8.026357	2.1085686	LXN	latexin
226290_at	3.97E-06	9.31E-08	7.7017931	8.026059	1.8576723	BDP1	B double prime 1, subunit of RNA polymerase III transcription initiation factor IIIB
201584_s_at	3.98E-06	9.36E-08	7.6995954	8.021418	1.678363	DDX39A	DEAD-box helicase 39A
215235_at	3.98E-06	9.37E-08	7.6988119	8.019763	1.2340246	SPTAN1	spectrin alpha, non-erythrocytic 1
213993_at	3.99E-06	9.40E-08	7.6974656	8.016919	2.7440977	SPON1	spondin 1

1556818_at	3.99E-06	9.43E-08	7.6959763	8.013773	1.4409886		
203886_s_at	3.99E-06	9.44E-08	7.6954163	8.01259	2.0214079	FBLN2	fibulin 2
215691_x_at	3.99E-06	9.46E-08	7.6942836	8.010197	1.4259064	HSPB11	heat shock protein family B (small) member 11
228754_at	3.99E-06	9.53E-08	7.6909569	8.003167	3.6663154	SLC6A6	solute carrier family 6 member 6
217886_at	3.99E-06	9.54E-08	7.6907304	8.002688	1.7468579	EPS15	epidermal growth factor receptor pathway substrate 15
200907_s_at	3.99E-06	9.54E-08	7.6906454	8.002508	2.5866755	PALLD	palladin, cytoskeletal associated protein

203935_at	3.99E-06	9.56E-08	7.689615	8.000331	1.7276205	ACVR1	activin A receptor type 1
200964_at	3.99E-06	9.57E-08	7.6890963	7.999234	1.1912407	UBA1	ubiquitin like modifier activating enzyme 1
221751_at	4.00E-06	9.61E-08	7.6869827	7.994767	1.3948068	PANK3	pantothenate kinase 3
208965_s_at	4.00E-06	9.62E-08	7.6865108	7.993769	2.8032602	IFI16	interferon gamma inducible protein 16
204048_s_at	4.00E-06	9.63E-08	7.6859948	7.992678	1.7422746	PHACTR2	phosphatase and actin regulator 2
208729_x_at	4.01E-06	9.70E-08	7.6830979	7.986553	1.809675	HLA-B	major histocompatibility complex, class I, B

218706_s_at	4.03E-06	9.77E-08	7.6793285	7.978582	1.6986202	GRAMD3	GRAM domain containing 3
213331_s_at	4.05E-06	9.88E-08	7.6743484	7.968047	1.5455604	NEK1	NIMA related kinase 1
230872_s_at	4.05E-06	9.92E-08	7.6725858	7.964318	1.1956698	ARPC4-TTLL3///TTLL3///ARPC4	ARPC4-TTLL3 readthrough///tubulin tyrosine ligase like 3///actin related protein 2/3 complex subunit 4

202760_s_at	4.05E-06	9.93E-08	7.671929	7.962928	2.1872145	PALM2-AKAP2///AKAP2	PALM2-AKAP2 readthrough///A-kinase anchoring protein 2
203047_at	4.05E-06	9.97E-08	7.6700474	7.958946	1.3687591	STK10	serine/threonine kinase 10
226136_at	4.05E-06	9.98E-08	7.669731	7.958277	2.107857	GLIPR1	GLI pathogenesis related 1
201965_s_at	4.06E-06	1.00E-07	7.6678764	7.954351	1.2024046	SETX	senataxin
201837_s_at	4.07E-06	1.01E-07	7.6657383	7.949825	1.0762325	SUPT7L	SPT7-like STAGA complex gamma subunit
203821_at	4.09E-06	1.01E-07	7.6628185	7.943644	2.8927059	HBEGF	heparin binding EGF like growth factor

219698_s_at	4.11E-06	1.02E-07	7.6593689	7.936339	1.1297159	METTL4	methyltransferase like 4
222453_at	4.11E-06	1.02E-07	7.6590775	7.935721	2.0448455	CYBRD1	cytochrome b reductase 1
212401_s_at	4.14E-06	1.03E-07	7.6535285	7.923967	1.218483	CDK11A///CDK11B	cyclin dependent kinase 11A///cyclin dependent kinase 11B
239277_at	4.16E-06	1.04E-07	7.6508636	7.918321	1.0650121		
239251_at	4.16E-06	1.04E-07	7.6500364	7.916568	1.6876739		
232213_at	4.18E-06	1.05E-07	7.64651	7.909094	1.9271575	PELI1	pellino E3 ubiquitin protein ligase 1

208789_at	4.20E-06	1.06E-07	7.6435886	7.902901	1.9941507	PTRF	polymerase I and transcript release factor
200762_at	4.20E-06	1.06E-07	7.6430128	7.90168	1.9039636	DPYSL2	dihydropyrimidinase like 2
221840_at	4.24E-06	1.07E-07	7.6376158	7.890235	2.3766216	PTPRE	protein tyrosine phosphatase, receptor type E
202729_s_at	4.27E-06	1.08E-07	7.6342379	7.88307	2.2757977	LTBP1	latent transforming growth factor beta binding protein 1
221773_at	4.27E-06	1.08E-07	7.6341908	7.88297	2.2680657	ELK3	ELK3, ETS transcription factor

224865_at	4.27E-06	1.08E-07	7.6336465	7.881816	1.2608946	FAR1	fatty acyl-CoA reductase 1
242059_at	4.27E-06	1.08E-07	7.6331386	7.880738	1.490253		
218085_at	4.27E-06	1.08E-07	7.6328265	7.880076	1.3428704	CHMP5	charged multivesicular body protein 5
226568_at	4.29E-06	1.09E-07	7.6303286	7.874776	2.1650246	FAM102B	family with sequence similarity 102 member B
207358_x_at	4.30E-06	1.09E-07	7.6271381	7.868005	1.33488	MACF1	microtubule-actin crosslinking factor 1
201939_at	4.30E-06	1.09E-07	7.6270749	7.867871	2.261047	PLK2	polo like kinase 2

36552_at	4.32E-06	1.10E-07	7.6233204	7.859901	1.1548721	C2CD3	C2 calcium dependent domain containing 3
228131_at	4.32E-06	1.11E-07	7.6217674	7.856604	1.0646134	ERCC1	ERCC excision repair 1, endonuclease non-catalytic subunit
212919_at	4.33E-06	1.11E-07	7.6199803	7.85281	1.5947098	DCP2	decapping mRNA 2
1559496_at	4.34E-06	1.11E-07	7.6190702	7.850877	1.2992505	PPA2	pyrophosphatase (inorganic) 2
236924_at	4.34E-06	1.12E-07	7.6183741	7.849399	1.1551148		
209627_s_at	4.36E-06	1.12E-07	7.6155552	7.843412	2.5865921	OSBPL3	oxysterol binding protein like 3

242918_at	4.36E-06	1.12E-07	7.6145425	7.841261	1.7051732	NASP	nuclear autoantigenic sperm protein
230913_at	4.36E-06	1.12E-07	7.6143397	7.84083	1.2002795	ABCG1	ATP binding cassette subfamily G member 1
213513_x_at	4.37E-06	1.13E-07	7.6136318	7.839326	1.5577661	ARPC2	actin related protein 2/3 complex subunit 2
240146_at	4.37E-06	1.13E-07	7.6123858	7.836679	1.8015337		
204567_s_at	4.39E-06	1.14E-07	7.6088707	7.829211	2.5552305	ABCG1	ATP binding cassette subfamily G member 1

227036_at	4.39E-06	1.14E-07	7.6083752	7.828158	1.8977114	RASAL2	RAS protein activator like 2
202778_s_at	4.39E-06	1.14E-07	7.6073501	7.825979	1.7874893	ZMYM2	zinc finger MYM-type containing 2
223300_s_at	4.40E-06	1.15E-07	7.6051351	7.821271	1.3487871	CCDC82	coiled-coil domain containing 82
203855_at	4.40E-06	1.15E-07	7.6047187	7.820386	1.4584618	WDR47	WD repeat domain 47
214988_s_at	4.41E-06	1.16E-07	7.6003836	7.811171	1.141702	SON	SON DNA binding protein
209748_at	4.41E-06	1.16E-07	7.6000389	7.810438	1.2139111	SPAST	spastin
218184_at	4.41E-06	1.16E-07	7.5995796	7.809461	1.0494984	TULP4	tubby like protein 4

209271_at	4.41E-06	1.16E-07	7.5992455	7.808751	1.149102	BPTF	bromodomain PHD finger transcription factor
219947_at	4.43E-06	1.17E-07	7.5962821	7.802449	2.199872	CLEC4A	C-type lectin domain family 4 member A

215921_at	4.43E-06	1.17E-07	7.5960102	7.80187	1.2219696	NPIPA5///NPIPB6///NPIPB8///NPIPB3	nuclear pore complex interacting protein family member A5///nuclear pore complex interacting protein family member B6///nuclear pore complex interacting protein family member B8///nuclear pore complex interacting protein family member B3

229141_at	4.43E-06	1.17E-07	7.5953004	7.800361	1.0716795	SFT2D3///WDR33	SFT2 domain containing 3///WD repeat domain 33
231716_at	4.45E-06	1.18E-07	7.592641	7.794704	1.3909464	RC3H2	ring finger and CCCH-type domains 2
240432_x_at	4.45E-06	1.18E-07	7.5912359	7.791715	1.8464789	KLF7	Kruppel like factor 7
225162_at	4.45E-06	1.19E-07	7.5901739	7.789455	1.4720657	SH3D19	SH3 domain containing 19
229802_at	4.45E-06	1.19E-07	7.5880612	7.78496	4.1633516	WISP1	WNT1 inducible signaling pathway protein 1

218224_at	4.46E-06	1.19E-07	7.5866645	7.781988	1.9807577	PNMA1	paraneoplastic Ma antigen 1
201792_at	4.47E-06	1.20E-07	7.5846148	7.777625	3.6221543	AEBP1	AE binding protein 1

214805_at	4.48E-06	1.21E-07	7.5825234	7.773174	1.5156118	SNORD10///SNORA48///SNORA67///EIF4A1	small nucleolar RNA, C/D box 10///small nucleolar RNA, H/ACA box 48///small nucleolar RNA, H/ACA box 67///eukaryotic translation initiation factor 4A1
218519_at	4.48E-06	1.21E-07	7.5824556	7.77303	1.3382005	SLC35A5	solute carrier family 35 member A5

228937_at	4.48E-06	1.21E-07	7.5824364	7.772989	2.0479014	LACC1	laccase domain containing 1
230292_at	4.49E-06	1.21E-07	7.5808923	7.769702	1.3237061	LOC100996412	uncharacterized LOC100996412
200037_s_at	4.49E-06	1.21E-07	7.5797541	7.767278	1.348498	CBX3	chromobox 3
208894_at	4.49E-06	1.21E-07	7.579056	7.765792	2.7405682	HLA-DRA	major histocompatibility complex, class II, DR alpha
202251_at	4.49E-06	1.22E-07	7.5787723	7.765188	1.5174114	PRPF3	pre-mRNA processing factor 3
221755_at	4.50E-06	1.22E-07	7.5768278	7.761047	2.2803113	EHBP1L1	EH domain binding protein 1 like 1

226641_at	4.52E-06	1.23E-07	7.5735429	7.754051	2.4852211	ANKRD44	ankyrin repeat domain 44
1567214_a_at	4.52E-06	1.23E-07	7.5735098	7.753981	1.7957321	PNN	pinin, desmosome associated protein
225988_at	4.52E-06	1.23E-07	7.5731388	7.75319	1.8440036	HERC4	HECT and RLD domain containing E3 ubiquitin protein ligase 4
231956_at	4.53E-06	1.24E-07	7.5702152	7.746962	2.3874454	RNF213	ring finger protein 213
223886_s_at	4.53E-06	1.24E-07	7.569773	7.746021	1.0261325	RNF146	ring finger protein 146
210809_s_at	4.54E-06	1.24E-07	7.5684802	7.743266	5.7580043	POSTN	periostin

225442_at	4.55E-06	1.25E-07	7.5652191	7.736317	2.0544912	DDR2	discoidin domain receptor tyrosine kinase 2
233898_s_at	4.56E-06	1.25E-07	7.5645693	7.734932	1.6144404	FGFR1OP2	FGFR1 oncogene partner 2
227386_s_at	4.56E-06	1.26E-07	7.5633378	7.732307	1.8942045	TMEM200B	transmembrane protein 200B
244197_x_at	4.57E-06	1.26E-07	7.5624869	7.730494	1.3772829		
242233_at	4.57E-06	1.26E-07	7.5619605	7.729371	1.0139512		
226609_at	4.59E-06	1.27E-07	7.5570726	7.718951	2.398925	DCBLD1	discoidin, CUB and LCCL domain containing 1
212982_at	4.59E-06	1.27E-07	7.5567298	7.71822	1.5510777	ZDHHC17	zinc finger DHHC-type containing 17

202994_s_at	4.63E-06	1.29E-07	7.5518008	7.707708	2.03225	FBLN1	fibulin 1
202548_s_at	4.65E-06	1.30E-07	7.5487082	7.70111	1.3876509	ARHGEF7	Rho guanine nucleotide exchange factor 7
226316_at	4.68E-06	1.32E-07	7.5417506	7.686263	1.6755541	RBM26	RNA binding motif protein 26
234762_x_at	4.69E-06	1.32E-07	7.5407893	7.684211	1.1218704	NLN	neurolysin
212791_at	4.71E-06	1.34E-07	7.5347691	7.671358	1.3549718	C1orf216	chromosome 1 open reading frame 216
232524_x_at	4.72E-06	1.34E-07	7.5337735	7.669232	1.1529613	ANAPC4	anaphase promoting complex subunit 4
210139_s_at	4.74E-06	1.35E-07	7.530022	7.661219	2.2722407	PMP22	peripheral myelin protein 22

206580_s_at	4.75E-06	1.36E-07	7.5282132	7.657355	1.3443486	EFEMP2	EGF containing fibulin like extracellular matrix protein 2
206101_at	4.75E-06	1.36E-07	7.5281547	7.65723	2.2672898	ECM2	extracellular matrix protein 2
239379_at	4.79E-06	1.37E-07	7.5222511	7.644616	1.3743023		
238430_x_at	4.85E-06	1.41E-07	7.5102543	7.618966	1.8721579	SLFN5	schlafen family member 5
225658_at	4.86E-06	1.42E-07	7.508504	7.615222	1.1548339	SPOPL	speckle type BTB/POZ protein like
212467_at	4.86E-06	1.42E-07	7.5078622	7.613849	1.7078014	DNAJC13	DnaJ heat shock protein family (Hsp40) member C13

239809_at	4.86E-06	1.43E-07	7.5052011	7.608156	1.8282136		
224842_at	4.86E-06	1.43E-07	7.5049664	7.607654	1.5373459	LOC101060386///BOLA2///SMG1	putative uncharacterized SMG1-like protein///bolA family member 2///SMG1, nonsense mediated mRNA decay associated PI3K related kinase
205250_s_at	4.87E-06	1.43E-07	7.5029193	7.603273	1.3502773	CEP290	centrosomal protein 290

213603_s_at	4.87E-06	1.44E-07	7.5016793	7.60062	2.4604432	RAC2	ras-related C3 botulinum toxin substrate 2 (rho family, small GTP binding protein Rac2)
218781_at	4.89E-06	1.44E-07	7.5001748	7.5974	1.7205952	SMC6	structural maintenance of chromosomes 6
227200_at	4.90E-06	1.45E-07	7.4981303	7.593024	1.4596482	ETV3	ETS variant 3
215116_s_at	4.90E-06	1.45E-07	7.4966329	7.589818	1.4484241	DNM1	dynamin 1
240061_at	4.91E-06	1.46E-07	7.4950807	7.586495	1.3051704		
228157_at	4.93E-06	1.47E-07	7.4905946	7.576889	2.275473	ZNF207	zinc finger protein 207

222630_at	4.94E-06	1.48E-07	7.4890251	7.573527	1.170255	RFX7	regulatory factor X7
235023_at	4.94E-06	1.48E-07	7.4881514	7.571656	2.1009352	VPS13C	vacuolar protein sorting 13 homolog C
1558111_at	4.97E-06	1.49E-07	7.4836941	7.562107	1.8648913	MBNL1	muscleblind like splicing regulator 1
226142_at	4.98E-06	1.50E-07	7.482322	7.559167	2.0206648	GLIPR1	GLI pathogenesis related 1
228478_at	4.98E-06	1.50E-07	7.4822259	7.558961	1.1989013	SPPL2A	signal peptide peptidase like 2A

207996_s_at	4.99E-06	1.50E-07	7.4807379	7.555772	1.8680504	LDLRAD4	low density lipoprotein receptor class A domain containing 4
228827_at	5.00E-06	1.51E-07	7.4786693	7.551339	1.8823841	RUNX1T1	RUNX1 translocation partner 1
210944_s_at	5.00E-06	1.51E-07	7.4784874	7.550949	1.5727375	CAPN3	calpain 3
235231_at	5.04E-06	1.53E-07	7.4722924	7.537668	1.4698316	ZNF789	zinc finger protein 789
233380_s_at	5.06E-06	1.54E-07	7.4697936	7.53231	1.0212757	RUFY1	RUN and FYVE domain containing 1

203301_s_at	5.06E-06	1.54E-07	7.4692874	7.531224	1.5195964	DMTF1	cyclin D binding myb like transcription factor 1
225594_at	5.10E-06	1.56E-07	7.4634683	7.518742	1.8541252	CREBZF	CREB/ATF bZIP transcription factor
214757_at	5.14E-06	1.58E-07	7.4576936	7.50635	1.2249898	PMS2P2	PMS1 homolog 2, mismatch repair system component pseudogene 2
202765_s_at	5.14E-06	1.58E-07	7.4568207	7.504477	2.5078918	FBN1	fibrillin 1

226753_at	5.15E-06	1.59E-07	7.4555782	7.50181	1.2974943	FAM76B	family with sequence similarity 76 member B
242853_at	5.16E-06	1.60E-07	7.4538359	7.498069	2.0697309		
224559_at	5.17E-06	1.60E-07	7.4517724	7.493639	2.0583041	MALAT1	metastasis associated lung adenocarcinoma transcript 1 (non-protein coding)
201153_s_at	5.17E-06	1.60E-07	7.4515869	7.493241	1.7064864	MBNL1	muscleblind like splicing regulator 1
221841_s_at	5.17E-06	1.61E-07	7.4508357	7.491628	2.8728491	KLF4	Kruppel like factor 4
243591_at	5.17E-06	1.61E-07	7.4504527	7.490806	1.9293779		

225512_at	5.20E-06	1.62E-07	7.4463058	7.4819	1.3240993	ZBTB38	zinc finger and BTB domain containing 38
230263_s_at	5.22E-06	1.63E-07	7.443784	7.476483	1.8417507	DOCK5	dedicator of cytokinesis 5
217762_s_at	5.22E-06	1.64E-07	7.4424163	7.473545	3.3394739	RAB31	RAB31, member RAS oncogene family
224698_at	5.23E-06	1.64E-07	7.4401833	7.468747	1.2892471	ESYT2	extended synaptotagmin 2
213416_at	5.23E-06	1.64E-07	7.4399291	7.468201	2.2359838	ITGA4	integrin subunit alpha 4
208966_x_at	5.23E-06	1.65E-07	7.4392005	7.466635	2.5622009	IFI16	interferon gamma inducible protein 16

229765_at	5.25E-06	1.66E-07	7.436846	7.461575	1.1834493	ZNF207	zinc finger protein 207
203182_s_at	5.25E-06	1.66E-07	7.436326	7.460458	1.0753448	SRPK2	SRSF protein kinase 2
225813_at	5.26E-06	1.66E-07	7.4342968	7.456096	1.3327525	RC3H2	ring finger and CCCH-type domains 2
223095_at	5.26E-06	1.67E-07	7.4341544	7.45579	1.9455425	MARVELD1	MARVEL domain containing 1
202396_at	5.26E-06	1.67E-07	7.4340429	7.45555	1.5939091	TCERG1	transcription elongation regulator 1
201088_at	5.28E-06	1.68E-07	7.4310237	7.44906	2.4910945	KPNA2	karyopherin subunit alpha 2

225610_at	5.31E-06	1.69E-07	7.4277515	7.442024	1.136442	UHRF2	ubiquitin like with PHD and ring finger domains 2
226016_at	5.33E-06	1.70E-07	7.4252476	7.436639	1.5862011	CD47	CD47 molecule
203298_s_at	5.35E-06	1.71E-07	7.4223419	7.430388	1.0037298	JARID2	jumonji and AT-rich interaction domain containing 2
201663_s_at	5.35E-06	1.71E-07	7.4211204	7.427761	2.1947398	SMC4	structural maintenance of chromosomes 4
233614_at	5.36E-06	1.72E-07	7.4190547	7.423316	1.7642866		
226077_at	5.36E-06	1.72E-07	7.4185109	7.422146	1.9838518	RNF145	ring finger protein 145

212887_at	5.37E-06	1.73E-07	7.4179682	7.420978	2.2846198	SEC23A	Sec23 homolog A, coat complex II component
244341_at	5.37E-06	1.73E-07	7.416995	7.418884	1.5896032		
241769_at	5.37E-06	1.73E-07	7.4161072	7.416974	1.735558		
221763_at	5.38E-06	1.74E-07	7.4148295	7.414224	2.2728139	JMJD1C	jumonji domain containing 1C
202380_s_at	5.39E-06	1.75E-07	7.4127376	7.409721	1.3906532	NKTR	natural killer cell triggering receptor
226677_at	5.41E-06	1.75E-07	7.4103912	7.40467	2.5766584	ZNF521	zinc finger protein 521
229285_at	5.42E-06	1.76E-07	7.4091796	7.402061	1.6531091	RNASEL	ribonuclease L

225144_at	5.46E-06	1.78E-07	7.4035189	7.389871	2.0805661	BMPR2	bone morphogenetic protein receptor type 2
230141_at	5.46E-06	1.78E-07	7.4025144	7.387708	1.2519046	ARID4A	AT-rich interaction domain 4A

204670_x_at	5.47E-06	1.79E-07	7.4011525	7.384774	2.5804732	LOC100996809///HLA-DRB4///HLA-DRB1	HLA class II histocompatibility antigen, DRB1-10 beta chain-like///major histocompatibility complex, class II, DR beta 4///major histocompatibility complex, class II, DR beta 1
208986_at	5.49E-06	1.80E-07	7.398499	7.379058	1.4309213	TCF12	transcription factor 12

203566_s_at	5.51E-06	1.81E-07	7.3952452	7.372046	1.6663266	AGL	amylo-alpha-1, 6-glucosidase, 4-alpha-glucanotransferase
238431_at	5.51E-06	1.81E-07	7.3949858	7.371487	1.3463986		
214686_at	5.53E-06	1.82E-07	7.3931593	7.367551	1.5678586	ZNF266	zinc finger protein 266
206332_s_at	5.53E-06	1.82E-07	7.3928018	7.36678	2.7925273	IFI16	interferon gamma inducible protein 16
1558620_at	5.53E-06	1.82E-07	7.3925085	7.366148	1.0145757	ZNF621	zinc finger protein 621
202633_at	5.56E-06	1.84E-07	7.3888301	7.358219	1.0952061	TOPBP1	topoisomerase (DNA) II binding protein 1

218217_at	5.57E-06	1.85E-07	7.3872103	7.354727	1.7601532	SCPEP1	serine carboxypeptidase 1
225710_at	5.59E-06	1.85E-07	7.3859491	7.352007	2.8677707	GNB4	G protein subunit beta 4
225159_s_at	5.59E-06	1.85E-07	7.3857139	7.3515	1.0757882	ELK4	ELK4, ETS transcription factor
203302_at	5.59E-06	1.85E-07	7.3854407	7.350911	1.4353566	DCK	deoxycytidine kinase
224929_at	5.59E-06	1.86E-07	7.3848418	7.349619	1.5363588	TMEM173	transmembrane protein 173
221044_s_at	5.60E-06	1.86E-07	7.3833626	7.34643	1.4405443	TRIM6-TRIM34///TRIM34	TRIM6-TRIM34 readthrough///tripartite motif containing 34

214684_at	5.60E-06	1.86E-07	7.3830015	7.345651	1.7954604	MEF2A	myocyte enhancer factor 2A
229366_at	5.61E-06	1.87E-07	7.3810085	7.341352	1.7514096		
236494_x_at	5.63E-06	1.88E-07	7.3791925	7.337435	1.3650286		
232174_at	5.63E-06	1.88E-07	7.378524	7.335993	2.1004784		
218669_at	5.63E-06	1.88E-07	7.3784479	7.335828	1.2896995	RAP2C	RAP2C, member of RAS oncogene family
219957_at	5.64E-06	1.89E-07	7.3763736	7.331353	2.1898975	RUFY2	RUN and FYVE domain containing 2
221478_at	5.64E-06	1.89E-07	7.3762792	7.331149	1.6780407	BNIP3L	BCL2 interacting protein 3 like
203620_s_at	5.66E-06	1.90E-07	7.3740536	7.326347	1.424105	FCHSD2	FCH and double SH3 domains 2

222476_at	5.66E-06	1.90E-07	7.3733759	7.324885	1.480465	CNOT6	CCR4-NOT transcription complex subunit 6
228812_at	5.66E-06	1.90E-07	7.3729164	7.323893	2.286683	REL	REL proto-oncogene, NF-kB subunit
1555411_a_at	5.66E-06	1.91E-07	7.3721066	7.322145	2.0956045	CCNL1	cyclin L1
220954_s_at	5.67E-06	1.91E-07	7.3714629	7.320756	1.6430254	MIR6840///STAG3L5P-PVRIG2P-PILRB///PILRB	microRNA 6840///STAG3L5P-PVRIG2P-PILRB readthrough///paired immunoglobin-like type 2 receptor beta

226395_at	5.67E-06	1.91E-07	7.3705877	7.318867	1.5555532	HOOK3	hook microtubule tethering protein 3
214093_s_at	5.67E-06	1.92E-07	7.3700422	7.31769	1.6451005	FUBP1	far upstream element binding protein 1
219885_at	5.67E-06	1.92E-07	7.3698463	7.317267	1.5198798	SLFN12	schlafen family member 12
220044_x_at	5.67E-06	1.92E-07	7.3688369	7.315088	1.930415	LUC7L3	LUC7 like 3 pre-mRNA splicing factor

201294_s_at	5.67E-06	1.92E-07	7.368368	7.314076	1.934507	WSB1	WD repeat and SOCS box containing 1
1558953_s_at	5.68E-06	1.93E-07	7.3672154	7.311587	1.1612761	CEP164	centrosomal protein 164
229419_at	5.68E-06	1.93E-07	7.366852	7.310803	1.2151661	FBXW7	F-box and WD repeat domain containing 7
238002_at	5.69E-06	1.93E-07	7.3661435	7.309273	1.6428343	GOLIM4	golgi integral membrane protein 4
209298_s_at	5.74E-06	1.96E-07	7.3600129	7.296034	1.4199673	ITSN1	intersectin 1

206853_s_at	5.76E-06	1.97E-07	7.3571964	7.28995	1.0432102	MAP3K7	mitogen-activated protein kinase kinase kinase 7
224914_s_at	5.77E-06	1.97E-07	7.3566935	7.288864	1.013453	SARNP	SAP domain containing ribonucleoprotein
207988_s_at	5.77E-06	1.98E-07	7.3564663	7.288373	1.4171248	ARPC2	actin related protein 2/3 complex subunit 2
212646_at	5.78E-06	1.98E-07	7.3545492	7.284231	2.1989788	RFTN1	raftlin, lipid raft linker 1
212709_at	5.79E-06	1.99E-07	7.3530628	7.281019	1.2341023	NUP160	nucleoporin 160

212671_s_at	5.79E-06	1.99E-07	7.3528432	7.280545	3.5178995	LOC100509457///HLA-DQA2///HLA-DQA1	HLA class II histocompatibility antigen, DQ alpha 1 chain-like///major histocompatibility complex, class II, DQ alpha 2///major histocompatibility complex, class II, DQ alpha 1
218395_at	5.79E-06	1.99E-07	7.3526305	7.280085	1.5612884	ACTR6	ARP6 actin-related protein 6 homolog

209970_x_at	5.79E-06	2.00E-07	7.3511574	7.276902	1.9072709	CASP1	caspase 1
225475_at	5.79E-06	2.00E-07	7.3499836	7.274365	1.4386964	MIER1	MIER1 transcriptional regulator
235190_at	5.79E-06	2.00E-07	7.3499437	7.274278	1.7631618		
213982_s_at	5.84E-06	2.03E-07	7.3438024	7.261003	1.7100591	RABGAP1L	RAB GTPase activating protein 1 like
227751_at	5.85E-06	2.04E-07	7.342384	7.257936	1.3002575	PDCD5	programmed cell death 5
219492_at	5.89E-06	2.06E-07	7.3374589	7.247284	1.3092189	CHIC2	cysteine rich hydrophobic domain 2
209007_s_at	5.90E-06	2.06E-07	7.3365485	7.245315	2.3006941	RSRP1	arginine and serine rich protein 1

209974_s_at	5.91E-06	2.07E-07	7.3353246	7.242667	1.4245011	BUB3	BUB3, mitotic checkpoint protein
230550_at	5.91E-06	2.07E-07	7.3352165	7.242433	2.2183613	MS4A6A	membrane spanning 4-domains A6A
225128_at	5.91E-06	2.08E-07	7.3338961	7.239577	1.1858661	KDELC2	KDEL motif containing 2
206335_at	5.91E-06	2.08E-07	7.3334818	7.23868	1.4278895	GALNS	galactosamine (N-acetyl)-6-sulfatase
221229_s_at	5.96E-06	2.10E-07	7.3283764	7.227633	1.131448	TRMT61B	tRNA methyltransferase 61B

208091_s_at	5.96E-06	2.10E-07	7.3278251	7.226439	1.3464307	VOPP1	vesicular, overexpressed in cancer, prosurvival protein 1
221645_s_at	5.96E-06	2.11E-07	7.3262061	7.222935	2.6722407	ZNF83	zinc finger protein 83
212031_at	5.96E-06	2.11E-07	7.3261226	7.222754	1.6172509	RBM25	RNA binding motif protein 25
230229_at	5.99E-06	2.13E-07	7.3231696	7.216361	2.334188	DLG1	discs large MAGUK scaffold protein 1
201163_s_at	5.99E-06	2.13E-07	7.3227494	7.215452	1.5409789	IGFBP7	insulin like growth factor binding protein 7

224916_at	5.99E-06	2.13E-07	7.3224761	7.21486	2.1639352	TMEM173	transmembrane protein 173
228531_at	6.02E-06	2.14E-07	7.3195454	7.208514	3.1063039	SAMD9	sterile alpha motif domain containing 9
212043_at	6.03E-06	2.15E-07	7.3185893	7.206443	1.0398804	TGOLN2	trans-golgi network protein 2
223595_at	6.03E-06	2.15E-07	7.3177165	7.204553	2.1828713	TMEM133	transmembrane protein 133
1556006_s_at	6.03E-06	2.15E-07	7.317108	7.203235	1.9037279	CSNK1A1	casein kinase 1 alpha 1
243993_at	6.03E-06	2.16E-07	7.3168544	7.202686	1.8359282		
226169_at	6.05E-06	2.17E-07	7.3145829	7.197766	1.5572075	SBF2	SET binding factor 2

207922_s_at	6.06E-06	2.17E-07	7.313569	7.195569	1.2112111	MAEA	macrophage erythroblast attacher
219286_s_at	6.06E-06	2.17E-07	7.3133392	7.195071	1.2723634	RBM15	RNA binding motif protein 15
225969_at	6.09E-06	2.19E-07	7.3100742	7.187997	1.2964548	ALKBH6	alkB homolog 6
236545_at	6.09E-06	2.19E-07	7.3098804	7.187578	1.7210718		
1556007_s_at	6.12E-06	2.21E-07	7.3053606	7.177782	1.8719663	CSNK1A1	casein kinase 1 alpha 1
226942_at	6.12E-06	2.21E-07	7.3053254	7.177706	1.2630459	PHF20L1	PHD finger protein 20-like 1
236322_at	6.15E-06	2.23E-07	7.3011744	7.168707	2.3804061		
233595_at	6.15E-06	2.23E-07	7.3010999	7.168546	1.6442898	USP34	ubiquitin specific peptidase 34
212632_at	6.15E-06	2.23E-07	7.3004391	7.167113	1.6030991	STX7	syntaxin 7

225929_s_at	6.16E-06	2.24E-07	7.2995997	7.165293	1.8053521	RNF213	ring finger protein 213
209356_x_at	6.18E-06	2.26E-07	7.2961781	7.157873	1.6935504	EFEMP2	EGF containing fibulin like extracellular matrix protein 2
215269_at	6.19E-06	2.26E-07	7.2947241	7.15472	1.401298	TRAPPC10	trafficking protein particle complex 10
224764_at	6.20E-06	2.27E-07	7.2929475	7.150866	1.576553	ARHGAP21	Rho GTPase activating protein 21
222843_at	6.20E-06	2.27E-07	7.2927769	7.150496	1.508718	FIGNL1	fidgetin like 1

225617_at	6.21E-06	2.28E-07	7.2920436	7.148905	1.1036664	ODF2	outer dense fiber of sperm tails 2
224973_at	6.22E-06	2.28E-07	7.2908222	7.146256	1.85627	FAM46A	family with sequence similarity 46 member A
204512_at	6.24E-06	2.30E-07	7.2879578	7.140041	1.5162432	HIVEP1	human immunodeficiency virus type I enhancer binding protein 1
204052_s_at	6.24E-06	2.30E-07	7.2878162	7.139733	3.7626514	SFRP4	secreted frizzled related protein 4

1555844_s_at	6.24E-06	2.30E-07	7.28659	7.137073	1.1204316	HNRNPM	heterogeneous nuclear ribonucleoprotein M
223218_s_at	6.25E-06	2.31E-07	7.2844653	7.132461	3.1058873	NFKBIZ	NFKB inhibitor zeta
205474_at	6.26E-06	2.32E-07	7.2835334	7.130439	1.3499723	CRLF3	cytokine receptor like factor 3
201908_at	6.29E-06	2.33E-07	7.2807393	7.124373	1.0789032	DVL3	dishevelled segment polarity protein 3
212780_at	6.30E-06	2.34E-07	7.2796979	7.122113	1.7060971	SOS1	SOS Ras/Rac guanine nucleotide exchange factor 1

225456_at	6.31E-06	2.35E-07	7.2782892	7.119054	1.4168127	MED1	mediator complex subunit 1
218181_s_at	6.31E-06	2.35E-07	7.2774815	7.1173	2.1087646	MAP4K4	mitogen-activated protein kinase kinase kinase kinase 4
218236_s_at	6.32E-06	2.35E-07	7.276851	7.115931	1.4897077	PRKD3	protein kinase D3
AFFX-HSAC07/X00351_3_at	6.32E-06	2.36E-07	7.276314	7.114765	1.5028732	ACTB	actin beta
227247_at	6.33E-06	2.36E-07	7.2758507	7.113759	1.2975464	PLEKHA8	pleckstrin homology domain containing A8

221039_s_at	6.34E-06	2.38E-07	7.2728197	7.107176	1.767157	ASAP1	ArfGAP with SH3 domain, ankyrin repeat and PH domain 1
201613_s_at	6.34E-06	2.38E-07	7.2720638	7.105534	1.2184746	AP1G2	adaptor related protein complex 1 gamma 2 subunit
220731_s_at	6.34E-06	2.38E-07	7.2720233	7.105447	1.3405163	NECAP2	NECAP endocytosis associated 2

204559_s_at	6.37E-06	2.40E-07	7.2675651	7.095761	1.4293221	LSM7	LSM7 homolog, U6 small nuclear RNA and mRNA degradation associated
221935_s_at	6.39E-06	2.42E-07	7.2644929	7.089086	2.260193	EOGT	EGF domain specific O-linked N-acetylglucosamine transferase
213373_s_at	6.40E-06	2.43E-07	7.2625812	7.084931	1.7517036	CASP8	caspase 8
201603_at	6.40E-06	2.43E-07	7.2624213	7.084583	1.9167461	PPP1R12A	protein phosphatase 1 regulatory subunit 12A

224833_at	6.40E-06	2.43E-07	7.2621647	7.084026	2.1686568	ETS1	ETS proto-oncogene 1, transcription factor
224701_at	6.42E-06	2.45E-07	7.2592368	7.077661	1.7125564	PARP14	poly(ADP-ribose) polymerase family member 14
212651_at	6.43E-06	2.45E-07	7.2587619	7.076629	1.6782509	RHOBTB1	Rho related BTB domain containing 1
233364_s_at	6.43E-06	2.45E-07	7.2581253	7.075245	2.6071238		
225731_at	6.43E-06	2.46E-07	7.2573158	7.073485	1.59718	ANKRD50	ankyrin repeat domain 50

226409_at	6.45E-06	2.48E-07	7.253566	7.065331	1.1070505	TBC1D20	TBC1 domain family member 20
217733_s_at	6.48E-06	2.50E-07	7.2501166	7.057829	1.8107393	TMSB10	thymosin beta 10
218652_s_at	6.48E-06	2.50E-07	7.2495761	7.056654	1.2282337	PIGG	phosphatidylinositol glycan anchor biosynthesis class G
227489_at	6.49E-06	2.51E-07	7.2486395	7.054616	1.3425652	SMURF2	SMAD specific E3 ubiquitin protein ligase 2
207850_at	6.50E-06	2.51E-07	7.2476011	7.052357	3.4485218	CXCL3	C-X-C motif chemokine ligand 3

223257_at	6.50E-06	2.51E-07	7.2473801	7.051876	1.0086159	G2E3	G2/M-phase specific E3 ubiquitin protein ligase
218628_at	6.50E-06	2.52E-07	7.2457607	7.048353	1.2704257	CCDC53	coiled-coil domain containing 53
239654_at	6.50E-06	2.52E-07	7.2457163	7.048257	1.1683521	CHD9	chromodomain helicase DNA binding protein 9
203569_s_at	6.50E-06	2.52E-07	7.2456946	7.048209	1.3506268	OFD1	OFD1, centriole and centriolar satellite protein
220974_x_at	6.50E-06	2.52E-07	7.2455232	7.047836	1.4691739	SFXN3	sideroflexin 3

1557080_s_at	6.50E-06	2.53E-07	7.2446269	7.045886	2.7138605	ITGBL1	integrin subunit beta like 1
214683_s_at	6.52E-06	2.54E-07	7.2429003	7.042129	1.8243255	CLK1	CDC like kinase 1
208812_x_at	6.54E-06	2.55E-07	7.2399531	7.035715	1.3317893	HLA-C	major histocompatibility complex, class I, C
225136_at	6.55E-06	2.57E-07	7.2378752	7.031192	1.5853254	PLEKHA2	pleckstrin homology domain containing A2
213077_at	6.56E-06	2.57E-07	7.2366372	7.028497	1.4233986	YTHDC2	YTH domain containing 2
226771_at	6.56E-06	2.58E-07	7.2355155	7.026055	1.7237125	ATP8B2	ATPase phospholipid transporting 8B2

210976_s_at	6.57E-06	2.59E-07	7.2339826	7.022717	1.0163323	PFKM	phosphofructokinase, muscle
226015_at	6.57E-06	2.59E-07	7.2328909	7.02034	1.5610407	ZNF12	zinc finger protein 12
224733_at	6.57E-06	2.60E-07	7.2324572	7.019396	2.1667539	CMTM3	CKLF like MARVEL transmembrane domain containing 3
224558_s_at	6.59E-06	2.61E-07	7.229932	7.013897	2.1423904	MALAT1	metastasis associated lung adenocarcinoma transcript 1 (non-protein coding)

204221_x_at	6.62E-06	2.63E-07	7.2275404	7.008688	1.588745	GLIPR1	GLI pathogenesis related 1
217763_s_at	6.63E-06	2.64E-07	7.2251706	7.003525	3.1109509	RAB31	RAB31, member RAS oncogene family
214124_x_at	6.64E-06	2.65E-07	7.2234109	6.999691	1.0639166	FGFR1OP	FGFR1 oncogene partner
201621_at	6.64E-06	2.66E-07	7.2216429	6.995839	2.41183	NBL1	neuroblastoma 1, DAN family BMP antagonist
201964_at	6.64E-06	2.66E-07	7.2214609	6.995442	1.3697905	SETX	senataxin
241893_at	6.64E-06	2.66E-07	7.2213623	6.995227	1.809025		
223215_s_at	6.65E-06	2.67E-07	7.2201841	6.99266	1.0074655	JKAMP	JNK1/MAPK8-associated membrane protein

209012_at	6.70E-06	2.70E-07	7.2155222	6.982499	1.8103886	TRIO	trio Rho guanine nucleotide exchange factor
227568_at	6.72E-06	2.71E-07	7.2133546	6.977773	1.6873484	HECTD2	HECT domain E3 ubiquitin protein ligase 2
223343_at	6.74E-06	2.72E-07	7.2117609	6.974298	3.0433016	MS4A7	membrane spanning 4-domains A7
202627_s_at	6.76E-06	2.73E-07	7.2099115	6.970266	2.9586384	SERPINE1	serpin family E member 1
212764_at	6.79E-06	2.75E-07	7.2067068	6.963277	2.3505038	ZEB1	zinc finger E-box binding homeobox 1

231940_at	6.79E-06	2.75E-07	7.2065896	6.963021	1.4029727	ZNF529	zinc finger protein 529
227266_s_at	6.79E-06	2.76E-07	7.2052555	6.960111	2.9668129	FYB	FYN binding protein
244659_at	6.82E-06	2.78E-07	7.201281	6.95144	1.2174125	TRIP12	thyroid hormone receptor interactor 12
40420_at	6.82E-06	2.79E-07	7.20096	6.95074	1.48907	STK10	serine/threonine kinase 10
221731_x_at	6.83E-06	2.79E-07	7.2000968	6.948856	3.9880498	VCAN	versican
212033_at	6.86E-06	2.81E-07	7.1972113	6.942559	1.3111139	RBM25	RNA binding motif protein 25
224691_at	6.87E-06	2.82E-07	7.1949897	6.937711	1.4760459	UHMK1	U2AF homology motif (UHM) kinase 1

37012_at	6.87E-06	2.82E-07	7.1946257	6.936916	1.5298841	CAPZB	capping actin protein of muscle Z-line beta subunit
219179_at	6.87E-06	2.83E-07	7.1939078	6.935349	2.201815	DACT1	dishevelled binding antagonist of beta catenin 1
239740_at	6.90E-06	2.85E-07	7.1911298	6.929284	1.4603629	ETV6	ETS variant 6
200797_s_at	6.90E-06	2.85E-07	7.1910445	6.929098	1.3862196	MCL1	BCL2 family apoptosis regulator
235078_at	6.91E-06	2.85E-07	7.1902708	6.927409	1.5912323		
212917_x_at	6.91E-06	2.86E-07	7.1889668	6.924562	1.9875245	RECQL	RecQ like helicase
207168_s_at	6.91E-06	2.86E-07	7.1887445	6.924076	1.4572991	H2AFY	H2A histone family member Y

204362_at	6.91E-06	2.86E-07	7.1884697	6.923476	2.0317877	SKAP2	src kinase associated phosphoprotein 2
212223_at	6.91E-06	2.86E-07	7.188381	6.923282	1.2866514	IDS	iduronate 2-sulfatase
202808_at	6.91E-06	2.87E-07	7.1879404	6.92232	1.4405911	WBP1L	WW domain binding protein 1-like
212765_at	6.94E-06	2.88E-07	7.1853183	6.916594	1.6634648	CAMSAP2	calmodulin regulated spectrin associated protein family member 2
239336_at	6.95E-06	2.89E-07	7.184521	6.914853	1.8720295	THBS1	thrombospondin 1
228335_at	6.95E-06	2.89E-07	7.1841248	6.913987	2.3616091	CLDN11	claudin 11

211784_s_at	6.98E-06	2.91E-07	7.1816588	6.9086	1.512498	SRSF1	serine and arginine rich splicing factor 1
222235_s_at	7.00E-06	2.93E-07	7.1783374	6.901344	2.0634693	CSGALNACT2	chondroitin sulfate N-acetylgalactosaminyltransferase 2
225612_s_at	7.00E-06	2.93E-07	7.1783257	6.901318	2.4618113	B3GNT5	UDP-GlcNAc:betaGal beta-1,3-N-acetylglucosaminyltransferase 5

207243_s_at	7.01E-06	2.93E-07	7.1775903	6.899711	1.1677857	CALM3///CALM2///CALM1	calmodulin 3///calmodulin 2///calmodulin 1
203570_at	7.03E-06	2.95E-07	7.1753216	6.894754	2.7430268	LOXL1	lysyl oxidase like 1
1555852_at	7.03E-06	2.95E-07	7.1751199	6.894313	1.8021471	PSMB8-AS1	PSMB8 antisense RNA 1 (head to head)
235352_at	7.05E-06	2.96E-07	7.1735454	6.890872	2.1254341	MR1	major histocompatibility complex, class I-related
211185_s_at	7.06E-06	2.97E-07	7.1717517	6.886951	1.1034429	SF3B1	splicing factor 3b subunit 1
202668_at	7.07E-06	2.98E-07	7.1708452	6.884969	2.8473016	EFNB2	ephrin B2

224796_at	7.07E-06	2.99E-07	7.1695922	6.88223	1.499442	ASAP1	ArfGAP with SH3 domain, ankyrin repeat and PH domain 1
1555827_at	7.08E-06	2.99E-07	7.1685884	6.880036	1.4943579	CCNL1	cyclin L1
223502_s_at	7.08E-06	3.00E-07	7.1681017	6.878971	2.8143448	TNFSF13B	tumor necrosis factor superfamily member 13b
228396_at	7.08E-06	3.00E-07	7.1678172	6.878349	1.4903641	PRKG1	protein kinase, cGMP-dependent, type I
202207_at	7.08E-06	3.00E-07	7.1671528	6.876897	2.9110263	ARL4C	ADP ribosylation factor like GTPase 4C

202273_at	7.08E-06	3.01E-07	7.1662236	6.874865	2.0670657	PDGFRB	platelet derived growth factor receptor beta
208747_s_at	7.08E-06	3.01E-07	7.1660186	6.874417	2.0456568	C1S	complement component 1, s subcomponent
225685_at	7.09E-06	3.01E-07	7.1653193	6.872887	2.0868934	CDC42EP3	CDC42 effector protein 3
226066_at	7.09E-06	3.03E-07	7.1637481	6.869451	1.6262198	MITF	melanogenesis associated transcription factor
224656_s_at	7.10E-06	3.03E-07	7.162901	6.867599	1.3209371	LUZP6///MTPN	leucine zipper protein 6///myotrophin

200899_s_at	7.14E-06	3.06E-07	7.1586083	6.858209	1.0775239	MGEA5	meningioma expressed antigen 5 (hyaluronidase)
213869_x_at	7.15E-06	3.07E-07	7.1576642	6.856143	2.4224671	THY1	Thy-1 cell surface antigen
218761_at	7.15E-06	3.07E-07	7.1567552	6.854154	1.4233359	RNF111	ring finger protein 111
202804_at	7.15E-06	3.08E-07	7.1561289	6.852784	1.4539218	ABCC1	ATP binding cassette subfamily C member 1
205330_at	7.15E-06	3.08E-07	7.1557697	6.851998	2.1618621	MN1	MN1 proto-oncogene, transcriptional regulator

209308_s_at	7.15E-06	3.08E-07	7.155498	6.851403	1.4600486	BNIP2	BCL2 interacting protein 2
201152_s_at	7.15E-06	3.09E-07	7.1543328	6.848854	1.662092	MBNL1	muscleblind like splicing regulator 1
216834_at	7.16E-06	3.10E-07	7.1533975	6.846807	3.8500252	RGS1	regulator of G-protein signaling 1
225464_at	7.17E-06	3.10E-07	7.152875	6.845663	2.3394337	FRMD6	FERM domain containing 6
203741_s_at	7.17E-06	3.10E-07	7.152477	6.844792	2.6267445	ADCY7	adenylate cyclase 7
233300_at	7.17E-06	3.11E-07	7.1516363	6.842952	2.1061023		
213939_s_at	7.18E-06	3.11E-07	7.1508617	6.841257	1.5582879	RUFY3	RUN and FYVE domain containing 3

1554149_at	7.18E-06	3.12E-07	7.1502527	6.839924	1.1041688	CLDND1	claudin domain containing 1
213817_at	7.18E-06	3.13E-07	7.1491818	6.83758	2.4871543	IRAK3	interleukin 1 receptor associated kinase 3
212647_at	7.18E-06	3.13E-07	7.1487694	6.836677	1.7508157	RRAS	related RAS viral (r-ras) oncogene homolog

226340_x_at	7.18E-06	3.13E-07	7.1485274	6.836147	1.0254759	MIR6859-1///MIR6859-2///LOC101930154///LOC100288778///WASH1///WASH7P///WASH2P///WASH3P	microRNA 6859-1///microRNA 6859-2///WAS protein family homolog 6-like///WAS protein family homolog 1 pseudogene///WAS protein family homolog 1///WAS protein family homolog 7 pseudogene///WAS protein family homolog 2 pseudogene///WAS protein family homolog 3 pseudogene

222018_at	7.18E-06	3.13E-07	7.1484868	6.836058	1.4844248	NACA	nascent polypeptide-associated complex alpha subunit
226575_at	7.20E-06	3.14E-07	7.1468677	6.832514	1.3715473	ZNF462	zinc finger protein 462
202411_at	7.20E-06	3.15E-07	7.1462623	6.831188	3.372323	IFI27	interferon alpha inducible protein 27
202611_s_at	7.21E-06	3.15E-07	7.1458807	6.830353	1.5263439	MED14	mediator complex subunit 14
224002_s_at	7.21E-06	3.15E-07	7.1457949	6.830165	1.6046282	FKBP7	FK506 binding protein 7

224567_x_at	7.22E-06	3.16E-07	7.1441287	6.826517	1.1726232	MALAT1	metastasis associated lung adenocarcinoma transcript 1 (non-protein coding)
201564_s_at	7.23E-06	3.16E-07	7.1435795	6.825314	1.9448693	FSCN1	fascin actin-bundling protein 1
203346_s_at	7.24E-06	3.17E-07	7.1421927	6.822277	1.3311554	MTF2	metal response element binding transcription factor 2
227300_at	7.28E-06	3.19E-07	7.1394059	6.816174	2.6861689	TMEM119	transmembrane protein 119

227529_s_at	7.29E-06	3.20E-07	7.138271	6.813688	2.2346727	AKAP12	A-kinase anchoring protein 12
226785_at	7.31E-06	3.22E-07	7.1358587	6.808403	1.9641525	ATP11C	ATPase phospholipid transporting 11C
224866_at	7.31E-06	3.22E-07	7.1358378	6.808358	1.7554227	FAR1	fatty acyl-CoA reductase 1
204552_at	7.33E-06	3.23E-07	7.1337554	6.803795	1.3238939	INPP4A	inositol polyphosphate-4-phosphatase type I A
217764_s_at	7.34E-06	3.24E-07	7.132493	6.801029	3.3542846	RAB31	RAB31, member RAS oncogene family

224800_at	7.36E-06	3.26E-07	7.130585	6.796848	1.2248054	WDFY1	WD repeat and FYVE domain containing 1
244286_at	7.36E-06	3.26E-07	7.1304727	6.796601	1.3084414		
212443_at	7.39E-06	3.28E-07	7.1279169	6.791	1.2247254	NBEAL2	neurobeachin like 2
225385_s_at	7.39E-06	3.28E-07	7.1274595	6.789997	1.2194788	HNRNPLL	heterogeneous nuclear ribonucleoprotein L like
211911_x_at	7.45E-06	3.31E-07	7.123064	6.780361	1.7405304	HLA-B	major histocompatibility complex, class I, B

226421_at	7.45E-06	3.31E-07	7.1229327	6.780073	1.351613	AMMECR1	Alport syndrome, mental retardation, midface hypoplasia and elliptocytosis chromosomal region gene 1
212792_at	7.46E-06	3.32E-07	7.1215338	6.777006	1.8549773	DPY19L1	dpy-19 like 1
200686_s_at	7.46E-06	3.32E-07	7.1214568	6.776837	1.2458289	SRSF11	serine and arginine rich splicing factor 11

207191_s_at	7.49E-06	3.35E-07	7.1182188	6.769736	2.8471771	ISLR	immunoglobulin superfamily containing leucine rich repeat
211864_s_at	7.51E-06	3.36E-07	7.1170379	6.767146	2.8591468	MYOF	myoferlin
238617_at	7.52E-06	3.37E-07	7.114994	6.762662	2.7736038	KIF26B	kinesin family member 26B
226633_at	7.52E-06	3.38E-07	7.1145825	6.76176	1.8628014	RAB8B	RAB8B, member RAS oncogene family
225295_at	7.52E-06	3.38E-07	7.1141236	6.760753	2.0384202	SLC39A10	solute carrier family 39 member 10

202594_at	7.53E-06	3.39E-07	7.1132313	6.758795	1.0703648	LEPROTL1	leptin receptor overlapping transcript-like 1
224872_at	7.54E-06	3.40E-07	7.1108513	6.753573	1.0532341	DIP2B	disco interacting protein 2 homolog B
232909_s_at	7.54E-06	3.40E-07	7.110831	6.753529	1.3378009	BPTF	bromodomain PHD finger transcription factor
212066_s_at	7.55E-06	3.41E-07	7.1095336	6.750682	1.0066457	USP34	ubiquitin specific peptidase 34

226311_at	7.56E-06	3.42E-07	7.1087846	6.749038	3.4236545	ADAMTS2	ADAM metallopeptidase with thrombospondin type 1 motif 2
213526_s_at	7.57E-06	3.44E-07	7.1060258	6.742983	1.3864425	LIN37	lin-37 DREAM MuvB core complex component
212412_at	7.57E-06	3.45E-07	7.1054313	6.741678	1.1136375	PDLIM5	PDZ and LIM domain 5
227995_at	7.58E-06	3.46E-07	7.1041155	6.73879	1.7989961	PLXDC2	plexin domain containing 2
202686_s_at	7.60E-06	3.47E-07	7.1016695	6.733421	2.4975841	AXL	AXL receptor tyrosine kinase

203375_s_at	7.61E-06	3.48E-07	7.1012689	6.732541	1.4657695	TPP2	tripeptidyl peptidase 2
229114_at	7.62E-06	3.49E-07	7.0999347	6.729612	1.2564836	GAB1	GRB2 associated binding protein 1
212959_s_at	7.63E-06	3.50E-07	7.0981946	6.725791	2.05541	GNPTAB	N-acetylglucosamine-1-phosphate transferase alpha and beta subunits
224810_s_at	7.64E-06	3.51E-07	7.0976465	6.724587	1.07847	ANKRD13A	ankyrin repeat domain 13A

225406_at	7.71E-06	3.57E-07	7.0897013	6.707135	1.8733109	TWSG1	twisted gastrulation BMP signaling modulator 1
221522_at	7.72E-06	3.58E-07	7.0888791	6.705328	1.3161288	ANKRD27	ankyrin repeat domain 27
203044_at	7.72E-06	3.58E-07	7.0879156	6.703211	2.5970513	CHSY1	chondroitin sulfate synthase 1
213140_s_at	7.73E-06	3.59E-07	7.0871309	6.701487	1.3773993	SS18L1	SS18L1, nBAF chromatin remodeling complex subunit
224587_at	7.75E-06	3.61E-07	7.084947	6.696687	1.0854836	SUB1	SUB1 homolog, transcriptional regulator
232356_at	7.76E-06	3.62E-07	7.0838368	6.694247	1.6983604		

222209_s_at	7.76E-06	3.62E-07	7.0835262	6.693565	1.0630341	TMEM135	transmembrane protein 135
202988_s_at	7.76E-06	3.62E-07	7.0830751	6.692573	3.7792373	RGS1	regulator of G-protein signaling 1
204806_x_at	7.77E-06	3.64E-07	7.0811788	6.688405	1.995958	HLA-F	major histocompatibility complex, class I, F
213199_at	7.79E-06	3.65E-07	7.0793261	6.684332	1.0633213	C2CD3	C2 calcium dependent domain containing 3
221875_x_at	7.81E-06	3.67E-07	7.076987	6.679189	2.0262934	HLA-F	major histocompatibility complex, class I, F

201531_at	7.81E-06	3.67E-07	7.0766415	6.678429	2.27905	ZFP36	ZFP36 ring finger protein
204163_at	7.81E-06	3.68E-07	7.0758827	6.67676	2.5547293	EMILIN1	elastin microfibril interfacer 1
226178_at	7.81E-06	3.68E-07	7.0755801	6.676095	1.3286427	SOCS4	suppressor of cytokine signaling 4
227577_at	7.82E-06	3.69E-07	7.0746042	6.673949	1.3648123	EXOC8	exocyst complex component 8
212753_at	7.83E-06	3.70E-07	7.0736428	6.671834	1.2947141	PCGF3	polycomb group ring finger 3
226280_at	7.83E-06	3.70E-07	7.0732007	6.670862	1.6824864	BNIP2	BCL2 interacting protein 2

224996_at	7.84E-06	3.71E-07	7.0729211	6.670247	1.9808723	ASPH	aspartate beta-hydroxylase
213649_at	7.84E-06	3.71E-07	7.0721757	6.668608	1.7011088	SRSF7	serine and arginine rich splicing factor 7
244433_at	7.89E-06	3.75E-07	7.0670159	6.657257	1.9492973		
226463_at	7.91E-06	3.77E-07	7.0657596	6.654493	1.4564543	ATP6V1C1	ATPase H+ transporting V1 subunit C1
201288_at	7.92E-06	3.78E-07	7.0644833	6.651684	2.1382248	ARHGDIB	Rho GDP dissociation inhibitor beta
216231_s_at	7.93E-06	3.78E-07	7.0636477	6.649845	1.2428446	B2M	beta-2-microglobulin
208999_at	7.93E-06	3.78E-07	7.0636436	6.649836	1.3941379	SEPT8	septin 8

225761_at	7.93E-06	3.79E-07	7.0633613	6.649215	1.4973436	PAPD4	poly(A) RNA polymerase D4, non-canonical
201069_at	7.94E-06	3.80E-07	7.0615667	6.645265	2.9819312	MMP2	matrix metallopeptidase 2
225508_at	7.94E-06	3.80E-07	7.0615155	6.645153	1.2640284	KIAA1468	KIAA1468
216194_s_at	7.98E-06	3.83E-07	7.0577302	6.63682	1.3915289	TBCB	tubulin folding cofactor B
209473_at	7.98E-06	3.85E-07	7.056316	6.633707	1.9328413	ENTPD1	ectonucleoside triphosphate diphosphohydrolase 1
213110_s_at	7.98E-06	3.85E-07	7.0561085	6.63325	2.1224504	COL4A5	collagen type IV alpha 5 chain

236155_at	7.98E-06	3.85E-07	7.0560718	6.633169	1.2238918	ZCCHC6	zinc finger CCHC-type containing 6
212867_at	7.99E-06	3.85E-07	7.0552371	6.631332	1.3290143	NCOA2	nuclear receptor coactivator 2
225074_at	8.00E-06	3.87E-07	7.0538659	6.628312	1.0335164	RAB2B	RAB2B, member RAS oncogene family
218649_x_at	8.00E-06	3.87E-07	7.0537801	6.628123	1.4965218	NEMF	nuclear export mediator factor
235151_at	8.02E-06	3.89E-07	7.051124	6.622274	1.068605	LOC283357	uncharacterized LOC283357

225893_at	8.02E-06	3.90E-07	7.0505493	6.621009	1.167512	RC3H1	ring finger and CCCH-type domains 1
208798_x_at	8.03E-06	3.91E-07	7.0490755	6.617763	2.4103764	GOLGA8A	golgin A8 family member A
230218_at	8.06E-06	3.94E-07	7.04545	6.609776	1.5620971	HIC1	hypermethylated in cancer 1
225102_at	8.08E-06	3.96E-07	7.0435871	6.605672	2.4885986	MGLL	monoglyceride lipase
207541_s_at	8.12E-06	3.98E-07	7.0406881	6.599283	1.089713	EXOSC10	exosome component 10
205907_s_at	8.12E-06	3.99E-07	7.0399394	6.597633	2.0105696	OMD	osteomodulin
204194_at	8.12E-06	3.99E-07	7.0398501	6.597437	1.2439455	BACH1	BTB domain and CNC homolog 1

218574_s_at	8.12E-06	3.99E-07	7.0396182	6.596925	1.5792654	LMCD1	LIM and cysteine rich domains 1
232431_at	8.13E-06	4.00E-07	7.0384771	6.59441	1.8902868	NR3C1	nuclear receptor subfamily 3 group C member 1
1556043_a_at	8.15E-06	4.02E-07	7.0370383	6.591239	1.0291727	TTN-AS1	TTN antisense RNA 1
1556053_at	8.19E-06	4.05E-07	7.0331383	6.582641	1.4426323	DNAJC7	DnaJ heat shock protein family (Hsp40) member C7
213186_at	8.19E-06	4.06E-07	7.0325473	6.581338	1.6840648	DZIP3	DAZ interacting zinc finger protein 3

203080_s_at	8.22E-06	4.08E-07	7.0295145	6.574651	1.4647868	BAZ2B	bromodomain adjacent to zinc finger domain 2B
201949_x_at	8.24E-06	4.11E-07	7.0270653	6.569249	1.1981888	CAPZB	capping actin protein of muscle Z-line beta subunit
232946_s_at	8.25E-06	4.11E-07	7.0268662	6.56881	1.177002	NADSYN1	NAD synthetase 1
239901_at	8.25E-06	4.11E-07	7.0262492	6.567449	1.1181643		
216903_s_at	8.25E-06	4.12E-07	7.0260867	6.567091	1.0385379	MICU1	mitochondrial calcium uptake 1
209583_s_at	8.27E-06	4.13E-07	7.0242208	6.562975	2.1218039	CD200	CD200 molecule

200625_s_at	8.28E-06	4.14E-07	7.0233809	6.561122	1.8118938	CAP1	adenylate cyclase associated protein 1
213505_s_at	8.29E-06	4.15E-07	7.0227449	6.559719	1.047063	SUGP2	SURP and G-patch domain containing 2
213596_at	8.33E-06	4.18E-07	7.0195959	6.552771	1.2385661	CASP4	caspase 4
214298_x_at	8.33E-06	4.18E-07	7.0192016	6.551901	1.9416896	SEPT6	septin 6
212587_s_at	8.33E-06	4.18E-07	7.0186785	6.550747	3.1251566	PTPRC	protein tyrosine phosphatase, receptor type C
222288_at	8.33E-06	4.19E-07	7.0184435	6.550228	2.4031016		
214163_at	8.34E-06	4.19E-07	7.0175549	6.548267	1.1616379	HSPB11	heat shock protein family B (small) member 11

219666_at	8.36E-06	4.21E-07	7.0156501	6.544063	2.214825	MS4A6A	membrane spanning 4-domains A6A
218014_at	8.36E-06	4.22E-07	7.0144896	6.541502	1.2699175	NUP85	nucleoporin 85
209436_at	8.36E-06	4.23E-07	7.0142775	6.541033	1.906187	SPON1	spondin 1
201068_s_at	8.36E-06	4.23E-07	7.0142148	6.540895	1.0477355	PSMC2	proteasome 26S subunit, ATPase 2
205168_at	8.38E-06	4.24E-07	7.0125292	6.537174	1.531462	DDR2	discoidin domain receptor tyrosine kinase 2
229422_at	8.38E-06	4.25E-07	7.012131	6.536295	1.2508402	NRDC	nardilysin convertase
230069_at	8.41E-06	4.27E-07	7.009132	6.529674	1.3727075	SFXN1	sideroflexin 1
204517_at	8.44E-06	4.30E-07	7.0065371	6.523944	1.1391552	PPIC	peptidylprolyl isomerase C

204749_at	8.44E-06	4.31E-07	7.0059141	6.522568	1.7540304	NAP1L3	nucleosome assembly protein 1 like 3
204620_s_at	8.45E-06	4.33E-07	7.003466	6.517161	3.8616461	VCAN	versican
239893_at	8.46E-06	4.34E-07	7.0028497	6.5158	1.5633941		
211946_s_at	8.46E-06	4.34E-07	7.0024211	6.514853	1.2437695	PRRC2C	proline rich coiled-coil 2C
1556821_x_at	8.53E-06	4.39E-07	6.9968775	6.502605	2.4270868	DLEU2	deleted in lymphocytic leukemia 2 (non-protein coding)
225040_s_at	8.55E-06	4.42E-07	6.9947226	6.497843	1.2467484	RPE	ribulose-5-phosphate-3-epimerase

225585_at	8.57E-06	4.43E-07	6.9936009	6.495364	1.8173609	RAP2A	RAP2A, member of RAS oncogene family
57715_at	8.62E-06	4.47E-07	6.9890609	6.485329	1.0309213	CALHM2	calcium homeostasis modulator 2
227139_s_at	8.62E-06	4.48E-07	6.9886482	6.484417	1.659163	HPS3	HPS3, biogenesis of lysosomal organelles complex 2 subunit 1
204202_at	8.64E-06	4.50E-07	6.9866778	6.48006	1.1893034	IQCE	IQ motif containing E
206584_at	8.65E-06	4.50E-07	6.9861516	6.478897	3.0431327	LY96	lymphocyte antigen 96

227776_at	8.68E-06	4.53E-07	6.9835623	6.473171	1.4019977	ACER3	alkaline ceramidase 3
230599_at	8.68E-06	4.53E-07	6.9832063	6.472384	1.954505		
203411_s_at	8.71E-06	4.55E-07	6.9812333	6.46802	1.7350741	LMNA	lamin A/C
224254_x_at	8.72E-06	4.56E-07	6.9803997	6.466177	1.5400443		
214475_x_at	8.72E-06	4.56E-07	6.9802523	6.46585	1.8409009	CAPN3	calpain 3
201307_at	8.72E-06	4.57E-07	6.9798586	6.46498	1.3770696	SEPT11	septin 11
214181_x_at	8.74E-06	4.59E-07	6.977626	6.460041	1.9056479	LST1	leukocyte specific transcript 1
219423_x_at	8.76E-06	4.60E-07	6.9760944	6.456652	1.8563898	TNFRSF25	TNF receptor superfamily member 25
209619_at	8.76E-06	4.61E-07	6.9755399	6.455426	2.1175195	CD74	CD74 molecule

207564_x_at	8.80E-06	4.65E-07	6.9719976	6.447587	1.7126402	OGT	O-linked N-acetylglucosamine (GlcNAc) transferase
212360_at	8.81E-06	4.66E-07	6.9711404	6.44569	1.0899921	AMPD2	adenosine monophosphate deaminase 2
205525_at	8.81E-06	4.66E-07	6.971105	6.445612	2.6407502	CALD1	caldesmon 1
218230_at	8.83E-06	4.68E-07	6.9684751	6.439791	1.1852677	ARFIP1	ADP ribosylation factor interacting protein 1

225321_s_at	8.85E-06	4.70E-07	6.9665372	6.435501	1.7692989	MIR6840///STAG3L5P-PVRIG2P-PILRB///PILRB	microRNA 6840///STAG3L5P-PVRIG2P-PILRB readthrough///paired immunoglobin-like type 2 receptor beta
226965_at	8.86E-06	4.71E-07	6.9659537	6.434209	1.2802786	DENND6A	DENN domain containing 6A
202351_at	8.89E-06	4.73E-07	6.9640197	6.429928	1.6312955	ITGAV	integrin subunit alpha V
224436_s_at	8.90E-06	4.74E-07	6.9629875	6.427642	1.1152545	NIPSNAP3A	nipsnap homolog 3A
212174_at	8.91E-06	4.75E-07	6.9625226	6.426613	1.501748	AK2	adenylate kinase 2

223451_s_at	8.91E-06	4.75E-07	6.9620856	6.425645	2.1760713	CKLF	chemokine like factor
209648_x_at	8.94E-06	4.78E-07	6.9599041	6.420814	1.4167589	SOCS5	suppressor of cytokine signaling 5
222186_at	8.97E-06	4.80E-07	6.9577402	6.416022	1.3254804		
244050_at	8.98E-06	4.81E-07	6.9568637	6.41408	1.9146321	HACD4	3-hydroxyacyl-CoA dehydratase 4
212907_at	8.99E-06	4.82E-07	6.9560928	6.412373	1.246673	SLC30A1	solute carrier family 30 member 1
1560199_x_at	8.99E-06	4.82E-07	6.9560314	6.412237	1.1404852		
1556060_a_at	8.99E-06	4.82E-07	6.9553301	6.410683	1.212795	ZNF451	zinc finger protein 451

225481_at	8.99E-06	4.83E-07	6.9551103	6.410196	2.593588	FRMD6	FERM domain containing 6
212410_at	8.99E-06	4.83E-07	6.9546404	6.409155	1.1886721	MICU2	mitochondrial calcium uptake 2
202880_s_at	9.05E-06	4.89E-07	6.9493449	6.397422	1.5330984	CYTH1	cytohesin 1
221613_s_at	9.11E-06	4.94E-07	6.9452387	6.388321	1.3238496	ZFAND6	zinc finger AN1-type containing 6
202391_at	9.11E-06	4.95E-07	6.9443329	6.386314	2.6498625	BASP1	brain abundant membrane attached signal protein 1
225782_at	9.11E-06	4.95E-07	6.9443232	6.386292	2.7509032	MSRB3	methionine sulfoxide reductase B3

228109_at	9.11E-06	4.95E-07	6.9442851	6.386208	1.6680827	RASGRF2	Ras protein specific guanine nucleotide releasing factor 2
221903_s_at	9.13E-06	4.96E-07	6.9427952	6.382905	1.3429923	CYLD	CYLD lysine 63 deubiquitinase
225019_at	9.14E-06	4.97E-07	6.942229	6.38165	1.0952061	CAMK2D	calcium/calmodulin dependent protein kinase II delta
225338_at	9.15E-06	4.99E-07	6.9402206	6.377197	1.2412171	ZYG11B	zyg-11 family member B, cell cycle regulator

212476_at	9.15E-06	4.99E-07	6.9400805	6.376886	1.6447932	ACAP2	ArfGAP with coiled-coil, ankyrin repeat and PH domains 2
205488_at	9.15E-06	5.00E-07	6.9399189	6.376528	2.2934859	GZMA	granzyme A
232090_at	9.19E-06	5.03E-07	6.9367845	6.369577	2.3332743	DNM3OS	DNM3 opposite strand/antisense RNA
215268_at	9.26E-06	5.09E-07	6.93161	6.3581	2.0370918	KIAA0754	KIAA0754
218051_s_at	9.28E-06	5.11E-07	6.9296095	6.353661	1.8691745	NT5DC2	5'-nucleotidase domain containing 2

224876_at	9.29E-06	5.13E-07	6.9283566	6.350881	1.22758	C5orf24	chromosome 5 open reading frame 24
239258_at	9.32E-06	5.16E-07	6.92518	6.343833	2.1935459		
223085_at	9.33E-06	5.18E-07	6.9238161	6.340805	1.4424971	RNF19A	ring finger protein 19A, RBR E3 ubiquitin protein ligase
211719_x_at	9.34E-06	5.20E-07	6.9217361	6.336189	3.6120827	FN1	fibronectin 1
221257_x_at	9.34E-06	5.21E-07	6.9215925	6.33587	1.1845629	FBXO38	F-box protein 38
226109_at	9.35E-06	5.21E-07	6.9211128	6.334805	1.171272	C21orf91	chromosome 21 open reading frame 91
241916_at	9.35E-06	5.22E-07	6.9204445	6.333322	2.2736359		

214459_x_at	9.37E-06	5.24E-07	6.9186722	6.329387	1.3665607	HLA-C	major histocompatibility complex, class I, C
203989_x_at	9.41E-06	5.28E-07	6.915315	6.321933	2.0771539	F2R	coagulation factor II thrombin receptor
213428_s_at	9.41E-06	5.29E-07	6.9146132	6.320374	2.1521821	COL6A1	collagen type VI alpha 1 chain
227415_at	9.45E-06	5.32E-07	6.9118905	6.314328	2.0647355	DGKH	diacylglycerol kinase eta
1559663_at	9.46E-06	5.33E-07	6.9109855	6.312318	2.1919564		

224710_at	9.46E-06	5.33E-07	6.9107888	6.311881	2.2744593	NARR///RAB34	nine-amino acid residue-repeats///RAB34, member RAS oncogene family
201731_s_at	9.46E-06	5.34E-07	6.9107168	6.311721	1.5591207	TPR	translocated promoter region, nuclear basket protein
216442_x_at	9.48E-06	5.36E-07	6.9090084	6.307926	3.5541063	FN1	fibronectin 1
210495_x_at	9.51E-06	5.39E-07	6.9060459	6.301344	3.4550045	FN1	fibronectin 1
201693_s_at	9.55E-06	5.42E-07	6.9039687	6.296729	2.2376296	EGR1	early growth response 1

213009_s_at	9.56E-06	5.42E-07	6.9033923	6.295448	1.0184252	TRIM37	tripartite motif containing 37
226873_at	9.56E-06	5.43E-07	6.9025838	6.293651	1.8580914	FAM63B	family with sequence similarity 63 member B
212535_at	9.58E-06	5.45E-07	6.9016721	6.291625	1.3644334	MEF2A	myocyte enhancer factor 2A
209451_at	9.60E-06	5.47E-07	6.8994276	6.286637	1.7823923	TANK	TRAF family member associated NFKB activator

215113_s_at	9.65E-06	5.52E-07	6.8954406	6.277774	1.1474454	SENP3-EIF4A1///SENP3	SENP3-EIF4A1 readthrough (NMD candidate)///SUMO1/sentrin/SMT3 specific peptidase 3
213517_at	9.65E-06	5.53E-07	6.894909	6.276592	1.0605923	PCBP2	poly(rC) binding protein 2
227013_at	9.70E-06	5.57E-07	6.8916937	6.269443	1.814643	LATS2	large tumor suppressor kinase 2
212464_s_at	9.71E-06	5.58E-07	6.890712	6.26726	3.8556252	FN1	fibronectin 1
219284_at	9.71E-06	5.59E-07	6.8904206	6.266612	1.3296164	HSPBAP1	HSPB1 associated protein 1

235410_at	9.72E-06	5.60E-07	6.8892779	6.26407	1.7147757	NPHP3	nephronophthisis 3 (adolescent)
212506_at	9.75E-06	5.63E-07	6.8870936	6.259212	1.136577	PICALM	phosphatidylinositol binding clathrin assembly protein
212628_at	9.76E-06	5.64E-07	6.8864481	6.257776	1.4283114	PKN2	protein kinase N2

225573_at	9.80E-06	5.66E-07	6.8842256	6.252832	1.1383375	NPHP3-ACAD11///ACAD11///NPHP3	NPHP3-ACAD11 readthrough (NMD candidate)///acyl-CoA dehydrogenase family member 11///nephronophthisis 3 (adolescent)
1556657_at	9.80E-06	5.67E-07	6.8837158	6.251698	1.4349943		
242695_at	9.81E-06	5.68E-07	6.8833043	6.250782	1.1714321		
212221_x_at	9.81E-06	5.68E-07	6.88271	6.24946	1.3535595	IDS	iduronate 2-sulfatase

212985_at	9.81E-06	5.69E-07	6.8824124	6.248798	2.029397	APBB2	amyloid beta precursor protein binding family B member 2
214719_at	9.82E-06	5.71E-07	6.8809852	6.245622	1.3489227	SLC46A3	solute carrier family 46 member 3
234723_x_at	9.84E-06	5.72E-07	6.8802842	6.244063	1.4554609		
227232_at	9.87E-06	5.76E-07	6.8767085	6.236105	1.2525748	EVL	Enah/Vasp-like
212486_s_at	9.87E-06	5.76E-07	6.8765145	6.235673	1.6278755	FYN	FYN proto-oncogene, Src family tyrosine kinase
235556_at	9.88E-06	5.79E-07	6.8747493	6.231744	1.1619093	CREBRF	CREB3 regulatory factor

235051_at	9.88E-06	5.79E-07	6.8744897	6.231166	1.1616268	CCDC50	coiled-coil domain containing 50
1560271_at	9.88E-06	5.80E-07	6.8741628	6.230438	1.5461986		
217028_at	9.89E-06	5.81E-07	6.8733576	6.228646	3.7345979	CXCR4	C-X-C motif chemokine receptor 4
228333_at	9.93E-06	5.84E-07	6.8707081	6.222747	2.05691	ZEB2	zinc finger E-box binding homeobox 2
224967_at	9.93E-06	5.84E-07	6.8705337	6.222359	1.5714771	UGCG	UDP-glucose ceramide glucosyltransferase
208961_s_at	9.93E-06	5.85E-07	6.8701091	6.221414	1.8173388	KLF6	Kruppel like factor 6

204299_at	9.94E-06	5.86E-07	6.8695347	6.220135	1.3812911	SRSF10	serine and arginine rich splicing factor 10
228164_at	9.94E-06	5.86E-07	6.8691834	6.219352	1.467675	AP4E1	adaptor related protein complex 4 epsilon 1 subunit
205582_s_at	9.99E-06	5.91E-07	6.8657258	6.211653	1.3099021	GGT5	gamma-glutamyltransferase 5
213798_s_at	1.00E-05	5.93E-07	6.8643633	6.208618	1.6159089	CAP1	adenylate cyclase associated protein 1
212067_s_at	1.00E-05	5.93E-07	6.8640419	6.207902	2.0410534	C1R	complement C1r subcomponent

210511_s_at	1.00E-05	5.94E-07	6.8628871	6.20533	4.513107	INHBA	inhibin beta A subunit
227594_at	1.00E-05	5.95E-07	6.8625279	6.20453	1.4922655	ZMYM6	zinc finger MYM-type containing 6
202763_at	1.00E-05	5.96E-07	6.8614875	6.202212	1.5682582	CASP3	caspase 3
201954_at	1.00E-05	5.98E-07	6.8603099	6.199589	2.3042414	ARPC1B	actin related protein 2/3 complex subunit 1B
209430_at	1.00E-05	5.98E-07	6.8599341	6.198751	1.9427334	BTAF1	B-TFIID TATA-box binding protein associated factor 1

219279_at	1.01E-05	6.02E-07	6.8574511	6.193219	2.2470111	DOCK10	dedicator of cytokinesis 10
239102_s_at	1.01E-05	6.07E-07	6.8536426	6.184732	1.7950552		
218940_at	1.01E-05	6.07E-07	6.8534088	6.184211	1.7897093	VCPKMT	valosin containing protein lysine methyltransferase
212923_s_at	1.01E-05	6.10E-07	6.8515181	6.179996	1.5189166	PXDC1	PX domain containing 1
219002_at	1.01E-05	6.11E-07	6.850853	6.178514	1.081385	FASTKD1	FAST kinase domains 1
235217_at	1.02E-05	6.12E-07	6.8503057	6.177294	1.069745	LINC01004	long intergenic non-protein coding RNA 1004

203884_s_at	1.02E-05	6.12E-07	6.8499612	6.176526	1.1097021	RAB11FIP2	RAB11 family interacting protein 2
225633_at	1.02E-05	6.15E-07	6.8482708	6.172757	1.0745825	DPY19L3	dpy-19 like 3 (C. elegans)
219678_x_at	1.02E-05	6.15E-07	6.8481406	6.172467	1.0436921	DCLRE1C	DNA cross-link repair 1C
212539_at	1.02E-05	6.16E-07	6.8474598	6.170949	1.0813988	CHD1L	chromodomain helicase DNA binding protein 1 like
217943_s_at	1.02E-05	6.17E-07	6.8465284	6.168873	1.473953	MAP7D1	MAP7 domain containing 1

212998_x_at	1.02E-05	6.17E-07	6.8464837	6.168773	3.420482	LOC101060835///HLA-DQB1	HLA class II histocompatibility antigen, DQ beta 1 chain-like///major histocompatibility complex, class II, DQ beta 1
243109_at	1.02E-05	6.23E-07	6.8423993	6.159665	1.4188939	MCTP2	multiple C2 and transmembrane domain containing 2
234994_at	1.02E-05	6.25E-07	6.8408486	6.156206	2.7293011	TMEM200A	transmembrane protein 200A

213532_at	1.02E-05	6.25E-07	6.8406705	6.155809	1.6097852	ADAM17	ADAM metallopeptidase domain 17
232544_at	1.03E-05	6.28E-07	6.8387032	6.151421	2.4033732		
238706_at	1.03E-05	6.29E-07	6.8377129	6.149212	1.225147	PAPD4	poly(A) RNA polymerase D4, non-canonical
219540_at	1.03E-05	6.32E-07	6.8361695	6.145769	2.0432993	ZNF267	zinc finger protein 267
225073_at	1.03E-05	6.32E-07	6.8358003	6.144945	1.3271718	PPHLN1	periphilin 1
235365_at	1.03E-05	6.35E-07	6.8340355	6.141007	1.1462504	DFNB59	deafness, autosomal recessive 59
239409_at	1.04E-05	6.37E-07	6.8327154	6.138061	1.5232148		

221830_at	1.04E-05	6.39E-07	6.8310886	6.134431	1.4601466	RAP2A	RAP2A, member of RAS oncogene family
213151_s_at	1.04E-05	6.42E-07	6.8292055	6.130228	1.7394912	SEPT7	septin 7
244383_at	1.04E-05	6.42E-07	6.8290815	6.129952	1.1088229		
228345_at	1.04E-05	6.44E-07	6.8276258	6.126702	1.3118738	CHIC1	cysteine rich hydrophobic domain 1
210105_s_at	1.04E-05	6.45E-07	6.8270162	6.125341	1.7269977	FYN	FYN proto-oncogene, Src family tyrosine kinase
208808_s_at	1.04E-05	6.47E-07	6.8256789	6.122356	1.7638598	HMGB2	high mobility group box 2

53720_at	1.04E-05	6.47E-07	6.825417	6.121771	1.5806561	C19orf66	chromosome 19 open reading frame 66
214016_s_at	1.05E-05	6.48E-07	6.8248891	6.120593	1.768983	SFPQ	splicing factor proline and glutamine rich
225643_at	1.05E-05	6.49E-07	6.8244806	6.119681	1.599483	MAPK1IP1L	mitogen-activated protein kinase 1 interacting protein 1 like
218802_at	1.05E-05	6.49E-07	6.8241111	6.118856	2.2625688	MCUB	mitochondrial calcium uniporter dominant negative beta subunit

209584_x_at	1.05E-05	6.50E-07	6.8238068	6.118177	1.0659575	APOBEC3C	apolipoprotein B mRNA editing enzyme catalytic subunit 3C
203615_x_at	1.05E-05	6.50E-07	6.8233051	6.117056	1.1648375	SULT1A1	sulfotransferase family 1A member 1
52164_at	1.05E-05	6.53E-07	6.8215361	6.113106	1.0403964	C11orf24	chromosome 11 open reading frame 24
202809_s_at	1.05E-05	6.53E-07	6.8213186	6.112621	1.1028359	INTS3	integrator complex subunit 3

203748_x_at	1.05E-05	6.57E-07	6.8188002	6.106997	1.6732455	RBMS1	RNA binding motif single stranded interacting protein 1
238455_at	1.06E-05	6.58E-07	6.8178512	6.104877	2.2598045	PLXDC2	plexin domain containing 2
205191_at	1.06E-05	6.61E-07	6.8162326	6.101262	1.5584439	RP2	retinitis pigmentosa 2 (X-linked recessive)
1561167_at	1.06E-05	6.66E-07	6.8130349	6.094119	2.0337846		

225611_at	1.06E-05	6.68E-07	6.8114294	6.090532	1.5576975	MAST4	microtubule associated serine/threonine kinase family member 4
1553117_a_at	1.06E-05	6.69E-07	6.8105331	6.088529	1.6141568	STK38	serine/threonine kinase 38
200959_at	1.06E-05	6.70E-07	6.8103028	6.088014	1.308922	FUS	FUS RNA binding protein
229206_at	1.06E-05	6.70E-07	6.8100327	6.087411	1.0133261		
238481_at	1.06E-05	6.70E-07	6.8099078	6.087132	3.3040838	MGP	matrix Gla protein
210017_at	1.07E-05	6.73E-07	6.8080785	6.083044	1.4533766	MALT1	MALT1 paracaspase
211367_s_at	1.07E-05	6.74E-07	6.8077231	6.08225	1.8646107	CASP1	caspase 1

212027_at	1.07E-05	6.75E-07	6.8068099	6.080209	1.4258927	RBM25	RNA binding motif protein 25
207691_x_at	1.07E-05	6.76E-07	6.8063634	6.079211	2.54585	ENTPD1	ectonucleoside triphosphate diphosphohydrolase 1
226155_at	1.07E-05	6.76E-07	6.8059543	6.078297	1.133712	FAM160B1	family with sequence similarity 160 member B1
202653_s_at	1.07E-05	6.81E-07	6.803287	6.072335	1.992343	7-Mar	membrane associated ring-CH-type finger 7
232095_at	1.07E-05	6.83E-07	6.8017316	6.068858	1.5432518		

201473_at	1.08E-05	6.85E-07	6.8002994	6.065656	2.0314545	JUNB	JunB proto-oncogene, AP-1 transcription factor subunit
226126_at	1.08E-05	6.87E-07	6.7994484	6.063753	1.36828	TBCK	TBC1 domain containing kinase
219848_s_at	1.08E-05	6.87E-07	6.7993566	6.063548	1.3682091	ZNF432	zinc finger protein 432
228220_at	1.08E-05	6.88E-07	6.7983644	6.061329	1.8950079	FCHO2	FCH domain only 2
1555778_a_at	1.08E-05	6.91E-07	6.7965246	6.057215	4.8716429	POSTN	periostin
209422_at	1.08E-05	6.94E-07	6.7949022	6.053587	1.275243	PHF20	PHD finger protein 20

211038_s_at	1.08E-05	6.94E-07	6.7944126	6.052492	1.423603	CROCCP2	ciliary rootlet coiled-coil, rootletin pseudogene 2
202163_s_at	1.08E-05	6.95E-07	6.7937614	6.051036	1.0132121	CNOT8	CCR4-NOT transcription complex subunit 8
1553955_at	1.09E-05	7.00E-07	6.7909887	6.044834	1.2237629	PPP1R21	protein phosphatase 1 regulatory subunit 21
209345_s_at	1.09E-05	7.01E-07	6.7903512	6.043407	1.1202514	PI4K2A	phosphatidylinositol 4-kinase type 2 alpha
225112_at	1.09E-05	7.04E-07	6.7886572	6.039618	1.0334196	ABI2	abl-interactor 2

208949_s_at	1.09E-05	7.04E-07	6.7885808	6.039447	1.8025786	LGALS3	lectin, galactoside binding soluble 3
212399_s_at	1.09E-05	7.05E-07	6.7878538	6.03782	1.3044675	VGLL4	vestigial like family member 4
235521_at	1.09E-05	7.05E-07	6.7876284	6.037316	2.3618288	HOXA3	homeobox A3
223279_s_at	1.10E-05	7.10E-07	6.784906	6.031224	2.4582398	UACA	uveal autoantigen with coiled-coil domains and ankyrin repeats
230233_at	1.10E-05	7.11E-07	6.7843059	6.029881	2.2730532		

222348_at	1.10E-05	7.11E-07	6.7841673	6.029571	1.9071757	MAST4	microtubule associated serine/threonine kinase family member 4
229287_at	1.10E-05	7.12E-07	6.7836299	6.028369	1.4714509	PCNX1	pecanex homolog 1 (Drosophila)
232024_at	1.10E-05	7.12E-07	6.7836037	6.02831	2.1605595	GIMAP2	GTPase, IMAP family member 2
1557302_at	1.10E-05	7.13E-07	6.7830741	6.027125	1.076082	ZNF585B	zinc finger protein 585B

224955_at	1.10E-05	7.14E-07	6.7821339	6.025021	1.3145643	TEAD1	TEA domain transcription factor 1
222371_at	1.10E-05	7.14E-07	6.7819688	6.024651	1.4887396		
209049_s_at	1.10E-05	7.17E-07	6.7806292	6.021653	1.0825261	ZMYND8	zinc finger MYND-type containing 8
204860_s_at	1.11E-05	7.22E-07	6.7775576	6.014777	1.5495916	NAIP	NLR family apoptosis inhibitory protein
201487_at	1.11E-05	7.24E-07	6.7760562	6.011415	1.621325	CTSC	cathepsin C
235791_x_at	1.11E-05	7.25E-07	6.7755326	6.010243	1.714922	CHD1	chromodomain helicase DNA binding protein 1
225256_at	1.11E-05	7.27E-07	6.7741213	6.007083	1.307905		

213056_at	1.11E-05	7.29E-07	6.7730323	6.004644	1.7414436	FRMD4B	FERM domain containing 4B
205745_x_at	1.11E-05	7.31E-07	6.7719272	6.00217	1.0669021	ADAM17	ADAM metallopeptidase domain 17
204773_at	1.12E-05	7.32E-07	6.7712033	6.000549	1.2783873	IL11RA	interleukin 11 receptor subunit alpha
230270_at	1.12E-05	7.33E-07	6.7708233	5.999698	1.7917307	PRPF38B	pre-mRNA processing factor 38B
224920_x_at	1.12E-05	7.35E-07	6.7693075	5.996303	1.5912427	MYADM	myeloid associated differentiation marker

203932_at	1.12E-05	7.37E-07	6.7685176	5.994533	2.0634984	HLA-DMB	major histocompatibility complex, class II, DM beta
212613_at	1.12E-05	7.38E-07	6.7678484	5.993034	1.3237029	BTN3A2	butyrophilin subfamily 3 member A2
201549_x_at	1.12E-05	7.38E-07	6.7674943	5.992241	1.0206271	KDM5B	lysine demethylase 5B
216268_s_at	1.13E-05	7.47E-07	6.7623114	5.98063	1.722237	JAG1	jagged 1
230078_at	1.13E-05	7.51E-07	6.7597508	5.974891	1.3841659	RAPGEF6	Rap guanine nucleotide exchange factor 6

221766_s_at	1.14E-05	7.53E-07	6.7590506	5.973322	1.5922829	FAM46A	family with sequence similarity 46 member A
237768_x_at	1.14E-05	7.53E-07	6.7588177	5.9728	1.3628711		
202995_s_at	1.14E-05	7.55E-07	6.7574026	5.969629	2.2031932	FBLN1	fibulin 1
203306_s_at	1.14E-05	7.58E-07	6.7559417	5.966354	1.4287268	SLC35A1	solute carrier family 35 member A1
227607_at	1.14E-05	7.61E-07	6.7540864	5.962195	1.8855163	STAMBPL1	STAM binding protein like 1
227041_at	1.14E-05	7.63E-07	6.7532598	5.960342	1.5116398	SESTD1	SEC14 and spectrin domain containing 1
215083_at	1.15E-05	7.65E-07	6.7519855	5.957485	1.3353514		

212572_at	1.15E-05	7.65E-07	6.7518987	5.957291	1.2650618	STK38L	serine/threonine kinase 38 like
226711_at	1.15E-05	7.68E-07	6.750286	5.953675	1.333632	FOXN2	forkhead box N2
212444_at	1.15E-05	7.68E-07	6.7501943	5.953469	3.4641286	GPRC5A	G protein-coupled receptor class C group 5 member A
226000_at	1.15E-05	7.68E-07	6.7501629	5.953399	1.8494577	CTTNBP2NL	CTTNBP2 N-terminal like
202258_s_at	1.15E-05	7.71E-07	6.7485391	5.949757	1.1746714	N4BP2L2	NEDD4 binding protein 2 like 2

242781_at	1.77E-07	1.63E-11	-12.3477661	16.360917	-1.0276718	FAM199X	family with sequence similarity 199, X-linked
1552893_at	3.43E-07	8.79E-11	-11.3267458	14.766589	-1.3494595	CAMK2N2	calcium/calmodulin dependent protein kinase II inhibitor 2
221186_at	5.41E-07	2.54E-10	-10.7172956	13.75566	-1.0015818	LOC100131532	uncharacterized LOC100131532
208486_at	5.41E-07	4.41E-10	-10.4090192	13.226535	-1.0344495	DRD5	dopamine receptor D5
217548_at	6.14E-07	6.85E-10	-10.1669389	12.802414	-1.3145289	ARPIN	actin-related protein 2/3 complex inhibitor

213014_at	6.24E-07	8.11E-10	-10.0754712	12.640161	-1.5057605	MAPK8IP1	mitogen-activated protein kinase 8 interacting protein 1
209567_at	6.29E-07	8.63E-10	-10.0415928	12.579784	-1.2819389	RRS1	ribosome biogenesis regulator homolog
216376_x_at	7.45E-07	1.19E-09	-9.869316	12.270391	-1.1833186		
239470_at	7.92E-07	1.41E-09	-9.780744	12.109774	-1.2814405	C15orf56	chromosome 15 open reading frame 56
203575_at	8.52E-07	1.66E-09	-9.6923625	11.948447	-1.1572384	CSNK2A2	casein kinase 2 alpha 2
1561314_at	9.08E-07	2.01E-09	-9.5922168	11.764364	-1.1058612	LOC101928658	uncharacterized LOC101928658

230070_at	9.11E-07	2.08E-09	-9.5738665	11.730485	-1.0940986	CNIH2	cornichon family AMPA receptor auxiliary protein 2
216986_s_at	9.52E-07	2.32E-09	-9.5168426	11.624912	-1.0141843	IRF4	interferon regulatory factor 4
205138_s_at	1.01E-06	2.64E-09	-9.4498835	11.500375	-1.2280127	UST	uronyl 2-sulfotransferase
234880_x_at	1.01E-06	2.81E-09	-9.4179539	11.440773	-1.0843705	KRTAP1-3	keratin associated protein 1-3
217087_at	1.01E-06	2.84E-09	-9.4118495	11.429361	-1.2093639	C1orf68	chromosome 1 open reading frame 68

214395_x_at	1.01E-06	2.89E-09	-9.4028809	11.412587	-1.0259202	EEF1D	eukaryotic translation elongation factor 1 delta
216382_s_at	1.10E-06	4.01E-09	-9.2345014	11.095592	-1.1670696	KMT2D	lysine methyltransferase 2D
228924_s_at	1.11E-06	4.33E-09	-9.1954859	11.021579	-1.1914612	UBE2J1	ubiquitin conjugating enzyme E2 J1
208221_s_at	1.13E-06	4.43E-09	-9.183381	10.998573	-1.0082123	SLIT1	slit guidance ligand 1

216351_x_at	1.13E-06	4.46E-09	-9.1793642	10.990934	-1.1667443	DAZ4///DAZ2///DAZ3///DAZ1	deleted in azoospermia 4///deleted in azoospermia 2///deleted in azoospermia 3///deleted in azoospermia 1
1566277_at	1.13E-06	4.48E-09	-9.1772661	10.986943	-1.0430782	OR5E1P	olfactory receptor family 5 subfamily E member 1 pseudogene

214171_s_at	1.13E-06	4.59E-09	-9.1648094	10.963236	-1.0185777	U2AF2	U2 small nuclear RNA auxiliary factor 2
216842_x_at	1.17E-06	4.82E-09	-9.1404661	10.916845	-1.3638034		
219138_at	1.17E-06	4.93E-09	-9.1289615	10.894892	-1.1062759	RPL14	ribosomal protein L14
235024_at	1.17E-06	4.99E-09	-9.1229063	10.88333	-1.2049812	JADE1	jade family PHD finger 1
1569465_at	1.20E-06	5.21E-09	-9.1002734	10.840068	-1.1603557	FAM160A1	family with sequence similarity 160 member A1
223157_at	1.20E-06	5.23E-09	-9.0982791	10.836253	-1.1292957	NOA1	nitric oxide associated 1

237536_at	1.20E-06	5.26E-09	-9.096173	10.832223	-1.3550552	MAPT-AS1	MAPT antisense RNA 1
216485_s_at	1.21E-06	5.42E-09	-9.0802264	10.801689	-1.0142536	TPSAB1	tryptase alpha/beta 1
1569644_at	1.21E-06	5.43E-09	-9.0798624	10.800992	-1.2004993		
206419_at	1.25E-06	5.85E-09	-9.0419454	10.728247	-1.2487164	RORC	RAR related orphan receptor C
213276_at	1.25E-06	6.06E-09	-9.0238762	10.693509	-1.2420129	CAMK2B	calcium/calmodulin dependent protein kinase II beta
224203_at	1.31E-06	6.67E-09	-8.9755143	10.600309	-1.0692548	SUFU	SUFU negative regulator of hedgehog signaling

227389_x_at	1.44E-06	8.34E-09	-8.8635949	10.383362	-1.095028	IRF2BP2	interferon regulatory factor 2 binding protein 2
210130_s_at	1.44E-06	8.37E-09	-8.8616137	10.379506	-1.679295	TM7SF2	transmembrane 7 superfamily member 2
229458_s_at	1.46E-06	8.97E-09	-8.8272108	10.312453	-1.1395289	GALK1	galactokinase 1
213087_s_at	1.46E-06	9.02E-09	-8.8242686	10.306711	-1.360415	EEF1D	eukaryotic translation elongation factor 1 delta
227118_s_at	1.46E-06	9.14E-09	-8.8181401	10.294747	-1.0186486	MRPS26	mitochondrial ribosomal protein S26
222831_at	1.47E-06	9.24E-09	-8.8126569	10.284037	-1.3053273	SAP30L	SAP30 like

244221_at	1.49E-06	9.54E-09	-8.7964496	10.252357	-1.033447		
1565843_s_at	1.50E-06	9.81E-09	-8.7827982	10.225645	-1.0470962	BRPF3	bromodomain and PHD finger containing 3
1557679_at	1.61E-06	1.16E-08	-8.7012373	10.065498	-1.15458	ERICH1-AS1	ERICH1 antisense RNA 1
1564706_s_at	1.67E-06	1.24E-08	-8.6683799	10.000716	-2.9936543	GLS2	glutaminase 2
1562836_at	1.67E-06	1.25E-08	-8.662766	9.989632	-1.5615545	DDX6	DEAD-box helicase 6
230706_s_at	1.68E-06	1.29E-08	-8.6460375	9.956577	-2.374872	CAMK2N2	calcium/calmodulin dependent protein kinase II inhibitor 2
212844_at	1.68E-06	1.31E-08	-8.6404266	9.945481	-1.1680098	RRP1B	ribosomal RNA processing 1B

229079_at	1.69E-06	1.32E-08	-8.6350385	9.934822	-1.0517461	EHMT2	euchromatic histone lysine methyltransferase 2
231475_at	1.70E-06	1.37E-08	-8.6169351	9.898977	-1.0543863	TBC1D21	TBC1 domain family member 21
207290_at	1.70E-06	1.37E-08	-8.6167519	9.898614	-1.3835146	PLXNA2	plexin A2
213651_at	1.72E-06	1.42E-08	-8.5986672	9.86276	-1.204815	INPP5J	inositol polyphosphate-5-phosphatase J
209978_s_at	1.82E-06	1.59E-08	-8.5429706	9.752043	-1.4731716	PLG///LPA	plasminogen///lipoprotein(a)
214556_at	2.02E-06	1.92E-08	-8.4526838	9.571628	-1.2011245	SSTR4	somatostatin receptor 4

201064_s_at	2.08E-06	2.02E-08	-8.4285878	9.523282	-1.8978221	LOC100996696///PABPC4	polyadenylate-binding protein 4 pseudogene///poly(A) binding protein cytoplasmic 4
241540_at	2.16E-06	2.21E-08	-8.3833099	9.432212	-1.0328966		
232491_at	2.17E-06	2.23E-08	-8.3793256	9.424184	-1.0824346		
1554151_at	2.18E-06	2.25E-08	-8.3758905	9.417261	-1.0941982	OGDH	oxoglutarate dehydrogenase
207341_at	2.18E-06	2.28E-08	-8.3685533	9.402468	-1.0055543	PRTN3	proteinase 3
1561019_at	2.23E-06	2.42E-08	-8.3391681	9.343145	-1.1621141		
211870_s_at	2.24E-06	2.43E-08	-8.3374915	9.339757	-1.0542339	PCDHA2///PCDHA3	protocadherin alpha 2///protocadherin alpha 3
234360_at	2.24E-06	2.46E-08	-8.3313996	9.327441	-1.0490477		

234002_at	2.25E-06	2.48E-08	-8.3279776	9.320521	-1.0572873	ABHD16B	abhydrolase domain containing 16B
224468_s_at	2.27E-06	2.53E-08	-8.3177809	9.299891	-1.6306989	SNORD88C///C19orf48	small nucleolar RNA, C/D box 88C///chromosome 19 open reading frame 48
212811_x_at	2.31E-06	2.61E-08	-8.3041904	9.272372	-1.3453018	SLC1A4	solute carrier family 1 member 4
215880_at	2.31E-06	2.63E-08	-8.2999557	9.263791	-1.1609791	NAGLU	N-acetyl-alpha-glucosaminidase
233941_at	2.34E-06	2.68E-08	-8.2908431	9.245319	-1.0114341	LRRC74A	leucine rich repeat containing 74A

207680_x_at	2.36E-06	2.73E-08	-8.2817969	9.226969	-1.0026323	PAX3	paired box 3
1553893_at	2.36E-06	2.73E-08	-8.2811603	9.225677	-1.2054479	CCDC105	coiled-coil domain containing 105
241839_at	2.36E-06	2.74E-08	-8.2799181	9.223156	-1.0115741	DLG3-AS1	DLG3 antisense RNA 1
208277_at	2.37E-06	2.79E-08	-8.2708567	9.204762	-1.0090066	PITX3	paired like homeodomain 3
213563_s_at	2.45E-06	2.96E-08	-8.242536	9.147195	-1.0378346	TUBGCP2	tubulin gamma complex associated protein 2
234717_at	2.49E-06	3.05E-08	-8.2293348	9.120322	-1.7279677		
244272_s_at	2.49E-06	3.07E-08	-8.2247721	9.111028	-1.0033537	TC2N	tandem C2 domains, nuclear

205531_s_at	2.51E-06	3.10E-08	-8.2200566	9.10142	-2.5081182	GLS2	glutaminase 2
217040_x_at	2.56E-06	3.35E-08	-8.1841984	9.028252	-1.7591698	SOX15	SRY-box 15
1558450_at	2.57E-06	3.39E-08	-8.1778222	9.015222	-1.0162245	A2M	alpha-2-macroglobulin
215819_s_at	2.61E-06	3.51E-08	-8.1616791	8.982207	-1.1718462	RHD///RHCE	Rh blood group D antigen///Rh blood group CcEe antigens
242411_at	2.70E-06	3.85E-08	-8.1176	8.891871	-1.1840516	ARL10	ADP ribosylation factor like GTPase 10
237263_at	2.86E-06	4.31E-08	-8.0634156	8.780444	-1.1412368		
207368_at	2.86E-06	4.34E-08	-8.0602225	8.773864	-1.0230864	HTR1D	5-hydroxytryptamine receptor 1D

1555165_a_at	2.87E-06	4.35E-08	-8.0590159	8.771378	-1.0411639	PGPEP1	pyroglutamyl-peptidase I
221162_at	2.87E-06	4.38E-08	-8.0559174	8.764991	-1.2613009	HHLA1	HERV-H LTR-associating 1
1568997_at	2.88E-06	4.45E-08	-8.0483701	8.749429	-1.2777636	POLR1E	RNA polymerase I subunit E
208466_at	2.90E-06	4.52E-08	-8.0407565	8.733722	-1.1475354	RAB3D	RAB3D, member RAS oncogene family
1565879_at	2.94E-06	4.63E-08	-8.029099	8.709657	-1.0629821		
238582_at	2.94E-06	4.64E-08	-8.0287744	8.708986	-1.2016114	C21orf2	chromosome 21 open reading frame 2

233325_at	2.94E-06	4.69E-08	-8.0232512	8.697577	-1.1228045	SLC35D2	solute carrier family 35 member D2
237853_x_at	2.94E-06	4.70E-08	-8.0227985	8.696642	-1.156092	KRTAP10-12///KRTAP10-7	keratin associated protein 10-12///keratin associated protein 10-7
224485_s_at	2.94E-06	4.71E-08	-8.0211995	8.693338	-2.7681727	SLC30A2	solute carrier family 30 member 2
220130_x_at	2.96E-06	4.77E-08	-8.0153866	8.681324	-1.1056796	LTB4R2	leukotriene B4 receptor 2
217723_x_at	3.03E-06	5.04E-08	-7.989326	8.627404	-1.2070377	RPS12	ribosomal protein S12

222705_s_at	3.04E-06	5.08E-08	-7.9854305	8.619335	-2.1192705	SLC25A15	solute carrier family 25 member 15
223653_x_at	3.04E-06	5.11E-08	-7.9829657	8.614229	-1.2812977	CELF4	CUGBP, Elav-like family member 4
224000_at	3.10E-06	5.35E-08	-7.9611065	8.568907	-1.1246402	C2orf16	chromosome 2 open reading frame 16
238196_at	3.15E-06	5.55E-08	-7.9435769	8.532512	-2.2888362	LOC285095	uncharacterized LOC285095
201848_s_at	3.20E-06	5.72E-08	-7.9292791	8.502795	-3.6802161	BNIP3	BCL2 interacting protein 3

223427_s_at	3.22E-06	5.84E-08	-7.9200672	8.483633	-4.0119102	EPB41L4B	erythrocyte membrane protein band 4.1 like 4B
AFFX-M27830_M_at	3.30E-06	6.07E-08	-7.9018501	8.445704	-1.0488614		
207873_x_at	3.32E-06	6.16E-08	-7.8950136	8.431457	-1.0065129	SEZ6L	seizure related 6 homolog like
207311_at	3.33E-06	6.21E-08	-7.8909314	8.422947	-1.0176532	DOC2B	double C2 domain beta
238858_at	3.37E-06	6.42E-08	-7.8752678	8.390272	-1.2441693	TIFA	TRAF interacting protein with forkhead associated domain

1556453_at	3.41E-06	6.61E-08	-7.8614927	8.361508	-1.1317825	LOC100506274	uncharacterized LOC100506274
211195_s_at	3.47E-06	6.92E-08	-7.8404881	8.317595	-1.0833798	TP63	tumor protein p63
235971_at	3.51E-06	7.04E-08	-7.8323972	8.300663	-1.5796832	TIFA	TRAF interacting protein with forkhead associated domain
1568649_at	3.63E-06	7.46E-08	-7.805271	8.243828	-1.3757337	LOC105370943	uncharacterized LOC105370943
223754_at	3.72E-06	7.86E-08	-7.7809352	8.19275	-1.4550987	C2orf88	chromosome 2 open reading frame 88

206999_at	3.79E-06	8.22E-08	-7.7598897	8.14851	-1.2494134	IL12RB2	interleukin 12 receptor subunit beta 2
230084_at	3.79E-06	8.26E-08	-7.7572846	8.14303	-3.2428211	SLC30A2	solute carrier family 30 member 2
221067_s_at	3.82E-06	8.43E-08	-7.7479779	8.123443	-1.9096236	SPX	spexin hormone
205947_s_at	3.82E-06	8.45E-08	-7.7471207	8.121638	-1.2220441	VIPR2	vasoactive intestinal peptide receptor 2
213809_x_at	3.91E-06	8.83E-08	-7.7264036	8.07799	-1.0089129	TCF3	transcription factor 3
221429_x_at	3.92E-06	8.86E-08	-7.725022	8.075077	-1.1562795	TEX13A	testis expressed 13A

231360_at	3.92E-06	8.89E-08	-7.723318	8.071484	-1.0517702	C20orf141	chromosome 20 open reading frame 141
1562581_at	3.92E-06	8.90E-08	-7.7227928	8.070376	-1.0263979	LOC254028	uncharacterized LOC254028
1566136_at	3.92E-06	8.94E-08	-7.7204841	8.065507	-1.0048093		
213990_s_at	3.93E-06	9.12E-08	-7.7116757	8.046923	-1.0039554	PAK5	p21 (RAC1) activated kinase 5
231707_at	3.96E-06	9.19E-08	-7.7078844	8.03892	-1.0995204		
209618_at	3.96E-06	9.21E-08	-7.7066932	8.036406	-2.3223577	CTNND2	catenin delta 2
214842_s_at	3.97E-06	9.30E-08	-7.7024249	8.027394	-3.7683216	ALB	albumin
228666_at	3.99E-06	9.43E-08	-7.6959319	8.013679	-1.309348	ARPIN	actin-related protein 2/3 complex inhibitor

203867_s_at	3.99E-06	9.44E-08	-7.6956751	8.013136	-1.490355	NLE1	notchless homolog 1
216848_at	3.99E-06	9.45E-08	-7.6951712	8.012072	-1.0749196		
210332_at	3.99E-06	9.50E-08	-7.6925851	8.006607	-1.0550934	ACHE	acetylcholinesterase (Cartwright blood group)
216567_at	3.99E-06	9.58E-08	-7.68883	7.998671	-1.0884873	MBP	myelin basic protein
239894_at	4.01E-06	9.70E-08	-7.6826409	7.985587	-1.3627223	NEBL-AS1	NEBL antisense RNA 1
237437_s_at	4.03E-06	9.77E-08	-7.679657	7.979277	-1.0149402	ADARB2	adenosine deaminase, RNA specific B2 (inactive)
1558586_at	4.03E-06	9.78E-08	-7.6791537	7.978212	-1.3566486	ZNF33B	zinc finger protein 33B

1561390_at	4.06E-06	1.00E-07	-7.6671477	7.952809	-1.0861259	FAM41AY2///FAM41AY1	family with sequence similarity 41 member A, Y-linked 2///family with sequence similarity 41 member A, Y-linked 1
227730_at	4.16E-06	1.04E-07	-7.6494503	7.915325	-1.4707955	DYM	dymeclin
207732_s_at	4.18E-06	1.05E-07	-7.6464274	7.908919	-1.0850105	DLG3	discs large MAGUK scaffold protein 3
241934_at	4.30E-06	1.10E-07	-7.6262844	7.866193	-1.12978	NTM	neurotrimin

220816_at	4.34E-06	1.12E-07	-7.6180629	7.848738	-1.5214275	LPAR3	lysophosphatidic acid receptor 3
221426_s_at	4.41E-06	1.15E-07	-7.6029652	7.816659	-1.0371359	OR3A3	olfactory receptor family 3 subfamily A member 3
231452_at	4.45E-06	1.18E-07	-7.5904571	7.790058	-1.216373	HRASLS5	HRAS like suppressor family member 5
235488_at	4.47E-06	1.20E-07	-7.5859519	7.780471	-1.0313798	RASL10B	RAS like family 10 member B
231211_s_at	4.48E-06	1.21E-07	-7.5820454	7.772156	-1.6618834	YIF1B	Yip1 interacting factor homolog B, membrane trafficking protein

237752_at	4.54E-06	1.24E-07	-7.5675955	7.741381	-1.1809893		
238347_at	4.54E-06	1.25E-07	-7.5668753	7.739846	-1.0777873	SLC38A10	solute carrier family 38 member 10
228420_at	4.57E-06	1.26E-07	-7.5609913	7.727305	-1.0679041	PDCD2	programmed cell death 2
1557680_at	4.64E-06	1.29E-07	-7.5505401	7.705019	-2.1898241	SAMD15	sterile alpha motif domain containing 15
242200_at	4.68E-06	1.32E-07	-7.5415285	7.685789	-1.0804073	ADAMTSL5	ADAMTS like 5
239127_at	4.70E-06	1.33E-07	-7.5378181	7.677868	-1.0887545	LOC100288860	uncharacterized LOC100288860

234554_at	4.74E-06	1.35E-07	-7.5306169	7.66249	-1.0313477	KCNK16	potassium two pore domain channel subfamily K member 16
232684_at	4.78E-06	1.37E-07	-7.5249407	7.650363	-1.2840605	ZNF503-AS1	ZNF503 antisense RNA 1
1559926_at	4.84E-06	1.40E-07	-7.5121997	7.623126	-1.0333418	LOC728353	uncharacterized LOC728353
1563327_a_at	4.86E-06	1.42E-07	-7.5059874	7.609838	-1.1005837	LINC01545	long intergenic non-protein coding RNA 1545
221382_at	4.90E-06	1.45E-07	-7.4978457	7.592414	-1.3492414		

1566989_at	4.92E-06	1.47E-07	-7.4926665	7.581326	-1.109753	ARID1B	AT-rich interaction domain 1B
237257_at	4.93E-06	1.47E-07	-7.4914425	7.578705	-1.0930532	RAB4B	RAB4B, member RAS oncogene family
242071_x_at	4.96E-06	1.49E-07	-7.485733	7.566475	-1.0356557	ITGA8	integrin subunit alpha 8
1562254_at	5.14E-06	1.58E-07	-7.4574354	7.505796	-1.1309004	STK35	serine/threonine kinase 35
233586_s_at	5.15E-06	1.59E-07	-7.454985	7.500536	-1.036423	KLK12	kallikrein related peptidase 12
1568986_x_at	5.16E-06	1.60E-07	-7.4530149	7.496307	-1.2086568	PIGT	phosphatidylinositol glycan anchor biosynthesis class T

233640_x_at	5.21E-06	1.63E-07	-7.4449963	7.479087	-1.0032568	KRTAP9-4	keratin associated protein 9-4
1560911_at	5.22E-06	1.64E-07	-7.4423452	7.473392	-1.1420395	LOC10013346 1	uncharacterized LOC100133461
209610_s_at	5.23E-06	1.64E-07	-7.439932	7.468207	-1.432995	SLC1A4	solute carrier family 1 member 4
241486_at	5.23E-06	1.65E-07	-7.4394438	7.467158	-1.0393446		
231686_at	5.23E-06	1.65E-07	-7.4389154	7.466023	-1.7443396	GATM	glycine amidinotransferase
234617_at	5.37E-06	1.73E-07	-7.4171926	7.419309	-1.0098168	OR52D1	olfactory receptor family 52 subfamily D member 1
71933_at	5.39E-06	1.75E-07	-7.4125509	7.409319	-1.3080541	WNT6	Wnt family member 6

220519_s_at	5.41E-06	1.76E-07	-7.4098001	7.403397	-1.0078905	LIM2	lens intrinsic membrane protein 2
203866_at	5.49E-06	1.80E-07	-7.399212	7.380594	-1.0604252	NLE1	notchless homolog 1
1554344_s_at	5.51E-06	1.81E-07	-7.3954062	7.372393	-2.34456	AQP12B///AQP12A	aquaporin 12B///aquaporin 12A
204943_at	5.55E-06	1.83E-07	-7.3901507	7.361066	-1.3973909	ADAM12	ADAM metallopeptidase domain 12
224501_at	5.56E-06	1.84E-07	-7.3889561	7.358491	-1.0287621	PERM1	PPARGC1 and ESRR induced regulator, muscle 1
241427_x_at	5.61E-06	1.87E-07	-7.3819413	7.343364	-1.3860514		
227963_at	5.64E-06	1.89E-07	-7.3762089	7.330998	-1.2160116		
244273_at	5.66E-06	1.91E-07	-7.3722046	7.322357	-1.0053493		

223373_s_at	5.67E-06	1.92E-07	-7.3683079	7.313946	-1.0713234	PLA2G12A	phospholipase A2 group XIIA
226870_at	5.76E-06	1.97E-07	-7.3576081	7.29084	-1.9970214	COMTD1	catechol-O-methyltransferase domain containing 1
1553478_at	5.79E-06	2.00E-07	-7.3517973	7.278284	-1.2473193	KIRREL3-AS3	KIRREL3 antisense RNA 3
234580_at	5.81E-06	2.02E-07	-7.3472963	7.268556	-1.0924754	TMEM106A	transmembrane protein 106A
230911_at	5.85E-06	2.04E-07	-7.3422078	7.257555	-1.1209543	SIX1	SIX homeobox 1

221409_at	5.95E-06	2.10E-07	-7.3294363	7.229926	-1.1219882	OR2S2	olfactory receptor family 2 subfamily S member 2 (gene/pseudogene)
218225_at	6.03E-06	2.16E-07	-7.316694	7.202339	-1.1501959	ECSIT	ECSIT signalling integrator
230184_at	6.05E-06	2.17E-07	-7.3146093	7.197823	-1.1473148	LINC01158	long intergenic non-protein coding RNA 1158
237775_x_at	6.18E-06	2.25E-07	-7.2965481	7.158676	-1.2606214		
204800_s_at	6.18E-06	2.26E-07	-7.296034	7.157561	-1.2809511	DHRS12	dehydrogenase/reductase 12
221124_s_at	6.23E-06	2.29E-07	-7.2891352	7.142595	-1.020688	VSX1	visual system homeobox 1

239265_at	6.25E-06	2.31E-07	-7.2849235	7.133456	-1.1116939	SLC35G1	solute carrier family 35 member G1
224393_s_at	6.37E-06	2.41E-07	-7.2660103	7.092383	-1.1434043	CECR6	cat eye syndrome chromosome region, candidate 6
220290_at	6.40E-06	2.43E-07	-7.2618723	7.08339	-1.1739371	AIM1L	absent in melanoma 1-like
206469_x_at	6.44E-06	2.47E-07	-7.2553193	7.069144	-2.6141236	AKR7A3	aldo-keto reductase family 7 member A3
213770_at	6.45E-06	2.48E-07	-7.253423	7.06502	-1.1877262	KSR1	kinase suppressor of ras 1
211235_s_at	6.50E-06	2.53E-07	-7.2451329	7.046987	-1.0650259	ESR1	estrogen receptor 1

217254_s_at	6.72E-06	2.71E-07	-7.2139448	6.97906	-1.2592187	EPO	erythropoietin
1568191_at	6.72E-06	2.71E-07	-7.2135993	6.978307	-1.0283095	MCAM	melanoma cell adhesion molecule
208474_at	6.76E-06	2.73E-07	-7.2099275	6.970301	-1.068177	CLDN6	claudin 6
242998_at	6.77E-06	2.74E-07	-7.2089709	6.968214	-1.5044007	RDH12	retinol dehydrogenase 12 (all-trans/9-cis/11-cis)
210684_s_at	6.77E-06	2.74E-07	-7.2083363	6.966831	-1.0719859	DLG4	discs large MAGUK scaffold protein 4
236219_at	6.81E-06	2.77E-07	-7.203411	6.956087	-1.0846802	SLC35G1	solute carrier family 35 member G1

1555697_at	6.83E-06	2.79E-07	-7.2003256	6.949355	-1.0791486	KLK4	kallikrein related peptidase 4
231463_at	6.83E-06	2.79E-07	-7.1997559	6.948112	-1.1093211	CNTD1	cyclin N-terminal domain containing 1
227890_at	6.90E-06	2.84E-07	-7.191606	6.930324	-1.0539737	TMEM198	transmembrane protein 198
214309_s_at	6.91E-06	2.86E-07	-7.1892868	6.92526	-2.352185		
237583_at	6.99E-06	2.92E-07	-7.179985	6.904944	-1.1374932		
212810_s_at	7.02E-06	2.94E-07	-7.1767358	6.897844	-1.3635209	SLC1A4	solute carrier family 1 member 4
234875_at	7.06E-06	2.97E-07	-7.1722911	6.88813	-1.1504462	RPL7AP10	ribosomal protein L7a pseudogene 10
217224_at	7.08E-06	3.01E-07	-7.1663634	6.875171	-1.0540562		

206716_at	7.09E-06	3.02E-07	-7.1643417	6.87075	-1.3540095	UMOD	uromodulin
227287_at	7.14E-06	3.06E-07	-7.1591404	6.859373	-1.046697	CITED2	Cbp/p300 interacting transactivator with Glu/Asp rich carboxy-terminal domain 2
231200_at	7.14E-06	3.06E-07	-7.1585013	6.857975	-1.0254136	LSM14B	LSM family member 14B
214630_at	7.17E-06	3.11E-07	-7.1515608	6.842787	-1.0164986	CYP11B2	cytochrome P450 family 11 subfamily B member 2

243261_at	7.39E-06	3.28E-07	-7.1275506	6.790197	-1.0921636	LINC00689	long intergenic non-protein coding RNA 689
231590_at	7.39E-06	3.28E-07	-7.1273092	6.789668	-1.2004496	GATM	glycine amidinotransferase
239128_at	7.48E-06	3.34E-07	-7.119379	6.77228	-1.0264368	TMEM221	transmembrane protein 221
244500_s_at	7.51E-06	3.36E-07	-7.1172897	6.767698	-1.1668691	EVI5L	ecotropic viral integration site 5 like

239754_at	7.51E-06	3.36E-07	-7.1169613	6.766978	-1.018163	SNORD65///SNORD49B///LRRC75A-AS1///SNORD49A	small nucleolar RNA, C/D box 65///small nucleolar RNA, C/D box 49B///LRRC75A antisense RNA 1///small nucleolar RNA, C/D box 49A
216381_x_at	7.54E-06	3.40E-07	-7.1107924	6.753444	-2.7635586	AKR7A3	aldo-keto reductase family 7 member A3

223018_at	7.62E-06	3.49E-07	-7.1000387	6.72984	-1.1141002	NOB1	NIN1/PSMD8 binding protein 1 homolog
241600_at	7.64E-06	3.51E-07	-7.097446	6.724147	-1.1150729	WIPF3	WAS/WASL interacting protein family member 3
206728_at	7.81E-06	3.67E-07	-7.0773092	6.679897	-2.1206525	ECE2	endothelin converting enzyme 2
207555_s_at	7.96E-06	3.81E-07	-7.0598834	6.641561	-1.1799361	TBXA2R	thromboxane A2 receptor
217095_x_at	7.96E-06	3.82E-07	-7.059474	6.640659	-1.0786375	NCR1	natural cytotoxicity triggering receptor 1

211172_x_at	8.00E-06	3.87E-07	-7.0534257	6.627343	-1.2187823	AKAP7	A-kinase anchoring protein 7
1564339_a_at	8.08E-06	3.96E-07	-7.0436829	6.605883	-1.7425493	CHRM3	cholinergic receptor muscarinic 3
225698_at	8.15E-06	4.02E-07	-7.0364539	6.589951	-1.4573589	EPB41L4A-AS1	EPB41L4A antisense RNA 1
217162_at	8.22E-06	4.08E-07	-7.0300254	6.575778	-1.0704007	TSPY1	testis specific protein, Y-linked 1
230572_at	8.24E-06	4.11E-07	-7.0271488	6.569433	-1.0417604	WWC2-AS2	WWC2 antisense RNA 2
235136_at	8.32E-06	4.17E-07	-7.0205679	6.554916	-1.2540841	ORMDL3	ORMDL sphingolipid biosynthesis regulator 3

235129_at	8.33E-06	4.19E-07	-7.0183183	6.549952	-1.3739666	PPP1R1A	protein phosphatase 1 regulatory inhibitor subunit 1A
241285_at	8.41E-06	4.28E-07	-7.008571	6.528435	-1.0165557		
1552473_at	8.48E-06	4.36E-07	-7.0007003	6.511052	-1.1967968	GAMT	guanidinoacetate N-methyltransferase
236188_s_at	8.53E-06	4.40E-07	-6.996437	6.501632	-1.0908111	NAP1L4	nucleosome assembly protein 1 like 4
242942_at	8.58E-06	4.44E-07	-6.9927406	6.493463	-1.0932496	SLC38A10	solute carrier family 38 member 10
237611_at	8.60E-06	4.45E-07	-6.9914902	6.490699	-1.0501918		

1555306_a_at	8.76E-06	4.60E-07	-6.9761847	6.456852	-1.3291682	ECE2	endothelin converting enzyme 2
240831_at	8.77E-06	4.62E-07	-6.9746395	6.453433	-1.1320548		
212246_at	8.91E-06	4.75E-07	-6.9624437	6.426438	-1.168595	MCFD2	multiple coagulation factor deficiency 2
223319_at	8.99E-06	4.83E-07	-6.9547297	6.409353	-1.1142284	GPHN	gephyrin
202753_at	9.01E-06	4.85E-07	-6.9529201	6.405344	-1.7040518	PSMD6	proteasome 26S subunit, non-ATPase 6
239000_at	9.01E-06	4.85E-07	-6.9529108	6.405324	-1.1557507	BRD4	bromodomain containing 4
205305_at	9.05E-06	4.89E-07	-6.9496195	6.398031	-4.7110136	FGL1	fibrinogen like 1
241125_at	9.11E-06	4.93E-07	-6.9460525	6.390125	-1.0609763		

220465_at	9.15E-06	4.97E-07	-6.9417238	6.380529	-1.1148359	CEBPA-AS1	CEBPA antisense RNA 1 (head to head)
1555616_at	9.15E-06	4.99E-07	-6.9403278	6.377434	-1.4393634		
208417_at	9.18E-06	5.02E-07	-6.9373733	6.370883	-1.0430066	FGF6	fibroblast growth factor 6
210328_at	9.31E-06	5.15E-07	-6.9264955	6.346752	-4.7996073	GNMT	glycine N-methyltransferase
238326_at	9.31E-06	5.15E-07	-6.926245	6.346196	-1.0509357	ODF3B	outer dense fiber of sperm tails 3B
213987_s_at	9.32E-06	5.16E-07	-6.9253884	6.344295	-1.0720043	CDK13	cyclin dependent kinase 13
1559877_at	9.33E-06	5.17E-07	-6.924627	6.342605	-2.4106082	LOC100289094	uncharacterized LOC100289094
229544_at	9.33E-06	5.18E-07	-6.9235351	6.340182	-1.9154455		

240670_at	9.33E-06	5.18E-07	-6.9234938	6.34009	-1.1420393		
239993_at	9.40E-06	5.26E-07	-6.916823	6.325281	-1.1098375	LOC390705	protein phosphatase 2 regulatory subunit B", beta pseudogene
233743_x_at	9.48E-06	5.36E-07	-6.9086193	6.307062	-1.2357796	S1PR5	sphingosine-1-phosphate receptor 5
217341_at	9.50E-06	5.38E-07	-6.9070555	6.303587	-1.0121514	DNM1	dynamin 1
223426_s_at	9.60E-06	5.47E-07	-6.8997936	6.28745	-1.8898775	EPB41L4B	erythrocyte membrane protein band 4.1 like 4B
215005_at	9.65E-06	5.53E-07	-6.8948589	6.276481	-1.0395391	NECAB2	N-terminal EF-hand calcium binding protein 2

1569452_at	9.70E-06	5.56E-07	-6.8920825	6.270307	-1.0045227	LOC692247	uncharacterized LOC692247
231011_at	9.71E-06	5.59E-07	-6.8904772	6.266737	-1.6753602	LARP1B	La ribonucleoprotein domain family member 1B
227432_s_at	9.80E-06	5.67E-07	-6.883641	6.251532	-1.8975946		
237900_at	9.82E-06	5.70E-07	-6.8813913	6.246526	-1.0151155	KLHDC4	kelch domain containing 4
221868_at	9.86E-06	5.74E-07	-6.8782059	6.239437	-2.9990161	PAIP2B	poly(A) binding protein interacting protein 2B
221462_x_at	9.87E-06	5.76E-07	-6.8767471	6.236191	-1.1055586	KLK15	kallikrein related peptidase 15

1555808_a_at	9.99E-06	5.90E-07	-6.8659041	6.21205	-1.0828955	EXD2	exonuclease 3'-5' domain containing 2
216597_at	1.01E-05	6.03E-07	-6.8567763	6.191715	-1.6308173		
1566168_at	1.01E-05	6.07E-07	-6.853994	6.185515	-1.0321073		
207567_at	1.01E-05	6.07E-07	-6.8534785	6.184366	-1.0046498	SLC13A2	solute carrier family 13 member 2
208550_x_at	1.01E-05	6.11E-07	-6.8507495	6.178283	-1.0431314	KCNG2	potassium voltage-gated channel modifier subfamily G member 2
204583_x_at	1.02E-05	6.20E-07	-6.8446105	6.164596	-1.132305	KLK3	kallikrein related peptidase 3
213861_s_at	1.02E-05	6.24E-07	-6.8413132	6.157242	-1.4246514	METTL21B	methyltransferase like 21B

221713_s_at	1.03E-05	6.27E-07	-6.8396964	6.153636	-1.1932418	MAP6D1	MAP6 domain containing 1
1566178_x_at	1.03E-05	6.31E-07	-6.8368035	6.147183	-1.1629073		
238209_at	1.03E-05	6.36E-07	-6.8330641	6.13884	-1.0459191		
231268_at	1.04E-05	6.39E-07	-6.831104	6.134465	-1.0657587	MYBL1	MYB proto-oncogene like 1
206014_at	1.04E-05	6.44E-07	-6.827495	6.12641	-1.046818	ACTL6B	actin like 6B
223285_s_at	1.05E-05	6.48E-07	-6.8250106	6.120864	-1.0931596	ST6GALNAC4	ST6 N-acetylgalactosaminide alpha-2,6-sialyltransferase 4
226597_at	1.06E-05	6.68E-07	-6.8112694	6.090174	-1.2034605	REEP6	receptor accessory protein 6
231135_at	1.07E-05	6.72E-07	-6.8091647	6.085471	-1.1030443	LOC151174	uncharacterized LOC151174

220161_s_at	1.07E-05	6.76E-07	-6.8060281	6.078462	-3.4758457	EPB41L4B	erythrocyte membrane protein band 4.1 like 4B
214325_at	1.07E-05	6.83E-07	-6.8015271	6.068401	-5.3307379	GP2	glycoprotein 2
216579_at	1.08E-05	6.89E-07	-6.7976783	6.059795	-1.0434296	GJB4	gap junction protein beta 4
221295_at	1.08E-05	6.96E-07	-6.7936264	6.050734	-1.192687	CIDEA	cell death-inducing DFFA-like effector a
1566990_x_at	1.10E-05	7.09E-07	-6.7852425	6.031977	-1.2925462	ARID1B	AT-rich interaction domain 1B

218653_at	1.10E-05	7.11E-07	-6.7837346	6.028603	-1.6838404	SLC25A15	solute carrier family 25 member 15
227032_at	1.10E-05	7.20E-07	-6.7785067	6.016902	-1.7185337	PLXNA2	plexin A2
223788_at	1.13E-05	7.43E-07	-6.7646229	5.985809	-1.0363048	GTPBP2	GTP binding protein 2
218725_at	1.14E-05	7.59E-07	-6.7554201	5.965185	-1.1937409	SLC25A22	solute carrier family 25 member 22
216535_at	1.14E-05	7.62E-07	-6.7535116	5.960907	-1.0185143	CADM3	cell adhesion molecule 3
220705_s_at	1.15E-05	7.74E-07	-6.7464844	5.94515	-1.0452248	ADAMTS7	ADAM metallopeptidase with thrombospo
